# Identification and validation of hub genes in uterine corpus endometrioid carcinoma: An observational study from TCGA and GEO

**DOI:** 10.1097/MD.0000000000042338

**Published:** 2025-05-02

**Authors:** Guoxian Luo, Caiying Bo, Jianqi Li

**Affiliations:** a Department of Gynecology, The Fourth Affiliated Hospital of Guangzhou Medical University, Guangzhou, China; b Department of Obstetrics and Gynecology, Guangdong Provincial Key Laboratory of Major Obstetric Diseases, Guangdong Provincial Clinical Research Center for Obstetrics and Gynecology, Guangdong-Hong Kong-Macao Greater Bay Area Higher Education Joint Laboratory of Maternal-Fetal Medicine, The Third Affiliated Hospital, Guangzhou Medical University, Guangzhou, China.

**Keywords:** hub genes, immunotherapy, prognosis, uterine corpus endometrioid carcinoma, weighted gene co-expression network analysis

## Abstract

Uterine corpus endometrioid carcinoma (UCEC) is a prevalent malignant tumor of the female reproductive system. Despite advancements in molecular biology and treatment strategies, the underlying molecular mechanisms of UCEC tumorigenesis remain incompletely understood. This study aimed to identify differentially expressed genes (DEGs) associated with UCEC pathogenesis, and to determine potential prognostic biomarkers and immunotherapy targets for UCEC. RNA expression datasets and clinical data from UCEC patients were collected from the UCSC Xena database and The Cancer Genome Atlas database. Principal component analysis and LIMMA methods were employed to screen 177 UCEC tissues and 24 normal endometrial tissues. Gene ontology enrichment analysis revealed that up-regulated DEGs were primarily involved in tissue development, cell cycle regulation, and epithelial development. Subsequently, weighted gene co-expression network analysis (WGCNA) identified DEGs in the blue modules that were significantly positively correlated with UCEC, while DEGs in the black modules were significantly negatively correlated with UCEC. Among the identified DEGs through WGCNA, 16 genes were selected, and further Kaplan–Meier analysis demonstrated that 5 of these genes (AURKA, CCNE1, IQGAP3, TTK, and UBE2C) were significantly negatively correlated with overall survival (OS) and considered as hub genes. The expression of these hub genes was validated using GEO datasets and immunohistochemistry (IHC) analysis from the human protein atlas. Additionally, the calculation of immune scores for immune infiltration, immune cell infiltration, and immune cell regulation across the 5 hub genes revealed potential immunotherapeutic targets and strategies. This comprehensive investigation provides insights into the molecular mechanisms underlying UCEC development, identifies 5 promising prognostic biomarkers and immunotherapy targets, and offers guidance for UCEC treatment approaches.

## 1. Introduction

Endometrial cancer is the second most common malignancy affecting the female reproductive system in China, while it holds the top position in the United States and other western developed nations.^[1,2]^ Notably, obesity, hypertension, and diabetes are established high-risk factors for endometrial cancer.^[3]^ Presently, endometrial cancer is categorized into 2 types based on distinct pathogenesis and biological behavior: type I, comprising predominantly endometrioid adenocarcinomas with a few mucinous adenocarcinomas, and type II, encompassing rare pathological subtypes like serous carcinoma, clear cell carcinoma, and carcinosarcoma.^[4]^ The prevailing type, endometrioid adenocarcinoma, typically exhibits slow progression and manifests early symptoms like abnormal uterine bleeding, leading to early diagnosis in most cases.^[5,6]^ Early-stage patients often achieve favorable clinical remission rates through surgery and supplementary radiation or chemotherapy, resulting in a 5-year survival rate as high as 80%. Nevertheless, some patients face challenges with poor pathological differentiation and tumor metastasis, leading to postoperative recurrence and eventual mortality.^[7,8]^ This underscores the need for improved insights into the mechanisms underlying endometrial cancer to identify novel candidate genes facilitating early diagnosis and treatment.

A promising and comprehensive approach in elucidating the occurrence and progression of endometrial cancer is the application of weighted gene co-expression network analysis (WGCNA). WGCNA is a well-established systems biology method that characterizes gene associations across different patients, enabling the identification of highly correlated gene sets. Through this methodology, potential biomarkers or therapeutic targets can be unveiled by exploring the interconnectivity between gene sets and their link to the phenotypic expression.^[9]^ In light of this, the primary objective of this study is to employ bioinformatics analysis, specifically WGCNA, to assess hub genes associated with endometrial cancer prognosis and immunotherapy. By leveraging this approach, we aim to shed light on key genes that can significantly impact disease outcomes and serve as potential targets for immunotherapeutic interventions.

## 2. Methods

### 2.1. Data collection and pre-processing

The ‘uterine corpus endometrioid carcinoma (UCEC)’ cohort RNA expression data and clinical information were downloaded on April 16, 2022 from The Cancer Genome Atlas (TCGA) (https://www.cancer.gov/about-nci/organization/ccg/research/structural-genomics/tcga) and UCSC Xena database (https://xenabrowser.net/datapages/) (Table S1, Supplemental Digital Content, https://links.lww.com/MD/O809). The RNA expression data were obtained from 201 patients, including 177 tumor samples and 24 normal tissue samples (Table S2, Supplemental Digital Content, https://links.lww.com/MD/O810). For principal component analysis (PCA), we use the R package stats (version 3.6.0) for analysis, specifically, we first perform z-score on the expression profile, and further use the prcomp function to perform dimensionality reduction analysis to obtain the reduced dimensionality matrix.

## 3. Identification of UCEC DEGs and GO functional enrichment

Limma^[10]^ (linear models for microarray data) is a differential expression genes screening method based on generalized linear models, here we use the R package limma (version 3.40.6) for differential analysis to obtain differentially expressed genes (DEGs) between UCEC and endometrium tissues. DEGs were defined as those showing |log2 (fold change) | > 2 and FDR < 0.05 (Table S3, Supplemental Digital Content, https://links.lww.com/MD/O811). Volcano plots of DEGs and the GO annotations of DEGs using the Sanger box tools, a free online platform for data analysis (https://vip.sangerbox.com/). *P*-values of <.05 and an FDR of <0.25 were considered statistically significant.

## 4. WGCNA and identification hub genes

A weighted adjacency matrix was constructed using a power function A mn=|C mn|^β (C mn = Pearson correlation between Gene m and Gene n; A mn = adjacency between Gene m and Gene n; β=soft-thresholding parameter). WGCNA analysis methods and procedures refer to published articles.^[11]^ Based on Hub gene screening from example (DOI: 10.3389/fonc.2018.00374),^[12]^ we calculated the expression correlation with genes to obtain gene significance (GS), and calculated the expression correlation of module feature vectors and genes to obtain module membership (MM). Setting the cutoff criteria (|MM| > 0.8 and |GS| > 0.6), those genes with high connectivity in the clinically significant module were identified as hub genes (Table S4 and Tale S5, Supplemental Digital Content, https://links.lww.com/MD/O812）.

## 5. Hub genes survival analysis

We used the R software package survival (version: 3.2-7), then integrated survival time, survival status and hub genes expression data, and evaluated the prognostic significance of each gene based on Cox regression analysis and Kaplan–Meier analysis (https://kmplot.com/analysis/).

## 6. Validation hub genes by mRNA expression and Immunohistochemical methods

The unified and standardized GEO dataset (GSE17025) downloaded from the Sanger box tools (https://vip.sangerbox.com/), and further extracted AURKA, CCNE1, IQGAP3, TTK, UBE2C, LOC134466, NPAS4, and TBC1D2B gene expression data in all patients. Finally, the expression data of 8 genes were obtained and drawn in Split violin plot.

The human protein atlas (HPA)is a Swedish-based program website with the aim to map all the human proteins in cells, tissues, and organs using an integration of various omics technologies (https://www.proteinatlas.org). The content and images of the HPA database are open access. In this study, the protein expression levels of 5 hub genes between normal endometrial tissues with UCEC tissues were compared and plotted.

## 7. Hub genes mutation landscape analysis

We mapped the genomes of 5 hub genes including mutations, copy number variants, and mRNA expression z-scores (log RNASeqV2 RSEM) using data from 373 UCEC samples of the UCEC dataset (TCGA, Nature 2013).^[13]^ Then further used the Mutation Mapper tool to map the mutation landscape of 5 hub genes. The data analysis using CBioPortal (https://www.cbioportal.org/).

## 8. Hub genes immune infiltration analysis

We downloaded the unified and standardized pan-cancer dataset from the Sanger box tools (https://vip.sangerbox.com/) (PANCAN, N = 19,131, G = 60,499), and further we extracted AURKA, CCNE1, IQGAP3, TTK, and UBE2C gene expression data in each sample. Then screened the sample sources, including primary blood derived cancer – peripheral blood (TCGA-LAML), primary tumor, metastatic of TCGA-SKCM, primary blood derived cancer – bone marrow, primary solid tumor, recurrent blood derived cancer – bone marrow samples, further log2(*x* + 0.001) transformation was performed on each expression value. And extracted the gene expression profile of each tumor from it, map the expression profile to GeneSymbol, and further use the R software package ESTIMATE (version 1.0.13, https://bioinformatics.mdanderson.org/public-software/estimate/, DOI: 10.1038/ncomms3612)^[14]^ to calculate the stromal, immune, and ESTIMATE scores of each patient in each tumor were obtained according to gene expression value. We used the corr.test function of the R package psych (version 2.1.6) to calculate the Pearson correlation coefficient between the hub genes and the immune infiltration score and 60 immune checkpoint pathway genes (including 24 inhibitory and 36 stimulatory; the Immune Landscape of Cancer, DOI:10.1016/j.immuni.2018.03.023)^[15,16]^ to determine the immune infiltration score of the hub genes.

Using CIBERSOR Method (CIBERSOR, DOI:10.1038/nmeth.3337)^[17]^ to evaluate the associations between hub genes expression and 22 types of immune cell infiltration scores of 178 UCEC samples.

## 9. Results

### 9.1. Data preprocessing

**Figure 1.**
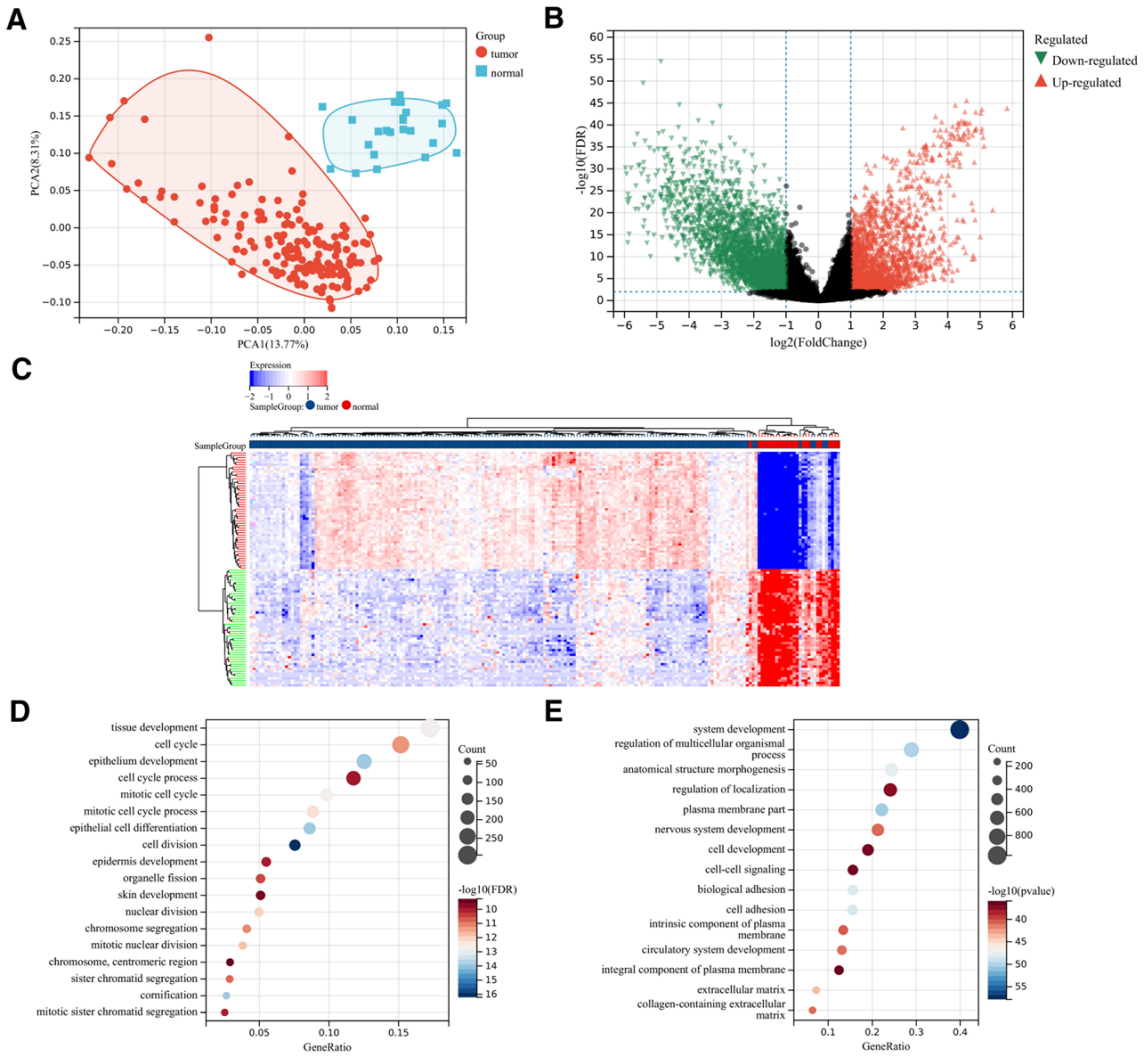
Identification of differentially expressed gene between 23 normal and 174 UCEC samples. (A) PCA distinguished UCEC samples from normal samples, PCA1 show 13.77% and PCA2 show 8.31%. (B) Volcano plot of differentially expressed gene by LIMMA. (C) Heatmap of differentially expressed gene by LIMMA. (D)GO analysis of functional enrichment of up-regulated genes. (E) GO analysis of functional enrichment of down-regulated genes. GO = gene ontology, LIMMA = linear models for microarray data, PCA = principal component analysis, UCEC = uterine corpus endometrioid carcinoma.

We utilized RNA sequencing data and clinical information from the TCGA UCEC cohort (Table 1). After implementing filtering procedures, 3 UCEC tumor samples and 1 normal sample were excluded (Fig. 1A). Subsequently, based on the PCA, 2 principal components were identified, with the first component (PCA1) accounting for 13.77% of the variance and effectively distinguishing tumor samples, while the second component (PCA2) accounted for 8.31% of the variance and distinguished normal samples. Consequently, the gene expression profiles of 24 normal samples and 177 tumor samples were retained for further investigation.

**Table 1:** The clinical information for TCGA UCEC dataset.

Characteristics	Living (N = 458)	Deceased (N = 91)	Total (N = 549)	*P*-value	FDR
Age					
Mean ± SD	63.20 ± 10.94	67.71 ± 11.43	63.95 ± 11.14		
Median (min–max)	63.00 (31.00–90.00)	68.00 (35.00–90.00)	64.00 (31.00–90.00)		
Stage					
Stage I	311 (56.65%)	32 (5.83%)	343 (62.48%)	2.60 × 10^−12^	2.90 × 10^−11^
Stage II	43 (7.83%)	9 (1.64%)	52 (9.47%)		
Stage III	91 (16.58%)	33 (6.01%)	124 (22.59%)		
Stage IV	13 (2.37%)	17 (3.10%)	30 (5.46%)		
Hypertension					
No	154 (34.00%)	27 (5.96%)	181 (39.96%)	.52	1
Yes	224 (49.45%)	48 (10.60%)	272 (60.04%)		
Diabetes					
No	255 (60.57%)	51 (12.11%)	306 (72.68%)	.51	1
Yes	92 (21.85%)	23 (5.46%)	115 (27.32%)		
Histological_type					
Endometrioid	363 (66.12%)	49 (8.93%)	412 (75.05%)	1.90 × 10^−06^	1.30 × 10^−05^
Mixed	16 (2.91%)	6 (1.09%)	22 (4.01%)		
Serous	79 (14.39%)	36 (6.56%)	115 (20.95%)		
Grade					
G1	97 (17.67%)	2 (0.36%)	99 (18.03%)	1.50 × 10^−06^	1.20 × 10^−05^
G2	108 (19.67%)	14 (2.55%)	122 (22.22%)		
G3	253 (46.08%)	75 (13.66%)	328 (59.74%)		
Peritoneal_wash					
Negative	308 (74.94%)	45 (10.95%)	353 (85.89%)	3.60 × 10^−08^	3.60 × 10^−07^
Positive	33 (8.03%)	25 (6.08%)	58 (14.11%)		

FDR = false discovery rate, TCGA = the cancer genome atlas, UCEC = uterine corpus endometrioid carcinoma.

## 10. Identification of DEGs in UCEC and GO enrichment analysis

Upon comparing 24 normal samples with 177 UCEC samples, we successfully identified 5476 DEGs with a false discovery rate (FDR) < 0.05. Among these DEGs, 2254 were up-regulated, while 3222 were down-regulated (Fig. 1B and C). Gene Ontology (GO) enrichment analysis revealed that the up-regulated DEGs were primarily associated with tissue development, cell cycle regulation, and epithelium development (Fig. 1D). Conversely, the down-regulated DEGs were found to be mainly involved in system development, regulation of multicellular organismal processes, regulation of localization, cell adhesion, and extracellular matrix functions (Fig. 1E).

## 11. WGCNA and identification of key modules and candidate hub genes

**Figure 2.**
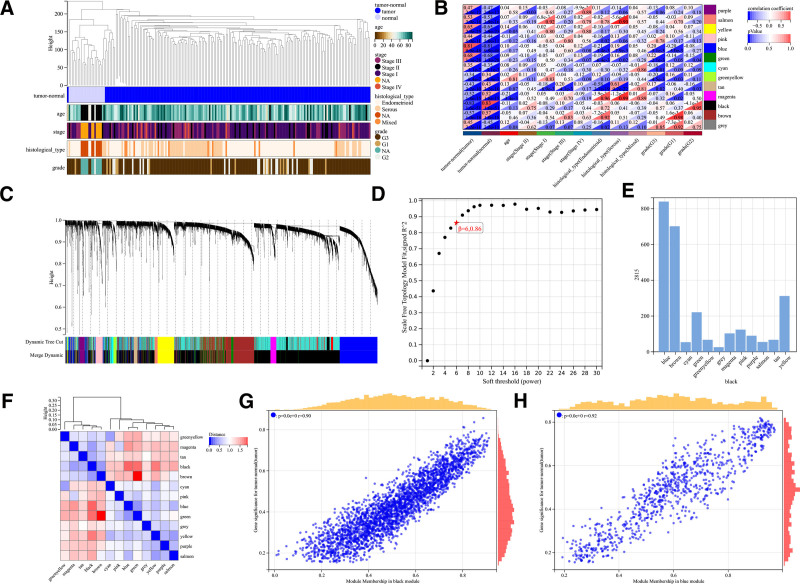
Weighted gene co-expression network analysis of differentially expressed gene in UCEC. (A) Clustering dendrogram of the clinical data from 197 samples. (B) Heatmap show the correlation between eigengenes module and clinical data of UCEC patients. (C) Dendrogram of 5476 differentially expressed gene. (D) Pick soft threshold power = 6 (E) Numbers of genes in the 13 modules. (F) Module eigengene dendrogram and heatmap of eigengene adjacency. (G and H) Scatter plots in the black modules (G) and blue modules (H). UCEC = uterine corpus endometrioid carcinoma.

We performed weighted gene co-expression network analysis (WGCNA) using a dataset comprising 5476 DEGs and clinical information from 549 UCEC patients. To ensure data quality, cluster analysis was conducted on the 549 samples using a height cutoff threshold of <200 (Fig. 2A). For the WGCNA, 6 clinical variables were considered, including disease status (tumor-normal), stage, age, histological type, and grade (Fig. 2A). A soft threshold power β of 6 and an independence degree of 0.86 were applied (Fig. 2D), leading to the identification of 13 co-expression modules, namely greenyellow, magenta, tan, black, brown, cyan, pink, blue, green, gray, yellow, purple, and salmon (Fig. 2C and E).

Eigengene analysis revealed that the results in the blue module exhibited a strong positive correlation with UCEC (correlation coefficient = 0.81, *P* = 9.9e−48), while the results in the black module showed a significant negative correlation with UCEC (correlation coefficient = −0.83, *P* = 1.6e−51) (Fig. 2B). These findings were further validated through hierarchical clustering, heatmaps, and adjacency relationships (Fig. 2F). Ultimately, a total of 289 genes within the blue and black modules were identified as candidate genes (Fig. 2G and H).

## 12. Hub gene expression and correlation with survival

**Figure 3.**
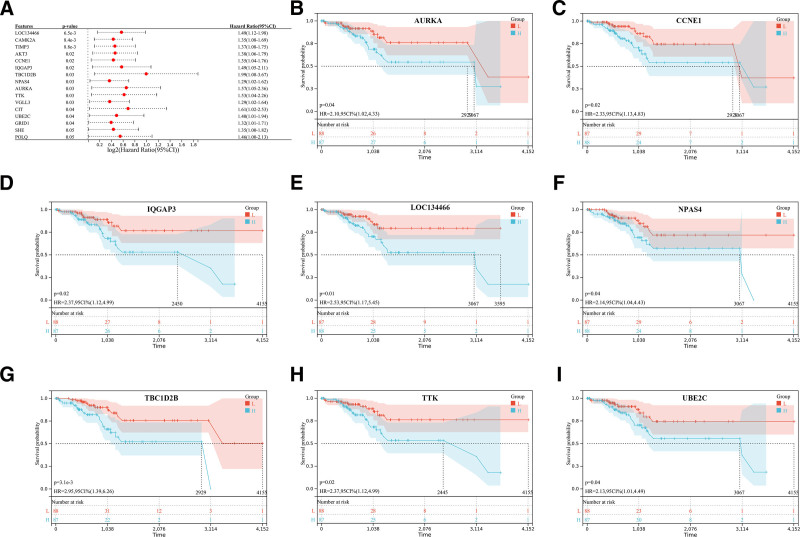
Survival analysis of hub genes. (A) Forest plot showing relationship between 16 hub genes expression levels with patients’ OS in UCEC. (B-I) Kaplan–Meier analysis of UCEC patients stratified by low or high expression of hub genes, including AURKA, CCNE1, IQGAP3, LOC134466, NPAS4, TBC1D2B, TTK and UBE2C, which *P*-Value < .05. OS = overall survival, UCEC = uterine corpus endometrioid carcinoma

Subsequently, we investigated the possible connections between gene expression and patient overall survival (OS) for the 289 genes present in the blue and black modules. This analysis led to the identification of 16 candidate genes (Fig. 3A). Notably, among these genes, AURKA, CCNE1, IQGAP3, TTK, and UBE2C from the blue module, along with LOC134466, NPAS4, and TBC1D2B from the black module, exhibited associations with prognosis (Fig. 3B–I).

## 13. mRNA expression and Immunohistochemical validated AURKA, CCNE1, IQGAP3, TTK, and UBE2C as hub genes

**Figure 4.**
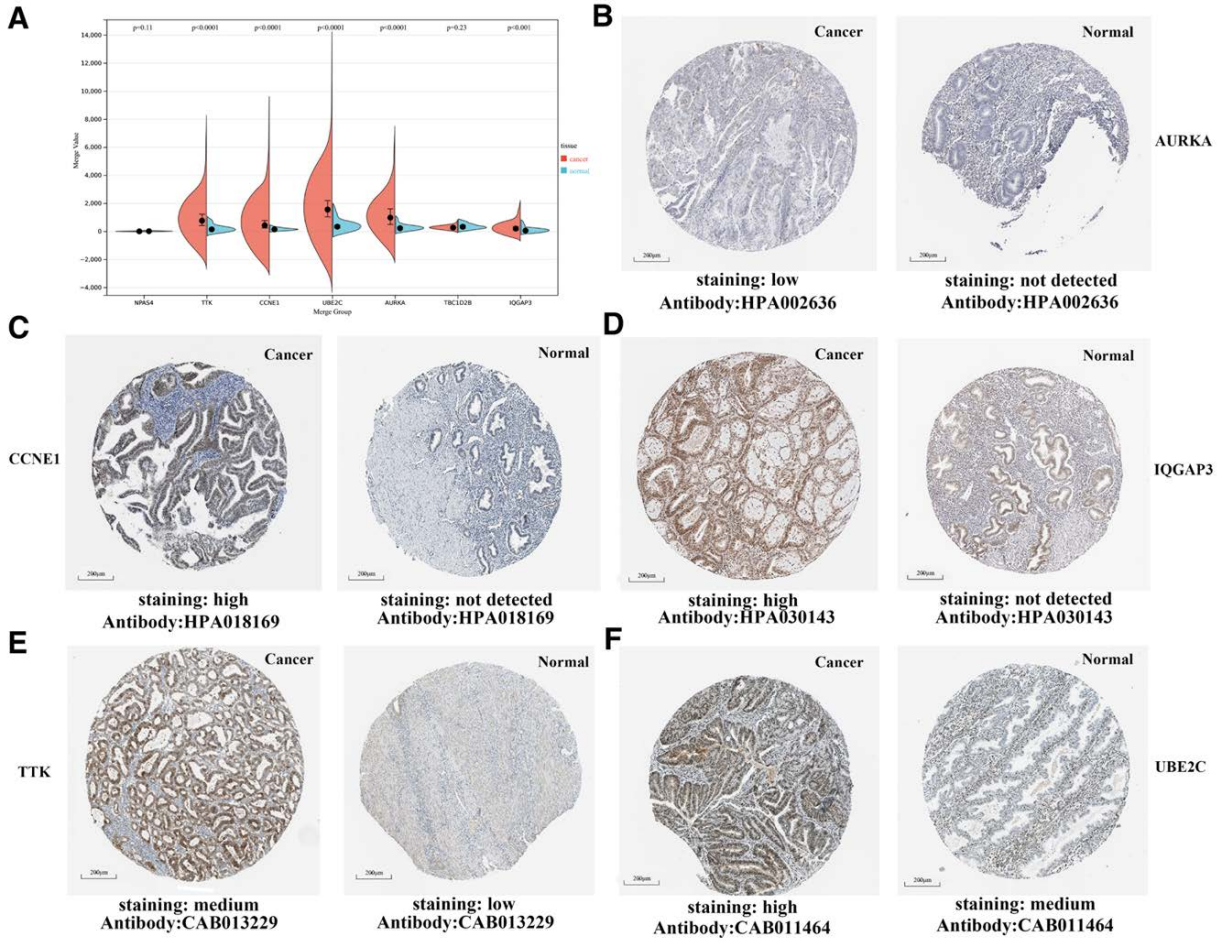
Differences expression of hub genes between normal and tumor tissues (UCEC) analyze via GEO data and IHC (human protein atlas). (A) Differences in expression of the 5 hub genes (including AURKA, CCNE1, IQGAP3, TTK and UBE2C) between normal and tumor tissues in split violin plot, *P* < .001. (B–F) Representative IHC images of distinct 5 hub genes (including AURKA, CCNE1, IQGAP3, TTK and UBE2C) in UCEC tissues and normal endometrium tissues (human protein atlas). UCEC = uterine corpus endometrioid carcinoma.

Utilizing the Sangerbox website and the Split violin plot tool, we validated significant differential expression of 5 hub genes (AURKA, CCNE1, IQGAP3, TTK, and UBE2C) between normal and UCEC tissues. Notably, AURKA, CCNE1, IQGAP3, TTK, and UBE2C were up-regulated in UCEC, suggesting their potential contribution to UCEC tumorigenesis (Fig. 4A).

Subsequently, we explored the immunohistochemical results of the 5 hub genes in UCEC tissues using the HPA. In endometrium tissues, AURKA, CCNE1, and IQGAP3 were not detected, while low and high expressions were observed in UCEC tissues (Fig. 4B–D). Similarly, TTK and UBE2C showed low or medium expressions in endometrium tissues, whereas medium and high expressions were observed in UCEC tissues (Fig. 4E and F). Collectively, these findings demonstrate the elevated expression of AURKA, CCNE1, IQGAP3, TTK, and UBE2C at both the transcriptional and translational levels in UCEC patients.

## 14. Mutation landscape of 5 hub genes

**Figure 5.**
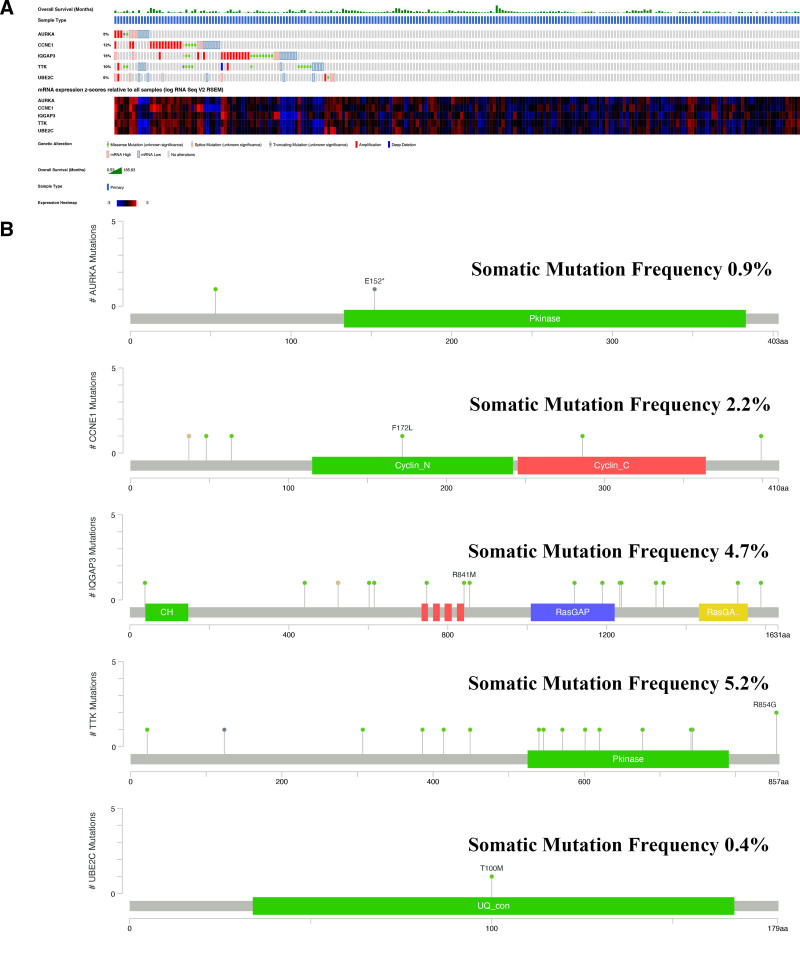
Mutations in the 5 hub genes of UCEC. (A) Landscape and heatmaps of the 5 hub genes mutations (including AURKA, CCNE1, IQGAP3, TTK and UBE2C). (B) Lollipop plots of 5 hub genes mutations (including AURKA, CCNE1, IQGAP3, TTK and UBE2C). UCEC = uterine corpus endometrioid carcinoma.

Subsequently, we utilized the OncoPrint view in the CBioPortal database to visualize mutations in the 5 hub genes. Notably, 48% of patients exhibited mutations in AURKA, CCNE1, IQGAP3, TTK, and UBE2C. IQGAP3 displayed the highest mutation rate (15%), encompassing both missense and splice mutations (Fig. 5A). Additionally, TTK showed the highest rate of somatic mutation (5.2%), with missense mutations and deletions being the most prevalent mutations observed (Fig. 5B).

## 15. Immune infiltration level of 5 hub genes

**Figure 6.**
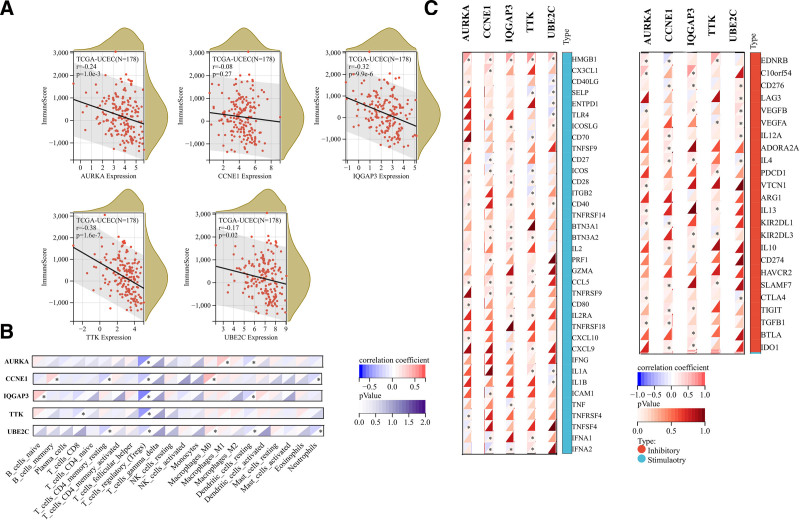
Correlation of 5 hub genes expression with immune infiltration level in UCEC. (A) AURKA, IQGAP3, TTK and UBE2C expression was significantly negatively correlated with immune infiltration in UCEC. (B) Correlation between 5 hub genes (including AURKA, CCNE1, IQGAP3, TTK and UBE2C) expression level and infiltration scores of 22 types of immune cell, calculated by CIBERSOR. (C) Correlation between 5 hub genes and 60 immune checkpoint pathway genes (24 inhibitory and 36 stimulatory). Spearman correlation test, *P* < .05 was considered significant. UCEC = uterine corpus endometrioid carcinoma.

Initially, we obtained immune infiltration scores for 177 UCEC samples and calculated Pearson correlation coefficients between the 5 hub genes and immune infiltration scores using the corr.test function of the R package psych (version 2.1.6). The results revealed significant negative correlations between AURKA (R = −0.24, *P* = 1.0e−3), IQGAP3 (R = −0.32, *P* = 9.9e−6), TTK (R = −0.38, *P* = 1.6e−7), and UBE2C (R = −0.17, *P* = .02) expressions with immune infiltration in UCEC (Fig. 6A).

Subsequently, we calculated the Pearson correlation coefficients between the 5 hub genes and 22 types of immune cell infiltration scores in UCEC, also using the corr.test function of the R package psych (version 2.1.6). We observed significant negative correlations between the 5 hub genes and T cells regulatory (Tregs) in UCEC. Specifically, AURKA expression was negatively correlated with dendritic cells resting but positively correlated with Macrophages M1. CCNE1 expression was negatively correlated with T cells CD4 memory resting and Neutrophils but positively correlated with B cells memory and Macrophages M0. IQGAP3 expression was negatively correlated with dendritic cells resting but positively correlated with B cells naive. TTK expression was negatively correlated with T cells CD8. UBE2C expression was negatively correlated with T cells CD4 memory resting, dendritic cells resting, and Neutrophils but positively correlated with Macrophages M0 (Fig. 6B).

Finally, we calculated Pearson correlations between the 5 hub genes and 60 immune checkpoint pathway genes (including 24 inhibitory and 36 stimulatory genes). Among the 24 inhibitory immune checkpoint pathways, we observed that at least 3 of the 5 hub genes showed significant positive correlations with HMGB1, ICOS, CCL5, and IFNA2. Similarly, among the 36 stimulatory immune checkpoint pathways, at least 3 of the 5 hub genes exhibited significant positive correlations with EDNRB, CD276, VEGFB, and IL4 (Fig. 6C).

## 16. Discussion

Endometrial cancer is a prevalent tumor within the reproductive system in China, and standard treatment approaches involve surgical resection, chemotherapy, and radiotherapy. While early-stage cases often respond well to treatment, there remains a lack of effective therapeutic options for locally advanced and progressive endometrial cancer, particularly in the context of molecular diagnosis and therapy. Therefore, understanding the molecular mechanisms underlying the onset and progression of endometrial cancer assumes crucial importance. In this investigation, we utilized Weighted Gene Co-expression Network Analysis (WGCNA) to identify potential hub genes associated with the prognosis and immunotherapy of UCEC.

Through WGCNA analysis, we identified a total of 5476 DEGs, 13 modules, and 16 candidate genes. Among these candidate genes, AURKA, CCNE1, IQGAP3, TTK, and UBE2C demonstrated significant associations with poor prognosis. Accordingly, these genes, namely AURKA, CCNE1, IQGAP3, TTK, and UBE2C, were designated as the “final” hub genes in our study.

Aurora kinase A (AURKA) is a member of the serine/threonine kinase family whose activation is required for the process of cell division by regulating mitosis. Based on the TCGA database, the expression of AURKA was significantly higher in cancer samples than in normal control samples. AURKA may promotes tumorigenesis via cell proliferation, epithelial-mesenchymal transition (EMT), metastasis, apoptosis and self-renewal of cancer stem cells.^[18]^ AURKA may play a role in impaired cell viability and enhanced apoptosis in UCEC.^[19]^ Further research found that AURKA inhibitor Alisertib may treat UCEC, revealing that AURKA is a promising therapeutic target.^[20]^ Liu found that AURKA is highly expressed in HCC and predicted 8 AURKA-related genes with prognostic value, and then successfully constructed a gene signature. These gene signatures are associated with immune cell infiltration and immune checkpoints shown potential therapeutic point in HCC patients.^[21]^

Interestingly, although this study and Yuan study^[20]^ analyzed different TCGA data using similar methods and tools, AURKA was screened and identified consistently as a key gene for predicting prognosis in UCEC. This study found that the expression of AURKA was significantly negatively correlated with immune infiltration, T cells regulatory (Tregs) and Dendritic cells resting in UCEC. HMGB1, CD40LG, TNFSF9, ICOS, CD28, CD40, IL2, CCL5, CD80, CXCL10, TNFRSF4, and TNFSF4 may be the stimulatory immune checkpoints of AURKA in UCEC. And EDNRB, C10ORF54, VEGFB, VTCN1, IL13, KIR2DL1, CTLA4, and TGFB1 may be the inhibitory immune checkpoint of AURKA in UCEC. The results of a Phase 2 randomized clinical trial named TBCRC041, which evaluated the efficacy of Alisertib (an Aurora A kinase inhibitor) alone or in combination with Fulvestrant (an estrogen receptor antagonist) in patients with endocrine-resistant advanced breast cancer. The study found that the group receiving the combination of Alisertib and Fulvestrant showed better progression-free survival compared to the group receiving Alisertib alone, suggesting that the combination therapy may be more effective in endocrine-resistant breast cancer.^[22]^

CCNE1 is a member of the highly conserved cyclin family. In CCNE1-overexpressing tumor cells, aberrant p53 further lost control of the G1-S checkpoint, increasing the dependence on S phase and, more importantly, survival dependence on the G2-M cell cycle checkpoint.^[23–25]^ CCNE1 is an oncogene in many tumors, and its elevated expression was further found to be associated with poor outcomes and platinum resistance.^[26–28]^ In this study, CCNE1 expression was negatively correlated with T cells CD4 memory resting and Neutrophils, but positively correlated with B cells memory and macrophages M0 in UCEC. HMGB1, CX3CL1, TLR4, ICOS, CD27, BTN3A1, BTN3A2, PRF1, CCL5, TNFRSF9, ICAM1, and IFNA1/2 may be the stimulatory immune checkpoints of CCNE1. And EDNRB, CD276, ADORA2A, IL12A, IL4, KIR2DL1, IL10, SLAMF7, TGFB1, TIGIT, and IDO1 may be the inhibitory immune checkpoint of CCNE1 in UCEC. A clinical trial investigated the impact of Cyclin E1 expression on the efficacy of Palbociclib, with a focus on patients with hormone receptor-positive metastatic breast cancer who had previously undergone treatment. The study found that patients with high Cyclin E1 expression had poorer outcomes with Palbociclib, as indicated by a shorter progression-free survival. This suggests that overexpression of Cyclin E1 may be a potential mechanism of resistance to Palbociclib.^[29]^

The protein encoded by IQGAP3 belong to the Rho family, which play critical roles in the development and progression of several cancers. Studies have found that the expression of IQGAP3 is increased in high-grade serous ovarian cancer, and the proliferation, migration and invasion of ovarian cancer cell are inhibited when IQGAP3 is silenced.^[30]^ Interestingly, high expression of IQGAP3 appears to be associated with tumor mutational burden, microsatellite instability, immune cell infiltration, and immunomodulatory agents, with integral roles in the progression and immune response of various human cancers.^[31]^ IQGAP3 expression was negatively correlated with dendritic cells resting, but positively correlated with B cells naive. HMGB1, ICOSLG, TNFSF9, ICOS, CD28, CD40, BTN3A1/2, IL2, CCL5, TNFRSF9, IL2RA, CXCL9, INF, and IFNA2 may be the stimulatory immune checkpoints of IQGAP3. And C10ORF54, CD276, VEGFB, IL4, KIR2DL3, IL10, TIGIT, BTLA, and IDO1 may be the inhibitory immune checkpoint of IQGAP3 in UCEC.

TTK encodes a dual-specificity protein kinase that is involved in cell proliferation. The expression of TTK promotes cell proliferation, migration and invasion, suggesting that TTK may be an oncogene.^[32,33]^ Inhibition of TTK expression caused chromosomal missegregation and tumor cell death, conversely, HER2-positive breast and hepatocellular carcinomas were associated with poor prognosis after overexpression of TTK.^[34,35]^ The abnormal expression of TTK is involved in breast cancer cell cycle pathway, DNA replication pathway, and P53 signaling pathway.^[36]^ TTK expression was also negatively correlated with T cells CD8. HMGB1, SELP, CD70, CD28, ICOS, BTN3A2, GZMA, CCL5, IL2RA, CXCL9, IL1A, and IFNA1/2 may be the stimulatory immune checkpoints of IQGAP3. And EDNRB, VEGFA, PDCD1, IL13, KIR2DL3, and SLAMF7 may be the inhibitory immune checkpoint of TTK in UCEC.

UBE2C (ubiquitin-conjugating enzyme E2C) encodes a protein that is associated with diseases such as methotrexate-associated lymphoid hyperplasia and complement component 7 deficiency. GO annotations reveals that the main functions of UBE2C are ligase activity and ubiquitin protein ligase binding.^[37]^ The abnormal expression of UBE2C gene has a clear relationship with the prognosis, clinical features, immunity, methylation of pan-cancer, suggest that UBE2C-encoded protein may contribute to cancer progression.^[38]^ Overexpression of UBE2C is associated with breast cancer recurrence and is involved in estrogen-dependent/independent proliferation in early HR+/HER2-breast cancer.^[39]^ UBE2C expression was negatively correlated with T cells CD4 memory resting, dendritic cells resting and Neutrophils, but positively correlated with Macrophages M0. HMGB1, CD40LG, SELP, ENTPD1, TLR4, ICOSLG, CD40, and IL1A/B may be the stimulatory immune checkpoints of UBE2C. And CD276, LAG3, VEGFA/B, IL12A, IL4, and CTLA4 may be the inhibitory immune checkpoint of UBE2C in UCEC.

## 17. Conclusion

In summary, the identified hub genes have shown significant involvement in tissue development and cell cycle regulation in different tumor types. In the specific context of UCEC, the 5 hub genes identified in this study are associated with prognosis and immune infiltration, suggesting their potential as indicators for prognostic assessment and immunotherapeutic interventions. Nevertheless, it is essential to acknowledge that the conclusions presented here are based solely on bioinformatics analysis. Further investigations exploring the carcinogenic mechanisms of UCEC are necessary to enhance the credibility and robustness of these findings.

## Acknowledgments

Thanks for the Sanger box tools, a free online platform for data analysis (https://vip.sangerbox.com/).

## Author contributions

**Conceptualization:** Jianqi Li.

**Data curation:** Guoxian Luo, Jianqi Li.

**Formal analysis:** Guoxian Luo, Caiying Bo.

**Funding acquisition:** Jianqi Li.

**Investigation:** Jianqi Li.

**Methodology:** Guoxian Luo, Caiying Bo.

**Project administration:** Jianqi Li.

**Resources:** Jianqi Li.

**Software:** Caiying Bo, Jianqi Li.

**Supervision:** Jianqi Li.

**Validation:** Jianqi Li.

**Visualization:** Caiying Bo, Jianqi Li.

**Writing – original draft:** Guoxian Luo, Caiying Bo.

**Writing – review & editing:** Guoxian Luo, Caiying Bo, Jianqi Li.

## Supplementary Material


