# Supplementary material for: Identification and validation of hub genes in uterine corpus endometrioid carcinoma: An observational study from TCGA and GEO
Source: Medicine (Baltimore). 2025 May 2;104(18):e42338. doi: 10.1097/MD.0000000000042338 (PMC12055170; doi:10.1097/MD.0000000000042338)
Supplement: Supplementary file 1 [file medi-104-e42338-s001.pdf]

```
{\rtf1\ansi\ansicpg936\cocoartf2639
\cocoatextscaling0\cocoaplatform0{\fonttbl\f0\fswiss\fcharset0
Helvetica;}
{\colortbl;\red255\green255\blue255;}
{\*\expandedcolortbl;;}
\paperw11900\paperh16840\margl1440\margr1440\vieww11520\viewh8400\vi
ewkind0
\pard\tx566\tx1133\tx1700\tx2267\tx2834\tx3401\tx3968\tx4535\tx5102\
tx5669\tx6236\tx6803\pardirnatural\partightenfactor0

\f0\fs24 \cf0 sample    TCGA-A5-A2K7-01 TCGA-FL-A1YI-11 TCGA-EO-
A3AS-01 TCGA-EY-A549-01 TCGA-EO-A3B0-01 TCGA-KJ-A3U4-01 TCGA-EY-
A2OQ-01 TCGA-PG-A7D5-01 TCGA-B5-A0JN-01 TCGA-FL-A1YU-11 TCGA-EO-
A3KW-01 TCGA-PG-A915-01 TCGA-D1-A3JQ-01 TCGA-AX-A2HH-01 TCGA-AJ-
A5DV-01 TCGA-5S-A9Q8-01 TCGA-DI-A2QY-01 TCGA-DI-A1BU-01 TCGA-FL-
A1YF-11 TCGA-B5-A5OE-01 TCGA-AX-A3G3-01 TCGA-PG-A916-01 TCGA-DI-
A2QY-11 TCGA-AJ-A3NC-01 TCGA-FL-A1YG-11 TCGA-DF-A2KY-01 TCGA-AX-
A3FV-01 TCGA-QS-A5YR-01 TCGA-DF-A2KZ-01 TCGA-A5-A2K2-01 TCGA-AX-
A3FZ-01 TCGA-B5-A0JR-01 TCGA-AJ-A3TW-01 TCGA-PG-A914-01 TCGA-AX-
A3GI-01 TCGA-D1-A2G0-01 TCGA-EY-A2OO-01 TCGA-A5-A7WJ-01 TCGA-AX-
A3G9-01 TCGA-BK-A4ZD-01 TCGA-B5-A3FC-01 TCGA-AJ-A3NE-11 TCGA-AP-
A3K1-01 TCGA-EY-A3QX-01 TCGA-AX-A0IZ-11 TCGA-AX-A05W-01 TCGA-AJ-
A3BI-01 TCGA-EY-A2ON-01 TCGA-AX-A3FS-01 TCGA-SL-A6JA-01 TCGA-B5-
A0K9-01 TCGA-AX-A2H4-01 TCGA-AJ-A3EJ-01 TCGA-AJ-A3NC-11 TCGA-EY-
A1GP-01 TCGA-EO-A3KX-01 TCGA-AJ-A3BH-01 TCGA-D1-A3JP-01 TCGA-AJ-
A3NF-01 TCGA-FI-A3PV-01 TCGA-AX-A05Y-11 TCGA-EY-A54A-01 TCGA-EO-
A22U-01 TCGA-D1-A3DH-01 TCGA-FL-A1YQ-11 TCGA-SJ-A6ZI-01 TCGA-DF-
A2KR-01 TCGA-AJ-A3NH-01 TCGA-EY-A1GL-01 TCGA-AX-A3G4-01 TCGA-SL-
A6J9-01 TCGA-BG-A3EW-01 TCGA-A5-A2K3-01 TCGA-B5-A5OD-01 TCGA-B5-
A5OC-01 TCGA-EY-A1GX-01 TCGA-E6-A8L9-01 TCGA-BG-A0MK-01 TCGA-AX-
A3GB-01 TCGA-E6-A2P8-01 TCGA-QF-A5YT-01 TCGA-5B-A90C-01 TCGA-AJ-
A3OK-01 TCGA-AX-A3G1-01 TCGA-SJ-A6ZJ-01 TCGA-K6-A3WQ-01 TCGA-EY-
A3L3-01 TCGA-AJ-A3BK-01 TCGA-KP-A3W0-01 TCGA-AX-A2IN-01 TCGA-BG-
A2AD-11 TCGA-AJ-A3QS-01 TCGA-EY-A210-01 TCGA-DI-A1C3-01 TCGA-AJ-
A3EL-01 TCGA-AX-A2HC-11 TCGA-A5-A3LP-01 TCGA-FL-A1YT-11 TCGA-EO-
A22Y-01 TCGA-AX-A3FT-01 TCGA-A5-A2K5-01 TCGA-DF-A2KN-01 TCGA-AJ-
A2QM-01 TCGA-AX-A3FX-01 TCGA-AJ-A3I9-01 TCGA-AJ-A3EM-01 TCGA-FL-
A1YH-11 TCGA-KP-A3W4-01 TCGA-PG-A5BC-01 TCGA-B5-A3FD-01 TCGA-E6-
A2P9-01 TCGA-B5-A3FB-01 TCGA-QS-A8F1-01 TCGA-FI-A2EY-01 TCGA-BK-
A139-02 TCGA-EO-A3KU-01 TCGA-B5-A11R-01 TCGA-B5-A1MS-01 TCGA-A5-
A3LO-01 TCGA-KP-A3W1-01 TCGA-A5-A1OH-01 TCGA-AJ-A3OJ-01 TCGA-A5-
A2K4-01 TCGA-AJ-A2QO-01 TCGA-AJ-A3NH-11 TCGA-EY-A4KR-01 TCGA-BG-
A3PP-01 TCGA-AX-A1C7-01 TCGA-AX-A0J0-11 TCGA-BK-A4ZD-11 TCGA-A5-
A7WK-01 TCGA-AX-A3FW-01 TCGA-AJ-A8CV-01 TCGA-AP-A5FX-01 TCGA-JU-
AAVI-01 TCGA-EO-A3AZ-01 TCGA-DF-A2KU-01 TCGA-BS-A0V7-01 TCGA-BK-
A6W4-01 TCGA-FL-A1YL-11 TCGA-EO-A1Y7-01 TCGA-BK-A13B-01 TCGA-QS-
A5YQ-01 TCGA-EO-A3AU-01 TCGA-AJ-A3NE-01 TCGA-BG-A3PP-11 TCGA-B5-
A3FH-01 TCGA-AX-A2HD-11 TCGA-B5-A3FA-01 TCGA-AX-A3G8-01 TCGA-A5-
AB3J-01 TCGA-DF-A2L0-01 TCGA-4E-A92E-01 TCGA-AJ-A3BF-01 TCGA-EY-
A2OP-01 TCGA-AJ-A23N-01 TCGA-FL-A1YN-11 TCGA-QS-A744-01 TCGA-2E-
A9G8-01 TCGA-PG-A6IB-01 TCGA-AJ-A3BG-01 TCGA-EO-A3AV-01 TCGA-B5-
A3F9-01 TCGA-QF-A5YS-01 TCGA-EY-A72D-01 TCGA-AJ-A5DW-01 TCGA-AX-
A3G7-01 TCGA-AJ-A6NU-01 TCGA-EO-A3L0-01 TCGA-AX-A3G6-01 TCGA-B5-
A1MW-01 TCGA-FI-A3PX-01 TCGA-D1-A3DG-01 TCGA-EY-A1GO-01 TCGA-BS-
```

A0V4-01 TCGA-AJ-A3OL-01 TCGA-FL-A3WE-11 TCGA-BG-A3EW-11 TCGA-AJ-
A3NG-01 TCGA-AJ-A8CT-01 TCGA-EY-A5W2-01 TCGA-DI-A2QT-01 TCGA-KP-
A3VZ-01 TCGA-EY-A548-01 TCGA-B5-A3S1-01 TCGA-PG-A917-01 TCGA-EO-
A3B1-01 TCGA-AJ-A3BD-01 TCGA-DF-A2KV-01 TCGA-EO-A22X-01 TCGA-AJ-
A3EK-01 TCGA-D1-A3DA-01 TCGA-KP-A3W3-01 TCGA-AJ-A3IA-01 TCGA-AJ-
A8CW-01 TCGA-H5-A2HR-01 TCGA-EY-A547-01 TCGA-FL-A1YV-11 TCGA-BK-
A6W3-01 TCGA-BK-A56F-01 TCGA-EO-A3AY-01\
ARHGEF10L 8.8300 10.0442 9.0378 9.1277 7.8386 9.7828
8.7956 8.6525 9.5438 9.4343 8.6945 9.4004 8.4709 9.6620
9.7271 8.9562 9.1745 9.8912 9.9266 10.6627 8.9249 8.8342
9.9831 10.0468 8.9394 7.9110 10.6914 9.7991 10.0220 10.1626
8.1840 7.9080 9.4941 8.2841 9.9867 8.5921 9.9720 10.1957
9.0466 5.2936 8.8252 9.0475 9.9141 10.9196 9.1883 10.2745
7.8657 10.5137 8.1691 9.5513 10.2161 9.2681 8.7083 9.3049
9.5947 8.3611 10.8642 9.0647 9.9099 10.4668 9.1852 9.2101
9.4700 9.6664 9.7225 9.9505 9.0817 8.5163 9.6237 9.4812
9.7531 9.1454 8.9074 8.7515 9.6568 9.0601 7.1892 9.6249
8.6867 9.6385 8.0483 9.0995 9.0073 9.0977 9.4639 8.3141
8.4827 10.0833 8.3954 8.6675 9.7827 8.7941 9.0462 9.7080
7.5710 8.6448 9.3313 10.0226 9.0585 10.1482 8.9030 10.0654
10.0746 9.0925 9.0271 8.0003 9.6648 8.7612 9.3460 9.6484
9.4012 8.9601 7.2019 10.9527 9.4452 9.5339 9.5445 9.4317
8.9484 9.5217 8.0408 9.5297 9.9085 9.3944 9.5942 9.2115
8.7335 7.6922 9.4317 8.9500 9.7998 10.6468 9.6505 9.9811
9.4232 7.4100 9.0842 9.2927 9.9193 9.9200 9.0363 9.3070
9.6741 9.8951 9.8407 8.8781 9.3975 10.2133 10.2706 7.9158
9.3120 7.2206 10.0796 9.7495 7.9782 9.8508 9.3022 9.5575
8.6884 7.9617 10.7432 10.7739 9.3804 10.0912 8.2211 8.5476
10.3331 10.2072 9.2618 10.7237 9.2337 8.5396 9.4424 8.2603
9.7826 9.6777 9.3002 9.9364 9.2787 9.5863 8.9040 9.8788
7.7158 8.7308 9.1798 9.3402 9.7130 9.7802 10.2786 7.4367
9.9822 7.6885 9.0104 11.1904 8.1013 7.2905 8.8030 9.8957
9.3525 9.7854 9.0527\
HIF3A 4.3316 5.7323 6.9491 2.4559 3.4218 6.5181 6.6199
10.1155 9.8901 5.5592 6.4812 7.6918 9.6736 4.5286 1.2358
2.1513 10.4556 6.7366 4.2822 4.8701 8.1285 1.5770 7.3230
1.6784 8.4802 4.5835 3.1387 4.6547 1.3857 6.3371 2.6553
3.7402 9.5180 10.6227 9.4240 7.2078 9.3519 3.3101 1.4957
2.0552 0.6649 5.8588 5.6793 5.2370 7.4857 4.7654 3.6457
9.2936 3.2025 4.5180 2.7077 5.2513 8.8572 9.2428 7.8903
1.9450 2.4376 8.4504 9.0025 9.3583 7.8960 9.4004 0.9583
8.6772 5.4184 2.1442 4.6221 8.5504 3.3716 6.9792 8.0452
1.9301 1.5110 11.3142 3.5065 3.6494 8.7629 1.8986 6.5658
4.6721 2.2758 2.9341 7.0210 7.2446 3.5316 10.2439 6.6093
1.8689 7.4606 2.8411 8.5378 9.8201 8.3895 3.4367 5.2920
8.1434 8.3385 7.5754 4.6504 3.0758 3.3101 4.2290 6.7712
4.7873 2.7166 4.7508 8.6736 8.5168 10.6818 7.2655 0.8916
7.5158 8.5933 9.0768 4.7835 8.7169 1.4679 10.7140 1.9462
2.9683 10.7984 0.5886 9.3911 6.5157 8.8122 9.4142 6.5949
10.9384 8.8562 7.7291 9.2258 3.9205 1.5194 3.6704 5.3024
6.4375 4.7107 7.5102 6.5841 8.9791 1.0240 1.5646 4.3077
2.3527 5.6139 10.2574 6.5787 9.5353 8.0254 5.1321 6.4453
0.0000 5.3760 10.7840 1.9146 8.1024 8.4674 5.9883 6.4192
1.2429 2.9615 6.4676 4.2771 5.3344 8.6210 4.2724 7.7480

```
7.0974  2.1803  9.1833  2.2163  8.2049  9.1238  6.5609  3.2253
4.8197  6.2094  8.4973  8.8196  5.7024  2.9186  9.2790  8.6268
2.4895  9.4741  3.6920  9.3217  8.4587  3.7742  6.8212  2.3837
5.7191  6.9473  8.3748  4.9601  5.7421  5.8436  8.5303  0.0000
3.1721  0.6959\
RNF17   0.5526  0.9197  1.1543  1.3959  0.0000  0.7223  0.0000
0.4748  0.0000  0.0000  0.4291  0.0000  0.4363  0.0000  0.0000
0.0000  0.0000  0.0000  0.0000  0.0000  0.0000  0.0000  0.0000
0.0000  0.0000  0.0000  0.0000  0.5990  0.0000  0.0000  0.4127
0.0000  0.5159  0.0000  0.0000  0.6819  0.0000  0.0000  0.0000
0.0000  0.0000  1.9303  1.0569  0.0000  1.0260  0.0000  0.0000
0.0000  0.0000  0.0000  0.0000  0.7768  0.0000  0.0000  0.4645
0.0000  0.0000  0.0000  0.4252  1.7785  0.0000  0.4969  0.5573
0.0000  0.0000  0.0000  0.0000  0.4832  0.0000  0.0000  0.0000
0.7001  0.0000  0.0000  0.5367  0.0000  0.0000  0.0000  0.6894
0.0000  0.5659  0.0000  0.0000  1.4748  0.5263  0.0000  0.5376
0.0000  0.0000  0.0000  0.0000  0.0000  0.0000  0.0000  0.5198
1.3960  0.0000  0.0000  0.0000  0.0000  0.0000  0.0000  0.0000
0.7358  0.0000  0.0000  0.0000  0.0000  3.7851  0.0000  0.0000
0.0000  0.0000  0.0000  0.5141  0.0000  0.0000  0.0000  0.0000
0.0000  0.0000  0.0000  0.0000  0.0000  0.0000  0.0000  0.0000
0.0000  3.6900  0.0000  0.0000  0.6684  0.0000  1.9109  0.0000
0.0000  0.0000  0.0000  0.0000  1.6315  0.0000  0.5748  0.0000
0.0000  0.4562  0.0000  0.0000  0.0000  0.0000  0.0000  0.0000
0.0000  7.9478  0.0000  0.0000  0.0000  0.5638  0.0000  0.0000
0.0000  0.0000  1.3735  0.0000  0.0000  0.0000  0.0000  0.0000
0.0000  0.0000  0.0000  0.0000  0.0000  0.9335  0.0000  0.0000
0.0000  0.0000  2.2714  9.2600  1.0692  0.5545  0.0000  0.0000
0.5055  0.0000  0.0000  0.0000  0.0000  0.0000  0.0000  0.0000
0.0000  0.0000  0.0000  0.0000  0.0000  0.6064  0.0000  0.0000
0.5077  0.0000\
RNF10   11.9095 11.8911 11.1028 11.8468 10.0470 12.5310 11.6893
12.0880 11.3735 11.8626 12.1964 11.8810 12.0969 11.7829 11.8618
11.7582 11.5812 12.1516 11.9590 11.7034 12.1662 12.1523 11.7012
11.8855 11.9736 11.4330 12.6924 11.7976 12.0080 11.9982 11.6908
11.8299 11.9705 12.3586 11.3259 11.6154 11.9922 12.0143 11.9144
11.3993 11.7994 11.8117 12.1897 11.7930 11.6689 12.4199 12.8583
12.0681 11.6890 12.0487 11.4817 12.2165 12.0889 11.7863 11.7720
11.9324 11.9169 11.9959 11.4259 12.2112 11.6204 11.7327 12.3733
11.9841 12.0414 12.0336 12.1298 11.4158 12.2777 12.8242 12.1345
11.9590 11.7472 11.7384 12.1887 12.3080 12.7313 11.8765 11.9196
11.8951 11.7992 11.4169 12.1763 12.5478 11.8675 12.0408 11.8398
12.0731 12.1381 11.9725 11.9481 11.6042 12.2264 12.2937 12.4221
11.8726 12.2451 11.7810 12.1091 11.7879 12.3145 12.0441 11.5329
12.4748 12.6078 12.3541 12.0796 12.2434 12.1221 12.0789 11.6812
11.7409 11.3478 12.1170 11.2396 11.8526 12.1899 12.0900 12.0159
12.1123 11.4124 12.1676 11.8798 12.0353 11.6604 11.4674 12.0527
11.7846 11.7058 11.6095 12.9322 12.2738 12.0047 11.9783 11.8338
11.1287 12.2903 11.7397 11.6234 11.7747 12.6207 12.0305 11.9442
11.9714 11.6244 11.7078 12.4730 11.8426 12.5678 11.8296 11.6730
11.6643 11.8927 12.3665 11.7258 12.0557 11.7013 11.7532 12.2045
11.5031 11.9560 12.1705 12.1382 11.7908 11.6569 12.1102 13.5746
11.7924 12.0670 12.1957 12.0318 12.2931 12.8779 12.0148 11.8944
11.9477 11.9498 11.9074 11.7666 11.7015 12.0446 11.6399 11.1085
```

```
11.9848 11.2523 12.1549 11.4469 12.1326 11.8475 11.9372 11.7168
12.1024 12.1521 11.9330 12.0105 12.1109 11.5813 11.7617 11.9848
11.6896 12.2219\
RNF11   9.8836  11.8090 11.4201 10.9072 10.4605 10.8193 9.9574
10.2801 10.6510 10.7374 10.5484 10.4815 10.4337 10.7915 10.4306
9.4293  9.9881  10.5752 11.3935 10.3942 10.0854 10.4019 10.4748
10.8906 11.6940 10.2231 9.9288  10.0730 10.8459 9.9520  10.9588
10.0925 9.8705  10.1968 9.8705  9.8013  10.5618 10.4598 11.1434
11.5909 11.1254 11.8199 10.7880 11.1019 12.0059 9.9276  9.4117
10.7962 10.5546 10.7523 10.3432 9.9117  10.5305 11.8751 10.3342
10.8173 10.4902 11.0064 10.8230 10.4312 11.9766 10.8463 10.3936
10.4578 11.1317 10.7941 10.1420 11.3673 10.5051 10.5289 9.7566
10.5649 9.6967  10.8049 11.3322 10.3522 10.8155 10.6223 10.6548
10.6715 9.8663  10.5566 11.0526 10.8820 9.8360  10.7168 11.0674
9.2652  10.8226 10.2333 11.4677 10.6111 10.5304 9.9953  10.1595
11.9630 11.7714 11.5374 10.1238 10.4890 8.3584  10.3017 11.0254
10.8517 10.7870 10.9024 10.4854 10.9025 11.1778 9.9067  10.3112
10.1744 9.8639  8.5196  10.6058 10.7834 10.6600 10.3215 10.9638
10.3925 10.4918 10.6504 10.8015 10.9232 10.8237 11.0443 10.8755
9.3074  11.7335 11.4265 10.8358 11.2313 10.0941 10.7775 10.3704
8.7862  8.9697  8.9660  10.7558 11.5496 10.5304 11.0951 10.1294
10.7940 10.7188 12.2152 10.9527 10.8807 11.0873 9.9053  10.7909
8.9992  10.5897 10.0338 9.8069  10.7173 11.2782 10.1760 10.3666
10.0125 10.4198 9.9521  10.6765 9.9478  9.9627  11.0138 10.4465
10.8314 10.1074 10.2244 10.7753 10.2932 10.2193 9.6096  8.7190
10.8971 10.9904 11.7429 10.5918 10.6879 10.5546 9.5295  10.2768
10.1690 10.5874 10.7701 10.1771 10.3155 9.9201  10.3164 10.6843
10.8511 11.2981 10.8610 10.3386 8.1794  10.4938 11.2273 10.3942
10.4805 8.5501\
RNF13   8.6590  10.1779 10.0697 9.9685  9.4533  9.5260  9.5457
9.8064  9.5096  9.3226  10.1993 9.7891  9.3959  10.0745 9.7085
9.0761  9.4088  10.1194 9.9347  9.3621  9.6001  9.8194  9.9178
9.9129  10.0260 9.2708  8.7974  9.2302  9.0868  9.2114  9.2932
9.3455  10.4745 9.8289  9.4907  9.3943  9.6319  9.8193  10.3127
9.8195  9.9244  9.9457  9.3475  10.0613 10.7852 9.4081  7.7885
9.7932  9.7768  10.2040 9.3986  10.9029 10.0164 10.6848 9.4305
9.9319  9.7336  10.6739 9.8032  9.8686  10.6011 10.2941 9.8644
8.9585  9.8707  9.5758  9.3390  9.6640  9.4488  9.1585  9.6398
9.3498  9.7566  9.7694  10.0952 9.8585  9.6092  9.6098  9.8299
10.0296 9.5906  10.1053 10.3169 9.2389  9.0633  9.8590  9.9003
8.9118  9.6604  9.2837  10.2740 10.0275 9.1248  9.6824  8.7026
10.3835 10.4640 10.0347 9.4013  9.9036  7.9177  9.7126  9.2247
9.3228  9.2919  10.8560 9.2828  9.7883  9.6539  9.3821  9.8817
9.5067  7.4426  8.6330  9.2581  9.5774  9.8300  9.4030  9.4197
10.2715 9.7667  9.9543  9.8140  9.6742  9.9943  10.6808 9.7771
8.4264  10.7234 10.2239 8.8942  10.3853 9.4311  9.1114  9.7807
8.0897  9.0722  8.1554  10.1246 10.2973 9.3260  9.7719  9.2462
9.7109  9.7313  10.0931 10.0252 9.8645  9.2930  8.6812  9.8576
6.8213  9.7744  9.2846  9.5690  9.1173  10.3104 10.1231 8.8161
9.3079  9.8076  9.1814  10.0493 9.4437  10.2695 9.7576  9.2651
10.2617 9.2906  9.2139  9.0895  9.7786  9.0176  9.6106  7.7966
9.2181  9.8258  10.3982 9.7974  9.6249  9.5712  9.4457  9.8608
10.0535 9.4673  9.7456  9.6530  9.2716  9.1222  9.3861  9.9548
9.7851  9.9596  9.4392  9.7808  7.9273  8.3417  10.2756 9.4923
```

10.1997 7.9706\
GTF2IP1 12.4235 13.3552 12.2797 13.2931 12.9195 12.4622 12.9959
13.2812 12.1190 13.3084 12.7948 13.5915 13.1124 12.5993 12.9169
12.8510 13.0076 13.7598 13.1449 12.7190 12.6814 12.8293 13.1714
13.1252 12.6387 11.8152 12.5373 13.1606 12.9456 12.7670 12.8240
13.0446 11.4635 13.5989 12.3626 12.0657 12.7131 13.5013 12.4093
12.2500 13.0015 12.8498 13.2244 12.9324 12.5251 13.7122 12.3469
12.2458 12.3846 12.7494 12.5744 13.1210 13.2836 12.5706 13.0402
12.8497 12.2039 13.1899 13.0694 12.6690 12.4763 12.8448 11.8101
12.5846 12.9880 12.7772 11.9158 12.5443 12.9445 13.1678 12.0906
12.9014 11.6865 13.0214 12.9990 13.0077 13.0385 12.8981 13.4570
12.2006 12.6791 12.9055 12.9310 12.1704 13.4885 13.0221 13.0859
12.4964 13.7276 12.6753 12.3644 12.5404 13.7825 13.3363 12.9682
12.3373 12.3947 12.8056 13.2018 12.0603 12.5803 12.6679 13.0320
13.3575 12.7502 12.5813 12.7837 13.1807 13.2689 12.7392 12.7364
12.7479 12.5131 13.1743 11.7820 12.0014 12.5347 12.9118 12.9778
12.1387 12.6586 13.2339 12.9498 11.8217 13.0615 12.9299 12.2889
13.1313 13.1788 12.6652 12.9524 13.4247 13.2067 13.0259 12.7503
11.1179 12.3159 12.5100 12.8241 13.3647 12.9473 12.9063 13.3375
12.8254 12.5431 12.9580 12.6661 12.8477 12.2400 11.8907 13.1788
10.9791 13.7001 13.1858 11.9340 12.9946 13.2172 12.6517 12.5733
12.4471 13.1537 12.3096 13.3218 13.0830 13.4825 13.4595 12.8939
12.6281 12.6062 13.2629 12.5884 12.1752 13.3005 13.6476 12.9795
12.9269 13.2666 12.4624 13.2763 13.1293 13.6724 12.3820 12.8057
12.9929 12.7379 13.1113 12.9068 12.4129 12.9853 12.7028 12.2013
12.9503 13.6493 12.9380 12.7852 12.8337 11.7267 13.4041 12.5244
13.2402 11.8191\
REM1 3.4941 7.4091 4.9555 1.9468 6.2070 3.3359 3.9659
9.0291 2.9992 7.3991 6.3464 3.5094 5.7913 2.6649 3.2604
5.7644 5.3913 3.7916 8.3803 5.5842 6.8955 4.4348 7.7506
3.1574 8.1715 6.9139 2.4493 6.5089 6.0514 2.9203 1.7306
5.2692 2.8112 4.2434 2.4602 3.7098 5.0418 1.7231 2.9681
1.2646 3.2902 5.3348 4.1496 2.7958 7.5268 4.3836 4.8007
3.4757 4.2626 2.1768 2.3384 4.3404 5.3703 6.6576 5.4816
2.2632 5.1162 5.1380 7.2627 5.2459 7.6529 3.7372 3.6224
3.4915 7.9292 3.4113 1.0195 4.3846 4.5630 7.6682 4.2110
3.4586 6.6030 5.1704 4.6108 2.0284 5.3363 4.8450 6.3002
5.0880 4.4992 2.1634 6.3681 3.4703 5.7383 4.5900 4.1826
4.5411 5.3155 6.3581 7.5985 4.0853 2.0170 6.9381 4.8463
8.1267 6.3166 7.6063 5.2882 4.4220 4.6421 4.7988 2.5704
5.5102 3.2265 3.9881 8.2636 4.9692 5.0823 3.8656 3.2557
2.0810 7.3621 4.8074 4.8706 3.6573 4.9959 2.8649 4.1541
4.5594 4.7335 2.7098 4.9129 4.8635 7.5032 4.2388 6.3631
4.3574 8.5864 8.5074 1.8110 2.1813 5.1321 3.5049 5.2745
5.1341 5.1119 8.5644 5.6992 7.2957 2.9483 3.2205 6.1164
3.6335 3.2560 4.9369 3.7541 8.9735 2.7981 3.5470 6.8195
5.9636 3.2708 4.0036 2.7093 6.5571 7.8549 3.2125 2.4961
4.8245 2.5111 4.0337 2.7999 2.7667 2.6724 2.7721 6.0395
4.7872 4.8266 2.6009 1.6727 3.0923 2.5860 4.6610 4.9830
2.5204 8.0404 7.8290 5.8030 7.5650 4.3035 5.9764 3.8265
3.2946 4.6429 3.3525 4.9889 4.6056 3.4329 3.8665 3.8580
2.9216 3.9503 6.8596 2.5735 6.0804 2.9620 6.8447 2.8496
6.1153 5.8893\
MTVR2 0.0000 0.0000 0.0000 0.0000 0.0000 0.0000 0.9367

0.0000	1.2625	0.4413	0.0000	0.4849	0.4363	0.4395	0.0000
0.0000	0.0000	1.2795	0.0000	0.0000	0.0000	0.4820	0.0000
0.9110	0.0000	0.0000	0.0000	0.0000	0.0000	0.0000	0.0000
0.0000	0.0000	1.5403	0.0000	0.6819	0.5555	1.1048	0.0000
0.4337	0.0000	0.0000	0.0000	0.0000	0.0000	0.9285	0.0000
0.0000	0.0000	0.0000	0.4530	0.0000	0.6494	0.0000	1.0973
0.0000	0.0000	0.8754	0.0000	0.0000	0.0000	1.4031	0.0000
0.0000	1.4936	0.3906	0.5980	0.4832	0.0000	1.0463	0.4334
0.3921	0.0000	0.0000	0.0000	0.0000	0.5548	0.0000	1.1539
1.0461	0.0000	0.0000	0.7204	1.7538	0.0000	0.0000	0.0000
0.0000	0.0000	0.0000	0.0000	0.0000	0.5215	0.0000	0.0000
0.0000	0.0000	0.0000	0.8845	0.5504	0.0000	0.0000	0.0000
1.2208	0.0000	0.0000	0.7314	0.0000	0.0000	0.5877	0.5136
0.0000	2.5153	0.0000	0.5141	0.0000	0.0000	0.7641	0.7085
0.0000	0.0000	0.0000	0.0000	0.4700	0.5970	1.2272	0.0000
0.0000	0.0000	0.0000	0.0000	0.0000	0.0000	0.7571	0.8107
0.0000	0.0000	0.0000	0.0000	0.5056	1.0240	0.0000	0.0000
0.0000	0.4562	0.5418	0.0000	0.0000	0.0000	0.0000	0.5019
0.0000	0.5414	0.0000	0.0000	0.7727	0.5638	0.3855	0.0000
0.0000	0.0000	0.0000	0.0000	0.4033	0.4418	0.8742	0.0000
0.7148	0.0000	0.5466	0.0000	1.4707	0.5410	0.0000	0.0000
0.0000	0.0000	0.6293	0.4012	0.0000	0.0000	0.0000	0.5257
0.0000	0.0000	0.6248	0.0000	0.0000	0.0000	0.0000	0.0000
0.0000	0.0000	0.4262	0.8682	0.0000	0.0000	0.6112	0.6701
0.5077	0.0000\
RTN4RL2	2.8211	4.5417	5.9465	6.2096	3.2314	1.8477	3.7864
5.7653	4.0653	5.0838	6.0243	5.4111	4.0781	2.8185	2.4639
4.0436	3.6848	7.9746	5.0628	4.9693	2.6649	7.4655	1.4101
2.9264	4.5522	5.0149	5.2406	6.7278	4.2890	6.7635	5.3603
5.8945	2.8112	8.2023	4.0452	4.2112	5.3714	9.2148	1.2418
7.1170	3.9118	5.0325	6.2187	7.0109	3.5062	5.6635	0.0000
9.3154	8.4829	5.8217	7.6558	5.9947	5.4662	4.6761	5.2564
5.4036	1.4095	5.2842	3.8451	7.1934	3.7837	2.7563	2.5147
8.2470	4.9383	4.1018	2.0292	5.9322	1.9829	4.5791	5.1715
8.6488	4.5926	2.4134	4.9209	3.9661	6.1570	6.6524	3.4332
2.5341	3.1186	4.9857	4.5648	2.9116	3.3567	2.2503	4.8352
6.9176	4.1146	3.5322	5.0503	3.4573	1.8528	4.3249	2.4164
5.2267	8.7362	4.9477	4.8328	1.9210	2.5890	4.0246	3.6120
2.8049	2.1962	3.6366	4.3789	3.1726	6.8589	4.1370	7.9845
3.2654	0.0000	2.5850	5.6627	5.9353	5.8679	4.2598	0.7085
2.5175	6.5395	6.3508	3.7448	10.3680	3.1191	3.3133	2.7753
0.0000	3.7927	3.4746	4.8997	4.2681	4.7867	2.7051	2.2542
0.8141	0.0000	0.0000	5.4051	4.0469	2.4986	2.7811	2.8615
1.9025	1.5157	3.6839	4.8260	4.4681	2.8976	5.4731	4.3617
0.0000	3.4009	3.5885	6.1250	4.1123	5.0462	7.3021	1.6149
2.5877	4.7329	0.0000	3.1622	2.8335	2.5889	1.9692	3.0493
4.1948	2.5502	4.4106	2.9193	3.5936	5.7366	5.9419	4.7036
6.7010	4.7436	3.9276	3.3644	3.8806	5.0179	4.2320	7.4039
5.5205	4.7892	3.4980	3.9892	5.3487	5.0314	5.0665	7.7560
5.7446	3.9503	3.2618	6.0308	0.0000	2.3729	4.1171	5.2461
4.3204	2.2387\
C16orf13		10.8142	9.8798	10.0023	9.7372	10.6045	8.8539
8.9502	9.5650	10.2465	9.9967	10.2751	9.0595	8.8222	8.7991
9.5918	11.1026	10.7801	10.5493	9.7878	9.7015	9.9772	9.8556

| | | | | | | | |
|---|---|---|---|---|---|---|---|
| 9.7695 | 9.8434 | 9.8107 | 10.8939 | 10.0018 | 11.1940 | 10.7779 | 9.9855 |
| 8.5633 | 11.0007 | 9.5736 | 9.9978 | 8.8370 | 11.2211 | 9.1791 | 9.3042 |
| 10.6838 | 8.6428 | 11.1303 | 8.8394 | 10.8711 | 9.7383 | 9.1598 | 12.1637 |
| 12.2704 | 10.8372 | 9.7266 | 11.1616 | 10.0443 | 10.3809 | 9.5752 | 9.3365 |
| 9.4712 | 10.5542 | 9.3303 | 8.4156 | 9.6330 | 10.1829 | 8.7149 | 8.3804 |
| 10.4300 | 9.7076 | 9.9846 | 9.7686 | 9.6171 | 9.8749 | 9.7093 | 10.9980 |
| 10.5183 | 9.2213 | 11.6600 | 9.4268 | 9.4585 | 10.5655 | 10.2544 | 10.0225 |
| 10.5754 | 9.7903 | 10.6722 | 9.3744 | 10.8309 | 10.6229 | 11.1986 | 10.5129 |
| 10.2736 | 11.1736 | 9.3165 | 10.3163 | 9.4333 | 9.3047 | 9.7870 | 11.5871 |
| 9.8323 | 9.8261 | 10.0688 | 9.9832 | 9.2946 | 10.6084 | 11.6059 | 10.5064 |
| 8.9368 | 12.1068 | 11.1440 | 11.0412 | 11.4067 | 8.5641 | 9.3420 | 10.7850 |
| 9.9655 | 9.7068 | 12.6630 | 12.6515 | 9.5295 | 9.9327 | 10.2940 | 9.4484 |
| 8.9260 | 9.9471 | 8.7542 | 10.3262 | 8.3522 | 10.4361 | 9.6514 | 9.5623 |
| 10.0239 | 12.4556 | 9.0592 | 8.2402 | 10.9367 | 11.0575 | 10.0280 | 11.9388 |
| 10.9752 | 12.0855 | 11.0194 | 12.7528 | 9.3499 | 9.3988 | 11.4132 | 10.2536 |
| 11.0253 | 8.9902 | 9.0412 | 9.7146 | 9.4839 | 10.1651 | 9.1691 | 11.9976 |
| 10.0990 | 13.0121 | 8.4162 | 9.5603 | 10.4444 | 9.1383 | 9.6127 | 9.2030 |
| 9.9311 | 10.9838 | 10.5456 | 9.6622 | 10.4841 | 10.3569 | 10.2598 | 8.4751 |
| 11.1579 | 9.1461 | 10.1154 | 9.6996 | 9.6880 | 10.1357 | 10.0124 | 10.2128 |
| 13.1772 | 9.3943 | 10.0771 | 9.6017 | 9.2148 | 10.2233 | 12.0367 | 10.0754 |
| 9.3172 | 10.5064 | 8.7573 | 10.3355 | 9.7156 | 11.3206 | 10.9099 | 10.0769 |
| 9.0829 | 9.2567 | 11.5146 | 10.5890 | 10.1349 | 11.2499 | 12.7097 | 9.7982 |
| 9.2858 | 9.0948 | 11.7013\ | | | | | |
| C16orf11 | | 0.9511 | 0.0000 | 0.0000 | 0.0000 | 0.9133 | 0.0000 |
| 0.0000 | 0.4748 | 0.4327 | 3.2610 | 0.0000 | 1.9247 | 0.7708 | 0.0000 |
| 0.0000 | 0.0000 | 0.0000 | 0.0000 | 0.5838 | 0.0000 | 0.0000 | 0.0000 |
| 1.6825 | 0.0000 | 0.0000 | 0.6062 | 0.4561 | 0.0000 | 0.0000 | 0.0000 |
| 0.0000 | 0.5612 | 0.0000 | 2.1491 | 0.5853 | 1.1431 | 5.7940 | 0.0000 |
| 0.5410 | 0.0000 | 0.0000 | 0.0000 | 0.6231 | 0.0000 | 0.0000 | 0.0000 |
| 0.0000 | 0.6741 | 0.0000 | 0.8339 | 0.7972 | 0.0000 | 2.1411 | 0.0000 |
| 1.3330 | 4.6839 | 0.0000 | 0.0000 | 0.4252 | 1.2977 | 0.0000 | 0.0000 |
| 0.0000 | 0.0000 | 0.0000 | 0.0000 | 0.0000 | 2.4264 | 0.9886 | 0.6160 |
| 0.7662 | 0.3921 | 0.0000 | 5.9898 | 0.0000 | 0.0000 | 0.0000 | 0.5408 |
| 0.6894 | 0.0000 | 0.0000 | 0.9627 | 2.6279 | 0.0000 | 0.0000 | 1.8814 |
| 2.7804 | 1.6425 | 0.9125 | 0.0000 | 0.0000 | 0.8470 | 4.6152 | 0.0000 |
| 0.0000 | 0.0000 | 0.5233 | 0.5454 | 0.0000 | 0.5504 | 0.0000 | 0.8684 |
| 0.0000 | 1.8724 | 0.0000 | 0.0000 | 0.0000 | 0.0000 | 0.0000 | 0.5877 |
| 0.5136 | 0.0000 | 0.0000 | 3.1699 | 0.5141 | 0.0000 | 0.5276 | 0.4321 |
| 0.3974 | 0.0000 | 2.2359 | 2.8168 | 0.0000 | 1.1079 | 3.2800 | 0.0000 |
| 1.0447 | 5.0345 | 0.0000 | 0.0000 | 2.0482 | 1.1234 | 0.5526 | 1.6184 |
| 0.8107 | 0.0000 | 0.0000 | 0.0000 | 0.5216 | 0.0000 | 0.0000 | 0.0000 |
| 2.6155 | 1.2444 | 1.5157 | 0.0000 | 0.0000 | 1.2499 | 1.7997 | 2.6654 |
| 0.0000 | 0.0000 | 0.0000 | 0.0000 | 0.0000 | 1.2732 | 0.0000 | 1.1539 |
| 0.0000 | 2.0666 | 0.0000 | 1.3735 | 0.0000 | 0.0000 | 0.7796 | 0.0000 |
| 0.0000 | 0.0000 | 0.5276 | 0.0000 | 2.3253 | 0.0000 | 1.2418 | 0.6229 |
| 0.0000 | 2.3068 | 3.0757 | 0.0000 | 0.0000 | 1.6765 | 0.0000 | 0.0000 |
| 1.2133 | 1.1756 | 0.7366 | 1.8914 | 0.9943 | 1.1775 | 0.0000 | 0.9785 |
| 0.0000 | 2.1004 | 2.7619 | 1.9066 | 0.8682 | 1.5946 | 7.3984 | 0.0000 |
| 0.3736 | 0.0000 | 1.5159\ | | | | | |
| FGFR1OP2 | | 7.8464 | 8.8861 | 8.6010 | 8.3705 | 8.7283 | 8.8620 |
| 8.4689 | 8.9920 | 8.7593 | 8.5788 | 7.9821 | 9.3970 | 8.5694 | 8.6801 |
| 8.4704 | 8.2229 | 7.4025 | 8.2962 | 9.2564 | 8.0105 | 7.9374 | 8.4998 |
| 8.7040 | 8.7923 | 9.0993 | 8.0137 | 9.7516 | 7.8457 | 7.6740 | 8.1291 |
| 9.1440 | 8.7732 | 8.6484 | 8.3748 | 8.8131 | 9.3048 | 8.3736 | 8.9224 |

8.4493 8.2937 9.1311 9.3943 8.0479 8.3634 9.4713 7.6222
6.8758 8.1341 9.1870 8.2748 8.1587 8.9769 8.1416 9.4369
8.8414 8.6543 8.6936 8.7479 8.3848 8.3519 9.8350 9.0038
8.9350 8.4296 8.9825 8.8781 10.0838 9.3215 8.4650 8.2432
7.9705 8.3981 8.9431 8.6220 9.3371 8.2604 8.5555 7.9878
7.5534 8.9778 7.9024 9.4024 8.2360 9.0835 7.5753 8.6816
9.2616 8.0159 8.4781 8.3311 9.3115 8.5849 8.4428 8.1692
8.7499 9.1450 8.0240 8.9918 8.0195 8.8083 7.2218 8.4826
7.4679 8.5272 8.1660 7.8840 8.7036 8.8587 8.8973 7.9388
8.5611 8.2020 6.8747 8.4838 9.1361 8.6138 8.8992 8.0704
8.1744 8.2774 9.4884 8.9089 8.5989 8.4838 8.8023 8.2312
9.2287 6.9515 9.3635 9.3389 8.9701 8.6244 8.2385 7.9745
8.3083 8.8445 8.1536 7.1415 8.9214 9.3743 7.9178 8.5240
7.4774 8.8531 8.5809 10.2622 9.0846 8.5751 8.5303 7.5238
8.8285 7.9799 8.7961 7.4426 8.5612 8.6924 9.0575 8.3979
9.1187 8.6926 8.0988 8.6450 8.2105 8.2939 9.5078 9.3226
8.0509 8.4492 8.3506 8.3718 8.7817 9.5108 8.3399 8.3632
6.3029 8.6850 9.0590 9.1842 8.6811 8.2795 7.9891 8.2856
9.2511 8.3025 8.7859 8.6840 9.4784 8.0824 7.6178 8.9680
8.9854 8.1100 7.8630 9.2599 8.5472 6.9569 7.9335 9.2545
8.7330 8.6961 7.6030\
TSKS 0.5526 2.0429 0.0000 1.3959 4.9962 4.6650 0.5431
4.4104 1.2625 4.5787 2.4603 1.3775 3.6205 0.0000 0.5377
0.0000 1.0553 2.7464 1.5827 1.1305 0.0000 1.7571 3.0325
0.0000 3.3728 2.3723 4.0218 3.1243 1.3857 1.3312 0.4127
0.5612 4.0424 2.3782 0.5853 0.6819 0.9556 1.4468 0.9336
0.7666 2.1741 1.5889 1.3900 0.0000 3.9549 0.0000 2.2746
4.0431 4.8494 1.7431 0.7972 1.9460 4.0312 5.0890 0.4645
0.0000 0.6343 0.8754 1.6122 3.9163 4.7791 2.4655 4.1678
1.0177 3.4971 2.0390 0.0000 2.3155 0.0000 3.0801 5.0239
0.3921 0.9449 0.4786 1.4868 2.1585 0.9546 3.0489 2.7035
2.9859 0.0000 0.3965 1.5572 1.9875 2.4335 1.3843 0.5376
1.0433 0.0000 1.2149 4.7570 2.6303 0.5215 2.5641 3.9773
4.9247 0.0000 2.2250 4.0206 1.5150 2.5890 1.6155 0.5352
4.8216 0.8449 0.4659 5.3438 0.7631 1.4990 4.6225 1.1907
3.3441 0.0000 0.0000 3.4950 0.0000 0.9131 2.4697 0.0000
1.3647 5.0885 1.0055 1.0557 5.3041 2.3506 1.6938 1.0447
2.2050 2.1156 2.7868 0.3935 0.0000 3.0001 6.4127 0.8107
3.6290 4.6477 0.0000 2.4209 1.9774 1.3507 0.9848 4.4648
0.0000 0.0000 2.3512 0.0000 3.2822 0.0000 2.6654 2.3678
5.8462 1.2425 1.8101 0.9437 1.4705 2.9440 0.3855 3.2097
3.2714 0.0000 2.3902 1.9935 0.9764 0.4418 2.2474 1.9676
1.9581 4.4721 1.2521 0.0000 0.9151 0.0000 0.0000 9.2278
0.8396 2.8318 4.7967 2.7595 2.9241 1.5234 1.1551 3.6356
0.5055 1.2218 0.0000 0.9943 2.9892 0.0000 1.9676 1.8202
0.5556 0.9737 1.4427 1.1623 0.0000 1.0320 3.2388 0.9160
1.8199 3.2749\
ATRX 9.5006 10.6817 10.3397 10.6833 9.8704 10.5772 10.1239
10.1594 9.8955 10.5662 10.1403 10.9791 9.7887 10.4700 10.4273
9.8697 9.8675 9.9287 10.4789 8.6736 9.5382 10.5837 9.8547
11.0566 10.8083 8.8602 10.2194 9.4199 9.7814 10.4682 9.9581
10.0479 8.6744 9.6958 9.8666 9.8013 10.4002 10.5851 10.0766
10.9924 9.9664 11.1260 9.6174 10.3818 11.2269 8.9855 8.6562
10.4756 11.2929 9.4875 10.1228 9.9815 10.6140 11.1859 10.3916

10.7255 10.7054 10.9230 10.8827 10.1350 11.1924 11.2591 9.6918
10.0125 10.4905 10.5740 9.1341 11.0549 9.9820 9.8147 9.2650
11.1849 9.8021 9.0798 10.7824 9.7073 9.9894 9.6827 10.0121
10.6015 9.4619 11.1551 10.3337 10.2490 9.6398 10.5050 11.4874
9.0825 10.4755 9.7654 10.5935 10.9933 9.4362 10.2435 9.9490
10.7255 10.6336 10.6613 11.1174 10.4759 8.6602 9.5513 10.4041
10.1371 9.9027 9.2613 10.0896 10.9437 10.7553 9.5856 10.9358
10.3495 9.8194 8.8392 11.2391 9.9704 10.4253 10.8123 10.8322
9.8747 10.4064 10.1797 10.6255 9.9506 10.5460 10.2190 10.5818
8.6529 11.1447 11.5624 9.6235 10.3276 9.8743 10.4282 8.9512
7.8213 8.8800 11.4196 10.9614 10.8488 9.4644 10.2297 9.6941
10.9874 10.8706 11.1512 10.7539 10.1175 9.6555 8.8005 10.6160
9.2275 11.3037 9.7103 10.6010 10.7036 10.8299 10.8647 9.0670
9.5594 9.0067 9.0110 10.6197 9.6570 10.3938 11.3016 9.2616
10.7765 10.1534 9.6811 11.1005 10.5242 9.2955 9.7618 10.6568
10.9685 10.7737 10.6832 11.3111 9.8031 9.2276 9.3794 9.5226
9.9080 10.9670 10.3306 10.6292 8.9254 9.2314 10.3576 10.5856
10.4628 10.5125 9.9241 10.5471 8.5023 8.6024 10.6375 10.7260
11.0037 8.0162\
PMM2 10.1255 9.4277 10.9791 9.9948 10.5993 8.9821 8.6215
9.9624 10.6659 10.2042 11.0721 10.6762 9.0140 8.8502 10.4020
10.0941 9.6715 10.8851 9.7163 9.0526 9.9869 10.3826 9.0118
9.8813 8.7148 10.4788 9.5508 10.3303 10.6562 10.7918 9.4003
10.8998 10.1512 9.7841 9.5958 10.7616 9.9570 10.7783 10.1983
8.9859 9.4590 9.5359 10.0382 9.7478 8.2767 10.8700 10.8790
10.7070 9.8966 10.1573 11.0128 10.0592 9.9454 8.7737 10.3001
9.3774 9.9464 9.7863 9.2603 9.8740 8.8527 10.6492 10.6679
9.3448 9.6996 9.9661 9.9543 8.9368 10.2060 11.3243 10.1552
10.1801 10.4768 10.2057 10.1455 9.9116 9.3823 10.7463 9.9908
10.4350 10.0159 10.4148 10.6388 11.0239 10.0661 9.8275 10.3621
10.9341 9.5988 9.5049 8.9297 9.6265 8.1182 10.3406 9.6909
9.1084 10.5907 8.8428 9.7182 9.8221 10.4889 10.2758 9.1037
10.6763 10.0479 10.0923 10.8311 8.7499 9.0324 10.6383 10.1913
10.0411 10.2674 11.0741 9.7501 10.6419 9.8525 10.3201 9.5648
10.7523 9.1583 9.9982 9.3614 10.8688 8.9022 10.5796 10.2629
10.5187 8.6548 8.9012 10.0157 10.1967 10.1696 11.6442 10.5062
10.5392 11.1430 10.1599 10.0264 8.8424 11.3223 10.5568 10.5166
10.1316 9.8767 8.5290 9.9651 8.9629 10.0096 9.9770 9.7503
11.1235 9.9587 10.3753 10.4193 9.7716 8.8860 9.4184 10.4845
10.3356 11.6971 11.0807 10.2362 9.7722 10.1842 10.0981 10.4337
10.1357 10.3307 10.0203 9.8053 9.7011 8.4062 9.7398 10.9865
9.3888 9.9219 8.7415 9.4554 9.9808 10.7035 10.8627 10.1668
10.4008 9.5735 9.7339 9.1470 11.0434 11.2121 10.0953 10.6702
10.3888 10.9649 10.2962 10.4519 9.7654 9.5715 8.7854 9.9254
9.7238 10.9976\
LOC100272146 3.8614 5.3462 5.7469 5.3275 5.2848 4.7194
5.7554 6.0376 5.8670 5.8190 5.6192 5.0601 5.1251 6.1673
4.8357 6.7278 5.2141 4.9861 5.6114 6.1651 4.3385 5.6147
4.5298 5.6898 4.4323 4.7607 4.3719 4.3973 5.8644 4.4741
5.1875 5.1194 5.1801 5.7462 6.1198 5.8225 5.8421 5.5904
4.0014 6.3821 5.6987 4.8436 4.7512 4.4349 3.8551 5.7628
6.1444 5.0253 3.7535 3.3772 4.5771 6.4944 5.5561 5.5467
6.0182 6.5120 4.9196 5.4857 4.4762 5.4839 4.3003 7.0729
5.5020 6.1946 5.3979 4.7613 5.1251 5.2776 4.4376 5.1546

4.7881	5.7490	3.8023	6.2280	6.0810	4.8243	6.0384	4.8676
5.5148	5.8171	5.3359	5.4705	5.5933	4.6279	5.9585	6.0897
5.3322	5.0829	5.8447	5.2477	4.0456	6.0809	4.8069	6.4429
5.1227	3.1952	5.0216	5.6745	4.3548	7.0134	5.8819	4.5672
5.3564	5.9896	3.9004	4.4321	6.4832	5.4892	5.1206	5.7894
6.4908	5.2126	7.1549	6.7682	4.5920	5.3805	4.4431	6.5146
6.3210	5.2976	5.9246	3.9172	4.9385	4.2555	5.9300	6.1980
3.9401	5.2955	4.3594	5.8867	4.9591	4.8128	4.5770	5.4972
5.3828	5.2246	5.6349	10.3547	6.3267	5.0762	5.6013	4.7518
5.7239	4.7557	5.3763	4.8710	5.7212	6.2369	3.0780	5.0761
5.2650	6.1533	5.4227	5.8618	5.9420	5.4075	5.5371	6.5933
4.6868	4.9574	2.2191	5.9036	5.3313	5.7862	6.3822	4.8942
4.5131	5.9221	4.2220	4.9298	6.0974	6.9243	5.3427	5.7381
7.6741	6.5017	5.7043	4.4449	5.2060	4.8583	4.0414	4.8070
6.1204	3.7191	5.8817	4.3198	6.4562	5.8046	5.6438	4.8408
4.4378	6.0303	5.4716	5.4804	4.9979	7.1743	2.9620	5.0290
5.6962	6.2251	4.1488\
ASS1	11.3145	11.1441	12.0781	10.5000	9.3921	13.9483	11.2873
12.9941	11.1899	10.6180	12.3891	10.7613	11.9439	11.1279	11.9120
7.6110	11.9195	9.1363	11.5912	12.0021	14.3371	7.9034	13.6012
10.9697	10.9417	12.1597	11.6656	10.8942	11.8318	12.1959	6.1609
6.6287	9.9894	13.8484	12.1156	9.9077	12.5889	10.0708	9.1951
6.3760	10.6643	10.9953	12.2614	11.3833	11.0443	12.0903	9.7072
12.6941	7.0121	9.2383	12.9302	13.2375	12.5399	11.0476	10.2322
10.6639	11.1368	9.9456	11.9074	11.0565	11.2069	10.9241	10.1856
10.4483	10.9808	12.2378	12.9345	12.2866	12.4203	11.7384	15.6387
12.5848	12.2314	12.5994	11.5139	8.9820	10.8819	9.0898	9.5130
9.2005	8.6200	9.5497	9.6242	11.8827	9.0891	12.0102	7.1208
11.4200	13.3963	9.4002	11.5827	11.1219	12.5552	9.2708	8.1446
12.1916	13.7014	10.9328	11.1737	11.8345	9.2186	11.9354	12.6288
10.1208	7.3024	13.0146	11.3912	12.2441	10.7029	11.5670	6.0159
13.5891	13.6773	13.2825	12.9858	12.5759	10.8685	11.0751	10.8459
12.7633	11.6749	12.5016	11.4074	10.7893	12.8534	11.1420	13.7720
13.9593	11.5648	10.4507	12.2885	11.1011	10.1378	11.8099	12.7721
10.1688	12.4213	10.8501	11.9809	10.6708	9.8224	7.8943	10.0448
10.3803	11.6648	10.0279	12.5891	13.2814	13.1947	11.5551	10.0646
12.4005	10.4735	11.9680	9.0538	9.9825	10.8047	9.5593	13.2806
10.5214	11.1255	14.0494	8.9814	12.2751	9.2665	11.8982	14.5100
12.4775	13.7986	11.1590	12.4897	9.0249	10.5769	7.0584	11.2226
11.5371	10.9543	11.3708	12.3052	9.2848	10.1277	12.4373	14.4844
7.4730	12.1425	11.3145	11.6142	11.4709	12.2580	5.7776	6.7849
12.7411	12.3504	10.4385	7.0425	15.0827	12.0054	11.0895	12.9946
9.1321	12.8226\
NCBP1	9.6243	8.9922	10.2947	9.8969	10.4740	10.2740	9.8548
9.8592	9.7973	9.7639	9.3262	10.0404	9.9201	9.3884	10.0317
9.1020	8.5006	9.9495	9.1336	8.4101	9.1532	9.8811	8.9168
10.1021	9.3869	9.5534	11.0217	9.5611	9.7493	9.6667	10.7720
9.8923	10.0306	9.1187	10.0319	10.4600	9.9869	10.4998	8.9543
9.7598	9.9905	9.5058	9.1336	10.3164	9.1441	9.1180	8.8060
9.3783	9.9845	9.0929	10.5072	9.3103	9.2697	9.1555	10.3224
9.8913	10.2056	9.6749	9.7859	8.8030	9.5260	10.2360	9.8648
9.4551	9.4309	10.2155	9.6152	9.6291	9.8791	8.8800	9.2771
10.6279	9.1916	9.5081	10.0749	9.7485	9.9511	9.5186	9.1111
10.1062	9.7671	9.8213	9.1058	9.5643	9.4648	9.3890	10.3821

| | | | | | | | |
|---|---|---|---|---|---|---|---|
| 9.5832 | 10.2625 | 10.3894 | 9.4100 | 9.9852 | 8.7575 | 9.1881 | 9.4139 |
| 9.5004 | 10.1673 | 9.0526 | 9.5537 | 9.8780 | 9.4706 | 10.2361 | 10.4435 |
| 9.3133 | 9.1531 | 9.5074 | 9.1962 | 9.2103 | 9.4014 | 9.1755 | 9.9825 |
| 10.2981 | 8.3577 | 9.5962 | 9.6303 | 10.5737 | 10.0430 | 10.2868 | 9.8609 |
| 9.0775 | 9.7363 | 10.0242 | 9.2284 | 9.8986 | 9.3543 | 10.0021 | 10.7791 |
| 8.0941 | 9.2291 | 9.8892 | 10.4191 | 9.9487 | 9.8679 | 9.3190 | 9.1339 |
| 9.2894 | 9.9323 | 8.6203 | 10.0768 | 9.0014 | 9.0220 | 9.9635 | 9.2133 |
| 10.0147 | 10.2638 | 9.0166 | 9.9701 | 8.9177 | 10.3127 | 9.2083 | 9.5774 |
| 8.5555 | 10.3441 | 9.2874 | 9.9864 | 10.9505 | 9.1570 | 10.1330 | 9.0746 |
| 9.7501 | 10.1245 | 9.9650 | 9.8963 | 9.4837 | 9.7962 | 10.5028 | 9.9160 |
| 10.0494 | 9.8811 | 9.3234 | 10.6197 | 9.6022 | 10.7040 | 10.4381 | 8.7888 |
| 10.4010 | 9.6502 | 9.1147 | 10.5250 | 9.1302 | 9.2151 | 9.3526 | 9.8157 |
| 9.5305 | 10.0608 | 9.4899 | 9.8671 | 8.9587 | 9.6949 | 10.1100 | 10.2583 |
| 10.1707 | 9.6636 | 9.0835 | 9.9383 | 10.2129 | 8.7268 | 8.9548 | 10.4248 |
| 9.8468 | 8.7272\ | | | | | | |
| ZNF709 | 7.3728 | 7.0305 | 6.4190 | 7.6905 | 6.7533 | 7.2049 | 8.1093 |
| 9.4770 | 6.4610 | 7.8851 | 6.2837 | 8.2319 | 8.7159 | 7.4583 | 7.3301 |
| 6.7739 | 7.9090 | 7.3740 | 7.2728 | 6.6310 | 6.2439 | 7.6363 | 7.7836 |
| 6.8564 | 7.1482 | 5.9564 | 6.8657 | 6.7895 | 6.9304 | 6.4828 | 6.6775 |
| 7.0209 | 6.3316 | 7.7970 | 7.8464 | 6.6711 | 7.2502 | 7.4083 | 7.6027 |
| 7.4906 | 8.0179 | 6.9342 | 5.1308 | 6.4719 | 7.0734 | 6.0453 | 7.8854 |
| 7.5083 | 7.6392 | 6.8535 | 6.7297 | 5.6704 | 8.0824 | 7.1673 | 6.2662 |
| 8.2641 | 6.2602 | 5.7321 | 8.5417 | 7.3691 | 7.2435 | 6.5661 | 7.6639 |
| 8.3750 | 7.7702 | 7.8387 | 6.9393 | 7.7267 | 6.4120 | 7.7310 | 4.8595 |
| 7.0462 | 5.4447 | 6.9045 | 7.0895 | 7.4256 | 8.8881 | 7.0977 | 7.4205 |
| 7.3803 | 6.3352 | 6.0131 | 7.5156 | 7.2333 | 7.1705 | 7.8232 | 9.5388 |
| 6.1176 | 7.1869 | 6.7433 | 6.7240 | 7.3780 | 6.9311 | 6.9235 | 6.7913 |
| 6.6201 | 5.5383 | 8.1309 | 7.0414 | 6.8429 | 6.8901 | 6.9254 | 8.7417 |
| 7.2856 | 7.7224 | 8.3258 | 7.0267 | 7.1690 | 7.4882 | 8.1140 | 8.1015 |
| 7.3871 | 6.9682 | 6.3576 | 7.0490 | 7.3166 | 6.9513 | 9.0460 | 6.4258 |
| 4.5594 | 7.8567 | 7.2057 | 6.4300 | 7.0728 | 8.0882 | 7.0732 | 7.2920 |
| 6.2904 | 7.8489 | 7.5686 | 7.1731 | 7.0360 | 6.9237 | 7.7753 | 5.2461 |
| 4.8977 | 7.6456 | 5.4591 | 7.6219 | 8.0497 | 7.0246 | 6.5621 | 7.5581 |
| 5.9685 | 6.4352 | 7.7860 | 7.5712 | 7.5614 | 7.5817 | 6.6225 | 7.1962 |
| 5.8705 | 7.4050 | 6.5812 | 6.0056 | 8.1005 | 7.6943 | 7.3105 | 8.4854 |
| 6.5038 | 6.2909 | 5.1910 | 7.2581 | 6.7984 | 6.8627 | 8.0157 | 6.6436 |
| 6.2362 | 8.2806 | 7.6504 | 7.0829 | 7.6744 | 7.6782 | 6.7823 | 4.6244 |
| 7.6858 | 7.8403 | 6.7128 | 7.7814 | 7.5354 | 6.6690 | 5.5168 | 8.3599 |
| 7.4104 | 8.0084 | 7.3964 | 7.9134 | 6.1632 | 6.8969 | 7.5191 | 7.1097 |
| 7.2813 | 8.6034 | 6.5723 | 7.1803 | 5.9450 | 7.1767 | 8.1079 | 7.1195 |
| 7.6322 | 6.0732\ | | | | | | |
| ZNF708 | 6.9347 | 7.5184 | 7.2915 | 7.6234 | 7.6420 | 7.3642 | 6.6333 |
| 7.2755 | 6.8503 | 7.6172 | 6.1703 | 8.5005 | 8.6247 | 7.4436 | 7.2424 |
| 7.0041 | 8.2931 | 7.4701 | 7.7813 | 7.7020 | 6.9712 | 7.4459 | 7.3671 |
| 7.9331 | 7.1624 | 6.2907 | 6.9021 | 6.9362 | 6.9587 | 7.1752 | 6.5958 |
| 7.8348 | 8.4030 | 5.8163 | 7.1557 | 6.7216 | 7.9789 | 7.9194 | 7.3922 |
| 8.0766 | 7.3503 | 7.3151 | 5.5850 | 8.1384 | 6.4771 | 6.6876 | 4.0317 |
| 6.9226 | 8.0362 | 6.5979 | 6.5079 | 7.0235 | 6.8840 | 7.0055 | 6.8732 |
| 8.5900 | 6.4991 | 6.1087 | 7.3350 | 6.8846 | 6.9239 | 7.0327 | 7.3420 |
| 8.0685 | 7.6837 | 7.9517 | 6.6308 | 7.7021 | 6.9603 | 7.5211 | 6.8038 |
| 7.4353 | 6.4280 | 7.3906 | 7.2174 | 6.6429 | 7.7878 | 6.8755 | 6.7246 |
| 7.7486 | 7.0459 | 6.7538 | 7.4583 | 8.5766 | 7.0749 | 7.2363 | 7.0488 |
| 6.5603 | 8.5579 | 7.3493 | 7.2239 | 7.2589 | 6.9155 | 7.4139 | 7.0062 |
| 6.1084 | 6.5432 | 7.7579 | 7.4881 | 7.3164 | 6.8349 | 6.7260 | 6.9807 |

7.3040  7.7862  7.3955  7.3639  7.7920  8.1199  7.5317  8.0697
7.2912  4.6197  6.7944  7.8564  7.9579  7.3136  7.0513  7.0438
6.2092  8.4257  6.9358  7.3181  6.5397  7.5315  5.6359  7.2372
4.1383  8.1468  7.6374  6.9787  8.0207  7.1107  7.2558  7.4958
5.5388  6.8547  6.0936  7.9385  8.4134  7.7092  6.3875  7.3919
7.0739  6.8476  8.6067  7.6502  7.1594  6.7442  6.1390  7.5850
5.5487  8.5520  7.6037  7.0151  7.9536  7.3666  7.9552  5.4530
6.4447  6.3004  6.5917  7.6170  7.5821  6.6162  8.1925  5.5942
7.6330  7.0314  7.5965  7.8885  7.8087  6.7659  6.5691  4.9830
7.9272  7.9048  6.7499  7.7408  6.7189  7.0262  5.7810  7.0915
7.5298  8.0121  8.3898  7.0384  5.5172  7.4802  7.4607  7.9918
7.2053  7.9915  7.2336  8.4484  5.0585  5.7627  8.3249  7.6220
8.0750  5.1333\
RBM14   10.6433 9.8229  10.4097 10.0926 10.7246 10.6622 11.0313
10.6847 10.8399 10.5385 10.6062 10.6151 10.4010 10.5824 10.3450
10.3610 10.2272 10.4747 9.9774  10.0236 10.3531 10.4320 10.5771
10.1652 10.3555 10.5284 10.3204 10.5797 10.1233 10.5175 10.7630
10.6805 10.4594 10.7316 10.5456 11.2486 10.2871 10.7109 9.7888
9.7928  10.9188 10.2943 9.9929  10.6725 9.6060  10.3061 10.6522
10.6185 10.6997 10.0066 10.2443 10.5163 10.4731 10.2314 10.4048
10.3211 10.1660 10.2576 10.2933 10.5287 10.0236 9.8338  11.7026
10.2025 10.2608 10.7549 10.0594 10.5706 10.0937 10.1820 10.4635
10.4860 10.2992 10.4307 9.9615  10.7082 10.4326 10.4014 10.4318
10.6743 10.4573 10.6386 9.9382  10.3415 10.5870 10.4132 10.3998
10.4803 10.2416 11.1851 10.0936 10.6311 10.4710 10.4166 10.7378
10.0985 9.9490  9.8759  10.5431 10.2076 10.7553 10.5016 10.6250
9.9959  10.5122 11.2794 10.5250 10.2965 10.3944 10.5630 10.6231
10.7737 10.7133 10.1808 10.0731 10.2603 10.7426 10.5116 10.6850
9.8287  10.7784 10.0958 10.2651 10.6091 10.5408 10.4545 10.4621
10.8636 9.8864  9.9889  10.6563 10.1554 10.5887 10.2587 10.4531
10.4795 11.3044 11.2005 10.5074 10.3200 10.3632 10.0913 10.4183
10.2291 10.2792 10.6210 10.9313 10.4819 10.5970 10.3460 10.2836
10.7709 10.3419 10.8087 10.4006 10.6501 10.3153 10.7498 10.5049
10.7542 10.2612 9.8717  10.4061 10.2838 10.3432 10.2597 9.7156
9.9589  10.5121 10.7512 10.8443 10.3964 10.6443 10.8942 10.1910
10.7283 10.6369 10.0052 10.8935 10.3877 10.5028 10.1267 10.5410
10.0200 10.3224 10.0437 10.5336 10.7256 10.5308 10.6453 10.7885
10.4682 9.8368  10.4633 10.3839 11.2882 10.5211 10.5216 10.2622
10.1529 10.4584\
NCBP2   10.8985 10.9208 12.1075 11.1357 10.7057 11.0762 10.8874
12.2594 11.6124 11.1779 11.8379 11.5988 11.3356 10.6816 10.8129
10.2046 12.6386 10.9624 10.8128 11.8128 10.1010 10.7498 10.8044
10.8499 11.0389 10.8341 11.5041 10.8157 10.8197 11.0899 11.1820
10.6280 12.5764 11.5583 11.1957 11.1968 10.3367 11.0134 11.0368
11.4962 11.5005 10.9057 11.1197 12.1165 10.6079 11.0846 11.8096
11.0000 11.6840 10.7349 11.2408 11.5478 10.9787 10.6789 10.8919
11.5015 10.5699 11.5093 10.7580 11.6277 10.7438 11.5902 11.2270
11.5917 11.1870 10.4997 11.2030 11.3306 10.5417 11.8703 12.0364
10.9491 10.6088 11.7160 11.0360 10.1422 11.5985 10.4967 10.5319
11.2184 10.9475 11.4313 10.7972 11.5381 10.9323 11.1857 11.4810
11.0117 12.5503 10.6819 10.5457 11.3256 10.7385 10.7114 10.9706
10.7458 11.4412 10.5517 11.3023 10.9815 10.2802 10.8968 11.3910
11.9267 11.3866 9.2111  10.8175 11.3115 12.4477 10.8187 10.8535
10.6481 11.6929 11.3987 11.3469 12.5635 11.4811 10.9605 11.0015

11.4905 13.1616 11.2734 11.1310 10.9278 10.8992 11.8823 11.6250
11.5146 10.9456 10.9726 10.6874 11.7654 10.8861 10.7837 10.6713
10.7943 10.7480 9.5373 10.9752 10.6206 11.4410 10.6624 10.7545
10.9005 10.6570 10.5329 10.3932 10.5392 11.0593 10.8596 10.5523
11.8175 10.7409 11.0723 11.0987 11.1879 10.6599 11.1999 11.5380
10.7925 10.5166 10.9330 11.5241 10.7695 11.6823 10.9392 10.9234
11.1199 10.7437 11.7074 11.1383 13.1747 10.6607 11.1045 10.4584
10.8899 11.3528 10.6309 10.8479 10.5291 10.8677 11.0515 13.2656
11.2454 11.1683 12.0940 11.1245 11.0105 10.6135 11.2073 10.8537
10.7560 12.1730 11.0148 11.5185 10.8450 11.8838 10.7421 10.5881
10.8635 10.4998\
DISC1 7.4247 7.4648 6.8298 7.4886 6.4450 7.3727 7.9505
7.1076 6.5077 7.4353 6.9590 6.7505 7.5366 7.1680 6.6386
9.7750 7.0595 7.6500 7.9796 5.0621 7.8297 7.4093 6.0843
9.0631 7.0241 5.5691 8.7309 6.2310 7.4124 6.4664 7.3717
9.9962 8.0119 7.0964 6.5737 4.9264 9.9440 8.3868 6.1697
8.0073 7.4742 7.6993 7.5077 8.0799 7.0734 6.0453 6.4171
7.2674 7.0892 6.4443 8.1036 6.9834 7.9272 7.5627 9.7910
6.7209 5.9168 8.1750 8.2385 6.5076 7.3400 8.4934 5.4081
7.4159 7.5871 7.7872 7.3053 8.5006 7.7738 7.1347 6.6000
8.8725 6.1285 5.8294 7.8951 7.0921 8.4164 7.1215 8.5312
6.1821 6.6842 8.9144 7.6886 9.2289 7.4097 7.8177 9.4835
6.5191 6.2140 8.2604 7.1859 7.5424 8.2081 7.6256 7.9609
6.8686 7.7968 8.1143 8.6175 6.2394 6.3933 6.6210 8.9996
6.9985 8.0620 7.1965 7.0699 8.1814 8.0142 6.9564 8.4178
7.7431 5.6813 6.8400 7.2744 7.6745 8.7404 7.2790 6.8028
7.2538 7.5239 6.1298 7.4794 6.2350 6.8684 7.4054 6.9204
4.7928 8.0228 7.5695 7.6652 6.4537 7.2263 7.9525 5.9026
6.1903 5.9928 5.8813 8.3877 7.3073 7.2680 7.9323 6.8234
6.5064 7.3097 8.5394 7.7453 7.1266 6.4011 6.8463 9.2277
5.3214 8.5129 6.5433 9.1902 7.4241 7.6559 7.9118 6.5109
5.3885 7.3225 4.7743 7.8305 6.7544 7.9824 6.7655 6.8937
8.9975 7.7403 7.5722 7.0061 6.0208 7.7255 8.6753 1.9200
8.8850 7.5219 6.3943 10.2065 7.3931 6.0058 5.5550 6.4692
7.1239 7.5516 8.0077 7.0919 6.0849 5.8881 6.7120 7.8774
10.2275 8.2183 7.8136 8.1835 5.6289 5.4882 7.4122 8.4875
7.8782 6.5639\
CAMK1 9.9136 9.1815 8.2931 8.4413 8.4135 8.0098 7.1596
8.8016 7.9610 9.0914 8.2919 7.6793 8.1083 7.6246 8.1480
8.6507 9.2839 8.7687 9.2307 10.8439 8.8940 7.8884 8.5311
7.7816 9.6945 9.8401 6.6836 9.2810 8.2674 8.2366 9.1416
8.1049 8.7195 8.7448 7.7426 7.7427 7.9375 7.9580 8.1629
7.6004 9.4983 8.8285 8.9204 8.6743 9.5510 10.0208 8.9913
7.6928 9.2335 8.9808 8.6897 9.4131 7.9044 9.8002 8.3510
8.8769 7.7203 8.6711 8.7673 8.5227 9.8153 8.4510 7.4028
8.5976 9.4443 8.0231 8.1996 7.7968 7.7742 9.5054 8.8823
9.1369 9.6802 8.6517 7.8380 7.9991 8.8858 8.0669 8.3387
8.0434 8.6163 8.8463 8.5344 7.8656 9.5424 8.6189 8.3001
9.2375 8.7649 9.7477 10.3106 8.6302 9.8974 9.3851 8.3817
9.6379 7.5655 9.4520 8.4421 9.0948 9.1135 9.3988 8.6918
9.3326 8.0245 9.1327 10.0843 8.4196 8.1264 9.3533 9.1632
7.7382 9.7314 9.6366 7.4317 9.4477 8.3908 8.9305 7.8043
8.9710 8.6197 8.4764 8.8068 6.9316 8.6962 8.1537 8.0407
8.6399 9.3239 9.3942 8.0693 8.8471 8.7596 8.3601 7.6864

10.0804 9.3409 9.9806 8.6800 9.3957 8.2462 7.9634 9.0843
7.3527 7.7739 10.5874 7.1972 10.1050 8.0784 8.8112 8.7722
9.1454 7.7376 7.7170 8.3380 8.2545 9.4550 8.1960 7.3480
10.6528 8.4269 8.5944 8.4147 8.9260 8.4310 7.5235 8.4789
8.2622 8.3479 9.1183 6.2339 9.0151 9.9239 8.0404 8.9183
8.3022 9.4769 10.4111 7.3903 8.7398 8.9367 9.4768 7.7653
8.5867 8.4779 9.0281 8.5671 10.4639 8.9581 7.8408 8.4325
7.8376 8.1874 7.7745 8.5560 9.3386 9.1925 9.5337 7.9402
8.0960 9.0287\
RPL37 14.0308 14.3657 12.6206 13.7093 13.3956 13.6671 14.7363
14.6741 14.5947 14.4483 14.0022 14.8587 14.8032 13.7097 13.8616
15.0348 14.8707 14.1349 14.0966 14.6263 14.5683 13.9772 14.2716
14.6533 14.1361 14.2518 14.2196 14.7540 14.4113 14.3964 12.8119
14.0445 15.3032 13.9748 13.5582 14.5757 13.5556 13.4684 14.5167
13.5161 14.6515 13.5361 14.8851 14.8663 13.7481 15.6555 14.2807
14.3684 14.1657 14.4930 14.1775 14.6926 13.7355 13.9994 13.2165
13.7580 13.6557 13.3074 14.3160 14.2679 13.8544 13.6282 13.7924
13.4585 14.5745 12.8265 14.4368 15.0158 13.8775 13.9319 14.9923
13.6839 14.9184 15.1561 13.4272 14.3031 14.5982 13.4078 14.9091
13.9406 14.6568 13.9709 14.5379 13.7807 15.5751 14.8307 13.6732
14.6566 14.4837 13.4445 13.9611 14.0119 15.8343 15.6519 14.0244
14.0326 14.1641 14.7110 13.7591 14.9569 14.8524 12.7180 12.6883
14.5080 14.5557 14.5478 15.0624 13.6380 14.5376 14.3912 14.3381
14.3905 16.3423 16.0668 13.2583 14.6301 14.9536 13.9578 12.8575
13.0516 15.1261 14.5567 12.4173 13.6143 14.3963 13.6093 15.3274
16.7553 13.9576 13.1226 14.0738 15.8987 14.0691 14.6663 15.6340
15.9042 14.7421 15.5508 14.3692 14.1647 15.3637 14.9698 15.4648
12.9388 13.5660 15.2494 13.3555 14.8083 13.0367 15.4954 14.7422
15.6954 13.2770 13.9417 13.9165 13.4282 14.3595 13.3682 14.4457
14.8083 13.8456 14.1710 14.7375 14.6564 14.3506 13.2447 14.9466
13.6643 13.7077 13.8774 13.1361 13.2436 13.6444 14.2209 16.3645
14.0658 14.4063 14.1582 13.5310 13.6168 15.4343 16.1177 15.6365
15.3003 14.0958 14.9481 14.1851 14.6504 14.7638 14.2665 13.3642
13.8668 14.2807 13.9180 13.9591 16.4689 16.2326 14.8434 13.1830
14.3430 14.9981\
SPR 9.1427 8.7745 10.9525 10.3507 8.5132 10.6219 6.4718
8.5225 8.6730 9.0434 10.3078 9.1188 9.3396 6.1601 9.6002
9.5031 9.6466 9.0340 8.8593 9.0069 9.9306 8.8956 10.5421
9.4538 8.4263 10.7931 9.9144 9.4867 9.2657 9.6356 10.0105
9.1697 9.2723 11.0116 8.9213 9.7657 8.7445 9.3334 10.0434
7.9797 9.5436 8.3590 11.1305 10.4644 8.8715 11.2436 9.1639
9.0398 8.9137 10.7747 11.0079 8.9521 8.4135 8.6407 8.7284
8.3590 10.4740 11.3590 8.9534 9.7564 8.8833 10.0986 10.5401
8.5169 8.9226 10.0457 10.5089 7.9144 8.5027 9.8062 10.1058
9.4684 9.8111 8.7711 9.5731 8.2550 8.1053 10.6438 9.4824
9.9588 7.5274 8.6205 10.5373 9.1054 9.0150 9.1509 9.0282
8.6870 8.2315 9.5114 9.1660 9.3653 10.2024 9.5241 7.1672
9.4601 8.9515 8.8907 8.8861 9.3357 10.1058 10.7018 8.8802
10.6449 9.5005 9.9842 7.9343 8.4479 9.1455 9.6015 6.2847
6.9135 11.3711 9.6402 8.5903 9.0078 9.3503 8.5666 8.5451
9.6999 9.8722 9.6861 9.7869 10.4449 10.0491 9.5520 8.6596
11.3674 8.5149 7.9219 9.1521 9.5267 9.6089 9.5154 9.7595
10.9113 10.7037 8.6407 8.7081 8.6728 8.8725 9.9452 9.7505
8.8156 9.2600 9.1591 7.9220 9.2078 9.3858 9.8911 9.2204

| | | | | | | | |
|---|---|---|---|---|---|---|---|
| 10.0897 | 8.5659 | 9.3548 | 8.1498 | 9.3792 | 9.5010 | 7.4151 | 9.2806 |
| 10.7550 | 10.4049 | 10.3094 | 9.5207 | 10.3214 | 10.3318 | 8.3032 | 10.9109 |
| 9.9542 | 8.9821 | 7.9984 | 8.4270 | 9.4834 | 9.9327 | 9.9076 | 10.9011 |
| 8.3998 | 8.8617 | 9.1637 | 8.1471 | 9.9545 | 10.9366 | 9.1924 | 9.6177 |
| 11.0024 | 9.0407 | 8.5084 | 9.2658 | 9.9013 | 9.5952 | 7.1615 | 8.5548 |
| 9.1581 | 8.4187 | 7.2103 | 9.1872 | 11.1079 | 10.4743 | 9.2520 | 7.3539 |
| 8.8062 | 7.5845\ | | | | | | |
| ZNF700 | 8.4745 | 7.8381 | 7.8884 | 8.7661 | 7.9092 | 7.9707 | 8.6089 |
| 8.6692 | 7.5367 | 8.6318 | 7.9441 | 8.6966 | 9.3030 | 8.0932 | 8.5210 |
| 8.7697 | 9.0871 | 8.6257 | 8.2866 | 6.8587 | 7.8894 | 8.0204 | 7.4052 |
| 8.5376 | 7.4899 | 7.9481 | 9.1162 | 8.1396 | 8.0394 | 7.6628 | 7.4585 |
| 9.2234 | 8.1901 | 8.2079 | 8.4272 | 8.7684 | 8.7555 | 8.6666 | 8.6007 |
| 8.5405 | 8.3117 | 7.8700 | 6.8656 | 9.4161 | 7.6057 | 7.6614 | 5.7914 |
| 8.0393 | 8.5337 | 8.6680 | 8.1305 | 9.0850 | 8.0300 | 8.3120 | 7.6766 |
| 9.2628 | 8.4305 | 7.2041 | 9.0967 | 7.7862 | 7.9288 | 7.8534 | 7.8957 |
| 9.0986 | 8.2720 | 8.7978 | 8.4028 | 7.9998 | 7.3392 | 8.9333 | 7.0789 |
| 8.0628 | 7.5535 | 7.8203 | 9.1655 | 7.9947 | 8.0374 | 7.6679 | 7.2910 |
| 8.3782 | 7.5863 | 7.0646 | 8.9649 | 8.9823 | 7.7299 | 9.1206 | 10.1790 |
| 7.6775 | 7.5550 | 8.8102 | 7.3400 | 8.5079 | 7.6309 | 8.6470 | 7.8184 |
| 7.0748 | 7.4586 | 7.7912 | 8.3980 | 9.2033 | 7.6756 | 8.0123 | 8.7791 |
| 9.4654 | 8.1337 | 8.2301 | 9.3746 | 8.2834 | 8.5419 | 9.3788 | 8.7620 |
| 7.9831 | 7.2019 | 8.0056 | 9.3669 | 8.2879 | 7.9403 | 10.0254 | 7.8450 |
| 8.5009 | 8.8443 | 8.2871 | 8.3038 | 7.5350 | 8.0663 | 9.2137 | 7.1427 |
| 6.0755 | 7.9826 | 8.3051 | 8.3593 | 8.8419 | 8.0532 | 10.3256 | 8.1978 |
| 7.3238 | 8.3667 | 8.6938 | 8.4343 | 8.0219 | 8.9133 | 8.1813 | 8.6038 |
| 8.3923 | 8.0173 | 8.0859 | 8.0952 | 7.6063 | 7.2839 | 8.0454 | 8.6397 |
| 7.1824 | 8.0380 | 7.2326 | 7.7372 | 8.1336 | 7.8844 | 8.3767 | 9.3609 |
| 8.3717 | 7.5382 | 7.9672 | 8.0275 | 8.0905 | 7.7258 | 8.6089 | 6.6330 |
| 8.2045 | 7.5324 | 8.0645 | 7.9809 | 8.7708 | 8.7017 | 7.9081 | 7.4853 |
| 8.4149 | 9.2221 | 7.6082 | 8.5820 | 8.6818 | 8.8522 | 7.4632 | 10.0539 |
| 8.4414 | 8.7617 | 8.1712 | 7.9104 | 7.1403 | 8.2073 | 8.6982 | 8.8107 |
| 7.8962 | 8.5093 | 7.8598 | 9.1872 | 7.6798 | 8.1400 | 8.1800 | 8.3508 |
| 9.0769 | 7.0149\ | | | | | | |
| ZNF707 | 8.1583 | 7.8097 | 8.3397 | 8.2578 | 8.9141 | 8.5152 | 9.3474 |
| 8.2690 | 7.8107 | 8.1767 | 7.9959 | 9.1607 | 8.1668 | 8.1156 | 7.7550 |
| 8.1284 | 9.3215 | 8.0269 | 8.0492 | 9.4845 | 9.2207 | 7.5195 | 7.7017 |
| 7.5278 | 7.6328 | 7.7766 | 8.1995 | 8.1103 | 7.7784 | 8.0266 | 7.8448 |
| 8.2429 | 8.8460 | 9.8853 | 8.2793 | 8.7882 | 8.6881 | 8.1862 | 7.3566 |
| 8.6301 | 8.5308 | 7.6308 | 8.0151 | 7.2484 | 7.3730 | 6.8769 | 8.2492 |
| 8.7714 | 7.6850 | 8.6708 | 8.0343 | 7.8674 | 8.8887 | 7.4761 | 8.8002 |
| 8.0762 | 8.6703 | 8.0322 | 7.7864 | 9.3678 | 7.2778 | 7.9063 | 7.8255 |
| 9.8116 | 7.9184 | 8.2189 | 8.7734 | 9.0884 | 8.0935 | 8.5606 | 10.0276 |
| 8.0796 | 7.4881 | 10.1050 | 7.7025 | 8.6292 | 9.0628 | 8.0235 | 7.5393 |
| 7.8978 | 8.3644 | 8.6469 | 7.0844 | 8.1821 | 7.9818 | 9.0167 | 8.1226 |
| 8.7524 | 9.1074 | 7.4796 | 7.6630 | 9.4294 | 8.2907 | 7.9926 | 7.8782 |
| 7.9154 | 8.2376 | 7.8698 | 8.8362 | 8.4175 | 7.3604 | 8.0146 | 8.5648 |
| 9.6900 | 8.4319 | 8.3530 | 8.2605 | 7.6238 | 8.2049 | 8.4196 | 8.1886 |
| 8.4599 | 8.2869 | 7.5925 | 7.3280 | 7.9610 | 8.0259 | 9.1103 | 8.5204 |
| 8.9744 | 8.7191 | 7.8752 | 7.9418 | 7.3396 | 8.0469 | 8.8597 | 7.5138 |
| 7.9498 | 7.6131 | 7.5525 | 9.3328 | 7.4692 | 8.0042 | 7.9586 | 8.6653 |
| 8.0977 | 8.4266 | 7.6644 | 8.0279 | 7.8905 | 8.8404 | 7.6560 | 8.3998 |
| 7.5380 | 7.8437 | 7.4372 | 8.1596 | 7.7852 | 8.3179 | 8.0992 | 7.9260 |
| 7.8939 | 7.9975 | 9.0206 | 7.9353 | 8.4538 | 7.7076 | 8.9673 | 8.8948 |
| 7.9774 | 7.8007 | 8.1125 | 8.0220 | 8.3606 | 8.6470 | 7.7927 | 7.5716 |

| | | | | | | | |
|---|---|---|---|---|---|---|---|
| 7.6071 | 8.7679 | 8.7978 | 8.9064 | 7.7416 | 8.8066 | 8.2398 | 7.9343 |
| 8.6669 | 7.9231 | 7.6001 | 8.7255 | 7.8523 | 8.5740 | 7.6816 | 8.1434 |
| 7.8979 | 8.8393 | 7.8907 | 9.2728 | 9.1757 | 7.9626 | 8.2212 | 7.7447 |
| 8.0652 | 8.3733 | 9.3037 | 8.7378 | 9.6347 | 9.7827 | 7.8681 | 8.4542 |
| 7.9842 | 7.9848\ | | | | | | |
| CAMK4 | 2.0932 | 3.1739 | 3.7938 | 2.6559 | 2.0327 | 3.2400 | 1.9040 |
| 3.4767 | 2.3778 | 5.3849 | 2.7843 | 1.3775 | 2.8079 | 0.0000 | 2.0573 |
| 5.1466 | 1.8857 | 2.1918 | 3.9510 | 0.6732 | 3.1720 | 0.8427 | 2.7060 |
| 4.8880 | 3.6666 | 1.8524 | 7.7316 | 3.0366 | 0.8532 | 2.0116 | 5.9759 |
| 3.1833 | 2.6227 | 3.3982 | 0.5853 | 3.4152 | 3.0901 | 3.4305 | 0.9336 |
| 0.0000 | 2.5069 | 3.7496 | 3.1865 | 4.0343 | 3.5062 | 0.0000 | 0.0000 |
| 1.1317 | 3.8278 | 0.8339 | 2.9335 | 3.7143 | 2.3160 | 4.3859 | 5.5416 |
| 2.2632 | 3.1261 | 3.8431 | 6.0989 | 2.1385 | 3.3396 | 4.8764 | 1.2717 |
| 4.6777 | 4.6903 | 5.0319 | 2.2002 | 2.5294 | 1.5697 | 2.6613 | 3.9742 |
| 3.2376 | 4.8913 | 3.2712 | 3.9040 | 0.0000 | 2.5085 | 2.4718 | 3.1642 |
| 2.5341 | 1.9568 | 2.1634 | 4.2084 | 3.6851 | 0.5263 | 0.6199 | 1.8912 |
| 2.7731 | 2.5491 | 7.8455 | 2.1125 | 3.6532 | 5.0726 | 4.4108 | 2.4164 |
| 3.5703 | 1.6718 | 4.1697 | 1.1822 | 4.2566 | 1.0805 | 3.2735 | 4.6324 |
| 3.1674 | 0.0000 | 2.1477 | 2.3106 | 4.4537 | 2.7941 | 1.3268 | 3.8760 |
| 1.0545 | 0.0000 | 0.0000 | 2.5132 | 4.1616 | 1.8676 | 2.1672 | 1.1815 |
| 3.3856 | 5.5887 | 2.0083 | 3.5592 | 1.8858 | 2.0269 | 3.7566 | 4.4414 |
| 1.2894 | 4.9093 | 3.4746 | 2.1537 | 1.7472 | 2.5026 | 2.7051 | 2.4659 |
| 2.2603 | 2.4535 | 4.6610 | 2.9132 | 3.5226 | 2.0359 | 2.9732 | 2.4747 |
| 3.8315 | 3.2560 | 2.5884 | 1.1308 | 1.9094 | 3.4496 | 1.2240 | 4.9717 |
| 0.0000 | 4.9984 | 2.9102 | 4.4446 | 2.1834 | 3.4012 | 5.0380 | 1.6149 |
| 3.5770 | 0.0000 | 2.7726 | 2.4523 | 0.7182 | 1.9510 | 2.7721 | 4.0254 |
| 4.9306 | 2.6550 | 2.2280 | 0.7903 | 0.9151 | 4.9765 | 5.9545 | 0.0000 |
| 5.7202 | 4.6208 | 2.0979 | 3.5337 | 4.7447 | 1.5234 | 0.6902 | 4.5763 |
| 1.9773 | 3.3684 | 2.9077 | 2.6907 | 2.2587 | 2.1566 | 4.9804 | 3.4758 |
| 1.2686 | 1.2903 | 2.7745 | 3.1443 | 1.5946 | 3.7591 | 3.3916 | 3.2137 |
| 2.5996 | 0.0000\ | | | | | | |
| ZNF704 | 6.3611 | 8.6976 | 8.4474 | 8.9475 | 9.1667 | 8.5925 | 9.3545 |
| 6.9331 | 6.9269 | 9.8490 | 6.3768 | 8.6108 | 8.1052 | 8.7723 | 8.4833 |
| 7.3315 | 7.4655 | 6.6875 | 8.2658 | 6.3394 | 6.4866 | 7.6534 | 7.8438 |
| 8.7173 | 8.2579 | 3.9655 | 8.2104 | 5.9114 | 4.5595 | 6.6591 | 9.2093 |
| 7.6861 | 6.4498 | 7.3132 | 7.7288 | 5.4159 | 8.3818 | 8.8460 | 6.1604 |
| 9.7103 | 7.7618 | 9.6407 | 6.0987 | 7.5362 | 9.1806 | 6.5131 | 4.8007 |
| 7.7228 | 6.9045 | 7.0495 | 7.0071 | 1.2789 | 5.9235 | 9.0602 | 8.3743 |
| 8.2279 | 7.0429 | 7.4511 | 8.7328 | 8.7027 | 9.3656 | 7.5039 | 7.9212 |
| 7.0739 | 9.5871 | 7.9875 | 4.6519 | 8.7246 | 7.2346 | 7.6906 | 5.9370 |
| 8.0287 | 3.0705 | 6.2280 | 7.4766 | 7.8336 | 5.8163 | 7.2944 | 6.8753 |
| 6.7624 | 6.9426 | 7.7849 | 7.5459 | 7.8819 | 6.3652 | 5.8689 | 6.7431 |
| 7.5567 | 7.2128 | 5.7389 | 7.9772 | 8.6988 | 5.8036 | 6.8175 | 5.7760 |
| 8.4318 | 6.9165 | 9.7069 | 8.4548 | 4.7554 | 5.3623 | 7.4665 | 8.2760 |
| 6.5049 | 8.2401 | 7.5964 | 4.9405 | 8.7260 | 8.2937 | 6.5930 | 8.9768 |
| 6.7571 | 4.0638 | 5.6724 | 5.7798 | 6.9298 | 8.3603 | 8.2114 | 8.4145 |
| 1.3647 | 6.4927 | 6.7857 | 7.6749 | 8.0233 | 8.7906 | 6.8676 | 8.1752 |
| 5.5832 | 9.6312 | 9.3028 | 6.7244 | 6.6940 | 6.8729 | 6.7266 | 4.6075 |
| 3.2234 | 4.6477 | 4.2995 | 8.8793 | 9.4568 | 6.9721 | 8.1166 | 5.7698 |
| 9.0044 | 8.9400 | 8.5180 | 7.8825 | 8.1683 | 5.1990 | 6.2696 | 8.7664 |
| 5.3214 | 9.5744 | 7.5850 | 9.2161 | 8.1696 | 9.1502 | 9.2202 | 7.6991 |
| 3.8290 | 7.0396 | 4.5073 | 8.2321 | 6.6854 | 9.0880 | 9.7597 | 7.1294 |
| 8.6628 | 8.3010 | 7.0323 | 9.7114 | 7.3805 | 7.4533 | 6.2993 | 5.2700 |
| 9.1635 | 8.8604 | 7.9099 | 8.8630 | 7.2111 | 5.9633 | 4.4501 | 7.6511 |

6.8938  6.9416  7.1924  8.1075  5.9293  4.7517  7.2607  7.9308
6.7812  6.8177  7.7139  9.3773  5.7421  8.4895  9.0429  7.8406
8.8021  5.0275\
LOC339240       0.0000  0.0000  0.0000  0.4935  0.0000  0.0000
0.0000  0.4748  0.4327  0.0000  0.0000  0.0000  0.0000  0.0000
0.0000  0.0000  0.6221  0.0000  0.0000  0.3755  0.0000  0.0000
0.0000  0.0000  0.0000  0.0000  0.8021  0.0000  0.0000  0.5901
0.0000  0.0000  0.0000  0.0000  0.0000  0.6819  2.2502  0.0000
0.5410  0.4337  1.4628  0.0000  0.0000  0.0000  0.0000  0.0000
0.0000  0.0000  0.0000  0.0000  0.0000  0.0000  0.0000  0.0000
0.4645  0.0000  1.0734  2.3708  0.0000  0.0000  0.5422  0.0000
0.0000  3.7330  0.0000  0.0000  0.0000  0.0000  0.0000  0.0000
0.0000  0.0000  0.0000  0.0000  0.0000  0.0000  0.5548  0.0000
0.0000  0.0000  0.0000  0.0000  0.0000  0.0000  0.0000  0.0000
0.0000  0.0000  0.0000  0.0000  0.0000  0.0000  0.9036  0.0000
0.0000  0.0000  0.0000  0.0000  0.0000  1.5150  0.0000  0.0000
0.0000  0.7358  0.0000  0.0000  0.0000  0.0000  0.0000  0.5877
0.0000  0.0000  2.8658  1.0000  0.0000  1.0399  0.0000  0.4321
0.0000  0.0000  1.5136  0.0000  1.0557  0.0000  0.0000  0.0000
0.0000  0.0000  0.0000  0.5074  0.0000  0.0000  0.0000  0.0000
0.0000  2.4722  0.0000  0.0000  0.0000  0.0000  0.0000  0.0000
0.0000  0.0000  0.0000  0.0000  0.0000  0.5454  0.0000  1.5869
0.0000  0.0000  0.0000  0.0000  0.0000  0.0000  0.5638  0.0000
0.0000  0.0000  1.6272  1.0431  0.0000  0.0000  0.0000  0.0000
0.0000  0.0000  0.0000  0.0000  0.0000  0.0000  3.5191  0.0000
0.0000  0.4801  0.0000  0.0000  1.6980  0.0000  0.0000  0.0000
0.9100  0.0000  0.0000  0.0000  0.0000  0.0000  0.0000  0.0000
0.0000  0.0000  0.0000  0.4262  0.0000  0.0000  0.0000  0.0000
0.0000  0.0000  0.0000\
GOLGA6B 0.0000  1.6912  1.1543  2.7464  2.0327  2.4722  0.0000
1.5600  0.4327  1.0519  0.0000  2.5346  1.2705  1.6493  1.4890
1.4442  0.0000  0.7772  1.9975  0.9198  0.0000  0.0000  1.0738
0.0000  0.5538  0.0000  1.3145  0.5990  0.8532  0.0000  1.2172
0.9642  0.0000  2.3782  0.5853  1.1431  0.5555  2.1545  0.0000
0.7666  0.0000  1.5889  0.0000  0.4326  0.0000  0.0000  0.0000
2.9168  0.4402  0.8339  0.7972  1.6507  0.0000  0.8853  0.8153
2.2632  0.6343  2.1170  0.7533  0.9799  1.2440  2.9220  0.9583
1.6085  0.6828  0.6977  1.6109  1.5796  0.0000  0.6160  0.0000
0.7001  0.0000  0.4786  2.2033  1.1076  1.5242  0.0000  1.1539
2.0686  0.5659  0.0000  0.7204  1.1284  0.0000  0.0000  0.5376
0.0000  1.6807  0.9113  1.0759  0.8470  0.0000  0.0000  0.0000
0.0000  0.9065  0.5454  1.1822  0.5504  0.0000  0.8684  0.9246
0.0000  0.0000  0.0000  0.7314  1.6282  0.5426  0.0000  0.8916
0.6215  0.0000  1.5850  1.8354  0.6116  1.2170  2.9970  0.0000
0.6089  1.8470  0.0000  1.8863  0.0000  2.9380  0.9214  1.0447
0.0000  0.5619  2.3827  0.7024  0.6684  0.5526  0.7571  0.0000
0.0000  0.0000  0.0000  1.4553  1.1757  0.6010  0.0000  0.0000
0.0000  1.6921  1.7133  2.1920  1.5047  1.5778  0.7381  1.4139
0.0000  1.2425  0.0000  0.9437  0.4374  1.5425  1.7867  1.3489
0.0000  0.0000  0.0000  0.0000  0.4033  1.4811  1.6241  0.0000
2.6158  0.0000  0.5466  1.8290  2.4406  1.8991  0.0000  0.0000
0.4801  2.0214  0.0000  2.0726  1.0692  1.2666  2.0241  1.8629
0.0000  0.7366  1.3929  0.0000  0.0000  0.0000  0.0000  0.5078
0.5556  0.5674  0.0000  0.0000  0.0000  0.0000  2.3840  1.1260

```
1.1797  0.0000\
RNF115  8.9245  8.9104  8.8281  9.1413  8.5477  9.0266  8.9028
8.9357  10.4757 8.6422  9.8655  10.5527 9.1374  8.8412  8.5970
7.5056  9.7782  8.5941  9.0465  9.6177  8.8815  8.1887  7.9572
9.3271  9.8114  8.1866  8.8413  8.1605  8.2336  8.6592  8.7143
9.3881  9.1067  10.5452 9.8243  8.3677  9.5411  9.8597  8.6555
9.4951  8.9397  9.3831  9.1463  8.8024  9.6059  8.8309  5.9491
10.5090 8.5365  8.6969  9.5311  8.9521  9.5880  9.6790  8.9595
9.2335  8.8241  8.4435  9.9130  10.6204 9.7641  10.0948 8.7034
8.5390  8.4221  8.7703  9.0073  9.5423  7.9430  8.6740  9.6499
9.6554  8.4578  8.4526  9.5385  9.1586  8.3654  7.8916  8.6321
8.3217  7.9168  10.5346 8.4273  9.2904  8.2741  8.6929  7.6643
8.0364  8.9204  9.0687  9.0365  9.1306  9.2068  7.4087  8.6475
9.6469  9.7367  9.1818  9.5496  8.1655  7.6558  9.0370  9.5239
9.1426  9.2547  8.5434  7.7868  9.0406  9.4599  8.7796  9.8243
9.1347  7.1864  9.2644  8.7234  9.5563  9.5758  10.5757 8.9062
8.7107  8.7653  8.8829  8.3330  8.5490  8.2524  9.0457  9.3985
8.5173  8.9453  9.2785  9.3919  8.9797  8.6372  9.3498  8.6572
6.5468  7.4791  7.0012  8.8346  9.0204  8.8404  9.1800  7.6914
8.3865  8.5879  7.9466  9.8325  8.2727  8.1617  8.4664  9.0302
6.6358  9.6458  8.4997  8.7118  9.3860  9.1046  8.6151  8.5444
8.1424  8.4826  8.5338  8.8019  8.0577  8.6249  9.1911  8.8186
9.6553  8.6524  7.9642  8.4647  10.5615 9.3152  8.6748  7.1314
9.1575  8.6799  9.4407  9.1190  8.5549  8.4499  8.4791  8.6694
7.8852  9.6339  9.4325  9.6170  8.7962  8.3441  8.1565  8.9538
9.3788  9.5854  8.5570  9.7936  8.4025  7.8673  8.5548  8.9760
8.8238  7.5129\
RNF112  1.7370  5.8146  4.2769  2.5594  2.3147  1.8477  4.5761
4.3006  4.3880  4.3699  4.3748  6.5742  3.3970  2.3988  2.0573
3.5017  6.0956  2.1918  5.7911  6.6180  3.4081  2.7123  2.9316
2.0289  5.4763  1.0317  4.5195  3.4974  3.6194  3.7147  0.7334
1.9460  3.8407  2.5759  2.3226  4.5826  4.0444  5.3571  2.4725
1.9280  2.8954  5.4847  2.0846  3.3350  4.4413  3.1906  0.0000
3.9349  1.4771  3.0763  1.6840  4.4386  4.8508  6.4842  5.6328
2.9356  4.5290  1.9711  4.3605  4.7423  5.9988  5.3679  3.9649
4.8669  4.6210  5.1377  4.4964  1.5796  4.5630  3.6688  3.8394
3.0326  3.2212  6.1015  3.1877  1.8854  5.7549  2.6909  3.7282
5.3739  4.0748  4.4721  3.5062  2.5223  4.7767  4.9320  4.9237
2.0646  3.4785  2.7496  5.5711  3.2907  4.5894  4.0850  5.2266
5.7040  4.1025  4.4069  3.5849  3.0758  3.0438  2.8465  3.8098
5.3547  1.9209  1.8753  5.6958  3.8324  3.0542  4.1370  3.0477
2.8996  6.3150  7.4998  3.1220  4.3226  2.6550  3.3823  1.9462
3.1499  4.7133  2.7098  2.7935  1.3451  4.2038  3.7081  5.2556
3.4738  4.7652  6.5633  2.5120  2.1813  1.7370  3.8189  5.2461
4.1440  2.2980  5.2624  4.5367  5.1632  3.9988  3.5569  3.4177
3.2040  1.0811  4.8710  1.1308  5.4654  4.2752  2.3242  5.2959
2.5542  2.5871  2.3250  1.9146  2.8119  5.1664  3.9154  5.0657
2.8833  1.0317  0.6138  3.4515  4.5982  3.5545  1.9692  0.0000
3.3186  2.9304  2.4870  2.4266  3.0154  5.5944  3.9615  1.2582
2.7064  5.3184  6.4405  3.0097  3.9334  3.0872  5.9904  4.0725
5.3077  3.6996  4.5034  3.9434  4.2224  2.6295  5.1673  0.8827
0.0000  5.1771  5.4693  1.4064  0.0000  6.1918  4.2826  2.7261
3.8150  3.3644\
ZC3H14  10.0935 10.1259 9.2530  11.0268 10.6461 10.5846 10.3244
```

| | | | | | | | |
|---|---|---|---|---|---|---|---|
| 10.8246 | 10.3390 | 10.2334 | 9.6011 | 10.4117 | 10.6027 | 10.2518 | 10.5942 |
| 11.0117 | 10.1926 | 10.8430 | 10.1058 | 9.8686 | 9.4407 | 10.7297 | 10.4534 |
| 10.7287 | 10.2382 | 9.6181 | 9.9679 | 9.9456 | 10.2935 | 10.5497 | 11.4259 |
| 10.8657 | 9.9225 | 10.4278 | 10.6511 | 9.7043 | 10.1808 | 10.2529 | 10.6244 |
| 13.2380 | 10.8270 | 10.0005 | 10.3959 | 9.7271 | 10.4351 | 10.0421 | 9.5828 |
| 10.4152 | 10.3303 | 10.0686 | 9.8596 | 10.3700 | 10.5710 | 10.2299 | 10.4593 |
| 11.0253 | 10.5133 | 9.9072 | 10.5878 | 11.0804 | 10.5642 | 9.5094 | 10.9864 |
| 10.0576 | 10.4003 | 10.5672 | 10.4094 | 9.7651 | 10.0412 | 9.6117 | 9.3580 |
| 10.6004 | 9.4858 | 9.4102 | 10.3280 | 10.0582 | 9.9679 | 10.8897 | 10.4426 |
| 10.3189 | 10.1650 | 10.4585 | 10.5029 | 10.8928 | 10.0872 | 10.5873 | 10.5024 |
| 9.9175 | 10.6436 | 9.8918 | 10.0985 | 10.4103 | 9.8611 | 10.1884 | 10.5330 |
| 10.1507 | 10.6762 | 10.2809 | 10.9252 | 10.6991 | 10.2004 | 10.3376 | 10.2923 |
| 10.6503 | 10.8808 | 10.6878 | 10.0800 | 10.6562 | 10.6165 | 10.3530 | 11.1572 |
| 10.4102 | 9.4166 | 11.3945 | 9.8795 | 10.4986 | 10.1033 | 10.8223 | 10.4952 |
| 9.9215 | 10.7355 | 10.6495 | 10.3132 | 10.3127 | 10.7095 | 10.0728 | 10.9664 |
| 8.2042 | 10.3974 | 10.6171 | 10.4957 | 10.6777 | 10.4256 | 10.2565 | 10.0111 |
| 9.2450 | 9.7257 | 9.8755 | 10.5009 | 10.4695 | 10.1614 | 10.1788 | 10.1806 |
| 10.3078 | 10.4793 | 10.8375 | 10.5915 | 10.0156 | 10.2108 | 10.1377 | 11.0499 |
| 9.4347 | 10.2972 | 10.0428 | 10.2199 | 10.2096 | 10.3011 | 10.3150 | 10.3237 |
| 9.8496 | 9.4389 | 9.7756 | 10.5372 | 10.2763 | 9.8213 | 10.8266 | 9.5280 |
| 10.6444 | 9.7210 | 10.7237 | 10.4162 | 10.3018 | 10.1400 | 9.2348 | 9.4422 |
| 10.6406 | 10.4834 | 10.1998 | 10.8028 | 10.5162 | 9.9477 | 10.2197 | 10.5494 |
| 10.6655 | 10.0061 | 10.2513 | 10.2553 | 9.7253 | 9.8606 | 10.3840 | 10.6370 |
| 10.3530 | 10.0410 | 10.8737 | 11.2510 | 9.0003 | 9.6762 | 10.2793 | 10.4881 |
| 10.7908 | 9.3731\ | | | | | | |
| SPN | 5.1886 | 6.8359 | 6.0164 | 6.1362 | 7.2059 | 7.1466 | 5.9060 |
| 5.1729 | 4.7487 | 6.3862 | 6.3341 | 4.6651 | 6.1263 | 2.3988 | 6.4771 |
| 4.9991 | 4.4253 | 6.6053 | 8.5872 | 3.7850 | 7.0135 | 6.3562 | 7.0008 |
| 5.4923 | 5.0969 | 6.5698 | 9.7696 | 7.3634 | 5.9609 | 4.2625 | 2.3146 |
| 7.2226 | 6.9736 | 6.3696 | 4.0452 | 7.7836 | 4.4684 | 4.3001 | 3.3999 |
| 3.8754 | 7.2317 | 6.7793 | 4.6617 | 6.0375 | 5.6942 | 7.1489 | 6.4701 |
| 5.7229 | 5.1395 | 3.5316 | 6.9175 | 8.6884 | 6.4684 | 6.5312 | 6.6348 |
| 5.0369 | 7.4643 | 7.9902 | 7.8086 | 4.2095 | 7.0682 | 9.2891 | 6.4631 |
| 3.5558 | 6.8953 | 8.4170 | 6.0605 | 4.7683 | 3.8855 | 4.8693 | 7.5952 |
| 4.5148 | 9.7317 | 7.4107 | 8.5561 | 1.7267 | 7.5521 | 6.5924 | 6.5564 |
| 7.8386 | 4.5296 | 4.9118 | 8.0299 | 7.2046 | 5.4783 | 3.7385 | 5.7012 |
| 7.4944 | 7.0350 | 6.7668 | 6.9606 | 7.8864 | 6.6786 | 7.2778 | 6.6110 |
| 6.5210 | 5.4254 | 6.6272 | 5.4223 | 9.2090 | 6.0491 | 7.8359 | 3.3828 |
| 6.3370 | 4.1145 | 3.2882 | 7.5734 | 5.9460 | 5.1764 | 5.3478 | 7.4012 |
| 6.3151 | 2.5153 | 3.7004 | 5.1394 | 6.8059 | 5.8570 | 5.8295 | 4.8371 |
| 8.2074 | 5.6108 | 6.2203 | 6.5428 | 5.9861 | 5.9300 | 7.1063 | 4.9921 |
| 5.5164 | 6.8787 | 6.5439 | 4.7396 | 4.0287 | 7.3728 | 6.9604 | 7.8936 |
| 8.3632 | 8.2416 | 7.6376 | 7.1464 | 6.7439 | 6.1022 | 5.3624 | 7.3217 |
| 6.5492 | 6.7006 | 4.6267 | 6.3025 | 6.7798 | 5.4652 | 3.6271 | 7.1076 |
| 6.3385 | 8.0872 | 7.2799 | 4.4749 | 6.0864 | 6.8372 | 3.1139 | 6.1526 |
| 8.2798 | 5.0833 | 7.2642 | 5.5306 | 6.4070 | 4.6235 | 6.1660 | 6.8119 |
| 8.1616 | 5.3793 | 5.6521 | 3.9624 | 3.3646 | 4.2283 | 3.6869 | 4.7036 |
| 2.3068 | 5.8205 | 5.6656 | 4.6831 | 8.5207 | 5.8968 | 4.3668 | 5.7363 |
| 6.4980 | 5.8487 | 7.4644 | 7.4724 | 6.3864 | 6.3215 | 6.0762 | 8.1502 |
| 4.7207 | 5.7680 | 7.2758 | 6.3556 | 6.4237 | 3.9171 | 6.5358 | 7.7041 |
| 7.0600 | 6.5827\ | | | | | | |
| HMGCLL1 | 0.0000 | 2.1912 | 0.6896 | 0.4935 | 0.0000 | 0.0000 | 4.4611 |
| 3.6208 | 1.6317 | 0.7786 | 1.2544 | 0.8472 | 0.7708 | 0.0000 | 1.2358 |
| 0.0000 | 3.8557 | 1.2795 | 2.9040 | 0.9198 | 4.8199 | 5.5181 | 3.1268 |

| | | | | | | | |
|---|---|---|---|---|---|---|---|
| 1.6784 | 2.9167 | 0.0000 | 0.4561 | 0.0000 | 0.0000 | 2.9203 | 1.2172 |
| 0.0000 | 0.0000 | 3.0448 | 0.5853 | 0.0000 | 0.0000 | 0.0000 | 0.0000 |
| 0.0000 | 3.8546 | 2.8124 | 3.4228 | 0.0000 | 3.6322 | 0.0000 | 0.0000 |
| 0.0000 | 0.4402 | 0.4764 | 0.0000 | 0.0000 | 2.4720 | 7.0796 | 1.3330 |
| 1.5362 | 0.6343 | 0.8754 | 4.2348 | 0.0000 | 5.3625 | 4.9357 | 0.0000 |
| 1.3432 | 1.7745 | 0.0000 | 0.0000 | 0.0000 | 0.5773 | 3.9922 | 0.7662 |
| 0.0000 | 0.0000 | 3.3288 | 1.2339 | 0.0000 | 0.0000 | 0.0000 | 1.7868 |
| 0.0000 | 0.9714 | 0.3965 | 3.1329 | 3.7501 | 2.5465 | 0.0000 | 0.5376 |
| 2.0646 | 0.9125 | 0.0000 | 5.3537 | 4.1837 | 3.5171 | 4.4518 | 0.0000 |
| 3.2784 | 0.5233 | 2.0758 | 3.0342 | 0.0000 | 0.0000 | 0.0000 | 0.0000 |
| 1.2208 | 0.0000 | 1.5391 | 1.5756 | 4.4306 | 0.5426 | 3.3293 | 0.5136 |
| 5.4443 | 3.5857 | 0.0000 | 0.5141 | 3.9314 | 5.8236 | 2.7195 | 4.4957 |
| 0.0000 | 3.6007 | 0.0000 | 4.1028 | 0.0000 | 4.7894 | 1.6938 | 5.2556 |
| 0.0000 | 3.3957 | 2.1283 | 0.7024 | 1.1234 | 0.5526 | 1.2511 | 2.9611 |
| 0.0000 | 0.0000 | 0.0000 | 2.2985 | 2.5936 | 3.5671 | 0.0000 | 1.0973 |
| 0.5424 | 0.4562 | 3.2722 | 5.6026 | 4.4378 | 0.0000 | 0.0000 | 3.0859 |
| 0.0000 | 2.3500 | 1.5875 | 0.0000 | 3.4011 | 2.4071 | 0.3855 | 0.0000 |
| 0.0000 | 0.0000 | 0.0000 | 3.0798 | 1.8400 | 0.4418 | 1.1696 | 0.0000 |
| 0.4012 | 0.5276 | 4.4717 | 0.4486 | 0.9151 | 2.9680 | 1.8874 | 0.0000 |
| 3.1531 | 1.2087 | 5.7115 | 4.3840 | 1.4046 | 0.0000 | 0.0000 | 0.0000 |
| 0.0000 | 1.2218 | 0.0000 | 1.7992 | 0.7058 | 0.0000 | 2.2874 | 0.5078 |
| 3.0084 | 0.0000 | 1.4427 | 1.1623 | 1.5946 | 0.0000 | 2.3840 | 0.0000 |
| 2.2618 | 2.9670\ | | | | | | |
| NACAP1 | 7.9316 | 7.9645 | 7.0393 | 7.6733 | 7.4070 | 7.1663 | 7.6817 |
| 7.8268 | 7.8352 | 7.5554 | 7.4504 | 8.1348 | 7.9532 | 7.1097 | 7.7122 |
| 7.2359 | 7.7911 | 7.6397 | 7.6223 | 6.9587 | 6.9834 | 7.7443 | 7.9149 |
| 8.2719 | 7.6463 | 8.0931 | 7.2857 | 7.5253 | 7.7022 | 6.9695 | 7.0244 |
| 7.1617 | 7.8437 | 7.4736 | 6.6944 | 7.6380 | 7.0294 | 6.8802 | 8.6209 |
| 8.2643 | 8.2212 | 7.2327 | 7.5868 | 7.3905 | 7.9937 | 8.1300 | 7.9725 |
| 7.4157 | 7.8547 | 7.5697 | 7.3350 | 7.7859 | 7.2661 | 8.0165 | 6.7462 |
| 7.5559 | 7.4822 | 7.3440 | 7.1558 | 7.5219 | 8.1899 | 7.3812 | 7.8698 |
| 6.6047 | 8.2173 | 6.3046 | 8.2554 | 6.8722 | 7.8568 | 7.1073 | 7.7464 |
| 7.1858 | 8.0630 | 7.1992 | 7.7088 | 7.4888 | 8.0955 | 6.7036 | 8.2722 |
| 7.8619 | 7.9758 | 6.6622 | 7.7155 | 7.3428 | 8.1897 | 8.1683 | 8.5152 |
| 7.6125 | 7.6244 | 7.0755 | 7.9548 | 7.4456 | 8.1001 | 8.6841 | 7.8129 |
| 7.3687 | 6.8634 | 8.0681 | 7.2280 | 8.6899 | 8.2648 | 6.7033 | 6.8797 |
| 7.9455 | 8.2306 | 7.8887 | 7.7297 | 7.2272 | 7.7523 | 7.9594 | 8.4455 |
| 8.1619 | 6.3150 | 7.4919 | 7.3126 | 8.0061 | 8.2433 | 7.7763 | 7.1377 |
| 7.1786 | 7.6404 | 8.1658 | 6.7082 | 7.0352 | 8.2621 | 7.2058 | 8.6952 |
| 8.6912 | 8.2200 | 7.3286 | 6.7583 | 9.5518 | 7.7683 | 7.9506 | 7.2887 |
| 7.1344 | 8.3396 | 6.2435 | 7.6283 | 8.1014 | 8.6437 | 8.2432 | 8.1822 |
| 7.2399 | 7.1567 | 8.8427 | 7.5066 | 7.9172 | 7.2654 | 8.4080 | 7.1880 |
| 8.2063 | 7.0360 | 7.5313 | 6.9733 | 6.7259 | 7.7595 | 7.0686 | 7.6991 |
| 8.1563 | 7.2473 | 7.1664 | 8.2651 | 7.5402 | 7.0183 | 7.4724 | 8.0229 |
| 7.3621 | 7.3255 | 7.3211 | 6.9531 | 7.4332 | 7.0984 | 7.8720 | 7.6741 |
| 7.4125 | 7.9257 | 8.1002 | 7.5712 | 6.6176 | 8.0129 | 8.4890 | 8.2305 |
| 8.8299 | 7.7999 | 7.8707 | 6.3196 | 8.0101 | 7.8373 | 7.7017 | 7.5984 |
| 7.0806 | 7.4510 | 6.2464 | 7.2207 | 9.0616 | 7.2627 | 8.4758 | 6.8870 |
| 8.1173 | 7.9813\ | | | | | | |
| LRRTM1 | 8.4516 | 6.4657 | 11.0124 | 3.6813 | 8.1368 | 4.4022 | 13.8737 |
| 8.7340 | 10.4000 | 7.2879 | 6.2045 | 12.5846 | 8.6452 | 8.2600 | 9.5048 |
| 10.7238 | 9.2619 | 3.7153 | 6.6187 | 0.0000 | 4.1342 | 5.7485 | 8.4655 |
| 10.4864 | 7.4824 | 1.0317 | 10.3810 | 7.2164 | 5.1569 | 9.8873 | 10.0110 |
| 9.4105 | 0.0000 | 5.7564 | 10.4573 | 1.7727 | 5.3044 | 8.1370 | 7.4867 |

2.7994 5.7149 5.0675 5.4309 8.0555 3.6322 6.4696 8.9379
9.3487 5.9543 6.6606 0.7972 0.0000 4.4985 4.1308 11.4291
11.1052 4.1347 4.5565 11.5449 9.2826 3.6327 9.2399 6.2826
11.0335 7.4933 9.5863 5.7385 12.6651 10.3710 8.2330 6.3565
3.8818 0.0000 5.3200 6.0617 2.9872 7.9525 4.1922 8.3796
6.1448 10.3711 7.9116 3.3316 10.1196 9.7074 13.5359 12.1838
6.2442 12.7925 4.7061 6.0161 8.5515 12.4886 8.2930 7.9150
3.4317 7.9391 6.7369 10.9002 0.9478 10.6365 8.2886 11.0072
7.0668 8.1560 9.7940 7.2307 10.1260 7.3537 5.5471 3.6974
7.8242 10.8299 3.8074 5.9291 8.3287 6.1177 12.0741 5.1902
3.4563 9.1638 5.0768 7.1142 0.8239 7.4868 6.4401 11.2788
14.7196 8.3625 6.0878 13.3258 5.1366 6.5309 8.0996 2.2542
2.4722 0.0000 7.0830 5.9019 5.9111 5.5544 8.7109 8.5211
8.2220 9.2362 3.3387 7.1676 8.1797 8.1914 5.0177 9.4829
7.3810 10.4131 10.7241 7.8741 12.6036 6.2853 9.4555 10.2973
1.7128 6.0392 1.3735 8.2905 10.1833 10.4273 8.6120 9.3955
5.7523 11.9612 11.9784 9.3719 6.2534 11.1403 6.6333 2.7155
10.1568 7.9792 5.2956 8.6166 5.0135 5.4936 4.1841 11.5846
6.3866 7.8999 10.9277 7.3060 7.7001 6.6707 10.0730 4.4094
3.9600 9.6993 5.3034 9.1426 9.8584 8.1899 7.9224 4.8474
9.0746 6.1126\
GRIN1 5.8795 0.9197 6.1344 7.6617 3.2977 4.1615 2.0702
1.8984 0.0000 3.5078 0.7594 4.0858 1.6409 2.3988 3.3269
6.4999 1.0553 5.4537 1.3199 4.2127 1.6493 5.6942 2.9316
3.7359 1.2653 8.1258 4.4959 4.8476 2.5456 1.0077 1.4094
8.8555 2.5183 1.5403 1.5856 5.5335 5.0622 4.3416 4.0014
6.7211 3.5284 1.1407 3.4228 0.0000 0.0000 3.4511 3.2199
1.1317 5.5108 5.7396 6.4420 4.1794 1.9420 0.0000 1.7133
5.9161 2.8225 3.0892 1.9041 0.9799 0.0000 2.7563 4.5039
0.0000 2.8183 3.6081 5.9207 1.9201 4.5017 1.3773 2.9368
1.8067 2.7116 3.6332 3.8599 6.0456 1.2673 3.9590 3.7934
4.8690 3.8042 1.9437 4.9445 2.7931 3.5855 0.6199 0.0000
3.4699 2.6540 4.0368 0.6361 0.8470 2.1644 2.5641 1.4518
0.0000 2.4256 0.9402 5.9124 1.9210 4.5420 4.3796 5.3082
4.3430 5.0023 0.8176 3.6874 0.7631 0.9361 3.9618 5.8224
4.2712 1.3631 2.3219 1.8354 1.0399 5.4683 2.4697 5.0454
2.6440 2.4470 1.5923 0.6224 7.2217 2.3506 1.8805 2.3928
0.0000 0.5619 0.8821 5.1239 3.8037 5.3292 5.9317 1.7059
3.1012 3.1433 0.0000 5.1635 0.8792 6.3780 5.8278 3.4927
2.5899 3.1391 0.5418 3.8760 1.9094 3.7449 4.1457 6.3455
2.2924 4.0429 2.0029 1.9146 1.2732 1.9521 4.1575 5.1932
4.2648 9.3836 3.0745 2.1631 2.5454 3.5979 4.1424 0.7889
1.5478 4.5826 0.5466 4.5835 0.0000 2.7896 1.0565 6.6007
4.4282 2.5381 0.0000 4.5112 3.7688 4.2689 1.1551 0.5257
4.7558 1.2218 2.2612 0.0000 1.1775 5.6929 1.5562 4.2586
1.7437 0.9737 1.9066 6.2024 0.0000 4.6756 1.0391 4.2945
5.1532 1.5159\
RBMY1A3P 0.0000 0.0000 0.0000 0.0000 0.0000 0.0000
0.0000 0.0000 0.0000 0.0000 0.0000 0.0000 0.0000 0.0000
0.0000 0.0000 0.0000 0.0000 0.0000 0.0000 0.0000 0.0000
0.0000 0.0000 0.0000 0.0000 0.0000 0.0000 0.0000 0.0000
0.0000 0.0000 0.0000 0.0000 0.0000 0.0000 0.0000 0.0000
0.0000 0.0000 0.0000 0.0000 0.0000 0.0000 0.0000 0.0000
0.0000 0.0000 0.0000 0.0000 0.0000 0.0000 0.0000 0.0000

```
0.0000  0.0000  0.0000  0.0000  0.0000  0.0000  0.0000  0.0000
0.0000  0.0000  0.0000  0.0000  0.0000  0.0000  0.0000  0.0000
0.0000  0.0000  0.0000  0.0000  0.0000  0.0000  0.0000  0.0000
0.0000  0.0000  0.0000  0.0000  0.0000  0.0000  0.0000  0.0000
0.0000  0.0000  0.0000  0.0000  0.0000  0.0000  0.0000  0.0000
0.0000  0.0000  0.0000  0.0000  0.0000  0.0000  0.0000  0.0000
0.0000  0.0000  0.0000  0.0000  0.0000  0.0000  0.0000  0.0000
0.0000  0.0000  0.0000  0.0000  0.0000  0.0000  0.0000  0.0000
0.0000  0.0000  0.0000  0.0000  0.0000  0.0000  0.0000  0.0000
0.0000  0.0000  0.0000  0.0000  0.0000  0.0000  0.0000  0.0000
0.0000  0.0000  0.0000  0.0000  0.0000  0.0000  0.0000  0.0000
0.0000  0.0000  0.0000  0.0000  0.0000  0.0000  0.0000  0.0000
0.0000  0.0000  0.0000  0.0000  0.0000  0.0000  0.0000  0.0000
0.0000  0.0000  0.0000  0.0000  0.0000  0.0000  0.0000  0.0000
0.0000  0.0000  0.0000  0.0000  0.0000  0.0000  0.0000  0.0000
0.0000  0.0000  0.0000  0.0000  0.0000  0.0000  0.0000  0.0000
0.0000  0.0000  0.0000  0.0000  0.0000  0.0000  0.0000  0.0000
0.0000  0.0000  0.0000  0.0000  0.0000  0.0000  0.0000  0.0000
0.0000  0.0000  0.0000\
DHX8    10.4733 9.6013  9.2223  9.5083  9.4378  10.0747 9.9850
9.6887  11.3791 9.8047  9.1544  10.5003 9.7771  10.7819 10.1093
9.2246  9.2783  9.9191  9.5464  9.6602  9.6247  9.6459  9.2588
9.7507  9.5119  9.8041  9.4565  9.5943  9.7344  9.6199  9.2802
9.9283  9.9560  10.0240 10.6913 10.6106 10.1936 10.3298 9.5330
9.2134  9.6589  10.1219 9.5399  9.5620  9.8392  9.3191  9.6055
10.1401 10.3686 10.0221 10.3025 9.3476  10.3976 9.8576  10.2876
9.4819  10.1786 10.4053 10.2455 9.9425  9.9951  10.1307 10.0284
9.3344  9.4251  10.3222 9.3819  9.5462  9.8590  9.3788  10.8061
10.6825 9.4727  9.2748  9.9737  10.0681 9.3978  9.4586  9.4625
10.0794 9.5542  9.3949  9.2039  9.5163  9.6759  9.6043  11.0271
9.8099  9.5543  9.8608  9.8843  10.6819 9.1636  9.0293  9.9877
9.7956  9.9159  9.7961  9.9291  9.4589  10.0896 10.2107 9.9937
8.5089  9.0938  10.7651 9.1040  9.2827  9.8501  9.8009  10.2461
10.0777 9.4132  8.9396  9.9215  10.3483 10.0876 9.9341  9.6861
9.3267  10.1788 9.8168  10.0259 10.3616 9.4473  9.4870  9.3871
9.1911  9.7137  10.1014 9.3999  9.1069  9.7695  8.8366  9.0030
10.5673 10.1419 9.2535  10.0453 9.7679  8.4334  9.9214  9.2380
10.4795 10.3488 9.3516  10.3024 9.3828  10.1698 9.8363  9.8348
9.5924  10.4891 10.5541 10.7577 10.0975 9.7798  10.3150 8.8144
9.7161  10.3785 9.3386  9.8430  9.5293  9.5677  10.4426 9.2685
10.5125 10.0604 10.2611 10.6023 9.8272  10.2876 10.1129 9.9943
10.2212 9.4465  9.6545  10.2615 9.1643  9.7932  9.4278  11.4160
9.1194  10.2056 9.3970  10.9292 10.2072 9.8898  10.5237 9.8902
10.5179 9.1430  10.0317 9.7774  9.4344  9.3041  9.7879  10.2338
10.0665 10.0120\
DHX9    11.9685 11.7262 12.0709 11.9736 12.2026 12.5970 12.6893
12.4272 12.6093 11.7936 12.0215 12.4497 12.2283 12.5727 12.0523
11.6120 11.3204 11.8025 11.7194 12.1195 11.7018 11.8534 11.7188
12.2815 12.0136 11.3558 12.4505 11.7110 11.3022 12.3270 12.3007
12.2748 12.2863 12.3821 12.6925 12.1622 12.2530 12.6833 11.5153
11.7436 11.7245 12.1407 11.5255 12.5010 11.7759 11.1149 10.5260
12.1267 12.7862 11.7297 12.2404 11.4917 12.5426 11.8901 12.2804
11.6437 12.1647 11.9120 12.3563 11.9373 12.0851 12.2512 12.1955
12.1808 11.6002 12.0431 11.9441 12.4742 11.7713 11.4864 12.2856
```

12.8665 11.2997 12.1301 12.2420 11.9657 12.2063 11.1043 11.3377
12.0743 11.9369 12.7487 11.0313 12.2139 11.5947 11.8185 13.2241
11.7313 12.3426 12.5306 11.8107 12.3964 11.8924 10.8144 12.2655
11.7926 12.0878 11.7569 12.4517 11.6135 11.4224 12.2203 12.3452
11.0449 12.1516 12.6200 10.9350 12.0168 12.0966 11.7854 12.4209
12.2583 9.7657 11.4009 12.1270 12.3359 12.5220 12.7863 12.1957
11.7004 12.4655 11.9514 12.1756 12.1352 12.0291 12.3549 12.3762
11.3642 11.8730 12.1573 12.3007 11.7418 11.8515 11.1832 11.7038
10.3185 11.8372 8.6475 12.2271 11.9505 11.2822 12.2742 11.5155
11.9564 12.3946 12.0663 12.4962 11.7772 11.9314 11.2796 12.0174
9.7927 12.5222 12.2379 12.5241 12.3479 11.8152 12.6636 12.1759
12.1006 11.8434 11.4432 12.0192 11.4121 11.9382 12.4709 11.3370
12.3739 12.0353 12.2099 12.8168 12.2750 12.2173 12.6203 9.0307
12.5953 11.8686 11.7799 12.9576 11.2616 11.7726 11.9118 12.6290
11.6585 12.5109 11.9227 12.1509 11.6467 11.3676 12.3765 12.1922
12.4576 11.9011 12.1258 12.4760 11.5715 11.2096 12.1512 12.0519
12.0600 11.1567\
LOC441204 4.8109 5.2980 5.3077 2.9880 2.6553 6.7665
5.1958 4.6307 4.6912 4.4896 4.2225 1.3775 4.3520 7.8521
5.4566 5.9364 5.7503 5.6511 4.5203 5.1732 4.4833 1.5770
6.3320 4.6558 5.8833 2.5109 5.7393 3.8461 4.7004 4.3722
7.5709 2.1139 3.6563 5.3057 4.2863 1.7727 5.8886 2.4102
4.5420 4.3424 0.6649 5.4586 5.1528 4.2351 5.7509 3.6717
0.0000 4.5627 0.0000 2.6056 4.9826 3.4576 3.6288 5.3207
6.3824 4.7618 4.5964 5.3740 4.2348 3.1339 6.3690 5.9412
0.5573 4.9648 5.3344 2.2422 4.9682 4.8895 3.1496 4.1302
3.4344 5.0786 3.0705 5.3200 4.9422 0.0000 3.3091 2.2135
3.7934 3.8392 5.0314 4.1251 4.3526 2.3651 4.0362 5.4752
3.9495 0.0000 7.8313 5.6906 5.2525 1.7639 4.8511 5.6301
0.0000 5.8472 4.2087 5.4351 2.8769 5.0054 3.5349 4.4630
2.5704 3.5417 1.1338 2.3768 2.7953 5.0166 6.2458 3.8149
5.0659 3.8543 7.0216 7.1085 6.6789 2.3852 2.3144 5.1382
4.7717 4.5269 8.4350 4.8686 6.2576 0.8239 7.4950 4.4003
5.7446 5.9961 5.7639 5.4592 6.3504 0.0000 3.9940 1.2511
4.6075 2.0117 2.7218 7.2159 4.8732 4.2514 1.3507 1.3036
5.2919 4.1236 4.8526 4.4574 0.6735 6.0694 3.7973 4.7216
5.3848 2.5542 4.9138 4.5886 6.1154 5.7958 6.1166 5.4146
7.2732 1.9005 1.3600 3.6012 4.6063 5.3112 5.6871 3.0869
4.0254 4.7703 2.1803 6.5411 4.2380 0.5290 3.6813 4.7791
0.0000 6.7547 4.2082 4.9060 6.8673 2.4311 4.0414 5.0401
6.4528 3.3550 5.4475 0.0000 1.3150 2.7395 3.5793 4.6588
3.0307 4.0446 4.9028 6.5619 1.7976 3.9204 0.0000 5.3973
3.7137 4.2897 4.3811\
TCOF1 11.1923 10.4884 10.6141 10.4330 11.5291 10.9570 12.0868
11.0916 11.2333 10.4000 11.1704 11.5983 10.8036 10.9484 10.2524
10.4709 10.6935 10.7281 10.6844 11.0691 10.9243 9.9126 10.3012
10.3510 10.7393 11.5058 11.0000 10.6963 10.3868 10.6584 10.4380
10.8998 11.5514 11.4706 11.8659 12.0036 10.1819 10.8863 10.4934
9.5528 10.6443 10.2536 11.5731 10.5974 9.7724 10.8903 11.7230
10.8145 11.4240 10.1344 10.7932 11.0358 10.5596 10.0617 10.1414
10.9947 10.7960 10.2214 10.3580 10.6942 10.0476 11.0071 11.2687
10.9774 10.2720 10.3028 10.4001 10.4297 10.0479 12.0381 12.0795
10.9906 11.1748 10.2113 10.3738 10.3820 10.7654 9.8470 10.3994
11.1649 11.0566 11.0269 9.9100 11.0512 10.9915 11.4055 11.0255

10.8507 10.9632 10.6896 10.3173 11.1196 10.8674 10.6192 11.3819
10.0425 10.6104 10.1383 10.6132 10.8468 12.0417 10.8556 10.5551
11.0728 10.9977 11.6349 10.9296 10.8072 11.1612 10.8712 10.7484
11.0090 11.9970 12.1431 10.4644 12.0358 10.2512 11.0194 9.8277
11.4104 10.5357 10.3964 11.1146 10.4588 10.2610 10.7674 11.3603
11.8354 9.9071 10.3246 11.0507 10.9601 10.3575 10.5971 12.8707
12.2323 11.6795 12.1985 10.7220 10.2197 11.1016 10.4034 10.9052
10.9043 11.0328 10.1756 10.1819 10.0702 10.8965 10.8893 10.1261
12.7260 10.3740 12.1426 11.6220 10.7604 10.1288 10.8772 11.2623
11.5611 10.6121 11.0660 10.7378 10.3566 10.5971 10.9490 11.0446
10.6284 10.7622 11.2735 11.4677 11.1070 11.7751 10.9057 11.4529
10.7606 10.8618 10.4340 11.1498 9.8356 11.2515 11.6644 11.5340
10.2211 11.1221 10.8141 11.2254 12.0940 10.8336 11.7616 10.8039
10.2516 10.5234 11.7453 10.8728 12.8742 11.8027 10.1453 10.4647
10.4953 11.8117\
LRRTM3 2.5026 0.0000 2.4032 0.0000 0.0000 0.4059 2.9738
1.7391 0.4327 0.7786 0.0000 0.0000 0.0000 4.7335 0.0000
0.0000 0.0000 0.0000 0.5838 0.0000 0.0000 0.4820 0.0000
2.8404 1.9289 2.0473 0.0000 0.0000 0.8532 0.0000 0.0000
0.0000 0.0000 0.0000 0.0000 0.0000 0.9556 1.2859 0.0000
0.4337 0.0000 4.1266 0.0000 0.0000 2.0387 0.0000 2.2746
0.6741 1.6510 0.0000 0.0000 0.0000 4.2191 1.4299 3.0048
0.5608 0.6343 0.0000 1.7655 0.0000 1.4980 0.0000 0.0000
0.5968 0.0000 0.6977 0.0000 4.3569 0.0000 0.0000 0.0000
0.9537 0.9449 3.8845 2.0547 0.0000 1.7421 0.0000 0.0000
6.6823 0.0000 0.3965 0.0000 0.6718 0.0000 0.6199 0.0000
0.0000 0.0000 6.1412 2.4416 0.4848 0.0000 0.6909 0.0000
1.8952 0.0000 1.9094 0.8845 0.0000 0.0000 0.0000 3.4436
0.0000 0.0000 0.0000 1.5756 1.2595 0.0000 0.0000 1.1907
0.0000 2.8658 0.0000 0.0000 3.3185 1.2170 0.7641 0.0000
0.0000 6.0074 0.0000 0.0000 0.0000 0.5970 0.0000 2.5319
1.2894 2.9388 2.1283 1.3606 0.0000 0.0000 0.0000 0.0000
0.0000 0.0000 0.0000 0.0000 1.1757 1.3507 0.5748 0.0000
1.4986 0.4562 6.7196 2.7968 0.5454 1.3155 2.1177 2.6803
0.0000 3.2708 0.0000 0.9437 0.0000 1.5425 0.0000 0.0000
2.6931 0.0000 0.0000 1.5791 0.0000 1.8107 0.0000 0.0000
0.4012 0.5276 2.8970 1.2980 0.0000 0.9335 0.0000 0.0000
2.7911 0.5232 1.0660 5.2033 0.0000 0.0000 0.0000 0.0000
0.0000 0.0000 0.6248 0.0000 0.0000 0.0000 1.2961 0.0000
3.1677 2.1288 0.7547 1.7976 0.0000 0.6064 3.3172 0.0000
0.0000 0.0000\
NUP98 10.9323 10.9614 10.2077 10.9827 10.8672 11.5029 10.3244
10.9016 10.8445 10.8743 11.1697 11.6976 11.2190 10.8871 11.0011
10.5123 9.7756 10.9900 10.8184 10.2781 10.5037 11.0717 10.5633
10.8595 10.8440 10.6513 11.0997 10.3259 10.3481 11.2740 11.0759
11.0617 11.4451 10.8749 11.4850 11.5723 10.8793 11.1641 10.8153
11.1654 11.3011 11.8148 11.0174 11.2665 10.9946 10.0811 9.9108
11.4128 11.3920 10.6587 11.0231 10.6896 10.7569 11.0787 10.5447
10.8256 11.4418 10.7838 10.4203 10.7185 11.2976 11.2191 11.6381
10.4323 10.7498 11.2418 10.4273 10.5935 11.0253 10.5925 10.1012
11.2551 10.2617 9.1424 11.0996 10.9824 10.6039 10.7189 10.5626
11.2938 10.5154 10.9309 10.6398 10.6756 10.5237 10.3480 10.8221
10.9044 11.2460 10.7914 10.9112 11.8861 11.2055 10.4797 10.9253
10.7801 11.2415 11.0168 10.7654 10.8292 10.5345 10.9667 11.1850

11.2321 11.1644 10.8836 10.9000 11.2749 10.6341 10.2273 11.0977
11.0864 10.4994 10.9880 11.3443 11.8555 11.1339 10.7942 11.7449
10.8462 10.9211 11.0282 11.1324 11.3785 10.7362 11.1499 11.3228
9.4632 10.9996 11.2999 10.3456 10.8447 10.8453 11.3297 10.4635
10.1839 10.2970 9.5006 11.3811 11.2161 11.1869 10.3856 10.3708
11.4576 11.3198 11.1288 11.7327 10.5714 11.0533 10.6350 11.0161
9.3184 11.2233 10.7917 11.3220 11.3061 11.0167 11.2017 11.1630
10.7258 10.7175 10.7800 11.0105 10.3930 10.8627 11.4970 11.1459
11.5542 11.0639 10.8796 11.3877 11.4017 10.4647 10.4864 9.3802
10.8132 10.8820 10.6687 11.4434 10.6680 10.4560 10.8317 11.2664
10.8047 11.1191 10.4433 10.5977 10.4627 10.4847 11.2901 11.4505
10.8970 11.4038 10.6368 10.9942 10.7551 10.6554 11.1513 11.3891
11.2145 10.4106\
XPC 10.0567 10.4622 10.3452 9.8403 10.4261 10.3152 10.2104
10.0180 9.5679 10.0720 9.3269 10.1538 10.4076 10.1520 10.0028
9.9270 10.6602 10.1249 10.5192 9.2031 10.1397 9.6622 10.5053
9.5803 10.1771 9.2262 9.3095 9.3636 9.9450 9.9483 10.3547
9.8650 9.4759 10.5690 9.2453 10.1533 10.4476 9.8198 10.0484
8.9748 10.0356 10.4374 9.1923 8.4380 10.9545 9.1320 8.4393
9.4194 9.9371 9.8203 9.7820 9.3789 9.6868 10.6673 9.6966
9.9164 10.0018 9.6127 10.2134 9.5035 10.6922 9.2310 10.0581
10.4297 10.1497 9.8956 9.8716 9.9939 9.7724 10.0255 8.9288
10.1642 9.4192 9.2830 9.9230 9.1488 10.1921 9.7903 10.0130
10.1775 9.4260 9.9208 9.5638 9.4712 9.7112 9.8540 9.6869
10.3551 10.6681 9.9508 10.2460 10.2103 10.3693 10.0313 9.7583
10.2299 9.4020 10.9298 9.7134 10.0612 9.9768 10.1884 10.1694
9.1861 9.4004 10.4188 10.2039 9.4042 9.6982 9.6547 10.0093
10.1472 7.9895 9.3015 9.9679 10.2124 10.1074 10.2043 9.5178
9.9378 9.9779 9.9321 9.9965 9.9551 10.5856 9.6010 9.3155
8.1684 10.6389 10.3999 10.0580 8.8785 9.8410 9.0601 9.1346
8.5114 9.8180 9.2446 10.4403 10.9603 8.7584 9.9155 9.5192
9.9888 10.2715 11.0400 9.7251 10.5818 10.2005 9.3390 10.2237
8.0130 10.3470 8.9954 10.6155 10.1275 10.8093 11.0499 9.1426
10.1362 9.9122 9.1695 9.8593 9.8753 10.1712 9.6733 9.1292
9.3391 9.5741 10.0863 10.7293 9.5750 10.9407 9.1428 8.2235
10.3051 9.9464 10.2692 10.4538 9.9680 9.9415 10.0572 10.1904
9.9660 10.0484 9.9491 10.3155 9.5747 9.4469 10.2258 9.8525
10.5701 9.0503 9.6352 10.1055 8.9716 8.0800 11.1517 9.9977
9.9853 9.5952\
SLC12A2 9.1534 8.5748 9.6385 10.3252 9.3653 8.8387 10.7113
9.1831 8.9561 9.1186 8.6504 8.7972 9.1643 9.0820 10.2150
9.5732 7.6276 10.1341 8.7813 5.9321 7.8092 11.0833 8.8767
10.9967 9.1525 10.7596 10.7442 9.3815 10.0028 8.3947 7.8857
10.7561 8.1730 7.9180 9.0302 8.4988 10.0139 10.7975 10.9505
8.3903 9.5871 9.8961 8.0030 8.6310 9.6512 7.3461 8.2123
8.3257 10.5063 9.5331 9.4197 7.5260 9.1449 9.1186 8.8710
9.4232 9.4422 10.3047 9.3306 8.3519 9.5837 9.0759 9.1892
8.0931 8.6304 10.8164 9.8613 9.2998 9.5327 8.5828 8.0661
9.6665 7.6826 9.3722 10.1088 9.1301 8.4549 11.4599 9.1582
10.7972 10.2517 9.4650 8.5570 9.3466 9.1088 8.6722 9.4916
8.1295 9.7904 8.9439 8.8568 8.8968 8.5236 7.9212 9.3842
9.4723 9.9854 8.8753 9.6818 8.6116 8.8482 9.3024 8.3982
7.9416 9.2937 8.7050 8.4657 9.1518 9.7686 8.8489 12.3489
10.1970 7.9163 7.1085 8.8358 9.0842 9.5590 9.4819 11.1110

```
5.9521  9.6623  9.6119  8.9068  9.2413  9.7388  9.1344  8.7407
7.5099  9.3456  9.9393  10.0799 8.6308  11.0623 9.3680  7.1346
7.7570  6.3602  7.4498  10.1868 8.6653  8.0333  10.8148 9.1482
11.5309 10.2658 8.6548  11.6136 8.3720  11.0543 11.1688 9.7467
8.2015  9.8539  8.8415  10.6311 9.0838  9.3077  9.2524  9.5623
10.6611 8.2175  10.1907 9.6760  8.9048  9.7152  9.7831  8.6060
13.1038 10.8476 8.3937  10.6542 9.1415  9.1297  9.3141  8.9231
9.7758  8.6251  9.0787  10.0390 9.9569  9.5094  7.4430  8.3245
8.8667  9.2158  8.9411  8.1882  7.8258  8.9976  11.1499 11.9433
9.6948  8.7688  7.7626  9.3272  8.3588  9.1731  8.6181  10.8536
11.0740 8.2368\
GRINA   12.7905 11.9534 12.6243 12.7698 11.9130 12.4220 13.4475
11.4784 11.5515 11.9982 12.8488 12.6650 12.2653 12.1645 12.1617
12.2538 12.6225 12.1858 11.8859 13.0703 12.8709 12.1799 11.7605
12.0284 11.7985 12.8826 12.1214 12.8859 12.8524 12.4026 12.7442
12.6419 12.8311 13.4517 11.3161 11.8679 12.2486 11.6003 11.4046
12.3840 12.7162 11.7650 12.4149 11.2682 11.2350 12.1761 12.8748
12.9039 11.8027 12.5848 12.1413 11.6659 13.1625 11.4739 12.2889
12.0881 12.4374 12.1421 11.7231 13.2824 11.3843 11.1988 11.9847
13.6438 12.0391 12.3607 12.8935 13.3462 11.5588 12.5947 13.2413
12.5580 12.4670 12.8873 12.3620 12.7793 13.2326 12.7044 12.1490
11.5332 12.1236 13.0506 11.8267 12.4596 13.0874 12.8614 11.6332
13.4199 13.0057 11.1737 11.9986 12.6228 13.0808 12.1404 12.3435
11.5858 12.9262 12.2688 13.1956 11.7485 12.5057 12.6111 12.2190
13.4864 13.2434 11.9108 11.1107 11.7593 12.0414 13.0462 12.5156
12.3524 13.6564 11.8106 11.3646 11.7993 12.8070 11.8373 12.5959
12.5458 12.0125 12.5734 12.0803 12.0130 11.7963 12.3577 11.9663
13.6680 11.5730 11.3601 12.9785 12.2281 12.6858 11.5697 12.3370
12.9888 12.0364 10.2764 11.8575 11.8351 13.8033 12.7952 12.9408
12.0956 12.1144 12.1740 11.6753 12.2099 12.8021 12.3454 11.8587
13.6177 12.3349 11.9560 12.5912 12.1081 11.7823 12.3990 13.1480
12.2604 13.2418 12.2932 12.5126 12.2048 12.3897 12.0742 12.5218
12.9895 13.4100 12.0949 13.0185 12.2328 12.2248 12.4370 11.5139
12.8559 11.6221 11.7842 12.1507 12.4284 13.1488 11.5653 11.3864
12.7721 12.1250 12.7248 12.7361 13.2679 12.9191 12.5405 12.1892
12.5324 12.7751 12.8788 12.5708 13.6779 13.7346 12.1882 12.1779
11.9099 12.7272\
LRRTM2  0.0000  3.1739  2.0229  1.3959  1.4681  1.2014  3.4058
0.8314  1.0352  2.0772  2.3666  1.3775  2.9465  5.3409  0.9286
1.4442  0.6221  0.0000  2.5821  0.0000  1.3800  1.9172  2.1093
1.2144  3.0035  1.3599  1.5155  1.0210  0.8532  3.6580  6.0785
2.2643  1.1952  1.7182  1.0004  1.1431  1.2685  2.4102  0.9336
1.4612  2.1741  3.7059  0.6231  0.7649  6.2206  0.9285  0.0000
1.9920  2.4952  0.4764  0.0000  2.1910  4.3455  4.2640  2.0139
1.9450  1.4095  1.8088  2.5360  2.2896  2.2170  1.9554  0.5573
2.0265  2.8183  1.5189  1.6109  0.0000  1.7910  2.6613  0.4334
1.1694  1.5110  1.7486  2.6798  1.7267  5.4018  0.0000  2.0226
4.9705  0.0000  3.8042  2.6279  0.6718  1.2145  2.2503  2.9586
2.3907  2.1793  5.6906  4.1393  3.1023  4.8292  2.4059  2.8225
3.6349  1.2090  3.7446  1.4288  0.9478  0.0000  0.0000  0.5352
2.8049  2.4273  1.1001  0.0000  2.7919  2.0692  0.5877  1.8341
1.3871  3.9213  0.0000  2.1456  3.5956  2.8443  2.2752  1.8223
2.7604  2.1175  1.3285  3.6855  0.8239  2.3506  0.5331  1.0447
0.0000  3.0257  3.1713  1.6759  1.1234  1.7370  2.7051  1.7059
```

2.9678 0.0000 0.0000 1.6679 2.5936 3.2906 0.9848 0.0000
2.5899 2.9437 2.7920 1.7568 3.9299 1.7997 2.3242 3.4021
0.0000 3.1278 1.0019 0.0000 0.7727 4.1872 2.5608 1.0225
0.9345 0.6063 1.0431 3.7992 0.7182 1.0531 1.4147 3.2830
2.0725 2.0324 0.5466 3.1737 1.8707 3.6292 2.4113 0.0000
1.9121 2.6427 3.3640 2.0726 1.6765 0.5545 1.5061 2.8387
0.5055 0.7366 4.7265 2.1610 1.1775 0.6567 0.9785 1.1800
2.1004 2.2796 1.4427 1.9594 2.3335 1.3603 2.2304 2.2759
3.1721 0.0000\
NUP93 10.6302 9.3176 9.6962 9.4564 10.8459 9.9716 9.6130
10.2967 10.4978 10.0198 9.7606 10.1457 10.2754 11.1802 10.1631
9.7451 9.4571 10.6171 9.4713 10.0902 10.1808 9.7486 9.8330
10.0929 9.7461 10.3675 10.3757 10.3692 10.0353 10.1934 11.1793
10.0944 10.9880 9.9679 10.2061 11.5533 10.1696 10.7808 10.7822
9.8669 10.3626 9.3805 10.5919 9.7217 9.1934 10.1955 10.9277
10.2309 10.8730 9.4330 9.9120 9.9170 10.6603 9.1339 10.5281
10.1754 10.4186 9.2900 10.3636 10.8776 9.6033 9.5839 10.6040
10.8458 9.4478 10.3482 11.2585 11.2600 9.7941 10.0233 10.4199
10.9728 10.4064 11.0441 10.1100 10.5159 10.0570 9.5437 9.5987
10.5435 10.9517 10.4127 9.4184 9.9291 10.4879 10.6043 10.2662
10.8445 10.7726 10.7362 9.4585 11.0725 9.7021 9.7994 10.7582
9.8131 9.9533 9.0960 10.4465 9.6063 10.3795 10.2937 9.8991
9.2802 11.3250 11.8552 9.3011 9.6710 11.6690 9.9933 10.0708
10.5416 9.3346 10.6970 10.6126 10.2960 10.2491 10.8156 10.5115
10.9589 10.7028 9.9164 9.8490 10.1403 9.6984 10.4706 11.1675
9.8346 9.5547 9.8246 10.8274 8.9746 10.4281 10.0247 11.1433
10.4234 10.6983 9.2222 9.8031 9.0954 10.8261 9.9910 10.4333
10.7381 10.5444 9.3884 10.0975 9.7045 10.6722 9.8384 9.4626
10.2668 10.0532 11.3476 11.6395 10.0692 9.3207 10.7333 10.9661
10.6187 9.8604 10.3130 10.2859 10.5894 10.7603 10.9547 8.9066
9.6977 10.4469 10.0175 11.0928 10.6388 10.3831 10.4956 9.5317
10.7448 9.7684 9.3188 11.0759 9.5328 10.3460 10.8302 9.4040
9.5780 10.1876 10.1668 10.7152 10.8237 10.2351 10.9863 10.6173
10.1371 10.0973 10.6021 10.4496 10.6949 9.6502 9.4214 10.3534
10.1443 11.1198\
HAP1 2.9134 7.2150 5.5906 5.9584 3.6419 2.4722 4.3361
4.7608 3.7647 5.0838 2.7099 8.1162 5.1396 2.1894 1.7043
6.4999 5.2955 3.5493 6.6761 5.9321 6.0481 1.1310 2.9316
2.5463 4.6630 7.5582 6.7359 4.8476 6.1530 6.5851 1.9932
2.4004 1.8398 4.7111 4.3584 8.1332 4.8198 2.6272 3.2698
3.1234 1.4628 7.4669 4.0589 1.6313 4.8570 3.3267 0.0000
4.0943 1.0498 7.0538 2.3384 7.3289 3.0688 5.2422 4.6616
5.1181 4.2647 4.4503 3.9448 4.2470 3.1300 3.7372 3.5661
6.5895 6.2142 2.0390 4.9682 5.5950 4.4044 2.3953 3.8743
1.5230 3.0705 3.3842 6.3437 8.1258 4.6393 4.9758 2.5610
8.4677 2.5369 3.5159 3.2356 3.5455 3.2271 4.4553 4.4740
2.3907 5.7533 3.8290 5.3341 3.2907 8.4100 6.0060 2.9894
3.0128 7.2129 8.3397 3.7299 5.5962 0.0000 3.7864 3.5019
3.6978 2.0651 2.0179 10.8570 4.0300 3.5239 1.8132 2.3994
2.7915 6.8747 4.6439 2.4008 5.0535 2.5502 6.6412 3.8712
4.0204 5.7565 1.8152 4.0092 1.7271 4.5578 3.4959 1.0447
4.3574 4.4565 5.0822 1.8110 1.4687 2.3786 2.5437 5.1575
6.3192 2.5939 4.9498 6.5643 7.2043 2.2071 3.2205 2.6155
5.7288 4.9426 3.0520 2.1920 4.3753 3.3826 5.4513 5.1848

0.9816 2.4734 3.4629 6.9519 4.5212 5.3124 3.6943 1.8394
4.8245 3.8658 4.9890 2.7999 3.7971 3.3158 2.3687 3.8177
5.1806 4.7810 0.0000 7.4358 6.0848 4.7992 3.4930 2.9928
7.4937 5.5648 2.5659 3.4932 4.5888 6.4699 3.7953 5.6879
1.8147 3.4586 6.9734 2.5754 4.6056 3.0864 3.4201 3.7263
3.6176 5.5026 2.3573 2.2371 5.2236 5.4882 6.1951 2.2759
2.4955 2.2387\
KLRA1 4.1539 6.4511 4.1331 5.8194 4.7813 4.7718 3.2756
4.7814 4.7673 6.9467 4.7905 4.6876 5.8276 6.9275 5.4860
6.5799 3.4200 6.0589 6.9392 2.7331 5.3598 5.5056 5.5671
5.7141 5.0569 2.0473 3.1984 3.8969 3.3032 3.4063 5.5760
4.9299 1.1952 6.1775 3.5244 5.0716 5.4510 6.0001 4.6241
4.7708 3.3740 5.1016 4.4989 6.1123 4.4413 4.5034 1.5457
3.4757 4.2892 4.2772 3.7981 3.0237 3.4897 6.1301 5.4317
5.2500 5.5663 5.3447 5.4089 4.0063 5.3784 5.7339 3.3826
4.3911 7.0216 4.8259 2.4912 5.8845 4.1077 5.8970 2.2792
5.4728 2.2328 5.4684 7.6773 5.0253 5.7039 4.5691 5.7465
4.9705 2.6516 5.3857 6.0674 4.7281 2.3108 6.5108 5.3483
2.9812 5.4083 4.0750 4.9243 4.8950 3.8136 6.7696 3.8089
3.5027 3.8194 5.8456 4.2938 7.3206 1.9211 4.5418 5.2413
5.0705 2.9575 2.8278 6.5760 5.5441 5.0627 4.5928 5.7554
4.6572 0.0000 5.3576 6.2785 4.8573 3.4214 5.3383 4.4546
6.4096 5.8707 4.5955 5.7219 1.5487 5.9894 5.0131 4.7852
0.7843 5.9753 6.4566 5.7549 5.2302 4.1912 4.8726 5.1575
0.0000 2.1237 0.0000 5.2813 5.9211 3.5028 3.4956 4.2656
5.1200 4.4232 5.4088 4.7642 5.0907 2.8976 1.2240 5.7730
0.0000 5.7255 2.8106 4.6174 6.0864 5.7571 4.9831 4.9012
3.6832 2.8614 3.4693 3.5156 5.0128 5.2029 5.7068 5.6157
4.8203 4.0011 5.4389 4.6687 5.3368 3.8727 5.3175 0.0000
6.2331 7.5247 4.6155 5.4688 5.9532 3.0054 5.8440 4.7977
4.8000 5.2596 4.0633 4.7438 2.4374 4.1471 4.6023 4.4094
4.2002 3.3443 4.8498 5.8568 0.0000 1.3603 5.9708 4.9884
6.3719 0.0000\
CAMKV 0.5526 0.0000 5.5345 3.7702 0.9133 1.3921 6.9852
7.6308 2.2773 0.4413 1.0281 1.9247 2.5713 2.4930 0.0000
0.0000 1.0553 0.7772 0.5838 2.0944 2.3988 0.8427 0.6346
0.9110 0.0000 3.8130 3.5122 1.3471 0.0000 0.5901 0.9954
0.5612 0.5159 2.2682 4.0008 5.9688 4.4987 0.6557 0.5410
0.0000 2.3500 0.0000 1.0569 1.7856 0.0000 0.0000 0.0000
5.6900 3.5921 0.0000 0.7972 1.2789 2.7409 0.0000 4.7860
3.7894 1.0734 1.9711 2.6972 1.7785 0.0000 2.8415 1.5290
2.7303 0.0000 0.6977 0.0000 3.9743 1.7910 0.0000 0.7662
0.0000 0.0000 0.8374 0.5367 4.1649 8.5447 0.9332 1.5048
2.0686 2.9495 2.5997 0.0000 2.1886 0.9110 2.7877 4.5868
0.0000 4.5272 5.4355 0.0000 1.1363 6.3799 0.6909 3.0661
0.0000 1.2090 0.0000 2.7914 0.0000 5.8404 0.0000 4.5239
6.9858 1.9209 4.9419 0.0000 4.0903 7.8781 3.2552 6.3542
5.0810 4.3124 2.5850 0.0000 4.6903 4.3210 4.5013 1.5372
1.3647 4.5853 0.0000 6.1077 2.5865 0.0000 3.4959 2.0665
5.0345 0.5619 0.0000 1.3606 0.0000 0.0000 5.6238 2.9611
0.0000 3.0489 0.0000 1.2057 0.0000 4.6605 0.0000 2.8615
0.5424 0.4562 0.0000 2.1920 0.0000 4.0773 3.6271 0.5019
0.0000 2.9691 6.1331 0.9437 3.4487 0.0000 4.4681 1.6149
3.5770 0.0000 0.0000 1.9935 6.3624 4.0980 2.1150 0.0000

| | | | | | | | |
|---|---|---|---|---|---|---|---|
| 0.7148 | 0.9131 | 0.5466 | 4.4211 | 2.3178 | 5.3427 | 3.0035 | 0.0000 |
| 5.0434 | 0.5232 | 0.0000 | 6.0464 | 1.6765 | 0.5545 | 1.7883 | 1.8629 |
| 0.8792 | 4.9533 | 1.0594 | 0.9943 | 3.2977 | 0.0000 | 3.7671 | 1.6366 |
| 0.9557 | 5.4559 | 1.2477 | 0.8682 | 6.4237 | 4.4868 | 0.0000 | 1.7506 |
| 1.1797 | 2.9670\ | | | | | | |
| SPPL3 | 10.2532 | 10.5455 | 9.6419 | 9.7906 | 10.3862 | 10.8156 | 10.5579 |
| 11.4494 | 9.9818 | 10.3989 | 10.0199 | 10.8405 | 10.7076 | 10.5795 | 10.0110 |
| 10.3002 | 10.5847 | 10.4079 | 10.5674 | 11.0549 | 10.1363 | 10.1467 | 10.3113 |
| 10.2720 | 10.6066 | 11.0860 | 10.6904 | 10.2657 | 10.4818 | 10.4459 | 10.4280 |
| 10.3322 | 11.0610 | 10.5037 | 10.5999 | 10.3900 | 10.6340 | 9.9543 | 10.4129 |
| 10.3391 | 10.4527 | 10.0904 | 10.7911 | 10.5707 | 10.4933 | 10.5530 | 11.1208 |
| 10.1947 | 10.4296 | 10.8537 | 9.9426 | 11.0610 | 10.7696 | 10.6039 | 11.2689 |
| 10.3862 | 10.3898 | 11.1452 | 10.5007 | 10.5049 | 10.6125 | 10.1168 | 10.2490 |
| 9.8490 | 10.5152 | 10.3980 | 10.0117 | 10.4193 | 10.8261 | 10.2217 | 10.8549 |
| 10.2418 | 10.6600 | 11.1337 | 10.6791 | 10.2308 | 11.1318 | 10.3217 | 10.5342 |
| 10.5430 | 11.1616 | 10.2238 | 10.5622 | 10.6153 | 10.4750 | 10.4857 | 10.5122 |
| 10.5428 | 10.5752 | 10.4934 | 10.5894 | 10.6045 | 11.0770 | 10.9238 | 10.5335 |
| 10.5424 | 9.9978 | 11.0494 | 10.1648 | 10.4531 | 10.3771 | 10.3518 | 9.6109 |
| 10.7406 | 10.4585 | 10.9488 | 10.1284 | 10.7262 | 11.1212 | 10.7563 | 10.2608 |
| 10.4136 | 10.6831 | 10.6375 | 9.7129 | 10.5997 | 10.8154 | 10.8990 | 10.3319 |
| 10.4800 | 10.3838 | 10.4342 | 10.3918 | 10.2156 | 10.2477 | 10.6816 | 10.7979 |
| 11.1295 | 10.8167 | 10.5538 | 10.6788 | 10.3967 | 10.2021 | 10.4511 | 10.1401 |
| 10.1410 | 10.3573 | 10.4977 | 10.2928 | 10.8563 | 10.8392 | 10.2777 | 10.7832 |
| 10.4642 | 10.1460 | 10.8716 | 10.5848 | 10.5853 | 10.7210 | 10.3505 | 10.3445 |
| 10.2097 | 10.1558 | 10.8907 | 9.8759 | 10.5627 | 10.6319 | 10.7973 | 10.6695 |
| 10.6476 | 10.0775 | 10.5931 | 10.6991 | 10.5109 | 10.1477 | 10.2709 | 11.0609 |
| 10.4570 | 10.6469 | 10.6769 | 10.5252 | 10.9978 | 11.5358 | 11.0600 | 10.5033 |
| 10.3766 | 10.3949 | 10.4248 | 10.3467 | 10.2456 | 10.6256 | 9.6621 | 9.3842 |
| 10.2348 | 9.8091 | 10.3391 | 10.0839 | 10.7851 | 10.4495 | 10.5426 | 10.0788 |
| 10.6787 | 10.2666 | 10.1477 | 10.1834 | 10.4626 | 11.1813 | 10.9025 | 10.2891 |
| 10.3620 | 10.4034\ | | | | | | |
| GOLIM4 | 8.2137 | 9.3769 | 10.6371 | 10.7463 | 11.5432 | 8.6682 | 9.7043 |
| 9.5090 | 11.8805 | 9.4417 | 9.8330 | 11.7845 | 9.3761 | 9.7529 | 9.9932 |
| 8.4740 | 8.1498 | 8.3974 | 9.4622 | 7.2454 | 7.9715 | 8.6355 | 8.3700 |
| 10.9477 | 9.5778 | 5.8945 | 9.5170 | 7.3315 | 7.7890 | 8.2002 | 10.6296 |
| 10.7825 | 8.4849 | 8.4141 | 9.7969 | 8.8348 | 10.3129 | 10.9376 | 8.4304 |
| 11.0171 | 7.7696 | 10.1974 | 7.3155 | 9.6145 | 10.3912 | 5.8331 | 4.5080 |
| 8.6864 | 10.6506 | 6.4572 | 9.6551 | 9.3866 | 8.7847 | 10.2436 | 10.5582 |
| 11.7587 | 9.0102 | 9.1349 | 10.2952 | 9.9361 | 10.3434 | 11.7717 | 10.3763 |
| 11.5492 | 8.7629 | 9.6795 | 6.3870 | 10.2716 | 8.0673 | 10.5920 | 6.5306 |
| 10.1465 | 6.6302 | 7.8353 | 9.5085 | 7.9550 | 8.9052 | 9.0055 | 7.4711 |
| 9.6250 | 7.2779 | 10.7015 | 9.1799 | 10.4403 | 7.7624 | 8.4466 | 9.0591 |
| 8.6417 | 8.7667 | 8.3815 | 9.5218 | 11.3474 | 6.7620 | 6.6064 | 7.9176 |
| 10.2788 | 8.4418 | 9.3719 | 10.9770 | 7.9109 | 7.6031 | 8.7101 | 10.1620 |
| 9.3987 | 10.4963 | 8.1968 | 6.3954 | 9.4321 | 11.8126 | 8.9536 | 10.2999 |
| 9.2818 | 6.0669 | 5.8826 | 9.0756 | 10.0145 | 9.3889 | 9.7800 | 10.4100 |
| 9.1829 | 11.9558 | 9.3318 | 9.3689 | 9.0575 | 9.3933 | 9.4085 | 10.4345 |
| 6.0909 | 10.8205 | 10.5453 | 10.9362 | 10.6151 | 8.9842 | 9.0620 | 6.0577 |
| 2.0117 | 5.7140 | 5.0346 | 10.7846 | 10.2454 | 8.7873 | 9.6222 | 8.2295 |
| 9.9679 | 9.5057 | 9.0836 | 9.3390 | 8.8753 | 8.4589 | 3.3718 | 10.3445 |
| 7.9289 | 13.0922 | 8.7770 | 10.9140 | 11.6460 | 9.5797 | 10.6743 | 7.2636 |
| 7.3862 | 9.0795 | 8.5502 | 10.2273 | 9.1241 | 6.4723 | 12.2816 | 7.2096 |
| 11.4250 | 9.8383 | 8.2152 | 12.0498 | 12.1272 | 9.4070 | 8.6862 | 1.2582 |
| 10.5853 | 9.4081 | 9.4361 | 9.3436 | 8.1654 | 7.3250 | 9.0527 | 9.9228 |

```
10.9498 10.3378 12.1155 10.9222 7.6329  7.0238  9.9333  10.2237
8.3197  8.7894  9.9792  12.3097 3.7138  5.5698  10.0487 9.0977
10.8566 3.2749\
ZNF646  8.9641  9.2819  9.6105  8.8489  9.3281  8.0221  10.2900
9.7585  9.9217  8.8172  10.1144 10.9185 10.9504 9.5375  8.7993
9.5634  8.5366  8.9386  9.2445  7.6813  8.7209  8.1848  9.1799
9.0136  8.7120  8.6075  7.5745  8.4339  9.0499  9.9579  8.6081
9.1685  8.7628  9.7208  9.8274  9.3020  9.6413  9.4160  8.0885
8.3113  8.5736  9.1733  8.4197  8.0984  9.0681  8.4648  8.5043
9.0737  8.9825  8.8721  9.0141  8.3244  9.3142  8.9802  9.6953
8.9830  9.4672  8.7674  9.2995  9.0189  9.0390  8.8125  8.9140
9.7951  8.7729  9.2345  8.9590  10.4092 9.5202  9.2113  9.7572
9.9220  8.2889  8.8221  9.0983  8.3753  8.8938  9.1407  8.6476
9.5326  8.4342  9.0752  8.5840  9.2474  8.8651  8.5514  9.9701
8.6777  8.7446  8.9808  9.1520  9.7232  8.4063  8.8469  8.8590
8.7542  8.9413  9.6552  8.5442  8.7565  9.0649  8.8535  8.7265
8.9070  8.7420  8.9769  8.7596  8.3419  8.1937  9.0095  9.1808
9.0884  8.7543  8.8234  8.1452  9.3253  8.6976  9.8162  8.6728
8.6943  9.3385  8.7321  9.0384  8.8347  9.2990  8.9075  8.5411
9.1142  9.1111  8.7820  9.3811  8.8044  8.8809  8.3490  8.3949
8.5845  8.3643  8.4611  9.4304  9.4645  8.9302  8.6436  8.5256
9.1482  9.1291  9.3293  8.0277  9.1749  9.3010  8.7700  9.2542
9.1652  9.6702  9.5737  9.2261  9.9020  9.4288  9.4636  8.6611
8.4790  9.1791  8.5482  8.6778  9.1481  9.4455  8.5363  8.5898
9.4095  8.6508  8.5455  9.5396  9.1539  8.7252  9.0355  8.6950
9.5098  8.6058  8.8551  9.2364  8.8815  8.7045  8.9419  9.4134
8.7005  8.8643  9.0140  8.5829  9.6343  8.7046  9.2188  8.9709
8.7821  8.0892  8.9123  9.1373  9.1566  8.9566  9.3310  9.4226
9.0610  8.6131\
OPA3    8.8708  8.9872  9.1306  8.1250  9.3350  7.6957  8.9961
10.3550 9.3181  8.9347  8.8778  9.4849  8.4979  8.1322  8.8678
8.2311  9.3682  8.9301  8.9450  9.6575  8.6010  8.2688  8.9671
8.7413  8.9841  9.6782  10.2413 9.0847  8.6875  9.5152  7.9853
8.6943  8.4935  8.4942  9.4050  9.6115  8.9475  9.2273  8.7285
8.9195  9.5527  9.2514  8.6408  8.4922  8.3320  8.8898  9.3995
8.9644  8.9651  9.1336  9.1336  9.1856  8.8341  8.3159  8.6731
8.4102  9.0781  9.3732  8.8692  8.7669  8.7687  8.4651  9.8579
9.2262  8.7749  9.4175  8.9560  8.6792  8.6407  9.3259  9.3580
8.9304  8.7978  9.1514  8.5953  8.6080  8.2809  8.9295  8.7741
9.4537  8.6445  8.7212  8.5244  9.2445  8.6029  8.0535  7.7991
9.2185  8.3783  9.1152  8.7588  9.6157  7.7140  8.1254  8.4522
8.7812  9.4748  8.8568  9.0711  8.3328  9.6424  9.2368  8.8636
8.3325  9.0662  9.0399  8.6213  8.9881  8.5751  9.2784  8.8164
9.7335  12.1620 8.8611  8.8745  8.1606  8.0716  9.1543  8.3045
9.5793  9.6550  8.5733  9.8848  9.3994  8.8187  9.9047  8.5656
9.1123  8.5575  9.1771  9.0431  8.2305  9.0248  9.0394  11.0809
10.7650 10.1462 8.5356  9.0935  8.6846  8.6136  8.4653  8.8764
8.5108  8.4474  8.6401  9.2974  8.5470  9.0807  9.1313  8.9824
9.5272  8.7109  10.3025 8.6046  9.8657  8.9303  8.7649  8.2344
9.7781  9.4236  8.8295  8.2651  8.9979  8.5648  8.4886  7.8964
8.4479  8.9177  8.9733  8.9988  8.6116  8.9726  8.9340  10.0293
8.3674  8.7592  8.6796  8.6822  8.8509  9.0361  9.2000  8.6818
8.8796  9.1257  8.8131  9.1509  9.4278  9.4374  9.2888  9.0431
9.0096  8.6561  10.1590 9.1963  9.7710  8.6369  8.8924  9.1750
```

8.6146  9.5301\
OPA1    9.8679  10.1895 10.6580 10.5277 10.4473 10.7230 10.2037
11.0679 11.0353 10.2256 10.4874 10.9182 10.4259 10.2565 10.3079
9.4472  11.1890 10.0809 10.1672 10.4251 9.2777  10.2353 9.9854
10.4654 10.5035 9.6494  11.5128 9.3431  9.6471  10.6018 10.4070
10.2608 11.0636 10.3082 11.0484 10.2286 10.0250 10.1327 10.4815
10.6869 10.5873 10.4065 9.9246  11.4603 10.4067 9.4609  9.6567
10.1371 10.9660 9.9116  10.3708 9.1980  10.5177 10.3033 10.5311
10.5501 10.8076 10.7865 10.7625 10.5421 10.3530 11.3153 11.0858
10.7426 10.1756 10.5681 10.2061 10.1830 10.1376 10.3602 10.2435
10.6021 9.6967  10.5126 10.5545 10.5216 9.8902  9.7694  9.4675
10.5961 9.7924  11.2883 9.9866  10.7346 9.3863  10.4878 11.2919
9.6070  11.2549 10.1208 10.0059 10.7544 9.5745  9.5633  10.1330
10.2813 11.0459 10.0522 10.8822 10.2380 9.2519  10.2361 10.9253
10.6616 10.1930 11.4963 9.7447  10.4920 11.3357 9.9403  10.2349
10.1486 9.4331  10.6439 11.5173 11.1571 10.8372 10.6556 10.5905
10.4132 12.0581 10.3806 10.6730 10.4920 10.1592 10.7670 11.3777
8.9595  10.3959 10.7954 10.1344 11.0141 9.7672  10.2000 10.0414
9.3792  9.5929  8.2381  10.5462 10.2044 10.2163 10.2748 9.5730
10.4492 10.7002 9.9220  10.6272 9.7281  10.2017 9.7027  10.2096
9.5386  10.3725 10.2339 10.9824 10.8934 10.1659 10.8089 10.5470
9.9534  10.1615 9.8816  10.2183 9.7096  10.8872 11.1501 10.4038
10.6225 10.0026 10.7882 10.7532 11.5618 10.1260 10.5387 8.8467
10.4719 10.3419 10.2018 10.5390 9.6498  9.6995  10.1190 11.2556
10.2302 10.5918 10.1790 10.7426 9.4987  9.6510  10.6220 10.4481
10.2461 10.7442 10.6082 10.5186 9.4963  10.1647 10.0189 10.3828
10.3620 8.3590\
RHEB    10.7398 10.7550 10.4424 11.2785 10.2933 10.7023 10.6983
9.6423  9.6021  10.4842 11.1384 10.4348 10.7590 10.1066 10.6181
10.7982 9.9500  10.3865 10.4416 11.1073 11.5920 10.3043 10.0739
10.8943 10.9220 10.2394 11.5345 10.0522 10.0197 9.5994  11.8264
10.5060 11.1740 11.0443 10.9386 10.0795 9.7538  10.1423 10.6331
11.3129 11.3478 10.7742 10.9489 10.6787 10.5149 10.7753 9.7720
9.6422  10.4400 10.2640 10.0683 10.6088 10.7597 10.5913 11.5530
10.6145 10.0010 10.8331 10.2480 9.5576  10.9151 10.8623 10.5447
9.7554  10.4856 10.2795 11.0566 10.1374 11.5358 10.7282 10.4915
10.1196 10.1938 10.4385 10.5053 11.2977 11.0352 9.7717  11.0804
10.2713 11.1199 10.7511 9.8261  11.1957 11.3223 10.5938 10.0723
10.5962 11.2533 11.2197 10.3185 9.8684  10.5887 11.2833 10.5096
11.0306 10.8359 9.9407  11.2606 10.4946 10.2388 10.5280 10.3892
10.9746 11.1312 10.9424 10.1104 10.5485 10.2063 11.4844 10.2668
10.1383 8.4054  9.6917  10.9986 9.7773  10.7797 10.4394 10.7195
11.3253 10.5055 10.1878 10.2867 10.2420 9.8367  10.7799 10.7564
9.7293  10.6106 10.4249 11.3088 10.6526 10.4671 10.2110 11.1860
8.7961  10.3161 8.9927  9.8515  10.3995 11.0992 11.1219 10.8426
10.2587 10.1587 10.0012 10.3636 10.2562 10.4695 10.5450 11.5311
8.4905  10.5297 10.3498 10.4444 9.9451  10.0167 10.3818 11.6759
10.2984 10.3546 10.0883 11.8174 10.5690 11.2941 10.6134 10.8104
10.1346 10.3517 11.0318 10.3724 10.2545 11.1841 10.8091 7.8498
10.4871 10.5928 10.7661 10.7613 9.7257  10.1764 10.1552 11.0795
11.1372 10.2056 11.4346 9.7575  10.0156 9.9490  10.2188 10.3250
10.3437 10.6100 10.6233 10.5649 10.3080 10.6365 10.1453 9.9639
10.4102 10.0267\
RAB40C  9.9327  9.4532  9.5946  9.5846  10.3210 8.7412  9.6932

| | | | | | | | |
|---|---|---|---|---|---|---|---|
| 8.7181 | 9.8007 | 9.6391 | 9.6523 | 9.4055 | 9.1109 | 9.9507 | 9.3831 |
| 10.0374 | 9.5040 | 9.8235 | 9.6113 | 9.4413 | 9.5362 | 10.2824 | 9.8118 |
| 9.2520 | 8.7781 | 9.7587 | 9.6044 | 9.7593 | 9.8674 | 10.2040 | 8.4841 |
| 10.1938 | 9.4863 | 9.7103 | 9.6953 | 10.1129 | 10.3811 | 9.7195 | 9.8066 |
| 9.5359 | 9.6046 | 8.7466 | 9.6604 | 8.5431 | 8.6287 | 9.4120 | 10.0531 |
| 10.2801 | 9.4685 | 10.2658 | 10.0281 | 9.2563 | 9.7415 | 8.8525 | 9.9330 |
| 10.1321 | 9.6014 | 7.9493 | 9.5868 | 9.5724 | 8.8735 | 8.6164 | 9.7098 |
| 9.6003 | 9.3292 | 9.7023 | 9.6644 | 9.2241 | 9.6914 | 10.7223 | 9.6718 |
| 9.5288 | 9.5117 | 9.1058 | 9.2974 | 10.0446 | 9.1511 | 10.2502 | 10.2535 |
| 9.8900 | 10.2465 | 9.7119 | 9.8465 | 10.3116 | 10.3157 | 9.4850 | 9.2250 |
| 10.0938 | 9.8523 | 9.8655 | 9.1408 | 9.3433 | 9.1636 | 9.9866 | 9.4102 |
| 8.8199 | 10.0475 | 9.4658 | 9.8723 | 9.8760 | 9.9911 | 9.8187 | 8.7996 |
| 10.9865 | 10.2294 | 10.2242 | 9.8371 | 8.5440 | 8.6800 | 10.6752 | 10.4119 |
| 10.1642 | 9.3693 | 10.2058 | 8.6157 | 9.8167 | 9.7772 | 9.7405 | 9.3681 |
| 9.5289 | 8.4442 | 9.6896 | 8.9436 | 10.2165 | 9.2488 | 9.6659 | 9.3559 |
| 10.1344 | 8.7097 | 8.4991 | 10.6719 | 9.9579 | 9.6796 | 11.7157 | 9.5792 |
| 8.9389 | 10.7119 | 9.6953 | 9.5881 | 9.2840 | 9.6969 | 9.5411 | 10.2824 |
| 9.6024 | 9.1320 | 9.3536 | 9.9104 | 9.5495 | 10.0343 | 10.8164 | 9.5436 |
| 11.0656 | 9.4408 | 9.4796 | 10.0625 | 9.7115 | 9.3580 | 9.9247 | 8.8177 |
| 9.5990 | 10.2075 | 9.8759 | 10.0281 | 10.1603 | 9.8292 | 9.0621 | 9.6956 |
| 9.4844 | 9.8996 | 9.4526 | 10.1287 | 9.4223 | 9.3529 | 10.0577 | 10.2131 |
| 10.1075 | 9.4402 | 9.0298 | 9.1479 | 9.9409 | 10.5800 | 9.8627 | 8.7889 |
| 9.8203 | 8.7880 | 10.0813 | 9.2923 | 9.8281 | 10.1232 | 9.6846 | 9.3194 |
| 9.7295 | 10.5125 | 10.2131 | 10.1238 | 8.8941 | 10.5335 | 9.1378 | 9.4606 |
| 9.6722 | 9.6859\ | | | | | | |
| RAB40B | 7.7465 | 8.1905 | 9.6573 | 8.7580 | 7.5801 | 8.1147 | 9.4405 |
| 8.5602 | 7.9313 | 8.7432 | 7.7764 | 10.0855 | 9.5009 | 8.7676 | 9.2599 |
| 7.5460 | 9.2708 | 8.6153 | 8.2981 | 10.3149 | 8.7444 | 9.2497 | 8.9750 |
| 8.4978 | 8.7949 | 8.4548 | 7.4845 | 8.9425 | 8.1377 | 10.0922 | 7.6032 |
| 8.2809 | 6.5916 | 7.8288 | 9.7441 | 8.6322 | 8.5155 | 8.2476 | 6.9787 |
| 8.3614 | 8.7798 | 7.6220 | 9.4278 | 7.6435 | 8.6770 | 9.3985 | 7.2956 |
| 10.0506 | 8.9264 | 9.2624 | 8.1305 | 10.3669 | 8.3521 | 8.9234 | 8.3560 |
| 8.2618 | 8.2950 | 9.9553 | 8.9303 | 9.5103 | 8.7071 | 9.9257 | 8.1847 |
| 7.0406 | 9.0842 | 9.4096 | 9.2111 | 9.4543 | 8.4650 | 7.8623 | 10.0391 |
| 8.5271 | 7.9595 | 9.6837 | 7.3508 | 8.1158 | 7.2571 | 8.3209 | 8.2722 |
| 7.9010 | 8.5329 | 8.8063 | 8.4489 | 8.0815 | 9.4201 | 9.1136 | 8.0134 |
| 8.7665 | 8.5663 | 8.7196 | 8.7195 | 8.7941 | 8.9743 | 8.5652 | 6.8358 |
| 8.4042 | 7.3850 | 8.8191 | 8.3123 | 8.1348 | 8.6975 | 8.1839 | 9.0438 |
| 7.9020 | 9.4373 | 7.8319 | 9.4623 | 8.1267 | 9.5989 | 9.1348 | 8.8845 |
| 8.7560 | 7.9256 | 7.8392 | 8.4631 | 8.7667 | 8.6024 | 9.2089 | 7.9888 |
| 7.6439 | 7.9614 | 8.0771 | 8.7691 | 8.2280 | 9.1072 | 9.0260 | 9.1526 |
| 7.9329 | 8.7389 | 8.3800 | 10.4998 | 9.8958 | 9.0492 | 8.6133 | 8.1866 |
| 6.7737 | 8.5153 | 8.7944 | 8.8068 | 9.0052 | 8.6842 | 8.9890 | 8.8833 |
| 7.4237 | 7.2964 | 9.2244 | 9.1720 | 9.1283 | 8.9200 | 8.2775 | 8.3302 |
| 7.9631 | 7.9263 | 9.2741 | 9.1031 | 7.9160 | 8.7369 | 8.3674 | 7.2391 |
| 7.6213 | 10.4373 | 8.7104 | 8.8632 | 9.0523 | 9.5086 | 7.7459 | 7.9096 |
| 8.6223 | 8.0380 | 9.2060 | 8.7924 | 8.3510 | 8.8110 | 8.3750 | 8.6050 |
| 8.5472 | 8.6845 | 9.0522 | 8.8620 | 8.6683 | 8.4027 | 9.3486 | 7.3191 |
| 9.1205 | 8.3165 | 9.7152 | 8.1858 | 9.2222 | 8.4597 | 7.5568 | 8.9452 |
| 8.5176 | 8.9070 | 9.7386 | 8.6619 | 6.9569 | 8.1795 | 9.2319 | 8.3258 |
| 9.0576 | 8.3508\ | | | | | | |
| RAB40A | 2.7225 | 3.5484 | 3.9734 | 2.9880 | 2.1806 | 2.7758 | 2.3543 |
| 3.0712 | 3.8014 | 4.9230 | 3.5532 | 3.5591 | 4.8876 | 3.7887 | 1.2358 |
| 2.1513 | 5.9269 | 3.0246 | 3.8548 | 2.5980 | 3.6109 | 2.0613 | 4.1354 |

3.1574 3.3061 2.8612 1.5155 3.5617 3.5215 1.8184 1.7306
1.7560 4.9784 1.5403 4.2103 4.2576 4.1243 1.1048 3.5753
2.2802 2.1741 3.2981 3.8038 3.0605 2.0387 3.0403 2.6132
3.7557 0.4402 2.1768 1.3077 2.5833 3.6288 3.4809 5.0894
3.2557 1.0734 2.7742 3.7004 2.4263 3.0528 3.3286 1.5290
0.0000 3.9568 0.9507 2.3530 5.7629 1.7910 5.3557 2.4738
3.4170 2.7116 3.9594 3.1877 1.1076 5.0804 2.8810 4.2764
2.2410 1.5467 2.5223 2.0835 3.3070 2.8405 5.4925 2.9586
3.0751 3.1604 3.0083 2.4416 3.6067 4.4237 5.3110 3.0661
4.8450 0.9065 2.3603 3.4794 3.7089 1.0805 0.4986 2.9516
4.2949 1.7606 2.0179 5.9786 3.5614 5.1764 3.4668 2.6161
2.4082 4.9483 4.0875 4.4295 4.6300 2.7528 4.1219 0.9644
1.6324 4.4201 3.7106 3.4207 0.4700 3.8406 3.0272 1.8708
3.5647 3.8415 5.3291 3.4641 3.0128 1.5194 4.5288 4.5622
4.3724 1.0837 5.5768 3.5714 3.8084 3.0418 1.3036 4.0343
0.9357 3.1391 4.4574 2.6677 3.6943 2.6913 2.3242 3.8810
2.5542 2.5871 4.3612 0.0000 4.1702 2.8514 4.1820 4.5974
2.7913 0.0000 2.0641 3.1622 4.0142 2.8261 2.5845 0.0000
2.4564 2.6550 3.2158 1.9700 4.1199 0.0000 3.5606 2.9928
3.6830 4.7899 4.1660 1.6980 4.2151 3.3748 4.6037 4.1813
2.3775 4.0288 2.9077 4.1184 4.3617 4.2814 3.6605 1.4263
3.5613 4.5038 4.3148 2.4698 3.9204 4.5843 3.3172 3.4270
2.7876 0.0000\
COL7A1 8.3292 11.3875 10.1038 10.4963 9.0149 8.6717 9.5118
9.3833 8.3531 12.6394 7.6602 8.6327 9.8785 6.8042 7.8709
6.0153 10.3991 9.0651 11.6949 11.7676 7.9311 8.0116 7.0316
8.0237 8.6120 7.2069 7.0225 11.3047 8.5138 9.7744 11.6987
5.8115 5.7285 10.4984 7.1758 8.7319 9.4433 8.0203 6.6790
8.5886 10.4461 8.7813 9.1129 5.7588 3.2157 7.6869 7.6063
9.9005 9.8399 6.8681 8.5594 8.7942 9.7756 7.0197 9.0768
10.4306 9.5983 9.4019 8.8208 9.0283 5.5427 8.5687 8.1917
10.6961 12.1035 9.5188 8.3007 9.0149 11.1125 9.4962 8.3463
7.0222 5.8895 9.4505 8.0722 9.3054 10.8858 9.6755 8.3005
8.4807 9.0521 9.4586 5.1426 5.9971 9.1619 9.7281 5.1967
8.5831 8.0938 9.4105 7.5266 8.1646 8.9755 11.6851 9.5834
6.4054 5.8540 9.4807 8.1173 9.3201 8.9296 9.0393 8.5768
9.1048 6.9589 4.9773 11.5387 8.6936 10.0529 9.9854 5.9476
7.9010 10.6066 11.2790 10.2665 9.7764 7.3839 9.6436 10.3597
7.6363 8.7567 7.3817 10.8886 7.1725 7.4702 10.2130 9.2986
7.6461 6.9190 10.2366 8.4436 6.3528 7.7958 7.8023 11.6196
8.8181 9.6948 11.9307 9.0562 9.2306 8.7704 3.8294 8.5995
9.3625 9.4190 8.4061 8.9349 6.3222 7.2514 6.8484 7.9982
7.4308 9.1623 8.2589 8.2185 9.3845 8.1958 11.8253 7.1051
8.3600 5.4537 9.5235 10.3911 10.2115 9.8383 9.4302 4.3164
8.9175 9.1350 7.9401 8.8664 7.9104 9.5168 9.0736 10.3594
8.6257 11.3375 7.6638 7.4809 9.1560 8.6432 10.7201 8.7244
9.0133 9.3036 11.2718 8.2786 8.1892 8.9429 8.7050 8.0108
7.8323 7.5321 8.7351 8.3165 8.9716 9.1900 8.9490 9.9236
8.7994 9.2855\
MDP1 8.0170 7.5174 6.1866 7.6219 8.0417 7.3711 7.0642
7.0556 6.9526 7.8571 6.9364 6.3411 7.5260 7.4862 7.1545
8.2856 8.7947 8.7766 7.5359 7.7588 8.6762 6.9961 7.9284
7.3125 7.5665 8.6562 8.4507 8.1737 7.8940 7.9961 7.6436
7.2684 8.2093 6.9598 5.2027 7.9166 7.0965 7.5457 8.6325

7.4065 8.1583 7.1660 7.9402 8.0647 7.8107 6.6581 9.2306
7.3467 7.8034 8.9746 7.2014 7.3450 7.4039 7.8632 8.2621
8.0914 6.9714 7.0754 7.8751 7.8887 7.4634 6.8982 6.4354
7.7079 7.9797 7.7471 7.9923 7.0636 7.7907 8.2345 8.5053
6.8866 8.3228 7.5017 6.7347 6.9124 7.7054 7.5478 8.3410
7.3443 8.2213 7.2543 7.7412 7.4199 8.3355 8.2028 7.0065
7.4698 7.6212 8.5547 7.8846 7.3464 7.7167 8.0848 7.4218
7.6156 7.6055 7.6784 7.0340 8.1906 8.4196 6.8187 7.0792
7.9531 8.4815 7.4830 7.6067 7.6162 7.2588 7.6886 7.0023
7.5307 9.5978 8.7842 7.2629 6.7849 6.9622 8.3886 7.0372
7.5141 8.9012 8.1758 6.5776 7.3422 7.7859 6.9363 8.0420
9.3950 7.5163 7.4435 7.5382 7.7465 7.5280 7.3089 7.7275
9.4733 8.1526 7.7627 7.0050 7.6991 8.4110 7.2250 8.3636
6.9776 6.8962 8.1896 6.5392 8.0733 7.5731 9.3943 8.0028
8.5730 7.2274 8.8545 7.4884 7.0264 7.8634 6.9171 8.0781
8.6356 8.1571 7.7831 7.5164 8.2310 7.8058 6.7364 7.1565
6.3731 7.5771 6.7549 7.1110 7.1351 7.8944 7.3226 9.0714
7.7319 7.9838 8.0217 7.0600 7.7373 8.9295 7.5878 7.8594
7.6652 7.4800 7.4215 7.4263 7.9404 7.6841 7.8704 7.2903
6.9176 7.6960 8.1299 6.9790 8.7749 10.6036 7.9113 7.3200
7.2743 8.6415\
GTSE1 9.7733 4.0142 8.7481 7.3262 9.9330 8.9443 9.0887
8.2745 9.5673 8.8832 9.3417 9.0078 8.2661 8.7922 8.4123
7.5723 8.7843 9.0535 5.4561 9.8129 9.8535 7.7967 3.5909
7.1615 4.3353 8.5623 10.4752 8.4531 6.6582 9.4088 10.1094
9.5661 8.7089 7.9089 10.2718 9.2312 7.1678 10.0424 8.6861
8.5075 8.9970 3.2379 9.6292 8.9817 1.0260 8.5297 7.4981
8.8273 9.9983 7.7484 8.8416 8.8286 6.9716 3.1083 7.7649
9.2494 7.6542 7.6349 8.9957 9.5520 3.5787 9.5215 9.3152
10.0443 7.4835 8.8580 7.3239 8.9397 5.6069 9.0276 8.3197
9.0993 8.9102 7.6779 7.7088 7.5552 7.9471 6.5228 5.5717
9.1144 8.7072 9.7774 5.9514 9.1558 8.1589 6.4142 7.4976
8.8402 8.3277 10.2163 3.8377 8.7849 7.5989 7.6876 9.6635
3.3571 7.7875 4.4977 8.2170 9.1453 8.7033 9.1514 9.9280
9.1976 7.8298 10.7991 6.6632 7.7599 8.7763 8.7189 8.8273
8.7141 7.4426 9.0251 8.3041 8.6215 8.3929 8.8082 9.2796
9.9894 9.4095 8.3990 9.5819 8.6326 3.8913 8.2818 8.1831
7.0092 4.4248 6.3857 8.5134 8.1003 8.7273 9.1592 8.2162
6.3328 8.9745 5.0346 8.0892 3.5743 8.7549 7.2079 7.4820
9.0597 8.8358 3.0520 8.2694 5.8910 8.3965 8.0122 6.5515
7.4550 7.9742 8.3099 9.5236 9.0514 3.7983 9.3141 7.8583
9.8150 8.6811 9.2942 8.0601 7.5625 7.6221 9.3422 7.3705
8.5661 9.1992 10.2486 9.7830 9.6155 9.3316 9.4858 5.1047
9.1525 6.8355 2.5659 9.1905 6.5088 9.0035 8.4515 9.3516
7.5845 8.9049 7.4421 9.8031 9.0881 8.9463 9.8100 9.7267
7.0906 8.4003 9.3986 8.8760 9.6709 8.2920 3.9300 8.8587
7.8873 8.3287\
FAM183A 0.0000 4.2006 5.9321 9.4062 3.7405 6.6217 7.8194
8.7100 3.8014 7.3868 4.3258 3.1684 9.7341 0.0000 9.2535
5.9764 7.4433 1.9468 4.1667 5.1249 0.6175 10.1948 10.8971
7.6403 3.8179 1.6270 2.7174 10.6541 1.7737 0.5901 1.8679
0.0000 0.0000 3.7652 1.0004 0.0000 4.5283 1.9549 0.5410
0.7666 2.3500 4.9599 6.0047 2.2769 0.0000 9.6689 10.5685
3.3964 2.7459 3.7571 3.3005 0.0000 4.5343 3.1083 3.9480

| | | | | | | | |
|---|---|---|---|---|---|---|---|
| 5.9718 | 2.4376 | 4.0389 | 2.9096 | 5.9259 | 3.7837 | 4.4024 | 0.0000 |
| 1.8326 | 7.5642 | 6.9339 | 0.0000 | 6.2438 | 2.6811 | 5.9723 | 3.5250 |
| 1.5230 | 1.9166 | 6.3373 | 0.5367 | 7.7406 | 8.0730 | 10.9093 | 11.2303 |
| 0.0000 | 6.9142 | 0.9627 | 12.2027 | 8.4105 | 10.5737 | 3.6793 | 2.0569 |
| 8.1512 | 0.5273 | 3.0083 | 2.9352 | 1.5834 | 1.2055 | 7.8462 | 2.6337 |
| 0.0000 | 1.2090 | 5.0312 | 8.8011 | 1.5150 | 7.6638 | 7.7989 | 3.1832 |
| 8.7307 | 9.2011 | 0.0000 | 5.7849 | 7.2864 | 6.4020 | 9.4207 | 8.1609 |
| 8.4264 | 3.1476 | 1.5850 | 3.3205 | 5.3987 | 10.5777 | 3.6861 | 0.0000 |
| 2.2252 | 2.9409 | 3.8176 | 3.2675 | 8.1243 | 10.5574 | 3.0272 | 1.6443 |
| 7.0011 | 1.2802 | 2.1283 | 0.0000 | 8.5355 | 10.1402 | 10.8106 | 6.3916 |
| 0.0000 | 3.8512 | 6.8690 | 6.4468 | 3.2317 | 9.8232 | 3.3646 | 10.8013 |
| 0.9357 | 0.4562 | 0.5418 | 8.1536 | 7.3744 | 0.9947 | 1.5869 | 5.4549 |
| 9.9770 | 5.6490 | 5.5273 | 1.7261 | 1.2732 | 7.4831 | 3.5127 | 10.0573 |
| 2.6931 | 1.8525 | 0.0000 | 8.4851 | 7.6204 | 0.4418 | 4.5282 | 8.1577 |
| 5.1678 | 7.3915 | 1.7238 | 4.0283 | 2.0357 | 0.0000 | 4.4275 | 2.9928 |
| 6.9925 | 5.0594 | 2.6934 | 7.6096 | 11.9049 | 5.4165 | 1.7883 | 5.4904 |
| 5.2129 | 4.3449 | 3.9157 | 0.5811 | 7.4420 | 8.3390 | 1.9676 | 0.8827 |
| 5.3879 | 4.6494 | 0.0000 | 4.0956 | 6.1640 | 6.6701 | 4.6286 | 4.9749 |
| 9.1408 | 6.3058\ | | | | | | |
| ARFRP1 | 10.0046 | 9.9800 | 9.3937 | 9.2075 | 9.8331 | 9.8458 | 9.6780 |
| 9.5120 | 9.9848 | 9.9707 | 9.9980 | 9.5316 | 9.4825 | 9.1730 | 9.3325 |
| 9.9142 | 10.1508 | 9.8572 | 9.8398 | 9.8168 | 11.6526 | 9.5260 | 10.0687 |
| 9.0452 | 9.9900 | 10.7678 | 9.4739 | 10.5677 | 10.1687 | 10.5252 | 9.7051 |
| 9.5841 | 9.2673 | 10.3565 | 9.7457 | 9.2951 | 9.7141 | 8.5080 | 9.1026 |
| 8.4142 | 10.3373 | 9.7191 | 10.1642 | 8.8080 | 9.7343 | 9.1390 | 12.1078 |
| 9.7951 | 9.3346 | 10.1997 | 9.1039 | 9.3586 | 9.9391 | 10.0537 | 9.7318 |
| 9.3378 | 9.8252 | 9.3271 | 9.6268 | 10.3454 | 9.5846 | 9.3126 | 8.9598 |
| 10.3212 | 9.9506 | 9.4390 | 10.8230 | 8.8679 | 9.4630 | 10.2698 | 11.2177 |
| 9.1962 | 11.0034 | 9.8467 | 8.8419 | 10.0170 | 9.8887 | 10.1341 | 9.8645 |
| 9.0287 | 10.2322 | 8.9265 | 9.7248 | 9.4640 | 10.4475 | 9.5751 | 10.0862 |
| 10.3351 | 9.5903 | 10.0204 | 10.2552 | 9.0793 | 9.7691 | 10.1808 | 9.5818 |
| 10.0929 | 9.1400 | 9.9897 | 9.9335 | 9.6080 | 10.4159 | 9.8526 | 9.2674 |
| 9.7986 | 10.2934 | 9.8928 | 10.5811 | 9.4086 | 9.3328 | 10.2831 | 9.1410 |
| 9.3090 | 12.5204 | 9.4858 | 9.1229 | 9.3823 | 9.0606 | 10.0099 | 9.6977 |
| 10.0370 | 10.8044 | 9.3675 | 9.9085 | 9.2431 | 10.1025 | 10.1006 | 9.1714 |
| 11.2559 | 9.4005 | 9.3124 | 9.4737 | 9.0064 | 9.7454 | 10.1907 | 10.6176 |
| 11.0535 | 10.5184 | 10.9959 | 9.5609 | 10.1532 | 10.3068 | 9.3756 | 10.3247 |
| 9.0817 | 9.2912 | 10.1111 | 9.4352 | 10.4325 | 10.5737 | 10.4971 | 9.7418 |
| 13.5243 | 9.0577 | 9.9115 | 9.3679 | 9.8053 | 9.9734 | 9.1363 | 10.0425 |
| 10.7343 | 9.7237 | 10.8513 | 10.0018 | 9.8823 | 10.2787 | 8.6618 | 8.6327 |
| 9.5163 | 9.3198 | 10.0065 | 9.4157 | 9.6485 | 10.2198 | 9.6016 | 11.3794 |
| 8.9918 | 9.6851 | 10.3403 | 8.8420 | 9.9505 | 10.2942 | 10.4434 | 9.6731 |
| 9.2906 | 9.7058 | 9.4039 | 10.3722 | 10.2026 | 10.3485 | 10.2064 | 9.1763 |
| 9.5540 | 10.3621 | 10.3576 | 9.2014 | 11.2355 | 10.4231 | 10.2396 | 9.3493 |
| 9.5589 | 11.0165\ | | | | | | |
| OVCH1 | 0.9511 | 0.9197 | 0.6896 | 0.8605 | 0.0000 | 0.7223 | 0.0000 |
| 0.4748 | 1.2625 | 1.6538 | 1.2544 | 0.8472 | 1.0422 | 0.7759 | 0.9286 |
| 0.0000 | 1.0553 | 0.0000 | 0.9983 | 0.0000 | 0.6175 | 0.0000 | 0.0000 |
| 0.9110 | 0.5538 | 0.0000 | 0.0000 | 0.0000 | 0.0000 | 1.0077 | 0.0000 |
| 0.0000 | 0.0000 | 0.8184 | 0.5853 | 0.0000 | 1.2685 | 1.5916 | 0.0000 |
| 0.0000 | 0.0000 | 0.8506 | 0.0000 | 0.0000 | 0.0000 | 0.0000 | 0.0000 |
| 0.0000 | 1.0498 | 0.8339 | 0.0000 | 0.0000 | 0.0000 | 1.4299 | 0.8153 |
| 0.0000 | 0.0000 | 3.1582 | 0.4252 | 0.5715 | 1.2440 | 1.9554 | 0.0000 |
| 0.0000 | 0.0000 | 2.1442 | 0.0000 | 1.1331 | 0.0000 | 0.0000 | 0.0000 |

```
0.0000  0.0000  0.4786  1.2339  2.5860  0.0000  0.0000  0.6894
0.0000  0.0000  0.7073  0.0000  0.0000  0.0000  2.2503  0.0000
0.0000  0.0000  0.5265  0.0000  0.4848  0.0000  0.6909  0.0000
0.0000  2.0217  0.0000  0.8845  0.0000  0.0000  0.4986  0.0000
0.0000  0.0000  0.0000  1.8642  0.0000  0.0000  0.0000  0.0000
0.0000  0.0000  0.0000  0.0000  0.0000  0.0000  1.4572  0.0000
0.0000  3.8806  0.0000  0.0000  0.0000  0.0000  0.0000  0.0000
0.0000  0.9653  0.5074  1.3606  0.0000  0.9511  0.0000  0.0000
0.0000  0.0000  0.0000  0.5216  0.8792  0.0000  0.0000  0.0000
1.2444  0.0000  0.5418  0.0000  0.0000  0.0000  0.0000  0.5019
0.0000  0.5414  1.3242  0.0000  1.2732  0.5638  0.9402  0.6000
0.0000  0.0000  0.0000  0.0000  0.4033  0.0000  0.0000  0.0000
0.4012  0.5276  1.5072  0.4486  1.6845  0.0000  0.0000  0.0000
2.0560  0.0000  0.0000  0.4012  0.0000  0.0000  0.0000  1.6771
0.0000  0.0000  1.8914  0.5811  0.0000  0.0000  0.5707  0.8827
0.0000  0.5674  1.0222  0.0000  0.0000  0.0000  0.0000  0.0000
0.0000  0.0000\
FAM183B 0.0000  0.0000  1.1543  0.8605  0.9133  2.7056  0.5431
0.0000  0.4327  1.0519  1.7761  0.0000  0.0000  2.0721  0.5377
1.4442  1.8857  1.6514  1.5827  1.9903  3.1720  0.8427  0.6346
0.0000  0.0000  1.0317  1.0809  0.0000  0.0000  0.0000  0.0000
2.1139  0.5159  0.4664  0.0000  2.8161  0.0000  2.7557  0.9336
0.0000  0.6649  0.4871  0.6231  0.7649  0.0000  1.4889  0.0000
1.4786  0.0000  4.3343  0.0000  0.0000  0.0000  0.0000  0.8153
0.5608  1.6820  0.8754  0.4252  1.2977  0.0000  2.5691  1.5290
3.2013  0.0000  1.3532  1.0195  0.4832  0.5773  0.0000  1.4604
0.7001  0.0000  1.3638  0.5367  4.4454  0.5548  1.4952  0.6894
0.0000  0.0000  0.0000  1.1987  0.0000  0.0000  2.2503  0.0000
0.0000  0.0000  0.9113  1.0759  0.4848  1.4549  2.2282  1.8492
0.0000  0.5233  0.5454  0.0000  0.9478  0.6391  0.8684  0.0000
0.7358  0.8449  1.1001  2.6511  3.7600  0.5426  0.0000  2.1442
0.6215  0.0000  2.3219  1.9988  0.0000  0.5276  0.4321  0.0000
1.0358  1.0794  0.0000  1.8863  0.4700  0.0000  0.0000  0.0000
0.0000  0.5619  0.5074  0.0000  1.7472  1.5194  1.6184  2.2542
0.0000  0.6413  4.2995  0.0000  0.0000  2.0359  0.5748  1.4380
0.9357  0.4562  0.0000  3.6209  1.2499  0.9947  0.7381  0.5019
4.0526  0.9341  1.0019  0.0000  0.0000  1.2837  1.1539  2.3578
0.9345  0.6063  1.0431  0.9957  0.0000  0.0000  0.0000  0.0000
0.9722  1.2170  0.0000  0.4486  0.0000  0.0000  0.0000  0.0000
0.0000  2.7403  1.0660  0.4012  2.4311  2.0978  4.4901  1.8629
1.4215  0.0000  0.0000  0.0000  0.7058  1.7250  0.5707  0.8827
0.0000  1.9604  1.0222  0.4985  2.8198  0.6064  0.0000  0.6701
1.6364  1.1634\
PSAP    14.7187 14.5733 16.0390 14.6965 14.7248 14.0642 15.5585
15.4562 14.8040 14.3216 15.0253 14.5552 15.2362 15.0366 15.1358
14.2129 14.6508 14.0315 14.5633 15.8361 15.0599 14.8132 14.2463
14.8073 14.7902 15.7238 15.6158 15.2173 15.3202 14.6623 14.2148
14.2948 15.2859 13.9379 14.2447 14.3229 15.4807 13.9440 13.1183
15.6406 15.5113 14.8575 14.7264 14.7227 15.1812 15.2255 15.8516
14.4924 13.7885 14.9223 14.2847 14.8900 15.9037 15.0226 15.1208
14.0269 14.7098 14.8997 14.8396 14.0803 14.8368 14.8661 14.8194
15.3866 14.1534 14.7465 14.6203 15.7103 14.3317 13.7431 15.7992
14.9333 16.0741 15.9073 15.0991 15.2829 15.2623 14.9468 14.4400
14.8821 14.9624 13.8048 14.4292 13.7153 15.1638 14.1318 15.6545
```

15.4077 15.4209 14.3504 15.4599 14.7751 15.9639 14.5951 14.8086
15.1849 14.9615 14.8835 15.3942 13.7984 14.7417 15.1101 14.3519
14.0169 15.1621 14.4428 13.5004 14.9664 14.8061 14.0315 15.0789
14.1438 16.2940 13.9857 14.1133 14.0003 15.1589 13.9540 14.3622
14.4702 13.7269 14.9220 14.8821 14.5301 14.1952 15.2587 14.4956
15.6540 14.6888 14.6942 14.8108 14.1468 14.9660 13.9002 15.2849
15.9075 15.4748 14.6911 14.4785 14.8640 15.4347 15.2399 15.1449
14.9516 14.2899 14.9723 15.4284 15.1157 15.4507 14.5610 14.6369
16.1262 15.1213 14.2266 14.9383 14.9902 14.7836 15.1715 14.5536
15.1275 15.7255 13.9304 15.0144 14.8535 14.7081 14.6210 14.4894
15.9480 14.5596 14.3129 14.9766 13.7830 14.7018 15.2873 14.8191
14.1343 13.6932 15.2481 14.0257 14.5963 14.8771 14.5284 14.0869
14.9297 14.5129 15.2248 16.6596 14.7464 14.6916 14.9247 14.9996
15.9894 14.7795 14.8785 14.7125 15.1318 14.9994 14.8861 14.8311
14.2951 15.2800\
KIAA0831 8.3725 9.7227 8.1781 9.0275 8.3645 9.4198
8.7641 9.5951 7.9042 9.4132 8.1760 9.4647 9.2753 8.5270
8.8282 9.2204 9.5545 9.1161 9.5084 8.9086 8.9733 8.8980
9.1023 9.2489 9.6536 8.1814 8.2302 8.1989 8.1377 10.0055
9.1799 9.7229 8.8634 9.3365 8.3975 8.8233 9.1072 8.5418
8.8487 10.3371 8.1693 9.4763 9.6814 8.6823 9.4839 8.7302
8.1745 9.2715 7.9823 8.6900 8.7432 9.5952 8.5792 9.9376
9.6979 8.9803 9.1458 9.0736 9.5404 9.5705 9.1311 8.5097
9.0778 9.3582 9.4377 9.0864 9.4544 8.4522 8.9034 9.5330
8.0773 8.8418 8.2278 8.5767 9.1700 9.1430 8.7998 8.7733
8.9648 8.6680 8.5515 8.9654 9.3570 8.1643 8.6240 10.0588
9.8319 8.0653 9.4787 7.9494 8.9886 8.9326 9.0270 9.8211
8.0176 8.8845 9.4748 9.6789 9.0779 9.3222 7.7259 8.7673
9.6622 9.7548 8.4985 8.9224 9.6376 9.4487 9.5032 8.9681
9.3186 8.5986 8.7384 10.0728 8.8559 9.0122 8.2641 10.1728
8.4105 8.9804 9.8619 9.0417 9.3900 8.4524 9.6394 8.6653
9.6616 8.1465 9.7500 9.7616 8.3974 8.9678 8.5908 9.5464
8.6759 8.6994 8.1677 9.8869 9.4167 9.6605 9.1110 8.3137
8.8920 9.2512 8.9649 9.4998 8.9575 8.9087 8.8158 8.0815
9.5517 7.7379 8.7572 9.0081 8.0875 9.0553 9.6180 9.5050
8.7450 7.8804 7.2172 7.9015 9.0425 8.7491 8.6990 9.0452
8.3883 8.3870 8.8475 8.9466 8.5682 9.3221 8.7725 7.9808
8.4003 8.5548 9.5041 9.4039 8.8500 9.1392 7.9595 8.6672
9.0361 8.9753 8.7506 8.9186 9.7600 8.2170 8.2625 8.6150
8.8336 8.4042 9.2621 8.1987 9.0445 6.9092 8.7567 9.7001
8.6820 9.5321 7.8595\
SPPL2A 8.6673 9.0407 8.5571 9.5991 9.6478 11.0758 8.3303
8.6650 8.8187 8.7275 9.5156 7.9040 8.9378 8.4716 9.8635
8.7410 7.6118 8.7093 9.3975 9.3686 9.4962 9.6763 8.2015
9.1617 8.5194 10.3058 7.6671 9.3061 8.7456 8.9783 8.3323
9.4255 9.1179 8.6393 8.3846 8.9971 9.0001 10.0923 9.2852
9.8340 9.8096 9.5109 8.4400 9.2463 8.9431 8.8593 8.4578
9.0381 9.0760 9.5796 9.5743 9.1169 8.5379 8.6346 8.1915
9.3703 9.5321 8.9181 8.6951 8.1642 9.1264 9.9397 9.4786
8.4080 8.8239 9.1085 8.7339 9.1422 8.8382 9.1517 9.5987
9.4271 9.3716 8.6799 9.7898 9.9779 8.3364 9.2728 8.5113
9.4013 8.9210 9.7195 8.8461 9.4748 8.5429 8.3068 8.6099
9.0086 8.8764 8.8980 8.7061 8.6072 8.1363 7.7174 8.9743
9.3489 9.0212 8.1285 9.1147 9.1536 8.4850 9.3661 8.6053

9.2833 9.5956 8.0405 7.8501 9.4365 8.2116 8.9135 9.6716
8.8381 8.1175 8.0000 9.8076 8.7650 9.3649 8.5929 9.6184
9.0984 8.3734 9.7036 8.5290 9.5924 8.2645 9.1841 9.6232
7.6821 8.8636 9.3427 8.5799 8.7402 9.7200 10.0569 8.2910
8.6431 8.5716 6.8221 8.9433 8.5682 9.2137 9.4572 8.9245
8.8359 9.2860 7.7190 9.9041 8.0828 8.9492 9.1803 9.1555
9.3872 9.4587 8.3459 8.4322 8.7241 8.6690 9.2268 9.3346
9.5340 10.0684 9.4456 8.7759 9.0887 8.9724 9.3189 8.5622
8.9346 9.4152 9.4306 8.4942 9.1095 7.7099 7.9465 7.3065
8.7043 8.4683 8.6425 8.9683 9.3995 9.3320 9.3390 8.7079
9.0953 8.3374 8.6897 8.2029 8.6038 9.0737 8.9051 9.7021
9.6141 8.5432 8.6176 9.1213 9.2505 8.6063 7.9098 10.1819
9.2617 8.8053\
SLC46A1 8.5129 8.3791 7.9854 10.1734 8.7947 9.3455 10.0644
8.0914 7.9254 9.1582 9.8137 8.8070 9.3839 9.0854 8.5897
8.3267 9.4639 7.8253 8.5580 8.0110 8.6848 9.2874 10.4866
8.9716 8.7587 9.7867 8.4551 9.2902 7.8288 9.3322 9.1265
9.2719 7.9012 8.1215 8.9587 7.9935 9.4932 9.0763 8.1640
6.4115 8.3792 8.0014 9.0622 9.2142 8.9938 8.6227 9.1452
9.1714 8.6409 9.1763 9.3866 9.4795 9.6012 8.9037 9.3513
8.4707 8.1457 8.9809 8.4057 8.8996 9.0853 8.4079 8.7017
8.6677 9.5568 7.6209 8.8954 9.8355 8.3133 9.5754 9.2849
8.5192 8.9202 7.9550 8.7363 7.8813 8.1548 10.1282 9.3607
9.1959 6.7045 8.2183 11.1069 8.2334 9.2077 9.5414 8.4636
8.5736 8.5549 9.2098 9.3380 8.5506 9.2714 9.0429 8.5462
8.0771 9.2975 9.4936 8.8769 8.2288 8.2885 8.9696 8.9110
7.8269 8.8517 7.8091 8.4080 7.8716 8.7273 9.5539 8.5200
8.7383 8.9504 9.8278 7.2090 7.9700 9.0574 8.9458 8.7476
8.2577 9.3605 8.2816 9.0578 7.2921 10.6583 8.0080 9.3658
9.4961 9.1435 8.5093 8.0676 7.7566 9.5292 9.8649 7.4418
9.2665 7.3490 8.4004 8.6016 9.5638 8.4296 6.3550 9.2818
7.1050 7.9991 9.6762 6.9701 9.7763 9.3886 8.8832 9.2096
8.5516 8.7036 7.4781 7.7162 8.7721 9.6708 8.0554 9.7254
9.5966 10.1015 8.5499 8.7506 8.6968 7.8162 7.8215 10.0374
9.1413 8.7928 7.8591 8.2856 8.7607 9.4878 8.0369 8.0681
9.3045 9.1058 8.9993 7.4421 10.2086 8.7273 9.1822 7.9468
9.3920 8.8195 9.4383 8.5877 9.0877 9.3632 8.1253 6.6270
8.8342 9.0860 8.7026 9.3369 8.2705 8.1132 9.7109 7.4926
8.9214 6.9048\
TGFBR2 9.4105 11.2778 10.8651 10.1110 10.1543 10.6255 9.7255
10.4241 9.8086 10.2291 9.5346 10.6772 10.1073 9.3845 10.7435
8.5355 10.4786 9.5365 11.6746 10.2784 10.2304 9.6315 9.8012
10.0159 11.6455 8.5742 7.3129 9.3591 10.6309 11.3152 8.1988
9.1780 9.6450 9.6513 8.9739 9.6380 10.4645 9.3753 9.1519
10.5451 11.0684 12.3059 9.9734 9.2329 13.7857 8.7695 8.6653
11.1176 8.4316 9.5039 9.6471 11.6722 10.9283 13.4413 9.9939
9.8906 10.1634 10.9543 11.6386 10.0719 13.3583 10.5857 8.7966
10.2825 10.2882 10.9302 9.4775 10.7885 10.3347 10.4119 8.4381
10.6945 10.3286 9.6572 11.4556 9.2922 10.1973 10.5710 9.8473
9.9021 9.6718 9.6041 11.5722 9.7055 9.2820 10.7015 8.8771
9.3344 10.5286 9.5720 13.2778 10.4321 9.2289 8.6841 10.3423
12.8053 10.6893 11.4558 9.0013 9.9739 7.9144 10.3923 10.4055
9.2299 8.4000 9.6495 10.1470 11.1670 10.4574 9.2405 8.4561
10.6882 8.4253 7.1293 10.6789 10.3577 8.3796 10.3061 11.2507

10.5443 9.8115 11.2284 10.9517 10.7726 10.6882 10.7974 10.5688
6.8377 12.5104 12.1857 9.0995 8.9247 9.5553 9.4008 10.1070
8.0776 8.4568 7.2693 11.8964 11.3866 7.3047 10.3487 8.9662
10.4586 11.4751 9.8019 10.9686 11.9245 10.3997 7.7010 11.6440
5.3214 10.0169 9.0578 9.3333 12.3261 12.3217 11.1088 8.7328
8.2798 9.5883 10.6456 9.5649 9.2060 10.6892 10.9254 8.5314
10.7277 9.6866 10.0334 9.9551 10.6943 9.4787 9.0837 6.9805
9.3566 10.8385 12.3154 9.6771 10.4767 8.9264 9.4845 9.8945
9.6240 9.7127 9.7834 10.9018 9.2994 8.3920 10.0924 10.2772
10.7291 9.6335 10.6691 9.5196 7.0256 8.9166 11.5529 11.8901
10.4843 9.8285\
ITGA8 0.9511 6.5298 3.3494 1.1529 1.4681 2.9066 0.0000
1.3555 3.4322 6.1005 0.7594 2.5346 3.7397 0.4395 3.3904
2.9791 0.0000 2.5841 6.9739 0.0000 0.6175 1.7571 1.9117
3.0845 7.3521 0.0000 2.2381 0.0000 1.7737 0.0000 3.3610
1.9460 0.5159 1.1010 1.3224 0.0000 3.0082 4.1678 0.9336
2.2802 4.0711 8.0579 1.6604 2.5595 8.2161 0.0000 0.0000
1.4786 5.5877 0.4764 2.1110 2.8913 1.9420 8.9959 5.1979
0.9635 3.7761 2.5866 6.8941 2.8718 7.8454 3.9050 3.1723
0.5968 5.1251 4.7613 0.0000 2.1953 3.3716 4.9951 1.7877
3.4586 0.9449 2.5162 7.1954 1.5483 1.2673 4.1186 1.5048
1.3771 0.9714 3.6673 5.9514 2.6641 1.4651 0.0000 3.7216
1.6425 3.5345 2.1773 7.5902 4.1516 0.0000 1.7895 3.7635
7.3591 1.8574 7.0586 1.4288 0.5504 3.1381 2.6706 1.8854
1.5830 0.4835 2.7482 2.9263 3.2273 4.4598 2.0061 2.7134
1.6572 1.3631 0.0000 2.2788 3.6573 2.5502 2.5579 1.5372
2.0533 6.5904 4.4368 1.3885 1.7271 4.7894 1.6938 2.7753
0.7843 7.8489 8.3945 2.5892 0.0000 1.5194 1.6184 1.3267
0.0000 0.0000 0.0000 7.7146 7.1960 2.9483 1.7855 0.0000
3.6335 6.0133 4.2656 3.4742 7.5579 0.9947 0.0000 7.5309
0.0000 4.7030 3.7040 0.0000 1.9388 8.0182 4.9115 1.8394
1.4969 2.2191 1.3735 2.4523 0.0000 5.5614 1.6241 0.0000
4.8527 1.2170 1.2521 1.9700 0.0000 0.9335 0.0000 0.0000
2.9470 5.7043 8.4257 3.7554 2.9241 0.0000 0.6902 0.9100
1.8147 0.0000 2.2612 2.7975 0.0000 0.0000 0.9785 2.4959
1.7437 0.9737 4.0109 1.9594 0.0000 0.6064 5.7482 3.9801
4.9712 0.0000\
ITGA9 6.5526 8.4589 7.8274 3.5366 7.3994 8.6118 5.0640
5.1572 4.0348 7.3556 6.7085 3.6988 6.8768 10.2987 7.5651
5.1466 7.2778 3.3616 9.0355 3.1743 5.6633 8.5556 3.8339
7.5752 8.8309 5.4187 6.2708 6.5804 4.0288 7.9132 6.8196
4.5744 2.9780 7.5012 6.3759 2.0076 8.0187 2.6272 3.2001
5.1585 5.8817 8.8457 3.8038 2.9987 8.4088 5.6635 4.7329
4.7659 8.4886 2.4071 3.5929 6.4001 8.1153 8.7890 6.9505
5.0982 6.2910 5.3740 8.4126 5.9259 9.0201 5.7114 4.2771
7.1435 7.5642 5.8478 5.8062 7.1478 5.0439 9.1008 6.2674
6.3445 5.2125 4.3134 7.3940 7.2759 7.7316 6.5788 5.8265
5.5807 7.7006 7.9710 8.3944 8.1703 5.4783 2.7877 4.7163
8.7345 9.1980 5.7150 8.3049 5.9379 3.7682 6.8175 6.1987
8.9854 4.3075 8.8132 8.8442 3.7592 4.3582 5.7617 8.7432
5.3547 5.1060 7.4181 6.1627 7.6490 7.4510 7.5741 7.3902
5.9632 5.0200 2.5850 4.9724 5.8847 8.4468 4.5013 8.7481
5.4088 7.4514 3.8683 6.2371 6.8012 4.2819 4.9519 5.5780
3.0394 9.5335 10.2376 5.0021 3.1145 7.1491 6.3161 4.3113

| | | | | | | | |
|---|---|---|---|---|---|---|---|
| 5.1341 | 5.9280 | 4.8598 | 9.2020 | 8.0881 | 5.1961 | 4.4625 | 4.3077 |
| 6.9029 | 7.1717 | 6.9551 | 8.8556 | 7.5823 | 5.1188 | 5.8357 | 8.7678 |
| 6.0079 | 7.1375 | 4.4299 | 4.7471 | 5.8936 | 9.2868 | 10.2859 | 5.2529 |
| 6.1061 | 3.1431 | 2.2364 | 6.5383 | 6.2136 | 10.0496 | 9.9424 | 5.3328 |
| 7.2358 | 8.5336 | 3.0652 | 7.1136 | 4.8750 | 5.7610 | 5.9545 | 4.3567 |
| 4.2895 | 7.8403 | 7.9583 | 7.9310 | 7.2841 | 3.4996 | 3.1660 | 3.3548 |
| 5.4667 | 3.4586 | 5.7724 | 5.3638 | 5.7034 | 3.8909 | 4.6308 | 5.3897 |
| 8.0137 | 6.9752 | 6.3412 | 8.8033 | 3.1828 | 7.4381 | 7.5403 | 8.9751 |
| 7.8950 | 6.9799\ | | | | | | |
| STK40 | 9.6630 | 10.2678 | 9.3685 | 9.9808 | 10.8569 | 10.4415 | 9.2038 |
| 9.8151 | 10.2683 | 9.5148 | 9.8154 | 10.3503 | 11.1521 | 10.2773 | 9.8086 |
| 9.3705 | 9.1432 | 10.1222 | 10.4302 | 10.8035 | 11.0232 | 10.3955 | 9.8929 |
| 9.9590 | 10.4885 | 10.9399 | 11.2416 | 10.9005 | 10.0141 | 9.5642 | 9.9436 |
| 10.0710 | 10.9600 | 10.9675 | 10.2875 | 9.2881 | 10.8096 | 9.9829 | 8.8401 |
| 9.8538 | 9.7810 | 11.0569 | 9.9492 | 10.3329 | 10.0848 | 9.6349 | 10.6535 |
| 9.9817 | 9.9902 | 10.2054 | 10.1502 | 9.8145 | 10.9190 | 10.1364 | 10.3498 |
| 9.8798 | 10.2365 | 10.1325 | 10.2496 | 9.4938 | 10.5625 | 10.1933 | 10.4918 |
| 10.0734 | 9.7027 | 10.2028 | 11.1209 | 11.1121 | 10.7568 | 10.4476 | 10.4817 |
| 10.4568 | 10.5432 | 11.4971 | 9.6895 | 9.9371 | 10.8772 | 11.5341 | 9.7572 |
| 11.0662 | 10.8588 | 9.8884 | 10.2510 | 10.5602 | 10.1405 | 10.5135 | 10.0479 |
| 10.2459 | 10.4185 | 10.2082 | 11.0157 | 9.5130 | 11.4164 | 8.9915 | 10.6393 |
| 10.6262 | 10.8717 | 10.2004 | 10.3294 | 8.9998 | 10.7943 | 10.4670 | 10.1190 |
| 9.2910 | 9.7389 | 10.7289 | 9.7590 | 10.0844 | 10.2344 | 10.1186 | 9.5261 |
| 10.5983 | 10.6678 | 9.1923 | 9.6389 | 10.4411 | 9.9559 | 10.0422 | 10.8728 |
| 10.1053 | 10.4802 | 9.4162 | 10.3560 | 10.4405 | 9.9680 | 10.6349 | 10.4803 |
| 10.1791 | 9.5737 | 10.1952 | 9.7967 | 9.4028 | 10.6118 | 10.7832 | 10.7069 |
| 9.8909 | 10.6589 | 9.2491 | 10.0672 | 10.1654 | 10.3991 | 10.4019 | 10.6907 |
| 10.3783 | 10.0711 | 11.6832 | 9.9351 | 10.4492 | 11.6663 | 11.0370 | 9.7839 |
| 10.6573 | 10.4637 | 11.5796 | 10.5316 | 10.5908 | 9.9422 | 10.9718 | 10.3789 |
| 10.7637 | 11.2365 | 10.5926 | 9.9527 | 10.1684 | 10.8417 | 10.1160 | 9.9062 |
| 9.6240 | 10.0075 | 10.0181 | 11.5532 | 9.5607 | 10.8300 | 10.5517 | 9.7940 |
| 9.6964 | 9.5257 | 10.9874 | 9.7584 | 10.8120 | 10.2779 | 9.7654 | 11.5003 |
| 10.0512 | 10.2418 | 9.6220 | 9.9207 | 9.7102 | 10.1888 | 10.8197 | 10.9290 |
| 10.6286 | 10.4807 | 9.6992 | 9.9145 | 10.5566 | 10.3683 | 9.9803 | 9.9851 |
| 9.8593 | 10.5508\ | | | | | | |
| MYO3B | 4.9910 | 4.4268 | 6.3877 | 3.9331 | 5.8573 | 6.4446 | 5.2488 |
| 5.0253 | 7.8571 | 4.8533 | 4.3987 | 6.1881 | 8.1381 | 5.9518 | 5.2495 |
| 8.1892 | 4.6587 | 3.6346 | 4.0412 | 6.0338 | 1.3800 | 4.9611 | 6.9820 |
| 8.6729 | 2.9167 | 0.0000 | 3.8464 | 4.0391 | 3.0458 | 5.8480 | 4.3606 |
| 6.5356 | 5.6565 | 6.7327 | 6.2488 | 0.6819 | 8.8467 | 7.3561 | 3.9598 |
| 6.3881 | 3.5998 | 4.4251 | 1.6604 | 6.3535 | 3.9549 | 4.2530 | 0.0000 |
| 7.7064 | 2.7459 | 8.3390 | 8.6978 | 0.7768 | 6.1012 | 3.6364 | 8.3842 |
| 5.8463 | 7.8255 | 8.0685 | 7.0664 | 5.9935 | 2.4755 | 5.1004 | 5.0053 |
| 3.6763 | 5.3770 | 6.6555 | 4.2461 | 7.0645 | 8.1793 | 5.3557 | 2.0543 |
| 8.5368 | 0.0000 | 6.9557 | 8.4032 | 6.0456 | 7.1081 | 8.6868 | 7.2216 |
| 2.8855 | 7.1617 | 7.6137 | 4.4413 | 3.4703 | 4.6305 | 9.3657 | 5.1607 |
| 3.6018 | 4.9541 | 1.8648 | 3.3023 | 5.5024 | 7.3250 | 4.6053 | 4.1980 |
| 1.0621 | 6.7624 | 4.6105 | 3.0342 | 2.6110 | 2.8342 | 5.6943 | 5.2918 |
| 2.6605 | 2.3164 | 6.6836 | 2.1046 | 8.5346 | 6.5215 | 3.3293 | 5.0842 |
| 7.5285 | 0.0000 | 5.3219 | 7.4066 | 5.7065 | 5.1829 | 6.2949 | 9.8477 |
| 1.8581 | 2.6311 | 5.4702 | 7.1085 | 5.9142 | 6.1121 | 7.2403 | 5.5619 |
| 4.7548 | 4.3594 | 3.8142 | 7.3162 | 7.2852 | 5.9682 | 5.0332 | 6.1216 |
| 0.0000 | 5.2456 | 4.8598 | 4.8297 | 4.3438 | 1.8416 | 7.8823 | 2.4747 |
| 6.8863 | 7.4638 | 0.0000 | 4.4078 | 5.6351 | 3.9441 | 4.4402 | 8.6397 |

1.5604  5.7502  5.1936  8.5595  6.0864  4.6662  5.7214  5.9982
0.5416  2.9615  0.0000  4.6920  3.9555  6.4309  4.2410  4.8408
8.0648  3.9608  4.7188  3.4869  6.4779  2.4724  6.8781  0.0000
4.8595  3.9471  3.2020  7.9941  6.2832  5.1759  5.5921  7.0102
1.9773  6.2783  5.0434  6.1981  4.9080  5.1297  6.6850  1.8202
8.5774  4.5038  7.1312  4.2600  2.3335  1.0320  3.7719  9.1140
5.6538  2.5752\
ATP2A1  4.7116  3.3098  4.1826  3.0603  4.6855  2.1935  2.7953
5.7756  4.0037  4.4896  3.3720  4.8563  5.4030  4.5286  4.5035
4.9193  4.4959  5.4300  3.2457  4.6656  4.2602  4.1746  3.9415
3.5315  1.9289  4.1033  3.4150  4.1691  2.8976  3.6580  3.8660
3.8394  3.8407  3.2898  4.3228  5.6422  4.3735  5.6163  4.0418
3.3879  3.5284  2.8926  6.3683  4.7101  1.0260  4.9870  3.7177
1.9920  5.6299  3.9151  4.7650  4.0611  3.4897  3.3066  5.9489
4.2519  3.8880  3.3475  3.2061  3.8691  2.3520  3.5473  5.1464
4.7618  4.3907  4.8259  4.7377  6.6729  3.4386  4.4087  6.0094
2.2472  2.4919  5.1704  3.3873  5.2315  4.7937  3.1261  4.4482
5.7329  2.5369  4.0444  4.2581  3.6851  4.4093  5.2495  3.8626
4.9406  3.7390  4.5790  2.4416  5.0246  4.3941  6.3040  4.3893
1.6671  2.7408  2.8937  1.8231  4.8280  4.8791  5.4842  2.8654
6.2001  4.1473  4.0223  4.1830  2.9303  4.0860  5.0954  2.9711
3.5571  4.1934  5.5245  4.3101  5.7915  2.0324  4.5665  2.9955
4.0663  7.6827  3.5337  2.0828  2.4909  3.7335  3.3133  4.2124
5.8776  0.9653  4.0156  3.0920  4.6196  4.2631  4.4850  4.0075
0.0000  4.8004  6.4797  3.5714  3.8923  6.1450  2.2974  4.9098
3.2040  3.1391  3.3387  4.5970  3.5880  3.5751  3.4621  3.4027
4.0526  2.8827  4.6475  4.1392  5.0851  2.6463  4.7567  4.8244
4.7036  3.2259  2.8804  3.7468  4.5789  4.0363  3.4032  3.6605
1.8339  4.2220  2.6009  3.6212  4.9389  5.2248  4.3532  4.4516
3.6379  4.0267  1.4008  3.9476  3.5152  3.0054  5.2171  3.9547
4.2192  2.9378  4.1094  3.4490  6.1884  3.1812  4.6308  3.1033
1.5256  1.5498  4.0717  5.3732  5.8471  3.8137  2.7661  3.2137
4.7628  2.2387\
ATP2A2  11.2631 12.1289 12.6294 12.5130 12.2336 13.5741 12.0577
12.6652 12.3894 12.1725 12.7445 12.0468 11.7373 12.6123 12.5646
11.7568 10.9243 12.0828 12.2103 12.0801 12.5788 12.7508 12.5969
12.7205 12.1584 11.8633 11.8176 11.3921 12.3921 13.0422 12.3104
12.5293 11.9076 12.8965 13.0046 12.1732 12.2666 12.9488 12.2248
12.4480 12.3373 12.6310 13.4664 12.5822 11.6480 12.2243 9.9718
12.4618 12.4805 11.4685 12.5634 12.9190 12.2536 11.9133 12.7013
12.0955 12.7690 13.0602 12.2518 12.1347 12.0317 12.6585 12.9583
11.3870 11.8754 12.8770 11.9385 11.9334 12.6675 12.5417 11.7783
13.0474 11.2778 11.0349 13.4900 12.3526 12.4916 12.9740 12.0639
12.7128 11.1880 12.7413 12.5987 13.0667 11.7126 11.6133 12.0599
11.5800 12.1654 12.3895 12.2530 13.0915 11.7207 11.7003 11.7930
12.2207 12.8422 11.9097 12.8068 12.0271 11.0531 12.5676 12.7711
12.0421 12.4600 12.7213 11.5632 12.5578 12.6372 12.4636 12.6071
12.3703 9.8294  12.2024 12.1217 12.3759 13.0746 12.2407 12.9127
12.7197 11.5532 12.4666 12.8909 12.4486 12.4245 12.5259 12.7418
11.5035 12.0267 12.6129 12.5120 12.0955 12.7416 12.6462 11.5018
10.0345 11.3193 9.8494  12.6720 11.9095 12.2873 12.2880 11.5468
13.1838 13.1609 11.7506 12.9819 11.8669 12.3649 11.0566 12.0065
10.1429 12.9852 12.9791 12.6064 12.4051 12.0421 12.7706 12.6131
11.4187 11.9077 12.8927 12.5555 10.9307 12.5898 13.4221 12.5341

```
13.2604 12.1547 11.8827 13.1265 12.7768 12.4664 12.4313 10.5590
12.3434 11.8857 12.1832 12.7302 12.6063 11.5677 11.7229 11.5867
11.5611 12.7525 12.6986 11.8281 12.1332 12.0746 11.6375 12.5485
12.9761 11.6976 12.7560 12.3748 10.9400 10.4422 11.5793 12.8158
12.6094 10.5188\
ATP2A3  8.3927  9.6771  10.0647 8.9747  8.7313  8.3178  6.7420
8.4869  6.5336  7.9111  7.1879  7.7757  8.4923  5.7754  7.7966
7.1336  7.9315  8.7264  10.0335 9.2775  9.1814  9.0568  8.0352
7.5312  9.4388  9.6085  11.9222 10.3420 9.2428  6.1132  8.5314
8.5806  7.8837  7.5163  6.5555  8.5203  8.8901  10.3031 8.3977
5.6765  9.0633  9.4063  7.0237  9.6897  9.4329  8.2158  9.1265
6.7092  7.1812  9.4820  10.7812 9.7903  7.7655  9.0874  9.9184
7.8484  8.3127  10.3316 9.0940  5.8670  10.1885 8.2617  8.4449
9.3582  8.1604  9.7588  10.0811 7.1233  6.8782  8.4594  9.4353
8.4259  11.6156 11.4933 10.3122 11.2871 7.7600  9.5507  8.8942
9.9093  8.3976  6.3628  9.8271  9.9578  8.1940  8.5201  9.1986
9.0683  8.1797  13.3114 9.3755  8.8413  9.2590  9.6856  8.6783
9.6731  10.1952 9.1784  7.1093  9.6226  8.2170  9.2986  11.3249
8.6964  8.2533  7.2331  8.7508  7.1655  8.5436  7.5395  8.7818
7.3590  10.0721 7.0334  6.4078  6.6926  7.5427  7.9432  10.9200
9.1816  9.0940  7.7625  7.9824  10.5023 7.0799  8.5970  10.0731
7.9498  8.9661  9.2327  7.2375  10.3316 8.6422  9.6859  9.3781
11.4883 9.7074  11.5431 8.6762  8.6698  7.8930  9.5449  8.8711
9.6911  7.8034  6.5474  11.2055 8.5559  13.7038 6.3188  10.2035
8.4589  8.4391  7.4132  8.3871  6.9491  9.9429  6.7818  7.4956
10.1782 10.3442 9.3087  7.0999  8.5174  9.1594  7.7091  9.7564
8.7363  8.2348  8.8987  6.8243  6.7394  12.0198 10.5133 11.2285
8.6315  7.8264  8.5697  10.8797 11.8591 8.9498  8.3053  7.5498
7.9106  8.8989  8.2114  9.6023  8.3457  11.7848 7.5343  12.5523
6.7182  8.7398  8.8110  7.7244  7.1743  9.4285  8.7137  11.1350
8.1216  8.5040\
ITGA1   7.8050  9.3496  10.0425 8.2866  8.5430  8.6368  7.8625
7.6222  8.4077  8.4419  7.8712  7.6293  7.5745  8.0994  7.9730
11.2545 7.0850  8.7956  9.8796  6.7790  8.3828  8.0182  5.8278
10.7853 10.4027 9.0167  6.7461  7.3634  10.0521 7.2393  7.0752
8.2587  7.8652  7.6567  7.3846  7.2712  8.8657  8.9966  7.8841
8.8872  8.4269  10.1391 8.3991  7.8934  10.5198 6.8879  7.1667
9.1772  8.4596  6.3915  8.2011  8.3530  8.7259  10.2322 9.9955
8.3150  10.3689 8.6837  9.2923  6.5448  10.7092 10.0664 7.5193
7.9805  8.7629  9.6095  7.2491  8.4199  10.0966 9.4844  6.4346
9.1696  8.3226  6.7761  10.2279 7.4544  10.0346 8.1068  7.9992
8.6432  11.5064 9.0955  9.2947  9.2999  7.3641  8.5312  8.3693
6.3973  8.1489  10.7403 9.6108  9.0010  7.3560  9.1053  9.0601
10.5695 8.9212  8.9211  8.0658  8.7872  6.6390  8.7133  9.0401
8.6754  6.6964  7.5265  8.6165  9.7453  8.5953  7.4004  8.0725
7.5327  6.1650  7.3038  9.5951  7.6520  7.7253  8.8893  8.8517
8.5168  7.9045  8.7406  9.3252  8.2354  7.0299  8.6878  8.4276
5.9961  10.1588 11.1163 9.0182  6.6523  7.6692  8.9637  8.2594
8.9741  7.3514  7.8037  9.0929  8.4602  6.3325  8.6024  7.1815
9.9140  9.4236  6.9143  9.1313  9.6348  7.8350  8.0743  10.7167
5.5784  10.6445 7.4591  8.3570  9.3713  9.3645  9.4739  6.9150
8.1882  7.0049  8.5184  6.7860  6.6301  9.3052  9.7428  6.5570
9.1236  8.2662  6.9858  7.6182  9.5574  6.5163  7.4678  7.8671
8.9927  9.3794  9.9068  9.1496  8.2718  7.4502  7.9803  6.9345
```

7.1065	8.4155	7.4150	9.5335	6.8559	6.3215	8.3011	9.3496
8.4908	7.8210	9.3994	8.7216	5.7421	5.7627	7.5239	9.5785
9.1943	7.1843\
ITGA2	6.7343	9.6771	8.9076	9.2409	6.6991	5.6077	7.8483
6.3925	8.0408	8.1874	5.8875	7.4396	6.1694	9.7748	5.8782
7.5192	4.8050	7.3553	8.4416	7.5293	5.8079	6.4311	7.3767
10.1787	8.6478	6.0729	5.0269	4.9230	8.9358	8.7602	5.2517
8.4494	5.9250	5.3737	3.7013	8.6540	8.9170	9.8561	8.8214
9.0349	7.9306	9.8691	7.8594	6.7217	7.5510	4.6139	2.0700
6.9083	6.2464	8.2491	8.4915	9.0831	7.6641	7.8857	8.9102
8.6879	11.3296	9.7897	7.8901	5.5602	8.1832	8.6057	6.0458
9.1580	7.1563	8.5594	6.6082	7.8977	9.5937	6.8183	4.5925
9.0686	6.1371	5.4028	10.1551	4.6361	7.2747	8.0682	7.0117
8.8472	9.1759	11.3797	7.5207	6.9516	7.4059	6.2407	6.9149
4.0803	7.2341	7.2937	7.9321	10.4934	8.7578	5.9354	4.3283
7.5844	8.0118	8.5470	8.0289	9.8952	4.5762	5.5619	8.9222
5.3547	3.0296	6.7413	5.2961	7.1655	7.6246	5.2747	8.8569
6.6758	2.8658	5.7004	7.3165	7.0323	8.7399	7.2263	8.8278
9.7933	6.1448	7.8596	10.6191	8.1752	8.5435	8.1128	7.2624
2.7607	9.3687	9.3942	8.7650	7.2409	7.5945	7.8023	7.2747
9.2946	3.0489	4.7638	9.3325	8.3646	5.3319	8.9666	5.1602
10.8187	10.8990	6.3439	6.9208	7.6761	8.3468	7.3923	9.0997
0.0000	10.1072	6.5352	8.5926	7.3540	8.8637	10.0328	4.5974
4.1947	8.2525	7.5356	7.8524	7.1261	6.8978	7.7736	6.4885
10.8739	5.5923	7.6668	10.3387	5.6985	4.9125	7.9210	5.7522
8.6655	7.0648	6.8284	8.6457	6.5174	7.3460	9.0775	6.5234
8.6120	8.1093	8.0378	6.9773	5.0794	6.0348	7.2143	8.9770
8.5933	6.2963	8.3138	7.7328	4.5374	7.3391	7.8517	8.5887
8.3155	5.4315\
ITGA3	10.4870	11.3370	11.9967	10.8459	9.3291	12.7984	12.9721
10.7725	10.3744	9.7227	14.0323	11.9906	12.2965	11.6287	10.2646
9.7188	10.9473	10.9290	11.1480	12.1552	13.0321	9.9220	12.5173
9.9742	10.7636	11.0284	10.9507	10.7299	12.1094	11.8716	7.4640
8.6793	11.5425	11.0196	11.5483	11.4421	11.9873	10.5319	11.2779
9.7677	8.7798	11.1156	13.9686	11.1144	10.9990	11.4542	9.1478
11.9670	8.4669	10.2130	11.2895	12.5548	11.1841	10.7583	10.4655
11.3308	14.5854	13.5017	11.4051	11.2368	11.2266	12.0069	9.7482
10.8998	9.3747	12.6470	12.0038	11.8225	10.9764	10.1498	11.2324
10.3250	11.4024	11.5146	9.5748	9.8945	10.6535	10.7432	11.3627
10.4614	10.5374	9.7913	11.2573	9.1558	10.2675	12.3120	10.2167
8.7417	11.0392	10.6636	11.0459	10.9107	11.0665	10.4400	10.4379
11.4557	12.1445	10.2616	9.6133	14.4373	10.6425	11.5968	10.9517
9.6854	7.6810	8.0674	10.6294	11.5522	11.3388	8.7479	8.6951
10.2706	11.5900	11.5187	10.6169	11.6287	8.9360	12.7040	10.4399
13.1234	12.0018	11.2643	13.0900	11.9314	11.8194	13.4925	10.3347
12.8383	11.1619	10.6024	10.7728	9.1776	10.4804	9.6279	14.1027
14.0667	7.6496	10.1936	10.2817	10.3586	7.7056	9.4247	10.4680
12.2319	12.0281	10.2265	10.2512	12.3662	11.8519	12.1563	10.0641
8.6177	11.0385	11.5166	9.6892	11.5922	10.7981	12.2016	10.9338
9.4898	11.2211	13.7355	9.8446	11.9506	10.5252	9.1567	12.4280
11.6391	9.9430	9.1083	11.1597	11.4582	10.7685	11.8481	11.9639
10.2458	9.6201	11.2089	9.8590	11.1976	10.1289	12.0232	11.7092
10.1815	10.8104	9.8552	12.9864	11.9380	10.7906	11.2170	10.0759
12.4243	10.1175	10.9943	9.9932	11.6046	8.1293	10.4657	12.0113

```
10.0946 13.7175\
ITGA4   7.8957  7.0342  9.8092  7.2233  6.8335  7.7707  7.3423
5.7280  4.8909  7.4703  7.1696  4.8859  7.1282  9.3413  8.0436
7.6245  4.7299  6.3544  7.6424  7.5069  7.1264  6.8099  6.9267
8.6087  6.1053  6.2319  4.5004  7.1553  7.5365  4.7097  5.8202
7.4510  6.9457  6.5463  4.7592  6.9007  7.6557  6.1752  8.4462
6.7698  8.0756  7.5502  6.1187  7.4559  6.3963  7.1904  4.9822
7.0684  7.6840  4.7374  6.5837  8.5447  7.2136  6.7149  7.8694
6.6569  7.5945  8.4613  8.3534  5.0606  7.4389  8.6282  5.8929
6.5394  7.4641  8.6515  6.7039  5.6888  6.5853  8.1686  6.8120
9.3962  9.3654  6.3644  10.0109 3.8939  6.8633  7.4691  7.0341
7.8017  6.7947  7.6332  8.6957  8.2996  5.8009  5.0578  6.0797
6.1868  7.8676  9.2503  6.7937  8.2045  6.2555  7.0034  7.1748
7.3407  6.5648  6.5378  6.5899  8.7316  4.6871  7.9509  7.4111
7.3033  5.8529  4.9376  7.5896  7.3993  7.4958  6.1407  7.4503
6.8324  0.0000  4.3547  7.6701  7.1899  7.0126  7.1619  5.6043
7.1919  8.0641  7.7931  8.2387  6.2204  6.5591  7.5771  6.9506
3.7565  8.4592  7.7391  6.9616  6.1728  7.4285  7.6553  6.8668
6.4642  6.2596  7.0748  7.0813  6.1262  5.1584  6.8703  6.7287
10.7973 8.5021  5.4173  7.0240  5.9921  6.6498  3.1800  8.7506
1.9723  9.2157  6.5802  7.4516  7.8690  7.0932  6.2693  6.2418
7.8842  5.7883  6.7728  6.6278  5.9793  7.0811  7.8607  4.2544
8.6977  6.0618  5.7598  5.3786  5.8515  5.8003  6.3977  5.2368
6.9976  6.9484  5.9267  7.9424  8.9120  6.9719  5.7021  6.1146
6.9013  7.1253  7.5334  7.3609  5.7631  6.3612  11.4132 8.5171
7.5179  6.7063  7.7114  7.0166  4.5248  4.7698  5.9568  8.5686
8.1454  5.7007\
ITGA5   9.8671  11.9312 11.1108 9.5684  9.8198  10.6791 9.5226
9.4825  9.7905  10.8607 9.9109  9.4977  9.5809  9.0021  9.6094
9.4365  10.3759 9.6304  12.0627 12.2025 11.6153 8.7356  7.7836
10.2492 11.6556 11.9231 10.1648 10.3357 11.3444 10.1193 11.3516
10.0988 11.6848 9.8511  9.0357  9.9914  10.4221 9.9205  10.6702
8.4992  10.0194 12.3940 11.1416 9.3426  11.5327 10.0396 10.6791
11.3177 10.5084 10.1803 10.8990 12.4148 11.4654 12.1836 11.3272
9.4606  10.5824 10.6244 11.1105 8.1593  11.6549 10.8111 9.3395
10.7863 10.9036 10.3088 10.5129 8.9595  11.3597 11.1314 9.8318
10.7220 10.9718 9.8361  11.1718 10.3064 10.9212 9.6307  9.4423
10.4636 9.8722  9.9965  10.8729 10.4242 9.1396  10.7427 9.1410
10.4841 9.5364  11.7756 11.7459 9.3317  9.6350  10.7066 10.5152
11.9864 10.5727 10.7092 9.6870  10.5038 10.3909 11.1289 9.6200
10.2929 7.7386  9.3725  12.2319 10.3670 9.8622  9.7471  9.2124
8.8931  10.3988 11.0841 10.9916 11.1217 9.2865  9.8245  10.0623
11.5759 10.7348 10.6377 11.7493 9.7151  8.2836  10.3945 10.0312
9.7354  10.9725 11.3080 9.9673  9.1937  9.2984  9.2239  10.1227
11.8768 9.5472  11.4584 10.3550 10.0115 8.5293  10.0192 9.2133
11.1443 10.0845 9.2602  9.5722  11.5987 11.4288 9.7085  11.0504
10.4589 10.1238 9.5603  9.5856  11.6233 11.0690 10.2465 9.0161
10.1703 9.6571  11.4394 9.1909  8.9619  9.6485  10.0185 10.4207
10.6095 9.8397  9.2601  9.8592  10.4572 8.9687  10.1429 12.1042
10.0230 10.4433 11.6079 10.0617 9.6389  10.1915 10.5279 8.5778
9.1602  10.2259 8.7973  11.3803 10.5588 10.2072 10.6718 10.6266
10.8823 10.4636 10.5448 8.9597  9.5633  10.0342 9.7028  9.5378
9.4518  9.8529\
ITGA6   11.1568 11.0608 12.0507 11.5559 9.2328  10.5830 10.1502
```

| | | | | | | | |
|---|---|---|---|---|---|---|---|
| 10.6881 | 11.7226 | 12.3011 | 10.3328 | 10.6095 | 10.3758 | 11.1932 | 12.2288 |
| 10.4779 | 10.3529 | 10.2570 | 10.8024 | 11.6598 | 9.3945 | 12.9704 | 10.8543 |
| 11.8835 | 11.9339 | 11.2727 | 11.3758 | 10.8571 | 11.7457 | 11.1466 | 9.4401 |
| 12.0320 | 9.0435 | 9.9508 | 11.3710 | 10.9178 | 11.1646 | 11.3102 | 11.6212 |
| 11.5741 | 10.9280 | 11.6351 | 10.8366 | 10.7292 | 12.2700 | 10.0568 | 9.0131 |
| 10.9600 | 11.3516 | 13.9895 | 11.4039 | 10.7535 | 10.3915 | 12.1426 | 11.0969 |
| 10.4365 | 12.5972 | 11.6729 | 11.1962 | 10.6477 | 12.2391 | 11.5044 | 11.9013 |
| 9.9578 | 12.4361 | 11.6631 | 11.2485 | 12.0357 | 10.9064 | 10.9311 | 8.8706 |
| 10.7072 | 9.9845 | 10.5052 | 12.3482 | 12.0724 | 11.6076 | 11.7867 | 11.2841 |
| 11.2538 | 11.9476 | 11.9391 | 11.5229 | 10.6450 | 10.6527 | 10.2548 | 10.4742 |
| 10.5530 | 10.5963 | 10.1122 | 11.7908 | 10.7032 | 9.2014 | 10.4143 | 11.6085 |
| 12.6600 | 10.1184 | 11.5790 | 10.9648 | 10.2341 | 10.3007 | 11.8270 | 10.4292 |
| 9.7380 | 10.3202 | 10.2655 | 10.2840 | 10.5925 | 12.3188 | 11.0580 | 11.6177 |
| 11.3469 | 8.8310 | 10.0444 | 10.4900 | 10.0737 | 11.1763 | 9.2673 | 10.7886 |
| 10.5000 | 10.9726 | 11.9631 | 11.4228 | 9.6258 | 10.8253 | 10.6276 | 11.4873 |
| 8.8755 | 12.2853 | 12.3647 | 10.7118 | 10.9378 | 11.5164 | 11.5033 | 10.2052 |
| 11.6031 | 8.1075 | 10.1516 | 11.7468 | 11.4790 | 9.1244 | 12.6808 | 11.1346 |
| 12.4071 | 12.8150 | 7.8243 | 12.2969 | 10.9880 | 10.7938 | 9.9854 | 12.0317 |
| 8.9114 | 11.9396 | 9.7402 | 11.4300 | 11.4912 | 12.2644 | 10.7958 | 10.5721 |
| 11.1993 | 10.6525 | 10.2173 | 11.1467 | 10.8835 | 11.9014 | 12.1675 | 10.2284 |
| 12.5279 | 10.8023 | 10.0814 | 10.7299 | 12.0440 | 11.3891 | 9.6086 | 9.8209 |
| 10.7458 | 12.1062 | 11.4913 | 11.0686 | 11.4206 | 10.9551 | 10.0580 | 10.2023 |
| 11.0819 | 11.0658 | 10.6852 | 11.3135 | 9.3094 | 10.0754 | 11.2898 | 11.1159 |
| 11.8115 | 10.0555 | 11.8743 | 10.3874 | 8.9889 | 10.4712 | 11.0933 | 12.4514 |
| 11.6894 | 10.6823\ | | | | | | |
| ITGA7 | 7.2631 | 8.6867 | 7.9823 | 8.2957 | 7.3766 | 9.0847 | 9.4490 |
| 9.4903 | 6.6437 | 8.6214 | 8.7302 | 7.6806 | 8.6591 | 5.9097 | 5.4417 |
| 7.3004 | 9.8347 | 7.2028 | 9.4018 | 7.5036 | 9.6537 | 6.8974 | 8.7491 |
| 6.5390 | 10.5649 | 9.6085 | 7.5461 | 7.4295 | 7.3135 | 5.5069 | 9.3868 |
| 6.6356 | 5.8081 | 9.0970 | 6.4187 | 7.7516 | 8.3838 | 6.7241 | 5.3906 |
| 4.9936 | 5.8381 | 9.6772 | 6.9116 | 6.7501 | 10.3620 | 5.3486 | 7.2172 |
| 5.4159 | 6.1698 | 5.9642 | 6.1957 | 7.1375 | 9.0522 | 10.7758 | 9.6137 |
| 7.6201 | 7.0369 | 8.6995 | 8.6116 | 5.9026 | 10.2744 | 9.8371 | 6.9980 |
| 9.7674 | 8.3274 | 5.5835 | 8.7058 | 6.5312 | 7.3392 | 9.4442 | 7.3692 |
| 6.9942 | 5.7226 | 7.6499 | 7.6517 | 7.2615 | 9.6187 | 6.6589 | 6.8448 |
| 9.0736 | 7.3896 | 6.1734 | 7.7636 | 6.1374 | 8.1765 | 10.6421 | 8.8448 |
| 6.1503 | 8.5028 | 8.5073 | 11.7210 | 6.9355 | 8.3283 | 8.5200 | 6.9617 |
| 11.3658 | 6.7624 | 8.3458 | 9.1398 | 8.1840 | 5.4197 | 7.4733 | 9.5586 |
| 8.9130 | 6.3259 | 6.4519 | 8.3825 | 7.4847 | 8.1340 | 8.2819 | 6.0063 |
| 4.9363 | 9.4199 | 6.9307 | 7.5844 | 8.6559 | 7.6701 | 10.1094 | 7.3119 |
| 5.0457 | 8.6421 | 4.7111 | 8.1858 | 4.3402 | 9.6850 | 7.0539 | 6.7165 |
| 8.9106 | 9.1802 | 10.7199 | 8.2654 | 6.5288 | 7.5910 | 7.3983 | 5.7289 |
| 7.6576 | 6.4086 | 9.0241 | 7.8890 | 8.2420 | 7.0188 | 6.1296 | 7.1059 |
| 7.3080 | 4.6538 | 7.6020 | 5.3734 | 11.5296 | 5.9894 | 5.5574 | 8.9800 |
| 7.2206 | 6.2067 | 9.3437 | 5.3471 | 9.5579 | 8.6690 | 7.7631 | 8.6049 |
| 8.5821 | 6.5540 | 6.1711 | 6.2105 | 9.8301 | 7.3983 | 5.4966 | 7.4805 |
| 6.9368 | 8.1499 | 6.6707 | 6.3440 | 7.4258 | 6.4652 | 7.9528 | 6.6007 |
| 7.4618 | 9.2104 | 12.6495 | 7.0668 | 7.5182 | 6.2828 | 7.4930 | 7.9181 |
| 5.4938 | 9.6555 | 7.2905 | 7.1635 | 8.8642 | 5.3898 | 6.7450 | 5.9484 |
| 7.3700 | 7.8267 | 7.5726 | 6.1019 | 8.6833 | 9.1731 | 8.2864 | 6.5336 |
| 8.2411 | 6.7909\ | | | | | | |
| TRHR | 0.0000 | 0.0000 | 0.0000 | 0.0000 | 0.0000 | 0.0000 | 0.0000 |
| 0.0000 | 0.0000 | 0.0000 | 0.0000 | 0.0000 | 0.4363 | 0.0000 | 0.0000 |
| 0.0000 | 0.0000 | 0.0000 | 0.0000 | 0.3755 | 0.0000 | 0.0000 | 0.0000 |

```
0.0000	0.0000	0.0000	0.0000	0.0000	0.0000	0.0000	0.0000
0.0000	0.0000	0.0000	0.0000	0.0000	0.0000	0.0000	0.0000
0.0000	0.0000	0.0000	0.0000	0.0000	0.0000	0.0000	0.0000
0.0000	0.0000	0.0000	0.0000	0.0000	0.0000	0.0000	0.0000
0.0000	0.0000	0.0000	0.0000	0.0000	0.0000	0.0000	0.0000
0.0000	0.0000	0.0000	0.0000	0.0000	0.0000	0.0000	0.0000
0.0000	0.0000	0.0000	0.0000	0.4698	0.0000	0.0000	0.0000
0.6159	0.0000	0.3965	0.0000	0.6718	0.0000	0.0000	0.0000
0.0000	0.0000	0.0000	0.0000	0.8470	0.0000	0.0000	0.0000
0.0000	0.0000	0.0000	0.0000	0.0000	0.0000	0.0000	0.0000
0.0000	0.0000	0.0000	0.0000	0.4315	0.0000	0.0000	0.0000
0.0000	0.0000	0.0000	0.0000	0.0000	0.0000	1.2610	0.0000
0.0000	0.0000	0.0000	0.0000	0.0000	0.0000	0.0000	0.0000
0.0000	0.0000	0.0000	0.0000	0.0000	0.0000	0.0000	1.7059
0.0000	0.0000	0.0000	0.0000	0.0000	0.0000	0.0000	0.0000
0.0000	0.0000	0.0000	0.0000	0.0000	0.0000	0.0000	0.0000
0.0000	0.5414	0.0000	0.0000	0.0000	0.0000	0.0000	0.0000
0.5416	0.0000	0.0000	0.0000	0.0000	0.0000	0.0000	0.0000
0.0000	0.0000	0.0000	0.0000	0.0000	0.0000	0.0000	0.0000
0.0000	0.5232	0.0000	1.3803	0.0000	0.0000	0.0000	0.0000
0.0000	0.0000	0.0000	0.0000	0.0000	0.0000	0.0000	0.0000
0.0000	0.0000	0.4262	0.0000	0.0000	0.0000	0.0000	0.0000
0.0000	0.0000\
LOC100132288	6.5382	6.3042	7.8771	7.5437	8.0648	8.2080
5.4432	9.6031	9.4284	8.4739	8.0326	8.1592	6.8594	7.8565
7.4127	5.4029	10.6133	8.5090	8.6441	8.8512	8.8617	9.0493
9.2692	7.3050	9.0932	7.1444	5.3759	3.3595	6.5382	8.5291
8.1608	4.9072	9.0965	7.3372	5.7822	3.6416	7.0449	6.9920
9.5357	5.4977	8.2603	7.8873	8.0508	6.5439	8.1440	7.8255
6.5312	8.9899	8.0105	11.0815	7.4460	7.1736	7.5329	8.0070
9.2684	2.8432	8.1427	7.2562	8.0062	8.4108	8.2365	5.0654
5.7497	8.2421	9.1746	2.6522	6.6006	9.1970	7.9715	8.8756
9.0347	7.7890	6.7590	7.2373	8.7193	9.7231	10.0307	4.1186
10.0309	9.2417	8.1639	6.7955	6.1486	8.2142	4.6559	6.4851
9.3972	7.9212	7.2799	8.8144	9.3573	8.3771	8.7578	6.9162
5.8430	7.2653	7.1342	8.2402	8.7005	8.7978	5.0097	4.5418
6.0761	10.3202	3.1638	10.2233	8.9430	7.7199	5.9796	6.3390
7.5469	6.1930	10.6354	6.9069	8.3080	5.9228	8.0974	8.9896
8.3117	7.3831	10.4100	9.5129	7.2935	3.3012	9.9907	2.9459
8.5534	10.8338	8.8695	6.2404	6.6942	6.6438	5.6167	9.3875
7.1953	8.1017	9.5929	8.4065	8.0565	8.4098	8.9783	8.9104
8.8283	5.9789	8.6666	8.9987	9.5461	9.9574	8.8030	10.2809
8.9255	9.6212	7.8963	10.0248	6.6004	8.4391	9.5689	9.5934
8.6830	9.3568	2.3724	6.0377	2.4523	8.9610	6.6003	7.1347
7.7134	3.3186	8.8196	8.8534	8.2465	6.3427	7.3881	5.6632
10.6329	7.8868	7.0601	8.1002	8.9619	5.7325	2.5076	9.3486
8.5976	9.4601	6.5278	7.9329	8.7748	5.4142	2.4884	5.0241
7.6509	3.2412	8.4329	7.2070	8.0280	10.2493	9.5731	7.1211
2.5185	7.8135	7.8401\
DENND4A	6.9347	8.5224	7.5471	8.0877	7.1331	8.7693	8.5219
6.9068	6.8131	8.1691	7.7760	7.5229	7.2823	7.7460	8.5155
7.6609	6.4649	6.6090	8.5044	6.1054	7.0717	8.2759	8.0322
8.3898	8.4971	6.8823	8.4722	7.0053	7.5046	7.2552	7.9853
7.9963	7.1025	6.7501	6.9138	7.2881	7.4837	8.1947	7.5892
```

8.3459 7.7580 9.8687 7.0237 8.1823 9.1891 6.7831 4.8968
8.1910 8.4841 6.8079 8.3841 7.4921 7.3886 9.3322 7.1719
8.1903 8.0429 8.1488 7.9436 7.4061 9.6440 8.8854 7.7003
6.0005 8.1001 7.9651 7.5892 6.7330 7.6915 7.1726 6.6885
7.6852 7.3146 7.1116 8.8461 8.0544 7.2571 8.0583 7.5814
8.2272 6.5714 7.7365 7.9247 7.9561 7.0726 5.6703 8.0956
6.2012 7.8618 7.7543 8.8019 7.8132 6.1951 7.4947 6.4970
8.7840 7.5523 8.9602 7.5739 7.9103 6.3545 7.7021 6.8064
7.5440 7.8867 7.0123 7.8625 8.0174 7.7493 6.5076 8.4313
7.7064 5.9976 6.5546 8.7705 7.8465 8.1713 7.4844 7.9399
7.3463 7.1747 7.9674 7.7345 7.7486 8.7853 7.6298 7.0287
5.1564 8.9381 9.4162 7.1350 7.6312 7.8168 7.2623 6.0246
6.2915 6.6608 6.2786 9.0944 8.8318 7.4652 8.0917 7.2747
8.1749 8.0047 9.1564 8.0884 7.8963 8.1385 6.2188 8.5983
6.9519 8.6566 6.6973 6.4636 6.6256 8.9414 7.7866 6.9028
7.0102 8.0226 7.7391 7.9966 7.4064 6.7471 8.8739 7.0603
8.8522 7.9583 7.1113 8.1164 7.8341 7.7890 6.5929 5.7889
7.5766 8.1683 8.5314 8.3351 8.4432 6.9734 7.4869 7.3773
7.8966 7.3697 7.7268 7.5735 6.6172 6.9382 6.8150 8.9235
8.0316 7.4144 7.2627 8.1791 6.2041 5.3286 8.6534 8.8588
8.6198 5.5884\
DENND4B 9.3246 9.7794 9.5575 10.1607 10.4149 9.6566 10.3212
10.0816 10.7572 9.8147 9.4289 9.6954 9.7143 9.9702 9.8048
11.0048 10.4709 9.6351 10.0794 9.7273 10.6410 8.7251 9.5906
10.8858 9.4610 8.7853 10.3784 9.3968 9.6630 10.3767 10.2645
11.2435 10.3474 10.6875 11.0475 9.8529 11.0948 10.8749 9.6097
10.2089 9.7711 9.7603 9.9690 10.2819 9.2583 10.0016 8.8963
9.8083 10.2293 9.8837 10.3594 10.4011 9.5312 9.8482 10.7056
10.0203 10.1239 9.9283 9.9956 10.0361 9.5956 10.0245 10.3225
10.3285 9.8035 9.7649 10.5460 10.1425 9.8294 10.3408 10.4811
10.6701 10.4171 10.0396 10.0866 9.3058 10.4870 8.8686 10.3206
10.2327 8.9590 10.2138 9.2927 10.1886 9.4305 10.2026 9.9722
8.7657 10.2884 10.8554 10.0206 10.3626 10.3315 10.3105 9.3869
8.9526 10.6457 9.8883 10.6110 10.0433 9.9998 9.8271 10.5108
11.1871 9.7462 10.4795 10.7180 9.6623 9.9860 10.3423 10.1990
9.9278 10.1540 11.3354 10.1517 9.7854 9.6447 11.7625 9.5877
10.3004 10.1190 8.7473 10.2684 9.0406 9.3554 10.3248 10.0326
9.6274 9.4593 9.9891 10.1961 9.6832 9.5215 10.2450 9.6417
9.4308 9.3608 11.2317 9.8225 9.7953 9.7939 10.3345 9.7945
9.6057 9.3249 9.9408 9.3440 9.3366 9.4900 9.6461 10.4033
9.1405 10.5359 10.4804 9.1141 11.0667 9.7974 10.5425 9.3873
10.0275 9.5088 9.9410 9.6590 9.7332 9.4036 9.7213 9.9246
10.1968 9.4799 10.8022 9.6613 11.1869 10.9030 10.6639 9.9645
10.3784 10.3272 9.5383 10.1312 9.6826 9.2585 10.4498 10.1505
9.6704 10.4931 10.1702 10.0257 10.2049 9.9872 10.7123 9.9118
10.1965 9.4194 11.3139 11.0817 10.1979 9.3406 9.6989 9.6922
9.9008 8.9837\
DENND4C 9.0377 10.0405 9.2134 9.6959 8.9377 10.2741 10.5500
9.0441 9.2534 9.9718 9.9197 9.5486 9.5527 9.4029 10.1597
9.0669 8.6338 9.1235 9.7532 8.4949 8.8367 9.4819 9.9496
9.7216 9.9726 8.4992 10.4424 8.8602 9.1434 9.9011 9.4214
8.9221 9.4742 9.3576 10.0943 9.3375 9.7741 10.2213 9.5017
9.4451 8.8215 9.9498 8.7150 10.1865 10.7211 8.5261 8.0088
10.8097 9.1468 9.4835 9.4776 9.0660 8.8572 10.6691 9.9127

| | | | | | | | |
|---|---|---|---|---|---|---|---|
| 9.3827 | 10.2979 | 9.9607 | 9.3848 | 9.5604 | 10.6535 | 9.4487 | 9.6910 |
| 8.7967 | 9.8281 | 10.0643 | 8.8889 | 8.8654 | 9.2957 | 8.2881 | 9.6052 |
| 10.1764 | 8.4878 | 8.7580 | 9.9476 | 9.0684 | 9.5171 | 9.1672 | 9.0705 |
| 9.8835 | 8.8295 | 8.7448 | 9.1653 | 9.6671 | 8.7190 | 8.3573 | 9.9882 |
| 8.0247 | 9.6737 | 8.5739 | 10.2584 | 9.5580 | 8.5739 | 9.1197 | 8.8027 |
| 9.9336 | 10.1958 | 10.2820 | 9.0631 | 9.7140 | 7.4823 | 9.1441 | 9.4265 |
| 8.4579 | 7.9965 | 8.6944 | 9.4838 | 9.6839 | 8.9028 | 8.0903 | 9.6325 |
| 9.1983 | 8.8008 | 6.9189 | 9.7861 | 9.6494 | 9.3218 | 8.9797 | 8.1520 |
| 8.5920 | 8.9115 | 9.8675 | 10.1170 | 9.2079 | 10.6408 | 9.2607 | 9.3652 |
| 7.1984 | 10.4529 | 10.6527 | 9.8299 | 9.3333 | 9.0694 | 9.9094 | 8.8952 |
| 8.9389 | 8.0987 | 9.8465 | 10.3313 | 10.3769 | 7.7444 | 9.4359 | 8.9580 |
| 10.0670 | 9.9374 | 10.7736 | 9.4731 | 10.0764 | 8.4891 | 8.7700 | 9.9957 |
| 8.4629 | 9.9414 | 8.3680 | 9.4214 | 8.8765 | 10.3335 | 9.4835 | 8.9500 |
| 9.0803 | 9.1180 | 9.1972 | 9.3322 | 9.1275 | 9.5683 | 9.9332 | 9.3055 |
| 10.5708 | 9.0982 | 8.9917 | 9.7467 | 10.6072 | 8.5921 | 9.0340 | 9.5425 |
| 9.4885 | 9.9960 | 10.3458 | 9.4572 | 9.2251 | 8.8047 | 8.7164 | 8.9086 |
| 9.2294 | 10.1158 | 9.3558 | 8.6785 | 8.2651 | 9.0503 | 9.5151 | 9.9276 |
| 9.4876 | 9.1368 | 9.2966 | 8.6337 | 8.1489 | 7.7739 | 10.3102 | 10.0278 |
| 10.0447 | 8.3091\ | | | | | | |
| SWAP70 | 9.9552 | 10.0838 | 10.3302 | 9.9086 | 9.9861 | 9.5858 | 8.9569 |
| 9.6139 | 8.7144 | 9.5056 | 10.4363 | 10.2201 | 10.0774 | 9.5811 | 11.0979 |
| 9.7451 | 8.4680 | 9.7560 | 10.2739 | 8.8951 | 9.2361 | 10.3629 | 9.6576 |
| 10.8186 | 10.0019 | 8.7955 | 10.8348 | 9.6020 | 9.8945 | 9.3474 | 9.6437 |
| 10.8825 | 11.0435 | 9.5572 | 9.5027 | 8.4448 | 10.1339 | 10.7092 | 10.4230 |
| 10.0369 | 9.4574 | 11.4194 | 8.9459 | 10.5724 | 11.4415 | 9.3574 | 8.1091 |
| 9.8987 | 10.3810 | 10.7869 | 9.9431 | 9.6979 | 9.3574 | 11.4180 | 9.5405 |
| 9.4895 | 10.6779 | 10.2980 | 9.8038 | 9.5103 | 11.5232 | 10.3816 | 10.1549 |
| 9.1160 | 9.5280 | 10.8169 | 9.5759 | 9.1921 | 9.9108 | 9.3903 | 8.5875 |
| 10.4352 | 9.0727 | 8.5929 | 11.0778 | 9.4577 | 9.0879 | 10.0500 | 10.0010 |
| 10.5112 | 9.3162 | 9.6570 | 10.5202 | 9.8927 | 9.3123 | 9.1261 | 9.0219 |
| 9.5499 | 10.5453 | 9.1072 | 10.6207 | 9.7239 | 9.5941 | 9.1434 | 9.0856 |
| 10.5613 | 10.0008 | 9.9995 | 9.4228 | 8.7125 | 7.9375 | 10.6084 | 10.0878 |
| 9.3873 | 9.5762 | 8.9359 | 9.1567 | 10.2936 | 9.8081 | 8.5000 | 9.9025 |
| 9.4175 | 8.3085 | 8.0334 | 9.9499 | 10.0623 | 9.5791 | 9.6253 | 10.5807 |
| 9.4936 | 9.1487 | 10.4893 | 9.7940 | 10.1476 | 9.9419 | 10.0698 | 10.1648 |
| 6.9430 | 10.9157 | 10.4394 | 9.1750 | 9.0820 | 9.9907 | 10.4318 | 8.5276 |
| 8.5381 | 8.5394 | 8.4988 | 10.4966 | 10.5083 | 8.9739 | 11.9168 | 8.9906 |
| 11.2600 | 10.9315 | 9.4952 | 10.9313 | 10.4093 | 9.7901 | 9.8820 | 10.7800 |
| 7.0076 | 10.7642 | 8.3007 | 10.2870 | 10.6547 | 10.7572 | 9.8058 | 8.8568 |
| 10.2409 | 9.8901 | 9.8612 | 10.1141 | 10.3520 | 10.2433 | 10.4809 | 9.4506 |
| 10.6887 | 9.6071 | 9.4752 | 9.9300 | 9.4672 | 8.6257 | 8.9733 | 8.9839 |
| 9.7804 | 9.4867 | 10.9121 | 10.4339 | 9.5145 | 9.8782 | 8.5134 | 9.4309 |
| 9.4558 | 9.8617 | 8.7850 | 9.5584 | 8.7292 | 8.8066 | 10.1096 | 10.8235 |
| 10.7536 | 10.2857 | 9.3037 | 10.1434 | 7.3273 | 8.2678 | 10.3287 | 10.7093 |
| 10.5828 | 8.6735\ | | | | | | |
| RARRES1 | 9.2305 | 10.0892 | 10.0263 | 10.6757 | 6.5439 | 5.4234 | 6.5085 |
| 9.5074 | 9.4226 | 6.3101 | 8.8048 | 7.1622 | 10.4472 | 4.3385 | 7.9652 |
| 9.1435 | 10.7528 | 6.4724 | 11.6774 | 4.4272 | 11.6895 | 6.8083 | 9.5627 |
| 11.3659 | 5.1549 | 4.9432 | 4.0863 | 7.6488 | 9.4926 | 9.5350 | 5.3603 |
| 6.8997 | 7.1234 | 7.0436 | 8.0884 | 7.1780 | 7.0190 | 9.3970 | 6.0258 |
| 3.8754 | 7.8002 | 9.3497 | 7.8527 | 6.5221 | 7.9029 | 7.0541 | 3.1173 |
| 9.9130 | 6.2121 | 3.6711 | 9.0971 | 8.6909 | 8.9481 | 8.2612 | 6.2733 |
| 4.7363 | 7.6971 | 7.9830 | 9.3722 | 10.0263 | 8.2012 | 7.3705 | 5.8939 |
| 11.2938 | 6.5487 | 7.9698 | 9.9122 | 7.1519 | 4.7593 | 6.4299 | 9.3495 |

6.8097 8.1120 9.2857 10.0591 2.0284 7.3841 5.0371 8.3609
8.1239 3.4069 7.2135 7.6426 10.3883 6.6445 10.9386 6.2557
5.5098 11.4941 7.5592 11.3038 11.8273 8.0863 7.1401 6.9667
6.0738 14.6223 7.4369 5.4365 8.0052 5.1522 8.0443 12.2596
7.5128 5.3080 6.2571 9.4851 8.9024 8.1665 5.6662 5.3031
4.9862 8.6843 6.5078 7.2162 9.9505 9.0304 12.2611 6.7947
8.1789 6.2257 7.0554 9.2111 5.1562 8.5553 10.2490 8.3984
4.9695 10.9238 5.7465 10.0550 6.1282 7.8522 5.4750 6.3522
6.5113 7.4284 5.5768 6.7567 7.9006 6.5629 8.9987 5.7082
8.5830 9.5776 9.4300 5.8754 10.1157 8.8901 7.6682 6.9319
4.1350 8.5572 7.0966 9.4057 10.3008 7.3583 6.5374 7.7449
5.5410 2.9615 12.1159 7.1461 10.3822 10.3969 6.2153 7.1219
8.8953 6.5906 9.1833 4.7492 5.8391 9.7535 7.5615 6.9805
8.0266 5.0782 10.2018 6.2363 9.6897 5.4323 7.9449 4.5763
7.0709 9.4288 5.9346 10.1003 9.7296 9.0936 7.0550 7.7560
7.9818 12.1661 11.0447 4.5414 8.5575 3.8663 7.6082 6.1268
7.8822 6.3935\
LOC100132287 9.2659 8.1905 7.1492 9.2888 8.7551 8.9376
7.5400 7.2755 8.2508 8.6266 8.8671 8.8024 9.2403 10.0864
9.0111 10.3881 10.0649 9.3768 8.4081 6.6694 8.2874 8.0270
8.6475 9.4264 7.5233 6.2217 8.5335 8.8377 9.8798 9.0055
8.8164 10.2257 9.4855 9.1648 8.6375 8.5155 9.5347 9.8552
8.4754 9.4972 7.7022 8.0644 10.0824 8.3355 6.0506 9.9396
7.1984 8.9331 8.8404 9.0991 9.5064 8.9417 8.7239 8.3649
9.4980 10.0779 7.8499 8.7110 8.2483 7.7894 6.6436 8.6551
7.5007 8.4572 9.0484 8.6346 8.6198 7.9795 8.5527 9.5617
7.5608 8.8920 7.5535 8.4297 8.2976 10.6796 9.1968 9.6107
8.9181 9.1473 8.7799 7.3623 9.6524 9.3868 9.2195 10.3937
8.9099 9.6342 8.4781 9.7566 7.6826 7.6256 8.5473 10.3397
7.1672 5.2882 7.6276 8.3620 8.7267 9.7580 9.9248 7.2799
8.6878 9.5343 8.5423 7.3987 9.4877 8.4436 8.6960 9.3908
9.1145 8.6478 7.9256 9.7022 9.8090 9.1845 7.4290 8.4916
9.5141 9.3466 9.2803 7.5959 8.5541 7.0604 8.8072 7.4613
7.6190 7.8268 7.6236 7.6251 8.1298 9.5507 9.2045 9.7658
7.7290 6.7935 9.3522 9.6690 8.1234 8.3161 9.3863 7.5355
9.1540 8.2042 8.4273 8.5982 8.9747 8.5093 9.6643 9.7968
8.6351 7.2393 8.4784 9.8788 8.4283 8.6395 8.3623 9.4088
6.0213 6.9275 7.8602 7.1066 8.5148 9.1522 7.1803 8.4886
7.5038 7.4307 10.1506 8.4744 9.3607 9.3389 7.2951 9.2127
9.3982 9.5011 8.4065 7.2881 9.8379 9.8075 7.3869 8.8981
8.9680 9.2993 8.7029 9.4891 8.0787 8.3679 9.4764 9.4223
8.2028 9.5817 7.5508 7.5830 9.4950 7.8153 7.6480 8.5888
8.6420 9.1874 6.4147\
PHLDA3 9.7977 10.5044 11.0251 10.9124 10.1374 10.6405 9.4310
9.5805 7.3327 9.6558 10.2907 8.1592 9.2563 10.5925 11.1956
11.8639 11.0378 9.9116 10.5877 11.7389 10.5696 9.7170 11.2397
11.2082 10.6602 9.8611 9.6525 10.8198 13.3861 11.0962 8.1116
10.7341 9.4820 9.8416 7.6591 9.8931 11.3408 10.0700 11.2366
7.9120 11.0313 9.9777 12.7708 8.9946 10.0200 12.9367 8.7210
10.3535 9.6934 13.2670 10.9665 10.6902 11.4065 9.8824 11.1517
10.3635 11.0620 9.1998 9.2932 11.3342 10.5281 7.7958 10.8861
11.3748 9.8300 10.2817 10.7296 9.1960 12.2094 9.8233 11.1863
10.3689 11.4044 9.5591 11.9983 10.4027 10.4097 10.4807 12.2208
11.0730 10.7568 9.3219 11.0174 8.6466 11.2438 9.5276 5.9214

11.4602 12.4037 11.1987 11.1780 7.5134 11.7736 10.6443 10.0140
10.4596 9.6639 10.3607 10.2939 10.5599 11.2198 11.3576 10.1260
8.4769 6.5508 8.9867 9.8225 10.3810 9.5680 8.3568 8.7648
9.9021 13.4092 9.2527 8.5130 9.6541 10.5525 8.0286 9.4780
10.3484 9.8416 10.5870 10.6509 11.9436 10.5371 8.7636 8.2602
11.7608 10.2135 9.7139 10.9480 5.3600 10.3713 8.1635 10.3522
12.2093 11.0829 11.1849 9.5684 10.6465 7.6548 11.7315 11.6050
11.2619 11.5303 9.0579 10.2756 11.2713 10.7529 14.1542 10.7137
9.3402 9.9110 9.8556 11.4733 10.3068 10.5147 11.4729 10.7118
10.7667 4.7609 11.4982 9.6548 11.0586 10.0550 8.1466 10.2510
8.1761 11.0837 9.4630 10.1183 9.0064 11.7465 11.0234 12.3582
10.1168 9.3762 10.6965 9.3228 9.5445 12.6078 8.4541 9.1352
10.9683 9.3005 10.0668 8.9644 9.8617 10.6589 10.6855 8.9661
12.3712 9.2868 10.3194 9.5675 11.4276 10.8529 10.7602 10.5745
9.4921 12.6688\
PHLDA2 8.3250 7.2991 7.4465 9.3112 6.6930 8.3740 8.0283
6.4579 5.8496 5.1288 9.1367 6.7125 6.6773 9.5656 6.7128
6.7964 6.5336 9.2708 6.4965 7.2255 6.2337 6.3562 6.2188
6.7185 5.6876 10.9725 9.0987 7.4338 11.2606 3.9213 3.9611
6.9449 6.6910 7.2746 4.5858 9.1008 9.0263 7.1755 10.3398
3.9094 7.8153 7.6965 9.7133 9.6343 3.5062 8.4940 6.6184
6.8499 3.7148 11.0648 10.0846 7.9818 8.5641 3.9449 8.2610
8.5666 9.9029 9.0534 5.8036 6.9877 4.7791 6.9678 9.8222
5.1427 4.2600 10.3578 8.7146 7.0984 11.2374 5.2384 8.4868
8.0846 9.8185 7.4372 7.1132 4.1016 6.9364 7.4712 6.4082
10.0318 8.8034 6.2109 4.6038 6.3827 6.8784 7.6795 3.6714
6.6845 7.2468 8.3032 5.4665 5.7686 8.6879 6.6946 8.6875
5.4937 7.5557 3.9728 7.5083 9.9258 8.9080 8.3574 6.9016
5.9120 3.9384 6.9335 6.3259 7.9448 8.1847 6.9447 3.6490
7.0487 6.7326 7.5314 6.9019 8.6079 6.0131 5.0799 10.4096
8.7179 5.5435 7.3990 8.2663 9.9557 4.5261 8.7827 7.3401
7.0173 4.5474 3.7250 8.7681 7.7156 6.5089 5.6438 10.4473
9.5826 6.7905 4.5503 7.6904 4.2193 5.0906 8.4472 6.0056
10.6666 9.9434 2.5884 7.3097 6.5373 10.9536 11.2263 5.9679
6.2302 7.1514 7.4259 7.8854 8.4912 4.2610 8.0780 8.0807
9.3488 9.7228 10.8380 5.2203 8.6274 8.1967 5.3123 7.8148
8.7122 9.1807 6.9050 9.2415 4.8530 7.4230 8.9148 9.2411
7.7347 4.0649 4.9321 6.5739 5.1757 9.3267 7.4582 6.8977
5.5850 7.5042 5.6686 6.4805 7.2580 8.7283 9.0809 8.0226
9.4060 5.9590 4.6471 6.7969 7.5458 9.6817 3.3172 10.0581
5.0650 9.7181\
TACO1 9.2450 8.2508 8.2291 8.5726 9.6533 10.6364 10.1274
9.9972 10.0178 8.7372 9.0097 9.5285 9.1687 8.9490 8.7562
8.8949 9.2182 9.1599 8.4354 9.6565 9.2689 8.5233 8.8919
8.3362 8.3885 9.8756 7.7909 9.6555 8.9640 9.0529 8.4088
8.9924 10.3611 9.5119 9.0065 10.7528 8.5700 8.5092 9.1239
7.7286 9.0821 7.9542 8.9895 8.9936 8.4733 8.8223 10.2479
9.5683 9.0384 9.2223 8.7295 8.1578 8.9280 8.1771 9.1099
8.5019 8.0998 9.4483 8.5925 9.2181 8.3817 8.6756 8.5079
8.6182 8.4672 8.7620 8.6789 9.1432 8.5066 8.5193 10.7964
8.6930 9.7143 9.5023 8.7177 8.7897 8.3364 8.6995 8.7802
8.6509 9.3399 8.6929 8.4701 9.3894 9.2749 9.2291 10.3146
9.4484 7.8996 9.1824 8.4354 9.4963 9.2435 8.7515 8.8989
8.6565 8.8223 8.4910 8.4408 8.7441 10.1517 8.8688 8.3788

9.0241 8.5299 8.8610 8.3302 8.7033 8.8134 9.2164 8.4561
8.9286 9.4751 9.2644 8.7190 9.2721 8.6284 8.9112 8.2856
8.9238 9.3865 9.0636 8.6444 9.2892 8.6669 9.7120 9.4008
10.4255 8.4960 8.0024 7.7599 8.6027 9.1175 7.3745 9.8197
10.9165 9.6987 7.8971 8.5478 8.3774 8.1270 8.7670 9.2175
8.4668 8.8181 8.4853 8.4618 8.7173 8.5303 9.4271 8.4535
10.2449 8.1965 9.4704 8.7740 8.3744 8.4963 8.3349 9.4698
9.8779 9.2855 9.2571 8.5912 9.3312 8.3344 8.5620 8.9022
8.9515 9.2377 9.1845 8.7268 9.6517 9.4952 9.1915 9.4012
8.6185 8.4772 8.4973 8.5490 8.5591 9.4908 9.8144 9.8573
8.9063 8.3813 8.7850 9.5688 10.0218 9.2857 9.2490 8.6564
8.9381 8.8509 8.6100 9.0812 10.7785 9.2825 8.7119 8.8742
8.3588 10.3365\
PMM1 9.3764 8.8335 9.3043 10.3977 10.1192 7.8316 7.5576
8.7576 8.7652 10.2217 7.6047 7.3470 8.6578 8.6587 10.4743
10.7282 9.4494 9.9009 9.3015 10.4611 8.8683 9.5151 10.3936
9.4931 9.1055 10.4836 9.4648 10.6729 9.7193 9.4594 9.1758
10.3921 8.7840 6.9487 7.9108 9.3747 7.8291 10.1580 9.3818
9.1902 10.5895 8.6432 9.9696 10.2616 9.7690 9.4385 10.8528
9.3394 9.2860 11.6684 9.2003 8.8709 8.6089 9.1490 8.0723
8.8333 9.5661 9.0980 9.2353 8.9525 9.2982 9.7530 8.1078
8.8806 9.8107 9.4710 8.8266 7.7240 8.5260 10.3661 9.0567
8.9830 9.6708 7.8697 9.6696 9.7185 7.6122 10.9076 10.7033
9.2928 8.8034 8.0420 10.3995 8.6273 10.6571 8.0880 7.3010
10.7565 8.7357 9.8015 9.6715 9.6852 7.8832 10.4803 8.3581
9.2736 8.3462 10.2568 8.9954 9.1142 10.6002 9.8422 9.4461
10.0218 10.0007 8.6690 10.1225 7.8477 7.9650 10.4711 10.2663
8.1968 9.5436 9.4594 7.9443 9.7712 9.5377 7.9513 7.9504
10.3513 8.0907 9.0362 9.9037 10.2327 10.0951 9.5119 7.6498
7.9071 9.4056 8.8600 8.0789 9.8226 10.5227 8.9191 8.9859
10.4673 10.8199 10.2210 10.0147 10.0249 8.9649 10.6324 10.8305
9.6622 8.5753 10.6415 8.9012 9.8744 8.6931 9.0573 9.5596
9.6824 9.3572 8.0400 8.3468 8.1318 9.9871 8.1972 9.6040
9.7959 11.0423 9.3609 9.5602 11.1558 8.0907 8.4751 7.0759
9.3008 9.3356 8.1628 8.7094 7.7386 10.0238 9.4847 10.2383
10.5316 9.4447 9.6749 7.8907 10.9434 11.7092 9.1924 8.7229
8.6381 9.4134 8.8626 8.4480 10.3076 10.4066 8.0175 9.6264
8.0241 8.6388 8.5358 10.7602 8.7321 9.6083 10.3855 8.9110
10.5720 9.8441\
GAP43 2.2429 3.7531 0.0000 0.4935 2.3147 0.9816 2.8873
2.8551 0.7652 1.9494 1.0281 2.3205 2.2881 7.7243 0.5377
2.1513 1.0553 2.5841 3.8042 0.3755 2.5380 0.4820 2.1093
2.1767 3.2362 1.8524 1.6918 1.8373 2.5456 2.5978 6.1340
2.2643 0.5159 0.0000 1.3224 0.0000 3.1675 1.7231 2.4725
0.7666 1.7407 2.9686 0.6231 0.7649 3.2157 0.0000 0.0000
0.0000 1.2792 0.0000 1.8408 1.2789 5.6732 2.7018 5.8573
0.5608 3.1261 3.4612 3.1515 0.5715 1.4980 3.5473 1.7473
2.8376 2.9371 1.6675 0.5980 4.8502 6.5319 5.2786 2.6452
1.3570 0.0000 3.4891 3.7189 0.0000 6.3518 1.4952 1.5048
2.9859 1.2875 2.5223 2.7716 0.6718 1.4651 2.6709 5.8441
0.0000 2.4361 9.8682 3.8377 1.1363 5.4769 1.7895 0.5198
1.8952 0.5233 2.0758 2.4992 3.4878 1.0805 2.2374 1.2310
2.1133 1.9209 3.4974 3.6115 2.7174 1.7151 0.0000 1.8341
0.6215 8.9732 0.0000 1.8354 6.4441 0.5276 5.0947 1.3703

```
1.8581  7.9288  1.0055  2.7935  1.1079  1.3435  2.0458  1.8708
2.4149  2.9388  3.1021  2.4304  0.6684  1.9260  2.5437  3.0945
3.6290  2.1237  5.0346  1.2057  0.0000  1.0240  0.9848  1.0973
2.4761  2.1200  2.2162  0.6735  2.3603  2.3133  3.7031  2.7711
0.0000  3.2708  3.5885  3.7009  1.9388  1.5425  3.8556  0.0000
4.0031  0.6063  1.6421  0.5821  0.9764  0.7796  1.1696  0.0000
0.7148  1.4679  0.5466  0.4486  1.8707  5.3905  2.2569  0.0000
4.9544  0.9063  4.8248  4.8687  1.4046  1.9305  2.0241  3.9132
0.0000  3.0580  2.5553  2.6907  1.8171  0.0000  3.0493  2.6970
3.7744  2.1288  2.0332  0.8682  0.0000  0.6064  1.0391  0.0000
3.8576  2.2387\
NENF    10.5862 10.5579 10.9773 11.2401 9.7031  9.8640  9.2050
10.6180 10.1740 10.7861 9.9764  9.0168  10.0646 9.6390  10.0453
11.6016 11.5687 10.7876 10.4338 10.1905 9.1369  9.7513  10.4414
10.7823 10.8437 11.3028 11.1865 11.0420 11.0550 8.9652  10.4496
11.3921 10.2611 9.4384  9.7108  9.6726  10.2586 10.6570 10.8338
10.2471 11.9239 9.7638  11.8086 10.6209 10.5934 12.8010 13.2738
9.7094  10.0443 12.4399 11.0768 9.3618  10.4728 10.6963 10.7390
10.7978 9.3524  10.7016 10.5878 10.8709 10.5742 9.9463  10.2552
9.9421  11.2795 9.6181  10.7581 10.7580 9.9523  11.8093 11.4840
10.7099 11.9086 9.2912  10.8000 12.2509 10.3917 10.5044 12.9079
9.8067  10.8210 10.0974 10.7882 10.8513 10.8032 11.1408 9.4231
11.3478 10.2666 11.6494 11.1029 9.3380  10.5875 11.4347 9.1051
10.7223 10.0664 11.0033 10.9393 10.5798 11.8785 10.0436 9.8031
11.1760 10.5669 11.7705 11.3845 9.4737  10.6328 11.5351 10.6005
9.2206  14.4199 10.8154 10.1060 9.2270  10.7760 8.6985  10.0263
9.6889  9.7256  10.2987 9.9965  9.8123  10.3521 9.6175  10.4548
11.3842 10.4267 9.8921  10.2082 10.4663 10.0580 10.6801 11.4965
13.0555 10.6008 12.4744 9.6252  10.7309 11.7582 12.0431 11.1353
9.3902  9.4158  11.2611 11.5669 11.0708 10.1877 13.3899 11.2898
13.0977 9.5297  10.1900 10.6908 8.6344  10.6591 9.4878  10.5692
11.6792 8.2275  10.4899 10.6338 11.0197 9.1439  8.9451  10.3701
9.8548  10.5848 11.3485 8.2551  10.7062 10.7137 10.9973 13.7066
11.0777 10.8005 11.2490 9.2471  10.1816 12.1045 9.8919  9.9886
11.1996 10.0051 10.8510 9.4356  11.1512 10.8041 9.7032  9.2003
10.5750 11.4102 11.4582 10.9310 11.1687 12.5809 11.0237 9.6203
9.7896  11.4956\
SQLE    9.8015  8.7403  13.3149 10.8806 11.6262 10.1314 11.5083
10.8779 12.0856 9.7264  10.4575 10.8253 9.8866  10.2125 10.1789
9.8266  9.9477  10.4775 9.0300  11.4438 9.2501  11.3556 9.7051
9.0948  9.4839  9.4322  11.6448 10.0881 11.0578 9.8873  11.1539
9.6775  10.9192 11.5745 11.9562 10.7386 9.8641  9.9722  9.9044
13.2797 9.8928  9.2825  10.8930 8.2717  8.4521  9.9436  9.0913
11.0291 10.8332 9.1985  9.8992  10.4531 10.5194 8.4266  10.0800
9.8422  10.9886 8.7211  11.0490 12.3255 9.8335  10.2903 10.0453
12.0519 9.7379  9.9073  10.5529 10.7653 10.9309 10.3217 9.3779
9.8637  9.2299  10.7423 9.3827  10.2460 11.1743 10.5775 10.6733
10.5522 11.3188 12.8357 10.2985 10.6120 10.5050 10.4573 10.1935
9.4451  12.2318 9.6828  7.6551  11.3314 9.3172  9.1037  10.0332
8.3547  10.4360 9.0316  11.2884 7.1423  9.4175  9.6825  10.8490
10.9703 10.9319 12.5930 8.2851  9.8909  10.3844 10.8919 10.4499
10.4249 10.1054 8.6689  10.2473 9.7197  11.7660 9.5432  10.7184
11.4741 10.8899 6.7389  9.9688  10.9736 9.9396  10.0983 11.4281
9.3512  9.3739  10.2822 12.3586 10.4687 10.4738 10.5042 9.9141
```

4.7015 10.2259 6.6448 10.4742 9.1938 11.2550 10.7087 10.3305
9.3534 9.6714 8.2202 10.9357 8.6480 10.1345 10.5011 10.0051
8.4945 10.1320 9.5907 10.3301 11.6439 9.5815 12.7126 11.5010
10.7324 6.8371 8.9458 10.7449 9.6979 12.2297 10.7911 11.0023
10.8490 12.5348 10.3488 10.9181 9.6831 10.4460 11.5337 6.4248
10.6782 10.6038 7.7688 12.1217 10.3658 9.7985 8.8238 9.8764
10.7617 9.7264 11.7340 9.8886 9.7113 8.8213 9.5815 9.6062
9.9052 10.0177 11.3520 10.3335 9.8709 11.1385 9.3648 10.5551
10.9610 9.7224\
MRPL28 11.3510 10.4060 9.6241 9.9262 11.2915 9.2722 9.4079
10.8785 11.0275 10.5750 10.8057 10.0934 9.8407 9.6958 10.4609
10.9313 11.1457 11.1136 10.4028 10.7932 10.8849 10.5382 10.8361
9.8749 10.5072 12.3816 10.6335 11.7236 10.9804 10.9361 9.6336
10.5876 11.2127 11.1296 9.9309 12.0712 10.5126 10.0037 10.8707
9.3170 10.9640 9.8071 10.7880 9.7808 9.8195 11.5482 13.2238
11.5609 11.1325 11.0632 11.1205 10.1486 10.5623 9.7114 10.7203
10.9135 10.5176 9.2161 10.1899 10.7872 9.7327 9.5962 10.3844
10.6872 10.4316 10.7347 10.6442 10.3789 10.1154 11.3224 11.7800
10.2555 11.7050 11.0678 9.9685 10.6422 10.9064 10.9069 10.8816
10.7565 11.6265 10.8741 10.3366 10.7835 11.4427 10.5721 9.9779
11.7975 9.7636 11.1467 10.1937 10.5613 10.2583 11.1602 11.3819
10.2312 10.6132 10.0554 9.9783 10.8905 12.5167 10.6957 9.1445
11.5775 11.5644 11.5889 10.6808 10.2876 9.6862 11.6732 10.3179
11.0604 13.0395 12.4239 9.4991 11.4365 10.4109 9.8575 9.8347
10.4752 10.2148 11.0365 9.3174 10.6586 10.2446 10.7165 10.7036
13.1437 9.7839 9.5717 11.0622 10.4989 11.0234 10.8925 11.8395
13.3214 11.9456 11.5395 9.9418 9.9872 10.5117 10.6069 11.4982
10.0138 10.1027 9.8568 9.8040 10.5512 10.6376 12.2076 9.9283
14.0167 9.5838 10.6423 11.0436 10.2467 10.0093 9.9054 10.3767
12.7020 10.9060 11.7014 10.9389 10.9298 10.5537 9.9521 10.6692
9.9983 11.1291 10.3048 10.1035 11.0918 10.7492 11.0193 12.4041
10.1039 10.3986 10.1451 10.2983 10.3470 11.9651 11.7068 10.6017
10.6928 9.5996 10.5075 10.5503 11.9200 11.7018 10.7998 10.2767
10.8132 11.6780 11.4134 10.4799 11.5444 13.5049 10.3251 10.1409
9.9569 12.9201\
LOC100289341 6.1211 6.0169 5.3299 5.5821 5.4542 4.6464
5.9278 5.1414 4.2883 6.1738 3.3229 5.9004 6.2452 5.9601
5.0864 5.8094 5.1930 6.4954 5.1465 5.7686 7.8058 5.2589
6.4461 5.2799 6.4290 5.1902 5.2825 5.3445 5.4241 6.0375
4.5762 5.2513 6.4569 6.3562 4.8082 5.3100 5.0622 4.1907
5.9322 4.3672 6.6599 5.9270 4.2348 3.8715 6.1381 5.0670
6.5312 4.6679 3.8986 5.4987 5.6456 3.7906 5.6242 6.4978
6.2733 5.1377 4.7523 4.3653 5.2490 5.3867 5.1565 4.8764
4.9403 6.2732 5.9990 4.7446 5.3045 6.1418 5.8479 5.1108
6.4228 5.5897 5.4750 6.0076 4.4416 5.2895 4.4967 5.8541
6.2430 4.0393 5.5283 4.8016 5.0887 4.2322 5.6398 5.8689
6.3060 5.0829 6.5893 5.7742 4.9760 6.1475 4.3016 5.5567
6.0835 5.9775 6.2601 5.5668 4.1292 6.1988 7.1214 4.0960
5.2751 5.2324 3.7378 5.4621 4.6683 4.1763 4.6813 5.9622
4.6652 5.6440 6.3432 5.4263 5.7798 5.2664 5.1829 6.8123
4.5158 5.0226 7.6484 5.8812 3.1844 4.4712 6.4277 3.6059
5.8809 4.8299 5.8136 5.9172 6.5325 4.6189 4.4898 5.4063
5.0301 5.8918 3.6728 3.8160 5.1281 6.1083 4.6896 4.4625
5.6439 4.8950 5.0598 6.2691 5.2435 6.6669 4.8225 4.4839

| | | | | | | | |
|---|---|---|---|---|---|---|---|
| 5.5218 | 5.0515 | 4.5146 | 4.0480 | 5.8980 | 5.4549 | 5.8911 | 5.0380 |
| 6.4788 | 5.7011 | 7.4375 | 5.1699 | 5.1813 | 6.1947 | 6.5068 | 4.9143 |
| 5.1673 | 5.0471 | 5.5109 | 4.1360 | 5.4606 | 5.0778 | 5.2595 | 4.1921 |
| 5.6358 | 5.2817 | 6.1749 | 5.5692 | 4.6831 | 5.6402 | 6.2207 | 6.0451 |
| 5.7001 | 5.2768 | 5.0140 | 5.3228 | 5.4150 | 4.4054 | 4.5535 | 6.4084 |
| 5.0286 | 4.8903 | 5.4075 | 5.5977 | 5.5488 | 7.6069 | 6.8766 | 6.2054 |
| 5.6288 | 5.7587 | 4.7896\ | | | | | |
| FBXL13 | 3.4330 | 3.5484 | 3.3494 | 5.9958 | 3.1619 | 6.0860 | 5.7796 |
| 3.1366 | 2.9347 | 4.2118 | 2.9870 | 4.0858 | 6.3613 | 4.3129 | 5.3804 |
| 5.5157 | 3.8010 | 2.4013 | 2.6976 | 3.1260 | 1.3800 | 6.1051 | 6.7793 |
| 4.5782 | 3.7692 | 0.6062 | 2.1197 | 5.0849 | 3.4165 | 1.8184 | 2.4950 |
| 3.0237 | 2.0034 | 4.9790 | 4.3584 | 1.4920 | 3.5025 | 3.5055 | 2.9681 |
| 3.1234 | 2.6483 | 4.0234 | 2.2575 | 2.3773 | 4.0482 | 4.4447 | 4.4245 |
| 2.6691 | 6.2799 | 2.1768 | 2.9335 | 2.1910 | 4.1282 | 4.3262 | 3.7586 |
| 4.1781 | 2.9310 | 3.5666 | 4.3115 | 1.5581 | 3.8307 | 2.9220 | 2.3905 |
| 1.6085 | 4.7884 | 5.4150 | 2.6173 | 4.5892 | 3.6228 | 3.9431 | 1.6333 |
| 3.2846 | 0.9449 | 2.8668 | 2.8697 | 4.7228 | 5.2677 | 6.2486 | 4.6376 |
| 2.6610 | 4.1153 | 3.5936 | 5.9960 | 4.4824 | 4.9304 | 4.4195 | 5.3322 |
| 2.6565 | 3.7390 | 1.8648 | 4.2273 | 3.1679 | 3.9820 | 4.2337 | 3.6683 |
| 3.5703 | 1.8574 | 4.2416 | 6.4268 | 3.9447 | 3.2267 | 3.8289 | 5.0195 |
| 4.8552 | 4.5170 | 2.8278 | 3.0465 | 5.6974 | 5.5300 | 4.2931 | 4.3392 |
| 4.8035 | 3.7633 | 3.3219 | 3.5487 | 3.6573 | 6.4949 | 5.0190 | 4.7205 |
| 2.3787 | 3.8806 | 4.2199 | 3.7448 | 4.8443 | 7.0412 | 3.2469 | 2.6587 |
| 1.9593 | 3.4596 | 4.4082 | 3.4641 | 3.8037 | 5.4573 | 6.3772 | 2.0060 |
| 3.2234 | 2.8392 | 0.0000 | 4.8945 | 4.4022 | 4.2150 | 2.2974 | 4.7356 |
| 2.8855 | 3.0118 | 4.9369 | 5.0498 | 4.9041 | 3.2383 | 2.6654 | 4.5008 |
| 2.7757 | 4.6516 | 3.2514 | 3.9366 | 2.8829 | 4.1488 | 4.3647 | 7.0443 |
| 1.7128 | 1.8525 | 2.8804 | 4.6920 | 4.4357 | 3.8695 | 4.2724 | 3.6605 |
| 5.1678 | 4.4431 | 4.5589 | 3.7441 | 2.9341 | 2.9680 | 3.9106 | 4.0279 |
| 4.2300 | 3.2842 | 4.6155 | 5.0472 | 7.0351 | 2.3835 | 2.2268 | 5.2616 |
| 3.2316 | 4.6039 | 3.8630 | 2.8969 | 3.3874 | 3.5079 | 3.6605 | 3.2385 |
| 3.9158 | 3.6515 | 3.0977 | 3.2101 | 2.8198 | 3.8663 | 4.3954 | 4.8023 |
| 5.3450 | 2.5752\ | | | | | | |
| FBXL12 | 8.6099 | 9.0970 | 8.6525 | 8.1292 | 8.6159 | 7.9576 | 8.8045 |
| 9.2649 | 9.3648 | 9.2507 | 9.0935 | 8.1950 | 8.3714 | 8.7136 | 8.1231 |
| 8.9411 | 9.4281 | 8.9003 | 9.2182 | 7.8130 | 8.7444 | 8.7714 | 8.6335 |
| 8.1895 | 9.2061 | 8.1840 | 8.5292 | 8.2752 | 8.1664 | 8.3839 | 8.2069 |
| 8.8391 | 8.7148 | 8.5422 | 8.2393 | 9.0654 | 8.9503 | 8.4687 | 8.5699 |
| 8.3721 | 8.5758 | 9.0843 | 8.4378 | 9.0201 | 8.8392 | 8.8452 | 7.6846 |
| 8.5371 | 9.1711 | 8.3459 | 8.2766 | 9.1786 | 9.5773 | 9.0648 | 8.5585 |
| 8.5846 | 8.5628 | 7.8168 | 9.5990 | 8.1788 | 8.9777 | 8.5935 | 8.3770 |
| 8.9762 | 9.4763 | 8.6085 | 8.3807 | 9.0160 | 8.3259 | 8.4746 | 7.9379 |
| 8.4937 | 8.1595 | 8.0454 | 8.2705 | 8.2182 | 9.3917 | 8.4466 | 8.8257 |
| 8.6812 | 8.2231 | 8.4920 | 8.6343 | 9.2445 | 8.7069 | 9.8338 | 9.3640 |
| 8.5344 | 7.8229 | 8.5264 | 9.1840 | 10.1329 | 8.8708 | 9.0411 | 8.9133 |
| 8.9382 | 7.1476 | 9.4788 | 9.0320 | 8.8910 | 8.2933 | 8.4231 | 9.7723 |
| 8.8013 | 8.0473 | 8.9812 | 9.8963 | 8.7117 | 8.5733 | 9.7993 | 8.7348 |
| 8.5225 | 5.9248 | 9.0112 | 8.9300 | 9.5048 | 8.3408 | 8.1382 | 8.0590 |
| 9.1379 | 9.9188 | 8.0361 | 9.2046 | 8.4555 | 8.7465 | 9.2267 | 7.9673 |
| 7.6252 | 8.9592 | 8.7156 | 7.9603 | 8.6563 | 8.7447 | 8.1425 | 8.5452 |
| 6.9163 | 8.5131 | 8.4457 | 8.6824 | 9.2703 | 8.5131 | 8.3048 | 8.2674 |
| 8.0783 | 8.3139 | 9.2878 | 8.3636 | 9.0189 | 8.7086 | 6.7262 | 8.7414 |
| 6.8702 | 8.3311 | 8.4217 | 8.4589 | 9.1724 | 9.0691 | 8.8458 | 10.2590 |
| 8.5456 | 8.6420 | 8.0589 | 8.6279 | 8.4670 | 8.3003 | 8.4650 | 8.4131 |

8.4663  8.3910  8.2107  8.3273  7.9980  9.0539  8.6918  7.5403
8.9042  9.6152  9.1004  8.3422  8.6407  7.9404  8.5037  10.6511
8.8030  8.2767  8.6802  8.9252  8.7798  8.5468  8.7447  8.3722
8.6651  8.3838  8.7513  9.0889  7.9744  7.4073  9.3900  8.5854
8.7967  7.8479\
FBXL15  9.2560  8.0831  7.3194  7.4626  8.7785  7.7248  7.8989
8.5632  8.1795  8.5693  8.0459  8.4271  8.3197  8.2398  7.7520
8.6696  7.6118  8.9280  8.0328  9.1542  8.5749  8.0997  8.6194
8.1895  8.1715  9.7837  8.0415  9.8189  9.0189  8.6319  7.1575
8.4236  7.9654  8.1929  7.6698  9.0325  8.8378  7.9633  7.9333
7.1811  9.4368  7.8171  8.6794  7.8235  7.8260  9.2351  10.0540
8.3860  7.4163  9.8597  8.5706  8.6454  8.4421  7.8142  8.3956
7.9872  7.8043  7.6679  7.5188  8.1345  7.1768  7.6290  7.9407
7.0957  8.2261  8.2909  8.1601  8.1366  8.0909  7.8119  9.6792
7.9045  10.1093 9.4586  7.4132  9.6302  8.7298  8.7074  8.6209
8.2504  8.9962  5.6264  8.0652  7.5117  10.0319 7.7314  6.5269
9.0611  7.8313  9.9851  8.5335  7.9669  9.8318  8.6602  7.6940
8.5641  7.1387  8.4799  8.5669  8.4883  9.3902  9.0404  7.3967
8.7079  8.7244  8.4786  8.2759  7.8542  7.3252  8.1192  8.6787
8.2411  10.4645 9.8826  7.2612  7.6405  8.5507  7.8436  7.9870
8.0128  7.8358  8.4076  7.5096  8.8837  8.4641  8.1244  7.5914
9.4121  7.1299  7.0221  7.2671  7.8321  8.8578  7.5965  9.1385
9.9738  10.6138 9.5264  7.9538  8.4996  8.8008  8.8413  9.6821
7.4764  8.0317  8.9517  8.0408  9.0863  9.0965  10.3520 8.2809
10.9985 7.1879  8.5831  8.3691  8.2012  8.6416  7.9228  7.9574
10.0748 9.5486  8.6003  8.1737  9.4303  8.1020  7.5674  6.3538
7.5490  9.3047  8.2174  7.3154  8.1017  7.9033  9.2931  9.9665
8.0310  7.7317  8.6326  7.2112  8.7274  10.0971 9.2119  7.8149
8.2318  8.0597  7.9955  7.4804  9.8220  9.4647  8.6274  7.5144
8.2300  7.6054  7.0698  8.5834  9.7677  10.3689 8.8812  8.1583
7.5534  10.8492\
CYP27B1 4.7116  4.2690  6.2080  7.3802  5.9006  6.1988  3.8334
5.1414  4.8396  5.1580  5.0539  5.0427  4.8703  5.1075  4.2186
6.6802  4.2727  5.3565  4.1261  2.9705  4.8199  3.6443  3.8339
5.7500  2.7258  4.8153  7.8245  4.6547  5.6494  4.0145  3.3610
5.2869  6.3545  4.9790  5.3932  5.0973  5.3214  5.7911  6.4047
4.5072  6.8622  4.5541  3.9622  5.5268  0.0000  4.3198  3.2199
5.5865  5.9543  4.8072  8.5818  5.2781  4.1282  2.3883  5.1369
6.4584  4.6290  6.0734  4.9462  3.4226  0.9353  4.5865  6.5011
4.4931  4.5104  4.0485  4.5606  4.4386  4.3705  4.9210  4.8595
5.5518  6.2641  4.1632  6.6717  4.5908  4.3715  4.0006  4.3213
5.4810  3.6471  4.1765  4.6417  5.9285  4.5515  4.6834  4.1826
5.1053  3.4203  5.2138  1.9147  5.0595  4.3941  6.4731  5.7413
2.2653  3.8194  3.2822  5.0510  5.1445  6.6390  6.9254  3.5580
4.5208  6.1830  2.8278  4.5913  3.4275  5.1764  4.4001  5.7668
4.8846  1.3631  5.8074  5.6869  5.6006  4.2220  4.8371  4.7548
5.1979  5.8523  3.0097  4.8603  6.0207  4.3194  5.7719  4.2929
2.2050  2.8462  3.8982  5.0301  4.8128  4.6049  5.9955  4.3651
6.3730  6.1260  3.3710  4.6412  4.0098  5.2362  3.9712  4.3884
4.7311  4.7763  3.2024  4.4476  2.5977  5.7708  3.0614  5.3111
4.1350  5.3278  5.3961  4.0615  5.5670  3.2643  5.0108  4.7434
4.8245  4.1886  4.9890  4.8984  5.9532  2.9650  4.9536  4.5584
4.9606  3.7402  4.2097  6.5768  3.8806  6.0457  5.5007  5.1619
4.4015  5.2702  1.0660  4.1171  5.0135  4.7174  5.3959  6.2088

| | | | | | | | |
|---|---|---|---|---|---|---|---|
| 6.2501 | 5.0723 | 3.5656 | 3.3819 | 5.3709 | 4.5176 | 5.6157 | 6.4229 |
| 4.7207 | 4.1198 | 3.3616 | 5.1731 | 5.1434 | 3.9171 | 3.0678 | 4.1579 |
| 5.1702 | 3.3644\ | | | | | | |
| FBXL17 | 8.4745 | 8.8697 | 8.3827 | 9.1107 | 8.4344 | 8.7823 | 9.1881 |
| 8.7820 | 8.1951 | 8.8477 | 9.7066 | 8.5132 | 9.9390 | 8.7160 | 8.5263 |
| 8.4740 | 7.4786 | 9.0022 | 8.7598 | 7.0650 | 8.4416 | 8.3270 | 8.7286 |
| 9.2686 | 9.3894 | 8.3979 | 7.1861 | 7.9181 | 8.6122 | 7.9955 | 7.4801 |
| 8.9107 | 8.2049 | 9.1677 | 9.1633 | 6.1017 | 8.9365 | 8.7710 | 9.2904 |
| 7.7824 | 9.0167 | 9.3192 | 7.9692 | 6.9793 | 9.7690 | 7.3777 | 7.8538 |
| 7.6129 | 9.0067 | 9.7656 | 9.2518 | 6.5613 | 8.4087 | 9.9451 | 7.9469 |
| 9.1000 | 9.0498 | 8.2534 | 9.1987 | 8.3837 | 9.5811 | 9.2957 | 8.0345 |
| 8.1695 | 8.8526 | 8.7222 | 9.0735 | 7.5964 | 8.2579 | 7.9332 | 8.6480 |
| 9.1596 | 8.0630 | 8.0730 | 9.2501 | 8.9019 | 7.7816 | 8.1187 | 9.0573 |
| 8.7398 | 8.2324 | 8.0105 | 9.0307 | 8.5311 | 8.1045 | 8.8114 | 9.6288 |
| 7.4986 | 8.4905 | 8.5367 | 9.2707 | 9.5755 | 8.1273 | 8.6359 | 8.4233 |
| 9.5653 | 8.8041 | 9.1164 | 8.3632 | 8.1228 | 7.4688 | 8.0261 | 7.8057 |
| 8.5010 | 8.4938 | 7.6218 | 8.4330 | 8.8069 | 9.1913 | 7.9911 | 8.7888 |
| 8.4173 | 8.2796 | 7.8009 | 8.8425 | 8.9703 | 8.3967 | 9.2649 | 7.5768 |
| 7.2045 | 8.4881 | 9.0717 | 8.6405 | 8.4412 | 9.2169 | 8.4572 | 8.2874 |
| 7.7849 | 9.7079 | 9.7679 | 8.2007 | 8.6626 | 8.0582 | 8.1944 | 6.9794 |
| 7.3576 | 8.4522 | 8.7944 | 8.7998 | 9.0459 | 7.8516 | 9.1153 | 8.5323 |
| 8.9090 | 9.2565 | 9.1336 | 8.9832 | 9.1057 | 8.6021 | 9.0445 | 8.9548 |
| 7.4867 | 8.9528 | 8.3961 | 8.6958 | 8.6032 | 9.1344 | 8.1821 | 8.6389 |
| 7.4018 | 7.9755 | 8.0561 | 8.9087 | 8.4853 | 7.8917 | 9.2603 | 8.5455 |
| 8.8668 | 7.8186 | 7.3980 | 8.6776 | 7.5053 | 8.1311 | 8.5577 | 8.7520 |
| 8.9486 | 8.8698 | 9.3816 | 8.8500 | 8.4658 | 8.8137 | 8.0421 | 9.0264 |
| 8.7618 | 8.5291 | 8.3781 | 8.1153 | 7.4782 | 8.1989 | 8.4843 | 8.4438 |
| 9.1366 | 7.3777 | 7.7373 | 8.9234 | 8.7925 | 7.9211 | 8.8601 | 8.8365 |
| 8.9850 | 7.4580\ | | | | | | |
| FBXL16 | 5.7603 | 6.9127 | 9.3937 | 8.6361 | 6.7651 | 5.9179 | 7.9290 |
| 6.5627 | 5.5811 | 5.4805 | 7.4533 | 7.7651 | 7.8532 | 5.4270 | 3.0405 |
| 5.8953 | 6.7729 | 7.7053 | 6.5044 | 7.8034 | 7.3332 | 3.1593 | 9.7194 |
| 3.7828 | 5.8602 | 9.4930 | 5.3098 | 4.5317 | 7.9991 | 8.8640 | 2.2152 |
| 6.6007 | 8.4011 | 8.9321 | 7.5971 | 4.7859 | 9.0198 | 6.0759 | 5.2767 |
| 4.5936 | 7.8633 | 6.2191 | 8.4400 | 4.2081 | 1.0260 | 3.8630 | 6.5706 |
| 8.1851 | 9.4656 | 8.0160 | 9.9053 | 6.5936 | 6.8201 | 2.6047 | 6.5729 |
| 8.2119 | 6.8214 | 6.3784 | 7.9271 | 8.3626 | 2.5892 | 5.5049 | 7.2377 |
| 5.8028 | 4.4716 | 6.2109 | 9.2807 | 7.7799 | 5.6918 | 6.7838 | 6.9662 |
| 4.6466 | 7.2459 | 7.5745 | 5.9816 | 7.1289 | 6.3601 | 4.2961 | 7.1300 |
| 4.4441 | 2.5369 | 7.5469 | 7.5359 | 7.4833 | 3.1576 | 8.5638 | 10.0687 |
| 4.2904 | 8.0961 | 9.8825 | 6.0036 | 4.6416 | 7.0870 | 5.5185 | 4.6358 |
| 5.4039 | 9.5930 | 7.2229 | 6.1371 | 6.1525 | 7.3157 | 6.2505 | 6.4688 |
| 7.9648 | 6.7129 | 8.0405 | 6.8315 | 3.5614 | 4.7564 | 6.3479 | 5.1552 |
| 3.6216 | 7.1389 | 6.7682 | 6.3932 | 8.1063 | 4.8708 | 8.5838 | 4.6311 |
| 8.5113 | 6.7874 | 6.2588 | 4.1474 | 7.7695 | 9.4270 | 6.4475 | 8.5877 |
| 8.1130 | 7.4019 | 3.4188 | 9.6797 | 8.0337 | 4.0357 | 9.5845 | 6.2415 |
| 5.9282 | 6.1027 | 1.5206 | 4.2370 | 7.6220 | 5.6906 | 6.0251 | 4.4271 |
| 4.9170 | 4.3722 | 2.5884 | 3.8163 | 9.4335 | 7.0608 | 7.1695 | 4.2713 |
| 4.1350 | 4.1205 | 5.3788 | 6.5586 | 7.3319 | 8.1060 | 5.5243 | 5.0877 |
| 4.4264 | 9.1985 | 6.2130 | 3.4515 | 6.8067 | 7.7856 | 3.3452 | 8.0627 |
| 5.6361 | 4.3210 | 3.7967 | 4.5835 | 5.9362 | 7.5369 | 1.8874 | 4.5406 |
| 7.8304 | 4.3386 | 6.3849 | 5.0332 | 6.0757 | 7.1409 | 8.0218 | 7.3621 |
| 6.8420 | 5.8487 | 6.9672 | 5.3286 | 7.1338 | 6.9980 | 5.1278 | 5.6175 |
| 4.3406 | 9.4822 | 8.4476 | 7.7660 | 6.4901 | 7.7670 | 8.5568 | 3.8967 |

4.4374 4.0443\
FAM169A 8.0758 8.4405 5.5153 7.7922 6.4450 7.0858 7.5222
8.5779 7.8527 9.7166 6.4007 8.0910 7.4635 8.5655 8.5439
9.1995 7.6039 8.7929 8.4375 5.4035 4.5832 6.6349 7.8983
8.9144 7.8444 8.5563 5.8080 6.9543 5.4509 6.8482 7.2166
7.0727 5.5546 6.8803 7.6303 8.0303 7.4953 8.6167 7.8071
5.1153 9.0805 9.1480 6.5862 7.0109 6.4938 7.5052 6.0500
4.0431 6.5006 7.5608 7.9291 4.9522 7.5936 6.8286 7.4288
8.9857 7.0247 7.5314 7.5429 8.2193 7.1631 8.0917 7.7643
7.0122 9.1594 7.3652 4.8447 7.6111 5.3870 7.0563 6.2071
8.3847 6.9219 6.1342 8.4707 5.4881 8.3343 6.8010 8.6867
9.2505 7.3434 7.6842 7.1451 6.6784 7.9070 8.6021 8.8895
6.7500 6.3683 7.1535 7.1243 8.1746 7.8911 8.1286 8.1335
5.9396 2.5385 8.0582 5.9622 7.2563 6.2421 7.9559 7.6975
7.2292 6.9168 6.9952 7.8747 7.7128 8.0936 7.3073 6.1171
7.8000 5.7671 5.6724 6.7146 7.7613 7.1211 7.1819 5.1522
5.1346 8.4701 8.0194 7.5966 4.5205 8.4259 6.8786 6.2161
5.0967 8.6998 9.3650 7.2847 6.4915 6.4174 7.0306 6.7152
5.1341 6.8823 6.4113 8.1007 8.8629 6.2069 8.2078 8.4071
8.7620 8.3894 7.8387 7.8117 8.4575 7.6511 6.9297 7.8237
3.4258 9.0428 7.8176 9.1508 7.8445 8.9373 7.5697 7.3390
7.3940 6.0619 6.5515 9.2767 7.8616 6.9861 9.7324 6.5345
7.8808 7.4869 6.6039 8.8171 8.5635 7.8867 6.3189 5.7144
8.8521 9.7139 6.9985 8.2510 7.8351 6.3256 5.8895 8.6363
8.1489 8.0306 8.6436 7.1784 6.6983 6.2793 9.5436 7.9432
6.7563 5.8785 6.8162 7.1301 6.5535 7.8960 9.0342 7.7602
8.4098 6.5827\
FAM116B 9.0719 7.7362 8.9222 9.0365 6.9923 8.1281 8.9806
8.4423 6.0684 8.7836 7.7923 6.9408 8.4272 8.3759 9.6897
9.6213 10.0627 9.5922 7.7549 10.4817 8.2036 10.5235 10.7833
8.6899 8.1479 9.2598 6.3453 11.1298 8.7099 8.1081 7.5532
8.7221 6.5591 8.1156 8.6193 7.7475 9.1035 8.0091 8.5854
7.1206 7.9648 7.5983 9.1171 7.2877 6.9089 10.4571 9.3237
8.2458 5.4649 9.2550 8.1735 8.0686 6.5407 7.9464 7.6276
8.6146 10.2549 8.4692 7.7081 9.2781 7.4682 8.9828 8.2753
8.8962 8.7055 8.7907 7.2864 7.8337 8.5432 8.2072 7.2076
7.6636 10.1392 8.8507 7.6868 9.2895 8.6712 11.3012 10.5313
7.8881 8.6340 7.7745 11.1031 8.4888 10.2442 7.1840 6.4912
10.3256 9.0461 8.7090 7.6351 6.7338 7.5427 9.5899 8.0224
7.4200 6.8634 7.6890 8.9293 9.4797 9.5435 9.2681 8.5407
8.9696 7.9647 8.1491 9.7970 7.8301 7.8498 9.5110 9.1366
8.9601 8.8655 10.7838 6.6728 6.8982 9.6226 8.1129 7.3404
10.0116 7.8544 8.7726 8.3828 9.1376 10.0856 6.6213 7.7060
8.7981 7.1876 7.7546 7.6628 7.9673 10.3930 9.6979 8.1716
8.7763 9.3852 10.7950 8.0470 7.8981 8.6155 7.6940 10.9479
8.3606 7.6675 7.3948 9.6448 8.8990 7.5929 9.5972 8.1176
9.6141 8.0872 7.8144 9.4282 7.0528 8.2489 7.3943 8.7205
8.6326 9.2575 8.5088 8.9323 10.1431 7.5577 6.7216 8.8553
7.4387 9.0854 7.5896 7.9408 6.9400 6.1694 9.1177 10.5616
8.6156 8.6998 7.8010 8.5013 11.6951 9.8293 8.3467 7.2624
8.1826 8.1505 8.9251 8.4190 8.9028 9.6817 7.5343 8.5451
7.9160 8.1443 7.3747 7.7046 5.6289 9.0605 8.1021 8.2840
9.4708 10.0146\
FAM116A 8.9162 9.5703 9.0628 9.2204 8.9377 9.2111 8.8594

| | | | | | | | |
|---|---|---|---|---|---|---|---|
| 8.9369 | 8.1585 | 9.4208 | 8.2220 | 9.3563 | 9.5065 | 8.4974 | 9.0620 |
| 9.1391 | 9.1084 | 8.8786 | 9.5034 | 8.9638 | 8.6517 | 8.8404 | 8.8902 |
| 8.9353 | 9.3599 | 8.6016 | 8.9720 | 8.7780 | 8.7804 | 9.4500 | 9.7734 |
| 8.7074 | 8.3297 | 9.2083 | 8.3738 | 8.7763 | 8.9156 | 8.6325 | 9.7244 |
| 9.0262 | 8.7817 | 9.4836 | 8.8713 | 8.6981 | 9.8699 | 8.1300 | 8.1552 |
| 8.5067 | 9.1431 | 8.9160 | 8.6952 | 8.6377 | 8.3036 | 10.0531 | 8.5672 |
| 9.0466 | 8.9210 | 8.9018 | 8.9564 | 8.7605 | 9.9905 | 8.8322 | 9.3614 |
| 9.3905 | 9.5138 | 8.7610 | 8.0637 | 8.9812 | 8.2810 | 9.3520 | 8.0336 |
| 8.5810 | 8.3100 | 8.7222 | 9.2308 | 8.3380 | 8.8239 | 8.2919 | 9.0523 |
| 9.5511 | 8.6922 | 8.7093 | 8.9798 | 9.4071 | 8.4677 | 8.7207 | 8.3433 |
| 8.0936 | 9.4352 | 9.2009 | 9.6812 | 9.3913 | 8.6556 | 9.0428 | 8.3523 |
| 9.1820 | 8.5834 | 9.8666 | 8.9281 | 8.7029 | 7.9144 | 8.7890 | 9.6892 |
| 8.8747 | 9.4063 | 8.8006 | 9.6984 | 8.5467 | 9.2487 | 9.2608 | 8.9841 |
| 8.3355 | 8.9867 | 8.2240 | 8.6142 | 8.7632 | 9.1663 | 9.7817 | 9.4722 |
| 8.1498 | 8.8640 | 9.3307 | 9.7522 | 8.2391 | 9.3800 | 8.9525 | 8.5817 |
| 8.3864 | 9.9953 | 9.5891 | 8.9872 | 8.7717 | 8.7177 | 8.4291 | 8.4141 |
| 7.9639 | 8.8312 | 9.4590 | 9.2605 | 9.9528 | 8.6714 | 8.6365 | 8.5278 |
| 9.5914 | 9.2513 | 10.4759 | 8.7677 | 9.5137 | 7.9579 | 8.5356 | 9.2143 |
| 8.5061 | 8.5397 | 7.8430 | 8.8423 | 8.9120 | 9.8666 | 9.7601 | 8.0557 |
| 9.3648 | 8.0197 | 8.3561 | 9.0493 | 8.8524 | 9.2056 | 8.7717 | 8.3372 |
| 8.4702 | 9.1774 | 9.7075 | 9.1666 | 9.1222 | 9.7110 | 8.7436 | 8.9679 |
| 9.3062 | 9.5452 | 9.8721 | 9.5843 | 8.7967 | 8.9388 | 8.5587 | 9.1194 |
| 8.7908 | 8.7749 | 9.0275 | 9.4346 | 8.3871 | 8.5311 | 8.8934 | 9.6613 |
| 8.7760 | 8.9399 | 8.3446 | 9.0218 | 8.7526 | 8.6538 | 9.7750 | 9.0390 |
| 9.1801 | 7.8595\ | | | | | | |
| FAM169B | 0.0000 | 3.9323 | 2.5614 | 0.4935 | 0.0000 | 0.0000 | 0.0000 |
| 0.8314 | 0.0000 | 0.4413 | 1.0281 | 0.0000 | 0.4363 | 0.0000 | 3.6198 |
| 0.0000 | 0.6221 | 0.0000 | 2.1673 | 0.3755 | 0.0000 | 1.3712 | 0.0000 |
| 1.8643 | 0.5538 | 0.0000 | 1.0809 | 0.0000 | 2.0792 | 0.0000 | 0.0000 |
| 0.0000 | 0.5159 | 0.0000 | 0.0000 | 0.0000 | 0.9556 | 0.0000 | 0.0000 |
| 0.4337 | 1.1184 | 3.4113 | 0.0000 | 2.6427 | 0.0000 | 0.9285 | 0.0000 |
| 0.0000 | 0.0000 | 0.0000 | 0.4530 | 0.0000 | 0.6494 | 1.4299 | 0.8153 |
| 0.0000 | 0.0000 | 6.4352 | 0.4252 | 0.9799 | 0.5422 | 1.7937 | 1.5290 |
| 0.5968 | 1.7745 | 2.4204 | 0.0000 | 0.0000 | 0.0000 | 0.0000 | 0.0000 |
| 0.7001 | 0.0000 | 0.4786 | 0.9270 | 0.0000 | 0.5548 | 1.4952 | 0.0000 |
| 0.6159 | 0.0000 | 0.0000 | 0.0000 | 0.6718 | 0.5263 | 0.6199 | 0.0000 |
| 0.0000 | 0.5273 | 0.0000 | 0.0000 | 1.1363 | 0.0000 | 1.7895 | 1.2026 |
| 0.0000 | 0.0000 | 0.5454 | 2.2653 | 0.0000 | 0.0000 | 0.8684 | 1.6984 |
| 1.5830 | 0.0000 | 0.0000 | 1.2143 | 0.0000 | 0.0000 | 1.3268 | 0.0000 |
| 1.3871 | 0.0000 | 1.5850 | 0.0000 | 2.5241 | 0.0000 | 0.7641 | 3.9911 |
| 1.6324 | 0.0000 | 1.3285 | 0.0000 | 0.8239 | 0.0000 | 0.0000 | 0.0000 |
| 0.0000 | 0.0000 | 0.0000 | 0.7024 | 1.1234 | 1.7370 | 1.2511 | 0.0000 |
| 0.0000 | 0.0000 | 0.0000 | 0.0000 | 0.0000 | 1.0240 | 0.0000 | 1.9445 |
| 0.0000 | 1.5157 | 0.0000 | 1.1308 | 0.0000 | 0.0000 | 0.0000 | 2.6803 |
| 0.0000 | 2.3500 | 1.5875 | 0.0000 | 0.4374 | 2.5317 | 1.3399 | 0.0000 |
| 0.0000 | 0.0000 | 0.0000 | 2.5777 | 0.4033 | 1.8107 | 1.1696 | 0.0000 |
| 4.5707 | 0.5276 | 1.7238 | 0.4486 | 0.0000 | 0.0000 | 0.0000 | 2.7155 |
| 0.0000 | 1.6715 | 0.0000 | 0.0000 | 2.1026 | 0.0000 | 1.5061 | 0.0000 |
| 1.4215 | 0.0000 | 1.6636 | 0.5811 | 0.0000 | 0.0000 | 0.0000 | 0.0000 |
| 0.0000 | 0.9737 | 0.0000 | 0.0000 | 0.0000 | 0.0000 | 0.0000 | 0.6701 |
| 1.8199 | 0.0000\ | | | | | | |
| RALGAPA2 | | 6.8262 | 6.8693 | 9.2831 | 7.5993 | 6.4595 | 9.6172 |
| 8.5573 | 6.9917 | 8.9571 | 7.7787 | 7.4504 | 7.5328 | 7.5311 | 8.1683 |
| 9.4592 | 6.4999 | 5.1277 | 5.3815 | 6.9155 | 6.0726 | 6.8428 | 9.1645 |

| | | | | | | | |
|---|---|---|---|---|---|---|---|
| 7.0255 | 8.8825 | 6.3150 | 3.3041 | 9.1675 | 6.4335 | 8.3487 | 9.1828 |
| 6.3776 | 8.5461 | 7.4451 | 8.6462 | 10.2834 | 7.3903 | 9.5682 | 9.9972 |
| 6.5164 | 7.5294 | 5.5801 | 9.1367 | 6.2187 | 8.7483 | 8.2849 | 6.6876 |
| 5.5566 | 8.9849 | 6.9552 | 6.8140 | 9.0457 | 7.6643 | 9.1318 | 8.8722 |
| 8.5513 | 7.0663 | 9.2224 | 8.6643 | 8.2319 | 7.2265 | 9.1704 | 9.0346 |
| 7.0858 | 7.6753 | 6.6125 | 9.6008 | 9.4534 | 7.7588 | 8.5715 | 6.9296 |
| 5.2400 | 10.5998 | 5.7723 | 5.7889 | 9.0228 | 9.0168 | 7.2615 | 8.3291 |
| 5.8109 | 7.6411 | 5.8500 | 7.8634 | 7.1251 | 7.0836 | 5.3902 | 6.8830 |
| 8.4515 | 5.9544 | 8.2251 | 6.3735 | 8.7495 | 8.9904 | 6.0698 | 5.0685 |
| 6.4698 | 7.6945 | 9.1083 | 7.3784 | 8.8977 | 6.5742 | 4.1922 | 8.5139 |
| 8.9976 | 6.1737 | 6.4946 | 7.6494 | 6.1085 | 8.5264 | 7.8640 | 6.0304 |
| 8.0697 | 7.7395 | 4.7091 | 7.7004 | 9.2531 | 8.1999 | 7.7814 | 8.8172 |
| 9.9953 | 7.8084 | 6.7128 | 9.2930 | 9.7576 | 8.8237 | 8.0187 | 7.3862 |
| 7.2064 | 4.4556 | 7.5740 | 8.9076 | 8.5355 | 5.6749 | 7.7744 | 7.3070 |
| 5.0630 | 4.7832 | 3.6080 | 5.6856 | 9.6745 | 6.9041 | 6.0693 | 8.2030 |
| 5.3735 | 10.4860 | 10.1007 | 7.5468 | 8.8685 | 7.6663 | 7.9637 | 6.5928 |
| 8.8403 | 5.4873 | 9.7849 | 8.4714 | 8.7417 | 8.4332 | 7.9859 | 8.4213 |
| 5.5829 | 3.4624 | 8.8126 | 7.7987 | 6.7858 | 5.6069 | 10.2253 | 9.3113 |
| 6.3279 | 10.6559 | 7.4582 | 7.0118 | 9.2054 | 8.9927 | 8.3680 | 8.1953 |
| 5.1047 | 7.2648 | 6.6533 | 7.1671 | 9.7864 | 7.4788 | 5.8739 | 6.5771 |
| 7.0822 | 6.4913 | 9.2728 | 6.0453 | 9.4602 | 6.5985 | 5.7089 | 6.1471 |
| 9.7043 | 9.6567 | 6.3139 | 8.8534 | 7.6102 | 3.4726 | 5.6012 | 7.3247 |
| 9.2364 | 9.1059 | 4.4234\ | | | | | |
| GHDC | 10.1040 | 9.9426 | 9.2646 | 9.3477 | 8.8171 | 8.3978 | 8.7610 |
| 9.3457 | 10.7774 | 10.2572 | 9.0871 | 10.0630 | 9.4376 | 8.7342 | 9.7310 |
| 10.5657 | 9.5082 | 9.5949 | 9.9325 | 9.9756 | 10.2852 | 9.8581 | 10.5554 |
| 9.7770 | 9.4222 | 10.5813 | 7.5632 | 10.3489 | 10.0562 | 9.9064 | 8.6680 |
| 10.0919 | 9.1554 | 9.0479 | 9.1133 | 10.6223 | 9.7538 | 9.8779 | 10.0539 |
| 8.0270 | 9.8077 | 9.7232 | 9.6282 | 10.1665 | 10.0415 | 9.8619 | 9.5888 |
| 10.0294 | 8.6939 | 10.3825 | 9.7477 | 9.1169 | 9.7511 | 9.9757 | 9.4416 |
| 9.3389 | 10.0447 | 10.0605 | 9.1173 | 9.4066 | 9.7784 | 9.0267 | 9.1126 |
| 9.5350 | 9.9698 | 9.8236 | 9.3640 | 8.7635 | 8.7797 | 9.1785 | 10.0046 |
| 9.6627 | 9.7795 | 9.8497 | 9.5058 | 9.8123 | 9.0526 | 10.6156 | 9.9836 |
| 9.7462 | 9.9667 | 8.5805 | 9.9866 | 8.2927 | 10.1816 | 9.6377 | 10.6475 |
| 10.5848 | 8.9796 | 9.4895 | 9.9785 | 9.9946 | 9.0485 | 10.3294 | 9.5310 |
| 9.6922 | 8.3939 | 10.4783 | 8.7195 | 10.0655 | 10.2626 | 9.6752 | 9.7164 |
| 8.4876 | 8.3427 | 8.9942 | 9.3530 | 8.6629 | 9.1980 | 9.4706 | 9.8399 |
| 9.6451 | 10.0075 | 9.4030 | 9.1129 | 8.9365 | 8.5893 | 9.4592 | 9.1430 |
| 9.3788 | 9.7344 | 9.7792 | 9.5122 | 9.9529 | 10.0484 | 8.8416 | 6.4879 |
| 11.2362 | 9.9508 | 8.8547 | 9.0754 | 8.4112 | 10.1568 | 8.3150 | 8.5335 |
| 10.0345 | 8.1336 | 9.4781 | 9.8073 | 10.2780 | 9.0574 | 9.5795 | 10.4927 |
| 9.6466 | 9.6813 | 9.9726 | 9.3796 | 10.5552 | 9.6749 | 9.3553 | 10.0127 |
| 9.8163 | 9.3227 | 10.3734 | 9.7243 | 8.8086 | 10.4146 | 9.1085 | 8.9500 |
| 10.2366 | 10.5319 | 10.0855 | 9.9892 | 10.7555 | 9.5505 | 8.7482 | 8.9687 |
| 9.7837 | 9.9280 | 9.1556 | 8.7068 | 8.0923 | 10.3662 | 9.9389 | 10.4339 |
| 9.2926 | 9.4963 | 9.6017 | 8.8339 | 9.7210 | 10.4155 | 10.6963 | 7.7516 |
| 10.7200 | 9.4942 | 10.1310 | 10.2370 | 10.0024 | 9.9790 | 10.1014 | 8.8548 |
| 6.6320 | 8.8065 | 8.3446 | 9.2225 | 10.1059 | 8.3347 | 9.7038 | 9.6849 |
| 9.6351 | 10.0673\ | | | | | | |
| H19 | 11.2501 | 15.0335 | 10.5233 | 9.1630 | 11.2129 | 17.7232 | 12.1552 |
| 16.5717 | 15.3337 | 14.4956 | 16.3095 | 11.7832 | 17.5780 | 16.3520 | 8.9465 |
| 10.1044 | 13.7806 | 13.8774 | 15.3978 | 8.7750 | 7.4168 | 9.3859 | 12.0812 |
| 11.6174 | 13.7350 | 12.9159 | 8.0313 | 12.4811 | 10.4712 | 10.1015 | 7.7458 |
| 9.2326 | 11.9877 | 14.2358 | 5.2488 | 18.3206 | 15.1222 | 10.6012 | 8.1728 |

```
9.9809  11.5958 12.6912 10.6975 10.7682 10.0806 10.0421 10.2532
14.1090 10.7249 7.1837  16.3274 10.7295 16.8460 11.2757 7.4971
12.6319 10.2031 11.0296 11.7171 15.9211 11.6306 10.8607 9.0082
5.7625  15.0252 11.3535 11.1348 8.1325  9.4478  12.6381 16.1695
13.3630 12.7203 12.6434 11.2962 6.8923  15.8508 11.1385 12.1128
15.1294 11.4718 12.4661 11.7177 10.7678 10.6735 16.6840 7.8423
10.9076 14.6370 14.7367 13.6681 14.2568 13.8336 10.6928 10.7521
12.7425 7.1342  13.1139 10.7342 11.3590 11.7008 10.9481 11.2566
10.0347 10.4731 9.3972  16.5375 15.6745 11.9975 12.2376 8.1822
9.3818  17.5591 14.0877 9.6726  16.7624 7.9088  11.1897 14.3862
11.7198 11.7794 11.7794 13.6713 13.2052 12.3046 13.8025 10.0105
5.8771  12.4982 13.0552 12.2887 9.5909  10.4799 10.4959 18.9201
15.8855 10.1087 13.8715 11.3400 13.3297 7.8156  10.7330 10.0702
12.2041 10.7515 13.5475 8.1108  14.3897 11.0015 14.2998 10.7790
8.9826  8.5075  13.2027 12.3314 13.4676 12.0299 15.7774 12.0047
10.6672 10.5330 9.9137  11.6980 10.6237 13.5221 11.0730 12.4851
11.6989 12.3354 7.7402  8.9926  15.9748 6.5019  10.6897 11.2125
12.0792 14.4213 14.4477 11.8030 10.0893 11.2852 14.0428 11.5500
9.4235  15.7203 9.8586  9.7300  14.7921 14.1019 13.9706 9.2401
14.2850 11.1594 9.4569  8.8722  10.9474 9.9041  13.8212 9.2172
9.0903  10.7310\
SDK2    5.6706  7.0597  9.7330  6.4235  4.0407  10.2213 11.2393
10.1982 8.2425  10.8841 8.5230  10.3874 7.9715  5.5083  5.3647
6.0901  7.4567  5.5667  6.4724  7.0227  6.4071  9.4787  8.4790
7.9016  8.6712  8.3246  6.7756  7.1075  7.0227  2.4720  8.9068
3.2569  5.3569  6.6583  8.1250  5.2193  8.6464  3.5768  3.9170
4.6144  6.7411  7.3151  4.6306  3.9388  3.7479  6.3786  1.1888
10.6402 11.0700 5.3817  9.1308  4.4386  10.6040 4.9177  7.6052
5.7089  4.8930  6.4000  7.9087  7.8755  4.9591  8.6389  4.3787
8.8900  11.4686 6.1741  8.6680  9.1228  5.6214  8.7931  8.0432
7.8760  4.9785  6.2873  4.0681  3.6494  8.7722  8.4910  7.4049
2.9859  7.1274  7.4897  9.9168  7.2836  6.7835  8.6337  10.0149
6.2542  5.4814  9.5018  5.9269  5.8407  7.6246  9.4512  8.0974
4.3633  9.6632  10.0053 6.6918  3.3631  4.8515  7.3325  8.1698
6.4943  3.8214  3.5917  6.2776  7.2306  8.6360  9.6672  8.2965
5.3898  7.4029  7.5622  4.7380  10.0807 8.8420  9.4769  2.8760
3.8730  10.9335 8.1684  7.6441  7.0352  8.5826  8.3546  6.8752
6.6006  5.9533  9.8353  4.9148  9.7114  6.1403  9.5845  4.6515
6.4625  5.5853  7.4498  7.0262  9.1042  5.1756  3.8294  10.3627
4.1236  6.7006  4.7294  4.3668  8.2136  6.6967  5.9493  5.3262
5.6916  8.5711  9.1800  4.1765  7.3414  9.4318  5.9813  7.7758
4.6782  4.6453  3.5368  9.1011  6.1231  5.4594  7.9971  9.7733
6.9741  6.3384  3.5915  3.7043  8.8421  7.8923  8.1105  6.9320
4.0353  11.2147 6.8004  4.5901  8.7625  4.1596  8.5279  10.5064
10.5027 4.4365  7.8841  4.0336  8.3706  5.5224  2.7703  2.3837
9.2500  8.3415  6.4586  4.6162  8.0424  7.3438  8.7600  6.7786
9.4014  3.6039\
GRP     6.9126  4.2006  6.8606  2.0919  2.8446  1.7112  5.0240
5.3209  5.4017  1.0519  0.7594  5.5772  1.0422  3.3079  2.5775
1.4442  4.7769  5.1689  6.3186  4.7636  5.0925  2.0613  0.0000
3.8282  0.5538  0.0000  0.8021  3.7390  5.0902  1.8184  3.6920
2.4004  1.8398  4.9963  1.0004  1.1431  4.8670  2.1545  6.4504
4.8254  4.1205  4.6262  6.8041  4.7852  0.0000  4.4447  5.0988
0.0000  2.8207  5.0984  5.6456  5.4290  4.3046  0.0000  6.7914
```

2.3993 7.7161 2.1170 2.6972 0.9799 0.5422 8.1047 5.0053
3.0309 0.0000 3.0811 1.8352 5.1701 6.9315 6.2699 3.3377
2.6575 3.4823 0.0000 2.4611 0.4698 4.5264 2.9674 3.6599
5.1968 4.9883 6.5252 2.2886 1.9875 4.2178 7.0266 5.4855
2.9812 3.0874 9.1965 1.4126 2.4311 3.6729 5.3976 8.2897
5.3853 6.8081 3.4734 4.0577 6.1143 7.6952 5.5105 6.4977
5.5721 1.5803 5.1421 4.6303 5.1358 2.3532 4.0076 3.1897
5.3711 6.4247 7.5314 3.1220 0.6116 3.5896 2.9970 6.1893
7.3228 8.1894 5.8812 3.8564 4.1655 0.0000 5.2348 4.8398
0.7843 2.7474 0.0000 6.8316 2.1813 4.2977 4.3451 7.3231
7.9726 3.1433 2.7241 1.2057 1.1757 1.3507 3.0604 1.9445
5.9789 6.4719 0.0000 2.3685 0.0000 6.1792 3.4621 3.2838
4.8222 5.2778 1.8101 2.2306 4.2259 2.2708 5.4249 3.5001
3.4015 0.0000 6.2130 2.1631 1.5535 3.2081 4.8739 4.1489
1.5478 5.0736 2.6009 2.9872 1.6845 5.8086 5.1521 3.2253
2.1868 0.0000 0.0000 5.8678 1.9052 5.9741 0.0000 2.9247
1.4215 6.3971 0.0000 7.0813 1.1775 3.9459 2.1363 5.0655
3.9158 3.5364 6.3654 4.4075 0.0000 2.0478 0.0000 7.6897
5.6778 7.2208\
TPRN 10.2220 8.1089 9.0191 9.3360 9.5627 8.7236 9.6430
9.0194 9.4809 8.2981 8.3561 9.9216 9.9112 9.0552 8.1916
9.5601 8.4414 9.6651 8.4416 9.1662 12.3381 8.5465 9.2756
8.8946 7.9922 9.9065 8.9429 9.5633 9.6644 9.8538 8.7591
9.5706 10.3517 10.2788 8.9405 9.7836 9.6413 9.2904 8.8529
8.8278 9.0362 7.3475 8.2912 8.8847 7.1803 8.8898 10.3045
8.7832 7.7244 10.6740 10.0393 8.4823 9.0413 7.3012 8.8100
9.6534 9.2838 8.0229 8.8021 8.6496 7.6364 9.5808 10.2580
9.1039 8.0527 8.7713 10.4765 9.1083 9.5479 8.4725 10.9743
8.8526 10.1057 10.7217 8.8061 9.6076 8.6376 8.8118 8.7290
9.2173 9.5579 8.2316 8.4569 8.4721 9.4764 8.9016 7.8952
9.9893 9.9149 9.1175 7.9580 9.1480 10.7118 8.6180 8.4875
7.7393 9.5666 8.3720 9.4906 8.8008 10.3178 9.0438 10.3475
8.7796 8.8934 9.4461 8.4024 8.0357 8.8538 9.0863 9.9930
9.0712 10.2669 10.2538 8.9121 8.6597 9.3027 9.3782 8.7755
8.7864 9.6878 9.1519 7.8638 9.3728 8.5375 9.3903 9.6672
11.3551 7.7439 7.0407 10.9525 9.7276 9.1187 8.6876 10.8111
11.1115 9.2981 9.1481 8.5711 8.0337 10.0518 9.0274 10.0771
8.0212 8.7018 8.9910 9.0894 9.0814 9.9507 10.9080 9.1940
11.0318 8.3433 8.5699 9.7570 9.4170 8.0730 8.0763 9.8415
10.3552 10.4330 10.4707 8.2392 9.8177 8.9369 8.1718 10.2977
9.0411 9.1156 8.7553 9.2134 8.9481 9.9145 9.3717 10.5997
9.1862 7.8756 8.1173 8.2480 8.9375 10.4258 10.4434 7.9722
9.7629 8.4378 10.2339 8.3851 9.6960 9.7730 9.0950 8.6729
8.9228 9.3595 8.8308 8.9858 11.3660 11.4812 8.3785 8.9590
8.1085 10.1956\
THSD4 6.9620 9.6316 9.1664 12.0568 7.9118 9.5266 11.0594
13.3457 12.1447 10.3340 13.9628 13.4900 10.8707 8.3078 12.5508
8.1807 12.6669 7.3740 10.0120 7.2679 12.9049 9.8389 12.0662
12.5523 10.7535 6.4456 7.4573 10.0908 8.3126 7.6447 5.8070
7.1424 5.5414 10.4282 13.1125 6.1759 9.2285 8.9470 6.8135
8.1026 8.3639 10.7916 5.8512 7.7737 9.9309 9.4609 9.0571
9.7688 10.9657 8.3108 10.8594 5.6704 7.2714 10.4190 8.4310
6.9929 7.9975 9.4638 11.7902 14.4483 10.8204 11.8568 8.6603
5.8028 10.3186 11.2670 12.8361 12.6650 9.3402 8.2758 9.6909

5.9224 5.3166 12.8534 8.8560 11.7279 8.0035 11.2832 9.8396
7.6903 11.1853 11.4328 10.7000 8.7615 11.6470 10.7899 8.1273
10.7902 9.4139 9.1014 11.1438 12.9119 6.1067 10.0431 12.1369
10.9748 7.1871 10.1383 10.3299 8.9162 6.3740 12.5644 8.7628
9.8701 8.9858 6.6889 9.2299 13.0538 12.0542 12.6927 10.1287
13.6570 12.3384 7.0768 8.6789 10.3524 12.3050 13.5041 8.3929
6.0120 11.8767 5.1807 9.2953 7.4893 11.8383 9.2878 14.3410
12.5411 11.3154 11.3737 8.9103 6.3735 12.3072 6.9920 8.4581
7.9942 8.0957 9.6423 10.5512 9.5877 7.8676 9.7523 12.6886
8.2395 8.3846 7.4672 13.3210 11.2382 6.8159 8.3560 9.9827
9.5499 11.2518 9.5737 9.7180 13.0525 11.3681 13.4858 14.2909
6.3672 4.8155 8.6450 11.6140 10.0360 10.1846 11.2081 9.1496
10.3247 11.6167 5.3439 6.7962 11.3583 9.4518 4.9145 5.3211
8.9940 9.9310 11.4913 10.5960 11.8742 5.7663 6.9930 10.5907
7.7899 7.8674 10.1240 13.6584 9.7956 8.7086 6.9514 9.3496
9.7478 10.4875 7.0549 8.0325 11.2595 9.2169 10.2552 8.8318
11.4009 11.7736\
KIAA0895L 10.0611 9.7633 7.7678 10.0304 8.1380 8.4768
6.8545 8.5199 9.3056 9.9925 7.6853 8.2915 8.5714 9.7434
9.2580 9.9392 10.1583 8.6951 9.5364 7.6769 7.7097 9.2680
8.6372 9.6156 8.7256 8.4421 8.7501 8.7091 9.3217 8.4702
9.3036 9.2428 7.9827 9.2546 8.2513 9.3005 10.0746 10.2827
9.8000 7.4509 9.0547 9.4430 9.2911 8.6690 5.9682 9.2036
9.0829 8.8499 8.4494 8.0438 8.9171 8.8906 7.8390 8.1374
9.8764 10.3595 8.7415 8.0350 7.8941 8.9754 6.9917 9.3756
9.1015 9.2303 9.7871 9.6499 8.7632 9.3047 8.6332 9.0663
7.6490 9.5601 7.5248 9.2550 9.0683 9.9739 9.5430 9.1395
8.4708 8.7326 8.4396 8.6153 8.7570 8.0124 9.8961 10.0083
9.4273 9.1767 8.4123 10.4819 8.8149 8.8465 8.2623 10.6738
9.2466 7.1206 7.0605 7.7656 9.3024 9.9412 9.4140 9.2090
9.0027 11.6355 8.7636 7.4394 10.7962 8.7606 8.5388 8.9045
9.6154 9.1859 9.4526 9.9441 9.2571 8.4026 8.2374 9.3119
8.4694 7.8084 9.0915 8.0608 8.6865 8.0041 9.3289 8.1666
8.9791 7.3884 7.3330 9.7476 8.4920 8.9352 9.4223 10.6102
8.0247 8.7240 10.0997 10.9089 9.0093 8.8898 9.4390 9.2504
9.5132 8.7077 8.5290 9.4518 8.8996 8.6502 9.0033 9.1861
9.6015 8.7991 10.2494 9.4291 9.4595 8.8896 8.3023 9.6432
8.9001 8.6081 8.8303 8.5511 8.9983 9.9121 9.0160 9.5800
7.9226 8.1532 8.9651 9.0185 9.5975 9.1966 8.3574 9.8309
9.4932 10.4516 10.6929 8.1678 9.4301 9.5116 8.9045 8.8888
7.0436 10.1203 8.7281 9.9708 7.1434 8.8990 9.9977 9.0228
8.8416 8.2854 9.1916 8.3751 9.6416 7.9393 8.5337 8.2840
9.3318 9.5797 7.9198\
CHST9 1.7370 4.7473 5.4152 2.9119 2.0327 4.2380 2.7953
6.1448 8.7779 5.5372 5.8094 3.8262 3.7011 5.6785 1.8915
3.8845 5.9011 3.4585 4.7781 4.8991 2.7816 8.5187 8.9559
5.7964 3.0035 1.8524 7.9642 2.3554 3.6194 4.4410 6.7636
0.5612 0.5159 0.8184 2.0007 0.0000 2.6245 5.8208 0.0000
4.5072 0.0000 5.1016 1.3900 1.2622 2.8515 0.0000 2.2746
1.1317 1.6510 0.8339 0.7972 2.7455 1.4359 2.9592 3.8373
3.1821 2.9310 5.9327 4.9779 3.8691 3.0528 7.8763 0.9583
3.2795 4.6210 0.6977 0.0000 6.2663 0.0000 3.6688 5.3921
2.1491 0.0000 4.6437 1.2339 0.4698 2.6228 1.4952 7.1550
7.7270 0.5659 3.4754 9.6395 7.0517 5.4201 3.8501 4.1091

| | | | | | | | |
|---|---|---|---|---|---|---|---|
| 3.7795 | 0.0000 | 7.2774 | 2.9352 | 6.3518 | 1.4549 | 0.6909 | 0.0000 |
| 0.6266 | 0.9065 | 4.1324 | 4.6259 | 4.8744 | 3.3101 | 0.0000 | 9.4664 |
| 5.8131 | 6.1348 | 4.1518 | 4.2346 | 3.4735 | 4.8043 | 5.1372 | 7.1766 |
| 5.8865 | 2.0513 | 2.3219 | 0.5141 | 4.9089 | 6.3463 | 4.3613 | 0.3974 |
| 0.6089 | 6.4320 | 0.0000 | 3.2675 | 2.7605 | 8.9665 | 5.3657 | 0.0000 |
| 1.6629 | 3.7927 | 3.8982 | 5.0575 | 4.4346 | 2.6167 | 7.1616 | 3.8654 |
| 2.2603 | 4.7412 | 0.0000 | 3.8581 | 4.6148 | 6.4308 | 4.4942 | 0.0000 |
| 2.6953 | 2.7177 | 0.5418 | 5.4733 | 5.8224 | 1.3155 | 2.1177 | 5.5087 |
| 6.2854 | 5.4380 | 4.2890 | 5.5021 | 3.1934 | 6.2586 | 2.7033 | 2.3578 |
| 2.4740 | 4.4522 | 0.0000 | 5.6919 | 4.8121 | 0.0000 | 0.5023 | 6.1019 |
| 7.2511 | 6.4299 | 3.5915 | 2.7730 | 2.1836 | 6.8593 | 5.0142 | 0.0000 |
| 4.3183 | 3.9471 | 3.9276 | 5.3491 | 9.1588 | 3.9568 | 1.1551 | 8.3769 |
| 2.4895 | 6.9648 | 3.0083 | 4.7703 | 4.4477 | 3.8909 | 2.9621 | 0.0000 |
| 4.3406 | 0.0000 | 1.0222 | 8.6982 | 2.3335 | 4.0136 | 3.5942 | 0.6701 |
| 8.3289 | 0.0000\ | | | | | | |
| CHST8 | 1.9260 | 5.3619 | 3.7938 | 1.3959 | 2.5505 | 0.0000 | 0.9367 |
| 2.6906 | 1.4589 | 3.9364 | 1.0281 | 0.0000 | 1.9354 | 2.1894 | 0.0000 |
| 0.0000 | 1.8857 | 1.6514 | 3.5205 | 8.8897 | 2.5380 | 3.5000 | 3.5909 |
| 0.9110 | 4.7408 | 8.4761 | 8.9330 | 2.3554 | 3.4165 | 0.0000 | 0.7334 |
| 0.5612 | 1.1952 | 1.1010 | 1.0004 | 5.9549 | 2.6245 | 1.5916 | 0.9336 |
| 0.7666 | 0.6649 | 3.6142 | 1.0569 | 0.0000 | 4.5728 | 1.4889 | 1.1888 |
| 3.3964 | 1.9464 | 8.9945 | 0.4530 | 1.2789 | 1.0956 | 3.8633 | 3.1038 |
| 1.2781 | 1.0734 | 5.0131 | 2.3545 | 3.7698 | 4.3333 | 0.4969 | 0.5573 |
| 1.0177 | 4.9383 | 1.3532 | 3.3568 | 2.8005 | 0.0000 | 2.7780 | 0.4334 |
| 0.3921 | 2.4919 | 3.1486 | 1.2339 | 1.3447 | 1.5242 | 3.5184 | 4.4071 |
| 2.7776 | 0.9714 | 0.7073 | 4.2581 | 1.4748 | 4.5242 | 1.3843 | 0.5376 |
| 1.0433 | 1.2163 | 8.3390 | 4.6655 | 1.3771 | 9.7955 | 3.5917 | 1.4518 |
| 5.8742 | 8.0020 | 5.9882 | 1.8231 | 0.9478 | 1.0805 | 0.4986 | 9.4274 |
| 2.3196 | 1.3743 | 1.1001 | 3.8282 | 2.6388 | 3.7365 | 3.0071 | 0.8916 |
| 0.6215 | 3.7633 | 6.5999 | 0.5141 | 1.8640 | 2.5502 | 2.0505 | 1.9462 |
| 0.0000 | 4.2893 | 1.3285 | 2.2557 | 2.9876 | 5.1008 | 0.0000 | 0.0000 |
| 0.7843 | 5.1017 | 4.7394 | 0.7024 | 0.6684 | 2.8211 | 4.7669 | 2.6505 |
| 2.2603 | 1.9254 | 4.6610 | 2.1648 | 4.4022 | 3.6878 | 1.3036 | 1.0973 |
| 2.3527 | 0.0000 | 6.2606 | 1.1308 | 4.6638 | 0.9947 | 2.3242 | 4.5271 |
| 0.0000 | 0.5414 | 2.1729 | 0.0000 | 1.0445 | 5.3967 | 1.5047 | 2.0337 |
| 1.4969 | 2.6375 | 1.0431 | 1.5791 | 0.4033 | 1.9510 | 0.0000 | 0.0000 |
| 4.0901 | 1.2170 | 0.0000 | 0.7903 | 0.0000 | 5.5390 | 1.3895 | 3.6013 |
| 5.0434 | 5.0782 | 4.0753 | 3.1725 | 2.2762 | 2.5076 | 1.5061 | 3.7342 |
| 1.1756 | 0.0000 | 0.6248 | 0.0000 | 0.0000 | 1.1063 | 1.5562 | 0.0000 |
| 0.0000 | 0.0000 | 2.3573 | 0.0000 | 4.8720 | 8.5471 | 5.0055 | 0.6701 |
| 0.8824 | 0.6959\ | | | | | | |
| COL4A5 | 3.7656 | 10.2828 | 9.6026 | 10.3326 | 10.1994 | 9.0899 | 7.2245 |
| 10.0681 | 10.5696 | 10.3336 | 9.7857 | 5.6932 | 8.1453 | 11.5388 | 10.5475 |
| 10.4010 | 10.5928 | 8.0348 | 9.2302 | 7.4555 | 9.7192 | 10.9368 | 9.5286 |
| 10.8805 | 12.8816 | 2.9613 | 9.2331 | 9.3937 | 9.9414 | 9.1727 | 7.8489 |
| 10.2785 | 1.8398 | 4.1534 | 9.2476 | 7.5936 | 10.7269 | 9.3373 | 11.5913 |
| 10.5101 | 9.8812 | 11.0125 | 7.3057 | 9.1277 | 10.6297 | 10.2650 | 7.7759 |
| 10.4051 | 11.8154 | 6.1742 | 9.3643 | 4.6583 | 10.3386 | 11.3146 | 10.0184 |
| 10.7480 | 8.8674 | 8.8571 | 9.9678 | 9.7302 | 10.9073 | 8.2225 | 9.5637 |
| 10.4398 | 10.7665 | 10.5406 | 6.0716 | 10.7527 | 7.6247 | 11.3556 | 6.1864 |
| 10.4079 | 3.8949 | 9.7365 | 9.6143 | 10.4002 | 10.6105 | 9.7151 | 10.3331 |
| 10.5332 | 9.9152 | 9.1582 | 9.7446 | 8.8513 | 10.1343 | 11.9642 | 9.3826 |
| 9.7516 | 6.6975 | 5.5620 | 10.5868 | 10.4889 | 8.5886 | 10.9356 | 9.9685 |
| 11.9530 | 7.4333 | 10.5294 | 10.2856 | 9.9679 | 7.9043 | 8.2656 | 4.6060 |

9.3787 9.7616 6.5373 11.5632 10.9278 12.1519 10.5742 10.4433
10.5566 6.5986 7.6511 10.5758 10.1079 9.8781 10.6680 10.5795
5.4088 11.0844 5.0107 10.1733 7.5169 9.9038 9.0651 10.4300
8.3581 10.4455 12.8768 10.3756 9.4388 9.8373 11.6751 7.2174
6.4871 3.7942 9.9510 10.7631 10.7997 10.4833 10.0482 9.2489
10.2715 10.3938 9.8246 11.2221 9.8430 10.0892 10.1040 11.0239
9.0794 11.3635 9.2858 10.5180 10.0759 10.5255 10.9109 9.2626
6.2251 2.8614 3.0745 10.4285 8.3928 6.9107 11.1564 10.0927
10.2286 9.6242 9.1298 8.8687 10.0340 8.5663 9.4617 5.7522
10.2425 11.0442 11.6561 10.7254 9.9553 8.6651 10.3400 8.5711
10.9244 10.5242 9.5117 9.5641 9.1551 9.7673 9.2865 10.3826
10.7481 9.9693 10.2748 10.6404 6.8088 8.4641 9.7845 9.4251
10.3193 6.6104\
COL4A4 3.4941 6.4511 8.5642 4.9049 3.0889 8.1464 3.1326
5.4679 9.2174 3.7185 4.6181 3.8262 6.3300 4.2331 2.8727
4.4366 6.8423 2.1918 6.4060 11.4615 8.9438 3.4486 4.6616
5.0691 4.3353 1.8524 5.3366 4.9711 3.8787 9.2040 2.1085
5.0590 3.0546 3.5933 3.6447 2.5445 7.6279 6.7742 3.6815
9.8329 4.2593 6.4815 9.1803 8.0517 8.3504 3.0403 3.3157
9.9334 2.8207 7.4457 2.7077 9.0583 3.7556 8.8000 5.3126
3.9309 9.4051 8.4709 5.6643 6.2174 8.9829 5.5308 3.7288
8.2830 3.7122 3.9062 3.7908 7.0298 3.0673 4.7882 3.1224
5.3456 6.3967 4.7317 4.2837 3.0555 4.8179 3.7310 4.2764
6.4206 2.7579 5.8166 5.8738 5.5073 3.9151 8.0994 4.6913
2.8809 4.0392 4.7773 7.4280 9.3362 3.7214 5.1203 4.0547
7.4562 4.3391 6.8330 4.6504 8.8393 2.8342 3.9875 6.3625
5.5518 1.3743 2.0179 4.5101 5.0922 6.4328 4.3653 4.0350
3.4186 5.8481 11.3242 4.9122 6.3723 3.2310 6.8780 3.3042
10.2610 4.4932 2.7098 11.2530 1.3451 5.1641 5.1646 8.0361
4.1963 8.9172 5.9071 4.2856 5.3180 3.6630 6.9116 8.2910
4.6149 3.0489 0.0000 5.5990 5.7176 3.8517 3.6157 3.5639
4.3980 2.2385 7.8784 3.3948 6.2103 2.6913 1.8766 5.0822
0.9816 4.8469 4.0911 6.8459 4.9756 4.8207 2.4027 3.5001
3.2017 2.2191 6.0142 5.2583 4.8449 3.3158 3.0144 5.5723
10.2871 2.5502 6.7156 4.1812 6.0942 1.7114 5.1521 3.6013
3.9641 4.8348 7.2780 4.5512 5.6402 3.5582 10.7379 6.1113
3.5742 5.8817 4.3960 4.4485 5.4354 2.7580 2.1363 4.3805
5.3216 6.6685 10.2222 1.6152 2.3335 5.3656 5.7900 2.9076
6.4366 2.4167\
COL4A3 0.9511 5.2980 6.9634 3.1292 0.9133 4.8057 0.9367
3.2591 7.0765 1.2815 2.1584 2.3205 4.2215 1.6493 1.8915
2.6237 5.0120 0.0000 4.8296 9.5077 8.4680 0.8427 3.9925
2.6512 2.0961 0.0000 0.4561 3.1243 0.0000 8.2562 0.0000
2.8443 0.8951 0.8184 2.0007 0.0000 6.3372 4.2355 0.0000
9.0569 0.6649 4.1593 8.4355 7.3415 5.0498 0.9285 0.0000
8.2027 0.7769 6.1184 0.4530 8.6273 1.0956 7.2459 3.9120
0.0000 7.4688 8.0662 2.5360 3.2855 6.8112 3.5473 1.5290
6.3477 2.3888 1.6675 2.0292 5.8046 1.5697 1.6464 1.4604
1.8067 4.0640 1.5690 3.3238 0.8237 3.0064 2.0646 1.1539
3.3294 0.9714 2.8095 3.5062 4.1859 1.4651 7.1462 0.9284
0.6140 0.5273 2.0295 4.2693 7.2437 0.5215 3.2630 0.9013
2.9121 2.3031 5.1291 1.1822 8.3838 1.9211 1.1625 2.4570
4.8552 1.3743 1.3363 2.1046 3.5614 3.6341 0.0000 2.1442
1.6572 1.3631 10.8963 2.7148 4.1184 1.4679 6.3577 1.1815

10.2279 2.5419 0.0000 9.8766 0.0000 4.1212 3.1042 4.9678
1.2894 6.9416 4.0527 2.7988 2.1813 2.0932 6.3772 5.5776
0.0000 0.0000 0.0000 3.9825 4.2829 3.6878 2.1461 1.4380
2.0686 0.4562 7.6420 0.0000 3.7446 0.9947 1.2240 3.0134
0.0000 1.7123 1.0019 4.4749 2.8829 3.1906 1.1539 2.2048
1.2429 0.6063 2.2364 2.4523 3.2305 1.6553 0.5023 0.7889
7.9422 0.0000 5.0550 1.9700 4.3251 0.0000 2.2569 0.0000
1.1272 2.7403 4.9829 2.0726 5.0853 0.0000 9.2704 4.7035
1.4215 4.1421 1.8914 1.5773 3.2020 0.0000 0.0000 2.6000
2.9216 2.4161 9.4197 1.1623 5.5688 2.8619 4.9573 1.3092
4.4925 0.0000\
COL4A2 12.8695 14.1969 13.5583 12.9437 13.0371 12.6808 12.4178
13.8797 14.5773 14.1611 12.5899 13.7803 13.3653 12.8362 12.8536
13.3678 13.7318 12.6765 14.7383 14.5337 13.5215 12.3891 11.2736
12.9988 14.9649 12.5929 11.3108 13.3334 13.5704 13.8322 14.0695
12.9136 11.0458 13.2669 13.3650 12.7273 13.6550 12.6448 12.1292
13.8930 13.4072 13.7232 13.7396 12.7046 13.5293 12.5174 12.6825
14.2822 12.8241 11.4249 12.2211 14.0798 14.3015 13.5910 14.3530
12.6187 14.5498 12.4931 14.2624 12.8382 13.8105 13.5733 12.1172
13.3221 14.4049 13.7341 11.8234 13.1812 12.6840 14.7054 13.2609
13.9312 13.1446 13.7779 13.5677 12.1925 14.3873 13.2896 11.9810
13.3590 14.1729 14.4622 13.2610 13.2952 13.0407 12.7547 12.2219
13.0074 12.5533 14.9660 14.0462 13.7074 13.4655 13.6892 13.3992
14.9455 12.7411 13.8639 13.6555 13.5390 12.7271 13.5320 13.1212
13.4493 11.8818 13.6337 14.5557 14.7197 13.5526 13.6476 13.4642
12.5923 12.9896 15.0538 13.4407 13.4536 12.4637 12.5676 12.9596
14.7293 13.4070 12.8275 15.0116 12.6729 11.5468 14.9622 13.1756
11.5567 13.6223 14.0501 12.9826 11.3927 13.1204 11.8805 14.4905
13.6044 11.0341 13.1629 12.7019 13.0059 11.5690 12.3702 12.5207
13.1212 13.0444 11.3995 12.0258 14.6569 13.1454 10.5957 13.4836
11.8706 13.6779 13.3667 12.4245 14.1108 13.3115 13.7666 11.8702
12.3320 12.2431 13.0588 12.9956 12.0783 13.6492 13.9418 12.2690
13.4874 13.2177 13.3393 13.9594 12.6858 12.6471 13.8953 13.5476
13.5893 13.8152 14.2892 13.5619 12.0574 12.1077 14.3433 12.3497
12.5004 13.1838 12.4116 14.8435 12.7143 12.4843 13.0743 13.8140
13.6169 12.8832 14.4066 13.1784 11.3200 11.7241 12.3322 13.5954
12.9449 12.9884\
COL4A1 12.2525 13.5033 13.3070 12.3055 12.4012 12.7535 11.9462
12.7075 13.9654 13.3220 12.2015 13.1905 12.4994 12.4895 12.1505
13.0003 12.6721 12.2084 13.8705 13.7587 12.6712 11.7097 9.2431
13.0150 13.9013 12.0154 11.4065 12.4050 12.6093 13.1075 13.2167
12.3971 10.9462 13.1596 12.5160 11.7064 13.6293 12.8152 12.0637
13.7323 13.0778 13.3345 13.1723 12.0762 12.6422 11.6024 11.0590
14.0938 12.9925 10.8967 12.0722 14.0566 13.2579 12.6893 14.1493
12.0829 13.4948 12.0128 13.5230 11.7777 13.1063 13.4353 11.7208
12.3317 13.6705 13.1380 11.0124 12.3273 12.4870 14.3042 11.4759
13.6004 12.8978 12.3773 13.5178 11.5374 13.9323 12.6797 11.2656
12.6565 12.8611 14.2345 13.1269 13.0396 11.6360 12.2965 12.2735
11.4956 11.6368 14.5600 12.7676 13.2224 12.0779 12.8717 12.7138
13.8502 12.4184 12.5888 12.7777 13.4027 11.2838 12.8644 12.5836
13.2702 11.3241 12.8217 14.0388 14.0187 13.0724 12.6181 12.6713
11.9290 10.4789 14.1714 13.8244 13.2408 12.4516 12.3901 12.2072
13.9877 12.9005 12.4402 14.4547 12.6920 9.6651 14.2685 12.2021
10.6599 12.7711 13.1291 12.5315 11.4455 11.9623 11.9996 13.4673

11.7447 9.7845 12.1453 12.0697 11.6570 10.9931 12.1330 11.3135
13.0015 12.5957 9.8122 11.8681 14.0431 12.2706 9.9420 13.0340
9.7045 13.3557 12.6583 12.0419 13.6102 12.2481 13.0170 11.2550
12.0301 11.7428 12.7116 11.9992 10.7491 12.8167 13.5120 12.1085
13.0839 12.3544 12.0203 13.0619 12.6898 11.2109 12.2355 12.6939
13.4204 12.9679 12.5455 13.0466 11.1603 11.5040 13.1863 11.5927
11.7134 12.4752 11.7734 14.5960 11.5622 11.2780 12.4893 13.5100
13.1442 12.3131 13.9474 12.6680 10.3863 10.2789 10.5019 13.3666
12.1833 11.0699\
SLC35D2 8.6030 9.0697 8.3315 9.8045 8.8821 9.6058 9.1410
8.6427 7.7392 9.3577 8.5790 8.5351 8.3775 9.4955 9.2331
9.7451 7.7042 9.0515 9.0519 9.0152 8.3782 9.6075 9.2017
9.2603 8.3743 9.5312 9.5610 8.7246 8.8118 8.1317 8.7211
8.6090 9.8718 7.6567 8.1957 8.9713 8.4969 8.7089 9.0316
11.4306 9.4894 8.6860 8.3734 9.7079 9.0428 8.8980 10.5109
7.9307 7.7910 9.0929 8.7420 9.1927 8.8232 9.1576 8.8112
8.8820 9.0377 8.7632 8.3181 7.8392 9.0785 9.1828 9.3098
7.4655 9.3211 8.9838 8.5345 8.2351 9.4519 9.5688 8.5993
8.6193 9.1639 8.8121 8.9352 8.9077 8.5769 9.8370 9.8415
10.0837 10.3377 7.6043 9.2180 9.4784 9.7538 7.4900 9.0219
9.6466 9.6326 8.2162 8.8465 8.5698 8.1781 9.5491 9.6141
8.8130 8.1268 8.9468 9.9197 9.2481 9.3489 9.0472 8.3962
8.0324 9.3218 9.9072 8.8553 8.6924 8.3950 9.3908 8.8910
9.5997 8.6050 8.1649 8.4114 8.8351 9.0936 8.4081 9.7271
8.8762 8.1333 8.7508 8.8130 9.2144 8.9381 8.5496 9.1019
7.7417 8.7707 8.3467 8.1992 8.9404 9.8060 9.2633 9.2974
9.1767 9.7036 8.5573 8.9572 8.9377 10.1284 9.2501 10.3073
8.9441 9.0135 9.2773 10.2742 8.8738 8.7722 9.9911 9.1386
9.5759 8.9515 8.3122 8.6974 8.9804 9.0600 9.4358 8.7861
10.2393 9.0023 9.3713 10.1555 9.8887 8.5386 9.5830 9.1951
9.2794 10.0835 8.6385 8.5943 7.5707 9.5936 9.7492 8.8726
8.4049 8.7809 9.0176 8.6166 8.9407 8.9594 8.7143 8.7482
9.4173 7.8382 10.0374 7.6751 8.3485 8.7746 8.8394 8.6119
8.9060 8.2348 8.2188 9.0501 9.9903 9.6341 9.2218 9.2899
9.3510 10.3427\
CHST1 4.1539 7.8409 7.3194 8.6984 9.2673 4.5295 8.8089
7.3737 6.0533 7.0476 10.2146 6.0118 6.8768 4.2868 9.5384
6.6558 6.7074 6.0589 8.1865 5.7448 7.4123 6.0106 9.3099
7.0317 6.0757 6.8369 10.2470 8.1888 8.4652 5.2048 4.0212
6.3547 4.6233 7.7995 4.5244 3.5700 8.6481 6.8154 4.6764
4.1564 7.0587 6.9055 4.9660 7.1665 6.1592 7.6742 6.8279
6.4973 4.5531 5.5478 10.3892 5.9785 11.3796 7.3127 8.3239
6.4189 6.6416 7.3141 6.8390 6.7990 7.8933 6.0792 5.4563
5.9295 7.4933 6.8663 5.7795 7.6082 7.5845 7.3242 6.7151
10.3424 5.1002 6.2128 5.7232 10.0287 10.2008 7.1587 8.4393
6.4296 6.2916 5.8565 6.0250 8.1127 7.0074 5.2697 4.6913
7.9799 6.4507 9.0107 7.4781 5.1757 6.1692 6.5413 10.0398
7.2956 8.8810 8.3899 7.9172 5.5540 9.8956 9.5959 5.9455
7.3693 6.1671 9.6739 8.4192 8.8673 6.7345 7.9768 4.9708
10.0863 9.5252 5.7549 8.4543 8.5255 6.8555 6.1463 4.9598
6.6549 7.9953 6.8183 7.2275 9.8922 7.2596 6.7102 7.3729
9.5253 7.9016 6.6567 8.0905 4.7510 6.2056 8.5187 6.1371
6.5351 7.3215 7.8157 8.5241 7.3890 3.7991 7.1193 6.1970
6.0700 5.1381 8.3945 9.3028 8.6774 8.3291 6.0399 7.5630

| | | | | | | | |
|---|---|---|---|---|---|---|---|
| 8.0872 | 9.6042 | 6.0261 | 3.5384 | 6.8329 | 8.7206 | 7.0587 | 10.3370 |
| 5.5689 | 5.9566 | 4.6359 | 6.2202 | 10.4363 | 6.7757 | 9.2023 | 7.2653 |
| 6.0532 | 11.4684 | 8.9600 | 6.6148 | 4.1199 | 5.9744 | 5.7526 | 7.0729 |
| 7.0750 | 6.5494 | 7.9959 | 6.6770 | 6.9921 | 9.4362 | 5.2868 | 7.2204 |
| 7.1152 | 6.0507 | 6.2337 | 7.0919 | 5.8683 | 7.2206 | 6.2791 | 4.8502 |
| 11.5478 | 8.9790 | 6.7160 | 9.3701 | 6.9332 | 7.9968 | 7.6237 | 12.1440 |
| 8.7150 | 6.5544\ | | | | | | |
| ZNF879 | 5.0733 | 6.6941 | 6.0164 | 6.2411 | 6.4450 | 6.4923 | 6.0818 |
| 7.7161 | 6.9878 | 6.0546 | 5.8183 | 6.7343 | 7.3742 | 6.2641 | 5.9326 |
| 6.1955 | 6.6773 | 5.6714 | 6.6761 | 6.0597 | 6.8225 | 4.9049 | 6.3613 |
| 6.2612 | 6.5420 | 5.3090 | 6.0538 | 5.9952 | 6.1530 | 6.6663 | 6.4395 |
| 5.7617 | 5.9352 | 6.0832 | 7.5362 | 8.0794 | 5.5699 | 6.5840 | 5.8885 |
| 6.6822 | 6.8476 | 6.0103 | 6.3957 | 5.2766 | 6.8191 | 5.2832 | 4.7329 |
| 6.4291 | 7.0436 | 5.9732 | 5.7080 | 5.9947 | 6.2049 | 7.2418 | 5.1054 |
| 7.7657 | 5.3490 | 5.3447 | 7.4183 | 7.6250 | 6.8966 | 5.8921 | 6.6440 |
| 6.8929 | 6.9243 | 3.1834 | 7.7494 | 6.2285 | 5.7719 | 7.1886 | 6.9000 |
| 6.3109 | 5.3821 | 6.9603 | 6.0122 | 6.0030 | 5.4947 | 5.3094 | 6.1834 |
| 6.1339 | 5.3865 | 4.7851 | 6.2130 | 7.2276 | 5.2302 | 7.1625 | 7.5576 |
| 4.4390 | 6.1880 | 6.2867 | 6.8191 | 6.3376 | 7.0496 | 6.1502 | 5.4144 |
| 6.2178 | 6.4452 | 7.1548 | 5.3790 | 6.4262 | 5.2399 | 6.1281 | 8.5037 |
| 6.9629 | 6.0593 | 6.5254 | 6.4179 | 6.3304 | 7.0147 | 6.3209 | 6.5902 |
| 5.2940 | 7.7784 | 5.2095 | 5.9393 | 7.0029 | 5.8123 | 7.4844 | 4.6854 |
| 4.6223 | 6.6923 | 5.7657 | 6.8832 | 5.3457 | 6.7220 | 5.8523 | 6.4533 |
| 6.5680 | 7.2656 | 6.4357 | 6.2230 | 6.0661 | 5.9895 | 6.8866 | 6.4549 |
| 2.6570 | 7.0980 | 7.2339 | 6.4611 | 7.5624 | 6.6692 | 4.9245 | 5.7394 |
| 5.2117 | 4.3195 | 6.7565 | 6.8028 | 6.4938 | 5.0770 | 5.4731 | 6.6018 |
| 5.2858 | 5.8099 | 6.5660 | 5.9742 | 7.9370 | 6.6846 | 5.8495 | 5.7988 |
| 4.8245 | 1.3600 | 2.9807 | 5.7988 | 6.3339 | 5.5170 | 6.3168 | 6.8579 |
| 6.6074 | 7.2975 | 7.5405 | 5.6506 | 7.1880 | 7.1730 | 5.6004 | 4.7036 |
| 6.7963 | 7.1338 | 6.7426 | 6.6950 | 5.7620 | 6.0779 | 5.7152 | 6.9854 |
| 5.6825 | 6.6088 | 7.2136 | 6.7435 | 5.8527 | 5.6102 | 6.3235 | 5.8250 |
| 5.7446 | 7.1952 | 6.5775 | 5.7053 | 6.4237 | 6.9995 | 7.7493 | 2.9076 |
| 6.4917 | 3.5284\ | | | | | | |
| CHST3 | 10.3794 | 10.2467 | 10.2777 | 9.7009 | 8.3510 | 6.8379 | 8.8707 |
| 8.1846 | 9.6272 | 10.1122 | 6.8581 | 8.9542 | 8.9656 | 7.9654 | 9.2222 |
| 8.3652 | 9.0227 | 9.5420 | 10.4065 | 10.6398 | 9.3667 | 10.1672 | 9.5732 |
| 10.1285 | 9.4848 | 8.8666 | 8.5679 | 9.8016 | 9.8982 | 8.3237 | 10.3098 |
| 11.2179 | 5.1801 | 7.6917 | 7.7013 | 8.8348 | 10.0514 | 11.3443 | 9.7057 |
| 12.4425 | 10.1238 | 9.7902 | 8.5952 | 7.6710 | 10.6789 | 9.7694 | 9.6555 |
| 9.0641 | 10.1792 | 9.9713 | 8.9326 | 8.8665 | 8.3868 | 10.6699 | 9.1148 |
| 8.9694 | 10.0211 | 8.8687 | 10.5807 | 8.4929 | 10.5559 | 9.0290 | 10.1125 |
| 7.5349 | 10.1397 | 9.6362 | 7.9334 | 8.3003 | 8.8930 | 10.7955 | 5.9287 |
| 9.4973 | 7.2282 | 7.3940 | 9.8023 | 9.7620 | 8.0167 | 9.7574 | 9.4688 |
| 8.8931 | 8.8567 | 10.3190 | 9.8445 | 8.3330 | 9.1429 | 7.4373 | 7.3774 |
| 8.3164 | 8.3454 | 9.9687 | 10.5737 | 9.7033 | 9.2195 | 8.9669 | 8.6490 |
| 10.1072 | 6.8849 | 10.3250 | 9.6548 | 8.7331 | 8.9703 | 9.2309 | 8.8326 |
| 11.2209 | 9.8483 | 9.0241 | 9.9023 | 8.0976 | 9.5697 | 8.5080 | 10.8953 |
| 8.9492 | 9.8928 | 9.6275 | 7.6505 | 8.5922 | 10.1154 | 8.3932 | 8.3765 |
| 8.5861 | 10.2273 | 9.9543 | 9.0575 | 7.4326 | 9.7628 | 9.5960 | 9.3512 |
| 8.0476 | 9.8125 | 10.2086 | 6.6854 | 9.6661 | 10.3789 | 8.3056 | 8.2665 |
| 9.8334 | 5.8601 | 8.8884 | 9.6996 | 9.9540 | 9.0727 | 10.7375 | 8.5760 |
| 10.2006 | 8.4132 | 11.3927 | 9.2837 | 10.1151 | 9.1326 | 9.3973 | 9.7627 |
| 8.0768 | 9.9260 | 8.7112 | 7.9622 | 10.0630 | 10.1095 | 9.9418 | 8.5524 |
| 8.6193 | 10.8613 | 7.7987 | 8.7743 | 8.3449 | 9.4305 | 9.1419 | 7.8008 |

| | | | | | | | |
|---|---|---|---|---|---|---|---|
| 8.4650 | 9.5907 | 6.9378 | 11.3006 | 8.3490 | 10.0420 | 7.9653 | 10.7852 |
| 9.7726 | 9.5786 | 10.8129 | 9.4703 | 9.2304 | 9.7349 | 9.2833 | 8.8819 |
| 9.3887 | 8.8160 | 7.3683 | 9.8182 | 9.5372 | 8.9597 | 8.3426 | 8.9854 |
| 9.0109 | 8.1855 | 8.3778 | 8.9716 | 7.7626 | 9.3568 | 9.9226 | 7.9202 |
| 10.0716 | 10.1702\ | | | | | | |
| CHST2 | 10.9731 | 9.4375 | 10.9640 | 10.6918 | 7.4032 | 7.8861 | 10.1191 |
| 8.5225 | 8.1847 | 9.6755 | 9.6696 | 8.1732 | 8.6413 | 11.9289 | 7.7790 |
| 8.8739 | 7.3124 | 10.3340 | 9.8295 | 9.5733 | 9.3853 | 9.4479 | 7.3279 |
| 8.9197 | 8.7328 | 8.5683 | 6.4655 | 8.5474 | 14.2777 | 9.0543 | 8.1185 |
| 10.0925 | 7.4593 | 7.4070 | 7.6411 | 7.9409 | 11.9532 | 10.3999 | 9.9496 |
| 5.7997 | 8.1277 | 9.9113 | 9.9727 | 10.1435 | 7.8650 | 9.7254 | 7.7968 |
| 10.3489 | 7.0319 | 10.1857 | 10.7546 | 10.7392 | 11.8086 | 8.6860 | 9.8624 |
| 8.9857 | 11.4735 | 8.9921 | 7.9393 | 7.9930 | 8.2430 | 8.2694 | 8.2687 |
| 10.6840 | 10.4703 | 10.2043 | 9.6246 | 8.8153 | 7.7781 | 7.9704 | 9.6556 |
| 13.0950 | 10.7371 | 8.1981 | 8.8320 | 8.7437 | 10.5422 | 8.6692 | 8.9595 |
| 7.1670 | 10.4042 | 8.2239 | 7.5999 | 8.4793 | 6.8064 | 6.3397 | 7.0924 |
| 6.7429 | 7.8480 | 9.2073 | 7.6551 | 7.3358 | 8.2786 | 7.8501 | 7.6788 |
| 10.8223 | 11.5531 | 8.6330 | 6.4126 | 8.8868 | 5.9223 | 9.6413 | 7.7345 |
| 8.4136 | 8.1680 | 8.0003 | 9.6706 | 7.8056 | 8.2049 | 7.5855 | 13.5544 |
| 7.9489 | 8.5576 | 7.4757 | 11.3732 | 7.9456 | 10.1991 | 7.4788 | 6.6009 |
| 8.0361 | 10.1294 | 9.1684 | 8.4622 | 9.4810 | 5.6498 | 7.9238 | 9.9896 |
| 6.3807 | 8.4476 | 8.8975 | 8.1136 | 9.9136 | 7.3399 | 8.8235 | 7.7645 |
| 7.3101 | 7.5016 | 7.4803 | 7.3372 | 8.3480 | 9.2955 | 11.1264 | 7.0512 |
| 10.2501 | 8.9400 | 8.3468 | 9.9910 | 7.4254 | 10.0567 | 13.7703 | 9.6257 |
| 4.7717 | 9.6823 | 8.3190 | 8.7571 | 7.5844 | 9.1992 | 9.4339 | 8.4412 |
| 7.9617 | 10.7749 | 7.4018 | 7.7021 | 7.6783 | 7.2934 | 6.4738 | 8.7324 |
| 7.4199 | 7.1617 | 9.1626 | 6.6932 | 9.0027 | 6.9038 | 5.9164 | 9.6489 |
| 10.1627 | 9.6039 | 7.7939 | 11.6667 | 8.2972 | 9.8975 | 8.8662 | 8.8103 |
| 7.3374 | 7.9239 | 6.5245 | 7.5121 | 8.3898 | 8.2544 | 11.4324 | 11.3189 |
| 12.9769 | 10.5450 | 8.5517 | 6.4942 | 7.3273 | 8.8331 | 7.1428 | 9.0797 |
| 10.2085 | 9.3376\ | | | | | | |
| CHST5 | 4.3001 | 3.8468 | 4.1880 | 5.5854 | 4.6155 | 3.2993 | 5.4474 |
| 4.6837 | 4.5807 | 5.4292 | 3.7884 | 4.3997 | 5.6003 | 4.3743 | 5.6432 |
| 4.9991 | 4.4253 | 4.4918 | 4.5347 | 1.7564 | 4.1360 | 6.1242 | 6.2978 |
| 4.6360 | 3.6719 | 3.9727 | 3.0161 | 7.1023 | 5.1569 | 2.4760 | 2.1107 |
| 0.5705 | 4.4140 | 3.8532 | 1.3224 | 4.6986 | 5.9578 | 3.4389 | 3.2719 |
| 1.7885 | 3.6678 | 2.5498 | 3.3483 | 2.3773 | 2.0460 | 5.2859 | 7.0036 |
| 3.0316 | 2.0766 | 3.2055 | 3.4657 | 3.3617 | 4.0873 | 3.9544 | 5.6261 |
| 4.8597 | 3.4529 | 0.8885 | 2.5386 | 3.3612 | 2.2227 | 3.5483 | 3.8779 |
| 3.5576 | 4.9133 | 4.5633 | 4.5926 | 4.5002 | 3.9884 | 4.2578 | 2.0543 |
| 3.0376 | 2.7197 | 5.0246 | 3.3935 | 5.0901 | 5.9513 | 6.3057 | 6.7346 |
| 3.7432 | 5.1596 | 3.7160 | 7.2309 | 4.4881 | 6.6779 | 4.2686 | 3.0440 |
| 5.7864 | 3.5948 | 3.4763 | 4.0023 | 2.7284 | 3.2938 | 5.6497 | 4.0933 |
| 3.0225 | 1.6738 | 4.3256 | 4.4711 | 4.2933 | 5.0097 | 4.6440 | 2.7793 |
| 5.8809 | 5.8061 | 2.5818 | 5.7849 | 4.4097 | 7.8223 | 5.3859 | 4.9383 |
| 4.9156 | 6.8355 | 4.2479 | 4.6443 | 3.8306 | 5.2963 | 4.0358 | 2.9465 |
| 2.8753 | 3.5573 | 2.9232 | 3.0979 | 5.0080 | 6.2037 | 2.5747 | 3.8924 |
| 5.6047 | 3.0274 | 3.9411 | 3.4442 | 4.2233 | 6.8021 | 7.8279 | 3.4335 |
| 5.5151 | 5.8740 | 5.6322 | 4.7095 | 4.5172 | 6.7988 | 2.5618 | 7.1207 |
| 2.3565 | 2.6364 | 3.7885 | 4.7046 | 4.8757 | 4.9700 | 4.7929 | 2.9433 |
| 5.9636 | 3.8917 | 2.7115 | 4.2186 | 3.2000 | 4.5652 | 4.0914 | 7.5588 |
| 4.5196 | 3.8207 | 3.3985 | 5.3219 | 6.5111 | 2.3081 | 3.4151 | 4.6023 |
| 2.3830 | 5.2225 | 3.9763 | 4.1274 | 4.1978 | 3.1267 | 4.5372 | 4.9180 |
| 4.7677 | 5.2567 | 3.9841 | 4.5616 | 8.4166 | 4.0906 | 4.1361 | 2.6576 |

```
4.3475  3.3795  4.9794  2.6918  4.3168  5.9586  4.8236  3.0366
4.0068  4.0384  3.0548  3.2749  7.7079  4.8685  4.5124  4.7210
6.1011  4.5434\
CHST4   1.7370  1.2250  4.9267  4.9049  6.0427  4.6464  2.5916
4.6754  6.3416  2.6705  4.9445  3.6538  4.8703  4.9667  6.2816
2.1513  3.1837  0.7772  1.3199  2.0944  6.1815  5.8709  7.2877
4.7532  3.6124  0.0000  6.1776  7.2798  4.8692  1.3312  0.7334
1.5370  0.5159  7.2603  6.2297  0.0000  4.0027  4.6698  0.0000
0.0000  4.7102  1.1407  2.0846  3.8009  0.0000  6.1951  0.0000
6.6420  1.2792  7.3930  4.7453  0.0000  3.1634  0.8853  2.4746
4.9729  6.7785  3.3475  4.0971  7.2861  1.7140  6.9726  3.9649
3.7330  3.8994  7.6394  1.8352  5.8046  3.3013  4.3345  5.1431
3.4990  0.0000  1.3638  6.7280  1.5483  2.6228  4.8899  5.1419
0.6159  3.5320  4.1510  6.4236  1.7538  1.4651  3.7954  0.9284
6.3973  3.4203  4.1122  5.0503  5.1757  1.4549  2.4059  3.5125
2.2653  3.9061  0.5454  3.9031  3.6567  6.0246  7.7078  5.4334
1.2208  0.4835  6.2996  3.0465  5.3358  3.7365  2.4661  2.2774
6.9827  4.1934  2.5850  5.4532  3.6573  5.9109  2.2752  6.1186
1.3647  2.6311  3.3319  4.2734  9.5239  7.1700  4.0178  4.2124
6.0123  2.2660  2.6959  3.3796  0.0000  6.2681  5.1217  3.3291
0.0000  4.8290  0.0000  5.1982  1.4215  2.7409  2.9732  2.1437
5.1573  4.3195  1.2434  7.5207  4.3430  3.8479  6.0399  5.7730
0.0000  4.7767  2.8106  5.0781  3.9564  2.4071  4.7890  5.8383
0.5416  0.0000  1.0431  3.4515  5.8918  0.7796  5.7068  5.4089
5.3826  7.2975  5.0748  5.0163  4.4472  0.9335  1.8874  0.0000
2.7064  2.6427  5.6021  6.0810  4.4865  3.9568  3.5147  5.4181
3.0239  7.3827  3.4980  4.0766  5.1060  5.9831  0.5707  2.4959
5.6397  3.3443  0.4262  5.8772  4.1011  1.0320  1.6369  9.2865
5.1009  6.3283\
CHST7   7.8581  7.4972  6.6122  5.7336  6.2156  6.3386  5.1220
5.9675  4.8039  8.0732  5.7825  5.4379  6.3237  9.7167  5.4566
5.8530  5.7063  5.7113  7.5816  4.8991  5.7374  5.8007  5.8137
5.2635  7.3016  8.0958  7.2719  7.2313  9.7561  7.0209  6.7811
6.4829  7.2347  4.3281  4.5555  6.1759  7.2591  4.4018  7.6943
3.8754  5.3918  7.0436  6.8454  6.5111  8.4219  5.5568  5.7055
5.7066  5.4414  5.3405  5.4766  6.5613  9.1564  7.0842  7.7724
6.5636  6.3508  6.4352  6.2033  4.6050  6.9293  5.5049  5.4407
8.5088  8.7447  7.4264  6.9088  7.7825  5.7055  5.4985  6.5141
8.2957  8.1168  6.3856  6.5795  6.8735  9.5364  6.5788  6.6822
6.5817  6.6292  5.8003  6.9920  5.7170  6.8177  6.7015  5.2490
5.5914  5.2832  8.1728  7.6108  5.0595  7.0731  6.6860  6.8193
7.9023  5.8209  8.3170  7.0414  6.7148  6.7021  6.2815  6.3782
5.5721  5.9145  5.8999  9.7819  5.2851  6.5915  6.0192  4.0721
5.4621  6.8939  6.3399  7.6257  9.1975  7.5324  5.6098  6.3267
5.9274  6.2257  8.1859  4.8333  6.5278  6.1952  5.3812  6.2467
5.6676  6.9360  6.9697  7.3443  4.4734  7.0561  5.6034  7.2747
8.1716  7.3415  6.4733  6.6544  7.6373  6.0469  5.3794  7.1644
7.6667  7.4849  10.0024 6.3767  7.8296  8.4260  8.3203  6.9466
5.5487  6.0862  7.0262  6.1250  5.5120  8.5852  6.9049  6.6589
7.4590  4.9916  8.1533  6.5612  6.5529  7.9112  4.3917  4.2627
6.5979  5.2680  4.0977  5.8734  5.5966  6.2917  5.7381  5.5527
7.0960  7.7841  7.5164  4.3160  7.1000  7.1020  5.1186  5.4181
4.8000  6.0651  5.2832  5.6892  6.1884  5.4671  7.9398  7.1701
8.1174  6.4393  5.8592  5.3294  4.8720  7.5788  7.2366  6.5834
```

4.9904  6.4962\
CHST6   9.9814  6.9856  9.3685  9.7030  7.1841  4.4022  5.8152
7.9727  9.6531  8.2981  7.4216  6.5742  9.5065  6.9732  9.3595
7.6609  6.2466  8.0963  6.9566  7.3647  8.4593  11.2167 8.9086
8.9661  4.6630  9.5812  7.3981  10.9783 7.9945  6.9341  7.0855
6.8470  10.4089 7.1828  8.6448  5.7433  10.1655 8.7480  8.3279
8.3614  6.8254  7.7132  9.0540  9.5324  1.0260  9.7254  9.6348
7.5363  8.9126  5.9642  10.1228 6.7259  6.5582  3.2435  7.7824
8.3422  7.2715  4.5565  5.2227  7.5181  6.0494  4.1585  10.2350
8.6107  8.0527  10.5532 9.9091  7.8611  8.8085  6.5980  7.3569
9.6158  6.8772  5.2767  6.6394  8.5350  7.4121  12.0454 10.8350
9.3749  9.1480  6.2232  10.4187 7.8151  9.8241  5.3287  4.5030
9.0131  5.4956  7.5660  6.3556  4.8755  6.2703  7.4243  9.3549
2.4199  7.4514  4.6897  7.8553  6.3625  9.2467  11.4227 8.3394
7.0668  8.1131  7.2549  7.0413  8.5346  10.1923 6.6447  8.9168
11.0782 7.6145  5.3219  9.6936  5.9600  9.9989  6.0123  9.3982
8.9938  7.0072  9.0375  6.7449  12.4489 7.6613  8.2610  8.5756
6.1653  6.3057  5.2985  7.8372  9.8570  11.3523 10.5500 4.6515
8.2201  10.6174 7.7420  8.9723  4.8217  9.1403  8.8612  9.5784
6.1086  6.5824  0.5418  10.6261 4.6374  10.4599 10.5648 7.8395
9.2322  8.6983  5.6185  6.8915  6.9241  8.3433  8.2749  7.4064
8.6638  6.7834  5.0823  9.0627  10.6535 7.5466  8.2250  6.7740
8.0989  9.5699  7.9669  7.7315  5.1694  8.7143  5.6164  10.7841
8.6836  6.2347  3.5773  9.6047  10.9251 8.8552  5.9336  8.0769
8.7322  9.1513  6.9734  6.7960  7.1722  10.4560 7.3874  9.4303
11.3868 5.6344  6.4586  6.4472  6.2041  9.2307  5.8572  10.9995
8.8491  11.0683\
ITGAX   7.3565  8.8321  7.2687  8.3829  9.2328  7.7446  6.9268
6.2146  5.5916  7.7088  8.4130  6.4279  8.1597  4.6358  9.2168
8.1635  6.3834  7.7869  10.9924 7.0841  9.7256  7.6899  7.6979
8.9223  5.0158  8.2719  5.2687  9.4329  7.6218  6.2344  4.6917
8.0622  9.0806  8.4076  5.0232  7.5384  6.3538  5.8499  4.1930
5.7814  8.5580  8.1341  7.2194  8.2635  4.9076  9.8106  7.6756
7.4056  5.8864  5.4479  8.7531  9.7774  8.4014  6.8866  7.3198
6.6818  7.6182  9.6611  8.0975  5.6882  6.4615  8.3425  7.1007
6.3835  7.2568  9.7531  9.0527  6.7708  5.7589  7.1833  9.2501
6.1489  9.5171  9.3739  9.0923  4.5675  9.2851  7.6019  8.0501
9.3887  5.5727  5.7160  8.7517  7.7045  7.8830  7.6870  4.3517
8.8234  9.2934  7.2937  6.9861  8.1944  8.1363  9.5180  7.2353
6.0144  9.2997  6.3222  7.0644  9.2645  8.5007  9.4149  5.5486
8.9581  6.9072  4.2124  10.9490 7.4068  6.1928  7.9652  7.1594
7.9075  6.0327  6.3923  8.6561  7.4445  5.4245  7.9411  7.3234
9.4237  6.0830  7.6763  6.4831  7.2180  6.5746  8.7944  7.2423
5.3218  6.0498  6.7613  7.0624  6.3213  9.1367  8.9716  8.5481
8.8774  9.6483  8.8125  8.0110  6.4505  7.8580  7.4930  8.6016
7.1029  7.0187  4.4870  8.4298  6.9977  6.6187  5.1593  7.6522
8.5138  8.2384  6.4714  6.0160  8.4553  6.2221  6.1020  6.9751
9.0422  6.4108  9.1985  7.5305  8.4351  7.0460  7.0780  7.6983
9.0869  7.2269  8.1697  5.0000  5.6861  6.4276  4.1488  6.0231
4.3743  8.5581  5.7849  5.9673  9.6399  7.5830  7.8290  6.6038
8.5568  7.0325  8.8252  8.6803  8.1577  9.9474  9.1601  8.8769
6.8177  7.7513  7.6949  7.8144  8.1282  6.4721  6.0302  8.4590
8.7980  7.5610\
OR8J1   0.0000  0.0000  0.0000  0.0000  0.0000  0.0000  0.0000

0.0000  0.0000  0.0000  0.0000  0.0000  0.0000  0.0000  0.0000
0.0000  0.0000  0.0000  0.0000  0.0000  0.0000  0.0000  0.0000
0.0000  0.0000  0.0000  0.0000  0.0000  0.0000  0.0000  0.0000
0.0000  0.0000  0.0000  0.0000  0.0000  0.0000  0.0000  0.0000
0.0000  0.0000  0.0000  0.0000  0.0000  0.0000  0.0000  0.0000
0.0000  0.0000  0.0000  0.0000  0.0000  0.0000  0.0000  0.0000
0.0000  0.0000  0.0000  0.0000  0.0000  0.0000  0.0000  0.0000
0.0000  0.0000  0.0000  0.0000  0.0000  0.0000  0.0000  0.0000
0.0000  0.0000  0.0000  0.0000  0.0000  0.0000  0.0000  0.0000
0.0000  0.0000  0.3965  0.0000  0.0000  0.0000  0.0000  0.0000
0.0000  0.0000  0.0000  0.0000  0.0000  0.0000  0.0000  0.0000
0.0000  0.0000  0.0000  0.0000  0.0000  0.0000  0.0000  0.0000
0.0000  0.0000  0.0000  0.0000  0.0000  0.0000  0.0000  0.0000
0.0000  0.0000  0.0000  0.0000  0.0000  0.0000  0.0000  0.0000
0.0000  0.0000  0.0000  0.0000  0.0000  0.0000  0.0000  0.0000
0.0000  0.0000  0.0000  0.0000  0.0000  0.0000  0.0000  0.0000
0.0000  0.0000  0.0000  0.0000  0.0000  0.0000  0.0000  0.0000
0.0000  0.0000  0.0000  0.0000  0.0000  0.0000  0.0000  0.0000
0.0000  0.0000  0.0000  0.0000  0.0000  0.0000  0.0000  0.0000
0.0000  0.0000  0.0000  0.0000  0.0000  0.0000  0.0000  0.0000
0.0000  0.0000  0.0000  0.0000  0.0000  0.0000  0.0000  0.0000
0.0000  0.0000  0.0000  0.0000  0.0000  0.0000  0.0000  0.0000
0.0000  0.0000  0.0000  0.0000  0.0000  0.0000  0.0000  0.0000
0.0000  0.0000  0.0000  0.0000  0.0000  0.0000  0.0000  0.0000
0.0000  0.0000\
SNORD115-17     0.0000  0.0000  0.0000  0.0000  0.0000  0.0000
0.0000  0.0000  0.0000  0.0000  0.0000  0.0000  0.0000  0.0000
0.0000  0.0000  0.0000  0.0000  0.0000  0.0000  0.0000  0.0000
0.0000  0.0000  0.0000  0.0000  0.0000  0.0000  0.0000  0.0000
0.0000  0.0000  0.0000  0.0000  0.0000  0.0000  0.0000  0.0000
0.0000  0.0000  0.0000  0.0000  0.0000  0.0000  0.0000  0.0000
0.0000  0.0000  0.0000  0.0000  0.0000  0.0000  0.0000  0.0000
0.0000  0.0000  0.0000  0.0000  0.0000  0.0000  0.0000  0.0000
0.0000  0.0000  0.0000  0.0000  0.0000  0.0000  0.0000  0.0000
0.0000  0.0000  0.0000  0.0000  0.0000  0.0000  0.0000  0.0000
0.0000  0.0000  0.0000  0.0000  0.0000  0.0000  0.0000  0.0000
0.0000  0.0000  0.0000  0.0000  0.0000  0.0000  0.0000  0.0000
0.0000  0.0000  0.0000  0.0000  0.0000  0.0000  0.0000  0.0000
0.0000  0.0000  0.0000  0.0000  0.0000  0.0000  0.0000  0.0000
0.0000  0.0000  0.0000  0.0000  0.0000  0.0000  0.0000  0.0000
0.0000  0.0000  0.0000  0.0000  0.0000  0.0000  0.0000  0.0000
0.0000  0.0000  0.0000  0.0000  0.0000  0.0000  0.0000  0.0000
0.0000  0.0000  0.0000  0.0000  0.0000  0.0000  0.0000  0.0000
0.0000  0.0000  0.0000  0.0000  0.0000  0.0000  0.0000  0.0000
0.0000  0.0000  0.0000  0.0000  0.0000  0.0000  0.0000  0.0000
0.0000  0.0000  0.0000  0.0000  0.0000  0.0000  0.0000  0.0000
0.0000  0.0000  0.0000  0.0000  0.0000  0.0000  0.0000  0.0000
0.0000  0.0000  0.0000  0.0000  0.0000  0.0000  0.0000  0.0000
0.0000  0.0000  0.0000  0.0000  0.0000  0.0000  0.0000  0.0000
0.0000  0.0000  0.0000  0.0000  0.0000  0.0000  0.0000  0.0000
0.0000  0.0000  0.0000\
BCL2A1  6.2327  6.3203  4.6732  6.2017  7.3766  5.5388  3.7378
3.7094  4.8219  3.3138  5.9535  4.3346  4.6847  1.6493  8.5139
5.0748  2.7928  5.6915  6.1766  6.1349  7.0430  5.2133  4.9204

| | | | | | | | |
|---|---|---|---|---|---|---|---|
| 8.0622 | 2.7258 | 7.6360 | 4.2920 | 8.1449 | 8.1294 | 5.0810 | 3.6161 |
| 5.6838 | 7.7575 | 6.4909 | 3.1707 | 5.8530 | 3.1675 | 3.6447 | 3.4608 |
| 3.6521 | 6.7092 | 6.9483 | 5.8101 | 6.7917 | 3.2157 | 6.7127 | 6.6089 |
| 5.5318 | 5.8422 | 3.3772 | 8.0159 | 8.4326 | 7.1438 | 6.1560 | 5.1677 |
| 5.6281 | 5.4797 | 6.2179 | 5.6837 | 2.5512 | 5.4704 | 6.9488 | 5.1464 |
| 3.7875 | 4.1163 | 8.5992 | 7.0441 | 4.5407 | 5.7589 | 4.7315 | 7.4838 |
| 4.8000 | 9.8155 | 7.1034 | 6.7821 | 1.1076 | 5.2140 | 4.6497 | 5.2619 |
| 6.8714 | 4.6992 | 3.8681 | 5.3855 | 6.6616 | 5.7383 | 6.5526 | 3.4508 |
| 6.9977 | 6.1256 | 6.6515 | 5.6997 | 6.8128 | 6.2784 | 6.9878 | 5.5408 |
| 5.0002 | 5.9793 | 4.1697 | 4.4694 | 7.4792 | 6.9170 | 7.2252 | 3.4436 |
| 5.5102 | 1.9209 | 2.0179 | 4.5513 | 5.4204 | 3.5239 | 5.8024 | 4.5903 |
| 6.4095 | 3.9213 | 4.5850 | 6.6173 | 5.7777 | 5.2003 | 4.3613 | 6.6834 |
| 7.7948 | 3.5559 | 6.8564 | 5.5664 | 6.9434 | 3.5563 | 7.6074 | 5.9445 |
| 3.7308 | 4.4874 | 2.8723 | 4.6523 | 3.4618 | 6.3611 | 5.2838 | 6.9708 |
| 8.3830 | 8.4909 | 6.7235 | 6.3805 | 4.5124 | 4.9530 | 6.4695 | 7.3622 |
| 5.7776 | 5.6677 | 2.8840 | 7.0879 | 4.0942 | 5.0119 | 4.1991 | 5.1514 |
| 5.6076 | 7.0901 | 4.5886 | 4.3821 | 5.5561 | 4.2610 | 4.8819 | 6.2438 |
| 7.4701 | 4.6751 | 9.0505 | 6.5227 | 6.4124 | 4.9916 | 4.9919 | 4.6449 |
| 6.5294 | 5.7545 | 5.5705 | 3.8574 | 4.6644 | 3.5191 | 3.0035 | 7.9425 |
| 2.6164 | 2.9177 | 4.8248 | 4.3840 | 6.2730 | 6.0373 | 4.6037 | 4.8645 |
| 5.3528 | 6.5485 | 6.4813 | 7.7699 | 6.6538 | 9.0706 | 6.4084 | 7.1616 |
| 7.3618 | 5.6758 | 5.5562 | 5.1228 | 5.9916 | 3.4498 | 3.1558 | 6.4820 |
| 5.8978 | 7.1782\ | | | | | | |
| GPSM2 | 9.3050 | 8.4326 | 9.5244 | 8.0379 | 8.8490 | 9.6099 | 8.9392 |
| 9.5396 | 9.5854 | 8.9836 | 9.8078 | 9.6802 | 8.8684 | 11.4097 | 8.5598 |
| 8.5454 | 8.3572 | 8.7443 | 8.3994 | 9.1175 | 9.3723 | 8.0098 | 6.1306 |
| 8.9733 | 9.1922 | 7.8603 | 10.9294 | 7.9875 | 6.9817 | 8.7486 | 9.2231 |
| 9.6295 | 9.5057 | 8.4982 | 10.2207 | 7.6520 | 7.9184 | 9.9275 | 9.5929 |
| 9.9016 | 9.4059 | 7.2962 | 8.7361 | 8.9675 | 8.6478 | 8.6114 | 6.6368 |
| 8.4703 | 6.6701 | 8.7172 | 9.1918 | 8.2177 | 7.7949 | 7.8800 | 10.4042 |
| 9.1743 | 9.2963 | 8.5998 | 8.9206 | 9.9837 | 8.4295 | 10.7900 | 9.5256 |
| 8.9945 | 8.7387 | 8.5148 | 7.5370 | 9.9646 | 10.7988 | 9.7037 | 9.0670 |
| 8.3386 | 6.7420 | 8.9291 | 9.0506 | 8.4903 | 8.1124 | 7.0634 | 6.4479 |
| 9.6058 | 10.2593 | 9.9896 | 6.3412 | 10.2870 | 7.8191 | 8.4844 | 8.5381 |
| 8.1586 | 8.5351 | 9.6767 | 8.1307 | 9.3185 | 8.0391 | 7.5136 | 9.3410 |
| 8.5990 | 7.9949 | 7.6654 | 8.3536 | 8.8262 | 8.0930 | 9.2371 | 10.1587 |
| 9.3075 | 9.5285 | 10.1386 | 7.3561 | 8.5245 | 10.1104 | 9.0533 | 9.0377 |
| 8.3672 | 6.1520 | 7.9840 | 9.3003 | 8.4877 | 8.9876 | 9.1562 | 8.7165 |
| 8.5518 | 9.1732 | 8.2958 | 9.0597 | 9.1732 | 6.7610 | 8.8674 | 9.5039 |
| 6.6588 | 7.4555 | 9.0987 | 10.4464 | 9.6250 | 8.3445 | 9.7428 | 8.9641 |
| 5.4525 | 7.7104 | 7.5689 | 8.1699 | 7.7401 | 9.8037 | 8.4806 | 7.5588 |
| 9.4296 | 9.3519 | 4.6227 | 8.6631 | 7.4750 | 8.1063 | 7.6373 | 8.8283 |
| 6.6912 | 7.8950 | 8.9722 | 9.3539 | 9.3124 | 7.5976 | 9.4979 | 8.5169 |
| 8.6332 | 8.4072 | 8.5280 | 7.9435 | 7.9796 | 8.6297 | 9.4061 | 7.9826 |
| 9.0487 | 9.0580 | 9.5765 | 9.1617 | 10.1158 | 9.6244 | 8.1508 | 6.2726 |
| 9.3628 | 8.4728 | 8.9006 | 10.0125 | 7.1410 | 9.0046 | 7.7408 | 10.2361 |
| 8.0289 | 9.3605 | 8.1445 | 8.6634 | 8.6711 | 8.1667 | 9.4383 | 9.5968 |
| 7.1707 | 8.9525 | 8.4740 | 9.0110 | 8.8837 | 7.7966 | 5.8757 | 8.7818 |
| 8.3177 | 7.1333\ | | | | | | |
| HLCS | 8.8001 | 7.8576 | 9.9098 | 10.0601 | 9.2104 | 9.4226 | 8.7256 |
| 9.4166 | 8.8007 | 8.9916 | 8.5066 | 9.0989 | 9.1580 | 9.2526 | 9.3546 |
| 9.5335 | 9.1307 | 9.7655 | 8.0999 | 8.4996 | 9.0436 | 9.2748 | 8.8354 |
| 9.3814 | 8.2267 | 9.2161 | 9.6690 | 9.7290 | 9.0008 | 9.2297 | 10.4318 |
| 10.1119 | 8.6028 | 9.1180 | 9.1907 | 8.6278 | 8.9747 | 9.2770 | 10.4124 |

| | | | | | | | |
|---|---|---|---|---|---|---|---|
| 9.9879 | 11.5371 | 8.4093 | 8.3875 | 9.1141 | 8.1440 | 8.9358 | 11.5901 |
| 8.7714 | 9.1239 | 9.8992 | 9.9555 | 8.7132 | 8.9922 | 8.0629 | 8.5902 |
| 9.0608 | 9.0796 | 8.8597 | 9.3018 | 9.0927 | 8.0872 | 8.4701 | 9.4487 |
| 9.5420 | 8.6908 | 8.9111 | 8.6570 | 9.5795 | 7.9800 | 9.2722 | 9.2037 |
| 9.6660 | 8.1595 | 9.1976 | 9.3056 | 10.4056 | 9.3498 | 10.1562 | 9.1348 |
| 9.3147 | 8.7171 | 9.0710 | 8.3748 | 11.5989 | 9.0680 | 9.0753 | 10.0330 |
| 9.1714 | 8.7783 | 9.5253 | 7.9930 | 9.1611 | 9.2661 | 8.7182 | 9.1107 |
| 8.7193 | 9.1049 | 8.0877 | 10.9673 | 9.0898 | 9.4069 | 9.0717 | 10.1265 |
| 10.0236 | 11.5881 | 9.9955 | 7.7695 | 9.1623 | 8.5187 | 8.4859 | 9.5859 |
| 9.3748 | 9.2477 | 8.8517 | 9.1590 | 9.5017 | 9.1829 | 9.8139 | 9.4501 |
| 8.1763 | 9.2682 | 8.7709 | 9.4221 | 9.2865 | 9.0258 | 8.9433 | 8.0877 |
| 8.8441 | 8.3223 | 9.0726 | 8.9701 | 9.8725 | 10.4427 | 9.7098 | 8.0331 |
| 8.9500 | 8.5652 | 7.8512 | 9.2717 | 8.2480 | 9.1482 | 9.6497 | 9.0179 |
| 9.1906 | 9.4454 | 8.0790 | 10.2270 | 8.5699 | 9.6186 | 9.3180 | 9.4929 |
| 8.2063 | 9.7248 | 9.2506 | 9.5968 | 9.8144 | 8.3644 | 9.0851 | 9.0219 |
| 9.3678 | 7.7802 | 9.2546 | 9.3707 | 9.0861 | 8.8656 | 9.4144 | 8.9895 |
| 9.4889 | 9.6624 | 8.2152 | 9.2125 | 9.7017 | 9.1132 | 8.8266 | 8.9558 |
| 9.5004 | 8.6457 | 7.8359 | 9.5259 | 8.8232 | 9.1571 | 8.7435 | 10.3903 |
| 9.3127 | 8.3581 | 9.2129 | 9.9089 | 9.1647 | 8.7574 | 9.2433 | 9.3645 |
| 9.1746 | 10.0960 | 9.0117 | 9.3254 | 8.7526 | 9.4854 | 8.2419 | 9.8885 |
| 9.7477 | 9.1566\ | | | | | | |
| NOG | 6.2327 | 4.0535 | 6.5284 | 6.8275 | 2.6553 | 2.5543 | 3.6354 |
| 5.8060 | 4.3637 | 7.6890 | 0.7594 | 4.8363 | 4.4249 | 6.5538 | 5.4114 |
| 8.5288 | 6.5831 | 7.3170 | 3.8042 | 3.8459 | 3.4081 | 6.4704 | 3.2986 |
| 4.0743 | 3.9548 | 7.1812 | 3.3107 | 7.2213 | 7.7942 | 5.6298 | 8.9881 |
| 6.7226 | 1.4434 | 7.4295 | 4.0008 | 4.5826 | 6.9329 | 6.6599 | 8.9344 |
| 4.0071 | 3.0046 | 4.0930 | 4.5330 | 5.2236 | 3.2157 | 5.3163 | 3.3157 |
| 7.9724 | 4.6990 | 3.7571 | 1.5081 | 1.9460 | 9.0842 | 2.3883 | 8.1399 |
| 7.9980 | 3.7168 | 1.1710 | 4.1257 | 5.5602 | 3.4032 | 2.2329 | 3.4464 |
| 4.3911 | 8.0295 | 6.0373 | 4.6811 | 4.8895 | 4.3002 | 2.3953 | 1.2639 |
| 6.9291 | 0.9449 | 3.0145 | 7.3548 | 5.3036 | 8.6926 | 5.7111 | 5.4556 |
| 4.5130 | 6.8068 | 7.3623 | 5.1426 | 4.7913 | 6.3497 | 4.5575 | 6.1496 |
| 6.1610 | 1.8667 | 4.1485 | 3.3811 | 3.0336 | 1.4549 | 7.4947 | 2.5291 |
| 1.0621 | 2.9993 | 4.8818 | 3.3656 | 5.0054 | 4.5420 | 4.1969 | 4.0633 |
| 2.5001 | 2.1962 | 3.2307 | 5.6958 | 4.5852 | 4.9393 | 5.3117 | 10.4805 |
| 1.0545 | 6.3150 | 2.0000 | 3.5487 | 5.7354 | 6.5840 | 5.6098 | 5.1262 |
| 0.0000 | 7.7711 | 4.4368 | 5.4105 | 2.8403 | 2.9380 | 3.8490 | 3.6659 |
| 4.2520 | 3.3289 | 5.0459 | 3.3355 | 3.2094 | 7.4785 | 4.1345 | 5.5313 |
| 3.7958 | 4.1530 | 3.0835 | 3.4616 | 4.7782 | 3.1296 | 6.1296 | 5.2276 |
| 6.5632 | 5.5694 | 1.4974 | 5.5302 | 3.7446 | 4.4160 | 8.3412 | 5.3111 |
| 1.5604 | 5.5821 | 5.2132 | 7.7025 | 5.5989 | 5.2773 | 7.1610 | 4.0419 |
| 4.1584 | 2.6375 | 0.6138 | 7.1461 | 5.5583 | 5.5504 | 3.9223 | 0.0000 |
| 2.1785 | 3.2973 | 2.4870 | 6.4319 | 4.5325 | 4.3936 | 5.5347 | 5.3705 |
| 5.6425 | 7.5785 | 2.6934 | 4.4277 | 4.6844 | 3.4385 | 5.3747 | 2.1752 |
| 4.7332 | 4.7540 | 0.6248 | 4.5740 | 4.1728 | 6.2023 | 6.5421 | 8.2110 |
| 5.3879 | 7.0817 | 3.3616 | 4.8815 | 0.0000 | 4.7048 | 4.5657 | 3.5042 |
| 6.4849 | 3.1794\ | | | | | | |
| LIPG | 10.0516 | 6.9856 | 11.6693 | 6.1109 | 6.0330 | 3.8725 | 7.9769 |
| 10.4819 | 7.5796 | 5.9666 | 6.4530 | 8.5335 | 6.2385 | 9.0668 | 7.4012 |
| 8.9002 | 7.0850 | 5.9795 | 6.4724 | 8.6871 | 5.6633 | 7.9527 | 4.8110 |
| 8.6635 | 4.1192 | 4.8934 | 7.8503 | 6.7066 | 8.6790 | 9.0989 | 5.4505 |
| 7.0522 | 8.9157 | 7.3775 | 5.4438 | 9.3681 | 9.3331 | 8.1734 | 9.7128 |
| 7.5937 | 6.9468 | 8.2452 | 10.4526 | 8.9717 | 2.8515 | 6.5131 | 8.1681 |
| 9.6888 | 6.0965 | 7.0452 | 7.5632 | 11.0807 | 8.4255 | 2.7018 | 6.6889 |

| | | | | | | | |
|---|---|---|---|---|---|---|---|
| 8.4203 | 9.7968 | 5.3595 | 8.0367 | 8.8947 | 4.3656 | 8.6639 | 9.7275 |
| 9.8223 | 6.7648 | 8.4520 | 5.5771 | 9.4770 | 9.7033 | 5.9600 | 5.4278 |
| 9.4446 | 9.3035 | 4.3966 | 8.2472 | 5.3856 | 7.7156 | 5.9945 | 5.8420 |
| 6.6972 | 9.8002 | 10.0654 | 3.8025 | 5.5984 | 7.0221 | 7.1571 | 9.0761 |
| 6.0728 | 8.6352 | 8.7014 | 4.3499 | 9.7616 | 9.3231 | 5.9208 | 7.7445 |
| 4.0206 | 8.6957 | 3.8858 | 7.2878 | 10.8494 | 6.4217 | 10.3200 | 10.2777 |
| 8.4079 | 6.5811 | 10.5279 | 7.1857 | 9.9083 | 8.9621 | 4.8152 | 9.4576 |
| 7.9583 | 7.4812 | 7.9944 | 11.4999 | 9.2843 | 5.6819 | 6.7293 | 8.0331 |
| 8.6591 | 10.1408 | 7.7692 | 9.7477 | 9.3338 | 4.3194 | 10.5246 | 8.3893 |
| 5.9797 | 4.7652 | 6.5374 | 10.0891 | 5.4009 | 6.3280 | 8.0702 | 9.3797 |
| 8.1979 | 5.5340 | 3.9958 | 7.1241 | 2.5936 | 7.3188 | 8.1934 | 6.1045 |
| 10.1865 | 10.2292 | 1.9011 | 9.7621 | 4.3753 | 8.3730 | 10.5787 | 6.9707 |
| 7.0401 | 8.4066 | 6.3875 | 7.3410 | 10.4245 | 4.7710 | 9.1619 | 5.4866 |
| 4.8011 | 4.6751 | 9.9482 | 6.2202 | 7.2133 | 7.7086 | 6.9862 | 1.6706 |
| 8.1056 | 11.6818 | 9.2709 | 8.7900 | 7.8229 | 7.3034 | 8.6556 | 4.0279 |
| 7.1770 | 5.5912 | 2.5659 | 7.6120 | 2.5710 | 6.8022 | 6.7350 | 6.9551 |
| 4.4583 | 9.2061 | 8.0846 | 7.7862 | 6.4811 | 4.8121 | 8.0067 | 8.3611 |
| 7.9765 | 5.9477 | 8.6188 | 6.1019 | 5.5060 | 6.3480 | 2.5229 | 10.7095 |
| 7.5469 | 10.5925\ | | | | | | |
| ITGAV | 9.8068 | 11.5079 | 13.0778 | 10.7858 | 10.9449 | 12.1090 | 11.1066 |
| 9.7310 | 11.3790 | 10.6418 | 11.7003 | 9.9963 | 11.0356 | 11.2089 | 10.8125 |
| 9.5535 | 9.7130 | 10.6673 | 11.9229 | 12.4106 | 10.4693 | 10.6077 | 10.9456 |
| 10.8772 | 11.3352 | 10.6920 | 9.8733 | 9.9702 | 10.4288 | 10.7210 | 10.9351 |
| 10.3669 | 11.6191 | 10.4358 | 10.3991 | 9.3668 | 11.9170 | 10.9578 | 11.0508 |
| 11.3710 | 11.2272 | 12.1794 | 11.7303 | 10.8981 | 12.7040 | 9.0360 | 8.7663 |
| 11.1588 | 12.2905 | 10.1725 | 11.2164 | 10.3016 | 10.6098 | 12.0359 | 10.2322 |
| 10.4311 | 13.5047 | 11.3874 | 12.1738 | 10.1925 | 12.2625 | 12.1066 | 10.7541 |
| 11.1263 | 10.8099 | 11.1861 | 10.7200 | 10.9358 | 10.4143 | 11.1693 | 9.6890 |
| 11.1895 | 10.8799 | 11.1121 | 11.5836 | 10.0116 | 10.1283 | 10.7862 | 10.1041 |
| 11.4303 | 10.2568 | 12.0174 | 10.4139 | 10.3541 | 9.2322 | 11.1823 | 9.5947 |
| 10.0797 | 11.3484 | 10.5286 | 11.3602 | 11.6512 | 9.8117 | 9.5515 | 10.0140 |
| 12.1259 | 13.2438 | 11.2753 | 9.9114 | 10.9032 | 8.5637 | 10.2870 | 10.5654 |
| 9.6559 | 10.0519 | 9.6739 | 9.9033 | 11.6586 | 12.1619 | 10.1310 | 10.6843 |
| 9.8117 | 9.9071 | 8.9744 | 11.8250 | 11.9904 | 10.1212 | 10.6559 | 11.2464 |
| 11.3048 | 10.5982 | 11.5159 | 11.9764 | 10.0159 | 11.0439 | 11.8854 | 10.4400 |
| 9.0069 | 11.9986 | 11.6637 | 11.5824 | 9.6556 | 10.1240 | 10.9302 | 12.3616 |
| 10.1037 | 8.8169 | 8.7637 | 10.7667 | 11.3798 | 9.2497 | 10.5406 | 9.2902 |
| 11.4475 | 11.7194 | 12.0046 | 11.9389 | 11.0229 | 10.7108 | 9.5860 | 10.7698 |
| 8.3206 | 11.2751 | 10.1012 | 10.4681 | 12.5249 | 11.1192 | 10.6306 | 10.7683 |
| 10.7611 | 10.3586 | 10.2955 | 10.3267 | 10.3689 | 11.7203 | 11.5331 | 10.7470 |
| 11.4112 | 10.3141 | 9.6458 | 10.3012 | 10.7484 | 9.3070 | 10.1970 | 9.1522 |
| 10.5154 | 10.5518 | 11.1776 | 11.0482 | 10.4344 | 10.1570 | 10.0655 | 10.7022 |
| 10.5866 | 11.1320 | 10.6747 | 11.9636 | 9.7977 | 9.5515 | 11.1086 | 11.9557 |
| 11.8200 | 9.8628 | 13.2161 | 10.7921 | 8.8056 | 9.4695 | 11.2769 | 11.9551 |
| 11.1182 | 9.3918\ | | | | | | |
| HIBCH | 8.4631 | 8.7191 | 10.0589 | 10.2682 | 9.4488 | 9.2926 | 8.1951 |
| 9.3748 | 10.6349 | 9.1598 | 9.3759 | 9.0322 | 9.9462 | 10.0616 | 8.3558 |
| 8.8739 | 9.8583 | 10.0704 | 8.7910 | 9.9790 | 8.7712 | 10.4432 | 9.5680 |
| 9.1979 | 9.2763 | 9.7315 | 9.1230 | 9.6481 | 9.6984 | 9.4077 | 8.9018 |
| 8.3913 | 9.7978 | 9.5269 | 8.9810 | 10.3504 | 9.3591 | 10.1368 | 10.8850 |
| 8.9006 | 10.6321 | 9.1551 | 10.0252 | 9.4227 | 9.2849 | 8.2589 | 9.8167 |
| 8.9331 | 9.2331 | 8.7371 | 9.9208 | 8.3498 | 9.6977 | 9.1262 | 8.7219 |
| 9.4905 | 9.2658 | 9.2451 | 9.5424 | 9.8113 | 9.0773 | 8.6522 | 9.5273 |
| 10.4957 | 9.0858 | 9.6390 | 9.8850 | 9.1694 | 8.8750 | 10.1266 | 9.3565 |

| | | | | | | | |
|---|---|---|---|---|---|---|---|
| 9.7247 | 9.1755 | 9.2348 | 9.2286 | 10.6477 | 8.8952 | 9.6090 | 9.7955 |
| 9.3784 | 9.8906 | 9.4766 | 9.1407 | 10.1119 | 9.5125 | 9.8936 | 9.4447 |
| 9.7542 | 9.3828 | 8.5789 | 8.8534 | 8.8198 | 9.6585 | 9.1117 | 9.2844 |
| 9.6042 | 9.5716 | 8.7376 | 8.8561 | 9.1594 | 9.4033 | 9.8775 | 9.3421 |
| 9.0479 | 11.0059 | 10.0899 | 8.0997 | 9.6979 | 10.1016 | 9.0982 | 9.8639 |
| 9.8168 | 9.8169 | 8.9159 | 10.8787 | 9.8835 | 8.8911 | 9.5486 | 9.6179 |
| 8.5683 | 9.8682 | 10.6086 | 8.8607 | 9.7702 | 10.1199 | 9.6002 | 9.4564 |
| 9.5643 | 9.4806 | 9.5320 | 10.0778 | 10.6932 | 10.4485 | 9.8019 | 8.8080 |
| 9.0400 | 9.6311 | 7.8743 | 9.1410 | 8.5856 | 10.7887 | 9.6836 | 9.6477 |
| 8.6169 | 9.3225 | 8.5863 | 11.2984 | 9.4142 | 9.8511 | 10.3819 | 9.0233 |
| 10.6815 | 8.9817 | 9.2506 | 9.8114 | 9.6543 | 9.2924 | 9.5528 | 9.6822 |
| 10.1097 | 9.2264 | 9.4235 | 9.8454 | 8.8554 | 9.5505 | 9.2216 | 8.8599 |
| 9.7826 | 9.5973 | 8.8663 | 10.4085 | 10.1306 | 7.8445 | 9.6382 | 8.6507 |
| 9.3320 | 9.0895 | 9.3992 | 9.4781 | 9.4615 | 9.1841 | 9.3114 | 10.1658 |
| 10.6292 | 9.8430 | 10.0344 | 9.7910 | 8.0891 | 9.5713 | 9.4695 | 9.1423 |
| 10.7481 | 10.2896 | 9.6402 | 9.3557 | 9.4175 | 9.9619 | 9.1215 | 8.4639 |
| 9.8769 | 9.3251\ | | | | | | |
| ZNF703 | 6.2855 | 8.4200 | 8.8646 | 8.8742 | 9.5277 | 9.0328 | 6.7296 |
| 8.7705 | 7.3390 | 9.4645 | 7.5551 | 6.2038 | 8.8278 | 7.6444 | 8.3154 |
| 9.2776 | 6.8423 | 6.9348 | 8.6095 | 4.3452 | 6.7669 | 7.3474 | 8.3457 |
| 8.8852 | 9.0157 | 5.8560 | 7.0266 | 7.5253 | 7.5177 | 5.6590 | 8.7458 |
| 8.6247 | 5.1099 | 6.7836 | 10.2712 | 7.2656 | 6.6920 | 7.1377 | 4.4246 |
| 9.7560 | 6.7568 | 8.2509 | 6.5699 | 7.4407 | 6.2987 | 7.8140 | 3.9741 |
| 6.0685 | 6.0738 | 5.6525 | 5.3648 | 4.6985 | 7.0292 | 7.2856 | 9.5427 |
| 7.4145 | 6.0357 | 7.3440 | 7.0061 | 6.7147 | 6.7876 | 6.2526 | 9.8185 |
| 3.6763 | 9.4565 | 8.8848 | 8.7269 | 8.0932 | 9.8459 | 8.2507 | 5.9857 |
| 6.5841 | 4.4163 | 5.1546 | 6.4590 | 6.7653 | 9.6958 | 8.6964 | 8.3928 |
| 6.6898 | 9.5319 | 7.0301 | 6.9011 | 8.0102 | 8.0239 | 7.8900 | 6.7672 |
| 7.6203 | 8.4401 | 7.6663 | 9.4217 | 5.2533 | 4.4237 | 8.4278 | 7.9381 |
| 7.9121 | 9.3195 | 10.3193 | 6.7553 | 6.9819 | 6.1086 | 8.5807 | 6.1509 |
| 6.8757 | 5.5722 | 5.8243 | 8.4440 | 7.1794 | 7.1415 | 8.7258 | 5.9375 |
| 7.9862 | 6.2862 | 4.9542 | 7.1525 | 5.3055 | 7.9609 | 7.8480 | 9.3138 |
| 5.7131 | 8.7933 | 5.3858 | 5.9902 | 8.0612 | 8.6356 | 6.8950 | 6.3061 |
| 6.3298 | 8.0015 | 8.0764 | 8.5787 | 4.2681 | 8.2672 | 6.9116 | 6.2840 |
| 5.0704 | 7.4517 | 6.2074 | 7.8996 | 9.5486 | 3.7445 | 8.3809 | 8.4547 |
| 8.2654 | 8.9606 | 8.6903 | 7.9638 | 10.3572 | 7.5439 | 0.7381 | 9.7034 |
| 2.5542 | 9.2651 | 9.1280 | 7.0406 | 8.5039 | 9.1611 | 9.2697 | 8.0862 |
| 6.6872 | 7.5939 | 7.7211 | 9.0452 | 8.0330 | 7.9577 | 9.5432 | 7.2789 |
| 7.4226 | 6.8052 | 8.3125 | 8.3693 | 4.6644 | 7.8978 | 8.1077 | 4.9830 |
| 8.7827 | 8.6536 | 8.6660 | 7.6790 | 8.1571 | 4.9969 | 5.3093 | 9.0683 |
| 8.6800 | 8.3928 | 7.1816 | 6.0548 | 5.2565 | 5.1765 | 7.2001 | 6.1245 |
| 5.8657 | 8.0789 | 6.5827 | 9.6349 | 5.3717 | 7.7566 | 9.4554 | 8.9313 |
| 8.0568 | 5.4723\ | | | | | | |
| ITGAL | 6.0106 | 7.6777 | 7.2042 | 8.4948 | 8.5562 | 8.7214 | 6.7964 |
| 6.5568 | 5.6828 | 7.8675 | 7.6402 | 6.0207 | 9.6984 | 2.6649 | 8.3873 |
| 6.6309 | 6.1731 | 8.5861 | 9.4134 | 6.2179 | 9.3035 | 8.8404 | 8.7875 |
| 7.0794 | 6.1152 | 8.5240 | 5.0432 | 8.4403 | 7.7408 | 5.8855 | 3.6161 |
| 9.0297 | 8.5932 | 7.7129 | 4.9782 | 8.8233 | 5.3214 | 5.1152 | 4.1194 |
| 4.8606 | 8.8889 | 7.6161 | 5.3943 | 7.5974 | 6.5427 | 8.7785 | 8.4604 |
| 7.8437 | 7.1848 | 5.4987 | 7.7153 | 10.4421 | 7.7917 | 7.3394 | 7.8386 |
| 7.1215 | 8.4142 | 9.2920 | 8.8295 | 5.7804 | 7.8597 | 8.1843 | 7.6436 |
| 5.4438 | 8.3382 | 9.7125 | 7.8362 | 5.4450 | 5.4534 | 6.5248 | 9.5368 |
| 5.4421 | 11.6258 | 7.9103 | 9.9230 | 5.1241 | 8.5875 | 8.5074 | 9.1582 |
| 9.3469 | 5.2105 | 6.0201 | 9.7126 | 9.2780 | 7.7179 | 6.2097 | 5.7012 |

```
8.7506  8.1797  7.7920  7.9451  9.2135  9.1236  9.4487  8.7943
7.9056  7.7203  7.8895  6.7326  10.5802 8.2250  9.4938  4.4368
8.4442  5.8660  4.0885  9.6671  7.8235  6.8589  7.1218  9.3224
7.6222  6.0327  5.4919  6.0176  6.0547  7.9325  6.0659  6.1830
10.1474 5.3602  7.4948  7.5266  7.0521  7.4827  8.8288  6.3998
5.3730  7.4616  7.7963  5.6506  5.9193  8.8403  9.5004  9.1728
10.4773 10.0166 10.8632 7.6843  7.5398  8.0671  7.2174  8.9363
8.2525  7.9603  5.6765  7.5891  7.4331  6.8473  4.7929  8.5464
7.7046  8.8211  6.9982  5.2454  7.9054  7.8786  4.0812  7.5952
9.8092  6.5134  8.9825  7.8770  7.4176  5.5394  7.8718  8.1540
9.2115  6.5504  6.3343  5.1258  5.4435  4.9555  4.0107  6.3773
4.5788  7.8430  6.8491  5.6730  10.9649 8.2578  6.3245  6.0835
7.8646  7.6113  9.2103  8.5238  8.4167  7.8079  7.6983  9.2779
4.6948  8.0712  8.6339  7.9313  8.0534  5.6620  7.4032  8.9658
8.5927  8.1291\
ITGAM   6.1965  6.9754  6.9845  7.4047  7.8661  7.3951  7.1146
4.6754  3.6488  5.6849  9.4130  5.4111  7.6777  3.5898  7.9067
5.5157  4.4610  5.9795  7.6584  6.0597  9.5851  7.1153  7.1983
6.7776  5.7765  7.0901  3.4150  7.6377  6.7036  4.6295  5.0209
6.4282  9.6129  7.7023  5.1506  5.0455  5.4354  4.5859  3.6815
4.8606  7.6818  7.0081  5.3376  6.8321  6.8711  7.7366  7.8092
7.5363  5.6090  4.4159  7.9572  8.4938  8.0579  7.6776  5.6656
4.1781  6.1183  8.5549  7.3071  4.6050  7.6691  7.6528  5.2749
5.3919  6.2259  8.0207  7.5816  5.2923  4.0659  5.9098  8.7205
5.0104  7.5176  7.7718  7.9275  2.1585  6.8743  7.2986  7.0916
8.9930  3.7537  5.4287  8.3635  6.9377  6.4763  5.0995  4.0708
8.0823  7.9312  5.2308  8.4330  6.9070  7.6529  6.8332  5.7058
6.7497  7.6116  6.5515  6.5162  7.0543  7.0745  8.8560  5.0973
6.8426  5.3787  3.6802  7.0340  6.0634  5.2986  7.0795  6.0992
6.6834  6.8552  4.2479  7.2970  6.0547  5.3945  5.6704  5.1777
8.1684  4.9909  8.4867  5.4105  6.0207  6.9242  8.5616  6.3816
5.8592  6.4928  7.3134  6.5994  5.2966  7.6323  7.5758  6.2415
8.2952  8.1761  7.5250  7.2325  6.1515  6.1238  6.3272  7.1815
5.2640  6.5018  5.9240  7.1557  7.4747  6.0979  2.8098  6.9466
7.0076  7.4952  6.0482  5.0781  8.5700  6.7046  4.4277  6.3387
8.2671  6.8437  7.7533  6.1513  7.0613  5.9852  6.4045  6.6850
10.0314 5.3007  6.9433  4.1812  4.3874  5.3265  3.4930  6.7006
4.2600  6.2922  6.8144  5.7524  8.1206  7.0053  5.2868  5.6250
7.5000  7.2627  7.3153  8.0813  7.1659  7.3587  8.0443  8.4298
6.3949  7.2871  7.0771  6.5651  7.3273  5.7627  6.2054  7.8275
7.8950  6.7499\
LOC286359       0.0000  0.0000  0.0000  0.0000  0.0000  0.0000
0.0000  0.0000  0.0000  0.0000  0.0000  0.4849  0.0000  0.0000
0.0000  0.0000  0.0000  0.0000  0.9983  0.0000  0.0000  0.0000
0.6346  0.0000  0.0000  0.0000  0.0000  0.0000  0.0000  0.5901
0.0000  0.0000  0.0000  0.0000  0.0000  0.0000  0.0000  0.0000
0.0000  0.0000  0.0000  0.0000  0.0000  1.0349  0.0000  0.0000
0.0000  1.7580  0.0000  0.0000  0.0000  0.7768  0.0000  0.0000
0.0000  0.0000  0.6343  0.8754  0.4252  0.0000  0.0000  0.0000
0.0000  0.0000  0.0000  0.0000  0.0000  0.0000  0.0000  0.0000
0.0000  0.0000  0.0000  0.0000  0.0000  0.0000  0.0000  0.5408
0.0000  0.0000  0.0000  0.0000  0.0000  0.0000  0.0000  0.0000
0.0000  0.0000  0.0000  0.0000  0.0000  0.8470  0.0000  0.0000
0.0000  0.0000  0.0000  0.0000  0.0000  1.2591  0.0000  0.0000
```

```
0.0000  0.0000  0.0000  0.0000  0.0000  0.0000  0.0000  0.0000
0.0000  0.0000  0.0000  1.0000  0.0000  0.0000  0.0000  1.2610
0.0000  0.0000  0.4553  0.0000  0.0000  0.0000  0.5970  0.0000
0.0000  0.0000  0.0000  0.0000  0.0000  0.6684  0.5526  0.0000
0.0000  0.0000  0.0000  0.0000  0.0000  0.0000  0.0000  0.0000
0.0000  0.0000  0.0000  0.0000  0.0000  0.0000  0.5814  0.0000
0.0000  0.0000  0.0000  0.0000  0.5476  0.0000  0.0000  0.3855
0.0000  0.0000  0.6063  0.0000  0.0000  0.0000  0.0000  0.0000
0.0000  0.0000  0.0000  0.0000  0.0000  0.5290  0.0000  0.0000
0.0000  0.0000  0.0000  0.0000  0.0000  1.0692  0.0000  0.0000
0.5257  0.0000  0.0000  0.0000  0.0000  0.0000  0.0000  0.0000
0.0000  0.0000  0.0000  0.0000  0.0000  0.0000  0.0000  0.0000
0.0000  0.0000  0.0000\
HLF     7.3232  8.4861  3.2600  5.5194  3.4800  3.8070  6.7664
5.3761  6.0908  8.2997  3.1079  4.8956  6.8812  6.6944  3.0405
4.0436  4.6892  4.9861  7.2540  8.6266  3.7887  4.2366  7.1542
6.6757  10.1255 1.3599  3.8832  4.3616  6.1854  7.8195  4.0789
6.4362  1.6552  4.4081  5.9316  5.3745  4.8670  8.2787  3.3999
6.4002  3.5998  8.6006  5.5355  4.9079  10.2100 0.9285  3.2199
7.3005  9.9600  5.3681  5.4766  5.3044  4.0805  11.3421 4.5951
3.7390  7.9975  5.9811  7.8130  6.6533  9.3746  6.2910  5.3435
3.9398  9.0167  3.8450  4.2065  5.1541  4.1483  6.0898  4.3661
7.2464  3.0705  4.9677  6.5241  6.8640  3.0883  4.9119  7.1612
3.8919  1.9568  4.9418  9.1341  5.0195  6.5323  5.7299  5.1424
2.0646  5.8667  6.7314  10.3538 6.1796  4.5362  5.3110  3.7167
9.4251  2.7408  10.6812 4.9755  7.2952  2.4487  4.4358  6.3937
4.7873  6.0156  5.7547  7.9343  6.5873  6.8702  3.7624  4.7593
3.9579  4.9483  6.6724  4.7835  7.4269  4.1521  4.7082  5.8035
3.4563  5.9334  4.9880  7.4396  1.5487  8.5845  4.4891  6.1529
4.1383  11.1652 10.3425 5.6596  4.6531  3.8143  5.4295  6.8628
5.0375  3.7348  6.6715  9.8192  10.9511 2.7409  6.9411  3.0717
5.6258  2.9437  11.6226 5.1499  9.6362  5.8223  4.5263  9.1171
1.5604  6.0470  4.6475  7.4555  3.1364  10.2956 1.5047  3.5644
0.0000  3.3786  2.3902  5.4833  4.2280  2.1964  2.5845  4.5131
8.4292  2.7528  3.6978  3.8206  3.6455  5.8660  8.4370  0.0000
4.3466  8.6551  10.4616 6.6217  7.4876  4.0819  7.7072  4.6286
5.6587  4.4802  5.7140  4.0766  4.4888  2.4884  4.8162  4.5972
4.5576  4.5038  4.5446  4.9979  2.3335  4.7615  10.6090 4.5526
5.1870  2.2387\
NOV     6.3611  8.7949  12.8992 8.4091  6.4004  8.1197  9.4927
9.4231  10.2268 7.4531  8.1481  8.7790  8.5120  7.0720  7.6323
8.8792  8.6279  7.2711  7.5202  7.7977  7.1115  8.4903  7.7980
7.4244  9.4591  3.3784  4.0544  8.9485  8.8602  6.8291  8.7777
6.7356  7.8491  8.0255  7.5284  9.5845  8.5118  7.6424  9.8464
6.9388  8.3588  8.4792  5.6793  8.1790  9.5924  8.5119  6.0219
6.2374  10.3686 5.7813  7.1261  5.8945  8.6173  9.1152  6.9725
9.1635  9.1429  9.9728  8.9899  9.7539  9.4971  8.8243  6.0964
8.3019  7.6096  8.4999  6.7526  9.3305  5.9998  7.4830  5.5940
7.5999  3.8023  11.5743 7.6678  4.1336  10.2599 9.3472  8.0026
5.2583  4.3712  8.9745  10.1286 8.3593  8.7679  9.1712  7.4685
8.1937  6.6790  6.2948  8.6575  7.2323  9.3798  7.6747  4.8679
7.6047  9.0333  8.0457  9.2249  11.5436 6.0969  6.2891  9.0487
7.6241  7.5400  5.5111  8.7836  8.9702  9.0909  8.1553  10.5372
5.8466  7.0216  4.5236  7.9911  8.5683  7.9088  9.1718  5.2027
```

6.8513  9.7790  6.3946  7.7455  7.5469  8.9061  10.1417 9.7363
8.2391  8.2223  8.2700  11.1690 8.7600  10.4480 10.4216 9.4806
7.3576  5.1351  5.3965  10.3560 7.9381  9.1350  8.0111  7.9122
8.8503  4.9940  4.4271  7.5484  9.2757  6.5362  4.8272  8.3358
7.2016  9.9021  8.2422  7.5963  9.0320  9.7295  6.0629  7.1737
4.1584  4.7329  7.5356  8.7243  7.4446  3.5545  5.4561  10.1722
9.6403  8.9399  8.5526  8.3485  5.4725  7.3034  4.9899  6.6615
7.0408  7.1996  10.2376 6.3139  8.8319  8.1877  8.3303  9.4419
8.0519  10.2717 8.4625  9.4693  9.0353  8.9122  5.5425  9.2828
8.1174  8.5395  10.4724 4.5917  9.3610  7.0345  7.3987  9.1919
9.4648  4.1488\
FAM134C 10.7019 10.8119 10.0229 10.8327 10.7302 9.9910  10.1084
10.1205 11.7020 10.8386 10.3176 10.6992 10.1732 11.5956 10.4230
11.4403 11.1847 11.2564 11.0577 11.6782 10.2640 10.7073 10.7373
10.6714 10.4852 11.3907 10.2351 11.3560 11.2260 10.2520 10.3173
11.0233 10.5562 10.1873 10.7658 11.2766 11.1311 10.5826 10.8753
10.1556 11.0746 10.1370 11.1868 11.3539 10.6241 11.2252 11.7995
10.7899 10.4740 11.8053 10.3067 10.2802 11.0574 10.2496 11.4018
11.1406 10.7711 11.3115 10.5932 11.6872 10.4929 10.3161 10.5194
9.8092  10.9226 10.7181 10.5276 10.3939 11.0064 10.4947 11.5473
10.5312 11.0419 11.0577 10.8649 11.2838 10.7743 11.0028 11.5251
10.7759 11.3671 10.0080 11.3500 10.2148 11.1152 10.7473 11.1623
10.9422 10.3278 10.6085 10.8164 10.7056 10.3225 11.4698 11.0337
10.7508 10.5748 11.2362 10.1984 10.8388 11.6121 10.6042 10.3628
10.4940 10.1159 9.7627  10.9360 10.2649 10.6320 10.7709 10.9235
10.3929 12.2939 11.1013 10.4653 10.0863 10.5436 10.7212 10.4140
10.9906 10.7063 10.9590 10.5692 10.9051 10.7646 9.8144  10.1138
11.0184 10.7647 9.9934  10.0870 9.6817  11.0446 10.6067 10.9385
12.7119 11.6315 12.2139 10.6942 11.0016 10.5203 10.9462 11.5446
10.7881 10.2049 10.8432 10.5289 11.1641 11.1017 11.9976 10.8445
12.0881 10.6762 10.6620 10.2432 9.9321  11.0951 11.0072 10.1128
11.0692 10.9313 10.6903 10.8018 11.0619 10.9718 9.9648  10.3858
10.6704 11.1196 11.0975 10.7342 10.8401 11.1959 11.3561 12.6170
10.6157 10.8580 10.7364 10.5572 11.0334 11.6671 9.7966  9.1797
11.2251 9.9624  10.8862 11.2704 10.9227 11.2192 10.7207 10.3940
11.2204 10.5073 10.5041 10.6463 10.3643 11.4503 10.9069 10.9651
10.5927 11.6800\
FAM134B 9.4291  8.2883  9.3924  9.0025  4.8712  5.8545  9.7340
8.1194  6.7413  8.2514  7.1177  8.6078  8.1543  7.5679  6.4624
6.2290  8.2141  4.6995  8.0492  6.2408  5.5362  7.8448  9.2627
7.8557  8.6172  6.9509  8.6206  7.0187  7.1346  7.8857  6.9019
6.1282  2.7201  4.6899  6.5087  4.9358  7.9158  7.0147  7.1732
8.8846  8.9311  8.1525  5.9925  8.5200  6.7514  6.7615  8.8366
6.3576  5.1395  8.0779  3.8726  5.0794  8.4038  7.1758  7.1098
8.6896  6.1746  6.8125  7.1136  7.0150  6.3121  6.9960  6.7740
9.1133  8.0328  7.7746  9.4006  9.5033  7.5546  7.7310  8.3401
8.0339  6.0592  10.2495 7.1865  9.8435  7.6463  9.3177  9.3370
6.8582  8.6235  7.7703  9.8737  8.1613  8.2005  9.6299  8.0823
6.1395  8.1797  6.6130  6.5894  6.6212  9.0837  8.3015  7.5002
5.9899  8.3129  8.1333  8.5556  6.1335  6.6390  3.7864  9.5431
8.5036  8.5500  7.3388  9.5539  7.5824  8.1127  8.3897  8.3674
8.0105  9.6320  5.8074  7.2772  7.6252  8.7006  8.5289  7.1214
6.8709  7.2052  7.9328  7.7418  6.7862  9.3154  6.6665  9.7390
6.0600  8.3542  7.9219  9.2220  7.8941  7.8551  8.5589  7.2535

5.6300  7.0622  8.0975  7.1330  7.7901  8.4095  7.5202  8.9041
6.2451  6.5541  7.5503  8.6573  7.3784  9.2769  7.7010  7.8577
6.7048  7.9344  4.3612  8.6267  7.2901  8.3283  8.2663  3.6260
3.9187  5.2510  3.4693  8.8336  7.4552  6.9107  8.4513  7.7480
9.0394  4.1875  6.8193  9.3193  7.1023  7.6326  4.7213  4.1460
8.8019  8.2413  7.9067  7.8965  8.8714  7.6653  5.6811  7.3812
9.5330  8.3463  8.6179  7.3329  6.8241  6.6293  4.5435  5.8569
8.1247  9.1219  7.3384  6.5588  7.7079  10.5022 8.5179  8.4914
8.1282  4.6541\
FAM134A 11.0839 11.3895 12.5259 11.2200 11.6061 11.9511 11.6360
11.3840 11.4362 11.2462 11.2348 11.7249 11.7825 11.8902 11.0767
11.3838 11.7996 11.5965 11.3420 12.5407 11.9124 11.1638 11.7694
11.1470 11.2345 11.5053 11.4605 11.3874 11.3036 11.4671 11.5528
11.3565 11.0607 10.7735 11.9355 10.9415 11.5714 10.9930 10.6520
11.5733 11.3107 10.8680 11.5221 11.2563 11.2856 11.4022 11.9276
11.2519 10.7576 11.9420 11.5711 10.7947 11.9557 11.2687 11.6007
11.0632 11.9231 12.1137 11.2992 11.2347 11.2194 11.0045 10.9841
11.5583 11.2371 11.2247 12.3165 11.5008 11.5060 11.5292 11.9122
11.2251 11.1492 12.0174 10.7446 10.9412 11.9129 11.6220 11.6414
11.2503 11.5543 11.1473 10.8971 10.7301 11.3744 12.0573 11.5567
11.9280 12.0041 11.6093 11.4837 10.9471 11.8529 11.1733 10.5788
11.1062 12.2625 12.0078 11.2358 10.9032 11.1365 11.3410 11.8208
10.3696 11.6022 11.3158 10.3187 11.4950 11.1523 11.6905 11.3858
11.1968 12.3440 11.0815 10.5919 11.4952 11.3223 11.5795 11.2004
11.4407 10.8235 11.2335 11.4871 11.7318 11.5591 11.8820 11.6378
12.8315 11.3151 10.9205 11.3723 10.7799 11.2379 10.7196 11.4358
11.4324 10.9145 10.1821 11.0354 11.7138 11.3006 11.3373 11.6301
11.1217 10.7859 11.9326 10.4198 11.8490 11.4194 11.5118 11.2448
11.6943 11.0307 11.7266 10.8868 12.1466 11.7059 11.4124 11.4709
11.0865 11.9601 11.0412 11.3641 11.5260 11.5587 11.1763 11.3865
11.1721 11.4059 11.7173 10.9088 12.0667 10.9343 11.8328 11.1109
11.1108 10.8689 11.3337 10.9440 10.8984 11.2990 11.6556 12.0009
11.5239 11.2511 11.5781 11.8719 11.0974 11.2133 11.1667 11.0739
11.3644 11.1736 11.0948 10.8233 11.6767 12.0241 11.9135 11.2015
11.2122 11.3765\
SERPING1        13.0355 14.5508 12.9810 12.2820 11.5025 13.3509
13.0076 12.7424 12.3831 11.6426 12.4224 11.9995 13.1666 12.3410
11.8623 12.9019 12.8291 11.3596 14.7052 13.0530 14.7339 12.4114
13.7189 11.9918 12.7064 12.6217 8.8401  12.2408 12.7030 13.8804
8.8572  11.3852 11.3537 12.0942 10.9085 9.9532  12.9653 7.6527
10.0030 10.5366 11.5950 14.3831 12.1976 10.4990 14.5254 12.3530
11.7795 14.4412 10.0453 8.9113  10.8723 13.3669 13.7946 14.3559
12.7277 10.8226 12.7299 12.5423 12.7010 11.4561 14.2337 11.4607
10.7434 13.2840 11.9646 13.1108 12.8246 13.8131 11.9452 12.4696
12.8777 12.2440 13.9466 12.3524 14.7252 7.2543  13.8105 12.2417
13.8315 12.6673 11.1379 12.9525 12.1763 12.4423 13.0177 14.3926
10.8268 13.2903 13.5707 12.4089 14.9824 12.1326 14.4251 13.7319
13.3288 13.9061 11.6953 13.8020 11.1876 13.3951 11.5693 12.1350
12.7467 11.4409 9.6869  9.9727  14.9121 14.0256 13.4151 12.1381
9.8830  11.9636 13.9700 14.2158 11.8976 12.5733 12.0524 12.4976
10.7565 13.5348 12.5411 12.0634 13.1213 9.9690  12.5910 12.8865
11.9118 14.4717 14.3158 12.5881 14.1302 9.8860  12.8545 11.6833
13.1011 14.3922 12.7031 12.7887 12.3200 13.8056 11.2574 10.3092
13.3496 12.5233 13.5469 14.6345 12.3958 14.2551 11.9179 10.3401

```
13.2016 11.6216 14.0358 11.4561 13.1299 13.6189 13.6586 11.7125
13.6673 12.0004 10.6426 13.3577 13.1902 14.0625 9.3233  11.1506
14.0933 12.7554 11.6279 11.1651 10.4898 12.0234 9.5033  11.4359
10.5294 11.1264 12.0521 14.4805 11.1349 13.5355 11.1285 13.7557
12.1392 13.5465 12.4403 13.5955 13.1258 12.7004 12.3499 12.3794
11.6258 13.2772 14.6462 13.2873 12.1528 11.8937 9.8594  13.7429
13.2712 12.7067 13.9177\
SMAD9   3.3026  6.2795  4.9555  5.5573  7.2856  3.0265  2.7953
3.3720  3.4322  8.2981  2.0419  3.5094  4.6647  3.2002  3.3269
7.2523  1.0553  5.1973  6.3365  1.6235  1.3800  8.7289  3.7175
5.9481  5.9720  2.5109  7.0183  4.1268  3.8787  4.1020  7.1709
4.1016  3.3260  4.4081  3.5244  0.6819  4.5574  6.4763  2.0653
7.3110  6.2468  7.3403  1.8880  4.7481  6.5586  4.9024  0.0000
3.9349  6.7457  1.5638  9.1088  1.6507  3.1634  7.0150  5.5533
5.0982  3.1261  4.1448  4.8465  4.5461  7.1069  3.5473  4.9403
4.3188  6.7729  6.1987  2.3530  4.8502  4.6784  5.2983  0.7662
5.8772  1.9166  2.7868  7.2347  5.3452  2.8273  6.4496  3.9730
2.7776  3.1963  5.4705  6.3794  5.7007  2.4335  3.1783  4.5030
3.2461  3.3596  6.2785  6.7760  4.2150  2.7354  4.7441  2.7312
6.6280  5.4400  6.8037  4.0206  1.7322  1.4180  2.3585  5.1347
5.1537  5.4195  4.9419  4.9092  4.9367  5.8139  3.0071  9.5369
5.1262  3.7633  0.0000  7.1995  5.6006  5.8123  3.9037  5.5538
3.1499  5.6959  3.4695  4.1906  5.2757  4.5261  1.8805  4.4414
0.7843  7.0018  8.6261  5.0977  1.1234  4.1539  5.1778  0.8107
0.0000  1.6954  0.0000  8.0399  7.4422  3.5671  5.1401  3.4177
6.4697  4.6749  6.1903  4.5970  5.0513  2.7981  1.8766  7.8629
2.9676  7.4050  5.1535  7.1384  4.0519  7.6469  6.0629  1.3489
0.9345  7.5178  2.0641  5.9456  1.9645  2.5889  8.6378  0.0000
6.1411  2.1803  2.7065  6.9700  0.9151  4.2967  2.9029  4.9180
6.1229  7.4291  6.1385  7.9367  6.1333  1.2666  6.8773  5.2943
6.6504  4.0866  3.4980  3.8962  1.1775  1.4486  6.4410  6.2577
3.9158  4.0375  5.4693  6.0579  0.0000  4.1891  7.3341  8.6685
7.2478  1.1634\
TRERF1  6.9181  8.7068  9.4718  7.6264  6.5371  9.3662  8.7350
8.0276  7.7058  8.5391  6.7875  8.1930  8.9368  3.4081  7.1988
5.4029  7.2728  7.6754  9.1117  5.7368  8.6304  6.7981  8.5230
7.7327  9.2366  6.9387  9.9886  7.0129  5.1569  5.0149  7.5759
6.0985  8.3827  8.5452  6.2393  6.9988  8.5467  8.4247  7.7624
9.0243  6.2798  8.2792  6.9995  7.0109  8.5148  6.7002  5.4123
5.1516  9.3226  5.1310  8.0056  7.3479  8.1902  9.0337  7.8694
7.0612  8.1370  8.5629  8.1385  7.0419  8.7039  8.7185  6.0040
7.7799  9.2193  8.9873  8.3077  9.2489  5.0222  8.2608  6.4918
7.5138  7.5029  7.8253  7.7425  9.0275  8.9578  7.9955  6.9770
9.0050  4.5593  7.6160  8.6154  7.5579  6.8891  8.8320  7.1301
7.0960  6.5007  8.1235  7.8269  7.6715  7.9148  8.9256  5.9277
8.0694  7.1207  9.7359  6.8483  8.5857  3.9007  8.1466  6.0664
8.0213  6.6797  6.6674  9.7076  7.3979  8.5454  7.1527  5.6114
5.7915  6.3432  6.4429  5.5748  7.6135  8.5041  8.5124  4.7031
7.3275  9.4087  7.1556  7.3801  7.5229  8.9885  8.0606  7.7984
7.1107  9.6049  9.4263  7.6695  8.1339  7.2892  8.2247  7.3231
6.8889  5.9147  8.4534  8.5892  9.1656  5.9658  5.9123  6.1970
7.0592  6.3257  9.0318  6.2695  8.1023  6.9200  6.9375  8.6957
4.8222  7.0108  8.6692  7.5580  7.9802  9.5190  7.6444  8.0360
9.2180  5.9073  6.5017  6.8493  6.4713  4.9588  6.2394  8.0390
```

```
8.1290  5.9527  7.9854  5.6081  8.0900  8.0640  8.4900  3.4255
8.1278  8.9084  8.4574  8.7875  8.6526  6.1174  5.8745  8.6363
8.0450  8.6750  6.0804  7.8954  7.3389  7.2966  5.9460  6.5838
7.7900  9.0397  7.9922  5.5988  7.2905  6.8702  9.0529  6.1450
7.9673  6.0599\
LOC653501       6.3611  6.7549  6.9775  7.1385  5.8127  7.4947
6.1107  7.4940  7.0765  7.3899  6.0166  6.4077  7.3242  7.6185
7.0145  6.2617  6.9150  6.9432  7.3409  5.2653  6.5122  6.9904
7.6668  7.7327  7.3807  5.8297  6.3714  6.2310  6.2931  5.6445
7.0642  7.2226  5.3569  7.4102  7.9839  7.0457  6.8764  7.5052
6.2144  6.4298  7.2317  6.2575  5.9427  5.9423  8.1334  5.8331
4.8335  5.8624  6.2996  6.5384  6.8727  4.6169  6.7069  7.8805
7.2351  7.3529  5.8130  6.7355  7.6279  6.9256  7.3522  7.0462
7.0503  6.8487  7.3400  5.4255  5.6962  6.5187  6.2993  6.7414
6.5141  7.5694  4.5926  6.4192  7.1594  6.4145  6.5161  6.5924
6.9979  7.8319  5.7506  5.8166  7.3763  6.7196  6.4181  6.4589
8.4936  6.0266  7.6049  7.1844  7.3419  7.2551  6.4677  6.6155
6.2653  7.5844  5.7406  7.2666  6.2853  7.5631  5.5107  6.6150
8.0792  5.0122  4.4130  5.7949  5.8358  6.9301  6.1470  5.6518
7.7554  7.4858  5.5901  5.3219  6.2382  7.1060  7.8326  8.1524
5.3669  5.4953  7.0155  6.7725  6.7156  6.2202  8.0636  6.8565
7.0121  4.1383  7.8608  8.0071  6.1924  4.6531  6.2505  6.5251
5.5547  2.9678  5.8034  4.1556  7.2029  7.4282  5.7333  6.2458
6.6211  6.0302  6.7809  8.0118  7.5666  7.8209  6.5818  5.8016
7.3366  4.2868  7.7144  6.7249  6.1057  6.5946  7.6015  6.9556
6.5756  5.5827  5.7482  5.3495  6.9800  7.1816  5.5831  7.1727
6.6436  7.3479  6.9201  6.2495  6.5823  8.0302  6.6916  5.7526
5.9274  8.3588  7.1382  6.7499  7.5217  7.0767  6.0678  5.4373
7.8783  7.0482  7.0759  7.0223  7.2600  5.8208  6.1447  5.8999
5.9185  7.0451  7.5396  6.2067  6.2810  6.2811  4.0595  7.6952
6.8762  7.2795  4.5045\
LRPAP1  12.4150 11.5849 12.6795 11.1422 12.2001 11.5667 12.1329
12.5332 12.4673 11.4911 11.7084 11.9638 12.1788 12.5191 11.6673
11.6032 11.5398 11.3517 11.8464 13.1791 12.4216 11.8045 12.0304
11.1318 11.6440 12.9318 9.9216  12.0590 12.2240 11.4904 11.4834
12.0439 10.8594 11.4958 12.1576 11.6996 11.8584 11.6277 11.0201
11.7090 11.7026 11.6186 11.1968 11.5387 11.6378 11.0189 11.9030
11.7497 10.8369 13.0980 11.2912 9.9815  12.5094 11.5868 12.1583
11.5840 11.6413 11.0169 11.3491 11.7175 11.3319 12.0308 11.9338
13.8634 11.5463 11.6299 11.7198 12.9236 12.1253 11.8717 12.5622
11.9603 12.6363 12.6608 11.4316 12.6791 12.8785 12.1092 12.0092
11.3698 13.6296 12.0618 11.2588 11.4355 12.5657 11.6992 10.8389
13.0115 11.2768 11.9285 11.8350 11.6311 12.4038 10.8015 12.1578
11.7676 11.9931 12.2739 11.3263 11.3403 12.3209 11.4845 12.7207
10.9237 11.4471 11.5126 11.0513 12.4607 11.3492 12.7586 11.3508
11.5926 13.2762 8.9915  10.9265 10.8992 10.9481 11.7646 10.7051
10.8686 10.9014 11.4702 11.0744 12.2149 11.6163 11.1567 11.5645
13.4683 11.5089 11.1535 12.8766 10.6124 11.7632 10.4589 11.9596
11.7555 12.4552 8.8363  11.6877 12.0970 11.1473 12.7401 12.3078
11.7425 12.0121 12.1733 12.9879 11.9419 12.2515 13.3019 11.9473
12.9935 11.6967 12.0910 12.8033 11.5643 12.0294 11.9421 11.9290
11.8506 12.2301 11.5822 11.5609 12.0939 12.4298 11.4524 11.5844
11.8954 11.6783 12.1040 11.6572 11.9538 12.8326 11.9273 10.9157
11.5874 10.7574 11.7397 10.4771 11.6905 12.4368 12.7773 10.7929
```

12.3926 12.3196 11.8233 12.5650 11.6622 11.8125 11.9407 11.0050
13.0826 11.8090 12.4019 11.8326 12.9887 12.7972 12.5747 11.8149
11.1931 13.2693\
LQK1 2.5026 4.3019 2.4032 5.0541 4.4435 4.2627 3.4058
4.2123 3.4782 4.6829 1.4500 4.5953 5.9967 6.5037 2.2060
5.9364 4.8861 5.6915 3.7518 4.5605 4.8469 4.8857 5.2274
3.8282 5.2467 4.3449 4.7362 2.4936 5.3114 5.1024 5.9910
7.2134 6.2360 4.2140 4.9316 3.7098 5.3214 6.7241 3.3363
2.0552 5.0532 3.7059 3.3483 5.2100 4.4413 2.6826 5.5368
3.3124 6.7264 3.4820 7.2326 2.4004 5.5028 2.2666 4.3485
5.3709 3.0318 4.9944 2.5360 2.9646 1.9018 2.2329 3.0129
5.6928 5.4587 3.2320 2.8406 5.5950 4.0229 4.6109 3.7294
3.3742 3.9820 4.5975 5.0831 3.7353 3.5007 5.5663 4.2300
5.8966 2.7579 5.2119 2.7716 5.9144 3.9151 5.9082 4.5868
4.4738 4.6840 7.2358 4.6337 3.5089 5.1977 4.6413 6.1289
4.3633 4.8130 4.2762 3.8617 4.2211 6.4587 4.4358 2.1993
3.8386 5.0377 4.7303 3.5314 4.0903 6.6712 3.3997 5.3493
2.5476 4.6197 4.7004 5.0303 5.9963 3.0116 5.0034 4.4546
4.6527 7.2268 3.1797 2.4101 4.3402 5.7631 4.4305 4.8127
6.0600 2.2660 1.8193 1.9345 3.8633 4.8811 6.2258 3.4335
6.4375 5.8178 0.0000 4.4532 1.4215 4.9769 4.4302 4.8540
4.1972 4.4963 3.6839 2.9153 4.8361 5.4813 7.0797 4.2078
3.4258 5.4227 4.1736 2.6037 4.5433 4.0271 2.7033 2.8458
4.4862 5.7482 5.9414 2.7999 3.6192 4.6023 4.8532 3.1709
5.1678 3.1615 6.3749 3.0521 4.7618 6.5163 3.3476 6.3773
3.7267 3.4082 1.6723 4.3390 3.4442 6.6490 3.0638 6.5095
3.3550 3.3684 6.9227 2.8969 4.7814 4.6560 4.3850 3.3620
4.3737 5.1771 5.5562 4.4353 3.4726 4.5200 4.3212 4.8325
4.8286 5.2786\
SMAD6 8.2824 8.5492 8.3985 7.1004 7.8274 9.1001 7.8018
7.8043 8.4645 8.3544 8.6454 8.0995 8.0791 8.5186 6.7796
6.7278 8.2881 8.2394 8.5646 8.4502 8.3642 6.5397 7.2463
7.5983 7.0395 6.2907 4.5195 7.3035 8.0082 7.1290 8.2309
7.6318 7.1673 8.3995 6.8526 6.1879 8.4336 7.2290 7.4831
7.2291 8.0212 7.6132 8.2509 7.4808 7.2210 8.5437 4.0317
6.1064 7.8186 6.7629 6.8406 7.4748 7.0292 7.7784 9.4588
7.5450 9.3390 8.2554 7.5718 8.2838 8.9540 8.2520 7.2777
8.7076 7.9991 7.6213 7.9870 8.2857 6.8415 8.4768 6.9127
7.6479 7.0881 8.4231 7.2433 4.6583 7.9850 6.3725 7.7714
7.3045 8.0870 8.0437 7.4476 7.0453 8.1831 8.3923 7.0089
7.9678 8.5630 6.9216 8.0117 7.0311 7.5121 8.0639 6.9617
8.4588 6.8360 8.5399 6.7036 8.2023 6.3933 8.0123 7.6913
8.5376 7.0484 4.9597 7.5121 8.4450 7.6112 7.6411 7.1067
7.4102 7.5311 8.0334 7.8213 7.7294 6.7706 8.4301 5.8909
8.5881 9.8192 8.0278 6.8018 8.1263 7.6130 8.0533 7.1854
8.2391 8.2223 6.9697 8.2771 7.5755 7.2455 6.9199 7.3433
5.8162 5.6829 7.3542 7.8917 8.2259 6.7477 7.1935 7.3821
7.1776 6.5768 8.1980 7.6881 8.7407 8.0116 6.7443 8.7733
5.5184 7.9371 7.8773 6.1533 8.2640 8.8048 8.5245 8.9944
7.6280 5.8691 7.3464 8.0109 7.8854 8.2905 7.0236 7.9139
6.9593 7.8603 7.7182 7.5636 7.2731 8.5044 7.2751 7.3192
8.3147 7.8729 8.2810 8.2751 7.2739 7.4921 7.9838 8.3402
7.6879 7.4557 7.4907 6.9193 7.6098 7.3587 7.5381 6.6214
7.4341 6.5097 7.9467 7.8354 8.0966 6.4114 8.0427 8.6096

7.5436 6.9294\
SMAD7 9.9098 10.1333 10.5119 8.6153 8.4121 8.3483 9.3635
8.4277 8.2640 10.3356 8.6529 9.4474 9.4132 9.7428 8.2290
8.3348 8.6338 9.6187 10.2440 7.7724 8.0434 9.1054 9.0892
8.9171 9.0170 5.1694 8.4835 8.2065 9.4909 8.9283 9.8468
8.6607 7.6930 9.3701 6.9375 6.4890 9.5637 7.8794 8.3522
8.8488 8.7309 10.0776 9.9484 8.3433 9.4393 8.6153 7.6801
6.7656 8.3769 7.4872 7.7228 9.6124 10.4448 9.3001 10.5658
9.2358 9.6518 8.3396 9.3655 9.6310 9.7093 9.0244 8.8223
10.3498 9.7349 9.0513 9.2334 9.6209 10.8425 7.8787 7.0333
10.0306 8.3878 8.9354 9.0923 6.8204 10.0589 8.5482 8.9144
8.5663 9.3388 9.5994 9.1140 7.2781 8.3364 8.9743 10.6455
8.6870 9.2903 8.3507 9.4017 8.5698 9.5620 9.2708 8.7146
9.1901 8.0926 9.6186 8.6283 9.5349 6.6470 8.8196 7.6007
9.5676 9.1785 8.9801 9.6049 9.8615 9.4831 8.4111 8.0465
8.4883 7.7887 7.6865 8.4150 9.3465 8.2157 10.2579 8.0470
8.2847 10.1034 9.5287 9.7504 8.2464 8.8382 9.4413 9.2452
8.6082 10.3918 9.2795 10.0310 8.4723 8.1297 7.9903 10.2235
7.3509 6.3602 7.4803 9.0761 10.0208 7.6361 7.3873 8.2806
9.5392 9.4561 8.6401 9.1601 9.4601 9.1478 8.4664 9.5533
5.3214 9.6143 8.5888 6.8226 10.2070 10.0754 10.6219 9.0233
6.9383 7.3979 8.6526 9.5640 8.9422 9.5057 8.9414 7.8831
8.5012 8.5826 8.2700 8.3009 8.3800 9.2649 10.0541 5.5092
9.1911 9.9604 9.6720 8.4506 8.9584 8.2468 8.6606 8.8103
9.6347 9.5530 8.8576 9.3059 7.9039 7.4802 9.3055 8.4508
9.6625 9.2700 9.8546 8.9645 7.5613 7.0629 9.4960 8.6183
9.0100 8.0334\
GOLPH3L 9.5154 9.6046 10.4831 10.3713 9.3091 10.7513 10.3739
10.3635 10.4589 9.4298 11.4315 9.6830 10.7323 9.6518 10.4609
10.0839 9.7541 9.6459 9.6021 8.7691 10.0974 10.2310 10.3805
11.4559 9.7087 8.7349 10.7776 10.0452 10.2464 9.8819 8.0854
10.1926 9.6960 9.8958 10.2055 9.2442 10.7849 11.2909 9.9934
9.6617 9.0167 8.8582 9.2976 9.8415 9.8921 10.4919 8.7319
10.8306 8.5043 10.7634 10.6372 8.7471 10.2838 9.6173 9.9302
9.5094 9.7148 10.2724 10.2928 10.9115 9.7873 10.7593 10.0395
10.2367 9.7990 10.2777 10.5938 10.4868 9.4294 9.4442 10.1353
11.0501 9.2972 10.1245 10.9942 9.5810 9.7289 10.3329 10.8345
9.2031 9.2184 10.3079 10.2873 10.7235 9.7987 9.9604 11.9707
9.5732 9.6869 9.4011 9.6145 10.2025 10.0442 9.4449 9.2824
9.9360 11.1941 10.0136 10.3506 9.5067 9.5020 9.9971 10.6677
9.1629 9.9359 11.2494 8.6595 10.2799 10.0473 9.9883 11.0255
10.3005 8.3577 10.3197 9.8564 10.6192 11.2304 10.8940 9.3873
9.4292 9.3590 10.2046 9.7567 9.9545 10.4189 10.6059 9.9025
9.8300 10.0527 9.6645 10.2648 10.2962 10.1697 9.8833 9.5197
8.4376 9.4397 8.9054 10.2309 9.8820 9.3237 11.0327 9.9077
9.7867 9.4244 10.0120 10.7146 9.8307 10.3407 10.6774 10.1933
9.2507 10.8048 10.4775 9.6062 10.1137 10.0496 9.8822 10.2462
8.5403 10.4849 9.1708 10.1018 9.9236 9.0049 9.7742 10.3436
11.3602 9.4719 10.1584 9.1010 10.3777 8.9057 10.2382 9.7245
10.1756 9.5435 9.4821 10.4607 10.9166 10.7828 10.0811 9.8811
10.1981 10.3730 10.5839 10.4779 10.1626 9.8362 9.4274 10.3126
10.8643 9.8873 9.5570 10.6899 10.2541 8.1076 9.9336 9.9754
10.1909 9.2869\
SMAD1 9.5941 10.3033 8.8085 9.5559 8.8270 10.0301 11.1285

9.3257 9.2047 9.7086 10.0793 8.9864 9.9159 10.7082 9.8926
9.5201 9.2858 9.3659 10.2451 8.0418 10.2703 8.9773 9.2808
10.4775 10.4781 9.5743 8.0231 9.0303 9.6296 9.2418 9.3382
8.4216 9.2663 9.0839 9.7583 8.7071 9.7221 9.9315 10.2569
10.0131 10.3189 10.0936 9.6164 8.4950 10.5492 8.3091 6.9374
9.2206 9.5009 8.9078 9.2066 8.1614 9.4257 10.5081 9.0018
10.0024 9.2697 8.9932 10.4407 8.9847 11.0538 10.6200 8.5042
9.4781 10.0337 9.8137 8.1393 9.7815 9.8529 9.3390 9.0896
9.8432 7.7019 9.0903 9.8664 8.7770 9.6014 8.8996 9.8861
9.4926 8.8567 9.6570 9.2583 9.8672 8.9238 9.0522 9.1444
8.6321 10.6524 8.6533 10.4597 10.5567 8.8105 8.8411 9.2984
10.7458 9.8046 10.6552 9.4709 9.0193 7.8426 9.3221 9.7086
9.2894 9.4488 10.0036 9.5153 10.2175 8.9381 9.0415 9.4479
9.2930 6.9316 7.8138 10.1583 9.6177 9.8081 10.1235 8.9503
9.0334 8.7063 9.2953 10.5387 9.0915 9.6422 10.3314 9.1377
7.9873 11.2193 11.0997 10.4698 9.8887 9.3509 8.9815 8.2665
6.6914 7.9854 6.8221 10.6358 9.8916 8.1819 9.7441 9.1336
10.4652 10.2685 8.3628 9.1480 10.1400 10.0343 8.7522 10.4726
6.5332 10.4698 9.2206 9.6803 9.6511 11.2487 9.3148 10.3564
7.9107 8.7048 8.9049 9.7553 9.3210 9.0544 10.4151 9.9236
9.4966 9.4889 10.0948 10.5688 9.2261 8.6779 8.6224 6.7387
10.2245 9.8279 9.8535 10.3446 8.9439 8.4748 8.6519 9.7808
9.5589 9.9787 9.9452 10.0898 8.8740 8.6205 9.3132 9.6333
10.3613 10.5776 9.4392 9.4022 8.0643 7.9086 10.3096 9.5902
9.7519 7.9988\
AFTPH 9.2350 9.8702 11.3206 10.6613 10.1209 11.0503 10.5929
10.3162 10.0256 9.5400 10.9435 10.2128 10.3286 10.1019 10.5661
9.8882 9.8931 9.8303 9.5853 9.5500 9.7365 10.1562 10.4926
10.3635 9.5339 8.6809 10.4505 9.5837 9.7880 10.6927 8.5352
10.3539 9.9000 9.7136 10.2648 9.7353 10.2148 10.5019 10.4042
10.5966 10.4814 10.2644 10.0745 10.7595 10.4144 10.1550 9.1575
10.3594 9.1202 10.5382 10.9601 9.4453 10.1032 10.0093 10.0332
10.3159 10.5892 11.0525 9.9943 10.4538 10.4432 10.5972 10.2168
10.0905 9.3428 10.7101 11.0391 10.4995 10.3456 9.8584 10.6771
10.1277 9.7946 9.6656 10.8231 10.5242 9.8773 10.4590 10.2636
10.0780 9.3695 9.7638 10.7840 10.5397 9.7420 10.1482 10.0130
9.6505 10.1918 9.1922 9.8353 10.7166 9.1157 9.7405 9.1410
9.7203 10.4093 10.0359 9.6958 9.6436 8.8289 10.0162 9.8704
10.8590 10.8962 9.9049 9.4596 9.9772 9.8565 10.1955 10.3500
10.0025 9.0394 9.2312 10.2845 10.3813 9.9050 10.4921 10.0631
9.2726 9.4313 10.6239 10.2954 10.3153 10.5059 10.3314 10.1893
9.6844 10.3665 10.0678 10.1540 11.2418 10.0124 10.2516 9.4897
8.6729 9.2955 9.8639 10.2309 10.2887 10.4118 10.2344 9.9697
10.3392 10.3389 10.0590 10.6412 9.8779 10.0323 9.2983 10.1876
8.7312 10.1309 10.0269 9.3135 10.4717 10.2137 9.8337 10.2152
8.9654 9.9523 9.5026 9.9997 9.8079 10.9565 10.4847 10.5670
10.7881 9.8515 10.1602 9.7170 10.3462 10.2215 9.9286 9.6713
9.7209 9.6743 9.9283 10.6229 10.5499 9.8653 9.6709 10.4572
10.3580 10.1652 9.7529 10.5140 10.0784 10.0482 9.6787 10.7316
10.0490 10.2061 9.6075 9.6799 8.7526 9.6436 10.2805 10.4878
10.3662 8.6177\
SMAD3 9.1826 11.0195 8.7274 9.5668 7.6953 9.8948 10.0102
9.4730 9.9165 10.6038 8.9452 8.7750 9.2646 10.0369 9.1135
10.1727 9.0640 10.0492 10.7898 11.5189 9.4025 8.7489 10.1276

| | | | | | | | |
|---|---|---|---|---|---|---|---|
| 9.9824 | 10.7978 | 8.8650 | 5.7790 | 9.0709 | 9.1475 | 10.9561 | 10.2313 |
| 8.2039 | 9.0977 | 9.4783 | 9.1645 | 9.9585 | 9.8466 | 9.7146 | 9.5125 |
| 6.1237 | 8.0723 | 12.0429 | 10.0882 | 9.5432 | 10.6420 | 9.3673 | 6.5995 |
| 10.3243 | 9.2893 | 9.0197 | 9.5553 | 11.4044 | 9.6444 | 10.8261 | 10.0009 |
| 9.1346 | 10.9657 | 11.1760 | 10.0467 | 9.9777 | 10.7330 | 10.6451 | 8.4233 |
| 9.8067 | 11.0541 | 9.1359 | 9.9546 | 9.9406 | 10.3407 | 9.4508 | 8.3633 |
| 9.3148 | 9.8521 | 9.8528 | 9.4796 | 7.9118 | 10.0633 | 9.8605 | 9.6807 |
| 9.1486 | 9.4920 | 9.6140 | 9.3297 | 9.0532 | 9.0489 | 9.2252 | 8.3809 |
| 8.8781 | 9.6999 | 9.1026 | 10.6813 | 9.7280 | 9.7319 | 10.1088 | 8.9094 |
| 10.3905 | 10.0552 | 10.9366 | 8.1584 | 10.6706 | 9.0036 | 9.4781 | 8.9355 |
| 8.8561 | 8.5743 | 8.2734 | 10.7599 | 10.4502 | 10.0460 | 9.5366 | 8.5545 |
| 8.7343 | 8.1814 | 10.7398 | 9.7099 | 10.3265 | 8.6316 | 10.1339 | 9.9672 |
| 11.5039 | 10.4799 | 8.7508 | 9.4209 | 8.8188 | 10.3633 | 10.3626 | 8.6114 |
| 9.1273 | 10.7627 | 11.2362 | 9.1820 | 9.3530 | 8.8979 | 8.4830 | 10.4735 |
| 9.3171 | 6.3204 | 9.4044 | 10.2696 | 10.8609 | 7.5631 | 8.6802 | 9.0099 |
| 9.4451 | 9.1128 | 11.4500 | 8.2098 | 10.6440 | 10.0053 | 8.3560 | 10.2663 |
| 7.5998 | 10.1138 | 9.4765 | 9.1508 | 9.7524 | 10.5496 | 9.7939 | 8.8194 |
| 8.3779 | 9.1369 | 11.1381 | 9.5040 | 9.5843 | 8.6624 | 9.3189 | 9.7195 |
| 10.0367 | 9.5428 | 9.5302 | 10.0097 | 9.6808 | 9.5382 | 10.2433 | 7.8234 |
| 10.0334 | 10.6143 | 10.6726 | 9.8525 | 9.5338 | 8.7158 | 11.8991 | 8.8846 |
| 8.7701 | 9.6602 | 9.7601 | 9.3138 | 8.6498 | 9.1057 | 8.7991 | 9.6432 |
| 9.9422 | 9.6968 | 9.3890 | 9.2811 | 8.7860 | 7.6022 | 10.8106 | 9.5117 |
| 9.5758 | 8.9443\ | | | | | | |
| MUS81 | 9.9265 | 9.6413 | 9.4416 | 9.2124 | 9.2909 | 9.1855 | 9.1949 |
| 9.2992 | 9.7448 | 9.5455 | 9.4866 | 8.9830 | 9.3692 | 9.2694 | 8.9869 |
| 9.6920 | 9.1280 | 9.8246 | 9.8460 | 9.9358 | 10.2590 | 9.1504 | 9.3212 |
| 9.0878 | 10.0472 | 9.9422 | 9.2143 | 9.6425 | 9.4648 | 9.1121 | 10.0147 |
| 9.7486 | 9.7985 | 9.4152 | 9.1821 | 9.4614 | 9.1674 | 9.9714 | 9.9985 |
| 10.0460 | 9.5414 | 9.4534 | 9.2914 | 9.4986 | 9.1414 | 8.9384 | 9.0536 |
| 9.1130 | 9.1376 | 9.5566 | 9.6411 | 9.9423 | 8.5727 | 9.9407 | 9.0007 |
| 9.8666 | 9.4593 | 8.4624 | 8.8745 | 9.5407 | 9.4281 | 9.1117 | 9.7648 |
| 9.2336 | 9.6152 | 9.2986 | 9.3482 | 9.7317 | 8.9828 | 9.8749 | 9.7169 |
| 9.5709 | 9.6593 | 9.5688 | 8.8377 | 9.1702 | 10.4436 | 9.0741 | 9.2009 |
| 10.0318 | 9.5496 | 8.2764 | 8.7143 | 9.6102 | 9.8199 | 9.6013 | 8.7944 |
| 9.2948 | 9.5140 | 10.2831 | 9.5348 | 8.8773 | 9.8483 | 9.6824 | 9.6773 |
| 9.5265 | 9.1840 | 9.5939 | 9.2370 | 9.9659 | 9.4864 | 9.7048 | 9.6813 |
| 10.6054 | 9.5664 | 10.1794 | 10.1925 | 9.1919 | 9.0254 | 9.9381 | 9.6286 |
| 9.5488 | 9.3098 | 9.6110 | 10.0679 | 8.9209 | 9.1938 | 9.5473 | 9.6800 |
| 9.3709 | 9.5539 | 9.3431 | 8.7366 | 9.1047 | 9.3611 | 9.1572 | 7.9142 |
| 9.7391 | 9.1247 | 9.6088 | 9.6863 | 9.6354 | 9.5941 | 9.9801 | 9.6675 |
| 8.6238 | 9.5023 | 9.7050 | 9.4431 | 9.8483 | 9.9264 | 9.1393 | 9.4421 |
| 9.1939 | 9.0774 | 9.2111 | 8.4667 | 9.4964 | 9.4218 | 9.7913 | 9.5919 |
| 9.5499 | 9.2811 | 9.7604 | 9.8688 | 9.7967 | 9.6012 | 9.4498 | 9.8153 |
| 10.0296 | 9.5596 | 9.5483 | 9.1024 | 9.9111 | 9.0515 | 9.0463 | 8.2224 |
| 9.1420 | 9.3158 | 9.9968 | 9.9860 | 9.3721 | 9.4022 | 8.6595 | 8.6457 |
| 10.0153 | 9.8376 | 9.7130 | 9.3627 | 9.5519 | 9.8360 | 9.1954 | 9.9917 |
| 9.3932 | 9.4454 | 9.8594 | 9.2969 | 9.7987 | 9.8021 | 10.0853 | 9.2106 |
| 8.9937 | 9.8777 | 9.6890 | 9.6609 | 9.5123 | 9.8561 | 9.9840 | 9.5176 |
| 9.3996 | 9.5032\ | | | | | | |
| AMMECR1L | | 9.0930 | 9.6021 | 9.6573 | 9.4969 | 9.5034 | 9.5919 |
| 9.7594 | 9.9845 | 10.1775 | 9.5964 | 9.2763 | 9.6781 | 9.7317 | 10.4128 |
| 9.3416 | 9.9064 | 9.5462 | 8.9656 | 9.8907 | 9.3634 | 9.0260 | 9.0835 |
| 9.0355 | 9.9169 | 9.4064 | 8.8455 | 9.3553 | 8.8490 | 9.2137 | 9.5565 |
| 10.2901 | 9.3685 | 9.0737 | 9.8392 | 10.3313 | 9.4314 | 9.9385 | 9.2956 |

9.2239 9.9988 9.3134 10.4231 9.8318 9.7169 9.5472 9.2502
8.6879 9.3735 10.0875 9.1215 9.6013 9.7053 9.5557 9.5586
10.1414 9.6241 10.3653 9.8127 9.8538 9.1751 9.7022 9.7224
9.2817 9.2063 9.5221 9.3036 9.3918 9.9917 9.4034 9.4962
8.6097 9.6732 8.6361 9.7279 9.1991 9.0549 10.0346 9.2297
9.3205 9.8489 9.2276 9.7800 9.2506 8.9957 8.9890 10.0442
9.2239 9.0540 9.7295 9.0663 9.4333 9.7334 9.1526 9.4141
9.3004 9.5018 9.5406 9.6917 8.8201 9.6512 8.4394 9.1282
9.4452 9.5266 9.1695 9.3278 10.5098 10.1110 10.2404 9.2004
9.4355 9.6383 9.6320 9.5812 9.9016 10.0933 8.9644 9.8586
9.3825 9.4488 9.6503 9.2731 10.1961 9.3820 9.1893 9.7485
9.8403 9.0089 9.7283 9.7553 9.5980 9.1450 9.0300 9.4879
9.8787 9.1767 8.8731 10.1681 9.6481 9.6673 9.2812 8.8838
9.0830 9.6711 9.7483 9.2255 9.4767 9.3374 9.4071 9.2500
9.6105 8.6141 9.8987 9.9167 9.6139 10.4156 9.4227 9.8916
8.4065 9.3837 8.6439 8.7357 9.3477 8.9721 8.9662 9.6163
9.0659 9.9001 9.1829 9.4658 9.8161 9.8024 8.4697 9.9524
8.9231 9.5353 9.8479 9.2071 9.7238 9.1051 8.7381 9.3701
10.0846 9.0015 10.2275 9.1316 9.7542 8.5045 9.0314 9.6406
9.2270 9.7013 9.3220 10.2412 9.3951 8.7321 8.9505 9.2256
9.5251 9.7227 8.7038\
IL1RAPL1 0.0000 0.9197 0.0000 0.0000 0.0000 0.0000
1.2456 0.4748 0.4327 0.4413 1.2544 0.0000 0.4363 0.0000
0.0000 0.0000 0.0000 0.0000 0.0000 0.0000 0.0000 0.0000
0.6346 0.9110 0.9530 0.0000 0.0000 0.0000 0.0000 0.5901
0.9954 0.0000 0.0000 0.0000 0.0000 0.0000 0.9556 0.0000
0.0000 0.0000 0.0000 0.4871 0.0000 0.0000 0.0000 0.0000
0.0000 0.0000 4.5310 0.0000 0.0000 0.0000 0.0000 1.1831
0.0000 0.0000 0.0000 0.0000 0.7533 0.0000 0.9353 4.2856
0.0000 0.0000 0.0000 0.0000 0.0000 1.5796 0.0000 0.0000
0.0000 0.0000 0.0000 0.0000 0.0000 0.4698 0.0000 0.0000
0.6894 4.3717 0.0000 0.0000 0.0000 0.0000 0.0000 0.0000
0.0000 0.0000 0.9125 0.0000 1.4126 2.3201 0.0000 0.0000
0.0000 0.0000 1.4589 0.0000 0.0000 3.7592 0.0000 0.0000
0.0000 0.0000 0.0000 0.0000 0.0000 1.2595 0.0000 0.0000
0.0000 0.0000 1.3631 1.0000 0.8924 1.0399 0.0000 0.0000
1.8223 0.0000 1.6899 0.0000 0.6224 0.0000 1.0180 1.2272
0.6149 0.0000 0.0000 0.5074 0.9567 0.0000 0.0000 0.0000
0.0000 0.0000 0.0000 0.0000 0.0000 0.8792 0.0000 0.0000
0.0000 0.0000 1.6921 0.9349 0.6735 1.2499 0.0000 0.0000
0.0000 0.0000 0.0000 0.5862 0.0000 1.0445 0.0000 0.0000
0.0000 0.0000 0.0000 0.0000 0.5821 0.4033 0.0000 0.0000
0.0000 0.4012 0.0000 0.0000 0.0000 0.0000 0.0000 0.6229
0.0000 0.0000 0.0000 1.0660 0.0000 0.0000 0.0000 1.1551
0.0000 0.0000 0.7366 0.0000 0.0000 0.0000 0.0000 1.2961
0.0000 0.0000 0.0000 0.0000 0.0000 0.0000 0.0000 0.0000
0.0000 0.0000 0.0000\
TMPRSS11B 0.0000 0.0000 1.5051 0.0000 0.0000 0.0000
0.0000 0.8314 0.0000 0.0000 0.0000 0.0000 0.0000 0.0000
0.0000 0.0000 0.0000 0.0000 0.0000 0.0000 0.0000 0.0000
0.0000 0.0000 0.0000 0.0000 0.0000 0.5990 0.0000 0.5901
0.0000 0.0000 0.0000 0.0000 0.0000 0.0000 0.0000 0.3648
4.4845 1.0370 0.0000 0.0000 0.0000 0.0000 0.0000 0.9285
0.0000 0.0000 0.0000 3.4820 0.0000 0.0000 0.0000 0.0000

0.4645 0.5608 0.0000 0.0000 0.0000 0.0000 0.0000 0.0000
0.0000 0.0000 0.0000 0.3906 0.0000 0.0000 0.0000 0.0000
0.0000 0.0000 0.0000 0.0000 2.7779 0.0000 0.0000 0.0000
0.0000 0.0000 0.5659 0.0000 0.0000 0.0000 0.0000 0.0000
0.0000 1.6425 0.0000 0.0000 0.0000 0.8470 0.0000 3.1676
0.0000 0.0000 0.0000 0.0000 0.0000 0.0000 0.0000 0.0000
0.0000 0.0000 0.0000 0.0000 0.0000 0.0000 0.0000 0.0000
0.0000 0.0000 0.0000 0.0000 0.0000 0.0000 0.0000 0.0000
0.0000 0.0000 0.0000 0.0000 0.0000 0.0000 0.0000 0.0000
0.0000 0.0000 0.0000 0.0000 0.0000 0.0000 0.0000 0.0000
0.0000 0.0000 0.0000 0.0000 7.4239 0.0000 0.0000 0.9848
0.0000 2.0686 3.9195 0.0000 1.9907 0.0000 0.0000 0.0000
1.4139 0.0000 0.0000 0.0000 0.0000 0.0000 0.0000 0.0000
0.6000 0.0000 0.0000 0.0000 0.0000 0.0000 0.4418 0.0000
0.0000 0.4012 0.0000 0.0000 0.0000 0.0000 0.0000 0.0000
0.0000 0.0000 0.0000 0.0000 0.4012 0.0000 0.0000 0.0000
0.0000 0.0000 0.0000 0.0000 0.0000 0.0000 0.6567 0.0000
0.8827 0.0000 0.0000 0.0000 0.0000 1.5946 0.0000 0.0000
1.1260 0.5077 0.0000\
IGF2R 10.6509 11.2358 13.1048 11.1777 10.5955 12.0348 11.8302
10.1910 11.9302 11.0293 11.6372 12.6328 11.3387 11.9732 11.7061
11.3726 10.4265 10.7852 11.0218 11.0623 10.7315 11.0545 10.3257
11.9069 10.9174 8.5802 10.7486 10.4903 12.4210 12.4372 13.4399
9.5117 11.7260 11.7205 12.4491 10.6459 11.6534 11.8560 11.0802
13.0363 9.9270 11.8018 11.6043 11.6040 11.8385 11.0894 10.1210
12.2364 11.2654 11.2794 11.4078 12.1266 12.1367 11.6502 12.2059
10.3335 11.5432 11.9933 12.0639 10.4573 11.5249 12.3333 12.1020
10.9013 10.8114 12.0421 11.7206 11.1390 12.5070 10.7898 10.7319
12.4455 10.9436 11.7855 11.8340 10.8164 10.8185 11.3318 10.3345
11.6969 11.2926 11.8294 10.5745 11.7696 10.7296 11.3214 11.7036
10.6085 10.8832 10.7201 11.3499 11.7894 11.9344 10.9889 10.9285
11.4167 13.2543 11.4930 12.2152 10.4079 10.4730 11.8478 11.9678
10.7727 10.3345 11.0824 10.2624 11.6010 11.8297 10.8715 11.6735
11.3889 10.2278 10.6777 11.8103 11.7970 12.0447 12.1130 11.8246
10.8995 10.8711 11.6172 12.6983 10.7046 10.4704 12.1283 11.3237
9.2959 11.3046 11.5442 11.4801 10.6912 11.3313 11.3339 10.3456
9.7494 10.0267 9.0599 11.6430 11.2946 11.0766 11.5831 10.1869
12.6756 11.4492 11.2313 11.2743 11.1754 10.8920 9.2782 11.4960
10.4286 12.6353 11.1971 12.3941 11.7844 11.3772 13.0753 10.1433
10.5676 12.8582 11.2070 10.8842 9.8999 11.5080 11.8290 10.4291
12.7839 11.2441 11.2554 12.3959 11.6498 11.3657 11.3671 8.9558
11.0055 10.6915 11.3591 11.4945 10.7258 10.2488 10.7518 11.0028
11.0782 11.8795 11.3580 12.1462 10.5756 10.4363 11.0398 12.1027
12.2738 11.2011 11.4268 11.7872 8.9658 8.8081 11.1723 12.3686
11.1937 9.9116\
TMPRSS11D 0.0000 0.5319 0.6896 0.4935 0.0000 0.4059
0.0000 0.8314 0.0000 0.7786 0.0000 0.0000 0.7708 2.8895
0.5377 0.0000 0.0000 0.0000 0.0000 0.0000 0.0000 0.4820
0.0000 0.9110 0.0000 0.0000 0.0000 0.5990 0.8532 1.3312
0.0000 0.0000 0.0000 0.0000 0.0000 0.0000 0.5555 1.7231
5.4519 3.4815 0.0000 0.0000 0.0000 0.0000 0.0000 2.8725
0.0000 0.0000 0.0000 4.4679 0.0000 0.0000 0.0000 0.0000
0.0000 1.2781 0.6343 0.0000 0.0000 0.0000 0.0000 0.8658
0.0000 0.0000 0.6828 3.5326 0.0000 0.4832 7.1671 0.0000

0.0000 0.9537 0.0000 0.0000 2.7779 0.0000 1.5242 2.5855
0.6894 2.5341 2.1251 0.0000 0.0000 0.6718 0.0000 0.0000
0.0000 0.0000 0.0000 0.0000 0.6361 0.4848 0.0000 2.4059
0.0000 0.0000 0.0000 0.5454 0.0000 0.0000 0.0000 0.0000
0.0000 0.0000 0.0000 0.0000 0.0000 0.0000 0.0000 0.0000
0.0000 0.0000 0.0000 0.0000 0.5141 0.0000 0.0000 0.0000
2.5250 0.0000 0.0000 0.0000 0.0000 0.0000 0.0000 0.0000
0.0000 0.0000 0.0000 0.5074 0.3935 0.0000 0.5526 0.0000
0.0000 0.0000 0.0000 0.0000 7.6843 0.0000 0.0000 5.2369
0.0000 6.6576 5.9797 0.0000 2.9153 0.0000 0.0000 0.7381
2.1141 0.0000 0.5414 0.5862 0.0000 0.0000 0.0000 1.6526
0.0000 0.0000 0.0000 0.0000 0.0000 0.0000 1.9510 0.0000
0.0000 1.9581 0.0000 1.5072 1.2980 0.5290 0.0000 0.6229
0.0000 2.6164 0.5232 0.0000 2.6160 0.0000 0.0000 0.0000
0.0000 0.0000 1.2218 0.0000 0.0000 0.0000 0.0000 0.0000
0.0000 0.9557 1.2903 0.0000 1.9594 0.0000 0.0000 0.6112
3.9250 1.1797 0.6959\
TMPRSS11F 0.0000 0.0000 0.0000 0.0000 0.0000 0.0000
0.0000 0.0000 0.0000 0.0000 1.0281 0.0000 0.0000 0.7759
0.0000 0.0000 0.0000 0.0000 0.0000 0.0000 0.0000 0.0000
0.6346 0.0000 0.0000 0.0000 0.0000 0.0000 0.0000 0.0000
0.0000 0.0000 0.5159 0.4664 0.0000 0.0000 0.5555 0.0000
0.0000 0.0000 0.0000 0.0000 0.0000 0.0000 0.0000 0.0000
0.0000 0.0000 0.0000 0.0000 0.4530 0.0000 0.0000 0.0000
0.0000 0.0000 0.0000 0.0000 0.0000 0.0000 0.0000 0.0000
0.0000 0.0000 0.0000 0.0000 0.0000 0.0000 0.0000 0.0000
0.0000 0.0000 0.0000 0.0000 0.0000 0.0000 0.0000 0.0000
0.0000 0.0000 0.0000 0.0000 0.0000 0.0000 0.0000 0.0000
0.0000 0.0000 0.0000 0.5265 0.0000 0.0000 0.0000 0.0000
0.0000 0.0000 0.0000 0.0000 0.0000 0.0000 0.0000 0.0000
0.0000 0.0000 0.0000 0.0000 0.0000 0.0000 0.5426 0.0000
0.0000 0.0000 0.0000 0.0000 0.5141 0.0000 0.0000 0.4321
0.0000 0.0000 0.0000 0.0000 0.0000 0.0000 0.0000 0.0000
0.0000 0.0000 0.0000 0.5074 0.0000 0.0000 0.0000 0.0000
0.0000 0.0000 0.0000 0.0000 0.0000 0.0000 0.0000 0.0000
0.0000 1.4986 0.8022 0.0000 0.0000 0.0000 0.5814 0.0000
0.0000 0.0000 0.9341 0.0000 0.0000 0.0000 0.0000 0.0000
0.0000 0.0000 0.0000 0.0000 0.0000 0.0000 0.0000 0.0000
0.0000 0.0000 0.0000 0.0000 0.0000 0.0000 0.0000 0.0000
0.0000 0.0000 0.0000 0.0000 0.4012 0.0000 0.0000 0.0000
0.0000 0.0000 0.0000 0.0000 0.0000 0.0000 0.0000 0.0000
0.5078 0.0000 0.0000 0.0000 0.0000 0.0000 0.0000 0.0000
0.0000 0.0000 0.0000\
ZC3H10 8.0683 8.3448 7.2042 7.3226 7.4107 8.8376 8.1792
8.5587 7.8658 7.9492 7.0995 7.6203 7.9817 7.1281 7.2467
7.6852 8.0653 7.7491 8.4478 7.8873 7.2697 7.3368 7.7506
7.3130 8.2776 7.2717 7.7861 7.5493 7.8658 7.6556 7.9815
7.1080 8.3354 7.7414 7.8306 8.4271 7.9298 7.6045 6.9994
8.3751 7.9236 8.0795 6.2701 9.0499 8.4479 7.0832 8.5145
8.0811 7.0935 8.1272 7.4490 8.1324 8.0944 8.4425 7.9646
7.8153 7.4598 8.2534 8.0081 7.8453 8.5226 7.1750 7.3294
9.0561 7.9111 7.9206 8.1181 8.1985 7.5125 7.8050 8.2844
7.8894 7.4196 7.9704 8.0649 6.2047 7.9250 7.7736 7.3733
8.1101 7.8937 7.1634 7.4530 7.9698 8.2198 7.1571 8.1686

| | | | | | | | |
|---|---|---|---|---|---|---|---|
| 7.8795 | 7.5718 | 7.6443 | 8.2028 | 8.3384 | 8.0794 | 7.5239 | 7.8621 |
| 8.0189 | 7.9288 | 8.8177 | 7.6396 | 7.4792 | 8.4255 | 7.4998 | 8.3253 |
| 7.9416 | 7.6093 | 8.4893 | 7.2806 | 7.6763 | 7.1227 | 7.7529 | 8.0063 |
| 7.6952 | 8.7595 | 7.4594 | 6.1277 | 7.5129 | 8.3467 | 8.1256 | 7.2711 |
| 6.9404 | 7.6350 | 7.6905 | 7.9098 | 7.4675 | 8.0187 | 7.1295 | 7.4538 |
| 9.5491 | 8.3223 | 7.9517 | 7.9029 | 8.5124 | 8.0198 | 6.7452 | 7.7794 |
| 7.5154 | 7.7241 | 8.1835 | 8.1786 | 8.9039 | 8.1560 | 6.5917 | 7.8565 |
| 7.8718 | 7.5777 | 8.4119 | 6.3767 | 8.2769 | 8.2839 | 8.0234 | 7.9432 |
| 7.9461 | 7.9155 | 7.4550 | 7.9078 | 7.4359 | 8.6774 | 7.9869 | 7.7414 |
| 7.0165 | 7.4462 | 6.8655 | 7.7462 | 8.0151 | 7.9701 | 7.2809 | 5.5942 |
| 7.7707 | 7.5185 | 8.2085 | 7.4599 | 7.6899 | 7.8157 | 7.9591 | 7.3192 |
| 7.9935 | 7.7168 | 8.0234 | 8.0281 | 7.6853 | 8.5086 | 7.5555 | 9.2014 |
| 7.9181 | 7.8959 | 7.8369 | 7.4602 | 8.4944 | 8.0593 | 8.2071 | 7.2602 |
| 7.3782 | 6.8115 | 6.7896 | 7.6010 | 8.5957 | 8.0174 | 9.0414 | 7.9636 |
| 7.7214 | 8.1732\ | | | | | | |
| RAD21L1 | 0.0000 | 0.0000 | 0.6896 | 0.0000 | 0.0000 | 0.0000 | 0.0000 |
| 0.4748 | 0.0000 | 0.0000 | 0.0000 | 0.0000 | 0.0000 | 0.0000 | 0.0000 |
| 0.0000 | 0.0000 | 0.0000 | 0.0000 | 0.0000 | 0.0000 | 0.0000 | 0.0000 |
| 0.0000 | 0.0000 | 0.0000 | 0.0000 | 0.0000 | 0.0000 | 0.0000 | 0.4127 |
| 0.9642 | 0.0000 | 2.1491 | 0.5853 | 0.0000 | 0.0000 | 0.0000 | 0.0000 |
| 0.0000 | 0.0000 | 0.0000 | 0.0000 | 0.0000 | 0.0000 | 0.0000 | 0.0000 |
| 0.0000 | 0.0000 | 0.0000 | 0.0000 | 0.0000 | 0.0000 | 0.0000 | 1.0973 |
| 0.0000 | 0.0000 | 2.3708 | 0.7533 | 0.9799 | 0.0000 | 3.0706 | 0.5573 |
| 0.5968 | 0.0000 | 0.0000 | 0.0000 | 0.0000 | 0.0000 | 0.0000 | 0.0000 |
| 0.0000 | 0.0000 | 0.0000 | 0.0000 | 0.0000 | 0.5548 | 0.0000 | 0.0000 |
| 0.6159 | 0.0000 | 0.0000 | 0.0000 | 0.0000 | 0.0000 | 0.0000 | 0.9284 |
| 0.0000 | 0.0000 | 0.0000 | 0.0000 | 0.0000 | 0.0000 | 0.0000 | 0.0000 |
| 0.0000 | 0.9065 | 0.0000 | 0.0000 | 0.5504 | 0.0000 | 0.0000 | 0.5352 |
| 0.0000 | 0.0000 | 0.0000 | 0.0000 | 0.0000 | 0.0000 | 0.0000 | 0.0000 |
| 0.0000 | 0.0000 | 0.0000 | 0.0000 | 0.0000 | 0.0000 | 0.0000 | 0.0000 |
| 0.6089 | 0.0000 | 1.3285 | 0.0000 | 0.0000 | 0.0000 | 0.0000 | 0.0000 |
| 0.0000 | 0.0000 | 1.1792 | 1.5269 | 0.0000 | 0.0000 | 0.0000 | 0.0000 |
| 0.0000 | 0.0000 | 0.0000 | 0.0000 | 0.0000 | 0.0000 | 0.0000 | 0.0000 |
| 0.0000 | 0.0000 | 0.0000 | 0.0000 | 0.0000 | 0.0000 | 0.0000 | 0.5019 |
| 0.0000 | 0.0000 | 0.0000 | 0.0000 | 0.0000 | 0.0000 | 0.0000 | 0.0000 |
| 0.0000 | 0.0000 | 0.0000 | 0.0000 | 0.4033 | 0.0000 | 0.0000 | 0.0000 |
| 2.9510 | 0.0000 | 0.0000 | 0.0000 | 0.0000 | 0.0000 | 0.0000 | 0.0000 |
| 0.0000 | 0.0000 | 0.0000 | 0.0000 | 0.0000 | 0.0000 | 0.0000 | 0.0000 |
| 0.0000 | 0.0000 | 0.0000 | 0.0000 | 0.0000 | 0.0000 | 0.0000 | 0.0000 |
| 0.0000 | 0.0000 | 0.0000 | 0.0000 | 0.0000 | 0.0000 | 0.0000 | 0.3736 |
| 0.0000 | 0.0000\ | | | | | | |
| IL1RAPL2 | | 0.0000 | 0.0000 | 4.6732 | 0.8605 | 0.9133 | 1.9724 |
| 3.3422 | 0.0000 | 4.6912 | 0.7786 | 0.4291 | 1.7643 | 2.7333 | 0.0000 |
| 0.0000 | 0.0000 | 0.6221 | 0.0000 | 0.0000 | 0.0000 | 1.6493 | 2.8772 |
| 1.4101 | 0.9110 | 0.0000 | 0.0000 | 1.5155 | 0.0000 | 0.0000 | 1.0077 |
| 2.2152 | 1.5370 | 0.0000 | 2.4804 | 3.0008 | 0.0000 | 3.0082 | 1.2859 |
| 1.2418 | 0.7666 | 0.0000 | 1.1407 | 1.3900 | 3.9055 | 0.0000 | 0.9285 |
| 0.0000 | 1.9920 | 4.9360 | 0.8339 | 0.0000 | 0.0000 | 1.0956 | 0.0000 |
| 3.9120 | 1.2781 | 2.5776 | 3.3475 | 3.0356 | 2.7727 | 0.0000 | 2.4655 |
| 0.0000 | 0.0000 | 0.6828 | 1.6675 | 0.0000 | 2.6255 | 0.5773 | 1.3773 |
| 0.4334 | 2.7936 | 0.0000 | 0.0000 | 0.0000 | 0.0000 | 0.5548 | 0.0000 |
| 0.0000 | 1.8728 | 2.1251 | 0.3965 | 0.0000 | 1.1284 | 0.5263 | 2.2503 |
| 0.0000 | 0.0000 | 0.5273 | 0.0000 | 0.0000 | 2.6303 | 2.1644 | 0.0000 |
| 0.5198 | 0.0000 | 3.6286 | 0.5454 | 1.4288 | 0.0000 | 0.0000 | 2.5739 |

| | | | | | | | |
|---|---|---|---|---|---|---|---|
| 0.9246 | 0.0000 | 0.4835 | 1.3363 | 0.0000 | 3.4735 | 4.2333 | 1.0041 |
| 2.7134 | 1.6572 | 2.0513 | 0.0000 | 1.4396 | 1.0399 | 1.6815 | 0.4321 |
| 0.7085 | 0.6089 | 2.4470 | 1.5923 | 3.4916 | 3.3012 | 1.6090 | 0.5331 |
| 0.0000 | 0.0000 | 0.0000 | 0.0000 | 2.8624 | 1.9805 | 0.0000 | 0.0000 |
| 0.0000 | 0.0000 | 0.0000 | 0.0000 | 1.8532 | 0.5056 | 1.3507 | 0.0000 |
| 0.0000 | 2.8855 | 1.8493 | 0.0000 | 1.7568 | 1.2499 | 0.9947 | 0.7381 |
| 0.5019 | 0.0000 | 2.3500 | 2.3250 | 1.5093 | 2.0663 | 1.2837 | 2.3166 |
| 0.0000 | 0.0000 | 3.1431 | 1.6421 | 0.9957 | 0.0000 | 3.8337 | 2.2474 |
| 0.0000 | 2.8261 | 0.5276 | 0.0000 | 0.0000 | 1.4707 | 2.2140 | 1.8874 |
| 0.0000 | 2.1868 | 0.9063 | 1.0660 | 2.2773 | 0.6314 | 0.5545 | 0.0000 |
| 0.9100 | 0.0000 | 3.4586 | 0.0000 | 2.5754 | 1.5325 | 0.6567 | 0.5707 |
| 4.2586 | 3.7744 | 0.0000 | 0.4262 | 0.0000 | 0.0000 | 1.0320 | 0.6112 |
| 2.5185 | 2.2618 | 2.7180\ | | | | | |
| SLC36A3 | 0.0000 | 0.0000 | 0.0000 | 0.0000 | 0.0000 | 0.0000 | 0.0000 |
| 0.0000 | 0.0000 | 0.7786 | 0.0000 | 0.0000 | 0.0000 | 0.0000 | 0.0000 |
| 0.0000 | 0.0000 | 0.0000 | 0.0000 | 0.0000 | 0.0000 | 0.0000 | 0.0000 |
| 0.0000 | 0.0000 | 0.0000 | 1.8490 | 0.0000 | 0.0000 | 0.0000 | 0.4127 |
| 0.0000 | 0.0000 | 0.0000 | 0.0000 | 0.0000 | 0.0000 | 0.0000 | 1.4957 |
| 0.0000 | 0.0000 | 0.0000 | 0.0000 | 0.0000 | 0.0000 | 0.0000 | 0.0000 |
| 0.0000 | 0.0000 | 0.0000 | 0.0000 | 0.0000 | 0.0000 | 0.0000 | 0.0000 |
| 0.5608 | 0.0000 | 0.0000 | 0.0000 | 0.0000 | 0.0000 | 0.4969 | 0.0000 |
| 0.0000 | 0.0000 | 0.0000 | 0.5980 | 0.0000 | 0.0000 | 0.0000 | 0.4334 |
| 0.3921 | 0.0000 | 0.0000 | 0.5367 | 0.0000 | 0.0000 | 0.9332 | 0.0000 |
| 0.0000 | 0.0000 | 0.0000 | 0.0000 | 0.6718 | 0.0000 | 0.0000 | 0.0000 |
| 0.0000 | 0.0000 | 0.0000 | 0.0000 | 0.0000 | 0.0000 | 0.0000 | 0.0000 |
| 0.0000 | 0.0000 | 0.5454 | 0.0000 | 0.0000 | 0.0000 | 0.0000 | 0.0000 |
| 0.0000 | 0.0000 | 0.0000 | 0.0000 | 0.0000 | 0.5426 | 0.0000 | 0.0000 |
| 0.0000 | 0.0000 | 0.0000 | 0.0000 | 0.0000 | 0.0000 | 0.4321 | 0.0000 |
| 0.0000 | 0.0000 | 0.0000 | 0.0000 | 0.0000 | 0.5970 | 0.0000 | 0.0000 |
| 0.0000 | 0.0000 | 0.0000 | 0.0000 | 0.0000 | 0.0000 | 0.0000 | 0.0000 |
| 0.0000 | 0.0000 | 0.0000 | 0.0000 | 0.8792 | 0.6010 | 0.0000 | 0.0000 |
| 0.5424 | 0.0000 | 0.0000 | 0.0000 | 0.0000 | 0.0000 | 0.7381 | 0.0000 |
| 0.0000 | 0.0000 | 0.0000 | 0.0000 | 0.0000 | 0.0000 | 0.3855 | 0.0000 |
| 0.0000 | 0.0000 | 1.3735 | 0.0000 | 0.0000 | 0.0000 | 0.5023 | 0.0000 |
| 0.0000 | 0.0000 | 0.0000 | 0.0000 | 0.0000 | 0.0000 | 0.0000 | 0.0000 |
| 0.0000 | 0.0000 | 0.0000 | 0.0000 | 0.0000 | 0.0000 | 0.0000 | 0.0000 |
| 0.5055 | 0.0000 | 0.0000 | 0.0000 | 0.0000 | 0.0000 | 0.9785 | 0.0000 |
| 0.0000 | 0.0000 | 0.0000 | 0.0000 | 0.0000 | 0.0000 | 0.0000 | 0.0000 |
| 0.0000 | 1.7990\ | | | | | | |
| CKS1B | 10.5381 | 7.8878 | 9.6218 | 8.9863 | 10.8962 | 10.4372 | 10.3818 |
| 10.9147 | 10.7911 | 9.1084 | 10.7528 | 9.9510 | 10.5446 | 9.7277 | 9.4629 |
| 9.1124 | 10.5211 | 9.8166 | 7.5965 | 11.5472 | 10.4622 | 9.0132 | 8.4790 |
| 9.3670 | 8.3162 | 10.8611 | 11.5851 | 10.2735 | 9.5086 | 10.5399 | 10.1193 |
| 10.3140 | 11.4240 | 10.8266 | 10.3905 | 10.4879 | 9.7189 | 10.4363 | 9.7985 |
| 9.3507 | 10.0121 | 7.9448 | 10.0643 | 10.1883 | 8.5229 | 8.8705 | 12.7848 |
| 9.9395 | 10.4418 | 10.4805 | 9.7508 | 9.7212 | 10.3774 | 8.7197 | 10.3669 |
| 9.5647 | 9.5640 | 8.5028 | 11.0797 | 10.1181 | 8.6377 | 10.8967 | 10.3279 |
| 10.4403 | 8.4672 | 9.5280 | 9.4083 | 10.2987 | 7.7781 | 10.3355 | 12.0008 |
| 10.2163 | 11.3810 | 10.5562 | 9.6704 | 9.3698 | 9.5688 | 7.7464 | 8.2864 |
| 10.2787 | 10.4706 | 10.8604 | 7.3819 | 10.1395 | 9.9392 | 10.4721 | 10.5100 |
| 10.2559 | 10.1532 | 10.9080 | 8.6888 | 10.6561 | 9.8047 | 7.5944 | 11.1307 |
| 8.3926 | 10.6006 | 8.2918 | 9.9083 | 8.8939 | 11.0054 | 9.5757 | 10.9468 |
| 9.3700 | 11.0283 | 11.9789 | 7.7070 | 9.7866 | 10.7411 | 10.5531 | 9.8606 |
| 9.7399 | 12.1350 | 9.2969 | 10.8908 | 10.4079 | 10.7760 | 10.1212 | 9.6009 |

| | | | | | | | |
|---|---|---|---|---|---|---|---|
| 9.9370 | 11.3543 | 9.6239 | 10.0392 | 9.4833 | 8.8154 | 11.1553 | 10.7919 |
| 12.5874 | 8.5447 | 8.3945 | 11.2200 | 9.5336 | 9.4466 | 8.9375 | 10.2639 |
| 12.6153 | 11.7490 | 7.1027 | 8.9984 | 8.2178 | 10.0927 | 9.9729 | 9.2299 |
| 9.7535 | 9.9790 | 8.3427 | 9.4302 | 8.9428 | 9.9449 | 12.1648 | 8.5073 |
| 11.7036 | 9.3820 | 10.4063 | 10.4159 | 10.8759 | 8.1600 | 10.4171 | 11.4517 |
| 11.4550 | 9.6298 | 9.6921 | 9.0933 | 9.6113 | 10.1107 | 9.7139 | 9.6804 |
| 10.3583 | 10.1562 | 11.5987 | 9.9305 | 11.4008 | 10.2933 | 11.5195 | 8.2369 |
| 9.7148 | 8.2640 | 8.9281 | 10.8858 | 7.5981 | 11.6397 | 10.8263 | 10.9630 |
| 8.6976 | 10.3341 | 9.5508 | 10.5013 | 11.1388 | 9.9532 | 11.1622 | 9.9628 |
| 10.1978 | 9.7607 | 10.8072 | 10.1023 | 13.6122 | 11.4807 | 8.8386 | 9.2350 |
| 9.0757 | 12.0455\ | | | | | | |
| SLC36A1 | 7.0561 | 7.3939 | 8.9671 | 7.4288 | 7.9092 | 7.8438 | 8.4633 |
| 8.2246 | 7.8701 | 7.4501 | 7.6871 | 8.1752 | 7.9917 | 6.6284 | 7.5477 |
| 7.8122 | 7.6930 | 8.2119 | 7.9825 | 6.3657 | 8.0199 | 6.9859 | 5.4805 |
| 7.6995 | 6.8241 | 8.4045 | 7.8102 | 7.5413 | 7.9148 | 6.7157 | 8.9028 |
| 8.6316 | 8.6744 | 8.1077 | 7.4521 | 7.7312 | 8.4119 | 9.2349 | 7.4757 |
| 7.8887 | 7.1976 | 7.5184 | 7.0237 | 7.7621 | 6.9698 | 7.3299 | 6.8033 |
| 7.6899 | 9.2585 | 7.2556 | 8.5033 | 8.6759 | 7.7389 | 7.3949 | 8.3493 |
| 8.0219 | 6.6809 | 7.7485 | 7.6746 | 6.9541 | 7.0534 | 8.1985 | 7.1966 |
| 8.1540 | 7.7742 | 8.0800 | 8.4649 | 8.2820 | 8.8750 | 7.8119 | 7.6888 |
| 7.9157 | 7.5677 | 7.5535 | 8.0029 | 7.2902 | 7.8241 | 7.3275 | 6.8137 |
| 8.7980 | 8.3152 | 7.3761 | 6.8207 | 7.5117 | 6.3881 | 8.8337 | 7.7785 |
| 6.9613 | 8.3474 | 8.8833 | 7.3566 | 8.0322 | 7.8234 | 8.0064 | 7.6228 |
| 7.8792 | 8.9180 | 6.8096 | 7.6396 | 7.3289 | 7.1156 | 7.9702 | 8.1494 |
| 6.7386 | 6.9543 | 7.3079 | 7.3465 | 8.1675 | 7.4660 | 6.8025 | 6.5247 |
| 7.9363 | 6.7747 | 6.4919 | 7.8457 | 8.0002 | 7.5427 | 8.1506 | 8.0625 |
| 7.9000 | 7.8145 | 7.4908 | 7.5392 | 8.3725 | 5.7494 | 8.4332 | 8.8427 |
| 6.9001 | 6.9019 | 7.6251 | 8.4706 | 7.3069 | 7.2849 | 8.7537 | 8.5625 |
| 6.0154 | 6.7758 | 7.6644 | 7.7176 | 6.8047 | 7.4695 | 7.8490 | 6.9748 |
| 8.2198 | 7.9143 | 6.5404 | 7.7808 | 6.6205 | 7.4610 | 6.5000 | 7.9260 |
| 6.6912 | 8.1230 | 8.2938 | 7.4440 | 8.2657 | 6.5364 | 9.0497 | 6.4954 |
| 7.9956 | 8.0284 | 7.7319 | 7.4832 | 7.7600 | 8.3247 | 7.7791 | 6.8119 |
| 8.4819 | 8.2908 | 7.2531 | 7.6236 | 7.9779 | 8.3680 | 7.8553 | 6.7758 |
| 7.9083 | 7.8403 | 7.0290 | 8.0683 | 7.2271 | 7.8348 | 7.8054 | 7.4076 |
| 7.9984 | 8.3639 | 7.6275 | 8.3679 | 7.4048 | 7.4424 | 8.1565 | 8.5104 |
| 7.7559 | 7.9042 | 8.3477 | 8.2574 | 6.6437 | 7.2379 | 6.3336 | 8.1583 |
| 7.4423 | 5.9339\ | | | | | | |
| ST13 | 12.1057 | 13.1291 | 12.7950 | 12.5455 | 12.5228 | 11.6100 | 12.0356 |
| 11.7122 | 12.9526 | 12.2769 | 12.1720 | 11.7988 | 12.1422 | 11.9200 | 12.4211 |
| 11.6120 | 11.9781 | 12.3358 | 12.6416 | 12.2150 | 11.2740 | 12.2451 | 12.7442 |
| 12.4966 | 13.1643 | 12.1364 | 11.4679 | 12.2755 | 11.9881 | 12.2746 | 12.5488 |
| 11.9470 | 11.4648 | 12.0368 | 12.2559 | 12.4191 | 11.7473 | 12.2560 | 12.1701 |
| 12.4095 | 12.9583 | 12.9110 | 12.3190 | 12.2986 | 13.9566 | 11.7871 | 11.5085 |
| 12.0271 | 12.8735 | 12.5505 | 11.8257 | 11.5781 | 11.1085 | 13.1937 | 11.2914 |
| 12.4702 | 12.8703 | 12.9727 | 12.1303 | 12.6824 | 13.4938 | 13.0772 | 13.5255 |
| 11.9988 | 12.5543 | 12.1941 | 11.9162 | 12.2639 | 12.1259 | 11.8635 | 11.5683 |
| 12.5706 | 11.8922 | 11.3246 | 12.4267 | 11.9021 | 11.9746 | 12.4768 | 12.1647 |
| 12.8147 | 12.2414 | 12.2648 | 12.2102 | 11.4237 | 12.4487 | 10.6147 | 12.3341 |
| 12.2616 | 12.6585 | 11.8136 | 12.8005 | 13.2940 | 11.5687 | 11.8389 | 12.0332 |
| 13.3652 | 12.0783 | 13.4435 | 12.1710 | 12.1189 | 11.9661 | 12.3024 | 12.1187 |
| 11.2563 | 11.7597 | 12.2334 | 11.9108 | 11.4265 | 12.0417 | 11.6180 | 12.4521 |
| 12.5127 | 11.4800 | 11.6439 | 12.7108 | 12.2780 | 12.5064 | 11.8013 | 11.5330 |
| 12.3460 | 12.7930 | 12.5269 | 12.4143 | 12.4551 | 12.8808 | 12.4089 | 11.8640 |
| 11.3993 | 13.3488 | 12.7502 | 12.0929 | 11.7275 | 12.5269 | 10.6820 | 11.5534 |

11.6003 12.3986 10.9172 12.7767 13.2345 11.4702 12.4818 12.3762
12.3263 12.0262 14.3602 12.2048 12.7751 12.1035 12.4499 12.6010
10.9244 11.9800 11.6352 12.0131 11.6304 13.1724 12.1306 11.5692
12.1528 12.3355 11.7935 12.5970 11.9163 12.1976 12.5799 11.8326
12.1390 11.9806 11.6544 12.4244 12.1029 11.2602 12.7567 11.0238
12.2560 12.4138 12.9608 12.5334 12.1800 12.2942 12.3670 11.8762
12.7787 12.4754 12.8064 11.8029 11.8269 12.3626 12.7777 12.4959
12.1888 11.7194 11.6521 11.8542 11.8541 11.3284 13.5808 11.9886
12.6451 11.7723\
RPL31P11 3.9940 2.8563 2.0229 1.9468 2.6553 2.1935
3.0554 3.7518 2.9992 2.8241 2.9870 2.8860 3.2969 3.0838
2.0573 3.5017 2.6760 1.6514 2.4567 2.5253 1.6493 2.3124
2.4383 2.3107 3.4365 5.0831 2.4493 1.3471 4.8692 2.0116
2.1085 0.5612 3.6563 2.2682 1.8080 3.8373 1.2685 2.1545
3.1268 2.2802 3.5284 3.0408 2.2575 1.6313 2.6279 1.8914
3.4056 4.9439 2.8207 2.7801 3.4541 2.7455 2.1411 2.8784
2.0139 3.3925 1.0734 2.2495 2.9096 3.2855 3.6848 2.6657
2.7350 1.8326 2.6888 2.0390 3.0340 2.8806 2.3036 1.0463
3.2341 2.4255 3.4823 2.7868 2.4611 2.0284 3.1657 2.5855
3.1642 2.9859 3.3401 1.3683 1.8442 2.7931 3.4757 3.1783
3.6714 3.1631 3.2962 2.1773 3.6594 6.1393 3.7214 2.5641
2.1605 2.9121 2.1693 2.9802 2.6033 3.7592 5.0344 1.6155
0.9246 2.9361 3.6025 2.2668 3.4466 2.5557 2.4767 3.0946
2.8045 2.2538 3.3832 3.1699 2.1456 3.2401 3.2310 2.8649
2.1659 3.2329 2.7943 3.4023 3.3462 2.9158 6.3573 1.2272
3.8366 5.0345 3.3289 2.2611 2.2519 3.3824 2.7225 2.5437
3.4335 3.1012 3.9590 4.5503 2.9132 3.2947 4.1743 3.3646
3.1663 1.7146 1.5157 4.2994 2.3685 3.6943 3.2383 3.7031
2.6803 5.4557 2.0661 0.5862 1.2540 3.1934 2.6463 3.0619
1.8394 3.2017 2.3724 1.3735 2.9924 3.0171 2.3049 1.9692
3.7412 1.3802 1.8676 1.7238 1.2980 2.3178 3.2697 2.9029
5.0451 3.3334 2.5381 3.4387 2.2773 2.4311 3.6686 3.3509
6.5642 3.6724 3.9060 3.3525 2.1610 2.4374 3.5793 2.5489
2.1293 3.5027 3.0407 1.9066 2.1049 3.1828 2.6380 3.0678
2.2759 2.2618 2.9670\
C14orf115 0.0000 2.3255 0.0000 0.0000 0.0000 2.3851
0.0000 0.4748 0.0000 0.0000 0.0000 0.8472 0.4363 0.0000
0.5377 0.0000 0.0000 0.0000 0.0000 0.9198 0.6175 0.4820
0.6346 0.0000 0.0000 1.0317 0.0000 0.0000 0.0000 0.0000
0.0000 0.0000 0.0000 1.5403 0.0000 0.6819 0.5555 0.0000
0.0000 0.0000 0.6649 0.0000 0.0000 0.0000 0.0000 0.0000
0.0000 0.0000 1.4771 0.4764 0.0000 1.2789 0.6494 0.0000
0.0000 0.0000 0.0000 0.0000 0.0000 0.5715 0.0000 1.4031
0.0000 0.0000 0.0000 0.0000 0.0000 0.0000 0.0000 0.0000
0.0000 0.0000 0.0000 0.4786 0.5367 0.0000 1.9314 0.9332
0.0000 1.0461 2.5369 0.0000 0.0000 0.0000 1.4651 3.2615
0.0000 1.6425 0.5273 0.0000 0.0000 0.4848 2.8268 0.0000
0.0000 0.6266 1.8574 0.0000 1.6394 0.0000 0.0000 0.4986
0.0000 0.0000 0.0000 0.4659 0.0000 3.1157 0.0000 0.0000
0.0000 0.6215 0.0000 0.0000 0.0000 0.6116 0.0000 1.9235
0.0000 0.0000 5.5320 0.0000 0.0000 0.0000 0.0000 0.0000
0.0000 4.0779 0.0000 0.0000 0.0000 0.0000 0.5526 0.0000
0.0000 0.0000 1.0837 0.0000 0.0000 0.0000 0.6010 0.0000
0.6506 0.5424 0.0000 0.0000 0.0000 1.2499 1.7997 1.2240

```
0.0000  0.0000  0.0000  1.0019  1.2540  0.4374  0.0000  0.6894
1.3489  1.7128  1.3600  0.0000  0.9957  0.4033  0.0000  0.0000
0.0000  2.0725  0.0000  0.0000  0.4486  0.9151  0.0000  0.0000
0.0000  0.0000  0.5232  0.0000  2.2773  0.0000  0.0000  0.0000
1.4637  0.0000  0.7366  0.0000  2.1610  1.5325  0.0000  0.0000
0.0000  0.0000  0.0000  0.0000  0.4985  0.0000  0.0000  0.0000
0.0000  0.5077  0.0000\
ZC3H15  10.3864 10.3043 11.1452 10.4910 10.2631 10.8827 10.3739
10.4704 11.6847 10.3316 11.0758 10.4791 10.9309 10.9785 10.6185
9.4962  10.3112 10.6642 10.2820 10.8477 10.3645 10.5516 9.9285
10.4355 10.3096 10.4799 10.8867 10.0431 9.9971  10.2592 10.5258
10.2342 11.4840 11.0550 11.6409 11.1071 10.2909 10.3598 11.0649
11.0314 10.4418 10.6816 10.7259 10.8135 10.2250 10.8011 8.8326
10.4426 11.4690 10.2277 11.0267 10.0443 10.7072 10.3178 10.6037
10.6940 10.9030 11.1311 11.6573 10.2988 10.4107 11.2911 11.0359
10.5941 10.5152 10.5941 10.3241 10.9469 10.3844 10.3977 10.4901
10.9362 9.8636  10.7196 10.9765 10.1714 10.7701 9.7360  9.8540
10.6015 10.2669 11.2554 9.7928  10.7411 10.1069 10.9519 11.1286
10.8511 10.7932 9.4291  10.0868 10.6889 10.1830 9.8142  10.8728
10.3143 10.9643 10.1193 10.3697 10.6305 9.9257  10.6042 10.5145
10.1431 11.0092 10.2855 10.2167 11.0268 10.7001 10.2802 10.2552
10.6510 9.3134  9.8750  11.2104 11.3935 10.7261 10.6453 10.8392
10.1914 10.9324 10.8087 10.6744 10.5217 10.1938 11.0923 11.0161
9.6077  10.3232 10.4783 10.7954 10.9716 10.5386 10.8586 11.4381
8.9299  10.2340 9.2579  10.5078 10.1447 10.4538 10.6154 9.8177
10.6317 10.8815 10.0385 11.3611 9.7013  10.6163 9.3830  10.0016
9.2872  10.6420 11.6389 10.5321 11.4040 10.1944 10.1588 11.1215
10.2300 10.5396 10.0706 10.5762 10.3605 10.7342 10.9520 10.6287
10.9080 10.7026 10.2288 10.9254 11.0604 9.3265  10.7397 8.6357
10.5382 10.7820 10.1084 10.8447 9.8164  10.1623 10.4267 11.3669
10.5172 11.4206 10.9708 10.7534 10.1386 10.2536 11.1533 10.6965
10.4599 11.0613 11.1696 10.9389 9.8956  9.3739  10.2154 10.4910
10.4271 9.5069\
TMEM213 4.4595  1.2250  4.8974  6.9617  4.0015  1.3921  0.0000
1.5600  1.0352  3.4617  1.6222  1.1366  0.7708  0.0000  4.5599
2.1513  2.4094  0.0000  0.5838  3.0760  1.6493  2.9530  0.0000
5.0112  2.0961  0.0000  1.0809  3.6823  1.3857  2.9298  0.0000
8.1243  0.0000  2.4804  1.0004  1.4920  1.7435  5.0288  4.5973
2.4747  5.8083  3.9124  0.0000  2.8667  0.0000  4.9870  4.1918
0.6741  0.4402  5.3405  2.2292  0.0000  2.8587  0.5094  0.8153
3.2557  0.6343  6.5229  1.2457  2.7727  0.0000  1.1594  0.5573
1.0177  3.0468  5.5456  4.4964  2.4264  0.5773  5.2587  1.2639
8.9521  0.9449  0.0000  5.4826  4.2837  1.2673  5.7604  2.4028
0.6159  0.5659  1.5351  5.0610  1.1284  3.8824  4.7711  1.2355
1.0433  0.9125  2.5470  0.6361  3.8257  0.9036  4.1858  3.2084
0.0000  5.0972  1.5047  5.1575  0.0000  2.9428  5.9176  3.0331
4.8552  7.9485  0.0000  1.5756  0.7631  3.5800  4.1776  5.8114
1.8846  2.0513  1.5850  0.5141  0.6116  4.7097  5.0947  3.2084
2.2252  1.6899  8.1784  4.7776  8.7246  1.6090  0.5331  4.6525
0.0000  0.9653  5.2354  1.1727  8.2783  4.4595  4.3933  0.0000
0.0000  1.4217  3.3710  1.2057  0.8792  3.8161  7.9018  6.8261
3.1308  1.8493  0.0000  8.3186  0.0000  3.7973  2.9411  6.3567
4.5495  5.2685  0.5862  0.5476  0.0000  1.7618  0.3855  1.3489
0.9345  4.3817  0.0000  5.7057  0.9764  2.1964  4.6766  0.0000
```

7.1601 0.5276 1.2521 0.0000 1.2195 1.2418 0.0000 6.5372
4.0865 4.3694 0.6293 3.1725 5.8743 5.4632 3.5901 0.5257
0.8792 4.1955 1.3929 0.5811 5.0245 1.9569 1.2961 6.9146
4.0446 4.8794 2.5389 1.9594 1.5946 4.6458 0.6112 3.6266
4.4374 0.0000\
C14orf118 6.7469 6.9702 6.6913 6.9563 7.8076 7.9856
6.7604 7.3839 6.8590 7.2375 5.7079 6.9870 7.5503 7.0293
7.5788 5.9364 6.3360 6.6775 7.0023 6.5509 6.7311 7.3154
5.9996 6.7125 7.4518 5.6613 7.1105 5.9836 6.3225 7.9222
7.6468 7.5241 7.2098 7.8019 8.3496 6.7940 7.0085 7.2207
6.3332 8.9195 6.8476 8.3105 6.4323 7.2283 7.5510 6.2981
5.2997 6.9436 8.2941 5.4987 7.2219 8.3717 6.4309 7.1021
7.6979 7.4658 7.1756 7.1542 7.2302 7.1789 7.6723 6.8182
7.3955 6.7628 7.1866 7.6815 7.2577 7.2189 6.9773 6.4477
6.5415 7.3903 6.2289 5.5674 7.4283 6.5212 6.4710 7.0441
5.5717 7.3940 6.0091 8.9095 6.3794 7.4247 6.4905 7.5491
7.2333 6.1928 7.8173 6.4698 6.8882 7.2886 6.2865 6.1251
6.6110 7.4695 7.7875 6.8446 7.7906 6.9606 5.7235 7.2989
7.0161 7.3980 6.9120 7.4214 6.5457 7.2103 7.5137 6.8660
6.5966 7.3684 5.7248 7.7347 7.3280 7.7648 7.1438 8.3257
7.7840 6.7634 7.8728 6.6834 7.1638 7.0811 7.0071 7.2947
8.2825 4.5915 7.2745 8.4125 7.7453 6.9073 6.4866 6.3772
6.1677 5.4665 6.1827 6.2435 7.6566 7.1668 7.4311 6.5546
5.9401 7.4083 7.5861 7.5746 6.3133 6.6205 7.1542 5.5574
7.8473 5.8943 7.6923 7.9405 7.3573 7.9862 7.3456 7.4932
7.1788 6.3350 6.0392 6.9911 6.9686 6.2323 7.0731 8.0592
4.6862 7.4199 6.3849 6.3343 8.0989 7.2312 6.3811 5.8903
4.4516 6.9284 7.0835 7.2104 7.7213 6.0990 6.0978 6.6582
7.4151 6.4503 7.0615 6.7002 7.3329 6.2614 5.5224 7.4447
7.9846 6.6791 5.9364 8.2271 7.3722 4.1011 5.5377 7.2813
7.5758 7.4388 5.5884\
ARTN 6.9013 4.6223 8.9706 7.9552 6.7053 6.9330 4.6568
4.8017 5.1691 5.5260 6.4125 5.2837 4.7238 6.8539 8.1371
7.4356 7.0146 6.8746 4.5513 6.8513 6.0481 6.6690 5.0938
8.4995 4.6086 6.2417 6.1850 6.2700 6.3370 5.1849 3.2649
7.7385 5.1099 5.7867 5.0232 5.5522 6.3032 6.8227 7.2268
6.4817 6.8329 4.6495 6.6341 5.2370 4.2967 7.8140 7.8377
5.2676 7.4869 8.2153 6.9219 7.5148 5.3096 4.0222 5.3399
7.1651 5.9221 6.0372 4.4310 5.7017 4.1601 2.9982 6.0040
5.9415 5.6094 5.3172 7.5621 6.7496 6.6498 7.7237 6.2071
5.4317 5.5338 4.4753 5.9609 7.2543 7.0884 6.1411 6.5843
6.5076 7.2554 6.5400 5.8086 7.6374 6.6189 7.6832 5.5143
7.0848 6.2310 7.5182 3.9963 5.0068 7.8617 7.2489 6.6046
3.1069 7.3888 4.1697 5.9022 6.1335 7.2110 6.8654 9.5335
8.4108 6.5323 7.8270 5.1428 5.7820 4.6022 7.1270 5.7554
5.3522 6.7952 6.4429 5.6869 4.7481 6.8389 6.9531 8.6145
6.5456 4.0191 5.5958 5.6617 7.0687 4.7624 4.2043 6.6107
7.0173 3.7423 4.8905 6.5422 6.8787 6.5089 8.1704 6.2415
5.9461 8.3618 4.9498 5.2152 4.0098 8.5886 6.7111 5.9927
6.3618 6.6156 2.2162 5.7212 4.9477 8.0116 7.8038 6.4034
7.4630 6.1431 6.4957 6.2527 5.9689 4.6108 6.3550 6.1315
3.4624 6.9015 4.1243 6.1812 8.5123 6.2390 5.7182 6.4649
5.5598 7.5798 7.7339 6.8879 6.9504 6.8422 5.8903 7.7414
7.1043 4.8783 4.7388 5.5697 4.7148 7.3829 4.5289 7.3309

| | | | | | | | |
|---|---|---|---|---|---|---|---|
| 6.5311 | 4.1421 | 6.1146 | 1.5773 | 7.4883 | 6.8616 | 6.2146 | 6.0802 |
| 5.4818 | 7.0766 | 5.4122 | 6.4057 | 6.6143 | 6.8830 | 3.9300 | 7.0407 |
| 5.5537 | 7.5372\ | | | | | | |
| ART1 | 0.0000 | 1.2250 | 0.0000 | 0.0000 | 0.5278 | 0.7223 | 0.0000 |
| 0.4748 | 0.0000 | 0.0000 | 0.7594 | 0.4849 | 0.7708 | 0.0000 | 0.9286 |
| 0.0000 | 0.0000 | 0.0000 | 0.0000 | 0.3755 | 0.0000 | 1.7571 | 0.0000 |
| 0.0000 | 2.2460 | 0.0000 | 0.0000 | 0.0000 | 0.0000 | 0.0000 | 0.4127 |
| 0.5612 | 0.0000 | 0.0000 | 0.0000 | 0.0000 | 0.0000 | 0.0000 | 0.0000 |
| 0.0000 | 0.6649 | 0.4871 | 0.0000 | 0.0000 | 0.0000 | 0.0000 | 0.0000 |
| 0.0000 | 0.0000 | 0.0000 | 0.4530 | 0.0000 | 0.0000 | 1.8243 | 0.0000 |
| 0.0000 | 1.0734 | 0.5030 | 0.4252 | 0.0000 | 1.7140 | 0.0000 | 0.0000 |
| 0.5968 | 0.0000 | 0.3906 | 0.5980 | 0.0000 | 0.0000 | 1.3773 | 0.0000 |
| 0.0000 | 0.0000 | 0.0000 | 0.9270 | 0.4698 | 0.5548 | 1.7110 | 0.0000 |
| 0.0000 | 0.0000 | 0.0000 | 0.7204 | 0.0000 | 0.0000 | 0.0000 | 0.0000 |
| 0.0000 | 0.0000 | 0.5265 | 0.0000 | 0.4848 | 0.0000 | 0.0000 | 0.0000 |
| 1.0621 | 0.0000 | 0.0000 | 0.0000 | 0.0000 | 0.0000 | 0.4986 | 0.0000 |
| 1.2208 | 0.0000 | 0.0000 | 0.0000 | 0.4315 | 0.0000 | 0.0000 | 0.0000 |
| 0.0000 | 0.0000 | 0.0000 | 0.0000 | 0.0000 | 1.6815 | 0.4321 | 0.3974 |
| 0.0000 | 0.0000 | 0.0000 | 0.0000 | 0.0000 | 0.0000 | 0.0000 | 0.0000 |
| 0.0000 | 2.2660 | 1.6358 | 0.0000 | 0.0000 | 0.0000 | 1.9109 | 0.0000 |
| 0.0000 | 0.0000 | 0.0000 | 0.9038 | 0.0000 | 0.0000 | 0.5748 | 0.0000 |
| 0.5424 | 0.0000 | 0.0000 | 0.0000 | 0.0000 | 0.0000 | 0.7381 | 2.2465 |
| 0.0000 | 0.0000 | 0.0000 | 0.0000 | 0.0000 | 0.0000 | 0.0000 | 2.2048 |
| 0.0000 | 0.0000 | 0.0000 | 0.0000 | 0.0000 | 0.0000 | 0.0000 | 0.0000 |
| 1.1906 | 0.5276 | 0.0000 | 0.0000 | 0.0000 | 0.5410 | 0.0000 | 0.0000 |
| 0.0000 | 0.0000 | 0.0000 | 0.0000 | 1.0692 | 0.0000 | 0.0000 | 0.0000 |
| 0.0000 | 0.0000 | 0.0000 | 0.0000 | 0.0000 | 0.0000 | 0.0000 | 0.5078 |
| 0.0000 | 0.0000 | 0.0000 | 0.4985 | 0.0000 | 0.0000 | 0.0000 | 0.0000 |
| 1.1797 | 0.0000\ | | | | | | |
| HRH3 | 3.1593 | 0.0000 | 0.0000 | 1.6038 | 0.5278 | 0.0000 | 3.4667 |
| 0.8314 | 0.0000 | 0.7786 | 0.0000 | 0.0000 | 0.4363 | 2.6649 | 0.0000 |
| 0.0000 | 0.6221 | 0.7772 | 0.0000 | 0.3755 | 0.0000 | 1.3712 | 0.0000 |
| 0.0000 | 0.0000 | 2.6374 | 1.6918 | 0.5990 | 0.8532 | 0.5901 | 2.2152 |
| 0.0000 | 0.8951 | 1.1010 | 1.0004 | 1.1431 | 4.7710 | 0.6557 | 0.5410 |
| 0.0000 | 0.0000 | 0.0000 | 1.0569 | 0.0000 | 0.0000 | 0.0000 | 0.0000 |
| 0.6741 | 0.0000 | 0.0000 | 0.4530 | 1.2789 | 0.0000 | 0.0000 | 1.3330 |
| 2.9356 | 0.6343 | 0.5030 | 0.0000 | 0.5715 | 0.0000 | 0.8658 | 0.5573 |
| 0.0000 | 0.0000 | 0.3906 | 0.0000 | 3.6479 | 1.5697 | 0.0000 | 0.4334 |
| 0.0000 | 1.9166 | 0.8374 | 0.5367 | 0.0000 | 1.7421 | 0.0000 | 0.6894 |
| 0.0000 | 2.1251 | 0.0000 | 0.0000 | 1.1284 | 2.8405 | 1.8814 | 0.9284 |
| 0.6140 | 2.5491 | 9.3485 | 0.0000 | 0.4848 | 3.0706 | 2.8364 | 0.9013 |
| 0.0000 | 1.2090 | 0.5454 | 2.2653 | 0.0000 | 0.0000 | 0.4986 | 0.0000 |
| 1.8724 | 1.1338 | 0.0000 | 0.0000 | 0.4315 | 1.7151 | 3.1772 | 0.5136 |
| 1.8846 | 4.8729 | 2.5850 | 0.5141 | 2.5241 | 3.6917 | 1.2610 | 0.7085 |
| 0.0000 | 3.0090 | 0.0000 | 0.0000 | 0.4700 | 0.0000 | 0.5331 | 2.5319 |
| 1.2894 | 0.0000 | 0.0000 | 0.3935 | 0.0000 | 1.7370 | 2.3620 | 0.0000 |
| 0.0000 | 0.0000 | 0.0000 | 0.0000 | 0.0000 | 1.3507 | 0.5748 | 0.0000 |
| 0.0000 | 1.3147 | 0.0000 | 0.6735 | 0.0000 | 4.9203 | 5.8691 | 0.0000 |
| 0.0000 | 0.9341 | 0.0000 | 0.0000 | 0.4374 | 0.0000 | 6.5656 | 1.3489 |
| 0.9345 | 0.0000 | 0.0000 | 0.9957 | 0.0000 | 5.7267 | 0.8742 | 0.0000 |
| 0.4012 | 0.5276 | 0.9420 | 1.2980 | 0.0000 | 7.7803 | 0.0000 | 0.0000 |
| 5.1273 | 0.0000 | 0.0000 | 5.5302 | 0.0000 | 0.5545 | 0.6902 | 0.5257 |
| 0.5055 | 1.2218 | 0.0000 | 0.0000 | 1.1775 | 0.0000 | 6.0017 | 0.5078 |
| 0.0000 | 1.2903 | 0.7547 | 0.8682 | 0.0000 | 2.3729 | 0.0000 | 0.6701 |

| | | | | | | | |
|---|---|---|---|---|---|---|---|
| 0.5077 | 0.0000\ | | | | | | |
| ART3 | 1.7370 | 1.8777 | 2.9525 | 2.8315 | 0.0000 | 0.9816 | 0.0000 |
| 0.0000 | 0.7652 | 2.5870 | 3.1647 | 1.1366 | 0.7708 | 1.4754 | 7.5302 |
| 6.4151 | 3.3455 | 0.0000 | 2.1673 | 0.9198 | 0.6175 | 5.6607 | 1.9117 |
| 5.4200 | 1.7397 | 1.6270 | 0.4561 | 4.1686 | 5.1569 | 1.8184 | 0.0000 |
| 5.0995 | 0.8951 | 0.4664 | 0.5853 | 0.0000 | 0.9556 | 5.4167 | 4.3622 |
| 4.4154 | 2.5069 | 2.9686 | 3.0042 | 2.1688 | 3.2157 | 3.6717 | 3.9741 |
| 0.0000 | 1.0498 | 2.9358 | 1.0749 | 0.0000 | 0.0000 | 3.6848 | 1.7133 |
| 0.0000 | 1.0734 | 0.0000 | 1.6122 | 2.1385 | 2.7929 | 0.4969 | 0.0000 |
| 4.7618 | 3.0468 | 3.9933 | 0.5980 | 1.5796 | 0.0000 | 2.3953 | 1.7877 |
| 2.4255 | 1.5110 | 0.4786 | 6.9440 | 1.1076 | 3.5007 | 3.0489 | 5.0109 |
| 0.0000 | 3.0365 | 4.3874 | 2.0835 | 7.0388 | 5.6270 | 3.7385 | 0.9284 |
| 1.3738 | 0.9125 | 3.3573 | 4.2273 | 2.7205 | 2.8268 | 3.9191 | 1.6641 |
| 0.6266 | 0.5233 | 1.2499 | 4.2938 | 0.0000 | 0.0000 | 1.9597 | 4.2789 |
| 2.1133 | 3.7378 | 1.3363 | 5.0878 | 2.8627 | 1.7151 | 2.3284 | 0.8916 |
| 2.2538 | 0.0000 | 2.0000 | 0.0000 | 1.0399 | 3.7871 | 1.0339 | 0.0000 |
| 1.0358 | 2.1175 | 3.2578 | 0.0000 | 0.0000 | 2.0269 | 1.4793 | 4.7571 |
| 1.2894 | 2.6412 | 0.5074 | 1.1727 | 1.1234 | 7.1680 | 4.3933 | 0.8107 |
| 1.3313 | 1.0837 | 5.1146 | 4.7148 | 1.9774 | 1.0240 | 6.4454 | 3.4177 |
| 5.6124 | 3.6893 | 2.6938 | 3.6209 | 1.9094 | 2.3133 | 1.2240 | 7.4576 |
| 0.0000 | 5.3918 | 0.0000 | 1.2540 | 0.4374 | 2.5317 | 1.5047 | 0.6000 |
| 2.4740 | 1.0317 | 1.6421 | 4.8742 | 2.1852 | 2.6724 | 0.8742 | 0.0000 |
| 4.2925 | 3.9193 | 1.2521 | 1.0663 | 1.2195 | 6.2578 | 6.2387 | 5.0451 |
| 0.4801 | 1.8570 | 5.7849 | 4.4277 | 5.0853 | 1.5234 | 0.0000 | 0.0000 |
| 2.9475 | 0.7366 | 4.0633 | 0.9943 | 0.0000 | 1.1063 | 1.2961 | 1.1800 |
| 3.4417 | 3.8086 | 3.7036 | 3.2101 | 0.0000 | 0.0000 | 1.3688 | 2.5185 |
| 3.8990 | 0.6959\ | | | | | | |
| HERC2P2 | 8.7305 | 10.7100 | 9.4630 | 10.1437 | 9.0561 | 10.4909 | 7.0463 |
| 9.0172 | 9.2691 | 10.7145 | 8.1983 | 8.2261 | 9.9483 | 8.5464 | 10.2672 |
| 11.0117 | 9.7667 | 9.8235 | 11.1214 | 9.3029 | 9.7091 | 9.9984 | 7.6550 |
| 10.9923 | 9.6965 | 8.0020 | 10.5600 | 9.0640 | 8.4150 | 10.4927 | 10.9756 |
| 10.0184 | 9.7503 | 9.2684 | 10.0163 | 9.4389 | 10.7852 | 9.7816 | 9.2810 |
| 8.6326 | 9.3694 | 9.8084 | 9.3026 | 9.9444 | 8.1011 | 9.8236 | 6.4596 |
| 10.7865 | 8.5976 | 5.6637 | 9.5930 | 10.6689 | 7.3788 | 10.8218 | 9.2178 |
| 11.2114 | 10.7257 | 10.3534 | 8.0739 | 10.3203 | 9.6799 | 9.4864 | 10.8248 |
| 12.6189 | 10.9560 | 8.3730 | 10.4148 | 8.6509 | 9.6750 | 9.2496 | 7.9564 |
| 11.3412 | 8.0975 | 10.0075 | 8.8191 | 11.3065 | 11.5665 | 9.8898 | 10.2272 |
| 9.9013 | 8.7768 | 9.8219 | 9.7532 | 10.5546 | 10.4570 | 10.9878 | 8.8729 |
| 9.8708 | 9.3095 | 10.2141 | 9.5327 | 9.6477 | 9.2195 | 10.9087 | 9.7121 |
| 7.6006 | 9.6404 | 9.7101 | 10.5140 | 11.6144 | 9.2009 | 10.1818 | 10.3589 |
| 14.3837 | 9.6279 | 7.4903 | 11.8704 | 9.7636 | 8.7581 | 9.6475 | 11.0382 |
| 9.8314 | 7.1707 | 11.3208 | 11.9460 | 9.3823 | 7.5116 | 11.1156 | 9.2942 |
| 10.5828 | 10.0177 | 8.9432 | 10.4580 | 6.1515 | 9.5196 | 8.5531 | 9.2616 |
| 6.0123 | 9.3190 | 9.5013 | 9.8195 | 11.2641 | 10.2220 | 10.9980 | 11.0241 |
| 4.4243 | 9.6178 | 9.3013 | 9.3907 | 10.3972 | 10.6132 | 7.1838 | 8.5995 |
| 9.4536 | 8.2849 | 9.8905 | 9.6101 | 9.5064 | 8.8221 | 7.4650 | 10.4371 |
| 8.3640 | 11.1087 | 10.8853 | 8.9227 | 10.4725 | 9.1429 | 9.0338 | 9.0600 |
| 8.9009 | 8.5625 | 9.4346 | 9.8218 | 9.9974 | 10.3254 | 9.8267 | 9.2058 |
| 9.5195 | 9.5454 | 10.1723 | 8.9874 | 10.8052 | 8.2218 | 9.1660 | 7.4627 |
| 10.9199 | 11.7878 | 10.4248 | 10.6566 | 10.6974 | 7.7047 | 10.4810 | 9.1148 |
| 10.2053 | 10.5275 | 9.0631 | 8.2670 | 9.0091 | 10.3182 | 9.6220 | 9.7566 |
| 8.8336 | 10.7932 | 7.9961 | 9.5838 | 8.1993 | 7.1019 | 9.4391 | 9.7845 |
| 9.8659 | 5.6431\ | | | | | | |
| ART5 | 4.0762 | 6.3123 | 2.4032 | 1.3959 | 2.0327 | 3.7731 | 3.9231 |

| | | | | | | | |
|---|---|---|---|---|---|---|---|
| 7.2536 | 2.5600 | 4.9230 | 1.7761 | 5.6364 | 6.4279 | 0.7759 | 2.3407 |
| 0.0000 | 6.0137 | 3.4585 | 4.7516 | 5.9180 | 3.6727 | 3.2820 | 2.2832 |
| 0.0000 | 4.8384 | 3.6425 | 5.6244 | 3.4301 | 3.1802 | 2.1819 | 1.8679 |
| 2.1139 | 3.3866 | 6.8849 | 5.2103 | 2.8161 | 2.7304 | 2.7557 | 2.6916 |
| 0.4337 | 3.3740 | 6.2191 | 3.1865 | 4.6104 | 7.7022 | 1.8914 | 4.9575 |
| 6.3989 | 3.0861 | 3.5316 | 1.0749 | 2.7455 | 4.7012 | 5.7647 | 3.2259 |
| 4.4531 | 2.4376 | 3.0892 | 4.3362 | 4.6889 | 5.9357 | 3.7810 | 4.3456 |
| 6.5740 | 5.1743 | 1.5189 | 1.0195 | 6.6272 | 1.9829 | 4.4444 | 5.8166 |
| 3.3742 | 3.0705 | 2.7868 | 3.8599 | 6.8056 | 4.0830 | 3.4600 | 0.6894 |
| 3.3294 | 4.0748 | 1.6845 | 3.5862 | 2.7931 | 4.4679 | 5.0995 | 5.9638 |
| 2.6565 | 4.7085 | 4.0368 | 5.7746 | 4.8755 | 4.5089 | 5.7000 | 4.5041 |
| 4.2849 | 3.0761 | 7.5649 | 5.2079 | 2.2375 | 1.9211 | 3.3338 | 3.3193 |
| 3.9669 | 4.3857 | 4.9946 | 3.4466 | 4.0300 | 5.1580 | 4.5314 | 0.8916 |
| 3.6834 | 7.2474 | 2.8074 | 3.7457 | 7.2973 | 2.6550 | 6.9160 | 2.3567 |
| 2.3787 | 6.5623 | 1.8152 | 3.3462 | 4.2261 | 4.2819 | 4.6260 | 5.4435 |
| 7.2404 | 6.8904 | 5.1001 | 2.3439 | 2.9034 | 1.5194 | 5.0928 | 3.4335 |
| 2.6570 | 1.9254 | 4.9498 | 4.6412 | 6.6685 | 3.2906 | 1.7855 | 1.9445 |
| 2.0686 | 1.5157 | 6.9121 | 0.0000 | 5.9565 | 4.0343 | 4.3951 | 4.8312 |
| 5.9178 | 0.5414 | 4.7041 | 0.5476 | 4.5433 | 6.2221 | 3.3916 | 7.4324 |
| 2.5877 | 1.8525 | 2.0641 | 4.5152 | 3.0735 | 1.6553 | 3.3452 | 4.6862 |
| 3.8546 | 5.7187 | 2.7065 | 1.6727 | 6.1582 | 4.7513 | 2.5508 | 0.0000 |
| 5.1109 | 4.1021 | 5.2754 | 3.8547 | 2.9241 | 2.0978 | 2.9539 | 5.5045 |
| 4.4304 | 5.0140 | 4.8671 | 4.7438 | 4.9674 | 3.0864 | 3.7671 | 0.5078 |
| 2.2504 | 7.0817 | 3.0977 | 1.6152 | 3.9204 | 10.0676 | 7.8318 | 1.4718 |
| 3.2381 | 4.6541\ | | | | | | |
| ART4 | 0.0000 | 0.9197 | 0.0000 | 0.0000 | 0.0000 | 0.0000 | 0.0000 |
| 0.0000 | 0.0000 | 0.7786 | 0.7594 | 0.0000 | 0.0000 | 0.4395 | 0.0000 |
| 0.0000 | 0.0000 | 1.2795 | 0.9983 | 0.0000 | 1.6493 | 0.0000 | 0.0000 |
| 0.5262 | 1.2653 | 0.0000 | 0.0000 | 0.0000 | 0.0000 | 0.0000 | 0.0000 |
| 1.5370 | 0.0000 | 0.0000 | 0.0000 | 0.0000 | 0.0000 | 0.0000 | 0.0000 |
| 0.0000 | 0.0000 | 2.0748 | 0.0000 | 0.0000 | 6.1381 | 0.0000 | 0.0000 |
| 0.0000 | 0.0000 | 0.0000 | 0.4530 | 1.6507 | 0.0000 | 5.0710 | 2.7436 |
| 0.0000 | 0.0000 | 2.1170 | 1.6122 | 0.5715 | 4.5454 | 0.8658 | 0.0000 |
| 0.0000 | 0.6828 | 0.3906 | 0.0000 | 0.8444 | 0.9886 | 0.6160 | 1.0365 |
| 0.3921 | 0.0000 | 0.8374 | 1.2339 | 1.1076 | 0.0000 | 0.0000 | 2.9521 |
| 0.6159 | 0.0000 | 0.0000 | 3.2356 | 0.0000 | 0.0000 | 0.6199 | 0.0000 |
| 0.0000 | 0.5273 | 0.0000 | 5.7891 | 0.8470 | 0.0000 | 0.0000 | 0.0000 |
| 4.9503 | 0.9065 | 0.9402 | 0.0000 | 0.0000 | 0.0000 | 1.4068 | 0.0000 |
| 0.0000 | 0.0000 | 0.0000 | 0.0000 | 1.0326 | 0.5426 | 0.0000 | 0.5136 |
| 0.0000 | 0.0000 | 0.0000 | 1.1918 | 0.6116 | 0.0000 | 0.7641 | 0.7085 |
| 0.0000 | 0.8008 | 1.0055 | 1.8863 | 0.0000 | 0.5970 | 0.9214 | 1.0447 |
| 0.0000 | 2.4022 | 2.8723 | 0.0000 | 0.0000 | 0.0000 | 0.0000 | 0.0000 |
| 0.0000 | 0.0000 | 0.0000 | 0.9038 | 0.8792 | 0.0000 | 0.0000 | 0.0000 |
| 0.0000 | 0.0000 | 1.9011 | 0.0000 | 4.9041 | 0.0000 | 0.0000 | 3.9983 |
| 0.0000 | 0.0000 | 1.3242 | 0.0000 | 0.0000 | 3.0310 | 0.0000 | 0.0000 |
| 0.9345 | 0.0000 | 1.3735 | 0.0000 | 0.0000 | 0.0000 | 0.5023 | 0.0000 |
| 1.9581 | 0.0000 | 0.0000 | 0.0000 | 0.5290 | 0.0000 | 0.0000 | 0.0000 |
| 0.4801 | 0.9063 | 5.2549 | 0.0000 | 1.4046 | 0.0000 | 0.0000 | 0.0000 |
| 0.0000 | 0.0000 | 0.0000 | 1.5773 | 0.0000 | 0.0000 | 0.9785 | 0.5078 |
| 0.5556 | 0.0000 | 2.0332 | 0.0000 | 0.0000 | 0.0000 | 1.6369 | 0.0000 |
| 2.2618 | 0.0000\ | | | | | | |
| RNF111 | 8.2910 | 8.7206 | 8.4600 | 9.0218 | 8.2520 | 10.2798 | 8.8523 |
| 8.5970 | 8.7582 | 8.7189 | 9.0038 | 8.3567 | 8.5341 | 8.9522 | 9.1380 |
| 7.8664 | 6.3551 | 8.9343 | 8.8884 | 7.9615 | 8.0951 | 8.9229 | 8.5615 |

| | | | | | | | |
|---|---|---|---|---|---|---|---|
| 8.9800 | 9.0531 | 8.3571 | 7.8386 | 7.9899 | 8.2102 | 8.7036 | 8.6716 |
| 8.6072 | 8.1514 | 8.6016 | 8.3781 | 8.7603 | 8.9544 | 9.2308 | 8.1100 |
| 8.4537 | 8.4389 | 9.4233 | 7.9378 | 8.8879 | 9.3016 | 7.5123 | 7.0669 |
| 8.6611 | 9.2072 | 8.4367 | 9.0707 | 8.4355 | 8.7754 | 9.1533 | 8.6917 |
| 8.8645 | 9.6289 | 9.2079 | 8.4709 | 7.9455 | 9.4176 | 9.3070 | 8.8343 |
| 7.8774 | 8.7097 | 8.8267 | 8.4028 | 8.8886 | 8.5451 | 8.1270 | 7.8442 |
| 9.4684 | 7.8881 | 8.2173 | 9.3149 | 9.0211 | 8.2278 | 8.6267 | 7.9392 |
| 9.3038 | 7.9024 | 8.7501 | 8.5743 | 8.3277 | 7.8057 | 8.2341 | 9.6337 |
| 7.3887 | 8.7237 | 8.0843 | 9.1990 | 8.9088 | 7.2933 | 7.5990 | 8.6661 |
| 9.0401 | 9.0188 | 9.1954 | 8.6190 | 8.7519 | 7.2850 | 9.1035 | 8.5970 |
| 8.6155 | 8.5159 | 8.0918 | 8.1970 | 9.2705 | 8.4450 | 8.2321 | 8.9902 |
| 8.9833 | 7.8091 | 7.5999 | 9.0938 | 8.9458 | 8.7473 | 9.4295 | 9.0038 |
| 8.9744 | 9.1192 | 9.1557 | 8.5035 | 9.3024 | 9.3988 | 9.0651 | 8.2170 |
| 7.5438 | 9.4357 | 9.5978 | 8.4821 | 8.6793 | 8.1201 | 8.2510 | 7.9607 |
| 6.5351 | 7.8021 | 6.7981 | 9.4503 | 9.3393 | 7.8867 | 8.2686 | 7.9325 |
| 9.3754 | 9.3881 | 9.2100 | 9.2088 | 8.9827 | 8.8503 | 7.6729 | 9.0337 |
| 7.6842 | 9.5579 | 8.2181 | 8.3832 | 8.9579 | 9.3250 | 9.2747 | 8.5404 |
| 7.8579 | 8.3058 | 8.2769 | 8.8632 | 7.9356 | 7.7691 | 9.7583 | 8.1053 |
| 9.0306 | 8.4882 | 8.0062 | 9.1947 | 8.7810 | 7.2134 | 8.1766 | 7.0729 |
| 8.8607 | 8.8725 | 8.8937 | 9.6100 | 8.6683 | 7.6619 | 8.2218 | 8.5761 |
| 8.4025 | 8.5977 | 8.5611 | 7.7534 | 7.8218 | 7.7892 | 8.7133 | 9.2551 |
| 9.2400 | 8.2371 | 8.3477 | 8.6156 | 7.8153 | 7.0964 | 9.2963 | 9.4804 |
| 9.2316 | 6.7248\ | | | | | | |
| TCEA2 | 9.9375 | 9.9082 | 8.7806 | 9.0354 | 7.6032 | 9.9627 | 8.9475 |
| 10.1697 | 8.8980 | 9.9136 | 8.3065 | 10.3959 | 9.9421 | 8.5884 | 8.8941 |
| 9.7099 | 11.1406 | 9.9805 | 9.8040 | 10.1298 | 10.2696 | 9.0929 | 10.4937 |
| 8.3382 | 10.2168 | 8.9724 | 8.7583 | 10.1844 | 9.2466 | 9.6457 | 9.8416 |
| 8.7732 | 9.7315 | 10.5820 | 8.8972 | 9.0719 | 9.8295 | 8.5183 | 7.4384 |
| 7.2853 | 8.5780 | 9.1418 | 10.8442 | 7.4378 | 9.3732 | 9.7438 | 10.9172 |
| 8.8610 | 6.9510 | 9.7308 | 9.4527 | 7.9023 | 9.8933 | 9.6435 | 10.0410 |
| 9.0058 | 8.2168 | 9.4170 | 10.0311 | 9.3593 | 9.3606 | 9.2545 | 8.3872 |
| 10.8732 | 10.0675 | 8.8867 | 9.7365 | 9.8614 | 8.6372 | 9.3102 | 10.3546 |
| 8.6810 | 9.6691 | 9.0576 | 8.1056 | 10.1776 | 10.1915 | 9.4198 | 10.1438 |
| 8.0989 | 9.4068 | 8.8114 | 9.3195 | 8.0495 | 10.5408 | 10.2693 | 10.4182 |
| 10.0410 | 9.8301 | 9.7020 | 9.7282 | 9.2115 | 10.2335 | 10.7658 | 8.5134 |
| 9.8821 | 9.8802 | 10.1737 | 9.7286 | 8.9176 | 9.7842 | 9.1430 | 9.7148 |
| 9.5638 | 8.6998 | 8.9996 | 10.5247 | 8.7402 | 9.7918 | 10.2237 | 8.3121 |
| 8.8832 | 11.1293 | 10.9484 | 7.8590 | 9.7341 | 9.5749 | 10.4816 | 9.2378 |
| 8.9982 | 10.2774 | 8.5029 | 9.1793 | 8.3161 | 10.1489 | 8.5148 | 9.3523 |
| 11.0871 | 9.5344 | 9.2103 | 9.8448 | 8.9813 | 9.2855 | 8.8774 | 9.3433 |
| 10.7856 | 7.4791 | 10.7250 | 8.8807 | 9.6080 | 8.4441 | 8.6347 | 9.7873 |
| 7.6924 | 7.8437 | 10.1376 | 9.6210 | 10.3379 | 8.4487 | 9.3815 | 9.2503 |
| 9.6087 | 8.6320 | 9.8176 | 8.5612 | 10.0233 | 9.6858 | 9.0211 | 10.6594 |
| 9.1293 | 8.2175 | 8.0958 | 9.6986 | 10.4282 | 9.6277 | 7.2097 | 9.7733 |
| 8.0819 | 9.3067 | 8.8361 | 8.6993 | 10.0966 | 9.8035 | 9.3799 | 8.5146 |
| 9.5619 | 9.7399 | 9.7529 | 8.5892 | 9.4354 | 9.0514 | 8.9562 | 8.8966 |
| 9.1644 | 8.7837 | 9.8820 | 10.3433 | 10.5874 | 9.5844 | 7.8456 | 7.4943 |
| 8.8380 | 10.2570 | 9.0336 | 8.7025 | 9.8709 | 7.8640 | 10.0226 | 7.1287 |
| 8.8265 | 10.1285\ | | | | | | |
| TCEA3 | 8.2092 | 7.5254 | 3.5887 | 10.1216 | 9.9524 | 8.9057 | 6.5085 |
| 8.6622 | 7.4325 | 8.9976 | 6.4066 | 7.8649 | 7.9241 | 9.2492 | 6.9045 |
| 10.8190 | 8.6436 | 6.7073 | 7.3050 | 10.9406 | 9.7164 | 2.8772 | 10.8812 |
| 9.5210 | 8.5929 | 10.0190 | 10.5515 | 6.4335 | 8.1294 | 8.3214 | 9.0748 |
| 7.2589 | 7.2912 | 7.1208 | 8.9652 | 7.9194 | 9.3694 | 10.5832 | 8.0568 |

9.7740 7.8450 8.3305 7.4239 10.5198 8.3777 8.0338 7.9239
9.7449 8.0224 12.0582 10.3205 7.7905 8.6026 9.4298 7.6136
9.5575 8.1623 9.9394 8.7696 9.3094 9.2537 8.5162 7.2104
8.1644 9.1564 5.6916 10.1719 7.9612 7.3479 7.7490 9.4728
7.3769 6.4280 8.4165 9.9244 9.5861 7.8270 8.0633 9.1489
7.0616 5.8973 7.6930 9.8915 9.6859 6.2279 8.0505 6.8084
6.6081 7.8173 8.2992 9.5594 5.3969 9.9628 9.1165 5.4720
8.0128 10.0921 9.6981 5.3936 5.4658 7.5906 6.0494 9.1863
7.8405 6.6797 7.7111 8.9176 6.7963 9.2563 7.5317 10.8232
5.5804 9.8760 10.2444 5.7456 8.7632 6.5436 10.4241 9.3422
9.0789 8.0670 8.8549 9.4907 9.7619 10.5686 5.9284 5.1078
9.7095 8.8368 8.4940 5.4058 9.5953 7.2366 6.1577 6.8055
6.2200 7.0562 9.6047 7.3565 9.8358 8.2423 11.0445 7.3165
9.1586 9.3234 11.5636 7.1314 10.8803 7.9230 9.2500 9.9708
5.0933 5.9757 9.2230 7.0356 6.2830 9.3643 7.0453 9.5672
10.9884 2.6375 8.9176 5.1000 9.3498 7.8587 5.3422 10.3126
8.6363 5.8010 10.7420 4.2380 7.8998 10.2060 5.1521 11.4268
9.2890 8.6219 10.4600 5.1934 8.5422 8.0181 10.6953 10.4160
7.7054 7.1454 5.3422 8.0546 8.6945 9.1865 7.3705 10.4093
6.8881 8.2599 10.7479 6.5900 9.7475 11.1274 10.2620 4.6911
8.1583 6.4458\
TCEA1 10.6148 10.4677 10.2062 11.3404 11.4175 10.5209 11.2815
10.5670 10.5285 10.2893 10.7093 10.8617 9.9925 10.5798 10.6317
9.9894 10.5989 11.2219 10.2698 11.4200 10.3674 10.4220 10.4596
10.6510 10.7543 9.9672 11.3481 9.7789 10.2026 10.4093 11.1723
10.5144 10.7466 10.5956 9.9529 11.6149 10.4057 10.9910 11.0916
11.4899 10.5175 10.1795 10.5726 11.5237 10.8547 9.9033 9.1942
10.6780 11.0385 10.2575 10.1972 10.7912 10.5839 10.7051 10.3119
10.9178 10.9637 10.8856 10.5720 10.8535 10.7727 10.8569 10.1045
11.2269 10.4672 10.7499 10.2074 10.9873 10.2107 10.0431 10.1353
10.8397 10.0476 10.0563 10.9323 10.8195 10.6481 10.4393 10.2184
10.8933 10.0995 11.4526 10.6024 11.0276 10.1230 10.7158 10.8207
10.2509 11.9330 10.2589 10.4483 11.2804 10.2496 10.2251 11.2034
10.4103 11.2210 10.4633 11.0855 11.0967 10.2126 10.1290 10.1444
10.4097 11.1949 11.2980 9.9112 11.4929 11.0116 10.7866 10.8252
10.3566 9.4783 10.4808 11.2854 10.3960 10.9651 10.6393 11.0757
10.8715 10.8431 10.9168 10.5986 10.8381 10.5985 10.4756 12.1600
9.5602 10.8420 10.5490 10.2911 11.3247 10.2382 11.1888 10.0558
9.5596 10.3123 8.3905 10.3526 10.5661 11.8088 10.8432 9.8076
10.6811 10.8390 10.5186 10.6657 10.2475 10.3385 9.9469 10.3028
8.6565 10.1995 10.6535 10.5365 11.2537 10.4706 10.7973 10.9181
10.3831 10.3078 10.1569 10.6882 9.8599 12.3689 10.9832 10.4238
10.0252 11.1750 10.5323 11.5794 10.4208 10.6011 10.8464 9.1451
11.5010 10.4782 10.7103 11.3504 10.3385 10.1875 11.0152 10.0793
10.4025 10.3694 10.7552 10.7177 10.7218 9.9641 10.7327 11.3336
10.3803 10.7667 10.5051 10.9617 9.9875 10.8410 10.5580 10.1315
10.5422 9.4247\
MGC57346 7.6187 6.8571 8.3544 8.3810 7.4219 8.2715
7.5291 9.3665 9.2657 7.0904 6.4913 7.8146 7.5908 7.9816
7.4242 7.9289 8.3180 7.1153 6.8890 7.2623 7.0075 6.6458
7.5911 7.5752 7.6564 7.4712 7.9125 7.4196 7.2613 6.9753
6.6106 7.4429 7.8025 7.0684 8.4060 8.0495 7.9127 7.5479
7.5436 7.8670 8.1693 7.0978 5.6793 7.1090 8.1254 6.5273
7.7288 6.8198 7.9284 8.1479 7.4086 7.5368 7.8514 7.7188

| | | | | | | | |
|---|---|---|---|---|---|---|---|
| 7.9798 | 7.4384 | 7.7387 | 7.8460 | 7.6986 | 7.6522 | 7.7560 | 7.7746 |
| 6.9807 | 7.5778 | 7.0873 | 7.7454 | 7.4980 | 7.5664 | 6.5459 | 6.0959 |
| 8.2779 | 7.9305 | 7.0584 | 7.3951 | 7.4427 | 7.5874 | 7.0035 | 6.7516 |
| 7.9414 | 8.0832 | 7.0448 | 6.5498 | 6.8030 | 6.7196 | 7.1438 | 7.7478 |
| 8.4092 | 7.4159 | 6.9135 | 7.8389 | 7.4691 | 8.6721 | 6.9562 | 6.9808 |
| 7.9483 | 7.1921 | 7.2266 | 8.1880 | 7.4432 | 7.5877 | 7.8628 | 7.4243 |
| 7.6197 | 6.7360 | 6.6669 | 7.7158 | 5.6958 | 7.2056 | 8.1256 | 7.7923 |
| 7.3939 | 7.7893 | 6.5266 | 6.8580 | 7.7569 | 7.7149 | 7.7843 | 8.7381 |
| 6.7272 | 6.4613 | 8.5304 | 7.1143 | 6.8347 | 7.0888 | 7.6582 | 7.5626 |
| 7.3360 | 7.4876 | 7.7581 | 7.7441 | 7.2194 | 6.8859 | 7.6758 | 6.7221 |
| 6.1048 | 8.4719 | 7.8589 | 6.6448 | 8.0588 | 8.1702 | 6.5406 | 7.2503 |
| 6.8014 | 6.7583 | 7.1416 | 8.8821 | 7.2409 | 7.8002 | 8.1969 | 7.5338 |
| 7.4715 | 7.6566 | 7.3731 | 8.9404 | 7.1525 | 7.5576 | 8.0129 | 7.7134 |
| 6.6736 | 6.8612 | 6.7276 | 6.3412 | 7.1612 | 7.6669 | 6.9527 | 7.9971 |
| 5.4334 | 7.9385 | 7.3000 | 7.6404 | 8.0083 | 8.1428 | 8.1357 | 8.2258 |
| 7.0275 | 7.7240 | 6.8629 | 7.7824 | 8.0485 | 6.4520 | 7.4958 | 7.0137 |
| 8.0702 | 7.1196 | 7.5874 | 7.6771 | 8.7501 | 7.4730 | 7.0428 | 8.2034 |
| 7.7333 | 7.4262 | 7.1567 | 7.6313 | 8.0094 | 8.6691 | 7.3157 | 8.1278 |
| 6.8166 | 7.5073 | 7.0758\ | | | | | |
| COX4I2 | 5.3292 | 4.5959 | 2.2256 | 2.7464 | 2.9307 | 2.7758 | 3.6354 |
| 6.6770 | 3.6488 | 5.0374 | 4.4453 | 3.9432 | 3.8140 | 3.7511 | 0.9286 |
| 4.1868 | 8.5027 | 2.8922 | 4.9740 | 4.7636 | 4.6765 | 1.5770 | 1.9117 |
| 4.1477 | 6.2544 | 2.0473 | 3.9883 | 5.1066 | 5.1240 | 1.0077 | 5.9375 |
| 2.8443 | 3.3260 | 3.1725 | 1.8080 | 0.0000 | 4.6413 | 3.0840 | 4.3622 |
| 0.4337 | 2.6483 | 5.8080 | 3.0042 | 4.5685 | 7.2990 | 3.6717 | 6.6646 |
| 5.4556 | 3.1455 | 3.4305 | 2.3384 | 4.9851 | 3.1634 | 6.9223 | 6.9192 |
| 1.9450 | 1.9111 | 3.1582 | 4.6037 | 3.1339 | 7.2260 | 3.1396 | 0.9583 |
| 7.1171 | 4.9096 | 2.5790 | 1.6109 | 6.8624 | 3.5027 | 5.7760 | 4.0060 |
| 3.6857 | 4.0640 | 5.8393 | 4.0681 | 2.9872 | 4.5555 | 3.5184 | 3.5882 |
| 3.4039 | 5.4826 | 3.4754 | 5.6125 | 4.0887 | 5.1249 | 11.2821 | 5.6240 |
| 3.3246 | 3.2962 | 4.8888 | 5.9137 | 2.0686 | 7.2444 | 5.5185 | 3.4566 |
| 7.3875 | 3.4086 | 5.3237 | 3.7299 | 3.0758 | 4.3582 | 2.1052 | 5.1889 |
| 5.0987 | 6.1183 | 3.6802 | 6.3140 | 4.8347 | 7.4919 | 2.7073 | 1.6498 |
| 2.4082 | 6.9129 | 6.7549 | 4.6174 | 4.2840 | 2.0324 | 4.9876 | 3.4784 |
| 2.2252 | 9.5002 | 2.8168 | 3.4207 | 3.0560 | 3.9403 | 2.9459 | 4.2929 |
| 5.3979 | 6.1881 | 6.2879 | 6.2470 | 2.5146 | 3.7656 | 4.6918 | 2.0060 |
| 7.7366 | 4.9118 | 7.0427 | 5.3133 | 4.7104 | 3.0418 | 4.3631 | 3.4927 |
| 2.3527 | 2.7971 | 3.6839 | 1.1308 | 6.5301 | 2.7981 | 5.0472 | 5.4686 |
| 7.3469 | 1.2425 | 5.7452 | 3.0681 | 3.7440 | 5.7698 | 4.4070 | 3.6260 |
| 3.0513 | 3.9166 | 2.8804 | 1.9935 | 1.8400 | 3.0916 | 1.9692 | 4.2069 |
| 2.2772 | 3.0116 | 2.4870 | 2.2163 | 4.0826 | 4.1929 | 1.8874 | 7.3927 |
| 2.3068 | 5.4102 | 6.6116 | 3.3644 | 3.9334 | 4.4027 | 4.0833 | 4.3148 |
| 2.4895 | 2.1147 | 2.2612 | 4.6614 | 3.8329 | 2.1566 | 4.5131 | 2.8735 |
| 3.8702 | 4.7562 | 3.8823 | 1.6152 | 5.0585 | 5.8952 | 5.0055 | 0.6701 |
| 2.3834 | 4.6541\ | | | | | | |
| COX4I1 | 13.0423 | 12.7175 | 11.5809 | 12.7814 | 13.1962 | 11.7351 | 11.6678 |
| 12.2737 | 13.4630 | 12.9112 | 12.6614 | 12.0007 | 12.4396 | 12.5420 | 12.6373 |
| 13.4656 | 12.6670 | 13.2549 | 12.6909 | 12.3395 | 12.8267 | 13.0635 | 13.5433 |
| 12.6237 | 12.7876 | 14.0046 | 13.0445 | 13.9537 | 13.5352 | 11.4264 | 12.2466 |
| 12.7920 | 13.3014 | 12.1756 | 11.8261 | 14.0187 | 12.8012 | 12.7930 | 13.5842 |
| 11.5171 | 13.4014 | 11.9176 | 13.6954 | 11.9970 | 12.1690 | 14.3307 | 15.4190 |
| 12.5594 | 12.6788 | 12.7503 | 12.9310 | 12.6734 | 12.8508 | 12.2108 | 12.7050 |
| 12.8769 | 12.6826 | 12.2056 | 11.9769 | 13.0199 | 12.1416 | 11.8224 | 13.1837 |
| 12.2419 | 13.0784 | 12.8891 | 12.8214 | 11.8444 | 12.9272 | 13.2650 | 13.5466 |

12.1743 14.1385 13.6317 12.3334 13.3688 12.5138 13.2586 13.4552
12.8796 13.6953 11.6565 13.1891 12.2926 13.7374 13.0238 11.7437
14.3094 11.8298 13.3503 12.7358 12.1889 12.4383 13.8357 12.9717
12.6900 12.1520 12.9189 12.3218 13.2041 14.5827 13.0184 11.5277
13.1837 13.6958 12.8322 13.5020 11.7741 12.1270 13.0051 12.9206
13.0957 14.6973 14.0702 12.1869 11.8896 12.5355 11.4153 11.8126
12.7053 12.1859 13.1695 11.6402 13.6297 12.7595 12.6281 13.4505
14.4083 12.2572 11.6896 11.7286 12.9011 13.1598 12.8858 14.7020
15.2331 14.5282 13.5071 12.4390 12.4689 14.3238 12.9419 14.0301
12.1377 11.9288 12.7228 13.1633 13.1707 13.0613 14.4731 12.8823
15.2727 11.9359 12.3089 13.2768 11.5836 12.7298 12.1936 13.3884
13.8163 13.4816 13.8609 13.2262 13.4754 11.9238 12.3602 13.6806
11.4376 12.9546 12.8001 12.2679 12.8255 12.5578 12.8466 14.4795
12.5993 13.0007 12.5052 12.2282 12.7766 14.1627 14.1741 12.3572
13.6596 11.9475 13.1073 12.3260 13.3650 14.0144 12.7542 12.3571
12.7625 12.3045 12.1760 12.3359 14.6239 13.9509 12.9822 12.6782
12.7267 14.7942\
BFAR 10.5354 10.3936 10.4544 10.1400 10.0548 9.4543 9.7917
11.0572 10.9189 10.2839 11.0428 11.6967 10.3838 10.2999 10.4711
9.7962 9.8775 10.8779 10.2475 9.7315 10.1554 10.5142 9.9775
10.3950 10.2287 10.2506 9.8344 10.1567 9.9800 11.2502 10.7466
10.3470 10.6258 10.5166 9.9782 10.7773 10.1495 10.5173 11.4010
9.7060 10.3183 10.3016 10.3672 10.2165 10.6297 10.5250 9.8905
10.4095 10.9437 10.1469 10.1185 10.2694 10.6598 10.3154 10.5266
10.3127 10.5061 10.3339 10.3504 10.6861 10.3615 10.6417 10.3619
10.5998 10.3887 10.3694 10.2017 10.0311 9.6522 10.2747 11.2029
10.6595 10.0324 10.1993 10.3515 9.3714 9.8987 9.8815 10.1929
10.5619 10.5574 11.0696 9.8485 10.4471 10.4785 10.5676 11.4657
10.4696 10.6029 10.0222 9.9879 10.8493 9.3212 10.3301 10.4674
10.4309 10.8582 10.6419 10.5073 10.4536 9.9454 10.3681 9.3757
10.4514 10.7598 10.2043 10.2342 9.8776 10.0714 10.7344 10.6551
10.7625 9.2736 10.6519 9.9260 11.1888 10.1573 11.0138 9.9461
10.3409 10.2705 10.4519 10.0251 10.6242 10.1618 10.9499 11.0404
10.5764 10.3524 10.0473 10.4039 11.3620 10.4813 10.0643 10.2936
9.7833 10.0359 9.4818 10.6908 10.3207 10.5638 10.0327 10.2470
10.2774 10.2780 10.1665 10.3017 10.1645 10.1698 9.7188 9.9072
10.2050 10.2228 11.1247 10.5185 10.5139 10.3373 10.5116 10.3530
10.4161 10.9231 10.0981 10.7449 10.1885 10.6889 10.8609 10.2817
10.7181 10.5873 8.9707 10.6665 10.4264 9.2755 10.4043 10.3686
10.3835 10.1845 10.0627 10.1173 10.0681 10.9052 10.4853 10.6452
10.7056 9.9084 10.3318 9.8833 10.8407 10.4060 10.9870 11.1344
10.5521 11.1682 10.2222 10.2168 10.0438 10.1502 10.3150 10.3207
10.4438 9.7723\
ZC3H18 10.2504 10.1578 9.5410 9.8157 10.6277 9.7705 8.9260
9.7177 10.4982 10.1527 9.4005 10.3818 9.7904 10.9198 9.7394
9.6432 9.5125 10.3679 10.0966 9.5380 9.7301 9.9483 10.1411
9.8531 10.1795 10.1253 10.0577 9.9012 9.8473 9.4769 10.1617
10.4803 10.0890 9.8487 10.1140 10.2380 10.2279 9.7521 8.8572
9.2287 9.3960 10.1452 10.3761 9.3372 9.8162 10.1911 8.7012
9.9281 10.2417 9.0541 10.1776 9.8281 10.6679 9.8168 10.7036
10.0874 9.8322 10.4308 9.3677 9.8042 9.9053 9.6695 9.8483
10.0401 10.2485 10.2518 9.6653 9.8810 10.2951 9.7403 9.9923
10.4568 10.0324 10.2768 9.9397 10.3513 10.1118 9.7679 9.8645
9.8704 9.9450 9.6451 9.0378 9.6427 10.0246 9.8952 9.9558

10.1243 9.5467 10.2088 9.9103 10.4905 9.2753 9.7457 10.4700
10.0403 10.1620 10.2825 10.1759 9.7345 10.1517 10.4670 8.8857
9.1324 9.9781 10.0655 9.7568 9.3975 9.2584 9.8147 9.7163
10.2023 9.0479 9.6618 9.7202 9.9311 9.8328 9.8240 9.5147
10.2770 9.9953 9.7659 9.6848 9.5852 9.8407 10.4792 10.1534
8.9637 9.7348 9.9412 9.5427 9.2024 10.2259 9.4052 10.9290
8.3665 10.2526 8.2019 10.1558 10.1002 10.0748 9.6258 9.6395
10.2073 10.0855 9.8209 10.2561 9.9346 9.5891 9.0113 10.0702
8.8117 10.1126 10.2846 10.2238 9.9389 10.2161 10.2617 10.2687
10.2774 10.6068 10.4056 9.8202 10.1006 10.1023 10.2873 9.2094
9.6339 9.8053 9.8547 10.5877 10.2998 10.1136 10.4761 8.4122
10.4926 10.1627 9.8738 10.5578 9.7125 10.2059 10.5110 9.7182
9.8149 9.6919 10.0544 10.1287 9.9890 9.7393 10.5992 10.4412
9.9490 9.5552 9.9826 10.3659 9.4046 8.7620 10.1002 10.4287
9.9439 9.7371\
TMEM212 0.0000 0.9197 0.6896 7.2393 1.2172 3.3816 0.0000
3.4253 0.4327 4.8533 1.4500 0.0000 5.8630 0.0000 7.2765
0.0000 1.0553 0.0000 1.3199 0.0000 0.0000 7.8423 8.5866
5.2130 0.9530 0.6062 0.4561 4.4321 0.0000 1.0077 0.0000
0.0000 0.5159 0.4664 0.5853 0.6819 1.5255 0.3648 0.0000
0.0000 0.0000 2.6372 0.0000 1.0349 0.0000 3.1906 4.2414
1.1317 0.4402 0.0000 0.4530 0.7768 1.4359 0.8853 0.0000
0.0000 0.6343 0.0000 1.4405 3.2117 0.0000 0.0000 0.0000
0.0000 4.2137 1.8022 0.5980 0.8444 0.5773 1.6464 0.0000
0.3921 0.0000 3.5387 0.0000 1.7267 0.5548 6.8530 5.6264
0.0000 1.7663 0.3965 7.7679 6.4913 5.7143 0.0000 0.0000
2.0646 0.5273 0.0000 2.5817 0.4848 0.5215 2.5641 0.0000
0.0000 0.5233 0.9402 5.8073 0.0000 1.6914 3.1447 0.5352
4.6791 5.0722 0.0000 1.5756 8.2606 2.3532 3.4668 5.4230
5.1483 0.0000 0.0000 0.0000 1.6379 6.6615 0.0000 0.0000
0.0000 0.0000 0.0000 0.0000 0.0000 8.4910 0.9214 0.0000
0.0000 0.9653 2.1283 0.0000 5.9608 5.4573 6.2391 0.8107
0.0000 0.0000 0.0000 3.0710 1.1757 2.6248 0.5748 5.0404
0.0000 0.0000 0.5418 2.1920 4.4378 0.0000 0.0000 1.8061
2.5542 2.3500 0.0000 0.0000 1.0445 3.3344 1.5047 5.9388
0.0000 0.0000 0.0000 4.2397 0.0000 0.4418 0.5023 0.7889
1.9581 2.0324 0.0000 0.0000 0.0000 0.0000 0.0000 0.0000
3.3334 0.9063 0.6293 2.9511 8.8267 0.0000 0.6902 1.6771
2.7813 1.2218 0.6248 0.0000 2.8698 2.6295 0.0000 0.0000
2.5104 0.9737 0.0000 0.8682 0.0000 0.0000 2.5229 1.1260
6.3938 0.6959\
SNORD115-13 0.0000 0.0000 0.0000 0.0000 0.0000 0.0000
0.0000 0.0000 0.0000 0.0000 0.0000 0.0000 0.0000 0.0000
0.0000 0.0000 0.0000 0.0000 0.0000 0.0000 0.0000 0.0000
0.0000 0.0000 0.0000 0.0000 0.0000 0.0000 0.0000 0.0000
0.0000 0.0000 0.0000 0.0000 0.0000 0.0000 0.0000 0.0000
0.0000 0.0000 0.0000 0.0000 0.0000 0.0000 0.0000 0.0000
0.0000 0.0000 0.0000 0.0000 0.0000 0.0000 0.0000 0.0000
0.0000 0.0000 0.0000 0.0000 0.0000 0.0000 0.0000 0.0000
0.0000 0.0000 0.0000 0.3906 0.0000 0.0000 0.0000 0.0000
0.0000 0.0000 0.0000 0.0000 0.0000 0.0000 0.0000 0.0000
0.0000 0.0000 0.0000 0.0000 0.0000 0.0000 0.0000 0.0000
0.0000 0.0000 0.0000 0.0000 0.0000 0.4848 0.0000 0.0000
0.0000 0.0000 0.0000 0.0000 0.0000 0.0000 0.0000 0.0000

| | | | | | | | |
|---|---|---|---|---|---|---|---|
| 0.0000 | 0.0000 | 0.0000 | 0.0000 | 0.0000 | 0.0000 | 0.0000 | 0.0000 |
| 0.0000 | 0.0000 | 0.0000 | 0.0000 | 0.0000 | 0.0000 | 0.0000 | 0.4321 |
| 0.0000 | 0.0000 | 0.0000 | 0.0000 | 0.0000 | 0.0000 | 0.0000 | 0.0000 |
| 0.0000 | 0.0000 | 0.0000 | 0.0000 | 0.0000 | 0.0000 | 0.0000 | 0.0000 |
| 0.0000 | 0.0000 | 0.0000 | 0.0000 | 0.0000 | 0.0000 | 0.0000 | 0.0000 |
| 0.0000 | 0.0000 | 0.0000 | 0.0000 | 0.0000 | 0.0000 | 0.0000 | 0.0000 |
| 0.0000 | 0.0000 | 0.0000 | 0.0000 | 0.0000 | 0.0000 | 0.0000 | 0.0000 |
| 0.0000 | 0.0000 | 0.0000 | 0.0000 | 0.0000 | 0.0000 | 0.0000 | 0.0000 |
| 0.0000 | 0.0000 | 0.0000 | 0.0000 | 0.0000 | 0.0000 | 0.0000 | 0.0000 |
| 0.0000 | 0.0000 | 0.0000 | 0.0000 | 0.0000 | 0.0000 | 0.0000 | 0.0000 |
| 0.0000 | 0.0000 | 0.0000 | 0.0000 | 0.0000 | 0.0000 | 0.0000 | 0.0000 |
| 0.0000 | 0.0000 | 0.0000 | 0.0000 | 0.0000 | 0.0000 | 0.0000 | 0.0000 |
| 0.0000 | 0.0000 | 0.0000\ | | | | | |
| TCEB3B | 0.0000 | 0.0000 | 0.6896 | 2.3445 | 0.0000 | 2.3851 | 0.0000 |
| 1.1171 | 0.7652 | 1.0519 | 0.0000 | 0.8472 | 1.2705 | 1.0485 | 0.5377 |
| 0.0000 | 0.0000 | 0.0000 | 0.0000 | 0.3755 | 2.5380 | 1.7571 | 1.0738 |
| 1.2144 | 0.0000 | 0.0000 | 1.3145 | 0.5990 | 0.0000 | 1.0077 | 1.4094 |
| 0.5612 | 0.8951 | 1.5403 | 0.5853 | 0.6819 | 1.5255 | 0.3648 | 0.0000 |
| 2.1720 | 1.1184 | 0.0000 | 0.0000 | 2.3773 | 0.0000 | 0.0000 | 0.0000 |
| 1.7580 | 0.0000 | 0.0000 | 0.0000 | 0.7768 | 3.1634 | 0.0000 | 1.7133 |
| 0.5608 | 0.0000 | 1.1710 | 0.0000 | 0.0000 | 0.0000 | 0.4969 | 0.0000 |
| 0.0000 | 0.6828 | 0.0000 | 0.0000 | 0.4832 | 0.0000 | 0.6160 | 0.4334 |
| 1.8067 | 0.9449 | 0.4786 | 0.5367 | 2.5860 | 3.1657 | 2.2135 | 0.0000 |
| 0.6159 | 0.0000 | 0.0000 | 0.0000 | 0.6718 | 0.0000 | 0.0000 | 0.5376 |
| 0.0000 | 0.9125 | 0.0000 | 0.0000 | 0.0000 | 0.0000 | 0.0000 | 0.5198 |
| 0.6266 | 0.5233 | 0.0000 | 0.0000 | 0.0000 | 0.0000 | 0.0000 | 1.2310 |
| 0.7358 | 0.0000 | 2.0179 | 0.0000 | 3.5614 | 2.3532 | 0.5877 | 0.5136 |
| 1.3871 | 1.3631 | 0.0000 | 3.2573 | 0.6116 | 0.0000 | 2.3757 | 0.7085 |
| 0.6089 | 3.0740 | 1.0055 | 1.6588 | 1.1079 | 0.0000 | 0.9214 | 1.3754 |
| 0.0000 | 0.0000 | 0.0000 | 2.0482 | 0.0000 | 0.0000 | 1.6184 | 0.0000 |
| 0.0000 | 0.0000 | 0.0000 | 0.9038 | 0.0000 | 2.3602 | 0.0000 | 0.0000 |
| 0.0000 | 0.4562 | 0.0000 | 1.4775 | 0.0000 | 0.9947 | 0.0000 | 0.5019 |
| 0.0000 | 0.9341 | 2.1729 | 0.0000 | 2.1834 | 0.5638 | 0.0000 | 0.6000 |
| 1.2429 | 0.6063 | 0.0000 | 0.0000 | 0.0000 | 0.0000 | 0.5023 | 0.0000 |
| 0.9722 | 0.9131 | 0.0000 | 1.0663 | 2.0357 | 1.4956 | 1.0565 | 0.0000 |
| 0.4801 | 0.5232 | 0.0000 | 1.9582 | 1.0692 | 0.0000 | 0.6902 | 2.7472 |
| 0.5055 | 0.0000 | 3.7513 | 0.5811 | 0.0000 | 0.0000 | 0.9785 | 0.0000 |
| 1.5256 | 2.6556 | 0.4262 | 1.7976 | 2.3335 | 0.0000 | 0.0000 | 0.0000 |
| 0.5077 | 0.0000\ | | | | | | |
| SIPA1 | 10.5061 | 10.5485 | 10.4290 | 10.2523 | 9.8690 | 10.1439 | 9.2359 |
| 9.5635 | 9.8944 | 10.4558 | 10.3164 | 9.3580 | 10.0382 | 9.2982 | 9.6889 |
| 10.5901 | 11.2576 | 11.1513 | 10.8899 | 12.0287 | 11.2036 | 9.3032 | 9.6556 |
| 9.8970 | 9.8189 | 11.7253 | 8.4547 | 11.0193 | 11.0885 | 9.7836 | 10.3717 |
| 11.3754 | 11.1665 | 10.4004 | 9.0923 | 9.3760 | 10.1626 | 10.0561 | 10.4401 |
| 11.1805 | 10.1853 | 9.6443 | 11.1794 | 9.7860 | 8.8810 | 10.8199 | 12.0127 |
| 10.1598 | 9.3283 | 11.0590 | 10.5688 | 10.8975 | 9.6779 | 9.2786 | 9.4423 |
| 10.0322 | 9.8278 | 9.9078 | 10.3443 | 10.5585 | 9.7821 | 9.8679 | 9.2530 |
| 9.9894 | 10.6430 | 9.7875 | 9.7598 | 11.3286 | 9.8483 | 11.0859 | 11.4546 |
| 9.5066 | 11.6279 | 11.0958 | 10.0227 | 10.8985 | 10.7612 | 10.2795 | 10.5372 |
| 10.2077 | 10.2148 | 8.9988 | 10.7600 | 10.9271 | 10.3843 | 8.7747 | 9.0207 |
| 10.7776 | 9.9994 | 10.4982 | 10.1395 | 9.1182 | 11.2834 | 11.0075 | 10.1497 |
| 9.9551 | 9.7404 | 10.2804 | 9.6078 | 10.4348 | 10.3335 | 10.4483 | 10.4994 |
| 12.2079 | 11.0569 | 12.2987 | 10.6592 | 10.0830 | 10.6664 | 10.8371 | 10.9477 |
| 9.5268 | 11.4421 | 11.4671 | 9.4075 | 9.1147 | 9.5065 | 8.9433 | 9.8660 |

| | | | | | | | |
|---|---|---|---|---|---|---|---|
| 10.8799 | 9.9885 | 9.7398 | 9.5438 | 9.1785 | 8.8953 | 10.1817 | 8.3381 |
| 10.9154 | 9.5409 | 9.4210 | 10.9948 | 10.7004 | 10.4183 | 11.1521 | 10.5246 |
| 12.2343 | 10.8078 | 11.6162 | 9.9056 | 9.6650 | 11.7161 | 11.1057 | 10.9095 |
| 9.7015 | 9.0695 | 9.0920 | 9.1646 | 10.3668 | 9.8542 | 11.7075 | 10.4025 |
| 12.4648 | 9.5958 | 10.3387 | 10.2913 | 10.3237 | 10.1574 | 9.2466 | 10.5066 |
| 11.8087 | 10.0642 | 10.8813 | 9.8399 | 11.0684 | 9.7152 | 8.8930 | 11.6778 |
| 10.1505 | 10.8885 | 11.2908 | 8.7829 | 9.9428 | 10.7659 | 8.7689 | 11.4812 |
| 10.6186 | 10.4047 | 10.0344 | 9.6218 | 10.4938 | 12.6873 | 9.6457 | 9.5269 |
| 10.4247 | 9.3791 | 9.1232 | 10.6215 | 10.8162 | 10.7698 | 10.3032 | 9.7824 |
| 9.8924 | 9.7430 | 10.2469 | 10.3998 | 12.2071 | 12.8511 | 9.6250 | 10.2539 |
| 9.6428 | 11.7366\ | | | | | | |
| CRTAM | 1.2631 | 4.0535 | 2.4032 | 3.3759 | 3.9196 | 4.1351 | 2.5916 |
| 3.7518 | 1.4589 | 3.7949 | 3.8242 | 2.0691 | 2.8079 | 0.0000 | 2.8727 |
| 2.1513 | 3.3455 | 4.0620 | 6.6260 | 1.6235 | 5.3598 | 2.7123 | 3.8887 |
| 2.9264 | 1.7397 | 3.5809 | 2.6334 | 3.7390 | 1.3857 | 3.1836 | 6.1065 |
| 4.5156 | 4.6969 | 4.0575 | 1.3224 | 5.7270 | 1.2685 | 0.6557 | 0.9336 |
| 2.0552 | 5.0014 | 4.6495 | 1.0569 | 2.0520 | 3.7479 | 2.2058 | 3.4056 |
| 3.3124 | 3.3102 | 2.4071 | 3.0015 | 7.0076 | 2.9676 | 3.4251 | 3.5868 |
| 2.8432 | 4.8384 | 3.7115 | 4.8465 | 1.2977 | 4.6007 | 4.3741 | 3.7792 |
| 0.0000 | 3.4175 | 5.3395 | 3.5590 | 2.1953 | 1.9829 | 2.3953 | 5.5623 |
| 1.5230 | 8.2189 | 3.3842 | 5.7108 | 0.0000 | 5.7549 | 1.7110 | 4.6017 |
| 4.7019 | 2.7579 | 2.2618 | 5.5340 | 4.4436 | 2.0290 | 2.5439 | 3.2600 |
| 4.9896 | 4.0392 | 3.6882 | 3.3023 | 5.5276 | 4.7613 | 4.1858 | 5.6065 |
| 3.6967 | 3.5228 | 2.9802 | 1.4288 | 6.7275 | 3.3101 | 5.6711 | 0.5352 |
| 1.2208 | 1.7606 | 1.5391 | 3.6874 | 4.0605 | 2.9726 | 1.0041 | 4.3084 |
| 2.7915 | 3.7633 | 1.5850 | 4.1453 | 1.8640 | 2.9304 | 4.0013 | 2.3567 |
| 6.2595 | 0.4553 | 3.8683 | 3.6237 | 2.5865 | 3.0314 | 5.1282 | 2.8832 |
| 0.0000 | 3.3289 | 3.8142 | 2.0482 | 0.6684 | 3.3026 | 3.1029 | 5.6002 |
| 6.3328 | 5.4441 | 3.6106 | 3.2795 | 4.0830 | 2.8483 | 3.8294 | 4.1313 |
| 4.8501 | 3.9195 | 1.4974 | 3.0248 | 3.8402 | 1.7997 | 1.8766 | 4.5782 |
| 4.7717 | 4.8469 | 2.0029 | 2.6037 | 4.0208 | 3.2643 | 0.6894 | 4.9012 |
| 6.1250 | 2.2191 | 4.8367 | 3.7468 | 3.3264 | 1.8107 | 4.8942 | 3.7412 |
| 4.0347 | 1.6815 | 2.0787 | 1.2980 | 0.5290 | 2.0651 | 0.0000 | 0.0000 |
| 0.0000 | 3.0757 | 3.2020 | 0.9723 | 5.7175 | 3.6686 | 2.0241 | 2.3092 |
| 3.6241 | 2.3211 | 5.5733 | 3.9434 | 4.3617 | 3.0864 | 3.7671 | 4.8074 |
| 1.2686 | 3.5364 | 4.1845 | 3.6503 | 2.8198 | 1.6276 | 3.5942 | 4.0069 |
| 3.0303 | 4.4645\ | | | | | | |
| EDA | 7.3105 | 6.9906 | 8.2053 | 8.2578 | 7.1885 | 6.1134 | 6.2983 |
| 8.8938 | 8.0350 | 7.9863 | 7.8987 | 7.7888 | 6.9237 | 8.1612 | 9.1357 |
| 7.2194 | 9.3927 | 7.0712 | 6.5124 | 7.8282 | 5.1802 | 8.1469 | 6.7865 |
| 6.8943 | 8.6974 | 7.5780 | 7.7690 | 7.4677 | 7.9576 | 9.9970 | 6.8858 |
| 4.7376 | 5.6442 | 7.2567 | 6.5478 | 6.7461 | 7.7682 | 7.3357 | 7.7472 |
| 8.4201 | 9.0378 | 7.4101 | 9.7527 | 7.8609 | 8.2262 | 7.8023 | 8.5641 |
| 8.3068 | 6.5834 | 9.5240 | 6.4723 | 9.4731 | 9.8098 | 8.6499 | 9.0335 |
| 7.3902 | 6.3893 | 5.0131 | 8.0120 | 8.7588 | 7.9043 | 8.8883 | 5.3096 |
| 8.5449 | 7.7460 | 2.6522 | 9.1314 | 9.1062 | 6.2628 | 8.0156 | 7.4114 |
| 6.9067 | 5.6447 | 8.0919 | 8.3682 | 9.6614 | 10.1576 | 7.9351 | 7.3839 |
| 6.7553 | 8.0819 | 7.3258 | 7.5951 | 8.2084 | 8.6320 | 9.5649 | 8.5762 |
| 8.6702 | 9.7555 | 3.8290 | 8.2871 | 8.0968 | 7.8752 | 7.1955 | 7.7210 |
| 6.4238 | 8.5144 | 7.7549 | 10.4997 | 5.9506 | 7.5266 | 8.4144 | 9.3200 |
| 8.2748 | 9.3995 | 7.8770 | 6.9671 | 8.1727 | 10.0183 | 9.1022 | 8.5095 |
| 8.9413 | 8.0592 | 7.2761 | 8.4223 | 7.6405 | 8.9884 | 8.6341 | 2.8123 |
| 10.2915 | 8.6515 | 8.6276 | 9.4266 | 8.1461 | 7.0242 | 6.5405 | 9.2973 |
| 7.2055 | 8.1945 | 8.1827 | 8.4694 | 6.9697 | 8.0353 | 8.5322 | 5.5776 |

```
5.9461  3.2319  6.2786  9.0935  7.5815  8.3096  7.6664  8.3851
7.6339  8.8134  8.1754  7.4089  8.0331  6.8473  7.8629  6.7486
8.9909  7.6667  8.7470  7.8250  9.3324  8.2781  9.4435  5.8252
4.2986  6.2117  2.9807  9.4112  8.8342  10.4514 8.8636  6.9710
7.8630  9.3745  6.5971  10.3564 8.5786  7.9358  3.0975  7.4397
7.3121  7.5281  8.5821  6.6725  6.5767  6.6218  8.0588  7.9897
8.4604  7.2501  8.0198  5.9545  7.3048  6.9584  7.7051  5.4041
8.7573  8.8158  7.0512  8.2728  4.7689  8.4274  8.3739  8.6259
9.0496  8.0470\
OR6P1   0.0000  0.0000  0.0000  0.0000  0.0000  0.0000  0.0000
0.4748  0.0000  0.0000  0.0000  0.0000  0.0000  0.0000  0.0000
0.0000  0.0000  0.0000  0.0000  0.0000  0.0000  0.0000  0.0000
0.0000  0.0000  0.0000  0.0000  0.0000  0.0000  0.0000  0.0000
0.0000  0.0000  0.0000  0.0000  0.0000  0.0000  0.0000  0.0000
0.0000  0.0000  0.0000  0.0000  0.0000  0.0000  0.0000  0.0000
0.0000  0.0000  0.0000  0.0000  0.0000  0.0000  0.0000  0.0000
0.0000  0.0000  0.0000  0.0000  0.0000  0.0000  0.0000  0.0000
0.0000  0.0000  0.0000  0.0000  0.0000  0.0000  0.0000  0.0000
0.0000  0.0000  0.0000  0.0000  0.0000  0.0000  0.0000  0.0000
0.0000  0.0000  0.0000  0.0000  0.0000  0.0000  0.0000  0.0000
0.0000  0.0000  0.0000  0.0000  0.0000  0.0000  0.0000  0.0000
0.0000  0.0000  0.0000  0.0000  0.0000  0.0000  0.0000  0.0000
0.0000  0.0000  0.0000  0.0000  0.0000  0.0000  0.0000  0.0000
0.0000  0.0000  0.0000  0.0000  0.0000  0.0000  0.0000  0.0000
0.0000  0.0000  0.0000  0.0000  0.0000  0.0000  0.0000  0.0000
0.0000  0.0000  0.0000  0.0000  0.0000  0.0000  0.0000  0.0000
0.0000  0.0000  0.0000  0.0000  0.0000  0.0000  0.0000  0.0000
0.0000  0.0000  0.0000  0.0000  0.0000  0.0000  0.0000  0.0000
0.0000  0.0000  0.0000  0.0000  0.0000  0.0000  0.0000  0.0000
0.0000  0.0000  0.0000  0.0000  0.0000  1.0531  0.0000  0.0000
0.0000  0.0000  0.0000  0.0000  0.0000  0.0000  0.0000  0.0000
0.0000  0.0000  0.0000  0.0000  0.0000  0.0000  0.0000  0.0000
0.0000  0.0000  0.0000  0.0000  0.0000  0.0000  0.0000  0.0000
0.0000  0.0000  0.0000  0.0000  0.0000  0.0000  0.0000  0.3736
0.0000  0.0000\
CCDC116 3.0001  3.3098  2.9525  2.4559  4.4435  1.8477  0.0000
2.0418  1.2625  3.2610  1.4500  2.0691  2.2881  0.0000  1.7043
3.2639  0.6221  1.9468  2.9970  4.6140  0.6175  2.0613  2.9316
2.1767  4.1575  1.8524  4.9936  3.0366  1.3857  2.1819  2.9920
4.0194  3.3866  0.4664  2.1706  1.4920  2.1003  3.4685  3.1268
1.9280  3.4533  3.3558  1.0569  1.0349  2.8515  0.0000  0.0000
1.4786  2.6671  3.5316  2.1110  3.4576  1.4359  4.1308  1.7133
3.9752  0.0000  0.0000  0.7533  1.9697  2.6946  3.7372  3.8751
0.0000  3.5724  3.0811  2.0292  1.5796  1.9829  2.7780  2.5620
1.1694  1.9166  3.6332  2.5746  3.4606  1.5242  2.2135  2.7035
1.6461  2.1251  0.0000  3.5862  1.9875  3.8284  2.5439  1.4887
3.9377  1.6807  2.5470  3.0361  0.4848  1.6676  4.4518  2.0132
1.8952  0.0000  3.2822  1.9860  2.4969  2.4487  2.7612  1.6984
3.9669  1.3743  0.4659  4.3789  1.4556  2.8861  0.5877  2.2774
0.0000  2.5153  3.5850  2.8060  1.6379  0.5276  3.7247  1.3703
3.1499  4.4932  1.3285  1.0557  1.5487  3.9403  1.4793  1.8708
2.7607  2.8462  3.4188  1.3606  1.1234  1.9260  3.1029  1.3267
7.5094  1.6954  4.9498  2.4209  2.7815  2.7409  0.5748  2.4747
2.2176  1.3147  3.5778  1.9907  2.9802  1.5778  3.6271  3.1549
```

| | | | | | | | |
|---|---|---|---|---|---|---|---|
| 3.4258 | 0.5414 | 3.5271 | 2.7093 | 1.6440 | 3.0310 | 1.5047 | 1.0225 |
| 2.9697 | 3.2259 | 3.0745 | 2.1631 | 1.5535 | 2.8972 | 2.8575 | 0.0000 |
| 1.3802 | 3.1615 | 1.7238 | 2.5213 | 0.5290 | 0.5410 | 1.0565 | 5.8248 |
| 0.8396 | 4.3386 | 4.1660 | 1.8340 | 3.9334 | 3.0872 | 1.5061 | 2.1752 |
| 2.3775 | 1.2218 | 2.5553 | 0.0000 | 3.2977 | 2.8760 | 3.4201 | 1.1800 |
| 1.2686 | 1.7696 | 1.6145 | 2.6702 | 5.7956 | 2.9620 | 3.6559 | 3.9528 |
| 2.5996 | 3.2749\ | | | | | | |
| CADPS2 | 7.8346 | 9.1990 | 8.5735 | 9.3514 | 6.7883 | 9.7346 | 8.3925 |
| 7.9016 | 7.0539 | 8.9347 | 10.0550 | 8.7323 | 9.5030 | 4.9502 | 9.7903 |
| 9.1079 | 5.4458 | 9.3845 | 9.5570 | 8.2840 | 8.9501 | 10.0816 | 8.8406 |
| 9.7646 | 9.7327 | 4.8153 | 11.7971 | 8.1657 | 8.5484 | 7.6842 | 7.5884 |
| 8.5931 | 4.7439 | 8.0896 | 6.9316 | 4.2112 | 8.6431 | 9.3915 | 9.2672 |
| 5.6364 | 6.6002 | 10.2296 | 6.9051 | 9.7205 | 10.6925 | 8.4940 | 9.2238 |
| 9.0184 | 6.1482 | 7.9717 | 9.8654 | 7.2364 | 7.8318 | 10.1536 | 8.7679 |
| 5.6281 | 6.7639 | 9.0703 | 8.9584 | 7.2951 | 10.1817 | 7.6410 | 7.9240 |
| 5.3563 | 8.9136 | 9.3483 | 7.4308 | 8.8813 | 9.3358 | 8.4811 | 9.1352 |
| 6.7772 | 6.4888 | 6.7549 | 8.9218 | 10.6799 | 5.9967 | 9.4006 | 8.5258 |
| 7.0788 | 8.9729 | 7.2618 | 9.4588 | 10.4305 | 8.0976 | 5.4925 | 8.4022 |
| 4.3666 | 6.4287 | 9.2735 | 9.7981 | 8.1424 | 8.3933 | 9.2621 | 8.9965 |
| 10.8036 | 5.0599 | 9.9141 | 8.5653 | 6.8371 | 7.3157 | 8.8573 | 8.9849 |
| 8.6636 | 10.0568 | 5.6922 | 10.6052 | 8.1900 | 7.0096 | 7.7155 | 9.9482 |
| 9.0182 | 6.3709 | 4.7549 | 8.9236 | 8.5603 | 8.4865 | 8.8438 | 10.5175 |
| 7.0416 | 6.0671 | 9.1734 | 7.5600 | 8.0040 | 9.1311 | 8.4144 | 8.4993 |
| 1.9593 | 10.2896 | 10.0188 | 8.3496 | 9.4739 | 8.9148 | 9.6191 | 7.6768 |
| 6.6484 | 7.0440 | 7.6376 | 9.7501 | 9.7018 | 9.5254 | 7.1544 | 8.8499 |
| 8.0832 | 10.3562 | 8.9044 | 9.3001 | 8.8352 | 8.5742 | 7.8629 | 10.1153 |
| 7.1431 | 10.0452 | 7.3347 | 6.1533 | 8.3542 | 10.2137 | 6.1211 | 9.5365 |
| 8.5279 | 9.6524 | 9.2088 | 9.1076 | 8.7886 | 4.9080 | 9.7024 | 9.0796 |
| 10.3066 | 9.2143 | 8.7630 | 5.9001 | 9.5454 | 8.1974 | 9.5265 | 8.4643 |
| 6.7173 | 8.9110 | 9.8155 | 11.6225 | 9.2720 | 8.6931 | 7.8054 | 8.2347 |
| 9.4288 | 8.7461 | 8.0584 | 8.0519 | 6.8401 | 8.9580 | 6.8639 | 8.4595 |
| 8.6416 | 5.5918 | 7.2336 | 8.1773 | 6.8088 | 7.1922 | 9.9140 | 9.8018 |
| 10.1459 | 4.9428\ | | | | | | |
| CCDC114 | 6.4639 | 5.0871 | 8.8965 | 9.6036 | 7.0654 | 9.4068 | 8.3506 |
| 9.3731 | 3.6885 | 7.8453 | 5.3147 | 5.8711 | 10.5346 | 2.3988 | 10.0015 |
| 7.1857 | 8.9632 | 8.8476 | 6.0628 | 8.0402 | 6.2026 | 11.1037 | 11.2347 |
| 8.3635 | 4.4935 | 7.5582 | 6.5073 | 11.2554 | 3.7111 | 7.6447 | 2.9920 |
| 6.9394 | 4.9784 | 7.0888 | 5.0883 | 4.6536 | 9.9057 | 6.2604 | 6.0055 |
| 6.7542 | 2.1741 | 5.4976 | 6.3487 | 3.3350 | 1.0260 | 9.7392 | 8.5834 |
| 7.9928 | 3.2025 | 7.0915 | 6.7692 | 9.6829 | 6.6427 | 4.4983 | 7.4096 |
| 6.7471 | 5.5323 | 6.5869 | 5.7858 | 7.6670 | 4.0452 | 7.3194 | 4.5039 |
| 7.3524 | 8.3274 | 8.8541 | 5.7523 | 9.1248 | 3.2274 | 6.2089 | 4.3414 |
| 5.9000 | 2.4919 | 6.4389 | 5.2632 | 9.8459 | 9.8794 | 11.5250 | 11.2039 |
| 4.9457 | 7.5047 | 5.9845 | 11.0556 | 9.2146 | 10.6444 | 8.4488 | 5.1788 |
| 7.5769 | 6.9135 | 6.5398 | 5.0977 | 5.7037 | 4.3331 | 7.7671 | 5.7058 |
| 3.1952 | 7.1741 | 5.9777 | 10.4482 | 6.6955 | 8.0869 | 8.7727 | 7.5218 |
| 9.9624 | 10.0712 | 3.9530 | 8.2943 | 10.2039 | 8.2402 | 9.1642 | 9.3253 |
| 9.4819 | 7.3059 | 6.7415 | 8.6561 | 7.4358 | 10.2559 | 8.9112 | 4.4957 |
| 7.0813 | 5.6325 | 7.0444 | 6.8898 | 9.4926 | 11.3290 | 6.5815 | 5.5940 |
| 8.7721 | 4.1815 | 5.0459 | 8.0103 | 9.3491 | 11.0990 | 11.9514 | 3.3291 |
| 7.5273 | 8.5219 | 4.4303 | 7.5567 | 5.5337 | 9.3593 | 4.7253 | 10.6473 |
| 5.0220 | 4.2924 | 6.5614 | 8.6141 | 7.9444 | 4.3473 | 6.6028 | 6.6742 |
| 10.2333 | 7.0108 | 7.8895 | 4.9330 | 6.0406 | 7.6977 | 5.3402 | 9.7364 |
| 6.1804 | 6.4544 | 4.2502 | 8.1836 | 10.4443 | 4.8554 | 5.0292 | 7.8287 |

6.2423 9.6450 2.2280 5.1704 5.9975 2.8815 5.7670 7.6543
7.8964 6.7850 4.9829 7.6539 12.7466 7.4694 4.6396 8.4015
6.1850 4.7179 3.8630 4.0336 7.8615 9.8561 5.6989 7.3893
6.6114 6.2605 3.2618 6.7862 6.6726 4.5525 6.6000 6.3207
9.8302 6.8998\
CCDC115 9.4978 10.1178 9.8236 10.1163 10.2305 9.9130 9.8400
9.8045 10.1692 10.1326 10.0621 9.6746 10.3227 9.9977 9.7062
9.7099 10.4338 10.7165 9.9140 9.4591 10.1768 9.6672 10.6860
9.9234 10.3998 9.1407 10.7819 9.4585 9.7748 9.6869 9.4483
10.1991 9.2673 11.1193 9.8628 9.4414 9.8228 9.6760 10.1168
10.3661 10.1366 9.5799 10.1284 9.3434 10.4984 10.2220 9.4266
9.6929 10.0937 10.5798 10.0332 8.6658 10.0715 10.4402 9.9799
10.3164 10.3030 10.5286 11.0133 9.8291 10.3971 10.2936 9.9508
9.3839 10.2392 9.9318 11.0096 9.9459 9.5403 10.2262 9.8072
9.4948 9.8273 10.4191 9.9103 9.7453 9.5918 9.5801 10.1808
9.9764 9.7046 9.9630 9.9866 9.1987 9.7412 10.7241 10.1603
10.2205 10.0210 9.8050 10.2773 9.8429 10.2397 9.7070 9.4376
10.1347 10.4010 10.6045 9.3804 9.7051 9.5218 9.7218 9.7476
9.8157 10.9103 9.7897 9.1766 9.5879 10.5117 10.0402 9.7603
9.2114 8.4120 9.2336 10.4900 9.9937 9.2090 10.2360 9.3259
9.6658 10.2596 9.7659 9.7728 9.8662 10.6656 9.4968 10.2282
10.0095 10.7189 10.1031 10.0435 9.9065 9.5499 9.9562 10.1583
8.3363 9.6372 8.7883 9.8576 10.5621 10.1601 10.3357 9.9094
9.4000 9.2714 10.7844 9.4755 10.4629 9.7018 9.4312 10.1601
7.8273 9.2800 10.3116 8.9580 10.2832 10.4702 9.1666 9.0418
8.9785 9.8548 9.3632 10.2761 9.8397 9.6715 9.1890 10.0308
9.5587 9.6290 9.6631 9.2010 10.7012 8.8697 10.4772 8.6998
9.9769 10.1366 10.6575 9.8000 9.4182 9.8507 9.4226 10.3066
10.2636 10.1534 10.3282 10.1268 9.1945 9.3534 9.5594 9.8689
9.3469 10.0659 11.0852 9.8560 9.2068 9.4589 10.8602 9.2086
9.6751 8.8736\
CCDC112 7.6289 7.6330 8.0671 8.1313 7.8104 8.0489 9.0605
8.7087 8.3392 7.6093 8.5365 8.4085 8.3512 7.9300 7.8054
6.6558 7.9969 8.2084 7.8762 6.8513 6.8428 7.6477 8.0563
8.3084 7.4557 7.0337 8.3567 7.3498 7.0754 6.2631 8.0498
7.8879 7.8026 7.7820 9.3058 6.7298 8.0394 7.9415 9.0676
7.9595 7.6653 6.9342 8.2585 7.6685 8.3550 7.6806 6.1703
7.2163 9.1376 7.6729 7.6426 7.0314 7.6681 7.4761 7.6793
8.8936 7.6343 8.1126 8.5443 7.6285 7.9844 8.5609 7.7036
7.3569 7.6185 7.9262 7.9395 8.3800 6.0964 8.0852 7.8661
8.1684 6.9865 7.4567 7.8251 6.8735 7.9361 7.5077 7.4256
8.3455 7.3132 8.6018 7.9414 8.4477 7.2808 8.3830 9.1525
7.1617 8.5426 7.9520 7.3370 8.1362 7.7611 6.9667 8.0272
7.2551 7.6371 7.3942 8.0613 7.8130 6.1545 7.1970 7.6435
8.4876 8.2190 7.7853 6.8808 8.2994 9.1582 7.4469 8.1544
7.8780 6.3432 6.4757 7.9010 8.3936 8.0501 8.5398 7.1507
6.4961 8.0549 8.5521 7.6518 7.6775 8.0272 8.1382 7.8950
5.5613 8.1588 7.8261 7.8072 9.2617 8.1653 8.5642 7.3829
6.0815 6.7309 3.6106 7.7500 7.3598 8.2472 7.2914 7.5450
8.3014 7.6137 6.8900 8.0857 6.7493 7.6439 7.1356 7.4881
5.8705 7.5732 7.9375 8.3631 8.7022 7.5136 7.8345 8.3346
6.7312 8.0541 6.5434 8.2767 7.3536 6.9021 7.3191 8.8756
7.8709 7.3413 8.1651 7.4718 8.4272 7.6360 7.3294 4.3567
8.5743 8.4719 7.8700 8.7749 8.1319 7.4502 7.3099 9.1576

```
8.0126  8.8053  8.3639  8.0247  7.2157  6.6293  6.9514  8.6714
7.2280  8.0451  7.1098  7.8065  5.8471  6.6994  7.5072  7.6024
8.2571  6.7828\
CCDC113 7.9261  7.0157  8.3232  10.5947 7.5901  9.1718  9.8598
9.6934  8.8756  9.1643  6.5993  8.4602  9.7631  9.7174  11.3589
8.2958  8.1361  8.8386  6.9215  7.2140  6.9019  11.4796 11.8904
9.7358  7.8948  6.6914  5.1223  10.9591 7.9292  6.2247  5.9452
8.4256  7.9327  8.2823  8.5146  8.1743  9.3735  8.4746  8.3682
4.9776  7.0776  7.3054  7.4102  6.8671  6.4938  9.9476  8.5094
9.0479  7.2369  6.7784  8.4556  4.8841  8.6866  6.4214  8.6744
9.0774  7.2029  7.9048  8.4848  8.2588  7.1994  8.0043  7.3996
7.6571  8.4398  9.7549  9.3504  9.1892  7.6247  7.1017  7.2246
8.8260  5.9979  8.0960  8.2599  8.9465  9.2299  12.0984 10.7374
6.7046  8.6130  8.1647  11.3550 8.6990  10.4264 8.5117  8.6166
9.3777  8.1146  6.4039  6.8332  8.7249  6.7677  7.8577  8.4752
7.1650  7.2298  7.2922  10.4164 8.1083  8.0095  9.2408  8.2357
7.2053  9.6595  6.9245  6.9285  8.5534  10.0782 8.7663  10.0277
10.3655 7.3350  8.9244  9.1579  8.6821  11.3724 8.2148  7.5624
6.3551  7.9657  9.1859  7.7161  10.5861 11.5552 6.5815  7.8190
7.3510  7.5883  8.2606  7.2405  8.2362  11.2032 10.2385 8.2162
7.1702  6.6779  5.0346  9.3286  7.8490  8.9409  8.9337  10.4615
7.9106  8.1479  6.9391  9.8288  8.5020  8.5206  7.3970  7.9529
7.2338  9.4170  9.1267  8.7213  7.9224  8.6925  8.2620  11.2425
6.2251  6.4800  7.3035  9.7115  9.5182  8.2019  8.6454  8.2399
8.5464  9.0510  6.9594  8.3290  8.2931  6.5019  8.2679  6.5018
9.4685  7.5178  6.2974  9.5505  12.3228 7.2995  8.5303  7.7807
8.4758  7.8632  8.2297  8.6993  7.9340  9.4046  7.9820  8.5613
8.0319  8.1418  8.0420  7.2523  7.3273  7.1183  7.9988  8.8878
10.6308 8.3063\
CCDC110 2.5026  4.0142  4.1331  2.8315  1.6818  7.2270  5.1029
4.4623  0.0000  3.4141  6.1772  0.0000  3.6614  3.2550  1.2358
4.7451  4.2319  4.1224  4.0412  4.6656  4.4127  2.0613  6.8842
3.8282  4.4935  2.9613  4.4719  2.7358  4.3473  4.2625  2.7292
0.9642  0.5159  2.2682  2.5857  2.6866  4.2719  4.3001  0.5410
3.5693  3.6678  3.7059  3.8038  5.1544  4.9566  0.0000  4.0317
3.8776  0.7769  3.9521  4.8036  3.9981  4.6034  4.6026  4.1467
2.3993  2.5776  6.5931  4.5832  3.2117  4.1964  6.9631  3.2459
4.3188  4.0120  5.7595  5.9207  5.4581  3.9785  2.7780  5.8695
3.4990  2.7116  4.1311  4.8773  2.1585  4.8416  4.7261  2.9521
3.7276  3.7014  0.3965  2.6279  2.9116  2.7490  5.0536  4.7650
0.0000  4.9946  3.9573  5.1437  2.7205  3.2790  4.9006  2.0132
4.9247  7.0027  5.0711  0.0000  4.3904  3.7293  4.1304  5.4334
4.3896  2.4273  4.0558  1.5756  4.8522  5.0428  2.4661  4.7593
4.9107  3.1476  4.4594  7.3319  5.1639  3.6416  4.0324  5.4106
2.8681  4.5168  5.2006  4.5965  3.1213  6.9544  3.4375  5.0854
2.9068  4.5474  5.1693  6.1291  2.6562  3.4941  3.2144  4.3113
0.0000  0.0000  3.9958  4.8074  5.1632  1.3507  4.3284  3.1663
3.2040  3.4648  6.3439  1.7568  6.3469  3.3826  2.6654  4.8521
0.9816  3.8739  3.4629  4.9110  4.7473  5.0670  4.6375  6.4279
0.9345  7.2223  0.0000  2.3149  4.0982  3.6808  2.7721  5.6579
4.7872  4.5005  5.5135  3.4392  4.5599  2.2140  4.7505  0.0000
2.6164  3.9471  5.7991  5.0472  4.1718  3.4385  5.7485  5.3420
0.5055  5.6333  2.4158  4.5126  5.1322  3.8909  3.5453  5.7018
4.7958  0.9737  2.2571  4.2600  3.4726  5.6320  5.9339  4.1816
```

```
4.2897  0.0000\
CCDC111 7.9807  7.6077  6.7747  7.0749  7.9146  8.0122  7.4285
6.8002  6.5801  7.8893  7.4001  7.0420  7.8435  7.3415  7.4315
7.0080  6.8233  7.1396  7.3121  6.6290  6.5849  7.2730  7.5662
7.5620  8.0926  6.8217  6.3163  6.7062  6.4338  6.7055  8.3456
7.2541  7.3160  7.8583  6.5442  6.5519  7.1726  7.8921  7.5108
6.1624  7.0235  7.7584  6.5577  6.8024  8.2333  5.4005  4.8473
7.3363  7.6785  7.2540  7.5446  6.5549  6.7609  8.4070  6.7347
7.5384  7.5204  7.9453  7.7022  6.6538  8.3738  8.3089  7.4772
7.4731  7.8790  6.9587  6.9209  7.1503  6.8604  6.1724  7.2961
7.7511  6.2374  6.1975  7.1431  6.9713  7.0501  6.2785  6.6558
6.9304  6.8277  7.6460  6.8801  6.7598  6.9776  7.2674  7.9384
7.1160  8.3159  8.0508  7.8881  7.0350  6.5388  7.2986  7.7212
7.8527  6.3527  7.8850  6.5293  7.5069  6.1299  7.1547  7.4418
6.1798  6.5047  7.0781  7.5715  7.3781  6.7958  6.2277  7.6776
7.2689  0.0000  6.3516  8.3548  7.7169  7.5678  6.6847  6.8399
6.6657  7.5387  7.5454  7.1316  6.6447  8.1426  5.9603  6.3461
5.5586  8.1212  7.8816  7.3625  6.1845  7.1953  5.9072  5.5067
4.1673  6.5497  7.0361  7.4238  8.3888  6.1162  7.4058  6.4283
7.2813  7.5079  8.6117  6.7101  7.9876  7.5948  6.5480  7.7707
0.0000  7.5774  7.4663  7.6093  6.9333  8.1034  7.3349  7.9885
6.9118  7.2847  6.0116  6.8047  8.1303  7.6244  6.5760  5.5408
6.6963  7.1240  7.5956  7.0165  6.8225  5.0027  7.0931  5.6390
8.3586  7.8759  8.3760  7.8616  7.0319  7.0292  7.1313  7.3045
7.8061  7.3855  6.5000  7.5002  6.3992  6.7395  7.6316  7.7951
7.1801  5.8691  5.8928  6.9681  5.0990  3.4149  8.5123  7.8391
7.4518  4.8686\
LEO1    9.1259  8.3606  8.2438  9.2612  9.4034  9.6711  8.8889
9.2071  9.1639  8.9400  8.7101  8.6137  8.6169  9.2046  9.2091
8.8526  7.8496  9.4195  8.7878  8.0548  8.0809  9.1302  8.4474
9.5331  8.5629  9.3766  8.4820  8.8522  9.0696  9.1689  9.1508
9.8934  8.9550  8.4076  8.0937  9.9364  8.7302  9.8475  9.4378
8.5978  9.4404  8.7372  8.2937  9.8853  8.8968  8.7606  8.0301
8.7870  9.8672  9.7564  9.1590  8.3244  8.8625  8.2269  8.5292
9.1125  8.9742  9.4231  9.1473  8.5539  8.6591  9.8345  8.6176
9.3515  8.9063  8.6593  8.3651  9.1073  8.8382  8.6204  8.6058
9.4073  8.6294  9.1830  9.6027  9.1222  8.9414  8.7507  8.4006
9.5043  9.1795  9.7437  8.5345  8.5426  8.8389  8.4399  9.8831
8.9276  8.3649  9.0921  8.4191  9.3610  8.3244  8.1723  9.2145
8.6584  9.1038  8.7110  9.0132  9.0347  9.6384  9.2496  8.2926
8.8457  9.6602  9.3592  8.7013  9.2915  8.1222  9.0956  9.9255
8.9570  7.2622  7.5314  9.3509  9.3001  8.8406  9.3515  9.4056
9.0915  9.2232  9.4108  8.2511  9.4995  8.7431  9.0518  9.3203
8.4711  8.8293  8.7214  10.3044 9.4631  9.3467  8.2180  8.6078
7.8828  8.7696  6.7488  9.1729  8.5228  8.9634  10.2980 8.8711
9.6075  9.4945  8.6235  9.4177  8.4404  8.8780  8.4987  9.2921
9.0129  9.3869  9.3235  9.0897  8.8222  9.0768  9.2268  9.2381
9.4051  9.6077  9.0403  8.8383  8.9860  9.5014  9.4012  9.0462
8.7999  9.2956  8.8847  9.5060  9.1393  8.7268  8.8128  7.8323
9.3356  8.9913  8.2580  9.6376  8.5251  9.5260  9.1322  8.9617
9.3447  8.9639  8.9203  8.8558  8.9859  9.0690  9.3746  8.9390
8.9682  9.0211  8.1313  9.4188  9.1668  8.0800  8.6973  9.1148
9.0496  8.5015\
ATP1A4  0.0000  0.9197  0.0000  0.4935  3.4800  0.4059  4.6035
```

```
0.4748  0.0000  1.4796  0.0000  0.0000  0.4363  10.1374 0.5377
0.0000  0.0000  0.0000  0.5838  1.1305  0.0000  0.8427  0.0000
0.9110  0.0000  0.6062  0.0000  1.0210  0.0000  0.0000  0.0000
0.9642  0.0000  0.4664  0.5853  0.6819  4.8901  0.0000  0.0000
0.0000  0.6649  2.4377  0.0000  0.0000  0.0000  0.0000  0.0000
0.0000  0.0000  0.0000  1.8408  0.0000  2.6127  1.8243  3.6317
0.0000  0.6343  0.8754  0.4252  0.0000  0.0000  0.4969  0.0000
1.6085  0.0000  0.3906  0.0000  6.4935  2.4407  0.0000  1.0365
1.9301  0.9449  0.8374  0.5367  0.0000  6.2665  0.0000  0.6894
0.6159  0.9714  0.0000  0.0000  2.3651  0.5263  0.0000  0.0000
0.0000  0.9125  0.9113  0.0000  0.4848  1.6676  0.6909  1.2026
0.0000  0.0000  3.8858  1.4288  0.0000  2.5890  0.8684  0.0000
0.7358  0.0000  0.0000  0.0000  1.4556  0.0000  0.0000  0.0000
1.3871  2.0513  1.0000  0.0000  0.0000  0.0000  0.0000  2.1659
0.0000  0.8008  0.5886  0.0000  1.3451  0.0000  0.0000  2.5319
0.0000  0.0000  1.6358  0.9567  0.0000  1.2631  0.7571  0.0000
0.8141  1.0837  0.0000  2.0174  0.0000  0.0000  0.0000  0.6506
0.0000  1.3147  0.0000  4.5970  1.2499  6.0874  3.1725  0.5019
0.0000  0.9341  0.5862  0.5476  0.0000  0.0000  1.1539  1.3489
1.2429  0.0000  0.0000  0.5821  0.9764  0.0000  2.4806  1.2961
1.3802  4.0786  1.2521  0.0000  0.0000  1.4956  0.0000  0.0000
0.0000  0.0000  1.6723  3.0661  0.0000  0.5545  0.0000  0.5257
0.0000  0.0000  5.0670  0.0000  0.0000  0.0000  6.9570  1.1800
3.9158  1.2903  0.4262  0.4985  1.5946  0.0000  1.3688  3.8967
1.4261  0.6959\
ATP1A3  1.9260  1.2250  3.7938  2.3445  3.2314  3.7029  0.9367
1.5600  3.4322  1.8091  2.5484  3.2311  1.6409  0.0000  0.9286
1.4442  1.6582  3.1459  3.3189  0.6732  7.7744  1.1310  2.9316
0.5262  1.5220  8.9888  6.2141  2.0314  1.7737  3.1836  0.0000
3.3269  2.5183  2.7497  2.4602  6.3989  1.9329  2.6272  0.5410
0.4337  2.6483  2.5409  0.6231  2.9342  1.6194  1.4889  1.8316
1.9920  8.9804  0.0000  2.3384  5.1679  1.4359  1.8243  2.3724
1.7550  3.7168  6.7855  4.2608  4.2470  2.0680  4.4302  1.2717
3.8401  1.7745  4.5477  4.9917  3.8203  1.5697  1.3773  7.6490
1.1694  6.8658  4.9122  4.8094  2.2778  2.8273  1.7110  3.2595
4.2955  0.5659  1.8200  3.9906  3.0210  1.6785  1.8814  4.7650
2.0646  2.3134  10.1139 1.9147  3.6983  4.2027  3.9765  2.5291
2.8039  6.9424  1.2499  2.8769  5.4506  2.5890  3.6025  2.7736
3.6218  1.7606  4.2987  3.3565  2.5557  3.5239  3.5924  2.7134
1.3871  5.3882  4.1699  2.1456  3.7164  1.4679  1.9235  0.9644
5.2976  6.2470  1.5923  3.8564  2.6761  1.6090  3.4959  2.7753
3.2729  2.2660  1.9820  0.9567  1.4687  2.9134  2.5437  6.5157
3.2234  3.1433  5.6322  0.9038  1.4215  6.4818  0.5748  1.9445
3.1308  2.4496  1.4974  1.9907  2.5977  2.8976  1.5869  2.5835
0.9816  2.8827  3.7584  2.3660  2.6584  1.2837  0.3855  2.6223
5.2442  1.0317  3.9862  0.0000  1.9645  2.6724  2.3687  7.4627
3.6487  0.9131  0.5466  2.4266  5.3987  6.6267  0.6229  0.0000
5.2219  0.9063  1.9008  4.4277  4.6214  3.0054  5.5360  3.3548
2.1234  2.3211  3.7513  2.9899  5.4142  1.4486  2.4241  3.3015
0.5556  1.9604  2.9126  2.2371  5.9450  7.2870  0.6112  2.8496
0.5077  2.2387\
ATP1A2  1.7370  6.7126  0.0000  3.9331  10.5324 2.7056  11.7144
5.1254  2.2773  8.7420  3.3720  2.0691  7.9715  12.8286 2.0573
5.2151  6.2870  6.8026  6.4313  1.3142  1.0485  3.9707  7.2410
```

6.9923  7.8648  1.8524  0.4561  5.5165  1.3857  1.5953  0.0000
0.5612  0.8951  3.5933  2.0007  0.6819  8.3592  5.0032  1.2418
0.4337  4.8631  7.5772  1.8880  0.0000  9.2312  2.8725  0.0000
1.7580  3.8278  2.9358  1.0749  1.9460  5.0120  9.4880  5.6217
2.1129  2.8225  3.5666  6.6792  7.3173  8.6801  1.6116  1.5290
0.0000  8.3964  3.9933  2.0292  11.0587 3.3716  3.9431  1.9272
0.7001  0.0000  4.2252  4.8993  1.8854  11.0339 4.9548  5.6084
2.2410  4.8022  2.2618  6.2380  10.0010 5.7501  3.0901  2.0569
7.0563  5.6807  9.2486  10.3550 4.0853  0.0000  4.4518  6.7040
8.0694  2.4256  11.4068 5.7742  0.9478  9.9090  3.9875  1.2310
3.7699  2.9575  4.2124  4.4676  6.5340  6.7771  8.5414  3.0477
7.5285  7.8588  2.0000  1.1918  3.7732  4.8708  3.8347  2.0603
0.0000  6.5904  0.5886  3.0021  4.6588  8.8686  3.1042  2.5319
2.5982  8.1149  8.7779  3.6912  0.0000  3.8143  5.3585  2.4659
0.8141  11.3496 4.8598  9.4521  8.9215  4.3672  4.9011  6.7792
5.7163  1.3147  10.6328 9.6394  9.9285  8.3686  4.1457  6.5579
3.6663  4.9984  3.8109  4.7471  1.0445  9.5255  4.9261  7.5875
1.4969  1.3600  1.6421  6.4008  7.9146  2.1964  11.7189 0.0000
6.8552  9.0641  7.7151  1.6727  2.8480  5.3747  1.3895  4.6244
4.8790  7.0742  11.1793 5.5201  5.7175  2.6218  1.7883  10.6017
3.3550  2.1147  12.4421 2.3128  3.2977  1.9569  10.5184 0.0000
2.3863  7.9071  3.0386  3.2731  4.2617  0.6064  8.5588  9.7587
8.9969  2.5752\
ATP1A1  13.0623 12.9286 14.4792 13.6774 14.1444 13.9859 13.2076
14.2070 14.7602 13.0603 13.5682 14.5023 13.5842 13.4347 13.5964
14.8988 12.8812 14.4086 12.8813 13.5119 13.7224 14.0954 13.5383
14.3631 12.5548 13.0192 17.2311 12.7668 13.9286 13.2443 12.4327
13.2881 13.2995 14.3109 13.7188 15.5154 14.0046 14.6186 13.5575
12.4198 14.2669 13.6695 13.4304 14.1541 12.7181 13.3360 12.9018
13.2521 12.7196 13.3103 13.0799 13.6927 14.5337 13.3592 13.5078
13.3482 14.6250 15.1961 13.3119 13.7957 12.8455 14.3998 13.2483
13.8234 12.6047 13.6639 14.6427 14.5143 16.0275 14.2275 13.3279
13.1412 12.7758 13.7551 13.6718 15.2517 14.0193 13.4989 14.0682
13.2424 14.8982 14.3394 13.0075 13.9932 13.4233 13.7378 13.4303
13.5844 13.5235 13.3457 13.1842 14.0056 14.3551 12.9068 12.5008
12.7649 13.6736 12.4429 13.1695 12.9152 14.4529 13.6896 13.9699
12.5239 13.9603 13.2314 11.5090 13.3869 13.5889 13.7160 13.4844
13.4062 12.3051 12.5901 12.6671 13.4068 13.8944 13.7071 14.2645
14.4071 13.5662 13.0624 13.9907 13.5929 13.3105 13.4351 14.1317
14.0448 12.9325 12.8805 14.3647 13.6676 13.5080 13.2161 12.9937
11.9455 13.0721 10.6072 13.3996 12.8060 13.6330 14.2335 13.4029
14.3964 14.0495 13.3918 14.4824 13.3523 14.5457 14.0252 13.9609
13.1472 13.9992 13.6537 13.6188 13.5467 13.0351 13.5688 13.6509
12.8123 14.0062 15.1885 13.6583 13.6305 14.3924 14.3377 14.0009
15.0492 13.6684 13.3612 13.2955 13.4395 14.6061 14.2629 13.2911
13.9098 12.5294 12.8874 13.5863 13.2669 13.2249 13.7063 14.3437
13.7297 13.8290 13.0774 12.8872 13.3505 13.1154 13.4703 14.2833
14.7909 12.8801 13.5041 13.9152 13.5360 13.3268 12.5266 14.1243
13.6287 13.5605\
KGFLP2  3.9633  6.3618  6.1807  6.7386  5.7653  4.5242  5.7232
6.5760  5.4739  4.8891  4.2147  6.7396  6.8388  6.8494  5.7445
6.2224  6.5589  5.6724  6.6675  5.2464  5.5658  5.4201  7.4955
7.2968  6.8567  4.2158  6.0968  3.7903  4.3184  6.7885  6.3724
6.5916  3.3984  6.4446  6.6118  5.5968  6.5363  5.4627  7.5436

4.8459 7.4572 6.2315 4.2060 5.3816 7.0657 5.0119 3.2199
5.2342 6.1913 6.1111 5.6129 6.0399 5.9806 7.3605 6.9404
5.6852 4.7393 5.6469 7.0503 5.9505 7.0508 6.1489 4.7575
6.4473 5.0767 5.7995 7.0989 6.3143 5.8125 4.9949 4.7439
6.3995 4.6200 6.8811 7.6332 5.8891 5.0911 6.1077 6.4606
6.9820 5.3908 6.8187 5.7026 5.6758 5.1378 6.5006 7.0516
4.3854 5.9490 5.7481 6.1991 6.1272 5.9363 6.6992 5.0106
5.9642 6.1107 6.8796 6.6632 6.9757 3.0438 5.3567 7.4751
5.4368 7.2056 4.3931 5.9780 6.6672 4.7581 5.2803 5.4882
6.2017 4.7091 6.9274 5.2990 5.2874 4.8185 7.7158 4.3234
4.4385 6.6081 6.5000 6.2153 3.3130 7.8864 6.5310 6.9786
4.0735 7.4254 7.2867 4.7930 5.9172 4.5551 6.0979 4.7735
2.5015 3.5207 5.6322 6.6342 6.6570 5.5243 5.0602 5.4068
4.3451 5.5348 6.6613 5.3337 6.2553 7.2589 5.5607 6.2320
3.9662 6.3717 5.6568 5.2505 4.9665 7.3439 5.7546 4.8530
5.2109 4.5423 3.2212 6.4957 6.2800 4.8914 5.9688 5.2246
6.7506 4.9531 5.2334 4.9615 6.7242 5.1043 5.7899 6.0231
6.0942 5.9886 6.8299 6.6169 6.4540 5.6885 6.0839 5.8232
4.4790 5.4754 5.8752 6.0485 5.2764 5.5166 5.2023 3.9838
5.8342 6.2624 5.8206 5.6143 5.1434 5.7255 7.6714 6.2600
7.2807 3.7440\
MGRN1 10.9694 10.9895 11.1338 10.9369 10.8190 10.2221 10.5315
10.8286 11.0737 11.1534 11.2888 10.1699 10.3091 10.7716 10.4711
10.6045 10.3421 11.1349 11.0152 9.8338 10.8444 11.0320 11.0850
10.6946 11.3210 10.7982 10.3680 10.6981 10.9601 12.1761 10.7214
10.7494 10.1404 11.3662 10.5107 11.2258 11.3736 11.0840 10.0923
9.7998 10.5370 10.9588 10.7756 10.1345 10.0186 10.5822 10.0738
11.2217 10.2498 10.8232 10.7806 10.5503 11.2425 10.6373 11.2553
10.5354 11.0285 10.2627 10.7338 10.3899 10.3888 11.2000 11.0381
10.3315 11.1089 11.1770 10.5346 10.5563 10.6433 11.2301 10.1726
11.4118 10.6325 11.1408 10.7155 11.0887 10.8794 11.2353 10.6814
11.0929 10.6348 10.3857 10.6245 11.0435 10.9981 10.2966 10.1767
10.8519 10.8237 10.9478 10.8725 11.4800 10.0328 10.7489 10.6841
10.8880 10.8527 11.2338 10.7692 10.9053 10.4141 11.2126 10.2551
10.7265 10.6197 10.7424 10.8159 10.5083 10.1442 11.0953 11.1626
11.0992 9.9328 10.4083 10.7426 10.9486 10.3276 11.7040 10.1040
11.4575 10.2864 10.4908 10.6557 10.9332 10.9203 10.4592 10.1324
10.5859 10.4431 10.9674 11.2934 10.4871 10.9596 11.2133 10.5090
9.4004 10.0843 9.5908 10.8979 11.1659 11.2890 10.6702 10.9227
10.2528 10.6999 10.8564 11.0262 11.0964 11.3229 9.8738 10.8323
9.9503 10.9916 11.7067 11.3473 10.8603 10.9038 10.6936 10.6745
11.0057 11.2634 11.2709 10.5492 10.7493 10.9177 10.9368 10.5213
11.2756 10.9016 10.3504 11.3693 10.4649 9.8196 11.4331 10.1079
10.8482 10.9757 10.6314 10.7901 11.0963 10.6084 11.4508 9.9442
10.4491 10.1441 10.9504 10.3974 11.3785 10.7025 11.0201 10.6480
11.0303 11.2572 10.8390 10.8088 8.9946 9.6170 11.0415 10.9785
10.6460 10.5418\
TCP1 12.1612 11.9515 12.0402 12.0677 11.7477 12.1884 11.7280
11.5416 12.3404 12.0362 12.3137 12.2513 11.8453 12.1808 12.0873
11.4778 11.1741 12.1937 11.8170 12.3956 11.2567 12.2707 11.9886
11.8254 11.7972 12.6765 12.3881 12.1220 12.1215 13.0223 12.2928
11.8161 12.2295 12.1898 12.7890 13.5195 11.5650 12.2740 12.2184
13.0061 12.7531 11.9344 12.5535 12.3175 11.8488 11.4141 12.4754
12.7030 13.1213 11.6426 11.8510 12.8234 12.1065 11.8329 12.2369

```
12.0976 12.4263 12.2723 11.8624 11.7035 11.8743 12.3784 13.5759
11.5265 12.0629 11.8991 12.3760 11.5941 12.2462 12.1045 12.6591
12.0660 12.2009 12.0770 12.0647 11.8833 11.7025 11.5548 11.4931
12.5851 12.3052 12.2070 11.5054 13.3869 12.0960 11.7354 12.5661
11.9865 11.5595 12.3562 11.7926 11.8386 12.2072 11.3674 12.2150
11.8605 11.7247 12.2620 12.8636 12.3532 12.3331 12.2527 11.6157
12.6222 13.0819 13.1402 11.8070 12.0379 12.3274 12.4088 12.2677
12.4516 11.7840 11.7455 12.6532 12.3649 13.2091 12.1072 12.3487
12.2639 12.6704 12.0868 12.8623 12.6612 11.9457 12.0520 12.1305
11.8022 11.9066 11.7382 12.2101 12.1596 12.4399 12.1774 12.5852
11.6058 12.6414 10.5574 12.1929 11.9730 13.1713 12.0561 11.6708
12.1012 12.2858 11.9842 12.0802 11.9207 12.3976 11.8750 11.4499
12.7054 11.8598 12.4932 12.1926 11.4485 11.8672 12.2530 12.9437
12.3707 12.0021 12.0600 12.0037 11.9808 12.2336 12.3371 12.0072
12.2788 11.7891 12.3171 12.5080 12.7073 12.0034 12.5552 10.5137
12.1345 12.0154 11.8538 12.5574 11.6476 12.5113 12.6979 12.7632
12.1541 12.6700 11.9089 11.8945 12.4709 12.0583 12.0471 12.3696
11.8814 12.2485 11.8135 12.7703 12.1772 11.9401 12.1125 12.0267
12.1198 12.0614\
CRTAP   11.5776 12.6301 12.4162 11.6864 11.4875 11.1634 11.9982
11.5661 11.6343 12.5544 11.2753 12.0500 12.2489 10.7586 11.6549
11.8336 12.5716 11.5717 12.5478 12.0155 10.8118 10.5332 11.9751
11.9174 13.0119 11.5709 10.4684 11.3332 11.1655 11.8992 13.4611
11.3790 11.6740 11.1730 10.6887 10.7024 11.3203 11.2731 11.3124
11.6935 11.7946 12.7207 11.7947 11.1242 13.5976 12.5017 11.1797
11.7204 11.5345 11.9582 11.3011 11.6509 11.1859 13.4884 11.2566
11.1718 11.7187 11.9113 12.2178 11.1073 13.4287 11.0556 11.2131
11.4725 12.6180 11.0061 11.4799 10.8560 11.5710 11.5398 10.8547
11.6140 11.7038 10.7075 11.4407 11.1934 11.7627 10.8312 11.9003
11.0901 12.0326 11.8122 11.7315 10.8218 12.2983 12.2029 10.6970
11.4026 11.6877 11.5958 13.4150 12.2517 11.8072 12.5081 11.3789
13.2979 12.2738 13.1931 11.4008 11.6976 11.2001 11.7055 11.6432
11.6957 11.4440 11.9423 12.4895 11.5092 12.4938 11.5766 11.9867
10.8747 11.8899 11.1007 11.6311 11.8346 11.8453 11.9032 11.0849
11.6984 12.2695 11.2441 12.2344 11.0042 12.4177 11.5234 11.1317
11.5646 12.8995 12.6498 12.1815 11.7410 11.4619 10.5606 11.5821
11.2459 10.7750 12.6061 12.3963 12.8784 11.3614 12.5654 11.9709
11.5462 11.6555 13.0137 10.3030 12.9969 10.7277 10.8207 12.4453
11.4782 11.7977 10.4781 10.8297 11.9972 12.9191 12.0435 10.7941
11.6449 11.2430 11.1401 12.6944 11.7110 11.6561 11.2499 11.7771
11.5298 11.1222 11.2073 9.8341  11.1096 11.3504 10.7895 12.2556
10.8839 12.4548 13.6959 10.9201 11.2551 11.5817 11.7126 11.6665
11.6942 10.9960 11.3790 10.9307 11.8906 11.2520 11.9717 11.6750
11.0627 11.1065 11.8295 11.6334 12.1376 10.7651 13.0356 11.1525
12.1479 10.9539\
CBLC    9.6787  7.2827  9.1954  8.4685  7.4107  7.9386  9.5422
9.5220  10.1823 7.9196  8.6896  9.6258  8.7822  0.4395  8.6418
7.4919  10.0355 9.5365  7.2774  10.2597 9.9909  8.6413  8.3481
9.0572  3.7188  10.1887 10.3075 9.8579  9.6486  10.2886 0.0000
8.8510  8.2545  8.8640  9.3607  8.5274  10.0898 9.2322  10.4013
8.8641  6.1550  7.7077  9.5304  7.9266  0.0000  9.8464  10.1835
9.3526  5.5332  11.2000 8.8532  10.3985 7.9055  1.6405  7.8457
8.0402  10.0150 8.5533  9.1084  7.9903  3.5225  8.7872  8.5357
9.7391  6.9738  9.4991  9.3730  8.0440  7.3866  9.1969  9.7420
```

| | | | | | | | |
|---|---|---|---|---|---|---|---|
| 8.4736 | 3.4823 | 9.7124 | 8.4979 | 9.0243 | 6.6022 | 8.4008 | 8.7962 |
| 10.3099 | 9.9541 | 9.3544 | 7.9900 | 8.7908 | 9.3478 | 8.7244 | 8.3005 |
| 10.1022 | 9.7650 | 6.6065 | 4.8150 | 8.8687 | 7.8372 | 8.3727 | 7.8648 |
| 0.0000 | 10.1795 | 6.2802 | 9.4484 | 9.4164 | 9.0160 | 8.9578 | 8.6767 |
| 9.0623 | 9.1735 | 7.1923 | 8.3033 | 8.4750 | 8.2271 | 8.8283 | 8.9346 |
| 9.7934 | 14.1168 | 11.1000 | 7.9666 | 7.9238 | 8.3719 | 9.3645 | 8.5104 |
| 9.7216 | 4.2617 | 9.7423 | 9.5880 | 9.8036 | 7.4868 | 10.0047 | 8.4972 |
| 10.5000 | 6.3652 | 4.9508 | 10.1505 | 9.8301 | 9.0040 | 7.8762 | 0.8107 |
| 11.8834 | 9.1750 | 9.3392 | 8.4913 | 6.3575 | 8.6769 | 9.0448 | 9.2783 |
| 9.2404 | 9.7998 | 0.5418 | 7.8079 | 8.0706 | 8.5929 | 11.8192 | 8.0169 |
| 8.7477 | 8.3433 | 10.0496 | 10.0792 | 9.4835 | 7.0290 | 9.6654 | 9.0544 |
| 6.1804 | 9.0934 | 10.7091 | 8.8305 | 9.6326 | 8.8183 | 7.0190 | 10.0278 |
| 8.2972 | 9.2079 | 9.4063 | 9.1029 | 9.5708 | 8.5098 | 8.5411 | 10.7401 |
| 8.9452 | 7.2707 | 4.5156 | 9.0066 | 7.2324 | 10.8392 | 9.5580 | 8.6074 |
| 9.3269 | 9.5206 | 8.2504 | 9.3690 | 10.4672 | 10.0196 | 8.2024 | 8.3025 |
| 9.0612 | 10.2427 | 5.5562 | 9.1106 | 10.8576 | 9.9057 | 6.7769 | 8.5653 |
| 8.6620 | 11.7374\ | | | | | | |
| SPHK1 | 8.1524 | 9.2699 | 7.9347 | 6.9246 | 6.8064 | 7.9293 | 7.1493 |
| 6.5497 | 5.1581 | 6.6401 | 6.1767 | 6.4811 | 5.9948 | 4.9929 | 5.0990 |
| 6.0666 | 6.1524 | 8.2640 | 9.3287 | 8.2994 | 9.0313 | 5.5432 | 5.7399 |
| 5.9839 | 6.6570 | 10.3534 | 5.3303 | 7.8839 | 8.6198 | 7.2857 | 7.6657 |
| 7.2314 | 8.9460 | 7.9707 | 5.3693 | 5.6689 | 7.6275 | 4.6929 | 6.4548 |
| 6.8300 | 6.5352 | 9.6732 | 7.2941 | 7.3079 | 7.4667 | 6.4983 | 7.7759 |
| 6.7748 | 11.6006 | 6.5284 | 9.3225 | 8.2172 | 8.6848 | 7.3655 | 8.1753 |
| 5.9610 | 7.5582 | 8.4942 | 6.8290 | 5.2871 | 7.0788 | 6.7817 | 4.9376 |
| 7.3661 | 5.1677 | 6.5160 | 6.7602 | 6.6151 | 7.3733 | 7.4361 | 8.5093 |
| 6.2674 | 8.7229 | 6.7999 | 7.5870 | 7.4063 | 7.7043 | 6.3207 | 6.1712 |
| 8.6458 | 6.7967 | 7.3249 | 5.0345 | 7.0275 | 6.7041 | 6.3104 | 5.3007 |
| 8.7801 | 6.8872 | 8.9072 | 8.7828 | 5.9514 | 10.3440 | 7.4996 | 7.9003 |
| 8.5102 | 9.9856 | 6.7913 | 5.0842 | 6.8193 | 8.1335 | 8.0190 | 6.9988 |
| 7.4308 | 4.9962 | 7.0711 | 8.1949 | 6.5027 | 7.1755 | 6.4304 | 5.0539 |
| 6.2756 | 8.6677 | 7.4757 | 8.6210 | 7.5586 | 5.7428 | 6.1869 | 9.0480 |
| 7.8872 | 7.5357 | 7.6558 | 6.3838 | 6.1117 | 5.8201 | 8.3272 | 7.2196 |
| 7.0802 | 6.3780 | 6.5967 | 6.0283 | 4.3021 | 7.0446 | 7.0615 | 11.5894 |
| 10.8516 | 8.0416 | 7.3472 | 6.6874 | 6.5160 | 5.4693 | 5.3211 | 7.3472 |
| 7.1028 | 7.1938 | 5.3877 | 6.6647 | 8.0558 | 6.8555 | 7.4038 | 6.5548 |
| 8.8669 | 6.3000 | 6.8295 | 5.6387 | 9.3348 | 7.0464 | 7.6345 | 5.4227 |
| 9.0867 | 5.0935 | 9.2068 | 5.5643 | 6.2795 | 6.4656 | 5.1787 | 5.9470 |
| 5.6351 | 6.3912 | 6.6779 | 7.0682 | 6.0265 | 8.4359 | 8.0105 | 7.6036 |
| 6.2885 | 6.0952 | 8.3156 | 6.5374 | 6.6099 | 8.5039 | 6.4979 | 5.7838 |
| 5.0760 | 7.1644 | 6.1180 | 7.6706 | 7.2520 | 6.7466 | 8.6702 | 8.3612 |
| 6.2763 | 7.8427 | 8.3910 | 7.7874 | 7.5301 | 8.3986 | 5.9855 | 6.4350 |
| 6.7250 | 9.1441\ | | | | | | |
| DPT | 3.6630 | 9.3456 | 0.0000 | 1.3959 | 4.0407 | 1.8477 | 3.4667 |
| 6.3722 | 2.0525 | 8.1129 | 0.7594 | 1.3775 | 3.7397 | 0.7759 | 2.3407 |
| 1.4442 | 3.1837 | 3.5493 | 10.6932 | 0.9198 | 2.0721 | 4.6091 | 7.6271 |
| 1.6784 | 9.8001 | 1.0317 | 3.2557 | 4.6250 | 5.8239 | 1.5953 | 2.9307 |
| 4.6029 | 0.8951 | 1.8766 | 0.5853 | 2.3867 | 1.5255 | 1.1048 | 2.0653 |
| 3.8405 | 5.1751 | 10.8386 | 3.0982 | 2.4713 | 13.0346 | 3.7705 | 7.0874 |
| 1.1317 | 5.9708 | 2.4071 | 3.7593 | 5.4990 | 5.5386 | 13.6630 | 5.1829 |
| 5.0577 | 2.8225 | 5.5123 | 7.2756 | 5.9259 | 13.0689 | 2.4655 | 2.5147 |
| 1.3432 | 9.1897 | 3.1834 | 0.0000 | 4.6590 | 3.5640 | 4.4793 | 2.1712 |
| 2.9181 | 7.0881 | 4.8932 | 8.1635 | 1.3447 | 7.5413 | 5.3898 | 5.7132 |
| 6.5492 | 5.7634 | 1.3683 | 7.8349 | 3.6170 | 7.0511 | 1.3843 | 2.6823 |

```
5.8521  6.5626  2.9271  13.2222 5.7037  3.8136  8.9915  5.4860
10.3284 0.9065  9.0851  7.7628  5.4196  6.2840  4.0960  0.0000
4.3430  6.3889  6.3135  7.9147  3.3309  6.8360  4.2172  2.8045
0.6215  4.8729  1.5850  8.5595  1.3697  5.5385  2.2752  5.5635
1.0358  2.5419  3.2578  5.1061  2.5865  11.1644 3.3768  5.7588
1.2894  11.4570 10.4249 1.6759  2.1813  1.9260  2.8502  4.1368
9.6617  6.7156  8.4988  8.5377  9.5217  2.0359  0.5748  4.1774
6.2794  4.0867  7.7860  2.5258  12.1281 0.0000  1.2240  9.3937
4.8711  6.2762  5.4131  0.9437  5.9525  9.1850  4.0007  1.8394
4.6523  1.0317  2.8804  5.0790  2.5454  3.0297  2.4806  2.9165
5.9523  4.0786  0.5466  1.9700  2.9341  0.9335  1.8874  6.5587
1.1272  8.9292  14.4639 4.7362  4.8854  3.4996  2.0241  2.3092
4.6147  3.0580  3.1906  3.2376  2.7395  0.6567  7.5038  5.8569
0.9557  1.2903  6.9665  4.7984  1.5946  0.0000  9.7601  2.5911
2.5996  4.2463\
BPESC1  0.0000  0.5319  0.0000  0.4935  1.2172  0.4059  0.0000
0.4748  0.0000  0.7786  0.0000  0.0000  0.4363  0.0000  0.0000
0.0000  0.6221  0.0000  0.5838  0.0000  0.6175  0.0000  0.0000
0.0000  0.0000  0.0000  0.0000  1.3471  0.0000  0.0000  0.0000
0.0000  0.0000  0.0000  0.0000  0.0000  0.0000  0.0000  0.0000
0.0000  0.0000  0.0000  0.0000  0.7649  0.0000  0.0000  0.0000
0.0000  0.0000  0.0000  0.0000  0.0000  0.0000  0.0000  0.0000
1.2781  0.0000  0.0000  0.4252  0.0000  0.0000  0.0000  0.0000
0.0000  0.0000  0.0000  0.0000  0.8444  0.0000  0.6160  0.0000
0.0000  0.0000  0.0000  0.0000  0.0000  0.0000  0.0000  0.0000
0.6159  0.0000  0.0000  0.0000  0.0000  0.9110  0.6199  0.0000
0.0000  0.0000  0.0000  0.0000  0.0000  0.0000  0.0000  0.0000
0.0000  0.0000  0.5454  0.5090  0.0000  0.0000  0.0000  0.5352
0.0000  0.0000  0.4659  0.0000  0.0000  0.5426  0.0000  0.0000
0.0000  1.3631  0.0000  0.0000  0.0000  0.0000  0.0000  0.0000
0.0000  0.0000  0.0000  0.0000  0.0000  0.0000  0.0000  0.0000
0.0000  0.0000  0.0000  0.0000  0.0000  0.5526  0.7571  0.0000
0.0000  1.4217  0.0000  0.0000  0.8792  0.0000  0.0000  0.6506
0.0000  0.0000  0.0000  0.0000  0.0000  0.0000  0.0000  0.0000
0.0000  0.0000  0.0000  0.5476  0.0000  0.5638  0.0000  0.0000
0.5416  0.0000  0.0000  0.0000  0.0000  0.4418  0.0000  0.0000
0.0000  0.0000  0.0000  0.0000  0.9151  0.0000  0.0000  0.0000
0.0000  1.6715  0.0000  0.0000  1.4046  0.0000  0.0000  0.0000
0.0000  0.0000  0.0000  0.0000  0.0000  0.0000  0.5707  0.0000
0.0000  0.0000  0.0000  0.4985  0.0000  0.0000  0.0000  0.0000
0.0000  0.0000\
CUEDC1  9.7056  8.1320  11.0051 9.1712  9.3251  9.7822  10.7902
8.9472  9.7814  9.1544  8.8999  9.8059  9.9369  10.4082 10.3698
8.2476  8.0997  9.3135  8.3911  9.1699  9.3169  11.5003 9.6693
9.0607  10.0293 7.2911  7.5660  10.5275 8.6561  7.9637  7.4961
10.3386 8.5242  10.8976 11.1379 7.9828  10.0942 9.6576  8.0909
8.2192  8.8854  8.8022  8.6152  8.7692  8.6660  8.4831  9.9261
8.7193  7.9680  9.5453  11.2526 7.1736  9.7914  8.8878  9.2879
8.6475  8.7667  9.5871  9.1456  11.1203 9.0226  9.1042  9.8775
9.2076  8.6171  9.7029  9.6293  9.7485  9.3522  8.8409  9.7241
10.5112 7.2282  9.5800  9.2275  12.4117 10.0621 10.3119 8.5914
8.7362  9.2675  9.6146  9.1927  10.2098 8.9264  8.5941  8.5931
10.5505 8.6871  8.3507  9.3240  8.7980  9.4344  7.4947  9.4759
9.2672  9.3450  8.5292  8.9990  7.6656  8.3060  10.3555 8.8397
```

7.2482 8.4101 8.8263 7.5431 9.3075 9.1291 9.8816 11.1882
10.3825 7.9625 6.7549 11.2353 8.6653 10.9322 9.6278 10.6880
7.5540 9.0030 9.5608 8.8302 11.5070 9.9731 10.1360 10.8290
8.5116 8.4653 9.6419 10.1913 9.5116 10.5936 8.4000 9.2192
6.4625 8.5948 6.3465 9.6019 8.7493 9.4335 10.0581 9.8783
9.3961 9.4197 7.8784 10.0342 9.9189 9.5049 8.3794 10.0545
9.2691 10.1905 9.2506 9.4691 9.1210 8.9089 9.5386 9.5799
7.7213 10.6482 8.7375 9.2996 9.5904 9.2707 9.5672 7.8831
9.2591 11.1497 10.7482 9.2605 8.6408 9.3876 9.0851 7.5617
9.6847 8.5631 9.7317 9.3228 10.3634 9.8594 8.6759 9.3301
9.0250 8.4461 9.1849 9.4723 8.7983 9.0675 10.3071 10.5377
10.1012 8.7479 9.3193 9.0646 8.0088 7.0573 9.3310 11.1831
10.0378 8.8812\
CUEDC2 11.7181 10.4907 9.0259 9.9141 10.1743 8.9212 10.7176
11.2361 10.4303 10.6715 10.2544 11.6843 10.1981 10.9554 10.1523
10.7658 10.8103 11.0693 10.4774 11.2383 10.0946 10.1426 10.5591
10.7279 10.7760 11.2779 10.0582 11.2346 10.8178 10.0161 11.1225
10.1486 10.1949 9.9580 9.4500 11.0988 10.5310 10.3194 10.6540
10.2232 10.7219 9.9897 10.5644 9.3231 9.9997 10.8688 12.6052
9.7064 10.2939 10.9604 9.7477 10.5198 10.4437 9.9599 10.5370
10.3499 10.3452 9.8379 10.1763 10.4986 9.9087 10.4477 10.6274
10.5251 10.7564 10.2337 9.4660 11.2333 10.1999 10.4872 11.4004
10.3137 10.8455 10.8944 9.9581 10.8688 10.8588 10.3706 10.4085
10.5383 11.5097 8.9312 9.8292 9.4172 12.0070 9.6551 8.6542
11.5423 10.4910 11.3799 10.5181 10.6196 11.8199 10.6553 10.8027
10.5191 9.2299 10.5909 10.6057 10.5385 11.7560 10.1766 9.4018
10.5131 11.1246 11.4950 10.4960 10.3712 10.6332 10.5656 10.9953
10.8493 11.0275 11.2697 10.4110 9.7651 10.8424 9.6773 9.9672
9.4170 10.3005 10.8408 9.8062 10.0577 10.3043 10.0835 9.9475
12.5274 10.1320 10.0305 9.6286 10.1660 10.6741 9.3483 10.9676
10.8821 11.3848 10.3485 10.2283 10.2935 11.8080 10.4722 11.0158
10.3021 10.3113 10.4601 9.6640 10.8164 10.6612 11.7756 10.1871
11.8766 10.1662 10.5027 12.0332 10.1506 10.3917 11.0831 10.1235
11.5528 10.9126 10.2466 10.7520 11.1326 10.2053 9.9833 10.2135
9.8623 11.1703 10.6511 10.7596 10.6076 9.5540 11.1305 10.6679
10.8346 10.3907 10.4904 10.4647 9.6239 11.4300 11.6014 10.3770
10.6565 10.9660 11.1511 10.5037 11.4811 11.1988 11.2102 10.2952
10.6363 10.2683 9.0992 10.3605 12.3871 11.4066 10.5029 9.9796
10.0727 11.8685\
CBLB 9.2725 9.3227 9.4003 9.2075 8.9260 9.7478 9.0128
8.9517 10.1088 8.5748 10.2605 8.2622 8.4316 8.7025 9.1700
8.7810 9.1126 8.8114 9.2170 7.7123 9.1369 9.2896 8.4519
9.3062 8.6844 7.0790 8.0190 8.2388 8.6152 8.5661 9.1516
9.3328 7.3907 7.7695 9.1094 7.8903 9.6250 9.8120 9.1014
9.0116 8.3037 9.2908 8.1828 8.5766 8.7023 8.6416 8.3144
9.3221 8.8682 8.1551 8.3305 9.8923 9.3385 9.1218 8.7578
9.3919 8.9406 8.6584 9.1368 8.6196 8.4900 8.7912 9.1175
8.8806 8.5347 9.3803 8.2456 9.4136 7.7220 8.7474 7.5661
9.6481 8.9673 8.1629 9.2459 9.1212 9.9022 8.7074 8.8315
8.3479 8.5753 9.3854 8.6828 9.0613 9.0549 8.7993 8.7497
9.2739 8.7100 8.5281 8.7252 9.7629 8.8640 9.2506 9.5428
8.1517 9.1981 8.5292 9.3804 8.7093 8.9815 9.2777 8.8369
8.8808 9.4846 8.8479 9.2107 11.2376 9.3652 9.6663 9.3435
8.8799 7.2172 8.5314 8.6637 9.0799 9.9050 9.1302 9.8879

| | | | | | | | |
|---|---|---|---|---|---|---|---|
| 8.4080 | 8.4132 | 8.6294 | 8.9325 | 8.7339 | 8.7379 | 9.1763 | 9.9137 |
| 7.4810 | 8.7485 | 9.0278 | 8.7012 | 9.4643 | 9.3332 | 9.6627 | 8.7983 |
| 6.0322 | 8.0508 | 9.4202 | 9.5808 | 8.6919 | 9.3353 | 8.9834 | 8.7369 |
| 8.8897 | 8.7146 | 8.6662 | 9.3377 | 8.2292 | 8.6204 | 8.8431 | 8.7774 |
| 8.0975 | 10.5708 | 8.4694 | 9.0688 | 9.7829 | 8.5415 | 9.9504 | 8.9211 |
| 6.4597 | 7.6625 | 7.0500 | 9.0109 | 9.1341 | 8.9295 | 10.7540 | 8.9603 |
| 9.0056 | 8.9605 | 8.5168 | 10.7900 | 8.6375 | 8.4188 | 7.4140 | 7.5617 |
| 9.6984 | 8.6598 | 8.9853 | 9.4755 | 9.0965 | 7.7743 | 8.5469 | 8.6057 |
| 9.1602 | 9.5683 | 8.8991 | 9.1484 | 8.2592 | 9.3226 | 9.1625 | 9.4339 |
| 9.6890 | 8.9832 | 9.2072 | 9.8694 | 6.7826 | 8.6331 | 8.7999 | 10.2285 |
| 9.7177 | 7.9599\ | | | | | | |
| CPEB4 | 7.1058 | 9.7090 | 8.9985 | 9.1382 | 8.4508 | 9.7236 | 7.1381 |
| 8.2246 | 7.6491 | 8.2649 | 7.9154 | 7.5234 | 8.9554 | 8.7050 | 9.3783 |
| 8.2719 | 7.6779 | 8.1075 | 9.0600 | 7.0095 | 7.8297 | 9.6373 | 9.1827 |
| 9.5158 | 9.4744 | 7.3337 | 9.6505 | 8.1237 | 8.6181 | 7.3191 | 7.2483 |
| 8.5239 | 7.3109 | 8.0733 | 7.4272 | 8.0033 | 8.8274 | 9.2294 | 8.6797 |
| 8.1099 | 8.1781 | 10.2388 | 7.8185 | 8.5923 | 9.8228 | 8.5507 | 5.2028 |
| 8.6801 | 7.5596 | 8.8901 | 9.0952 | 8.4737 | 8.2069 | 9.1164 | 8.3050 |
| 8.9911 | 9.0040 | 9.2717 | 8.6129 | 8.9152 | 9.4742 | 9.3747 | 8.7796 |
| 7.5428 | 8.3676 | 9.5974 | 9.5395 | 7.9795 | 9.4201 | 8.1488 | 7.3131 |
| 9.0466 | 8.4232 | 8.2344 | 9.8788 | 8.6597 | 7.9634 | 9.6397 | 8.6498 |
| 9.0765 | 7.2325 | 7.2073 | 9.7026 | 9.3295 | 7.2890 | 8.4598 | 8.4195 |
| 6.8396 | 8.1215 | 7.4103 | 8.8307 | 8.7889 | 7.0590 | 8.5628 | 8.3826 |
| 9.6262 | 8.9843 | 8.5166 | 7.7286 | 8.5593 | 5.0587 | 8.4759 | 8.1585 |
| 8.8457 | 7.9647 | 7.3114 | 8.4738 | 9.6119 | 9.1788 | 7.7394 | 9.1223 |
| 8.9986 | 6.7111 | 6.7814 | 8.5231 | 7.9017 | 8.0716 | 7.7809 | 8.7640 |
| 8.0619 | 7.7809 | 9.2470 | 8.6770 | 8.6929 | 8.9561 | 7.8752 | 7.8918 |
| 5.1853 | 9.4825 | 9.8280 | 8.3839 | 8.1430 | 8.6557 | 9.5675 | 7.6391 |
| 4.6588 | 6.5095 | 6.2074 | 9.0215 | 8.9791 | 8.0809 | 9.7887 | 8.0581 |
| 9.4145 | 9.4056 | 8.9356 | 9.8540 | 7.9842 | 8.3965 | 7.6200 | 9.5062 |
| 6.2854 | 9.4937 | 8.3007 | 7.8396 | 8.9309 | 9.2477 | 8.1404 | 7.2828 |
| 6.6018 | 7.9725 | 8.4509 | 8.8508 | 7.2039 | 8.0405 | 9.6315 | 8.5650 |
| 9.9519 | 7.9246 | 7.2043 | 8.0659 | 7.6072 | 7.0146 | 7.2239 | 5.7144 |
| 8.2051 | 9.0158 | 9.3175 | 9.1031 | 9.4536 | 7.8079 | 7.4930 | 7.0395 |
| 8.6918 | 8.3871 | 7.9676 | 8.2412 | 7.3501 | 6.9246 | 7.5456 | 8.9026 |
| 8.8527 | 8.3437 | 8.3355 | 8.2901 | 3.4726 | 5.9327 | 8.1933 | 9.0095 |
| 9.2357 | 6.1881\ | | | | | | |
| CPEB1 | 0.0000 | 5.3462 | 2.8336 | 0.0000 | 0.0000 | 2.9678 | 2.2192 |
| 2.4024 | 0.0000 | 4.0023 | 0.0000 | 1.1366 | 4.3520 | 0.0000 | 1.4890 |
| 0.0000 | 4.8326 | 0.7772 | 4.2063 | 4.0921 | 4.2194 | 0.8427 | 6.6263 |
| 0.5262 | 4.6894 | 0.0000 | 3.1387 | 0.0000 | 0.8532 | 1.8184 | 4.5356 |
| 1.2789 | 0.0000 | 3.4495 | 3.3931 | 1.7727 | 1.9329 | 0.3648 | 0.5410 |
| 0.4337 | 0.6649 | 4.7818 | 1.0569 | 0.4326 | 6.1381 | 0.0000 | 0.0000 |
| 0.0000 | 0.7769 | 0.0000 | 0.7972 | 0.7768 | 2.4720 | 6.0401 | 2.1436 |
| 0.0000 | 2.2825 | 5.7546 | 3.9448 | 2.9646 | 5.5568 | 2.9982 | 0.0000 |
| 4.2032 | 4.6210 | 0.3906 | 0.0000 | 3.9373 | 3.2274 | 1.3773 | 0.4334 |
| 0.0000 | 1.5110 | 1.7486 | 2.8697 | 0.4698 | 3.1657 | 2.2135 | 1.5048 |
| 0.0000 | 0.0000 | 0.7073 | 5.0610 | 1.4748 | 1.4651 | 1.6542 | 2.3403 |
| 1.8689 | 2.3134 | 2.6518 | 6.3360 | 1.9243 | 4.2027 | 1.1561 | 0.5198 |
| 4.3633 | 3.5228 | 7.0930 | 1.4288 | 2.9940 | 0.0000 | 1.7978 | 1.2310 |
| 2.3196 | 1.1338 | 1.5391 | 4.2843 | 3.4275 | 4.8043 | 0.5877 | 0.5136 |
| 0.0000 | 0.0000 | 1.0000 | 1.6510 | 5.2465 | 2.0324 | 1.4572 | 0.3974 |
| 0.0000 | 7.3369 | 0.5886 | 3.6237 | 0.0000 | 6.6053 | 1.2272 | 0.0000 |
| 0.0000 | 6.6986 | 5.9071 | 0.0000 | 0.0000 | 0.0000 | 0.7571 | 2.8142 |

| | | | | | | | |
|---|---|---|---|---|---|---|---|
| 0.0000 | 0.0000 | 0.0000 | 5.1982 | 7.1878 | 0.0000 | 0.0000 | 0.0000 |
| 0.9357 | 0.4562 | 8.4061 | 0.0000 | 5.3725 | 0.5814 | 0.0000 | 4.8931 |
| 0.0000 | 1.4965 | 2.5882 | 0.0000 | 3.4487 | 5.8676 | 1.3399 | 2.4961 |
| 0.0000 | 0.0000 | 0.0000 | 1.3167 | 0.0000 | 1.0531 | 0.0000 | 3.1709 |
| 3.4085 | 0.0000 | 0.0000 | 0.4486 | 0.9151 | 8.1612 | 0.0000 | 1.2582 |
| 0.4801 | 3.9875 | 6.3849 | 1.5479 | 5.4540 | 0.5545 | 0.6902 | 0.5257 |
| 0.0000 | 1.5843 | 1.3929 | 3.6333 | 1.5325 | 0.0000 | 0.0000 | 1.8202 |
| 0.0000 | 2.7619 | 1.9066 | 0.0000 | 0.0000 | 0.0000 | 7.5606 | 0.0000 |
| 2.3834 | 0.0000\ | | | | | | |
| ZNF45 | 8.1079 | 7.5393 | 7.6583 | 7.7131 | 8.4743 | 7.5926 | 8.4910 |
| 9.2731 | 8.2003 | 7.9842 | 7.9092 | 8.1389 | 7.2790 | 8.1914 | 8.0635 |
| 7.9186 | 7.5958 | 7.7443 | 7.8327 | 6.9308 | 6.0940 | 7.2455 | 7.6149 |
| 7.7593 | 7.9076 | 6.8369 | 8.8587 | 7.5922 | 7.1994 | 7.9490 | 7.3975 |
| 8.2609 | 7.1887 | 7.0098 | 8.8495 | 7.8644 | 8.0650 | 8.1805 | 7.5720 |
| 7.8951 | 7.2260 | 8.1238 | 6.1545 | 7.2148 | 8.4983 | 6.6229 | 5.6877 |
| 7.6857 | 8.8471 | 7.7510 | 8.2594 | 6.7831 | 8.0914 | 8.5060 | 8.1263 |
| 8.2072 | 7.3172 | 7.7316 | 7.6254 | 7.6179 | 8.6195 | 8.5483 | 7.6705 |
| 7.9450 | 7.9111 | 8.2413 | 7.2912 | 8.3433 | 7.1270 | 7.6143 | 8.8200 |
| 8.4220 | 6.8544 | 8.3096 | 8.1770 | 7.1911 | 7.1752 | 7.0977 | 7.0916 |
| 7.6295 | 7.5349 | 7.9764 | 7.7021 | 8.3116 | 7.5176 | 7.1297 | 8.3005 |
| 7.2090 | 7.6720 | 7.7484 | 8.0117 | 8.2428 | 6.8243 | 7.3004 | 7.3586 |
| 7.6716 | 8.0504 | 8.4056 | 8.2415 | 7.8483 | 7.3555 | 7.4733 | 7.8736 |
| 6.8088 | 8.1234 | 7.6576 | 7.2371 | 8.4877 | 8.1982 | 7.5159 | 8.0087 |
| 7.8345 | 6.8939 | 7.3663 | 8.0552 | 7.7401 | 7.4905 | 8.4605 | 7.6216 |
| 7.7880 | 8.5693 | 7.7659 | 8.2460 | 7.5439 | 8.2860 | 8.6169 | 7.6267 |
| 6.7240 | 8.5187 | 8.3671 | 7.6762 | 7.6911 | 7.6391 | 7.6766 | 8.9578 |
| 6.3054 | 7.4378 | 7.8971 | 8.3882 | 8.3312 | 6.5066 | 7.5048 | 7.4162 |
| 7.9242 | 8.0662 | 8.4761 | 8.0983 | 7.9763 | 7.5207 | 6.8567 | 8.2097 |
| 5.1734 | 8.5694 | 8.7503 | 7.3614 | 7.6495 | 8.2714 | 7.9640 | 6.3015 |
| 7.3979 | 6.9632 | 7.1121 | 7.7158 | 7.7856 | 7.2086 | 8.4323 | 5.2246 |
| 7.6923 | 7.7462 | 7.4933 | 8.2942 | 7.4805 | 8.2863 | 7.0348 | 6.8298 |
| 8.7070 | 8.1642 | 8.2082 | 8.3408 | 7.1677 | 7.4921 | 5.1931 | 9.1498 |
| 7.2285 | 8.5002 | 8.1216 | 7.8023 | 7.0810 | 7.4848 | 7.2331 | 8.1221 |
| 8.0499 | 8.6817 | 9.0468 | 9.2452 | 6.3542 | 6.3572 | 8.6259 | 8.2771 |
| 8.2167 | 6.2003\ | | | | | | |
| CPEB3 | 5.3458 | 6.7003 | 6.0435 | 7.5656 | 7.1576 | 7.4578 | 5.6673 |
| 6.4058 | 5.8756 | 5.9332 | 6.8581 | 6.7125 | 7.6901 | 6.9233 | 7.1399 |
| 6.1612 | 6.1838 | 6.6158 | 6.1362 | 5.3624 | 5.7229 | 8.0071 | 7.2037 |
| 7.1923 | 6.9108 | 5.4535 | 7.6907 | 6.1286 | 5.1240 | 6.5775 | 5.3487 |
| 6.9670 | 6.1513 | 5.9736 | 6.4438 | 5.2654 | 7.2368 | 6.8117 | 6.6466 |
| 7.6997 | 6.8329 | 6.4620 | 5.7242 | 7.1128 | 7.5187 | 6.7615 | 5.9341 |
| 6.7968 | 6.2733 | 6.8873 | 6.5311 | 6.9915 | 6.2912 | 7.3507 | 5.7896 |
| 6.7598 | 5.6794 | 6.5869 | 6.3500 | 7.5670 | 7.3880 | 6.5020 | 6.0964 |
| 5.8160 | 6.2831 | 7.1163 | 6.5852 | 4.9832 | 5.5168 | 6.4030 | 5.7986 |
| 5.9807 | 6.0791 | 7.2485 | 6.9387 | 8.6638 | 5.3194 | 7.6679 | 7.3189 |
| 6.0082 | 5.6015 | 8.2009 | 7.7807 | 6.7831 | 6.2531 | 5.9082 | 5.5564 |
| 5.8118 | 6.1616 | 6.0483 | 7.3320 | 6.6898 | 5.8802 | 6.4015 | 5.1928 |
| 6.7999 | 6.8795 | 6.9977 | 6.5229 | 6.6955 | 3.0438 | 6.6271 | 5.3082 |
| 7.7894 | 8.0495 | 7.7422 | 5.6396 | 7.0103 | 6.8925 | 5.7495 | 7.4549 |
| 6.4721 | 4.0638 | 4.2479 | 6.7779 | 7.1814 | 6.5367 | 6.1675 | 7.3656 |
| 6.3826 | 5.9765 | 7.2684 | 6.3748 | 8.4961 | 7.5032 | 5.8298 | 6.2668 |
| 3.2729 | 7.5595 | 7.2981 | 6.4828 | 7.8506 | 6.7216 | 7.5914 | 5.1267 |
| 4.3186 | 5.9543 | 5.1146 | 7.0643 | 7.2165 | 7.4695 | 6.6700 | 6.2193 |
| 6.7338 | 6.1556 | 7.9964 | 8.2328 | 6.3387 | 6.3644 | 6.3779 | 7.6522 |

| | | | | | | | |
|---|---|---|---|---|---|---|---|
| 5.4873 | 6.8662 | 5.4467 | 4.3168 | 5.7490 | 7.4907 | 5.9813 | 6.0993 |
| 5.4686 | 6.1705 | 6.2834 | 7.0788 | 6.0893 | 7.5355 | 6.6397 | 8.1760 |
| 7.2480 | 6.7706 | 6.5553 | 4.7878 | 8.3113 | 4.8683 | 6.0865 | 6.8818 |
| 5.8041 | 6.4520 | 7.4859 | 6.2846 | 7.8558 | 6.2387 | 7.1320 | 6.3477 |
| 7.5970 | 5.9610 | 6.5159 | 6.1076 | 6.3534 | 5.0314 | 4.6023 | 7.4287 |
| 5.9558 | 6.5324 | 5.2523 | 6.8129 | 4.2617 | 7.1715 | 7.0133 | 6.9085 |
| 7.4458 | 5.4315\ | | | | | | |
| CPEB2 | 5.9465 | 8.2801 | 9.2104 | 9.4904 | 7.6292 | 8.7236 | 7.2156 |
| 6.7467 | 7.3850 | 7.9720 | 8.0906 | 7.4296 | 7.8707 | 8.7233 | 8.5543 |
| 8.7697 | 6.7586 | 8.2731 | 8.7582 | 5.5202 | 7.7885 | 7.9034 | 8.4220 |
| 8.9788 | 9.6188 | 5.4706 | 5.0912 | 6.5491 | 7.4124 | 8.3440 | 7.1642 |
| 8.7981 | 6.2027 | 7.4643 | 7.5362 | 8.6669 | 8.3195 | 8.5997 | 9.1086 |
| 9.4880 | 8.0660 | 8.9606 | 7.5205 | 5.6424 | 9.1520 | 7.1941 | 6.4062 |
| 7.4748 | 9.1994 | 7.8275 | 7.8013 | 6.8108 | 7.4509 | 8.4582 | 7.3638 |
| 7.9953 | 9.3609 | 8.4366 | 7.9613 | 8.7161 | 9.4069 | 8.3204 | 7.1873 |
| 6.9598 | 7.9148 | 8.5305 | 7.0883 | 8.1549 | 7.6102 | 8.5987 | 6.2740 |
| 7.7809 | 6.8428 | 6.0765 | 9.0290 | 8.5425 | 7.0429 | 8.5092 | 8.3822 |
| 7.5902 | 6.3352 | 8.0210 | 10.0004 | 7.9071 | 6.8730 | 7.0676 | 7.5782 |
| 5.8913 | 7.2634 | 6.3735 | 8.8568 | 7.5384 | 6.8019 | 7.7507 | 5.4435 |
| 9.3822 | 7.9797 | 8.4076 | 7.3149 | 9.4891 | 6.1317 | 7.2092 | 6.9652 |
| 9.2678 | 9.3405 | 8.1779 | 8.2574 | 9.3930 | 8.3310 | 7.9796 | 8.5918 |
| 6.3536 | 7.1864 | 5.9069 | 8.8263 | 7.9207 | 6.9869 | 7.9935 | 7.9130 |
| 7.2249 | 7.3034 | 7.6620 | 7.8870 | 8.2206 | 8.7117 | 7.2145 | 7.7811 |
| 5.2136 | 9.0643 | 9.3380 | 7.8934 | 9.3451 | 7.7744 | 8.9026 | 7.6004 |
| 6.3192 | 4.4402 | 7.3542 | 8.4307 | 8.5745 | 7.3095 | 8.6629 | 7.4065 |
| 8.7205 | 7.7193 | 8.8989 | 8.8849 | 8.2358 | 6.9200 | 7.6053 | 9.0975 |
| 6.5781 | 8.5659 | 6.6833 | 7.5258 | 8.0589 | 8.7124 | 5.4146 | 8.3755 |
| 6.2162 | 7.0339 | 8.3230 | 8.2056 | 7.1160 | 6.1325 | 7.8972 | 8.6194 |
| 8.3364 | 7.1119 | 6.2141 | 6.1502 | 7.8618 | 7.4192 | 5.8231 | 6.3529 |
| 7.8178 | 8.6937 | 9.4475 | 9.1207 | 8.6876 | 6.7654 | 7.4379 | 6.0928 |
| 8.7562 | 7.0103 | 8.1874 | 7.2460 | 6.6356 | 6.8032 | 5.4654 | 8.6397 |
| 8.4161 | 8.6212 | 7.9527 | 8.3732 | 5.1434 | 7.3345 | 8.0224 | 6.3313 |
| 8.4098 | 3.9890\ | | | | | | |
| BEGAIN | 0.9511 | 6.4945 | 5.5153 | 2.4559 | 3.9611 | 4.1351 | 2.8873 |
| 4.4366 | 3.1202 | 4.8353 | 1.9151 | 2.5346 | 2.9465 | 4.7162 | 0.5377 |
| 2.9791 | 5.6141 | 3.1459 | 5.8422 | 2.9147 | 5.5362 | 1.5770 | 5.8418 |
| 3.0076 | 6.4446 | 7.2814 | 5.7790 | 2.8432 | 2.5456 | 1.3312 | 0.7334 |
| 3.2569 | 1.8398 | 2.1491 | 0.5853 | 3.2417 | 4.5574 | 1.7231 | 1.7115 |
| 1.0370 | 5.6321 | 5.7337 | 3.0042 | 3.3350 | 5.4765 | 4.6662 | 0.7135 |
| 0.0000 | 6.9798 | 2.2965 | 6.3074 | 2.5833 | 5.0620 | 5.8089 | 6.0350 |
| 2.5237 | 3.7168 | 5.1550 | 6.3740 | 3.2117 | 5.3302 | 5.0295 | 3.4464 |
| 4.3554 | 4.3484 | 3.4933 | 4.3588 | 3.8203 | 4.2262 | 5.7476 | 4.8939 |
| 2.4255 | 3.3577 | 5.0894 | 3.2574 | 1.8854 | 1.7421 | 3.0489 | 4.6017 |
| 5.2381 | 1.9568 | 2.3540 | 5.0610 | 3.3909 | 4.0744 | 4.1842 | 5.4561 |
| 4.5411 | 4.1146 | 8.1371 | 7.9451 | 2.6303 | 7.4982 | 6.4731 | 3.3984 |
| 5.2680 | 2.1693 | 7.0335 | 4.4694 | 4.7304 | 2.5890 | 4.6876 | 3.3828 |
| 5.0416 | 2.1962 | 4.8685 | 4.9092 | 4.2574 | 4.3017 | 4.6802 | 1.8341 |
| 1.3871 | 5.6813 | 4.6439 | 2.9726 | 4.5671 | 1.6815 | 3.3332 | 2.4433 |
| 3.0620 | 7.3793 | 1.3285 | 3.0961 | 3.2437 | 6.9722 | 3.9774 | 4.4057 |
| 4.3574 | 6.3230 | 4.8277 | 2.1537 | 3.3824 | 3.6089 | 2.9821 | 3.7078 |
| 5.8355 | 4.1977 | 3.8160 | 5.7815 | 6.7939 | 3.4355 | 1.5646 | 3.5639 |
| 3.3403 | 1.6921 | 6.8103 | 1.1308 | 6.9556 | 4.4491 | 2.6654 | 4.8521 |
| 1.9723 | 2.7908 | 2.3250 | 7.7435 | 4.6284 | 6.4123 | 3.9444 | 3.0392 |
| 5.0984 | 1.6272 | 3.4693 | 2.6931 | 3.4163 | 2.0789 | 2.1150 | 4.6023 |

5.1678 3.6917 2.3633 4.6893 1.6845 6.4652 4.8072 5.0451
4.8982 5.1687 7.1169 2.9511 4.4865 2.7277 1.5061 3.8265
2.1234 3.3684 3.8082 4.1184 3.6991 2.7580 1.2961 1.9830
1.2686 3.2005 3.7777 2.6702 5.3717 6.9084 7.1101 3.0687
3.6791 3.5284\
RBMXL3 0.0000 0.0000 0.0000 0.0000 0.0000 0.0000 0.0000
0.0000 0.0000 0.0000 0.0000 0.0000 0.0000 0.0000 0.0000
0.0000 0.0000 0.0000 0.0000 0.0000 0.0000 0.0000 0.0000
0.0000 0.5538 0.0000 0.0000 0.0000 0.0000 0.0000 0.0000
0.0000 0.0000 0.0000 0.0000 0.0000 0.0000 0.0000 0.0000
0.0000 0.0000 0.0000 0.0000 0.0000 0.0000 0.0000 0.0000
0.0000 0.0000 0.0000 0.0000 0.0000 0.0000 0.0000 0.0000
0.0000 0.0000 0.0000 0.0000 0.0000 0.0000 0.0000 0.0000
0.0000 1.1445 0.0000 0.0000 0.0000 0.0000 0.0000 2.2792
0.0000 0.0000 0.0000 0.0000 0.0000 0.5548 0.0000 0.0000
0.0000 0.0000 0.0000 0.0000 0.0000 0.0000 0.6199 0.0000
0.0000 0.0000 0.0000 0.0000 0.0000 0.0000 0.0000 0.0000
0.0000 0.0000 0.0000 0.0000 0.0000 0.0000 0.0000 0.0000
0.0000 0.0000 0.0000 0.0000 0.0000 0.0000 0.0000 0.0000
0.0000 0.0000 0.0000 0.0000 0.0000 0.0000 0.0000 0.0000
0.0000 0.0000 0.0000 0.0000 0.0000 0.0000 0.0000 0.0000
0.0000 0.0000 0.0000 0.0000 0.0000 0.0000 0.0000 0.0000
2.0117 0.0000 0.0000 0.0000 0.0000 0.0000 0.0000 0.0000
0.0000 0.0000 0.0000 0.0000 0.0000 0.0000 0.0000 0.0000
0.0000 0.0000 0.0000 0.0000 0.0000 0.0000 0.0000 0.0000
0.0000 0.0000 0.0000 0.0000 0.0000 0.0000 0.0000 0.0000
0.0000 0.0000 0.0000 0.0000 0.0000 0.0000 0.0000 0.0000
0.0000 0.0000 0.0000 0.0000 0.0000 0.0000 0.6902 0.0000
0.0000 0.0000 0.0000 0.0000 0.0000 0.0000 0.0000 0.0000
0.0000 0.0000 0.0000 0.0000 0.0000 0.0000 0.0000 0.0000
0.0000 0.0000\
OSGEP 9.6589 8.9616 7.4616 8.6508 9.8394 8.7280 7.4674
9.1309 9.0562 9.3772 8.8126 7.9443 8.9399 8.8254 8.8368
9.1610 10.2384 10.0781 8.9710 9.4573 9.5537 8.6138 8.6963
9.1651 9.0013 10.1280 10.3227 9.6508 9.3957 9.8703 9.2380
9.4649 9.4281 7.7845 8.3716 9.3615 8.8139 9.0453 9.5161
9.3249 10.0508 8.7056 10.0614 8.9247 8.5865 9.1344 9.9608
8.5371 9.0684 9.5536 8.5222 9.9579 9.6154 8.9171 9.0553
9.6854 9.0796 7.8615 9.0840 9.0404 8.3005 8.2540 8.5173
10.3543 9.1731 8.8714 9.3707 8.0199 8.6120 10.2300 9.0124
8.8379 9.3307 8.7329 8.8205 8.9476 9.1015 9.2066 8.9877
9.2903 9.0586 10.1381 9.0885 8.8494 9.6983 9.7309 8.7707
9.2107 9.4158 9.6704 8.9126 8.5969 9.5487 9.6430 9.0764
8.9075 9.7790 8.8294 9.7357 9.3399 9.5912 9.2593 9.5124
9.6619 9.6814 9.7753 9.6280 9.2098 8.2249 9.1768 9.1022
9.1499 9.7072 9.9986 8.2864 8.3708 8.7279 9.2916 9.2663
9.5523 8.7970 9.2470 8.2587 9.3103 8.8253 8.2291 9.8734
9.3287 8.7864 8.9202 8.8517 9.0293 8.9735 9.5343 9.1424
8.3764 10.2028 10.2721 8.9149 8.9512 9.9568 9.0608 10.0533
8.7741 8.9046 9.0468 8.8085 8.7157 9.1453 9.8416 9.1418
9.1676 8.6086 9.8055 8.9145 8.7611 8.8843 8.9673 9.3552
10.1115 9.3970 9.8447 9.3008 10.2398 9.4477 9.0789 8.1540
7.6789 9.5665 8.8171 8.9400 8.0544 10.2204 8.5093 9.3952
8.8045 9.4348 9.0463 9.1920 9.2962 9.8300 9.6567 8.1208

8.8111  8.2982  8.9315  9.4683  8.8894  9.5042  9.0744  9.4984
9.0599  9.5291  10.0898 9.3328  7.9153  9.9010  9.1672  9.4070
9.1256  10.2198\
ABAT    5.0120  7.4428  6.1839  8.5941  6.7651  6.5232  5.0240
6.6437  4.5038  8.3902  7.3095  8.0306  6.8983  6.7669  8.3378
7.4499  6.3360  7.6754  7.7040  4.7793  8.7676  9.1980  8.9939
8.5596  6.7756  7.8005  3.7300  6.9721  8.3053  6.4415  8.0802
6.6494  4.1158  6.6692  5.6447  7.1720  7.9736  9.2910  5.6348
3.7304  7.7103  7.9330  4.4640  8.0033  8.2655  6.8204  4.7672
6.8647  10.5242 7.0915  8.4841  5.7490  9.5080  8.1855  8.9395
6.6134  6.6574  5.8825  6.6841  7.0890  7.9180  7.5104  6.2913
8.1252  7.8058  7.6394  8.1366  7.3757  8.3036  5.6893  8.7108
7.5479  5.6711  6.7230  7.7395  5.8185  8.1678  8.7778  7.3464
7.2450  5.1137  3.4338  7.5258  5.3057  8.4035  7.5899  9.2732
7.0155  6.4652  7.8810  7.1582  6.4139  8.3707  7.7832  5.3236
6.4238  8.5057  8.3211  8.8362  7.0941  5.6928  7.1970  5.7182
6.9318  7.6894  4.1824  7.9028  6.6883  6.8475  6.6154  8.6498
5.5476  6.8155  6.3399  7.3165  9.0327  9.0831  7.7129  7.2290
7.0416  9.4876  7.2350  5.5332  7.7847  8.6957  5.5695  5.4258
5.0967  7.8930  6.9697  5.7549  7.8762  6.7901  8.4690  5.5313
3.1012  7.1156  6.2435  7.9564  7.8930  8.3283  9.3258  7.2533
7.2224  4.1179  8.8837  4.9422  7.9842  7.4851  5.6563  7.8629
5.3562  8.8738  9.0900  6.2174  6.3782  7.7847  4.9549  8.1426
6.9167  8.7821  5.3495  7.4711  7.2413  4.4451  7.5353  8.5285
6.6678  6.6552  7.5369  7.7706  6.2865  5.4814  7.3342  5.9274
8.0135  7.3150  7.2157  7.1043  7.8896  6.2297  5.9480  10.0933
9.0446  6.9258  9.3183  6.9601  6.2254  5.2217  4.6588  8.1264
7.6350  8.9200  6.7941  8.2632  5.6289  6.8702  7.8582  6.3884
8.5397  3.8093\
LOC100129637    7.7738  8.1528  7.0992  8.1783  8.1707  6.3616
5.2742  7.1386  8.5726  8.5886  7.3702  7.5940  7.4024  8.2185
7.7980  9.4083  8.0738  7.7423  8.4363  7.2857  7.4986  7.1707
7.9862  8.0703  7.2910  7.7854  7.8392  7.8612  7.2988  7.1390
7.8705  8.6798  7.8223  7.1568  8.0802  8.2325  8.2316  7.2295
8.1600  7.8563  7.7111  7.8410  8.2845  7.6014  6.8058  7.9378
8.0295  7.6539  7.8371  6.0880  7.5418  8.7285  7.3341  7.8425
8.9292  8.6137  7.6952  7.7461  7.0408  7.0345  7.4691  6.8371
8.1506  8.2292  8.4226  7.5574  8.0647  7.0960  7.6400  8.2129
7.9578  7.8774  7.8663  8.2902  8.3404  8.2775  7.7585  7.9551
8.4266  8.5051  6.7826  7.2319  8.1802  7.8294  7.8518  8.2766
7.0008  7.8165  7.5615  8.1612  7.7430  7.8711  6.9887  9.5120
7.8622  6.4353  7.0640  8.0731  8.1953  8.4908  8.1185  8.1470
7.0516  8.0722  8.0657  7.1946  8.7662  6.9115  9.6580  8.3165
7.8895  7.9956  7.5650  9.3925  8.5624  8.0539  7.0598  7.6177
7.8107  8.2022  7.5263  6.2722  7.7919  6.7213  8.5245  8.6162
8.0874  7.3497  7.3962  7.8113  6.3635  7.4061  7.9536  8.5170
9.5100  8.8108  8.5797  10.2070 8.3715  8.1644  8.9858  7.4235
9.1311  7.5647  7.0068  7.9516  7.7291  8.1876  7.7661  7.1384
8.5836  8.5949  8.0673  8.1527  8.0541  7.7540  8.0378  8.3215
8.3829  8.3156  7.3496  7.7462  7.5875  8.5162  7.6764  7.9037
7.6043  7.5179  8.1355  7.6822  8.1432  8.2767  6.7072  7.9808
8.5320  7.8057  8.9658  7.4548  7.8746  8.2282  8.0813  8.6092
7.5565  7.6672  8.5010  8.2908  7.3717  7.6831  8.2175  8.4309
7.8155  6.8864  7.4255  7.5225  9.0175  7.9628  7.0590  8.4735

7.6302	8.4898	8.1066\
ZNF780A	7.3020	8.2592	8.1903	8.1909	8.1835	7.7115	8.3964
10.0283	8.3298	8.5196	8.2648	8.7565	7.7947	8.2192	8.5085
8.4386	7.9908	8.0773	8.0920	6.2179	6.8293	7.6305	7.7506
8.2595	8.2131	7.8206	8.5248	8.6209	8.3235	8.4972	7.1168
8.2743	8.8270	7.2091	8.8759	8.0336	8.6122	8.0644	8.2546
8.6529	8.4143	7.9019	7.8860	7.5551	8.5591	7.6354	7.1858
7.6982	8.9609	8.3717	8.3502	7.1736	8.3395	8.6484	8.3875
8.8393	8.2512	8.7082	7.8348	10.6429	8.6211	8.4718	7.8727
8.1012	8.6950	8.5281	7.8651	9.0778	7.9168	7.6794	8.4598
8.2741	7.9966	9.1130	9.4343	7.2831	7.7284	8.3654	8.2019
8.4656	7.4777	8.0350	8.3406	8.1127	7.7477	7.4595	8.1109
8.0129	7.8942	7.5423	8.2613	10.6100	6.4748	8.2442	7.9407
7.9883	8.2376	8.6822	7.6966	8.4938	8.4255	8.0008	8.1765
7.6577	7.9577	6.9952	7.7868	8.5386	9.9040	8.3524	8.3044
8.2462	9.2515	8.6366	7.6004	7.8760	7.7492	8.1364	7.4570
8.6497	8.4701	8.7097	9.1007	7.8726	8.7046	8.4125	7.7950
7.1698	8.8156	8.7098	7.9236	8.1034	8.1630	9.1024	9.4715
8.1867	8.2037	8.9714	8.8578	8.5424	7.1134	7.6205	8.4119
7.4005	8.2324	8.2743	8.6487	8.3539	7.8596	7.6682	8.4206
7.5101	8.8326	8.2612	6.8632	8.3605	8.5635	8.0145	6.7857
8.1260	6.9572	7.6428	8.3356	8.1358	7.5046	8.5950	8.5650
7.7189	8.0951	7.3822	8.2007	8.0923	8.2968	7.5615	8.4700
8.2489	8.4139	7.9392	8.2203	8.6760	8.4672	8.0218	7.4445
8.3291	8.6174	8.0875	8.3570	8.6768	8.0593	7.3957	8.3331
7.8500	8.3156	8.2743	8.3857	7.9032	7.9149	8.7100	8.6484
8.8345	7.2504\
ZNF780B	7.3565	7.9463	8.1781	8.4281	7.9224	7.4200	7.1660
6.9917	7.9881	8.5639	8.3065	7.3328	7.6526	8.6549	8.1759
8.5355	8.1608	7.8816	7.9595	5.7765	6.4606	7.9642	8.3110
8.2595	8.2356	6.9870	7.4755	7.2703	7.3708	8.1395	7.1168
8.6125	8.5055	6.8614	8.0065	8.4867	8.4119	7.4421	7.7533
9.0272	7.2204	8.2471	7.0056	7.7806	7.8650	7.3620	5.6330
7.8806	9.0186	6.9203	8.0035	8.0532	7.6761	8.8150	8.4262
8.9843	7.8465	8.3433	7.4638	9.4831	8.1718	7.9621	7.8581
8.2687	8.6392	8.5257	9.0145	8.6736	7.7288	8.0037	7.6690
8.0746	6.6302	9.3705	7.9851	6.8592	7.2213	7.8266	7.7794
8.5884	7.4463	7.7106	7.9414	8.0300	7.4429	4.5900	7.0089
6.8921	8.0488	8.0511	8.2001	10.5567	7.1054	8.1063	5.8646
7.5803	6.7968	8.5417	7.7933	9.1850	6.6146	7.8122	7.9251
7.5128	7.9554	6.2059	8.3213	8.5799	8.9314	7.2417	8.0902
7.9426	8.0335	8.0112	9.3471	8.3891	6.8719	8.1874	7.1635
8.3968	8.7824	7.7924	8.8740	6.9115	8.9650	8.0387	5.9194
6.3168	8.6647	8.9984	6.8630	8.2305	7.4785	8.2412	8.6134
6.5929	7.0068	7.5395	8.9723	8.9001	6.9481	7.1741	7.5494
8.0212	7.6137	8.7030	8.0602	8.2181	6.7172	6.5107	8.8559
5.5784	8.4784	7.9897	7.5894	7.6897	8.6242	8.3322	6.4450
7.2802	4.4863	7.2442	8.2007	7.9010	7.8982	7.9900	8.1242
7.5515	7.6544	7.7308	8.0421	8.6263	7.9833	7.6456	6.4248
8.4032	8.5928	7.9359	8.6154	7.8962	7.2064	7.9556	8.3596
7.9256	8.7859	8.1549	7.7434	7.8338	8.1233	7.4686	8.5884
7.5399	8.1541	8.2791	7.3971	6.9802	7.3895	8.9257	8.3311
9.0678	5.5884\
TLN2	9.4524	8.5389	9.2689	8.5956	9.1171	9.1339	8.4331

```
8.9265  10.6826 9.4117  7.8840  7.8919  8.7624  9.6620  8.9959
8.5488  9.1473  9.2336  9.0828  10.5347 8.1931  7.8070  9.4464
8.9521  8.8498  7.9235  10.6566 9.2240  8.9021  9.6984  8.8736
8.7685  6.1858  7.9043  9.0937  9.0073  9.3653  9.7612  9.7544
9.9638  9.3227  9.3674  9.7281  9.2479  9.4922  9.0210  8.8823
9.5165  10.0932 8.6157  8.4691  8.4967  8.7791  8.9778  7.8857
8.5393  9.8137  8.2235  8.7047  9.9181  8.9447  9.4247  6.8643
8.8050  9.3667  8.5378  9.4555  9.0082  9.4107  10.2926 8.7769
10.5235 8.0173  8.8396  8.9325  9.6267  8.9276  8.0633  9.4663
8.7612  8.7220  10.3466 8.7944  9.0724  9.7807  8.4174  8.4681
9.9588  9.1472  8.1706  9.4584  9.9240  8.4590  9.6296  8.8844
8.1563  9.0357  8.9944  10.2992 8.3838  7.7485  9.6398  8.2988
9.7062  9.7207  8.8311  8.7662  10.0821 9.1582  9.7352  8.4579
9.2374  10.0380 10.7863 9.0338  9.3743  9.5849  9.2309  7.5575
9.4714  10.4264 9.1506  9.4711  8.9582  9.6505  9.0124  7.5714
8.9741  9.1611  9.1687  7.9567  9.0439  10.2789 8.4690  8.4519
7.8405  8.3618  9.2446  9.5906  8.7661  9.0277  8.1493  9.3354
8.9863  9.4492  9.2709  9.8335  9.7621  8.5966  8.9849  8.7664
8.5741  9.9620  8.6386  8.8950  9.3212  9.2408  9.1796  7.8994
8.3700  9.1207  7.0615  10.0067 9.2692  8.6091  9.5735  8.7024
8.6164  10.4170 7.8989  9.3193  9.2024  9.5893  6.8648  8.6998
8.9613  9.0436  9.2479  9.7979  9.1629  7.5033  10.4396 8.5150
7.8954  8.2069  10.4870 7.9457  9.6938  9.8801  8.7833  8.6833
10.5040 10.0251 9.9266  9.1604  8.8941  9.1091  9.5366  10.1560
9.3181  10.4973\
FRG1    9.9987  9.4894  8.4600  8.9125  9.1353  8.7855  10.4616
9.4116  8.8231  9.4185  9.5393  9.3197  9.5211  9.5202  9.2589
9.0341  9.0885  9.7843  9.5182  8.6566  8.7372  9.1754  9.1395
9.6428  9.4782  9.8250  9.0859  9.9857  9.5969  8.8656  9.3847
8.9897  9.3187  8.9276  8.3251  9.7727  8.5665  9.0287  9.4935
10.1885 9.6827  9.2543  9.2676  8.9636  10.0286 9.4347  10.8883
9.1330  9.4016  9.9034  8.9976  8.7058  9.0782  9.9345  8.8930
9.7280  9.1764  9.3308  9.5913  9.4261  10.0098 10.1322 9.4516
9.2736  9.2739  9.1573  9.1195  9.0821  9.3522  8.9552  10.0487
9.4543  10.2790 8.8458  9.2629  9.0275  9.3120  8.6267  9.1904
9.0154  10.1510 8.3635  9.2149  9.2931  9.5638  9.2407  9.5870
10.8511 9.2120  9.7907  9.4413  9.2028  10.3171 9.4062  9.9883
9.6390  8.6525  9.5767  8.8495  9.4085  10.4135 9.3202  8.5055
8.6825  9.4505  9.7665  9.2488  9.7314  8.9515  8.6610  9.2061
9.4084  10.7306 9.0715  9.9068  9.5069  9.6386  9.0526  9.1798
9.2442  9.4360  9.5979  8.5787  9.0617  9.4808  8.8031  9.4008
10.4899 9.9136  9.3557  8.9809  9.2110  9.7168  8.7444  9.5707
10.6756 10.8721 9.1096  9.4295  9.5233  9.2607  9.8420  9.6385
9.1237  9.2038  9.8188  9.2488  9.6042  9.1704  10.7779 8.8905
11.3117 8.6582  9.5017  8.5417  9.0114  9.5414  9.1658  9.5455
9.9300  8.9935  9.7286  9.4711  9.9928  7.6912  8.9304  8.8093
8.2387  9.3057  9.1638  8.9390  9.3615  8.9044  9.7600  10.2969
9.5851  9.3667  9.9878  9.8046  8.9472  10.1611 9.4342  9.3053
9.5693  9.7962  9.7012  9.2149  9.9277  9.6479  9.7568  9.1910
9.2567  8.4823  8.9468  9.2657  10.3621 10.9124 9.9132  8.8262
9.1947  10.5791\
FRG2    0.5526  0.0000  0.0000  1.3959  2.1806  0.0000  0.0000
0.4748  0.0000  1.0519  0.4291  0.4849  0.0000  0.0000  0.0000
0.0000  0.0000  0.0000  0.0000  0.3755  0.0000  2.4232  0.0000
```

| | | | | | | | |
|---|---|---|---|---|---|---|---|
| 0.5262 | 0.0000 | 0.6062 | 0.4561 | 1.3471 | 0.0000 | 0.0000 | 0.0000 |
| 0.0000 | 0.0000 | 0.0000 | 0.0000 | 0.0000 | 0.9556 | 0.3648 | 0.0000 |
| 6.7542 | 0.0000 | 0.0000 | 0.0000 | 0.0000 | 0.0000 | 0.0000 | 1.8316 |
| 0.6741 | 4.5310 | 0.0000 | 5.1568 | 0.0000 | 0.0000 | 0.0000 | 0.0000 |
| 4.2155 | 0.0000 | 0.0000 | 0.0000 | 0.0000 | 0.0000 | 0.0000 | 0.0000 |
| 0.0000 | 0.6828 | 0.0000 | 0.0000 | 0.4832 | 0.0000 | 0.0000 | 0.0000 |
| 0.0000 | 0.0000 | 0.0000 | 0.0000 | 0.4698 | 3.8681 | 1.2414 | 0.0000 |
| 0.0000 | 0.0000 | 0.0000 | 0.0000 | 1.1284 | 0.0000 | 0.0000 | 0.0000 |
| 0.0000 | 0.0000 | 0.0000 | 0.0000 | 0.0000 | 0.0000 | 0.0000 | 1.2026 |
| 0.0000 | 0.5233 | 0.0000 | 0.8845 | 0.0000 | 0.0000 | 4.0607 | 0.0000 |
| 0.0000 | 0.0000 | 0.0000 | 0.0000 | 0.0000 | 0.0000 | 0.0000 | 0.0000 |
| 0.0000 | 0.0000 | 0.0000 | 0.0000 | 0.0000 | 6.2985 | 0.0000 | 0.9644 |
| 0.0000 | 0.4553 | 0.0000 | 0.6224 | 4.3940 | 0.0000 | 0.0000 | 0.0000 |
| 0.0000 | 0.0000 | 0.0000 | 0.0000 | 0.0000 | 1.5194 | 0.0000 | 0.0000 |
| 0.0000 | 0.0000 | 0.0000 | 0.0000 | 0.0000 | 0.0000 | 0.0000 | 0.0000 |
| 0.5424 | 0.0000 | 0.0000 | 0.6735 | 0.0000 | 0.0000 | 0.0000 | 0.0000 |
| 0.0000 | 0.0000 | 2.9102 | 0.0000 | 0.0000 | 0.0000 | 0.0000 | 0.0000 |
| 1.7128 | 0.0000 | 0.0000 | 0.0000 | 0.0000 | 0.0000 | 0.8742 | 0.0000 |
| 0.0000 | 0.0000 | 0.0000 | 0.0000 | 0.0000 | 0.0000 | 1.0565 | 0.0000 |
| 0.4801 | 0.5232 | 0.0000 | 2.8900 | 0.0000 | 0.0000 | 0.6902 | 0.5257 |
| 0.0000 | 0.0000 | 1.6636 | 0.0000 | 0.0000 | 0.0000 | 0.5707 | 1.8202 |
| 0.5556 | 0.0000 | 0.4262 | 0.4985 | 0.0000 | 0.0000 | 0.0000 | 0.6701 |
| 0.0000 | 0.0000\ | | | | | | |
| RHEBL1 | 4.9910 | 3.8896 | 3.8562 | 6.1775 | 2.6553 | 6.2795 | 6.4718 |
| 6.8816 | 3.7271 | 4.5787 | 4.8610 | 4.9525 | 6.5196 | 4.5938 | 5.9432 |
| 5.7644 | 5.8880 | 6.2604 | 4.7516 | 6.3605 | 6.5862 | 6.4574 | 6.0243 |
| 4.1115 | 4.8618 | 7.6131 | 6.9462 | 5.5644 | 3.6194 | 4.9218 | 7.3544 |
| 5.1390 | 7.0783 | 4.4081 | 4.1708 | 4.6185 | 3.7742 | 4.7796 | 2.8817 |
| 4.3424 | 5.0275 | 4.0586 | 4.3916 | 6.2512 | 4.9566 | 5.3803 | 5.8079 |
| 4.1916 | 5.9037 | 3.0763 | 4.5771 | 5.9287 | 5.1570 | 4.6761 | 5.8761 |
| 4.9059 | 3.9918 | 5.1550 | 5.8036 | 4.1711 | 5.5285 | 4.4302 | 5.0675 |
| 5.8801 | 4.0120 | 4.6584 | 4.9201 | 4.3846 | 2.7877 | 4.1734 | 5.9534 |
| 3.4586 | 6.8998 | 4.5499 | 5.2632 | 5.0422 | 6.1664 | 6.4867 | 7.0048 |
| 5.7615 | 4.4043 | 4.9566 | 6.2004 | 5.7962 | 5.4783 | 5.4577 | 6.1948 |
| 4.4390 | 5.5778 | 6.7433 | 3.5947 | 4.3052 | 6.6350 | 5.2188 | 5.4720 |
| 5.5618 | 4.9417 | 3.2124 | 5.2882 | 5.5106 | 4.4713 | 5.2847 | 6.2046 |
| 5.2066 | 5.1712 | 4.0223 | 4.3324 | 5.0774 | 4.1979 | 5.2367 | 4.9510 |
| 4.0546 | 4.5244 | 4.8580 | 4.6909 | 6.0200 | 6.8052 | 6.3140 | 5.2395 |
| 5.7835 | 6.0751 | 4.2584 | 3.4207 | 4.8825 | 5.9541 | 5.6359 | 4.2532 |
| 6.2904 | 4.1431 | 4.2252 | 5.2834 | 4.5480 | 5.7352 | 6.7545 | 5.3024 |
| 4.5695 | 5.0401 | 0.0000 | 3.2133 | 3.0966 | 3.6287 | 4.0586 | 5.1135 |
| 3.7845 | 2.7177 | 2.8840 | 3.8163 | 4.4378 | 4.1191 | 5.1593 | 3.5631 |
| 3.8724 | 3.4618 | 5.2132 | 3.7512 | 4.7282 | 4.4622 | 5.5050 | 6.6065 |
| 5.9553 | 4.4174 | 5.6681 | 4.5462 | 5.5876 | 4.0044 | 2.9381 | 3.4840 |
| 5.0610 | 4.6348 | 6.5902 | 3.7043 | 7.0161 | 6.5163 | 6.0865 | 3.7579 |
| 5.2521 | 3.2842 | 5.2549 | 4.1171 | 7.6968 | 4.6108 | 3.1660 | 5.6879 |
| 4.8000 | 5.7978 | 5.1574 | 4.8218 | 4.4054 | 4.6226 | 4.2808 | 5.2367 |
| 3.5613 | 4.5341 | 4.9005 | 3.7421 | 6.5535 | 7.7177 | 3.3916 | 3.5775 |
| 5.1186 | 3.6757\ | | | | | | |
| ZNF41 | 6.3280 | 7.7512 | 7.6362 | 7.6588 | 7.4550 | 8.3768 | 7.9505 |
| 7.6616 | 7.0462 | 7.8742 | 8.6429 | 7.6778 | 7.0712 | 7.7460 | 7.6158 |
| 7.3004 | 7.2423 | 6.6674 | 7.7347 | 5.5480 | 5.8882 | 7.3010 | 7.0008 |
| 8.1408 | 7.6862 | 5.9443 | 7.1526 | 5.8612 | 6.8214 | 8.9505 | 7.0855 |
| 7.5754 | 6.7670 | 6.7584 | 7.5087 | 7.3374 | 8.0394 | 8.0814 | 6.7232 |

```
7.2492  6.4537  7.7647  6.2701  7.7125  7.6878  6.7495  6.2206
7.7389  8.2044  7.3521  7.6794  7.4748  7.8282  7.5497  7.9668
7.7467  7.1756  7.8274  7.7569  9.4363  7.9740  8.0587  7.3874
7.9599  7.7173  7.9517  7.4351  8.0110  6.6778  7.1404  6.6052
8.2131  6.4587  6.4192  7.9221  7.1210  7.0274  7.1448  6.8601
6.9480  5.8011  7.4087  7.8102  8.0846  6.0380  7.7674  8.6380
5.7420  7.8060  7.1219  7.0895  8.4580  6.3571  6.9667  7.0867
7.3006  8.0551  7.7610  8.1456  7.4978  5.7082  7.3027  7.7646
7.9571  7.6181  7.9938  7.6748  7.9850  7.4585  6.5703  8.2075
7.4639  5.7671  6.4263  10.6430 10.3542 7.6256  7.9795  7.9833
7.3416  7.2481  7.5501  7.7382  7.4707  7.5513  6.4401  7.4139
5.0660  7.8638  8.3089  7.7068  7.5531  6.7532  8.2313  6.4425
5.5388  4.9645  5.3310  8.0753  7.8647  7.2122  6.8644  6.3153
7.5902  7.4237  7.4065  7.3732  6.9715  6.7837  7.0141  8.0914
6.0296  8.4505  6.9047  7.6896  7.2095  7.8903  7.7913  6.6141
5.5689  7.1446  6.8390  7.4832  6.3047  7.6711  8.0771  7.8102
7.8788  7.0933  7.3377  7.7438  8.2006  5.9951  6.6644  5.7522
7.9060  8.3650  7.1997  7.9119  7.1732  6.7467  5.9480  7.5430
7.0888  7.8756  7.2704  7.4562  6.4913  6.2468  7.1016  8.0319
7.5651  7.8450  7.7808  7.5980  6.1640  6.1605  7.5280  8.1189
8.0885  5.5508\
IFT80   8.5967  9.3767  10.3687 9.9413  8.7540  9.7023  8.9075
9.6766  9.5122  9.8228  9.2495  9.4964  10.6287 9.5398  10.1573
10.4492 10.1506 9.3175  9.4517  7.7354  9.4719  10.2298 9.8434
10.0689 9.1500  7.0860  8.8802  8.9294  8.0984  9.0783  9.8770
9.1067  9.2116  9.2749  9.6198  8.3569  9.5263  9.5802  9.5781
9.2359  9.3747  9.3009  9.3241  9.7521  8.9439  8.9648  6.4701
9.2536  9.3765  8.6755  8.9774  9.9448  8.6239  9.5484  9.6522
10.0668 8.6359  9.7006  9.3824  9.9136  8.9861  10.9668 8.7335
9.8605  9.7587  9.3754  9.4612  9.6446  8.7592  9.7654  7.5629
9.0047  6.9361  10.0135 9.8271  8.9877  9.4928  10.0642 9.9713
8.4539  8.3242  9.2092  10.1594 9.5260  9.2944  10.2842 10.5157
7.8062  9.6253  9.4205  8.3474  9.3469  8.2193  10.2785 9.0115
8.6285  9.0482  9.1338  9.0405  10.0847 7.3157  8.8345  9.8575
9.9212  9.1577  7.8258  10.1343 9.7235  10.1857 9.1132  9.6282
9.5726  6.9863  10.4757 10.0892 10.3366 10.3812 10.2396 8.8668
8.9975  10.0531 9.2100  9.5762  8.8097  10.2287 9.5171  9.5617
6.6533  9.4348  9.6927  9.4047  10.3571 9.3871  10.4116 7.8553
5.5851  7.7593  7.6511  9.3958  9.5251  8.9694  8.7977  9.3819
8.5657  8.8376  9.7964  9.3547  8.6341  9.1508  7.4815  9.5150
5.8943  9.5118  8.6858  8.5852  9.3687  9.2335  9.7265  9.8641
8.0245  8.7152  7.4810  9.7606  9.7415  8.8853  9.6065  8.5257
9.4525  8.9895  9.4290  8.9424  9.3161  7.2967  8.9721  6.8473
10.0486 10.3221 8.5334  9.9065  10.6625 8.0949  9.1099  9.5616
9.6951  9.3681  9.5676  9.4998  8.7018  9.0038  8.5532  9.2912
8.8246  9.5983  9.4270  9.3727  7.1124  8.5511  9.3867  9.1868
10.4531 6.0996\
FBRS    10.7590 11.1695 10.9640 10.8671 10.9312 10.5110 11.6558
11.0632 11.2618 10.8602 11.6950 12.1851 12.7151 11.0933 10.8430
11.4421 11.0929 10.1801 11.2326 10.3633 11.0265 11.0361 11.2570
10.9486 10.8166 10.6457 10.0349 11.1927 11.1589 11.2751 10.0867
11.0694 10.5208 11.4811 11.0212 10.6540 11.7083 10.7827 10.5783
9.9107  10.8358 10.8338 11.7487 9.7722  10.1107 11.7226 11.1669
10.7320 10.4127 11.0582 10.6335 10.5008 10.8199 10.6284 11.4349
```

```
10.6345 10.6305 10.4789 11.2337 10.8884 10.3700 10.0663 11.2042
10.7868 11.1487 11.2052 10.9195 11.6655 11.3513 11.5087 11.3467
10.8434 10.8968 11.2092 10.9525 11.2840 11.0252 11.2442 11.4235
10.7458 10.7636 11.0226 11.4045 11.3897 11.3111 11.1490 11.4191
11.0939 11.0090 10.7580 10.8009 11.0077 10.5414 11.5986 10.7762
10.3693 11.1647 11.1452 10.9417 11.2666 11.3257 10.4147 9.9353
12.2463 11.1098 11.0857 11.5483 10.3900 11.0432 11.4962 10.7971
11.0184 11.0034 11.7237 10.7655 11.0215 10.7057 11.9779 10.4582
10.8141 11.0058 10.6975 10.2873 10.6896 10.8496 11.0012 10.7533
11.2754 10.6458 10.4429 10.9321 11.0968 11.1773 12.0914 10.7568
10.8838 11.3098 11.8453 10.5806 11.1563 12.5102 10.6750 11.5328
10.8230 10.7546 10.8698 11.9292 10.8361 11.0377 11.1764 10.8542
11.4357 10.7228 11.3139 10.7048 12.0037 10.7620 10.7519 11.3775
10.9457 9.3482  10.6829 10.8853 11.0441 10.6807 10.4125 11.3411
10.5875 11.1697 10.2431 10.8596 11.0195 9.8268  11.0541 10.7715
11.0620 11.0979 10.7534 10.9146 11.6026 10.9961 10.8743 10.2849
10.8191 10.0302 10.8172 10.2991 11.8505 11.3521 11.0291 10.4987
10.6988 10.5717 10.8061 10.6291 11.1250 11.0634 10.8524 10.7986
10.8177 10.4985\
ZNF414  8.8106  8.5337  7.3949  7.1551  8.4527  7.7025  8.7837
9.0237  8.4283  8.8172  8.9623  7.6946  7.2592  7.4553  7.1899
8.0925  8.8447  8.6335  8.3140  7.4845  7.7207  8.4318  8.3185
7.4020  8.5395  9.3935  7.0711  8.8570  8.8426  8.2052  7.2004
7.9080  7.3380  9.1503  7.3803  8.6409  8.1438  8.4499  7.6590
7.3946  7.8116  7.8450  6.9625  8.3937  6.6508  8.5507  7.7675
7.0118  6.6920  8.9541  7.7279  7.6335  9.1303  7.6776  7.4634
7.5668  7.7161  6.7964  8.4371  8.1119  7.3360  6.6746  8.1029
8.6031  8.8316  7.9805  7.8265  7.9863  7.9459  7.6906  8.1976
7.7788  8.4540  8.5841  7.4545  8.3173  7.9305  8.4541  8.3337
8.3097  8.9880  7.6090  7.5756  7.3533  9.2831  7.4418  8.5434
8.5052  7.0006  8.4951  7.7019  8.8895  9.1821  8.6733  8.2137
8.1069  7.1565  8.6839  8.3595  8.2537  7.8426  8.5540  7.4825
7.9140  8.0646  9.4318  8.8655  7.9909  6.9629  9.8653  7.9845
7.8345  7.7258  8.6760  8.1099  8.3754  7.5461  7.4329  7.7840
7.4838  8.3124  7.9058  7.8231  7.9887  8.0385  7.7580  8.6690
7.2744  7.7245  7.5750  7.5848  8.1095  8.5554  6.9524  8.2307
7.0789  8.5263  7.5964  7.9257  8.5840  8.6896  7.3489  9.1554
7.6438  7.4299  8.5552  7.5891  8.7623  8.4446  9.3494  8.2077
7.7897  7.4583  7.7620  7.9542  8.1994  8.6842  8.0482  9.9432
9.5017  8.7701  7.9363  7.7759  8.6344  7.6937  7.4893  6.4649
7.6901  8.1875  9.0901  8.3629  7.6491  8.7680  8.6459  7.3564
8.2489  8.4772  7.9392  7.8965  8.1034  9.3320  6.6928  7.6094
7.9842  6.2279  8.0903  7.0866  8.7942  8.4761  8.0989  7.4636
7.5723  7.5508  7.1661  8.6954  7.6798  7.6022  9.0515  8.4444
7.5073  8.8640\
IFT88   7.5194  8.4125  7.7678  9.3550  7.8851  7.9038  8.4445
8.4758  8.0078  8.8149  7.8519  7.9606  8.6156  7.7532  9.7039
8.6325  7.6664  8.2154  8.4250  7.2312  6.0597  9.3583  9.5971
9.0428  8.5947  7.8503  5.7984  9.2908  7.1011  6.9868  8.6044
8.5625  8.1859  7.7207  7.7117  7.9013  8.5718  8.3542  8.5473
7.3142  7.9546  8.3921  8.7696  8.2911  8.5310  8.3009  6.4278
7.6562  8.0105  7.9120  7.8108  6.4001  7.7312  8.9410  8.0120
8.8979  7.1421  8.0617  8.0885  7.6497  8.5009  9.1256  7.3378
7.1277  8.6887  8.9055  8.1181  8.1120  8.7203  7.1184  6.9000
```

| | | | | | | | |
|---|---|---|---|---|---|---|---|
| 8.2116 | 6.9329 | 9.0264 | 8.2663 | 8.1337 | 8.4646 | 9.7679 | 9.7242 |
| 7.2195 | 8.5951 | 8.5805 | 9.5613 | 7.3792 | 9.0260 | 8.5327 | 8.5521 |
| 7.8366 | 8.6778 | 7.9900 | 7.9930 | 8.2951 | 7.9017 | 8.6948 | 8.3883 |
| 8.2555 | 6.1927 | 8.5238 | 8.8106 | 8.5716 | 7.8908 | 7.9583 | 7.5147 |
| 8.3206 | 7.5057 | 6.8785 | 8.8050 | 9.3505 | 8.2027 | 8.3722 | 9.0391 |
| 8.9206 | 7.1389 | 9.3038 | 7.4175 | 7.9081 | 9.5231 | 8.4902 | 8.0102 |
| 8.0216 | 7.7711 | 8.9477 | 7.6825 | 8.3108 | 10.0044 | 6.9271 | 7.1696 |
| 5.1564 | 8.5410 | 8.6670 | 7.3637 | 8.2502 | 9.2238 | 8.6285 | 8.3556 |
| 5.2536 | 7.8094 | 7.8157 | 8.7699 | 8.9718 | 7.3783 | 7.7574 | 9.2435 |
| 8.1566 | 7.5972 | 8.8470 | 8.4047 | 8.3376 | 7.7804 | 7.4974 | 8.3451 |
| 4.6083 | 8.4277 | 7.9580 | 8.1684 | 7.6419 | 8.5653 | 8.4493 | 8.5895 |
| 8.2413 | 7.4419 | 7.7987 | 8.8461 | 8.9915 | 8.1771 | 8.3352 | 6.9959 |
| 7.6789 | 8.6868 | 7.3044 | 7.7315 | 6.4268 | 7.2867 | 9.4031 | 5.7522 |
| 8.7749 | 8.8766 | 8.2911 | 8.8135 | 10.1617 | 8.0337 | 7.5365 | 7.2950 |
| 8.2968 | 8.0883 | 7.7561 | 8.4106 | 7.2218 | 8.1441 | 7.9081 | 8.5597 |
| 8.2613 | 8.0556 | 7.6338 | 7.8276 | 6.4573 | 6.6701 | 8.8940 | 8.2990 |
| 9.1158 | 7.7137\ | | | | | | |
| IL10RA | 7.1866 | 7.6134 | 8.0770 | 7.8341 | 8.2520 | 8.1415 | 7.2463 |
| 6.5508 | 6.0226 | 7.3366 | 7.9339 | 5.9290 | 8.3371 | 3.6318 | 8.3617 |
| 6.8186 | 6.0842 | 7.6449 | 9.2457 | 6.3605 | 9.8275 | 7.1520 | 8.2882 |
| 6.9155 | 6.3893 | 8.9221 | 6.3649 | 8.3408 | 7.8658 | 5.5229 | 6.5608 |
| 8.7058 | 9.7373 | 8.0815 | 5.7424 | 8.1050 | 5.9336 | 5.6741 | 4.1567 |
| 5.3690 | 8.9636 | 7.9658 | 6.1213 | 7.8888 | 7.0397 | 7.5123 | 8.2822 |
| 7.9894 | 7.4754 | 6.3505 | 8.2818 | 10.3314 | 8.2721 | 7.2459 | 8.2538 |
| 6.5195 | 8.3051 | 9.0397 | 9.4112 | 5.4995 | 8.1065 | 8.1090 | 7.1685 |
| 5.3381 | 7.4639 | 9.0162 | 8.0187 | 5.3497 | 6.0964 | 6.2498 | 9.5584 |
| 6.4254 | 11.5297 | 8.2826 | 9.6855 | 5.5005 | 7.9553 | 7.9643 | 8.5210 |
| 9.1926 | 5.7376 | 6.1154 | 8.6272 | 9.1255 | 6.7367 | 6.3009 | 5.7744 |
| 8.5178 | 8.7855 | 8.1860 | 9.2511 | 8.6219 | 9.0390 | 9.0208 | 8.3561 |
| 7.9089 | 7.6839 | 7.4407 | 7.3034 | 9.5149 | 7.6558 | 9.6890 | 5.4031 |
| 8.2307 | 6.4689 | 6.6346 | 7.4911 | 7.7436 | 6.7095 | 6.9029 | 8.1082 |
| 7.4283 | 7.2019 | 5.1699 | 7.2490 | 6.7000 | 7.0887 | 6.0045 | 6.6699 |
| 9.4788 | 5.9246 | 8.5560 | 7.7050 | 7.1255 | 6.9955 | 8.9961 | 7.1153 |
| 5.9631 | 7.7974 | 7.8838 | 6.4573 | 5.6582 | 8.4763 | 8.6259 | 8.9335 |
| 9.5581 | 9.7427 | 9.5227 | 7.3058 | 7.6127 | 7.7617 | 7.3049 | 8.1185 |
| 8.1144 | 7.6833 | 6.6427 | 7.3575 | 8.7774 | 6.8720 | 4.8936 | 8.4030 |
| 8.7112 | 8.5985 | 7.1536 | 6.9411 | 8.7845 | 7.3750 | 5.2959 | 7.2732 |
| 9.3738 | 6.2219 | 8.3086 | 7.8430 | 6.8150 | 6.4665 | 7.4312 | 8.2399 |
| 9.7879 | 6.4446 | 7.4407 | 5.9772 | 6.4634 | 6.4199 | 4.8348 | 6.2512 |
| 4.8982 | 6.5562 | 8.1287 | 6.0878 | 9.4615 | 7.4424 | 6.4613 | 6.3399 |
| 7.5396 | 8.0121 | 8.4758 | 9.2016 | 7.8536 | 7.6920 | 8.4546 | 9.0465 |
| 6.7053 | 8.7102 | 8.9225 | 7.8561 | 7.5143 | 7.1767 | 7.1428 | 8.8177 |
| 8.0863 | 7.9777\ | | | | | | |
| POU5F1B | 5.6907 | 6.4584 | 4.4523 | 9.5942 | 5.1832 | 3.9351 | 4.3030 |
| 5.1572 | 8.1393 | 8.0616 | 5.0235 | 5.1276 | 6.4572 | 2.7437 | 6.7313 |
| 8.4062 | 7.8895 | 5.9465 | 5.6114 | 6.8661 | 7.2085 | 3.2220 | 6.2067 |
| 6.6057 | 4.5231 | 1.4370 | 6.2076 | 6.9897 | 6.8007 | 6.9161 | 0.9954 |
| 6.0985 | 4.3467 | 5.3604 | 5.2488 | 6.4092 | 4.8901 | 6.0820 | 7.0449 |
| 4.4482 | 6.1668 | 6.3947 | 7.9153 | 4.5471 | 3.3683 | 7.4011 | 6.1574 |
| 6.6505 | 4.8494 | 6.2681 | 7.1413 | 6.4829 | 3.5609 | 4.8767 | 3.2142 |
| 6.6480 | 5.8821 | 4.6067 | 5.9468 | 6.7864 | 3.9204 | 6.1561 | 2.6291 |
| 6.5107 | 6.7879 | 5.4150 | 5.4472 | 3.9743 | 4.5327 | 7.1991 | 5.2665 |
| 6.1678 | 5.8895 | 6.9293 | 7.8049 | 4.9007 | 3.9753 | 7.3152 | 8.7332 |
| 3.6067 | 2.0684 | 7.5113 | 6.4665 | 4.1381 | 7.7118 | 5.7444 | 5.2490 |

6.7357 3.0533 3.0852 4.1839 6.6036 5.4769 9.2219 6.0365
2.6524 4.6110 5.6987 5.1229 8.6201 6.9821 6.4131 4.2451
7.5946 5.9427 3.3434 9.1831 5.7070 6.3705 7.1374 6.6892
4.7603 5.1534 9.5275 5.0859 6.1861 5.0384 5.3131 2.1214
7.7603 5.4608 6.1089 8.5121 4.0694 5.8807 6.7587 3.7819
5.9797 5.4549 5.6769 5.2834 6.8272 7.5158 6.1296 3.7078
2.2603 5.0644 7.0427 5.9124 5.1366 7.1611 7.0833 7.5273
5.6476 6.2073 2.6938 3.9884 5.2029 5.1594 2.9411 7.5857
5.3214 6.5176 7.2230 6.4279 5.3954 7.1397 4.4681 5.2723
2.2684 4.7977 5.9163 6.8599 7.2258 4.0070 5.8386 6.3538
4.7360 4.5005 5.1516 5.9001 6.8653 5.0575 3.3786 5.6358
6.6106 8.7650 4.5497 4.3390 6.3332 7.1167 7.3592 3.7342
4.8000 5.0723 6.8979 6.2271 6.9694 6.7807 3.6040 5.8034
4.4376 6.4151 5.4804 7.1470 3.7138 5.1138 5.3973 5.5399
7.4458 7.0010\
SP1 10.7144 11.3904 11.5803 11.2685 10.8825 12.2250 11.2834
11.3555 10.4571 11.2752 11.4489 10.8119 11.8556 11.2259 11.7233
11.6056 10.7734 11.2606 11.4126 10.3594 10.6647 11.3777 10.9863
11.8857 11.6140 10.1090 10.6233 10.6476 10.8647 11.4779 10.9013
11.6079 10.7567 11.3886 11.3930 10.9678 11.3075 11.8146 11.0726
11.1823 10.5740 11.6331 11.0427 11.2959 11.5661 11.7160 9.6805
11.4014 11.2065 10.5137 11.0066 11.3071 10.8830 12.0248 10.9981
11.4298 12.1320 11.6478 11.0217 11.2690 11.8289 11.3246 11.2431
9.8744 11.1180 11.4655 12.4488 10.2349 10.8666 11.2294 10.2818
11.4230 10.5610 10.8621 11.9440 11.4020 10.6760 10.6918 11.2066
11.4860 10.2870 11.0566 11.6264 11.3698 10.6476 11.1850 10.8131
10.3702 10.7591 11.0233 11.4104 11.2431 10.9997 11.6585 10.9175
11.2066 10.9352 11.6487 10.7049 11.6504 10.1860 11.3385 11.4867
11.5875 10.9204 11.2891 11.0103 11.4644 10.9064 10.6814 11.7944
10.8374 10.1157 11.3454 10.7009 11.3958 11.3577 12.5098 11.4641
11.3537 10.9904 11.2205 11.4792 11.6454 11.5861 11.2793 11.0476
10.1138 11.8723 11.8951 11.0571 11.8533 10.9470 11.2861 10.6773
9.6698 10.2117 11.3469 11.6333 11.6892 10.9168 11.4226 10.5623
11.5793 11.4768 11.6319 11.6367 11.1990 10.3413 10.4020 11.5041
9.3935 11.6071 10.9013 10.9778 11.4115 11.5401 11.2882 10.5901
10.7238 10.3190 11.3154 11.3075 10.8346 11.2580 11.2826 12.0185
11.7393 11.1859 11.0706 11.0426 11.3002 10.9815 11.2914 10.9115
11.4189 11.6822 11.4762 11.2404 11.0458 10.8735 10.7793 11.3738
11.0877 11.2958 10.5097 10.3851 10.8462 11.0153 10.9849 11.1975
10.7315 10.7635 10.4530 11.0635 10.7123 9.8923 11.4871 11.3550
11.8528 9.5265\
SLC27A4 10.6725 9.0625 10.0372 9.1773 10.6540 9.8973 10.1239
9.8509 9.8398 9.4528 9.2641 9.2228 8.6679 10.8093 9.4330
9.8321 9.4494 10.6502 9.4197 11.2475 9.7091 9.5507 8.7230
9.5037 9.4495 11.9122 9.8727 10.4820 9.6208 9.9819 10.1236
10.2211 10.2149 9.6458 9.9544 11.0707 10.3001 10.0008 10.0088
9.3058 10.4689 8.5333 9.6233 10.4644 8.6697 10.1109 10.6057
10.6402 9.4605 11.0100 9.8975 10.2719 9.2684 8.5458 11.4921
9.8526 10.2704 9.2785 9.5149 9.3668 8.9881 9.0176 10.2952
9.8083 9.1731 8.8018 9.0259 9.6914 10.5293 9.9009 11.0249
10.1922 10.2231 9.8790 9.4390 9.8061 10.3982 9.7717 9.7021
10.7319 10.8707 10.1065 8.9816 8.8605 10.0240 8.9257 9.3461
10.6572 10.1170 10.3561 9.5783 9.9643 9.4534 9.4813 10.3676
9.4601 10.9278 8.6128 9.7514 9.8472 11.2535 10.3568 10.6871

8.7352  8.9006  10.4352 9.7491  9.6680  8.5524  9.6347  10.1243
10.5592 11.5959 10.0674 9.4871  9.4005  9.1350  9.4945  9.2810
10.6527 9.5561  10.0145 10.3513 11.1223 8.2907  9.5809  10.4267
10.9056 8.5935  8.9389  9.8903  9.2224  10.0286 9.0805  10.6806
11.8444 10.8798 10.1277 9.5543  8.5919  9.3807  10.1223 10.3905
10.5227 10.3513 8.5269  10.0991 8.9211  10.1301 10.9909 9.3603
11.3445 9.7217  10.6535 10.8924 9.4684  8.8186  10.0891 8.6974
10.9964 10.7719 10.0606 9.5837  9.9140  9.4506  10.1241 9.1825
9.3074  10.1816 10.1941 10.1230 9.7500  10.8970 9.6596  10.4060
9.4401  9.2688  8.7763  9.7298  8.9212  10.7720 11.0796 8.3826
9.7622  9.0534  9.6230  9.7367  10.2756 10.5349 11.0898 10.1474
9.9387  9.3711  9.3278  9.9902  10.5796 11.2236 8.8436  9.9394
9.3594  11.7329\
SP2     8.8447  8.8208  9.0987  9.0925  9.3409  10.0413 10.0580
9.5731  8.6668  9.1462  9.0852  9.9997  8.5973  9.4718  8.8254
8.0462  8.0448  8.5342  8.7498  8.0851  8.3070  9.2877  8.7983
8.8798  9.3224  8.3821  9.0079  8.3545  8.8677  9.3429  8.5404
8.6142  8.2886  8.9644  9.4787  9.3721  9.1779  9.4561  8.4342
9.1815  8.9449  9.6822  7.0995  9.2904  9.1728  7.6222  7.8418
9.2826  8.8737  8.2803  8.6680  8.6247  9.4865  9.6820  9.6074
8.8105  9.0071  9.5124  9.0405  8.9998  9.4565  9.3163  8.9707
8.4614  8.5557  9.4030  9.0858  8.8089  9.0620  8.2124  8.5809
9.3683  8.0926  8.1064  8.8405  9.5480  8.7266  8.6756  8.4992
9.1172  8.5698  9.6087  8.4192  8.7693  8.7295  8.4264  10.2583
8.4184  8.6917  9.3436  9.3278  9.4963  9.5053  7.5045  9.4285
9.1085  9.6863  9.5636  9.4948  8.3938  8.0840  9.1100  9.2398
7.4970  8.5001  9.2993  7.7782  9.6661  8.6210  8.9389  9.1988
9.4288  6.9129  7.9830  9.2930  8.9688  9.0948  9.2988  9.3138
8.6591  9.2725  8.8706  8.9933  9.2998  9.1126  9.3583  9.4759
7.8940  9.3614  9.4767  8.3744  9.8459  9.2538  8.0813  8.4857
7.0789  7.7655  6.7981  9.4875  9.5372  8.9272  8.8211  8.0015
9.5802  9.4675  9.4713  8.7197  9.2367  8.9521  7.8167  9.4017
6.6776  9.3207  9.4132  8.5909  9.3076  9.3548  9.2917  8.9180
8.3029  9.5294  8.7500  8.5931  8.1878  8.6249  9.6024  8.4526
9.1927  9.1110  8.8052  9.3160  8.2684  9.0151  9.4134  6.6415
8.7801  8.6425  9.6368  9.5483  8.6780  7.6075  7.7775  9.4573
9.2623  9.0678  8.8457  9.0261  8.6247  8.4286  9.2730  9.1889
9.0161  8.2958  8.9588  9.1224  7.8410  7.5225  9.6983  9.2871
9.2061  7.5705\
OLA1    11.2778 10.6066 11.0702 10.9637 10.7826 10.5769 11.0079
10.9676 11.7132 10.8624 11.0926 11.2815 11.1066 12.5441 11.0153
10.9553 11.3398 11.1169 10.4824 11.2276 10.3639 10.6716 10.8133
11.3399 10.3634 11.0991 11.3768 10.3704 10.9270 10.8679 11.4219
10.8156 11.5500 11.3022 11.3609 11.5183 10.4611 10.7030 11.8578
11.7926 11.1694 9.7177  11.6455 11.2550 10.3923 11.7276 10.4282
11.0279 11.4261 10.6266 11.3713 10.6102 11.2789 10.2576 10.7226
11.2349 11.9765 11.1154 11.7055 10.4943 10.3002 11.2091 12.2026
12.1834 11.1113 10.8218 10.6960 11.3241 11.4901 11.4805 10.5866
11.1781 10.5548 10.3390 11.1593 10.5995 11.5232 10.0506 10.6001
11.0576 11.4696 11.7154 10.1575 10.9738 11.0793 11.5609 11.5113
11.3803 11.4961 10.7777 10.1238 10.1684 11.4690 10.7805 11.4798
10.4618 11.3951 11.1656 10.5367 11.4064 11.0003 10.8398 10.8651
10.6909 11.2883 11.7640 10.0949 11.1607 11.7401 11.0799 11.2826
11.4013 10.6439 11.2372 11.4597 11.5371 10.9293 10.3430 11.8307

| | | | | | | | |
|---|---|---|---|---|---|---|---|
| 10.3473 | 11.4288 | 11.4470 | 11.1355 | 10.9479 | 10.7890 | 11.0843 | 12.2903 |
| 10.8154 | 10.6930 | 10.0294 | 11.3104 | 12.5143 | 11.0039 | 10.0681 | 11.9917 |
| 10.7613 | 10.8613 | 9.9455 | 11.0966 | 10.6133 | 11.4079 | 11.2839 | 10.7640 |
| 11.2837 | 11.4430 | 10.0291 | 10.6163 | 10.8164 | 11.5711 | 10.9140 | 10.4530 |
| 10.8555 | 10.8157 | 11.5975 | 11.1914 | 11.6032 | 10.2651 | 10.8838 | 11.5729 |
| 11.1226 | 10.5622 | 10.9416 | 11.1974 | 11.0839 | 11.1498 | 11.0913 | 11.0565 |
| 10.9439 | 10.4414 | 11.4795 | 11.2163 | 11.0942 | 10.5185 | 11.1556 | 10.5020 |
| 11.4945 | 10.7909 | 10.1555 | 11.0707 | 9.8983 | 10.6711 | 11.0088 | 11.5949 |
| 11.3966 | 11.6007 | 11.4973 | 11.2320 | 10.5509 | 11.0385 | 12.1414 | 11.2065 |
| 10.9755 | 11.4890 | 11.7007 | 11.0396 | 10.7802 | 10.9967 | 10.5939 | 10.8343 |
| 10.8916 | 10.6741\ | | | | | | |
| PNOC | 5.1321 | 0.9197 | 3.5887 | 2.3445 | 3.2314 | 3.0265 | 7.8654 |
| 8.6979 | 7.6912 | 1.8091 | 4.9924 | 8.5974 | 7.6601 | 2.8185 | 3.1176 |
| 0.0000 | 9.9238 | 1.9468 | 2.8044 | 1.1305 | 10.4128 | 2.1923 | 8.9069 |
| 0.5262 | 0.9530 | 2.7536 | 1.0809 | 6.1909 | 3.9557 | 0.0000 | 1.5789 |
| 2.4004 | 2.8970 | 8.4173 | 5.1708 | 3.8373 | 9.5228 | 3.3101 | 1.7115 |
| 0.7666 | 6.0569 | 1.1407 | 6.5367 | 4.3632 | 1.6194 | 3.1906 | 1.8316 |
| 8.8178 | 3.0861 | 0.8339 | 1.0749 | 6.3121 | 8.4770 | 0.8853 | 10.3431 |
| 0.5608 | 2.5776 | 3.7567 | 6.7267 | 7.0205 | 0.5422 | 10.7070 | 2.9261 |
| 2.8376 | 0.6828 | 3.5708 | 3.0340 | 9.3125 | 0.0000 | 2.5345 | 5.2129 |
| 5.4317 | 9.3369 | 8.5421 | 4.7860 | 0.4698 | 5.2140 | 0.5408 | 2.2252 |
| 4.0393 | 1.5467 | 2.1634 | 3.0222 | 6.6784 | 0.9110 | 11.9754 | 0.5376 |
| 1.3738 | 11.1301 | 6.9629 | 2.1125 | 9.7768 | 12.5918 | 3.0654 | 4.9911 |
| 3.8130 | 9.5906 | 1.7212 | 0.0000 | 4.0697 | 2.7168 | 2.7612 | 1.4835 |
| 3.5417 | 0.0000 | 9.5056 | 1.5756 | 11.3682 | 11.1661 | 0.5877 | 3.4934 |
| 2.0810 | 11.3072 | 4.6439 | 1.6510 | 8.0238 | 3.0885 | 9.4192 | 1.3703 |
| 3.3113 | 11.4321 | 2.0083 | 9.5480 | 2.6761 | 8.5710 | 4.8437 | 2.2388 |
| 10.4547 | 2.6412 | 1.9820 | 13.2333 | 2.9034 | 1.2631 | 4.8382 | 4.1973 |
| 3.4404 | 5.9412 | 0.0000 | 1.2057 | 0.0000 | 1.0240 | 1.3036 | 2.8615 |
| 3.4648 | 2.8723 | 2.8840 | 2.9153 | 11.0803 | 1.9919 | 3.6271 | 2.8564 |
| 3.1371 | 1.4965 | 9.6558 | 5.8980 | 2.6584 | 1.7618 | 6.8901 | 5.3655 |
| 7.6247 | 0.0000 | 4.9890 | 4.5152 | 2.6967 | 4.1571 | 1.6241 | 4.3164 |
| 1.6979 | 1.2170 | 1.5072 | 2.0984 | 9.7859 | 1.7114 | 11.1224 | 0.0000 |
| 4.2600 | 1.2087 | 1.9008 | 7.0389 | 5.1084 | 1.9305 | 7.6063 | 7.8783 |
| 1.1756 | 10.0384 | 3.7513 | 4.0336 | 7.8924 | 2.1566 | 1.9676 | 4.4929 |
| 11.0987 | 9.2335 | 5.2781 | 1.6152 | 6.9332 | 0.6064 | 2.0583 | 2.7261 |
| 1.9827 | 5.0275\ | | | | | | |
| CNTF | 1.9260 | 2.9189 | 2.2256 | 3.9913 | 3.5697 | 2.2319 | 2.9738 |
| 1.7391 | 5.0045 | 3.5525 | 2.7734 | 2.3205 | 3.4906 | 3.7887 | 4.6918 |
| 1.4442 | 0.0000 | 3.1248 | 1.8049 | 1.6235 | 1.8761 | 4.9611 | 6.6343 |
| 3.8282 | 2.5058 | 2.5730 | 3.9621 | 2.2025 | 1.7737 | 3.4734 | 2.4077 |
| 2.8357 | 2.9003 | 3.5470 | 2.4745 | 3.9544 | 1.9329 | 3.6287 | 2.2142 |
| 2.1090 | 1.1184 | 2.8124 | 1.8880 | 1.6313 | 0.0000 | 1.8914 | 2.4538 |
| 3.0263 | 3.5413 | 1.9026 | 1.8408 | 2.7455 | 1.8987 | 1.9872 | 2.5699 |
| 2.6383 | 2.7114 | 2.1170 | 2.1468 | 2.5512 | 2.3520 | 3.0706 | 1.9369 |
| 1.6085 | 2.9371 | 3.0271 | 3.2826 | 2.4381 | 2.5659 | 2.2365 | 1.2639 |
| 3.1920 | 0.0000 | 0.8374 | 3.2574 | 2.9153 | 3.3759 | 6.3324 | 2.0226 |
| 2.3950 | 1.9568 | 3.8052 | 3.5862 | 2.1886 | 2.7490 | 1.3843 | 0.9284 |
| 0.6140 | 2.1793 | 3.0083 | 1.4126 | 2.8054 | 0.9036 | 0.0000 | 1.8492 |
| 2.0921 | 0.9065 | 1.9094 | 3.8617 | 1.7322 | 0.0000 | 2.3185 | 2.1993 |
| 0.7126 | 4.2862 | 2.7449 | 1.2143 | 3.6441 | 3.2744 | 1.3268 | 4.4841 |
| 3.7426 | 0.0000 | 2.5850 | 2.9726 | 2.9231 | 2.4371 | 3.7623 | 3.8914 |
| 2.0533 | 3.1959 | 2.0083 | 2.0828 | 3.8470 | 5.8594 | 2.2776 | 2.2388 |
| 0.0000 | 1.9477 | 2.6959 | 2.2519 | 2.6562 | 2.9134 | 4.3933 | 2.2542 |

3.5378 1.4217 0.0000 2.8272 2.1235 2.9483 1.5646 1.4380
2.4761 3.4648 2.3512 1.9907 2.3603 3.7449 2.3242 2.2465
0.0000 2.7017 1.8101 2.7154 2.6584 2.7525 3.6833 3.2241
2.0666 1.6272 2.9807 1.8011 1.9645 1.4811 3.2219 3.7412
2.6757 0.7061 1.9122 3.3899 2.4406 0.0000 1.0565 4.1460
2.7012 1.2087 1.9008 3.6047 6.6653 1.7413 2.5627 3.1558
1.4215 2.5017 1.6636 2.1610 2.0548 0.6567 3.5453 2.8974
3.1677 2.9536 3.0608 2.2371 0.0000 2.2195 1.6369 2.5185
4.6462 2.2387\
MCM10 8.8196 1.4768 6.1839 6.1856 9.7023 8.2986 8.8959
9.3125 8.6316 7.5108 8.4914 7.9112 7.9532 8.8106 7.5267
5.8094 7.4917 7.9868 3.0845 8.4721 7.9561 6.5641 2.7060
7.0414 2.6200 7.7277 10.4140 7.0129 6.0159 9.0433 9.2277
8.0442 9.7936 8.3698 8.8558 9.9356 6.8295 8.7870 8.0369
8.0075 8.0179 3.2379 8.3545 8.3877 1.6194 7.3699 7.3419
8.9366 9.7501 7.1562 8.1381 7.9405 8.0205 2.9592 8.2502
8.5300 7.9272 6.3784 8.0461 8.4128 2.3520 9.0890 8.4566
9.0380 6.2605 7.2608 6.3050 9.3116 4.5017 7.6299 8.5106
8.0729 8.2760 6.9694 7.6485 7.0682 6.4786 3.4600 3.5128
9.0302 7.8613 9.8797 3.6619 8.2954 7.3641 7.9255 8.5240
7.4469 7.7714 8.3072 2.2864 9.3047 8.2867 5.0418 9.0352
2.5596 8.9102 2.2250 7.3713 8.1108 7.4551 8.0600 8.3192
7.5440 8.5112 9.8642 4.5913 7.8168 8.3289 8.0607 8.2867
8.2152 7.3483 7.6147 9.0064 9.8517 8.2391 8.8250 8.6249
9.1298 8.8149 7.2155 8.9165 7.6910 3.0314 8.5820 8.6153
8.2077 3.1077 5.4171 8.2565 8.3655 7.1586 7.5548 7.7031
7.0950 8.4841 5.1146 7.2696 1.4215 8.7075 6.8213 5.5060
8.4118 8.8672 1.2434 6.4073 3.8858 8.0172 7.5903 5.4821
6.7447 6.7970 8.9566 9.1729 8.7670 2.5317 8.7659 8.2901
9.2711 7.4505 8.0474 7.0788 6.6672 8.2037 8.7854 6.9877
7.9347 8.5041 8.9263 9.0344 9.6180 7.9833 9.1247 5.0451
8.1135 5.8966 1.4008 9.2295 4.6214 7.8288 8.4840 9.2344
5.2768 8.1806 6.3911 8.8818 8.6723 7.1106 9.3339 8.8495
6.4029 7.4863 9.3831 8.5280 8.9658 7.5866 2.3840 8.0123
7.3997 7.6704\
PDIA5 10.2881 8.8711 11.1322 10.6166 9.1205 8.7114 9.3834
10.0234 9.7774 9.8920 10.6797 8.6771 8.8489 9.7464 10.0914
9.8908 9.7595 10.2895 8.6076 10.1062 10.0783 10.1973 9.9046
10.4663 9.1092 10.3851 8.7219 9.1528 11.0561 9.6520 8.4974
10.8631 8.0660 9.1791 10.2776 9.7806 8.6996 10.6386 9.6031
9.0541 8.8764 9.1128 10.3848 10.9584 8.9672 9.9476 9.3926
10.3323 10.1446 10.4364 9.1747 10.0177 11.6962 8.7153 9.6394
9.6285 8.7722 9.7567 9.2805 9.9064 7.9844 8.7886 10.7596
12.2808 9.3252 8.8999 9.6844 11.3015 10.4050 9.6647 9.2476
9.9014 9.4765 8.9989 9.8153 10.1646 10.1670 9.5836 10.7054
8.9330 9.9216 10.9471 8.8582 10.2913 10.2220 10.5050 9.8797
9.9269 10.5897 8.7075 9.5615 9.0994 8.8600 9.7048 9.0600
8.8661 10.2229 8.8907 10.6913 8.6654 10.6983 9.3304 10.0902
8.5983 9.9009 9.6773 8.8135 8.8982 9.5167 9.4511 10.8235
9.3642 8.2575 9.1724 9.0160 8.8250 9.2261 8.6642 10.3763
11.1404 9.0206 8.8783 10.4497 9.2367 9.4043 8.8331 10.2466
8.4172 9.4661 9.3613 11.4463 9.8661 9.7825 8.8837 9.8879
7.5033 9.4443 6.6715 9.7768 9.7721 9.6559 12.2654 10.2382
10.9809 10.0286 8.8341 9.6619 10.3961 10.4487 8.3051 10.2721

| | | | | | | | |
|---|---|---|---|---|---|---|---|
| 9.3935 | 11.4312 | 9.9249 | 12.4913 | 8.7983 | 9.5851 | 9.5865 | 10.2171 |
| 9.1363 | 10.5602 | 9.2819 | 10.0674 | 10.5669 | 10.7281 | 10.3159 | 8.9196 |
| 10.3455 | 9.8145 | 9.3268 | 10.4850 | 9.6994 | 9.7813 | 8.8920 | 8.6306 |
| 9.8542 | 9.2831 | 9.2322 | 9.0227 | 8.9114 | 10.3635 | 10.4754 | 9.2396 |
| 10.6250 | 9.8176 | 9.4891 | 9.9376 | 8.6316 | 9.6448 | 10.1291 | 10.2349 |
| 10.5847 | 11.1215 | 11.1617 | 10.9373 | 8.0534 | 8.2848 | 9.9336 | 9.4220 |
| 9.6336 | 9.5565\ | | | | | | |
| SP4 | 7.4518 | 8.0076 | 7.4616 | 7.5872 | 7.8469 | 8.3726 | 7.8368 |
| 7.4039 | 8.5383 | 7.1088 | 8.0943 | 9.0926 | 7.9283 | 8.4468 | 7.2638 |
| 6.6309 | 6.1731 | 7.1746 | 7.7280 | 5.6455 | 6.9588 | 7.0391 | 7.6747 |
| 7.8882 | 7.7346 | 5.4875 | 8.5087 | 6.0964 | 5.9038 | 7.4332 | 7.5809 |
| 7.8820 | 7.6749 | 8.8305 | 8.4603 | 6.4194 | 8.1652 | 7.9245 | 6.9735 |
| 8.3365 | 8.0597 | 8.2065 | 6.4854 | 7.9287 | 8.8359 | 5.6889 | 4.8968 |
| 7.1930 | 8.5869 | 6.7784 | 6.8863 | 6.7548 | 8.2853 | 8.3575 | 7.9245 |
| 8.3058 | 6.6574 | 7.4373 | 8.0272 | 7.4191 | 8.7718 | 8.1218 | 6.6372 |
| 8.1566 | 7.3237 | 7.6967 | 6.3602 | 8.9549 | 7.2204 | 7.0271 | 6.6909 |
| 8.1902 | 6.7955 | 6.8998 | 7.4471 | 6.8544 | 7.9053 | 6.5510 | 6.6296 |
| 7.8285 | 6.6081 | 8.3524 | 7.1841 | 7.9071 | 6.6445 | 8.0593 | 9.1953 |
| 6.3228 | 8.4510 | 6.8736 | 7.9451 | 9.2900 | 6.6908 | 6.4120 | 7.3586 |
| 7.4695 | 7.6212 | 8.7438 | 7.6118 | 7.5488 | 4.6095 | 6.4061 | 8.4929 |
| 7.1161 | 7.2730 | 7.8295 | 6.6818 | 8.1321 | 7.5208 | 7.5473 | 7.5859 |
| 7.6915 | 5.0200 | 5.7814 | 8.1669 | 7.9017 | 7.2438 | 8.1594 | 8.0470 |
| 7.0358 | 8.0946 | 7.8627 | 7.6284 | 7.3868 | 8.2789 | 7.4392 | 8.1239 |
| 5.3730 | 9.1814 | 7.9905 | 8.1586 | 7.5256 | 7.1396 | 7.1546 | 6.3522 |
| 2.9678 | 5.9147 | 4.1556 | 8.5461 | 9.0111 | 6.5066 | 6.9919 | 6.6460 |
| 7.5764 | 7.5721 | 9.4098 | 7.8494 | 7.6857 | 7.4487 | 6.0974 | 8.3725 |
| 4.9641 | 8.2981 | 7.7917 | 7.1895 | 7.9202 | 8.6364 | 8.4366 | 7.2876 |
| 6.6086 | 6.6547 | 6.5351 | 8.1360 | 6.7942 | 7.6273 | 8.6868 | 7.1369 |
| 8.0699 | 7.0265 | 8.0369 | 7.8365 | 8.3549 | 7.1821 | 7.1869 | 1.9200 |
| 8.2071 | 7.3268 | 7.6243 | 9.4069 | 7.2111 | 6.4699 | 6.0316 | 8.1117 |
| 7.2565 | 7.9159 | 8.5105 | 8.0109 | 5.6681 | 5.6271 | 6.8335 | 8.2355 |
| 8.0345 | 7.9527 | 8.4702 | 7.6552 | 3.7138 | 6.0166 | 9.2077 | 7.8387 |
| 8.0795 | 4.3811\ | | | | | | |
| TMEM38B | 5.3947 | 7.7392 | 7.4261 | 7.1675 | 8.0837 | 7.3325 | 6.1760 |
| 5.7960 | 5.8231 | 6.2706 | 6.0396 | 7.0708 | 6.0515 | 7.6597 | 4.1831 |
| 4.9991 | 4.3886 | 7.0006 | 6.7448 | 5.8527 | 4.8469 | 7.1195 | 7.3029 |
| 6.6443 | 7.9020 | 7.3706 | 8.9699 | 3.8969 | 5.2818 | 6.0152 | 9.2412 |
| 7.6894 | 6.9736 | 7.7155 | 7.1708 | 7.8866 | 4.0849 | 8.0488 | 7.2268 |
| 4.4848 | 6.3947 | 7.6161 | 6.3099 | 7.6384 | 8.4900 | 3.8630 | 3.4901 |
| 6.0426 | 8.1780 | 6.2724 | 5.2436 | 6.3630 | 6.8346 | 7.6321 | 7.8926 |
| 6.2126 | 5.0456 | 7.2601 | 6.3069 | 5.3698 | 8.3315 | 7.5741 | 7.5378 |
| 4.9167 | 6.6303 | 5.3172 | 6.0039 | 4.9277 | 7.0647 | 5.0190 | 7.0677 |
| 7.0530 | 6.3319 | 7.1875 | 6.7341 | 4.6136 | 6.2399 | 5.6207 | 4.0814 |
| 8.5335 | 5.4512 | 6.8793 | 7.1582 | 5.4692 | 3.1576 | 5.9588 | 7.5918 |
| 5.0135 | 5.7882 | 6.2703 | 7.2239 | 7.3106 | 5.4909 | 2.4059 | 6.0649 |
| 6.6438 | 7.9468 | 7.8698 | 3.1069 | 6.7894 | 4.1475 | 6.4747 | 7.6173 |
| 5.9120 | 6.6684 | 6.0150 | 4.3789 | 5.8877 | 6.3053 | 5.7084 | 6.1435 |
| 2.6747 | 5.9976 | 5.2479 | 7.1039 | 7.4225 | 3.1615 | 6.5898 | 7.0193 |
| 6.4007 | 7.9763 | 5.5654 | 6.8018 | 7.4517 | 7.5275 | 5.9494 | 6.5869 |
| 1.9593 | 8.0228 | 7.7742 | 6.3111 | 7.6911 | 4.3316 | 5.2580 | 5.9908 |
| 3.3360 | 5.5340 | 0.0000 | 7.1014 | 7.7315 | 6.0693 | 7.1983 | 6.6864 |
| 7.2744 | 6.6746 | 8.5180 | 7.1854 | 6.0595 | 5.6333 | 6.5928 | 6.7980 |
| 1.5604 | 6.2332 | 6.0806 | 7.2476 | 5.4665 | 7.2400 | 5.8186 | 5.5515 |
| 7.5318 | 6.8952 | 7.2791 | 6.7056 | 5.9457 | 6.6057 | 6.7216 | 4.9804 |

| | | | | | | | |
|---|---|---|---|---|---|---|---|
| 7.4493 | 3.3607 | 6.7093 | 6.3375 | 7.2354 | 7.0595 | 7.0048 | 4.3567 |
| 5.5844 | 6.9056 | 7.0229 | 6.5346 | 5.4540 | 5.2840 | 6.6928 | 6.5165 |
| 7.4967 | 6.5174 | 7.2551 | 4.6894 | 3.8329 | 2.8760 | 5.5122 | 8.2615 |
| 6.8237 | 6.2695 | 5.7802 | 6.5268 | 4.7689 | 5.6471 | 7.4989 | 5.7447 |
| 5.3893 | 3.8093\ | | | | | | |
| THUMPD3 | 9.8068 | 9.0358 | 10.0058 | 10.0233 | 9.8980 | 10.9253 | 9.6520 |
| 10.0180 | 9.8997 | 9.6999 | 9.4392 | 10.4138 | 10.4337 | 10.0659 | 9.7417 |
| 9.7962 | 10.3481 | 10.0942 | 9.2266 | 9.8277 | 9.5781 | 9.7795 | 9.5468 |
| 9.8640 | 9.3245 | 9.7154 | 9.7535 | 9.5943 | 9.9485 | 9.7339 | 9.9142 |
| 10.2602 | 9.8678 | 10.1305 | 9.5274 | 10.2184 | 9.4798 | 10.2928 | 10.0539 |
| 9.5615 | 10.7984 | 9.0199 | 9.7143 | 8.7186 | 9.2036 | 10.2000 | 9.1815 |
| 9.7053 | 10.2034 | 10.0916 | 10.4190 | 9.4260 | 9.1230 | 9.1522 | 9.1796 |
| 10.1783 | 9.7760 | 9.2920 | 10.0039 | 9.9819 | 9.3576 | 9.1920 | 10.2784 |
| 10.1167 | 9.4957 | 9.6221 | 9.5990 | 9.9123 | 10.0466 | 10.4591 | 9.5817 |
| 9.8525 | 9.2410 | 9.2930 | 10.1129 | 9.5576 | 10.0079 | 9.2823 | 9.4613 |
| 9.6672 | 9.4240 | 11.1864 | 9.6754 | 9.8235 | 9.4950 | 10.2084 | 9.7578 |
| 9.6303 | 10.1499 | 9.9012 | 9.2031 | 10.1976 | 9.4489 | 9.3812 | 9.9040 |
| 9.4534 | 9.7248 | 9.1530 | 9.8546 | 9.6046 | 9.9462 | 10.1242 | 9.7446 |
| 9.9528 | 10.7024 | 10.1453 | 9.2935 | 9.9383 | 9.9252 | 10.0869 | 10.0170 |
| 10.0399 | 9.9258 | 9.5333 | 10.4477 | 10.7107 | 10.1489 | 10.2064 | 10.1115 |
| 9.4682 | 9.8043 | 10.2578 | 9.9988 | 9.9674 | 9.4756 | 9.8945 | 9.3745 |
| 8.4019 | 9.5574 | 9.7369 | 10.4460 | 10.2191 | 9.6926 | 9.3680 | 9.4852 |
| 9.0504 | 9.8036 | 9.2884 | 9.6512 | 9.3634 | 10.9045 | 10.0178 | 9.6498 |
| 10.1712 | 9.7250 | 9.2473 | 10.0698 | 9.2969 | 10.1396 | 9.7415 | 9.7397 |
| 9.6230 | 9.9028 | 9.0151 | 9.8283 | 9.5306 | 9.3044 | 10.3732 | 9.4896 |
| 9.7036 | 9.9644 | 9.3039 | 9.9870 | 9.7577 | 10.3934 | 10.2743 | 9.7994 |
| 10.0924 | 9.8453 | 10.0517 | 10.2840 | 10.3845 | 9.9764 | 8.9918 | 10.2350 |
| 9.9431 | 9.6400 | 9.1387 | 10.1849 | 9.1903 | 10.0621 | 10.0312 | 10.7049 |
| 10.0259 | 9.4523 | 9.9428 | 9.9362 | 10.1442 | 9.6059 | 10.0514 | 10.0658 |
| 9.8125 | 9.4929 | 9.4434 | 10.1746 | 9.7509 | 9.6890 | 9.4069 | 10.1653 |
| 9.9550 | 9.3945\ | | | | | | |
| IGFBPL1 | 0.5526 | 2.8563 | 1.5051 | 4.0443 | 1.8679 | 5.6917 | 0.0000 |
| 7.0480 | 3.3846 | 2.1946 | 1.0281 | 3.1684 | 3.1323 | 6.5150 | 4.2186 |
| 8.5880 | 2.6760 | 0.7772 | 2.8044 | 2.8567 | 2.6649 | 3.7755 | 0.0000 |
| 6.6632 | 2.0961 | 2.2189 | 6.7805 | 3.9459 | 1.3857 | 2.0116 | 2.4950 |
| 0.0000 | 0.0000 | 2.8293 | 6.9551 | 0.6819 | 2.7304 | 2.6929 | 0.5410 |
| 0.0000 | 1.9736 | 1.5889 | 1.0569 | 0.0000 | 0.0000 | 5.4112 | 2.4538 |
| 0.0000 | 1.6510 | 0.4764 | 0.7972 | 0.0000 | 4.9603 | 0.8853 | 8.0925 |
| 0.5608 | 1.4095 | 6.0644 | 2.8422 | 3.3557 | 4.1964 | 1.6116 | 0.0000 |
| 4.0331 | 1.7745 | 1.6675 | 1.3453 | 5.8451 | 5.9661 | 3.2513 | 5.4511 |
| 2.0437 | 0.9449 | 1.5690 | 3.1146 | 3.4606 | 5.7674 | 2.6909 | 5.3294 |
| 1.0461 | 7.1617 | 1.6845 | 2.6279 | 5.8565 | 4.6559 | 1.0522 | 3.7216 |
| 4.5079 | 0.5273 | 7.3532 | 2.8267 | 3.2907 | 1.8528 | 2.4059 | 3.9370 |
| 3.6967 | 4.3699 | 0.5454 | 6.1882 | 0.0000 | 3.6674 | 0.0000 | 3.8993 |
| 2.5001 | 4.9288 | 0.4659 | 1.5756 | 2.0486 | 4.3017 | 2.0061 | 3.0477 |
| 3.0001 | 2.0513 | 2.5850 | 1.8354 | 4.7481 | 5.7895 | 3.1748 | 1.8223 |
| 0.0000 | 4.9219 | 0.5886 | 2.0828 | 1.1079 | 1.3435 | 2.3286 | 1.0447 |
| 1.6629 | 1.5385 | 3.0295 | 3.9473 | 1.9805 | 3.7656 | 3.7466 | 1.3267 |
| 4.8224 | 0.0000 | 0.0000 | 2.5337 | 1.4215 | 6.2467 | 3.6721 | 1.9445 |
| 2.2176 | 0.4562 | 1.2434 | 5.2206 | 1.2499 | 1.5778 | 0.0000 | 7.0178 |
| 0.0000 | 7.2497 | 3.8616 | 5.1170 | 2.5751 | 2.4071 | 3.2125 | 4.4001 |
| 1.4969 | 0.0000 | 0.6138 | 2.5777 | 0.9764 | 0.0000 | 4.4752 | 3.0493 |
| 6.9330 | 2.6550 | 0.5466 | 1.0663 | 7.5774 | 7.0645 | 0.6229 | 0.0000 |
| 4.7994 | 1.6715 | 3.8748 | 5.9374 | 2.4311 | 0.5545 | 0.6902 | 5.4181 |

| | | | | | | | |
|---|---|---|---|---|---|---|---|
| 1.1756 | 2.5017 | 2.0881 | 1.3150 | 1.1775 | 2.3320 | 0.0000 | 1.4263 |
| 2.1004 | 1.7696 | 1.0222 | 0.0000 | 0.0000 | 8.9306 | 0.0000 | 2.5185 |
| 4.7628 | 0.0000\ | | | | | | |
| SP5 | 4.1155 | 8.8711 | 7.0325 | 5.6423 | 9.9040 | 8.0908 | 3.4058 |
| 5.4679 | 4.3880 | 6.8955 | 2.2662 | 2.2003 | 3.0112 | 11.2485 | 7.8170 |
| 13.2471 | 5.0359 | 4.7766 | 9.3476 | 9.2879 | 4.4485 | 6.6233 | 6.9883 |
| 10.3441 | 5.4611 | 4.7327 | 6.1627 | 3.1243 | 10.2926 | 6.2913 | 9.9514 |
| 8.3728 | 1.6552 | 0.8184 | 3.1707 | 1.4920 | 9.5868 | 6.4289 | 3.9170 |
| 7.9996 | 5.4890 | 6.5009 | 3.1865 | 5.5268 | 6.9817 | 8.7606 | 4.5480 |
| 4.6336 | 2.1914 | 8.4832 | 7.3995 | 6.2728 | 6.9183 | 5.9638 | 12.6252 |
| 3.2557 | 3.5225 | 6.5163 | 4.1813 | 5.7546 | 4.8942 | 3.4956 | 10.2479 |
| 6.5584 | 7.3561 | 5.1377 | 3.4274 | 8.2465 | 11.2740 | 6.3568 | 3.3869 |
| 4.5537 | 2.4919 | 4.7102 | 7.9381 | 3.2432 | 5.7798 | 9.9633 | 10.8041 |
| 5.3553 | 11.9625 | 5.7921 | 7.4476 | 4.7601 | 10.7470 | 2.9962 | 3.4508 |
| 6.8058 | 4.6077 | 4.6312 | 5.7301 | 3.1023 | 5.5985 | 10.3815 | 5.9069 |
| 3.8677 | 4.9822 | 8.8767 | 3.1760 | 1.2591 | 8.7894 | 4.0960 | 4.9160 |
| 4.4784 | 6.7507 | 6.9017 | 8.0322 | 5.0166 | 4.6022 | 8.3722 | 6.5838 |
| 3.1823 | 5.5901 | 6.5699 | 5.7685 | 4.6604 | 8.6316 | 1.6299 | 8.1440 |
| 5.1346 | 8.1875 | 3.7106 | 5.7938 | 3.9666 | 6.4532 | 4.2043 | 4.9921 |
| 4.0148 | 7.8158 | 5.6650 | 7.1188 | 5.0104 | 3.7656 | 5.7024 | 5.7694 |
| 2.9678 | 6.8265 | 7.2517 | 6.1784 | 8.5440 | 4.2546 | 10.6793 | 7.9426 |
| 9.0695 | 9.1772 | 5.4394 | 4.3245 | 7.7487 | 4.4491 | 5.3836 | 11.4482 |
| 2.2924 | 9.5570 | 3.5271 | 7.9676 | 5.7299 | 7.8095 | 6.2311 | 6.6953 |
| 8.5474 | 2.7538 | 4.0337 | 8.4196 | 8.6344 | 6.0568 | 6.4805 | 5.1076 |
| 5.6080 | 8.8125 | 4.9727 | 3.6634 | 3.5936 | 8.5646 | 5.1952 | 6.4013 |
| 5.5962 | 6.5223 | 5.6021 | 2.6160 | 7.3680 | 6.3086 | 4.5289 | 7.5666 |
| 4.8844 | 4.4365 | 10.7606 | 3.4490 | 4.9381 | 3.3538 | 8.0310 | 6.0252 |
| 8.2919 | 4.5929 | 7.0549 | 8.2179 | 4.4061 | 6.1393 | 8.7844 | 8.5743 |
| 8.3754 | 6.4458\ | | | | | | |
| ATG4C | 7.0408 | 7.1349 | 7.7637 | 7.2276 | 7.5362 | 7.8801 | 7.6752 |
| 7.5340 | 7.4119 | 7.4021 | 7.3251 | 7.5605 | 7.6826 | 7.1428 | 7.5923 |
| 6.3557 | 7.0793 | 7.1531 | 7.5589 | 7.0521 | 6.8955 | 7.1520 | 7.3428 |
| 7.5483 | 7.6993 | 6.6694 | 8.1663 | 7.1289 | 6.3079 | 7.2101 | 8.1219 |
| 7.4278 | 7.9973 | 7.0141 | 7.4187 | 7.7596 | 6.6789 | 7.3176 | 7.7982 |
| 7.9595 | 7.4837 | 6.9103 | 6.7760 | 7.6511 | 8.4132 | 6.5827 | 6.4385 |
| 8.1031 | 8.0224 | 7.3896 | 7.6075 | 6.6865 | 7.1669 | 8.0923 | 6.7815 |
| 7.4064 | 6.9494 | 7.4373 | 7.7034 | 7.9455 | 8.0726 | 8.0807 | 7.3081 |
| 7.2542 | 7.1254 | 7.4701 | 7.3053 | 7.5389 | 7.2157 | 6.8386 | 7.5062 |
| 7.4327 | 7.2103 | 7.2373 | 7.8675 | 7.2973 | 6.8801 | 6.4720 | 6.6207 |
| 7.5415 | 7.0719 | 7.8357 | 7.2714 | 7.2219 | 6.6508 | 6.7015 | 7.7903 |
| 6.8985 | 7.7059 | 7.3257 | 7.7658 | 7.4588 | 7.4013 | 7.0086 | 7.4863 |
| 7.9696 | 7.4693 | 7.5823 | 6.5750 | 7.3496 | 6.0369 | 7.5324 | 7.6337 |
| 6.7476 | 7.2951 | 7.3114 | 6.8231 | 7.4186 | 7.6246 | 6.6301 | 6.9358 |
| 7.5116 | 5.3330 | 6.3576 | 7.4318 | 8.0061 | 7.3492 | 7.4703 | 7.1794 |
| 7.2350 | 7.4391 | 7.9388 | 7.3613 | 6.8975 | 7.6613 | 7.2360 | 7.4139 |
| 6.0755 | 8.0949 | 7.9761 | 7.9980 | 8.0141 | 7.1348 | 6.9524 | 6.7048 |
| 5.9461 | 6.8050 | 6.4426 | 7.7703 | 7.5941 | 6.6765 | 7.6061 | 6.5172 |
| 6.9247 | 7.4392 | 7.3512 | 7.5757 | 7.4138 | 7.3816 | 6.0103 | 7.4947 |
| 5.0933 | 7.2880 | 7.2181 | 6.2614 | 7.5951 | 7.7847 | 6.8901 | 7.4832 |
| 7.7150 | 6.9511 | 5.7126 | 7.3879 | 7.3336 | 7.0063 | 7.7819 | 6.5232 |
| 7.7854 | 6.1539 | 6.8134 | 6.8519 | 8.0900 | 7.3524 | 6.5359 | 3.6013 |
| 6.9835 | 7.5586 | 7.6792 | 7.4809 | 6.9607 | 6.3507 | 7.2817 | 7.6192 |
| 7.5970 | 8.0741 | 7.1267 | 7.6216 | 7.2034 | 6.4121 | 7.0014 | 7.0650 |
| 7.1538 | 7.6337 | 6.9506 | 7.6463 | 6.6726 | 6.9758 | 7.5925 | 7.5258 |

7.5140  6.4762\
ATG4B   10.7124 10.3280 9.2223  10.6170 10.8151 10.3283 10.1227
10.5528 11.4798 10.6130 9.9382  10.5003 10.5739 10.9859 10.1268
10.9984 11.5559 10.9018 10.4312 11.4464 10.9471 10.4644 10.3337
10.0396 9.8925  10.8912 9.3732  11.3161 10.2056 9.7399  10.7785
10.7159 9.9340  11.1066 10.8732 10.9765 10.7507 10.4005 9.8535
11.0244 10.5147 9.9811  10.5504 10.6372 9.3845  11.0677 10.7124
10.3449 10.2637 10.8803 10.3342 10.4728 10.8326 9.9220  11.2058
10.7460 10.7955 10.5676 9.6528  10.9791 9.6466  9.5256  10.4818
11.1859 10.3484 10.3021 11.2774 10.9757 10.3817 11.8620 10.9485
9.6913  11.2259 11.9359 9.8775  10.6867 11.4019 11.0437 10.6543
10.3639 10.7798 10.2022 9.9922  10.3632 11.1872 11.5579 10.1996
11.9996 10.2161 10.6703 9.9407  10.0806 10.7723 10.9723 10.8601
9.7688  9.8298  10.1092 10.4128 10.4908 11.5616 10.7050 10.2886
11.5221 9.9241  10.2592 11.1660 10.5519 10.6717 11.4650 10.3997
10.4551 11.4372 11.6952 10.5319 10.3378 9.8543  10.3399 10.4373
10.8943 10.5048 10.0527 10.0922 9.8828  10.0463 10.6941 10.8441
11.4723 9.3862  9.8179  10.9167 11.2765 10.8435 11.0125 11.6408
10.6922 11.8469 11.7070 10.3769 10.1538 10.9854 9.8611  11.4197
10.0596 9.6334  9.9700  10.4787 10.3338 10.8339 10.9530 10.3422
11.6695 10.6858 10.9223 9.8879  11.2822 10.0671 10.1791 11.1772
11.0111 10.3632 10.2547 10.4946 11.2500 10.8619 10.3691 10.4053
9.9164  10.5216 11.5804 10.5681 10.4948 11.2446 11.1631 10.8300
10.2681 10.6964 9.8355  10.2186 11.2970 11.0526 10.7144 10.2717
10.3206 10.6438 10.5054 10.7776 11.1911 10.8322 10.9343 10.1050
10.1311 10.6162 9.7146  11.3793 11.1053 11.4605 10.3215 10.5119
10.4350 11.3995\
ATG4A   7.9288  7.3552  7.8506  7.8596  8.0016  7.7380  6.1576
7.4815  8.2409  7.6329  8.4319  7.9560  8.6130  7.6899  8.3357
7.3469  7.7805  8.1696  7.4060  6.4661  8.1174  8.7071  7.5695
8.2471  7.6764  8.1041  9.4020  8.3814  8.4978  7.6300  6.5250
8.1127  8.5738  7.0310  7.8835  7.8303  7.5721  8.5340  7.7195
8.0577  8.7289  7.3151  6.6262  8.5131  7.7994  7.8140  8.4864
7.7023  8.4727  8.3717  8.5776  8.2350  8.2694  7.7756  8.4326
8.3961  7.1021  8.5549  7.7569  8.4190  7.7416  8.5530  9.2135
7.6125  7.5961  8.8148  7.7355  8.3039  8.2486  8.2807  7.8310
8.4127  8.1312  7.0657  8.5492  8.6761  7.6259  8.5001  8.3689
7.7270  7.5199  7.0440  8.5014  9.1362  7.9070  8.0299  8.5898
7.9890  7.0733  7.9850  7.7172  8.4645  8.1273  7.3930  7.7876
8.3035  8.2788  7.1733  9.4718  7.6457  8.6622  8.4591  8.1030
8.2841  8.7725  7.3114  7.8167  8.6505  8.2618  8.2078  8.1865
8.3014  7.5311  6.1293  9.0160  8.5005  8.3719  8.0150  8.8238
7.3602  7.5490  8.1195  7.7382  8.4775  7.8235  8.6732  8.5349
6.5006  7.6409  8.1273  7.8352  8.1370  8.4224  9.3437  7.4279
7.4851  8.9255  8.2821  7.9690  7.5135  8.9783  8.9250  7.7801
7.7789  7.6328  7.0574  8.7479  7.5151  8.6949  7.8421  7.8290
8.6842  8.3574  7.7112  6.5300  8.1373  7.3956  7.2126  6.6589
6.5676  8.6921  8.3134  8.2440  7.9432  6.2321  8.3463  6.9025
8.1967  7.6353  7.4330  7.4895  8.3991  8.8795  8.3867  7.9748
7.8796  7.7168  7.8968  7.8571  8.2692  7.9182  8.0387  6.3785
8.1868  8.3813  7.9456  7.9134  8.1954  8.4691  8.0443  8.5353
8.7698  8.6405  7.4101  7.9434  7.2718  8.1185  7.4431  8.6810
8.0175  7.7137\
THUMPD2 8.2584  7.8631  8.1410  8.1438  7.4550  8.4789  8.9714

| | | | | | | | |
|---|---|---|---|---|---|---|---|
| 8.3896 | 7.6057 | 8.2599 | 7.7604 | 7.9040 | 8.3988 | 8.6485 | 7.8765 |
| 8.2799 | 8.8001 | 8.0773 | 7.7482 | 8.3734 | 7.5584 | 7.2455 | 7.5269 |
| 8.3921 | 7.9896 | 7.7663 | 7.5518 | 7.6076 | 7.7189 | 7.5310 | 8.2467 |
| 8.2721 | 8.2504 | 7.9848 | 8.3160 | 8.4042 | 8.2916 | 7.9583 | 8.6668 |
| 8.6691 | 8.2930 | 7.9163 | 8.6152 | 8.8237 | 7.6732 | 8.0288 | 7.4827 |
| 8.1031 | 8.4713 | 8.0737 | 8.2242 | 8.0571 | 7.7235 | 8.3741 | 7.6846 |
| 8.3838 | 8.2539 | 7.8795 | 7.9473 | 8.3583 | 7.8165 | 8.7615 | 8.0110 |
| 7.8584 | 8.1451 | 7.8090 | 8.4028 | 8.3363 | 7.3738 | 8.6627 | 8.7711 |
| 8.5525 | 7.6630 | 7.8253 | 8.1725 | 7.8361 | 8.4968 | 6.8416 | 8.4770 |
| 8.1806 | 7.7848 | 7.7849 | 7.4898 | 8.2707 | 8.3026 | 8.5719 | 7.8309 |
| 8.5549 | 7.6211 | 8.0049 | 7.5136 | 8.0256 | 8.3007 | 8.7349 | 8.1223 |
| 6.7497 | 7.9365 | 8.1333 | 7.5349 | 8.5160 | 7.5738 | 7.9487 | 7.3378 |
| 8.6446 | 8.5127 | 7.2295 | 8.8998 | 8.4129 | 8.6622 | 7.4552 | 8.4223 |
| 7.8242 | 7.7887 | 7.8074 | 9.2203 | 7.9579 | 7.8739 | 8.0686 | 7.6053 |
| 8.0128 | 8.8890 | 7.7389 | 8.0718 | 6.6097 | 8.3531 | 8.2334 | 8.4074 |
| 8.6215 | 7.9301 | 8.3089 | 8.2536 | 9.2153 | 7.6118 | 8.9815 | 7.7844 |
| 7.2752 | 8.0293 | 9.1290 | 8.1699 | 8.2795 | 8.6287 | 8.0056 | 8.2074 |
| 7.4276 | 6.7262 | 8.5464 | 8.2694 | 7.5684 | 7.9522 | 8.4691 | 7.9793 |
| 6.7707 | 7.8515 | 8.7283 | 8.0121 | 8.2756 | 7.9612 | 7.8365 | 8.3999 |
| 8.7775 | 7.6437 | 7.5763 | 8.4891 | 8.3803 | 8.1501 | 8.0134 | 8.6274 |
| 7.8124 | 7.7522 | 8.8720 | 7.9642 | 8.5293 | 8.4053 | 8.2805 | 7.7692 |
| 8.6083 | 8.6614 | 7.9132 | 8.0458 | 7.7749 | 8.3498 | 8.2307 | 7.8373 |
| 8.5308 | 8.4323 | 8.6859 | 7.7434 | 7.9039 | 7.8409 | 8.2560 | 7.9726 |
| 8.3387 | 8.7316 | 8.5264 | 8.4484 | 8.0859 | 8.8397 | 8.2444 | 7.4375 |
| 8.4556 | 7.5080\ | | | | | | |
| LOC441208 | | 4.4286 | 5.8707 | 6.0435 | 5.6304 | 5.5112 | 4.6275 |
| 6.7482 | 4.1817 | 6.3354 | 5.9416 | 5.0539 | 3.5591 | 5.3416 | 5.7848 |
| 5.4417 | 4.1868 | 2.9009 | 5.5000 | 5.6114 | 5.3624 | 3.9961 | 6.2993 |
| 6.0843 | 4.9509 | 5.6477 | 4.5518 | 6.2498 | 4.5635 | 3.3032 | 5.0373 |
| 5.1204 | 5.0794 | 4.8975 | 4.8321 | 6.1608 | 4.6185 | 4.8198 | 6.3947 |
| 2.5862 | 4.3173 | 4.6775 | 6.4220 | 3.6259 | 5.6015 | 5.6353 | 3.8630 |
| 3.0068 | 5.6392 | 4.9360 | 3.9881 | 5.1568 | 4.5744 | 5.4662 | 5.6838 |
| 4.8444 | 3.6325 | 5.5323 | 6.1432 | 4.6037 | 3.1339 | 5.7647 | 4.6821 |
| 5.0263 | 3.7875 | 5.2219 | 6.0097 | 5.0820 | 4.4386 | 6.2158 | 4.7601 |
| 3.6908 | 5.5614 | 3.3577 | 4.5006 | 3.5626 | 5.0919 | 4.6662 | 6.0250 |
| 3.4332 | 5.2583 | 3.9901 | 4.7346 | 4.6038 | 4.5570 | 4.3167 | 3.9537 |
| 3.8626 | 4.9653 | 4.6590 | 5.3289 | 5.5022 | 4.1188 | 4.4526 | 2.9555 |
| 5.0106 | 6.0265 | 3.4668 | 6.2544 | 5.4782 | 4.3581 | 4.3969 | 6.0937 |
| 5.1711 | 3.3666 | 4.0114 | 4.8495 | 4.1830 | 4.9692 | 3.9218 | 4.1370 |
| 4.8041 | 5.2741 | 4.1934 | 5.0875 | 5.2245 | 4.0739 | 5.5248 | 5.1093 |
| 4.8531 | 4.2367 | 4.2335 | 5.0331 | 6.1077 | 6.1119 | 5.5896 | 4.8880 |
| 4.7285 | 4.5476 | 5.1017 | 5.6887 | 4.9148 | 3.2985 | 5.4876 | 3.6704 |
| 4.6075 | 2.8207 | 3.5403 | 2.2444 | 6.1521 | 5.8176 | 3.4355 | 3.9712 |
| 4.6725 | 4.8727 | 5.3633 | 4.9582 | 5.9186 | 5.6081 | 4.8965 | 3.4621 |
| 5.4954 | 1.9723 | 5.1725 | 5.1330 | 5.6685 | 4.4293 | 5.8438 | 5.1788 |
| 5.4360 | 4.7286 | 6.4800 | 5.0595 | 4.3840 | 4.8610 | 6.1471 | 5.3570 |
| 0.0000 | 6.6121 | 5.1293 | 3.8438 | 5.8823 | 5.2217 | 5.1712 | 5.5178 |
| 4.0279 | 4.9359 | 4.8348 | 5.3738 | 5.5986 | 4.5888 | 4.0414 | 5.3093 |
| 4.6027 | 4.4304 | 3.9060 | 4.6351 | 5.5123 | 4.5288 | 3.6472 | 4.9580 |
| 5.8034 | 5.1604 | 3.1228 | 6.5775 | 4.5668 | 2.8198 | 4.2299 | 5.7482 |
| 6.8834 | 5.5921 | 4.2463\ | | | | | |
| ATG4D | 9.3438 | 8.5588 | 8.2720 | 8.7175 | 9.8693 | 8.7294 | 9.3535 |
| 9.2158 | 9.4233 | 8.5513 | 9.6184 | 10.0650 | 12.7960 | 8.5993 | 8.1874 |
| 9.4341 | 9.9471 | 9.3649 | 8.7001 | 10.6699 | 9.4352 | 8.8980 | 8.6051 |

| | | | | | | | |
|---|---|---|---|---|---|---|---|
| 8.5488 | 7.9789 | 10.3496 | 10.3564 | 9.4715 | 9.2594 | 8.4703 | 8.1776 |
| 9.5324 | 9.3876 | 9.3538 | 9.9282 | 9.5376 | 9.8880 | 8.9519 | 9.0782 |
| 8.2013 | 9.2022 | 8.1977 | 8.9210 | 9.9742 | 6.8794 | 9.2448 | 10.8344 |
| 7.8267 | 7.7200 | 9.5857 | 8.6730 | 9.3359 | 9.2014 | 7.7748 | 8.9334 |
| 8.8585 | 8.7897 | 7.3275 | 9.4839 | 8.4820 | 7.1392 | 8.4074 | 9.5470 |
| 8.5799 | 8.3139 | 9.5274 | 8.9928 | 9.0206 | 9.2458 | 8.6746 | 9.0939 |
| 8.9606 | 9.3868 | 9.7897 | 8.3399 | 9.2128 | 10.1756 | 9.6448 | 9.1373 |
| 9.2789 | 9.6790 | 8.8474 | 9.0537 | 9.6522 | 9.2385 | 9.0203 | 9.8771 |
| 9.9027 | 7.8884 | 10.4147 | 7.8406 | 8.8760 | 9.7520 | 9.2549 | 9.3064 |
| 7.0022 | 6.9600 | 8.5057 | 9.3766 | 8.9733 | 10.4522 | 9.2812 | 10.2334 |
| 9.2209 | 9.1870 | 10.0918 | 9.0341 | 9.0828 | 8.4021 | 10.5514 | 8.3963 |
| 9.2549 | 10.3548 | 9.8310 | 9.1320 | 8.9861 | 8.0113 | 11.4698 | 8.1456 |
| 8.9729 | 9.1783 | 8.6696 | 9.2308 | 9.5837 | 8.2116 | 10.5807 | 8.5650 |
| 10.7083 | 7.7717 | 7.3020 | 8.5012 | 9.2655 | 9.5749 | 8.9384 | 8.6384 |
| 10.0233 | 10.6413 | 10.2010 | 8.3350 | 8.4160 | 10.3384 | 8.5342 | 9.4535 |
| 8.5442 | 8.4497 | 8.1093 | 9.0312 | 8.0534 | 9.7072 | 9.4335 | 8.5707 |
| 10.8778 | 8.2093 | 8.3454 | 9.1016 | 8.9542 | 8.1879 | 8.9231 | 10.2100 |
| 9.9811 | 9.6382 | 9.7724 | 8.7275 | 8.8893 | 9.1670 | 8.5842 | 10.2363 |
| 8.1377 | 9.0520 | 9.3442 | 9.0244 | 9.0582 | 10.8286 | 9.6595 | 9.6308 |
| 9.2629 | 8.5064 | 8.2576 | 8.8295 | 8.8680 | 9.9242 | 8.5483 | 10.3989 |
| 9.0186 | 9.0774 | 9.0417 | 9.2313 | 9.4155 | 9.8253 | 9.3505 | 8.6455 |
| 8.6687 | 9.1374 | 8.1539 | 8.8822 | 10.9348 | 10.4844 | 8.4251 | 8.8271 |
| 8.2672 | 10.0346\ | | | | | | |
| PHOX2A | 0.0000 | 0.0000 | 0.0000 | 0.4935 | 1.4681 | 1.2014 | 2.0702 |
| 0.4748 | 0.0000 | 1.0519 | 0.0000 | 3.0341 | 4.6647 | 0.0000 | 0.0000 |
| 0.0000 | 0.6221 | 0.0000 | 0.0000 | 5.3414 | 7.4032 | 0.4820 | 0.0000 |
| 0.5262 | 0.9530 | 0.0000 | 3.8086 | 0.0000 | 0.0000 | 0.0000 | 0.0000 |
| 0.0000 | 3.1967 | 2.7497 | 3.6447 | 0.0000 | 0.0000 | 0.0000 | 0.0000 |
| 6.2014 | 1.1184 | 0.0000 | 0.6231 | 0.0000 | 0.0000 | 0.0000 | 0.0000 |
| 0.0000 | 1.6510 | 0.0000 | 0.0000 | 0.0000 | 0.0000 | 0.0000 | 0.0000 |
| 0.5608 | 0.0000 | 0.0000 | 0.0000 | 0.9799 | 0.0000 | 0.0000 | 0.0000 |
| 1.0177 | 1.4936 | 0.3906 | 0.0000 | 4.2096 | 0.0000 | 0.0000 | 0.7662 |
| 0.0000 | 0.0000 | 0.8374 | 0.0000 | 0.0000 | 3.9579 | 0.0000 | 0.0000 |
| 0.0000 | 0.0000 | 0.3965 | 0.0000 | 0.6718 | 0.0000 | 0.0000 | 0.0000 |
| 1.6425 | 0.0000 | 0.5265 | 0.0000 | 1.1363 | 0.0000 | 0.6909 | 0.0000 |
| 0.0000 | 1.4589 | 0.0000 | 0.0000 | 0.0000 | 0.0000 | 0.0000 | 0.0000 |
| 0.0000 | 0.0000 | 0.0000 | 0.0000 | 0.0000 | 0.5426 | 0.5877 | 0.0000 |
| 0.0000 | 5.0200 | 1.0000 | 0.0000 | 0.0000 | 0.0000 | 1.7841 | 0.0000 |
| 0.0000 | 3.8438 | 0.0000 | 0.0000 | 0.0000 | 1.6090 | 0.5331 | 0.0000 |
| 1.6629 | 0.0000 | 0.0000 | 0.9567 | 0.0000 | 0.0000 | 0.0000 | 0.0000 |
| 0.0000 | 0.0000 | 0.0000 | 0.0000 | 0.0000 | 0.6010 | 0.0000 | 0.6506 |
| 0.0000 | 0.4562 | 0.0000 | 0.0000 | 2.5977 | 0.0000 | 0.0000 | 0.0000 |
| 0.0000 | 0.0000 | 4.4299 | 0.0000 | 0.0000 | 0.0000 | 0.0000 | 4.7709 |
| 0.0000 | 0.0000 | 0.0000 | 0.0000 | 0.0000 | 0.0000 | 0.0000 | 0.0000 |
| 0.0000 | 0.0000 | 0.0000 | 0.0000 | 0.0000 | 0.0000 | 0.0000 | 0.0000 |
| 0.0000 | 2.0214 | 0.6293 | 3.3644 | 0.0000 | 0.0000 | 2.0241 | 8.8341 |
| 0.0000 | 0.0000 | 0.0000 | 0.0000 | 0.0000 | 0.0000 | 0.0000 | 0.0000 |
| 0.0000 | 1.2903 | 1.2477 | 0.4985 | 0.0000 | 0.0000 | 0.0000 | 0.3736 |
| 0.0000 | 0.0000\ | | | | | | |
| PHOX2B | 0.0000 | 0.0000 | 0.0000 | 0.0000 | 0.0000 | 0.0000 | 0.0000 |
| 0.0000 | 2.1693 | 0.0000 | 0.0000 | 0.0000 | 0.0000 | 0.0000 | 0.0000 |
| 0.0000 | 0.0000 | 0.0000 | 0.0000 | 2.8567 | 0.0000 | 0.4820 | 0.0000 |
| 0.0000 | 0.0000 | 0.0000 | 0.0000 | 0.0000 | 0.0000 | 0.0000 | 0.0000 |
| 0.0000 | 0.0000 | 0.0000 | 0.0000 | 0.0000 | 0.0000 | 0.0000 | 0.0000 |

```
0.0000  0.0000  0.0000  0.0000  0.0000  0.0000  0.0000  0.0000
3.7557  0.7769  0.0000  0.0000  0.0000  0.0000  0.0000  0.0000
1.2781  0.6343  0.0000  0.0000  0.0000  0.0000  0.0000  0.0000
0.0000  0.0000  0.0000  0.0000  0.0000  0.0000  2.0689  0.0000
0.0000  0.0000  0.0000  0.0000  0.0000  0.5548  0.0000  0.0000
0.0000  0.0000  0.0000  0.0000  0.0000  0.0000  0.0000  0.0000
0.0000  0.0000  0.0000  0.0000  1.1363  0.0000  0.0000  0.0000
0.0000  0.0000  0.0000  0.0000  0.0000  0.0000  0.0000  0.0000
0.7358  0.0000  0.0000  0.0000  0.0000  0.0000  0.0000  0.0000
0.0000  0.0000  0.0000  0.0000  0.0000  0.0000  0.0000  0.0000
0.0000  0.4553  0.0000  2.0828  0.0000  0.0000  0.0000  0.0000
0.0000  0.0000  0.0000  0.0000  0.0000  0.0000  0.0000  1.3267
0.0000  0.0000  0.0000  0.0000  0.0000  0.0000  0.0000  0.0000
0.0000  0.8022  0.0000  0.0000  0.0000  0.0000  0.0000  0.5019
0.0000  0.0000  0.0000  0.0000  0.0000  0.0000  0.0000  0.0000
0.0000  0.0000  0.0000  0.0000  0.0000  0.0000  1.1696  0.0000
0.0000  0.0000  0.0000  0.0000  0.0000  2.2140  0.0000  0.0000
4.5303  0.0000  0.0000  3.7208  0.0000  0.0000  0.0000  0.0000
0.0000  0.0000  0.0000  0.0000  0.0000  0.0000  0.0000  0.0000
0.0000  0.5674  0.0000  0.0000  0.0000  1.0320  0.0000  0.0000
0.0000  0.0000\
SNORD114-31     0.0000  0.0000  0.0000  0.0000  0.0000  0.0000
0.0000  0.0000  0.0000  0.0000  0.0000  0.0000  0.0000  0.0000
0.0000  0.0000  0.0000  0.0000  0.0000  0.0000  0.0000  0.0000
0.0000  0.0000  0.0000  0.0000  0.0000  0.0000  0.0000  0.0000
0.0000  0.0000  0.0000  0.0000  0.0000  0.0000  0.0000  0.0000
0.0000  0.0000  0.0000  0.0000  0.0000  0.0000  0.0000  0.0000
0.0000  0.0000  0.0000  0.0000  0.0000  0.0000  0.0000  0.0000
0.0000  0.0000  0.0000  0.0000  0.0000  0.0000  0.0000  0.0000
0.0000  0.0000  0.0000  0.0000  0.0000  0.0000  0.0000  0.0000
0.0000  0.0000  0.0000  0.0000  0.0000  0.0000  0.0000  0.0000
0.0000  0.0000  0.0000  0.0000  0.0000  0.0000  0.0000  0.0000
0.0000  0.0000  0.0000  0.0000  0.0000  0.0000  0.0000  0.0000
0.0000  0.0000  0.0000  0.0000  0.0000  0.0000  0.0000  0.0000
0.0000  0.0000  0.0000  0.0000  0.0000  0.0000  0.0000  0.0000
0.0000  0.0000  0.0000  0.0000  0.0000  0.0000  0.0000  0.0000
0.0000  0.0000  0.0000  0.0000  0.0000  0.0000  0.0000  0.0000
0.0000  0.0000  0.0000  0.0000  0.0000  0.0000  0.0000  0.0000
0.0000  0.0000  0.0000  0.0000  0.0000  0.0000  0.0000  0.0000
0.0000  0.0000  0.0000  0.0000  0.0000  0.0000  0.0000  0.0000
0.0000  0.0000  0.0000  0.0000  0.0000  0.0000  0.0000  0.0000
0.0000  0.0000  0.0000  0.0000  0.0000  0.0000  0.0000  0.0000
0.0000  0.0000  0.0000  0.0000  0.0000  0.0000  0.0000  0.0000
0.0000  0.0000  0.0000  0.0000  0.0000  0.0000  0.0000  0.0000
0.0000  0.0000  0.0000  0.0000  0.0000  0.0000  0.0000  0.0000
0.0000  0.0000  0.0000  0.0000  0.0000  0.0000  0.0000  0.0000
0.0000  0.0000  0.0000\
SNORD114-30     0.0000  0.0000  0.0000  0.0000  0.0000  0.0000
0.0000  0.0000  0.0000  0.0000  0.0000  0.0000  0.0000  0.0000
0.0000  0.0000  0.0000  0.0000  0.0000  0.0000  0.0000  0.0000
0.0000  0.0000  0.0000  0.0000  0.0000  0.0000  0.0000  0.0000
0.0000  0.0000  0.0000  0.0000  0.0000  0.0000  0.0000  0.0000
0.0000  0.0000  0.0000  0.0000  0.0000  0.0000  0.0000  0.0000
0.0000  0.0000  0.0000  0.0000  0.0000  0.0000  0.0000  0.0000
```

```
0.0000  0.0000  0.0000  0.0000  0.0000  0.0000  0.0000  0.0000
0.0000  0.0000  0.0000  0.0000  0.0000  0.0000  0.0000  0.0000
0.0000  0.0000  0.0000  0.0000  0.0000  0.0000  0.0000  0.0000
0.0000  0.0000  0.0000  0.0000  0.0000  0.0000  0.0000  0.0000
0.0000  0.0000  0.0000  0.0000  0.0000  0.0000  0.0000  0.0000
0.0000  0.0000  0.0000  0.0000  0.0000  0.0000  0.0000  0.0000
0.0000  0.0000  0.0000  0.0000  0.0000  0.0000  0.0000  0.0000
0.0000  0.0000  0.0000  0.0000  0.0000  0.0000  0.0000  0.0000
0.0000  0.0000  0.0000  0.0000  0.0000  0.0000  0.0000  0.0000
0.0000  0.0000  0.0000  0.0000  0.0000  0.0000  0.0000  0.0000
0.0000  0.0000  0.0000  0.0000  0.0000  0.0000  0.0000  0.0000
0.0000  0.0000  0.0000  0.0000  0.0000  0.0000  0.0000  0.0000
0.0000  0.0000  0.0000  0.0000  0.0000  0.0000  0.0000  0.0000
0.0000  0.0000  0.0000  0.0000  0.0000  0.0000  0.0000  0.0000
0.0000  0.0000  0.0000  0.0000  0.0000  0.0000  0.0000  0.0000
0.0000  0.0000  0.0000  0.0000  0.0000  0.0000  0.0000  0.0000
0.0000  0.0000  0.0000  0.0000  0.0000  0.0000  0.0000  0.0000
0.0000  0.0000  0.0000  0.0000  0.0000  0.0000  0.0000  0.0000
0.0000  0.0000  0.0000\
LRGUK   2.2429  3.0238  3.5132  7.4956  3.4800  6.0791  6.8374
6.1289  4.3637  5.3346  3.8242  3.1028  7.3274  1.2777  7.4280
5.7180  3.7440  1.9468  3.7518  2.7331  3.6109  7.5412  7.2568
5.7964  2.0961  1.6270  3.8086  7.5253  2.8976  2.5978  3.6920
6.3208  3.1274  6.5272  5.2677  2.9348  5.0210  4.3210  1.4957
2.1720  3.0046  3.2379  1.3900  2.4713  0.0000  7.1023  5.0988
3.7557  3.7148  3.2043  4.3349  3.6337  3.2521  1.1831  5.4938
6.1367  2.2825  4.4776  3.0947  4.0911  1.4980  5.4651  3.8280
2.4883  4.8197  6.3327  2.2002  5.0881  3.8367  4.5791  3.5250
2.1491  2.4919  1.5690  3.7675  7.0431  6.6022  8.2708  8.1184
2.0686  5.1916  3.1030  8.0721  6.5745  7.1393  6.7598  5.3955
6.6315  1.8667  1.8648  1.0759  4.4441  4.0594  7.0560  4.1281
0.6266  2.0217  2.8937  7.1004  3.8079  5.2819  4.7106  5.1347
6.0047  6.0156  1.1001  5.2215  7.0374  6.7095  6.4174  6.4420
6.4367  3.5857  6.3038  3.4391  3.2401  6.8719  5.8987  3.5189
1.8581  4.0518  4.3681  4.6595  5.5493  6.6428  3.1042  1.6443
2.7607  2.7474  3.4188  4.8219  5.7558  7.6425  8.6335  2.0060
2.2603  3.7348  4.2995  5.5596  3.3552  5.6761  4.3631  7.5581
2.3527  1.5157  1.4974  6.3559  2.9802  5.2563  2.5049  6.1731
4.3571  5.0784  3.7040  2.3660  3.3519  3.8471  3.5881  8.0389
2.2156  2.2191  2.5292  6.2488  6.4341  2.7513  4.2724  4.6449
4.0347  5.9932  2.6009  3.9957  2.0357  3.1267  4.0582  5.2700
4.0002  5.9587  1.0660  6.2667  8.8423  4.7174  2.8349  3.9950
5.7958  4.7892  4.5374  5.0763  3.0995  5.9432  2.2874  2.9542
3.1677  1.7696  3.3126  3.7421  3.1828  5.7204  2.7661  5.1771
7.1739  3.0771\
MPHOSPH8        8.4914  9.5550  9.6037  9.6709  9.5243  9.3583
9.2172  9.0313  9.4978  9.2676  9.3002  9.8308  9.2941  9.6370
9.1217  8.6757  7.1793  8.9280  9.5084  8.1164  8.4658  9.4298
9.4521  9.3408  9.4734  8.5503  7.9534  8.4990  8.7936  9.5709
9.2593  9.0901  8.9015  8.9025  9.1808  8.9326  9.0648  9.7516
9.3082  8.6876  9.1945  10.2009 8.8780  8.7186  10.6373 8.3536
7.1602  9.5466  8.4479  8.9989  9.1524  9.0793  9.1274  10.5737
8.7791  8.8182  9.2158  8.9932  9.5417  8.5779  10.6918 9.7364
9.8267  9.6295  9.2583  9.5065  9.7547  8.8629  9.2322  8.6047
```

| | | | | | | | |
|---|---|---|---|---|---|---|---|
| 8.0471 | 9.7411 | 8.7208 | 8.8070 | 9.6536 | 9.1508 | 8.5910 | 9.1988 |
| 8.3116 | 9.4092 | 8.6871 | 9.1638 | 8.7386 | 8.6970 | 8.6448 | 8.6140 |
| 9.8651 | 8.1779 | 9.6596 | 9.0045 | 10.0473 | 9.5481 | 8.8063 | 8.0405 |
| 8.9416 | 9.8322 | 8.6634 | 10.1163 | 9.8022 | 8.8868 | 8.1703 | 9.1825 |
| 8.5700 | 8.4688 | 8.4745 | 8.5624 | 8.9641 | 9.1340 | 8.8509 | 8.3788 |
| 9.6526 | 9.3980 | 8.0507 | 9.5546 | 9.0836 | 9.8141 | 9.2865 | 9.9722 |
| 8.7682 | 9.4650 | 8.4610 | 9.0771 | 9.8149 | 9.3268 | 9.9169 | 8.4068 |
| 8.9271 | 5.9463 | 10.0764 | 9.7297 | 8.3074 | 8.9695 | 9.1223 | 8.1978 |
| 8.3287 | 7.0869 | 8.2732 | 8.4914 | 10.3646 | 10.3623 | 7.1506 | 9.3498 |
| 8.5477 | 9.4713 | 9.2291 | 10.9525 | 9.4327 | 9.6211 | 9.0540 | 7.4974 |
| 9.9091 | 7.4867 | 9.4956 | 9.4332 | 8.8379 | 9.9295 | 10.2470 | 9.2668 |
| 7.9454 | 8.6243 | 9.2587 | 9.0036 | 9.2285 | 9.1407 | 9.3615 | 9.8743 |
| 7.8380 | 9.1518 | 9.2207 | 8.8141 | 9.3916 | 7.5940 | 8.5044 | 9.2781 |
| 7.4046 | 9.4111 | 9.3052 | 10.4345 | 9.7584 | 9.0115 | 8.7206 | 9.0410 |
| 8.7319 | 8.9717 | 9.5896 | 9.5726 | 9.5325 | 8.0925 | 7.9890 | 9.4466 |
| 9.2178 | 9.1426 | 8.0738 | 9.9564 | 9.4498 | 7.3453 | 5.8171 | 10.3186 |
| 9.6289 | 9.8698 | 7.2027\ | | | | | |
| UPK2 | 3.7656 | 1.8777 | 2.4032 | 3.4850 | 2.0327 | 1.8477 | 3.2756 |
| 10.7748 | 8.4759 | 2.4985 | 2.0419 | 7.8172 | 4.7429 | 7.6289 | 3.1176 |
| 0.0000 | 7.5715 | 5.3565 | 1.9975 | 5.8151 | 1.8761 | 2.8772 | 1.4101 |
| 3.1574 | 1.7397 | 4.8934 | 3.1984 | 3.3595 | 3.3032 | 1.5953 | 1.2172 |
| 1.5370 | 1.6552 | 6.6638 | 1.8080 | 1.1431 | 5.5981 | 1.9549 | 4.3299 |
| 2.4747 | 0.0000 | 1.5889 | 4.3153 | 0.0000 | 0.0000 | 5.7140 | 1.8316 |
| 1.9920 | 1.0498 | 4.1558 | 2.1110 | 2.1910 | 3.8722 | 1.1831 | 6.2301 |
| 3.9752 | 2.8225 | 3.6165 | 10.0886 | 9.1118 | 0.5422 | 2.9220 | 5.3267 |
| 1.3432 | 2.8183 | 2.9126 | 3.7908 | 4.1134 | 8.2533 | 3.0801 | 2.2792 |
| 1.8067 | 2.9022 | 4.3134 | 3.8144 | 3.7353 | 3.9579 | 2.0646 | 2.2252 |
| 5.2979 | 4.6992 | 2.5997 | 2.9023 | 1.4748 | 3.9566 | 12.2608 | 8.3170 |
| 3.4699 | 4.7564 | 1.6790 | 1.9147 | 6.2942 | 3.7682 | 3.3524 | 2.1605 |
| 0.0000 | 5.0408 | 2.4839 | 2.9577 | 3.4878 | 4.5420 | 3.0033 | 1.6984 |
| 7.1968 | 3.5544 | 2.0179 | 0.0000 | 4.7249 | 5.1016 | 1.3268 | 2.3994 |
| 1.8846 | 9.6118 | 5.7004 | 3.1220 | 6.0547 | 4.4431 | 10.3573 | 6.5383 |
| 1.0358 | 5.8707 | 3.5951 | 5.2764 | 2.5865 | 2.6149 | 3.1042 | 2.2388 |
| 6.8467 | 1.9477 | 1.1792 | 2.2519 | 3.8633 | 1.9260 | 3.2144 | 3.0945 |
| 3.1012 | 4.0100 | 5.2624 | 1.2057 | 0.8792 | 3.6287 | 2.6745 | 2.3186 |
| 3.7359 | 7.7816 | 1.4974 | 3.1266 | 1.7212 | 3.5137 | 6.5000 | 2.6803 |
| 3.4258 | 2.5871 | 10.0740 | 4.5336 | 5.0392 | 0.9683 | 5.4454 | 4.7154 |
| 3.6832 | 2.6375 | 3.2456 | 2.1631 | 2.3765 | 1.2829 | 1.4147 | 4.2627 |
| 2.8261 | 3.9193 | 1.7238 | 3.7829 | 8.0399 | 0.0000 | 2.2569 | 2.3719 |
| 4.7994 | 1.6715 | 1.4008 | 4.7187 | 2.4311 | 2.7277 | 3.2614 | 3.8705 |
| 3.2316 | 4.2968 | 4.0633 | 6.4724 | 4.3168 | 2.7580 | 1.7765 | 2.4959 |
| 4.0851 | 6.9697 | 3.7777 | 1.7976 | 4.4061 | 2.7543 | 1.6369 | 7.7464 |
| 3.1721 | 3.6039\ | | | | | | |
| CFH | 7.8839 | 6.8132 | 12.4683 | 7.8772 | 8.5995 | 11.5143 | 8.8206 |
| 10.2158 | 8.3156 | 8.3794 | 11.0675 | 6.7558 | 10.1068 | 5.6684 | 7.5053 |
| 10.9364 | 9.1224 | 8.3665 | 8.1311 | 7.4033 | 9.3084 | 12.8931 | 9.1758 |
| 12.2343 | 8.2153 | 8.6661 | 6.9154 | 8.4079 | 10.3561 | 6.7364 | 7.2101 |
| 7.4710 | 7.9276 | 7.4611 | 9.0316 | 7.1476 | 9.4462 | 7.2483 | 6.7780 |
| 6.5534 | 10.4931 | 8.8100 | 7.3542 | 9.9253 | 10.4458 | 6.8432 | 7.4514 |
| 8.2877 | 9.8787 | 6.4048 | 7.4551 | 10.2254 | 12.8753 | 10.4694 | 9.9993 |
| 7.9818 | 7.7609 | 11.9211 | 9.3089 | 6.9371 | 10.2003 | 9.9684 | 7.1007 |
| 6.7898 | 8.3027 | 11.2750 | 8.0498 | 10.3293 | 9.7025 | 11.1232 | 10.7596 |
| 10.5421 | 9.5406 | 11.4165 | 10.0796 | 3.6045 | 10.7478 | 8.6708 | 11.0390 |
| 8.4393 | 8.0587 | 6.6395 | 11.2178 | 11.5837 | 10.7971 | 10.1338 | 6.6292 |

| | | | | | | | |
|---|---|---|---|---|---|---|---|
| 10.0819 | 7.3902 | 11.8125 | 11.5564 | 9.8265 | 7.2194 | 11.4487 | 9.0833 |
| 10.6861 | 7.6435 | 8.6595 | 11.9818 | 8.9383 | 7.5135 | 8.2694 | 9.4265 |
| 8.4688 | 6.2674 | 6.4891 | 6.2403 | 11.6665 | 10.9268 | 9.2513 | 6.7649 |
| 9.8203 | 9.5191 | 6.0875 | 10.2361 | 9.2819 | 10.5918 | 10.1789 | 8.2364 |
| 10.0182 | 8.2504 | 8.6585 | 9.8515 | 7.6528 | 9.2955 | 10.6337 | 10.1847 |
| 4.0148 | 10.6616 | 8.1229 | 6.5900 | 8.3961 | 9.8083 | 10.7000 | 8.4237 |
| 7.9058 | 8.2785 | 9.4320 | 8.8659 | 9.5051 | 7.8090 | 9.7441 | 8.2158 |
| 8.8771 | 10.5700 | 11.9985 | 11.8266 | 9.8850 | 7.1492 | 7.3036 | 11.9654 |
| 10.6140 | 12.6788 | 7.7654 | 6.5656 | 10.7625 | 9.1815 | 7.9391 | 8.7537 |
| 7.4326 | 6.9872 | 10.6480 | 11.8245 | 10.9525 | 8.3344 | 8.2898 | 11.6135 |
| 11.3180 | 10.3121 | 8.7782 | 6.0897 | 7.9263 | 9.6412 | 7.7028 | 7.7875 |
| 8.0462 | 8.4486 | 11.0909 | 9.6872 | 11.1436 | 7.9704 | 8.6497 | 6.8213 |
| 9.5354 | 11.0627 | 11.0541 | 8.8246 | 8.6812 | 6.4882 | 10.2153 | 9.0222 |
| 11.2385 | 9.2358 | 9.4420 | 10.1098 | 8.4111 | 4.3456 | 9.1242 | 10.8464 |
| 11.4459 | 7.5178\ | | | | | | |
| CFI | 11.6481 | 11.7106 | 11.5517 | 10.7998 | 8.4815 | 11.7747 | 11.2738 |
| 10.4661 | 12.8502 | 11.5472 | 12.7154 | 9.3269 | 12.3651 | 9.8750 | 12.8303 |
| 11.7922 | 11.9289 | 8.0229 | 11.6953 | 11.4461 | 12.3815 | 11.6581 | 11.8033 |
| 13.1018 | 8.9841 | 7.3196 | 5.7493 | 12.0104 | 11.9465 | 11.7137 | 5.3603 |
| 6.7799 | 7.2952 | 8.7950 | 10.4771 | 8.8964 | 11.8839 | 9.5615 | 7.3283 |
| 10.5679 | 11.1318 | 11.9342 | 9.9734 | 10.7331 | 9.4329 | 11.7226 | 7.8215 |
| 12.7641 | 8.2676 | 7.4130 | 9.9946 | 8.7638 | 10.9002 | 10.6533 | 10.9154 |
| 9.1112 | 10.3671 | 10.3113 | 11.6553 | 10.3905 | 10.2766 | 12.4827 | 7.3336 |
| 10.0966 | 11.0434 | 12.6930 | 9.5769 | 10.6539 | 11.2071 | 10.6486 | 8.5875 |
| 11.6908 | 8.3918 | 11.4120 | 11.6781 | 5.8283 | 11.6902 | 10.1423 | 13.2952 |
| 6.8179 | 11.0769 | 13.0644 | 10.5653 | 10.9957 | 12.8765 | 12.7646 | 6.2894 |
| 12.7747 | 11.5300 | 9.9585 | 11.5001 | 12.8850 | 8.6352 | 12.3142 | 10.7633 |
| 9.7742 | 10.5183 | 10.5114 | 10.9290 | 11.4195 | 7.7963 | 11.9912 | 12.8674 |
| 10.0209 | 7.6950 | 6.9425 | 11.0780 | 13.5556 | 12.2974 | 12.1284 | 6.3005 |
| 11.2993 | 10.0337 | 10.2715 | 8.9338 | 11.0281 | 8.9490 | 12.3882 | 8.2041 |
| 10.3030 | 9.6369 | 11.4878 | 12.4028 | 7.2621 | 11.6663 | 11.3757 | 11.1138 |
| 11.6298 | 12.4464 | 10.2441 | 13.5831 | 12.2063 | 12.5323 | 10.7119 | 10.6238 |
| 7.1954 | 7.7949 | 9.8494 | 11.7845 | 11.7249 | 8.1690 | 11.2966 | 13.2728 |
| 10.4633 | 12.0853 | 9.2189 | 10.4593 | 11.3884 | 12.9290 | 9.2453 | 11.3279 |
| 7.1431 | 13.3645 | 10.6075 | 11.5588 | 11.1967 | 11.4616 | 11.6498 | 12.5596 |
| 7.2460 | 7.6992 | 10.1113 | 13.5307 | 12.9897 | 11.2253 | 12.7323 | 12.6193 |
| 12.9258 | 10.7916 | 9.4836 | 11.2927 | 8.6926 | 9.9653 | 10.4793 | 9.3712 |
| 9.9774 | 11.1323 | 10.2635 | 10.7078 | 11.9520 | 7.2691 | 13.7029 | 8.3883 |
| 12.3547 | 12.3655 | 12.5638 | 10.8581 | 11.8234 | 11.9697 | 8.9037 | 7.6569 |
| 12.8981 | 11.8311 | 11.3970 | 10.6963 | 11.0705 | 4.8425 | 11.2097 | 11.9639 |
| 11.8720 | 11.9887\ | | | | | | |
| PNP | 11.0858 | 9.4430 | 10.6153 | 10.5970 | 10.5280 | 10.9919 | 10.8065 |
| 10.9538 | 10.2469 | 9.3226 | 10.4947 | 8.8779 | 9.6478 | 11.4081 | 11.2534 |
| 9.3170 | 10.1583 | 9.5544 | 9.5221 | 11.7836 | 9.9416 | 10.6744 | 8.2547 |
| 10.4802 | 7.9896 | 10.1951 | 12.3033 | 10.8627 | 12.4579 | 12.7558 | 9.7200 |
| 10.7883 | 11.5805 | 10.1339 | 9.5612 | 11.4311 | 10.4892 | 11.7553 | 10.9207 |
| 10.5596 | 10.7413 | 11.2669 | 11.4254 | 10.7354 | 8.1597 | 9.9685 | 10.5920 |
| 11.9112 | 10.4859 | 11.8625 | 11.2689 | 12.2812 | 11.2802 | 9.1447 | 12.0601 |
| 10.8938 | 11.0959 | 9.0925 | 9.8766 | 10.4830 | 9.1098 | 10.1543 | 11.3413 |
| 11.7488 | 8.5856 | 10.4412 | 10.3830 | 10.8247 | 11.8685 | 10.8876 | 9.6417 |
| 10.8229 | 12.0925 | 8.9238 | 11.4309 | 11.0966 | 11.0856 | 9.8124 | 9.5999 |
| 10.4964 | 11.9209 | 12.5558 | 9.1637 | 10.4193 | 10.3207 | 10.0862 | 11.5213 |
| 10.7715 | 11.2904 | 11.0864 | 9.5783 | 10.7049 | 11.7545 | 8.9219 | 11.9690 |
| 8.7451 | 11.2357 | 7.7942 | 11.5064 | 10.6804 | 11.6683 | 11.3737 | 11.1889 |

9.8178 11.5589 11.3481 9.2441 10.7477 9.2924 10.9868 9.7417
11.1855 10.1914 10.8696 11.0545 11.3810 11.5083 9.2459 11.8361
11.9421 9.0732 11.8414 11.5134 10.4943 8.8591 9.8265 12.5789
10.8765 9.0094 8.5437 10.2718 11.1886 10.8306 10.0810 10.4181
11.0254 10.4045 8.9927 10.5620 7.9626 10.6118 11.5160 10.1827
10.8773 12.0145 5.6241 11.2569 8.5872 12.2306 12.2418 10.5511
10.8863 10.6653 10.6978 11.5988 10.6916 8.7529 11.0884 10.4657
11.1075 11.0236 10.1333 10.1334 11.2545 12.5244 10.3050 10.1099
9.4034 11.5302 9.6956 11.9562 10.9668 11.6989 10.8979 10.4368
10.2164 8.6090 9.2976 11.5354 9.7154 11.6790 11.5899 9.9849
10.4282 10.4003 10.4229 9.5479 11.6828 10.5774 11.5698 11.1355
11.8054 10.2979 10.6581 11.0348 10.1295 11.8040 7.9224 11.2229
10.0015 11.7187\
ANKFY1 10.2107 10.0719 10.7033 10.4708 10.4326 10.2205 9.9745
9.7250 10.3205 10.6737 9.8396 9.9493 10.3872 9.5561 10.4244
9.8430 9.5441 9.9455 10.5251 10.0458 9.9879 10.2728 10.4613
10.2700 10.4523 10.2769 10.1033 10.3888 10.2339 10.4248 10.1020
10.2416 10.0178 9.7683 9.8980 10.6909 10.8989 10.9737 10.1221
9.9674 10.3056 10.2320 9.5028 10.8747 10.7105 9.5865 8.9769
10.8656 11.4897 10.0957 10.7432 11.3826 10.2144 10.8136 10.9247
10.5211 10.1211 9.9135 10.1655 9.3973 11.2473 9.6266 10.9573
11.3441 10.4972 10.9061 11.1229 10.1636 10.3987 10.0684 9.8362
10.8077 9.7519 10.1997 10.7244 10.4899 10.7172 10.5856 9.8597
10.6573 10.2882 9.7119 10.0965 10.2033 9.8532 9.8855 10.4043
10.1008 10.0063 10.6850 10.7778 10.4338 11.0695 9.8093 10.3111
10.8053 11.2967 10.6253 10.1145 10.2275 9.7170 10.6678 11.0709
10.2311 9.2909 10.6968 10.1868 10.9880 9.5697 9.4207 10.4609
10.5576 8.6224 9.2021 9.9948 9.6570 10.1640 9.8162 10.7045
10.3000 10.4186 10.4689 10.1787 10.9763 10.5544 9.4357 9.7327
8.3708 10.5491 10.9598 10.7164 9.2714 10.5950 9.7829 9.7123
9.7982 9.8853 9.1529 10.6354 10.5211 9.1948 9.9602 10.3445
10.9825 10.7923 10.9604 10.6935 10.4430 10.4806 9.4312 10.4179
9.0846 11.0482 10.0081 10.8002 10.2572 10.4990 10.7851 9.5524
10.1720 10.2594 10.1312 10.0438 10.0602 9.9838 10.8580 10.3533
10.2162 10.5143 9.9672 11.1214 9.9454 10.4442 10.4248 8.8069
10.3796 10.6485 10.3250 10.7398 10.7031 9.9449 11.2568 10.0758
10.2982 10.0430 10.3421 10.7898 10.0868 10.5411 10.2932 10.7437
10.5211 10.3462 10.9307 10.0711 9.0117 8.5829 10.4446 11.0308
10.6828 10.0912\
C1orf14 0.0000 0.0000 0.0000 0.0000 0.5278 0.0000 0.0000
0.4748 0.0000 0.4413 0.0000 0.0000 0.0000 0.4395 0.0000
0.0000 0.0000 0.0000 0.0000 0.0000 1.6493 0.0000 0.0000
0.0000 0.5538 0.0000 4.8889 0.0000 0.0000 0.0000 0.0000
0.0000 0.0000 1.7182 0.0000 0.0000 0.0000 0.0000 0.0000
0.0000 0.0000 1.1407 0.0000 0.0000 0.0000 0.0000 0.0000
0.0000 0.0000 0.0000 0.0000 0.7768 0.0000 0.0000 0.0000
0.5608 0.0000 0.0000 0.0000 0.0000 0.0000 0.0000 0.0000
2.1974 0.0000 0.3906 0.0000 0.0000 0.0000 0.0000 0.0000
0.0000 1.9166 0.0000 0.0000 0.0000 0.0000 0.0000 0.0000
0.0000 0.0000 0.0000 0.0000 0.0000 0.0000 0.0000 0.0000
0.0000 0.0000 0.5265 0.0000 0.0000 0.0000 0.0000 0.0000
0.0000 0.0000 0.5454 0.0000 0.0000 0.0000 0.0000 0.0000
0.0000 0.0000 0.0000 0.0000 0.0000 0.0000 0.5877 0.0000
0.0000 0.0000 0.0000 0.0000 0.0000 0.0000 0.4321 0.0000

```
0.0000  0.4553  0.0000  5.0127  0.0000  0.0000  0.0000  0.0000
0.0000  0.0000  0.0000  0.0000  0.0000  0.0000  0.0000  0.8107
0.0000  0.0000  0.0000  0.0000  0.0000  0.0000  0.0000  0.0000
0.0000  0.0000  0.0000  0.0000  0.0000  0.0000  0.0000  0.0000
0.0000  0.0000  0.0000  0.0000  0.0000  0.0000  0.3855  0.0000
0.0000  0.0000  0.0000  0.0000  0.0000  0.0000  0.0000  0.0000
0.0000  0.0000  0.0000  0.0000  0.0000  0.0000  0.6229  0.0000
0.0000  0.0000  0.6293  0.0000  0.0000  0.0000  1.1551  0.0000
0.0000  0.0000  0.0000  0.0000  0.0000  0.0000  0.0000  0.0000
0.0000  0.0000  0.0000  0.0000  0.0000  0.0000  0.0000  0.0000
0.0000  0.0000\
CFB     12.8408 10.9125 12.3355 12.7646 7.7513  14.4032 11.3340
11.8591 11.4517 8.7080  14.6875 10.8943 13.2175 9.2826  13.5740
14.7202 11.9607 7.6754  11.0228 11.4288 15.3596 10.0614 12.2624
12.0810 7.0918  10.4002 2.8719  12.7713 11.6301 12.3494 4.1344
7.4037  12.1172 14.9564 14.4166 9.0797  12.8783 10.8592 6.9525
5.6262  8.9929  10.9682 11.9226 12.6631 9.5089  13.0278 11.6056
14.0827 8.2875  4.5180  12.8820 12.1799 12.2691 9.1348  12.0859
8.0863  9.9380  12.2664 11.9660 12.1305 9.7195  12.1338 6.6456
13.5807 8.3686  13.6251 15.6291 12.7001 15.1776 10.1183 16.2373
11.5120 10.8735 14.9036 13.0185 5.0590  11.3592 9.5358  13.0583
7.8635  10.3457 11.8480 10.0111 13.0935 12.6831 14.5010 9.7018
14.3937 14.1800 7.3143  11.8071 13.8841 13.3667 12.2037 9.5777
9.3521  15.1412 10.1471 13.3743 12.3724 9.9314  11.6532 11.2953
12.1349 9.0027  7.3557  10.3656 11.8290 10.2453 12.9838 6.2428
11.5860 14.4994 14.4767 11.0843 12.3927 11.7600 12.9086 11.4719
11.8332 9.3841  13.6271 12.5494 8.2950  10.7624 12.5665 8.0031
14.3778 11.5965 8.2002  13.5306 10.7298 12.2470 10.7758 11.5234
11.7232 8.5285  12.9201 10.7350 11.7765 9.8646  10.2261 13.1461
11.8780 13.4608 7.1758  11.3994 11.5470 12.0702 8.3966  11.7481
12.9647 13.6555 12.0262 11.8071 10.6216 11.3176 8.3707  14.4489
8.5034  11.8372 15.2838 12.0637 14.0931 8.3231  10.7362 14.9761
14.3017 12.3226 12.5437 10.2481 11.9383 10.9584 10.1809 13.1049
8.8583  7.1382  9.1722  10.9749 12.7127 7.6282  13.3576 11.9747
12.7379 12.1875 12.9700 13.0039 12.0795 13.3007 8.6591  7.6754
13.1396 13.2195 10.4191 11.0079 14.6689 10.0704 10.9912 13.2581
11.4369 13.9214\
OR5P3   0.0000  0.0000  0.6896  0.0000  0.0000  0.0000  0.0000
0.0000  0.0000  0.0000  0.0000  0.0000  0.0000  0.0000  0.0000
0.0000  0.0000  0.0000  0.0000  0.0000  0.0000  0.0000  0.0000
0.0000  0.0000  0.0000  0.4561  0.0000  0.0000  0.0000  0.0000
0.0000  0.0000  0.0000  0.0000  0.0000  0.0000  0.0000  0.0000
0.0000  0.6649  0.0000  0.0000  0.0000  0.0000  0.0000  0.0000
0.0000  0.0000  0.0000  0.0000  0.0000  0.0000  0.5094  0.0000
0.0000  0.0000  0.0000  0.0000  0.0000  0.0000  0.0000  0.0000
0.0000  0.0000  0.0000  0.0000  0.0000  0.0000  0.0000  0.0000
0.0000  0.0000  0.0000  0.0000  0.0000  0.0000  0.0000  0.0000
0.0000  0.0000  0.0000  0.0000  0.0000  0.0000  0.6199  0.0000
0.0000  0.0000  0.9113  0.0000  0.0000  0.0000  0.0000  0.0000
0.0000  0.0000  0.0000  0.0000  0.0000  0.0000  0.0000  0.0000
0.0000  0.0000  0.0000  0.0000  0.0000  0.5426  0.0000  0.0000
0.0000  0.0000  1.5850  0.0000  0.0000  0.0000  0.0000  0.0000
0.0000  0.0000  0.0000  0.0000  0.0000  0.0000  0.0000  0.0000
0.0000  0.0000  0.0000  0.0000  0.0000  0.0000  0.0000  0.0000
```

```
0.0000  0.0000  0.0000  0.0000  0.0000  0.0000  0.0000  0.0000
0.0000  0.0000  0.0000  0.0000  0.0000  0.0000  0.0000  0.0000
0.0000  0.0000  0.0000  0.0000  0.0000  0.0000  0.0000  0.0000
0.0000  0.0000  0.0000  0.0000  0.0000  0.0000  0.0000  0.0000
0.0000  0.0000  0.0000  0.0000  0.0000  0.0000  0.0000  0.0000
0.0000  0.0000  0.0000  0.0000  0.0000  0.0000  0.0000  0.0000
0.0000  0.0000  0.0000  0.0000  0.0000  0.0000  0.0000  0.0000
0.0000  1.2903  0.0000  0.0000  0.0000  0.0000  0.0000  0.0000
0.0000  0.0000\
OR5P2   0.0000  0.0000  0.0000  0.0000  0.0000  0.0000  0.0000
0.0000  0.0000  0.0000  0.0000  0.0000  0.0000  0.0000  0.0000
0.0000  0.0000  0.0000  0.0000  0.0000  0.0000  0.0000  0.0000
0.0000  0.0000  0.0000  0.0000  0.0000  0.0000  0.0000  0.0000
0.0000  0.0000  0.0000  0.0000  0.0000  0.0000  0.0000  0.0000
0.0000  0.0000  0.0000  0.0000  0.0000  0.0000  0.0000  0.0000
0.0000  0.0000  0.0000  0.0000  0.0000  0.0000  0.0000  0.0000
0.0000  0.0000  0.0000  0.0000  0.0000  0.0000  0.0000  0.0000
0.0000  0.0000  0.0000  0.0000  0.0000  0.0000  0.0000  0.0000
0.0000  0.0000  0.0000  0.0000  0.0000  0.0000  0.0000  0.0000
0.0000  0.0000  0.0000  0.0000  0.0000  0.0000  0.0000  0.0000
0.0000  0.0000  0.0000  0.0000  0.0000  0.0000  0.0000  0.0000
0.0000  0.0000  0.0000  0.0000  0.0000  0.0000  0.0000  0.0000
0.0000  0.0000  0.0000  0.0000  0.0000  0.0000  0.0000  0.0000
0.0000  0.0000  0.0000  0.0000  0.0000  0.0000  0.0000  0.0000
0.0000  0.0000  0.0000  0.0000  0.0000  0.0000  0.0000  0.0000
0.0000  0.0000  0.0000  0.0000  0.0000  0.0000  0.0000  0.0000
0.0000  0.0000  0.0000  0.0000  0.0000  0.0000  0.0000  0.0000
0.0000  0.0000  0.0000  0.0000  0.0000  0.0000  0.0000  0.0000
0.0000  0.0000  0.0000  0.0000  0.0000  0.0000  0.0000  0.0000
0.0000  0.0000  0.0000  0.0000  0.0000  0.0000  0.0000  0.0000
0.0000  0.0000  0.5466  0.0000  0.0000  0.0000  0.0000  0.0000
0.0000  0.0000  0.6293  0.0000  0.0000  0.0000  0.0000  0.0000
0.0000  0.0000  0.0000  0.0000  0.0000  0.0000  0.0000  0.0000
0.0000  0.0000  0.0000  0.0000  0.0000  0.0000  0.0000  0.0000
0.0000  0.0000\
MLLT4   11.1955 11.4415 12.2043 12.2353 10.9857 10.7697 11.9224
10.6929 11.9122 11.4549 11.5286 11.4994 11.7859 11.7631 12.3637
11.9436 10.7383 11.2172 11.4134 11.7626 10.9572 12.7094 12.3669
11.8564 10.5242 9.6239  10.8389 11.1142 11.3493 11.4741 10.1129
12.4288 10.8153 11.7393 13.1463 12.6760 12.1267 12.0327 11.1054
13.1622 11.0513 11.7211 11.8220 11.7886 10.5798 10.6332 9.5779
11.9329 9.5206  12.2081 11.2395 12.3486 11.2227 11.0441 11.9855
12.5166 11.4518 11.5602 11.6495 11.6401 11.2008 12.1157 10.5838
10.9667 11.2097 11.7746 12.2349 10.6863 11.6773 11.3774 11.0632
12.2110 10.0923 11.2566 12.3969 11.7327 10.5221 12.6766 11.6428
11.1732 11.2084 11.3943 12.4178 11.9048 11.1661 11.1092 11.5825
10.5027 11.1945 10.0112 10.4124 11.2372 10.4559 11.8014 10.6278
9.9794  11.0631 10.9913 11.4367 11.0555 9.7378  11.3734 11.5423
11.6193 10.3180 11.2424 11.3841 11.7621 12.0026 11.2584 12.4148
11.5450 10.7856 11.6013 11.5205 11.1976 11.9607 11.3146 11.4263
11.1088 11.9066 11.8833 12.3293 11.3060 12.5312 11.3689 10.9502
9.3967  11.3905 11.3652 12.3327 12.0095 11.6723 12.7743 10.5640
9.2998  9.5771  10.6020 11.9288 11.1951 11.2350 12.1157 11.4834
12.0544 12.2870 10.7153 11.7907 11.7382 10.5240 10.1497 12.1352
```

```
9.4509  12.3220 11.2861 12.5747 11.3346 11.5688 12.3520 11.3453
9.6644  11.1096 9.9255  11.8687 11.6889 11.3669 11.8774 11.5147
11.9228 11.1979 11.1063 11.9597 11.4977 10.2791 11.1678 10.1615
11.4210 11.6971 10.4937 12.2058 12.5966 11.2190 11.4536 11.8519
11.5409 11.9093 11.2291 11.4604 10.8152 11.3049 9.6485  11.1159
11.1657 11.0973 12.0521 11.6867 10.0575 9.6015  11.3565 11.6159
12.7192 9.6816\
SNORD115-6      0.0000  0.0000  0.0000  0.0000  0.0000  0.0000
0.0000  0.0000  0.0000  0.0000  0.0000  0.0000  0.0000  0.0000
0.0000  0.0000  0.0000  0.0000  0.0000  0.0000  0.0000  0.0000
0.0000  0.0000  0.0000  0.0000  0.0000  0.0000  0.0000  0.0000
0.0000  0.0000  0.0000  0.0000  0.0000  0.0000  0.0000  0.0000
0.0000  0.0000  0.0000  0.0000  0.0000  0.0000  0.0000  0.0000
0.0000  0.0000  0.0000  0.0000  0.0000  0.0000  0.0000  0.0000
0.0000  0.0000  0.0000  0.0000  0.0000  0.0000  0.0000  0.0000
0.0000  0.0000  0.0000  0.0000  0.0000  0.0000  0.0000  0.0000
0.0000  0.0000  0.0000  0.0000  0.0000  0.0000  0.0000  0.0000
0.0000  0.0000  0.0000  0.0000  0.0000  0.0000  0.0000  0.0000
0.0000  0.0000  0.0000  0.0000  0.0000  0.0000  0.0000  0.0000
0.0000  0.0000  0.0000  0.0000  0.0000  0.0000  0.0000  0.0000
0.0000  0.0000  0.0000  0.0000  0.0000  0.0000  0.0000  0.0000
0.0000  0.0000  0.0000  0.0000  0.0000  0.0000  0.0000  0.0000
0.0000  0.0000  0.0000  0.0000  0.0000  0.0000  0.0000  0.0000
0.0000  0.0000  0.0000  0.0000  0.0000  0.0000  0.0000  0.0000
0.0000  0.0000  0.0000  0.0000  0.0000  0.0000  0.0000  0.0000
0.0000  0.0000  0.0000  0.0000  0.0000  0.0000  0.0000  0.0000
0.0000  0.0000  0.0000  0.0000  0.0000  0.0000  0.0000  0.0000
0.0000  0.0000  0.0000  0.0000  0.0000  0.0000  0.0000  0.0000
0.0000  0.0000  0.0000  0.0000  0.0000  0.0000  0.0000  0.0000
0.0000  0.0000  0.0000  0.0000  0.0000  0.0000  0.0000  0.0000
0.0000  0.0000  0.0000  0.0000  0.0000  0.0000  0.0000  0.0000
0.0000  0.0000  0.0000  0.0000  0.0000  0.0000  0.0000  0.0000
0.0000  0.0000  0.0000\
CREBBP  10.5098 11.5918 11.4693 10.4967 11.2583 10.9017 11.3726
6.8624  11.2572 11.2192 10.9654 11.3044 10.8215 11.3298 10.6255
10.5965 10.5463 10.6469 11.5027 10.1121 10.5887 10.6779 10.8904
10.9243 11.5700 9.8200  10.0999 10.0346 10.2683 11.7914 11.3418
10.9325 10.0320 11.8904 11.8062 10.5445 11.5607 11.1443 10.3734
10.7505 10.9820 11.9193 10.5321 6.6582  11.5973 10.0775 9.7899
11.3298 11.4614 10.0270 10.8867 10.4384 11.5727 11.8066 11.5654
10.7380 11.3822 10.1207 11.5145 11.0618 11.7841 11.2053 10.2557
10.3458 11.4988 11.2648 10.7213 11.0150 10.5872 11.2969 9.7531
11.6826 9.8707  10.9588 10.9260 10.8729 10.8371 10.6886 10.5667
11.2255 9.8692  11.1847 10.7935 11.6424 10.3843 11.0266 10.2107
10.0826 11.4291 10.5500 11.4845 11.8606 10.2246 11.0674 10.4464
11.3611 11.7280 12.2163 10.8123 10.5679 10.0107 10.9065 10.5641
11.3015 10.6274 11.2629 10.9842 10.8814 10.7252 10.8548 11.2307
10.9927 9.8784  10.7254 10.3470 11.2990 10.7684 11.7850 10.7956
10.6903 11.1228 10.7128 10.5548 11.2718 11.2807 11.1354 10.9930
10.7315 11.6763 11.9356 11.0950 10.7886 10.5897 11.6864 9.4374
9.4652  10.2340 11.5313 11.7201 11.9175 10.0714 10.0076 10.3838
11.1320 10.9367 11.8310 11.2343 11.2939 10.5910 9.8342  11.5090
9.3575  11.5144 10.7258 10.8684 11.5852 11.8643 11.2455 10.7338
10.2496 10.0831 10.3926 10.9436 10.2271 11.0786 11.4225 10.7616
```

```
11.5123 10.8069 10.1267 11.2990 11.0942 10.2664 10.8922 10.1754
11.1264 11.2791 11.3355 11.5433 10.6864 9.8404  10.4940 10.2316
10.2196 10.4165 10.8094 11.0053 10.3325 10.4121 10.8401 10.7736
10.8888 11.5340 11.2347 10.9516 9.2883  10.2099 11.7600 11.3564
11.2904 9.5589\
PNN     11.2075 11.1093 10.3880 12.4430 11.2091 11.8282 11.1986
10.9609 10.9928 11.8705 11.4502 11.5052 11.6700 11.4494 11.3009
12.0352 11.2275 11.1576 10.8971 11.2376 11.5666 11.4142 10.7452
11.6091 10.8999 10.3405 11.7637 10.5270 10.3238 11.4390 11.9514
11.7547 10.5334 12.1498 11.1904 11.3904 11.0501 11.4364 11.1916
12.6146 12.2999 11.8658 10.5520 12.2638 10.5759 10.4071 9.8650
10.6643 12.0172 10.4939 10.9129 11.4701 10.4466 11.7372 11.6231
12.0544 11.0783 11.2092 11.1910 11.8757 11.0500 11.3211 11.5024
11.5438 11.4664 11.2919 11.0178 11.3788 10.6905 12.6126 10.5611
11.9878 10.5459 10.2932 11.5975 11.7707 10.7947 10.6009 11.3644
11.2613 10.3574 10.9989 10.4710 11.7309 11.7862 12.0524 11.9373
10.5340 11.4705 11.2653 10.6038 11.4428 11.4049 11.5917 11.6937
10.0778 11.6353 11.1452 12.3476 11.5447 10.5312 11.1819 10.8434
12.4325 11.9888 11.5698 11.2073 11.9694 12.2992 11.4281 11.6615
11.2475 10.4254 12.3368 11.8955 12.0491 10.5525 12.0419 11.6389
11.1064 12.3081 11.0822 11.4995 10.5491 11.8444 11.0858 11.8506
9.8162  11.1978 11.7798 10.3844 11.9067 11.2233 12.2406 10.8957
9.8286  10.6504 10.2786 11.7435 11.3275 12.5695 11.3557 10.8802
11.3452 11.4363 11.5204 10.9083 10.7510 10.3637 10.0229 12.0480
10.2286 11.7861 11.5249 11.3460 11.6238 11.1142 11.7381 11.2193
10.7454 9.9831  10.9358 11.5197 11.1966 11.7499 12.5647 9.9704
10.8117 11.3489 11.1252 11.7184 11.5593 10.9606 10.4678 9.0036
11.8329 11.8799 11.5406 11.7434 11.3526 10.5145 11.4403 12.0252
11.7540 12.1081 11.7652 11.3625 10.4175 11.1026 11.9420 11.2988
10.6882 11.5790 11.7031 12.2294 10.1756 12.0277 11.3213 12.0334
12.2290 10.1436\
PHYHIPL 5.1819  8.0022  4.5035  6.5630  5.2303  3.7423  3.0968
6.0646  10.2424 6.2885  5.8968  5.2017  6.1246  5.3720  9.7555
8.4457  7.0907  4.0620  7.4196  7.8544  4.4833  9.7649  8.0465
8.8942  3.6002  0.0000  7.4220  9.2893  4.6550  8.0046  4.5467
4.1008  1.8518  5.1863  6.7888  4.2530  5.5011  8.4686  6.2174
3.3156  8.0277  6.8897  4.4661  4.2423  4.5268  8.7062  7.9651
6.7652  3.2579  3.9381  3.3193  5.2637  4.1272  5.1749  6.2981
8.1128  6.9540  4.1654  5.0137  8.8302  5.6584  9.8669  2.4314
3.5918  6.5161  8.7870  2.2462  7.5467  3.2970  4.6399  4.1267
3.9609  3.3732  4.6910  8.7182  8.3024  6.1785  7.4019  7.5803
2.7709  6.6004  5.1239  10.3589 5.8392  9.9160  3.9745  4.8612
6.8864  5.5377  9.0484  6.0215  9.9169  4.7143  8.5532  8.1318
2.4618  0.9431  5.3900  6.0309  2.8948  6.8419  9.1555  4.9827
6.2732  7.3579  6.2067  9.5088  6.3651  7.2616  9.2605  11.1376
5.9043  5.2756  8.2830  0.0000  5.0420  10.5006 3.7009  3.8444
6.7029  7.8250  1.1696  7.3009  1.7287  7.8888  4.0329  5.8859
4.5024  5.7017  5.0090  5.6665  10.7674 10.4444 9.7694  4.2555
0.0000  2.8027  4.7737  7.6626  4.8585  9.4330  3.9771  9.3436
6.2430  5.8415  5.0581  8.7441  4.9563  4.4475  4.7577  6.5652
5.6641  9.8048  4.0277  7.2354  8.0463  6.8161  9.5434  7.8130
2.7401  6.0731  6.4550  8.9616  7.6481  3.6127  9.8051  6.9060
6.2573  9.6582  3.4715  8.2811  3.5658  8.0072  2.7596  3.6013
7.7534  5.3629  6.5867  5.7433  9.1695  6.6952  6.9507  4.9097
```

7.3772  7.7574  4.1947  4.7065  8.9459  10.2859 5.9219  3.3375
4.2675  7.9318  4.9480  4.1653  3.9204  6.1009  4.5010  4.6973
9.2028  7.6168\
ATP6V0E1        11.5770 12.0237 11.9655 11.6748 11.2421 11.6237
10.9653 11.3148 11.7846 11.7142 12.1421 11.2246 11.8362 10.8778
12.3746 11.1891 11.5463 12.0687 11.9474 11.3661 12.0924 11.4541
11.5557 11.5474 11.3680 12.1524 11.5604 12.8691 12.9760 12.8647
11.3834 11.3559 11.6505 11.5676 10.4303 11.4807 11.4342 11.4016
11.9445 10.9422 12.2846 11.5490 12.2531 11.1608 11.7505 12.6360
13.0654 12.2906 11.1790 12.8596 11.3204 12.0520 11.9376 11.4364
11.2404 11.9762 11.1852 11.7145 11.8042 11.3313 11.5554 11.6424
11.9629 11.2837 11.4796 12.0532 12.4014 11.7176 12.0946 12.0977
11.8969 11.7575 13.2555 11.7750 12.5441 11.7400 10.9813 12.1947
12.1412 11.9151 11.8703 10.9803 11.9478 12.3047 11.8996 11.8606
11.2598 12.2787 11.7479 11.7369 11.8244 10.9468 12.4989 11.5700
11.5715 11.8809 12.1789 11.4940 11.2897 11.6186 12.3103 12.3328
11.1323 11.2293 12.1076 10.1884 11.1697 12.0500 11.6411 11.8802
11.2594 11.5364 11.7341 10.3565 11.7836 11.0318 11.6802 11.0252
11.1895 11.6874 10.5501 12.2708 11.9373 12.3371 11.4219 11.4742
12.6427 12.6541 11.6857 10.9435 12.0228 12.3943 12.2538 11.0212
12.0250 12.4110 12.3082 11.5359 11.0503 11.5189 12.2741 12.4406
12.0316 11.6898 11.4503 11.8576 11.9855 11.6371 12.3207 12.6067
11.5604 13.3577 11.1202 11.6925 10.9136 12.4783 11.6326 11.2331
11.4967 11.7875 12.5552 12.0257 11.9341 11.6737 11.5390 11.1659
12.1729 11.5589 12.2237 11.6651 11.1290 10.9005 11.9099 11.4712
12.2223 11.1133 11.4222 11.7739 10.9687 12.0091 12.6412 11.7218
10.5940 12.6077 10.9157 12.3612 11.1866 12.1202 12.1813 11.5296
11.5970 12.1642 11.3528 11.5154 11.5114 11.8557 12.4146 11.6988
11.6449 11.1918 12.3705\
ATP6V0E2        10.4446 7.7613  9.9954  12.8340 9.8656  8.7991
9.3655  9.6131  10.1727 9.5863  8.4362  10.2069 10.0029 11.1070
9.3566  11.6503 10.7237 10.4348 8.6608  9.6807  10.7673 10.2333
10.9092 11.0790 8.6606  11.1203 11.7777 11.2756 10.2935 7.8730
11.9123 11.4548 9.3833  10.6808 9.7697  9.5171  9.9474  10.7306
9.6819  12.1510 11.3871 7.8897  10.1395 10.8843 8.3996  11.2153
13.4413 7.6813  9.1403  11.3675 10.9156 9.5765  10.4968 8.3919
12.0469 11.1980 7.7424  10.5822 9.4469  9.3962  8.3373  9.4435
12.2750 8.9117  8.8747  10.5766 11.2816 10.5482 11.4208 11.9254
10.8841 10.1599 9.6510  10.2169 9.8580  13.8388 9.5741  11.4923
12.4847 9.8278  11.5323 10.8876 10.9267 10.2191 11.8671 10.2991
10.6365 11.3598 10.2042 11.0870 9.2310  10.2282 11.0089 10.9545
11.0994 8.8960  9.6901  9.3188  12.1147 9.8370  12.3865 9.9266
10.8830 11.1954 12.7355 12.2530 7.0770  9.5224  9.1616  12.0571
11.6276 10.5618 11.8232 9.4939  8.8941  8.2382  11.4771 9.9929
11.5558 8.9190  9.3565  8.9638  8.9640  10.7773 10.1078 8.8500
12.2063 10.7576 9.1344  8.6289  10.7039 10.8982 10.5431 11.8994
9.4040  11.6362 11.4873 8.9497  10.1683 9.3418  13.4829 11.8215
11.8609 9.0423  8.5194  8.8006  11.6316 10.1771 10.9397 11.7565
12.2617 12.9015 11.9697 11.2123 9.7481  8.9525  8.7778  10.6056
11.0695 10.3997 11.8509 10.2635 10.6650 10.9402 10.3575 10.5293
9.9683  9.4889  12.3775 10.6565 10.9835 11.0015 11.3761 10.2258
10.9281 10.9044 8.2137  9.4108  11.7252 10.3842 9.6457  6.4206
9.9544  12.1397 9.8078  10.7292 9.9096  10.5851 10.6620 8.0310
10.1949 9.7430  10.1907 10.3612 11.4218 11.1276 12.8069 9.7714

| | | | | | | | |
|---|---|---|---|---|---|---|---|
| 9.3739 | 9.8508 | 9.8721\ | | | | | |
| RNF128 | 2.7225 | 2.5617 | 6.7984 | 4.6709 | 2.7531 | 2.1935 | 3.5251 |
| 5.6356 | 2.8671 | 1.0519 | 3.7142 | 1.5838 | 2.3888 | 3.0838 | 1.4890 |
| 0.0000 | 2.2551 | 4.2361 | 1.5827 | 3.0242 | 4.4833 | 0.4820 | 4.5298 |
| 10.7819 | 0.5538 | 5.5531 | 8.4954 | 1.3471 | 3.0458 | 3.6580 | 2.7292 |
| 1.7560 | 2.2837 | 3.0448 | 0.0000 | 2.5445 | 4.7205 | 11.1375 | 2.9681 |
| 1.6341 | 1.7407 | 1.9303 | 2.9036 | 3.8009 | 2.3632 | 1.4889 | 0.7135 |
| 1.1317 | 2.6671 | 8.4252 | 3.8726 | 1.6507 | 4.9066 | 3.3066 | 3.6752 |
| 0.9635 | 9.1819 | 9.8438 | 3.3585 | 0.9799 | 1.7140 | 10.0850 | 0.0000 |
| 4.0331 | 2.0096 | 0.6977 | 0.0000 | 3.7367 | 5.7324 | 4.4793 | 1.4604 |
| 8.2828 | 2.4919 | 3.7219 | 9.1991 | 0.8237 | 5.8163 | 2.3485 | 1.1539 |
| 7.4031 | 3.0365 | 3.9880 | 7.1251 | 2.9116 | 0.5263 | 4.1410 | 2.3403 |
| 0.0000 | 0.5273 | 4.6566 | 5.4112 | 1.9243 | 2.9938 | 1.1561 | 2.2941 |
| 0.6266 | 2.4256 | 1.9094 | 0.0000 | 7.8307 | 2.5890 | 0.8684 | 1.2310 |
| 4.9200 | 0.0000 | 2.3768 | 1.2143 | 2.1653 | 2.3532 | 4.2556 | 3.2557 |
| 1.3871 | 3.1476 | 3.5850 | 3.9985 | 4.9587 | 3.6416 | 2.4697 | 2.8123 |
| 2.5175 | 3.8060 | 1.3285 | 3.5592 | 3.7359 | 5.3748 | 2.3286 | 3.2484 |
| 2.4149 | 4.1037 | 0.0000 | 3.1441 | 0.0000 | 1.5194 | 1.9109 | 4.1368 |
| 6.8796 | 0.0000 | 0.0000 | 5.7232 | 3.0966 | 4.9040 | 6.5242 | 0.0000 |
| 2.9720 | 2.3479 | 6.3276 | 6.1294 | 5.8224 | 2.8976 | 5.9801 | 1.1689 |
| 3.1371 | 7.4879 | 2.4626 | 3.1454 | 4.9092 | 1.5425 | 3.9728 | 1.0225 |
| 6.8892 | 11.9722 | 5.1699 | 0.5821 | 4.3921 | 0.7796 | 1.9692 | 4.0884 |
| 10.6988 | 2.1803 | 0.5466 | 1.2980 | 4.8081 | 0.0000 | 3.3476 | 2.7155 |
| 5.0085 | 0.0000 | 3.4387 | 3.3188 | 2.4311 | 6.1932 | 9.2342 | 3.8705 |
| 0.0000 | 1.2218 | 0.0000 | 2.4500 | 2.9892 | 1.4486 | 3.5453 | 4.4378 |
| 7.9872 | 1.5498 | 1.0222 | 1.1623 | 0.0000 | 0.6064 | 3.7719 | 1.6179 |
| 2.1290 | 1.1634\ | | | | | | |
| CFP | 3.6089 | 5.2980 | 4.2769 | 4.9814 | 5.3949 | 3.7029 | 3.5251 |
| 5.9765 | 3.4322 | 5.5917 | 4.5343 | 2.9620 | 4.9549 | 3.5018 | 6.5761 |
| 5.2804 | 4.3124 | 4.3414 | 5.4061 | 3.9044 | 5.7229 | 7.7228 | 5.8964 |
| 5.3900 | 4.4007 | 6.3286 | 9.9430 | 8.2065 | 5.7822 | 3.8723 | 3.2143 |
| 5.2149 | 5.3720 | 4.0575 | 2.8081 | 4.4292 | 4.5574 | 4.3620 | 3.5753 |
| 3.0024 | 4.6775 | 5.7553 | 4.2756 | 4.9411 | 5.8054 | 6.9837 | 7.4671 |
| 5.2898 | 3.6750 | 3.8383 | 5.1266 | 6.6865 | 3.3357 | 5.7870 | 5.6656 |
| 3.7894 | 4.8384 | 5.6743 | 6.1762 | 2.6662 | 5.6383 | 4.9162 | 3.7792 |
| 4.3188 | 5.4587 | 5.6113 | 4.7097 | 4.2703 | 3.8855 | 5.6743 | 5.7430 |
| 3.7541 | 7.3395 | 8.2058 | 5.4080 | 4.7437 | 4.7185 | 9.8710 | 8.2977 |
| 5.3364 | 4.8983 | 2.5223 | 6.5486 | 6.0497 | 6.4692 | 4.3067 | 2.5771 |
| 6.1286 | 5.3627 | 4.7061 | 7.8127 | 3.9797 | 6.3494 | 7.0692 | 4.2317 |
| 6.7053 | 3.4086 | 4.8127 | 5.0874 | 5.7030 | 5.2185 | 6.0849 | 4.5239 |
| 5.8634 | 3.4531 | 2.9033 | 5.9634 | 5.5866 | 4.4598 | 5.8535 | 4.9903 |
| 6.5067 | 5.7248 | 4.3923 | 6.5726 | 5.5199 | 6.7706 | 3.5636 | 4.4122 |
| 6.3826 | 4.7335 | 4.1804 | 4.5307 | 7.1878 | 4.6193 | 5.8854 | 3.0773 |
| 4.1383 | 4.2553 | 4.1587 | 4.3086 | 3.3824 | 6.4866 | 7.2427 | 5.2172 |
| 7.0626 | 6.6203 | 8.4457 | 5.6748 | 4.0098 | 4.5050 | 4.7253 | 6.6704 |
| 4.3011 | 3.4154 | 3.5778 | 6.2583 | 6.9177 | 4.3821 | 3.9096 | 5.3111 |
| 7.6842 | 4.8005 | 4.9345 | 4.1392 | 3.5827 | 5.1855 | 4.1325 | 3.5644 |
| 6.3020 | 3.8132 | 6.6690 | 6.3922 | 4.9540 | 3.3669 | 3.2219 | 3.9594 |
| 4.9153 | 4.7339 | 5.6119 | 4.1220 | 3.8806 | 5.2595 | 4.7213 | 6.9484 |
| 3.0876 | 4.5949 | 6.3173 | 3.8547 | 10.6245 | 4.7174 | 3.2614 | 3.8705 |
| 5.1965 | 3.1691 | 4.2393 | 7.3417 | 5.8683 | 7.7001 | 4.3850 | 3.6795 |
| 5.6261 | 5.9590 | 4.7242 | 4.4353 | 5.4404 | 4.4868 | 4.3588 | 3.9250 |
| 4.7403 | 6.1881\ | | | | | | |
| AAAS | 10.5436 | 9.6079 | 9.3871 | 9.5434 | 10.5843 | 9.6285 | 9.6577 |

10.7453 10.3378 10.0744 9.5869 10.0913 10.2522 9.9517 9.7233
11.0462 10.4600 9.9816 9.6664 9.6001 10.0052 9.7938 9.9862
9.7381 9.6686 10.2574 9.7848 10.1311 9.6355 9.6592 10.2317
10.1749 10.2881 10.1645 9.8338 10.6621 9.7864 10.0073 10.3694
9.3186 9.5750 9.1652 9.7207 9.7247 9.0457 10.5452 10.5032
9.6162 10.0993 10.1096 9.6257 10.4502 9.8915 9.4057 10.3089
9.9971 9.5630 9.8012 9.0368 10.0276 9.0699 9.4897 9.9125
9.6536 9.9355 9.5722 10.9576 9.3975 9.5517 10.5434 9.8554
9.6402 10.2085 10.2625 9.6632 10.4821 10.2959 9.6065 10.2998
10.1172 10.2230 9.9166 9.7115 9.8581 10.4602 10.8389 10.1489
9.8039 9.2141 10.3754 9.3779 9.8665 10.1873 11.2234 10.4343
9.4898 8.6540 9.6110 9.2084 10.3552 10.7571 10.1322 10.0919
10.6443 10.5212 11.2489 10.6021 9.6323 9.1431 10.5030 9.8439
10.0509 10.0805 10.8431 9.3965 9.9620 9.4701 11.1044 9.8738
9.6536 10.7377 9.6984 9.5910 9.0216 10.0315 9.7387 9.7718
11.3379 9.3541 9.3686 10.0749 11.0451 10.1354 9.9672 9.9096
9.8393 10.5212 11.2034 9.8617 9.6149 10.3132 9.5124 10.1877
9.8735 9.8278 9.5232 9.6221 9.9601 10.0039 10.1283 9.8069
10.1565 9.5864 11.0513 9.8188 9.7984 9.5321 9.6719 10.2252
10.4898 10.2119 9.5856 10.1238 10.2718 9.9169 9.5585 11.1834
9.3590 9.6466 10.1804 9.7931 10.0919 10.1424 10.3324 10.6802
9.5430 10.2839 9.3039 10.2622 9.7535 10.6765 10.0630 10.5989
9.9155 9.7309 9.7474 9.5772 10.6724 10.1665 10.7709 9.9157
9.3312 9.7258 9.5688 10.0198 11.4548 9.8006 9.8337 9.5874
10.0413 10.3330\
HAMP 1.9260 5.5245 3.7286 3.5366 6.3544 3.5512 2.4779
1.7391 4.1811 0.0000 6.5307 1.3775 5.8365 0.4395 7.2120
6.2938 3.9084 5.2523 4.5817 4.8847 4.2194 3.3397 6.1191
5.2962 1.2653 3.3784 3.3638 7.0014 5.8842 2.0116 0.9954
4.9959 4.6233 3.4991 1.8080 2.3867 1.5255 0.0000 0.5410
1.0370 6.3540 4.8027 4.4989 4.9079 0.0000 8.3009 4.3808
2.5272 3.3102 1.1202 2.4400 5.1390 4.4985 1.4299 3.7985
0.0000 4.7225 6.6601 4.1257 2.6662 2.0680 6.4074 3.0948
0.0000 4.0651 5.1629 4.7377 3.2856 3.3013 2.3953 6.0250
0.9537 7.8654 4.5006 2.0547 0.8237 5.0191 5.7111 6.7078
5.9348 3.2700 1.8200 5.2932 4.0375 6.3881 4.3453 0.9284
6.9489 6.1705 3.5861 4.3886 3.7845 5.8479 7.6660 4.9308
0.6266 2.8322 2.2250 3.6349 4.4220 7.3846 5.1402 0.0000
1.8724 2.6265 0.8176 4.4676 4.4765 3.5800 5.7762 1.4385
2.5476 5.4414 3.8074 0.5141 2.7673 3.0116 1.7841 3.1581
5.5927 1.5136 1.0055 2.7935 3.3565 4.5889 4.0953 5.4783
3.0394 4.3594 2.1283 4.9879 3.0128 5.6167 3.8878 7.1576
8.3090 6.7385 6.7235 3.7687 3.4131 3.5028 5.1199 6.7792
4.0055 1.8493 4.8253 2.9153 5.1479 3.6905 0.7381 4.9131
7.7640 5.7005 3.8616 1.2540 4.2529 3.8471 0.6894 4.2122
6.2683 3.0551 4.0797 7.6279 6.6016 4.0044 2.2474 4.6023
4.5312 1.4679 2.3633 2.2163 0.0000 0.0000 0.0000 3.2253
0.8396 3.5224 1.9008 1.6980 5.5245 3.3081 4.4501 0.5257
4.9834 3.4586 7.1654 5.2926 5.9143 6.6209 3.6040 3.3015
1.7437 3.7581 6.4586 4.8196 7.3807 1.3603 2.7661 5.5028
5.5276 3.8093\
ZNF394 8.8106 9.2175 8.8203 9.1235 8.6871 9.2292 8.4800
9.4149 8.6162 8.7754 8.6391 8.6122 9.1536 8.5144 8.3814
9.2615 8.7825 9.0766 9.0505 9.0127 9.8266 8.4085 8.6944

| | | | | | | | |
|---|---|---|---|---|---|---|---|
| 8.4854 | 8.7579 | 8.7921 | 9.2491 | 8.7610 | 8.9021 | 8.1160 | 8.5066 |
| 9.2731 | 8.8661 | 9.4424 | 9.0316 | 8.9886 | 8.5718 | 8.7748 | 8.7994 |
| 8.2791 | 8.9987 | 9.1763 | 9.4985 | 8.6208 | 9.1832 | 9.4956 | 9.4077 |
| 8.4852 | 8.5268 | 9.4322 | 8.6965 | 9.1997 | 8.8214 | 9.1164 | 8.9715 |
| 8.7208 | 8.6387 | 9.1740 | 9.0657 | 8.3039 | 9.0846 | 9.0061 | 9.0903 |
| 8.3772 | 8.9063 | 8.8434 | 9.2603 | 8.0483 | 9.4252 | 9.3078 | 9.3893 |
| 8.3311 | 9.4519 | 9.3047 | 8.7315 | 8.9699 | 9.0628 | 8.2940 | 8.8811 |
| 8.4656 | 8.6323 | 8.2749 | 8.7603 | 8.9288 | 8.8222 | 9.2496 | 8.8191 |
| 8.6926 | 9.7730 | 8.6753 | 8.9995 | 8.9606 | 9.0998 | 9.4588 | 8.7858 |
| 8.7954 | 8.5669 | 9.2367 | 8.5987 | 9.0141 | 9.3340 | 8.6357 | 8.6020 |
| 9.3510 | 8.9327 | 8.4220 | 9.4129 | 8.8113 | 9.5452 | 8.8410 | 8.8232 |
| 8.6323 | 10.6368 | 10.1189 | 9.2802 | 8.5723 | 8.5285 | 9.0966 | 8.6502 |
| 9.7026 | 9.0216 | 9.0649 | 8.8043 | 8.5957 | 8.6886 | 8.4970 | 8.6366 |
| 9.7095 | 8.9960 | 8.3467 | 9.0014 | 8.7303 | 8.6788 | 8.5483 | 9.8305 |
| 9.6399 | 9.2136 | 9.8406 | 8.8082 | 9.2595 | 8.9783 | 8.8459 | 8.6759 |
| 8.2525 | 8.2918 | 9.1230 | 8.8740 | 8.9074 | 8.9793 | 9.3420 | 9.1837 |
| 9.6565 | 8.3910 | 9.2624 | 8.4627 | 8.1079 | 9.1257 | 8.6628 | 9.4855 |
| 8.6293 | 8.5242 | 8.9658 | 8.8162 | 9.0024 | 9.0994 | 8.1551 | 9.2410 |
| 8.5012 | 8.5776 | 9.4335 | 8.3788 | 9.0027 | 8.8556 | 8.9148 | 10.4824 |
| 8.4082 | 8.8481 | 9.1233 | 8.8410 | 8.8747 | 9.1618 | 9.1051 | 9.9384 |
| 9.0145 | 8.7190 | 8.6514 | 8.7991 | 8.7652 | 9.1936 | 8.6693 | 8.2851 |
| 8.9186 | 9.1577 | 8.7374 | 8.6824 | 9.6163 | 9.5908 | 9.2382 | 8.6085 |
| 8.6589 | 9.1314\ | | | | | | |
| SIDT1 | 3.3026 | 5.6060 | 7.8274 | 8.5430 | 3.6921 | 8.4048 | 3.2059 |
| 5.3625 | 5.4136 | 5.5482 | 4.8610 | 4.9148 | 5.4963 | 7.9878 | 9.4292 |
| 6.7739 | 2.7928 | 5.1689 | 6.6333 | 2.1915 | 5.3969 | 8.7714 | 6.9043 |
| 7.4354 | 4.7153 | 3.1429 | 3.5585 | 5.7274 | 7.6218 | 2.4720 | 2.9920 |
| 6.6764 | 2.7201 | 3.8818 | 5.0009 | 4.1139 | 8.7125 | 7.9532 | 1.7115 |
| 7.6067 | 5.2436 | 5.9646 | 1.3900 | 8.7599 | 4.6343 | 7.9361 | 8.8547 |
| 3.8179 | 4.2356 | 4.3620 | 8.1809 | 5.6294 | 8.2069 | 5.0710 | 6.3758 |
| 5.6281 | 4.3453 | 5.8093 | 5.7495 | 5.1894 | 5.6902 | 5.0654 | 4.6734 |
| 3.9398 | 6.1294 | 8.8048 | 7.0042 | 4.5159 | 6.4531 | 5.6439 | 6.3809 |
| 5.0104 | 6.7590 | 4.6208 | 6.8400 | 9.7290 | 8.1176 | 8.3871 | 7.5201 |
| 5.0880 | 7.6737 | 5.8003 | 8.6082 | 8.5608 | 7.5141 | 2.7877 | 2.0569 |
| 5.8784 | 4.8028 | 4.2851 | 5.5197 | 5.5523 | 5.7345 | 7.0493 | 8.5100 |
| 4.5756 | 2.8322 | 6.2103 | 9.2350 | 5.4658 | 8.9783 | 4.4630 | 6.3307 |
| 8.2717 | 9.1484 | 1.7170 | 6.8564 | 6.9301 | 5.6131 | 7.5741 | 8.4718 |
| 7.7431 | 1.3631 | 1.0000 | 4.1100 | 5.1639 | 6.0899 | 4.0013 | 10.1888 |
| 5.5120 | 6.1448 | 3.4023 | 5.0366 | 5.9234 | 7.6392 | 4.7510 | 6.0867 |
| 2.4149 | 6.0393 | 5.3441 | 2.7322 | 7.7904 | 8.5465 | 10.0200 | 4.8150 |
| 5.4910 | 4.8290 | 8.7387 | 7.3333 | 6.5181 | 7.9871 | 7.1091 | 7.9122 |
| 7.2399 | 7.6110 | 3.8753 | 7.5160 | 6.6734 | 6.2655 | 7.2790 | 7.3477 |
| 6.8213 | 8.1417 | 5.8741 | 2.2306 | 4.8215 | 5.9812 | 5.1292 | 4.9975 |
| 4.5437 | 2.9615 | 5.2724 | 7.0735 | 6.0477 | 8.1410 | 6.8470 | 7.7770 |
| 7.2662 | 8.4136 | 7.5757 | 5.7320 | 4.6893 | 6.5517 | 6.4315 | 8.7095 |
| 6.1712 | 4.9412 | 5.4114 | 9.4572 | 8.6106 | 3.8670 | 4.4901 | 4.9284 |
| 6.6920 | 6.2406 | 7.7231 | 4.8715 | 4.8774 | 7.1981 | 4.5732 | 7.4001 |
| 6.9387 | 5.2151 | 4.5865 | 7.9957 | 4.5374 | 4.5843 | 7.1156 | 10.0898 |
| 7.6229 | 6.1508\ | | | | | | |
| SERPINA10 | | 0.0000 | 0.0000 | 1.7871 | 0.0000 | 0.0000 | 0.0000 |
| 0.0000 | 0.0000 | 0.0000 | 1.2815 | 0.0000 | 0.4849 | 0.4363 | 0.0000 |
| 0.0000 | 0.0000 | 0.6221 | 3.8641 | 0.5838 | 0.6732 | 0.0000 | 2.0613 |
| 0.6346 | 0.0000 | 0.0000 | 0.0000 | 0.0000 | 0.0000 | 0.0000 | 0.0000 |
| 0.0000 | 0.0000 | 0.0000 | 1.7182 | 0.5853 | 0.0000 | 0.0000 | 0.0000 |

```
0.0000  0.0000  0.0000  0.8506  0.0000  0.0000  3.6322  1.4889
2.4538  0.0000  0.0000  0.0000  0.0000  0.0000  0.0000  0.0000
0.0000  0.0000  0.0000  3.4055  0.0000  0.5715  0.0000  0.0000
0.9583  0.5968  1.1445  0.0000  0.5980  0.0000  1.3082  0.0000
0.0000  0.0000  0.0000  0.0000  2.4611  0.0000  0.5548  0.9332
1.7868  0.6159  0.0000  0.0000  1.8442  0.0000  0.5263  0.6199
0.0000  0.0000  1.2163  0.9113  0.0000  0.0000  0.0000  1.1561
0.0000  0.0000  1.8574  0.0000  0.0000  0.0000  0.0000  2.3585
0.0000  0.7358  0.0000  0.0000  0.0000  0.0000  1.2448  0.0000
0.0000  0.0000  0.0000  0.0000  0.5141  0.0000  0.0000  0.0000
0.3974  0.0000  0.0000  0.0000  0.0000  0.0000  1.8330  0.0000
0.0000  0.0000  0.9653  0.0000  0.3935  0.0000  0.0000  0.0000
0.8107  0.0000  0.0000  0.0000  0.5216  0.0000  0.0000  0.0000
0.0000  0.0000  0.0000  2.2162  1.1308  0.0000  0.0000  0.0000
0.0000  0.0000  0.9341  0.0000  0.0000  0.0000  0.0000  0.3855
0.0000  0.0000  0.0000  0.6138  0.5821  0.0000  0.0000  0.0000
0.0000  0.0000  0.0000  0.0000  0.0000  0.0000  0.0000  0.0000
0.0000  0.0000  0.9063  0.0000  0.7148  1.0692  0.0000  0.0000
1.2133  0.8792  1.2218  0.0000  1.3150  0.0000  0.6567  0.0000
0.0000  0.0000  0.5674  0.4262  0.0000  2.8198  0.0000  2.0583
0.0000  1.8199  0.0000\
SIDT2   8.5693  10.0078 8.9533  9.7274  9.7731  9.5896  10.2176
9.8963  8.9016  10.0097 9.0223  9.7247  10.1883 9.3975  9.3232
9.6794  9.4835  10.1042 10.1323 10.6429 10.4175 9.3038  9.6510
9.3602  10.2212 10.6578 7.7289  9.2175  8.8636  9.6715  8.9871
9.2075  9.3874  9.8546  8.8347  8.9011  10.1628 9.2371  8.6835
11.1517 9.6413  10.2376 9.7532  7.6291  10.4245 8.5565  8.9606
10.1719 9.1185  9.8970  9.3983  10.4986 9.4006  10.3782 9.3155
9.6583  9.2200  9.1412  9.5394  9.9835  10.3027 9.1576  9.1499
9.6503  10.4810 9.1058  9.6211  9.2100  9.5778  9.7510  9.3297
9.3164  10.3126 9.8233  9.7301  9.5690  9.4028  10.2578 9.7599
9.7446  9.4404  8.3688  9.7292  9.0899  9.5537  10.3563 8.3458
9.1919  10.2994 9.3238  10.6384 9.4905  10.2725 9.7677  8.7640
10.5626 10.5380 10.9402 9.9629  9.4098  8.9453  9.2027  8.7743
9.7100  9.9985  9.0179  10.8777 9.1851  9.7994  9.0276  9.4088
9.6678  10.7411 10.6349 9.4479  9.3028  9.1135  9.6210  9.7294
9.6897  10.1355 9.5127  9.3388  9.3093  9.9085  8.8409  8.6172
10.3317 10.2994 10.3193 9.1579  8.7789  9.4406  8.7430  8.4887
10.1108 9.7458  9.6472  10.0903 10.9082 9.8548  9.6745  9.8063
9.1938  9.2521  11.1934 8.8645  10.4797 9.5414  10.2069 9.9823
10.0228 9.5595  9.5308  8.2709  10.1625 10.4105 9.7770  9.1931
10.7389 9.7232  9.0830  9.7357  9.5385  9.2605  9.4058  8.0000
9.5991  9.1649  8.8038  9.1260  9.8391  9.1404  8.8222  9.0012
8.7346  9.9252  10.3973 9.2799  9.9215  9.1417  10.1189 9.3296
9.9183  9.4122  9.5768  10.2611 8.9381  8.5465  10.0125 8.9929
9.4709  9.2092  8.9552  9.8327  9.0444  9.4768  10.5562 9.0132
9.6568  8.9018\
SNORD115-7      0.0000  0.0000  0.0000  0.0000  0.0000  0.0000
0.0000  0.0000  0.0000  0.0000  0.0000  0.0000  0.0000  0.0000
0.0000  0.0000  0.0000  0.0000  0.0000  0.0000  0.0000  0.0000
0.0000  0.0000  0.0000  0.0000  0.0000  0.0000  0.0000  0.0000
0.0000  0.0000  0.0000  0.0000  0.0000  0.0000  0.0000  0.0000
0.0000  0.0000  0.0000  0.0000  0.0000  0.0000  0.0000  0.0000
0.0000  0.0000  0.0000  0.0000  0.0000  0.0000  0.0000  0.0000
```

| | | | | | | | |
|---|---|---|---|---|---|---|---|
| 0.0000 | 0.0000 | 0.0000 | 0.0000 | 0.0000 | 0.0000 | 0.0000 | 0.0000 |
| 0.0000 | 0.0000 | 0.0000 | 0.0000 | 0.0000 | 0.0000 | 0.0000 | 0.0000 |
| 0.0000 | 0.0000 | 0.0000 | 0.0000 | 0.0000 | 0.0000 | 0.0000 | 0.0000 |
| 0.0000 | 0.0000 | 0.0000 | 0.0000 | 0.0000 | 0.0000 | 0.0000 | 0.0000 |
| 0.0000 | 0.0000 | 0.0000 | 0.0000 | 0.0000 | 0.0000 | 0.0000 | 0.0000 |
| 0.0000 | 0.0000 | 0.0000 | 0.0000 | 0.0000 | 0.0000 | 0.0000 | 0.0000 |
| 0.0000 | 0.0000 | 0.0000 | 0.0000 | 0.0000 | 0.0000 | 0.0000 | 0.0000 |
| 0.0000 | 0.0000 | 0.0000 | 0.0000 | 0.0000 | 0.0000 | 0.0000 | 0.0000 |
| 0.0000 | 0.0000 | 0.0000 | 0.0000 | 0.0000 | 0.0000 | 0.0000 | 0.0000 |
| 0.0000 | 0.0000 | 0.0000 | 0.0000 | 0.0000 | 0.0000 | 0.0000 | 0.0000 |
| 0.0000 | 0.0000 | 0.0000 | 0.0000 | 0.0000 | 0.0000 | 0.0000 | 0.0000 |
| 0.0000 | 0.0000 | 0.0000 | 0.0000 | 0.0000 | 0.0000 | 0.0000 | 0.0000 |
| 0.0000 | 0.0000 | 0.0000 | 0.0000 | 0.0000 | 0.0000 | 0.0000 | 0.0000 |
| 0.0000 | 0.0000 | 0.0000 | 0.0000 | 0.0000 | 0.0000 | 0.0000 | 0.0000 |
| 0.0000 | 0.0000 | 0.0000 | 0.0000 | 0.0000 | 0.0000 | 0.0000 | 0.0000 |
| 0.0000 | 0.0000 | 0.0000 | 0.0000 | 0.0000 | 0.0000 | 0.0000 | 0.0000 |
| 0.0000 | 0.0000 | 0.0000 | 0.0000 | 0.0000 | 0.0000 | 0.0000 | 0.0000 |
| 0.0000 | 0.0000 | 0.0000 | 0.0000 | 0.0000 | 0.0000 | 0.0000 | 0.0000 |
| 0.3736 | 0.0000 | 0.0000\ | | | | | |
| TAF8 | 9.3925 | 8.6519 | 8.9475 | 8.6017 | 9.0657 | 9.7385 | 9.6205 |
| 9.7150 | 8.9732 | 8.6665 | 9.0451 | 9.6045 | 9.2802 | 8.9584 | 8.6962 |
| 8.5686 | 9.4224 | 8.7952 | 8.7927 | 8.6704 | 9.8198 | 8.4887 | 8.7983 |
| 8.6744 | 8.3204 | 8.9143 | 8.4820 | 8.4552 | 8.2142 | 9.3751 | 9.0907 |
| 8.3872 | 9.4371 | 9.4256 | 9.8774 | 8.6167 | 9.0749 | 9.2963 | 8.7377 |
| 8.9174 | 8.5444 | 8.6035 | 8.1908 | 9.8393 | 8.4176 | 8.1439 | 8.8745 |
| 9.1663 | 8.3754 | 8.8612 | 8.4495 | 8.4995 | 8.9580 | 8.8813 | 9.2613 |
| 8.2119 | 8.7146 | 8.7701 | 8.9693 | 9.0310 | 8.4660 | 9.2652 | 9.1586 |
| 9.1437 | 8.5627 | 8.9167 | 8.9738 | 10.1189 | 8.8305 | 8.9644 | 9.2054 |
| 8.8662 | 8.6856 | 9.4399 | 8.7208 | 8.5749 | 9.5454 | 8.1923 | 8.1958 |
| 9.0949 | 8.5045 | 9.5630 | 8.5520 | 8.7174 | 8.6652 | 8.9400 | 10.5630 |
| 8.3496 | 8.7696 | 8.8671 | 8.7402 | 8.9881 | 8.8761 | 8.4252 | 9.2218 |
| 8.2734 | 7.8544 | 8.6145 | 8.4959 | 8.5468 | 8.9296 | 8.6987 | 9.3708 |
| 8.3620 | 8.7606 | 9.0199 | 7.9382 | 8.9851 | 8.7243 | 9.0011 | 8.6451 |
| 9.1403 | 8.6281 | 8.6257 | 8.0176 | 9.3336 | 8.4988 | 9.4332 | 8.7104 |
| 8.4946 | 9.4673 | 8.8392 | 9.0340 | 8.5007 | 8.4682 | 8.9872 | 8.6424 |
| 9.3400 | 8.4960 | 8.6878 | 7.9512 | 8.5917 | 8.9397 | 8.3520 | 8.6706 |
| 9.6778 | 9.5590 | 7.1604 | 9.0384 | 8.8784 | 8.5374 | 7.8521 | 8.7233 |
| 8.9226 | 8.8252 | 8.6134 | 8.8831 | 8.4289 | 8.5610 | 8.5173 | 8.8803 |
| 8.1480 | 9.0100 | 9.7950 | 8.2733 | 9.2407 | 8.9486 | 9.0851 | 8.8838 |
| 8.9759 | 8.6737 | 8.8660 | 8.7192 | 8.6850 | 8.4062 | 8.7786 | 8.3851 |
| 8.9710 | 8.6220 | 9.4383 | 9.0700 | 9.2795 | 8.8385 | 8.9372 | 8.0825 |
| 8.7775 | 8.3897 | 8.4141 | 8.9214 | 8.8663 | 8.4460 | 8.7497 | 9.2656 |
| 8.4552 | 9.4687 | 9.0018 | 9.4127 | 9.8908 | 8.9763 | 9.5510 | 8.3666 |
| 9.0988 | 9.5515 | 9.5116 | 9.3800 | 8.8879 | 8.6576 | 8.5709 | 8.9666 |
| 8.9460 | 8.0936\ | | | | | | |
| PISRT1 | 0.0000 | 0.0000 | 0.0000 | 0.0000 | 0.0000 | 0.0000 | 0.0000 |
| 0.0000 | 0.0000 | 0.0000 | 0.0000 | 0.0000 | 0.0000 | 0.0000 | 0.0000 |
| 0.0000 | 0.0000 | 0.0000 | 0.0000 | 0.0000 | 0.0000 | 0.0000 | 0.0000 |
| 0.0000 | 0.0000 | 0.6062 | 0.0000 | 0.0000 | 0.0000 | 0.0000 | 0.0000 |
| 0.0000 | 0.0000 | 0.0000 | 0.0000 | 0.0000 | 0.0000 | 0.0000 | 0.0000 |
| 0.0000 | 0.0000 | 0.0000 | 0.0000 | 0.0000 | 0.0000 | 0.0000 | 0.0000 |
| 0.0000 | 0.0000 | 0.0000 | 0.0000 | 0.0000 | 0.0000 | 0.0000 | 0.0000 |
| 0.0000 | 0.0000 | 0.0000 | 0.0000 | 0.0000 | 0.0000 | 0.0000 | 0.0000 |
| 0.0000 | 0.0000 | 0.0000 | 0.0000 | 0.0000 | 0.0000 | 0.0000 | 0.0000 |

```
0.0000  0.0000  0.0000  0.0000  0.0000  0.0000  0.0000  0.0000
0.0000  0.0000  0.0000  0.0000  0.0000  0.0000  0.0000  0.0000
0.0000  0.0000  0.0000  0.0000  0.0000  0.0000  0.0000  0.0000
0.0000  0.0000  0.0000  0.0000  0.0000  0.0000  0.0000  0.0000
0.0000  0.0000  0.0000  0.0000  0.0000  0.0000  0.0000  0.0000
0.0000  0.0000  0.0000  0.0000  0.0000  0.0000  0.4321  0.0000
0.0000  0.0000  0.0000  0.0000  0.0000  0.0000  0.0000  0.0000
0.0000  0.0000  0.0000  0.0000  0.0000  0.0000  0.0000  0.0000
0.0000  0.0000  0.0000  0.0000  0.0000  0.0000  0.0000  0.0000
0.0000  0.0000  0.0000  0.0000  0.0000  0.0000  0.0000  0.0000
0.0000  0.0000  0.0000  0.0000  0.0000  0.0000  0.0000  0.0000
0.0000  0.0000  0.0000  0.0000  0.0000  0.0000  0.0000  0.0000
0.0000  0.0000  0.0000  0.0000  0.0000  0.0000  0.0000  0.0000
0.0000  0.0000  0.0000  0.0000  0.0000  0.0000  0.0000  0.0000
0.0000  0.0000  0.0000  0.0000  0.0000  0.0000  0.0000  0.0000
0.0000  0.0000  0.0000  0.0000  0.0000  0.0000  0.0000  0.0000
0.0000  0.6959\
SIX4    8.1273  7.0305  8.5453  9.4904  8.4689  9.6207  9.0692
9.3090  8.3972  8.2154  9.4305  9.0692  9.1155  8.6434  9.3018
7.9691  8.8328  8.9717  7.3276  7.9905  8.7191  9.6971  8.5928
8.8485  5.2467  8.1736  6.6583  8.1888  7.5553  9.1395  5.2517
8.8346  9.0668  9.3296  8.4145  6.3989  9.3918  8.9069  8.5172
10.5263 9.5871  7.8476  8.6330  9.4168  5.3355  7.9985  8.1904
8.0029  8.7320  8.0160  8.7080  8.7327  8.1732  6.4142  9.0188
9.3024  8.8188  7.1710  8.3412  9.6124  6.1078  6.9245  9.6193
8.5936  7.9470  9.6260  9.8420  9.8099  7.8750  9.7872  7.7859
8.7380  7.5956  8.7356  8.7959  9.6552  9.2949  9.4322  9.2350
9.1241  8.8965  9.6497  9.1927  9.5291  8.8527  9.5883  10.2216
8.6981  9.3353  8.1029  6.5387  9.0173  8.1474  8.7161  9.1597
3.0128  10.1047 7.1171  10.1927 8.1490  8.6936  9.1947  9.8522
10.1078 9.9229  9.9987  5.9169  10.0950 9.3968  9.7164  9.0214
9.6246  7.9984  7.6582  9.0326  9.0515  9.5496  10.5129 9.9056
8.3742  9.8710  9.3262  8.1341  9.5389  8.4430  9.5943  10.3477
8.0515  7.8309  7.0361  8.3662  9.8579  8.7097  9.8534  6.6409
6.4748  8.4313  6.8457  8.3938  7.1710  9.5848  8.5506  9.4134
7.6139  8.2090  6.9917  9.8651  6.8561  9.6295  8.6503  7.8395
8.7213  8.9830  9.3503  7.8338  9.3853  6.7178  9.5303  9.6547
6.9383  7.7172  7.4551  9.2985  8.8951  9.2939  10.4525 8.4705
7.6260  9.5965  9.7405  8.2841  9.1828  8.8154  8.4144  7.6741
8.6199  7.7727  6.0929  10.3238 9.6209  8.2061  7.6378  9.7859
9.2375  8.3344  9.0125  9.6206  7.4472  8.7283  8.1904  8.3886
9.1840  8.7846  7.2434  9.5548  7.7491  9.2630  7.1904  9.7724
9.3659  7.3470\
LOC283070       5.7603  9.0887  7.9179  5.7878  6.4303  7.7358
8.9233  8.7406  7.2052  7.3899  6.7555  7.0361  7.9675  8.6253
8.2225  6.3557  7.7840  5.9958  9.0176  6.8060  8.8584  7.4557
8.0771  8.1021  7.3479  4.6451  9.3002  7.6227  8.6033  8.8483
10.6257 5.5584  8.3374  8.5628  6.9198  4.6536  8.6157  8.0139
4.5699  8.8122  6.1668  7.0171  7.4284  8.1859  7.9215  6.6492
7.5679  8.6136  6.3945  8.5067  7.5604  7.1154  7.7504  7.8384
8.0925  7.1507  5.7843  8.0929  8.7718  6.2268  7.8223  7.7247
7.0908  7.4487  7.7006  6.2760  8.2865  9.8195  7.0647  7.7525
7.0295  6.7813  7.7083  9.8442  7.8507  8.5350  7.3921  5.5242
7.2097  7.4478  7.1857  5.1870  8.6530  8.6657  6.1406  8.8010
```

| | | | | | | | |
|---|---|---|---|---|---|---|---|
| 7.5611 | 6.1610 | 8.0295 | 10.6780 | 7.9386 | 5.9658 | 10.0250 | 8.4329 |
| 5.9069 | 7.7430 | 8.9297 | 7.5754 | 6.0384 | 6.8075 | 8.7033 | 7.8280 |
| 9.5976 | 7.6672 | 5.2638 | 8.1646 | 7.1659 | 7.6813 | 8.2661 | 7.4469 |
| 5.3792 | 9.2180 | 7.4938 | 4.9069 | 8.6469 | 8.4578 | 8.2949 | 6.7528 |
| 9.1947 | 6.8579 | 8.4319 | 6.6974 | 7.2119 | 7.1802 | 7.7567 | 9.2878 |
| 9.3465 | 7.7896 | 8.1000 | 7.2349 | 9.4705 | 8.6958 | 5.7849 | 7.7007 |
| 7.3298 | 5.9983 | 8.2601 | 8.3905 | 6.8685 | 7.3149 | 7.8419 | 7.5694 |
| 6.9551 | 6.9462 | 6.0216 | 6.5262 | 9.0559 | 7.6199 | 8.0527 | 6.9840 |
| 8.4346 | 7.5023 | 7.3376 | 6.5274 | 7.0507 | 7.8445 | 7.9886 | 5.6961 |
| 8.4326 | 6.0276 | 4.7609 | 6.1711 | 6.5612 | 8.3690 | 5.7461 | 6.7273 |
| 9.4729 | 8.0474 | 8.5959 | 9.7051 | 7.8133 | 7.2895 | 9.4480 | 7.5239 |
| 6.4013 | 9.3285 | 6.6843 | 8.0051 | 9.9984 | 8.6207 | 5.9415 | 6.2796 |
| 8.4657 | 5.7174 | 8.2422 | 7.2241 | 7.6106 | 7.7304 | 7.7356 | 5.6989 |
| 6.4648 | 5.8423 | 6.8115 | 6.9586 | 6.2734 | 7.8282 | 8.7268 | 7.9034 |
| 8.3124 | 8.2249 | 6.2482\ | | | | | |
| SIX6 | 0.0000 | 0.0000 | 0.0000 | 0.0000 | 0.0000 | 0.0000 | 0.5431 |
| 1.7391 | 0.0000 | 0.0000 | 0.4291 | 0.0000 | 0.0000 | 0.0000 | 0.0000 |
| 0.0000 | 0.0000 | 0.0000 | 0.0000 | 0.0000 | 0.0000 | 0.0000 | 0.0000 |
| 0.0000 | 0.0000 | 0.0000 | 0.4561 | 0.0000 | 0.0000 | 0.0000 | 0.0000 |
| 0.0000 | 0.0000 | 0.0000 | 0.0000 | 0.0000 | 1.2685 | 0.0000 | 0.0000 |
| 0.0000 | 0.0000 | 0.0000 | 0.0000 | 0.0000 | 0.0000 | 0.0000 | 0.0000 |
| 0.0000 | 0.0000 | 0.0000 | 0.0000 | 0.0000 | 0.6494 | 0.0000 | 0.0000 |
| 0.0000 | 0.0000 | 0.0000 | 0.0000 | 0.0000 | 0.0000 | 0.0000 | 0.0000 |
| 0.5968 | 0.0000 | 0.0000 | 0.0000 | 2.1953 | 0.0000 | 0.6160 | 0.4334 |
| 0.0000 | 0.0000 | 0.0000 | 0.0000 | 0.0000 | 3.4396 | 0.0000 | 0.0000 |
| 0.0000 | 0.0000 | 0.0000 | 0.0000 | 0.0000 | 0.0000 | 0.0000 | 0.0000 |
| 0.0000 | 0.0000 | 0.0000 | 0.0000 | 0.0000 | 3.2128 | 0.0000 | 0.0000 |
| 0.0000 | 0.5233 | 0.0000 | 0.0000 | 0.0000 | 0.0000 | 0.0000 | 0.0000 |
| 0.0000 | 0.0000 | 0.0000 | 0.0000 | 0.4315 | 0.0000 | 0.0000 | 0.0000 |
| 0.0000 | 0.0000 | 0.0000 | 0.0000 | 0.0000 | 0.0000 | 0.4321 | 0.0000 |
| 0.0000 | 3.9165 | 0.0000 | 0.6224 | 0.0000 | 0.0000 | 0.0000 | 0.0000 |
| 0.0000 | 0.0000 | 0.0000 | 0.0000 | 0.6684 | 0.0000 | 0.7571 | 0.0000 |
| 0.0000 | 0.0000 | 0.0000 | 0.0000 | 0.0000 | 0.0000 | 0.0000 | 0.0000 |
| 0.0000 | 0.0000 | 0.0000 | 0.0000 | 0.5454 | 0.9947 | 0.7381 | 0.0000 |
| 0.0000 | 0.0000 | 0.0000 | 0.0000 | 0.0000 | 0.0000 | 0.0000 | 0.6000 |
| 0.0000 | 0.0000 | 0.0000 | 0.0000 | 0.0000 | 0.0000 | 0.0000 | 0.0000 |
| 0.0000 | 0.0000 | 0.0000 | 0.0000 | 0.0000 | 3.3997 | 0.0000 | 0.0000 |
| 1.3669 | 0.9063 | 0.0000 | 0.4012 | 0.0000 | 0.0000 | 0.0000 | 0.0000 |
| 0.0000 | 0.0000 | 0.6248 | 0.0000 | 0.0000 | 0.0000 | 0.0000 | 0.0000 |
| 1.2686 | 0.0000 | 0.0000 | 0.0000 | 0.0000 | 0.0000 | 0.0000 | 0.0000 |
| 0.0000 | 0.0000\ | | | | | | |
| PM20D1 | 0.0000 | 3.9738 | 4.4886 | 0.4935 | 4.1161 | 0.7223 | 0.0000 |
| 5.2635 | 0.4327 | 1.6538 | 0.7594 | 0.0000 | 3.0112 | 0.4395 | 0.0000 |
| 0.0000 | 0.0000 | 1.2795 | 2.3192 | 7.3036 | 1.0485 | 1.1310 | 3.1268 |
| 1.4650 | 3.5562 | 1.6270 | 0.4561 | 2.0314 | 3.7973 | 0.0000 | 0.0000 |
| 0.5612 | 0.0000 | 0.0000 | 0.0000 | 0.6819 | 1.5255 | 0.6557 | 0.0000 |
| 0.0000 | 2.3500 | 0.0000 | 1.0569 | 0.0000 | 3.0451 | 1.8914 | 0.0000 |
| 5.7709 | 0.7769 | 0.0000 | 0.4530 | 2.5833 | 0.6494 | 2.1337 | 0.4645 |
| 0.5608 | 0.6343 | 1.1710 | 1.7655 | 1.7785 | 3.2731 | 1.7937 | 0.0000 |
| 0.5968 | 2.0096 | 1.6675 | 1.6109 | 0.0000 | 0.5773 | 0.6160 | 0.4334 |
| 0.7001 | 4.3524 | 3.9224 | 2.7779 | 0.0000 | 1.2673 | 0.5408 | 1.7868 |
| 0.0000 | 0.0000 | 0.3965 | 0.0000 | 2.3651 | 1.4651 | 2.6709 | 2.9586 |
| 1.0433 | 2.1793 | 0.0000 | 0.0000 | 1.7639 | 2.5333 | 0.0000 | 0.5198 |
| 2.6869 | 0.9065 | 4.0548 | 0.0000 | 0.9478 | 1.0805 | 0.4986 | 1.4835 |

```
1.8724  0.4835  0.4659  3.6115  1.4556  0.5426  0.5877  0.5136
0.0000  4.5244  0.0000  0.0000  0.0000  0.0000  0.4321  0.3974
2.7604  0.4553  0.0000  0.0000  0.4700  6.1227  2.1941  0.6149
0.0000  3.2589  3.1713  1.6759  0.0000  0.0000  0.0000  2.0060
0.0000  2.5939  0.0000  1.8532  2.9476  0.0000  0.5748  1.7133
1.7146  0.8022  4.3962  0.6735  4.0144  0.0000  0.0000  2.9370
1.5604  0.5414  1.0019  0.0000  0.7727  2.4071  0.6894  0.6000
0.5416  0.0000  1.0431  0.5821  0.0000  0.4418  0.5023  0.0000
2.9510  1.6815  0.5466  0.0000  0.5290  0.0000  0.6229  0.0000
0.4801  2.8318  2.9187  0.7148  2.4311  0.5545  7.6554  0.0000
1.1756  0.0000  1.6636  0.5811  0.7058  0.6567  7.1468  2.3837
0.0000  0.0000  0.0000  0.0000  0.0000  2.6380  4.1603  0.3736
1.1797  0.0000\
MR1     6.7343  6.2795  8.4373  8.0603  6.5843  7.9386  5.5170
6.5921  7.1097  6.8153  6.9580  6.9028  7.9345  6.9359  7.5267
6.2290  5.7503  3.9331  6.4145  4.4072  7.1500  6.3280  6.4277
7.7652  6.4979  6.1807  2.5443  6.6486  7.1994  6.2050  4.6731
6.5936  6.2195  5.9470  6.3407  3.8970  8.3530  8.3930  6.4504
6.7066  5.7308  7.1535  3.9114  7.4261  6.7652  6.7373  5.6877
7.3059  5.5770  6.9841  8.5720  6.3755  8.1093  6.9423  7.7973
5.4977  6.6255  7.3403  7.8901  5.2273  6.7575  8.2828  3.4464
6.6931  6.4321  8.0016  6.5697  7.0516  6.1869  7.5295  8.1872
8.4021  8.3677  5.0894  8.1793  6.8496  5.7297  6.5440  7.6268
6.6972  5.6575  6.1607  5.4077  8.1158  6.9314  7.2465  7.3774
7.2142  6.0411  7.1310  6.9282  7.5902  7.2361  5.4991  6.2241
7.4472  7.1476  6.6137  6.6377  6.5952  4.9062  6.2427  7.0556
5.6696  6.0933  6.6942  4.8108  6.4387  7.6511  5.4998  7.8086
7.0777  4.1934  4.6439  8.7609  6.4265  7.8548  7.7272  7.4673
5.8242  4.8679  6.5808  6.7303  6.4409  6.6279  7.2360  6.1202
4.4556  7.6096  7.3362  8.5269  5.3180  7.7244  6.5251  5.8470
6.1134  6.2372  3.9958  6.7034  6.7268  5.9658  8.4185  6.0435
7.1125  7.2548  7.2979  8.1952  6.7554  7.2561  7.0868  7.3440
6.1533  7.9128  6.1837  6.6608  7.3633  6.7502  6.2545  6.0213
6.2768  6.5540  7.3370  6.3664  7.4970  3.9044  6.7273  4.3164
8.0955  6.6168  6.3987  6.5657  6.4342  3.7802  7.1044  6.7574
7.6437  5.9789  6.6356  7.2143  6.0874  7.0314  7.3965  5.2450
6.9634  6.5990  7.8163  7.4439  6.5796  6.4789  5.3500  5.5798
9.1330  5.8182  7.0771  8.0003  5.6289  4.7334  6.7976  8.4493
6.6756  6.0191\
MGC4473 2.0932  0.9197  3.0624  0.8605  1.4681  0.4059  1.7160
3.9097  3.0610  4.8885  0.4291  1.1366  0.4363  2.4930  2.4639
2.6237  2.0822  0.0000  0.5838  0.3755  0.0000  4.6553  0.0000
4.4677  1.9289  0.0000  0.4561  3.0366  0.0000  3.9686  0.7334
0.0000  0.0000  0.0000  0.0000  0.6819  9.5583  2.6272  2.0653
2.9378  1.1184  0.0000  0.0000  2.5595  0.0000  2.6826  0.0000
1.7580  6.7215  2.0462  0.0000  1.9460  3.3357  0.5094  7.9177
6.1751  0.0000  0.8754  1.0204  2.2896  0.5422  2.6657  4.1678
3.2013  4.8197  3.7482  1.8352  5.6417  5.7324  4.5133  0.4334
0.7001  0.0000  0.0000  0.9270  0.4698  8.3280  2.3485  1.7868
0.0000  9.2998  7.5396  0.0000  3.0210  2.6513  5.8689  3.8172
1.6425  0.0000  2.3114  0.0000  3.6983  0.0000  3.5917  4.2646
0.6266  1.2090  0.9402  2.7914  0.0000  2.2933  7.4116  4.7574
1.5830  3.2265  3.5453  0.0000  5.3968  2.5905  3.1772  0.0000
0.6215  5.0883  3.7004  0.8924  0.6116  1.6815  7.7763  9.6552
```

| | | | | | | | |
|---|---|---|---|---|---|---|---|
| 0.6089 | 5.7859 | 5.4702 | 1.0557 | 0.4700 | 0.0000 | 1.2272 | 8.0184 |
| 2.4149 | 1.5385 | 1.9820 | 5.5187 | 2.6562 | 1.2631 | 9.4472 | 0.8107 |
| 0.0000 | 0.0000 | 0.0000 | 0.9038 | 0.0000 | 1.0240 | 0.9848 | 3.7587 |
| 3.5232 | 2.1200 | 0.5418 | 1.4775 | 0.5454 | 5.5744 | 5.1593 | 1.4139 |
| 2.5542 | 2.4734 | 1.0019 | 0.0000 | 3.9889 | 1.2837 | 5.1542 | 1.3489 |
| 0.5416 | 0.0000 | 0.0000 | 2.6931 | 2.6230 | 8.1896 | 5.5360 | 0.0000 |
| 3.0096 | 1.8676 | 5.5135 | 0.4486 | 0.5290 | 5.2423 | 5.9545 | 0.0000 |
| 0.4801 | 4.6208 | 0.0000 | 1.9582 | 4.3764 | 0.0000 | 3.2614 | 3.3548 |
| 2.2560 | 0.0000 | 3.2738 | 0.0000 | 3.2020 | 1.1063 | 3.6605 | 0.0000 |
| 7.6984 | 3.5951 | 1.2477 | 5.7280 | 0.0000 | 0.0000 | 0.6112 | 0.6701 |
| 1.1797 | 1.7990\ | | | | | | |
| SIX3 | 2.6167 | 0.0000 | 6.6741 | 3.3759 | 0.5278 | 4.8224 | 5.4880 |
| 6.7096 | 2.7963 | 0.4413 | 4.9606 | 6.0295 | 8.9626 | 0.0000 | 4.2186 |
| 0.0000 | 2.5489 | 0.7772 | 0.5838 | 7.4965 | 0.0000 | 4.3249 | 0.6346 |
| 2.3107 | 0.0000 | 3.5809 | 0.4561 | 1.0210 | 0.0000 | 0.0000 | 0.0000 |
| 0.0000 | 0.0000 | 3.3450 | 6.5323 | 0.0000 | 4.2000 | 0.0000 | 0.9336 |
| 0.0000 | 0.6649 | 0.0000 | 0.0000 | 6.5000 | 0.0000 | 5.2147 | 2.8871 |
| 0.0000 | 1.0498 | 0.0000 | 0.0000 | 5.8771 | 9.4564 | 0.0000 | 1.0973 |
| 0.9635 | 4.8930 | 4.3056 | 1.0204 | 6.2361 | 0.0000 | 6.2132 | 0.5573 |
| 2.4883 | 0.0000 | 3.0811 | 3.1217 | 6.8820 | 0.0000 | 1.3773 | 1.0365 |
| 0.0000 | 0.9449 | 2.9425 | 0.5367 | 2.2778 | 10.0384 | 4.9335 | 3.5882 |
| 4.4789 | 0.9714 | 3.1552 | 3.3316 | 4.1381 | 0.5263 | 2.0776 | 0.0000 |
| 5.1705 | 5.6930 | 6.8407 | 1.4126 | 0.4848 | 2.9127 | 0.0000 | 0.0000 |
| 0.0000 | 3.6286 | 0.5454 | 1.6394 | 4.6790 | 0.0000 | 0.4986 | 6.4242 |
| 3.1674 | 2.7166 | 1.8753 | 1.5756 | 2.9948 | 2.0692 | 3.3293 | 0.8916 |
| 4.5285 | 1.3631 | 0.0000 | 1.9988 | 3.9314 | 3.6416 | 0.4321 | 4.2534 |
| 0.0000 | 7.2161 | 4.0104 | 1.8863 | 7.9667 | 1.6090 | 4.3056 | 2.8832 |
| 0.7843 | 0.0000 | 0.0000 | 1.9345 | 3.2094 | 2.2429 | 1.9109 | 2.6505 |
| 6.8228 | 0.6413 | 0.0000 | 1.6679 | 0.0000 | 2.7409 | 0.5748 | 2.4747 |
| 0.9357 | 0.4562 | 0.0000 | 7.1314 | 0.5454 | 3.3826 | 4.3485 | 0.0000 |
| 3.2886 | 0.0000 | 0.5862 | 0.0000 | 6.7640 | 0.0000 | 0.0000 | 5.9747 |
| 7.7672 | 0.6063 | 0.6138 | 0.5821 | 0.0000 | 3.0916 | 1.1696 | 4.0254 |
| 5.8834 | 2.0324 | 4.6411 | 1.8290 | 2.3178 | 7.5405 | 1.8874 | 0.0000 |
| 2.7064 | 1.4586 | 1.0660 | 4.6650 | 6.3233 | 0.0000 | 3.0638 | 4.5495 |
| 0.0000 | 0.7366 | 0.0000 | 1.9914 | 2.2587 | 1.9569 | 1.2961 | 0.8827 |
| 6.5763 | 0.5674 | 8.6339 | 0.0000 | 3.1828 | 5.7627 | 0.0000 | 2.6602 |
| 2.8731 | 2.0355\ | | | | | | |
| SNORD115-4 | | 0.0000 | 0.0000 | 0.0000 | 0.0000 | 0.0000 | 0.0000 |
| 0.0000 | 0.0000 | 0.0000 | 0.0000 | 0.0000 | 0.0000 | 0.0000 | 0.0000 |
| 0.0000 | 0.0000 | 0.0000 | 0.0000 | 0.0000 | 0.0000 | 0.0000 | 0.0000 |
| 0.0000 | 0.0000 | 0.0000 | 0.0000 | 0.0000 | 0.0000 | 0.0000 | 0.0000 |
| 0.0000 | 0.0000 | 0.0000 | 0.0000 | 0.0000 | 0.0000 | 0.0000 | 0.0000 |
| 0.0000 | 0.0000 | 0.0000 | 0.0000 | 0.0000 | 0.0000 | 0.0000 | 0.0000 |
| 0.0000 | 0.0000 | 0.0000 | 0.0000 | 0.0000 | 0.0000 | 0.0000 | 0.0000 |
| 0.0000 | 0.0000 | 0.0000 | 0.0000 | 0.0000 | 0.0000 | 0.0000 | 0.0000 |
| 0.0000 | 0.0000 | 0.0000 | 0.0000 | 0.0000 | 0.0000 | 0.0000 | 0.0000 |
| 0.0000 | 0.0000 | 0.0000 | 0.0000 | 0.0000 | 0.0000 | 0.0000 | 0.0000 |
| 0.0000 | 0.0000 | 0.0000 | 0.0000 | 0.0000 | 0.0000 | 0.0000 | 0.0000 |
| 0.0000 | 0.0000 | 0.0000 | 0.0000 | 0.0000 | 0.0000 | 0.0000 | 0.0000 |
| 0.0000 | 0.0000 | 0.0000 | 0.0000 | 0.0000 | 0.0000 | 0.0000 | 0.0000 |
| 0.0000 | 0.0000 | 0.0000 | 0.0000 | 0.0000 | 0.0000 | 0.0000 | 0.0000 |
| 0.0000 | 0.0000 | 0.0000 | 0.0000 | 0.0000 | 0.0000 | 0.0000 | 0.0000 |
| 0.0000 | 0.0000 | 0.0000 | 0.0000 | 0.0000 | 0.0000 | 0.0000 | 0.0000 |
| 0.0000 | 0.0000 | 0.0000 | 0.0000 | 0.0000 | 0.0000 | 0.0000 | 0.0000 |

```
0.0000  0.0000  0.0000  0.0000  0.0000  0.0000  0.0000  0.0000
0.0000  0.0000  0.0000  0.0000  0.0000  0.0000  0.0000  0.0000
0.0000  0.0000  0.0000  0.0000  0.0000  0.0000  0.0000  0.0000
0.0000  0.0000  0.0000  0.0000  0.0000  0.0000  0.0000  0.0000
0.0000  0.0000  0.0000  0.0000  0.0000  0.0000  0.0000  0.0000
0.0000  0.0000  0.0000  0.0000  0.0000  0.0000  0.0000  0.0000
0.0000  0.0000  0.0000  0.0000  0.0000  0.0000  0.0000  0.0000
0.0000  0.0000  0.0000  0.0000  0.0000  0.0000  0.0000  0.0000
0.0000  0.0000  0.0000\
WASH3P  9.2866  9.6054  8.4775  9.1610  9.6517  8.9135  8.9192
9.3240  10.1613 9.6533  9.2698  9.1291  9.6157  8.7221  8.8124
9.8484  11.0707 9.1831  9.1552  9.7605  9.6689  8.7316  9.8047
8.8192  9.5734  9.0597  8.9220  10.1111 8.9871  9.3630  8.8270
9.6783  8.9550  10.3761 8.9739  9.1522  9.7285  8.3376  9.2511
8.7347  9.0961  8.9049  9.4779  9.2487  8.6362  9.5153  10.6016
9.2206  8.3598  10.0485 8.7617  10.2736 8.6483  9.2194  9.8374
9.7804  9.3992  8.7296  8.8262  9.0243  8.3558  9.4056  8.7935
10.4361 9.8214  8.4924  8.7919  9.2685  8.8840  10.3060 10.1099
9.5679  10.4249 10.4058 8.7084  9.1291  10.3305 9.5402  9.6202
8.5582  9.6013  8.3923  9.2915  8.3877  10.0166 9.8081  8.3251
9.7754  9.0958  9.3328  9.0011  9.0481  9.6877  10.0780 9.3597
8.0926  8.2101  9.4383  9.1441  9.5768  10.7992 8.6726  9.1774
10.5673 8.6789  8.6223  11.1033 8.9571  8.5612  9.7318  8.6297
8.3427  11.5535 11.1997 8.9662  9.5132  8.6945  8.5851  8.5179
9.9555  9.7790  8.2731  8.5437  9.0248  9.6587  8.8722  8.5056
11.0507 8.7597  8.6276  8.7618  10.2262 9.3436  9.1293  10.3313
11.0719 10.8464 13.0241 9.2263  9.2486  9.9149  8.4712  10.6767
8.1612  8.7747  9.6949  8.4859  10.1199 9.5486  9.6888  9.4778
11.1936 9.0540  9.5963  9.0183  9.2280  9.3820  9.2075  9.4540
9.8303  9.0126  10.2231 9.1321  10.8280 8.7940  8.8016  9.4551
7.9775  8.9412  10.4112 8.2688  9.3290  9.7118  9.5547  12.5368
9.0140  9.6304  9.9013  8.6283  9.5392  10.7720 9.4808  9.4078
9.8593  8.4788  9.2621  9.4095  10.1594 9.4682  9.4223  8.6366
9.1245  10.0197 9.2728  9.3133  10.7719 9.7498  9.8085  8.4961
8.6224  10.1164\
CNPY4   8.8519  9.3913  8.4701  9.1843  8.9583  8.0891  7.9344
9.4213  8.2037  10.1734 8.9613  8.7036  9.2156  8.8891  8.9036
10.1021 8.6610  8.5426  9.8914  8.2192  7.9592  9.1096  8.2728
9.1133  9.6677  8.0425  9.3086  9.5335  8.9710  6.8355  12.1481
8.9639  7.2306  7.3940  8.3474  7.6070  8.1245  7.9765  6.7232
8.7641  9.3253  8.8381  9.5472  7.5004  9.4415  9.4196  8.3961
7.3954  9.1255  7.4294  8.2153  8.5304  8.5877  9.5073  11.4289
9.2403  7.6656  8.8827  9.2304  7.8331  9.3506  9.7829  7.0296
8.7648  10.4196 8.3064  7.7180  9.1246  9.9393  9.0554  7.8221
8.5268  7.9649  8.6632  8.5282  8.0977  9.6585  8.7338  10.4527
6.9418  10.8430 8.5553  9.4786  8.4526  9.5073  9.8928  7.8952
9.5158  8.9707  9.4373  9.3302  8.8363  8.4500  9.7934  8.5770
9.5767  8.0669  9.4094  8.4721  8.3369  9.8914  8.3318  9.1863
8.5908  8.1316  8.2283  9.3011  8.3860  9.6473  9.0661  8.8040
7.3495  8.4384  8.5925  8.8704  7.3956  8.5249  9.1045  8.6408
8.3719  9.9678  8.3686  8.3898  8.2094  8.4660  8.3166  8.4276
9.9570  9.1623  9.4535  9.1305  8.1248  8.7447  8.2180  7.8329
7.4164  8.3958  8.5926  8.4913  9.3751  8.2811  9.2220  9.0754
8.2909  7.9364  9.5392  7.7493  9.0936  8.5458  7.3866  9.6066
```

```
7.4389  9.3186  8.7685  8.4049  8.7278  9.4789  9.2369  8.7446
9.1305  8.9635  7.7391  9.5109  9.4878  8.3533  7.6202  8.3011
7.9118  8.3853  7.9100  8.4747  8.7471  8.8837  9.2844  8.5843
9.3008  10.1128 9.3493  8.6271  8.8406  9.2948  8.5819  7.3659
9.4358  9.5337  8.4260  9.1152  8.8525  9.1297  8.6078  8.4013
8.4378  10.2524 8.3013  7.9288  7.8282  8.3601  9.1725  7.8067
8.4762  8.2486\
CNPY3   11.9807 10.9075 12.4933 10.6925 11.0738 11.7285 12.0152
12.1925 11.5511 11.1694 12.1475 11.7731 11.5238 10.9859 11.5242
11.8309 13.0258 11.1494 10.9743 12.5419 13.3203 11.8711 11.3276
11.0473 10.8019 12.3372 11.6555 12.4136 11.4584 11.4133 11.6171
11.3502 12.0634 11.4114 11.8403 11.1083 11.2091 10.9580 10.9814
11.0915 11.6656 10.3575 11.6503 12.8493 10.3767 12.8947 12.9271
11.9325 10.6192 12.0596 10.8540 11.0284 11.5678 10.3457 11.5968
11.0230 11.3816 11.7030 11.4113 11.8180 10.4261 11.5213 11.2715
12.3684 11.4371 11.2461 11.3952 12.9499 10.9313 12.4651 12.9758
10.9086 12.2175 12.9303 10.6672 10.7121 12.2056 12.1052 11.9422
10.7934 12.5147 12.1620 11.9313 11.1247 12.5311 12.2410 11.1543
12.9172 10.9397 11.2666 10.6348 11.0969 11.9026 12.1182 11.8366
10.6342 10.3189 10.9653 11.3241 11.3503 12.0684 11.7152 11.0075
10.9603 10.6259 11.0334 11.0527 11.1965 12.1071 12.6019 11.2455
10.4939 13.4794 13.1235 10.2207 11.1380 11.4684 11.2610 10.6358
11.5890 11.9624 11.4342 11.8548 10.6892 10.8710 11.2645 11.6354
14.3792 10.6548 10.1102 11.4347 10.9855 11.6086 10.8935 11.3789
12.9057 11.8114 11.7436 10.8226 10.6926 11.3676 11.5688 12.5108
10.9460 10.8869 10.9568 10.6789 11.0225 11.1494 12.4670 11.1998
12.4661 11.0952 12.0267 11.2781 11.3301 10.9772 10.9667 11.5300
11.9312 12.0283 11.5651 11.7430 12.3831 11.0523 10.4235 12.4570
10.8633 11.3088 12.0241 10.5928 11.5272 11.7193 12.3715 12.1164
11.1090 11.2003 10.5400 10.3129 11.4886 12.4910 12.1767 11.7183
11.9672 11.3272 11.5231 11.5371 12.6754 11.9380 11.1918 11.0015
11.2018 11.7756 11.0655 11.6970 12.5181 12.3296 11.0448 11.2028
10.9060 12.7479\
CNPY2   11.8571 10.4862 11.5780 11.5259 11.4424 11.7713 10.8748
11.8941 11.7147 11.0366 11.3377 10.8929 11.2895 11.0563 11.8346
11.5429 12.3783 11.4051 10.4323 11.8226 11.4981 11.9184 10.8348
11.5552 10.4329 11.6790 12.0638 11.8543 12.0423 11.1327 11.3705
11.3975 11.1863 10.9008 10.7499 11.3280 11.0932 11.3411 11.4669
11.5525 12.0436 9.9679  11.4656 10.9359 9.9924  12.0576 12.9874
11.7883 10.8891 11.9200 10.8557 11.3305 11.8164 10.1728 12.1600
11.3356 11.2737 11.6199 11.2415 10.6963 9.9775  10.9632 11.8097
12.5579 10.9993 11.3653 12.6856 12.5261 11.4394 11.4966 11.6461
11.3870 12.0781 11.3239 11.7923 11.3823 12.1835 11.2665 12.0715
11.1072 12.5021 9.9933  11.1242 11.5521 12.4255 12.1531 11.3636
12.3640 10.5491 11.3729 10.2440 11.4308 12.0211 12.0055 11.8128
10.0529 11.1200 10.5366 11.1149 11.2471 12.6703 11.1568 11.2912
12.2778 12.0190 11.3923 10.1843 11.0405 11.0475 11.7280 11.3480
11.3490 12.3832 10.8486 10.4290 10.7310 11.8945 11.1430 11.1981
10.8727 11.0545 11.4183 11.2201 11.0999 10.7310 11.0769 11.9160
13.7887 10.2641 9.9627  11.4378 11.4311 11.7519 10.8935 11.1879
11.7228 12.0445 10.7282 11.0213 10.4062 12.3943 12.1178 12.3903
11.0019 11.1753 10.5562 10.8397 10.6941 11.4791 12.5776 11.3816
13.5878 11.3034 11.2461 11.4251 10.9015 10.7564 11.3508 11.6026
11.6539 12.0306 11.7506 11.9068 11.6318 11.4131 11.0420 11.1319
```

```
10.8754 11.6520 11.3228 11.0749 11.4423 11.5571 12.1568 11.9308
11.1325 10.9173 10.2921 10.4475 10.8554 12.1164 12.2453 11.0956
12.3117 11.7257 11.8989 10.3200 12.4253 12.0932 11.6784 10.8952
11.8633 11.2473 11.0334 11.3804 12.5106 12.9770 10.7815 11.0678
11.1541 12.5509\
CNPY1   0.0000  0.0000  0.0000  0.0000  0.0000  0.0000  0.0000
0.0000  0.0000  0.0000  0.0000  0.0000  0.0000  8.2566  0.0000
3.2639  0.0000  0.0000  0.0000  0.0000  0.0000  0.0000  0.0000
0.5262  1.5220  0.0000  6.6530  0.0000  0.0000  0.0000  0.0000
0.0000  0.0000  0.0000  0.0000  0.6819  1.2685  0.6557  0.0000
0.0000  0.6649  0.0000  0.0000  0.0000  0.0000  0.0000  0.0000
0.0000  0.7769  0.0000  0.4530  0.0000  0.0000  0.5094  2.1436
0.0000  1.6820  0.8754  0.0000  0.0000  1.2440  5.2325  1.2717
0.0000  0.0000  0.0000  0.0000  0.0000  3.0673  0.0000  0.0000
0.9537  0.0000  0.0000  0.0000  0.0000  6.4007  0.0000  0.0000
0.0000  3.8042  0.0000  0.0000  0.0000  1.6785  0.0000  0.0000
0.0000  0.0000  0.0000  0.6361  0.0000  1.2055  4.0850  0.0000
0.0000  0.0000  0.5454  0.0000  0.0000  0.0000  0.0000  0.0000
0.0000  0.0000  0.4659  0.0000  0.0000  0.0000  0.0000  0.0000
0.0000  2.0513  1.0000  0.8924  0.6116  0.9131  0.0000  3.1059
0.0000  4.1754  0.0000  0.0000  0.0000  1.3435  0.5331  0.0000
0.0000  0.0000  1.8193  0.3935  0.0000  0.0000  0.0000  0.8107
0.0000  0.0000  0.0000  0.0000  0.0000  1.0240  1.3036  0.0000
0.0000  0.0000  0.9349  0.0000  0.9402  0.0000  0.0000  0.5019
0.0000  0.0000  0.0000  0.0000  0.0000  0.0000  0.0000  0.0000
0.0000  0.0000  0.6138  0.9957  0.4033  0.0000  0.0000  0.0000
0.0000  0.0000  0.0000  0.0000  0.9151  0.0000  0.0000  0.0000
0.0000  1.2087  0.0000  0.4012  0.6314  0.0000  0.0000  0.0000
0.0000  0.7366  0.0000  0.0000  0.0000  0.0000  1.5562  0.0000
0.0000  0.0000  0.0000  0.0000  2.3335  0.0000  0.6112  0.0000
0.0000  0.0000\
NODAL   2.7225  3.2435  0.6896  0.4935  3.7874  1.3921  1.2456
2.6906  4.0653  3.1490  2.3666  2.0691  2.7333  3.9636  0.5377
4.9193  3.0015  1.2795  3.6973  2.0944  2.6649  1.5770  1.9117
3.4755  1.9289  0.0000  3.3638  0.0000  1.3857  2.0116  1.5789
2.4004  2.1504  1.3374  3.5858  2.8161  3.8229  2.0582  1.7115
1.4612  1.7407  3.1095  1.8880  1.6313  2.8515  3.1906  0.0000
1.1317  2.6671  1.7431  1.5081  1.2789  1.0956  3.1083  3.1038
2.2632  2.7053  0.5030  2.7715  1.7785  2.7929  1.9554  1.7473
1.8326  2.8183  1.6675  2.3530  3.4521  3.9328  1.8731  2.8693
2.5842  0.0000  2.7868  3.6166  1.8854  2.3844  0.9332  2.9521
1.8728  7.5047  2.5223  0.7204  2.1886  2.7490  1.3843  0.9284
2.0646  1.8667  2.0295  3.1304  2.3201  3.5171  3.5917  1.4518
2.2653  1.4589  3.0618  1.8231  0.5504  4.4713  1.9597  0.0000
3.2704  0.4835  1.7170  3.8282  3.8673  3.9218  2.0061  1.4385
1.3871  0.0000  3.1699  3.8776  2.6508  2.0324  3.4758  1.5372
2.5175  3.8806  0.0000  2.0828  0.4700  1.8330  0.9214  1.3754
0.7843  2.1156  2.2611  0.7024  0.6684  1.2631  1.9109  0.0000
0.0000  3.7942  2.7241  2.9132  3.2947  3.6878  1.5646  1.7133
2.0686  1.6921  3.9194  0.0000  2.0758  2.8976  0.0000  4.5529
0.0000  2.2151  3.3254  3.7009  2.0663  2.1203  3.6244  1.0225
2.5877  0.6063  0.0000  1.8011  1.3855  0.7796  2.4806  3.2830
2.3696  1.2170  3.1425  2.6102  3.7442  3.2000  1.0565  0.0000
1.5724  1.8570  3.0195  1.6980  1.0692  0.5545  2.2268  4.3463
```

0.8792  2.5017  1.3929  1.3150  2.8698  1.7250  1.2961  1.1800
0.9557  1.2903  3.0977  2.8461  0.0000  2.2195  2.8738  1.3092
3.1721  0.6959\
SYNJ1   5.9574  7.7026  8.3586  7.2740  7.4766  8.0418  5.0640
6.3169  6.9597  7.5636  6.6450  6.1881  6.9026  7.6237  7.2723
7.5192  6.5993  6.7653  7.8040  6.2855  6.7238  7.1520  7.4469
7.6780  7.7721  7.0956  8.1550  6.6187  6.5382  6.7835  8.2514
7.8936  6.5191  6.3222  6.7491  7.1537  7.5028  7.8741  6.6068
8.2969  7.8153  8.1217  6.8723  8.2519  8.3366  6.5827  4.7672
7.8248  8.5113  6.2869  7.6663  7.9236  7.6841  8.2849  7.3967
7.9980  7.1365  8.4226  7.0774  7.0787  8.8408  7.4059  7.2908
5.9535  7.9363  7.9715  7.0497  7.0473  6.2993  7.2903  6.7006
7.4940  6.6569  7.2485  8.1971  7.7638  7.3800  7.1215  7.1173
7.0326  5.8011  6.4011  7.9184  7.7209  6.2279  6.5023  7.5403
6.0499  7.2257  7.7125  7.9484  7.6343  6.3261  7.4294  6.6551
8.0723  7.6212  7.7549  6.8746  8.1561  5.3226  7.4046  7.3258
7.9533  7.0266  6.9558  7.7386  8.0047  6.6386  5.8906  7.7836
6.5816  5.6364  5.9542  7.1394  7.7329  6.7291  6.9367  8.3847
7.4582  7.3303  7.6834  7.9162  7.2328  7.5748  7.1838  6.4533
4.0779  8.3956  8.8665  6.7995  7.6536  6.9181  7.9014  6.9708
5.7769  6.4086  6.8457  8.1743  8.0724  7.2762  7.0676  6.4530
6.5632  7.2229  7.8867  7.9809  7.3132  7.0227  6.2444  8.2809
5.8462  7.5523  6.9754  5.0381  7.4738  7.9886  6.8214  5.8383
6.8385  5.2311  7.4286  7.1103  6.9752  6.5294  7.1175  6.0710
7.9885  6.5571  6.2319  6.4916  7.9930  7.2698  7.2187  6.1416
7.1809  8.3025  7.9741  7.6493  7.4519  6.6218  7.3592  6.8044
6.8785  7.0971  7.1816  7.6994  6.8480  6.4882  6.0234  7.7131
7.5970  7.6406  7.9667  7.6284  4.9682  5.1059  7.7351  7.5188
7.5886  4.5045\
KNDC1   2.8211  7.0789  7.4716  6.2566  3.6419  1.3921  5.9386
3.3166  1.4589  7.9637  1.0281  3.2912  5.2101  0.0000  7.1583
3.5017  5.1498  3.1459  7.6296  3.3103  1.6493  9.5756  11.0523
4.5240  8.4419  7.8697  1.0809  6.8993  3.7973  0.5901  0.4127
2.6394  1.4434  0.8184  0.5853  5.4362  10.4017 0.6557  0.9336
2.6461  2.1741  6.8463  3.0042  0.4326  6.6055  4.6662  5.5761
1.4786  3.3102  0.4764  8.7666  1.6507  2.8587  8.1601  0.8153
4.9286  5.2478  3.6648  4.4982  5.5896  7.5962  6.3357  1.2717
1.0177  8.2319  3.4113  6.9810  1.5796  3.8367  6.6138  0.7662
2.3391  2.4919  3.3288  5.8299  3.2432  3.6155  6.7036  7.5487
2.2410  2.5369  1.8200  9.4746  3.1228  8.9277  1.6542  1.4887
4.4032  3.1604  3.5861  8.0753  3.1023  10.4965 5.8763  2.2941
8.2658  6.5159  8.1567  6.6680  2.6110  1.0805  6.7920  2.1993
5.7609  4.7904  4.1518  7.9953  4.7438  6.0208  5.8660  6.5966
1.3871  4.1934  1.0000  1.9988  1.0399  8.6139  1.2610  0.3974
5.7417  4.3949  5.4371  2.7935  6.7192  10.9306 2.4516  2.6587
0.7843  8.1174  8.2138  1.9345  5.2966  5.2606  4.2436  2.4659
5.8355  1.4217  7.7546  6.5443  8.4433  2.0359  4.5556  6.4992
2.9720  1.8493  10.2038 4.2362  8.7820  1.3155  1.2240  6.7430
1.9723  3.7814  3.7040  0.0000  2.4867  8.2028  2.0225  5.8121
5.3924  0.0000  4.8367  4.6063  2.3765  1.4811  2.5845  4.8770
9.4675  3.6416  2.6009  0.4486  1.8707  0.5410  2.7948  4.3567
3.0876  7.3463  6.4037  2.9511  11.6583 3.8199  3.0638  1.2133
2.6905  1.8738  3.9157  1.3150  2.5964  3.3538  2.5489  1.8202
0.5556  1.2903  3.4995  2.1049  4.8720  9.5681  8.4455  2.2759

6.6756 4.2926\
HMG20A 9.6404 9.5291 10.1142 9.8537 9.7652 10.1726 9.3893
9.8822 9.5988 10.2572 9.7385 9.3116 9.8936 9.8624 9.4730
9.4754 9.3154 9.3391 9.3487 8.7241 8.9407 9.3176 9.1338
10.0366 10.2527 9.2098 9.2429 9.1331 8.6648 9.2669 10.1155
9.5220 9.6310 10.1582 8.9753 9.8876 9.6539 9.6760 9.5576
9.3154 9.4622 9.9874 8.6408 9.7814 9.8937 8.6019 8.0371
8.9348 10.9026 9.2870 9.6662 9.0191 9.5469 10.2671 9.2879
9.7600 9.2077 9.9883 9.8246 10.2426 10.2514 9.9251 9.8775
8.6182 10.2656 9.0283 8.8347 9.6202 9.0009 9.6322 9.2642
10.2003 8.5282 9.7472 9.7721 9.9246 10.1487 9.1522 9.6436
10.0942 9.1554 9.3571 9.5148 9.8339 9.1134 9.4367 10.3465
8.9710 9.2205 9.6284 9.7092 9.8360 9.8496 9.2197 9.5209
9.6380 9.1106 10.3005 9.3573 9.3726 8.0265 9.6150 8.8397
9.4324 9.7118 9.1271 9.1212 9.9931 9.8699 9.7471 9.9500
9.4467 7.5793 8.9972 9.9010 11.3692 9.4152 10.0636 9.1583
9.0334 10.7730 9.9969 9.0100 9.3526 9.8543 9.4056 8.9751
8.8213 10.3413 10.5310 8.9501 10.0320 9.2260 9.6652 9.4776
6.9609 9.0931 9.2491 10.0748 10.1388 9.6288 9.5609 9.0631
9.7420 9.8313 10.4294 9.8816 9.5610 9.5524 8.7589 9.9265
7.5178 9.8631 9.1459 9.6045 9.5055 10.0926 9.8220 9.6436
10.0784 8.9098 8.8023 10.0150 9.0689 9.5415 10.0697 8.4556
9.2749 9.5539 9.3515 9.7571 10.0094 9.1587 9.0176 8.1895
10.1060 10.3150 10.0193 10.3749 9.2605 9.0616 9.4771 8.6694
9.6109 9.2713 9.9719 8.9310 8.0415 9.0074 9.2764 9.4840
9.4951 9.6757 10.2102 9.6491 8.7526 7.6518 10.1824 9.6440
9.9152 7.6483\
HMG20B 12.0138 11.1720 10.5119 11.0319 11.8230 11.3495 11.2204
9.9406 10.4119 11.8451 11.4103 10.3009 10.4181 10.9102 10.7123
12.1391 11.5043 11.6889 11.1846 11.1742 11.6643 12.3756 11.2430
10.6904 11.2554 12.7634 10.4672 12.2944 11.3604 11.3040 10.3333
12.7554 10.4320 9.7884 10.7314 10.8509 11.3644 11.9793 10.8850
11.0379 11.0048 10.3592 10.8598 9.8965 10.0513 11.8453 13.3286
11.3160 10.9261 12.0455 10.6637 10.6914 10.1407 10.3649 10.5806
11.5352 10.7679 10.5912 9.8175 10.6530 9.9970 9.8332 11.0933
12.0204 11.7620 11.4054 11.6272 12.1495 10.9038 11.5058 11.2032
10.8446 11.2605 11.1151 10.7109 12.1583 10.8061 11.8401 11.4226
11.6682 11.6221 9.8057 11.9487 10.2249 12.0918 10.9358 10.1252
12.2563 9.9706 11.4990 10.4984 9.8628 11.6020 11.8574 10.9848
10.7854 10.0210 10.7953 11.9358 11.4449 12.4894 11.4100 10.1817
11.9468 11.3814 10.9438 11.7465 10.7184 10.2572 10.8669 12.1514
11.0469 11.9588 11.8800 11.3762 9.5462 11.6504 9.9087 10.6877
10.4842 10.1399 11.4365 10.8402 13.0625 10.8824 10.5669 10.7059
11.4716 10.0802 10.2526 10.5974 11.9772 11.4652 11.1045 11.0779
12.7299 12.5314 12.4725 10.8545 10.6578 12.2571 11.9741 11.9752
10.9589 10.6456 10.0410 11.8254 11.1922 10.6639 12.0061 11.2231
12.8126 10.6886 10.3105 11.2058 10.4871 10.9727 10.8270 10.7756
11.9801 12.3239 11.3157 11.0757 11.2907 10.8253 10.7302 10.5004
9.7816 11.3908 11.2059 10.8147 9.8845 10.5893 11.1154 12.6141
11.1831 11.6498 10.8701 10.8748 11.5998 13.1603 11.1883 10.0746
11.6865 10.1441 10.7470 11.2197 11.7367 12.1896 11.4240 10.8080
10.5576 10.7651 10.5980 11.7279 11.2172 12.3476 10.6942 10.6975
11.2104 12.1619\
PHOSPHO2 6.4756 5.3619 6.9563 5.9584 6.3388 6.4082

```
5.9568	6.2222	5.9180	5.9162	5.6192	5.8063	6.5811	5.8880
6.3147	4.5469	5.3726	5.6714	4.9275	6.6665	6.1052	6.3976
6.2295	6.2859	4.9598	5.7619	5.2894	7.1604	5.5782	5.7700
4.7282	6.2991	6.6359	5.4762	7.2753	4.8535	5.7188	5.4644
5.7490	6.0272	7.7383	5.0721	4.7512	6.0528	6.4059	3.8449
6.2452	5.0771	5.2734	6.6717	6.2596	3.0237	6.7533	6.3332
6.2259	4.9509	5.1883	6.6560	5.5633	4.6595	5.2632	4.5971
6.6319	5.7760	5.3979	5.5395	6.7584	6.3104	5.0439	5.1951
5.3679	6.5338	5.1002	6.8544	5.3771	5.4632	5.5097	5.0569
5.9460	5.9962	6.6222	5.9320	4.8282	5.3897	6.0087	6.6561
6.4912	6.6468	6.6303	6.5330	5.2676	5.7191	6.8019	3.9765
7.3472	5.3391	6.1810	5.8278	6.0277	3.2964	6.5559	5.1736
5.4408	4.0848	6.9289	5.3040	4.3324	5.9862	5.6398	5.5315
5.6732	6.8437	6.5510	4.0875	5.5927	5.7777	6.3696	6.3995
5.8803	3.6413	6.2865	5.2396	5.7166	5.0428	6.5353	6.3029
6.9958	6.3427	5.6388	5.7701	5.9760	5.3806	6.1403	4.2436
6.2840	5.3903	5.8740	4.6610	6.0325	6.3262	5.4216	6.3006
5.5418	4.3341	4.6538	5.4545	6.1665	6.4023	5.8474	5.8188
5.5218	6.2302	5.9333	5.7528	5.8948	5.9714	5.7717	5.9996
7.1265	5.1912	4.8155	4.1675	6.4216	6.5529	6.5446	5.5826
0.0000	5.6442	6.2407	4.8347	5.6188	5.5833	6.9786	6.3189
4.4516	6.2516	5.4059	5.1746	6.4311	4.5888	6.6286	5.1931
8.2180	5.8493	6.9416	6.7620	6.1981	5.2078	5.2656	5.7761
6.2239	6.1722	6.4614	6.2331	5.9339	6.8088	7.9488	6.3242
6.1649	6.2552	6.0191\
AAGAB	10.3965	9.1272	9.4390	10.1504	10.5710	10.6171	10.2816
10.6741	10.3725	9.8876	9.6133	9.7832	10.0358	10.4326	10.4456
9.5335	8.9349	10.4600	9.3954	11.0311	9.4790	10.5512	9.4047
10.1748	9.2388	10.6915	9.7454	10.7890	10.5587	9.4152	8.9275
9.9876	10.1255	10.2191	10.4028	10.7066	10.4102	10.1613	10.1408
9.2881	10.5612	9.9498	9.4244	10.7866	9.2337	10.0739	9.7518
9.3992	10.2370	10.1862	10.6673	9.0486	9.8941	9.1555	10.2744
10.3990	10.0119	10.5577	9.6952	10.2999	9.4649	10.5919	10.3557
10.1107	9.2178	9.8047	10.4300	10.5235	10.4149	9.6351	9.9738
10.7297	9.9157	11.1508	10.4417	10.1279	10.0410	10.0450	9.8492
10.2947	10.8625	10.4320	9.7204	10.4636	10.1990	9.4187	10.8899
10.1841	10.0001	10.3063	9.3573	10.1907	9.3618	9.2593	10.9182
9.8804	9.7412	8.7078	9.8690	9.4006	10.6100	10.4615	9.4265
9.4366	10.0792	9.5808	8.9255	10.4325	9.7911	10.2053	9.9381
10.3376	10.1095	9.6511	10.7620	9.8632	10.2899	9.8769	10.7649
9.7456	10.4708	10.8018	9.5459	10.8829	9.5903	10.5745	10.1965
10.7211	9.2049	9.7811	9.7038	9.7768	10.4402	9.8296	10.4173
9.8571	10.6624	9.1903	10.0483	8.8472	10.0384	10.5831	10.2538
10.3274	10.3717	8.7140	10.3720	9.0542	10.7078	10.5057	9.4634
10.6310	10.4946	9.6423	10.5284	10.1298	8.9132	10.4720	10.9996
10.6253	10.4917	10.4157	10.3488	10.4613	9.4328	10.7134	9.9461
9.3534	10.5790	9.6835	10.6460	9.9595	11.2200	10.1675	10.4281
10.3494	9.3800	9.2322	10.3100	10.1415	10.4253	10.0892	10.1311
10.1579	9.9528	9.9252	9.6414	9.7719	10.4643	11.1039	10.3140
10.8004	9.5863	9.3309	10.0267	10.3731	9.7819	8.9734	10.5011
10.0806	10.9660\
C4orf19	8.2864	8.6337	7.6318	7.8344	4.0015	8.2113	2.4063
8.2076	10.2675	8.3685	7.5958	6.2576	7.1233	7.5868	9.8049
8.3876	7.2053	6.3765	8.2872	4.7724	4.9251	8.8975	8.3408
```

9.6454 9.7787 1.5076 8.1395 9.2943 9.8102 9.8308 5.5905
9.4313 0.5961 6.6084 7.5096 8.2409 6.5690 10.1285 7.9790
2.4946 8.8042 8.9059 8.4265 8.6860 6.3727 9.5117 9.3352
9.2047 2.7459 7.0725 9.2030 3.0587 6.6752 6.4076 6.2987
6.3108 5.3382 8.4296 8.5673 9.3313 6.8460 9.6156 6.4708
8.0720 8.2776 9.8345 8.8118 7.7887 5.4366 7.7141 8.3786
9.3192 5.0492 8.5754 9.8170 8.5811 4.2283 8.6669 9.2834
4.6416 9.1672 9.2181 9.0084 8.3970 8.9989 7.4545 6.8372
7.4502 4.5272 4.1122 6.4899 10.1952 7.2678 7.9990 8.1156
9.1156 8.3693 7.7779 7.7375 7.3460 8.9296 8.5600 7.8801
7.7724 9.0457 7.9895 6.2776 7.9827 8.2288 8.9975 8.4882
8.1456 4.6197 8.6294 10.8526 8.9489 8.5181 9.3706 7.9624
8.5855 6.1472 9.1416 8.7708 2.1288 7.3754 8.9672 7.9950
7.4413 8.6981 9.5291 8.7790 9.1877 9.3369 9.3951 6.9459
2.9122 2.4237 8.1927 8.5069 8.6698 9.1115 9.6990 8.6362
8.0964 9.4712 0.9349 9.3430 5.4561 7.3971 7.1957 8.5496
9.8893 9.6748 7.6903 5.9386 8.9751 8.5906 7.7651 4.8102
4.8724 10.0206 10.4531 9.1359 9.0315 9.2965 9.4037 8.6821
9.2101 9.3503 9.5481 6.6093 9.0237 7.7742 8.5115 10.6580
6.0646 8.6315 6.4405 7.6591 8.3184 7.3597 9.4393 6.9033
9.7362 8.9981 8.3616 8.6447 9.1375 9.3316 3.7881 8.2048
9.5422 6.9885 7.0698 8.5568 7.1300 7.4029 8.5372 8.5265
8.4205 8.5152\
C3orf79 0.0000 0.0000 0.0000 0.0000 0.0000 0.0000 0.0000
0.0000 0.0000 0.0000 0.0000 0.0000 0.0000 0.0000 0.0000
0.0000 0.0000 0.7772 0.0000 0.0000 0.0000 0.0000 0.0000
0.0000 0.0000 0.0000 0.0000 0.0000 0.0000 0.0000 0.0000
0.0000 0.0000 0.0000 0.0000 0.0000 0.0000 0.0000 0.0000
0.0000 0.0000 0.0000 0.0000 1.2622 0.0000 0.0000 0.0000
0.0000 0.0000 0.0000 0.0000 0.0000 0.0000 0.0000 0.0000
0.0000 0.0000 0.0000 0.0000 0.0000 0.0000 0.4969 0.0000
0.0000 0.0000 0.0000 0.0000 0.0000 0.0000 0.0000 0.0000
0.0000 1.9166 0.0000 0.0000 0.0000 0.0000 0.0000 0.0000
0.0000 0.0000 0.3965 0.0000 0.0000 0.0000 0.0000 0.0000
0.0000 0.0000 0.0000 0.0000 0.0000 0.0000 0.0000 0.0000
0.0000 0.0000 0.0000 0.0000 0.0000 0.0000 0.0000 0.0000
0.0000 0.0000 0.0000 0.0000 0.4315 0.0000 0.0000 0.0000
0.0000 0.0000 1.0000 0.5141 0.0000 0.0000 0.0000 0.0000
0.0000 0.0000 0.0000 0.0000 0.0000 0.0000 0.0000 0.0000
0.0000 0.0000 0.0000 0.0000 0.0000 0.0000 0.0000 0.0000
0.0000 0.0000 0.0000 0.0000 0.0000 0.0000 0.0000 0.0000
0.0000 0.0000 0.0000 0.0000 0.0000 0.0000 0.0000 0.0000
0.0000 0.0000 0.0000 0.0000 0.0000 0.0000 0.0000 0.0000
0.5416 0.0000 0.0000 0.0000 0.0000 0.0000 0.0000 0.0000
0.0000 0.0000 0.0000 0.0000 0.5290 0.0000 0.0000 0.0000
0.0000 0.0000 0.0000 0.0000 0.0000 0.0000 0.0000 0.0000
0.0000 0.0000 0.0000 0.0000 0.0000 0.0000 0.0000 0.0000
0.0000 0.0000 0.0000 0.0000 0.0000 0.0000 0.0000 0.0000
0.5077 0.0000\
SLC7A6OS 7.0151 6.8744 6.7062 7.3912 8.0132 7.2104
7.1505 7.5019 8.3842 7.3337 6.6124 7.4437 7.4708 8.0629
7.3538 6.6773 5.6410 7.9962 7.0856 7.2451 7.5458 7.7433
7.4184 7.3251 7.6677 6.8284 7.3223 7.0641 6.7714 6.7690
7.5822 7.3329 8.4133 8.3257 7.8943 7.9772 7.4312 7.3439

```
6.6398  6.2787  7.4462  7.0641  7.1999  7.0713  7.4455  6.7412
4.8165  7.9046  8.6913  5.7475  7.3057  7.2204  7.6618  7.1326
8.2708  7.3255  7.6595  7.2033  7.2375  7.6359  7.6118  6.9303
7.9052  6.8973  7.3828  7.7448  8.5399  7.1321  7.9889  6.8035
7.5044  7.5181  7.2491  8.1952  7.1317  7.4667  7.5608  7.6613
7.7237  7.6626  7.3833  7.3534  6.9867  7.0473  7.0145  7.5118
7.3996  7.7089  7.0665  7.6847  7.5549  7.7563  6.8903  5.5994
7.8073  7.3999  7.0633  7.5361  7.1970  6.8658  6.7263  8.3650
6.8908  6.1083  7.3568  6.4507  5.8484  6.3990  7.0923  6.5429
7.4610  7.6568  0.0000  6.8536  7.4543  7.5825  7.7222  7.2758
7.1387  7.3642  8.1713  7.2349  6.9471  7.7892  7.4577  7.3150
7.7150  5.6030  7.3946  7.5790  7.7483  6.1416  7.5008  6.0700
8.2561  4.3468  7.5339  3.6325  7.8284  7.5588  6.5273  7.1376
6.9637  7.7586  7.1605  7.6941  7.5752  7.2188  7.6250  6.2806
7.5862  0.0000  7.8137  8.5033  7.3136  7.5702  7.6199  7.5307
7.5657  7.4675  7.9159  7.9520  7.5724  7.2593  7.7467  7.6577
6.5651  7.0158  8.2169  6.9850  7.7222  7.5960  7.1175  7.3654
0.0000  8.2139  7.3298  7.2012  7.7028  7.0494  7.1145  7.5664
6.9536  7.3492  7.0318  7.5404  7.5974  7.6956  6.9437  8.1154
7.4267  7.0839  6.5636  8.1035  7.3233  6.6751  4.8179  7.7215
7.7555  7.5464  6.7227\
C3orf77 0.0000  0.0000  0.0000  0.0000  0.0000  0.0000  0.0000
0.0000  0.0000  0.0000  0.0000  0.0000  0.0000  0.0000  0.0000
0.0000  0.0000  0.0000  0.0000  0.0000  0.0000  0.0000  0.0000
0.0000  0.0000  0.0000  0.0000  0.0000  0.0000  0.0000  0.0000
0.0000  0.0000  0.4664  0.0000  0.0000  0.0000  0.0000  0.0000
0.0000  0.0000  0.0000  0.0000  0.0000  0.0000  0.0000  0.0000
0.0000  0.0000  0.0000  0.0000  0.0000  0.0000  0.0000  0.0000
0.0000  0.0000  0.0000  0.0000  0.0000  0.0000  0.0000  0.0000
0.0000  0.0000  0.0000  0.0000  0.8444  0.0000  0.0000  0.0000
0.0000  0.0000  0.0000  0.0000  0.0000  0.0000  0.0000  0.0000
0.0000  0.0000  0.0000  0.0000  0.0000  0.5263  0.0000  0.0000
0.0000  0.0000  0.0000  0.0000  0.0000  0.0000  0.0000  0.0000
0.0000  0.0000  0.0000  0.0000  0.0000  0.0000  0.0000  0.0000
0.0000  0.0000  0.0000  0.0000  0.0000  0.5426  0.0000  0.0000
0.0000  2.0513  0.0000  0.0000  0.0000  0.0000  0.0000  0.0000
0.6089  0.0000  0.0000  0.0000  0.0000  0.0000  0.0000  0.0000
0.0000  0.0000  0.0000  0.0000  0.0000  0.0000  0.0000  0.0000
0.0000  0.0000  0.0000  0.0000  0.0000  0.0000  0.0000  0.0000
0.0000  0.0000  0.0000  0.0000  0.0000  0.0000  0.0000  0.0000
0.0000  0.0000  0.0000  0.0000  0.0000  0.0000  0.0000  0.0000
0.0000  0.0000  0.0000  0.0000  0.0000  0.0000  0.0000  0.0000
0.0000  0.0000  0.0000  0.0000  0.0000  0.0000  0.0000  0.0000
0.0000  0.0000  0.0000  0.0000  0.0000  0.5545  0.0000  0.0000
0.0000  0.0000  0.0000  0.0000  0.0000  0.0000  0.0000  0.0000
0.0000  0.0000  0.0000  0.0000  0.0000  0.0000  0.0000  0.0000
0.0000  0.0000\
C3orf75 9.8150  8.7716  8.3232  9.2075  9.7333  9.1563  8.9462
9.5951  8.4106  9.5897  8.8236  8.9875  9.4428  8.7979  9.0287
9.3014  9.1758  9.7039  8.6919  9.8229  8.8417  9.2174  9.0700
8.8178  9.2654  10.0399 8.3033  9.9372  9.3660  8.9041  9.7177
9.3005  10.1149 9.6984  8.4787  9.6138  9.2431  9.4737  9.4807
8.3827  9.7272  8.4463  9.0063  8.6872  8.7023  9.5242  10.3902
8.9729  9.3097  9.6807  9.2535  9.0112  8.6173  8.6978  8.9650
```

| | | | | | | | |
|---|---|---|---|---|---|---|---|
| 9.4232 | 8.6447 | 9.5902 | 9.3159 | 9.7539 | 8.5562 | 8.1282 | 9.4281 |
| 9.4656 | 9.5066 | 8.8772 | 8.3874 | 9.2213 | 8.8116 | 9.6742 | 9.0318 |
| 9.1156 | 9.3595 | 9.7583 | 8.6722 | 9.4951 | 9.7635 | 9.4313 | 9.4360 |
| 9.0521 | 10.0123 | 9.5875 | 9.1718 | 9.0565 | 9.7457 | 9.1738 | 9.0030 |
| 9.6899 | 8.5443 | 10.4694 | 8.7970 | 9.2964 | 9.9406 | 9.4763 | 9.8330 |
| 9.2875 | 8.1994 | 8.9197 | 9.2521 | 9.0398 | 10.0014 | 9.3670 | 9.2768 |
| 9.2600 | 9.7234 | 10.0685 | 8.9038 | 8.5995 | 9.2158 | 9.6654 | 9.5093 |
| 9.3453 | 9.5221 | 9.5333 | 9.3864 | 9.1270 | 9.4291 | 9.4527 | 9.4547 |
| 9.5391 | 9.7680 | 9.4827 | 9.2164 | 9.5064 | 8.9214 | 8.3644 | 8.9659 |
| 9.6090 | 8.9729 | 8.7400 | 9.0119 | 9.3620 | 9.5209 | 8.6972 | 8.6545 |
| 9.7392 | 10.0390 | 9.5264 | 8.8740 | 8.8180 | 9.1652 | 9.1115 | 9.8896 |
| 8.9267 | 8.9531 | 9.4357 | 9.1661 | 9.0617 | 9.1926 | 9.7062 | 9.0118 |
| 9.7162 | 8.8225 | 8.3392 | 8.6435 | 8.9225 | 9.0313 | 9.5463 | 8.8584 |
| 10.0176 | 10.1687 | 9.4456 | 9.5526 | 9.7507 | 8.9548 | 8.9005 | 9.0950 |
| 7.9720 | 9.5170 | 9.7789 | 8.9141 | 9.6373 | 10.1354 | 8.8266 | 9.4651 |
| 9.4294 | 9.3752 | 8.6680 | 9.4800 | 8.8371 | 9.8790 | 9.5138 | 9.9176 |
| 9.3428 | 9.3005 | 8.7991 | 9.2400 | 10.1281 | 9.8650 | 9.5861 | 9.0104 |
| 9.3238 | 8.7894 | 8.5411 | 9.2265 | 10.1373 | 9.5918 | 8.9689 | 8.9452 |
| 9.0064 | 9.7662\ | | | | | | |
| C3orf74 | 0.0000 | 0.0000 | 0.6896 | 0.0000 | 0.0000 | 0.9816 | 0.9367 |
| 0.4748 | 0.0000 | 0.4413 | 0.0000 | 0.0000 | 1.2705 | 0.4395 | 0.5377 |
| 0.0000 | 1.3881 | 0.0000 | 0.0000 | 0.0000 | 0.0000 | 0.4820 | 0.0000 |
| 0.0000 | 0.0000 | 0.0000 | 1.0809 | 0.0000 | 0.0000 | 0.0000 | 0.9954 |
| 0.0000 | 0.0000 | 1.1010 | 1.0004 | 0.0000 | 1.5255 | 1.8437 | 0.0000 |
| 0.0000 | 0.0000 | 0.0000 | 0.0000 | 0.0000 | 0.0000 | 0.0000 | 0.0000 |
| 0.0000 | 3.8636 | 0.0000 | 0.0000 | 0.0000 | 0.0000 | 0.0000 | 0.4645 |
| 0.9635 | 0.0000 | 0.0000 | 0.0000 | 0.0000 | 0.0000 | 0.0000 | 0.0000 |
| 0.0000 | 0.6828 | 0.3906 | 1.3453 | 0.0000 | 0.0000 | 0.6160 | 0.0000 |
| 0.0000 | 0.0000 | 0.0000 | 0.0000 | 0.0000 | 0.0000 | 0.0000 | 0.0000 |
| 0.0000 | 0.0000 | 0.7073 | 0.0000 | 0.6718 | 0.0000 | 1.0522 | 0.5376 |
| 0.0000 | 0.5273 | 0.0000 | 0.0000 | 0.4848 | 0.0000 | 0.0000 | 0.0000 |
| 0.0000 | 0.5233 | 0.5454 | 0.0000 | 0.0000 | 0.0000 | 0.0000 | 0.0000 |
| 0.7358 | 0.0000 | 0.0000 | 0.0000 | 1.0326 | 0.5426 | 0.0000 | 0.0000 |
| 0.6215 | 0.0000 | 0.0000 | 0.5141 | 0.0000 | 0.0000 | 0.4321 | 0.0000 |
| 0.6089 | 0.4553 | 1.0055 | 0.6224 | 0.0000 | 0.0000 | 0.0000 | 0.0000 |
| 0.0000 | 0.5619 | 1.1792 | 0.0000 | 0.0000 | 0.5526 | 0.0000 | 0.0000 |
| 0.0000 | 0.0000 | 0.0000 | 1.4553 | 0.5056 | 0.0000 | 0.0000 | 0.0000 |
| 1.7146 | 0.0000 | 0.0000 | 0.0000 | 0.0000 | 0.0000 | 0.7381 | 0.5019 |
| 0.0000 | 0.0000 | 0.0000 | 0.0000 | 0.7727 | 0.0000 | 1.5047 | 0.0000 |
| 0.0000 | 0.0000 | 0.0000 | 0.0000 | 0.0000 | 0.0000 | 0.0000 | 0.0000 |
| 0.0000 | 0.0000 | 0.0000 | 0.4486 | 0.9151 | 0.0000 | 0.0000 | 0.0000 |
| 0.8396 | 0.0000 | 0.0000 | 0.0000 | 0.0000 | 0.0000 | 0.0000 | 0.5257 |
| 0.0000 | 0.0000 | 0.0000 | 0.0000 | 0.7058 | 0.0000 | 0.5707 | 0.0000 |
| 0.5556 | 0.0000 | 0.0000 | 0.0000 | 0.0000 | 0.0000 | 1.0391 | 0.3736 |
| 0.5077 | 0.0000\ | | | | | | |
| C4orf10 | 7.9920 | 7.8558 | 8.4117 | 7.9556 | 8.7320 | 8.5051 | 8.2460 |
| 7.8239 | 9.1741 | 8.1506 | 7.6573 | 8.6498 | 8.0572 | 8.8416 | 7.3625 |
| 8.3096 | 7.4282 | 8.5611 | 7.6634 | 9.0160 | 8.9520 | 7.7997 | 8.2102 |
| 7.7989 | 8.2860 | 8.4396 | 6.4934 | 8.0965 | 8.7681 | 7.7414 | 8.4962 |
| 8.5915 | 9.7920 | 8.3981 | 8.2584 | 8.4154 | 8.3606 | 7.7569 | 8.3039 |
| 8.4597 | 7.8511 | 8.0786 | 7.4888 | 8.4488 | 8.0584 | 7.9364 | 8.9994 |
| 7.9417 | 8.2514 | 8.9523 | 8.7901 | 6.5336 | 7.4153 | 8.6106 | 7.9345 |
| 7.9883 | 8.2718 | 7.1346 | 7.9303 | 8.1544 | 8.0421 | 8.4252 | 7.2461 |
| 9.2986 | 8.2924 | 8.1404 | 7.6093 | 7.8720 | 8.1977 | 8.5735 | 9.7773 |

8.1215 7.9081 8.3145 8.3082 6.9526 9.0430 7.8538 7.8765
8.1267 7.9678 7.9870 7.7055 8.2472 8.0962 7.7195 8.1281
7.9690 8.0250 8.2511 8.4064 7.9787 8.1136 8.0443 8.3174
8.2211 7.3981 8.5015 7.3517 8.2156 7.6857 7.6939 8.2978
8.1985 8.0351 8.1996 7.8391 8.4314 7.3880 8.2204 7.9581
7.8216 9.0314 8.0772 7.8493 7.9432 7.7720 8.8891 7.7591
7.1837 7.6900 8.1838 6.8909 7.0017 8.3500 7.5687 7.1319
9.3635 8.2228 8.2466 8.6438 7.2204 8.1397 8.0078 8.2242
9.3736 7.1647 7.8177 8.0325 8.6641 7.5471 8.2403 8.3106
9.0176 7.7464 8.6165 6.1393 8.3498 8.3930 8.2433 8.3870
8.8065 8.1950 8.3421 8.2551 9.1655 8.3233 7.9373 8.8196
8.0918 8.3690 8.2501 7.5605 7.9257 8.6172 8.2347 8.1666
8.5373 7.8555 8.2862 8.2755 7.9250 8.3232 7.4234 8.3892
8.3417 7.9262 8.1776 7.9959 7.9287 8.7041 7.7943 8.6604
8.0373 8.5711 7.8467 8.9033 7.9839 7.8855 7.9079 7.8208
8.1963 7.3483 8.1842 8.0043 8.6759 9.7143 8.7541 8.1851
7.8796 7.9961\
C3orf72 3.8614 8.8461 6.2894 5.4266 3.8769 3.7384 5.2999
5.3487 5.5916 10.4613 2.9226 3.9053 7.5958 1.2777 5.1972
3.5017 6.6310 6.1487 8.7279 1.7564 2.7816 5.1177 7.0854
4.6304 9.8918 5.6613 3.0765 7.0526 6.0514 2.8206 8.1150
3.1833 2.0034 3.1101 1.5856 3.5700 4.6138 3.9701 3.6815
6.3327 6.9806 8.8203 6.1435 2.9987 4.1359 3.8630 2.8871
2.9168 4.3409 3.4305 2.9335 2.8913 4.3853 8.2731 5.3263
4.3560 5.9352 6.8702 7.6203 6.3087 6.2701 5.8921 4.3117
2.4883 10.4801 6.3548 2.8406 5.4711 6.9773 8.2807 1.4604
4.4749 3.9820 4.7317 6.9227 1.8854 5.7798 6.9357 6.1834
6.0667 5.7376 5.0560 6.6911 4.2770 5.9380 3.9537 4.0708
4.6365 7.1781 6.9163 9.1194 5.3553 0.5215 7.9502 5.7296
8.9075 3.0761 9.8681 5.0693 4.2912 3.6027 6.3118 5.3082
5.5921 4.8906 5.2900 8.6310 7.7829 7.7339 6.7762 3.4375
3.6834 2.0513 4.0875 1.1918 4.9830 2.1803 5.7095 3.1059
5.5448 6.8304 5.8182 6.7007 5.1405 8.4408 5.9070 3.7819
0.0000 9.3425 11.0193 4.2856 3.5369 5.2250 5.1500 5.0630
3.2234 2.4535 7.5395 8.3957 9.6909 2.2071 3.6157 5.2919
5.6523 5.4270 11.0062 4.2811 7.6029 5.1793 1.5869 7.8732
2.2924 6.2846 5.1737 1.9146 0.0000 8.6858 4.6196 2.2048
4.1211 1.0317 4.5073 7.3132 3.6930 6.9447 7.4102 5.0454
7.1570 5.7427 1.9122 3.9282 3.1654 0.5410 4.0107 3.7579
3.8892 10.2880 8.4348 6.7774 7.5857 4.6915 5.0129 2.4318
6.1428 6.0936 5.5896 5.9999 4.6785 4.3236 0.5707 4.8502
2.5104 3.8574 5.5349 5.6110 5.2995 3.5172 9.2495 6.0322
5.9281 2.7180\
C3orf71 5.6323 5.2834 4.9299 5.8906 4.3867 6.0033 4.7643
5.6966 4.2105 5.5809 4.6229 4.9725 6.2113 5.6291 4.6405
7.0977 6.4997 6.4038 5.2821 5.6550 5.7082 5.6147 6.2918
5.5801 6.2021 5.2309 4.5195 5.5002 5.1892 4.9019 5.0227
6.1795 5.3417 6.1132 4.6843 4.7578 4.5052 4.6799 6.3891
4.9286 6.0569 4.5570 5.3403 3.9713 6.2206 4.5034 5.2520
4.9157 4.8132 6.4890 5.3792 4.1794 4.8790 5.9273 5.2274
6.1367 4.7225 4.2450 5.3126 6.3105 5.1380 4.5616 5.3602
6.4860 6.2142 5.1333 5.3597 5.0905 5.2467 5.8444 6.2034
5.5141 5.7965 5.0035 4.8094 5.6535 4.5057 5.5801 6.3977
4.2978 5.5283 5.9173 5.5340 5.2865 6.3957 6.2020 4.8603

| | | | | | | | |
|---|---|---|---|---|---|---|---|
| 5.6862 | 5.9514 | 5.9709 | 5.4131 | 5.1434 | 5.2164 | 6.4631 | 6.3212 |
| 5.6146 | 4.8788 | 6.2591 | 5.3357 | 6.1525 | 7.2164 | 5.4316 | 4.9464 |
| 5.3311 | 5.1871 | 4.2417 | 6.1890 | 4.4537 | 5.3956 | 5.8282 | 5.4794 |
| 5.0810 | 7.0010 | 6.0875 | 4.7147 | 4.8037 | 5.3183 | 6.2585 | 4.5357 |
| 4.8581 | 6.4007 | 5.3506 | 4.3536 | 4.3459 | 6.1851 | 4.0633 | 4.7852 |
| 6.3555 | 5.6454 | 5.3608 | 5.2441 | 4.8128 | 5.2953 | 5.6636 | 4.5192 |
| 5.4415 | 5.8349 | 6.0124 | 5.4675 | 6.3882 | 5.2559 | 4.8825 | 6.2663 |
| 5.2979 | 4.4803 | 6.3852 | 4.6996 | 6.1211 | 5.7443 | 5.4291 | 6.0108 |
| 5.5487 | 4.8533 | 4.1329 | 5.2801 | 4.0587 | 6.0779 | 5.2629 | 4.9587 |
| 5.2091 | 5.6763 | 4.9647 | 5.1000 | 6.4373 | 5.6521 | 4.6999 | 5.4334 |
| 4.3923 | 5.7208 | 6.1025 | 5.2314 | 4.0849 | 5.9124 | 4.1042 | 6.0836 |
| 6.2592 | 5.6551 | 5.7265 | 5.3620 | 5.8193 | 6.7813 | 5.3959 | 5.5593 |
| 5.8281 | 4.1421 | 6.1615 | 6.0331 | 5.6858 | 6.1210 | 5.0665 | 5.2040 |
| 4.5338 | 4.2351 | 4.5260 | 5.7168 | 6.2041 | 6.6019 | 6.8381 | 5.3685 |
| 5.1532 | 4.9716\ | | | | | | |
| C3orf70 | 4.1155 | 8.0736 | 5.3732 | 2.9880 | 6.1363 | 4.4242 | 2.7953 |
| 7.3290 | 4.1243 | 7.8587 | 3.2721 | 3.5094 | 4.4249 | 5.1659 | 3.8631 |
| 4.4366 | 5.5981 | 4.3913 | 8.4039 | 2.0944 | 3.1720 | 2.5262 | 5.5157 |
| 4.7995 | 11.4073 | 2.7536 | 8.6014 | 4.3616 | 6.2325 | 2.5978 | 6.3309 |
| 5.4050 | 3.0546 | 4.8702 | 5.8713 | 3.0446 | 6.2946 | 4.3001 | 4.0014 |
| 3.3387 | 4.1682 | 8.9205 | 5.0150 | 4.2877 | 9.1066 | 1.8914 | 3.1173 |
| 4.2379 | 4.8314 | 3.1417 | 3.0665 | 4.2351 | 6.0526 | 9.1447 | 5.9130 |
| 2.5237 | 5.5150 | 3.8006 | 8.1936 | 3.8203 | 9.0452 | 6.4823 | 3.8280 |
| 2.8376 | 8.0949 | 4.7446 | 2.7333 | 4.7471 | 3.5640 | 8.1407 | 4.0676 |
| 4.3479 | 5.7476 | 3.7643 | 6.7032 | 3.3560 | 6.8915 | 2.3485 | 3.8558 |
| 6.1229 | 5.5132 | 5.0283 | 6.5977 | 3.7501 | 6.1315 | 4.2262 | 7.1161 |
| 2.2368 | 5.1990 | 8.2033 | 10.0397 | 5.1270 | 1.2055 | 4.9006 | 5.2920 |
| 10.8784 | 3.6787 | 8.4687 | 5.6581 | 4.5983 | 3.3101 | 3.6025 | 7.0702 |
| 5.1804 | 1.5803 | 6.6836 | 7.7607 | 6.5715 | 4.2679 | 4.4001 | 3.1897 |
| 1.8846 | 5.5901 | 4.6439 | 5.5486 | 5.5848 | 4.0011 | 5.4346 | 5.5827 |
| 3.9729 | 7.7238 | 5.7113 | 3.8564 | 3.6050 | 6.5432 | 4.1326 | 5.1298 |
| 1.6629 | 8.9633 | 10.7346 | 4.3755 | 2.3575 | 4.6856 | 5.3585 | 4.9964 |
| 5.4161 | 4.1530 | 4.2995 | 7.6059 | 8.0858 | 2.3602 | 4.0156 | 2.6155 |
| 5.3792 | 4.1485 | 7.5712 | 3.0248 | 9.0802 | 3.5751 | 2.6654 | 8.3743 |
| 5.4873 | 4.7767 | 3.5271 | 2.0813 | 5.3078 | 9.0034 | 4.9115 | 3.4328 |
| 3.5208 | 2.6375 | 4.4732 | 4.1210 | 2.8974 | 4.9080 | 5.2971 | 5.6157 |
| 5.2059 | 5.2680 | 2.6009 | 3.0521 | 3.4836 | 5.7366 | 4.6610 | 3.6013 |
| 4.8397 | 9.0134 | 10.1897 | 5.8914 | 5.5585 | 4.9756 | 4.5289 | 4.7276 |
| 3.3550 | 5.2343 | 5.2832 | 5.8835 | 3.8329 | 1.9569 | 3.8177 | 5.1014 |
| 4.6415 | 4.1593 | 5.4804 | 3.3334 | 2.3335 | 8.1157 | 7.6391 | 4.4587 |
| 5.9381 | 4.5045\ | | | | | | |
| ZNF285 | 0.9511 | 6.6753 | 6.0164 | 1.7854 | 5.2848 | 5.8786 | 6.8258 |
| 8.1981 | 1.7860 | 6.9592 | 6.3526 | 6.8979 | 5.8891 | 5.3535 | 5.8446 |
| 2.1513 | 7.7080 | 2.5841 | 6.6691 | 7.1185 | 5.4330 | 5.5056 | 6.8976 |
| 6.2021 | 6.6669 | 2.2189 | 7.1221 | 6.0855 | 2.5456 | 6.8097 | 5.9139 |
| 2.7455 | 4.6233 | 6.7482 | 6.9782 | 1.4920 | 1.2685 | 1.8437 | 1.4957 |
| 7.8737 | 3.3740 | 5.7337 | 4.0114 | 6.1625 | 6.2004 | 4.6139 | 4.8007 |
| 7.5501 | 6.1409 | 1.3590 | 1.5081 | 7.0854 | 6.4021 | 6.3180 | 6.3955 |
| 0.9635 | 2.7053 | 5.6861 | 5.2875 | 8.0418 | 6.3286 | 4.9550 | 1.2717 |
| 7.0848 | 6.4321 | 5.5360 | 6.6158 | 7.0298 | 2.3036 | 6.7768 | 6.4974 |
| 1.3570 | 1.9166 | 7.4240 | 2.4611 | 0.8237 | 6.6022 | 6.7951 | 4.4071 |
| 5.1543 | 6.4819 | 6.7581 | 7.3875 | 6.4913 | 6.5185 | 5.6396 | 6.5056 |
| 1.3738 | 7.8508 | 2.7496 | 6.4501 | 7.7161 | 1.8528 | 6.8017 | 1.2026 |
| 6.2283 | 3.9476 | 6.5085 | 6.3615 | 5.9066 | 2.1192 | 2.9270 | 6.2307 |

| | | | | | | | |
|---|---|---|---|---|---|---|---|
| 3.3666 | 6.7346 | 6.5491 | 7.1049 | 6.3975 | 6.9786 | 5.7495 | 6.7070 |
| 5.5309 | 5.9616 | 6.2479 | 2.1456 | 6.1861 | 5.7663 | 7.2889 | 6.1120 |
| 7.5460 | 6.9905 | 6.3147 | 6.3271 | 1.5487 | 7.3163 | 7.2360 | 5.7446 |
| 6.9430 | 6.6240 | 6.5569 | 7.0488 | 6.2998 | 2.6167 | 6.9443 | 9.2852 |
| 2.4722 | 6.2265 | 7.5539 | 7.0068 | 6.8316 | 6.8481 | 1.9771 | 5.8575 |
| 4.3980 | 1.3147 | 7.9782 | 7.0299 | 7.3256 | 3.8968 | 6.1526 | 6.1309 |
| 0.0000 | 7.0658 | 8.0833 | 0.0000 | 5.7202 | 6.7243 | 2.4027 | 6.2438 |
| 4.7036 | 1.6272 | 4.3660 | 5.7596 | 6.9453 | 6.2795 | 3.6624 | 6.6224 |
| 5.6454 | 1.6815 | 4.5304 | 5.5754 | 7.3996 | 5.9744 | 4.9401 | 0.0000 |
| 6.7335 | 7.1382 | 6.5455 | 4.7534 | 6.5431 | 2.3835 | 4.6396 | 6.5095 |
| 6.3124 | 2.1147 | 2.4158 | 5.4482 | 5.1580 | 5.5224 | 5.3671 | 6.2892 |
| 1.7437 | 7.7577 | 7.4332 | 2.1049 | 6.2431 | 6.4973 | 7.2959 | 5.6962 |
| 6.4713 | 5.3240\ | | | | | | |
| MRO | 4.7116 | 2.6668 | 2.8336 | 2.0919 | 4.1161 | 3.2887 | 2.8873 |
| 2.1723 | 2.4718 | 1.8091 | 4.5975 | 3.1028 | 3.6614 | 2.0721 | 4.6918 |
| 2.6237 | 2.5489 | 3.7153 | 2.5821 | 2.8567 | 4.4485 | 3.3952 | 2.2832 |
| 3.2932 | 3.0853 | 4.3449 | 5.7393 | 3.5617 | 3.1802 | 0.0000 | 2.7292 |
| 3.1057 | 4.2844 | 2.3782 | 1.3224 | 4.4691 | 3.1675 | 4.7179 | 1.8992 |
| 2.7248 | 4.0199 | 1.1407 | 3.0042 | 4.5253 | 2.6279 | 4.5034 | 0.0000 |
| 4.5259 | 2.2999 | 2.0462 | 2.6239 | 3.2569 | 5.0120 | 2.6047 | 3.7985 |
| 2.2632 | 2.4376 | 4.0751 | 3.2061 | 2.2896 | 2.0680 | 4.8764 | 5.3267 |
| 1.0177 | 2.5466 | 1.6675 | 2.8406 | 4.7471 | 2.7877 | 3.2513 | 5.3679 |
| 4.5537 | 3.7033 | 2.9425 | 4.2499 | 1.8854 | 3.3759 | 2.8810 | 2.0226 |
| 3.0798 | 3.7014 | 2.3540 | 3.5062 | 4.2322 | 3.3567 | 3.6793 | 2.6823 |
| 4.4738 | 2.6540 | 4.7302 | 5.6207 | 2.8855 | 4.9150 | 3.6634 | 2.8225 |
| 2.2653 | 4.7674 | 2.2250 | 2.7004 | 2.0879 | 2.7168 | 4.5418 | 2.6756 |
| 2.9361 | 5.1060 | 0.4659 | 2.3106 | 4.1482 | 2.5905 | 4.8152 | 2.3994 |
| 3.7426 | 4.5244 | 2.3219 | 4.2135 | 3.5311 | 3.9608 | 2.9970 | 3.0518 |
| 3.3113 | 5.6645 | 5.3683 | 3.2675 | 2.2783 | 2.8381 | 3.9359 | 3.5397 |
| 2.2050 | 2.1156 | 3.5282 | 3.5045 | 3.1145 | 5.0733 | 5.4525 | 2.2542 |
| 3.8725 | 2.1237 | 6.0536 | 3.5714 | 2.5936 | 3.9514 | 5.4778 | 3.6318 |
| 3.4039 | 2.5446 | 8.4724 | 3.6890 | 5.3564 | 2.5760 | 2.5049 | 4.0716 |
| 5.8705 | 3.8284 | 2.9102 | 5.1360 | 3.9889 | 1.9521 | 3.0080 | 3.0392 |
| 3.5770 | 7.5939 | 3.6629 | 4.8245 | 2.6230 | 3.3158 | 2.3687 | 0.0000 |
| 4.0627 | 1.4679 | 3.9335 | 3.4392 | 0.0000 | 3.9596 | 1.3895 | 0.0000 |
| 2.8712 | 1.6715 | 3.2020 | 2.0726 | 1.4046 | 3.8670 | 3.1660 | 3.0828 |
| 4.0096 | 4.1421 | 3.4271 | 2.6907 | 3.5517 | 2.6295 | 4.0467 | 3.6312 |
| 3.5613 | 4.6214 | 5.2781 | 3.3334 | 5.2236 | 4.7048 | 3.1558 | 3.4270 |
| 4.0536 | 3.1794\ | | | | | | |
| ZFP82 | 5.6706 | 6.9599 | 6.5475 | 6.7960 | 5.7898 | 6.3898 | 4.2346 |
| 9.1359 | 6.6687 | 7.2133 | 5.7642 | 7.1501 | 5.8976 | 6.2026 | 6.4771 |
| 2.1513 | 7.2728 | 7.2098 | 7.2012 | 6.5092 | 6.0364 | 5.2737 | 6.5518 |
| 6.3260 | 7.3888 | 7.3523 | 7.4934 | 6.6111 | 6.5001 | 6.4991 | 7.2546 |
| 5.6155 | 7.5044 | 5.9910 | 8.1068 | 7.9999 | 3.3110 | 2.0582 | 5.6610 |
| 7.1279 | 7.2760 | 6.9670 | 6.1976 | 5.6323 | 7.7022 | 3.0403 | 5.9787 |
| 6.6675 | 7.8300 | 1.9026 | 6.3922 | 5.8417 | 6.8274 | 7.5787 | 6.3758 |
| 6.6616 | 6.0835 | 6.3042 | 6.9828 | 7.6944 | 7.8738 | 5.4917 | 6.1643 |
| 6.8358 | 7.2104 | 6.4451 | 7.1780 | 6.8525 | 4.9778 | 6.7269 | 7.0601 |
| 5.7984 | 6.7590 | 7.9727 | 5.0043 | 2.4904 | 6.0890 | 4.3929 | 5.6264 |
| 7.5941 | 5.8739 | 6.5002 | 6.9993 | 6.9585 | 5.9276 | 5.7444 | 5.9106 |
| 6.5684 | 5.8990 | 7.3801 | 7.7510 | 6.6502 | 6.4606 | 6.4929 | 3.8529 |
| 7.1867 | 6.6970 | 7.8209 | 5.4782 | 7.1705 | 6.6866 | 6.7204 | 7.1407 |
| 6.2001 | 6.8684 | 7.1269 | 6.8646 | 6.6183 | 7.8412 | 6.4838 | 6.8205 |
| 5.7774 | 8.5933 | 6.8704 | 6.7437 | 6.7853 | 3.0116 | 7.2431 | 5.8439 |

```
4.7684  8.0120  5.2006  6.8088  6.0794  7.3530  5.8411  6.6107
7.5931  7.9826  7.5362  6.0697  5.5168  5.0932  6.2125  6.9178
6.0322  6.7684  6.8919  7.3216  7.5688  6.1659  2.5594  6.3956
5.0422  4.7958  7.4783  6.6574  7.3744  5.4489  7.6777  6.1897
5.4233  6.6543  7.3257  0.5476  7.2233  7.7175  6.3388  6.3109
6.6419  3.3043  3.6629  6.7595  6.5076  3.2630  6.3318  6.2193
7.5140  6.5706  7.5333  5.9689  7.7175  6.0054  5.1738  7.0275
6.9925  7.5075  7.3904  7.2174  6.3624  6.6954  5.5550  9.4096
5.1461  6.2906  6.7002  7.0330  5.4354  6.4975  5.9346  3.5812
5.3385  7.7153  7.0990  2.6702  6.7558  7.2379  7.7135  3.8382
7.0462  5.3679\
CHFR    9.4350  8.9655  7.7637  9.0925  8.5612  9.0355  8.1655
8.7909  8.7802  9.3267  8.7290  9.3116  9.3038  9.7923  9.1869
8.9761  8.8328  9.5585  9.5769  9.3090  8.9423  9.7724  8.3506
9.5167  8.7949  9.3270  9.4240  9.5365  8.7320  8.8243  9.8127
9.6598  9.0102  9.5940  9.5708  8.6648  9.7060  9.0127  5.3429
9.8390  7.4931  8.6490  9.2395  9.6469  7.9029  9.1867  8.0336
8.9436  9.2535  8.8380  9.6067  9.9031  8.9059  8.2966  9.7998
7.4104  9.1174  9.2110  9.2410  9.1641  8.3817  9.1859  9.8402
8.8759  9.2977  9.8894  8.9059  8.5194  9.5696  9.9256  9.1325
9.5548  9.7811  9.8368  9.4353  9.4734  9.6392  9.4660  9.1979
9.4841  9.5606  8.6729  9.4535  9.5303  9.4603  8.2160  8.6607
9.3451  8.8791  9.7756  8.4074  9.1929  9.7059  9.8004  8.8897
8.5620  8.2542  8.0229  9.2043  9.6063  9.1395  9.8920  8.5648
9.7188  8.5683  9.2914  10.1276 9.5983  9.3551  9.2761  9.7221
9.8734  8.4835  9.0928  8.2443  8.8483  9.7685  8.9886  9.8044
9.4926  8.5565  8.6817  9.7861  9.3602  7.8494  9.2310  9.1977
7.6719  7.9244  8.7848  9.2482  9.3174  9.6260  9.8534  9.9253
8.2812  9.6968  9.4241  9.1945  8.2834  9.9048  8.6853  9.7742
6.4992  9.0145  7.5039  10.0456 7.9578  9.2560  8.9068  9.0210
8.8365  9.7487  9.6314  8.5237  9.5429  8.0807  9.8259  8.6220
9.8136  8.9362  9.9255  9.4761  9.1031  8.7694  9.4882  8.7075
9.3399  9.2419  10.2821 9.2953  9.3241  10.5141 9.7973  7.8671
9.4893  9.5033  8.1399  9.3499  9.6973  9.5703  8.3922  8.4476
9.7046  8.7506  7.0223  8.7959  8.8146  9.5898  9.5906  9.6659
9.1617  8.9012  9.4313  6.5461  8.5023  8.6463  7.8810  9.6803
9.1059  9.4884\
EFHA1   9.9868  9.5593  9.5123  9.9999  9.7386  9.5055  9.5492
9.9672  9.9612  8.9284  10.0783 10.3014 10.1082 9.7923  10.3688
9.3131  9.1446  9.0785  9.5560  9.3204  8.8847  10.1722 9.8946
10.2700 9.5403  9.4762  8.6395  9.8546  9.5803  9.4728  9.8807
9.4135  9.3351  9.0164  9.1746  9.7577  9.9092  9.8765  10.4575
9.7036  10.3930 9.7294  9.4061  9.8480  9.7995  8.8871  8.0821
9.2715  8.8835  9.9157  9.5231  8.8949  9.2777  9.7446  9.5624
9.9389  9.3537  9.2862  9.8060  9.1244  9.8776  11.1063 9.1927
9.9814  9.7225  10.3399 9.7675  9.2289  10.1608 9.2734  9.0652
9.8646  9.3245  10.2079 10.1240 10.1402 9.8431  9.7762  9.8588
9.4126  10.2025 10.3308 9.6731  9.2431  9.7082  9.9705  10.7017
9.0234  9.9769  9.7618  9.3340  10.0532 9.3212  9.4664  10.1612
9.4844  9.1478  9.5592  10.0906 9.6089  8.9751  9.3760  9.2062
9.4695  8.7791  8.7414  9.0866  9.9921  9.8059  9.9067  9.9051
10.0325 7.6374  10.2432 9.7649  10.1760 9.7073  9.5989  9.4157
9.0928  9.8115  10.4766 9.0085  9.6742  10.1489 9.4524  9.8767
7.6092  10.0331 9.7290  8.9419  9.9493  10.1738 9.2698  10.0629
```

| | | | | | | | |
|---|---|---|---|---|---|---|---|
| 7.5566 | 9.1454 | 8.3415 | 9.7295 | 9.6635 | 7.9332 | 9.5951 | 9.9844 |
| 9.9639 | 10.2189 | 9.7463 | 9.8011 | 9.5371 | 9.3740 | 9.5910 | 9.7241 |
| 7.7046 | 9.7037 | 9.3691 | 9.3669 | 10.4245 | 9.6866 | 9.8156 | 8.8932 |
| 9.7509 | 10.0372 | 10.0126 | 10.2510 | 9.9516 | 9.4964 | 10.4843 | 9.3719 |
| 8.9050 | 10.0628 | 9.2763 | 9.5523 | 8.3646 | 9.1792 | 10.5064 | 7.5510 |
| 9.8865 | 9.8574 | 9.6652 | 10.2924 | 9.7808 | 9.8925 | 9.9323 | 9.1148 |
| 10.0767 | 10.0905 | 10.1864 | 9.8543 | 9.5200 | 9.6898 | 9.1153 | 9.5267 |
| 10.1862 | 9.9257 | 9.8689 | 9.8617 | 9.1869 | 7.9730 | 9.8337 | 8.8356 |
| 10.0845 | 8.6909\ | | | | | | |
| RTBDN | 1.2631 | 0.0000 | 1.5051 | 0.4935 | 1.2172 | 3.9654 | 1.4999 |
| 4.7814 | 1.2625 | 0.7786 | 2.0419 | 3.5591 | 4.1940 | 0.0000 | 0.9286 |
| 0.0000 | 0.0000 | 1.2795 | 0.0000 | 8.8614 | 1.3800 | 0.8427 | 0.0000 |
| 0.0000 | 0.5538 | 8.8666 | 1.6918 | 1.8373 | 1.3857 | 8.9041 | 0.7334 |
| 0.0000 | 4.5979 | 1.1010 | 1.8080 | 3.2417 | 3.0082 | 2.0582 | 0.9336 |
| 0.7666 | 0.6649 | 0.0000 | 3.0042 | 0.4326 | 0.0000 | 0.0000 | 2.2746 |
| 2.5272 | 1.0498 | 1.5638 | 4.2812 | 3.1450 | 2.6127 | 0.0000 | 0.0000 |
| 1.9450 | 1.0734 | 4.0389 | 0.4252 | 2.5512 | 0.0000 | 2.1008 | 1.9369 |
| 7.7459 | 1.1445 | 0.6977 | 0.5980 | 7.9589 | 0.5773 | 2.5345 | 2.1712 |
| 0.0000 | 0.9449 | 0.0000 | 1.8890 | 0.0000 | 3.8681 | 1.2414 | 0.0000 |
| 2.8855 | 0.0000 | 0.9627 | 0.7204 | 2.1886 | 3.6375 | 2.4045 | 1.8912 |
| 1.8689 | 0.9125 | 7.9545 | 0.0000 | 1.5834 | 2.6379 | 0.6909 | 0.5198 |
| 0.0000 | 3.5228 | 0.0000 | 0.5090 | 1.5150 | 2.7168 | 5.0168 | 3.7143 |
| 4.7873 | 2.1962 | 1.8753 | 0.0000 | 1.0326 | 2.6959 | 3.2552 | 2.1442 |
| 1.0545 | 3.3832 | 3.3219 | 2.1456 | 7.1656 | 2.9304 | 2.1672 | 1.3703 |
| 4.6223 | 5.1799 | 0.0000 | 6.5004 | 3.3565 | 0.0000 | 7.5187 | 1.3754 |
| 3.4738 | 0.0000 | 0.0000 | 0.0000 | 1.1234 | 1.9260 | 3.1029 | 1.3267 |
| 3.3360 | 3.2319 | 0.0000 | 0.0000 | 0.0000 | 3.2906 | 0.5748 | 1.4380 |
| 1.2444 | 1.6921 | 0.0000 | 0.0000 | 0.0000 | 0.0000 | 2.6654 | 0.0000 |
| 2.5542 | 0.0000 | 3.0034 | 0.5476 | 2.6584 | 0.5638 | 1.1539 | 3.2880 |
| 8.2628 | 1.0317 | 2.3902 | 0.0000 | 2.6230 | 0.7796 | 0.8742 | 0.0000 |
| 0.4012 | 0.9131 | 0.5466 | 1.4975 | 3.4253 | 5.1893 | 1.6598 | 4.3567 |
| 3.2758 | 0.5232 | 0.0000 | 0.7148 | 0.0000 | 0.0000 | 4.6747 | 4.5222 |
| 2.7813 | 1.2218 | 1.0594 | 0.0000 | 6.7070 | 0.0000 | 2.9621 | 0.0000 |
| 0.0000 | 1.7696 | 0.7547 | 0.4985 | 0.0000 | 2.8619 | 0.0000 | 0.0000 |
| 4.1251 | 0.0000\ | | | | | | |
| EFHA2 | 0.9511 | 8.0324 | 4.8974 | 7.4424 | 3.7405 | 6.7094 | 2.3543 |
| 4.7399 | 3.6080 | 7.3270 | 3.7142 | 2.0691 | 6.2250 | 6.4341 | 6.3787 |
| 6.7042 | 6.1404 | 6.4954 | 7.8168 | 1.4771 | 3.1720 | 3.5000 | 7.0194 |
| 7.7797 | 7.6328 | 1.8524 | 1.9907 | 4.3616 | 3.5215 | 2.3343 | 1.5789 |
| 2.9368 | 1.1952 | 2.1491 | 3.8082 | 1.7727 | 6.0197 | 1.9549 | 3.6815 |
| 3.8754 | 4.6099 | 7.5953 | 4.6306 | 2.8667 | 8.1492 | 0.0000 | 2.7566 |
| 3.6225 | 2.4952 | 1.9026 | 1.3077 | 3.5483 | 6.2270 | 8.2907 | 7.0531 |
| 6.1656 | 6.0952 | 5.8200 | 5.8036 | 6.0790 | 8.1278 | 6.8849 | 2.7350 |
| 1.3432 | 8.1327 | 4.2268 | 2.8406 | 3.3432 | 4.7853 | 5.8310 | 1.9272 |
| 4.8939 | 1.5110 | 4.0305 | 7.1319 | 0.4698 | 4.7440 | 4.7507 | 5.0381 |
| 3.9918 | 2.9495 | 3.5159 | 6.6264 | 5.5260 | 5.1249 | 3.9028 | 2.5771 |
| 5.9420 | 4.1863 | 5.6405 | 6.9924 | 3.8257 | 5.2808 | 7.7671 | 4.1635 |
| 6.7786 | 5.9592 | 8.0432 | 5.9622 | 7.0134 | 3.4638 | 4.4630 | 5.4483 |
| 6.7022 | 2.3164 | 4.9597 | 8.8177 | 6.6537 | 7.5028 | 6.8660 | 2.8045 |
| 3.0001 | 3.1476 | 2.0000 | 4.9920 | 5.4344 | 3.4214 | 5.6303 | 6.3605 |
| 4.1108 | 6.6070 | 3.0097 | 6.4026 | 1.7271 | 7.9183 | 3.7566 | 4.1705 |
| 0.0000 | 8.0202 | 7.9905 | 1.9345 | 3.9205 | 3.9940 | 5.0928 | 5.1575 |
| 0.0000 | 0.6413 | 5.5192 | 6.7625 | 8.3847 | 1.6169 | 2.8803 | 6.3153 |
| 4.1236 | 6.0783 | 7.7127 | 5.3314 | 6.5933 | 2.3133 | 2.1177 | 7.3805 |

| | | | | | | | |
|---|---|---|---|---|---|---|---|
| 4.2868 | 6.6673 | 5.4467 | 0.9437 | 4.8215 | 7.6191 | 6.5134 | 1.3489 |
| 3.7334 | 0.6063 | 2.2364 | 7.5188 | 4.4989 | 3.3669 | 6.6397 | 5.4575 |
| 7.1731 | 4.4431 | 4.0977 | 2.9193 | 3.1654 | 6.0849 | 6.0280 | 0.0000 |
| 7.6091 | 8.3650 | 7.9034 | 7.6969 | 6.0157 | 2.6218 | 3.6618 | 4.9284 |
| 4.5386 | 6.4748 | 4.6351 | 4.7703 | 4.7136 | 2.8760 | 5.2976 | 3.9396 |
| 5.6666 | 3.5951 | 5.0725 | 3.5005 | 2.8198 | 4.6756 | 7.6730 | 3.6472 |
| 7.3143 | 0.6959\ | | | | | | |
| AAA1 | 0.0000 | 0.0000 | 0.0000 | 1.3959 | 0.0000 | 0.0000 | 0.0000 |
| 0.0000 | 0.0000 | 0.0000 | 0.4291 | 0.0000 | 0.4363 | 0.0000 | 0.5377 |
| 0.0000 | 0.0000 | 0.0000 | 0.0000 | 0.0000 | 0.0000 | 0.0000 | 0.0000 |
| 0.5262 | 0.0000 | 0.0000 | 0.0000 | 1.6129 | 0.0000 | 0.0000 | 0.0000 |
| 0.0000 | 0.0000 | 0.4664 | 0.0000 | 0.6819 | 0.0000 | 0.0000 | 0.0000 |
| 0.0000 | 0.0000 | 0.0000 | 0.0000 | 0.0000 | 0.0000 | 0.0000 | 0.0000 |
| 0.0000 | 0.0000 | 0.0000 | 0.4530 | 0.0000 | 0.0000 | 0.0000 | 0.0000 |
| 0.0000 | 1.4095 | 0.0000 | 0.0000 | 0.0000 | 0.5422 | 0.4969 | 0.0000 |
| 0.0000 | 1.4936 | 0.9507 | 0.0000 | 0.0000 | 0.0000 | 0.6160 | 0.0000 |
| 0.0000 | 0.0000 | 0.0000 | 0.0000 | 1.3447 | 0.0000 | 0.5408 | 0.0000 |
| 0.0000 | 0.0000 | 0.0000 | 0.0000 | 0.0000 | 0.9110 | 0.0000 | 0.0000 |
| 0.0000 | 0.0000 | 0.0000 | 0.0000 | 0.0000 | 0.0000 | 0.6909 | 0.0000 |
| 0.0000 | 0.0000 | 0.0000 | 0.0000 | 0.0000 | 0.0000 | 0.4986 | 0.0000 |
| 0.0000 | 0.0000 | 0.0000 | 0.0000 | 0.0000 | 0.0000 | 0.5877 | 1.4385 |
| 0.0000 | 0.0000 | 0.0000 | 0.0000 | 0.0000 | 0.5276 | 0.0000 | 0.3974 |
| 0.0000 | 0.0000 | 1.0055 | 0.0000 | 0.4700 | 0.0000 | 0.0000 | 0.0000 |
| 0.0000 | 0.0000 | 0.0000 | 0.3935 | 0.0000 | 2.6167 | 0.0000 | 0.0000 |
| 0.0000 | 0.0000 | 0.0000 | 0.0000 | 0.0000 | 0.6010 | 0.0000 | 1.4380 |
| 0.0000 | 0.0000 | 0.0000 | 0.0000 | 0.0000 | 0.0000 | 0.0000 | 0.0000 |
| 0.0000 | 0.0000 | 0.0000 | 0.0000 | 0.0000 | 0.0000 | 0.0000 | 0.0000 |
| 0.0000 | 1.6272 | 0.0000 | 0.0000 | 0.4033 | 0.0000 | 0.5023 | 0.0000 |
| 0.4012 | 0.0000 | 0.0000 | 0.0000 | 0.5290 | 0.0000 | 0.0000 | 0.0000 |
| 0.0000 | 0.0000 | 0.0000 | 0.0000 | 0.0000 | 0.0000 | 0.0000 | 0.0000 |
| 0.0000 | 0.0000 | 0.6248 | 0.0000 | 0.0000 | 0.0000 | 0.0000 | 0.0000 |
| 0.0000 | 0.0000 | 0.0000 | 0.4985 | 0.0000 | 0.0000 | 0.0000 | 0.0000 |
| 0.8824 | 0.0000\ | | | | | | |
| GPR75 | 1.2631 | 3.4341 | 4.8674 | 3.5865 | 4.5288 | 4.4459 | 2.2192 |
| 3.8329 | 3.8371 | 3.6379 | 2.5484 | 5.0942 | 3.4445 | 3.6727 | 3.1176 |
| 1.4442 | 2.2551 | 1.6514 | 3.8042 | 3.8459 | 2.5380 | 2.9530 | 4.4224 |
| 2.1767 | 3.9106 | 1.0317 | 4.0863 | 1.0210 | 0.0000 | 3.9213 | 5.5959 |
| 3.1057 | 3.8834 | 3.8818 | 3.2487 | 2.5445 | 4.2364 | 4.0723 | 2.0653 |
| 3.7304 | 3.0046 | 5.6668 | 0.6231 | 4.7101 | 2.0387 | 2.8725 | 0.0000 |
| 3.8179 | 3.5041 | 0.4764 | 4.1673 | 3.3607 | 3.6936 | 4.0222 | 4.6398 |
| 3.6325 | 2.5776 | 3.5149 | 2.8422 | 3.1339 | 3.5225 | 2.5691 | 3.9207 |
| 2.3502 | 3.3334 | 4.2026 | 3.8434 | 4.4906 | 2.5659 | 3.1682 | 2.6452 |
| 3.1890 | 0.0000 | 1.3638 | 3.8599 | 2.8398 | 3.5007 | 2.3485 | 1.5048 |
| 2.0686 | 1.7663 | 3.8365 | 3.1329 | 3.2178 | 1.6785 | 3.6175 | 4.9237 |
| 2.0646 | 3.2962 | 3.0083 | 3.3023 | 4.7948 | 2.9318 | 2.0255 | 1.4518 |
| 2.5596 | 1.6718 | 4.2061 | 2.6033 | 2.0879 | 1.6914 | 2.2374 | 3.9837 |
| 3.1674 | 2.7166 | 3.9170 | 1.8642 | 3.6838 | 3.3409 | 3.0071 | 3.5471 |
| 3.5571 | 2.5153 | 2.8074 | 3.6006 | 3.3929 | 1.2170 | 3.9369 | 5.0998 |
| 1.6324 | 3.5559 | 1.3285 | 4.7198 | 1.8858 | 4.6782 | 2.4516 | 2.8832 |
| 0.7843 | 4.1037 | 5.6650 | 3.1944 | 1.7472 | 2.5026 | 4.3451 | 2.6505 |
| 2.0117 | 0.0000 | 0.0000 | 4.5636 | 4.1183 | 3.7991 | 1.5646 | 2.7437 |
| 3.1308 | 3.5588 | 4.4574 | 2.9153 | 3.4947 | 2.4506 | 0.7381 | 4.1413 |
| 0.0000 | 4.9777 | 2.3250 | 2.7093 | 4.1415 | 4.0271 | 3.6943 | 3.3322 |
| 0.9345 | 1.6272 | 2.0641 | 2.7999 | 1.3855 | 3.6808 | 3.4589 | 4.5131 |

| | | | | | | | |
|---|---|---|---|---|---|---|---|
| 4.5511 | 3.4214 | 3.9763 | 4.0283 | 5.8722 | 2.5860 | 2.7948 | 0.0000 |
| 2.5204 | 3.8190 | 3.1136 | 4.1695 | 3.3695 | 0.5545 | 3.1660 | 4.5495 |
| 2.5935 | 3.6236 | 2.5553 | 2.6907 | 3.0995 | 1.9569 | 2.5489 | 3.5295 |
| 3.0902 | 4.1977 | 3.9155 | 4.0603 | 2.3335 | 1.6276 | 4.1603 | 4.4194 |
| 4.3506 | 0.6959\ | | | | | | |
| NUDT9P1 | 0.5526 | 1.6912 | 2.7039 | 1.3959 | 0.5278 | 2.0872 | 0.9367 |
| 2.0418 | 1.2625 | 3.3648 | 2.0419 | 3.6072 | 3.4445 | 2.0721 | 0.0000 |
| 3.2639 | 1.0553 | 3.0246 | 2.9040 | 2.2825 | 3.6109 | 1.3712 | 1.9117 |
| 2.8404 | 2.5058 | 1.3599 | 4.0218 | 1.6129 | 0.0000 | 2.5978 | 4.6731 |
| 0.0000 | 0.8951 | 3.3450 | 2.8081 | 1.4920 | 1.5255 | 1.2859 | 0.9336 |
| 0.4337 | 4.2593 | 3.5163 | 1.6604 | 1.4585 | 3.5062 | 0.0000 | 0.0000 |
| 2.6691 | 1.4771 | 1.1202 | 1.0749 | 0.0000 | 3.1634 | 3.4251 | 3.3386 |
| 0.5608 | 2.2825 | 1.1710 | 2.6972 | 2.2896 | 3.5225 | 1.9554 | 0.0000 |
| 1.6085 | 2.9371 | 1.5189 | 2.2002 | 2.8005 | 1.9829 | 1.8731 | 1.2639 |
| 1.6718 | 3.0705 | 1.1245 | 1.4868 | 0.0000 | 3.7219 | 0.0000 | 2.4028 |
| 1.3771 | 2.2758 | 3.5553 | 2.4682 | 0.6718 | 2.6513 | 2.9962 | 1.8912 |
| 1.8689 | 3.8754 | 4.6312 | 2.2864 | 3.4573 | 2.9127 | 4.1363 | 3.3378 |
| 2.4199 | 2.0217 | 2.8016 | 0.0000 | 2.3730 | 0.0000 | 0.4986 | 1.6984 |
| 2.5001 | 0.0000 | 0.4659 | 3.3565 | 2.7174 | 3.7365 | 2.1763 | 0.0000 |
| 1.6572 | 0.0000 | 3.0000 | 1.8354 | 4.6604 | 1.8676 | 3.2823 | 0.0000 |
| 2.0533 | 3.7274 | 1.3285 | 2.9016 | 0.4700 | 2.9380 | 1.8805 | 0.6149 |
| 1.2894 | 3.6900 | 2.8723 | 0.9567 | 1.1234 | 0.0000 | 0.7571 | 2.4659 |
| 0.0000 | 1.0837 | 4.8598 | 3.0710 | 3.4131 | 0.6010 | 0.0000 | 1.7133 |
| 0.5424 | 1.6921 | 3.3387 | 0.0000 | 2.7033 | 2.4506 | 0.7381 | 2.2465 |
| 0.0000 | 2.6925 | 2.0029 | 2.7093 | 2.2918 | 2.6463 | 2.8937 | 1.6149 |
| 2.0666 | 0.0000 | 2.2364 | 3.4515 | 2.1852 | 3.2630 | 1.6241 | 4.0254 |
| 2.3696 | 2.7528 | 1.5072 | 2.0984 | 3.4253 | 1.4956 | 0.6229 | 0.0000 |
| 2.5204 | 3.0757 | 2.8104 | 0.9723 | 2.6986 | 0.5545 | 3.5147 | 2.5448 |
| 1.8147 | 2.8065 | 3.1906 | 0.0000 | 2.8698 | 1.1063 | 1.5562 | 1.1800 |
| 0.5556 | 3.2742 | 2.5389 | 0.0000 | 3.4726 | 3.6432 | 3.2388 | 2.4421 |
| 2.4955 | 0.6959\ | | | | | | |
| SYPL2 | 3.1593 | 6.1036 | 3.3494 | 2.6559 | 5.3010 | 2.5543 | 3.4058 |
| 3.9097 | 2.2773 | 5.3600 | 3.0487 | 2.8059 | 3.1893 | 1.9445 | 2.7808 |
| 6.2290 | 2.7928 | 4.2361 | 6.8358 | 2.4488 | 3.0838 | 3.8568 | 2.1093 |
| 4.4677 | 8.3034 | 8.2768 | 2.2381 | 3.0366 | 4.0984 | 2.8206 | 2.4077 |
| 3.9764 | 2.5183 | 3.4991 | 1.0004 | 4.0627 | 5.4354 | 2.9827 | 1.4957 |
| 4.1845 | 3.7950 | 6.1226 | 3.7469 | 2.8667 | 7.5268 | 2.2058 | 4.5480 |
| 3.1281 | 2.6671 | 2.6955 | 3.0665 | 4.3904 | 3.6288 | 6.9122 | 3.3917 |
| 2.8432 | 3.5225 | 4.8967 | 5.3854 | 7.0042 | 8.4790 | 3.7810 | 4.8953 |
| 1.8326 | 5.6962 | 4.1276 | 3.4274 | 3.5036 | 3.3013 | 6.7838 | 2.1712 |
| 3.7541 | 3.5970 | 2.7868 | 4.6631 | 1.5483 | 3.7723 | 4.2623 | 3.9155 |
| 6.9166 | 2.7579 | 3.3468 | 6.2256 | 3.7501 | 3.2933 | 3.6793 | 1.8912 |
| 2.9812 | 3.0105 | 4.8003 | 6.1000 | 2.8855 | 4.7380 | 6.2468 | 3.9773 |
| 7.6444 | 2.5385 | 6.1741 | 4.2938 | 3.1532 | 3.6674 | 3.9875 | 2.9516 |
| 5.8131 | 6.6908 | 3.6802 | 5.3201 | 5.1919 | 4.3986 | 3.3997 | 2.5118 |
| 1.8846 | 2.5153 | 4.2479 | 3.7457 | 5.4863 | 1.6815 | 2.7941 | 2.9370 |
| 3.9729 | 7.2726 | 3.8176 | 4.0567 | 3.6935 | 3.8406 | 3.5519 | 4.0367 |
| 0.7843 | 6.9303 | 7.5940 | 2.4304 | 3.1145 | 3.5526 | 4.1901 | 3.6220 |
| 3.7958 | 3.6728 | 0.0000 | 5.6500 | 5.9606 | 3.1296 | 2.4344 | 3.8753 |
| 3.2040 | 3.5588 | 6.9121 | 2.9153 | 4.4977 | 2.6913 | 2.3242 | 6.2222 |
| 4.2868 | 3.2708 | 2.7037 | 2.4897 | 4.5433 | 5.7052 | 3.6244 | 2.0337 |
| 8.7805 | 1.6272 | 2.9807 | 2.9924 | 1.7039 | 3.9385 | 3.3452 | 4.6449 |
| 3.8222 | 4.9554 | 1.7238 | 2.4266 | 3.0154 | 2.2140 | 3.1858 | 5.9600 |
| 2.6164 | 6.4805 | 5.9471 | 3.7893 | 4.2572 | 3.6145 | 4.8383 | 3.0828 |

| | | | | | | | |
|---|---|---|---|---|---|---|---|
| 3.0965 | 2.6622 | 2.5553 | 4.2370 | 6.6718 | 1.9569 | 2.8694 | 4.1255 |
| 2.6247 | 2.7619 | 4.0413 | 3.9100 | 1.5946 | 5.5856 | 4.9075 | 2.5911 |
| 5.2523 | 2.8479\ | | | | | | |
| RFX8 | 0.9511 | 0.5319 | 1.1543 | 2.3445 | 1.6818 | 0.9816 | 0.5431 |
| 1.7391 | 0.4327 | 1.4796 | 0.7594 | 1.3775 | 1.6409 | 0.4395 | 1.8915 |
| 0.0000 | 2.2551 | 1.2795 | 1.5827 | 1.3142 | 2.2447 | 1.5770 | 0.6346 |
| 2.7488 | 0.5538 | 1.0317 | 1.5155 | 1.6129 | 0.8532 | 0.5901 | 3.5766 |
| 0.9642 | 0.5159 | 0.8184 | 1.0004 | 0.0000 | 1.2685 | 0.6557 | 0.5410 |
| 0.4337 | 0.0000 | 0.4871 | 1.0569 | 1.6313 | 1.6194 | 3.7705 | 1.5457 |
| 1.7580 | 0.0000 | 0.8339 | 1.3077 | 2.1910 | 2.1411 | 2.5005 | 2.6594 |
| 0.9635 | 0.6343 | 3.0892 | 1.2457 | 1.2977 | 1.9018 | 0.4969 | 0.0000 |
| 1.6085 | 1.1445 | 0.6977 | 0.0000 | 0.8444 | 4.0229 | 1.0463 | 0.4334 |
| 0.9537 | 0.0000 | 1.5690 | 1.2339 | 0.4698 | 3.3759 | 0.9332 | 3.4332 |
| 0.6159 | 1.5467 | 0.3965 | 2.6279 | 0.6718 | 1.4651 | 1.8814 | 0.5376 |
| 3.8342 | 1.6807 | 2.4340 | 1.9147 | 0.8470 | 0.5215 | 2.8364 | 1.2026 |
| 1.0621 | 1.4589 | 1.2499 | 1.8231 | 2.4969 | 0.0000 | 0.4986 | 0.5352 |
| 5.6506 | 0.0000 | 0.0000 | 0.7314 | 1.7824 | 0.0000 | 1.0041 | 0.0000 |
| 1.3871 | 0.0000 | 2.3219 | 5.7107 | 1.6379 | 1.4679 | 1.0339 | 1.6868 |
| 1.8581 | 3.0090 | 1.3285 | 1.3885 | 0.0000 | 2.0269 | 1.8805 | 1.0447 |
| 0.0000 | 1.5385 | 1.9820 | 0.7024 | 0.6684 | 2.8211 | 3.1029 | 0.8107 |
| 0.0000 | 0.6413 | 0.0000 | 1.6679 | 0.0000 | 6.0469 | 1.9771 | 2.3186 |
| 2.0686 | 1.8493 | 3.8298 | 0.6735 | 2.4839 | 0.0000 | 0.0000 | 1.4139 |
| 0.0000 | 0.9341 | 0.5862 | 1.2540 | 5.5342 | 0.9683 | 1.5047 | 1.8394 |
| 1.2429 | 0.0000 | 0.0000 | 1.8011 | 0.4033 | 0.4418 | 1.1696 | 0.0000 |
| 0.7148 | 0.9131 | 0.9420 | 0.4486 | 0.9151 | 0.9335 | 0.0000 | 0.0000 |
| 1.1272 | 0.5232 | 2.9187 | 0.4012 | 1.4046 | 0.0000 | 0.6902 | 0.0000 |
| 0.0000 | 0.0000 | 0.0000 | 0.0000 | 0.0000 | 0.6567 | 2.5489 | 0.8827 |
| 2.2504 | 1.2903 | 1.4427 | 0.0000 | 0.0000 | 1.3603 | 1.0391 | 0.6701 |
| 1.6364 | 0.0000\ | | | | | | |
| ZNF222 | 6.0748 | 6.1415 | 6.3118 | 5.5392 | 5.9732 | 5.4451 | 6.1011 |
| 8.0211 | 6.1201 | 6.3120 | 6.0861 | 6.0383 | 5.8227 | 6.3796 | 5.5843 |
| 6.5252 | 5.9895 | 5.6490 | 6.1124 | 5.1851 | 5.2221 | 5.6991 | 6.0335 |
| 5.7381 | 5.8602 | 5.7166 | 6.6229 | 5.4501 | 6.2151 | 6.7118 | 5.6303 |
| 6.6373 | 6.0891 | 5.9381 | 6.3318 | 6.1822 | 5.8866 | 6.3136 | 6.2494 |
| 6.8475 | 5.5801 | 6.5221 | 4.2664 | 5.4998 | 6.6656 | 5.5003 | 4.6978 |
| 5.4003 | 7.2706 | 5.5353 | 5.9982 | 4.7376 | 5.9235 | 6.6627 | 6.7012 |
| 6.7511 | 4.6608 | 6.2812 | 5.8460 | 6.0397 | 6.7017 | 6.6910 | 5.7623 |
| 6.4943 | 6.1407 | 6.1344 | 5.2857 | 6.7767 | 5.6012 | 5.8804 | 6.6258 |
| 6.1803 | 5.2827 | 6.1287 | 6.1923 | 4.5908 | 6.1901 | 5.4054 | 6.1055 |
| 5.3924 | 5.9543 | 5.8154 | 5.7082 | 6.2117 | 5.7826 | 5.1992 | 6.5127 |
| 5.2744 | 6.2338 | 5.6376 | 5.9658 | 6.5307 | 6.2815 | 6.1627 | 6.2153 |
| 5.7485 | 5.9893 | 6.0562 | 6.0236 | 6.4303 | 6.1317 | 5.6276 | 5.9869 |
| 4.7521 | 5.7865 | 5.4369 | 5.9763 | 6.8399 | 6.1033 | 5.6662 | 6.1924 |
| 5.8247 | 3.5857 | 3.8074 | 6.3381 | 5.5924 | 5.8193 | 6.1953 | 5.4735 |
| 6.1254 | 6.6316 | 5.5178 | 6.1077 | 5.2757 | 6.2127 | 6.8720 | 3.9824 |
| 5.9631 | 6.9075 | 6.3338 | 6.1801 | 6.3213 | 5.9529 | 5.7908 | 8.2514 |
| 4.5695 | 6.1937 | 3.9958 | 6.1275 | 6.1685 | 4.9842 | 6.7689 | 5.9401 |
| 5.5150 | 5.7297 | 6.5119 | 5.9043 | 6.2631 | 6.0768 | 1.8766 | 6.1867 |
| 4.2129 | 6.5104 | 7.0482 | 5.4421 | 5.7865 | 6.3405 | 6.2037 | 4.9740 |
| 4.8684 | 3.9971 | 4.7190 | 5.5264 | 6.3803 | 5.2847 | 5.0475 | 3.0782 |
| 5.3261 | 5.6306 | 6.1320 | 6.4135 | 6.0583 | 6.5794 | 5.3555 | 4.1460 |
| 7.2570 | 6.1764 | 6.1557 | 6.4513 | 5.4646 | 6.4358 | 3.0638 | 6.0165 |
| 6.2628 | 7.0251 | 6.6294 | 5.7701 | 6.0022 | 6.2358 | 5.8199 | 6.5775 |
| 6.2162 | 6.3654 | 6.8399 | 7.3904 | 6.2811 | 5.4017 | 6.6463 | 6.1901 |

| | | | | | | | |
|---|---|---|---|---|---|---|---|
| 6.4217 | 4.9135\ | | | | | | |
| RFX4 | 0.0000 | 0.0000 | 0.6896 | 0.0000 | 0.5278 | 1.8477 | 0.0000 |
| 5.7126 | 0.0000 | 0.0000 | 0.0000 | 0.0000 | 0.7708 | 0.0000 | 0.0000 |
| 0.0000 | 0.0000 | 2.1918 | 0.0000 | 0.0000 | 0.0000 | 2.0613 | 2.1093 |
| 1.2144 | 0.0000 | 0.0000 | 1.0809 | 0.5990 | 1.7737 | 0.0000 | 9.8312 |
| 0.0000 | 0.0000 | 0.0000 | 0.0000 | 0.0000 | 3.3110 | 2.0582 | 0.9336 |
| 0.0000 | 0.0000 | 0.0000 | 0.0000 | 2.4713 | 0.0000 | 0.0000 | 0.0000 |
| 2.7982 | 1.0498 | 0.0000 | 0.0000 | 0.0000 | 1.0956 | 0.0000 | 4.8252 |
| 0.9635 | 0.0000 | 0.0000 | 0.0000 | 0.0000 | 0.0000 | 2.5691 | 0.0000 |
| 0.0000 | 0.0000 | 1.3532 | 0.0000 | 6.5800 | 0.0000 | 0.6160 | 0.7662 |
| 0.3921 | 0.9449 | 1.1245 | 0.9270 | 0.0000 | 2.6228 | 2.3485 | 0.0000 |
| 0.0000 | 1.2875 | 0.0000 | 0.0000 | 6.4528 | 0.0000 | 0.0000 | 1.2355 |
| 1.3738 | 0.0000 | 7.5592 | 0.0000 | 0.8470 | 2.1644 | 0.0000 | 0.0000 |
| 0.0000 | 0.5233 | 0.5454 | 0.5090 | 0.0000 | 6.8489 | 0.4986 | 0.5352 |
| 0.7358 | 0.0000 | 0.0000 | 0.0000 | 2.3737 | 3.3409 | 1.0041 | 0.0000 |
| 1.6572 | 5.4414 | 0.0000 | 1.4396 | 2.9753 | 0.5276 | 2.0505 | 0.3974 |
| 0.0000 | 4.8495 | 0.0000 | 3.6855 | 1.3451 | 1.3435 | 1.2272 | 2.5319 |
| 0.0000 | 0.0000 | 0.0000 | 0.9567 | 0.0000 | 0.5526 | 4.0766 | 0.0000 |
| 0.0000 | 2.2980 | 3.9958 | 0.0000 | 0.0000 | 2.2071 | 0.0000 | 1.9445 |
| 0.0000 | 0.0000 | 0.0000 | 4.4476 | 0.0000 | 4.3473 | 0.0000 | 0.0000 |
| 1.9723 | 0.0000 | 2.3250 | 1.2540 | 1.0445 | 0.0000 | 0.3855 | 1.3489 |
| 2.9697 | 0.0000 | 0.0000 | 0.0000 | 1.5535 | 0.7796 | 5.4966 | 0.0000 |
| 0.4012 | 1.6815 | 0.5466 | 0.0000 | 0.0000 | 0.0000 | 0.0000 | 0.0000 |
| 0.4801 | 0.0000 | 0.0000 | 4.2926 | 0.0000 | 0.0000 | 2.0241 | 0.9100 |
| 0.0000 | 0.0000 | 3.1906 | 0.0000 | 0.0000 | 0.0000 | 0.0000 | 0.0000 |
| 2.3863 | 0.0000 | 0.0000 | 0.0000 | 0.0000 | 0.0000 | 0.0000 | 0.0000 |
| 0.5077 | 0.0000\ | | | | | | |
| OR2C3 | 0.0000 | 0.5319 | 0.0000 | 0.0000 | 0.0000 | 1.9724 | 0.0000 |
| 0.0000 | 0.0000 | 1.2815 | 0.0000 | 0.4849 | 1.2705 | 0.0000 | 0.0000 |
| 0.0000 | 0.0000 | 0.0000 | 0.0000 | 0.0000 | 2.3988 | 0.0000 | 0.0000 |
| 0.0000 | 0.0000 | 0.0000 | 0.0000 | 0.0000 | 0.0000 | 0.0000 | 0.0000 |
| 0.0000 | 0.0000 | 2.3363 | 0.0000 | 0.0000 | 0.0000 | 0.0000 | 0.0000 |
| 0.0000 | 0.0000 | 0.0000 | 0.0000 | 0.0000 | 0.0000 | 0.0000 | 0.0000 |
| 0.0000 | 0.0000 | 0.0000 | 0.0000 | 0.0000 | 0.0000 | 0.0000 | 0.0000 |
| 0.0000 | 0.0000 | 0.0000 | 0.4252 | 0.0000 | 0.5422 | 0.0000 | 0.0000 |
| 0.0000 | 0.0000 | 0.0000 | 0.0000 | 0.0000 | 0.0000 | 0.0000 | 0.0000 |
| 0.3921 | 0.0000 | 1.3638 | 0.0000 | 0.0000 | 0.0000 | 0.0000 | 0.0000 |
| 0.0000 | 0.0000 | 0.0000 | 0.0000 | 0.0000 | 0.0000 | 0.0000 | 0.0000 |
| 0.0000 | 0.0000 | 0.0000 | 0.0000 | 0.0000 | 0.0000 | 0.0000 | 0.0000 |
| 0.0000 | 0.0000 | 0.0000 | 0.0000 | 0.0000 | 0.0000 | 0.0000 | 0.0000 |
| 0.0000 | 0.0000 | 0.0000 | 0.0000 | 0.0000 | 0.0000 | 0.0000 | 0.0000 |
| 0.0000 | 0.0000 | 0.0000 | 0.0000 | 0.0000 | 0.0000 | 2.0505 | 0.0000 |
| 0.0000 | 0.0000 | 0.0000 | 0.0000 | 0.0000 | 0.0000 | 0.0000 | 0.0000 |
| 0.0000 | 0.0000 | 0.0000 | 0.0000 | 0.0000 | 0.0000 | 0.0000 | 0.0000 |
| 3.2234 | 0.0000 | 0.0000 | 0.0000 | 0.0000 | 0.0000 | 0.0000 | 0.0000 |
| 0.0000 | 0.0000 | 0.0000 | 0.0000 | 0.0000 | 0.0000 | 0.0000 | 0.0000 |
| 0.0000 | 0.0000 | 0.0000 | 0.0000 | 0.0000 | 0.5638 | 0.0000 | 0.0000 |
| 0.0000 | 0.0000 | 0.0000 | 0.0000 | 0.0000 | 0.0000 | 0.0000 | 0.0000 |
| 0.0000 | 0.0000 | 0.0000 | 0.0000 | 0.0000 | 0.0000 | 0.0000 | 0.0000 |
| 0.0000 | 0.0000 | 0.0000 | 0.4012 | 0.0000 | 1.2666 | 0.0000 | 0.0000 |
| 0.0000 | 0.0000 | 0.6248 | 0.0000 | 0.0000 | 0.0000 | 0.0000 | 0.0000 |
| 0.0000 | 0.0000 | 0.0000 | 0.4985 | 0.0000 | 0.0000 | 0.0000 | 0.0000 |
| 0.0000 | 0.0000\ | | | | | | |
| AVPR2 | 0.9511 | 6.1036 | 1.5051 | 2.0919 | 4.6606 | 3.3816 | 1.4999 |

| | | | | | | | |
|---|---|---|---|---|---|---|---|
| 3.9827 | 5.4487 | 4.4896 | 2.1584 | 4.9338 | 5.2508 | 3.4557 | 2.0573 |
| 0.0000 | 4.1466 | 2.8922 | 6.2540 | 7.5818 | 6.8495 | 2.8772 | 4.9973 |
| 1.6784 | 6.5275 | 4.3815 | 4.2361 | 3.9933 | 2.7323 | 2.1819 | 2.5774 |
| 2.5248 | 5.8836 | 3.9547 | 3.1707 | 2.5445 | 7.9436 | 1.7231 | 3.3363 |
| 1.2646 | 3.1062 | 5.6895 | 1.3900 | 0.7649 | 7.6210 | 0.9285 | 0.0000 |
| 6.2145 | 2.4952 | 2.4071 | 2.1110 | 6.8823 | 3.1634 | 7.3766 | 4.5015 |
| 1.5362 | 3.8331 | 5.2687 | 5.8643 | 5.0692 | 8.0554 | 2.6657 | 0.5573 |
| 0.5968 | 4.1163 | 1.8022 | 3.0340 | 2.9564 | 1.5697 | 5.0884 | 3.6908 |
| 0.9537 | 3.2212 | 6.8267 | 4.6631 | 3.3007 | 2.6228 | 3.5184 | 4.3648 |
| 3.0798 | 2.9495 | 2.1634 | 5.8416 | 1.4748 | 4.7997 | 3.8501 | 4.5315 |
| 2.6565 | 4.1509 | 3.2939 | 7.9898 | 3.6532 | 3.6228 | 4.4518 | 3.2084 |
| 7.8692 | 2.6431 | 6.6734 | 3.1760 | 6.2079 | 2.7168 | 3.6974 | 1.2310 |
| 3.5417 | 3.0982 | 3.0433 | 5.1695 | 3.3800 | 3.3409 | 4.2931 | 1.8341 |
| 1.0545 | 6.3432 | 6.8455 | 0.5141 | 5.6469 | 1.8676 | 7.9058 | 2.1659 |
| 3.5882 | 6.2541 | 1.8152 | 7.9607 | 7.4769 | 5.8807 | 7.5081 | 1.3754 |
| 10.3413 | 7.1543 | 6.3929 | 0.0000 | 1.4687 | 2.7225 | 3.6704 | 3.6220 |
| 10.0715 | 3.6080 | 4.5503 | 7.0019 | 7.0575 | 2.3602 | 2.2974 | 3.7587 |
| 1.2444 | 5.0758 | 2.2162 | 1.1308 | 7.8412 | 1.7997 | 2.8098 | 6.4034 |
| 3.2886 | 2.0661 | 5.8741 | 1.7261 | 5.8763 | 8.1308 | 1.5047 | 3.6850 |
| 3.4624 | 2.5111 | 2.9807 | 2.4523 | 1.9645 | 2.8261 | 1.4147 | 3.5749 |
| 3.0096 | 2.9304 | 2.6009 | 1.2980 | 7.5332 | 3.5752 | 3.4930 | 8.6050 |
| 1.5724 | 5.2538 | 7.4230 | 2.1786 | 4.7148 | 2.8263 | 9.4188 | 5.9359 |
| 2.8668 | 4.8571 | 4.3960 | 3.7442 | 3.6273 | 1.4486 | 2.5489 | 1.8202 |
| 2.7306 | 3.0407 | 3.8823 | 2.7609 | 5.7421 | 3.3048 | 6.3892 | 1.4718 |
| 1.9827 | 3.6039\ | | | | | | |
| RFX7 | 8.7000 | 8.3992 | 8.0965 | 8.6898 | 9.7258 | 9.8294 | 9.2238 |
| 8.6829 | 8.6692 | 8.8500 | 8.5513 | 8.7881 | 8.6362 | 9.3729 | 9.1541 |
| 8.1105 | 6.8218 | 8.3974 | 8.4724 | 7.2762 | 8.0608 | 8.5976 | 8.7380 |
| 9.2478 | 9.0492 | 7.9753 | 6.8519 | 8.1475 | 8.1001 | 8.2538 | 8.6166 |
| 8.4395 | 7.6442 | 8.6016 | 8.9536 | 8.3808 | 8.7711 | 9.2218 | 8.7757 |
| 8.2676 | 8.8746 | 8.4446 | 7.1607 | 9.1096 | 8.8999 | 7.9092 | 7.2418 |
| 8.4756 | 10.3849 | 7.7085 | 8.9628 | 7.9236 | 8.6866 | 8.9619 | 8.4870 |
| 9.3100 | 9.7167 | 9.1740 | 8.8414 | 7.7830 | 9.1897 | 9.1383 | 8.0551 |
| 7.7765 | 8.9529 | 8.5946 | 7.6310 | 8.4958 | 7.7942 | 8.5109 | 7.8639 |
| 9.5367 | 7.9270 | 8.0814 | 9.2839 | 7.8041 | 8.4855 | 7.9108 | 8.5449 |
| 9.1621 | 7.8790 | 9.6371 | 8.4435 | 8.3116 | 8.3732 | 8.3478 | 9.3208 |
| 8.1753 | 8.1351 | 8.6470 | 8.7624 | 8.9501 | 7.5859 | 8.4071 | 8.8318 |
| 8.6408 | 8.3520 | 9.1032 | 8.6694 | 9.0824 | 7.8035 | 8.6741 | 7.9620 |
| 8.3994 | 8.4599 | 8.1316 | 7.9498 | 8.9901 | 8.8395 | 8.6823 | 8.9878 |
| 8.9444 | 7.9534 | 7.9658 | 9.2772 | 9.0884 | 9.1730 | 9.5878 | 8.9128 |
| 7.8050 | 8.9978 | 8.9926 | 8.2714 | 8.8564 | 9.1753 | 8.6874 | 8.5736 |
| 7.8719 | 9.3520 | 9.6111 | 8.0173 | 8.8364 | 8.4263 | 8.4058 | 7.7443 |
| 6.8420 | 8.5989 | 8.8480 | 9.5015 | 9.1217 | 7.9782 | 8.1910 | 8.3578 |
| 9.2447 | 9.3865 | 8.8626 | 8.7197 | 8.8366 | 8.2908 | 7.6151 | 8.7511 |
| 8.1128 | 9.5761 | 8.2962 | 9.5136 | 8.3354 | 9.0074 | 9.5267 | 8.8503 |
| 8.9811 | 7.4676 | 7.7745 | 9.2273 | 8.2069 | 7.2431 | 9.9209 | 8.0469 |
| 9.0852 | 8.2929 | 7.8821 | 9.4567 | 9.0432 | 7.5615 | 7.8178 | 8.3209 |
| 9.2872 | 8.8899 | 8.3671 | 9.2787 | 8.1819 | 8.0844 | 8.4105 | 8.7079 |
| 8.4638 | 8.6076 | 9.0674 | 8.6032 | 7.9561 | 8.3079 | 9.3798 | 8.8950 |
| 8.9464 | 8.7119 | 7.9785 | 8.8748 | 8.3676 | 7.4162 | 9.0939 | 8.9030 |
| 9.4613 | 8.0470\ | | | | | | |
| NRXN3 | 2.5026 | 6.3123 | 7.8235 | 2.2237 | 9.1455 | 1.9724 | 5.3813 |
| 7.9016 | 6.0609 | 6.3166 | 7.2808 | 5.4246 | 8.5355 | 9.8750 | 2.0573 |
| 6.4151 | 7.8663 | 3.7153 | 5.6114 | 6.5509 | 5.9765 | 2.8772 | 7.9795 |

| | | | | | | | |
|---|---|---|---|---|---|---|---|
| 8.2554 | 6.6400 | 4.2292 | 5.8643 | 3.9459 | 2.7323 | 7.4162 | 6.5759 |
| 4.0194 | 5.4450 | 6.4155 | 8.0121 | 5.0973 | 5.6664 | 6.3240 | 2.6916 |
| 4.9615 | 5.5261 | 7.3757 | 8.6983 | 2.4713 | 7.3457 | 5.2832 | 5.0157 |
| 7.8019 | 3.9327 | 1.9026 | 2.4400 | 7.5091 | 8.0142 | 5.6956 | 8.6514 |
| 3.9752 | 3.9408 | 5.8093 | 8.0404 | 9.4464 | 5.9885 | 7.9644 | 8.3811 |
| 5.6784 | 5.4979 | 6.4139 | 3.6795 | 7.7158 | 10.0236 | 5.9966 | 4.7697 |
| 3.4990 | 3.3577 | 4.5254 | 4.5566 | 1.8854 | 6.5235 | 3.4600 | 7.1425 |
| 3.9427 | 1.7663 | 5.5111 | 7.7066 | 8.0846 | 7.4682 | 7.4900 | 8.1179 |
| 8.0393 | 3.0105 | 6.8898 | 7.8513 | 10.0565 | 5.7579 | 8.9562 | 10.0097 |
| 9.5297 | 8.6649 | 6.5157 | 6.3909 | 1.9210 | 5.3024 | 7.0874 | 6.7593 |
| 7.3635 | 7.1204 | 2.8278 | 7.2496 | 8.6328 | 9.4036 | 8.4090 | 3.6490 |
| 9.0697 | 7.6823 | 4.1699 | 7.3126 | 5.7210 | 4.4431 | 7.8868 | 4.5158 |
| 4.5269 | 9.3590 | 1.0055 | 7.4261 | 4.9197 | 8.2230 | 5.7837 | 7.1153 |
| 5.9797 | 6.6786 | 7.6736 | 7.7304 | 1.7472 | 2.0932 | 8.0702 | 4.4670 |
| 2.6570 | 9.5244 | 10.3893 | 6.6356 | 6.9242 | 6.5785 | 6.5165 | 7.0007 |
| 5.4105 | 2.4496 | 2.4747 | 9.3324 | 6.7369 | 5.8474 | 5.9493 | 7.6129 |
| 7.8458 | 8.0992 | 6.2422 | 1.2540 | 5.4549 | 7.6086 | 3.8858 | 2.4961 |
| 5.5125 | 5.1271 | 2.8804 | 8.5837 | 9.0904 | 0.7796 | 11.9439 | 6.3666 |
| 6.6632 | 9.0096 | 6.9486 | 3.8574 | 5.2045 | 4.5697 | 3.1858 | 5.1619 |
| 5.2066 | 6.8189 | 9.5213 | 8.4898 | 7.3193 | 1.2666 | 3.5147 | 7.8944 |
| 5.5205 | 7.0251 | 6.1033 | 3.0773 | 8.0587 | 5.2217 | 8.7641 | 2.4959 |
| 2.2504 | 5.7292 | 5.1016 | 5.3145 | 4.1011 | 3.7591 | 6.9158 | 9.8700 |
| 8.5117 | 4.1488\ | | | | | | |
| GPR77 | 3.3026 | 2.1912 | 4.1826 | 3.8127 | 4.9141 | 5.6268 | 1.4999 |
| 0.8314 | 2.1693 | 6.1376 | 4.0522 | 0.8472 | 2.2881 | 1.4754 | 6.4989 |
| 3.7058 | 1.0553 | 1.2795 | 2.5821 | 1.7564 | 2.8895 | 6.2773 | 2.4383 |
| 4.7995 | 5.6342 | 0.6062 | 5.8917 | 5.1280 | 4.2890 | 3.2617 | 0.9954 |
| 5.9060 | 3.8834 | 2.9048 | 1.8080 | 0.6819 | 5.4354 | 2.4862 | 1.2418 |
| 2.6461 | 4.7421 | 4.0234 | 0.0000 | 2.1688 | 1.0260 | 5.3803 | 0.7135 |
| 3.9900 | 1.2792 | 4.3892 | 6.6992 | 3.7906 | 3.9272 | 2.2666 | 5.6656 |
| 2.5237 | 2.4376 | 2.6835 | 3.4964 | 2.1385 | 1.4980 | 3.3286 | 5.7112 |
| 0.0000 | 4.6561 | 5.3939 | 5.6818 | 1.7598 | 3.7337 | 4.1734 | 4.1553 |
| 5.5614 | 2.9022 | 1.1245 | 5.0638 | 1.8854 | 0.9546 | 5.1522 | 3.7282 |
| 2.3950 | 4.4366 | 0.0000 | 3.5062 | 3.9844 | 5.1430 | 1.3843 | 1.2355 |
| 6.3790 | 2.3134 | 1.8648 | 3.1304 | 2.5341 | 1.6676 | 4.1858 | 5.0863 |
| 2.0921 | 1.2090 | 4.1697 | 6.7210 | 2.0879 | 2.4487 | 4.6643 | 0.9246 |
| 1.5830 | 4.1473 | 1.3363 | 0.0000 | 0.7631 | 2.8861 | 4.1776 | 5.0096 |
| 4.1884 | 0.0000 | 0.0000 | 5.3956 | 2.0594 | 5.5657 | 1.0339 | 4.4122 |
| 3.0620 | 1.0794 | 6.0983 | 1.8863 | 4.8443 | 3.1191 | 2.4516 | 1.8708 |
| 0.7843 | 2.5267 | 5.8969 | 1.5269 | 1.4687 | 4.8811 | 3.9535 | 0.8107 |
| 1.3313 | 1.9254 | 0.0000 | 4.0599 | 3.7645 | 2.6248 | 5.0995 | 4.5374 |
| 3.6335 | 7.1902 | 3.1292 | 4.7323 | 0.9402 | 1.9919 | 3.1725 | 5.9774 |
| 4.7717 | 5.7982 | 1.5875 | 0.5476 | 2.8829 | 4.8449 | 2.4027 | 0.6000 |
| 2.2156 | 4.1886 | 2.2364 | 5.9687 | 3.5809 | 2.0789 | 3.5642 | 0.0000 |
| 3.4085 | 3.8766 | 1.2521 | 1.4975 | 1.2195 | 0.9335 | 0.0000 | 4.7036 |
| 0.8396 | 4.7198 | 3.0195 | 1.5479 | 3.9334 | 4.1970 | 2.2268 | 0.9100 |
| 5.9009 | 1.8738 | 5.8007 | 1.9914 | 1.1775 | 2.1566 | 3.6605 | 4.1599 |
| 5.7696 | 3.7058 | 2.0332 | 6.2501 | 1.5946 | 3.4498 | 4.1171 | 5.3954 |
| 4.6217 | 1.5159\ | | | | | | |
| NRXN1 | 0.0000 | 2.1912 | 0.6896 | 1.9468 | 0.0000 | 0.0000 | 1.2456 |
| 2.7752 | 1.2625 | 8.2599 | 2.8551 | 1.1366 | 4.5606 | 0.0000 | 0.0000 |
| 0.0000 | 4.3886 | 2.7464 | 4.3891 | 0.0000 | 0.0000 | 2.0613 | 0.6346 |
| 0.5262 | 2.8244 | 2.0473 | 1.9907 | 1.3471 | 0.0000 | 0.0000 | 0.7334 |
| 0.0000 | 0.0000 | 0.0000 | 0.0000 | 0.0000 | 0.5555 | 2.6929 | 0.9336 |

```
1.4612  0.0000  2.8926  1.0569  0.4326  5.4765  0.0000  0.0000
1.1317  7.5097  1.3590  0.4530  2.1910  3.6936  6.2553  4.2936
1.2781  0.0000  4.9168  3.6618  5.3867  5.0612  3.5972  0.0000
0.0000  8.0756  0.6977  0.0000  2.4264  0.0000  2.2414  0.4334
0.3921  0.0000  4.8545  3.3238  1.7267  0.0000  0.0000  4.2764
3.0798  0.9714  0.0000  3.9906  3.0210  0.9110  0.0000  2.0569
1.3738  1.6807  8.9336  6.9150  1.1363  1.4549  0.0000  0.0000
2.4199  0.0000  4.2416  1.9860  0.0000  0.0000  0.8684  3.5019
1.8724  9.2434  1.7170  3.3565  3.2800  6.3625  0.5877  2.7134
2.2538  5.1534  0.0000  1.6510  2.5241  2.0324  2.7195  0.0000
0.0000  8.9070  0.0000  5.2561  0.0000  2.8381  1.2272  3.2484
0.0000  5.2170  4.0889  2.1537  0.0000  0.0000  2.7051  1.3267
0.0000  1.6954  5.9700  2.0174  4.7782  3.8517  0.5748  0.0000
1.2444  0.4562  4.5998  0.6735  4.8127  0.9947  3.0614  4.3021
1.5604  0.5414  3.9105  2.2306  0.0000  4.6662  1.1539  0.0000
3.9615  0.0000  0.0000  2.4523  0.9764  0.0000  0.8742  0.0000
6.7517  0.5276  1.2521  0.0000  0.5290  5.8772  2.7948  0.0000
4.2600  7.6742  7.4138  7.2603  3.2075  0.0000  0.6902  0.5257
0.0000  0.0000  1.8914  1.7992  0.7058  0.0000  2.1363  0.5078
0.5556  0.5674  1.2477  0.0000  0.0000  0.0000  4.2022  0.6701
0.0000  0.0000\
RFX3    6.0313  4.9272  7.1367  7.9740  7.0366  7.8721  8.5183
7.8513  6.9144  6.4817  6.3647  6.2038  7.6244  6.1015  8.6852
5.9364  5.7212  5.9795  5.5203  4.0409  5.5852  9.5260  7.5778
7.1437  5.1738  4.3815  6.6689  7.0526  4.5595  5.8225  6.1272
6.7291  5.9653  6.0504  7.2817  4.0627  7.0602  6.6680  6.1967
7.9996  5.1515  6.9900  5.0624  5.7306  4.5085  6.9098  3.3157
6.7656  6.2259  5.3681  7.0875  5.8236  6.4117  5.6956  6.5497
6.6885  5.3684  5.3595  6.2625  6.3087  5.1749  7.1626  3.8751
5.3919  5.7459  7.8640  4.7377  6.2513  4.7853  5.3743  6.0633
7.7706  4.2855  5.8393  6.6329  6.8448  5.5244  8.3433  6.4492
6.7973  5.7114  6.6978  7.2834  7.2781  6.6571  5.4752  6.8654
5.3501  7.2921  5.8199  4.7570  6.3015  6.5097  5.4794  6.8737
4.3247  6.6475  5.3564  7.9222  5.2713  4.3582  6.3920  7.3734
7.7045  5.9520  6.2427  5.3670  6.9094  6.9629  5.2174  8.3199
7.8208  4.6197  4.9069  5.9790  7.2827  8.9695  6.5950  6.4097
3.9729  6.1822  7.8783  5.8756  7.4831  7.6055  5.4418  8.2476
5.2955  5.2535  6.1405  7.0624  6.7893  7.6758  8.3732  4.5622
1.7113  3.5403  5.7370  7.6628  5.5337  6.2565  6.6420  6.0183
6.9935  6.6693  4.0443  8.5965  4.7402  6.4240  5.8855  5.9582
3.8724  7.7964  5.3961  7.1049  6.2565  6.0453  7.9100  7.7927
5.1912  5.3832  5.1910  6.7987  5.2876  6.7180  7.6384  4.2069
7.3677  6.6803  6.4065  7.3768  7.9157  3.7802  5.4834  3.7579
7.2196  6.0376  4.7680  7.8370  8.9789  6.1558  4.8989  6.5371
5.2291  7.0103  5.8422  6.2366  5.2325  6.2793  7.1168  7.0328
6.6114  6.0351  6.0878  5.9561  4.2617  6.5784  5.7051  7.9025
7.9938  5.5316\
SNORD104        0.0000  0.0000  0.0000  0.0000  0.0000  0.0000
0.0000  0.0000  0.0000  0.0000  0.0000  0.0000  0.0000  0.0000
0.0000  0.0000  0.0000  0.0000  0.0000  0.0000  0.0000  0.0000
0.0000  0.0000  0.0000  0.0000  0.0000  0.0000  0.0000  0.0000
0.0000  0.0000  0.0000  0.0000  0.0000  0.0000  0.0000  0.0000
0.0000  0.0000  0.0000  0.0000  0.0000  0.0000  0.0000  0.0000
0.0000  0.0000  0.0000  0.0000  0.0000  0.0000  0.0000  0.0000
```

| | | | | | | | |
|---|---|---|---|---|---|---|---|
| 0.0000 | 0.0000 | 0.0000 | 0.0000 | 0.0000 | 0.0000 | 0.0000 | 0.0000 |
| 0.0000 | 0.0000 | 0.0000 | 0.0000 | 0.0000 | 0.0000 | 0.0000 | 0.0000 |
| 0.0000 | 0.0000 | 0.0000 | 0.0000 | 0.0000 | 0.0000 | 0.0000 | 0.0000 |
| 0.0000 | 0.0000 | 0.0000 | 0.0000 | 0.0000 | 0.0000 | 0.0000 | 0.0000 |
| 0.0000 | 0.0000 | 0.0000 | 0.0000 | 0.0000 | 0.0000 | 0.0000 | 0.0000 |
| 0.0000 | 0.0000 | 0.0000 | 0.0000 | 0.0000 | 0.0000 | 0.0000 | 0.0000 |
| 0.0000 | 0.0000 | 0.0000 | 0.0000 | 0.0000 | 0.0000 | 0.0000 | 0.0000 |
| 0.0000 | 0.0000 | 0.0000 | 0.0000 | 0.0000 | 0.0000 | 0.0000 | 0.0000 |
| 0.0000 | 0.0000 | 0.0000 | 0.0000 | 0.0000 | 0.0000 | 0.0000 | 0.0000 |
| 0.0000 | 0.0000 | 0.0000 | 0.0000 | 0.0000 | 0.0000 | 0.0000 | 0.0000 |
| 0.0000 | 0.0000 | 0.0000 | 0.0000 | 0.0000 | 0.0000 | 0.0000 | 0.0000 |
| 0.0000 | 0.0000 | 0.0000 | 0.0000 | 0.0000 | 0.0000 | 0.0000 | 0.0000 |
| 0.0000 | 0.0000 | 0.0000 | 0.0000 | 0.0000 | 0.0000 | 0.0000 | 0.0000 |
| 0.0000 | 0.0000 | 0.0000 | 0.0000 | 0.0000 | 0.0000 | 0.0000 | 0.0000 |
| 0.0000 | 0.0000 | 0.0000 | 0.0000 | 0.0000 | 0.0000 | 0.0000 | 0.0000 |
| 0.0000 | 0.0000 | 0.0000 | 0.0000 | 0.0000 | 0.0000 | 0.0000 | 0.0000 |
| 0.0000 | 0.0000 | 0.0000 | 0.0000 | 0.0000 | 0.0000 | 0.0000 | 0.0000 |
| 0.0000 | 0.0000 | 0.0000 | 0.0000 | 0.0000 | 0.0000 | 0.0000 | 0.0000 |
| 0.0000 | 0.0000 | 0.0000\ | | | | | |
| RFPL2 | 0.5526 | 1.4768 | 5.8880 | 4.7820 | 0.0000 | 1.7112 | 0.5431 |
| 1.5600 | 1.2625 | 0.4413 | 0.7594 | 2.5346 | 1.6409 | 1.6493 | 0.9286 |
| 1.4442 | 2.9009 | 1.2795 | 0.5838 | 1.9903 | 3.0838 | 0.0000 | 2.2832 |
| 1.2144 | 0.9530 | 3.2258 | 3.0115 | 0.0000 | 0.0000 | 1.8184 | 0.7334 |
| 1.5370 | 1.6552 | 3.4495 | 2.3226 | 3.9544 | 4.5574 | 5.1507 | 2.4725 |
| 0.7666 | 3.3740 | 0.4871 | 0.0000 | 4.2877 | 1.0260 | 0.0000 | 0.0000 |
| 2.7982 | 3.4580 | 1.3590 | 3.4047 | 2.1910 | 1.9420 | 1.4299 | 1.3330 |
| 1.2781 | 2.1087 | 5.2371 | 2.1468 | 0.5715 | 0.9353 | 3.2053 | 3.3159 |
| 1.6085 | 2.0096 | 1.1659 | 0.5980 | 1.7598 | 0.5773 | 1.8731 | 1.9272 |
| 2.6575 | 0.9449 | 4.1945 | 4.3805 | 3.1208 | 2.6228 | 0.9332 | 3.5128 |
| 2.5341 | 0.5659 | 0.7073 | 4.5648 | 0.6718 | 0.5263 | 5.5096 | 2.9586 |
| 2.0646 | 1.2163 | 3.5861 | 1.6854 | 2.6303 | 2.4205 | 3.5917 | 1.8492 |
| 0.0000 | 2.9181 | 0.5454 | 0.8845 | 3.7592 | 2.2933 | 2.5739 | 3.5019 |
| 2.5001 | 0.8449 | 2.0179 | 0.7314 | 1.6282 | 1.7151 | 2.0061 | 1.1907 |
| 0.0000 | 0.0000 | 3.8074 | 0.8924 | 3.0691 | 0.9131 | 2.7195 | 0.9644 |
| 0.0000 | 4.0191 | 1.0055 | 0.6224 | 0.0000 | 1.0180 | 1.4793 | 1.0447 |
| 1.6629 | 0.5619 | 1.1792 | 4.0327 | 3.1145 | 0.9511 | 0.0000 | 0.0000 |
| 0.0000 | 1.0837 | 0.0000 | 0.5216 | 2.1235 | 1.0240 | 2.2974 | 0.0000 |
| 0.9357 | 0.0000 | 1.2434 | 0.0000 | 0.0000 | 2.9906 | 2.1177 | 2.5835 |
| 0.0000 | 0.5414 | 1.3242 | 0.0000 | 0.7727 | 1.5425 | 0.3855 | 0.6000 |
| 1.9005 | 6.0731 | 2.2364 | 1.8011 | 0.9764 | 0.4418 | 1.6241 | 0.0000 |
| 1.9581 | 0.5276 | 1.5072 | 2.4266 | 5.1870 | 2.3490 | 0.0000 | 0.0000 |
| 1.7522 | 0.5232 | 1.0660 | 2.1786 | 0.6314 | 1.2666 | 2.0241 | 1.6771 |
| 2.5935 | 2.3211 | 0.6248 | 1.3150 | 6.3309 | 1.1063 | 1.7765 | 1.1800 |
| 2.1004 | 3.7581 | 1.4427 | 1.1623 | 0.0000 | 0.0000 | 1.3688 | 0.6701 |
| 1.6364 | 0.0000\ | | | | | | |
| VPS37D | 8.8636 | 7.9040 | 6.9202 | 8.1501 | 7.0415 | 8.0185 | 7.7719 |
| 8.7019 | 8.0292 | 7.8698 | 8.1778 | 8.6167 | 7.7338 | 2.3988 | 7.4763 |
| 7.5460 | 9.9911 | 8.4834 | 7.5778 | 10.1545 | 8.3167 | 7.2379 | 8.6173 |
| 7.5174 | 7.5914 | 9.2902 | 7.4450 | 9.2669 | 8.5761 | 9.4604 | 7.2917 |
| 9.0685 | 5.5414 | 8.7547 | 8.5650 | 7.6640 | 8.5948 | 8.4605 | 7.1503 |
| 6.9633 | 7.6818 | 6.4152 | 8.2962 | 9.5507 | 7.0052 | 8.1206 | 9.2843 |
| 8.3337 | 8.1402 | 9.9492 | 8.3404 | 7.9488 | 7.7917 | 6.6757 | 7.4971 |
| 8.5170 | 7.8079 | 9.4466 | 8.9484 | 8.0889 | 6.8574 | 8.4751 | 6.2292 |
| 7.6717 | 7.9715 | 8.8028 | 6.9926 | 8.3130 | 6.0109 | 7.9142 | 8.3771 |

8.4999	8.5136	10.0185	7.5719	8.8280	8.1584	8.0731	7.6838
7.8115	7.4303	8.1711	6.7443	6.8588	8.5799	8.0447	8.3312
8.4581	8.4993	7.6443	7.4280	8.7863	8.9917	7.2076	7.5906
6.8278	8.1087	8.3559	9.1908	8.0789	9.7397	8.7369	8.3493
7.3221	5.9705	7.3987	7.0980	7.6565	8.0040	9.0525	8.5447
7.4147	8.7062	8.6257	6.1967	6.8593	6.7764	8.3872	6.2203
8.9065	7.8002	8.7924	8.3498	8.1303	8.3025	7.4054	5.6712
9.8312	7.0492	6.4286	8.3510	8.1580	8.2932	5.0928	7.6979
8.3928	8.4796	7.5250	7.0019	8.0858	7.6949	8.7176	8.4406
7.2529	6.9937	7.5148	7.5757	9.0086	6.7240	8.0886	7.6068
7.5998	6.9215	7.7351	8.3262	8.1079	7.7338	7.5296	7.0330
8.0782	8.2987	6.4589	7.5725	7.8086	6.1177	7.6771	5.9057
8.4116	7.2227	9.5355	8.3961	7.0929	9.3973	9.9934	8.5091
8.7968	6.7266	7.1561	7.6331	6.7113	9.1910	8.7037	8.7259
7.4832	8.2611	8.2984	8.2125	9.2023	9.4576	6.8759	7.2721
7.7081	8.4469	8.2839	7.7688	9.5517	7.3391	8.2864	6.7979
7.2834	8.3370\
NLRP13	0.0000	0.0000	0.0000	0.0000	0.0000	0.0000	0.0000
0.8314	0.0000	0.0000	0.0000	0.0000	0.0000	0.0000	0.0000
1.4442	0.0000	0.0000	0.0000	0.0000	0.0000	0.0000	0.0000
0.0000	0.0000	0.0000	0.0000	0.0000	0.0000	0.0000	0.0000
0.0000	0.0000	0.0000	0.0000	0.0000	0.0000	0.0000	0.0000
0.0000	0.0000	0.0000	0.0000	0.0000	0.0000	0.0000	4.4245
0.0000	0.0000	0.0000	0.0000	0.0000	0.0000	0.0000	0.0000
0.0000	0.0000	0.0000	0.0000	0.0000	0.0000	0.0000	0.0000
0.0000	0.0000	0.0000	1.6109	0.0000	0.0000	0.0000	0.0000
0.0000	0.0000	0.0000	0.0000	0.0000	0.0000	0.0000	0.0000
1.0461	0.0000	0.0000	0.0000	0.0000	0.0000	0.0000	0.0000
0.0000	0.0000	0.0000	0.0000	0.0000	0.0000	0.0000	0.0000
0.0000	0.0000	0.0000	0.0000	0.0000	0.0000	0.0000	0.0000
0.0000	0.0000	0.0000	0.0000	0.0000	0.0000	0.0000	0.0000
0.0000	0.0000	0.0000	0.0000	0.0000	0.0000	0.0000	0.0000
0.0000	0.0000	0.0000	0.0000	0.0000	0.0000	0.0000	0.0000
0.0000	0.0000	0.0000	0.0000	0.0000	0.0000	3.5049	0.0000
0.0000	0.0000	0.0000	0.0000	0.0000	0.0000	0.0000	0.0000
0.0000	0.0000	0.0000	0.0000	0.0000	0.0000	0.0000	0.0000
0.0000	0.0000	0.5862	0.0000	0.0000	0.0000	0.0000	0.0000
0.0000	0.0000	0.0000	0.0000	0.0000	0.0000	0.0000	0.0000
0.0000	0.0000	0.0000	0.0000	0.0000	0.0000	0.0000	0.0000
0.0000	0.0000	0.0000	0.0000	0.0000	0.0000	0.0000	0.0000
0.0000	0.0000	0.0000	0.0000	0.0000	0.0000	0.0000	0.0000
0.0000	0.0000	0.0000	0.0000	0.0000	0.0000	0.0000	0.0000
0.0000	0.0000\
APC	8.5044	9.0272	9.9577	8.8127	9.4561	8.9203	9.7820
9.2278	9.4745	9.0981	8.9124	9.2693	9.4508	9.2433	9.0448
8.7293	7.4208	8.9758	9.1951	7.2567	7.8829	8.4597	8.5803
9.2289	9.5823	8.5861	9.1487	7.9604	8.3308	8.8593	9.4228
9.0072	8.1901	8.7264	9.4978	8.3834	9.1072	9.2555	9.2272
9.2529	8.7069	9.0266	8.1392	8.5553	9.9657	7.6354	7.2418
9.2076	10.2216	8.4318	8.7884	8.1722	8.8922	9.4527	8.6514
9.3755	9.3850	8.9218	9.3451	8.9006	9.7519	9.3421	9.2008
8.4822	9.1533	9.1454	9.0707	8.7083	8.9123	8.4551	7.9379
10.0659	7.1922	7.9772	9.5559	8.2182	8.2232	8.8001	8.2693
9.3350	8.1518	8.8012	9.0145	9.3740	8.1137	9.0108	9.0419

7.6924 9.0316 8.7242 9.3779 10.0511 8.4518 8.2731 8.6127
9.7054 9.2946 9.2033 8.7195 8.6269 7.2432 8.7781 9.0389
8.9288 8.6984 8.4505 8.2002 9.8086 9.4287 7.9477 9.1882
8.9879 7.4812 7.6073 9.0983 9.0729 8.8210 9.4670 8.7213
8.1710 8.7776 9.0278 9.1913 8.9771 9.0400 9.1252 9.1833
7.2196 9.6822 10.1245 9.1401 8.9212 8.4046 8.6900 7.9475
6.3192 7.2596 7.7916 9.7428 9.3402 8.4226 8.6006 7.8279
9.3032 9.5699 9.3617 9.5369 8.8147 9.1567 7.9590 9.3475
6.6638 9.6507 9.0913 8.9937 9.2600 9.4337 9.5546 7.8994
7.9800 8.0485 8.4222 8.9072 8.4643 8.6650 9.7721 8.1167
10.0015 8.5181 7.8537 9.5794 9.2197 8.3601 8.7050 6.0537
9.1635 9.2420 9.2084 9.8915 8.7643 8.0968 8.5820 9.2687
8.5160 9.2142 8.5486 9.1845 8.2915 7.9117 8.7753 9.5325
9.1546 7.8896 8.5102 8.8152 7.2905 8.4726 9.2841 9.5713
9.3075 7.2563\
TMEM179B 10.7051 10.2135 10.2562 10.3408 10.3120 10.3363
9.8804 10.5438 11.3553 10.1340 10.8500 10.0636 10.5264 10.0047
10.0607 10.3562 10.5279 10.7366 10.2981 10.7167 10.7845 10.2504
10.4664 10.0836 9.7895 11.0623 10.3628 10.4031 10.4589 10.1651
9.7717 10.5506 11.4268 11.0151 9.4028 11.0518 9.8850 9.9734
10.5079 11.7197 10.6549 9.8308 10.9166 10.7656 9.9124 10.7475
10.6051 9.9822 9.7803 10.4959 10.3176 10.1441 9.8226 9.8025
9.4486 10.6588 10.1211 10.4944 9.7611 10.9231 9.6743 10.6275
9.9189 10.0082 10.3410 9.9546 10.3505 11.1118 10.7723 10.9715
11.2417 10.1445 10.8006 10.6319 9.9502 10.0324 9.4538 10.7317
10.8301 10.2836 10.3825 10.7598 10.7245 10.6299 11.1625 10.6363
9.8989 11.0910 10.7368 10.4613 10.1188 9.6361 10.5903 10.5751
10.1601 9.8937 10.4888 10.1418 9.7585 10.3465 11.0073 10.5567
9.9353 10.3381 10.5239 9.3347 10.1504 10.4001 10.5675 11.1316
10.2028 10.1884 10.4369 10.7895 10.1792 9.3937 9.9629 10.0929
10.5480 10.1738 10.4361 10.6345 9.5940 10.0143 10.1072 9.8533
11.5307 9.5309 9.9355 9.1644 10.5095 10.3322 10.5980 10.3654
10.9184 10.0786 10.2055 9.6822 9.6228 10.1388 11.6286 10.1788
10.5176 10.2100 10.0756 9.6015 9.6523 10.1318 10.5069 10.7553
9.8176 11.3406 9.8411 10.0713 10.6006 9.8880 10.3563 9.7114
10.5430 10.6999 11.2910 10.5488 10.4118 10.4276 9.9797 9.6292
10.4622 9.4902 10.5402 10.1219 9.9278 9.7787 9.2238 9.4999
10.6518 9.9239 10.1455 9.8381 9.6805 10.6965 11.0230 10.5476
10.6892 10.4964 10.2933 10.4028 10.7126 10.3353 10.6319 10.7852
9.8857 10.5016 10.4002 10.3478 9.5233 10.5643 10.2771 10.1824
10.0601 9.9684 11.2270\
ABCE1 10.1767 10.1578 9.7649 10.5456 10.7991 10.5116 10.7293
10.1949 10.3319 10.5566 10.7452 11.0463 10.7988 10.3097 10.4807
9.8101 9.8758 10.3834 10.1227 9.1137 10.3468 10.0195 9.8512
10.6687 10.2493 10.3058 10.5969 9.7369 9.8089 10.3037 10.8217
10.6013 10.7390 9.9865 10.8159 11.3382 9.9426 10.3679 11.3137
10.8858 10.9345 10.6894 11.1734 10.2698 10.1664 10.2176 9.0640
10.7724 11.7381 9.6704 10.2856 10.4076 9.6849 10.2258 9.9799
10.5285 10.6808 10.7005 10.4069 9.7018 10.5263 11.0458 11.0443
9.6490 10.5400 10.2198 9.7709 9.8359 10.4804 10.0365 10.1943
10.7086 9.9403 10.4062 10.6767 10.1943 10.3446 9.6149 9.8683
10.9803 9.8203 10.7423 10.0629 10.5264 9.6595 10.6724 10.1449
9.9348 10.9872 9.8614 10.0511 11.6350 9.9349 9.7228 10.6302
10.1008 10.7410 10.1288 10.0714 11.0980 9.6802 10.3816 10.1518

10.4793 11.0807 11.2535 10.4278 10.3912 10.2759 9.4521 10.6363
10.8989 8.2575 9.9571 11.1358 10.9147 10.4714 11.5633 10.1888
10.3646 10.6584 10.7145 10.2165 10.5730 10.1502 11.1610 10.5314
9.1142 10.3435 10.7823 10.5405 11.2586 10.2129 10.6175 10.2559
9.7671 9.9290 8.6938 10.7667 10.1916 10.4686 10.3433 9.6008
10.8846 11.0075 10.1469 10.4557 9.8161 10.4866 9.5784 10.1245
8.7736 10.4813 10.8700 10.7619 9.9357 10.0799 10.5656 10.7768
10.4746 10.1798 10.2572 10.5178 9.8936 10.1943 10.9992 10.6153
10.4461 10.2853 10.4587 11.2724 10.6579 9.0977 10.0267 8.4239
10.5201 10.7603 9.7996 10.9424 9.7535 10.1594 10.7482 10.6240
10.4689 11.1792 10.4483 11.7727 10.0024 10.0839 10.9083 11.2918
10.3364 11.0294 10.4395 10.9079 10.5013 9.6332 10.1613 10.4008
10.6173 9.3932\
UQCRC2 11.4704 11.0257 11.0132 11.7695 11.6258 10.6964 11.5006
11.9643 12.2260 11.4542 11.9157 12.6312 11.7588 11.1688 12.0460
10.7384 9.9374 11.3994 10.8271 10.1282 11.6071 11.8508 11.6855
12.0148 11.7521 11.7572 11.0580 11.3498 11.2453 11.8366 10.5899
11.1279 10.3422 11.6438 11.3496 12.8861 11.2227 11.5241 11.5218
10.2703 11.4355 11.4240 11.0501 10.5912 11.9218 10.3119 10.5710
11.4437 12.1254 11.0182 11.4896 10.1932 11.4212 11.8220 11.1466
11.1833 11.1519 11.0769 11.6407 11.3983 11.8184 10.6305 11.7686
12.0513 11.1445 11.8375 11.6873 10.9483 11.2755 11.0230 12.0744
11.4857 11.1972 10.6914 11.2772 12.1866 10.4157 11.2609 10.7797
11.6197 11.4465 11.4300 10.8024 11.1335 11.6101 10.9871 12.7092
12.0344 11.8899 11.3823 11.5751 11.6562 10.6097 10.5309 11.6537
11.6901 12.1660 11.7161 11.1803 10.7083 12.1142 11.8391 10.4174
10.9033 12.4594 11.4345 10.3886 10.9355 10.9793 11.2148 11.7478
12.0574 9.6462 10.9958 11.2594 11.7180 11.4880 12.2776 10.9509
10.9771 11.5414 11.8540 11.0049 12.5946 11.9046 12.1004 11.7105
10.8497 11.5985 11.7630 11.4720 11.4122 12.0633 10.4071 10.3770
10.1506 11.3262 9.0241 12.0499 11.4900 11.3414 11.9421 11.2046
11.6484 11.5376 11.8992 11.7665 12.0168 11.6252 11.0310 11.4324
11.1342 11.6628 11.5252 12.0382 11.0693 11.6697 11.3016 11.9279
11.6667 10.4264 11.2125 11.6878 11.0993 11.5106 12.0958 11.1987
11.8227 11.3381 10.2354 12.0320 12.4290 10.6733 11.1433 9.9402
11.4860 11.0186 11.8594 11.5728 10.9170 11.2277 12.5170 11.4461
12.2114 11.2515 11.8521 11.1133 12.0666 11.4732 11.5647 11.6951
11.4899 10.5671 10.8562 11.7034 11.9978 11.4281 11.9317 11.6441
11.8882 10.9740\
XPNPEP1 10.2854 9.9129 9.9070 10.6234 11.0424 9.7335 9.9928
10.6047 9.7928 10.4646 10.1723 10.2235 10.4355 10.8519 10.7495
10.5997 9.3938 10.3578 10.0321 10.0068 9.6737 10.4972 10.0493
11.2239 9.9692 10.4019 11.0628 10.3277 10.6072 10.1562 10.9216
10.3370 10.1996 10.1310 9.9168 11.0764 10.5177 10.6070 10.3832
10.9765 10.5735 9.7941 10.1709 9.4951 9.6438 9.8408 11.1789
9.9412 10.4920 10.2069 10.3354 10.1932 10.2877 9.7056 10.6738
10.3867 10.8514 10.2454 10.2581 9.8513 9.6719 10.8021 10.7231
10.3008 10.0821 10.8452 9.5632 10.5266 11.1022 9.9287 10.5156
9.8559 10.3234 9.9455 10.3446 11.1037 9.8987 10.4019 10.4483
10.5916 10.4862 8.4417 9.9110 10.0567 11.2362 9.2623 8.8234
10.6318 10.6106 10.1985 10.2591 10.5632 10.9640 10.0689 11.0671
9.8771 9.6962 9.7109 11.2541 9.7400 11.0633 10.0542 9.3970
10.3927 10.9918 11.3993 10.0949 10.0078 10.4668 10.0470 11.1613
11.6423 9.6881 10.0056 9.6869 10.1568 10.9922 9.6342 10.8355

9.9494 10.2890 10.1416 10.5197 10.5293 9.9321 10.2357 10.2447
9.9528 9.7596 10.1386 9.5986 10.1048 10.5167 9.7964 9.9705
10.0766 11.9267 10.1982 10.3584 9.4799 11.3989 10.8070 10.8278
10.4721 10.5006 9.7975 10.8161 10.2437 10.5443 10.4469 10.1331
11.5932 10.7348 10.3647 10.6189 10.2242 9.5019 10.7871 9.8870
10.2726 10.7013 10.2721 10.4906 10.3078 9.8417 11.5598 9.7294
10.7499 11.2863 10.1538 10.8266 10.1504 9.7565 10.7757 9.2642
10.3667 9.9936 10.0193 10.7221 10.2612 10.1605 10.7170 10.6707
11.2787 10.3910 11.0035 9.7526 10.1658 10.0607 10.7624 10.5229
10.1757 9.8418 9.9363 10.4833 10.6574 10.7759 9.5879 11.2892
10.3840 10.2965\
XPNPEP2 0.0000 1.6912 0.0000 0.8605 7.0984 2.4722 1.2456
1.3555 0.0000 4.3947 2.2662 0.4849 1.0422 1.9445 2.6827
2.1513 2.5489 1.2795 3.3189 0.0000 0.0000 1.5770 0.6346
0.9110 4.1192 2.6374 0.0000 1.8373 1.3857 0.0000 2.8666
2.8443 1.1952 0.4664 0.0000 3.7749 0.9556 0.0000 0.0000
0.7666 4.4257 3.3558 0.0000 0.0000 6.1800 0.9285 0.0000
0.6741 1.0498 0.0000 1.0749 2.4004 3.0688 6.5246 3.4925
2.2632 0.0000 3.0892 2.2544 2.4263 5.3137 1.1594 0.5573
0.0000 5.0213 0.3906 0.0000 3.0286 0.5773 3.0801 1.6333
0.0000 1.5110 1.1245 4.0681 2.3880 0.9546 1.2414 1.5048
3.1679 2.5369 0.3965 4.8177 2.3651 1.8644 7.2208 1.2355
1.0433 2.6540 0.9113 7.5567 1.1363 0.0000 5.2424 1.6641
6.0384 1.8574 4.3430 2.1324 1.2591 1.4180 0.0000 0.0000
1.2208 0.4835 1.5391 3.4466 2.8627 2.2182 1.0041 2.1442
0.6215 3.1476 1.0000 1.8354 3.6573 0.9131 1.7841 2.1659
1.0358 6.4505 0.0000 1.8863 4.5681 2.1978 0.9214 2.5319
1.2894 2.8462 4.1242 0.3935 1.1234 1.9260 0.7571 0.0000
2.9678 4.2410 5.7867 1.2057 3.7193 1.0240 1.3036 3.5639
2.2176 3.3111 3.2024 0.0000 7.1733 0.0000 1.2240 3.8396
0.0000 0.5414 2.0029 0.0000 1.2732 2.6463 1.5047 0.6000
2.6931 0.0000 0.0000 4.3492 0.9764 1.0531 1.4147 1.6706
2.0725 1.4679 0.0000 1.0663 0.0000 1.7114 2.5508 9.6083
1.9121 4.3386 5.6182 0.7148 1.4046 0.5545 2.8349 2.6495
2.1234 1.2218 2.9077 2.3128 0.0000 0.0000 0.5707 0.8827
2.6247 1.7696 3.5427 0.4985 0.0000 1.8529 2.9741 1.3092
2.5996 2.2387\
TIGD4 2.6167 3.9323 4.7735 5.9297 5.0549 5.0934 4.9619
3.7518 4.3637 5.0056 3.7518 4.9525 5.4030 5.1075 5.5430
5.1466 4.1466 3.6346 3.9510 0.9198 4.0896 6.2170 6.4911
6.0184 3.3061 0.6062 2.5443 4.0836 4.1647 3.3358 3.4512
4.7881 2.7201 5.2048 4.0008 2.0076 4.8436 4.5331 3.4608
4.6349 4.4257 3.6608 3.7469 3.6483 3.2157 4.7166 0.0000
1.1317 3.2025 3.8772 4.0111 3.7906 3.4897 4.3262 5.3263
4.5143 2.2825 4.7478 5.0393 3.8691 2.4755 5.0476 3.9207
1.8326 5.0480 4.8259 4.0364 5.0012 3.3716 3.7280 1.4604
4.4950 0.0000 1.7486 5.7354 4.3668 6.8095 6.0842 4.8363
2.3950 3.4069 4.4304 5.7228 4.9099 4.6305 5.5761 5.4412
3.3991 4.8028 3.1582 3.5269 4.6645 3.9820 4.6764 4.4479
2.0921 2.9181 4.3430 4.8957 4.4220 3.9007 3.2105 4.6837
5.2577 3.8615 2.1477 3.5314 5.3482 3.2744 3.6514 5.5203
5.0810 0.0000 2.3219 3.8776 4.5346 5.9527 7.7367 5.2027
1.3647 2.8695 3.3319 2.5495 4.3674 6.3752 3.6579 3.0773
0.7843 4.7397 4.2252 3.7596 5.0365 5.2069 5.4295 2.2542

| | | | | | | | |
|---|---|---|---|---|---|---|---|
| 0.0000 | 1.0837 | 4.7638 | 5.0160 | 4.8429 | 4.5374 | 3.2205 | 5.0652 |
| 4.6807 | 3.9545 | 3.9194 | 5.4142 | 4.2762 | 4.1191 | 2.6654 | 5.6588 |
| 0.0000 | 5.8216 | 3.9578 | 4.4137 | 4.1702 | 5.2950 | 5.6262 | 6.1526 |
| 0.9345 | 3.8658 | 2.3902 | 5.0576 | 4.2768 | 6.2661 | 3.8821 | 5.0454 |
| 4.3839 | 4.4431 | 3.9763 | 4.8978 | 5.0966 | 0.9335 | 4.4981 | 0.0000 |
| 5.4605 | 4.8567 | 2.9187 | 5.2912 | 6.5431 | 3.9997 | 3.7301 | 4.6286 |
| 6.5635 | 3.8404 | 3.8082 | 2.8969 | 2.0548 | 4.4043 | 3.6040 | 4.4929 |
| 4.4989 | 4.4088 | 2.2571 | 4.7549 | 1.5946 | 1.6276 | 5.1193 | 6.4963 |
| 5.8669 | 2.2387\ | | | | | | |
| ZNHIT3 | 9.8224 | 9.0809 | 7.7550 | 9.1379 | 9.4496 | 9.9037 | 8.5544 |
| 9.3047 | 9.9124 | 9.1778 | 8.9119 | 9.6806 | 9.2864 | 9.9617 | 9.5110 |
| 9.2122 | 9.2683 | 10.0479 | 8.9048 | 10.0793 | 8.5106 | 9.1367 | 9.2004 |
| 9.2092 | 9.1395 | 9.4301 | 9.8554 | 9.3757 | 9.4468 | 8.8807 | 8.8547 |
| 9.2315 | 10.3955 | 9.2245 | 9.5391 | 10.7520 | 8.9980 | 9.2179 | 9.7845 |
| 8.6940 | 9.6415 | 8.6692 | 9.2380 | 8.9390 | 9.5715 | 9.1406 | 9.1232 |
| 9.6387 | 9.0950 | 9.7290 | 8.7996 | 10.1255 | 9.4397 | 9.1988 | 9.6470 |
| 9.8378 | 9.1155 | 9.6480 | 9.9407 | 9.6247 | 9.3123 | 9.9207 | 9.9393 |
| 8.4589 | 9.1138 | 9.0546 | 8.8404 | 9.1303 | 9.2364 | 8.5871 | 9.9396 |
| 9.3363 | 9.6362 | 8.8589 | 9.3458 | 9.1754 | 8.8694 | 8.8859 | 9.2007 |
| 9.4350 | 9.9503 | 8.8451 | 8.8242 | 8.7978 | 9.9398 | 9.2928 | 10.6269 |
| 9.4119 | 8.9211 | 9.8082 | 9.0609 | 10.0285 | 10.0423 | 8.9933 | 9.4544 |
| 8.9796 | 8.9807 | 9.8139 | 9.4280 | 9.6608 | 10.1073 | 9.3392 | 9.0509 |
| 8.6659 | 9.2078 | 8.5916 | 8.3408 | 9.6378 | 9.1457 | 9.8967 | 9.1713 |
| 9.3445 | 8.6338 | 9.5046 | 8.0686 | 9.2849 | 9.4954 | 9.4789 | 8.9444 |
| 9.2231 | 9.2342 | 9.9023 | 9.4117 | 9.4397 | 9.3800 | 9.6941 | 9.2989 |
| 10.2816 | 9.2841 | 8.5085 | 8.2418 | 8.5521 | 9.5260 | 8.3321 | 9.5675 |
| 9.6805 | 10.5352 | 7.9196 | 9.4554 | 9.2152 | 9.0456 | 9.3379 | 9.4613 |
| 9.1156 | 9.2402 | 9.5147 | 9.3176 | 9.3936 | 9.8137 | 10.1313 | 9.1584 |
| 9.2228 | 9.0114 | 8.8096 | 8.8766 | 8.7031 | 9.0983 | 9.0588 | 10.5141 |
| 9.6598 | 9.2312 | 9.2564 | 9.5509 | 9.6104 | 9.1586 | 9.2134 | 8.3517 |
| 9.0432 | 9.4054 | 9.2114 | 8.9164 | 9.7810 | 10.1313 | 9.7415 | 8.5201 |
| 9.7152 | 8.8590 | 9.3185 | 9.2374 | 9.0734 | 9.7334 | 10.0282 | 10.6097 |
| 9.7491 | 9.1730 | 9.6088 | 8.8404 | 10.4795 | 9.5695 | 10.0430 | 9.5733 |
| 9.4527 | 8.9408 | 8.6587 | 9.3963 | 9.4779 | 9.3065 | 9.4327 | 9.2191 |
| 9.2064 | 10.0551\ | | | | | | |
| GGCX | 8.8136 | 8.6836 | 10.3150 | 8.9688 | 9.2339 | 8.9241 | 9.0765 |
| 10.5648 | 10.1754 | 9.0686 | 9.3293 | 9.1797 | 9.8329 | 9.9729 | 9.0969 |
| 9.0150 | 9.5943 | 8.4366 | 8.9581 | 9.9093 | 9.4983 | 9.2193 | 9.0730 |
| 9.3162 | 8.7873 | 9.6324 | 10.0002 | 8.9797 | 9.4282 | 9.7054 | 9.9349 |
| 9.4985 | 9.7161 | 9.0655 | 9.3738 | 9.7806 | 9.4058 | 9.2837 | 8.8894 |
| 9.5548 | 9.1668 | 8.9241 | 9.7724 | 9.7594 | 8.4350 | 8.8760 | 9.5888 |
| 10.3102 | 8.9578 | 9.5972 | 9.2191 | 8.9646 | 10.0859 | 8.8339 | 9.3837 |
| 9.0975 | 10.1096 | 10.5424 | 9.2605 | 9.9310 | 9.0087 | 9.8089 | 9.3830 |
| 9.7434 | 9.0566 | 8.9543 | 9.9426 | 9.9359 | 9.3833 | 9.4801 | 9.4132 |
| 9.4979 | 9.2758 | 9.6961 | 9.7413 | 9.0869 | 10.5531 | 8.6083 | 8.6738 |
| 9.0679 | 9.2276 | 9.7893 | 8.5345 | 9.1101 | 9.1575 | 10.3132 | 9.1975 |
| 9.7875 | 9.4742 | 8.9139 | 9.0182 | 9.4151 | 9.4957 | 8.8020 | 9.0232 |
| 8.9573 | 9.3643 | 8.7252 | 8.6917 | 9.4503 | 9.0976 | 9.6005 | 9.5168 |
| 8.9754 | 9.2845 | 9.4957 | 8.2035 | 9.6228 | 9.4803 | 8.5395 | 8.9446 |
| 9.1608 | 9.6990 | 9.1059 | 9.8663 | 8.9989 | 8.7839 | 9.8569 | 9.5034 |
| 10.1434 | 9.8186 | 9.4959 | 10.6230 | 9.0227 | 8.8859 | 9.7066 | 9.5717 |
| 10.3580 | 9.0384 | 9.0544 | 10.7880 | 9.3646 | 8.9628 | 10.9323 | 9.4295 |
| 10.2393 | 8.8169 | 9.0899 | 9.1696 | 9.0344 | 9.3571 | 9.6391 | 9.0051 |
| 9.3843 | 9.6137 | 8.7541 | 9.0332 | 9.0492 | 10.4435 | 9.4941 | 9.1429 |

9.5960 9.6392 9.7762 9.3639 9.6209 9.0167 9.5587 9.2455
9.2933 10.1017 9.4021 9.0840 8.9554 9.4986 9.4933 8.4586
10.9205 9.4683 9.2601 8.6239 10.4425 9.0440 9.8421 8.2032
8.9289 9.1044 8.5655 9.5006 8.4364 9.1003 10.6191 9.8614
9.0342 9.6919 9.0365 9.9133 9.8370 9.1964 9.9489 8.5900
9.5826 8.9900 10.0979 9.2393 9.4260 9.7972 8.7600 9.6519
9.1958 9.7631\
PAPLN 9.0731 10.4749 9.0661 7.4825 7.5362 6.4871 7.0363
10.0229 7.1097 9.1307 8.4056 8.0106 10.2978 8.7438 5.5843
6.1612 9.1154 6.7934 10.4713 11.2834 8.6421 7.5685 8.0977
5.9887 8.7039 6.7900 3.8464 8.4866 7.1511 11.1075 10.2153
6.6218 10.3000 6.7786 10.0724 6.9988 8.9975 5.0288 7.1641
9.9603 7.9783 9.7876 9.6323 9.2718 9.7707 6.3786 6.7783
9.2936 5.5877 5.4987 6.6890 10.6278 8.8766 9.1175 9.7130
8.9884 9.2502 9.5949 8.8338 8.7653 9.0440 8.4516 6.1832
8.1592 9.9175 7.3460 4.8703 7.3193 10.3576 8.6927 6.9662
7.7971 7.2103 9.5621 7.7636 5.0081 9.8103 8.2877 8.9631
7.4997 8.3021 8.9617 8.9630 7.2945 7.3525 10.0574 4.8801
7.5238 9.0145 7.2443 9.6297 9.1151 9.2661 8.9383 7.7416
8.6030 8.9128 10.7651 7.3639 7.7270 5.6613 8.8117 9.0861
8.0176 6.3401 6.1215 11.9704 10.5337 9.1765 7.5159 6.9756
7.0010 10.0972 10.9607 6.6789 9.0472 6.3066 8.6605 6.2325
11.8444 9.5039 8.4745 11.6097 4.3674 7.6867 8.9693 8.2701
8.9214 9.3340 8.8415 8.0121 9.5743 7.9426 7.7979 10.3447
7.4729 7.1671 9.2086 9.2357 10.2721 6.8351 4.0586 8.0826
7.4985 5.7195 9.5533 6.3663 8.6578 6.7575 4.5676 9.6801
2.9676 7.7202 7.2278 3.3552 8.6069 9.8799 7.4383 8.4109
8.2713 2.3724 5.8248 8.0109 7.7164 7.0499 7.3228 8.4616
10.2447 6.2658 6.6836 7.4747 5.8166 8.1907 5.7381 5.9919
7.8528 8.8563 10.1389 9.0535 9.4660 5.9305 9.9628 8.6957
6.6684 8.6272 6.1808 9.6324 9.4436 7.7779 5.5869 7.1869
9.0764 7.6337 9.9996 7.0425 6.1640 7.5788 10.0094 8.2675
8.0923 6.6104\
ADNP 10.6277 10.8626 11.2728 11.0641 11.2991 11.8082 12.2624
11.5244 11.7142 11.2742 11.7161 11.9627 11.3911 11.9370 11.1808
10.4409 10.7112 10.9173 10.7983 10.6701 10.9761 10.7021 10.7549
11.2484 11.1809 9.4463 10.9256 9.7585 10.4212 11.9916 11.5863
11.0446 10.9231 11.9738 12.2630 10.8608 11.6458 11.5828 10.4153
11.7306 10.5735 11.3974 10.6000 10.5510 11.1127 10.0007 8.1681
11.4781 11.6514 9.8253 10.6650 11.0649 11.5866 11.2630 11.6106
11.0253 11.1814 10.8259 11.5831 11.3726 11.2746 11.6472 10.9878
11.2501 11.1152 11.1871 11.1983 12.0825 10.7131 11.5172 10.5958
11.5675 9.8303 10.2103 11.1239 11.0155 12.7524 10.4355 10.0568
11.4289 9.8906 11.8989 10.7904 11.4904 10.1000 11.1193 12.4209
9.9348 11.3050 10.7609 10.8138 11.7424 10.9574 10.5482 10.8486
10.8822 10.6843 11.2371 11.5788 10.9901 9.2677 11.0369 11.6760
11.4540 11.0417 11.4261 10.5228 11.5547 11.3922 10.8021 11.7462
11.2424 8.8059 10.2842 11.1256 11.6991 11.4414 12.2791 11.5582
10.8828 12.0652 11.3406 11.3767 10.5918 11.1789 11.5596 11.4417
10.4406 11.3743 11.6606 11.1862 11.3646 10.8243 10.9029 10.3304
7.3970 9.7661 8.9821 11.3776 11.2209 10.6236 10.3417 9.7836
11.2629 11.0547 11.3330 11.4833 11.0986 10.8920 8.7160 11.1660
8.7079 11.7700 11.6932 10.8677 12.1365 11.0719 11.7819 11.3035
9.8645 10.9569 10.5539 11.0929 9.9525 11.4555 11.7998 11.1524

11.7571 10.7912 11.3882 11.7949 12.3808 11.3065 11.1228 7.5828
11.5238 11.4206 10.6129 11.7792 10.7136 10.0328 10.8013 11.9874
10.6576 12.4117 11.2111 11.4548 10.0968 10.3162 11.1431 10.9964
11.3891 11.8975 11.9263 11.3355 9.5824 8.1212 11.3072 11.4134
11.5557 8.5308\
TMEM218 8.5820 7.8039 7.8921 8.7769 7.6420 8.0613 7.7506
8.8748 7.8851 8.6266 7.5147 7.7625 8.3497 8.4571 8.4686
8.0273 8.5533 7.7539 7.9566 7.8819 7.7388 8.5753 8.9300
8.2678 8.5249 8.7750 8.4033 8.8996 8.4252 6.3006 8.6704
8.3561 7.9353 7.5695 8.0937 8.1999 7.4840 8.4211 8.0344
8.5820 8.3665 8.1647 3.9622 7.3133 8.4564 7.8596 7.7332
7.3697 7.9368 8.6042 7.7869 7.6592 7.1895 8.3593 7.6712
8.2641 7.2664 8.2885 8.4751 8.2004 8.2646 8.3875 7.4354
8.0351 8.6014 8.1793 8.4158 7.6715 7.7153 7.0563 8.1288
7.5575 8.3392 8.1410 8.4853 9.0632 7.7506 8.4745 9.1411
8.3573 8.6905 7.9545 8.2329 7.7490 9.2269 8.3760 7.5611
8.2750 8.4327 8.9503 8.2190 8.2896 7.2933 8.1127 7.7762
8.5092 7.4186 8.2511 8.2968 8.2906 8.2830 8.1695 6.5881
6.7653 8.2533 7.0870 8.4738 7.9221 8.0936 7.5039 8.3785
8.4687 7.6260 6.8704 8.3653 7.5418 8.9871 8.3506 7.8588
7.0701 8.4716 8.4807 7.4663 7.9667 9.0924 7.3785 7.6267
8.5483 8.3436 8.4071 8.4332 7.7789 8.6739 7.2228 7.4467
7.3774 9.1368 8.1554 8.3617 8.5294 6.9481 8.9666 8.8174
7.8232 7.7446 8.4254 8.2184 8.7031 8.5704 8.0992 8.3358
8.2158 8.3871 8.4026 7.3161 8.0003 8.5671 7.7426 9.2032
8.4919 8.0878 7.0442 8.5893 9.3927 7.8430 8.1274 6.2333
8.2417 8.2114 7.3822 8.2413 8.4530 7.1127 8.1384 8.0296
8.6528 8.3859 8.4708 5.4156 8.7734 8.9876 6.6046 8.5778
8.9424 8.1706 8.1159 8.5219 7.9451 8.5016 8.0989 8.2694
7.8173 7.8113 6.7113 8.1876 7.6511 8.4811 8.7723 8.0658
8.5016 7.6922\
STXBP6 10.9108 7.3354 8.1963 10.4754 9.3624 8.0942 10.9012
9.1724 9.3996 8.6016 10.7829 10.3423 9.3792 9.0523 9.9517
9.9796 11.7805 9.9444 7.1052 8.5091 11.0060 9.9518 7.6668
10.3392 5.6744 3.4491 7.8126 10.0557 9.8994 9.4850 9.3796
11.6690 10.9323 7.1169 9.5516 8.6605 10.1260 11.0090 10.6434
9.9789 11.0281 7.7674 8.3991 9.4846 6.4602 9.3109 11.6921
9.3194 6.9798 10.4093 10.2076 8.3435 10.3712 7.6135 9.6018
10.5974 4.5290 6.2179 9.7571 10.2033 6.9076 8.3241 9.6106
9.1541 7.5503 9.8477 9.7339 9.7485 8.1988 8.4832 8.9329
9.5104 7.6564 9.3943 9.8566 10.8229 9.9022 9.0874 9.6524
1.0461 10.7703 10.6505 6.2380 10.3664 10.9949 11.0898 12.0101
11.0812 9.7302 5.4502 6.3458 10.1375 8.8437 8.3512 11.2964
6.0969 10.0635 8.2876 11.8234 5.8496 10.7664 10.0099 8.5648
9.0315 10.4478 11.0763 5.6958 10.7977 11.1902 10.2561 11.2854
10.4334 9.3659 8.5850 10.3507 8.8955 10.9069 11.8593 11.1728
5.1771 9.3704 10.4597 5.9655 10.3713 6.8810 7.2317 11.6800
7.6972 8.5828 7.2825 10.6788 6.3106 10.7132 9.5819 5.8087
7.2682 10.3530 6.8221 10.1986 8.7647 10.1083 10.8800 10.1042
10.0074 8.9745 7.4635 10.6684 7.1640 9.5020 11.8396 10.1115
11.7345 9.9593 9.9660 8.9989 8.3912 8.9345 10.8111 10.8141
3.6832 10.9554 6.2334 9.7718 10.2134 11.3155 10.3192 6.7252
7.6561 11.4106 10.6223 9.3503 11.6285 9.4897 7.9808 9.3374
11.5768 7.4401 6.9861 9.6076 8.1460 11.3147 7.7365 8.7904

9.8947 9.0019 11.2664 11.0278 10.7984 10.1150 10.1203 9.6770
10.6629 11.2143 9.5217 9.8450 9.2648 12.3359 8.8731 10.1856
9.9064 11.0087\
KHDRBS1 12.5677 12.6391 11.1271 11.8650 12.5274 12.2453 12.1249
12.4622 12.4102 12.3959 12.6468 12.9756 12.5157 12.5064 12.2623
11.3056 12.4559 11.9957 12.1363 11.8530 11.7993 12.0137 12.1500
12.3465 12.5121 12.0052 12.4528 12.2979 11.9792 12.1477 12.3991
12.1086 12.3501 12.0234 12.2552 12.5024 11.8765 12.2209 12.2934
12.3215 12.2399 12.3943 12.1816 11.8503 12.3532 12.2357 12.2254
11.6295 12.8168 11.6895 11.8058 11.6794 12.2778 12.3917 11.7088
12.4479 11.8074 11.7982 12.3739 11.8522 12.4545 12.5787 11.8927
12.1663 12.3165 12.4120 11.7320 12.2347 11.7818 11.9606 12.3187
12.2683 11.9691 12.1824 12.1936 11.6970 12.3735 11.2382 12.0376
12.0741 12.4991 12.5374 11.6231 12.4186 12.3140 11.9177 13.2897
12.0283 12.3036 12.4657 11.8586 12.3382 12.2280 11.8350 13.0074
12.0184 11.7360 12.6387 12.3647 12.2135 12.3130 11.9294 12.3366
11.8903 12.1671 12.2376 11.4995 12.1864 12.5155 12.4089 12.3119
12.3540 12.4516 11.2101 12.7675 12.5369 12.3708 12.1621 12.3371
12.0919 12.5354 12.2652 12.2059 11.8548 12.0885 12.3804 12.6121
12.5104 12.5589 12.3240 12.1194 12.7084 12.3573 11.7904 12.1144
11.9147 12.2935 11.6111 12.2626 12.5710 12.1583 12.2161 12.0344
12.3041 12.1610 12.5126 12.0949 12.0771 11.9294 12.3193 11.9190
12.1562 12.1281 12.4973 12.1858 12.5219 12.2231 12.3628 12.4500
12.6100 11.6471 11.9542 12.5331 12.2997 11.8614 12.3806 12.4811
11.7269 12.2165 12.6189 12.2227 12.3527 12.1037 12.1390 11.7549
12.7102 12.3522 12.0398 12.5856 11.6519 12.2366 11.7911 12.7077
12.1169 12.3982 12.2699 12.3159 12.1857 12.2159 12.6143 12.2677
12.2617 12.5163 11.6826 12.1289 12.6608 12.6055 12.5612 12.0456
12.2192 12.2146\
RUNDC2C 1.2631 2.9001 3.9745 4.6275 2.9490 4.0868 2.6133
3.2407 3.3514 4.0782 3.5647 4.0536 5.6357 3.1720 3.7620
3.3901 2.9165 1.4700 4.1878 1.1305 2.6649 4.7309 4.8056
4.3719 3.8459 1.0317 3.3814 1.8373 1.3857 4.1845 3.3209
3.9109 2.2837 4.5568 2.9726 2.6866 4.0266 4.0402 2.2142
4.5671 2.3500 5.0208 3.8391 3.3841 3.5967 1.9667 4.2414
3.8463 3.0038 0.0000 3.3478 4.5657 3.9272 4.8922 4.5104
3.2756 3.6300 4.1335 3.8260 3.0167 4.3656 4.4882 4.7180
3.2572 4.3827 4.2783 4.3838 3.7653 3.3716 2.7451 1.3075
2.8528 3.4823 2.2101 5.2708 5.9553 2.3343 5.3741 3.8280
3.5972 0.0000 4.1747 4.0029 4.3829 2.9265 3.6793 2.9238
0.6140 4.7695 0.9113 4.2866 4.4475 2.5333 3.8943 2.6735
2.5596 3.7905 4.5684 4.0511 3.5764 0.0000 3.0736 3.5917
5.3771 3.3739 2.5744 4.0702 3.0061 4.1186 3.4510 3.7948
1.4651 2.0513 4.3327 5.1414 4.2813 2.4868 6.4103 4.0558
4.0663 3.0097 3.0758 4.1059 2.2400 5.6310 2.5452 2.9767
2.2050 4.6045 5.6472 3.2487 4.6568 3.4946 5.2856 1.3267
0.0000 3.1433 4.5503 5.3166 4.5212 4.0123 1.3036 2.8717
3.0544 2.8875 7.1834 3.5287 3.6511 2.2134 1.8766 4.8488
3.5510 4.4275 3.3254 3.3552 4.4760 4.0872 4.1644 3.4328
1.9005 1.0317 2.5503 2.6931 1.9940 4.0868 4.5078 6.0852
4.6909 2.5150 3.1205 2.9865 3.9537 0.0000 0.6229 0.0000
3.4651 4.1698 4.4807 3.2229 7.1348 1.2666 3.1972 3.2915
2.7866 2.4250 2.4591 3.3819 2.9379 1.4486 2.4136 3.0754
3.1503 2.7066 3.6701 4.0914 0.0000 3.4156 4.4966 3.1802

5.0512  1.5159\
KHDRBS3 6.2056  7.8381  10.5257 8.4685  7.7466  3.1372  9.1468
7.8342  8.8938  7.8631  9.4020  7.8967  6.4101  8.4644  5.1612
4.1868  8.2554  7.8146  7.3890  6.6353  8.0837  7.3297  7.7908
8.1386  7.8561  5.4875  9.0510  6.1909  7.0926  6.7567  11.0563
5.0794  5.4162  5.0135  8.5186  7.6811  5.8771  7.6630  10.4467
9.6993  9.0883  7.2172  4.8626  5.4249  7.8194  5.9001  9.3309
3.8776  7.1417  8.3337  6.4298  4.5305  7.5151  7.5399  8.5336
8.6094  9.3957  8.5897  7.6600  9.7343  7.7385  7.8170  10.2789
7.8868  8.5811  4.1531  6.7663  9.0171  7.3696  6.4030  6.9127
7.0152  4.6992  9.0067  6.5520  7.6606  5.5244  7.7736  8.5637
7.3617  7.8004  7.7514  7.3987  9.1331  8.2721  6.6251  6.8084
8.9007  8.4745  9.4886  8.1475  7.0999  7.8067  8.0270  9.9834
6.9470  6.8025  7.8267  10.0945 4.8042  8.2331  6.2891  4.7087
8.5272  10.0734 9.2800  8.1609  9.4071  7.8383  8.8155  7.7294
9.3200  7.9442  5.8329  8.3913  7.0611  8.8544  8.6617  7.7547
4.1540  7.7339  4.6830  8.1697  6.5096  7.6430  6.3716  9.2300
4.5476  7.8219  7.7405  8.1554  9.3633  8.2069  7.8376  8.3014
7.9149  9.6831  6.8457  6.7798  7.0160  9.8339  6.0251  8.4895
7.6504  4.9072  7.8097  8.5674  7.9868  5.7309  8.5891  7.4981
7.3031  7.9478  6.1837  6.7062  8.8109  7.5060  6.2428  5.6732
4.3955  7.7420  4.5073  7.4751  8.0489  5.0078  8.3013  6.3279
8.6651  10.3448 6.9270  10.6633 7.9263  8.1027  9.0191  4.6244
9.4491  8.2985  8.2135  10.6634 6.1219  4.6108  4.0301  6.8491
6.5245  8.4042  8.9819  8.5603  6.1759  7.7434  7.1219  5.3897
6.1874  8.4065  7.6187  8.5053  7.4820  9.7498  7.3435  8.8082
8.1260  9.2725\
GGCT    11.0630 9.3317  10.5034 11.9334 11.4463 10.7442 10.8971
10.0528 12.0977 10.5223 10.4255 10.2379 10.2675 10.9251 10.9477
10.6485 11.7040 10.6956 9.2505  10.7190 9.8443  11.0014 10.0318
11.1018 9.3150  10.3890 11.9601 11.4983 11.3906 10.0245 10.5809
11.6465 11.0525 11.0860 10.5289 11.1330 10.5181 10.3588 11.6281
10.7668 10.7609 9.7889  10.3755 11.4130 9.0737  11.9164 12.1841
10.9220 10.3941 12.1355 10.8395 10.9500 11.3873 9.4289  10.7095
11.8442 9.5619  11.7427 10.8274 10.4850 9.2867  10.8038 11.6775
10.1405 9.9974  10.9186 9.9350  11.8304 11.4784 11.1579 10.8145
10.4991 10.0552 11.1924 11.2548 11.6551 10.0934 10.3676 10.3609
10.9607 12.0208 11.3073 10.2662 10.9627 11.3332 11.1162 10.0981
11.0352 11.3030 10.5963 9.2206  11.4368 10.8206 10.5025 11.0394
9.1914  10.0210 9.3043  11.0217 10.7325 11.4384 10.6084 10.3445
10.5282 12.0308 11.3479 9.1483  10.5452 10.2317 11.5017 11.0511
11.2678 10.1238 10.7674 11.7442 10.4639 10.9184 10.1356 12.2154
10.6208 10.9811 11.3071 10.0576 11.9384 10.0188 10.4249 11.4587
10.9532 9.2907  9.2941  11.2007 12.1156 11.1975 11.4633 10.1602
10.5411 11.6942 8.9551  11.0363 9.0076  12.3003 11.1367 11.1721
10.6230 11.2163 8.5482  11.3167 9.4936  10.6709 12.2725 10.5277
10.6239 10.2184 11.4377 10.9558 10.1501 9.2431  10.9494 11.2762
10.4205 10.5726 10.4379 11.4347 11.0920 12.2666 11.0138 11.4377
10.3632 11.4548 11.0497 10.5366 11.2055 12.5953 11.3706 11.2443
11.2038 10.1840 9.1076  11.6717 9.7163  11.3681 10.4921 10.1751
11.4940 10.5793 10.7896 10.1703 10.9575 11.5781 11.2208 11.6271
11.0435 11.0652 10.6900 10.4737 11.4212 11.6064 9.2420  10.7245
10.5875 11.6278\
VPS37C  9.5526  9.8819  9.9850  9.5846  9.7644  9.5431  10.2731

| | | | | | | | |
|---|---|---|---|---|---|---|---|
| 9.1656 | 10.3823 | 9.8827 | 9.8292 | 10.1034 | 9.3198 | 9.2635 | 9.8438 |
| 9.5234 | 9.4460 | 9.1689 | 9.8585 | 9.7962 | 9.5921 | 10.2847 | 9.5057 |
| 9.8681 | 9.7818 | 9.2237 | 9.2925 | 9.7679 | 9.7520 | 9.3237 | 9.3345 |
| 9.5948 | 10.2787 | 10.5460 | 8.9493 | 9.5536 | 9.6463 | 9.8712 | 9.1496 |
| 9.0301 | 9.5772 | 9.6594 | 9.3075 | 8.6662 | 9.2287 | 9.7544 | 9.2604 |
| 9.5420 | 9.2918 | 9.6745 | 9.8107 | 10.4428 | 9.3611 | 9.2538 | 8.9125 |
| 9.5996 | 9.3463 | 9.4135 | 9.9624 | 9.7441 | 9.3435 | 9.3854 | 10.0160 |
| 9.1424 | 9.8339 | 9.6569 | 10.2771 | 9.7828 | 9.2752 | 9.4596 | 9.3734 |
| 9.5421 | 8.7302 | 9.7551 | 9.3371 | 10.1728 | 9.1945 | 10.5779 | 9.6623 |
| 9.7182 | 9.5861 | 9.2836 | 9.7822 | 9.8311 | 9.8241 | 9.2773 | 9.2130 |
| 9.9191 | 9.6564 | 9.6555 | 9.3052 | 9.4746 | 9.7568 | 9.5830 | 9.7203 |
| 9.4789 | 9.0200 | 9.3808 | 9.7134 | 9.1094 | 9.1622 | 9.8383 | 9.9557 |
| 9.9244 | 9.7543 | 9.0315 | 9.3339 | 9.4744 | 9.7435 | 9.7352 | 9.6617 |
| 9.6739 | 9.0307 | 9.5018 | 9.1847 | 9.8368 | 9.6426 | 9.4214 | 10.4160 |
| 9.0145 | 9.5047 | 9.2458 | 9.3736 | 9.7842 | 9.3543 | 9.6051 | 9.5277 |
| 9.5630 | 9.3038 | 9.3408 | 9.6586 | 9.4822 | 9.7625 | 10.5007 | 9.0834 |
| 9.6094 | 10.1211 | 9.0650 | 9.3237 | 9.3308 | 10.3232 | 9.2208 | 9.8412 |
| 9.2447 | 9.4064 | 9.2973 | 9.9732 | 9.2367 | 10.1377 | 9.4200 | 9.5652 |
| 10.1254 | 9.6243 | 9.5926 | 9.5398 | 9.8330 | 9.6048 | 9.9031 | 9.6921 |
| 9.0669 | 10.1542 | 10.1078 | 9.7652 | 9.5752 | 9.4155 | 9.1398 | 9.0776 |
| 9.7721 | 9.8314 | 9.5779 | 9.5863 | 9.3865 | 9.2819 | 9.4793 | 8.5036 |
| 9.4261 | 9.7458 | 9.6545 | 10.1060 | 9.6694 | 9.8293 | 9.4633 | 9.5676 |
| 10.3314 | 9.6094 | 9.8408 | 10.1553 | 9.6480 | 10.0228 | 9.7397 | 9.8105 |
| 9.5603 | 10.2643 | 8.7175 | 9.3235 | 9.3742 | 8.9520 | 9.3497 | 9.8405 |
| 9.4299 | 9.6158\ | | | | | | |
| STAC2 | 11.9379 | 4.3966 | 4.3653 | 7.7326 | 5.9633 | 4.4242 | 1.9040 |
| 6.7778 | 3.6885 | 2.8952 | 6.5890 | 3.3489 | 6.5687 | 6.6127 | 1.8915 |
| 2.6237 | 2.7928 | 0.7772 | 4.4562 | 3.6548 | 8.4615 | 1.3712 | 3.0325 |
| 2.4333 | 5.7887 | 6.0617 | 7.2891 | 2.9431 | 8.3162 | 3.1011 | 3.1074 |
| 5.8595 | 1.4434 | 4.3819 | 7.8179 | 2.2096 | 9.5033 | 2.8159 | 2.5862 |
| 1.0370 | 2.6483 | 5.1995 | 1.3900 | 3.1197 | 5.3355 | 1.8914 | 0.0000 |
| 10.9785 | 3.1455 | 3.6261 | 5.5579 | 2.5833 | 5.4846 | 7.3318 | 2.2625 |
| 2.5237 | 1.9111 | 2.3708 | 4.0971 | 2.1385 | 5.8240 | 2.3538 | 4.7257 |
| 2.4883 | 2.0096 | 2.5790 | 6.1467 | 6.8574 | 8.9842 | 3.4751 | 7.4291 |
| 11.3574 | 0.0000 | 2.4134 | 3.0375 | 6.2709 | 5.4018 | 1.7110 | 4.3648 |
| 2.7776 | 2.2758 | 1.6845 | 2.6279 | 1.9875 | 4.0362 | 8.5285 | 4.3517 |
| 3.1631 | 2.0314 | 4.6053 | 6.4773 | 3.4573 | 6.8571 | 3.2630 | 2.0132 |
| 5.3472 | 7.8517 | 6.6001 | 2.8769 | 1.2591 | 6.1990 | 3.6025 | 8.5528 |
| 3.3666 | 0.4835 | 4.0885 | 6.7275 | 2.5557 | 6.8010 | 2.0061 | 1.8341 |
| 3.4186 | 4.9483 | 5.8826 | 6.3784 | 5.9722 | 4.0011 | 3.6464 | 1.5372 |
| 2.5175 | 6.4505 | 4.5652 | 3.8017 | 7.9262 | 4.2433 | 3.7566 | 9.6823 |
| 9.6459 | 6.1107 | 5.4729 | 3.1441 | 1.9805 | 2.0932 | 3.3178 | 1.3267 |
| 2.8207 | 5.5165 | 0.0000 | 6.2127 | 6.3868 | 1.6169 | 3.8783 | 2.7437 |
| 4.3341 | 2.2385 | 4.5160 | 7.4488 | 6.6927 | 12.2864 | 8.0491 | 4.5529 |
| 0.0000 | 2.0661 | 2.0029 | 1.5093 | 2.2918 | 5.2229 | 2.2251 | 5.6136 |
| 2.3506 | 1.8525 | 4.2502 | 1.5791 | 5.4020 | 1.6553 | 2.8575 | 6.1019 |
| 1.9581 | 2.6550 | 7.0822 | 1.6727 | 1.2195 | 4.6763 | 1.6598 | 3.6013 |
| 6.8317 | 3.4664 | 7.6831 | 7.4442 | 2.5710 | 3.4996 | 7.6063 | 7.9722 |
| 1.4215 | 8.8096 | 2.0881 | 2.1610 | 6.1376 | 2.1566 | 1.2961 | 3.1033 |
| 7.7338 | 6.0457 | 3.5846 | 1.1623 | 4.9682 | 3.3791 | 7.1641 | 5.1413 |
| 7.0183 | 2.7180\ | | | | | | |
| LOC283404 | | 2.3786 | 0.5319 | 0.0000 | 1.1529 | 0.5278 | 0.9816 |
| 0.0000 | 1.5600 | 0.0000 | 0.0000 | 0.4291 | 0.4849 | 1.6409 | 1.4754 |
| 1.2358 | 1.4442 | 2.7928 | 1.2795 | 1.3199 | 3.1743 | 2.7816 | 1.3712 |

| | | | | | | | |
|---|---|---|---|---|---|---|---|
| 2.1093 | 0.0000 | 0.0000 | 0.0000 | 2.1197 | 1.3471 | 0.8532 | 0.0000 |
| 0.0000 | 1.5370 | 1.4434 | 0.8184 | 1.3224 | 0.6819 | 0.0000 | 0.0000 |
| 0.5410 | 0.7666 | 3.1062 | 0.8506 | 0.0000 | 1.4585 | 2.0387 | 2.4638 |
| 1.5457 | 0.0000 | 0.0000 | 2.1768 | 1.0749 | 1.6507 | 0.6494 | 0.5094 |
| 2.3724 | 1.9450 | 1.4095 | 2.6835 | 1.7655 | 0.9799 | 0.0000 | 1.7937 |
| 2.3905 | 0.5968 | 0.0000 | 0.6977 | 1.6109 | 1.5796 | 1.3082 | 2.0689 |
| 2.6452 | 1.1694 | 1.9166 | 2.8668 | 1.2339 | 3.8164 | 0.5548 | 0.0000 |
| 0.0000 | 1.8728 | 0.9714 | 0.7073 | 1.5572 | 2.1886 | 2.4335 | 2.4045 |
| 1.2355 | 2.3907 | 0.9125 | 0.0000 | 0.0000 | 0.0000 | 1.2055 | 3.0654 |
| 0.0000 | 0.0000 | 2.3031 | 0.0000 | 0.8845 | 0.9478 | 3.5349 | 0.8684 |
| 0.5352 | 1.8724 | 0.0000 | 1.5391 | 0.7314 | 1.0326 | 1.2448 | 1.8132 |
| 0.5136 | 1.8846 | 2.0513 | 2.8074 | 0.8924 | 0.0000 | 1.2170 | 1.4572 |
| 1.1815 | 1.3647 | 1.6899 | 1.0055 | 0.6224 | 1.7271 | 2.1978 | 1.2272 |
| 1.0447 | 3.7308 | 0.0000 | 0.5074 | 0.9567 | 3.0128 | 0.9511 | 2.3620 |
| 0.8107 | 0.0000 | 2.5939 | 0.0000 | 0.5216 | 0.5056 | 0.0000 | 0.0000 |
| 0.6506 | 2.0686 | 1.8493 | 0.0000 | 0.6735 | 2.0758 | 0.0000 | 3.0614 |
| 0.5019 | 3.8724 | 0.0000 | 1.5875 | 2.6037 | 0.4374 | 0.0000 | 0.0000 |
| 1.6149 | 0.0000 | 0.0000 | 0.0000 | 1.3167 | 0.4033 | 0.4418 | 0.5023 |
| 0.0000 | 0.9722 | 1.6815 | 1.5072 | 1.2980 | 0.5290 | 1.4956 | 0.0000 |
| 0.0000 | 0.4801 | 0.0000 | 1.0660 | 5.9069 | 1.9052 | 2.0978 | 1.1551 |
| 2.5448 | 0.0000 | 0.0000 | 0.0000 | 0.0000 | 0.0000 | 0.6567 | 0.9785 |
| 1.8202 | 0.5556 | 1.7696 | 1.0222 | 0.4985 | 0.0000 | 0.0000 | 0.0000 |
| 1.4718 | 1.9827 | 1.7990\ | | | | | |
| ZNF395 | 9.8475 | 10.1504 | 10.0438 | 10.9031 | 11.6677 | 9.9579 | 10.5865 |
| 10.0623 | 9.4879 | 10.2421 | 10.8017 | 9.5977 | 10.0596 | 10.8185 | 10.2364 |
| 9.9647 | 9.5545 | 9.9384 | 10.0431 | 10.4772 | 9.5931 | 9.7789 | 11.2515 |
| 10.6608 | 9.9746 | 9.2089 | 9.3348 | 9.9951 | 10.2766 | 10.1363 | 10.9963 |
| 11.6224 | 9.3990 | 9.8045 | 11.4481 | 10.7538 | 11.5610 | 11.0012 | 10.0744 |
| 10.7693 | 10.0675 | 9.8660 | 9.7671 | 9.6482 | 11.0027 | 9.1912 | 10.4248 |
| 9.5814 | 9.5696 | 10.3430 | 10.4332 | 8.9164 | 11.2420 | 10.8040 | 10.3409 |
| 10.9211 | 10.2064 | 9.9614 | 10.1276 | 8.9791 | 10.9647 | 10.6305 | 9.7662 |
| 9.5830 | 9.5984 | 10.9901 | 9.2518 | 10.9545 | 9.8971 | 8.5421 | 9.4749 |
| 10.9922 | 9.5135 | 10.0277 | 9.4717 | 10.1910 | 10.7813 | 10.1238 | 10.1304 |
| 10.6410 | 9.8256 | 9.0275 | 9.6547 | 10.3774 | 9.9837 | 9.6534 | 9.7781 |
| 10.7182 | 10.1215 | 10.3944 | 11.2288 | 9.2826 | 10.7589 | 9.6162 | 9.6697 |
| 10.0661 | 11.4739 | 11.0292 | 11.3638 | 10.3100 | 10.2072 | 10.0015 | 10.8243 |
| 9.9421 | 9.9792 | 11.3180 | 9.8523 | 9.8667 | 9.9298 | 10.8090 | 10.7959 |
| 10.3664 | 10.7465 | 9.1898 | 8.8612 | 10.0175 | 10.9885 | 10.1210 | 11.3230 |
| 8.1202 | 9.9581 | 9.9913 | 10.0116 | 10.4075 | 11.4628 | 9.5868 | 10.1984 |
| 11.0531 | 10.7762 | 9.9832 | 11.9144 | 9.6139 | 9.8594 | 10.8330 | 9.2229 |
| 8.3090 | 9.9835 | 11.6705 | 11.2279 | 11.3224 | 9.6278 | 11.4275 | 10.3430 |
| 10.6702 | 9.5425 | 12.4163 | 10.4686 | 11.7226 | 10.4400 | 10.2103 | 10.2723 |
| 10.7919 | 10.7029 | 10.7312 | 10.2026 | 9.6606 | 11.0160 | 11.7161 | 9.8191 |
| 10.3330 | 10.5751 | 9.8743 | 10.2223 | 10.7253 | 10.0065 | 10.6106 | 10.2179 |
| 10.4460 | 10.5280 | 11.0272 | 11.9741 | 9.3860 | 10.9599 | 10.5117 | 11.5410 |
| 11.8684 | 9.6273 | 11.0224 | 11.7105 | 9.8248 | 9.5916 | 9.4188 | 9.9238 |
| 9.7605 | 10.2910 | 10.3638 | 9.1242 | 10.2771 | 10.0218 | 10.4530 | 10.6472 |
| 10.7162 | 10.4913 | 9.8880 | 11.0377 | 9.8584 | 9.2382 | 11.5741 | 10.5355 |
| 10.5644 | 10.1522\ | | | | | | |
| STAC3 | 7.2038 | 5.6060 | 5.4958 | 5.3850 | 6.8309 | 5.9908 | 4.4907 |
| 5.1560 | 4.4904 | 5.7702 | 6.3028 | 5.8188 | 6.1400 | 5.3093 | 6.5829 |
| 7.7406 | 5.9011 | 5.9105 | 5.7589 | 5.1001 | 8.0723 | 6.1568 | 6.2918 |
| 6.7886 | 2.9167 | 7.4759 | 7.4968 | 8.1293 | 6.6697 | 6.7543 | 3.6161 |
| 5.9174 | 7.3559 | 5.6617 | 2.9077 | 5.9791 | 6.6002 | 5.9671 | 5.6867 |

| | | | | | | | |
|---|---|---|---|---|---|---|---|
| 3.6521 | 6.9464 | 4.7718 | 4.3916 | 6.3269 | 2.8515 | 8.1110 | 6.8520 |
| 5.5318 | 4.8494 | 6.0427 | 7.1288 | 7.4158 | 5.9350 | 3.5346 | 6.5300 |
| 6.2129 | 5.2267 | 6.6482 | 5.7678 | 4.6021 | 3.2731 | 6.0347 | 5.7994 |
| 6.2461 | 5.2904 | 6.8757 | 9.5850 | 5.6803 | 5.3175 | 4.7601 | 8.0490 |
| 5.1718 | 8.9938 | 6.7014 | 6.6717 | 5.2414 | 6.0690 | 6.1265 | 6.2889 |
| 6.4116 | 6.4089 | 4.3432 | 6.5682 | 5.9700 | 7.0303 | 6.8192 | 2.9586 |
| 8.3456 | 6.0314 | 7.2799 | 5.9911 | 5.6124 | 7.3053 | 8.1568 | 6.7395 |
| 3.7560 | 5.4963 | 5.3434 | 5.0081 | 7.3158 | 7.7068 | 6.8545 | 5.5940 |
| 6.2244 | 6.1738 | 5.2839 | 6.3726 | 6.3456 | 4.5695 | 6.3911 | 5.6861 |
| 6.0293 | 6.6218 | 7.9244 | 4.6423 | 5.5364 | 5.8655 | 5.6683 | 5.4165 |
| 7.2796 | 8.4911 | 7.2376 | 5.2824 | 6.0834 | 5.7817 | 6.2149 | 5.1941 |
| 4.5915 | 3.8415 | 3.7250 | 5.6452 | 5.6915 | 7.7303 | 7.5803 | 6.6625 |
| 7.6357 | 8.0809 | 8.9222 | 5.3077 | 4.0830 | 6.6001 | 5.9698 | 7.6754 |
| 6.4965 | 5.9618 | 2.6938 | 6.2604 | 6.1701 | 6.3632 | 6.4009 | 5.6707 |
| 7.6285 | 6.0169 | 4.8507 | 5.2010 | 6.0864 | 4.9065 | 5.3064 | 7.2586 |
| 8.0880 | 5.3974 | 7.4810 | 6.2194 | 7.7093 | 6.1922 | 5.3860 | 6.4530 |
| 6.0376 | 6.8801 | 6.4144 | 5.1875 | 3.6455 | 6.4800 | 5.4481 | 6.8473 |
| 6.0377 | 6.0280 | 4.2093 | 5.4546 | 7.8734 | 6.9017 | 7.3099 | 4.9232 |
| 7.9592 | 5.7087 | 6.6848 | 5.7701 | 8.9323 | 6.7407 | 6.0858 | 6.2564 |
| 5.1884 | 6.9417 | 5.5562 | 5.9073 | 7.4982 | 6.6326 | 4.5881 | 7.8048 |
| 6.4545 | 9.1441\ | | | | | | |
| TBL1Y | 0.0000 | 0.0000 | 0.0000 | 0.0000 | 0.0000 | 0.0000 | 0.0000 |
| 0.4748 | 0.0000 | 0.0000 | 0.0000 | 0.0000 | 0.0000 | 0.0000 | 0.0000 |
| 0.0000 | 0.0000 | 0.0000 | 0.0000 | 0.0000 | 0.0000 | 0.0000 | 0.0000 |
| 0.0000 | 0.0000 | 0.0000 | 0.0000 | 0.0000 | 0.0000 | 0.0000 | 0.0000 |
| 0.0000 | 0.0000 | 0.0000 | 0.5853 | 0.0000 | 0.0000 | 0.0000 | 0.0000 |
| 0.0000 | 0.0000 | 0.0000 | 0.0000 | 0.0000 | 0.0000 | 0.0000 | 0.0000 |
| 0.0000 | 0.0000 | 0.0000 | 0.0000 | 0.0000 | 0.0000 | 0.0000 | 0.0000 |
| 0.0000 | 0.0000 | 0.0000 | 0.0000 | 0.0000 | 0.0000 | 0.0000 | 0.0000 |
| 0.0000 | 0.0000 | 0.0000 | 0.0000 | 0.0000 | 0.0000 | 0.0000 | 0.0000 |
| 0.0000 | 0.0000 | 0.0000 | 0.0000 | 0.0000 | 0.5548 | 0.0000 | 0.0000 |
| 0.0000 | 0.0000 | 0.0000 | 0.0000 | 0.0000 | 0.0000 | 0.0000 | 0.0000 |
| 0.0000 | 0.0000 | 0.0000 | 0.0000 | 0.0000 | 0.0000 | 0.0000 | 0.0000 |
| 0.0000 | 0.0000 | 0.0000 | 0.0000 | 0.0000 | 0.0000 | 0.0000 | 0.0000 |
| 0.0000 | 0.0000 | 0.0000 | 0.0000 | 0.0000 | 0.0000 | 0.0000 | 0.0000 |
| 0.0000 | 0.0000 | 0.0000 | 0.0000 | 0.0000 | 0.0000 | 0.0000 | 0.0000 |
| 0.0000 | 0.0000 | 0.0000 | 0.0000 | 0.0000 | 0.0000 | 0.0000 | 0.0000 |
| 0.0000 | 0.0000 | 0.0000 | 0.0000 | 0.0000 | 0.0000 | 0.0000 | 0.0000 |
| 0.0000 | 0.0000 | 0.0000 | 0.0000 | 0.0000 | 0.0000 | 0.0000 | 0.0000 |
| 0.5424 | 0.0000 | 0.0000 | 0.0000 | 0.0000 | 0.0000 | 0.0000 | 0.0000 |
| 0.0000 | 0.0000 | 0.0000 | 0.0000 | 0.0000 | 0.0000 | 0.3855 | 0.0000 |
| 0.0000 | 0.0000 | 0.0000 | 0.0000 | 0.0000 | 0.0000 | 0.0000 | 0.0000 |
| 0.0000 | 0.0000 | 0.0000 | 0.0000 | 0.0000 | 0.0000 | 0.0000 | 0.0000 |
| 0.0000 | 0.5232 | 0.0000 | 0.0000 | 0.0000 | 0.0000 | 0.0000 | 0.0000 |
| 0.0000 | 0.0000 | 0.0000 | 0.0000 | 0.0000 | 0.0000 | 0.0000 | 0.0000 |
| 0.0000 | 0.0000 | 0.0000 | 0.0000 | 0.0000 | 0.0000 | 0.0000 | 0.0000 |
| 0.0000 | 0.0000\ | | | | | | |
| LHCGR | 0.0000 | 0.0000 | 0.0000 | 0.0000 | 0.0000 | 0.0000 | 0.0000 |
| 0.0000 | 0.4327 | 2.6705 | 0.0000 | 0.4849 | 0.0000 | 0.0000 | 0.0000 |
| 0.0000 | 0.0000 | 0.0000 | 0.0000 | 0.3755 | 0.0000 | 0.0000 | 0.6346 |
| 0.0000 | 1.9289 | 0.0000 | 0.0000 | 0.0000 | 0.0000 | 0.0000 | 0.4127 |
| 0.0000 | 0.0000 | 0.0000 | 0.0000 | 0.0000 | 1.9329 | 0.0000 | 0.0000 |
| 0.0000 | 0.6649 | 0.0000 | 0.0000 | 0.0000 | 0.0000 | 0.0000 | 0.0000 |
| 0.6741 | 0.0000 | 0.0000 | 0.0000 | 0.7768 | 1.4359 | 0.8853 | 0.0000 |

0.0000 0.0000 0.0000 0.4252 0.5715 0.0000 0.4969 0.0000
0.0000 1.1445 0.0000 0.0000 0.4832 0.0000 0.6160 0.0000
0.0000 0.0000 0.4786 0.0000 0.0000 0.0000 0.0000 0.0000
0.0000 0.0000 0.0000 0.0000 0.0000 0.0000 0.0000 0.0000
0.0000 0.0000 0.0000 1.0759 0.0000 0.0000 1.1561 0.0000
0.0000 0.0000 0.0000 0.0000 0.0000 0.0000 0.0000 0.0000
0.0000 0.0000 0.0000 3.8282 0.0000 0.0000 0.0000 0.0000
0.6215 0.0000 0.0000 0.0000 0.6116 0.0000 0.4321 0.0000
0.0000 1.6899 0.0000 0.0000 0.0000 1.0180 0.5331 0.0000
0.0000 0.0000 3.8568 0.3935 0.0000 0.0000 0.0000 0.0000
0.0000 0.0000 0.0000 0.0000 0.0000 0.0000 0.0000 0.0000
0.0000 0.0000 4.4870 0.0000 0.9402 0.0000 0.0000 0.0000
0.0000 0.5414 0.5862 0.0000 0.0000 0.0000 0.0000 0.6000
0.0000 0.0000 0.6138 0.0000 0.0000 0.0000 0.5023 0.0000
0.0000 0.0000 0.0000 0.0000 0.0000 0.0000 0.0000 1.2582
0.0000 3.2180 1.0660 0.0000 0.0000 0.0000 0.6902 0.0000
0.0000 0.0000 0.0000 0.0000 0.0000 0.0000 0.0000 0.0000
0.0000 0.0000 0.0000 0.0000 0.0000 0.0000 0.0000 0.0000
0.5077 0.0000\
TMEM211 5.2953 4.0917 6.4396 6.1109 5.1296 0.7223 4.6035
1.5600 2.7963 5.0374 4.7353 2.6308 1.4675 4.8822 5.4114
6.1261 2.7928 2.5841 1.9975 1.6235 0.0000 5.8312 3.8339
6.5526 2.6200 0.0000 1.6918 1.3471 8.4387 1.5953 0.0000
6.2860 0.0000 1.7182 1.5856 0.0000 6.5032 6.6721 4.9556
0.4337 4.0711 3.8734 1.0569 5.2100 0.0000 1.8914 0.7135
1.4786 0.0000 5.0309 2.5349 1.9460 3.2521 0.0000 2.8986
4.3891 3.3767 2.2495 3.8103 2.8718 0.9353 2.6657 7.0244
4.7343 3.8994 3.4113 3.4274 0.8444 4.8605 3.8922 0.4334
4.0544 0.0000 5.0556 5.0831 5.2608 2.8273 3.1994 7.0522
1.3771 6.4104 4.7685 2.9023 2.5223 4.8665 1.0522 1.4887
5.4053 3.0105 0.0000 0.6361 2.1998 1.2055 3.8593 2.1605
0.0000 2.6431 3.0618 0.0000 0.0000 2.8342 3.3338 4.0633
0.7358 3.1638 3.9170 0.7314 4.0300 1.2448 2.5918 3.7895
0.6215 3.5857 1.0000 3.5487 2.3852 1.2170 2.3757 1.8223
1.0358 0.4553 5.9996 3.6855 7.4486 2.7308 2.6700 4.8127
2.9068 3.4596 2.3827 4.7226 5.9051 3.0001 0.0000 0.0000
0.0000 5.3680 0.0000 3.6233 5.2453 0.6010 6.9121 5.2919
5.9039 2.8723 0.0000 2.9153 2.0758 4.9203 8.5120 6.9021
3.4258 6.2846 0.5862 6.4787 1.6440 5.7571 5.6351 3.2880
0.0000 6.4630 0.6138 2.5777 5.8840 6.2183 1.8069 7.2377
1.5478 6.4592 2.8049 4.6479 0.5290 2.8815 2.0840 7.0120
1.1272 4.5418 0.0000 3.2229 3.8806 7.5830 3.2614 2.5448
5.8387 2.8065 2.4158 1.9914 4.0681 5.1058 3.4840 4.6707
5.4199 4.3071 0.0000 3.5522 3.7138 0.0000 4.9816 3.6128
3.8990 5.4924\
OR1Q1 0.9511 0.0000 1.5051 0.0000 1.6818 1.2014 0.0000
0.0000 0.4327 0.7786 1.2544 0.4849 1.2705 0.7759 0.0000
1.4442 1.0553 0.0000 0.0000 1.1305 0.6175 0.0000 0.0000
0.0000 0.0000 0.0000 0.4561 0.0000 0.8532 0.5901 0.0000
0.5612 0.8951 1.3374 0.0000 0.0000 1.5255 0.0000 0.0000
0.0000 0.6649 0.0000 1.3900 0.0000 0.0000 0.0000 0.0000
0.0000 0.0000 0.0000 0.0000 0.0000 0.0000 0.0000 0.0000
0.0000 0.6343 0.0000 1.0204 0.0000 0.0000 1.4031 0.0000
0.0000 1.4936 0.6977 0.5980 0.0000 0.0000 0.0000 0.0000

```
0.0000  0.0000  0.4786  1.2339  0.4698  0.0000  0.0000  1.7868
0.0000  0.0000  0.0000  0.7204  0.0000  0.0000  0.6199  0.0000
0.0000  0.9125  1.2149  0.0000  2.5341  0.5215  0.6909  0.0000
0.0000  0.5233  0.0000  1.6394  1.5150  0.0000  1.4068  0.0000
0.0000  0.0000  0.4659  3.4466  0.4315  0.0000  0.0000  0.8916
0.0000  0.0000  2.3219  2.2788  2.2315  0.0000  0.7641  0.0000
1.8581  1.5136  1.3285  3.3462  0.0000  0.5970  0.0000  1.3754
0.0000  0.0000  0.0000  0.3935  0.0000  0.9511  1.2511  0.0000
0.0000  1.4217  0.0000  0.0000  0.0000  1.3507  0.0000  1.4380
0.0000  0.0000  0.0000  0.0000  0.5454  0.0000  0.0000  0.0000
0.0000  0.0000  0.0000  0.5476  0.4374  0.0000  0.3855  0.0000
0.0000  0.0000  0.0000  0.9957  0.4033  0.0000  1.4147  0.0000
0.7148  0.0000  2.0787  0.0000  1.4707  0.0000  0.0000  0.0000
0.8396  1.4586  0.0000  1.9582  0.6314  0.0000  0.0000  0.5257
0.8792  0.0000  0.6248  0.0000  0.0000  1.1063  0.0000  0.0000
0.0000  0.0000  0.0000  0.8682  0.0000  0.0000  0.0000  0.3736
0.5077  0.0000\
TMEM127 10.6080 11.2708 11.5235 10.7304 10.9860 11.4727 11.1620
10.9493 10.7595 10.7145 11.2106 10.9324 11.2083 11.2525 10.6411
10.4426 10.9406 10.7729 11.3608 11.0962 11.2750 10.5071 10.8146
10.5864 10.9227 11.0194 10.5607 10.4831 11.0263 11.2120 10.6233
10.6610 11.1796 11.0737 11.4007 10.5102 11.2987 10.4042 10.1067
10.6951 10.6464 11.2249 11.2371 10.8112 11.1175 10.6487 10.6683
10.8088 10.1707 10.4295 11.4917 10.7565 11.1372 10.7319 11.3763
10.3425 11.3690 11.8201 11.0747 11.1723 11.0247 11.3200 10.6471
10.4205 10.6512 11.3008 11.8944 11.4120 11.3344 11.4421 11.1445
10.8039 10.7324 11.0497 10.6984 10.8824 11.3127 11.1661 10.7696
10.7247 10.6269 10.8920 10.9453 10.8517 10.4144 10.8274 10.7407
10.9028 10.8989 10.4995 11.2238 11.3388 10.7118 10.4394 10.6939
11.3014 11.1266 11.1595 10.4513 10.2469 10.6053 11.0843 11.0898
10.0153 10.1847 9.9738  10.1604 11.0249 10.5081 10.4307 10.3490
10.6896 11.3763 10.4929 10.7325 11.1901 10.3206 11.0255 10.6242
11.5010 10.6801 10.6746 11.5716 11.6082 10.5386 11.1719 10.6739
11.8717 11.0383 10.9073 10.8204 9.8851  10.7212 10.5990 11.0551
11.0016 10.8777 10.4211 10.6720 10.9673 10.4597 10.8168 10.3400
10.8849 10.7887 11.2555 10.7721 10.9103 11.2261 10.2496 10.7642
10.8061 10.9309 10.9950 10.1635 11.5737 10.9891 10.8111 9.5325
10.4778 10.9231 10.9720 10.6329 10.5957 11.5688 11.0696 10.7939
10.9309 11.0302 10.8076 10.6081 11.0658 10.8275 10.8272 10.8465
10.3397 10.4887 10.9296 10.5621 10.6165 10.5667 10.4748 11.5314
10.7610 10.9413 10.3965 11.2980 11.0167 10.8404 11.0445 10.6961
10.8526 10.7747 11.3384 10.4282 10.4057 9.8970  10.7376 11.3247
10.5290 10.4961\
GPR78   0.0000  0.0000  0.0000  0.0000  0.0000  1.9724  0.5431
0.0000  0.0000  0.0000  0.4291  0.0000  0.0000  0.7759  0.0000
0.0000  0.0000  0.0000  0.0000  2.7370  1.8761  0.0000  0.0000
0.0000  0.0000  0.0000  0.0000  0.0000  0.0000  0.0000  0.0000
0.0000  0.0000  0.8184  0.0000  0.0000  1.2685  0.0000  0.0000
0.0000  0.6649  0.0000  0.0000  0.0000  0.0000  1.8914  0.0000
0.0000  0.0000  0.0000  0.0000  1.2789  0.0000  0.0000  0.0000
0.0000  0.6343  0.0000  0.0000  0.0000  0.0000  0.0000  0.0000
0.0000  0.0000  0.0000  0.0000  0.0000  1.3082  0.0000  0.4334
0.0000  0.0000  0.0000  0.0000  0.0000  0.5548  0.0000  0.0000
0.0000  0.0000  0.0000  0.0000  0.0000  0.0000  0.0000  0.0000
```

| | | | | | | | |
|---|---|---|---|---|---|---|---|
| 0.0000 | 0.5273 | 0.0000 | 0.0000 | 0.0000 | 0.0000 | 0.0000 | 0.0000 |
| 0.0000 | 0.0000 | 0.0000 | 0.0000 | 0.5504 | 0.0000 | 0.0000 | 0.5352 |
| 0.0000 | 0.0000 | 0.0000 | 0.0000 | 0.0000 | 0.0000 | 0.0000 | 0.0000 |
| 0.0000 | 0.0000 | 0.0000 | 0.0000 | 0.0000 | 0.0000 | 1.6299 | 0.7085 |
| 2.5293 | 0.4553 | 0.0000 | 1.0557 | 0.0000 | 0.5970 | 0.0000 | 0.0000 |
| 0.0000 | 0.0000 | 0.0000 | 0.0000 | 0.0000 | 0.0000 | 0.7571 | 0.8107 |
| 0.0000 | 0.0000 | 0.0000 | 1.7874 | 0.0000 | 0.0000 | 0.0000 | 0.0000 |
| 1.2444 | 1.9541 | 0.0000 | 0.0000 | 0.5454 | 0.0000 | 0.0000 | 0.0000 |
| 0.0000 | 0.0000 | 1.0019 | 0.0000 | 0.0000 | 0.0000 | 0.3855 | 0.6000 |
| 0.0000 | 0.0000 | 0.0000 | 0.0000 | 0.0000 | 1.0531 | 0.0000 | 0.0000 |
| 0.0000 | 0.0000 | 0.0000 | 0.0000 | 0.0000 | 0.0000 | 0.0000 | 0.0000 |
| 0.4801 | 0.0000 | 0.0000 | 0.0000 | 0.0000 | 0.0000 | 0.6902 | 0.5257 |
| 0.0000 | 0.0000 | 0.0000 | 0.0000 | 0.0000 | 0.0000 | 0.0000 | 0.0000 |
| 0.0000 | 0.0000 | 0.4262 | 0.0000 | 0.0000 | 0.0000 | 0.0000 | 1.5598 |
| 0.0000 | 0.0000\ | | | | | | |
| TRPA1 | 1.2631 | 0.0000 | 0.0000 | 1.1529 | 0.9133 | 0.7223 | 1.4999 |
| 1.7391 | 0.0000 | 1.2837 | 0.4291 | 0.0000 | 0.0000 | 0.0000 | 0.5377 |
| 1.4442 | 2.0822 | 0.0000 | 2.1737 | 1.0290 | 1.4005 | 0.0000 | 0.6346 |
| 1.4901 | 0.9530 | 0.0000 | 5.7692 | 1.4155 | 3.6194 | 0.0000 | 2.2204 |
| 1.2789 | 0.0000 | 0.0000 | 0.0000 | 0.0000 | 0.0000 | 0.3808 | 0.0000 |
| 0.0000 | 1.1184 | 0.4871 | 1.0569 | 0.0000 | 2.0387 | 2.8725 | 0.0000 |
| 0.0000 | 1.2792 | 0.0000 | 0.4530 | 3.4576 | 2.4808 | 2.1406 | 3.8373 |
| 4.4395 | 0.6343 | 0.5030 | 1.0204 | 0.9799 | 2.0680 | 3.7810 | 0.0000 |
| 2.2070 | 1.7745 | 1.1659 | 0.0000 | 1.3736 | 7.1690 | 0.0000 | 0.4334 |
| 0.0000 | 5.1760 | 2.4647 | 1.2339 | 0.0000 | 0.0000 | 1.7289 | 0.0000 |
| 2.0686 | 1.9568 | 0.0000 | 2.3133 | 0.6931 | 0.9110 | 0.0000 | 2.0569 |
| 4.4738 | 0.9125 | 5.7625 | 3.9155 | 0.0000 | 0.5215 | 2.2282 | 0.0000 |
| 2.4199 | 0.5233 | 1.2610 | 0.5090 | 2.3730 | 1.6914 | 0.4986 | 0.0000 |
| 2.8049 | 0.0000 | 0.4659 | 0.0000 | 2.1653 | 4.2724 | 1.3268 | 1.8341 |
| 0.0000 | 0.0000 | 0.0000 | 6.5095 | 3.7164 | 1.4679 | 2.7430 | 1.1996 |
| 1.0358 | 6.6374 | 0.0000 | 2.4246 | 1.1079 | 1.3435 | 2.4516 | 0.6149 |
| 0.0000 | 0.9653 | 1.4300 | 0.3935 | 1.1234 | 0.5526 | 0.0000 | 0.8107 |
| 0.0000 | 1.0837 | 0.0000 | 0.9105 | 0.0000 | 1.0240 | 0.0000 | 1.0973 |
| 4.3203 | 0.4562 | 1.4974 | 2.3685 | 0.5544 | 0.5814 | 3.7753 | 5.1946 |
| 0.0000 | 2.7006 | 2.0029 | 3.1454 | 4.4988 | 0.9683 | 3.6376 | 0.6000 |
| 0.9345 | 0.0000 | 0.0000 | 0.0000 | 0.4033 | 0.0000 | 0.5023 | 0.0000 |
| 1.5541 | 0.9131 | 0.0000 | 0.7903 | 0.5290 | 0.9335 | 1.3895 | 0.0000 |
| 0.4801 | 0.0000 | 2.9187 | 2.6895 | 1.9052 | 2.0978 | 0.0000 | 0.0000 |
| 0.5055 | 0.0000 | 0.0000 | 0.0000 | 0.0000 | 0.0000 | 0.9785 | 2.7054 |
| 0.5648 | 0.9737 | 0.4335 | 2.7609 | 2.8198 | 0.0000 | 1.6539 | 3.6809 |
| 0.5077 | 0.0000\ | | | | | | |
| VPS37B | 10.8675 | 10.1333 | 9.8987 | 10.3475 | 10.2210 | 10.1052 | 10.4049 |
| 10.9527 | 10.4145 | 10.3199 | 10.1354 | 9.9399 | 10.1003 | 9.9580 | 10.6710 |
| 10.8217 | 10.0414 | 9.8944 | 10.2073 | 10.8678 | 9.4025 | 10.9632 | 10.2615 |
| 10.5390 | 9.4715 | 10.5911 | 11.2541 | 11.4826 | 10.5671 | 10.4459 | 10.2620 |
| 10.9822 | 10.5896 | 10.5154 | 10.5873 | 9.9275 | 10.9980 | 10.6933 | 11.0600 |
| 9.5959 | 10.0745 | 10.0224 | 10.4554 | 10.7055 | 9.1093 | 10.9987 | 10.3610 |
| 9.9552 | 9.5810 | 11.2805 | 10.7472 | 10.5647 | 10.1371 | 9.3194 | 9.9972 |
| 10.2617 | 10.7919 | 11.1004 | 10.2337 | 11.4626 | 9.0834 | 10.1775 | 10.2816 |
| 10.3594 | 9.9966 | 11.0215 | 9.5275 | 9.8706 | 11.2386 | 10.7232 | 9.2551 |
| 10.3645 | 9.9565 | 11.3965 | 10.1546 | 10.6910 | 10.6165 | 11.4007 | 10.5187 |
| 10.9390 | 10.5304 | 9.5251 | 10.7605 | 10.6434 | 10.8321 | 8.9113 | 9.5475 |
| 10.9927 | 10.4054 | 9.8992 | 9.5456 | 9.8794 | 10.5095 | 10.8232 | 10.7228 |
| 9.6132 | 9.5766 | 9.4927 | 10.3953 | 10.4899 | 11.2459 | 10.5622 | 10.0638 |

```
11.1467 10.3727 10.0084 10.9179 10.5675 10.9258 10.4042 10.7289
11.5124 10.7306 11.7125 9.3631  10.5096 11.0369 10.9293 10.2876
10.0614 9.2957  10.3658 10.4256 11.4211 9.3711  10.6067 10.8082
11.1094 9.4652  9.1127  10.6162 9.6910  11.1464 10.8814 11.2839
10.7549 11.5847 10.8961 9.9424  9.5421  11.0235 11.7692 10.8114
10.5840 10.6121 8.8254  10.6369 10.0059 11.1500 10.8843 10.2086
10.6832 10.6519 11.0056 9.7852  11.6049 9.7814  10.4048 9.3978
11.9208 11.5785 10.5298 10.2738 10.7545 10.1121 10.0848 10.2493
10.2771 10.9781 11.5278 11.4697 9.6253  11.1864 11.1539 10.9946
10.0372 9.6697  9.7270  10.4643 10.6275 10.8147 9.8378  8.5992
10.2822 10.1063 10.4235 10.6667 10.7304 11.0396 10.3970 10.1016
11.3416 9.7846  9.6445  10.3870 9.9961  10.6112 9.4147  10.8578
10.2395 11.7772\
TTC7A   9.3795  9.1957  9.0512  10.2538 9.7949  9.4328  9.3515
9.2767  9.2242  9.4791  9.1907  8.9483  10.1210 10.2663 9.9835
9.4996  9.6043  9.9431  9.5363  10.3749 10.7151 11.1827 9.9004
8.8608  8.3098  10.0014 8.7521  10.6733 8.8702  9.9106  7.5014
9.8186  10.3407 9.8928  9.6230  9.5337  9.7452  9.5229  9.2704
9.6115  9.5527  8.9085  10.9700 10.2046 8.6586  10.5232 9.8739
10.1264 8.6789  8.9031  10.0178 11.7111 9.9282  8.8968  10.3010
8.9079  10.7610 9.9209  9.7450  9.3411  8.6060  9.9672  9.2707
9.2734  9.1971  9.2233  10.7048 9.0629  10.9393 9.4220  10.2229
8.9851  10.4003 9.1150  9.2199  10.5767 9.6958  11.5604 10.2942
9.7291  8.9962  8.5803  11.1409 8.9217  9.4330  10.2692 8.2453
10.1742 9.7316  9.4965  9.3340  9.2982  10.1217 9.4838  9.1263
8.1899  9.9866  8.4650  8.7735  9.4849  8.8218  9.5632  9.7438
9.6234  9.6973  9.3002  10.1395 9.1754  9.0812  9.0039  10.3504
9.3151  9.7683  9.6938  8.7974  9.1270  10.3438 8.9515  9.8585
10.2075 8.9946  8.5918  9.9987  10.7703 9.8623  9.8437  9.5868
10.3793 8.3330  8.6394  9.0048  9.5461  10.7659 10.4483 10.3572
10.5615 10.1709 10.1301 8.7376  8.4942  9.6156  9.7791  9.9664
9.4451  9.2120  8.5252  9.0799  9.7690  9.6931  9.4907  9.0782
8.9375  9.1097  8.9122  8.7476  10.2439 9.0324  8.9935  9.5643
10.5392 10.4300 10.1854 9.2905  9.7529  9.2002  8.6058  10.0821
9.3726  9.0341  9.1167  8.9697  10.4531 10.1029 8.8800  10.5226
9.4629  9.2305  9.3372  9.0691  10.4469 9.9613  10.0933 9.5626
9.5330  9.0696  8.9235  9.8206  10.0949 9.8552  8.6055  9.9437
9.2895  9.0916  10.6645 8.9049  8.6619  10.7336 8.6469  10.2956
10.2773 10.6420\
UQCRHL  9.2294  8.3781  8.9295  9.5861  8.8058  9.9130  6.9049
9.1577  4.4586  9.2584  8.9693  4.1842  8.1363  9.1185  8.7993
9.7595  5.5491  10.0857 8.2517  4.6656  9.1287  9.9344  8.1686
9.1338  8.8675  10.5035 10.3269 9.9523  9.4043  5.2244  10.1550
9.5060  10.8301 5.7666  5.7822  11.7526 8.0901  9.8412  9.6986
9.8072  10.1389 8.2698  6.1435  10.2804 8.2010  8.7023  10.0462
7.8915  9.8258  10.2301 10.1218 6.3121  8.8323  8.5540  8.4246
9.0362  9.5745  9.5426  6.9710  6.3087  8.0380  9.3051  9.8768
8.7934  9.0234  9.4835  9.1602  10.0850 9.1633  10.4465 11.5626
4.9533  8.7736  9.0155  8.8741  9.7855  9.9800  8.3614  9.0869
9.2044  10.3711 4.8340  8.3720  10.1828 10.1618 10.5060 9.4297
9.4319  9.7242  9.5442  8.4628  8.6379  11.6243 9.2152  9.7446
8.4787  9.1786  9.2800  8.8389  9.0812  10.0778 9.1493  9.0178
9.4072  10.7916 10.2352 8.6262  9.2615  6.5287  5.3117  9.1988
9.7873  8.5516  7.6294  9.0994  10.1943 9.7953  8.6973  9.4632
```

| | | | | | | | |
|---|---|---|---|---|---|---|---|
| 6.0237 | 7.4361 | 10.0758 | 6.1631 | 10.2221 | 8.7414 | 8.4845 | 9.6943 |
| 11.5452 | 8.2542 | 7.8367 | 10.1076 | 10.5257 | 9.6959 | 8.1068 | 11.1288 |
| 8.3665 | 9.9882 | 7.7793 | 9.8303 | 8.4928 | 10.6246 | 10.1083 | 9.3891 |
| 8.5323 | 9.0705 | 8.9044 | 9.2102 | 8.8511 | 9.1004 | 10.2825 | 9.4246 |
| 10.5517 | 8.2834 | 8.9449 | 9.2194 | 9.3212 | 8.7465 | 9.6105 | 10.7887 |
| 10.4574 | 9.1568 | 9.6345 | 9.4404 | 9.0672 | 9.3957 | 9.1578 | 9.1084 |
| 10.5097 | 9.5899 | 4.5589 | 9.3735 | 3.6957 | 10.3477 | 9.3323 | 8.6607 |
| 9.6652 | 8.8536 | 8.6911 | 9.4239 | 8.4226 | 10.1694 | 10.4654 | 11.3937 |
| 10.2053 | 9.5721 | 9.8594 | 5.7701 | 9.5286 | 9.4659 | 9.2582 | 8.6773 |
| 9.5212 | 5.3744 | 6.0805 | 9.3746 | 12.1480 | 10.3892 | 8.8747 | 9.5469 |
| 8.9571 | 9.6283\ | | | | | | |
| ZBED4 | 9.4154 | 9.2346 | 10.2166 | 9.3799 | 9.9646 | 8.2731 | 10.0739 |
| 9.3054 | 8.8485 | 9.4535 | 9.2187 | 9.3439 | 9.0688 | 10.0650 | 9.7132 |
| 9.4221 | 9.3105 | 9.0102 | 9.1914 | 9.8239 | 8.3287 | 9.6430 | 9.0476 |
| 10.0287 | 8.7296 | 8.7171 | 8.9098 | 8.9620 | 9.2919 | 9.9594 | 9.8847 |
| 9.7558 | 8.0567 | 9.0583 | 10.8342 | 9.9611 | 9.6472 | 10.1186 | 9.4264 |
| 9.7000 | 8.5846 | 9.1833 | 9.5145 | 8.5871 | 7.6585 | 8.8166 | 8.1648 |
| 9.4219 | 9.9186 | 9.3831 | 9.5390 | 8.7732 | 7.9021 | 8.1558 | 9.0092 |
| 9.6180 | 10.3017 | 9.9734 | 9.8740 | 9.3313 | 8.3417 | 10.0679 | 9.6844 |
| 9.6536 | 9.2526 | 9.6158 | 7.5621 | 9.5214 | 9.8536 | 8.5421 | 7.9705 |
| 10.1915 | 8.6090 | 8.7930 | 9.3924 | 8.7605 | 9.1747 | 9.5153 | 9.1567 |
| 10.8106 | 9.4609 | 10.4543 | 9.1959 | 9.1468 | 9.0513 | 7.3307 | 8.0159 |
| 8.8699 | 10.0609 | 9.6774 | 8.2587 | 9.4386 | 8.3970 | 8.9634 | 9.3954 |
| 8.0547 | 8.2521 | 8.5328 | 9.7379 | 9.2448 | 9.0843 | 9.4288 | 10.3102 |
| 8.5758 | 8.6086 | 8.4490 | 9.0918 | 8.6455 | 8.8236 | 8.7224 | 9.9299 |
| 10.0258 | 7.5188 | 8.6795 | 8.4132 | 9.1092 | 10.0351 | 9.1727 | 9.3394 |
| 10.1567 | 9.0268 | 9.2848 | 10.2424 | 9.6817 | 8.8463 | 8.8555 | 9.0142 |
| 8.1756 | 8.7080 | 9.1570 | 8.6184 | 8.7968 | 9.5854 | 9.0750 | 8.6300 |
| 8.2670 | 9.4068 | 8.0474 | 9.6543 | 8.5840 | 8.8661 | 9.0948 | 9.2230 |
| 9.6092 | 9.7871 | 8.3366 | 9.7261 | 8.6545 | 9.2217 | 9.1686 | 9.2911 |
| 8.5061 | 10.2462 | 9.4866 | 10.1985 | 9.5511 | 8.6416 | 10.4914 | 7.9484 |
| 9.2290 | 9.4647 | 8.5684 | 9.5060 | 9.0947 | 8.5204 | 9.9147 | 8.7904 |
| 10.1333 | 9.6434 | 9.6333 | 10.3264 | 9.4825 | 8.7360 | 10.2529 | 8.2956 |
| 9.9286 | 9.3676 | 7.5336 | 10.3527 | 9.1392 | 8.8508 | 9.0309 | 8.7274 |
| 9.4209 | 10.5463 | 9.9531 | 9.9572 | 8.4113 | 9.1445 | 9.9368 | 9.6918 |
| 9.5531 | 9.2508 | 8.6301 | 9.7896 | 7.8410 | 7.9180 | 8.5986 | 9.6948 |
| 9.7227 | 8.6669\ | | | | | | |
| C11orf2 | 11.6270 | 11.6667 | 10.8238 | 9.9802 | 11.1088 | 10.5126 | 11.8779 |
| 11.3865 | 11.0139 | 11.6486 | 10.8012 | 11.2339 | 10.9473 | 10.6608 | 10.4623 |
| 11.4287 | 11.2513 | 11.3271 | 11.6648 | 11.2669 | 11.2062 | 10.6834 | 11.7829 |
| 10.9582 | 11.9730 | 11.6368 | 9.0479 | 11.8505 | 11.3635 | 10.8984 | 10.3521 |
| 11.0915 | 11.2858 | 11.8180 | 10.7246 | 11.6088 | 10.7148 | 10.0949 | 10.6786 |
| 11.3289 | 11.5317 | 11.1742 | 10.5023 | 12.2966 | 11.4687 | 11.1905 | 12.1057 |
| 10.9827 | 10.9021 | 11.7861 | 11.0302 | 11.7135 | 11.1096 | 11.7437 | 10.3011 |
| 10.3898 | 10.8621 | 10.0412 | 10.6808 | 11.8302 | 11.5024 | 10.2470 | 11.2515 |
| 11.4372 | 11.6830 | 10.9546 | 10.9168 | 10.8132 | 11.3844 | 11.3901 | 12.0728 |
| 10.4938 | 11.3685 | 11.5170 | 10.2154 | 10.8363 | 11.2053 | 11.4832 | 11.1349 |
| 11.3301 | 11.8575 | 10.0270 | 10.8724 | 10.7092 | 11.8148 | 10.9259 | 10.6402 |
| 11.6713 | 11.6298 | 11.0861 | 11.8158 | 10.4462 | 11.8444 | 11.6977 | 10.4967 |
| 12.0533 | 11.0197 | 12.5637 | 10.8584 | 10.6221 | 11.5453 | 10.9806 | 11.3923 |
| 11.0344 | 11.5708 | 10.8426 | 11.4955 | 10.6766 | 10.8804 | 11.3502 | 10.7517 |
| 11.7614 | 12.4218 | 11.1984 | 10.0627 | 9.9497 | 11.0335 | 10.7601 | 10.9534 |
| 10.1177 | 11.2390 | 10.0952 | 10.3363 | 11.0905 | 11.3779 | 10.7100 | 11.5622 |
| 12.2209 | 11.3065 | 10.7097 | 11.3615 | 11.0059 | 11.3370 | 11.1198 | 11.5119 |

12.2327 11.7690 12.0901 11.5863 12.2738 11.7461 11.1978 11.9151
10.7641 10.4089 12.7947 10.4097 12.4371 11.4081 11.9132 11.4544
12.2947 10.6400 11.8423 10.1253 11.4789 12.2606 10.8241 11.0039
12.0014 11.9986 11.2765 11.1906 11.3452 10.7630 10.1878 10.2622
11.0542 11.1791 11.4480 10.9967 9.7581 10.6964 10.7952 12.3900
10.7639 11.0561 11.6473 11.0023 10.8571 12.1263 10.9823 10.8230
11.1962 10.7278 11.0058 11.3011 11.3135 11.7504 11.6464 10.9083
10.9657 10.6642 11.1982 10.8778 13.4422 11.7604 12.7655 10.7345
10.7794 12.3039\
C11orf1 8.8015 8.3289 7.6451 8.2845 7.8821 7.5740 8.2171
8.7727 7.9456 8.4678 7.2012 8.8381 8.8233 9.4660 8.0085
7.7787 8.2529 9.7853 7.6405 8.1557 6.8694 8.1768 8.7965
9.3534 8.4987 7.5859 7.7087 7.9088 8.1294 5.6848 7.1193
7.9668 8.6313 7.6591 6.6662 8.2750 7.9239 7.1282 9.3520
7.1494 8.2127 7.7183 9.5451 6.8667 7.8838 9.5171 8.2154
7.6562 8.3249 8.7580 7.9044 6.8470 7.7076 7.9313 8.3677
9.0490 8.2193 8.3579 8.6476 7.7569 7.8104 8.8085 8.1933
7.7459 8.7095 7.6677 7.5893 8.5131 9.2273 7.8420 8.7335
8.5691 7.8539 8.2134 8.7344 8.1288 8.0374 7.7815 8.9770
7.9387 8.8877 7.4030 8.6034 6.7264 9.0524 9.6180 8.6509
8.3355 8.8747 8.2162 7.7248 8.3558 7.1935 9.2950 8.4214
7.8588 6.4296 8.7234 7.1303 9.7626 8.8941 8.0510 5.4280
7.9528 7.8222 7.6756 7.6841 7.7958 9.7295 7.1979 7.8547
8.1888 8.4054 8.4429 8.4366 8.4026 8.4189 7.5003 8.4849
7.0757 9.5046 8.9564 7.2533 8.0813 8.9485 7.1614 8.4276
9.4423 8.4358 8.1181 8.0426 8.6090 7.7465 7.4391 8.6051
8.0572 8.2680 7.4343 7.8334 8.3026 8.1189 8.3553 8.7153
8.2654 8.1922 8.4119 7.8117 8.4385 7.7201 9.1450 8.3184
6.7048 7.8507 8.4755 8.6728 8.2043 8.3131 8.2617 8.9254
7.7460 8.8176 6.5917 8.4567 9.3033 8.4714 7.1685 8.2503
7.4984 7.7403 6.7156 8.0826 8.8954 5.1893 7.8918 9.8341
8.8132 8.5379 7.8357 7.8502 8.0621 9.0086 7.3753 9.4011
9.7559 8.2069 8.8774 7.6890 7.7261 7.8554 8.6431 7.5174
8.4397 7.8113 5.8823 7.9284 8.9303 8.2555 8.8840 7.7830
8.4221 6.8149\
DPYSL5 0.5526 0.5319 0.6896 0.0000 1.8679 2.8426 0.5431
3.6208 3.5228 2.8241 1.2544 2.3205 1.4675 8.5298 0.0000
0.0000 2.2551 2.5841 0.9983 1.3142 3.4081 0.0000 0.6346
0.0000 1.7397 6.9325 9.8920 1.6129 0.0000 0.0000 2.5774
0.5612 0.0000 2.5759 0.0000 3.0446 4.1626 1.1048 0.0000
0.0000 0.0000 0.4871 0.0000 0.0000 0.0000 1.8914 0.0000
1.7580 5.1100 0.0000 0.4530 2.1910 8.0110 0.8853 7.2816
1.5362 2.7053 1.1710 2.1468 0.0000 0.9353 4.5865 3.3159
0.5968 1.4936 0.0000 0.0000 5.2016 0.5773 2.7780 0.4334
0.9537 4.5926 3.4891 0.5367 0.0000 10.0085 1.2414 0.0000
1.3771 0.0000 0.0000 0.7204 9.8112 1.2145 2.4045 6.3782
2.0646 0.5273 11.8869 1.4126 1.1363 8.7061 1.1561 1.2026
1.3960 1.8574 0.0000 0.8845 1.2591 0.0000 0.0000 5.0589
3.1674 0.0000 0.0000 0.0000 1.7824 2.3532 1.8132 0.0000
1.0545 5.0883 3.9069 0.0000 6.0662 0.0000 4.7462 0.0000
0.6089 8.6286 0.0000 2.0828 3.2437 1.6090 0.0000 1.0447
8.1317 0.5619 0.5074 0.9567 0.0000 0.0000 2.7051 7.3764
0.0000 0.0000 3.8160 0.9038 0.5056 0.6010 0.0000 0.0000
0.0000 0.4562 4.7294 0.0000 0.0000 4.4491 0.0000 0.0000

```
0.0000  0.5414  3.2514  0.5476  1.6440  0.0000  1.7867  3.6850
3.7334  0.0000  0.0000  0.0000  0.7182  3.8337  3.0144  5.2797
0.0000  0.5276  0.0000  3.4869  0.5290  7.5890  1.6598  0.0000
5.5113  1.4586  0.6293  8.4145  0.0000  0.0000  2.7052  8.0603
0.8792  2.5017  2.4158  0.0000  2.2587  0.6567  1.5562  0.0000
0.0000  2.5408  1.0222  0.0000  0.0000  7.6291  0.0000  1.1260
0.0000  0.0000\
DPYSL2  8.7352  11.5413 10.6608 8.0669  10.7622 7.9632  9.3136
10.1624 7.5822  11.1669 10.7766 8.6543  8.9430  11.6785 8.1601
8.6998  8.7683  8.5726  11.7085 7.2369  9.1369  8.0004  10.2860
8.2366  10.9601 8.6493  10.6120 9.3545  9.2078  6.4075  11.9798
8.2743  8.4284  8.3051  5.9316  10.7253 8.9868  9.4963  7.2180
9.6517  9.5300  10.7217 8.6964  9.7883  12.1259 8.6252  9.4636
8.1822  12.2627 7.0915  9.4505  9.5327  10.2936 11.8864 10.8634
10.9593 10.0049 10.3386 10.2682 7.3477  11.5576 10.1066 9.0292
8.9269  11.0438 9.3428  8.5365  11.7614 11.1929 9.8601  8.7618
9.8838  9.7047  9.2263  9.9450  7.9684  11.1837 8.9376  9.5094
9.2083  11.0474 8.8613  10.5080 9.3894  9.2615  9.7227  9.4475
8.2196  8.8912  12.2135 12.0419 8.2560  11.0782 10.1670 8.4805
11.7013 9.8360  11.5523 11.0897 10.1907 8.5161  9.0302  10.3169
9.4856  7.6613  11.3466 11.1274 9.1482  9.8874  10.0173 8.0015
7.5030  9.9282  7.4179  10.1781 7.5900  10.2169 8.9936  11.5394
8.9863  10.7244 9.6165  9.7468  10.1064 10.3928 8.6234  10.5011
8.9490  11.2788 10.7123 8.6548  6.8642  8.9162  10.2450 9.0794
9.0064  10.0436 9.5802  10.6448 11.4016 8.4138  8.0328  8.5323
8.9199  8.5440  12.0486 11.6109 12.0669 7.7608  7.4650  10.7687
9.5814  9.2587  8.6019  7.0202  9.7921  11.5278 11.3350 7.5039
9.0546  9.4529  9.2534  10.4332 9.2418  8.0869  10.9817 8.7665
11.5270 8.7954  7.1770  8.0200  7.7944  8.8125  10.3928 8.7474
11.4070 10.5710 11.7927 10.6088 9.6973  8.5579  8.2395  9.0624
8.6135  9.3112  8.9057  9.2065  9.2161  7.8732  8.3997  9.7481
9.3100  10.0788 9.8286  8.8556  9.1617  10.1002 11.0933 9.6719
8.8411  7.4882\
DPYSL3  10.2158 13.3704 11.9721 7.6762  11.4436 10.5049 10.5636
9.4141  8.8463  11.0909 9.4035  7.7862  8.6078  9.8962  7.5822
12.1728 9.1998  9.2691  13.6945 7.6581  12.0716 10.5432 10.9573
11.2387 13.7727 6.5132  13.8021 8.5924  12.1257 5.8978  6.4988
10.8064 6.4211  7.7845  8.3341  7.0391  9.1436  9.7779  12.5512
8.3843  9.2799  12.5951 10.0342 11.7816 13.7228 7.3540  8.8244
8.2115  8.8280  7.0367  10.9784 9.0349  11.1873 13.9848 11.2504
9.4272  10.5704 9.2392  12.3751 7.7798  13.7456 9.4546  8.7468
11.9763 11.5266 8.6526  5.8454  8.2408  10.4705 11.9003 9.9937
11.2548 8.3309  8.2039  11.6747 10.4883 8.3469  8.8249  11.0024
11.4916 11.8828 8.9114  11.3529 8.7888  10.1190 9.1358  8.4496
9.2948  11.4298 13.3925 13.5136 8.7439  10.9099 11.2491 9.0220
14.7350 9.5922  12.2375 10.1380 9.1057  12.0730 10.2302 8.3454
9.9768  10.2582 9.7294  11.0149 10.3601 9.0666  9.4365  11.2519
6.1054  10.8692 8.8329  11.2536 7.7401  9.0701  8.7368  12.6137
10.0985 10.4519 10.6495 9.2753  8.0757  10.6436 9.2438  11.5059
12.5570 13.4218 13.3715 11.7091 7.3596  9.7841  9.1679  8.9201
11.3085 9.5950  10.4714 11.4959 11.7649 11.8386 10.3547 7.6346
11.1530 10.8065 12.6111 8.1566  12.5719 8.5438  6.3188  12.4811
10.8654 11.1615 9.1899  6.9303  12.2467 12.3911 8.7689  11.5829
9.2838  7.3662  9.1332  8.7628  10.0262 9.1782  8.9776  11.9176
```

| | | | | | | | |
|---|---|---|---|---|---|---|---|
| 12.5227 | 9.7337 | 8.7164 | 7.7090 | 6.9085 | 10.7023 | 8.6125 | 10.6430 |
| 10.8785 | 12.1297 | 13.8333 | 13.4854 | 10.3434 | 10.0691 | 9.5259 | 11.6290 |
| 8.2318 | 11.2018 | 9.7068 | 9.8535 | 8.2047 | 9.5898 | 11.9414 | 11.5776 |
| 10.3838 | 7.7800 | 11.4476 | 11.0506 | 8.3137 | 11.4685 | 11.6466 | 8.9709 |
| 12.4283 | 9.3918\ | | | | | | |
| OSTM1 | 8.0173 | 9.8968 | 9.4680 | 9.5583 | 8.7102 | 8.2269 | 7.3948 |
| 7.5431 | 8.1118 | 8.3763 | 8.9381 | 8.8406 | 8.6503 | 8.1949 | 8.8900 |
| 8.1105 | 7.2163 | 9.1990 | 9.8816 | 8.5859 | 7.9530 | 8.3932 | 8.3796 |
| 9.1156 | 10.0421 | 10.4863 | 7.6618 | 8.9575 | 9.2581 | 8.4645 | 10.8695 |
| 8.5698 | 8.5106 | 7.8288 | 8.1733 | 7.5384 | 8.5878 | 8.6656 | 8.9871 |
| 9.4232 | 8.9585 | 10.0771 | 8.9140 | 9.0191 | 10.4808 | 8.0388 | 8.1422 |
| 8.7812 | 9.3529 | 9.6634 | 8.9628 | 9.6111 | 8.4538 | 9.8608 | 9.2640 |
| 8.9446 | 8.4534 | 9.2746 | 9.0168 | 7.8635 | 10.5061 | 9.2081 | 9.3088 |
| 8.3571 | 8.9877 | 8.9406 | 10.3241 | 8.6030 | 8.4810 | 8.8308 | 8.7803 |
| 8.7683 | 9.6903 | 7.6527 | 9.6568 | 8.2107 | 8.5227 | 9.0508 | 8.5210 |
| 9.4137 | 8.3581 | 8.9672 | 9.4334 | 9.7598 | 8.2177 | 8.2160 | 9.2847 |
| 8.4559 | 8.8628 | 9.0532 | 9.9285 | 8.5819 | 8.0226 | 8.6359 | 8.4700 |
| 10.5013 | 9.5641 | 9.3719 | 8.8106 | 8.8276 | 7.3798 | 9.1260 | 7.8873 |
| 9.3062 | 8.4269 | 8.3614 | 8.8135 | 8.9732 | 8.5716 | 8.3546 | 8.5806 |
| 8.3805 | 7.8091 | 7.5078 | 9.1995 | 8.7456 | 8.9425 | 8.3351 | 8.5921 |
| 9.2848 | 9.3385 | 9.6728 | 9.2190 | 8.4122 | 8.5845 | 8.6921 | 8.6896 |
| 5.8947 | 9.8576 | 9.9976 | 7.7347 | 8.5961 | 8.7177 | 9.7276 | 8.4735 |
| 8.2164 | 7.9499 | 7.7420 | 8.8360 | 9.7328 | 8.6604 | 9.1343 | 8.1565 |
| 9.2780 | 8.8335 | 9.2698 | 8.8703 | 8.8337 | 9.1529 | 8.0048 | 9.0910 |
| 7.9687 | 8.6856 | 7.7385 | 8.5309 | 8.4003 | 9.3346 | 8.1341 | 7.6811 |
| 11.1253 | 8.2401 | 9.2101 | 8.9012 | 8.1108 | 8.0581 | 8.6528 | 7.8102 |
| 8.6105 | 8.7234 | 8.1371 | 8.0989 | 8.9273 | 8.2041 | 8.0963 | 8.4812 |
| 8.3022 | 9.2126 | 10.1234 | 8.3207 | 8.8998 | 8.7971 | 8.0981 | 7.6946 |
| 8.7646 | 8.5549 | 9.7254 | 8.6680 | 8.6768 | 8.3052 | 8.6847 | 9.5308 |
| 8.5933 | 8.9735 | 8.7547 | 9.1074 | 7.7355 | 7.6667 | 9.2508 | 8.8833 |
| 8.9299 | 7.5515\ | | | | | | |
| RUNX2 | 5.6167 | 7.5149 | 7.3896 | 5.3990 | 7.9638 | 6.9931 | 5.5170 |
| 7.0775 | 4.8396 | 6.9128 | 5.8094 | 5.2688 | 6.9279 | 7.2580 | 6.5204 |
| 10.9070 | 7.2006 | 5.8427 | 7.4103 | 6.4758 | 5.9393 | 6.4311 | 5.1169 |
| 9.6332 | 6.1818 | 4.4520 | 10.8977 | 6.1909 | 6.4208 | 4.8222 | 10.9958 |
| 6.1574 | 5.6317 | 6.0420 | 3.6447 | 5.4362 | 5.4967 | 5.2079 | 5.8205 |
| 5.3192 | 6.2798 | 6.3169 | 6.1762 | 7.2080 | 6.2405 | 6.7971 | 4.4245 |
| 3.9349 | 9.2357 | 3.3772 | 6.0746 | 7.2701 | 7.5417 | 6.2473 | 10.5589 |
| 6.6412 | 7.0062 | 7.5249 | 7.1383 | 5.7546 | 6.0394 | 6.7147 | 8.9267 |
| 5.0565 | 6.0787 | 7.2283 | 7.8459 | 6.5062 | 10.9023 | 6.1984 | 5.6555 |
| 5.2303 | 7.7950 | 7.6779 | 7.8194 | 2.5860 | 7.4705 | 6.4193 | 9.9590 |
| 6.9788 | 9.4638 | 5.5408 | 7.6841 | 6.6616 | 7.6625 | 7.7495 | 4.6913 |
| 5.9667 | 7.9749 | 8.3910 | 6.1794 | 6.7921 | 4.2364 | 10.2689 | 6.9158 |
| 4.9247 | 5.4970 | 7.3624 | 5.1403 | 7.7490 | 6.9236 | 6.6028 | 10.0232 |
| 6.4290 | 4.8313 | 5.4241 | 7.1323 | 7.7784 | 6.7032 | 6.7960 | 5.6240 |
| 5.4443 | 6.5986 | 4.9542 | 8.6637 | 6.8918 | 4.3833 | 6.6259 | 9.3618 |
| 7.7845 | 7.1906 | 6.7044 | 6.5676 | 4.7442 | 5.5269 | 7.0922 | 6.3443 |
| 6.3931 | 7.8066 | 5.3738 | 7.3162 | 4.2681 | 5.6706 | 5.8128 | 7.7893 |
| 5.6519 | 5.4987 | 9.2841 | 6.3267 | 7.1456 | 4.2931 | 7.2687 | 7.5538 |
| 8.4195 | 7.7938 | 7.5815 | 5.7212 | 5.0312 | 4.6621 | 4.4839 | 10.0127 |
| 4.4241 | 9.8793 | 7.6222 | 8.0453 | 9.1733 | 6.6229 | 7.8111 | 7.3663 |
| 5.7508 | 3.0551 | 6.0024 | 9.2368 | 6.4233 | 6.9278 | 6.0974 | 6.6010 |
| 7.0838 | 6.4664 | 4.9727 | 7.6989 | 4.0826 | 6.9576 | 7.5574 | 3.8992 |
| 6.7912 | 6.3319 | 4.9829 | 8.5140 | 7.5940 | 5.5085 | 5.8594 | 6.5165 |

4.9641  6.8192  5.0670  6.6397  4.8774  4.1471  8.7447  6.8706
6.3374  6.9862  7.4013  7.2327  3.9204  5.5698  7.4562  6.6739
8.5250  4.4645\
C11orf9 7.5664  9.1979  8.8281  10.1416 5.5112  10.5670 9.8248
8.6856  11.7022 8.9134  12.5321 8.9308  8.4168  6.0559  11.3695
5.7644  9.2338  9.5337  9.2493  9.9345  9.4790  3.3952  10.0590
9.7536  7.7282  8.0764  9.5133  9.1837  8.4047  7.9192  8.7245
7.4318  7.9851  10.2632 9.2333  12.4226 7.7806  11.5854 9.5912
7.0498  6.3745  9.2323  11.7397 9.0566  9.0457  10.3098 4.6978
10.4184 7.8072  10.4278 10.3029 9.9693  9.7386  8.6129  6.1003
7.1459  11.4251 10.8179 10.1882 10.3378 9.6134  13.0432 6.3425
5.4094  7.8019  10.4151 4.3588  9.2213  3.7861  8.1889  10.1145
6.8610  8.9620  8.5527  9.5174  5.7684  6.6091  9.3682  7.9247
6.9849  4.2670  7.0916  5.4295  8.0783  7.6874  9.2823  7.4648
5.1053  9.2459  6.0862  10.0275 10.9561 9.9508  8.3182  6.5981
8.8977  5.7756  7.6266  7.6518  11.3963 7.0625  10.3708 8.6136
8.8889  4.7694  7.9960  7.6794  9.1447  9.9095  9.8900  3.4934
6.7713  11.1191 11.0875 5.3048  10.8908 3.5896  9.0268  7.5355
11.1811 12.2023 11.1684 10.5815 9.0585  9.6904  10.4819 5.6712
10.6068 9.2060  8.8769  5.4988  10.0697 9.5589  5.8478  8.9468
10.9844 5.7140  8.1648  8.6405  8.3217  4.7738  7.9265  9.2862
10.9799 11.2488 10.3034 3.3108  10.9376 4.9896  5.6175  7.3805
5.6361  6.6673  10.2175 7.5580  10.3857 7.6977  4.6726  10.6121
6.0476  9.5696  11.2083 9.4911  10.3847 2.8972  6.8312  11.0751
12.4980 7.9583  8.1228  7.1841  9.0661  7.8812  9.5885  8.3020
7.5312  9.5085  8.8482  7.0840  7.5481  10.8429 10.5056 10.6885
5.9309  10.6771 8.2166  9.5688  10.7044 10.0364 5.6717  10.5141
7.1491  8.6698  8.3507  9.8732  9.6310  8.9881  8.8118  10.2207
7.8026  9.7569\
KRTAP10-12      0.0000  0.0000  0.0000  0.0000  0.0000  0.0000
0.0000  0.0000  0.0000  0.0000  0.0000  0.0000  0.0000  0.0000
0.0000  0.0000  0.0000  0.0000  0.0000  0.0000  0.0000  0.0000
0.0000  0.0000  0.0000  0.0000  0.0000  0.0000  0.0000  0.0000
0.0000  0.0000  0.0000  0.0000  0.0000  0.0000  0.5555  0.0000
0.0000  0.0000  0.0000  0.0000  0.0000  0.0000  0.0000  0.0000
0.0000  0.0000  0.0000  0.0000  0.0000  0.0000  0.0000  0.0000
0.0000  0.0000  0.0000  0.0000  0.0000  0.0000  0.0000  0.0000
0.0000  0.0000  0.0000  0.0000  0.0000  0.0000  0.0000  0.0000
0.0000  0.0000  0.0000  0.0000  0.0000  0.0000  0.0000  0.0000
0.0000  0.0000  0.0000  0.0000  0.0000  0.0000  0.0000  0.0000
0.0000  0.0000  0.0000  0.0000  0.0000  0.0000  0.5215  0.0000
0.0000  0.0000  0.0000  0.0000  0.0000  0.0000  0.0000  0.0000
0.0000  0.0000  0.0000  0.0000  0.0000  0.0000  0.0000  0.0000
0.0000  0.0000  0.0000  0.0000  0.0000  0.0000  0.0000  0.0000
0.0000  0.0000  0.0000  0.0000  0.0000  0.0000  0.0000  0.0000
0.0000  0.0000  0.0000  0.0000  0.0000  0.0000  0.0000  0.0000
0.0000  0.0000  0.0000  0.0000  0.0000  0.0000  0.0000  0.0000
0.0000  0.0000  0.0000  0.0000  0.0000  0.0000  0.0000  0.0000
0.0000  0.0000  0.0000  0.0000  0.0000  0.0000  0.0000  0.0000
0.0000  0.0000  0.0000  0.0000  0.0000  0.0000  0.0000  0.0000
0.0000  0.0000  0.0000  0.0000  0.0000  0.0000  0.0000  0.0000
0.0000  0.0000  0.0000  0.0000  0.0000  0.0000  0.0000  0.0000
0.0000  0.0000  0.0000  0.0000  0.0000  0.0000  0.0000  0.0000
0.0000  0.0000  0.0000  0.0000  0.0000  0.0000  0.0000  0.0000

```
0.0000  0.0000  0.0000\
KRTAP10-11      0.0000  0.0000  0.0000  0.0000  0.0000  0.0000
0.0000  0.0000  0.0000  0.0000  0.0000  0.0000  0.0000  0.0000
0.0000  0.0000  0.0000  0.0000  0.0000  0.0000  0.0000  0.0000
0.0000  0.0000  0.0000  0.0000  0.0000  0.0000  0.0000  0.0000
0.0000  0.0000  0.0000  0.0000  0.0000  0.0000  0.0000  0.0000
0.0000  0.0000  0.0000  0.0000  0.0000  0.0000  0.0000  0.0000
0.0000  0.6741  0.0000  0.0000  0.0000  0.0000  0.0000  0.0000
0.0000  0.0000  0.0000  0.0000  0.0000  0.0000  0.0000  0.0000
0.0000  0.0000  0.0000  0.0000  0.0000  0.0000  0.0000  0.0000
0.0000  0.0000  0.0000  0.0000  0.0000  0.0000  0.0000  0.0000
0.0000  0.0000  0.0000  0.0000  0.0000  0.0000  0.0000  0.0000
0.0000  0.0000  0.0000  0.0000  0.0000  0.0000  0.0000  0.0000
0.0000  0.0000  0.0000  0.0000  0.0000  0.0000  0.0000  0.0000
0.0000  0.0000  0.0000  0.0000  0.0000  0.0000  0.0000  0.0000
0.0000  0.0000  0.0000  0.0000  0.0000  0.0000  0.0000  0.0000
0.0000  0.0000  0.0000  0.0000  0.0000  0.0000  0.0000  0.0000
0.0000  0.0000  0.0000  0.0000  0.0000  0.0000  0.0000  0.0000
0.0000  0.0000  0.0000  0.0000  0.0000  0.0000  0.0000  0.0000
0.0000  0.0000  0.0000  0.0000  0.0000  0.0000  0.0000  0.0000
0.0000  0.0000  0.0000  0.0000  0.0000  0.0000  0.0000  0.0000
0.0000  0.0000  0.0000  0.0000  0.0000  0.0000  0.0000  0.0000
0.0000  0.0000  0.0000  0.0000  0.0000  0.0000  0.0000  0.0000
0.0000  0.0000  0.0000  0.0000  0.0000  0.0000  0.0000  0.0000
0.0000  0.0000  0.0000  0.0000  0.0000  0.0000  0.0000  0.0000
0.0000  0.0000  0.0000  0.0000  0.0000  0.0000  0.0000  0.0000
0.0000  0.0000  0.0000\
KRTAP10-10      0.0000  0.0000  0.0000  0.0000  0.0000  0.0000
0.0000  0.4748  0.0000  0.0000  0.0000  0.0000  0.0000  0.0000
0.0000  0.0000  0.0000  0.0000  0.0000  0.0000  0.0000  0.0000
1.0738  0.0000  0.0000  0.0000  0.0000  0.0000  0.0000  0.0000
0.0000  0.0000  0.0000  0.0000  0.0000  0.0000  0.0000  0.0000
0.0000  0.0000  0.0000  0.0000  0.0000  0.0000  0.0000  0.0000
0.0000  0.0000  0.0000  0.0000  0.0000  0.0000  0.0000  0.0000
0.0000  0.0000  0.0000  0.0000  0.0000  0.0000  0.0000  0.0000
0.0000  0.0000  0.0000  0.0000  0.5980  0.0000  0.0000  0.0000
0.0000  0.0000  0.0000  0.0000  0.0000  0.0000  0.0000  0.0000
0.0000  0.0000  0.0000  0.0000  0.0000  0.0000  0.0000  0.0000
0.0000  1.3738  0.0000  0.0000  0.0000  0.0000  0.0000  0.0000
0.0000  0.0000  0.0000  0.0000  0.0000  0.0000  0.0000  0.0000
0.0000  0.0000  0.0000  0.0000  0.0000  0.0000  0.0000  0.0000
0.0000  0.0000  0.0000  0.0000  0.0000  0.0000  0.0000  0.0000
0.0000  0.0000  0.4553  0.0000  0.0000  0.0000  0.0000  0.0000
0.0000  0.0000  0.0000  0.0000  0.0000  0.0000  0.0000  0.0000
0.0000  0.0000  0.0000  0.0000  0.0000  0.0000  0.0000  0.0000
0.0000  0.0000  0.0000  0.0000  0.0000  0.0000  0.0000  0.0000
0.0000  0.0000  0.0000  0.0000  0.0000  0.0000  0.0000  0.0000
0.0000  0.0000  0.0000  0.0000  0.0000  0.0000  0.0000  0.0000
0.0000  0.0000  0.0000  0.0000  0.0000  0.0000  0.0000  0.0000
0.0000  0.0000  0.0000  0.0000  0.0000  0.0000  0.0000  0.0000
0.0000  0.0000  0.0000  0.0000  0.0000  0.0000  0.0000  0.0000
0.0000  0.0000  0.5674  0.0000  0.0000  0.0000  0.0000  0.0000
0.0000  0.0000  0.0000\
IGFBP2  14.1634 11.6781 15.8924 11.6303 13.4574 10.9459 12.9439
```

14.1373 15.2566 11.6142 14.2684 13.3409 13.7620 13.8939 12.7172
9.8402 15.3104 10.7782 12.1440 11.1985 13.7944 13.4754 11.5282
11.2028 12.2744 14.0087 11.2912 14.0254 15.1742 12.6254 10.2368
8.5295 10.7770 13.1816 14.2748 13.5639 13.7603 11.2957 11.2976
8.2158 14.1817 11.2037 14.4779 15.0496 10.4405 15.0672 12.2015
12.0024 13.7595 12.1072 11.5908 7.9939 14.8864 10.2182 13.7758
14.1927 10.3695 12.9917 13.2327 13.2172 10.9233 13.6088 9.1880
15.1138 11.9722 13.5787 11.3111 16.0100 11.7595 14.1777 15.0907
13.9529 9.9871 15.6263 9.4530 11.0583 15.7348 13.8712 11.3246
11.7572 15.2643 14.2797 11.9143 12.2859 14.2005 16.4070 11.5981
15.2347 13.6719 13.4956 11.2040 12.8904 13.2199 12.2033 14.2758
13.5345 13.9031 11.1621 12.5015 11.0191 11.3762 11.3403 11.7766
9.5638 10.3636 12.6345 14.0658 14.1793 14.5329 11.8887 9.3726
13.7244 15.8664 12.1542 13.9974 13.1190 13.4219 11.0111 13.6988
9.4248 13.8758 12.7794 12.8199 13.1806 11.2580 14.0004 15.6449
15.9341 12.1603 11.3332 16.3647 10.3069 13.5075 12.2023 11.0678
10.3846 14.0594 11.4784 11.0058 10.7355 13.6120 8.2150 13.9871
11.6118 11.8255 13.2122 13.9625 12.2097 14.9361 15.3847 10.6339
13.8381 13.1468 13.7402 11.4768 15.4652 11.6631 13.6237 13.4493
13.3377 9.2439 9.7340 12.8066 13.2014 10.8284 13.4888 13.5082
11.6869 13.5066 14.0524 12.8866 14.1135 12.5846 15.1137 10.0216
12.7363 11.7719 10.8256 12.9294 12.6884 10.7372 12.0185 15.0323
11.9238 13.8890 10.6408 14.4512 14.3682 13.7982 16.3209 8.8455
15.3050 12.6277 14.5630 8.7125 15.1085 12.8191 10.4386 13.5819
11.5613 16.1652\
IGFBP3 13.2568 15.8263 14.3810 13.0089 10.8059 10.8988 11.6531
13.8371 12.8613 12.1453 12.7296 12.5249 11.5001 11.9198 12.7720
13.2483 13.4705 11.8132 15.9226 15.1667 12.4903 12.4331 10.0296
13.1864 13.4942 11.5290 9.5309 12.6165 13.8473 13.3479 12.6677
13.0942 11.6161 11.8330 11.2137 11.9688 13.6455 12.9201 12.5416
11.0374 12.2787 14.9753 14.8859 12.6907 14.3460 14.0891 12.3484
13.8196 12.6562 12.1937 13.5354 14.5530 11.8365 14.8466 13.5349
13.9056 11.7625 12.3346 11.6769 12.9099 14.6939 13.0131 11.3551
13.8711 13.9492 14.6433 12.2151 15.4965 13.1983 13.6599 11.2282
14.6132 14.9457 12.4207 13.9991 12.6856 14.2652 12.0411 12.9941
11.6413 12.1510 12.2730 14.5327 13.5595 12.2148 14.2253 11.8580
12.3592 13.1488 13.7445 14.0302 13.2369 11.1748 15.0120 12.1749
12.2950 15.7556 14.7632 11.8499 13.6163 12.2399 14.6359 14.0482
13.4479 11.2056 12.0251 13.2458 12.5600 13.9851 12.2107 11.5825
14.5424 12.4076 15.1011 12.3361 12.3263 12.4879 12.4981 10.4625
13.9965 14.6596 13.9322 13.4523 11.7181 11.5113 13.1043 12.9524
13.4499 15.2761 12.7457 14.7190 11.4072 12.8495 11.5578 13.6406
12.9496 10.2420 13.8810 14.0001 15.8422 11.3273 14.4302 11.1768
13.7941 11.9459 11.0512 14.8940 12.8168 13.6017 14.1616 14.0275
10.8433 11.9167 11.9113 12.2954 13.9217 14.3453 13.0726 12.4188
11.6822 12.0286 16.4806 11.9075 13.3448 10.8730 12.5625 13.3274
12.3984 13.1046 15.4173 13.8247 13.9010 11.1591 11.7642 14.0244
13.7114 13.2561 12.4322 12.1075 13.6949 13.0506 14.6073 11.8226
11.4639 12.0941 11.1089 12.5850 13.0661 13.0173 11.5079 12.2744
13.7583 12.9341 13.5779 11.2965 10.8146 12.1288 15.7895 14.3729
13.0162 14.5997\
IGFBP1 1.5194 10.5749 1.5051 1.1529 0.0000 0.4059 0.5431
3.0712 0.0000 0.0000 1.2544 0.8472 1.4675 0.7759 2.6827
0.0000 0.6221 5.4300 12.2619 7.3803 1.8761 0.8427 5.6646

1.2144  0.9530  0.6062  1.5155  2.3554  4.4034  7.1650  1.5789
0.5612  0.5159  0.0000  0.0000  0.6819  1.9329  1.2859  0.0000
0.4337  1.1184  11.7305 5.3568  2.1688  5.2595  2.2058  0.0000
10.6070 1.6510  1.7431  0.4530  8.6221  0.6494  1.6405  0.0000
0.5608  4.5631  3.0892  0.4252  3.0518  0.9353  2.5691  0.9583
1.0177  1.4936  2.9126  3.2826  3.0286  1.3082  0.0000  0.4334
2.5842  7.1738  0.4786  3.7189  0.0000  9.6711  3.8721  1.1539
0.0000  2.1251  0.0000  3.8679  1.1284  3.0077  1.3843  0.0000
0.0000  1.2163  1.2149  2.7094  2.3201  0.5215  2.9555  0.9013
0.6266  2.4256  3.7446  0.5090  4.5983  2.7168  3.0033  0.9246
1.5830  0.0000  1.3363  5.3438  0.7631  2.5905  2.0061  0.0000
3.4186  10.5025 8.2336  2.2788  2.6508  0.9131  8.7810  0.0000
10.3024 3.0740  0.5886  12.0026 0.4700  5.0791  3.1042  0.0000
1.2894  4.2553  0.5074  0.9567  1.9805  0.5526  3.1029  2.9611
9.3136  0.0000  0.0000  5.6374  6.7211  0.0000  1.3036  0.6506
2.8855  1.0811  4.9369  0.6735  3.7932  0.5814  1.8766  1.9683
0.0000  1.4965  0.5862  1.2540  0.4374  5.3124  0.0000  1.8394
0.0000  0.6063  8.1720  3.3844  0.4033  0.0000  0.0000  0.0000
2.9510  1.2170  0.9420  3.1737  0.0000  2.2140  1.0565  0.0000
1.1272  0.5232  2.6934  1.1907  3.9843  0.9540  7.4069  3.9950
0.5055  2.1147  0.6248  0.0000  2.8698  1.1063  0.9785  0.0000
1.5256  3.7058  1.4427  0.0000  3.1828  0.6064  4.8293  3.0170
1.1797  1.5159\
BOP1    10.3975 7.7512  9.6151  9.0719  10.6583 8.1710  10.5553
7.7917  8.2105  7.9720  8.2203  8.4569  8.6426  9.1102  6.6231
5.5157  6.4560  7.8772  6.8854  9.1958  8.8400  7.4060  7.4651
8.7472  8.9517  9.7674  10.2763 8.9871  8.8652  9.0625  7.9682
9.8368  7.6504  9.4853  8.8163  11.9136 6.8706  7.8882  8.4973
9.0926  8.9380  7.3439  7.7729  9.1178  6.1592  9.0896  5.5368
8.9331  8.6927  7.4746  8.7282  9.1481  8.2587  5.7190  8.5873
7.5596  9.7129  9.5482  8.3259  8.5256  7.6331  8.6284  8.5023
10.3361 8.2319  7.9946  8.6255  9.3674  9.0078  9.0754  10.3391
9.3226  8.2455  7.8153  6.4590  10.7570 8.4099  8.0058  7.0321
8.9361  8.9195  9.4534  7.8308  6.6950  8.9739  7.5979  8.9487
10.3315 10.0799 6.0578  8.0453  9.5177  9.3836  6.1750  8.6860
7.5131  7.9027  7.5823  9.4649  8.2493  9.9584  8.8815  9.1571
11.0076 10.7441 9.6739  8.3996  8.0902  8.5888  9.0593  8.9371
8.9971  2.8658  7.1898  6.6423  8.7876  9.6298  8.7297  7.7652
10.7706 9.7802  7.4988  9.1980  9.1937  7.1283  8.9577  8.7317
7.4396  7.2249  7.1772  9.4775  9.0195  9.2626  4.8726  9.2499
5.2246  9.6503  8.5137  8.7305  8.0383  9.0630  8.5506  9.6219
8.4099  8.7929  6.3759  8.8369  8.7925  10.0171 6.9609  7.4090
11.7792 7.7049  10.6075 9.9254  8.5053  6.6299  9.5339  9.3518
9.0030  8.8877  8.8920  9.2356  7.5625  9.9559  6.7665  7.8562
9.4531  9.4301  10.0091 10.8220 8.7110  9.1191  8.4965  0.0000
8.4315  8.4737  5.9343  9.3228  6.1445  9.6046  9.7796  8.8493
7.9527  8.8224  8.8389  10.3161 9.5735  7.9792  9.8416  8.1502
8.3553  9.2020  10.1677 8.4855  11.6615 10.9841 6.6387  7.5980
8.8425  10.8223\
IGFBP7  13.3166 14.8765 13.1425 11.6539 11.9366 10.9963 12.2643
13.5937 11.6699 13.6746 11.7524 10.7013 12.6814 10.5507 12.9198
12.5958 12.5667 12.9287 15.0372 13.1822 11.5650 12.9780 13.2377
12.4470 13.4326 11.6609 10.7724 13.7178 13.4043 13.2842 11.6376
12.0572 10.3771 11.3429 9.4675  11.4027 11.8120 11.7001 11.2547

11.2015 13.4950 14.1076 14.3641 11.6830 14.1318 13.4969 10.9734
13.6893 10.8899 10.6133 11.1425 13.1249 11.8979 14.1661 13.2897
12.0280 13.5497 12.5930 13.2729 11.6130 14.5062 12.7117 10.8944
10.7129 14.0102 12.2840 10.1853 11.7256 12.5341 13.4698 10.3206
12.5206 13.7776 11.7849 13.2316 11.1192 13.1828 14.2707 13.9908
12.1233 12.9054 11.5724 15.1099 12.8949 13.4157 12.2158 11.0057
12.3842 12.0324 13.8031 13.9131 11.4898 11.1824 14.6192 12.9220
14.7019 11.9223 13.9676 11.8132 14.5975 11.0890 13.1108 10.7644
13.4320 11.5244 10.9047 14.0636 13.2904 12.8893 12.9681 11.2950
11.0056 11.0975 12.4221 12.5157 11.2316 12.1210 11.5033 10.9458
14.3764 11.6952 13.0674 13.2112 11.8880 12.9998 13.5109 12.0269
10.8782 14.2962 13.0125 12.1247 11.7516 13.7567 12.3824 14.1215
12.1220 11.2815 12.4534 12.3907 13.4646 10.7739 12.0870 12.9052
11.7013 11.1602 12.0995 11.9887 14.5707 11.1560 9.6497 13.5196
10.2995 12.7295 11.9418 11.7173 12.2132 13.8426 11.9532 11.2305
11.9645 10.6000 13.1633 12.9831 11.1861 11.8803 11.6460 12.0913
12.2715 12.2240 9.9798 10.7345 11.6909 10.6011 11.2886 11.4770
12.0628 13.5208 14.1137 11.6346 14.3541 12.3946 13.8773 11.5724
12.4006 11.5509 11.7427 12.4355 11.7208 11.5859 12.1690 11.7758
12.3925 12.4404 13.0567 11.8351 10.6700 10.7376 12.5882 11.8143
13.2233 11.4410\
IGFBP4 11.7362 15.0254 11.9628 12.4485 11.6470 10.0161 11.5848
12.3839 11.6920 15.4419 10.7294 10.4460 11.6864 11.6476 12.9603
13.6403 11.6081 11.3894 15.5773 12.7240 13.0685 13.8985 12.6923
12.4478 15.5556 11.3188 11.2522 14.1531 13.3280 11.5582 11.2414
11.5268 11.1265 10.8637 9.9867 9.7546 11.6475 9.1425 11.0738
10.1658 12.7665 15.0315 11.3898 12.3449 14.3967 14.5232 12.8053
10.9631 13.9244 10.8313 11.1298 11.7750 12.0550 15.1275 14.1908
10.6690 12.0143 13.4939 13.4695 12.3957 14.5742 11.8659 10.8789
10.9956 15.6413 11.3068 10.6676 11.2374 13.6579 12.9033 10.9918
11.0154 14.0340 11.9505 13.0101 11.2118 13.6325 15.5937 13.8070
12.2431 14.3964 10.4794 16.1551 10.9686 13.3041 13.4694 9.6097
11.5330 11.8390 12.6022 15.7759 10.8710 10.4379 15.5627 11.9591
15.5449 11.8909 14.5951 11.8677 11.3137 11.6484 11.7302 10.7362
11.7508 10.2722 11.6392 15.4437 12.3556 13.2864 13.2231 10.8276
9.9911 15.6335 13.1490 12.1913 11.5167 11.2799 11.1940 11.2710
13.3740 14.2661 13.3656 12.2875 11.6989 13.2326 12.0677 12.1226
14.4236 14.4603 15.2313 11.0664 11.1419 12.9606 10.8799 12.7035
14.8512 11.8745 15.0870 13.7195 14.2532 10.4356 11.7040 14.9202
12.6798 13.0801 15.0522 10.9145 14.6426 10.0546 11.4956 14.5003
12.0441 12.0913 11.9298 11.1908 11.9172 15.0049 12.6194 9.9523
12.1242 9.7102 11.6399 14.6363 11.2526 11.1349 10.7159 11.4554
11.9857 11.6476 9.7947 11.0611 12.6139 12.7544 11.4289 15.7917
10.4631 15.5482 14.8431 11.5351 14.2670 13.7905 11.0139 11.2618
15.0027 11.6463 11.7334 11.7801 11.2352 11.3383 13.0629 11.4650
11.6337 11.6594 12.9353 12.2281 11.1750 13.4229 14.1731 11.3692
13.6592 12.6392\
IGFBP5 13.0478 14.8968 15.6613 11.8028 11.5977 10.4656 12.6964
13.2231 11.3435 15.5534 10.5387 10.6944 11.7386 9.1259 11.4781
12.5086 11.0056 10.9955 14.1470 8.0867 9.0995 12.6443 12.4675
10.4281 17.2170 9.7630 8.6820 11.7766 14.4059 9.9469 14.1654
11.8077 7.1367 8.9798 11.9093 8.7278 13.8562 10.3464 10.6219
10.8257 10.7119 15.3389 12.1882 12.0086 15.0102 14.6559 11.9620
12.6162 11.1701 13.8099 14.0491 12.1195 13.9412 16.2901 12.5827

| | | | | | | | |
|---|---|---|---|---|---|---|---|
| 13.0869 | 12.3975 | 12.1258 | 14.0205 | 10.3054 | 14.9662 | 11.4234 | 11.1272 |
| 14.2744 | 16.3737 | 12.0437 | 11.6828 | 15.3043 | 13.4767 | 13.7659 | 10.8682 |
| 15.9432 | 10.2387 | 11.9976 | 13.5307 | 12.5192 | 14.4198 | 13.5586 | 11.3243 |
| 13.7926 | 11.1809 | 12.0461 | 13.8472 | 13.2345 | 11.0169 | 13.2163 | 11.3242 |
| 11.1953 | 11.2384 | 16.0761 | 16.9782 | 10.2619 | 10.1025 | 12.1315 | 13.2322 |
| 16.8933 | 14.8773 | 13.8575 | 13.3396 | 11.7715 | 12.2731 | 11.7881 | 13.2270 |
| 11.8264 | 12.5484 | 14.0898 | 14.1501 | 12.2799 | 14.1752 | 12.0110 | 14.2719 |
| 13.6533 | 13.6728 | 8.2336 | 15.2674 | 11.3114 | 13.7599 | 11.5661 | 12.9009 |
| 11.0759 | 14.0386 | 12.9232 | 11.7715 | 14.3396 | 14.4109 | 10.5047 | 15.3303 |
| 12.9797 | 14.0915 | 17.0263 | 15.6010 | 11.0358 | 11.5033 | 13.6658 | 11.3871 |
| 14.3891 | 10.1751 | 15.0720 | 13.5632 | 13.8782 | 13.5038 | 10.5964 | 10.4761 |
| 12.6312 | 10.2527 | 17.1794 | 15.2597 | 15.3041 | 10.6608 | 14.5274 | 14.7714 |
| 11.8837 | 13.1703 | 12.3908 | 9.4850 | 14.5066 | 14.4112 | 14.0233 | 11.9606 |
| 10.2630 | 10.0394 | 11.3604 | 11.1109 | 9.0409 | 8.6483 | 11.3373 | 12.2683 |
| 11.1255 | 11.9850 | 13.2545 | 14.1815 | 8.1698 | 9.8717 | 11.0504 | 10.4031 |
| 13.1519 | 16.0715 | 16.8453 | 14.0451 | 13.4085 | 11.3392 | 11.5833 | 14.4113 |
| 10.2542 | 14.5781 | 10.5054 | 11.0576 | 10.2960 | 12.6255 | 13.9198 | 12.3094 |
| 14.3979 | 11.7344 | 14.3107 | 11.3712 | 8.6547 | 9.2194 | 13.1971 | 12.4780 |
| 13.3270 | 12.5955\ | | | | | | |
| SKP2 | 7.6954 | 5.4529 | 6.6566 | 7.5872 | 8.4344 | 8.0347 | 7.3551 |
| 8.3946 | 8.2933 | 7.3899 | 7.7441 | 8.4781 | 7.9490 | 8.3588 | 7.8513 |
| 5.3429 | 5.2955 | 6.8658 | 5.9854 | 6.5185 | 7.1928 | 7.8448 | 6.6971 |
| 7.3755 | 7.0498 | 6.6914 | 8.3762 | 6.1909 | 4.7873 | 7.9372 | 8.7853 |
| 7.6592 | 7.5281 | 7.2817 | 9.0950 | 8.0923 | 7.3027 | 8.7833 | 6.8193 |
| 7.0383 | 7.1268 | 5.2460 | 5.8911 | 8.3997 | 6.4431 | 5.9001 | 6.5609 |
| 7.3114 | 9.2686 | 5.7710 | 8.3729 | 6.1035 | 8.1123 | 6.4773 | 6.9855 |
| 8.2234 | 7.4416 | 6.0372 | 7.9771 | 7.2861 | 6.6238 | 7.7106 | 8.8042 |
| 9.3055 | 7.2511 | 7.4977 | 6.5221 | 8.9397 | 5.8601 | 8.0216 | 6.5415 |
| 8.6589 | 5.3166 | 5.7991 | 6.7156 | 7.5874 | 8.5374 | 6.6060 | 5.6441 |
| 7.9792 | 7.1422 | 9.3696 | 6.0674 | 8.3062 | 6.8177 | 6.4051 | 7.9621 |
| 6.9113 | 7.1649 | 8.0463 | 6.7240 | 8.1706 | 6.4677 | 5.8146 | 7.4366 |
| 6.3391 | 7.4369 | 6.7554 | 7.5050 | 7.3121 | 7.5483 | 7.7388 | 8.5528 |
| 6.7296 | 7.3348 | 8.7103 | 5.5408 | 6.9260 | 7.5387 | 7.1930 | 8.2827 |
| 7.3731 | 7.2019 | 7.9307 | 8.8519 | 8.7242 | 8.7983 | 7.9370 | 8.3331 |
| 5.7835 | 9.4921 | 7.3597 | 7.4707 | 8.2736 | 6.8168 | 6.9944 | 8.2324 |
| 7.7172 | 6.7570 | 7.5230 | 7.4619 | 7.2632 | 7.4823 | 6.4812 | 5.8280 |
| 5.0375 | 8.1307 | 6.4733 | 8.1612 | 6.6863 | 7.6658 | 6.4932 | 6.3660 |
| 8.3466 | 8.1760 | 6.7806 | 8.0920 | 6.5656 | 7.1340 | 6.8315 | 7.0270 |
| 5.8705 | 8.1807 | 8.4175 | 9.3760 | 8.1696 | 6.4987 | 8.9298 | 7.3436 |
| 7.1796 | 6.9141 | 6.3317 | 7.2628 | 6.2073 | 8.8919 | 8.9043 | 6.3279 |
| 7.7255 | 7.2055 | 7.9669 | 8.7600 | 7.9626 | 7.9544 | 7.7793 | 5.0451 |
| 8.5818 | 6.7147 | 6.1607 | 8.5341 | 6.2730 | 6.9017 | 7.5972 | 8.9981 |
| 6.8630 | 7.7156 | 7.3871 | 7.6925 | 5.2565 | 5.8739 | 8.5304 | 8.4156 |
| 7.3863 | 7.6785 | 8.7921 | 8.1957 | 6.8599 | 7.6291 | 6.5602 | 8.0926 |
| 7.7999 | 6.9440\ | | | | | | |
| TTC4 | 9.2822 | 8.6770 | 9.5080 | 8.9104 | 9.4213 | 9.5230 | 9.3011 |
| 9.6990 | 10.1512 | 9.1517 | 10.2562 | 9.5787 | 9.4796 | 9.2743 | 9.0886 |
| 9.0102 | 9.4330 | 9.4419 | 8.7440 | 10.0009 | 9.5121 | 8.8279 | 9.3508 |
| 9.0485 | 8.9854 | 9.2902 | 9.1083 | 9.0612 | 8.9940 | 9.3214 | 9.1330 |
| 9.1657 | 9.6435 | 9.8919 | 9.1355 | 10.1843 | 8.9511 | 8.9579 | 9.2661 |
| 9.1498 | 9.3617 | 9.0580 | 9.6014 | 10.0810 | 8.9186 | 9.0410 | 8.9511 |
| 10.0368 | 9.9688 | 9.0686 | 8.9754 | 9.3397 | 9.5215 | 9.1417 | 8.6203 |
| 9.3359 | 9.0707 | 9.5753 | 9.5371 | 9.5912 | 9.3221 | 9.1867 | 9.6118 |
| 9.4140 | 9.1063 | 8.8856 | 9.1872 | 9.8916 | 9.3398 | 9.3185 | 10.2321 |

| | | | | | | | |
|---|---|---|---|---|---|---|---|
| 9.2107 | 9.2694 | 10.1623 | 8.9813 | 8.9100 | 9.4374 | 8.7748 | 9.1411 |
| 9.1781 | 9.1578 | 9.4900 | 9.1604 | 9.3506 | 9.0668 | 8.7671 | 10.1426 |
| 9.1980 | 9.3347 | 9.3009 | 9.1351 | 9.3128 | 10.0105 | 8.8276 | 9.5883 |
| 8.6329 | 9.4488 | 9.1772 | 8.5876 | 9.1512 | 8.9378 | 9.1715 | 9.3226 |
| 9.0563 | 9.4529 | 9.6120 | 9.2488 | 9.0634 | 9.5077 | 9.6964 | 9.0140 |
| 9.3022 | 8.7490 | 9.4819 | 9.4171 | 9.7998 | 9.0426 | 9.5504 | 9.0224 |
| 9.5684 | 9.8507 | 9.1891 | 9.6061 | 9.2116 | 9.4546 | 10.0976 | 9.3131 |
| 10.1528 | 9.3318 | 9.1435 | 9.4610 | 9.4165 | 9.2746 | 9.0860 | 10.0831 |
| 8.5673 | 9.5654 | 9.0750 | 9.1038 | 9.1990 | 9.2752 | 9.0138 | 9.1219 |
| 8.9760 | 9.0793 | 9.4690 | 8.9370 | 9.3129 | 9.3282 | 9.3887 | 8.9123 |
| 8.8179 | 8.8808 | 9.9652 | 9.0094 | 9.0572 | 9.1891 | 9.1365 | 9.6506 |
| 9.7051 | 9.0851 | 9.1126 | 9.1946 | 9.5369 | 8.2872 | 8.8161 | 9.4141 |
| 9.4818 | 9.2279 | 9.8562 | 9.2641 | 9.1472 | 9.5844 | 8.5803 | 9.3832 |
| 9.1198 | 9.2965 | 9.1034 | 9.1215 | 8.9040 | 9.2916 | 9.8176 | 10.1663 |
| 9.0008 | 9.7467 | 9.1860 | 9.9261 | 9.8685 | 8.8666 | 9.7454 | 9.1868 |
| 9.1370 | 10.0117 | 9.3606 | 9.4370 | 10.5566 | 9.5830 | 9.5932 | 8.9434 |
| 9.0403 | 9.2725\ | | | | | | |
| CYP2R1 | 6.6630 | 7.1303 | 6.7824 | 8.0735 | 7.8851 | 7.9097 | 7.4373 |
| 8.1749 | 7.0115 | 7.4531 | 7.6402 | 7.8649 | 7.9532 | 8.0039 | 3.5096 |
| 8.1977 | 7.6078 | 6.8391 | 7.4396 | 6.5509 | 6.7950 | 7.4943 | 6.9437 |
| 7.6749 | 7.1858 | 6.9263 | 7.0142 | 5.4501 | 6.9956 | 7.2774 | 6.9567 |
| 7.9051 | 5.1628 | 8.7458 | 7.6303 | 6.5273 | 7.9736 | 7.9798 | 8.3938 |
| 7.5779 | 6.7172 | 6.8463 | 7.3253 | 7.9695 | 7.0510 | 7.0243 | 7.7375 |
| 7.3328 | 6.5006 | 8.3494 | 7.2432 | 6.3378 | 6.7759 | 7.5787 | 7.4540 |
| 6.4506 | 6.5253 | 7.4511 | 7.1418 | 7.7605 | 6.9561 | 7.7770 | 7.5523 |
| 7.5547 | 7.4589 | 4.2739 | 6.7526 | 7.1438 | 7.1270 | 7.0563 | 8.8813 |
| 7.5694 | 7.2013 | 6.9373 | 7.6223 | 8.0813 | 7.3841 | 4.2961 | 7.9356 |
| 7.8046 | 5.1336 | 6.5643 | 7.7111 | 7.0646 | 4.3483 | 7.1242 | 9.1200 |
| 7.3089 | 7.8754 | 7.8971 | 6.9347 | 8.9571 | 7.7669 | 8.2616 | 8.3732 |
| 6.1421 | 6.9008 | 7.2050 | 7.2839 | 7.7950 | 7.9243 | 6.4814 | 8.2947 |
| 7.5076 | 7.9693 | 7.4562 | 8.0138 | 7.4506 | 7.3211 | 7.4959 | 8.4701 |
| 7.4010 | 8.2723 | 6.8948 | 4.9526 | 9.9877 | 7.4834 | 7.5150 | 7.2799 |
| 5.7697 | 7.9222 | 6.8438 | 6.9602 | 2.7605 | 7.7291 | 7.7309 | 7.6986 |
| 8.1935 | 7.1970 | 7.6127 | 7.1918 | 7.6064 | 5.6167 | 8.6779 | 6.7958 |
| 7.6080 | 8.0599 | 7.0221 | 7.5798 | 7.4935 | 7.5866 | 7.4891 | 7.2261 |
| 3.4648 | 7.2443 | 7.2726 | 7.7332 | 7.2752 | 6.7509 | 7.9158 | 8.1644 |
| 7.0507 | 7.2235 | 7.0206 | 7.7773 | 7.8643 | 7.4597 | 7.7405 | 6.9270 |
| 4.9574 | 6.7696 | 5.4752 | 7.4589 | 8.3349 | 3.6808 | 6.8831 | 7.9052 |
| 7.2900 | 7.5151 | 7.8508 | 6.6148 | 8.1676 | 7.6556 | 7.1869 | 6.8987 |
| 8.0823 | 7.8890 | 7.4903 | 7.6401 | 7.4654 | 7.8728 | 6.8622 | 8.1412 |
| 4.7558 | 7.9896 | 3.8630 | 7.1980 | 7.5430 | 7.7278 | 7.2968 | 4.4094 |
| 7.2725 | 8.0212 | 6.6632 | 8.3678 | 9.0779 | 7.7705 | 7.8183 | 6.9049 |
| 7.8924 | 6.5639\ | | | | | | |
| CKLF | 8.7849 | 7.8381 | 7.7759 | 8.5586 | 8.7117 | 8.6142 | 7.7476 |
| 8.6692 | 8.3791 | 8.3192 | 7.9762 | 7.2669 | 8.3528 | 9.0716 | 8.7486 |
| 8.7057 | 8.1716 | 8.7553 | 8.3050 | 8.9525 | 8.5256 | 8.1510 | 8.1491 |
| 8.5680 | 7.6531 | 9.1750 | 10.5171 | 9.5682 | 9.2078 | 7.0921 | 8.5223 |
| 8.6264 | 9.4829 | 8.7700 | 7.6627 | 8.1790 | 7.8527 | 8.7908 | 8.8487 |
| 6.9552 | 9.0633 | 7.6425 | 9.2877 | 7.7644 | 7.6659 | 9.5295 | 9.4158 |
| 7.8399 | 8.7137 | 7.7999 | 8.9151 | 8.1899 | 8.4515 | 7.8805 | 8.4840 |
| 7.9461 | 8.6644 | 8.6359 | 8.7559 | 7.6462 | 8.0279 | 7.8813 | 8.2009 |
| 8.0904 | 8.0593 | 8.7917 | 8.6716 | 7.4918 | 6.5847 | 7.3098 | 8.6225 |
| 8.1747 | 10.1428 | 8.8083 | 8.3059 | 8.6193 | 7.9025 | 8.1210 | 8.8717 |
| 8.7612 | 8.0587 | 7.7869 | 8.3860 | 8.2199 | 9.1328 | 8.5432 | 7.5576 |

9.1460  8.8723  9.6626  8.2482  8.6779  8.7851  8.1031  9.0459
7.9348  7.8241  7.9090  8.0195  9.2381  9.2858  8.8828  7.6723
8.7239  8.7527  8.4300  8.1135  8.1446  7.8725  8.4939  8.2282
7.7027  8.6395  7.2288  8.3578  7.6745  8.7384  7.4531  7.9652
9.0435  7.9419  8.1859  7.9130  9.0761  8.2131  8.6905  7.7565
8.3130  7.9272  7.5097  7.6981  6.2448  8.4631  8.0553  9.0954
9.5064  10.0918 7.4954  7.4636  8.0947  8.0643  8.9792  8.4240
8.3805  7.6543  8.0320  8.5469  8.4947  8.8749  9.5440  8.4206
8.1179  8.2876  8.6459  8.8510  8.1227  8.2444  8.1575  9.5071
10.0619 7.9422  9.0903  7.8368  9.6460  9.4245  7.2887  8.8845
8.2202  8.7606  8.4781  7.2718  8.0735  9.2776  8.3867  8.8939
9.1485  7.9792  8.0770  8.1241  8.6288  9.8925  8.1173  7.7338
8.5584  7.5095  8.6745  8.2341  8.5991  9.0936  9.0482  9.0974
8.5268  8.1220  9.2832  8.5896  9.2600  9.3852  8.5158  8.4711
7.6474  10.1237\
ZNF193  8.5100  8.5039  7.6183  8.4109  7.7197  7.9557  8.4254
8.7154  8.4283  7.8112  8.1268  8.5613  8.6247  9.0877  8.0635
9.3966  9.1126  8.1444  8.5143  7.3828  7.5295  7.9318  7.3671
8.6914  7.9519  8.1657  7.8479  7.8392  8.0527  7.3904  7.5353
8.6037  7.6627  7.0888  8.7134  8.2118  8.7318  8.2626  8.1659
9.2261  8.5399  8.4710  8.1420  8.3846  7.6585  7.6485  7.4302
7.8399  7.6673  7.9352  7.3868  7.8090  7.6519  7.8648  8.9802
9.0988  7.6932  7.7980  7.8152  8.4948  7.7870  7.7824  8.0110
8.6201  8.2084  7.9769  7.8491  8.3731  8.0433  8.2758  7.8747
8.6622  6.9653  9.3593  8.2621  7.0090  8.7137  7.6419  8.5566
7.8848  7.8731  8.3759  8.3776  8.4453  8.7429  8.7976  8.3513
8.0129  8.1665  8.1947  7.6865  7.6659  9.5250  9.5035  8.6768
7.1759  7.5422  8.0050  7.8658  8.8231  8.6582  7.3102  8.0624
9.2787  7.7547  7.6979  8.8978  8.9171  7.6674  8.4531  8.5838
7.7825  7.2915  9.8564  7.9036  7.8465  8.3110  8.2299  7.4174
8.3558  8.4381  8.0771  8.0308  6.8882  7.6974  8.0288  7.9457
8.7261  8.2632  8.0987  7.3304  8.6222  7.8925  8.9109  8.7736
7.2963  7.8414  9.0650  7.9179  7.9962  8.3444  8.0166  8.4618
7.6306  7.6754  7.8615  7.8002  7.6165  8.0005  7.7240  8.3815
7.2206  8.9247  8.4468  7.8911  8.2690  7.9529  8.6453  7.8291
7.2417  6.3288  7.0672  8.4771  8.4709  6.8403  7.7846  6.8212
7.4361  7.6256  8.6698  7.8661  8.6942  8.9194  8.3983  7.6342
9.3008  9.1102  7.9359  8.1208  8.1571  7.8900  8.2248  8.8466
8.6652  9.0223  8.5837  8.5697  8.0519  8.7555  8.5659  7.7728
8.3820  8.9300  8.6289  8.8059  7.4153  6.7210  8.1268  8.0737
8.6932  7.4932\
C15orf26        0.5526  3.3733  6.2080  9.3922  2.7531  6.2673
6.2297  6.5746  1.4589  5.8990  2.3666  0.8472  7.4343  0.0000
8.5543  3.8845  4.6587  0.7772  2.9970  0.6732  1.0485  8.8823
8.7097  6.6506  2.5058  0.0000  1.0809  8.5434  0.0000  0.0000
2.7995  2.8443  0.0000  3.6818  0.0000  0.0000  3.9598  0.8977
1.7115  0.0000  1.7407  3.7496  2.9036  1.0349  1.0260  7.6156
4.1918  1.1317  1.2792  1.1202  0.7972  2.1910  2.6127  2.1337
1.3330  5.7349  1.9111  0.5030  1.6122  3.1339  4.2319  2.2329
1.7473  2.3502  5.5360  1.1659  0.5980  4.3286  1.9829  2.6613
1.9272  1.1694  0.0000  1.7486  0.9270  5.1241  5.6510  9.8413
8.8508  1.8728  5.3865  4.2503  9.6570  6.3199  7.6874  2.5439
3.6193  6.9176  1.8667  2.5470  0.0000  1.3771  0.0000  5.8303
0.9013  1.3960  0.9065  2.9802  7.9348  0.9478  5.2399  1.4068

| | | | | | | | |
|---|---|---|---|---|---|---|---|
| 2.9516 | 5.7068 | 7.2878 | 2.1477 | 3.3565 | 5.3482 | 5.1016 | 7.6375 |
| 6.4133 | 5.6283 | 0.0000 | 1.0000 | 2.9726 | 5.0304 | 9.3494 | 4.0927 |
| 3.0518 | 2.0533 | 3.0090 | 3.0972 | 1.0557 | 6.3070 | 8.6669 | 2.0458 |
| 1.8708 | 3.5647 | 2.2660 | 2.5988 | 1.1727 | 7.1055 | 8.9036 | 8.0628 |
| 0.8107 | 0.0000 | 1.0837 | 3.8160 | 5.7468 | 3.1658 | 6.3417 | 2.1461 |
| 8.0111 | 1.4986 | 1.0811 | 0.5418 | 6.3559 | 4.7152 | 2.8976 | 0.0000 |
| 4.5529 | 3.5510 | 5.5403 | 3.0909 | 1.5093 | 0.4374 | 4.8207 | 2.6338 |
| 7.5758 | 1.7128 | 1.0317 | 0.6138 | 7.2105 | 5.8030 | 1.4811 | 4.3917 |
| 4.7265 | 4.2687 | 5.4968 | 1.2521 | 4.2380 | 0.5290 | 0.9335 | 0.0000 |
| 0.0000 | 6.4176 | 3.0757 | 2.8104 | 6.9103 | 10.1024 | 3.1647 | 1.1551 |
| 4.4369 | 4.7332 | 1.5843 | 0.6248 | 0.5811 | 2.4374 | 7.1810 | 0.9785 |
| 1.1800 | 3.8702 | 1.2903 | 1.6145 | 3.7421 | 2.3335 | 2.3729 | 4.5657 |
| 4.2504 | 8.3420 | 2.8479\ | | | | | |
| C15orf27 | | 1.2631 | 2.5617 | 4.5653 | 3.3181 | 5.7311 | 6.0225 |
| 3.4667 | 5.9854 | 1.7860 | 3.5525 | 4.1679 | 4.8761 | 4.8171 | 2.5815 |
| 2.4639 | 2.9791 | 4.9630 | 3.7153 | 2.1673 | 5.4919 | 3.7319 | 2.7971 |
| 4.1354 | 2.3107 | 3.4365 | 6.0275 | 5.7493 | 3.0366 | 2.7323 | 4.5997 |
| 2.6553 | 3.9764 | 1.6552 | 3.6382 | 5.0453 | 3.7098 | 4.4684 | 4.0968 |
| 1.7115 | 3.2351 | 3.0046 | 2.8124 | 2.6786 | 3.1766 | 0.0000 | 4.1830 |
| 3.7177 | 3.5510 | 5.2518 | 3.2642 | 4.9826 | 5.8945 | 3.0688 | 3.3671 |
| 3.4925 | 2.2632 | 3.2979 | 3.8006 | 3.6618 | 4.2470 | 1.7140 | 2.9220 |
| 3.1723 | 5.8290 | 3.9568 | 2.5019 | 1.8352 | 6.1255 | 1.9829 | 3.6688 |
| 6.2934 | 2.7936 | 1.9166 | 3.0145 | 4.6108 | 3.4092 | 4.3046 | 3.2691 |
| 2.4028 | 4.1299 | 3.0365 | 1.9437 | 5.1426 | 4.3627 | 3.9970 | 5.0536 |
| 4.8122 | 2.9812 | 2.0314 | 3.4180 | 2.8267 | 2.8054 | 8.9805 | 3.8593 |
| 3.2084 | 2.0921 | 4.8572 | 2.9802 | 3.7299 | 2.0879 | 3.1381 | 3.0757 |
| 3.8098 | 4.5620 | 3.9384 | 4.3536 | 3.3565 | 5.0010 | 2.8861 | 4.8905 |
| 3.3794 | 3.6834 | 4.7091 | 4.2479 | 3.9985 | 6.7574 | 4.4431 | 4.6289 |
| 3.2571 | 2.9683 | 6.4320 | 3.1797 | 2.7935 | 1.8858 | 4.0781 | 3.1773 |
| 2.7753 | 5.1564 | 2.4022 | 3.7703 | 4.5973 | 4.8721 | 2.8211 | 4.3451 |
| 3.6220 | 0.0000 | 5.7293 | 4.5503 | 2.1648 | 2.6906 | 4.5692 | 1.9771 |
| 4.7044 | 2.3527 | 2.2385 | 1.2434 | 2.5258 | 2.9802 | 2.5760 | 1.8766 |
| 3.5631 | 4.9641 | 3.2011 | 4.2516 | 2.7093 | 2.8829 | 3.1906 | 2.8937 |
| 5.1727 | 3.9615 | 7.0958 | 1.6421 | 4.2397 | 2.2840 | 1.4811 | 3.4589 |
| 5.4334 | 4.7360 | 2.3144 | 4.3136 | 2.3253 | 5.7587 | 6.3732 | 2.6780 |
| 6.5798 | 3.5915 | 3.5762 | 2.6934 | 4.6092 | 5.0378 | 2.2477 | 6.3464 |
| 5.6378 | 4.7103 | 3.5434 | 2.7995 | 4.3110 | 3.8329 | 3.9459 | 3.5453 |
| 1.6366 | 1.2686 | 3.2005 | 2.9770 | 4.1636 | 6.4901 | 5.1701 | 3.9300 |
| 2.8496 | 3.8150 | 3.0771\ | | | | | |
| C15orf24 | | 9.9940 | 10.3210 | 9.5493 | 10.1983 | 10.5285 | 10.0660 |
| 9.4500 | 10.2619 | 9.7507 | 9.9055 | 10.1153 | 9.4425 | 9.4825 | 9.8908 |
| 10.1968 | 10.3081 | 9.7871 | 10.0473 | 10.3819 | 9.3692 | 10.0819 | 10.1617 |
| 10.0219 | 10.1516 | 10.0208 | 11.2057 | 9.1383 | 10.1037 | 10.4296 | 9.6691 |
| 9.6269 | 10.1456 | 10.5912 | 8.5873 | 8.9536 | 10.4310 | 9.1977 | 10.1194 |
| 10.7313 | 9.5629 | 11.0159 | 10.0078 | 9.6371 | 9.9753 | 10.1388 | 10.7753 |
| 9.9755 | 9.9843 | 10.2340 | 10.4990 | 10.1840 | 10.0177 | 9.6196 | 9.8759 |
| 9.7738 | 10.3531 | 10.0619 | 10.1878 | 9.4421 | 9.9623 | 10.0668 | 10.6744 |
| 10.8308 | 10.3177 | 10.3062 | 9.6031 | 9.8094 | 10.6614 | 10.6331 | 9.7546 |
| 10.0975 | 9.8824 | 10.8721 | 10.0483 | 10.4695 | 10.5680 | 10.5101 | 9.9983 |
| 10.4937 | 9.9991 | 10.7487 | 10.0357 | 10.3609 | 10.5911 | 10.5237 | 9.9744 |
| 10.0193 | 10.3702 | 9.2333 | 10.5631 | 10.0735 | 9.3879 | 9.6824 | 10.5809 |
| 10.6726 | 10.1882 | 9.5633 | 9.9474 | 9.5913 | 10.9712 | 10.7147 | 9.9370 |
| 8.9656 | 10.5692 | 10.6575 | 9.0729 | 10.2593 | 9.6253 | 9.6572 | 10.3886 |
| 9.8107 | 9.7198 | 8.5086 | 9.8265 | 9.8041 | 10.1501 | 9.7187 | 9.3250 |

9.6214 10.1593 9.7059 10.1753 9.3571 10.1977 9.6859 10.3532
9.7008 10.3871 10.2890 9.6942 9.9130 10.4304 10.1420 9.0878
10.7082 9.9727 10.7007 9.0850 9.6996 9.9662 11.3382 10.6646
10.1087 10.0151 10.1610 9.9179 10.2370 9.7545 10.9878 10.1471
10.0460 10.9467 9.7456 10.2921 9.3840 10.0844 9.9316 9.8457
10.1842 10.4649 10.3054 10.9086 10.3115 10.2983 9.9323 9.8826
10.5705 9.2232 10.1958 9.6516 9.5670 10.1760 9.4339 9.2744
9.7985 9.7296 10.3689 10.1382 9.4820 9.9970 10.4822 11.0549
9.4770 10.9207 9.3262 10.1593 9.1980 10.2924 10.3240 10.0969
10.1464 10.3327 9.8762 9.6776 10.0777 10.3510 9.6595 9.8528
9.8631 9.7875 10.5697\
GJA3 0.5526 5.6321 5.3517 2.3445 2.0327 3.1372 4.0479
4.2718 1.2625 2.8241 2.3666 5.5156 1.7957 0.4395 1.4890
2.9791 1.8857 2.1918 4.6972 0.3755 1.6493 1.7571 4.1354
0.0000 3.6666 2.7536 0.8021 0.5990 1.3857 1.3312 1.9932
1.2789 1.1952 2.4804 1.8080 2.2096 2.6245 1.2859 1.8992
1.2646 2.1741 6.0013 1.3900 2.5595 1.0260 1.8914 0.7135
1.9920 1.0498 0.4764 2.7870 1.9460 2.7409 3.3066 6.2154
3.3257 2.1087 4.9944 2.5360 1.2977 3.8762 4.5363 4.3117
1.3432 3.2441 3.3244 4.8447 2.0643 6.3524 5.4109 2.4738
1.8067 3.4823 2.7021 2.7779 0.0000 5.6644 2.8810 0.0000
4.1731 3.4069 2.6730 3.8025 2.1886 1.2145 2.2503 2.2055
1.3738 2.6540 1.6790 2.4416 1.5834 0.9036 0.0000 1.6641
5.8197 3.5228 4.4977 0.5090 1.2591 0.0000 3.3338 2.6756
3.9042 0.0000 1.5391 2.6511 4.2834 3.9646 1.8132 0.8916
4.1884 0.0000 3.1699 1.1918 4.3226 2.1803 2.1672 2.6758
6.4273 4.7927 1.0055 3.8017 1.8858 1.3435 1.2272 0.6149
1.2894 5.2714 2.3827 4.0048 1.1234 2.3786 0.7571 3.9382
0.0000 0.0000 0.0000 2.8272 6.8316 0.6010 1.3036 1.7133
6.9301 3.0768 4.7294 1.4775 1.7212 4.1191 3.5470 3.3441
0.0000 0.9341 2.8106 0.9437 1.6440 5.2773 4.0549 1.6149
9.9829 1.0317 4.2899 0.0000 1.5535 1.8107 2.1150 4.0884
1.6979 1.8676 3.6456 3.8932 1.6845 0.5410 4.7791 0.0000
1.7522 2.5381 1.9008 8.2373 1.9052 2.7277 3.0638 2.7472
1.8147 3.9687 1.3929 2.5754 1.1775 2.1566 2.1363 2.6000
2.3863 1.9604 2.1495 0.0000 6.2811 1.3603 4.7467 4.1338
2.1290 3.8717\
APLNR 8.2802 8.2317 8.3532 4.9814 7.2938 7.8621 3.9659
6.9005 5.4942 9.9856 6.6697 5.9195 7.3494 7.9384 5.9432
8.0082 7.1467 7.9705 9.0505 6.6778 6.8428 7.0819 4.2234
6.3101 8.8160 6.8035 7.3163 7.7626 8.2755 5.3188 6.6203
6.5936 7.4593 7.6890 5.4766 5.8828 9.1662 7.3019 7.4232
6.9794 7.7696 9.3000 6.8249 6.2041 9.7827 6.2128 8.9323
6.8499 7.5786 7.5060 7.6292 8.8376 7.5636 8.7807 9.8776
5.0781 6.9101 7.4614 9.3580 6.9140 10.1245 7.4267 6.2383
6.3477 9.0890 7.1515 5.2857 6.2132 7.7153 10.4243 6.3379
8.4621 6.0987 8.0281 9.1957 5.7582 7.9769 7.0929 5.8420
6.9970 11.6111 7.4871 8.6987 7.3843 7.7741 6.3492 6.3305
7.2598 6.9449 8.8299 9.5929 7.1083 6.8840 8.2413 5.4720
9.9817 6.0090 8.8366 7.9323 7.3821 6.7098 6.4270 6.2479
7.3751 6.7560 7.9208 7.8868 7.9075 7.7089 8.1905 7.1935
5.2539 10.1177 7.7482 7.7136 6.5536 7.1347 10.6015 8.0031
7.8352 6.7874 6.7658 6.7787 6.7761 5.5428 5.9284 4.7571
6.2082 9.9178 9.0872 6.6362 5.4792 7.0510 7.8289 6.2699

| | | | | | | | |
|---|---|---|---|---|---|---|---|
| 9.0688 | 7.1894 | 8.8655 | 8.9623 | 6.2503 | 5.7747 | 7.0885 | 7.4353 |
| 8.4137 | 7.4729 | 2.2162 | 6.4764 | 10.4922 | 6.9200 | 7.2665 | 8.6694 |
| 9.2917 | 7.8023 | 8.0913 | 6.7252 | 8.5249 | 8.7025 | 7.1011 | 5.3103 |
| 7.5600 | 7.8505 | 7.1664 | 6.6638 | 6.0824 | 7.1875 | 6.1235 | 5.9403 |
| 8.4858 | 7.3647 | 6.4375 | 7.5994 | 4.6389 | 6.9202 | 6.7245 | 10.2283 |
| 7.3580 | 9.1879 | 9.3992 | 8.0440 | 5.8193 | 7.9070 | 5.1687 | 5.7715 |
| 6.5635 | 5.9455 | 6.5074 | 8.3172 | 7.1010 | 5.7862 | 8.3187 | 8.5088 |
| 8.5139 | 6.8115 | 8.4896 | 7.3321 | 6.8847 | 6.3105 | 4.0266 | 8.3165 |
| 7.4140 | 6.8149\ | | | | | | |
| C15orf23 | | 9.8650 | 6.8966 | 7.7391 | 8.2976 | 9.6854 | 11.0662 |
| 9.1115 | 9.4272 | 9.6544 | 8.5761 | 9.8121 | 9.2237 | 8.8177 | 8.7700 |
| 8.9623 | 8.3193 | 8.8481 | 8.8386 | 7.2398 | 9.2096 | 9.6680 | 8.3324 |
| 7.1259 | 8.2657 | 7.3186 | 9.2199 | 9.2891 | 8.6945 | 7.3778 | 9.2716 |
| 9.2855 | 9.3983 | 11.3214 | 7.8264 | 8.9953 | 9.8198 | 7.6981 | 9.0111 |
| 9.6537 | 8.5714 | 9.3910 | 7.3114 | 8.9601 | 10.0187 | 6.9579 | 9.2308 |
| 8.3989 | 9.2688 | 9.7224 | 7.9260 | 9.3610 | 9.0171 | 8.3769 | 6.6021 |
| 8.2285 | 9.7166 | 8.2512 | 7.9248 | 8.6305 | 9.4484 | 6.6104 | 9.8824 |
| 9.5904 | 9.1398 | 8.2114 | 8.1160 | 7.8134 | 9.1041 | 8.0134 | 9.4062 |
| 9.2781 | 9.0952 | 8.9074 | 8.5314 | 8.3263 | 8.3686 | 8.7024 | 7.5256 |
| 7.4049 | 8.7718 | 8.9907 | 10.4791 | 7.5459 | 9.6755 | 8.6094 | 8.3549 |
| 9.7305 | 8.8452 | 8.2916 | 10.0295 | 7.4326 | 8.9664 | 8.4908 | 8.2760 |
| 9.8716 | 6.3868 | 8.4935 | 7.6400 | 8.5227 | 9.7179 | 9.0694 | 8.9421 |
| 8.8691 | 10.7534 | 9.8736 | 10.1069 | 7.3925 | 8.2308 | 9.0751 | 9.6973 |
| 8.6817 | 8.9142 | 7.2172 | 9.3772 | 10.8260 | 10.2758 | 9.2451 | 9.2326 |
| 9.3907 | 9.4226 | 10.0300 | 9.1872 | 8.9887 | 8.8837 | 7.0634 | 9.5155 |
| 9.9467 | 9.1785 | 7.1299 | 7.2708 | 8.5952 | 9.8404 | 8.8564 | 9.6279 |
| 9.1691 | 7.8018 | 8.8294 | 7.1027 | 8.3249 | 7.2327 | 10.6865 | 8.1764 |
| 8.1067 | 8.8388 | 9.2783 | 6.6944 | 7.8494 | 7.1075 | 8.3859 | 9.1902 |
| 7.6098 | 8.8426 | 8.0204 | 9.4927 | 9.3476 | 9.8804 | 6.8963 | 9.1573 |
| 9.6630 | 9.6108 | 8.3823 | 8.7606 | 8.3019 | 8.1878 | 9.1457 | 8.9168 |
| 9.1160 | 8.0734 | 9.7292 | 10.1677 | 9.4018 | 10.0742 | 8.5224 | 8.6575 |
| 7.1876 | 9.3417 | 8.1376 | 6.9354 | 9.5340 | 7.1566 | 9.3476 | 9.3768 |
| 9.7837 | 8.4344 | 8.7394 | 8.4867 | 7.6106 | 9.5212 | 8.9087 | 10.1440 |
| 9.8360 | 8.1222 | 9.1961 | 8.3200 | 9.0056 | 9.6922 | 9.0562 | 7.6199 |
| 8.5055 | 8.4417 | 8.7102\ | | | | | |
| C15orf21 | | 6.4408 | 5.4081 | 3.2600 | 5.5321 | 5.9734 | 6.9894 |
| 4.6568 | 7.0565 | 6.2974 | 7.2945 | 5.9854 | 6.5374 | 5.9480 | 6.3547 |
| 6.0454 | 5.1466 | 5.2349 | 5.0804 | 5.0411 | 7.1789 | 5.3969 | 6.2247 |
| 5.4443 | 5.2130 | 5.7263 | 5.2309 | 6.5987 | 7.4844 | 5.8644 | 5.8096 |
| 6.5456 | 6.3547 | 6.6359 | 6.6028 | 6.0009 | 7.2542 | 4.6946 | 7.2151 |
| 6.5093 | 5.4052 | 6.4633 | 5.9739 | 5.9553 | 6.0679 | 5.4765 | 5.2832 |
| 6.0912 | 4.6679 | 5.7009 | 6.4247 | 6.6577 | 5.2513 | 4.9066 | 6.0308 |
| 5.2988 | 6.3783 | 4.6608 | 5.6625 | 6.2495 | 6.2268 | 5.1749 | 6.1139 |
| 3.8280 | 5.2631 | 6.3596 | 6.9005 | 4.5289 | 6.5919 | 5.1070 | 4.5466 |
| 7.4320 | 6.1102 | 6.0791 | 6.8710 | 5.3926 | 5.1081 | 6.1282 | 5.1887 |
| 5.4353 | 4.3717 | 7.1569 | 6.9475 | 5.6125 | 6.3827 | 6.4329 | 5.0767 |
| 5.7980 | 5.8254 | 5.7767 | 7.2274 | 5.2525 | 6.0201 | 5.6114 | 5.0946 |
| 5.6694 | 6.2073 | 4.9822 | 6.4641 | 5.5581 | 5.1061 | 4.4713 | 5.6711 |
| 5.8019 | 4.9200 | 6.3612 | 6.0319 | 5.6204 | 5.8092 | 5.9156 | 5.7894 |
| 4.2448 | 5.7915 | 4.9483 | 6.0444 | 6.4857 | 5.8187 | 6.6615 | 6.4119 |
| 6.9872 | 5.5120 | 6.1748 | 5.9651 | 5.1506 | 6.3688 | 6.4532 | 5.7481 |
| 7.1208 | 5.1564 | 6.0393 | 5.5785 | 6.7707 | 6.4139 | 6.9181 | 5.4525 |
| 5.6873 | 4.7429 | 6.1260 | 4.9498 | 6.9161 | 5.8494 | 6.3044 | 6.3184 |
| 6.7335 | 5.1388 | 6.2987 | 6.0385 | 4.1422 | 6.5230 | 3.9441 | 5.5777 |

5.6469  5.2119  6.3424  5.9925  5.8527  5.7585  6.1461  6.3278
7.5443  7.6898  5.6161  5.9036  5.8369  7.1557  6.5680  5.8068
5.4812  5.7263  6.8828  6.6576  6.8519  5.5427  5.7118  6.0750
4.9830  5.7628  5.8859  5.7849  6.6450  6.9350  7.6917  4.8070
6.8380  5.4251  5.8319  6.4548  4.6328  4.9674  6.8969  5.0024
5.5670  5.7061  5.5627  5.9819  6.4335  4.7689  6.4721  6.4687
7.1317  6.8172  5.7958\
GJA8    0.0000  0.0000  1.1543  0.0000  0.0000  1.9724  0.0000
0.0000  0.4327  0.4413  0.0000  0.0000  0.4363  0.0000  0.0000
0.0000  0.0000  0.0000  0.0000  0.0000  1.3800  0.4820  0.0000
0.5262  0.0000  0.0000  0.0000  0.0000  0.0000  0.0000  0.0000
0.5612  0.0000  4.2140  0.0000  0.0000  0.0000  0.0000  0.0000
0.4337  0.0000  0.0000  0.0000  0.0000  0.0000  0.0000  0.0000
0.0000  0.0000  0.0000  0.0000  0.0000  0.0000  0.8853  0.4645
0.0000  0.0000  0.0000  0.0000  0.0000  0.9353  0.0000  0.0000
0.0000  0.0000  0.0000  0.0000  0.8444  0.0000  1.0463  0.4334
0.0000  0.0000  0.0000  0.0000  0.0000  1.2673  0.0000  0.0000
0.0000  0.0000  1.9437  0.0000  0.0000  0.0000  0.0000  0.0000
0.0000  0.0000  0.0000  0.0000  0.0000  0.0000  0.0000  0.0000
0.6266  0.9065  0.9402  0.0000  0.0000  0.0000  0.0000  0.0000
0.0000  0.0000  3.5453  0.0000  0.0000  0.0000  0.0000  0.0000
0.0000  2.8658  1.5850  0.0000  0.0000  0.0000  0.4321  0.0000
0.0000  4.9041  0.0000  0.0000  0.0000  0.0000  0.0000  0.0000
0.7843  0.0000  0.0000  0.3935  0.0000  0.0000  0.0000  0.8107
0.0000  0.0000  0.0000  0.5216  0.0000  0.0000  0.0000  0.0000
0.0000  0.0000  0.0000  0.0000  0.0000  0.0000  0.0000  0.5019
0.0000  0.0000  0.5862  0.0000  1.2732  0.0000  0.0000  2.0337
0.0000  0.0000  0.0000  0.0000  0.0000  0.4418  0.0000  0.0000
0.0000  0.0000  0.0000  0.0000  0.0000  0.0000  0.0000  0.0000
0.0000  0.0000  0.0000  0.0000  0.0000  0.0000  0.6902  0.9100
0.5055  0.0000  0.0000  0.0000  0.0000  0.0000  0.5707  0.0000
0.0000  0.0000  0.7547  0.0000  0.0000  0.0000  0.0000  0.0000
0.0000  0.0000\
CYP8B1  2.3786  3.7046  2.2256  3.2578  2.7531  3.0829  0.5431
4.6972  4.5258  2.5870  1.2544  2.9620  5.0033  2.9572  1.4890
0.0000  6.2670  6.0895  3.3893  4.7636  1.6493  3.5498  3.2986
2.3107  2.5058  0.0000  0.0000  5.4501  3.5215  5.8855  0.0000
0.9642  4.2522  4.6899  2.1706  3.5700  4.7956  0.3648  4.1930
4.0071  1.9736  3.7496  4.6306  1.2622  4.0482  3.1906  2.6132
5.4159  0.4402  3.4820  3.7195  5.7298  1.7111  2.7018  0.0000
2.6383  4.1793  1.9711  5.1549  5.1104  3.1300  1.4031  6.1738
2.6144  1.7745  4.2026  6.0268  3.2255  1.9829  3.1682  2.6452
3.2846  3.0705  4.3694  5.1022  1.1076  3.3091  3.9590  3.3490
1.3771  0.5659  4.2741  2.7716  3.8720  2.3108  5.9712  0.0000
2.5297  4.7798  0.0000  3.7806  4.6645  1.8528  2.2282  3.6683
3.0128  4.1025  3.0618  1.9860  3.5463  2.2933  4.1969  3.8552
5.0416  2.0651  2.4789  1.5756  3.8324  1.2448  1.8132  2.2774
5.7915  4.3124  4.3219  7.0350  2.6508  1.4679  3.4298  5.2516
6.1140  1.3128  2.7098  6.0145  3.8937  2.1978  5.7361  3.0773
1.2894  1.2802  2.1283  2.0482  3.6084  4.8109  5.5191  4.4670
0.8141  3.7942  0.0000  1.8532  1.1757  4.7738  2.7811  0.0000
2.0686  1.6921  0.5418  2.9153  2.7033  5.4813  2.6654  2.8564
3.5510  1.7123  3.0034  1.2540  2.8119  3.0310  2.2251  1.6149
0.9345  2.0474  0.6138  1.9935  0.9764  3.8337  2.1150  0.0000

| | | | | | | | |
|---|---|---|---|---|---|---|---|
| 3.7553 | 5.2680 | 5.6254 | 1.4975 | 2.0357 | 0.0000 | 1.3895 | 1.9200 |
| 1.1272 | 2.0214 | 2.2714 | 4.6281 | 3.8257 | 1.9305 | 3.8577 | 1.8629 |
| 1.1756 | 1.5843 | 1.0594 | 3.6333 | 6.2132 | 4.1471 | 1.9676 | 1.4263 |
| 3.7744 | 0.9737 | 1.6145 | 2.1049 | 2.3335 | 0.0000 | 3.8265 | 2.7892 |
| 2.8731 | 4.5812\ | | | | | | |
| C15orf28 | | 1.2631 | 1.8777 | 1.1543 | 1.3959 | 0.0000 | 1.3921 |
| 0.9367 | 1.5600 | 1.6317 | 2.0772 | 0.4291 | 0.4849 | 0.4363 | 1.4754 |
| 1.2358 | 2.6237 | 0.6221 | 0.7772 | 0.5838 | 0.0000 | 0.0000 | 1.9172 |
| 1.4101 | 1.4650 | 1.2653 | 0.0000 | 0.0000 | 1.6129 | 0.8532 | 2.7135 |
| 0.4127 | 1.9460 | 0.5159 | 3.0448 | 2.1706 | 1.1431 | 2.8291 | 2.2449 |
| 0.5410 | 1.6341 | 1.1184 | 1.1407 | 0.0000 | 1.6313 | 0.0000 | 0.0000 |
| 0.0000 | 2.5272 | 0.4402 | 0.4764 | 1.8408 | 2.4004 | 0.6494 | 1.4299 |
| 1.8714 | 2.2632 | 2.7053 | 1.6258 | 0.7533 | 1.5581 | 1.4980 | 1.7937 |
| 0.5573 | 1.0177 | 3.1488 | 1.5189 | 1.8352 | 0.4832 | 0.5773 | 0.0000 |
| 0.7662 | 3.0867 | 0.0000 | 0.0000 | 2.0547 | 0.4698 | 0.5548 | 0.9332 |
| 2.5610 | 2.0686 | 0.0000 | 1.8200 | 0.7204 | 0.6718 | 0.0000 | 0.6199 |
| 0.5376 | 0.0000 | 0.9125 | 1.4655 | 0.0000 | 2.5341 | 1.2055 | 0.0000 |
| 1.8492 | 0.6266 | 1.2090 | 1.2499 | 1.6394 | 0.5504 | 0.0000 | 1.6155 |
| 2.4570 | 2.5001 | 1.1338 | 0.4659 | 0.0000 | 1.4556 | 0.5426 | 1.0041 |
| 2.1442 | 1.6572 | 0.0000 | 1.5850 | 2.7148 | 1.8640 | 1.2170 | 2.7195 |
| 1.1815 | 1.3647 | 0.0000 | 0.5886 | 1.3885 | 0.4700 | 2.6149 | 0.5331 |
| 0.0000 | 2.2050 | 1.2802 | 2.8723 | 0.7024 | 1.1234 | 1.5194 | 2.1540 |
| 0.8107 | 0.0000 | 0.6413 | 1.5206 | 1.8532 | 1.6315 | 2.0359 | 0.0000 |
| 2.3186 | 1.7146 | 1.3147 | 0.5418 | 2.5258 | 1.2499 | 0.5814 | 1.8766 |
| 2.3678 | 0.0000 | 3.2011 | 1.5875 | 1.2540 | 1.9388 | 1.9521 | 3.1640 |
| 0.6000 | 1.9005 | 1.0317 | 1.3735 | 0.0000 | 0.7182 | 2.5889 | 2.7721 |
| 0.0000 | 2.8261 | 0.0000 | 1.7238 | 1.0663 | 1.8707 | 0.0000 | 0.0000 |
| 0.0000 | 1.1272 | 0.9063 | 0.6293 | 1.6980 | 1.6765 | 0.0000 | 2.2268 |
| 0.5257 | 0.5055 | 0.0000 | 1.3929 | 0.0000 | 1.1775 | 1.4486 | 1.2961 |
| 0.5078 | 0.0000 | 0.5674 | 1.9066 | 2.4698 | 2.3335 | 0.6064 | 2.5229 |
| 0.6701 | 2.7876 | 0.6959\ | | | | | |
| C15orf29 | | 8.9479 | 8.4843 | 7.8313 | 9.0882 | 8.2624 | 8.8066 |
| 8.1307 | 8.6027 | 8.6442 | 8.9305 | 8.5751 | 8.2453 | 8.3760 | 8.6561 |
| 9.1345 | 8.8949 | 9.1932 | 8.4891 | 8.6332 | 8.1378 | 7.3236 | 9.1803 |
| 8.5636 | 9.2910 | 8.5230 | 8.8795 | 7.0429 | 8.7069 | 8.5546 | 8.3440 |
| 8.1271 | 8.6876 | 8.5916 | 7.3201 | 8.2128 | 7.7394 | 8.7835 | 8.4723 |
| 9.5655 | 8.4652 | 9.3148 | 8.5081 | 7.8425 | 8.9278 | 8.1334 | 8.7117 |
| 7.2234 | 8.1125 | 8.5323 | 8.7009 | 8.3206 | 8.0990 | 7.9225 | 8.7384 |
| 8.1989 | 9.5281 | 8.5501 | 8.6673 | 8.3228 | 8.6249 | 8.9945 | 9.1245 |
| 9.2937 | 8.3638 | 8.9704 | 8.1160 | 8.3171 | 9.5315 | 8.5602 | 8.2124 |
| 7.7721 | 8.6409 | 8.3515 | 8.8070 | 9.0780 | 8.3886 | 9.0126 | 8.1855 |
| 8.8237 | 8.5356 | 8.7220 | 9.0531 | 8.5244 | 8.9498 | 8.8555 | 8.3359 |
| 8.7958 | 8.5031 | 8.8655 | 8.5722 | 8.4121 | 8.2428 | 7.4452 | 9.5396 |
| 9.0423 | 8.5495 | 7.6992 | 7.9282 | 8.7762 | 9.3573 | 8.4208 | 8.5155 |
| 7.9928 | 9.5575 | 8.9173 | 7.6521 | 9.8711 | 8.3665 | 8.5751 | 8.9550 |
| 9.0261 | 8.8055 | 7.5188 | 8.8704 | 9.0420 | 8.3776 | 9.0365 | 8.3382 |
| 8.8157 | 8.6212 | 8.1199 | 8.6728 | 8.3640 | 8.1655 | 8.9605 | 8.7245 |
| 8.5035 | 8.0278 | 8.6287 | 9.2052 | 8.7784 | 9.3000 | 8.7273 | 9.2970 |
| 8.8883 | 6.3991 | 8.8936 | 9.6321 | 8.6639 | 8.5440 | 9.1961 | 8.8117 |
| 8.5630 | 8.7158 | 8.7311 | 8.4196 | 8.9243 | 8.5184 | 8.2999 | 8.6599 |
| 8.5639 | 8.1628 | 8.8668 | 8.9197 | 8.6149 | 9.0988 | 8.3581 | 8.8506 |
| 9.0104 | 8.7654 | 7.7734 | 8.3013 | 8.8663 | 8.7578 | 9.0160 | 9.5024 |
| 8.6821 | 8.0262 | 8.6300 | 8.8303 | 8.7983 | 9.4362 | 6.8365 | 7.8553 |
| 7.3065 | 9.1425 | 9.4302 | 8.6757 | 8.9022 | 8.5527 | 8.2754 | 7.9158 |

8.5661	8.9474	8.5952	8.9601	7.4480	8.2261	9.1193	9.1128
8.7597	8.6433	9.1818	8.3733	9.1800	8.4942	7.5428	8.5828
8.9172	9.0892	8.0058\
STMN1	14.4132	12.7116	10.7797	11.7166	13.4019	12.7895	12.7356
13.8315	14.1645	12.9757	13.2521	13.8500	12.9550	12.9503	12.5182
12.4806	14.2883	12.9495	12.3005	13.3938	12.2854	12.4925	9.9775
12.4616	11.5383	13.2691	14.3274	13.2571	13.0538	13.1730	14.6444
13.3665	13.0375	12.3977	12.9839	13.2319	12.5947	12.9821	13.8531
13.9303	13.0688	12.0219	14.2466	12.3700	11.8142	12.6310	13.1965
12.0844	14.1792	12.3582	11.6786	12.0332	12.9828	11.2593	12.8356
13.8633	11.6775	11.9521	13.8564	12.9033	11.5420	13.6368	13.1134
13.2586	12.3379	12.1913	10.4230	13.2842	10.9218	13.4220	13.2083
13.2873	12.3573	13.0425	12.4294	11.4973	13.5198	11.5005	12.4844
13.2684	13.6381	13.1966	10.9000	13.6286	13.4138	13.2176	13.2890
13.3073	13.5707	15.0597	10.7031	12.5880	13.3410	12.6924	13.9830
10.8485	12.3056	12.0227	13.3546	13.4316	13.9077	12.4737	14.3400
12.6664	13.7655	14.2954	10.8436	13.1980	14.4449	13.5345	12.8722
12.7133	12.7009	12.3049	13.0025	13.5941	12.9678	12.5900	13.5062
12.8033	13.8133	13.2180	12.8334	12.3651	10.4157	12.8198	13.8258
13.0697	12.0554	11.3833	12.9572	13.7255	12.8226	13.2841	12.6654
11.7370	13.9830	11.0990	12.0578	11.3529	13.7973	12.9251	11.9469
12.9713	12.6953	12.2271	12.4719	11.1534	12.4483	12.8931	11.3775
12.3320	12.5051	13.4259	13.3554	13.8897	11.2425	13.4365	13.1807
13.7086	11.3904	12.1809	13.4533	12.8229	11.6301	12.5187	13.1209
10.8737	13.8239	14.8757	13.3755	12.8373	13.2088	13.3970	10.9466
14.3972	12.1657	10.7253	14.5549	11.0417	12.9067	12.2882	14.4906
12.2562	12.9664	12.8686	13.5981	13.1511	13.2752	13.2277	12.8404
12.6577	13.3280	13.0746	12.9679	12.3811	13.6268	11.5466	12.1666
12.7163	13.2158\
S1PR2	8.9695	10.0134	9.5946	8.6391	8.0384	7.1857	10.3789
9.6274	9.3678	9.9646	9.9912	12.0316	8.2121	9.0171	9.2821
9.3668	9.8089	8.3974	10.3404	9.9398	8.6049	10.1948	7.6550
8.5797	8.8790	8.8907	9.5975	9.6313	9.6222	9.3134	8.5339
8.0776	9.2129	9.4822	7.8083	7.5290	8.2024	7.3636	7.6784
9.6957	9.8883	10.0235	8.4755	8.8215	7.2310	9.4385	10.7979
8.5623	8.4256	7.9784	8.5265	10.5551	10.2302	7.9339	9.2160
8.6863	7.5476	7.5762	9.5081	8.7194	8.2387	7.5169	9.8637
11.0017	10.1528	8.4887	9.7287	8.7608	9.7478	8.7348	7.9109
7.4687	9.5334	8.4065	8.2386	7.8933	10.1717	9.6699	9.1904
8.4282	10.4813	9.8259	8.9479	9.6911	9.7390	9.5963	9.4633
10.4430	8.6288	8.5588	9.0151	8.6205	9.5411	10.3587	9.7091
7.9914	8.1381	9.3648	9.5149	7.6490	9.0098	8.8183	10.4822
8.8623	7.9647	9.6800	10.1301	10.0904	9.5257	10.2953	8.2220
9.5796	10.8992	8.2527	9.0490	9.7395	8.3642	10.7182	9.2400
9.4564	8.9777	8.9432	9.7110	9.7484	7.2303	10.5428	8.6328
9.3858	8.3976	8.5997	9.6148	10.3440	9.5098	8.7901	8.1295
10.2192	9.4350	9.7338	8.0255	8.7195	8.9164	9.1958	9.9631
8.3323	9.3309	7.9572	7.7925	8.9101	9.2360	9.5721	8.5205
10.4204	9.4587	8.6185	9.0080	9.2575	8.3789	10.2458	10.6899
8.6573	6.7902	7.9142	9.9764	9.9473	9.7078	9.0900	9.2854
8.8943	9.2659	8.7461	9.5304	7.8285	9.6388	9.6692	9.5798
7.7347	9.4173	9.4247	8.1969	8.6895	8.4576	8.0621	9.6622
9.7097	9.5530	9.0320	9.2149	8.7673	9.3458	8.4264	8.3064
8.3387	9.6517	8.7081	8.4755	9.6420	10.2029	8.4520	9.5819

8.6635  10.4007\
KDM3B   10.9195 10.8483 11.2223 11.1391 11.0442 11.6023 11.8108
11.3356 11.2931 11.0021 11.3870 11.3151 11.4974 11.4186 11.3207
10.4444 9.9262  11.2531 10.7652 9.6345  10.7165 11.1900 11.1597
11.1392 11.0312 9.7163  11.1677 10.2542 10.7066 9.8000  10.7552
10.9548 10.1031 12.3059 12.0494 10.8490 11.5025 11.5185 10.8707
10.4909 10.0823 11.1471 10.4708 10.4253 11.1262 10.2066 8.9323
11.1188 11.1814 10.8436 10.9329 9.8179  10.9917 11.2258 10.7164
11.3762 11.4924 10.7708 11.0368 10.9371 11.1368 11.2823 10.9810
10.5069 10.8811 11.4599 11.1882 10.6863 10.7457 10.9879 10.3387
11.7593 9.9858  10.3560 10.7465 10.8124 10.4196 10.7535 10.7436
11.4464 10.3690 11.4331 10.5454 11.0173 10.5124 10.3265 11.5651
10.3214 11.2812 10.0832 10.8928 11.3403 10.6853 10.2725 10.3086
10.7186 11.1830 11.2817 10.9424 10.9371 9.6140  10.5404 11.0059
10.3616 10.6843 10.8221 10.1104 11.2211 11.3525 10.2970 11.2422
11.0903 7.5793  9.9873  10.6607 10.8243 11.0010 11.6487 10.7114
10.3167 10.6798 10.6866 11.5195 11.3456 11.5151 10.6891 10.0602
10.0602 11.2702 11.3782 11.2827 10.8067 10.7912 10.6219 10.1099
8.1371  9.8117  8.3415  11.4456 11.2989 10.4517 10.4761 10.2491
11.5743 11.4698 11.4666 10.8822 11.1222 10.8969 10.1022 11.1455
7.9056  11.6155 12.2937 11.7563 11.0258 11.1535 11.5456 10.9007
10.2377 10.5741 10.2566 11.1961 10.3934 11.0201 10.9617 10.7622
11.6391 10.9125 10.9377 11.6441 10.6203 10.8990 11.1172 8.7888
11.2765 10.7763 11.1266 12.0714 10.9417 10.5038 10.3863 11.3173
10.8175 10.9271 11.0559 11.1364 10.1894 10.3956 11.2507 11.2106
11.7204 10.2155 10.7939 11.2834 9.5083  9.5441  11.3530 11.1217
11.3285 9.0644\
REG1P   0.0000  0.0000  0.0000  0.0000  0.0000  0.0000  0.0000
0.4748  0.0000  0.0000  0.0000  0.0000  0.0000  0.0000  0.0000
0.0000  0.0000  0.0000  0.0000  0.0000  0.0000  0.0000  0.0000
0.0000  0.0000  0.0000  0.0000  0.5990  0.0000  0.0000  0.0000
0.0000  0.0000  0.0000  0.0000  0.0000  0.0000  0.0000  0.0000
0.0000  0.0000  0.0000  0.0000  0.0000  0.0000  0.0000  0.0000
0.0000  0.0000  0.0000  0.0000  0.0000  0.0000  0.0000  0.0000
0.0000  0.0000  0.0000  0.0000  0.0000  0.0000  0.0000  0.0000
0.0000  0.0000  0.0000  0.0000  0.0000  0.0000  0.0000  0.0000
0.0000  0.0000  0.0000  0.0000  0.0000  0.0000  0.0000  0.0000
0.0000  0.0000  0.0000  0.7204  0.0000  0.0000  0.0000  0.0000
0.0000  0.0000  0.0000  0.0000  0.0000  0.0000  0.0000  0.0000
0.0000  0.0000  0.0000  0.0000  0.0000  0.0000  0.0000  0.0000
0.0000  0.0000  0.0000  0.0000  0.0000  0.0000  0.0000  0.0000
0.0000  0.0000  0.0000  0.0000  0.0000  0.0000  0.0000  0.0000
0.0000  0.0000  0.0000  0.0000  0.0000  0.0000  0.0000  0.0000
0.0000  0.0000  0.0000  0.0000  0.0000  0.0000  0.0000  0.0000
0.0000  0.0000  0.0000  0.0000  0.0000  0.0000  0.0000  0.0000
0.0000  0.0000  0.0000  0.0000  0.0000  0.0000  0.0000  0.0000
0.0000  0.0000  0.0000  0.0000  0.0000  0.0000  0.0000  0.0000
0.0000  0.0000  0.0000  0.0000  0.0000  0.0000  0.0000  0.0000
0.0000  0.0000  0.0000  0.0000  0.0000  0.0000  0.0000  2.3719
0.0000  0.0000  0.0000  0.0000  0.0000  0.0000  0.0000  0.0000
0.0000  0.0000  0.0000  0.5811  0.0000  0.0000  0.0000  0.0000
0.0000  0.5674  0.0000  0.0000  0.0000  0.0000  0.0000  0.0000
0.0000  1.7990\
STK17A  10.0699 8.2801  10.0860 9.3259  9.6270  8.5088  8.9233

| | | | | | | | |
|---|---|---|---|---|---|---|---|
| 8.3471 | 9.9018 | 8.4281 | 8.8788 | 9.3764 | 8.8533 | 8.4889 | 8.9185 |
| 8.7583 | 8.9317 | 8.6516 | 8.9288 | 9.2482 | 9.3818 | 9.1065 | 9.0416 |
| 9.1717 | 8.0366 | 9.8007 | 9.9780 | 9.3658 | 10.7045 | 8.3169 | 8.1791 |
| 8.7221 | 8.3469 | 8.9589 | 8.6411 | 8.9415 | 8.7142 | 8.4746 | 10.1073 |
| 9.8793 | 9.7733 | 8.6447 | 8.2146 | 9.6958 | 8.4436 | 8.9062 | 9.0467 |
| 9.5270 | 8.8538 | 8.2210 | 8.5265 | 9.2781 | 8.9547 | 7.9612 | 9.8084 |
| 9.4655 | 8.2007 | 9.7028 | 9.1173 | 7.9624 | 8.8551 | 8.9639 | 9.6761 |
| 8.6406 | 8.7406 | 9.6975 | 9.0073 | 9.6893 | 10.9562 | 9.1203 | 9.3975 |
| 9.5732 | 10.0714 | 8.5662 | 9.5671 | 8.8891 | 8.8372 | 9.5340 | 9.1828 |
| 8.7736 | 9.8033 | 9.5527 | 9.0590 | 9.3995 | 8.9471 | 8.7133 | 8.8349 |
| 8.5073 | 9.2417 | 8.9946 | 8.2897 | 9.0273 | 8.8091 | 9.0344 | 9.5858 |
| 8.9302 | 9.4968 | 8.4366 | 8.9167 | 9.1370 | 8.7803 | 8.7072 | 9.9544 |
| 8.8290 | 9.0906 | 9.4485 | 8.5596 | 10.0151 | 8.6991 | 8.7680 | 9.1377 |
| 9.4434 | 9.0132 | 7.9009 | 10.3945 | 8.6691 | 8.8294 | 8.8150 | 10.0691 |
| 8.8890 | 10.3534 | 8.8643 | 9.4700 | 9.5989 | 8.7257 | 10.1100 | 9.1899 |
| 7.7514 | 8.7754 | 8.3782 | 9.9144 | 8.1370 | 9.1331 | 8.9933 | 9.1424 |
| 10.0060 | 9.7399 | 8.4301 | 8.5941 | 8.4187 | 9.3073 | 9.0368 | 8.8972 |
| 9.9265 | 9.5571 | 8.7077 | 8.5002 | 8.3107 | 10.5854 | 7.8167 | 9.2581 |
| 8.3162 | 9.5631 | 9.5737 | 8.3447 | 9.0447 | 8.3810 | 8.9487 | 9.7979 |
| 9.2236 | 8.5056 | 8.8660 | 8.8586 | 8.9164 | 10.0966 | 9.5024 | 8.3110 |
| 9.4557 | 9.1179 | 8.7813 | 10.7425 | 9.3807 | 7.7036 | 8.7901 | 8.1757 |
| 8.4641 | 8.4930 | 8.4552 | 9.2202 | 9.4637 | 8.5846 | 8.6207 | 8.8173 |
| 8.5292 | 8.9069 | 9.6051 | 9.0667 | 8.8701 | 8.4383 | 9.1039 | 8.9770 |
| 8.9937 | 8.9243 | 10.0870 | 8.7831 | 7.8410 | 8.9041 | 8.0282 | 9.9368 |
| 9.6034 | 9.1169\ | | | | | | |
| KDM3A | 9.3825 | 10.1538 | 10.3031 | 11.2457 | 10.6212 | 10.4402 | 12.0136 |
| 10.6512 | 9.9853 | 10.2635 | 10.5259 | 11.0392 | 10.9136 | 11.0085 | 10.7994 |
| 10.5208 | 10.3852 | 10.1783 | 10.0966 | 10.2040 | 10.6562 | 10.5188 | 10.1572 |
| 10.7966 | 10.1133 | 9.1420 | 9.8321 | 9.8016 | 9.1760 | 10.3746 | 11.0065 |
| 10.3705 | 9.8518 | 11.0057 | 11.2515 | 11.0229 | 10.6164 | 8.2893 | 10.0989 |
| 11.1521 | 9.3306 | 10.0028 | 10.1277 | 10.7951 | 10.5220 | 10.0966 | 8.6744 |
| 10.4296 | 9.5394 | 9.6883 | 10.4773 | 9.8584 | 11.3416 | 10.6430 | 10.9881 |
| 10.8507 | 11.2875 | 10.7875 | 10.5628 | 10.8847 | 10.5998 | 10.9393 | 10.8471 |
| 10.3961 | 9.9803 | 11.1120 | 10.7907 | 11.8896 | 10.4770 | 10.3343 | 9.0951 |
| 10.8410 | 9.6343 | 9.9655 | 9.7758 | 10.4574 | 11.5287 | 10.1265 | 9.8692 |
| 10.9818 | 10.2061 | 10.3083 | 10.1493 | 10.6283 | 10.0566 | 10.6441 | 10.7091 |
| 9.8013 | 10.6325 | 9.9151 | 9.8970 | 11.1665 | 10.2542 | 10.3148 | 10.3012 |
| 9.6644 | 10.7735 | 10.2665 | 10.8261 | 10.3765 | 9.4964 | 10.7132 | 10.9840 |
| 10.7667 | 10.3796 | 10.9943 | 9.9560 | 10.5472 | 11.1230 | 10.5541 | 10.9358 |
| 10.5237 | 7.9625 | 10.0224 | 10.7179 | 10.7773 | 10.9204 | 10.9331 | 10.6833 |
| 8.9774 | 10.9033 | 10.4786 | 10.5224 | 10.8170 | 10.4162 | 10.7322 | 11.5956 |
| 9.8628 | 10.4602 | 10.5595 | 11.2131 | 10.7222 | 10.3503 | 10.4855 | 9.4958 |
| 9.1030 | 9.4455 | 9.5908 | 10.8710 | 10.3981 | 10.6738 | 10.5297 | 10.3073 |
| 11.0949 | 10.2737 | 10.3358 | 10.9899 | 9.8094 | 10.1648 | 9.1602 | 10.4002 |
| 8.6355 | 11.0673 | 10.5627 | 10.3399 | 11.5940 | 10.5469 | 11.4539 | 11.0942 |
| 9.1008 | 10.2402 | 8.9080 | 10.7378 | 9.7695 | 11.3208 | 10.7833 | 10.0908 |
| 10.5257 | 9.7471 | 10.6655 | 11.7383 | 11.0403 | 10.4503 | 10.7965 | 9.2378 |
| 10.9843 | 10.1522 | 10.2551 | 11.4240 | 10.2567 | 9.0386 | 10.3650 | 11.1420 |
| 10.5304 | 11.2511 | 8.6649 | 10.0109 | 8.9532 | 10.2712 | 10.8933 | 10.2728 |
| 10.4449 | 10.6033 | 9.7328 | 8.0211 | 9.1256 | 9.3987 | 10.3024 | 10.6807 |
| 11.0002 | 8.8428\ | | | | | | |
| LOC401387 | | 1.7370 | 1.6912 | 0.0000 | 2.1815 | 2.9808 | 0.4059 |
| 0.0000 | 2.0418 | 0.0000 | 2.3031 | 2.0419 | 5.2307 | 1.3445 | 2.3094 |
| 1.4890 | 2.9791 | 3.1837 | 0.7772 | 2.2115 | 0.9198 | 2.2769 | 0.5848 |

| | | | | | | | |
|---|---|---|---|---|---|---|---|
| 1.6825 | 0.0000 | 2.6568 | 0.0000 | 1.0809 | 0.5990 | 0.8532 | 1.0077 |
| 0.9954 | 2.4004 | 4.9338 | 0.8184 | 1.4389 | 2.0076 | 1.2685 | 0.8977 |
| 1.4957 | 1.8074 | 0.6649 | 0.8949 | 5.5230 | 1.0349 | 1.6194 | 0.0000 |
| 4.7672 | 1.4786 | 1.4771 | 1.9026 | 1.0749 | 0.0000 | 1.7111 | 1.0088 |
| 1.0973 | 1.9627 | 1.0734 | 0.0000 | 1.0204 | 0.5715 | 1.4980 | 0.4969 |
| 0.9583 | 0.0000 | 2.0096 | 0.9507 | 0.5980 | 1.1331 | 0.9886 | 1.0794 |
| 1.0365 | 1.3570 | 0.0000 | 1.3638 | 1.4868 | 4.8596 | 3.6155 | 1.7110 |
| 1.5048 | 1.6461 | 0.0000 | 0.3965 | 2.7716 | 1.9875 | 1.2145 | 3.0901 |
| 1.2711 | 0.0000 | 2.9410 | 1.8648 | 0.0000 | 1.5834 | 1.4549 | 2.5641 |
| 0.5198 | 0.6266 | 0.9065 | 2.1964 | 1.8265 | 2.2905 | 2.8342 | 5.5148 |
| 1.8854 | 3.5417 | 5.4909 | 0.5053 | 2.3106 | 2.1301 | 1.7151 | 1.5904 |
| 0.0000 | 0.8798 | 2.0513 | 2.8074 | 5.0705 | 1.3697 | 0.9131 | 1.4572 |
| 0.5076 | 1.0358 | 1.8720 | 0.8689 | 0.0000 | 0.8239 | 2.6149 | 0.0000 |
| 1.3754 | 1.6629 | 0.0000 | 1.9681 | 1.9345 | 2.1813 | 0.0000 | 3.3367 |
| 0.8107 | 0.0000 | 6.7960 | 0.0000 | 1.4553 | 1.4921 | 6.3446 | 1.5646 |
| 1.8519 | 0.9357 | 1.3147 | 0.5731 | 0.0000 | 1.2499 | 0.9947 | 0.0000 |
| 1.1689 | 3.9653 | 1.2425 | 0.0000 | 1.2540 | 0.7727 | 1.5425 | 1.2757 |
| 1.0225 | 0.0000 | 1.0317 | 0.0000 | 5.5199 | 2.6967 | 4.5663 | 1.1696 |
| 0.0000 | 0.4012 | 6.6231 | 2.0787 | 1.8290 | 0.0000 | 2.4724 | 0.0000 |
| 0.0000 | 0.8396 | 2.9774 | 0.7654 | 2.0085 | 3.1408 | 2.7277 | 1.1551 |
| 5.8870 | 0.8792 | 1.5843 | 1.3929 | 2.4369 | 1.5325 | 0.0000 | 0.5707 |
| 0.0000 | 1.5256 | 4.7713 | 1.2477 | 1.2575 | 0.0000 | 0.0000 | 1.0391 |
| 0.3736 | 1.6364 | 0.0000\ | | | | | |
| S1PR1 | 6.5015 | 10.0295 | 8.4600 | 7.2815 | 6.4953 | 7.6333 | 6.6982 |
| 7.8243 | 6.8148 | 8.5612 | 6.9898 | 6.0644 | 7.3337 | 8.4542 | 7.6091 |
| 6.9847 | 5.9522 | 6.6572 | 9.8072 | 6.5139 | 7.1059 | 9.4528 | 6.5687 |
| 7.9383 | 9.8278 | 6.4370 | 7.5461 | 8.2165 | 7.8457 | 6.4331 | 6.6634 |
| 7.4787 | 6.2360 | 7.4798 | 6.1907 | 6.1269 | 8.9296 | 7.0306 | 7.1687 |
| 6.4180 | 7.8487 | 11.2786 | 7.1607 | 6.6879 | 11.6151 | 7.2380 | 5.6515 |
| 7.9962 | 7.0358 | 6.6662 | 7.3740 | 8.7033 | 7.8604 | 12.0697 | 9.0778 |
| 7.5919 | 7.2767 | 8.0299 | 9.6565 | 5.5896 | 11.6072 | 8.5656 | 7.0757 |
| 5.9535 | 8.5038 | 7.9928 | 7.2184 | 7.7402 | 6.9488 | 8.4241 | 7.3600 |
| 7.5163 | 7.5029 | 6.8515 | 8.8560 | 8.6292 | 8.3797 | 7.9108 | 8.4162 |
| 7.9390 | 7.1762 | 7.1016 | 10.4756 | 8.5403 | 7.7359 | 6.6484 | 6.6226 |
| 6.6081 | 7.5173 | 8.4042 | 10.5727 | 8.0056 | 7.0637 | 7.6440 | 7.5444 |
| 11.4644 | 8.1041 | 9.7156 | 8.2129 | 7.5981 | 5.4930 | 8.1403 | 7.4196 |
| 8.0028 | 6.1751 | 7.1230 | 7.6370 | 8.6802 | 8.5647 | 7.1322 | 8.6802 |
| 6.2755 | 5.7671 | 5.1293 | 7.8775 | 6.9729 | 8.4322 | 6.9612 | 7.0780 |
| 8.9300 | 7.8217 | 8.1480 | 7.9065 | 6.8062 | 7.6613 | 7.0732 | 8.3801 |
| 4.7548 | 10.5655 | 10.5791 | 6.8276 | 5.9744 | 7.6187 | 7.8463 | 8.3048 |
| 6.0652 | 5.5853 | 7.3868 | 10.0748 | 9.5671 | 6.1238 | 8.2732 | 6.7716 |
| 8.9172 | 8.8358 | 6.9551 | 8.3078 | 10.2228 | 6.9318 | 8.0850 | 10.5407 |
| 4.6083 | 8.0628 | 7.2846 | 7.4818 | 6.6465 | 10.6697 | 7.0653 | 6.3015 |
| 7.7429 | 6.7902 | 7.2289 | 7.3222 | 5.9829 | 6.9901 | 7.4859 | 8.3437 |
| 8.6200 | 7.2438 | 5.5423 | 6.4380 | 7.2895 | 6.7719 | 6.7465 | 6.8298 |
| 8.7433 | 8.6815 | 10.6339 | 7.5467 | 8.8127 | 7.1020 | 6.5490 | 6.6297 |
| 6.8473 | 6.4962 | 7.1211 | 8.4190 | 6.7751 | 5.7089 | 8.0706 | 8.1177 |
| 8.0110 | 8.0917 | 8.0211 | 6.7916 | 4.8720 | 4.5525 | 8.7670 | 7.6177 |
| 8.0750 | 5.4521\ | | | | | | |
| ZNF642 | 6.8083 | 6.6370 | 6.8376 | 7.0391 | 6.1896 | 6.5831 | 5.4880 |
| 6.9873 | 7.0539 | 6.9042 | 6.2708 | 6.9219 | 7.9756 | 6.5037 | 6.1316 |
| 6.6056 | 7.1412 | 7.0558 | 6.8670 | 7.1848 | 6.2641 | 5.0328 | 6.0843 |
| 6.3955 | 6.8001 | 6.7486 | 6.6742 | 6.2603 | 4.3473 | 5.7566 | 7.0244 |
| 6.6007 | 6.9437 | 6.3084 | 7.1808 | 5.9265 | 6.1009 | 3.1787 | 7.2784 |

```
6.5426  6.3745  5.9459  6.4049  5.6625  6.3906  6.2128  6.0912
5.4359  7.2023  2.6955  6.6890  6.3755  6.6262  6.5574  6.6567
7.1555  6.0478  6.6301  7.1696  6.1792  7.3360  6.6393  6.9383
6.7696  7.1624  6.3327  6.3420  6.9761  5.2065  6.8114  7.0525
6.8764  6.5035  6.6739  6.5451  4.1016  6.7973  5.8654  5.6962
6.2595  6.1718  6.2109  6.7268  6.9024  5.9988  7.7423  6.9834
6.0499  6.4795  7.2896  6.8541  6.7174  7.3132  7.0889  6.4839
6.6594  6.2183  6.3550  6.2295  4.7554  7.1272  6.6271  6.8742
6.4178  5.9049  6.7566  7.2434  5.7636  6.5499  6.8025  6.1609
6.2141  4.7091  5.1293  6.8758  6.9360  6.7351  7.1923  6.3881
6.0808  6.7777  3.8176  7.1475  5.4511  6.6129  7.1063  6.3061
5.9293  6.9910  6.8796  6.1611  7.2010  6.2768  7.2228  7.2100
6.6265  4.8572  7.3376  6.7392  6.6685  5.9172  3.2205  6.4901
5.4259  6.2712  6.6228  5.7049  7.0183  4.8722  2.3242  5.7284
4.3571  5.8560  6.6620  6.9839  7.4535  6.6229  6.1649  5.7443
6.5254  0.6063  5.5092  6.6134  6.5329  6.3318  6.1991  6.7644
7.3788  5.9832  7.4483  6.2979  7.1792  7.4719  5.6937  5.7144
7.4423  7.6680  6.8896  6.2606  6.3624  5.4323  5.8129  6.8100
6.8785  7.2438  4.1973  6.5811  7.5479  5.8739  6.7120  4.3208
5.9002  7.9750  5.9741  3.1443  5.6866  6.6019  6.3050  5.9926
6.3793  5.7130\
TMC4    10.9419 9.7273  11.6029 11.1021 10.1476 10.6021 10.6883
11.0241 10.4430 10.8828 11.9973 11.4173 11.3099 10.4973 11.2809
11.5537 11.4386 10.8078 9.3004  9.6479  11.4319 11.9863 12.1810
10.8615 7.3644  9.5034  6.1701  11.7343 11.5364 11.5497 2.3146
10.3983 10.7097 11.2257 10.2671 10.1192 11.8415 11.3419 10.0017
9.8739  10.4937 9.9214  9.4492  9.5014  5.2199  10.5685 10.3936
11.7291 5.8422  12.8664 11.6106 9.5724  11.0365 5.1591  10.3805
10.5903 12.0719 11.6365 10.3031 12.0863 6.7330  11.1761 10.6205
11.7023 9.7225  11.4981 11.6523 10.7596 10.5307 10.8208 11.6340
11.1790 10.2409 11.5450 10.6519 11.0240 9.0113  12.0370 11.4834
9.7345  10.7767 10.5288 10.7039 10.7575 11.7595 10.4905 10.3742
11.0910 11.0274 8.9374  8.6693  10.9143 9.2036  10.8985 8.4396
5.5451  11.5472 9.4927  11.1931 10.5313 10.3625 11.0843 8.5700
10.0301 10.1092 8.4505  9.5901  10.6196 10.4825 11.2066 12.0052
11.5865 10.7131 11.2866 10.1441 10.6428 10.9377 10.7227 9.8522
10.7588 7.8544  11.1800 9.9477  11.2666 11.9625 10.3893 10.7309
11.9346 9.2072  8.6044  10.5708 10.5463 11.4516 11.1468 10.7166
9.9352  10.0806 10.0686 10.5847 9.6553  9.6051  11.6611 11.8922
10.5633 10.5186 3.6318  10.8308 11.7323 10.9554 11.4436 10.6928
11.0968 11.5110 10.0095 10.4672 8.6124  10.5114 10.3340 11.1128
6.6744  11.7739 10.5534 11.1931 11.8216 10.4265 10.3411 10.2622
10.6613 10.4156 8.8244  8.4722  9.4186  11.1662 11.1753 11.2706
10.3292 9.6720  8.7870  9.9898  11.8302 11.0828 11.5102 10.1469
11.3195 10.2194 10.7989 10.3984 11.5173 11.3189 8.7463  10.1746
11.6450 10.1972 11.3678 11.5550 10.9086 9.8795  10.0465 11.1238
11.1228 9.9654\
MYO3A   0.0000  2.1912  4.0286  0.0000  0.0000  0.4059  0.0000
0.8314  0.0000  5.6545  6.1841  0.4849  0.0000  2.1894  0.0000
0.0000  0.6221  0.0000  1.3199  0.0000  4.5174  0.4820  5.6003
0.0000  2.0961  0.0000  6.8840  1.3471  1.3857  0.0000  8.2085
0.0000  0.0000  0.0000  0.0000  0.0000  0.0000  0.0000  0.0000
0.0000  0.0000  2.8124  0.0000  0.4326  3.5062  0.0000  0.0000
0.0000  0.4402  0.0000  0.0000  0.0000  0.0000  2.6047  9.0778
```

0.0000 0.0000 0.8754 0.0000 2.5512 2.0680 4.6821 0.0000
0.0000 3.0468 0.3906 0.0000 0.4832 0.0000 1.3773 0.0000
0.0000 0.0000 3.5868 0.0000 0.4698 0.5548 0.5408 2.4028
0.6159 0.0000 0.0000 1.5572 0.6718 0.0000 0.0000 0.9284
0.0000 1.4671 4.1839 4.2273 0.4848 0.5215 2.7067 0.0000
0.0000 6.0666 2.8016 0.0000 0.0000 0.0000 0.0000 0.0000
0.7358 0.0000 0.0000 0.0000 1.4556 4.6291 3.0071 0.5136
0.6215 0.0000 0.0000 0.5141 0.6116 0.5276 0.7641 0.0000
0.6089 5.1501 0.0000 4.6283 0.0000 4.4936 0.9214 0.0000
0.0000 2.1156 2.9531 0.3935 0.0000 0.5526 0.0000 1.7059
0.0000 0.0000 1.5206 1.6679 2.2561 0.6010 0.0000 1.7133
1.2444 0.0000 1.4974 0.0000 4.2762 0.0000 0.0000 0.8737
0.0000 4.5146 5.9226 0.0000 2.2918 2.6463 1.6526 2.7383
0.5416 0.0000 0.0000 1.5791 5.7171 1.0531 0.0000 0.0000
0.9722 0.0000 0.0000 0.0000 0.0000 0.0000 9.0296 0.0000
0.0000 5.5912 4.2093 8.0335 0.6314 0.0000 0.0000 5.7001
0.0000 0.0000 0.0000 0.0000 0.0000 0.0000 0.0000 0.0000
0.0000 0.0000 0.0000 0.0000 6.9802 0.0000 3.5298 0.3736
0.0000 0.0000\
TMC6 9.6910 8.6535 11.3575 9.3559 10.1880 11.0276 10.5243
11.3458 10.5486 8.3369 10.4164 10.6398 10.5952 11.7200 9.8795
9.2974 11.3328 10.4868 9.6414 11.3165 11.2728 8.2558 10.0820
9.5943 7.6157 11.5722 9.5603 10.3804 10.5052 9.7459 8.1807
8.4727 10.2975 10.4777 10.6613 9.1150 11.4189 8.9258 10.6331
9.9133 10.4937 8.9217 10.9762 10.1224 7.6950 10.9885 10.1530
10.2444 8.2372 10.4393 11.1272 10.7524 9.6506 8.5814 11.7520
9.7843 11.5024 10.7906 9.9816 10.6963 8.6656 9.8830 9.3921
9.3526 8.7283 10.9329 10.3282 10.3888 11.1041 12.0126 11.9722
9.7795 12.0214 11.6215 10.0270 9.5081 10.2807 9.7352 9.1411
9.8778 10.5481 10.1299 8.9366 10.7210 10.0313 10.6231 11.1385
11.0050 10.3129 8.2520 9.1674 10.1002 10.7733 9.7570 9.4750
8.8147 10.1979 8.1119 9.7528 10.3475 8.8827 11.1482 9.4691
10.9945 9.0402 7.8001 9.5864 11.1844 8.9069 10.1088 9.1223
10.1622 12.5254 10.6812 9.5535 10.4384 9.1259 11.0692 9.3507
11.0175 9.9033 10.4669 11.1121 9.7368 9.4921 11.2894 9.8013
11.8921 8.2246 8.2857 10.8425 10.1489 10.4818 11.2204 10.6183
12.1533 10.2124 11.1569 9.1966 8.1123 9.2716 10.3638 10.1570
10.0639 10.7251 5.8346 10.9631 9.2541 9.8878 11.3990 9.3964
10.3649 9.8041 12.1112 9.9302 11.1291 8.7561 10.4440 9.8744
10.2080 9.5160 11.2563 8.9686 10.0719 10.0408 8.5314 10.4215
10.7980 10.0952 10.6499 9.3695 10.1711 11.0322 12.3560 11.0132
9.8518 8.5191 8.5759 9.5070 10.1443 9.9565 10.9496 9.6614
8.8125 9.7354 9.5838 9.9347 11.0978 10.6256 9.1179 9.7685
11.0209 9.9672 10.5034 9.7916 11.4775 10.5300 7.9898 10.1541
8.9497 11.2773\
KIAA1958 4.8109 5.2138 5.1171 5.6423 7.0559 6.7793
6.3558 5.7445 5.8409 5.5482 4.6778 5.9756 5.2641 5.7470
6.2217 4.1868 3.0015 5.2251 4.8296 5.6283 4.5506 6.3702
5.4259 5.2799 5.7263 4.3449 5.7393 4.3250 4.2282 6.2050
4.7461 5.8476 5.9352 5.2197 7.2677 5.6767 6.8706 6.4575
4.1567 6.5642 3.2011 6.0370 4.1496 6.4020 4.7499 4.6139
2.8871 6.3576 4.9360 3.3218 6.6149 5.3805 5.2676 4.3564
6.3625 4.8594 5.7403 4.8348 5.9062 4.5758 4.7303 6.5535
5.1464 5.3381 5.5360 6.7832 4.3588 6.1499 5.0002 3.3298

| | | | | | | | |
|---|---|---|---|---|---|---|---|
| 5.0996 | 7.0564 | 4.2154 | 4.8740 | 5.3294 | 5.3588 | 5.3194 | 5.9842 |
| 4.2300 | 6.1118 | 4.3026 | 6.4437 | 4.6038 | 5.4300 | 4.4389 | 4.4553 |
| 5.5285 | 4.1248 | 5.8111 | 3.8730 | 4.0456 | 6.0809 | 4.3331 | 4.0318 |
| 5.1227 | 5.6889 | 5.9284 | 5.2732 | 5.4782 | 4.9194 | 3.8458 | 5.6114 |
| 6.4467 | 5.0122 | 4.1793 | 5.0620 | 2.9263 | 5.4321 | 5.2647 | 3.0946 |
| 6.2365 | 5.8865 | 4.5244 | 5.9307 | 4.9122 | 6.3538 | 6.5229 | 5.6605 |
| 5.8198 | 3.6498 | 5.5663 | 5.8812 | 5.0366 | 6.5576 | 4.9889 | 4.0178 |
| 6.6570 | 4.5024 | 5.1216 | 6.3784 | 6.1737 | 5.1122 | 4.9480 | 4.2436 |
| 3.0945 | 3.7958 | 4.6477 | 3.6106 | 6.5841 | 5.6708 | 3.9988 | 3.5569 |
| 4.1774 | 6.3212 | 6.6798 | 6.7866 | 5.9327 | 4.4681 | 4.9438 | 5.0472 |
| 5.8163 | 3.1371 | 6.9536 | 4.4955 | 6.7808 | 6.1453 | 4.9822 | 6.7938 |
| 4.1296 | 4.2986 | 6.5779 | 4.9890 | 5.2203 | 4.3471 | 6.6475 | 7.1601 |
| 4.4181 | 6.5787 | 4.5826 | 5.1328 | 6.7772 | 5.4435 | 4.9972 | 5.2978 |
| 3.4255 | 5.2066 | 4.8348 | 3.5773 | 6.7303 | 5.2609 | 3.1647 | 5.3314 |
| 6.0456 | 3.8508 | 4.7540 | 5.1791 | 5.6469 | 4.5288 | 3.7742 | 6.0866 |
| 6.3200 | 5.9338 | 4.4411 | 5.0123 | 5.3732 | 3.7138 | 4.8425 | 5.1625 |
| 6.6315 | 5.7587 | 4.4645\ | | | | | |
| APIP | 9.5148 | 8.4152 | 7.2971 | 9.3553 | 8.9854 | 8.3834 | 8.3331 |
| 9.0406 | 8.3380 | 8.6514 | 8.8600 | 9.0950 | 8.5997 | 8.3558 | 9.6701 |
| 7.6296 | 9.3380 | 8.2930 | 8.1204 | 8.1199 | 7.7841 | 8.2677 | 8.7208 |
| 9.3591 | 8.9323 | 8.3678 | 8.8363 | 8.8482 | 8.9815 | 8.8130 | 8.6182 |
| 7.9006 | 8.7235 | 8.5650 | 8.4086 | 8.4485 | 7.9074 | 9.4763 | 9.7383 |
| 8.4986 | 9.2393 | 8.6096 | 7.5664 | 8.9401 | 8.9434 | 8.4727 | 6.7442 |
| 8.5133 | 8.6868 | 8.7718 | 7.7135 | 8.2974 | 8.4574 | 8.9769 | 7.6133 |
| 9.0757 | 8.2840 | 8.3132 | 7.8154 | 8.7877 | 9.0885 | 8.8922 | 8.9085 |
| 8.6082 | 8.6161 | 8.8734 | 9.3106 | 7.9601 | 8.2061 | 7.9617 | 8.7424 |
| 8.8725 | 8.2499 | 8.2676 | 8.9637 | 9.0482 | 7.6139 | 8.1827 | 8.5799 |
| 8.3274 | 8.7957 | 8.6514 | 8.2416 | 9.5134 | 8.8411 | 7.8369 | 8.2234 |
| 9.1509 | 8.8444 | 9.0611 | 8.5399 | 8.1540 | 8.3708 | 7.5713 | 9.2573 |
| 8.5892 | 6.2699 | 8.6132 | 8.2062 | 8.3576 | 7.7927 | 8.6849 | 9.1753 |
| 9.0562 | 9.4187 | 8.2505 | 7.7297 | 8.8630 | 8.3250 | 7.7580 | 8.3787 |
| 9.0821 | 6.5750 | 6.8459 | 8.4756 | 9.3243 | 8.4161 | 7.9948 | 9.5773 |
| 7.7623 | 8.4856 | 8.5258 | 7.8437 | 8.5250 | 8.9244 | 8.1958 | 9.6543 |
| 7.7801 | 8.9552 | 8.8592 | 8.2911 | 8.7090 | 9.2953 | 8.9476 | 7.2232 |
| 6.3991 | 8.4289 | 5.4591 | 8.4423 | 8.7430 | 8.7545 | 9.6102 | 8.8821 |
| 9.2352 | 9.2224 | 8.3373 | 8.8904 | 8.5571 | 8.6090 | 8.0600 | 9.7218 |
| 8.4266 | 8.1535 | 7.4811 | 9.6521 | 8.7245 | 8.6341 | 8.6749 | 9.4166 |
| 9.2878 | 9.3142 | 8.5942 | 9.1058 | 8.5261 | 8.7450 | 8.5646 | 7.7913 |
| 9.1227 | 8.8958 | 9.0571 | 9.1983 | 8.8569 | 6.4072 | 7.6996 | 6.9484 |
| 8.4782 | 8.7056 | 8.9654 | 8.8384 | 8.4801 | 8.4191 | 8.7028 | 9.3035 |
| 8.9998 | 9.1009 | 9.7727 | 8.1256 | 8.6339 | 8.7106 | 9.0769 | 8.5979 |
| 8.6413 | 9.8278 | 8.4452 | 8.6599 | 8.5183 | 8.0883 | 9.0055 | 7.9207 |
| 8.5529 | 8.4453\ | | | | | | |
| TMC1 | 0.5526 | 1.6912 | 2.0229 | 5.2979 | 1.4681 | 4.4022 | 1.2456 |
| 0.0000 | 1.2625 | 3.6379 | 2.8551 | 0.0000 | 1.6409 | 2.4930 | 3.6198 |
| 0.0000 | 0.0000 | 0.0000 | 1.9975 | 0.0000 | 1.6493 | 4.6091 | 2.5783 |
| 2.1767 | 1.2653 | 0.0000 | 1.8490 | 3.2853 | 2.0792 | 0.0000 | 0.0000 |
| 1.2789 | 0.5159 | 1.3374 | 1.8080 | 0.0000 | 3.3777 | 1.7231 | 0.9336 |
| 3.2351 | 4.3859 | 2.2062 | 0.6231 | 0.7649 | 1.0260 | 3.0403 | 6.3843 |
| 0.0000 | 0.0000 | 4.1871 | 1.0749 | 0.0000 | 1.9420 | 2.8784 | 0.8153 |
| 2.7443 | 0.0000 | 1.8088 | 1.9041 | 0.0000 | 1.7140 | 1.6116 | 0.9583 |
| 0.5968 | 3.3334 | 2.1442 | 0.0000 | 0.0000 | 1.3082 | 2.3953 | 0.0000 |
| 3.1387 | 0.0000 | 1.1245 | 2.9560 | 1.1076 | 1.7421 | 4.5412 | 2.4028 |
| 1.3771 | 0.5659 | 1.9437 | 1.5572 | 7.1024 | 0.9110 | 0.0000 | 0.5376 |

2.0646 1.4671 0.0000 0.6361 0.0000 0.0000 2.0255 0.0000
0.0000 0.0000 2.9802 5.0693 0.0000 0.0000 0.4986 0.5352
1.2208 6.6964 5.5469 4.8108 2.2732 0.5426 0.0000 2.7134
3.4895 0.0000 2.5850 2.5132 0.6116 2.7528 1.9235 2.7457
0.0000 0.4553 0.5886 0.6224 2.1590 2.1978 1.2272 3.6042
0.0000 1.7575 1.6358 0.3935 1.7472 3.0819 5.1217 0.0000
2.0117 1.6954 0.0000 1.8532 3.5743 4.8008 1.5646 0.0000
2.7936 1.9910 0.0000 3.1266 0.5454 0.5814 4.0325 2.8564
2.5542 1.4965 0.5862 0.9437 0.0000 2.6463 0.0000 2.2048
1.4969 3.2259 1.0431 0.5821 1.1954 2.7513 3.2849 3.1709
0.7148 3.4214 0.9420 1.2980 0.0000 0.0000 0.0000 0.0000
1.7522 2.0214 0.6293 2.8900 1.9052 0.5545 0.0000 1.6771
3.2946 0.0000 1.3929 0.0000 0.0000 0.0000 0.0000 1.8202
3.8231 0.9737 0.7547 6.2734 0.0000 0.0000 2.0583 4.0069
3.4196 0.0000\
GPR15 0.0000 0.5319 2.0229 0.4935 1.8679 0.9816 0.0000
0.0000 0.0000 1.4796 0.4291 0.0000 0.4363 2.2979 0.9286
1.4442 0.0000 0.0000 0.9983 0.0000 0.0000 0.8427 0.0000
0.9110 0.0000 0.0000 0.0000 0.5990 0.0000 1.3312 0.0000
0.0000 0.0000 0.4664 1.3224 0.0000 0.0000 1.1048 0.0000
0.7666 0.6649 0.8506 0.0000 0.0000 0.0000 0.9285 1.5457
1.1317 0.0000 0.0000 2.3384 0.7768 0.0000 0.0000 4.5723
0.0000 1.0734 0.0000 0.7533 0.0000 0.0000 0.8658 1.5290
0.0000 0.0000 4.0754 0.5980 0.0000 1.3082 0.0000 0.0000
0.0000 0.0000 0.0000 0.9270 0.0000 1.5242 0.0000 0.0000
1.0461 0.0000 0.0000 0.0000 1.4748 0.0000 0.6199 0.0000
0.0000 0.9125 1.2149 1.0759 1.5834 0.0000 0.6909 0.0000
0.0000 0.5233 0.9402 0.0000 0.5504 0.0000 1.6155 0.0000
4.9514 0.0000 0.0000 0.0000 0.0000 0.0000 0.0000 2.7134
0.6215 0.0000 0.0000 0.8924 0.6116 0.5276 0.4321 0.0000
1.0358 0.4553 0.0000 1.0557 0.0000 0.5970 3.0272 0.0000
0.0000 1.7575 1.8193 0.7024 1.1234 0.5526 1.6184 0.0000
0.0000 0.0000 0.0000 0.0000 0.0000 0.0000 0.0000 0.6506
0.9357 0.8022 0.0000 0.6735 0.0000 0.0000 0.0000 2.1141
0.0000 1.9000 0.0000 0.0000 0.4374 1.5425 0.0000 0.6000
0.0000 0.0000 0.0000 0.9957 0.4033 0.4418 0.5023 0.0000
1.1906 0.5276 2.3633 1.0663 1.2195 0.0000 0.0000 0.0000
0.0000 0.0000 0.0000 0.4012 0.6314 0.0000 0.6902 0.0000
0.0000 0.7366 1.3929 0.0000 0.0000 0.0000 0.9785 0.0000
0.0000 0.0000 1.2477 0.4985 0.0000 0.0000 1.3688 0.3736
1.1797 0.0000\
TMC3 0.0000 0.0000 1.1543 3.3759 0.0000 2.1935 0.0000
1.1171 0.4327 1.6538 0.4291 0.0000 1.0422 0.0000 1.8915
0.0000 0.6221 0.0000 0.0000 0.0000 0.6175 2.4232 4.6298
0.9110 0.5538 1.3599 1.0809 0.5990 2.3312 0.0000 0.0000
0.5612 0.0000 0.4664 0.5853 0.0000 4.2000 2.9292 0.5410
0.0000 0.0000 1.1407 0.0000 0.0000 0.0000 1.4889 0.0000
0.0000 1.0498 0.4764 1.0749 0.0000 0.0000 1.4299 0.4645
0.9635 2.7053 0.0000 1.2457 0.5715 0.0000 1.6116 0.5573
0.5968 1.1445 0.0000 0.5980 1.1331 0.0000 0.6160 0.7662
0.7001 1.9166 0.4786 1.2339 1.1076 2.2486 4.3614 0.6894
0.0000 0.5659 0.0000 3.2356 0.6718 2.1768 2.2503 1.2355
0.6140 0.9125 0.5265 0.6361 0.8470 3.9003 0.6909 0.0000
0.0000 0.9065 0.9402 0.8845 0.5504 2.4487 0.4986 0.5352

| | | | | | | | |
|---|---|---|---|---|---|---|---|
| 1.2208 | 1.1338 | 0.8176 | 1.2143 | 1.0326 | 0.5426 | 0.5877 | 3.1204 |
| 1.0545 | 0.0000 | 1.0000 | 1.4396 | 3.0691 | 1.6815 | 1.7841 | 0.0000 |
| 0.6089 | 0.4553 | 0.0000 | 0.0000 | 0.0000 | 4.9415 | 0.5331 | 0.6149 |
| 1.6629 | 0.5619 | 1.1792 | 0.0000 | 0.6684 | 1.5194 | 2.7051 | 0.8107 |
| 0.0000 | 0.0000 | 0.0000 | 1.4553 | 0.5056 | 1.0240 | 1.3036 | 1.4380 |
| 1.4986 | 0.0000 | 1.9011 | 0.0000 | 2.0758 | 1.5778 | 1.2240 | 1.8061 |
| 1.5604 | 1.2425 | 1.0019 | 1.7261 | 0.0000 | 0.5638 | 0.6894 | 0.6000 |
| 0.0000 | 1.8525 | 0.6138 | 0.0000 | 0.4033 | 0.0000 | 1.1696 | 0.0000 |
| 1.3802 | 1.4679 | 0.0000 | 0.0000 | 0.0000 | 0.0000 | 4.3908 | 0.0000 |
| 1.9121 | 0.9063 | 0.0000 | 1.5479 | 6.0520 | 0.0000 | 0.6902 | 0.0000 |
| 0.5055 | 0.7366 | 2.0881 | 0.0000 | 0.0000 | 0.6567 | 0.0000 | 0.8827 |
| 1.2686 | 0.9737 | 0.7547 | 0.8682 | 0.0000 | 1.8529 | 0.6112 | 1.1260 |
| 2.6966 | 0.0000\ | | | | | | |
| PKLR | 0.0000 | 0.0000 | 0.7902 | 0.8638 | 1.1895 | 1.6528 | 0.0000 |
| 1.2317 | 0.0000 | 0.0000 | 0.7858 | 3.1906 | 1.8362 | 1.4846 | 0.0000 |
| 2.1899 | 3.1261 | 3.4118 | 0.7385 | 0.7517 | 0.0000 | 0.6409 | 0.0000 |
| 1.4511 | 0.7552 | 0.0000 | 0.7583 | 0.0000 | 0.0000 | 0.0000 | 0.0000 |
| 0.9430 | 0.0000 | 0.6032 | 0.0000 | 1.9791 | 1.2797 | 4.1754 | 1.0523 |
| 0.5557 | 2.4889 | 0.0000 | 0.6231 | 0.0000 | 3.3242 | 0.0000 | 6.8030 |
| 0.0000 | 0.6015 | 4.2053 | 1.4586 | 0.0000 | 0.0000 | 0.7096 | 1.7609 |
| 1.0017 | 3.7028 | 0.0000 | 0.0000 | 0.8767 | 0.0000 | 1.1513 | 0.0000 |
| 1.5288 | 0.0000 | 0.0000 | 2.1559 | 1.6100 | 0.0000 | 1.8773 | 2.1244 |
| 0.0000 | 2.9479 | 0.0000 | 1.2144 | 1.1024 | 0.0000 | 0.0000 | 2.1634 |
| 0.0000 | 0.9854 | 1.1117 | 1.8827 | 0.8002 | 1.3174 | 2.2936 | 1.9208 |
| 2.0572 | 0.0000 | 0.9147 | 0.0000 | 0.0000 | 1.1068 | 2.1405 | 0.0000 |
| 1.3132 | 0.6693 | 0.0000 | 0.6863 | 0.9373 | 3.8141 | 0.6805 | 0.0000 |
| 2.6267 | 2.6030 | 0.5734 | 0.0000 | 0.0000 | 0.0000 | 0.0000 | 0.6338 |
| 0.0000 | 7.6682 | 0.0000 | 0.7853 | 0.0000 | 1.4885 | 1.7260 | 0.0000 |
| 1.3909 | 1.9764 | 0.0000 | 0.0000 | 0.0000 | 0.0000 | 0.0000 | 0.0000 |
| 4.1282 | 0.7534 | 0.6537 | 0.5836 | 1.5348 | 0.0000 | 2.2132 | 0.0000 |
| 7.9572 | 2.0554 | 9.0599 | 0.0000 | 0.6932 | 0.0000 | 0.0000 | 4.2292 |
| 0.0000 | 0.0000 | 0.7281 | 1.8878 | 0.0000 | 1.6571 | 5.5928 | 1.1555 |
| 5.3783 | 1.7223 | 0.0000 | 1.6686 | 0.5638 | 0.7922 | 0.5302 | 0.0000 |
| 4.0092 | 0.0000 | 0.0000 | 0.0000 | 4.0889 | 0.0000 | 0.0000 | 0.0000 |
| 1.1578 | 0.0000 | 0.0000 | 1.2720 | 1.6825 | 0.0000 | 0.0000 | 7.4964 |
| 0.7028 | 1.7163 | 0.0000 | 0.0000 | 1.6106 | 3.6971 | 0.0000 | 2.3333 |
| 1.4416 | 0.0000 | 0.0000 | 0.0000 | 3.0388 | 2.2043 | 0.8868 | 0.6030 |
| 1.1965 | 2.5468 | 0.0000 | 0.7207 | 5.2506 | 5.6908 | 2.3855 | 0.0000 |
| 0.0000 | 0.0000\ | | | | | | |
| TECRL | 0.0000 | 0.0000 | 0.0000 | 0.0000 | 0.0000 | 0.0000 | 0.0000 |
| 0.4748 | 0.0000 | 0.0000 | 0.4291 | 0.0000 | 0.0000 | 0.0000 | 0.0000 |
| 0.0000 | 0.0000 | 0.0000 | 0.0000 | 0.0000 | 0.0000 | 0.0000 | 0.0000 |
| 0.0000 | 0.0000 | 0.0000 | 0.0000 | 0.0000 | 0.0000 | 0.0000 | 0.4127 |
| 0.0000 | 0.0000 | 0.0000 | 2.8081 | 0.0000 | 0.0000 | 0.0000 | 0.0000 |
| 0.0000 | 0.0000 | 0.0000 | 0.0000 | 0.0000 | 0.0000 | 0.0000 | 0.0000 |
| 0.0000 | 0.4402 | 0.0000 | 0.0000 | 0.0000 | 0.0000 | 0.0000 | 0.0000 |
| 0.0000 | 0.0000 | 0.0000 | 0.0000 | 0.5715 | 0.0000 | 0.0000 | 0.0000 |
| 0.0000 | 0.0000 | 0.0000 | 0.0000 | 0.4832 | 0.0000 | 0.6160 | 0.0000 |
| 0.0000 | 0.0000 | 0.0000 | 0.0000 | 0.0000 | 0.0000 | 0.0000 | 0.0000 |
| 0.0000 | 0.0000 | 0.0000 | 0.0000 | 0.6718 | 0.0000 | 0.0000 | 0.0000 |
| 0.0000 | 0.0000 | 0.0000 | 0.0000 | 0.0000 | 0.0000 | 0.0000 | 0.0000 |
| 0.0000 | 0.0000 | 0.0000 | 0.0000 | 0.0000 | 0.0000 | 0.0000 | 0.0000 |
| 0.0000 | 0.0000 | 0.0000 | 0.0000 | 0.0000 | 0.0000 | 0.0000 | 0.0000 |
| 0.0000 | 0.0000 | 0.0000 | 0.0000 | 0.0000 | 0.0000 | 0.4321 | 0.0000 |

0.0000 1.5136 0.0000 0.0000 0.0000 0.5970 0.0000 0.0000
0.0000 0.0000 0.0000 0.0000 0.0000 0.0000 1.9109 0.0000
0.0000 0.0000 0.0000 0.0000 0.0000 0.0000 0.0000 0.0000
0.0000 0.0000 0.0000 0.0000 0.5454 0.0000 0.0000 0.0000
0.0000 0.0000 0.0000 0.0000 0.0000 0.0000 0.0000 0.0000
0.0000 0.0000 0.0000 0.0000 0.0000 0.0000 0.0000 2.9165
0.4012 0.0000 0.0000 0.0000 0.0000 0.0000 0.0000 0.0000
0.0000 0.0000 0.0000 0.0000 0.0000 0.0000 0.0000 0.0000
0.0000 0.0000 0.0000 0.0000 0.0000 0.0000 0.0000 0.0000
0.0000 1.9604 0.0000 0.0000 0.0000 0.0000 0.0000 0.0000
0.0000 0.0000\
EIF2B4 10.2669 9.6284 9.3765 10.3353 10.5934 9.7080 10.2149
10.3622 10.0737 9.9050 9.8143 9.7793 9.9693 10.2678 9.7015
10.7165 10.5953 10.5397 9.7117 10.5393 9.9306 10.0635 9.4981
9.7095 9.7477 10.4635 9.4473 10.5335 9.9367 9.7001 10.2577
10.4941 10.1492 10.1897 9.6439 10.6551 9.8950 10.1573 10.1621
10.2699 10.3811 9.6687 10.3866 9.7676 9.5210 10.1446 11.7938
9.5501 10.1509 11.0015 10.4249 10.0979 9.8007 9.5504 10.1410
10.1130 9.9456 9.8516 9.7189 10.3225 9.4834 9.9123 10.0278
10.7943 9.9039 9.4754 10.4326 10.0965 10.2433 10.5053 10.9289
9.9824 10.3192 9.9706 9.5076 9.6784 11.1215 9.9731 10.2528
9.7453 10.4944 9.8656 9.6454 9.7968 10.5085 10.1179 9.9213
10.8176 9.8964 9.8710 9.8034 9.6887 9.9729 10.2566 10.2691
9.5612 9.4452 9.5442 9.4837 9.8245 11.4462 9.9358 10.3746
10.6663 10.5243 9.5583 10.4934 9.5100 9.7550 9.5000 10.6639
9.9453 10.7544 11.4528 10.3398 10.0116 9.6014 10.1871 9.8655
10.3397 10.4922 10.0004 10.0236 10.2697 9.5710 9.4292 10.2108
11.4616 9.3308 9.7000 9.9427 10.6988 10.2805 10.5474 11.4814
11.3535 10.6302 11.1039 9.7391 9.6769 10.4952 10.3476 10.6907
9.7428 9.7187 9.6057 9.1082 9.5495 9.9528 10.9344 9.9672
11.4111 9.7737 10.3859 9.8702 10.1729 9.6302 9.7172 10.1255
10.6365 10.7869 9.6181 9.9476 10.4904 10.0914 9.9564 9.6868
9.2629 9.8522 10.0845 10.1244 11.1638 9.7458 9.5875 10.6317
9.8805 10.1128 9.7952 9.7701 10.1088 11.1501 9.7906 10.3329
10.2602 10.2283 9.9316 9.9014 10.5134 10.0553 10.1335 9.7425
9.7013 10.2341 10.8530 9.9347 10.5272 10.9093 9.6451 9.4405
9.9697 10.6291\
EIF2B5 10.3606 9.5230 10.6226 10.3165 10.5609 10.3922 9.9272
10.4720 10.8137 10.1859 10.5275 10.3477 10.3456 10.1977 10.1979
10.6140 11.9881 10.2952 9.7673 10.8906 10.4962 10.4023 9.8803
10.3874 9.3793 10.4565 11.5628 10.3477 10.5243 10.4007 10.7812
10.5524 11.3454 10.2912 10.2146 10.9088 9.9330 10.3413 10.1961
10.2446 10.8714 9.4763 10.3156 11.8233 9.6212 9.9866 12.4100
10.1023 10.1685 10.7569 10.0861 10.3121 10.4895 9.7012 11.0356
9.9375 9.5006 10.2538 9.7982 10.7868 9.4871 10.8848 10.1615
10.6046 9.9094 10.0751 10.6237 9.8749 10.3019 11.3840 11.1768
10.0922 10.3379 11.6285 10.0332 10.4933 10.2660 10.0307 10.5754
10.1788 10.8573 10.9016 9.7980 11.3371 10.7247 9.8739 10.6685
10.7329 10.7376 10.1265 9.6236 10.1927 10.2002 10.5116 10.3948
9.7407 10.1325 9.6732 10.7395 10.1941 11.4250 10.0424 11.1389
11.2567 10.8992 10.8863 10.3757 9.9902 10.6262 10.7138 10.5594
10.2516 10.8789 11.0451 10.4938 10.3336 10.4364 9.6736 10.4204
10.3680 11.0971 9.8983 10.0844 10.3326 9.9907 10.3926 11.5990
10.8832 9.5437 9.8360 10.2128 10.7962 10.4523 10.2368 10.3803

```
10.7300 10.7883 10.1587 10.2177 9.7769  10.7333 10.4552 10.4899
10.1021 9.9678  9.7524  9.9299  9.9936  10.2192 10.5209 10.1239
12.1715 10.1155 10.2535 9.8136  10.2344 9.7782  10.5962 10.8108
10.3973 10.7471 10.2918 10.3367 10.3795 11.0399 10.3262 9.6015
10.1587 10.6144 11.2972 10.4158 10.3194 11.1872 10.5815 10.6304
10.0464 10.0847 9.7409  10.3460 10.0510 10.4419 10.4598 10.8107
10.3763 10.1089 10.1338 10.4764 10.8273 10.6839 10.4027 10.1666
10.0687 10.7254 9.6314  10.7800 11.2049 12.0825 9.9689  10.2005
10.4093 10.8113\
EIF2B2  10.0318 9.2943  8.6326  8.8450  9.9356  9.4503  9.2705
10.0586 9.3070  9.0117  8.9703  9.5041  9.7578  8.8733  9.2320
8.7979  9.3191  9.7994  9.3231  9.3204  8.9717  9.2317  9.1167
9.0000  9.4524  8.7853  9.8881  9.7738  9.4958  10.1947 9.3061
9.6080  9.9181  9.6898  9.5660  10.5415 8.7727  8.6293  9.6073
9.0758  9.4705  9.1284  9.3665  9.3364  8.9879  8.8871  9.2902
9.4529  9.9831  9.4820  10.0698 9.9713  8.9229  8.9778  9.6673
8.6679  9.3204  9.3073  9.3617  10.0933 9.1110  9.0682  10.2237
9.6416  9.2511  9.5334  9.2036  9.4320  9.2821  9.2280  10.2830
9.6549  9.6493  8.9238  9.1723  9.3167  8.9727  9.2043  8.9524
9.1554  9.8707  9.7389  9.1390  9.6983  9.3605  9.6893  9.7876
9.1365  9.4288  9.8648  8.9933  9.6390  9.6877  8.7719  10.0980
9.0667  9.7566  9.1260  10.0347 9.3614  9.5392  9.8054  9.5943
9.2025  9.7008  9.7340  8.9274  9.6821  9.5601  9.4333  8.9853
9.5152  9.5826  9.5736  8.8398  10.2485 8.8224  9.6637  9.3646
9.3172  10.4561 9.5198  9.5849  9.9545  9.1855  9.9717  9.6587
9.3140  9.0384  8.7751  9.2879  9.6429  9.5880  8.4320  10.1659
10.5062 9.7726  9.0344  9.0538  8.9178  9.3695  9.0700  9.2876
9.3270  9.4056  8.7556  9.3796  9.2112  10.4568 9.4002  9.0059
9.4183  8.9368  10.0652 8.9567  9.5090  9.0181  8.9673  9.7477
9.8055  9.6802  8.6752  9.2545  9.5300  8.8229  9.2913  8.3502
8.6034  9.3257  8.4688  9.3838  9.5282  9.7274  9.2617  8.7843
9.2558  9.1987  9.0773  9.0448  9.1280  9.5551  9.8900  10.4146
9.5661  9.2572  9.0365  9.7053  9.9527  9.0425  9.9956  9.5682
9.5322  8.7316  9.5518  9.1160  9.0993  9.1414  8.8353  9.3021
8.9620  9.8196\
EIF2B3  9.5436  8.2676  8.7806  8.7648  9.2412  8.9115  8.1815
9.4523  9.3701  8.9016  9.5115  9.0234  9.7789  8.6239  9.1449
8.4599  8.2781  9.0804  8.6441  8.8438  9.4093  8.9773  8.4496
8.4712  9.3934  10.4619 9.2942  9.2178  8.7374  8.3773  8.9548
8.1875  9.0102  8.8012  9.0777  9.6249  8.3979  8.4908  8.3642
8.7168  9.4159  8.5255  8.6907  9.7419  9.0257  8.3214  10.4969
8.8830  8.6317  9.1296  8.9903  8.3372  8.8555  8.9083  8.4262
8.7240  8.0555  8.6037  8.7763  8.9181  8.9095  8.9193  9.5199
9.1173  8.8316  8.7579  8.9228  9.1289  9.0659  9.1231  9.6718
8.9927  9.3696  9.5673  8.9576  9.7930  9.0501  9.0296  8.9684
8.8100  9.6938  9.0262  8.8682  9.4796  9.4567  9.2483  10.0349
9.2984  8.6871  9.2745  8.9535  8.8337  10.0904 8.3099  9.4872
9.2402  9.0806  8.4096  8.9761  8.7932  10.1225 8.7970  8.7523
9.0186  9.6388  9.7753  9.0330  8.7367  8.6442  9.7326  8.6981
9.1416  10.2278 9.1293  9.3319  9.4612  9.4512  8.9555  9.4632
9.2329  8.9735  9.2941  8.8043  8.8002  8.6651  9.5784  9.4143
9.4438  8.8651  9.1127  8.7472  8.8839  9.2238  8.7257  10.4910
10.3952 9.4764  8.1459  8.9005  8.5392  9.0277  9.1703  9.3481
8.8009  9.0873  9.0430  8.6509  8.6213  9.3621  9.9585  8.4874
```

| | | | | | | | |
|---|---|---|---|---|---|---|---|
| 11.3353 | 8.6086 | 9.5546 | 8.7432 | 8.2920 | 8.7861 | 8.3308 | 9.3530 |
| 10.0613 | 8.6363 | 8.8791 | 9.1037 | 9.0204 | 8.4319 | 9.1183 | 9.1735 |
| 9.3793 | 8.8965 | 9.4161 | 8.8471 | 8.9312 | 9.7397 | 8.7455 | 9.1905 |
| 8.0223 | 9.0555 | 9.2710 | 8.9070 | 8.4040 | 8.7413 | 8.8850 | 9.1442 |
| 8.9226 | 9.3777 | 8.5444 | 8.7028 | 9.6205 | 8.8896 | 9.0201 | 9.0198 |
| 9.0549 | 10.2461 | 8.3401 | 8.4365 | 10.7652 | 10.2746 | 8.5116 | 9.0946 |
| 8.4659 | 10.3330\ | | | | | | |
| EIF2B1 | 10.2902 | 9.9550 | 9.0792 | 9.6074 | 10.3929 | 10.4171 | 9.7510 |
| 10.1286 | 9.6730 | 10.1169 | 10.1261 | 10.3908 | 10.4421 | 9.8635 | 9.8309 |
| 9.8074 | 9.5483 | 10.0434 | 10.0580 | 10.3865 | 9.7274 | 9.9507 | 9.9995 |
| 9.8434 | 10.0260 | 10.0801 | 9.3205 | 10.1645 | 9.9753 | 9.9948 | 10.0239 |
| 10.3962 | 11.2880 | 10.7779 | 10.2857 | 10.7242 | 9.9190 | 10.0720 | 10.1414 |
| 10.3410 | 9.9932 | 9.6165 | 10.0838 | 10.0163 | 10.1053 | 9.6609 | 10.5746 |
| 10.0961 | 10.2464 | 10.4051 | 10.5772 | 10.2744 | 10.1378 | 10.1850 | 10.3859 |
| 10.3175 | 9.7815 | 10.5743 | 10.0001 | 10.1913 | 10.0326 | 10.4527 | 10.5406 |
| 9.6943 | 10.0675 | 9.1860 | 9.4428 | 9.6612 | 10.0236 | 10.5005 | 10.6459 |
| 9.9731 | 10.1777 | 10.6171 | 10.4487 | 10.2736 | 10.4250 | 9.7009 | 10.0921 |
| 9.9688 | 10.2160 | 9.2131 | 9.7629 | 10.4532 | 9.9699 | 9.6270 | 10.2773 |
| 9.8798 | 9.7832 | 10.0661 | 9.9120 | 10.4375 | 10.3502 | 10.1823 | 10.2326 |
| 10.2351 | 9.3899 | 9.9554 | 9.5488 | 10.2764 | 10.4105 | 10.3252 | 9.1840 |
| 10.9679 | 10.5976 | 10.5479 | 10.4083 | 9.9270 | 10.2844 | 10.5526 | 9.6949 |
| 10.1244 | 9.6853 | 10.1599 | 10.1824 | 11.1625 | 10.3902 | 10.5246 | 9.9099 |
| 9.4746 | 10.6937 | 9.5069 | 9.8558 | 9.9148 | 10.0301 | 10.3077 | 10.6252 |
| 9.7182 | 10.0694 | 9.9976 | 10.8582 | 10.4155 | 9.9748 | 10.1796 | 10.1659 |
| 9.4292 | 10.5559 | 9.4241 | 9.9846 | 10.0044 | 10.2872 | 10.0818 | 10.0740 |
| 9.7565 | 9.6994 | 9.9389 | 9.9978 | 10.0751 | 10.3341 | 10.1724 | 9.9564 |
| 9.9937 | 9.6556 | 10.8355 | 9.1832 | 10.3117 | 10.0147 | 9.7784 | 10.1629 |
| 10.1605 | 10.1661 | 10.1729 | 10.0053 | 10.4907 | 9.7396 | 9.9363 | 10.2553 |
| 10.1759 | 10.1783 | 10.4886 | 10.0460 | 10.8908 | 11.1614 | 10.5202 | 9.0153 |
| 10.0852 | 10.2387 | 10.1857 | 9.9156 | 9.6547 | 10.3789 | 9.9241 | 10.9719 |
| 10.3492 | 9.7161 | 10.2326 | 9.5460 | 10.8323 | 9.9499 | 10.3576 | 10.3245 |
| 9.9449 | 10.0204 | 9.6715 | 10.2947 | 10.2589 | 10.6591 | 10.2282 | 9.8114 |
| 10.0856 | 10.2024\ | | | | | | |
| PRKAR2A | 5.6439 | 6.4511 | 7.4766 | 6.9937 | 7.8330 | 8.0021 | 7.7071 |
| 7.2238 | 7.1600 | 7.2133 | 6.7180 | 7.5483 | 7.4165 | 7.2945 | 7.8569 |
| 3.8845 | 4.0085 | 5.4300 | 6.3095 | 6.4515 | 5.5528 | 8.0808 | 7.2568 |
| 7.3289 | 6.6264 | 5.1269 | 6.1996 | 5.4501 | 5.0202 | 8.4226 | 6.5146 |
| 6.5430 | 6.6044 | 8.8793 | 8.1456 | 7.1720 | 7.8556 | 8.1117 | 5.6739 |
| 7.9349 | 5.6150 | 7.8222 | 5.1308 | 5.9986 | 7.5980 | 5.0276 | 2.2746 |
| 8.2766 | 8.5708 | 4.9419 | 8.0424 | 7.0156 | 6.9318 | 7.2297 | 6.6942 |
| 6.5195 | 7.7237 | 7.8897 | 7.7105 | 6.7672 | 8.1967 | 6.6629 | 8.1963 |
| 7.3018 | 5.9146 | 7.8426 | 6.7731 | 7.0900 | 7.6915 | 5.9476 | 5.2400 |
| 8.2491 | 4.2855 | 3.9224 | 7.2217 | 7.4223 | 5.8980 | 7.8266 | 4.3648 |
| 7.6411 | 5.5283 | 7.8944 | 6.4344 | 7.2891 | 4.8881 | 4.6529 | 7.3377 |
| 5.1273 | 7.3750 | 5.9592 | 7.6432 | 8.3331 | 4.7843 | 3.5917 | 5.8210 |
| 7.4915 | 8.1492 | 6.9925 | 7.8260 | 5.0865 | 4.8515 | 7.6378 | 8.2206 |
| 4.6411 | 7.1368 | 6.9154 | 5.2716 | 6.6637 | 6.7831 | 5.1778 | 7.5600 |
| 7.5243 | 3.5857 | 4.5236 | 7.6099 | 7.7648 | 8.6540 | 7.6440 | 7.6963 |
| 6.3081 | 6.3943 | 6.7524 | 8.7637 | 8.6381 | 7.6867 | 6.3638 | 7.0531 |
| 4.1963 | 7.0388 | 8.3146 | 7.5563 | 5.1366 | 6.7594 | 5.8650 | 4.1973 |
| 4.0146 | 5.0401 | 0.0000 | 8.4361 | 7.0160 | 6.9602 | 6.0783 | 5.1602 |
| 7.6667 | 8.3256 | 7.4485 | 7.4586 | 7.0586 | 6.6828 | 4.1457 | 7.4335 |
| 5.6641 | 8.5093 | 6.8176 | 7.7833 | 7.7191 | 7.3750 | 8.6737 | 5.3474 |
| 5.8792 | 6.6916 | 6.3784 | 6.3751 | 5.1632 | 8.4244 | 8.4903 | 5.9403 |

| | | | | | | | |
|---|---|---|---|---|---|---|---|
| 7.9479 | 6.6168 | 5.9169 | 8.4751 | 6.9607 | 6.2406 | 5.2371 | 3.8992 |
| 7.0835 | 5.8752 | 7.0648 | 4.0063 | 7.2111 | 4.5543 | 6.0977 | 6.7583 |
| 5.8701 | 7.2814 | 6.1481 | 7.5959 | 5.7034 | 4.1933 | 6.6850 | 7.9774 |
| 7.7591 | 4.5638 | 7.5516 | 7.6844 | 3.9204 | 4.4528 | 7.0133 | 8.3364 |
| 7.8666 | 3.6039\ | | | | | | |
| CSNK2A1P | | 7.2410 | 7.4091 | 9.2703 | 7.8545 | 8.5780 | 9.5145 |
| 9.2938 | 9.0063 | 9.6774 | 7.9303 | 8.5107 | 9.4961 | 8.2070 | 9.1222 |
| 8.4704 | 7.2194 | 5.3913 | 7.9540 | 7.2255 | 7.1759 | 6.7880 | 8.2633 |
| 7.4875 | 8.4090 | 8.2221 | 7.0790 | 8.9879 | 6.2310 | 7.2461 | 8.5852 |
| 8.8817 | 7.9885 | 8.4675 | 9.7097 | 9.8774 | 8.8058 | 8.5485 | 8.7499 |
| 6.8874 | 9.5139 | 7.3962 | 8.5931 | 6.4679 | 8.4234 | 8.2361 | 6.0649 |
| 6.4805 | 8.8848 | 9.5002 | 6.8873 | 8.8497 | 7.8227 | 8.2721 | 7.9163 |
| 8.7854 | 7.7689 | 9.2528 | 8.3321 | 8.9160 | 8.1119 | 8.4918 | 9.1351 |
| 8.6935 | 8.7800 | 7.4639 | 8.7600 | 8.1261 | 9.4419 | 8.2718 | 6.7557 |
| 7.5173 | 9.0432 | 6.9865 | 8.0856 | 8.3263 | 8.1158 | 8.7250 | 7.0441 |
| 5.6264 | 8.6471 | 6.8793 | 9.3717 | 6.8124 | 7.7766 | 6.1406 | 8.0937 |
| 10.1082 | 7.2142 | 8.7696 | 7.7246 | 8.0842 | 8.9571 | 7.5427 | 5.5377 |
| 7.8377 | 8.3427 | 8.9743 | 8.0730 | 8.5749 | 7.1376 | 6.4678 | 8.0600 |
| 8.9862 | 6.7296 | 8.0538 | 8.7530 | 6.2529 | 8.6341 | 7.7026 | 7.0742 |
| 8.5414 | 8.4376 | 4.7091 | 7.5925 | 8.5513 | 9.4858 | 8.9190 | 8.4447 |
| 9.1202 | 8.3833 | 9.9667 | 8.3664 | 8.1534 | 8.6794 | 8.3280 | 8.2714 |
| 9.0723 | 5.6260 | 8.2108 | 8.9525 | 9.5851 | 6.7184 | 7.8168 | 6.7266 |
| 8.0780 | 6.3054 | 6.3602 | 0.0000 | 8.7913 | 8.2500 | 7.0015 | 7.7143 |
| 5.7394 | 8.8229 | 9.0392 | 8.1338 | 8.9047 | 8.1023 | 8.2930 | 5.9801 |
| 8.1132 | 5.9859 | 9.1839 | 9.1713 | 8.7510 | 9.5347 | 8.0730 | 9.3766 |
| 7.1158 | 6.7798 | 8.1607 | 7.6694 | 7.7192 | 6.4070 | 8.4717 | 9.4807 |
| 7.1143 | 9.5980 | 7.9455 | 7.7739 | 9.3349 | 8.9233 | 7.9491 | 8.0174 |
| 5.2700 | 8.6141 | 7.5109 | 7.5920 | 9.3514 | 7.1399 | 6.5369 | 7.4831 |
| 10.5086 | 7.2762 | 9.2525 | 7.9070 | 8.7199 | 5.8046 | 6.0221 | 8.6078 |
| 8.7820 | 8.5968 | 8.1195 | 9.3741 | 8.5036 | 6.6437 | 4.0595 | 8.1241 |
| 8.7350 | 8.5880 | 5.2078\ | | | | | |
| XRRA1 | 8.9834 | 8.6807 | 9.4433 | 8.5794 | 8.7958 | 9.2480 | 8.7766 |
| 8.7753 | 8.5095 | 9.1577 | 9.1358 | 8.3285 | 9.2280 | 8.5980 | 8.6686 |
| 9.2135 | 9.7009 | 8.4283 | 9.2702 | 9.0052 | 9.2041 | 9.0692 | 9.4412 |
| 9.2143 | 9.3108 | 9.4200 | 7.7827 | 9.5430 | 8.8091 | 8.2848 | 7.8949 |
| 8.6528 | 8.6605 | 8.5121 | 8.9634 | 8.3090 | 8.0420 | 9.0010 | 9.0435 |
| 8.5914 | 8.9139 | 8.2098 | 8.7132 | 10.4194 | 8.8155 | 8.9040 | 9.5455 |
| 9.0487 | 8.9252 | 9.2040 | 8.7478 | 8.4193 | 8.1456 | 9.3387 | 7.4798 |
| 9.1873 | 7.9969 | 8.8262 | 8.5397 | 9.2169 | 9.2636 | 7.9454 | 8.6378 |
| 8.0569 | 9.5791 | 8.6462 | 8.6348 | 9.1959 | 8.2288 | 9.2337 | 9.0370 |
| 8.5101 | 8.9024 | 8.3610 | 8.2197 | 9.7212 | 8.5175 | 9.0125 | 9.6953 |
| 8.5065 | 8.6375 | 8.2950 | 9.3981 | 9.5070 | 9.6918 | 9.4159 | 7.9981 |
| 8.9141 | 9.0604 | 8.8034 | 9.1464 | 8.1037 | 9.5271 | 9.2948 | 9.3666 |
| 9.2871 | 8.2853 | 9.1070 | 8.0920 | 8.8430 | 9.3220 | 9.1008 | 7.6106 |
| 9.7229 | 8.7076 | 9.0051 | 8.6545 | 8.8250 | 9.5107 | 10.0766 | 9.1069 |
| 8.9871 | 10.5170 | 9.1685 | 9.0942 | 8.3664 | 8.6845 | 8.4150 | 8.5516 |
| 8.1237 | 8.6429 | 8.8275 | 7.8157 | 8.1469 | 9.2232 | 8.6486 | 9.8786 |
| 10.0179 | 8.7452 | 8.2673 | 8.4078 | 9.2289 | 8.7477 | 9.8818 | 8.7690 |
| 10.0055 | 10.1730 | 10.2397 | 8.5293 | 9.4984 | 9.2463 | 8.7889 | 9.1029 |
| 8.2588 | 7.5105 | 9.3580 | 9.2208 | 8.8911 | 8.9754 | 9.6572 | 8.5885 |
| 10.3997 | 8.3123 | 9.0786 | 8.9325 | 9.4995 | 9.3899 | 7.9752 | 9.3162 |
| 9.3279 | 8.1314 | 9.1967 | 8.9369 | 8.8808 | 7.4956 | 8.1262 | 9.3777 |
| 8.2969 | 8.6207 | 9.0819 | 8.8533 | 9.2434 | 8.3387 | 7.9954 | 10.2976 |
| 9.4857 | 9.3152 | 8.7451 | 8.2692 | 9.4615 | 9.5505 | 8.4967 | 8.9729 |

8.6776	9.1188	8.7292	9.2444	8.8422	9.0573	8.7368	8.8906
9.0301	8.8538	9.7498	8.4749	9.6398	10.1696	9.0722	9.0207
8.9151	9.6644\
C20orf56		1.9260	6.3522	5.9749	11.3958	12.1771	1.7112
5.4126	1.8984	1.2625	9.8952	7.0132	0.0000	2.4830	4.0280
8.2865	10.1812	6.2771	7.9204	5.2821	0.3755	1.0485	4.6091
3.0325	8.1021	5.9933	0.6062	0.0000	6.7418	5.3967	1.0077
0.4127	10.3102	0.5159	0.8184	0.5853	1.4920	2.3861	7.3911
8.7003	9.8680	9.4894	6.3669	3.0982	8.8326	2.0387	7.5888
3.4901	0.0000	6.1189	10.3339	8.0504	0.0000	3.4148	4.0593
7.2568	13.3116	1.4095	10.4413	1.4405	6.7990	1.4980	2.2329
8.8238	0.5968	8.8695	4.8879	8.1601	1.9201	2.8871	7.4526
1.7877	4.3479	0.0000	0.4786	7.7486	6.8829	4.7440	7.4223
10.3829	9.3563	4.9663	0.7073	7.9145	8.2142	9.7604	3.4854
9.1942	10.3829	0.0000	5.8966	0.6361	3.3484	0.0000	10.5623
8.1644	0.0000	1.2090	8.2982	10.9553	0.5504	12.6337	4.8200
0.0000	12.4561	7.8449	5.3852	5.3670	2.6388	4.4598	10.5661
12.3770	0.6215	0.0000	0.0000	3.1220	7.6782	9.8024	9.0074
10.2588	1.0358	1.5136	3.1797	1.8863	8.6284	4.4264	0.5331
0.6149	2.9068	8.4077	8.6565	1.5269	6.8197	7.2805	5.4295
0.0000	2.0117	9.9528	7.5824	7.0358	9.7040	8.9118	10.1610
8.9126	7.2615	5.5351	0.0000	3.3108	2.7033	5.0981	4.4839
10.6076	10.7990	11.2456	6.0591	7.6636	0.7727	8.3602	3.2594
3.7417	12.4860	7.6437	5.0129	9.7857	7.3015	7.3610	7.2178
0.0000	6.3652	9.6282	0.0000	6.4858	1.2195	7.0297	7.8918
8.9639	10.2077	10.2049	1.0660	3.8547	7.0471	7.3829	0.0000
0.5257	11.0537	6.0794	12.6279	1.9914	0.0000	5.4095	9.0269
11.1214	3.4417	3.4112	3.2618	10.7630	2.3335	8.9896	9.3107
7.5780	10.1825	1.5159\
C20orf54		9.2538	8.1905	9.3001	8.8127	6.0714	9.2393
6.3638	8.5009	8.9107	8.8376	8.7231	9.4482	8.0486	6.6794
8.4996	10.7954	9.6601	6.2469	8.3760	6.0662	7.0890	9.3808
7.9604	10.0036	5.6342	4.5193	9.2071	8.0695	8.8092	9.6673
2.7292	8.9417	8.5900	9.2631	7.3496	5.2425	10.5037	8.8701
9.0127	3.9427	7.6982	9.2197	7.7111	8.3417	5.3721	9.0606
6.9672	8.1341	4.4858	8.3302	9.3258	7.6283	5.9502	6.7285
10.1283	5.6958	8.4119	8.5127	9.2138	9.2587	7.2735	8.2809
8.8521	10.9013	7.6580	9.0614	9.8077	7.6743	10.0987	7.4438
8.3260	7.0799	5.4447	9.8595	8.6003	4.9908	6.6434	7.9564
9.1317	6.9229	8.5951	8.4125	8.3464	7.0059	8.1206	8.8404
8.5381	8.5630	9.9110	5.5886	5.9137	8.5668	3.0706	7.4799
5.7413	6.2594	7.5690	6.7181	9.7479	8.9707	5.8955	9.2279
9.3351	7.2795	6.5323	3.3434	6.7094	7.6665	8.7227	8.3524
8.7663	7.1341	8.0507	8.6366	8.2645	8.2231	7.7433	8.4676
9.1438	9.1446	8.9092	8.2964	8.0128	7.3014	7.2450	8.2205
7.9673	9.6563	7.9073	6.3110	10.7883	7.8171	8.6030	8.3398
6.4425	9.6657	4.8004	6.5033	7.0310	6.8579	8.8091	9.8405
8.1991	9.3053	9.7675	4.2994	10.9392	7.0930	8.9665	9.4002
9.6551	7.5779	9.0540	8.9925	8.5488	9.9728	7.9940	8.4644
7.3798	4.1947	8.1868	9.4609	8.2604	9.9942	8.8700	7.1131
8.1868	8.0887	10.2281	8.3125	5.7221	6.3427	5.5808	7.0701
9.2312	6.6789	7.9842	5.5525	6.6542	7.8660	8.7413	6.8698
8.7918	6.9389	8.6960	4.7844	8.5101	7.1208	8.4877	5.5122
7.4497	10.2511	11.0241	4.7794	8.1078	7.7626	5.2712	7.0596

6.8431	6.6815	8.1355\
TXNIP	12.5687	14.1528	13.4569	13.6002	11.8992	13.9251	12.0086
12.5277	10.6774	13.8809	10.5610	13.1486	13.0485	13.0153	11.7883
12.7213	10.5582	11.4577	13.7687	11.1085	11.9683	13.2900	12.8594
12.7681	15.5354	9.5138	10.5749	12.5951	13.6265	14.4339	8.8374
12.8968	11.9395	13.9588	11.9237	12.6858	13.0876	12.9022	9.4510
12.3594	12.0380	14.1981	13.0820	11.7942	16.0971	11.2268	11.5451
14.4415	9.9902	14.3260	13.3356	12.1967	12.9546	16.0464	12.4275
12.5132	11.6480	12.5888	13.4131	12.3561	14.8532	13.8074	11.4527
11.7516	14.3238	12.6273	11.3688	12.1349	12.1069	12.7304	12.6496
12.6486	12.4810	12.8671	13.8431	15.0090	12.2344	13.4774	13.8891
11.9932	10.5860	9.0375	14.7147	12.8166	13.0066	11.9038	12.7295
11.2777	13.3955	11.8503	15.1752	13.5657	13.7929	12.1114	12.8349
15.1235	10.7116	15.0452	12.3726	12.3761	12.0375	12.3362	13.9619
11.7112	11.5403	12.2939	13.9055	12.4588	12.5412	11.9860	14.3190
12.6270	10.0008	11.9061	10.5790	13.9627	12.4704	14.5938	12.3196
13.3746	12.3521	11.7117	13.8135	12.7398	14.3364	12.2414	10.5683
9.6691	15.3016	15.1687	12.4180	12.1301	12.3632	12.4346	11.9683
11.4252	11.6215	11.6743	14.5225	15.7899	11.0181	14.1763	11.8591
12.1415	12.3314	13.8117	14.1670	14.5198	12.0216	12.6752	14.9795
9.8022	12.9302	12.2241	11.3084	12.1591	15.6212	12.2044	10.1938
10.6716	12.3852	13.0380	12.2368	12.3971	11.7773	11.2576	12.0816
13.9102	11.1477	12.0483	11.9424	13.3306	11.3793	12.0658	11.3619
12.8170	14.2801	15.2284	11.6429	14.0490	13.2628	12.7376	10.4554
12.2499	11.2440	13.1765	13.3313	12.6942	11.9270	11.5459	11.8055
12.2249	12.2405	11.8634	12.6480	10.3217	10.5517	15.6549	12.9566
12.8783	12.1992\
SLC36A4	6.1965	7.9227	6.8221	5.6540	8.3970	7.3556	6.9483
8.1593	7.1315	6.2021	7.8454	3.6538	7.1450	6.9691	3.8176
4.5469	5.3346	8.3445	7.7515	7.3225	6.3889	3.5000	6.6503
3.7359	7.5122	4.3074	9.4739	6.7418	6.4610	6.4664	7.9567
3.3269	6.9234	6.8279	8.9924	2.9348	3.5611	2.6272	6.9578
5.2004	7.6230	7.9866	7.4239	8.3182	7.1906	6.2981	7.1143
7.0512	6.5834	5.9460	7.8551	6.8290	6.9251	7.3394	6.9505
4.7363	6.7192	7.0491	7.5402	5.5149	7.3278	7.9810	5.0263
6.8991	6.3596	4.4890	5.8062	6.4807	7.1270	8.0710	6.4462
7.7561	6.7955	5.6713	7.9248	1.5483	6.6834	7.1860	4.5270
7.4740	4.8268	7.5639	6.9993	8.3224	3.9970	8.3141	6.8654
5.1705	6.2562	9.2518	6.9083	7.2091	7.4161	6.6687	7.5344
7.4739	7.8841	6.3631	7.2280	6.0751	4.9328	4.6876	6.7230
7.3100	8.1110	6.8785	6.5559	7.6910	7.6803	7.2842	6.4420
7.6063	7.6712	6.6439	7.5353	6.8127	5.7187	6.9612	7.5206
5.7835	7.8311	6.9358	6.8226	6.9658	6.6204	7.2698	7.5096
3.7308	6.9692	7.8234	6.7244	7.0931	6.0210	8.9026	5.1876
7.0789	7.3315	6.6176	6.4250	6.7607	1.8416	5.1401	4.6065
6.3212	7.9276	7.2073	5.0498	5.5807	6.8031	3.2756	7.0634
7.3119	6.1707	7.0591	6.6004	7.3571	6.6643	6.9012	5.7988
7.0955	7.0958	6.3967	5.2769	6.9752	4.5591	8.2860	6.8488
7.5540	7.1026	7.0674	2.6939	6.1311	6.5931	3.0035	7.2283
8.0266	6.8520	7.3132	4.2446	6.7922	6.9626	5.7152	8.1344
3.7644	8.0196	4.0158	4.7963	5.8527	2.4884	7.5305	4.1936
7.3944	6.6548	8.3185	3.3334	5.2236	7.2228	6.8579	6.5104
6.5185	5.9339\
LOC389791		3.3026	2.6668	3.5887	4.0443	0.9133	4.1615

| | | | | | | | |
|---|---|---|---|---|---|---|---|
| 1.7160 | 3.5743 | 2.2773 | 2.7493 | 2.2662 | 4.1842 | 5.0191 | 1.4754 |
| 2.6827 | 5.5690 | 3.6848 | 2.8922 | 2.3192 | 2.5253 | 4.5506 | 2.8772 |
| 3.2153 | 4.7053 | 1.5220 | 2.5109 | 2.5443 | 2.9431 | 2.5456 | 2.5978 |
| 3.4068 | 3.3937 | 2.9780 | 4.8321 | 2.7012 | 2.5445 | 2.9214 | 3.3513 |
| 3.8729 | 1.4612 | 3.8546 | 2.4377 | 3.0042 | 2.5595 | 0.0000 | 1.4889 |
| 0.7135 | 3.0263 | 1.0498 | 2.9358 | 2.9335 | 2.7455 | 1.7111 | 2.6047 |
| 3.2833 | 2.9356 | 2.7053 | 2.6835 | 3.2061 | 1.7785 | 0.5422 | 5.3242 |
| 1.7473 | 3.3537 | 2.5466 | 3.2320 | 4.0808 | 1.9201 | 3.4386 | 1.6464 |
| 3.1224 | 2.7272 | 0.0000 | 1.1245 | 2.7779 | 2.9153 | 2.3844 | 2.4718 |
| 3.4332 | 1.8728 | 0.9714 | 1.1797 | 0.0000 | 2.7931 | 2.9265 | 4.9571 |
| 3.7216 | 2.7731 | 5.0145 | 1.8648 | 1.4126 | 2.4311 | 5.0155 | 3.5917 |
| 2.6337 | 0.0000 | 3.9476 | 2.9802 | 2.2653 | 4.2912 | 1.6914 | 3.0757 |
| 3.9837 | 1.5830 | 2.4273 | 1.1001 | 2.1046 | 2.2732 | 1.4990 | 2.3284 |
| 2.1442 | 2.8996 | 3.9213 | 5.2095 | 3.3810 | 4.0279 | 0.9131 | 5.4691 |
| 1.6868 | 2.0533 | 3.4619 | 2.5942 | 2.2557 | 0.8239 | 5.1641 | 3.3768 |
| 2.0665 | 2.7607 | 1.7575 | 2.9531 | 4.0327 | 2.5146 | 2.8211 | 3.1029 |
| 3.2165 | 0.0000 | 2.2980 | 1.5206 | 2.0174 | 2.6906 | 1.8416 | 2.4344 |
| 2.8615 | 0.9357 | 3.2560 | 1.7133 | 2.6677 | 3.3488 | 2.8976 | 0.7381 |
| 3.0859 | 0.0000 | 3.5764 | 1.3242 | 2.6037 | 2.7372 | 3.4651 | 2.9520 |
| 3.0392 | 2.3506 | 1.3600 | 2.6560 | 2.1631 | 5.1370 | 4.0363 | 2.3687 |
| 3.2830 | 4.2445 | 2.5502 | 2.2280 | 2.5213 | 2.6586 | 4.0012 | 1.3895 |
| 1.9200 | 2.3068 | 2.6427 | 1.6723 | 2.5385 | 3.0248 | 1.9305 | 3.4351 |
| 0.0000 | 3.3550 | 3.1691 | 3.9666 | 3.1597 | 2.2587 | 2.8760 | 1.7765 |
| 2.3837 | 0.0000 | 2.4161 | 2.0332 | 2.6702 | 4.1011 | 2.7543 | 3.1558 |
| 2.1850 | 2.5996 | 1.1634\ | | | | | |
| ZNF876P | 4.8109 | 4.1289 | 1.7871 | 1.1529 | 3.9196 | 4.1875 | 1.4999 |
| 4.3006 | 5.1264 | 5.4689 | 4.8610 | 2.4315 | 4.1940 | 3.8254 | 4.5035 |
| 3.8845 | 4.6587 | 5.3565 | 4.1667 | 5.3624 | 5.8352 | 3.8957 | 3.2153 |
| 3.5854 | 4.7153 | 3.0550 | 2.7174 | 3.3595 | 2.7323 | 4.2240 | 4.0789 |
| 1.5370 | 1.8398 | 2.0192 | 4.0452 | 3.2417 | 3.3777 | 2.8159 | 3.9170 |
| 5.0859 | 1.4628 | 4.8027 | 4.6617 | 5.9007 | 3.9549 | 4.1094 | 6.1703 |
| 2.1933 | 3.1455 | 1.7431 | 4.5546 | 1.2789 | 3.8151 | 5.0527 | 3.7985 |
| 5.9161 | 0.0000 | 2.4827 | 4.8292 | 3.7174 | 3.9204 | 2.1008 | 4.7762 |
| 3.2795 | 5.2219 | 3.9933 | 3.8434 | 1.1331 | 0.0000 | 5.9600 | 2.9368 |
| 3.3742 | 0.9449 | 3.1486 | 3.9468 | 4.5439 | 4.0008 | 1.2414 | 3.2595 |
| 4.1299 | 4.7771 | 5.3072 | 4.2581 | 3.3909 | 4.0362 | 2.8957 | 3.9911 |
| 3.4699 | 1.4671 | 4.3489 | 3.1304 | 2.7205 | 2.9938 | 2.8364 | 2.5291 |
| 3.8677 | 1.8574 | 4.4977 | 3.8191 | 4.7049 | 1.0805 | 1.4068 | 4.3757 |
| 4.3896 | 4.0114 | 5.9979 | 5.1695 | 6.0407 | 3.9218 | 5.5626 | 3.1897 |
| 0.6215 | 2.8658 | 4.6439 | 6.1712 | 4.5989 | 2.5502 | 5.4229 | 2.0603 |
| 2.6440 | 2.7943 | 4.4368 | 4.6283 | 4.0360 | 5.0791 | 2.5649 | 3.2484 |
| 0.0000 | 4.6873 | 5.2673 | 1.5269 | 3.1145 | 3.4941 | 3.7466 | 3.7887 |
| 0.0000 | 4.6795 | 0.0000 | 4.2032 | 4.3438 | 4.4377 | 1.5646 | 4.1313 |
| 3.7359 | 4.7763 | 5.0603 | 0.0000 | 4.0942 | 0.9947 | 4.9877 | 4.0354 |
| 0.0000 | 3.3374 | 2.8106 | 3.4189 | 4.8572 | 4.3651 | 2.7696 | 3.8489 |
| 3.9615 | 1.8525 | 2.2364 | 3.6924 | 4.1515 | 0.4418 | 3.1560 | 0.0000 |
| 3.3643 | 3.0885 | 3.6978 | 3.4392 | 5.9152 | 2.0651 | 2.6780 | 2.9928 |
| 4.6933 | 5.2538 | 5.0076 | 4.7705 | 3.4442 | 3.7712 | 3.5147 | 5.5727 |
| 2.4895 | 1.2218 | 2.9077 | 3.8473 | 1.8171 | 1.9569 | 1.5562 | 3.2385 |
| 1.2686 | 3.1228 | 6.1864 | 1.7976 | 3.4726 | 3.6432 | 4.4660 | 2.9633 |
| 4.0165 | 3.8093\ | | | | | | |
| MLF1 | 3.3026 | 7.8548 | 10.2402 | 9.9638 | 8.6918 | 10.9845 | 10.7428 |
| 11.6967 | 9.9803 | 8.1054 | 9.5646 | 9.3466 | 11.6954 | 6.1238 | 10.3238 |
| 8.2061 | 11.1142 | 3.7916 | 7.7975 | 10.1148 | 10.3427 | 10.4984 | 11.0178 |

```
9.2022  8.1855  7.1966  9.4829  9.8320  8.8501  8.7636  8.7755
2.9368  9.0621  9.5403  10.6848 2.9348  9.6598  7.5301  7.0248
8.9143  7.9375  8.6403  9.2839  5.7115  8.3180  0.9285  9.1231
10.4726 3.3612  8.0589  2.9335  9.8639  10.7053 8.5877  7.6739
2.8432  7.7009  9.0881  9.4918  10.8581 8.5736  10.2703 2.5147
12.3187 8.3782  10.3194 7.6569  10.0388 4.8359  8.3293  8.1785
8.4999  7.6364  9.2206  6.1740  8.0482  11.2883 9.1683  10.3629
7.7943  4.7516  8.6962  11.4079 10.5183 4.8881  9.5203  10.2872
8.3355  9.6541  7.8971  8.0664  9.4746  9.3817  7.1895  9.4148
7.5762  9.7144  8.1190  6.6499  7.4373  9.8086  3.0033  10.4390
9.4722  9.7342  8.4026  7.9990  11.1115 10.6157 10.4803 9.1872
10.0010 9.2477  9.8234  9.3594  10.9206 11.5033 10.1565 7.0814
8.6610  9.9260  9.0568  9.6180  2.6761  10.6004 9.7250  9.3779
9.5533  8.5738  8.3070  9.7978  9.7894  3.6089  10.7413 7.8565
9.3188  8.5609  5.6322  7.8514  8.2239  10.0278 7.7210  7.4682
4.0853  3.8468  8.4368  10.4544 8.4328  7.8158  9.8225  6.8971
8.5061  3.4009  10.4180 8.7861  9.3883  8.5653  8.6050  12.7631
3.8746  4.6751  6.6309  8.9042  10.6959 7.8520  6.2314  10.7513
9.0279  9.1133  10.0498 2.6102  10.1329 10.0764 9.5855  5.0451
8.9521  7.9283  8.5613  9.2425  11.2564 7.7743  7.6901  10.9422
8.6976  9.2271  3.8082  9.8023  8.6430  8.1989  7.9341  3.9396
7.9411  10.9192 9.8028  2.4698  10.1243 11.5870 8.4257  6.7191
9.4130  8.6713\
QPRT    6.1965  9.0321  9.8922  7.6054  9.0916  10.7960 6.5442
10.5114 11.6771 10.6929 10.6779 9.5548  12.3703 9.3899  10.7216
8.8949  8.8549  12.5929 8.6405  10.1367 11.6533 10.7438 11.1663
10.9119 10.5183 10.9574 10.3672 12.0143 11.6450 13.1920 11.2798
8.7841  11.1649 12.2572 10.4649 10.1479 9.6913  11.2591 8.2927
8.1901  7.9982  9.4852  10.8914 10.1233 8.9522  10.4403 12.1991
12.5115 7.9721  8.9632  11.1680 10.6638 10.7008 9.5577  10.6670
7.0405  11.0631 10.1575 10.5710 10.9874 8.8971  10.9210 11.3750
11.2622 10.7442 11.1838 11.6724 9.9069  7.1522  11.2072 12.6147
11.1652 10.7550 9.8741  10.3158 8.9599  9.7352  11.5871 8.6164
7.8685  10.7588 11.7520 9.9765  9.1423  10.2389 10.1813 11.7541
11.4616 8.5272  7.6942  9.9864  11.6295 9.3780  9.8993  9.8446
9.9134  11.9933 10.6349 6.4268  9.4722  10.8969 11.6801 9.1639
9.9777  8.8788  11.0923 8.8449  10.5856 10.3500 10.1180 9.9419
9.7651  10.0315 7.4919  11.0323 11.7328 9.3736  10.4470 10.3259
9.9916  11.1161 9.7842  10.0576 10.8631 11.3221 11.3708 10.1020
10.9116 9.5629  9.9510  11.0855 10.9062 11.4704 9.1060  8.4766
9.6399  10.7620 9.8347  10.4425 10.1210 9.9303  10.6814 11.4013
7.9404  5.7093  10.3480 9.3873  10.6768 11.9572 12.0332 9.6112
11.4280 10.6067 11.9285 7.7742  10.9665 10.0568 9.2509  11.3909
8.2564  12.2488 11.8408 10.2744 11.0102 10.7152 9.6163  10.4053
11.9883 10.6826 9.0176  7.2786  9.7253  8.5026  10.5090 9.9685
8.6300  10.1309 10.3434 7.1955  10.9955 10.4893 11.9394 11.1618
7.6791  8.2829  8.8909  8.5866  11.6892 11.6736 8.8069  11.4254
10.6836 8.8342  8.5264  6.2264  9.4922  5.8697  10.5645 9.7147
10.2264 11.1441\
IFT140  9.1710  9.8633  9.6660  10.0125 9.7175  9.3504  10.0758
10.3003 9.7181  10.1094 9.5217  9.2627  10.3997 9.6575  10.6948
10.1834 9.7773  9.4770  9.7665  9.3096  8.8667  11.2292 11.7233
10.1274 9.5321  8.1943  8.9098  10.9278 9.5711  9.9941  8.9038
9.6792  8.5997  10.0403 9.3452  9.0191  10.5823 9.7492  8.4531
```

8.1935  7.8523  9.7039  9.4855  7.7644  9.4522  10.3160 8.1389
10.1523 8.9296  9.9022  9.2544  7.9236  9.4482  9.6542  9.8529
9.9064  9.7833  8.1660  9.7770  9.5237  9.3805  9.2091  9.3184
9.1053  10.0437 9.9991  9.5165  9.4485  9.3336  9.6590  7.6765
9.9224  7.7399  8.7515  9.0549  9.6004  10.1373 11.5462 10.6825
8.8588  9.8180  9.7108  10.9505 9.0820  10.8535 9.5182  9.4221
10.2503 9.9851  9.2745  9.5573  9.8079  9.1832  10.3708 9.2994
9.4909  8.8083  10.0168 9.7119  10.2291 9.6545  10.0710 8.8298
10.1311 9.6524  8.6195  10.1728 9.7136  9.0849  10.3085 10.6069
10.5715 8.6166  10.7065 8.7190  9.5553  9.4809  10.3180 7.8390
8.7950  9.2759  9.8248  8.6637  10.5315 11.3255 8.8416  8.7865
10.3235 9.4475  9.5229  10.2358 9.6556  10.9897 11.2765 8.1099
7.0789  9.1093  9.9591  9.8234  10.2034 8.8962  9.3585  10.7440
8.9172  9.0695  10.1164 10.1596 10.5723 9.8519  8.7251  9.5668
8.0020  10.3666 9.4765  9.5208  9.0969  10.0812 9.9409  10.9564
8.7349  9.9223  9.0213  9.9146  10.8684 9.6394  9.3860  9.8182
9.8153  9.2926  8.8448  9.6281  9.1118  9.1460  9.7929  9.3952
10.0789 9.7363  9.2863  9.9346  12.2258 9.2094  9.5882  10.6131
9.3577  8.4949  9.7152  8.7894  9.3463  10.1973 10.1222 9.2944
9.7782  9.6655  10.4045 9.4335  9.4132  8.3040  10.2707 9.9784
10.7301 9.6032\
MLF2    12.7306 12.0936 12.5074 12.2702 12.8385 12.5701 12.2265
12.4111 12.6262 12.1702 12.4180 12.6320 12.2020 12.1841 12.1981
12.2493 12.6734 12.9550 12.2269 12.9723 13.3672 12.2246 11.8878
11.9941 12.2522 13.4206 13.2091 12.5927 13.0175 12.0050 12.4870
12.6356 12.8265 13.4201 12.5066 13.1489 12.2507 12.1524 12.8952
11.2664 12.8936 11.8330 12.5451 12.4152 11.4640 12.8837 13.1543
12.6515 12.4191 12.9403 12.0235 12.0971 12.6538 11.6661 12.6341
12.6204 12.2696 12.6042 12.3803 12.5068 11.6557 12.1240 12.6594
12.9976 12.0784 12.5993 12.2143 13.8671 12.3115 12.6943 13.2531
12.4210 12.9307 13.9421 12.8097 12.7373 12.8962 11.8814 12.6241
12.7461 12.5809 13.0445 11.8382 12.9897 12.5897 12.6162 13.3050
12.4078 12.1471 12.8358 11.9113 11.7837 13.2383 12.5715 12.6466
11.9169 13.8118 11.8104 12.2385 12.6029 13.2513 12.8033 12.4422
13.1142 13.1875 12.8804 12.2422 11.6565 12.9280 13.1193 12.0732
12.9539 12.6731 13.8652 12.7111 12.4281 12.4041 11.9131 12.4836
12.3887 12.7336 12.5548 11.8800 12.2874 11.8594 12.8021 12.8894
13.1868 11.7116 11.5243 12.5883 12.7573 12.3445 12.2794 13.0090
13.0253 12.3393 11.9237 11.5142 12.0839 13.3510 12.7849 12.3653
12.4836 12.6031 11.7380 11.7973 11.9602 12.3385 13.2756 11.7472
14.3264 11.5045 11.7801 12.6956 12.1065 11.7030 12.0505 12.6752
13.1578 12.9816 12.7564 12.2885 12.1300 12.9023 12.1857 13.0857
11.7711 12.9506 12.6874 12.4636 12.9072 12.5687 12.9446 12.7521
12.1629 12.3926 12.1258 12.2775 11.0876 13.0051 12.9636 13.3770
12.6290 12.6345 12.2414 12.7282 13.3934 12.8991 12.4143 12.3146
12.4759 12.4945 13.0862 12.0476 13.2896 12.6335 11.9445 11.8644
11.8170 12.6159\
TMEM14C 11.4561 11.2129 10.3774 11.2566 10.8875 10.5203 11.1831
11.9199 11.1324 11.0959 11.4257 11.1966 11.9289 10.7058 10.8683
11.1372 11.5201 11.8251 11.0986 11.8409 12.3027 10.6610 11.3844
10.7509 11.3069 11.1344 11.1682 11.0926 11.2074 10.3283 11.1791
10.6110 12.4946 11.9809 11.4797 11.3675 10.7702 10.9999 11.7421
11.0333 11.5920 10.8987 11.2212 9.6177  11.2037 11.1899 10.5007
11.1141 10.8512 11.8428 10.8737 10.8097 10.6770 11.1044 11.4485

11.2349 10.9561 11.3036 11.2826 11.7935 11.1568 11.0203 11.3971
11.5210 11.3548 10.9754 10.6076 11.5997 11.1999 11.0493 11.2092
10.3774 11.0215 11.8753 11.0307 10.9669 11.5926 10.8650 11.4682
11.1433 11.3288 11.3471 10.7090 10.7031 11.4511 11.7076 11.3076
10.9372 10.9552 11.3294 11.2773 11.1181 11.4685 11.2808 11.0205
11.3174 11.5965 11.2202 10.9265 11.1748 11.3475 10.9225 10.7093
10.9569 11.2402 10.9717 10.9154 10.5702 10.9613 11.8001 10.6269
10.9715 11.9077 11.8941 10.9647 10.8410 11.3509 11.4268 10.6930
10.2690 11.7835 11.4425 10.4778 10.9558 11.2840 11.0034 10.7949
13.3396 11.2956 10.5396 10.2537 11.0670 10.1808 10.8039 11.5552
11.1169 11.2838 10.5985 10.6413 11.0912 11.1364 11.0697 11.3881
10.5178 11.0604 11.0694 10.7853 11.4581 10.5873 11.1813 10.9083
10.1822 10.0655 11.1153 10.7654 10.8215 11.1772 10.8353 11.8508
10.6456 10.7392 11.1666 11.0319 11.5315 10.7843 10.4396 11.1973
10.5696 11.1333 11.1569 10.3664 10.9962 11.9230 11.1196 11.0572
11.0454 11.2537 11.5233 10.4302 10.7183 11.7415 11.4861 11.6526
11.4388 11.2372 11.0005 11.3250 11.0228 11.2066 11.3048 10.9356
10.8970 11.3784 11.3855 11.4261 12.1030 11.7947 11.2177 10.8947
10.5942 10.6381\
FAM157A 0.5526 2.4485 3.9160 5.0891 3.7405 6.6500 2.2192
2.2919 4.6516 4.1257 3.5952 5.0942 3.5785 6.4460 4.1095
2.1513 2.2551 1.9468 0.5838 0.3755 1.6493 1.5770 1.6825
4.4961 1.7397 1.3599 2.1197 2.4936 0.0000 3.1011 2.9920
4.5156 0.8951 5.0135 5.8713 3.1465 6.1108 6.2819 2.6916
5.0094 1.4628 2.4377 2.2575 3.1197 1.0260 1.8914 0.0000
4.2379 3.5488 2.6955 4.4851 2.4004 3.0688 2.2666 5.9840
4.8594 3.8331 4.0018 3.3095 0.0000 2.4755 5.4516 2.1044
3.0309 1.1445 5.4868 3.2826 1.9201 0.9886 1.8731 2.5620
6.0298 0.0000 1.1245 2.6798 5.8668 3.0064 3.2691 3.8558
2.5341 0.0000 3.7712 1.8442 4.4436 0.5263 3.1783 4.4142
1.0433 4.2208 1.8648 1.0759 3.0336 4.3639 2.2282 3.0661
1.3960 3.2184 3.0618 3.6349 2.3730 1.4180 2.1052 3.9837
3.2704 1.9209 0.8176 0.0000 4.6060 3.7851 1.3268 4.4841
2.4082 1.3631 2.0000 4.3710 3.9805 3.7402 4.7649 6.2325
3.2329 4.2893 0.5886 3.0021 2.7605 2.0269 1.4793 2.2388
1.2894 1.5385 1.9820 4.1897 2.1813 3.6630 1.2511 1.3267
0.0000 0.6413 0.0000 3.4034 1.9774 2.3602 1.5646 2.3186
3.1308 5.1682 3.4632 4.1900 2.8937 0.9947 1.5869 2.7711
0.0000 5.7623 3.7584 3.5384 4.2259 2.4071 4.7402 1.8394
2.5877 2.3724 0.6138 1.3167 3.0171 3.3669 3.2849 4.2069
5.7945 4.9554 2.8049 3.7441 5.6227 4.4547 3.5606 0.0000
4.5055 1.8570 1.0660 5.4583 2.9241 0.5545 2.0241 3.9547
3.0965 3.2722 5.3422 4.9196 0.7058 1.1063 2.4241 2.3837
3.1677 0.0000 2.5389 4.7549 0.0000 1.8529 4.2022 3.0687
5.1009 1.1634\
FAM157B 0.0000 0.0000 1.1543 2.3445 2.1806 2.4722 1.2456
0.8314 1.2625 0.4413 0.7594 1.5838 1.0422 2.9572 3.0405
0.0000 0.0000 0.0000 0.5838 0.0000 0.0000 2.1923 1.0738
2.6512 0.5538 0.0000 0.4561 1.6129 0.0000 1.8184 0.0000
2.4004 0.5159 0.8184 1.8080 0.0000 3.4415 4.4967 0.9336
3.4354 0.0000 0.0000 1.3900 0.4326 0.0000 2.4638 0.0000
0.6741 0.0000 1.7431 3.7593 0.7768 1.4359 0.0000 2.4746
0.9635 0.0000 1.1710 0.4252 0.0000 0.0000 1.7937 1.2717
0.5968 0.6828 1.9254 2.3530 0.0000 0.0000 0.6160 0.4334

| | | | | | | | |
|---|---|---|---|---|---|---|---|
| 1.9301 | 0.0000 | 0.4786 | 0.5367 | 3.4606 | 1.9314 | 2.4718 | 1.5048 |
| 1.0461 | 0.5659 | 0.7073 | 0.0000 | 0.0000 | 0.0000 | 0.0000 | 1.4887 |
| 0.0000 | 0.0000 | 0.5265 | 0.0000 | 0.4848 | 1.2055 | 0.6909 | 1.2026 |
| 0.0000 | 0.9065 | 1.2499 | 1.4288 | 1.2591 | 1.6914 | 0.4986 | 1.4835 |
| 0.0000 | 0.0000 | 0.0000 | 0.0000 | 1.4556 | 0.0000 | 0.0000 | 2.5118 |
| 0.0000 | 0.0000 | 1.0000 | 2.2788 | 0.6116 | 1.6815 | 1.4572 | 2.7457 |
| 0.6089 | 0.8008 | 0.0000 | 1.0557 | 1.7271 | 0.0000 | 0.0000 | 0.0000 |
| 0.0000 | 0.0000 | 0.8821 | 1.6759 | 0.0000 | 0.9511 | 0.7571 | 0.0000 |
| 0.0000 | 0.0000 | 0.0000 | 2.2985 | 0.8792 | 0.0000 | 0.0000 | 0.6506 |
| 1.7146 | 1.0811 | 0.0000 | 2.9153 | 0.5454 | 0.9947 | 0.0000 | 1.8061 |
| 0.0000 | 1.2425 | 1.8101 | 1.2540 | 0.4374 | 0.0000 | 3.0619 | 0.0000 |
| 0.5416 | 2.0474 | 1.8685 | 0.0000 | 1.7039 | 0.4418 | 0.5023 | 0.0000 |
| 2.8899 | 2.1803 | 0.0000 | 1.0663 | 2.5538 | 0.0000 | 1.3895 | 0.0000 |
| 0.4801 | 0.0000 | 0.0000 | 3.1725 | 2.8157 | 0.0000 | 1.1551 | 0.0000 |
| 0.0000 | 0.0000 | 2.6826 | 0.0000 | 0.0000 | 0.0000 | 2.1363 | 3.5295 |
| 2.1004 | 0.0000 | 0.4262 | 2.4698 | 0.0000 | 0.0000 | 0.6112 | 2.0881 |
| 1.9827 | 0.0000\ | | | | | | |
| FANCF | 8.6656 | 8.5680 | 8.4948 | 9.5203 | 9.2264 | 8.5165 | 9.7502 |
| 9.6274 | 9.4802 | 9.2346 | 9.2690 | 8.5429 | 9.2908 | 9.7343 | 8.8066 |
| 9.4543 | 9.2169 | 8.9676 | 8.8184 | 7.3854 | 8.1692 | 9.7592 | 9.8408 |
| 9.6943 | 8.7104 | 7.9961 | 8.5219 | 8.9664 | 8.9382 | 7.8949 | 8.9834 |
| 9.7000 | 7.0269 | 8.8781 | 9.2782 | 5.1957 | 9.1520 | 8.8886 | 9.0737 |
| 8.7156 | 9.0805 | 7.9751 | 7.9878 | 9.2118 | 8.2311 | 9.1157 | 7.7843 |
| 8.6114 | 9.6004 | 8.9912 | 9.0962 | 6.8910 | 9.0614 | 8.0235 | 8.8474 |
| 9.3755 | 8.6064 | 8.8980 | 8.5323 | 9.2368 | 8.2452 | 9.0726 | 8.7099 |
| 8.3660 | 9.2540 | 9.2210 | 8.7902 | 8.8318 | 9.2096 | 8.5828 | 8.9298 |
| 9.6215 | 7.9325 | 8.4783 | 8.4279 | 8.1956 | 8.9714 | 9.6883 | 9.4512 |
| 8.8388 | 9.0151 | 7.0782 | 9.8526 | 8.5495 | 8.5032 | 8.4967 | 9.9152 |
| 8.8166 | 9.3895 | 8.8479 | 8.0453 | 8.7140 | 8.7794 | 9.7186 | 5.6320 |
| 8.0128 | 7.9747 | 8.8893 | 9.3480 | 8.3244 | 8.9378 | 9.0991 | 9.1191 |
| 9.1911 | 9.0402 | 7.0166 | 8.2417 | 9.3757 | 8.4279 | 8.9565 | 9.8379 |
| 9.3899 | 8.3646 | 8.1293 | 8.4419 | 9.4503 | 9.7149 | 8.5479 | 9.4370 |
| 7.8549 | 8.9313 | 9.4373 | 8.2096 | 7.0852 | 9.5999 | 9.1020 | 8.9567 |
| 9.6130 | 9.2118 | 8.8987 | 8.4213 | 9.4929 | 8.8880 | 9.9207 | 8.7023 |
| 7.7417 | 8.5065 | 7.5102 | 9.3383 | 8.7209 | 9.2275 | 8.5335 | 9.2941 |
| 9.0938 | 7.7841 | 8.6466 | 9.1631 | 8.8879 | 8.7172 | 8.5146 | 9.1375 |
| 8.0075 | 9.6791 | 8.7999 | 8.6250 | 9.6644 | 9.1635 | 8.4443 | 8.7978 |
| 7.8493 | 9.2489 | 8.3746 | 9.7209 | 9.3210 | 9.3289 | 9.0632 | 9.2076 |
| 9.0438 | 9.2293 | 9.1730 | 8.6569 | 9.3060 | 8.1612 | 8.7831 | 8.4529 |
| 9.8105 | 9.3847 | 7.2575 | 9.4446 | 9.7960 | 9.3278 | 7.7198 | 9.4031 |
| 9.5500 | 9.6339 | 9.6747 | 8.8481 | 8.3541 | 9.0472 | 4.4181 | 7.9726 |
| 9.1699 | 10.1181 | 7.9346 | 8.6634 | 8.8121 | 9.0320 | 9.1526 | 9.5029 |
| 9.8435 | 8.0705\ | | | | | | |
| MECOM | 11.2001 | 10.8166 | 12.2734 | 12.0932 | 11.9266 | 11.9787 | 12.3429 |
| 12.1132 | 12.7063 | 11.5759 | 13.7360 | 12.2708 | 11.9959 | 10.8963 | 12.6610 |
| 9.2246 | 11.4702 | 9.6777 | 10.6178 | 11.6901 | 10.0996 | 11.9851 | 11.1047 |
| 11.9391 | 9.4466 | 6.9630 | 5.3233 | 11.4249 | 11.0643 | 12.4274 | 5.8155 |
| 11.2807 | 10.1880 | 11.8664 | 12.5670 | 11.3642 | 12.2942 | 11.2255 | 11.2534 |
| 12.2389 | 12.5249 | 11.0245 | 12.1757 | 10.9079 | 9.7060 | 11.3185 | 10.9489 |
| 12.6882 | 8.7149 | 9.1908 | 11.1454 | 12.3927 | 12.8117 | 9.3553 | 10.7381 |
| 12.0829 | 10.2542 | 10.7892 | 11.3704 | 12.4276 | 10.3271 | 13.4580 | 10.8475 |
| 11.3404 | 11.0635 | 11.9468 | 11.5827 | 13.0290 | 9.2855 | 12.2852 | 10.6566 |
| 12.3024 | 10.1510 | 12.4293 | 10.4234 | 11.5028 | 12.3724 | 11.5636 | 11.9125 |
| 8.0876 | 11.3267 | 12.1583 | 11.7910 | 12.6338 | 11.9321 | 11.5319 | 12.4723 |

11.4858 12.1166 7.3532 9.1977 11.8229 12.4363 11.6694 12.4576
8.9962 9.7419 10.1823 12.3753 10.5916 9.6212 11.5939 12.0443
12.6007 12.4824 12.4729 9.6012 13.2767 12.1255 11.8860 11.5190
12.2933 11.5106 10.9447 11.6462 11.7756 11.7678 12.1226 11.4437
12.2249 12.1912 12.4589 12.8414 11.7433 11.3903 11.2553 13.2552
11.3201 11.2376 10.5294 12.1732 13.0684 11.9475 12.8089 5.9207
4.8605 10.7717 10.4290 11.7579 10.4397 11.7009 11.1070 12.4963
11.0481 11.6494 5.4242 13.2728 10.6211 11.4435 10.2152 10.6316
10.9396 12.7917 10.9548 9.2317 13.0953 10.9092 12.8847 11.7557
10.4248 9.7519 10.4522 13.2535 11.6343 11.5157 13.0274 12.2166
11.9144 12.1851 12.1852 11.0938 12.1734 11.3741 11.3788 9.3952
11.7452 11.5677 8.8737 13.1738 11.7854 10.1770 12.0046 12.5293
13.2695 12.1826 12.5769 12.0936 11.4319 11.2584 11.0345 10.1127
12.3848 12.0954 11.7390 12.1621 10.6896 11.3583 11.1137 12.8338
12.2040 10.8094\
ITGA2B 4.1155 5.5104 5.2852 7.9810 6.8225 4.6275 3.3422
7.0899 7.7392 6.3984 1.4500 5.0073 4.7989 5.5852 5.0671
5.8953 8.2681 6.6875 3.8042 7.3621 5.4682 2.3124 5.7411
3.8723 4.5522 5.5849 7.1144 7.0470 5.1569 3.0135 4.6917
2.6394 4.6482 4.4081 7.0937 4.0627 7.1101 6.3446 4.6764
5.1585 6.1068 5.2152 5.0389 7.7363 3.5062 5.7140 6.0912
3.9900 3.9985 8.6456 6.9862 5.6294 4.6368 4.7232 6.5843
7.5413 4.4216 7.5411 6.4382 7.3901 2.7929 4.9927 5.1075
6.6123 5.3979 5.5647 6.9984 6.5374 1.3082 6.8046 7.0563
4.1077 3.0705 7.2373 3.5065 5.2752 5.5679 3.2691 4.5648
4.4441 4.8983 2.5997 5.0887 4.5201 4.4963 7.6177 7.8536
5.9042 5.4671 3.5322 4.7570 5.9285 6.0415 5.9922 3.8956
1.8952 3.1490 6.2717 4.4416 3.5463 6.5897 6.0037 6.2393
8.5858 5.0201 6.6179 6.1222 2.7174 5.9900 8.3832 6.7420
5.8865 7.2474 6.5546 5.5748 5.5032 3.2310 7.3824 5.7704
4.9752 7.5143 3.7651 5.7366 8.6437 6.8747 4.8660 3.9892
7.4575 4.3594 5.3884 6.8316 7.2521 5.3292 5.0332 0.8107
5.6948 6.5358 7.5250 5.9737 6.8991 4.8533 4.2189 6.7489
3.2040 2.1200 6.2259 1.4775 6.9122 4.5443 4.8608 4.9907
3.6663 3.1278 6.8304 4.0211 3.6656 5.5810 6.5278 5.4530
5.4537 6.7205 3.7789 3.8987 8.1259 5.0078 4.9536 5.4812
6.7301 4.9959 7.0919 3.7043 6.4489 9.4339 6.6489 7.8585
8.2051 5.9383 5.1253 4.1435 3.7688 6.5001 4.3233 6.9241
5.7738 5.1556 6.0453 4.8218 7.2096 6.6124 5.3839 7.0281
3.9158 5.7423 5.2393 7.8302 7.1943 7.7978 7.2466 3.3454
7.1948 3.1794\
RSPH1 4.9038 6.7844 7.9032 11.2455 5.6189 9.9993 9.0642
10.5696 6.9102 9.4298 6.5034 7.6026 11.3379 7.5211 10.9089
8.7351 9.3093 7.4466 7.0841 7.5154 6.6719 12.3524 12.7602
9.9416 6.7126 5.6005 6.3519 12.0087 6.8418 6.7157 3.3137
7.5573 6.1426 7.4798 7.5783 6.0368 8.3152 7.7939 7.1503
4.7522 7.1802 7.5057 6.3864 7.0871 3.9549 11.1007 10.4011
8.6801 2.2999 7.5183 7.7404 4.9522 7.6801 6.0947 6.2080
8.9348 7.4999 9.0363 7.4008 8.0760 6.8574 7.6820 6.3509
8.5878 9.3588 9.3128 6.8522 9.5591 8.4448 7.2198 5.4278
6.8415 3.5970 9.0155 7.0459 9.6337 9.9268 13.1792 12.4157
4.3341 9.4697 5.7503 12.7607 10.3928 12.0254 7.1186 7.8338
9.8065 7.0350 7.4796 7.2131 6.2342 6.3339 8.8755 6.4627
4.4376 6.4307 7.1027 11.5032 8.1678 9.6130 9.8628 9.7469

9.0223 10.7802 4.4064 7.1725 9.5353 7.5137 9.7655 10.5558
11.3611 6.9500 7.4179 6.5596 8.0267 11.1191 7.7367 8.4594
5.8108 6.3879 9.5637 6.4390 10.0224 12.5270 6.7527 7.3867
7.8036 6.3143 6.4840 8.3552 10.2033 12.2672 11.4961 5.6002
7.0295 3.2319 5.7867 9.0819 6.6566 10.9161 7.9553 11.6708
7.2786 7.4268 5.8114 9.6808 9.3466 6.7837 7.2091 7.9674
10.1590 7.7816 7.0753 4.8660 5.8936 8.4615 7.4203 12.1743
4.8475 7.4591 8.0789 10.2189 9.9960 5.2714 6.7102 9.6001
8.8378 9.2143 6.5482 7.4982 6.0369 5.7366 8.4878 6.7940
9.0585 7.2419 6.3271 9.3350 13.1698 9.3662 7.0871 8.2199
8.8339 7.5664 6.9483 8.7650 8.6316 10.7302 6.0866 7.7189
8.3026 7.7832 5.7802 6.0579 8.1177 8.1769 7.1265 8.9869
11.1536 8.5236\
IFI16 10.8857 11.9335 11.3470 9.2603 9.2275 13.1051 10.9245
10.2769 11.0874 10.8713 12.0344 10.9056 10.8164 7.5406 10.7186
10.0015 9.9294 9.1831 12.2951 7.1580 12.3226 10.1446 11.4107
11.8966 11.3895 9.3010 10.0374 10.1613 11.9010 9.8090 7.8898
9.3317 9.4820 11.6731 11.7083 8.5532 11.5308 11.8518 9.1263
8.5296 9.9201 12.9006 8.7927 11.8623 12.4557 10.4496 6.4908
11.9412 10.9655 6.5262 9.4603 10.8697 11.8611 12.6437 12.0120
8.4597 9.6065 11.6440 12.1364 9.2204 12.1839 11.8273 8.1468
9.8736 11.4365 11.7651 10.6685 11.0606 9.5789 10.3806 11.9318
10.5278 11.0202 8.5004 12.3620 6.4968 11.0199 10.0401 11.2936
10.0435 8.6427 12.1763 11.1267 12.0523 10.0203 11.5981 10.9438
10.8065 10.6090 10.5756 12.1214 10.9474 10.6572 11.0045 10.4347
11.9652 9.8449 11.6265 10.4320 11.4824 7.8600 10.0940 11.7900
9.7233 7.6726 7.8770 11.6722 12.5887 10.8414 9.5550 8.9206
9.5067 7.5554 9.8750 11.6188 12.1402 11.2237 13.1628 11.6118
11.5226 10.3195 10.2918 11.4671 8.1675 11.1912 11.4012 9.0921
9.7976 11.9591 11.5430 11.7587 10.5401 10.6555 11.9070 10.7836
10.0937 9.0094 10.1034 10.4909 11.6526 8.5151 10.4008 9.8853
11.1941 10.8358 11.3026 11.0523 11.1563 10.5942 6.2317 10.8704
8.5443 12.0907 9.4775 10.0240 11.7681 11.6473 7.6729 11.7403
9.6083 7.6809 12.2888 10.7628 10.9695 9.5283 9.0922 12.2783
13.3914 8.8252 11.7916 9.8632 13.1666 9.7573 11.7961 8.2302
11.6736 11.0770 11.2874 8.9061 10.9542 8.7029 11.0983 11.2307
10.7131 12.0950 11.2696 10.8166 10.5146 9.9219 9.3241 9.7789
11.6410 12.6338 11.6720 10.8632 10.6502 5.0615 11.4315 10.9054
9.8652 10.1116\
SOX6 8.8680 6.2030 8.1842 6.5732 8.0977 6.0652 5.1029
8.2544 8.8746 6.6809 7.3157 9.1177 8.8763 5.3153 6.7066
3.2639 8.7377 7.4348 6.8421 5.4435 6.9401 8.6795 9.2232
8.0041 4.7153 6.3002 1.0809 8.0887 4.1647 6.7018 2.7995
7.7385 7.0783 7.9673 7.1250 5.1226 8.5266 9.2099 8.4827
7.3047 7.9648 6.7846 7.8047 8.7588 5.3721 7.6089 8.0650
8.3469 7.2639 4.4159 9.2579 4.2351 9.2017 6.2148 5.2420
8.4675 5.1614 8.9230 9.0922 9.0337 5.7029 9.2584 6.0964
6.1433 6.9386 8.8375 6.6677 9.1543 4.1077 7.9643 2.3798
10.4520 5.0203 8.0128 7.6773 5.5005 7.7411 7.8004 8.1311
6.4026 6.5202 8.4456 9.0747 6.5381 6.5662 6.6013 5.6240
6.8463 5.2667 6.2785 6.2121 10.4450 3.7682 7.9102 8.4504
3.9714 4.4586 6.7977 4.8750 5.4658 7.6519 8.4792 8.1607
6.8342 8.0473 5.7949 5.7499 9.1500 10.3394 5.2367 10.0714
9.5724 6.9129 7.9009 8.7117 8.0150 8.2200 9.6748 2.9370

3.9238  8.7405  5.5186  7.0392  7.5821  9.6633  7.8249  8.4074
7.1625  7.7049  5.1693  4.8378  9.5898  8.7415  9.2222  4.8903
4.0146  8.4998  8.5063  7.6123  7.2487  8.5111  7.6974  7.8243
5.8132  4.3979  5.0991  10.0626 8.6026  7.2372  6.8896  5.8686
6.1923  8.1920  6.4047  6.7564  9.8341  6.7502  8.4109  6.9452
2.8833  7.8308  7.7604  7.6459  6.2929  6.6731  10.2032 6.7836
8.9487  8.8012  5.9059  8.8910  10.1821 6.9681  3.3476  8.3642
10.6775 6.2094  7.0589  8.6376  8.5207  4.6651  8.5493  8.8327
7.9818  8.9129  4.0633  8.5603  7.7218  9.1312  6.6782  6.3200
9.5026  9.1540  5.0725  8.5248  6.0366  6.7423  7.2111  7.9618
8.7592  7.9706\
LOC440944       7.4671  7.5667  9.0841  8.8501  7.8797  8.8458
7.0760  8.2997  8.0234  8.3144  8.0192  9.0056  9.1035  8.4932
7.8456  9.2816  8.4724  8.0269  7.4187  8.1133  8.2625  8.2502
7.4919  8.3303  6.8121  7.0452  8.6342  7.9181  7.5920  7.5928
8.0114  8.6759  7.2388  9.3063  8.2225  7.8940  8.7302  8.1834
8.1475  8.0859  9.2676  7.9211  7.8560  7.6308  6.5586  7.3299
6.3164  8.2115  7.4133  7.7949  7.2219  8.1433  7.1086  8.2208
8.1458  8.7077  7.0550  8.0183  7.2465  8.8947  6.9347  7.7985
7.6299  8.7766  7.7622  7.9317  8.0101  9.0160  7.4731  8.6909
6.7437  7.7073  6.9865  8.5767  8.9641  7.9505  8.7784  7.6808
8.1153  7.6295  7.2599  9.1590  7.4846  8.0135  8.2240  9.5903
7.2591  7.0036  8.9516  8.1573  6.4951  8.4214  7.9226  8.6315
8.4664  5.7630  8.0167  7.2795  8.1040  8.6099  8.0810  7.5484
8.0005  9.0872  8.0232  7.2869  8.7285  8.6060  9.2691  8.4260
8.3391  8.0925  8.4120  9.5450  9.9074  8.6040  7.5630  9.5574
8.2914  7.7914  8.4926  7.3464  8.5437  5.8578  8.6393  8.5148
7.7530  6.9001  7.5990  8.3070  8.8517  9.0195  8.0016  9.6931
8.5683  7.0708  7.7468  9.5227  8.1301  7.9257  9.0032  7.4733
8.1420  7.5057  7.0992  7.6154  7.4290  7.2580  7.6582  7.8167
8.3340  7.1231  8.3493  6.7852  7.2344  8.4436  7.6366  9.3699
7.6029  7.2929  7.1068  6.6075  7.6987  8.4591  8.5246  8.0727
8.4940  7.8369  7.7313  8.3537  7.9557  8.7080  7.9142  7.0289
8.7567  7.7240  8.4139  7.1054  8.1324  8.5840  7.7176  8.7227
8.7079  8.5520  8.2549  8.1355  8.2855  8.4193  8.2407  8.1091
8.1587  7.7049  7.9186  7.9245  8.8811  8.6691  7.7842  7.8810
7.6979  9.0252  6.4662\
TTC1    10.1515 10.1201 10.0820 9.9226  10.0721 9.9924  10.3146
10.8071 10.3751 9.9210  11.1560 10.1023 10.8642 9.9314  9.8760
10.0953 10.3057 10.1222 10.1253 9.1307  10.6199 10.0474 10.0545
10.0468 10.1619 10.4485 9.5431  10.6458 10.6265 9.2114  9.5455
10.1618 10.4212 9.7550  10.1276 10.2052 9.9807  9.5055  10.4575
9.1719  9.9209  9.6609  10.1059 9.6125  10.2375 10.3016 11.3903
9.7559  10.0375 10.6764 10.2580 9.5354  10.5796 9.9175  9.1843
10.2036 9.5289  9.8607  10.2113 10.4791 10.0917 9.9814  10.4330
10.7533 9.9880  9.6834  10.9650 10.0079 10.1993 10.0826 10.9436
10.0220 10.3369 10.2089 10.3229 9.6225  9.8511  10.1242 10.4451
9.7318  10.5257 9.5257  10.4664 10.7401 10.1467 10.7934 10.6919
10.3644 10.1482 10.2264 10.1000 9.6332  10.6158 10.0048 10.5469
10.1256 10.3055 10.0040 9.8683  10.0327 11.1397 10.0052 10.8543
10.1658 10.7157 9.2773  9.6753  10.4545 10.1804 10.1856 10.0645
10.1744 11.3081 9.7245  10.2417 10.5252 10.0723 10.2009 10.0342
9.3490  10.2229 10.3108 9.5880  9.8446  9.9650  9.7334  11.2030
11.4393 10.0979 9.7560  10.4022 10.6383 10.1119 9.6292  11.0062

10.9984 11.0120 10.5181 9.7768 9.9399 10.8534 10.2326 10.5165
10.0278 9.7626 10.0485 10.1061 9.9753 10.5424 11.1562 9.8621
12.4811 9.8490 10.4699 9.5136 10.1984 9.9658 9.5715 10.4793
10.2716 10.0872 9.9295 10.2321 10.5061 9.7268 9.5861 10.3266
10.3359 10.2190 9.9903 9.5863 9.7888 10.2032 9.9625 10.9936
10.0032 10.1157 10.3226 9.8915 10.0828 10.6158 9.8788 10.3941
10.2572 10.0209 10.2749 10.2635 10.5454 10.5321 10.3469 10.0196
10.3206 10.2225 9.6782 10.1549 10.9882 11.5006 10.0393 9.8876
9.8376 10.7514\
C9orf128 1.5194 0.5319 2.5614 3.1292 0.0000 3.4689
0.0000 2.5050 1.0352 2.1946 0.0000 1.1366 1.6409 0.7759
2.8727 2.6237 0.0000 0.0000 0.0000 0.0000 1.3800 3.6443
2.7060 4.4387 0.5538 0.6062 1.6918 1.8373 2.0792 0.5901
0.0000 0.9642 0.0000 0.8184 0.0000 0.0000 2.5102 1.5916
0.0000 0.7666 0.6649 0.8506 0.0000 2.4713 0.0000 3.4511
0.0000 0.6741 0.4402 2.0462 2.1110 0.0000 0.6494 0.0000
0.4645 0.0000 1.0734 3.2241 0.0000 2.2896 0.0000 0.0000
0.0000 0.0000 0.6828 2.6522 4.6811 0.0000 0.0000 1.3773
1.2639 1.6718 0.9449 3.8845 1.7019 3.3560 2.8273 3.7796
1.7868 0.0000 0.5659 0.3965 2.2886 2.1886 3.3567 2.2503
1.4887 2.2368 0.0000 1.4655 0.6361 0.0000 0.5215 3.3524
0.5198 0.0000 0.0000 1.2499 2.8769 0.5504 0.0000 1.1625
0.0000 3.1674 1.7606 0.0000 0.7314 1.2595 0.0000 1.8132
2.1442 0.6215 3.1476 0.0000 0.0000 1.0399 3.2310 3.3332
2.0603 1.6324 0.8008 0.0000 0.6224 2.5865 3.4920 0.0000
1.3754 0.0000 1.7575 1.1792 0.9567 3.3824 2.3786 4.0766
1.3267 0.0000 0.0000 3.9958 1.8532 1.8148 3.0418 1.9771
3.2550 1.4986 1.0811 0.0000 2.9153 1.2499 1.3155 2.6654
3.1549 1.5604 2.0661 0.0000 0.5476 0.7727 0.0000 1.1539
3.4328 0.0000 2.3724 0.0000 2.1631 1.7039 1.0531 1.1696
3.1709 3.4931 1.8676 1.5072 0.7903 1.8707 0.0000 1.6598
5.4645 1.5724 1.2087 0.0000 2.8900 4.4865 1.5234 0.6902
0.0000 2.7813 0.7366 2.5553 0.0000 0.0000 3.4329 0.9785
3.3015 1.2686 0.5674 0.4262 0.4985 0.0000 2.2195 1.6369
1.1260 3.1721 0.6959\
C9orf129 3.5816 5.3009 6.1471 5.7868 6.3361 6.9993
6.1283 5.2297 5.4198 5.8264 4.5669 6.5197 5.3716 6.1052
6.3157 3.1677 0.7616 5.5286 5.2403 4.0315 4.5414 5.9795
5.7424 6.1445 5.7497 2.2075 5.9085 4.0044 3.7957 6.0666
5.4389 6.2610 4.6363 5.5467 6.1037 5.2520 6.1182 7.5265
4.7134 6.5690 4.5211 6.1735 3.9350 6.2832 4.5216 4.1086
0.0000 5.8538 5.5122 4.5400 7.0727 5.0733 5.3441 5.6802
5.8308 4.8375 6.8523 6.0204 5.4306 4.8590 5.6313 5.8682
5.8742 4.9323 5.4553 6.8772 5.2352 5.0578 5.4364 4.6999
3.6631 7.4510 1.8808 3.1556 5.7251 6.0084 5.2564 6.6089
2.6254 5.9011 3.4488 6.6266 4.6588 4.7352 2.3083 3.3223
4.9008 3.2234 5.6950 4.1104 5.4380 5.8222 2.0852 2.5415
4.6610 6.0208 6.5600 5.8460 5.4246 3.2472 2.4399 6.0031
6.4203 3.2453 4.4906 5.5514 4.7792 5.0484 5.0288 3.0921
6.4125 6.0086 2.0837 3.6264 5.3197 5.6575 6.2680 5.7203
5.3702 3.7410 4.3428 5.8049 5.2144 7.2176 6.2931 5.8277
5.7961 0.9226 5.3052 6.4541 5.2268 5.6764 5.8624 4.0335
1.5660 0.0000 1.8975 0.0000 7.1648 5.6227 4.4974 5.3838
3.0882 6.3798 6.7221 5.5387 5.7906 5.3554 5.4036 0.8493

| | | | | | | | |
|---|---|---|---|---|---|---|---|
| 5.9424 | 0.0000 | 7.0374 | 4.8018 | 6.6426 | 6.0131 | 5.7998 | 6.2690 |
| 3.5937 | 3.1721 | 6.3039 | 5.6562 | 5.0777 | 3.0613 | 6.2312 | 6.6924 |
| 3.7599 | 7.1256 | 4.5473 | 4.6557 | 7.0419 | 4.7356 | 4.8520 | 4.4264 |
| 0.0000 | 5.0799 | 5.3103 | 5.2242 | 6.8723 | 5.5231 | 3.2338 | 4.4053 |
| 5.4326 | 5.2458 | 5.2536 | 5.0961 | 5.4774 | 3.0075 | 3.6492 | 5.1681 |
| 6.9059 | 5.7897 | 3.6956 | 5.2620 | 6.0482 | 0.0000 | 0.0000 | 5.6611 |
| 6.9190 | 6.3549 | 1.6330\ | | | | | |
| MYT1L | 0.5526 | 0.5319 | 0.0000 | 0.0000 | 0.5278 | 0.4059 | 0.9367 |
| 0.8314 | 0.0000 | 1.4796 | 0.0000 | 0.0000 | 0.0000 | 0.0000 | 0.0000 |
| 0.0000 | 0.0000 | 0.0000 | 0.5838 | 0.3755 | 0.0000 | 0.0000 | 1.4101 |
| 0.0000 | 0.5538 | 0.0000 | 3.4644 | 0.5990 | 0.0000 | 0.0000 | 0.0000 |
| 0.0000 | 0.0000 | 0.0000 | 0.0000 | 0.0000 | 0.0000 | 0.0000 | 0.0000 |
| 0.0000 | 0.0000 | 1.1407 | 0.0000 | 0.0000 | 1.6194 | 0.0000 | 3.5700 |
| 0.6741 | 0.0000 | 0.0000 | 0.0000 | 0.0000 | 0.0000 | 3.4251 | 0.0000 |
| 0.0000 | 0.6343 | 0.5030 | 1.2457 | 0.0000 | 3.2731 | 0.0000 | 0.0000 |
| 0.0000 | 1.1445 | 1.1659 | 0.0000 | 0.4832 | 0.0000 | 1.3773 | 6.1442 |
| 0.0000 | 0.0000 | 0.0000 | 0.0000 | 0.0000 | 0.0000 | 0.0000 | 0.0000 |
| 0.6159 | 0.9714 | 0.0000 | 6.7788 | 0.0000 | 0.5263 | 1.0522 | 0.0000 |
| 0.0000 | 0.0000 | 7.1756 | 0.0000 | 0.0000 | 0.5215 | 2.4059 | 0.0000 |
| 1.0621 | 0.5233 | 1.7212 | 0.0000 | 0.9478 | 0.0000 | 0.0000 | 1.2310 |
| 0.0000 | 0.0000 | 0.0000 | 0.0000 | 0.4315 | 0.0000 | 0.0000 | 0.0000 |
| 0.0000 | 0.0000 | 0.0000 | 0.5141 | 0.0000 | 1.2170 | 0.4321 | 0.0000 |
| 0.0000 | 2.8695 | 0.0000 | 0.0000 | 0.0000 | 1.6090 | 0.0000 | 0.0000 |
| 0.7843 | 0.0000 | 2.1283 | 0.3935 | 0.0000 | 0.0000 | 0.0000 | 0.0000 |
| 0.0000 | 0.0000 | 0.0000 | 0.0000 | 0.0000 | 0.0000 | 0.0000 | 0.0000 |
| 0.0000 | 0.0000 | 0.5418 | 0.0000 | 0.0000 | 0.0000 | 0.7381 | 0.0000 |
| 0.0000 | 0.0000 | 0.0000 | 0.0000 | 0.0000 | 0.0000 | 0.3855 | 4.2518 |
| 0.0000 | 0.0000 | 0.0000 | 0.0000 | 0.0000 | 0.0000 | 0.0000 | 0.0000 |
| 0.0000 | 0.0000 | 0.0000 | 0.0000 | 0.5290 | 1.4956 | 0.0000 | 0.0000 |
| 0.8396 | 0.5232 | 0.0000 | 0.7148 | 0.6314 | 0.0000 | 0.0000 | 0.0000 |
| 0.5055 | 0.0000 | 0.6248 | 0.0000 | 0.0000 | 0.0000 | 0.0000 | 0.8827 |
| 0.0000 | 0.0000 | 0.0000 | 0.0000 | 0.0000 | 0.0000 | 0.0000 | 0.3736 |
| 0.0000 | 0.0000\ | | | | | | |
| C9orf125 | | 3.1593 | 7.5769 | 7.8734 | 8.0087 | 3.4800 | 1.5605 |
| 9.1398 | 8.7260 | 1.4589 | 8.4854 | 6.7693 | 9.2419 | 8.1015 | 2.7437 |
| 2.2060 | 3.2639 | 5.9895 | 3.3616 | 7.4145 | 2.9147 | 8.3287 | 1.7571 |
| 9.4567 | 2.5463 | 8.4554 | 3.0550 | 2.7967 | 4.0391 | 4.2890 | 2.9203 |
| 7.1134 | 4.3573 | 3.3260 | 6.8614 | 6.9018 | 6.3463 | 3.7742 | 2.3299 |
| 9.2446 | 2.7994 | 3.9669 | 7.2745 | 2.5514 | 7.0495 | 8.5310 | 0.9285 |
| 3.6457 | 3.1281 | 8.6852 | 1.9026 | 3.3536 | 4.2351 | 7.7079 | 8.3649 |
| 6.3288 | 3.4563 | 5.7253 | 7.7145 | 7.5429 | 3.8203 | 8.6111 | 9.7700 |
| 2.2545 | 5.9050 | 8.2748 | 3.6444 | 2.2002 | 9.1000 | 5.0222 | 6.3188 |
| 5.6853 | 3.2846 | 2.2328 | 7.4470 | 5.2967 | 7.5523 | 6.5235 | 4.3614 |
| 7.1977 | 4.8690 | 3.3401 | 6.5153 | 6.7001 | 6.5655 | 4.7296 | 6.5023 |
| 8.2734 | 0.0000 | 5.9097 | 4.5790 | 9.0135 | 7.4815 | 5.5855 | 6.0197 |
| 3.5125 | 8.1899 | 4.0271 | 8.7592 | 3.7299 | 3.5463 | 3.3890 | 4.4358 |
| 7.1725 | 3.6978 | 2.6265 | 7.0377 | 7.3288 | 5.9941 | 4.9606 | 6.1879 |
| 2.2774 | 8.5246 | 7.2019 | 3.7004 | 3.3810 | 7.4314 | 4.8489 | 10.1534 |
| 8.3416 | 3.5238 | 7.6905 | 4.4368 | 5.1937 | 2.9158 | 9.3200 | 4.9930 |
| 5.6254 | 6.3036 | 8.2787 | 8.3374 | 7.4999 | 2.9034 | 3.9070 | 6.4812 |
| 3.7078 | 3.8725 | 2.2980 | 7.4954 | 8.9786 | 8.6860 | 2.3602 | 3.2205 |
| 6.2193 | 3.3403 | 5.6677 | 9.9063 | 7.7332 | 9.2574 | 2.3133 | 0.7381 |
| 7.1715 | 4.9184 | 5.6359 | 7.1382 | 2.0813 | 6.7451 | 8.4497 | 4.3860 |
| 6.0774 | 3.1284 | 1.8525 | 4.4027 | 4.6063 | 1.5535 | 2.8972 | 8.3555 |

7.2096 7.4546 3.9608 5.3274 5.0485 7.2690 7.9624 8.3680
5.0451 6.7388 8.0570 8.8703 4.7873 7.1787 3.2382 6.3355
7.7398 2.4895 2.5017 3.1906 4.0766 3.2020 7.6718 7.4326
3.7715 2.7306 7.6648 5.5771 3.1443 8.9658 7.7106 8.8202
6.8689 8.3270 3.4488\
C9orf122 5.8091 4.7231 6.0701 7.1469 2.1806 6.9215
1.4999 5.7233 5.4136 6.0777 5.1833 5.6594 5.9314 4.4602
6.1953 6.5270 5.7790 7.4988 4.8794 4.4272 6.4430 6.0106
7.1486 6.1223 3.2362 5.5849 7.1861 4.6839 5.2207 5.2244
0.0000 5.2149 0.0000 4.2722 6.1708 2.9348 4.2364 6.4432
6.4948 0.7666 6.2016 4.2836 2.2575 6.7780 0.0000 4.0318
5.1775 0.0000 2.6671 1.5638 6.5652 2.5833 4.4617 0.5094
6.2374 5.1950 6.5079 6.7070 3.3095 6.1695 2.4755 4.3452
6.1738 5.0786 5.7459 0.3906 5.7523 3.7791 0.9886 4.4444
5.6555 7.0012 3.5970 4.8740 5.7834 0.0000 4.0008 6.2994
5.6962 5.8034 0.9714 6.9363 6.0812 5.1213 6.9622 2.0776
5.2490 0.6140 0.9125 6.9527 3.7213 5.7686 5.3285 7.0824
0.0000 0.0000 4.3075 4.1697 5.7173 6.6496 1.4180 0.0000
5.0195 2.3196 5.0023 1.5391 5.2961 1.6282 5.9156 4.8407
3.3189 5.8600 0.0000 3.8074 5.5877 5.7915 6.6361 6.8780
5.2273 0.6089 4.5628 4.7655 5.1723 0.4700 7.4236 6.0007
1.8708 1.9593 4.5177 4.0156 1.1727 4.1770 5.9245 5.6438
5.5547 5.4665 4.9645 0.0000 5.8701 4.8217 5.7747 5.5549
6.8234 4.0055 5.3502 1.9011 3.8163 6.3222 3.7449 1.2240
5.9869 0.0000 6.2505 5.2891 3.8469 4.3563 4.3982 4.8516
0.6000 0.5416 6.3100 5.7837 6.7124 6.9186 3.7206 6.1576
1.9676 6.0532 3.8325 0.5466 0.0000 8.8069 6.2917 5.0142
4.1460 6.5812 5.9485 4.0278 3.1203 5.7765 6.1174 4.3233
7.2033 6.2179 5.2343 5.3803 3.2376 3.8954 6.0847 0.5707
4.9122 3.6176 5.0777 3.3616 0.4985 5.7956 6.8244 5.1836
3.6128 6.7453 0.6959\
CEPT1 8.5044 8.8036 9.6184 9.8745 8.2707 9.4409 8.6941
9.2184 8.1690 8.8722 9.6171 8.5974 8.9106 8.2071 9.6334
8.6450 8.8897 8.8205 8.9523 8.7789 8.8896 9.2544 9.3324
9.4511 8.8309 9.1487 9.0909 8.7211 9.3766 8.7802 9.1633
9.0634 9.0621 9.0100 8.5323 8.8039 8.4139 8.7757 9.4944
9.7030 9.5346 8.9289 9.3900 8.2218 9.2873 8.5918 8.3657
9.2881 9.3576 8.9518 8.9671 10.1959 8.4998 9.2508 8.1204
9.2595 8.8961 8.9944 8.1420 9.3049 9.3894 9.6511 9.2795
9.1489 9.2266 8.9434 9.2771 8.7896 8.8613 8.7897 9.1262
8.9972 8.9990 9.2609 9.2286 9.2148 8.7073 8.7042 9.4512
9.2755 8.6358 8.7722 9.2825 9.4109 9.0405 9.2848 9.5887
8.5406 9.6541 8.7211 9.1590 8.9267 9.2507 9.2794 8.7175
8.8457 8.6509 9.0505 9.1092 9.4349 8.2958 8.9956 9.0560
9.7119 9.5717 8.0468 9.5296 8.7225 8.4317 8.8923 9.0624
9.2062 7.4426 8.5736 8.9070 9.3835 9.1818 9.2517 8.4010
8.4541 8.7468 9.4999 9.1467 8.7049 9.8607 8.9472 9.2839
8.4203 9.3393 9.1538 9.0610 9.7124 9.2648 9.8748 8.1753
7.3576 9.0031 8.7883 9.5709 9.1272 8.4987 9.1178 9.1453
9.0523 9.0259 9.7387 9.6587 9.1576 9.1642 8.9522 8.9883
8.5405 9.2480 8.3658 7.9702 8.7822 9.3809 8.7900 9.4207
9.2964 8.3130 8.5805 9.6468 9.0724 8.5945 9.3094 9.1515
9.0137 8.8196 8.8112 9.1712 8.5084 9.0808 8.1847 8.2763
9.1842 9.5909 9.3026 9.0785 9.4251 8.8390 8.3494 9.3965

9.5444  9.1681  9.1105  8.3894  8.6293  9.3496  8.8163  9.3337
9.2874  9.8219  8.3013  8.8375  9.0285  8.6520  9.3427  9.4076
9.2794  7.7390\
KIAA1267        9.4252  10.2200 10.0680 10.1912 9.7783  11.0561
10.1485 10.1982 9.7501  9.9846  9.4057  10.2403 9.7933  10.9624
10.0292 10.0492 9.3799  9.9846  9.9938  9.1207  9.3667  10.0447
9.9383  10.2643 10.4251 8.3151  9.6671  9.2596  9.0974  9.9848
9.7469  10.3804 9.1312  9.7051  10.9471 9.9646  10.4864 10.2859
9.6414  10.1937 9.4221  10.1446 10.1831 9.8464  10.3321 9.1755
7.8657  10.2158 10.6458 9.4182  9.8458  9.8771  10.5160 10.6464
10.4507 10.2169 10.2536 10.7534 10.1933 10.5682 10.5011 10.3727
9.7640  9.6230  10.0102 10.1967 9.5245  9.9701  9.5441  9.5749
9.6568  10.5864 8.3351  9.3029  10.1287 10.2331 9.8223  9.8857
9.6936  10.1735 9.0983  9.3544  10.0176 9.7539  9.3103  10.2898
10.8688 9.1978  9.9572  9.8798  10.1564 10.6359 8.7950  10.0168
9.8116  9.8442  9.7544  10.8501 9.9057  10.1895 8.4601  9.8551
10.0295 9.9634  9.1464  9.6584  9.9560  9.8203  10.4691 10.1598
10.4070 10.0727 8.2501  9.2216  10.2463 10.2666 9.7699  11.1730
9.7239  9.9691  10.6713 10.0500 9.8947  10.0245 10.3834 9.5425
9.5047  7.5269  10.6251 10.5542 9.3285  9.3978  9.6828  10.0736
8.6865  7.6357  8.7566  10.2097 10.5612 10.6705 9.1257  9.3563
9.7984  9.9306  9.7706  10.8143 10.5786 10.1908 10.0700 8.4934
10.3661 6.8940  10.2112 10.7786 9.4850  9.9316  10.7809 10.2941
8.7346  8.9022  8.9591  8.7886  10.0827 9.6326  9.5269  10.6249
9.2094  10.3055 9.9718  10.1291 10.2047 10.6838 9.6113  10.2407
8.5255  10.7214 10.3125 10.0737 10.4970 9.6606  9.0829  8.7206
10.1597 9.7926  9.9234  9.9404  10.6640 9.5519  8.9547  9.9756
10.2555 9.9255  9.7258  10.0294 9.9426  7.8410  7.8608  10.6322
10.1653 10.4516 7.7762\
RTCD1   9.0955  8.7523  9.7536  9.5203  9.4174  9.8283  9.6594
9.2391  9.7073  8.9220  10.1831 9.5277  9.2588  8.1504  9.5960
9.3817  9.0001  9.6121  8.7943  9.7797  9.1079  9.3987  8.8441
9.5271  8.7827  9.3879  9.2640  9.6029  9.6730  9.0855  9.4740
9.4493  9.5120  9.4440  9.7724  9.0342  8.8018  9.3552  9.9895
10.3623 10.2387 8.9745  9.4931  9.9097  8.6733  9.4253  9.0077
9.7094  9.7387  9.6261  9.3699  9.1169  9.4126  8.8813  8.9261
9.7905  9.5017  9.7945  8.6735  9.3900  8.4863  9.9607  10.0304
8.9806  8.9669  9.3128  9.6798  9.8421  9.3424  9.4780  9.8782
9.3676  9.5549  9.5007  10.0301 9.8289  9.1295  9.3849  9.3946
9.5336  9.7971  10.1586 9.3151  10.1960 9.4612  9.4753  9.6500
8.9922  9.1074  9.1095  8.7934  9.4690  9.4957  9.5180  9.5080
8.7759  9.9443  8.6676  9.4405  9.3481  9.3126  9.4897  9.0754
9.2569  9.9534  9.9152  9.4031  9.7832  9.5221  9.6366  9.7969
9.5826  8.9777  8.6653  9.8590  9.8133  9.4871  9.2599  10.1111
9.3196  9.2742  9.5238  9.9085  9.9450  8.9620  9.1662  9.3335
8.0671  8.6059  8.7185  9.5685  9.7145  9.6647  10.0484 10.4340
8.3056  9.6997  9.0241  9.0179  8.6013  10.0292 9.9459  9.4503
9.5905  9.7858  8.5499  8.8407  8.6280  9.7043  9.8405  9.2853
9.4245  9.3522  9.5478  9.3456  9.0163  8.6842  9.4301  10.1261
9.6183  9.5109  9.4941  9.4415  9.1870  9.2299  10.0329 9.4294
10.3494 9.4096  9.8147  9.3325  8.8752  9.7671  8.7813  8.5999
8.9124  8.9638  8.7853  9.4151  9.3408  9.3023  8.6497  9.6377
9.5314  9.8272  9.6641  8.5121  10.2126 9.4788  7.5305  9.5865
9.3227  10.0090 9.3076  9.4540  9.7339  9.6945  8.6084  9.5639

9.4005  8.7688\
CFHR2   0.0000  0.0000  0.0000  0.0000  0.0000  0.0000  0.0000
0.4748  0.0000  0.0000  0.0000  0.0000  0.0000  0.0000  0.0000
2.6237  0.0000  4.4396  0.0000  0.3755  0.0000  0.0000  0.0000
4.4092  0.0000  0.0000  0.0000  0.0000  0.0000  0.0000  0.0000
0.0000  0.0000  0.0000  0.0000  0.0000  0.0000  0.6557  0.0000
0.0000  0.0000  0.0000  0.0000  0.0000  4.2185  0.0000  0.0000
0.0000  0.0000  0.0000  0.0000  0.0000  0.0000  0.0000  0.0000
0.0000  0.0000  0.0000  0.0000  0.0000  0.0000  0.0000  0.0000
0.0000  0.0000  0.0000  2.0292  0.0000  0.0000  0.0000  0.0000
0.3921  0.0000  0.0000  3.3238  0.0000  0.9546  0.0000  4.4071
0.0000  0.0000  0.0000  0.0000  0.0000  0.0000  0.0000  0.0000
0.0000  0.0000  0.0000  0.0000  0.0000  0.0000  0.0000  0.0000
0.0000  0.0000  0.0000  0.0000  0.0000  0.0000  1.6155  0.0000
0.0000  0.0000  0.0000  0.0000  0.0000  0.0000  0.0000  0.0000
0.0000  0.0000  0.0000  1.1918  0.0000  0.0000  0.0000  0.0000
0.0000  0.0000  0.0000  0.0000  0.0000  0.0000  0.0000  0.0000
0.0000  0.0000  0.0000  0.0000  0.6684  0.0000  0.0000  0.0000
0.0000  0.0000  0.0000  0.0000  0.0000  0.0000  2.2974  0.0000
0.0000  0.0000  0.5418  0.0000  0.0000  0.0000  0.0000  5.5218
0.0000  2.6925  0.0000  0.9437  0.0000  0.0000  0.0000  0.0000
0.0000  0.0000  0.0000  0.0000  0.0000  0.0000  0.0000  0.0000
0.0000  0.0000  0.0000  0.0000  0.0000  0.5410  0.0000  0.0000
0.0000  0.0000  0.0000  0.0000  0.0000  0.0000  0.0000  0.0000
0.0000  0.0000  0.0000  0.0000  0.0000  0.0000  0.0000  0.0000
0.0000  0.0000  0.7547  0.0000  3.4726  0.0000  1.0391  0.0000
0.0000  0.0000\
CFHR3   2.3786  0.0000  3.5887  2.5594  1.6818  4.5072  0.0000
3.1366  0.0000  0.4413  1.4500  0.8472  2.6546  0.0000  0.0000
0.0000  3.3455  0.7772  0.5838  0.9198  1.6493  3.3397  1.6825
4.0361  0.0000  0.0000  3.7300  2.0314  1.3857  0.0000  0.4127
0.0000  2.5183  0.0000  1.0004  1.1431  2.1003  0.3648  0.5410
0.0000  2.5069  0.4871  3.0982  1.9249  3.0451  2.4638  0.0000
0.0000  1.4771  0.0000  0.0000  0.0000  0.6494  1.1831  1.0973
0.0000  1.4095  5.0316  2.3545  0.0000  1.2440  1.4031  0.5573
0.5968  1.1445  2.1442  1.6109  3.6014  0.9886  2.2414  2.5620
3.4170  0.9449  2.3027  3.5626  0.0000  0.9546  0.5408  2.4028
0.0000  0.5659  0.0000  3.7339  3.4703  2.1768  3.0901  2.0569
2.0646  0.0000  1.4655  2.7094  1.1363  0.0000  3.6634  0.5198
0.0000  0.0000  0.0000  2.2653  0.5504  1.9211  0.8684  0.5352
1.5830  0.0000  0.8176  0.0000  4.7438  1.7151  0.0000  0.0000
1.0545  0.0000  0.0000  1.9988  3.5956  0.9131  1.0339  0.0000
1.0358  0.4553  0.0000  2.0828  0.4700  0.5970  0.0000  1.6443
0.0000  0.9653  0.0000  0.0000  3.3824  1.5194  0.7571  0.0000
0.0000  0.0000  0.0000  0.5216  1.1757  1.0240  0.5748  0.0000
1.2444  1.5157  2.3512  4.0927  0.5454  0.0000  0.0000  1.6233
0.0000  4.0429  1.5875  2.0813  1.9388  1.2837  0.6894  1.0225
0.0000  0.6063  2.3902  1.5791  0.4033  0.0000  1.4147  4.1489
3.0660  2.8443  2.0787  0.0000  1.2195  0.0000  0.0000  0.0000
2.1868  1.2087  1.4008  1.9582  2.2762  2.7277  1.5061  4.0725
0.0000  2.5017  1.0594  3.0773  1.8171  0.6567  0.9785  0.8827
4.2366  0.5674  0.0000  1.4064  2.3335  0.0000  1.0391  0.7736
3.1029  0.0000\
CLIC4   11.0366 13.4388 11.2033 10.4737 11.4314 11.6865 12.1144

| | | | | | | | |
|---|---|---|---|---|---|---|---|
| 10.5180 | 11.6036 | 12.0820 | 11.8724 | 11.5433 | 11.1818 | 11.6463 | 11.6569 |
| 9.8402 | 10.4954 | 10.1854 | 13.1441 | 11.5806 | 10.5491 | 11.2203 | 10.0886 |
| 10.9649 | 14.2180 | 10.2237 | 9.7824 | 10.5876 | 11.3095 | 10.9151 | 11.8053 |
| 10.6102 | 10.8667 | 11.1428 | 10.6312 | 11.1322 | 10.8789 | 10.7260 | 11.2588 |
| 12.3565 | 11.0102 | 13.6382 | 11.3241 | 11.0185 | 13.7009 | 11.0025 | 9.8310 |
| 11.2781 | 11.7714 | 9.9004 | 11.2191 | 11.4574 | 12.2076 | 13.4283 | 11.5612 |
| 10.9100 | 10.7825 | 11.4316 | 12.4236 | 9.5576 | 13.9973 | 12.0741 | 11.7600 |
| 11.5460 | 12.5229 | 11.3249 | 11.1818 | 11.1937 | 11.6212 | 11.7352 | 10.7185 |
| 12.2603 | 10.8657 | 9.5896 | 11.9933 | 10.7935 | 11.3370 | 10.5684 | 10.0937 |
| 12.0074 | 10.9628 | 11.7406 | 11.7452 | 11.9548 | 10.4232 | 10.8703 | 10.5077 |
| 10.8315 | 11.7769 | 11.4556 | 13.2543 | 10.5764 | 10.2887 | 10.8580 | 12.4147 |
| 14.3444 | 11.7452 | 13.1420 | 11.1022 | 11.1840 | 9.1295 | 11.0923 | 11.6494 |
| 10.9646 | 11.2518 | 11.7711 | 12.3906 | 11.8415 | 11.9929 | 10.8422 | 10.1865 |
| 11.3682 | 10.2889 | 9.9600 | 12.3845 | 11.8254 | 11.3825 | 10.6509 | 12.3537 |
| 10.5407 | 11.9660 | 11.6285 | 11.6307 | 10.6448 | 10.9397 | 11.7461 | 11.8127 |
| 9.2470 | 13.3363 | 14.1482 | 11.3594 | 11.7710 | 10.9195 | 10.9941 | 10.6079 |
| 9.6937 | 10.6812 | 10.0227 | 12.0650 | 13.1430 | 10.7912 | 11.3484 | 9.7128 |
| 11.8642 | 11.7343 | 11.4946 | 11.2997 | 12.4900 | 10.9111 | 10.3468 | 12.4107 |
| 8.9826 | 11.4293 | 10.1813 | 11.2218 | 12.4773 | 12.9744 | 11.6913 | 12.0550 |
| 11.2506 | 7.9081 | 10.0549 | 10.9650 | 10.4194 | 11.6931 | 11.9530 | 11.3463 |
| 11.3797 | 11.6707 | 11.9080 | 12.2123 | 11.4109 | 10.4304 | 10.3782 | 9.4012 |
| 11.8543 | 12.7181 | 13.5023 | 12.4376 | 10.6315 | 9.9436 | 10.7353 | 10.7215 |
| 9.9624 | 11.1030 | 11.0221 | 11.6818 | 10.5762 | 10.4399 | 12.2120 | 11.8907 |
| 11.2739 | 11.2671 | 11.2451 | 10.6622 | 9.4882 | 10.9695 | 12.6982 | 11.9557 |
| 11.5795 | 10.1277\ | | | | | | |
| CLIC5 | 3.3693 | 5.9352 | 8.1689 | 9.0741 | 5.8352 | 9.7651 | 4.0872 |
| 9.1975 | 8.1045 | 8.3575 | 7.7348 | 6.3452 | 9.1705 | 9.1568 | 7.1491 |
| 10.5392 | 8.1580 | 7.0480 | 7.0894 | 4.3662 | 12.1274 | 10.2499 | 8.7398 |
| 10.4560 | 7.4167 | 4.2688 | 11.0656 | 7.0244 | 4.2890 | 5.7294 | 10.0435 |
| 6.0055 | 5.4162 | 5.8644 | 6.0121 | 8.1050 | 9.2817 | 7.8218 | 9.3389 |
| 8.5241 | 7.3555 | 7.5953 | 4.1496 | 6.4198 | 8.6733 | 6.5691 | 8.8745 |
| 6.0163 | 6.1482 | 8.0695 | 5.3122 | 6.9670 | 5.3301 | 8.0070 | 9.1383 |
| 6.1846 | 10.7163 | 6.0094 | 9.1852 | 9.7868 | 8.5787 | 7.9763 | 6.3341 |
| 4.1206 | 8.0788 | 10.1740 | 10.3253 | 6.6673 | 9.5992 | 5.4109 | 9.2902 |
| 6.2231 | 7.4807 | 8.0709 | 8.2299 | 5.3314 | 5.8980 | 8.3271 | 9.7965 |
| 7.3895 | 10.4579 | 5.7755 | 9.1157 | 6.7033 | 7.4465 | 7.3498 | 6.2386 |
| 6.1395 | 8.1753 | 5.4931 | 8.8671 | 11.0778 | 3.2790 | 8.3781 | 8.7470 |
| 6.8208 | 7.1476 | 7.1548 | 9.6892 | 6.7212 | 5.7537 | 7.3834 | 5.7547 |
| 6.9858 | 9.9453 | 4.4321 | 6.0378 | 9.2754 | 9.5086 | 9.0849 | 7.2432 |
| 9.6167 | 9.9047 | 8.3576 | 3.4391 | 9.6168 | 9.0854 | 9.5084 | 9.2370 |
| 7.7982 | 9.3960 | 5.5027 | 6.4656 | 7.6856 | 8.8906 | 10.8082 | 5.7868 |
| 9.4967 | 8.3976 | 7.7742 | 6.4521 | 6.1760 | 7.4747 | 7.1120 | 5.7694 |
| 6.7228 | 5.9147 | 7.6776 | 7.0829 | 7.8199 | 8.0587 | 7.9946 | 7.0697 |
| 8.8417 | 9.6130 | 3.4632 | 11.5865 | 8.4965 | 6.3381 | 9.4914 | 9.6219 |
| 3.4258 | 9.9770 | 9.7629 | 8.9885 | 7.2301 | 8.1911 | 7.7611 | 9.0247 |
| 5.1548 | 3.5167 | 7.8854 | 8.5304 | 9.2201 | 9.4521 | 8.4667 | 8.4705 |
| 7.7145 | 10.4276 | 6.8134 | 7.6989 | 4.0444 | 8.0907 | 6.3479 | 5.5527 |
| 8.7124 | 7.5981 | 8.1566 | 8.2327 | 10.0966 | 5.9955 | 4.4091 | 11.9465 |
| 8.9681 | 6.1214 | 5.7288 | 10.1010 | 7.4679 | 6.7807 | 6.9285 | 6.7400 |
| 7.8203 | 5.7161 | 4.8150 | 9.1779 | 7.2141 | 9.2801 | 7.6121 | 9.7850 |
| 8.9211 | 6.8998\ | | | | | | |
| CLIC2 | 5.8680 | 9.6120 | 6.2780 | 7.1675 | 5.7430 | 7.3868 | 5.1029 |
| 6.1840 | 5.9753 | 8.1803 | 7.8235 | 5.9100 | 6.9026 | 5.1370 | 6.5897 |
| 5.6700 | 6.9903 | 7.1888 | 9.3242 | 5.7920 | 7.0075 | 6.7024 | 6.2081 |

| | | | | | | | |
|---|---|---|---|---|---|---|---|
| 7.0121 | 9.4951 | 5.8030 | 5.1974 | 7.2654 | 6.3513 | 5.1024 | 5.5251 |
| 6.9111 | 6.6359 | 6.7584 | 4.9078 | 6.1017 | 7.3780 | 6.2926 | 6.1326 |
| 5.1007 | 7.8040 | 9.6494 | 6.4854 | 8.5485 | 10.4639 | 6.7971 | 5.8880 |
| 6.0030 | 6.2865 | 6.3226 | 6.4111 | 7.9858 | 7.3739 | 10.5845 | 7.0736 |
| 6.7471 | 7.0728 | 7.9639 | 8.7662 | 5.9143 | 10.3650 | 7.4336 | 6.5815 |
| 5.9050 | 9.3773 | 7.2132 | 6.0268 | 6.7172 | 6.1067 | 7.4958 | 7.1939 |
| 6.4769 | 8.2323 | 6.9093 | 8.1244 | 5.7479 | 7.6362 | 7.2039 | 7.8539 |
| 7.7663 | 5.9983 | 6.2353 | 9.1767 | 7.4396 | 6.8998 | 6.4323 | 5.4109 |
| 6.1286 | 7.2634 | 7.1127 | 9.9729 | 7.3358 | 7.0916 | 8.1845 | 7.2681 |
| 9.6711 | 6.7797 | 9.7203 | 7.4149 | 8.0336 | 5.6292 | 7.5291 | 5.3564 |
| 7.7045 | 6.0764 | 6.1909 | 8.9812 | 8.4144 | 7.6078 | 6.5930 | 6.4840 |
| 5.0105 | 6.2269 | 5.8074 | 8.1797 | 6.6553 | 5.8460 | 6.4924 | 5.7192 |
| 7.6439 | 6.8767 | 6.2301 | 7.1254 | 5.7992 | 7.8998 | 7.2446 | 6.3816 |
| 4.3574 | 10.1712 | 9.8233 | 6.6407 | 6.4139 | 6.9181 | 8.0702 | 7.3500 |
| 6.9254 | 7.0683 | 9.0137 | 8.9484 | 9.6583 | 6.8738 | 6.6700 | 6.9485 |
| 6.9569 | 5.5235 | 9.1219 | 7.0688 | 8.6496 | 6.1187 | 6.0103 | 8.8740 |
| 5.4557 | 6.8549 | 6.1837 | 5.4421 | 6.4675 | 9.8592 | 6.0429 | 6.1630 |
| 7.1659 | 4.7885 | 5.7975 | 7.2056 | 6.1626 | 5.8750 | 6.7775 | 7.8241 |
| 7.8329 | 6.4076 | 5.5565 | 4.9834 | 6.1492 | 5.1343 | 5.2577 | 6.9155 |
| 5.9587 | 8.9625 | 9.4373 | 6.7039 | 8.6045 | 6.6624 | 5.2639 | 5.0673 |
| 7.0344 | 6.6573 | 8.2140 | 7.0654 | 6.0983 | 6.1909 | 5.8643 | 7.1994 |
| 7.4064 | 7.5946 | 7.3475 | 5.9172 | 5.4404 | 5.4370 | 9.4522 | 6.0708 |
| 6.9706 | 4.5812\ | | | | | | |
| CLIC3 | 5.6968 | 4.0917 | 5.9175 | 3.9712 | 7.4513 | 3.2887 | 4.4907 |
| 7.1972 | 4.3880 | 6.9425 | 5.2765 | 6.1068 | 6.7262 | 9.4601 | 3.5096 |
| 5.8094 | 7.2677 | 7.1238 | 5.5045 | 9.6875 | 8.3501 | 9.5651 | 6.1531 |
| 4.2844 | 4.9735 | 8.6152 | 1.3145 | 8.1396 | 6.8520 | 5.4745 | 1.5789 |
| 7.0366 | 6.4284 | 7.4359 | 5.5244 | 6.6010 | 7.8498 | 7.2673 | 6.9943 |
| 2.8702 | 6.7877 | 3.4648 | 8.3159 | 4.7293 | 3.9549 | 8.4122 | 7.9907 |
| 8.4039 | 2.0741 | 9.1595 | 10.6538 | 6.8108 | 7.3640 | 3.4251 | 8.4947 |
| 7.8632 | 5.8956 | 3.3475 | 7.4008 | 7.8575 | 4.3003 | 3.2053 | 9.6744 |
| 4.7888 | 7.2280 | 7.2313 | 6.5141 | 3.8604 | 10.9207 | 7.1727 | 6.2207 |
| 6.3665 | 9.8463 | 7.2107 | 5.3771 | 1.1076 | 5.5961 | 7.7945 | 6.4793 |
| 7.3382 | 8.8892 | 1.9437 | 6.9244 | 3.4703 | 5.4201 | 8.9619 | 5.3799 |
| 7.3041 | 6.6155 | 4.9310 | 6.4862 | 3.9427 | 7.5393 | 6.1872 | 3.8956 |
| 4.9755 | 7.9595 | 6.0987 | 3.6832 | 8.6371 | 5.9090 | 7.1135 | 8.7493 |
| 7.1503 | 2.7166 | 7.1772 | 5.3670 | 5.8534 | 2.4767 | 6.9854 | 10.3599 |
| 3.6834 | 10.2334 | 7.0000 | 6.9919 | 6.8982 | 5.5923 | 4.5665 | 9.1349 |
| 7.5780 | 6.2750 | 5.7251 | 6.0502 | 9.4841 | 5.8165 | 7.1433 | 5.4783 |
| 7.7658 | 4.4874 | 4.1923 | 6.0964 | 3.7414 | 5.3292 | 4.6528 | 8.3884 |
| 6.3991 | 6.8616 | 6.6977 | 8.1524 | 4.9249 | 4.6307 | 7.2959 | 6.3858 |
| 8.9967 | 8.9191 | 0.9349 | 8.1354 | 7.7762 | 6.6828 | 10.0043 | 6.1814 |
| 3.7730 | 5.3918 | 9.4735 | 5.3140 | 6.3904 | 5.4291 | 7.5103 | 3.8996 |
| 8.7021 | 3.5811 | 6.9296 | 4.2397 | 7.5124 | 3.7970 | 3.6142 | 5.6157 |
| 4.3389 | 8.1182 | 5.9920 | 5.4843 | 5.3049 | 3.2000 | 3.6252 | 7.5617 |
| 6.0214 | 7.1111 | 5.1698 | 5.6820 | 6.7633 | 8.5380 | 7.1877 | 2.9247 |
| 3.9324 | 5.5740 | 4.9453 | 7.9829 | 6.6356 | 6.9449 | 5.1278 | 5.9873 |
| 6.1592 | 7.2601 | 7.4977 | 6.9684 | 5.9450 | 9.2568 | 5.1411 | 7.3143 |
| 7.5469 | 7.6168\ | | | | | | |
| CFHR4 | 0.0000 | 0.0000 | 0.6896 | 0.0000 | 0.0000 | 1.7112 | 0.0000 |
| 0.0000 | 0.4327 | 0.0000 | 1.4500 | 0.0000 | 0.4363 | 0.0000 | 0.0000 |
| 0.0000 | 0.0000 | 1.2795 | 0.0000 | 0.0000 | 0.0000 | 0.0000 | 0.0000 |
| 0.5262 | 0.0000 | 0.0000 | 1.3145 | 0.0000 | 0.0000 | 0.5901 | 0.0000 |
| 0.0000 | 0.5159 | 0.0000 | 0.0000 | 0.0000 | 0.0000 | 0.0000 | 0.0000 |

0.0000 0.0000 0.0000 0.6231 0.0000 0.0000 0.0000 0.0000
1.4786 0.0000 0.0000 0.0000 1.2789 0.0000 0.0000 0.0000
0.0000 0.0000 1.1710 0.0000 0.0000 0.0000 0.0000 0.0000
0.5968 0.0000 0.0000 0.0000 1.7598 0.0000 0.6160 0.4334
0.0000 0.0000 0.0000 1.2339 0.0000 0.0000 0.0000 0.0000
0.0000 0.0000 0.9627 0.0000 0.0000 0.0000 0.0000 0.0000
0.0000 0.5273 0.0000 0.0000 0.0000 0.0000 0.0000 0.0000
0.0000 0.5233 0.0000 0.0000 0.0000 0.0000 0.4986 0.0000
0.0000 0.0000 0.4659 0.0000 0.0000 0.0000 0.0000 0.0000
0.0000 0.0000 2.0000 0.8924 0.0000 0.0000 0.0000 0.0000
0.0000 0.8008 0.0000 0.0000 0.0000 0.0000 0.0000 0.0000
0.0000 0.0000 0.0000 0.0000 0.0000 0.0000 1.6184 0.0000
0.0000 0.0000 0.0000 0.0000 0.0000 0.0000 0.0000 0.0000
0.0000 0.0000 0.0000 1.1308 0.0000 0.0000 0.0000 0.0000
0.0000 0.9341 0.0000 0.0000 0.0000 0.0000 0.0000 0.0000
0.0000 0.0000 0.0000 0.0000 0.0000 0.0000 0.0000 0.0000
0.0000 0.0000 0.0000 0.7903 0.0000 0.0000 0.0000 0.0000
0.0000 0.0000 0.0000 0.7148 0.0000 0.0000 0.0000 1.2133
0.0000 0.0000 0.0000 0.0000 0.0000 0.0000 0.0000 0.0000
0.0000 0.0000 1.0222 0.0000 1.5946 0.0000 0.0000 0.0000
0.0000 0.0000\
CFHR5 0.0000 0.0000 0.0000 0.4935 0.0000 0.0000 0.0000
1.5600 0.0000 0.0000 0.0000 0.0000 0.0000 0.0000 0.0000
2.6237 0.0000 0.7772 0.0000 0.0000 0.0000 0.0000 0.0000
2.7488 0.0000 0.0000 0.0000 0.0000 0.0000 0.0000 0.0000
0.0000 0.0000 0.8184 0.0000 0.6819 0.5555 1.1048 0.0000
0.0000 0.0000 0.0000 1.0569 0.7649 2.3632 1.4889 0.0000
0.0000 0.0000 0.0000 0.0000 0.0000 0.6494 0.0000 1.7133
0.0000 0.0000 0.8754 0.0000 0.0000 0.0000 0.0000 0.0000
0.0000 0.6828 0.0000 1.0195 0.4832 0.0000 0.0000 1.0365
0.3921 0.0000 0.0000 2.2033 0.0000 0.0000 0.0000 1.5048
0.0000 0.0000 0.0000 0.7204 0.0000 0.0000 0.0000 0.0000
0.0000 0.0000 0.9113 0.0000 0.0000 0.0000 0.0000 0.0000
0.0000 0.0000 0.0000 2.1324 0.0000 0.0000 1.6155 0.0000
0.0000 0.0000 0.0000 0.0000 0.4315 0.0000 0.0000 0.5136
0.0000 0.0000 0.0000 0.0000 1.3697 0.0000 0.0000 0.0000
0.0000 0.0000 0.0000 0.6224 0.0000 0.0000 0.0000 0.0000
0.0000 0.0000 0.0000 0.0000 0.0000 0.0000 0.7571 0.0000
0.0000 0.0000 0.0000 0.5216 0.0000 0.0000 3.0604 0.0000
0.0000 0.0000 0.9349 0.0000 0.0000 0.0000 0.0000 4.9717
0.0000 3.2708 0.0000 0.5476 0.0000 0.0000 0.0000 0.6000
0.0000 0.0000 0.0000 0.0000 0.0000 0.0000 0.0000 0.0000
0.0000 0.0000 0.0000 0.0000 0.5290 0.0000 0.0000 0.0000
0.0000 0.0000 0.0000 0.0000 0.0000 0.0000 0.0000 0.5257
0.0000 0.0000 0.0000 0.0000 0.0000 0.0000 0.0000 0.5078
0.0000 0.0000 0.0000 0.8682 2.8198 0.0000 1.0391 0.0000
0.8824 0.0000\
KRTAP2-2 0.0000 0.0000 0.0000 0.0000 0.0000 0.0000
0.0000 0.0000 0.0000 0.0000 0.0000 0.0000 0.0000 0.4395
0.0000 1.4442 0.0000 0.0000 0.0000 0.6732 0.0000 0.0000
0.0000 0.5262 0.0000 0.0000 0.0000 0.0000 0.0000 0.0000
0.0000 0.0000 0.0000 0.0000 0.0000 0.0000 0.0000 0.0000
0.0000 0.0000 0.0000 0.0000 0.0000 0.0000 0.0000 4.1830
0.0000 0.0000 0.0000 0.0000 0.0000 0.0000 0.0000 0.5094

2.7436  0.0000  0.0000  0.0000  0.0000  1.5581  0.0000  0.0000
0.0000  0.0000  0.6828  0.0000  0.0000  0.4832  0.0000  0.6160
0.0000  0.0000  0.0000  0.4786  2.0547  0.0000  0.0000  0.0000
0.0000  0.0000  0.0000  0.0000  0.0000  0.0000  0.0000  3.5530
0.0000  0.6140  1.2163  0.0000  0.0000  0.0000  2.4205  0.0000
0.5198  0.6266  0.0000  0.0000  0.5090  0.0000  0.0000  0.0000
1.8854  0.7358  0.0000  0.0000  0.0000  0.4315  0.0000  0.0000
0.0000  0.0000  0.0000  2.5850  0.0000  0.6116  0.0000  0.0000
0.0000  1.0358  0.0000  0.0000  0.0000  0.0000  0.0000  0.5331
0.0000  0.0000  0.0000  0.0000  0.0000  0.6684  0.0000  0.0000
3.8654  0.0000  0.0000  0.0000  0.0000  0.0000  0.0000  0.5748
0.6506  2.4761  0.0000  0.0000  0.0000  0.0000  0.0000  0.0000
0.0000  0.0000  0.0000  0.0000  0.0000  0.4374  0.0000  0.3855
0.0000  0.0000  0.0000  0.0000  0.0000  0.0000  0.0000  0.0000
0.0000  0.0000  0.0000  0.0000  0.0000  0.0000  0.0000  0.0000
0.0000  0.0000  0.0000  0.0000  0.0000  0.6314  0.0000  0.6902
0.5257  0.0000  0.0000  0.0000  0.0000  0.0000  0.0000  0.0000
0.0000  0.9557  0.0000  1.0222  0.0000  0.0000  0.0000  0.0000
0.0000  0.0000  0.0000\
KRTAP2-1        0.5526  0.0000  0.0000  0.0000  1.2172  0.0000
0.0000  1.5600  2.9347  0.0000  0.4291  1.3775  0.0000  0.4395
0.0000  1.4442  1.6582  0.7772  0.0000  3.3103  0.0000  0.0000
0.6346  0.5262  0.0000  0.0000  0.0000  0.0000  0.8532  1.0077
0.0000  0.0000  2.5183  0.8184  1.0004  0.0000  0.0000  0.3648
0.0000  0.0000  0.0000  0.4871  2.5514  0.0000  0.0000  4.4447
0.0000  0.0000  0.4402  0.0000  0.7972  1.2789  0.6494  0.0000
3.2259  0.9635  1.6820  1.4162  2.4481  1.2977  0.0000  0.0000
0.0000  0.0000  0.6828  0.0000  0.0000  0.8444  0.9886  0.0000
0.0000  0.0000  0.0000  2.7868  1.8890  0.0000  0.9546  0.0000
0.0000  0.6159  0.5659  1.9437  0.0000  0.0000  0.9110  2.7877
0.0000  0.6140  0.9125  0.0000  0.0000  0.8470  1.4549  1.5073
0.0000  0.0000  1.4589  0.0000  0.5090  1.2591  0.0000  1.6155
2.4570  0.0000  0.0000  0.0000  0.0000  4.3090  1.7151  0.0000
0.0000  0.0000  0.0000  4.4594  2.7148  2.5241  0.0000  2.0505
0.7085  1.3647  0.0000  0.0000  2.0828  0.8239  0.0000  3.8490
0.0000  0.0000  0.0000  0.5074  1.6759  0.0000  0.0000  0.0000
7.4342  1.3313  0.0000  0.0000  0.0000  0.0000  0.0000  0.5748
0.6506  2.5899  0.4562  0.0000  0.0000  0.0000  0.0000  0.7381
0.8737  0.0000  0.0000  1.3242  0.9437  2.0663  0.0000  0.0000
0.0000  0.0000  0.0000  0.0000  0.0000  0.7182  0.0000  0.0000
0.0000  0.0000  0.0000  0.0000  2.3253  0.0000  0.0000  0.0000
0.0000  0.0000  0.9063  0.0000  0.0000  0.0000  1.7413  0.6902
0.5257  0.0000  2.5017  0.6248  0.5811  0.0000  0.0000  0.5707
0.0000  1.2686  0.0000  3.2091  0.4985  0.0000  0.0000  0.0000
0.6701  0.8824  0.0000\
CXorf1  0.0000  0.0000  3.7286  0.4935  0.0000  0.0000  0.5431
0.0000  3.2846  0.0000  1.2544  0.0000  0.0000  0.4395  0.5377
0.0000  0.0000  0.0000  0.0000  0.0000  0.6175  0.0000  1.6825
0.0000  0.0000  0.0000  0.0000  0.0000  0.0000  1.5953  0.0000
0.0000  0.0000  0.0000  0.0000  0.0000  0.0000  1.1048  0.0000
0.0000  0.0000  0.0000  0.6231  0.0000  2.3632  0.0000  0.0000
0.0000  0.0000  0.0000  0.0000  0.7768  2.3160  0.8853  0.0000
1.7550  0.0000  0.0000  0.0000  0.9799  0.0000  1.7937  0.0000
0.0000  0.0000  0.6977  0.0000  2.4264  0.0000  0.0000  0.0000

0.3921  0.0000  1.3638  0.9270  0.0000  0.0000  0.0000  0.0000
0.0000  0.0000  0.0000  0.7204  0.0000  0.5263  0.0000  0.0000
0.0000  1.8667  0.0000  1.0759  3.1679  0.5215  0.0000  0.0000
0.6266  0.5233  0.9402  0.0000  0.0000  0.0000  0.0000  0.9246
0.0000  0.0000  0.0000  0.0000  0.7631  2.4767  0.0000  0.0000
0.6215  1.3631  0.0000  0.5141  0.6116  0.0000  2.7941  0.0000
0.0000  0.8008  0.0000  3.0961  0.0000  3.1191  0.0000  0.0000
0.0000  0.0000  0.0000  0.0000  0.0000  0.0000  2.3620  0.0000
0.0000  0.0000  0.0000  0.0000  0.0000  0.0000  0.0000  0.0000
0.0000  0.0000  0.5418  0.0000  3.1391  2.1615  1.2240  0.0000
0.0000  0.5414  0.0000  0.0000  0.4374  0.0000  0.3855  0.0000
0.0000  0.0000  2.3902  0.0000  0.0000  0.0000  0.0000  0.0000
3.2228  0.5276  2.2280  0.0000  0.0000  2.2140  0.0000  0.0000
1.7522  0.0000  0.6293  0.0000  0.0000  0.0000  0.0000  2.6495
0.0000  2.6622  0.0000  0.9943  1.5325  3.1812  0.0000  0.0000
1.2686  0.0000  0.0000  0.0000  0.0000  0.0000  0.0000  0.0000
0.0000  0.0000\
KIAA0020          9.6320  8.8363  9.6341  9.4781  9.7311  10.1595
10.6408 10.3716 9.7276  9.2826  10.2891 9.4123  9.4139  9.2884
9.6086  8.8845  7.7191  9.5267  8.7894  9.1714  9.9657  9.2154
8.0742  9.8441  8.8309  10.1213 10.4296 9.3992  9.1618  10.5560
9.8248  9.7090  10.6305 10.0256 10.1564 10.1858 9.1709  9.3621
9.2978  9.9154  9.8595  9.6779  9.3795  10.9230 8.7758  9.0160
9.1575  10.0603 10.5484 9.2522  9.9075  9.8281  9.1905  9.0487
9.7338  9.5729  9.4939  8.8055  9.2908  9.1678  9.0870  9.1160
9.2283  9.4946  9.1394  9.1383  8.5504  9.1523  9.6183  8.7775
11.1081 9.8250  9.3349  9.4929  9.7913  9.2957  10.1337 8.7853
8.9613  9.9085  10.0773 9.7027  8.7581  9.6124  9.3371  8.9400
10.3069 9.6973  10.2416 9.3707  9.0120  9.8934  9.4362  9.0512
9.6620  8.0399  9.4095  8.6693  9.7422  9.5847  9.7312  9.4814
8.5785  8.6707  9.3746  10.2552 9.5765  9.5662  8.9219  8.7763
9.3548  9.8883  9.0479  8.5736  9.9215  10.8477 10.0550 8.7238
9.3597  9.7640  9.6956  9.8054  9.5594  8.9649  9.2777  9.9026
10.4256 8.3927  9.0094  9.3705  9.9880  9.9925  9.4705  10.0466
9.6102  9.6818  8.8936  9.3056  9.9010  8.8861  9.2692  9.6302
9.4966  10.0049 10.3168 9.3077  9.1480  8.5958  9.5686  8.9925
9.1608  9.0210  9.4064  9.1498  10.4812 9.1053  9.0260  10.0571
10.2102 10.1256 9.5576  9.8865  9.4851  10.0623 9.8069  9.6360
8.8644  10.1416 9.4171  9.1959  9.9829  10.7054 9.5069  9.3810
8.9190  9.5345  9.6828  9.2322  9.9223  8.8091  9.9353  9.1353
10.7275 9.4191  10.3701 9.2986  8.7959  9.5163  9.1649  10.5764
9.9313  9.6381  10.1405 9.5170  9.6893  10.4831 9.8939  9.0108
9.3255  9.4193  10.4961\
KRTAP2-4          0.0000  0.0000  0.0000  0.0000  0.0000  0.0000
0.0000  0.0000  0.0000  0.0000  0.0000  0.0000  0.0000  0.0000
0.0000  0.0000  0.0000  0.0000  0.0000  0.0000  0.0000  0.0000
0.0000  0.0000  0.0000  0.0000  0.0000  0.0000  0.0000  0.0000
0.0000  0.0000  0.0000  0.0000  0.0000  0.0000  0.0000  0.0000
0.0000  0.0000  0.0000  0.0000  0.0000  0.0000  0.0000  3.5656
0.0000  0.0000  0.0000  0.0000  0.0000  0.0000  0.0000  0.0000
1.8714  0.5608  0.0000  0.0000  0.0000  0.0000  0.0000  0.0000
0.0000  0.0000  0.0000  0.0000  0.0000  0.0000  0.0000  0.0000
0.0000  0.0000  0.0000  0.0000  2.6798  0.0000  0.0000  0.0000
0.0000  0.0000  0.0000  0.0000  0.0000  0.0000  0.0000  0.0000

0.0000  2.0646  0.0000  0.0000  0.0000  0.0000  0.0000  0.0000
0.0000  0.0000  0.0000  0.0000  0.0000  0.0000  0.0000  0.0000
0.0000  0.0000  0.0000  0.0000  0.0000  0.0000  0.0000  0.0000
0.0000  0.0000  0.0000  0.0000  0.0000  0.0000  0.0000  0.0000
0.0000  0.0000  0.0000  0.0000  0.0000  0.0000  0.0000  0.0000
0.0000  0.0000  0.0000  0.0000  0.0000  0.0000  0.0000  0.0000
0.8107  0.0000  0.0000  0.0000  0.5216  0.0000  0.0000  0.0000
0.0000  3.9640  0.0000  0.0000  0.0000  0.0000  0.0000  0.0000
0.5019  0.0000  0.0000  0.0000  0.0000  0.0000  0.0000  0.0000
0.0000  0.0000  0.0000  0.0000  0.0000  0.0000  0.0000  0.0000
0.0000  0.0000  0.0000  0.0000  0.0000  0.0000  0.0000  0.0000
0.0000  0.0000  0.0000  0.0000  0.0000  0.0000  0.0000  0.0000
0.0000  0.0000  0.0000  0.0000  0.0000  0.0000  0.0000  0.0000
0.0000  0.0000  0.0000  0.0000  0.0000  0.0000  0.0000  0.0000
0.0000  0.0000  0.0000\
TMEM147 10.9620 10.7535 10.3184 10.6609 11.0047 9.8102  11.0222
12.4603 11.1429 10.7844 11.2542 10.8221 10.2864 10.5026 10.5925
11.1405 11.4223 11.2877 10.7821 12.5419 10.7333 10.8112 10.5157
10.4473 10.5256 11.9673 11.6124 11.5828 10.6035 10.7471 10.0105
10.7470 12.0961 11.3729 11.2756 11.7767 9.9495  10.6216 10.8857
11.5574 11.5994 10.2330 11.2582 9.7894  9.7587  11.5165 11.7098
10.3711 10.7352 11.6899 10.3624 10.8670 11.2138 10.0034 10.6721
10.9273 10.4394 10.9923 10.4707 11.6295 9.9638  10.2084 11.0837
10.9040 10.8464 10.4759 11.2030 12.1960 11.1003 11.5717 12.0911
10.8139 12.0291 12.5325 10.0450 11.1202 10.8423 10.5454 11.4018
11.3396 11.9423 11.0219 10.3517 11.2568 11.5249 10.6537 9.9259
11.2447 11.1129 10.8528 10.4654 10.5203 11.4877 11.2275 10.8232
10.5019 11.5855 10.4363 11.3315 11.0123 12.2359 10.3523 10.3950
10.8340 11.3892 11.5549 10.7096 10.4875 11.7105 11.4226 10.4490
10.8195 12.7030 11.9549 10.2412 11.0913 10.7673 10.8513 10.3742
11.3277 11.5266 10.9055 11.5538 10.8074 10.2700 10.4788 10.7670
13.2506 10.0751 9.7790  12.3017 11.3174 10.8559 10.0634 13.3848
12.5334 11.4480 9.7589  10.3102 10.2223 11.4115 11.0798 11.2431
10.4661 10.5997 10.5316 10.6331 10.4713 11.0425 12.1288 10.5804
11.7116 10.1314 11.9066 11.1462 10.6569 10.5024 10.6381 11.6534
12.2150 10.9768 10.7696 11.1906 11.1268 11.1459 10.2057 11.6378
11.8339 11.4686 10.6366 10.3984 11.1506 11.1409 11.2069 11.2835
10.3920 10.8135 10.4242 9.9775  10.0966 11.5848 11.4185 12.4331
11.2526 11.1052 10.8878 10.5707 11.1884 11.4162 11.1655 10.5356
10.6696 11.5344 11.5694 10.7660 12.5051 12.2138 10.5559 10.3386
10.4084 12.1673\
ASGR1   3.8143  5.2311  3.9734  3.5865  4.5829  3.5106  3.8334
4.8217  1.9254  5.2147  3.5100  5.2386  5.1396  1.6493  2.8727
3.5017  5.8345  5.9127  5.4885  4.9555  5.5362  2.9530  3.8339
3.7828  4.4323  6.5214  5.0432  3.8969  4.9467  3.4063  4.9608
3.3937  5.5678  3.2898  3.1707  3.7749  6.7994  2.2449  2.8817
1.4612  4.5390  4.9784  4.7220  3.4777  4.5728  3.1906  5.7576
3.6906  3.4104  5.5598  3.8726  4.6169  5.8543  4.1989  6.9985
4.6016  3.5225  4.5565  4.8117  2.1385  4.8029  4.2235  2.1044
6.2539  5.3344  4.6225  4.5289  5.2627  3.6228  4.5466  5.2129
3.0867  7.0979  4.2552  4.7622  4.1336  4.1607  3.7796  4.0814
4.9705  3.3401  1.9437  2.9023  4.2322  4.8446  5.5432  4.1826
5.9295  4.3821  5.3908  5.5878  2.4311  7.7230  6.6860  2.8225
5.4223  5.0216  4.9690  3.3051  5.4658  5.7832  5.1736  8.8369

| | | | | | | | |
|---|---|---|---|---|---|---|---|
| 5.5721 | 0.8449 | 2.6639 | 5.5202 | 3.4735 | 3.7851 | 4.4001 | 3.2557 |
| 3.5571 | 6.1963 | 4.7549 | 3.8350 | 5.1850 | 3.0116 | 3.3332 | 2.8760 |
| 5.4953 | 5.9765 | 4.3681 | 3.5592 | 2.5865 | 4.2038 | 4.5175 | 3.3270 |
| 5.4465 | 4.5177 | 4.2888 | 4.4806 | 2.5146 | 4.1912 | 3.6704 | 5.1267 |
| 6.9784 | 5.6349 | 6.5616 | 3.2795 | 3.8923 | 2.6248 | 2.4344 | 5.2054 |
| 3.9211 | 3.5126 | 2.2162 | 3.2216 | 6.1556 | 5.5593 | 3.7031 | 4.4467 |
| 5.3214 | 5.1356 | 4.9810 | 3.8924 | 4.9756 | 4.3312 | 3.2594 | 2.8458 |
| 6.4672 | 2.0474 | 5.4227 | 3.4515 | 4.0707 | 2.6724 | 3.3452 | 3.1709 |
| 4.1948 | 4.6089 | 3.9335 | 4.1220 | 2.3178 | 7.9115 | 5.4659 | 2.3719 |
| 4.2300 | 5.1687 | 5.5185 | 4.7362 | 3.9843 | 3.7712 | 5.7319 | 3.9547 |
| 2.6905 | 3.4586 | 3.9157 | 6.5585 | 6.6628 | 4.6226 | 4.5435 | 4.2586 |
| 3.3779 | 3.5364 | 3.8823 | 3.0753 | 5.8969 | 7.9519 | 4.1603 | 2.1850 |
| 3.5808 | 6.0996\ | | | | | | |
| ASGR2 | 3.7656 | 1.6912 | 0.6896 | 3.1949 | 2.0327 | 0.4059 | 0.9367 |
| 0.0000 | 0.7652 | 1.4796 | 2.1584 | 0.8472 | 3.1323 | 0.4395 | 1.8915 |
| 3.2639 | 0.0000 | 5.2523 | 1.5827 | 1.6235 | 3.6727 | 1.1310 | 2.5783 |
| 2.1767 | 1.2653 | 3.3784 | 1.3145 | 3.9933 | 3.1802 | 1.3312 | 1.4094 |
| 0.5612 | 1.4434 | 2.2682 | 0.0000 | 2.3867 | 0.9556 | 0.3648 | 0.5410 |
| 0.7666 | 2.5069 | 3.1095 | 3.2696 | 2.2769 | 3.9549 | 4.9870 | 4.8968 |
| 0.0000 | 2.0741 | 1.5638 | 2.7870 | 4.8841 | 2.6127 | 2.6047 | 2.2625 |
| 0.5608 | 1.9111 | 6.3042 | 3.3585 | 1.2977 | 2.4755 | 2.2329 | 0.5573 |
| 1.3432 | 2.2116 | 2.4204 | 3.3568 | 0.0000 | 2.1522 | 1.0463 | 3.6511 |
| 1.3570 | 6.1371 | 5.6824 | 3.7675 | 1.1076 | 2.0987 | 2.2135 | 4.0282 |
| 2.3950 | 0.9714 | 0.9627 | 3.5062 | 2.9116 | 2.5465 | 0.0000 | 0.5376 |
| 3.6635 | 5.5778 | 2.7496 | 3.7213 | 1.5834 | 4.6152 | 5.1704 | 1.2026 |
| 2.0921 | 1.2090 | 1.5047 | 1.1822 | 3.7089 | 3.7293 | 4.5418 | 0.5352 |
| 3.6218 | 0.4835 | 1.3363 | 1.2143 | 2.7919 | 1.2448 | 2.1763 | 1.6498 |
| 1.6572 | 2.0513 | 1.5850 | 1.9988 | 2.3852 | 2.3144 | 1.4572 | 1.3703 |
| 5.0457 | 1.9886 | 1.3285 | 0.6224 | 1.5487 | 1.6090 | 4.4003 | 0.6149 |
| 1.6629 | 1.5385 | 1.8193 | 0.7024 | 1.1234 | 2.9134 | 2.7051 | 3.0945 |
| 5.6948 | 4.6151 | 5.3310 | 1.4553 | 0.0000 | 1.0240 | 2.1461 | 2.7437 |
| 1.4986 | 3.1987 | 1.4974 | 1.7568 | 3.6421 | 2.3133 | 0.7381 | 1.9683 |
| 4.5495 | 4.2979 | 2.0029 | 1.2540 | 3.9564 | 0.0000 | 1.3399 | 1.0225 |
| 5.2615 | 1.3600 | 4.3660 | 1.3167 | 3.2305 | 0.4418 | 1.4147 | 1.9676 |
| 3.2228 | 1.4679 | 5.4692 | 0.4486 | 0.9151 | 2.3490 | 0.6229 | 0.0000 |
| 0.4801 | 1.2087 | 2.4262 | 1.5479 | 3.8257 | 0.9540 | 1.5061 | 0.5257 |
| 2.1234 | 1.2218 | 2.4158 | 4.8218 | 1.5325 | 5.5042 | 3.8177 | 3.1725 |
| 1.7437 | 2.6556 | 2.6218 | 2.2371 | 1.5946 | 1.3603 | 3.0678 | 1.6179 |
| 2.5996 | 3.2749\ | | | | | | |
| PCDHA1 | 6.1911 | 3.7402 | 2.1677 | 6.8773 | 2.0107 | 4.6306 | 6.5008 |
| 5.5749 | 3.2975 | 4.0849 | 4.7582 | 6.1419 | 2.8706 | 6.4625 | 3.3823 |
| 7.1236 | 8.0383 | 7.3662 | 2.8229 | 0.9711 | 6.4470 | 6.4842 | 3.5783 |
| 6.9474 | 1.3942 | 4.9933 | 0.0000 | 5.9763 | 0.8787 | 4.3740 | 0.0880 |
| 4.9569 | 0.0000 | 2.4333 | 2.7012 | 4.7471 | 7.8138 | 5.8923 | 0.3687 |
| 2.4656 | 1.2940 | 3.3284 | 5.8647 | 0.0000 | 4.4086 | 7.9173 | 0.8882 |
| 2.4460 | 5.3490 | 7.4021 | 0.0000 | 5.9312 | 6.3078 | 4.0622 | 7.2809 |
| 2.7717 | 3.1160 | 2.8030 | 4.6961 | 5.9390 | 4.4499 | 3.8919 | 2.3787 |
| 5.3642 | 5.9995 | 1.2811 | 0.0000 | 6.0885 | 2.1425 | 5.4660 | 7.4885 |
| 6.4711 | 0.0000 | 4.3910 | 7.2642 | 3.2136 | 7.3585 | 5.1649 | 0.0000 |
| 6.1051 | 0.0000 | 3.9149 | 5.6077 | 3.6884 | 6.8962 | 8.6768 | 6.8893 |
| 0.0000 | 2.8200 | 7.2971 | 2.1615 | 4.7244 | 6.8355 | 7.2826 | 3.3134 |
| 3.9513 | 5.6440 | 4.3972 | 7.6574 | 7.4623 | 0.0858 | 2.3689 | 5.3812 |
| 0.7358 | 1.4871 | 5.6772 | 0.0000 | 8.7699 | 6.5206 | 6.7483 | 0.0000 |
| 4.7418 | 6.7360 | 0.0000 | 2.9639 | 4.5164 | 0.0865 | 5.5497 | 5.9473 |

| | | | | | | | |
|---|---|---|---|---|---|---|---|
| 4.0776 | 5.7859 | 8.0034 | 5.4829 | 3.6658 | 4.8720 | 6.5021 | 2.4444 |
| 5.4858 | 4.2452 | 3.8714 | 4.6609 | 0.0826 | 5.2761 | 0.7571 | 4.6794 |
| 0.0000 | 0.0000 | 0.0000 | 3.0239 | 4.9457 | 1.8561 | 6.7770 | 3.8327 |
| 2.8320 | 5.3124 | 2.5349 | 4.0627 | 3.8808 | 3.1515 | 8.4155 | 4.0054 |
| 0.1846 | 5.6467 | 2.8563 | 1.4025 | 6.2841 | 3.5958 | 6.4062 | 0.0796 |
| 4.8860 | 5.9523 | 3.2047 | 0.0142 | 3.8484 | 0.0000 | 2.3154 | 5.2721 |
| 2.1897 | 7.8736 | 5.0275 | 1.4578 | 3.4721 | 5.6034 | 6.1770 | 1.2832 |
| 1.1035 | 3.9666 | 2.9291 | 4.6016 | 5.5599 | 0.0000 | 4.9565 | 5.2390 |
| 0.0000 | 3.5327 | 4.2732 | 1.7701 | 5.6931 | 7.4423 | 6.6857 | 1.2301 |
| 7.4193 | 5.6335 | 4.8346 | 0.0000 | 0.9481 | 3.7779 | 4.0139 | 2.9441 |
| 3.5859 | 2.6611\ | | | | | | |
| FOXL2 | 4.6856 | 10.0683 | 6.1594 | 5.1400 | 4.0407 | 4.0807 | 6.1576 |
| 5.6468 | 6.3785 | 11.2267 | 3.4195 | 4.8563 | 8.0658 | 1.4754 | 4.9665 |
| 4.1868 | 8.5616 | 6.9093 | 9.7254 | 2.8567 | 3.5464 | 5.5181 | 7.9411 |
| 4.4092 | 10.3244 | 8.1788 | 4.0218 | 8.6764 | 6.7475 | 2.8206 | 8.5921 |
| 3.5187 | 1.8398 | 3.9898 | 1.8080 | 4.0095 | 6.0197 | 3.7410 | 4.1194 |
| 6.0569 | 7.3760 | 8.5113 | 6.5617 | 3.7266 | 4.5728 | 5.5842 | 6.0639 |
| 2.5272 | 4.4858 | 4.9601 | 3.0665 | 4.3904 | 4.6368 | 7.4022 | 5.5059 |
| 4.8114 | 6.0835 | 6.9503 | 7.5161 | 6.2546 | 6.0091 | 5.2943 | 4.8953 |
| 3.7875 | 11.7423 | 6.6466 | 4.6221 | 5.4450 | 6.6498 | 8.5987 | 4.2110 |
| 3.7871 | 5.7226 | 6.4125 | 6.1919 | 4.6136 | 6.4786 | 7.8439 | 7.7094 |
| 6.6055 | 7.3045 | 5.2363 | 6.8207 | 4.1381 | 7.6719 | 4.7132 | 3.7216 |
| 5.3687 | 6.9806 | 6.5466 | 9.2511 | 5.2533 | 2.9127 | 8.8622 | 6.4485 |
| 8.3714 | 3.6286 | 10.8676 | 5.1403 | 4.6526 | 4.7356 | 7.1306 | 5.5896 |
| 5.8468 | 4.6372 | 5.8048 | 10.2748 | 7.0025 | 7.5064 | 7.7016 | 3.3794 |
| 4.4595 | 6.9682 | 4.6439 | 0.8924 | 4.0279 | 3.5357 | 5.4917 | 3.3042 |
| 5.2976 | 7.0442 | 5.1605 | 6.6474 | 5.4125 | 8.4042 | 6.3161 | 4.0367 |
| 0.0000 | 9.6155 | 10.3007 | 5.1873 | 3.1145 | 5.5320 | 4.7669 | 6.0413 |
| 8.1640 | 4.4769 | 11.0503 | 8.2505 | 10.5669 | 3.1296 | 3.4956 | 6.5873 |
| 5.1938 | 4.8526 | 11.9088 | 4.9970 | 8.3740 | 6.7575 | 1.2240 | 7.8834 |
| 5.7965 | 6.0470 | 5.9580 | 2.2306 | 2.1834 | 9.5227 | 4.7402 | 2.7383 |
| 6.0674 | 1.0317 | 4.7254 | 8.3964 | 5.2391 | 6.9447 | 6.5463 | 5.8339 |
| 7.0906 | 6.3619 | 3.4768 | 4.2380 | 3.4836 | 0.9335 | 5.0382 | 7.2283 |
| 4.0353 | 11.6490 | 8.4775 | 6.5544 | 7.7493 | 5.9955 | 6.1485 | 1.6771 |
| 5.9797 | 5.4694 | 5.6531 | 6.2460 | 6.9909 | 5.8453 | 0.9785 | 3.8154 |
| 2.2504 | 4.7817 | 5.8677 | 5.5231 | 7.9628 | 5.9327 | 10.3233 | 5.8371 |
| 4.9516 | 5.8588\ | | | | | | |
| RIT1 | 8.9021 | 9.3277 | 9.4120 | 10.3248 | 8.9583 | 10.0859 | 9.4415 |
| 9.3423 | 9.6412 | 9.2371 | 9.5513 | 8.8050 | 9.4924 | 9.4359 | 9.4647 |
| 9.3287 | 9.4616 | 9.1617 | 9.3242 | 10.1541 | 9.2826 | 8.9428 | 8.0322 |
| 10.2203 | 9.1406 | 8.9452 | 9.2464 | 9.5769 | 10.5164 | 9.4406 | 9.9003 |
| 9.6027 | 9.0874 | 9.7018 | 9.0219 | 9.0073 | 10.4300 | 9.8321 | 9.7680 |
| 9.6902 | 9.2731 | 9.4616 | 10.0687 | 9.6100 | 9.1520 | 11.1606 | 8.5444 |
| 9.3566 | 9.5401 | 9.3924 | 10.2383 | 9.6602 | 10.1099 | 9.1479 | 10.5447 |
| 9.9500 | 8.9406 | 10.3712 | 9.7271 | 9.4899 | 9.3405 | 10.4228 | 8.9378 |
| 9.2299 | 9.3481 | 9.8042 | 9.2223 | 10.4964 | 9.1657 | 8.8341 | 9.7085 |
| 10.9296 | 8.9780 | 9.6754 | 9.9561 | 9.6737 | 10.5607 | 9.4093 | 9.6904 |
| 8.5376 | 8.8863 | 9.6244 | 9.3528 | 10.2672 | 8.7650 | 9.1080 | 9.5484 |
| 8.6147 | 9.5330 | 9.7028 | 9.0785 | 9.1591 | 8.5821 | 9.3132 | 9.3162 |
| 9.2467 | 11.0983 | 9.3344 | 10.1271 | 9.1827 | 9.0739 | 8.9275 | 10.5756 |
| 10.8533 | 9.3502 | 9.5678 | 9.0294 | 10.1065 | 10.1148 | 8.7878 | 10.4183 |
| 8.6264 | 8.1578 | 9.1923 | 9.9074 | 9.4644 | 10.3226 | 10.6519 | 10.0670 |
| 9.2603 | 9.4559 | 9.2398 | 9.8931 | 10.4102 | 8.3621 | 9.4870 | 9.2616 |
| 7.9708 | 9.4005 | 9.1549 | 10.6591 | 9.7836 | 9.9272 | 10.3586 | 8.4109 |

8.8397 9.2459 8.8004 8.8833 9.0367 10.0292 10.3482 8.8552
9.4879 8.8911 8.8655 10.7379 8.7820 10.1402 9.4752 9.4685
8.3725 9.9103 9.7974 8.1937 9.9774 9.1980 10.1638 9.0684
8.9254 9.0429 8.5845 8.9235 8.9029 8.3863 10.0461 9.0262
10.3862 9.3208 10.0385 8.9184 10.2871 9.1690 9.6692 8.7705
10.3945 9.1204 8.9977 9.9332 9.0268 9.4908 9.5450 8.7468
9.0707 10.1760 9.5298 9.4064 9.4721 9.0706 9.0963 9.9822
10.5768 10.1685 9.8368 9.7263 8.5957 8.3986 8.7775 9.2136
9.0017 8.0638\
TMEM141 10.2203 9.1858 9.7247 9.8518 10.4927 9.5525 8.8293
9.9627 9.8590 10.3049 9.4138 8.8356 9.7008 9.3998 9.5128
11.4151 9.8964 11.6814 8.9035 10.0369 11.2320 9.6243 10.6884
9.7580 9.1489 11.3893 10.9101 11.0247 12.0006 10.0329 9.8715
10.2934 10.4538 10.2366 8.7888 12.0815 9.9760 10.4146 11.2097
8.8706 10.5129 8.7520 10.2389 10.1309 8.6020 11.0906 12.1394
9.8019 8.5240 11.5654 10.5737 10.1826 10.1907 8.7455 9.7631
11.1560 10.1550 9.3759 9.8236 10.6306 8.6623 9.5231 9.6951
9.4296 10.1013 10.1382 10.0492 9.1911 10.2421 10.2785 11.6770
9.3070 10.8051 10.6599 9.8254 9.0092 9.0204 10.8688 10.4489
10.4172 10.7443 9.1542 10.4471 8.9772 10.7031 9.7916 8.5779
10.4621 10.2305 10.3697 9.2511 9.7075 9.4380 10.2825 9.6620
8.9870 9.8249 9.5146 8.4976 10.8233 10.5060 10.2072 8.8566
9.9768 10.3614 9.8289 9.8183 9.6833 9.1913 9.1743 10.3790
10.2599 9.9351 12.0077 9.2642 9.7079 9.7794 9.4720 9.8566
9.7736 9.8186 10.4827 8.5121 10.9974 10.2471 9.4320 9.7568
11.1080 9.0345 8.5174 11.1800 10.6756 10.1420 9.8674 9.6323
11.0096 9.5277 9.6888 9.4867 9.3789 10.6874 9.9722 10.3708
9.9020 9.6422 9.7371 7.8117 10.1312 10.6295 12.3123 9.5152
10.8900 9.2382 9.5813 9.9308 8.9974 9.5524 9.2305 10.0842
11.1013 11.3105 12.5615 9.5640 10.2177 9.5792 8.7341 10.1704
9.5872 10.5823 9.5866 8.9634 9.2100 10.3806 9.6778 11.0722
9.9892 9.9655 9.2672 9.1560 10.4078 11.0884 11.1211 9.0659
10.7307 8.2230 9.7031 9.1433 10.0340 10.4297 9.7479 10.6780
9.6617 9.5459 10.0388 8.9246 11.8462 12.4617 9.8610 9.6646
9.3751 11.3195\
FOXL1 3.0819 4.4564 5.0652 3.0603 3.3611 1.2014 0.9367
0.8314 0.0000 3.6379 3.5532 0.0000 1.0422 2.0721 5.1055
6.3557 0.0000 2.1918 3.8042 1.6235 2.9899 1.1310 1.6825
4.5240 4.8848 1.0317 5.6135 3.3595 3.4165 0.5901 2.8666
3.7906 0.0000 0.4664 0.0000 3.7749 2.5102 4.5150 1.2418
1.2646 5.8817 3.2981 6.5534 2.8667 5.6049 7.8197 3.6457
0.6741 1.2792 4.7455 6.6682 2.4004 3.5609 5.8306 8.0120
6.3280 7.4688 3.5666 3.4964 1.9697 4.4879 1.6116 4.9403
1.3432 3.9568 3.0271 3.4947 0.4832 6.5623 4.3345 1.9272
4.2086 5.2125 0.0000 5.1395 1.1076 0.0000 5.3741 5.1911
4.1299 4.7771 0.7073 4.1036 2.6641 4.5242 0.6199 0.5376
6.6919 3.2299 5.3908 4.8431 1.7639 1.2055 8.1349 3.2084
3.9205 2.3031 1.9094 0.8845 7.0990 4.8515 4.6166 4.9160
6.3606 3.0982 1.8753 3.6874 1.2595 1.2448 2.4661 2.6161
0.0000 2.5153 3.9069 3.1912 0.6116 2.0324 1.7841 4.8998
3.3113 5.2519 3.7106 3.0961 4.0017 3.0314 5.0908 2.2388
1.2894 3.1853 4.0156 0.0000 3.7414 2.6167 2.8502 1.3267
8.3532 6.0549 6.7235 2.5337 4.1183 3.6287 4.8530 5.6924
4.8727 5.1977 2.7920 6.1416 4.1697 2.8976 2.6654 6.4590

3.2886 4.9567 2.0029 2.0813 2.5751 3.6413 1.9094 1.8394
5.2268 3.3043 7.6428 5.4672 4.3698 6.9614 0.5023 0.0000
1.8339 6.8828 0.0000 1.8290 1.6845 2.8815 1.3895 6.2245
2.5204 2.9988 5.8817 2.1786 3.4442 3.5582 1.5061 0.5257
3.5224 0.7366 0.0000 1.7992 1.5325 0.0000 4.9353 5.8983
1.7437 0.0000 2.4509 6.3113 4.4061 6.0397 2.9741 6.8320
5.8459 5.3014\
SF1 12.4733 12.1320 11.9915 11.9603 12.4689 12.5548 12.5644
12.0832 12.4174 12.4231 12.0168 12.5688 12.1341 12.4778 11.9844
12.1973 11.9729 11.9911 11.8939 11.8817 12.0291 11.9867 12.0511
11.9891 12.1153 12.0653 11.4910 12.0888 12.1402 11.7809 12.1480
12.6343 11.9343 12.2746 12.0812 12.3956 12.2805 12.3976 11.4581
12.5582 12.4373 12.8323 11.9956 12.8478 11.8779 11.9362 11.8412
11.9134 12.2954 11.7391 11.9121 11.9514 12.2423 12.5028 11.9087
12.1103 11.9431 11.7579 12.3154 11.8495 12.3792 11.8819 12.1256
11.6700 12.0435 12.1157 12.2464 12.2723 11.9708 12.1765 12.2052
12.5693 12.0234 12.2173 12.1172 12.2374 12.8122 11.8949 12.2765
12.4514 11.9993 11.8961 11.9865 12.0673 12.2499 12.3106 12.5100
12.2524 12.3015 12.5247 12.6375 12.4766 12.7662 12.2194 12.1737
12.1294 12.1302 12.5627 12.1099 12.1125 12.6399 12.0482 12.2693
12.1166 12.1635 12.5219 12.5321 12.1664 12.1110 12.3713 12.3444
12.3757 12.5638 12.1206 12.0384 11.8468 12.2701 12.2841 12.7696
11.5709 12.6512 11.8614 11.9389 12.1497 12.5490 12.1943 13.0232
12.1077 11.9391 12.4106 12.4043 11.9254 12.1223 12.1474 12.2371
12.0738 12.4036 12.3147 12.5462 12.5532 12.2548 11.9439 12.1552
12.1641 12.0952 12.6431 12.3179 12.5268 12.7264 12.1765 12.2739
12.2880 12.2838 12.4617 12.4014 12.9085 12.2284 12.4011 12.4940
12.2005 12.0277 11.6162 12.2886 12.3913 11.9072 12.3548 11.8176
12.2850 12.1818 11.9655 12.7128 11.6396 12.1309 12.2693 12.2865
12.3174 12.3982 12.5208 12.6576 12.1569 12.3036 11.5184 12.2760
12.1269 12.3512 12.1330 12.0957 11.7929 12.0921 12.5841 12.4813
12.2818 12.0079 12.1536 12.4125 12.5495 12.0980 12.5858 12.1278
12.4510 12.1949\
PCDHA6 4.8375 5.9309 4.1855 7.7160 5.8279 3.3699 9.0974
4.1714 3.8540 6.9223 3.8792 8.7009 6.4227 3.3187 5.7264
4.1112 0.0000 5.0179 2.9970 0.0000 5.8403 8.9642 5.2702
4.1420 7.4288 0.0000 0.0000 4.1691 2.6042 3.0527 0.0000
4.6731 0.0000 5.5128 3.0883 3.4815 9.0648 4.3706 5.1258
2.9199 0.0000 6.2282 4.9229 4.2643 5.9075 5.6391 0.0000
2.4093 3.1024 0.0000 2.9139 7.0274 2.7458 5.5618 9.2945
0.0000 4.0759 4.5639 5.8937 4.6868 5.6052 5.2153 2.2715
0.0000 6.0252 3.2734 0.0000 4.2127 5.4186 6.1017 4.7028
2.9031 0.0000 3.3810 3.9904 3.6939 8.2803 5.8342 0.0000
6.1345 4.9318 2.3132 6.3378 3.8479 3.7940 6.3281 9.0264
3.1153 3.5927 5.5365 4.2264 3.4458 4.7497 0.0000 2.3820
5.8959 4.1512 6.1669 2.6536 5.5778 0.0000 3.2802 9.3560
4.8237 0.0000 7.4562 3.9747 9.2466 8.2913 1.7840 1.1907
4.4844 0.0000 3.2095 3.7919 7.0634 0.0000 7.7373 4.7587
6.3965 4.5425 5.1903 5.5057 3.2057 4.9185 3.4020 5.6275
4.6840 6.0942 6.5648 3.4730 0.0000 5.2912 5.5926 4.3369
0.0000 0.0000 0.0000 3.9801 5.7184 0.0000 5.0402 0.0000
3.3507 2.8664 5.0203 4.8882 5.3313 0.0000 0.0000 6.0202
0.0000 4.7195 4.2143 1.3873 3.8219 6.0019 3.8974 0.0000
4.2500 3.7872 6.0187 5.2337 3.5735 0.0000 1.1454 0.0000

| | | | | | | | |
|---|---|---|---|---|---|---|---|
| 3.7577 | 5.4919 | 2.0787 | 0.0000 | 5.9953 | 1.8885 | 5.0476 | 0.0000 |
| 0.0000 | 6.1516 | 5.1030 | 4.9695 | 3.8939 | 4.6308 | 0.6902 | 4.1445 |
| 3.6715 | 5.2673 | 4.7909 | 7.1688 | 2.0548 | 0.0000 | 3.7911 | 2.0769 |
| 3.8483 | 3.2842 | 7.8441 | 3.5873 | 0.0000 | 0.0000 | 6.0843 | 4.8032 |
| 5.3795 | 0.0000\ | | | | | | |
| PKIB | 5.9021 | 6.3203 | 5.5534 | 5.6185 | 4.0407 | 5.1206 | 4.8747 |
| 4.8609 | 3.3355 | 5.6749 | 6.7739 | 6.9594 | 5.6049 | 4.6965 | 5.5148 |
| 3.7058 | 5.5491 | 6.4252 | 5.4061 | 3.3103 | 6.4162 | 5.5912 | 6.7720 |
| 5.7850 | 5.0158 | 5.4535 | 2.7174 | 6.7962 | 3.3032 | 7.0265 | 2.8666 |
| 5.6294 | 8.1316 | 5.0304 | 4.3932 | 4.1139 | 4.5283 | 4.8676 | 3.6815 |
| 3.6113 | 5.4509 | 5.6895 | 5.3182 | 4.1238 | 4.0482 | 7.1581 | 5.1255 |
| 5.1024 | 1.8062 | 5.8317 | 5.5120 | 8.3082 | 5.2023 | 4.8129 | 4.5015 |
| 6.1560 | 7.0787 | 7.8973 | 7.4038 | 5.4198 | 4.5454 | 6.8182 | 3.1723 |
| 5.1427 | 7.3772 | 5.1878 | 6.2181 | 5.8148 | 3.8367 | 3.8922 | 7.3194 |
| 5.7151 | 4.9356 | 7.6527 | 6.0122 | 2.4904 | 5.7549 | 6.0351 | 6.3872 |
| 5.7895 | 3.9901 | 3.1552 | 7.8226 | 6.2086 | 5.3124 | 5.2082 | 5.9742 |
| 5.5098 | 7.0301 | 8.4004 | 5.2939 | 4.9705 | 6.4606 | 5.5567 | 3.2084 |
| 3.5703 | 5.0020 | 8.7157 | 6.0841 | 5.1633 | 3.6674 | 6.8448 | 3.7143 |
| 7.6906 | 4.2414 | 3.7224 | 4.0742 | 6.4329 | 6.7529 | 6.1165 | 3.3794 |
| 4.2712 | 5.8481 | 4.8074 | 5.7910 | 5.2263 | 6.1177 | 3.8347 | 7.3262 |
| 5.1979 | 6.3815 | 6.5732 | 4.6899 | 3.8169 | 6.6937 | 4.8880 | 3.4722 |
| 8.7432 | 6.8130 | 5.7465 | 4.5595 | 4.3538 | 5.8091 | 8.9991 | 5.3024 |
| 8.4533 | 5.4807 | 5.5768 | 5.9737 | 7.7143 | 4.3672 | 6.1496 | 5.5593 |
| 5.3950 | 4.3979 | 2.3512 | 5.1255 | 5.3564 | 5.1188 | 5.9801 | 5.7056 |
| 5.9178 | 5.6878 | 5.3961 | 4.2483 | 6.3597 | 7.1746 | 4.3860 | 5.6287 |
| 6.2768 | 6.3100 | 4.6962 | 5.1208 | 6.8954 | 3.3669 | 2.6813 | 7.2167 |
| 5.5200 | 5.2844 | 6.9160 | 4.1220 | 2.7564 | 6.0945 | 3.3476 | 1.2582 |
| 4.1993 | 6.7266 | 3.5773 | 4.8367 | 6.9155 | 5.9955 | 4.5289 | 5.7946 |
| 5.2768 | 6.2658 | 6.1481 | 5.9076 | 5.5370 | 6.7578 | 3.0493 | 1.4263 |
| 5.7820 | 7.0558 | 5.3034 | 4.7768 | 6.0366 | 4.8941 | 7.2715 | 5.2235 |
| 6.1922 | 3.1794\ | | | | | | |
| TMEM149 | 8.7880 | 6.9235 | 7.1736 | 6.5403 | 8.2722 | 7.5031 | 7.2032 |
| 7.5281 | 7.1082 | 6.6059 | 8.1582 | 6.8476 | 7.0467 | 6.7684 | 7.2810 |
| 8.2308 | 6.6099 | 7.7385 | 7.7838 | 8.0889 | 8.2887 | 6.7588 | 6.7439 |
| 7.1987 | 5.6317 | 9.4261 | 7.2041 | 9.1564 | 8.8247 | 8.0873 | 5.9094 |
| 7.7275 | 8.9339 | 7.9446 | 6.7391 | 7.4081 | 7.4020 | 7.9149 | 5.8679 |
| 5.4129 | 8.9113 | 6.8949 | 6.8408 | 6.6704 | 5.9211 | 8.1206 | 7.2956 |
| 7.3752 | 5.7866 | 7.1965 | 8.2322 | 8.5325 | 7.6800 | 6.0690 | 7.8435 |
| 6.9958 | 7.3950 | 7.9837 | 7.8408 | 6.9082 | 5.7558 | 7.4421 | 6.5900 |
| 8.5700 | 6.8448 | 8.1514 | 8.0213 | 7.9554 | 6.1185 | 6.8882 | 9.9403 |
| 7.8213 | 11.7035 | 9.4210 | 8.6571 | 6.2656 | 7.6580 | 6.9144 | 7.6618 |
| 7.9430 | 7.3238 | 8.1214 | 7.5308 | 8.0897 | 8.1030 | 6.6875 | 5.7312 |
| 8.7337 | 7.9229 | 7.8891 | 7.4643 | 8.2162 | 9.5445 | 8.1051 | 8.1560 |
| 6.7771 | 7.7595 | 6.2521 | 6.7427 | 8.8603 | 8.1675 | 8.3225 | 6.6791 |
| 7.3852 | 6.7839 | 7.0810 | 7.6812 | 6.7742 | 7.5996 | 7.7120 | 7.0392 |
| 7.0526 | 9.8736 | 8.3760 | 7.0431 | 7.3658 | 7.1644 | 7.3441 | 5.8369 |
| 8.6176 | 7.1310 | 7.9389 | 7.6991 | 6.2642 | 6.1994 | 7.9076 | 6.0319 |
| 9.1561 | 6.4889 | 5.1863 | 8.0584 | 6.5670 | 8.0532 | 7.9484 | 9.3447 |
| 11.1668 | 10.3101 | 9.6457 | 6.6655 | 6.1119 | 7.4746 | 7.9731 | 8.8605 |
| 6.8329 | 7.3680 | 6.2829 | 7.7234 | 6.3168 | 7.1075 | 9.4626 | 7.7294 |
| 8.8241 | 7.7955 | 7.5799 | 8.4407 | 7.6251 | 6.0944 | 5.5027 | 8.2560 |
| 9.9303 | 7.8257 | 9.3937 | 7.1537 | 8.5869 | 6.9665 | 6.4997 | 7.6117 |
| 7.7520 | 6.7183 | 7.4313 | 5.7765 | 7.6812 | 7.0724 | 7.7739 | 9.0761 |
| 6.6057 | 6.2903 | 5.8068 | 5.5765 | 8.4669 | 9.0845 | 8.8779 | 8.0601 |

| | | | | | | | |
|---|---|---|---|---|---|---|---|
| 7.6390 | 6.8229 | 8.4850 | 7.5663 | 9.3508 | 7.7705 | 7.4380 | 7.7069 |
| 8.1138 | 8.4637 | 8.9126 | 7.7829 | 8.8445 | 8.3724 | 6.2670 | 7.9492 |
| 7.3214 | 9.3494\ | | | | | | |
| API5 | 11.2021 | 10.8612 | 10.3721 | 11.2814 | 11.2206 | 10.8911 | 11.5220 |
| 11.4141 | 10.7118 | 11.1925 | 11.5729 | 11.6456 | 11.1588 | 10.8852 | 11.2790 |
| 10.2623 | 10.6326 | 11.0215 | 10.9091 | 13.5538 | 10.9255 | 10.9514 | 10.8387 |
| 11.1256 | 11.2852 | 10.8150 | 11.0469 | 10.7499 | 10.9029 | 10.7058 | 11.2788 |
| 11.0335 | 10.7702 | 10.8257 | 11.2604 | 11.2071 | 10.6970 | 11.2895 | 10.9713 |
| 11.4607 | 11.6476 | 11.1453 | 10.5623 | 11.3417 | 11.3845 | 10.3829 | 10.6558 |
| 10.7446 | 11.5435 | 10.8961 | 10.8401 | 10.9374 | 11.2343 | 11.1809 | 10.5000 |
| 11.8669 | 11.2817 | 10.8932 | 11.4343 | 11.1416 | 11.3727 | 11.4444 | 11.9844 |
| 10.2263 | 10.9599 | 11.1096 | 11.1199 | 10.9065 | 11.1638 | 10.2797 | 10.9913 |
| 11.3755 | 10.5985 | 10.0337 | 11.3435 | 10.8031 | 10.5526 | 10.4632 | 10.6537 |
| 11.3788 | 10.9216 | 10.6414 | 10.6268 | 10.5732 | 10.8736 | 9.9965 | 11.3359 |
| 10.6284 | 11.3550 | 11.1922 | 11.0470 | 11.4719 | 10.7520 | 10.4420 | 11.1739 |
| 11.3388 | 10.9899 | 10.8694 | 11.1473 | 10.7447 | 11.0379 | 11.2948 | 11.4701 |
| 10.9147 | 11.6557 | 10.6174 | 10.4559 | 11.1206 | 10.2306 | 10.4154 | 11.4107 |
| 11.2465 | 11.1830 | 10.1749 | 11.1849 | 11.6830 | 11.3587 | 11.5649 | 11.9122 |
| 10.2273 | 10.6759 | 11.3820 | 10.7436 | 11.0659 | 11.0826 | 11.1201 | 11.6318 |
| 9.0780 | 11.3347 | 11.4437 | 10.2059 | 11.1419 | 11.2180 | 11.1716 | 11.1083 |
| 10.9909 | 11.3586 | 10.1516 | 11.2177 | 11.0198 | 11.1939 | 11.1327 | 10.7105 |
| 11.3207 | 11.4144 | 10.7508 | 11.2527 | 10.8747 | 11.2519 | 11.2565 | 10.8088 |
| 11.0916 | 11.1947 | 9.8336 | 11.2304 | 11.3955 | 10.9836 | 11.3753 | 10.8902 |
| 11.0238 | 10.6976 | 10.6688 | 11.2306 | 10.7751 | 10.9965 | 11.7783 | 10.6556 |
| 11.1721 | 11.1747 | 10.7590 | 11.6017 | 11.4086 | 11.0897 | 10.1600 | 10.4712 |
| 11.3887 | 11.0597 | 11.0576 | 11.3085 | 10.4739 | 10.8426 | 10.3365 | 11.2556 |
| 10.9641 | 11.5595 | 11.5663 | 11.3414 | 10.6556 | 10.9559 | 11.3246 | 11.4321 |
| 11.3587 | 11.6894 | 10.8360 | 11.2613 | 10.8872 | 11.0722 | 11.0874 | 11.1769 |
| 11.1937 | 10.6548\ | | | | | | |
| KRTCAP3 | 10.2549 | 8.3527 | 9.4094 | 10.0853 | 9.8184 | 8.6945 | 9.1904 |
| 9.8051 | 9.5061 | 8.7079 | 10.8358 | 9.3598 | 8.9264 | 9.2177 | 9.5021 |
| 8.9462 | 9.3348 | 9.0457 | 7.9479 | 10.5131 | 9.3550 | 9.1291 | 10.1579 |
| 9.5054 | 4.6630 | 9.4046 | 9.6518 | 9.8926 | 9.0846 | 9.8796 | 4.2890 |
| 9.2951 | 9.3129 | 10.1963 | 8.9609 | 10.0979 | 8.9156 | 9.6406 | 10.7189 |
| 9.2312 | 7.7579 | 8.0579 | 10.9382 | 9.9702 | 1.0260 | 10.8653 | 12.3192 |
| 9.4964 | 5.5108 | 10.2931 | 11.4610 | 9.1280 | 8.9744 | 4.1653 | 7.9863 |
| 9.5429 | 9.1387 | 8.7632 | 8.6129 | 9.7302 | 3.5787 | 9.4529 | 8.7734 |
| 4.9648 | 7.9039 | 8.7569 | 10.5094 | 8.7233 | 8.6389 | 9.6370 | 11.6756 |
| 9.1245 | 7.5747 | 8.2058 | 8.1725 | 9.9246 | 8.7967 | 9.1042 | 9.9731 |
| 6.2291 | 9.0534 | 9.3219 | 8.3944 | 9.4591 | 9.6891 | 7.7921 | 8.4954 |
| 9.2640 | 9.0046 | 6.7668 | 6.3161 | 8.9756 | 8.8077 | 9.3435 | 9.5302 |
| 0.6266 | 7.7702 | 7.5331 | 9.1676 | 8.3737 | 7.9473 | 9.2796 | 9.2579 |
| 9.5330 | 10.9501 | 9.9733 | 6.4938 | 8.7426 | 7.6835 | 8.6876 | 9.8386 |
| 9.5163 | 9.5943 | 10.1849 | 10.1866 | 8.2732 | 9.4319 | 9.3522 | 9.3880 |
| 9.7126 | 4.6074 | 9.7567 | 9.6210 | 10.3991 | 9.6877 | 9.5066 | 7.2723 |
| 11.9257 | 7.6375 | 6.0608 | 6.7749 | 10.3661 | 9.6320 | 9.9218 | 9.4545 |
| 10.0050 | 10.4520 | 8.1742 | 8.8483 | 7.9282 | 11.4776 | 9.7999 | 9.6518 |
| 2.6953 | 9.1781 | 4.0836 | 8.2887 | 9.0948 | 9.1224 | 12.1698 | 8.9043 |
| 10.8264 | 8.6615 | 9.6674 | 9.3740 | 8.1300 | 8.5340 | 8.6364 | 9.6676 |
| 5.4080 | 10.3436 | 8.9049 | 9.2451 | 10.1841 | 5.6361 | 8.6528 | 9.3071 |
| 10.2200 | 10.0622 | 10.0104 | 7.3481 | 9.6824 | 9.8412 | 8.6125 | 10.5643 |
| 8.9463 | 7.4070 | 6.6747 | 8.9720 | 9.8013 | 10.9941 | 10.3420 | 10.3495 |
| 10.5373 | 8.2860 | 9.5075 | 7.5810 | 8.7860 | 9.2507 | 9.3426 | 9.2391 |
| 9.2071 | 9.1207 | 9.7815 | 9.9779 | 10.9101 | 11.0000 | 8.2394 | 9.0259 |

9.2911	10.9067\
NUMBL	8.1273	10.5770	8.9899	9.0861	8.4380	8.8929	9.9830
8.5482	8.3639	10.3403	8.7089	9.6533	8.6578	8.0969	8.2247
10.3120	9.4122	9.8325	10.3476	9.7501	9.1801	9.1645	8.9462
9.0560	10.3619	9.1579	10.4520	8.9871	8.4978	8.2915	9.0335
9.5867	9.9136	8.5555	10.4547	7.6336	10.4769	8.4004	8.4991
9.3813	8.4558	10.3776	9.4164	8.1131	9.7238	9.5206	6.7442
9.0365	10.0173	7.3347	8.9151	8.8906	9.2017	9.9824	10.5849
8.8227	8.6043	9.8927	9.2549	8.3879	10.0765	8.7764	8.6836
9.4446	10.9195	8.0515	9.0471	9.8571	8.3522	9.7039	9.6600
9.0844	8.8008	9.8583	8.4448	9.3167	9.5226	9.1113	9.2247
9.5043	8.1784	8.6684	9.3078	9.0772	9.0501	9.5842	8.2776
8.7788	9.2480	9.7756	10.5611	8.1686	8.7166	10.4735	8.3427
9.7203	10.4822	10.5534	9.3348	9.1106	8.3995	8.6461	9.4123
10.0374	8.4101	8.3224	11.0282	8.9970	10.5657	9.6841	9.1723
8.4219	9.6603	10.3498	9.0408	9.1700	8.6976	9.8117	8.9859
9.8811	9.6923	8.1784	9.2791	8.7261	9.6514	9.2639	8.5996
8.3390	9.6484	10.0053	9.0005	8.7775	8.8491	9.1714	11.1558
8.8657	7.8966	10.6498	8.9773	10.5103	8.8404	8.1982	9.1612
8.4041	8.4179	10.2015	8.8666	9.8050	8.0687	8.0418	9.6669
6.2672	8.5219	9.8872	9.9818	8.4273	9.6874	9.1803	8.1994
9.2225	9.2289	7.8854	8.0920	8.8311	8.1076	7.8871	9.7294
8.0102	8.4136	8.7005	9.4249	8.6165	9.2412	8.2654	8.2369
9.5058	10.7567	10.4063	8.8440	10.2862	7.7272	8.6519	8.5577
9.2819	9.1814	8.6258	8.9629	8.4352	7.8590	7.9847	8.7695
8.0293	10.1972	9.9836	9.4031	8.9363	8.3440	10.1181	8.4296
8.8103	7.9272\
RTKN2	8.7956	6.5228	8.5118	7.1047	9.0811	7.5127	8.0998
9.1782	8.5025	7.9842	9.0545	8.6472	8.1033	9.0329	7.8285
6.8619	9.1041	8.2962	6.5816	7.8964	6.9401	7.8597	4.6928
7.8721	4.8384	7.7628	9.5301	7.5413	5.8644	8.0679	8.6586
8.3435	7.6002	7.1599	8.4766	7.9049	7.2635	8.7861	9.3926
8.7156	8.0660	5.2612	8.2277	8.9278	3.6322	8.1253	7.3756
6.8794	7.8811	6.7472	7.0836	7.7905	8.6686	3.2435	7.8576
8.5356	7.4867	6.7909	9.1970	7.5446	3.5787	8.8541	7.3955
8.1899	7.4033	7.6167	5.7659	9.0661	5.1070	7.2147	7.1695
7.9938	6.7834	7.6499	7.1998	7.1642	7.4121	6.5370	6.0564
9.5573	7.8004	8.9377	6.7703	8.3250	8.1567	7.9909	8.0260
7.8500	7.8229	8.6101	2.9352	8.6087	8.1518	7.1773	8.7470
2.9121	6.5633	6.0987	8.3521	7.8913	7.1272	7.1005	8.0816
8.5453	8.4713	9.2083	4.7765	8.5865	8.9055	7.0955	8.8608
8.0105	7.7258	7.6724	8.1210	8.5481	8.5181	8.8492	8.8879
8.2478	8.2609	7.5694	8.3593	7.6501	5.8165	8.2334	11.3898
8.2596	6.2068	6.4145	7.4438	9.6106	7.9535	8.9109	7.7494
5.7364	8.2092	6.1326	7.5229	5.5206	9.2570	5.9006	7.0450
8.1519	8.7747	2.4747	7.4876	4.9261	7.1134	8.2960	6.6975
7.5925	7.6072	8.0038	9.4906	8.9109	5.3297	8.9979	7.2490
8.9915	6.3475	7.1066	7.6917	6.4765	7.2901	9.0083	7.5437
8.2387	8.9541	9.3666	8.8788	9.0213	8.6634	8.3200	5.7522
8.4528	7.3846	2.2714	9.2448	6.2627	7.4149	8.1109	8.1876
7.2994	8.9229	7.5504	9.0879	8.5778	7.8978	9.1879	8.2172
7.4341	8.3586	8.5451	7.7796	8.0751	8.3509	5.2249	7.3356
8.0338	7.7925\
RNF180	9.0769	8.5127	7.2801	8.1102	3.7874	3.0829	7.0760

6.8232	8.4530	9.2584	7.7787	7.7166	8.4003	7.0450	8.3357
7.7787	8.7522	7.9990	8.0301	4.3240	4.2194	8.9229	7.7690
8.7633	8.6974	2.9613	4.9422	8.4361	8.4718	7.4951	5.8155
5.2149	3.5006	5.4638	8.2249	3.5700	8.4473	7.1726	8.0015
5.4052	7.2204	8.2182	6.4854	3.8009	9.1598	6.6229	7.3131
8.0811	4.2892	7.0664	6.1589	6.3505	8.8341	9.1555	6.2874
9.4430	6.6011	4.0751	7.1765	8.3816	9.0490	7.2856	5.1271
6.9478	8.9172	8.8078	5.0599	7.9772	6.0109	6.1773	4.3661
9.2378	4.4775	6.8515	7.3667	5.9228	6.1758	8.1765	7.9888
7.1345	6.9256	8.4548	8.1399	5.5260	7.3795	6.0317	7.1804
7.8697	8.3802	5.2970	8.1248	8.9560	6.0604	7.1401	8.1861
8.2244	5.0972	8.4134	7.9147	4.4529	6.2207	8.3336	8.4893
6.9397	7.2430	7.0667	7.6226	8.2606	7.3737	7.3918	9.1616
8.0868	7.7151	6.6724	7.2531	5.5199	9.2936	7.9615	6.0853
4.1540	5.8052	7.2589	8.2070	3.3565	8.1905	5.6743	8.0877
8.2494	9.1766	9.0255	9.5798	9.1908	8.9273	7.6570	4.8532
5.4910	4.7107	6.2786	7.6597	8.5872	6.5549	6.1496	7.8601
6.9883	7.2964	8.4023	10.6157	8.0998	6.7308	8.1407	7.9061
5.4233	8.9634	3.9105	6.6476	7.5274	8.5888	7.6311	3.8996
3.1284	1.0317	4.0337	8.9250	7.0683	5.7169	8.4936	5.0769
7.5416	6.5436	7.7151	7.7115	8.1609	8.0541	5.3175	3.8992
6.4176	9.4484	8.9853	7.5417	8.2692	6.5296	8.8002	7.2119
8.5011	6.3971	9.1246	9.2923	7.9303	8.6703	6.7708	4.0169
8.5541	8.7365	6.9506	6.8902	6.2041	6.6994	8.5629	8.9057
7.8403	9.3636\
P2RX7	2.2429	5.1787	4.6381	3.8540	4.8712	5.9179	3.1326
3.8718	1.6317	4.2930	3.1647	2.2003	3.5785	1.0485	4.1831
2.9791	1.0553	0.7772	5.1465	1.6235	3.8962	4.3809	4.6928
3.3566	5.5646	1.6270	1.6918	2.3554	3.0458	2.4720	2.9307
4.1016	4.0424	3.3982	2.5857	2.6866	3.3110	1.7231	0.0000
1.7885	3.1062	5.8080	0.6231	3.2841	6.7091	3.1906	1.5457
3.7557	2.4952	1.5638	3.6785	5.5215	4.8221	6.4142	3.4925
2.6383	3.9918	7.9273	5.6837	1.7785	6.2443	4.8354	3.3826
0.5968	4.6210	4.8417	5.2076	3.1628	2.1522	3.9922	3.5683
2.9765	3.8023	2.4134	5.2115	0.0000	3.1657	3.6807	2.7035
5.5482	0.9714	1.5351	5.2692	4.9929	2.4335	2.6709	2.2055
2.2368	4.1146	2.3114	5.8734	4.6871	2.8268	3.5163	3.5125
6.3488	3.5228	6.5301	2.9577	5.1819	3.3101	4.1304	2.6756
3.8386	1.1338	2.1477	4.0742	4.6265	2.0692	1.8132	3.8760
2.5476	2.8658	1.5850	3.1220	3.2401	4.0011	4.6888	3.2084
4.5912	2.2359	3.3319	3.0021	2.9876	5.1641	4.9309	3.2484
1.2894	6.3818	6.7444	2.9233	1.4687	3.1593	4.0766	2.8142
3.8725	2.5939	5.7370	6.1521	6.5572	3.5671	2.6745	2.6155
3.4648	3.8090	8.1126	3.1266	4.8818	2.3133	0.7381	5.8890
2.5542	5.1725	2.5882	2.6037	5.1585	6.6507	2.1273	2.9457
2.5877	2.7538	2.8804	3.1622	2.1852	3.0916	4.4201	6.0710
6.5194	2.3144	2.6009	2.5213	2.1836	2.5860	0.6229	0.0000
1.9121	4.2749	4.8794	2.9511	5.4358	1.2666	2.4045	2.3092
3.2946	3.4586	4.3198	3.2376	2.4374	0.6567	3.4840	5.6900
1.7437	2.4161	5.1016	4.1300	0.0000	0.0000	6.2259	5.6459
4.9904	0.6959\
SECISBP2L		8.2759	10.7085	9.9353	10.6503	9.3624	11.0503
9.8313	9.3336	9.8326	9.7415	9.2763	9.4250	9.6749	10.2827
10.3654	10.8190	8.4006	9.7631	10.4975	8.3072	8.7354	9.9385

10.2340 10.7491 9.9523 7.3196 7.5940 8.7438 9.1920 10.8792
10.1020 9.7613 8.4338 9.6138 8.8728 8.8310 10.0679 11.0193
9.6455 9.8900 9.0852 11.4039 9.1936 9.2050 10.9492 8.8815
4.4668 9.6401 9.2399 9.5752 9.7594 9.3973 9.2550 10.6627
10.2050 10.0084 9.9293 10.3137 9.5569 9.0148 10.9011 10.0310
8.9041 9.3194 10.1234 10.1258 9.1718 9.5214 9.8787 9.5036
7.7107 9.8574 8.0580 9.1273 10.9904 9.6448 9.9854 10.3951
9.7684 10.1480 8.9437 9.7027 10.9785 9.1781 8.4222 9.7309
10.7211 7.7924 9.8823 8.5178 10.3580 10.0075 8.1228 9.4128
8.1925 10.2358 9.5848 10.6126 9.1698 9.9497 7.8214 9.1483
9.6892 9.9204 9.3607 8.3597 10.0022 10.5889 9.6133 8.9344
10.7846 9.4209 7.0390 8.5507 9.7854 9.3994 9.4466 10.4738
9.4580 10.0109 9.1795 10.1143 10.1247 10.0707 10.5990 9.5102
9.3860 5.9797 10.9556 10.7206 10.6868 9.9908 9.3744 9.8263
8.0121 5.0038 7.1098 7.2867 10.6131 10.5529 9.2411 10.5472
8.8920 10.6158 10.9010 10.3824 10.3958 9.7766 8.8332 7.7194
10.8869 6.4869 10.6292 9.3865 9.9397 9.4349 10.6105 10.3781
9.1466 8.4809 8.7351 8.9719 9.4007 8.8229 10.7162 10.1991
9.8752 9.8102 9.6047 8.9680 9.7872 9.7988 8.4715 8.6691
6.8120 9.8105 10.0350 10.2883 10.3481 10.7983 9.2632 8.5634
8.7846 9.5297 8.9715 9.6161 8.9133 8.2503 8.5176 9.2165
9.7927 9.7430 9.4508 8.7786 9.9378 6.6143 5.9078 10.1593
10.1616 10.5551 6.0732\
SBF1P1 0.9511 1.4768 1.1543 0.8605 1.8679 0.4059 0.0000
0.0000 1.0352 1.4796 1.2544 0.0000 1.2705 1.0485 0.0000
2.1513 1.0553 0.0000 0.9983 1.1305 0.6175 0.8427 1.0738
1.2144 1.2653 2.2189 1.0809 1.3471 0.8532 1.3312 0.9954
0.0000 0.0000 0.8184 1.5856 1.7727 0.0000 0.0000 0.0000
0.0000 0.6649 0.8506 1.0569 0.4326 0.0000 0.0000 0.0000
2.1933 1.4771 0.8339 0.4530 1.2789 0.6494 0.0000 0.4645
2.1129 0.6343 0.5030 0.0000 0.5715 1.7140 1.1594 1.7473
1.0177 0.6828 0.3906 0.5980 0.4832 1.5697 1.3773 0.7662
0.9537 1.9166 0.4786 0.5367 1.1076 1.2673 1.2414 0.0000
0.6159 0.9714 0.7073 0.7204 1.1284 0.9110 0.6199 0.0000
0.6140 0.5273 0.5265 1.6854 1.1363 0.5215 1.5073 0.5198
0.6266 0.5233 0.0000 0.8845 1.7322 0.6391 0.8684 0.5352
1.2208 0.4835 0.4659 1.5756 1.0326 0.0000 0.0000 0.8916
1.6572 0.0000 0.0000 0.0000 0.6116 1.2170 0.7641 0.9644
2.8681 0.4553 0.0000 1.6588 1.3451 1.3435 0.0000 1.0447
0.0000 0.5619 0.5074 0.3935 0.0000 1.2631 0.7571 0.0000
0.0000 0.6413 1.5206 0.5216 1.8148 1.0240 1.3036 0.0000
0.5424 1.3147 1.2434 1.9907 1.2499 1.5778 1.2240 0.8737
0.0000 0.9341 0.5862 1.2540 1.0445 0.5638 2.4027 0.0000
1.2429 0.0000 1.0431 0.0000 1.3855 0.4418 0.0000 4.0884
0.7148 1.2170 0.5466 1.4975 1.2195 1.2418 0.0000 2.3719
1.1272 0.0000 0.6293 0.9723 0.6314 1.5234 0.6902 0.9100
0.0000 1.2218 0.0000 0.5811 0.7058 0.6567 1.5562 0.8827
1.5256 0.0000 0.4262 1.1623 0.0000 0.0000 1.3688 1.6179
0.5077 0.0000\
LOC100128811 0.0000 0.0000 0.0000 0.0000 0.0000 0.0000
0.0000 0.0000 0.0000 0.4413 0.0000 0.0000 0.0000 0.0000
1.0750 0.0000 0.0000 0.0000 0.0000 0.3755 0.0000 0.0000
0.0000 0.0000 0.0000 0.0000 0.0000 0.0000 0.0000 0.0000
0.0000 0.0000 0.0000 0.0000 0.0000 0.0000 0.0000 0.0000

| | | | | | | | |
|---|---|---|---|---|---|---|---|
| 0.0000 | 0.0000 | 0.0000 | 0.4871 | 0.0000 | 0.0000 | 0.0000 | 0.0000 |
| 0.0000 | 0.0000 | 0.0000 | 0.4764 | 0.0000 | 0.0000 | 0.0000 | 0.0000 |
| 0.0000 | 0.0000 | 0.0000 | 0.0000 | 0.0000 | 0.0000 | 0.0000 | 0.0000 |
| 0.0000 | 0.0000 | 0.6828 | 0.0000 | 0.0000 | 0.0000 | 0.0000 | 0.6160 |
| 0.0000 | 0.0000 | 0.0000 | 0.0000 | 0.0000 | 0.0000 | 2.1575 | 0.0000 |
| 1.5048 | 0.0000 | 0.0000 | 0.0000 | 0.0000 | 0.0000 | 0.0000 | 0.0000 |
| 0.0000 | 0.0000 | 0.0000 | 0.0000 | 0.0000 | 0.0000 | 0.0000 | 0.0000 |
| 0.0000 | 0.0000 | 0.0000 | 0.5454 | 0.0000 | 0.5504 | 0.0000 | 0.0000 |
| 0.0000 | 0.0000 | 0.5320 | 0.0000 | 0.0000 | 0.0000 | 0.0000 | 0.0000 |
| 1.4385 | 0.0000 | 0.0000 | 0.0000 | 3.4300 | 0.0000 | 0.0000 | 0.0000 |
| 0.3974 | 0.0000 | 0.9357 | 0.0000 | 0.0000 | 0.0000 | 0.0000 | 0.0000 |
| 0.0000 | 0.0000 | 0.0000 | 0.0000 | 0.0000 | 0.0000 | 0.0000 | 0.0000 |
| 0.0000 | 0.0000 | 0.0000 | 0.0000 | 0.0000 | 0.0000 | 1.1436 | 0.0000 |
| 0.0000 | 0.0000 | 0.0000 | 0.0000 | 0.0000 | 0.0000 | 0.0000 | 0.7381 |
| 0.5019 | 0.0000 | 0.0000 | 0.0000 | 0.0000 | 0.0000 | 0.0000 | 0.3855 |
| 0.0000 | 0.0000 | 0.0000 | 0.0000 | 0.5821 | 0.0000 | 0.0000 | 0.0000 |
| 0.0000 | 0.0000 | 0.0000 | 0.9420 | 0.0000 | 0.0000 | 0.0000 | 0.0000 |
| 0.0000 | 0.0000 | 0.0000 | 0.6293 | 0.0000 | 0.0000 | 0.0000 | 0.0000 |
| 0.0000 | 0.0000 | 0.0000 | 0.0000 | 0.0000 | 0.0000 | 0.0000 | 0.2849 |
| 0.0000 | 0.0000 | 0.0000 | 0.0000 | 0.0000 | 0.0000 | 0.0000 | 0.0000 |
| 0.0000 | 0.0000 | 0.0000\ | | | | | |
| HAS2 | 8.2459 | 6.2460 | 4.1826 | 5.1640 | 5.2179 | 2.1935 | 3.6354 |
| 3.3418 | 3.1202 | 3.1490 | 2.2251 | 3.6072 | 2.4830 | 2.9572 | 1.2358 |
| 5.2151 | 1.3881 | 3.2578 | 7.1208 | 1.3142 | 4.3761 | 3.3952 | 2.5783 |
| 6.7469 | 4.6191 | 2.8612 | 4.8052 | 2.7358 | 3.6194 | 2.0116 | 5.5726 |
| 3.2569 | 1.4434 | 1.5403 | 1.0004 | 1.4920 | 4.6682 | 2.1545 | 2.8817 |
| 2.3543 | 1.9736 | 6.8459 | 5.1528 | 2.0520 | 5.4077 | 2.4638 | 3.0068 |
| 2.3699 | 3.9327 | 2.2965 | 3.7593 | 5.3028 | 6.5662 | 6.8277 | 7.3890 |
| 4.9227 | 5.6949 | 5.0131 | 5.5408 | 0.5715 | 4.7224 | 4.8964 | 3.9207 |
| 3.5355 | 3.4175 | 4.0485 | 2.2002 | 4.2998 | 8.0041 | 4.2562 | 1.2639 |
| 2.5691 | 3.2212 | 0.8374 | 6.2001 | 0.8237 | 5.5097 | 1.7110 | 4.2478 |
| 5.4981 | 3.6471 | 3.6276 | 4.6680 | 3.9844 | 4.0744 | 2.5439 | 2.4635 |
| 3.3991 | 4.1509 | 5.9787 | 7.0049 | 2.6303 | 2.5333 | 6.9355 | 3.1390 |
| 4.8450 | 2.6431 | 5.1291 | 2.3870 | 3.8550 | 1.4180 | 3.9104 | 8.2605 |
| 4.1937 | 1.9209 | 3.8802 | 5.8924 | 4.2038 | 4.3895 | 2.5918 | 2.6161 |
| 1.3871 | 2.8813 | 0.0000 | 7.3237 | 5.7915 | 5.2844 | 3.7990 | 3.8073 |
| 2.5175 | 6.6316 | 3.6436 | 5.2148 | 2.7605 | 4.0336 | 4.2388 | 4.3162 |
| 0.0000 | 5.5894 | 5.0247 | 7.7096 | 2.5146 | 1.7370 | 4.5119 | 2.4659 |
| 6.1134 | 1.9254 | 5.3965 | 5.7804 | 4.7560 | 2.0359 | 4.6709 | 3.6966 |
| 6.2538 | 4.6958 | 1.9011 | 3.6209 | 4.6897 | 5.6333 | 1.5869 | 6.3997 |
| 0.0000 | 7.3746 | 2.9102 | 7.4773 | 6.7000 | 5.7265 | 6.8357 | 2.5351 |
| 3.8290 | 1.3600 | 4.2502 | 6.2860 | 2.7667 | 3.4637 | 6.8994 | 0.7889 |
| 2.6158 | 3.2310 | 4.6898 | 4.5614 | 2.1836 | 5.4814 | 4.1488 | 2.7155 |
| 4.3619 | 3.5224 | 7.7523 | 9.1457 | 3.3695 | 3.4102 | 3.5901 | 4.3375 |
| 3.1656 | 4.4802 | 2.9077 | 3.0773 | 1.8171 | 1.1063 | 4.8888 | 4.6221 |
| 3.0084 | 5.9591 | 5.1556 | 4.2600 | 3.9204 | 4.1084 | 4.3903 | 4.1816 |
| 4.5174 | 3.7440\ | | | | | | |
| KIAA1199 | | 6.2056 | 10.4618 | 9.1664 | 6.6572 | 7.6983 | 5.4664 |
| 6.8828 | 9.8448 | 7.9519 | 5.1139 | 7.4186 | 5.6364 | 9.0343 | 10.3769 |
| 9.2190 | 11.5118 | 8.4097 | 5.3312 | 9.0478 | 6.2633 | 6.4430 | 6.2550 |
| 8.4220 | 12.1278 | 6.4829 | 4.7327 | 4.9766 | 8.9500 | 8.5042 | 4.5997 |
| 3.0509 | 8.8964 | 4.1856 | 6.4155 | 7.5516 | 5.4562 | 7.8731 | 6.5401 |
| 9.6755 | 5.4171 | 7.9783 | 8.9940 | 4.9408 | 7.0571 | 2.6279 | 8.1622 |
| 3.7862 | 5.4556 | 6.3822 | 8.4689 | 6.6094 | 5.5872 | 7.9191 | 4.2954 |

```
10.8233 8.6829  8.7975  7.6649  7.4412  4.8915  4.3972  5.8204
11.9959 5.4607  5.9990  7.8269  4.7377  8.1346  10.7253 7.2705
8.2096  5.7406  7.2013  4.3134  9.5201  3.1208  9.4673  8.9680
9.4037  6.3564  8.5197  8.5455  7.5509  3.9844  8.2680  5.8952
4.9237  7.4901  6.4579  8.0967  5.9004  6.6898  4.6656  7.9926
7.3888  6.8619  7.2129  11.0818 5.0325  5.5962  8.3706  7.0202
6.9177  5.2577  3.9384  7.6792  6.6725  8.3619  7.3657  8.2127
6.1082  7.3060  5.7248  4.9542  8.9451  7.5499  5.3793  10.4980
8.0710  5.0909  6.9347  5.0551  7.2223  8.3265  9.0073  8.0214
5.5455  8.5059  9.4327  6.5761  7.2613  5.0874  5.5750  4.4399
2.2542  0.0000  6.1027  6.6977  9.1663  10.3409 4.5692  10.7011
7.2425  11.6639 11.0202 9.3046  5.8157  9.8996  5.9667  5.9493
10.0167 3.5510  11.7675 7.4957  11.2491 10.9232 8.5780  9.5917
9.5514  3.2017  4.0131  4.9400  8.7677  7.0683  8.9391  7.7515
7.5493  6.9777  5.7895  5.3439  9.5572  4.0052  10.7946 6.1092
7.2013  7.3821  5.0594  6.5789  5.5599  9.0161  7.3666  5.5550
10.6660 6.7381  7.6879  5.8285  6.2271  4.7814  4.0500  10.9271
7.1869  7.9076  7.0080  8.6574  7.8536  7.0031  3.2264  8.6065
10.4252 9.0723  4.8216\
HLA-DPA1        12.9236 12.1919 14.4486 12.0528 11.0363 12.0333
10.7767 9.9879  8.3251  10.1312 12.8324 10.8511 11.7431 6.5862
11.3429 12.3325 9.2260  11.0344 12.6509 8.7572  13.8366 11.5337
13.4506 10.3120 11.3373 11.7002 9.1961  11.4146 11.0832 10.3197
7.8857  11.4209 12.8725 11.7288 9.9767  10.7763 9.9281  8.9703
8.5297  9.4039  12.2761 12.1142 9.4199  10.5634 12.2787 11.9804
11.3050 12.7725 10.0043 9.4930  11.4629 12.7132 11.2082 12.2897
9.9558  8.2416  11.0162 12.2963 13.0898 8.8315  12.3826 12.5222
10.1327 8.2026  10.4617 13.0050 12.7500 9.4657  11.1135 9.7706
13.5088 10.5631 14.7464 12.0743 13.8411 7.3390  11.0589 11.1451
12.2395 12.6128 10.1895 9.0121  13.4211 11.8190 10.8593 11.2002
8.9113  12.5894 13.1456 12.3984 12.7830 12.3758 12.4462 12.3313
10.8851 12.3645 13.4174 11.6013 9.7090  13.1241 11.4124 12.2219
8.1100  10.5046 9.8370  9.0575  9.7480  10.9665 9.3929  10.4037
10.7398 11.9568 9.1149  7.8009  10.6845 11.0737 11.8143 12.0292
9.0692  12.5451 9.6596  11.8986 10.1008 9.9388  10.7844 11.9541
10.4543 9.1010  12.2760 11.5408 9.2973  9.8066  12.6285 11.7248
11.7586 12.0927 13.0322 11.2136 11.4611 10.9475 10.4270 11.9281
11.3949 12.5063 13.7797 10.3307 10.2299 12.2761 10.8261 9.5746
11.6297 10.1254 12.9829 10.0366 11.5488 12.0118 13.3672 7.2126
11.1698 12.2083 12.7268 12.5476 11.0611 12.4730 8.8963  10.7952
14.3071 13.7984 10.1483 9.9758  8.7673  8.8560  9.3204  7.3956
9.2804  7.9083  10.3864 11.8852 9.8733  12.6039 11.3737 11.0126
9.0683  12.0399 10.7027 14.0467 11.2314 13.3787 11.3670 11.9660
11.7636 9.9903  12.2887 11.8502 11.7250 10.5527 8.0144  11.6986
13.6963 12.0342 12.2986\
ZSWIM3  6.3111  6.0848  7.5445  6.2411  6.1453  6.0225  6.0120
7.0649  7.1458  6.3613  6.3154  7.5234  7.0328  6.6794  6.6165
6.5270  7.2627  7.0402  7.0134  6.2744  7.6799  5.4543  6.5433
6.8564  6.5128  6.8303  7.4694  6.9721  6.8317  8.1752  6.8572
7.0574  6.9437  7.3440  6.1094  6.1143  7.1958  6.3997  7.4039
6.5801  6.7646  5.9922  6.1869  6.5918  6.8838  5.7386  7.0669
6.9781  7.1634  6.6381  6.5020  7.1807  7.4742  6.9122  7.4540
6.2670  6.3508  6.5097  7.0883  7.1594  7.0682  6.6452  6.0763
7.3569  6.4818  6.5542  6.7108  7.8236  6.2442  6.8114  6.8658
```

6.4920  6.7590  6.7706  6.5311  6.6813  8.1151  7.2303  7.0522
6.1981  6.3179  6.2947  6.7091  6.7517  6.5862  7.6334  8.5311
7.2038  6.1705  7.0325  7.1070  7.6285  7.2973  6.5973  6.2572
6.9470  6.1489  7.2752  6.2213  6.6760  7.0382  6.3118  7.1635
7.5128  6.7560  7.0749  6.5559  6.9424  5.7783  7.2127  6.9001
6.8065  8.7437  6.4919  6.4078  6.7991  6.4373  8.4860  7.0334
6.6321  6.8993  6.2397  7.3987  5.9684  6.6721  7.5874  6.2058
7.7943  7.2294  6.7387  6.3615  6.7893  6.6158  6.3652  6.9533
8.2091  7.3164  6.7235  6.7912  7.0978  6.8738  5.9123  6.7792
5.5150  5.4640  7.2511  6.7157  7.1870  7.1340  6.2571  6.6917
7.2941  7.0755  7.3392  5.2454  7.4300  7.0131  6.6778  6.4620
5.5689  7.0847  6.8256  6.3577  7.1459  6.4488  5.2817  6.6436
6.9140  6.4446  6.4065  6.4380  7.9779  5.5248  7.5069  7.2149
6.5204  6.8300  6.6514  6.4418  6.4646  6.9353  7.4782  6.3631
6.4082  7.3537  6.0688  7.4562  7.5479  6.4411  6.1861  6.5123
7.1538  8.0212  7.0475  6.7590  7.4820  8.2231  7.1101  7.2569
6.5960  5.8588\
FAM32A  11.4229 10.9494 10.8267 10.4360 10.8045 10.6326 12.3519
11.2007 11.6967 10.9996 11.4741 12.5680 11.7795 10.7574 10.8223
10.5509 11.9438 11.0358 11.2149 12.3730 10.8812 10.8474 10.8820
10.7279 10.6673 10.9703 11.4557 11.4911 11.2496 10.9681 10.4734
10.8171 13.0072 11.8123 10.9108 11.4728 10.7636 10.6518 11.3755
10.6324 11.1639 10.4386 10.2136 11.1881 10.6807 11.3993 12.2336
10.9553 11.1414 11.2918 11.0343 10.5268 11.2680 10.5401 10.5799
11.0782 10.6662 10.5712 12.1264 11.3633 10.5405 10.5619 11.0018
11.3914 10.9586 11.0738 11.1508 11.4198 10.8930 11.1980 11.8634
10.8771 10.9910 11.8121 10.7548 10.7894 11.5492 10.9097 10.8821
10.8033 11.4774 11.1386 10.9827 11.5569 11.1700 11.5813 11.7956
11.3884 10.0429 11.2366 10.6701 11.6235 11.5005 10.8976 11.1181
10.9284 10.9980 10.7285 11.0728 11.0198 11.6486 11.1708 12.4992
10.8871 11.2150 11.2919 10.5485 11.1637 11.7269 11.2620 10.6977
10.8681 12.3527 10.1548 10.7124 12.3374 10.7493 12.1645 10.5774
10.4979 11.8363 11.0240 10.9033 11.2589 10.5401 11.6278 10.5184
11.8614 10.7761 10.2541 11.0043 11.2220 11.1998 11.0431 11.1911
11.7821 11.8963 10.7813 10.4733 10.4501 11.1869 10.9275 11.1955
10.6926 10.8611 10.5033 11.0319 10.8562 11.1529 11.1913 10.3320
11.8306 10.6846 11.2206 10.8028 11.5204 10.5203 10.6497 12.5558
11.4115 11.0734 11.0834 11.1451 11.0174 10.4782 10.5026 11.2305
10.6380 11.2031 11.0866 10.8281 11.4200 11.2386 11.1522 11.2310
11.0610 10.7816 10.4877 10.6040 10.5797 11.4211 10.5547 13.5695
11.1988 11.5509 10.8468 11.6254 11.3284 11.3565 11.5531 10.6571
10.9090 11.3745 10.4360 11.2182 11.6988 11.7258 10.5874 10.8914
10.6327 11.6421\
KIAA1191        10.8703 11.5493 11.4140 11.8349 10.9587 11.0301
11.5347 11.2124 10.7965 10.9267 11.5680 11.3362 11.4772 10.7645
11.0363 10.7701 10.7264 11.0812 11.2493 10.6406 10.9015 11.0748
11.6013 11.0632 11.5546 10.9919 10.4378 10.3815 11.2808 10.9565
10.7703 10.7621 10.5004 11.5425 10.3200 10.9024 11.2461 11.0150
11.5492 11.0405 11.2890 11.4240 11.6578 9.9033  11.2244 10.9229
10.4956 11.2597 10.6212 11.2741 10.7238 10.6739 11.1827 11.1444
10.7670 11.4937 11.3505 10.4593 11.0338 10.9009 11.2889 11.2961
11.8270 11.1378 11.1746 11.3929 11.8513 11.1762 11.6171 11.2172
10.1452 11.2268 10.4452 10.5828 11.5795 12.3043 11.1388 11.2396
11.4044 11.6800 10.9358 10.4572 11.1470 11.3097 11.1246 11.4348

11.7771 10.7216 11.6018 10.7860 11.3596 10.7591 11.7572 10.8585
10.9317 11.3473 10.9681 11.6438 11.4698 11.1915 11.0398 11.3869
11.8602 11.3219 11.1356 10.5744 10.7466 11.5770 10.9295 11.0340
11.0937 10.8289 10.8607 9.5392 10.6781 11.0794 11.5272 11.1539
10.5857 10.3438 10.7152 11.4789 11.7011 11.2845 11.5349 10.1955
11.5622 10.7980 11.6132 11.1433 11.5451 11.1306 11.0019 10.6655
11.1019 9.6427 11.0544 9.9455 10.9864 11.6693 11.7868 10.6231
10.8155 11.3307 11.3232 11.8874 11.3334 11.4558 11.4833 10.4837
11.0484 10.2861 11.3089 10.9186 10.9403 11.9210 11.4550 11.4780
10.6948 10.5840 11.0039 10.4878 11.2589 10.7753 11.2601 11.4945
11.6411 11.4637 11.0884 10.7389 11.5281 10.4203 11.7488 11.1450
10.2416 11.6080 11.0544 11.3912 11.1470 10.8934 10.8950 10.6872
10.7611 11.5235 10.8253 11.5389 10.8492 10.6185 10.5878 11.7358
11.2668 11.0897 10.7635 10.8495 11.1197 10.1321 11.0110 11.5904
11.3566 11.2156 9.6158\
FER1L6 0.5526 0.9197 1.1582 2.2237 0.9133 4.2627 1.2456
1.4675 1.4589 0.0000 1.7761 1.1366 0.4363 1.0485 2.8727
0.0000 0.0000 0.0000 0.0000 0.0000 0.0000 4.0762 4.2654
2.0289 0.9530 0.0000 1.0809 1.0210 0.0000 0.5901 4.0212
0.5612 0.5159 2.1491 1.8080 0.0000 0.9556 5.7454 0.0000
0.0000 1.1184 2.0748 0.0000 0.4326 0.0000 3.5656 0.0000
0.6741 0.0000 0.0000 4.0756 0.0000 1.9420 0.8853 1.0973
0.0000 1.6820 1.6258 0.7533 0.5715 1.2440 2.4655 0.0000
0.5968 0.0000 5.4968 1.6109 0.4832 1.9829 0.0000 0.0000
1.1694 0.0000 0.0000 2.2033 1.3447 1.7421 5.5663 0.6894
1.3771 0.5659 0.3965 5.3168 1.7538 0.0000 1.0522 1.2355
0.0000 0.0000 0.0000 0.6361 0.4848 0.0000 0.0000 0.5198
0.0000 0.5233 0.5454 2.3870 0.0000 0.0000 1.6155 0.5352
0.7358 1.1338 2.2656 0.0000 1.4556 0.5426 1.3268 1.1907
1.6572 0.0000 0.0000 0.8924 0.0000 1.2170 0.7641 5.3219
0.0000 4.3164 0.0000 1.3885 1.5487 1.6090 2.8596 0.6149
0.0000 1.5385 1.8193 0.0000 1.4687 2.3786 0.7571 0.0000
0.0000 0.0000 0.0000 2.6791 0.7520 1.6169 0.9848 0.0000
3.5232 2.4496 2.0673 3.8760 0.5454 0.5814 0.0000 3.1549
0.0000 2.3500 1.3242 0.0000 1.7989 1.2837 1.1539 0.0000
0.0000 1.6272 0.0000 0.5821 0.0000 0.0000 0.0000 0.0000
2.8899 1.2170 0.0000 2.6230 0.0000 0.0000 0.0000 0.0000
1.3669 0.0000 0.0000 1.8340 5.2181 0.0000 0.0000 1.8629
0.5055 1.8738 0.6248 0.0000 0.0000 0.0000 0.0000 3.8994
1.9330 0.0000 0.7547 1.6152 0.0000 0.0000 0.0000 4.4392
2.3834 0.0000\
ALOX12P2 4.6592 1.4768 1.1543 0.4935 6.4080 7.8581
5.1408 5.2189 5.8231 3.4617 7.2251 2.3205 6.4514 1.0485
5.5004 2.6237 5.1715 1.9468 1.3199 5.5572 7.3140 2.6223
5.2899 0.5262 1.2653 0.6062 7.4327 6.7066 0.0000 6.9578
0.0000 0.5612 5.7858 6.3291 6.7491 2.5445 7.7526 0.8977
0.9336 0.0000 6.5104 1.3821 7.5949 7.9855 0.0000 0.9285
5.9491 7.5774 5.0017 4.3061 0.4530 8.2754 6.1012 0.5094
6.7462 1.7550 0.6343 5.8825 3.1515 2.7727 1.2440 7.2703
0.9583 7.9599 2.5466 5.1377 7.6496 5.7200 4.6504 7.8885
5.3556 1.5230 4.4163 5.3620 0.5367 0.0000 5.9967 0.9332
3.3490 0.6159 0.5659 6.8400 3.5862 1.7538 2.1768 7.6795
7.1067 1.3738 4.8254 1.2149 2.1125 1.7639 2.0170 2.4059
7.7060 0.0000 6.6093 0.5454 4.7445 6.9224 1.9211 1.7978

```
2.8654  8.2498  3.6490  1.1001  1.5756  7.6910  2.7941  2.7073
1.1907  1.3871  5.6813  5.7814  8.1647  6.3256  1.6815  7.4093
3.8712  6.6993  6.3943  6.5345  6.2977  5.2613  6.0465  5.3341
6.2366  4.1383  0.5619  0.0000  7.4899  1.1234  1.7370  8.4175
4.5622  4.8605  5.8877  4.1556  0.5216  1.4215  6.1763  0.9848
4.3486  4.3980  2.5446  0.5418  0.0000  2.9802  2.1615  5.4731
0.5019  3.1371  5.8332  6.8430  0.0000  5.2146  0.5638  3.7610
6.6736  1.4969  0.0000  0.0000  3.3141  5.8278  6.0175  0.8742
5.9741  1.1906  5.2680  1.5072  1.2980  4.7139  0.5410  4.4981
4.2552  0.4801  2.6427  2.0979  5.9598  6.4556  0.5545  2.9539
6.0647  0.5055  3.4586  7.1816  0.0000  6.3645  0.0000  0.5707
6.1590  7.0140  6.2785  6.7806  6.2963  4.9682  0.0000  2.3840
6.1084  0.5077  0.0000\
FER1L4  6.7779  6.5228  7.4963  9.3612  6.1543  8.5791  4.6035
5.6910  4.3139  6.6708  6.2708  7.4659  6.7117  7.6132  7.8054
6.9649  9.6251  7.6189  6.1156  8.4575  5.2827  8.1530  4.6616
5.9685  4.3683  6.8303  5.7089  8.8586  7.6040  8.6060  4.0789
7.1569  5.5678  6.1074  5.1708  7.2599  8.0624  6.6433  8.0690
7.2325  8.6700  5.1350  7.0824  5.4131  1.6194  8.4794  8.8940
6.5894  3.7911  6.1974  8.7207  10.6852 6.0892  5.5203  8.8402
9.1260  5.7551  9.4064  4.6239  7.4314  3.5787  6.0880  7.0244
8.8017  6.0787  7.3097  8.5661  8.2578  6.3172  8.9919  7.5661
6.3334  9.8962  7.9384  5.0244  6.8448  8.9981  7.8237  6.1465
8.2828  6.1718  5.7332  5.9960  8.1403  8.0953  8.3643  6.2212
9.0366  6.2728  6.8240  4.8707  5.9658  6.4171  8.5054  7.7120
3.3571  8.4777  4.7890  9.2936  9.7455  5.4008  6.9830  8.1585
11.5697 7.0441  7.9366  7.4317  6.8669  7.7339  9.3843  8.3878
7.1669  6.4508  10.7895 8.7161  8.0470  5.5109  6.9652  9.2581
10.1129 6.7481  6.3328  8.9197  5.3593  4.9415  7.4054  6.8488
4.7928  3.8886  6.5374  7.6403  7.7436  7.6425  9.6012  8.0037
8.4470  6.4736  8.7512  6.7095  6.1515  9.7764  6.5546  8.9313
7.5415  7.3368  6.8677  6.0279  5.0711  6.0118  9.1399  5.9290
9.2133  6.6737  5.5116  7.1001  6.5297  4.8449  8.9761  9.2937
7.1099  3.5811  9.8735  8.5986  8.9860  6.7471  7.7571  5.7583
5.3826  8.5642  4.7188  5.9001  7.4553  6.0849  7.3676  6.1698
8.4082  7.0881  5.2549  7.5712  8.6837  5.7786  10.1505 5.6505
8.0055  6.6284  6.9672  6.5122  8.3429  9.2397  6.4649  6.3502
5.4818  7.5208  3.8135  6.6564  5.7421  7.5589  5.7340  7.7324
7.3888  6.2244\
FER1L5  2.0932  0.0000  0.6896  1.1529  2.1806  4.4459  2.3543
4.3837  2.1693  1.9494  5.8359  3.8262  4.9712  0.0000  3.5096
0.0000  0.6221  1.2795  1.9975  0.6732  2.7816  2.0613  3.2153
0.9110  0.5538  0.0000  0.0000  3.5617  1.3857  1.3312  0.4127
0.0000  3.3260  5.3469  2.5857  0.0000  5.7188  0.0000  0.9336
0.4337  0.6649  2.0748  0.0000  2.8667  0.0000  0.0000  0.0000
1.4786  1.8062  0.0000  0.4530  2.1910  3.4148  1.8243  5.5533
0.5608  1.4095  3.9247  2.0305  0.5715  1.4980  7.0861  0.9583
0.0000  1.7745  3.5326  4.7922  1.9201  0.5773  2.7780  2.6452
0.0000  0.9449  7.4823  1.2339  1.1076  5.1770  4.3929  1.1539
1.0461  1.7663  0.7073  1.5572  1.4748  4.9094  6.0783  4.6400
0.0000  2.8432  0.5265  1.0759  1.1363  2.1644  2.2282  3.4566
0.6266  0.5233  0.9402  2.6033  0.5504  0.6391  0.0000  1.2310
1.8724  0.0000  0.0000  2.1046  1.2595  0.9361  0.0000  0.5136
4.1884  2.0513  2.5850  1.8354  4.8037  0.5276  4.9392  0.0000
```

| | | | | | | | |
|---|---|---|---|---|---|---|---|
| 1.0358 | 4.7732 | 0.0000 | 1.0557 | 1.1079 | 3.0314 | 3.4959 | 1.6443 |
| 3.9489 | 1.2802 | 1.4255 | 0.9567 | 1.4687 | 2.8211 | 5.6831 | 1.7059 |
| 2.6570 | 0.6413 | 4.6610 | 0.5216 | 1.1757 | 1.3507 | 0.5748 | 1.9445 |
| 0.5424 | 0.4562 | 2.0673 | 2.1920 | 3.2124 | 4.5443 | 0.0000 | 0.0000 |
| 0.0000 | 0.9341 | 5.0698 | 0.0000 | 3.7440 | 1.5425 | 0.9402 | 2.2048 |
| 1.2429 | 0.0000 | 0.0000 | 1.5791 | 2.5454 | 0.0000 | 2.4806 | 4.1489 |
| 1.1906 | 0.5276 | 1.9122 | 0.0000 | 1.6845 | 1.2418 | 1.3895 | 0.0000 |
| 0.0000 | 1.4586 | 2.2714 | 2.1786 | 4.4507 | 0.5545 | 2.2268 | 6.0165 |
| 1.1756 | 3.4586 | 1.8914 | 0.5811 | 3.3874 | 0.0000 | 0.0000 | 0.0000 |
| 1.9330 | 0.0000 | 1.6145 | 0.0000 | 2.3335 | 0.6064 | 2.9741 | 3.7459 |
| 2.1290 | 0.6959\ | | | | | | |
| CA5B | 6.5058 | 8.4785 | 6.8412 | 8.7153 | 7.7081 | 7.6238 | 6.0501 |
| 7.3404 | 6.1989 | 8.7841 | 7.2270 | 6.9654 | 6.3629 | 8.3024 | 7.5330 |
| 7.8738 | 7.7135 | 6.4749 | 8.2920 | 6.8588 | 5.5214 | 8.4016 | 8.7359 |
| 7.5649 | 8.9381 | 6.3178 | 8.3485 | 7.7655 | 8.0186 | 7.0737 | 6.9001 |
| 6.7811 | 5.1328 | 6.6479 | 6.6961 | 5.6037 | 7.5401 | 8.5145 | 8.2892 |
| 6.7129 | 7.4126 | 8.7833 | 7.2961 | 6.6819 | 8.8022 | 6.5598 | 6.8900 |
| 6.6260 | 6.1875 | 8.0267 | 7.6234 | 7.0068 | 7.6970 | 8.8832 | 8.4012 |
| 8.4325 | 7.4734 | 7.4053 | 8.2054 | 6.8945 | 8.5361 | 7.5314 | 8.0831 |
| 6.4501 | 9.1567 | 8.0103 | 7.6529 | 7.8743 | 7.8610 | 7.6834 | 5.7240 |
| 5.6818 | 7.0382 | 5.8625 | 7.7011 | 7.5258 | 8.4428 | 8.9627 | 7.7682 |
| 7.4576 | 7.3298 | 7.6056 | 8.7533 | 6.7120 | 7.3478 | 7.6493 | 6.2496 |
| 6.5873 | 7.3041 | 7.1020 | 8.2168 | 6.3780 | 5.9463 | 8.0154 | 7.1372 |
| 8.2591 | 6.2325 | 8.6008 | 8.2427 | 7.1968 | 7.7961 | 7.2857 | 8.2212 |
| 7.7211 | 7.1048 | 7.7340 | 7.8869 | 7.8974 | 7.6441 | 6.7224 | 7.3879 |
| 7.3016 | 6.7048 | 6.3764 | 7.6989 | 6.1204 | 6.6829 | 7.5859 | 8.0545 |
| 6.7213 | 6.4807 | 7.2592 | 7.0358 | 8.0268 | 8.9580 | 5.9863 | 7.4957 |
| 6.5680 | 8.7165 | 9.0341 | 7.5133 | 6.5074 | 8.0516 | 7.9189 | 6.7684 |
| 6.5815 | 7.2408 | 8.7260 | 8.3901 | 8.5803 | 7.6485 | 7.2628 | 7.3752 |
| 8.3354 | 7.9563 | 8.8490 | 9.2679 | 7.7565 | 7.3536 | 9.5857 | 8.6623 |
| 7.5090 | 7.8199 | 6.3200 | 9.5725 | 7.0958 | 8.8771 | 7.9889 | 7.7316 |
| 6.3536 | 6.6296 | 7.1614 | 7.9229 | 7.7356 | 8.0945 | 8.8267 | 7.2492 |
| 6.7383 | 7.0319 | 5.3499 | 7.1014 | 6.3622 | 6.6655 | 6.3522 | 8.8683 |
| 7.1931 | 8.2320 | 8.2020 | 7.1872 | 8.7091 | 7.8955 | 6.2952 | 6.5574 |
| 7.1267 | 6.5558 | 7.3011 | 7.1168 | 5.8803 | 6.4575 | 8.2656 | 6.8109 |
| 6.9136 | 7.5733 | 7.4220 | 7.6509 | 5.0619 | 6.8104 | 8.3994 | 8.2289 |
| 7.9685 | 5.9456\ | | | | | | |
| ZNF559 | 7.9535 | 8.8162 | 7.4616 | 8.4349 | 7.7402 | 7.5127 | 7.8534 |
| 8.3765 | 9.1768 | 8.9781 | 8.1197 | 7.2058 | 7.9772 | 7.3007 | 7.9672 |
| 8.2913 | 8.5718 | 7.6602 | 8.8284 | 6.9131 | 7.7277 | 7.6429 | 8.4008 |
| 7.6522 | 8.6568 | 7.4546 | 8.6744 | 7.9119 | 7.3135 | 7.3507 | 7.6367 |
| 8.2743 | 7.3109 | 8.6384 | 6.6229 | 8.1462 | 7.5220 | 6.9253 | 7.5371 |
| 8.0708 | 8.9534 | 9.0611 | 7.0881 | 8.6948 | 9.1066 | 7.0092 | 6.5995 |
| 6.7968 | 8.9368 | 7.3029 | 6.9350 | 8.0377 | 7.9789 | 9.5697 | 7.2816 |
| 8.5703 | 7.0846 | 7.3985 | 8.8093 | 7.7911 | 9.1068 | 7.1804 | 7.2689 |
| 7.4099 | 9.2324 | 7.6122 | 7.6657 | 8.4212 | 6.5847 | 8.0965 | 7.4408 |
| 7.9133 | 6.7466 | 8.5669 | 7.7595 | 7.6319 | 9.3791 | 7.3875 | 8.1343 |
| 7.4769 | 7.6161 | 8.4206 | 8.8612 | 9.2697 | 7.7149 | 7.9516 | 9.5960 |
| 7.3564 | 8.5391 | 8.9073 | 8.4830 | 9.2112 | 8.2847 | 8.9827 | 7.7120 |
| 8.5241 | 6.5963 | 9.3455 | 7.6687 | 8.6818 | 7.6195 | 6.8655 | 8.1150 |
| 7.9140 | 7.6731 | 8.3763 | 8.9831 | 8.1760 | 7.9304 | 8.9029 | 5.1724 |
| 7.5989 | 6.7747 | 8.1497 | 8.7607 | 8.1173 | 7.4141 | 8.7314 | 7.4330 |
| 8.0872 | 8.8879 | 7.4958 | 7.7902 | 7.0906 | 9.2104 | 7.8834 | 7.4607 |
| 7.2744 | 9.3259 | 8.6370 | 6.7913 | 8.2474 | 7.4010 | 8.3672 | 7.4342 |

7.1188 8.3042 9.0032 8.5309 8.9454 8.1350 6.4373 8.0364
7.5867 7.0371 9.7919 8.1892 8.9721 6.8803 5.7125 8.4760
6.6358 7.6755 6.9286 6.7056 7.8578 9.0876 8.1622 8.9530
7.5634 6.1913 7.3605 8.4238 7.8775 7.2567 7.9720 7.0041
8.3347 9.2197 8.9420 7.8239 8.4128 7.3611 7.3050 7.3192
8.6709 9.3761 9.1787 8.5086 8.1235 6.5658 6.7491 8.9655
7.9414 8.0489 8.2559 7.8626 7.3389 7.7120 7.9111 8.0924
7.3371 8.7398 7.1861 4.5917 7.7218 7.4162 9.7035 7.9862
8.4648 7.4681\
VWA1 12.1100 11.4859 11.2130 11.7450 9.9073 11.9356 11.5394
12.0195 11.4725 10.6863 11.7917 10.5999 10.9493 11.1500 12.2886
11.7416 11.8549 9.9381 11.8340 12.5833 11.0648 12.7840 11.3510
11.7637 10.8864 12.1765 10.3834 12.4993 12.5405 12.9251 9.7508
10.9589 10.2661 13.3861 11.6907 10.0526 11.2013 10.4791 9.3540
10.1202 9.6786 11.1712 13.0236 11.4432 9.9078 12.3777 10.4501
12.3811 8.1886 11.3019 12.3128 12.2126 10.7818 10.6230 11.3874
10.5257 10.9408 10.0762 11.1600 12.0531 11.0516 10.8769 9.4261
11.8392 10.8793 12.0587 11.4097 10.4553 11.7583 12.2855 13.1326
11.2696 10.5441 10.6728 10.8760 11.1671 11.2105 11.6072 11.4908
9.0822 12.2366 12.3362 12.0835 12.0961 12.5458 11.6912 8.7175
12.3566 12.0424 10.9123 11.2731 10.3825 9.0318 11.5244 9.8776
11.5909 9.6978 10.8350 11.8020 11.4875 11.0663 12.4302 12.1973
12.5751 10.6234 10.6445 11.6193 11.3797 12.8675 12.9218 10.4389
11.4535 12.7682 12.8595 11.6591 10.6076 10.9151 10.8780 10.4625
12.0653 9.6623 12.2167 12.3689 11.7784 10.4921 11.2206 11.9707
12.9354 11.1417 9.8479 12.2068 11.0595 11.8309 11.6406 11.9225
11.2373 9.9132 11.1896 11.8070 11.0767 11.6877 12.4683 12.2117
10.0664 11.5390 9.4943 11.4991 12.0515 11.3588 12.3646 11.7709
12.3459 12.2963 11.9498 11.2738 10.0505 11.1826 11.8522 12.0374
11.4144 12.3996 12.3234 11.6584 12.2454 11.5808 11.3519 11.9789
10.9027 12.4188 11.6128 11.2069 9.5725 10.9018 11.5481 13.5299
10.8457 10.5623 11.3315 11.1041 11.4180 10.8270 11.9815 9.1498
11.8209 11.3042 11.0164 11.9996 12.0921 12.5713 9.1204 10.5876
11.8531 11.0446 11.5722 11.3909 10.5133 11.6973 10.7536 12.0701
11.0049 12.7153\
C19orf22 11.3453 11.2820 11.0072 10.8220 11.3551 10.1640
11.2884 10.0115 11.2681 10.9800 11.1880 10.5599 10.3071 10.3957
11.2400 11.1891 11.6576 11.6294 11.2678 10.6861 10.2065 11.3516
10.9760 10.9399 10.7698 12.1118 10.4017 11.8641 11.9981 11.0744
9.8280 11.3457 11.8584 9.9113 10.3607 10.9739 11.3150 10.6459
11.5325 9.7259 10.4551 11.1869 11.4180 10.3089 9.2162 12.2710
12.0430 12.6124 10.8015 11.3453 11.6007 11.2179 10.3285 9.8168
10.2189 11.1114 11.0713 11.2984 10.7456 10.7039 9.8429 10.1831
10.4452 11.8856 10.8704 11.8753 10.8028 10.9038 11.2746 10.6293
10.7847 10.6257 11.3690 10.8899 10.8572 11.2394 11.1973 11.4645
11.4177 11.4059 11.0792 9.9095 11.3868 10.1478 11.1633 10.8723
9.7601 11.3668 10.1244 11.2364 10.4939 10.4052 10.8509 11.2999
11.0885 10.4337 10.1280 10.0801 11.4237 11.6950 11.3632 11.7952
9.6421 11.0039 10.6961 10.1203 11.7258 11.0149 10.4476 10.8532
10.7300 11.3448 11.2488 11.7616 11.2391 10.3501 10.6377 9.6956
10.8122 11.1676 10.2340 11.5854 11.6536 11.5776 10.0931 10.4638
10.1493 10.7080 9.8702 10.0077 9.6869 11.3303 11.2001 10.6667
11.5134 12.6801 11.5876 11.9648 10.5143 9.7328 11.3774 11.4303
11.2518 11.3653 11.0873 9.2548 11.7131 10.3260 10.5952 11.8101

10.5785 11.5142 10.2952 10.3364 10.7391 9.8536 9.9281 10.1080
10.1982 11.4168 11.1286 12.2609 11.2300 11.0041 11.1023 10.4943
10.9341 9.7427 11.1851 11.0390 10.9077 10.2281 10.4647 11.2223
12.8945 10.7231 10.8058 10.0449 10.8093 10.9421 11.4514 11.1841
11.7899 11.0794 9.7634 10.9345 11.4252 11.1246 12.0531 10.6580
11.0071 11.5158 10.6676 10.4277 11.0205 10.4057 11.3453 9.3486
10.8844 10.5336 11.9685\
C19orf23 5.2953 4.2352 5.0914 4.3630 5.4099 4.4883
3.7864 4.2718 4.8915 4.3191 5.4118 4.0858 3.2969 4.6358
4.3522 5.2151 4.1466 6.3641 4.4230 3.7850 3.1720 4.6091
4.3063 4.4677 2.5058 7.4928 3.1984 5.3445 6.1031 4.4070
4.5762 5.0172 3.5006 4.4591 4.2103 6.2465 4.4058 6.4143
4.2631 4.5072 7.1328 4.1914 3.4937 4.0647 2.0387 4.9453
6.3507 4.3262 3.7535 6.5920 6.3667 5.7103 3.9801 3.5346
4.9545 6.0358 4.6920 4.6067 2.2544 4.1317 2.6946 3.5473
6.7802 4.4259 4.6903 3.7482 3.2044 4.5159 4.8848 6.3938
5.1715 4.8787 4.4775 4.2252 5.7354 3.1208 5.7039 5.4954
4.9551 7.4869 5.1725 4.0444 4.9445 4.5570 4.8881 4.2262
3.9495 4.9653 3.9596 6.5669 3.3023 3.2907 4.2364 5.9922
4.5582 2.5596 3.5228 2.8937 4.5498 5.7659 6.2840 4.7554
3.9837 5.3780 4.6372 3.9530 4.6303 5.0774 3.6341 4.9148
4.4279 4.7754 4.1934 6.2479 5.8021 4.4674 3.3607 5.3507
5.0315 3.3856 3.8806 5.3506 2.6767 3.9306 3.6178 3.8036
4.1705 4.4556 3.1077 2.6959 3.1441 5.6915 5.0328 4.3933
4.1973 7.1109 3.9061 4.9498 3.9825 4.0098 5.7747 4.4302
6.4436 5.6391 5.6571 3.8298 2.6677 3.5318 6.3203 5.8357
4.8312 6.3728 4.7030 4.8617 4.6443 4.5865 3.5262 4.0280
3.5644 5.8453 6.5540 4.5405 4.1617 5.6446 2.9650 4.4201
2.2138 3.7207 3.6416 4.9078 5.7711 4.0826 4.9765 5.1738
2.9928 4.4802 4.2082 2.5659 3.8863 4.5888 6.8847 3.8577
4.4073 5.0212 3.2722 5.9088 4.4153 4.9080 4.7517 5.4171
4.3208 4.4376 3.8574 4.2897 4.7327 5.5688 5.1059 3.5942
4.2278 4.3506 4.7896\
C19orf20 8.7525 8.2676 6.5755 5.3850 6.3069 5.9023
8.0853 7.2573 7.2323 8.6344 6.4300 6.8736 6.6572 8.0218
5.3960 8.6635 6.0137 8.9131 7.8884 7.0521 5.8352 6.8634
7.4378 7.0934 7.3766 8.5861 5.5347 9.1977 7.8150 6.8670
6.6821 8.4669 7.9604 4.6245 5.2103 7.4613 7.3861 6.9321
5.6867 4.9286 7.2650 7.0081 6.6104 4.7101 6.3545 8.5012
4.8007 7.8286 5.0177 8.5510 7.9070 4.3404 7.3081 6.4214
7.1058 6.2309 6.3988 6.3784 6.0697 6.5221 5.3625 5.2482
5.3602 6.2732 8.3543 6.5252 3.9905 6.9061 8.1345 6.0556
7.2842 7.1703 8.6294 7.7924 5.7716 7.7038 7.2571 7.4300
7.4359 7.4031 9.4240 5.9480 6.9772 6.2653 9.2056 6.7526
6.5687 8.7840 6.7517 8.6753 7.1969 5.3832 9.0521 7.3172
5.8320 8.1097 6.3086 8.3107 7.6759 7.7207 7.2325 8.3150
5.4631 5.9744 5.9613 5.8048 8.0285 5.8962 4.8277 6.7894
7.2514 7.0070 8.4319 6.7279 6.2217 6.1650 7.1165 7.0128
4.8844 6.6245 7.9178 6.9825 4.9129 7.0646 6.7497 6.1074
6.9202 6.3168 5.1412 5.1001 7.7537 6.6857 8.2974 5.5619
8.2451 4.7015 8.0839 8.0060 7.1901 8.0836 6.3232 7.3094
9.1307 6.3618 7.1828 7.2810 6.3348 8.6094 8.2747 9.3988
7.5502 6.9291 6.4058 6.8367 7.5963 6.7966 8.1035 7.5296
5.5356 9.6358 7.6286 6.0260 6.7595 8.6576 6.2390 6.2631

0.7889  6.0876  7.9141  7.2133  7.3351  5.8830  6.7292  8.6361
7.3192  7.3821  6.8300  7.0470  6.2667  7.5816  9.5306  6.4613
7.5362  4.8215  4.8235  7.2500  6.6325  8.5244  8.2680  7.0068
4.8290  6.5974  4.8556  6.8638  7.7103  5.9450  8.0911  8.0513
6.8906  6.1922  9.8787\
C19orf21        9.7818  8.2863  10.3459 9.1329  10.4477 9.7689
11.9651 10.7658 11.6546 7.1638  10.7060 10.7439 9.8606  9.1595
9.5714  8.4528  9.7782  12.0494 8.4478  8.9023  10.0869 10.0272
11.1436 8.2719  2.7258  10.7006 6.1093  11.3546 8.7910  10.4131
0.0000  3.4576  9.1346  9.3399  9.7904  11.4521 11.6906 11.2703
11.3817 6.1876  6.0945  8.5840  10.1074 10.2300 0.0000  10.4828
10.9390 12.4168 6.2259  10.8101 11.5298 10.9757 9.2252  2.7928
9.3804  10.5060 12.3013 11.1270 10.4478 10.8136 6.3368  9.4444
10.5045 9.6572  5.9146  10.0722 9.4555  10.4735 11.5543 10.0017
7.2178  9.6402  10.9949 11.9065 9.5816  7.7942  9.7297  10.9537
10.0249 11.3354 11.3055 9.7882  10.4769 8.2483  8.7737  10.1060
10.1345 8.5031  10.3401 8.2268  6.6857  10.4475 11.7031 6.4429
10.0604 1.6671  9.4416  6.2717  10.5872 11.1567 12.6788 11.4015
8.5700  9.4762  6.7454  10.0169 6.2277  10.5883 10.6782 9.1680
6.2608  11.9856 11.8307 11.2064 10.2045 9.2181  9.4880  10.4585
10.8998 9.9042  7.6827  11.3730 10.6304 12.8527 10.2250 9.6459
10.9607 11.0899 7.4380  3.4188  10.4046 10.0641 10.3073 9.3483
10.9285 13.6694 9.7512  7.5824  8.5138  4.5387  7.0807  6.5165
9.4886  9.7389  10.8308 3.5216  10.9802 8.6887  12.2212 11.7213
6.4726  10.4132 8.8283  9.8734  7.8770  9.5518  7.0396  10.3655
9.6566  8.4827  11.7657 12.5955 7.3312  10.6074 4.6850  8.3296
10.4859 10.0520 10.9925 10.8583 11.1958 9.7001  11.0354 9.4248
12.0587 9.0860  5.5912  5.6500  9.9046  11.4041 10.8576 10.7006
12.5254 8.6681  9.7831  5.8007  11.1752 11.8829 12.5006 8.2719
10.9076 10.4833 10.4428 6.2464  10.3985 12.0883 10.1816 6.6233
11.1950 7.4212  12.4405\
C19orf26        5.1886  4.9059  5.5345  5.7113  6.5975  0.0000
6.2384  2.9308  4.5476  4.9731  2.3666  3.5591  3.0112  2.8185
1.2358  4.9193  3.5585  6.0118  4.7781  6.2005  4.7642  4.1425
1.0738  2.9264  2.5058  6.1598  3.0765  2.9431  6.1693  4.8729
7.7767  7.2454  4.6727  4.5791  2.3226  6.0500  4.8670  4.6368
3.7318  6.3010  4.1205  6.9900  5.2786  2.2769  0.0000  3.9499
0.0000  4.7659  7.2058  6.2650  5.4030  6.9834  3.8722  1.8243
4.5723  5.8463  6.2287  2.7742  2.9740  3.9163  2.5892  4.5865
6.0250  6.7831  4.1163  4.6225  3.4947  5.6068  5.1275  3.5426
1.0365  5.1310  3.4823  3.6783  6.2008  2.9872  6.2128  3.9162
2.4028  6.9909  4.4992  5.4497  2.2886  2.1886  3.9566  4.9818
5.5838  4.6054  6.1527  5.6906  3.4559  2.8855  5.4046  3.1676
4.3283  1.8952  4.9210  2.9802  5.5581  6.2437  3.4638  5.7284
2.0509  4.7160  3.1638  2.7482  4.4239  5.4204  4.1615  4.2556
3.9969  4.4238  3.3832  5.0000  3.1220  5.9843  4.4721  4.8546
5.3998  5.7417  5.7762  5.2396  5.0602  5.1087  1.6090  1.2272
1.6443  0.0000  2.5267  2.5988  3.0379  3.7414  4.2977  2.5437
3.3291  2.0117  4.1069  0.0000  4.5094  3.1658  3.2906  6.3184
1.9445  5.6391  4.7163  2.6938  3.9333  2.8937  6.4971  3.6271
4.8727  0.0000  4.4257  6.2132  7.5825  3.4948  1.7618  4.8668
2.8458  6.1895  7.4930  3.9371  3.2401  3.4592  6.9820  3.6142
3.2830  3.3186  3.5357  0.5466  6.6414  5.0199  4.3297  5.3936
2.3719  5.5604  4.2749  2.0979  4.5512  1.6765  6.0475  4.1841

1.6771 4.6633 4.1421 6.8296 3.5744 3.3874 2.3320 5.8154
6.9903 3.8231 3.6515 5.4353 6.9052 0.0000 2.8619 1.3688
4.1093 3.9392 3.1794\
ZMAT4 2.0932 3.3733 3.3494 0.8605 0.5278 0.4059 6.4188
4.7399 2.7217 2.3031 0.7594 3.6538 3.4906 0.4395 0.9286
1.4442 5.2141 1.2795 1.9975 1.8781 1.8761 0.0000 1.0738
1.2144 5.3330 0.6062 9.1278 1.8373 1.3857 0.0000 0.4127
0.0000 0.0000 3.1101 0.0000 1.1431 0.9556 0.3648 0.0000
0.7666 1.4628 0.8506 2.4120 0.0000 1.0260 0.9285 0.0000
1.1317 3.0861 2.4071 0.0000 0.0000 1.0956 1.6405 2.8986
0.9635 0.6343 3.1582 4.4310 2.1385 0.9353 5.0654 0.9583
2.1974 3.9568 0.6977 0.0000 6.7442 0.0000 3.0801 1.4604
1.6718 0.9449 7.1640 0.9270 0.4698 2.6228 1.2414 2.8331
0.6159 0.9714 1.1797 1.5572 0.0000 2.9265 5.6855 3.1171
1.3738 3.0874 7.7602 1.0759 0.8470 6.6724 4.8400 0.9013
3.0128 4.0271 5.0907 0.5090 0.9478 0.0000 0.4986 3.1832
1.2208 0.0000 2.5744 3.3565 4.7058 4.2679 2.5918 4.4841
1.6572 6.5750 3.1699 0.8924 3.0691 1.4679 3.2296 0.0000
0.6089 8.2184 1.5923 2.7935 0.4700 1.6090 1.6938 2.5319
3.3768 3.3957 4.0156 5.7715 0.0000 0.0000 0.7571 2.0060
0.0000 0.6413 6.2786 2.1648 3.4689 1.6169 0.5748 1.7133
0.9357 1.0811 4.0836 0.0000 2.0758 0.9947 2.6654 1.6233
0.0000 1.2425 2.5882 5.3954 2.1834 4.0271 0.3855 0.0000
1.2429 0.0000 0.0000 2.3149 1.9645 1.2829 1.1696 0.0000
2.1785 0.9131 0.5466 0.7903 0.9151 0.9335 0.6229 0.0000
2.8712 3.8630 2.4262 9.4813 1.0692 1.7413 0.0000 1.2133
1.4215 3.3684 2.4158 3.3819 4.4477 1.1063 5.1866 0.5078
0.5556 2.7619 1.2477 0.0000 1.5946 1.0320 3.5298 0.3736
1.9827 2.9670\
C19orf24 10.0898 9.5720 10.6017 9.6888 10.0017 9.2000
9.4310 8.8236 9.5599 9.5141 9.9912 8.9414 8.7388 8.4305
9.5065 9.9371 9.8296 10.1110 9.4642 9.5223 9.6080 9.4931
9.9407 9.5202 8.5876 11.7946 9.4979 11.2994 11.1761 9.4375
8.5871 9.7024 9.5938 9.0510 9.0830 10.3926 9.6781 9.7361
10.0290 9.1971 10.8768 8.6804 8.9864 9.8293 8.2752 10.8744
12.9581 8.9401 9.4605 11.7796 10.4559 10.4061 9.1127 8.4925
8.6757 9.8028 9.9799 9.8006 8.2154 8.9861 8.4397 8.4190
10.4875 10.5316 9.4490 9.1422 9.3951 9.7344 9.3248 10.6058
10.5688 9.4517 11.1829 10.3214 9.5662 9.9773 10.1869 10.4005
10.2815 10.7955 10.8427 8.5721 10.5246 9.5926 10.6373 9.1805
8.4440 10.7570 8.9490 10.2953 9.1812 8.0841 10.1207 10.6220
9.5964 9.3271 8.4865 9.0073 9.7002 10.0403 10.8748 10.1119
8.8538 10.6048 10.6805 10.2832 10.2271 10.1074 8.5436 10.1541
9.5510 9.7280 12.3959 10.9447 9.7762 8.3799 9.8251 8.9248
9.1774 9.1473 8.8197 10.2572 8.6978 9.9910 8.7923 9.0913
9.6701 11.5224 8.2015 7.8992 8.8547 10.3732 10.2416 10.0026
10.3237 13.2837 11.3190 11.7816 9.2554 8.9014 11.4036 10.0548
11.1488 9.4068 9.4546 9.0051 10.1369 9.4191 10.3100 12.3905
9.2304 13.6905 8.6680 10.7189 10.2036 9.1044 9.0869 8.5659
9.2638 11.7620 11.1911 10.9397 9.3631 10.4540 9.1439 8.5428
9.3301 8.5126 10.1618 9.9166 9.2818 8.5969 10.3325 10.4026
12.4174 8.8970 9.4840 8.6699 8.8640 10.2779 12.0978 9.6490
8.4201 10.3272 8.5369 9.8322 9.4612 10.8770 10.8899 10.4314
8.7413 10.2851 9.3961 9.3689 9.9133 11.9285 11.8606 9.1566

```
9.1905  8.8464  11.7408\
C19orf25        9.8195  8.9897  9.3510  8.5895  9.3536  8.2563
9.4603  8.2265  8.6914  8.8342  9.2511  7.9278  8.1809  8.6789
8.5228  9.7990  9.3492  10.1148 8.9853  9.3324  8.4372  8.9170
9.4836  8.6960  8.9747  10.6013 8.5407  10.0466 9.8372  8.5966
9.0408  8.9305  9.7746  7.0766  8.6284  9.8958  9.4413  7.8545
8.7727  8.0425  10.0698 8.3160  7.9908  8.7090  8.2992  9.2416
11.3996 8.5137  8.7112  9.9724  9.5687  8.9164  8.4538  8.7398
7.8903  10.0051 9.6147  8.3763  8.2499  8.6824  8.4753  7.4267
10.0965 9.9836  9.2104  9.1422  8.8747  8.4910  8.7068  9.0539
9.6543  9.3449  9.9792  9.5007  8.7797  9.3176  8.8432  10.1271
9.5382  8.9330  9.6828  6.9326  9.6992  8.3515  9.8360  8.4488
8.5931  10.1400 7.5685  9.8601  8.9373  8.2840  9.1897  9.5597
9.8842  8.5867  7.6403  9.2757  9.0203  9.2915  11.1624 9.6760
7.7970  8.9503  9.2909  8.6676  9.3383  9.8781  7.5943  8.9492
9.0356  9.2818  10.9204 9.2456  7.9138  8.3936  8.6171  8.2366
8.2559  8.1472  8.9925  9.0264  8.6188  9.5448  9.1573  8.5531
7.9269  9.9167  8.8141  8.1009  7.6739  9.1554  9.4931  8.8322
9.4232  11.1500 11.0112 9.8552  8.9572  9.1845  9.2668  8.3978
10.0141 8.6937  8.2234  8.6516  8.7176  9.3199  10.2102 9.8830
8.6184  11.2855 8.3105  8.6222  8.7740  8.5011  9.0819  8.1897
8.7046  10.6956 9.3154  9.6884  9.2778  9.9130  9.3662  8.4701
7.7961  7.7079  9.8849  8.8833  8.9781  7.6299  9.8932  9.7347
11.0619 9.5299  9.0895  8.7343  8.6318  9.4400  11.2224 8.8737
8.9230  8.7250  8.5549  8.7921  9.7633  9.9435  9.9574  9.9867
9.2886  8.9144  8.8112  8.4841  8.5377  9.7810  10.7577 9.4358
8.7716  8.6843  10.8608\
MYBL1   7.0911  5.2980  6.3339  6.4098  7.9250  7.2762  4.8290
6.3722  4.7857  6.8471  7.4418  5.8210  6.7358  6.0636  6.8715
6.1261  7.3933  6.3515  6.3542  7.8376  6.3133  5.9190  5.6167
5.8078  5.6068  6.0504  7.9425  6.0068  4.6080  6.6152  8.5404
7.1520  6.9234  6.5684  6.9198  5.6596  4.8670  7.4587  5.0773
5.8087  7.2976  5.3058  7.4056  5.8046  4.8044  5.2147  4.5869
6.5348  6.8113  5.6525  6.5764  7.8452  5.2023  6.0214  6.1856
6.5120  5.6480  6.0644  6.2299  6.9023  5.8240  6.2681  7.1204
7.0629  6.3489  6.0097  6.1150  6.8917  4.5928  6.2192  6.7531
5.0380  5.5047  5.7472  5.6473  6.6649  6.0384  4.3291  5.5901
6.0319  5.4826  5.8407  5.2692  6.7196  5.7263  6.9905  8.0895
6.5923  7.7450  7.8756  4.9504  7.1249  4.5362  6.3372  6.9516
4.9755  7.2256  5.5527  6.5814  8.2906  5.3426  6.2505  6.8179
7.2227  6.6456  6.6782  7.3049  6.4899  7.3737  6.3027  5.8967
5.4621  5.8481  4.9069  8.2665  6.4528  7.0603  6.5634  7.4039
6.5374  7.7389  5.9057  7.1198  6.0958  5.7900  7.3785  6.5133
5.4935  5.6036  6.4704  6.6766  4.5112  4.8109  7.5281  8.0901
5.0704  6.3404  6.3130  6.2462  5.8599  7.6985  5.3099  5.5060
6.2100  6.8051  5.5133  5.8004  5.8224  5.3293  6.1526  5.9869
4.0526  5.5403  5.7851  6.1345  6.6311  5.4130  6.9663  6.6292
6.4065  5.6465  5.2118  6.0248  6.6254  6.9572  5.7182  4.9467
5.9523  7.4762  8.1109  7.0275  8.0759  7.1956  5.7086  3.7579
6.7652  6.4229  6.1045  7.2812  5.9143  6.0165  6.5771  7.0346
5.8281  5.7270  5.7724  6.6891  6.0849  5.5582  6.8699  6.1245
4.1245  6.1375  7.1867  7.4627  6.0366  7.5866  6.1951  6.5966
6.6516  4.4234\
NR1D1   7.3497  9.8210  8.0476  9.0882  7.7104  9.1892  8.5803
```

7.3184 9.2235 9.5147 7.5669 8.4025 8.5181 7.9579 7.6270
9.1402 8.4913 8.4240 9.4310 7.7279 7.9552 8.7586 9.5559
8.0646 9.9774 8.5556 6.7553 7.6738 9.1398 8.1330 7.2645
7.9880 7.7823 8.0921 8.2307 7.4991 8.6751 7.8240 8.0630
6.9591 7.6618 9.7788 8.6571 8.3547 7.9621 8.4960 7.7234
8.2294 7.0644 10.1145 7.5650 7.5542 8.8668 10.4260 8.3764
8.4573 9.3485 9.2649 8.6904 8.9257 8.9176 9.4889 8.7443
7.7092 9.2176 9.3221 8.3431 7.1059 9.2229 8.7744 7.4324
8.0778 9.4892 8.0335 7.8108 10.2273 9.1515 9.3593 8.7705
8.9790 8.0851 7.4095 8.2627 7.3031 9.2587 7.7391 8.1431
7.9987 7.8848 8.8411 10.3472 9.1163 8.0392 9.4252 8.8240
9.4909 8.6376 10.8865 8.3301 8.5419 8.9836 8.6425 7.5200
7.9031 7.6096 7.6526 10.5237 8.2033 8.9810 8.6169 7.7507
7.6498 8.6293 9.3945 7.9309 8.5984 6.6668 8.2524 7.9740
7.6092 8.0921 8.3475 7.8804 8.7713 8.5204 8.8176 7.9997
8.9418 11.3107 9.7410 10.2823 7.7104 7.6041 8.2012 8.3052
9.0793 7.5512 9.7664 9.2445 10.7292 7.3716 8.4721 8.7111
9.2424 9.1386 11.2577 8.6787 10.2501 9.4236 8.7836 9.5537
7.5489 8.2492 8.0906 8.4872 8.2352 10.4389 7.5466 8.4899
7.3407 10.3742 9.8263 8.3566 8.4481 7.4388 8.2078 9.5954
9.1082 6.8954 7.3525 7.8230 8.3253 9.0493 8.4404 9.6695
8.2515 8.8997 10.0010 7.7962 8.4720 8.6555 10.3607 7.4214
9.1941 8.2181 8.1428 8.1844 8.4112 7.2288 8.0862 8.3630
8.8355 7.8586 8.7623 8.2180 9.6107 7.4294 11.2944 10.0068
7.9643 8.7585\
C19orf28 10.7597 10.2987 10.0548 10.1036 10.5177 9.7418
10.9095 9.3187 10.1350 11.0680 10.6767 9.6661 9.7898 9.2270
11.0109 10.6983 10.6674 11.2539 10.6620 11.0482 11.1901 11.1832
11.1750 10.1022 9.6299 12.4511 9.8881 11.5726 11.0385 11.7219
9.2341 10.2274 10.4030 9.8131 9.4928 11.3171 10.5329 9.7953
10.2761 10.3658 11.0134 9.9040 10.8569 8.9514 7.7859 11.5147
12.3018 11.9018 9.5276 10.6854 10.1739 12.0145 10.0784 8.2946
9.7357 10.4030 11.2035 9.8555 9.6699 10.0229 8.3935 9.7468
11.0349 11.3886 10.3584 10.5713 10.7226 10.9876 10.5322 10.5648
11.1774 10.0254 12.1329 10.5805 10.1477 10.5276 10.6863 11.2320
10.2801 11.1048 12.0093 9.5708 10.7702 10.0615 11.5649 9.9162
8.7360 12.0534 9.9241 11.3943 9.4833 9.9091 10.9504 11.0932
11.2463 8.9494 9.7293 9.0703 10.8594 11.1490 11.4910 11.1387
9.5638 10.4542 10.3942 9.7013 10.3562 10.6934 9.6006 10.2831
10.0063 10.9704 12.0326 11.8525 10.2351 9.7052 10.0321 9.5773
10.2074 11.5282 9.3736 10.8647 11.7332 12.1257 10.5822 10.9671
10.2579 10.8716 8.8651 9.0452 9.3654 9.9250 11.3134 10.2735
11.6466 13.1226 11.4209 11.1234 9.8805 9.1369 11.5895 10.7296
12.2298 10.2117 10.7670 8.3508 10.5904 10.1846 11.2478 10.6827
9.9624 13.2411 10.2250 9.9631 10.7092 10.0853 9.4673 10.4258
9.7987 12.1030 11.4503 11.8008 10.6973 10.6077 10.1131 9.9424
10.1354 9.3569 11.0075 10.2681 10.4949 9.3090 9.6753 10.5192
12.0484 9.7856 10.4144 8.9020 9.9853 10.6850 11.9328 12.1150
9.2982 10.9341 9.9034 10.8716 10.4382 11.1778 11.2777 10.6265
9.8683 9.6381 10.0224 10.4107 10.8674 10.9504 12.0744 9.2395
10.5143 10.0839 12.8807\
C19orf29 9.7594 9.5081 9.1003 8.7292 9.8567 8.7619
9.7441 9.0008 8.9304 9.7135 8.9176 9.2189 8.7495 9.2743
8.9584 9.1566 9.5545 9.6239 9.5358 9.1943 9.4714 9.0887

9.4969 8.6335 9.4611 9.5940 8.8448 9.4784 9.1290 8.9898
8.5746 9.8789 8.3583 7.9935 9.5507 10.0795 9.3735 9.3251
8.6636 8.8899 9.8058 9.2148 8.6564 8.4880 8.7927 9.3791
9.5924 8.7929 9.3902 9.2042 8.7751 9.2863 8.8087 8.8123
9.0325 9.6652 9.3192 8.9043 8.4513 8.9722 8.9855 8.2520
9.4022 10.1372 9.6352 9.2742 9.0748 10.4516 9.3036 9.2061
9.3245 9.2906 9.3431 9.5101 8.6269 9.1061 9.4325 9.5533
9.1348 9.6775 9.5514 8.7049 9.3063 8.7536 9.8360 8.8488
8.7206 9.7319 8.3178 9.3239 9.3463 8.6336 9.4444 9.7712
9.3908 9.4205 8.3049 9.7367 9.6855 9.4036 9.9165 9.3778
8.3454 9.5029 9.2973 9.0586 9.9957 9.2923 8.3269 9.1843
9.3301 9.6605 9.1231 9.5098 9.3956 8.3000 9.0606 8.6900
8.7071 8.6778 9.0693 8.8168 9.0487 9.4864 9.3072 8.6429
8.4453 8.9762 9.0121 8.9708 8.8980 9.1268 9.4847 9.0261
9.8636 9.6738 10.3530 9.6690 9.4893 9.5067 9.6419 9.0260
9.7515 8.9468 9.0135 9.9043 9.0607 9.6059 10.0634 9.0536
9.3466 9.3489 9.1380 9.4374 9.4223 8.9702 9.5779 9.2675
8.7010 9.9868 9.4764 9.1359 9.2928 9.6012 9.2048 9.0744
8.0310 8.6387 9.2514 9.6705 9.4856 8.6263 8.6340 9.3810
9.4508 9.4270 9.7184 9.1937 9.2802 9.3818 9.8558 8.8081
9.1731 8.8111 8.6890 9.0469 9.7071 9.8220 9.3886 9.8553
9.1195 8.9505 8.6544 9.4441 9.8560 9.0173 9.6945 9.8287
9.6089 9.1212 9.9690\
ITGB1BP1 9.6028 10.4378 9.9362 9.0554 9.5977 9.3133
10.1456 9.5951 9.4851 9.6031 9.8553 9.9434 9.6686 9.6946
8.6054 9.1696 10.4214 9.9641 10.7602 10.6743 8.4483 9.2327
9.8399 9.2582 10.0788 10.3789 9.9780 9.7386 9.7735 9.5795
9.8908 9.4697 9.9378 8.7925 9.1443 9.9284 9.3204 9.5697
9.5339 9.7127 10.4719 10.0984 10.4515 9.8475 10.4682 10.1296
11.1125 9.5165 10.3748 9.1937 9.9904 10.6016 9.9274 10.4759
9.8488 9.4606 9.5953 10.0978 9.3321 10.4860 10.1789 9.6962
9.8579 9.8490 9.7359 9.0292 10.8147 9.8201 9.6496 9.6882
10.3972 8.9099 10.2265 9.8687 9.2912 9.5824 10.9142 9.4445
9.6292 9.7998 9.9094 9.1836 9.8576 9.7419 9.4585 10.0154
9.1375 9.4185 10.1216 10.3218 10.3335 10.5037 10.1798 9.4763
9.7001 10.5555 9.7911 10.0953 9.3081 10.2671 10.2045 9.6825
9.6526 10.2415 9.8483 9.5415 10.8190 9.8281 9.2755 9.0025
9.3021 9.1139 11.3608 10.0265 9.8782 9.7616 9.4475 10.0099
9.7395 9.9184 9.7911 9.4215 9.8988 9.3329 9.8844 10.1497
9.6999 11.6407 10.1700 9.7714 10.1532 10.4118 9.0300 10.1101
11.6838 10.8296 10.1793 10.1373 9.3907 9.9050 9.9001 9.4562
9.2704 9.1342 8.8889 9.7849 9.7552 10.3572 9.4901 10.2871
9.7012 10.1906 8.7511 9.6903 8.7981 9.7753 9.8330 9.0778
9.7511 10.4884 9.7941 9.6432 8.8242 10.0284 9.9601 8.9230
9.6203 9.1231 9.8376 9.9539 8.4422 10.4006 10.8152 9.7023
9.3982 9.5597 9.8676 10.3847 9.5891 9.7097 9.6583 9.2960
10.4902 9.1549 9.4232 9.6290 9.7501 9.9463 8.8983 9.2141
9.3089 9.2798 10.3017 9.1385 9.4136 10.1956 10.1888 10.0299
9.8028 9.0970 10.0172\
ITGB1BP3 0.0000 2.1912 0.0000 0.0000 0.9133 0.0000
0.0000 0.0000 0.0000 0.7786 0.0000 0.0000 0.0000 0.4395
0.0000 0.0000 0.0000 0.0000 1.5827 2.8567 0.0000 0.0000
0.0000 0.5262 4.4007 4.8934 0.0000 0.5990 0.0000 0.5901
2.4077 0.0000 1.1952 0.0000 0.0000 3.2417 0.5555 0.0000

| | | | | | | | |
|---|---|---|---|---|---|---|---|
| 0.0000 | 0.0000 | 0.0000 | 2.9686 | 0.0000 | 0.0000 | 2.8515 | 0.0000 |
| 0.0000 | 0.0000 | 0.0000 | 0.0000 | 0.0000 | 0.7768 | 0.0000 | 0.0000 |
| 0.0000 | 0.0000 | 0.0000 | 0.0000 | 1.0204 | 0.0000 | 0.0000 | 0.0000 |
| 0.5573 | 0.0000 | 0.0000 | 0.0000 | 0.0000 | 0.4832 | 0.0000 | 0.0000 |
| 0.0000 | 0.0000 | 0.0000 | 0.0000 | 0.0000 | 0.0000 | 0.5548 | 0.0000 |
| 0.0000 | 0.6159 | 0.0000 | 0.0000 | 0.7204 | 0.0000 | 0.0000 | 0.0000 |
| 0.0000 | 0.0000 | 0.9125 | 2.1773 | 2.4416 | 0.0000 | 2.6379 | 1.1561 |
| 0.0000 | 1.0621 | 0.0000 | 0.5454 | 0.0000 | 0.0000 | 0.0000 | 0.0000 |
| 0.0000 | 0.7358 | 0.0000 | 0.0000 | 1.8642 | 0.4315 | 0.0000 | 0.0000 |
| 0.0000 | 0.0000 | 2.5153 | 2.3219 | 1.1918 | 0.0000 | 0.0000 | 0.0000 |
| 0.0000 | 1.0358 | 3.3082 | 0.0000 | 0.0000 | 0.0000 | 0.0000 | 0.0000 |
| 0.0000 | 0.0000 | 0.0000 | 1.1792 | 0.3935 | 0.0000 | 0.0000 | 0.0000 |
| 0.0000 | 0.0000 | 1.9254 | 0.0000 | 0.5216 | 0.8792 | 0.0000 | 0.5748 |
| 0.0000 | 0.0000 | 0.0000 | 0.0000 | 0.0000 | 0.5454 | 0.9947 | 0.0000 |
| 0.5019 | 0.0000 | 1.7123 | 0.0000 | 0.0000 | 0.0000 | 0.9683 | 0.0000 |
| 0.0000 | 4.1211 | 0.0000 | 0.0000 | 0.0000 | 0.0000 | 0.0000 | 0.0000 |
| 0.0000 | 0.0000 | 0.0000 | 0.0000 | 0.4486 | 0.0000 | 5.6079 | 0.0000 |
| 0.0000 | 0.0000 | 0.9063 | 2.8104 | 0.0000 | 0.6314 | 0.0000 | 2.0241 |
| 0.0000 | 0.0000 | 0.0000 | 0.6248 | 0.0000 | 0.0000 | 0.0000 | 0.5707 |
| 0.0000 | 0.0000 | 0.0000 | 0.0000 | 0.0000 | 1.5946 | 1.6276 | 0.0000 |
| 0.0000 | 0.0000 | 0.0000\ | | | | | |
| ITGB1BP2 | | 4.9910 | 5.1964 | 1.5051 | 2.0919 | 6.2156 | 1.7112 |
| 0.9367 | 4.0521 | 2.1693 | 4.9059 | 2.2662 | 0.4849 | 2.5713 | 2.9572 |
| 2.4639 | 3.8845 | 4.4959 | 3.4585 | 6.4643 | 4.7636 | 3.1720 | 1.5770 |
| 1.6825 | 3.0076 | 8.5947 | 1.8524 | 4.2074 | 3.7390 | 4.2890 | 2.8206 |
| 2.7292 | 4.6029 | 0.8951 | 1.3374 | 1.0004 | 4.1139 | 3.8700 | 3.5416 |
| 4.0811 | 1.2646 | 3.2902 | 6.0719 | 3.4937 | 4.3385 | 7.6732 | 3.0403 |
| 5.2997 | 1.1317 | 2.5837 | 3.1417 | 2.6239 | 4.2351 | 4.3046 | 7.5066 |
| 4.1154 | 4.9286 | 2.8225 | 3.6648 | 5.8812 | 1.9697 | 7.7539 | 2.4655 |
| 3.0948 | 2.8376 | 5.1251 | 2.3341 | 1.8352 | 4.3846 | 1.3082 | 6.6294 |
| 3.7294 | 2.5071 | 2.7116 | 4.4233 | 4.2499 | 3.2432 | 4.4664 | 2.7891 |
| 3.1642 | 3.7276 | 3.4708 | 4.6093 | 3.2356 | 2.3651 | 5.1249 | 4.8538 |
| 1.7039 | 2.6565 | 5.9718 | 5.1437 | 6.1571 | 3.9797 | 3.5709 | 4.2337 |
| 4.1635 | 9.2337 | 2.6431 | 5.7380 | 5.0693 | 4.2566 | 4.5071 | 2.3585 |
| 3.1101 | 4.6411 | 3.6941 | 3.5453 | 5.2215 | 3.4735 | 2.8861 | 3.6514 |
| 2.8902 | 2.0810 | 5.7671 | 4.5236 | 1.8354 | 2.0594 | 0.9131 | 3.6861 |
| 5.1130 | 4.5594 | 3.2531 | 2.1785 | 2.6767 | 0.8239 | 3.2018 | 3.4959 |
| 4.5436 | 3.2729 | 7.2790 | 8.1009 | 2.6625 | 3.2985 | 2.9134 | 4.5713 |
| 3.7887 | 6.0815 | 3.7942 | 5.4591 | 5.0160 | 5.4392 | 3.8517 | 2.6745 |
| 2.3186 | 2.6953 | 2.1200 | 5.4088 | 2.3685 | 5.4951 | 2.6913 | 1.2240 |
| 5.4821 | 5.4557 | 2.6925 | 4.4299 | 2.9864 | 4.4054 | 6.4443 | 4.1575 |
| 2.8458 | 4.0828 | 0.0000 | 3.6012 | 2.6931 | 4.4141 | 3.3158 | 2.5845 |
| 3.1709 | 0.9722 | 3.9608 | 4.6675 | 2.6102 | 3.1654 | 3.5752 | 3.6252 |
| 4.7787 | 4.2895 | 6.3628 | 7.6442 | 4.1171 | 3.5152 | 4.0819 | 5.1931 |
| 0.5257 | 2.2560 | 3.0580 | 2.9077 | 0.5811 | 5.7208 | 4.4431 | 4.1290 |
| 2.6000 | 3.1677 | 3.0407 | 3.6254 | 3.2731 | 3.9204 | 3.3048 | 5.2451 |
| 2.1850 | 4.2582 | 3.7440\ | | | | | |
| ZNF7 | 8.7763 | 8.3341 | 9.1347 | 9.1899 | 9.0523 | 9.0070 | 9.8551 |
| 8.6116 | 8.3109 | 8.7543 | 8.3092 | 9.0466 | 8.7811 | 8.9126 | 8.3980 |
| 9.1828 | 9.3443 | 8.8644 | 8.2513 | 9.1209 | 8.8941 | 8.1055 | 8.2486 |
| 8.4274 | 8.5774 | 8.2887 | 9.1583 | 8.1464 | 8.6062 | 8.7112 | 9.0005 |
| 8.8143 | 9.1773 | 9.9521 | 8.4987 | 9.6772 | 8.6838 | 8.5847 | 8.1418 |
| 9.3231 | 8.7358 | 8.9213 | 9.0362 | 7.7230 | 8.7370 | 7.7313 | 8.6855 |
| 8.6266 | 8.9186 | 8.6405 | 8.5943 | 7.9201 | 8.6736 | 9.2202 | 9.2004 |

| | | | | | | | |
|---|---|---|---|---|---|---|---|
| 8.8499 | 8.8776 | 8.2859 | 8.4745 | 9.3529 | 8.8515 | 8.4838 | 8.6800 |
| 9.6634 | 8.4978 | 8.3466 | 8.5041 | 8.9933 | 8.2620 | 8.9958 | 9.2445 |
| 8.7121 | 8.0056 | 9.3898 | 8.7025 | 9.0451 | 9.1912 | 8.2488 | 8.3247 |
| 8.3825 | 8.1984 | 9.0386 | 8.4061 | 8.8027 | 8.6691 | 9.3281 | 9.0069 |
| 8.5401 | 9.9964 | 8.3234 | 8.3395 | 9.6338 | 8.7980 | 9.2175 | 8.4494 |
| 8.1772 | 8.3593 | 8.8392 | 8.9838 | 9.4599 | 8.6294 | 8.3797 | 8.6065 |
| 9.9670 | 9.4102 | 8.5712 | 9.1936 | 8.2766 | 8.5128 | 9.0565 | 8.8462 |
| 8.6319 | 8.8551 | 8.6797 | 8.3550 | 8.9314 | 8.6767 | 9.4206 | 8.8517 |
| 9.1633 | 9.1082 | 8.2320 | 8.8031 | 7.7889 | 9.0500 | 9.1501 | 8.0998 |
| 9.3445 | 8.8434 | 8.9363 | 9.5059 | 8.2961 | 8.2848 | 8.7415 | 8.6274 |
| 9.2377 | 8.5776 | 9.6624 | 8.9994 | 8.7996 | 9.3732 | 8.1648 | 8.6584 |
| 8.4073 | 8.6592 | 8.8968 | 8.3250 | 8.6326 | 8.2670 | 7.9395 | 8.7737 |
| 8.5778 | 8.6085 | 9.3438 | 8.3901 | 8.8417 | 8.7774 | 9.3988 | 9.7318 |
| 8.1803 | 7.8316 | 7.4369 | 8.7149 | 8.6593 | 9.1399 | 8.8728 | 8.8041 |
| 9.2296 | 9.3205 | 8.6013 | 9.6230 | 8.6571 | 8.6810 | 8.7470 | 8.3209 |
| 9.5277 | 8.9682 | 8.7321 | 9.7154 | 8.4816 | 8.5685 | 7.8651 | 9.4868 |
| 8.7730 | 8.9908 | 8.3980 | 8.8422 | 9.0626 | 8.3334 | 8.9547 | 8.5110 |
| 8.4082 | 8.6873 | 9.3589 | 9.3466 | 9.8615 | 9.3723 | 9.1365 | 8.5646 |
| 9.0294 | 7.8164\ | | | | | | |
| ZNF2 | 6.8786 | 6.7126 | 6.8530 | 6.8275 | 6.9670 | 6.8663 | 7.7749 |
| 7.9433 | 7.0914 | 7.2646 | 7.0132 | 7.5877 | 7.6347 | 7.7532 | 6.7796 |
| 7.6972 | 7.4299 | 7.3299 | 7.1413 | 6.6862 | 6.6490 | 6.4704 | 6.8292 |
| 7.2725 | 7.2404 | 7.0337 | 7.0429 | 6.7760 | 6.3795 | 7.0096 | 7.6056 |
| 7.3668 | 7.2388 | 7.0183 | 7.8651 | 7.2599 | 7.1052 | 7.5256 | 6.8481 |
| 7.7357 | 7.2595 | 6.8106 | 7.1332 | 6.9305 | 7.0397 | 7.2466 | 7.3645 |
| 6.9296 | 7.5181 | 6.7367 | 7.2915 | 6.8016 | 7.1438 | 6.9571 | 7.0239 |
| 7.5884 | 7.1079 | 7.1584 | 7.0022 | 7.8575 | 7.4344 | 6.7371 | 7.4393 |
| 7.4359 | 7.0744 | 6.9844 | 7.3285 | 7.9168 | 6.9257 | 7.6415 | 6.8218 |
| 6.8802 | 6.5753 | 7.5565 | 6.7156 | 6.4275 | 7.7348 | 6.5992 | 7.3572 |
| 7.0443 | 6.7756 | 6.7955 | 7.0981 | 6.7517 | 6.9571 | 7.5241 | 7.6643 |
| 7.0036 | 7.7271 | 7.1579 | 6.9542 | 7.3917 | 7.1370 | 7.2489 | 7.4257 |
| 6.9909 | 6.8902 | 7.6630 | 6.8053 | 7.0186 | 7.5696 | 6.6745 | 7.3967 |
| 7.2419 | 7.2315 | 5.5702 | 6.8727 | 7.3370 | 6.6969 | 7.0525 | 7.4049 |
| 7.2812 | 7.4938 | 7.1189 | 7.0994 | 7.5212 | 6.6361 | 7.7105 | 6.8473 |
| 6.7281 | 7.9506 | 6.9535 | 6.9093 | 7.0479 | 7.0412 | 6.9740 | 7.3066 |
| 7.9832 | 7.5000 | 7.3512 | 7.2933 | 7.3282 | 6.8321 | 7.3070 | 7.1803 |
| 7.0042 | 7.4971 | 7.9307 | 7.4203 | 7.3338 | 7.3557 | 6.7510 | 6.7412 |
| 7.0343 | 6.7160 | 7.0867 | 7.3151 | 6.9177 | 7.1237 | 6.8484 | 6.9270 |
| 6.8337 | 7.2367 | 7.5156 | 6.0364 | 7.4448 | 7.5022 | 7.1203 | 6.0098 |
| 6.4597 | 6.2518 | 5.6222 | 7.0412 | 7.2747 | 6.8671 | 7.5256 | 6.9285 |
| 7.0423 | 6.9358 | 7.7525 | 7.1400 | 7.6868 | 6.3167 | 7.1325 | 7.4964 |
| 7.6120 | 7.5315 | 6.9224 | 7.2049 | 6.5431 | 6.6954 | 6.9999 | 7.5766 |
| 7.2285 | 7.7292 | 7.1654 | 8.0081 | 7.3832 | 6.9108 | 7.3012 | 6.8176 |
| 6.6922 | 7.8389 | 7.2270 | 7.4251 | 7.0697 | 7.2279 | 7.4734 | 6.9744 |
| 7.2558 | 6.1881\ | | | | | | |
| ZNF3 | 9.6374 | 10.2278 | 9.4869 | 11.1971 | 9.7696 | 9.9183 | 10.2882 |
| 10.7652 | 9.6666 | 10.4798 | 9.4752 | 9.7333 | 11.1962 | 9.5005 | 10.5301 |
| 10.0208 | 10.2357 | 9.7581 | 9.9214 | 9.3649 | 9.4206 | 10.0400 | 10.5925 |
| 10.0797 | 9.8656 | 8.9703 | 9.9090 | 10.8159 | 9.9845 | 8.7265 | 10.3351 |
| 10.8018 | 10.0659 | 10.1671 | 10.5954 | 9.7231 | 9.8403 | 9.6169 | 9.3160 |
| 9.7764 | 9.8306 | 10.0615 | 9.7246 | 10.0923 | 9.3518 | 10.3841 | 10.6184 |
| 8.6946 | 10.1405 | 9.5028 | 9.6217 | 9.5557 | 10.0370 | 9.6016 | 10.1247 |
| 10.2943 | 9.4850 | 10.2236 | 9.9277 | 9.4552 | 9.3890 | 10.5893 | 9.7692 |
| 8.9367 | 10.4899 | 9.7974 | 9.8118 | 9.5897 | 9.7368 | 9.8555 | 9.8267 |

| | | | | | | | |
|---|---|---|---|---|---|---|---|
| 9.9375 | 9.0426 | 10.3869 | 9.7631 | 9.9632 | 10.4835 | 10.8823 | 10.6819 |
| 9.5786 | 9.8501 | 9.6661 | 10.4573 | 10.1607 | 10.5424 | 10.2077 | 10.3707 |
| 10.2811 | 10.6412 | 9.7837 | 9.2633 | 10.6954 | 10.1848 | 10.5419 | 9.8272 |
| 8.8978 | 9.0835 | 10.0355 | 10.0946 | 9.7233 | 10.1037 | 9.7554 | 10.1208 |
| 10.4188 | 10.5750 | 9.4268 | 9.6562 | 10.2922 | 9.8695 | 10.3976 | 10.0807 |
| 10.4163 | 11.1004 | 10.1189 | 10.3366 | 9.3350 | 10.4026 | 10.1538 | 10.0809 |
| 9.2814 | 10.0938 | 9.9157 | 9.5369 | 9.5768 | 10.7609 | 9.7421 | 9.7499 |
| 10.3545 | 9.8245 | 9.9603 | 10.0288 | 10.2159 | 10.5454 | 10.4099 | 9.7995 |
| 10.1151 | 9.9222 | 11.0627 | 10.4710 | 10.1655 | 10.1967 | 9.4327 | 10.8025 |
| 9.6234 | 9.2577 | 9.7363 | 9.9772 | 9.8717 | 10.1667 | 9.8335 | 10.4357 |
| 10.4620 | 10.3623 | 10.2740 | 9.3263 | 9.2700 | 9.8774 | 9.7953 | 11.5698 |
| 9.2625 | 9.9317 | 8.6335 | 10.3147 | 10.3271 | 10.3446 | 9.4878 | 10.2952 |
| 9.7908 | 9.9915 | 10.3692 | 9.7183 | 9.5880 | 9.9491 | 10.5527 | 10.4665 |
| 10.1387 | 10.5926 | 9.3218 | 10.6676 | 11.0981 | 10.6933 | 8.9259 | 11.3349 |
| 10.2895 | 10.9960 | 9.6864 | 9.8229 | 9.6715 | 10.2924 | 10.0077 | 9.6884 |
| 9.5923 | 10.8546 | 9.8939 | 9.8139 | 10.7257 | 9.8795 | 10.2425 | 9.6805 |
| 10.4690 | 9.8068\ | | | | | | |
| CTCFL | 4.1790 | 3.4872 | 3.6645 | 3.5712 | 6.5391 | 12.7603 | 6.8166 |
| 8.8871 | 5.3535 | 4.6027 | 11.0642 | 6.9363 | 10.1539 | 4.7228 | 4.4386 |
| 2.6907 | 5.5586 | 4.5780 | 3.7777 | 6.2586 | 10.6611 | 4.4556 | 3.8411 |
| 4.0866 | 4.5668 | 3.7794 | 5.4247 | 4.1131 | 4.2126 | 4.0827 | 6.2755 |
| 4.3023 | 5.0662 | 11.1230 | 11.0263 | 7.6380 | 4.1367 | 4.3422 | 4.4024 |
| 4.1524 | 3.0715 | 3.7543 | 4.1456 | 4.3090 | 3.4139 | 3.6428 | 1.5457 |
| 5.9276 | 11.5272 | 3.2858 | 4.0866 | 2.8534 | 4.1771 | 3.7036 | 5.2714 |
| 3.9466 | 4.0970 | 4.0843 | 10.0217 | 8.3792 | 3.5537 | 8.4154 | 4.0707 |
| 4.0936 | 4.0583 | 4.4456 | 3.5413 | 7.0360 | 4.6856 | 4.1649 | 10.4361 |
| 4.4530 | 2.7773 | 6.5493 | 3.6429 | 3.7898 | 4.2240 | 1.7756 | 2.7409 |
| 4.7110 | 4.2460 | 5.8456 | 3.7184 | 4.3058 | 4.0265 | 4.7046 | 5.4317 |
| 3.8104 | 8.4182 | 4.3158 | 3.3926 | 6.3398 | 4.6122 | 2.8647 | 4.0867 |
| 4.1060 | 4.6822 | 3.2117 | 4.2744 | 4.9293 | 3.2068 | 3.1995 | 2.8230 |
| 3.9372 | 3.9563 | 4.2987 | 3.3759 | 5.9363 | 9.3829 | 4.3005 | 3.7961 |
| 3.4402 | 4.8729 | 8.0558 | 5.5214 | 10.2798 | 4.1047 | 10.1484 | 3.3813 |
| 3.6131 | 10.9716 | 4.5922 | 5.6403 | 4.0339 | 3.8019 | 10.3331 | 5.8375 |
| 5.8947 | 4.2671 | 4.2999 | 4.1060 | 4.7178 | 4.7623 | 2.9821 | 3.4234 |
| 8.3993 | 10.6565 | 0.0000 | 4.1052 | 4.2748 | 3.7545 | 4.5295 | 3.4007 |
| 4.2887 | 4.6631 | 4.5540 | 4.0099 | 3.5736 | 3.6235 | 2.5384 | 4.2573 |
| 0.0000 | 3.8757 | 11.7460 | 3.7019 | 7.2677 | 3.9732 | 4.2655 | 6.5766 |
| 4.4617 | 3.4320 | 3.6225 | 3.6439 | 3.9977 | 4.9580 | 4.5225 | 7.6591 |
| 3.8018 | 4.4761 | 4.2452 | 5.2530 | 10.4568 | 3.5619 | 9.2352 | 0.0000 |
| 4.8090 | 4.0539 | 3.6280 | 4.9434 | 3.7577 | 4.0073 | 4.1928 | 11.2191 |
| 3.5793 | 8.8053 | 3.8689 | 8.3178 | 3.9798 | 3.8055 | 4.6873 | 4.4582 |
| 3.7358 | 4.8114 | 7.3720 | 4.5643 | 9.8172 | 0.0000 | 4.5564 | 4.3054 |
| 3.7595 | 3.2776\ | | | | | | |
| PHLDA1 | 10.3216 | 11.1504 | 11.7142 | 9.6859 | 9.9199 | 10.3074 | 9.0052 |
| 8.6650 | 8.1830 | 10.1590 | 10.3436 | 7.7545 | 8.6052 | 11.8691 | 11.1511 |
| 13.5146 | 8.6358 | 8.5315 | 10.9421 | 8.0072 | 9.3956 | 10.1602 | 7.5226 |
| 13.2036 | 11.1756 | 9.9399 | 10.4755 | 8.8554 | 12.2228 | 7.0432 | 8.9777 |
| 8.8256 | 8.2826 | 7.7259 | 5.9078 | 7.6026 | 10.9179 | 9.6159 | 11.8561 |
| 7.4793 | 9.3134 | 12.1794 | 9.6104 | 10.8277 | 10.1806 | 10.3005 | 8.9677 |
| 7.9059 | 8.2097 | 9.1713 | 9.0884 | 9.6163 | 10.1738 | 10.8143 | 11.8860 |
| 10.5947 | 11.6080 | 10.9458 | 9.1120 | 7.0150 | 8.9473 | 10.3615 | 12.7632 |
| 10.2664 | 10.8995 | 10.6868 | 9.6534 | 9.2366 | 13.3173 | 9.0128 | 7.5419 |
| 9.5452 | 9.7856 | 7.7456 | 11.3407 | 8.6883 | 11.2204 | 9.5304 | 12.2230 |
| 9.2442 | 12.9360 | 8.9246 | 8.8782 | 10.9009 | 10.8099 | 9.9805 | 7.2071 |

```
11.1672 10.3528 12.4096 10.2727 8.5622  7.4982  12.0311 10.4875
9.7580  9.0151  9.5601  10.1412 12.5711 11.4306 9.8869  10.9193
9.1845  8.3725  7.9633  10.6503 10.3056 10.3111 10.0416 7.2913
10.9904 8.0928  8.0224  10.8609 10.0856 9.2906  9.1481  12.3111
9.0942  10.4245 9.4436  10.4919 8.9638  7.6466  10.9143 8.4822
7.8313  9.9905  10.5343 9.4192  7.8057  8.0456  9.2487  10.8135
12.2810 11.6376 10.4250 9.5865  9.7755  7.5027  12.2787 10.0564
13.0615 12.8287 11.0466 11.2300 10.1007 11.2330 11.5125 12.2356
8.8938  12.8663 11.1283 12.0220 9.9930  9.6136  10.5143 9.8276
10.8338 7.6475  11.4183 10.9664 10.9293 12.4541 9.7211  9.6829
9.9810  9.5665  9.0493  12.2254 7.6930  9.2966  13.5083 8.8510
11.3235 10.3916 10.3585 10.9641 8.7274  9.3885  8.1613  8.4603
8.4326  10.9276 10.1844 8.9413  8.0346  9.9210  12.2136 9.7481
11.7731 9.2393  8.8253  10.1307 8.2481  8.1717  9.2669  11.5032
11.0287 10.5078\
RPL31   13.8464 14.0966 12.4007 13.6411 13.4860 13.4261 14.0348
15.0784 14.6805 14.2311 13.6475 14.9422 14.4958 14.4533 13.6446
14.9124 14.4051 13.9990 14.1709 14.0276 14.2909 14.5432 14.8652
14.4970 14.2723 14.4034 14.2113 14.3848 14.2969 13.8731 13.5636
13.8332 15.1539 13.4635 13.6690 14.8655 13.5557 13.4660 14.5607
14.7208 14.7525 13.6946 14.8481 14.1057 14.0006 15.6644 15.2390
13.3522 14.2384 14.0173 14.5928 14.2063 13.7279 14.5559 13.6479
13.5139 13.8609 14.0446 14.1766 14.1283 14.1684 14.0939 13.9684
13.5168 14.7039 13.0250 14.6261 14.1596 14.3057 13.7919 14.7037
13.4143 14.7735 14.5147 13.4281 14.1098 14.9745 13.2280 14.5174
14.2579 14.8264 13.8532 14.2288 13.4712 15.7899 15.0849 14.1478
14.9784 14.7047 13.7016 14.0471 14.7973 15.5716 14.9952 14.1129
14.0777 13.2367 15.1738 13.9696 14.8106 14.5297 12.8567 12.7007
14.4919 14.5738 13.0928 14.6860 13.8718 14.5716 14.2587 14.1923
14.5108 15.7601 15.2537 13.7895 14.5703 14.8204 14.1753 13.3418
13.4782 14.8520 14.6242 13.0661 14.0951 15.1064 13.5787 15.2444
16.7098 14.1435 13.5931 13.9200 15.5980 14.3691 14.2836 15.2305
15.1466 14.6417 15.0027 14.7536 14.6218 15.5752 14.4802 15.2262
12.8608 13.3710 15.5417 12.9046 14.9984 13.0377 15.1647 14.6567
15.8962 13.7556 13.8767 13.8616 13.8169 14.4234 13.9314 13.9878
14.5804 14.1841 13.5486 14.8581 14.5446 14.1587 13.7362 14.1967
13.8798 13.7077 12.9775 13.3719 12.9103 13.9871 14.2950 15.7703
14.0704 14.1627 14.7293 13.9202 13.7033 15.0293 15.2885 14.8715
14.9554 14.6900 15.0443 13.2796 13.8084 14.3819 14.0882 13.5515
13.9862 14.4423 13.6846 13.9110 16.2334 15.4729 15.3340 13.5598
14.6207 14.7841\
ZNF8    6.4994  6.3070  6.3284  6.6363  6.2693  6.2766  6.7032
7.4544  8.1295  6.3392  6.4133  7.1444  6.3992  7.0486  6.7154
5.2804  6.4370  6.3486  6.2467  6.5521  5.9946  6.4715  6.1662
6.6808  5.6699  5.9773  6.5987  5.9928  6.7381  7.0960  6.7290
6.3003  7.2668  6.5909  7.8585  7.7179  7.4468  7.2824  6.1283
6.7142  6.2061  7.0866  6.4563  5.9356  5.4077  5.5209  4.3808
7.5759  7.3703  5.6959  6.3459  5.9959  6.6452  6.1952  7.6206
6.4176  6.4180  6.8990  6.6764  6.7396  6.0586  7.0404  6.2628
6.5904  6.3278  6.8053  5.1951  7.1162  6.2770  5.9578  6.4731
7.0964  6.1563  7.4636  6.2787  6.1748  6.5584  6.0900  6.4489
6.5851  6.1312  6.8514  5.7228  6.7467  6.2778  6.7556  6.3170
6.1733  6.9300  6.4225  5.6764  7.1314  5.9418  6.2942  5.7790
5.5373  6.5657  6.1216  6.3085  6.9259  6.2090  6.5259  8.4060
```

| | | | | | | | |
|---|---|---|---|---|---|---|---|
| 6.8069 | 5.9383 | 6.5952 | 6.1454 | 6.9028 | 6.9754 | 6.1460 | 7.4000 |
| 7.0024 | 6.3665 | 8.6461 | 6.5333 | 6.6377 | 5.9272 | 7.5205 | 5.7594 |
| 6.5723 | 6.9589 | 5.9648 | 6.3118 | 6.3598 | 6.1025 | 7.6034 | 7.0077 |
| 7.0232 | 6.2870 | 6.8927 | 6.4185 | 5.8983 | 6.1921 | 6.5114 | 6.8692 |
| 7.3980 | 6.6703 | 6.8966 | 6.8569 | 6.2503 | 6.0457 | 5.5546 | 6.3550 |
| 5.8859 | 6.5101 | 5.5015 | 6.7958 | 5.8125 | 6.0467 | 6.0914 | 6.3432 |
| 5.6460 | 7.6485 | 8.5107 | 6.9523 | 6.7014 | 6.1586 | 7.4219 | 5.6705 |
| 6.3027 | 6.1405 | 5.8108 | 6.0857 | 6.3037 | 5.4416 | 7.7279 | 4.4181 |
| 6.5754 | 6.2799 | 6.3805 | 6.7439 | 7.7204 | 7.3325 | 6.7740 | 6.3700 |
| 7.4472 | 6.4442 | 5.3874 | 7.1536 | 6.4833 | 5.5521 | 7.3105 | 6.6532 |
| 5.6240 | 7.1016 | 6.8662 | 6.7804 | 6.9035 | 6.2908 | 7.1142 | 6.6514 |
| 6.5686 | 6.1487 | 6.9950 | 7.6819 | 6.3352 | 6.5269 | 6.2437 | 6.5981 |
| 6.7724 | 5.7291\ | | | | | | |
| PCP4L1 | 0.5526 | 2.7646 | 7.3630 | 7.1385 | 2.4374 | 3.0265 | 3.4667 |
| 3.1991 | 2.1693 | 4.2118 | 2.5484 | 2.3205 | 1.4675 | 8.9239 | 3.5658 |
| 3.7058 | 0.0000 | 0.0000 | 2.9040 | 1.8781 | 2.0721 | 5.1341 | 1.0738 |
| 7.4609 | 6.9275 | 2.3723 | 4.4719 | 0.0000 | 0.0000 | 2.9203 | 0.4127 |
| 2.4004 | 2.8112 | 4.4338 | 1.5856 | 1.4920 | 4.6413 | 0.3648 | 2.6916 |
| 3.9094 | 1.9736 | 4.0930 | 0.0000 | 5.3892 | 4.7499 | 3.1906 | 3.4901 |
| 3.7557 | 0.7769 | 6.1345 | 4.8412 | 0.7768 | 6.4868 | 5.0890 | 6.3955 |
| 3.3925 | 0.0000 | 3.2241 | 2.1468 | 1.7785 | 3.9204 | 2.8415 | 2.2545 |
| 4.2813 | 3.4971 | 6.0097 | 7.0992 | 5.5950 | 6.4286 | 2.9862 | 3.2868 |
| 4.5916 | 1.5110 | 1.5690 | 3.1877 | 7.0176 | 5.5244 | 4.1559 | 2.2252 |
| 3.0798 | 5.4353 | 3.6673 | 1.8442 | 3.6170 | 2.5465 | 5.0536 | 4.3195 |
| 2.7731 | 3.6404 | 4.4395 | 5.0503 | 1.1363 | 3.4030 | 1.1561 | 2.6337 |
| 5.1403 | 7.5986 | 3.4734 | 5.6581 | 0.5504 | 0.0000 | 7.1889 | 4.8944 |
| 2.6605 | 0.8449 | 0.0000 | 3.0465 | 4.4071 | 1.4990 | 2.0061 | 0.5136 |
| 3.8543 | 0.0000 | 7.5314 | 4.5398 | 4.3968 | 2.7528 | 3.7623 | 8.1760 |
| 0.6089 | 3.8806 | 2.0083 | 1.6588 | 2.0289 | 3.2018 | 0.5331 | 2.5319 |
| 2.4149 | 1.2802 | 7.3362 | 3.1441 | 1.7472 | 4.0357 | 3.8189 | 1.3267 |
| 2.6570 | 6.4086 | 0.0000 | 4.2699 | 3.4131 | 3.5671 | 0.5748 | 0.6506 |
| 4.9599 | 6.6851 | 4.4271 | 9.3562 | 5.2732 | 3.0780 | 7.8588 | 4.8727 |
| 0.0000 | 3.4618 | 4.9106 | 4.1392 | 2.5751 | 3.9842 | 3.8556 | 3.1270 |
| 0.0000 | 0.0000 | 1.8685 | 2.1631 | 2.3765 | 8.7187 | 2.3687 | 0.0000 |
| 3.0096 | 4.6089 | 2.0787 | 5.4365 | 3.4253 | 6.9202 | 3.9615 | 0.0000 |
| 4.7788 | 3.9057 | 5.1025 | 7.0874 | 1.4046 | 1.2666 | 3.7953 | 3.7811 |
| 0.8792 | 4.2968 | 3.4271 | 1.7992 | 3.2020 | 1.9569 | 0.0000 | 1.1800 |
| 6.3705 | 3.1228 | 3.8483 | 0.4985 | 0.0000 | 4.1472 | 2.2304 | 3.1187 |
| 1.9827 | 1.5159\ | | | | | | |
| C11orf17 | | 8.8979 | 8.5509 | 6.8453 | 7.9995 | 8.4344 | 7.8520 |
| 6.7845 | 8.3121 | 7.6691 | 8.4070 | 7.9380 | 7.4949 | 8.1826 | 8.4585 |
| 8.1090 | 7.9186 | 7.4830 | 8.6335 | 8.5928 | 6.9060 | 7.8894 | 7.9176 |
| 8.2363 | 8.2699 | 8.8690 | 9.4794 | 9.0490 | 8.5454 | 8.2755 | 7.1902 |
| 9.6686 | 8.7302 | 8.9247 | 7.5520 | 7.0562 | 8.1940 | 8.3757 | 8.9069 |
| 8.3053 | 7.4821 | 7.9883 | 8.1070 | 7.8594 | 9.3348 | 8.6325 | 8.2245 |
| 8.6097 | 8.3783 | 8.6952 | 8.7594 | 8.8715 | 7.1593 | 7.8282 | 8.4720 |
| 7.8144 | 8.1649 | 8.3883 | 7.9972 | 8.2037 | 7.5446 | 8.2922 | 7.9210 |
| 7.9380 | 7.0122 | 8.1264 | 8.7244 | 7.5621 | 7.7049 | 8.2108 | 8.0097 |
| 7.9359 | 8.3506 | 8.5987 | 7.8203 | 8.6416 | 7.5254 | 7.7693 | 7.4859 |
| 7.9748 | 8.1911 | 8.4639 | 7.4846 | 8.0617 | 7.0646 | 8.1809 | 6.9026 |
| 7.2885 | 8.2400 | 7.9850 | 9.5786 | 8.5270 | 8.1885 | 8.6368 | 7.9645 |
| 9.0087 | 8.5703 | 7.6529 | 8.5907 | 7.4641 | 8.8611 | 8.8003 | 8.8815 |
| 8.2697 | 7.9020 | 7.8696 | 8.4755 | 7.9186 | 8.5548 | 7.5064 | 7.4999 |
| 8.5397 | 8.5225 | 8.6788 | 7.1898 | 8.2320 | 8.4026 | 7.9351 | 7.0999 |

| | | | | | | | |
|---|---|---|---|---|---|---|---|
| 8.9623 | 7.6171 | 8.0469 | 8.6440 | 7.7599 | 8.3808 | 8.3822 | 8.2161 |
| 7.9703 | 7.5604 | 8.5593 | 8.4160 | 7.1155 | 7.3334 | 8.1606 | 7.8506 |
| 7.8424 | 8.7837 | 9.1581 | 7.9742 | 8.2360 | 8.5903 | 8.1586 | 8.9973 |
| 7.8636 | 8.5287 | 8.5252 | 8.7723 | 8.3159 | 8.8525 | 8.6168 | 8.8494 |
| 8.3096 | 7.9687 | 7.7175 | 7.6404 | 7.8483 | 8.4741 | 8.6347 | 7.9318 |
| 8.2145 | 9.3618 | 8.4973 | 9.1945 | 7.6566 | 8.5350 | 8.4423 | 7.5578 |
| 8.6796 | 7.8886 | 8.2641 | 7.9401 | 7.7682 | 8.3839 | 8.2926 | 7.9081 |
| 8.8811 | 8.4976 | 7.8621 | 8.6641 | 7.9666 | 7.9644 | 9.2253 | 7.3099 |
| 7.3309 | 8.1056 | 7.8424 | 8.1103 | 7.2740 | 8.5849 | 8.3720 | 8.6555 |
| 8.6119 | 8.4397 | 8.2139 | 7.4895 | 8.2119 | 7.8282 | 8.6761 | 8.8535 |
| 8.6009 | 7.9477 | 9.1830\ | | | | | |
| C11orf16 | | 0.5526 | 2.6668 | 2.0229 | 7.5933 | 5.1477 | 6.2612 |
| 3.4667 | 7.1185 | 2.7963 | 5.7630 | 5.7175 | 2.3205 | 8.7159 | 0.0000 |
| 8.4162 | 4.5469 | 5.0120 | 1.6514 | 2.6976 | 0.0000 | 2.6649 | 8.9111 |
| 8.9703 | 5.7500 | 1.9289 | 0.0000 | 3.5585 | 8.8871 | 2.0792 | 0.0000 |
| 0.9954 | 1.2789 | 1.6552 | 1.7182 | 4.5244 | 4.3459 | 6.5902 | 1.2859 |
| 1.4957 | 0.0000 | 1.4628 | 3.9873 | 0.0000 | 4.4581 | 0.0000 | 6.4696 |
| 6.1047 | 5.6392 | 5.3810 | 1.1202 | 4.6210 | 1.6507 | 3.3357 | 1.8243 |
| 1.3330 | 4.4840 | 0.6343 | 4.8557 | 1.0204 | 4.2095 | 2.7929 | 5.4243 |
| 0.0000 | 0.0000 | 5.2904 | 7.0090 | 2.2002 | 1.7598 | 0.9886 | 1.6464 |
| 0.4334 | 1.1694 | 0.0000 | 3.0145 | 0.9270 | 3.2432 | 4.9767 | 9.5269 |
| 8.2491 | 0.0000 | 5.7887 | 0.7073 | 8.1266 | 5.8266 | 8.1962 | 1.0522 |
| 2.0569 | 6.6392 | 0.5273 | 4.7302 | 1.9147 | 2.1998 | 1.2055 | 5.3110 |
| 0.9013 | 0.6266 | 2.7408 | 1.7212 | 8.2949 | 2.6110 | 3.5349 | 7.1600 |
| 2.0509 | 6.6265 | 7.7005 | 1.5391 | 5.1957 | 4.0903 | 4.3348 | 6.0080 |
| 7.1935 | 8.0195 | 1.3631 | 1.5850 | 1.1918 | 3.9805 | 8.7174 | 0.0000 |
| 1.6868 | 1.3647 | 2.9409 | 6.4539 | 1.8863 | 7.2943 | 8.5593 | 0.5331 |
| 2.2388 | 1.2894 | 1.7575 | 2.1283 | 0.3935 | 5.2526 | 9.0415 | 9.0823 |
| 0.8107 | 7.0461 | 2.7218 | 4.4303 | 5.9434 | 2.3776 | 6.4394 | 1.5646 |
| 8.2780 | 0.5424 | 1.6921 | 0.5418 | 6.4956 | 4.8591 | 0.0000 | 4.1457 |
| 1.8061 | 6.6776 | 4.7526 | 3.3958 | 0.0000 | 2.8829 | 5.1076 | 3.5881 |
| 7.7553 | 1.2429 | 0.0000 | 0.0000 | 5.9801 | 4.4357 | 0.0000 | 3.3452 |
| 4.6023 | 4.7360 | 4.1158 | 4.6142 | 2.9872 | 0.5290 | 1.7114 | 1.6598 |
| 7.8323 | 5.5844 | 3.5762 | 1.4008 | 5.2185 | 10.0995 | 5.4006 | 0.6902 |
| 5.5184 | 3.8508 | 2.1147 | 0.6248 | 1.3150 | 4.2704 | 7.1285 | 0.5707 |
| 5.9484 | 7.1960 | 3.4112 | 0.0000 | 3.9491 | 5.5688 | 1.0320 | 2.6496 |
| 5.6629 | 8.2944 | 2.8479\ | | | | | |
| ZAP70 | 4.2276 | 6.7003 | 4.8055 | 6.6572 | 8.0648 | 6.9558 | 4.0872 |
| 5.1572 | 4.4814 | 6.6809 | 5.7825 | 4.8563 | 7.1012 | 5.4624 | 5.1429 |
| 8.9208 | 7.5384 | 7.1531 | 8.9552 | 4.3868 | 7.9088 | 5.9378 | 6.3806 |
| 8.7323 | 4.1575 | 6.2810 | 4.3975 | 7.9057 | 5.8443 | 4.1438 | 11.7891 |
| 7.5861 | 6.6044 | 5.5368 | 3.3227 | 7.6204 | 4.2364 | 4.8389 | 3.3999 |
| 3.5693 | 8.0341 | 5.7006 | 5.3568 | 6.2245 | 3.9549 | 5.9641 | 7.0179 |
| 4.6679 | 9.7070 | 4.4159 | 6.3406 | 8.8035 | 5.0372 | 5.9638 | 9.6940 |
| 6.0358 | 7.1079 | 7.5950 | 6.7862 | 4.2470 | 5.4704 | 6.4689 | 6.0146 |
| 3.9398 | 6.5855 | 8.3838 | 5.6527 | 4.9649 | 6.8353 | 5.2786 | 8.5502 |
| 3.9417 | 10.4034 | 7.0487 | 8.3760 | 2.4904 | 8.2939 | 6.1411 | 8.1343 |
| 7.4912 | 7.8553 | 4.3874 | 7.2408 | 7.2836 | 6.9469 | 5.7004 | 3.6714 |
| 8.3355 | 6.5626 | 6.3029 | 5.7451 | 7.5508 | 7.6899 | 9.4321 | 7.8993 |
| 5.7485 | 4.3699 | 5.7130 | 4.0577 | 9.5376 | 6.1990 | 8.0465 | 3.5580 |
| 7.8572 | 4.5170 | 1.8753 | 8.8449 | 6.5448 | 4.7068 | 5.7084 | 7.4337 |
| 6.1823 | 5.3330 | 4.1699 | 5.3357 | 4.6300 | 5.6314 | 5.6405 | 4.8531 |
| 8.6648 | 6.1748 | 5.2202 | 6.3654 | 5.7065 | 4.4936 | 7.5535 | 4.8663 |
| 4.0148 | 5.5751 | 5.4866 | 4.8219 | 5.2748 | 7.6391 | 7.7007 | 8.4488 |

| | | | | | | | |
|---|---|---|---|---|---|---|---|
| 8.5233 | 10.6464 | 9.5873 | 6.1344 | 5.8283 | 6.8610 | 5.7772 | 8.3223 |
| 6.7025 | 6.4475 | 4.1592 | 5.9467 | 6.0192 | 5.4157 | 3.9096 | 8.4311 |
| 5.4557 | 8.6253 | 5.7452 | 4.7221 | 5.9276 | 5.7052 | 2.8937 | 6.4192 |
| 9.3172 | 4.4863 | 7.6955 | 6.9743 | 7.0332 | 5.0238 | 6.2631 | 4.2069 |
| 7.1763 | 5.5923 | 4.8855 | 3.6634 | 3.3012 | 3.0495 | 3.0975 | 5.6358 |
| 2.3068 | 6.7850 | 5.0319 | 4.4908 | 9.2835 | 6.4155 | 4.9853 | 5.9565 |
| 6.3794 | 6.2906 | 8.3520 | 7.3284 | 7.5283 | 6.3523 | 5.7388 | 7.4251 |
| 2.3863 | 5.9135 | 6.8381 | 7.4828 | 6.1228 | 4.3456 | 6.1422 | 7.3303 |
| 6.0798 | 7.0824\ | | | | | | |
| SNORD115-9 | | 0.0000 | 0.0000 | 0.0000 | 0.0000 | 0.0000 | 0.0000 |
| 0.0000 | 0.0000 | 0.0000 | 0.0000 | 0.0000 | 0.0000 | 0.0000 | 0.0000 |
| 0.0000 | 0.0000 | 0.0000 | 0.0000 | 0.0000 | 0.0000 | 0.0000 | 0.0000 |
| 0.0000 | 0.0000 | 0.0000 | 0.0000 | 0.0000 | 0.0000 | 0.0000 | 0.0000 |
| 0.0000 | 0.0000 | 0.0000 | 0.0000 | 0.0000 | 0.0000 | 0.0000 | 0.0000 |
| 0.0000 | 0.0000 | 0.0000 | 0.0000 | 0.0000 | 0.0000 | 0.0000 | 0.0000 |
| 0.0000 | 0.0000 | 0.0000 | 0.0000 | 0.0000 | 0.0000 | 0.0000 | 0.0000 |
| 0.0000 | 0.0000 | 0.0000 | 0.0000 | 0.0000 | 0.0000 | 0.0000 | 0.0000 |
| 0.0000 | 0.0000 | 0.0000 | 0.0000 | 0.0000 | 0.0000 | 0.0000 | 0.0000 |
| 0.0000 | 0.0000 | 0.0000 | 0.0000 | 0.0000 | 0.0000 | 0.0000 | 0.0000 |
| 0.0000 | 0.0000 | 0.0000 | 0.0000 | 0.0000 | 0.0000 | 0.0000 | 0.0000 |
| 0.0000 | 0.0000 | 0.0000 | 0.0000 | 0.0000 | 0.0000 | 0.0000 | 0.0000 |
| 0.0000 | 0.0000 | 0.0000 | 0.0000 | 0.0000 | 0.0000 | 0.0000 | 0.0000 |
| 0.0000 | 0.0000 | 0.0000 | 0.0000 | 0.0000 | 0.0000 | 0.0000 | 0.0000 |
| 0.0000 | 0.0000 | 0.0000 | 0.0000 | 0.0000 | 0.0000 | 0.0000 | 0.0000 |
| 0.0000 | 0.0000 | 0.0000 | 0.0000 | 0.0000 | 0.0000 | 0.0000 | 0.0000 |
| 0.0000 | 0.0000 | 0.0000 | 0.0000 | 0.0000 | 0.0000 | 0.0000 | 0.0000 |
| 0.0000 | 0.0000 | 0.0000 | 0.0000 | 0.0000 | 0.0000 | 0.0000 | 0.0000 |
| 0.0000 | 0.0000 | 0.0000 | 0.0000 | 0.0000 | 0.0000 | 0.0000 | 0.0000 |
| 0.0000 | 0.0000 | 0.0000 | 0.0000 | 0.0000 | 0.0000 | 0.0000 | 0.0000 |
| 0.0000 | 0.0000 | 0.0000 | 0.0000 | 0.0000 | 0.0000 | 0.0000 | 0.0000 |
| 0.0000 | 0.0000 | 0.0000 | 0.0000 | 0.0000 | 0.0000 | 0.0000 | 0.0000 |
| 0.0000 | 0.0000 | 0.0000 | 0.0000 | 0.0000 | 0.0000 | 0.0000 | 0.0000 |
| 0.0000 | 0.0000 | 0.0000 | 0.0000 | 0.0000 | 0.0000 | 0.0000 | 0.0000 |
| 0.0000 | 0.0000 | 0.0000 | 0.0000 | 0.0000 | 0.0000 | 0.0000 | 0.0000 |
| 0.0000 | 0.0000 | 0.0000\ | | | | | |
| C11orf10 | | 11.3771 | 10.9284 | 10.5554 | 10.6000 | 10.5224 | 10.2741 |
| 10.5180 | 11.5739 | 11.9479 | 11.0916 | 11.3204 | 11.1272 | 10.8274 | 10.6747 |
| 11.1212 | 11.4014 | 11.0926 | 11.4827 | 10.8937 | 11.0115 | 10.1914 | 11.5115 |
| 10.5654 | 10.7060 | 10.5720 | 11.5842 | 10.8619 | 11.6328 | 11.6688 | 9.9483 |
| 10.7883 | 10.8010 | 11.8663 | 11.6419 | 9.3329 | 11.3652 | 9.8347 | 11.0659 |
| 11.5625 | 10.0931 | 10.7109 | 10.0904 | 11.4129 | 9.8365 | 9.8458 | 11.7444 |
| 11.4529 | 10.5405 | 10.3323 | 11.6363 | 11.0719 | 10.8091 | 10.4740 | 10.0520 |
| 10.2885 | 11.0200 | 10.2653 | 10.7352 | 10.5550 | 11.3099 | 10.1304 | 10.3936 |
| 10.8904 | 9.8166 | 11.1070 | 10.9512 | 11.0718 | 11.9677 | 11.2489 | 10.6690 |
| 11.5331 | 10.6953 | 11.9069 | 11.4759 | 10.6487 | 11.1977 | 11.3731 | 10.5567 |
| 11.7791 | 10.6691 | 11.6996 | 10.1681 | 11.4586 | 11.3104 | 12.2301 | 11.6927 |
| 10.3309 | 10.7120 | 10.4152 | 11.4080 | 10.5553 | 10.3076 | 11.5839 | 11.6017 |
| 11.2804 | 10.3290 | 10.0234 | 10.9799 | 10.1664 | 11.0123 | 12.0980 | 10.8553 |
| 10.4069 | 11.7040 | 11.2390 | 10.1593 | 10.8446 | 10.7783 | 10.9278 | 11.5735 |
| 10.8858 | 11.0187 | 12.3819 | 11.3515 | 10.8030 | 10.3466 | 11.3832 | 10.5202 |
| 11.0311 | 10.1921 | 10.9933 | 11.4454 | 9.7486 | 10.9714 | 10.7713 | 10.7518 |
| 11.5541 | 14.2097 | 10.2273 | 9.6350 | 11.3217 | 11.2450 | 11.4361 | 10.2169 |
| 11.3282 | 11.3269 | 12.1282 | 10.4522 | 10.6318 | 10.5828 | 12.1176 | 11.4642 |
| 11.4573 | 10.6766 | 10.7983 | 10.8691 | 10.8284 | 10.7394 | 11.0116 | 12.6120 |

```
11.0241 12.8073 10.7278 10.8680 10.3176 10.6076 10.7213 10.9095
11.5908 11.4641 11.3909 10.3948 11.1461 11.1595 10.4034 10.1893
10.6903 10.5358 11.3354 10.4788 10.4387 10.4329 9.9803  11.3225
11.9799 10.8411 10.9386 10.2946 10.5698 11.1313 12.5313 10.5780
11.4882 11.5707 10.7261 11.3037 10.5503 11.1428 11.6409 11.0933
10.6994 11.2706 11.4749 10.4227 11.0570 12.1947 11.4611 10.8354
10.6033 10.7994 11.2766\
ZCCHC13 0.0000  0.0000  0.0000  0.0000  0.0000  0.0000  0.0000
0.0000  0.0000  0.0000  0.0000  0.0000  0.0000  0.0000  0.0000
0.0000  0.0000  0.0000  0.0000  0.6732  0.0000  0.0000  0.0000
0.0000  0.0000  0.0000  0.0000  0.0000  0.0000  0.0000  0.0000
0.0000  0.0000  0.0000  0.0000  0.0000  0.0000  0.0000  0.0000
0.0000  0.0000  0.0000  0.0000  0.0000  0.0000  0.0000  0.0000
0.6741  0.0000  0.0000  0.0000  0.0000  0.0000  0.0000  0.0000
0.0000  0.0000  0.0000  0.0000  0.0000  0.0000  0.0000  0.0000
0.0000  0.0000  0.0000  0.0000  0.0000  0.0000  0.0000  0.0000
0.0000  0.0000  0.0000  0.0000  0.0000  0.0000  0.0000  0.0000
0.0000  0.0000  0.0000  0.0000  0.0000  0.0000  0.0000  0.0000
0.0000  0.0000  0.0000  0.0000  0.0000  0.0000  0.0000  0.0000
0.0000  0.0000  0.0000  0.0000  0.0000  0.0000  0.0000  0.0000
0.0000  0.0000  0.0000  0.0000  0.0000  0.0000  0.0000  0.0000
0.0000  0.0000  0.0000  0.0000  0.0000  0.0000  0.0000  0.0000
0.0000  0.0000  0.0000  0.0000  0.0000  0.0000  0.0000  0.0000
0.0000  0.0000  0.0000  0.0000  0.0000  0.0000  0.0000  0.0000
0.0000  0.0000  0.0000  0.0000  0.0000  0.0000  0.0000  0.0000
0.0000  0.0000  0.0000  0.0000  0.0000  0.0000  0.0000  0.0000
0.0000  0.0000  0.0000  0.0000  0.0000  0.0000  0.0000  0.0000
0.0000  0.6063  0.0000  0.0000  0.0000  0.0000  0.0000  0.0000
0.0000  0.0000  0.0000  0.0000  0.0000  0.0000  0.0000  0.0000
0.0000  0.0000  0.0000  0.0000  0.0000  0.0000  1.5061  0.0000
0.0000  0.0000  0.0000  0.0000  0.0000  0.0000  0.0000  0.0000
0.0000  0.0000  0.0000  0.0000  0.0000  0.0000  0.0000  0.0000
0.0000  1.1634\
UFSP2   9.2559  9.0995  7.8729  8.9657  9.0218  8.6933  10.1747
8.5213  7.9401  9.3093  8.8632  8.6282  8.9431  8.7780  8.9143
8.9473  8.3618  9.0060  8.8694  8.4193  8.1810  8.9288  8.9904
8.9481  9.8555  9.5739  8.2666  8.9643  8.8202  8.1561  8.3013
8.8024  8.2995  8.5866  8.4661  8.8799  8.5027  8.6691  9.2486
8.9325  9.1525  9.0145  9.2265  7.9570  10.2533 8.5563  10.0736
8.8767  8.9567  9.6771  8.7559  8.5047  8.8442  10.0221 8.0705
9.0752  8.6138  8.9610  9.1128  8.1575  9.9102  9.6671  8.6666
8.3665  9.4051  9.0143  8.0164  8.3816  8.8276  8.0426  9.1532
8.9119  9.1315  8.1852  8.7366  8.7697  9.0435  8.3225  8.6780
8.8538  9.5709  8.0101  8.5638  8.1963  9.1989  9.0684  9.0009
8.4117  8.9092  9.0118  9.5180  8.6129  9.3570  8.6057  9.3518
9.4986  8.8761  9.5459  8.9096  8.6205  9.1136  8.7844  8.6411
8.7156  9.3048  8.9936  9.0214  9.0010  8.4569  7.9394  8.9880
9.0519  8.7438  8.3666  9.8087  8.6387  8.8051  8.5721  8.3887
8.8603  8.8241  8.7790  8.4599  8.7170  9.1655  8.1297  8.5443
9.2739  9.8978  9.7572  9.4667  8.3792  8.9757  8.0581  8.6115
9.5262  9.9341  8.7482  9.1925  9.6713  8.7450  9.4946  8.9997
9.2136  9.1277  10.5032 9.3023  9.6568  9.0760  9.6252  9.0417
9.5775  8.7740  8.9470  8.8176  8.6972  9.7231  8.7988  9.2539
9.0565  9.2560  8.8638  8.7605  9.6538  8.2591  9.0481  8.4664
```

8.3888 9.3124 8.9543 9.1786 8.5517 8.5315 8.8536 8.5048
8.8882 9.0268 10.0747 9.2083 8.5912 9.2690 9.3399 8.2837
8.9039 9.0557 8.9437 8.9115 9.0273 8.9402 8.9865 8.8523
9.1125 7.5319 8.0796 9.2829 8.4667 9.6190 10.0397 8.8140
8.9173 9.5635\
OGFR 11.1819 10.6430 10.6321 10.0066 10.0877 10.8684 10.5188
10.1124 10.9808 10.4692 10.5092 10.4924 10.2766 10.0250 9.7886
10.9499 11.0671 10.6999 10.8123 10.8614 12.1021 9.9851 11.1033
9.6695 10.0471 11.7186 10.1375 11.2871 10.9383 11.3525 10.7202
10.3882 10.9655 11.0244 10.4442 10.7269 10.5148 10.1829 9.5992
9.2419 10.6125 10.2381 11.3231 9.6308 9.8624 11.3959 11.9448
11.4578 11.0661 10.9165 10.0321 11.2375 11.1693 10.0537 11.1267
10.3193 10.3772 10.4044 10.1303 10.7448 9.8663 10.7095 9.7433
10.6445 10.6211 10.5897 11.4684 10.5096 10.4426 11.0455 12.6671
10.1293 12.7157 11.2566 10.4286 10.6878 11.1384 10.6731 10.7772
10.6080 10.3556 10.3023 10.3513 10.8249 10.9558 10.5081 10.7109
11.7748 10.7181 10.7216 10.6784 10.6953 11.7743 11.1719 11.2019
10.1928 10.3775 10.9483 10.2584 11.1521 10.9813 10.8962 10.6566
11.1845 10.0170 11.2158 11.3442 10.5666 10.0833 11.2595 9.9312
10.6794 12.3571 11.7243 9.5469 10.4832 9.8321 11.0006 10.6618
11.6639 10.4345 9.8768 10.7316 10.2608 10.4467 10.7703 9.7796
11.7329 9.8800 9.4543 10.8295 10.5122 10.9084 11.0358 11.7815
12.0965 11.3594 11.8379 10.1433 10.7510 11.1118 10.0220 11.3011
10.2381 9.9713 10.6626 10.7356 11.0506 11.2987 11.0227 10.2260
13.2127 10.2102 11.8708 10.7364 10.3771 10.5860 9.2171 11.4701
11.9290 10.7519 12.0268 10.4645 10.9695 11.1204 9.4750 10.2364
10.6408 10.4325 11.4556 10.4505 11.4235 11.3209 10.5718 11.0944
10.4248 10.0987 10.2306 9.7123 11.4567 11.1400 11.4670 9.9889
10.3911 10.3454 11.0988 10.7780 11.8999 11.0065 9.3597 9.7467
9.7781 11.7524 11.1766 10.5777 11.5078 10.5622 10.9535 10.6139
10.0482 12.2810\
EPM2AIP1 4.6592 10.0915 10.1301 8.7634 8.8437 10.3353
9.5091 10.1633 9.1083 10.5039 9.6461 10.0098 10.3716 10.9269
9.4064 6.7739 11.2719 9.8538 9.9214 8.4600 9.1190 9.9541
9.8021 8.7278 10.6036 5.8297 9.8072 6.6633 6.3225 9.4860
10.4568 6.3878 8.7222 9.4495 9.5391 5.1718 10.0552 4.6200
5.5946 9.8010 9.3081 9.7928 9.5699 9.0045 10.0640 9.9236
8.2492 9.4541 5.6299 3.6711 5.5120 9.3680 9.6749 10.4118
9.4344 5.5425 9.1130 9.0038 10.5900 10.3894 10.1663 9.2131
10.0472 8.5310 10.6463 8.9121 9.7348 10.3069 9.5546 9.5566
8.4308 5.1438 8.0173 9.2692 8.0305 9.5487 10.1100 8.0109
8.9053 6.7046 8.9894 10.2941 9.9578 9.7804 9.2259 10.9609
9.6662 8.4775 10.4301 7.1712 9.8826 10.2108 9.4685 10.0894
10.3883 9.8157 10.1697 10.4049 9.4255 10.6373 9.3611 6.7033
7.7616 8.4661 9.5005 6.9017 10.2916 9.7261 10.4006 10.2649
5.6972 9.8143 8.9777 10.0512 10.6864 9.7625 5.8348 10.6590
10.1069 9.4521 11.1640 10.0389 9.1699 5.2321 10.4854 8.9923
8.7117 8.2112 10.6922 10.6591 10.0886 10.3342 5.8680 10.2557
8.9201 6.8609 8.2940 10.1982 9.7811 10.6457 10.3428 4.8530
9.0273 6.3046 9.1472 10.6619 9.5124 9.9427 8.5648 3.7753
9.7084 8.2531 9.9165 8.8112 3.1454 10.4197 10.0945 9.7370
8.7995 5.8567 8.8109 8.3062 10.0567 9.1822 9.8029 8.6603
9.7793 8.7801 9.5100 6.1870 9.3639 10.4105 9.5644 4.9652
5.1619 4.4015 11.0352 10.0246 9.7974 9.7257 5.0386 9.0241

| | | | | | | | |
|---|---|---|---|---|---|---|---|
| 10.1729 | 9.3778 | 9.8419 | 6.4003 | 10.1395 | 8.6014 | 8.8878 | 6.6918 |
| 6.5452 | 9.6158 | 9.9879 | 9.4786 | 5.8361 | 9.0173 | 3.4498 | 10.5539 |
| 9.9002 | 9.9253 | 5.7958\ | | | | | |
| CDC5L | 9.8628 | 9.9122 | 10.4303 | 9.7584 | 10.0967 | 10.5493 | 10.5153 |
| 10.5134 | 10.2344 | 9.7577 | 10.5363 | 10.6680 | 10.4588 | 10.2216 | 10.0637 |
| 9.3743 | 10.3313 | 9.7332 | 9.7431 | 9.5912 | 10.5305 | 9.9320 | 9.5318 |
| 10.1560 | 9.9813 | 9.4013 | 10.6566 | 9.4595 | 9.7880 | 9.9424 | 10.7964 |
| 9.9149 | 10.4239 | 10.0424 | 10.5579 | 10.2308 | 9.9232 | 10.3705 | 10.3418 |
| 10.4346 | 9.6765 | 10.0101 | 9.5822 | 10.9441 | 10.2523 | 9.2651 | 8.9511 |
| 10.1983 | 10.5536 | 10.1725 | 9.7272 | 9.0679 | 10.2686 | 9.8927 | 10.0290 |
| 10.2959 | 10.1232 | 9.7664 | 10.4651 | 9.7892 | 10.0698 | 10.7644 | 9.6365 |
| 9.9959 | 9.8557 | 9.9614 | 9.4858 | 9.9182 | 9.8405 | 9.4118 | 10.0329 |
| 10.3521 | 9.5062 | 10.3890 | 10.3045 | 9.6455 | 10.3394 | 9.5646 | 9.4322 |
| 9.9109 | 10.0190 | 10.8469 | 9.2840 | 10.5048 | 9.6108 | 10.0139 | 11.1580 |
| 9.5802 | 9.9565 | 10.0661 | 9.6236 | 10.4857 | 10.3112 | 8.9401 | 10.2772 |
| 9.8348 | 9.3643 | 9.9285 | 10.3012 | 10.0498 | 9.0253 | 9.9069 | 10.2386 |
| 9.2025 | 10.1911 | 9.9249 | 9.2378 | 10.2912 | 9.8798 | 10.3435 | 10.1165 |
| 9.9500 | 9.0000 | 9.0471 | 10.6031 | 10.4949 | 10.8562 | 10.3611 | 10.5109 |
| 9.9324 | 10.5794 | 10.2064 | 10.0965 | 9.9701 | 9.7509 | 10.1560 | 10.0559 |
| 9.6995 | 10.1813 | 9.9868 | 9.3865 | 10.2297 | 9.8964 | 9.4386 | 9.3748 |
| 9.0317 | 9.2813 | 7.6907 | 10.0153 | 9.9043 | 9.6410 | 10.0374 | 9.3681 |
| 10.3901 | 10.3256 | 10.3261 | 10.1633 | 9.5706 | 10.1447 | 9.8901 | 9.8705 |
| 8.4945 | 10.2506 | 9.9272 | 10.0310 | 10.4953 | 9.8828 | 10.0809 | 10.1348 |
| 9.9266 | 9.7299 | 9.6716 | 9.9374 | 9.8694 | 9.2555 | 10.3845 | 9.8707 |
| 10.1428 | 9.9488 | 10.3771 | 10.4402 | 10.3705 | 10.2215 | 10.1783 | 8.1045 |
| 10.5340 | 9.8568 | 9.7538 | 10.5174 | 9.5047 | 9.9172 | 9.7986 | 10.4063 |
| 9.7255 | 10.6171 | 10.0885 | 10.6391 | 10.1386 | 9.3873 | 9.6432 | 10.2899 |
| 9.9586 | 10.6343 | 10.2660 | 10.5555 | 9.3068 | 9.3336 | 9.7974 | 10.2605 |
| 9.9841 | 8.4019\ | | | | | | |
| RAC2 | 7.9777 | 8.5162 | 9.9180 | 8.1869 | 8.9506 | 10.0355 | 8.1494 |
| 7.9838 | 8.8287 | 8.0343 | 9.5359 | 8.7445 | 8.4652 | 4.3637 | 8.6708 |
| 7.5723 | 8.3609 | 9.1290 | 10.3106 | 7.5154 | 10.0187 | 8.0893 | 9.2152 |
| 7.8775 | 7.3186 | 9.5624 | 11.2136 | 9.3981 | 9.4665 | 8.4120 | 5.1477 |
| 9.3571 | 9.4145 | 8.4957 | 5.4927 | 9.8178 | 9.7679 | 6.6012 | 5.5809 |
| 5.8356 | 10.0940 | 8.2547 | 9.2613 | 7.9079 | 8.3227 | 10.2575 | 8.8101 |
| 9.1315 | 7.3003 | 7.3029 | 9.7459 | 10.9279 | 9.1863 | 8.2043 | 9.1777 |
| 7.5776 | 9.6025 | 11.0927 | 9.4428 | 6.7412 | 8.7024 | 10.1365 | 8.1963 |
| 8.9821 | 8.7222 | 10.3424 | 9.0980 | 7.0254 | 7.0700 | 7.6025 | 10.3650 |
| 7.3823 | 13.1113 | 9.2024 | 10.3066 | 3.3007 | 9.8880 | 8.0633 | 8.9108 |
| 9.5190 | 6.8734 | 6.3903 | 9.8516 | 9.8782 | 7.7239 | 9.9744 | 9.0869 |
| 10.6065 | 8.8858 | 9.3358 | 9.3377 | 9.5481 | 9.9779 | 10.2125 | 10.1469 |
| 9.0609 | 8.4652 | 8.7880 | 7.1571 | 11.4808 | 8.7840 | 10.2928 | 9.5239 |
| 9.2139 | 6.1909 | 5.8048 | 10.2946 | 9.0959 | 7.7736 | 7.9477 | 9.3060 |
| 8.1808 | 7.4556 | 8.9159 | 7.6443 | 9.4405 | 8.4847 | 8.0686 | 6.8232 |
| 11.0860 | 8.6883 | 8.7372 | 9.4733 | 8.1713 | 8.0691 | 10.3854 | 6.9577 |
| 8.0357 | 8.6356 | 7.8941 | 7.5659 | 6.8568 | 9.0467 | 8.9067 | 10.1819 |
| 11.6967 | 10.5783 | 10.3547 | 8.1916 | 8.4519 | 8.2954 | 7.4930 | 9.2516 |
| 8.9267 | 8.6771 | 6.8161 | 8.7559 | 8.6128 | 9.1926 | 6.3308 | 9.1236 |
| 7.7960 | 9.3869 | 8.1357 | 6.5372 | 8.8981 | 8.1332 | 5.8495 | 8.6463 |
| 10.7431 | 7.5463 | 10.5971 | 8.5497 | 8.2549 | 6.5571 | 7.9947 | 9.6042 |
| 10.4156 | 6.9148 | 8.0191 | 6.2224 | 5.8391 | 6.2578 | 8.6459 | 7.6443 |
| 5.3391 | 8.1286 | 7.5378 | 6.8312 | 10.6053 | 8.6090 | 9.5580 | 7.3582 |
| 8.3882 | 9.3978 | 9.9082 | 9.7886 | 10.4902 | 9.0721 | 7.9708 | 9.4557 |
| 6.8766 | 9.2055 | 8.8641 | 8.3515 | 9.5321 | 6.7633 | 8.4499 | 9.5076 |

8.2229 9.4064\
RAC3 9.3405 4.9689 7.8921 8.3970 9.8879 6.6546 9.9380
8.7366 9.0524 7.5609 6.3587 9.5448 8.6691 8.1395 6.3310
8.8472 9.3880 9.9846 5.6114 10.5433 9.3108 8.5061 7.2091
7.3090 6.4524 9.9635 6.1400 8.4361 8.4586 9.2927 9.1873
8.8361 9.1961 9.4883 8.7508 9.0022 8.1074 8.1221 7.8673
9.6454 9.2988 5.1514 8.9412 10.9354 3.7479 6.7495 10.1787
9.1452 10.8578 8.6708 8.4434 8.8577 8.8661 3.7315 9.4720
9.0032 7.0669 5.8723 9.1165 8.7360 1.7140 8.3241 9.7980
9.0575 6.1785 6.2229 6.4220 9.3864 6.1067 10.3006 9.7537
7.6922 7.5535 9.1604 6.4590 9.4788 8.0219 7.4300 6.8059
8.2853 9.5366 9.5864 6.7356 8.5101 8.6111 9.2200 8.7222
7.8664 8.6191 9.6284 3.5269 7.9010 10.3478 7.8385 7.9883
4.2441 9.3936 5.1848 7.6579 7.1890 9.9503 8.4109 9.7819
10.0655 9.2538 10.6818 5.7675 7.7898 7.8412 9.1335 8.9768
9.3296 10.2106 8.1085 6.6484 8.5358 8.7694 9.3171 8.4966
7.3921 9.3500 8.1117 7.3661 9.2847 6.6354 9.2278 10.4706
11.0861 5.4071 5.4313 10.8076 8.4937 8.2092 9.6292 8.3489
10.4650 10.6988 5.6856 7.3098 4.7104 9.2112 9.1115 7.7498
7.8858 6.2000 5.6373 7.6964 7.9604 6.4569 10.1171 7.7447
9.4489 7.0559 11.3133 9.1068 8.2270 5.3124 8.4582 8.8806
9.5905 9.6885 7.3835 7.0081 8.2910 8.0405 7.6049 6.0395
7.5341 8.5076 9.7170 8.6686 9.1673 9.4433 8.7418 8.4414
9.2817 6.4448 5.6811 9.0192 7.2165 8.0024 8.9018 9.0493
8.8431 7.8999 9.6191 8.1255 8.5944 8.3364 9.3945 8.2131
7.3822 8.6265 9.0947 8.7993 8.3228 10.9237 5.7340 7.6242
7.5598 9.9591\
RAC1 12.8327 12.8422 12.0471 12.8288 12.4421 12.4259 11.4581
11.8172 12.4964 12.2960 13.0356 12.3840 12.8311 12.0623 12.7615
11.9612 11.7043 12.6060 12.7418 12.8574 12.5080 12.7429 12.2471
12.4109 12.8979 13.4761 12.5025 13.1670 12.8712 12.6165 13.1362
12.6687 13.2816 13.3247 12.2427 12.2700 12.0039 12.1907 12.7180
11.1788 13.2247 12.4679 12.9485 12.8389 12.8992 13.3092 13.1274
12.4032 12.3892 13.0410 12.3076 11.8286 12.7270 12.8057 12.2512
12.7554 11.5985 13.0004 12.7836 12.0517 12.8908 12.8868 12.3243
12.8138 12.6459 12.6871 12.0576 12.6871 13.1314 12.9678 13.0916
12.3463 12.4456 12.9667 12.3626 13.1564 12.0243 12.5944 12.4603
12.7449 12.8778 11.9597 12.7439 12.6288 12.8453 12.7102 12.4263
12.5649 11.9095 12.5532 12.5273 12.2362 12.3865 12.1960 12.8509
12.8182 12.3157 12.5807 13.0078 12.5484 12.6349 12.9267 12.1948
12.2246 12.6426 12.6138 13.2302 12.6945 12.1881 12.8580 12.3230
12.4119 13.0513 12.1665 12.9846 12.5405 12.0949 12.5699 13.1567
12.4293 11.8313 12.8241 12.5887 12.7343 12.3290 12.8897 12.6720
11.6938 12.7460 12.4518 12.3554 12.5676 12.5897 12.0042 12.4635
12.9191 12.8496 13.3701 12.2293 12.4070 12.8297 12.5483 13.0383
12.7690 12.4940 12.4048 12.8159 12.6785 12.3499 12.8756 12.4571
12.1746 12.0359 12.7883 12.2304 12.6908 12.5404 12.0406 13.1592
12.9912 12.4405 12.8500 12.6745 12.7904 12.8779 12.6242 13.6190
13.1350 12.5326 12.8253 12.6993 12.3054 12.2880 12.9319 12.2299
12.2769 12.8860 12.7123 12.7585 12.3978 13.2135 12.4989 11.9391
12.4672 12.3636 12.5399 12.5686 12.9842 12.8876 12.8001 12.4636
12.7562 12.7016 12.0028 11.9459 13.4301 13.1442 12.3472 12.2603
12.4723 13.0482\
SBDS 9.6672 10.8532 9.6173 10.2018 9.7586 9.9706 9.5036

```
10.0256 9.0797  9.8355  10.1085 10.5067 10.3681 9.7795  9.4647
9.1169  9.6896  10.7811 10.8624 10.0122 10.2045 9.4479  9.9831
9.8414  11.0337 10.3658 11.2331 9.0874  10.0779 10.5222 10.5886
10.3643 9.7831  9.9690  9.7390  10.5670 9.4207  9.7400  10.6528
10.2918 10.8884 11.3043 10.6303 10.1296 12.0019 9.5153  8.9566
10.8334 10.7364 10.1288 9.8869  10.3186 10.1530 11.6745 10.4981
10.0735 10.1943 10.9350 10.4535 9.3508  11.9359 10.7871 11.0443
9.3240  10.2847 9.8241  9.7810  8.9891  11.3540 10.2383 9.3119
9.4998  9.6360  9.8117  10.5008 10.2373 9.6810  9.4006  9.6989
10.1602 9.4775  9.8514  10.4276 10.2163 9.3449  9.9146  10.1857
9.0867  10.7697 10.4381 11.4421 10.0689 10.2978 9.5253  10.0581
11.8837 10.3713 10.8083 9.6078  10.8051 9.1747  10.4385 9.3431
9.9214  10.0492 9.9901  10.8327 10.4857 10.8043 9.5268  9.5469
9.6729  8.0160  9.9425  10.4779 9.6370  8.9834  9.9956  10.4270
11.0518 10.1783 9.9969  10.4222 10.1815 10.3142 11.2897 9.9850
8.6658  11.4154 11.1186 9.7000  9.7904  9.2725  9.5181  10.9088
9.7392  9.4978  8.4611  10.3454 11.2031 10.1351 10.2777 9.2147
10.2592 10.3045 11.2673 10.1468 10.8008 10.4327 9.6759  10.5910
7.6566  9.3642  10.2535 9.1164  11.0256 10.8253 9.5171  9.2638
10.5436 10.0163 10.5534 9.6468  9.5468  10.1952 9.8438  10.1465
9.8801  9.3287  9.1404  9.8660  9.6405  9.7565  10.1762 7.8757
9.8042  10.4821 11.8198 9.9916  9.6655  9.6253  10.5345 9.6723
9.7871  9.6732  9.9252  9.6950  9.0002  9.0519  10.0587 9.9924
9.8860  10.3339 10.2539 9.9231  9.7167  8.5390  10.9983 9.8872
9.6313  9.0827\
RCL1    8.0633  7.7924  8.3532  8.4161  8.7010  9.1112  9.7216
9.5508  8.3837  8.1442  8.6138  8.7269  8.7892  8.8378  8.0562
7.9491  7.2576  8.0658  7.8515  7.4108  8.7570  7.9411  8.7803
8.2943  8.1832  8.4310  9.6222  7.6340  8.2830  8.8780  8.3113
8.3974  8.6850  9.3288  9.1858  8.8909  8.4199  8.2173  8.5561
8.4023  8.6215  8.4743  7.8493  9.6526  7.9937  7.4391  7.4032
10.3257 9.4656  7.8300  8.1493  8.4995  8.6625  8.5669  9.1119
8.1932  8.3276  8.4067  8.4126  8.2217  8.5456  7.6110  8.2124
8.2125  8.0852  7.9644  7.0553  8.2634  8.3237  7.5085  9.2983
8.2986  8.0018  8.0018  8.4467  8.2677  9.1283  7.8121  8.3061
8.6258  8.6685  8.5983  8.0996  8.8457  8.5815  7.5241  8.7752
8.3567  8.8912  8.1484  8.2324  8.7030  8.6493  7.8501  8.1491
7.8214  8.3655  8.6611  8.4267  8.2755  8.2464  8.1592  9.6126
7.0668  8.1378  9.2102  7.8988  8.1552  8.2402  7.8283  8.1822
9.5152  6.3150  6.4429  7.6505  9.3903  8.8140  7.1574  7.8229
8.4389  8.4731  8.1351  8.1964  8.0339  8.6632  9.4423  8.9796
7.7514  8.7469  8.8148  8.7691  8.6114  8.3271  9.2239  8.6997
7.3238  7.1558  4.5503  9.2949  9.0274  7.2953  8.3248  7.9258
8.3466  8.3668  9.1183  7.8494  8.4706  8.2816  7.2852  8.2198
7.8273  8.1671  8.3258  8.5000  7.6264  8.3893  8.3594  9.1453
8.5974  8.3341  8.3792  8.6513  8.1974  8.3295  8.3070  8.0900
8.9802  8.1273  7.9562  9.2459  9.6785  8.3480  8.4527  7.3192
8.5907  8.0618  8.3011  8.5613  8.5293  8.3142  8.2941  8.6203
8.8821  9.1257  8.6764  7.5043  8.3925  7.9988  9.5285  8.7198
8.1606  8.3670  8.2887  8.6427  9.2409  8.3159  8.8812  8.1342
8.3954  7.4832\
SBK2    0.5526  2.1912  0.0000  0.0000  0.5278  0.0000  0.5431
0.0000  1.0352  0.0000  0.0000  0.0000  0.0000  0.4395  0.0000
0.0000  0.0000  0.0000  1.9975  0.0000  2.2447  0.0000  0.0000
```

0.5262 0.5538 1.0317 0.0000 0.0000 0.0000 0.0000 0.0000
0.0000 0.5159 0.8184 0.5853 0.0000 0.5555 0.0000 0.0000
0.0000 0.0000 0.4871 0.0000 0.0000 0.0000 0.0000 0.0000
2.1933 0.0000 0.0000 0.4530 1.6507 1.9420 0.0000 0.4645
0.5608 0.6343 1.8088 2.2544 0.0000 0.0000 0.0000 0.0000
0.0000 1.1445 0.6977 0.0000 3.2255 0.0000 1.0463 0.0000
0.0000 0.0000 0.8374 0.0000 0.0000 0.0000 0.0000 0.0000
1.8728 0.0000 0.7073 0.0000 0.0000 1.2145 0.0000 0.0000
0.0000 1.2163 0.0000 0.6361 0.4848 0.0000 0.0000 0.0000
2.6869 4.2424 2.2250 0.8845 0.0000 0.0000 0.4986 0.0000
0.0000 0.0000 0.0000 0.0000 0.4315 0.0000 0.0000 0.0000
0.0000 0.0000 0.0000 0.5141 0.0000 0.0000 0.4321 0.0000
0.0000 5.0567 0.0000 0.0000 1.5487 0.0000 0.5331 0.0000
1.6629 0.9653 1.4255 0.0000 0.0000 0.0000 0.0000 0.8107
0.0000 4.4769 0.0000 1.8532 3.0966 0.0000 0.0000 0.0000
0.5424 0.0000 0.0000 0.0000 1.5047 0.0000 0.7381 0.0000
0.0000 0.0000 0.0000 0.0000 0.0000 2.8514 0.0000 0.6000
3.7334 0.0000 0.0000 0.0000 0.0000 0.0000 0.5023 1.2961
0.0000 0.5276 0.0000 0.0000 0.0000 6.6200 0.0000 0.0000
0.4801 1.4586 0.0000 0.9723 0.6314 0.0000 5.8745 0.0000
0.0000 0.0000 0.0000 0.0000 0.0000 0.0000 0.5707 0.0000
0.9557 0.0000 0.0000 0.0000 0.0000 0.0000 1.0391 0.0000
0.0000 0.0000\
MRPS18A 10.1889 9.3769 10.0942 9.5018 9.8415 10.0256 10.0361
10.5558 10.3063 9.6130 9.9422 9.8982 10.1532 9.2551 9.5297
9.5990 10.2872 10.0188 9.4764 10.3034 10.9549 9.2384 9.8701
9.3408 9.6248 10.2357 10.0184 10.4648 9.8785 10.3390 9.1708
9.3402 10.6352 9.7651 9.4644 10.6755 9.6682 9.2824 9.8795
9.7139 9.9723 9.2917 10.2083 11.1533 9.1467 9.5557 11.5342
10.6499 9.7083 10.2282 9.6491 9.9444 10.0076 9.1704 9.7176
9.4905 9.3353 9.7350 9.5595 10.3032 9.2825 9.5715 10.3337
10.0324 9.4314 9.5298 10.2223 9.1911 9.4710 9.6984 11.1378
9.4316 11.0947 11.1632 9.2459 9.8591 9.8445 9.5525 9.4550
9.3574 10.5001 9.4138 9.1190 9.7736 10.0074 9.9017 10.0662
10.0013 9.5079 9.4966 9.7073 10.2444 10.2562 9.0019 10.1628
9.6072 8.8930 9.5290 9.5698 9.1896 10.4954 10.0282 9.6574
9.3407 10.0508 10.1179 9.3602 9.6266 9.4372 9.8082 9.5093
9.9672 11.5841 10.7134 8.4543 10.2277 9.7634 9.6680 9.8660
10.2653 10.6259 9.8937 9.7294 10.4078 9.6440 9.8350 9.9813
12.5120 9.3697 9.0983 8.4770 9.5765 9.8808 9.3205 10.4635
11.8530 10.8221 8.5429 9.2626 9.5861 10.1404 9.9609 10.1770
9.7882 9.7526 9.0920 9.6745 9.9521 10.3229 11.4335 9.1397
11.7635 9.0772 10.4730 9.4223 9.9115 9.6110 9.3217 9.9242
10.3411 10.3331 10.1016 9.5178 9.9753 8.9713 9.2913 10.2311
9.6099 10.1410 9.8782 9.5849 10.0677 10.1591 10.3013 9.9725
9.4637 9.2263 9.7529 9.4245 9.2145 10.3527 10.3420 10.3021
9.9612 9.6908 9.1769 10.0905 11.0315 10.2461 10.3049 9.7720
9.8838 10.2008 9.6752 10.0521 11.6333 11.0259 9.7327 9.5435
9.1343 11.1820\
CPLX2 1.5194 5.1245 3.5887 2.0919 9.7213 0.9816 7.2507
3.6658 2.2773 3.3648 4.3006 0.0000 4.3012 8.7486 9.5515
6.6558 3.0015 9.7392 3.5205 0.0000 2.2447 0.0000 1.9117
5.5878 0.0000 4.7327 0.0000 4.8732 4.5595 3.0135 0.4127
1.5370 0.0000 0.4664 5.3228 0.0000 4.7460 2.8736 1.8992

```
0.0000  3.9118  4.9029  3.0982  1.0349  0.0000  11.3427 1.1888
0.6741  3.6750  4.3061  0.0000  2.5833  4.4617  0.0000  6.3955
5.7604  4.3055  10.1841 1.2457  0.5715  0.0000  5.0295  4.3787
6.1008  2.8183  3.2320  2.7333  4.2998  6.7255  4.1734  5.1993
5.8145  2.2328  1.7486  6.3754  0.8237  6.5952  6.4193  3.6599
1.3771  5.6575  2.9341  1.5572  6.2653  5.6398  5.1219  2.4635
5.2531  6.0980  11.6101 0.6361  0.8470  5.1094  7.1834  0.0000
0.0000  7.1116  1.5047  1.8231  1.9210  8.8912  4.4630  3.1101
4.6411  0.8449  4.2417  0.0000  3.2273  3.9218  3.7624  1.6498
1.3871  9.0860  2.0000  4.5920  8.0413  3.4214  1.2610  5.9439
1.3647  4.5400  3.6540  5.6306  10.1939 2.3506  3.8931  2.3928
7.3402  1.9477  2.5988  5.8907  0.0000  5.3947  6.8781  3.3291
8.5233  10.4317 7.4343  0.0000  2.3776  6.5390  6.0678  3.6318
7.8633  7.4968  2.7920  0.6735  3.2124  7.5050  2.8098  6.6742
8.6910  6.1888  9.7292  9.1188  5.9852  2.4071  5.8186  6.7377
0.0000  12.5014 0.6138  2.8993  4.8610  2.4059  1.1696  10.7525
2.5383  1.4679  0.9420  2.6939  0.5290  10.5482 1.0565  4.7787
5.4078  2.5381  1.9008  7.3481  1.0692  6.1558  2.8349  9.1621
2.5935  5.4253  3.1906  0.0000  4.9962  0.0000  9.4314  10.4412
0.0000  0.0000  6.7985  4.7984  6.5221  0.0000  3.3172  5.8593
6.8009  3.6757\
CPLX3   2.3786  0.5319  6.9540  9.9643  5.1832  0.9816  0.0000
0.0000  1.7860  1.6538  1.7761  0.8472  0.0000  6.2123  0.5377
5.1466  1.3881  1.2795  0.0000  2.1640  0.6175  0.0000  0.0000
4.5015  1.5220  0.6062  6.4822  2.4936  5.9795  0.0000  0.0000
7.2649  0.5159  1.1010  0.5853  1.7727  6.8458  4.8095  5.3258
0.4337  1.4628  1.5889  0.6231  1.0349  0.0000  1.4889  0.0000
0.0000  2.1914  8.5620  2.1110  3.4519  0.6494  0.8853  2.8232
4.1397  0.6343  2.1170  0.4252  2.2896  0.0000  1.6116  0.5573
2.8376  2.3888  0.3906  2.3340  2.0643  4.8527  0.0000  1.0365
0.0000  1.5110  1.5690  6.7285  4.7161  0.9546  2.6909  0.6894
1.5339  2.8152  1.8200  0.0000  1.9875  1.2145  1.3843  0.0000
0.0000  0.9125  3.9976  0.0000  0.0000  1.4549  2.0255  1.4518
0.0000  0.5233  1.2499  3.4236  1.2591  0.0000  0.0000  4.3063
3.5417  2.3164  0.0000  5.0653  1.7824  1.7151  1.0041  9.3075
0.6215  1.3631  2.9764  2.8060  1.8640  2.1803  1.5937  1.8223
1.0358  3.3613  0.0000  0.0000  2.5678  0.0000  0.9214  3.4014
1.2894  1.5385  0.5074  1.3123  3.8462  1.5194  7.2607  2.4659
0.8141  4.3638  0.0000  0.5216  1.6315  1.8416  7.4157  1.0973
4.2673  1.0811  0.5418  0.6735  0.0000  8.5540  6.3427  5.3415
0.0000  3.7204  0.0000  1.7261  0.4374  0.0000  3.7440  2.4961
4.5672  4.0590  0.0000  2.8993  3.8626  3.7895  0.8742  0.0000
0.7148  3.4214  0.9420  2.8480  0.5290  8.3233  0.0000  10.5075
6.1274  2.8318  0.6293  6.7327  1.6765  3.6145  1.7883  0.5257
1.3638  0.0000  1.0594  1.3150  2.0548  0.0000  1.2961  5.5802
0.9557  6.4662  0.0000  1.3327  2.3335  0.0000  1.6369  1.3092
0.0000  0.0000\
ORC3L   8.6202  9.0721  9.2341  8.7152  9.2455  8.7214  8.9435
8.8808  9.2335  9.1065  9.7513  9.6858  9.5789  9.3745  9.1100
9.0622  9.4360  9.7843  8.8279  8.7027  9.0797  8.7948  9.3099
9.0595  9.4094  8.4505  8.8201  8.3881  8.4487  9.4901  9.4781
8.9319  8.4797  9.8045  9.4705  9.1886  9.0849  9.0985  9.3926
10.2164 9.5311  8.6605  9.4233  9.8752  9.2656  9.0508  8.2792
9.8096  9.8567  8.4753  8.5565  8.4650  9.5762  9.0807  9.6311
```

9.5827 8.8276 9.3129 9.6748 9.4076 9.2899 9.7061 9.1358
9.2616 9.4403 8.7682 9.1615 9.9499 8.6443 9.6495 9.1034
9.0635 8.8304 9.1293 9.3621 8.2385 8.9373 8.9241 9.0067
9.0184 8.6375 9.1381 9.2274 9.5970 8.9764 9.7409 10.1489
8.5569 9.2077 8.8914 8.9110 9.0793 9.8679 9.5372 9.5277
9.0550 9.6253 9.4162 8.8925 9.3917 8.9329 8.9482 8.9368
9.2971 9.2669 9.1142 9.3703 10.0064 9.8867 9.2176 9.1078
9.1222 7.7784 9.9054 10.2428 10.3565 9.7352 9.6208 9.6949
9.2139 10.4108 9.0771 9.4322 8.5856 9.5316 9.2660 9.8880
8.0788 9.5223 9.3787 9.7444 9.3749 8.8795 9.9870 9.3649
7.1344 8.6819 7.8512 9.1977 9.4316 9.5887 8.9090 8.7636
9.2949 9.1215 9.5938 8.2526 9.2256 9.1778 8.2107 8.9205
7.1231 9.0378 9.3983 9.0311 9.0330 9.1767 9.0168 9.7709
8.6243 8.5921 8.0034 9.1836 8.9155 9.0794 9.5041 9.6571
9.7438 9.0783 9.2997 8.9141 9.3507 9.2314 9.3927 7.0579
9.6137 9.6120 8.7343 9.2743 9.0628 8.5863 9.2268 9.9911
9.1205 9.7755 10.3361 9.3003 9.3265 8.5266 8.7944 8.9733
8.9423 10.0659 9.4125 9.4387 8.5881 8.6706 9.4489 9.2129
9.1568 8.2720\
CPLX1 8.2295 8.1905 9.0020 7.7436 5.0927 6.9368 5.7796
5.5779 6.7030 8.1979 5.0539 7.3220 6.1336 2.8895 5.7388
6.2617 8.1082 8.8698 7.4313 6.9343 7.3140 7.2974 6.9175
4.8222 6.9550 8.7225 4.3191 8.0860 6.1366 7.1549 6.3309
4.8607 4.7439 6.3359 7.0065 6.1759 6.3866 5.8499 7.1364
6.3074 6.7646 7.4570 5.3182 6.7687 5.8318 9.3494 5.3683
4.4094 3.7535 7.1681 8.2153 5.9287 4.6368 8.3741 6.0923
7.5116 4.9713 7.4614 6.2299 6.3263 6.4313 4.9357 5.9610
7.7320 7.6185 5.5929 6.1043 4.7037 3.8367 6.3938 4.9765
6.5841 7.4657 7.6329 5.1760 6.2122 4.6662 5.5099 7.8073
5.9221 5.6575 4.5714 7.3931 5.9425 7.6874 7.0733 5.9533
6.9796 5.6556 8.7045 7.3419 5.5767 8.3106 8.1906 5.0299
7.0689 7.0125 7.7852 7.7767 6.9279 6.7098 8.3628 5.6424
8.1247 6.2970 4.9946 8.9774 5.1500 5.5996 7.5039 4.7130
5.9632 7.7989 8.6147 7.1173 5.7637 5.1829 7.0050 5.5342
5.3542 7.8475 6.9767 4.2326 6.3416 7.3575 4.9519 5.8007
5.2955 6.5599 6.5439 5.6416 7.5485 6.0719 8.8646 6.5966
8.2635 5.3483 9.8347 6.9571 7.6433 6.6838 5.7381 7.3572
5.2293 5.1977 7.5326 6.6142 7.9524 6.7172 6.6611 7.5756
5.8705 6.1244 6.1331 6.5228 5.8321 8.3433 6.7286 4.4001
6.9753 7.7630 5.3495 6.6918 6.9263 7.0381 6.8780 3.9594
6.7517 6.8221 4.2097 5.7614 7.2312 6.9310 6.1092 8.7047
8.0375 8.0283 6.9354 6.2485 8.1846 5.7909 6.6928 5.3420
8.2774 6.8021 8.5423 6.7435 6.5410 8.0278 6.2881 4.9122
5.3216 6.9073 6.3103 8.6838 5.9450 8.5450 8.2469 5.9518
6.6210 5.5508\
AP2A1 11.0737 11.1178 10.6356 10.8869 11.7015 10.9584 10.9664
11.2925 11.0037 11.5346 10.9720 10.6065 10.9413 11.3936 10.7895
10.7993 10.6139 11.1110 11.3717 9.8601 11.0754 11.1095 11.1575
10.5791 11.5421 12.1857 11.4529 10.8487 11.4562 10.5645 10.7843
11.0554 10.7813 11.6377 10.9531 11.5680 11.7408 10.9191 10.2557
11.2840 10.8832 10.8672 10.7548 10.4319 11.1200 11.0485 10.1497
11.0466 11.1812 11.2720 11.3533 10.6813 11.8485 11.0873 11.5977
10.3200 11.1937 10.7955 10.7850 10.8981 10.9823 11.0245 11.3940
11.4686 11.6297 11.2629 10.7239 11.1354 11.1536 11.2136 11.9486

11.1155 11.0432 12.0426 10.6036 11.5892 10.8134 11.3728 10.5310
11.4170 10.8281 11.0149 10.6534 11.4020 11.1387 10.6740 10.3918
11.1696 9.0083 11.3498 11.5616 11.0579 10.7338 10.9601 10.8739
11.9070 11.7483 11.3816 11.7932 10.6515 10.7552 11.1816 10.8586
10.5007 10.5858 11.2845 11.6537 11.0770 11.1214 11.2550 11.1161
11.3196 10.2820 11.0681 10.8170 10.4548 10.7918 10.8627 10.7289
11.1455 11.2233 9.8714 10.3247 11.8726 10.8553 11.1056 10.6676
11.2078 10.7471 11.2995 11.5551 10.4045 11.2760 11.3313 11.6080
11.4007 10.5790 10.1495 10.9091 11.2942 11.1932 11.0019 10.6129
11.0415 11.0688 11.6918 10.9635 11.6399 11.5467 10.8716 11.0235
11.4355 10.8404 12.3487 11.2163 10.7937 11.1304 11.2844 10.2714
11.3914 11.0705 11.4555 10.5987 10.8385 10.3802 11.0985 10.1628
10.6880 11.0900 10.6307 10.9191 10.3561 11.7693 11.4183 10.0334
10.7815 11.1460 11.0380 10.8625 10.9272 10.7496 10.9986 10.4211
10.7218 11.6703 10.5228 11.3485 10.9548 10.7488 11.3116 10.7000
11.2824 10.9601 12.0293 11.0554 10.9546 10.5829 11.0818 11.0714
10.6266 11.6547\
CPLX4 0.0000 0.5319 1.7871 0.0000 0.0000 0.0000 0.0000
1.5600 0.0000 1.0519 2.1584 0.0000 0.0000 0.7759 0.0000
0.0000 0.0000 0.7772 0.9983 0.6732 0.6175 0.4820 0.0000
0.9110 0.0000 1.3599 0.0000 1.0210 0.0000 0.5901 1.7306
0.5612 0.0000 0.4664 0.5853 2.2096 0.0000 2.5584 0.0000
0.0000 0.6649 0.4871 0.0000 0.4326 0.0000 0.0000 0.0000
1.1317 0.0000 2.1768 0.0000 0.0000 0.0000 0.0000 0.4645
0.5608 0.0000 0.0000 0.0000 0.9799 0.0000 2.4655 0.9583
0.0000 0.0000 0.6977 0.0000 4.0455 1.3082 0.6160 0.7662
0.3921 0.0000 0.8374 0.9270 0.0000 0.9546 0.0000 0.6894
0.0000 0.0000 0.9627 1.1987 0.6718 0.0000 0.0000 0.9284
1.3738 0.0000 0.0000 0.6361 0.8470 0.0000 0.0000 0.5198
0.0000 0.5233 0.0000 0.0000 0.9478 1.6914 0.8684 0.0000
0.0000 0.4835 0.4659 0.7314 1.6282 1.2448 0.0000 0.0000
0.0000 0.0000 0.0000 0.5141 0.6116 0.5276 0.0000 0.0000
0.6089 2.7150 0.0000 0.0000 0.0000 0.0000 0.0000 1.3754
0.0000 0.0000 0.0000 0.3935 1.1234 0.0000 0.0000 0.0000
0.0000 0.0000 0.0000 0.5216 0.5056 0.6010 0.0000 0.0000
0.0000 0.0000 0.0000 1.4775 0.0000 0.0000 0.7381 0.0000
0.0000 0.0000 0.5862 0.0000 0.7727 0.0000 0.0000 0.0000
0.9345 3.2259 0.0000 0.0000 0.0000 0.0000 0.8742 0.0000
0.0000 0.9131 0.9420 0.0000 0.0000 0.0000 0.0000 0.0000
0.0000 0.9063 0.0000 0.0000 0.0000 1.2666 1.1551 0.9100
0.0000 0.7366 0.6248 1.9914 0.0000 0.0000 0.5707 0.0000
0.9557 0.9737 0.0000 0.0000 0.0000 0.0000 0.0000 0.9160
1.4261 0.0000\
AP2A2 11.3250 10.8825 9.7288 10.9315 11.3985 10.6216 10.1036
10.6329 11.2116 10.9026 10.5197 10.3560 10.6396 10.8424 10.9004
10.9134 10.6769 11.4318 10.8124 11.6078 11.1177 10.5409 11.2587
10.8956 10.9052 11.7769 10.3858 11.6925 11.7868 12.0860 10.8730
11.3438 10.9776 10.3540 10.2297 12.2319 10.8432 11.6124 10.3322
10.6193 11.1476 10.7920 11.7323 11.9140 10.7690 10.8478 11.3769
11.7888 10.7692 11.0865 10.9923 11.0086 10.7253 10.9409 10.2489
11.2931 10.6279 10.7063 10.0904 11.2766 10.8942 10.5841 10.5124
10.9243 10.8383 10.9923 9.8692 9.8440 10.7106 10.8531 10.5300
12.1082 11.7653 9.9017 10.7757 11.0341 10.1032 11.4781 10.9829
10.9822 10.5851 10.2629 10.9519 9.9509 11.3376 9.9519 10.1518

11.4686 11.0611 10.8229 11.3047 11.5528 10.9402 10.8373 10.8815
11.0257 11.3382 11.0133 10.6026 11.2747 11.6986 11.7758 10.5896
10.8058 10.9725 10.5189 11.6888 10.8339 10.4334 10.9244 11.8506
10.9564 12.3212 11.9915 10.3031 10.5830 10.6745 10.0186 10.9320
11.1309 10.6804 11.7061 11.7284 11.2443 11.0270 10.9863 11.1995
9.0819 10.4106 10.8740 10.0482 10.9425 11.2284 10.9220 10.7829
12.1198 11.3015 12.3052 11.3728 10.9036 11.0356 11.1517 11.3238
9.7754 10.3285 10.7516 11.0827 11.5088 10.6470 11.3957 11.0801
10.9538 11.1258 10.7025 11.3013 10.9865 10.9891 10.2649 9.9835
11.4950 11.7711 11.0639 10.4378 11.0075 10.4137 11.2785 9.9311
11.2560 10.9400 10.4416 11.7616 10.8240 11.5327 10.4535 11.0852
10.6979 10.6111 11.0598 10.9313 11.3373 11.8013 12.8124 9.7780
10.3473 10.7464 10.6452 10.7028 12.0066 11.0193 10.2971 10.9544
11.3543 10.7663 10.2656 10.7616 10.3034 11.3160 11.0285 10.7885
11.0967 12.4766\
LOC389333 4.1912 6.0069 4.6021 5.3565 4.1878 6.3616
5.0442 5.4551 4.8915 6.2021 4.5343 5.9100 4.8703 5.0621
4.7168 5.5157 3.1837 3.0246 5.3717 3.1743 6.0481 5.8211
5.8278 4.9302 6.0144 3.2258 3.3638 4.2875 4.7873 5.6875
3.5360 4.2887 3.6062 5.3604 6.4521 4.0627 5.9987 5.7760
2.2142 3.7681 4.9748 6.3313 3.8038 4.1805 5.2199 4.1830
0.0000 5.0253 3.0861 3.1417 3.9777 4.7376 5.8111 5.2741
5.7797 3.7894 4.1793 4.6790 5.1127 4.6335 5.6250 5.2482
4.6998 4.5255 6.3164 6.0777 6.3781 3.7367 6.1771 4.7882
4.8063 5.3567 3.8023 3.7643 4.9839 7.0557 4.9551 6.8473
5.2848 4.8950 3.9458 3.4754 5.0887 4.5201 4.6047 4.4902
5.7012 3.3246 4.1509 4.1839 5.7150 4.8558 3.8576 3.6634
3.8529 5.4762 4.8788 7.3784 5.6581 3.7089 1.4180 4.9413
5.4031 4.9821 4.4397 3.8802 4.5101 4.9692 4.7318 4.6517
5.2392 5.6440 0.0000 3.7004 5.4811 4.9089 4.5826 6.0045
4.4122 4.3149 4.6074 3.4695 5.2963 4.8635 6.7634 4.9309
2.5319 1.6629 5.9421 6.5504 4.5973 3.6084 5.2953 5.2580
3.4335 0.0000 3.3943 0.0000 6.0797 6.8991 4.7738 4.1410
4.9896 4.0055 4.4963 3.2722 4.0927 6.6339 4.9896 0.0000
6.2382 0.0000 5.4073 5.0037 3.8469 5.5342 6.9916 5.6789
5.4012 3.7820 4.5837 3.3985 4.2397 5.0554 3.8695 5.3273
3.5749 5.4046 3.3607 3.7967 4.5835 5.2217 5.5390 4.3146
0.0000 4.5788 5.1151 5.1477 5.5599 6.1219 3.2382 5.0929
5.7599 4.9446 5.5330 4.6662 5.4150 4.0128 2.8760 4.0467
5.4041 4.6140 2.6556 4.9968 5.2995 1.5946 0.0000 6.3430
5.7367 6.0887 2.9670\
LILRB3 5.6916 3.5015 5.1630 5.4719 7.0756 4.5311 2.6971
4.1450 4.7720 3.6900 5.5282 4.1544 5.6579 2.8082 6.4846
4.6494 4.5300 6.2191 5.4188 5.6736 6.6626 4.1262 5.0420
6.4969 3.4594 8.2555 4.2955 6.7147 6.6756 3.8911 4.4670
6.0181 7.8177 6.0362 3.3227 5.6300 3.1675 2.8129 3.5753
3.4028 7.1439 5.1239 5.3530 5.8776 2.8515 7.5806 0.0000
6.3601 5.7637 3.2765 5.8312 7.9505 6.2666 5.1603 5.4723
4.2705 6.0954 5.5780 5.8538 2.7727 4.2322 5.5432 4.9961
3.1186 2.9908 8.0291 6.2206 4.7484 4.9574 3.8124 7.5112
4.4166 9.6553 6.7849 6.7687 3.1833 4.3162 3.4925 6.1073
7.7858 4.4059 2.8606 6.4445 5.8089 5.6738 5.7972 2.2055
5.7546 5.8998 6.3503 6.5417 5.4572 6.0489 7.6607 6.2438
3.9462 5.6286 4.0448 4.7415 7.4761 7.2411 7.0947 3.1552

| | | | | | | | |
|---|---|---|---|---|---|---|---|
| 5.6984 | 4.5066 | 2.1477 | 3.1574 | 5.7643 | 3.7851 | 6.2748 | 4.7641 |
| 5.6846 | 6.3432 | 5.1371 | 4.8036 | 4.2443 | 3.8145 | 3.9842 | 5.3796 |
| 8.4438 | 3.5426 | 6.7890 | 3.5285 | 5.4037 | 4.2292 | 7.2982 | 5.2087 |
| 4.1963 | 3.9243 | 3.2398 | 5.1727 | 3.2985 | 6.5454 | 6.5796 | 6.5610 |
| 9.0517 | 8.6105 | 8.8941 | 4.7709 | 4.1183 | 4.8918 | 4.2044 | 6.5171 |
| 5.2202 | 5.0082 | 2.4900 | 6.1821 | 5.6699 | 5.0981 | 4.1991 | 5.1585 |
| 7.4470 | 6.5403 | 4.3870 | 3.4941 | 6.6166 | 4.3233 | 3.9134 | 4.8440 |
| 7.9974 | 4.0810 | 7.9526 | 5.7014 | 4.6594 | 3.6333 | 4.5891 | 5.5854 |
| 5.0252 | 5.3741 | 5.9158 | 3.6844 | 5.1334 | 4.4732 | 2.5508 | 7.1595 |
| 2.1868 | 3.3382 | 5.1089 | 3.6921 | 5.3966 | 6.0475 | 5.3879 | 4.1743 |
| 4.7319 | 5.6588 | 4.7546 | 5.4782 | 6.5434 | 7.2707 | 6.0628 | 6.4151 |
| 5.3904 | 6.2236 | 5.5736 | 4.5617 | 6.2431 | 4.8654 | 2.5229 | 5.0863 |
| 6.0854 | 6.2557\ | | | | | | |
| CADPS | 0.5526 | 2.8563 | 4.8055 | 6.6396 | 0.5278 | 2.7758 | 3.7378 |
| 3.5263 | 6.1978 | 7.6381 | 4.0217 | 3.1028 | 5.7540 | 4.0280 | 4.1468 |
| 2.1513 | 6.9150 | 6.2195 | 1.8049 | 2.7331 | 2.6649 | 4.0067 | 1.9117 |
| 4.6558 | 4.5231 | 1.3599 | 5.4141 | 6.2506 | 3.3032 | 2.8206 | 1.7306 |
| 2.9368 | 1.6552 | 2.9765 | 1.3224 | 2.6866 | 2.5102 | 3.1787 | 1.2418 |
| 3.8754 | 5.9516 | 3.2981 | 7.1221 | 2.0520 | 3.0451 | 3.7705 | 2.7566 |
| 1.7580 | 1.2792 | 3.2642 | 2.6239 | 1.2789 | 3.8722 | 2.3883 | 4.7457 |
| 4.2155 | 5.3684 | 3.8431 | 5.7678 | 3.5478 | 1.4980 | 6.4074 | 2.7350 |
| 2.7303 | 6.0787 | 5.9957 | 1.0195 | 2.3155 | 3.5640 | 4.9210 | 2.0543 |
| 1.6718 | 2.9022 | 2.8668 | 3.6166 | 0.0000 | 3.3759 | 5.3094 | 4.1326 |
| 1.0461 | 6.6570 | 4.9418 | 5.1426 | 4.0375 | 4.9094 | 4.0964 | 2.6823 |
| 3.6635 | 4.3513 | 5.6405 | 3.7213 | 4.8558 | 2.9938 | 8.3860 | 6.0270 |
| 2.4199 | 1.4589 | 3.2124 | 3.4794 | 4.5129 | 0.0000 | 3.8702 | 6.2981 |
| 5.7431 | 2.1962 | 1.1001 | 3.7595 | 7.3617 | 4.8957 | 6.2560 | 2.6161 |
| 3.4186 | 6.1650 | 3.9069 | 1.8354 | 3.3929 | 3.5357 | 2.3757 | 1.3703 |
| 4.5912 | 6.8491 | 5.4702 | 3.4916 | 4.6588 | 1.8330 | 5.2853 | 1.3754 |
| 1.6629 | 3.7927 | 4.5447 | 4.4806 | 3.5369 | 4.8811 | 2.3620 | 5.6002 |
| 0.0000 | 1.4217 | 6.0124 | 2.2985 | 2.3776 | 2.8483 | 0.9848 | 4.8253 |
| 3.6335 | 4.0867 | 7.1984 | 5.0498 | 1.7212 | 4.0773 | 0.7381 | 5.0280 |
| 0.0000 | 7.0360 | 3.9105 | 0.0000 | 2.0663 | 2.9440 | 5.0646 | 3.0392 |
| 2.4740 | 0.6063 | 3.7221 | 6.0789 | 4.8449 | 7.4105 | 7.3153 | 0.0000 |
| 3.8546 | 5.5522 | 1.2521 | 1.9700 | 2.1836 | 7.2090 | 4.4632 | 6.4480 |
| 0.0000 | 6.8300 | 4.2513 | 5.5500 | 6.2730 | 3.3081 | 6.9999 | 1.2133 |
| 5.0024 | 5.0723 | 4.3198 | 5.1386 | 2.5964 | 4.1933 | 1.9676 | 1.9830 |
| 1.9330 | 3.7058 | 2.3573 | 4.2600 | 2.3335 | 2.5115 | 2.0583 | 3.6472 |
| 2.9539 | 1.5159\ | | | | | | |
| FIGNL1 | 7.9453 | 7.0694 | 8.3613 | 8.3217 | 8.3818 | 8.4457 | 8.5678 |
| 8.0637 | 8.8627 | 7.8938 | 8.6945 | 8.3229 | 8.5894 | 8.4409 | 8.2225 |
| 7.4356 | 8.9397 | 7.9828 | 7.4925 | 7.8376 | 7.7603 | 7.0607 | 7.5526 |
| 8.2302 | 7.9437 | 7.7062 | 9.7738 | 7.7523 | 7.1914 | 8.2514 | 9.6449 |
| 9.1175 | 7.9678 | 8.6366 | 8.8987 | 9.0342 | 7.2413 | 9.1233 | 7.7288 |
| 8.6986 | 6.9536 | 7.0081 | 7.4553 | 9.0404 | 8.0791 | 7.9146 | 6.9598 |
| 8.3601 | 9.3386 | 7.3206 | 7.8108 | 7.8542 | 8.1034 | 8.2472 | 8.4436 |
| 9.1186 | 7.6739 | 8.9143 | 8.7375 | 8.0418 | 8.3598 | 8.5097 | 8.0293 |
| 8.4102 | 7.8481 | 7.8640 | 7.6385 | 9.3044 | 8.8750 | 8.1863 | 7.9985 |
| 8.3779 | 6.9329 | 7.6471 | 8.4634 | 7.0304 | 7.4590 | 6.8586 | 6.9910 |
| 8.5764 | 8.2370 | 8.7448 | 7.8950 | 8.8642 | 6.7603 | 8.6021 | 8.8617 |
| 6.9302 | 8.9335 | 8.4630 | 7.5004 | 8.7692 | 7.3211 | 8.0303 | 8.4979 |
| 7.7021 | 7.9236 | 8.2684 | 7.4256 | 8.9287 | 6.7992 | 7.5579 | 8.5717 |
| 8.2871 | 8.5592 | 8.6891 | 7.7997 | 8.5786 | 8.1056 | 8.2102 | 8.1104 |
| 8.0014 | 7.0730 | 8.8297 | 9.4156 | 8.3913 | 8.1020 | 8.8806 | 9.3625 |

| | | | | | | | |
|---|---|---|---|---|---|---|---|
| 8.3231 | 8.3734 | 8.1299 | 8.4644 | 7.5587 | 8.0441 | 8.0940 | 8.1211 |
| 6.9260 | 8.3667 | 8.4178 | 8.7131 | 9.0867 | 7.5341 | 8.0923 | 7.5078 |
| 6.3861 | 7.7803 | 7.4954 | 8.2526 | 8.0104 | 7.7617 | 6.9696 | 6.8663 |
| 8.5482 | 8.4642 | 8.2464 | 7.6714 | 7.7364 | 7.9608 | 7.9316 | 8.5512 |
| 6.4552 | 8.3513 | 8.4447 | 7.7497 | 9.0648 | 8.1840 | 8.2947 | 7.8583 |
| 7.8776 | 7.0107 | 7.4507 | 7.9913 | 8.7152 | 8.0639 | 8.6288 | 7.8652 |
| 8.4130 | 8.4192 | 9.3112 | 8.7031 | 9.5195 | 8.9647 | 8.2033 | 6.1698 |
| 9.0702 | 8.1620 | 7.5755 | 9.3773 | 6.7038 | 7.9784 | 7.7072 | 8.4293 |
| 6.9537 | 8.6656 | 7.8573 | 8.7517 | 7.4679 | 7.8264 | 8.5266 | 8.8402 |
| 7.2053 | 9.0891 | 8.4218 | 8.5615 | 7.7626 | 8.0772 | 8.4235 | 8.4996 |
| 8.0408 | 6.4762\ | | | | | | |
| KDM4DL | 0.0000 | 0.9197 | 0.0000 | 0.0000 | 0.0000 | 0.7223 | 0.0000 |
| 0.0000 | 1.7860 | 0.7786 | 0.0000 | 0.0000 | 0.0000 | 0.0000 | 0.0000 |
| 0.0000 | 0.0000 | 0.0000 | 1.3199 | 0.3755 | 0.0000 | 0.0000 | 0.0000 |
| 0.5262 | 0.0000 | 0.0000 | 0.0000 | 0.5990 | 0.0000 | 0.5901 | 0.4127 |
| 0.0000 | 0.0000 | 0.0000 | 0.5853 | 0.0000 | 0.0000 | 0.0000 | 0.0000 |
| 0.4337 | 0.0000 | 0.0000 | 0.0000 | 0.0000 | 0.0000 | 0.0000 | 0.0000 |
| 1.1317 | 3.4580 | 0.0000 | 0.0000 | 0.0000 | 0.0000 | 0.8853 | 0.0000 |
| 0.9635 | 0.0000 | 0.0000 | 0.7533 | 0.5715 | 0.5422 | 0.0000 | 0.0000 |
| 0.0000 | 0.6828 | 0.0000 | 0.0000 | 0.4832 | 0.0000 | 0.6160 | 0.4334 |
| 0.0000 | 0.0000 | 0.0000 | 0.0000 | 0.0000 | 0.0000 | 0.0000 | 0.0000 |
| 0.0000 | 0.0000 | 0.0000 | 0.0000 | 0.0000 | 0.0000 | 0.0000 | 0.0000 |
| 0.0000 | 0.5273 | 0.0000 | 1.0759 | 0.0000 | 0.9036 | 0.6909 | 0.0000 |
| 0.0000 | 0.5233 | 1.2499 | 0.0000 | 0.0000 | 0.0000 | 0.0000 | 0.0000 |
| 0.0000 | 0.0000 | 0.0000 | 0.0000 | 0.0000 | 0.0000 | 0.0000 | 0.5136 |
| 0.0000 | 0.0000 | 0.0000 | 0.0000 | 0.6116 | 0.0000 | 0.0000 | 0.0000 |
| 0.0000 | 0.0000 | 0.0000 | 0.0000 | 0.0000 | 0.5970 | 0.0000 | 0.0000 |
| 0.0000 | 0.9653 | 1.4255 | 0.0000 | 0.0000 | 0.0000 | 0.0000 | 0.0000 |
| 0.0000 | 0.0000 | 0.0000 | 0.0000 | 0.0000 | 0.6010 | 0.0000 | 0.0000 |
| 0.0000 | 0.4562 | 0.5418 | 0.0000 | 0.5454 | 0.0000 | 0.0000 | 0.5019 |
| 0.0000 | 0.0000 | 2.0029 | 0.0000 | 1.0445 | 0.5638 | 0.0000 | 0.0000 |
| 0.0000 | 0.0000 | 1.3735 | 0.0000 | 0.4033 | 0.0000 | 0.0000 | 0.0000 |
| 0.0000 | 0.0000 | 0.0000 | 0.0000 | 4.9389 | 0.0000 | 0.0000 | 0.0000 |
| 0.0000 | 0.5232 | 0.0000 | 1.6980 | 0.0000 | 0.0000 | 2.2268 | 0.0000 |
| 0.5055 | 0.0000 | 0.0000 | 0.0000 | 0.0000 | 0.0000 | 1.7765 | 0.0000 |
| 0.0000 | 0.0000 | 0.0000 | 0.0000 | 0.0000 | 0.0000 | 0.0000 | 0.0000 |
| 1.1797 | 0.0000\ | | | | | | |
| PBX3 | 6.0106 | 9.1466 | 9.7257 | 5.0362 | 7.5014 | 8.3237 | 6.0818 |
| 6.4895 | 4.5038 | 8.1369 | 6.8880 | 8.0568 | 6.0046 | 3.9303 | 5.4860 |
| 4.9193 | 6.3740 | 5.4060 | 9.2493 | 5.7920 | 7.6572 | 5.2439 | 8.4150 |
| 4.8444 | 8.1408 | 8.2273 | 4.4719 | 5.9599 | 5.7609 | 7.4416 | 6.1609 |
| 8.4650 | 7.9924 | 7.7870 | 7.0562 | 4.2112 | 7.9950 | 4.3416 | 7.9963 |
| 6.6167 | 6.4440 | 8.2829 | 6.3294 | 5.6424 | 9.1441 | 5.9641 | 3.9741 |
| 5.8021 | 9.5525 | 9.1367 | 5.8439 | 7.4397 | 6.5140 | 9.2225 | 6.5613 |
| 9.2795 | 7.1920 | 7.5950 | 8.2037 | 4.5158 | 9.0515 | 8.3733 | 5.5752 |
| 7.2542 | 8.0494 | 6.0441 | 5.7104 | 5.0012 | 6.6212 | 6.7342 | 7.6030 |
| 5.1939 | 8.1918 | 5.7889 | 11.0720 | 3.6930 | 7.3637 | 5.8427 | 5.9024 |
| 9.5491 | 4.8022 | 5.3637 | 7.7200 | 5.9700 | 5.5880 | 7.1840 | 7.5576 |
| 5.5755 | 6.4866 | 7.0613 | 9.8008 | 5.9932 | 5.5724 | 7.7507 | 6.1018 |
| 8.8355 | 7.5149 | 8.3780 | 5.3191 | 6.8134 | 5.8955 | 6.6919 | 7.0750 |
| 6.7205 | 4.2714 | 7.0830 | 8.3243 | 8.5413 | 6.3625 | 5.2367 | 4.9309 |
| 4.6877 | 5.5901 | 5.2479 | 8.0176 | 4.1616 | 4.5005 | 6.9971 | 5.9062 |
| 7.1994 | 9.5496 | 10.1326 | 6.5758 | 7.8844 | 8.4766 | 6.2916 | 9.1872 |
| 5.3218 | 8.2564 | 8.7958 | 8.5121 | 3.3824 | 5.6706 | 6.1716 | 7.8798 |

6.8131 7.7543 7.6511 7.3098 7.8091 5.2163 5.5246 5.7547
6.3457 5.9100 11.6482 5.1974 8.5075 7.2793 5.9648 7.8808
5.5487 5.9757 6.2892 3.2188 8.6292 8.4117 5.5434 5.9982
9.0171 4.1467 6.3317 6.1911 4.6912 8.2177 5.7851 6.3666
9.0199 5.5248 8.6166 5.2692 6.9400 8.7329 8.9733 8.0898
4.8790 7.9767 9.2164 9.7417 6.9285 5.4323 8.2482 4.7035
5.7958 4.9840 6.2848 6.0548 5.1580 5.2656 7.2470 6.5322
6.0912 5.6894 8.0532 5.3294 6.3542 3.5816 7.6276 6.5609
6.4506 4.0443\
PBX1 11.1417 12.8253 10.3513 10.6911 10.5575 12.3367 11.5820
11.1080 11.3820 11.9855 12.0337 11.8404 11.7762 11.5720 10.5777
10.1683 11.0233 10.2679 11.9539 8.2321 10.7531 11.3494 11.7343
11.8536 14.5008 7.9931 9.9381 10.2141 9.6369 9.2346 10.3098
12.0231 10.7748 11.4743 11.2984 7.2769 12.2684 11.5695 11.2421
12.2410 10.9929 13.1083 9.4210 10.2363 13.6047 10.1073 10.2585
10.1762 10.4573 11.7690 10.5412 7.2899 12.0074 14.1670 11.6113
10.6920 9.5797 10.5044 12.4310 10.9086 13.2938 11.0673 9.2597
11.0154 12.5294 10.7639 11.0408 12.5087 10.3192 11.2166 11.8280
10.0448 7.3477 10.6171 11.1344 11.8771 11.4307 10.3546 10.9345
10.7512 10.8438 10.9591 10.9069 12.2348 10.5804 10.9682 11.9203
9.7797 11.9067 11.1476 13.2544 10.3686 10.5611 10.2251 9.9369
14.0437 9.6349 13.0066 12.3908 7.9220 9.7796 10.5340 13.2266
9.6524 10.6965 11.7953 11.1167 11.7697 11.5973 10.6929 11.5078
11.9228 11.0819 9.6618 10.8622 11.7888 12.5403 12.7529 10.8059
7.9158 12.3830 10.3828 10.1093 11.2921 12.3935 10.4583 11.1608
10.8870 13.4689 14.1927 10.9251 10.7924 10.4222 11.2175 8.3622
8.1487 11.0717 10.3731 12.5662 12.9895 10.3650 11.3977 10.4238
11.1205 10.7875 11.7447 12.7004 12.4107 10.5933 10.8217 12.8016
10.3903 11.8241 11.4047 12.3283 11.3022 12.9535 11.8584 11.3680
9.3728 9.3107 6.8915 11.2610 10.4054 10.4886 11.7776 10.9873
11.0546 11.0426 12.6706 11.5850 12.7032 9.7336 11.1491 10.0197
11.6387 12.4651 13.2023 13.2313 10.7885 10.4617 9.1787 12.8787
10.8715 11.5633 11.1497 11.5042 9.9628 9.8659 10.6782 10.2032
10.7747 11.5330 10.9668 11.6296 10.8686 8.8544 12.8595 10.4839
11.5068 9.7569\
AIRE 0.0000 0.0000 0.0000 0.0000 0.0000 0.0000 0.5431
0.4748 0.0000 3.0897 0.4291 0.8472 0.4363 0.0000 0.9286
0.0000 0.0000 0.0000 2.3192 0.3755 0.6175 0.4820 0.6346
0.0000 0.5538 0.0000 0.0000 1.3471 0.0000 0.5901 0.7334
0.0000 0.0000 0.4664 0.0000 1.4920 0.0000 0.0000 0.0000
0.4337 0.6649 0.8506 1.0569 0.0000 0.0000 0.0000 0.0000
0.0000 0.0000 0.4764 0.4530 0.0000 0.0000 0.0000 1.5357
0.0000 0.0000 0.5030 1.0204 0.5715 0.0000 0.4969 0.0000
4.1206 2.3888 0.0000 0.0000 1.3736 0.0000 0.6160 0.0000
0.0000 1.5110 2.6121 0.0000 0.0000 0.9546 1.8986 0.6894
0.0000 0.5659 0.0000 0.0000 0.0000 0.0000 0.0000 1.4887
1.0433 0.5273 0.9113 0.0000 0.0000 2.4205 3.1676 0.5198
0.0000 0.9065 0.5454 0.8845 0.0000 0.0000 0.0000 0.0000
1.2208 0.4835 0.0000 2.1046 0.0000 0.9361 0.0000 0.0000
0.6215 0.0000 2.0000 1.1918 2.2315 0.0000 1.2610 0.0000
1.0358 2.8695 0.0000 1.3885 0.4700 0.0000 1.2272 0.0000
0.7843 0.0000 0.8821 0.7024 0.0000 0.9511 0.0000 0.0000
0.0000 0.0000 3.8160 0.5216 0.5056 0.6010 0.5748 0.6506
0.5424 0.8022 0.5418 1.1308 0.0000 0.0000 0.7381 0.8737

| | | | | | | | |
|---|---|---|---|---|---|---|---|
| 0.0000 | 0.0000 | 1.3242 | 0.0000 | 0.0000 | 0.9683 | 0.3855 | 0.0000 |
| 1.4969 | 0.0000 | 0.0000 | 0.5821 | 0.7182 | 0.4418 | 0.0000 | 0.0000 |
| 1.1906 | 0.0000 | 0.0000 | 0.0000 | 0.5290 | 0.0000 | 0.0000 | 0.0000 |
| 0.8396 | 2.3027 | 0.0000 | 3.9773 | 2.4311 | 0.0000 | 0.0000 | 0.0000 |
| 0.0000 | 1.5843 | 0.0000 | 0.5811 | 0.0000 | 0.0000 | 0.0000 | 0.0000 |
| 0.0000 | 0.5674 | 0.0000 | 0.4985 | 0.0000 | 1.0320 | 0.0000 | 0.3736 |
| 0.5077 | 0.0000\ | | | | | | |
| TMOD2 | 6.4330 | 8.3586 | 7.3304 | 7.8672 | 4.8043 | 6.3842 | 4.8521 |
| 6.2812 | 4.2086 | 7.2977 | 5.0688 | 5.5893 | 5.4504 | 4.7923 | 5.2323 |
| 5.2804 | 5.4984 | 4.7766 | 8.1464 | 3.9605 | 6.4252 | 5.6030 | 7.6940 |
| 5.7850 | 8.4125 | 7.2120 | 6.3649 | 5.3074 | 6.7367 | 4.7428 | 6.7144 |
| 5.0794 | 5.9045 | 6.3084 | 3.2487 | 3.9544 | 6.7686 | 6.3292 | 5.3429 |
| 5.1442 | 7.1147 | 8.1586 | 4.6306 | 5.1825 | 9.6916 | 5.1053 | 1.5457 |
| 6.1674 | 8.8051 | 5.1470 | 7.9550 | 6.8381 | 6.8417 | 9.7317 | 7.8694 |
| 5.2856 | 5.9863 | 8.0275 | 7.0627 | 4.9150 | 9.6959 | 8.2225 | 5.5609 |
| 6.3567 | 8.0262 | 6.4807 | 6.9513 | 4.9277 | 5.7849 | 6.2397 | 5.6148 |
| 6.6839 | 5.7226 | 6.0424 | 7.7576 | 3.6494 | 7.6017 | 5.5938 | 5.9171 |
| 7.2651 | 5.3011 | 5.5890 | 7.5207 | 5.6160 | 5.4925 | 5.6396 | 8.9341 |
| 5.0370 | 7.4016 | 9.4922 | 9.8622 | 6.1556 | 6.3104 | 6.4631 | 4.2968 |
| 8.5827 | 5.8864 | 8.5699 | 6.1020 | 6.0850 | 3.2267 | 5.8573 | 7.5289 |
| 6.2639 | 4.3297 | 5.0118 | 7.0910 | 6.2600 | 6.3784 | 4.8658 | 5.4932 |
| 3.6834 | 7.0898 | 5.2479 | 7.1611 | 7.1549 | 4.8489 | 7.0627 | 5.2151 |
| 5.5769 | 7.1422 | 6.2870 | 6.6474 | 4.7648 | 8.3910 | 5.9494 | 5.1298 |
| 3.0394 | 8.7770 | 9.3924 | 7.5989 | 4.2681 | 5.2953 | 5.6034 | 7.4591 |
| 5.7157 | 4.5127 | 6.1705 | 8.4307 | 8.5809 | 3.6287 | 5.6138 | 5.1370 |
| 6.6179 | 5.4640 | 9.4214 | 6.4073 | 8.9279 | 7.3380 | 5.7488 | 8.5734 |
| 3.1371 | 6.9954 | 6.7112 | 7.8309 | 7.0296 | 8.6381 | 5.3182 | 4.2903 |
| 5.2953 | 3.8658 | 5.8647 | 5.8862 | 5.0968 | 4.6648 | 4.8323 | 4.5584 |
| 7.8590 | 5.3007 | 5.6521 | 6.1868 | 7.9905 | 6.1965 | 6.6796 | 5.4645 |
| 8.2803 | 7.6143 | 10.1213 | 6.3986 | 6.8483 | 4.6651 | 6.6406 | 5.1939 |
| 6.5376 | 7.3942 | 8.4802 | 6.2175 | 4.3617 | 5.0814 | 6.8397 | 6.3427 |
| 4.8438 | 7.7926 | 6.2333 | 5.2218 | 4.2617 | 5.2906 | 8.3739 | 5.9587 |
| 6.3497 | 2.8479\ | | | | | | |
| MFSD11 | 8.0544 | 8.3622 | 9.5112 | 8.7599 | 8.9977 | 8.3628 | 8.4342 |
| 9.1224 | 7.8992 | 8.6577 | 8.9102 | 8.2138 | 8.8401 | 9.7819 | 8.4132 |
| 8.7801 | 8.1767 | 9.0842 | 8.2164 | 7.9270 | 8.4397 | 8.4623 | 8.2093 |
| 8.7236 | 8.2026 | 8.4947 | 6.7499 | 7.9286 | 8.1279 | 7.9694 | 8.0778 |
| 8.3137 | 8.7860 | 8.3978 | 8.4301 | 8.7985 | 8.6878 | 8.8419 | 9.0693 |
| 8.8159 | 8.9048 | 8.5771 | 8.2808 | 8.4240 | 8.9216 | 8.1047 | 8.8606 |
| 8.0758 | 8.2698 | 9.2511 | 8.9633 | 8.1845 | 9.9167 | 9.0156 | 10.3012 |
| 7.8253 | 7.9733 | 9.9375 | 8.6394 | 8.1863 | 8.9065 | 8.3614 | 8.8092 |
| 7.9299 | 8.4172 | 8.5170 | 8.2966 | 9.1941 | 10.0619 | 9.1576 | 9.3021 |
| 8.5809 | 8.4224 | 8.7526 | 8.8056 | 9.5646 | 8.2193 | 8.2276 | 8.0605 |
| 8.6256 | 9.4566 | 8.4428 | 8.4914 | 9.0762 | 7.9677 | 9.3270 | 8.7852 |
| 7.9850 | 7.9094 | 9.0127 | 8.3590 | 8.6636 | 8.5093 | 8.4729 | 7.9308 |
| 8.9825 | 9.1574 | 8.4933 | 7.7786 | 8.2069 | 8.3562 | 8.6057 | 8.6325 |
| 9.3747 | 9.2680 | 8.4973 | 7.9531 | 8.7659 | 8.6733 | 8.5132 | 8.2392 |
| 8.2550 | 8.7332 | 6.9696 | 8.8278 | 7.9411 | 8.4193 | 9.3179 | 9.1106 |
| 8.0369 | 9.1707 | 8.5608 | 9.7260 | 9.1356 | 8.3086 | 9.4232 | 8.3425 |
| 8.0252 | 8.7680 | 8.5956 | 8.6964 | 8.7367 | 8.3884 | 9.2178 | 7.3721 |
| 8.8637 | 8.2973 | 11.0566 | 8.4055 | 8.7273 | 8.1586 | 8.5626 | 8.4735 |
| 9.0386 | 9.0731 | 8.0172 | 8.4685 | 8.5659 | 8.3684 | 8.6215 | 8.8015 |
| 8.5030 | 8.5858 | 8.8666 | 8.4141 | 8.4953 | 8.5910 | 8.9041 | 8.8824 |
| 8.6338 | 8.3418 | 8.3073 | 8.2927 | 8.7409 | 7.9734 | 8.2368 | 7.3655 |

| | | | | | | | |
|---|---|---|---|---|---|---|---|
| 8.9431 | 8.2551 | 8.7932 | 8.3244 | 8.7966 | 8.8101 | 8.9993 | 11.1005 |
| 8.6352 | 8.1005 | 8.5424 | 8.6555 | 8.3597 | 8.2445 | 8.5444 | 8.6392 |
| 8.3711 | 8.5409 | 8.4725 | 8.0701 | 8.5079 | 8.4359 | 8.8013 | 8.9906 |
| 8.9609 | 7.3892 | 8.7254 | 8.3631 | 8.1720 | 7.6377 | 8.4549 | 8.3170 |
| 8.5319 | 7.0662\ | | | | | | |
| NSUN2 | 10.2061 | 10.0671 | 10.7154 | 10.3294 | 10.2908 | 11.0352 | 10.4871 |
| 10.5847 | 10.7235 | 10.2057 | 10.1399 | 10.7932 | 10.7269 | 10.4884 | 10.2128 |
| 10.1683 | 10.5024 | 10.5299 | 9.9443 | 10.7470 | 10.6413 | 10.3598 | 10.1985 |
| 10.4828 | 10.2242 | 10.7756 | 11.9391 | 10.1051 | 10.4497 | 10.6388 | 10.0552 |
| 10.6298 | 10.8510 | 10.5304 | 11.6085 | 10.9916 | 10.5442 | 10.4636 | 10.7608 |
| 9.7090 | 10.7007 | 10.4854 | 10.4948 | 11.1113 | 9.8763 | 10.0359 | 9.0726 |
| 11.1249 | 11.2106 | 10.2178 | 10.7571 | 10.2560 | 10.0669 | 9.9757 | 10.1849 |
| 10.5588 | 10.6209 | 10.0795 | 10.6193 | 10.1750 | 10.1091 | 10.4006 | 11.2571 |
| 12.3660 | 10.1210 | 10.3589 | 10.4728 | 11.4105 | 10.4242 | 10.9630 | 10.6043 |
| 10.5478 | 10.1261 | 10.7243 | 10.7623 | 10.0825 | 10.7576 | 10.0543 | 10.3968 |
| 10.9370 | 10.1824 | 11.5827 | 9.9802 | 10.5362 | 9.9164 | 11.3379 | 10.2107 |
| 10.2326 | 10.5588 | 10.4581 | 10.0013 | 10.6983 | 10.2751 | 10.1930 | 10.4370 |
| 9.9239 | 11.1445 | 9.9447 | 10.1493 | 10.3871 | 10.1728 | 10.5420 | 10.6942 |
| 10.3066 | 10.2856 | 11.2769 | 10.2207 | 10.2827 | 10.4981 | 10.2339 | 10.3053 |
| 10.5776 | 9.5344 | 11.2390 | 11.8141 | 10.8252 | 10.2349 | 10.3025 | 10.2270 |
| 10.1966 | 11.4047 | 10.5400 | 10.8508 | 10.9406 | 10.2188 | 10.8705 | 10.7345 |
| 9.3670 | 9.9136 | 10.3208 | 10.4535 | 10.0562 | 10.3513 | 10.6692 | 11.0979 |
| 9.6495 | 10.3920 | 8.7387 | 10.6632 | 10.0445 | 10.5539 | 10.3771 | 9.6250 |
| 10.6179 | 10.7534 | 9.9857 | 10.4465 | 9.9107 | 10.8292 | 9.7793 | 10.1347 |
| 8.6461 | 10.5167 | 10.9843 | 10.9318 | 10.7134 | 9.9912 | 10.4402 | 10.8624 |
| 10.7607 | 10.3785 | 10.6020 | 10.1977 | 10.0809 | 10.8378 | 10.6654 | 9.8696 |
| 10.7936 | 10.2996 | 10.3022 | 10.9536 | 10.4757 | 10.4238 | 10.4612 | 9.0798 |
| 10.3109 | 10.3982 | 9.9776 | 10.7682 | 9.9861 | 10.4822 | 11.5072 | 11.6060 |
| 10.4092 | 10.6251 | 10.3559 | 11.3921 | 10.4360 | 10.4091 | 11.2211 | 10.7760 |
| 10.4332 | 10.6573 | 11.6180 | 10.4691 | 10.3148 | 10.8208 | 10.0609 | 10.4668 |
| 10.4805 | 9.9310\ | | | | | | |
| NSUN5 | 10.5290 | 9.1455 | 9.6049 | 9.7536 | 10.5860 | 9.8201 | 9.5745 |
| 10.3826 | 9.1692 | 9.6077 | 9.7925 | 10.3077 | 10.1442 | 9.5751 | 9.2461 |
| 9.9038 | 10.0822 | 10.5999 | 9.2516 | 10.7547 | 10.9298 | 9.5284 | 9.8154 |
| 8.9585 | 9.5109 | 10.9159 | 11.1609 | 10.4069 | 9.8128 | 10.5118 | 9.7188 |
| 10.6495 | 9.6474 | 10.6762 | 10.1746 | 10.6888 | 9.6362 | 9.8792 | 9.7213 |
| 8.7722 | 10.1132 | 8.9313 | 10.3813 | 10.3485 | 8.5025 | 10.2964 | 11.6907 |
| 9.9342 | 9.2055 | 10.2145 | 10.1333 | 10.2493 | 10.1963 | 8.6377 | 9.9761 |
| 9.5863 | 10.1929 | 9.7897 | 9.5457 | 9.3551 | 8.5992 | 9.7210 | 10.5134 |
| 10.1134 | 9.2045 | 9.4454 | 9.5808 | 9.6955 | 9.8689 | 10.1606 | 11.1485 |
| 9.4715 | 10.9638 | 10.4290 | 9.3571 | 10.2368 | 9.5409 | 9.6182 | 9.7116 |
| 9.7551 | 10.0888 | 9.6157 | 9.1555 | 10.1523 | 10.1595 | 10.0697 | 9.9152 |
| 10.6006 | 9.8796 | 9.4564 | 9.1280 | 9.7019 | 10.6638 | 9.8871 | 10.2005 |
| 9.0191 | 8.7842 | 9.1507 | 10.0288 | 9.8074 | 10.6695 | 9.6391 | 9.6962 |
| 9.8924 | 10.0589 | 11.2943 | 9.6387 | 9.0977 | 10.0126 | 10.5228 | 9.8666 |
| 9.5775 | 12.2550 | 11.6466 | 8.8599 | 9.4623 | 9.3227 | 9.6649 | 10.3191 |
| 10.7558 | 10.1299 | 9.6174 | 9.7504 | 9.6216 | 9.4409 | 9.9484 | 9.4630 |
| 11.3893 | 8.8141 | 8.7667 | 9.8269 | 9.9285 | 9.6655 | 9.2730 | 11.1366 |
| 11.9397 | 10.8163 | 10.4977 | 9.1729 | 9.4237 | 10.1002 | 9.5618 | 10.2725 |
| 9.4545 | 9.5586 | 9.0280 | 9.0910 | 9.6295 | 9.7508 | 10.6732 | 9.6295 |
| 11.5214 | 8.9674 | 10.3048 | 10.1693 | 10.1564 | 9.3164 | 9.0134 | 10.4219 |
| 10.5458 | 10.4088 | 10.4566 | 9.5304 | 10.2548 | 10.1145 | 8.7171 | 9.7661 |
| 9.5623 | 9.6804 | 10.4714 | 9.9364 | 9.9950 | 12.1054 | 10.1129 | 10.5253 |
| 9.6461 | 9.4954 | 9.0373 | 9.9469 | 9.4849 | 10.8717 | 10.3529 | 10.7125 |

| | | | | | | | |
|---|---|---|---|---|---|---|---|
| 9.8156 | 9.3734 | 9.6191 | 9.6967 | 10.6674 | 10.3491 | 10.2380 | 9.4170 |
| 9.2611 | 10.4181 | 10.1560 | 9.7459 | 11.8126 | 11.0183 | 9.6536 | 9.2830 |
| 9.2133 | 11.6583\ | | | | | | |
| NSUN4 | 9.6002 | 8.6098 | 9.8782 | 9.3441 | 9.7008 | 9.9753 | 7.4599 |
| 10.0728 | 9.7917 | 9.0394 | 9.6392 | 9.4744 | 9.6711 | 9.2635 | 9.4236 |
| 8.9761 | 8.5840 | 9.3983 | 8.6021 | 9.7214 | 9.7091 | 9.1872 | 8.6750 |
| 9.6789 | 8.4145 | 9.3081 | 8.6220 | 9.1709 | 8.9547 | 9.2089 | 9.0444 |
| 9.3189 | 9.6520 | 10.1052 | 9.2793 | 9.8848 | 9.0237 | 9.3571 | 9.1793 |
| 9.7259 | 8.9259 | 9.3101 | 9.3818 | 10.4128 | 8.5551 | 9.3653 | 9.5670 |
| 10.1016 | 9.3963 | 9.4016 | 9.2803 | 8.6377 | 8.8991 | 8.9766 | 8.4389 |
| 9.1358 | 9.9129 | 9.6196 | 9.2877 | 9.4302 | 8.8594 | 9.7625 | 9.3738 |
| 9.3503 | 8.7447 | 9.0454 | 8.8052 | 9.2517 | 9.0378 | 9.5402 | 9.9576 |
| 9.7723 | 8.8304 | 10.2345 | 9.1392 | 8.8902 | 8.7218 | 8.8162 | 9.1348 |
| 9.1144 | 9.3675 | 9.3942 | 8.4273 | 9.1811 | 9.1088 | 9.7382 | 9.6362 |
| 9.9214 | 9.5313 | 8.7226 | 8.2217 | 9.5035 | 10.6525 | 8.6270 | 9.4573 |
| 8.2910 | 9.8955 | 8.6463 | 8.7310 | 9.0089 | 9.8614 | 9.5943 | 9.8339 |
| 9.3333 | 9.3642 | 9.5612 | 8.6286 | 9.4688 | 9.2584 | 9.3788 | 9.6686 |
| 9.7908 | 9.3098 | 9.2021 | 9.8517 | 9.8673 | 8.9230 | 9.3698 | 9.3825 |
| 9.8120 | 9.1777 | 9.9616 | 9.8320 | 9.7092 | 8.8072 | 9.9333 | 9.2641 |
| 9.5602 | 8.8428 | 8.9525 | 10.1623 | 9.1835 | 9.4543 | 9.2088 | 9.4988 |
| 8.7177 | 9.5023 | 9.3640 | 9.5873 | 8.7813 | 9.0906 | 9.3258 | 9.3072 |
| 9.2447 | 9.3124 | 8.7218 | 9.0783 | 8.8014 | 9.2419 | 9.1535 | 9.1096 |
| 9.8518 | 9.6902 | 9.5411 | 9.5053 | 9.2533 | 8.8593 | 9.2846 | 9.0999 |
| 8.8801 | 9.3858 | 9.3632 | 9.3932 | 8.9087 | 8.2194 | 9.9034 | 9.4064 |
| 10.2692 | 9.6022 | 10.2513 | 10.1575 | 8.6911 | 9.2892 | 8.7706 | 9.5850 |
| 9.2162 | 8.9071 | 8.5841 | 9.6709 | 8.5400 | 9.5343 | 10.1481 | 9.5883 |
| 9.4013 | 9.8229 | 9.3558 | 9.1191 | 9.9127 | 9.6479 | 9.3756 | 9.4763 |
| 8.9060 | 8.9694 | 9.1010 | 9.2935 | 10.5853 | 9.0562 | 8.9194 | 9.5532 |
| 9.4844 | 9.6351\ | | | | | | |
| NSUN7 | 8.2092 | 6.8303 | 6.8982 | 8.8140 | 6.0903 | 8.9135 | 7.9099 |
| 8.3315 | 8.0861 | 7.9239 | 7.4447 | 8.7605 | 8.9838 | 7.5787 | 9.0250 |
| 8.4948 | 7.9505 | 7.7004 | 6.4229 | 6.8698 | 7.6572 | 9.5049 | 8.9607 |
| 8.6222 | 6.9441 | 3.3041 | 8.2672 | 7.6599 | 6.4208 | 8.1108 | 5.4396 |
| 7.3915 | 8.1404 | 8.9105 | 8.3228 | 5.3533 | 8.2070 | 7.4253 | 7.0646 |
| 8.7872 | 5.7623 | 7.1535 | 5.7678 | 7.7598 | 4.6932 | 7.5265 | 7.9390 |
| 7.5317 | 2.9595 | 5.3124 | 6.8636 | 6.0108 | 7.2977 | 6.3998 | 7.4788 |
| 5.1377 | 6.0112 | 7.4750 | 8.0253 | 8.2496 | 5.1001 | 8.0408 | 6.7169 |
| 8.1592 | 8.1358 | 1.9254 | 8.1393 | 8.4440 | 7.2673 | 8.7757 | 6.8571 |
| 7.5647 | 5.6178 | 8.0106 | 7.8675 | 7.5042 | 8.6102 | 9.2121 | 8.5983 |
| 7.5039 | 7.8097 | 6.7831 | 9.0467 | 8.8843 | 8.5715 | 7.9319 | 6.5340 |
| 7.8533 | 6.1437 | 7.5626 | 5.5542 | 8.2951 | 7.8011 | 8.2702 | 7.1889 |
| 4.7315 | 7.4149 | 7.5042 | 9.2320 | 1.5150 | 7.3157 | 7.9096 | 9.0681 |
| 7.9990 | 8.3863 | 8.4662 | 7.3049 | 8.7284 | 8.1550 | 9.1718 | 7.6333 |
| 7.6143 | 5.0200 | 2.3219 | 5.9691 | 7.8096 | 8.9758 | 8.9464 | 7.2137 |
| 4.2763 | 7.1038 | 6.7114 | 7.0915 | 7.6693 | 9.4356 | 8.0987 | 7.5180 |
| 6.9848 | 7.2017 | 7.2349 | 8.3455 | 8.9847 | 8.5100 | 9.1042 | 6.5625 |
| 5.2246 | 5.0153 | 6.9800 | 7.6628 | 7.3598 | 8.5394 | 6.6278 | 9.0616 |
| 6.8582 | 6.5598 | 7.2029 | 7.6840 | 7.3090 | 6.9608 | 8.2397 | 7.2989 |
| 5.9178 | 6.9903 | 7.4047 | 8.0503 | 8.3851 | 7.5728 | 7.5950 | 8.9349 |
| 6.4522 | 1.6272 | 7.6080 | 8.4049 | 8.0785 | 8.3003 | 8.5540 | 8.3566 |
| 8.1999 | 8.0787 | 7.9880 | 6.5489 | 7.4877 | 8.9882 | 8.3490 | 5.1047 |
| 7.5885 | 7.5981 | 6.5707 | 7.6049 | 10.0710 | 6.6954 | 7.6901 | 8.6787 |
| 8.2156 | 8.0883 | 7.4994 | 8.3701 | 7.6098 | 6.9039 | 6.5345 | 6.2092 |
| 5.9558 | 7.1664 | 6.7624 | 6.4608 | 6.8088 | 8.4317 | 7.5806 | 8.0172 |

| | | | | | | | |
|---|---|---|---|---|---|---|---|
| 9.2989 | 5.6959\ | | | | | | |
| NSUN6 | 7.8048 | 7.7749 | 7.8313 | 7.4923 | 8.0408 | 7.9576 | 7.1842 |
| 8.0447 | 7.6715 | 8.3305 | 7.8367 | 7.1980 | 7.8663 | 8.5682 | 7.4690 |
| 9.4221 | 8.1635 | 8.0465 | 7.6901 | 6.7829 | 7.5169 | 7.9224 | 8.0830 |
| 8.8346 | 7.5844 | 6.7555 | 7.0183 | 7.4968 | 6.9956 | 7.3099 | 7.4748 |
| 8.1875 | 8.5575 | 8.4076 | 7.2724 | 7.6554 | 8.2544 | 7.7153 | 7.8785 |
| 8.0952 | 6.6765 | 7.8450 | 6.8386 | 8.0421 | 7.2990 | 7.9624 | 7.0874 |
| 8.1249 | 8.2372 | 7.2333 | 7.8436 | 7.3479 | 8.0733 | 8.0070 | 8.3324 |
| 7.8513 | 7.0308 | 7.0938 | 7.7034 | 7.7670 | 7.3801 | 8.2866 | 7.1685 |
| 8.7058 | 8.6059 | 7.6439 | 7.6422 | 8.3748 | 7.5845 | 7.6568 | 8.3022 |
| 7.6746 | 6.6831 | 8.2494 | 7.7726 | 8.1572 | 7.7600 | 7.7915 | 8.4368 |
| 7.6564 | 7.2140 | 8.2071 | 8.0227 | 7.3895 | 8.4713 | 8.3238 | 8.3632 |
| 6.8726 | 8.0123 | 7.5286 | 6.9282 | 8.0989 | 8.1585 | 9.3530 | 7.9483 |
| 6.6201 | 7.0700 | 7.7113 | 7.7628 | 8.8157 | 7.4505 | 7.6820 | 7.9037 |
| 8.8034 | 7.9807 | 8.0024 | 9.1005 | 7.5559 | 8.5259 | 7.8815 | 8.6467 |
| 7.5862 | 6.2569 | 7.5622 | 8.8358 | 10.2746 | 8.0621 | 8.0983 | 7.6170 |
| 7.1891 | 8.4458 | 7.9269 | 7.4216 | 7.0437 | 8.6687 | 7.8531 | 8.1538 |
| 7.1625 | 7.6444 | 8.2582 | 7.8312 | 7.4547 | 7.6659 | 8.3305 | 7.3895 |
| 6.0975 | 7.8308 | 9.0032 | 8.1234 | 7.9381 | 8.6567 | 7.4970 | 8.3478 |
| 7.3449 | 7.7546 | 8.4042 | 7.4780 | 7.4969 | 7.2747 | 6.0974 | 8.1831 |
| 6.4712 | 7.6859 | 7.9434 | 7.9022 | 7.5135 | 7.5211 | 8.1451 | 8.4196 |
| 6.8781 | 7.2122 | 7.7462 | 8.3019 | 8.5892 | 7.9004 | 8.0365 | 7.6469 |
| 7.6856 | 7.8848 | 7.7402 | 7.8411 | 8.3193 | 7.7162 | 7.9146 | 6.8818 |
| 7.7401 | 9.1727 | 7.7469 | 8.1340 | 7.9258 | 7.8049 | 8.3412 | 8.6772 |
| 8.2656 | 8.5651 | 7.7194 | 8.3328 | 8.1545 | 7.7080 | 7.3492 | 7.6779 |
| 7.3494 | 8.1245 | 7.8142 | 8.1566 | 7.1124 | 6.9877 | 7.8284 | 7.5646 |
| 8.3175 | 6.6555\ | | | | | | |
| PRAMEF4 | 0.5526 | 0.0000 | 0.0000 | 0.0000 | 0.0000 | 0.0000 | 0.0000 |
| 0.0000 | 0.0000 | 0.0000 | 0.0000 | 0.8472 | 0.0000 | 0.0000 | 0.0000 |
| 0.0000 | 1.0553 | 0.0000 | 0.0000 | 0.0000 | 2.3988 | 0.0000 | 0.0000 |
| 0.0000 | 0.0000 | 0.6062 | 0.0000 | 0.0000 | 0.0000 | 0.0000 | 0.0000 |
| 0.0000 | 0.5159 | 0.0000 | 1.3224 | 0.0000 | 0.0000 | 0.0000 | 0.0000 |
| 0.0000 | 0.0000 | 0.0000 | 0.0000 | 0.0000 | 0.0000 | 0.0000 | 0.0000 |
| 0.6741 | 4.9692 | 0.0000 | 0.0000 | 0.0000 | 0.0000 | 0.5094 | 0.0000 |
| 0.9635 | 0.0000 | 0.0000 | 0.4252 | 3.7174 | 0.0000 | 0.0000 | 0.0000 |
| 0.0000 | 0.0000 | 0.0000 | 0.0000 | 0.4832 | 0.0000 | 0.0000 | 4.7130 |
| 0.0000 | 0.0000 | 0.0000 | 0.0000 | 0.0000 | 0.5548 | 0.0000 | 0.0000 |
| 0.0000 | 0.0000 | 0.0000 | 0.0000 | 0.0000 | 0.0000 | 1.0522 | 0.0000 |
| 0.0000 | 0.0000 | 0.0000 | 0.0000 | 0.0000 | 0.9036 | 0.0000 | 0.0000 |
| 0.0000 | 3.7740 | 0.0000 | 0.0000 | 0.0000 | 0.0000 | 0.0000 | 0.0000 |
| 0.0000 | 0.0000 | 0.0000 | 1.5756 | 0.0000 | 0.0000 | 0.0000 | 0.0000 |
| 1.3871 | 0.0000 | 0.0000 | 0.0000 | 0.0000 | 1.6815 | 1.2610 | 0.0000 |
| 0.0000 | 0.0000 | 0.0000 | 0.0000 | 0.0000 | 0.0000 | 2.0458 | 0.0000 |
| 3.4738 | 0.0000 | 0.0000 | 0.0000 | 0.0000 | 0.0000 | 0.7571 | 0.8107 |
| 0.0000 | 1.9254 | 0.0000 | 0.0000 | 0.0000 | 0.6010 | 0.0000 | 0.0000 |
| 0.0000 | 0.0000 | 0.0000 | 0.0000 | 0.0000 | 0.0000 | 0.7381 | 0.0000 |
| 0.0000 | 0.0000 | 1.8101 | 0.0000 | 0.0000 | 0.0000 | 0.0000 | 0.0000 |
| 0.0000 | 0.0000 | 1.8685 | 0.0000 | 0.0000 | 0.0000 | 0.0000 | 0.0000 |
| 0.0000 | 0.5276 | 0.9420 | 0.0000 | 7.1570 | 0.0000 | 0.0000 | 0.0000 |
| 0.0000 | 0.0000 | 0.0000 | 2.9511 | 0.0000 | 0.0000 | 3.3509 | 3.8705 |
| 0.0000 | 0.0000 | 0.0000 | 0.0000 | 1.8171 | 0.0000 | 0.9785 | 0.0000 |
| 0.0000 | 0.5674 | 0.0000 | 0.0000 | 1.5946 | 0.0000 | 0.0000 | 0.0000 |
| 0.5077 | 0.0000\ | | | | | | |
| PRAMEF5 | 0.0000 | 0.0000 | 0.0000 | 0.0000 | 0.0000 | 0.0000 | 0.0000 |

0.0000 0.0000 0.0000 0.0000 0.0000 0.0000 0.0000 0.0000
0.0000 0.0000 0.0000 0.0000 0.0000 1.8761 0.0000 0.0000
0.0000 0.0000 0.0000 0.0000 0.0000 0.0000 0.0000 0.0000
0.0000 0.0000 0.0000 0.0000 0.0000 0.0000 0.0000 0.0000
0.0000 0.0000 0.0000 0.0000 0.0000 0.0000 0.0000 0.0000
0.0000 1.8062 0.0000 0.0000 0.0000 0.0000 0.0000 0.0000
0.0000 0.0000 0.0000 0.0000 0.0000 0.0000 0.0000 0.0000
0.0000 0.0000 0.0000 0.0000 0.0000 0.0000 0.0000 3.2868
0.0000 0.0000 0.0000 0.0000 0.0000 0.0000 0.0000 0.0000
0.0000 0.0000 0.0000 0.0000 0.0000 0.0000 0.0000 0.0000
0.0000 0.0000 0.0000 0.0000 0.0000 0.0000 0.0000 0.0000
0.0000 2.5385 0.0000 0.0000 0.0000 0.0000 0.4986 0.0000
0.0000 0.0000 0.0000 0.0000 0.0000 0.0000 0.0000 0.0000
0.0000 0.0000 0.0000 0.0000 0.0000 0.5276 0.0000 0.0000
0.0000 0.0000 0.0000 0.0000 0.0000 0.0000 0.0000 0.0000
1.2894 0.0000 0.0000 0.3935 0.0000 0.0000 0.0000 0.0000
0.0000 2.2980 0.0000 0.0000 0.0000 0.0000 0.0000 0.0000
0.0000 0.0000 0.0000 0.0000 0.0000 0.0000 0.0000 0.0000
0.0000 0.0000 0.5862 0.0000 0.0000 0.0000 0.0000 0.0000
0.0000 0.0000 1.0833 0.0000 0.0000 0.0000 0.0000 0.0000
0.0000 0.0000 0.0000 0.0000 4.2127 0.0000 0.0000 0.0000
0.0000 0.0000 0.0000 0.4012 0.0000 0.0000 1.5761 3.4738
0.0000 0.0000 0.0000 0.0000 0.0000 0.0000 0.0000 0.0000
0.0000 0.0000 0.0000 0.0000 0.0000 0.0000 0.0000 0.0000
0.0000 0.0000\
PRAMEF6 0.0000 0.0000 0.0000 0.0000 0.0000 0.0000 0.0000
0.0000 0.0000 0.0000 0.0000 1.1366 0.0000 0.0000 0.0000
0.0000 0.0000 0.0000 0.0000 0.0000 2.8895 0.0000 0.0000
0.0000 0.0000 0.6062 0.0000 0.0000 0.0000 1.0077 0.0000
0.0000 0.0000 0.0000 0.0000 0.0000 0.0000 0.0000 0.9336
0.0000 0.0000 0.0000 0.0000 0.0000 0.0000 0.0000 0.0000
0.0000 4.0304 0.0000 0.0000 0.0000 0.0000 0.0000 0.0000
1.9450 0.0000 0.0000 0.4252 0.0000 0.0000 0.0000 0.0000
0.0000 0.0000 0.0000 0.0000 0.4832 0.0000 0.0000 4.1834
0.0000 0.0000 0.0000 0.0000 0.0000 0.5548 0.0000 0.0000
0.0000 0.0000 0.0000 0.0000 0.0000 0.0000 0.0000 0.5376
0.0000 0.0000 1.2149 0.0000 0.0000 0.9036 0.0000 0.0000
0.0000 3.5228 0.0000 0.0000 0.5504 0.0000 0.0000 0.0000
0.0000 0.0000 0.0000 0.0000 0.0000 0.0000 0.0000 0.0000
1.0545 0.0000 0.0000 0.0000 0.0000 1.6815 0.0000 0.0000
0.0000 0.0000 0.0000 0.0000 0.0000 0.0000 0.5331 0.0000
1.2894 0.0000 0.0000 0.3935 0.0000 0.0000 0.0000 0.0000
0.0000 1.0837 0.0000 0.0000 0.0000 0.0000 0.0000 0.0000
0.0000 0.0000 0.0000 0.0000 0.0000 0.0000 0.0000 0.0000
0.0000 0.0000 0.0000 0.0000 0.0000 0.0000 0.6894 0.6000
0.0000 0.0000 2.6426 0.0000 0.0000 0.0000 0.0000 0.0000
0.0000 0.0000 0.0000 0.0000 5.5344 0.0000 0.0000 0.0000
0.0000 0.0000 0.0000 1.1907 0.0000 0.0000 3.1431 3.6857
0.0000 0.0000 0.0000 0.0000 0.0000 0.0000 1.9676 0.0000
0.0000 0.0000 0.0000 0.0000 0.0000 0.0000 0.0000 0.0000
1.1797 0.0000\
TUBG2 8.3623 8.5267 6.8063 7.8080 7.8770 7.1399 6.0322
7.1545 8.7453 8.6109 6.8495 8.0173 7.6826 8.2123 7.1719
8.1195 9.6093 8.6769 8.7548 9.0293 8.1878 7.6704 8.2702

```
7.2476  8.9130  8.9329  9.0490  8.0500  8.0527  6.6300  7.9834
8.2743  7.4522  7.9451  7.5047  8.7175  8.6088  7.8273  8.3197
7.1242  7.8413  8.3269  8.5770  8.4696  9.0428  7.9467  7.4032
7.7708  8.1126  8.3477  7.4551  8.6454  8.0914  9.2737  8.3050
7.7403  7.5261  8.4120  8.2757  8.1617  8.8636  7.3451  7.8315
8.7766  8.8450  8.3744  8.2481  7.6715  7.2252  7.8917  8.5257
8.0994  7.7273  7.4599  7.3862  8.5865  7.9361  8.0159  8.4992
8.0591  7.2779  6.5643  8.0617  7.3585  8.3285  9.1094  9.7381
7.4203  7.7271  8.9126  9.3561  7.4423  7.8372  8.9722  8.5083
9.1533  8.1041  8.8949  7.8070  8.5324  8.3262  7.8437  7.8201
8.5606  6.5073  8.0364  9.4002  7.6788  7.7462  8.2842  7.1289
7.4987  7.3759  8.5038  8.2382  7.4225  7.2141  8.6802  8.2057
8.1841  8.6960  7.5846  7.4707  7.2872  9.0747  6.5125  7.1098
7.1913  8.8899  8.6075  7.9291  6.6523  7.8018  7.7753  7.6554
8.2420  8.0262  8.8941  8.1324  8.9622  7.0015  7.7409  7.9393
7.5021  7.0104  9.2836  7.3937  9.5010  8.8094  7.5903  8.6276
7.1629  7.2410  8.4917  8.0503  7.4910  8.7893  7.5533  6.3661
8.9281  7.2020  8.2139  7.7694  8.2610  7.6403  7.5418  6.8488
7.6307  7.9403  7.6636  7.3863  8.6567  10.0389 8.2704  7.8927
8.5578  8.9863  9.4732  7.9367  8.1516  7.8139  6.5107  6.7757
8.0102  7.3770  7.9676  9.0137  9.1615  7.9083  8.1267  7.2200
8.1677  8.5357  8.3613  7.7742  7.1333  7.0054  9.1764  7.4001
7.6625  7.7390\
FAM71F1 1.5194  0.5319  2.4032  0.4935  1.6818  2.7056  0.0000
0.0000  1.2625  1.2815  3.1647  0.0000  0.7708  1.6493  2.6827
2.9791  4.8596  1.6514  0.5838  1.3142  2.0721  1.7571  0.6346
2.0289  0.9530  2.0473  5.0594  3.6823  2.8976  1.0077  0.7334
1.2789  0.0000  8.1275  1.8080  1.1431  3.3777  4.7491  1.8992
0.0000  4.1205  0.8506  0.0000  1.0349  0.0000  2.8725  0.0000
1.4786  1.6510  4.4931  1.6840  1.2789  1.0956  0.0000  1.5357
3.7894  1.6820  1.1710  1.2457  2.5512  0.5422  0.4969  0.5573
2.1974  0.0000  1.8022  2.6173  0.4832  0.5773  1.3773  1.0365
1.8067  0.0000  1.3638  3.2574  2.4904  0.9546  1.8986  2.2252
0.6159  0.0000  2.9927  2.4682  0.6718  2.0290  3.2615  0.9284
2.2368  0.5273  0.5265  0.0000  1.5834  0.0000  2.2282  3.6683
0.0000  1.6718  0.0000  0.5090  1.9210  1.4180  0.4986  1.6984
2.1133  0.0000  0.0000  3.6115  3.5614  1.7151  0.0000  1.9975
0.6215  3.1476  6.7004  1.9988  0.6116  0.0000  3.9695  0.9644
0.0000  2.6311  2.5942  0.6224  0.8239  0.0000  0.0000  0.6149
0.0000  0.0000  1.6358  3.4225  2.9034  1.9260  6.0715  1.3267
0.0000  0.0000  5.9263  0.5216  0.5056  2.3602  0.9848  2.9704
0.0000  2.3479  0.0000  2.9153  0.0000  3.3826  4.7929  2.4796
0.0000  2.9691  1.3242  0.5476  1.2732  0.0000  1.5047  1.6149
0.5416  2.0474  0.0000  0.9957  3.5414  0.0000  1.9692  0.0000
0.0000  3.0885  0.5466  0.7903  0.5290  2.9680  0.6229  3.8992
0.0000  1.8570  0.6293  3.1725  5.8332  3.9997  3.3509  2.1752
0.8792  1.2218  1.3929  0.9943  2.2587  2.8760  0.0000  0.0000
2.8293  1.9604  0.0000  0.0000  0.0000  1.6276  0.0000  3.1187
0.8824  1.1634\
PRAMEF1 0.0000  0.0000  0.0000  0.0000  0.0000  0.0000  0.0000
1.1171  0.0000  0.0000  0.0000  1.7643  0.0000  0.0000  0.0000
0.0000  1.8857  0.0000  0.0000  0.0000  1.0485  0.0000  0.0000
0.0000  0.0000  0.0000  0.0000  0.0000  0.0000  0.0000  0.0000
0.0000  0.0000  0.4664  0.5853  0.0000  0.0000  0.0000  0.0000
```

| | | | | | | | |
|---|---|---|---|---|---|---|---|
| 0.0000 | 0.6649 | 0.0000 | 0.0000 | 0.0000 | 0.0000 | 0.0000 | 0.0000 |
| 0.0000 | 2.0741 | 0.0000 | 0.0000 | 0.0000 | 0.0000 | 0.0000 | 0.0000 |
| 1.9450 | 0.0000 | 0.0000 | 0.0000 | 0.0000 | 0.0000 | 0.0000 | 0.0000 |
| 0.0000 | 0.0000 | 0.0000 | 0.0000 | 0.0000 | 0.0000 | 0.6160 | 0.0000 |
| 0.0000 | 0.0000 | 0.0000 | 0.0000 | 0.0000 | 0.0000 | 0.0000 | 0.0000 |
| 0.6159 | 0.0000 | 0.0000 | 0.0000 | 0.0000 | 0.0000 | 1.0522 | 0.0000 |
| 0.0000 | 0.0000 | 0.0000 | 0.0000 | 0.0000 | 1.2055 | 0.0000 | 0.0000 |
| 0.0000 | 3.6286 | 0.0000 | 0.0000 | 0.0000 | 0.0000 | 0.0000 | 0.0000 |
| 0.0000 | 0.0000 | 0.0000 | 0.0000 | 0.0000 | 0.0000 | 0.0000 | 0.0000 |
| 0.6215 | 0.0000 | 0.0000 | 0.5141 | 0.6116 | 1.8676 | 3.6464 | 0.0000 |
| 0.0000 | 0.0000 | 0.0000 | 0.0000 | 0.0000 | 0.0000 | 0.0000 | 0.0000 |
| 0.7843 | 0.0000 | 0.0000 | 0.0000 | 0.0000 | 0.0000 | 0.0000 | 0.0000 |
| 0.0000 | 0.0000 | 0.0000 | 0.5216 | 0.0000 | 0.6010 | 0.0000 | 0.6506 |
| 0.0000 | 0.0000 | 0.0000 | 0.0000 | 0.0000 | 0.5814 | 0.0000 | 0.0000 |
| 0.0000 | 0.0000 | 1.5875 | 0.0000 | 0.4374 | 0.0000 | 0.3855 | 0.0000 |
| 0.0000 | 0.0000 | 1.0431 | 0.0000 | 0.0000 | 0.0000 | 0.0000 | 0.0000 |
| 0.0000 | 0.0000 | 0.0000 | 0.0000 | 5.6227 | 0.0000 | 0.0000 | 0.0000 |
| 0.0000 | 0.0000 | 0.0000 | 2.3697 | 0.0000 | 0.0000 | 3.2614 | 0.0000 |
| 0.0000 | 0.0000 | 0.0000 | 0.0000 | 0.0000 | 0.0000 | 1.5562 | 0.0000 |
| 0.0000 | 0.0000 | 0.0000 | 0.0000 | 0.0000 | 0.0000 | 0.0000 | 0.0000 |
| 0.5077 | 0.0000\ | | | | | | |
| PRAMEF2 | 0.0000 | 0.0000 | 0.0000 | 0.0000 | 0.0000 | 0.0000 | 0.0000 |
| 0.0000 | 0.0000 | 0.0000 | 0.0000 | 0.8472 | 0.0000 | 0.0000 | 0.0000 |
| 0.0000 | 0.6221 | 0.0000 | 0.0000 | 0.6732 | 0.0000 | 0.0000 | 0.0000 |
| 0.0000 | 0.0000 | 1.0317 | 0.0000 | 0.0000 | 0.0000 | 0.5901 | 0.0000 |
| 0.0000 | 0.5159 | 0.0000 | 0.5853 | 0.0000 | 0.5555 | 0.0000 | 0.0000 |
| 0.0000 | 0.0000 | 0.0000 | 0.0000 | 0.0000 | 0.0000 | 0.0000 | 0.0000 |
| 0.0000 | 2.2999 | 0.0000 | 0.4530 | 0.0000 | 0.0000 | 0.0000 | 0.0000 |
| 0.9635 | 0.0000 | 0.0000 | 0.7533 | 0.0000 | 0.0000 | 0.0000 | 0.0000 |
| 0.0000 | 0.0000 | 0.0000 | 0.0000 | 0.0000 | 0.0000 | 0.0000 | 0.0000 |
| 0.0000 | 0.0000 | 0.0000 | 0.0000 | 0.0000 | 0.0000 | 0.0000 | 0.0000 |
| 0.0000 | 0.0000 | 0.0000 | 0.0000 | 0.0000 | 0.0000 | 0.6199 | 0.0000 |
| 0.0000 | 0.0000 | 0.5265 | 0.0000 | 0.0000 | 0.5215 | 0.0000 | 1.2026 |
| 0.0000 | 3.7271 | 0.0000 | 0.0000 | 0.0000 | 0.0000 | 0.0000 | 0.0000 |
| 0.0000 | 0.0000 | 0.0000 | 0.0000 | 0.0000 | 0.0000 | 0.0000 | 0.0000 |
| 0.0000 | 0.0000 | 0.0000 | 0.0000 | 0.0000 | 1.2170 | 0.0000 | 0.0000 |
| 0.0000 | 0.0000 | 0.0000 | 0.0000 | 0.0000 | 0.0000 | 0.0000 | 0.0000 |
| 0.0000 | 0.0000 | 0.0000 | 0.0000 | 0.0000 | 0.0000 | 0.0000 | 0.0000 |
| 0.0000 | 0.0000 | 0.0000 | 0.0000 | 0.0000 | 0.0000 | 0.0000 | 0.0000 |
| 0.0000 | 0.0000 | 0.0000 | 0.0000 | 0.0000 | 0.9947 | 0.0000 | 0.0000 |
| 0.0000 | 0.0000 | 0.0000 | 0.0000 | 0.0000 | 0.0000 | 0.0000 | 0.0000 |
| 0.0000 | 0.0000 | 1.0431 | 0.0000 | 0.0000 | 0.0000 | 0.0000 | 0.0000 |
| 0.0000 | 0.0000 | 0.0000 | 0.0000 | 6.1582 | 0.0000 | 0.0000 | 0.0000 |
| 0.0000 | 0.0000 | 0.0000 | 0.7148 | 0.0000 | 0.0000 | 3.0638 | 0.0000 |
| 0.0000 | 0.0000 | 0.0000 | 1.3150 | 0.0000 | 0.0000 | 2.8694 | 0.0000 |
| 0.0000 | 0.0000 | 0.0000 | 0.0000 | 0.0000 | 0.0000 | 0.0000 | 0.0000 |
| 0.0000 | 0.0000\ | | | | | | |
| FAM71F2 | 1.5194 | 3.6544 | 3.1646 | 3.4850 | 2.4374 | 4.0242 | 1.2456 |
| 0.4748 | 3.5228 | 3.9364 | 3.3720 | 1.3775 | 2.7333 | 3.1432 | 3.5096 |
| 5.1466 | 3.0015 | 3.5493 | 3.4564 | 2.5253 | 4.5174 | 3.3952 | 1.4101 |
| 3.0845 | 1.9289 | 1.6270 | 6.2777 | 3.2071 | 1.7737 | 0.5901 | 3.4068 |
| 4.5453 | 0.5159 | 3.2323 | 4.2863 | 3.5700 | 3.5611 | 4.2133 | 2.0653 |
| 1.2646 | 3.9669 | 3.0408 | 2.9036 | 4.0647 | 0.0000 | 4.8582 | 0.0000 |
| 2.7982 | 0.7769 | 1.7431 | 3.1287 | 3.3607 | 2.4720 | 2.1337 | 3.7985 |

| | | | | | | | |
|---|---|---|---|---|---|---|---|
| 4.5731 | 0.6343 | 3.1582 | 1.4405 | 2.9646 | 0.9353 | 2.4655 | 2.1044 |
| 3.4242 | 4.0651 | 3.9648 | 5.7929 | 2.0643 | 4.8359 | 4.0857 | 2.2792 |
| 3.4170 | 0.9449 | 1.3638 | 4.5003 | 4.7016 | 3.0883 | 3.6286 | 2.7035 |
| 1.3771 | 1.7663 | 3.4754 | 3.2356 | 3.6170 | 3.2933 | 3.9028 | 1.8912 |
| 1.6425 | 3.7390 | 2.0295 | 2.2864 | 2.8054 | 1.8528 | 3.9765 | 2.2941 |
| 0.6266 | 2.8322 | 1.7212 | 2.1324 | 2.0879 | 1.4180 | 0.8684 | 3.1832 |
| 3.9042 | 1.1338 | 1.7170 | 5.9634 | 3.8673 | 3.7365 | 2.4661 | 3.3794 |
| 2.4082 | 3.9213 | 5.9069 | 4.4579 | 1.3697 | 0.9131 | 6.2493 | 3.5583 |
| 3.5238 | 3.0090 | 3.0097 | 3.9092 | 2.5865 | 3.2018 | 1.6938 | 1.6443 |
| 0.0000 | 0.9653 | 2.8723 | 2.9817 | 3.4618 | 3.4330 | 5.5828 | 5.2172 |
| 0.0000 | 1.6954 | 3.9958 | 3.4616 | 2.5936 | 3.5671 | 1.5646 | 1.7133 |
| 1.9025 | 3.3642 | 2.2162 | 4.5970 | 2.4839 | 2.1615 | 2.6654 | 5.3262 |
| 0.0000 | 3.5764 | 1.8101 | 2.8999 | 1.2732 | 3.3344 | 4.0812 | 2.2048 |
| 1.2429 | 1.6272 | 0.6138 | 3.2401 | 4.3240 | 5.1161 | 3.2219 | 0.0000 |
| 2.5383 | 3.0885 | 3.5915 | 1.0663 | 0.0000 | 2.7896 | 1.8874 | 2.7155 |
| 2.5204 | 4.7899 | 2.2714 | 3.8547 | 4.5214 | 3.0872 | 3.4351 | 2.8387 |
| 2.4895 | 3.0580 | 2.4158 | 1.5773 | 0.7058 | 1.9569 | 1.9676 | 2.4959 |
| 4.1245 | 2.6556 | 3.4550 | 1.6152 | 1.5946 | 3.3791 | 3.5298 | 5.1888 |
| 4.3506 | 0.0000\ | | | | | | |
| PTCH2 | 8.3684 | 6.6241 | 4.6381 | 6.4372 | 6.3230 | 4.7888 | 5.9493 |
| 6.7778 | 4.4119 | 10.0830 | 4.3258 | 5.2386 | 5.7820 | 4.9166 | 5.0671 |
| 7.1158 | 5.4984 | 6.6366 | 7.1156 | 4.5785 | 4.0896 | 6.7133 | 7.0194 |
| 4.8880 | 8.3682 | 5.9443 | 3.3638 | 6.0411 | 3.9557 | 2.4720 | 5.0927 |
| 5.6838 | 3.1967 | 4.0240 | 5.0883 | 5.1226 | 9.7949 | 5.9132 | 3.4608 |
| 4.9936 | 6.5560 | 6.3947 | 3.0042 | 4.9411 | 6.4938 | 5.1053 | 0.0000 |
| 4.9157 | 2.5837 | 5.9460 | 5.1716 | 4.3904 | 6.1712 | 7.3432 | 7.1137 |
| 5.3874 | 5.3875 | 4.9944 | 5.0393 | 4.2095 | 7.5121 | 3.7810 | 5.5464 |
| 7.2396 | 10.1257 | 5.7260 | 4.7922 | 5.2776 | 8.2440 | 8.2507 | 3.2868 |
| 8.7817 | 1.9166 | 4.8932 | 6.3357 | 5.1556 | 7.1468 | 8.1116 | 6.3002 |
| 6.3184 | 5.9765 | 3.0489 | 7.7021 | 4.4824 | 6.3497 | 4.5575 | 7.3133 |
| 4.5411 | 4.7327 | 6.5934 | 7.1969 | 4.1516 | 7.8290 | 8.6820 | 5.3545 |
| 5.7338 | 3.2184 | 8.0127 | 5.4365 | 5.3056 | 2.9428 | 6.6028 | 5.3082 |
| 6.4620 | 4.6140 | 5.0620 | 7.1857 | 6.8408 | 4.6022 | 7.6154 | 5.9674 |
| 5.7488 | 2.5153 | 4.6439 | 4.8277 | 5.3435 | 6.0992 | 7.0702 | 3.1059 |
| 5.0457 | 5.6433 | 5.1807 | 5.5332 | 5.5253 | 7.1283 | 3.8931 | 6.0292 |
| 2.2050 | 6.9303 | 8.0605 | 3.5821 | 5.7400 | 6.6828 | 7.5548 | 5.6002 |
| 1.7113 | 1.0837 | 4.9498 | 7.3756 | 8.0497 | 4.7463 | 2.7811 | 5.3932 |
| 5.6258 | 4.4479 | 8.6532 | 5.7212 | 6.1923 | 7.9170 | 2.3242 | 7.1589 |
| 0.0000 | 4.9567 | 4.1736 | 4.2129 | 2.5751 | 7.1457 | 4.4681 | 4.7979 |
| 5.8453 | 2.5111 | 4.9149 | 6.5838 | 5.2267 | 5.9184 | 5.7960 | 3.5749 |
| 4.9306 | 5.5248 | 6.4604 | 3.2858 | 4.5047 | 5.9104 | 9.7645 | 2.7155 |
| 6.0646 | 9.3997 | 6.5623 | 5.2060 | 8.2355 | 5.1571 | 4.8989 | 6.8269 |
| 6.2421 | 5.4909 | 6.4369 | 4.0766 | 4.0681 | 4.2380 | 3.3532 | 4.1255 |
| 7.0604 | 6.2050 | 3.9480 | 5.3874 | 2.3335 | 1.0320 | 7.4562 | 5.4743 |
| 7.3888 | 2.2387\ | | | | | | |
| OR7G1 | 0.0000 | 0.0000 | 0.0000 | 0.0000 | 0.0000 | 0.0000 | 0.0000 |
| 0.0000 | 0.0000 | 0.0000 | 0.0000 | 0.0000 | 0.0000 | 0.0000 | 0.0000 |
| 0.0000 | 0.0000 | 0.0000 | 0.0000 | 0.0000 | 0.0000 | 0.0000 | 0.0000 |
| 0.0000 | 0.0000 | 0.0000 | 0.0000 | 0.0000 | 0.0000 | 0.0000 | 0.0000 |
| 0.0000 | 0.0000 | 0.0000 | 0.0000 | 0.0000 | 0.0000 | 0.0000 | 0.0000 |
| 0.0000 | 0.0000 | 0.0000 | 0.0000 | 0.0000 | 0.0000 | 0.0000 | 0.0000 |
| 0.0000 | 0.0000 | 0.0000 | 0.0000 | 0.0000 | 0.0000 | 0.0000 | 0.0000 |
| 0.0000 | 0.0000 | 0.0000 | 0.0000 | 0.0000 | 0.0000 | 0.0000 | 0.0000 |
| 0.0000 | 0.0000 | 0.0000 | 0.0000 | 0.0000 | 0.0000 | 0.0000 | 0.0000 |

0.0000  0.0000  0.0000  0.0000  0.0000  0.0000  0.0000  0.0000
0.0000  0.0000  0.0000  0.0000  0.0000  0.0000  0.0000  0.0000
0.0000  0.0000  0.0000  0.0000  0.0000  0.0000  0.0000  0.0000
0.0000  0.0000  0.0000  0.0000  0.0000  0.0000  0.0000  0.0000
0.0000  0.0000  0.0000  0.0000  0.0000  0.0000  0.0000  0.0000
0.0000  0.0000  0.0000  0.0000  0.0000  0.0000  0.0000  0.0000
0.0000  0.0000  0.0000  0.0000  0.0000  0.0000  0.0000  5.0395
0.0000  0.0000  0.0000  0.3935  0.0000  0.0000  0.0000  0.0000
0.0000  0.0000  0.0000  0.0000  0.0000  0.0000  0.0000  0.0000
0.0000  0.0000  0.0000  0.0000  0.0000  0.0000  0.0000  0.0000
0.0000  0.0000  0.0000  0.0000  0.0000  0.0000  0.3855  0.0000
0.0000  0.0000  0.0000  0.0000  0.0000  0.0000  0.0000  0.0000
0.0000  0.0000  0.0000  0.0000  0.0000  0.0000  0.0000  0.0000
0.0000  0.0000  0.0000  0.0000  0.0000  0.0000  0.0000  0.0000
0.0000  0.0000  0.0000  0.9943  0.0000  0.0000  0.0000  0.0000
0.0000  0.0000  0.0000  0.0000  0.0000  0.0000  0.0000  0.0000
0.0000  0.0000\
PCDHGA2 0.9511  6.2931  3.0624  1.7854  2.8446  3.5906  3.0554
3.0712  2.9347  5.9410  3.8242  3.8262  3.2969  2.9572  2.7808
0.0000  0.0000  2.1918  5.5513  0.0000  3.0838  4.1097  3.4522
1.8643  6.9759  1.0317  1.6918  0.0000  2.8976  3.4734  2.1085
2.4004  0.8951  3.8818  3.8082  1.1431  6.1196  3.2676  0.9336
4.0985  0.6649  7.4457  2.4120  2.6427  6.8593  0.0000  0.0000
1.9920  5.3686  1.3590  1.5081  2.8913  2.3160  7.7163  4.5491
3.2557  4.2480  3.6648  5.5014  2.4263  7.6264  2.7563  3.3885
2.0265  6.8177  3.1332  4.6221  3.2255  3.6228  4.6109  2.2792
3.7871  0.9449  1.7486  4.7305  1.3447  3.4396  2.7891  1.5048
4.7311  1.7663  2.7429  3.8679  3.0210  1.4651  2.0776  3.8172
0.6140  3.2272  2.6518  7.3949  2.7205  0.0000  0.6909  2.9084
6.9844  2.3031  7.5378  5.4074  1.2591  0.0000  3.4473  3.9421
2.1133  1.3743  2.6639  3.6874  5.0320  6.3107  0.0000  1.6886
1.6572  0.0000  1.0000  4.3101  3.7732  0.9131  3.9369  1.9462
1.6324  3.9165  2.3308  3.4916  1.8858  4.6491  4.3694  2.7753
1.2894  7.5776  8.2093  2.0482  0.6684  2.3786  1.2511  2.0060
0.0000  0.0000  0.0000  6.5423  8.0509  1.3507  0.9848  1.4380
2.6953  3.0118  7.5118  2.2759  6.3600  2.0927  1.2240  6.5838
0.0000  3.5764  3.0034  2.4670  4.6076  7.3171  4.0172  0.6000
0.9345  2.3724  1.3735  3.2401  0.9764  4.2838  3.3256  1.6706
4.6805  4.3833  2.0787  3.1141  1.6845  2.4724  3.8579  0.0000
0.4801  5.3146  6.8877  6.7270  2.9241  1.5234  0.6902  2.2963
2.1234  3.1691  1.8914  2.7975  1.8171  0.0000  2.8694  2.8735
2.8293  1.2903  3.7411  2.1049  0.0000  0.0000  6.7641  2.8496
3.8990  0.0000\
PRAMEF9 0.0000  0.0000  0.0000  0.0000  0.0000  0.0000  0.0000
0.0000  0.0000  0.0000  0.0000  0.0000  0.0000  0.0000  0.0000
0.0000  0.0000  0.0000  0.0000  0.0000  1.0485  0.0000  0.0000
0.0000  0.0000  0.0000  0.0000  0.0000  0.0000  0.0000  0.0000
0.0000  0.0000  0.0000  0.0000  0.0000  0.5555  0.0000  0.0000
0.0000  0.0000  0.0000  0.0000  0.0000  0.0000  0.0000  0.0000
0.0000  3.5921  0.0000  0.0000  0.0000  0.0000  0.0000  0.0000
0.5608  0.0000  0.0000  0.0000  0.0000  0.0000  0.0000  0.0000
0.0000  0.0000  0.0000  0.0000  0.0000  0.0000  0.0000  2.4738
0.0000  0.0000  0.0000  0.0000  0.0000  0.0000  0.0000  0.0000
0.0000  0.0000  0.0000  0.0000  0.0000  0.0000  0.0000  0.0000

| | | | | | | | |
|---|---|---|---|---|---|---|---|
| 0.0000 | 0.0000 | 0.0000 | 0.0000 | 0.0000 | 0.0000 | 0.0000 | 0.0000 |
| 0.0000 | 1.2090 | 0.0000 | 0.0000 | 0.0000 | 0.0000 | 0.0000 | 0.0000 |
| 0.0000 | 0.0000 | 0.0000 | 0.0000 | 0.0000 | 0.0000 | 0.0000 | 0.0000 |
| 0.0000 | 0.0000 | 0.0000 | 0.0000 | 0.6116 | 0.5276 | 0.4321 | 0.0000 |
| 0.0000 | 0.0000 | 0.0000 | 0.0000 | 0.0000 | 0.0000 | 0.0000 | 0.0000 |
| 0.0000 | 0.0000 | 0.0000 | 0.0000 | 0.0000 | 0.0000 | 0.0000 | 0.0000 |
| 0.0000 | 0.0000 | 0.0000 | 0.0000 | 0.0000 | 0.0000 | 0.0000 | 0.0000 |
| 0.0000 | 0.0000 | 0.0000 | 0.0000 | 0.0000 | 0.0000 | 0.0000 | 0.0000 |
| 0.0000 | 0.0000 | 0.5862 | 0.0000 | 0.0000 | 0.0000 | 0.0000 | 0.0000 |
| 0.0000 | 0.0000 | 1.0431 | 0.0000 | 0.0000 | 0.0000 | 0.0000 | 0.0000 |
| 0.0000 | 0.0000 | 0.0000 | 0.0000 | 4.7139 | 0.0000 | 0.0000 | 0.0000 |
| 0.0000 | 0.0000 | 0.0000 | 0.7148 | 0.0000 | 0.0000 | 1.1551 | 2.0275 |
| 0.0000 | 0.0000 | 0.0000 | 0.0000 | 0.0000 | 0.0000 | 0.5707 | 0.0000 |
| 0.0000 | 0.0000 | 0.0000 | 0.0000 | 0.0000 | 0.0000 | 0.0000 | 0.0000 |
| 0.0000 | 0.0000\ | | | | | | |
| PRDM9 | 0.0000 | 0.0000 | 0.0000 | 0.0000 | 0.0000 | 0.0000 | 0.0000 |
| 0.4748 | 0.4327 | 0.0000 | 2.4603 | 0.0000 | 0.0000 | 0.0000 | 0.0000 |
| 0.0000 | 0.0000 | 0.0000 | 2.1673 | 0.0000 | 0.0000 | 0.0000 | 0.0000 |
| 0.0000 | 0.0000 | 0.0000 | 0.0000 | 0.5990 | 0.0000 | 1.3312 | 0.0000 |
| 0.0000 | 0.5159 | 0.0000 | 0.5853 | 0.0000 | 0.9556 | 0.0000 | 0.0000 |
| 0.0000 | 0.0000 | 0.4871 | 0.0000 | 0.0000 | 0.0000 | 0.0000 | 0.0000 |
| 0.0000 | 0.0000 | 0.0000 | 0.0000 | 0.0000 | 0.0000 | 0.5094 | 0.0000 |
| 0.5608 | 0.0000 | 0.5030 | 0.0000 | 0.5715 | 0.0000 | 0.0000 | 0.0000 |
| 0.0000 | 0.0000 | 0.0000 | 0.0000 | 1.5796 | 0.0000 | 0.6160 | 0.4334 |
| 0.7001 | 0.0000 | 0.0000 | 0.0000 | 0.0000 | 1.2673 | 0.0000 | 1.1539 |
| 0.0000 | 0.0000 | 0.3965 | 0.0000 | 0.0000 | 1.2145 | 0.0000 | 2.2055 |
| 0.0000 | 0.0000 | 0.0000 | 0.0000 | 0.8470 | 0.0000 | 0.0000 | 0.0000 |
| 0.0000 | 0.0000 | 0.0000 | 0.0000 | 0.0000 | 0.0000 | 0.0000 | 0.0000 |
| 0.0000 | 0.0000 | 0.0000 | 0.0000 | 0.0000 | 0.5426 | 0.0000 | 0.0000 |
| 0.0000 | 0.0000 | 0.0000 | 2.8917 | 2.0594 | 0.0000 | 1.2610 | 0.0000 |
| 0.6089 | 3.5096 | 0.0000 | 0.6224 | 0.0000 | 0.0000 | 1.2272 | 1.3754 |
| 0.0000 | 0.0000 | 0.0000 | 0.0000 | 0.0000 | 0.0000 | 5.9640 | 0.0000 |
| 0.0000 | 0.0000 | 0.0000 | 0.5216 | 0.0000 | 1.0240 | 0.0000 | 0.0000 |
| 0.0000 | 0.0000 | 0.0000 | 0.0000 | 0.0000 | 0.0000 | 0.0000 | 0.0000 |
| 0.0000 | 0.0000 | 0.5862 | 0.0000 | 0.0000 | 0.0000 | 0.3855 | 0.0000 |
| 0.0000 | 0.0000 | 0.0000 | 0.0000 | 0.0000 | 0.0000 | 0.0000 | 0.0000 |
| 0.0000 | 0.0000 | 0.0000 | 0.0000 | 0.0000 | 0.0000 | 0.0000 | 0.0000 |
| 0.0000 | 0.0000 | 0.0000 | 0.4012 | 0.0000 | 0.0000 | 1.1551 | 2.5448 |
| 0.0000 | 0.0000 | 0.0000 | 0.0000 | 0.7058 | 0.0000 | 0.0000 | 0.0000 |
| 0.0000 | 0.0000 | 0.0000 | 0.0000 | 0.0000 | 0.0000 | 0.0000 | 0.0000 |
| 0.0000 | 0.0000\ | | | | | | |
| SGK269 | 9.1534 | 10.4736 | 10.9667 | 9.6483 | 9.1733 | 10.2786 | 9.6363 |
| 10.2234 | 8.6226 | 10.1289 | 9.5231 | 8.1450 | 9.4087 | 10.0177 | 9.6301 |
| 10.3524 | 8.7045 | 9.3999 | 10.7061 | 7.8432 | 8.7939 | 9.4626 | 9.1494 |
| 9.9817 | 10.4033 | 8.0736 | 8.1737 | 8.6134 | 8.4553 | 8.1081 | 9.9993 |
| 8.9897 | 8.9575 | 9.2277 | 8.0562 | 7.8265 | 10.4500 | 9.1038 | 9.1181 |
| 8.9748 | 8.5216 | 10.3203 | 8.6053 | 9.0413 | 11.1053 | 8.7903 | 6.9374 |
| 9.2406 | 11.4608 | 8.6900 | 9.1608 | 9.5765 | 9.2671 | 10.7772 | 9.5624 |
| 9.3578 | 9.1274 | 9.1895 | 9.5582 | 8.7621 | 10.9481 | 9.6738 | 8.2420 |
| 7.8931 | 10.2420 | 9.2233 | 8.1627 | 9.3125 | 9.3281 | 10.3672 | 9.1504 |
| 10.3538 | 7.4274 | 8.6730 | 9.9210 | 9.5135 | 10.2054 | 9.8463 | 10.5811 |
| 9.4227 | 8.4322 | 8.5971 | 10.4913 | 9.6859 | 8.6495 | 8.6929 | 8.5693 |
| 7.4944 | 8.7161 | 9.9819 | 10.2604 | 8.3926 | 10.2240 | 9.8946 | 8.8924 |
| 10.7770 | 9.1243 | 10.2584 | 9.2002 | 8.9491 | 8.2411 | 9.1639 | 8.0935 |

9.6198 8.9161 8.9758 9.3191 9.8978 9.5861 8.9521 9.0356
9.1702 7.1549 8.2715 9.8584 10.2492 9.5615 8.7614 8.8085
8.8535 9.5912 9.1872 8.9841 8.7521 8.9486 8.8232 8.8377
8.4172 10.7460 10.9993 9.0829 9.4798 8.9533 9.6774 8.2523
7.1188 7.6130 9.2491 9.8977 9.5591 9.0177 8.3873 8.4803
9.9150 9.8343 9.6082 10.1878 9.5539 8.5108 7.5186 10.4751
6.9058 9.7789 9.1687 8.7054 9.2987 10.1108 9.8962 9.3967
8.0610 7.8074 7.6001 9.1076 8.3392 9.3321 9.9833 9.9322
9.4838 8.9334 9.1833 8.4928 9.2293 9.3807 7.9338 8.7979
9.6013 10.2794 10.0546 10.3446 10.2173 7.9210 8.8605 8.0267
8.4414 8.4378 8.5339 8.5977 7.8615 8.2517 8.4080 9.4746
9.7913 8.6370 9.2181 8.9585 8.4615 6.9698 9.1445 9.4051
9.5861 6.3171\
FAM108B1 8.4185 8.2986 8.6101 8.6841 8.5680 8.4933
8.4388 8.6114 8.1443 8.1406 7.9175 8.4437 8.6052 9.2867
9.0399 7.9186 7.8895 8.6257 8.3629 7.5478 7.8161 8.5738
8.4033 8.6914 7.8027 8.0680 8.5306 8.5051 8.1294 8.6282
8.6227 7.9502 7.6411 8.0276 8.0037 8.5108 8.3046 8.9419
8.1265 8.0952 8.6172 8.7249 6.6419 8.2503 8.5551 7.2380
7.0321 8.8102 8.7283 8.5852 8.0704 8.0648 7.9886 8.5941
9.2150 8.0350 8.3668 8.3925 8.6723 7.5104 8.7290 9.0380
8.7468 7.4402 7.7980 9.1190 7.8903 8.4056 8.2556 7.6337
7.4203 8.7285 7.3229 8.4132 8.7053 7.8116 9.0204 8.8350
8.3282 9.1952 8.3873 8.2823 8.5142 7.6713 8.1544 7.2208
8.4864 7.5028 8.4419 8.2479 8.2271 9.0857 6.9717 7.0692
8.5639 8.4226 8.0551 8.6263 8.4249 7.8219 7.9731 8.5090
8.5424 6.9782 7.8770 7.8270 7.4372 8.0453 7.8498 7.5627
8.8286 8.8227 5.9248 6.8074 7.8643 8.5297 8.5743 8.8514
8.7724 7.4753 8.1503 8.9388 7.5885 8.8151 8.7327 8.4627
8.4844 6.3168 8.7276 8.4736 9.3661 7.0993 8.6372 8.0174
6.8721 6.7434 7.9499 5.3965 8.7598 8.5277 7.1663 8.7075
8.4757 8.8910 8.7031 8.3508 9.3337 8.0974 7.8030 8.1745
8.4030 6.3897 8.6069 7.9405 8.8292 8.5993 8.6433 8.4443
7.7994 8.9562 8.6268 7.5148 8.6152 8.3662 8.3816 8.9800
7.4863 8.6663 8.3467 7.9045 8.7527 8.1452 8.7800 8.2006
6.1129 9.0162 7.7553 7.9897 9.0272 8.9375 7.8814 8.1077
9.1079 8.3645 8.2891 8.5297 7.9998 7.8137 8.3052 8.4666
9.1041 8.6916 8.1220 7.9204 8.2456 7.4323 6.6477 8.2693
8.5172 8.8305 7.2737\
TBC1D1 10.3216 12.0075 9.8602 10.0329 10.0726 11.0868 9.3515
10.3839 10.4671 11.0064 10.4898 9.8910 10.3727 9.9713 10.4517
11.1677 9.5462 10.6546 12.1123 10.2604 9.5330 11.2947 11.3886
9.4949 12.4956 9.7429 10.0282 11.0039 10.2767 9.8252 10.1987
8.3329 10.7581 10.6883 9.6257 11.1484 10.0742 9.9755 10.4537
11.6523 11.5231 12.5123 9.1573 9.5317 12.7466 10.4946 10.9134
9.9334 10.4938 8.0355 10.1108 10.5836 10.2672 12.7740 9.7899
9.9870 10.6642 9.9394 10.9076 10.7680 12.5369 10.6166 10.3875
10.6736 11.0810 10.6671 10.6493 11.1451 9.5211 11.1177 10.3511
9.1939 10.4113 10.5056 10.5515 10.6574 10.4314 10.6686 10.6488
10.5070 10.4057 9.7426 11.6849 11.1151 11.2558 10.5387 9.1525
12.0925 10.6979 11.2218 11.8533 10.2406 11.5792 11.1976 10.1850
12.8483 10.9557 12.3863 10.4133 10.5199 11.2269 10.7885 10.8782
10.6152 11.1939 10.9187 11.1337 10.5170 10.2290 11.5589 11.0143
10.1568 9.8712 11.1092 10.5509 10.2239 10.4874 10.1113 10.6134

| | | | | | | | |
|---|---|---|---|---|---|---|---|
| 10.1265 | 10.1667 | 10.0082 | 10.0634 | 10.2461 | 11.3790 | 10.6836 | 10.1668 |
| 10.1555 | 11.9561 | 12.0229 | 11.0660 | 10.7317 | 10.8157 | 10.8613 | 10.8587 |
| 10.3855 | 9.9835 | 10.9625 | 11.8473 | 12.7275 | 11.7948 | 8.8117 | 11.8386 |
| 10.4823 | 10.5569 | 10.1217 | 10.5876 | 11.8025 | 10.2976 | 8.8967 | 11.3917 |
| 10.1737 | 10.7770 | 10.5116 | 10.6278 | 10.7161 | 12.4833 | 10.1541 | 10.9758 |
| 10.3021 | 9.0740 | 10.5105 | 10.9599 | 10.6047 | 11.1477 | 10.4014 | 10.2493 |
| 11.3721 | 11.1139 | 10.7548 | 10.3050 | 10.0778 | 11.5158 | 10.7470 | 10.2646 |
| 10.5405 | 10.9186 | 11.9999 | 10.0275 | 11.0432 | 9.8233 | 9.6719 | 10.6042 |
| 11.4923 | 9.8668 | 11.0236 | 10.8845 | 10.0209 | 10.5072 | 9.8325 | 9.6217 |
| 9.7335 | 9.7446 | 10.6596 | 10.3923 | 10.2707 | 10.2352 | 12.3917 | 11.1581 |
| 11.0070 | 11.3213\ | | | | | | |
| ZXDA | 4.2977 | 5.5927 | 5.9608 | 4.7820 | 4.5009 | 5.0086 | 4.7081 |
| 5.1254 | 5.2509 | 5.2692 | 5.5462 | 5.6820 | 5.2904 | 4.7356 | 4.8127 |
| 3.5017 | 3.6230 | 4.9861 | 5.2821 | 4.4470 | 3.9470 | 4.8061 | 6.0725 |
| 5.2635 | 6.1725 | 4.2292 | 4.8889 | 3.6823 | 4.3473 | 6.3640 | 3.6546 |
| 4.7881 | 4.1856 | 4.3004 | 5.9316 | 4.3882 | 5.1790 | 5.6659 | 4.1930 |
| 5.8444 | 2.6483 | 5.9459 | 4.2348 | 4.9079 | 6.3727 | 2.8725 | 3.9141 |
| 5.3542 | 1.4771 | 0.8339 | 5.5351 | 4.6985 | 5.7050 | 6.3706 | 4.7860 |
| 4.7363 | 5.6794 | 5.6264 | 5.6545 | 4.3876 | 6.2701 | 4.7932 | 4.6734 |
| 5.2241 | 5.5732 | 5.2000 | 5.6527 | 5.3776 | 4.1878 | 3.7848 | 4.7322 |
| 5.8772 | 4.0640 | 5.0894 | 5.0244 | 4.4707 | 2.6228 | 3.6286 | 4.2300 |
| 5.2381 | 2.9495 | 4.8340 | 4.3976 | 4.9381 | 4.0362 | 4.2262 | 6.2386 |
| 3.6018 | 5.2667 | 4.5790 | 6.2013 | 6.2033 | 4.3639 | 3.5163 | 4.0165 |
| 5.9138 | 5.5247 | 6.4338 | 5.1229 | 3.8079 | 3.9535 | 4.6166 | 5.7305 |
| 3.3666 | 4.7904 | 4.4574 | 4.2346 | 4.7249 | 4.7068 | 3.6514 | 5.5601 |
| 4.8581 | 4.1934 | 3.4594 | 5.4948 | 4.5346 | 5.3007 | 6.0584 | 4.6675 |
| 3.5882 | 4.0518 | 4.4368 | 5.2561 | 4.8635 | 6.1646 | 5.3180 | 4.9179 |
| 3.7308 | 6.3057 | 6.5633 | 4.9148 | 4.6189 | 3.4941 | 3.5049 | 5.9736 |
| 4.2046 | 4.3240 | 0.0000 | 6.0420 | 6.4225 | 4.0892 | 4.7778 | 4.4648 |
| 1.7146 | 4.5199 | 6.5614 | 4.5242 | 5.8224 | 3.2383 | 3.8440 | 5.7056 |
| 2.9676 | 6.2332 | 5.1123 | 5.7200 | 5.5989 | 6.0966 | 5.4454 | 4.7709 |
| 3.6311 | 5.0151 | 3.5368 | 4.0792 | 4.9082 | 6.2861 | 5.5993 | 0.0000 |
| 5.9818 | 3.7402 | 4.5868 | 5.1557 | 5.3049 | 4.1192 | 4.6298 | 2.9928 |
| 5.3807 | 5.1687 | 5.9725 | 5.2060 | 4.7148 | 4.2689 | 5.2407 | 5.1046 |
| 4.2192 | 5.6333 | 4.8671 | 4.6328 | 3.6991 | 4.4808 | 4.8888 | 4.7855 |
| 5.0624 | 4.5341 | 4.6070 | 1.1623 | 2.3335 | 2.3729 | 6.1314 | 5.0541 |
| 5.1532 | 3.6039\ | | | | | | |
| PAMR1 | 6.8023 | 12.9565 | 9.3564 | 7.7922 | 5.6446 | 6.5683 | 9.2896 |
| 8.7006 | 8.8938 | 12.4910 | 7.3251 | 7.0921 | 10.7076 | 5.1943 | 7.3502 |
| 2.6237 | 9.6515 | 9.3502 | 12.7149 | 5.8074 | 6.9526 | 11.1886 | 9.4725 |
| 7.7476 | 9.7366 | 6.5698 | 7.6458 | 10.6015 | 9.1017 | 5.8855 | 3.4068 |
| 7.4864 | 4.4638 | 5.9736 | 4.0883 | 5.7270 | 7.1678 | 6.0448 | 6.6466 |
| 9.1754 | 9.7213 | 11.8851 | 9.0288 | 5.4597 | 12.5592 | 6.7735 | 6.5312 |
| 6.9296 | 7.0200 | 6.9388 | 4.3867 | 5.7868 | 8.1416 | 10.1062 | 8.8907 |
| 6.6134 | 9.4145 | 10.3802 | 10.8721 | 8.7244 | 11.0011 | 9.5376 | 7.4626 |
| 7.2347 | 12.4703 | 9.3449 | 5.8325 | 8.1346 | 11.2720 | 11.5473 | 4.6540 |
| 6.8571 | 7.5535 | 7.8793 | 8.9667 | 3.5581 | 10.2851 | 12.2141 | 10.5875 |
| 8.1266 | 9.3622 | 8.0210 | 11.4972 | 6.7596 | 9.4117 | 8.2721 | 7.1486 |
| 7.8828 | 8.5747 | 10.1541 | 11.0500 | 8.6379 | 5.8259 | 11.4998 | 9.9458 |
| 11.2365 | 5.8540 | 12.4759 | 8.4355 | 7.7706 | 6.3933 | 8.6872 | 8.2528 |
| 8.4108 | 7.6810 | 7.7878 | 9.5889 | 10.3276 | 10.2475 | 9.8877 | 6.8635 |
| 7.6725 | 9.0943 | 6.2095 | 6.8863 | 6.6852 | 7.4290 | 9.1275 | 6.6103 |
| 8.2353 | 8.5749 | 9.1835 | 9.9182 | 8.4284 | 9.3002 | 8.9302 | 9.2802 |
| 7.4751 | 12.4084 | 10.9302 | 7.9365 | 7.4054 | 9.6796 | 7.7197 | 8.3786 |

8.0240 5.3874 11.6085 9.2929 10.7591 6.4818 6.0032 8.9563
8.8827 8.1346 6.1903 8.4422 10.2589 6.8411 4.3951 10.1255
7.1629 9.1985 9.1280 5.5167 5.6609 11.5135 8.0196 6.9810
7.6738 5.1696 7.7675 10.7557 7.4392 9.4462 8.8980 7.5493
9.7715 8.3030 5.2066 6.4135 6.5882 5.2072 8.1384 8.6507
5.5962 11.8412 10.5068 8.4612 11.4631 8.0337 7.7734 6.3631
9.0583 9.4218 8.8893 9.2353 8.1923 7.7356 5.2976 6.1418
6.2599 7.6920 9.9554 8.2139 8.0534 5.5698 10.8906 8.7179
8.8635 7.5563\
RAD9A 9.2222 8.2555 7.6408 8.6348 9.1022 8.5174 8.2240
9.1069 8.7373 8.8312 9.5871 8.0196 9.2098 7.8210 8.1943
9.0562 10.2378 9.2344 8.4440 9.9101 10.0901 9.0169 8.8880
8.2417 7.7348 8.6565 9.1638 9.3255 8.6564 8.0578 8.8707
9.2488 8.8947 8.7470 8.4792 9.2575 8.7796 8.5459 8.9682
8.5037 8.0642 7.6229 8.2373 10.0013 7.2088 8.8889 9.7536
8.4709 8.0370 10.0408 7.6893 9.3831 7.8245 7.5148 7.9844
9.4229 7.8783 8.1621 8.1262 8.6987 7.3213 8.1894 9.1649
8.1445 8.6446 8.3658 9.1299 9.5255 7.7880 10.2369 9.1280
7.6522 9.3867 9.8620 7.6159 8.4430 9.4107 9.5241 9.2927
8.7731 8.5826 8.6049 8.9118 9.2257 9.3963 9.3335 7.3509
9.1844 8.1896 8.4974 7.7720 7.9742 10.1089 9.9529 9.0538
7.3580 7.3017 8.0794 8.4972 9.3979 9.6523 8.6074 8.8267
11.2258 9.3254 8.5311 9.8315 9.8352 8.1981 10.5528 8.4440
8.3184 11.0981 9.8961 8.9329 8.0842 8.2247 8.9267 8.9880
8.6585 9.2834 7.6471 8.2773 8.0851 9.0240 8.1481 6.9765
9.8354 7.2951 7.5095 8.4833 10.1036 8.9879 10.4857 9.1170
9.3218 9.6513 11.9065 8.1584 8.3358 10.6287 7.6606 9.9506
7.9884 7.5757 7.9928 7.4254 8.4746 8.4244 8.6574 8.1808
10.6333 8.0448 9.5657 7.9225 8.3636 8.0198 7.9156 9.8047
9.3278 8.3887 9.1398 8.5769 9.5990 8.7899 8.0162 9.4557
7.9993 8.3168 9.2833 8.0863 9.4787 8.5851 8.4046 10.9081
8.4382 8.9845 7.8878 8.1784 11.1294 9.8344 8.2334 8.7822
8.8360 8.9675 8.6575 8.6089 9.7553 8.9820 8.8560 8.5890
7.5660 9.7947 10.0487 9.0106 9.8929 10.8492 8.5206 8.3956
8.9620 8.9951\
ERGIC2 9.3885 9.3004 9.8372 9.7900 9.0834 9.9095 9.3178
9.4722 10.4947 9.2600 9.6708 9.3893 9.7537 9.7112 9.8691
8.8526 9.5104 9.5721 9.2622 8.8531 9.7210 9.5575 8.3383
9.7433 9.3516 9.5910 10.4531 9.6039 9.5619 9.2466 9.8928
9.5823 10.0585 9.3924 9.3183 9.0540 9.1460 9.7111 10.1826
9.9562 9.9907 9.4550 8.7535 9.8827 9.2137 9.5865 9.6669
9.6184 9.9972 10.0843 9.3626 8.9354 9.5546 9.2296 9.8723
9.8226 9.4962 9.9327 8.9976 9.3486 9.2644 9.5715 9.9426
9.3582 9.4465 9.6641 11.5689 10.3182 9.4800 9.5319 9.2334
9.4349 9.6140 9.9357 10.5327 9.1331 9.2254 8.8016 8.9631
9.3171 9.8476 10.2558 9.2674 10.2988 9.1088 10.3563 11.0224
9.3177 9.5720 9.5253 9.0991 9.2057 10.0448 9.0760 9.5964
9.3320 9.3747 8.6742 8.7748 9.2392 9.0619 9.4646 8.9195
9.5226 10.1390 8.5639 8.7419 9.7403 10.0866 9.4270 9.6510
9.3876 9.8344 9.0251 10.2869 10.0566 9.8088 9.0929 9.6620
9.3149 9.9473 9.9840 9.5111 9.5246 8.8072 9.9921 10.7210
8.7481 9.2337 9.5426 9.8129 9.7691 9.6413 9.5948 9.6459
10.0827 9.1539 8.5210 9.6116 8.9426 9.6742 10.2024 9.3879
9.7896 9.8313 8.9450 9.6057 8.5075 10.1364 10.0266 9.1868

9.4549  9.5744  8.7670  8.7510  9.6726  9.2454  9.6093  9.9872
8.8376  10.0221 9.6763  9.7726  9.5654  11.4485 9.8034  9.0079
9.5245  9.5808  9.3070  9.4226  10.2424 9.0489  9.5506  9.5290
9.7094  9.3686  9.3212  9.5612  9.1037  9.5324  9.6988  10.1768
10.1909 9.6020  9.5909  8.1307  10.2497 9.5144  9.6335  9.6770
9.5826  9.5431  10.4234 9.5908  9.0725  10.3131 9.0443  9.7071
9.4691  9.1234\
ERGIC3  12.8075 11.8506 12.6102 12.6269 12.6349 12.4280 12.7707
13.5861 13.3160 12.6195 12.7199 13.0237 13.0848 12.3365 12.7271
12.8105 13.1433 12.1026 11.9205 13.1946 12.4979 13.3209 13.3457
12.3092 12.3358 13.2554 12.1438 13.2517 13.1037 13.1241 12.2443
12.3867 13.1025 13.5950 12.7261 12.2257 12.3617 11.7088 12.4683
12.7025 12.6464 11.8708 13.4826 11.5501 11.7874 13.2120 14.3233
13.6049 12.7429 13.1183 12.2076 12.2172 13.6563 12.0654 12.6521
12.3878 12.0480 12.2999 12.1516 13.2538 11.9745 12.3420 12.6713
13.4406 12.4505 12.2099 12.8886 13.8454 12.5863 13.0665 13.5398
12.3354 13.4309 14.9789 12.1785 13.3652 13.6942 13.2901 13.0706
12.3184 13.5322 12.5472 12.8752 12.6779 13.6602 13.4893 13.4589
13.4575 12.6251 12.2202 12.0732 12.6460 12.6995 12.9342 12.7251
12.0769 12.9622 12.4867 13.0997 12.2416 13.6144 12.1375 12.3183
13.8946 13.0460 13.3147 12.0893 12.8275 12.8326 13.4707 12.8107
12.6412 14.2639 12.4476 12.0935 12.4574 12.4640 12.8429 12.7613
12.6330 12.6435 12.3806 12.9153 12.3507 13.0235 12.2071 12.9234
14.7745 11.8802 11.8223 13.2597 12.5962 12.8684 12.5740 13.0306
13.3920 13.0182 12.6151 12.3458 12.1150 13.7735 13.1508 13.5528
12.3687 12.2657 12.3668 12.7446 12.9288 11.6579 13.8994 12.3000
14.9444 12.2276 12.9537 12.3291 12.3452 12.2092 12.4667 13.9787
12.7148 12.6210 12.7987 13.1750 12.8516 12.6468 12.3283 12.6957
12.7411 12.6211 12.5904 12.0241 12.4225 12.4936 12.9090 13.8162
12.3009 12.0025 12.0433 12.2256 12.8959 13.1984 14.1796 12.7886
13.2618 13.0417 12.3872 12.6530 13.0787 13.1219 12.7956 12.0174
12.9198 13.2464 12.3381 12.1403 14.8835 13.9718 12.5766 12.1392
13.1260 13.4702\
CDR2L   10.0849 11.1035 10.6857 9.6059  9.9697  12.3997 10.8642
10.7651 11.7696 9.6793  12.2705 11.2308 11.1575 10.6471 9.8551
8.3270  11.4381 11.6625 11.2344 12.5651 11.9748 9.8470  8.9086
9.2520  9.7864  9.5684  7.7566  9.8230  10.4041 10.3094 9.3558
9.0257  9.9666  10.3548 10.9175 11.4190 10.8713 9.9455  10.3408
10.1881 7.9816  10.2910 12.4761 9.6958  8.5511  11.1324 9.7226
11.5549 9.7626  9.5774  10.1118 11.7864 8.9347  8.4460  9.9081
9.8688  11.2753 12.1186 10.7932 12.4947 9.4602  10.7031 9.6910
9.8474  9.9785  10.2400 10.5245 10.1571 9.7084  11.3136 13.2663
9.8150  11.5247 9.9847  8.6497  10.1814 9.3088  9.5577  8.3392
10.3796 9.7472  10.9618 8.5718  9.5544  9.3816  9.7615  10.5971
10.3286 9.3195  9.2052  9.1093  10.8023 9.3883  8.8641  8.8139
9.1994  10.8059 10.5909 9.6847  10.6297 10.1623 11.1970 9.5177
10.2062 8.9432  10.0767 10.2640 11.1755 10.3800 9.3777  9.8406
9.6087  9.5496  10.6421 10.0845 11.6388 9.3917  10.4991 9.9903
11.8727 9.7432  10.6612 12.5974 11.9498 8.7064  11.9918 10.9358
10.3933 9.7556  9.0532  9.2182  10.7322 10.6328 10.1579 10.5647
10.1961 9.9658  8.5786  9.7740  11.5073 9.4431  8.6347  9.1797
9.2716  10.1824 6.8277  10.0706 9.0189  9.7836  9.2421  9.2374
9.9259  9.4122  11.8336 8.9282  11.9874 10.5123 9.7535  9.7206
9.5673  10.8933 10.6889 8.8430  10.0921 10.5132 9.0531  10.7329

10.1982 11.3223 10.1861 9.7492 11.6006 9.6881 10.5154 8.8640
10.3869 9.4645 8.5964 9.8560 9.1109 8.5722 11.1120 11.6459
8.4043 9.9332 9.0125 10.4903 11.0189 10.7491 9.6948 10.0365
9.3737 9.4617 10.8597 10.2480 11.8651 8.6407 10.0487 9.9914
9.1180 11.8383\
ERGIC1 12.0503 11.7839 13.0088 13.6892 12.0669 12.3184 12.4683
12.4595 12.6063 12.6144 14.0833 12.6074 12.1474 12.2817 12.7757
12.1473 11.3082 12.8010 11.9155 11.0029 12.6063 12.9605 12.2548
12.8899 11.7452 11.3344 10.7380 12.5005 12.8238 12.4792 10.2257
13.8313 11.6396 11.7372 11.9669 12.0889 12.4782 14.0883 12.7036
11.4126 12.7112 12.0813 11.7797 12.9204 11.6392 12.2963 12.2523
12.6992 11.9113 13.6757 12.4729 11.2871 13.0095 11.5361 11.3843
12.1250 12.1612 12.7183 12.3639 12.5479 11.8242 12.6101 12.6758
12.2377 11.9938 12.9767 14.0289 12.4212 12.5286 11.9909 11.8206
12.4874 11.1551 12.3267 12.5444 13.2706 12.3179 12.7725 12.6423
11.4180 12.4049 12.8742 11.9399 11.8816 12.3186 12.8231 13.0394
12.3988 12.1463 11.0160 12.0606 12.7489 11.9521 11.1563 11.7018
12.0319 12.4861 11.8523 12.7991 11.4854 12.2656 12.6593 11.7828
12.1619 12.3743 11.4558 11.1874 12.2952 12.7408 11.2364 13.2170
11.9224 11.3321 11.2603 11.8522 12.2314 12.0482 12.6282 12.6808
11.6504 11.6513 13.4194 12.8849 13.4152 12.2394 11.9551 12.5629
12.1757 11.7761 12.0533 12.3575 14.1690 13.0821 10.5360 11.4255
9.6964 11.2903 10.2074 12.5774 11.8364 12.7261 13.4262 11.2215
12.9183 11.9837 11.6373 12.6274 12.4255 12.7957 11.1826 12.8871
11.8241 12.4655 12.3600 11.5302 13.4019 12.2440 12.0625 12.2762
10.7431 14.6336 12.0198 11.4152 11.9453 13.1862 12.0800 12.9159
13.5814 12.4714 11.4695 11.9957 11.5382 11.9926 13.2961 12.4023
11.8688 12.0484 12.0669 11.9953 11.7655 13.4974 12.4359 11.7073
14.0784 11.7599 12.3885 12.2850 12.1922 12.6444 11.6414 13.7785
12.4455 11.8284 11.2941 12.0004 11.1636 10.9860 11.7197 13.0132
12.3026 11.4051\
CLEC14A 7.5593 10.6782 7.9858 7.5085 7.8019 8.3208 7.3389
8.1295 7.7765 9.6261 8.2713 7.6693 8.4680 7.6341 7.5788
8.0178 7.6158 8.0849 10.3225 8.0914 8.5607 7.7890 6.6739
8.4202 11.0469 7.7971 7.0791 9.5740 8.9070 7.8702 6.7680
8.3158 7.6349 8.0255 7.6733 6.8854 9.4510 7.4540 7.7411
7.1915 9.3253 10.2383 8.1447 7.1839 11.6921 6.8432 8.5345
9.4964 7.6236 7.9896 7.5405 8.7872 8.4326 11.0832 9.9875
6.4893 8.3546 8.3996 10.3572 7.2635 11.8276 8.7464 7.2332
6.6859 9.5126 8.3798 8.1524 8.1907 7.6707 10.0351 8.4012
7.6569 8.5744 8.7994 9.1404 8.3568 9.3560 8.7152 9.6818
7.9515 8.7971 7.8496 10.6893 8.8587 9.1203 7.5156 8.0605
8.2501 8.0464 9.2601 10.8253 8.4983 7.7755 8.5344 7.5971
11.7837 8.8649 10.8612 8.5244 7.9789 8.3995 8.9590 7.2167
9.0497 7.3133 8.2228 9.0312 8.9212 8.1480 8.8473 7.0932
7.3495 8.3228 6.7142 7.8403 6.8528 7.6256 7.9288 7.8249
8.9191 7.5025 7.8087 8.6326 7.5229 7.8203 7.7400 7.8886
7.9790 11.3326 10.5567 7.7827 6.8495 8.5732 7.9903 8.0121
8.4186 8.3594 9.0949 10.8267 9.9169 6.4134 8.7927 8.3249
8.5036 6.7058 6.7747 7.8419 10.7701 7.5966 7.3808 11.1955
8.0924 7.4952 8.2869 6.7929 6.8770 11.8791 7.1672 7.8193
8.0262 8.1893 8.1667 7.4301 7.4176 7.8430 7.5546 8.9518
8.7832 8.4340 7.8961 7.4658 8.7781 8.2174 8.0705 9.9422
8.2167 9.4366 10.5516 8.1650 8.9343 8.2884 8.3790 6.9653

8.5357 6.9099 8.0903 9.2483 8.5195 7.8409 7.7642 8.0911
8.6534 9.5394 8.4855 7.5606 8.0424 6.5546 9.8552 8.1672
7.8744 7.9418\
PDF 8.3474 6.4984 7.3541 7.6126 8.3546 6.6071 6.8696
7.4886 9.1291 7.8857 7.2878 7.3735 7.8473 7.7621 7.5966
7.7551 8.0970 8.1421 6.6605 8.2670 7.8321 6.9994 7.1643
7.2178 7.6682 9.7821 8.4340 8.8939 8.5535 7.9269 6.6802
8.0626 9.0855 8.1778 8.1024 9.8092 7.8065 7.5387 8.7980
6.0047 8.7422 6.8005 7.9799 7.6098 4.9450 7.9540 9.6359
8.2384 8.2345 7.3534 8.5627 7.5699 7.5036 5.8396 8.0085
7.4538 7.2119 7.4113 7.9581 7.9269 6.5570 7.3264 7.3119
8.1796 7.1990 7.8002 8.7745 7.2406 6.8904 8.3940 9.7605
7.7014 9.1140 8.1764 8.0668 7.9853 9.4486 6.8612 8.2257
7.8714 8.8307 8.0368 7.2731 7.3392 8.3442 8.0644 7.5120
8.8624 7.4812 8.4260 7.3525 8.6045 7.0770 7.4698 8.7664
6.7524 8.7618 6.3570 7.0161 8.0545 9.2376 8.2280 7.0244
7.5224 9.0299 8.5318 6.7452 6.9786 7.2313 7.7816 7.7842
8.8264 8.2364 8.0293 8.3359 8.2504 8.1965 6.9563 8.2355
8.1889 7.9832 7.8099 7.9132 8.4936 7.0830 9.2985 8.4366
8.7712 6.2764 7.1195 7.9885 7.3432 8.3699 6.9736 10.4603
9.5722 9.3056 7.5397 7.5946 5.9615 8.6908 8.2204 8.5130
7.5363 7.9600 6.2277 7.5094 6.8447 8.1786 9.8973 6.9197
10.1597 7.4006 8.3572 8.4115 7.6955 6.7291 7.7831 9.3790
9.7650 8.5598 8.6196 7.9299 8.2803 7.7066 7.4407 7.8380
7.2433 8.1557 7.4658 8.0631 8.6482 7.6968 7.8144 8.1821
7.5637 7.3515 6.3221 7.5660 7.0613 9.4603 9.6283 7.7080
7.6341 7.1065 7.3043 7.2860 8.4250 8.4610 8.7744 7.7069
8.5581 8.1091 8.5454 7.7188 10.3230 9.0800 6.6098 7.6650
7.2035 9.9763\
B3GAT3 11.2565 9.5257 10.3953 10.4807 10.7807 9.4157 10.9976
9.9469 10.4653 10.2487 10.7321 10.2667 9.8200 10.2773 9.6432
10.5787 10.3659 10.9194 9.8708 11.1017 11.2821 10.3219 10.4602
9.9685 9.5421 12.2192 9.8943 10.9512 11.4629 10.2021 10.9177
10.2758 10.4762 11.0113 9.3889 10.6151 10.0892 9.8294 10.2337
11.3369 10.6150 9.5220 10.5530 10.2800 8.1959 10.6839 12.0326
10.1925 9.2728 10.4809 10.3428 11.7463 9.8663 9.0863 9.4611
10.1878 9.6508 9.9511 9.0895 9.5816 8.7413 10.4796 10.5939
9.8815 10.1681 9.7380 10.7120 10.4351 10.4242 11.1951 11.9376
10.1953 10.9369 10.4137 9.4446 10.7768 10.2980 10.6118 10.8858
10.8050 11.4435 9.7680 10.1084 10.3735 11.3445 10.6566 9.4513
11.0695 9.4760 11.2977 9.7515 9.2518 11.5122 10.8938 10.8062
9.0357 9.9947 9.2067 9.8539 10.2925 11.8561 10.5480 9.9537
10.6704 10.9007 9.8529 10.7411 9.9234 10.8950 10.9623 9.6043
10.2837 11.9639 10.6165 10.1813 9.9443 10.0847 9.5773 9.8762
9.6742 9.9348 9.9790 9.7992 9.9131 9.8945 10.4860 10.7091
9.5630 9.1173 9.0567 10.1536 9.5754 10.4039 10.7012 10.3031
11.5719 11.5295 11.1780 9.5476 9.4086 11.2756 10.3798 11.2420
9.8220 9.7883 9.1289 9.5380 9.9786 11.0112 12.0277 9.6758
13.5023 9.9679 10.4831 10.7979 9.7595 9.3464 10.4443 11.5894
11.7310 11.0002 10.7533 10.7348 11.2921 9.9621 9.2064 10.2951
9.5302 10.7822 10.6011 10.5618 9.5265 9.9042 10.2075 11.7503
9.9763 9.9843 9.7391 9.5606 9.8425 11.7142 10.5123 10.2829
11.0596 9.7523 10.0269 11.0506 10.2354 10.7882 10.2724 9.9213
10.4030 10.5201 9.5662 10.2985 11.1547 12.2089 9.6909 9.8400

9.5710 11.6488\
B3GAT2 1.9576 2.4983 2.0488 4.3017 1.7074 3.1709 0.9572
4.5359 2.3234 2.3403 3.0839 4.9198 3.2569 2.6028 2.6226
3.5764 1.6827 3.4964 3.4306 2.2332 2.8104 2.5769 2.8946
2.8823 3.9275 1.3975 3.8155 1.8803 0.0000 2.8502 8.2137
3.3687 1.7093 3.4898 3.9173 3.2745 2.4067 3.6088 1.7413
4.0982 1.4991 3.2623 2.8624 2.8231 2.0675 1.9262 5.6367
2.9461 2.5285 1.9146 2.4672 2.6553 2.1724 3.3504 6.2433
3.9515 1.9508 3.4397 1.9303 3.3189 2.5198 4.8860 4.2242
1.9035 2.2488 3.2831 3.0872 2.6595 2.8313 3.5790 2.8427
2.6081 0.0000 3.5191 3.9822 3.9110 1.7938 2.8595 2.0781
3.2962 1.5956 3.0177 1.2349 3.3462 1.5390 3.6895 8.2062
1.7142 3.3915 3.2796 2.3300 3.4055 2.8909 2.0768 2.0424
2.3040 3.4258 3.4608 3.7631 3.8485 1.1217 2.0049 2.3919
2.9683 2.4622 0.8454 1.9029 3.4139 4.4175 1.0220 2.1623
2.6087 6.8345 3.8268 5.1031 3.9118 3.6411 5.9731 3.4189
3.7674 4.5931 3.3498 3.7994 1.7520 3.5258 2.2440 2.4288
0.0000 3.1400 3.7676 3.7505 3.2430 2.1519 3.2399 2.8895
6.7067 0.6669 7.6387 3.6228 3.6597 3.8511 0.0000 1.1576
3.0803 2.2876 2.8488 2.7076 2.2807 1.8221 0.0000 4.4324
5.8707 2.1124 2.2249 2.7905 2.2146 3.5722 2.1741 2.5274
3.0353 1.0645 0.6337 0.5964 2.1192 2.6119 3.1267 0.0000
3.4128 1.7169 1.5372 2.7363 3.4542 3.1116 1.9573 8.4511
3.3598 3.3105 1.9486 4.4767 2.3229 1.3163 3.7747 4.3042
2.1685 4.3983 3.0410 2.5585 0.7334 0.6828 2.0012 2.7166
2.6476 2.2967 2.2808 3.7390 0.0000 0.0000 1.3776 3.2727
3.2800 0.0000\
B3GAT1 0.9511 4.9059 8.5595 4.6476 3.5899 3.2400 5.6673
3.3166 4.3390 4.8171 7.2516 4.2463 3.9188 4.9831 1.2358
6.0153 6.7000 3.2578 5.3186 4.7948 6.1052 2.1923 6.7200
4.5240 2.5058 4.8678 5.6025 2.3554 8.3735 1.8184 8.0837
3.0237 2.7201 5.4130 0.0000 4.3459 9.4949 2.8736 0.5410
1.2646 7.6941 5.5603 0.6231 3.3350 2.0387 6.7735 1.1888
1.1317 2.8917 7.6949 4.2254 5.7681 5.6732 3.7315 6.3758
5.4824 3.8331 7.7400 7.0737 4.7683 2.5892 6.0072 6.5743
1.8326 3.4971 4.6761 7.5061 2.3155 3.2274 2.0689 3.0632
6.8532 5.6971 3.7643 8.0576 0.0000 3.3091 3.3356 5.6441
3.2509 2.2758 4.4304 4.7147 2.5223 5.3749 7.7710 2.8722
0.6140 5.0724 8.5655 5.2733 3.2907 8.5821 5.0946 3.2745
4.2441 9.4497 5.8107 1.8231 5.0666 1.4180 5.7943 3.6120
4.1402 2.7166 1.7170 3.2604 5.2722 9.0280 7.0137 3.7441
1.8846 5.8481 3.4594 0.8924 4.8307 2.1803 4.5234 1.8223
4.9752 7.7809 2.8168 6.5511 3.3012 6.1014 4.0953 1.8708
2.7607 6.0393 3.8142 2.7322 2.5146 3.0001 4.1901 4.4170
4.3186 4.4769 6.8690 4.5899 7.3413 3.2124 4.8772 4.7356
3.1308 2.2385 2.0673 4.2362 6.6137 4.3117 0.0000 5.5731
3.4258 8.2770 2.4626 0.5476 6.6156 5.6236 1.7867 2.6223
3.6311 1.0317 3.3241 4.3136 8.4471 4.8193 5.1184 7.2377
6.1799 1.8676 0.0000 0.7903 2.9341 7.9755 5.5679 0.0000
4.8595 4.5418 4.3319 1.3803 6.1333 3.3748 0.0000 2.0275
3.2946 4.2470 3.3525 5.3811 4.5288 1.1063 2.2874 2.6970
4.9572 4.1198 4.3148 2.4698 1.5946 8.9275 7.4343 2.8496
2.4955 4.1984\
FAM66E 0.0000 1.2250 0.0000 2.3445 0.0000 0.4059 0.0000

| | | | | | | | |
|---|---|---|---|---|---|---|---|
| 2.2919 | 1.4589 | 0.4413 | 0.7594 | 2.6308 | 0.0000 | 1.2777 | 0.5377 |
| 0.0000 | 0.0000 | 1.2795 | 0.0000 | 0.3755 | 0.0000 | 1.1310 | 1.6825 |
| 1.2144 | 2.0961 | 0.0000 | 0.8021 | 1.0210 | 0.8532 | 0.0000 | 0.9954 |
| 1.7560 | 0.0000 | 1.3374 | 0.0000 | 0.0000 | 1.5255 | 0.3648 | 0.0000 |
| 0.7666 | 1.1184 | 1.1407 | 0.0000 | 0.4326 | 1.6194 | 0.9285 | 0.0000 |
| 0.0000 | 1.9464 | 0.4764 | 0.0000 | 0.0000 | 1.7111 | 2.2666 | 0.0000 |
| 0.5608 | 0.0000 | 0.5030 | 1.0204 | 0.5715 | 0.5422 | 0.0000 | 0.0000 |
| 0.5968 | 1.1445 | 0.3906 | 0.0000 | 1.5796 | 0.0000 | 0.0000 | 0.7662 |
| 2.1491 | 0.9449 | 0.0000 | 0.9270 | 1.8854 | 1.5242 | 1.4952 | 0.0000 |
| 0.6159 | 0.0000 | 0.0000 | 0.7204 | 0.0000 | 2.0290 | 1.3843 | 1.7039 |
| 0.0000 | 0.5273 | 1.2149 | 1.6854 | 0.0000 | 0.9036 | 1.1561 | 0.5198 |
| 1.8952 | 0.5233 | 1.5047 | 1.9860 | 0.5504 | 0.0000 | 0.0000 | 1.4835 |
| 0.0000 | 0.0000 | 0.0000 | 0.0000 | 0.0000 | 0.5426 | 1.3268 | 1.4385 |
| 1.3871 | 0.0000 | 0.0000 | 0.0000 | 1.8640 | 1.8676 | 0.0000 | 0.3974 |
| 0.0000 | 0.0000 | 0.0000 | 0.6224 | 0.0000 | 0.5970 | 0.0000 | 0.0000 |
| 0.7843 | 0.5619 | 1.9820 | 0.3935 | 0.0000 | 1.2631 | 1.2511 | 0.8107 |
| 0.0000 | 1.0837 | 0.0000 | 0.9038 | 2.2561 | 1.3507 | 0.9848 | 1.0973 |
| 0.0000 | 1.0811 | 2.3512 | 0.6735 | 0.0000 | 0.9947 | 0.0000 | 1.6233 |
| 0.0000 | 2.9691 | 2.7037 | 1.5093 | 0.7727 | 2.7525 | 2.0225 | 0.0000 |
| 0.0000 | 0.0000 | 0.0000 | 0.5821 | 1.3855 | 0.4418 | 0.0000 | 0.0000 |
| 1.5478 | 0.9131 | 1.7238 | 1.2980 | 1.2195 | 0.5410 | 2.5508 | 0.0000 |
| 0.4801 | 1.4586 | 1.4008 | 0.4012 | 1.0692 | 0.0000 | 0.0000 | 0.5257 |
| 0.8792 | 0.7366 | 1.8914 | 2.7975 | 0.7058 | 0.0000 | 0.0000 | 0.0000 |
| 0.5556 | 1.2903 | 0.7547 | 1.4064 | 0.0000 | 0.6064 | 2.6496 | 0.0000 |
| 1.1797 | 0.0000\ | | | | | | |
| FAM66C | 2.9134 | 5.1059 | 5.1423 | 6.2254 | 2.3147 | 5.7797 | 2.9738 |
| 5.4422 | 4.8571 | 5.7147 | 3.4655 | 5.7691 | 5.2904 | 5.4029 | 3.3269 |
| 3.7058 | 4.6892 | 4.8137 | 4.3543 | 3.7214 | 4.9992 | 2.6223 | 5.3101 |
| 4.8444 | 5.7390 | 2.8612 | 4.6746 | 3.8969 | 2.3312 | 1.5953 | 4.8820 |
| 3.9322 | 0.5159 | 4.1534 | 4.1708 | 2.2096 | 4.4987 | 4.3620 | 2.3491 |
| 2.5630 | 4.2593 | 5.4586 | 1.8880 | 5.2236 | 5.3721 | 3.7705 | 3.8516 |
| 1.7580 | 3.9985 | 3.0077 | 3.5481 | 2.4004 | 5.3503 | 6.0858 | 6.4213 |
| 2.9356 | 3.2145 | 6.5359 | 4.2083 | 3.8203 | 5.6383 | 3.8235 | 3.0129 |
| 3.4915 | 4.9942 | 2.2422 | 3.5590 | 6.5860 | 1.5697 | 4.4087 | 4.1553 |
| 4.6466 | 2.2328 | 4.2846 | 4.4114 | 2.6757 | 5.5244 | 3.1994 | 5.0381 |
| 4.1731 | 2.2758 | 2.9927 | 4.9445 | } | | | |
