# Supplementary material for: Identification and validation of hub genes in uterine corpus endometrioid carcinoma: An observational study from TCGA and GEO
Source: Medicine (Baltimore). 2025 May 2;104(18):e42338. doi: 10.1097/MD.0000000000042338 (PMC12055170; doi:10.1097/MD.0000000000042338)
Supplement: Supplementary file 2 [file medi-104-e42338-s002.pdf]

```
{\rtf1\ansi\ansicpg936\cocoartf2639
\cocoatextscaling0\cocoaplatform0{\fonttbl\f0\fswiss\fcharset0
Helvetica;}
{\colortbl;\red255\green255\blue255;}
{\*\expandedcolortbl;;}
\paperw11900\paperh16840\margl1440\margr1440\vieww11520\viewh8400\vi
ewkind0
\pard\tx566\tx1133\tx1700\tx2267\tx2834\tx3401\tx3968\tx4535\tx5102\
tx5669\tx6236\tx6803\pardirnatural\partightenfactor0

\f0\fs24 \cf0 Tag	GSM425837	GSM425838	GSM425839
	GSM425840	GSM425841	GSM425842	GSM425843
	GSM425844	GSM425845	GSM425846	GSM425847
	GSM425848	GSM425849	GSM425850	GSM425851
	GSM425852	GSM425853	GSM425854	GSM425855
	GSM425856	GSM425857	GSM425858	GSM425859
	GSM425860	GSM425861	GSM425862	GSM425863
	GSM425864	GSM425865	GSM425866	GSM425867
	GSM425868	GSM425869	GSM425870	GSM425871
	GSM425872	GSM425873	GSM425874	GSM425875
	GSM425876	GSM425877	GSM425878	GSM425879
	GSM425880	GSM425881	GSM425882	GSM425883
	GSM425884	GSM425885	GSM425886	GSM425887
	GSM425888	GSM425889	GSM425890	GSM425891
	GSM425892	GSM425893	GSM425894	GSM425895
	GSM425896	GSM425897	GSM425898	GSM425899
	GSM425900	GSM425901	GSM425902	GSM425903
	GSM425904	GSM425905	GSM425906	GSM425907
	GSM425908	GSM425909	GSM425910	GSM425911
	GSM425912	GSM425913	GSM425914	GSM425915
	GSM425916	GSM425917	GSM425918	GSM425919
	GSM425920	GSM425921	GSM425922	GSM425923
	GSM425924	GSM425925	GSM425926	GSM425927
	GSM425928	GSM425929	GSM425930	GSM425931
	GSM425932	GSM425933	GSM425934	GSM425935
	GSM425936	GSM425937	GSM425938	GSM425939\
FAM95B1 /// LOC101928261 /// LOC102724314	8.2	244.2
106.6	14.2	24.6	6.7	11.3	22.8	17.0	20.2	63.5
	58.6	8.1	18.7	25.6	18.6	15.4	70.6	55.2
	10.4	329.5	89.8	80.2	5.0	43.2	27.9	12.2
	103.4	71.3	15.4	42.0	209.1	165.5	13.1	43.4
	8.0	37.6	12.3	17.8	19.3	25.3	7.9	32.9
	17.1	90.7	37.9	19.1	21.4	507.9	8.4	24.9
	88.3	23.3	108.9	66.3	24.8	28.4	16.8	21.5
	41.8	87.5	43.7	52.0	99.7	87.0	88.9	6.3
	80.9	30.3	51.3	195.8	112.5	72.0	8.7	11.9
	48.1	52.0	22.5	79.5	22.8	220.5	240.3	8.3
	20.4	301.5	444.3	123.3	143.5	53.8	72.8	10.1
	97.1	90.0	25.4	115.2	44.1	229.9	20.8
133.2	89.8	64.1	27.2	13.1\
HTR4	54.666666666666664	142.83333333333334
80.76666666666667	54.400000000000006	127.83333333333333
	85.93333333333334	75.10000000000001
52.2000000000001	130.9333333333333	155.5666666666667
```

	92.36666666666667	96.26666666666667	20.2	40.4
	149.66666666666666	59.166666666666664
61.06666666666666	108.16666666666667	87.5
79.96666666666667	291.1333333333333	134.00000000000003
	243.03333333333333	63.6	69.33333333333333
112.33333333333333	98.96666666666665	343.5666666666666
	173.63333333333333	164.4	106.89999999999999
92.73333333333333	152.06666666666666	79.86666666666666
	136.63333333333333	152.29999999999998
17.366666666666664	231.0333333333334	37.36666666666667
	102.63333333333333	82.10000000000001
85.36666666666667	170.0666666666667	66.43333333333334
	41.0	123.89999999999999	80.8	90.33333333333333
	93.8	158.93333333333334	143.4	94.39999999999999
	108.63333333333334	169.79999999999998
133.5666666666667	219.8666666666667	134.79999999999998
	139.86666666666665	84.53333333333333
240.0666666666663	199.70000000000002	70.89999999999999
	69.23333333333333	76.73333333333333
98.89999999999999	110.0	124.2	42.666666666666664
141.6	111.33333333333333	215.36666666666665
215.10000000000002	101.26666666666665	79.39999999999999
	112.36666666666666	22.366666666666664	71.1
99.96666666666668	85.56666666666666	33.333333333333336
	181.73333333333335	52.86666666666667
85.43333333333334	142.29999999999998	184.13333333333335
	89.89999999999999	90.89999999999999	182.0
229.20000000000002	173.5666666666667	44.9
95.76666666666667	101.33333333333333	85.2
82.13333333333334	111.33333333333333	84.73333333333333
	112.2	64.63333333333334	161.36666666666665
144.0	100.33333333333333	102.23333333333333\
LOC100133669	59.8	44.4	90.9	26.6	231.2	119.9	7.5
	177.2	80.5	53.4	51.9	34.9	45.2	63.5	61.2
	55.4	96.6	101.1	90.1	20.7	171.9	140.4	90.0
	40.9	52.8	64.8	90.5	31.5	125.4	44.3	30.4
	24.7	128.8	15.2	20.7	26.5	7.7	77.3	44.6
	58.0	80.0	53.3	16.0	78.1	84.0	36.4	76.7
	60.6	166.0	34.0	31.8	73.0	26.8	71.9	19.6
	23.6	11.0	93.6	35.0	40.7	49.3	26.6
260.6	5.7	73.9	173.7	15.2	9.3	16.7	75.7
236.8	157.8	99.7	24.6	112.8	21.2	177.3	14.3	6.6
	128.7	118.6	87.7	163.0	112.3	79.9	123.0
136.9	21.8	80.3	34.2	15.3	263.4	95.0	43.9	95.0
	31.6	122.4	39.7	137.2	24.2	78.4	106.4
78.8\
CRHBP	57.4	61.9	5.9	41.9	105.0	3.1	33.8	71.1
	6.1	81.8	23.1	3.2	21.9	8.8	130.3	59.0
	26.6	4.9	31.1	4.6	9.0	10.4	7.0	4.3
	27.1	9.5	59.8	137.3	73.0	28.0	7.7
115.5	9.4	76.7	93.8	65.6	39.5	71.4	38.8	19.5
	71.2	27.1	117.6	41.0	23.2	15.2	32.3	5.7
	103.0	62.5	9.2	103.8	5.8	168.0	54.9	20.6
	17.2	85.4	85.4	80.1	713.5	57.3	14.8	11.9

11.5 117.1 54.1 25.5 9.8 18.0 83.8 25.0
72.8 8.6 4.1 29.0 7.0 54.0 74.0 18.9
133.3 106.5 8.4 25.0 7.2 315.5 97.6
117.6 47.5 7.9 4.3 37.6 57.5 17.1 13.8 46.5
73.4 87.0 52.6 20.3 26.6 48.7 97.3\
HTR6 56.2 47.95 61.5 28.85 49.7 47.2
47.050000000000004 107.95 83.1 132.25 65.85 17.85
42.650000000000006 71.7 131.05 39.1 109.45 73.45 19.5
63.800000000000004 114.8 86.95 132.2
28.200000000000003 59.35 32.35 49.55 149.55 52.0 41.8
20.25 17.15 88.65 76.1 192.65 88.85 21.65 37.4
61.150000000000006 28.599999999999998 75.6
56.699999999999996 26.9 89.95 9.35 64.65
82.19999999999999 43.25 47.6 41.449999999999996
49.050000000000004 70.05 131.95000000000002
149.54999999999998 75.1 75.85 23.35 108.75 75.45
29.35 396.75 26.450000000000003 83.95 74.4
75.89999999999999 78.45 51.75 19.0 58.6 33.2
31.25 49.099999999999994 109.19999999999999 35.9
22.45 32.050000000000004 49.45 38.75 71.0 87.25
125.55 28.45 19.3 64.3 132.1 52.699999999999996
77.05 27.6 88.10000000000001 40.05 62.550000000000004
123.35000000000001 48.75 31.5 19.0 24.85 54.1
26.6 26.25 64.75 85.95 47.9 41.7\
HTR7 36.733333333333334 22.166666666666668
27.96666666666667 8.666666666666666 87.36666666666666
20.26666666666667 33.300000000000004
69.89999999999999 24.166666666666668 94.0 73.7 21.8
15.6 28.433333333333334 40.766666666666666 28.3
26.033333333333335 20.433333333333334 21.8
35.699999999999996 52.833333333333336 52.03333333333334
42.199999999999996 21.866666666666664 12.1
34.96666666666667 42.93333333333334 71.6
41.166666666666664 59.46666666666666 48.800000000000004
39.666666666666664 39.266666666666666 9.0
86.13333333333333 52.0 36.699999999999996 46.9
38.03333333333333 15.766666666666666 24.133333333333336
30.733333333333334 46.93333333333334
11.933333333333332 53.166666666666664 87.59999999999998
52.1 7.099999999999999 49.1 24.5
42.699999999999996 26.7 29.23333333333333
50.03333333333333 28.333333333333332 51.800000000000004
13.1 71.06666666666666 13.6 57.06666666666666
295.43333333333334 54.06666666666666 57.4
34.733333333333334 14.4 60.36666666666667 30.8
22.266666666666666 37.666666666666664 29.7
40.26666666666666 84.56666666666666 43.79999999999999
21.833333333333332 40.23333333333333 36.6
46.333333333333336 62.43333333333334 33.0
14.233333333333334 67.56666666666666 34.46666666666667
31.73333333333333 45.366666666666674
158.03333333333333 117.33333333333333 49.199999999999996
50.76666666666667 72.43333333333334
13.733333333333334 27.266666666666666 55.699999999999996

43.8666666666667	20.3	53.666666666666664	32.4
88.56666666666666	30.8	35.96666666666667
49.96666666666667	20.066666666666666	66.53333333333335
13.066666666666668\
LURAP1L	411.1	281.8	358.8	101.3	651.8	111.4	132.1
487.3	272.8	361.5	309.7	298.0	256.8	348.9	211.2	75.6
438.9	392.3	161.9	376.0	248.0	267.9	479.0
405.7	591.0	238.2	625.4	260.5	386.9	539.8	196.5
379.8	52.8	291.4	1275.0	293.4	153.0	117.6	438.8
219.0	127.9	243.4	170.9	210.2	144.9	259.8	140.7
1638.9	414.6	219.3	262.9	307.0	346.4	403.3	298.5
305.1	271.8	393.5	228.6	383.8	460.2	164.0	358.4
771.5	203.0	302.1	298.1	517.7	268.3	243.1	439.9
247.1	148.6	281.5	286.2	194.5	332.1	170.6	201.3
154.0	149.5	284.2	231.6	723.6	266.2	23.6	150.8
366.2	218.0	285.3	234.8	207.8	137.5	180.5	125.2
166.4	283.4	228.6	193.0	137.9	199.0	157.7	169.6\
APITD1 /// APITD1-CORT	1386.9	304.9	686.8	1366.4	435.0
1300.8	1194.5	613.2	1257.5	1164.3	933.2	1344.9	1371.4
1012.0	891.7	429.5	371.8	2705.9	1580.0	716.1	398.0
2162.1	988.7	1027.2	1720.6	1268.1	2549.9	720.4	704.8
1239.4	648.4	1164.5	1015.0	902.5	1499.0	774.4	1813.2
1023.8	672.1	506.9	741.2	662.9	1392.7	1029.1	1401.0
2489.3	1709.2	1961.0	505.6	479.7	1585.2	1942.0	1352.2
832.6	1146.1	637.5	1192.2	727.4	1452.5	1221.6	1158.9
1259.5	943.5	1116.5	435.7	848.1	875.0	644.0	927.8
1715.5	717.5	1797.0	234.5	1182.3	523.5	878.1	1703.7
1071.0	785.2	787.3	1813.3	1779.5	1489.3	651.9	2242.7
808.9	1758.4	1186.6	1091.7	904.7	999.1	865.6	1266.3
583.7	483.4	924.6	1371.2	672.5	610.8	886.8	643.8
1034.2	655.0\
FAM19A2	192.6	25.6	110.6	174.0	47.4	55.8	151.1	66.4
290.2	75.4	92.3	32.3	46.6	30.2	38.0	61.6
63.2	137.7	26.2	159.1	64.5	48.3	159.7
232.0	39.6	38.4	38.1	258.9	71.0	205.1	584.3
128.6	45.6	227.3	136.4	227.9	38.3	3.8	175.0
532.5	108.9	15.3	346.6	115.9	77.9	99.5	150.9
121.5	44.2	244.4	100.9	60.0	42.6	218.7	146.2
119.1	64.0	26.4	21.5	363.0	598.4	31.6	556.0
764.1	79.7	29.6	246.3	144.9	163.2	210.7	162.0
171.5	127.1	244.8	291.3	171.2	298.9	287.4	322.1	17.8
43.0	93.9	73.9	25.7	64.8	135.7	145.3	65.5
32.4	54.8	113.7	64.4	180.7	221.1	207.6
337.7	270.0	200.1	82.2	350.2	191.6	84.9	192.8\
BBX	1116.457142857143	3260.3571428571427
1203.185714285714	1589.1000000000001	1444.7428571428572
999.9857142857144	1096.9428571428573
2167.2142857142853	1077.257142857143	1676.2285714285715
1372.314285714286	1901.1	1609.7428571428572
1332.3142857142855	1814.3857142857144	2069.6571428571433
1366.5571428571427	1419.3142857142855
1199.457142857143	935.6857142857144	924.342857142857
1886.8714285714286	1536.2571428571428
1291.5428571428572	1523.9428571428573	2033.3714285714289

        2008.714285714286   1652.0 1609.4142857142858
1459.4142857142858   1550.6571428571428   2908.4000000000005
        1691.7857142857142   2323.7714285714287
2817.0142857142855   2179.1857142857143   910.9142857142857
        834.9   1069.2714285714287   1561.5428571428572
1166.0857142857142   1779.5142857142855   1373.542857142857
        1366.5285714285715   1441.1 1812.5 2299.614285714286
        899.9428571428572   1946.6714285714284
1311.1857142857145   1070.4857142857143   2492.5857142857144
        1529.8999999999999   1728.1142857142856
1374.6000000000001   2307.442857142857   1789.6285714285714
        1547.8714285714284   1469.9857142857145
1736.3285714285714   1206.7714285714285   2145.6285714285714
        1574.6285714285714   1507.3285714285716
1238.4714285714285   1782.8999999999999   1253.6285714285714
        1419.428571428571   1666.3999999999999
1728.114285714286   1635.1142857142859   1550.185714285714
        1322.4571428571428   1000.2571428571429
1032.8285714285714   1367.742857142857   1702.6142857142856
        1270.6714285714286   1555.3000000000004
7709.742857142858   1415.1142857142859   2021.2571428571428
        1257.5571428571427   1837.142857142857   2151.0
1038.1142857142856   1211.3285714285714   2094.0714285714284
        1487.2428571428572   2898.128571428571
1402.1142857142856   1243.2714285714285   1311.4714285714288
        1609.5142857142855   1805.9714285714285
1436.0714285714282   1818.1000000000001   1546.6571428571428
        1739.2714285714285   1489.4142857142856
1660.9428571428573   1691.3857142857141   1741.3714285714286\
FAM19A1 8.7   6.8   8.9   56.6   29.0   8.4   2.3   39.9
        2.5   6.8   5.3   28.1   2.1   8.2   8.1   42.9
        5.9   4.5   7.0   3.6   20.5   4.9   7.5   23.9
        8.0   4.4   2.3   8.2   12.1   7.3   5.4   10.9
        5.3   7.1   4.9   4.4   5.3   10.1   18.9   8.6
        2.9   9.2   28.9   4.0   4.2   39.9   3.2   4.9
        3.4   40.8   69.0   17.5   44.1   20.4   3.7   26.8
        47.9   18.9   10.0   3.5   207.6   3.5   24.0   34.9
        11.9   4.1   3.9   17.3   40.0   4.5   5.2   3.2
        10.5   4.1   11.4   13.3   2.7   3.4   9.8   39.0
        7.9   8.6   51.5   14.9   4.4   5.3   4.2   4.4
        25.0   11.4   6.7   10.3   5.4   38.0   6.4   2.7
        27.3   8.9   54.4   6.7   46.8   4.7   51.2\
LINC01000 /// LINC01001 /// LINC01002 /// LOC100132062 ///
LOC100133182 /// LOC100133331 /// LOC101929819 /// LOC729737
1166.2 5552.3 4312.2 1152.0 7297.5 799.0   576.9   7130.2
689.4   1352.6 905.7   3725.3 1061.4 1335.9 2287.5 1462.7
1355.3 1710.0 644.4   2593.7 8634.4 1443.0 3657.8 1446.2
616.0   5664.9 1146.9 6555.4 3958.9 1603.0 1080.8 3388.4
8424.0 700.7   3350.7 3497.3 734.3   4789.0 1372.4 644.1
599.4   1089.8 911.7   514.6   705.9   1983.7 4824.8 955.5
16982.9 3545.2 1370.9 1336.5 3185.0 15834.2 2840.0 7518.7
1816.1 4014.1 3317.9 3295.9 2552.6 1724.2 2180.5 1303.3
2056.8 4477.9 917.0   2075.9 8430.5 1395.8 6337.7 4954.6
2039.7 1757.1 1859.1 956.6   3698.2 1945.9 2362.9 1449.1

3745.4 3019.4 1026.5 2131.5 16523.4 11081.3 8423.6 3546.6
634.3 3311.3 524.9 3584.3 1519.8 2121.7 2840.5 1530.4
1877.7 2138.9 4284.8 2077.8 3790.5 1577.5 2241.4\
FAM19A5 14.6 15.666666666666666 159.1 83.33333333333333
93.5 64.96666666666667 236.20000000000002
86.69999999999999 236.4333333333333 19.233333333333334
27.333333333333332 73.83333333333334
43.03333333333333 1417.9333333333334 32.56666666666667
37.699999999999996 226.23333333333335
260.6333333333334 22.566666666666666 201.4333333333333
196.80000000000004 39.00000000000001
49.20000000000001 642.1 27.466666666666665
20.566666666666666 62.73333333333333 237.36666666666667
98.33333333333333 87.13333333333333
195.20000000000002 38.333333333333336 60.03333333333333
166.03333333333333 24.866666666666664 119.8
776.0333333333334 22.5 15.366666666666667
111.83333333333333 368.43333333333334 472.0333333333333
423.7666666666667 1542.5 40.3 175.76666666666665
115.5 219.5666666666667 43.63333333333333 67.7
109.10000000000001 59.800000000000004
27.100000000000005 201.5 289.1333333333333
31.133333333333336 179.36666666666667 142.83333333333334
40.9 140.29999999999998 136.20000000000002
99.93333333333334 56.199999999999996 57.53333333333334
290.6 100.46666666666665 137.5 1058.5666666666666
293.8666666666666 126.89999999999999
76.93333333333334 37.13333333333333 122.63333333333333
466.4666666666667 111.73333333333333
94.16666666666667 807.4 104.66666666666667 193.6
231.1 118.33333333333333 46.1 63.133333333333326
109.33333333333333 173.0 77.63333333333334
11.366666666666667 548.0 104.56666666666666
44.800000000000004 408.7666666666667 661.1333333333333
381.8333333333333 248.13333333333333
393.79999999999995 192.79999999999998 149.9333333333333
160.1666666666666 143.29999999999998
288.23333333333335 181.4 152.70000000000002
128.2666666666668\
FAM19A4 3.3 10.1 3.4 4.1 5.9 0.9 13.4 4.9
8.8 58.0 29.0 5.6 45.9 2.0 28.8 87.5
39.3 15.2 22.0 7.4 66.3 61.1 99.7 10.1
3.3 13.8 12.6 55.5 59.6 5.4 30.8 74.9
4.7 29.4 16.1 10.2 8.4 40.2 4.6 28.7
3.4 11.8 8.9 42.7 20.3 53.6 40.8 36.3
13.6 23.6 13.9 9.9 7.7 5.5 8.7 41.1
9.2 8.8 21.9 9.5 163.3 8.9 58.1 6.3
1.5 10.4 5.7 19.7 4.2 11.4 6.8 83.2
35.3 46.7 18.4 4.9 49.2 3.9 7.3 5.1
10.7 50.9 2.5 2.1 28.4 125.1 7.7 3.9
53.7 51.0 25.6 71.4 11.4 17.5 1.4 28.8
33.9 2.4 29.6 30.5 32.7 4.1 29.1\
WDR83OS 1798.1 2295.8 1062.2 2071.2 1043.9 2784.4 2024.6
842.4 1384.0 1272.3 4709.4 1715.9 1840.1 1683.6 2107.3

2155.7	2007.7	840.8	1899.6	2021.7	881.0	1484.0	1178.5	
1404.1	979.4	2366.0	3233.8	1149.7	1796.4	2455.5	3559.9	
884.3	1587.8	3383.1	3791.0	2207.8	1608.7	985.1	2864.2	
3683.9	2583.8	2636.9	1212.3	2136.1	1648.6	1282.3	1889.5	
1863.2	2477.8	2345.0	1455.6	2539.4	2372.2	981.5	2252.4	
3744.0	1092.8	1090.1	1611.7	1122.8	861.6	1539.5	1349.8	
1912.4	1947.9	1970.6	2032.8	2796.2	1008.1	2865.0	804.7	
927.5	1695.1	2501.9	1454.5	3881.1	1359.5	1789.6	2176.8	
1056.1	1857.7	848.7	2998.7	2281.8	710.1	602.5	1728.4	
1016.7	1246.2	922.0	4723.0	1313.3	2825.7	3298.4	2583.8	
2754.0	2575.4	2036.3	1986.4	1678.1	2027.3	1194.0	3507.2\	
GSTK1	1865.2	3394.9	5386.9	1838.0	3023.7	2789.8	2518.5	
777.4	1802.2	3368.5	1958.9	2715.5	2261.9	2816.9	802.2	
1797.1	1840.1	3330.8	4419.2	2715.3	946.9	2454.3	2224.4	
2929.4	1254.1	3483.8	3150.2	1802.6	1674.9	2318.2	2514.2	
2936.0	2931.9	3038.5	2957.0	2945.2	2456.2	1971.4	3979.3	
2133.2	1636.0	2565.0	2088.7	1896.9	6068.3	2877.9	1304.2	
912.0	2326.9	5795.7	2629.9	2977.0	3988.1	752.9	2837.3	
1450.2	2702.2	2107.6	2408.8	2216.8	1050.1	3709.1	2177.2	
2443.6	1998.1	2799.4	3271.8	1803.2	2862.6	3585.3	2192.0	
2253.9	2102.9	2391.9	2923.4	3162.0	1971.6	2932.3	1624.3	
483.1	924.3	815.6	2618.8	2594.2	1325.5	576.1	744.6	
1704.5	2617.9	815.0	3261.7	1231.0	2941.4	2502.0	1868.5	
2549.9	2423.1	2571.0	3811.5	2872.4	2431.7	1910.7	2874.5\	
DHDH	88.2	98.0	23.0	43.5	90.5	184.0	12.0	31.7
	9.0	72.3	7.1	12.6	63.8	25.6	32.9	58.7
	93.3	19.2	42.5	46.9	31.5	13.2	38.4	26.1
	12.4	54.0	13.3	27.7	28.2	13.8	72.9	65.5
	14.8	9.9	44.5	48.0	9.5	28.3	8.7	33.6
	15.5	181.4	64.6	40.0	8.0	27.2	15.4	
219.2	159.2	11.0	74.7	13.1	104.3	30.6	8.1	
168.2	51.0	9.7	20.5	87.7	50.2	83.6	14.7	11.4
	6.3	77.6	4.0	12.2	31.9	11.4	17.1	17.0
	6.9	52.6	6.9	6.3	23.7	61.3	10.0	20.6
	67.0	127.0	122.2	35.3	61.0	47.2	17.0	24.6
	8.3	25.2	11.7	11.6	60.3	11.2	32.3	11.6
	58.6	13.1	94.7	40.3	18.0	16.5	17.5\	
SAA2-SAA4 /// SAA4			67.5	172.4	3177.9	139.4	197.9	
132.6	72.8	223.9	193.1	1356.8	133.2	56.7	391.5	
271.9	193.7	232.8	104.6	467.9	138.6	602.3	619.0	
154.8	618.3	301.5	114.9	102.6	794.2	611.5	532.7	
223.4	67.3	326.2	3308.8	133.7	426.8	271.8	29.4	
489.4	646.1	177.4	87.7	86.7	640.0	106.9	1038.2	
184.9	1203.0	72.1	890.1	239.4	141.0	434.8	598.6	
406.4	228.1	235.5	85.4	225.2	762.3	297.4	834.4	
1488.9	746.2	2226.6	250.3	1652.9	138.7	110.0	1125.8	
1593.1	273.7	138.6	150.4	105.6	22.3	870.4	958.0	
104.4	483.0	13.2	134.8	109.3	70.6	213.1	408.2	
642.2	154.3	610.0	418.5	401.5	17.1	94.5	84.1	84.1
	83.9	69.9	148.2	80.6	32.8	113.3	53.1	65.2
	74.8\							
BCR	455.3333333333333			494.8333333333333				
551.4666666666667			438.90000000000003			591.6666666666666		
	638.9	1177.0333333333335			440.8666666666666			

546.3666666666667	488.6666666666667	582.5333333333333
	803.6999999999999	490.76666666666665
693.0333333333334	391.8	638.5333333333333
1149.9333333333332	511.5666666666666	982.8666666666668
	468.0	468.90000000000003	624.6666666666666
448.3333333333333	480.4333333333334	358.9666666666667
	474.5333333333333	563.4	425.43333333333334
612.5666666666666	464.93333333333334	535.0333333333333
	426.1000000000001	532.7666666666667
594.8333333333334	268.43333333333334	438.6000000000001
	306.1	550.3333333333334	790.2333333333332
491.2	789.7666666666668	457.09999999999997
377.4666666666667	447.2	843.6666666666666	341.5
921.5666666666666	309.73333333333335	1221.6333333333334
	515.6	450.6666666666667	440.93333333333334
523.6666666666666	676.8333333333334	607.0333333333333
	764.2000000000002	557.9666666666667
425.5333333333333	408.4666666666667	330.40000000000003
	541.0666666666666	604.0666666666666
510.9666666666667	487.6666666666667	578.6999999999999
	859.4	546.6333333333333	509.8	618.4333333333334
	543.7333333333333	701.6	423.93333333333334
674.7666666666667	736.6333333333333	719.6	571.6
792.6666666666666	703.0333333333333	653.6999999999999
	477.20000000000005	343.1666666666667
561.7666666666667	412.9333333333334	452.9666666666667
	756.2333333333332	2933.2333333333336
1005.8333333333334	663.8000000000001	884.9000000000001
	281.1666666666667	548.1666666666666	656.1
338.0	444.76666666666665	412.76666666666665	335.8
253.19999999999996	357.5333333333333	410.7333333333333
	341.93333333333334	452.5333333333333	465.7
438.23333333333335\
ORAI3	276.9	493.1	485.4	913.7	796.7	1398.0	1432.9
572.7	826.8	1609.5	1205.1	746.5	330.0	1283.1	1546.0
1529.0	451.8	662.1	1379.7	807.1	969.7	789.0	651.5
1094.2	402.9	2283.4	1462.2	1595.6	1666.7	1462.7	1532.8
534.6	1256.1	2440.9	2693.7	1585.4	860.8	799.5	1288.3
870.8	1218.0	584.9	1235.1	1443.5	802.9	916.6	1670.6
744.7	1178.9	1424.2	1463.1	1458.4	1928.9	496.1	1280.1
977.9	1514.7	713.4	923.8	753.6	913.3	1252.3	1066.8
750.0	1299.1	1615.1	1172.4	1733.8	1955.4	1681.6	788.3
1103.5	693.5	1661.4	1097.6	2794.5	1582.7	1118.8	1552.9
435.6	1511.6	533.7	2151.2	2327.5	1116.3	971.6	1049.2
1856.5	600.7	920.8	1052.5	1155.3	2290.0	1593.1	1550.0
2019.6	2224.3	1537.7	1314.7	1621.8	2017.9	1859.5	1689.6\
AGTR1	60.7	134.8	57.3	42.85	165.45	21.75
110.14999999999999	76.15	60.25	81.0	69.75	46.6
53.449999999999996	37.85	118.15	84.0	69.9	84.65
42.45	49.3	97.5	40.95	140.60000000000002	73.55
34.65	61.349999999999994	24.900000000000002	90.4	94.5
	45.25	91.35000000000001	181.05	105.2	65.95
99.14999999999999	136.7	39.35	56.35	51.349999999999994
	44.75	95.45	22.3	77.5	34.65	71.65	64.6

```
67.15	52.449999999999996	141.6	57.0	78.3	79.2	57.6
	172.79999999999998	42.85	98.94999999999999
59.099999999999994	75.55	54.6	66.9	232.0	31.0
34.949999999999996	50.85	66.25	65.3	25.35
34.599999999999994	77.75	113.85000000000001	134.65
71.85	54.300000000000004	45.5	72.25	33.95	60.15
51.599999999999994	52.5	42.05	129.14999999999998
71.75	13.35	40.0	71.9	114.05000000000001	41.75	53.2
	91.6	103.44999999999999	24.2	131.04999999999998
	65.85	51.300000000000004	41.7	27.2	49.75	83.7
	110.95	57.05	94.15	119.05	80.15\
ORAI2	238.35	923.4000000000001	1030.2666666666667
458.1666666666667	1041.6333333333334	206.48333333333335
	574.9333333333333	616.2333333333332
483.5166666666667	860.75	318.3833333333333	747.35
277.7666666666667	482.6333333333334	696.2833333333334
	586.6666666666666	457.98333333333335	801.0
325.3	636.6166666666667	1809.8	499.8666666666673
1091.1499999999999	338.6666666666667	368.93333333333334
	490.0833333333334	320.45	847.7833333333333
583.9666666666668	396.5666666666666	325.34999999999997
	579.5500000000001	1092.5333333333335
414.3166666666666	686.3333333333334	1625.5166666666667
	172.89999999999998	1302.9166666666667
430.53333333333336	257.46666666666664	604.2333333333333
	419.84999999999997	611.85	702.6333333333332
456.6666666666667	569.4333333333333	566.4833333333333
	288.55	815.7333333333332	645.9	473.0333333333333
	273.3166666666666	376.98333333333335
803.1833333333333	525.9	640.85	518.8833333333333
1267.6333333333332	605.8833333333333	873.4666666666667
	362.95	2145.1833333333334	700.35	477.75000000000006
	767.1833333333334	942.1999999999999
357.66666666666674	476.40000000000003	864.1666666666666
	340.8666666666667	994.25	959.7833333333333
700.0666666666667	467.68333333333334	843.0333333333333
	275.5833333333333	616.1666666666666	537.65
463.43333333333334	658.6	836.7166666666666
825.3499999999998	461.1000000000001	419.5
1019.7833333333333	778.3000000000001	520.8666666666667
	614.85	584.3000000000001	811.1166666666667
449.1166666666666	751.3333333333334	300.7333333333333
	355.5833333333333	485.66666666666674
261.71666666666664	306.5666666666666	363.83333333333326
	527.25	389.1833333333332	346.0	572.6833333333334
	294.03333333333336\
ORAI1	249.9	194.7	33.5	227.2	116.9	261.1	98.8
331.0	244.8	385.9	210.9	65.8	160.4	313.5	148.8
119.9	148.3	158.6	308.1	209.7	266.2	257.9	197.6
250.5	363.1	91.7	375.7	135.4	160.0	262.1	157.4
305.8	141.7	280.9	374.8	139.8	311.8	229.6	245.2
277.7	296.3	224.2	234.7	202.1	297.7	298.9	345.5
350.7	239.8	71.9	302.9	257.7	273.7	87.4	264.2
232.2	272.3	141.5	243.0	220.4	735.4	191.8	187.1
```

189.7	156.2	228.8	260.2	190.8	132.5	283.3	132.8
214.1	62.9	212.3	187.8	331.3	240.9	199.6	182.8
136.5	139.0	283.8	285.2	156.6	310.7	331.4	217.0
274.1	306.9	170.9	241.6	307.9	199.3	148.9	103.3
203.4	142.8	196.2	110.2	110.0	26.6	204.0	169.7\
VOPP1	3270.8	1355.3	2895.4	4185.6	2720.3	4790.7	6203.1
3912.0	5553.2	5881.9	5081.9	4999.5	1285.2	5697.6	2397.4
6543.2	4308.2	3261.6	4497.9	5744.5	1811.7	4757.0	2476.4
7033.8	5036.2	5087.0	8365.7	5152.4	3549.3	4835.3	7292.7
2877.9	2297.8	7797.5	6602.9	3618.3	5975.1	1560.7	5939.1
4917.6	3416.1	4258.1	6697.2	9082.6	5843.7	5152.9	5078.5
7751.6	2596.0	4088.1	5682.9	4172.8	3391.3	1328.3	4635.7
3354.0	4642.8	1495.5	3222.1	3475.8	1915.8	3041.5	4735.0
5077.7	5063.0	2639.3	8414.7	7474.0	3400.3	6741.0	2226.3
2560.5	5831.6	6530.7	5933.8	10772.7	3607.3	6754.8	6089.6
2152.6	2136.4	3132.5	5483.9	7247.2	742.1	207.7	1602.7
2573.0	2164.6	940.7	40627.9	1888.7	3413.2	3507.3	2487.4
3061.3	2492.7	3180.7	2991.1	2622.9	2850.3	2260.4	3522.9\
AGTR2	9.733333333333333	49.6	826.8666666666667
33.06666666666667	68.63333333333334	39.199999999999996
	30.2	231.8666666666667	22.86666666666667
10.266666666666666	47.96666666666667	10.9
3041.7666666666664	54.233333333333333	58.46666666666667
	42.333333333333336	54.43333333333334
29.166666666666668	38.86666666666667	80.60000000000001
	62.6	3335.1	76.06666666666666	115.03333333333335
	518.3	35.0	69.3	12.4	42.53333333333333
5.033333333333333	45.699999999999996	13.633333333333333
	62.766666666666666	172.73333333333335
58.93333333333333	3355.266666666667	1107.2
52.666666666666666	17.833333333333332	23.333333333333332
	31.900000000000002	13.933333333333332
54.16666666666668	48.400000000000006	75.83333333333333
	22.833333333333332	47.099999999999994	273.0
67.00000000000001	22.566666666666666	44.63333333333333
	174.4	21.900000000000002	247.79999999999995
48.03333333333333	14.666666666666666	92.83333333333333
	160.9	36.03333333333333	72.3	432.23333333333335
	49.5	30.733333333333334	92.63333333333334
11.366666666666667	47.5	20.566666666666666
32.800000000000004	40.833333333333336	133.8666666666667
	65.53333333333335	71.6	49.800000000000004
18.433333333333334	23.633333333333336	25.866666666666664
	199.70000000000002	1459.1333333333334
52.13333333333333	50.333333333333336	130.23333333333332
	167.70000000000002	7.3999999999999995
16.666666666666668	32.300000000000004	90.23333333333333
	70.43333333333334	585.6	55.333333333333336
25.266666666666666	38.199999999999996	42.43333333333334
	395.3333333333333	5.466666666666668
65.33333333333333	99.23333333333333	267.2
120.43333333333334	383.76666666666665	149.26666666666665
	47.06666666666667	165.03333333333333
145.1666666666666\

COL13A1 19.166666666666668	110.8	78.26666666666667
116.66666666666667	452.6666666666667	76.86666666666667
	577.4666666666666	107.53333333333335
392.43333333333334	1635.8333333333333	48.733333333333333
	116.16666666666667	56.733333333333333
63.699999999999996	656.4666666666667	1427.4666666666665
	114.63333333333333	406.09999999999997
88.53333333333335	222.5	2013.9333333333334
116.56666666666668	132.23333333333332	417.46666666666664
	56.266666666666667	75.43333333333334	87.0
578.3	692.3000000000001	291.96666666666664
102.39999999999999	199.0	250.63333333333335
115.03333333333335	83.7	81.6	63.6	211.6
157.53333333333333	39.53333333333333	542.3000000000001
	219.70000000000002	2520.5333333333333
334.0666666666666	445.1333333333334	184.0666666666667
	143.93333333333334	63.50000000000001
310.0333333333333	1011.6999999999999	176.0
65.43333333333334	113.5	204.33333333333334
562.6999999999999	348.1666666666667	233.5	129.0
105.53333333333335	359.63333333333327	200.76666666666665
	241.96666666666667	450.29999999999995
182.79999999999998	120.93333333333334	334.46666666666664
	104.03333333333335	401.6666666666667
352.6666666666667	83.63333333333334	256.96666666666664
	297.09999999999997	323.26666666666665
33.43333333333333	119.23333333333333	745.5666666666667
	197.9	339.3333333333333	432.03333333333336
251.0333333333333	92.03333333333335	211.33333333333334
	914.5999999999999	5316.966666666666	278.5
94.56666666666666	200.0666666666667	104.2	87.0
120.3	303.6333333333333	311.53333333333336
116.06666666666668	83.66666666666666	152.06666666666666
	60.63333333333333	122.0	130.56666666666666
434.96666666666664	133.5	134.73333333333332	44.6
53.56666666666666\
SHMT2	1844.6000000000001	1169.2	772.8000000000001
1452.0666666666666	701.3333333333334	2785.066666666667
	1985.0	771.3000000000001	1953.4666666666665
1589.4666666666665	4125.133333333333	1377.2	1381.7
814.3333333333334	3622.6000000000004	1119.0333333333335
	2479.8333333333335	1477.3999999999999	2146.1
1131.3999999999999	604.7	1227.5333333333335
659.8666666666667	806.0	1306.8999999999999
2110.5333333333333	938.3666666666667	1089.8333333333333
	1108.7666666666667	1113.6000000000001
1129.1000000000001	1667.6000000000001	890.8333333333334
	1086.5	3360.6999999999994	1874.7333333333333
2306.8333333333335	843.6	1173.6333333333332
1374.1333333333332	1854.1333333333334	3061.2666666666664
	963.7666666666668	1065.8	1237.3999999999999
2237.866666666667	1465.9666666666665	2266.6666666666665
	887.3333333333334	845.9666666666666
1500.1000000000001	2386.2000000000003	2389.5	1485.8

1164.8333333333333	1383.7	1104.4666666666667
1427.1333333333332	951.4666666666667	896.5
3657.366666666667	912.2666666666668	1119.7333333333333
1449.5	607.4666666666667	883.6999999999999
1007.2333333333332	664.6	1353.5666666666666	1142.7
524.2666666666667	1133.5333333333335	539.4666666666667
1634.6333333333332	809.3333333333334
1405.3666666666668	921.6666666666666	893.3333333333334
1085.8	1072.6666666666667	794.5666666666666
836.6666666666666	4899.433333333333	1505.3
753.3333333333334	721.1333333333333	845.8666666666667
1336.4333333333334	1006.9000000000001
1139.2666666666667	2828.366666666667	499.0333333333333
1087.8666666666666	1232.6333333333334
673.2666666666667	1185.9666666666665	1046.0	940.1
968.8666666666667	1409.6666666666667	782.0333333333333
1078.8999999999999	892.0\
TARSL2	223.05	508.40000000000003	387.79999999999995
191.75	311.84999999999997	416.5	62.65	135.55	206.75
118.5	184.55	511.95	216.15	300.95000000000005	115.7
530.9	140.6	292.1	297.59999999999997	339.79999999999995
699.0	206.45	522.85	427.95	282.25	454.0	174.75
417.79999999999995	328.09999999999997	162.1	474.15
197.9	336.15	383.1	136.89999999999998	330.95	304.3
187.0	117.8	234.70000000000002	427.45	228.7
288.29999999999995	474.35	233.95	325.45	141.85	125.7
441.65	511.45	341.59999999999997	120.9	413.8	79.35
263.2	321.6	271.9	339.59999999999997	365.6	685.15
218.9	246.3	252.25	215.14999999999998	547.2	369.1
497.45	304.5	257.65	186.9	474.2	348.09999999999997
608.6	391.2	290.15000000000003	210.0	144.85	237.25
368.0	436.54999999999995	146.1	95.45	369.95	230.6
246.65	315.85	104.15	221.15	205.6	574.1	167.45	338.25
316.59999999999997	479.65	581.0500000000001	555.85
391.0	494.25	442.4	520.9	626.05	328.54999999999995
535.6\
SHMT1	57.7	124.14999999999999	63.55	44.65
99.60000000000001	16.25	53.35	88.1	69.85	82.6
66.75	65.45	35.4	37.199999999999996	19.1	38.9	68.6
40.0	54.800000000000004	86.65	128.75	50.3
126.3	56.8	75.15	35.25	52.599999999999994
57.349999999999994	25.9	34.25	8.05	143.5	48.0
57.650000000000006	83.3	44.1	53.2	136.5	35.85
27.25	4.75	37.8	52.699999999999996	52.95	28.65	78.9
148.05	52.05	113.39999999999999	78.75	84.25
54.400000000000006	93.65	74.3	41.7	60.05
53.449999999999996	135.1	48.45	95.15	750.15	73.65
77.55	59.05	74.4	59.9	4.3	43.7	23.0	47.75	84.7
66.85000000000001	55.55	27.05	52.150000000000006
23.3	101.4	32.8	44.349999999999994	169.2
163.15	178.7	31.65	53.45	230.0	106.05000000000001
173.60000000000002	55.75	25.75	68.15	16.75	114.25
44.45	43.65	31.45	90.8	91.2	44.4	94.5	65.2
48.849999999999994	59.25	69.85\

ORM1 /// ORM2   37.65   47.0   20.7   29.0   56.400000000000006
        41.9   3.75   25.05   130.35 6311.45 21.05   40.35
70.15   66.8   27.95   44.599999999999994   16.85   56.8   5.75
        35.6   130.1   20.85   263.85 13.2   38.699999999999996
        48.9   59.349999999999994   40.45   65.6   62.45
136.6   23.099999999999998   248.8   8.3   250.65 120.85
15.85   51.05   15.1   19.15   6.95   31.1   99.5   20.6
359.8   57.95   124.55000000000001   16.65   108.8   485.95
53.75   20.099999999999998   63.45   52.75   38.6
32.849999999999994   11.65   284.4   124.05 56.0   43.05
26.200000000000003   37.5   41.75   309.0   147.55 245.25
13.899999999999999   55.45   51.0   76.35   31.05   76.75
17.45   32.0   18.05   24.400000000000002   20.9   1206.95 22.7
        33.65   32.800000000000004   8.95   25.05   68.2
82.44999999999999   78.65   57.45   42.45   87.7   18.35
49.15   192.55 12.549999999999999   27.650000000000002
147.65 69.3   183.95 180.35 74.30000000000001   155.6
28.35   117.25\
FOXP1-IT1   366.2   1077.7 1054.3 189.9   688.1   165.3
298.0   672.5   260.3   622.1   227.6   544.1   301.8   429.7
398.9   489.0   386.4   719.7   269.3   478.2   1316.9 186.9
1081.3 147.7   328.1   370.9   188.2   830.5   915.5   323.6
214.1   639.6   654.5   298.4   899.6   455.9   129.6   621.9
304.4   170.7   244.3   251.4   515.3   330.8   163.2   441.1
248.1   146.9   849.3   438.7   281.9   298.2   278.0   672.8
643.6   858.5   327.7   679.9   529.7   873.1   742.6   287.9
475.9   304.1   526.8   640.7   221.3   297.8   384.0   344.0
1135.5 890.3   610.7   294.3   755.0   150.8   441.3   299.8
374.9   783.9   648.0   559.4   241.9   203.6   1022.2 1987.3
477.2   269.4   562.2   812.0   150.8   776.2   372.6   260.0
653.5   364.5   681.1   380.5   709.0   845.7   717.0   198.1
399.6\
LOC643711   14.133333333333333   16.066666666666666   31.8
        17.96666666666667   62.033333333333324
27.400000000000002   8.9   30.633333333333336
15.433333333333332   6.533333333333334   16.8   22.3
9.433333333333334   21.099999999999998   52.26666666666667
        27.899999999999995   18.333333333333332
43.699999999999996   12.700000000000001   3.466666666666667
        43.86666666666667   20.53333333333333   13.4   15.4
        7.5   40.76666666666667   35.13333333333333
42.666666666666664   48.866666666666674   37.4
12.200000000000001   36.53333333333333   63.13333333333333
        8.033333333333333   14.466666666666669
30.333333333333332   27.333333333333332   62.0
16.633333333333333   8.733333333333333   14.166666666666666
        1.633333333333333   25.933333333333334
19.833333333333332   32.36666666666667   24.733333333333334
        6.866666666666667   6.0   45.699999999999996
29.600000000000005   20.333333333333332   20.733333333333334
        26.133333333333336   13.799999999999999
35.266666666666666   45.1   16.7   24.866666666666664
22.633333333333336   35.300000000000004   77.7
30.96666666666665   8.233333333333333   37.06666666666666

22.166666666666668	32.800000000000004
24.133333333333336	27.333333333333332	44.79999999999999
32.5	38.199999999999996	58.93333333333333
31.633333333333336	33.13333333333333	4.466666666666667
16.066666666666666	78.8	30.866666666666664
21.166666666666668	26.833333333333332	62.63333333333333
6.1000000000000005	5.633333333333333
17.566666666666666	84.5666666666666	45.5666666666666
50.0666666666666	36.03333333333333
5.833333333333333	51.23333333333333	19.266666666666666
33.5666666666667	7.266666666666667
10.933333333333332	9.1	16.500000000000004
20.233333333333334	17.333333333333332	58.5666666666666
37.800000000000004	37.666666666666664
41.76666666666667	16.133333333333336\
LINC01001 /// LINC01347 /// LOC101060494 /// LOC101926894 ///
LOC101929038 /// LOC101929819 /// LOC101930127 /// LOC101930567 ///
LOC388572	9.4	32.5	15.4	51.8	20.4	31.4	74.5
162.4	25.1	128.5	13.4	42.4	15.4	39.0	11.7
19.0	12.1	84.5	22.9	15.4	30.4	57.0	51.7
12.2	82.5	20.3	10.9	74.9	60.2	10.0	29.9
93.1	19.4	9.3	40.2	19.1	52.5	70.2	11.0
24.6	6.7	51.6	45.7	6.1	22.1	53.1	26.7
54.2	36.7	72.0	14.9	27.6	33.9	123.2	71.9
74.8	37.7	81.4	128.1	41.1	188.4	53.7	37.3
30.2	75.6	44.9	47.6	29.1	36.6	9.7
224.4	52.9	15.6	18.0	27.6	36.7	54.0	18.2	23.0
51.2	85.6	34.3	19.6	75.5	35.0	137.0	45.5
24.3	35.4	73.7	11.4	26.2	15.7	7.3	30.6
14.5	13.0	7.1	12.9	19.0	37.2	89.1
24.4\
RDH12	81.1	85.2	179.6	8.3	154.3	61.5	58.9	99.6
67.0	21.1	16.7	15.3	24.0	72.2	115.6	41.1
28.1	128.2	24.4	6.0	148.3	110.4	195.4	20.2
40.2	20.7	61.1	47.0	95.3	24.1	5.8	31.4
84.5	13.0	61.9	198.6	46.8	51.9	13.6	80.8
71.2	6.1	36.0	69.3	13.7	50.0	6.2
125.8	36.7	11.2	39.3	130.3	60.5	115.1	78.2	25.6
13.4	30.8	122.9	63.3	460.7	17.8	79.8
129.6	100.2	31.4	34.2	89.6	217.2	129.4	23.1	33.1
6.7	48.3	16.2	16.7	150.7	13.9	107.3	91.7
13.0	18.0	35.1	13.5	68.4	50.8	80.3	11.3
11.1	18.0	55.2	87.9	18.0	10.9	19.0	19.2
195.6	11.0	134.2	14.6	15.1	13.6	21.7\
T	158.5	138.0	83.1	98.4	447.9	132.6	82.5
144.7	81.4	29.8	5.9	33.3	74.6	134.1	200.4	22.1
101.2	169.4	16.3	132.3	221.6	87.0	160.1	69.1
79.9	103.5	22.9	307.9	110.2	132.3	86.6	51.6
147.3	61.5	96.1	84.1	121.2	32.5	88.7	97.3
468.9	70.4	84.0	17.0	89.8	92.8	25.6
165.5	68.1	72.4	89.1	145.9	107.5	108.9	24.9	29.1
90.2	105.5	7.3	118.7	242.9	110.0	109.1	87.7
138.4	200.8	119.1	31.8	97.5	24.2	124.9
223.8	16.7	134.0	130.6	84.3	89.7	82.4	52.2

133.7 93.8 55.9 104.4 52.4 36.0 333.7 157.0 35.3
25.1 79.7 32.0 30.2 81.6 80.4 99.6
107.9 70.8 145.5 112.2 151.4 67.9 186.8 46.4\
RDH11 3984.975 1704.5249999999999 1202.225
3273.5 1907.0749999999998 1864.5500000000002 2412.55
1702.25 1923.05 1546.1000000000001 1797.8749999999998
2037.6 2514.975 1842.1250000000002 1206.1000000000001
1170.9750000000001 1347.8000000000002
2035.0750000000003 2710.8 1956.325 1296.525
1674.8500000000001 1387.7499999999998 2717.4500000000003
2984.2499999999995 2060.9500000000003 3381.325
1309.75 2176.275 1592.9499999999998 1143.475
2217.9 1068.85 1791.5 1379.2749999999999 1388.375
2994.7 1721.575 1547.35 2042.0249999999999
1286.5249999999999 2042.7 1965.225 2195.975
1983.65 1662.3249999999998 3297.275 2711.05 1568.25
1146.95 1446.05 2702.125 1439.95 2277.9750000000004
1850.125 2067.825 1902.9250000000002 1458.325
2010.375 1593.0 1161.95 1224.825 1659.125
1533.8 1213.7250000000001 1314.3 1854.35 1241.725
1487.9250000000002 1966.675 1513.125
1458.775 1434.875 1731.55 1955.425 3692.725
1286.55 1321.675 1670.0000000000002 1559.225
1271.475 2184.85 1032.975 960.4 1603.475
1263.9 2181.4 1387.3500000000001 1120.7749999999999
1190.575 1189.65 1218.75 1052.4499999999998
1321.1249999999998 1254.075 1548.5249999999999
1504.2500000000002 1201.125 1303.425
1179.7250000000001 1293.2500000000002 1781.3249999999998
1240.8\
RDH10 563.25 378.67499999999995 2747.2750000000005
911.55 865.375 746.125 266.675 5163.299999999999 1249.0
622.325 1161.325 726.5749999999999 496.825
2593.9249999999997 174.125 3142.1749999999997
1873.5500000000002 1734.9499999999998 2139.425
697.3249999999999 231.975 1958.975 731.375 2585.35
1849.7499999999998 911.3249999999999 1382.025
1222.3 570.5 869.55 636.7750000000001 261.075 605.025
690.125 4224.775000000001 755.2249999999999
1154.5500000000002 381.975 1161.0749999999998 3079.325
1154.05 1420.125 1873.425 664.4250000000001
5744.9 381.675 1495.175 1072.8500000000001
1751.5000000000002 714.8499999999999 369.375
2587.4249999999997 1562.8 283.3 2963.6000000000004
239.72500000000002 729.525 564.7499999999999
235.79999999999998 503.475 843.925 731.75 2338.475
1869.05 764.625 1200.6249999999998 611.5500000000001
513.25 1804.875 2156.475 1330.2749999999999
847.8 284.42499999999995 1919.125 818.0 5014.525
2536.2750000000005 2788.375 1305.7
333.27500000000003 299.3 319.325 123.9 924.9749999999999
1628.65 4835.475 146.39999999999998 436.5
4092.2999999999997 4280.8 2811.075 234.8 260.55
828.075 157.29999999999998 197.27499999999998

215.07500000000002	446.825 366.22499999999997	323.225
207.9	157.425 228.775\
KRT8P17 /// KRT8P17	41.7	134.6	17.3	59.4	69.3	6.5
	13.1	15.4	122.5	7.7	57.4	10.5	63.4	6.5
	14.1	5.8	67.7	94.4	28.7	69.9	13.2	15.8
	25.4	49.5	9.4	60.2	2.2	8.4	13.9	76.2
	59.2	83.8	69.5	5.6	18.9	133.9	55.6	13.7
	100.3	24.2	7.6	86.4	45.9	8.3	45.4
114.9	69.0	44.0	101.4	10.6	20.4	11.8	8.6	6.2
	65.5	10.2	16.6	14.6	68.0	91.1	18.6	7.1
	11.6	7.2	31.5	3.9	4.7	8.7	98.1	93.0
	167.0	129.5	9.7	9.5	88.1	20.9	8.0	11.6
	57.0	4.3	80.3	52.8	12.1	8.5	13.9	42.7
	11.9	81.3	11.2	18.5	36.8	38.8	42.3	37.4
	4.7	32.5	10.9	8.1	34.0	10.7	20.7	69.2
	74.6\
RDH16	9.7	67.6	164.8	13.2	51.4	31.2	20.1
100.8	66.9	38.6	65.6	92.3	33.9	116.8	212.8
167.6	163.7	43.2	47.3	189.0	318.3	64.5	55.5	34.0
	33.8	29.7	19.5	67.0	168.7	18.4	40.0
260.7	171.0	40.6	118.3	25.1	18.9	64.5	73.6	17.9
	150.0	60.8	144.8	115.3	12.4	148.6	12.8	10.6
	34.4	53.3	17.9	25.6	39.7	1149.1	67.3	54.3
	33.6	46.2	123.5	21.7	54.6	145.4	19.6
114.7	69.1	86.2	35.7	31.6	134.4	32.6	65.8	97.1
	105.3	43.8	125.0	13.1	20.8	84.4	37.2	30.3
	196.1	11.7	166.9	30.8	172.4	336.7	16.2	84.8
	13.7	60.8	8.3	71.1	94.6	31.6	143.7	91.3
	87.7	95.3	116.3	221.6	89.5	30.4	84.5\
LOC100132352	788.2	1276.1	548.9	381.9	384.1	324.1
260.7	285.7	210.2	218.3	262.5	445.3	163.3	315.2
426.1	396.8	99.6	218.2	74.1	550.1	253.8	398.9
279.2	98.8	126.3	313.8	62.5	677.1	335.6	227.0
370.6	873.8	582.1	141.5	55.7	677.2	333.6	210.3
390.4	258.8	234.4	719.6	583.2	277.6	121.8	197.8
345.8	128.4	325.6	562.6	494.7	133.7	132.7	432.2
312.2	408.1	465.0	537.5	376.6	674.4	451.9	422.5
407.4	270.4	522.7	607.7	260.2	357.9	521.0	298.0
711.8	418.1	714.1	434.0	616.3	183.1	381.8	195.1
465.4	163.6	300.6	572.6	170.4	581.0	374.4	90.7
266.5	325.4	582.2	429.0	295.7	593.4	636.6	599.5
606.9	614.6	576.5	664.8	668.6	561.6	876.1	489.3
654.1\
LOC100132354	73.2	16.4	9.9	10.8	112.7	136.4	2.6
	83.1	141.5	16.6	31.8	5.4	7.4	26.8
197.0	281.6	35.4	57.1	69.3	77.3	1309.7	5.7	86.4
	45.7	41.7	157.2	7.0	30.2	124.3	186.5	94.1
	93.7	99.7	45.1	238.8	104.9	7.6	28.2
101.0	107.8	50.0	90.6	48.8	401.8	62.8	99.9	7.7
	456.9	467.7	361.9	62.6	73.9	94.4	209.8	58.8
	27.4	166.3	88.4	7.4	259.8	476.1	18.4	19.5
	3.2	873.4	138.7	435.3	588.5	118.5	62.7
136.0	30.1	123.5	52.8	120.6	42.2	52.8	55.3
203.1	26.5	93.5	17.6	72.5	31.0	72.2	384.3	22.4

	12.0	131.5	58.7	47.1	124.5	33.0	3.0	54.7
	2.9	30.4	20.4	83.1	153.3	38.1	127.0
72.7\
RDH13	366.425 1000.175	663.325 319.7	696.4000000000001
	287.075 238.70000000000002	848.35	227.42499999999998
	354.275 534.0	576.8	368.325 567.75	204.275 328.95
361.05	419.375 329.05	437.9	824.175 436.65	675.025 356.35
295.7	725.2750000000001	399.325 428.2	591.85	322.225
218.225 883.95	1518.1999999999998	292.1	84.30000000000001
	802.5999999999999	295.475 1100.825	302.025
334.425 505.62499999999994	705.05	271.325 431.075 423.175
271.125 631.225 367.65	611.7	454.1	295.425 538.9000000000001
	554.575 904.5250000000001	261.29999999999995
262.35	415.32500000000005	553.825 1105.525	259.85
478.82499999999993	873.9000000000001	495.525 540.925
420.90000000000003	921.4749999999999	373.025
414.92499999999995	476.925 383.9	575.65	613.125
416.17500000000007	405.07500000000005	326.09999999999997
	317.17499999999995	996.5	335.4	356.725 332.375
1527.0749999999998	1444.3249999999998	622.825
971.4749999999999	783.625 1184.75 452.525 1219.1999999999998
	672.125 983.0	322.925 960.125 622.1	1193.375
917.425 455.8	553.075 581.6750000000001	1322.875
524.7	653.45	438.725 647.8\
LOC100132356	91.2	653.5	405.4	216.8	318.1	205.8	74.7
	516.1	108.2	197.0	116.0	423.5	88.0	87.1
139.1	160.8	143.8	208.2	221.2	86.4	779.8	157.3
287.7	246.0	113.6	111.9	140.2	894.4	649.4	135.2
112.1	953.0	460.6	136.7	349.4	343.0	49.7	562.9
148.3	39.3	145.3	139.0	376.0	146.7	62.5	375.4
299.1	129.5	776.3	358.7	248.6	61.7	172.1	672.6
222.5	564.8	268.7	122.8	354.4	588.0	651.1	152.6
429.7	85.2	588.7	737.9	530.2	378.6	380.0	109.4
1203.2	898.5	234.5	89.8	383.3	206.5	380.2	64.4
145.0	194.8	455.2	182.8	351.8	141.9	1112.9	2461.9
346.2	609.0	192.6	305.8	114.2	743.2	368.1	257.2
546.2	514.7	691.5	712.9	354.2	476.9	536.7	388.7
733.3\
GTF2IRD2B	27.9	7.9	74.9	63.7	130.0	64.0	59.8
	75.5	44.4	98.4	23.8	117.9	78.9	26.0	7.4
	63.1	8.1	92.7	53.2	20.8	25.6	26.7
194.0	46.5	10.3	36.1	17.2	134.3	17.8	59.4	25.6
	37.9	114.0	14.4	9.6	53.5	47.8	66.7	33.2
	46.4	9.3	9.6	96.4	33.3	57.0	106.8	41.0
	95.3	154.3	101.0	23.9	3.5	30.5	90.5	71.1
	124.2	14.5	80.5	55.8	80.2	208.5	62.9	81.4
	56.3	28.9	98.5	92.5	33.3	82.1	26.7
364.9	205.5	83.0	32.8	111.3	5.6	113.9	53.0	60.3
	27.5	12.3	97.7	24.6	69.3	187.0	190.6	41.2
	82.7	35.1	72.7	54.6	98.7	67.6	47.3	31.1
	15.5	15.2	16.6	95.0	95.7	57.2	75.8
59.7\
GSTM4	132.25	51.050000000000004	90.14999999999999
64.45	89.9	131.1	32.05	90.25	67.45	80.60000000000001

67.05 62.0 89.15 279.65 177.5 173.29999999999998
79.60000000000001 124.35000000000001
105.30000000000001 68.1 97.9 50.8 55.1 45.95 21.4
46.1 97.8 109.89999999999999 46.25 55.95
126.10000000000001 109.75 94.8 161.14999999999998
97.10000000000001 109.75 254.0 159.15 87.30000000000001
89.35 168.6 147.85000000000002 86.95 86.75
123.89999999999999 80.9 64.9 113.65 181.0 85.7
114.75 132.95 99.30000000000001 102.75 61.75
169.04999999999998 61.65 50.5 54.75 103.10000000000001
325.20000000000005 156.1 75.10000000000001
39.45 101.95 65.2 150.20000000000002 86.0 115.1 97.7
265.9 135.9 93.2 91.25 93.30000000000001
126.95 75.75 31.55 50.85 117.80000000000001 118.7 27.5
86.15 113.8 125.75 216.75 54.3 85.45 88.25
109.5 127.5 169.85 78.0 74.75 128.25 111.85 89.2
82.85 135.4 155.35000000000002 143.95 89.3 80.5\
GSTM3 1235.3 99.55 409.45 723.35 1121.6 3236.9
1412.3999999999999 2227.5 600.45 519.35 1437.2 808.85
336.55 1138.45 346.35 269.55 949.8499999999999
1233.3999999999999 816.4 885.8 713.2 460.25 1098.3
1746.65 1139.4 140.25 1361.75 532.55 265.3 401.95 515.0
575.65 481.04999999999995 556.35 1513.0 6359.1
1645.4499999999998 3369.25 542.25 501.95 2147.6 1083.75
416.79999999999995 599.8000000000001 1258.9 804.0
2118.1 3953.1000000000004 554.2 840.1 1459.8999999999999
1617.6999999999998 1414.35 396.95000000000005
355.09999999999997 576.9000000000001 532.95 691.05
529.9 397.65000000000003 639.3000000000001 1573.2
609.9 910.0 765.9 680.3 1451.6 658.8 812.1500000000001
1517.65 186.0 1136.3500000000001 272.85 1516.9
542.2 1159.65 695.25 433.7 587.9 1751.9 939.6 1268.45
247.64999999999998 493.45 293.05 676.65 224.3
2612.8999999999996 1069.8 1653.4 1573.5 428.25 1778.75
1447.4 1529.6 1448.25 1124.9 1425.1999999999998 1731.15
718.0999999999999 1224.0500000000002 1218.1000000000001
1386.5\
GSTM2 571.0 354.7 276.6 291.9 507.3 1705.4 229.3
681.7 1345.2 829.5 474.0 908.0 231.2 4916.8 1000.8
707.9 396.6 921.7 364.5 378.9 1154.0 397.0 482.9
315.0 221.6 395.8 792.1 364.7 245.5 505.6 543.6
868.9 835.1 708.0 1055.5 4978.8 735.5 810.6 340.8
979.3 1939.3 1143.2 922.1 235.3 371.0 704.6 555.1
2711.3 832.2 2390.3 1549.7 614.2 561.5 669.5 391.4
1654.9 444.1 279.8 431.7 434.9 2331.5 441.5 707.3
400.5 677.8 553.3 2020.4 325.4 542.3 942.8 1313.9
3336.9 780.7 549.7 405.8 783.9 372.0 275.2 328.7
879.1 1093.4 492.2 1028.9 996.2 517.8 950.3 345.8
737.9 651.9 624.6 498.0 1811.2 1498.7 816.5 2628.5
1843.3 1891.9 703.5 2357.5 2613.0 2508.2 1469.1 1471.3\
GSTM1 288.85 111.80000000000001 113.55000000000001
218.6 461.15 1072.1 171.15 372.6 748.1 711.55 263.15
395.05 142.6 2392.3 471.05 387.15 235.25 523.45 265.75
215.35000000000002 591.35 293.35 322.15 257.4 190.05

339.9	586.8	190.85	236.5	193.2	308.2	429.54999999999995
	471.0	600.8	533.55	3743.25	386.6	398.0
168.85000000000002	650.8	1640.6999999999998	703.9
515.4	200.2	247.55	306.29999999999995	588.2
1949.8000000000002	510.20000000000005	1239.1	810.6
574.0	319.75	446.6	202.3	942.3	330.0	216.45	197.05
352.95	1408.05	271.5	364.65	191.6	336.35	444.7	1538.1
164.5	280.79999999999995	741.85	611.55	1694.0	443.7
444.95000000000005	250.8	501.75	310.15	224.85	157.95
671.95	650.3	193.95	587.55	841.5	323.2	631.15
316.04999999999995	694.75	501.65	540.4000000000001
372.0	1048.25	936.5500000000001	522.3	1160.75	1085.25
827.9	483.75	1207.65	1152.6	1688.0	871.8499999999999
1013.85\
NEBL	529.1	120.36666666666667	139.0	87.94999999999999
	247.26666666666665	23.416666666666668
32.56666666666667	877.6999999999999	157.01666666666665
	434.3666666666666	1126.5666666666668
26.03333333333333	268.2666666666666	864.0666666666667
	318.33333333333337	563.5166666666667
150.03333333333333	127.35000000000001	754.3333333333335
	233.01666666666662	239.76666666666668
48.03333333333333	464.74999999999994	136.74999999999997
	30.0	57.11666666666667	46.449999999999996
97.21666666666668	145.29999999999998	517.2166666666666
	186.54999999999998	80.73333333333333
64.31666666666666	292.68333333333334	545.7166666666667
	301.7666666666667	42.46666666666666
91.23333333333333	383.75	70.10000000000001
177.35000000000002	482.51666666666665	168.08333333333334
	101.78333333333332	50.26666666666667	68.85
114.11666666666666	150.1	457.83333333333326
84.16666666666666	59.583333333333336	69.73333333333333
	40.35	67.39999999999999	60.98333333333334
282.34999999999997	90.33333333333333	131.23333333333332
	141.1	48.54999999999999	408.56666666666666
411.98333333333335	390.8833333333334	46.9
96.48333333333333	81.06666666666666	216.91666666666666
	86.16666666666667	151.81666666666663
68.38333333333333	162.66666666666666	92.51666666666667
	280.9166666666667	34.36666666666667
163.33333333333334	168.16666666666666	342.9333333333334
	166.46666666666667	504.7666666666666
162.71666666666667	318.0	202.70000000000002
414.7166666666664	257.93333333333334	87.75
383.78333333333336	50.699999999999996	495.71666666666664
	83.13333333333333	77.86666666666667
198.7166666666667	247.23333333333332	405.13333333333327
	211.16666666666666	155.6	419.7833333333333
253.01666666666668	218.33333333333334	528.5666666666666
	508.53333333333336	247.11666666666667
780.9499999999999	499.18333333333334\
LINC00869 /// LOC100996741	65.0	57.1	89.3	17.6
139.0	54.7	107.5	84.6	86.3	107.7	82.2	7.0	67.8

66.2 89.4 15.5 64.0 62.2 16.0 112.7 49.5
65.9 150.7 69.2 76.8 46.7 44.7 151.4
113.7 5.4 50.9 273.0 88.1 50.3 14.4 32.6 56.9
65.5 72.1 21.1 71.3 80.4 90.1 58.7 85.5
143.0 92.4 89.7 18.1 105.3 15.9 72.1 93.1
53.5 68.3 80.4 108.5 68.7 23.3 150.8
421.3 59.5 156.2 80.6 45.8 63.7 104.9 59.2
173.5 51.3 76.5 65.8 191.9 94.6 32.1 58.4 84.8
45.5 64.3 97.8 220.6 54.9 61.1 137.2
204.1 150.4 60.1 73.3 65.3 45.0 102.2 208.5 84.3
72.3 53.4 145.2 101.9 55.8 5.7 149.5 50.6
59.3 138.1\
TMEM254-AS1 28.6 92.2 15.3 97.4 114.4 18.4
127.5 115.8 14.9 14.7 87.7 83.6 12.8 59.7 23.0
79.8 53.0 17.3 87.6 101.9 91.0 14.2
184.2 54.9 7.1 37.4 15.3 161.4 141.0 97.6 35.4
101.3 251.0 148.7 154.6 14.7 25.3 124.2 82.0
18.8 115.1 26.3 98.9 69.9 23.8 92.6 28.1
14.0 138.5 94.3 90.3 118.4 44.9 173.9 83.0
25.2 123.1 115.5 132.9 167.3 52.0 119.4
153.5 41.5 186.6 142.1 69.9 31.9 37.6 84.7
117.2 141.8 118.1 37.7 38.9 17.8 109.3 25.6 57.4
47.6 96.5 73.1 87.4 43.6 30.7 114.6 88.3
129.2 67.5 94.4 13.6 121.2 121.6 120.1
107.0 123.5 108.1 79.2 110.1 39.3 84.7 124.3
97.7\
SPAG5-AS1 43.449999999999996 129.55 8.2 27.0
88.75 77.1 56.349999999999994 32.5 82.2 66.4
61.55 90.4 19.9 60.9 108.55 31.25 81.55 90.1
24.75 60.7 168.1 27.45 157.60000000000002 42.2
51.15 70.4 30.1 279.95 23.0 120.75 43.4 129.1
134.05 48.949999999999996 84.89999999999999 90.75
28.05 35.0 49.25 48.8 23.05 22.7 58.65 50.05 22.4
62.849999999999994 50.349999999999994 42.7
179.5 86.65 127.5 109.10000000000001 81.19999999999999
185.60000000000002 40.800000000000004 49.8 93.4
78.1 26.650000000000002 56.2 581.75 81.75
37.25 44.25 101.65 109.0 14.0 100.2 97.9 48.2
43.150000000000006 127.80000000000001 42.15 57.85
116.19999999999999 55.3 59.300000000000004
31.150000000000002 42.400000000000006 48.6
68.14999999999999 65.15 15.15 59.4 91.05000000000001
162.39999999999998 84.14999999999999 118.35
32.699999999999996 85.5 30.0 87.75 93.2 57.7
8.850000000000001 100.7 96.8 33.05 89.85000000000001
126.95 73.35 23.65 24.85\
DHFR 1386.475 371.85 650.6750000000001 2240.7
1055.375 1635.6 1806.375 289.525 1351.225
1149.8999999999999 1185.975 1270.7 1524.725
994.85 579.8 706.0749999999999 542.1 883.45
1197.9250000000002 1028.875 603.75 1292.875
582.1249999999999 865.525 2413.5499999999997
758.3000000000001 2345.4249999999997 836.025 603.575
996.475 707.275 829.8000000000001 719.75 1056.675

```
484.2	429.225	1536.0249999999999	1200.525	1274.5
823.05	798.6500000000001	509.02500000000003
1050.3000000000002	934.6	893.6	1667.5249999999999
2073.5750000000003	1047.55	599.725	658.575	1540.575
841.25	825.05	1022.2500000000001	974.3500000000001
386.94999999999993	749.025	1018.325	876.5500000000001
	1286.825	841.375	752.275	1263.875	867.0
432.4	755.8000000000001	592.8500000000001	902.425
505.1	649.7249999999999	510.175	876.5250000000001
399.075	658.625	996.05	729.5	625.6	943.825	521.6750000000001
	2201.2749999999996	1132.6000000000001
1716.4750000000001	966.0	738.075	893.7750000000001
563.35	1668.3	1047.8	1180.0749999999998	583.95
602.9000000000001	551.725	415.75	503.575	309.675	514.65
300.9	474.32500000000005	374.34999999999997	271.7
257.22499999999997	520.6	320.84999999999997\
PKIB	20.3	45.099999999999994	113.7	90.5	41.9
212.4	157.2	232.10000000000002	43.75	95.75	57.95
24.25	66.15	86.85	53.75	63.4	107.6	98.25	224.75	77.6
	138.2	123.75	27.25	168.7	68.6	35.75	57.4
110.25	75.15	64.15	111.65	45.5	69.5	110.95
25.049999999999997	89.3	22.75	40.7	65.75	241.55
31.35	52.75	65.25	9.6	293.70000000000005	55.55	47.7
	23.4	25.3	107.2	63.75	52.45	97.25	32.0
43.849999999999994	53.95	99.75	31.65	78.05	63.3
178.3	108.44999999999999	131.15	172.65	95.45	74.55
167.75	21.1	115.69999999999999	105.8	116.7	115.35
62.75	430.8	99.55	141.45	118.25	137.3	118.0	188.05	90.4
	694.1	75.0	92.5	127.75	77.3	39.3	158.6	54.5
	13.549999999999999	188.6	402.45	275.5	450.0
120.55	181.35	407.15	208.8	128.15	254.1	298.15	109.2
400.55\
PKIA	32.949999999999996	6.45	38.1	84.75
290.09999999999997	15.05	744.1500000000001	86.75
175.65	67.75	345.95000000000005	49.5	8.1	56.6
737.6	34.199999999999996	99.85	11.200000000000001
229.39999999999998	53.5	62.4	60.8	108.8	157.15	82.9
	38.35	41.25	79.5	42.55	115.0	291.05	83.9
51.75	49.4	80.35	51.349999999999994	40.25
73.64999999999999	18.150000000000002	462.35	8.8
5.050000000000001	117.75	15.350000000000001	37.35
89.55000000000001	53.15	26.1	115.2	17.95	64.25
33.949999999999996	7.5	98.89999999999999
32.550000000000004	25.95	54.75	55.75	29.0	55.95
121.10000000000001	44.75	277.4	40.449999999999996	47.1
	34.1	15.299999999999999	42.9	34.95
39.800000000000004	24.4	57.35	38.9	30.55	51.45
33.75	65.2	37.4	71.25	153.3	770.3499999999999
905.6500000000001	32.25	234.25	95.95	12.100000000000001
	26.0	298.35	52.6	14.65	283.4	82.2	173.35
87.05	66.6	65.9	125.44999999999999	31.1	25.75
123.55000000000001	150.55	53.85	201.8\
AX746627	89.7	99.1	196.1	106.2	127.6	97.6	35.8
	53.4	64.7	5.8	48.5	18.0	78.0	41.0
```

```
102.8	167.3	149.8	77.8	100.1	119.5	95.4	73.9
144.6	82.4	5.8	80.1	56.4	82.3	134.0	68.5	49.6
	23.9	54.9	33.0	61.8	8.1	33.1	85.4	60.6
	47.6	74.0	65.0	91.0	61.8	52.9	15.0
110.7	10.7	130.3	254.3	26.2	49.5	35.5	49.6	59.2
	121.5	56.7	123.5	100.5	62.4	138.3	49.4	3.5
	24.8	67.1	130.5	71.6	95.0	102.4	157.2
390.5	7.3	121.9	87.1	173.6	60.1	81.2	110.3	46.7
	34.3	89.2	53.4	28.3	65.9	152.0	22.0	15.2
	141.5	41.7	48.8	37.6	287.7	87.7	102.9
145.5	148.5	190.1	171.6	79.7	94.6	209.1	102.9
127.8\
STRN	707.97	644.9000000000001	645.1099999999999
417.9	525.49	387.11	462.84	1074.5900000000001
459.21999999999997	425.41999999999996	544.05	667.25
485.91	543.4499999999999	634.4399999999999	386.96
721.73	374.63	449.64	508.2900000000001	367.39
382.42999999999995	540.72	366.10999999999996
559.3300000000002	718.9300000000001	494.75	684.05
673.84	441.85999999999996	507.43	961.0099999999999
377.92	376.94	678.94	455.85	492.56000000000006
441.49000000000007	471.82	470.21999999999997	594.99
628.2099999999999	471.25	367.83000000000004
312.62000000000006	513.89	570.78	270.03000000000003
637.33	480.02	514.96	595.1000000000001	773.7099999999998
	782.6999999999999	404.89	541.0	408.88
475.80999999999995	597.23	454.83000000000004	670.48
502.78000000000003	604.6	497.58000000000004	693.01
542.2100000000002	470.61	513.6899999999999	407.83
447.19000000000005	373.04	529.2899999999998
526.4599999999999	389.17999999999995	432.43
333.62000000000006	496.14	399.31	530.55	1413.31	766.55
740.99	738.26	547.18	537.48	808.8700000000001	756.09
729.0400000000001	536.59	727.3800000000001
388.96999999999997	766.6400000000001	456.48	456.43
525.2700000000001	500.12999999999994	487.2799999999999
	470.5	464.05	674.7700000000001	628.99	681.28
533.72\
L1TD1	39.35	87.9	61.65	20.950000000000003	69.2
28.950000000000003	62.7	39.050000000000004	34.1
46.45	43.65	61.5	52.099999999999994	66.4	63.65
44.55	87.05	67.55	64.19999999999999	60.05	92.15	64.5
	117.4	38.75	63.5	50.3	73.8	44.2
27.799999999999997	46.65	36.6	159.4	84.15	32.5	53.4
	91.8	57.85	58.85	12.299999999999999
63.650000000000006	28.45	42.099999999999994	94.55	28.4
	49.8	48.7	6.3	10.9	32.7	85.75
52.449999999999996	44.1	82.85	91.3	76.30000000000001
	73.4	41.45	20.9	31.950000000000003	57.4
394.5	43.15	35.4	5.05	80.5	43.3	60.1
22.200000000000003	12.4	33.35	107.75	69.05	43.75
72.44999999999999	104.44999999999999	32.45	17.7	60.7
	34.55	27.45	59.5	49.55	21.25	33.85	169.7
103.45	33.45	126.45	58.5	21.4	36.2	67.35	47.6	14.0
```

41.05 32.25 28.7 9.950000000000001 100.0
93.30000000000001 79.94999999999999 28.55 43.9\
AC007557.2 /// BC031974 10.9 136.8 44.2 25.5 78.2 46.8
29.1 52.8 61.0 77.5 9.3 87.6 20.9 96.2
106.4 60.1 7.7 32.7 26.7 33.1 111.3 69.6
10.2 38.1 61.6 60.4 8.7 186.3 118.5 10.5
20.4 115.0 37.8 68.0 19.7 59.0 39.5 15.9
7.2 51.0 23.0 14.9 49.8 11.2 64.5 41.7
34.5 6.4 244.2 94.4 34.7 60.7 45.9 6.7
55.6 20.7 60.5 7.3 77.0 24.4 258.5 51.4
14.1 45.5 86.1 97.9 31.9 55.9 14.1 70.7
108.3 13.2 60.1 2.1 73.0 11.1 49.6 58.7
73.1 45.7 139.5 73.2 79.9 83.4 149.2
218.0 80.1 7.0 11.1 11.8 41.8 159.8 83.8 55.3
40.5 32.4 70.8 5.5 65.1 15.8 17.5 68.8
55.6\
PKIG 712.2 510.8 1523.4 2389.4 751.3 1140.5 952.7
1239.4 2116.0 1754.0 1683.7 835.6 1075.2 1289.5 3413.4
832.8 1757.8 1624.9 602.8 1168.2 1039.4 1974.7 1741.5
3002.5 1262.3 1202.3 1695.0 2126.6 1738.2 1911.7 1936.3
1546.7 1394.4 2742.4 2444.7 905.4 1508.7 763.0 896.1
1405.8 1646.5 695.7 2272.3 1658.2 1628.1 1751.5 975.6
2701.1 764.0 943.6 1963.5 2132.1 1528.5 1226.6 1322.3
1541.6 1908.0 1119.5 1427.7 1697.0 1937.9 1803.7 1215.0
2085.5 1594.7 1061.2 1483.2 2410.4 5736.0 2559.7 1442.6
1582.4 1523.8 1542.3 791.1 3274.4 2649.1 1653.9 2036.0
1553.9 1991.4 820.0 2144.8 1690.9 1180.5 1261.4 1247.2
2310.3 1862.6 976.6 1260.1 2030.4 2392.8 3625.8 2995.0
2410.5 2809.1 4112.3 1313.9 2175.8 1639.2 1460.8 1765.5\
ARHGAP22-IT1 44.2 81.1 91.45 12.850000000000001
50.95 5.949999999999999 53.15 48.349999999999994
78.15 67.9 53.949999999999996 38.400000000000006 30.9
40.2 38.35 44.55 32.15 50.099999999999994 21.8
75.45 52.8 33.05 113.3 23.35 26.5 16.5 48.9
124.39999999999999 46.4 60.2 30.95 106.8
10.35 58.0 86.9 49.4 19.6 59.849999999999994
10.65 7.25 45.25 18.400000000000002 27.7 26.6
21.15 64.6 13.2 36.0 62.9 49.9 35.65 66.6
31.75 37.2 49.55 73.85 98.0 21.15 58.8 86.2
298.90000000000003 67.75 35.699999999999996
29.549999999999997 76.8 88.25 47.45 12.6 70.2
51.300000000000004 71.4 31.05 68.9 29.799999999999997
42.55 18.45 20.55 52.0 8.05 39.800000000000004
102.6 19.55 69.8 12.4 133.9 34.9
79.89999999999999 73.10000000000001 36.5 54.55
33.95 66.0 36.85 22.1 46.75 56.150000000000006
34.35 28.65 55.45 43.5 30.450000000000003 14.2
110.0\
GSTM5 129.7 401.3 233.9 246.1 220.6 139.0 146.0
436.7 534.5 292.0 210.9 184.3 199.1 285.6 354.4
232.9 215.5 183.9 135.3 428.2 580.5 193.9 407.9
424.8 264.5 293.7 136.4 422.0 293.5 254.3 301.9
345.3 189.9 562.1 369.5 316.6 230.8 288.4 138.0
339.4 216.8 142.6 533.4 112.0 107.9 419.9 629.2

112.6	378.4	360.5	241.0	237.2	168.8	670.4	237.3
317.1	238.1	252.5	243.3	222.9	885.8	293.4	150.4
164.8	341.0	354.7	239.4	180.0	339.5	260.2	387.3
368.0	307.0	284.8	571.8	294.2	465.9	226.8	386.9
164.0	525.1	490.4	206.4	276.2	485.5	750.9	211.6
1589.8	437.4	431.1	104.2	3903.2	1309.1	842.9	1923.4
981.7	1135.6	458.6	540.2	694.3	1078.5	1581.1	833.6\
KCNC1	6.55	68.85	4.95	10.15	93.9	12.2	24.25	45.2
	42.5	42.55	11.5	16.0	30.7	15.700000000000001
	28.45	58.95	6.8500000000000005	50.0	20.4	27.5
	17.45	23.95	96.69999999999999	34.85	45.65	9.7
	9.65	80.7	36.55	16.4	5.949999999999999
13.100000000000001	53.5	8.85	106.55	25.9	5.9	10.8
	10.95	24.35	69.75	9.9	9.9	6.0	22.65
65.55	46.75	20.75	81.14999999999999	87.75	32.85
52.75	9.8	69.35000000000001	10.85	75.19999999999999
	31.25	32.0	6.05	12.25	271.95000000000005
11.05	18.65	17.7	20.099999999999998	15.1	4.55	8.95
	45.35	15.25	153.85	66.10000000000001	6.9
35.65	16.15	16.4	43.05	21.6	42.35	15.100000000000001
	19.200000000000003	28.6	25.6	12.95
11.350000000000001	31.2	15.65	29.049999999999997	73.0
	16.6	3.15	10.299999999999999	45.6
21.950000000000003	33.650000000000006	13.95	60.05
23.75	14.1	27.7	13.35	64.5	47.9\
KCNC2	28.1	22.35	45.1	33.25	56.05	11.0	7.6
61.75	61.35	88.60000000000001	9.65	27.099999999999998
	10.1	29.95	15.2	8.2	17.55	67.64999999999999
	22.2	18.1	82.15	17.3	27.1	11.65	17.5
13.899999999999999	13.2	73.14999999999999	8.3	43.4
	6.050000000000001	20.45	20.450000000000003	14.5
	18.05	37.4	16.2	17.700000000000003	21.25	8.95
	12.95	35.85	5.6499999999999995	29.099999999999998
	10.3	45.300000000000004	11.95	17.4
62.599999999999994	12.25	19.95	9.8	22.85
41.400000000000006	16.4	24.0	27.400000000000002
46.949999999999996	20.6	48.099999999999994	61.5
51.95	32.95	7.3	13.850000000000001	39.05
20.349999999999998	8.4	23.4	8.0	47.7	14.45
20.650000000000002	10.5	51.199999999999996
14.899999999999999	11.0	23.65	29.45	13.75	36.65	26.9
	15.0	31.75	23.650000000000002	77.95
30.450000000000003	15.2	40.0	119.05	9.6	22.3
15.85	38.349999999999994	8.9	8.25	33.6	12.75
37.25	20.05	13.05	25.450000000000003	13.600000000000001\
KCNC3	64.23333333333333	131.4	67.56666666666666
67.23333333333333	53.9	34.766666666666666667	37.5	78.1
	118.60000000000001	73.96666666666667
65.43333333333334	32.266666666666666	92.46666666666665
	67.5	61.9	51.166666666666664	66.8	122.5
45.13333333333333	54.86666666666667	140.73333333333335
	125.86666666666667	200.79999999999998
56.833333333333336	105.93333333333334	49.699999999999996
	44.1	136.76666666666665	79.86666666666666

39.8666666666667	51.93333333333334	119.3	136.6	97.7
	77.83333333333333	118.53333333333335
22.233333333333334	76.96666666666665	86.09999999999998
	69.8	47.199999999999996	57.7666666666666
100.83333333333333	46.23333333333333	97.73333333333333
	137.03333333333333	22.133333333333336
29.833333333333332	125.73333333333333	87.93333333333332
	62.23333333333333	73.2	48.3666666666667
114.39999999999999	121.4666666666665	78.9666666666668
	73.63333333333334	85.43333333333334	98.0
153.8	98.5666666666668	122.13333333333333
52.63333333333333	41.1	59.4	60.9666666666666
78.03333333333333	40.8666666666667	192.5666666666667
	141.73333333333335	165.83333333333334	144.4
99.43333333333334	57.5666666666666	53.73333333333334
	48.833333333333336	131.93333333333334
49.7666666666667	53.5666666666666	39.5666666666667
	47.9666666666667	83.2	68.33333333333333
94.59999999999998	95.83333333333333	230.53333333333333
	50.0666666666666	100.10000000000001
112.33333333333333	169.6	7.066666666666667
93.43333333333334	76.4666666666667	50.400000000000006
	34.5	24.5	81.3666666666666	81.89999999999999
	149.6666666666666	70.43333333333334
104.93333333333332	164.9	77.8\
KCNC4	70.3666666666666	65.1666666666667
55.83333333333334	48.9666666666667	135.1
60.2666666666667	53.1	134.5	60.73333333333333
56.800000000000004	41.4666666666667	61.8666666666667
	36.3666666666667	53.5666666666667	119.7
71.2666666666667	68.39999999999999	136.9333333333333
	60.4	179.1666666666666	111.9666666666665
105.4666666666665	145.1	58.8666666666667
72.93333333333332	35.53333333333334	207.7666666666665
	180.0	129.2666666666668	193.83333333333334	80.3
	151.79999999999998	223.19999999999996
94.23333333333335	114.43333333333334	181.2666666666668
	47.599999999999994	480.33333333333326	127.5	78.8
	94.23333333333333	120.3666666666666
101.5666666666666	70.63333333333333	68.0
119.53333333333332	51.3666666666667	52.3666666666667
	154.23333333333335	120.5666666666666
153.93333333333334	110.13333333333334	74.23333333333333
	74.5666666666666	127.43333333333334
182.8666666666665	88.33333333333333	90.6666666666667
	119.13333333333333	78.3	162.73333333333332
115.2	165.6666666666666	144.70000000000002
96.8666666666667	209.70000000000002	102.73333333333335
	102.03333333333335	171.0	70.5	119.5
102.03333333333335	83.9666666666665	50.333333333333336
	131.9666666666667	86.9666666666665	117.7
60.9666666666666	119.33333333333333	169.5666666666667
	97.23333333333335	105.2666666666665
197.2666666666665	86.73333333333333	419.2666666666665

	230.9	83.96666666666665	200.63333333333333
571.5333333333334	225.36666666666665	85.06666666666666
	208.63333333333335	309.00000000000006	225.1
154.6	268.23333333333335	235.63333333333335
185.29999999999998	273.7666666666667	192.89999999999998
	180.13333333333333	102.39999999999999
236.16666666666666\
RP11-629E24.2	137.3	354.5	183.6	126.3	218.3	124.5	84.1
	201.2	156.9	188.5	133.2	122.8	135.3	81.5
260.7	226.1	205.2	135.7	68.9	419.7	148.6	201.8
267.1	108.2	133.7	194.7	164.4	392.1	311.9	181.1
128.1	273.9	289.4	201.2	388.1	234.3	120.6	269.7	50.1
	137.8	185.2	199.2	322.7	135.9	160.9	149.8
188.6	22.9	223.6	245.6	250.6	269.6	255.7	334.4
222.8	176.5	258.9	219.0	282.4	296.2	670.1	146.3
129.0	278.0	223.5	261.5	143.2	132.5	230.7	295.7
417.4	180.4	254.4	62.0	308.2	149.3	268.7	141.0
139.3	184.8	152.1	161.3	215.4	182.7	469.6	323.6
177.2	229.6	243.4	358.7	104.8	513.6	158.9	135.1
114.6	62.4	136.7	111.4	206.8	491.1	195.8	284.9
122.2\
LOC643733		27.75	29.4	125.89999999999999		139.15
70.75	19.55	66.5	137.15	60.25	83.1	16.2	69.7
27.049999999999997		104.85000000000001		14.5	117.6
105.25	38.7	26.4	52.85	466.75	37.650000000000006		88.7
	138.4	82.0	34.65	47.9	145.29999999999998		74.2
	99.2	77.8	75.55	88.45	48.349999999999994
81.85	83.15	74.6	89.3	7.75	68.35	58.449999999999996
	51.2	137.7	47.0	53.599999999999994
107.44999999999999		90.4	14.55	81.65	62.05	60.45
41.550000000000004		55.300000000000004		163.85000000000002
	56.45	109.85	71.6	94.9	70.05	68.4	136.4
78.65	86.85000000000001		65.35	72.80000000000001		53.1
	46.699999999999996		30.55	59.449999999999996		59.0
	194.89999999999998		80.89999999999999
192.64999999999998		47.7	203.25	52.15	29.349999999999998
	66.6	105.0	26.85	126.80000000000001		95.7	17.6
	134.75	170.9	28.549999999999997		105.5	123.65	88.2
	111.6	44.65	116.1	105.25	118.89999999999999
88.15	91.65	87.0	86.10000000000001		66.35	120.95
93.95	98.25	85.5\
LOC79999		53.1	69.4	50.5	46.4	121.6	10.3	6.9
	6.9	5.0	89.1	35.5	29.8	9.9	79.8	92.3
	95.8	49.7	146.1	55.1	6.3	25.1	53.9
114.7	5.7	14.9	73.0	2.8	229.1	54.5	78.9	16.9
	77.6	150.3	18.8	66.9	86.1	23.3	88.9	5.3
	7.1	4.3	55.9	10.2	16.2	14.8	26.9	51.9
	33.4	80.6	12.5	17.3	14.5	33.3	183.4	47.0
	83.9	5.9	56.2	93.7	183.1	14.3	24.0	77.0
	7.3	6.5	128.5	17.7	58.9	26.0	73.3
124.7	146.8	63.6	59.8	69.5	20.7	126.1	11.3	3.5
	6.7	115.2	80.4	3.9	8.5	335.1	45.2
112.9	58.2	44.1	38.5	6.4	8.8	78.2	4.3	6.0
	4.9	7.6	114.5	61.7	68.7	60.0	15.4

45.4\
LOC283682 13.1 56.0 28.4 12.4 43.7 41.4 7.4
65.5 58.2 8.5 22.4 45.3 68.9 30.2 19.7
29.3 17.4 39.7 13.3 92.6 16.8 31.0 4.3
11.0 20.5 14.9 46.4 52.2 14.4 109.2 43.7
69.3 15.0 86.5 82.7 13.4 7.4 165.4 17.0
31.0 40.4 38.0 62.6 5.3 65.3 35.2 3.6
12.9 113.0 63.9 14.1 11.0 13.0 16.0 13.0
80.7 52.4 94.9 33.7 104.2 56.9 33.3 18.9
7.3 18.1 92.3 47.3 8.7 67.6 13.7 61.4
8.8 50.8 10.3 63.6 7.1 4.6 30.9 40.9
52.0 74.2 45.9 6.2 9.7 77.8 33.4 72.5
13.6 12.1 132.1 8.2 27.1 31.0 6.3 61.3
45.1 65.0 63.8 14.8 88.6 17.2 110.6 7.4\
LOC101928669 /// LOC101930100 /// LOC644450 4.5 377.5 12.9
6.2 137.7 35.1 71.2 89.8 25.7 113.0 5.2
13.1 26.2 49.1 159.5 33.3 15.6 14.7 51.1
28.9 61.5 40.5 8.0 6.2 26.0 12.5 46.1
102.5 143.5 34.2 52.9 115.3 89.0 46.3 10.7
10.9 49.7 97.4 73.4 15.1 62.9 74.0 33.6
29.5 8.1 43.1 43.2 38.9 96.1 74.3
106.9 15.4 39.2 70.7 93.1 45.5 15.4 46.9 75.9
216.4 73.5 70.9 78.4 53.7 152.0 114.7 46.2
110.8 16.4 27.3 223.6 196.8 80.2 27.4 93.5
3.6 62.1 41.1 94.7 2.9 103.8 39.8 34.8
97.1 185.4 53.7 24.2 62.6 44.6 138.3 9.5
214.3 23.3 31.0 49.4 73.9 53.5 104.8 47.2
12.5 41.6 155.9 43.1\
LOC100288181 3.7 37.9 6.2 5.4 19.1 7.6 2.2
8.3 5.0 4.4 5.2 3.5 7.1 8.7 39.5
14.9 3.8 11.2 26.8 3.1 25.7 9.7 16.5
3.5 2.8 42.7 4.8 7.7 17.2 6.8 4.5
15.5 51.3 5.5 12.4 4.3 5.5 5.1 9.4
6.1 6.9 6.5 6.9 10.7 4.2 6.2 11.1
7.0 10.6 30.0 5.7 7.3 9.7 172.5 13.1
139.7 8.0 17.0 2.8 3.6 14.2 3.7 16.7
3.4 14.4 70.8 4.6 5.3 4.2 6.4
108.0 16.1 4.7 7.8 3.7 3.7 13.8 6.4 4.3
15.4 27.7 42.5 2.7 14.1 8.4 27.8 10.9
22.6 6.0 18.3 7.8 12.6 9.8 5.0 36.3
31.3 24.4 7.5 6.1 8.8 9.8 9.4
11.9\
NPAP1 4.3 155.6 22.4 33.0 96.5 9.6 64.2 11.7
70.7 23.3 43.1 5.5 11.4 4.3 202.8 23.5
38.2 52.3 20.4 12.9 20.0 23.5 130.5 53.3
36.6 84.4 51.9 50.6 69.8 65.1 26.2 7.0
19.6 61.0 26.4 100.7 95.5 24.6 41.6 6.6
52.8 68.6 11.7 23.9 15.4 135.8 4.3 19.5
24.1 55.1 19.9 41.0 44.4 38.5 51.8 74.0
33.9 85.0 53.7 84.7 37.0 81.7 107.6 21.7
81.5 20.0 7.3 9.9 74.1 58.7 166.4 43.9
39.7 8.1 52.6 86.7 51.3 11.2 39.2 6.8
61.9 7.0 43.8 56.7 47.1 129.0 99.8 15.9
28.7 192.9 53.5 4.5 4.7 2.4 15.7 4.0

82.9 92.3 24.4 33.3 26.8 12.6 17.7\
MAGI2-IT1 26.0 15.6 9.9 4.7 73.3 5.4 15.6
1.5 1.4 6.5 2.1 24.2 29.2 2.6 3.8
2.1 4.2 34.5 3.6 18.5 6.0 25.2 73.9
6.4 19.3 6.6 23.7 10.7 7.9 3.5 10.7
9.4 7.9 10.2 2.6 23.6 10.1 21.2 1.7
2.2 1.5 23.5 36.6 3.6 4.7 49.4 3.7
12.6 6.3 59.9 10.7 2.7 6.7 38.4 2.0
8.2 28.9 27.9 3.0 6.5 13.8 9.4 2.4
22.8 4.7 31.5 27.3 1.8 74.0 1.8 12.3
51.5 22.1 2.0 3.7 15.8 1.8 14.9 4.5
31.4 38.4 4.6 1.3 1.3 9.0 17.5 20.0
58.3 13.8 33.7 29.3 24.9 42.7 2.1 1.7
3.0 29.9 7.8 38.5 52.7 14.5 3.6 5.5\
GLT1D1 69.7 11.8 134.1 117.8 59.7 14.2 17.2 77.6
97.2 105.0 39.9 67.8 38.6 53.0 18.7
151.9 53.3 12.3 14.7 79.8 21.2 84.5 265.6 61.1
15.4 25.5 14.3 32.0 30.8 27.9 89.5 43.0
157.1 62.9 56.2 29.4 72.1 22.4 64.6 9.9
37.3 24.8 75.4 6.9 53.2 33.4 90.9 55.0
26.0 72.4 73.9 81.7 274.0 117.6 35.7 14.7
54.1 15.3 60.8 93.1 264.1 48.5 8.8 19.5
37.5 22.3 187.3 128.2 191.6 91.4 36.0 9.0
23.0 13.8 48.8 16.5 147.2 8.3 50.9
1157.6 155.8 8.6 18.8 92.8 81.2 24.8 38.2 36.3
123.5 9.8 50.2 384.8 248.1 221.5 394.7 61.8
272.9 48.1 31.7 25.5 152.5 230.3 65.9\
NEFL 16.566666666666666 70.96666666666667
74.33333333333333 16.0 109.8 22.866666666666664
43.20000000000001 78.3 41.93333333333333
55.43333333333333 21.7 30.400000000000002
28.46666666666667 29.766666666666666 27.166666666666668
29.03333333333333 35.166666666666664
39.266666666666666 43.5 42.46666666666667 95.8
31.133333333333336 42.76666666666667 32.03333333333333
34.23333333333333 38.03333333333333
55.699999999999996 65.76666666666667 10.933333333333332
51.333333333333336 46.93333333333334
50.33333333333334 37.333333333333336 51.06666666666666
70.89999999999999 31.633333333333336
43.79999999999999 17.099999999999998 25.166666666666668
35.800000000000004 57.53333333333333
23.16666666666667 60.70000000000001 31.400000000000002
42.93333333333334 30.46666666666667
51.93333333333334 20.8 58.1 54.26666666666667
37.266666666666666 18.133333333333333 38.0 35.4
37.300000000000004 49.70000000000001 51.83333333333334
47.96666666666667 13.833333333333334
49.13333333333333 175.76666666666665 47.1
37.06666666666666 34.56666666666667 13.600000000000001
15.766666666666666 39.7 53.4 114.83333333333333
54.53333333333333 65.39999999999999 51.5
38.63333333333333 78.83333333333333 45.63333333333333
39.43333333333334 47.4 31.233333333333334

64.93333333333332	37.63333333333333	52.833333333333336
	44.79999999999999	10.3	27.900000000000002
73.36666666666666	74.26666666666667	16.46666666666667
	93.63333333333334	38.733333333333334	45.9
42.43333333333333	51.76666666666667	28.700000000000003
	18.633333333333333	33.766666666666666
22.46666666666667	19.5	25.566666666666666
87.39999999999999	64.3	33.13333333333333
51.49999999999999	34.63333333333333\
ZNF32-AS3	25.4	122.2	29.85	28.349999999999998	74.0
	26.8	16.15	60.650000000000006	21.15	12.85	2.9
	5.1	12.9	15.4	11.4	35.0	25.45	5.05	5.55
	3.9000000000000004	107.8	15.65	13.05	21.05
24.95	19.45	4.199999999999999	54.9	39.300000000000004
	37.15	1.65	15.75	92.1	3.45	37.65	18.1
21.55	45.4	12.8	42.85	16.099999999999998	14.45	52.9
	31.65	3.75	21.799999999999997	19.45	9.3
17.85	17.25	13.149999999999999	29.200000000000003	21.7
	28.1	30.9	37.65	11.45	22.9	16.55	38.25
141.05	22.25	6.550000000000001	4.4	15.55	35.05
10.95	5.45	97.25	11.649999999999999	72.8	47.3
12.149999999999999	14.7	8.4	12.350000000000001	23.5
	14.799999999999999	4.95	4.6	94.1	35.2
28.85	28.1	25.9	123.0	32.2	15.299999999999999
19.75	64.6	12.299999999999999	26.7	12.600000000000001
	15.5	12.05	3.0999999999999996	12.6	21.45
43.95	32.75	3.45	29.35	36.2\
NEFM	13.5	22.2	32.05	17.65	32.85	22.5	27.6
30.15	200.9	17.5	19.4	28.4	24.95	24.200000000000003
	15.7	7.5	34.400000000000006	22.75	5.2	77.3
	102.8	45.449999999999996	65.10000000000001
47.300000000000004	17.85	8.25	57.25	56.900000000000006
	45.25	89.1	38.45	17.5	37.7	10.7
13.399999999999999	9.25	30.1	25.599999999999998
23.349999999999998	20.900000000000002	62.699999999999996
	4.55	36.35	348.4	42.65	54.35	13.0	31.8
54.45	35.8	89.35	47.65	16.6	43.3	44.05	30.5	35.1
	79.75	11.1	47.9	40.95	9.95	13.35
58.550000000000004	7.949999999999999	35.699999999999996
	5.7	41.55	31.75	9.15	15.6	86.35	75.7	61.0
	7.85	13.0	18.55	53.75	46.900000000000006	30.0
	40.45	30.25	21.7	42.15	81.3	134.65	79.5
119.75	46.25	24.7	19.05	72.39999999999999
49.800000000000004	73.1	58.25	30.349999999999998
31.15	12.65	10.45	10.8	31.85	66.75	14.899999999999999\
AK025975 /// RP1-74M1.3	26.9	11.2	55.9	56.4	94.0	54.6
	71.7	124.2	36.3	36.9	74.2	78.3	103.7	56.2
	289.9	88.2	53.6	76.7	9.9	98.7	343.8	46.5
	196.2	35.1	61.9	75.7	36.4	131.2	74.0	20.6
	69.0	34.1	166.5	41.9	59.5	92.7	74.3
168.4	43.8	41.0	90.4	8.4	57.7	69.3	47.3	26.3
	26.1	5.0	88.5	58.5	42.2	28.4	4.4
160.2	76.4	148.2	77.5	32.7	3.2	13.1	73.7	35.3
	171.4	72.6	192.7	47.6	54.4	201.0	74.2	10.1

77.0 27.9 33.4 41.8 68.6 29.3 52.3 7.9
68.1 4.8 126.5 131.5 218.2 55.9 185.9
156.3 95.3 190.7 142.5 164.1 65.3 147.5 53.7 38.3
38.0 55.6 99.3 96.7 214.4 61.3 68.4 43.7
87.2\
NEFH 38.75 23.95 51.25 27.35 66.3 141.8 28.55
458.75 30.150000000000002 753.4000000000001 557.65
76.85 45.8 58.3 1154.25 51.3 44.55 35.9 20.75
238.7 127.39999999999999 58.0 63.95 117.75 36.35
42.95 50.1 41.75 103.65 77.45 31.9 709.15
220.60000000000002 96.95 52.849999999999994
378.54999999999995 26.549999999999997 210.9 37.1 26.3
777.3 42.85 85.4 928.2 88.9 403.4 39.55
68.75 41.550000000000004 26.75 43.599999999999994
46.65 26.95 41.75 574.3 58.4 32.75 110.7 33.65
11.55 225.25 863.7 217.6 756.65 71.95 14.2 63.75 55.6
102.25 49.5 72.2 25.7 94.2 24.6
56.699999999999996 38.2 87.05 24.75 41.55 867.75
80.25 27.45 1265.15 1307.85 43.6 362.75 165.15 136.25
59.550000000000004 62.150000000000006 911.8499999999999
80.75 123.1 43.2 68.30000000000001 20.35 41.7
79.0 96.6 23.75 68.44999999999999 135.3
48.1\
MYBPC3 172.4 53.4 23.4 94.8 56.1 45.0 72.3
125.4 124.9 170.7 62.9 32.2 32.3 134.2 80.3
146.6 147.2 42.6 27.0 152.5 266.3 61.9 155.3 42.3
112.2 85.7 20.2 239.1 115.4 150.5 72.7
135.8 79.6 73.5 88.2 141.3 24.8 35.9 91.4 62.5
104.6 37.0 72.9 41.9 26.5 89.3 36.9
133.5 196.8 99.1 210.0 53.0 55.7 62.4 99.1
124.1 143.4 159.8 50.2 73.2 1012.8 208.5 198.5 87.8
96.2 56.9 84.1 109.8 84.8 179.8 45.2
275.5 79.6 45.9 88.2 163.9 184.4 122.2 49.9
140.4 53.2 107.0 56.2 78.8 195.5 137.2 207.9 71.2
65.5 125.8 113.6 71.5 82.4 121.3 41.7
107.9 173.9 98.6 50.5 67.9 117.6 42.5 90.6\
MYBPC1 51.6 8.5 34.7 22.3 147.8 7.7 9.4 94.4
53.9 57.7 43.2 21.3 46.0 11.7 60.9 3.2
21.7 52.4 36.1 25.0 32.9 68.7 193.8 21.9
13.8 33.2 25.0 5.1 4.4 2.3 20.6 70.0
17.1 38.3 49.5 122.3 24.5 30.3 2.2 50.3
5.7 23.6 32.7 22.6 2.6 12.7 5.4 26.2
64.8 47.3 13.9 31.5 1.9 47.2 13.8 22.1
73.4 6.8 53.1 87.6 310.6 41.6 44.5 53.5
36.1 35.5 23.0 28.5 17.6 45.6 113.2
144.2 39.2 15.7 49.1 40.8 79.9 35.1 3.4 53.9
38.5 31.0 95.7 44.1 45.6 19.9 24.0 72.7
69.4 11.7 3.8 29.1 10.8 2.8 8.2 50.3
21.4 21.5 7.3 99.4 22.9 103.0 21.7\
KCND1 91.3 186.8 73.6 92.1 34.1 12.9 57.0
141.5 7.3 15.1 35.3 123.2 70.0 60.9 46.2
147.8 85.3 67.2 61.1 77.7 54.6 6.2 17.3 95.6
19.9 30.4 10.9 16.6 114.9 60.2 62.9 23.8
192.1 80.8 20.9 43.7 9.6 183.2 65.7 13.8

162.7 127.8 32.3 36.9 67.5 86.1 65.4 23.2
416.5 63.9 33.3 13.8 158.5 368.7 86.7
129.0 51.2 5.0 14.6 36.9 41.2 56.6 202.2 24.1
79.8 63.5 63.5 127.7 18.1 74.3 111.8 41.9
7.4 18.9 6.3 35.4 47.0 48.8 109.3 22.2
138.4 25.0 84.2 36.8 273.5 79.4 12.7
157.6 80.4 8.0 35.4 130.9 33.7 112.5 112.6 51.3
7.9 105.2 16.2 7.3 54.4 64.6 10.1\
MYBPC2 35.7 24.1 10.0 8.0 18.6 39.7 288.7 3.6
1450.2 373.0 110.1 78.8 77.8 238.4 47.8
105.1 44.0 60.9 4.6 187.6 151.3 6.4 37.6
196.9 3.8 62.8 4.5 59.2 10.2 182.4 43.2 71.0
10.7 247.7 18.9 45.3 3.0 8.2 105.8 11.6
65.1 100.6 204.1 454.5 557.4 13.9 4.5 6.5
7.2 41.6 32.8 6.4 20.0 66.8 228.3 15.9
89.2 5.4 15.6 194.2 279.1 29.0 92.2 47.2
49.6 87.2 101.5 8.4 371.4 20.7 146.3 33.8
26.2 94.4 81.4 434.2 7.8 289.7 5.8 66.8
18.5 31.1 5.3 11.6 12.5 9.8 6.8 98.4
11.0 15.5 6.5 12.0 3.6 76.5 19.4 47.2
92.9 16.1 5.7 121.4 43.0 6.1 4.4\
DQ573539 /// SNX18P3 /// SNX18P3 5.8 8.8 31.3 1.8
22.1 4.6 9.3 38.1 2.6 13.8 6.1 2.5
3.9 33.8 7.7 6.5 14.9 8.8 47.4 4.9
41.8 6.0 75.3 4.4 3.4 9.2 7.1 14.1
9.4 7.1 8.6 82.3 3.2 16.0 15.2 12.7
4.2 5.6 3.1 6.3 5.2 4.3 4.4 3.1
5.0 16.6 10.6 3.2 38.8 6.5 6.4 6.8
8.2 32.8 4.0 12.9 3.6 40.9 6.7 51.2
13.6 3.7 13.5 2.1 27.5 8.3 46.8 5.9
7.1 10.6 44.7 121.8 3.0 39.2 2.6 5.7
30.7 4.4 17.8 12.6 8.9 4.1 8.6 8.2
30.3 16.3 9.9 8.5 4.6 51.8 5.7 5.7
5.0 1.9 36.3 6.7 8.3 7.2 9.5 12.7
9.1 13.3 12.5\
GSTO2 786.75 331.9 656.85 709.85 742.9 1116.6 367.25
895.9000000000001 739.1500000000001 660.9499999999999
157.1 808.7 538.65 535.25 415.0 891.0999999999999
916.2 372.2 576.1500000000001 957.8 253.7
779.5500000000001 312.9 267.25 341.9 684.85
1215.8500000000001 943.2 409.65 305.3 792.7 636.6
377.1 1301.15 650.5999999999999 594.9 876.0999999999999
462.55 1116.25 889.25 473.6 76.65 783.15 605.6
657.5 105.85 350.4 280.25 503.45 1452.4 300.35 578.95
1093.5 200.7 778.85 939.85 671.95 688.65 609.8 1110.8
431.85 282.7 758.5500000000001 646.8000000000001
346.95 699.55 424.85 746.75 308.1 668.3 223.75
509.20000000000005 379.70000000000005 611.1 851.75
127.19999999999999 470.8 517.55 1042.75 167.25 397.9
94.25 541.15 524.1500000000001 430.6 193.8 763.5
483.7 860.55 793.2 103.6 499.59999999999997 1113.55
615.5 588.55 1157.8 1270.8 1164.85 886.0 1038.05 1189.35
441.5 1267.1\
KCND2 8.9 133.5 99.1 66.7 60.2 19.0 4.9 66.9

130.2 122.4 57.6 68.8 7.2 59.5 55.9 40.2
142.5 228.9 13.3 65.1 77.7 110.9 74.1
184.0 54.6 47.5 122.0 63.0 83.9 76.9 252.1
241.4 135.5 66.3 22.1 37.4 93.4 7.3 5.0 72.5
32.8 50.6 31.2 67.3 68.7 39.2 31.8 85.7
39.4 10.8 80.9 150.0 11.6 53.1 161.3 56.4
30.4 257.1 44.2 31.6 31.8 42.1 106.4 56.3
135.6 6.7 76.0 25.5 116.2 35.6 160.8 6.8
34.1 132.2 49.7 54.2 455.1 60.7 31.0 18.5
40.0 4.2 51.2 147.8 73.9 90.0 47.1 75.4
4.4 80.1 64.0 38.5 77.7 167.6 69.5 20.4
2.0 91.4 54.1 90.0 130.1 79.9 21.0\
FLJ20021 145.9 178.8 196.7 94.3 83.8 70.0
114.4 108.9 112.0 164.8 217.7 133.4 183.5 35.6
188.4 265.8 22.5 83.8 157.1 118.2 281.7 69.4
196.5 81.9 31.0 175.7 138.0 94.5 186.6 171.4
137.8 214.9 106.5 166.6 99.6 322.4 14.4 173.1 93.6
65.7 33.2 16.0 152.3 50.3 52.4 97.0
209.1 95.5 192.1 144.8 90.9 175.6 216.1 506.0 71.1
285.0 201.7 219.5 220.7 200.5 108.1 290.2 46.1
91.2 174.1 206.3 51.4 160.6 230.5 127.9
149.1 173.5 101.2 117.7 151.4 71.8 268.5 87.4 94.6
87.6 47.1 91.5 158.6 186.0 119.0 188.9
280.0 297.9 185.7 88.8 6.2 34.8 137.1 185.7
327.1 321.2 382.6 179.1 295.9 452.3 111.7 248.8
302.9\
GSTO1 4247.05 479.1 4189.5 6718.2 2395.7999999999997
4274.85 3547.0 2184.25 4689.65 4847.05 3292.95 3705.25 3612.55
6865.4 1578.05 3388.6 3413.55 3788.9 5281.7 3598.75
1665.8999999999999 4393.25 2867.7 4581.85 3784.05 3741.6
7546.0 4165.650000000001 2720.5 3373.3500000000004
4239.15 3323.55 2376.9 6751.8 3862.95 4206.95 5080.150000000001
1810.3 2908.3 3479.5 4059.95 2700.75 6294.95
3090.4500000000003 4028.25 7564.7 3502.1499999999996
5490.1 1395.1 2111.5499999999997 5367.0 5319.15 3548.15
2613.5499999999997 4147.849999999999 1808.85 3673.1
3085.15 2992.6000000000004 3682.25 4754.55 2803.1499999999996
2936.95 2675.15 1694.8 3217.0 2847.45 2960.4500000000003
3739.95 4370.5 1222.8 5298.5 2284.35 4496.5 5245.65
6146.55 2897.6 5110.25 3910.7 3674.4 1800.1000000000001
2345.7000000000003 2140.3 3502.5 2855.2999999999997
1749.3500000000001 3204.0 3065.1 3133.15 4402.75
4061.8999999999996 1627.55 4804.75 3915.8 3011.0499999999997
4842.05 4686.05 4684.85 3482.85 4238.1 3520.3500000000004
3662.55 5048.45\
KCND3 32.9 53.275000000000006 113.67500000000001 73.2
79.75 20.575 34.55 67.3 57.275000000000006
68.025 35.699999999999996 93.125 50.15 105.85 28.1
24.15 121.125 87.22500000000001 96.125 58.87499999999999
174.975 37.25 101.85000000000001 205.95000000000002
44.375 14.600000000000001 60.7 102.325 33.65
72.02499999999999 166.375 64.10000000000001 78.375
89.825 27.875 131.04999999999998 32.775 56.4 385.225
46.52499999999999 38.150000000000006 30.475 147.7 27.1

	29.05	71.95	61.449999999999996	18.75
102.22500000000001	70.37500000000001	78.1
55.14999999999999	182.54999999999998	44.099999999999994
	139.475 60.72500000000001	151.32500000000002
60.074999999999996	72.72500000000001	108.625 221.875
63.824999999999996	173.85000000000002	74.575
210.04999999999998	121.475 209.725 93.75000000000001
85.85000000000001	110.625 121.4	108.3	106.77499999999999
	53.8	111.54999999999998	70.65	137.15	111.475
349.675 9.399999999999999	64.32499999999999
42.00000000000001	100.725 33.275	152.5	86.725
58.92500000000001	81.57499999999999	104.85	79.25
27.825	136.07500000000002	221.6	118.125 134.05	49.875
65.775	70.05	104.32499999999999	67.77499999999999
81.375	250.925 78.32499999999999\
BGN	286.3666666666666	405.8333333333333
549.8666666666667	1170.9666666666667	687.9
371.5666666666666	654.9333333333333	1587.3333333333333
	1757.8333333333333	2103.1	114.3	863.5666666666666
	392.93333333333334	407.0666666666666	272.3
1860.7333333333336	2721.0333333333333	710.7333333333332
	223.16666666666666	1517.8999999999999
2430.4333333333334	584.7666666666667	749.0666666666666
	1712.7666666666664	367.6666666666667
215.66666666666666	347.2333333333333	825.5666666666666
	523.1333333333333	665.9666666666666
1683.3333333333333	956.4333333333334	727.0333333333333
	692.3333333333334	494.5333333333333
183.9333333333333	379.0	1025.1666666666667
356.26666666666665	514.5333333333333	630.9666666666666
	313.5	698.7333333333332	1732.1666666666667
335.3666666666667	280.5333333333333	1418.5
291.73333333333335	222.9666666666667	851.7333333333332
	1447.8666666666668	360.56666666666666
469.5333333333333	2123.0	1027.7333333333333
512.1999999999999	2349.5333333333333	423.0333333333333
	260.8333333333333	1114.3666666666666	1234.8
677.6666666666666	417.3	442.8666666666666
992.7666666666668	330.6666666666667	1203.2
945.0666666666666	2135.366666666667	225.73333333333332
	942.4666666666667	480.2	1046.0666666666666
506.9666666666667	796.0999999999999	273.03333333333336
	1729.0333333333335	414.09999999999997
1405.1333333333332	1112.0666666666666	1186.2333333333333
	857.1666666666666	419.40000000000003	506.0
2155.7333333333336	431.0	3316.5	2470.9	1407.8666666666668
	635.4333333333334	418.6666666666667
2890.8333333333335	206.03333333333333	713.4333333333334
	379.06666666666666	156.0666666666667	190.0
261.8666666666667	146.73333333333332	163.79999999999998
	185.73333333333335	340.56666666666666
180.26666666666665\
RPL7AP71 /// RPL7AP71	15.6	3.4	133.7	99.8	82.4	88.8
	62.6	49.9	50.1	6.1	98.8	21.5	38.4	55.2

154.1	90.8	50.6	47.4	15.2	42.1	30.0	76.8
91.0	90.7	63.9	75.7	137.7	103.1	82.8
131.1	56.5	38.2	19.9	207.0	15.4	23.4	31.5	30.0
35.3	35.2	77.8	11.3	20.5	60.6	63.5	80.3
86.5	77.2	147.0	77.8	100.3	148.5	112.9	18.9
34.8	101.8	164.5	173.1	139.2	15.7	227.0	60.1
65.5	78.2	15.7	109.4	55.2	5.7	12.2
108.6	27.7	22.9	106.8	69.6	90.3	97.9	11.6	88.7
14.7	133.6	78.6	21.9	69.7	29.2	66.2	64.3
46.7	82.4	114.4	104.3	54.1	91.4	121.5
102.3	70.1	184.2	81.5	139.9	138.5	98.2	163.1	50.6
195.8\
LINC00921 /// ZNF263	439.2	443.25	547.25	698.5	305.85
587.35	461.70000000000005	91.85000000000001	362.15
334.55	435.09999999999997	595.0	513.05	491.6	359.45
284.25	226.6	417.15	425.75	283.9	456.84999999999997
461.70000000000005	438.2	401.79999999999995	490.2
564.55	540.0	409.59999999999997	315.6	369.65
690.3000000000001	380.5	398.59999999999997	272.85
329.75	458.70000000000005	516.65	424.15000000000003
387.0	213.5	415.5	345.04999999999995	370.9	371.9
415.75	382.8	245.4	733.7	514.7	417.65000000000003
333.8	255.75	399.85	308.95000000000005	340.40000000000003
301.3	356.5	301.15000000000003	295.75	498.05
568.8	343.5	342.25	336.85	301.4	450.45	284.5	360.35
390.75	404.70000000000005	619.95	504.75	325.85	419.8
302.45	309.9	142.2	327.65000000000003	366.2	147.9
141.1	180.75	817.4	383.2	538.45	352.90000000000003
190.85000000000002	281.25	273.8	381.5	322.65	300.05
503.65000000000003	604.4	581.65	754.5	689.85
734.6999999999999	466.55	583.8	556.3	361.6
731.5500000000001\
LOC283692	6.1	11.7	17.1	2.3	66.9	7.5	13.3
4.1	4.9	17.4	15.7	10.4	7.3	3.1	11.2
10.7	14.1	13.3	11.1	13.6	26.7	8.3	27.1
5.8	17.0	24.8	3.0	25.2	27.7	32.7	4.1
24.1	10.3	19.6	23.3	7.3	5.0	8.3	14.6
6.0	7.9	5.1	9.2	3.0	12.1	16.6	17.8
14.7	23.7	11.1	9.0	11.8	20.1	24.6	8.2
13.8	8.7	9.7	4.2	22.2	59.3	37.4	5.4
9.1	39.3	7.4	14.1	8.9	6.4	5.7	10.5
13.4	25.9	9.1	8.9	8.9	23.5	10.6	8.4
4.5	16.8	8.7	10.9	7.3	21.6	33.6	16.7
35.9	7.7	10.2	11.6	13.3	4.2	5.1	3.0
16.1	25.3	10.0	13.8	10.5	32.3	20.7	5.3\
LOC283693	39.93333333333333	173.20000000000002	40.5
26.433333333333337	63.03333333333333
43.53333333333334	18.066666666666666	58.93333333333334
41.43333333333333	15.366666666666665
28.799999999999997	32.96666666666667	35.6	18.7
43.43333333333334	24.0	38.46666666666666	70.7
43.366666666666674	51.73333333333333	161.83333333333334
32.63333333333333	59.73333333333333
46.16666666666664	67.6	67.1666666666667

36.46666666666667	38.699999999999996	49.9
45.03333333333333	33.0	74.5	63.53333333333334
23.600000000000005	66.03333333333335	63.0
31.46666666666667	73.60000000000001	9.6	56.6
42.666666666666664	29.033333333333335	51.800000000000004
	49.333333333333336	23.96666666666667
29.599999999999998	18.933333333333334	40.43333333333333
	91.5	84.13333333333334	28.53333333333333
64.43333333333334	20.466666666666665	47.6
28.200000000000003	66.13333333333333	56.866666666666674
	55.0	46.133333333333326	54.0	156.73333333333332
	64.0	52.03333333333333	22.433333333333334
77.13333333333333	99.10000000000001	24.233333333333334
	44.1	99.60000000000001	23.366666666666664
88.26666666666667	93.93333333333332	48.03333333333333
	23.76666666666667	41.166666666666664	48.9
66.13333333333334	27.299999999999997	50.23333333333333
	44.0	18.4	99.73333333333335	38.93333333333334
	46.93333333333334	153.26666666666665	136.0
42.86666666666667	48.43333333333333	53.73333333333334
	91.3	10.933333333333332	106.8	13.033333333333331
	22.7	21.233333333333334	31.433333333333337
52.63333333333333	35.93333333333334	68.16666666666667
	30.433333333333334	23.46666666666667
74.43333333333334	38.699999999999996\
TMEFF2	148.46666666666667	374.63333333333327
49.800000000000004	196.13333333333333	122.16666666666667
	162.20000000000002	67.9	2550.6	133.83333333333334
	132.26666666666668	133.5	175.20000000000002	84.7
	109.63333333333333	205.89999999999998	144.6
173.9333333333333	182.43333333333337	106.96666666666665
	125.93333333333332	203.9333333333333
144.6666666666666	221.29999999999998	156.8
148.33333333333334	153.6666666666666	152.03333333333333
	138.96666666666667	201.3666666666667
179.06666666666663	119.2	2232.5333333333333
320.93333333333334	116.76666666666667	76.8
288.73333333333335	162.83333333333334	179.83333333333337
	97.23333333333333	290.53333333333336
114.43333333333334	321.1666666666667	155.36666666666667
	407.90000000000003	105.39999999999999
361.0666666666666	1336.7666666666667	263.1333333333333
	168.70000000000002	227.1666666666667
194.53333333333333	131.86666666666665	224.43333333333337
	2997.7666666666664	166.2	139.29999999999998
389.0666666666666	217.79999999999998	265.46666666666664
	223.79999999999998	8810.266666666668
234.43333333333337	148.29999999999998	193.1
268.43333333333334	165.63333333333333	75.23333333333333
	169.83333333333334	444.0333333333333
145.86666666666667	284.5666666666666	235.20000000000002
	113.89999999999999	245.86666666666667	149.3
259.23333333333335	1194.3666666666666	128.4
173.7666666666665	893.1666666666665	2579.2333333333336

2986.933333333334	180.53333333333333
380.00000000000006	3842.2000000000003	4549.3	3091.5
1505.0333333333335	161.1666666666666	206.96666666666667
84.6	2238.1666666666665	289.7666666666665
261.03333333333336	279.4666666666664	280.9666666666667
411.7666666666665	274.6666666666667
449.7666666666667	520.6	387.7	647.6	193.8666666666667\
EIF1B-AS1	56.96666666666666	188.70000000000002
151.23333333333332	48.53333333333333	239.0666666666667
83.13333333333334	90.13333333333333	115.0
87.43333333333334	96.7666666666665	90.2666666666665
101.5	123.23333333333335	94.23333333333333
229.0666666666667	141.33333333333334	97.7666666666667
218.7666666666665	93.83333333333333
109.3666666666666	456.7666666666665	66.9
186.9666666666667	65.33333333333333	87.9666666666665
124.06666666666668	45.699999999999996
234.9666666666667	143.5	114.7	66.23333333333333
218.2666666666665	182.5	102.0666666666666
198.6666666666666	87.5666666666666	62.133333333333326
158.5666666666666	17.366666666666664	84.8
49.0666666666667	65.4	111.6666666666664
73.7666666666667	97.63333333333333	109.9666666666665
98.23333333333333	45.866666666666674	147.5
141.6	96.10000000000001	124.4666666666665	115.2
287.3666666666673	87.53333333333332	181.4666666666667
68.7666666666667	205.03333333333333
112.1666666666667	126.93333333333334	342.8666666666673
143.33333333333334	83.43333333333334	96.0
194.63333333333333	146.9	77.83333333333333
83.0666666666666	154.33333333333334	204.9666666666667
174.63333333333335	167.4	157.63333333333333
110.23333333333335	158.3666666666665	82.6666666666667
105.39999999999999	111.06666666666668
83.53333333333335	127.2666666666665	138.3666666666667
161.3666666666665	130.13333333333333
104.73333333333333	203.0333333333333	172.5
169.83333333333334	122.56666666666668	177.0
188.20000000000002	61.93333333333334	187.73333333333335
94.09999999999998	79.5	99.53333333333335
128.6666666666666	153.1666666666666	165.53333333333333
166.76666666666668	150.9	149.70000000000002
178.73333333333332	151.1666666666666\
GTF2H2B	206.1	4766.5	1857.5	632.1	886.0	118.1	363.9
109.6	168.1	445.9	266.9	1127.1	289.3	149.7	173.9
762.1	208.3	839.0	238.3	319.9	372.4	699.3	1297.7
134.6	136.6	582.8	200.3	1336.1	1276.5	97.6	588.4
185.6	895.5	373.0	319.7	410.7	111.7	1492.5	528.3	46.9
274.3	160.0	791.2	453.9	194.2	475.4	171.0
291.0	1233.4	1121.5	219.4	207.5	341.0	529.5	636.4
1038.7	517.7	754.1	528.4	2104.4	539.5	709.5	530.4
139.0	1306.2	1226.2	735.9	625.1	390.9	431.0	1112.2
1211.2	974.8	290.9	452.1	63.8	143.7	469.6	457.5
335.3	371.0	306.3	339.5	494.6	570.4	657.5	185.6	81.0

	187.4	748.8	97.6	566.5	168.9	477.0	522.4
308.0	325.0	497.0	592.7	185.0	1147.7	462.2	240.8\
LEFTY1 /// LEFTY2	76.2	151.6	62.6	367.7	215.2
123.2	778.1	124.7	378.8	1519.9	17.8	330.4	257.1	29.8
	110.0	32.4	1319.7	117.7	69.0	161.0	16.7
113.6	123.7	1255.2	47.6	114.1	21.9	156.0	35.6	22.2
	1444.4	33.7	30.3	98.3	92.8	166.8	185.6	28.9
	29.6	34.9	140.5	47.1	158.2	12.6	69.4
110.5	140.0	12.2	1099.8	86.7	153.6	95.5	61.2
120.3	883.7	1170.9	176.5	109.9	108.3	113.6	300.2
788.7	186.1	443.2	156.0	75.9	11.1	5.1	191.0
194.9	31.9	114.6	1402.0	75.1	35.8	65.9	149.6	74.2
	1924.9	158.3	59.2	189.2	16.7	69.8	93.7
172.4	16.5	217.7	11.4	35.9	27.2	811.9	309.6
1353.4	1295.0	273.9	535.8	520.5	30.1	300.5	526.0
215.4	536.5\
PKN3	499.7	83.8	194.2	629.5	336.0	850.4	295.4
326.2	354.0	232.6	310.6	358.3	415.9	615.5	973.5
288.8	114.2	367.0	573.9	391.9	335.0	247.7	463.0
315.0	356.1	709.1	512.2	277.3	186.8	109.1	157.9
267.2	498.3	171.6	119.5	295.6	433.5	624.9	247.0	37.3
	230.9	888.8	142.1	87.6	121.8	584.7	721.4
342.7	622.1	191.6	467.4	195.8	360.7	931.7	527.5
840.3	265.0	506.1	252.5	535.4	194.5	209.2	228.5
241.3	174.3	669.4	139.2	197.0	334.6	149.6	453.9
792.2	245.1	293.8	353.6	148.2	238.8	268.0	204.6
624.2	1115.8	408.1	177.7	206.5	1030.6	1057.9	565.5
950.4	420.4	133.4	227.8	610.7	410.5	220.6	231.3
247.7	196.8	192.3	168.0	360.3	180.5	295.1	227.1\
LOC101060405 /// RRN3P3	601.9	1956.1	514.8	444.8	745.0
228.8	459.6	667.1	212.7	746.2	150.7	784.5	588.4
603.0	184.7	333.4	419.8	814.9	366.3	627.6	2048.9
582.4	593.1	216.8	291.2	529.4	524.0	881.0	817.1
372.2	368.0	414.8	1234.3	28.8	711.6	531.4	126.9
1903.5	924.6	65.0	643.2	658.4	585.0	327.2	399.5
472.8	166.6	186.9	1324.2	1394.5	432.8	43.0	403.3
1107.2	955.4	626.4	301.9	726.9	904.4	818.1	59.7
1379.4	1008.1	642.3	654.7	920.5	619.2	275.3	592.8
289.7	1412.5	998.6	724.3	241.5	786.1	133.6	653.4
328.3	866.5	444.2	404.1	472.6	307.4	541.0	1018.2
297.9	630.9	171.7	811.1	799.7	442.9	748.3	242.1
270.0	265.6	241.7	130.1	300.2	340.8	28.7	458.4
447.3	468.3\
SYNPO2	210.3	136.25714285714287	369.14285714285717
695.5857142857142	425.99999999999994	201.25714285714284
	207.4857142857143	473.7428571428572
696.3000000000001	274.0142857142857	113.67142857142858
	395.47142857142853	123.70000000000002
339.90000000000003	69.41428571428573	1556.685714285714
	1216.9857142857143	428.35714285714283
322.2857142857143	463.11428571428576	634.1285714285714
	200.97142857142856	379.37142857142857
2288.0285714285715	276.87142857142857	80.42857142857143
	640.0571428571428	732.2857142857143

309.5142857142857	624.2714285714286	1683.857142857143
	419.84285714285716	347.6285714285714
762.2285714285714	347.8285714285715	218.31428571428572
	251.6285714285714	234.7857142857143
206.05714285714288	534.5428571428572	273.6857142857143
	40.05714285714286	1224.9428571428573
456.0571428571429	209.82857142857142	172.57142857142858
	358.40000000000003	98.44285714285716
115.92857142857143	251.85714285714286	727.7285714285715
	273.7	132.5142857142857	422.2	712.8714285714286
	143.5857142857143	334.04285714285714
534.3428571428572	325.2142857142857	552.8571428571429
	662.6	435.1857142857143	218.92857142857147
259.31428571428575	365.77142857142854	143.5285714285714
	334.41428571428565	344.62857142857143
814.6000000000001	200.48571428571427	653.9428571428572
	423.35714285714295	671.1142857142858
855.4571428571427	463.5142857142857	383.6142857142857
	413.4857142857143	437.24285714285713
440.7142857142857	146.5	467.5571428571428
136.02857142857144	503.0142857142858	262.34285714285716
	298.22857142857146	176.20000000000002
107.82857142857142	744.4428571428572	357.68571428571425
	481.1714285714285	503.2714285714286
1817.3714285714289	882.7428571428571	1972.5142857142855
	2123.9285714285716	384.6714285714285
836.0714285714284	604.0857142857142	219.7142857142857
	605.0571428571429	738.6857142857143
1705.5285714285712	498.6142857142857\
KCNA1	27.45	70.2	10.55	24.549999999999997	32.35
14.799999999999999	32.7	58.349999999999994	46.0	49.2
	52.65	50.55	19.65	54.849999999999994	72.75
31.65	38.0	64.95	36.6	53.35	23.0	79.19999999999999
	98.44999999999999	56.300000000000004	8.8
25.55	46.55	101.65	132.85	28.1	19.1	58.5	79.95
34.55	101.15	101.05	37.599999999999994	72.7	8.65	44.3
	25.599999999999998	60.150000000000006	36.2
45.099999999999994	41.5	78.4	58.349999999999994
35.95	58.900000000000006	54.2	56.25	69.25	10.05
137.4	48.5	69.85	66.39999999999999	37.7	78.65	94.0
	278.25	65.9	55.900000000000006	44.0	52.65
109.35	56.65	41.4	70.45	58.0	38.8	48.8
56.099999999999994	17.95	51.5	40.199999999999996	42.0
	27.349999999999998	42.0	64.95	47.900000000000006
	81.19999999999999	59.1	45.099999999999994
59.85	123.10000000000001	79.39999999999999
61.349999999999994	80.85	76.6	21.450000000000003	52.3
	65.75	18.65	10.7	39.849999999999994
46.849999999999994	14.850000000000001	78.19999999999999
	64.9	23.9	109.0	46.85\
GSTP1	1838.0	288.8	757.7	1534.7	518.0	2304.0	2031.0
811.5	1543.1	977.7	1188.4	1023.4	2403.0	1088.7	827.8
1780.0	1107.2	1406.3	2249.1	1050.2	365.9	1363.7	1025.9
1419.7	2459.2	479.9	2874.3	768.3	1193.2	1347.5	539.2

1661.1	1243.7	1442.3	891.5	1468.1	1659.7	1091.7	1311.7
4278.9	2327.4	1970.7	982.5	1734.5	2421.2	2402.0	395.8
1169.7	550.1	692.7	1499.9	2366.4	1119.5	454.8	1876.1
850.1	1066.5	895.8	1148.2	658.1	1393.5	1523.8	1324.0
1110.4	456.3	1019.7	961.1	1310.2	983.7	1799.2	413.8
640.3	590.6	1280.1	1087.1	1462.0	996.4	699.9	963.3
712.9	798.3	817.4	2198.4	936.8	990.1	767.4	964.9
1684.3	2707.8	1840.7	1262.8	501.8	1555.6	1471.8	809.9
838.5	818.5	1353.9	1247.4	605.2	676.6	642.9	1074.8\
PTAFR	183.66666666666666	290.9666666666667
410.53333333333336	579.0333333333334	322.73333333333335
	182.73333333333335	305.1	576.4	360.20000000000005
	586.0333333333333	323.03333333333336
258.8333333333333	252.0333333333333	373.9666666666667
	318.4	358.03333333333336	353.63333333333327
474.79999999999995	411.8	270.5666666666666
350.4666666666667	460.43333333333334	531.4333333333334
	387.63333333333327	367.5	348.9666666666667
616.0666666666667	555.1999999999999	422.0666666666666
	370.5	200.26666666666665	361.5	765.3000000000001
	317.03333333333336	315.3	188.46666666666667
323.8666666666666	935.3333333333334	226.76666666666668
	506.5333333333333	529.3333333333334
284.46666666666664	427.1666666666666	213.93333333333337
	443.8999999999999	288.06666666666666
429.03333333333336	190.66666666666666	338.03333333333336
	285.5	413.43333333333334	321.7666666666667
230.93333333333337	464.3333333333333	464.76666666666665
	298.8666666666666	247.83333333333334
299.29999999999995	369.53333333333336	193.86666666666667
	542.2666666666667	458.36666666666673	212.9
352.79999999999995	301.26666666666665	807.1999999999999
	374.09999999999997	242.26666666666668
566.5999999999999	442.86666666666673	349.0	287.1
410.1000000000001	368.0666666666666	366.43333333333334
	372.1333333333334	731.1666666666666
412.73333333333335	428.90000000000003	162.53333333333333
	390.23333333333335	466.46666666666664
416.40000000000003	543.4	1034.4666666666665
423.86666666666673	429.4666666666667	665.0666666666667
	702.3000000000001	315.3666666666667
325.1666666666667	236.69999999999996	190.16666666666666
	227.26666666666668	164.23333333333332
173.43333333333337	221.0666666666667	266.2
276.6333333333333	205.0	188.5	218.76666666666665
241.5\
RPA3OS	882.8	354.1	312.5	460.1	529.8	506.2	597.6
276.4	625.6	364.7	408.0	391.3	379.8	344.3	263.2
518.5	160.6	360.0	440.5	445.4	236.7	582.6	305.2
931.6	411.6	474.6	248.8	71.7	407.2	488.2	795.4
305.4	140.8	407.9	256.6	543.5	771.5	258.1	370.8
702.8	273.4	131.2	590.5	259.6	216.2	437.1	451.8
533.9	406.2	301.8	250.6	818.6	731.2	251.6	207.9
245.9	363.6	206.7	363.2	305.5	338.0	256.6	309.5

```
443.0	454.8	568.4	728.8	283.0	237.6	560.0	174.7
312.1	498.4	597.6	525.1	782.3	344.9	514.5	504.4
340.6	808.9	322.3	303.0	393.9	96.6	341.7	501.1
357.4	385.2	380.6	679.9	416.5	722.2	727.8	573.0
483.5	734.7	722.5	415.1	418.3	664.2	734.8	722.4\
KCNA2	51.55	23.65	39.15	25.8	29.25	6.35	22.7
32.25	153.29999999999998	154.14999999999998	8.2	46.4
	25.0	85.65	62.5	17.049999999999997	87.5	22.2
	144.35	114.69999999999999	190.35	85.65
98.80000000000001	24.3	50.65	64.5	143.64999999999998
	64.55	85.25	180.95	179.5	196.95	48.3	140.95
125.65	58.4	8.3	82.5	40.0	59.45	16.6	57.2	48.9
	288.35	82.25	97.8	46.35	29.55	152.8	142.15
61.300000000000004	55.55	73.44999999999999	138.55
34.45	70.9	117.19999999999999	46.599999999999994
23.25	86.4	107.15	67.6	80.5	28.9	71.64999999999999
	106.2	184.35	448.8	24.25	12.8	32.150000000000006
	114.8	131.05	27.450000000000003	159.2	8.6
107.15	6.65	200.3	56.25	115.45	64.55	71.1
29.200000000000003	70.0	94.44999999999999	49.65
53.15	77.75	50.550000000000004	35.05	94.55	131.2	36.5
	62.6	223.3	202.5	48.05	94.7	83.25	121.5	25.8
	128.4\
ADAM22	22.950000000000003	35.4	22.8625	58.112500000000004
	51.262499999999996	17.637500000000003	48.95
42.925	40.05	31.8	33.5125	38.512499999999996	24.7125
23.2375	78.3	32.0125	54.82499999999999	47.8
33.099999999999994	34.1625	82.8125	42.3625	27.4	36.325
33.1375	53.925	56.55	79.6875	35.275000000000006
90.43750000000001	104.925	64.19999999999999	40.4125
58.6875	41.2375	41.1375	34.1125	80.15	25.362499999999997
43.650000000000006	46.212500000000006	29.4375
48.949999999999996	51.925000000000004	40.599999999999994
	22.8	21.875000000000004	45.825	62.162499999999994
	49.412499999999994	82.30000000000001
22.037499999999998	40.14999999999999	77.67500000000001
	35.875	25.4375	26.35	80.14999999999999
45.862500000000004	61.099999999999994	180.76249999999996
	52.262499999999996	55.5125	35.712500000000006
62.849999999999994	32.9125	30.25	30.275000000000002	54.0
	39.975	86.64999999999999	52.3875	64.0125
57.56250000000001	33.15	20.674999999999997	62.5625
20.1375	41.2125	98.4	80.3375	69.375	79.4625	32.625	77.825
97.275	70.325	58.300000000000004	40.587500000000006
87.3625	25.574999999999996	99.75	54.8625	59.5	55.2875
67.11250000000001	59.175	57.8875	60.39999999999999	90.1
	68.4	115.15	48.45\
KCNA3	25.6	50.4	15.6	104.7	28.8	27.5	7.7
169.5	89.1	48.2	14.5	99.9	21.6	56.5	8.3	17.3
	97.3	15.4	39.8	103.5	116.2	88.1	117.6
295.2	8.4	46.2	22.4	49.3	28.8	16.0	94.6	20.1
	26.1	17.8	183.5	68.6	121.3	120.1	9.8
182.9	92.1	68.2	86.0	164.8	26.7	27.3	61.1	19.8
	22.8	22.4	17.5	5.7	3.8	17.6	11.5	61.8
```

	79.5	59.7	116.8	10.4	73.5	153.6	14.7
109.0	158.0	18.5	109.8	64.0	392.9	15.0	142.9
280.3	306.8	88.7	41.4	28.3	66.7	25.4	69.6	25.7
	121.8	17.2	3.7	115.0	11.3	7.3	43.4	12.3
	80.6	159.3	53.8	20.5	77.2	109.4	68.4	16.2
	66.0	5.7	38.3	32.8	85.2	82.3	137.5\
KIAA2022		145.70000000000002		363.26666666666665
140.56666666666666		24.666666666666668		253.06666666666667
	30.233333333333334		26.633333333333336
210.23333333333335		30.799999999999997		97.83333333333331
	306.7	244.93333333333337		180.83333333333334
196.86666666666665		14.5	51.1	653.5666666666667
86.83333333333333		106.96666666666668		142.53333333333333
	29.266666666666666		88.36666666666667
116.33333333333333		134.9333333333333		19.233333333333333
	22.633333333333333		35.03333333333333
139.23333333333332		98.5	179.56666666666663
85.5666666666666		50.333333333333336		99.8
105.96666666666665		41.6	41.666666666666664
466.9000000000001		62.199999999999996		44.0
62.366666666666674		27.933333333333334		209.93333333333337
	110.8	34.4	27.8	14.4	185.4666666666667
34.699999999999996		41.43333333333333		131.16666666666666
	422.8333333333333		29.933333333333334
265.8333333333333		67.10000000000001		53.400000000000006
	70.63333333333334		80.16666666666667
32.36666666666667		242.73333333333332		25.733333333333334
	350.7	83.96666666666667		205.26666666666668
154.1	91.0	168.20000000000002		99.43333333333334
37.63333333333333		50.5	169.46666666666667
112.13333333333334		17.366666666666667		24.666666666666668
	47.06666666666666		68.2	164.13333333333333
78.33333333333333		103.60000000000001		80.1	1009.1
44.13333333333333		48.53333333333333		24.900000000000002
	45.733333333333334		14.766666666666666
80.26666666666667		60.46666666666667		329.0
43.7666666666667		53.5666666666666		43.53333333333333
	104.2	252.79999999999998		206.89999999999998
174.5	134.03333333333333		212.0	118.3	268.50000000000006
	234.76666666666668		109.26666666666667
83.63333333333333		168.63333333333333\
KCNA4	18.5	121.8	19.8	7.5	48.2	25.1	11.0	13.1
	53.5	9.0	14.0	21.5	13.2	8.0	35.7	9.8
	6.3	12.0	23.5	14.2	26.5	30.7	18.9	36.7
	7.8	18.1	12.9	6.6	15.6	27.2	11.0	18.9
	87.5	9.0	26.3	6.7	6.4	20.6	5.8	36.9
	22.0	5.6	17.1	20.8	11.1	10.9	30.1	10.3
	29.1	20.3	11.1	10.4	65.6	22.6	22.4	12.6
	12.6	32.7	18.1	21.7	158.9	19.1	10.6	46.3
	7.5	23.5	9.8	9.3	36.2	17.8	10.1	90.3
	7.4	10.4	11.2	11.9	13.9	14.8	12.0	76.2
	4.3	12.7	147.2	11.7	91.3	32.1	83.3	22.5
	17.7	37.5	63.0	29.0	10.6	16.0	10.1	6.4
	4.1	44.2	15.2	14.3	22.4	17.5	37.2\

ADAM21 46.15 66.05 57.449999999999996 28.1 70.55 20.1
22.5 36.35 13.1 35.3 56.15 38.75
69.19999999999999 64.7 83.6 26.549999999999997 47.2
52.95 27.75 78.69999999999999 124.45 36.65
59.349999999999994 40.4 108.0 38.1 20.0 102.6 45.8
67.25 63.6 81.15 50.8 76.15 67.35
41.150000000000006 39.25 28.35 17.65 127.35 10.75
32.35 68.65 46.35 67.7 38.6 112.45 43.949999999999996
35.9 40.650000000000006 57.95 28.25 100.85 67.3
54.3 62.45 39.05 44.45 53.75 116.15 332.5
31.95 51.75 40.900000000000006 45.349999999999994 32.0
15.65 25.0 56.0 38.5 111.0 98.9 27.6 32.1
40.35 55.150000000000006 50.55 34.699999999999996
50.1 35.35 176.55 39.65 34.900000000000006
22.25 56.75 78.14999999999999 69.19999999999999
57.15 58.65 83.7 93.6 86.8 76.69999999999999 28.0
35.35 47.25 42.95 56.400000000000006
21.950000000000003 60.75 27.400000000000002 103.4
65.05\
KCNA5 21.1 12.8 33.6 2.5 116.1 3.1 21.9
148.2 96.2 15.7 5.8 23.1 5.4 22.2 35.6 6.3
9.3 60.7 4.1 132.3 310.8 13.1 37.8 64.8
6.8 7.7 41.4 27.5 8.0 137.9 4.0 35.0
138.1 37.2 10.0 10.2 8.6 45.7 43.0 9.8
211.4 9.1 59.9 115.6 4.3 21.2 101.7 4.7
52.6 37.0 15.7 12.8 6.0 154.9 18.4 26.4
64.6 26.5 50.6 14.4 458.3 8.0 18.1
107.7 129.3 6.9 4.6 67.7 12.4 9.5 37.4 84.4
253.0 104.9 50.0 5.7 4.7 4.0 98.2 6.8
30.0 12.7 109.5 15.2 26.0 13.0 59.4 27.6
187.0 74.8 37.0 16.1 7.3 14.9 32.7 68.1
11.3 3.2 4.6 5.1 6.8 20.7 7.3\
ADAM20 14.299999999999999 50.2 35.25 44.650000000000006
55.2 20.650000000000002 38.9 11.2 14.5
27.05 47.9 16.2 45.75 22.2 76.45 28.25 52.7
17.35 42.2 88.15 124.05 38.65 93.55 16.6 48.1
53.55 70.8 44.25 73.4 30.45 29.25 138.85 16.4
42.25 132.7 34.75 13.55 145.0 32.050000000000004
27.85 17.05 53.85 12.6 29.0 32.25 41.25 43.7 27.0
78.65 13.75 42.949999999999996 49.6 23.25
116.45 39.15 45.15 6.6 45.2 21.799999999999997
101.55 318.75 34.65 54.050000000000004 17.849999999999998
44.150000000000006 39.1 9.5 9.25 134.6
63.900000000000006 127.25 30.450000000000003
33.900000000000006 10.25 65.7 30.75 9.55 10.6
48.25 56.75 100.64999999999999 101.95 19.950000000000003
23.7 138.1 159.5 72.55 29.599999999999998
98.65 175.7 38.2 78.75 41.0 5.45 41.55 22.85 45.4
34.75 106.5 29.950000000000003 24.6 39.3
37.6\
KCNA6 12.4 131.5 18.7 9.5 275.4 13.1 5.8 15.5
10.8 55.9 10.4 3.3 2.9 12.8 24.9 92.5
27.4 88.2 45.3 32.6 41.3 8.2 27.7 6.2
56.3 36.7 7.8 145.7 71.1 46.2 5.4 7.1

	23.1	29.6	70.1	23.1	27.9	76.4	11.2	26.9
	22.8	26.3	22.2	66.5	79.4	102.5	56.9	15.5
	113.1	70.8	55.4	73.2	12.9	23.8	12.6	40.5
	13.0	89.0	7.9	127.9	31.3	57.2	13.3	10.0
	5.3	89.1	49.1	16.7	27.3	56.9	148.8
121.9	53.8	50.8	11.4	63.8	12.7	37.5	45.1	42.9
	125.0	37.8	12.3	44.3	18.1	17.5	56.4
110.1	15.0	130.7	38.9	16.4	64.8	9.1	6.6	19.8
	8.2	13.3	36.4	15.6	4.7	87.2	9.8\
SDAD1	975.6333333333333	377.2666666666667
519.6999999999999	1039.3999999999999	734.3333333333334
	1046.7666666666667	1503.3999999999999
416.1666666666667	1439.2666666666667	650.1666666666666
	794.2333333333332	963.8000000000001
1273.1333333333334	1268.4333333333334	789.4333333333334
	463.5333333333333	695.8000000000001
984.9666666666667	1172.3	516.9666666666667
423.3999999999999	905.6333333333333	749.5666666666667
	1087.3999999999999	1619.6333333333332
1021.1999999999999	930.6666666666666	525.1333333333333
	572.7	501.2666666666667	1009.1333333333333
219.33333333333334	602.7666666666667	745.6666666666666
	837.8333333333334	743.4666666666667
778.1666666666666	588.6999999999999	1220.3999999999999
	470.7666666666667	649.8000000000001	1321.3
413.7333333333333	871.5	1401.7	865.8000000000001
925.9666666666667	577.9666666666667	667.6999999999999
	618.2333333333333	898.7333333333332
564.4333333333334	645.5	884.4666666666667
569.9666666666667	662.0666666666667	724.6999999999999
	599.5333333333333	793.7999999999998
558.3333333333334	902.5	547.9	627.6666666666666
511.5	667.6666666666666	578.1666666666666
758.4666666666667	600.8666666666667	694.1999999999999
	403.43333333333334	505.1666666666667
670.7666666666668	643.6999999999999	1276.2333333333333
	652.5	826.0333333333333	398.4666666666667
763.9333333333334	614.3000000000001	962.5
501.6666666666667	551.9333333333333	615.9333333333333
	1034.2	443.8333333333333	445.6333333333334
567.2	346.0333333333333	1281.7333333333333
690.7333333333332	1049.6000000000001	402.8
505.90000000000003	641.4	495.1333333333334
531.4333333333333	516.0	680.8333333333334
441.43333333333334	574.2666666666667	502.3333333333333
	499.8666666666666	666.9333333333334\
KCNA7	3.8	30.0	15.0	5.9	13.6	10.3	3.7	7.2
	11.1	7.7	15.0	19.9	17.4	35.0	22.2	10.4
	12.4	10.0	4.4	6.4	7.4	22.3	10.3	11.9
	6.1	7.1	5.2	14.5	19.4	10.5	8.2	28.8
	15.2	4.4	27.0	10.8	5.1	10.0	6.2	11.5
	12.8	6.0	17.0	15.6	3.1	36.1	19.3	3.8
	16.6	10.0	7.6	6.7	5.4	10.7	6.5	18.5
	19.0	9.1	10.4	10.3	56.0	8.7	4.5	14.6

11.6 10.8 5.1 6.5 14.9 21.9 10.5 7.3
10.5 9.7 23.2 9.7 9.5 5.5 3.6 3.0
12.9 8.1 10.6 3.5 7.4 12.3 9.4 11.9
15.6 13.4 16.3 6.0 7.1 5.3 11.3 4.3
11.1 12.7 5.6 12.7 4.2 7.6 12.3\
RP11-31K23.2 16.6 57.9 4.2 2.4 27.1 4.6 45.2
23.4 47.9 31.7 2.1 27.0 47.1 13.2 12.3
31.6 4.1 16.2 14.9 5.2 11.4 49.0 7.2
5.2 13.0 18.3 5.4 11.7 23.3 58.0 1.9
35.4 3.9 2.7 10.8 2.9 3.1 35.4 2.6
5.4 8.0 4.5 2.5 5.2 8.9 6.8 8.1
5.4 42.7 35.4 38.1 41.2 36.6 11.6 8.1
17.1 24.0 29.5 6.9 156.3 34.0 7.4 7.3
6.6 50.0 57.0 25.6 4.0 13.3 24.7 94.0
20.4 4.7 16.1 7.0 5.4 27.7 2.3 6.5
4.5 3.4 7.5 4.6 4.7 12.9 11.9 32.3
4.9 4.4 15.5 1.7 3.9 5.7 1.4 4.8
8.1 63.5 20.0 2.8 7.6 44.2 10.1
18.1\
LOC642426 0.9 36.3 2.9 0.5 52.8 5.4 10.3
29.5 0.7 50.0 0.3 3.2 21.6 1.0 1.9
1.8 20.6 0.6 10.8 19.0 0.9 1.3 3.0
7.1 1.4 1.3 2.6 3.4 0.8 1.2 7.5
58.1 35.2 14.1 1.9 13.2 7.3 1.5 1.2
1.3 0.5 0.7 2.3 28.5 17.0 16.7 22.9
1.1 51.5 3.4 3.5 18.0 7.9 0.9 3.4
44.4 5.1 0.3 0.7 16.5 160.8 49.4 0.6
1.4 12.3 3.6 15.4 1.5 1.4 12.1 1.4
24.6 27.1 0.4 2.8 12.7 3.6 0.7 2.3
0.1 40.1 21.5 0.3 24.1 5.7 3.0 29.4
0.8 18.1 21.6 17.2 28.4 17.6 6.2 1.7
19.1 4.9 29.3 0.9 11.5 8.3 54.5 3.1\
PAXBP1-AS1 71.45 109.15 63.3 74.95 177.7 25.45
104.75 102.10000000000001 39.0 82.35 35.55
58.550000000000004 9.55 81.25 46.199999999999996 93.2
81.45 25.7 65.3 64.65 96.0 38.800000000000004
35.0 50.95 27.299999999999997 48.849999999999994
82.95 90.4 30.7 66.85 58.7 13.65 148.55 48.9
110.35 44.650000000000006 61.15 130.2
74.10000000000001 52.75 87.6 53.35 47.95 24.1 73.2
117.3 122.69999999999999 57.95 27.35 49.65
67.65 41.7 53.55 103.1 32.6 59.8 110.4
68.05000000000001 39.5 67.9 445.15 59.85 92.25 41.5
53.05 67.65 59.1 90.0 46.599999999999994 73.0
82.2 105.75 100.75 105.95 13.0 31.200000000000003
125.9 42.6 81.7 142.65 182.3 94.1 8.25
67.35 111.44999999999999 176.6 154.75 64.8
44.849999999999994 39.3 41.65 120.9 88.15
91.80000000000001 161.75 98.15 189.75 87.44999999999999
98.80000000000001 116.30000000000001 137.35
160.05 123.75\
ADAM28 3800.95 2772.25 1249.525 156.89999999999998
639.85 118.875 56.775000000000006 487.825 1325.65 418.35
216.52499999999998 543.5 289.1 496.425 712.1999999999999

660.325 2072.375 1197.2 567.7249999999999
860.375 797.025 407.475 749.6 444.8 555.425 3470.075
557.95 1298.275 570.325 1270.075 975.3 667.125
1476.975 560.65 59.375 282.5 531.075 349.625 1882.6
187.65 1861.625 717.5 764.9749999999999 613.875
523.325 318.6 644.575 46.85 2181.8 3073.6 938.1999999999999
253.45 522.625 398.34999999999997 738.075 691.075
1664.65 155.725 973.3 1263.975 467.875 411.275 1634.025
564.125 1830.475 630.475 1041.025 932.2
628.825 639.525 940.35 238.875 1726.4 1073.625 1059.85
173.6 1009.2499999999999 554.0 1434.0 17.475 155.2
514.55 734.05 529.6750000000001 342.625 93.2 153.9
128.22500000000002 743.2750000000001 136.5 142.1
2272.1 676.5999999999999 649.5250000000001 1234.45
770.9000000000001 965.0500000000001 1080.75
843.8000000000001 2584.15 1930.1499999999999
351.67499999999995 2327.6\
INO80B /// INO80B-WBP1 18.2 137.95000000000002 48.35 98.1
122.30000000000001 90.80000000000001 70.5
99.25 83.9 157.75 126.94999999999999 53.5 128.15
176.89999999999998 25.5 85.0 52.6 139.2 124.7 67.6
72.4 108.1 86.35 94.7 90.5 27.0 61.55 43.2
52.75 102.10000000000001 91.5 37.1 60.9
73.75 133.95 138.14999999999998 42.9 54.05
101.80000000000001 152.35 64.1 33.949999999999996
41.95 92.2 142.45 209.5 44.3 63.95 69.6 84.3
108.3 40.1 70.75 111.45 120.3 20.85 118.55 52.3
80.35 71.95 45.5 132.15 126.35 52.25 53.05
108.39999999999999 138.64999999999998 81.30000000000001
165.2 77.6 87.8 98.75 40.1 83.55000000000001
81.3 95.8 86.55 50.0 51.65 28.85 19.6 13.6
94.15 78.1 42.35 94.25 55.3 67.94999999999999
176.3 154.35 79.6 36.85 81.6 103.35
103.14999999999999 51.2 65.7 33.1 133.95 39.0
111.35 42.25 69.4\
LOC100132319 15.6 11.2 18.6 64.3 83.5 26.1 13.1
170.4 17.8 33.0 20.6 48.7 44.9 57.5
147.0 48.9 14.7 14.9 50.6 20.9 142.9 33.2 73.6
28.2 51.4 39.5 61.5 38.4 70.7 24.1 32.4
89.5 82.6 14.5 90.7 74.7 17.0 105.5 18.1
60.4 70.2 15.4 80.5 5.5 46.6 17.0
103.7 48.4 127.6 12.1 39.5 56.0 53.1 49.9 7.2
238.7 129.1 39.7 14.5 93.6 449.4 8.2 78.4
76.8 77.9 13.4 25.8 38.1 94.8 9.6 85.9
42.2 7.6 24.2 111.5 86.1 23.1 100.8 40.3
61.5 99.3 144.9 9.2 43.1 192.5 168.0 92.0
96.1 68.8 19.1 10.7 106.3 48.2 41.1 23.9
51.5 25.7 46.8 12.3 102.2 31.9 15.3 6.6\
KIAA2018 470.73999999999995 386.28000000000003
490.1 553.8599999999999 369.24 496.8 1066.28 673.96
634.78 483.64 561.92 603.8 396.02000000000004
534.4399999999999 773.4 538.76 611.5 642.72
631.3199999999999 455.3 311.12 480.2200000000001
645.38 571.5600000000001 557.1400000000001

532.8599999999999	689.88	810.4599999999999
797.0400000000001	570.4200000000001	607.6	320.0
381.12	1482.78	1049.08	647.8399999999999	567.3399999999999
	345.7	483.48000000000013	989.46	712.1200000000001
	465.4	552.12	586.34	435.02	635.64	695.12	272.72
332.56	617.6	652.72	416.64000000000004	549.76	573.78
441.74000000000007	575.42	904.1400000000001	526.48
695.02	594.86	685.6000000000001	493.7999999999999
558.88	669.6800000000001	587.6400000000001	676.64
626.3199999999999	503.5	428.3	578.76	484.55999999999995
	585.3799999999999	558.1800000000001	869.1
708.4799999999999	634.5	538.6999999999999	919.64
571.7800000000001	721.4599999999999	460.14	530.72
276.94	610.52	347.1	426.02	622.26	982.46	402.64	993.26
569.62	450.48	576.98	529.5799999999999	724.42	659.5
718.3800000000001	625.98	461.88	693.54	810.48	415.98
768.0400000000001\
ADAM23	325.97499999999997	44.05	36.775000000000006
23.775	74.85	18.925	26.799999999999997	41.599999999999994
	363.72499999999997	27.074999999999996	45.275
130.4	18.875	68.75	50.7	85.975	90.8	46.125	171.275
427.025	633.125	39.45	64.1	63.65	33.150000000000006	19.0
	65.275	86.65	27.975	294.45	311.9	67.5	89.15
27.549999999999997	46.025	47.75	41.825	37.45
411.00000000000006	68.075	39.550000000000004	26.475
53.699999999999996	1277.05	37.525000000000006	38.25
35.75	913.9000000000001	30.05	533.8000000000001
117.30000000000001	23.724999999999998	19.299999999999997
	26.225	65.975	20.8	42.025	26.575	24.075
70.60000000000001	220.225	51.425	50.75	40.050000000000004
	1046.2	28.625	170.77499999999998	1895.9250000000002
	86.82499999999999	55.150000000000006	64.0
53.35	755.825	40.2	45.55	31.6	71.625	21.825000000000003
	902.875	325.825	31.299999999999997	58.775
18.974999999999998	17.85	68.925	244.35000000000002
70.35	97.24999999999999	67.375	391.225	51.35
78.19999999999999	40.025000000000006	38.724999999999994
	27.424999999999997	34.125	58.849999999999994
65.325	41.675	32.375	72.15	84.82499999999999
54.224999999999994\
STX6	518.98	679.66	386.93999999999994	639.4200000000001
	1060.24	1149.5400000000002	987.7	664.9
754.3799999999999	740.28	830.5600000000001	743.76
729.58	571.02	552.1000000000001	901.7	1153.4	715.1
677.8000000000001	760.04	429.4	766.24	654.24	816.76
693.9800000000001	604.64	802.02	698.14	1010.3
841.6600000000001	1076.0	764.4200000000001	471.2
1007.6	1899.42	392.52	1118.1600000000003	486.65999999999997
	879.1200000000001	1539.6599999999999	547.36
1301.38	812.64	919.96	903.1600000000001	579.2	655.68
1516.0400000000002	1178.1200000000001	826.74	917.1
891.5199999999999	926.0	553.1600000000001	638.22
1169.0	1364.3	445.34	420.2	661.8599999999999	284.5
546.74	873.32	621.66	513.8	811.38	1333.06	834.5	486.84

679.1000000000001 854.9399999999999 720.78 407.9
1435.76 849.32 1365.7400000000002 499.0 1261.7399999999998
901.6200000000001 618.16 399.85999999999996
513.76 485.8 790.3199999999999 402.58000000000004
311.43999999999994 490.84000000000003 401.38
433.78000000000003 353.71999999999997 859.54 404.32
526.12 399.8 355.3 570.98 500.36 488.08000000000004
615.78 468.94000000000005 509.6 304.96000000000004
457.38\
FLJ21369 34.4 54.3 146.7 41.2 201.2 24.7 15.6
44.1 84.2 43.3 55.1 137.5 50.2 47.5
137.5 84.7 34.8 147.7 54.9 141.3 348.6 47.3
150.1 25.3 85.8 33.7 46.0 100.6 83.3 38.6 34.4
10.3 205.1 58.1 96.6 12.5 3.3 180.1 24.0
64.1 2.2 19.9 76.2 38.4 42.8 51.4 5.1
3.7 72.6 105.4 6.1 44.9 1.5 80.4 85.4
102.0 27.9 144.4 56.0 48.0 26.9 72.7 67.7
40.4 75.5 79.4 24.7 38.7 35.2 36.5 34.5
129.6 110.4 48.4 89.2 50.4 64.4 75.3 5.3
2.0 101.1 38.6 45.3 44.7 59.9 87.0 29.0
83.1 59.0 125.1 19.9 37.4 3.7 28.0 85.7
40.4 69.1 4.1 126.6 58.9 62.4 68.9
36.8\
ACAD11 /// NPHP3 /// NPHP3-ACAD11 2622.3 3868.2 4592.8
2398.0 1579.7 1358.0 902.3 1910.2 947.2 1563.2 3348.7
3126.8 975.0 5034.1 3317.1 2909.8 1132.1 2680.6 1240.1
1983.0 6592.8 1672.7 5825.0 1177.8 1126.2 6892.2 1479.8
3382.0 6486.4 1848.0 1107.7 2658.0 4790.1 1497.4 2103.0
3975.9 1294.1 2913.0 1768.0 965.1 1066.5 1330.9 1776.6
2181.3 1338.7 2670.7 1772.7 1150.0 13705.8 3107.7 2336.6
1383.3 2548.0 4055.1 5452.3 6734.3 2176.3 7702.9 4253.9
3784.6 1709.5 3450.6 1336.3 2490.8 3941.5 2170.7 1221.7
1480.3 2645.7 3918.0 5696.1 5358.5 3337.4 1283.3 3062.1
1235.6 3569.9 1771.8 1761.3 1378.6 1662.3 1801.1 3350.5
2909.6 5204.0 2327.4 2480.1 2665.1 1878.6 10321.2 768.7
7295.2 3518.3 2744.3 2853.1 2658.4 3475.2 1838.3 3574.3
3645.9 6236.8 8524.9 3053.4\
STX5 212.8 157.3 272.5 168.3 242.1 244.1 322.6 90.9
325.8 91.0 316.4 266.1 181.7 273.8 57.9
175.1 233.9 142.8 262.9 267.0 289.4 284.5 204.8
245.6 192.8 183.9 205.3 192.6 246.1 246.8 162.2
197.1 209.7 275.9 244.7 176.1 492.4 237.3 332.4
247.0 275.4 247.9 219.0 171.8 310.1 88.0 168.1
133.6 290.7 213.0 249.0 301.5 141.0 38.5 167.7
100.6 204.5 207.4 346.9 150.1 346.8 206.2 206.7
328.7 233.6 265.4 278.2 153.3 308.8 224.6 225.9
185.0 296.7 233.3 296.3 412.3 278.4 439.3 177.0
112.0 214.5 75.8 217.7 150.5 51.2 454.9 116.7
136.8 332.2 323.6 313.8 128.7 211.5 221.5 196.5
155.1 209.2 186.9 152.4 124.0 213.7 155.2 279.5\
STX4 611.0 1388.0 512.85 624.15 762.0 676.15 895.5
557.1 499.85 473.8 398.9 1056.0 628.75 832.6500000000001
278.35 399.35 386.7 480.15 800.7 516.6 774.65
439.65 509.40000000000003 714.75 394.05 456.29999999999995

665.3499999999999	512.0	440.04999999999995
402.6	893.65	730.85	800.6999999999999	488.59999999999997
720.95	610.1	869.75	821.8	682.5	570.85	554.55
778.55	561.3	803.3499999999999	619.3	404.45000000000005
662.15	432.2	654.95	762.1500000000001	597.05
516.8	516.05	677.0	543.0999999999999	389.35	738.8
528.35	633.85	715.6500000000001	227.5	575.9000000000001
662.7	428.90000000000003	585.1	752.6500000000001
852.4000000000001	779.6	713.7	492.6
738.5500000000001	848.3	758.95	745.0999999999999
613.35	485.9	418.9	760.45	556.4000000000001
345.20000000000005	268.5	525.45	502.54999999999995
720.85	583.05	705.3	336.15	528.1	617.7	659.85	1108.65
620.8	562.8499999999999	581.75	568.5	462.1
622.5999999999999	807.8	842.8	662.55	727.6500000000001
537.3	688.15\
STX3	788.1666666666666	601.4666666666667
578.2666666666667	864.6333333333333	477.5666666666666
577.4333333333334	568.6666666666666
656.4333333333333	390.40000000000003	452.8333333333333
513.2	632.2333333333332	444.8666666666666
405.5	323.73333333333335	382.5333333333333
659.6333333333333	527.9	813.7333333333332
465.5666666666666	221.0	451.43333333333334
616.4333333333333	422.9666666666667	334.53333333333336
861.8333333333334	905.4666666666667	543.5
808.5	660.7666666666667	444.3666666666666
575.4666666666667	645.5	504.5333333333333
416.2333333333333	569.1333333333333	435.09999999999997
736.6333333333332	905.1666666666666
558.3000000000001	615.2	624.7333333333332
508.26666666666665	596.8333333333334	561.2666666666667
740.2333333333332	570.0666666666666
780.2666666666665	449.3	763.0333333333333	587.6
728.0	537.8666666666667	599.8333333333334
580.5666666666667	501.3666666666666	421.8999999999999
633.6999999999999	755.1999999999999
658.4333333333334	460.23333333333335	700.8666666666667
526.1666666666666	678.5666666666667	605.5
769.4333333333334	502.6000000000001	564.7333333333333
476.3	586.7666666666667	444.0666666666666
680.5666666666666	830.5333333333333	789.1333333333333
500.3	557.1	455.23333333333335	537.5666666666666
697.0333333333333	219.36666666666667	554.0
599.3333333333334	688.6333333333333	718.2666666666668
990.4666666666667	818.5	465.8	583.4	812.9
859.8666666666667	350.5	450.1333333333334
490.06666666666666	519.9666666666667	662.3666666666667
565.5	483.20000000000005	414.2	534.9333333333333
517.0	556.0333333333333	507.3	512.9666666666667\
STX2	135.45	361.45000000000005	802.05	342.84999999999997
250.55	249.95	520.65	260.55	225.4	302.9	391.0
133.15	148.89999999999998	227.9	141.20000000000002
372.25	242.3	383.85	471.85	159.14999999999998	318.8

384.34999999999997	721.0	280.05	273.0	357.45	162.85
580.0	440.4	366.05	252.20000000000002	106.9	554.05
326.9	188.85	355.6	119.0	245.2	114.44999999999999
175.54999999999998	391.25	650.0999999999999	447.3
315.05	359.0	632.4	142.1	301.15	180.8	379.59999999999997
	255.75	265.25	362.3	272.45	427.1	595.8000000000001
	282.7	478.7	335.95000000000005	455.90000000000003
	428.25	621.5	74.25	672.8499999999999	307.45
481.15	389.95	210.2	481.65	931.45	525.3499999999999
454.4	223.04999999999998	261.85	351.0	417.15000000000003
	596.65	313.3	314.8	478.0	337.45	512.1	227.6
226.85	783.5	715.9	187.1	313.95	349.7	663.85	198.05
696.3000000000001	257.65	387.75	333.65	267.15	159.7
322.85	177.35	299.9	246.95	512.5	261.70000000000005\
BID	500.3666666666666	83.7	353.0333333333333
383.5333333333333	445.2	973.1666666666666
426.9333333333334	670.9666666666667	531.9333333333333
	650.5333333333333	533.7333333333332	364.2
471.6666666666667	292.8333333333333	567.8333333333334
	540.1333333333333	380.06666666666666
692.1666666666666	562.1333333333333	325.6333333333334
	138.96666666666667	568.4	294.7	422.8	470.0
409.56666666666666	1508.1999999999998	407.20000000000005
	313.3333333333333	465.3	391.3666666666666
352.23333333333335	529.2333333333333	439.1666666666667
	721.1333333333333	319.6666666666667
761.9333333333334	512.3333333333334	416.1333333333334
	232.4333333333333	696.3333333333334
276.1666666666667	367.5	473.5666666666666
1159.3999999999999	494.5	1054.7	1453.5	370.79999999999995
	307.06666666666666	491.6000000000001
766.8333333333334	417.8333333333333	569.9333333333333
	418.73333333333335	366.73333333333335
392.1333333333334	411.1000000000001	204.79999999999998
	391.0333333333333	789.9666666666667
316.40000000000003	515.6333333333333	901.5333333333333
	325.8333333333333	437.56666666666666
334.7333333333333	285.0	616.4	653.7666666666667
220.70000000000002	342.0333333333333	260.26666666666665
	482.36666666666673	226.63333333333333	690.1
665.6999999999999	393.9333333333334	436.0333333333333
	634.4333333333333	551.4333333333334	986.5
314.40000000000003	942.4	424.9666666666667	223.6
630.1	576.6666666666666	269.40000000000003
163.36666666666667	633.6	379.3666666666666
502.6000000000001	468.56666666666666	238.4333333333333
	407.5333333333333	292.5	537.9333333333333
455.73333333333335	514.9333333333333	315.40000000000003
	339.8666666666666	391.3666666666666\
SPDEF	153.06	2000.2400000000002	682.86	106.46	520.86
1038.2400000000002	68.80000000000001	221.84	685.76
1062.7	247.58	1032.54	59.64000000000001	1813.72
89.02000000000001	124.54	537.2	713.26	642.7	1041.72
388.48	622.12	398.9	753.48	259.98	1855.1599999999999

1644.58 1729.78 1461.14 658.36 318.28 1658.6 244.82
1100.1200000000001   1580.1599999999999   196.33999999999997
        298.76 195.28000000000003   565.0600000000001
986.2199999999999   137.34 161.64 1506.0400000000002
153.7   1238.3799999999999   654.98 1402.3799999999999   48.0
        1977.44 1641.4 1216.0400000000002   232.3   1078.94
826.2599999999999   916.5   1284.6 1290.84 220.98000000000002
        2240.22 789.08 1202.98 677.16 494.16 645.02 831.78
1048.6799999999998   977.0799999999999   69.92
1024.5400000000002   1324.1399999999999   1308.62
1547.8200000000002   542.74 694.4399999999999   748.76
1671.36 943.2400000000001   880.9599999999998
673.8599999999999   96.12   359.42 174.82 365.7
114.48000000000002   278.15999999999997   180.07999999999998
        103.32000000000001   328.14 145.85999999999999
103.97999999999999   170.88 308.88 1422.2199999999998
335.46000000000004   504.08000000000004   654.5799999999999
        708.42 778.74 1493.86 796.4   1061.3 1414.22 790.6\
MBD3   332.3333333333333   350.3999999999999   258.4
553.8000000000001   653.6999999999999   395.2666666666667
        368.73333333333335   318.6333333333334   552.0
529.6333333333333   379.70000000000005   361.3333333333333
        367.7666666666667   270.8666666666667
579.4666666666667   432.0   525.9333333333333   407.0
345.1333333333334   407.3   755.5333333333333
387.36666666666673   437.9666666666667   390.9666666666667
        359.8   556.5   439.76666666666665   502.4333333333334
        553.1333333333333   323.79999999999995
191.36666666666665   501.3333333333333   594.1
480.56666666666666   356.0333333333333   405.90000000000003
        294.7333333333333   560.4666666666667   379.0
239.63333333333335   296.23333333333335   389.7666666666667
        289.0   502.5333333333333   228.63333333333333
446.5333333333333   431.6333333333334   285.9
950.3666666666667   386.5333333333333   453.90000000000003
        354.9666666666667   347.36666666666673
672.2666666666667   439.76666666666665   272.8
388.43333333333334   413.79999999999995   348.90000000000003
        442.1333333333334   627.5666666666667
388.1333333333334   277.90000000000003   400.9666666666667
        389.86666666666673   475.5   296.43333333333334
591.2   804.6999999999999   421.40000000000003
342.20000000000005   405.6333333333334   329.9666666666667
        301.3666666666667   335.43333333333334
486.09999999999997   347.0333333333333   265.96666666666664
        379.76666666666665   303.90000000000003   576.5
377.5   279.6   395.5333333333333   827.4333333333334
437.59999999999997   535.5666666666667   367.9333333333334
        269.7   377.6666666666667   221.83333333333334
460.06666666666666   398.0   385.6333333333334
499.3666666666666   323.5333333333333   330.43333333333334
        390.1666666666667   445.5   333.40000000000003
292.40000000000003   374.3666666666666   361.06666666666666\
MBD4   1810.4000000000003   5574.749999999999   4835.625

2374.4999999999995	3308.6749999999997	2246.625
2722.2250000000004	3289.9	2139.4249999999997
2903.675	1391.5	1747.85	2140.9249999999997	3542.475
3198.0	2479.7500000000005	2716.15	2743.8	2917.0
2578.4249999999997	3324.1	2767.275	3063.85
2479.2000000000003	2344.0	2678.55	2469.7999999999997
3802.225	4609.45	2099.425	1366.2250000000001
3285.725	6931.025	2618.25	4569.725
2947.0249999999996	2405.025	3482.875	3828.325
2623.225	2318.925	4437.4	4299.975
2975.775	5746.875	4126.6	1643.0500000000002
5333.700000000001	5256.7	2510.7999999999993	2047.7
2297.2250000000004	1614.9499999999998	1594.3000000000002
3984.05	4599.0	3743.8999999999996	3211.05	3474.475
5461.85	1941.8749999999998	3266.25	3299.3499999999995
2373.6000000000004	3434.6750000000006	4132.25
1982.925	2969.0	5686.475	2596.625
5279.900000000001	5077.875	2612.55	1911.3250000000003
2208.975	2555.675	5098.2	3035.5	2317.125
2373.15	1359.2	1786.0	2472.6499999999996	3226.125
1524.6000000000001	1150.9499999999998	1559.95
1554.775	2260.05	4358.775	3923.725
2193.1749999999997	2057.6000000000004	2790.175
2942.65	1874.625	3112.475	2255.05	5636.950000000001
3232.2500000000005	2443.075	2681.075
2623.925\
MBD5	176.63333333333333	236.33333333333334
175.76666666666668	192.29999999999998	317.6333333333333
174.4333333333333	284.3333333333333
267.09999999999997	177.1	202.13333333333333
151.70000000000002	227.4333333333333	203.23333333333335
154.16666666666666	320.5	232.20000000000002
189.9333333333333	158.0	313.09999999999997
202.60000000000002	214.23333333333335	145.79999999999998
218.63333333333335	234.1	178.9333333333333
177.70000000000002	276.76666666666665	266.26666666666665
242.26666666666668	274.1666666666667
386.1333333333334	255.23333333333335	142.79999999999998
305.0333333333333	261.73333333333335
300.9666666666667	137.06666666666666	273.5
181.20000000000002	309.3	319.7666666666667
145.03333333333333	290.09999999999997	343.29999999999995
158.63333333333335	199.5	145.8666666666667
255.5666666666667	167.63333333333333	258.53333333333336
287.40000000000003	179.0666666666667
312.76666666666665	206.16666666666666	178.26666666666668
232.53333333333333	270.86666666666673
152.03333333333333	148.0666666666667	131.7	404.7
222.99999999999997	323.6333333333333	169.33333333333334
252.23333333333332	136.13333333333335
233.16666666666666	319.2666666666667	224.33333333333334
227.86666666666667	234.79999999999998
153.23333333333332	204.26666666666665	239.5666666666667
247.3666666666667	200.0	142.76666666666668

139.93333333333334	209.26666666666668	256.93333333333334
	248.43333333333333	278.96666666666664
164.93333333333334	368.6666666666667	286.40000000000003
	259.6333333333333	198.6	318.53333333333336
181.3666666666667	325.79999999999995	284.5
192.56666666666663	245.16666666666666	235.26666666666665
	185.4	260.7333333333333	248.73333333333335
245.4	338.40000000000003	346.7666666666665
254.29999999999998	265.7	311.6666666666667\
MBD6	575.4666666666667	1041.3333333333333
1207.9666666666665	366.73333333333335	674.2333333333333
	316.8666666666667	314.90000000000003
1799.8000000000002	557.9666666666666	537.1666666666666
	549.6666666666666	1248.8999999999999
420.3666666666666	1023.2666666666668	1253.0666666666668
	1124.8	415.40000000000003	833.9666666666667
491.09999999999997	553.1	2484.233333333333
690.8666666666667	956.3666666666668	394.73333333333335
	931.2666666666668	784.0	518.2666666666667
1045.1	1497.033333333333	804.1666666666666
425.3666666666666	1091.6000000000001	1297.5
440.1666666666667	900.0	1124.1000000000001	579.0
1045.5	722.2666666666668	421.3333333333333
456.8999999999999	579.3666666666667	624.2333333333333
	588.0999999999999	471.73333333333335
970.7333333333335	779.8666666666667	337.9666666666667
	4031.8333333333335	897.8000000000001
553.6333333333333	505.73333333333335	1713.2666666666667
	1149.3	1846.2666666666667	2487.9666666666667
714.9333333333333	963.3666666666668	1702.2666666666667
	943.0333333333334	1093.3333333333333
806.1333333333333	1120.3999999999999	627.2666666666667
	1250.7666666666667	1158.8999999999999
697.8666666666667	1009.5333333333333	870.5	565.5
1786.266666666667	1154.7	622.9666666666666
426.03333333333336	535.6666666666666	327.59999999999997
	1179.1666666666665	838.9	1035.2333333333333
503.1333333333334	667.9333333333334	572.9666666666667
	405.5333333333333	1741.3000000000002
1169.1000000000001	1839.6333333333332	1282.5666666666668
	611.1666666666666	702.1	853.5333333333334
660.0666666666666	1116.2333333333333	516.8	706.5
1401.2333333333333	685.3666666666667	855.0999999999999
	441.2	1509.5333333333335	696.1333333333333
969.3666666666667	1600.6666666666667	886.0333333333332\
GATSL3	57.8	72.9	96.95	85.30000000000001	25.25
203.85	60.75	160.3	115.35000000000001	72.65	82.3
100.8	95.0	90.75	67.95	159.05	135.8	60.2	153.9
142.5	115.4	37.949999999999996	153.35	88.0	83.2
114.0	59.099999999999994	56.150000000000006
241.89999999999998	104.5	91.05	94.69999999999999
128.45	213.9	168.1	220.85	313.35	23.4	287.45	81.35
177.4	66.35	102.4	84.6	202.25	103.15	89.6	8.25	34.4
	83.15	204.85	108.1	121.69999999999999	154.6

```
106.80000000000001	120.75	58.25	118.85	194.05	76.35
232.4	190.5	123.14999999999999	95.75	107.5	221.55
146.6	165.5	93.94999999999999	49.55	210.5	136.8	90.3
	115.75	134.15	164.2	213.70000000000002	95.95
95.85	171.3	103.7	156.7	131.0	296.04999999999995
166.3	359.05	326.25	201.45	111.60000000000001	216.45	68.0
	148.55	142.35	284.9	104.4	138.8	157.2	156.0
146.4	147.79999999999998	217.75	183.7	180.4\
PKLR	10.666666666666666	40.56666666666667	35.6
22.433333333333334	71.26666666666667	6.133333333333334
	29.200000000000003	28.0	18.866666666666667
41.733333333333334	15.366666666666667	16.333333333333332
	6.599999999999999	24.26666666666667
16.133333333333333	30.366666666666667	16.333333333333332
	30.600000000000005	8.633333333333333
8.933333333333332	25.666666666666668	59.23333333333333
	25.53333333333333	25.066666666666663
8.366666666666665	19.433333333333334	15.299999999999999
	34.333333333333336	19.433333333333334
42.63333333333333	7.1000000000000005	99.83333333333333
	10.200000000000001	19.133333333333336	18.8	21.2
	25.799999999999997	20.433333333333334	4.7
6.266666666666667	9.5	9.1	20.3	12.266666666666667
	6.966666666666666	35.800000000000004
40.733333333333334	33.53333333333334	87.60000000000001
	22.366666666666664	40.766666666666666
13.966666666666667	21.666666666666668	122.43333333333334
	17.233333333333334	45.20000000000001	41.2
27.266666666666666	48.5	13.399999999999999
58.400000000000006	38.36666666666667	15.966666666666669
	6.466666666666666	28.233333333333334	41.6
8.666666666666666	38.6	29.100000000000005
20.96666666666667	151.83333333333334	23.099999999999998
	8.966666666666667	13.366666666666667	36.7
13.833333333333334	20.1	7.033333333333332
12.866666666666665	27.233333333333334	18.3
28.599999999999998	11.1	14.200000000000001	32.4
96.26666666666667	51.0	39.266666666666666
18.333333333333332	43.6	12.266666666666667
28.433333333333337	11.366666666666667	28.966666666666665
	11.033333333333333	13.733333333333334
15.666666666666666	13.200000000000001	32.06666666666667
	50.26666666666667	29.933333333333334
19.066666666666666	13.233333333333334\
KCNB1	50.45	86.225	134.925	47.4	133.05	34.55	27.9
73.075	88.625	110.725	77.55	54.05	50.25	63.525
93.07499999999999	47.65	87.14999999999999	68.9
26.049999999999997	44.1	113.75	37.425	230.50000000000003
	118.9	66.22500000000001	41.575	58.9	97.175
157.47500000000002	91.275	114.92500000000001	49.5
80.625	81.275	101.04999999999998	152.475	49.225
55.324999999999996	61.150000000000006	65.075	56.8	54.6
	82.625	58.14999999999999	38.7	103.67500000000001
	53.75	56.0	107.875	66.77499999999999	92.225	78.0
```

34.275000000000006 155.7 38.775 141.175
88.32499999999999 162.575 113.9 99.22500000000001
252.25 64.725 43.0 53.1 124.32499999999999 77.575
60.900000000000006 48.099999999999994 114.32499999999999
160.6 290.75 94.9 156.35 74.85000000000001
52.375 88.65 93.15 43.125 100.075 108.05 100.35000000000001
65.85 97.125 77.875 123.24999999999999 268.0
78.025 115.0 68.1 96.97500000000001 25.975
154.27499999999998 152.525 74.125 169.425 68.525 75.05
170.65 71.10000000000001 173.82500000000002 76.175
413.95000000000005 74.825\
BIK 229.6 38.6 717.3 234.3 50.0 782.7 17.8
439.3 220.3 306.6 607.7 150.7 112.1 599.8 182.6
138.2 163.5 863.6 1253.3 387.6 236.1 841.1 1265.6
249.3 746.1 834.4 490.8 164.3 792.5 668.7 235.5
758.9 404.6 309.9 1254.0 543.1 291.7 690.5 171.3 86.1
48.8 502.6 415.9 299.7 187.7 548.8 556.4
417.8 422.9 312.1 554.0 1562.2 1857.9 39.7 440.5
501.1 840.0 327.8 1131.5 139.7 38.5 214.7 363.8
675.3 1155.8 642.4 657.2 637.3 383.5 1097.3 760.2
837.0 652.0 418.5 253.5 751.3 867.7 246.4 1161.3
922.3 180.4 595.4 804.5 381.7 1271.5 1110.3 353.6
387.3 921.4 49.4 277.1 22.1 279.3 660.6 247.0 89.7
112.8 695.6 793.6 595.7 439.0 428.1 436.0\
MBD1 564.48 617.3999999999999 681.94 535.5 609.78
629.78 518.7 404.0 428.0000000000001 466.06000000000006
641.0999999999999 518.52 345.38000000000005
656.6800000000001 494.06000000000006 696.18 356.7
469.9 344.04 587.68 1057.58 839.9199999999998 653.72
639.24 385.98 779.86 621.5799999999999 580.6199999999999
809.58 524.3 755.3199999999999 459.66 755.78
537.9399999999999 650.34 567.8 513.44 700.72
469.64000000000004 467.48 594.2199999999999 457.2
513.6400000000001 471.82 519.1200000000001 626.3
613.86 547.3000000000001 1034.8799999999999
679.8800000000001 563.7199999999999 510.88
424.55999999999995 869.8199999999999 593.28 782.88
536.74 614.5600000000002 785.9200000000001 720.84
416.30000000000007 615.26 553.0 482.7399999999999
658.98 659.9399999999999 530.5 618.1999999999999
592.4799999999999 710.22 810.0600000000001 784.78
586.88 467.7999999999999 517.58 513.1600000000001
642.42 526.1999999999999 623.64 768.02 502.2999999999999
640.5799999999999 401.0400000000001
1050.3799999999997 873.64 1324.8200000000002 710.24
652.84 507.76000000000005 464.76000000000005
453.2200000000001 807.46 919.8 747.8399999999999
973.4199999999998 1062.2600000000002 943.8399999999999
767.6 935.5799999999999 1013.3 918.3599999999999
737.78 841.6800000000001\
KCNB2 10.9 58.9 97.4 26.9 81.2 46.65 46.7
52.75 64.1 50.45 45.25 57.25 22.05 42.3 92.15 75.0
75.5 24.55 30.0 38.3 110.6 9.7 114.85 41.7
16.35 33.9 11.399999999999999 113.05000000000001

40.05	31.5	71.75	53.35	69.35	71.45
82.64999999999999	63.25	36.599999999999994	68.05	64.7
42.099999999999994	55.85	11.45	51.1	31.05	19.5
67.75	32.949999999999996	67.30000000000001
20.85	64.8	33.75	41.849999999999994	13.6	51.0
65.65	33.5	91.30000000000001	54.35	37.1
138.64999999999998	420.79999999999995	17.35	37.8
68.95	106.2	93.65	104.5	62.95	52.900000000000006
69.94999999999999	28.5	74.85	41.85	28.25
31.049999999999997	13.3	26.5	18.75	35.05	71.9
104.25	37.25	32.05	56.25	73.0	177.5	19.9
15.350000000000001	38.45	38.45	4.15	47.4	35.75
77.05	21.4	52.6	22.8	50.45	95.2	73.1	55.8	19.3
43.2\
RP4-657D16.3	123.8	124.5	143.0	125.3	230.9	123.1
153.5	220.6	57.4	149.6	134.6	274.3	134.2	16.7
209.5	68.1	124.5	143.4	74.4	162.5	300.1	31.0	34.7
45.3	174.5	162.1	145.5	328.2	216.3	128.1	61.6
24.8	159.5	140.9	32.9	190.1	108.5	225.7
112.9	64.8	124.4	166.4	162.0	115.6	57.3	92.3
279.8	74.3	221.0	193.5	79.8	92.5	77.5	390.2
167.2	259.4	78.4	208.2	168.8	310.2	561.9	190.8
161.8	131.7	198.3	206.6	93.0	136.3	302.2	123.5
198.0	212.2	182.0	147.7	77.3	41.8	176.3	93.6
141.1	158.5	210.5	199.8	90.7	146.0	518.4	380.0
282.4	67.6	26.3	159.4	67.1	377.7	108.2	131.4
163.2	174.5	229.5	142.0	147.0	142.3	191.2	235.0
158.7\
MBD2	773.125	386.15000000000003	686.75	1120.05	651.025
491.05000000000007	758.95	372.72499999999997	663.1
800.6	784.25	877.4499999999999	1084.9	701.6249999999999
494.65	723.225	906.225	814.3000000000001
763.2499999999999	1039.3999999999999	446.90000000000003
507.22499999999997	709.4499999999999
765.5499999999998	738.5749999999999	904.75
1348.2250000000001	536.825	777.8000000000001	1251.975
765.075	1155.1250000000002	616.5500000000001
659.3249999999999	653.25	766.5749999999999	671.975
935.4	929.9749999999999	636.025	877.5250000000001
668.85	775.9999999999999	795.6000000000001	571.95
739.925	665.3750000000001	920.775	524.5	640.825	935.225
1238.1749999999997	751.025	894.1750000000001	770.025
461.875	666.525	682.775	791.4499999999999	685.5999999999999
610.775	699.15	1125.9250000000002	989.7499999999999
711.0999999999999	486.15000000000003	643.7
864.725	586.0250000000001	841.5250000000001	406.575
735.1999999999999	730.275	746.725	599.0500000000001
930.0	758.725	798.425	786.4249999999998	759.2750000000001
674.9250000000001	971.3249999999999	503.7
887.95	928.5500000000001	837.125	639.875	763.325	1540.35
1724.9250000000002	685.7	479.425	751.425	876.875
606.1999999999999	709.7	601.7749999999999	850.15
788.825	673.025	569.4250000000001	602.8	607.6000000000001\
ADAM33	616.25	1352.35	935.8499999999999	452.3

1383.1000000000001 231.4 256.35 856.1500000000001
326.8 825.1999999999999 230.8 599.8 388.35 169.85
1022.2 595.1 585.35 836.2 384.09999999999997 822.2
2331.7999999999997 482.90000000000003 1449.4 398.95
393.7 508.6 203.0 1291.55 1077.25 295.1 441.55 895.1
1402.7 326.55 621.8 620.35 227.85 3516.95 410.9
244.35000000000002 391.1 376.3 966.75 509.9 161.0
459.34999999999997 417.0 178.4 1106.3 835.1
485.04999999999995 417.04999999999995 371.3 1234.25
886.25 913.35 671.55 1154.65 850.9499999999999 1304.25
715.1 991.85 633.0 548.0500000000001 915.2 1025.0
246.60000000000002 328.2 1592.2 513.4 2342.1 1232.2
1164.2 299.5 813.85 256.84999999999997 1007.9 399.65
584.3499999999999 880.15 995.9499999999999
619.9499999999999 505.75 525.65 1327.6499999999999
1272.45 1189.55 1689.55 526.6500000000001 1139.55 192.9
3670.45 1528.9499999999998 1248.2 2907.0 963.5999999999999
1444.0 1207.7 1595.2 1100.05 1365.3 2581.8500000000004
1179.75\
ADAM32 72.1 114.5 39.4 75.9 261.8 80.6 38.8
242.4 193.6 105.8 147.7 207.2 5.2 16.2 230.5
229.8 74.8 174.5 199.3 151.4 300.2 104.8 25.9
154.1 14.7 83.9 52.7 290.0 98.3 156.1 349.2 86.8
205.3 255.9 114.7 103.2 2.7 176.3 60.5 95.3
192.4 100.0 186.9 164.1 62.7 72.6 80.0 96.1
395.7 399.9 10.4 45.0 160.4 11.8 191.4
316.4 337.6 272.5 18.9 313.3 508.3 112.8 112.7 81.6
56.0 118.3 184.5 119.3 52.6 154.4 157.6
192.5 163.4 83.8 104.0 30.1 112.5 163.6 109.7 44.6
161.7 11.1 198.7 8.4 237.1 34.7 153.2
273.8 59.5 59.8 155.8 187.4 411.2 177.6 182.5
239.2 330.9 269.4 249.9 414.5 213.3 163.8 133.2\
KIAA2013 1185.5 666.8 566.3000000000001 931.1
459.85 1634.1499999999999 1147.8 759.65 1211.6 921.05
1137.55 966.6999999999999 734.05 1170.2 1129.35
1344.3500000000001 705.5 616.4 956.3 1029.0
429.15000000000003 1130.25 569.75 1010.5 1495.75 913.85
1266.3500000000001 897.3 706.95 1313.75 794.7 675.35
866.85 1195.3 939.55 1206.1000000000001 1635.95 556.3
1233.75 770.75 1266.8 1869.8999999999999 550.55
1257.3000000000002 1098.0 804.9 1154.1 1588.7 620.2
921.9499999999999 979.15 1314.7 1440.3 610.6 1000.9
921.1500000000001 868.15 558.15 795.5500000000001
556.35 721.45 1106.1 593.85 896.45 760.0999999999999
659.05 986.3 1121.55 488.09999999999997 1326.7 456.65
671.3 666.0 1115.55 1161.45 1558.85 1128.9499999999998
1358.75 1270.7 1285.65 671.75 1147.5 1220.3000000000002
2484.5499999999997 543.7 494.04999999999995
1491.3500000000001 765.65 1262.8999999999999
420.79999999999995 1209.8500000000001 787.95 924.25
1204.5 965.1999999999999 1054.25 897.1999999999999
1248.55 896.7 836.25 885.85 1313.55 1101.6000000000001\
ADAM30 10.4 14.75 63.7 16.55 23.85 41.25 4.85 33.7
31.299999999999997 104.95 27.450000000000003 6.4

49.5 41.0 34.15 44.2 9.2 12.05
18.799999999999997 37.05 36.25 24.799999999999997 53.6
17.849999999999998 50.45 41.6 46.0 82.0
78.60000000000001 17.9 28.15 73.45 30.5 8.0
145.75 22.25 14.0 34.55 13.0 19.75 69.35 25.4
43.05 34.7 17.75 45.25 72.3 44.15 25.85
13.899999999999999 60.550000000000004 10.25 65.05 9.9
15.6 27.55 22.6 43.85 6.5 45.900000000000006
312.84999999999997 52.400000000000006 70.9
29.75 36.85 39.25 8.850000000000001 23.4
47.150000000000006 48.599999999999994 35.75 13.3 47.2
42.050000000000004 31.35 52.6 54.300000000000004
24.75 38.45 25.15 13.350000000000001 42.35 66.4
48.95 84.1 115.45 57.050000000000004 71.7 39.5
64.85 19.85 90.7 14.1 21.549999999999997 18.7
38.15 8.9 38.4 45.65 46.55 28.1 41.8
34.05\
HNRNPA1P3 /// HNRNPA1P3 5888.3 1132.3 1805.6 3978.2 1734.7
5418.1 17162.6 672.2 5550.4 2831.8 5839.7 2482.5 4950.7
2598.2 5082.1 3998.3 3478.4 3940.2 1881.0 3061.6 1654.1
3641.0 1662.3 4012.6 13109.4 2350.5 3822.5 2728.6 1740.0
3874.2 5448.6 1922.7 1082.7 3997.1 2431.7 1391.4 3202.8
2697.6 3564.7 6244.3 4932.0 5183.3 2962.5 4238.2 3878.3
2641.5 853.1 2117.8 1623.8 2584.8 4906.9 3152.7 3492.1
665.2 3563.2 1731.2 2846.4 2119.2 2882.8 1881.5 1137.7
1930.4 2651.4 2079.2 2280.2 1319.7 4613.3 1826.2 2865.3
1160.0 1280.0 1689.7 1910.2 5569.7 4890.1 3159.0 664.1
5271.8 2634.9 961.2 1279.1 796.2 4569.2 1600.5 707.0
713.7 1906.1 1089.1 4092.2 1896.9 4228.1 999.5 2217.3
2344.7 2559.6 2731.2 2273.1 2065.2 1353.5 1263.5 2168.3
1381.2 3152.5\
PRKRIR 6859.9 1296.1 1515.5 2924.4 1067.2 3682.6 3731.5
2203.3 3623.4 1303.1 3316.0 2383.7 2425.2 1963.9 4534.9
9567.1 3012.8 1231.4 3284.9 3233.9 1589.3 2677.8 731.6
3000.7 4520.5 2670.4 2106.2 1714.0 1493.6 3771.6 4595.7
1525.0 836.3 4055.4 1429.5 2312.4 3349.0 1248.2 4023.4
14910.5 4955.0 4264.0 1627.9 3740.4 3250.9 1945.6 2482.6
4287.9 3636.0 4468.3 2102.1 2168.4 1375.2 1975.0 2918.8
2431.1 2929.5 1908.7 2137.7 2367.4 1043.9 1271.8 1773.7
2421.1 2781.8 1464.3 4075.0 2696.1 1522.6 2922.3 1013.7
1258.6 2501.9 5026.7 2741.3 2569.5 1309.9 3968.1 5381.8
2268.6 17413.0 3247.1 5255.5 2022.0 1674.9 1794.6 2456.2
1737.9 2105.0 1383.1 3736.3 3226.5 3363.9 2961.3 2582.9
2772.1 3550.7 3875.4 2026.6 3081.5 3259.8 2681.2 4650.9\
PKN2 3307.6666666666665 2486.2000000000003
3170.6666666666665 2857.3000000000006 2796.4
1566.466666666667 2411.5333333333333 950.6333333333333
2211.5000000000005 2028.2333333333336
1867.6999999999998 2895.233333333333 2147.4666666666667
2392.6666666666665 2387.266666666667
2002.6666666666667 1980.7666666666664 2716.1666666666665
2665.9666666666667 2165.9 2947.033333333333
2169.1 3467.8333333333326 1863.8333333333333
2830.633333333333 3111.3333333333335 1921.4999999999998

2531.866666666667	2895.7000000000003
1577.5666666666666	1761.6666666666667	2727.0333333333333
2861.933333333334	1857.5999999999997	2063.0
2595.266666666667	2272.633333333333	3188.5	2240.4
2152.633333333333	2602.0666666666666	3004.166666666666
2297.3333333333335	2004.3000000000002
2143.7999999999997	2518.8333333333335	1105.1666666666667
2860.7999999999997	3978.2000000000003
2005.1000000000001	2562.1666666666665	1983.0333333333335
1941.5666666666666	1776.4999999999998
2110.733333333333	2768.5	3383.2333333333336
2902.2000000000007	2758.4	2929.633333333333
1016.8333333333334	2327.2000000000003	2343.0
2147.866666666667	2016.7666666666664	2160.1
1587.4666666666665	1286.3999999999999	3244.2000000000003
2013.8999999999999	4497.233333333334
3049.566666666667	2026.6000000000001	1720.2666666666667
2436.0666666666666	1472.7333333333333
1289.6000000000001	1696.3000000000002	2047.3666666666666
1385.7	1040.4666666666665	1724.6666666666667
2078.2333333333336	2451.566666666667	1694.8	1732.2
998.1333333333332	1313.9333333333334	3080.566666666666
2502.1	2512.3333333333335	1759.3	2450.1
2519.7000000000003	2825.8333333333335	2912.566666666667
3163.633333333333	2516.9666666666667
3532.066666666667	3553.633333333333	3090.266666666667
2455.0333333333333	3066.7999999999997\
ADAM3A	9.9	12.0	91.9	7.6	44.8	5.4	3.7	5.3
31.1	56.6	4.7	3.3	2.7	21.9	11.5	23.5
57.8	47.4	28.3	51.9	44.6	39.2	101.6	11.5
2.3	73.4	6.8	11.4	11.3	52.0	5.6
129.3	6.1	3.1	3.3	93.3	5.3	11.7	1.0	1.3
2.4	36.7	8.2	2.0	9.8	47.6	6.5	5.1
18.4	25.5	61.4	19.8	39.8	3.7	9.0	10.8
13.4	5.8	55.2	3.6	53.1	1.7	8.2	4.6
71.4	2.3	7.3	43.6	45.8	77.6	14.9	2.8
9.9	5.5	8.3	7.5	2.0	5.0	10.4	2.1
9.4	74.6	5.6	33.8	7.3	155.9	13.6	4.6
16.0	6.3	1.8	74.4	3.3	17.0	7.9	6.2
3.8	2.5	30.4	54.3	7.5	53.9	3.3\
MITD1	1115.4	1075.4	1515.0	895.8	1012.8	465.1	888.3
523.2	632.4	501.5	571.4	609.7	480.9	778.1	520.9
529.5	902.4	484.1	616.4	617.4	744.3	447.9	606.6
702.0	812.1	243.6	942.6	792.8	719.5	761.3	920.5
498.9	536.8	215.7	215.7	505.0	637.1	612.8	663.8
920.7	515.7	268.1	711.4	635.8	392.8	759.8	441.8
732.2	536.8	729.5	464.8	468.0	787.4	505.3	743.6
443.1	812.1	715.9	589.2	1007.7	266.8	527.3	1039.5
597.8	955.4	744.9	635.9	524.1	753.3	502.9	840.4
494.1	1006.2	886.6	873.6	465.5	416.5	588.8	556.2
364.6	362.3	283.4	399.8	704.5	292.9	161.6	347.6
291.5	663.9	952.2	565.2	378.0	552.2	479.3	475.7
940.3	511.2	1042.2	506.0	536.8	723.7	478.9	566.2\
GPR108 /// MIR6791	512.6	746.4	482.3	546.2	690.0

964.3 926.2 438.2 735.9 313.5 487.8 917.5 722.9
951.4 666.7 698.2 751.3 655.2 1096.2 572.1 494.4
572.9 488.2 734.5 495.7 945.7 698.9 428.9 643.0
384.4 699.7 253.7 502.3 732.0 458.0 1015.3 920.4
585.3 888.5 361.0 378.8 837.4 474.9 700.8 656.6
367.0 349.3 346.6 730.4 753.8 572.9 625.7 728.5
386.5 632.0 800.8 612.3 479.6 860.5 275.1 471.9
946.2 512.7 587.1 436.4 957.0 1094.1 558.2 528.6
618.8 482.5 510.9 959.3 669.8 595.3 605.5 550.9
614.1 685.2 283.2 247.1 425.1 834.8 686.3 481.0
519.0 505.9 509.8 770.6 373.4 484.0 627.1 724.5
934.1 1013.8 865.7 950.7 975.1 776.0 690.7 718.5
804.1 1239.4\
PKN1 252.9 113.6 210.4 363.4 173.7 527.4 527.1
107.1 322.6 453.5 261.6 514.0 285.0 16.1 307.5
308.6 300.2 191.9 491.8 398.1 390.2 409.0 363.9
317.3 256.2 221.8 261.9 112.8 212.8 257.8 320.1 21.7
244.1 217.9 334.2 136.0 299.0 310.4 417.5
536.5 469.5 653.1 411.0 469.3 428.6 299.3 186.7
276.6 156.5 218.7 310.5 158.4 300.6 28.4 250.0
228.0 351.9 285.4 339.9 167.1 208.7 229.4 130.3
274.9 370.9 322.2 386.9 346.6 372.2 150.5 239.8
192.3 362.3 350.0 562.0 206.5 96.6 330.6 301.8
257.4 340.5 26.4 544.1 341.7 260.7 256.6 128.7
345.1 420.3 438.3 861.4 21.2 188.0 224.6 176.7
168.4 94.9 190.7 106.9 103.9 217.7 192.2 230.7\
FAM45A /// FAM45B 1625.2 1287.6000000000001
778.1666666666666 2042.3666666666668 2042.7
1678.6333333333332 1547.3333333333333 1648.1666666666667
888.7333333333332 1746.9000000000003
1507.5666666666668 1064.8999999999999 1571.1000000000001
999.0 1330.6000000000001 1272.8333333333333
1313.6666666666667 1105.3333333333333 2384.133333333333
1237.7333333333333 949.1333333333332
1200.6666666666667 1015.7666666666668 1409.8333333333333
1292.6999999999998 2165.066666666667
2428.9333333333334 1443.5333333333335 1746.6333333333334
1468.0666666666668 1021.7666666666668
1714.9333333333334 1158.3999999999999 2467.6333333333337
2228.2000000000003 1183.533333333333
2244.4666666666667 1261.6666666666667 1449.2333333333333
1072.5666666666666 1127.8 887.4333333333334
1889.3 734.0333333333333 1399.6333333333334
1568.0333333333335 2896.7999999999997 1636.1333333333332
2900.4666666666667 1122.2333333333333
1247.6666666666667 2387.8333333333335 2182.7000000000003
2325.2333333333336 1889.6333333333332
851.0333333333334 1103.7333333333333 1027.1333333333334
1266.2666666666667 1702.1666666666667
1372.1666666666667 977.3666666666667 1434.4333333333334
1294.8999999999999 757.0666666666666 1908.7
1023.3333333333334 1437.7 934.7666666666668
1499.4666666666665 852.4666666666666 1695.6333333333332
1194.2 1536.8 840.6333333333333 2339.166666666665

1231.9666666666665	758.1999999999999
1835.7666666666667	1637.1333333333332	1740.1333333333332
1283.3333333333333	1839.2	1464.1999999999998
1679.1333333333334	2536.6333333333337	1756.6333333333332
1491.3333333333333	1256.2666666666667
2304.566666666667	1246.1666666666667	1598.3999999999999
1650.5	1918.4333333333334	2150.4	2037.5
2357.5666666666666	1996.9666666666665	1921.7333333333333
1947.1333333333332	1626.1000000000001
1174.3666666666666	1528.3999999999999\
KCNG1	15.3	19.700000000000003	65.3	24.450000000000003
21.6	89.14999999999999	24.6	71.55	91.85	43.0
69.2	46.45	697.65	94.14999999999999	216.85
16.35	49.25	76.55	41.95	36.6	68.8	18.15	476.9
51.25	46.95	18.549999999999997	31.1	20.6	107.9	71.2
12.25	22.299999999999997	388.9	38.75	84.05
15.75	89.25	102.55	29.15	134.75	189.5	25.25	10.25	52.0
56.65	20.15	9.2	29.299999999999997
26.950000000000003	16.5	17.4	19.4	14.3
51.800000000000004	18.35	21.2	59.95	748.35	125.45	26.1
234.85000000000002	200.8	130.35	56.75
68.39999999999999	41.55	7.25	31.099999999999998	95.6
39.7	32.1	20.1	13.25	28.05	12.95	272.35
16.25	28.25	25.049999999999997	97.0	27.799999999999997
38.25	52.15	33.2	23.25	186.64999999999998
104.75	98.2	33.0	62.9	30.2	134.0	140.0	84.65
131.65	78.0	129.25	47.75	241.95	24.75	27.4	278.5
8.15\
MED24 /// MIR6884	510.3	353.8	304.7	458.7	1449.0
1642.6	566.7	395.7	576.3	581.0	581.0	557.0	680.0
677.8	648.3	507.2	442.8	771.5	889.6	597.6	623.0
698.9	545.6	715.1	520.7	664.7	980.9	1246.1	857.5
481.7	558.4	412.1	714.7	985.2	675.9	512.6	608.6
610.3	728.9	975.7	848.9	2066.6	989.7	768.9	1837.1
510.0	582.9	311.2	760.3	738.2	493.9	369.9	572.9
419.3	732.2	792.1	1067.4	298.8	549.2	1014.3	1349.1
736.8	676.7	494.1	482.3	299.7	817.3	810.0	2029.2
651.9	421.1	1174.4	422.1	785.3	880.5	2024.5	797.0
1363.0	563.9	530.4	406.2	495.4	965.5	320.4	447.9
593.7	290.1	389.6	358.8	361.6	474.6	521.8	1067.2
531.0	468.0	429.6	547.4	641.4	2131.1	406.1	559.7
179.7	526.1\
KCNG2	4.5	8.0	5.4	3.0	9.9	3.0	1.9	4.2
7.2	24.0	5.2	4.2	5.4	7.3	10.4	3.8
2.8	3.8	3.4	4.9	10.9	2.5	7.3	2.3
7.1	6.0	3.0	10.9	5.4	2.8	6.0	11.8
6.1	4.3	5.1	4.4	4.4	10.3	6.2	3.6
3.6	2.3	8.0	18.4	2.7	4.9	5.0	5.9
7.5	7.7	7.5	6.5	8.1	6.9	3.8	3.8
4.9	8.0	4.7	14.2	40.7	5.0	4.0	2.5
5.4	9.2	3.2	13.1	10.5	4.6	12.8	11.5
5.4	2.1	4.4	3.2	7.2	4.5	2.9	3.0
8.9	2.9	3.0	3.9	6.1	11.6	3.9	8.1
10.0	7.8	2.4	4.1	4.1	2.2	2.2	4.2

	7.7	3.0	4.5	5.5	3.2	5.4	6.7\
KCNG3	128.95	198.10000000000002	88.1	89.4
112.55000000000001	41.1	83.95	194.0	25.75	30.85
63.25	35.4	306.79999999999995	55.050000000000004
45.400000000000006	10.25	77.19999999999999	171.4
24.95	47.25	161.65	121.6	145.7	26.700000000000003
98.05	62.8	24.75	59.6	68.5	54.699999999999996	69.7
	147.35000000000002	56.099999999999994	46.7	16.4
	105.7	32.05	68.6	50.75	56.5	61.15	107.8
41.15	27.9	9.950000000000001	39.45	55.65
294.90000000000003	32.35	36.6	13.799999999999999
477.0	79.6	7.05	65.55	36.2	25.65	485.7	19.65
83.15	127.94999999999999	84.3	33.5	53.15	146.8
23.25	45.8	33.95	82.15	29.9	70.2	58.849999999999994
	35.6	62.8	42.3	43.4	55.75	62.949999999999996
	99.39999999999999	274.55	259.05	108.2	27.7	14.8
	30.65	1330.8	82.65	585.6999999999999	215.95
334.2	135.85000000000002	137.35	82.9	92.05
35.199999999999996	48.1	69.45	73.55	126.35000000000001
	60.5	65.65	213.39999999999998	63.0\
KCNG4	56.3	10.7	8.7	13.2	32.5	29.4	44.2	11.7
	41.5	9.4	38.2	16.1	52.5	15.1	70.7	81.0
	10.0	12.8	37.4	26.0	39.3	78.2	13.2	20.0
	36.2	98.5	10.4	159.1	133.1	6.1	11.3	28.5
	18.3	90.4	19.7	16.4	54.1	64.0	7.6	89.8
	59.4	31.7	94.5	27.6	7.2	28.6	50.9	59.5
	75.7	6.0	9.4	8.5	43.0	101.0	28.6	27.3
	48.7	11.3	17.8	24.5	63.2	90.9	18.5	56.3
	14.4	34.6	23.8	5.3	28.8	41.7	14.3	15.3
	22.9	68.5	121.9	13.6	15.1	11.6	71.7	95.7
	17.4	15.2	77.0	23.5	15.5	9.9	40.6	65.2
	11.5	8.4	2.0	34.5	72.6	54.5	5.4	81.0
	57.6	70.7	61.1	38.3	48.5	8.4	29.1\
KRTAP4-6 /// LOC101930568	65.3	22.7	13.9	35.9	25.3
	49.3	6.7	26.3	26.1	20.8	21.6	7.1	39.2
	31.2	25.7	94.6	85.2	161.9	20.8	165.9
189.4	11.9	43.2	12.7	98.8	21.0	48.3	196.7
107.7	21.0	63.8	156.9	17.6	15.6	20.5	99.1	51.8
	100.1	8.9	13.0	19.1	9.6	150.3	56.9	41.2
	16.6	15.9	8.3	132.6	80.5	42.7	126.1	60.1
	15.3	79.7	9.9	18.2	148.2	112.3	37.6
119.8	12.8	35.6	43.6	15.9	120.2	15.3	54.7	18.4
	21.3	30.8	19.6	10.8	17.3	8.7	74.7
121.9	42.0	14.7	78.2	87.2	32.9	28.6	13.0	60.2
	44.1	17.4	18.1	100.2	150.3	23.7	11.2	68.0
	13.6	6.0	14.2	18.7	44.4	16.3	10.9	37.2
	51.7	12.2\
RASL11B 174.95	232.3	301.59999999999997	186.1
444.65000000000003	217.8	226.04999999999998	195.45
929.6500000000001	498.25	148.4	139.1	335.79999999999995
	121.4	324.65	939.3	392.5	420.55	95.75
1028.3999999999999	4229.25 252.75	235.85000000000002
210.95	378.2	35.25	79.39999999999999	504.90000000000003
	258.05	2029.4	1385.0500000000002	330.3	321.85

786.95	113.94999999999999	156.2	66.4	351.5	624.1
298.95000000000005	72.8	232.20000000000002	232.15
4972.05	352.05	377.9	240.1	431.3	507.35	2450.5	297.9
131.15	107.35000000000001	345.1	153.15	228.15	1535.9
315.75	114.10000000000001	516.1999999999999	807.05
348.90000000000003	296.05	136.7	5554.05	80.05	2994.6
7974.400000000001	263.3	174.65	216.1	77.6	3442.05
529.0	316.0	245.8	260.8	381.5	3783.25	87.55000000000001
502.35	298.0	227.35	186.20000000000002	133.3
413.04999999999995	436.9	433.4	276.2	544.25
145.20000000000002	518.9	210.8	441.0	370.5	258.6
283.3	403.5	357.6	413.15	155.05	416.6	331.25\
FBXO27	55.5	164.9	40.8	262.0	82.1	47.6	16.5
476.6	49.5	62.3	15.8	72.1	86.8	153.1	36.6	74.6
83.4	127.7	73.5	21.4	67.6	141.8	44.8
135.3	71.9	52.3	112.0	46.7	141.4	269.1	46.2
113.4	165.0	77.5	505.6	178.7	25.2	59.7	10.8
137.1	28.4	136.9	120.9	37.0	22.9	42.9	184.9	61.3
258.4	189.5	54.7	21.7	38.0	945.6	158.8	98.7
95.3	71.6	294.2	191.4	117.1	34.6	78.3	6.4
27.2	147.6	20.5	16.3	187.3	169.0	154.1
278.5	86.4	26.0	49.0	34.7	423.1	78.1	38.0
764.1	665.2	962.7	237.6	60.0	717.0	2510.0	532.9
681.7	201.8	886.7	195.8	327.8	137.5	85.4	31.2	81.6
171.6	107.1	41.6	91.5	102.5	49.3	53.0\
FBXO28	1506.6999999999998	946.9399999999999	649.42
863.8800000000001	966.9200000000001	931.4799999999999
1576.5800000000002	1313.0399999999997
899.3800000000001	884.82	1199.3	736.54	1436.22
1060.8200000000002	868.3799999999999	820.5199999999999
1184.7400000000002	628.14	1070.7199999999998
989.9	554.26	731.5799999999999	664.28	793.0600000000001
974.72	776.22	1144.6799999999998	806.5200000000001
939.9399999999999	1808.0	2477.7	454.58000000000004
557.6600000000001	804.3199999999999
1242.0800000000002	1318.7400000000002	871.4400000000002
642.76	1937.9	1372.4399999999998	1086.82	752.82
843.1600000000001	918.32	736.9200000000001
652.3199999999999	1137.1	1185.6	709.52	1336.18	692.38
1247.94	1442.66	807.08	604.8	1322.48	906.4399999999999
766.26	626.6400000000001	589.78	704.0400000000001
924.8	1249.04	1016.8399999999999	1114.06	1035.66
1364.3999999999999	1061.08	483.4	903.8799999999999
451.5200000000001	723.9599999999999	710.18
1244.5200000000002	907.8600000000002	1002.6800000000001
684.44	925.4599999999998	1406.2199999999998
1199.68	1176.66	1221.72	762.9599999999999	1136.7400000000002
1279.4399999999998	888.78	1151.56	863.0600000000001
665.4399999999998	534.04	1598.62	768.4
927.1600000000001	910.5	901.3199999999999
1012.1600000000001	934.8399999999999	817.6800000000001
725.9	843.68	771.5400000000001	1184.0399999999997
926.76\
RASL11A	52.6	159.8	114.9	66.2	27.4	64.0	70.0	98.6

```
	29.4	144.6	64.0	55.4	66.9	74.5	138.4	27.7
	97.7	113.1	55.6	4.9	224.7	17.8	50.6	5.0
	103.8	15.2	26.6	79.9	98.3	134.9	45.6
244.2	52.6	101.3	185.3	41.7	6.1	181.6	57.7	68.0
	48.3	51.7	33.8	44.2	44.9	83.2	121.7	62.4
	153.1	110.3	67.9	72.6	135.5	194.3	88.7	68.7
	115.8	100.3	92.5	194.5	529.8	55.7	20.5	68.7
	98.5	99.5	2.7	124.4	7.7	58.8	44.6
157.3	94.7	88.0	60.1	63.1	116.4	58.4	19.1	30.5
	41.2	52.6	14.1	146.4	131.2	74.4	86.0
125.0	104.3	21.0	6.6	72.8	59.0	2.0	119.6
142.8	52.1	69.9	29.4	73.0	16.0	157.7	105.1\
FBXO25	668.1	503.5	425.4	615.7	262.6	714.2	473.5
152.0	650.5	559.9	914.1	411.7	1174.4	694.4	189.1
444.2	653.1	611.8	655.3	526.8	361.5	483.6	387.1
732.9	731.9	1072.3	1043.0	383.3	491.2	762.4	1022.5
429.9	297.5	699.4	515.4	618.8	942.3	238.7	522.9
607.7	825.8	1100.6	701.5	848.6	923.0	521.1	522.8
414.8	152.6	420.8	475.9	1450.3	1066.5	481.6	485.3
676.5	515.0	419.5	714.8	378.6	557.9	461.8	555.8
873.1	529.7	522.8	862.9	705.7	427.6	567.2	123.2
412.6	510.2	946.3	497.3	1069.3	390.6	1119.2	641.6
306.5	183.6	627.3	773.1	301.6	300.9	161.4	552.8
478.1	304.3	232.5	689.3	367.0	582.2	618.3	448.1
712.4	616.3	553.6	393.0	521.4	539.3	412.2	876.8\
GOLIM4	3527.0333333333333	949.8666666666668
509.6666666666667	793.9666666666667	829.9
1739.7333333333333	961.5666666666666	1250.7666666666667
	1377.4333333333334	818.1666666666666
1132.8999999999999	7109.0	535.2666666666667	464.3
918.7333333333332	781.5333333333333	2700.0333333333333
	1781.8666666666668	1404.3000000000002
1725.2666666666667	2770.4	1079.2	1279.8333333333333
1267.8	2942.2666666666664	1420.3666666666668	740.5
1701.9333333333334	1434.1666666666667	1711.6000000000001
	2520.366666666667	1330.5666666666666
815.2999999999998	2483.9	817.3666666666667
868.7333333333332	1055.9666666666665	1319.1666666666667
	1408.7333333333333	1925.466666666667	1327.0
1918.7666666666667	946.0	1822.3	599.8333333333334
2839.866666666667	1512.0	1849.1000000000001
3797.6666666666665	3204.633333333333	2680.6
649.8000000000001	695.6	2220.2333333333336	1190.8
1276.2666666666667	6107.366666666666	724.8333333333334
	1496.5	2943.133333333333	2703.1	646.3333333333334
	1255.4666666666667	968.7333333333332	2665.9
521.7666666666668	3627.7999999999997	2602.0333333333333
	1451.3333333333333	1496.2	698.7666666666668
1752.8333333333333	1239.3	4145.366666666667
1208.3999999999999	833.2333333333335	694.3666666666667
	2050.9	2023.2333333333336	537.6333333333333
4877.766666666666	1478.2666666666667	649.9
780.9666666666667	1880.6000000000001	1132.3333333333333
	2086.233333333333	2532.8333333333335
```

482.40000000000003	1012.9	1263.2333333333333
2217.566666666667	3408.9666666666667	2053.7999999999997
	2197.8999999999996	2268.866666666667
3324.933333333333	2632.3333333333335	671.0
4257.099999999999	3496.6	1365.7666666666667
1780.7666666666664\
LOC102723684	3.5	7.4	3.9	2.7	6.9	11.3	18.9
	2.1	3.0	8.9	7.4	68.2	27.3	7.9	10.4
	3.9	7.3	5.0	69.9	5.0	11.2	26.5	5.1
	3.3	79.4	5.9	5.9	26.5	31.9	3.8	4.4
	13.2	13.9	11.4	60.2	7.9	33.6	18.6	15.6
	4.3	5.4	4.5	48.8	19.2	6.9	21.7	20.2
	41.3	46.1	10.6	1.0	66.6	19.8	15.6	3.0
	70.9	3.7	13.5	5.3	4.1	251.1	5.8	26.8
	9.1	8.7	3.3	3.9	4.8	1.9	16.8	65.4
	5.2	3.0	5.7	12.1	3.9	9.3	6.0	8.1
	4.2	7.2	11.3	2.2	38.0	12.8	5.5	2.1
	3.3	18.4	4.7	1.9	10.2	2.9	3.2	4.2
	2.5	4.1	39.3	10.4	2.6	3.4	10.3
21.7\
LOC100289473	60.9	96.1	8.8	28.3	255.8	32.2	25.3
	72.0	143.0	127.2	57.1	17.6	40.2	58.9	75.4
	128.7	127.6	70.1	54.2	31.5	274.8	79.2
233.8	30.2	118.7	64.7	78.3	154.8	163.7	50.3	33.3
	135.1	170.5	85.5	254.5	92.9	77.7	97.2	44.5
	50.2	60.7	60.2	118.0	76.1	87.8	101.4	92.4
	4.1	87.1	212.4	87.0	79.4	91.7	64.4	84.0
	149.7	130.6	136.2	56.1	148.0	351.2	75.5	85.9
	37.9	137.5	134.1	76.0	73.3	16.3	93.3	46.2
	146.8	91.7	42.0	110.6	104.4	129.3	80.6	62.0
	77.1	123.2	59.1	72.2	87.4	106.7	385.6	62.3
	108.1	64.0	64.6	53.6	161.6	29.2	75.7	65.6
	102.4	41.2	44.4	41.4	64.0	69.2	152.9
64.6\
FBXO24	26.5	82.5	45.45	71.4	106.6	32.5	73.6
44.95	74.0	54.8	68.25	58.15	73.35	52.6	36.05	78.2
	44.35	109.55000000000001	75.55	54.5	36.35	11.8
	128.25	45.95	30.55	40.15	62.05	65.15
90.64999999999999	65.55	24.6	26.95	65.5	67.35
91.55000000000001	81.55	84.55	96.25	46.3	40.95
24.65	43.5	96.5	24.650000000000002	31.1	41.75
62.75	76.6	140.25	94.5	96.6	29.4	115.9	135.15	69.0
	191.3	103.55000000000001	39.050000000000004
81.95	136.65	174.05	58.8	24.2	59.75	105.95	129.8
48.75	67.35000000000001	115.25	50.35	136.9
60.300000000000004	67.25	51.5	34.45	40.400000000000006
	80.4	33.9	59.300000000000004	21.25
40.599999999999994	117.15	63.1	85.75	121.94999999999999
	82.8	104.3	135.6	43.3	76.7	37.650000000000006
	81.55	134.05	90.8	90.75	123.5	80.25	30.55
160.95	119.9	129.20000000000002	154.15	62.150000000000006\
MCHR2-AS1	43.35	26.049999999999997	85.1
33.550000000000004	44.5	11.25	43.0	63.2
51.400000000000006	74.64999999999999	38.5	39.95

17.45	38.15	79.7	11.1	18.549999999999997
92.60000000000001	44.05	41.75	202.25	73.55	90.85
61.599999999999994	58.25	59.45	53.099999999999994
84.05	26.35	48.85	15.25	183.15	70.85	15.850000000000001
	18.5	26.45	16.549999999999997	55.0	11.7	34.1
	16.25	30.9	106.85	17.35	10.5	16.55	45.2	74.1
	56.05	68.65	81.9	19.2	40.85	141.65	58.0
21.099999999999998	60.5	81.2	12.55	95.55000000000001
	178.05	8.6	78.7	72.5	33.3	90.25	31.7
14.850000000000001	146.55	27.1	145.35	65.39999999999999
	58.35	26.099999999999998	45.449999999999996
48.099999999999994	26.099999999999998	29.1	13.1
61.25	35.199999999999996	38.35	46.85	34.7	75.5	58.7
	112.8	71.2	56.300000000000004	37.4	9.25
23.349999999999998	28.900000000000002	9.299999999999999
	19.4	41.9	68.1	40.0	129.5	26.1	42.85
29.45	70.75\
FBXO21	1025.6333333333332	1025.0	934.7666666666665
1082.0666666666666	587.9666666666667	1284.5
1442.7666666666667	973.9	1699.8333333333337
863.2333333333335	2163.0	811.6666666666666
1304.4333333333334	572.5333333333334	5248.000000000001
	2015.533333333333	1487.9666666666665	590.1
1805.8333333333333	1094.5333333333333	716.6333333333333
	1209.3	1048.5666666666668	1846.8333333333333
1183.4666666666667	1008.6999999999999	999.6333333333333
	1177.9333333333334	794.3000000000001
1298.5333333333333	3117.8333333333326	874.6	801.1
2358.7666666666664	1216.0666666666666	1920.1333333333334
	2096.5333333333333	576.2666666666667
1139.2333333333333	1516.8	1671.6000000000001
1666.9333333333334	976.6666666666666	1705.3666666666668
	1424.4333333333334	1124.3	912.7000000000002
1775.2	867.6333333333336	1354.8	1142.8666666666668
2045.8666666666668	1645.3333333333333	718.5	864.9
1224.2666666666667	1341.7333333333333	1033.1333333333332
	873.8333333333334	1065.6	1869.1666666666667
597.6	1297.7666666666667	1550.1333333333332
1225.5666666666666	747.9000000000001	2662.6
2827.533333333334	718.9666666666666	1497.233333333333
	799.2333333333335	590.8000000000001
1302.1000000000001	1111.1000000000001	1317.0333333333333
	1520.8000000000002	627.5333333333334
2349.1666666666665	2779.633333333333	819.2333333333332
	1108.0	1602.3000000000002	10123.6	3865.5333333333333
	529.4666666666666	706.2333333333335
990.3666666666667	2223.733333333333	948.9999999999999
	684.6999999999999	2518.5	928.8333333333334
2164.0333333333333	2693.233333333333	2225.133333333333
	2980.033333333333	2773.9	3356.833333333334
2274.7	1908.7666666666667	2226.4	1537.2333333333336
3606.6000000000004\
FBXO22	644.5200000000001	240.76	349.98	472.84	264.64
531.48	563.9399999999999	338.4	394.78	333.62	625.3

351.74 427.52 355.72 355.44 333.94 287.28000000000003
554.14 280.04 367.86 118.96 567.0600000000001 261.8
514.02 456.46000000000004 351.7 490.9999999999999
276.7 270.32000000000005 391.84000000000003 478.64
425.93999999999994 162.18 434.82000000000005 153.38
327.78 565.46 326.84000000000003 393.0 262.88 361.14
577.8199999999999 339.91999999999996 461.71999999999997
474.44000000000005 381.44 697.8999999999999
613.9200000000001 248.68 288.5 369.42 505.84000000000003
282.41999999999996 276.88 275.21999999999997
313.2 253.67999999999998 267.58000000000004
337.84000000000003 310.2 337.88 247.2 295.84 344.08
319.99999999999994 246.38000000000002 437.62
269.91999999999996 264.19999999999993 433.4799999999999
240.04000000000002 322.84000000000003 286.86
419.5 306.76000000000005 559.8799999999999 325.74
474.86 344.1 547.78 264.26 382.7 405.76 293.84000000000003
230.1 198.94 494.12 220.2 491.5400000000001
161.68 552.0600000000001 213.08 357.41999999999996
323.18 216.32 422.78000000000003 325.25999999999993
370.8 252.14000000000004 256.76 278.64 286.4 278.98\
FADS3 142.35 455.6 168.75 358.5 363.3 700.0
638.4000000000001 353.5 241.8 233.2 255.1 399.4
316.35 229.3 336.05 693.95 267.9 326.15 253.20000000000002
142.6 431.95 303.5 363.6 243.95 205.2 218.5
322.0 376.3 426.29999999999995 210.5 209.10000000000002
333.9 363.4 161.6 377.65 227.89999999999998
249.45000000000002 385.4 128.55 147.4 560.2
555.9000000000001 169.45 662.0999999999999 267.85
486.65 316.25 264.45000000000005 334.95 294.0 191.8
225.39999999999998 300.85 444.29999999999995 283.3
378.90000000000003 179.10000000000002 254.1 354.55
440.95000000000005 888.7 414.6 272.1 200.9 243.45
463.15 215.95000000000002 451.4 496.65 229.70000000000002
465.04999999999995 343.1 169.4 225.25 173.95
248.7 497.3 404.04999999999995 263.05 182.45 160.75
233.5 674.8 424.35 593.05 620.95 165.85 261.6 284.0
821.0 210.95 375.4 321.3 630.4 459.0 359.29999999999995
439.65000000000003 387.5 511.45000000000005
326.55 402.0 415.85 350.1\
FADS2 173.25 307.5 113.45 251.45000000000002 197.9
202.95 144.2 252.45000000000002 223.05 347.70000000000005
128.85 238.45000000000002 233.45 189.2
213.95000000000002 219.55 263.3 245.10000000000002 54.2
156.4 409.85 274.45 481.65 171.45 281.1
127.19999999999999 211.89999999999998 360.9 286.55
182.15 92.39999999999999 526.5 298.3 209.8 373.4
252.5 205.5 206.55 190.8 205.14999999999998 215.5
311.85 143.65 191.2 206.45 395.9 414.65 229.8 577.25
156.45000000000002 223.2 187.9 96.3 431.79999999999995
336.75 273.1 364.70000000000005 252.35 443.15
344.05 725.5 313.3 227.20000000000002 127.19999999999999
110.55000000000001 275.85 237.55 92.2 363.15
234.04999999999998 316.79999999999995 324.65

102.05000000000001	152.25	204.25	232.25	167.85000000000002
	188.4	109.1	155.9	247.39999999999998	270.4
189.8	196.7	257.65	393.15	263.1	333.3	201.35	227.85
152.95	137.95	181.2	109.80000000000001	173.2
104.85000000000001	133.55	12.899999999999999	147.7
156.85	89.05	245.65	107.35\
POTEM	56.3	25.2	483.1	259.4	194.2	166.1	85.9
413.9	82.4	23.2	360.0	1519.0	61.9	1134.3	6435.0	35.0
	201.8	283.1	197.2	158.8	59.4	72.2	466.9	74.2
	86.1	500.7	86.7	622.6	377.1	136.4	110.4
529.3	1120.4	199.1	1104.1	290.8	1624.4	359.1	112.2
284.8	90.5	95.3	142.0	170.5	108.3	294.4	192.6	9.4
	528.6	267.2	66.7	436.0	36.0	417.3	222.9
10106.0	3546.8	142.7	233.6	301.0	672.1	22.2	256.1
337.7	153.1	167.8	130.7	203.8	785.2	198.2	251.3
4301.6	203.8	605.8	7505.5	98.3	311.6	106.8	49.2
922.4	550.5	76.0	535.8	1429.7	286.3	673.2	163.0	60.6
	391.8	1675.6	187.5	169.3	123.9	132.5	135.7
138.5	105.4	133.0	355.2	113.7	145.9	105.5	83.2\
POFUT2	132.8	169.025	222.2	96.625	217.925	115.32500000000002
	89.8	175.17499999999998	125.15	216.8	121.65
144.875	71.25	186.95	184.175	149.52499999999998	119.675
218.5	107.975	196.925	294.17499999999995	127.275
244.02499999999998	79.85	111.89999999999999	192.25
138.875	173.2	245.525	140.45000000000002	116.45	324.05
271.3	143.1	224.0	221.7	84.725	209.9	84.22500000000001
	150.275	86.35	79.375	117.925	126.8	93.57499999999999
	183.675	227.79999999999998	69.575	347.57500000000005
	142.0	146.4	143.175	138.625	224.0	197.2	265.85
174.375	171.29999999999998	132.975	196.475	341.075	194.125
112.64999999999999	110.8	118.5	120.925	92.7	109.125
290.275	188.225	301.275	144.02499999999998	172.25	118.025
154.65	148.32500000000002	232.525	149.55	99.075	123.8
259.84999999999997	140.1	111.94999999999999	154.925
283.3	519.225	199.675	225.05	209.64999999999998	213.825
107.14999999999999	213.9	219.85	209.02499999999998
168.775	168.45	186.775	172.275	228.8	201.2	208.45000000000002
	293.97499999999997	195.75\
PTAR1	2482.2	3736.6666666666665	2995.2000000000003
1705.6333333333332	1364.4	1619.1000000000001	1027.0
1441.3333333333333	1454.2666666666667	1193.0666666666666
	1148.2333333333333	2268.3333333333335
935.6666666666666	1814.2333333333333	2116.2333333333336
	2827.7000000000003	1501.5	1756.466666666667
1268.4666666666667	1655.0333333333335	1676.6000000000001
	1764.766666666667	2271.7	1567.2666666666667
1521.5333333333335	1954.5666666666666	1436.1666666666663
	2659.233333333333	2361.9666666666667
2106.4666666666667	2577.9	3523.6666666666665
1255.8666666666666	1746.1666666666667	1067.9999999999998
	1841.9333333333332	1275.8999999999999
1387.9666666666665	1866.8333333333333	1288.9
1276.7333333333333	2031.7333333333333	1940.1333333333334
	2416.566666666666	1171.7666666666667	2024.8

1513.3666666666666	918.8666666666668	1872.6666666666667
	1838.9333333333334	1568.5	1752.0000000000002
1493.3000000000002	1652.9666666666665	1402.3000000000002
	2759.1000000000004	2103.133333333333
2654.2000000000003	1712.966666666667	1928.5999999999997
	1198.1333333333334	2212.2666666666664
1697.466666666667	1911.3999999999999	3058.2999999999997
	2148.5	2019.3000000000002	1979.9333333333334
1388.2	2080.2000000000003	1776.3333333333333
1702.3333333333333	3002.6000000000004	1807.7666666666667
	2494.633333333333	969.6999999999999
1528.6000000000001	1193.966666666667	2471.5333333333333
	487.8333333333333	1559.2333333333333
1430.466666666667	1030.7666666666667	2412.9666666666667
	1435.6000000000001	1184.5666666666668
1898.1333333333332	1875.2666666666664	1024.2
1431.2666666666667	1270.1000000000001	3858.7999999999997
	2808.566666666667	1959.5	2835.933333333333
3119.0	3035.7666666666664	2052.1666666666665
1986.9333333333334	3633.1999999999994	3868.5333333333333
	2786.866666666667	3737.0333333333333\
FNTA	4767.133333333333	7796.299999999999
5872.966666666666	5836.799999999999	6597.266666666666
	5484.7	5482.866666666666	9403.933333333332
6378.433333333333	6308.5999999999985	6454.133333333334
	5702.833333333333	7382.299999999999	5118.3
5823.099999999999	6571.133333333332	6905.066666666667
	7741.599999999999	5665.466666666667
5197.066666666667	4139.833333333333	4250.866666666667
	6574.399999999999	6387.166666666667	5732.0
8727.666666666666	5379.933333333333	5505.900000000001
	5076.966666666666	5089.9	6192.100000000001
5491.5	4872.966666666666	5084.633333333333
10891.199999999999	5520.933333333333	4415.599999999999
	5212.5	6903.366666666666	4830.333333333333
6493.5	6025.566666666667	5308.3	4906.3	5505.5
9852.866666666667	7833.400000000001	10443.0
6381.966666666666	7345.266666666666	7461.799999999999
	5600.5	6018.666666666668	4737.2	6665.966666666666
	7567.400000000001	6933.666666666667
6807.133333333334	7039.0	4646.066666666667
1832.1000000000001	6859.666666666667	5358.633333333333
	5741.066666666667	7197.200000000001
6891.733333333334	4411.466666666666	4628.933333333333
	6941.766666666667	5271.833333333334
4815.633333333333	6386.0	5814.766666666666	4285.0
5109.3	4726.933333333333	5638.633333333334
5818.933333333333	7030.0999999999985	5508.666666666667
	7636.7333333333345	8590.533333333333
5825.733333333334	4969.366666666667	8737.966666666667
	28594.300000000003	6015.233333333334
7707.333333333332	7276.733333333333	4523.266666666666
	5648.633333333332	6202.466666666667
5207.633333333333	6597.666666666667	6095.3

7436.133333333332	5584.933333333333	5564.333333333333
	6499.8	6089.0	7748.333333333333	5781.733333333334
	7048.533333333333\
AX746699	61.1	95.1	51.0	7.6	64.3	4.5	2.9
	8.2	99.8	11.9	34.2	42.8	6.0	23.7	12.7
	30.1	6.6	13.8	4.2	4.7	83.5	7.8
105.7	8.6	4.7	6.4	2.3	78.2	11.9	21.4	20.2
	74.7	27.4	4.3	87.7	35.2	16.5	107.0	23.0
	49.8	17.0	2.6	5.8	4.1	3.3	49.5	8.5
	14.2	50.8	6.7	57.1	53.4	12.8	12.1	46.7
	81.2	17.7	3.1	8.4	13.5	58.4	22.3	34.0
	37.9	41.4	17.9	1.9	43.0	73.5	47.7
106.9	103.8	114.5	2.6	4.3	15.4	75.7	38.7	42.0
	9.6	11.6	4.6	7.0	13.7	12.8	4.5	36.0
	37.3	24.8	19.2	5.4	61.3	71.9	3.1	23.3
	38.4	25.1	3.2	38.3	70.7	54.2	5.2
36.5\
POFUT1	215.0	227.10000000000002	239.95	219.25	233.65
265.7	459.84999999999997	187.65	356.0	306.8	462.0
857.4	767.1500000000001	165.2	329.75	220.3	314.45
380.25	314.54999999999995	235.05	96.6	447.40000000000003
	419.90000000000003	288.90000000000003	498.75
225.15	246.85	221.45	209.25	295.5	359.7	343.15	279.35
353.0	227.85	232.15	281.4	302.75	426.2	225.04999999999998
	252.39999999999998	338.3	231.45	480.65000000000003
	376.15	301.1	271.55	330.35	242.8	378.4	359.7
83.80000000000001	228.89999999999998	251.8	324.0
335.79999999999995	430.55	285.15	224.39999999999998
174.95	441.65	565.6	314.3	265.3	313.05	316.5	282.1
318.85	361.65	143.5	188.0	236.7	218.75	409.65	366.55
157.65	168.29999999999998	327.95	233.35000000000002
94.95	141.1	128.2	736.3000000000001	194.9	188.35
289.4	174.75	315.45	289.59999999999997	623.05
996.5999999999999	172.10000000000002	303.29999999999995
	305.2	193.7	205.65	214.95	292.40000000000003
243.8	273.65	221.20000000000002	256.25	215.0\
C18orf32 /// RPL17 /// RPL17-C18orf32	4129.4	264.3	1081.1
3290.7	1333.1	4139.6	2576.5	1157.6	2258.4	1873.6	3606.9
2372.9	3771.8	3366.8	1361.5	1999.5	2009.1	1604.7	3037.4
1970.5	284.9	2023.3	1030.7	2670.8	2506.9	2426.1	4029.0
1267.7	1414.8	1865.4	1994.3	1172.9	1133.2	2856.5	1891.7
3443.0	4891.6	843.0	2635.6	2366.8	2507.1	1898.2	2397.0
2841.0	2977.0	1687.5	2487.6	5432.8	369.1	1451.1	2081.0
3378.5	3250.5	829.2	1950.6	923.9	1871.5	1044.2	2258.7
996.9	611.3	2055.4	2452.1	2648.7	1753.1	1719.8	2606.3
3306.1	903.2	3384.6	334.8	1828.3	1754.0	3473.1	1757.7
4997.2	2150.9	2140.4	3062.3	3450.2	1993.3	1814.1	1993.7
2877.2	627.6	851.0	2355.0	1892.4	1481.1	2192.5	2119.4
1145.8	2659.6	2175.7	1500.0	2108.6	2002.2	2714.4	1170.3
1357.4	1662.8	1847.3	2366.7\
NEK1	194.07999999999998	1201.8799999999999	535.4
626.1800000000001	977.54	511.64000000000004
477.56000000000006	610.6400000000001	654.8199999999999
	679.3	498.64	688.98	515.48	157.28	205.5

425.25999999999993	319.38	935.36	521.8599999999999
395.06	617.6600000000001	1037.66	1376.6	714.8599999999999
	614.4799999999999	446.68	385.21999999999997
701.92	662.12	320.18	387.14	564.72	1073.92	841.86
483.0800000000001	7522.780000000001	498.32000000000005
	990.9399999999999	540.08	290.56000000000006
314.32	603.74	1135.34	499.88	703.76	1318.1799999999998
824.1	516.28	833.9400000000002	540.56	789.7
234.61999999999998	469.73999999999995	887.2599999999999
	606.5400000000001	1156.88	948.6800000000001
635.58	706.74	1277.7600000000002	873.3	1221.7400000000002
	713.54	698.76	638.98	428.02	544.06	571.0200000000001
	1089.2999999999997	670.22	2160.4399999999996
1329.56	697.34	543.06	471.7200000000001	492.64000000000004
	985.9800000000001	601.6	673.5	348.76000000000005
	647.66	1039.8600000000001	300.15999999999997
297.36	1196.24	1319.56	768.9399999999999	882.1600000000001
	451.4799999999999	683.0000000000001	411.86
916.74	745.8599999999999	584.26	750.1600000000001
752.4399999999999	775.68	743.0000000000001
863.4599999999998	864.8	772.6600000000001
1238.6399999999999	739.1200000000001\
TNFRSF8	66.2	95.6	174.8	98.7	155.9	24.8	101.2	93.9
	87.9	33.4	47.6	68.1	36.5	143.7	95.4	74.0
	140.4	25.5	56.9	15.5	279.1	41.3	25.9	49.3
	48.7	55.7	62.5	152.4	140.0	247.4	66.0
149.9	232.3	114.2	48.7	165.6	57.5	230.2	58.5	32.8
	69.1	35.4	63.2	110.9	184.2	24.5	75.1	49.9
	37.6	131.4	46.4	63.4	34.5	171.8	65.1	86.8
	61.4	34.2	19.4	49.4	321.4	108.3	51.4	54.2
	90.1	126.8	97.2	92.2	70.4	91.7	94.6	98.9
	16.6	49.6	109.7	78.4	196.0	80.5	92.9	87.6
	121.9	99.5	164.6	60.8	254.6	136.4	118.0
124.2	52.7	207.3	73.0	97.9	71.8	100.8	118.0	79.4
	102.3	61.5	103.1	14.5	71.3	117.8	35.8\
NEK2	1244.15	472.45	160.25	502.65	399.7	786.4	682.25
528.4000000000001	599.55	677.4	770.75	551.9000000000001
	672.6	540.95	937.8	137.65	990.35	914.3	511.25
420.7	95.0	1219.15	185.2	360.4	1147.8	277.4	682.2
548.75	476.6	688.4	324.40000000000003	884.8000000000001
	205.35	289.3	668.0500000000001	102.6
695.9499999999999	390.0	400.40000000000003	1085.5
715.2	558.1	237.35	91.85	632.0	1245.4	996.1999999999999
	1576.2	299.15	225.65	555.75	1560.05	462.4	714.65
403.1	742.4	526.6	378.8	507.3	197.65	327.5	330.3
257.45	652.85	97.05	218.0	385.55	71.55	111.5	456.7
121.95	728.2	47.8	300.25	1160.55	1058.2	294.04999999999995
	536.3000000000001	247.7	1935.3	982.0	1211.3
580.4	1010.8499999999999	727.0	374.90000000000003
1198.95	738.0500000000001	468.0	218.9	1167.8	295.1
52.05	14.299999999999999	33.9	128.85	49.15	74.15	97.4
	83.95	87.6	377.29999999999995	32.05\
RPL36A /// RPL36A-HNRNPH2	6804.9	2335.1	4836.7	11660.4
2117.4	15504.0	12605.1	1397.8	8752.1	8360.9	9418.9	3219.0

```
8552.4	9058.3	4846.4	5166.4	7561.1	7998.9	14163.2	7287.2
1268.6	6848.7	5825.6	8594.3	8839.2	4161.3	9049.1	6653.2
4173.1	6051.4	16101.6	4048.0	2743.6	12087.4	7629.7	5979.7
8179.4	3940.8	10262.6	20191.7	6622.0	2904.5	9884.4	6043.4
6638.4	10251.0	1823.3	12433.6	1960.7	11103.5	10218.4	7079.0
8746.2	663.6	6794.9	2418.7	14790.3	5454.4	5926.9	7001.8
10367.2	4851.1	7001.9	5147.5	3565.9	6049.6	7901.0	3188.0
9416.2	7330.9	2300.7	4437.8	3630.6	13506.3	7120.8	6591.1
1913.0	5774.1	5883.8	1458.6	3235.0	3034.8	6958.3	5080.4
2020.0	2627.0	2023.4	2389.5	7920.3	8632.0	8813.6	994.6
10181.6	8437.3	6958.2	11487.6	11002.7	10221.1	7353.2	7982.5
11036.1	4666.7	11411.8\
NEK3	276.90000000000003	364.2	351.95	350.45	510.55
196.05	405.75	160.45000000000002	168.3	194.15
252.45000000000002	442.35	228.0	215.6	698.8	268.2
124.15	271.5	413.3	112.65	583.55	219.04999999999998
380.15	130.9	135.65	565.7	258.4	327.75	438.84999999999997
	239.15	362.54999999999995	586.1	602.35	241.2
179.5	417.90000000000003	240.6	597.4	193.9
95.19999999999999	184.05	285.0	302.05	182.9	176.6
267.6	162.39999999999998	146.45	729.6	334.95	178.05
232.35000000000002	410.7	338.65	384.15	390.75	312.45
695.25	293.15000000000003	488.25	268.15	474.9	332.85
187.2	477.59999999999997	379.4	280.6	368.9	174.55
263.90000000000003	340.35	687.0	518.4	238.45000000000002
	318.4	166.5	142.0	225.75	268.40000000000003
357.8	351.75	202.95	205.45000000000002	329.4	538.05
509.6	254.75	196.5	193.35000000000002	623.15
239.39999999999998	380.55	382.3	462.2	546.9	303.35
329.45	497.0	502.40000000000003	499.90000000000003
379.95	570.1500000000001	382.45\
TNFRSF4	14.0	49.1	54.900000000000006	41.75	82.6
19.95	72.8	76.65	27.0	52.7	33.35	93.2	39.7
90.45	68.85	32.5	67.55	66.6	45.199999999999996
34.85	181.4	43.0	80.05	65.8	79.60000000000001
67.45	85.05	78.65	80.1	101.75	48.15	190.2	58.9	63.0
	71.15	28.35	44.45	68.2	48.15	35.3	19.85	13.2
	123.0	48.2	38.1	40.0	84.6	16.85	83.15	54.4
	49.800000000000004	25.65	51.3	60.6	57.8
62.849999999999994	32.099999999999994	54.8	29.05	58.3
	270.35	157.5	91.4	35.1	81.30000000000001
53.45	47.45	85.85	100.85	21.849999999999998	195.3
57.45	64.9	41.15	70.5	54.45	53.650000000000006	25.3
	105.05	59.75	73.85	44.45	20.599999999999998
116.0	43.5	93.05	55.5	103.0	61.150000000000006
108.7	32.75	96.65	44.6	107.7	41.65	37.0	34.75	66.4
	80.1	30.200000000000003	58.95	44.4	61.5\
SPDYE1 /// SPDYE2 /// SPDYE2B /// SPDYE5 /// SPDYE6	173.6
2261.8	344.2	269.5	883.7	415.5	142.6	1021.2	224.4
482.3	182.0	566.5	265.9	159.9	564.3	349.4	509.4
450.0	231.1	236.0	472.7	280.6	589.3	177.5	235.6
322.4	166.4	463.1	249.5	317.3	376.4	601.7	800.7
201.9	170.0	482.7	177.1	494.7	360.3	131.7	398.6
375.8	539.8	209.4	238.6	534.7	370.4	195.3	474.0
```

741.0 358.3 217.4 140.3 1385.2 198.6 421.2 834.4
671.8 593.3 467.4 499.4 434.3 250.1 342.6 515.6
652.7 318.8 216.4 582.3 192.5 659.6 791.3 402.8
350.6 320.1 179.9 426.5 249.4 340.3 501.8 834.3
497.6 455.5 314.0 815.1 583.5 475.2 257.4 272.4
198.5 357.3 845.9 496.6 345.9 638.0 954.8 700.4
487.9 741.4 670.7 843.8 575.4 498.4\
KCNH1 3.2 58.8 5.3 7.2 21.8 10.7 9.3 20.6
4.9 15.7 9.0 7.1 9.8 5.5 12.3 13.2
6.9 34.4 31.5 4.6 12.4 22.9 18.4 9.9
3.8 52.5 5.4 114.4 42.0 7.2 17.4 80.0
30.0 16.0 11.6 8.2 3.7 8.3 4.4 12.0
3.5 5.4 40.8 5.6 13.1 21.7 22.0 9.2
113.1 7.5 10.2 8.2 8.3 48.6 5.2 11.7
5.9 23.3 5.5 8.8 34.6 6.7 3.3 3.5
22.2 45.8 35.9 5.8 81.3 9.8 10.8 15.8
3.9 20.0 33.8 48.3 24.8 2.5 5.6 30.0
7.3 3.4 13.5 14.5 16.6 14.0 4.9 21.5
7.6 27.7 10.5 6.7 6.1 4.7 6.1 24.6
16.6 4.1 25.4 11.4 7.2 9.6 5.4\
KCNH2 209.6 155.5 69.2 131.9 324.55 455.15000000000003
82.35 826.1 175.65 77.3 240.35 101.95 87.55 83.7
468.2 378.59999999999997 236.05 221.35 434.85
161.25 679.1999999999999 81.2 220.4 280.90000000000003
104.4 178.4 82.10000000000001 207.9 163.25
171.4 231.45 278.15000000000003 279.95 185.1
1259.3999999999999 114.0 472.55 133.0 68.6
307.59999999999997 381.05 416.3 181.35 158.35
79.89999999999999 834.5 135.89999999999998 1041.5
135.5 409.0 130.3 84.75 67.75 691.05 183.0 839.75
352.75 121.3 186.6 149.0 356.40000000000003 22.15
241.55 218.5 319.65000000000003 153.10000000000002
156.7 223.95 371.25 117.85 221.85 429.3 170.1 222.75
218.35 179.10000000000002 355.1 57.949999999999996
186.3 1275.1499999999999 585.6 265.90000000000003
581.95 366.95000000000005 150.9 332.4 180.89999999999998
575.8000000000001 114.0 159.55 440.65 303.65
303.5 142.85000000000002 248.15 165.75 212.65 138.35
143.25 252.1 126.0 451.1 195.39999999999998\
PIAS4 262.9 580.6 275.4 305.85 486.8 363.45000000000005
406.55 171.95000000000002 317.79999999999995
376.95000000000005 321.0 604.1 342.35 400.0
434.59999999999997 385.95000000000005 428.8 358.4
454.9 436.8 542.0 398.7 360.65 299.65 283.45 561.15
359.8 512.65 485.95000000000005 400.15 319.70000000000005
391.65000000000003 541.55 332.25 424.5 206.05
279.6 557.5 492.59999999999997 219.64999999999998
278.85 405.75 298.04999999999995 379.45 283.35 371.7
194.55 348.0 629.7 459.85 393.0 267.35 254.85 274.25
385.25 623.7 296.35 439.25 351.20000000000005 563.65
343.45 415.75 406.70000000000005 277.65 527.2 596.7
395.05 493.9 489.4 201.0 461.85 480.5 482.15 284.1
330.3 265.85 254.55 398.29999999999995 353.15 108.45
159.45 250.1 206.10000000000002 253.35 321.1 100.85

266.55 161.6 316.9 456.65000000000003 230.65 160.05
243.75 305.20000000000005 387.25 249.05 214.75
209.54999999999998 331.8 266.65 275.75 249.1 342.9\
PIAS3 1600.0 3759.9 3826.2 1666.3 4533.4 2473.5 2030.8
3854.9 1408.0 3807.9 5186.2 3622.7 2720.2 2014.1 2566.7
1321.3 1952.7 2391.2 1454.8 2260.2 3865.8 3298.7 3193.7
1480.2 2379.0 3325.4 1228.9 3380.2 3532.1 1552.9 2381.0
6347.0 2327.7 2718.8 3914.7 3271.3 3158.4 2503.7 2130.9
3743.0 1610.6 3547.5 1585.7 1504.5 2836.9 2189.1 3342.4
1436.5 7142.6 4227.7 2887.2 1618.7 2434.5 3804.3 3894.6
6085.4 4789.4 3213.0 2621.3 2516.8 1204.5 3295.8 2527.4
1616.1 4449.9 2808.3 4155.8 1788.7 3297.5 3594.5 4222.2
2316.7 1662.8 2104.7 2637.5 2813.0 5014.0 2437.5 5067.4
3786.5 3955.8 2079.6 1155.1 865.3 2358.8 4263.9 2558.8
3555.1 2052.0 2080.7 1553.4 5696.0 3463.1 1955.1 2688.9
2739.0 3433.2 2534.6 3540.9 3997.8 4364.6 5049.2 2546.1\
KCNH3 21.9 27.5 68.7 6.5 397.9 44.7 56.6 74.9
48.6 278.7 315.9 563.2 113.1 39.7 47.6 6.9
123.0 63.4 21.1 117.7 53.2 336.0 50.7 10.2
39.2 280.4 399.5 190.3 81.2 207.7 22.1
574.1 62.7 26.7 43.1 51.0 81.8 196.2 19.4
100.3 170.4 58.2 108.7 18.5 17.6 43.1 24.3 17.7
88.4 47.3 200.2 19.6 9.6 40.9 22.2
151.0 113.2 17.0 132.7 85.9 94.9 203.0 65.6 56.7
78.9 37.0 61.6 23.1 76.5 47.1 178.3
150.4 41.5 65.4 101.8 53.2 106.7 8.6 59.4 8.0
35.4 25.5 224.7 107.0 27.5 87.1 75.2 19.4
12.0 14.6 137.5 30.1 15.3 13.2 18.1 37.4
90.1 131.7 35.6 24.7 34.4 18.4 89.4\
KCNH4 27.4 34.6 18.6 59.3 227.7 27.5 8.8 9.9
36.1 129.6 11.0 5.7 18.1 8.4 42.6 19.5
15.8 88.2 40.4 42.0 108.9 98.5 72.1 10.1
42.4 83.9 70.6 210.2 51.3 105.4 9.0
193.7 26.0 26.9 43.9 49.6 30.1 96.2 15.4 10.6
35.4 37.4 157.4 11.7 17.5 64.6 126.4 87.7
141.8 149.9 33.0 55.6 72.4 139.8 44.4 51.5
143.4 25.0 49.9 46.4 202.7 93.2 68.8 75.4
75.6 25.7 22.3 67.2 31.6 43.9 217.3
155.1 61.1 23.8 47.2 52.9 47.5 16.6 6.8 72.8
72.4 136.6 104.9 105.3 107.2 35.9 69.4 31.3
77.0 56.5 16.7 112.1 35.2 5.8 80.1 6.6
61.5 39.4 36.6 33.3 35.3 102.0 10.4\
DGAT2 278.75 29.55 116.25 82.25 77.45 206.15 46.3
242.39999999999998 80.85000000000001 99.7 94.05 42.0
31.25 73.25 265.65 94.7 74.9 66.85 165.25 96.2
40.1 316.75 150.8 77.4 79.15 92.45
86.94999999999999 162.3 162.6 118.95 118.55 109.5
162.3 52.55 98.7 130.75 48.550000000000004 60.5
68.75 619.0 203.8 101.6 43.05 56.1 58.0 122.5
116.3 171.8 137.2 173.5 25.1 148.39999999999998
146.95 190.64999999999998 123.65 224.2 61.35 136.65
42.85 117.6 273.75 118.19999999999999 118.55000000000001
124.45 131.4 234.60000000000002 68.2 67.75
125.9 162.05 28.1 179.4 41.05 64.05 82.55000000000001

75.8 201.95 101.5 103.30000000000001
117.69999999999999 191.7 63.15 121.75 71.0 51.35
36.95 118.69999999999999 129.85 45.949999999999996
133.15 95.10000000000001 123.9 110.80000000000001
84.95 75.55 182.89999999999998 59.25 96.25 83.0
190.0 82.45 72.89999999999999 118.85000000000001\
KCNH5 27.766666666666666 126.33333333333333
65.43333333333334 12.833333333333334 67.66666666666667
38.1 22.833333333333332 39.53333333333334
8.133333333333335 174.33333333333334 51.833333333333336
39.666666666666664 75.53333333333333
34.733333333333333 45.43333333333334 26.2
38.199999999999996 46.400000000000006 16.099999999999998
41.4 88.39999999999999 131.6 68.03333333333333
32.93333333333333 40.333333333333336
29.900000000000002 37.300000000000004 53.833333333333336
40.266666666666666 29.200000000000003
10.566666666666668 52.7666666666667 72.8666666666666
58.166666666666664 68.5 49.26666666666667
34.166666666666664 91.0666666666666 28.399999999999995
58.833333333333336 45.366666666666674
24.4666666666667 81.60000000000001 48.833333333333336
11.633333333333333 41.833333333333336
61.7666666666667 26.7 69.53333333333333
30.266666666666666 28.666666666666668 29.96666666666667
52.53333333333333 59.833333333333336
58.633333333333326 68.39999999999999 68.36666666666666
103.10000000000001 36.43333333333334
55.06666666666666 326.93333333333334 80.53333333333335
27.0 44.79999999999999 30.433333333333334
68.83333333333333 19.8 14.6 78.56666666666666
41.46666666666667 70.89999999999999 101.66666666666667
31.03333333333333 34.833333333333336 32.4
26.766666666666666 37.666666666666664 13.633333333333333
27.066666666666666 60.833333333333336
16.333333333333332 34.333333333333336 22.900000000000002
37.8 140.03333333333333 84.2 36.03333333333333
45.833333333333336 49.76666666666667
21.033333333333335 25.53333333333333 80.36666666666666
31.066666666666666 47.333333333333336
36.76666666666667 28.366666666666664 29.26666666666667
41.266666666666666 25.733333333333334
62.166666666666664 52.199999999999996 30.5
34.800000000000004\
DGAT1 742.2 1445.4 330.0 377.6 611.7 762.1 346.8
346.1 346.7 219.8 345.5 541.4 348.3 437.4 412.9
165.3 517.1 422.1 624.1 333.2 932.7 264.7 645.7
396.5 258.9 632.4 394.4 434.8 589.2 326.5 198.0
816.5 806.7 262.7 139.0 647.8 465.9 692.8 711.5
377.3 425.5 998.3 333.5 510.3 696.1 436.9 448.6
578.5 568.9 520.2 374.0 363.7 519.3 615.5 377.4
806.5 450.2 559.4 499.7 474.0 60.0 462.6 429.0
311.2 406.4 827.4 503.6 614.3 508.2 390.0 612.9
506.5 368.5 255.6 233.3 392.5 457.9 356.0 478.5

775.2   297.6   176.7   856.5   1466.0 622.1   702.1   404.9
356.4   524.3   412.7   557.9   769.8   389.3   739.5   560.9
335.2   336.6   559.4   1004.5 469.0   452.2   712.5   208.5\
KCNH6   10.799999999999999   52.133333333333326
22.133333333333336   14.433333333333332   38.93333333333333
        35.36666666666667   31.7   10.499999999999998
7.833333333333333   25.13333333333333   6.866666666666667
        31.900000000000002   25.099999999999998   16.4
55.96666666666667   9.833333333333334   24.066666666666666
        88.33333333333333   29.866666666666664
48.76666666666667   80.2   5.566666666666666
75.86666666666666   22.599999999999998   29.766666666666666
        30.066666666666663   23.133333333333333
11.633333333333335   11.466666666666667   17.46666666666667
        24.033333333333333   39.66666666666667
30.53333333333333   24.933333333333334   29.366666666666664
        38.3   8.5   26.96666666666667   25.8
14.100000000000001   8.066666666666666   14.466666666666667
        6.466666666666666   58.93333333333334   6.7
24.666666666666668   30.666666666666668   22.599999999999998
        60.13333333333333   30.633333333333333   9.5
18.133333333333336   22.3   78.80000000000001
23.666666666666668   43.5   30.366666666666664
62.633333333333326   26.73333333333333   56.166666666666664
        40.1   26.833333333333332   65.03333333333333
18.666666666666668   65.89999999999999   59.366666666666674
        26.899999999999995   20.366666666666667
28.166666666666668   22.8666666666667   56.6
50.76666666666667   55.9   17.366666666666667
26.599999999999998   16.966666666666665   55.366666666666674
        38.96666666666667   11.433333333333332
28.16666666666667   48.79999999999999   41.73333333333333
        27.3   40.9   42.5   23.833333333333332
34.93333333333334   50.43333333333334   52.43333333333334
        16.9   7.666666666666665   45.46666666666667
24.166666666666668   10.5   17.566666666666666   19.0
27.96666666666667   9.933333333333334   16.866666666666664
        46.86666666666667   9.233333333333333
10.733333333333334   5.5\
FBXO18 354.15 761.9   434.65 391.55 455.1   500.5999999999997
        529.6   305.85 470.75 568.05 344.2   504.90000000000003
        395.20000000000005   423.0   616.85 328.20000000000005
        442.95 438.55 391.7   281.05 692.9   300.5   795.25
540.8   378.45 398.5   239.04999999999998   517.7   277.25
362.2999999999995   463.05 581.6   477.75 506.25 344.6
436.3   482.65000000000003   548.1   455.25 560.85 521.1
411.45 452.6   627.6   461.95000000000005   475.75 362.95
489.95000000000005   614.05 339.95000000000005   299.55
284.1   390.0999999999997   494.90000000000003   627.65
447.35 402.8   428.7999999999995   350.5   669.2   418.8
273.35 324.75 287.75 441.7   520.5   486.65 406.95 492.85
181.75 554.05 566.3000000000001   500.5   529.35 356.4
330.9   392.75 549.65 463.85 378.75 262.55 337.05 783.1
480.75 404.20000000000005   550.5   397.85 342.15 460.1

613.1 415.2 327.1 431.45000000000005 445.1 356.2
262.95 137.25 202.10000000000002 283.0 417.0 419.7
352.9 352.0\
KCNH7 38.25 56.0 49.75 53.75 52.45 21.3 26.35 44.4
68.5 84.55000000000001 31.150000000000002 87.6
15.65 44.8 29.6 10.65 46.85 76.65 13.9
54.75 133.1 69.5 106.65 24.0 35.55 56.949999999999996
15.45 73.7 56.75 7.9 22.1 43.949999999999996
50.65 34.8 91.3 48.800000000000004
12.100000000000001 58.55 20.9 34.4 35.550000000000004
68.55000000000001 35.85 22.0 29.0 38.65
37.35 36.4 67.25 55.35 20.65 17.2 42.5 45.3
71.80000000000001 94.3 59.85 78.85000000000001 53.6
73.95 27.049999999999997 17.55 36.5 17.4
49.300000000000004 90.7 30.700000000000003 22.0
32.55 33.3 51.05 49.95 86.30000000000001
53.550000000000004 70.80000000000001 35.2 62.6
35.65 34.55 47.7 73.1 64.15 54.6 54.3 46.3
119.2 63.75 79.05000000000001 78.4 45.95 23.55
54.949999999999996 36.1 31.8 39.1 40.8 41.4
53.900000000000006 28.9 53.25 82.85 34.5 24.8\
KCNH8 35.7 13.8 75.0 25.6 118.6 6.9 1.7 28.4
60.6 3.0 41.1 42.2 30.5 69.5 326.0
499.8 13.3 37.2 85.3 52.7 88.6 8.0 11.8 61.4
78.5 37.0 28.2 75.3 11.7 8.1 70.9 51.2
141.3 52.0 12.7 41.8 32.3 6.5 2.3
182.9 32.2 50.8 35.5 131.3 37.6 7.8 52.1 5.9
37.8 45.2 15.8 3.7 15.1 47.2 26.7 4.4
11.0 8.9 7.8 129.8 207.9 13.1 4.0 22.8
199.8 101.1 97.8 72.9 36.0 6.9 104.3 53.5
111.2 22.2 68.3 5.9 15.8 20.4 93.0 22.5
580.7 30.2 294.3 28.2 56.8 28.5 52.5 17.2
42.5 7.3 20.8 204.0 217.1 187.6 252.9
246.1 283.6 214.1 360.5 460.0 176.9 58.0 312.5\
FBXO16 441.1 934.9 454.3 586.4 826.4 368.9 276.0
320.9 373.7 818.4 475.5 695.5 664.8 854.4 281.8
203.7 648.8 1359.7 348.2 399.6 557.5 245.5 685.8
407.9 446.0 661.9 660.7 699.6 853.7 481.8 250.1
1306.7 401.7 806.1 291.2 360.5 290.5 794.2 330.5
249.3 87.4 332.9 952.2 361.0 526.7 1070.0 407.5
542.8 830.5 658.5 850.5 437.1 517.7 542.1 377.8
721.0 818.7 510.3 574.8 814.8 819.6 652.5 789.4
438.5 501.7 463.4 349.7 527.4 1317.5 628.6 1180.8
1514.7 417.6 664.0 627.9 645.9 614.8 374.9 495.4
247.0 547.0 307.1 346.1 263.5 910.0 460.1 258.0
200.7 347.0 426.9 242.4 457.5 443.1 187.2 391.2
409.5 442.7 396.3 713.9 574.4 340.7 303.2 482.5\
STX8 1571.5 681.4499999999999 579.3 1058.5500000000002
536.6 1034.5 956.05 307.7 755.5 1275.1000000000001
688.75 1126.45 548.25 780.0 414.1 704.9 556.4
718.6 421.8 940.85 635.8 1187.0 656.25 912.45
873.8499999999999 791.7 955.65 680.8 705.35 802.05
600.8499999999999 1506.0 499.1 1120.1499999999999
628.1500000000001 1701.25 1213.75 611.1999999999999

911.3000000000001 664.85 640.4 529.75 852.9
802.5500000000001 759.85 1204.1 696.3 605.8 338.8
530.35 1027.3 748.85 740.15 475.15 750.45 975.15
1092.6499999999999 480.25 878.05 863.1 425.65 899.05
755.75 1055.3 621.3499999999999 603.15 666.85 779.65
1110.5 967.75 354.09999999999997 1537.15 741.45 1068.9
1103.2 980.7 500.1 1178.85 944.6 760.7 283.55 382.25
991.0 579.9 263.40000000000003 267.15 791.0999999999999
707.75 528.5 598.1 590.6500000000001 468.6
1157.1 672.6999999999999 811.35 805.6 922.5 991.1
854.6 844.6500000000001 814.1 984.3 644.75\
STX7 1638.75 1208.425 1322.275 944.1500000000001
758.2 960.2 854.7 1305.0749999999998
1220.8000000000002 779.375 1206.925 993.075 635.7
1613.625 1188.3249999999998 1239.225 1200.775
598.575 1400.9 1135.925 774.7750000000001
1412.5 749.675 1412.25 1606.3249999999998 1523.275
1116.775 964.4250000000001 664.175 1369.6 1402.425
630.7750000000001 738.5 892.85 806.1500000000001
936.675 1371.6 695.25 673.975 1476.4 715.2750000000001
1117.925 993.425 1397.975 1803.0249999999999
849.55 1119.225 1178.9 393.225 813.6999999999999
859.2249999999999 1075.725 1239.1499999999999
531.8 935.8249999999999 855.75 796.3499999999999
641.875 867.9000000000001 658.075 729.15 761.225
1030.6 1400.3 1282.175 494.29999999999995 1338.65
1976.175 809.7249999999999 1204.325 586.1
605.25 1403.35 967.5 1028.2 1005.075 977.1000000000001
1181.4 995.2 1003.95 1158.0 1045.975 2072.975
1052.6 1138.025 884.525 1373.3000000000002
1427.0500000000002 1352.65 1320.9 1469.0500000000002
823.825 1170.75 901.8 960.9750000000001 1232.525
1283.3 936.375 989.575 1248.7250000000001 1264.375
1301.825 1269.2\
FBXO17 6.9 137.1 40.6 552.7 54.6 8.9 20.6 59.3
7.8 7.8 4.5 5.1 4.3 10.1 773.2 5.6
20.5 6.6 331.6 117.8 100.0 12.1 68.9
233.1 4.8 10.9 3.9 27.5 11.0 5.6 167.2 24.7
485.1 32.6 110.6 7.3 5.8 53.7 8.1 5.2
520.3 332.1 151.7 47.7 6.3 43.9 26.3 9.4
32.9 7.2 8.6 6.3 5.0 703.2 8.3 17.9
13.4 13.4 17.9 143.4 554.9 48.5 26.6 4.7
6.9 12.8 30.7 6.3 18.2 10.1 157.1 25.2
28.4 4.3 12.6 14.4 8.9 5.6 26.4
1680.8 35.4 313.6 1453.0 5.6 18.6 3875.0 60.1
362.0 44.7 15.1 293.7 497.0 145.7 274.5 501.2
121.9 220.8 138.1 109.0 10.6 294.0 171.7 215.0\
FBXO15 40.9 215.7 606.2 110.8 627.2 109.4 80.7 88.2
351.0 285.1 324.7 40.8 59.6 172.6 128.1
434.2 12.6 349.8 152.5 82.9 946.4 585.2 452.2
323.7 278.0 90.4 98.3 780.3 514.2 414.7 209.5
1013.3 638.4 764.5 150.6 333.0 31.3 100.5 146.4
116.3 107.4 75.2 1087.6 333.1 115.8 1065.4 157.5 82.0
290.6 559.0 496.2 286.5 90.5 21.2 282.9

625.0 422.7 376.8 519.2 687.0 1795.5 1091.9 137.3
962.1 292.5 424.6 758.0 174.4 391.0 1251.0 649.9
1566.1 195.3 210.7 785.0 753.2 1311.9 296.2 1166.4
273.2 307.9 356.7 177.0 158.9 603.3 297.7 106.2
269.8 57.8 363.7 19.9 1075.0 833.9 438.2 504.2
1385.0 965.6 1530.7 586.9 1824.7 788.3 439.6 754.0\
LOC100288152 104.2 1791.5 130.9 131.7 395.0 153.0 33.9
297.9 22.1 67.5 160.7 39.9 99.2 45.3
7770.5 522.1 208.3 74.0 117.9 6.8 474.1 239.3
100.9 118.0 119.9 21.7 60.4 163.1 477.0 39.4 96.9
162.6 327.8 145.6 41.2 14.1 175.4 184.3
209.1 77.6 99.6 324.0 50.1 85.6 106.8 149.1
162.9 301.9 289.4 302.8 124.9 191.7 91.7 378.1
215.6 4276.2 743.4 124.0 170.2 230.2 292.4 176.4
255.5 135.8 57.0 623.9 185.8 250.0 252.1 236.7
533.6 156.5 208.9 90.1 87.7 30.9 588.7 215.0
244.9 6.9 448.3 379.4 59.2 11.7 1577.7 3557.7
159.0 307.4 115.0 119.5 63.4 819.2 331.7 362.2
459.0 732.9 737.0 322.0 407.2 559.6 387.2 358.8
712.5\
LOC102723697 5.2 18.6 3.2 21.1 40.9 3.4 4.2
112.1 4.5 18.4 5.2 44.9 47.4 29.6 20.1
4.0 24.4 3.7 16.2 11.4 16.8 72.6 9.2
3.8 33.7 4.8 2.2 29.0 79.8 10.6 8.4
168.1 10.9 5.4 68.3 75.8 130.3 32.1 16.6
10.2 19.5 29.9 10.7 26.1 10.4 33.1 10.5
31.1 32.7 9.1 12.0 21.5 29.9 11.5 29.9
32.3 12.4 58.1 30.4 17.3 418.5 3.7 10.3
3.8 7.3 47.1 6.2 2.5 46.8 3.5 77.7
102.2 49.2 8.3 65.0 32.0 7.1 5.8 27.3
2.6 36.0 19.0 3.5 5.4 107.6 97.0 43.5
45.9 80.4 6.2 2.7 66.1 41.2 3.5 5.9
4.0 36.6 6.5 7.7 12.9 10.5 34.3
18.4\
FBXO10 72.9 131.64999999999998 78.5 73.45 139.2 81.8
58.400000000000006 147.85000000000002 75.95 87.6
87.09999999999998 63.425000000000004 47.1
80.475 114.9 119.7 81.675 100.625 55.725 75.4
396.44999999999993 73.64999999999999 92.0
31.224999999999998 35.325 131.125 70.775 172.575 98.6
74.225 35.575 114.4 107.425 79.55 107.24999999999999
113.52499999999999 51.025 119.37500000000001
83.80000000000001 64.25 53.35 52.77499999999999
19.799999999999997 88.37499999999999 10.95
102.82499999999999 53.599999999999994 68.2
117.77499999999999 54.75000000000001 37.85
40.900000000000006 55.225 162.75 58.699999999999996 88.0
38.875 173.75 126.725 147.14999999999998 312.0
109.07499999999999 40.25 56.75 121.8 123.625 71.025
117.15 74.32499999999999 57.975 120.125 48.175000000000004
131.85 74.67500000000001 73.725 72.94999999999999
117.4 81.09999999999998 104.4 148.29999999999998
113.025 27.549999999999997 56.37500000000001
50.275 164.475 238.575 99.72500000000001 154.2 36.425

176.54999999999998	35.275	144.125	65.2	66.52499999999999
	56.775000000000006	63.725	69.85	58.75	38.575
50.925	63.475	82.975	82.775\
FBXO11	1980.3	1319.6333333333334	1913.0	1351.7
1688.0333333333335	933.0	1371.1000000000001
2534.2000000000003	1155.3	1086.8	1286.5000000000002
1008.4	1168.1333333333334	1327.8	2500.7	1779.3999999999999
	1870.9333333333332	1415.2	934.6999999999999
1115.8333333333333	1898.1999999999998	1186.0666666666666
	1481.0666666666668	1503.7333333333333	1782.7
918.5666666666667	1293.6000000000001	1581.1333333333332
	1915.7	1198.2666666666667	1114.3333333333333
2524.0333333333333	1749.6333333333334	1372.1333333333332
	1591.3666666666666	1054.7666666666667
1189.7666666666667	1271.0333333333333	915.5333333333333
	1491.1000000000001	1786.3	1318.4666666666667
1537.5	890.9000000000001	1080.3666666666666
1673.3333333333333	1458.6666666666667	2458.2
2438.3333333333335	1127.8	1417.2666666666667
1174.5333333333333	1268.3	2724.5666666666666
1481.5333333333335	2007.4333333333332	1729.5999999999997
	1542.3666666666666	1614.0000000000002	1674.0
2485.3	1012.2666666666668	1488.8	1129.8999999999999
1449.2666666666664	1364.8999999999999	840.0	1333.3
1688.6666666666667	1243.6	1675.2	1332.6000000000001
1155.5000000000002	1165.8333333333333	1671.033333333333
	998.1	1457.8	1113.7333333333333	1017.5
3771.3000000000006	2452.6666666666665	2781.566666666667
	1286.0666666666666	1465.4000000000003	2066.2
3791.7999999999997	2330.2000000000003	1730.9666666666665
	889.4	1567.0666666666666	1062.2333333333333
2801.866666666667	1452.8	1489.1666666666667
1842.0666666666668	1663.6666666666663	1710.8666666666668
	1732.3666666666666	2094.266666666667
2082.6666666666665	1534.966666666667	1455.1666666666667
	1085.7\
PIAS2	339.6875	487.05000000000007	504.5875
363.125	353.04999999999995	546.7125	297.9375
240.98749999999998	333.65	440.01250000000005	427.7125
	301.975	299.01249999999993	470.5625
633.7125000000001	293.66249999999997	363.575	393.3375
	236.51249999999996	403.34999999999997
364.73749999999995	473.025	643.8	288.1	394.6
348.52500000000003	219.88750000000002	480.1	716.5375
	348.8125	254.95	703.6249999999999	618.25
331.125	471.5124999999999	360.7125	309.22499999999997
	558.5375	325.15000000000003	369.86249999999995
	474.87500000000006	615.4875	338.1875
421.65	284.625	538.5625	323.5250000000001	599.0625
	589.9625000000001	463.75	391.8625
361.7875000000001	534.6624999999999	625.1125
327.7375	693.0000000000001	419.12499999999994
459.95	459.6125	572.1375	609.3625	280.9625
	375.63750000000005	329.425	410.30000000000007

589.2625	285.2875	271.7875	475.48750000000007
	308.51249999999993	528.2750000000001	591.8125
	305.77500000000003	369.1	507.77500000000003
328.9	414.3375	355.1125	306.275	496.4
1002.1250000000001	452.5625	408.075	442.01249999999993
	412.3374999999999	542.075	558.375	615.1125
217.02500000000003	522.5875000000001	356.925	581.3875
	313.25	229.48749999999998	276.875	372.56250000000006
	351.15	321.5375	443.11249999999995	431.2375
	411.85	344.2875000000001	330.2625\
PIAS1	1278.3333333333333	505.4333333333334
872.2666666666668	940.5	708.3666666666668
826.6333333333333	1132.5	816.8000000000001
871.2666666666668	792.7000000000002	987.1333333333333
	949.5666666666666	629.1333333333333
800.5333333333334	1154.8333333333333	1252.8999999999999
	714.5333333333334	761.9	918.2666666666668
664.6	837.6	900.1	1254.2	1217.9333333333334
850.2333333333332	947.3333333333334	807.2333333333332
	1041.7	981.5666666666666	730.6666666666666
872.6999999999999	1108.9333333333332	1296.2666666666667
	1179.0666666666666	547.8666666666667
1463.1333333333332	805.7666666666668	1006.6
866.7666666666668	694.9666666666666	597.8
837.6666666666666	875.9333333333334	974.4
722.0666666666666	839.3666666666667	740.3666666666668
	698.0	861.1666666666666	932.5666666666666
949.3000000000001	1088.3333333333333	757.8666666666667
	916.5666666666667	750.2666666666668
937.3333333333334	1091.0666666666666	857.0999999999999
	1082.8666666666666	938.4	1033.4	932.3666666666668
	1140.3666666666668	967.4333333333333
1073.4333333333334	992.1	859.9	919.7333333333332
1614.5333333333335	843.9666666666667	910.6999999999999
	760.1666666666666	1327.5666666666666
711.2666666666668	1148.6000000000001	863.5666666666666
	752.4000000000001	1075.0666666666666
907.5333333333333	650.9333333333334	683.7333333333332
	1033.6000000000001	1257.7666666666667	1522.0
737.4666666666667	1045.7666666666667	756.3666666666668
	817.7333333333332	901.5333333333334
1087.2666666666667	986.3666666666667	1016.8666666666667
	1187.2666666666667	1522.5	1815.6000000000001
1499.0	1354.3666666666668	1383.8999999999999
1248.0333333333333	1241.6000000000001	1384.6333333333332
	875.6333333333332	1519.4333333333332\
LOC102723692	82.5	95.8	39.3	61.7	16.4	47.6	6.5
	65.9	61.1	29.1	64.6	113.1	20.8	43.2	68.5
	44.8	39.4	117.0	19.9	70.5	287.7	49.3
105.2	15.2	28.8	40.2	49.6	80.8	20.5	144.3	57.5
	178.1	131.2	41.8	47.7	47.4	55.2	37.6	46.6
	41.8	46.1	25.8	97.0	47.0	41.0	10.7	55.1
	18.9	21.9	152.7	58.4	19.2	67.8	6.6	14.2
	7.8	4.0	73.0	17.4	11.4	92.8	80.3	6.2

13.2 517.2 58.0 251.8 148.1 74.6 25.9 42.6
34.9 64.7 32.2 78.4 55.9 19.3 47.9 39.4
32.4 13.1 40.2 36.3 28.4 31.9 31.0 53.9
23.7 14.1 67.6 4.3 104.1 24.7 36.4 59.0
13.6 8.8 74.2 21.8 43.5 11.5 149.7
57.2\
LENEP 66.2 154.3 41.8 35.5 216.5 34.9 16.3
124.3 5.9 78.7 45.1 49.7 7.3 78.7 94.6 92.0
11.8 47.4 9.9 14.6 149.9 7.0 51.4 32.0
22.9 71.6 7.0 111.9 163.3 35.3 12.8 97.6
215.4 85.5 143.9 87.4 19.8 73.1 59.6
177.7 80.2 56.0 26.9 8.4 15.3 72.7 95.2 32.1
261.4 69.7 111.5 46.1 122.4 80.8 8.2
191.4 7.4 44.3 13.7 15.2 455.6 10.0 112.5 20.2
27.2 34.0 60.4 6.7 9.8 6.2 121.5 23.0
62.2 6.0 11.1 8.3 16.2 34.3 13.7 10.8
139.8 82.2 22.9 51.0 151.2 304.4 91.3 26.9
113.0 29.6 42.9 11.1 7.5 67.0 21.3 27.8
13.8 64.2 17.4 17.4 8.5 71.2 17.5\
NPAS4 4.25 17.75 6.5 15.1 18.7 9.95 8.7
25.65 8.4 16.8 11.1 24.599999999999998 10.3 7.25
26.65 26.549999999999997 11.149999999999999
12.15 14.5 9.5 58.5 26.75 15.25 8.6 8.15 7.7
10.15 25.9 16.05 7.65 25.25 17.05 13.7
11.05 16.45 13.700000000000001 5.800000000000001 34.7
6.05 13.9 8.25 9.2 8.3 6.800000000000001
23.299999999999997 16.65 9.45 25.349999999999998
19.7 32.15 12.25 21.85 26.6 30.85 14.25
19.65 8.05 49.45 9.899999999999999 15.75 54.7 9.1
62.449999999999996 29.45 11.3 41.9 5.2 20.9
42.75 8.95 33.35 12.950000000000001 8.65
5.800000000000001 11.8 6.85 15.1 20.15
8.100000000000001 10.7 9.2 18.25 5.1 9.8
30.65 55.9 15.15 32.6 14.0 29.4 5.3500000000000005
43.9 68.65 8.55 31.2 7.800000000000001
51.75 8.3 13.0 19.25 37.7 36.75 11.899999999999999\
LENG1 125.4 186.2 161.1 113.5 97.7 139.8 210.3 50.3
103.1 157.8 73.4 315.5 243.8 140.4 90.9
166.6 243.5 216.0 237.0 189.5 204.7 63.1 182.2
179.9 91.2 178.0 120.6 139.2 161.7 190.0 204.3
270.4 219.5 166.1 250.6 260.4 116.7 126.5 124.0
119.4 119.1 118.1 168.3 209.9 121.1 47.0 130.6 93.2
57.9 108.6 296.6 43.0 173.9 118.8 159.4
328.2 248.4 208.2 211.7 89.6 34.3 199.0 189.9 89.4
131.7 279.9 146.3 157.3 368.3 111.7 158.9
133.0 203.5 241.8 80.9 110.3 96.8 84.1 140.1 84.7
98.6 104.0 112.8 126.5 182.6 146.1 89.4
217.7 256.9 192.9 126.5 104.2 71.5 161.2 168.9
145.0 82.9 52.9 65.3 102.9 152.2 169.6 239.8\
MINPP1 1304.4 280.1 691.7 1690.9 735.0 525.4 1603.0
813.5 1163.3 425.2 1393.0 902.3 1207.1 657.1 934.8
1325.3 826.9 529.4 1030.9 865.8 167.4 874.2 168.5
1208.7 1235.7 591.3 1019.6 739.7 688.6 1494.9 1556.1
325.6 410.0 1341.0 632.7 673.5 1391.6 595.1 1146.2

```
1103.5	1569.0	986.4	790.5	1012.8	522.4	730.7	1155.5
2058.3	532.0	316.7	501.9	1252.5	1118.9	525.4	806.6
415.9	659.6	584.0	640.8	591.5	605.1	260.2	782.3
1096.5	497.9	610.0	748.0	799.5	410.2	869.6	284.3
788.9	628.0	849.3	633.5	1522.6	559.6	552.4	573.5
626.2	518.2	919.8	466.2	573.9	414.2	404.4	1002.0
484.6	457.7	576.4	533.6	739.3	815.2	1069.8	764.3
953.7	1093.1	844.7	793.9	1036.9	834.6	723.0	961.1\
DMTF1	1928.1	3913.0	3702.8	1658.4	3447.1	1446.2	1446.0
2645.6	1060.3	1857.3	1668.6	1534.8	1511.0	1953.4	4170.0
2575.7	2132.9	1903.8	1141.4	1951.7	3720.2	1885.0	2813.3
1531.5	1471.1	2827.2	1139.0	2371.6	4434.7	1342.7	1355.2
2123.2	2280.2	850.9	1253.3	2947.4	1360.0	2864.6	1260.0
794.1	2053.7	1739.8	2057.3	1695.3	1425.1	2711.9	2255.3
1266.5	5202.0	2243.8	1602.7	1041.3	1604.7	5401.7	2282.8
3403.8	2429.4	3676.6	2393.4	2981.0	1362.5	1906.1	1795.1
1449.8	3487.0	2495.2	1431.7	1251.8	2115.3	2072.2	4354.3
2769.2	2780.4	1218.9	2939.1	758.1	2763.3	1566.9	2049.5
2506.6	3831.2	2541.4	1933.7	1941.2	4765.9	1508.7	2443.6
3059.2	2294.0	1588.4	1512.9	5892.3	3121.7	2172.8	4310.9
3402.9	3693.6	2878.0	5261.5	4535.7	3888.5	5385.9	2655.5\
GRASPOS	8.7	92.2	13.7	9.5	20.1	11.0	6.7	9.8
	9.2	16.1	20.9	9.9	70.0	17.4	42.7	2.5
	16.8	4.6	9.9	13.1	100.2	46.3	136.2	26.2
	11.7	12.0	3.5	35.4	21.5	39.7	22.0	20.7
	5.1	6.4	23.1	54.1	5.5	13.7	20.9	6.3
	20.4	41.9	29.1	2.4	6.4	17.7	17.3	9.9
	21.0	57.1	15.9	21.4	22.2	17.2	8.9	22.9
	4.5	1.9	7.4	129.6	194.2	52.6	36.4	11.9
	15.8	2.9	54.0	8.2	33.1	39.6	166.2	6.6
	9.6	2.0	2.4	5.4	23.7	6.5	8.5	22.0
	55.5	9.3	63.3	15.6	99.7	33.2	12.3	36.8
	7.4	11.9	5.7	19.5	6.7	53.1	18.6	17.0
	43.4	33.4	16.9	35.8	8.1	35.9	4.1\
PKP2	297.15	228.15	240.29999999999998	545.65	244.0
202.9	259.9	277.75	316.59999999999997	173.85	548.45
432.0	625.5	186.0	332.7	370.2	201.95	272.59999999999997
	476.8	138.45	159.4	518.55	484.6	417.59999999999997
	851.15	872.9	657.7	84.60000000000001
487.09999999999997	145.7	328.34999999999997
303.34999999999997	98.1	604.15	210.7	370.70000000000005
	533.2	171.45	159.7	484.2	86.6	438.7	71.45
278.55	324.3	743.3	290.55	235.25	386.29999999999995
237.2	293.95	928.2	415.45	249.35	488.25	249.1
172.70000000000002	529.25	156.25	257.3	178.7	356.5
131.75	221.95	286.45	68.5	407.25	193.85	209.4
635.0500000000001	159.75	265.3	134.45000000000002
594.55	454.0	528.35	154.45000000000002	565.8	317.55
238.35	747.0	478.54999999999995	762.85	104.2	229.4
289.35	832.6	279.2	159.64999999999998	108.35
424.09999999999997	195.35	293.40000000000003	422.6
133.75	483.1	291.79999999999995	407.29999999999995
344.2	278.2	393.84999999999997	424.4	321.25\
PKP1	28.7	29.4	30.85	17.1	45.949999999999996
```

5.949999999999999	5.25	8.6	10.4	24.1	9.95	9.45
	5.6	22.25	38.5	33.949999999999996	8.1
35.050000000000004	4.55	59.05	40.75	7.25	38.2	6.1
	60.65	23.0	19.15	23.4	73.35	24.5	8.0	57.4
	9.65	9.6	94.14999999999999	41.6	22.6	54.3
	123.2	20.650000000000002	51.4	23.95	5.8	15.4
	8.65	30.15	15.3	15.65	33.900000000000006
17.05	13.05	55.55	11.0	92.2	15.6	13.05	49.75
10.15	23.349999999999998	7.449999999999999	412.25
34.55	10.3	20.95	18.25	16.05	30.950000000000003
182.0	57.8	25.15	52.349999999999994	23.450000000000003
	11.75	6.8	25.5	14.850000000000001	11.85	10.5
	14.35	18.65	25.3	23.85	31.0	9.2	25.9
139.35	9.75	16.9	88.65	24.35	44.3	42.050000000000004
	33.0	8.0	7.0	26.35	17.349999999999998	4.55
	20.05	70.5	17.75	46.949999999999996	20.0\
PKP4	935.6	1535.8200000000002	1590.48	583.42
1777.8400000000001	874.04	1337.98	1109.1	1645.86	1365.1
837.04	1790.2	919.6200000000001	1238.08	1109.66	1297.04
1383.6	1420.7	921.2	1531.7	2155.84	2194.1400000000003
1077.3	1005.9599999999998	2449.1800000000003	1324.6
1565.42	1876.7	1258.42	1585.62	2407.7400000000002	1449.4
1896.2599999999998	2615.2799999999997	1038.3600000000001
	634.68	1034.3400000000001	2597.88	1895.64
1359.6399999999999	1544.86	1181.28	1457.5	2661.46	1385.08
3112.0	1853.3200000000002	1641.48	2512.34	2404.04	2099.06
1496.6799999999998	2021.72	1439.1	2224.94	1068.8
3434.8199999999997	892.9399999999999	1306.3600000000001
	2623.42	2508.84	936.7	1674.8	2040.5	1815.9	1197.08
2294.58	3112.5	2149.56	1732.72	2275.98	1436.78	1720.36	1268.1
1226.42	1356.52	1451.22	1854.6599999999999	2121.2599999999998
	1636.24	1120.7	1605.5	771.0799999999999	1465.9
2345.18	1407.78	742.0	988.5599999999998	1650.28
1798.7800000000002	1158.6799999999998	2307.1800000000003
	1903.36	1283.44	1380.3600000000001	2510.7400000000002
	2214.7200000000003	1707.8399999999997	3415.56
2314.98	3244.8599999999997	913.86	2761.9199999999996\
LOC401463	33.05	114.75	14.950000000000001
11.600000000000001	106.95	24.35	25.95	89.55000000000001
	30.65	61.4	9.2	93.85	49.099999999999994	42.8
	131.95	84.5	53.85	33.95	20.2	44.85	257.05	16.1
	39.0	27.700000000000003	24.55	36.05	23.4
40.35	98.85	35.65	35.15	90.4	100.8	70.85	64.9
122.05	40.400000000000006	69.6	17.700000000000003
33.15	56.099999999999994	30.5	42.45	11.350000000000001
	53.599999999999994	103.2	114.85000000000001
12.45	66.1	40.900000000000006	41.900000000000006	28.7
	67.05	107.25	95.0	127.25	17.55	41.5
96.44999999999999	32.8	337.1	102.3	52.75	92.05
46.599999999999994	97.2	30.5	100.75	10.8	51.3	83.3
	76.85	59.0	44.3	77.15	30.7	10.65	21.7	31.2
	38.849999999999994	64.2	42.6	19.049999999999997
	80.5	111.05	122.65	62.5	89.65	99.8	122.3
20.85	88.69999999999999	117.3	27.65	22.1	23.85

28.85   36.5   82.2   91.45   41.05   56.9   43.45\
PKP3   106.80000000000001   98.60000000000001   95.15
169.70000000000002   108.85 200.35 84.8   55.3   158.95
222.75 127.8   142.65 187.39999999999998   115.25 136.15
116.6   71.85   243.89999999999998   313.35 238.29999999999998
        101.0   88.85   220.2   112.1   94.8   167.20000000000002
        255.95 105.05 92.55   179.10000000000002   145.05
183.75 176.9   149.95 265.0   83.4   135.35000000000002
157.3   134.85 190.5   108.6   159.79999999999998   24.75
107.64999999999999   150.85 144.2   97.05   86.55000000000001
        207.10000000000002   61.35   164.0   210.45000000000002
        174.6   104.5   97.85000000000001   103.4   74.0
131.9   58.75   69.35000000000001   195.04999999999998
113.75 134.55 163.45 20.75   188.7   171.1   238.85000000000002
        223.35 139.79999999999998   161.65 106.5   73.3
106.05000000000001   75.95   151.3   60.25   82.5
45.349999999999994   63.95   183.35000000000002   85.1
145.5   228.39999999999998   25.2   65.39999999999999
123.19999999999999   207.8   244.8   139.6   122.2
54.050000000000004   174.6   163.95 102.65 115.8   155.25
128.8   90.7   116.2   104.75 48.4   167.05\
MIR210HG   335.1   328.05 621.4000000000001   292.4
310.05 560.35 158.4   94.94999999999999   164.45 611.75
237.39999999999998   447.15 483.15000000000003   563.55
303.45000000000005   201.9   125.05 510.54999999999995
499.4   420.2   436.7   259.9   431.70000000000005   190.1
265.05 575.4   353.4   311.15 1026.45 317.0   70.75
1613.0500000000002   1812.1 157.8   276.95000000000005
578.2   527.8   1671.2 590.8   38.95   93.1   652.0   309.75
508.55 330.1   309.5   760.65 220.5   683.6500000000001
555.1   593.0   446.5   849.35 1555.65 205.75 263.8   342.55
507.45 638.2   350.75 69.75   380.7   526.35 249.35 368.1
712.05 194.95 414.35 1023.0999999999999   213.35 1338.6
564.3   203.15 33.2   276.15 174.15 759.1   317.95 88.2
227.0   114.75 805.85 567.2   133.75 1131.5500000000002
3390.55 555.8   277.95 734.0   938.7   158.05 293.0   229.5
280.3   113.95 161.2   190.60000000000002   316.55 566.05
455.65 232.95 785.2   166.9\
CR936796   127.13333333333333   147.79999999999998
758.3666666666667   193.73333333333335   141.4
46.199999999999996   36.50000000000001   339.53333333333336
        165.0   234.1   524.8666666666667   109.7   160.9
657.3666666666667   3297.933333333333   990.4000000000001
        182.0333333333333   94.83333333333333
176.73333333333335   447.3333333333333   285.03333333333336
        129.20000000000002   265.59999999999997
184.46666666666667   47.13333333333333   128.26666666666665
        22.46666666666667   485.5333333333333
969.9333333333334   236.13333333333335   201.9333333333333
        411.9333333333334   136.86666666666667
638.6333333333333   1793.3 328.73333333333335
67.76666666666667   312.90000000000003   225.46666666666667
        168.83333333333334   263.93333333333334   442.7
770.1666666666666   335.2666666666665   109.16666666666667

406.7666666666665	341.5666666666666	575.1
579.8333333333334	869.2999999999998	215.7666666666665
60.1	80.93333333333334	624.4333333333334
287.7666666666665	564.9666666666667	258.09999999999997
128.4	73.33333333333333	379.9666666666667
210.73333333333335	236.1666666666666	860.7666666666668
154.2666666666665	812.0333333333333	1337.2
43.53333333333333	296.7666666666667	803.9	219.4
955.8333333333334	472.43333333333334	243.23333333333335
583.6666666666666	444.8666666666666
152.5666666666666	234.70000000000002	148.0
481.0666666666666	464.1333333333334	1409.4333333333334
223.6	909.0	1457.5333333333335	74.7
417.8666666666666	37.0666666666667	1999.3666666666668
100.46666666666665	100.13333333333333
695.0666666666666	1151.3	582.2333333333333	361.2
570.7666666666668	982.5666666666666	338.66666666666663
1310.9333333333332	1230.4666666666665
1736.2333333333336	834.5333333333333	521.3666666666667
1172.5\
BLK	26.0	24.35	43.65	10.8	118.5	30.349999999999998
4.550000000000001	55.050000000000004	33.4	13.7
48.2	50.45	24.8	38.95	78.8	12.6	15.45
78.15	22.25	16.8	51.3	40.650000000000006	69.6
52.55	39.45	35.75	48.2	91.1	37.9	25.200000000000003
29.450000000000003	107.0	64.8	10.95
67.94999999999999	12.649999999999999	25.8
99.94999999999999	20.0	10.1	29.9	40.15	58.35
21.95	16.65	37.25	21.75	5.5	108.35	24.7	45.85	15.2
47.3	118.35	51.05	16.2	29.5	102.0	21.5
95.25	189.25	84.15	30.85	51.099999999999994	65.55	75.5
41.05	13.850000000000001	38.6	37.4	157.45
80.75	76.95	30.650000000000002	53.849999999999994
44.199999999999996	46.85	50.3	35.25	78.85000000000001
40.650000000000006	12.0	36.9	64.45	89.95
50.900000000000006	23.75	27.05	32.75	19.05
52.349999999999994	87.35	69.60000000000001	60.35
47.25	41.099999999999994	42.0	41.25	60.0	45.45	50.0
47.15	10.049999999999999\
DNMBP /// LOC101927371	21.4	84.7	47.2	68.5	221.6	1.7
21.1	55.5	36.5	6.2	29.5	159.8	80.7	23.6
11.8	28.5	42.2	17.7	94.1	16.8	21.8
100.2	176.9	13.6	48.5	48.2	100.4	35.2	59.4	21.6
22.9	72.8	167.8	17.6	2.8	44.3	3.1
387.5	39.7	38.0	14.0	44.2	15.5	101.4	3.2
195.5	24.8	4.5	137.9	67.6	94.1	31.7	86.2	7.2
33.6	2.9	143.3	41.2	67.5	231.0	48.9	10.7
76.3	34.8	77.9	76.8	40.0	81.4	20.6	49.2
164.3	91.5	77.5	20.7	4.3	30.4	43.3	43.6
54.1	7.9	50.2	38.0	75.0	2.3	91.3
103.3	38.4	6.0	117.2	96.9	31.1	32.4	39.5	26.0
5.2	5.9	29.4	19.3	43.7	123.2	10.4	22.5
25.2\
LOC100505716	47.0	91.7	42.3	39.4	60.5	8.2	12.5

	188.3	8.2	136.1	6.8	19.6	27.6	8.6	30.4
	38.7	33.6	61.3	11.2	8.3	57.3	14.1	31.4
	12.8	47.4	9.0	67.0	42.0	29.1	63.1	7.0
	20.3	14.6	16.7	11.4	13.0	20.8	109.5	25.2
	38.8	38.9	56.0	9.8	15.4	4.8	47.0
125.4	83.3	108.7	56.5	73.4	8.7	23.5	51.6	17.1
	68.3	50.2	11.5	82.2	3.7	14.6	41.1	5.4
	70.6	85.6	13.6	37.5	62.5	26.7	14.1	66.0
	8.8	34.3	49.2	11.6	33.0	14.9	5.9	37.8
	38.5	40.9	58.1	30.8	47.7	20.6	93.8	47.1
	19.3	22.8	39.5	19.3	69.4	31.0	33.1	10.9
	9.2	43.8	28.0	8.9	12.1	9.6	54.3	6.3\
LOC100505715	15.3	198.5	140.1	36.2	137.8	426.2	72.0
	287.1	29.6	149.4	39.2	178.6	94.8	1806.1
694.1	662.7	610.4	214.5	135.6	125.7	771.4	76.7
186.5	84.4	37.7	95.1	46.2	270.7	232.2	602.0
248.9	228.8	315.3	284.1	541.2	34.3	121.3	289.6
105.3	107.6	90.9	139.1	128.4	153.2	211.8	172.0
201.7	358.5	303.0	196.9	37.0	78.4	74.8	476.3
255.6	196.8	95.3	1467.8	88.0	293.7	676.1	200.6
717.5	305.0	361.1	191.0	321.9	358.9	120.2	141.8
285.0	2147.1	147.7	80.8	407.3	181.5	189.3	53.9	80.6
	22.6	484.0	89.8	712.0	196.2	168.9	195.2
181.0	239.0	786.0	420.7	65.8	421.7	395.0	162.1
376.8	291.3	250.3	181.9	210.9	921.0	567.9	472.5
437.9\
KCNE1	4.3	70.05	299.6	72.55	43.65	36.8	10.25
42.400000000000006	106.75	584.3	475.25	8.7	338.05
87.85000000000001	64.95	28.7	29.25	294.34999999999997
	64.94999999999999	10.399999999999999	65.2
326.09999999999997	491.75	89.65	74.05	46.9	10.7
205.79999999999998	379.75	9.35	22.15	145.0	228.3
302.0	102.7	158.9	135.45	62.7	114.65	152.5	7.6	32.4
	266.05	11.149999999999999	443.55	242.25	89.95
46.15	78.55000000000001	57.3	219.0	247.64999999999998
	47.849999999999994	126.55000000000001	289.65
93.95	103.4	78.9	185.25	78.95	640.45	243.3	78.35
478.84999999999997	52.1	89.55000000000001	94.35	5.2
	441.0	776.75	190.10000000000002	279.15	79.5	54.8
	128.35	595.35	914.0999999999999	76.95	161.75	62.2
	67.35	36.400000000000006	30.0	32.099999999999994
	94.7	108.35000000000001	51.35	119.7	67.15
72.69999999999999	39.0	58.9	83.65	213.54999999999998
	30.400000000000002	102.64999999999999	27.5
299.84999999999997	42.5	85.5	36.7	20.8	21.7\
BLM	1680.7	947.9	279.2	705.6	1107.8	683.9	744.4
548.0	1069.6	792.5	689.9	501.5	1002.9	756.4	1410.8
310.8	499.3	573.4	587.7	736.0	722.1	1043.6	551.3
576.6	1332.5	724.5	899.2	717.8	670.5	482.2	231.9
872.9	691.1	384.0	612.7	282.7	906.2	858.3	521.4
530.5	998.7	397.6	537.9	348.0	920.1	1195.7	725.5
1011.2	978.5	330.6	922.2	976.4	532.1	2022.4	887.2
520.2	632.0	1174.9	688.0	838.0	1155.9	821.8	377.2
619.9	424.6	429.3	344.7	293.5	942.1	626.1	638.2

752.7	365.6	358.4	771.4	415.0	364.6	570.9	405.3
959.4	1347.4	656.9	1709.3	800.6	965.4	779.2	878.9
818.1	1130.7	990.8	983.7	881.6	425.8	175.0	186.2
191.0	363.3	176.3	305.4	328.2	260.3	571.0	310.5\
KCNE2	7.8	51.1	120.6	16.1	67.0	48.6	18.2	26.5
	25.2	18.7	18.9	16.9	11.7	73.6	126.8	24.0
	12.6	8.6	8.0	14.5	22.3	32.3	10.7	13.5
	8.9	10.0	12.6	31.3	23.4	10.1	10.9	40.4
	11.6	64.9	113.8	17.1	29.3	15.2	39.6	11.5
	4.9	52.6	8.8	11.9	9.2	23.0	17.3	15.8
	44.0	20.1	10.0	42.3	15.1	135.0	14.1
207.3	13.3	30.8	17.1	11.0	418.2	31.2	17.2	25.7
	37.4	24.1	7.2	20.7	21.7	25.5	31.8	20.2
	73.6	14.0	57.8	13.7	129.8	11.3	9.2	8.0
	28.1	19.3	10.1	13.7	30.0	165.3	104.5	16.8
	23.6	34.0	12.2	12.2	18.4	9.8	20.9	27.0
	23.6	12.3	25.0	24.4	9.8	10.1	14.7\
PDZRN3-AS1	105.2	12.0	6.9	62.2	172.9	10.1	5.5
	14.1	52.2	17.8	7.2	292.2	21.5	3.5	31.3
	211.1	25.7	95.3	76.0	93.5	230.0	82.6	44.3
	102.0	62.1	16.9	77.9	65.5	41.6	282.5
168.6	24.2	135.2	178.9	84.5	64.9	16.8	34.2
209.3	44.7	8.4	7.1	129.0	4.8	22.3	13.4	23.4
	24.2	42.6	261.7	63.2	32.2	29.9	24.3	29.8
	17.4	161.8	43.0	92.5	102.7	157.3	17.3
113.1	21.3	118.6	26.0	237.6	173.0	7.3	35.6	38.8
	34.8	139.2	16.2	28.0	51.0	131.6	26.0	22.9
	24.9	8.6	9.7	9.7	12.9	15.3	33.1	25.9
	28.3	7.7	22.3	27.1	122.5	25.1	126.6
148.7	147.8	45.7	80.8	15.4	269.3	108.1	73.7
88.5\
KCNE3	561.0333333333334	594.8	977.6	186.0333333333333
	84.39999999999999	54.56666666666667	332.8
4253.7	203.46666666666667	1124.8333333333333	540.7
516.8000000000001	135.16666666666666	2938.233333333333
	423.79999999999995	505.7	927.0666666666667
197.53333333333333	94.56666666666668	319.3666666666667
	165.63333333333335	576.0333333333333
284.2333333333333	340.6	81.7	65.1	335.5666666666666
	467.46666666666664	661.9333333333333
218.36666666666667	89.56666666666668	850.2666666666668
	280.1666666666667	206.69999999999996	1358.0
472.0666666666666	3507.033333333333	369.03333333333336
	157.03333333333333	772.9333333333333
437.5666666666666	137.2	871.4	313.03333333333336
1971.0666666666668	513.8333333333333	654.6
897.3000000000001	259.8	138.46666666666667	541.4
522.5	329.86666666666673	928.3666666666667
201.10000000000002	200.9666666666667	357.5
785.7333333333335	1099.6	85.63333333333333
566.6333333333333	747.1	500.53333333333336
239.16666666666666	151.20000000000002	156.53333333333333
	143.86666666666667	355.43333333333334
314.8666666666667	2874.7999999999997	1362.966666666667

175.73333333333335 142.73333333333332
84.10000000000001 155.86666666666665 138.63333333333335
1004.4999999999999 198.9333333333333
296.53333333333336 327.9666666666664 1022.6333333333332
373.0333333333333 961.3666666666668
1108.0333333333333 1086.9666666666665 251.9666666666666
398.8333333333333 598.6333333333333 289.7
744.4333333333333 229.70000000000005 197.2666666666665
421.5666666666666 123.80000000000001
260.03333333333336 229.8666666666665 235.63333333333333
157.83333333333334 440.1666666666667
285.03333333333336 179.5 205.7666666666665
132.1666666666669\
KCNE4 52.8666666666667 178.83333333333334
60.5666666666666 78.03333333333335 169.0 126.7
108.2 572.5333333333333 139.83333333333334
216.3666666666667 28.7 117.8 90.13333333333333
107.13333333333333 83.0 52.93333333333334 219.6
92.13333333333333 16.599999999999998 102.8
123.5666666666666 57.0666666666666 243.6
336.03333333333336 28.666666666666668 41.3
42.833333333333336 231.73333333333335 83.89999999999999
168.76666666666668 421.3 109.5 89.3 370.0
147.0666666666666 64.63333333333334 408.6000000000001
201.0 108.3666666666667 476.0666666666666
82.63333333333334 42.9 1387.3333333333333
184.53333333333333 94.43333333333334 114.0
181.20000000000002 133.8666666666667 74.8
138.70000000000002 84.89999999999999 112.10000000000001
136.9666666666667 48.5 157.83333333333334 58.5
117.3 56.73333333333333 81.03333333333333
206.13333333333333 270.9666666666664 95.7666666666667
73.33333333333333 75.0666666666666
138.63333333333333 45.23333333333333 173.6 93.5
193.4333333333333 47.6 277.4666666666664
99.7666666666667 265.7 129.4 170.23333333333332
88.93333333333334 221.9333333333333 180.9333333333333
157.93333333333334 1000.6999999999999
101.13333333333333 52.13333333333333 35.16666666666664
68.93333333333334 139.3666666666665
42.9666666666667 103.3666666666667 461.8666666666666
159.23333333333332 44.53333333333333 17.0
558.1666666666666 199.4333333333333 142.1
163.33333333333334 60.366666666666674 60.53333333333334
136.70000000000002 47.53333333333334 55.4 71.7
771.1666666666666 29.933333333333334\
ZC4H2 278.7 134.13333333333333 113.43333333333332
146.13333333333333 200.63333333333333 20.833333333333332
137.33333333333334 201.4 165.9 166.9666666666667
117.0 172.53333333333333 116.09999999999998
39.73333333333333 175.8666666666667 46.199999999999996
113.3 109.5 72.89999999999999 111.39999999999999
167.7 30.899999999999995 177.3666666666665
122.60000000000001 70.0 19.033333333333335 183.0

147.9	60.7666666666667	52.63333333333333
180.26666666666665	41.233333333333334	22.600000000000005
	248.53333333333333	46.53333333333334
74.7666666666667	26.733333333333334	130.9
119.1666666666667	268.23333333333335	14.9
306.40000000000003	82.3666666666667	187.4333333333333
	60.6	16.133333333333333	111.43333333333334
26.400000000000002	115.0666666666666	209.4
77.73333333333333	26.03333333333333	110.6666666666667
	159.0666666666667	127.60000000000001
36.699999999999996	171.03333333333333	41.5666666666666
	129.3666666666667	125.33333333333333
104.13333333333333	179.1666666666666	139.63333333333335
	37.9	162.63333333333333	188.70000000000002
173.1666666666666	94.43333333333332	46.9666666666667
	114.2666666666665	224.9	19.566666666666666	92.5
	34.63333333333333	41.4	87.8	128.63333333333333
	44.9	79.13333333333333	166.2666666666668
163.20000000000002	49.9666666666667	103.5
14.266666666666666	28.4666666666667	53.9666666666667
	17.866666666666664	193.5666666666663
29.566666666666666	81.63333333333333	179.29999999999998
	178.1	153.1666666666666	136.1	155.1	131.1
137.3666666666667	154.9666666666667	81.2666666666667
	173.13333333333333	189.80000000000004	131.9
199.23333333333332\
LOC100505718	28.6	84.7	16.1	18.6	6.6	4.7	27.3
	5.8	22.4	12.6	15.2	4.5	19.3	42.3	7.1
	28.9	20.5	4.2	3.7	6.3	123.1	44.9	6.5
	4.8	19.9	58.2	33.0	22.2	60.4	5.2	27.1
	18.4	29.2	14.0	5.1	4.1	42.1	51.7	36.9
	11.1	30.2	3.3	54.6	25.2	38.5	71.8	8.2
	21.0	18.3	24.6	78.9	59.1	6.2	46.7	5.0
	42.0	17.7	6.0	4.5	14.0	13.9	4.4	5.3
	68.8	51.6	42.2	2.4	18.8	36.4	37.8	27.3
	52.1	29.8	15.5	13.8	11.1	35.8	4.7	25.5
	18.5	14.7	13.8	7.0	6.0	101.4	19.4	9.8
	24.1	10.1	76.7	31.8	26.0	3.9	1.5	7.9
	19.6	7.9	4.6	16.4	4.3	7.5	11.8
29.7\
LOC283665	44.3	35.2	59.9	69.8	42.4	22.6	21.4
	48.6	34.1	60.8	19.5	22.3	43.7	87.0	22.4
	12.8	19.8	57.1	3.9	9.4	26.2	5.5
118.0	15.7	59.1	116.7	8.5	130.8	46.7	24.5	26.3
	171.0	21.0	19.4	31.6	154.9	18.3	74.2	74.1
	31.2	36.6	3.7	52.3	7.4	81.4	108.1	20.8
	44.6	96.6	76.7	11.2	23.8	12.2	63.1	70.8
	58.2	3.7	21.9	15.5	89.8	359.9	133.5	89.0
	16.6	68.9	16.3	16.2	52.2	16.8	70.1	39.4
	133.8	9.3	2.7	43.3	70.8	73.0	9.5	16.7
	60.7	54.5	15.8	14.0	49.9	26.2	139.7	35.9
	46.8	97.6	32.9	7.2	9.5	63.5	7.6	12.2
	34.4	69.6	8.7	81.3	75.4	46.7	18.2
89.9\

IREB2 905.275 232.02499999999998 735.325 859.875 571.05
1013.6999999999999 944.7 326.5 620.125 307.95 849.25
533.775 643.325 549.1500000000001 494.72499999999997
631.025 327.84999999999997 508.5 604.5999999999999
528.525 315.5 546.1 369.525 890.725 743.2000000000002
457.9 852.1500000000001 585.7 683.9499999999999
553.2500000000001 608.3249999999999 451.45000000000005
556.3000000000001 413.425 148.5 396.7 664.975
703.05 663.0749999999999 385.84999999999997 767.825
578.8249999999999 705.3249999999999 561.4 438.3
427.75 596.7499999999999 468.42500000000007 345.75
596.05 579.875 673.1499999999999 501.45000000000005
468.65 422.57499999999993 446.95 377.525 733.4000000000001
667.8249999999999 518.7750000000001
421.59999999999997 565.85 608.375 617.425 583.8 475.625
449.15 565.35 387.15 466.70000000000005 434.75
531.6000000000001 557.6 811.475 458.0250000000001
549.925 443.22499999999997 564.9999999999999 540.45
553.45 362.325 663.525 562.0749999999999 613.95 483.975
434.1 509.925 300.075 414.575 245.45 530.15 438.75 540.225
718.1 885.5999999999999 791.075 754.75 882.55 741.675
670.6249999999999 756.1249999999999 507.725
690.7250000000001\
GSTT2 102.0 157.0 118.3 31.1 214.9 170.2 122.3 10.8
8.4 5.5 10.0 7.0 94.6 10.1 11.8
362.4 24.8 6.7 73.2 90.4 51.1 3.8 95.2 74.2
12.4 175.1 12.2 17.2 106.6 9.5 46.2 16.8
392.3 62.5 14.1 97.2 72.3 7.4 64.5 11.1
595.3 149.3 16.7 172.0 54.0 11.9 12.4 97.7
197.7 97.3 8.6 10.3 13.3 92.8 75.7
171.3 15.6 10.3 197.0 177.9 121.9 8.4 44.1 21.0
57.6 214.9 88.0 132.5 146.9 7.7 205.4
448.3 80.9 110.0 136.9 126.1 32.3 5.1 207.2 91.8
231.3 133.0 113.7 94.6 127.3 244.0 199.4
110.1 9.1 66.3 159.6 218.7 368.5 596.0 320.6
133.1 129.4 650.5 184.1 602.5 164.7 116.3 195.1\
LOC100289495 23.5 25.8 15.8 8.7 17.2 60.4 9.7
37.5 68.3 37.5 31.8 19.3 5.0 34.8
129.8 107.0 19.5 92.8 66.7 21.1 60.8 44.1
161.5 17.2 42.6 18.8 61.1 26.2 38.9 30.9 15.9
50.1 16.6 55.2 47.0 35.4 23.4 137.8 10.0
82.3 96.4 14.0 59.9 14.1 15.5 102.1 6.3
11.9 45.1 15.2 176.9 42.1 73.6 40.4 27.2
93.0 100.4 30.5 43.5 49.0 91.5 19.5 21.1
13.3 27.4 27.4 10.3 14.8 89.4 25.3 87.6
24.4 13.2 13.1 10.8 61.0 31.1 17.9 28.0
25.0 25.2 22.0 77.2 44.4 190.2 36.6 53.5
39.4 24.5 82.3 51.5 77.5 20.5 14.4
109.9 29.2 108.8 47.5 7.7 43.0 36.4 28.8
86.5\
FBXO45 1014.5666666666667 1757.3999999999999
353.0333333333333 945.0666666666666 695.2666666666668
1502.7 922.3333333333334 2980.0333333333333
1618.066666666666 1172.7 838.0333333333333

1282.5666666666666	1037.3	878.3000000000001
2294.5666666666666	458.3666666666666	1158.7333333333333
	973.0999999999999	795.9333333333334
728.5666666666666	356.76666666666665	1692.0666666666666
	587.8000000000001	934.8333333333334
1519.5333333333335	822.0333333333334	1049.9666666666667
	631.6666666666666	492.93333333333334
1409.6000000000001	1011.1666666666666	781.9333333333334
	677.8333333333334	815.8000000000001
1944.0333333333335	666.6999999999999	1507.2333333333333
	490.56666666666666	1331.8999999999999
1213.2666666666667	1504.5	1692.8666666666668	578.5
1162.6666666666667	1153.5	830.4	1383.4333333333334
2907.0	419.76666666666665	740.9666666666667
1129.5333333333335	1011.6999999999999	864.0666666666666
	1392.3999999999999	667.5666666666666	474.0
859.7666666666668	463.3333333333333	820.4333333333334
	514.0666666666666	334.59999999999997
815.6333333333333	703.7000000000002	686.1666666666666
	577.4	652.3666666666667	1013.1333333333333
1000.3000000000001	419.6666666666667	604.8000000000001
	285.8	904.0	557.6	751.4333333333334
416.40000000000003	781.0666666666666	608.2666666666668
	1127.3333333333333	875.4333333333334
3648.2999999999997	611.5	3075.1	941.1333333333333
1288.4333333333334	566.0666666666667	989.7666666666668
	1965.2333333333336	587.1666666666666
1708.2333333333333	1579.7666666666667	1916.8666666666668
	522.4	587.5	437.0	505.26666666666665
393.7666666666667	548.9666666666667	660.5
323.0666666666666	419.40000000000003	475.7333333333333
	664.5333333333333	653.7666666666667\
GSTT1	129.3	50.550000000000004	304.4	182.2	304.4
791.45	9.6	477.2	251.85	425.0	192.25	367.0	206.7
44.15	37.2	522.5999999999999	431.6	188.85000000000002
	545.75	266.5	487.45	405.5	225.4	36.1	355.85
290.85	294.55	361.65	246.39999999999998	250.2
659.6999999999999	215.75	314.35	483.2	463.45	371.75
677.2	223.0	275.8	25.55	261.05	6.949999999999999
477.85	210.0	629.8499999999999	282.35	13.55	366.1
308.9	573.5	514.95	303.5	464.85	327.85	468.05	349.95
71.25	215.05	297.1	21.700000000000003	83.65	291.25
288.55	297.65000000000003	338.9	346.65	323.65000000000003
	418.1	13.8	630.4	474.2	280.95	418.75	301.15
75.25	403.85	339.7	563.8	321.45	343.95000000000005
87.95	44.4	73.19999999999999	249.35000000000002
268.5	171.45	473.7	288.45000000000005	40.45
134.35000000000002	160.9	349.6	46.0	767.0	522.7
808.0	404.8	55.6	853.2	41.05	456.05	480.40000000000003
	498.5\
C6orf25	31.2	13.2	79.1	66.3	205.7	3.1	8.7
126.8	15.7	93.4	61.0	14.2	54.1	16.1	27.1	60.9
	60.1	16.2	48.4	55.8	112.9	21.3	151.9	46.6
	57.6	5.9	15.3	77.1	7.0	7.1	3.2

100.1 64.6 15.5 47.9 40.2 4.5 11.1 5.3 20.9
39.7 13.9 18.7 2.8 9.3 27.5 27.7 54.1
58.4 53.6 10.2 84.6 41.7 51.3 72.1 8.6
11.8 16.9 58.8 82.9 80.8 87.9 13.7 35.6
13.2 124.4 9.2 5.8 11.8 24.9 31.6 33.8
10.4 15.3 26.2 49.2 106.2 31.2 39.5 31.1
47.0 12.2 15.9 47.9 120.0 49.3 17.2 25.5
54.9 109.1 3.9 151.3 61.9 68.0 11.5 6.5
6.2 40.3 20.7 48.1 45.0 60.8 41.9\
FBXO46 1051.6 1594.8 1071.5 810.9 1149.8 1102.8 640.1
1389.3 1279.8 1255.8 1582.9 1003.5 959.6 1472.6 11740.0
937.1 1219.6 1516.3 464.8 1273.3 2770.5 987.9 1804.4
1241.2 1213.1 1043.3 996.4 1304.7 816.1 1306.8 989.2
1395.8 901.3 1069.9 1461.9 1028.4 921.6 1751.1 1052.6
928.2 1372.2 975.7 598.1 611.2 656.4 979.6 1284.7
1106.9 1526.4 1525.4 1209.9 1076.8 1511.5 2860.2 1413.4
1269.6 937.0 1292.3 1199.8 636.8 1725.5 941.9 835.1
1086.5 1172.7 1620.1 945.1 605.9 1361.1 627.7 1016.5
886.9 1126.3 856.7 911.5 1016.9 1903.8 1054.3 1266.7
1086.5 1301.1 1254.5 3602.5 1944.5 2648.2 2361.7 2315.8
1807.9 1791.1 1358.1 956.2 2214.4 957.5 1167.0 1300.6
1273.3 1161.6 754.0 879.1 1049.3 1343.0 1455.3 1252.7\
FBXO43 63.7 1042.5 167.0 92.6 222.5 243.1 18.1
285.0 77.5 137.7 25.7 64.3 101.5 116.9 320.9
208.9 86.4 220.0 100.9 67.2 211.4 142.6 406.0 85.2
133.6 181.3 30.4 424.0 124.8 87.0 109.3
529.0 351.7 96.5 56.5 134.8 111.3 187.9 26.0
404.3 131.1 73.4 168.7 58.7 202.0 291.9 144.0 73.3
270.4 221.1 114.6 265.7 141.2 178.6 350.1
216.0 58.1 234.3 153.5 344.0 141.4 256.3 75.9
118.1 147.2 160.3 103.9 79.6 137.1 291.2 350.8
486.7 75.6 53.2 184.7 84.8 234.3 105.0 133.8
891.9 602.7 217.0 139.5 52.3 213.3 241.2 833.5
292.1 117.4 85.5 65.9 442.5 91.9 63.8 58.1
113.8 191.7 106.7 106.7 231.5 129.4 317.0 106.8\
FBXO44 105.5 100.0 124.94999999999999 52.2 174.5
20.799999999999997 34.7 43.05 21.75 98.69999999999999
55.45 80.5 67.05 70.95 106.35 107.15 84.8
109.05000000000001 40.85 145.0 128.25 14.5 86.8 29.2
64.35 71.25 67.6 55.400000000000006 39.65
46.300000000000004 25.35 117.45 168.55 66.2 113.75 88.0
53.95 116.95 29.6 38.5 42.15 37.3 79.6 51.0
42.9 41.15 60.2 74.35 60.5 100.5 63.65 28.6
29.45 278.75 120.6 119.15 37.3 83.35 33.75 59.4
223.45 142.45 77.9 25.75 76.5 86.55 77.4 85.7
138.55 47.2 206.55 39.9 115.35 56.7 130.7 59.5
72.55 45.75 38.1 86.2 100.25 81.65 27.65 23.7
173.14999999999998 201.3 81.55 184.25
140.60000000000002 116.25 13.45 80.4 43.5 70.45
63.95 68.8 62.85 30.6 157.6 75.65 27.05
57.449999999999996 56.599999999999994\
NPAS1 71.6 19.25 9.0 39.1 89.1 21.3
57.449999999999996 69.35 11.45 73.65 25.799999999999997
48.2 6.199999999999999 37.55 24.6 21.95

18.15	14.05	12.55	34.45	193.04999999999998	13.1	13.1
	8.05	11.7	21.05	6.25	55.35	42.900000000000006
	15.15	62.55	182.45	19.3	38.150000000000006
23.65	30.9	5.0	64.30000000000001	34.25	8.15	13.3
	37.85	12.45	26.15	32.1	138.55	7.35	12.2	25.2
	24.35	69.85000000000001	38.45	32.9	23.4	12.6
	95.95	78.65	14.9	17.0	42.8	148.85	55.05
30.55	20.95	86.75	77.15	38.5	40.949999999999996
25.549999999999997	50.300000000000004	22.15	86.75
31.75	18.35	19.15	18.2	26.9	10.3	41.85	47.7	54.3
	40.0	28.3	33.75	72.05000000000001	44.35	68.8
	22.799999999999997	22.05	87.30000000000001	36.0
	72.85	38.5	18.45	30.2	80.85	45.550000000000004
	19.450000000000003	16.95	18.95	52.599999999999994
	37.95	17.25\
NPAS2	55.8	102.96	536.58	64.58	203.95999999999998
229.12000000000003	111.62	257.76	111.35999999999999
233.50000000000006	171.4	77.58000000000001	154.96
293.22	320.18	207.51999999999998	270.04	180.95999999999998
	157.06	525.24	328.67999999999995	173.35999999999999
	304.5	105.14000000000001	61.32000000000001
268.76000000000005	244.88000000000002	120.11999999999998
	377.86	376.2	98.14	597.1800000000001
601.5600000000001	159.27999999999997	164.46
314.82000000000005	164.88	667.1800000000001
230.36000000000004	107.72	325.34000000000003
251.12000000000003	162.9	171.8	80.94000000000001
267.36	139.7	127.24000000000001	135.76	232.64000000000001
	172.64000000000001	245.64000000000001
672.4200000000001	397.68	175.44	565.24	156.58
123.75999999999999	329.12	219.61999999999998
444.58000000000004	119.97999999999999	298.32
258.34000000000003	161.40000000000003	1179.02	164.54
236.56	246.56	187.44	226.45999999999998	125.94000000000001
	119.25999999999999	126.32000000000001	117.88
112.24000000000001	184.44	99.97999999999999	357.5
155.44	69.36	106.36000000000001	69.54	288.82
1065.2199999999998	549.04	155.4	165.66	870.8199999999999
	1235.32	97.64	164.57999999999998	112.32000000000001
	152.68	149.33999999999997	134.16	240.82
121.55999999999999	194.66000000000003	132.38	149.8
116.75999999999999	98.16000000000001\
CCDC144A	8.0	95.95	87.25	9.25	14.7	32.65	18.3
	76.45	30.2	44.3	17.95	70.25	12.35	41.85	47.8
	2.05	28.1	111.3	6.75	6.7	91.69999999999999
	27.1	208.8	14.65	22.9	25.35	34.9
108.69999999999999	82.55	19.4	4.55	162.2	77.15
8.549999999999999	54.3	36.4	27.75	83.0	26.55	14.8
	25.6	56.95	51.5	20.1	19.3	59.2	25.0	38.6
	117.8	63.699999999999996	30.95	31.1	36.15
60.050000000000004	50.75	26.700000000000003	79.6
27.35	21.799999999999997	97.45	148.1	59.349999999999994
	83.0	26.1	44.449999999999996	99.05	22.85
28.35	83.4	24.0	397.75	255.3	35.05	17.2	56.55	2.95

33.199999999999996	29.200000000000003
5.300000000000001	30.45	128.0	51.3	23.2
26.849999999999998	68.25	38.45	39.75	83.6	53.65
19.25	4.35	25.0	15.3	28.450000000000003	56.25
19.15	21.95	52.85	64.55	11.049999999999999	25.05	9.9
17.0\
NPAS3	33.44444444444444	1158.2444444444445
577.1222222222223	40.51111111111106	103.5666666666666
3413.933333333333	823.3000000000001
323.9222222222222	706.9777777777778	332.00000000000006
2438.5333333333338	191.0666666666667
572.0666666666667	971.4000000000001	444.3333333333333
102.71111111111111	227.60000000000002
1750.5222222222221	45.077777777777776	283.1555555555556
176.85555555555553	1043.9222222222224
1138.4777777777776	893.4999999999999	1151.3222222222223
25.811111111111114	216.16666666666666
2303.822222222222	3739.511111111112	400.3777777777778
1111.2333333333333	658.3111111111111
177.33333333333334	2658.2444444444445	1874.1999999999998
61.12222222222223	1886.4555555555553
57.433333333333344	40.75555555555556	3699.644444444444
47.677777777777784	2768.2777777777774	1896.0
63.61111111111112	2233.588888888889	1699.8111111111111
337.08888888888885	94.6888888888889
178.3111111111111	1459.8444444444442	1012.2555555555557
109.35555555555555	42.62222222222222
874.6888888888889	1470.822222222222	1970.7777777777778
1311.3222222222223	324.74444444444447
173.9888888888889	1538.333333333333	1104.8555555555556
243.4777777777778	320.21111111111117
234.56666666666672	77.48888888888888	53.42222222222222
836.2333333333332	47.12222222222221
1236.6111111111113	1379.2555555555555	2387.044444444445
3858.7999999999997	148.42222222222222
2422.022222222223	1368.911111111111	3309.6
928.1333333333334	2416.4888888888886	281.6222222222222
29.211111111111116	412.06666666666666
187.2777777777778	254.7111111111111	843.6333333333333
85.24444444444445	98.58888888888889
101.14444444444445	334.08888888888885	34.13333333333333
37.96666666666667	966.4222222222221
673.5111111111111	1366.8444444444442	414.79999999999995
357.14444444444445	1378.6111111111109
1437.4333333333332	926.3444444444443	1627.7
1078.722222222222	1700.1444444444446	581.2333333333332
1049.8666666666663\
FBXO42	640.85	547.75	690.625	593.625	1349.4750000000001
719.175	479.925	672.35	533.1	662.65	539.8	804.05
573.4250000000001	607.9000000000001	884.0	821.6
529.0	637.825	716.7249999999999	794.0	782.55	524.3
1052.8	541.125	545.075	547.625	751.725	1208.9250000000002
1256.0	777.75	441.67499999999995	1251.9	920.1999999999999
1330.85	789.25	2482.75	451.825	1072.625	1320.925

	493.5	664.175	682.5	535.125	715.05	536.05	646.275
853.7750000000001	408.875	1435.6000000000001
974.9000000000001	647.4	550.175	601.4	1929.9
1219.5749999999998	970.325	812.3	2553.0249999999996
715.775	1177.6	613.675	2156.7	669.775	629.8	884.6	988.7
639.7	711.5	834.45	711.925	1016.15	791.4499999999998
882.025	1069.25	1092.1	784.2750000000001	887.5
816.9000000000001	761.25	558.15	1039.975	893.875
566.2	664.4	727.75	1409.65	773.0	1018.0	617.9749999999999
	668.4	523.875	1656.7250000000001	946.4750000000001
	861.7500000000001	922.0749999999999	774.5
766.675	736.1999999999999	1134.2	851.4	943.4250000000001
	859.25	914.725\
FBXO40	29.099999999999998	14.100000000000001	9.65
5.050000000000001	63.349999999999994	5.35	14.75	15.3
	7.8	7.2	9.8	20.2	26.5	11.85	14.9
36.699999999999996	7.4	12.6	6.4	8.850000000000001
	31.299999999999997	15.85	14.45	5.6	30.85	11.7
	5.35	54.05	18.3	7.3500000000000005	5.1
10.45	13.899999999999999	8.75	16.6	10.55	5.1	13.8
	10.85	25.05	21.8	4.05	44.349999999999994	10.6
	23.650000000000002	14.799999999999999	8.65	5.3
	16.45	13.9	16.75	9.45	3.8	27.15	9.4
17.099999999999998	38.0	9.6	6.5	14.8	46.5	31.0
	11.1	7.8	11.8	11.25	9.2	11.3	7.6	26.3
	42.35	15.0	5.75	7.800000000000001	10.45
10.25	12.3	6.45	6.1	16.5	10.45	35.8	7.15	7.45
	13.700000000000001	11.7	6.949999999999999
13.35	12.7	33.4	10.350000000000001	16.15	8.1
10.25	8.6	10.350000000000001	10.8	41.45
14.899999999999999	9.55	3.6	6.800000000000001
35.0\
LOC100134937	57.4	115.6	111.6	111.7	33.8	103.9
104.5	96.9	70.5	143.1	75.6	94.9	7.7	132.2
129.8	74.8	114.3	76.0	146.2	101.8	253.4	85.5	45.2
	149.3	102.4	130.0	41.4	186.5	84.1	128.3	92.2
	141.0	129.2	127.4	204.7	105.0	116.3	99.8	63.4
	52.2	74.9	68.1	20.2	88.0	86.6	102.3
136.8	61.3	198.0	19.9	67.7	32.4	103.4	149.9	58.5
	127.3	102.0	95.4	84.1	113.0	98.8	82.5
132.9	33.7	50.5	78.6	104.9	26.0	142.2	89.6
128.6	93.8	236.0	144.3	78.9	19.6	130.2	82.3	77.7
	80.4	86.7	56.2	105.2	75.7	116.2	30.5
172.8	109.1	162.0	129.3	96.8	76.4	50.4	94.6
194.1	170.3	199.2	198.0	107.3	128.1	181.7	55.9
198.9\
MBIP	1028.5	129.6	809.7	1095.1	293.2	531.8	2945.1
349.2	486.9	613.8	506.5	460.4	96.9	1326.6	863.6
524.1	432.5	325.0	435.9	428.0	663.0	624.2	453.2
581.1	1108.8	744.1	870.7	634.8	732.3	487.8	660.1
573.1	417.5	383.3	636.8	652.2	728.0	955.2	338.4
725.3	1042.9	115.7	579.9	328.7	412.1	247.7	273.4
607.3	338.5	425.3	718.6	564.0	578.2	678.2	569.7	92.0
	448.0	885.2	796.9	399.0	428.0	508.8	582.7

462.6 880.5 1195.3 383.1 323.5 395.0 572.3 611.3
252.2 1001.9 628.4 721.3 370.0 586.8 508.0 491.8
404.0 917.9 595.8 346.4 518.4 465.9 504.6 2321.3
753.1 439.4 473.9 565.0 574.8 1272.4 1078.5 905.4
1779.7 1038.7 1261.1 1271.8 1605.0 1445.3 1033.5 1167.0\
BMF 434.9 200.9 260.7 187.5 346.1 110.2 488.3
303.0 308.3 424.7 1134.1 933.8 619.4 185.5 115.7
119.6 547.0 132.3 760.0 171.7 279.8 170.5 193.5
391.4 126.3 298.7 743.7 275.4 678.4 177.6 172.9
363.4 1048.3 604.5 193.7 122.3 37.6 421.6 331.6
267.5 426.4 255.7 313.4 425.8 609.4 165.3 132.9
290.0 280.1 340.8 330.2 436.9 200.5 202.0 292.1
250.9 416.4 152.1 43.6 267.3 344.4 713.8 345.9
188.0 507.5 423.8 359.7 634.5 784.3 234.3 63.3
260.2 310.7 394.7 527.0 465.0 313.3 225.5 402.0
106.9 239.6 248.5 348.2 259.7 33.8 159.8 160.1
546.1 154.6 249.6 365.8 455.9 517.5 393.8 529.9
353.2 312.4 558.3 602.6 949.6 267.2 120.5 425.1\
CHMP3 /// RNF103 /// RNF103-CHMP3 1130.4 393.2 378.5
1096.1 552.7 1051.7 1059.6 1082.5 1093.5 1037.3 991.6
784.3 1089.4 833.1 454.8 420.3 1068.1 630.1 1659.8
883.4 14.2 1153.6 726.1 1246.3 995.9 615.7 1681.1
525.3 471.1 1025.9 1186.2 789.0 343.3 1193.1 517.1
1014.9 1337.3 409.3 803.4 1081.0 605.3 452.4 917.3
1174.3 1372.7 954.8 769.6 1070.7 286.8 690.0 1694.6
770.3 1747.1 175.8 774.3 265.8 1005.5 285.9 763.1
338.7 890.4 639.6 1225.5 1229.5 572.1 659.1 1267.2
1403.7 846.9 837.8 96.2 223.2 742.9 858.0 474.2
1175.0 777.9 1316.7 889.5 306.8 431.0 542.0 551.0
1001.5 357.7 501.2 897.1 730.4 853.8 658.7 1363.4
359.9 738.4 859.4 489.6 1111.7 639.4 1034.1 383.7
515.5 778.4 527.0 997.6\
LOC100505711 61.0 305.8 124.6 25.2 98.2 184.8 49.4
81.5 151.9 153.0 262.6 27.8 22.6 212.1
164.8 234.1 216.0 340.7 134.5 204.1 358.1 83.7
325.0 48.8 171.1 66.7 37.4 447.8 258.1 9.7 23.9
320.7 275.3 193.6 498.0 184.8 176.0 109.9
143.6 52.3 132.8 57.8 168.6 116.6 80.1 164.2
231.7 51.0 391.9 266.0 207.3 252.2 273.0 429.1 38.9
296.1 270.3 167.0 162.0 264.4 1257.4 248.3 94.9
214.5 228.7 272.0 59.9 57.4 50.1 310.2
118.3 312.3 164.5 211.4 49.3 165.6 199.0 135.6
164.9 206.2 220.7 68.0 33.8 40.8 226.6 369.5
125.4 322.9 274.6 282.2 22.0 78.2 255.9 141.7
158.1 259.5 317.2 49.5 160.4 64.7 152.5 298.2
221.3\
LOC100505710 131.7 53.9 34.2 145.0 235.4 124.1
329.6 232.3 106.7 37.4 113.2 37.3 17.2 267.8
375.8 137.7 218.6 158.7 155.3 51.5 269.2 134.6
179.2 150.3 107.0 132.0 61.6 347.7 283.2 112.2
176.0 59.4 470.6 248.1 279.5 48.6 82.5 35.6 17.7
176.9 221.7 147.8 208.5 83.2 105.8 185.8
135.7 129.3 138.0 224.7 132.0 199.5 189.8 115.3
114.2 87.1 183.9 186.4 47.0 128.4 623.0 264.5 68.4

223.4 90.8 231.4 90.9 84.5 308.1 148.7
485.4 164.9 38.1 161.9 82.3 130.0 143.4 111.4 86.1
22.2 269.0 149.4 252.9 34.8 12.6 250.2
240.4 113.7 275.1 470.4 38.6 231.1 410.8 150.9
154.6 249.3 517.8 287.7 412.2 255.0 222.1 53.1
298.9\
KIR2DL5A 81.5 74.0 78.7 40.2 58.5 38.1 20.4
115.9 23.5 202.3 191.0 73.8 29.1 63.4
100.3 67.5 117.8 24.6 19.2 39.7 57.9 25.2 82.4
21.7 44.3 51.2 65.6 461.7 68.4 21.7 21.4
46.2 221.0 35.0 84.4 101.5 14.6 164.1 19.4
39.0 13.8 57.0 191.8 49.1 20.5 110.1
155.9 169.5 270.1 53.8 35.6 38.0 27.7 92.8 17.5
47.8 21.3 93.2 89.4 61.0 117.5 36.6 39.9
142.4 49.3 43.0 18.1 133.7 112.7 91.2 74.5
67.9 42.4 37.1 86.1 62.3 253.4 101.8 30.1
31.8 86.2 31.0 64.3 59.6 425.5 179.9
190.8 174.2 148.7 32.8 34.6 46.3 41.8 105.2 79.8
33.6 110.1 9.7 32.9 48.4 44.8 156.9
101.3\
NEK8 191.55 739.65 837.5 378.15 1725.2 324.1
202.85000000000002 396.9 236.5 423.95 235.95 1205.75
306.15 314.35 709.5999999999999 329.6 224.9 631.6
695.5999999999999 354.05 2062.9 201.4 1033.9 203.2
138.6 673.9 478.2 1113.2 751.8 405.6 249.5 1174.95
2042.9 400.6 531.75 659.1500000000001 172.1 1900.1
219.64999999999998 169.5 409.15 141.05 574.55 289.25
105.8 385.65 766.25 106.89999999999999 2003.15 1001.6
386.1 172.55 598.05 1159.7 395.6 600.5 593.65 916.05
858.25 940.55 110.4 852.55 573.0 389.70000000000005
938.45 971.65 274.9 456.6 979.45 423.55 1112.4 941.35
505.34999999999997 310.75 307.05 213.95 1190.5500000000002
186.95 369.5 327.04999999999995 2062.45
907.9000000000001 333.45000000000005 194.35000000000002
1309.05 2413.4 893.0 1061.8 296.75 1150.5 144.8
1275.35 860.25 628.3 842.4 997.25 1070.35 787.75 1184.3
1377.5 713.95 783.7 922.7\
NEK9 1593.7666666666667 1549.033333333333
2992.9666666666667 1554.7666666666667 1056.0
2667.8666666666663 2261.3333333333335 925.1999999999999
1981.6666666666667 1209.6 1827.0666666666668
2376.3666666666663 701.0333333333333 2353.3666666666663
1472.3 2421.2000000000003 1135.2666666666667
2297.0 1396.3666666666668 2011.8999999999999
3732.866666666667 2637.4666666666667 1974.2 1874.8
2544.5333333333333 3034.4666666666667 803.5333333333332
1664.8333333333333 1576.3666666666666
1226.2666666666667 2982.7000000000003 1186.6000000000001
2208.866666666667 1601.8 762.7666666666668
1532.1999999999998 1360.2 2368.9 2328.3 1146.4666666666667
1880.3666666666668 202.13333333333333
1373.6333333333332 1843.1999999999998 1758.5666666666668
1619.966666666667 1380.4333333333334
866.8333333333334 3671.2999999999997 2131.2000000000003

1449.4333333333334	859.5666666666666
1451.7333333333333	1765.3333333333337	2121.766666666667
523.8333333333334	1468.0333333333335	2150.0
1907.3000000000002	2428.366666666667	445.1666666666667
1663.166666666667	1547.5666666666668
1682.6333333333332	1950.6666666666667	2013.0666666666666
1639.4333333333334	848.1333333333333
1830.1000000000001	1217.8333333333333	2827.7666666666664
1788.0666666666666	2016.2666666666667
737.9333333333333	3379.1999999999994	851.9
972.7666666666665	2189.266666666667	2346.0666666666666
193.6666666666666	1076.8666666666666
941.7000000000002	3104.633333333333	931.2999999999998
1507.6333333333334	2004.5666666666666
1688.8333333333333	1338.5	1898.0666666666668
1840.1333333333332	572.5666666666667	1597.5666666666668
1672.8666666666666	1650.4999999999998
2485.9333333333334	1582.0666666666666	1577.8666666666668
1173.0666666666666	1438.1666666666667
1476.4666666666665	3462.4	2275.9	2234.8\
KCNF1	117.1	12.8	42.9	58.0	17.9	81.6	22.0	19.9
53.8	68.7	78.4	5.4	6.2	55.6	28.3	12.6
11.5	23.5	10.3	47.7	303.3	9.9	79.4	11.1
9.8	10.7	20.4	12.3	22.5	58.0	44.1
186.1	13.1	22.5	141.8	13.9	5.4	53.0	5.0	5.2
10.1	12.4	85.6	33.6	504.2	72.8	12.7	66.9
11.9	18.9	20.4	10.9	17.8	34.7	10.7	24.4
22.2	48.6	9.8	83.1	52.5	14.9	5.7	13.6
8.4	9.1	66.8	21.5	42.9	15.5	60.1	17.5
14.7	9.2	99.8	30.4	22.4	107.0	35.1	11.6
25.3	26.9	6.3	6.1	38.1	119.6	39.5
147.0	44.7	10.0	3.2	19.3	10.6	9.6	26.7	39.1
8.0	22.6	192.7	7.1	6.6	9.5	77.9\
SDF2L1	701.3	762.4	298.0	903.5	513.3	1589.5	544.1
202.7	996.9	646.3	764.1	594.6	636.4	797.6	238.9
418.3	725.5	357.6	772.3	485.3	135.6	366.6	395.3
419.2	509.5	740.6	557.9	627.3	313.8	432.4	365.7
424.5	413.9	721.2	728.6	1004.7	544.0	296.7	678.9
568.4	1113.9	337.2	355.6	255.6	431.6	334.4	465.7
569.2	406.1	1111.6	726.9	391.4	515.3	276.0	570.2
572.7	533.1	451.3	929.3	454.2	534.8	503.4	296.3
495.6	567.7	1407.0	1126.3	1491.2	405.5	936.5	264.1
748.1	621.5	629.6	491.8	377.0	238.1	1635.7	678.8
237.5	76.2	191.1	287.8	241.4	372.1	357.9	224.7	64.6
465.1	319.0	192.2	302.5	176.0	336.5	199.3
118.3	200.8	232.3	382.7	196.9	131.9	333.4	311.8\
HTR1E	409.9	6.1	88.3	1.5	53.3	34.3	41.8	42.0
45.0	26.2	25.1	51.1	21.5	22.6	92.4	76.3
43.7	101.4	44.7	71.5	10.3	49.3	14.6	22.8
8.2	59.5	24.8	15.5	71.8	38.7	33.5
133.7	98.2	28.9	25.2	13.5	61.8	66.6	28.7	35.1
446.8	91.9	93.3	73.3	87.2	75.3	108.9	30.6
150.8	33.1	84.5	33.1	29.2	86.5	157.3	69.2
139.9	49.7	39.8	81.4	453.1	140.4	12.1	51.5

92.9 53.3 77.4 10.7 77.1 57.2 58.5
102.9 104.1 13.3 99.1 54.8 52.5 54.8 71.0
124.3 460.5 30.5 80.1 22.9 51.5 215.1 71.1
177.1 55.1 14.1 163.0 149.2 89.5 47.0 64.1 32.7
57.6 73.9 112.3 24.5 4.0 262.2 63.0\
RASL10A 81.9 70.0 181.4 11.3 200.0 104.0 6.7
169.9 47.0 213.2 78.1 63.1 53.4 137.2 27.6 69.3
19.3 34.1 207.3 30.2 706.9 137.1 57.7 40.4
25.1 134.8 116.4 21.3 187.6 133.2 16.5
389.6 587.5 52.8 57.2 50.4 15.4 177.9 40.3 28.8
287.3 11.8 90.7 305.3 14.8 64.6 96.4 34.9
368.6 123.9 81.2 120.5 12.2 28.9 31.9
268.4 56.0 218.6 263.7 270.6 254.1 196.9 180.5 20.2
64.1 157.6 177.5 534.5 222.9 151.4 58.9
462.1 150.8 23.1 156.9 84.0 135.9 77.2 55.7
146.2 332.0 116.1 26.4 43.9 44.4 515.3 143.1
142.1 48.9 53.2 65.8 125.5 78.2 9.4 29.8 23.8
95.4 13.7 74.8 38.2 15.4 247.0 82.3\
NEK4 1937.2 1144.4 1492.85 1003.6 855.55 791.5500000000001
1421.45 658.6 698.85 579.6 1016.75 1012.7
638.4499999999999 1292.65 1152.95 855.25 853.9 1215.45
1011.9 955.95 584.3 1238.75 823.9000000000001
644.1999999999999 1369.3 1273.25 1035.25 856.05
1299.4499999999998 1048.65 890.7 1226.0 604.55 732.15
766.4 746.5 908.4499999999999 880.6999999999999
775.0 1338.3999999999999 647.3000000000001 1284.25
768.9 866.4 853.45 980.0 748.95 731.1 1094.0 755.9
664.75 1655.3999999999999 660.0 1260.35 925.15 1053.1
896.65 1456.05 949.5 716.1 1305.1 733.05 855.1999999999999
1574.3 1031.15 673.95 1016.0500000000001 534.3
486.45000000000005 1361.3 762.5999999999999 1014.35
840.6500000000001 1274.8500000000001 1134.3999999999999
1527.8999999999999 717.1 1231.45 877.6 675.25
1116.95 1565.2 1114.1 711.0 807.1500000000001 569.75
752.4000000000001 822.5 607.8000000000001 736.2
1225.15 1077.35 1062.95 683.3 841.9000000000001 972.95
966.65 1215.95 859.5999999999999 962.9499999999999
930.15 1105.35 748.0\
RASL10B 41.1 45.35 42.05 24.65 47.9 25.05 53.15
102.6 56.2 86.25 59.5 71.95 23.099999999999998
66.55 104.0 106.5 121.55000000000001 25.650000000000002
19.95 78.55 142.8 26.1 138.15 55.75 70.3 62.8
56.75 59.3 120.25 56.35 77.45 129.05 40.25
71.75 114.75 58.3 20.35 64.6 52.4 88.6 72.5 14.2
26.85 26.0 28.2 174.75 127.95 156.25 69.6
34.55 58.0 26.549999999999997 53.650000000000006
154.15 34.35 51.15 58.599999999999994 55.3 38.2
52.05 162.64999999999998 70.15 49.25 23.25 39.75
38.25 32.35 42.0 94.39999999999999 48.45 82.3
48.349999999999994 43.8 57.35 44.2 56.15
73.14999999999999 34.85 49.45 101.25 114.95 55.35
56.75 89.6 112.8 398.2 174.7 185.6 153.70000000000002
104.15 75.8 170.85 81.65 84.85000000000001
61.150000000000006 66.25 70.55 83.94999999999999

102.0	47.900000000000006	77.7	72.19999999999999
87.10000000000001\
HTR1F	149.7	4.8	93.0	43.7	120.5	4.8	63.3
142.2	83.7	74.5	310.0	11.8	24.0	33.3	278.0	66.9
	253.2	123.6	41.1	50.5	332.4	46.5	79.9	50.9
	22.6	60.0	28.6	104.7	19.6	5.2	42.1	11.5
	116.0	46.1	154.2	27.3	52.5	53.5	60.4	86.8
	91.3	12.9	64.5	58.9	36.2	104.0	81.9
186.6	156.6	103.4	40.3	30.8	70.9	194.0	136.5	70.9
	10.8	117.1	63.5	70.3	386.8	24.8	61.7	68.1
	86.6	9.5	29.9	9.3	150.1	41.6	80.7	29.0
	103.8	65.3	31.9	53.1	59.4	24.1	45.7
188.2	186.3	82.5	72.4	62.9	24.9	89.1	91.4
112.3	91.8	81.7	22.5	88.0	44.6	58.7	19.5	38.8
	9.7	31.0	136.8	86.3	37.6	15.1	117.9\
NEK5	293.35	1283.5500000000002	4538.15 379.75	1345.05
172.85	295.35	283.75	346.65	1620.95 937.3499999999999
397.25	84.80000000000001	660.15	199.55	70.55	287.95
2184.8999999999996	487.55	156.14999999999998
759.1500000000001	1503.5	3044.95 557.55	875.55	698.3
375.3	1407.55 1152.35 327.05	242.4	1342.65 1571.9499999999998
	696.5	214.4	580.4000000000001	352.3	817.95
217.7	119.9	117.6	65.3	1404.8	194.20000000000002
1395.05 1471.75 598.6	101.35	1930.9	748.85	902.2
502.29999999999995	842.95	998.65	1268.6999999999998
1700.3	885.6	3112.45 2205.55 1939.8500000000001	1207.65
1640.9	1680.8	1586.8000000000002	578.55	954.45
565.0999999999999	36.45	1414.6	2094.85 4269.5	1807.75
233.65	584.45	965.8	696.8	1780.15 405.45000000000005
864.15	241.85000000000002	1001.25 533.3	448.04999999999995
	286.9	828.0	456.85	330.0	226.0	179.05
103.14999999999999	562.7	634.6	395.0	639.6
440.70000000000005	467.20000000000005	413.5	1527.55
514.45	821.9000000000001	381.15	649.3499999999999
313.05\
TNFRSF9 30.633333333333336	55.666666666666664
50.4666666666667	104.10000000000001	43.5666666666667
	21.666666666666668	53.7666666666667
63.63333333333333	34.3666666666667	69.2666666666667
	60.43333333333334	82.03333333333335
40.9666666666667	121.59999999999998	38.4666666666667
	35.7666666666667	38.03333333333333	70.8	47.6
	63.7666666666667	111.10000000000001
52.46666666666666	74.33333333333333	100.46666666666665
	56.300000000000004	61.2666666666667	85.5
148.4666666666667	78.2	72.23333333333333
30.399999999999995	161.73333333333332	134.1
41.333333333333336	75.1666666666667	69.10000000000001
	27.4666666666667	60.9	26.633333333333336
42.233333333333334	44.300000000000004	34.733333333333334
	95.8666666666667	22.166666666666668	69.5	71.2
	21.333333333333332	47.333333333333336	112.3
27.666666666666668	64.53333333333335	41.8
51.73333333333333	56.73333333333333	39.63333333333333

41.733333333333334	84.56666666666668	76.3
92.96666666666668	68.6	41.733333333333334
93.53333333333335	60.30000000000001	43.699999999999996
50.599999999999994	49.233333333333333
56.96666666666667	49.9	123.76666666666667	80.9
129.03333333333333	95.03333333333332	105.63333333333334
54.93333333333334	55.93333333333333
59.36666666666667	60.46666666666666	43.96666666666667
36.766666666666666	63.03333333333333
61.46666666666666	26.933333333333334	48.93333333333334
82.26666666666667	108.96666666666665
68.89999999999999	73.66666666666667	122.63333333333333
115.06666666666668	98.83333333333333	34.5
34.36666666666667	22.333333333333332	44.76666666666667
48.5	27.599999999999998	57.633333333333326
47.833333333333336	19.033333333333335	60.466666666666676
43.96666666666667	69.93333333333334
48.833333333333336\
NEK6	259.65	396.70000000000005	211.15	386.95	390.625
1536.8	199.25	329.425	328.85	414.0	449.67500000000007
390.02500000000003	450.075	356.54999999999995
410.65000000000003	302.15	175.25	450.6499999999999
398.00000000000006	339.425	280.2	378.15000000000003
347.7	327.72499999999997	291.7	329.15	598.5749999999999
304.25	322.575	319.72499999999997	236.97500000000002
394.975	393.52500000000003	387.02500000000003
401.375	335.425	639.675	495.57500000000005	368.925	243.575
226.64999999999998	251.37499999999997	238.975	297.75
419.825	227.625	477.82499999999993	213.62499999999997
224.9	233.15000000000003	467.25000000000006
553.4499999999999	334.6	533.15	343.3	303.27500000000003
226.89999999999998	479.84999999999997	499.575
270.57500000000005	202.15	467.55	397.05	355.825	309.725
382.62499999999994	191.72500000000002	141.075	325.95
392.84999999999997	328.175	275.175	238.8	357.45	251.175
569.725	341.6	342.72499999999997	255.225	204.575	214.05
351.375	140.75	267.22499999999997	222.275	432.075	425.45
364.6	311.9	219.95000000000002	409.05	257.95	244.675
267.95	212.725	267.34999999999997	206.875	304.0
240.77499999999998	209.1	218.525	414.57500000000005
275.975\
FBXO38	1077.9333333333332	939.4000000000001
1402.5666666666666	1052.3666666666666	601.9666666666667
702.0	1065.2	698.0666666666667	891.0	598.8
1126.6	1120.1666666666667	1033.466666666667
809.3333333333334	852.8333333333334	1258.1000000000001
1083.2666666666667	981.0333333333333
980.9666666666667	1219.7	1397.8666666666668
864.5666666666667	1113.5	1086.3999999999999	798.5
1108.1333333333332	1290.8666666666666	1056.0666666666666
1401.1666666666667	1383.2666666666667
1316.0333333333333	1416.5	1198.9666666666665	963.1
696.2333333333335	1392.5333333333335	834.0333333333334
1092.1666666666667	1424.8000000000002

```
729.6999999999999	1310.4666666666667	93.96666666666668
	1059.6666666666667	1065.1333333333334
725.6666666666666	1035.8999999999999	788.0
829.9000000000001	1486.6666666666663	1117.7
1050.9666666666665	930.0666666666666	956.1666666666666
	1006.3666666666668	1364.2333333333333
888.5333333333334	1242.0	1416.2	1413.6000000000001
1015.9000000000001	1208.3	1155.3333333333333
968.4000000000001	1189.9666666666665	1172.9
1117.5333333333333	1128.2	1103.8666666666666	1022.1
1228.9666666666667	1232.0333333333333	1075.6333333333334
	1407.7333333333336	1447.3000000000002
1095.7333333333333	1083.1333333333334	1150.7333333333333
	1207.8999999999999	1016.9	631.2666666666667
1308.6333333333334	1053.3333333333333	695.1
1259.8333333333333	1375.8000000000002	1086.0333333333333
	1172.1333333333334	874.4333333333333
622.9333333333333	896.2666666666668	950.2333333333335
	1338.3	1409.0666666666666	1309.2333333333333
1402.2666666666667	1596.1000000000001	1379.6333333333332
	1421.033333333333	1921.1333333333334	2160.0
1486.0666666666666	2221.7666666666664	1250.5666666666666\
STYX	319.29999999999995	327.02500000000003
184.54999999999998	561.8249999999999	385.825	443.25
423.17500000000007	243.9	506.27500000000003
488.32499999999993	314.975	354.95000000000005
396.90000000000003	302.975	190.775	309.72499999999997
314.22499999999997	418.375	449.57500000000005
272.52500000000003	210.29999999999998	418.425
236.12499999999997	477.87500000000006	533.25
295.99999999999994	328.65	263.92499999999995
288.95000000000005	315.75	258.025	593.4250000000001
196.625	264.575	217.725	254.42499999999998	394.2
375.67499999999995	287.37499999999994	726.7249999999999
	233.6	268.5	385.2	346.1	366.35	437.02500000000003
	353.875	406.84999999999997	224.55	261.3	601.625
409.20000000000005	375.3	319.1	259.07500000000005
217.175	357.9	356.57500000000005	290.2	322.775	570.375
267.95	392.65	318.45000000000005	254.02499999999998
281.15	300.94999999999993	327.15	326.47499999999997
346.59999999999997	285.95	375.1	219.17499999999998
389.825	192.575	302.775	232.125	232.07500000000002
255.47499999999997	204.9	279.675	285.125	195.52499999999998
	245.275	276.45	372.45000000000005	205.825
287.95000000000005	637.45	239.02500000000003
269.27500000000003	313.77500000000003	407.07500000000005
	268.675	254.175	383.025	403.7	300.975	405.72499999999997
	547.8	264.775	403.57500000000005	308.72499999999997\
FBXO39	10.6	139.2	51.6	74.1	93.5	51.0	9.1	84.3
	74.6	6.3	27.3	49.1	5.8	18.7	80.2	47.8
	42.6	83.1	51.8	18.1	28.5	56.6	109.2	8.9
	66.8	96.0	35.4	137.2	92.1	90.0	21.5	46.2
	85.4	61.7	12.3	89.1	47.8	23.1	19.6	53.6
	21.6	55.4	56.0	52.1	4.5	81.0	70.2	45.0
```

91.4	33.6	79.7	58.7	33.0	147.4	25.0	80.2
81.2	88.6	36.9	44.0	65.5	77.8	73.9	8.2
107.6	47.5	83.3	45.5	181.8	92.9	123.6
211.8	19.2	35.6	25.5	29.0	71.7	28.4	50.9
118.6	101.3	91.5	39.2	97.8	184.4	13.0	14.5
110.1	71.0	206.3	3.1	86.4	23.7	43.3	61.0	80.0
107.2	72.8	75.7	113.9	116.4	60.5	57.6\
NEK7	5170.1	1747.6	1829.1	2135.6	1651.9	2054.5	3022.0
1683.3	1075.3	1070.2	2218.7	1179.2	2480.5	2196.5	1096.8
2238.7	2173.7	930.1	3123.2	1348.5	575.8	1373.3	1558.3
2396.0	1960.6	2163.2	1929.7	2336.3	1579.5	2091.7	4835.3
887.6	1928.8	1758.1	1378.4	1545.8	2589.8	1295.7	2185.2
2962.8	2339.8	2218.0	2569.5	1254.7	985.0	1773.9	2715.2
3927.0	1178.6	2418.1	1739.5	2457.0	2787.6	1012.2	1417.9
4690.2	2327.7	1191.2	1553.5	1394.0	867.9	1801.9	2119.8
2063.6	2190.3	2203.3	3153.9	1703.3	1146.5	2511.7	1561.0
2248.1	1791.6	2336.0	2334.1	1794.7	2159.7	1334.3	2827.7
2069.8	1882.6	3859.1	2127.2	2272.6	1200.2	1051.1	1413.1
2160.0	1101.7	739.6	3517.8	1807.8	2523.9	3404.1	3178.0
2983.4	4079.8	3289.6	2635.8	4504.0	2539.7	2403.9	3228.4\
DCTN1 /// SLC4A5		195.2	117.7	87.7	283.9	126.7
421.8	385.2	179.9	311.6	195.0	366.7	269.6	249.5
340.4	321.5	166.3	300.7	164.4	391.5	230.9	10.1
197.3	104.8	308.1	200.8	249.9	214.7	124.3	153.0
207.0	345.5	67.0	173.2	291.3	137.3	128.8	263.5
115.4	233.1	419.8	266.0	294.6	163.2	277.2	393.6
299.6	166.8	227.6	144.8	160.8	194.2	209.6	332.8	63.9
272.1	167.1	264.1	83.0	261.9	143.1	15.0
180.3	201.2	206.2	124.9	106.8	486.1	287.8	204.2
144.3	68.4	132.2	163.9	304.1	303.6	235.2	234.2
258.6	192.9	214.6	111.2	95.1	128.2	143.9	119.1
183.0	37.9	174.4	223.9	18.1	292.1	81.4	144.2
157.9	188.2	108.8	79.2	126.3	116.5	26.6	146.0	72.4
133.3\
HTR1D	77.5	8.9	17.2	9.5	14.8	46.3	6.2	8.8
5.2	11.6	4.5	44.1	5.9	7.8	14.8	15.1
16.3	6.0	3.1	13.5	28.1	4.0	8.3	5.7
10.4	5.5	6.1	6.2	40.3	6.6	10.5	23.8
4.9	5.1	27.1	16.4	7.1	9.9	32.2	10.6
6.7	21.0	5.6	2.3	15.2	7.2	4.4	4.5
23.4	7.7	6.9	8.6	7.9	64.1	6.7	7.4
7.4	6.5	2.9	4.7	61.0	6.3	5.6	7.2
6.9	88.8	3.7	6.4	27.5	23.4	9.1	6.1
4.8	8.7	25.0	3.9	56.3	7.7	41.6	18.3
19.6	46.2	4.2	4.1	16.9	16.0	8.5	11.6
14.9	6.0	1.9	6.0	6.2	4.5	9.4	7.2
8.0	31.8	8.3	9.9	18.3	22.1	8.7\
ACTG1P4 /// AMY1A /// AMY1B /// AMY1C /// AMY2A /// AMY2B
471.0	958.0	25184.9	383.0	2188.3	236.1	2228.6	1105.9
149.1	1305.7	2524.7	3478.6	1789.3	37571.7	3829.7	1373.2
267.4	639.8	842.9	274.8	1760.8	194.8	4111.3	1075.2
440.5	1119.0	158.2	664.2	3811.0	227.9	470.4	3184.4
3824.2	51.4	787.5	90219.8	6464.2	4789.0	584.1	156.7
226.7	147.6	830.0	403.9	6676.0	1587.9	495.1	75.6

```
4219.4  2328.0  662.0   5670.4  735.2   1972.6  473.1   14482.3
17482.9 2681.0  1252.0  1371.4  322.0   1441.4  2017.9  1023.9
2350.4  2495.4  1313.0  1033.4  2578.1  8469.1  4047.3  2561.8
882.9   433.3   1735.9  190.1   1804.8  269.5   1653.8  55.1
4303.3  627.4   2128.7  1955.9  2247.5  816.7   280.0   1635.0
163.0   1742.3  471.7   3422.5  2362.3  2566.7  4414.7  3628.7
2723.3  2350.2  3314.4  4444.8  4689.2  962.3   3297.3\
FBXO36  53.25   123.45  146.6   45.25   150.65  58.099999999999994
        57.300000000000004      69.75   68.25   144.8
65.14999999999999       76.0    25.599999999999998      646.5   55.2
        25.65   55.699999999999996      184.45  80.69999999999999
        16.25   71.25   220.35  139.4   40.0    66.7    103.0   60.4
        86.89999999999999       80.55   52.7    40.15   93.35   97.5
        96.2    151.5   108.64999999999999      60.900000000000006
        77.7    70.35   72.0    64.6    59.199999999999996
249.54999999999998      40.8    114.5   182.20000000000002
46.15   19.05   145.1   74.5    87.3    240.6   73.45   117.4
89.94999999999999       99.3    103.75  57.0    99.25   95.55
126.60000000000001      81.15   94.85000000000001       176.6
77.75   34.4    42.55   90.35   162.95  151.0   155.35  536.3   70.2
        57.0    83.05000000000001       156.45000000000002
108.75  74.6    45.8    50.150000000000006      77.1    54.5
25.799999999999997      17.1    19.75   32.2    27.55   65.6    61.0
        92.0    51.75   18.2    66.75   20.3    36.849999999999994
        63.449999999999996      57.85   76.39999999999999       88.4
        55.0    53.5    90.2    37.849999999999994\
HTR1A   20.1    34.0    49.1    62.2    13.4    30.8    7.8     67.0
        5.0     12.3    21.3    10.3    4.9     78.0    23.4    6.2
        14.6    23.4    38.6    34.9    21.7    37.9    9.8     19.2
        13.3    4.9     9.4     48.9    20.3    5.9     5.4     84.0
        34.3    21.7    7.0     8.1     5.8     27.1    4.6     63.3
        4.6     11.7    4.1     47.1    5.8     11.9    55.7    3.7
        20.8    25.2    30.1    14.3    11.7    10.4    5.5     10.0
        17.5    11.5    10.1    19.9    397.3   77.7    4.9     6.7
        21.6    86.6    6.5     12.6    59.2    5.4     147.1   14.3
        7.7     20.8    18.6    5.2     31.5    4.4     6.7     5.1
        10.4    82.8    73.7    63.1    13.5    132.0   22.4    15.9
        11.9    67.3    9.1     29.3    14.6    19.9    8.9     7.4
        8.0     9.0     4.9     10.7    12.1    54.4    11.0\
LOC283674       15.05   108.75  46.05   35.849999999999994      12.1
        23.25   4.25    13.1    23.7    9.7     28.450000000000003
        11.75   5.9     7.949999999999999       10.7    26.65   31.8
        23.55   15.4    5.05    77.75   19.3    54.349999999999994
        8.65    19.2    12.05   26.5    12.25   58.0
47.599999999999994      10.7    48.550000000000004
58.949999999999996      20.950000000000003      86.95
20.349999999999998      13.85   101.1   32.75   14.85   16.3
29.35   43.349999999999994      31.1    38.7    9.25    18.1
10.649999999999999      18.8    8.4     14.45   51.95   6.9
66.05   15.4    30.200000000000003      28.45   69.05   43.15
47.050000000000004      254.8   19.8    28.849999999999998
16.75   53.6    12.45   3.2     5.75    60.3    8.7     24.75   55.9
        9.95    19.95   7.800000000000001       44.95   5.85    10.9
        12.5    43.099999999999994      23.4    7.15    6.0     5.95
```

	36.05	41.7	5.65	55.6	37.6	64.05	4.05
28.200000000000003	26.200000000000003	17.7	5.3	33.8
	16.6	10.95	5.75	4.7	8.95	7.55	11.3\
LOC100288175	7.6	169.8	39.2	4.4	166.8	18.3	61.5
	21.8	12.7	224.8	16.9	13.9	7.8	70.1
175.1	22.3	16.8	146.2	58.7	50.6	206.1	82.4	55.4
	8.1	53.3	20.9	13.7	41.7	33.3	19.2	11.7
	51.3	36.0	74.3	44.1	34.7	15.4	16.2	31.0
	17.2	13.9	50.0	73.9	31.9	15.1	68.6	27.4
	18.5	209.4	116.3	37.0	26.9	110.7	25.2	11.3
	39.5	121.0	14.3	33.2	33.0	279.8	18.8	16.6
	33.6	33.4	33.9	21.6	11.2	121.4	71.1
158.4	69.3	31.9	14.4	59.0	19.4	69.0	37.8	21.8
	21.4	206.2	32.5	64.8	75.1	52.8	569.2	25.8
	13.7	73.3	39.1	54.0	53.4	99.7	7.1	44.8
	11.4	15.1	13.7	113.6	97.7	23.0	49.7
26.7\
HTR1B	5.4	237.9	76.8	70.4	7.9	66.5	65.4
115.3	130.3	33.7	55.1	30.1	114.0	119.1	157.9	76.8
	92.1	35.8	23.4	105.6	303.7	41.8	187.6
113.0	93.3	69.7	20.3	165.4	54.4	10.2	64.5	41.0
	5.6	202.8	247.2	45.1	75.4	25.3	71.4	61.9
	90.2	49.4	40.2	21.3	42.5	50.1	53.3
119.9	222.2	116.5	21.3	124.8	121.5	119.6	112.9	40.7
	139.8	63.2	93.5	36.3	157.9	42.2	111.1
109.9	95.8	95.6	53.1	9.8	65.0	58.5	141.2
116.3	21.4	122.3	24.5	101.1	95.5	92.0	108.9	53.4
	114.2	17.9	67.3	95.6	62.5	329.7	18.2
160.0	28.9	87.6	4.3	22.1	37.9	48.1	79.2	19.1
	112.3	34.4	23.4	46.0	36.6	26.5	100.0\
CSNK1G2-AS1	17.3	40.4	48.8	15.3	82.0	101.7	79.2
	114.0	86.6	98.3	10.8	29.0	5.8	22.1
175.2	28.8	204.6	126.2	18.5	27.5	232.2	5.3
213.3	28.3	64.0	54.4	53.9	23.4	168.1	97.9	8.5
	119.5	28.9	45.8	128.1	29.3	136.1	128.4	72.4
	150.3	79.9	103.6	8.6	11.5	7.1	30.3	8.7
	36.2	107.0	94.4	28.2	141.2	66.9	61.9	66.8
	21.0	12.2	74.8	137.9	129.0	705.3	18.8	8.2
	84.5	25.8	129.4	9.1	88.9	118.7	160.5
209.6	9.8	146.1	74.1	86.5	65.4	109.5	59.6
120.0	83.1	40.0	152.9	105.4	163.4	211.2	136.0	25.8
	68.5	199.9	268.3	127.2	155.2	65.9	21.5	18.7
	53.7	110.3	108.5	136.4	71.7	14.7	23.4
95.8\
FBXO34	2915.0	841.0	1549.8	1045.3	1068.3	1737.7	1718.6
2682.2	1884.9	1794.0	4579.4	2880.4	2018.6	1478.2	2025.3
2148.4	1165.4	1544.8	4010.4	1903.5	948.0	2408.7	1676.6
1636.1	2752.6	2062.5	2110.4	1002.1	2252.6	1861.9	1786.2
1690.3	1493.7	2507.8	1150.8	2125.1	2159.4	1208.9	2630.9
3346.3	1434.6	2209.1	1135.6	1304.2	2589.1	1708.7	1747.8
3061.0	1985.9	2529.0	1853.4	5252.6	3351.5	1610.3	2344.6
2289.1	1800.6	2346.5	1403.0	995.2	1021.3	1483.1	2228.5
2914.9	1500.3	1650.1	2175.0	989.0	1044.5	2986.0	493.2
1515.0	1426.2	1860.8	1723.5	2460.1	1481.4	2684.3	2658.6

```
1231.7  2323.7  1542.8  5588.8  2758.9  2342.7  1970.3  1865.1
2176.4  1963.7  1574.9  1710.2  1463.4  2659.7  2277.1  2095.2
2863.8  1996.2  2972.2  1420.7  1714.7  2639.0  2081.9  2560.5\
RGMB-AS1        55.8    342.2   105.0   4.9     185.0   3.8     18.2
        223.9   65.6    9.2     82.3    173.0   54.6    86.9    80.0
        50.6    36.5    46.9    90.3    82.1    415.0   52.4
222.1   40.7    6.2     94.2    3.2     37.1    165.1   35.7    9.3
        34.9    55.0    32.6    261.1   140.7   15.8    102.7   53.2
        37.2    39.3    48.6    53.1    38.8    10.7    61.5    57.9
        30.3    153.3   30.6    55.4    54.6    7.6     128.1   86.1
        50.8    30.9    111.0   174.5   75.7    80.1    180.1   69.6
        119.8   63.8    31.7    9.6     75.9    19.9    93.8
148.9   52.3    34.4    50.2    96.6    27.4    40.7    45.2    66.3
        72.2    59.8    5.1     55.8    48.6    106.4   140.1   87.9
        6.1     26.8    59.9    36.1    63.0    18.9    62.2    33.7
        76.7    72.4    40.9    99.4    92.6    91.9    143.3
14.7\
IGH /// IGHA1 /// IGHG1 /// IGHG2 /// IGHG3 /// IGHM ///
LOC102725526    57.5    5.8     14.4    1.6     54.5    1.7     9.8
        88.3    28.2    5.5     18.3    26.2    4.0     6.3     5.4
        6.3     44.3    1.9     2.3     10.5    7.9     24.4    4.2
        0.8     29.7    13.2    5.4     12.1    12.7    37.4    3.7
        15.3    55.3    2.9     4.8     31.0    36.7    8.9     16.0
        23.0    2.7     4.4     3.0     10.6    3.8     8.7     3.6
        45.1    8.1     2.5     2.7     11.7    2.0     20.0    2.6
        48.2    9.4     55.7    28.7    5.3     238.0   1.9     6.2
        2.3     15.4    6.3     1.8     15.2    8.3     7.2     15.4
        33.6    4.6     3.6     7.5     6.8     6.4     5.6     16.1
        6.9     3.5     35.6    15.3    14.3    34.5    14.0    11.4
        10.1    7.7     28.9    10.7    4.4     17.4    3.0     7.3
        7.2     6.4     17.1    7.8     4.3     6.7     46.9
17.5\
BMX     24.8    13.0    5.3     38.5    18.5    12.4    6.8     8.6
        20.9    43.3    65.8    6.8     77.3    36.2    11.1    80.7
        209.9   33.1    8.9     77.5    91.2    24.9    20.1    34.2
        19.5    16.5    86.8    13.0    104.4   52.7    29.6    31.0
        4.8     33.5    118.7   5.9     243.2   11.5    7.0     14.9
        174.6   6.9     64.7    7.2     9.5     108.0   7.9     8.1
        214.6   10.2    13.5    33.8    26.3    5.8     50.2    55.6
        15.0    43.7    18.0    134.0   159.5   16.5    14.6    97.6
        22.7    8.2     8.3     9.8     62.7    51.5    62.5
105.4   103.4   57.7    114.0   32.7    12.8    39.2    56.1    8.6
        26.8    18.5    5.0     4.8     6.7     237.0   105.3   23.4
        7.2     80.4    1.8     107.5   12.8    84.7    45.0
126.5   23.6    22.4    54.8    112.9   80.4    28.0    148.1\
C6orf15 20.3    160.5   37.2    95.0    127.0   32.2    24.9    55.7
        42.2    46.0    22.1    21.7    93.9    22.9    67.2    29.1
        26.9    194.8   23.2    442.2   73.6    24.7    206.8   15.2
        63.9    26.0    32.6    63.5    43.6    464.5   88.1    43.1
        105.0   41.0    41.2    42.4    28.3    48.0    325.9   56.8
        28.3    161.5   51.8    366.1   125.2   31.8    46.8    30.0
        98.6    295.9   62.9    40.0    33.8    64.8    34.5    77.0
        20.0    48.1    50.5    16.8    170.6   46.3    95.0    37.6
        110.9   66.7    253.6   292.5   60.1    45.0    83.8    50.8
```

37.9	30.3	38.6	27.6	525.8	36.2	75.6	71.6
	44.8	49.7	39.2	53.2	51.6	113.8	53.7	61.0
	31.7	62.5	17.1	61.0	40.8	23.9	28.4
108.5	30.6	31.6	53.6	45.2	29.1	67.5	36.2\
FBXO32	39.028571428571425	282.0285714285714
129.55714285714288	99.44285714285715	325.12857142857143
	72.57142857142857	108.94285714285715
352.35714285714283	141.48571428571427	610.3285714285714
	117.35714285714288	221.7	754.2571428571429
306.4428571428571	229.58571428571426	198.29999999999998
	724.3428571428573	217.95714285714286
215.45714285714286	241.24285714285716	442.40000000000003
	137.7	234.21428571428572	710.0428571428572
70.34285714285714	178.31428571428572	207.78571428571428
	265.6	86.94285714285714	333.1857142857143
796.2142857142856	176.97142857142856	351.0142857142858
	115.1857142857143	175.41428571428568
194.3142857142857	75.47142857142856	322.2857142857143
	88.25714285714285	148.3857142857143
74.38571428571429	146.3142857142857	223.24285714285716
	278.3	103.32857142857144	71.08571428571429
98.18571428571428	44.271428571428565	148.27142857142857
	223.64285714285714	365.42857142857144
207.94285714285712	1316.6714285714286	73.62857142857142
	140.07142857142858	114.28571428571429
118.05714285714285	160.90000000000003	269.5571428571429
	186.31428571428572	241.35714285714286
250.2857142857143	256.35714285714283	168.57142857142858
	665.1571428571427	1262.0	289.6428571428571
447.54285714285714	252.17142857142858	153.21428571428572
	321.85714285714283	178.4857142857143
397.8142857142857	109.84285714285714	76.51428571428572
	106.77142857142857	170.7	85.77142857142857
394.8571428571428	81.25714285714287	78.48571428571428
	106.51428571428573	779.4571428571429
474.08571428571423	759.7428571428571	482.7857142857143
	153.1142857142857	232.54285714285717	346.2
252.95714285714283	287.3	197.7	294.5	293.35714285714283
	951.5571428571429	309.9857142857144
491.19999999999993	247.09999999999997	419.0142857142856
	173.08571428571426	296.22857142857146
149.95714285714286	311.2571428571428\
FBXO33	1075.6	1310.45	919.0	1098.85	923.8	662.5	1257.95
846.0	673.5	651.2	977.75	745.1999999999999	858.35
1508.4	950.15	975.8	831.2	801.3	857.8499999999999
837.9000000000001	634.55	722.8	698.15	1002.8	1354.8
951.2	1143.6	1081.45	980.05	692.95	929.75	645.65	821.85
1021.8	1074.4	1118.5	784.95	802.2	791.3	2070.45	586.65
801.75	939.9	1055.45	1012.7	951.25	1052.6	759.5	640.3
695.2	778.1	1401.4	625.8	964.05	893.3499999999999
739.8	727.95	831.95	869.2	1092.7	752.8499999999999
755.8499999999999	884.0500000000001	879.6	741.5
830.0	735.85	718.3	699.95	1094.8000000000002	567.05
1168.75	845.1500000000001	1534.65	1008.0	1307.55	728.6

962.0	993.5	750.75	1260.0	835.55	1077.75	872.45	595.25
812.3000000000001	994.25	829.45	615.75	896.05	1110.7
792.0999999999999	902.3	901.2	801.7	987.6500000000001
	1042.0	854.2	942.1	1179.45	957.5999999999999
998.9000000000001	1146.85\
FBXO30	1776.4	721.85	584.15	696.95	689.25	858.95	1261.25
1066.25	1039.35	535.35	1021.7	519.5	1105.7	631.45
858.9000000000001	625.65	1684.3500000000001	673.1
815.3000000000001	852.8	228.1	693.8000000000001
454.35	1190.05	1624.95	368.4	438.4	364.3	526.05	779.2
965.6999999999999	431.25	258.0	661.25	719.0999999999999
	349.2	1044.1	307.6	874.1999999999999	1381.35
737.75	957.4499999999999	542.95	1008.85	1666.45	980.65
832.65	2358.15	387.90000000000003	562.75	660.3
861.3499999999999	639.35	911.0999999999999	634.15
331.55	600.5500000000001	557.65	483.45000000000005
351.04999999999995	287.1	390.95	464.55	460.8	496.9
463.1	951.1	812.55	259.85	809.15	275.4	400.5	498.3
1078.6	555.7	856.75	313.25	770.75	500.45	2513.7999999999997
	979.45	691.25	1304.3999999999999	633.5500000000001
	386.45000000000005	413.85	1044.8	392.1	503.4
633.3000000000001	1658.35	882.8000000000001	1027.7
680.45	612.3	638.3	602.1	693.3	339.9	518.15	689.85
858.65	581.15\
SMR3A /// SMR3B	18.0	10.3	17.9	2.2	25.2	2.6	10.8
	3.8	4.7	21.9	8.6	8.1	12.3	11.2	13.5
	24.1	19.3	7.6	1.8	10.4	24.5	4.0	27.4
	2.8	9.1	52.0	8.1	34.3	11.3	6.4	9.6
	26.7	10.1	8.0	49.0	14.8	36.7	5.4	4.5
	14.6	8.2	54.5	8.5	6.7	4.2	9.7	16.1
	15.0	26.4	6.3	8.3	53.5	14.2	8.1	11.1
	8.2	11.7	14.0	21.8	9.1	56.6	15.9	14.5
	10.7	14.5	8.4	3.0	4.8	112.1	6.0	13.8
	10.8	11.0	11.7	22.9	7.5	11.9	2.8	24.3
	6.8	18.1	22.2	5.5	7.9	11.5	17.1	12.8
	10.4	12.7	78.8	11.3	12.9	10.6	10.7	6.4
	11.4	19.5	6.0	8.6	20.9	4.3	7.5	6.3\
FAM90A1	5.6	29.1	180.4	82.8	11.3	13.0	25.7
122.2	5.2	21.3	6.0	17.7	82.7	6.5	126.7	62.4
	5.2	15.4	124.3	21.8	19.7	28.8	25.1	13.7
	6.9	51.1	3.0	23.6	10.6	80.7	83.4	8.9
	30.4	18.6	228.0	21.0	68.8	179.0	5.6	94.6
	246.2	10.5	20.8	4.0	81.7	32.2	19.5
286.5	33.8	35.4	3.4	25.4	21.2	9.5	21.5	19.7
	60.6	9.9	15.6	98.9	64.3	69.3	159.3	10.7
	7.8	176.3	82.5	10.2	141.4	141.6	33.6	9.8
	15.0	2.6	6.7	7.2	7.0	56.4	21.4	95.4
	44.6	10.6	389.8	302.0	31.3	30.9	29.7
100.3	12.5	12.5	121.3	29.1	95.1	63.2	14.2	66.5
	27.2	17.0	38.7	28.1	23.0	21.7	85.6\
FBXO31	228.60000000000002	750.6	552.4	394.54999999999995
	558.75	381.75	362.20000000000005	262.15000000000003
	379.5	450.8	533.55	580.45	495.9	314.6
216.85000000000002	389.25	290.85	511.1	433.7	365.65

```
1585.7	486.4	830.0	555.25	259.3	457.75	337.3	894.1
481.55	513.15	602.4000000000001		471.5	804.85	506.2
234.35	692.35	258.1	527.65	301.4	229.1	414.05	408.1
508.7	478.0	368.85	322.15	327.55	303.34999999999997
1430.7	524.8	428.0	491.05	404.7	531.3	494.90000000000003
	927.8	450.35	604.25	531.25	612.05	373.0	818.6
545.1	529.25	401.5	529.25	432.75	866.8	816.5	408.2
971.4000000000001		757.0999999999999		513.1
371.70000000000005	421.84999999999997		371.75	692.25
345.6	516.8499999999999		182.3	265.35	331.7	222.75
302.6	397.35	516.95	462.9	385.0	415.65	377.35
80.69999999999999		529.7	376.7	534.35	805.8499999999999
	512.25	619.55	701.5	444.4	451.0	494.8	563.0
415.85\
DRAP1	236.2	358.4	97.3	352.9	109.0	442.1	463.1
150.0	188.0	341.3	133.7	420.0	281.9	226.2	202.3
406.9	325.2	332.9	386.2	237.1	186.8	163.6	146.4
211.5	264.3	205.1	312.0	190.7	286.2	148.0	206.9
213.5	234.1	233.6	367.3	189.4	253.3	192.1	270.7
692.3	379.8	387.0	251.7	481.5	214.9	312.2	79.8
241.2	123.0	205.0	208.0	190.8	135.8	20.7	182.7
188.8	284.0	232.7	247.5	275.7	455.8	285.2	124.2
150.4	174.5	302.2	202.9	314.5	261.1	136.2	174.2
351.4	296.4	230.8	190.3	204.4	163.9	151.1	190.5	87.8
	162.9	94.1	256.1	222.0	144.3	157.6	81.8
162.3	322.1	397.1	254.5	73.9	196.3	193.2	188.2
151.7	207.5	163.2	157.6	121.4	174.2	225.3	217.5\
AF086125 /// RP11-61A14.1	27.0	4.5	28.8	34.4	5.1
	16.9	2.5	11.2	4.1	21.7	11.3	5.1	9.9
	42.6	20.6	7.9	70.8	3.1	3.3	32.7	83.6
	5.8	18.1	7.1	7.1	6.4	4.5	8.8	13.1
	48.2	3.0	28.8	6.7	19.1	9.0	19.6	4.3
	79.0	5.3	4.0	4.2	45.7	4.9	33.3	8.9
	3.0	20.4	6.7	36.4	7.7	12.2	20.0	17.7
	12.2	34.0	19.1	5.8	8.3	34.1	11.3	18.0
	5.0	14.6	29.5	50.4	6.6	2.9	2.0	5.9
	24.0	9.3	14.0	16.2	12.2	70.1	18.3	8.8
	39.8	62.1	7.9	11.2	7.1	5.9	5.0
100.2	93.4	32.2	20.3	54.6	13.8	7.1	15.2	36.0
	4.4	7.1	10.0	15.5	38.2	5.9	55.6	33.7
	11.7	18.0\
GLULP4 /// GLULP4	11.7	67.2	42.3	68.7	141.6	21.0
	50.4	19.9	10.9	98.8	69.8	123.9	99.8	57.0
	128.9	72.1	61.1	58.2	44.2	57.0	248.5	81.6
	222.3	36.6	88.9	23.4	37.0	100.5	73.6	34.6
	104.2	136.6	87.1	70.1	122.6	70.6	41.6	14.9
	5.0	40.5	86.1	18.0	183.0	65.4	59.0
113.7	125.3	55.7	216.3	92.0	91.4	50.4	85.5	33.5
	28.6	54.1	64.9	18.5	48.9	71.4	29.7
115.4	54.2	8.7	66.0	74.1	40.8	11.1	72.4	52.0
	120.9	60.9	41.2	59.8	72.0	19.6	141.7	8.6
	69.4	18.8	69.9	95.2	19.7	58.6	15.3	59.6
	75.7	15.8	112.7	127.8	44.9	125.0	13.1	17.4
	12.7	9.4	11.3	36.5	17.8	67.5	7.6	17.1
```

55.5\
ND3 /// SH3KBP1 29404.3 102237.8 63410.2 55721.1 103526.2
38872.3 34656.1 19832.4 47044.7 78268.5 35435.1 50783.8
58190.8 34937.6 83121.7 50120.6 49659.3 91219.9 47730.5 45470.6
95788.1 53746.7 118619.2 33471.8 41492.6 50252.5 30576.7
72056.7 63603.2 32131.8 36784.9 92905.1 83784.4 55669.2 70671.4
68891.1 36419.0 77793.1 44355.3 46427.4 36117.4 39735.9 74032.5
44706.2 55844.7 65370.6 34521.2 51731.2 87079.8 62506.4 57165.0
45196.5 42770.4 25785.7 63790.3 83620.5 64278.6 71547.5 49380.4
100811.4 155372.5 78906.8 66796.9 48652.3 62115.5
65049.7 42393.1 47226.1 103160.1 53765.9 113210.9
110099.4 62139.0 38453.6 46562.7 39757.3 18724.6 33423.1
41313.0 5547.2 1707.4 8670.5 45180.8 33027.1 62144.6 59862.3
39625.4 21979.2 64420.6 91128.7 47858.9 19182.5 40677.3 29263.4
38793.8 53039.5 61286.4 34253.7 76440.6 53431.6 49345.4 64863.5
47172.3\
C6orf10 19.1 59.2 35.1 1.9 101.7 4.3 40.2 10.2
3.4 73.3 31.6 21.2 8.7 5.0 8.4 7.1
13.3 11.4 31.8 71.9 103.9 5.8 55.6 7.5
23.7 4.3 2.7 20.5 33.3 60.6 6.5 5.9
10.3 7.0 11.5 33.2 10.6 15.7 37.0 2.3
27.7 41.0 67.1 34.9 13.7 12.7 3.5 15.6
18.1 135.2 64.6 14.1 19.6 4.0 3.8 14.1
5.4 78.7 23.4 12.1 95.1 45.4 29.8 10.7
73.2 54.2 16.1 45.9 56.2 25.5 7.3 63.3
26.5 10.2 44.5 7.3 5.6 1.0 10.0 26.0
68.2 8.7 61.8 43.0 4.3 43.9 8.7 6.3
12.3 54.8 35.6 3.2 26.4 2.6 7.1 55.6
7.5 11.6 3.4 5.1 45.3 15.0 8.7\
LOC220077 22.299999999999997 9.9 18.8 10.75 46.2
38.65 7.550000000000001 14.25 33.95 44.5
18.65 49.900000000000006 37.45 16.95 19.099999999999998
14.649999999999999 74.9 40.2 39.449999999999996
19.2 34.15 19.9 47.650000000000006 10.25 16.0
24.25 19.849999999999998 32.15 28.15 32.75 10.5
131.4 34.35 12.95 14.25 30.4 12.45 17.5 7.0
41.45 10.05 19.05 13.850000000000001 11.2
49.15 22.2 34.75 25.75 37.35 21.55 30.8 20.4
25.35 40.25 49.900000000000006 55.2 24.1 20.4
23.549999999999997 66.75 188.3 72.6 28.05 11.05 29.5
45.5 35.3 31.55 24.5 16.35 99.5 44.95
17.15 9.15 20.4 67.89999999999999 75.05 28.35
22.35 20.55 16.2 21.75 14.5 11.55 30.0 76.15 76.1
27.45 15.15 31.8 16.0 21.599999999999998
12.950000000000001 8.0 14.05 44.050000000000004 10.0
24.15 26.400000000000002 21.650000000000002
11.899999999999999 41.7 18.700000000000003\
CELP 4.7 46.1 6.2 12.8 20.5 7.6 6.9 22.5
63.8 139.9 106.8 5.5 4.2 5.3 74.0 20.6
7.5 30.6 3.8 7.2 15.5 13.1 69.8 5.9
14.4 19.3 11.6 24.8 20.9 5.5 7.6 16.5
9.6 8.2 10.2 26.4 12.2 22.4 6.4 8.6
9.1 48.3 28.2 32.8 16.7 32.2 4.8 63.5
21.9 283.4 205.8 11.0 4.7 11.1 28.0

231.8	11.5	17.6	6.7	17.1	99.4	221.8	9.4	15.8
	22.4	85.8	18.9	5.2	17.5	18.0	30.5	51.0
	7.1	8.1	44.4	26.3	15.6	5.6	97.0	7.2
	26.3	12.9	16.4	43.5	14.5	43.9	6.1	10.7
	20.4	25.2	70.5	14.8	17.1	29.8	39.0	54.2
	49.7	8.4	17.4	33.9	16.6	26.7	54.6\
MNAT1	395.1	760.4	423.4	327.3	233.1	505.5	335.0
474.7	251.6	334.7	487.8	332.6	712.6	587.6	433.3
316.5	321.7	339.7	298.3	423.2	212.6	313.8	369.2
146.3	218.8	367.3	384.8	308.0	402.0	287.1	432.8
235.5	426.1	372.8	283.7	409.7	484.8	349.3	252.2
1095.1	308.7	548.5	311.5	385.1	356.0	354.2	227.7
462.0	273.6	247.2	333.4	474.5	351.1	330.6	479.6
472.2	241.7	300.7	431.8	345.4	389.9	401.1	471.7
466.2	270.7	240.2	322.9	302.0	435.2	526.3	465.5
475.2	194.0	741.6	633.4	492.1	374.1	525.0	351.6
416.7	312.7	483.8	398.0	292.6	368.1	307.0	253.0
374.6	273.1	323.3	270.4	178.1	562.4	426.3	379.1
363.1	461.8	353.4	683.7	410.8	333.1	270.6	320.3\
PIWIL2	12.6	75.96666666666667	44.96666666666667
43.166666666666664	118.86666666666667	11.866666666666665
	10.766666666666666	41.5	60.666666666666664
21.333333333333332	34.63333333333333	35.56666666666666
	24.299999999999997	22.299999999999997	119.8
27.53333333333333	61.03333333333333	57.73333333333333
	23.3	32.03333333333333	127.33333333333333
49.96666666666667	78.76666666666667	39.56666666666667
	69.1	32.6	8.066666666666666	63.70000000000001
	82.26666666666667	66.03333333333333
58.56666666666666	73.36666666666666	25.299999999999997
	37.76666666666667	73.03333333333335
478.06666666666666	34.733333333333334	22.099999999999998
	8.433333333333335	27.933333333333334
24.933333333333334	43.4	31.066666666666663
49.73333333333333	21.8	42.800000000000004	78.6
69.23333333333333	30.96666666666667	58.633333333333326
	45.46666666666667	66.1	23.933333333333334
17.633333333333333	50.06666666666667	52.56666666666666
	50.666666666666664	56.56666666666666
16.73333333333333	29.666666666666668	105.96666666666665
	86.0	26.599999999999998	23.866666666666664
59.70000000000001	41.93333333333333	52.56666666666666
	43.599999999999994	50.96666666666667
54.866666666666674	190.93333333333337	97.13333333333334
	116.83333333333333	25.900000000000002
42.46666666666666	59.20000000000001	63.86666666666667
	57.56666666666667	79.5	39.233333333333334
59.76666666666666	30.133333333333336	70.7	45.0
61.099999999999994	93.60000000000001	43.0
63.43333333333333	27.8	24.733333333333334	19.0	69.0
	67.5	50.666666666666664	68.03333333333335
71.33333333333333	81.76666666666667	53.96666666666667
	33.56666666666667	48.166666666666664	121.7
163.1	78.43333333333334\

KCNK5 60.9 419.2 339.8 320.2 640.1 106.3 815.1
615.7 374.8 424.7 545.2 293.6 270.9 270.6 747.5
311.6 547.9 374.3 585.4 613.0 374.4 316.4 484.4
456.0 399.3 478.6 628.9 399.5 370.1 367.5 271.9
752.6 374.5 281.4 848.7 429.3 222.5 609.2 495.9
336.5 872.2 510.7 563.1 206.5 477.8 322.9 412.6
386.3 833.2 521.2 582.1 474.7 873.1 493.6 315.3
271.6 291.0 273.8 235.6 203.9 217.3 401.8 542.2
794.3 340.0 802.1 303.9 287.8 261.9 475.7 368.8
303.4 274.8 366.1 278.9 416.9 186.6 288.2 425.3
337.3 317.1 138.4 988.2 903.9 1252.9 584.5 844.9
415.9 879.8 302.4 571.6 378.6 299.7 646.1 432.1
640.0 509.2 782.8 328.2 549.8 377.9 216.0 455.0\
BCAR3 83.1 159.1 398.8 186.4 284.7 357.9 59.9
194.0 880.9 356.1 1047.9 127.2 706.9 337.8 548.6
248.1 670.8 183.2 207.1 556.3 319.0 377.0 248.2
394.2 228.9 142.2 329.5 803.6 291.3 627.9 227.5
112.4 304.2 799.6 199.4 219.2 465.9 245.1 868.1
567.5 391.7 199.4 748.1 948.1 672.7 307.5 199.7
594.0 62.9 336.6 358.6 301.9 190.4 169.6 1034.1
396.8 221.9 267.7 326.0 502.4 447.3 208.7 299.6
250.8 330.5 374.3 311.8 1015.4 255.7 621.4 477.7
190.4 289.1 489.9 299.5 839.3 265.3 516.1 214.9
281.3 273.3 826.4 439.9 159.5 988.8 271.8 173.1
255.8 780.7 321.9 415.1 339.4 254.5 432.7 153.9
373.1 196.6 363.2 177.2 352.6 251.6 249.4 332.2\
PIWIL1 49.8 93.6 92.9 9.5 81.4 54.2 8.9 58.9
27.5 136.1 22.4 92.8 1.9 255.0 394.4
126.0 57.1 91.0 213.1 101.1 114.8 19.7 99.0 33.0
57.9 155.1 33.4 29.9 207.4 336.0 42.8 85.0
84.1 47.9 17.7 61.9 71.2 50.6 56.1
111.0 242.2 2.6 10.4 92.6 42.5 17.5 131.5 42.5
162.1 147.5 78.1 58.9 243.3 66.2 123.7 43.9
79.2 76.5 76.7 189.0 349.9 75.8 141.6 28.1
133.9 99.6 55.3 60.9 128.7 86.6 131.4
219.6 136.5 34.9 74.5 22.4 138.4 40.2 113.2 35.3
16.9 51.1 60.3 26.7 136.1 108.5 37.1 84.9
34.1 77.3 56.7 92.7 76.6 92.0 41.6
125.1 113.2 59.0 189.5 120.9 125.5 72.9 124.9\
KCNK6 149.5 231.4 98.4 89.0 164.2 185.4 68.4
183.2 271.8 293.1 88.7 218.0 165.3 390.9 289.6
166.0 126.3 327.2 166.8 196.7 278.3 131.5 241.5
470.4 112.5 257.2 313.6 361.3 433.5 179.8 181.3
207.6 251.2 340.7 204.4 354.8 87.9 135.8 403.7
171.0 38.8 162.4 345.5 191.3 554.8 263.8 303.0
150.6 264.1 277.4 191.3 133.7 156.2 221.7 300.3
126.8 295.7 172.0 193.1 159.6 602.6 237.0 161.8
261.7 300.3 231.4 194.0 132.1 383.7 352.2 282.7
383.0 188.4 312.1 257.9 448.9 425.9 1155.5 175.8
201.2 256.0 68.1 236.6 165.4 193.4 269.6 132.0
202.8 198.7 80.5 83.4 230.6 225.7 190.5 124.4
111.8 146.7 136.6 379.7 195.5 126.2 167.5 194.0\
KCNK7 23.866666666666664 15.433333333333332
9.066666666666666 29.733333333333334 24.033333333333335

50.133333333333326	16.099999999999998	22.2
10.299999999999999	14.566666666666668	10.9
18.833333333333332	9.266666666666666	27.600000000000005
9.233333333333333	10.833333333333334
7.233333333333334	42.333333333333336	5.5
26.96666666666667	35.06666666666667	14.866666666666667
14.866666666666665	33.833333333333336
6.933333333333334	57.43333333333334	24.96666666666667
19.866666666666664	46.13333333333333	16.1
24.866666666666664	17.96666666666667	16.599999999999998
21.933333333333334	15.733333333333334	40.9	8.9
12.033333333333331	7.666666666666667	30.5
34.63333333333333	25.333333333333332	15.9	21.5
44.166666666666664	10.799999999999999	9.0
10.833333333333334	23.03333333333333	33.96666666666667
35.833333333333336	46.333333333333336
12.833333333333334	55.96666666666666	11.666666666666666
58.1	21.3	43.23333333333333	12.266666666666667
8.766666666666667	132.56666666666666	20.7
7.800000000000001	25.96666666666667	13.033333333333333
26.833333333333332	12.466666666666667
25.666666666666668	24.166666666666668	26.03333333333333
23.833333333333332	26.233333333333334
11.700000000000001	28.433333333333337	38.86666666666667
32.333333333333336	8.5	8.333333333333334
11.733333333333334	8.833333333333334	41.699999999999996
16.433333333333334	41.43333333333333
43.166666666666664	56.13333333333333	43.6	42.5
16.166666666666668	35.06666666666667	13.033333333333331
18.233333333333334	26.73333333333333	48.4
28.566666666666666	54.9	55.300000000000004
16.23333333333333	36.166666666666664	21.46666666666667
12.866666666666665	35.0	15.666666666666664
62.20000000000001\
ALAS2	21.2	12.4	37.15	14.1	141.6	36.65
13.600000000000001	29.85	32.85	64.7	48.2	33.65	29.5
27.0	51.199999999999996	26.0	32.1	41.75	36.6
40.9	88.95	39.9	106.8	37.65	45.900000000000006
31.35	5.75	93.8	76.6	34.75	22.400000000000002
52.5	31.35	67.9	10.8	40.35	5.699999999999999
64.3	22.200000000000003	16.200000000000003	23.0
37.35	73.8	26.799999999999997	43.199999999999996
73.3	34.2	40.15	67.4	39.5	108.4	10.3
41.45	95.4	59.0	62.699999999999996	36.8	52.35
24.15	14.1	28.15	35.4	49.449999999999996	11.45	48.6
50.35	17.5	26.650000000000002	30.65	38.45
29.45	55.15	48.2	4.1	49.7	29.25	12.05	37.9
34.550000000000004	30.0	26.450000000000003
23.650000000000002	15.1	30.950000000000003	21.75
74.35	32.699999999999996	51.5	37.95	65.05	32.6
55.099999999999994	22.5	5.9	5.55	33.5	8.55
30.25	39.75	51.849999999999994	65.5	11.0	34.85\
PIWIL4	92.6	60.5	586.6	114.1	377.1	180.3	41.4
237.4	117.3	150.6	152.0	78.8	90.8	130.8	520.5	84.8

115.9 300.4 577.2 79.3 567.8 198.4 589.4
234.8 99.1 155.4 106.7 386.4 247.1 120.8 205.6
392.3 345.0 135.3 10.7 382.5 74.5 232.6 140.6
122.2 557.2 94.8 135.4 141.7 50.0 81.6 176.2 93.2
316.9 256.8 200.3 282.2 191.2 193.3 148.3
208.7 138.9 275.9 337.3 153.6 165.2 324.6 274.6
286.4 111.9 352.5 151.1 189.2 201.2 154.4 297.1
258.1 132.1 7.5 123.3 76.3 267.2 102.1 154.2 75.5
205.5 279.1 117.7 165.0 581.6 1552.0 350.8
172.1 733.8 610.6 111.8 347.1 123.0 211.6 178.5
140.5 244.7 206.7 207.8 304.9 213.8 274.0 244.8\
BCAR4 33.5 9.3 12.7 2.2 3.9 49.8 3.6 48.1
5.1 7.6 52.4 38.7 15.7 42.6 4.1 49.2
27.3 18.0 36.3 49.3 116.4 46.3 108.1 4.7
29.5 8.6 4.7 70.5 39.9 3.3 3.1 11.0
17.9 55.9 66.7 90.3 19.3 33.0 31.8 62.2
22.3 2.5 73.7 6.3 17.4 5.1 32.3 2.6
8.3 7.4 12.8 28.7 6.4 44.3 28.1 10.0
3.9 54.7 7.9 23.1 235.5 2.9 66.0 10.6
18.9 38.8 8.1 35.3 81.4 16.7 6.1 54.0
10.0 51.5 4.6 43.3 32.9 3.4 3.5 46.2
62.7 36.7 7.0 6.1 60.6 94.3 10.2 3.5
3.6 2235.4 35.1 9.5 6.9 2.3 3.7 25.8
11.4 5.6 44.7 10.8 8.4 96.4 26.4\
ALAS1 1804.2 770.2 885.7 1903.2 767.5 3098.7 2773.4
2475.0 1514.0 1748.8 1694.9 1258.0 1952.4 2797.7 604.0
1129.9 3338.3 1455.4 1831.8 2157.4 573.5 1442.1 603.1
1779.7 1265.3 895.6 2456.6 730.7 1085.5 1558.9 1094.6
793.9 594.9 1659.1 3318.8 1632.6 2714.7 613.6 2773.1
2154.7 1185.3 1691.0 1108.7 1684.3 1999.7 1106.0 2559.9
1312.6 368.2 1040.1 1536.0 2407.8 1349.9 846.2 1016.7
1495.1 1108.0 1284.9 3380.8 548.7 585.0 3286.0 1442.3
2244.7 644.3 1686.0 1766.6 1785.3 933.8 1281.2 364.7
472.5 1251.8 2992.2 1123.5 2221.7 1104.2 2479.6 1159.7
1324.2 541.8 816.5 1294.0 1457.8 729.2 525.7 1448.8
1250.3 1314.4 746.2 3027.7 477.1 681.2 1002.3 776.2
700.6 606.7 838.7 773.0 550.9 652.4 688.4 907.8\
LOC284933 66.8 30.5 101.0 16.5 17.2 28.4 14.3
17.1 20.6 15.7 69.6 19.2 14.1 48.2 61.6
111.1 29.1 88.2 5.0 108.7 217.4 104.7 44.2
51.7 90.7 65.9 30.0 55.0 91.4 25.1 15.2
210.2 29.2 89.5 89.4 37.2 104.6 49.0 26.0
32.8 17.1 113.1 12.8 22.8 12.6 19.0 30.1
100.8 246.1 42.8 21.0 102.6 18.3 49.2
132.0 40.7 34.0 100.0 23.7 61.0 199.9 152.4 16.2
35.1 14.0 41.2 9.7 11.0 31.9 82.9 51.9
96.2 36.6 29.6 303.3 53.9 35.4 18.2 16.8
13.2 40.9 15.1 27.5 20.7 151.8 82.8 27.2
48.1 23.5 47.0 8.8 21.4 95.3 17.5 16.4
16.6 35.7 19.4 33.2 26.9 70.5 76.6
134.1\
ONECUT2 55.480000000000004 105.1 95.05999999999999
33.94 126.85999999999999 35.959999999999994
225.14000000000001 23.240000000000002 41.720000000000006

	91.46000000000001	50.3	74.14	28.98	47.22
939.9199999999998	106.94000000000001	149.64000000000001
	97.76	57.379999999999995	143.88	85.78	22.92
135.84	33.56	104.82000000000001	125.62	61.48
117.03999999999999	53.38000000000001	67.53999999999999
	52.1	57.67999999999999	94.44000000000001
66.16	71.16	45.38	98.96000000000001	33.22
24.880000000000003	19.72	156.11999999999998
160.76000000000002	64.98	96.67999999999999	35.94	56.4
	56.68000000000001	76.82000000000001	160.02
137.94	59.4	33.480000000000004	86.5	96.23999999999998
	30.680000000000007	51.94000000000001	96.76
61.239999999999995	37.62	113.92	151.57999999999998	58.1
	74.02	49.519999999999996	40.28	178.37999999999997
	45.6	40.42	80.78	46.64	62.67999999999999
139.3	54.620000000000005	67.14	84.22	20.540000000000003
	131.54	64.48	23.66	79.53999999999999
80.96000000000001	1011.36	33.739999999999995
114.48000000000002	2398.0600000000004	52.580000000000005
	90.05999999999999	55.0	188.82	52.42	37.42
55.419999999999995	41.56	55.43999999999998	49.0
55.720000000000006	16.580000000000002	15.960000000000003
	42.4	51.7	47.98	32.62	35.92\
KCNK9	18.549999999999997	81.4	25.15	10.75	26.15	52.2
	13.1	25.25	47.7	33.599999999999994
29.450000000000003	41.4	61.099999999999994	13.75
108.05	92.55	34.4	53.2	72.6	25.05	75.05	58.05
54.349999999999994	11.85	34.6	60.099999999999994
14.05	69.15	133.35	43.5	11.45	29.549999999999997
43.45	116.95	66.75	74.9	70.55000000000001	29.85
34.35	36.25	15.8	24.75	65.4	12.6	10.7	77.9	89.5
	30.4	89.1	39.75	48.05	127.0	11.350000000000001
	95.6	19.6	234.65	22.45	31.35	20.95
59.199999999999996	512.35	26.65	59.3	57.6	19.3
26.85	23.2	21.549999999999997	27.45	30.2	69.95
30.55	44.8	23.0	25.65	46.1	105.80000000000001	33.7
	13.55	28.4	107.0	37.05	177.89999999999998
118.3	84.4	116.4	25.3	94.55	53.2	50.5	38.95
114.65	50.0	20.05	82.9	17.6	59.599999999999994	50.2
	22.75	78.8	55.0	45.55	59.65\
ONECUT3	3.3	131.5	18.8	53.6	55.7	45.0	42.0	84.1
	76.6	63.7	86.2	52.5	71.6	82.0	279.2
141.2	95.8	141.4	21.7	57.1	29.0	160.0	281.5	18.8
	136.4	63.2	129.0	180.9	194.4	84.4	69.9
161.3	81.4	175.1	211.3	71.6	125.2	129.9	49.1	96.1
	55.8	112.8	130.8	76.9	114.3	113.2	67.5	96.7
	220.7	109.9	65.4	143.4	67.6	53.4	113.9
115.5	133.1	66.1	129.7	17.8	78.6	101.6	85.9	64.9
	52.7	176.7	11.2	42.3	65.3	165.0	155.0
181.8	157.5	111.1	72.9	98.6	206.1	71.4	52.2	81.7
	43.0	192.2	71.6	27.7	200.0	207.0	105.8	94.0
	115.1	133.0	12.1	95.6	67.3	43.9	58.2
153.5	89.7	129.6	110.1	151.0	51.2	153.1	137.4\
LRRC37BP1		25.6	32.5	10.9	7.1	75.4	20.2	2.9

51.6 6.5 7.0 21.5 66.5 4.2 11.7
125.5 75.5 32.1 61.7 17.0 72.5 48.3 11.5 17.9
15.4 9.9 101.7 22.4 13.4 26.2 4.0 5.6
82.7 82.7 11.2 11.0 4.9 74.9 18.8 24.1
42.0 34.0 35.0 50.6 37.7 48.1 24.4 12.8
11.5 29.5 28.3 60.0 27.1 55.8 113.7 30.0
16.0 8.8 120.8 37.8 15.7 67.3 30.7 42.2
40.0 48.5 73.1 18.5 5.1 27.0 22.5 85.6
27.6 108.5 3.8 89.2 17.8 9.3 34.6 35.8
22.4 100.7 65.2 9.6 7.0 83.5 28.7
112.6 9.6 65.3 64.9 10.9 64.4 8.9 32.6 9.9
44.5 33.6 12.6 51.8 13.9 9.9 46.8
55.6\
ONECUT1 2.4 81.5 10.8 63.9 121.4 5.1 66.4
108.5 63.5 25.8 50.4 66.0 81.5 41.8 151.5 58.3
85.2 186.4 123.5 46.3 212.1 90.3 165.5 49.2
87.9 77.8 39.8 116.7 58.4 11.1 9.7 77.2
15.1 29.0 65.3 54.9 9.6 135.6 68.5
101.5 80.0 4.2 94.7 55.9 84.9 15.7 120.5 41.3
108.5 25.6 32.2 72.5 9.3 151.2 139.0
216.2 107.8 160.4 74.7 29.9 181.7 74.1 49.1 18.0
67.5 44.1 55.5 72.2 77.3 62.6 20.9
180.1 38.5 70.1 10.9 66.2 57.9 78.9 37.2 67.5
146.2 134.1 56.2 104.9 30.0 400.0 224.6 12.8
86.0 122.1 32.3 135.2 19.2 53.4 43.5 54.9
79.7 88.8 82.2 79.9 36.8 89.8 67.4\
LOC284930 30.3 20.0 11.3 7.7 14.8 5.8 36.8
90.0 18.7 21.1 11.2 24.2 6.6 30.0
104.6 12.2 13.6 32.5 30.7 15.0 49.2 2.7 18.1
4.9 11.3 8.5 5.3 29.1 12.5 34.4 17.0
101.6 10.2 26.8 26.6 79.4 51.5 23.9 4.6
6.9 26.3 2.2 16.3 9.5 20.5 7.8 11.4
16.2 74.7 15.1 13.9 12.3 5.7 10.7 64.5
66.4 17.6 17.8 16.8 16.4 57.8 161.9 10.9
11.6 46.9 11.8 8.0 21.4 57.6 6.9 27.0
12.8 3.5 105.4 28.9 15.2 16.5 7.6 7.5
41.2 109.8 99.1 61.3 16.5 37.0 17.7 97.0
10.7 40.1 11.8 53.8 118.9 8.8 33.6 42.5
7.9 16.8 77.6 45.4 13.0 8.3 81.7
52.6\
HTR2B 109.4 105.0 69.7 719.3 120.3 171.3 342.7
287.9 2394.2 157.2 38.3 276.5 75.4 261.6 7.8 41.5
696.1 218.8 228.9 356.2 172.1 206.2 259.0
3607.6 198.1 12.5 238.1 165.7 206.2 522.3 2530.2
153.1 58.4 281.4 15.8 133.3 400.7 34.8 70.2
599.2 200.7 90.7 809.6 641.2 415.0 79.7 26.2 77.5
81.0 63.1 536.8 226.6 18.5 118.3 362.9 9.9
144.4 287.0 120.1 274.4 38.3 322.6 271.8
167.0 307.9 45.5 250.1 144.6 369.2 92.3 113.2
140.0 695.8 482.2 285.0 295.0 168.5 311.2 1298.1 50.1
138.0 74.2 17.1 189.0 26.7 15.3 503.9
192.7 167.2 83.0 420.4 913.1 553.6 1087.3 4343.0
1200.5 1195.4 753.7 110.2 603.4 1178.5 908.9 418.3\
PPWD1 367.5 874.9 483.6 490.45 490.45000000000005

271.55 414.54999999999995   176.15 221.0   260.55 319.4
370.0   374.25 373.0   236.45000000000002   460.95000000000005
        239.85 378.45000000000005   234.29999999999998
274.85 603.8   390.6   477.5   312.40000000000003
201.29999999999998   396.34999999999997   291.45 513.55
508.3   358.95000000000005   448.45 291.90000000000003
494.4   338.25 158.10000000000002   474.8   296.85
347.59999999999997   388.2   188.75 275.5   503.1   544.45
353.35 321.3   487.6   234.89999999999998   239.2   570.85
643.0999999999999   305.65 194.8   395.7   420.05 547.5
470.85 583.95 509.0   521.2   782.3499999999999   237.1
658.25 413.35 296.54999999999995   591.4000000000001
590.1   431.5   372.1   414.65 344.65 533.55 666.8   757.8
516.25 342.4   218.95000000000002   403.55 444.1   415.9
194.0   250.6   224.75 224.60000000000002   510.54999999999995
        454.90000000000003   531.5   254.1   88.9
309.04999999999995   295.5   297.85 622.8   414.75 626.95
717.25 727.2   642.15 635.1   842.55 837.3499999999999
768.5   356.25 598.0\
HTR2C   34.8   6.8999999999999995   4.5   10.7
70.80000000000001   3.8   18.400000000000002   10.45
23.349999999999998   26.75   47.849999999999994   24.35
13.05   19.099999999999998   190.20000000000002
22.400000000000002   21.900000000000002   17.85   3.8
15.05   10.35   10.9   54.25   1.7000000000000002   9.55
19.35   4.5   27.65   20.0   34.5   9.5   446.29999999999995
        31.05   20.35   27.2   61.95   37.2   39.3   3.5
18.299999999999997   19.15   5.050000000000001   23.25   27.6
        27.65   5.3   31.55   28.1   31.35   28.299999999999997
        3.65   3.8   14.7   4.6   28.0   43.8   15.95
3.5999999999999996   22.9   19.700000000000003   209.7
17.95   104.75 13.899999999999999   13.45   43.9   10.1
11.85   18.400000000000002   19.599999999999998
52.599999999999994   69.5   26.15   19.05   26.95   16.9
37.15   4.3   4.95   44.75   32.65   12.55   98.3
84.10000000000001   14.55   19.1   38.05   5.15   4.55   32.1
        16.25   54.25   18.0   23.3   12.299999999999999   11.3
        32.0   35.25   42.199999999999996   57.45   19.05
2.9000000000000004   14.700000000000001\
NENF   321.7   313.53333333333336   101.05 262.84999999999997
        293.5333333333333   288.18333333333334
123.91666666666667   432.75 187.38333333333333
268.90000000000003   204.64999999999998   231.25
162.26666666666665   179.28333333333333   329.90000000000003
        296.9833333333333   328.3   168.79999999999998
188.63333333333333   227.91666666666666   314.1666666666667
        178.86666666666667   175.51666666666665
184.58333333333334   204.70000000000002   261.2   314.55
343.8333333333333   222.8166666666667   453.7333333333333
        318.03333333333336   166.4   160.0   336.78333333333336
        477.45 344.7333333333333   214.4   219.48333333333332
        230.63333333333333   335.16666666666663
303.4166666666667   214.01666666666665   157.51666666666665
        128.33333333333334   158.0666666666667

255.14999999999998	451.90000000000003	624.7833333333333
	304.4166666666667	470.06666666666666
312.56666666666666	286.8333333333333	595.5833333333334
	434.65000000000003	172.94999999999996
545.6833333333333	272.55	252.11666666666665
222.0666666666667	181.4	895.8166666666666	228.1
212.3666666666667	209.2166666666667	299.5
347.83333333333326	261.51666666666665	162.28333333333333
	228.20000000000005	268.59999999999997
183.73333333333335	292.2666666666667	215.61666666666667
	284.58333333333337	207.94999999999996
313.26666666666665	336.05	202.5	337.56666666666666
560.3166666666667	341.0166666666667	351.1166666666666
	167.4	299.93333333333334	506.8666666666666
231.10000000000002	370.93333333333334	245.1833333333333
	264.90000000000003	173.20000000000002
273.3333333333333	398.93333333333334	636.4666666666667
	428.2833333333333	450.15000000000003	543.25
553.2166666666666	419.7	443.8833333333334
495.6166666666666	327.76666666666665	397.1666666666667
	385.51666666666665\
HTR2A	44.4	109.0	59.333333333333336	43.9
85.80000000000001	23.733333333333334	35.43333333333333
	79.76666666666667	104.63333333333333
38.36666666666667	17.766666666666666	42.16666666666667
	37.800000000000004	59.26666666666667
59.46666666666667	67.46666666666665	73.39999999999999
	59.06666666666666	41.766666666666666
25.633333333333336	167.7	57.5	118.36666666666666
43.70000000000001	63.76666666666667	35.733333333333334
	26.899999999999995	87.53333333333335
36.46666666666667	45.06666666666666	48.73333333333333
	78.23333333333333	76.73333333333333
31.966666666666665	52.1	59.26666666666667	33.5
49.133333333333326	30.600000000000005	53.26666666666667
	44.23333333333333	52.76666666666666
74.63333333333333	39.96666666666667	36.53333333333333
	99.66666666666667	91.39999999999999
55.633333333333326	37.400000000000006	69.7
62.76666666666667	42.70000000000001	43.5
80.69999999999999	40.53333333333333	20.400000000000002
	80.7	55.300000000000004	94.83333333333333
102.0	174.76666666666665	70.86666666666667	68.2
61.43333333333334	65.8	54.76666666666667
38.56666666666667	49.73333333333333	73.86666666666666
	79.96666666666667	106.46666666666665
103.93333333333334	48.633333333333326	32.1
11.966666666666667	45.53333333333334	79.06666666666666
	38.9	58.96666666666666	66.2	57.4	63.0	60.1
	39.666666666666664	98.36666666666667
44.23333333333333	88.03333333333335	49.96666666666666
	57.199999999999996	86.63333333333334
41.13333333333333	92.5	74.1	54.800000000000004
40.300000000000004	37.36666666666667	39.4

34.13333333333333	57.46666666666667	47.96666666666666
	54.36666666666667	77.39999999999999
74.33333333333333\
TNFSF13B	240.15	26.8	632.45	3095.3	769.4	186.65
638.25	1378.6	244.75	470.5999999999997	302.2999999999995
	978.35	192.45	1218.2	276.85	893.6	743.05	277.65
905.15	445.25	1503.45	308.9	740.1500000000001	1425.1
215.6	197.25	324.95	1114.2	229.79999999999998	275.4
441.25	2463.0	818.1	398.7	384.2999999999995	498.35
597.3	397.65	559.5999999999999	879.75	165.39999999999998
	196.3	835.0	358.2	646.05	281.15	501.7	146.8
945.75	971.2	563.8499999999999	363.7	189.2	548.8
344.0	785.5	770.95	330.9	588.1	389.35	609.3	1218.85
517.55	252.6	1215.7	814.55	574.85	881.95	756.7	409.9
571.9	617.4	1039.9	719.35	926.05	373.95	358.35	541.5
811.3499999999999	82.8	219.25	618.3499999999999
78.55	1914.0	590.7	474.8	277.65	433.20000000000005
414.70000000000005	545.35	619.05	294.65	325.95	555.2
437.75	445.0	815.25	389.0	830.6999999999999	838.3
617.4	434.15	462.5\
LOC102724975	8.2	25.6	14.9	17.2	26.9	53.2	34.1
	11.8	10.7	20.7	6.4	25.3	54.6	7.4	12.8
	10.4	8.1	14.9	16.5	100.9	79.0	13.8	87.1
	11.9	8.1	16.7	9.4	61.8	10.6	30.5	5.3
	34.4	16.3	7.3	77.8	60.1	8.8	97.7	11.2
	8.3	6.1	19.2	8.4	5.7	27.7	20.4	24.9
	12.0	36.1	8.6	15.2	10.7	8.5	24.9	12.0
	22.8	20.7	9.2	94.3	35.9	15.0	18.9	7.6
	5.4	8.3	19.6	3.0	29.1	56.2	12.3	21.0
	107.4	10.0	42.7	8.1	6.4	51.9	3.7	9.4
	30.9	96.2	19.5	15.8	12.4	40.3	23.6	20.1
	31.4	13.4	11.6	9.4	15.0	14.5	3.5	7.3
	10.1	44.0	7.3	12.6	13.5	11.9	10.4
12.2\
BOC	294.8333333333333	641.2333333333335
238.63333333333333	256.0	1088.7333333333333
110.23333333333333	726.6	478.4666666666667
219.96666666666667	643.1666666666666	474.3333333333333
	129.9333333333333	238.5	378.4333333333334
1696.7	438.1666666666667	966.9333333333334
424.5333333333333	79.43333333333334	254.9	358.3
149.26666666666665	325.6	321.3666666666666
123.1666666666667	47.333333333333336	241.9666666666667
	417.90000000000003	218.89999999999998	283.2
343.7666666666665	718.9333333333334	279.8666666666667
	177.96666666666667	89.33333333333333	1572.2
319.5999999999997	173.0	251.9333333333333
192.60000000000002	653.0666666666667	180.0
265.8333333333333	179.1666666666666	215.73333333333335
	130.5	254.4	84.2	452.23333333333335	361.8
399.8	203.13333333333335	226.5	899.9666666666667
362.9666666666667	487.7	415.56666666666666	1036.7
442.2333333333333	283.3333333333333	426.2
293.93333333333334	666.2333333333333	228.03333333333333

178.63333333333335	294.8	101.8	152.53333333333333
660.9666666666667	292.1333333333334
713.3333333333334	132.06666666666666	200.70000000000002
128.6	201.33333333333334	208.0666666666667
221.4	60.166666666666664	160.9666666666667
264.93333333333334	433.4666666666667	738.6666666666666
334.53333333333336	301.90000000000003
155.9666666666667	297.8	1136.6333333333334	449.8
64.26666666666667	39.266666666666666	315.1666666666667
392.8333333333333	529.7333333333333	437.3
684.1666666666666	323.3666666666667	334.2333333333333
703.4666666666667	1489.1000000000001
791.5666666666666	364.6666666666667	329.3333333333333
302.93333333333334\
CKAP2 /// IGLC1 /// IGLV3-1 /// IGLV3-1 /// IGLV@	16.7	64.1
71.2	106.2	20.8	78.2	57.4	17.6	53.7	10.4
9.0	18.9	22.4	141.5	125.3	12.8	58.5	18.6
67.3	19.7	12.6	5.5	25.3	61.7	7.7	7.8
36.5	53.5	29.0	527.3	23.9	175.5	6.0	25.2
100.9	17.1	103.1	56.3	5.4	295.0	32.7	4.2
31.5	5.5	6.1	15.8	17.4	16.8	20.2	26.2
90.3	18.6	16.2	17.0	50.8	22.2	17.6	16.1
60.9	128.6	189.3	15.3	114.3	26.7	12.5	50.4
177.7	12.3	92.8	19.9	33.7	229.4	88.5	48.1
123.9	91.0	27.7	41.8	67.6	9.6	16.9	44.2
7.4	346.5	13.6	17.7	22.3	22.5	47.5	13.3
28.0	19.0	57.8	71.2	69.6	35.0	11.3	90.2
40.6	21.5	11.2	22.3	8.2\
LOC102723645	3.3	8.6	2.8	5.1	63.5	76.7	21.3
8.1	31.8	64.4	66.7	76.8	6.9	7.2	13.2
8.3	15.1	14.0	145.7	54.9	131.1	26.4	26.1
6.7	3.4	12.3	54.8	49.3	7.3	7.8	16.9
88.4	2.9	8.4	33.7	25.5	5.3	82.7	10.5
61.0	119.6	13.5	171.5	71.4	4.6	52.8	13.6
2.7	153.6	9.1	63.0	31.7	58.2	59.3	43.1
6.0	76.6	51.9	13.0	11.5	314.6	3.4	3.9
17.1	47.2	11.1	24.5	8.7	45.8	41.0
119.5	19.6	9.2	47.0	10.1	75.4	14.1	31.6	3.0
49.7	102.9	41.4	20.0	60.6	165.1	21.6	5.4
93.7	7.4	5.3	51.5	120.2	11.1	7.3	40.2
57.7	43.6	36.9	43.4	47.9	18.7	63.2
18.4\
SPDL1	2244.5	586.3	379.0	1443.1	1133.1	1495.2	1792.6
284.1	908.1	613.6	1124.0	688.4	1442.9	1529.8	1473.4
607.4	1434.1	620.6	644.9	719.1	315.2	1351.2	366.2
906.3	1904.1	807.4	1631.3	195.5	450.8	779.0	486.7
199.1	386.3	442.3	441.0	278.1	1844.6	1558.9	777.5
1569.6	1599.9	856.0	514.4	623.1	937.0	905.7	431.3
695.4	1014.9	393.3	618.8	1502.5	559.0	1075.5	973.3
422.9	417.5	754.5	523.1	498.6	25.2	459.6	387.0
333.3	206.4	329.0	558.3	351.0	412.9	732.0	194.1
519.9	141.4	679.7	555.9	978.2	294.3	342.3	398.7
1017.0	690.1	929.2	1235.9	885.6	1358.0	1388.6	976.6
357.1	3131.6	1491.2	1630.6	380.1	544.8	576.9	594.2

731.3   482.6   437.1   293.5   387.1   448.3   299.7   447.3\
NEO1   124.46666666666665   468.0   558.4333333333333
298.2   422.2   523.0666666666667   691.6333333333333
578.9333333333333   759.7666666666668   666.3666666666667
        714.7999999999998   348.59999999999997
67.23333333333333   507.6333333333334   1120.5
712.2666666666665   591.0   391.3   648.9666666666667
523.5666666666667   820.5666666666666   495.93333333333334
        730.6333333333333   782.2333333333332
340.1333333333333   90.16666666666667   346.8333333333333
        890.6   725.0333333333333   260.8   651.1   113.7
762.2666666666668   778.0   516.1999999999999
683.6333333333333   719.9000000000001   496.6666666666667
        565.7333333333333   672.4   773.0   631.6333333333333
        526.6999999999999   595.6666666666666
324.3333333333333   476.7666666666667   367.06666666666666
        524.0333333333333   289.8333333333333
738.7666666666668   614.4666666666667   518.4333333333333
        179.0   456.70000000000005   673.1333333333333
963.6666666666666   886.0666666666666   472.9666666666667
        350.0333333333333   619.3   986.7999999999998
579.6   444.90000000000003   542.8666666666667
607.0666666666667   528.6   710.8333333333334
370.43333333333334   820.2000000000002   289.73333333333335
        757.6999999999999   612.8333333333334
673.3000000000001   561.8   743.6   691.4666666666667
807.0666666666666   810.7666666666668   866.1   629.4
945.5   599.2666666666668   903.2333333333332   712.9
769.3333333333334   1010.2333333333332   536.0333333333334
        863.6   601.2333333333332   379.43333333333334
482.0   855.8333333333334   703.8000000000001
711.8333333333334   631.6   684.2333333333332   624.6
603.5333333333333   680.7333333333332   816.3666666666668
        972.0333333333334   885.1999999999999
1006.4333333333333\
DQ581328   36.2   59.6   6.2   23.5   133.5   33.1   35.9
        29.9   27.9   24.4   17.3   78.3   43.4   48.9
121.6   39.1   39.4   47.4   11.6   6.1   68.6   83.7   30.2
        14.3   91.1   55.9   38.3   169.8   43.1   13.7   11.5
        20.3   52.0   11.0   41.2   62.7   3.8   22.2   7.2
        41.7   73.2   54.5   47.1   51.2   9.6   95.3   20.9
        10.7   58.5   77.4   16.8   14.5   9.9   121.3   11.8
        78.6   62.5   19.4   119.9   70.7   324.7   28.8
126.3   12.6   24.7   20.2   12.0   59.1   30.5   29.1   24.9
        35.5   91.9   15.8   3.6   9.5   12.4   5.8   20.8
        60.5   46.4   18.0   13.6   93.1   29.9   12.5   21.4
        50.0   18.9   23.9   10.2   36.2   24.4   38.4   20.9
        55.3   119.7   8.9   15.6   94.2   9.5   9.4
10.7\
RP3-497J21.1   2.3   49.8   26.6   30.3   118.0   5.7   7.6
        64.8   18.3   63.8   7.8   56.4   75.2   48.6   38.9
        33.0   31.5   57.9   50.4   17.6   257.1   18.0
111.7   7.0   30.0   24.6   6.1   45.2   69.3   99.8   27.7
        154.6   117.2   10.5   45.6   10.4   7.3   133.1   11.7

	5.6	42.3	63.0	99.8	61.2	10.7	8.5	21.9
	6.0	77.9	37.5	35.0	4.7	3.0	91.6	23.0
	117.8	58.0	52.1	8.8	112.5	21.6	34.7	57.0
	40.8	87.4	44.2	6.9	69.7	74.0	5.2	
232.1	195.9	11.9	4.8	21.7	31.9	51.7	46.4	28.3
	49.2	62.2	8.9	48.6	20.2	25.1	100.8	33.9
	9.9	41.6	80.3	1.8	18.3	14.3	29.9	22.0
	60.6	15.6	6.4	51.7	22.3	12.9	28.1	4.7\
BCAS2	6177.5	567.1	801.8	4991.1	1736.6	3516.2	4836.0	
4896.7	4011.6	2486.6	4583.2	3027.2	4316.7	3373.6	3048.8	
2653.8	4213.2	2012.2	4116.4	3094.0	919.2	3733.9	1194.9	
4544.7	3815.8	2312.6	4141.9	1302.5	1701.4	3838.6	3843.7	
1088.6	1132.4	3729.8	2571.0	3191.6	4746.3	1090.4	3352.2	
6196.2	2790.9	3181.7	2892.7	3683.3	4156.3	1948.6	4359.9	
10612.2	567.3	1499.6	3461.7	3705.3	2723.7	1435.8	2244.8	
1312.5	1524.5	1493.0	2139.5	962.7	1724.5	1709.5	2863.1	
3850.5	1903.6	1145.7	2939.0	2450.9	1458.5	3492.3	568.4	
1161.8	1693.9	4328.5	1665.0	4845.3	1858.9	3877.4	3798.4	
5284.9	2124.9	3433.7	3703.8	2666.1	805.6	1093.3	4594.1	
2124.5	2088.1	2093.4	4995.4	1776.6	4666.5	3294.5	2828.0	
3643.6	3977.1	3611.4	1108.6	2638.3	2754.9	2672.3	3599.1\	
AX746755 /// RP11-77403.3				1422.1	1604.4	2723.3	372.7	
1580.1	481.9	957.7	1679.1	1460.4	1572.2	2103.5	2226.0	
1247.4	821.8	1609.2	3797.5	3232.9	996.3	1890.3	2641.5	
2549.8	628.6	5416.4	1730.2	956.4	436.3	248.7	1649.4	
4530.7	1175.9	2245.8	1331.1	1088.9	2218.9	681.1	3719.8	
1284.7	317.0	3514.1	825.2	973.6	635.3	1135.9	1584.7	
1990.7	2128.0	592.2	187.5	5551.5	1919.2	932.4	537.5	
197.4	2629.0	1188.2	2393.1	3915.5	1624.1	660.8	6303.5	
829.1	3232.8	2957.8	267.2	3125.3	1586.1	1936.0	4611.8	
2715.2	1562.4	1882.1	4002.3	4431.6	2783.4	4249.9	1337.1	
1756.6	1354.8	3356.6	837.4	1455.5	744.5	1331.7	767.7	
938.7	1945.0	580.9	598.6	688.1	1104.9	711.0	4489.6	
1525.1	2061.0	3676.7	2818.4	2507.8	1975.7	1710.1	3849.3	
3180.5	1601.0	2506.3\						
BCAS1	188.3	53.7	109.9	41.3	99.5	27.5	76.3	96.5
	124.0	269.4	125.9	83.5	44.4	71.3	88.7	69.8
	73.4	199.1	122.8	190.6	291.5	173.2	264.3	
182.2	89.5	254.2	229.9	163.9	87.8	239.4	55.3	
334.5	112.2	45.3	159.5	203.8	99.5	126.3	164.3	98.4
	94.5	54.6	232.9	69.0	117.2	152.2	106.7	51.8
	234.4	110.7	211.2	68.2	172.4	218.2	48.3	89.1
	170.7	98.7	196.7	169.1	494.9	153.7	264.0	
148.2	250.1	330.7	75.1	82.7	257.6	176.4	566.3	
196.5	89.0	119.9	184.6	160.1	254.2	161.0	80.2	33.3
	180.9	86.0	34.7	134.5	259.4	113.3	95.1	
274.7	162.4	295.3	43.6	164.0	124.8	61.4	84.2	
117.4	43.3	163.0	182.5	176.5	15.9	246.0	104.1\	
MBL2	1.9	3.6	7.9	1.9	6.1	1.8	1.9	1.9
	3.6	5.9	1.7	5.1	1.5	4.9	12.4	1.9
	3.1	1.6	10.5	7.5	12.0	3.9	38.0	12.9
	2.1	6.7	4.0	3.3	5.1	44.4	1.6	9.0
	51.6	2.7	4.4	5.9	5.5	13.0	23.2	3.1
	2.8	1.6	11.4	1.4	45.7	2.9	3.2	20.4

```
        4.4     1.7     29.9    6.1     6.8     6.4     10.3    7.8
        58.3    7.5     3.6     4.6     12.5    26.0    16.2    6.2
        13.9    6.1     9.5     18.2    4.1     7.0     74.8    15.2
        2.9     26.1    8.4     3.9     4.6     4.1     2.0     3.2
        39.4    19.3    2.4     4.9     30.2    63.2    2.6     5.2
        4.7     10.3    5.2     19.8    36.5    8.3     1.3     5.7
        6.1     11.2    1.6     4.2     4.0     8.2     3.2\
BOK     194.9   467.9   349.7   465.4   391.5   2123.3  332.1
178.7   547.5   516.6   970.3   824.9   507.4   321.6   55.9
186.8   325.3   468.3   1006.3  255.4   123.7   401.6   303.8
536.3   67.2    495.8   629.4   779.3   542.2   18.7    419.7
656.3   394.6   1376.3  115.7   464.1   517.9   259.2   300.7
372.5   560.7   720.0   616.4   556.7   732.9   681.8   334.3
349.1   126.3   530.6   704.2   580.2   359.6   223.8   694.5
348.7   575.6   111.4   458.0   375.0   25.6    291.8   548.5
516.2   257.0   390.9   494.3   970.4   711.3   552.0   39.1
708.2   276.1   1019.7  628.3   1303.4  492.1   893.3   671.8
190.0   60.3    300.5   247.9   241.1   207.5   262.1   178.3
940.2   233.8   105.3   374.6   477.8   1351.1  1153.2  913.4
1127.3  1381.0  1256.5  1385.0  918.2   1158.3  922.2   1444.8\
LOC100505727    31.2    106.6   92.69999999999999       77.55
112.4   44.95   36.2    104.44999999999999      41.05   23.6    57.8
        38.1    48.050000000000004      86.30000000000001
131.05  58.75   62.4    67.75   71.35   86.2    35.7    50.0
29.15   55.1    30.25   76.7    28.65   107.45  75.6    98.35   39.0
        132.85  127.10000000000001      34.2    96.44999999999999
        98.94999999999999       59.1    70.55   45.650000000000006
        5.8     36.05   53.2    24.6    56.85   48.4    92.05
19.650000000000002      84.85   115.05  43.3    61.75   34.15   50.5
        90.35000000000001       31.75   92.3    17.55
55.199999999999996      66.45   40.55   365.3   28.1
107.69999999999999      26.6    44.5    27.6    34.95   43.15
101.75  110.35  170.05  142.7   20.05   58.9    83.5    64.3    58.0
        84.1    78.1    908.05  159.64999999999998
57.650000000000006      74.7    73.05   115.2   329.8   94.75
255.0   45.05   110.35  31.75   79.1    40.85   52.35   30.75   72.9
        68.4    90.64999999999999       58.55   9.6     54.3
107.35  45.449999999999996\
BCAS4   110.85999999999999      142.44  88.5    149.38  96.66
85.16   86.35999999999999       165.44  72.32000000000001
107.84  79.72   111.72  73.64000000000001       126.48000000000002
        198.14000000000001      140.16  104.44000000000001
137.45999999999998      133.76  139.4   182.57999999999998
119.94000000000001      168.32  97.25999999999999
75.88000000000001       164.5   59.279999999999994      101.04
87.16   77.14000000000001       76.14   228.32  117.88000000000002
        114.16  218.28000000000003      73.62   83.24000000000001
        180.18  46.9    81.4    120.46  77.0    61.620000000000005
        80.38   97.9    120.01999999999998      103.94000000000001
        108.25999999999999      148.6   72.34   138.24
156.92000000000002      67.14   129.56  77.11999999999999
50.28   98.66   86.64000000000001       135.82  130.18  215.42
60.86   80.02000000000001       135.33999999999997
56.879999999999995      129.61999999999998      46.620000000000005
```

```
	95.28	168.94	129.4	116.61999999999998	98.4	60.0
	88.7	114.82000000000001	105.13999999999999
127.92	68.42	66.3	166.04	125.08	114.1	298.78000000000003
	111.7	175.8	170.64000000000001	174.16
166.54000000000002	74.04	161.11999999999998
92.75999999999999	116.1	97.52	93.55999999999999
109.24000000000001	110.46	141.68	84.8	132.16	93.1
124.51999999999998	97.2	107.17999999999999\
SLC36A1	378.0	927.8	261.9	306.5	149.65	365.84999999999997
	542.05	888.25	228.4	268.20000000000005	433.0
539.5500000000001	290.05	293.95	247.95	432.15	299.35
569.05	404.15	369.15	716.5	250.8	236.25	280.45	379.55
229.1	338.85	489.55	221.14999999999998	404.29999999999995
	322.7	291.4	1225.6	149.5	392.5	506.0	113.2
249.2	140.15	346.55	342.55	841.2	267.7	755.65	439.05
247.05	620.3000000000001	244.60000000000002	511.6
247.65	121.9	386.20000000000005	333.25	953.75	252.9
454.65000000000003	154.89999999999998	487.35	247.6
216.35	265.45	334.35	333.15	252.55	331.2	477.40000000000003
	232.5	564.9	399.6	133.4	350.85	276.1	539.25
425.2	253.1	210.5	453.35	308.2	310.84999999999997
589.85	422.75	478.65000000000003	174.55	319.34999999999997
	606.1	666.0	686.85	392.54999999999995	240.9
382.1	210.85000000000002	334.75	153.8	112.69999999999999
	123.30000000000001	103.30000000000001	117.05
266.8	123.1	156.25	310.4	148.75	131.85\
BCAS3	129.3	508.9	87.3	128.4	411.6	368.4	218.6
290.6	227.9	367.6	266.3	179.0	147.0	215.0	135.1
244.0	174.2	146.8	298.8	131.6	444.9	216.3	420.0
438.3	130.0	155.7	309.3	324.3	235.6	193.1	510.5
245.2	186.9	300.6	230.9	440.4	299.4	50.0	278.5
234.8	166.9	159.6	220.7	177.9	304.7	139.6	210.3
104.4	255.4	131.6	256.5	184.9	362.3	33.9	179.8
297.3	178.7	119.2	243.7	306.0	237.0	207.7	203.2
159.2	212.1	108.7	288.7	230.1	149.1	244.3	73.1
172.3	245.6	314.3	92.0	332.8	327.6	380.4	494.0
329.9	212.8	411.8	973.6	538.4	253.5	280.3	269.3
313.8	127.5	55.5	233.6	301.3	196.8	422.6	269.7
346.5	270.1	477.5	292.5	327.4	189.9	193.3	630.2\
PIANP	43.25	131.45	31.05	51.65	85.25	65.3	41.2	46.0
	35.95	38.95	68.2	69.45	26.6	67.4	153.0
132.05	97.0	79.85	11.6	100.19999999999999	69.15	69.2
	72.25	102.7	18.799999999999997	34.7	25.3
164.35	37.3	62.55	53.599999999999994	88.15	107.1
28.15	64.3	52.25	50.4	68.15	53.2	46.15	79.25
138.05	77.6	50.9	16.55	65.69999999999999	35.7
49.05	70.35	28.200000000000003	88.25	13.950000000000001
	17.45	143.95	85.1	30.5	37.2	53.300000000000004
	89.25	115.3	135.15	142.85	19.95	52.400000000000006
	95.75	46.2	28.45	52.550000000000004	207.05
58.300000000000004	233.15	128.75	59.599999999999994	54.5
	108.9	37.949999999999996	168.4	11.75	64.05
69.25	107.19999999999999	45.75	174.39999999999998
57.75	34.45	215.14999999999998	101.8	36.65	187.0	93.9
```

23.6	147.75	114.05	95.7	17.55	89.85	78.95	36.4
	33.449999999999996	98.95	68.45	123.7	80.85\
VPS13C	2269.3	5114.3	5274.35	1750.925	3171.0499999999997
	850.3249999999999	1108.275	3100.425
1173.5249999999999	1492.7999999999997	2520.2999999999997
	2977.125	1511.6000000000001	2707.175
833.5	2048.325	1811.35	2771.975	2018.2500000000002
	2817.125	2354.475	1893.9	4273.55	1880.325
	2234.675	3507.95	1543.525	4094.175
4809.749999999999	2369.075	1703.475
3401.8499999999995	3133.9249999999997	2537.1249999999995
	1748.775	3024.875	1632.5	3552.875
2304.5	1265.7749999999999	1561.475	2207.525
2815.725	1909.7	1572.3249999999998	2103.65	2336.2
598.75	2901.0	3267.55	1861.8999999999996	2388.7	3002.35
2726.875	3201.5499999999997	2325.75	2428.275
3311.45	2378.625	4098.2	4072.1749999999997	3508.625
	2739.5	3081.05	3516.2499999999995	2924.5499999999997
	2164.8	2045.6249999999998	2725.125
2910.6499999999996	3703.6	3350.225	3870.5499999999997
	1959.675	3650.85	2247.325	3229.0249999999996
	3105.3	3029.0	1236.625	2836.425	2089.6
761.15	1274.75	3936.2500000000005	7912.374999999999
1820.125	2310.225	2782.775	6064.500000000001
	1148.55	3680.15	2918.7999999999997	2662.525
3370.225	3255.525	3624.8499999999995
3690.0750000000003	4745.325000000001	3939.1000000000004
	4263.325	2983.1	2935.0000000000005\
VPS13D	376.97999999999996	617.42	1267.76	284.08	638.5
852.6	312.65999999999997	1158.64	422.0	584.0200000000001
	473.16	711.1600000000001	315.36	667.46
598.5999999999999	606.8	386.44000000000005
677.2800000000001	487.94000000000005	585.9	1188.42
535.92	1068.42	484.8	428.91999999999996	648.9199999999998
	401.68	766.64	478.52	376.92	382.0	830.0400000000002
	652.76	503.5400000000001	894.2799999999997
650.0799999999999	1449.1200000000001	678.3199999999999
	417.43999999999994	480.8	379.6	539.24	738.78
476.93999999999994	453.43999999999994	519.16	461.14
227.35999999999999	836.9399999999999	902.86	469.64
370.52	539.7	691.02	588.8	928.9599999999998	553.0
1126.0	666.0200000000001	594.64	261.18	742.6
545.8399999999999	527.74	1180.14	619.3000000000001
530.0	438.16	753.58	407.12	1053.28	872.9199999999998
794.1800000000001	606.4	634.62	324.17999999999995
489.36	719.9200000000001	489.14	311.48	471.7	314.7
354.7	798.78	531.16	780.0600000000001	302.8	652.76
553.8	873.2400000000001	836.3	500.03999999999996
601.6000000000001	517.7	630.42	505.4599999999999
438.0	512.7	872.64	671.2	756.4399999999999	736.34
572.5400000000001\
DHPS	277.1333333333334	384.0	237.29999999999998
376.5666666666666	197.66666666666666	706.9333333333334
	472.0	171.79999999999998	447.3999999999999

368.59999999999997	832.6666666666666	364.7
519.5666666666667	462.7666666666667	845.9333333333334
	459.2	262.93333333333334	233.5	260.4666666666667
	500.1333333333334	390.90000000000003
345.5333333333333	288.7666666666667	291.26666666666665
	192.03333333333333	388.93333333333334	585.9
199.80000000000004	197.5	382.2	676.5666666666667
214.9333333333333	209.13333333333333	464.40000000000003
	684.0	458.23333333333335	349.5	265.6666666666667
	376.09999999999997	839.2000000000002
451.6666666666667	380.3999999999999	262.59999999999997
	408.3999999999999	367.8999999999999
246.0666666666667	438.46666666666664	171.5
159.63333333333335	395.6000000000001	349.3
541.0666666666667	310.6666666666667	214.03333333333333
	294.09999999999997	268.4666666666667
307.53333333333336	257.43333333333334	344.6333333333334
	185.5666666666667	303.96666666666664	295.4
348.93333333333334	252.33333333333334	224.16666666666666
	303.46666666666664	451.0	387.8333333333333
281.2666666666667	341.3666666666666	217.4333333333333
	281.2	357.3666666666666	386.26666666666665
456.40000000000003	440.6000000000001	269.56666666666666
	382.40000000000003	338.53333333333336
224.46666666666667	237.23333333333335	268.2
918.1666666666666	335.03333333333336	136.4333333333333
	115.43333333333334	272.8	294.2333333333333
406.26666666666665	255.4333333333333	1155.8666666666666
	272.3333333333333	254.76666666666665
417.9666666666667	252.23333333333335	292.8666666666666
	267.7	349.2	246.33333333333334	266.09999999999997
	325.40000000000003	269.6666666666667
373.3666666666673\
HTR3C	12.4	19.3	12.8	4.6	47.4	10.8	3.9	17.0
	10.1	6.0	11.6	7.4	7.6	16.4	19.2	7.8
	74.6	105.8	8.8	5.5	8.5	6.7	70.2	5.2
	18.0	7.7	4.7	21.5	6.5	20.1	8.0	85.3
	20.6	22.5	25.3	35.7	4.0	16.1	3.4	9.8
	10.5	5.1	4.9	5.0	9.6	22.6	17.8	6.4
	18.2	16.3	6.5	18.0	8.3	28.8	8.7	18.2
	13.6	10.9	6.9	9.5	51.5	14.9	7.9	25.3
	21.8	26.0	11.3	6.6	36.8	10.9	21.3	13.2
	7.8	14.3	5.9	9.8	23.6	8.7	5.2	12.2
	15.1	8.5	12.2	23.2	20.4	6.8	8.2	14.6
	13.7	31.2	10.0	11.3	15.8	4.1	10.6	7.8
	6.8	6.5	13.4	18.5	13.8	7.3	14.5\
VPS13A	848.5833333333334	1082.5666666666668
1720.6000000000001	839.3000000000001	914.7166666666667
	207.25	265.73333333333335	536.0000000000001
400.8666666666666	478.0833333333333	688.9000000000001
	850.15	421.34999999999997	564.9499999999999
1047.7666666666667	679.4833333333333	520.5333333333333
	763.0	669.3666666666667	539.0833333333334
675.9499999999999	765.0833333333334	818.7333333333332

478.43333333333334	909.9833333333335	2113.35
784.1833333333333	619.7666666666668	929.2999999999998
551.5666666666666	342.6000000000001	1185.0
1220.3999999999999	251.15	374.95000000000005
735.5666666666666	271.3333333333333	1249.3166666666666
541.0833333333334	227.01666666666668
300.03333333333336	389.03333333333336	527.5333333333334
344.1333333333334	401.6333333333334
673.3000000000001	832.3666666666668	116.10000000000001
2179.0499999999997	514.4500000000002
578.3333333333334	920.9833333333332	519.65
1616.1499999999999	605.0166666666667	138.98333333333332
310.73333333333335	1181.1166666666668
692.2166666666667	630.2333333333333	550.3166666666666
772.9499999999999	720.8666666666667
760.9833333333332	739.9833333333332	812.6833333333334
237.95000000000002	428.3333333333333
630.9833333333333	840.8000000000001	941.9166666666665
701.8333333333334	717.3499999999999
481.4166666666667	483.20000000000005	396.45000000000005
651.1833333333334	709.2166666666667
576.6999999999999	611.9	1040.0166666666667
856.2833333333333	198.21666666666667	873.8333333333334
2690.3166666666666	1280.3333333333335
737.7833333333333	648.8333333333334	584.75
568.9333333333333	357.29999999999995	766.1500000000001
472.1666666666667	530.7666666666667
562.8000000000001	474.0166666666667	445.26666666666665
605.0666666666667	669.8000000000001
525.9833333333333	652.65	682.1	426.05\
VPS13B	472.55	3553.3250000000003	529.1500000000001
892.075	1608.575	316.4	528.0500000000001	1259.05
452.22499999999997	684.6750000000001	529.825	974.525
736.1000000000001	428.175	1059.5000000000002	876.05
746.625	1051.05	908.3500000000001	511.925	1024.825
643.625	1067.025	798.8	416.37500000000006
1247.1499999999999	552.65	858.6250000000001
1203.4249999999997	464.35	712.4749999999999
807.8499999999999	1534.275	746.4250000000001
993.475	759.8000000000001	452.7750000000001
1347.1750000000002	467.175	745.775	490.875	889.775
942.6500000000001	525.2	631.6999999999999	1178.075
530.025	620.0	1946.5	824.65	648.975	640.65	661.725
787.6250000000001	736.6	1980.0	767.475	872.9	724.25
966.5500000000001	511.725	713.7	858.15	581.6500000000001
803.95	801.625	587.375	671.6250000000001
1694.5749999999998	647.5999999999999	2070.25	1538.15
902.8499999999999	616.275	541.2500000000001	632.3
1118.0249999999999	579.1	788.1	1406.15	1045.6000000000001
863.2249999999999	543.625	747.2249999999999
1041.95	2394.025	927.3250000000002	1290.35
645.8999999999999	1096.8	557.775	1764.0500000000002
1108.625	1394.9250000000002	2174.25	1060.2
1409.6999999999998	946.525	876.8	935.625	1126.5249999999999

1084.45 1193.6\
HTR3A 43.15 20.5 78.15 29.4 36.4 18.099999999999998
28.95 81.1 38.4 137.45 36.3 21.25 52.05
138.95 59.05 52.5 36.95 70.65 84.0 30.55 87.0
47.95 190.75 17.65 47.65 81.8 49.1 127.35 99.4
100.9 17.3 91.44999999999999 98.25 40.199999999999996
116.2 95.15 43.1 20.45 48.3 96.14999999999999
58.5 54.800000000000004 37.4 54.75
43.449999999999996 51.099999999999994 69.25 67.1
131.6 45.05 90.9 72.05 60.25 50.7 43.85
73.89999999999999 87.8 102.85 45.9 18.9
349.04999999999995 128.39999999999998 44.8 28.3 35.4
150.95000000000002 55.4 61.400000000000006
130.2 52.150000000000006 115.44999999999999 48.8
41.599999999999994 54.0 54.25 52.7 57.85 48.55
91.25 71.4 72.65 44.95 102.9 412.79999999999995
179.9 190.39999999999998 48.7 65.75 88.5 49.55
40.65 71.55 38.45 40.199999999999996 38.6 14.8 70.7
41.699999999999996 63.1 89.5 38.2 80.0
55.4\
HTR3B 58.5 155.1 30.6 40.5 62.6 46.0 9.0 15.8
19.5 7.4 4.6 7.8 5.0 15.5 34.2 22.3
6.2 16.4 42.1 24.3 209.4 81.8 39.7 29.6
37.8 24.5 92.3 12.8 58.9 25.6 15.6 38.1
31.8 19.9 41.6 40.8 5.0 65.1 8.9 12.9
4.4 58.4 10.7 32.9 13.6 84.6 59.9 38.6
38.5 30.0 10.0 120.8 18.4 24.4 26.5 20.2
35.3 138.4 56.3 19.6 330.8 107.4 14.1 75.5
29.7 83.6 11.1 18.9 20.6 93.4 214.7 10.3
10.6 50.5 126.2 8.8 28.5 9.6 18.7 60.9
47.9 39.5 7.5 22.6 16.8 129.1 31.6 99.1
23.2 180.8 11.8 56.1 85.4 9.9 17.0 25.7
9.6 2.5 18.7 71.6 38.7 55.2 23.7\
RABGGTB 2723.4 1981.0 2220.2 2098.8 1901.2 3387.0 2420.7
804.1 2181.0 1274.6 2430.4 1255.7 2627.9 1201.5 2615.4
2191.0 2063.3 1947.3 2384.9 1331.6 934.3 1461.3 989.6
1855.1 1536.3 1719.0 1702.3 1447.0 1349.1 2135.2 2349.1
546.0 2019.7 1257.2 491.3 1162.3 1798.6 1787.4 3714.5
4000.2 2047.4 3531.3 1677.2 2912.2 2486.0 1477.9 1054.7
3031.9 763.6 1415.6 1400.2 1918.7 1685.6 805.4 2252.0
912.5 1018.1 1895.2 1382.5 1239.4 72.7 1811.2 1564.4
1243.3 1540.1 2062.4 2543.2 1124.7 739.6 989.7 1185.1
1363.1 2442.5 2437.3 1640.1 1496.1 785.3 2308.3 1618.3
1710.0 757.0 460.6 1107.5 1411.0 711.6 851.9 917.1
633.5 2321.2 1886.3 4174.8 489.4 965.6 928.5 606.6
799.4 553.9 995.5 581.0 537.4 912.9 850.6 1364.0\
RABGGTA 220.3 139.5 290.6 311.6 278.1 719.1 341.9
284.2 378.9 400.1 415.6 334.0 345.1 205.9 425.9
288.5 441.6 262.0 335.0 306.1 525.1 350.4 301.4
262.1 209.1 236.6 498.1 328.6 285.8 397.3 319.7
133.1 584.7 337.5 367.9 784.6 258.9 358.5 368.8
496.8 272.5 250.7 241.0 449.1 321.4 260.9 472.1
445.7 418.7 398.4 296.5 417.9 434.0 167.7 288.0
601.6 417.1 406.6 445.7 260.0 333.1 251.1 347.5

```
397.6	349.4	696.2	404.0	431.6	441.4	340.3	355.3
206.2	290.4	221.1	217.6	293.7	649.7	378.6	403.3
467.2	215.9	166.7	379.5	559.0	236.7	155.9	235.4
297.0	361.4	280.0	299.3	176.5	341.6	416.7	326.6
526.7	368.4	445.0	316.4	405.9	555.3	328.3	401.9\
NEMF	2639.7250000000004	2869.9750000000004	3767.275
	2716.8499999999995	4739.0	1380.1499999999999
2044.875	2651.45	1235.425	1639.125
2554.2000000000003	3130.15	2562.3500000000004	2861.975
	2648.525	2090.6499999999996	1845.2	3054.625
	2101.35	2180.125	7052.474999999999
1898.9250000000002	2911.0750000000003	1588.3999999999999
	1610.1499999999999	2363.625	2030.95	3867.3
2576.625	2084.8500000000004	1353.55	5056.775
5128.875000000001	1394.3	1330.9999999999998	4404.25
1702.9499999999998	7477.6	1999.875	1924.1750000000002
	1256.875	1475.6499999999999	2686.375
2068.0499999999997	1054.4750000000001	2187.05
1908.5749999999998	1916.1750000000002	4747.874999999999
	2735.9500000000003	2159.0750000000003	1364.625
	2091.625	4425.025	2461.775	4250.125
	2413.55	5166.0	2908.25	3458.075	1741.175
2543.425	3211.4750000000004	2290.625	3413.875
	2950.4500000000003	1562.05	1917.95	2586.2999999999997
	2016.95	6149.974999999999	4265.625	2299.875
	1916.8	1507.05	1318.9	2852.0	1724.8999999999999
2335.7000000000003	2034.1	2795.4	2503.2000000000003
2500.1	2139.325	4257.525	4724.325
3094.0750000000003	1866.675	1453.175	3368.325
	1581.125	3171.65	3162.175	2713.75	3677.95
3394.15	3925.7	3199.375	4497.974999999999	4165.025
	3415.2	3712.7	2676.725\
C1D	2412.2	325.8	883.8	1376.7	800.5	1963.0	1727.4
1100.8	1356.8	416.6	1652.8	742.8	1251.2	1614.8	682.2
815.4	1168.8	587.2	2333.6	961.7	282.5	1635.8	535.1
1900.4	1553.3	590.8	2030.6	538.4	495.1	1148.7	2114.3
647.2	363.3	1096.6	482.9	629.2	3484.5	538.9	1238.8
2159.0	1180.3	991.7	962.1	1062.5	1185.0	665.0	882.8
3494.6	291.8	497.2	1235.1	1479.1	1268.6	304.9	640.1
266.2	718.1	519.7	1069.8	386.3	456.1	414.3	1493.3
1872.3	700.1	476.6	1390.1	1102.9	379.4	1289.3	418.6
407.6	749.0	1897.6	698.7	1826.9	583.4	1456.9	916.7
1895.2	583.6	1187.3	1184.6	1633.3	207.5	211.6	819.1
533.0	625.3	405.1	2571.3	813.0	1576.9	1675.3	1391.9
1972.2	1659.7	2073.8	740.0	1398.5	1176.6	1025.8	1575.2\
LOC257396	197.9	51.0	140.1	127.9	85.2	96.5
109.9	15.0	148.6	157.6	134.9	72.4	160.9	75.1
153.8	52.6	100.4	133.6	179.2	40.9	107.8	192.7
123.7	145.8	149.9	97.8	55.8	77.5	74.1	172.6
166.6	57.0	167.2	148.1	63.9	177.2	174.2	49.2
112.5	50.3	112.1	82.0	74.4	90.2	123.6	185.9
163.9	62.1	74.0	215.1	73.3	116.0	76.8	14.7	70.0
	179.5	54.0	90.3	67.7	46.3	226.1	71.6	84.8
	78.8	118.6	173.5	122.3	117.1	48.6	145.4	28.0
```

167.8	153.8	152.2	172.6	45.2	132.5	96.3	93.1
65.1	103.1	81.3	90.2	36.5	9.3	230.6	82.6
23.4	127.4	166.4	44.3	110.5	193.9	130.6
214.8	268.2	274.2	299.4	168.0	383.8	265.2	165.6
286.3\
BCAT1	157.15	513.7750000000001	5013.424999999999
6120.675	2296.325	1474.6	726.775	748.7	6122.4
817.75	3236.35	578.175	548.4250000000001	338.95
5312.575000000001	209.72500000000002	9918.1	5059.25
203.925	5706.825000000001	1312.05	4368.950000000001
5666.725	5089.275	9513.8	172.55	1436.5749999999998
6136.799999999999	4474.725	10902.25
7680.424999999999	5378.7	3866.525	11276.525000000001
718.0	20732.1	7136.3	4004.8250000000007	657.875
3347.825	1360.275	1416.525	2064.475
2076.975	2368.075	1103.725	3652.15
3196.3999999999996	302.82500000000005	8717.125
9941.3	2117.575	415.375	1592.7	3309.825	6296.275
13047.275000000001	8429.275	4817.125
5534.099999999999	10715.2	1718.8249999999998	11640.825
6564.225	812.7750000000001	716.85
3349.6000000000004	4939.25	2228.5750000000003	8431.05
634.5	107.875	2442.575	7198.55	9170.425
7291.775000000001	1185.875	8642.375	3408.75
1300.775	8377.85	1769.25	1414.25	5317.049999999999
126.275	120.375	495.70000000000005	1894.9250000000002
158.15	77.625	18162.649999999998	1422.825
8739.199999999999	5656.875	3316.075
11646.224999999999	9588.425	8183.8	4921.075
11626.8	12397.225000000002	1127.9249999999997
8437.974999999999\
BCAT2	360.4	274.85	248.35	438.34999999999997	331.2
727.25	407.95	326.3	211.1	250.8	462.25	414.2	151.85
318.85	392.85	490.35	257.95	191.1	521.2	174.75	350.25
154.54999999999998	233.65	212.70000000000002	182.15
390.45	457.59999999999997	134.15	348.25	178.3	334.5
143.9	314.0	290.3	608.6	569.15	276.35	225.64999999999998
213.60000000000002	371.5	440.54999999999995
349.15	234.7	192.55	258.6	248.25	566.0	278.15
388.20000000000005	217.25	314.84999999999997	422.4
322.2	283.8	254.3	572.1	131.05	391.95000000000005
618.9499999999999	120.15	136.1	218.85	236.3	273.85
343.15	273.2	306.34999999999997	240.79999999999998
334.0	253.6	338.4	348.0	287.0	353.25	262.05
419.79999999999995	423.75	245.15	347.5	205.7	585.8
467.85	604.5	248.95000000000002	359.2	455.7
490.65000000000003	376.9	275.15	487.20000000000005
249.4	461.15	455.95000000000005	332.0	368.15	371.5
408.05	382.95	383.15	395.6	316.65	249.1	372.0\
IGH /// IGHA1 /// IGHA2 /// IGHD /// IGHG1 /// IGHG3 /// IGHG4 ///
IGHM /// IGHV3-23 /// IGHV4-31	13.1	16.0	17.4	115.7	28.5
10.0	8.5	48.6	146.0	19.2	69.8	162.8	31.5
405.1	56.4	98.4	138.0	47.8	101.9	111.9	29.3
58.4	148.6	260.6	18.9	15.4	57.3	175.4	41.1

```
	129.4	54.4	237.4	12.5	22.8	57.4	109.2
105.3	17.1	100.1	301.8	75.9	32.0	200.1	49.6	11.5
	24.2	81.7	10.2	49.6	91.2	85.8	27.1	10.7
	16.6	38.3	17.6	20.3	74.4	88.1	35.8	56.1
	97.6	56.8	13.5	40.8	13.4	238.2	104.5	41.7
	14.7	57.1	37.8	65.5	335.4	127.9	200.0
111.7	82.1	53.7	24.7	64.1	171.6	84.9	202.2	92.9
	162.6	16.2	73.5	191.8	56.5	243.4	19.8	37.5
	60.5	55.4	22.8	25.2	16.1	21.3	83.5	62.5
	22.0	121.8\
LOC653160	50.3	16.6	71.4	9.1	98.7	28.3	41.3
	5.9	9.3	33.0	11.4	9.9	9.4	14.7	16.6
	7.6	4.9	13.9	64.1	71.7	67.5	11.1	23.0
	9.8	6.5	18.8	49.9	11.3	9.7	4.2	13.5
	14.3	46.0	7.6	22.9	15.2	8.6	11.4	7.2
	12.0	94.3	71.6	14.1	24.5	31.4	25.8	15.3
	14.8	18.5	13.3	68.8	8.3	8.4	14.2	9.3
	12.7	8.3	11.1	133.2	17.1	19.0	15.3	21.9
	4.7	12.1	11.1	47.9	12.3	8.0	10.4	33.5
	11.1	110.4	19.7	14.1	46.1	68.6	5.2	14.0
	6.6	10.6	134.1	43.0	9.3	14.8	90.5	11.5
	15.7	12.4	36.6	67.3	88.7	15.0	53.3	50.6
	41.5	11.1	16.0	68.4	11.9	7.2	28.7
39.5\
C1S	268.7	154.9	885.9	2445.7	552.6999999999999
235.95000000000002	892.75	2580.4500000000003	2313.05
1343.25 377.75	1486.1	2520.6	2430.7000000000003
272.90000000000003	6462.8	3615.7	664.8000000000001
562.8	1067.2	1239.6	588.0	909.5999999999999	3589.6
467.1	224.85	780.5	1814.0	1095.65 1743.25 2048.5	691.65
1511.1000000000001	2957.7000000000003	1739.0500000000002
	1713.25 872.9	449.25	651.65	6320.0	644.6999999999999
	309.6	3190.6	1023.45 3010.3500000000004
1020.9499999999999	902.1	1226.25 633.55	677.95
1386.8000000000002	1201.9499999999998	553.8	725.1
2515.7000000000003	482.05	810.15	1467.7	680.75	980.05
554.5500000000001	1666.1	1131.2	1001.0500000000001
1283.9	1149.8	2399.3	2740.3500000000004	1348.4	988.4
1137.15 1160.6000000000001	2020.45 1885.5	1334.35 902.45
1245.8	1681.1	2446.05 240.5	634.75	370.45	592.95	2441.95
2431.55 645.25	315.84999999999997	1997.65 2770.1000000000004
	2855.95 1141.05 1871.5500000000002	696.1	2050.0
2611.1000000000004	1039.2	1225.65 1422.8	604.4	673.65
1446.5	1604.9	907.75\
C1R	230.3	191.5	318.0	1128.7	614.5	320.8	755.3
1457.4	1212.7	1150.5	269.1	575.9	820.0	712.8	444.4
2600.0	907.6	559.7	440.4	579.8	702.1	344.4	639.2
1581.9	247.0	270.9	523.1	754.3	571.3	641.9	997.1
481.1	686.3	1399.9	729.1	1039.7	510.7	289.8	508.7
1883.8	284.8	436.8	1283.3	499.0	541.3	693.1	619.0
238.9	495.8	431.6	754.8	724.6	449.8	583.1	1024.3
656.0	690.0	776.7	542.4	787.4	531.9	889.4	868.2
626.2	411.6	578.3	1596.1	1648.2	1494.0	483.8	719.5
406.3	802.8	776.3	885.2	425.5	586.0	923.4	1462.4
```

321.5	540.9	696.3	477.4	1484.8	1651.3	612.2	281.9
1596.2	1477.2	585.8	829.4	1067.4	562.8	1184.6	1173.2
618.5	595.0	616.0	506.4	464.7	581.7	808.5	651.3\
LRTM2	13.4	35.2	6.6	5.7	59.4	3.7	3.1	6.7
	5.2	26.6	14.5	6.8	4.8	26.7	21.3	21.3
	15.5	8.4	6.4	17.4	43.3	4.9	39.4	6.1
	7.0	7.5	4.3	5.3	29.9	14.0	4.1	96.7
	10.1	7.6	10.4	28.1	55.9	12.4	66.9	20.6
	27.6	23.7	5.9	17.1	5.1	28.4	9.6	10.4
	47.7	9.0	27.1	13.9	5.3	14.3	11.0	8.9
	25.0	11.1	11.6	12.4	123.6	77.8	6.9	4.8
	15.6	15.8	41.4	24.0	44.3	22.8	68.5	17.9
	5.5	4.9	20.5	59.3	24.6	5.1	5.8	5.2
	10.9	7.8	5.0	16.3	19.8	14.9	19.7	31.1
	10.7	49.2	4.0	132.2	9.0	5.1	12.5	8.1
	8.4	6.9	14.6	7.7	5.0	89.3	11.2\
LRTM1	25.85	64.0	26.45	22.95	30.4	27.2	35.65	58.4
	54.0	106.5	58.45	57.25	62.8	30.75	54.95
26.45	27.6	32.349999999999994	33.6	47.1	203.95	51.0
	17.7	34.7	59.6	37.85	70.9	56.55	52.0
76.30000000000001	25.5	98.3	35.85	69.1	91.15	45.3
	10.25	103.2	24.65	51.85	70.45	6.9	60.45	47.0
	44.1	78.65	50.7	34.3	37.15	67.85	89.35	50.5
	45.0	154.95	68.25	69.30000000000001	57.6
61.95	71.55	28.15	285.85	63.85	47.349999999999994	66.1
	81.1	62.85	71.8	111.4	97.65	5.65	161.75	52.2
	50.85	61.65	81.0	30.0	74.25	46.400000000000006
	34.7	77.5	85.5	89.85000000000001	19.05	80.3
	98.25	217.9	36.199999999999996	62.650000000000006
	65.9	108.2	56.5	108.45	62.0	31.700000000000003
	28.2	28.05	88.8	84.8	27.400000000000002
55.15	42.400000000000006	5.15	68.4\
LENG9	15.7	41.9	102.9	109.6	12.7	31.8	36.9	40.6
	101.0	102.1	69.9	70.9	20.7	11.0	99.0	33.7
	75.8	119.1	103.0	15.7	199.9	107.2	235.9	29.3
	51.6	132.3	102.0	95.1	165.2	156.0	70.1	21.6
	77.4	123.9	47.4	74.3	16.4	94.8	79.8	70.9
	9.3	52.3	67.8	60.5	4.9	11.1	168.4
120.4	32.1	123.7	45.6	120.4	76.1	31.9	85.2	88.6
	129.8	46.4	64.2	140.8	76.1	87.8	78.2	99.0
	42.7	119.1	50.8	92.5	148.7	125.3	197.7	31.2
	15.8	72.0	15.3	49.6	26.0	94.2	87.2	83.4
	95.4	143.0	69.5	30.2	161.6	46.3	111.5
236.5	39.0	99.6	17.2	18.8	22.7	28.4	33.8
140.7	121.1	86.8	161.0	41.9	194.1	126.6	130.4\
LOC284950	22.2	5.1	44.9	21.4	7.7	0.7	2.0
	2.5	14.1	3.4	1.6	2.2	39.6	7.1	4.6
	15.7	4.9	6.6	1.0	43.6	96.3	22.6	7.6
	1.7	39.7	7.2	3.6	8.0	16.6	0.9	28.8
	8.6	6.0	2.8	5.6	7.0	4.4	6.4	5.0
	34.7	1.9	6.5	2.7	1.4	1.5	3.8	29.1
	2.2	4.5	9.2	20.5	13.4	1.4	2.8	0.5
	67.4	3.2	18.9	16.4	3.2	8.3	35.4	1.5
	2.9	42.1	3.1	14.7	25.1	57.9	43.6	57.2

10.2 29.6 31.1 4.0 2.8 0.8 1.9 0.9
18.6 12.1 2.9 4.6 2.6 7.9 18.7 8.1
7.4 19.8 11.5 26.0 3.2 3.1 2.3 14.1
5.9 3.3 44.9 83.4 6.6 16.4 1.4 6.1\
LOC102723661 78.7 174.6 157.3 68.2 248.4 80.6
109.1 216.9 82.1 205.3 123.4 171.8 159.7 131.4
209.4 173.1 76.7 239.3 130.0 113.1 366.6 139.2
159.4 107.0 126.8 180.8 57.5 278.9 214.0 190.9 87.7
263.4 159.0 110.4 260.5 160.9 80.8 22.1
149.8 168.3 126.0 83.6 144.5 121.8 83.6 133.6
157.4 127.6 255.5 191.9 150.9 119.3 69.2 392.1
241.3 244.1 138.6 227.2 132.2 181.9 279.4 181.5
161.7 160.8 212.3 262.0 143.5 96.8 300.3 186.4
218.6 214.1 261.5 149.9 209.9 150.1 171.1 182.1
145.7 168.1 193.7 213.5 104.2 199.7 308.1 271.6
185.5 198.0 161.3 262.3 73.8 171.7 131.2 72.7
140.0 173.0 150.9 116.4 209.2 280.2 184.6 185.0
106.7\
LENG8 163.5 1037.6 339.4 297.9 421.8 174.1 149.2
308.7 175.9 152.0 165.9 339.8 345.5 288.3 396.0
341.6 198.3 480.2 227.7 157.7 695.8 306.5 307.3
347.2 118.6 404.9 205.4 330.6 361.8 170.9 120.8
931.6 974.4 186.3 97.4 373.1 190.2 535.8 324.6
135.6 334.7 563.8 387.4 203.7 262.9 454.5 254.9
221.1 649.9 345.1 346.5 176.2 327.6 346.7 489.8
448.6 328.1 411.2 395.0 546.7 1019.3 550.9 269.3
315.9 314.8 353.6 314.9 258.9 709.1 293.0 647.7
525.9 421.6 225.0 195.9 92.7 244.3 396.3 300.4 69.4
473.5 420.7 200.4 536.2 376.7 485.3 374.9
189.2 332.4 328.4 252.8 496.0 714.1 561.5 966.0
624.1 838.4 629.9 596.6 593.0 896.4 522.8 562.6\
U2AF1 1318.4250000000002 1569.9499999999998 1303.125
1907.2499999999998 915.6750000000001 1564.375
1631.875 802.9 1110.375 1079.425
1023.4499999999999 1864.6250000000002 1222.1 1478.175
1078.225 1044.975 661.9000000000001
1299.975 742.55 1610.8249999999998 1154.75 1151.325
980.675 922.425 892.075 1512.8 1657.975
1291.9249999999997 918.5 1434.05 967.65 1264.2250000000001
2283.175 619.5 604.975 1168.775
719.0500000000001 1898.95 2007.9 698.9749999999999
1417.075 2163.2 998.95 1710.65 807.8000000000001
1452.9 806.6500000000001 350.475 1077.875
1421.9250000000002 708.85 757.9499999999999 853.65
645.3499999999999 1544.925 1067.225 863.375
2029.375 1153.975 1622.3249999999998 227.725
2106.125 785.35 1572.475 1152.1499999999999
1555.5499999999997 1370.3500000000001 1259.425
776.225 1236.5000000000002 763.75 1227.5 1283.0
1185.4250000000002 1808.0750000000003 746.6500000000001
840.8499999999999 1570.7749999999999 1047.625
2656.95 724.2 1094.9 546.425 1192.925 586.625
698.45 565.3249999999999 813.5 1648.25 2533.6 806.95
910.95 1209.6000000000001 1371.75 1565.0500000000002

931.575 1249.375 760.9000000000001 1114.65
867.6500000000001 1376.325 894.6999999999999
1270.2\
IL2RG 40.8 178.6 29.6 208.2 192.4 94.0 64.4
214.0 153.7 134.9 116.8 222.6 40.1 249.9 238.3
128.1 77.9 46.0 88.7 80.3 140.9 138.7 174.4
163.7 62.5 109.9 54.1 287.9 95.8 160.0 59.5 33.0
235.1 96.2 411.9 190.1 122.9 86.3 27.5
200.9 13.3 47.5 204.8 105.4 140.6 168.0 138.2 75.7
165.1 128.7 124.0 190.4 111.6 186.1 153.3 32.7
116.1 12.8 32.1 62.4 656.6 229.0 81.9
157.4 154.2 21.2 117.6 69.5 163.2 149.9 86.6
187.0 286.6 141.8 228.1 26.3 225.7 130.1 145.9
107.4 162.1 113.8 11.9 122.5 41.2 270.8 188.6
213.3 322.6 79.8 99.1 125.5 124.7 110.4 76.8
100.6 9.0 70.3 96.2 27.9 93.8 124.2 135.8\
RP13-20L14.1 13.9 248.0 8.6 11.0 9.2 36.6 51.0
6.8 11.6 9.1 11.3 130.6 10.1 11.7 15.8
101.4 8.9 5.1 100.6 10.2 253.0 93.1 10.3
8.5 56.7 72.2 21.8 15.4 15.3 66.1 32.6
274.4 113.5 6.2 17.4 9.4 10.0 234.4 4.6
13.1 17.0 6.9 42.5 10.4 30.6 25.4 7.5
6.1 503.0 20.8 10.3 7.4 14.0 14.6 10.5
226.3 88.3 48.0 101.3 74.6 19.0 15.3
104.1 3.9 151.6 6.5 6.6 125.9 11.9 23.3
116.3 133.2 8.7 23.7 61.4 8.7 16.0 70.5 53.9
9.3 194.0 12.3 19.6 16.4 28.2 21.9
158.4 25.4 6.8 12.4 19.7 190.4 14.4 105.2
258.1 107.3 209.8 58.8 683.5 88.1 134.4 112.2
128.5\
RP1-286D6.5 57.8 81.7 68.1 32.0 93.3 81.6 87.2
76.4 11.6 94.2 26.3 83.5 47.9 50.3 84.6
97.3 16.3 23.4 34.5 53.3 93.1 40.5
105.1 43.6 10.1 80.3 13.5 104.3 14.0 92.9 65.4
109.3 52.5 62.3 147.6 125.1 29.8 65.1 22.1
38.9 69.2 71.6 59.3 6.7 56.6 11.1 83.8
57.9 77.4 84.2 51.3 55.4 29.6 165.3 38.0
59.0 22.1 8.8 44.0 19.8 127.4 61.6 54.4
34.3 46.5 131.8 30.3 31.6 2.9 84.9 29.7
160.6 67.4 96.0 55.3 11.6 30.1 58.7 37.2
84.7 109.0 15.2 107.8 11.6 21.1 8.2
104.0 79.0 27.5 27.7 52.8 140.9 59.1 100.8 77.2
118.2 139.2 139.7 20.9 99.9 165.2 56.5
178.9\
BC034423 /// RP11-966I7.2 8.4 102.9 6.8 5.7 7.0
2.9 4.0 2.4 5.4 6.9 10.7 16.6 4.9
6.0 7.3 8.8 5.0 6.3 3.9 8.1 9.7
60.5 8.9 2.4 10.4 6.5 9.9 49.0 12.7
7.3 5.5 10.7 3.6 8.4 12.2 4.9 14.7
7.4 15.5 8.8 3.7 6.1 2.5 2.8 5.2
8.3 21.5 50.8 83.0 6.0 7.2 12.3 7.6
15.5 7.8 9.7 7.0 17.0 33.8 21.5 55.3
3.2 4.0 4.9 7.3 16.7 3.2 2.9 5.0
8.5 41.2 53.3 3.6 8.5 9.4 27.5 50.8

4.9	6.5	4.7	26.6	9.1	1.2	9.3	54.4
6.4	10.1	8.0	38.0	16.1	2.8	12.0	2.8
3.0	3.9	6.5	15.0	13.9	6.6	4.6	12.6
7.2	6.2\
AKAP2 /// PALM2-AKAP2	372.73333333333335	345.5666666666666
1163.3999999999999	1476.3999999999999	825.0
1336.4333333333334	1146.0333333333335	1842.6999999999998
1888.4666666666665	2105.4666666666667
799.8000000000001	1589.5	1498.8	1297.1999999999998
1241.8666666666668	3538.633333333333	4592.66666666667
1051.3999999999999	946.5	1949.3333333333333
1324.7666666666667	835.6333333333332	1690.6999999999998
3110.2666666666664	1231.4333333333332
985.0666666666666	592.0666666666666	1261.8333333333333
718.5333333333333	1876.3000000000002
2388.0666666666666	1389.2666666666667	846.2333333333335
973.8000000000002	1824.9000000000003
265.8666666666667	740.0333333333333	1222.0
524.3333333333334	2360.2000000000003	1090.1333333333332
1414.8666666666668	1411.5	2639.6333333333337
977.5666666666666	448.8666666666666	728.8000000000001
199.1666666666666	376.1333333333334
471.6666666666667	1585.7333333333333	716.4333333333333
874.2333333333332	763.3333333333334
1069.0333333333333	773.1	768.7666666666668
1457.3666666666668	630.2666666666667	901.9666666666666
967.4	1157.5666666666668	822.4333333333333
527.5	1460.4333333333334	703.9	1341.5666666666666
994.4666666666667	1295.9666666666667	484.29999999999995
1199.3666666666668	923.0	1815.3333333333333
1016.6666666666666	1233.3666666666668	364.8666666666666
1046.2666666666667	957.2666666666668
841.2333333333335	413.40000000000003	1123.3999999999999
1087.9	2481.266666666667	1388.5	1507.733333333333
292.3	638.2	1660.266666666667	1857.5333333333335
1339.8999999999999	1153.2333333333333
1458.166666666667	360.5666666666666	1475.3333333333333
1028.3333333333333	355.3999999999999
447.20000000000005	568.5333333333333	220.03333333333333
305.2	498.20000000000005	823.3333333333334
240.79999999999998\
DRAM1	3135.2	190.1	1114.0	1218.8	841.5	522.4	1931.6
1110.7	1679.9	1040.2	916.7	1034.5	959.9	2002.9	213.6
1049.1	3050.1	1547.0	728.5	1447.3	766.5	2128.5	759.6
1797.7	2238.4	1079.0	849.4	1149.9	385.4	670.6	940.5
438.1	523.0	996.7	868.0	508.6	1305.7	775.5	415.3
1613.5	617.8	1984.9	1108.4	731.8	1806.2	2270.6	689.5
2037.0	650.2	343.0	1249.6	449.2	1098.6	595.2	630.8
442.1	1162.1	684.0	791.5	447.1	963.0	434.7	265.5
460.9	620.3	611.7	1067.7	363.6	1044.9	572.0	1254.0
826.0	840.0	818.0	1973.5	750.1	494.9	1193.2	1519.1
735.2	262.0	594.7	56.5	459.5	1083.8	962.4	474.1
940.4	3185.4	1747.6	601.6	443.6	673.8	854.0	921.4
992.2	983.7	964.8	808.1	888.7	661.0	546.6	880.7\

AWAT1 9.4 32.6 15.6 18.0 16.0 14.2 3.1 13.5
14.4 9.1 11.9 9.9 7.1 12.9 49.9 19.7
14.4 13.7 15.2 17.9 165.5 13.1 30.8 8.4
13.8 11.5 11.7 10.9 16.8 16.8 2.8 22.1
4.9 20.9 11.0 11.8 56.5 23.4 14.8 12.7
4.2 9.7 18.2 8.9 6.1 119.4 4.6 16.5
11.3 45.7 16.8 16.0 15.5 12.3 23.6 14.8
24.6 9.8 14.5 64.9 51.5 13.1 3.7 10.9
15.3 4.8 29.1 47.7 21.7 7.4 21.0 5.3
24.3 19.7 12.5 7.4 7.6 22.5 9.3 51.1
5.0 4.6 18.9 15.6 39.2 39.9 8.4 5.2
14.4 14.5 17.9 18.9 11.3 12.5 3.7 14.6
9.3 12.7 24.2 6.6 7.1 23.5 20.7\
U2AF2 615.4499999999999 967.3750000000001
601.8000000000001 875.1 738.35 1027.3999999999999
947.4499999999999 1128.3499999999997 850.9 751.35
737.6 1079.3 657.45 948.6 1040.4750000000001
668.5249999999999 719.175 912.6999999999999 698.55
761.0749999999999 1027.95 978.5000000000001 712.425
800.575 1110.1000000000001 1339.0 1149.7749999999999
722.05 1057.45 798.1750000000001 555.85 1113.3 1014.4
504.45 1524.175 916.075 869.575 953.05 752.0 695.475
1170.675 958.675 572.625 701.5999999999999 637.7
1219.1 1359.025 1374.5 1094.8 936.75 997.125 976.75
1128.8249999999998 1809.975 1251.775
888.8249999999999 1167.225 1029.25 605.825 839.175
766.2249999999999 1054.05 585.0999999999999
720.9499999999999 659.3249999999999 1034.375
610.6500000000001 664.475 1036.05 742.3500000000001
683.875 756.15 656.475 685.6750000000001 885.45
1083.1499999999999 1229.55 1119.525 662.375 949.075
1547.325 2032.5999999999997 896.5500000000001
1429.3500000000001 1317.45 1214.875 1517.4250000000002
1408.425 971.875 844.6500000000001 748.95
1585.975 1270.3749999999998 696.875 829.775
762.8500000000001 742.425 892.0999999999999
875.0250000000001 940.425 1145.4499999999998 1570.55
776.7250000000001\
LOC100288123 10.0 10.0 15.9 3.0 57.1 5.7 5.0
48.2 42.4 6.8 17.5 10.3 1.9 10.6 7.5
8.9 4.9 12.3 20.7 9.1 64.0 4.6 37.2
5.3 8.5 5.4 2.4 83.8 84.8 24.7 2.6
45.0 7.4 7.5 11.3 6.6 4.2 8.1 2.4
8.3 4.0 1.8 9.0 36.2 6.8 2.9 68.6
39.6 16.4 64.9 8.2 7.4 41.5 14.2 3.1
19.9 4.8 8.4 2.9 58.6 45.1 24.7 60.9
6.6 8.5 10.9 5.8 1.7 13.5 4.3 16.9
34.1 53.9 4.3 77.9 5.2 68.4 42.6 3.7
13.1 89.2 9.3 6.4 7.7 28.3 11.1 68.0
3.1 53.0 8.9 5.6 70.6 10.9 5.1 30.4
34.5 5.7 13.2 6.5 7.4 2.7 3.3
97.0\
DRAM2 957.3 315.7 1207.65 948.3499999999999 526.65
725.9 861.65 896.3 555.15 608.8 1196.3000000000002

612.95	730.65	1219.8000000000002	573.3	673.5	777.7
956.2	817.35	767.5999999999999	400.70000000000005
782.75	618.1	905.25	811.05	1039.55	1171.9	870.95	1055.75
699.75	966.9499999999999	762.9	1095.1	1330.4	395.1
680.6	1189.0	751.8	1387.95	777.2	662.8499999999999
754.9499999999999	1024.75	736.8499999999999	1539.45
875.75	1417.3	1306.8	790.55	904.5999999999999
1009.5999999999999	1328.75	1363.95	631.4	1014.5	502.8
866.9000000000001	789.55	1178.45	1046.9	870.4
708.1500000000001	776.6999999999999	921.1	645.85
857.1	1028.0	481.4	983.05	1452.35	797.1	887.0	950.4
1184.6	887.15	1540.25	1291.25	1099.6	780.45	645.15	583.6
831.7	1102.0	860.0	914.45	785.5500000000001	664.65
647.45	634.05	876.75	915.95	737.2	657.9000000000001
1010.05	664.45	991.75	835.05	1263.75	925.7	606.8	951.25
696.4	1083.55\
KCNJ1	16.2	54.6	7.1	2.55	23.900000000000002
23.75	39.15	50.400000000000006	58.05	35.45	8.0
19.35	8.95	5.65	21.75	8.35	25.65	28.4
30.900000000000002	11.85	31.05	63.550000000000004	9.1
	20.35	22.650000000000002	25.4	16.5	51.1	48.4
	43.050000000000004	14.899999999999999	44.25	62.4
	39.0	13.3	20.700000000000003	16.0	146.6	25.9
	5.65	79.19999999999999	6.199999999999999
24.299999999999997	9.55	16.9	23.349999999999998	6.9
	34.699999999999996	10.85	23.15	29.8	39.15	2.45
	39.6	26.0	44.75	8.75	36.75	66.5	3.85	49.0
	24.25	21.1	23.55	45.1	55.0	23.85	19.1
16.25	67.35	73.85	31.45	4.45	5.35	5.75
26.349999999999998	39.15	44.2	5.949999999999999
37.35	107.8	18.2	29.95	19.25	69.75	63.550000000000004
	58.5	58.5	10.8	110.64999999999999	17.95	10.3
	27.85	19.700000000000003	20.65	12.3	34.7	6.4
	49.2	17.35	20.7	13.7	8.5\
SLC36A4	244.35	27.0	374.1	385.34999999999997	249.7
75.75	251.3	283.0	146.45	318.4	433.55	77.65
190.10000000000002	345.0	318.75	173.10000000000002
222.0	319.55	251.35000000000002	294.3	257.55	161.6
267.7	134.55	38.5	4.0	324.6	103.45	124.8	180.4
139.7	1017.55	112.65	64.7	305.65	381.20000000000005
168.55	239.85000000000002	11.85	162.1	147.35	24.55
144.35	101.0	30.75	356.6	157.7	255.7	200.10000000000002
	140.0	139.5	313.25	272.3	735.6	95.0	128.05
198.5	493.8	291.45	118.4	239.55	145.35	426.2
444.65000000000003	402.70000000000005	398.0	161.8
89.05	69.1	267.8	244.75	355.95	286.05	67.25	178.2
140.95	146.55	59.05	245.95000000000002	712.4
326.45000000000005	254.35	179.85	116.30000000000001
93.15	424.4	276.9	595.15	201.4	287.9	44.15	292.15
269.79999999999995	178.1	191.35000000000002	183.5
345.0	193.95	91.44999999999999	244.55	210.45	210.45
181.95\
KCNJ2	12782.4	137.0	2342.5	1364.9	1291.9	111.8	1470.0
3425.2	580.1	3247.1	1829.7	223.4	211.9	2767.3	208.1

601.7	5437.4	875.8	386.5	2029.3	943.0	1436.2	1120.0
552.1	735.8	526.6	321.0	648.2	1580.6	1164.7	576.0
202.8	811.0	1234.4	1509.0	247.0	465.1	3482.3	504.7
715.8	1510.8	277.3	1240.5	9731.9	1044.8	884.1	1345.3
802.2	418.6	523.4	1481.2	666.6	1825.5	2044.9	522.1
590.5	418.3	2298.7	257.2	780.5	5393.1	2553.8	2410.2
1566.5	1418.2	648.8	520.3	1974.5	933.7	284.7	820.6
457.1	1781.8	423.6	1115.1	740.7	2105.2	839.1	495.8
5141.8	2217.8	1199.7	1139.4	1602.9	1862.4	1205.6	4591.0
3203.8	1327.4	1724.8	363.0	1302.5	184.8	481.8	171.1	96.3
	188.8	277.7	68.2	114.4	258.8	3969.7	126.1\
KCNJ3	172.96666666666667	90.63333333333333
8.299999999999999	50.666666666666664	97.26666666666667
	32.5	13.533333333333333	46.333333333333336
6.533333333333334	45.0	19.0	55.666666666666664
34.300000000000004	27.266666666666666	76.93333333333332
	31.400000000000002	56.633333333333326
86.36666666666667	90.63333333333333	61.46666666666667
	114.2	31.266666666666667	95.43333333333334
12.866666666666667	23.833333333333332	15.533333333333331
	40.833333333333336	99.8	56.29999999999999
70.53333333333333	26.966666666666665	80.16666666666667
	107.23333333333335	21.233333333333334
55.43333333333334	25.566666666666666	7.066666666666666
	117.33333333333333	28.46666666666667
51.699999999999996	53.1	28.866666666666664
34.800000000000004	33.13333333333333	16.900000000000002
	84.53333333333333	28.933333333333334
34.56666666666667	64.39999999999999	54.23333333333333
	38.333333333333336	19.7	50.6	113.06666666666666
	31.833333333333332	69.03333333333333
42.53333333333333	56.5	30.866666666666664
55.666666666666664	303.2666666666667	35.400000000000006
	50.333333333333336	22.96666666666667
48.43333333333334	54.699999999999996	22.133333333333336
	19.266666666666666	24.666666666666668
35.86666666666667	59.199999999999996	68.13333333333333
	46.53333333333333	22.966666666666665
17.599999999999998	54.300000000000004	44.46666666666667
	27.3	42.699999999999996	28.166666666666668
61.46666666666667	38.833333333333336	28.46666666666667
	33.199999999999996	70.33333333333333
128.5666666666667	50.56666666666667	46.866666666666674
	45.0	80.5	25.5	45.833333333333336
27.266666666666666	37.26666666666667	41.86666666666667
	37.9	38.166666666666664	30.866666666666664
53.06666666666666	43.1	35.199999999999996
11.533333333333331	52.666666666666664\
KCNJ4	26.049999999999997	57.0	116.85	78.95	15.0
54.75	104.55	5.75	5.800000000000001	14.600000000000001
	154.04999999999998	12.399999999999999
26.950000000000003	15.35	2439.05	10.55	9.55	25.45	67.7
	7.949999999999999	46.0	36.6	19.45	26.9	7.75
	36.15	29.55	122.85	10.95	16.3	6.550000000000001

64.65	199.0	46.5	25.4	47.0	25.8	136.3
21.15	25.15	10091.25	54.15	31.35	40.7	25.1
39.050000000000004	39.099999999999994	10.7	17.7	6.9
24.2	30.799999999999997	42.9	46.1	93.3
114.6	14.1	16.1	38.15	32.8	57.5	40.7
55.300000000000004	75.64999999999999	34.4	26.8
47.75	10.15	59.4	54.8	74.15	16.15	51.35	116.25	9.15
19.15	11.65	49.35	46.05	65.6	27.450000000000003
53.9	395.59999999999997	84.75	76.15
53.150000000000006	108.30000000000001	85.65	45.5	58.5
22.1	51.449999999999996	46.699999999999996	23.0
47.45	42.4	18.4	48.0	46.800000000000004
10.25	9.65	26.45	7.25\
KCNJ5	40.81666666666666	69.93333333333334
106.78333333333335	49.13333333333333	110.69999999999999
54.06666666666666	242.75	136.53333333333333
251.86666666666665	155.98333333333335	43.98333333333333
42.06666666666666	37.51666666666666
87.06666666666666	162.55	367.8	108.53333333333335
105.86666666666667	62.43333333333334	128.24999999999997
411.3666666666666	81.41666666666667
216.0666666666667	106.14999999999999	60.449999999999996
68.58333333333333	62.366666666666674
128.01666666666665	79.1	129.41666666666666
132.70000000000002	131.06666666666666	145.03333333333333
113.05000000000001	81.99999999999999
108.51666666666667	58.73333333333333	510.61666666666673
115.63333333333333	94.96666666666668
113.06666666666668	43.41666666666666	59.78333333333333
247.38333333333333	41.91666666666667
126.31666666666668	66.45	45.35	78.2	142.21666666666667
85.48333333333333	50.833333333333336	55.4
87.93333333333334	35.73333333333333	72.98333333333333
154.8166666666667	118.14999999999998
59.300000000000004	155.03333333333333	155.33333333333334
59.68333333333334	132.04999999999998
87.18333333333334	178.68333333333333	83.25	145.35
444.1333333333334	93.38333333333333	52.76666666666667
132.66666666666666	94.03333333333335
155.08333333333334	66.21666666666667	206.8166666666667
35.76666666666667	60.86666666666667
68.53333333333332	245.03333333333333	70.88333333333333
64.63333333333333	72.16666666666667
76.03333333333333	39.21666666666667	166.83333333333334
201.23333333333335	98.28333333333335	42.5
57.76666666666667	84.96666666666667	77.58333333333333
86.46666666666665	97.66666666666667
147.28333333333333	188.79999999999998	148.15
198.2833333333333	76.3	69.10000000000001	141.75
190.45000000000002	68.45	202.85000000000002\
KCNJ6	24.15	15.149999999999999	21.2	14.8	44.55
22.650000000000002	21.2	114.85000000000001	8.05	7.3
16.35	46.35	28.9	90.3	16.0	20.15	25.0	9.85
8.95	43.7	76.6	13.100000000000001	17.45

8.149999999999999	8.25	36.25	15.9	103.0	8.8	15.4
	13.850000000000001	20.0	11.8	13.45	31.1
30.95	23.15	23.700000000000003	8.85	48.05	4.95
21.65	72.95	30.5	26.25	8.4	25.049999999999997
35.75	40.900000000000006	28.55	30.950000000000003	34.8
	39.7	42.85	36.4	60.65	83.39999999999999	8.75
	3.65	18.2	69.9	9.15	64.25	29.1	8.4
42.75	10.7	40.55	7.2	9.1	49.199999999999996
14.45	31.85	21.299999999999997	17.950000000000003	7.4
	8.8	13.85	11.15	9.85	44.95	19.2	20.4	12.0
	48.15	47.75	40.35	6.85	12.049999999999999
29.65	8.4	21.7	11.9	8.65	4.45	11.649999999999999
	25.05	10.9	83.8	8.350000000000001	4.75
25.35	45.300000000000004\
COL26A1	44.449999999999996	23.0	25.25	55.6
26.349999999999998	93.3	468.95000000000005	22.3	72.9
	58.45	88.7	8.8	10.55	16.3	30.650000000000002
	406.95	13.25	28.65	17.3	139.65	71.3	51.4	36.4
	34.599999999999994	29.9	14.5	10.600000000000001
	27.25	23.25	109.05	9.05	82.35000000000001	50.2
	100.3	33.75	28.2	50.55	140.05	10.75	18.25
29.549999999999997	35.05	39.75	261.05	58.15	44.5
16.95	58.55	27.450000000000003	76.65	18.1
15.049999999999999	14.1	56.4	98.85	28.5	58.75
16.45	13.05	21.75	93.3	16.0	76.25	64.75	24.55	21.3
	15.149999999999999	113.0	34.05	51.75	26.1
117.25	13.1	122.44999999999999	44.45	53.8
27.299999999999997	32.65	16.299999999999997	31.0
29.450000000000003	30.8	28.65	15.700000000000001
42.45	86.6	33.8	114.15	34.4	36.599999999999994	59.9
	37.0	33.55	89.55	33.15	28.7	18.7	46.8	15.4
	18.6	13.149999999999999	34.0	30.45\
AK091729	195.4	537.5	186.3	118.7	276.0	121.6
110.9	167.6	73.4	240.1	148.4	141.0	86.6	208.2
193.5	142.4	228.2	133.4	65.4	175.6	329.6	110.7
165.2	88.2	130.7	200.6	144.0	185.3	146.7	197.7
140.1	574.6	312.9	59.0	205.4	185.3	116.5	133.9
127.5	188.7	191.7	159.3	205.4	115.2	98.6	224.3
132.2	243.9	246.0	240.4	172.9	126.4	102.0	146.1	85.2
	226.0	197.5	203.5	195.8	189.9	396.7	139.6
223.3	108.9	196.4	158.5	137.0	291.2	186.2	161.8
195.9	136.4	166.8	118.5	228.4	146.9	236.8	110.5
115.0	205.8	153.5	115.4	278.5	331.1	224.0	103.4
204.9	147.9	230.2	179.8	99.6	298.6	429.1	102.2
359.2	389.0	371.7	282.1	305.9	536.6	297.0	252.3
382.0\
KCNJ8	46.65	50.3	117.9	160.85	17.65	90.4
436.54999999999995	243.35	267.0	304.95	87.7
116.69999999999999	135.7	152.0	97.0	24.8	366.7
114.45	74.4	209.95	92.35	94.60000000000001	77.6
531.9	118.9	120.3	148.8	170.2	265.3	265.0	582.6
183.15	168.35	461.79999999999995	614.35	132.25	89.6
311.25	98.0	205.45	259.25	64.2	318.7	185.0	93.55	47.1
	453.8	72.85	42.15	121.3	272.95	155.95	429.4

169.2 319.3 51.25 133.4 206.05 14.2 99.55000000000001
479.9 180.25 110.89999999999999 114.35 291.05 56.7
150.25 145.05 300.95 99.7 151.29999999999998 92.6
429.55 215.9 192.39999999999998 161.7 247.75
260.1 170.85 598.8 174.4 115.89999999999999 223.15
282.65 252.15 121.25 391.85 383.9 203.55 207.8 137.45
669.5 356.9 417.35 518.25 154.55 317.25 252.4
69.80000000000001 208.25 237.95000000000002 338.45
144.85\
GPKOW 1114.5 684.4 438.9 557.1 711.9 1549.2 1059.1
528.1 612.0 437.0 479.7 779.6 867.4 844.5 137.5
476.5 655.2 448.4 1438.8 616.6 385.6 729.4 526.8
916.3 655.8 604.4 1417.0 306.3 324.8 444.6 966.7
717.6 301.7 716.1 392.1 598.6 877.8 410.7 867.4
914.1 959.8 815.5 390.2 795.1 1203.8 380.9 560.9
583.4 620.3 820.2 550.7 426.6 647.7 740.6 812.5
394.5 569.0 475.9 693.7 417.9 196.8 452.0 673.3
954.4 814.6 418.9 995.9 698.4 501.9 413.0 495.9
525.0 771.0 843.1 607.6 834.2 506.3 1093.6 632.9
636.5 457.3 352.1 1871.1 522.5 330.2 520.1 159.6
409.9 822.4 485.4 1445.0 339.3 447.6 888.9 557.6
507.2 398.2 832.6 449.6 424.6 487.7 267.0 685.9\
KCNJ9 19.45 24.65 30.5 30.9 54.1 27.4 23.0 13.6
49.85 38.4 53.699999999999996 33.6 27.65
23.35 32.550000000000004 32.7 9.5 28.75
30.849999999999998 28.1 261.5 5.9 31.0 11.9
17.85 44.1 23.75 68.4 47.599999999999994
31.349999999999998 13.9 33.7 16.3 30.2 41.5
60.599999999999994 35.05 13.350000000000001
39.300000000000004 46.55 19.2 32.35 34.4 7.8 8.7
20.15 38.1 12.0 74.85 71.2 21.6 45.35 43.1
59.8 20.799999999999997 41.9 13.45 54.25
59.050000000000004 42.6 45.35 14.4 10.75
23.150000000000002 101.25 75.15 70.25 85.80000000000001
73.64999999999999 65.45 9.4 20.15 54.3 12.4
12.649999999999999 40.800000000000004
27.799999999999997 21.0 39.4 30.400000000000002
21.650000000000002 34.349999999999994 60.35 22.3
37.300000000000004 38.65 41.599999999999994 17.0
40.95 21.2 5.15 35.300000000000004 5.55 13.35
37.900000000000006 48.5 6.6 26.400000000000002 19.2
18.9 25.35 31.0 36.8\
FAM156A /// FAM156B 2850.4 17886.3 4534.2 2502.8 2861.4
3853.7 1865.2 3703.4 1456.2 2817.3 2398.2 3948.8 2367.7
3504.1 2714.2 4085.7 3150.9 3273.5 4131.2 3856.1 9142.3
1976.0 3400.7 1791.5 1227.0 5020.7 2100.5 5009.7 4532.7
3215.6 3778.8 5411.1 8912.5 2093.1 4434.8 4521.1 1970.6
5777.4 3856.3 2402.2 3169.9 5273.9 2578.3 5019.3 1515.0
2679.9 2632.9 1682.9 11943.4 7024.3 1566.2 2299.9 4916.7
5089.5 5664.3 8054.0 2896.6 5635.9 4729.2 4808.9 1587.8
3902.6 2118.7 2868.1 4285.1 8065.1 3249.8 4744.2 2521.2
3611.7 4900.9 5995.6 5853.6 2542.1 4394.5 1685.7 5075.4
2369.5 2941.8 3749.2 5503.0 2246.3 3516.0 5787.1 8528.0
6985.2 2790.3 2362.0 3925.9 5776.1 1572.1 9471.0 8322.4

4223.5 7132.5 6341.8 6509.0 4948.4 7316.7 7576.0 7487.2
8988.6 6393.5\
HTR5A 7.0 13.0 19.6 3.6 7.8 10.4 7.0 8.6
39.1 14.9 5.2 9.8 5.5 10.4 16.6 19.2
6.7 5.6 9.6 16.4 124.0 7.6 26.3 5.0
23.1 10.2 13.1 10.4 23.3 4.0 5.3 10.6
39.7 10.0 26.6 109.6 5.7 10.9 11.4 7.3
8.6 8.5 6.5 38.5 11.9 27.6 18.7 12.9
13.0 7.2 9.4 6.7 7.3 25.0 8.9 17.5
27.8 77.7 12.9 17.5 92.5 52.1 4.9 5.6
7.5 8.2 8.6 5.3 18.0 9.3 21.7 11.9
15.9 5.2 24.1 7.7 15.0 18.3 9.5 19.7
20.6 6.4 16.0 8.8 28.3 34.9 15.0 14.6
17.7 17.3 24.3 14.0 4.6 14.2 13.3 7.8
5.8 5.6 5.3 6.6 12.8 8.2 18.9\
FLJ35934 47.85 254.04999999999998 152.35000000000002
81.3 593.8000000000001 61.300000000000004
32.45 115.39999999999999 22.25 113.25 25.75 177.8
57.95 45.4 77.75 129.55 95.60000000000001 212.6
119.85 134.2 430.20000000000005 156.95 614.65 128.1 92.3
166.9 44.050000000000004 299.5 334.1 163.05
200.25 750.3000000000001 385.65000000000003 71.5
157.75 84.0 65.25 266.55 60.6 44.2 94.7 92.45
243.5 55.3 50.6 122.7 109.85 75.5 548.1 305.85
85.65 66.8 183.25 229.4 91.35000000000001 208.0
273.75 335.05 195.45 320.95 311.25 145.85000000000002
209.20000000000002 78.5 306.15000000000003 309.65
111.35 131.35 82.89999999999999 65.0 710.4499999999999
355.70000000000005 131.05 94.4 67.3 58.85
139.2 73.89999999999999 105.0 218.25 110.65 110.15 73.9
40.2 169.25 207.4 119.9 71.35 61.0 211.45 22.0
173.85 89.45 94.05 237.8 113.5 183.45 73.65
159.0 210.25 109.35000000000001 209.65 96.2\
GSTZ1 575.4 177.4 214.9 280.9 316.9 709.2 297.0
170.2 464.2 253.1 445.6 347.5 210.7 260.8 149.1
204.6 385.4 390.9 520.2 206.5 15.6 290.1 438.2
257.3 185.0 524.4 606.1 260.4 372.7 444.9 208.3
300.7 247.4 477.5 366.6 482.9 309.6 199.7 258.5
354.4 267.4 182.5 343.0 251.5 463.4 178.4 199.2
287.4 327.3 430.8 291.7 418.7 287.0 306.3 274.7
268.1 207.4 201.4 284.1 280.4 406.5 375.9 292.0
336.9 166.6 167.1 333.2 279.6 391.2 265.0 498.6
239.4 206.7 347.7 141.3 440.6 124.7 234.2 233.0
190.7 252.1 99.1 440.8 57.4 174.0 316.0 290.3
119.7 255.9 193.6 109.3 182.2 378.0 330.2 223.7
399.9 295.6 247.9 434.5 64.1 297.4 288.9 392.4\
BRE 974.0666666666666 1121.2666666666667
722.7999999999998 566.5 581.5 785.8000000000001
962.5666666666666 1209.1333333333334 805.4 640.0
1184.4333333333334 937.2333333333332 1192.0333333333335
1578.6000000000001 1255.7333333333333
884.9333333333334 872.8666666666667 624.6666666666666
1460.6333333333332 1179.5666666666666
729.3333333333334 864.6 774.6666666666666

1064.0666666666666	715.0333333333333	1043.7
1102.6666666666667	936.5666666666666	1035.8666666666668
	902.1333333333332	1771.2333333333333
608.7666666666667	779.5	1882.8666666666668
1413.1333333333332	1196.2666666666667	1015.2333333333332
	857.0666666666666	969.9333333333334
1252.3333333333333	2055.1666666666665	1516.3333333333333
	712.9	808.7666666666668	874.8333333333334
1018.4	731.1333333333333	532.8666666666667	835.5
1336.0000000000002	1015.4666666666667	1508.7666666666667
	972.2666666666668	653.9666666666667	931.1
1105.7	696.3000000000001	628.2666666666667
1159.7333333333333	623.9333333333333	714.5666666666666
	990.5	889.7666666666665	1053.7333333333333
740.8666666666668	953.7333333333332	1805.7666666666664
	1246.8	950.7333333333332	1078.1666666666667
857.1666666666666	688.9333333333334	1027.5666666666666
	1803.8999999999999	1825.6666666666667
1210.2333333333333	941.9666666666666	1249.5	1097.3
1513.7333333333333	850.2333333333332	641.3666666666667
	1908.3	597.0	990.9333333333334	1551.1000000000001
	946.1666666666666	1051.7666666666667
928.0666666666666	1776.6666666666667	933.5333333333333
	1078.7	1226.8	927.9	840.1666666666666
854.5666666666666	915.3333333333334	657.8000000000001
	744.7000000000002	816.6666666666666
966.2999999999998	894.8666666666667	1101.8999999999999\
TCR- alpha V 33.1 /// TRAV40 /// TRAV40	96.6	182.5	114.3
106.7	107.3	32.5	74.8	118.5	97.1	128.6	94.9	58.3
	20.7	81.2	94.1	76.3	79.2	163.0	56.6	28.8
	277.3	92.5	126.9	47.1	105.6	90.3	143.2	81.5
	130.6	90.7	44.8	161.6	38.0	50.8	163.6	8.7
	77.8	163.9	122.1	83.8	64.1	66.4	106.6	65.7
	81.7	154.1	99.4	46.9	151.7	94.8	68.4
108.8	79.5	146.7	102.2	53.3	80.3	171.4	75.6
101.1	467.6	99.9	51.4	97.1	109.2	71.6	108.1	32.6
	50.1	79.6	146.2	73.7	114.8	86.5	49.8	59.4
	153.5	54.0	95.3	136.2	122.7	72.0	44.9	83.9
	183.3	200.0	119.8	46.4	68.5	133.1	6.1
102.5	102.4	54.0	36.8	78.6	82.6	38.7	123.6
170.2	90.9	74.2	105.9\
LOC644172 /// MAPK8IP1	116.6	584.3	210.9	165.1	531.1
165.0	35.1	80.5	100.5	276.7	119.6	362.4	19.6	91.8
	100.8	222.0	88.5	210.6	167.4	173.7	117.7
149.5	102.3	126.3	116.9	236.2	60.6	171.7	172.7
146.4	154.8	18.6	61.0	156.0	193.2	138.8	128.8	46.4
	108.2	255.1	143.0	126.7	166.0	103.4	185.6
125.9	51.1	152.5	125.3	485.2	103.4	97.1	26.2	16.9
	10.9	292.7	223.1	273.7	198.0	34.1	540.8
536.8	238.3	192.1	417.3	189.1	209.5	113.9	160.3
173.2	48.0	145.5	127.1	114.5	132.1	156.2	149.3
118.1	96.9	47.6	15.0	29.2	186.7	127.5	153.2
380.6	27.5	36.8	45.1	65.5	195.3	44.3	83.7
115.1	109.5	86.4	111.4	134.8	178.6	28.0	75.9

115.8	217.1\
IL2RA	80.94999999999999	24.45	51.949999999999996
88.65	17.549999999999997	25.6	50.05	94.2	59.6
73.75	18.5	79.9	56.05	137.4	58.9	91.25	109.8
106.9	62.0	21.200000000000003	310.55	50.35
71.39999999999999	149.5	59.35	57.449999999999996	34.9
	99.35	85.4	11.350000000000001	60.75	23.7
114.75	58.099999999999994	27.25	61.75	87.25	36.15
59.25	136.8	39.95	111.25	178.85000000000002	48.9
31.200000000000003	84.55000000000001	120.19999999999999
	21.4	51.6	46.5	141.60000000000002	166.5	80.4
	93.89999999999999	59.650000000000006
31.450000000000003	86.44999999999999	84.85	73.95
55.05	248.15	140.55	74.95	91.85	108.6	115.05	70.25
257.04999999999995	170.20000000000002	106.5
170.54999999999998	110.8	63.349999999999994	92.75	79.4
	75.85000000000001	99.30000000000001	161.7
38.75	50.0	38.85	177.5	10.100000000000001
54.300000000000004	168.75	87.14999999999999	114.25
220.0	150.25	109.35	68.65	58.550000000000004
95.94999999999999	71.8	34.7	12.25	15.3	30.45
52.65	148.3	50.15	106.05000000000001	17.15\
IL2RB	201.5	264.5	325.7	753.5	194.0	171.9	240.6
302.3	310.7	388.5	234.6	241.6	308.7	946.6	229.1
632.7	311.5	225.0	302.6	259.4	207.9	99.2	214.4
735.9	173.6	234.8	168.9	2163.2	336.5	843.2	142.3
764.4	751.2	362.5	428.6	1036.6	248.9	363.1	169.5
572.5	376.0	219.7	938.6	154.9	315.2	415.3	325.7
199.5	421.6	377.1	263.4	321.3	281.2	344.8	325.2
167.2	245.3	485.6	389.2	198.3	512.4	832.9	336.2
365.2	389.8	514.9	232.8	436.2	631.7	450.5	440.5
364.4	696.2	494.4	803.2	213.1	350.1	519.2	421.7
322.1	377.5	458.7	56.9	773.6	711.1	294.2	315.1
661.8	540.5	271.9	116.5	793.6	362.0	298.9	392.5
214.5	309.1	319.3	178.8	392.9	248.8	448.4	233.5\
LOC401442	23.2	31.3	11.7	67.0	21.7	37.0	5.5
	24.9	74.9	56.0	6.4	66.8	54.3	40.0	66.0
	51.2	3.9	13.9	31.2	76.1	10.9	49.9	10.4
	3.0	8.5	37.8	52.0	120.9	79.5	91.9	3.3
	36.0	45.3	6.5	101.0	75.9	45.9	37.9	62.7
	38.6	8.9	41.2	12.9	7.8	70.7	37.8	50.6
	34.1	150.0	58.1	76.0	45.9	51.3	156.6	45.9
	86.9	61.5	137.1	78.4	143.7	331.1	96.1	66.1
	41.9	121.9	69.0	14.1	17.2	33.1	11.4	64.5
	115.8	55.4	21.4	6.0	89.9	79.6	53.2	72.0
	11.5	78.6	13.8	51.8	63.8	12.5	170.5	18.9
	6.6	13.7	61.7	23.5	96.8	23.9	35.9	18.9
	40.7	63.5	4.5	7.8	45.3	66.2	55.8
53.1\
KCNK1	336.67499999999995	52.175000000000004
242.70000000000002	598.6999999999999	651.625
208.47500000000002	173.225	783.85	619.775	594.3249999999999
	478.2	442.2	1965.25	227.25	286.475	298.275	353.375
655.525	905.4999999999999	380.125	228.29999999999998

591.575 800.95 517.55 744.575 359.34999999999997
508.19999999999993 603.5 390.15 461.92500000000007
1322.775 360.025 488.525 979.775 325.2 1046.625
1025.9499999999998 331.17499999999995 1017.875
852.85 389.6 431.8 848.725 296.50000000000006 516.025
529.825 721.25 624.6999999999999 491.82499999999993
459.625 532.275 1308.2250000000001 963.45 477.825 347.5
351.925 955.0500000000001 346.35 602.675 529.25 1049.3
618.2 938.55 1266.1499999999999 313.15 899.55
740.8500000000001 242.425 504.075 828.75 426.775 616.275
155.725 1002.5500000000001 223.50000000000003
1334.2749999999999 846.3750000000001 956.1999999999999
960.2750000000001 738.475 575.8000000000001
768.6999999999999 870.8 464.325 420.675 501.70000000000005
563.25 598.525 789.525 214.4 1056.1 242.75
451.79999999999995 1217.9499999999998 424.3499999999999
771.1 847.35 882.825 356.75 548.775 666.7
407.04999999999995 1287.375\
KCNK2 32.0 124.0 14.2 46.6 11.7 20.9 22.4 3.7
11.0 4.7 15.1 47.6 3.6 4.3 27.9 44.7
56.3 31.7 4.7 3.3 157.1 4.6 50.0 7.1
24.9 38.4 7.2 94.3 37.4 49.9 26.6 36.0
6.2 12.3 29.4 7.3 2.2 29.4 2.8 24.1
6.2 40.3 17.5 3.5 12.9 14.2 22.3 6.2
50.3 13.6 11.6 12.9 26.4 85.4 33.2 4.8
21.4 99.4 61.8 71.8 44.9 55.6 5.9 6.8
7.3 15.9 13.1 14.5 20.6 40.4 12.5 8.3
6.9 2.1 18.9 5.7 12.5 31.3 27.1 3.3
93.3 29.1 4.9 9.9 59.5 12.1 66.4 10.2
11.5 75.6 19.4 12.2 3.2 6.6 3.2 4.3
3.8 8.4 8.3 5.4 9.1 9.9 54.4\
BCAR1 93.15 209.75 305.1 91.8 219.45 53.65 107.5
148.2 119.8 135.45 53.050000000000004 246.5 66.95
157.45 28.2 210.2 56.65 245.35 151.25 298.40000000000003
517.95 58.3 227.75 103.15 28.6 186.0 138.15
248.15 180.5 104.64999999999999 96.8 208.8 321.65
180.1 161.95 217.05 54.55 334.55 102.15 11.75 140.6 76.2
191.70000000000002 217.75 114.75 68.94999999999999
84.95 157.6 203.79999999999998 120.6
144.64999999999998 150.39999999999998 148.14999999999998
175.05 163.95000000000002 177.15 102.6 169.25
75.65 261.3 61.900000000000006 164.6 198.8 188.15
252.95 271.05 110.4 190.75 168.4 62.7 157.1
216.89999999999998 190.20000000000002 128.35 195.9
123.0 107.6 181.75 178.8 114.60000000000001 143.15
89.15 89.25 119.1 355.9 55.0 159.60000000000002
128.1 255.0 430.15 69.4 172.5 143.65 208.25 220.8
65.25 133.7 174.35 169.95000000000002 93.0 147.6
165.45000000000002 53.1\
KCNK3 63.166666666666664 95.23333333333333 68.5
50.833333333333336 45.599999999999994 41.699999999999996
47.36666666666667 94.03333333333335
82.76666666666667 136.33333333333334 84.10000000000001
55.800000000000004 61.26666666666667 19.5

71.43333333333334	46.5	36.699999999999996
128.76666666666668	134.33333333333334	44.03333333333333
	157.26666666666668	44.666666666666664
98.63333333333333	111.36666666666667	82.83333333333333
	57.9	61.9	140.03333333333333	24.100000000000005
	215.06666666666667	46.599999999999994
76.56666666666666	195.06666666666667	29.366666666666664
	67.76666666666667	70.73333333333333
52.70000000000001	82.4	67.2	69.10000000000001
157.36666666666667	28.0	91.89999999999999
98.63333333333333	40.36666666666667	57.800000000000004
	119.03333333333335	90.23333333333333
69.39999999999999	54.56666666666667	47.36666666666667
	72.43333333333334	64.5	143.9	52.46666666666666
	33.300000000000004	84.36666666666667
175.79999999999998	85.2	103.33333333333333
207.70000000000002	116.96666666666665	60.06666666666666
	49.06666666666666	42.699999999999996	125.3	19.2
	151.13333333333333	92.86666666666667
72.39999999999999	95.56666666666666	92.60000000000001
	45.73333333333333	36.1	36.833333333333336
56.666666666666664	107.76666666666665	70.39999999999999
	49.0	54.699999999999996	68.0	24.900000000000002
	140.86666666666667	35.199999999999996
168.23333333333332	170.33333333333334	111.36666666666667
	125.10000000000001	276.4	119.43333333333334
73.10000000000001	100.13333333333333	85.39999999999999
	80.26666666666667	85.23333333333333
40.666666666666664	45.56666666666666	55.6
86.46666666666665	47.1	75.2	175.20000000000002
70.96666666666665\
CDRT15	73.3	119.7	53.4	45.1	99.0	14.1	54.9	83.6
	22.6	32.5	56.1	73.0	21.5	29.9	177.5
143.0	102.2	28.4	10.4	150.2	265.6	21.0	155.8	6.5
	84.5	161.4	55.1	57.6	70.9	63.3	59.5
207.1	203.1	108.9	170.3	14.1	52.2	114.0	85.8	8.0
	127.0	21.9	25.1	17.5	20.1	58.3	93.0	80.3
	58.7	106.7	121.9	137.1	51.7	161.3	100.0
209.9	23.1	110.7	48.7	77.4	142.2	164.3	110.3	91.7
	26.6	34.9	60.3	73.9	146.5	62.4	267.2	31.7
	53.7	129.5	92.9	45.4	159.5	98.5	25.9
182.8	240.2	68.1	117.1	116.6	119.2	225.2	76.4
101.8	150.4	172.1	70.8	195.6	29.1	60.2	41.8	22.4
	90.9	87.1	163.9	154.3	43.3	139.6	126.3\
KCNK4	7.3	27.1	9.1	6.2	19.9	9.7	4.2	7.8
	7.3	22.3	10.4	4.9	7.3	8.2	18.6	10.4
	14.9	5.7	6.5	6.1	25.6	7.8	56.8	24.9
	6.8	11.2	10.6	26.9	23.9	6.4	4.4	27.1
	10.5	19.5	26.3	14.8	14.4	12.7	9.9	9.4
	6.3	7.4	13.4	19.4	10.4	18.4	10.7	5.2
	22.8	19.9	27.2	16.6	5.8	12.5	16.7	30.7
	18.6	9.9	4.0	102.8	71.9	15.0	4.1	13.7
	72.2	13.1	4.2	4.9	103.9	14.8	30.3	70.2
	35.2	12.9	26.0	11.1	7.1	8.8	10.4	9.1

8.4 11.1 6.9 19.8 67.4 21.7 11.2 15.9
8.8 9.4 31.2 11.7 15.0 4.9 13.3 5.4
5.5 32.0 10.2 77.1 4.5 9.1 9.4\
SLC23A3 169.89999999999998 345.90000000000003 344.9
137.75 223.45 103.5 135.4 181.85 162.4 189.25 156.9
211.89999999999998 232.70000000000002 281.95
160.70000000000002 263.0 223.20000000000002 240.85
189.45 277.0 331.90000000000003 149.9 250.05 99.05
171.1 384.3 165.65 363.9 450.40000000000003 185.1
198.95 357.4 368.6 135.0 231.64999999999998 439.8
175.20000000000002 344.15000000000003 250.60000000000002
119.85 93.60000000000001 332.45000000000005
156.39999999999998 200.4 104.5 292.05 82.60000000000001
158.15 419.34999999999997 248.9 238.8
165.54999999999998 214.89999999999998 342.70000000000005
351.0 383.70000000000005 185.75 232.5 232.4
199.2 123.60000000000001 179.05 274.8 142.4
210.45000000000002 455.15000000000003 162.2 344.2
228.25 291.3 405.20000000000005 339.2 191.64999999999998
177.8 271.95 166.4 271.45 154.35 287.4 98.45
270.7 448.90000000000003 132.55 410.4 240.0 267.35
322.7 188.6 277.55 285.95 40.35 441.34999999999997
274.15 265.25 531.6 329.65000000000003 334.0 182.8
408.1 551.5500000000001 252.9 329.0 251.25\
SLC23A2 103.25 338.72499999999997 185.375 134.525 237.95
136.225 105.15 141.29999999999998 170.57500000000002
140.5 131.6 109.65 141.14999999999998 123.275 107.4
183.32500000000002 97.42500000000001 169.45 111.1
135.725 251.89999999999998 125.12499999999999 214.675
114.125 185.35 113.05000000000001 82.2 130.025 145.25
134.9 276.9 100.875 302.95 170.525 156.47500000000002
136.3 110.19999999999999 293.875 205.25 106.175 114.325
108.975 91.3 169.95000000000002 107.45 95.05
97.32499999999999 328.40000000000003 213.325 156.05
133.625 189.375 136.7 148.725 155.275 274.32500000000005
190.75 138.4 94.425 172.375 264.0 98.425 176.875 180.05
190.7 215.12500000000003 153.3 155.67499999999998
254.45 155.32500000000002 324.225 225.62499999999997
183.32500000000002 113.75 208.07500000000002 71.25
154.25 141.14999999999998 142.0 195.45 143.82500000000002
152.15 147.95 90.67500000000001 263.425 137.125
129.425 226.375 174.275 218.57500000000002 120.44999999999999
190.55 93.1 224.075 209.65 153.125 152.4 131.05
158.725 130.5 189.1 136.05 208.25000000000003\
LOC100505658 3.8 6.2 5.4 2.1 16.2 5.3 4.6
11.4 6.7 19.1 4.2 5.5 4.6 4.6 12.0
5.7 6.7 7.3 17.2 6.9 111.7 6.9 28.4
1.6 11.3 8.2 2.8 21.8 7.1 66.1 2.1
53.5 9.6 7.1 11.3 48.1 6.4 34.9 21.4
7.2 4.0 77.8 6.7 2.2 69.6 4.7 4.0
9.9 17.6 10.6 3.8 44.1 4.6 10.9 10.7
5.2 49.6 8.5 5.7 5.2 29.0 1.8 2.9
3.5 4.4 87.5 13.2 4.3 7.6 7.0 4.3
11.5 4.7 2.6 11.7 23.2 2.7 1.6 5.4

```
	14.3	4.0	4.2	1.8	5.1	6.4	20.3	21.6
	13.6	9.4	7.0	25.6	8.9	4.0	5.2	3.6
	42.0	3.0	26.2	3.8	6.8	2.7	6.1	4.6\
CERK	1004.1	370.4	875.5	1337.4	888.9	968.3	1704.5
808.5	951.4	708.8	912.8	1080.2	862.5	1050.9	1531.3
2533.8	1051.7	504.5	1516.9	1675.4	1491.0	862.9	837.8
713.5	1051.8	884.5	1213.9	1119.1	869.7	1407.7	2008.6
570.6	1447.2	829.4	788.9	755.9	600.0	686.9	1795.9
1003.4	971.0	717.7	1042.5	4326.5	733.1	599.4	583.2
988.9	876.8	1079.6	904.8	693.3	1011.9	920.8	984.8
910.5	928.5	729.6	773.4	721.9	1159.4	667.8	1142.7
873.9	1424.2	586.4	1413.6	3635.0	1086.1	667.4	629.4
802.1	1701.0	1093.5	1162.3	1222.5	810.2	667.1	1145.4
965.6	744.8	649.4	1408.8	1315.1	801.4	765.0	901.5
984.8	1020.4	563.6	845.4	779.8	1215.4	1255.7	1804.1
1384.8	1137.5	1709.3	711.8	1422.1	1524.1	1285.3	1404.6\
SLC23A1	17.6	79.6	304.2	56.6	68.8	33.4	1.8
107.9	78.0	1059.3	538.6	87.7	66.6	228.5	22.9	8.7
	47.0	375.7	217.6	71.8	59.2	235.6	1117.6
160.8	140.7	128.3	164.1	188.5	231.2	9.6	22.7
148.5	197.4	200.2	23.5	369.4	124.3	118.3	42.5	27.7
	24.7	58.9	333.6	2.8	199.9	146.9	258.8	13.7
	213.0	31.3	236.7	221.0	153.8	86.3	128.2
610.7	111.3	275.7	429.7	251.9	1029.7	319.2	76.7
120.6	45.4	64.2	84.0	32.1	236.4	800.2	1451.7
247.5	36.4	55.5	185.2	367.8	315.5	233.6	97.9	73.8
	198.4	109.4	45.5	45.6	229.0	286.5	76.9	92.4
	92.9	126.3	9.5	256.3	228.0	115.3	95.8
248.7	156.4	964.3	73.9	294.8	28.0	158.1	25.8\
LGALSL	1057.15	229.6	390.5	949.9	657.5	991.25	766.8
1184.3	1486.4499999999998	598.6999999999999	864.25
848.3	751.85	435.2	210.35000000000002	1078.4	893.85
738.05	999.75	2945.5	1377.1	502.7	444.3	833.1
716.0500000000001	1049.9	1393.05	861.55	823.5	2172.05
1654.5	464.1	326.55	978.7	874.5	764.1	898.8
403.95000000000005	2568.85	742.9	167.1	726.0
1241.3999999999999	6134.2	1137.75	1154.4	1413.2	2741.25
688.5500000000001	709.7	900.1	598.0999999999999
1135.75	741.0500000000001	951.1999999999999	530.35
817.2	354.05	1113.5	796.0	686.8499999999999	310.6
980.35	1006.2	2457.7999999999997	819.9000000000001
1555.8500000000001	6725.95	651.55	730.4	527.2	699.1
2364.5	730.8000000000001	1107.45	1303.7	500.1
1132.3000000000002	1248.8	2241.15	628.9	735.65
173.14999999999998	702.5500000000001	373.75	526.1
147.85	891.4499999999999	402.45	999.35	1228.8000000000002
	866.8	1232.3500000000001	794.25	815.05	1343.4
1094.95	921.55	1042.7	760.0	914.4499999999999	1058.3
1001.8\
CCP110	951.4000000000001	1185.7	1561.35	647.45	669.1
577.45	609.55	309.3	358.54999999999995	594.65	604.7
558.25	431.5	763.85	672.75	639.35	568.0	1008.8
491.04999999999995	818.4000000000001	581.0999999999999
	899.8	989.4	561.75	653.1	885.7	495.09999999999997
```

1157.65 980.95 796.4000000000001 933.85 1028.4
909.95 489.75 783.8 986.45 469.7 492.34999999999997
920.65 735.15 744.3 1010.3 1115.55 763.25 531.5 974.5
411.84999999999997 1068.15 620.3 1104.5 594.1 1022.8
408.45000000000005 365.40000000000003 932.75 1204.35
723.55 1000.6 963.6 1122.05 1055.45 998.7 833.6 1432.4
1352.75 1484.4 750.7 854.95 656.8 1898.0 702.65
1669.0500000000002 1002.7 708.4 796.05 1074.0 508.85
846.35 1108.65 398.90000000000003 319.34999999999997
398.1 745.55 785.1 505.2 441.5 320.09999999999997
396.0 431.29999999999995 479.5 396.65 655.1 760.1
695.05 1008.6 1145.5 1053.6 981.35 604.5500000000001
1047.15 1076.1 715.55 856.4000000000001\
PEX26 136.9333333333333 376.8333333333333 161.4
342.8666666666666 448.76666666666665 283.40000000000003
193.03333333333333 159.46666666666667
401.9333333333334 341.4666666666667 244.53333333333333
309.1333333333333 270.0 190.20000000000002
225.53333333333333 133.76666666666668 288.59999999999997
289.1333333333334 334.4666666666667
221.26666666666665 430.8333333333333 361.23333333333335
282.8 258.76666666666665 339.2333333333333
113.06666666666666 207.83333333333334 359.1000000000001
213.20000000000002 234.4333333333333 193.1
394.9666666666667 294.46666666666664 81.06666666666666
288.73333333333335 229.36666666666665 104.3
300.96666666666664 210.9666666666667 144.26666666666668
311.7 236.76666666666665 161.3 342.0
515.7333333333333 257.06666666666666 164.86666666666667
198.46666666666667 313.43333333333334
274.6333333333333 331.5333333333333 203.80000000000004
156.23333333333332 203.20000000000002 185.9
287.8666666666667 230.5 180.23333333333332
284.1333333333333 303.53333333333336 644.1666666666666
258.23333333333335 232.46666666666667
175.5666666666667 148.5666666666667 329.2
306.93333333333334 170.1 402.90000000000003
113.59999999999998 355.5 381.0333333333333
232.26666666666665 270.43333333333334 287.6666666666667
162.66666666666666 136.86666666666667
222.76666666666665 156.70000000000002 149.46666666666667
207.66666666666666 196.83333333333334
107.16666666666667 157.86666666666667 292.3333333333333
353.76666666666665 195.30000000000004 159.9
445.3666666666673 280.40000000000003 290.93333333333334
223.1 214.83333333333334 169.53333333333333
94.03333333333335 112.13333333333333 148.56666666666663
99.76666666666667 119.8 172.4 169.6 259.0
165.96666666666667\
LGALS4 47.9 89.2 85.0 45.2 73.3 113.5 3.6 20.8
3.6 19.2 38.9 16.8 54.7 6.0 7135.2 19.7
6.7 53.3 277.7 24.0 119.7 4.9 27.4 4.4
52.0 57.9 49.0 40.5 125.3 33.6 2.4 22.3
104.4 5.2 7.2 80.3 45.8 6.0 3.5 5.0

22.0 209.7 13.4 11.2 34.5 81.4 4.4 5.2
146.7 31.4 94.8 64.1 51.4 12.4 13.9 74.4
42.3 44.4 28.7 22.8 259.8 116.2 13.7 83.9
47.7 72.1 24.6 53.0 60.9 59.9 136.6 14.3
55.6 5.5 30.8 26.6 69.5 8.8 10.7 13.5
33.9 30.4 39.9 177.9 21.7 164.8 157.0 6.9
36.9 8.8 39.9 83.3 126.1 199.6 237.4
165.1 235.8 142.5 224.7 109.8 166.1 44.2 83.6\
LGALS3 6035.5 4250.55 12071.05 8761.95 3772.3500000000004
10370.4 2943.4 20597.25 14036.55 22827.65
16089.6 14063.65 19800.7 11202.1 1643.55 17074.2
7573.7 12796.849999999999 10383.25 12733.45
4252.65 16371.699999999999 20775.4 12822.45 14728.55
10383.25 8785.3 5094.2 7383.3 15597.25
5119.15 8013.95 6862.75 6048.3 9417.5 34850.75 17409.65
12077.4 13486.1 8581.199999999999 3082.8
11797.150000000001 10733.85 13604.65
7784.099999999999 5096.8 11646.35 9080.2 2082.65
4272.700000000001 22077.300000000003 11145.5 12470.7
8153.2 11404.65 8802.85 5014.599999999999
12504.650000000001 17090.9 3746.8 2805.0499999999997
15165.55 11778.349999999999 18458.899999999998
11238.699999999999 16676.7 8425.5 7892.8 11649.45
10992.1 7486.2 6094.45 13282.95 9919.599999999999
6781.0 6982.35 23157.8 13379.85 10040.9 2155.5 11674.5
22666.6 15523.35 7487.900000000001 23648.95
29287.5 10067.85 12904.449999999999 28711.15
29160.2 9091.7 10023.550000000001 5342.85 11592.800000000001
10137.2 6568.8 8197.15 10991.4 14831.650000000001
7330.400000000001 6741.950000000001 10707.949999999999
10619.35\
LGALS2 11.4 26.3 28.2 109.3 165.6 35.5 4.3 54.4
44.0 99.4 48.6 55.5 19.1 226.3 14.8
110.7 9.2 43.5 13.9 17.1 93.1 39.2 41.3 64.5
17.6 6.2 17.8 44.1 26.8 10.9 14.7 27.7
249.6 43.1 28.0 28.4 91.8 17.9 39.0 5.6
16.5 5.7 191.1 12.0 77.5 16.8 10.4 8.7
37.1 33.2 25.1 9.3 13.1 30.1 54.0 14.5
7.3 31.0 15.8 165.0 53.9 105.4 25.7 12.3
66.1 16.9 92.1 12.5 278.2 19.6 61.5 39.3
136.1 50.5 22.2 51.0 24.9 71.4 31.6 16.8
7.2 26.9 10.2 158.0 37.0 41.6 21.2 84.0
117.4 6.9 108.9 28.5 14.7 180.4 24.4 11.0
9.1 12.2 16.0 102.9 13.6 19.7 34.1\
LGALS1 3027.8 683.8 617.9 5169.7 1731.3 1692.5 1118.6
184.3 3724.0 6289.4 1181.7 1940.4 4756.5 6843.8 446.4
6444.2 4531.8 713.4 5144.7 5827.2 1516.3 929.2 1376.1
5728.7 999.4 2117.1 9090.6 2240.5 1541.7 5599.6 2962.0
522.1 2632.0 2032.4 1965.2 3743.6 2004.6 3127.6 1902.4
3596.6 2149.9 2235.8 6347.6 4002.4 1816.9 1257.1 335.5
1166.3 455.7 588.8 3845.9 1608.0 1533.7 424.4 2241.9
1840.1 1082.6 2246.4 3450.0 1304.0 855.8 4075.9 4355.9
810.9 946.9 2507.8 1481.9 3099.2 3970.3 1429.5 602.4
851.8 1127.2 1515.8 1719.5 1946.3 438.1 915.1 1679.3 99.3

	202.7	396.3	524.0	877.8	303.1	290.8	277.1
922.8	1327.6	1514.7	2427.9	567.7	3099.2	4674.7	4824.5
1216.8	2435.2	942.7	911.1	1008.6	1371.0	2910.9	1620.0\
HMGN2P46		47.1	125.2	275.3	156.5	162.1	14.3	0.7
	388.1	103.2	241.6	179.3	1.2	70.5	104.5	46.5
	116.2	78.5	130.2	130.4	180.6	37.7	387.7
513.8	266.1	103.4	236.1	190.4	334.7	283.2	63.7	11.0
	1066.9	350.5	79.8	134.6	124.7	124.1	195.1	41.7
	53.9	4.3	62.0	75.3	28.3	85.9	95.7
232.7	66.3	311.4	5.6	164.4	204.6	261.0	187.7
210.9	136.1	180.7	397.2	134.4	269.3	553.0	155.7
265.2	353.6	141.9	317.1	47.4	54.9	423.4	473.8
262.1	281.3	171.3	31.2	113.8	168.6	419.8	159.6
169.5	22.4	52.7	138.5	31.8	95.3	529.7	492.9	91.4
	80.0	1116.9	115.8	46.3	114.5	16.3	142.9
150.7	70.7	54.2	269.3	102.4	124.1	12.0	79.1
140.8\
IL36A	53.8	110.0	102.1	44.8	131.9	50.7	82.4	76.8
	71.2	137.9	107.3	9.0	20.9	90.2	166.1	80.0
	67.7	79.2	23.0	100.7	226.9	79.8	29.9	49.8
	107.2	16.5	62.6	105.6	188.7	62.8	49.7
162.3	71.6	61.1	84.4	53.1	85.5	205.0	64.9	78.4
	31.6	18.6	92.9	39.9	89.1	161.3	97.8	18.1
	51.3	83.5	32.4	108.9	127.3	51.2	109.1
213.2	20.2	166.5	55.4	42.8	40.1	57.7	54.5	79.5
	87.6	24.5	19.8	89.0	24.8	155.2	154.0	97.8
	132.1	64.1	74.1	6.3	49.7	95.6	60.0	77.8
	106.6	94.4	85.5	69.6	163.0	212.3	117.3	23.5
	18.4	114.0	52.5	144.7	101.9	39.8	154.9	20.5
	22.5	105.8	168.8	132.0	87.4	143.8	47.9\
IL36B	4.6	15.6	17.25	23.0	10.4	6.3	18.1
26.299999999999997	11.65	11.65	12.549999999999999
17.65	4.55	19.95	44.150000000000006	22.05	26.7	5.25
	7.800000000000001	14.85	21.950000000000003	6.95
	26.75	2.9499999999999997	11.9	11.8	7.25	75.0
	44.1	52.2	6.800000000000001	9.2	55.6
26.85	72.05	21.0	4.0	15.3	30.1	34.4	7.45
11.35	8.8	3.5500000000000003	4.75	10.8	6.95	9.25
	29.8	8.7	38.9	31.55	24.2	10.8	8.8	56.5
	7.45	6.5	8.7	13.9	173.6	20.65	26.95	31.9
	25.15	40.400000000000006	18.900000000000002	17.9
	4.8	33.95	28.599999999999998	10.75	21.0
17.900000000000002	9.35	6.550000000000001	31.3	6.25
	13.450000000000001	20.05	52.75	9.899999999999999
	26.45	16.650000000000002	117.25	9.55	14.05
31.799999999999997	13.850000000000001	56.35	5.7	14.9
	43.1	10.600000000000001	6.449999999999999	5.95
	28.85	9.8	62.8	32.949999999999996	15.4
11.45	9.75\
FOXD3-AS1		6.7	7.6	18.6	5.4	23.4	4.3	18.7
	9.4	7.6	21.1	10.4	4.0	26.2	126.6	11.6
	6.1	4.2	43.8	114.3	81.8	391.0	4.5	21.9
	14.9	32.6	18.0	8.4	8.4	17.7	7.9	21.6
	11.1	20.4	10.4	9.5	12.2	6.1	54.4	11.6

7.9 20.1 125.3 13.0 65.2 51.1 41.8 8.0
72.5 17.8 6.8 10.1 10.8 85.7 33.5 6.0
24.6 15.2 9.5 29.0 177.5 380.7 3.9 55.5
6.7 19.9 42.1 40.0 3.5 59.0 7.4 17.9
16.8 23.7 12.5 71.0 5.4 15.7 14.2 6.7
196.1 9.9 170.0 39.6 6.8 85.1 21.6 28.7
27.6 9.5 262.9 2.7 15.9 75.9 7.7 8.0
4.3 4.4 3.1 5.8 9.7 9.2 13.1 9.1\
BSG 1116.6 1215.3 472.0 606.1 679.3 2441.0 2879.5
459.7 1973.0 1960.0 960.7 2280.6 1454.5 739.6 642.0
587.8 1778.9 599.9 717.2 1238.5 226.3 810.9 541.7
1257.7 785.2 194.7 1130.5 658.8 1114.2 1572.6 630.1
181.3 742.7 2285.6 551.7 378.2 804.4 448.2 1988.3
1915.9 716.2 1606.3 1345.6 1378.2 821.4 786.4 570.3
711.7 336.8 1369.3 915.4 1710.6 381.0 331.8 1021.8
1918.8 964.6 392.9 1068.0 915.6 777.3 780.3 1141.4
739.1 531.8 705.6 1942.8 2037.8 976.3 686.7 421.7
431.4 464.5 796.7 636.2 1162.4 442.9 1279.2 728.8
214.7 269.0 348.8 836.5 385.8 258.2 370.6 275.5
428.9 954.9 586.2 512.6 209.8 366.8 693.4 348.9
438.4 324.8 467.8 441.1 250.0 402.5 297.4 528.1\
TUNAR 9.1 15.9 41.2 3.3 8.4 5.7 2.6 5.0
124.1 6.4 2.9 6.5 2.3 3.5 9.5 6.6
3.3 20.0 2.3 10.3 12.1 104.6 10.6 68.3
100.9 9.5 11.9 171.6 84.5 47.9 30.6 9.8
5.3 436.1 12.6 114.6 10.2 1035.9 3.0 42.0
5.3 3.3 438.9 20.2 6.6 11.8 3.8 8.5
66.5 10.4 5.1 573.8 9.7 8.2 50.5 4.7
12.1 839.2 61.5 12.0 62.8 81.9 5.0 2.7
5.8 6.7 6.1 6.5 75.0 61.2 11.7 42.6
40.3 68.3 80.2 41.5 6.4 80.7 110.9 6.9
223.5 9.3 43.0 4.4 6.6 35.5 31.9 22.4
2352.7 14.3 3.4 14.5 44.9 60.6 223.8 88.9
149.9 75.0 171.5 58.4 28.9 257.0 230.6\
LGALS9 305.1 281.4 478.0 311.5 1089.5 145.0 219.2
418.9 566.8 466.3 270.9 215.6 239.4 490.3 339.8
581.6 222.4 666.3 1237.5 205.4 669.9 335.8 511.9
824.4 491.5 219.8 295.0 750.8 542.4 299.7 139.3
529.9 952.2 403.6 408.6 343.3 262.4 317.0 201.8
215.3 194.6 219.1 699.6 321.2 1200.6 538.3 231.2
259.1 833.7 449.2 380.0 498.6 211.3 359.6 525.3
334.0 464.1 469.6 186.1 360.1 629.3 698.4 551.8
374.2 322.8 661.4 353.6 341.5 850.0 422.9 785.1
817.2 210.3 339.2 661.6 456.4 408.5 445.4 387.5
156.5 489.2 231.5 121.4 375.9 275.0 541.3 323.6
479.0 305.7 480.1 270.0 380.3 339.1 216.0 299.9 72.0
80.6 216.7 494.8 312.2 246.5 270.4 185.5\
ATXN7L1 98.675 101.475 228.65 93.175 214.57500000000002
170.40000000000003 70.875 194.325 139.05 522.975 90.575
145.125 100.57499999999999 202.35000000000002
121.00000000000001 96.65 156.15 219.75 204.625 144.875
167.7 150.45000000000002 80.27499999999999
179.47500000000002 126.825 105.74999999999999
138.02499999999998 265.7 329.72499999999997

129.32500000000002	95.375	238.22500000000002	199.6
113.85	142.1	175.05	169.55	195.54999999999998
84.47500000000001	235.6	113.42500000000001	177.875
321.025	89.3	434.04999999999995	364.625	85.5	122.5
549.8	233.7	132.025	76.65	132.35	172.525	220.475
232.37500000000003	207.74999999999997	165.60000000000002
	149.32500000000002	201.75	218.57500000000002
214.9	172.17499999999998	74.125	98.2	190.775	135.0
81.55	386.875	277.25	436.45	418.65	94.55	134.925	145.15
179.82500000000002	223.55	169.27499999999998	136.65
119.64999999999999	213.65	107.22500000000001
60.99999999999999	85.7	242.725	133.75	287.675	164.05
94.75	92.525	148.375	180.55	266.4	165.27499999999998
276.84999999999997	314.55	340.35	233.14999999999998
319.075	281.2	273.675	119.5	292.375\
ATXN7L2	5.8	6.2	96.5	85.5	36.2	52.0	42.7
129.1	70.0	145.2	68.7	106.1	28.2	47.5	113.4
113.4	10.8	48.2	43.9	7.0	119.0	54.8	186.1	66.1
	18.2	55.5	9.9	139.8	239.2	109.0	32.4
160.4	109.2	70.2	84.6	95.2	25.4	150.7	70.0	64.4
	53.0	81.8	59.0	29.6	61.6	105.7	100.9	57.5
	344.3	10.2	95.9	57.3	46.4	176.7	71.3
178.8	86.6	14.8	42.5	27.3	121.8	100.8	40.2	63.5
	54.6	171.6	35.0	13.4	86.7	83.1	150.2	89.8
	94.5	12.8	56.3	50.6	39.0	36.1	30.8	48.1
	239.6	155.6	46.7	60.0	268.6	290.1	99.6	99.3
	23.2	79.6	25.9	209.3	69.0	36.3	62.0	87.1
	32.1	124.2	159.8	137.8	34.0	61.0	96.3\
LGALS8	1303.966666666667	472.99999999999994
1105.6999999999998	785.0333333333334	831.1666666666666
	391.18333333333334	1203.6000000000001
1646.4833333333333	474.3	596.7833333333333	289.85
770.5333333333334	602.15	840.9499999999999
735.0166666666668	1005.1	1017.1333333333332
536.0666666666667	537.0666666666667	692.4666666666666
	1089.8999999999999	622.05	1021.1666666666666
826.5333333333334	572.9499999999999	1036.35
803.3166666666666	768.0500000000001	765.3166666666667
	555.9833333333332	1177.216666666667
1820.6000000000001	737.4666666666666	556.25
704.0499999999998	529.5333333333333	983.0333333333333
	586.2833333333333	870.6833333333334
806.3666666666667	723.9333333333334	817.7833333333333
	696.85	894.15	475.48333333333335	566.6166666666667
	1180.2333333333333	927.5333333333333
1472.1833333333334	1823.5166666666667	812.4833333333332
	850.2666666666668	3154.0333333333333	1100.2
453.81666666666666	1046.0333333333333	1170.35
635.3666666666667	1179.3500000000001	913.9833333333335
	651.8833333333333	627.3833333333333
1412.1000000000001	749.2833333333333	956.8000000000001
	1510.8166666666666	1558.3833333333332
745.0333333333334	1286.6833333333334	695.9166666666666
	1118.9333333333332	854.8000000000001

639.6833333333333	721.2000000000002	617.5833333333334
	302.1	841.4666666666667	593.1166666666667
980.3333333333335	750.0500000000001	678.7666666666667
	636.6333333333333	912.6666666666666
497.6333333333334	778.8166666666666	772.9666666666667
	760.4833333333335	469.06666666666666
553.4166666666666	694.75	518.9666666666667
725.9333333333334	615.6166666666667	948.5333333333333
	1091.8166666666664	858.5833333333334
1124.5166666666667	858.4	951.9	1016.1833333333333
900.8833333333333	601.6833333333334	994.3999999999997\
BSN	45.7	124.6	21.5	61.9	29.5	43.9	165.8
113.8	112.4	30.6	100.8	109.8	102.5	23.4	42.5	40.5
	84.9	36.1	13.4	206.1	174.4	73.2	59.2	48.8
	40.2	14.4	18.5	233.8	29.3	29.9	19.1	27.9
	149.3	25.7	95.6	42.3	13.3	82.2	13.7	67.8
	71.7	155.1	66.0	87.8	27.6	97.4	103.7	52.2
	167.5	30.5	138.0	64.5	8.7	61.5	61.2	28.3
	86.9	30.2	50.9	23.6	486.5	29.0	25.1	26.1
	21.8	31.0	93.8	77.6	126.4	103.8	75.8	22.9
	22.8	62.7	174.8	91.5	29.7	25.1	26.9	34.3
	107.0	93.4	168.7	104.6	30.7	89.0	41.1
190.2	197.0	95.6	107.1	162.0	152.4	83.7	123.8	88.6
	111.8	52.4	12.7	32.5	35.1	126.2	89.9\
INHBA-AS1	19.25	33.95	38.75	10.75	47.45	3.65
14.55	26.1	10.2	14.35	6.5	8.05	8.15	24.05
23.75	34.55	22.3	6.05	33.4	8.2	49.65
2.9499999999999997	23.5	8.35	11.05	30.35	35.1
23.25	22.85	108.25	13.9	84.65	22.75	6.45	5.7	17.3
	23.7	52.05	13.45	3.3	30.15	25.950000000000003
	19.200000000000003	5.95	5.15	24.9
3.6999999999999997	11.45	23.7	46.9	6.0	22.9
14.450000000000001	33.35	39.199999999999996	9.35
14.35	5.5	12.55	34.099999999999994	34.1	35.0	11.9
	22.400000000000002	70.85000000000001	20.55
15.35	24.85	20.6	24.9	42.35	8.25	30.849999999999998
	2.5999999999999996	31.5	20.55	13.7	9.35
11.05	22.05	35.35	10.950000000000001	16.7	10.3
22.65	29.55	18.55	34.15	12.45	21.05	5.1000000000000005
	43.5	11.600000000000001	14.950000000000001	19.9
	18.65	26.5	24.25	25.299999999999997	33.95	17.7
	3.5	64.85\
LOC102724938	14.0	84.7	3.0	5.1	6.1	4.0	2.8
	2.2	2.2	29.1	4.2	35.2	1.3	18.6	28.3
	9.8	5.9	15.9	3.2	7.5	13.6	1.7	5.8
	17.2	38.7	4.2	8.7	57.4	20.2	1.9	5.8
	7.6	57.1	7.7	9.8	6.3	3.5	8.8	3.4
	48.0	5.3	2.2	5.2	2.3	5.7	1.9	4.3
	2.3	37.4	7.4	12.8	5.2	11.5	10.8	32.6
	42.0	29.3	4.5	10.0	14.3	107.5	3.5	3.4
	4.2	3.4	2.9	5.6	3.6	2.4	9.3	53.5
	2.2	13.5	1.5	24.8	4.3	24.2	12.2	13.6
	4.5	45.3	26.2	1.7	6.6	49.9	24.0	7.9
	111.0	8.0	79.5	6.8	2.7	53.3	5.8	32.7

10.0 2.7 57.3 72.2 4.3 6.7 18.5
13.3\
ATXN7L3 458.8 1166.1 286.2 690.6 838.5 529.6 791.2
471.5 938.8 1076.4 363.3 554.4 497.8 723.6 611.2
476.2 471.8 509.0 519.0 343.4 651.0 503.6 223.4
511.5 713.6 521.9 466.1 897.5 764.3 665.1 567.1
465.5 1081.6 766.1 672.9 371.2 382.0 574.9 580.7
408.5 706.9 493.9 366.3 556.1 540.7 578.9 720.5
377.3 1026.6 720.0 689.4 300.6 343.0 724.7 605.0
663.3 491.8 529.6 713.2 411.6 112.1 784.2 382.7
578.3 619.9 854.2 509.8 610.7 783.8 320.7 560.6
931.8 324.9 380.4 611.9 753.3 484.2 389.7 582.9
371.5 111.5 775.6 624.1 472.1 413.8 1388.4 307.2
952.2 611.0 537.0 456.1 400.4 586.2 263.1 327.0
422.3 345.3 429.6 721.4 589.7 368.3 538.6 235.9\
CER1 61.8 159.4 12.2 64.6 94.3 15.2 39.1
104.1 63.8 49.6 78.9 91.6 78.3 31.2 78.3 79.4
18.8 52.7 50.0 65.3 157.5 52.5 145.5 63.5
21.9 47.9 15.8 129.8 159.9 73.1 19.5
263.1 132.5 43.6 170.5 20.0 88.3 204.6 42.8
101.7 111.2 73.2 209.3 42.6 64.9 21.3 175.8 47.6
80.3 47.2 58.1 109.5 40.6 175.9 124.8
147.8 38.9 61.3 170.4 29.8 233.4 28.2 147.7
107.1 158.9 177.5 50.9 12.8 28.0 131.5 122.2
160.9 113.8 59.5 27.9 42.0 61.1 132.3 101.3 53.3
208.4 213.8 30.6 139.5 71.6 398.2 23.8
112.9 25.7 125.8 14.9 281.6 108.9 58.0 51.7
125.5 67.4 23.1 65.9 98.9 32.5 156.7 95.1\
RP11-506E9.3 7.3 6.8 3.4 2.3 43.1 8.7 2.3
18.5 43.2 30.1 5.4 5.7 4.3 27.3 8.0
47.6 3.6 27.3 2.8 2.5 64.3 41.4 6.7
6.0 48.9 6.6 18.1 19.7 17.3 103.5 18.0
5.1 94.2 11.3 8.7 9.7 7.3 12.4 4.0
5.5 3.5 11.8 18.5 4.2 5.5 8.9 17.6
2.3 44.9 4.5 13.3 3.6 16.4 55.0 19.6
13.9 21.8 8.2 46.5 9.1 60.1 5.8 9.1
9.1 9.9 11.3 16.9 10.5 16.8 7.3 20.9
128.0 8.6 2.4 13.4 32.9 3.0 3.7 5.3
18.2 12.6 5.3 48.6 28.0 79.0 122.4 6.0
81.7 24.4 15.1 4.0 10.6 64.4 5.5 28.8
81.6 27.7 10.2 3.3 11.0 9.8 23.0 6.0\
LIMCH1 970.5749999999999 658.6 1795.9750000000001
1415.05 1430.65 264.1 444.5 1045.875 481.775 1666.75
944.05 1191.475 2320.7 943.175 1406.15 287.85 837.175
1402.1 141.175 459.625 689.6500000000001 1262.975
1661.475 967.0 2329.15 1765.725 564.575 1032.05
783.25 867.9000000000001 490.5 1247.25 587.925 1100.225
415.525 1611.6750000000002 1512.25 1815.775
411.225 204.625 1084.325 572.5 810.9749999999999
255.9 664.8 712.45 1160.1 405.45 364.825 910.025 1163.475
789.7 1262.0 721.4 936.0 1129.0 1269.5 998.95
1664.65 1358.625 858.9 1016.075 1838.025
1189.35 699.55 895.175 715.05 200.89999999999998 1135.7
1285.175 1821.3 190.35 639.45 295.35 1348.875

856.675 729.9499999999999 444.425 870.8 490.82500000000005
1116.275 1682.15 100.15 1004.375 921.575
1513.5500000000002 2382.725 3489.1000000000004
878.5 1961.275 742.825 672.425 1081.375 1160.275
856.375 1149.6999999999998 1016.8499999999999
1277.8 1977.625 1187.1 1102.3 537.05 1051.4\
TTTY9A /// TTTY9B 18.8 123.0 65.0 40.1 85.3 80.2
49.7 85.2 82.0 28.6 109.0 5.3 43.4 76.3
133.8 98.3 120.6 83.1 73.6 59.2 110.4 91.9
94.6 35.0 51.0 30.1 49.8 131.0 109.7 77.0
40.4 103.1 163.3 59.1 216.9 58.2 79.9 96.2
92.1 92.2 48.8 41.8 120.3 49.2 98.0
127.1 54.8 53.4 138.3 138.4 65.1 90.3 52.7 72.4
96.8 165.5 137.0 140.3 67.0 27.5 350.0 58.7
65.3 5.9 94.8 107.7 55.3 24.9 6.8 91.8
23.3 33.2 119.5 67.8 51.3 78.0 111.0 82.3
22.8 84.0 90.5 73.6 28.1 85.2 149.9 60.0
125.9 92.0 119.9 167.0 28.9 107.1 66.7 42.9
44.3 98.2 44.0 19.2 60.7 123.9 60.8 86.5
90.7\
GOLPH3L 1489.4 253.1 1896.5 969.1 2085.9 1889.6 2079.7
2124.9 2230.1 2550.4 2513.5 1083.4 2792.8 2534.2 1954.3
1442.0 1846.0 1415.7 2891.2 1265.5 169.9 3013.4 1861.5
3394.9 4852.3 2662.3 1550.6 1732.3 1149.0 1396.7 2862.7
1763.6 425.5 2798.9 2969.9 1635.0 2598.8 534.5 1923.3
2957.9 1495.7 1132.1 1146.7 1033.6 3802.8 1331.8 2967.4
2840.7 554.2 2214.8 1878.4 3188.8 5229.7 427.1 1595.0
2193.8 2083.0 1059.4 1312.9 787.3 2603.0 1435.3 2253.8
2274.4 1976.1 2538.4 4499.9 1001.3 619.1 2985.6 603.8
1035.2 784.5 2082.2 1956.0 3273.7 1401.8 3081.6 3390.4
5173.4 1269.9 1383.7 2010.6 1828.5 437.6 203.0 1941.0
1872.0 2501.8 639.3 2433.0 539.2 1610.1 1726.0 1066.0
1949.1 2270.2 2366.9 1425.6 1705.0 1533.6 762.4 2177.9\
BTC 663.1999999999999 108.4 446.4 982.8
182.39999999999998 7.55 34.6 471.25 272.25 349.5
398.75 189.85000000000002 128.95 147.15 162.25 217.7
175.05 219.55 487.2 200.95000000000002 97.75 340.4
482.9 324.05 231.15 67.8 391.15 242.8 329.95 217.75
284.90000000000003 275.55 211.75 325.0 70.0 240.75
48.05 376.0 473.45 291.0 18.95 88.7 468.84999999999997
201.9 410.70000000000005 133.35 617.75 20.3
203.3 530.2 263.25 434.85 215.04999999999998
83.60000000000001 242.0 172.8 345.54999999999995
279.2 218.7 171.55 650.55 492.2 285.0 546.5 459.15
401.5 214.04999999999998 213.25 217.70000000000002
623.95 327.9 237.05 412.3 100.6 354.15 708.0 601.65
284.3 392.54999999999995 28.6 497.04999999999995
143.14999999999998 25.25 538.0 252.95000000000002
92.19999999999999 560.0 347.15 240.95000000000002
194.60000000000002 360.65000000000003 298.79999999999995
452.70000000000005 391.9 382.3 679.3000000000001
471.45 687.75 538.95 795.5 555.7 316.25 618.3\
ZNF542P 1109.0 476.25 850.45 452.84999999999997 201.1
625.0 891.8 616.65 561.25 164.7 258.45 420.20000000000005

452.1 1746.8000000000002 911.1999999999999
404.55 764.6 1081.0 94.6 655.25 751.0 876.0 512.45
525.8499999999999 347.25 795.75 139.25 550.35 699.55
850.4 347.75 861.3 679.7 724.95 1257.25 471.95 810.85
525.7 626.8 1354.7 228.75 336.35 732.0 689.45
440.45000000000005 524.2 944.7 451.7 343.2 868.5
705.7 549.1500000000001 818.55 1924.1999999999998
524.0 600.5 930.4499999999999 587.4499999999999
876.75 858.0999999999999 1255.7 349.35 1001.6999999999999
687.7 596.95 273.5 693.2 320.6 712.0 800.1
616.1 1476.95 251.8 390.3 994.0999999999999 767.25
894.2 935.05 651.5 625.6 1084.65 630.6 517.4
429.29999999999995 78.85 171.4 743.3499999999999
742.9499999999999 678.0999999999999 442.90000000000003
136.75 973.5 985.95 587.9 704.45 826.4 766.3
655.75 1001.0 774.5500000000001 763.75 705.05 830.2\
BTD 218.07500000000002 162.325 98.39999999999999
139.7 114.92500000000001 126.65 133.575 128.2 188.95
220.77499999999998 138.3 137.5 187.9 172.6
59.474999999999994 102.77499999999999 115.275 130.225
168.575 141.2 118.44999999999999 225.77499999999998
102.57499999999999 135.025 121.225 99.875 221.675 135.925
135.7 237.55 206.475 90.75 39.1 191.325 286.95000000000005
133.5 213.89999999999998 106.6 225.0 228.625 57.6
94.17500000000001 180.325 230.8 265.425 124.9
115.1 116.875 97.44999999999999 108.475 139.525 174.15
132.25 66.975 107.35 161.5 169.65 82.80000000000001
235.35 110.07499999999999 301.975 116.44999999999999
225.52499999999998 126.57499999999999 143.2 104.825
200.47500000000002 282.6 136.0 161.175 171.075 99.85
149.6 144.2 169.55 197.925 146.8 210.575 176.8 111.05
48.625 52.525000000000006 158.72500000000002 103.425
75.025 86.30000000000001 112.375 60.275 141.5 157.875
115.3 118.60000000000001 113.575 95.22500000000001
163.225 201.85000000000002 150.8 159.5 163.39999999999998
114.1 140.72500000000002 115.12500000000001
183.85\
CFDP1 969.9 1323.1999999999998 1156.05 952.1500000000001
978.8499999999999 1538.6 1422.3500000000001
478.9 1485.1499999999999 1291.5 1039.75 1648.05 1125.25
2423.5 620.75 1133.35 1256.1499999999999 1352.8 1305.7
1540.3 1163.25 915.35 1275.85 964.6999999999999 1444.2
1900.8 1703.65 1253.35 1126.1 1297.95 1347.7 832.7 1352.55
1635.9 1847.3 1053.05 1194.75 1170.4 1123.65 842.25
1842.3500000000001 899.1 1480.1 1349.25 1192.3999999999999
822.95 524.5 1223.6499999999999 1677.2 1206.95
1359.2 1354.05 1525.55 585.75 1417.75 1387.55 1362.35 1103.55
1095.35 1182.45 666.25 997.5 999.55 1142.3999999999999
697.35 1228.95 1094.0 1029.35 1268.3 1301.1 1298.0 1397.4
1395.65 969.75 1300.6999999999998 1210.1499999999999
700.85 1209.75 1317.65 780.0 700.05 746.35 746.6 512.0
784.75 708.9499999999999 415.25 586.85 1338.4499999999998
1386.75 650.1 797.5999999999999 1796.8 1567.4
1884.6 1556.45 1422.2 2123.7000000000003 2016.9499999999998

1494.15 1349.9 1117.25 1476.05\
CRISPLD1 1204.5 40.2 828.1 462.7 760.6 76.7
1576.1 550.7 5074.0 1982.5 2348.4 193.6 2619.4 3895.7
640.2 3443.7 780.6 751.8 145.1 2720.3 346.1 499.9
356.0 1969.4 3841.2 39.8 161.8 933.4 1539.7 3732.6
3814.4 379.0 943.2 2779.3 290.0 372.4 2144.3 369.6
3471.2 6840.1 1028.2 4296.3 2441.7 2245.0 3562.9 2060.5
204.9 4203.8 90.1 1289.7 1737.8 737.6 72.2 633.7
2134.4 394.5 1499.3 247.3 180.5 1227.9 1253.0 302.3
761.5 1654.1 287.9 239.7 2630.2 521.9 2355.7 2496.5
1455.7 1257.9 225.7 6348.9 5358.4 12898.7 1898.4 6082.7
946.0 3439.9 773.8 834.2 865.4 1096.2 7.9 160.8
625.0 972.6 86.7 126.0 2477.2 1492.9 5616.5 4546.4
1426.3 6442.8 4256.8 6263.2 1749.5 3958.8 3669.5 815.6
5689.1\
SOAT1 919.0333333333333 457.59999999999997
1119.3333333333333 1167.2666666666667 1674.3999999999999
615.5666666666666 321.53333333333336 1050.2
992.4666666666667 525.8000000000001 598.5666666666667
1587.1666666666667 838.1999999999999
665.4999999999999 244.0666666666667 798.1666666666666
810.8666666666667 1021.5333333333333 2024.2
510.6666666666667 321.5 2309.633333333333
914.8333333333334 1382.0333333333335 1982.7333333333336
1116.2333333333333 2614.5333333333333
1351.5333333333333 822.3000000000001 827.8000000000001
1396.7666666666667 1122.5333333333333
563.3333333333334 1386.8333333333333 863.7333333333332
1915.6333333333334 913.9333333333334
709.6666666666666 840.6666666666666 947.4333333333333
903.4000000000001 742.8000000000001
1077.2666666666667 419.0333333333333 1200.7333333333333
680.6 1332.1333333333334 984.9333333333334
1056.6000000000001 1989.2333333333333 822.4666666666667
1254.1333333333334 4274.566666666667
680.3000000000001 912.1 1431.2333333333333
1495.6666666666667 745.3000000000001 951.2666666666668
936.3000000000001 1054.5333333333333
1268.2333333333333 734.9333333333334 1366.6666666666667
1229.7666666666667 2091.9333333333334
1127.1666666666667 479.3666666666673 775.7333333333335
2140.3333333333335 606.6 1007.7999999999998
716.8000000000001 963.1 1142.4999999999998
1028.6666666666667 792.9666666666667 1239.0666666666666
1526.7333333333333 1788.0666666666666
678.4666666666667 850.6999999999999 281.9
311.1333333333334 978.2666666666665 677.2666666666667
1013.4333333333334 440.5 748.2666666666668
360.3666666666666 503.26666666666665 627.9
948.7666666666668 1030.7666666666667 796.7000000000002
1153.4333333333334 1166.2 1405.8666666666666
771.9666666666667 1350.1 1015.0666666666666
1251.3666666666666 1192.3999999999999\
CRISPLD2 784.9 140.75 364.25 510.1 590.0 228.65

1323.8999999999999	514.8	955.25	494.3	174.4	434.75
445.1	171.5	152.8	116.2	788.35	182.25	341.4	458.75
614.15	300.35	428.5	817.9	433.0	249.6	339.4
664.8499999999999	432.3	1066.1	730.6500000000001
251.7	355.6	708.8	625.4499999999999	1285.0
275.84999999999997	390.15000000000003	289.0	228.9
818.0	225.45	612.6500000000001	281.59999999999997
409.45000000000005	554.65	753.6	301.79999999999995
388.65000000000003	505.55	506.55	297.15	410.25
573.0500000000001	377.6	323.75	224.75	1078.65	300.45
289.45	413.2	1106.8	368.95	437.35	477.0	530.25
891.5999999999999	1125.1	475.55	204.95	464.75
508.40000000000003	739.4	451.8	325.84999999999997
249.9	593.25	347.1	878.6	156.6	730.8	526.35	253.6
472.65	301.2	383.9	979.85	2399.25	619.9000000000001
342.55	199.9	1490.25	614.4	1105.8	1747.35	389.59999999999997
729.65	542.25	616.15	251.5	526.75	1066.3
443.04999999999995\
SOAT2	6.2	21.5	83.5	51.2	21.5	4.0	8.0	6.3
27.0	28.8	12.0	8.7	8.3	17.5	18.3	24.5
25.7	9.5	11.6	60.3	177.8	7.4	24.4	13.9
21.3	14.5	22.5	12.0	25.7	14.5	5.5	27.9
27.2	11.5	85.8	12.1	8.9	22.7	7.2	21.8
26.5	18.5	15.6	14.3	9.8	15.1	8.0	22.8
50.0	19.2	19.7	18.8	16.2	26.3	18.4	19.5
21.0	13.5	16.7	13.0	25.1	16.8	13.0	14.4
26.3	16.8	13.5	4.3	50.2	21.2	25.8
154.5	58.4	33.8	99.0	19.1	15.3	12.8	23.1	18.2
14.9	11.6	9.1	61.0	17.2	174.3	12.7	14.2
21.3	18.9	17.6	53.3	55.5	17.6	61.1	11.7
16.7	24.6	12.6	12.7	16.2	39.7	18.8\
SUB1	5322.283333333333	1403.0666666666666
2020.4666666666665	5436.25	1926.9166666666667
5588.166666666667	4476.683333333333	3800.8333333333335
3663.116666666667	2154.2	5095.716666666666
3498.8333333333326	3115.0	3971.633333333333
2819.5500000000006	3168.7833333333333	6690.116666666666
2388.75	4976.499999999999	3461.7000000000003
997.4500000000002	2758.2666666666664	1500.1333333333334
4333.2	3528.066666666667	2921.2333333333336
6051.483333333334	5136.066666666667	2679.55
4373.366666666666	3979.4833333333336	2812.6166666666663
1422.1000000000001	4633.400000000001
5938.116666666668	2540.95	4414.366666666667
1617.6833333333334	3484.2000000000003	4871.616666666666
3900.633333333333	2826.7000000000003	6520.8
3201.9166666666674	2884.15	2692.2999999999997
5484.016666666666	8588.366666666667	1164.2333333333333
3086.633333333333	3045.4166666666665
4101.883333333333	4831.849999999999	1490.533333333333
2799.0333333333333	2086.683333333333
3229.9166666666674	2608.3833333333337	3802.15	2444.0
2902.616666666667	2928.1666666666665	3916.5499999999997
3397.4833333333336	3005.4166666666665	2115.9

5417.416666666667 3058.033333333333 2201.1666666666665
4250.983333333334 999.3166666666666 2716.5
2554.566666666666 4575.116666666667 2819.7833333333333
8105.516666666666 3125.983333333333
3972.566666666667 3375.2000000000003 3920.1499999999996
3641.0833333333335 3729.5166666666664
2846.283333333333 3584.316666666667 1400.9333333333332
1425.5 2972.666666666666 4240.216666666667
1715.55 1557.8000000000002 4394.933333333333
1755.8166666666666 5038.933333333333 4041.483333333333
2835.316666666667 3960.283333333333
3271.2166666666667 4527.499999999999 2414.3833333333337
3060.3666666666663 2663.116666666667
3058.7333333333336 3230.183333333334\
NINJ2 338.1 30.8 214.9 210.3 30.7 157.5 37.7
808.1 360.7 346.9 54.5 61.6 11.6 142.3 145.1
508.5 259.4 8.4 142.6 31.4 140.3 322.0 29.4
311.0 215.1 154.2 54.8 19.9 110.5 656.0 201.5
330.0 4.7 380.0 166.3 54.4 19.0 15.4 3.5
478.4 345.3 27.6 128.3 147.3 242.6 407.7 123.8
590.8 27.4 116.5 189.6 270.1 13.1 278.9 299.0
113.6 287.4 115.9 233.0 117.6 16.0 135.2 91.1
306.6 110.7 32.0 432.6 134.3 86.1 483.9 46.0 13.5
292.6 144.1 112.3 136.2 525.4 27.8 239.7 69.5
467.6 146.5 258.5 275.8 22.5 369.7 250.3
305.5 178.2 216.4 182.8 93.3 193.2 229.1 106.1
148.5 131.6 204.6 140.1 92.1 44.9 88.6 129.1\
KCNQ1 61.1 67.65 225.75 112.19999999999999 153.2
171.2 156.60000000000002 193.7 129.55 90.5 34.0
114.39999999999999 68.35 89.19999999999999 177.55
216.1 83.8 171.4 158.4 103.35 106.95 100.15 136.2
94.75 70.4 76.64999999999999 142.8 225.79999999999998
187.70000000000002 335.95000000000005 142.35
173.10000000000002 328.9 278.25 202.64999999999998
216.79999999999998 148.8 137.6 102.1 123.39999999999999
106.75 272.15 82.7 217.05 100.8 102.15 123.9
138.65 95.05 163.75 152.7 151.25 324.4 55.8 136.7
314.29999999999995 225.70000000000002 110.65
157.70000000000002 153.25 148.55 131.3 262.65
554.8499999999999 106.9 352.0 120.15 177.70000000000002
191.95000000000002 157.0 114.85 161.0 185.1 29.0
197.45 226.0 203.6 211.5 264.15 26.8 143.7
128.85 63.3 70.05 155.5 291.5 196.8 172.25
76.44999999999999 548.4 141.10000000000002 139.85
332.6 249.45000000000002 114.05 260.55 224.65 445.5
594.45 286.65000000000003 176.3 119.5 249.05\
NINJ1 320.2 275.0 131.5 401.4 88.7 802.2 391.1
1018.6 434.3 329.7 457.7 395.3 271.1 177.5 297.0
786.4 309.6 209.2 530.5 298.2 163.3 411.0 263.4
621.5 286.6 637.4 1002.4 547.4 487.9 515.4 284.8
490.6 482.3 828.6 803.0 417.7 660.7 282.8 127.9
246.4 304.4 556.7 612.6 503.3 403.3 559.3 732.4
796.9 299.0 421.5 466.2 507.9 192.0 75.7 239.3
486.0 431.0 171.4 361.2 311.5 845.8 311.2 251.6

556.5 265.3 745.5 354.1 649.8 1032.9 558.2 172.7
464.7 486.1 429.7 312.2 925.4 1321.5 226.7 596.1
416.1 130.6 499.5 439.5 671.8 527.5 626.9 1225.4
862.3 222.7 742.7 396.1 522.0 793.3 668.9 863.3
825.6 1003.1 1113.4 751.9 478.7 810.0 167.2 738.4\
KCNQ2 6.766666666666666 24.566666666666666 33.1
23.26666666666667 139.76666666666668 8.633333333333333
21.900000000000002 27.5 13.566666666666668
32.666666666666664 5.566666666666666 10.266666666666667
14.166666666666666 40.03333333333333
41.96666666666666 19.666666666666668 17.433333333333334
64.2 71.23333333333333 30.53333333333333
27.766666666666666 9.4 52.1 14.333333333333334
32.5666666666667 10.4 22.566666666666666 97.0
25.166666666666668 25.599999999999998 29.96666666666667
45.733333333333333 61.06666666666666
13.733333333333333 18.966666666666665 55.23333333333333
22.7 462.5333333333333 20.000000000000004
27.100000000000005 38.13333333333333 24.166666666666668
12.6 16.3 35.93333333333333 27.833333333333332
28.266666666666666 29.0 34.43333333333333
47.8666666666667 18.0 16.166666666666668 26.7
115.2 31.26666666666667 18.866666666666667
27.266666666666666 36.96666666666666 12.366666666666667
74.43333333333332 85.36666666666666
57.833333333333336 167.29999999999998 14.833333333333334
15.566666666666665 22.2 16.466666666666665
25.266666666666666 71.89999999999999 7.966666666666668
18.7 37.666666666666664 16.933333333333334
14.166666666666666 42.5 12.433333333333335
30.8666666666667 8.766666666666666 12.266666666666666
50.43333333333334 15.933333333333332
19.633333333333336 20.03333333333333 11.333333333333334
92.33333333333333 62.0 34.4 77.7
789.6333333333333 474.0 13.666666666666666
50.20000000000001 25.8 18.333333333333332
18.833333333333332 33.1 49.9666666666667
13.433333333333332 16.7 35.3666666666667 22.2
39.3666666666667 12.233333333333334\
BTK 42.3 23.6 19.8 171.0 36.2 24.6 75.3 64.3
16.3 119.8 9.9 67.3 47.9 143.9 18.9
100.8 78.8 92.8 83.8 15.2 219.2 24.6 46.0
157.8 20.7 17.5 94.4 167.4 36.6 54.8 15.2 23.7
254.5 111.9 78.1 133.1 21.3 69.5 21.4 97.2
16.5 7.8 160.7 16.6 113.1 116.7 75.8 26.2
31.2 20.9 29.6 29.4 12.6 42.8 116.8 48.5
69.0 21.1 30.9 90.0 276.1 152.9 21.7 55.1
51.4 14.5 64.6 49.0 56.9 49.5 64.1 59.0
149.2 84.6 171.4 39.6 48.3 51.2 23.3 17.8
21.7 39.3 14.2 170.1 25.4 43.6 10.4 56.6
15.5 51.4 49.9 65.9 36.9 41.1 18.2 62.2
54.6 12.0 162.8 17.9 33.4 25.1 18.3\
KCNQ3 31.0 117.10000000000001 84.16666666666667
36.800000000000004 139.23333333333332 81.4666666666665

61.699999999999996	93.53333333333335
104.66666666666667	155.56666666666666	78.13333333333333
231.03333333333333	57.5	32.0	181.53333333333333
71.16666666666667	101.53333333333335
107.83333333333333	86.53333333333335	91.06666666666666
88.26666666666667	83.0	148.76666666666668
99.53333333333335	47.73333333333333	325.366666666667
79.63333333333334	141.5	82.93333333333332
134.46666666666667	75.73333333333333	121.63333333333333
144.16666666666666	71.0	91.63333333333334
119.83333333333333	59.26666666666667	154.76666666666668
77.53333333333335	82.96666666666665	81.1
62.900000000000006	94.66666666666667	53.199999999999996
82.03333333333335	83.73333333333333
120.46666666666665	82.06666666666666	139.20000000000002
114.76666666666667	96.60000000000001	145.0
99.26666666666667	118.3	76.13333333333334	105.5	91.2
42.03333333333333	51.900000000000006
79.86666666666666	198.73333333333335	115.76666666666665
89.83333333333333	68.89999999999999
104.83333333333333	161.23333333333335	67.26666666666667
63.56666666666666	130.76666666666668
73.26666666666667	177.06666666666667	436.1000000000001
83.3	91.63333333333333	56.46666666666667
69.43333333333334	80.56666666666666	63.86666666666667
112.2	128.83333333333334	73.7	76.73333333333333
201.03333333333333	104.06666666666668
156.43333333333337	53.43333333333334	231.83333333333334
148.73333333333332	85.23333333333333
54.53333333333333	60.4	113.73333333333335
73.76666666666667	77.1	34.2	71.10000000000001
79.89999999999999	90.2	32.26666666666667
85.03333333333333	82.63333333333333	124.06666666666666
127.03333333333335\
KCNQ4	43.199999999999996	73.65	212.25	73.85000000000001
263.4	42.050000000000004	17.4	155.0	8.7
51.95	43.9	72.6	106.95	114.3	69.85	26.6	79.7
164.75	73.75	15.4	140.4	25.950000000000003	184.55
42.35	27.049999999999997	26.299999999999997	98.85
58.45	70.2	31.349999999999998	38.2	196.75	110.7
15.35	59.3	40.65	32.55	274.75	12.350000000000001
34.15	61.95	52.05	56.3	14.850000000000001	68.55
86.85	41.3	83.19999999999999	82.5	16.85	29.05	61.0
46.65	58.7	52.7	169.0	82.80000000000001
71.05	25.2	183.55	168.95	47.75	188.9	54.95	20.85
91.85	13.25	29.25	118.55	31.1	57.55	131.4	41.4
27.95	39.0	47.45	63.3	42.35	34.15	98.35000000000001
135.3	29.4	67.4	39.95	138.55	100.3	70.65
66.10000000000001	16.1	41.95	53.050000000000004
78.15	49.449999999999996	68.9	98.05	31.4
37.199999999999996	83.85	110.85	14.899999999999999
49.699999999999996	29.35	15.149999999999999\
KCNQ5	3.0	32.55	5.35	17.05	18.55	39.2	2.1
23.75	25.35	7.1000000000000005	30.849999999999998	8.9

6.2	22.25	38.1	14.7	23.900000000000002	5.25
9.55	5.35	10.3	5.45	25.15	38.75	19.4	16.3
12.450000000000001	43.95	38.8	5.45	27.7	29.7
33.3	8.45	22.75	9.9	7.65	4.45
5.8500000000000005	32.45	28.15	12.3	71.44999999999999
11.149999999999999	40.45	8.5	13.5	4.25
11.149999999999999	27.65	7.4	19.75	9.45	18.9	6.9
4.6	27.05	4.949999999999999	8.05	10.7
76.85	5.35	21.650000000000002	4.55	62.45
6.050000000000001	20.349999999999998	14.0	2.85	5.8
7.3	29.400000000000002	23.05	21.549999999999997
42.5	4.4	2.65	13.200000000000001	3.5
31.75	56.35	4.9	8.2	21.85	8.0	37.75	42.4	8.3
33.4	16.05	3.0500000000000003	26.799999999999997
9.45	22.049999999999997	32.25	10.9	20.55
4.949999999999999	75.3	7.95	10.55	6.35	15.45\
CES3	368.90000000000003	51.349999999999994	627.6
227.1	587.75	147.7	117.45	243.95	305.9	263.9	130.05
744.6	204.4	184.4	496.85	136.85	115.5	512.65	351.05
436.95	1526.8999999999999	193.6	730.65	425.35	156.55
3468.8	402.55	633.4	1115.15	752.8000000000001	496.7
738.0	647.5	1045.1	173.8	461.54999999999995	166.0
193.25	729.75	108.80000000000001	355.5	69.6	133.4
103.64999999999999	120.95	300.05	192.75	137.35
6541.900000000001	2257.9	293.0	171.39999999999998
2286.85	345.95	97.15	235.0	718.35	798.2	419.45000000000005
1019.3499999999999	529.95	396.20000000000005
477.5	399.55	909.8	721.6	703.9	852.1	840.25
483.20000000000005	82.45	539.9	587.6	993.95	407.75
183.0	139.5	253.5	332.6	63.1	418.4	122.64999999999999
138.15	228.55	1007.95	277.25	317.65	182.7	88.95
124.5	105.75	335.75	157.5	254.5	254.3	342.59999999999997
375.5	355.05	128.0	504.65	472.9	293.15	624.75\
CES2	253.23333333333332	538.4666666666667
1248.6333333333334	749.7666666666668	634.5333333333333
623.0	674.6	470.06666666666666	804.1666666666666
953.6999999999999	655.6999999999999
706.1666666666666	730.4	703.0333333333333	949.5
588.5333333333334	442.8	1016.7666666666668
954.0666666666667	767.3666666666668	903.2666666666668
875.9	853.9333333333334	893.4666666666667
1142.4333333333334	763.5666666666666	760.5999999999999
739.0	742.5333333333333	610.3	427.8666666666666
858.2333333333332	1018.9333333333334
841.4333333333334	953.9333333333334	1497.6333333333332
619.9666666666666	792.6	619.5	399.8
690.5666666666666	524.0	514.6666666666666
525.0333333333333	872.2333333333332	932.0333333333334
554.6666666666666	561.8333333333334
1046.5333333333335	671.2333333333332	885.2666666666668
725.6666666666666	528.8000000000001
743.3333333333334	753.9666666666667	602.7666666666667
887.3666666666668	1562.9666666666665
739.3000000000001	552.2333333333333	1371.966666666665

841.4666666666666	638.8	811.0666666666666
392.5333333333333	532.1666666666666	570.1999999999999
484.5333333333333	1254.4666666666667
784.6999999999999	908.6999999999999	687.7333333333332
491.0333333333333	552.4	916.0	726.5666666666666
1288.8666666666668	639.0666666666667	702.9
385.0	851.7000000000002	584.6	492.43333333333334
407.7666666666665	1433.7	699.8666666666668
1094.7333333333333	800.5	1193.2666666666667	676.5
424.2	917.2000000000002	912.5666666666666	782.6
852.8000000000001	844.4666666666667	1038.3666666666666
902.7666666666665	766.0	814.3333333333334
827.5333333333334	617.0	895.0666666666666\
CETP	3.1	86.3	9.6	2.0	54.1	10.1	54.2	29.3
24.1	124.6	6.7	49.6	4.8	155.6	82.8	30.2
7.2	7.7	7.5	5.0	155.1	60.2	12.2	7.7
43.6	60.9	47.1	29.3	93.3	5.7	2.8	44.2
65.0	52.9	162.6	8.0	9.2	140.9	4.3
180.9	31.4	14.7	20.6	72.0	14.5	11.3	11.5	71.1
52.5	62.9	16.0	13.8	56.5	129.3	10.4	17.2
93.2	152.4	28.2	21.5	30.3	67.1	9.6	30.1
36.3	9.8	16.3	41.3	13.8	83.9	27.7	23.6
7.0	10.5	14.4	20.0	23.8	41.8	4.4	23.7
14.8	54.8	5.5	48.4	23.4	28.4	107.9
107.1	19.9	29.8	7.7	15.4	11.6	34.0	52.0	9.8
50.4	11.6	8.2	7.5	4.3	18.3	10.9\
GRB7	75.3	926.6	83.6	148.9	430.1	213.5	231.5	92.8
161.7	246.2	262.8	181.9	70.8	116.2	353.1
189.7	82.0	83.6	357.7	86.6	373.2	41.5	195.3
134.7	163.7	295.6	201.9	41.2	91.6	218.2	193.0
104.6	434.8	48.9	74.8	142.3	96.5	322.8	303.5
155.2	473.3	167.9	159.1	163.1	25.1	86.9	27.1	25.1
132.7	206.6	45.5	290.9	115.2	207.6	168.5	92.5
185.7	177.1	243.0	151.1	18.9	311.0	418.7
223.4	245.6	476.8	186.8	243.4	248.2	153.6	214.5
144.7	175.5	27.8	56.8	91.1	51.3	103.6	193.3	68.1
187.2	232.0	587.8	246.0	291.6	335.2	248.8	73.2
110.0	401.6	227.1	39.3	107.7	205.3	88.5
248.9	180.0	198.3	332.9	329.2	161.6	224.3	187.5\
DHX8	248.13333333333333	345.40000000000003
214.23333333333335	221.4666666666667	398.03333333333336
376.5	354.43333333333334	202.9	230.7666666666665
277.6666666666667	362.0333333333333
256.73333333333335	229.9666666666667	202.79999999999998
219.7666666666665	424.0666666666666
286.1666666666667	210.26666666666665	300.2
204.79999999999998	263.7333333333333	227.33333333333334
201.13333333333333	216.26666666666665	216.9
357.6666666666667	386.1666666666667	340.4666666666667
369.59999999999997	273.3666666666666
496.6666666666667	148.86666666666667	320.0333333333333
400.1333333333334	466.76666666666665	383.3
158.5666666666666	322.2	321.1333333333334
255.23333333333335	353.59999999999997	348.0666666666666

168.93333333333334	329.2666666666665
232.4666666666667	262.06666666666666	203.4
182.13333333333333	368.7666666666665	224.20000000000002
230.4	324.7666666666665	303.59999999999997
160.5	347.0	482.9666666666667	294.1	205.4333333333333
346.40000000000003	313.6333333333333
162.16666666666666	281.7666666666665	220.9666666666667
303.3666666666667	294.0	305.5666666666666
269.1666666666667	329.1333333333334	270.93333333333334
291.5	234.89999999999998	254.10000000000002
234.4666666666667	352.23333333333335	253.16666666666666
367.8666666666666	158.26666666666668
244.63333333333335	226.5	372.4666666666667	127.8
199.69999999999996	226.79999999999998	309.8333333333333
217.69999999999996	167.9	271.0	296.90000000000003
216.9666666666667	95.43333333333334	225.1
170.4	232.13333333333335	280.03333333333336
326.73333333333335	192.73333333333332	204.83333333333334
174.1	232.0666666666667	164.93333333333334
196.8666666666667	224.5	273.90000000000003\
RRAD	33.13333333333333	72.33333333333333
1428.4333333333332	44.03333333333333	68.3666666666666
25.03333333333333	96.26666666666665
71.06666666666666	113.09999999999998	219.20000000000002
220.8666666666667	44.06666666666666
75.3666666666666	54.0	21.099999999999998
55.666666666666664	55.03333333333334	600.7666666666668
28.9666666666667	25.866666666666664	42.2
595.1666666666666	652.5333333333333	166.33333333333334
59.333333333333336	27.8	45.366666666666674
320.0666666666666	190.29999999999998	106.10000000000001
70.7666666666667	29.266666666666666
534.5333333333333	227.20000000000002	72.5666666666668
1284.1000000000001	27.066666666666666
143.1666666666666	27.633333333333336	68.1666666666667
65.83333333333333	55.166666666666664
1007.9333333333334	59.23333333333333	456.7666666666665
13.233333333333333	46.23333333333334
45.13333333333333	115.6666666666667	82.03333333333333
191.5	78.93333333333334	39.699999999999996	73.3
484.6333333333334	262.5	130.33333333333334
796.3333333333334	259.2333333333333	213.63333333333335
358.1666666666667	339.43333333333334
91.60000000000001	2425.566666666667	44.70000000000001
108.43333333333332	163.79999999999998
13.966666666666667	729.7666666666668	934.3666666666668
600.0333333333333	375.93333333333334
160.96666666666667	46.333333333333336	131.43333333333334
666.0333333333333	1851.9333333333332
76.3666666666666	141.73333333333332	32.63333333333333
64.03333333333335	53.13333333333333
219.13333333333333	28.9666666666667	192.23333333333335
94.4666666666665	37.800000000000004
93.10000000000001	181.9666666666667	116.0

14.300000000000002	63.733333333333333	156.53333333333333
	255.19999999999996	199.80000000000004
109.66666666666667	85.76666666666667	420.666666666667
	145.1	209.93333333333333	109.63333333333333
90.89999999999999	181.33333333333334\
DHX9	1117.3999999999999	1238.6999999999998
522.8000000000001	1247.766666666667	1221.3000000000002
	1625.766666666667	1824.9333333333334	371.7
1625.533333333333	1390.166666666667	1223.166666666667
	1431.0	1942.6333333333332	634.866666666667
1077.466666666667	1012.0	949.6333333333333
1062.5000000000002	1574.6000000000001	883.366666666668
	687.666666666666	1844.1999999999998	811.9
1505.4333333333334	1751.866666666668	921.6
785.366666666667	619.2333333333332	468.73333333333335
	933.1999999999999	1337.466666666667	648.0
774.1	991.066666666666	592.7333333333333
648.466666666666	1599.3000000000002	1402.966666666667
	1666.8	1070.8	2653.866666666663	1686.5
801.866666666667	1667.4333333333332	2382.766666666664
	1348.3999999999999	511.2	2023.6000000000001
831.1333333333333	1396.466666666665	1349.3333333333335
	665.4333333333333	1414.3333333333333
657.966666666667	1162.366666666666	1131.8333333333333
	1681.3	700.9	817.0333333333333	966.366666666668
	660.9333333333334	1128.066666666668	1370.3
856.8000000000001	747.166666666666	1179.7
2057.633333333333	1248.566666666668	1251.366666666666
	501.966666666667	1054.8999999999999
1047.9333333333334	811.566666666666	913.7000000000002
	702.4	785.766666666665	550.6	1068.3333333333333
	1383.766666666667	790.0333333333333
481.766666666667	736.1	1285.366666666668
582.4333333333333	690.666666666666	430.966666666667
	761.866666666667	640.3333333333334
1206.466666666665	672.0333333333333	2009.1333333333334
	575.6333333333333	617.5	615.2333333333333
769.166666666666	547.966666666667	611.266666666667
	759.866666666667	583.4333333333333
636.8333333333334	799.3333333333334	584.866666666667
	928.5999999999999\
ZBTB1	1775.5500000000002	1783.0	1859.9499999999998
921.7	1082.05	793.0	1057.8	1151.0	881.5	943.25	928.75
1372.8000000000002	751.5999999999999	1363.05	777.05
878.75	709.5	863.8	916.15	1218.6499999999999	964.9
1382.85	1057.0	709.45	890.4000000000001	705.4	617.05
1098.65	1161.8	951.15	1000.85	1156.6999999999998	915.6
545.0	430.75	1125.9	1103.8	1023.1	1188.25	1016.8000000000001
	1176.8	1246.3500000000001	822.6	1339.6000000000001
	2057.15	776.75	1044.95	779.75	1751.6	993.0	920.35
771.9	1104.55	1807.1999999999998	826.6	1584.05
788.0500000000001	1559.05	1223.2	786.35	238.95	752.8
863.2	863.3	1317.3	1174.3	876.6	1201.95	593.15	616.75
1398.0	988.4000000000001	1141.85	679.3000000000001

1192.3 621.4000000000001 1295.75 574.1 1217.6 662.15
702.0 1229.7 1181.9 642.8 1900.3999999999999 1125.7
1084.2 704.05 1623.8999999999999 1859.5 1225.0 1576.3
531.1 896.1 616.5 648.55 635.0 725.9 770.5 1140.55
894.0999999999999 1351.0500000000002 726.5\
LOC102723620 27.7 96.1 108.4 86.0 385.3 109.1 64.9
138.0 45.9 154.3 56.2 96.3 94.0 52.6
139.0 163.7 121.7 52.5 49.9 56.4 685.4 59.8
129.2 19.5 175.1 115.8 53.9 459.1 174.9 59.9 20.7
113.1 271.9 119.6 389.0 150.4 53.6 253.8 73.7
79.1 84.1 92.4 174.1 52.0 148.3 55.9 11.1
35.0 211.7 221.0 154.3 122.4 69.9 395.2
137.7 86.7 52.3 192.6 96.5 160.5 1037.1 145.5 37.8
52.0 64.3 52.0 37.1 69.9 305.5 160.2
389.3 153.9 68.9 24.0 69.7 75.4 95.0 89.8
133.3 105.1 302.1 238.5 21.1 160.4 151.1 356.4
176.7 166.6 123.1 155.4 59.4 171.3 191.1 73.2 70.8
30.0 104.2 60.9 57.6 41.3 26.3 159.0
126.9\
ZBTB5 3946.0 1487.4 1517.2 1377.7 1534.5 2508.1 2678.6
1108.6 1661.3 1102.4 2689.8 937.5 1649.6 2865.7 2766.4
1655.3 1832.1 1018.2 981.6 2741.7 1003.8 1612.0 1599.5
1575.1 1313.9 1385.5 1814.2 2138.5 1477.5 1575.8 2822.4
1159.2 1125.4 1616.3 1225.9 1567.5 1579.5 1584.2 2414.2
1954.4 2207.1 2305.8 1288.1 1762.2 1632.6 1061.7 1190.2
2062.9 1869.6 2053.6 1364.3 1474.3 1087.8 2000.0 1260.3
1509.7 1166.4 1759.4 1661.1 1046.4 1331.4 1514.5 1566.4
1797.4 1990.6 2115.4 1804.2 2007.3 1234.0 2076.2 1124.4
1138.2 1895.8 2001.7 3181.8 1706.7 1298.4 1738.8 1841.3
1672.9 1594.4 1166.1 2504.1 1741.6 1541.6 1898.0 1380.2
991.4 1844.9 1464.5 2014.7 1417.0 3196.7 2408.6 1878.7
2301.0 1910.2 2303.4 1642.7 2343.3 2432.8 2068.3 2926.3\
ZBTB4 1435.95 1764.8 2602.1000000000004 2067.4500000000003
2857.35 1578.6499999999999 1231.55 3058.85 2197.95
3561.05 1508.85 3222.65 2353.55 2535.5 2613.5 3599.3
2018.8000000000002 2570.8999999999996 1460.4 2574.65
5131.700000000001 2146.4 2385.5 2318.15 2058.8
3240.6499999999996 2606.95 3274.1 3113.8999999999996
2182.95 2175.5 2885.0499999999997 3125.7999999999997
3317.6 2299.0 3382.7000000000003 1807.5 4343.85 1586.65
1271.25 926.2 1915.3000000000002 2974.8500000000004
2270.75 2401.8 2375.25 3687.45 1337.2 2885.45 2688.9 2618.0
1822.5500000000002 3171.95 4133.55 2321.65 2834.7 2567.55
2148.05 2815.4 2293.2999999999997 2721.2 2795.8 3391.6
2168.6 2983.7999999999997 4061.2999999999997 1837.5
2872.8500000000004 3970.2000000000003 1905.15 2900.75
2816.45 3117.2 2514.25 3065.65 1857.1499999999999 3810.5
2339.25 2676.4 1645.35 3482.7999999999997 2127.25 2569.0
1831.5500000000002 3375.15 6435.8 3453.5 4395.5
4073.0499999999997 2993.6499999999996 1316.85
4786.049999999999 3239.6 3810.6 6191.75 3053.75 3763.65
3527.65 5433.85 2724.0 3669.4500000000003 2591.15 3435.65\
ZBTB3 202.2 148.85 286.2 187.5 294.8 192.25
245.29999999999998 171.7 207.4 138.05 156.25 268.75

138.0 308.0 216.4 134.1 239.8 112.4 247.35 262.65
407.75 141.45 128.3 118.3 116.7 223.70000000000002
306.79999999999995 270.85 293.9 282.1 184.60000000000002
373.9 223.9 298.54999999999995 336.0 331.9
126.55 377.85 126.15 91.6 185.5 70.6 129.8
217.35000000000002 122.7 209.15 137.14999999999998
134.1 444.55 187.45 233.75 149.2 170.3 227.75 156.35
164.35000000000002 199.8 286.70000000000005 242.5
223.1 267.25 227.95000000000002 302.55 150.5 276.0
227.2 112.55000000000001 236.25 208.7 195.10000000000002
197.64999999999998 294.35 216.75 294.6 193.5
268.15 235.95 155.5 151.64999999999998 102.8 226.45
135.4 161.5 239.5 240.75 391.4 75.6 339.85 126.65
267.70000000000005 115.65 172.0 285.9 235.15
190.35000000000002 299.75 267.29999999999995 306.0
425.9 397.55 268.95 231.25 213.45\
ZBTB2 1188.9 459.9 855.6 1000.6 992.2 956.4 2179.9
819.9 1493.3 672.4 644.3 699.3 769.4 1193.7 3333.1
1702.5 1140.7 772.3 779.8 1164.2 565.2 1048.0 576.8
1034.5 1556.2 728.2 639.0 548.3 782.7 1417.6 985.7
724.0 384.7 1036.5 1520.5 415.3 814.5 628.0 888.7
939.1 1115.7 1078.4 580.8 941.0 1017.1 1256.0 792.7
973.1 878.7 758.1 1107.0 874.9 608.5 914.1 1064.6
820.6 1348.3 608.0 538.4 672.9 1488.9 437.0 686.3
578.0 837.7 475.1 879.9 969.9 505.5 756.8 588.1
587.8 803.5 1202.5 1363.0 709.1 720.9 1162.9 917.4
1116.5 950.4 920.0 1006.7 569.5 938.0 777.1 669.1
1038.5 797.3 705.9 1847.2 712.6 1084.0 719.9 973.5
1213.7 1051.9 1094.5 856.8 947.6 1252.5 753.9 808.0\
NEU2 20.4 60.3 28.6 67.0 17.2 15.4 7.8 66.6
8.2 9.7 41.0 10.5 7.2 9.3 17.2 16.8
5.4 25.8 51.6 13.5 38.7 4.4 22.1 40.9
8.0 11.3 30.1 12.3 23.6 22.0 4.9 22.7
15.6 15.3 32.0 24.7 5.7 29.8 12.5 49.9
13.0 5.6 13.7 10.7 12.1 17.0 18.4 5.0
14.2 73.8 16.5 7.1 13.4 22.7 10.6 15.8
6.0 66.0 21.6 25.0 73.7 15.4 12.1 11.4
7.7 20.2 54.6 13.2 20.5 4.6 15.1 94.2
36.7 5.2 14.0 6.4 31.2 29.8 8.9 15.2
11.4 46.0 18.0 18.6 154.8 34.6 52.4 22.1
14.8 131.9 49.8 22.5 16.3 12.1 28.5 19.1
13.4 11.7 37.9 22.9 15.5 20.8 8.6\
PKHD1 74.50000000000001 115.23333333333335
109.76666666666667 12.300000000000002 263.1
21.433333333333337 40.333333333333336 50.26666666666667
51.199999999999996 51.76666666666667
22.53333333333333 62.06666666666666 25.666666666666668
55.03333333333334 125.06666666666666
27.933333333333337 42.9 45.46666666666667
64.43333333333334 32.9 106.26666666666667
104.43333333333334 56.76666666666666 40.43333333333333
75.5 91.66666666666667 68.2 72.3
72.86666666666666 43.93333333333334 42.86666666666667
181.4333333333333 96.36666666666667

79.23333333333333	89.53333333333335	58.166666666666664
	48.166666666666664	157.53333333333333
46.03333333333333	43.666666666666664	62.0
23.766666666666666	17.066666666666666	68.39999999999999
	23.433333333333334	71.39999999999999
49.86666666666667	31.599999999999998	324.23333333333335
	159.20000000000002	49.96666666666667
59.96666666666666	112.43333333333334	86.89999999999999
	70.76666666666667	66.0	44.800000000000004
52.46666666666667	37.833333333333336	61.300000000000004
	102.16666666666667	92.86666666666666
78.03333333333333	67.16666666666667	87.03333333333332
	130.03333333333333	102.23333333333333
89.53333333333335	44.0	96.63333333333334
205.06666666666663	47.79999999999999	54.9	39.0
101.46666666666665	39.833333333333336	69.76666666666667
	41.6	74.3	61.76666666666667	131.03333333333333
	42.9	201.03333333333333	35.93333333333334
360.4666666666667	679.6	53.199999999999996
41.36666666666667	523.2333333333333	196.26666666666668
	12.4	75.56666666666666	69.03333333333333
91.36666666666667	46.1	81.73333333333333
145.23333333333332	42.93333333333334	96.63333333333333
	75.73333333333333	50.03333333333334
62.46666666666667	112.0\
NKAIN2	21.85	36.55	13.25	11.6	10.55	8.5	14.65	18.4
	25.1	34.65	10.5	37.2	3.55	8.4
63.050000000000004	29.849999999999998	12.350000000000001
	50.7	17.8	6.8	13.9	17.1	30.049999999999997
	19.95	32.6	5.6	16.35	39.099999999999994	7.1
	45.949999999999996	29.900000000000002	81.45	33.0
	5.5	58.5	29.25	13.25	36.4	13.850000000000001
	83.4	8.649999999999999	30.0	60.4	37.2	24.9
	6.55	54.199999999999996	16.25	47.2	9.45
23.299999999999997	34.35	15.2	50.25	25.05	67.7	5.75
	10.95	23.5	47.949999999999996	297.45	9.4	7.25
	5.25	14.15	48.550000000000004	13.450000000000001
	57.1	32.4	5.45	32.8	17.8	40.550000000000004
	13.350000000000001	21.4	1.75	4.15	21.45	2.5
	23.05	55.150000000000006	13.9	914.5	25.05	34.9
	10.35	32.15	22.25	24.65	51.5	4.7	13.3
37.400000000000006	30.35	11.9	14.15	13.200000000000001
	3.65	25.8	41.900000000000006	7.35	24.55
35.85\
NEU3	98.32500000000002	356.0	222.60000000000002
196.775	253.72499999999997	141.75	212.1	118.5	222.0
102.975	175.95000000000002	210.14999999999998	129.65
100.7	182.45000000000002	111.80000000000001
102.77499999999999	253.35000000000002	181.825
120.25000000000001	296.975	183.475	241.775	119.82499999999999
	192.1	107.775	124.35000000000001	153.775
217.92499999999998	98.5	180.29999999999998
191.82500000000002	234.0	189.22500000000002	169.975
116.0	76.15	198.4	135.2	472.425	195.10000000000002

154.05	90.37499999999999	146.54999999999998	163.9
189.09999999999997	246.89999999999998	128.025	312.2
168.92499999999998	133.54999999999998	266.875
87.07500000000002	535.075	194.15	241.2	174.125	189.1
185.2	222.2	378.19999999999993	193.625	162.29999999999998
	105.22500000000001	158.29999999999998	230.65
199.05	147.67499999999998	226.79999999999998	165.325
164.35	142.27499999999998	195.325	169.575	197.75000000000003
	130.275	180.525	181.24999999999997	137.775	152.125
507.6	193.07500000000002	133.825	129.2	356.525
296.07500000000005	184.62499999999997	154.45	286.2
465.90000000000003	135.1	129.85	117.775	104.775
96.89999999999999	140.57500000000002	138.17499999999998
	142.475	182.42499999999998	173.25	156.57500000000002
	184.07500000000002	168.52499999999998\
NKAIN1	27.1	136.0	14.1	61.6	37.5	96.0	112.5
327.4	93.1	88.4	13.4	50.5	14.0	10.6	199.2	78.2
	15.3	29.1	11.8	34.7	42.8	74.2	195.6	75.6
	22.3	51.3	23.3	54.8	37.0	29.0	8.7	22.2
	207.8	18.0	50.2	144.7	48.7	155.8	33.6	53.9
	324.6	113.1	102.9	42.8	154.4	127.1	24.9
393.6	46.0	23.0	156.5	16.7	114.1	15.3	70.8	28.0
	200.0	20.5	25.2	24.0	68.5	17.3	127.0	37.6
	23.9	115.2	40.6	15.7	141.6	54.4	49.9
168.4	130.2	38.7	18.4	69.7	150.5	40.9	15.7
143.1	64.2	74.2	44.6	23.0	82.5	58.0	198.3	81.3
	18.9	121.3	74.7	59.0	84.2	10.0	85.5	81.4
	49.4	31.7	172.2	95.6	68.5	27.6	20.5\
NKAIN4	6.0249999999999995	94.67500000000001	29.15	24.5
	24.5	23.125	85.05	17.975	37.575	159.92499999999995
	36.825	21.925	4.025	29.1	125.5	52.075
35.800000000000004	24.125	72.82499999999999	21.525
85.25	10.475	28.0	30.975	45.725	18.425	25.325000000000003
	32.3	15.05	46.25	17.25	40.949999999999996
457.225	28.15	40.45	68.325	26.675	835.125	15.100000000000001
	31.025	67.375	18.950000000000003	20.6	26.825
15.899999999999999	34.075	234.875	38.875	44.3
63.925000000000004	21.025	34.325	44.2	42.8
51.675000000000004	57.400000000000006	34.175	38.25
11.775	40.4	83.2	38.75	127.39999999999999
49.400000000000006	13.125000000000002	86.275
28.875000000000004	23.125	68.1	38.2	86.32499999999999
	37.025	23.450000000000003	17.175	40.849999999999994
	24.1	53.55	13.3	52.15	40.99999999999999
114.825	45.3	26.225	35.375	635.15	192.75	30.675000000000004
	870.5	227.7	119.525	84.875	128.125	44.6	31.225	28.0
	42.4	58.02499999999999	37.525	60.95	35.65
40.475	79.35000000000001	28.075\
MC2R	35.1	80.06666666666666	31.733333333333333
46.733333333333334	132.29999999999998	31.900000000000002
	42.46666666666667	46.86666666666667
57.833333333333336	44.233333333333333	94.06666666666666
	49.26666666666667	43.666666666666664
50.43333333333334	83.43333333333334	73.8666666666666

59.6 40.9 20.333333333333332 14.666666666666666
131.9 63.46666666666667 139.13333333333333 46.5
60.233333333333334 50.5 35.06666666666667
138.0 95.90000000000002 59.93333333333334
27.833333333333332 168.4333333333333 77.26666666666667
27.433333333333337 57.96666666666667
73.83333333333333 19.366666666666664 84.96666666666667
42.56666666666667 57.9 24.833333333333332
24.633333333333336 25.666666666666668 15.666666666666666
8.633333333333333 90.2 57.1 60.5666666666666
88.3 63.5 64.33333333333333 60.666666666666664
36.26666666666667 107.93333333333334 36.4
127.8 97.8 110.63333333333333 39.6 51.96666666666667
351.0 66.60000000000001 45.633333333333326
22.700000000000003 32.733333333333334 53.0
25.766666666666666 31.766666666666666 41.96666666666667
72.93333333333334 116.03333333333335
62.13333333333333 55.9 20.066666666666666
72.36666666666666 36.4 78.23333333333333
41.03333333333333 27.5 70.03333333333333
83.26666666666667 11.100000000000001 37.46666666666667
29.96666666666667 136.76666666666668
109.93333333333334 82.36666666666666 65.13333333333334
72.1 63.0 44.26666666666667 76.2
35.333333333333336 18.533333333333335 29.099999999999998
32.06666666666667 61.9 39.46666666666667
47.76666666666667 62.166666666666664 73.23333333333333
95.73333333333333 84.76666666666667\
NEU4 79.5 147.0 78.5 15.6 43.1 48.7 69.4
194.5 27.8 137.4 91.2 123.6 116.5 137.0 172.6 67.0
155.8 59.4 18.7 81.2 210.3 23.8 61.8 12.8
60.2 95.5 24.0 64.4 64.0 14.3 27.7
267.5 139.5 18.7 182.3 86.9 57.5 173.9 23.5
190.8 80.9 23.2 32.8 64.6 132.2 44.6 188.0 22.1
135.4 161.9 137.5 24.0 43.2 112.9 26.5
180.6 50.8 98.1 29.6 56.0 124.8 184.5 104.5 62.5
190.1 15.6 52.0 130.4 110.2 129.9 37.8 56.6
146.6 78.5 200.3 113.5 69.3 52.3 143.5 25.6
116.1 41.8 135.5 119.9 149.0 66.2 17.3 73.7
86.5 190.5 59.4 99.5 72.8 42.7 120.3 21.4
111.2 21.2 19.4 235.2 144.1 30.9 175.6\
NKAIN3 47.1 31.5 110.0 64.7 134.3 107.7 43.3 45.7
108.1 162.4 26.1 91.2 122.7 25.5 30.4 22.8
55.0 145.7 123.6 25.5 39.2 75.5 303.2 46.8
63.7 130.4 79.0 34.4 49.2 84.6 86.1
229.0 34.7 23.5 41.6 16.5 10.5 103.3 60.4
193.7 47.8 47.9 107.6 12.9 16.3 208.9 112.5 82.1
272.7 29.7 119.9 21.8 79.9 180.2 22.4 25.7
24.7 25.4 31.3 27.5 372.6 30.1 61.4 49.9
192.2 63.5 93.2 10.2 176.3 22.9 219.1
168.6 131.9 76.3 9.0 18.4 27.4 113.1 62.7
141.9 103.6 70.8 151.1 32.0 49.4 321.6 210.4
206.1 31.8 105.7 48.6 26.4 22.0 12.4 19.2 21.4
120.2 124.7 23.5 32.4 90.7 132.6 18.5\

RACGAP1 2918.6 2198.8 446.3 1933.2 1272.8 2516.3 1132.7
1820.1 1486.9 1128.2 1646.0 1002.1 1885.3 1668.3 4222.3
873.9 1824.6 1038.3 2232.1 1035.9 556.1 2094.2 534.6
758.6 2641.0 1020.9 2478.4 496.2 673.8 1133.4 1057.7
1203.4 713.2 509.1 2673.2 378.1 2587.1 2608.2 1009.6
1822.6 1852.8 1481.3 742.1 346.2 1212.6 2560.1 1681.7
4288.0 1542.6 449.2 1380.1 2437.8 1737.3 2171.2 1198.6
897.2 668.9 1222.9 1093.2 757.5 816.7 797.5 811.5
807.0 421.8 449.2 767.2 575.2 438.3 1176.0 256.3
1346.3 358.0 785.0 871.2 1461.5 484.8 1020.6 612.4
4393.5 1779.7 2899.4 1325.4 2635.9 1122.0 1072.6 2791.6
1585.6 1612.4 808.1 3681.7 1105.9 646.1 358.8 370.7
858.0 633.5 570.0 660.6 1137.6 519.6 2736.5 448.2\
RRAS 252.5 108.9 192.8 393.1 231.4 102.9 325.1
163.0 231.4 188.6 246.4 313.8 108.6 647.8 80.5
260.6 333.4 202.1 1302.3 166.5 62.0 169.6 324.0
263.7 243.4 368.4 396.5 241.8 179.7 357.0 395.1 93.9
272.2 309.4 289.3 407.4 196.1 309.3 262.6
378.8 429.8 253.3 309.6 268.1 351.0 235.2 98.0
188.1 55.7 92.8 313.9 296.1 322.1 135.1 293.0
230.3 173.7 248.5 269.3 127.7 592.4 345.9 232.7
177.4 277.3 337.2 570.6 258.0 459.5 319.2 423.9
247.5 188.3 217.3 337.0 380.3 173.7 191.0 298.0
118.0 232.8 225.3 1097.2 353.3 350.2 412.2 92.1
372.8 574.5 393.7 153.7 184.9 120.7 703.5 421.9
420.3 329.8 478.2 381.0 257.7 329.8 161.0 412.4\
LOC100287610 /// ZNF717 5.4 153.7 7.5 16.8 90.9 7.9
5.4 4.0 108.0 50.4 6.2 10.1 3.9 12.1
38.3 27.4 3.7 7.5 5.9 8.0 160.7 12.3
26.7 3.5 4.1 19.2 4.8 101.0 55.7 3.7
35.0 97.0 19.6 5.5 32.8 18.7 7.0 11.4
11.0 9.4 5.7 10.3 15.0 6.6 15.1 75.0
101.3 10.8 14.5 31.5 9.8 5.1 4.6 14.8
45.4 17.1 18.2 32.3 28.7 12.1 266.6 7.2
8.0 33.0 12.8 27.5 2.8 28.7 58.4 10.2
36.2 43.6 3.8 62.6 8.0 9.1 3.1 16.9
9.3 13.3 76.3 17.4 40.2 13.1 15.9 48.6
60.6 5.7 16.6 13.5 28.7 10.0 8.3 34.1
29.3 37.3 6.4 35.3 11.0 26.9 12.9 8.8
14.4\
NEU1 564.5 233.0 90.4 612.4 545.0 805.4 911.1
359.0 446.8 313.8 482.5 765.0 379.5 523.4 310.1
313.0 406.4 277.7 1510.9 330.4 44.1 302.9 298.9
497.4 305.8 356.2 825.4 169.2 199.2 385.5 429.4 66.7
195.5 952.0 782.7 263.3 672.6 217.0 1199.5
462.1 520.6 431.8 296.4 490.7 459.4 198.2 698.2
951.3 83.6 427.4 299.2 595.8 984.4 155.1 289.9
185.9 318.8 207.4 413.6 240.9 489.3 315.9 418.9
348.8 266.4 470.1 828.2 885.8 148.0 456.4 10.5
137.8 328.8 1096.2 408.2 729.6 294.5 795.5 581.1
760.5 127.0 202.2 1671.7 588.1 116.1 385.9 257.4
174.1 299.5 290.6 679.4 171.3 333.8 419.4 312.3
316.9 396.7 512.0 239.2 307.6 311.1 200.3 437.5\
KCNN1 153.4 155.7 30.7 42.0 68.6 122.7 73.3 98.5

63.4 147.9 43.0 114.5 85.8 170.8 132.3 63.8
44.6 80.4 106.4 114.5 206.5 73.1 44.1 93.7
116.9 205.2 220.7 134.9 130.6 106.9 50.6 37.6
29.1 81.7 53.0 144.5 118.7 59.5 123.5
234.8 71.7 227.1 49.4 228.0 275.1 28.8 136.3
183.5 13.7 64.3 57.5 100.2 59.5 239.1 30.0 61.9
42.5 178.0 53.8 113.7 469.0 35.7 30.8
128.6 150.5 156.9 129.5 226.0 444.6 61.3 24.8 46.5
175.9 172.1 123.4 254.4 224.5 205.0 149.2
233.2 82.7 136.2 224.2 194.5 169.4 344.7 107.9
120.3 49.8 282.6 166.5 69.1 143.6 131.4 113.1 94.2
136.7 149.7 94.7 41.8 158.1 205.3 244.3\
FAM192A 1169.6 1824.0 1264.675 1093.875 1367.075
1443.925 1391.4499999999998 1128.7250000000001
1167.75 1604.025 1562.925 1719.8249999999998
1102.1750000000002 1303.725 1194.85 1233.4
972.275 1572.4499999999998 1754.325 1220.75
1865.7749999999999 1306.075 1362.3000000000002
1021.55 1437.45 1345.775 1532.975 1556.2000000000003
1504.4 1315.6999999999998 1117.6750000000002
1615.35 1705.175 1270.625 1917.8 1680.175
1145.825 2010.0000000000002 1354.625 952.95
1542.0249999999999 1268.225 1284.625 1366.625
1353.025 1468.1999999999998 1698.5749999999998
1423.85 2480.15 1780.1 1619.75 1621.725
1505.0749999999998 2228.625 1401.7 1389.3 1308.625
1562.4 1728.675 1810.025 1719.175
1739.0249999999999 1106.1 1193.125 908.275
1723.2250000000001 1366.85 1552.3 1995.0249999999999
1334.6 1433.175 1595.775 1713.275
1279.2749999999999 1376.075 1355.075
1829.1499999999999 1289.975 1141.075 737.65
1909.375 982.95 1346.35 1188.5 1957.4500000000003
1111.9499999999998 1679.5749999999998 1342.825
1148.8 1477.65 971.0249999999999 1576.85 1557.45 909.175
1189.15 1222.325 1147.15 1219.8249999999998 1377.1
1319.875 1339.525 1420.0 1455.475\
KCNN2 394.9 297.3 132.1 164.4 87.0 15.1 73.0
135.2 281.0 116.2 242.4 466.9 115.4 66.2 55.1 84.6
234.5 113.5 66.4 310.1 220.3 127.0 51.7
282.2 335.3 378.6 514.5 13.8 186.5 161.1 301.2
265.4 7.6 150.5 73.1 12.7 8.4 263.4 425.9
160.8 52.4 64.3 18.1 133.3 150.1 240.5 83.2 79.5
547.7 269.6 479.6 143.8 257.3 353.0 156.6 21.9
297.6 267.7 196.4 139.0 86.0 115.9 328.9 43.5
317.1 180.1 207.4 142.1 203.8 207.8 318.0 15.4
105.0 176.4 127.8 151.6 266.6 194.5 157.1
158.7 582.4 100.9 8.2 102.1 184.5 43.7 230.0
233.4 172.9 21.8 57.7 214.4 266.5 243.1 172.1
506.7 371.1 249.3 197.0 439.0 355.4 264.1 208.7\
KCNN3 18.4 29.8 96.375 39.275000000000006
61.675000000000004 40.474999999999994 17.400000000000002
39.475 30.15 164.525 45.0 36.050000000000004
34.45 26.5 26.975 27.225 55.35 37.550000000000004

```
30.025  16.9    111.4   29.55   172.3   39.65   9.6     22.3
16.325  95.3    29.35   41.275  39.825  45.300000000000004
44.45   61.05   53.9    76.725  21.9    44.45   27.8
45.849999999999994      47.3    18.025  102.82500000000002
22.175  43.5    49.724999999999994      42.075  15.775  38.625
128.875 40.2    8.6     11.525  18.8    28.425  215.85  58.6    95.1
        45.35   71.825  106.87500000000001      201.65  64.975
101.27499999999999      41.725  46.475  128.275 34.775000000000006
        226.025 81.44999999999999       344.8   107.5   51.325
30.075  56.175000000000004      43.225  77.92500000000001       41.4
        46.475  22.125  35.05   36.25   55.35000000000001
59.800000000000004      50.12499999999999       16.875  9.1
77.125  47.12500000000001       24.425  55.52499999999999
53.425  32.550000000000004      34.7    38.4    29.125  8.075
80.225  92.45   19.599999999999998      28.599999999999998
31.475  22.15\
OR7E47P 346.9   390.6   610.0   226.4   409.2   265.9   266.4
1492.3  368.1   311.0   522.8   214.0   219.2   738.0   285.1   12.7
        426.2   826.3   231.7   277.7   885.8   676.3   1216.6
328.7   431.4   96.4    372.8   792.0   616.2   402.1   95.3
851.8   582.7   395.6   1222.1  591.7   381.2   364.5   123.0
506.9   284.9   198.6   1034.5  311.3   733.4   895.3   1032.0
213.2   56.4    427.5   600.9   472.7   707.3   522.3   301.3
394.3   749.0   408.3   651.9   845.4   1336.1  579.9   216.0
881.6   525.6   689.5   495.0   405.7   640.0   1292.1  641.5
745.2   246.0   280.2   410.1   862.1   1683.3  363.8   304.8
321.9   833.2   367.1   390.1   432.7   790.2   60.9    442.4
531.5   386.5   278.2   244.1   367.8   281.0   240.8   46.7
347.5   210.3   341.2   225.5   372.5   151.0   242.8   187.5\
KCNN4   150.1   189.0   210.8   117.1   171.1   280.9   70.6
117.5   483.8   245.9   26.6    178.4   68.8    126.9   30.7
312.9   246.3   37.2    102.9   594.6   266.5   80.9    29.5
468.3   22.8    1617.7  306.8   492.2   361.0   936.4   298.9
384.2   179.2   176.5   242.5   106.1   214.1   203.9   726.1
108.1   229.9   170.7   239.4   329.1   410.5   64.6    319.5   45.0
        1242.5  1005.8  752.5   26.5    2736.3  33.4    364.1
262.3   523.6   122.4   796.1   101.3   50.3    46.9    219.2
606.4   355.6   1695.2  548.0   197.2   629.0   295.8   373.8
103.7   240.7   160.7   200.0   172.8   486.8   431.1   259.4   20.9
        20.1    150.2   42.5    250.0   209.8   20.9    126.3   27.6
        200.6   81.0    378.4   148.1   400.3   276.1   272.7
322.8   256.0   258.8   573.8   158.7   328.7   83.4    265.3\
NTNG1   65.65   41.150000000000006      50.8    41.85   48.6    42.4
        20.3    77.65   57.95   78.2    294.70000000000005
100.7   158.4   50.35   47.45   95.45   137.85  40.95   37.15
35.85   54.4    40.55   70.69999999999999       51.599999999999994
        22.2    25.75   10.55   47.35   61.2    154.7   9.5     58.0
        19.3    58.45   58.65   41.3    59.1    69.05   24.75   13.1
        365.6   49.55   71.9    27.0    9.5     67.65
42.400000000000006      36.95   67.65   105.7   43.5    33.3
33.55   59.7    40.400000000000006      23.450000000000003      54.3
        17.4    13.65   19.450000000000003      79.1    51.1
210.35000000000002      16.3    29.15   30.35   47.1    31.55
15.850000000000001      41.25   44.599999999999994      18.75   56.7
```

	39.400000000000006	17.6	23.55	21.200000000000003
	29.95	37.2	56.55	31.35	105.9	12.35	59.05	69.6
	20.4	160.70000000000002	94.3	35.05	12.75
39.55	87.15	57.35	87.55	61.550000000000004	66.6	35.4
	27.0	8.95	36.75	39.55	58.4	70.65\
SLC10A4	41.4	8.6	48.5	21.8	110.5	1115.0	127.6	52.3
	114.2	41.7	166.0	55.7	15.7	40.4	217.8	38.5
	17.5	107.5	2.1	51.7	70.6	198.7	7.1
234.4	4.5	5.9	33.0	37.6	79.1	26.2	37.5
185.1	120.4	171.5	60.7	4.6	314.9	14.8	4.5	84.8
	647.4	150.9	60.6	90.4	28.1	201.5	52.4
277.7	15.2	28.2	13.4	48.1	26.7	682.7	113.0
136.3	135.5	67.9	41.1	63.1	23.8	54.4	28.4
102.1	83.8	46.0	1.3	43.2	38.2	48.4	58.2	81.7
	1.7	165.4	85.8	48.5	45.8	75.6	6.9
203.6	107.3	77.5	61.6	11.4	82.5	175.6	897.4
198.8	24.0	193.8	379.0	72.1	40.3	79.4	41.2	25.3
	53.2	29.6	103.3	32.9	19.2	4.1	3.7\
LOC284926	211.7	74.5	184.7	252.3	366.3	179.6
122.9	194.5	168.1	237.1	214.5	169.9	98.6	113.8
354.3	127.9	53.7	122.6	295.3	93.9	93.4	199.3
124.3	179.8	363.6	63.9	115.7	87.1	108.4	133.8
139.7	115.0	150.5	108.3	7.0	23.2	422.4	142.7
148.6	131.0	191.6	86.0	232.1	158.2	181.0	163.2
298.4	314.9	66.0	100.7	155.0	69.4	179.8	29.8
101.1	31.7	198.2	93.3	98.6	315.8	248.8	166.6
184.8	168.5	144.4	289.4	223.1	88.8	241.2	97.6
105.6	109.5	121.9	211.7	193.7	124.6	157.9	174.8
115.7	206.1	261.3	282.7	374.8	171.9	229.6	288.4
275.2	281.8	201.1	51.2	123.9	211.9	122.0	79.4
130.0	222.2	136.6	190.7	139.5	198.9	116.3	211.8
119.5\
BBS2	1861.2	2652.0	2149.9	1489.9	1672.4	1338.4	1195.4
1381.5	1028.9	1323.2	1253.5	1673.4	1032.1	1086.1	1869.9
2456.7	748.6	2101.9	1054.1	2049.5	3358.9	2147.8	1368.5
1104.1	1414.0	1573.9	1736.9	1986.6	2112.0	2273.0	2286.0
1495.5	1661.1	1806.5	869.8	2036.5	2235.6	1905.9	3196.3
1079.3	1624.7	2363.8	1670.9	4168.7	2146.3	1969.7	1375.5
1185.9	2385.2	2590.7	1376.3	1046.8	1230.0	2556.7	1657.8
2568.5	2318.4	1790.7	1754.2	3101.1	1118.1	2036.4	1300.6
1704.0	1374.2	1565.6	2178.3	4432.0	2706.4	1690.7	2095.8
1630.1	2481.1	2526.1	1841.2	1929.2	2921.5	1272.0	2088.6
1247.5	2618.6	1743.5	1597.3	915.9	2450.8	1615.9	1989.7
1361.5	1534.6	1554.1	892.9	2496.7	2520.5	2688.8	2927.1
2369.5	2719.2	2624.6	2697.8	3121.3	2307.8	2075.9	3120.8\
BBS1	368.23333333333335	495.7333333333333
706.9666666666667	241.4333333333333	539.1	279.8
377.1666666666667	625.3333333333334	427.6333333333334
	642.0	530.2333333333333	409.93333333333334
303.5	626.6666666666666	962.8666666666667
1262.4666666666667	362.93333333333334	519.9
369.5333333333333	473.76666666666665	580.4666666666667
	624.6333333333333	506.5	505.3333333333333
447.20000000000005	521.6	359.4666666666667

586.6666666666666	568.2333333333333	302.53333333333336
	385.5	768.4666666666666	612.5	640.6666666666666
	420.06666666666666	593.6333333333333
266.8666666666667	363.03333333333336	379.6666666666667
	462.8333333333333	465.1666666666667
320.96666666666664	640.8333333333334	424.0
576.0666666666667	624.8666666666667	487.7
133.46666666666667	708.0333333333333	413.5
519.7666666666667	593.4	314.90000000000003
814.7666666666668	602.0333333333333	506.3333333333333
	582.5333333333334	480.5333333333333
464.8999999999999	582.9	568.6333333333333
421.23333333333335	385.5666666666666	532.2666666666667
	451.59999999999997	452.06666666666666
367.76666666666665	501.8	759.0999999999999
699.7333333333332	632.7666666666667	507.26666666666665
	371.0	414.40000000000003	469.8333333333333
588.1666666666666	888.3666666666667	483.96666666666664
	585.4	309.6	1024.8333333333333	469.5
693.4333333333333	405.8	571.0	682.2333333333332
658.1666666666666	675.7666666666668	501.3
464.40000000000003	346.56666666666666	777.0999999999999
	753.6666666666666	712.2333333333335
849.9000000000001	863.7666666666665	736.0	825.6
575.3666666666667	689.5	698.9	503.43333333333334
818.0666666666666\
NTNG2	28.5	82.9	32.0	78.7	51.1	62.7	84.2	87.1
	166.4	73.2	20.0	69.1	19.2	44.3	11.7
216.5	56.3	191.9	99.2	73.8	372.7	8.2	176.3
128.6	95.7	53.6	50.0	97.6	101.6	217.4	59.4	97.2
	223.0	123.3	101.8	59.9	31.7	100.4	44.2	13.7
	60.7	82.0	107.3	236.2	22.9	52.5	35.7	84.2
	166.7	34.8	88.8	67.3	10.9	85.7	85.1
130.6	110.1	27.0	61.1	17.2	226.8	120.7	117.4	16.8
	103.1	94.1	94.8	461.6	90.0	74.5	126.8
180.3	221.3	91.0	7.3	4.5	87.9	15.2	87.7	95.2
	165.9	127.7	175.3	48.4	28.7	70.2	402.0	77.6
	56.0	19.4	32.0	223.4	56.5	86.2	110.2	96.6
	131.4	29.8	64.6	81.9	31.3	118.2	85.4\
XBP1	875.2	1039.1999999999998	1138.5	966.05	1284.0
2693.5499999999997	417.3	851.8	1003.15	1734.75	2269.25
2158.5	1330.1	2662.1	895.45	1312.25	2451.3	1369.1	1275.2
1912.1999999999998	1381.3500000000001	2359.05	2016.35
2540.3	1437.15	2142.8	2160.6	1893.1999999999998
1927.4499999999998	2138.4500000000003	729.25	2034.55
1358.0	2130.9	2640.1	1366.5	1326.1	1805.95	1597.1	1982.65
631.5999999999999	388.45	2144.5	793.65	1897.75	1486.75
2588.3	643.85	2085.3	2081.85	1790.1	1460.9499999999998
5263.3	948.1500000000001	2305.0	2515.3	1363.05	1813.15
4116.5	1921.15	667.35	1484.2	1371.0	2226.75	4294.95
1364.9499999999998	2029.5	957.6999999999999	1176.65
4545.650000000001	3508.95	3214.2	2394.25	1591.45	1285.35
5598.7	2335.95	4623.3	2577.85	461.55	744.4000000000001
807.0	1331.6499999999999	2144.85	549.35	1901.1499999999999

779.0999999999999 424.59999999999997 788.4
1040.5 1555.3 598.75 2024.7 1201.35 1297.75 1881.3 2152.15
2675.4 1592.15 1247.5 1954.5500000000002 1732.8000000000002
1461.5\
SLC10A7 566.2333333333332 265.2 563.7666666666667
194.23333333333335 224.4666666666667 257.5
159.7666666666665 270.8333333333333 245.13333333333333
251.9 178.69999999999996 233.83333333333334 91.2
549.3666666666667 153.9333333333333
136.9333333333333 182.9666666666667 369.5666666666666
221.63333333333333 242.23333333333335
218.13333333333333 175.7666666666665 266.3333333333333
358.2 383.73333333333335 268.93333333333334
134.70000000000002 341.8666666666673 348.23333333333335
280.4 162.9333333333333 482.3333333333333
191.5666666666667 229.2666666666668 217.13333333333335
281.6333333333333 250.1 140.66666666666666
159.73333333333332 152.3 214.5333333333333
201.9333333333333 276.8 135.16666666666669
113.63333333333333 228.03333333333333 627.466666666667
110.39999999999999 244.36666666666667
382.0666666666666 294.09999999999997 204.16666666666666
281.06666666666666 341.5 181.6 181.73333333333335
283.93333333333334 286.8666666666667
398.43333333333334 237.03333333333333 264.2
207.46666666666667 224.23333333333332 235.79999999999998
341.6666666666667 348.23333333333335
203.0333333333334 177.5666666666667 200.56666666666663
257.09999999999997 177.53333333333333
426.23333333333335 264.40000000000003 243.03333333333333
378.0333333333333 204.29999999999998
294.50000000000006 295.8333333333333 286.46666666666664
240.13333333333333 161.8666666666667
198.96666666666667 113.5 182.20000000000002
428.666666666667 254.29999999999998 188.56666666666663
265.1333333333333 186.6 175.16666666666666 91.7
376.8 245.0666666666667 153.5666666666666
281.7 270.2 440.76666666666665 320.8333333333333
320.40000000000003 342.4666666666667 237.4666666666667
399.4666666666667 247.5\
YES1 3253.86666666667 760.3333333333334
1826.233333333333 3316.433333333334 1875.6000000000001
3211.3999999999996 3249.266666666667
1646.7666666666667 2959.2000000000003 2873.7000000000003
2180.2000000000003 2771.1999999999994
2833.8333333333335 2511.0666666666666 2653.5666666666666
1961.9333333333336 3185.633333333333
2721.566666666667 2135.2 2714.7666666666664
792.9333333333334 4533.766666666666 1020.1666666666666
3013.1 4070.2999999999997 3355.633333333333
3090.6666666666665 2098.3333333333335 2327.4 2578.1
2568.4666666666667 2767.8333333333335 957.8000000000001
2179.9 4801.166666666667 1359.5333333333333
3400.7999999999997 1689.4666666666665 2459.16666666667

3785.3999999999996	2632.7000000000003
2648.4333333333334	3041.7999999999997	2516.4666666666667
2713.6333333333337	2373.733333333333
3209.8999999999996	4268.766666666666	1282.9333333333332
1557.2333333333333	3149.2000000000003
3587.2666666666664	2845.4666666666667	2020.8	2574.6
1368.6000000000001	2257.6333333333337	2010.0
1810.5666666666666	1690.7	2045.2	1314.8	2619.4666666666667
2959.766666666667	1443.1333333333332
1515.5333333333335	1980.2666666666667	1794.9333333333334
1508.3000000000002	2286.0333333333333
919.1666666666666	1748.6333333333334	1146.6666666666667
3523.5333333333333	2380.866666666667
2372.566666666667	1601.6333333333332	3049.9
2309.7999999999997	7006.566666666667	2422.2000000000003
2838.366666666667	1351.2	2724.9	1806.466666666667
1185.4333333333332	2630.133333333333
2103.6666666666665	1751.8	1883.5333333333335
4011.1000000000004	1451.0666666666666	2087.9666666666667
2134.733333333333	1244.6000000000001
1729.1999999999998	1551.6333333333332	2098.133333333333
1567.1000000000001	2652.7	1720.0	2649.5333333333333
1640.2333333333336\
SLC10A1 54.0	220.9	115.2	87.8	158.7	76.4	120.9
232.0	127.2	59.2	45.5	142.2	75.7	250.1	182.5
123.6	28.8	213.5	38.7	86.8	65.1	112.2	403.6	49.8
41.9	23.3	22.7	43.9	68.3	25.2	23.8
140.3	25.9	175.9	449.7	38.2	99.4	56.8	67.2
137.1	50.8	45.1	51.7	62.4	39.1	124.9	24.2	99.3
85.5	37.3	21.1	129.0	18.0	157.6	119.1	62.2
91.1	201.7	182.8	182.5	797.1	219.1	59.0
117.4	287.8	75.1	111.4	25.4	215.6	133.4	230.5
227.9	74.6	37.9	146.0	35.7	58.9	44.7	35.8
109.1	266.5	28.1	92.0	117.0	86.1	407.6	97.8
250.2	214.6	312.5	55.2	184.3	98.9	20.4	20.6	40.7
110.6	21.9	48.4	200.8	163.8	273.2	136.0\
RP11-173B14.4	100.2	402.6	178.1	26.9	171.8	157.2
150.6	196.9	149.3	260.5	172.2	139.4	70.2	117.2
314.8	300.8	111.7	182.7	96.1	155.6	162.9	131.8
177.5	170.2	158.4	138.4	92.0	286.7	185.3	118.2
136.3	150.4	282.5	174.3	222.9	235.8	120.0	124.4
115.4	171.8	106.3	164.0	216.3	125.7	88.3	169.4
132.0	118.6	281.1	169.7	118.6	52.0	129.1	75.4
191.3	144.2	143.3	222.1	134.7	99.1	552.5	138.0	57.5
131.9	263.0	243.5	146.4	215.2	128.3	182.1
213.0	155.2	337.4	229.9	340.2	130.4	189.2	173.9
138.5	230.6	32.0	193.9	199.4	160.5	254.0	297.0
221.1	286.7	229.9	116.1	169.9	294.0	229.4	297.0
301.4	239.0	236.0	269.1	173.1	236.9	253.2	207.8
232.6\
TRDV3	32.45	145.8	82.5	83.75	48.6	24.299999999999997
14.899999999999999	60.05	160.55	111.10000000000001
50.05	40.35	32.55	75.5	45.349999999999994
58.45	55.7	62.45	59.650000000000006	51.85	142.05

```
48.85   185.25  55.35   60.650000000000006      33.15   37.95
136.2   45.949999999999996      194.5   81.3    24.25
86.55000000000001       177.10000000000002      86.85   125.45
197.14999999999998      66.25   59.400000000000006
80.30000000000001       35.25   54.400000000000006      112.8
79.94999999999999       33.95   77.05   26.049999999999997      38.8
        66.3    427.0   94.35   38.8    23.65   81.85
87.19999999999999       30.400000000000002      147.9   99.65
42.35   200.5   126.80000000000001      72.5    74.5    31.55
109.0   67.25   171.75  767.5   59.9    46.900000000000006
114.75  77.25   130.35  88.35   190.35000000000002      29.4
48.25   59.05   340.4   37.6    38.25   59.9    47.900000000000006
        87.8    48.8    27.299999999999997      81.25   90.8    85.4
        83.5    10.2    53.55   20.85   84.4    53.099999999999994
        60.5    26.95   83.4    95.95   75.75   92.05000000000001
        84.19999999999999       108.25\
SLC10A2 81.1    93.6    101.1   86.3    22.8    17.2    35.0
135.9   108.0   93.8    26.6    51.0    42.8    71.0    160.7   35.1
        41.4    108.7   81.7    30.0    443.8   36.1    56.8    51.1
        113.8   55.7    47.7    73.0    82.0    69.1    58.2    31.4
        144.9   152.9   192.3   73.9    78.6    157.0   82.0    82.0
        21.2    33.0    122.1   82.6    75.1    108.6   33.4
104.1   194.5   108.0   78.3    112.7   61.6    153.8   26.2    80.8
        16.5    97.7    119.2   161.5   489.6   98.9    127.3   68.3
        201.8   133.3   44.3    50.1    126.2   142.1   166.6   97.1
        78.5    65.6    69.7    96.4    142.4   62.9    50.8    58.0
        123.1   179.6   85.7    56.0    196.4   89.9    341.9
111.4   128.5   188.4   41.0    86.8    24.1    17.9    42.0
133.2   78.1    98.4    38.3    148.4   111.4   130.6   66.4\
SLC10A3 527.4   111.5   158.8   512.2   310.0   627.6   571.7
415.2   633.5   471.3   505.6   767.7   544.4   638.2   60.8
707.4   498.9   326.4   1198.2  518.3   83.0    350.7   312.9
363.8   146.6   443.7   520.7   285.6   415.9   111.4   624.2
345.4   155.0   566.4   547.5   458.8   941.4   261.9   740.0
409.6   507.6   407.7   290.2   573.7   453.8   387.9   425.3
556.5   479.2   721.8   513.5   444.3   174.1   561.4   484.8
564.8   488.0   386.9   255.3   152.7   205.8   365.6   283.7
360.5   521.4   222.0   392.6   459.8   282.7   393.7   415.7
351.9   347.3   567.7   522.7   656.0   465.9   504.0   532.1
555.7   426.2   582.9   519.3   761.4   343.7   336.7   524.3
157.4   583.3   273.0   348.1   366.4   883.3   663.6   660.6
743.7   654.8   665.5   298.5   333.0   723.0   511.1   601.2\
SUCO    2730.5  4962.6  1535.9  1804.7  3675.3  2194.9  3063.5
2657.1  1803.1  2180.7  1860.6  3246.9  2631.4  2954.2  2299.4
2342.5  3127.8  828.4   1531.4  2317.9  2271.0  1304.9  1675.8
1573.8  1755.2  3399.9  1852.9  3623.8  2663.9  2371.0  3263.1
1734.4  1774.1  2778.9  2195.3  3658.3  2293.8  2077.5  3242.0
3198.9  2657.0  2768.9  2232.6  2678.8  1378.1  1677.4  1970.0
5513.7  4053.8  5156.4  1586.6  1351.0  4909.3  1716.7  2453.5
5215.2  2721.4  3538.2  2199.0  2392.6  1355.3  3133.5  2484.6
1932.3  4452.0  4201.5  3835.5  3643.0  1391.4  2469.0  2185.0
2436.6  2461.0  3433.5  2243.0  2670.3  1902.5  2240.0  5171.5
7688.9  2123.5  1934.6  2718.1  2104.8  2096.3  1614.9  1211.2
1599.4  966.8   1625.5  1913.2  2196.0  4834.1  3085.4  2838.9
```

```
3773.3  4146.5  3552.1  3230.1  4624.9  3349.0  4026.2  3137.9\
IL36G   40.5    20.6    42.0    74.4    56.7    38.1    18.9    70.2
        131.9   145.5   65.2    8.1     73.0    15.6    139.6   36.2
        33.4    72.2    9.3     607.1   51.3    5.3     140.3   19.9
        35.1    52.2    88.2    124.5   35.2    68.0    3.0
101.2   89.0    3.5     88.1    74.1    81.2    102.0   76.5    23.5
        9.3     71.0    6.3     61.5    46.8    65.0    67.0    11.1
        81.0    27.5    49.8    40.8    77.3    95.3    49.6    65.5
        32.7    8.8     110.7   22.8    422.7   5.3     71.7    45.7
        61.8    152.9   47.5    108.9   9.7     83.1    134.2   9.6
        9.0     68.3    70.3    51.3    69.8    61.7    42.7    47.3
        45.2    125.7   43.1    36.3    91.4    83.2    150.4   12.9
        53.1    21.5    64.6    130.4   45.9    33.0    44.9    20.4
        31.1    71.5    45.1    34.7    58.4    102.9   64.9\
NET1    6968.55 4728.3  3689.8999999999996      1705.0  2785.65
3018.3  5001.5  4712.9  3148.5  3200.05 2548.2  6847.6
7412.099999999999       3008.25 2333.9  18197.35        4671.5
3448.15 3681.85 5121.8  3438.5  5072.3  2013.2  3448.1499999999996
        6230.75 6629.45 6618.7  2857.7  1717.55 3950.65 2988.7
4203.25 2842.9  3039.0  1022.5  928.0   5850.4  4986.349999999999
        5307.5  2189.2  8790.95 5231.549999999999
2823.6000000000004      10752.35        5406.35 3448.2  2240.7
10221.45        1520.1999999999998      2985.9  3957.25 5080.85
6030.1  2978.35 4897.35 1703.9499999999998      2819.5
2011.1499999999999      4572.3  2112.25 1717.9  2418.5499999999997
        2539.65 3586.85 4126.45 3453.65 4616.95 8558.1  1625.1
2714.45 1669.8  1400.1999999999998      9591.15 2749.35 2413.3
2331.2  3454.9  2071.85 3213.8500000000004      3073.55 1724.85
6207.9  10287.650000000001      2291.0  1571.05 960.2   9092.2
1342.6  2676.35 865.15  4539.65 1771.35 2246.95 2280.3  2971.0
4686.4  2981.2  2499.1000000000004      1276.0  3087.6  2814.95
2571.8  3786.3\
ZBTB9   263.3   255.9   211.4   406.7   566.9   559.9   493.8
218.5   529.9   369.4   405.7   325.4   301.9   268.5   482.1
299.5   449.3   520.9   257.6   267.4   471.9   424.5   390.3
275.7   465.4   411.6   395.9   433.8   200.7   737.6   448.8
402.1   448.6   391.7   589.7   218.2   291.5   147.7   426.9
507.9   479.5   345.8   271.5   456.6   382.5   283.9   371.3
593.2   490.4   251.6   458.7   387.4   378.9   546.2   313.6
829.1   380.8   255.5   250.0   328.9   310.7   423.0   329.9
310.3   171.8   243.8   331.2   400.7   429.8   388.0   245.3
734.7   194.5   338.7   192.8   525.8   425.5   402.0   409.1
542.0   609.0   402.2   833.6   558.4   314.9   273.0   461.4
404.4   495.1   322.2   489.4   333.6   358.2   405.7   408.6
463.6   321.9   418.7   331.2   303.6   315.0   208.4   545.5\
LOC102724967    152.0   461.8   162.5   306.4   464.0   254.4
172.5   209.7   301.1   291.2   127.9   336.8   312.3   235.5
366.6   359.8   206.4   374.1   109.7   297.9   212.9   256.1
397.4   102.3   263.5   331.1   136.7   333.1   579.5   298.9
178.2   334.4   292.5   258.9   608.9   197.6   203.6   301.6
332.9   248.4   141.7   133.2   103.2   209.8   240.2   504.3
212.2   197.1   558.6   447.6   400.7   336.8   215.8   416.4
265.0   519.7   375.5   328.9   238.1   402.6   404.3   278.5
203.2   268.3   293.6   490.2   160.1   204.7   259.0   287.7
```

632.7	528.2	221.3	215.6	193.4	255.2	302.3	93.3
240.0	183.5	336.9	385.1	206.6	301.3	663.5	712.6
403.8	457.6	554.0	260.8	131.6	368.3	306.3	227.1
245.8	257.1	259.1	209.7	315.4	273.5	257.0	343.6
331.5\
PEX5L	28.7	18.175	10.625	14.549999999999999	48.0
4.9750000000000005	5.0	23.700000000000003	17.625
9.875	13.65	5.975	17.049999999999997	17.375	45.15
20.75	14.774999999999999	49.099999999999994	16.9
18.975	60.025	13.374999999999998	54.55	11.450000000000001
	22.775	12.925	21.275	43.575	39.175000000000004
39.275	11.7	38.175	7.8999999999999995	21.875000000000004
	48.05	43.575	10.65	26.125	16.3	5.025	18.375
7.6000000000000005	12.225000000000001	15.0
13.024999999999999	44.625	12.125	17.525000000000002
11.325000000000001	22.425	22.225	21.724999999999998
3.475	10.2	16.3	44.224999999999994	14.649999999999999
	22.025	19.8	25.075	310.275	35.3	41.949999999999996
	17.775	20.125	20.975	40.95	25.0	51.224999999999994
	29.424999999999997	33.175000000000004	28.25
36.300000000000004	11.475	11.275	17.275000000000002
21.425	8.625	24.7	511.67499999999995	33.050000000000004
	15.099999999999998	16.65	5.975	50.949999999999996
	79.925	35.925	53.5	13.674999999999999
23.150000000000002	24.425	44.225	17.1	10.1	13.65	21.4
	41.425	18.85	39.025	41.4	27.8	39.025	18.325\
LOC100506990	425.8	5236.85	3823.85	629.6	1784.75	1107.5
1439.5	330.4	431.65	1157.7	1989.0	532.3	728.9	780.0
1216.5	1725.15	1082.7	1462.6499999999999	1421.1499999999999
	513.9499999999999	762.2	687.8000000000001
3378.7	960.25	358.55	1152.15	706.5	2585.2999999999997
2462.05	360.84999999999997	1075.9	1823.6999999999998
3647.3	2567.25	282.9	1030.0	1687.55	742.1500000000001
440.55	826.3	864.1999999999999	763.0500000000001
3100.8	711.3499999999999	1125.8000000000002	1801.45
623.4499999999999	454.7	1644.35	1177.95	906.3499999999999
	624.9	723.05	1772.25	1547.1499999999999	2791.9
1201.15	730.9000000000001	2243.5	2824.2000000000003
598.4	1648.9499999999998	1061.65	2479.65	1285.95	1676.8
1385.8999999999999	1068.3999999999999	3086.4
2455.7000000000003	3925.45	3478.2000000000003	1399.85
1359.1	1476.8999999999999	1419.7	3161.25	772.7	1270.7
1433.9	766.1999999999999	793.5	2192.7	297.90000000000003
	1135.9	613.6	808.2	1026.9	180.05	694.0	189.5
2069.0	1504.7	2747.6499999999996	1501.95	2777.6
3384.7999999999997	3575.2	2080.45	4103.65	3366.25	2445.05
2338.3500000000004\
MC1R	107.8	550.0	129.3	228.0	429.7	104.8	64.8	21.7
	215.6	288.4	141.4	193.8	71.2	176.0	205.3
351.8	193.2	129.8	85.6	183.3	903.2	201.3	202.6
180.3	209.0	129.9	79.7	168.4	449.9	293.8	228.0
206.9	457.8	140.0	173.6	330.4	63.5	460.1	252.2
170.2	187.0	227.4	130.6	470.8	215.4	151.4	188.2
198.7	760.1	173.6	220.2	139.8	103.0	684.9	400.4

295.5 169.8 387.2 230.8 346.0 644.1 267.6 243.0
146.3 268.4 268.3 203.8 491.7 469.3 183.8 329.0
499.7 282.4 194.6 130.3 185.8 262.2 173.4 242.3
127.4 411.5 87.8 147.9 144.8 236.8 690.1 48.1
214.5 435.2 547.0 81.4 215.5 153.0 196.5 160.4
170.5 154.6 128.8 303.0 244.4 139.1 177.0 217.7\
ZBTB6 71.7 4.2 59.3 22.2 80.9 4.5 19.3 2.9
47.3 54.7 32.0 29.8 67.9 37.0 24.0 32.6
26.3 54.4 3.6 2.0 7.3 40.0 18.7 56.5
63.0 64.8 49.5 13.1 38.3 47.1 62.0 7.3
56.7 20.6 10.9 39.2 45.8 2.9 62.9 20.4
6.3 33.7 15.2 37.5 65.8 20.2 0.8 15.3
4.7 1.4 60.3 17.2 13.3 8.7 32.9 10.1
44.1 12.9 29.4 49.4 24.1 3.1 39.5 40.2
1.7 18.2 25.1 32.2 49.1 38.0 31.5 7.1
3.4 53.0 5.4 31.3 5.7 35.3 35.3 36.0
35.4 35.3 74.5 1.3 100.4 11.3 17.3 3.7
38.0 3.2 82.2 1.4 13.8 27.1 39.1 38.0
14.9 17.2 3.7 22.2 13.4 40.6 23.3\
TTTY4 /// TTTY4B /// TTTY4C 5.4 89.5 42.1 6.3 20.2
12.2 4.1 17.1 58.2 46.9 12.2 7.9 6.2
6.8 35.0 55.6 24.7 24.4 17.4 20.5 31.7
52.2 12.1 35.3 11.9 13.9 14.2 138.9 29.7
4.4 12.2 26.9 26.7 18.2 23.7 83.2 27.3
95.5 21.5 8.6 15.5 9.6 18.1 12.1 40.6
4.1 118.3 23.7 44.6 59.5 15.0 10.1 20.3
128.8 14.1 16.7 21.3 12.5 31.0 19.3 78.4
17.4 42.0 13.2 21.9 14.5 8.0 13.1 47.8
27.4 172.9 40.1 68.8 6.7 8.6 8.1 10.7
35.8 64.8 36.9 24.2 4.6 13.8 13.2
228.3 130.7 5.0 18.5 20.5 66.7 34.8 18.1 37.1
18.6 72.0 74.9 130.3 14.5 116.8 11.2 34.5
13.9 18.5\
LOC102724965 4.0 5.9 14.7 8.1 8.5 2.8 1.9
65.2 5.5 12.0 3.0 46.1 12.1 56.6 8.0
34.4 12.6 14.2 4.8 12.8 13.7 3.5 28.6
2.8 10.3 16.8 3.1 9.1 7.9 5.2 3.1
7.2 14.2 4.3 8.6 17.9 9.7 6.5 2.9
4.1 2.3 16.4 22.9 13.2 5.3 6.2 4.7
5.8 31.1 5.0 15.3 9.3 3.5 13.9 9.1
10.8 5.7 9.7 4.7 14.6 33.5 10.0 16.4
3.5 4.8 61.3 22.3 5.1 9.0 5.8 14.4
6.7 16.5 4.5 25.6 3.8 5.1 3.1 3.7
4.9 25.4 27.6 3.3 49.6 17.9 14.8 4.3
16.4 6.3 12.1 2.6 4.7 5.6 5.9 17.3
3.8 4.3 9.6 9.2 10.4 3.3 31.0 6.9\
LOC100505664 10.8 29.1 8.2 8.4 17.2 11.1 6.2
8.2 9.5 22.2 9.7 6.3 5.2 8.1 16.7
11.6 5.9 11.4 7.1 6.8 21.4 4.4 8.9
6.9 9.6 11.5 6.2 13.3 18.4 20.0 6.8
11.9 34.1 13.5 15.9 23.4 6.7 29.2 36.9
11.5 8.5 6.4 18.6 3.6 8.0 15.0 16.9
9.3 25.5 14.7 14.1 25.0 7.9 12.9 22.9
7.0 13.0 10.3 8.7 27.1 69.4 15.5 8.6

4.5	12.2	39.9	12.5	6.9	11.4	10.7	25.2
7.4	15.9	7.6	7.6	8.8	16.2	8.7	6.7
7.1	11.5	13.4	7.3	15.0	26.4	16.7	10.1
11.3	14.3	13.3	5.5	12.4	7.6	4.8	4.5
8.1	46.8	4.3	10.8	11.7	10.7	14.6	7.8\
SPDYA	142.6	168.6	272.1	27.2	280.9	2.3	90.1	87.1
3.6	98.4	28.7	231.3	68.3	47.0	57.5	28.3
181.5	49.3	28.6	108.7	127.4	63.0	446.0	33.6
22.4	110.2	65.5	166.1	77.0	59.4	12.2
185.3	18.3	39.6	63.6	94.9	19.2	83.0	78.8	34.7
19.7	36.5	75.7	50.4	119.7	36.9	88.8	31.4
398.8	33.4	86.4	32.1	17.7	37.8	104.5
145.5	80.6	125.1	222.1	20.4	244.9	200.7	9.9	92.4
131.0	88.8	33.0	35.9	77.5	112.4	218.9	64.5
62.3	111.3	66.1	29.4	149.2	58.8	42.0	63.9
6.2	55.9	33.6	82.6	106.6	10.8	35.7
106.7	51.0	184.3	12.3	57.7	30.1	15.1	37.2	7.8
21.1	2.7	7.5	50.3	48.8	57.2	63.6\
GRB2	903.7333333333332	947.7333333333335
585.2333333333333	1450.8999999999999	1014.9
1310.5666666666666	1263.9666666666667	1944.2
1148.4999999999998	1283.8000000000002	1119.8333333333333
1510.3333333333333	1012.5666666666666	1287.8
613.8666666666667	1237.8333333333333	1135.3999999999999
861.0666666666667	1597.4333333333334
810.6999999999999	525.6	1107.7333333333333
860.2666666666668	1205.5666666666668	1146.5
1143.9333333333334	1595.2666666666667	921.5666666666666
889.0666666666667	1161.6333333333332	998.0
784.1999999999999	908.4000000000001	1154.5
1719.4000000000003	1062.2	1248.8	730.3666666666667
1082.9666666666665	1425.4666666666665	793.7000000000002
926.0333333333333	867.5333333333332
1164.5333333333333	1330.4666666666667	1131.5666666666666
2115.6666666666665	862.6999999999998
717.0666666666667	825.6999999999999	1235.1333333333332
1562.7666666666664	977.5	1075.9666666666667
1013.8666666666667	944.0	1044.1333333333334
716.8666666666668	990.4333333333334	748.1666666666666
1352.8999999999999	1386.9333333333334
941.8666666666667	1185.8999999999999	856.9000000000001
1085.0666666666668	1102.5666666666666	1362.4
1051.0666666666666	1153.5666666666666	801.5666666666667
928.1666666666666	1009.4	1120.9	998.5999999999999
1430.6666666666667	1475.8666666666668
1414.7333333333333	1052.0666666666666	3484.0	1418.8
2008.7666666666667	1563.5000000000002	2274.9
1128.3666666666666	1580.5333333333335	1835.3
1904.5999999999997	1589.6999999999998	1148.4
2293.866666666667	792.7666666666668	934.5666666666666
959.4	833.8000000000001	765.4666666666667
749.5666666666666	780.6333333333333	661.0000000000001
591.3666666666667	631.7666666666668
743.3666666666667	970.9666666666668\

GRAP 7.05 75.4 29.1 32.65 44.6 34.55 11.15 42.8
96.35 47.0 53.0 27.65 52.15 22.349999999999998
13.8 12.3 28.35 75.35000000000001 12.8 34.1
92.94999999999999 21.4 58.25 6.75 26.8
14.95 14.75 154.5 61.25 78.3 9.35 49.15 74.75
80.44999999999999 43.6 67.65 20.700000000000003 88.3
46.599999999999994 21.9 17.55 66.6 29.15
80.95 36.650000000000006 90.55 21.25 12.25 69.65
102.95 48.35 14.25 13.1 104.15 44.4 58.0 52.7
25.15 102.85 155.8 313.70000000000005 63.65 53.7
32.65 113.25 49.150000000000006 120.65 79.64999999999999
91.10000000000001 8.35 213.6 83.6 113.85 60.4
43.7 40.05 33.45 129.5 108.5 42.2 32.1 46.3
8.0 53.0 93.1 53.1 97.55 138.75 15.45
55.099999999999994 10.9 33.45 54.85 49.25 94.05
27.450000000000003 13.05 26.299999999999997
63.800000000000004 24.6 39.85 61.0 88.55\
LOC100506999 1.5 11.8 10.8 25.8 59.0 24.4 16.6
2.5 20.8 8.2 16.0 14.8 9.0 51.8 12.1
5.8 11.5 37.7 13.6 4.2 13.3 30.2 6.0
1.0 38.4 39.6 2.6 4.5 2.7 2.4 38.2
2.7 4.6 32.0 5.0 11.3 14.3 19.9 45.5
11.1 1.6 0.6 5.6 3.7 5.7 72.6 50.0
24.7 4.1 3.4 29.4 6.0 2.0 38.1 7.7
1.6 24.8 6.3 14.6 1.9 7.7 2.2 0.9
17.9 24.6 28.0 2.1 41.8 3.1 10.6 18.7
8.0 12.9 2.3 1.0 0.8 4.6 24.6 4.9
6.7 7.7 32.7 1.0 2.2 17.7 53.7 3.8
33.7 19.0 1.8 2.5 5.8 21.8 4.0 1.4
5.7 25.2 13.6 17.3 43.2 2.8 2.3
24.2\
LOC100505666 166.4 190.3 72.4 120.6 206.9 74.9 68.4
92.6 78.5 170.3 99.9 103.3 51.2 54.0
145.7 87.8 58.5 159.2 93.3 132.4 210.4 94.4
221.6 47.2 111.4 84.4 49.1 142.4 84.0 131.8 10.1
237.9 152.8 19.0 123.7 267.2 101.6 128.9
113.7 59.1 39.6 63.1 140.5 40.7 117.3 74.0
105.6 109.3 149.0 122.4 125.5 108.0 98.8 170.4
108.4 118.6 122.4 140.5 173.2 255.1 324.2 113.8 65.2
95.6 179.1 88.8 104.9 135.3 19.7 112.4 57.8
138.4 96.8 110.9 112.5 78.6 25.3 43.2 85.4
93.3 124.0 77.7 107.1 115.0 187.3 305.1 72.9
160.4 99.9 138.7 68.9 170.2 94.1 54.2 51.2
18.4 74.1 57.8 51.4 164.0 105.4 184.7
104.5\
LOC100506403 /// LOC101928269 /// RUNX1 261.0076923076923
132.93076923076924 482.7384615384616 133.27692307692305
203.23076923076923 251.03076923076924
852.6692307692307 180.0307692307692 239.86153846153852
189.33076923076925 129.2307692307692
160.06923076923073 166.05384615384617 127.90000000000002
82.4846153846154 739.5230769230768
353.57692307692315 181.03846153846155 122.78461538461535
554.6769230769231 757.9153846153845

136.23076923076925 308.0615384615385 91.05384615384615
111.66923076923078 199.01538461538465
213.42307692307696 358.7461538461539 333.38461538461536
346.79999999999995 176.30769230769235
359.2307692307692 195.8615384615385 94.74615384615383
144.48461538461538 112.82307692307693
135.08461538461538 145.55384615384614 433.9384615384615
225.2692307692307 220.23076923076923
490.29230769230765 203.79230769230767 1001.446153846154
201.76153846153846 118.42307692307693
227.3461538461538 352.0692307692308 337.87692307692305
350.5846153846154 194.25384615384615
133.58461538461538 323.94615384615383 92.82307692307693
150.25384615384615 306.0769230769231
348.69999999999993 83.15384615384617 261.63846153846157
222.8 170.64615384615385 106.4923076923077
189.1769230769231 341.2307692307693 774.8615384615384
549.5076923076922 500.0692307692308
706.3846153846152 291.44615384615383 206.39230769230764
249.39230769230764 197.3846153846154
261.1692307692308 176.0 140.9461538461539
131.27692307692308 380.66923076923075 209.76923076923077
155.64615384615388 75.95384615384616
52.70769230769231 290.1923076923077 59.7
155.93846153846155 352.59230769230766 217.94615384615378
144.5 98.20000000000002 202.2 149.06923076923078
178.66153846153844 100.5153846153846
45.58461538461539 96.47692307692306 67.26923076923077
33.91538461538462 26.08461538461539
82.95384615384616 189.8230769230769 57.00769230769231
30.276923076923076 83.71538461538461
59.83076923076923\
AC079305.10 14.0 16.9 16.6 3.5 62.0 15.8 48.6
16.4 102.2 20.8 36.3 43.7 3.8 5.8 10.7
28.6 16.1 13.5 102.4 7.8 301.6 49.7 12.8
25.9 14.5 27.9 70.5 33.0 40.0 33.1 56.7
32.9 8.1 16.5 81.2 17.2 9.2 40.0 3.4
67.6 27.2 57.6 87.0 47.8 25.2 14.1 28.2
10.9 30.0 21.4 98.2 22.9 52.0 36.4 9.4
20.4 99.0 19.3 32.1 6.9 213.3 75.8 16.7
76.8 52.0 44.2 56.2 50.0 89.6 88.0 67.1
63.5 5.3 81.2 35.8 85.6 64.0 32.5 23.9
42.7 20.4 62.8 5.2 10.3 115.9 76.4 6.8
97.6 36.8 36.2 4.5 8.4 33.7 93.1 10.9
15.5 125.4 107.9 58.0 92.4 39.6 62.9
11.9\
LOC100506472 /// TAB1 20.5 230.4 136.6 45.7 254.9 61.1
13.6 60.3 11.7 80.2 144.7 132.9 44.8
113.4 231.7 89.8 109.7 32.2 103.8 39.9 133.4 99.8
44.9 115.3 34.1 53.6 126.4 231.6 67.2 17.8
94.2 28.2 150.5 87.3 147.1 106.1 143.1
134.7 119.3 88.4 127.5 27.7 168.9 107.5 50.7 27.1
12.3 49.7 248.5 120.6 30.3 7.5 145.6
169.4 154.5 161.6 111.8 112.5 116.1 133.8 14.7

```
109.7   57.5    13.7    56.4    150.7   131.0   109.8   215.8   56.9
        89.8    132.7   160.0   108.6   131.1   78.4    133.8   87.8
        71.4    20.1    172.1   86.9    43.4    60.3    170.7
239.7   236.3   227.5   70.2    139.0   31.0    198.3   126.3
174.4   335.5   292.6   278.9   239.0   136.6   215.2   181.7
116.4   185.1\
TFB1M   560.425 1269.55 738.725 586.425 1123.0500000000002
772.8   582.95  673.55  752.2   488.20000000000005      638.7
604.0   679.875 561.35  382.025 350.775 646.75  960.95  607.65
812.4749999999999       1232.6  645.425 520.3   500.9   526.325
723.0   851.775 717.975 833.3   655.875 633.8499999999999
1143.35 924.6   502.725 1090.55 664.7   423.79999999999995
1453.45 686.325 499.625 502.6749999999995       593.7249999999999
        666.9749999999999       697.85  513.5   706.575 1171.225
        811.55  1061.925        635.9000000000001       687.2
956.5   597.0250000000001       1231.85 642.625 626.45  768.875
838.625 772.5   1016.4749999999999      1030.95 658.25  600.25
665.35  632.4000000000001       824.575 577.2   783.35  1050.9
494.84999999999997      964.325 1098.7250000000001      590.45
507.35  727.725 653.3   785.55  722.55  444.825 616.875 1163.425
        915.35  252.89999999999998      352.1   1350.9
962.9000000000001       863.075 950.825 590.4   695.4000000000001
        575.275 799.7   702.2   546.675 556.225 616.7750000000001
        693.175 598.9   866.1   783.55  636.95  707.675 540.825\
C8B     90.3    59.4    116.4   17.8    146.8   12.6    26.8    96.2
        115.7   26.7    33.7    13.3    11.9    15.3    93.2    88.9
        20.4    117.3   44.6    15.5    190.2   121.9   270.8
105.3   74.4    43.5    13.4    186.7   58.6    110.6   45.8
190.8   51.8    107.4   131.7   22.0    86.6    134.7   12.2
109.3   18.5    61.4    20.1    77.3    106.5   34.7    62.7    8.3
        124.4   24.8    101.3   208.6   108.9   18.3    20.5
206.2   111.7   86.6    72.1    110.8   235.4   74.8    74.5    55.1
        28.8    181.1   64.2    33.2    17.3    165.9   119.6   57.4
        157.8   32.0    168.6   90.2    11.9    58.5    94.8    22.4
        146.1   28.0    15.0    27.4    97.8    328.6   54.0    31.2
        37.0    303.7   28.4    73.1    95.1    65.9    55.0    22.5
        90.8    48.0    20.6    33.4    18.8    45.8    115.7\
C8A     30.7    46.4    74.2    65.2    30.8    35.2    6.2     97.5
        20.8    103.2   15.2    22.0    52.1    39.9    29.3    53.4
        21.1    28.2    86.9    85.1    275.9   4.7     240.1   25.8
        24.5    27.0    16.8    148.7   17.1    32.5    6.3     12.0
        118.5   20.9    144.5   133.6   19.5    45.8    80.9    10.4
        12.6    37.1    26.0    9.9     17.1    48.9    50.5    49.5
        331.2   112.7   15.2    92.6    47.8    152.6   71.9    12.1
        91.3    124.6   21.5    8.7     553.6   53.0    24.6    70.8
        167.0   52.9    27.0    4.6     35.3    13.6    163.6   32.9
        53.2    46.5    55.5    26.8    110.7   12.0    110.9   49.3
        92.2    18.1    10.2    22.2    113.1   125.6   52.1    36.6
        31.2    385.9   27.6    68.1    19.9    14.9    44.9    12.9
        25.1    13.5    71.3    22.8    20.1    27.1    60.8\
RP1-187B23.1    40.0    21.6    131.0   84.6    14.1    5.6     42.0
        47.8    75.8    87.5    48.6    66.8    84.7    73.9    72.2
        36.8    37.8    157.2   72.6    17.0    38.6    8.1     73.6
        43.3    61.0    64.5    8.0     73.1    73.6    50.5    2.6
```

```
	103.6	148.8	9.1	102.6	58.9	44.8	155.6	39.8
	72.2	12.8	79.2	29.6	44.6	106.5	66.0	8.9
	42.6	71.1	81.0	58.0	130.8	76.2	145.5	55.9
	28.4	129.9	99.8	16.9	66.5	25.7	49.8	21.2
	38.0	65.3	99.0	37.8	46.0	161.6	53.8	99.1
	104.7	63.9	42.5	44.2	13.4	80.5	78.5	18.8
	42.7	49.0	18.9	6.7	102.3	41.8	22.8
169.1	28.9	71.2	16.8	21.5	11.3	45.5	41.2	56.9
	38.6	102.0	47.7	101.5	83.4	17.3	103.9
36.5\
MC4R	35.8	52.5	52.9	40.7	112.9	40.3	44.8	78.8
	9.5	32.4	55.8	59.8	10.4	44.1	37.5	16.3
	50.0	93.8	70.8	69.4	77.2	85.3	17.3	34.0
	55.5	163.7	36.3	16.4	78.5	114.7	23.5	41.4
	154.1	76.0	117.5	89.8	35.3	120.9	22.7	14.8
	27.1	9.1	19.3	51.9	49.0	85.3	48.9	18.0
	78.3	6.8	21.6	51.5	69.9	221.6	45.0	79.5
	46.3	130.7	6.1	92.5	63.4	80.7	51.6	55.8
	14.5	26.5	24.8	2.1	74.9	15.6	28.2
135.6	128.8	52.4	10.3	59.1	51.6	71.5	58.8	91.9
	17.0	84.2	110.1	23.3	108.7	99.9	56.9
131.9	31.3	110.9	35.3	11.6	25.3	52.0	28.3	10.8
	50.3	40.6	21.0	56.4	20.0	10.3	59.9\
KCNT1	49.46666666666667	22.633333333333336
58.56666666666667	32.5	57.166666666666664
19.433333333333334	14.9	23.433333333333334
38.36666666666667	15.766666666666666	12.5
55.23333333333333	42.1	25.53333333333333	24.0
12.833333333333334	24.96666666666667	31.999999999999996
	36.56666666666667	25.166666666666668
150.33333333333334	13.5	35.1	53.43333333333334
18.933333333333334	57.56666666666666	16.8
32.13333333333333	46.56666666666667	16.400000000000002
	38.2	16.599999999999998	56.666666666666664
18.033333333333335	41.5	21.63333333333333
34.333333333333336	42.233333333333334	19.900000000000002
	26.833333333333332	30.599999999999998	28.3
22.399999999999995	8.766666666666667	38.333333333333336
	49.53333333333333	8.566666666666666
14.333333333333334	82.2	28.0	13.733333333333334
45.53333333333333	27.3	59.0	41.333333333333336
54.73333333333333	27.333333333333332	35.86666666666667
	17.8	43.86666666666667	58.26666666666667
51.866666666666674	35.26666666666667	30.366666666666664
	32.6	30.299999999999997	23.333333333333332
31.96666666666667	19.4	16.23333333333333
57.300000000000004	40.833333333333336	35.96666666666666
	5.8999999999999995	64.66666666666667
24.566666666666663	42.1	36.4	51.93333333333334
18.166666666666668	31.833333333333332	35.3
41.166666666666664	55.76666666666667	75.7	58.9	45.9
	38.43333333333334	13.666666666666666
37.900000000000006	26.96666666666667	21.733333333333334
	8.033333333333333	16.166666666666668	25.2
```

18.766666666666666 10.633333333333335 14.266666666666666
14.533333333333333 39.46666666666667
34.96666666666667 23.733333333333334 27.600000000000005\
C8G 8.4 61.4 41.4 5.8 13.9 8.5 23.7 53.3
7.0 5.2 9.5 5.6 6.9 65.5 53.0 6.5
5.7 16.4 25.5 17.5 23.6 5.5 5.7 4.3
9.6 54.8 9.2 10.6 9.9 77.1 3.8 20.7
4.7 7.6 6.6 40.6 3.5 7.5 6.2 10.2
10.3 45.1 18.6 12.3 3.0 6.9 16.3 19.2
79.6 16.1 24.8 12.0 3.8 5.9 15.4 4.6
6.1 8.6 2.7 23.8 46.2 15.5 74.7 4.7
11.8 15.7 7.3 38.0 71.9 32.4 27.4 15.1
29.7 5.3 32.0 6.2 34.7 5.0 10.1 5.8
12.9 20.0 40.5 27.3 240.7 21.5 7.5 26.8
6.6 47.1 3.3 10.2 9.5 2.8 33.8 3.4
12.4 38.7 34.1 5.1 8.5 46.9 6.3\
KCNT2 23.5 75.65 15.4 72.55 308.70000000000005
2.8499999999999996 154.35 361.65 19.75 22.75 12.3 33.2
31.0 61.599999999999994 35.95 513.1 153.8 39.8
43.95 54.75 157.15 18.0 36.8 54.8
41.650000000000006 21.55 42.65 69.75 68.75 66.0
143.5 55.15 59.7 325.5 48.45 3.9000000000000004
32.900000000000006 33.35 32.15 66.15 12.850000000000001
54.949999999999996 74.25 58.05 16.4 38.2 35.0
51.45 23.5 63.1 37.25 25.450000000000003 17.5
125.05 56.95 53.15 102.95 73.85 37.5 292.0
468.75 50.849999999999994 14.5 68.35 94.85 73.6
72.35 45.05 123.75 17.8 296.05 80.89999999999999 72.1
26.1 7.35 4.6 135.0 39.4 26.35
24.950000000000003 769.35 36.95 24.0 29.0
87.55000000000001 107.7 33.2 91.3 41.949999999999996
39.5 287.65 119.25 40.85 551.5 290.5 141.7 85.4
228.8 89.15 175.05 134.3 48.8 146.6\
SUFU 41.150000000000006 144.125 43.625 73.05000000000001
121.775 129.65 123.8 74.225 136.175 123.9 92.1
113.49999999999999 89.875 122.82499999999999 121.225
45.325 104.325 125.94999999999999 136.125 80.7
127.94999999999999 77.375 48.5 85.05 80.6 120.375
102.125 194.10000000000002 177.025 145.8 149.0 143.775
122.6 237.75 129.5 146.5 67.67500000000001
175.77499999999998 91.15 95.22500000000001
99.54999999999998 114.725 151.45 136.65 106.64999999999999
132.05 99.1 97.5 113.55000000000001 207.725
192.02500000000003 77.0 111.6 139.325 122.04999999999998
156.375 143.375 119.20000000000002 105.675 137.55
308.325 112.65 140.75 106.05 106.25 172.675 115.19999999999999
89.05 180.375 69.875 206.125 112.375 192.85 57.375
127.04999999999998 106.15 90.42500000000001 103.5
180.85 63.3 106.67500000000001 89.1 72.65 89.325
295.2 242.04999999999998 109.55000000000001
288.29999999999995 133.9 78.725 99.275 113.35 119.275
57.349999999999994 108.92499999999998 163.325 100.725
164.875 79.60000000000001 171.55 146.2 234.575
271.65000000000003\

BBS9	165.16	189.4	296.46	137.52	178.74	221.58
113.18000000000002	135.94	176.45999999999998	250.74
200.38000000000002	116.61999999999998	75.18	149.5
121.44000000000001	158.18	125.96000000000001
227.97999999999996	98.4	154.74	128.78	228.04000000000002
	200.32	209.0	198.56	259.12	222.5	284.3
212.95999999999998	174.52	401.96	173.54000000000002
190.2	314.12	190.9	213.78000000000003	134.7	100.4
142.61999999999998	328.14	239.42	143.84	260.12
183.85999999999999	144.4	275.20000000000005
87.36000000000001	233.83999999999997	176.5
223.51999999999998	180.16000000000003	186.95999999999998
	147.8	88.44	129.66	167.0	233.84	155.9
205.03999999999996	237.3	278.56000000000006	199.36
230.22000000000003	332.86	307.67999999999995	145.72
217.66	246.66	257.48	298.96	255.56000000000003	211.1
166.74	217.3	100.88	218.9	306.3	202.5	261.52000000000004
	167.2	207.16000000000003	109.4	243.04000000000002
	111.97999999999999	195.92	146.32	135.38000000000002
	273.3	59.44	103.41999999999999	136.78	197.78
396.21999999999997	353.46000000000004	291.58000000000004
	415.66	317.54	411.52	308.84000000000003	362.4
419.17999999999995	204.8	241.45999999999998\
FCER1A	35.25	122.85	77.69999999999999	51.599999999999994
	103.1	18.7	99.85	51.05	29.700000000000003
49.65	56.45	56.35	34.05	83.65	63.599999999999994
119.69999999999999	68.35	150.45	48.15	109.9
173.85000000000002	111.45	139.35	36.599999999999994
81.19999999999999	38.849999999999994	45.6	74.1
91.35	120.19999999999999	44.5	63.0	94.5	236.5
82.25	202.9	45.8	69.6	65.6	54.4	60.8	22.55
146.5	29.1	99.6	80.95	61.85	44.849999999999994
50.599999999999994	84.3	50.35	63.55	53.849999999999994
	20.35	75.05	35.75	58.5	114.65	21.7	99.95
185.55	93.30000000000001	79.65	143.14999999999998
165.05	74.65	80.2	66.25	160.89999999999998	170.95
126.8	121.1	57.35	39.65	38.55	80.7	74.75	40.2
104.6	70.35	79.6	84.14999999999999	73.80000000000001
	60.0	78.5	143.35000000000002	76.05000000000001
	65.5	65.6	64.95	67.2	86.5	72.8	219.9
65.35	73.44999999999999	99.85	131.85	98.64999999999999
	117.6	96.2	108.8	66.55000000000001\
LOC101929373 /// SLC9B1 15.366666666666665	34.733333333333334
	29.033333333333333	22.066666666666666
45.666666666666664	34.53333333333333	10.233333333333334
	22.5	32.233333333333334	43.1	56.4
13.200000000000001	23.0	68.13333333333334
89.56666666666666	25.73333333333333	10.366666666666667
	56.9	40.833333333333336	15.266666666666666
98.26666666666665	22.066666666666666	33.766666666666666
	44.9	23.26666666666667	30.7	50.46666666666667
	82.0	39.73333333333333	81.93333333333334	41.9
	41.76666666666667	38.333333333333336
57.76666666666667	32.233333333333334	60.23333333333334

	24.066666666666666	34.666666666666664
60.633333333333326	22.566666666666666	32.666666666666664
	14.266666666666667	44.800000000000004
14.466666666666667	21.066666666666666	44.96666666666667
	29.099999999999998	49.333333333333336
83.36666666666667	42.76666666666667	41.5
19.733333333333333	29.600000000000005	53.16666666666668
	12.5	35.199999999999996	61.36666666666667
40.766666666666666	63.166666666666664	42.13333333333333
	185.86666666666667	61.13333333333333
31.566666666666666	23.76666666666667	19.733333333333334
	47.5	45.5	32.76666666666667	99.76666666666667
	22.399999999999995	85.76666666666665
21.53333333333333	41.93333333333333	11.233333333333334
	37.86666666666667	28.599999999999998
58.699999999999996	32.766666666666666	53.9
18.233333333333334	34.56666666666667	33.233333333333334
	13.066666666666668	29.03333333333333
75.89999999999999	29.666666666666668	53.73333333333333
	39.666666666666664	33.23333333333333
31.599999999999998	25.8	44.5	58.43333333333334	43.5
	41.96666666666667	84.46666666666667	71.0
67.83333333333333	34.53333333333333	61.166666666666664
	94.43333333333332	93.93333333333332
81.96666666666667\
BBS7	300.55	325.4	395.65	470.1	417.75	449.25
354.40000000000003	220.7	431.2	237.14999999999998
434.8	187.85000000000002	267.3	322.09999999999997
285.05	264.05	236.05	401.95	400.90000000000003	336.0
414.9	304.40000000000003	327.0	366.85	454.95	466.3
367.29999999999995	447.2	393.15	333.70000000000005
427.6	425.75	294.6	350.40000000000003	235.3	277.6
282.0	313.25	330.15	263.15	160.4	279.65	634.7	308.25
251.25	370.85	383.15000000000003	436.15	362.35	311.25
363.25	285.05	320.7	520.55	259.45	272.85	378.84999999999997
	391.04999999999995	455.25	515.5	1025.2	355.65
258.45	484.65	304.0	264.1	294.3	277.8	329.6	406.9
414.9	468.25	432.0	479.85	444.54999999999995
304.40000000000003	446.79999999999995	348.34999999999997
	383.5	433.05	723.95	454.9	247.6	301.6	347.55
193.85000000000002	466.09999999999997	502.6	160.8
457.95	189.3	636.0	474.55	549.8	626.85	628.95	632.85
617.45	478.1	848.0	606.95	499.3	489.54999999999995\
MRPL51 /// SPTLC1	242.3	157.4	44.1	143.2	208.1
391.3	230.7	31.1	107.0	61.6	331.7	116.4	295.9
149.0	198.7	198.5	258.9	291.9	198.8	341.1	260.9
385.2	298.0	98.9	230.8	203.3	140.3	46.8	288.3
177.1	313.3	173.4	166.4	84.0	188.7	317.7	213.4
133.6	91.0	318.3	320.9	259.6	215.3	251.4	440.8	95.7
	71.6	248.7	29.4	142.2	179.2	222.7	276.4
166.5	211.7	44.6	40.6	91.4	166.6	207.8	353.1
105.6	124.3	252.5	237.0	212.8	247.7	205.3	160.9
312.6	102.8	231.5	252.8	295.1	41.4	162.7	24.6
298.0	235.9	180.1	45.8	148.2	506.0	142.6	160.1

230.0 73.8 77.6 247.9 245.1 242.2 34.1 146.3
186.5 183.7 144.5 196.9 111.1 104.8 115.9 146.4
122.2 178.1\
BBS5 212.1 258.0 159.5 67.7 26.1 68.4 53.8 48.3
27.3 168.7 89.8 63.7 47.7 38.0 149.9 79.5
74.4 223.7 67.7 71.0 47.3 240.5 104.9 85.3
46.1 19.9 99.7 198.1 94.7 94.9 75.6
189.4 212.4 107.0 34.3 127.1 72.3 127.0 137.1
165.4 24.3 92.3 153.2 56.4 180.8 218.2 143.1
101.9 102.8 51.8 139.1 201.3 97.6 31.3 88.9
200.9 99.9 136.3 292.5 128.7 56.8 180.5 235.0
189.0 15.3 60.8 13.0 148.5 22.4 161.8 269.8
200.3 16.2 90.8 110.2 158.3 176.6 112.0 126.8
126.9 58.2 66.3 59.6 113.0 66.7 139.0 65.3 30.0
48.9 212.3 200.2 151.8 126.1 62.9 106.6
138.5 97.0 114.4 108.7 201.1 124.7 212.5 138.4\
LOC400043 1073.5 87.5 468.5 49.1 23.2 56.7
1106.7 3383.2 31.4 105.0 171.0 411.3 26.8 110.4
3577.8 1545.0 134.3 3.9 825.0 133.2 336.4 79.8 24.6
128.6 112.1 277.7 72.4 170.0 30.1 319.2
348.1 89.4 135.9 183.5 67.8 127.7 100.3 110.6 85.9
36.8 146.2 101.4 142.1 502.9 54.1 804.8
100.1 77.5 600.1 674.8 75.1 2800.2 416.0 104.6 44.2
761.5 587.0 8.8 9.0 169.8 26.8 94.7
155.5 82.0 282.9 48.0 960.5 790.1 174.2 101.9 32.1
207.1 312.9 119.6 257.5 102.5 60.3 73.7 69.8
4848.1 568.1 988.3 2712.8 4104.0 55.2 2569.7
5095.4 1363.5 78.8 1322.6 113.2 22.0 139.1 14.6
288.0 390.5 270.2 126.5 439.7 255.6 438.5 100.1
240.0\
LOC727820 668.75 290.1 2168.8 1113.3 1595.0 77.2
1088.75 281.45 529.0500000000001 924.75 369.20000000000005
1348.3 726.65 790.6 1673.95 1437.8999999999999
874.5500000000001 1083.35 1096.1 812.8499999999999
997.9 744.5 1569.9499999999998 429.3 497.95 1499.8
383.1 1501.6000000000001 1454.25 635.0 958.5999999999999
754.3 2101.15 467.2 813.55 938.25 120.4 1763.95
776.85 400.5 1257.8 705.25 998.8499999999999 793.65
326.7 440.4 178.2 136.4 1478.45 2221.5499999999997
439.65 611.1 672.6 374.55 810.05 572.65 964.75 1556.45
997.75 2027.75 843.95 1485.15 1911.75 619.3 1701.8
2378.2000000000003 1329.3 1048.55 1231.8500000000001
582.8000000000001 1746.25 1038.8500000000001 1821.25
421.6 1825.15 176.85000000000002 405.65 577.6999999999999
1574.05 178.1 602.05 362.8 304.95 745.75 439.85
641.9000000000001 183.5 267.2 1069.95 1211.25 455.7
428.20000000000005 363.3 443.1 826.2 486.9 459.25
540.5 1165.25 689.8000000000001 802.25 290.2
638.1500000000001\
BBS4 372.95000000000005 997.05 655.75 905.4000000000001
966.5500000000001 2004.8000000000002 1208.0
589.15 589.7 936.4 744.8499999999999 1403.6999999999998
718.25 367.55 371.6 701.1500000000001 293.0
1048.8 630.9000000000001 607.1 837.25 1791.55 1065.1

930.1	541.1500000000001	900.9	685.4	783.2	606.05
523.25	988.6	618.6	1210.5	708.2	265.2	969.5	1266.5
1726.5	1469.75	195.35000000000002	1084.6999999999998
784.25	997.0500000000001	825.35	953.5	1166.9499999999998
953.25	403.0	683.05	1134.75	1085.9	618.0	641.95
1288.65	621.05	602.05	1328.35	562.55	1496.6999999999998
976.0	637.1500000000001	817.25	980.25	828.3	531.2
1318.6	953.5	855.45	856.45	856.35	1824.85	1297.4499999999998
650.65	849.95	609.05	693.8	1651.6	984.0999999999999
1027.1	768.25	455.45	935.6	1171.7	394.9	1201.25
1297.05	1280.1	583.2	596.55	289.85	385.35	1281.3000000000002
1300.85	1266.6999999999998	1978.5	2065.95	2000.8
1699.85	1402.1999999999998	2411.95	2643.2	1008.95	2285.45\
LOC101927809	20.4	60.8	79.3	58.9	196.2	106.9	37.1
178.1	112.4	79.5	84.4	55.9	81.2	173.1	78.9
74.9	24.7	127.2	260.4	122.9	135.3	54.0
168.1	131.9	163.6	123.6	87.3	186.3	274.6	162.3
105.3	159.2	96.9	258.4	285.1	430.3	121.8	117.7	98.5
60.3	31.1	79.8	158.9	66.2	85.5	152.0
144.1	73.8	227.2	48.2	60.6	188.9	108.4	193.3
167.0	66.6	201.7	125.1	91.5	155.9	165.8	79.5
140.4	72.3	42.9	172.6	26.7	48.8	84.4	333.1
216.2	111.2	9.2	102.1	57.7	189.2	121.1	74.6
109.7	122.5	420.8	129.0	120.4	89.0	120.1	143.8	80.8
115.9	126.7	171.6	56.3	63.7	233.3	211.5	94.4
227.7	176.7	225.0	261.9	197.6	133.7	121.5
187.6\
PCYT1B	58.833333333333336	141.5	37.333333333333336
8.233333333333333	89.63333333333334	31.8
24.766666666666666	32.5	45.766666666666667
47.666666666666664	121.90000000000002	16.366666666666664
68.43333333333334	37.93333333333333
75.39999999999999	64.33333333333333	52.20000000000001
164.16666666666666	20.13333333333333
64.03333333333335	191.13333333333335	102.13333333333334
133.63333333333333	59.6	88.89999999999999
38.733333333333334	41.199999999999996	86.83333333333333
58.833333333333336	43.03333333333334
50.79999999999999	154.33333333333334	57.800000000000004
83.8	63.70000000000001	61.099999999999994
26.766666666666666	42.46666666666666	17.433333333333334
112.43333333333334	42.93333333333334
27.866666666666664	111.33333333333333	74.43333333333334
102.16666666666667	171.93333333333333
35.06666666666667	57.23333333333333	77.6
119.56666666666668	92.43333333333334	166.53333333333333
15.4	148.46666666666667	61.26666666666667	97.7
79.03333333333332	73.86666666666666
99.73333333333335	70.93333333333334	202.9
75.56666666666668	40.6	95.69999999999999
92.26666666666665	51.26666666666666	64.56666666666666
71.93333333333332	95.8	129.3	75.46666666666665
161.29999999999998	36.699999999999996
54.23333333333333	98.66666666666667	122.0

139.8666666666665	70.93333333333334	27.033333333333335
	32.9	62.06666666666666	21.233333333333334
45.96666666666667	22.433333333333334	77.03333333333333
	81.86666666666667	20.73333333333333
25.433333333333334	56.36666666666667	70.26666666666667
	27.633333333333336	28.7	41.7	57.4
39.199999999999996	60.166666666666664	54.333333333333336
	56.866666666666674	32.833333333333336	19.0
43.400000000000006	58.5	41.73333333333333\
ZHX2	699.6999999999999	91.3	398.5	308.8	749.95
520.85	1447.15	505.1	512.55	724.9499999999999	745.65
555.7	782.6	608.1	2428.75	809.25	553.45	436.9	433.0
810.0500000000001	1207.05	308.84999999999997	357.8
446.45	472.5	1186.45	663.6	601.0999999999999	778.15
721.4	744.25	1043.1499999999999	732.35	403.5
356.45000000000005	418.65	349.25	857.1999999999999
445.8	532.85	585.4000000000001	380.0	418.90000000000003
	327.09999999999997	167.65	240.3	425.85
359.40000000000003	540.2	853.0500000000001
314.54999999999995	549.8000000000001	609.7	515.35
714.6	524.05	235.4	328.7	610.55	452.7	696.05	507.8
376.85	1063.25	434.2	1002.6	1050.85	802.75	629.15	559.25
742.4	324.05	798.5999999999999	416.04999999999995
666.75	448.70000000000005	479.45000000000005	334.45
613.05	259.5	537.15	365.59999999999997	1336.8	1240.95
1157.7	775.95	353.5	642.0999999999999	557.85	642.9
329.90000000000003	502.7	1298.3999999999999	1354.85
1044.7	575.75	896.6	850.6999999999999	653.25
1184.1000000000001	731.65	426.05	744.5\
ZHX3	135.7	228.0	155.65	242.25	226.95	80.3	164.4
76.10000000000001	117.0	172.64999999999998
98.55000000000001	115.89999999999999	184.85	85.5
92.55	122.85	98.0	145.05	180.85000000000002	149.55
282.8	105.45	119.14999999999999	106.55	79.95	78.05
104.85	163.0	85.25	89.85	121.65	107.4	193.0	141.4
138.2	115.05000000000001	52.349999999999994	338.0
75.15	81.75	108.94999999999999	85.15	82.5	167.6	83.5
	131.25	137.70000000000002	134.4	158.2	47.8
74.45	130.6	156.15	83.19999999999999	132.2
240.64999999999998	113.6	199.75	102.9	194.95000000000002
	233.95	243.75	117.55000000000001	110.1	106.25
201.2	108.6	142.85000000000002	279.3	130.25	142.6
91.05	109.44999999999999	93.55	82.75	56.3	98.85
89.64999999999999	128.65	142.75	142.29999999999998
167.65	155.45	83.05	267.35	359.25	102.05	191.25	521.4
217.35	70.7	78.05	122.05	89.0	135.45	148.1	134.6
88.60000000000001	91.05	36.6	116.85	150.25	127.55\
FCER1G	117.0	118.55	163.10000000000002	509.05	166.2
233.75	277.3	190.55	174.70000000000002	254.35	163.7
179.54999999999998	98.7	413.95	94.75	243.45	279.85
134.5	220.3	174.4	169.05	197.14999999999998
206.35000000000002	263.45	166.3	106.35	111.8	315.75
156.15	162.2	142.85	155.1	314.4	136.45	164.7	161.0
231.05	137.0	88.4	381.0	109.75	147.20000000000002

234.89999999999998	83.55	637.7	139.35	122.2	121.2
104.2	143.5	221.89999999999998	219.15	61.2	122.8
192.2	83.35	204.29999999999998	150.15	99.94999999999999
	130.5	166.1	308.9	173.35	115.2	194.45000000000002
	142.0	271.45	308.15000000000003	243.95000000000002
	177.04999999999998	179.79999999999998	138.75
292.9	221.45	476.7	194.05	106.4	158.3	167.4	85.95
32.300000000000004	101.60000000000001	44.9	204.85
161.45	142.0	142.6	143.65	264.7	140.15	174.3	130.7
88.55	124.65	78.05	58.1	98.75	124.15	79.75	120.25
94.75	82.35	151.65\
PCYT1A	749.6	1312.9333333333332	667.1	673.3666666666667
	675.0	662.1	532.2333333333333	1146.7666666666667
	494.3666666666666	664.7666666666667
801.3000000000001	766.4666666666667	674.9333333333333
	1050.4666666666665	964.5	710.3000000000001
970.0	924.0	692.3000000000001	752.1999999999999
1327.8	632.2666666666667	707.8666666666667	696.1
502.0666666666666	792.9333333333333	638.5	1325.3
856.4333333333334	502.1666666666667	768.6666666666666
	762.6666666666666	973.5	1048.8666666666668
1396.4333333333334	543.9333333333334	543.4	898.6
658.0	817.7000000000002	903.9	724.9	805.6
665.6666666666666	765.5666666666666	840.5666666666666
	594.9666666666666	567.6999999999999
1164.3333333333333	655.7666666666667	535.6
755.2333333333332	681.9333333333333	949.5333333333333
	921.6999999999999	1048.3333333333333	577.1
662.6333333333333	625.8	768.6333333333333
567.2666666666668	706.6666666666666	445.0333333333333
	618.2333333333333	665.7666666666667
675.4666666666667	807.9666666666667	608.8333333333334
	764.7666666666668	798.2666666666668	950.5
1022.1999999999999	732.3666666666667	898.8333333333334
	815.2666666666668	691.5999999999999
964.7999999999998	762.8000000000001	748.0666666666667
	766.0333333333333	547.4	493.3	607.0333333333334
	904.5999999999999	718.6333333333333
743.6666666666666	624.5666666666667	630.5333333333333
	820.6666666666666	1428.7	1157.5666666666666
648.7333333333332	798.9333333333334	592.7666666666667
	663.5666666666667	566.4333333333333
603.6666666666666	568.3333333333334	658.9
473.5333333333333	669.6999999999999	612.3666666666667
	653.6\
LOC101927814	28.55	24.0	45.650000000000006	40.55	9.9
	28.7	4.75	10.15	21.549999999999997	49.4	6.55
	44.65	12.45	38.3	21.25	13.85	31.4
52.599999999999994	9.1	29.15	8.3	12.25
36.550000000000004	10.75	14.0	39.5	12.5	16.15
10.95	69.65	38.05	32.25	19.35	75.1	37.0	65.4
21.25	7.949999999999999	26.85	20.45	32.2	28.9	5.25
	47.099999999999994	5.45	40.05	24.299999999999997
	34.5	81.05	39.35	73.4	6.0	30.2	87.3

12.05	9.85	11.75	20.9	21.4	15.600000000000001
387.54999999999995	18.7	48.300000000000004	32.45	23.9
	38.85	16.2	4.25	33.05	7.699999999999999
23.25	13.7	12.850000000000001	9.25	31.1	14.5
16.25	19.8	9.0	23.6	8.25	7.2	12.5	15.45	54.4
	17.45	13.85	63.5	31.8	17.25	34.4	64.7
13.45	24.349999999999998	30.450000000000003	35.3
26.950000000000003	57.849999999999994	20.6	71.65
32.55	69.6	13.35\
LOC101927811	156.9	340.5	248.0	74.8	369.9	132.1
136.5	664.2	365.3	650.9	161.1	284.3	21.6	77.9
162.6	471.2	171.9	123.5	13.0	395.8	152.6	115.5
364.7	175.4	28.6	441.1	408.8	202.8	132.8	59.4
457.0	594.0	61.1	323.5	544.7	458.1	175.5	296.0
223.1	303.1	280.4	227.7	340.5	77.2	123.8	485.4
200.8	202.6	212.5	410.8	308.9	158.6	685.6	107.4
302.4	113.1	236.8	316.8	249.8	230.3	1189.2	332.3
641.0	415.5	259.9	58.4	300.7	280.5	396.9	337.0
133.1	179.2	399.1	266.1	72.7	501.1	257.4	186.1
355.4	273.1	501.3	190.5	155.6	470.8	380.2	191.6
699.2	28.8	116.2	16.1	138.8	369.2	534.3	544.6
832.5	1055.4	929.1	668.9	649.2	528.0	550.9	644.6
905.6\
IGLC1 /// IGLV2-5 /// IGLV2-5	9.0	18.3	100.6	2.8	14.9
	17.5	5.8	16.4	9.1	33.8	4.4	52.9	32.4
	8.8	6.0	45.0	39.6	3.0	10.0	62.0
215.5	105.6	190.1	116.3	43.0	103.5	19.3	40.7	63.5
	70.2	40.4	105.2	3.8	6.3	153.7	16.7	59.7
	168.4	36.7	86.2	43.5	5.5	23.7	2.8	5.1
	26.8	13.7	7.0	17.8	11.4	67.7	8.3	21.5
	88.1	7.8	17.0	37.0	97.8	85.7	11.1
149.9	66.9	3.0	7.8	8.5	13.9	50.3	33.4
116.3	50.3	112.0	77.3	57.3	41.2	81.6	51.4	7.6
	6.8	83.7	14.8	92.2	96.6	3.6	38.1	39.5
	54.3	76.8	12.9	153.9	105.9	30.4	16.3	9.3
	26.5	9.6	44.4	18.4	21.0	4.5	13.4	42.4
	4.3	10.5\
AGTRAP	237.46666666666667	292.06666666666666	330.8
227.5	422.8999999999999	355.8	269.2666666666665
223.0	252.70000000000002	300.9666666666667
265.1333333333333	289.1333333333334	292.9	480.5
360.26666666666665	267.23333333333335	285.1333333333334
	361.5666666666666	579.4666666666667
346.3333333333333	398.1666666666667	398.9666666666667
	348.7	297.1666666666667	323.4	287.46666666666664
	432.6666666666667	389.5333333333333
436.90000000000003	282.43333333333334	198.66666666666666
	296.1333333333334	707.5666666666666
317.1333333333334	517.0	309.93333333333334
246.26666666666665	391.63333333333327	373.43333333333334
	393.0333333333333	367.5666666666666
262.8333333333333	388.56666666666666	274.3666666666667
	429.9666666666667	340.06666666666666	261.8
267.09999999999997	286.73333333333335	375.8666666666673

	220.79999999999995	574.0333333333333	218.0
377.79999999999995	399.0333333333333	410.1666666666667
	242.30000000000004	346.8333333333333
363.8666666666673	330.43333333333334	87.33333333333333
	307.43333333333334	316.7333333333333
449.5666666666666	220.29999999999998	402.5666666666666
	305.3333333333333	273.0666666666666
506.23333333333335	406.43333333333334	497.566666666668
	382.0	237.5666666666667	341.73333333333335
423.6000000000001	627.8000000000001	434.3666666666666
	308.3333333333333	312.7	225.99999999999997
257.23333333333335	268.2666666666665	622.3000000000001
	467.7666666666665	479.5	459.40000000000003
298.5333333333333	320.9666666666664	501.9666666666667
	353.90000000000003	310.5	244.26666666666665
253.39999999999998	273.7333333333333	190.5
216.79999999999998	251.9666666666666	307.9666666666667
	328.59999999999997	288.1333333333334	227.4
224.33333333333334	277.5\
CACYBP	3518.725	2218.225	1057.95	3438.05	2140.825
	4719.8	4314.925	2777.625	2881.3500000000004
	2620.3	2743.7749999999996	4300.975	3520.125
	3386.0999999999995	2495.3	2206.5	3370.4	2826.3
2327.675	2306.95	832.9	3585.3	1585.85	3434.1000000000004
	5361.15	2329.175	2731.2	2329.325	1589.525
	2468.525	4219.05	1766.5500000000002
973.6750000000001	2333.425	3004.425	1904.875
	4404.975	1294.25	2863.725	4992.35
2423.2749999999996	3011.925	1731.75	1788.975
3280.85	2790.2	3607.3500000000004	6838.599999999999
1177.75	2338.8250000000003	2321.7999999999997	3225.175
	3728.95	2639.0249999999996	2265.375	2806.95
2311.4	1916.325	2237.2000000000003	1383.65	3090.15
2029.525	2094.775	1675.1	1579.3249999999998
2113.325	3216.375	1907.5500000000002	1053.25
2406.8	589.15	1577.225	1268.9	3190.475	2154.975
	3227.875	1399.975	3272.8	2780.075
14468.525	2919.725	2816.025	2110.925
1976.725	2023.675	1277.3	2834.9	2417.325
2226.25	1322.5	3010.075	1875.875	2963.625
1541.275	1567.925	2622.8	2074.225	2539.825
	1406.525	1827.025	2069.5750000000003
1955.75	2050.95\
TFB2M	496.575	220.27499999999998	183.62500000000003
310.00000000000006	271.55	572.7	866.925	345.8	377.4
177.125	810.9499999999999	252.575	531.325	254.05
478.92500000000007	354.87499999999994	332.09999999999997
	277.47499999999997	487.82500000000005	276.325
173.675	538.4999999999999	132.82500000000002
380.79999999999995	362.325	293.375	419.12499999999994
149.775	204.775	348.575	1233.375	161.79999999999998
86.17500000000001	430.34999999999997	411.475	197.6
688.6999999999999	159.125	714.475	603.6750000000001
549.225	299.87500000000006	186.35000000000002	169.95

574.475	209.45	680.575	718.25	266.65	425.175	367.99999999999994
	614.6	812.5	196.65	258.67499999999995	421.65
368.475	291.1	275.725	249.89999999999998	243.77499999999998
	224.0	362.57500000000005	382.9	296.75	265.55
711.2750000000001	237.47500000000002	112.125
395.70000000000005	197.05	214.7	226.65	600.9	187.825
684.8	274.45	488.0	631.4250000000001	510.775	292.05
343.1	1088.625	407.55	196.575	390.7	361.075
234.45000000000002	247.29999999999998	126.875	1235.325
	127.775	473.90000000000003	358.34999999999997
322.325	534.9	351.95	536.0500000000001	283.37500000000006
	363.67499999999995	471.54999999999995	252.725
587.8\
ZHX1	595.0	1353.25	857.0	886.95	655.35	526.95	981.5
485.4	627.2	621.8	586.75	528.95	683.0	983.65	586.65
715.25	900.95	885.3499999999999	1304.15	572.5	579.5
513.55	561.0	673.35	464.8	845.1	655.7	851.4	790.5
858.2	972.0999999999999	496.15	330.95	818.25	645.95
958.8499999999999	686.95	395.8	394.95	1140.9	734.25
581.5	831.4	592.75	450.05	839.95	327.15	606.5	255.25
533.85	678.6	748.2	560.35	264.8	573.65	943.8	749.05
585.55	631.6500000000001	718.5500000000001	720.15
487.75	685.95	584.2	661.8	446.65	418.6	655.75	486.2
619.45	619.85	600.8000000000001	761.5	735.25
828.4499999999999	767.25	353.20000000000005	603.5
616.0999999999999	686.6	270.85	407.70000000000005
491.3	1172.0	273.95	251.3	454.9	468.05	411.70000000000005
	468.1	661.8499999999999	493.4	932.5999999999999
	895.0500000000001	906.05	1027.35	1029.55
835.8499999999999	739.55	888.55	895.1	899.05	879.65\
RNF144A	255.35000000000002	33.85	152.1	207.9	454.3
257.4	153.60000000000002	422.85	408.09999999999997
141.39999999999998	370.8	109.9	76.85	115.35	339.6
291.45	396.15	192.0	202.5	210.95	175.2	179.8	212.95
312.4	223.25	215.5	269.40000000000003	436.8	315.5
250.65	444.95000000000005	120.05	176.75	386.65000000000003
	249.9	205.1	168.75	315.65000000000003	220.45
585.95	413.84999999999997	233.4	348.75	249.7
368.15000000000003	245.2	263.04999999999995	315.2	59.7
	194.6	240.54999999999998	148.29999999999998
154.0	324.95	278.1	177.1	173.2	286.65	109.85
113.69999999999999	795.65	454.09999999999997
230.70000000000002	171.15	112.05000000000001	253.95
112.80000000000001	160.5	222.64999999999998	183.4
127.7	204.25	318.25	155.6	262.59999999999997	333.2
184.9	212.5	467.0	725.9	231.35000000000002	264.1
490.75	245.6	133.1	784.4000000000001	373.25	332.95
223.85000000000002	204.7	324.15000000000003
531.4499999999999	133.25	381.8	443.04999999999995
183.5	422.55	265.95	162.0	275.25	274.95	303.95	370.0\
MC3R	7.7	22.7	59.9	77.6	86.1	10.3	1.7	39.8
	51.6	4.2	52.2	49.1	34.1	30.7	34.9	12.3
	5.1	50.8	11.6	15.8	40.4	39.5	115.1	65.6
	19.7	7.1	11.4	19.7	54.6	62.0	70.1	98.9

9.3	20.7	11.7	64.6	12.1	58.3	15.9	2.8
30.2	56.9	13.6	30.8	14.2	12.2	5.7	6.8
69.5	29.8	37.2	20.5	26.0	40.6	25.0	41.3
14.3	15.2	14.2	38.2	122.6	15.6	7.8	5.0
38.2	6.2	50.5	33.2	18.8	88.7	105.0	33.3
8.5	6.9	3.9	42.2	7.1	6.2	2.0	55.0
11.0	21.5	4.9	16.0	56.7	65.4	103.6	79.9
38.8	95.9	3.5	29.9	35.8	6.6	61.2	2.1
10.5	56.7	82.6	7.8	14.3	17.2	16.2\
RNF144B 678.5749999999999	753.825 1682.625	1527.0
618.0500000000001	743.45	3367.425	1364.0750000000003
2021.75 1077.425	1481.075	1851.85 1291.1
788.1	671.8499999999999	4249.45 1150.15 1584.3999999999999
2042.6000000000001	3130.475	1197.2	1702.375
1301.8750000000002	1104.7749999999999	2526.925
940.3499999999999	1385.7499999999998
611.9000000000001	828.675 2637.7	2275.2	526.075 917.7
1447.4499999999998	1392.8	1730.2250000000001
1539.1000000000001	1288.7749999999999	3205.325
718.825 3311.475	633.5250000000001	816.975 2186.4
1411.45 719.1750000000001	1177.4750000000001
3232.0750000000003	1187.2	2929.3250000000003
2372.2749999999996	1919.175	2245.8250000000003
693.6	1027.4	812.6999999999999	1402.225
1649.4250000000002	1756.725	820.1	675.025 1356.125
2133.8999999999996	2464.7499999999995	2168.05
1429.25 2305.9750000000004	1794.4500000000003
830.8000000000001	2165.875	760.125 360.62500000000006
2143.575	1776.45 979.05	1151.7250000000001
1555.875	1546.4499999999998	3205.9750000000004
547.625 2248.7000000000003	1486.375	393.34999999999997
2243.9249999999997	1664.4250000000002	1809.95
3359.9	2281.65 3527.65 427.99999999999994	2336.3749999999995
1003.6999999999999	1556.7250000000001	2407.725
1392.675	2290.6	1866.6499999999999	2820.125
897.15	1673.4	1408.2500000000002	1298.475
2627.7\
KCNU1	7.9	59.9	14.8	10.3	17.4	4.4	7.2	4.2
17.0	36.8	13.7	16.6	58.2	10.5	30.8	6.8
24.3	4.8	9.8	5.5	133.4	34.7	61.0	5.9
6.9	12.1	6.3	78.0	65.5	28.4	26.2	54.8
11.9	7.7	11.6	27.7	36.2	4.1	7.0	4.6
3.7	25.7	5.8	32.9	39.4	2.3	7.1	10.4
102.4	49.2	4.2	48.1	2.2	15.8	2.4	68.5
2.1	6.4	14.3	6.9	228.1	31.0	9.7	8.5
11.6	14.7	6.6	55.4	41.5	49.5	11.8	3.5
5.5	22.4	66.9	28.7	10.6	22.4	56.2	4.7
24.9	51.0	17.6	8.0	97.2	60.7	10.9	11.7
0.9	2.7	3.8	13.3	4.6	2.5	13.9	8.3
74.0	3.8	61.5	50.2	7.0	10.7	7.8\
AC007292.3	43.9	219.4	136.3	52.6	237.6	17.9	45.8
38.5	55.2	120.9	95.1	86.7	65.6	181.2
269.2	120.9	65.0	251.4	96.0	243.8	352.0	126.8
378.6	31.3	124.1	59.2	76.5	232.0	139.4	157.9	44.9

118.7 20.4 13.0 168.6 138.2 84.3 421.8 19.2
92.6 30.7 100.5 206.4 13.9 48.1 176.8
144.8 12.0 288.5 163.3 108.8 152.8 73.2 186.1 9.5
159.4 172.8 292.1 172.1 190.6 206.5 50.7 96.3
109.5 62.6 285.7 17.6 81.7 47.9 72.3
318.7 327.3 154.3 24.0 44.8 139.4 89.0 126.1 8.5
123.5 146.7 226.4 45.9 183.9 133.3 66.5
222.2 22.9 33.4 212.4 77.8 277.8 118.4 151.7
263.4 91.3 57.1 73.1 122.2 108.5 153.8 116.9
189.5\
PITPNM1 205.1 411.1 266.5 186.4 227.5 235.9 235.3
175.4 280.5 541.0 412.6 318.8 386.1 198.7 242.0
438.6 201.9 606.7 275.5 266.1 414.8 417.0 564.2
119.4 294.5 333.0 208.9 267.7 333.4 157.1 120.2
230.2 613.5 211.4 67.2 391.1 35.9 519.7 262.5
874.3 353.5 204.1 198.1 224.3 306.3 307.6 140.3
204.3 366.1 315.7 458.1 591.4 211.5 438.4 352.6
520.1 367.6 330.9 386.9 467.9 664.3 555.6 281.2
479.9 170.0 477.7 211.5 314.3 377.1 458.9 557.3
337.1 230.3 119.8 272.0 300.1 281.7 319.3 238.6
155.3 255.9 240.8 549.8 34.3 671.7 687.5 120.3
265.5 532.8 420.4 132.1 338.6 155.7 246.2 226.2
220.6 217.6 281.4 270.0 304.1 158.6 133.7 226.8\
PITPNM3 373.45 343.3 503.3 67.75 492.5 164.75 247.4
234.85 211.25 230.45 498.25 364.2 311.8 178.85 724.15
391.34999999999997 225.14999999999998 291.2 170.95
299.75 451.9 245.6 337.9 172.2 690.0500000000001
369.6 190.9 201.25 407.5 331.65 194.3 520.0
289.09999999999997 233.35 73.19999999999999 982.9
383.75 181.20000000000002 600.9 162.2 508.79999999999995
168.45 166.4 988.25 127.9 244.2 188.6 189.0
290.9 298.6 318.09999999999997 289.6 334.84999999999997
370.2 271.25 389.95 191.29999999999998 734.1
770.3 221.04999999999998 165.5 501.85 290.6 244.25
507.40000000000003 299.5 166.75 856.1 186.3 262.95
226.2 305.04999999999995 511.55 378.8 267.2
165.70000000000002 202.2 211.6 186.05 66.0
201.60000000000002 169.2 393.59999999999997 307.1
149.35 210.95 348.15000000000003 251.45 340.9 169.8
139.55 391.8 413.6 333.3 505.5 505.75 555.0500000000001
424.9 473.45 491.15 433.7 914.75 427.55\
PITPNM2 43.43333333333333 101.66666666666667
79.06666666666666 74.86666666666666 158.23333333333332
63.69999999999999 55.43333333333334
116.33333333333336 64.66666666666667 120.36666666666667
96.60000000000001 64.76666666666667
93.39999999999999 67.60000000000001 138.73333333333332
90.03333333333335 47.1 149.89999999999998
113.60000000000001 85.13333333333334 245.23333333333332
87.89999999999999 217.6 50.46666666666667
102.3 81.53333333333335 71.66666666666667
65.43333333333334 165.03333333333333 71.2 59.0
142.33333333333331 147.76666666666668 47.86666666666667
90.86666666666667 141.13333333333335 64.0

270.3	52.46666666666667	41.6	79.06666666666666
99.26666666666665	62.4	66.46666666666668
94.83333333333333	135.83333333333334	57.03333333333333
	87.19999999999999	163.06666666666666	118.3
95.60000000000001	93.46666666666665	57.833333333333336
	158.26666666666668	100.13333333333333	159.0
149.33333333333334	57.333333333333336	89.3
152.16666666666666	342.1666666666667	77.10000000000001
	72.2	69.8	82.63333333333334	125.69999999999999
	70.76666666666667	112.26666666666665
140.03333333333333	83.7	103.10000000000001
204.23333333333332	131.3666666666667	29.933333333333334
	89.16666666666667	31.96666666666667
70.06666666666666	78.83333333333333	106.10000000000001
	89.80000000000001	106.66666666666667
126.56666666666665	68.73333333333333	81.1
285.6666666666667	314.53333333333336	129.6
89.13333333333333	161.9	195.63333333333333
73.06666666666666	234.0	74.06666666666666
73.63333333333334	84.03333333333335	75.96666666666668
	107.96666666666665	83.0	111.2	95.26666666666667
	95.3	81.13333333333334	98.36666666666666\
TAX1BP1 4345.466666666666	3270.5	4780.599999999999
3401.2666666666664	3894.7666666666664	3889.7666666666664
	4078.7666666666664	6298.3	2712.5666666666666
5519.400000000001	5296.766666666666	3567.4
3018.933333333333	4379.766666666666	2256.5333333333333
	4068.966666666667	6039.033333333333
5189.166666666667	3101.133333333333	3731.566666666667
	2961.433333333333	3343.7000000000003	2439.3
3404.3333333333335	2707.066666666667	3379.0333333333333
	4479.566666666667	4817.733333333334	3964.5
4204.8	4099.400000000001	3658.3333333333335
2895.4666666666667	5105.733333333334	5548.533333333333
	8226.533333333333	5305.266666666666
3317.1666666666665	3391.2666666666664	4672.833333333333
	2502.1	2064.5	5816.599999999999	4382.166666666667
	1872.8	4621.766666666666	2975.1666666666665
5746.833333333333	2007.2333333333333	3191.6666666666665
	3857.6666666666665	4865.7	3995.066666666667
1764.3999999999999	3536.7666666666664	3335.8333333333335
	4663.133333333333	3984.0333333333333
4943.866666666667	3158.933333333333	4655.033333333333
	5548.866666666666	4394.866666666667
5466.933333333334	4474.233333333334	2957.3333333333335
	3490.9666666666667	4523.766666666666
4543.933333333333	6255.333333333333	2649.066666666667
	4326.233333333334	4000.7666666666664
5006.400000000001	2852.633333333333	5392.233333333334
	5492.466666666667	5313.266666666666	4676.8
4026.9666666666667	3649.1	3751.4	3210.1333333333337
5775.766666666666	1887.0	3563.5	2819.3666666666663
3656.7333333333336	2650.9	5541.666666666667
3652.7999999999997	1991.7666666666664	3946.6

```
3783.766666666664	2952.7000000000003	3372.3333333333335
	3312.6666666666665	4481.8	4701.633333333333
3821.133333333333	2926.5333333333333	4916.400000000001
	3291.5\
KDM4B /// LOC100507022	113.7	1089.1	213.3	70.5	309.8	76.8
	90.3	187.7	125.9	491.4	87.8	325.7	179.8	70.1
	222.4	277.8	163.1	266.1	80.3	527.1	484.3
409.4	335.6	88.2	100.8	519.5	99.0	136.5	29.6	87.9
	104.4	236.1	434.9	90.0	175.6	194.7	93.9
428.8	49.2	123.9	97.9	203.7	288.5	307.3	114.4	89.4
	199.8	8.7	267.2	285.8	203.5	90.7	173.7
503.1	420.0	517.2	231.1	290.4	357.1	695.3	573.8	78.6
	169.7	111.4	344.5	277.3	184.6	163.4	377.4
170.2	827.5	276.8	238.8	115.7	199.7	42.6	275.9
304.5	107.3	144.2	176.0	154.1	95.4	99.2	172.3
354.4	45.2	28.3	81.2	306.2	49.2	189.2	82.7
150.7	231.9	76.2	193.8	200.9	264.8	146.2	229.2
183.4	203.9\
RP11-330M19.1	8.5	140.1	11.9	44.4	32.0	37.2	9.7
	60.0	39.7	83.8	55.3	52.5	5.1	13.9	4.5
	8.6	15.8	37.8	63.5	6.6	191.9	68.7
133.0	8.6	12.5	6.8	29.8	76.3	58.0	20.1	7.4
	11.3	20.0	7.7	30.9	11.3	6.0	4.9	73.6
	72.7	9.7	10.5	6.3	29.3	27.0	7.3	69.5
	9.2	15.0	117.3	13.4	10.3	50.8	33.6	46.5
	100.7	65.2	6.6	75.4	51.0	25.4	27.1	10.9
	54.7	15.9	56.1	16.9	50.0	63.5	8.2	28.2
	35.8	30.6	21.1	29.9	23.8	43.7	8.5	16.2
	8.3	9.6	83.1	52.2	4.1	201.5	26.2	26.8
	19.6	105.1	13.6	9.6	130.1	5.9	6.1	12.1
	15.7	22.7	6.2	49.2	10.6	110.5	5.3	4.9\
LOC100505685	2.3	52.6	3.2	39.8	107.4	65.0	17.9
	11.4	47.0	6.6	38.9	60.7	6.4	35.5	33.4
	44.6	30.7	3.7	66.2	26.4	12.1	74.4	9.2
	28.6	7.5	24.9	2.8	45.0	98.9	100.8	15.9
	70.7	58.1	11.2	8.8	20.6	13.7	32.9	21.8
	16.7	31.6	3.2	49.2	14.3	51.6	7.6	31.5
	28.3	68.6	10.3	55.0	35.2	4.8	13.7	5.3
	9.7	3.2	4.9	9.9	74.8	63.2	2.2	29.2
	61.9	8.0	54.5	10.5	30.8	18.1	38.4	68.2
	17.3	5.9	5.2	38.6	6.9	9.3	37.1	29.7
	27.4	87.8	12.6	17.0	25.9	19.1	134.8	51.5
	72.6	64.0	66.6	22.9	100.9	6.1	29.3	11.6
	18.1	49.3	10.0	71.8	12.6	11.6	9.4
75.4\
FAM193B	138.85	725.5	393.6	146.25	280.45	275.05	122.25
347.0	225.95	290.95	228.55	404.15	220.29999999999998
194.8	405.85	345.85	213.0	337.35	206.15	358.4	1130.35
256.8	508.34999999999997	201.20000000000002
204.85000000000002	713.6	208.1	316.15	408.34999999999997
	365.05	131.8	644.85	579.55	217.25	378.0	501.1
200.8	635.8	256.8	186.5	258.35	324.8	355.2	192.0
170.65	388.0	403.7	148.5	860.95	453.35	415.65000000000003
	256.65	376.8	1025.3999999999999	522.45	650.25
```

350.59999999999997 339.09999999999997 397.75 364.45
626.3 351.4 238.95 238.3 347.95000000000005 349.4
217.0 220.75 580.35 262.5 882.5 358.4 299.2 199.75
236.9 169.35 629.0 217.7 380.85 210.3 386.95 350.8
262.55 419.25 806.3000000000001 943.5500000000001
444.4 663.4499999999999 314.8 336.65000000000003
114.05 814.25 400.5 429.34999999999997 574.4
387.40000000000003 568.65 469.15 588.0 502.5 605.7
490.84999999999997 635.9\
FAM32A 2211.3 1087.8 902.8 2018.1 850.6 2082.8 2344.4
1109.7 2494.8 1546.9 1839.2 1914.0 1722.2 1882.0 1709.7
1300.3 1902.1 1593.0 2755.7 1642.6 1296.9 2208.7 1035.4
2279.7 2902.6 1541.2 2937.2 1104.5 1068.1 1574.3 2321.6
1204.1 1077.0 1805.1 3037.2 1568.1 2701.9 992.5 2156.8
3055.4 2087.0 1795.8 1275.1 2332.3 2874.6 1205.6 1474.9
1590.2 977.0 1132.4 1727.7 1738.4 1330.8 1104.1 1658.8
1070.4 1339.3 883.9 1638.6 969.1 1111.1 1852.0 1582.5
1579.6 1065.8 966.7 1696.7 2972.4 1386.8 1729.9 511.3
1009.6 1521.0 1736.8 1605.3 2508.7 1408.1 1899.9 1449.2
1426.2 1702.7 1895.7 4298.1 1849.1 1120.2 1760.2 1921.1
1284.5 1644.6 1157.6 5405.4 916.1 1785.4 1740.5 1405.9
1696.9 1421.6 1574.4 1544.6 1425.0 1212.3 1039.9 1783.0\
LOC101927815 3.6 67.9 25.5 42.1 81.6 16.7 20.9
90.3 61.4 28.0 10.7 27.4 12.5 46.2
103.8 34.9 40.4 11.5 44.0 77.2 239.5 32.6 75.9
43.1 41.4 45.4 56.8 152.8 8.0 2.7 18.4
203.8 89.9 3.9 145.9 33.9 59.9 6.5 3.6
31.6 38.9 42.6 107.0 42.7 28.7 106.2 21.1
22.5 51.7 86.6 36.5 40.7 3.4 10.6 14.6
87.2 26.1 76.0 25.5 46.2 63.2 6.6 10.8
36.2 15.9 56.0 42.0 6.9 9.7 33.8
114.0 53.5 4.0 8.7 30.8 48.3 42.3 11.7 57.0
4.6 15.3 4.4 74.2 38.1 236.7 28.8 60.5
29.4 12.8 15.6 63.0 3.1 61.8 15.9 66.9
32.8 64.5 24.8 35.9 19.0 8.8 16.9 8.8\
FAM193A 1003.0 1470.9 1690.5 1048.8 1866.4 2011.1 1549.1
756.0 1548.3 1412.1 1743.5 1913.9 1301.9 1771.1 2654.6
2089.9 1484.4 1532.0 1393.3 2002.7 3324.9 1456.7 1555.4
1074.4 1205.9 2444.1 2042.7 2390.5 2628.5 1670.8 1736.9
2334.7 3124.7 2051.0 1178.7 1385.1 922.3 2945.1 2241.8
1151.1 1293.2 2085.9 1592.7 1626.9 1385.0 1059.8 1732.9
536.2 6934.2 2235.8 1467.1 1255.9 1946.8 1857.7 2575.8
3799.8 1648.6 2344.4 1603.6 2202.5 1246.7 2051.4 1804.9
1606.1 2332.7 2145.3 1590.4 1884.1 1755.2 1444.6 2243.2
2194.3 2055.0 1902.8 2161.2 1598.4 1032.6 1502.1 1806.3
634.2 982.3 1040.9 761.2 1600.9 2091.4 2632.3 1413.1
1189.3 1730.9 1990.1 1427.2 1289.3 1098.8 1300.2 1637.2
1126.9 1206.3 1143.6 1316.2 1014.2 1599.4 1312.9 1395.0\
LOC102724905 44.5 103.3 53.4 3.4 7.2 50.2 25.3
30.5 9.3 41.3 40.0 63.5 26.8 24.6 91.1
24.7 27.2 80.8 43.3 36.6 35.3 52.8 50.0
36.4 22.8 26.8 52.0 156.3 93.5 14.6 4.9
137.8 7.2 32.5 99.6 61.3 56.6 4.5 13.0
36.2 61.1 24.8 70.5 34.2 23.1 28.9 49.2

23.8	42.2	10.7	129.0	61.0	36.8	5.0	88.6
60.0	76.8	43.6	16.9	7.9	348.8	55.5	1.6
30.4	11.6	26.5	44.7	63.3	83.4	10.0	63.4
4.2	32.9	82.2	12.6	40.7	9.0	26.8	39.2
59.9	7.5	48.8	71.1	52.2	70.5	164.0	61.3
91.0	4.4	77.3	5.5	109.8	52.7	8.1	40.7
47.0	23.0	45.0	97.9	11.4	20.2	71.0
21.8\
LOC101927820	5.1	73.2	7.2	14.9	4.0	4.4	1.5
3.9	7.9	20.0	4.4	1.8	21.8	60.4	54.5
123.8	6.4	11.0	25.2	12.5	143.3	5.8	10.2
5.5	25.8	7.0	4.8	83.6	3.4	50.9	3.6
16.7	12.0	3.8	5.3	27.3	1.9	9.5	4.1
3.8	62.9	23.7	99.1	18.5	5.1	6.5	29.5
37.5	46.7	18.4	10.3	9.6	2.4	13.0	37.0
36.9	9.8	7.1	5.5	14.3	17.9	5.0	5.6
3.9	2.9	7.0	42.4	1.7	10.1	3.0	20.5
4.5	38.0	7.1	6.5	5.1	3.2	7.2	29.0
3.3	4.2	12.8	73.0	43.2	7.9	9.7	6.6
132.6	7.5	15.5	2.2	45.9	8.6	21.6	3.2
104.3	70.8	34.9	61.9	24.8	47.7	5.2
76.4\
CCDC142 /// MRPL53	1459.9	706.4	1166.1	2362.8	1510.7
2870.6	2555.7	767.9	1205.7	1207.0	1583.0	1827.9	2219.8
2748.0	1458.3	1139.6	1452.8	1353.6	1947.2	1361.4	1404.8
1466.5	730.9	969.9	1167.5	2036.0	3790.6	1310.2	1182.7
1314.4	2596.0	999.6	1155.0	1761.0	3483.6	2840.8	1477.5
946.6	2045.9	1660.4	1832.3	1328.5	1185.9	1450.7	1872.1
1540.2	1121.9	1752.1	2031.1	1793.5	1912.5	2139.0	4252.4
1307.0	1332.9	707.3	1418.4	1050.1	2653.0	772.6	562.5
2312.5	1745.9	1249.3	1169.6	1886.4	2136.9	3165.9	956.0
1862.6	481.5	783.0	1119.2	940.6	1174.2	2028.6	885.4
985.4	1551.5	1001.7	479.9	441.6	2295.7	1118.7	940.3
911.5	753.5	540.8	1383.9	2096.7	1711.6	925.1	1699.7
1807.6	1705.8	2141.4	2198.6	2485.4	1921.8	1607.1	1630.9
1304.4	2797.8\
LOC101927824	6.3	32.5	189.3	38.4	40.2	120.7	17.6
28.7	44.9	22.1	69.7	49.6	12.2	33.8	20.6
77.2	13.1	33.8	66.3	44.1	103.0	11.3	47.9
21.6	24.5	21.0	8.4	109.9	164.0	21.4	22.9
138.5	33.3	11.1	20.1	46.7	7.7	32.9	53.3
14.8	105.8	41.0	25.6	25.2	17.7	13.9	21.3
6.8	79.0	17.8	9.7	42.2	14.3	217.5	25.2
43.6	94.2	22.2	14.1	153.3	59.9	29.7
108.6	21.8	28.3	56.0	24.7	59.8	108.9	21.2
298.3	32.8	25.3	21.0	89.4	9.5	33.2	42.7	27.9
8.4	100.3	18.6	538.7	18.7	115.0	38.6	25.9
23.8	123.4	189.0	96.9	197.9	88.3	52.3	59.8
21.4	33.8	199.9	93.0	27.6	94.3	213.8
157.7\
BC024027 /// RP11-268G12.1	9.2	107.9	8.0	51.0	39.1
48.6	87.7	10.6	9.3	156.8	59.2	655.5	24.0
129.7	420.3	8.9	18.2	18.1	28.8	70.4	93.2
6.7	53.1	55.6	19.5	10.7	7.0	25.8	15.8

9.2 35.2 19.6 11.5 23.2 23.4 19.7 20.8
115.8 12.6 33.4 48.7 60.4 16.5 9.0 4.5
13.8 7.6 52.7 16.2 50.9 6.7 102.7 6.4
86.6 76.9 14.5 16.7 16.5 47.2 60.5
102.1 104.2 116.1 37.7 77.4 76.9 21.1 10.1 4.8
12.8 16.2 16.2 18.6 12.5 15.3 11.4 12.4
9.9 12.9 93.8 61.0 9.3 62.4 155.5 11.1
151.1 7.0 19.8 31.9 525.1 48.7 26.3 40.1
8.9 7.8 12.4 15.5 9.0 55.6 100.9 7.9
12.1 6.6\
RPS17P5 /// RPS17P5 554.2 249.6 567.4 615.1 241.6
817.7 398.4 422.0 729.7 407.2 963.8 376.5 425.8
627.2 627.3 810.0 513.3 1196.9 423.9 562.3 298.0
461.0 557.1 769.9 468.5 338.8 640.8 657.6 638.8
827.7 684.4 434.8 383.6 900.4 505.9 259.0 658.1
596.9 475.6 568.4 577.4 364.9 617.3 465.8 414.8
442.6 403.3 603.3 351.3 421.8 807.8 701.2 848.3
458.2 536.3 287.6 696.8 889.9 629.5 418.5 696.6
397.1 558.1 394.3 366.4 355.6 447.1 390.0 639.5
546.6 432.9 535.4 444.5 725.4 582.8 953.3 566.4
570.7 579.5 541.1 620.3 734.5 616.7 762.2 690.9
368.6 480.1 440.9 519.9 499.5 589.5 642.1 1175.5
768.1 717.7 1301.6 847.4 1049.9 1051.3 1203.2 777.5
681.1 923.7\
SSNA1 602.8 664.8 316.0 944.2 342.0 943.2 347.3
357.1 790.3 631.8 502.3 1005.7 684.1 464.0 849.6
350.4 425.2 718.2 499.5 416.5 231.8 475.0 487.0
494.5 436.3 496.9 1016.5 348.8 443.0 685.5 307.3
465.0 382.4 643.3 621.3 813.5 465.4 487.0 581.7
453.1 420.6 569.7 420.6 526.2 741.1 657.1 702.1
500.0 349.5 592.9 719.7 621.4 522.9 334.7 553.9
558.4 736.9 552.8 709.5 497.1 475.1 622.7 519.9
521.6 506.6 823.6 436.2 464.8 709.6 366.2 328.6
396.6 412.0 408.6 474.7 780.9 544.8 548.8 563.3
339.7 441.0 484.4 361.4 298.2 431.9 669.2 334.0
580.7 743.8 352.8 1020.8 252.0 407.7 421.8 269.5
301.6 230.8 388.8 459.9 391.9 338.2 532.4 436.2\
FAM35A 4453.8 3217.5 2579.3 3673.6 3732.0 5284.0 1363.9
3396.6 2122.9 2411.6 4665.1 2812.0 2944.2 3155.2 1529.0
4131.1 5375.8 2839.3 4241.3 3890.5 4161.7 2707.8 2007.1
1662.9 1826.5 7544.7 11541.2 4293.3 4518.2 3824.7 3036.3
4428.1 4201.6 4135.6 1482.7 2073.9 3252.9 3032.6 1892.0
3245.5 1365.0 3960.5 5020.0 2574.5 3749.3 2408.9 5216.5
3104.3 7869.6 2284.4 3257.9 1991.1 4554.3 2775.6 2475.4
2227.8 1747.3 2782.9 3047.4 3331.1 3608.3 2475.0 1496.5
7374.5 3883.0 2766.7 1369.9 1411.4 1321.3 4294.1 2262.5
2560.0 5815.6 5717.8 2018.6 4017.6 2559.9 2631.5 3044.6
5345.6 9061.1 2018.5 2583.7 2205.8 4019.8 4747.6 2736.8
2058.7 2110.4 4312.1 2312.5 3363.2 5442.2 5310.4 3755.4
3933.6 7198.2 6054.2 6297.5 8214.1 2576.4 8751.0 5743.2\
NEXN 215.0 209.14999999999998 186.85 494.35 293.05
128.25 154.45 651.75 242.95000000000002 141.55 60.1
398.15 219.10000000000002 234.1 120.85 144.3 868.25
162.9 84.65 212.10000000000002 500.7 132.3 146.25

1396.35 127.1 39.25 118.3 190.5 164.55 166.7
449.90000000000003 172.5 232.29999999999998 210.45
201.35 91.7 95.6 324.8 71.6 325.0 114.80000000000001
68.30000000000001 316.5 258.45 87.80000000000001
126.1 141.5 148.45 177.05 131.1 242.95 151.6
35.65 557.95 229.8 87.30000000000001 139.6 144.3
124.3 209.75 258.5 260.0 65.25 68.95 141.45 143.1
136.05 159.5 145.4 93.65 282.45 275.75 141.1 260.7
128.6 69.05 166.05 130.35 99.55000000000001 359.5
212.64999999999998 195.9 28.799999999999997
256.45000000000005 416.95 990.25 103.9 255.6
186.35000000000002 458.45 86.25 975.5 306.15 591.8
585.3499999999999 79.0 107.0 192.65 153.60000000000002
135.75 89.7 637.3 24.200000000000003\
LOC101930608 /// LOC102723581 41.6 86.7 250.7 74.7
476.0 138.0 38.3 257.4 236.7 71.7 254.7 261.8
174.9 73.7 200.3 299.4 216.7 109.1 65.5 134.7
874.8 49.6 129.7 44.5 30.8 214.7 79.1 433.9
255.3 332.8 272.2 12.0 220.0 149.8 168.0 158.1
163.3 77.7 152.0 53.6 52.8 188.3 196.3 294.8 80.3
150.9 216.3 55.4 346.3 756.1 88.9 70.3
207.3 175.7 108.7 219.8 241.0 97.4 111.0 269.2
627.8 189.1 210.6 177.4 734.0 324.0 352.3 628.0
226.3 123.7 317.4 255.6 600.1 43.0 178.3 119.9
333.3 101.9 186.5 57.7 389.1 83.5 48.7 114.3
852.1 57.6 61.8 183.0 71.2 158.1 61.8 288.3
366.8 207.0 247.2 349.2 300.9 261.7 404.5 308.9
298.6 150.9 270.6\
RP11-1277A3.1 51.4 222.4 60.1 55.8 233.5 174.2 20.3
182.5 97.6 86.2 88.9 99.5 94.1 177.4 79.2
97.0 302.6 23.5 112.2 143.0 599.5 46.2
297.6 84.4 27.4 74.9 12.0 14.2 72.3 47.3 11.0
227.0 130.1 55.0 125.9 19.1 20.9 35.9 75.5
110.8 123.6 224.5 20.8 61.2 72.1 36.2
158.0 78.0 584.3 208.9 163.4 24.7 50.4 408.2 67.4
199.6 153.4 101.0 107.3 8.5 62.6 187.4
105.5 70.0 238.5 329.5 164.6 192.7 14.8 78.3
291.1 271.9 39.2 87.7 84.7 80.3 117.9 76.1
183.4 98.9 202.2 71.1 221.1 153.4 232.3 231.8
278.0 38.0 157.0 272.6 50.4 92.4 112.2 156.8
181.7 247.8 210.2 7.6 218.3 363.4 249.5 133.8
179.4\
RP11-1277A3.3 55.8 148.0 78.0 72.1 93.0 76.1 67.3
60.2 29.3 19.5 51.9 73.9 6.8 51.1 14.8
26.2 17.7 86.9 24.1 127.3 12.3 75.1
181.9 36.2 10.4 75.8 70.6 9.1 65.0 9.3 80.1
24.7 14.3 69.7 20.2 14.5 59.2 20.2 49.5
37.4 74.5 42.3 63.5 53.1 75.9 14.8 96.6
42.2 128.3 197.7 92.0 31.3 132.6 85.2 28.0
45.2 46.3 65.5 97.2 141.2 329.3 134.5 61.0
64.4 94.1 112.6 54.1 40.7 101.2 73.7
194.0 156.6 58.9 30.7 40.6 47.3 128.3 30.1 87.9
58.0 77.4 24.6 22.3 110.1 164.7 154.2 81.8
238.8 119.0 7.2 63.8 112.0 133.3 46.3 76.0

130.5 104.9 101.2 66.5 154.5 92.7 53.9
99.2\
FAM196A 5.1 8.2 18.8 3.1 2.5 54.8 56.9 5.7
1.9 75.9 17.4 26.4 89.5 3.7 6.2 31.8
3.2 33.2 6.1 29.0 87.6 23.3 2.6 4.3
20.5 41.3 4.7 13.2 8.8 1.9 1.2
101.0 33.0 43.8 40.4 9.3 5.0 7.2 4.0 33.8
39.7 19.0 49.3 1.6 4.3 101.8 23.6 1.6
114.8 8.3 4.1 3.9 5.9 12.8 24.5 4.5
12.8 3.6 4.7 65.9 370.7 34.7 47.5 9.7
4.4 51.6 3.4 5.2 3.0 18.2 5.9 45.6
60.9 1.5 7.9 5.5 5.5 9.6 40.6 41.0
7.7 31.6 3.2 2.1 5.1 16.2 14.1 91.9
3.4 29.0 29.4 9.3 2.1 1.4 4.4 28.6
51.2 2.5 35.1 0.7 50.3 5.4 4.6\
LOC101927829 1.6 4.7 32.8 3.7 9.1 2.3 1.5
70.7 4.9 8.7 3.4 4.3 21.7 1.6 8.1
7.1 17.2 1.9 5.6 4.8 7.2 6.1 9.9
24.9 3.8 2.7 2.1 7.3 7.6 6.5 1.5
5.6 5.2 9.6 3.9 9.3 2.8 1.7 3.8
3.2 2.0 1.8 32.8 1.3 4.0 9.8 3.4
1.7 13.2 9.0 2.8 5.3 5.6 11.3 26.8
8.9 5.5 5.3 3.5 3.0 13.7 2.4 5.2
16.8 3.2 3.3 3.5 5.1 4.9 8.2 51.5
9.1 3.4 3.6 20.3 4.1 12.8 5.1 4.6
4.1 5.7 4.9 1.6 2.5 5.0 5.8 8.2
12.1 1.9 13.7 0.9 5.3 2.1 1.7 2.9
16.2 1.5 1.5 65.0 5.2 5.5 4.0
32.2\
PEX16 575.8000000000001 799.3000000000001 537.4
834.9666666666667 588.5 959.9333333333334
1001.0666666666666 619.5 1177.1333333333332
1003.3333333333334 908.0 647.1333333333333
538.4666666666667 1016.6999999999999 688.5333333333333
589.0333333333333 993.9333333333334
808.1666666666666 782.5 690.3666666666667
899.4333333333334 925.8333333333334 985.6999999999999
803.9666666666667 432.93333333333334
688.4333333333334 804.1333333333332 930.3333333333334
598.1999999999999 740.2333333333332
717.9333333333333 894.8000000000001 713.7666666666668
685.1 643.0 1041.9666666666667 849.8666666666667
776.7333333333332 817.3333333333334 853.5
661.0 815.2666666666668 509.3999999999999
734.1666666666666 890.4333333333334 730.3000000000001
515.9 617.0666666666667 594.6333333333333
676.0333333333333 939.7333333333332 977.1
859.0999999999999 494.23333333333335 653.0333333333333
617.3666666666667 918.7333333333332
979.7000000000002 1248.8666666666666 541.4333333333333
615.4 1218.0 650.9 878.6333333333333
637.0333333333334 772.9666666666667 888.6
730.9666666666667 857.6666666666666 707.3333333333334
656.4333333333334 690.5333333333333

677.9333333333333	1230.3999999999999	548.2666666666667
	906.1	668.1333333333333	793.9333333333334
734.8333333333334	368.3	324.59999999999997
278.8333333333333	1175.1000000000001	683.2666666666668
	411.90000000000003	904.0666666666666	292.2
579.7666666666667	1014.7333333333335	736.1
634.9666666666667	423.7666666666665	641.0
658.4666666666666	650.4666666666666	665.6333333333333
	899.6	659.1333333333333	896.7666666666668
935.9	741.2999999999998	1084.6666666666667
848.6333333333333\
AC018766.6	6.3	6.7	8.4	5.2	93.1	2.3	9.7
	33.2	24.1	9.5	26.6	10.3	23.9	14.8	95.0
	12.9	6.1	44.9	24.2	13.1	37.3	76.6
127.3	3.9	86.6	46.1	56.5	6.0	16.0	83.8	2.0
	10.8	54.7	26.9	31.3	26.7	34.6	13.7	6.1
	55.3	4.8	11.9	6.9	13.4	24.6	5.4	7.1
	7.6	387.4	9.3	5.6	20.8	58.2	30.4	12.9
	5.7	10.8	6.0	21.3	7.5	73.3	32.8	16.7
	5.2	7.4	52.7	3.8	59.2	96.8	5.7	17.6
	119.0	17.4	6.2	16.5	9.2	10.0	69.3	34.5
	5.7	62.6	14.8	48.0	34.4	21.5	17.8	6.6
	118.8	56.9	13.2	9.1	36.1	14.7	8.4	15.6
	18.8	38.7	49.1	8.0	17.8	34.1	43.4
14.8\
PEX19	438.5	289.25	298.95	452.20000000000005	176.6
1595.25	811.7	596.8	533.65	647.55	1043.6	281.8	885.0
883.05	1467.1	533.25	663.45	266.95	655.75	255.6
82.35000000000001	565.15	270.29999999999995	651.25
414.54999999999995	306.5	533.3	152.75	273.20000000000005
	577.2	849.1999999999999	127.7	103.2
515.4000000000001	627.8	487.8	1287.9499999999998
107.80000000000001	781.75	953.5999999999999	656.7
436.2	217.9	369.0	601.9	330.15	469.4	914.55	145.5
512.3499999999999	546.2	582.9	599.1	225.35	256.45
354.25	622.6500000000001	147.7	373.1	114.7	437.35
529.3499999999999	628.05	407.4	589.75	339.04999999999995
	1068.55	681.75	416.8	465.75	84.55	124.1	281.85
873.3	493.35	936.3000000000001	386.85	525.4	746.3
881.65	213.0	306.2	779.8	826.25	197.25	253.85000000000002
	311.1	374.95	311.2	299.15	1010.8	143.55	634.25
645.7	514.75	629.3	567.3	555.9	334.8	292.85
473.79999999999995	324.25	661.7\
KLHL1	42.75	13.7	23.75	4.55	32.0	27.45
5.550000000000001	39.7	9.700000000000001	6.65
39.35	35.6	4.2	11.15	26.450000000000003	12.25
13.700000000000001	23.599999999999998	7.1	12.0
41.800000000000004	43.6	43.25	3.95	10.549999999999999
	32.4	3.45	82.85	101.2	18.45	9.5	105.0	42.2
	26.45	14.15	12.95	20.599999999999998	25.95
36.150000000000006	29.1	7.05	7.8	19.049999999999997
	43.4	43.3	7.0	20.7	23.200000000000003
17.25	28.7	7.35	6.85	7.0	8.05	33.65
22.200000000000003	42.949999999999996	49.699999999999996

28.15 12.65 34.9 11.8 25.45 10.75 9.3 10.6
40.599999999999994 40.5 9.6 5.85 55.85 80.1
22.45 4.25 44.900000000000006 27.85 12.7 7.4
6.949999999999999 9.25 52.85 20.5 14.2
40.349999999999994 121.39999999999999 60.95 7.65 61.6
65.25 22.85 23.900000000000002 32.15 24.35 5.85
8.200000000000001 11.25 7.95 10.25 24.1 12.6
21.75 45.25 7.7\
BC028044 62.6 59.0 67.2 51.3 165.0 78.2 15.2
143.9 55.2 79.4 36.7 8.0 26.8 38.7 38.0
31.7 48.6 15.8 61.7 16.0 27.1 50.2
127.6 63.6 59.3 7.7 53.7 159.4 133.7 6.4 43.0
225.7 130.1 143.1 215.1 38.0 55.2 59.7 52.2
181.1 34.4 110.4 82.0 9.5 17.8 103.9 86.7
49.7 22.3 59.4 75.5 54.0 43.5 58.3 2.0
119.4 103.0 117.1 26.8 71.9 247.3 44.4 56.8
71.2 49.8 5.9 50.3 55.6 95.9 61.3
224.1 105.6 60.2 52.9 62.8 37.7 85.8 39.7 26.5
98.2 79.1 67.5 96.5 60.0 185.3 146.2
127.7 32.8 15.5 74.7 5.3 90.7 70.3 32.4 37.4
90.9 78.4 105.3 54.6 78.4 55.5 42.7
73.7\
KLHL2 1630.1 1888.2 3022.1 1691.2 1770.7 575.2 2726.1
1384.5 1535.0 1691.0 1949.9 1482.7 804.9 929.6 1265.7
3605.9 2050.7 1474.0 5218.3 2683.5 1724.2 1540.7 1600.1
1892.0 1802.8 8045.3 2442.8 2610.0 3181.1 2872.7 2666.1
2424.0 2292.8 2061.4 571.8 2354.3 1136.5 1198.7 3751.3
613.3 1587.9 2811.0 2107.3 3690.5 1303.9 1504.7 2746.6
1239.8 1269.4 2387.0 1797.8 1786.0 1952.1 1592.9 2615.2
4041.0 3190.3 2540.5 2523.5 2741.2 1775.6 2334.3 1319.3
3143.6 4141.1 2089.2 2401.9 2850.8 1480.0 1927.6 1416.5
2169.5 3907.9 2082.8 2498.6 1787.1 2226.4 2602.6 3359.1
1622.3 1207.8 3145.8 1734.4 3869.8 1533.0 2040.7 3457.0
1268.8 841.0 1408.4 2089.4 2441.0 1556.3 2737.0 1587.4
1783.0 2041.7 2445.4 1828.3 3087.5 1490.3 5501.3 1751.7\
KLHL3 758.4 292.7999999999995 1197.6499999999999
949.6500000000001 2360.0 64.4 561.6 747.95 371.5
310.79999999999995 363.2 703.15 306.05 225.5 1049.0
771.1 1287.0 2139.7000000000003 65.0 1062.75 1887.8
1380.6 1664.2 508.1 789.8499999999999 1071.4 751.1
2186.2999999999997 2108.25 679.35 540.45 1660.8500000000001
1689.45 1342.1 151.85 1703.4 396.05 3574.75 1630.95
279.0 1173.5 892.15 989.9499999999999 515.1 686.5
2183.2999999999997 1486.3 216.70000000000002 5728.6
1193.6000000000001 151.64999999999998 626.1999999999999
633.2 2513.25 1290.75 460.6 507.45 3203.4 345.3
2398.8 724.3000000000001 2655.6 783.5 672.6
299.84999999999997 524.1 761.65 518.7 747.6 1760.4
2989.0 1425.55 1124.15 1217.1499999999999 2209.65 408.05
670.8 1309.2 1404.75 601.6999999999999 596.5 1232.8
990.3000000000001 477.15 816.0 3729.0499999999997
2026.5 683.4 245.95000000000002 1395.3999999999999
208.75 2790.65 667.3000000000001 523.15 849.5 712.15
1330.75 441.09999999999997 1106.5 1131.55 1209.25

670.3000000000001	868.75\
PEX10	95.65	164.05	168.14999999999998	282.15	132.15
198.85	139.05	242.25	311.65	207.15	233.5	236.7
254.54999999999998	283.85	236.2	248.4	143.25	318.35
258.35	232.1	276.25	214.2	504.7	168.7	96.35	324.5
298.8	181.7	243.9	149.75	194.3	227.7	278.75
196.64999999999998	542.35	317.7	179.45	373.5	177.1
194.5	234.75	116.8	188.6	233.0	226.10000000000002
399.75	343.7	316.95	385.95	309.59999999999997	243.95
153.95	217.35000000000002	200.05	219.75	53.5	342.45
220.65	248.3	296.9	196.29999999999998	473.84999999999997
163.25	226.14999999999998	305.0	334.29999999999995
164.29999999999998	252.54999999999998	396.15
131.3	260.6	237.79999999999998	166.8	145.35
121.85000000000001	165.6	428.8	270.2	177.6	78.7
389.95000000000005	216.35	216.7	167.1	443.85
476.95000000000005	208.5	382.6	358.9	335.75	237.1
199.85	344.95	207.35	250.4	301.35	381.25	292.75	568.25
386.05	276.25	214.8	257.75\
KLHL4	17.1	37.6	6.95	13.299999999999999	16.75
6.6499999999999995	17.25	9.25	20.799999999999997	5.05
11.75	4.7	6.800000000000001	18.700000000000003
37.0	10.3	41.0	8.55	13.700000000000001
23.45	24.75	31.700000000000003	79.65	12.850000000000001
9.8	35.199999999999996	21.549999999999997
14.05	42.2	108.55	69.65	9.2	59.650000000000006
51.599999999999994	28.65	8.9	32.55	21.75	4.9
18.15	16.55	10.5	12.9	24.6	2.35	51.55	30.25
11.85	46.599999999999994	29.349999999999998	15.3
10.05	14.55	43.65	19.4	44.25	10.25	15.25	37.35	14.9
54.4	33.2	13.7	19.05	73.0	62.0	9.9	4.7
6.45	7.0	87.9	20.150000000000002	49.0	27.1
61.6	20.8	7.25	6.45	42.05	178.14999999999998
13.450000000000001	19.1	21.9	42.25	27.65
33.199999999999996	32.0	10.7	7.4	11.799999999999999
3.85	47.150000000000006	18.9	13.5	7.1	21.7
30.05	38.6	16.0	10.2	7.0	74.30000000000001
8.55\
KCNRG	147.5	251.1	931.4	275.7	213.3	117.3	263.2
216.7	411.2	606.2	735.7	334.7	136.3	168.4	22.7	89.5
154.4	1281.8	238.1	95.1	472.8	645.6	1737.5
243.0	369.0	177.6	386.1	1314.4	1209.3	226.8	108.3
603.4	558.3	700.2	167.5	368.7	127.2	108.4	155.3
256.9	65.9	195.1	1123.9	110.0	264.3	676.9	391.0
303.8	989.1	441.1	959.7	376.0	149.9	297.7	419.9
772.8	992.5	446.1	649.6	830.3	501.7	1001.0	164.9
1040.9	244.4	18.7	571.2	158.4	868.1	2126.7	2521.9
706.3	198.3	265.4	906.3	627.8	1219.3	251.1	425.5
228.8	412.7	174.8	135.8	105.3	511.5	174.7	159.7
187.1	172.6	145.5	99.9	395.3	182.6	317.3	213.1
458.1	240.5	969.2	357.0	482.0	17.9	151.2	141.5\
PEX12	837.4	440.3	300.7	562.4	463.8	603.4	786.4
382.4	721.5	441.0	617.2	780.9	475.0	623.3	449.9
775.8	532.2	623.4	867.5	428.3	249.5	729.5	632.4

787.1	826.7	550.9	633.8	513.7	650.3	757.5	870.0
503.8	298.8	722.2	750.0	777.1	653.8	417.7	507.3
450.7	602.4	461.6	698.1	531.7	730.3	517.1	445.1
436.4	652.3	506.3	565.7	520.9	541.8	269.5	470.2
636.7	585.6	507.4	479.3	283.0	850.9	409.6	577.1
603.3	568.8	436.6	591.9	614.0	315.6	819.0	291.1
400.4	651.9	938.4	615.9	781.3	402.0	500.5	423.8
467.2	400.1	484.9	634.4	522.4	228.0	310.1	450.4
578.5	565.0	335.9	692.6	571.8	826.2	923.7	604.9
972.4	728.7	804.4	334.8	868.3	847.0	589.2	882.5\
PEX13	1633.58 734.74 727.5600000000001	637.6800000000001
	859.3199999999999	916.76	983.1199999999999
1724.2	934.82	940.8399999999999	1065.54 736.5600000000001
	655.7800000000001	1358.3200000000002	766.8
585.0799999999999	1151.8600000000001	1110.76 777.96
794.24	401.61999999999995	1237.72 534.0	931.6	1602.58
830.9399999999999	893.4	680.6800000000001	505.98
818.6	966.8	1080.4800000000002	306.66	574.0200000000002
	897.4599999999998	727.1199999999999	1078.92
621.98	772.0	1095.6000000000001	861.5200000000001
902.86	701.5999999999999	721.9	1082.6	1016.4799999999999
	1458.3799999999999	1118.86 578.9000000000001
622.8800000000001	963.5400000000002	1040.8	1233.9
1082.0000000000002	682.46	385.52000000000004
495.2800000000001	753.16	1107.48 383.26000000000005
470.19999999999993	560.46	866.0199999999999	1033.54
757.9399999999999	652.5200000000001	1007.3
723.5600000000001	362.64	758.56	332.96	491.75999999999993
	410.84	1138.84 604.4399999999999	1019.1200000000001
	970.4599999999998	896.9400000000002
621.4200000000001	1759.36 988.1600000000002	1160.92
528.1	740.1600000000001	718.9199999999998
837.3399999999999	977.0600000000001	795.22
844.6600000000001	882.4399999999998	812.4	697.0
618.56	458.6	388.31999999999994	685.9599999999999
540.74	610.74	419.56000000000006	631.98	654.96	756.28
572.52\
PEX14	288.46666666666664	227.33333333333337
235.1666666666666	313.7333333333333	347.59999999999997
	350.3666666666666	270.5	385.46666666666664
340.40000000000003	345.3666666666666	280.1333333333334
	393.73333333333335	294.3666666666666
325.50000000000006	452.8666666666673	321.03333333333336
	227.39999999999998	497.70000000000005
467.2666666666665	300.53333333333336	351.90000000000003
	479.76666666666665	282.06666666666666
376.13333333333327	470.20000000000005	364.6000000000001
	485.06666666666666	351.06666666666666
436.1666666666666	338.59999999999997	310.46666666666664
	572.5666666666666	427.8666666666666	390.2
448.2333333333333	390.8	337.40000000000003
537.3333333333334	195.5	262.7666666666667
356.40000000000003	294.06666666666666	360.0333333333333
	274.8333333333333	277.73333333333335

428.3666666666666	418.7666666666665	359.3333333333333
	340.23333333333335	411.7	354.3666666666666
411.2	375.79999999999995	376.9333333333334
450.7666666666665	623.8333333333334	625.2
402.1666666666667	423.49999999999994	425.3666666666666
	708.7000000000002	483.59999999999997
301.0666666666666	354.8666666666673	374.8666666666666
	434.09999999999997	300.90000000000003
310.6666666666667	509.3666666666666	380.0333333333333
	311.4	423.90000000000003	306.1	310.73333333333335
	329.1333333333333	454.7666666666666
524.3333333333334	290.03333333333336	431.5666666666666
	359.23333333333335	761.9333333333333
533.2666666666667	346.6333333333334	630.4666666666667
	703.5666666666666	960.1666666666666
573.8000000000001	773.9	438.6666666666667
487.23333333333335	353.8	484.8333333333333
469.9666666666664	490.8	557.8000000000001
448.40000000000003	401.6666666666667	615.5
599.8333333333334	579.1666666666666	405.5666666666666
	413.59999999999997	475.93333333333334\
KLHL9	1021.8000000000001	545.775 805.425 777.8249999999999
	667.2500000000001	748.8500000000001
785.2499999999999	461.67499999999995	857.075 586.8
1068.375	644.775 340.7	722.125 732.0000000000001
827.75	750.9749999999999	556.4	687.05	664.05	667.65
585.325 515.425 681.2249999999999	696.2249999999999
627.65	842.625 678.45	872.5250000000001	663.6750000000001
	1162.1999999999998	473.20000000000005
464.4000000000001	891.725 893.025 545.275 692.3000000000001
	537.275 753.15	677.925 648.6750000000001	496.425
786.225 729.5999999999999	829.225 482.4	578.075
767.3000000000001	584.0	748.4	636.8499999999999
801.125 658.15	501.85	687.2249999999999	394.59999999999997
	602.5500000000001	604.625 679.1	516.425 637.35
643.0500000000001	669.025 805.75	1000.2	728.0250000000001
	804.4	780.7249999999999	535.075 840.275 471.5
355.475 762.5250000000001	478.7	885.725 1131.9	485.5
748.325 870.1	613.65	602.6	342.325 825.95	889.275 641.925
621.8	545.8000000000001	662.6000000000001	708.0
763.6500000000001	1204.575	449.375 1001.9000000000001
	1023.9000000000001	672.7499999999999	1116.7
879.6999999999999	1010.0250000000001	541.0	784.8
862.8499999999999	618.8499999999999	1183.125\
ETAA1	1114.6	794.6	905.7	1050.1	684.5	441.8	548.7
485.0	547.2	462.6	722.1	516.9	530.1	523.7	974.0
773.7	622.8	446.1	1984.7	656.3	853.0	592.2	938.5
292.9	778.2	1105.2	663.7	553.6	1164.4	224.0	927.1
718.0	492.2	657.5	412.3	1235.7	526.6	975.1	581.5
436.6	588.6	581.9	623.8	927.2	547.7	1534.9	462.6
739.6	2496.3	667.4	736.9	491.0	1130.4	878.6	821.1
982.4	731.0	1795.1	1145.8	818.6	632.6	595.1	655.3
617.2	877.4	567.6	426.3	771.8	631.8	1271.4	765.1
758.8	670.9	803.3	648.5	408.5	498.8	209.3	632.1

526.8 214.1 1335.5 433.5 476.5 735.7 782.5 1283.4
531.4 434.9 1248.2 67.5 849.3 1378.5 1388.8 928.3
2322.2 1875.9 886.8 1034.9 2547.6 1545.3 1488.6 1088.2\
TFAP4 124.4 179.4 79.6 28.2 24.5 46.5 89.9 91.0
29.4 49.3 30.0 40.8 132.6 70.3 294.2
108.4 170.0 130.5 145.3 133.2 87.0 120.4 242.8 83.4
162.9 31.0 54.7 46.6 32.9 52.8 24.5 21.9
30.9 85.5 118.4 100.6 64.0 49.9 31.1 75.0
111.3 94.0 10.4 29.3 165.3 101.1 179.0 61.1
32.1 101.8 86.5 87.8 27.2 58.6 90.0 22.0
16.3 31.5 67.9 41.2 98.3 26.7 96.3 9.1
83.6 83.4 114.0 125.9 36.5 73.4 78.5 51.0
11.5 97.8 98.6 97.8 120.4 11.5 47.0 78.6
241.0 24.0 52.2 10.2 28.3 29.9 176.3 48.7
173.3 44.6 117.7 68.4 186.6 8.0 25.7 68.4
111.1 88.9 79.5 132.4 11.0 25.0 59.3\
KLHL5 1006.725 159.65 574.9 878.5 575.45 452.475
462.62500000000006 875.7 524.775 400.32500000000005
710.05 306.42499999999995 377.65000000000003 793.45
446.55000000000007 736.1750000000001 688.0250000000001
555.3000000000001 640.55 568.3 869.0250000000001
728.05 515.75 931.75 497.17499999999995 78.5
677.3 626.3 327.2 730.175 784.2 351.27500000000003
686.75 523.525 583.3 154.525 219.05 769.625 355.17499999999995
518.425 403.35 904.9 754.2249999999999
984.2250000000001 903.4250000000001 400.725 558.9
490.42499999999995 479.5 261.725 320.55 322.70000000000005
589.65 700.025 487.45000000000005 290.425 278.275
329.75 266.425 531.2750000000001 538.425 385.45 529.275
591.4000000000001 387.65 830.275 444.07500000000005
369.05 457.875 288.35 527.15 509.75 531.825 343.55 648.025
362.95 685.4749999999999 378.79999999999995 408.85
879.275 756.05 636.2 119.3 705.75 525.95 539.775 642.875
684.875 421.325 629.0500000000001 714.725 640.3 441.55
629.0 440.15 327.225 335.975 358.075 268.05 235.875 296.45
307.575 221.27499999999998\
KCNS1 240.3 827.9 180.7 44.7 610.2 271.8 37.1
683.1 191.6 232.9 73.1 198.6 354.4 353.2 1211.7
228.2 187.9 232.3 35.6 222.6 1127.3 411.1 895.7
140.8 239.6 208.8 156.7 309.0 271.5 142.5 86.1
420.1 494.3 311.4 987.9 446.7 55.6 400.3 188.6
162.2 154.6 140.4 216.8 38.5 154.1 476.3 699.9
232.0 816.7 629.0 311.8 44.6 143.6 788.3 264.9
338.0 420.2 290.3 107.8 492.2 1129.1 302.1 57.8 89.3
270.6 459.9 158.9 215.4 745.1 265.1 712.4
266.3 260.4 120.8 428.4 143.2 571.6 206.1 272.6
723.2 1003.7 418.3 531.1 503.0 1846.8 2311.8 797.3
247.7 349.1 509.4 193.6 519.7 505.2 145.0 158.1
296.1 377.3 243.2 307.3 389.6 286.5 274.6 279.7\
FAM195B 172.8 190.9 149.8 509.7 329.3 863.8 359.7
388.9 550.6 604.3 686.6 336.4 263.8 273.9 337.3
331.4 212.4 154.3 176.6 366.2 661.7 352.9 166.5
573.0 187.2 291.2 219.8 547.3 382.4 591.6 334.2
528.0 461.2 730.9 570.6 454.4 872.5 496.1 802.6

971.4	310.3	337.5	303.0	552.1	257.6	394.7	258.5
293.3	533.2	244.2	406.7	284.9	142.3	316.9	400.5
286.2	585.5	245.8	215.6	644.2	117.9	780.1	259.3
186.5	427.3	242.1	585.9	506.7	682.1	396.1	598.1
235.9	142.9	252.8	632.8	518.6	342.8	528.0	559.3
356.5	495.7	407.8	1254.4	587.4	355.2	460.8	403.9
269.6	338.2	339.9	243.9	150.4	554.1	612.0	310.1
419.2	297.2	405.6	285.2	224.4	444.2	357.5	333.8\
KLHL6	42.925	69.3	291.59999999999997	254.225	50.875	30.5
	62.824999999999996	255.22500000000002	73.6
125.075	216.325	117.775	19.3	193.45	79.15	80.1	74.425
239.4	71.25	57.525	231.75000000000003	95.92500000000001
	380.475	253.15	51.8	71.45	37.05	248.45	132.2
67.325	40.650000000000006	120.8	274.625	133.3	156.075
183.925	90.175	84.05	42.675	172.375	26.25	20.525000000000002
	331.47499999999997	27.574999999999996	77.625
100.65	94.0	44.875	88.925	92.575	229.1	141.12499999999997
	34.5	56.3	76.925	365.7	162.85	119.075
223.52499999999998	161.29999999999998	274.95	240.825
84.55	144.65	95.875	61.74999999999999	176.60000000000002
	59.875	377.075	304.475	841.1249999999999	516.525
220.99999999999997	98.35000000000001	152.475	172.65
469.34999999999997	85.125	129.075	27.875	85.72500000000001
	131.17499999999998	128.575	123.325	96.10000000000001
	125.17499999999998	71.75	153.95	82.15	46.75
78.05	91.80000000000001	72.75	118.75	51.64999999999999
	54.525	86.425	264.4	68.80000000000001
114.62499999999999	49.875	80.975	59.800000000000004\
FAM195A	413.70000000000005	1681.5	501.9	750.65	597.5
2377.9	1132.45	465.04999999999995	579.05	685.9	1147.5
835.45	669.5	848.25	1273.3	533.35	757.0	827.1999999999999
	1144.9499999999998	654.9	375.35	547.9	629.15
483.6	486.95	796.45	1110.8	415.55	876.1500000000001
460.90000000000003	467.3	690.55	822.25	760.1	3054.25
799.55	1574.8	720.7	556.15	678.4	567.4	666.4	621.15
434.9	633.55	752.7	1089.65	1340.05	650.5	994.8	712.5
1126.2	1718.3500000000001	429.85	574.7	955.3
936.8499999999999	783.25	1191.55	711.5999999999999
484.7	524.35	480.15	608.25	561.0	1139.5	625.3	444.05
651.9000000000001	767.55	320.05	1128.25	337.05	957.8
780.9000000000001	1085.5	558.1500000000001
719.5999999999999	628.45	338.5	719.7	387.59999999999997
	1582.1999999999998	1168.0	473.2	244.25	303.8
857.55	408.54999999999995	764.35	551.95	309.75	1057.25
593.05	394.7	593.05	586.95	842.1500000000001	737.05
618.9	581.1	660.15	553.55\
KCNS2	78.73333333333333	134.13333333333335
72.33333333333333	50.633333333333326	74.13333333333334
	38.2	30.7	85.5	77.66666666666667	29.7
67.60000000000001	59.0	39.800000000000004	39.7
122.36666666666667	59.13333333333333	44.300000000000004
	52.13333333333333	54.73333333333334
128.26666666666668	120.83333333333333	44.866666666666674
	129.2	53.800000000000004	56.833333333333336

52.36666666666674	50.26666666666667	124.83333333333333
	71.7	135.36666666666667	44.133333333333326
141.06666666666666	94.60000000000001	54.43333333333334
	95.46666666666665	90.96666666666665
40.166666666666664	86.89999999999999	44.26666666666667
	62.6	57.23333333333333	16.73333333333333
50.06666666666666	58.166666666666664	35.4
70.06666666666666	50.76666666666667	25.600000000000005
	127.89999999999999	43.333333333333336
68.60000000000001	82.06666666666666	76.66666666666667
	66.26666666666667	54.0	45.70000000000001
59.833333333333336	83.96666666666667	65.46666666666665
	75.83333333333333	315.8333333333333
78.16666666666667	44.36666666666667	60.333333333333336
	106.36666666666667	76.73333333333333
56.26666666666667	50.36666666666667	96.43333333333334
	85.16666666666667	131.7	80.33333333333333
121.16666666666667	72.36666666666666	113.76666666666665
	54.6	62.800000000000004	41.166666666666664
52.76666666666667	57.800000000000004	71.96666666666667
	52.73333333333333	33.56666666666666
48.333333333333336	71.60000000000001	109.3
57.76666666666667	59.93333333333334	50.63333333333333
	28.866666666666664	30.53333333333333	151.6
64.33333333333333	49.23333333333334	44.76666666666667
	63.06666666666667	58.43333333333334	45.5
118.89999999999999	57.26666666666667	76.03333333333333
	99.66666666666667	47.36666666666667\
KLHL7	1545.6333333333332	1153.6000000000001
726.3333333333334	1416.3333333333333	1065.0333333333333
	1125.5	1809.9666666666665	918.3000000000001
1004.7333333333335	1034.8999999999999	899.9333333333334
	743.0333333333333	604.6999999999999
832.6666666666666	923.0666666666666	596.2333333333332
	1290.5333333333335	777.3333333333334
759.6666666666666	976.2666666666668	461.4333333333334
	1289.5333333333335	628.9000000000001
1071.1000000000001	1425.0999999999997	900.3000000000001
	1256.8999999999999	940.9333333333334
1128.1333333333332	1147.8666666666666	1327.8
1108.4666666666665	403.3333333333333	1018.0
956.1666666666666	674.9666666666667	1136.3333333333333
	666.7666666666668	831.4	1689.1333333333332
1235.8666666666668	953.7333333333332	1040.0666666666666
	1405.7666666666667	689.2666666666665
1980.7666666666667	889.1	2358.1666666666665
537.2666666666668	846.0333333333333	955.5	1837.8
694.7333333333332	870.4666666666666	850.1
679.3333333333334	769.0	925.3000000000001
747.1666666666666	742.9333333333333	667.8000000000001
	825.6666666666666	1249.4	1054.2	861.7666666666668
	629.8333333333334	916.7666666666668
1000.2666666666668	769.6666666666666	1233.5333333333333
	499.43333333333334	1255.5666666666666

809.5999999999999	1250.3666666666666	936.6999999999999
1600.6333333333332	941.5666666666666
944.8333333333334	785.5333333333333	1060.5666666666666
1135.1333333333334	982.9666666666667	991.0
965.4666666666666	477.8333333333333	480.73333333333335
940.2999999999998	932.2333333333332
480.0333333333333	574.6666666666666	1475.3333333333333
792.1	1387.966666666667	1108.2	1102.4666666666665
1488.1333333333334	1332.5666666666668
1005.7666666666668	1084.8	1385.3333333333333
1189.6000000000001	863.1	950.3000000000001\
KCNS3	178.5	1121.9	235.6	199.8	595.5	966.7	2720.8
544.0	345.9	212.7	242.4	808.7	371.7	323.5	143.9
1043.5	653.0	565.7	676.7	415.6	790.5	52.2	496.2
756.3	189.6	179.8	359.8	446.2	372.9	1337.4	629.9
334.6	141.3	587.4	702.0	190.7	1033.4	345.5	386.0
1421.0	2304.9	2018.9	824.5	584.1	311.5	1557.1	332.7
1497.7	201.4	572.7	379.1	314.6	1323.0	1010.7	193.6
828.7	466.7	377.8	431.9	163.0	834.4	255.7	514.0
218.9	464.2	980.7	954.0	181.9	852.7	276.1	571.4
234.4	325.3	322.5	278.0	585.9	527.5	795.6	514.2
249.8	394.0	275.9	1330.7	1700.4	249.1	291.3	1990.3
301.0	402.6	549.2	205.1	757.0	2214.0	1648.8	879.5
2091.4	2031.0	1205.6	209.3	1189.2	2289.5	386.3	1716.0\
MC5R	19.9	40.5	80.0	29.8	134.4	17.6	2.7	17.2
56.4	17.3	36.0	14.1	56.3	19.2	64.1	51.6
32.6	14.2	10.2	14.1	142.3	86.0	18.9	4.3
25.5	12.9	51.7	92.4	18.1	28.4	25.8
208.1	15.5	17.0	66.6	82.7	9.7	19.8	12.3	36.0
63.6	12.0	5.1	17.1	46.4	64.8	10.3	67.0
75.8	133.2	7.4	7.6	64.7	38.4	19.9	81.4
13.4	117.3	18.3	83.0	47.6	42.2	24.0	24.7
11.6	15.8	15.5	43.9	14.0	50.0	9.3	49.9
75.3	7.1	20.6	12.4	44.6	24.4	12.0	14.6
19.8	16.1	31.1	26.2	135.6	126.1	84.6	35.7
7.2	104.9	14.7	21.0	15.0	61.0	11.2	20.1
10.8	65.4	105.8	30.3	44.2	50.8	46.2\
KLHL8	773.5333333333332	839.5666666666666
1170.1333333333332	1406.8333333333333	1930.5	1821.2
1272.0666666666666	755.7000000000002	1021.9666666666667
2162.6666666666665	1203.8333333333333	1459.2
1828.5	2161.9333333333334	949.1999999999999	1417.3
961.9666666666668	1237.5000000000002	1349.2666666666667
1340.2	628.0333333333333	1551.3999999999999
870.7333333333332	722.6666666666666	1042.0333333333333
1121.0	1461.4999999999998	936.2666666666665
967.4666666666667	755.5333333333334	1096.1666666666667
1794.8333333333333	837.5333333333333
1042.1000000000001	3170.066666666667	1135.2666666666667
645.6	1654.3666666666668	1240.2333333333333
922.8333333333334	1045.6333333333332	587.2666666666667
815.9333333333334	999.1999999999999
737.6333333333333	719.3666666666667	1234.6666666666667
1203.7333333333333	1295.5666666666668

908.4333333333334	1198.7333333333333	1155.7666666666667
	799.8666666666667	1491.5666666666668	794.4
1191.7	946.5	1603.966666666667	1237.0333333333333
1016.1666666666666	1029.0666666666666	1116.4
2403.8666666666663	1568.466666666667	841.6333333333333
	1302.8666666666666	678.1333333333333
1307.4333333333334	842.6666666666666	988.1999999999999
	943.9333333333334	1068.4	1138.3999999999999
1030.8666666666666	937.3000000000001	922.5
1005.6666666666666	718.6999999999999	932.4000000000001
	1087.6999999999998	1224.6000000000001
1585.4333333333334	271.8	1001.8333333333334	1299.2
479.3333333333333	1204.5000000000002	1257.3666666666666
	1770.8333333333333	2342.7999999999997
1401.033333333333	1186.2333333333333	1214.8333333333333
	931.9666666666666	1021.7000000000002
1024.6333333333332	1088.5666666666668	772.8333333333334
	916.8000000000001	1150.3333333333333	1084.9
1077.3999999999999	938.4\
LOC101927839	6.4	38.4	10.7	9.2	22.2	4.1	3.0
	17.2	13.1	29.8	4.5	24.0	46.6	8.7	10.1
	5.7	12.1	42.5	7.8	65.0	78.0	10.9	41.6
	5.1	12.7	10.2	12.9	98.0	17.9	31.2	7.0
	20.7	28.6	8.1	27.9	14.0	11.0	25.7	8.6
	9.2	4.2	7.2	30.0	7.9	6.9	20.3	19.6
	6.3	11.5	22.3	13.6	7.0	5.4	24.2	10.3
	12.7	16.6	7.6	15.4	15.9	18.0	14.5	6.0
	19.0	23.9	73.0	6.0	7.7	11.8	30.7	28.3
	24.1	6.9	11.0	9.1	7.9	27.8	15.5	7.4
	5.9	22.5	8.7	10.3	6.8	12.3	21.2	9.1
	14.5	21.2	21.6	2.9	13.0	9.7	10.7	11.5
	12.4	14.7	5.4	8.5	8.9	5.1	27.7	7.0\
TRIM43 /// TRIM43B	14.649999999999999	29.099999999999998
	420.6	22.349999999999998	94.4	27.450000000000003
	6614.7	1462.45	29.6	131.75	2991.7999999999997
28.25	298.95	22.799999999999997	197.45000000000002
11.350000000000001	54.550000000000004	157.85000000000002
	24.05	269.2	13.7	105.8	350.85	14.200000000000001
	172.3	5.45	8.85	45.949999999999996	1107.15
95.15	18.5	52.900000000000006	31.8	42.4	249.4
497.5	75.65	73.05	17.2	402.40000000000003	74.4
47.15	24.5	6.15	57.55	26.7	31.65	29.05	92.75	16.4
	14.9	26.25	24.35	60.7	60.349999999999994
53.85	27.95	54.599999999999994	15.65	60.45	35.8	42.6
	44.900000000000006	23.7	32.949999999999996	46.8
	28.65	33.55	12.4	54.0	96.75	10.850000000000001
	28.549999999999997	50.300000000000004
37.050000000000004	51.2	10.8	48.2	39.7	52.5
125.6	72.64999999999999	54.5	60.050000000000004
52.300000000000004	53.8	1048.85	38.7	54.05	39.2	49.5
	30.05	27.299999999999997	41.05	43.5	55.5
53.650000000000006	17.45	32.3	10.3	37.45	18.25
20.0\
LOC102724927	2.0	209.9	33.8	32.9	2.3	60.8	9.4

259.7 29.0 19.7 38.7 2.4 41.9 267.2 21.1
55.9 26.9 39.6 47.1 38.7 132.6 11.9 36.7
69.5 18.1 41.5 3.9 42.8 26.3 17.4 29.3
124.9 110.8 76.9 46.0 2.7 60.6 31.4 1.3
14.8 21.0 1.1 85.2 28.6 1.1 94.1 31.6
38.9 33.7 64.5 25.5 4.5 28.8 83.1 59.9
30.1 42.2 65.9 80.7 102.3 272.0 26.5 34.5
32.8 47.5 8.9 20.0 37.2 123.4 24.2 33.1
97.9 73.6 62.7 25.1 1.7 62.7 54.3 23.0
313.2 106.2 67.8 23.5 42.1 146.4 151.3 46.5
9.3 50.9 60.1 21.4 79.3 62.1 83.9 90.8
55.0 61.8 57.7 76.5 30.4 70.5 122.4
57.7\
AF086288 /// RP11-768F21.1 6.5 124.7 70.8 50.6 30.8
27.6 31.9 116.7 18.9 22.3 11.4 55.8 26.8
123.4 78.5 79.4 17.1 16.9 9.3 51.2 54.1
27.9 76.0 33.8 10.9 20.3 54.4 73.1 32.8
4.9 46.2 88.9 37.1 105.5 129.9 47.7 10.4
126.5 59.5 39.9 321.4 8.4 104.3 196.9 26.5
52.1 10.5 8.7 67.4 70.0 132.4 18.4 12.1
17.2 45.4 10.6 14.1 7.3 62.8 79.1
321.4 156.2 18.3 18.4 159.5 147.7 6.4 99.5
157.7 65.7 26.6 34.9 23.2 7.9 80.4 17.1 14.7
58.4 51.7 8.2 50.1 76.2 28.1 94.2 32.4
28.5 12.3 191.2 74.3 57.2 14.4 21.4 21.4
115.0 36.6 87.0 82.5 133.2 120.5 32.7 16.0
90.6 119.1\
LOC101927843 26.4 69.0 23.0 3.3 4.9 1.3 1.7
44.5 5.0 11.0 7.2 7.6 8.7 3.4 10.2
12.7 3.9 13.1 6.9 4.7 30.3 30.3
119.4 3.6 10.7 5.3 2.2 65.6 77.2 68.6 5.8
4.7 20.5 3.1 9.5 11.8 47.3 14.1 4.0
3.0 5.7 19.8 57.6 10.6 31.1 20.3 37.0
27.7 18.7 21.9 54.3 51.1 10.8 9.5 11.0
79.0 32.8 9.8 9.8 161.4 13.1 8.2 78.0
24.9 10.4 12.3 25.3 1.7 4.2 29.7 57.3
68.8 65.5 41.0 6.6 3.1 5.7 9.5 6.3
11.2 28.2 59.5 15.1 52.8 107.7 18.0 2.8
6.6 55.2 10.7 43.2 22.8 23.4 1.4 16.5
4.8 9.0 34.2 25.3 11.5 23.1 62.0 8.0\
LOC101927841 12.1 6.7 8.6 91.9 20.3 68.6 8.3
18.2 185.8 60.0 116.9 10.9 86.0 18.2 54.3
100.0 8.1 170.1 12.0 5.7 33.7 10.1 16.0
32.5 2.7 9.2 12.1 31.7 9.0 12.9 13.0
10.2 23.7 12.2 15.1 278.3 59.9 19.8 6.5
420.2 10.6 2.5 26.7 18.8 10.3 32.1 6.7
9.1 9.4 47.9 4.1 92.3 98.9 25.8 15.6
3.0 12.4 397.8 4.8 14.8 495.4 17.9 8.9
11.4 10.3 61.7 6.6 9.9 43.9 62.8 34.3
12.7 62.0 109.8 4.9 99.0 26.2 111.5 4.3
9.9 187.6 12.1 100.6 14.5 91.5 42.5 31.5
13.9 11.6 23.9 8.0 14.6 200.1 138.1 92.9
675.9 83.8 146.5 15.9 85.1 24.6 50.0
197.1\

GK-AS1 /// GK-AS1 61.5 218.2 118.4 23.7 93.2 61.8
55.2 81.5 81.8 126.1 17.8 65.3 5.4 50.2
137.7 67.8 108.5 47.4 33.5 15.0 255.0 38.2
180.0 14.2 118.8 16.7 111.1 61.0 125.1 28.6
60.4 119.1 173.5 70.7 236.2 27.3 51.5
106.2 59.3 84.5 82.4 11.8 89.0 32.2 11.1 93.4
142.0 101.1 181.2 81.0 149.2 18.2 73.8 97.8
19.4 75.4 129.0 84.3 177.1 16.1 99.0 89.6
68.7 93.0 218.6 101.9 79.9 17.4 156.7
113.1 69.7 104.5 88.8 87.1 53.4 85.8 223.9 86.6
102.3 91.0 85.7 127.7 12.5 77.5 362.2
138.6 205.3 55.5 93.9 112.1 104.8 34.9 84.3 83.4
49.6 122.9 113.1 26.2 163.3 123.3 104.8 90.7
42.4\
INPPL1 421.2 920.5 543.7 485.4 831.9 1121.7 544.1
763.7 535.5 847.8 585.1 782.5 364.2 546.2 1507.6
1759.1 466.7 672.0 708.0 479.3 1283.0 797.9 1307.5
549.2 365.7 836.0 493.7 930.3 795.9 519.2 304.7
529.4 1257.0 585.1 240.4 669.9 622.6 707.9 403.4
974.3 799.8 785.1 242.9 487.4 539.5 546.7 551.9
526.1 1639.8 708.4 789.0 574.5 458.3 906.5 732.3
926.9 772.7 549.2 1129.7 880.2 153.0 695.5 379.7
580.9 627.8 780.4 910.8 659.0 819.4 803.0 897.7
646.8 557.5 273.0 436.3 508.8 668.9 600.2 636.3
581.4 360.2 573.6 1102.0 1959.3 943.2 1534.1 445.4
393.4 717.1 528.8 421.5 696.2 615.9 710.9 577.5
528.4 753.3 540.9 551.7 771.7 845.6 931.7 619.2\
PLAT 118.0 98.9 329.4 407.9 560.8 188.4 1269.4
395.1 2574.5 236.8 278.0 362.2 281.3 435.4 303.8
112.4 966.8 378.2 281.7 1902.6 1309.4 114.0 401.9
721.5 359.3 160.3 493.4 454.9 276.7 1502.0 753.3
409.1 261.3 383.4 1017.5 388.3 164.5 212.5 190.9
262.8 655.0 226.6 492.3 2873.8 75.8 493.8 620.0
512.5 205.8 214.4 742.9 661.9 145.1 598.0 569.6
246.9 941.7 452.2 139.4 635.9 1511.1 495.9 303.3
1012.0 1986.2 273.6 1647.6 3608.5 1897.9 220.9 312.1
410.8 297.9 561.4 333.6 423.5 565.8 339.8 784.1
651.6 907.9 605.3 5165.7 441.7 288.5 281.5 115.8
2342.3 816.1 605.8 557.5 703.2 449.9 758.8 598.0
387.5 227.3 535.7 292.3 334.8 366.4 235.5 260.9\
PLB1 52.0 59.35 64.1 39.55 45.6 22.25 85.9 77.8
70.25 57.85 70.0 25.2 29.15 40.9 31.45
111.14999999999999 22.5 18.85 39.0 47.95 163.0
68.75 52.349999999999994 15.25 26.1 30.15 9.85 53.0
34.9 53.1 31.3 141.9 13.3 65.5 40.45 61.7
26.849999999999998 67.15 35.7 8.95 76.6 43.1
26.8 16.0 21.45 54.5 26.450000000000003
34.45 192.4 102.45 58.699999999999996 52.95 32.65
27.599999999999998 14.5 51.2 80.65 14.399999999999999
33.55 10.2 56.800000000000004 60.849999999999994
18.55 28.95 30.15 38.0 29.85 31.6 51.0
44.45 48.8 8.25 32.05 56.5 70.05 66.5 50.8
58.099999999999994 30.7 20.049999999999997
48.900000000000006 50.95 22.15 60.75 125.60000000000001

	94.45	76.15	56.95	6.65	67.7	16.8	52.0
111.45	8.8	70.75	54.1	51.550000000000004	24.9
34.45	63.25	46.55	30.8	32.55\
IGF1R	425.96000000000004	434.91999999999996
1000.5800000000002	143.35999999999999	315.1
730.8399999999999	825.6600000000001	422.75999999999993
	1007.14 343.58000000000004	556.6600000000001
241.92	501.30000000000007	489.78000000000003	1676.6
1308.5799999999997	653.6	842.1800000000001	179.78
646.9799999999999	500.12	557.1200000000001
1108.6200000000001	619.8800000000001	663.6400000000001
	587.74	115.32000000000001	1280.8999999999999
1772.64 901.68	856.5199999999999	881.5600000000001
762.3199999999999	1118.72 687.9799999999999
873.1800000000001	842.26	749.46	781.08	1175.6200000000001
	1493.54 722.58	1243.7599999999998	650.1800000000001
	619.42	825.3799999999999	137.49999999999997
390.26	690.28	789.72	467.5999999999999	545.6800000000001
	790.8	364.49999999999994	725.0800000000002
935.5999999999998	1238.68 1032.26 711.62	1098.24
1460.4799999999998	826.76	580.38	700.3199999999999
616.96	743.2	449.38	663.6600000000001	1170.8799999999999
	813.24	1919.8999999999996	454.36	491.41999999999996
	1037.7800000000002	841.5200000000001	1216.56
1422.74 1414.14 715.0600000000001	319.00000000000006
838.5600000000001	1138.0399999999997	2904.62
216.77999999999997	214.07999999999998	1260.54 84.24
985.04	404.84000000000003	944.5400000000002
405.09999999999997	881.78	1082.94 1220.6	1235.1000000000001
	1580.4599999999996	1558.8	1520.98 2441.04
1861.7600000000002	1490.26 944.3599999999999
1541.7400000000002\
TXN2	611.25	513.3	683.65	1058.1	571.2	1644.35
944.0999999999999	579.0	864.75	776.0	967.2	842.5
905.9	911.7	595.8	771.3	1067.25 902.5	1196.65 610.8
854.3	680.05	540.35	856.6500000000001	578.75	906.85
1310.45 769.5999999999999	1091.3500000000001	997.65
1215.75 908.7	835.55	1275.1999999999998	1438.3999999999999
	1390.5	1024.25 732.1500000000001	906.9499999999999
	1019.25 1016.05 682.0	769.6	626.7	762.9000000000001
	865.0	1703.85 829.1	626.6	1141.3999999999999
916.5	1671.35 1316.05 614.3499999999999	770.6
1324.3500000000001	1292.1999999999998	843.25	1106.5
590.05	573.6	910.8	599.8	952.3	823.3	933.7
758.5500000000001	800.5999999999999	1242.3500000000001
	1047.25 291.1	545.6500000000001	553.4
1016.6999999999999	713.75	1500.05 772.15	879.45
797.0500000000001	1137.4	619.55	1154.9	576.5	1007.35
769.4	536.3000000000001	1093.15 1132.75 674.0	642.45
575.05	731.8499999999999	1385.0500000000002	1074.1
702.75	1529.1	1043.45 1267.3500000000001	1519.85 1096.65
1157.0	972.5	1345.3\
FAM198A 6.9	54.1	16.6	36.4	102.7	195.7	19.6
101.5	107.7	125.8	89.1	3.2	41.7	247.2	21.9	72.8

127.6	13.4	5.9	23.6	163.7	15.6	77.8	82.2
71.1	9.7	7.9	52.7	21.0	43.7	148.3	15.6
46.8	131.3	255.0	199.6	130.9	13.8	9.9
341.8	32.7	127.0	277.2	43.0	252.0	58.9	11.3	51.2
64.3	86.0	79.2	24.1	67.5	14.1	146.0	44.9
51.9	45.9	71.6	54.9	15.3	71.2	5.8	69.1
90.0	93.4	172.7	4.7	59.1	171.7	256.1	84.7
26.0	141.4	138.2	195.6	13.7	120.5	90.6	84.5
87.3	44.4	46.1	51.9	15.6	199.3	56.1
153.1	139.2	62.3	124.7	87.3	124.8	89.4	127.9
128.9	68.0	127.8	92.9	52.1	149.4	130.9	74.6\
FLJ33360		22.4	269.5	128.7	81.7	103.6	85.3	47.4
167.9	112.2	152.1	89.1	37.9	115.1	132.4
226.3	225.7	424.9	81.9	121.0	145.1	297.8	91.8
251.0	110.4	89.0	132.9	160.8	254.6	457.1	34.7
115.9	215.5	157.3	105.3	229.4	90.0	67.6	127.5
118.5	138.3	42.2	95.5	229.6	137.2	114.4	131.5
146.9	70.4	389.8	207.9	91.6	136.8	105.5	297.9
265.4	238.3	91.8	170.5	55.4	56.2	293.6	173.4
140.8	117.5	152.6	149.7	98.0	95.3	147.5	153.6
207.1	585.8	212.3	49.0	161.9	137.0	174.4	115.1	55.0
64.6	295.0	150.5	147.3	101.4	239.2	234.7
140.4	373.5	150.4	222.5	77.3	236.8	183.0	139.2
408.1	155.1	218.0	116.7	267.0	86.7	139.4	170.1
194.6\
RP11-805I24.3	14.9	307.7	164.9	25.1	275.8	264.7
123.2	222.4	183.3	110.6	120.0	145.1	178.5	78.2
354.0	233.9	245.2	134.4	213.6	233.1	480.0	94.5
224.1	137.4	123.7	180.1	89.7	471.6	469.9	99.3
168.3	113.4	306.6	143.9	380.1	173.5	138.9	223.2
108.5	233.3	104.2	324.6	314.7	123.3	108.7	106.9
238.4	85.7	426.9	245.5	135.3	223.4	249.5	469.7
109.9	260.2	272.2	208.4	168.5	163.3	857.6	139.2
223.3	58.0	345.2	315.3	179.6	144.2	353.1	258.2
169.4	203.9	220.6	97.6	118.3	138.6	245.4	141.1
165.1	203.7	332.9	138.7	105.0	245.0	346.8	740.3
221.4	200.8	279.8	231.0	142.7	251.1	68.4	105.1
138.8	131.2	308.1	174.7	231.0	198.2	152.3	240.1
243.0\
PPIP5K1	250.4	197.6	317.8	207.75	357.65	287.75
230.39999999999998	179.1	182.0	254.89999999999998
258.3	412.8	155.05	392.05	261.5	278.9	204.05	382.7
176.45	191.5	364.9	221.05	404.15	157.65	214.35000000000002
392.45	317.05	191.89999999999998	220.9	186.15
210.39999999999998	199.1	871.1	154.85	156.85	273.45
216.95	349.6	286.6	139.2	186.85	496.34999999999997
279.75	183.3	255.55	206.5	173.4	252.85	569.5	435.6
179.25	200.35000000000002	243.5	279.7	154.55	388.0
287.35	271.4	291.6	245.15	362.85	274.25	289.75	185.05
262.1	585.9	236.4	381.15	203.2	214.0	414.3	366.25
234.7	203.5	169.4	157.9	278.5	237.54999999999998
244.85000000000002	55.650000000000006	178.05	182.75
166.89999999999998	159.65	258.05	163.5	209.35000000000002
66.3	256.55	200.9	206.45	195.25	157.35	211.75

186.5	159.05	166.95	159.5	156.1	214.85	233.45	193.55
149.85000000000002\
PLAU	287.34999999999997	129.9	516.0	461.4
364.40000000000003	278.7	665.1	752.2	865.6	1809.0
776.6999999999999	463.65000000000003	1372.1	1099.75
51.75	423.4	1415.5	1457.3	151.7	1555.1	1563.8	1026.2
489.55	861.0500000000001	360.40000000000003	859.5
1849.2	432.35	326.7	2932.75	462.55	293.5	1020.5
734.1500000000001	519.35	234.25	546.35	768.0999999999999
	261.75	246.15	626.4	845.95	374.75	1386.3500000000001
	405.0	327.1	233.9	302.90000000000003	346.3
377.84999999999997	1030.95	636.6999999999999	513.4
365.59999999999997	1982.95	302.25	687.9499999999999
355.25	469.75	653.4000000000001	429.55	1197.4499999999998
	483.0	1749.05	602.85	684.1999999999999
769.0999999999999	1899.8000000000002	919.35	778.5
234.15	290.65	802.5	909.1	705.2	762.0500000000001
1069.5	560.7	421.4	147.65	796.75	1705.8500000000001
108.4	436.5	539.5	277.3	567.3000000000001	667.9
2365.95	1157.55	333.90000000000003	1007.65	197.2	1048.75
451.45	109.7	195.85000000000002	288.95	102.35	462.9
220.9	274.75	170.35\
PPIP5K2	1447.2	2669.8	1995.0	1767.4	1228.2	1564.6	1357.0
1370.7	890.9	644.6	1435.4	1212.5	1025.5	1313.0	100.1
1266.7	1031.9	1338.3	991.7	1193.5	1303.5	756.3	1038.0
949.8	1255.4	1279.3	1009.9	836.6	2242.0	1331.6	1479.5
1497.9	1131.9	1087.0	474.8	1637.1	1272.9	709.7	904.0
1258.1	1498.5	1035.2	1653.3	970.6	954.7	1042.3	2065.5
585.4	1631.9	1446.8	1099.9	1564.6	897.2	1251.4	1294.5
1279.9	981.1	2319.9	1547.6	1174.8	922.8	1419.5	1497.8
1437.6	1834.0	1053.8	1755.4	939.0	489.2	1361.3	1042.1
976.6	1556.1	1919.2	1785.7	1097.8	1848.4	1328.7	1355.3
1361.1	3471.3	1876.1	719.4	1295.2	2097.2	1124.0	1449.4
2054.3	595.4	929.5	928.7	3350.5	1924.9	1611.5	2058.3
2137.8	2403.4	2892.2	2361.9	3767.4	2544.2	2406.4	1724.9\
LINC00599 /// MIR124-1	42.45	100.15	51.7	79.55	186.3	44.0
	48.6	105.4	20.75	157.4	33.5	41.0	44.9	77.6
	186.3	56.9	17.8	119.6	72.95	39.75	192.65
25.85	176.1	28.5	41.150000000000006	45.800000000000004
	29.75	98.05	58.65	12.75	35.6	128.7	115.65
50.25	103.25	96.0	10.899999999999999	90.6	26.45	33.8
	24.15	36.85	40.0	22.25	51.300000000000004	48.9
	33.15	45.199999999999996	91.30000000000001	48.6
	97.7	46.35	66.19999999999999	103.55	37.35	97.4
	97.6	54.7	46.7	119.45	350.25	101.55000000000001
	35.65	44.35	54.099999999999994	52.05	50.75	33.5
	114.0	77.2	132.1	66.75	55.35	50.6
96.80000000000001	36.5	52.05	16.6	42.05	43.05
170.35	97.7	55.9	39.0	85.05	186.7	33.15	180.0
106.95	131.7	22.15	125.14999999999999	34.4	17.55	39.3
	22.4	43.45	34.2	35.3	33.4	61.4
56.150000000000006	36.25\
FAM198B	3930.7000000000003	182.0	559.1	1132.0
361.29999999999995	1245.7	1462.8	1932.3	2780.75	392.6

217.15 1287.5 192.9   468.65 85.25   968.15 1951.25 677.4
399.1   1647.25 732.2   1467.1 978.5500000000001   2980.35
454.15 5446.75 1023.15 4983.75 1881.7 766.15 4827.200000000001
        3059.9 422.54999999999995   3387.5 237.75 378.2
1551.95 264.7   350.8   1327.3999999999999   283.1   165.0
4152.95 923.3   259.85 2273.8999999999996   2018.0 6117.3
305.55 2506.95 1260.1499999999999   833.4000000000001
987.0500000000001   1026.85 1388.3500000000001
172.35000000000002   1414.7 781.15 3405.6499999999996
1788.45 1071.95 590.1   749.2   3408.4 6591.0 125.9   2045.95
360.75 1124.75 1609.8 1102.1 534.2   4571.1 1595.15 2049.25
1632.75 2780.3 3130.6499999999996   4008.9 107.6   1622.7
468.59999999999997   792.3   781.6   273.0   146.45 153.9
4050.15 128.95 239.25 536.45 4858.1 4029.9500000000003
3703.65 4554.85 3129.75 3015.0 4299.099999999999   4775.45
5704.55 4513.400000000001   8537.25 2386.5\
ENPP2   39.7   1135.75 971.9000000000001   1038.75 462.55
362.25 1137.8 2236.8 1088.85 470.65 235.8   593.25 471.3
1212.85 169.55 853.5   2372.6 170.0   201.05 290.40000000000003
        403.15 260.1   493.2   4351.6 235.45000000000002   61.4
        194.2   1303.75 691.9499999999999   855.2   4660.25
289.84999999999997   408.54999999999995   4345.65
1504.1000000000001   415.20000000000005   1082.85 297.2
439.59999999999997   1284.0 269.25 273.15 3915.3 1347.3
435.25 389.65 2209.85 101.0   202.85000000000002   523.7
464.55 3548.5 220.35 387.34999999999997   1604.3 495.0
229.05 479.54999999999995   233.95 712.4000000000001
1892.1 1476.3 766.2   847.55 688.4   142.14999999999998
808.2   1961.15 1367.15 4785.400000000001   812.95 438.7
3911.3999999999996   792.6   519.7   486.55 1574.3 1940.8
2083.55 1555.7 5967.25 268.1   210.15 2236.65 145.45 186.45
290.65 2452.9 255.2   945.75 476.79999999999995   2638.25
2403.4 5796.0 5087.85 1532.1999999999998   3818.0 2282.25
1748.75 2470.8999999999996   4670.8 2414.3 2189.25\
ENPP1   384.25 79.525 80.9   41.675 231.25 160.9
38.699999999999996   134.0   72.75   425.225 47.925
349.19999999999993   112.14999999999999   31.075 77.55
3515.0000000000005   253.89999999999998   52.725 78.575
129.79999999999998   134.39999999999998   50.85   126.525
116.9   68.8   107.62500000000001   88.125 57.775
79.69999999999999   491.475 220.275 111.42499999999998
87.625 93.35   480.8   204.875 32.0   65.7   43.675 83.05
51.15   79.55000000000001   85.77499999999999
208.77499999999998   44.95   132.275 92.22500000000001
310.35 163.15 30.2   138.35 30.725 233.29999999999998
122.89999999999999   72.125 203.65 67.89999999999999
151.675 74.075 62.37500000000001   122.625 132.6
54.69999999999999   85.54999999999998   95.8   126.4
61.074999999999996   80.05   77.32500000000002   47.175
134.04999999999998   91.92500000000001   97.95   58.25
70.325 52.099999999999994   28.7   51.8   141.25
177.07500000000002   175.29999999999998   58.3   91.375
64.55   58.77499999999999   248.15 51.925000000000004
129.1   42.74999999999999   104.44999999999999   132.475

292.92499999999995	185.65	382.7	332.05	126.05	174.175
217.07500000000002	187.5	228.175	170.7	297.475	200.625\
ENPP4	2725.8999999999996	2617.1000000000004	3110.8
445.54999999999995	2103.75	2405.0	1251.25	6169.15	1092.5
1302.3	2183.8	2100.35	1518.95	3806.95	1518.6999999999998
982.5999999999999	2434.15	609.85	1027.0	1203.25	209.45
1249.3	2041.8999999999999	1599.7	1362.9	1278.25
1478.9499999999998	1408.5	1772.8	2045.6	1301.5	1198.75
473.25	947.3000000000001	1059.25	2130.9500000000003
3373.7	293.54999999999995	3088.6000000000004	2827.5
1327.7	2548.6	1895.3	1060.65	2827.85	1038.55	4129.15	2358.3
445.45000000000005	1554.5	1713.4499999999998	947.75
2000.0	1039.25	1542.45	1242.75	1003.6	643.85	2025.45	520.4
584.35	1926.2	1280.05	1978.0	1648.0	1821.35	1555.05	827.95
1482.8	3362.55	966.8499999999999	824.1500000000001
1759.1	2206.5	1473.5	2885.35	4190.099999999999
2688.3999999999996	1599.25	1759.15	1101.65	1930.3000000000002
	1449.05	1609.75	752.75	126.3	4171.75	998.0999999999999
	279.75	664.0	1327.7	685.6	1681.1	1260.35	609.65
2116.8500000000004	1182.4	1914.25	1791.6999999999998
1634.95	1505.9	1323.6	1522.45\
ENPP3	117.25	3159.1	3954.2	73.75	261.35	233.55	36.35
1864.65	924.0	585.8	410.05	215.65	1073.1	4875.8	121.75
9570.0	1536.35	372.35	40.25	190.8	11458.05	186.8
16531.15	80.0	244.1	157.7	115.6	443.95000000000005
	785.1	1037.3	2161.95	154.05	127.35	180.85000000000002
	308.5	50045.65	211.05	98.75	97.45
375.20000000000005	89.65	88.5	548.6	1651.1	80.3
193.39999999999998	139.9	383.75	247.04999999999998
1654.3	507.9	151.5	4164.25	287.95	273.75	1581.75
2007.6999999999998	25246.95	17699.05
1244.1000000000001	556.0	38364.75	359.5	130.45
18056.5	134.15	1991.0500000000002	4509.85	213.9	383.6
674.8	172.55	15297.05	1571.15	172.0	181.95	1606.2
496.7	4064.4	71.0	161.0	110.25	87.35	84.05
126.30000000000001	320.5	85.9	157.10000000000002	84.1
	77.05	66.75	1561.05	252.0	376.70000000000005
1499.4	446.0	467.65	1056.85	274.4	634.25	412.8	24794.2
1305.95\
LOC101927849	2.7	60.4	9.5	4.5	24.5	8.1	4.0
	63.9	11.0	73.7	102.5	12.2	45.3	81.3	12.1
	75.0	12.8	59.0	45.4	107.7	14.8	65.4	25.2
	61.8	41.9	50.5	3.9	173.5	13.2	7.4	23.8
	127.1	12.1	68.5	68.7	91.9	25.1	14.6	47.2
	14.2	112.8	7.1	75.3	36.4	3.0	153.5	7.2
	3.5	107.4	52.6	107.2	13.8	4.5	49.3	16.5
	8.0	102.3	104.3	33.5	36.3	21.6	7.6	27.3
	75.9	92.9	83.1	10.3	43.1	39.2	19.4	15.6
	11.2	43.9	132.7	10.2	15.6	103.3	5.1	8.8
	4.4	20.2	5.5	13.7	31.2	22.5	180.9	18.6
	133.2	46.7	8.2	7.6	98.7	16.8	18.8	11.5
	11.5	13.7	4.0	17.1	32.3	41.1	78.8
46.0\
ENPP6	4.7	6.0	49.3	15.2	124.2	4.0	4.6	7.3

```
	61.3	10.6	44.1	2.3	0.3	7.7	9.8	32.5
	70.7	3.9	6.5	4.7	32.7	2.5	52.1	2.0
	16.8	1.5	1.5	2.4	53.9	5.1	23.7	9.6
	42.5	6.6	58.1	54.9	5.6	71.9	20.0	1.4
	28.7	13.5	7.6	5.3	23.6	5.7	16.0	11.4
	49.9	74.7	1.7	9.2	15.9	13.9	17.3	62.2
	36.5	7.5	28.2	25.4	586.0	4.6	4.8	22.4
	11.3	57.4	2.6	35.4	74.9	7.3	120.0	2.6
	10.0	59.8	5.9	5.0	0.9	4.9	5.6	35.5
	7.8	21.6	1.6	1.9	86.1	17.6	2.4	33.9
	2.2	12.0	12.7	13.0	2.8	25.1	16.2	42.7
	30.0	2.9	1.4	37.5	20.0	37.5	34.2\
ENPP5	841.45	838.05	643.5	185.89999999999998	526.75
904.65	545.7	3559.55	323.35	578.05	1030.4499999999998
349.79999999999995	443.05	2987.3999999999996	1239.75
612.0	678.25	312.45000000000005	952.4000000000001
306.3	135.55	374.35	590.05	542.45	95.7	437.6	600.15
381.6	487.90000000000003	527.5	590.25	351.3
173.39999999999998	780.45	996.75	610.1	816.35	158.1
607.2	1530.05	427.95	890.1999999999999	1201.0	339.1
560.95	406.54999999999995	361.95	694.1	140.8	324.65
299.45	724.6	915.3499999999999	156.3	290.6	1025.85
362.25	157.6	606.95	330.85	582.5	639.7	827.8	957.0
359.6	895.3	453.75	593.1	443.6	1652.25	258.4	337.25
379.9	1233.5	546.0	887.8000000000001	1426.45	469.75
650.55	178.8	658.2	302.20000000000005	807.4000000000001
	1115.95	200.7	59.75	679.25	260.4	11.55	314.05
439.40000000000003	167.9	1411.6	611.75	393.4	1431.8
1126.25	1527.65	635.95	1102.95	822.05	146.25	1098.4\
ENPP7	101.4	8.8	92.5	66.9	25.9	30.2	65.9	44.0
	97.9	103.1	125.4	75.7	45.0	7.1	118.9	84.0
	17.6	75.6	20.5	44.2	86.2	87.0	50.6	92.0
	73.8	138.6	50.5	154.6	159.5	41.6	21.8	39.7
	13.2	137.3	112.4	37.9	64.1	39.0	11.1	75.4
	6.7	58.6	49.0	19.6	45.3	168.2	84.5	69.3
	153.5	21.5	95.6	97.6	49.6	125.1	125.3
104.5	167.5	148.5	110.5	25.3	100.7	7.5	33.4	12.2
	22.2	33.5	41.0	4.9	8.5	142.3	31.9	96.4
	17.0	84.6	91.8	93.6	86.8	31.4	71.5	19.9
	123.9	16.5	32.7	33.3	71.2	96.3	132.0	73.8
	89.8	69.1	26.0	103.8	126.5	84.0	45.5	18.2
	57.5	105.2	16.7	119.0	89.3	179.0	131.6\
CARD10	516.1	233.13333333333333	557.9333333333333
390.3333333333334	473.3999999999999	1636.533333333333
	1119.8	465.3333333333333	1537.5	405.3333333333334
	981.1666666666666	1034.7	423.73333333333335
665.4333333333334	264.1333333333333	294.70000000000005
	720.1333333333333	1401.8666666666668	1221.6
708.9666666666667	709.5666666666666	2821.1000000000004
	439.3666666666666	1458.8666666666668
1371.5666666666666	3316.066666666667	3423.066666666667
	1145.5333333333333	1787.5999999999997
610.5666666666666	329.46666666666664	3436.2999999999997
	1551.8	889.8666666666667	1001.3666666666667
```

463.3666666666666	2777.433333333334	2881.1333333333337
	494.1666666666674	274.6333333333327
1290.6666666666667	1427.2333333333333	1588.3666666666666
	676.2333333333333	2642.5333333333333
1505.0666666666666	3573.8999999999996	310.6666666666667
	2345.233333333333	500.70000000000005
1989.9333333333332	793.8000000000001	409.8999999999999
	2801.9666666666667	2498.3333333333335
977.0666666666666	849.6333333333333	372.7666666666665
	994.1999999999999	1614.7666666666667	506.5
422.2666666666665	946.7000000000002	1313.0
376.7666666666665	859.0666666666667	663.1666666666667
	507.0666666666666	1735.1333333333332
1822.4333333333332	2264.5	2126.6	470.3666666666666
1439.9333333333332	906.1666666666669	2229.9333333333334
	3458.866666666667	2199.5666666666666
795.4666666666667	656.6999999999999	345.6333333333334
	2227.8333333333335	1135.6	290.4666666666664
5104.0	2228.566666666667	5890.2	1070.3666666666666
1727.0333333333335	641.2666666666667	1199.8
1603.9333333333336	1086.0	1130.5333333333335
698.9666666666667	695.0333333333333	845.9333333333334
	858.5666666666667	1492.1000000000001	550.4
762.6999999999999	376.1000000000001	641.5333333333334\
LOC389247	48.6	56.7	34.0	57.1	33.5	15.9	40.0
	30.6	39.5	14.3	69.8	17.3	16.9	26.9	86.0
	13.4	123.4	110.8	22.1	38.9	142.2	27.2
120.8	93.4	37.5	34.4	48.3	29.1	43.5	62.8	24.6
	178.4	123.8	14.1	8.8	5.6	7.6	74.8	36.3
	68.6	7.4	75.9	88.5	4.7	85.5	57.1	61.1
	104.1	166.9	43.8	149.0	63.3	55.7	63.0	7.5
	104.5	62.6	15.1	118.2	94.2	711.5	48.0	55.8
	49.2	16.7	60.4	48.4	11.1	72.1	10.5	61.6
	131.8	86.1	11.6	96.5	61.1	58.2	12.0
101.0	69.5	88.1	51.7	51.7	11.0	125.1	36.4	80.8
	57.4	40.4	58.0	54.4	159.8	38.6	9.2	74.6
	67.6	39.8	67.0	38.6	71.8	72.6	104.6
65.4\
ADAM11	31.55	180.85	82.5	44.2	121.8	59.15	15.5	88.6
	15.350000000000001	94.10000000000001	25.45	35.9
	51.7	54.25	76.05	18.45	56.599999999999994
35.15	21.5	30.95	40.1	51.3	146.45000000000002
41.35	35.4	46.35	32.35	86.95	75.95	46.400000000000006
	50.449999999999996	149.65	47.35	14.049999999999999
	72.5	35.8	57.650000000000006	49.6	18.2	35.5
	20.9	47.6	69.35	20.95	31.0	38.05	37.8
23.200000000000003	57.55	86.35	18.75	62.55	24.75
223.45	17.7	109.9	82.45	33.400000000000006	29.55
29.65	297.45	51.3	46.85	32.849999999999994	39.9	32.3
	7.75	41.050000000000004	95.2	18.9	148.75
30.450000000000003	21.65	34.15	80.60000000000001
28.700000000000003	69.0	64.05	77.4	131.6	128.5
17.049999999999997	79.0	42.45	156.0	142.65	83.05
31.900000000000002	77.3	28.950000000000003	14.4

89.15	32.15	37.199999999999996	87.0	87.8	64.9	61.8
	80.4	88.85000000000001	58.7	141.45	101.15\
RP11-521O16.2	15.4	68.7	7.8	35.7	56.5	4.3	21.3
	32.1	37.2	119.1	38.0	15.7	2.0	24.3	5.5
	2.1	44.0	5.4	25.9	5.8	30.1	15.1	29.6
	5.3	2.8	47.3	22.2	11.5	70.8	15.3	51.5
	124.3	35.7	5.1	4.2	9.1	12.8	49.9	19.8
	3.3	48.7	10.9	29.9	27.2	4.3	2.7	9.2
	37.1	48.0	26.0	1.9	30.0	62.0	7.0	27.7
	38.6	32.8	2.7	9.0	15.8	183.4	28.2	38.1
	26.1	70.7	5.7	6.2	6.7	22.7	24.5	10.0
	58.9	3.3	39.9	64.9	23.9	104.7	4.0	32.0
	68.5	57.0	6.7	7.5	5.9	13.8	162.7	56.6
	11.2	68.2	5.3	12.3	39.9	10.8	36.0	44.4
	42.6	23.2	10.0	28.7	3.3	5.2	99.7
20.5\
ADAM10	3809.066666666666	892.5	1541.4999999999998
4166.166666666667	1648.333333333333	2136.0
3905.866666666667	1433.8	2413.6666666666665
1544.5333333333335	2826.3333333333335	3200.8333333333335
	4583.833333333333	2549.633333333333
2934.7666666666664	3701.8333333333335	1741.4333333333334
	1786.8	3668.533333333333	2514.7999999999997
1130.2333333333333	1814.2333333333333	1434.8333333333333
	2404.9666666666667	2961.2000000000003
3239.3000000000006	4490.7	2247.9666666666667
1893.8666666666668	3846.7666666666664	2579.8
1432.8999999999999	1622.5999999999997	2546.3666666666663
	1359.4	2951.6666666666665	2619.9333333333334
2763.6	3888.7999999999997	2191.4	2474.6	2959.0333333333333
	2831.4	3359.633333333333	2627.6666666666665
1564.2	2013.6333333333334	2832.2666666666664
1131.0333333333333	1749.5999999999997	2482.0
3862.6333333333337	2534.5333333333333	1237.0666666666666
	2524.4666666666667	796.1333333333332
1626.5333333333335	1602.2	1779.1000000000001
1466.033333333333	1120.2333333333333	1915.1000000000001
	3850.133333333333	2678.5666666666666
2512.0666666666666	1968.8666666666666	2568.3666666666663
	3999.4666666666667	1200.2333333333333
2309.2000000000003	1176.4333333333332	1706.5333333333335
	2267.8333333333335	3601.1000000000004
1704.8666666666668	2886.3000000000006	2013.7333333333336
	2403.566666666667	2224.0333333333333
1383.4333333333334	1578.0666666666668	2954.8666666666663
	1709.3333333333333	2667.6	2988.8000000000006
764.0333333333333	1720.666666666667	2243.933333333333
	2999.933333333334	2623.5333333333333
2985.0333333333333	1650.5666666666666	2861.066666666666
	3358.366666666667	2277.0333333333333
3604.033333333333	3207.766666666667	3496.3333333333335
	2751.866666666667	2984.4666666666667
2560.8333333333335	1785.0333333333335	2995.1000000000004\
DHDDS	495.8	227.9	362.55	283.95	285.3	435.15	367.4

297.2	339.05	295.15	387.05	587.05	458.29999999999995
459.65	310.34999999999997	321.75	403.5	428.40000000000003
	541.2	411.29999999999995	309.15	406.95	372.5
481.35	248.79999999999998	414.84999999999997	357.5
344.65	543.95	293.29999999999995	293.95	680.3	424.0
312.09999999999997	432.1	399.65	402.5	359.5	387.3
421.2	418.54999999999995	417.15	468.4	398.15000000000003
	373.7	399.40000000000003	416.1	369.1
685.3499999999999	299.2	531.8000000000001
391.40000000000003	398.65	387.54999999999995	294.3
279.25	296.35	379.09999999999997	549.35	225.89999999999998
	395.5	445.04999999999995	428.5	358.9
430.29999999999995	502.7	455.75	388.85	442.05
272.79999999999995	269.55	529.25	363.95	384.0	473.7
651.5	382.0	421.15000000000003	452.34999999999997
373.5	331.7	230.7	326.7	562.85	483.35	194.2	407.95
367.45000000000005	303.75	486.2	367.8	413.25	404.45
283.2	336.9	385.15	356.3	343.55	302.09999999999997
292.75	244.9	460.7	304.15\
ADAM19	55.4	225.15	99.85000000000001	197.25	210.8
151.39999999999998	78.7	202.2	271.25	289.85	147.95
93.15	202.5	168.75	144.85	331.15	162.39999999999998
165.6	46.800000000000004	243.10000000000002	615.75
160.15	219.3	181.79999999999998	101.85	143.5	99.4
274.65	147.75	270.85	160.7	280.04999999999995	191.55
193.65	244.35000000000002	139.75	82.3	175.35	86.3
117.7	93.85	231.1	182.89999999999998	212.4
250.04999999999998	160.55	126.4	109.75	204.7	230.7
107.0	140.6	133.85000000000002	295.85	379.8	152.2
254.95000000000002	170.1	112.85	206.95000000000002
1025.45	121.69999999999999	136.0	128.4	252.65	103.5
200.6	1114.05	312.34999999999997	160.25	220.05	174.8
261.95	124.7	170.35000000000002	141.0	217.05	107.0	97.8
	108.65	110.1	119.1	176.3	169.0	248.9
254.60000000000002	141.7	238.4	289.84999999999997
227.04999999999998	83.45	185.4	86.7	125.69999999999999
	80.3	81.4	64.35	92.85	143.65	137.89999999999998
	96.5	195.75	102.65\
ADAM18	29.15	85.8	36.8	8.95	84.4	28.9	31.0
14.149999999999999	52.05	7.15	25.8	53.400000000000006
	23.85	12.7	75.7	11.399999999999999	50.15	16.3
	26.75	29.900000000000002	28.8	58.7	72.0
50.75	68.2	51.949999999999996	36.2	92.15	74.65
111.75	5.75	28.65	65.8	24.1	74.15	16.35	44.75	29.4
	27.2	37.5	9.8	47.1	14.3	14.25	21.75	28.1
	22.2	7.35	18.2	12.149999999999999	32.25	42.0
	40.85	55.75	39.8	12.95	48.150000000000006
42.65	11.149999999999999	19.4	178.05	43.650000000000006
	71.44999999999999	30.15	10.55	67.1
7.949999999999999	31.450000000000003	28.400000000000002
	28.7	35.6	103.1	82.44999999999999
28.099999999999998	14.55	29.5	39.0	8.5
20.200000000000003	25.35	13.799999999999999	10.25	9.4
	42.099999999999994	18.55	167.75	34.2

59.599999999999994	59.150000000000006	96.19999999999999
	45.25	39.45	39.95	12.0	32.2	71.05
52.900000000000006	37.05	44.75	53.35	26.5	41.5
11.9\
ADAM17	936.3333333333334	589.9	1017.7333333333332
923.7999999999998	1000.3333333333334	690.5666666666666
	1109.8666666666666	2207.066666666667
730.0666666666666	648.2333333333333	783.5666666666666
	652.0666666666666	1025.4666666666667
1246.3666666666666	1032.6333333333332	1085.6666666666665
	943.9333333333333	772.8666666666667
752.3333333333334	899.8000000000001	727.1
742.4333333333334	638.8	752.1333333333333	688.5
928.5333333333333	729.1333333333333	657.3333333333334
	834.6	636.4333333333334	925.6999999999999
700.8333333333334	702.9333333333333	710.1666666666665
	1422.6333333333332	922.2666666666668
675.7333333333333	828.6666666666666	816.6
1026.5666666666666	1121.7333333333333	895.0333333333334
	706.6333333333333	923.8000000000002
899.3333333333334	898.5999999999999	708.1333333333333
	899.0999999999999	868.3666666666667
602.8666666666667	759.9666666666667	1034.1
733.8666666666668	782.1	1002.2333333333335
704.7333333333332	746.4	659.4333333333333	601.0
695.5	941.8000000000001	802.0666666666667	1003.4
944.2333333333332	573.9666666666667	626.7666666666667
	570.1666666666666	945.8333333333334
787.5333333333333	852.0	612.1666666666666
650.2333333333333	535.9333333333333	1032.6666666666667
	722.8666666666667	844.3333333333335
932.3666666666664	855.5	611.5666666666666
1466.3999999999999	1177.7333333333333	1424.8000000000002
	1110.5333333333333	1138.3999999999999
963.3000000000001	1633.1000000000001	895.9666666666667
	1358.0333333333333	544.9666666666666
2458.6666666666665	1497.8333333333333	847.8333333333334
	745.3000000000001	852.5333333333333	744.5
823.7999999999998	937.6999999999999	786.8333333333335
	1068.5333333333333	1080.0666666666666
839.9000000000001	616.5666666666666	710.2666666666668\
BRMS1	899.2	517.9	563.5	1077.0	516.8	1699.9	1330.8
302.5	1234.1	907.0	812.4	937.5	1091.0	1286.0	515.6
1276.0	1208.5	1273.3	1140.3	1180.1	710.9	958.3	526.2
854.2	1072.4	500.1	802.1	462.7	587.5	854.8	546.3
244.3	513.2	868.2	871.1	527.7	1093.1	796.8	1072.5
1792.3	908.0	1002.3	577.1	1106.9	1407.6	736.5	378.9
725.6	520.1	680.0	1008.1	792.4	837.1	492.7	680.5
435.7	790.7	598.6	588.2	615.1	49.9	824.8	594.7
847.4	302.1	742.8	1044.8	648.5	603.0	667.6	53.9
567.1	730.8	843.8	836.8	854.0	336.2	806.9	475.7
362.6	36.8	218.9	942.5	687.5	277.5	249.1	246.8
301.4	699.8	451.2	683.6	165.0	504.6	515.0	444.6
509.5	442.1	510.3	532.2	513.6	480.9	528.2	559.7\

ADAM15 61.25   345.6   65.15   44.6   249.15 68.4
187.79999999999998   181.04999999999998   167.2   218.1
186.05 141.65 135.2   67.9   175.85 112.35000000000001
110.5   84.2   188.4   281.7   205.85 122.05000000000001
238.29999999999998   147.6   58.7   194.29999999999998
100.15 228.54999999999998   217.15 204.39999999999998
76.35   258.95 100.80000000000001   139.75 365.20000000000005
        107.55000000000001   153.05 279.55 207.8
290.40000000000003   132.45 89.5   185.45 108.0   100.8
271.7   176.8   235.6   336.3   254.8   243.45 211.95 191.8
259.55 193.10000000000002   283.70000000000005   223.8
166.0   204.8   70.7   387.95 229.8   116.55000000000001
136.85 207.1   191.0   119.15 149.05 127.2   111.55000000000001
        290.95 65.1   105.95 189.95 94.55000000000001
133.1   168.75 172.55 151.95000000000002   95.75   151.5
151.20000000000002   77.3   172.45000000000002   301.65
223.1   243.85 187.25 204.75 251.3   87.55   241.8   83.1
153.64999999999998   189.2   88.7   65.95   143.25 303.3
147.25 145.4   216.35000000000002   179.0\
LOC91548   435.4   1094.5 1969.7 877.4   2157.0 942.3
408.8   741.5   1248.5 795.2   551.0   718.1   448.6   867.4
1421.8 545.1   727.1   1275.6 1297.3 539.8   1661.6 1205.4
1602.1 773.0   1006.2 649.6   1099.3 1428.3 1285.8 571.7
870.7   1419.1 979.1   1754.7 1316.2 510.5   406.3   877.1
537.9   1368.4 497.4   338.5   907.8   686.3   1090.6 821.5
678.0   367.1   1971.1 1270.5 1609.9 716.0   838.1   1054.7
628.9   1419.5 1529.3 1559.5 1052.0 1908.0 1478.6 1744.6
975.2   516.5   1428.8 1638.4 1195.1 676.4   1211.3 1493.4
1556.3 2288.4 964.6   495.4   1791.4 334.0   569.8   741.5
981.3   417.3   1013.9 450.7   1145.9 613.2   667.6   522.9
461.7   952.1   1394.7 1218.0 1021.4 605.9   840.0   688.2
821.5   1186.5 852.5   1259.9 1268.1 1207.6 1216.6 1162.8
1064.1\
ADAM12 119.34 153.01999999999998   166.35999999999999
245.48000000000002   169.52 54.3   113.52000000000001
190.46 581.2599999999999   287.65999999999997   64.62
175.46 94.53999999999999   94.74000000000001   59.06
244.72000000000003   294.28 256.14 40.66   152.16
243.77999999999997   142.11999999999998   164.8   98.88
91.38   60.55999999999999   236.32 399.36 457.7   125.34
346.06 396.06000000000006   161.94 544.7   159.12 67.4
55.279999999999994   164.7   175.45999999999998   352.88
185.00000000000003   55.779999999999994   680.6000000000001
        270.41999999999996   103.66 106.34 113.4   85.08
43.42   81.94000000000001   351.2799999999999
67.88000000000001   87.29999999999998   210.68 323.9
67.52   94.02000000000001   154.99999999999997   93.78
247.02000000000004   230.03999999999996   103.38000000000002
        109.4   132.8   189.92 125.86000000000001
46.17999999999999   54.0   526.88 56.379999999999995
288.36 159.4   171.1   457.55999999999995   255.43999999999997
        346.46 384.71999999999997   375.02 128.73999999999998
        72.70000000000002   136.07999999999998   226.44
37.699999999999996   156.32 270.41999999999996

```
96.38000000000001	157.76	273.0400000000001	165.04
152.35999999999999	95.85999999999999	448.87999999999994
	148.16	145.68	143.32	92.02000000000001	87.9
177.18	260.42	98.72	72.82000000000001	188.04000000000002
	54.959999999999994\
PLAA	362.9666666666667	270.53333333333336
230.53333333333333	427.4333333333334	247.23333333333335
	295.03333333333336	523.4000000000001
383.7666666666665	438.2666666666665	396.4333333333334
	404.0333333333333	278.03333333333336
308.43333333333334	311.1	424.6333333333334
244.29999999999998	227.9333333333333	234.7666666666665
	354.5	352.8666666666666	306.43333333333334
350.5	273.9	478.0333333333333	617.3666666666667
268.40000000000003	445.43333333333334	315.7
213.3666666666667	366.2333333333333	464.0666666666666
	266.73333333333335	212.5666666666667
361.8666666666666	539.6	337.8666666666667
567.8000000000001	403.5666666666666	518.2333333333333
	302.5	231.53333333333333	251.1666666666667
298.59999999999997	412.2666666666665	637.1333333333333
	403.93333333333334	589.8	638.1666666666666
256.53333333333336	231.6666666666666	369.5666666666666
	449.8	364.20000000000005	333.1	258.3666666666667
	208.7666666666665	394.3666666666666	296.3
271.73333333333335	267.2	353.3666666666666
268.1666666666667	369.5333333333333	274.5666666666666
	181.79999999999998	251.9666666666667
421.43333333333334	494.3666666666673	240.80000000000004
	338.43333333333334	251.6	323.7666666666665
246.4666666666667	327.0666666666666	305.90000000000003
	357.0333333333333	290.93333333333334
360.0666666666666	345.09999999999997	872.0666666666667
	488.3333333333333	393.5333333333333
204.4666666666667	438.43333333333334	364.0666666666666
	304.0666666666666	465.0333333333333
476.5666666666666	363.3333333333333	527.0666666666667
	465.7	236.66666666666666	302.2	269.06666666666666
	233.23333333333335	203.9666666666667
215.1666666666666	285.7666666666665	211.83333333333334
	209.7666666666665	180.1666666666666	277.2
221.4333333333333\
CARD18	6.4	257.6	13.7	25.9	43.8	15.8	1.5	13.5
	23.6	4.1	33.3	2.1	4.5	56.6	4.2	38.7
	9.1	23.7	42.7	59.0	19.8	23.4	8.9	2.5
	4.8	9.6	5.4	21.2	73.9	52.5	27.8	96.7
	13.5	69.8	60.0	658.2	36.7	10.5	1.7	5.5
	20.0	15.7	19.5	40.8	23.0	22.9	35.2	61.1
	17.5	13.4	30.0	63.5	35.1	61.2	26.1	53.5
	8.8	60.2	9.8	79.8	38.9	2.7	66.9	29.9
	22.8	13.1	26.3	47.8	61.8	23.1	40.9	42.9
	3.9	38.8	22.5	64.5	15.6	57.9	2.9	46.9
	51.5	27.5	2.5	89.3	93.3	22.8	3.5	10.1
	4.8	51.2	37.2	9.9	70.6	12.5	15.0	24.3
```

	8.6	30.1	2.6	79.7	39.9	49.5	11.0\
COX6CP1 /// COX6CP1	168.5	588.0	112.0	160.6	90.1
243.8	142.6	131.0	314.2	171.9	187.0	168.2	143.8
229.1	98.1	98.9	235.6	250.2	152.1	85.4	119.9
131.6	73.6	117.5	215.9	174.1	278.4	58.2	218.3
188.1	129.2	138.9	192.0	55.4	187.6	106.7	134.4
101.7	152.7	256.5	196.7	106.7	274.4	81.3	178.0
168.5	157.7	98.1	33.9	126.0	149.1	228.1	303.6
117.6	230.9	202.5	182.6	179.3	182.2	192.5	446.6
281.7	170.5	159.3	155.8	169.3	196.8	110.8	161.6	87.7
	44.5	63.2	156.3	152.3	171.8	296.3	138.4
166.2	66.2	287.7	115.6	65.6	185.5	269.9	29.2	28.6
	125.7	271.5	152.9	74.7	153.3	80.0	111.3
168.6	75.5	109.4	129.6	239.3	93.1	174.3	98.0
123.5	68.3\
CARD16	148.79999999999998	43.1	363.9	787.5	107.25
67.45	209.15	360.1	313.55	522.45	198.25	288.09999999999997
	150.79999999999998	1020.3000000000001	16.95
461.05	155.95	267.2	855.5	344.5499999999995	243.85
205.4	231.45000000000002	562.1999999999999	474.75
121.65	313.55	385.84999999999997	220.25	384.3	267.85
192.0	190.85	412.7	419.3	300.5	242.25	242.85	222.9
399.15000000000003	156.54999999999998	14.75	506.35
305.65000000000003	354.59999999999997	154.15	204.65	51.9
	69.45	130.20000000000002	350.8	473.95
29.799999999999997	95.6	199.8	38.85	339.25	157.25	91.1
	177.35	461.35	500.90000000000003	389.05	406.55
352.55	248.85000000000002	354.85	490.0	646.4000000000001
	275.59999999999997	153.6	160.7	654.5	355.85
525.1	264.45	254.20000000000002	320.70000000000005
289.95	6.9	106.0	177.7	73.5	575.55	108.9
198.20000000000002	69.65	282.65000000000003	420.0
450.6	320.25	149.1	247.9	326.4	247.25	79.85000000000001
	225.04999999999998	200.7	111.95	199.1	182.85
175.60000000000002	339.3\
CTD-2251F13.1	27.7	19.6	18.7	1.6	37.4	9.8	5.5
	106.3	64.3	92.2	92.0	37.9	49.7	5.1
103.1	57.7	44.6	73.8	45.0	11.9	123.0	73.7
125.4	32.9	37.9	38.2	6.6	94.9	13.4	108.8	57.6
	97.8	92.9	64.6	36.4	12.4	19.9	65.7	31.6
	21.3	31.7	6.2	34.2	55.7	1.9	148.4	9.3
	18.7	78.9	18.1	80.4	9.5	60.0	103.2	68.8
	87.9	39.0	74.7	12.3	5.8	63.7	74.2	64.5
	31.9	34.9	144.7	18.5	40.8	85.2	38.3	54.2
	112.9	79.7	26.4	38.7	30.5	9.3	58.8
104.9	20.5	47.6	13.2	19.0	29.4	111.2	80.6	5.7
	21.7	8.8	135.1	16.5	132.0	11.6	33.7	38.4
	79.1	50.6	24.5	38.2	8.1	62.0	18.0
43.2\
CARD14	39.81666666666666	72.88333333333333
52.01666666666667	71.35000000000001	137.23333333333332
	33.2	27.183333333333334	182.26666666666665
61.23333333333333	99.64999999999999	55.68333333333334
	29.53333333333333	68.68333333333334

47.916666666666664	84.3166666666668	28.266666666666667
	90.53333333333332	70.16666666666667
54.550000000000004	57.550000000000004	309.98333333333335
	62.20000000000001	136.89999999999998	24.75
67.38333333333334	133.71666666666667	52.550000000000004
	96.03333333333335	91.25	98.64999999999999
35.55	123.58333333333333	93.11666666666667
31.283333333333333	89.26666666666665	108.21666666666668
	41.833333333333333	123.43333333333334
68.18333333333334	40.516666666666666	91.43333333333334
	40.983333333333334	25.983333333333333
48.366666666666667	70.64999999999999	88.76666666666667
	92.366666666666667	34.31666666666667
164.29999999999998	39.6	45.0	49.46666666666667
77.43333333333334	113.05	57.583333333333336
97.68333333333332	33.300000000000004	93.18333333333334
	61.68333333333334	50.366666666666667
83.633333333333333	102.01666666666667	92.14999999999999
	64.266666666666667	120.76666666666665
167.95000000000002	30.816666666666666	65.06666666666666
	49.083333333333336	38.283333333333333
171.0666666666667	100.94999999999999	128.73333333333335
	41.800000000000004	46.1	39.433333333333333
89.11666666666667	19.233333333333333	75.833333333333333
	29.433333333333334	110.98333333333335
48.516666666666667	64.566666666666666	117.58333333333331
	188.11666666666667	383.73333333333335
118.18333333333334	90.40000000000002	196.20000000000002
	185.783333333333333	41.416666666666664
103.61666666666667	47.033333333333334	31.233333333333334
	58.783333333333333	39.583333333333336
32.083333333333336	22.433333333333334	44.166666666666664
	52.083333333333336	59.683333333333333
99.71666666666665	49.550000000000004\
RP11-216L13.19	177.5	1499.3	152.9	174.9	283.4	104.0	46.6
	114.7	69.1	107.3	78.1	284.3	85.4	105.4
343.3	124.4	59.6	189.3	79.3	79.0	347.1	154.3
120.9	70.2	136.5	784.9	147.8	163.7	263.5	97.9
114.8	1015.0	150.1	18.8	182.4	169.1	312.6	54.3
124.8	85.5	43.0	181.8	247.2	115.4	83.7	395.3	91.5
	147.6	1051.2	118.4	370.6	67.3	400.1	435.2
162.5	343.6	110.4	68.0	213.4	266.0	191.4	98.7
185.2	18.8	131.1	169.8	83.3	97.8	198.2	152.5
280.3	809.9	278.5	166.1	77.4	70.6	111.9	83.2
158.0	572.1	212.6	218.8	198.2	174.4	294.3	254.8
232.2	203.3	66.7	93.3	156.7	717.1	307.0	280.4
530.1	616.7	583.6	472.8	384.2	941.8	998.5	814.3
526.4\
CARD11	26.1	73.9	34.95	52.55	68.15	34.1	27.5
10.85	59.2	111.65	23.8	39.95	49.05	45.95	25.9	59.3
	21.5	45.2	32.849999999999994	23.7	150.05
48.95	134.8	37.7	72.15	21.5	18.799999999999997
57.95	26.25	52.599999999999994	12.9	57.400000000000006
	137.85	11.9	121.35	34.0	10.85	36.5	24.0

20.05	101.2	13.5	57.55	40.65	43.2	63.650000000000006
	43.900000000000006	26.799999999999997	81.65	94.6
	19.6	26.299999999999997	34.75	104.35000000000001
	23.450000000000003	45.400000000000006	56.8
26.25	70.65	56.0	92.95	65.55	17.4	31.85	40.7
43.15	40.85	49.45	191.14999999999998	16.2	137.25	76.8
	85.80000000000001	26.85	91.44999999999999	27.9
	58.0	60.55	25.35	36.6	21.5	74.6	50.2
12.25	173.75	161.75	39.2	38.1	60.75	201.3	14.4
20.95	47.4	30.95	41.35	23.75	37.95	13.8	19.15
20.700000000000003	56.0	69.5	47.599999999999994\
RP11-350F4.2	949.2333333333332	711.1999999999999
887.8666666666667	678.5	487.8666666666666
370.4333333333334	617.7666666666665	963.2333333333335
	434.93333333333334	721.5666666666667
473.7333333333333	618.0666666666667	267.5666666666666
	604.8000000000001	1364.0	798.0666666666666
516.3666666666667	498.23333333333335	380.4666666666667
	722.7999999999998	383.06666666666666
464.5333333333333	581.0333333333333	493.7666666666666
	931.1666666666666	419.40000000000003
500.40000000000003	745.9666666666666	794.0333333333333
	514.1999999999999	385.0333333333333
1017.8666666666667	354.26666666666665	627.2333333333332
	839.0333333333333	466.2666666666667	372.7
349.56666666666666	518.0333333333333	544.8000000000001
	549.1	542.0333333333333	504.1666666666667
496.59999999999997	480.93333333333334	1024.6000000000001
	928.4666666666667	531.2333333333332
541.9333333333334	992.1666666666666	739.2666666666668
	395.40000000000003	538.1666666666666
1309.2333333333333	549.4333333333334	623.2666666666667
	701.6999999999999	587.9666666666667	572.0
752.6666666666666	360.8666666666666	449.06666666666666
	382.1666666666667	453.4666666666667
767.8666666666667	682.5	734.6999999999999
377.1666666666667	861.3333333333334	497.1000000000001
	423.5666666666666	704.0	860.2000000000002
816.1666666666666	980.1333333333336	392.8
617.1666666666666	520.3666666666667	506.4333333333332
	771.9000000000001	1320.3666666666666
675.6666666666666	482.8666666666673	383.56666666666666
	849.9666666666667	309.6333333333333	1258.0
584.3333333333334	798.2999999999998	310.1
787.1999999999999	1357.3666666666668	745.3333333333334
	318.3333333333333	466.03333333333336
723.7666666666668	684.2666666666665	471.1000000000001
	361.90000000000003	563.9333333333334
884.7666666666668	853.5666666666666	482.7\
LOC101927865	5.2	27.9	18.9	42.0	86.2	38.7	25.4
	7.5	6.8	45.1	40.1	38.8	29.8	39.3	18.1
	12.4	19.0	6.6	1.9	7.8	77.1	31.8	83.4
	10.2	54.2	5.1	35.4	59.8	13.7	9.3	15.9
	13.7	7.0	56.2	10.1	6.5	8.1	26.2	9.6

	10.1	12.7	15.8	16.6	60.7	30.7	4.9	7.2
	14.2	49.1	65.3	47.6	11.9	19.7	5.8	28.8
	42.7	40.9	53.8	5.8	16.7	183.0	47.3	36.5
	4.9	55.9	15.7	84.6	24.7	11.3	26.7	68.8
	13.0	5.9	55.8	3.2	9.7	9.8	5.0	2.9
	2.5	9.7	6.7	50.5	2.5	79.4	9.5	48.1
	9.6	56.2	8.6	22.2	62.7	28.3	6.6	7.6
	41.8	15.4	35.9	26.9	85.2	34.0	11.8
41.4\
LOC101927869	13.1	44.7	53.6	53.5	53.0	2.8	20.1
	5.3	33.2	89.4	5.9	6.6	14.2	56.2	52.3
	23.9	4.8	22.9	38.9	5.7	110.0	25.2	83.6
	10.7	26.4	27.3	23.9	86.3	20.3	44.3	31.0
	46.3	5.1	42.2	97.4	28.6	23.1	2.3	31.9
	25.8	26.1	36.4	31.8	7.9	10.8	62.3	39.0
	18.3	35.4	31.5	44.7	19.3	34.5	38.5	54.4
	22.8	61.0	32.5	61.1	45.2	237.3	26.5	49.0
	21.8	15.1	7.4	3.5	2.5	33.4	1.7	51.6
	89.3	2.7	6.2	24.1	4.5	64.5	40.0	6.6
	27.7	15.2	39.5	22.2	3.4	52.9	59.2	61.7
	9.7	5.2	99.0	3.0	50.7	28.4	32.3	12.7
	40.8	34.2	35.4	82.9	44.3	23.8	75.6
59.5\
GOLGA6A /// GOLGA6B /// GOLGA6C /// GOLGA6D	18.3	97.7	88.4
	7.3	171.0	99.2	36.3	136.9	76.1	62.2	16.8
	70.7	78.2	8.5	85.1	58.6	47.1	47.4	21.4
	91.0	182.6	12.5	95.3	53.0	59.7	71.9	89.2
	69.2	103.0	70.3	42.4	68.1	84.5	16.6	15.3
	129.0	58.8	191.7	21.1	88.2	44.5	50.8	78.3
	15.3	85.0	61.1	112.9	39.2	52.3	66.2	21.1
	92.3	42.9	151.1	51.9	90.8	127.9	62.0	50.9
	177.2	402.9	150.2	61.8	71.8	113.3	95.3	59.5
	35.6	137.3	87.2	188.9	48.0	67.0	19.4
106.3	37.4	87.9	53.5	36.8	7.6	140.4	56.5	25.1
	17.7	130.9	137.4	77.6	186.4	136.8	191.9	35.1
	100.6	33.7	15.9	11.7	107.8	46.7	75.1
103.4	18.3	68.7	70.9	91.8\
HTRA3	119.85	101.85	176.55	98.55000000000001
242.70000000000002	171.7	94.45	158.4	142.05	270.0	77.1
	197.35000000000002	161.15	130.55	169.4
109.44999999999999	240.75	155.5	61.35	314.75
414.04999999999995	143.5	254.3	218.5	83.9
182.10000000000002	85.45	334.05	203.5	171.25	207.8
255.8	286.0	192.75	242.4	174.75	81.05	137.64999999999998
	68.45	177.05	82.69999999999999	128.0	127.2
198.85	106.8	197.0	144.25	186.4	195.39999999999998
204.25	278.4	159.39999999999998	89.3	242.5	218.0
173.75	224.95	174.89999999999998	61.099999999999994
158.7	318.79999999999995	245.55	187.45	109.7	284.15
157.2	117.25	231.9	429.29999999999995	196.0
94.44999999999999	154.15	141.6	176.3	201.8	152.3
241.5	150.95	226.75	187.35	124.1	259.8	91.85	228.3
268.0	294.55	261.6	239.45	174.85000000000002
158.60000000000002	137.6	240.04999999999998	182.5

225.8	230.65	192.95	234.25	217.45	256.9	246.3	228.85
172.2	170.45\
HTRA4	15.0	318.7	99.2	613.2	130.1	65.8	85.3
1149.3	79.2	182.5	20.0	304.5	55.1	392.9	16.1
398.2	493.4	133.0	234.2	14.4	387.2	153.0	468.3
366.3	68.1	106.3	3.9	211.3	76.1	154.5	15.3
501.6	2915.8	12.7	48.5	166.6	114.7	227.8	72.1	61.5
	17.7	83.3	186.1	82.2	78.8	176.7	214.4	11.7
	202.2	84.0	104.8	64.6	4.1	201.0	47.0
412.0	156.7	108.7	95.0	208.2	31.4	494.8	479.1
149.2	203.8	163.3	207.3	229.9	602.1	86.3	901.0
683.6	1787.4	109.3	147.0	44.9	494.7	154.0	168.3	15.3
	123.9	221.5	33.9	261.0	320.5	877.6	346.4
268.5	186.8	71.5	172.7	168.2	44.1	61.9	81.6
109.8	152.9	65.0	205.5	104.5	39.4	165.2	174.8\
PLD5	9.25	15.5	3.05	27.65	106.95	34.8	14.95	41.2
	32.150000000000006	60.9	27.85	50.9	9.55	19.9
	67.8	31.85	17.95	37.050000000000004	37.25
18.400000000000002	7.05	65.0	132.65	47.65	38.25
47.199999999999996	107.6	52.15	51.599999999999994
53.75	81.25	50.75	9.399999999999999	66.2
39.699999999999996	65.05	30.4	39.900000000000006
146.25	45.95	47.849999999999994	30.85	22.05	32.95
37.349999999999994	40.55	79.9	34.25	33.7	79.25
70.55	20.299999999999997	27.9	35.25	28.55	46.0
17.35	238.65	32.4	30.900000000000002	44.0
726.9499999999999	128.05	18.3	16.15	23.95	160.65	2.5
	29.35	52.199999999999996	63.85	29.55	4.3	14.2
	46.05	37.3	19.85	37.25	33.75	26.55	90.35
31.299999999999997	4.35	35.900000000000006	7.9	65.2
	55.1	74.44999999999999	34.75	36.2	30.85	45.4
	52.45	38.1	53.05	52.650000000000006	115.45	43.9
	26.45	28.0	49.1	60.15	43.900000000000006\
HTRA1	1368.1	699.0	1661.9	1666.5	1154.1	3074.8	1271.9
1876.5	5980.3	3102.7	1031.0	2839.5	5926.4	1737.2	381.9
1860.0	4285.7	1179.0	676.3	8857.6	2016.2	1105.2	1048.6
3313.8	860.5	496.4	1700.0	2506.9	5340.7	6522.7	2624.3
1775.9	1509.5	12865.3	2507.0	1393.8	2733.2	845.1	5634.6
4002.9	1398.7	3010.8	2610.8	5932.4	2530.4	644.0	2938.0
974.1	1230.3	1748.3	3382.7	1430.4	831.5	1660.4	4119.9
628.3	2492.6	1066.8	958.6	1835.7	3234.5	1186.2	1577.4
2579.8	1887.4	842.5	3040.9	2895.5	3672.0	829.4	1144.6
3627.0	3032.1	6366.2	4578.6	8308.9	4570.8	6611.8	6267.9
2079.4	979.7	994.1	1419.3	1262.4	1530.5	318.1	2439.9
2153.5	1833.3	933.1	1838.3	3005.3	1905.1	2942.1	4593.5
1535.0	2143.8	1429.0	379.3	953.3	2197.2	1394.7	1626.6\
PLD4	58.5	56.9	60.900000000000006	8.05
86.14999999999999	30.85	30.35	43.7	41.849999999999994
	49.199999999999996	38.85	45.3	25.7
31.450000000000003	57.650000000000006	66.0	16.85
121.30000000000001	43.95	58.25	154.2	50.349999999999994
	131.35	29.6	14.05	54.650000000000006	8.25
63.95	51.849999999999994	23.0	23.45	24.950000000000003
	56.3	55.6	59.849999999999994	18.2

9.149999999999999	25.299999999999997	25.65
12.850000000000001	39.35	7.15	57.9	21.9	62.35
20.55	56.75	13.7	124.85000000000001	22.400000000000002
	56.300000000000004	41.45	76.25	70.44999999999999
	109.8	89.89999999999999	36.15	47.5	36.2
46.85	132.3	48.949999999999996	83.4	35.75	43.85
16.950000000000003	24.95	48.150000000000006	50.65
11.95	20.85	113.35	24.35	21.8	76.15	38.25	54.95
12.35	95.35	31.1	24.549999999999997	71.35	45.95
60.900000000000006	15.05	73.5	37.65	74.5	48.9	14.0
	54.35	104.85	58.65	40.699999999999996	66.3
70.35	56.4	18.45	82.8	118.45	16.55	75.75	44.3\
DAPP1	72.3	415.875	1046.7749999999999	268.025	431.625
151.3	119.375	689.375	265.7	1261.775	373.42499999999995
	210.8	443.54999999999995	1087.6999999999998
242.35	375.95	217.325	738.875	165.125	125.375	1096.075
354.25	1206.7	425.225	259.09999999999997	118.225	318.275
636.075	466.9	321.975	137.375	701.0	658.825	260.7	258.0
514.525	250.0	834.425	184.925	208.9	96.875	108.30000000000001
	494.32500000000005	150.875	297.925	236.3
323.15000000000003	107.5	398.325	240.975	307.65	435.75
375.425	288.42499999999995	415.45000000000005	371.175
210.625	708.425	870.9749999999999	352.325	732.35	914.975
724.7	575.225	248.425	459.95	169.8	214.77499999999998
592.7249999999999	571.85	729.7	512.575	441.0
215.29999999999998	433.025	305.35	747.475	249.125	305.225
157.07500000000002	303.2	343.3	114.175	653.1	1214.95
567.075	262.975	225.825	577.8	385.675	133.575	149.225	188.0
369.70000000000005	94.125	145.4	163.60000000000002
279.475	284.175	194.425	218.975	235.4	187.85\
HTRA2	481.9	419.55	356.35	483.2	456.75	711.65	680.7
411.2	627.6	283.04999999999995	672.5	549.9	472.6
561.15	514.3	702.75	448.65	592.75	547.7	319.15	164.1
470.95	600.4	536.55	365.4	763.4	511.35	488.85	254.8
451.15	738.1	527.95	463.1	518.4	454.45000000000005
391.15	618.7	269.3	655.35	874.25	788.6500000000001
639.6	401.8	395.45	605.5	425.15	687.7	612.55	703.75
543.65	607.3	638.75	447.0	395.75	475.5	391.29999999999995
	588.95	329.15	466.75	430.35	327.1	365.45	353.05
417.15	424.75	382.15	671.5999999999999	784.4	427.1
517.85	372.3	560.6	435.9	440.15	397.20000000000005
872.75	509.9	602.8	481.0	887.2	273.4	339.95000000000005
	684.5	542.45	113.15	463.05	394.65	324.20000000000005
	488.20000000000005	334.5	538.8	403.0	570.55
861.6500000000001	693.45	709.45	691.15	913.9	641.3
645.25	770.55	642.85	705.75\
FAM223A /// FAM223B	129.2	783.5	310.1	125.0	110.6	41.5
	109.8	294.8	55.8	150.0	73.1	163.5	142.8
167.0	59.6	246.5	138.1	194.8	75.1	71.5	751.8	61.6
	159.8	117.2	105.4	135.6	218.7	234.7	233.8	78.6
	190.5	206.6	374.9	65.6	193.0	88.1	134.7
163.4	176.1	52.7	135.7	210.7	167.7	201.5	124.5
322.1	90.1	63.8	428.9	274.8	47.5	41.5	127.6
518.4	241.3	334.6	194.3	203.6	74.3	235.7	316.9

```
196.2	157.1	80.1	246.1	145.5	31.3	48.9	252.2
130.7	436.1	176.7	262.1	187.1	130.6	63.8	163.0	82.8
	169.4	50.1	355.1	274.1	108.9	196.2	200.8
588.0	402.1	156.7	72.2	143.8	39.8	282.8	233.4
245.0	409.4	349.6	540.4	282.2	530.2	237.8	337.9
302.6	236.5\
PLD6	55.4	135.7	128.7	85.5	97.3	26.4	31.5
102.3	89.4	84.9	82.6	75.0	23.6	110.6	155.1	19.9
	91.6	94.2	45.3	172.9	274.9	107.7	182.0	4.9
	68.1	104.6	76.3	165.7	139.3	89.4	9.9
296.3	83.9	68.4	69.4	83.5	62.6	141.4	42.3	49.4
	32.4	65.1	25.4	77.7	15.1	121.3	26.4	83.5
	135.8	82.7	136.1	101.1	104.1	234.6	54.5
239.1	52.8	91.3	119.6	61.5	40.6	100.9	19.8	92.7
	104.1	91.3	118.0	64.5	160.3	78.1	83.9	90.0
	125.5	32.4	128.6	35.0	87.0	72.6	118.2	31.2
	107.9	81.8	63.2	88.1	207.7	299.5	96.5	8.2
	129.7	90.5	45.8	124.5	86.1	55.7	36.7	49.1
	116.7	74.6	81.2	93.6	112.0	119.6	80.1\
PLD1	170.44285714285712	18.642857142857142
142.22857142857146	85.54285714285716	142.5
70.11428571428571	41.21428571428571	294.3142857142857
	92.15714285714286	139.94285714285712
41.24285714285714	212.32857142857142	103.98571428571427
	97.7142857142857	43.885714285714286
73.87142857142858	125.32857142857145	78.78571428571429
	116.61428571428573	188.94285714285712
124.2857142857143	67.68571428571428	112.27142857142859
	94.81428571428572	60.814285714285724
93.54285714285713	79.05714285714286	117.58571428571427
	108.14285714285714	151.14285714285714
66.72857142857143	220.62857142857143	140.81428571428572
	75.89999999999999	103.44285714285714
97.37142857142855	42.800000000000004	84.42857142857143
	126.10000000000001	124.21428571428571	70.5
46.92857142857143	148.6142857142857	86.74285714285713
	63.08571428571428	82.14285714285714	110.0
107.77142857142857	107.7	93.14285714285714
93.57142857142858	152.08571428571426	139.94285714285715
	94.61428571428571	84.94285714285715
82.84285714285714	62.01428571428571	100.72857142857143
	154.0142857142857	90.17142857142858
126.17142857142858	84.71428571428571	147.57142857142858
	154.87142857142857	98.02857142857142
130.28571428571428	51.6	99.55714285714285
134.71428571428572	113.62857142857145	204.59999999999997
	104.62857142857142	94.2857142857143
48.8142857142857	68.54285714285713	42.65714285714286
	193.68571428571428	56.22857142857142
69.74285714285715	140.35714285714286	64.9857142857143
	109.77142857142857	38.15714285714285
74.22857142857141	274.7	737.8571428571429
71.18571428571428	117.95714285714284	124.11428571428571
	333.3285714285715	87.85714285714286
```

83.58571428571429 81.25714285714285 111.49999999999999
111.88571428571429 86.78571428571426 86.7
70.87142857142858 88.34285714285714 77.91428571428571
70.91428571428573 43.32857142857142
64.05714285714286\
PLD3 258.5 300.1 249.6 1774.3 825.8 1777.8 2916.3
669.2 1502.3 1731.0 697.4 970.0 783.4 939.0 186.1
757.2 818.7 746.6 1410.0 1354.2 773.3 902.9 631.2
991.2 525.5 781.1 887.8 445.7 697.7 1542.7 788.7
168.6 770.6 1352.1 463.5 395.9 748.4 589.7 1679.9
2219.1 6213.8 1076.7 516.9 2702.6 1522.9 485.5 510.0
352.9 762.4 735.5 665.2 543.8 336.9 385.5 529.0
347.7 530.0 263.1 687.3 875.3 333.8 1017.2 1272.0
618.2 675.0 728.1 1421.1 1591.7 737.1 388.2 441.0
740.6 558.7 778.5 658.0 573.8 845.0 470.3 679.8
511.4 227.4 581.3 728.5 563.2 399.2 424.0 327.3
771.3 849.8 699.2 1340.2 356.8 425.4 617.9 520.5
381.8 401.4 255.3 526.2 253.4 593.8 360.4 742.2\
PLD2 234.9 431.7 382.9 221.9 305.4 272.8 402.3
213.5 232.3 323.0 261.5 172.0 109.4 345.7 244.2
377.0 207.0 248.7 265.1 450.6 763.4 140.7 260.1
148.7 239.0 443.3 169.2 280.3 324.0 454.7 227.3
274.2 534.8 179.4 305.2 277.1 117.5 375.8 275.5
178.1 310.0 244.7 305.7 172.9 163.3 269.3 136.2
247.1 449.8 260.3 295.9 147.7 302.7 220.6 222.0
560.4 249.6 186.0 408.6 269.4 568.5 301.2 285.0
212.7 185.9 493.0 153.0 174.1 515.6 212.5 141.7
345.8 299.0 214.0 342.8 139.1 188.6 220.1 238.0
198.8 233.2 164.6 381.5 259.7 248.5 305.0 201.6
200.5 211.4 238.3 133.9 186.4 149.6 366.6 315.4
175.1 266.2 277.0 402.5 368.4 323.4 210.6 230.5\
GRK1 54.6 109.5 56.2 64.6 141.2 54.3 67.2
113.5 76.4 116.6 38.6 11.8 37.3 16.0 153.8 75.7
68.4 9.1 55.5 46.4 32.3 30.6 167.1 31.9
28.4 14.8 20.9 116.8 76.7 42.7 20.8 12.7
10.4 40.7 98.9 73.3 13.5 103.2 24.6 15.3
14.1 42.2 109.3 60.5 45.6 137.7 84.4 64.0
113.2 81.1 44.4 70.5 99.5 120.8 137.0 33.3
17.1 98.7 53.6 17.9 447.2 95.7 43.4 64.8
28.9 63.0 24.5 81.4 101.4 18.1 93.9
120.3 95.2 59.2 6.1 16.4 72.5 41.9 97.0 81.9
88.7 69.0 64.4 59.2 163.6 288.8 16.8
125.8 90.0 148.1 5.5 112.9 76.3 47.0 10.8 69.7
11.0 84.4 69.2 25.7 48.5 79.7 22.8\
KCNV1 33.3 58.550000000000004 8.45 3.6 46.6 56.5
15.0 45.9 11.600000000000001 3.8
38.550000000000004 4.65 9.5 7.699999999999999
38.25 29.4 4.85 22.05 23.55 16.6 61.55
5.050000000000001 48.650000000000006 13.399999999999999
4.9 41.099999999999994 7.2 77.6 12.4 34.1
2.55 18.75 36.1 4.2 10.6 40.75 2.5
11.55 2.9 8.0 63.150000000000006 47.95 77.15
11.95 5.35 19.5 19.7 20.5 7.5 10.55 7.3 24.7
3.75 82.05 9.2 12.85 42.400000000000006

7.550000000000001	7.6	51.15	68.7	16.55	58.2	11.5
	34.15	8.25	39.1	33.55	5.8500000000000005
5.449999999999999	27.3	14.649999999999999
27.450000000000003	20.650000000000002	35.800000000000004
	11.15	32.55	45.55	43.400000000000006	56.35
11.85	28.599999999999998	34.85	20.099999999999998	49.4
	78.6	44.2	78.8	90.75	26.9	5.55	33.35	33.2
	18.3	18.1	6.5	37.75	5.550000000000001	44.9
	20.2	7.1	10.0	12.2\
KCNV2	41.5	110.3	101.3	8.6	5.5	16.8	2.0	71.5
	18.6	104.0	4.9	39.7	37.5	37.7	78.4	22.3
	2.2	4.6	43.8	41.0	9.3	45.2	98.8	10.2
	24.0	45.1	10.7	114.7	33.9	30.2	6.9
125.4	78.3	12.4	15.3	35.4	6.0	64.9	28.7	52.8
	57.0	10.2	56.4	78.2	53.0	52.3	10.7	17.9
	90.3	17.3	55.1	74.3	51.3	36.0	33.8	19.7
	1.4	75.5	34.0	8.9	261.2	53.0	58.2	7.5
	77.5	73.3	45.5	35.8	89.0	3.7	33.7	33.3
	41.1	36.0	18.1	48.1	53.6	1.9	68.9	8.1
	38.5	11.1	5.0	32.1	24.2	106.4	19.8	52.2
	14.3	19.5	7.3	40.4	30.3	22.5	38.4	11.4
	23.8	37.8	27.0	70.2	13.8	20.0	68.8\
RBBP4P4 /// RBBP4P4	21.8	27.6	13.6	4.6	4.3	2.4
	1.9	3.3	9.4	4.3	4.5	4.6	3.1	7.0
	5.3	22.3	2.6	14.8	2.0	31.9	164.0	38.1
	9.7	2.9	18.4	12.7	7.4	68.5	68.2	2.7
	5.7	3.9	9.4	21.9	6.8	4.1	37.7	12.2
	2.3	40.7	11.6	25.4	3.7	36.2	11.9	6.8
	3.8	5.9	76.3	18.3	62.6	4.1	2.2	9.5
	3.0	6.4	10.0	6.9	2.7	5.0	391.4	4.0
	3.9	2.0	2.6	13.6	38.4	5.4	6.0	14.4
	5.9	96.2	29.4	20.2	4.2	4.3	28.5	2.3
	19.4	3.3	8.0	4.4	8.1	7.3	4.4	5.8
	4.0	89.1	10.2	3.8	20.7	10.0	20.6	1.8
	1.7	2.0	39.4	3.6	3.4	4.0	6.9	3.3
	4.0\
GRK5	96.55	359.8	79.35	96.85000000000001	126.4
132.95	99.1	352.5	156.9	176.70000000000002
73.57499999999999	117.57499999999999	77.25	121.375
116.85	199.59999999999997	126.69999999999999
113.49999999999999	44.925	109.6	466.225	98.025	182.5
191.85	94.17500000000001	121.5	93.675	241.02499999999998
	181.29999999999998	174.70000000000002
123.64999999999999	138.04999999999998	103.875	135.45
183.575	109.07499999999999	110.025	122.475	60.6	114.85
135.325	80.15	324.0	111.35	73.525	151.775	271.04999999999995
	69.3	175.67499999999998	184.325	191.425	97.6
215.0	234.92499999999998	132.75	109.725	83.94999999999999
	158.125	126.85	89.875	420.02500000000003
115.57500000000002	124.42499999999998	147.875	201.45
200.025	110.20000000000002	137.875	155.925	123.35	334.1
169.625	214.4	68.0	126.575	96.125	267.77500000000003
120.475	155.65	155.15	213.875	195.475	76.75	178.75	293.6
161.67499999999998	822.575	218.475	116.30000000000001

111.375 103.30000000000001 348.6 151.675 324.32500000000005
327.675 132.4 136.725 135.27499999999998 97.75
179.325 119.275 163.375 112.225\
GRK4 17.05 170.25 101.94999999999999 60.525 216.775
54.70000000000001 54.925 36.65 44.0 72.275 38.75
37.925 39.35000000000001 60.5 68.625 75.85000000000001
56.4 65.9 44.075 62.375 209.3 59.89999999999999
60.175 47.475 54.275 68.725 38.75 152.9
37.650000000000006 29.925 52.3 72.55000000000001
86.125 69.3 61.35000000000001 46.25 24.400000000000002
33.55 55.39999999999999 53.6 71.175 81.675
128.95000000000002 33.475 61.5 148.075 51.15 23.625
220.875 48.800000000000004 68.825 12.399999999999999
12.625 55.275 53.4 91.9 77.8 64.325 95.67500000000001
200.89999999999998 75.9 81.05 44.79999999999999
45.775 21.525 83.79999999999998 59.25
43.675000000000004 127.775 39.025 249.2 75.60000000000001
57.425 67.8 71.67500000000001 47.9 50.3
48.75 42.300000000000004 12.8 110.575 41.375
31.575000000000003 15.225000000000001 107.52499999999999
127.69999999999999 59.55 87.675 54.575 123.05
55.875 112.55 29.05 43.525 58.8 52.025000000000006
47.15 44.375 72.5 83.15 93.89999999999999 70.1
94.7\
GRK7 45.7 128.7 47.9 26.8 96.9 17.4 8.0 4.2
38.0 23.1 30.8 31.6 1.3 9.3 90.3 61.2
54.8 47.8 18.0 17.2 69.2 35.9 105.0 15.8
3.1 39.9 2.1 83.6 87.5 9.7 25.2 6.2
34.9 59.5 62.1 14.6 3.3 59.1 11.0 14.8
2.3 30.4 2.5 19.3 73.7 45.8 51.8 23.1
5.5 56.3 3.2 47.5 2.5 21.7 99.8 80.9
85.0 6.8 4.3 8.4 211.0 6.0 46.2 18.6
4.8 3.7 44.7 35.6 77.0 33.5 27.3 50.3
63.1 23.0 67.2 6.5 55.2 10.9 79.6 14.9
49.0 60.3 47.8 15.8 4.9 160.0 44.1 90.1
16.8 30.5 17.4 8.0 24.2 43.6 9.1 32.0
62.3 34.3 4.7 34.8 40.3 65.8 49.1\
GRK6 220.60000000000002 249.25 123.875 298.7
215.97500000000002 311.22499999999997 277.95 166.35
311.5 209.70000000000002 329.425 233.2 300.45000000000005
278.725 258.025 309.675 298.92499999999995 206.675
373.125 202.75 455.47499999999997 213.25 326.95000000000005
146.6 224.45000000000002 377.025 385.42500000000007
281.72499999999997 253.27500000000003 275.8
226.575 336.875 207.375 314.5 261.175 194.60000000000002
236.475 299.925 387.6 259.275 290.97499999999997 232.475
235.35000000000002 615.775 271.2 261.65000000000003
263.125 239.2 276.8 221.57500000000002 247.425 235.9
225.67499999999998 282.25 252.10000000000002 195.55
317.65 157.0 273.675 232.875 364.12500000000006 290.275
182.75000000000003 250.25 250.9 266.9 259.475 547.175
478.99999999999994 132.925 170.2 377.1 392.85 233.375
217.275 258.025 326.0 196.575 274.7 212.4 167.125 333.8
377.95 323.2 418.35 313.25 245.39999999999998 348.325

```
290.77500000000003	217.60000000000002	244.65	238.75
220.2	234.375	195.25000000000003	175.075	225.175	282.7
271.15	180.70000000000002	174.75	219.64999999999998
245.3\
PIBF1	461.1	145.2	699.5	740.0	528.2	314.5	225.5
251.3	504.8	398.4	339.2	304.6	474.7	393.3	535.7
318.8	314.9	568.9	678.7	379.0	410.9	510.4	313.8
327.8	477.5	703.1	502.7	588.3	840.7	474.0	862.9
562.2	419.5	453.9	169.7	689.6	520.2	292.1	370.4
307.7	137.9	266.5	1001.6	469.0	417.9	763.5	237.7
337.6	681.7	394.6	611.6	691.1	238.2	197.3	406.0
437.2	563.3	605.9	413.0	522.4	842.2	407.7	603.1
734.2	579.0	305.6	219.8	517.4	486.6	682.3	547.5
626.9	428.6	622.1	385.1	520.4	595.2	246.0	545.8
390.3	653.3	308.3	304.1	341.9	472.2	250.5	293.5
350.7	220.6	257.5	595.2	449.0	596.3	661.2	524.5
635.8	665.8	387.3	579.4	838.6	494.2	471.9	636.9\
LOC101927876	7.7	67.1	6.2	3.6	87.2	12.2	6.3
	34.7	54.5	62.2	41.0	20.6	3.6	8.8	14.3
	5.3	121.8	16.4	2.6	32.6	17.8	2.7	18.6
	22.1	7.7	7.4	4.7	109.9	35.2	10.8	5.1
	65.4	27.7	6.7	10.6	5.2	31.1	12.6	6.6
	42.8	34.9	11.6	54.7	10.7	2.6	7.6	9.3
	36.3	39.7	4.4	59.4	33.2	35.9	30.8	32.6
	29.7	48.3	15.1	28.3	6.5	280.5	3.2	38.4
	9.1	3.1	13.1	33.4	46.7	51.5	36.2	41.1
	13.2	37.6	31.9	37.0	23.6	47.1	64.2	2.4
	52.8	60.5	55.6	41.7	27.2	7.8	94.9	4.5
	63.3	5.6	107.7	65.7	69.1	4.1	22.7	3.0
	62.4	55.5	3.6	9.4	11.1	9.3	3.3
42.7\
LOC101927877	39.3	157.9	96.1	11.6	182.0	19.2	3.6
	107.7	18.3	88.6	60.1	14.0	13.7	44.4
105.1	38.5	21.3	33.6	77.3	9.8	33.2	32.3
200.1	9.2	132.6	64.9	8.6	74.1	131.6	35.2	19.5
	29.0	33.4	23.7	41.6	54.9	60.3	90.1	23.7
	92.7	95.0	13.1	92.8	10.4	30.6	107.2	16.4
	20.8	94.5	51.9	41.3	18.1	22.8	185.6	25.1
	21.7	54.4	27.5	17.5	54.8	61.7	68.7	25.1
	19.1	99.5	102.3	18.4	20.8	140.6	61.8	20.2
	199.7	72.1	26.9	13.3	11.4	86.5	21.6	43.9
	22.2	143.4	184.5	19.7	79.2	169.7	287.1
164.8	26.3	19.0	54.4	26.3	23.6	20.4	18.0	46.2
	15.1	17.8	63.1	15.2	15.9	57.9	25.7
131.5\
CHCHD10	309.7	568.6	411.1	495.7	188.5	1611.9	409.6
653.7	609.5	798.6	538.4	337.5	394.8	546.2	275.9
270.5	395.9	681.0	679.1	227.4	240.2	519.2	705.4
465.8	416.8	392.0	544.4	501.3	903.2	274.3	144.4
682.0	818.7	481.1	627.0	1941.5	884.4	722.9	138.5	79.0
	378.3	68.9	774.9	323.3	291.4	691.8	820.0
218.5	337.4	405.4	432.0	528.1	1436.8	550.4	326.0
737.8	305.7	574.3	816.1	428.5	1162.4	649.2	343.4
306.9	258.0	790.3	282.4	280.4	613.4	268.2	190.4
```

873.0	259.1	308.7	390.9	396.9	924.2	323.5	395.3
502.2	659.9	365.3	418.1	2633.6	889.8	3236.2	634.1
2359.6	845.1	734.4	281.6	542.4	782.5	627.8	657.9
513.7	408.2	1127.1	914.3	343.1	618.9	377.8	419.0\
FAM199X 1018.6666666666666	813.9	836.1333333333333
1004.4333333333334	906.3000000000001	712.0666666666666
	920.7666666666668	896.4666666666667
663.3000000000001	406.0666666666666	994.9
2275.366666666667	839.6	924.6666666666666
999.0666666666666	1302.9	990.3333333333334	834.5
1425.4666666666665	945.7999999999998	718.9666666666666
	794.6333333333333	554.9	851.4	955.3666666666668
	1482.3999999999999	1824.8666666666668
1116.4666666666667	1180.8333333333333	1062.4333333333334
	984.3333333333334	1330.3999999999999
608.1333333333333	1337.3666666666666	1144.6000000000001
	1064.3999999999999	840.2333333333332	977.4
1148.4333333333334	1032.7333333333333	1072.2666666666667
	1157.8	867.9	858.3333333333334	851.1
663.3333333333334	1353.4666666666667	1211.6333333333334
	1732.1666666666667	960.6666666666666
824.3000000000001	1220.8666666666666	859.1999999999999
	955.4	740.6	982.6333333333333	801.6666666666666
	1423.9000000000003	822.6333333333333	713.9
1571.8666666666668	1018.7999999999998	950.7000000000002
	1048.0	1142.7666666666667	1090.9333333333334
949.1333333333333	787.4	717.2000000000002	1046.0
738.1	774.1333333333333	1057.7666666666667
1434.7666666666667	1370.1333333333332	1140.0	836.5
1555.5	1194.7	1433.8333333333333	1246.6333333333334
1278.3	473.7	1230.0	2965.566666666667	1473.1333333333332
	890.7333333333332	1042.5666666666666	879.0
1181.6333333333334	707.7666666666668	988.4333333333333
	1308.0333333333333	933.9333333333334
881.7000000000002	1168.7333333333333	1042.4666666666667
	1065.5	1646.6666666666667	1332.2333333333333
1120.1333333333334	1474.5666666666668	932.3666666666668\
IGF2R	1329.3999999999999	2011.1	1068.65	1754.05	1304.25
2089.4	3011.65	3615.4	3188.1	1795.8	1545.15	2111.7	1407.05
1982.75	1003.4	1732.4499999999998	3169.05	2602.3
1667.6999999999998	2805.4500000000003	1289.7	1881.7
1409.0	771.25	2683.95	1731.15	964.5500000000001	819.05
877.8	2877.7999999999997	1833.1999999999998	960.95
1194.0500000000002	1469.7	1677.15	1155.25	1345.8500000000001
	1264.65	4110.3	2994.75	4277.349999999999	4322.25
1192.75	3761.1000000000004	2997.0	756.05	1288.75
2317.7000000000003	979.55	1576.4499999999998	1338.95
1590.2	1399.3000000000002	2765.4	1664.75	871.0	1310.7
1622.5500000000002	1487.8000000000002	849.75	777.05
1645.8500000000001	1490.35	2039.25	1495.4	2337.7	2736.75
4234.65	892.35	982.55	870.15	1180.0500000000002	3042.55
1598.8000000000002	1476.1999999999998	1026.95	1201.25
3141.4	1183.55	2967.05	863.3	1288.7	757.3	441.35	701.8
935.65	2231.7	1121.35	3281.85	2621.6	1943.85	1687.1999999999998

357.70000000000005	858.1500000000001	727.0
462.4	618.0	627.8499999999999	749.3	410.0	612.0
517.35	868.1\
BSPRY	723.9499999999999	1846.4499999999998	545.9
492.35	508.8	1052.35	237.55	495.6	410.5	776.35	1030.85
716.65	1255.05	771.95	296.45	212.35	694.55	525.0	658.2
753.95	501.8499999999997	521.9	805.55	477.45000000000005
424.75	687.2	986.2	555.35	1053.9	968.8	482.65
635.3	585.25	738.7	510.5	1036.0	1525.0	460.6	1343.8
408.5	309.85	1416.5	373.05	900.35	1032.2	683.45	931.2
886.5999999999999	380.1	590.45	768.7	955.1	688.05
457.15	473.35	540.45	515.85	371.45000000000005	698.7
300.55	797.3	579.4	591.3	838.85	761.75	436.75
756.3499999999999	1136.6	586.15	1092.85	302.4	416.4
701.1	836.5999999999999	767.5	1124.25	550.55	771.65
772.3	106.80000000000001	156.85	518.65	403.3	436.45
222.4	323.65	319.85	316.04999999999995	580.25	319.65
386.79999999999995	305.7	597.4	380.0	429.95000000000005
669.5	418.40000000000003	649.7	849.05	672.6
404.55	426.7	556.7\
LOC101927870	865.4000000000001	127.3	4.25	10.6
32.95	4.35	113.4	13.85	2.85	38.300000000000004	4.25
51.5	15.149999999999999	22.4	23.25	3.5	6.55
2.35	4.0	9.25	7.5	18.200000000000003
47.75	1.0	2.1	73.75	2.75	8.3	36.949999999999996
29.5	6.6499999999999995	44.05	41.0	11.85
38.75	37.55	2.05	4.6	2.8	29.15	24.9	5.65	3.1
0.85	2.45	36.75	28.3	16.35	29.4	30.75	54.6
11.5	34.15	5.4	6.699999999999999	24.4	24.1
18.7	45.25	28.45	16.3	9.549999999999999
5.050000000000001	2.0	150.3	63.7	45.0	19.5
36.349999999999994	14.8	71.85000000000001
24.299999999999997	10.5	14.899999999999999	2.75	3.4
52.45	3.0	8.2	43.65	24.549999999999997	4.15
31.4	3.9	33.85	33.0	450.45	7.3999999999999995
11.7	28.6	3.25	3.7	15.2	17.6
12.850000000000001	13.3	21.85	22.400000000000002
3.6999999999999997	59.800000000000004	38.4	8.55
14.350000000000001\
RPS6KA2-AS1	6.3	24.5	46.6	11.4	11.3	17.8	7.2
11.9	42.7	69.6	64.8	73.6	31.7	40.0	7.0
17.3	12.2	116.0	9.1	99.6	17.3	37.6	31.1
11.3	34.4	49.9	10.1	8.6	32.1	14.9	6.5
25.1	12.7	39.0	135.2	29.6	29.9	8.7	43.4
5.9	7.6	10.2	26.1	11.5	9.3	12.4	4.3
66.3	33.9	5.7	13.0	84.2	25.0	17.6	11.1
27.3	15.6	21.2	6.9	12.8	59.9	53.4	12.8
46.6	12.3	14.7	10.3	12.1	11.8	9.2	21.9
31.3	14.0	11.5	22.7	9.8	21.3	7.8	5.5
55.0	9.9	14.7	4.8	13.0	38.3	40.6	10.0
20.4	20.1	68.4	3.2	44.3	12.1	3.2	15.0
13.0	15.1	4.9	21.3	46.2	17.0	15.9
12.7\
SPINK13	218.2	22.6	60.1	11.0	11.3	10.6	55.4	66.1

123.4 116.8 13.9 24.7 68.0 58.6 130.9 13.7
30.0 68.7 86.7 137.9 114.3 76.2 122.7 47.0
8.8 23.3 91.5 119.8 39.9 10.5 31.2 60.7
123.8 25.4 84.4 161.6 16.2 71.9 319.1 78.4
49.5 58.1 77.3 9.2 26.5 14.9 12.7 55.7
175.0 165.3 45.5 14.7 10.9 23.9 9.9 11.5
155.7 69.7 334.9 68.5 27.1 24.8 215.2 84.2
230.5 210.5 55.1 201.8 132.3 43.6 13.3
139.2 154.1 13.1 37.0 11.2 98.4 8.1 502.7 60.3
19.4 52.7 84.4 50.1 134.2 37.4 36.6 24.2
12.5 49.6 17.9 148.6 13.2 105.0 59.5
101.1 18.3 38.9 82.1 33.9 86.1 545.7 54.2\
IGHV7-81 /// IGHV7-81 8.0 54.3 26.9 5.0 64.6 14.3
3.0 56.9 7.3 4.7 4.1 2.6 14.5 9.1
22.2 38.6 11.1 6.6 18.9 2.7 18.5 6.7
26.3 4.2 6.9 26.6 37.0 34.2 65.9 15.5
22.4 13.8 22.4 5.4 5.1 23.2 3.7 10.5
18.8 4.1 7.0 21.3 12.3 4.5 51.5 29.3
7.6 4.5 3.1 13.8 10.7 5.1 22.2 15.9
39.1 45.4 53.2 10.3 2.2 5.6 20.8 6.5
10.1 31.3 27.4 64.2 18.3 18.1 7.0 5.0
95.5 64.8 10.4 7.6 14.6 4.8 23.7 29.9
2.6 3.3 6.2 3.6 1.8 38.9 10.3 6.1
9.1 13.8 6.9 9.2 21.5 10.5 35.9 9.2
18.4 35.2 44.9 6.4 29.9 39.5 45.4 39.8
40.1\
RP11-161D15.1 30.9 4.8 3.2 1.9 15.9 4.3 2.5
5.0 3.5 4.6 7.4 7.6 6.1 46.1 68.1
6.8 3.9 10.2 15.5 58.2 43.7 4.1 6.0
7.0 42.7 35.9 2.8 24.7 27.8 3.0 3.5
5.7 89.5 23.5 10.5 12.6 11.2 3.3 2.2
4.4 10.8 2.3 8.7 19.8 50.7 36.5 8.3
5.3 5.5 8.7 7.7 4.1 32.0 29.3 7.2
5.2 12.4 32.5 4.6 35.3 39.2 11.4 8.2
13.5 8.1 12.2 2.1 2.0 6.0 29.4 18.1
3.9 6.2 10.8 5.0 5.0 5.7 3.0 18.9
22.4 66.8 10.3 19.0 9.9 15.3 8.2 20.6
11.0 26.1 8.1 3.8 3.7 3.6 6.9 9.6
8.8 5.2 36.9 9.8 22.3 3.1 74.4
38.1\
NUDT4 /// NUDT4P1 1213.8 181.4 401.1 438.3 246.7
775.8 1059.9 318.4 677.9 405.7 2159.4 265.1 308.9
905.3 669.0 895.7 1275.7 194.5 320.8 1295.8 338.2
465.8 413.8 431.8 481.2 270.8 468.7 318.0 220.6
2811.7 955.8 275.0 209.3 506.7 360.5 503.3 950.9
294.8 1352.1 937.0 917.2 182.2 409.7 1482.7 458.4
568.2 274.5 2272.1 121.8 825.1 365.2 493.8 350.3 42.8
198.4 113.8 412.4 475.2 852.2 300.7 267.4
223.8 1053.4 495.6 1217.9 392.9 1189.5 3195.8 261.2
501.8 266.8 94.9 692.4 747.6 441.7 732.5 357.6
357.0 568.7 629.9 222.4 591.7 705.0 449.1 110.5
372.0 654.3 183.9 278.1 418.5 679.2 387.8 990.8
1172.0 1035.9 932.3 1157.7 792.5 443.7 976.0 602.5
501.7 830.6\

LOC101927886		184.0	217.0	83.2	318.8	156.1	164.4	
866.7	156.0	503.7	119.0	325.1	107.8	177.8	133.6	
219.4	306.9	186.9	209.2	200.0	255.9	256.2	120.4	
152.8	106.8	131.1	234.9	324.0	304.6	232.7	299.9	
157.1	125.1	156.9	168.6	214.7	91.5	72.6	296.5	33.9
	197.5	301.1	174.4	232.2	165.7	146.9	144.7	58.4
	287.6	385.7	228.2	342.4	120.5	165.8	318.6	
147.0	226.5	442.2	145.6	89.9	196.6	326.6	145.4	
296.9	154.8	183.8	233.8	242.8	287.9	228.5	237.4	
174.9	187.3	139.2	278.2	290.3	243.8	131.8	192.2	
211.0	486.1	370.5	220.1	70.9	94.8	127.0	251.5	
331.8	406.3	164.9	72.2	86.0	69.5	149.2	79.3	69.1
	79.5	91.5	69.7	136.9	28.1	123.1	127.2	
101.7\								
ZMYND15	11.2	31.7	87.7	10.1	57.9	14.3	3.1	18.6
	10.1	53.4	10.7	19.1	15.8	29.1	31.1	32.3
	21.6	19.4	38.7	54.6	93.2	42.9	131.0	16.7
	12.7	11.8	9.8	23.6	31.3	13.9	16.0	45.6
	55.1	37.8	21.5	54.6	14.0	115.0	8.5	13.5
	20.0	161.0	16.1	16.3	27.6	37.6	12.7	27.0
	40.5	11.5	22.0	18.9	16.6	30.1	17.3	
113.9	51.4	105.7	22.5	21.4	22.6	90.8	39.6	19.7
	161.2	17.5	29.1	20.5	38.3	51.2	25.5	
170.0	22.3	60.3	35.8	9.0	148.6	22.9	17.1	53.2
	12.2	9.2	14.3	93.3	35.1	89.8	21.3	36.8
	41.5	87.6	10.9	32.0	23.4	54.1	17.5	28.0
	10.4	70.4	9.5	13.7	16.8	28.3	36.0\	
LOC101927884		14.7	243.4	81.2	40.9	145.3	73.7	40.9
	106.4	43.1	82.2	60.5	53.9	29.6	53.4	80.5
	5.7	94.1	13.6	101.7	11.2	47.8	36.9	71.9
	37.3	5.8	56.8	98.6	109.2	105.6	59.9	43.1
	108.9	19.4	71.5	27.4	80.5	56.3	88.1	68.8
	49.8	32.6	49.9	140.8	126.5	59.6	41.2	
108.8	62.9	169.7	182.6	13.2	52.6	80.9	264.2	
103.5	96.9	103.0	174.9	98.2	96.9	128.1	89.6	83.6
	83.3	88.4	28.5	59.5	39.1	120.0	46.6	
136.3	127.3	151.5	27.3	169.5	34.8	120.1	70.4	74.7
	47.0	138.7	146.8	83.6	29.3	34.7	211.5	
123.7	136.6	81.4	20.6	3.5	106.3	20.8	83.6	47.5
	56.5	40.9	90.6	68.8	108.5	123.2	22.6	
135.9\								
ZMYND19	583.9	680.2	363.5	837.5	558.6	677.0	392.7	
269.3	324.3	502.5	250.2	352.1	523.1	329.2	711.5	
427.0	263.4	669.8	295.7	298.7	362.3	388.9	527.0	
232.6	584.2	269.5	762.3	372.9	378.5	380.6	328.3	
384.7	469.9	286.0	627.6	447.8	383.7	666.2	558.6	
344.2	230.2	597.2	221.7	302.2	369.3	353.2	312.9	
711.9	375.5	293.9	361.5	309.7	361.0	651.9	431.6	
521.3	505.3	480.4	537.2	289.5	89.7	433.3	220.4	
184.7	263.6	441.7	309.0	449.8	431.5	409.9	385.8	
440.4	311.7	352.8	363.5	340.3	433.5	180.0	315.9	
299.2	361.1	423.4	392.7	599.8	485.8	814.8	406.5	
330.4	743.9	495.7	360.7	285.8	297.1	335.8	241.0	
376.2	200.8	269.5	464.0	378.7	273.0	442.2	437.8\	

DIAPH2-AS1	30.35	65.95	35.05	34.75	63.25	14.95	25.3
	52.05	41.5	58.3	31.1	27.5	24.900000000000002
	26.8	36.449999999999996	28.9	34.85	62.0	8.7
	32.45	104.55	41.65	14.4	9.85	39.9	30.8
33.55	61.5	56.800000000000004	28.200000000000003
23.700000000000003	134.35	85.75	33.8	80.1
23.549999999999997	30.700000000000003	66.95	15.05
36.05	42.8	30.05	37.949999999999996	14.700000000000001
	21.400000000000002	55.650000000000006	9.6	26.2
	61.05	42.1	41.25	59.85	40.75	56.8	56.95
39.550000000000004	21.299999999999997	82.45
50.199999999999996	42.15	350.8	64.95	42.050000000000004
	34.45	28.8	36.95	15.299999999999999	13.7	7.5
	86.6	110.85	18.15	72.3	55.650000000000006	75.5
	61.849999999999994	66.05	48.9	31.1	53.95
22.75	12.05	26.25	73.55	59.8	85.45	31.450000000000003
	38.2	41.55	36.25	13.55	71.6	10.100000000000001
	27.3	58.05	35.55	71.5	12.75	54.9	74.2
45.15	51.45	54.95\
PLEK	36.7	221.85000000000002	227.45	455.90000000000003
	112.5	102.45	282.9	494.0	154.5	267.45
124.94999999999999	143.9	83.05	412.75	73.95	368.25
137.35	201.65	244.7	116.85	56.099999999999994
247.35000000000002	235.6	442.35	137.6	149.55	106.2
361.05	254.95000000000002	190.2	109.95	176.6	315.1
213.39999999999998	220.45	197.25	379.45000000000005
199.45	62.650000000000006	318.4	120.0	113.75	213.2
164.35	269.3	122.25	215.75	81.15	276.8	167.64999999999998
	153.85	65.55000000000001	85.85	201.85000000000002
	251.60000000000002	136.6	232.35	66.9	190.6
206.64999999999998	220.7	270.5	170.3	80.25	174.2
122.9	283.29999999999995	180.7	237.29999999999998
231.15	201.85	211.4	330.75	182.9	186.4	199.75	373.9
168.3	194.7	116.25	121.6	246.7	60.2	215.55	140.1
133.35000000000002	191.4	155.05	322.4	102.95	261.2
155.55	161.3	158.4	39.8	97.35	96.25	76.75	98.45	87.7
	61.85	112.4	166.7\
PITPNC1	309.43999999999994	709.28	431.21999999999997
478.23999999999995	290.28000000000003	649.62	158.6
404.8	636.5000000000001	446.3	440.5799999999999
661.9399999999999	1298.8400000000001	319.55999999999995
	812.04	864.78	917.4200000000001	440.52
285.96000000000004	867.7	991.04	710.54	407.91999999999996
	461.26000000000005	500.16	1154.8400000000001
1769.6200000000001	677.58	657.42	1087.6	649.58	1440.7
655.2	1173.5	525.94	383.34000000000003	1006.3999999999999
	656.6400000000001	992.5	550.12	535.5
414.64000000000004	774.0000000000001	663.06
499.08000000000004	266.43999999999994	305.26
703.0400000000001	378.96	813.6200000000001	898.7
1020.5799999999999	1163.86	436.1	604.2	719.6	1013.54
670.24	610.62	878.9399999999999	431.62000000000006
1185.66	719.4200000000001	923.5799999999999	687.24
990.1199999999999	614.24	1748.86	653.96	669.0400000000001

558.1999999999999	296.71999999999997
663.5400000000002	792.42	671.68	735.3	638.1199999999999
516.1600000000001	992.5799999999999
230.63999999999996	267.16	513.4	1517.6399999999999
265.0	505.88	645.88	656.12	409.68	145.72000000000003
116.42	288.46000000000004	355.6	408.62	415.58000000000004
471.52	579.0	589.54	302.78000000000003
432.28000000000003	786.4	759.9799999999999
354.7200000000001	624.1400000000001\
GRM1	27.5	36.800000000000004	26.033333333333333
20.533333333333333	30.899999999999995	28.166666666666668
31.366666666666664	42.9	32.96666666666667	19.2
30.866666666666664	8.033333333333333
40.800000000000004	37.766666666666666	64.26666666666667
28.100000000000005	8.799999999999999
68.16666666666667	19.5	9.933333333333334
20.166666666666668	29.5	79.16666666666667
14.966666666666667	17.0	42.800000000000004	23.7
37.733333333333333	13.233333333333334	35.9
14.233333333333334	87.3	7.099999999999999
35.300000000000004	58.6	28.5	37.833333333333336	23.5
22.266666666666666	24.76666666666667	7.3
11.833333333333334	19.166666666666668	28.166666666666668
43.06666666666666	82.76666666666667
11.266666666666666	48.53333333333333	97.59999999999998
30.8	63.46666666666667	31.599999999999998
17.733333333333334	123.66666666666667	34.8	34.7
37.43333333333334	27.76666666666667	40.9
92.63333333333333	201.06666666666663	74.6
47.166666666666664	11.533333333333331	48.5	38.6
18.266666666666666	10.700000000000001	42.199999999999996
30.066666666666663	57.866666666666674
121.43333333333334	57.233333333333333	9.200000000000001
28.0	42.666666666666664	38.5	15.733333333333334
53.06666666666666	59.96666666666666
33.96666666666667	63.866666666666674	17.866666666666664
40.800000000000004	27.766666666666666
40.06666666666667	43.43333333333334	29.533333333333333
34.133333333333333	44.4	18.733333333333334	56.6
35.633333333333333	6.466666666666666
34.96666666666666	49.733333333333333	64.66666666666667
31.366666666666664	48.733333333333334
44.033333333333333	66.89999999999999	35.833333333333336
13.600000000000001\
GRM3	82.2	138.7	4.3	34.9	52.7	6.6	3.9
206.8	69.7	18.5	54.7	73.1	60.5	46.9	150.8	88.4
72.7	86.0	40.8	109.5	387.3	47.2	11.0	42.9
53.8	61.4	25.2	70.5	25.9	59.3	45.2
127.2	100.1	128.5	64.2	54.2	11.4	66.8	6.4	10.8
27.3	46.9	34.1	66.3	16.6	61.8	30.5	46.7
77.9	150.4	10.3	8.1	6.8	29.7	10.3	7.8
198.6	133.2	72.3	206.7	331.0	8.8	74.7	9.6
183.6	64.3	187.3	75.3	8.6	8.0	73.5	17.2
22.2	44.1	136.7	28.8	134.4	41.4	118.9	65.7

72.7	71.1	34.1	26.7	17.4	269.9	58.5	59.7
24.8	9.9	4.7	45.2	42.4	27.2	68.3	5.9
60.2	50.6	69.9	74.2	6.1	13.7	102.2\
GRM2	104.0	21.65	20.85	30.5	26.85	34.45
43.150000000000006	76.75	49.2	21.950000000000003	38.8
116.35	58.35	61.15	70.25	31.200000000000003	11.7
41.75	38.1	51.2	95.55	55.349999999999994
47.75	10.6	25.7	35.0	55.05	17.85	22.2	44.65	12.6
76.10000000000001	107.45	30.599999999999998
69.85	46.0	22.9	62.25	7.95	48.45	34.1	16.95
39.85	14.45	43.05	11.5	32.95	59.400000000000006
90.94999999999999	71.95	47.1	14.799999999999999
20.55	94.1	22.25	31.5	48.0	112.75	47.75	81.7
132.35	13.65	32.849999999999994	53.65	50.7	47.0
49.95	32.6	33.05	107.85	85.45	18.05	13.5	55.65	66.6
38.75	52.849999999999994	21.6	8.5	25.65
16.299999999999997	21.3	36.4	50.0	39.95	106.55
40.35	21.25	12.55	13.3	31.65	46.5	51.45
36.400000000000006	32.699999999999996	8.649999999999999
12.25	64.4	30.7	105.2	18.15	33.75	14.55\
GRM5	70.76666666666667	31.733333333333338
113.03333333333335	16.133333333333336	52.300000000000004
69.93333333333334	53.43333333333333
56.03333333333333	99.63333333333333	143.20000000000002
130.29999999999998	39.5	50.833333333333336	32.1
193.5666666666667	44.5	50.86666666666667
69.93333333333334	25.13333333333333	34.03333333333333
75.93333333333334	111.53333333333335
222.60000000000002	32.666666666666664	102.33333333333333
28.166666666666668	31.633333333333336
102.76666666666667	44.699999999999996	39.53333333333334
46.4	66.43333333333332	35.93333333333333
72.56666666666668	33.300000000000004	78.36666666666667
71.56666666666666	42.06666666666667
19.933333333333334	62.26666666666666	79.3
15.866666666666665	79.43333333333332	28.166666666666668
36.13333333333333	58.666666666666664
60.76666666666666	71.36666666666666	66.7
56.66666666666666	51.4	103.13333333333333	73.7
23.133333333333336	80.10000000000001	77.43333333333334
160.46666666666667	101.23333333333333
27.866666666666664	43.900000000000006	396.56666666666666
132.8	33.833333333333336	62.33333333333332
23.53333333333333	47.9	55.099999999999994
20.23333333333333	70.43333333333332	61.333333333333336
76.7	49.73333333333333	18.400000000000002
54.43333333333334	48.56666666666667	76.93333333333332
92.36666666666667	42.76666666666667
49.900000000000006	44.5	32.1	16.466666666666665
57.26666666666667	26.333333333333332	27.600000000000005
35.233333333333334	41.53333333333333
39.56666666666666	74.8	69.83333333333333
28.966666666666665	60.26666666666667	78.03333333333333
62.96666666666667	96.63333333333333

61.166666666666664	53.43333333333333	100.93333333333334
	22.933333333333334	114.2	53.13333333333333	89.7
	70.30000000000001\
CA1	50.75	149.60000000000002	109.05	42.7	157.9
36.25	18.5	99.30000000000001	90.89999999999999	77.3
	72.05	83.35	39.35	71.0	125.15	57.4	49.5
11.149999999999999	47.25	57.75	155.75	82.6	97.75	55.4
	31.85	107.05	40.55	85.1	79.5	92.0	35.7
160.6	86.69999999999999	42.05	129.95	67.80000000000001
	32.0	54.75	55.2	73.45	51.35	85.95	164.55
46.05	10.2	93.85000000000001	54.449999999999996	62.5
	133.5	101.2	109.65	34.150000000000006	14.8
171.45	62.85	118.85	14.350000000000001	120.80000000000001
	13.65	115.35	563.3	40.55	39.3	41.5	136.1	50.7
	26.0	27.950000000000003	53.599999999999994	34.7
	126.95	63.45	44.95	43.75	89.35	18.1	129.1
110.9	65.6	44.3	78.25	51.85	28.45	101.65	127.8
177.05	107.85000000000001	144.2	77.65	45.95	59.55
68.95	88.2	47.65	19.700000000000003	81.05
51.849999999999994	40.5	51.25	141.1	46.75	131.05
113.55\
GRM4	176.1	189.6	56.8	36.1	82.3	79.3	24.6
108.5	93.8	73.1	24.3	49.7	32.7	22.7	134.3
120.1	61.6	97.1	52.5	185.1	338.7	45.6	128.3	45.3
	77.4	20.7	85.2	251.6	67.8	31.0	94.5
239.6	82.0	122.9	159.3	91.1	84.7	191.5	54.5
124.7	168.2	19.0	38.4	15.8	31.2	147.4	123.6
109.8	156.1	207.2	83.3	47.9	105.8	70.6	86.8
203.4	121.8	73.8	132.5	28.2	304.7	31.2	123.7	69.7
	142.6	42.5	106.8	83.0	180.5	107.1	284.7	40.5
	140.0	98.2	104.3	34.5	151.9	43.4	110.7
130.3	49.3	85.9	89.0	173.0	141.3	400.3	84.6	34.8
	131.0	229.0	73.5	42.8	40.5	15.8	12.2	44.6
	123.4	90.9	22.8	31.5	90.2	155.7	48.0\
BC040624 /// FOXP1-AS1 /// FOXP1-AS1	48.2	18.5	83.9	62.7
	53.7	6.5	9.8	10.6	87.7	13.4	23.7	5.3
	9.3	7.8	17.5	11.0	15.6	7.4	68.7	90.2
	56.5	9.9	147.9	81.2	57.9	70.9	10.5
108.6	96.2	60.9	6.0	88.1	84.9	49.6	37.5	31.4
	51.1	14.0	42.3	82.9	4.1	62.6	57.8	24.7
	10.7	142.4	53.7	48.5	166.9	18.3	48.3	47.5
	23.5	112.2	90.6	71.8	72.6	81.4	31.1	18.6
	23.1	39.1	13.7	8.5	63.1	71.2	9.9	10.2
	7.9	18.3	14.0	68.5	11.7	47.5	107.9	12.9
	107.2	13.9	35.3	12.4	64.7	89.1	7.9	10.3
	163.8	185.9	92.3	21.3	77.4	161.4	10.0	9.4
	3.7	9.5	68.8	98.6	7.1	7.7	12.5	7.6
	49.3	51.1	26.8\
CA3	35.2	119.3	135.1	5.3	196.5	35.6	78.7
160.1	22.4	85.5	34.7	58.6	5.4	15.6	126.4	16.5
	40.7	61.4	35.6	4.8	142.1	58.9	53.9	38.6
	29.9	40.6	2.3	90.7	108.3	26.6	62.8
109.8	103.8	49.4	4.7	225.4	34.7	32.0	9.7	30.3
	46.3	63.2	73.7	51.0	3.3	114.5	52.6	62.4

	47.2	100.6	59.3	24.3	56.2	62.0	5.8	
114.5	149.0	101.6	4.0	70.7	30.1	79.4	141.2	28.5
	180.3	154.1	44.2	63.2	70.3	55.5	89.8	2.9
	97.9	45.0	81.2	20.8	120.6	27.6	60.8	13.8
	181.8	47.9	63.6	52.4	109.9	172.7	24.2	33.2
	3.2	6.5	3.9	136.6	158.2	78.7	122.7	
249.1	209.6	178.7	464.9	338.4	206.9	258.7	115.6\	
ZIC2	152.6	566.5	3.8	32.8	20.5	18066.4	7557.6	1.7
	4680.4	5956.9	19230.5	20.2	15971.4	262.5	14453.6	2.2
	9344.8	137.1	58.3	64.9	11.2	229.1	5.1	40.1
	277.4	20.2	6.5	81.8	329.4	1608.2	17.7	62.6
	13.8	28.8	666.1	7.5	10025.0	5.8	50.2	71.2
	238.0	7294.5	91.9	28.8	84.5	159.6	67.7	7.4
	20.4	4.3	1124.3	63.0	103.7	8.1	3855.5	8.8
	67.7	40.0	246.8	53.3	300.3	47.3	4210.7	39.7
	88.2	9.0	63.7	69.0	9.6	110.0	60.1	7.5
	40.4	5994.3	116.6	1712.8	2.7	122.5	41.6	
112.0	42.2	3634.5	11127.8	2.6	520.9	602.2	13.3	33.2
	1570.9	2162.9	581.5	17.4	3.1	8.4	28.1	64.4
	48.2	54.6	12.6	43.9	4.5	67.8	5.6\	
CA2	33.2	251.2	93.9	244.9	151.3	84.5	360.7	
226.6	596.2	2463.6	575.7	208.5	12543.8	230.5	76.3	50.9
	433.1	167.5	78.4	1006.7	260.7	118.8	74.4	34.4
	210.7	96.1	1147.9	156.1	98.0	554.5	596.1	55.5
	217.6	48.1	171.8	128.2	155.1	1297.0	2808.1	
281.6	84.8	619.2	241.0	649.9	780.2	74.4	227.1	
464.4	53.6	144.2	208.7	687.0	1273.7	108.5	86.9	54.4
	128.3	209.2	70.5	146.8	31.8	56.3	208.1	
198.9	256.7	195.1	195.0	471.4	95.6	34.1	84.5	83.0
	123.5	72.0	59.4	84.5	142.9	64.3	49.7	86.6
	326.0	899.7	25.1	144.2	957.7	717.1	239.5	93.9
	650.6	141.4	138.1	73.6	44.9	21.2	7.9	58.3
	42.4	63.4	27.4	41.2	2.0	477.6	74.4\	
GRM6	62.0	68.7	43.2	81.3	103.6	54.9	32.4	
128.6	72.2	32.6	117.4	122.9	46.8	404.3	99.0	51.7
	95.8	52.8	70.1	87.9	191.0	37.6	121.0	35.5
	68.8	95.7	100.7	113.7	101.2	68.3	39.0	
123.2	82.9	38.1	123.8	86.3	91.6	138.3	73.5	
102.8	72.5	47.0	54.8	29.8	50.2	83.8	87.6	73.5
	154.1	111.1	134.2	80.0	122.1	189.9	65.3	52.7
	120.6	77.9	87.4	98.5	237.8	80.9	65.7	
101.5	99.3	106.2	55.6	34.7	131.0	73.3	39.6	
163.0	140.0	55.8	141.3	119.5	78.1	37.1	22.2	95.4
	125.8	68.4	91.7	99.2	248.0	361.3	88.1	
203.7	31.3	111.8	49.1	132.3	66.6	61.8	24.9	41.0
	92.3	199.2	50.0	59.5	48.8	61.9	86.7\	
ZIC3	55.6	59.8	24.6	22.2	7.4	18.6	23.0	15.5
	26.1	71.9	68.8	43.0	34.7	52.8	28.5	4.8
	37.7	84.1	28.6	44.1	23.9	25.6	14.7	24.7
	68.5	30.0	23.5	50.3	94.4	49.7	38.4	16.6
	26.2	21.7	60.7	112.9	65.7	133.0	22.0	51.2
	7.7	6.4	119.1	21.0	50.7	61.0	30.4	51.8
	73.9	9.8	57.8	19.0	25.9	148.6	62.4	30.6
	5.7	16.3	9.8	62.1	54.6	3.6	61.0	12.7

```
	58.1	102.4	65.3	10.7	5.9	75.5	20.1	25.2
	14.7	22.7	83.2	65.7	8.8	61.0	26.6	36.6
	39.0	90.1	50.0	36.6	59.9	39.2	140.8	15.0
	14.0	42.2	22.8	121.3	37.6	43.2	31.6	30.4
	12.5	36.6	40.5	78.8	31.2	34.0	4.2\
ZIC1	49.46666666666666	1866.8666666666668	71.9
40.666666666666664	2985.566666666666	19.4
26.233333333333334	230.0	156.66666666666666	1299.7
92.73333333333333	257.2666666666667	1006.9666666666666
	955.2666666666665	5063.633333333334	21.5
934.0	44.06666666666666	115.60000000000001
2322.733333333333	1339.4666666666667	18.766666666666666
	499.93333333333334	10.0	7.8999999999999995
274.1333333333333	71.13333333333334	222.66666666666666
	28.733333333333334	119.76666666666667
4316.466666666666	21.933333333333334	19.8
450.1666666666667	14.0	25.366666666666664
789.8333333333334	64.13333333333334	21.7666666666667
	1799.1999999999998	16.000000000000004
72.2666666666667	7.466666666666666	323.6333333333333
	25.066666666666666	9.666666666666666
209.53333333333333	24.233333333333334	183.5	1775.8	16.9
	22.333333333333332	4043.1000000000004
87.46666666666665	76.46666666666665	3590.1999999999994
	465.43333333333334	8.666666666666666
37.333333333333336	565.5333333333333	70.23333333333333
	34.43333333333333	906.6666666666666
53.833333333333336	425.5999999999997	66.9666666666667
	963.3000000000001	2060.066666666667
79.5666666666666	311.6333333333333	99.93333333333334
	13.200000000000001	1216.2	120.03333333333335
102.2	13.933333333333332	43.6	77.3	1204.4666666666667
	2581.7	100.13333333333334	281.1333333333333
30.233333333333338	53.166666666666664	23.166666666666668
	24.666666666666668	207.29999999999998
45.4666666666666	23.933333333333334	55.99999999999999
	134.2666666666668	27.4666666666667
27.033333333333335	22.7	46.833333333333336
18.066666666666666	106.8666666666667	61.366666666666674
	7.8	23.2	39.2	75.83333333333333
43.8666666666667\
GRM8	26.033333333333335	16.5	75.96666666666668
20.566666666666666	63.0	34.9	10.200000000000001
17.900000000000002	89.1666666666667	57.8666666666667
	18.3	47.5666666666666	33.63333333333333
37.833333333333336	24.9666666666667	32.166666666666664
	7.566666666666666	59.5666666666667	21.7
73.93333333333332	104.3	27.599999999999998
35.03333333333333	27.03333333333333	54.4
35.43333333333334	29.599999999999998	56.2666666666667
	30.099999999999998	91.83333333333333
94.60000000000001	31.8	39.166666666666664	45.6
90.96666666666668	8.133333333333333	28.166666666666668
	78.23333333333333	25.133333333333336
```

44.53333333333333	23.266666666666666	34.2
25.966666666666665	244.29999999999998	47.233333333333334
	14.366666666666667	29.3	61.43333333333333
54.46666666666666	59.266666666666667	41.400000000000006
	28.7	24.366666666666667	29.13333333333333
16.166666666666668	48.06666666666666	80.86666666666667
	21.1	34.03333333333333	14.866666666666667
140.0	32.599999999999994	22.733333333333334
34.533333333333334	71.7	47.666666666666664
134.83333333333334	397.0333333333333	47.766666666666667
	34.00000000000001	146.5666666666667	45.9
192.73333333333332	31.566666666666663	11.9
38.50000000000001	44.866666666666674	20.7
65.433333333333334	29.333333333333332	13.399999999999999
	35.96666666666666	28.599999999999998	36.4
64.933333333333334	67.1	39.0	51.833333333333336
49.199999999999996	39.733333333333334	49.199999999999996
	30.866666666666664	33.46666666666666	18.3
6.066666666666667	27.2	23.166666666666668
30.233333333333334	12.766666666666666	34.5666666666667
	35.266666666666666	47.333333333333336
30.566666666666666\
CA4	15.2	25.95	36.65	27.1	40.0	24.099999999999998
	63.9	64.6	64.0	43.2	34.85	23.65	27.2
31.45	34.55	33.15	40.2	14.05	38.650000000000006	46.3
	44.7	31.45	137.65	51.0	55.65	46.75
65.80000000000001	61.15	104.80000000000001	34.85	45.5
	162.39999999999998	98.8	44.3	79.55	40.3	6.75
	125.80000000000001	20.5	45.6	104.95	62.5	89.3
	34.35	26.650000000000002	67.1	34.05
27.049999999999997	57.050000000000004	59.150000000000006
	85.35	48.1	18.25	116.6	50.2	65.3	85.7
76.89999999999999	26.45	111.39999999999999	206.85	56.9
	67.4	32.95	33.15	64.75	29.05	59.349999999999994
	85.5	96.15	100.0	43.800000000000004	85.3	36.3
	84.05	24.4	27.7	22.9	63.900000000000006
30.349999999999998	76.4	54.9	52.0	49.15	189.3
184.35	72.8	107.0	95.45	117.85	45.05	115.3	40.45
29.25	60.85	64.5	35.1	30.1	125.25	59.2	55.0	98.9
	13.55\
SPATA31C2	3.9	8.2	9.5	1.5	9.5	1.8	1.8
	8.7	7.6	89.1	2.6	1.9	3.4	31.3	40.4
	2.4	2.8	27.0	7.8	38.3	53.2	3.2	66.1
	15.4	17.6	3.5	21.8	70.4	17.8	15.6	23.1
	16.6	41.1	2.5	21.5	52.4	1.9	30.4	17.4
	2.6	1.9	7.2	5.5	3.6	83.9	21.9	2.3
	2.1	6.1	42.1	33.0	3.7	4.8	46.0	6.4
	21.7	31.4	32.0	4.5	55.8	19.9	69.0	13.4
	12.9	10.0	4.4	3.0	4.4	3.3	7.6	34.5
	54.3	8.6	5.9	29.0	1.7	19.3	20.3	6.5
	12.4	10.6	21.2	41.6	36.5	3.9	26.9	2.8
	134.0	14.6	2.5	38.9	21.1	1.4	27.6	11.1
	2.3	2.0	4.4	47.7	54.6	1.7	59.9
45.1\

CA7 17.6 247.4 95.4 43.0 104.5 42.7 32.3
110.1 25.5 56.7 19.6 32.3 12.1 16.3 38.6 76.4
31.3 66.3 65.8 21.7 80.2 27.2 108.4 11.6
52.0 81.0 83.5 200.5 156.8 12.2 17.6 25.9
54.9 17.3 88.7 113.0 14.0 329.3 91.2
123.1 19.8 26.6 10.7 37.3 22.6 17.2 18.5 17.5
237.0 171.4 124.8 14.9 68.5 183.5 12.1
167.3 72.2 151.8 160.8 145.9 83.8 67.0 30.8
108.6 69.4 147.5 18.3 132.6 208.6 28.2 147.6
148.0 170.8 54.2 93.9 28.6 17.7 78.0 28.6 49.5
175.8 76.6 75.2 105.7 35.3 45.5 15.2
140.9 25.9 185.9 41.6 76.8 32.5 76.6 111.0
140.6 78.4 144.7 106.1 89.0 144.6 87.1 69.2\
CA6 24.5 25.4 31.099999999999998 11.7 68.4
16.75 37.0 16.3 113.55 141.25 10.600000000000001 27.1
74.75 15.45 54.5 22.9 30.799999999999997
58.85 11.85 49.65 953.75 17.5 31.55 38.6 14.35 57.9
44.0 77.1 27.1 256.5 118.85000000000001
123.2 29.45 18.7 76.0 20.85 23.95 68.8 66.2
74.35 57.1 40.1 75.95 617.6 11.2 26.75 22.95
56.800000000000004 157.89999999999998 243.15
14.600000000000001 16.25 60.949999999999996
24.200000000000003 13.6 20.2 45.55 64.05 44.55
59.65 58.150000000000006 16.7 36.7 14.25
1195.6000000000001 108.25 574.8 120.75 99.65 51.45 44.2
16.65 538.15 16.7 73.25 14.5 27.650000000000002
4.75 656.75 35.45 25.0 14.600000000000001
20.099999999999998 31.8 105.25 142.9 63.25 17.95
99.55000000000001 79.65 6.5 49.0 16.8 18.45 21.8
19.45 60.4 21.299999999999997 92.19999999999999
16.8 91.0 48.7 82.3\
CA9 304.0 443.9 120.6 126.1 55.6 256.5 149.4
160.8 273.6 175.0 173.6 71.3 98.1 510.8 566.2
216.1 248.8 209.7 426.3 192.8 505.5 64.5 235.0
103.6 261.5 344.8 85.3 307.2 274.9 434.9 127.7
420.3 2134.6 215.0 618.2 407.7 633.1 356.0 126.8
281.6 25.5 1081.4 208.5 291.0 225.8 263.3 283.0
142.6 256.2 278.7 249.2 641.1 638.9 302.6 318.3
250.9 255.2 235.8 298.9 202.6 397.5 21.5 266.5
460.1 228.7 322.4 131.0 202.5 602.8 229.7 303.7
483.5 280.7 38.7 191.0 125.0 521.3 107.3 241.6
205.1 369.3 288.8 143.8 188.3 310.0 312.3 285.2
173.6 197.1 157.2 222.0 265.6 242.2 134.1 270.1
202.6 213.1 167.4 64.7 294.6 238.2 128.7 181.7\
MYBL2 562.2 69.3 38.4 710.4 286.3 439.7 543.8
434.9 322.8 376.1 381.1 363.5 481.4 312.4 576.0 27.0
420.3 316.0 175.5 256.6 18.3 450.3 122.1
207.9 567.0 349.8 803.0 49.3 170.9 307.7 18.5 72.6
49.8 62.4 496.7 151.5 542.0 242.0 483.7
438.8 637.1 282.6 111.4 143.9 389.3 362.6 439.8
1265.6 70.0 48.3 69.4 473.5 263.1 276.0 282.2
213.9 78.2 326.3 207.7 59.5 72.5 30.1 164.8 54.6
13.8 118.6 196.9 90.7 54.7 157.6 21.2 51.8
35.2 146.5 210.2 377.1 57.3 182.6 48.6

1373.3	308.9	399.4	491.1	308.7	196.3	126.5	585.4
219.2	354.2	48.6	702.8	172.8	11.8	25.9	27.8	28.4
	21.2	64.1	25.1	13.6	30.3	201.4	26.6\
CA8	58.46666666666667	34.36666666666667
160.70000000000002	68.8	224.33333333333334
133.86666666666667	97.16666666666667	214.20000000000002
	139.5	210.80000000000004	83.56666666666666
136.63333333333333	212.4	83.8	211.26666666666665	80.6
	286.9666666666667	262.6666666666667	54.0
211.0	156.1	94.3	130.5666666666667	114.53333333333335
	48.6	221.4	274.8	263.8333333333333
207.79999999999998	170.70000000000002	256.90000000000003
	273.90000000000003	127.2	138.36666666666665
243.9	103.40000000000002	88.10000000000001
182.83333333333334	304.26666666666665	252.63333333333333
	89.7	64.89999999999999	185.13333333333335	61.4
	291.26666666666665	299.40000000000003
212.69999999999996	300.1333333333333	286.8666666666667
	293.06666666666666	148.16666666666666
166.29999999999998	1075.7	199.8666666666667	107.7
123.46666666666665	158.16666666666666	136.13333333333333
	182.3666666666667	212.53333333333333
375.1666666666667	119.7	261.1333333333333
214.80000000000004	264.23333333333335	403.06666666666666
	193.73333333333335	161.06666666666663
114.60000000000001	198.63333333333333	230.30000000000004
	110.40000000000002	168.83333333333334
174.4333333333333	140.13333333333333	183.9333333333333
	122.73333333333335	222.16666666666666
271.73333333333335	106.43333333333334	87.93333333333334
	98.10000000000001	51.0	181.5	63.766666666666667
	316.5666666666666	104.03333333333335
312.6333333333334	16.033333333333335	45.833333333333336
	132.4	110.2	225.8666666666667	168.73333333333332
	138.1666666666669	374.4666666666667
262.3333333333333	230.16666666666666	159.43333333333334
	233.8666666666665	237.06666666666663	114.7
356.76666666666665\
MYBL1	536.45	845.25	124.64999999999999	100.7
329.15000000000003	347.5	136.45	260.8	85.85	425.6
170.54999999999998	169.6	373.84999999999997
602.5999999999999	281.35	300.95	353.15000000000003
124.89999999999999	141.65	242.8	120.8	220.29999999999998
	47.95	135.55	315.84999999999997	404.7	241.15
232.0	102.9	192.8	81.35000000000001	129.3	156.15
81.25	220.7	252.35000000000002	449.55	516.95	120.9
340.05	249.64999999999998	358.9	234.04999999999998
56.25	201.35	510.34999999999997	109.35	514.7	265.3
95.69999999999999	297.8	162.4	151.55	429.5
225.14999999999998	88.9	107.55000000000001	286.0
316.4	173.1	37.0	206.15	53.45	107.0	80.25	138.8
79.95	48.1	125.8	220.4	260.75	209.2	124.19999999999999
	100.0	201.35000000000002	132.3	134.75	115.85
122.8	1422.0	245.75	441.95	146.1	495.15000000000003

```
229.75  313.15  478.15  473.65  225.1   197.3   156.5   269.8
137.25  144.05  182.70000000000002      150.55  210.15  110.05  91.1
        153.45  120.25  213.39999999999998      172.4\
SUN3    33.1    10.4    8.0     20.5    9.6     6.3     5.3
653.2   16.7    135.0   8.7     12.1    11.3    11.1    18.8    7.1
        1.7     54.4    21.1    20.6    4.7     15.6    14.9    12.7
        18.5    7.2     7.4     15.8    10.5    7.1     5.9
124.4   12.0    9.3     21.2    10.6    5.8     49.5    8.6     20.8
        11.5    2.8     46.2    10.2    19.5    26.2    10.0    10.7
        20.2    11.6    15.4    48.6    6.2     37.2    5.5     5.9
        6.5     16.5    5.7     12.7    29.1    8.1     26.0    5.5
        21.8    3.4     33.0    9.5     8.6     17.3    79.3    15.1
        65.1    6.3     18.5    4.7     12.7    1.6     7.7     54.3
        10.0    33.9    5.3     4.1     4.0     21.5    12.2    10.9
        17.1    23.8    31.1    13.3    12.3    1.5     1.8     20.5
        2.3     3.4     3.3     11.9    11.1    8.3     8.1\
SUN2    867.7   821.7   470.4   987.6   602.8   1062.3  932.2
607.2   1804.3  1053.8  560.2   1447.3  1097.9  948.1   488.6
1088.0  902.4   797.2   902.3   1126.7  1407.4  736.3   828.8
994.6   713.3   1147.6  825.2   806.5   527.2   1478.8  785.2
769.8   714.1   501.4   879.2   1100.3  860.1   605.0   1283.0
770.9   1252.5  1054.9  564.7   1727.6  813.2   381.6   742.6
439.6   561.9   748.3   1222.0  437.6   611.3   695.2   871.4
519.2   622.9   509.5   647.1   587.8   115.3   1187.4  913.5
851.9   973.2   1152.8  1549.0  1708.2  729.5   423.1   208.1
554.3   2626.6  1046.3  733.4   637.4   722.7   1079.5  1403.0
640.5   375.3   548.3   614.1   577.6   401.0   415.0   784.9
777.6   1290.3  621.1   1042.5  822.5   437.3   845.2   786.1
422.8   639.2   896.6   497.3   408.4   502.6   1108.0  754.2\
SUN1    1898.1750000000002      4081.1000000000004      5452.25
2352.1  4183.025        1726.5249999999999      2181.425
2582.525        1746.2  3404.625        2494.1  1977.575
1491.5500000000002      3253.25 3498.475        2172.875
1779.6750000000002      2331.5  1595.55 2185.75 2677.55 2455.8
2619.3250000000003      1869.825        2433.4750000000004
3085.4249999999997      1358.725        3118.025        3828.65
1886.9750000000001      1821.4999999999998      2538.325
3189.1249999999995      1636.275        1425.8000000000002
1852.975        2194.1499999999996      3434.625        1491.375
        1947.2500000000002      1865.9  2306.675
2811.4249999999997      1599.525        549.5000000000001
2978.1  1529.175        2551.1250000000005      3658.975
2053.4  2720.475        2203.5  1413.575        4358.275
3041.0  3763.15 2644.0750000000003      4461.799999999999
3036.4750000000004      3090.6  974.0250000000001
2373.2250000000004      1906.5249999999999      2976.625
2113.15 2485.275        1361.4250000000002      1145.1  2431.775
        3294.975        4368.750000000001       3823.075
2114.6  2530.3500000000004      2252.525        1988.2
3314.9500000000003      2182.0750000000003      2013.1  2127.075
        3830.9750000000004      2728.6000000000004      1900.225
        3133.375        3056.275        2558.8  1924.375
2286.9249999999997      1644.975        2124.8500000000004
1867.1249999999998      3863.25 2800.7749999999996
```

2476.5249999999996	4326.225	3778.1249999999995
4216.175	2742.8999999999996	3893.175	4918.85
4015.55 3072.7999999999997	3276.0499999999997\
ATP6AP1 2505.0	1709.2	268.8	1220.7	498.1	3912.1	1858.4
2667.8	2643.3	1845.6	1556.9	1891.1	2246.6	2222.1	456.7
2436.3	2484.3	1027.4	1600.1	1599.8	349.7	1551.9	686.9
2140.3	1197.5	1557.6	1806.8	934.5	983.4	2485.6	1880.8
373.6	1197.6	3550.1	1557.6	1877.9	2710.4	888.8	2689.2
2244.0	1796.6	2945.9	1055.5	2180.5	3521.9	899.6	2391.0
1643.3	811.2	1531.5	962.5	1510.5	1546.7	630.1	1429.2
1198.6	1608.9	442.1	1827.4	865.1	1492.5	1776.1	2147.5
1443.0	993.9	1363.9	3628.0	3933.8	1485.1	1427.8	560.6
521.4	1993.8	2401.0	1883.0	2622.0	2315.8	1963.6	2410.6
1706.3	758.0	891.5	1006.5	2662.0	292.4	1197.5	1453.6
2153.2	3122.5	950.0	1402.6	783.0	967.9	909.2	895.9
1097.4	854.5	1194.3	688.8	541.1	990.9	396.6	1237.9\
CAD	207.6	359.45	207.85	292.75	386.5	350.35	448.5
448.3	247.65	238.65	452.09999999999997	366.25	313.05
174.9	614.8	413.34999999999997	303.5	243.9	461.25
269.1	506.0	273.95	165.6	148.14999999999998	134.55
685.1	424.4	193.85	372.2	307.5	530.65	363.8
464.95000000000005	300.25	206.04999999999998	151.45
221.4	356.29999999999995	384.70000000000005	488.3
695.5500000000001	224.79999999999998	161.45	244.35
265.75	407.8	298.0	286.1	1429.6	312.85	177.75	289.25
354.70000000000005	544.4499999999999	537.0500000000001
	528.0	334.0	279.75	193.9	255.75	56.7	273.4
229.79999999999998	150.0	249.1	325.8	305.8	556.1
265.45000000000005	264.3	304.15	250.15	220.75	203.45
173.3	161.95000000000002	292.0	231.29999999999998
314.95000000000005	649.75	420.2	618.25	904.8
317.04999999999995	740.5500000000001	661.55
312.79999999999995	225.0	223.64999999999998
340.59999999999997	396.54999999999995	578.45
301.59999999999997	460.8	357.05	333.0	359.3
401.45000000000005	388.09999999999997	320.35	279.95
336.59999999999997	203.95\
ATP6AP2 5545.733333333333	4442.733333333333
2702.633333333333	5296.733333333333	1988.8666666666668
	4414.9	5079.599999999999	7753.066666666667
4743.366666666666	4158.733333333334	3788.2000000000003
	6730.966666666667	4668.933333333333	5161.7
4016.866666666667	4696.599999999999	5787.700000000001
	2993.8000000000006	4857.733333333333
4297.633333333333	1686.8333333333333	5272.366666666667
	1903.3666666666666	5823.266666666666
7192.766666666666	3549.266666666667	4504.766666666667
	3860.3666666666663	2550.4	4849.0	4751.533333333334
	2602.4666666666667	1298.2666666666667
5980.666666666667	6270.566666666666	2720.3666666666663
	6151.866666666666	1969.2	4362.066666666667
7345.933333333333	3915.433333333333	6048.7
4421.900000000001	3727.166666666666	6244.5
2866.766666666667	6747.899999999999	4762.16666666667

	1054.7333333333333	2675.4666666666667	4550.8
4712.633333333333	3588.3666666666663	2229.4666666666667
	3801.1333333333337	2943.133333333333
4134.233333333333	2946.633333333333	3557.433333333333
	2226.166666666667	1860.6000000000001
2935.2000000000003	3703.066666666667	4656.233333333333
	3381.4	1508.0	4397.233333333334	3386.066666666667
	3057.6000000000004	5398.833333333333
1194.5333333333335	2380.3333333333335	4205.3	6910.7
4170.833333333333	5603.633333333332	5340.599999999999
	5359.866666666667	4387.599999999999
5625.966666666667	3495.8666666666663	5351.133333333332
	5138.433333333333	5032.733333333334
2283.1666666666665	1423.8333333333333	5880.366666666668
	4302.133333333333	5619.566666666666
3903.066666666667	6968.866666666668	3171.433333333333
	4125.633333333333	3606.7000000000003	2727.4
4290.933333333333	3780.3333333333335	5106.633333333333
	3280.3666666666663	3954.2333333333336
3309.966666666667	3636.433333333333	3641.6999999999994\
HUNK	150.5	98.60000000000001	65.55000000000001	78.9
	310.1	155.55	60.4	141.10000000000002	667.05
300.9	155.95	124.30000000000001	221.75	151.6	236.5
880.9499999999999	295.75	91.25	100.55	720.4	956.0
102.1	85.0	177.5	109.25	62.1	40.3	111.25	73.1
682.6	501.59999999999997	90.65	87.80000000000001
330.6	111.2	174.7	492.20000000000005	172.2	1742.6
193.45000000000002	260.7	223.45000000000002	175.3
1909.35	224.65	177.65	44.4	778.6	132.95	725.8000000000001
	174.25	57.650000000000006	128.9	244.14999999999998
	181.65	139.45	186.8	165.1	151.1	175.70000000000002
	453.1	145.15	229.25	136.35000000000002	644.4	83.9
	739.55	2059.2000000000003	160.15	188.35	257.5
134.85	748.15	356.95000000000005	299.9	92.5	104.85	90.3
	521.5500000000001	319.20000000000005	104.75
261.5	241.20000000000002	65.2	242.70000000000002
274.8	203.85000000000002	101.64999999999999	428.6
40.45	279.65000000000003	124.0	79.15	163.7	114.55
59.75	137.8	119.7	24.299999999999997	106.85
69.85000000000001	80.45	44.75\
SUN5	56.3	121.44999999999999	70.475	76.725	57.125
49.625	32.675	93.675	38.1	54.55	51.349999999999994
75.225	60.900000000000006	36.775000000000006	134.4	78.8
	88.75	73.95	17.775	78.8	167.05	67.675	107.275	22.8
	49.0	46.525	53.099999999999994	147.575	75.45	84.0
	30.125	112.025	103.55000000000001	62.824999999999996
	128.7	98.7	73.1	111.2	64.65	74.67500000000001
	74.32499999999999	86.6	96.025	57.9	39.3
89.57499999999999	55.9	94.07499999999999	131.7
111.675	50.324999999999996	95.1	70.125	163.15	87.85
108.32499999999999	87.975	108.0	72.30000000000001	87.0
	203.57500000000002	59.925000000000004
95.69999999999999	42.025	97.3	120.375	53.025000000000006
	49.825	102.39999999999999	73.625	143.625

136.07500000000002	95.3	90.725	88.52499999999999
49.875	37.574999999999996	66.125	54.15	77.525	98.475
61.15	63.05	102.1	118.625 139.925 56.25	102.625
120.82500000000002	113.57500000000002	39.375
135.52499999999998	57.85	31.474999999999998	68.9
49.150000000000006	106.95	69.225	78.175	82.25
111.30000000000001	135.07500000000002	79.625\
RP5-991G20.4	145.1	212.0	207.8	117.3	87.0	216.6
241.5	73.0	146.3	226.4	389.5	152.0	78.4	179.5
141.8	195.9	97.2	142.3	295.9	280.4	287.6	231.0
164.6	189.0	361.9	185.4	395.7	228.1	241.7	167.1
143.9	227.7	99.9	285.8	378.3	106.7	99.7	177.8
224.6	121.2	228.6	105.7	194.3	151.5	257.7	204.4
138.7	281.9	330.5	228.7	243.7	394.7	87.5	373.0	94.6
	104.4	171.3	148.9	371.9	352.9	422.0	279.3
279.8	308.5	166.1	98.6	199.6	444.8	164.8	153.6
163.8	222.9	212.7	123.0	221.4	287.4	213.9	221.4
213.6	379.7	139.4	363.5	305.7	106.0	325.5	42.8
310.4	101.8	80.0	104.4	117.9	208.5	144.6	151.5
121.6	373.6	298.0	188.6	266.7	100.7	205.9	241.4
268.9\
ELF1	1294.5500000000002	666.0	798.35	1963.0	1326.55
722.6	2093.1499999999996	670.35	1712.5	2100.9	2179.5
2258.1	2479.45 1145.5	865.2	2047.8	1035.35 2074.1	4164.4
1224.8000000000002	510.45000000000005	3127.9	1915.3
2431.15 2191.75 2114.55 1601.25 940.15	1556.4499999999998
1749.8999999999999	1912.05 664.35	1494.6	2070.25 988.8
1829.6000000000001	1122.5	1686.9	2808.8	1104.4	1153.35
1601.75 1806.8	1918.6999999999998	2885.6000000000004
1732.3	1012.5999999999999	832.6500000000001	623.5
1599.05 2700.85 2173.85 1430.55 616.7	2132.8	960.8	2333.95
1454.5500000000002	2279.35 1002.15 442.7	1767.9
1989.5500000000002	2419.55 1621.0	1356.9	3132.9500000000003
	2650.95 1195.3000000000002	2357.8	1116.25 902.3
2107.3	2062.3	1635.8	2222.5	1098.3	2054.1	1890.0
333.54999999999995	821.3	1265.15 2683.65 1235.5	727.35
682.8	1022.25 797.75	1826.35 1612.5	2409.05 589.15	1102.05
1982.9499999999998	1448.05 914.95	858.45	1696.0	1158.35
1266.7	1207.0	1240.15 1776.9\
ELF2	822.1999999999999	1489.9666666666665
758.2333333333335	529.1	599.5666666666666
737.3666666666668	648.4333333333333	863.9333333333334
	681.9	569.6666666666666	775.5666666666666
856.7333333333335	553.0	816.3666666666667
877.2666666666668	730.3666666666668	646.8000000000001
	444.3666666666666	626.0	723.3666666666668
892.9	792.9	886.2333333333332	652.5000000000001
867.3333333333334	810.6333333333333	706.1333333333333
	854.0333333333334	1030.4333333333332
854.1999999999999	992.1	1101.5666666666666
655.2666666666668	524.7333333333332	580.7666666666667
	868.9333333333333	621.6666666666666	884.9
898.0999999999999	516.5666666666667	943.8666666666667
	932.3333333333334	596.3333333333334

488.73333333333335	473.73333333333335	619.8333333333334
	1071.1333333333332	531.4666666666667	1349.4
969.1	769.6	719.9666666666666	947.0	1136.1666666666667
	974.4666666666667	1252.5666666666666
973.9333333333334	974.1333333333333	673.7333333333333
	691.9333333333334	1267.8666666666668
1129.5333333333333	578.0333333333333	644.7333333333332
	1050.6666666666667	984.2666666666668
917.2333333333332	642.1666666666666	608.4333333333334
	966.0333333333334	900.9333333333334
797.3666666666667	776.6	731.8333333333334
639.7333333333332	737.0	937.8666666666667
921.6999999999999	922.4	796.3333333333334
1055.1666666666667	856.3000000000001	876.4
1096.7333333333333	1228.1666666666667	1311.6333333333332
	899.2333333333332	1038.4333333333332
601.2333333333332	667.8666666666667	353.23333333333335
	1344.8333333333333	1318.1666666666667
903.9666666666667	1108.8	1137.8	1228.0333333333333
1080.9333333333334	1139.1333333333332	1403.8333333333333
	1104.2333333333333	1177.6000000000001	1030.7\
ELF3	1780.1666666666667	1796.2	2840.2999999999997
807.9	5251.366666666666	354.5	691.6	2897.3000000000006
	818.2000000000002	1687.8	1017.4333333333334
3556.366666666667	3017.7666666666664	1844.4666666666665
	645.0666666666667	541.1	1323.9	2483.9
4085.6999999999994	1386.0666666666666	1996.2333333333336
	2104.5	2031.1333333333332	1289.5333333333335
1984.1666666666667	2674.7333333333336	2178.4
1299.7666666666667	1872.5666666666666	1299.7666666666667
	553.9	2873.7333333333336	4646.0	979.8666666666668
	1817.5333333333335	1782.7	1632.5666666666668
6182.066666666667	3775.033333333333	487.5	1011.6
3338.1666666666665	881.5666666666666	754.3666666666667
	2072.366666666667	1000.7000000000002
3502.2000000000003	814.0666666666666	5142.166666666667
	2909.7333333333336	927.2000000000002
3053.1333333333337	4528.099999999999	1191.6666666666667
	3886.1	4475.099999999999	2704.0333333333333
2026.5666666666668	1022.9666666666666	1417.3
1277.7333333333333	3994.7000000000003	2515.2999999999997
	3373.566666666667	2776.5333333333333
3029.7999999999997	1957.1333333333332	651.1
2231.1666666666665	2851.7999999999997	3580.133333333333
	1319.8666666666668	2082.4333333333334	645.2
1077.8333333333333	985.4000000000001	3615.2999999999997
	1864.0666666666666	2019.2666666666667
47.96666666666667	1990.2333333333336	2196.0666666666666
	3119.2666666666664	6237.166666666667	5228.7
4332.000000000001	1108.8999999999999	1381.3666666666666
	2275.3666666666663	1220.0666666666666
1396.2333333333336	981.5	2896.1666666666665
2745.7000000000003	2364.633333333333	1129.2333333333333
	1600.6666666666667	1694.2666666666664	2563.0

3799.6333333333337	2420.6000000000004	856.9333333333334
	1406.3\
GFOD1	122.1	282.95	228.89999999999998	102.0
305.84999999999997	179.0	150.0	167.45000000000002
165.79999999999998	550.05	211.4	205.6	1234.25	627.15
328.34999999999997	585.85	275.25	401.2	112.4	191.45
403.70000000000005	227.70000000000002	425.04999999999995
	192.89999999999998	489.45	99.39999999999999
81.15	326.45	191.75	291.05	204.5	250.55	328.9	192.35
361.1	430.95	115.75	219.55	118.05	179.25	150.75	180.75
244.3	146.1	97.1	221.25	120.14999999999999	345.3
119.1	161.0	160.25	249.7	157.35	92.85	380.45
283.84999999999997	279.4	445.7	102.65	320.65	228.15
452.40000000000003	333.05	271.59999999999997	153.05
348.90000000000003	155.85	345.85	459.5	323.0	441.8
387.95	212.8	295.05	207.4	126.05	164.64999999999998
182.9	204.05	128.85	112.65	188.5	110.5	1173.95	309.85
258.7	106.25	373.4	766.85	640.8000000000001	303.35
133.9	86.95	269.65000000000003	136.6	136.5	66.7
103.95	108.85	151.55	90.75	165.15	129.0\
ELF4	796.15	71.3	599.2	879.25	627.65	925.75
283.15000000000003	1285.0	841.85	771.55	392.20000000000005
	955.8	639.25	941.75	805.3000000000001	820.95
534.9	964.9499999999999	1446.6	490.6	1052.5
641.9000000000001	803.9000000000001	1108.9
1667.1499999999999	1106.9	820.05	1528.85	897.05	825.1
946.1	844.75	1690.6000000000001	767.85	1464.75	1617.6
775.15	1247.15	550.55	628.1	1749.35	1894.5500000000002
800.2	470.45	1618.1000000000001	1318.6	877.05	1199.45
1252.85	732.75	1119.1	1075.6	401.2	1782.4	1581.8	998.6
876.4499999999999	1054.9	557.85	683.75	415.05	1462.7
693.75	793.4499999999999	656.5999999999999	606.9
846.35	397.15	1453.65	709.15	1531.85	1106.45	715.8	1095.6
1225.3999999999999	1074.9	1188.45	2757.1000000000004
565.25	3576.1000000000004	1658.15	1630.8500000000001
1121.25	2598.4	1806.4	1898.8	687.85	1363.65	1516.1	1760.5
894.6500000000001	974.6999999999999	565.25	901.8
776.8499999999999	718.8	563.5	786.95	967.85	634.35
843.6999999999999	695.6	884.7\
ELF5	800.0	65.0	95.60000000000001	10.6
63.949999999999996	27.75	30.8	1699.05	71.2
256.65000000000003	461.55	3790.6	2306.35	29.8
27.549999999999997	4.699999999999999	1769.2	280.0	3.4
	979.0500000000001	152.7	73.7	259.1
30.950000000000003	54.55	121.1	193.5	95.65	1560.65
295.65000000000003	18.4	71.2	96.25	62.6
479.09999999999997	3394.9	61.400000000000006	57.85
201.95	404.34999999999997	72.45	53.75	206.05	464.15
108.65	80.60000000000001	37.9	271.95	1955.85	86.25
116.2	52.6	506.9	68.95	86.55	41.25	183.55	16.9
171.2	82.65	37.5	271.5	250.20000000000002	107.15
78.95	48.5	27.25	519.25	146.85	300.65000000000003
114.39999999999999	31.700000000000003	180.8	243.9
13.55	112.75	177.45000000000002	23.35	58.05	12.55	5.35

	10.4	19.25	3.5	31.9	24.35	225.95000000000002
	12.7	5.4	66.15	9.85	12.65	8.0	6.85	17.4
	2.6	13.8	14.5	25.85	9.25	5.65	36.2
9.75\
CAT	1393.8666666666668	3806.8333333333335
1337.4333333333334	2216.1	3986.7666666666664
3584.7666666666664	6875.366666666666	4278.833333333333
	1496.1999999999998	3559.5	1641.1666666666667
6787.566666666667	2249.866666666667	944.5666666666666
	3236.266666666667	2074.4666666666667
1664.3666666666668	2430.8666666666663	6648.700000000001
	1393.5	567.4666666666667	2712.7999999999997
2010.0999999999997	1966.2333333333333	1116.3666666666668
	2139.4	4393.366666666667	1669.5	2651.3
1542.6333333333334	1698.033333333333	1956.6333333333332
	3815.133333333333	2972.2666666666664
1847.3666666666668	1559.6666666666667	2670.6
1514.3333333333333	2425.633333333333	1335.0666666666668
	3225.2000000000003	2366.6666666666665	2285.7
1768.6333333333332	2702.933333333333	2225.0333333333333
	4924.833333333333	918.9666666666667
2250.9333333333334	3015.4666666666667	3097.7999999999997
	4491.4	6567.966666666667	3021.466666666667
1853.466666666667	2578.8666666666663	3117.0	1321.5
3619.8000000000006	2161.5333333333333	1345.8
2712.2000000000003	3011.7000000000003	2193.9	1235.2
3950.4666666666667	2305.633333333333	2099.3333333333335
	2651.6666666666665	3259.1	1935.3333333333333
2182.4	1254.5	1251.2	1532.7	2877.0333333333333
3845.6666666666665	2730.0	1622.1666666666667
1748.3666666666666	1584.8333333333333	3447.2666666666664
	2049.3333333333335	678.5333333333333
1247.0666666666666	2376.2666666666664	8043.966666666667
	3283.366666666667	1750.2	3546.066666666666
826.7666666666665	2398.4	2640.0666666666666
2628.366666666667	3678.0333333333333	2671.5333333333333
	2536.3333333333335	2872.6	5107.2	2692.9	2099.8
1798.2	2826.5333333333333\
GFOD2	165.7	541.35	294.4	272.45	489.85	259.65	292.1
240.15	359.04999999999995	423.1	262.55	222.15
217.70000000000002	252.89999999999998	434.90000000000003
	325.7	198.10000000000002	396.2	164.6	327.05
422.65	290.05	342.20000000000005	200.35000000000002
250.7	221.2	244.25	384.3	466.35	296.45	176.3	209.25
397.65	295.75	381.1	219.89999999999998	169.14999999999998
	317.6	320.9	205.65	315.95	294.9	358.55	360.4
342.35	341.55	265.8	279.35	374.05	332.15	351.0	278.35
270.29999999999995	681.8000000000001	243.85000000000002
	430.95000000000005	244.9	324.5	365.15	545.4
59.15	279.35	301.25	269.7	256.65	499.9	261.05	267.25
409.15	286.25	435.85	337.04999999999995	252.55	238.65
263.55	209.2	390.4	375.55	287.15	235.05	381.09999999999997
	247.04999999999998	181.15	262.05	429.15	359.0
273.4500000000005	334.05	290.70000000000005	544.25

158.85000000000002	294.8	207.9	156.55	158.6	225.75
249.29999999999998	225.89999999999998	235.15	207.35
251.15	279.35	167.05\
ZMYND10	38.15	23.45	277.0	97.2	76.39999999999999
121.75	88.35	42.650000000000006	134.4	378.04999999999995
	304.65	19.45	38.25	100.8	85.1	41.349999999999994
	51.0	477.54999999999995	42.2	79.0	101.65
543.4	295.15000000000003	85.5	118.95	13.850000000000001
	73.15	292.05	274.4	43.05	71.35	219.8	184.2
409.55	54.8	163.64999999999998	85.6	54.45	32.05
355.35	31.9	38.7	273.3	39.6	496.54999999999995
395.2	95.6	19.85	175.85	159.75	290.95	505.95000000000005
	21.1	128.35000000000002	253.79999999999998
307.35	150.4	162.6	411.3	257.95	208.79999999999998
253.6	123.6	627.55	62.7	190.60000000000002	271.1	40.6
	299.5	618.1	393.1	215.0	29.6	106.1
179.35000000000002	585.35	360.75	252.45	291.15	16.35	20.8
	108.10000000000001	292.05	104.30000000000001
123.8	102.55	57.3	135.75	64.3	108.05000000000001
136.2	61.75	41.0	115.15	69.25	207.2	30.45
327.04999999999995	70.6	170.25	17.45	67.5
36.699999999999996\
ZMYND11	2008.675	2101.7999999999997	1356.5	2248.65
2127.125	2757.25	2176.4	3065.275	2240.65	2701.45
2706.975	2554.4500000000003	2675.8999999999996
1286.325	3010.625	2706.1	2297.625
1923.5249999999999	2024.475	1580.3249999999998
960.4	2042.65	1501.6	2227.075	2526.15	1934.8500000000001
	2879.3	2588.65	2198.025	2225.775	2228.1
1556.55	2191.6749999999997	3812.65	1599.875	2749.475
	2521.475	3138.675	2674.15	2699.7000000000003
	3289.1000000000004	2121.225	2657.325
2689.5750000000003	2056.875	1945.9	2661.25	2641.1
1971.2	2790.0	2667.825	1711.55	2330.1	2759.4	3426.625
	2378.375	2390.5499999999997	2001.1000000000001
	2000.7749999999999	2298.975	1850.475
2231.2999999999997	2019.1000000000001	2332.95
2239.0499999999997	2604.4	2402.2999999999997	2614.2
1512.725	2468.2999999999997	1884.1499999999999
1862.175	2911.65	2288.6	1692.8	1797.625	2672.75
2407.7999999999997	2482.85	3390.8	1810.5249999999999
3701.125	4263.525	2815.675	2340.7
4010.7999999999997	3752.4	2550.125	1717.825
2374.1000000000004	2244.925	4217.8	2598.45	2324.625
	2757.825	2045.175	2576.125
1846.2749999999999	2742.625	2148.225	2623.475
	2284.0750000000003	2920.275\
SHANK3	25.650000000000002	101.19999999999999	236.35
264.05	482.35	119.3	75.9	312.65000000000003	169.1
201.25	148.75	204.15	139.4	99.05000000000001	184.4
247.75	138.55	297.7	115.3	157.15	1717.4	149.15	467.7
300.35	90.5	55.65	75.25	413.45	377.55	96.75	173.1
249.6	396.55	226.95	38.45	20.1	89.05000000000001
438.04999999999995	35.3	85.15	165.35	138.45	372.25

137.45	131.35	260.05	259.45	428.25	243.5	190.4	140.65
78.30000000000001	125.0	1275.8	332.59999999999997
847.15	254.54999999999998	253.5	180.85	524.65	335.6
163.85	396.75	194.70000000000002	223.95	210.25	64.45
237.2	632.5	120.55	735.05	413.2	199.79999999999998
72.45	199.75	175.7	671.5999999999999	142.55	204.3
624.5	560.15	504.45	156.15	202.55	1343.55	1835.85	414.55
725.45	270.90000000000003	269.35	155.85	841.0	90.05
304.75	330.35	156.6	109.15	108.25	182.1	104.0
104.85000000000001	164.25	81.45\
ZMYND12	241.0	99.2	700.3	245.5	121.3	157.3	277.5
305.8	241.8	556.9	433.8	143.6	178.1	354.7	452.3
270.9	206.2	251.2	339.4	229.1	293.9	402.9	456.6
320.5	190.8	365.0	236.6	462.9	572.4	278.0	358.7
687.7	446.5	793.2	506.1	418.5	136.0	122.9	370.2
256.4	183.0	204.9	652.9	129.6	506.2	255.5	219.0
133.9	353.1	783.4	596.4	417.9	390.4	14.7	454.2
898.3	611.6	382.6	557.2	430.5	1061.4	832.4	249.7
819.2	341.0	196.3	470.0	193.8	498.2	1371.0	680.4
590.3	161.8	401.7	195.3	635.3	880.2	204.2	695.6	91.0
	346.8	15.8	513.4	207.3	164.2	452.1	471.1
135.6	196.1	184.1	77.9	263.7	647.4	441.7	460.9
876.0	794.2	844.3	581.1	817.6	686.5	394.6	653.1\
SHANK2	637.28	598.64	908.3800000000001	190.46	434.5
279.26	417.8	874.9199999999998	272.5	454.84
200.98000000000002	412.5	30.440000000000005
210.98000000000002	132.48000000000002	598.46	697.32
778.7	253.34	434.35999999999996	456.66	660.4600000000002
	770.3600000000001	443.4	619.34	453.02	341.56
1313.8200000000002	1743.2	248.72000000000003
299.58000000000004	1509.0	678.8199999999999	1195.56
269.00000000000006	306.4	382.6	300.21999999999997
268.94	5978.620000000001	483.71999999999997	855.1
1187.9599999999998	228.68	588.5400000000001	1322.06
742.88	155.8	978.54	953.26	687.1199999999999
239.62000000000003	318.6	1016.32	1003.3399999999999
2448.4399999999996	1219.6799999999998	374.99999999999994
	978.5199999999999	1346.2	708.7	473.4	167.84
802.1800000000001	916.04	643.9	766.64	327.72	868.9
1011.5	3057.2200000000003	866.8399999999998
362.65999999999997	206.5	1168.7	733.12	998.4399999999999
	603.6400000000001	474.26000000000005	233.8
1172.8200000000002	197.06	340.88	114.84	820.1	1291.7
182.71999999999997	785.4200000000001	579.96	309.82
213.74	1590.22	1329.44	632.58	799.74	987.5199999999999
991.9999999999998	932.4399999999999	1247.56	1239.78
1081.6	631.3599999999999	839.4399999999999\
PLEC	71.75	585.5	218.1	249.45	346.2	117.6	191.35
249.85000000000002	202.54999999999998	306.8	79.95
225.2	221.70000000000002	32.15	179.25	291.34999999999997
	258.15	340.8	206.5	146.54999999999998	296.5
158.35000000000002	396.55	159.95	166.75	376.45
174.45000000000002	360.6	188.45	159.79999999999998
107.55	229.39999999999998	788.0999999999999	216.9

300.1	382.95	80.45	285.90000000000003	282.95	101.3
172.0	242.35	213.54999999999998	303.40000000000003
136.4	290.5	240.7	98.65	340.79999999999995	276.05
249.35	206.89999999999998	226.29999999999998	406.75
304.29999999999995	559.9	149.85	317.95	204.39999999999998
	199.7	471.35	416.25	271.95000000000005	242.95
241.54999999999998	533.25	220.3	296.2	416.45
182.04999999999998	547.6	325.6	394.75	85.45	124.55
118.60000000000001	304.2	153.85	221.9	203.0	320.6
377.85	295.0	367.34999999999997	491.25	696.6500000000001
	270.5	342.95	335.1	251.29999999999998	60.45
234.65	153.15	174.35	126.19999999999999	173.75	169.0
118.05	205.75	206.4	180.2	264.05	180.5\
SHANK1	20.0	60.6	24.1	97.7	153.9	6.3	42.9	59.0
	41.9	49.5	8.4	33.9	61.0	92.9	74.9
101.1	43.1	16.4	53.7	71.2	157.1	57.4	35.9	45.4
	75.6	112.9	26.6	61.9	66.0	54.1	36.7
104.5	11.6	21.4	26.5	108.2	69.6	62.3	52.8	69.1
	31.8	47.4	88.3	32.6	5.5	41.3	35.7	54.9
	220.4	18.8	52.6	12.5	11.9	66.9	150.2	77.5
	60.2	16.8	5.5	132.4	35.5	80.7	97.3	8.4
	98.2	46.0	11.7	51.8	53.0	94.2	211.8
172.8	112.3	11.4	85.0	82.0	17.0	88.1	10.6	84.6
	44.3	126.8	11.9	24.7	25.9	120.5	149.1
132.4	16.4	122.0	48.9	127.0	47.9	67.1	50.1	7.9
	44.4	20.2	129.3	16.3	8.3	87.8	44.0\
RRN3	1643.9	1047.7	1010.9	1527.9	727.4	2305.5	2202.5
290.1	1687.6	792.2	1343.0	1213.0	1214.2	880.6	802.9
1212.1	961.5	886.5	1041.1	1385.7	427.0	1119.9	329.9
1463.1	1578.3	884.3	1212.8	652.5	704.5	1037.9	2606.3
409.3	535.3	883.1	1407.2	930.5	1799.2	684.8	1409.5
958.8	1612.1	2149.5	821.2	1125.5	1145.6	597.0	895.7
1294.0	733.9	763.5	827.8	1265.7	968.2	553.1	771.2
639.4	740.5	897.9	819.8	717.4	615.3	602.1	769.5
1766.8	918.5	604.6	1377.0	1198.4	406.9	638.5	554.3
849.6	1161.1	1864.5	975.7	1035.3	398.8	1273.8	1239.1
459.3	633.7	818.1	2089.5	1338.8	728.3	535.3	751.1
579.7	749.4	476.9	2385.8	395.5	739.2	1144.7	696.2
920.8	764.8	1056.6	695.6	503.5	800.4	626.3	1016.7\
DAPL1	3270.4	112.8	205.9	41.1	97.0	93.6	204.8
735.3	283.0	572.3	86.5	1129.0	232.3	186.9	1265.8	49.9
	535.1	87.3	21.7	218.6	418.7	193.2	119.4
108.0	40.2	99.1	438.8	25.4	187.8	217.7	25.5
585.8	255.1	136.4	206.4	406.7	261.5	29.8	175.3	64.5
	70.9	123.4	112.0	322.1	87.9	557.8	118.5
194.7	135.5	86.5	709.9	591.7	174.9	498.5	24.1
208.5	144.3	329.6	135.0	147.2	370.0	654.4	582.3
287.0	401.1	22.3	88.9	2011.4	83.1	211.0	177.6
109.0	192.3	59.7	153.5	87.9	103.7	57.9	226.3
559.2	305.3	136.6	3133.8	1056.2	98.8	39.3	205.6
125.4	40.5	59.9	185.6	68.6	164.7	66.9	62.1
214.0	169.1	87.4	640.1	419.2	14.4	77.6	50.1\
RP11-379F4.6	179.1	196.6	470.4	123.4	424.8	88.6	58.2
	118.3	212.5	195.9	79.8	182.0	36.3	211.2	67.6

68.5 109.1 127.4 53.8 74.8 460.9 75.1
121.8 94.7 102.2 68.5 9.9 189.9 187.6 237.1 57.2
45.1 135.3 41.6 259.2 113.2 45.0 194.8 94.2
95.8 31.8 79.7 29.0 51.7 92.8 208.6 74.7
37.6 154.6 103.5 146.8 103.8 60.9 75.2 89.0
183.3 180.2 434.8 82.2 240.5 164.9 64.2
152.0 135.5 167.8 206.2 68.1 104.7 45.1 38.8
140.4 150.1 143.9 100.1 193.5 68.9 48.7 77.3 41.0
34.8 36.7 21.6 128.5 92.2 99.1 178.4
143.6 113.1 74.3 126.4 209.5 51.5 62.3 79.1 32.3
72.6 55.4 66.5 122.6 81.2 104.2 118.9
118.7\
PLSCR3 /// TMEM256-PLSCR3 2234.25 1427.75 784.8000000000001
2105.4500000000003 1402.55 2358.75 3415.65 2219.0
2460.75 2791.0 1985.1999999999998 1501.6 1093.3 1771.6
3264.3500000000004 2769.75 1741.5 1527.85 1170.45 2630.5
2457.35 1237.25 1368.35 1951.9499999999998 1685.25 2810.55
2559.75 2410.65 1854.9499999999998 2657.35 2203.75 1864.65
1590.75 2162.9 1761.5 1734.25 1218.9 1547.9 1494.2
1928.3000000000002 1755.8 1310.1 1753.4499999999998
2225.25 1500.2 1781.3 2313.3999999999996 2179.35 1589.15
1636.55 2912.8999999999996 1274.75 1469.15 4463.45 2103.5
1042.45 1804.75 1285.45 1877.2 1426.25 2973.55 1853.45 1433.25
1417.3500000000001 1954.75 2459.0 1653.3500000000001
3245.25 2367.7 1566.3500000000001 1175.85 1422.25 1878.5
2162.5499999999997 1484.15 2276.55 3262.05 1570.6999999999998
2001.1 1852.1000000000001 2774.6 2332.2 1871.3
1783.95 2661.95 2419.3 4141.05 4038.7 1926.6499999999999
1490.5 1295.35 3357.7000000000003 2452.95 2404.45 2531.8
1570.7 1924.2 1356.6000000000001 1816.5500000000002
2243.45 1686.65 1804.0 1345.25\
CBL 491.46000000000004 821.5600000000001 384.32
505.73999999999995 498.93999999999994 329.5 980.9
502.03999999999996 594.28 610.1200000000001 500.48
616.14 460.8 522.32 966.0200000000001 783.4799999999999
624.24 420.73999999999995 644.6200000000001
517.9 440.7 661.04 492.6 617.1600000000001 716.36
606.3799999999999 384.53999999999996 433.66
336.43999999999994 650.8 498.64 458.88 541.94 539.12
418.06000000000006 264.96 375.96000000000004
443.0200000000001 526.28 421.7 890.82 671.3
504.96000000000004 909.18 556.12 414.1 505.8 449.12
354.82 416.26000000000005 516.8399999999999
447.46000000000004 356.46000000000004 462.41999999999996
351.8 594.08 432.65999999999997 368.1
413.78000000000003 420.8 540.2 403.54 491.7
471.0200000000001 472.68 467.82 641.86 909.8399999999999
522.58 323.84 427.9599999999999 360.06000000000006
638.14 475.17999999999995 460.32 387.50000000000006
296.2 621.3 422.5 1356.14 347.28000000000003
851.4 436.08000000000004 533.74 503.6 389.44
769.1600000000001 648.6600000000001 637.32
624.1600000000001 328.8 531.2 252.76 399.48 320.36
262.86 219.88000000000002 326.36 247.08 316.3

320.41999999999996	238.84	301.7\
FLJ44087	31.0	24.3	19.5	5.3	51.8	61.6	40.6
	59.3	27.5	12.5	5.7	20.3	5.3	5.5	63.4
	76.7	8.8	68.3	9.7	45.8	32.5	10.7	58.6
	62.3	10.1	38.1	71.7	33.6	19.8	73.4	36.4
	99.1	12.1	93.6	55.4	10.4	39.0	36.9	3.2
	45.3	6.1	8.2	11.9	18.5	26.3	16.2	74.0
	59.8	96.0	14.0	15.4	2.7	54.8	17.0	10.9
	26.6	81.1	64.2	8.5	30.3	396.0	13.8	78.3
	12.0	13.9	95.6	5.2	10.2	89.4	6.1	26.1
	140.4	5.1	13.9	76.0	35.4	15.8	30.2	6.0
	6.3	68.0	11.6	15.8	30.9	130.8	13.7	18.0
	120.6	100.3	8.5	11.9	11.4	17.9	22.3	99.4
	39.8	6.0	10.1	73.0	89.4	38.8	29.0
72.8\
AC005606.14	19.8	108.9	77.2	28.7	145.3	59.0	50.2
	33.8	21.0	99.6	53.7	28.4	30.6	11.2	74.9
	52.2	67.8	66.7	57.6	10.5	148.3	53.6	19.2
	42.7	69.8	38.9	122.5	21.1	12.3	5.9	13.1
	143.6	75.9	67.0	17.2	12.5	33.0	74.4	25.2
	80.1	73.0	9.6	13.8	67.2	7.6	84.2	75.9
	54.5	23.7	85.4	65.7	44.0	65.2	65.6
120.8	58.4	76.1	56.5	124.9	106.1	317.1	76.7	52.3
	82.9	115.6	91.3	10.8	43.2	58.0	96.7	27.9
	18.8	21.4	28.1	42.4	17.5	26.9	102.6	67.7
	58.6	77.5	52.3	46.9	38.1	304.7	300.6	67.7
	111.3	5.9	45.4	30.3	13.7	75.8	33.0	54.0
	78.1	52.7	6.9	168.7	67.9	88.8	52.0
10.5\
KRT17P5	57.7	14.7	77.3	38.5	80.6	50.8	31.5	9.5
	81.5	132.0	26.7	101.4	73.0	30.9	31.3	67.4
	82.4	29.6	5.1	37.5	13.1	55.9	78.3	47.6
	71.3	99.6	81.5	51.3	93.6	82.6	8.4	28.4
	103.2	16.2	74.2	46.5	66.8	28.2	83.8	37.5
	4.3	17.9	18.5	54.4	63.0	3.9	44.6	62.0
	126.9	53.5	23.7	50.3	55.5	22.1	83.5	25.3
	8.3	59.6	57.0	102.9	21.5	62.6	16.3	59.9
	46.0	83.9	70.0	22.1	85.6	92.8	24.1	5.3
	91.8	10.6	20.0	17.4	187.9	31.2	23.3
1317.1	73.8	15.6	117.9	80.6	255.3	148.1	5.9	28.4
	100.7	25.2	70.0	181.4	112.1	84.7	69.9	13.7
	74.0	23.5	100.5	17.4	43.7	14.0	44.2\
LINC00511 /// LINC00673	1131.65	226.75	425.15	169.75	903.45
381.9	651.5	397.35	352.5	353.95	333.79999999999995
325.15	495.15	296.65	357.5	267.6	597.3	497.15000000000003
	662.85	678.3	478.5	554.3	429.9	295.9
361.95000000000005	490.4	680.9000000000001	302.65
371.29999999999995	509.84999999999997	425.95	435.0
764.55	504.4	712.85	249.3	493.5	476.45	234.0	424.1
491.55	489.29999999999995	287.45000000000005	484.05
203.9	405.29999999999995	242.35	472.55	754.35	492.15
407.54999999999995	1160.9499999999998	801.5	492.45
471.05	294.75	444.95	357.15	495.35	373.29999999999995
684.4	276.55	658.3000000000001	769.1	417.1	1040.8

546.45 443.09999999999997 490.05 973.45 354.75
396.45000000000005 234.0 480.4 334.75 547.25 852.75
310.0 535.1 124.95 476.5 323.70000000000005 2084.2
1059.9 558.5 571.3000000000001 243.64999999999998
383.54999999999995 669.6 422.20000000000005 215.25
267.65 317.45 461.65000000000003 294.0 540.2 560.45
571.15 197.4 621.4 478.8 196.15 699.85\
SV2C 9.25 69.35 39.4 29.65 71.7 15.5 53.65 65.1
52.15 37.45 47.05 26.6 47.0 58.9 367.55
24.450000000000003 22.35 52.9 84.69999999999999
45.55 62.95 69.35 102.55 54.1 41.05 32.050000000000004
40.5 42.75 72.75 86.15 81.15 100.6 266.45
237.5 160.5 410.3 54.75 94.25 34.1 29.45 39.15 51.4
90.75 156.35 7.199999999999999 51.9 37.7
267.2 44.4 46.65 169.75 109.25 60.6 30.4 77.45
48.400000000000006 58.199999999999996 101.0
43.699999999999996 280.8 301.5999999999997 32.25 52.4
45.65 67.25 22.4 49.0 59.7 188.45 38.25
51.55 106.25 35.75 58.1 141.95 55.3 36.55
30.599999999999998 39.6 90.65 81.35000000000001 37.9
535.0 125.05 29.599999999999998 105.14999999999999
96.5 194.85000000000002 56.35 55.95 73.6
115.6 76.1 156.20000000000002 72.05 31.6
14.700000000000001 113.05 129.45 94.1 27.15 95.1
62.95\
SV2B 48.800000000000004 33.0 62.13333333333333 67.8
91.03333333333335 108.3 38.03333333333333 75.0
78.96666666666665 87.66666666666667
12.899999999999999 16.866666666666664 19.466666666666665
111.66666666666667 86.33333333333333 42.0
96.86666666666667 66.8 31.233333333333334
95.43333333333332 119.09999999999998 28.5
124.16666666666667 54.36666666666667 91.06666666666668
59.1 45.166666666666664 76.5 77.96666666666667
82.10000000000001 66.23333333333333
70.26666666666667 82.43333333333334 48.5
38.53333333333333 34.46666666666667 41.800000000000004
67.63333333333333 29.166666666666668
69.76666666666667 73.43333333333334 24.599999999999998
65.83333333333333 35.96666666666667
32.13333333333333 93.86666666666667 119.09999999999998
34.699999999999996 70.36666666666666
78.43333333333332 61.0 16.233333333333334
120.33333333333333 67.26666666666667 39.8 75.3
32.300000000000004 101.63333333333333 39.13333333333333
89.66666666666667 216.73333333333335
47.13333333333333 52.633333333333326 31.066666666666666
77.5 66.56666666666668 31.866666666666664 31.3
51.1 65.7 33.63333333333333 45.699999999999996
102.06666666666668 13.5 127.76666666666667
25.666666666666668 19.433333333333334 39.1
49.166666666666664 116.56666666666666 46.699999999999996
51.1 34.1 45.0 52.666666666666664
78.5666666666666 70.03333333333335 103.60000000000001

32.13333333333333	69.5666666666666	51.6	84.2
26.899999999999995	102.3	35.800000000000004
31.833333333333332	55.666666666666664	41.1666666666667
17.966666666666665	61.9	20.933333333333334	72.3
50.733333333333333\
CBS	326.23333333333335	74.1666666666667
97.10000000000001	480.0333333333333	334.8666666666667
1026.7333333333333	1636.6000000000001
189.4666666666667	127.03333333333335	212.8666666666665
346.3333333333333	151.7	275.2333333333333
146.8666666666667	733.8333333333334	80.4666666666667
191.73333333333332	204.03333333333333
104.1666666666667	144.5666666666666	384.49999999999994
106.8666666666666	239.60000000000002
136.2666666666665	104.9666666666665	19.733333333333334
146.93333333333337	94.53333333333335
111.73333333333333	18.7	26.3	229.4	104.03333333333332
93.23333333333333	168.10000000000002
301.1666666666667	159.1	388.8333333333333
22.599999999999998	79.1666666666667	217.23333333333332
437.9666666666667	132.23333333333332
104.10000000000001	268.03333333333336	92.0
379.7666666666665	221.53333333333333	84.3
72.2666666666667	165.33333333333334	267.6	25.0
914.1666666666666	113.9666666666668	60.366666666666674
67.53333333333333	196.9	119.89999999999999
134.4	163.3666666666667	188.9333333333333
156.2666666666665	287.3666666666667	63.800000000000004
63.23333333333333	72.93333333333332
65.4666666666667	137.5666666666666	61.300000000000004
102.89999999999999	115.89999999999998
104.0666666666666	165.63333333333333	93.4666666666665
46.833333333333336	139.6666666666666	158.4
78.60000000000001	766.5	194.83333333333334
191.3666666666667	240.80000000000004	190.6666666666666
285.3	1211.1333333333334	273.7666666666665
358.0	528.6333333333333	479.8	334.2666666666667
171.3666666666667	208.5333333333333	313.0666666666666
83.73333333333333	211.39999999999998
124.43333333333334	319.09999999999997	449.6000000000001
130.13333333333333	196.53333333333333	142.8
229.23333333333335\
TBX22	842.4	1.7	25.1	1.6	159.1	4.0	1.4	11.1
11.1	20.5	5.6	49.2	33.4	2.3	5.9	3.7
5.1	1.5	16.5	11.7	2.6	27.8	4.2	16.6
1.8	13.9	3.5	4.6	8.1	2.6	20.9	6.4
9.9	1.0	3.0	46.8	4.7	4.5	3.1	0.9
11.0	5.1	2.2	0.7	5.4	38.5	2.0	3.2
74.7	7.6	59.7	83.8	17.4	5.6	4.7	1.2
3.4	1.8	1.9	5.4	9.4	5.4	39.3	22.9
4.3	2.1	31.1	2.7	14.7	11.5	48.1	9.1
2.2	3.0	14.1	3.6	3.0	2.1	2.3	23.9
91.9	11.3	18.7	3.4	6.0	42.0	5.3	2.7
30.8	7.3	9.5	8.5	6.9	3.5	2.1	23.0

	3.8	40.9	51.8	7.1	40.7	8.6	3.3\	
SV2A	401.4	54.8	122.3	269.6	1111.9	14.3	889.6	
321.0	607.0	58.5	41.2	33.7	132.3	50.5	575.1	
468.2	325.0	121.5	36.2	67.3	664.5	9.2	110.4	
236.6	23.7	317.0	22.9	169.1	100.1	218.1	439.2	24.1
	127.8	169.7	165.5	110.9	13.2	22.5	7.0	
141.5	360.8	470.3	227.5	519.5	230.2	193.1	174.0	
704.3	265.3	306.9	190.4	46.9	26.4	751.4	185.5	
444.2	241.8	109.4	21.6	283.4	336.9	80.2	79.3	72.7
	232.2	79.3	204.3	1225.0	846.7	86.8	40.8	
324.8	227.6	124.3	170.7	85.2	185.4	20.9	147.0	
7257.4	449.5	326.6	212.4	78.9	139.9	24.8	738.9	
509.0	123.2	93.4	359.7	397.1	201.7	310.6	270.9	
143.2	291.2	138.0	198.4	105.3	218.0	177.1	203.6\	
AX746710		10.0	62.3	22.9	0.7	10.7	33.2	1.8
	0.5	18.0	49.0	14.1	35.7	3.4	3.0	14.7
	8.7	25.5	9.3	3.7	37.1	102.6	25.8	30.2
	5.9	4.4	29.9	20.2	2.5	42.7	44.2	21.2
	8.7	81.1	6.6	46.5	11.5	0.8	5.8	2.9
	0.7	1.2	1.0	13.3	16.4	2.8	1.3	4.6
	49.0	70.5	54.7	6.4	7.0	13.2	21.4	34.7
	4.0	18.9	10.8	14.9	56.3	115.0	87.4	8.0
	29.1	47.4	15.1	8.5	46.9	1.9	23.8	56.3
	69.2	35.7	10.5	3.4	22.8	13.9	3.6	32.0
	38.8	41.0	0.9	10.7	12.2	80.6	180.2	3.3
	17.8	13.9	6.3	10.3	15.3	44.7	0.5	12.2
	10.9	2.3	22.7	11.7	48.2	24.0	51.6	
13.6\								
TBX21	55.3	102.8	60.4	166.7	110.1	55.3	58.6	
130.1	98.3	60.0	13.9	91.6	20.2	180.6	99.3	48.8
	72.7	40.0	33.4	25.5	216.1	83.7	8.0	73.7
	68.9	51.7	54.2	104.4	138.9	118.2	80.7	
188.5	115.1	121.5	157.0	104.1	102.9	147.9	95.2	
124.0	39.4	67.5	5.8	47.3	6.1	68.1	105.5	20.1
	101.0	99.3	57.7	104.7	87.8	156.8	23.5	
103.6	25.0	157.6	124.5	78.4	140.5	145.5	91.7	57.0
	51.5	96.7	58.7	78.6	168.3	9.3	64.8	
232.3	153.8	56.7	187.9	47.2	37.0	111.6	75.6	86.0
	14.0	53.0	51.5	129.1	107.6	33.7	126.0	
121.1	129.0	13.1	58.7	193.1	84.6	54.1	74.3	24.8
	54.8	81.5	74.7	98.0	59.7	88.4	41.6\	
TBX20	19.6	35.6	63.3	27.2	51.5	35.3	6.7	46.2
	9.1	110.8	12.6	6.7	18.9	3.6	62.1	14.8
	55.2	18.3	4.7	43.0	39.5	45.0	20.1	25.2
	9.5	8.7	6.1	129.8	65.6	120.0	46.1	62.1
	11.9	23.7	63.7	9.9	9.0	35.9	56.4	54.2
	12.7	14.9	52.2	15.5	55.8	77.0	9.1	33.1
	37.0	6.8	42.7	48.9	31.9	115.2	3.3	50.8
	48.1	3.8	5.6	68.9	67.1	36.5	26.3	3.3
	68.4	14.5	5.7	48.8	37.6	22.2	68.3	16.9
	115.4	21.7	11.0	50.3	3.4	49.5	8.5	28.6
	17.3	59.9	33.9	20.9	6.1	132.8	86.5	6.3
	59.8	9.0	28.3	16.9	46.9	10.1	8.7	22.3
	76.1	29.1	11.9	52.8	7.4	12.5	8.8\	

```
CLVS2   83.1    95.6    5.2     29.9    100.7   63.3    11.1    57.1
        38.0    14.6    40.6    25.9    63.0    7.9     14.0    54.6
        42.7    99.1    20.1    11.2    216.8   52.2    12.9    50.4
        77.3    39.8    77.2    82.2    135.2   191.6   36.8    69.9
        108.1   61.6    75.2    105.9   59.6    74.4    32.6    48.2
        33.4    46.5    34.4    12.8    7.7     36.9    51.9    35.2
        12.4    44.0    40.8    68.8    27.9    134.7   16.2    76.0
        4.7     96.8    2.5     73.1    341.7   9.5     17.2    16.8
        76.6    68.6    38.9    11.7    67.6    55.0    153.0   72.1
        10.0    53.9    63.3    80.9    92.1    69.0    39.4    48.3
        102.3   73.0    43.0    63.4    55.6    109.7   66.5
102.6   49.3    171.7   22.8    80.5    47.8    35.8    48.0    52.5
        94.9    15.6    24.4    78.2    7.5     67.4    91.6\
SPATA31E1       3.25    9.5     5.4     4.65    7.8999999999999995
        9.45    3.2     3.9000000000000004      4.4
5.050000000000001       5.6     3.75    3.95    5.75    7.75    4.85
        3.5999999999999996      5.4     4.35    8.85    9.45
5.949999999999999       43.95   3.25    7.7     8.3     6.2     8.3
        7.45    3.1     3.4     13.899999999999999      5.25    4.65
        13.3    5.25    3.25    10.75   3.4000000000000004      4.9
        3.8     4.0     6.15    3.05    8.55    11.65   9.4     4.05
        8.55    5.45    7.1     8.35    3.8499999999999996      8.4
        7.1     11.45   6.8500000000000005      7.3     3.0     8.55
        16.2    5.3     7.4     3.65    5.35    10.55   3.25    3.45
        9.0     8.15    9.0     5.25    5.4     3.3     6.75    4.7
        7.05    4.6     2.8499999999999996      7.4     8.4     8.25
        6.1     4.0     6.3     20.35   8.45    30.05   4.55
6.949999999999999       7.199999999999999       16.75   4.5     2.3
        4.75    6.5     4.55    7.1     7.15    11.15   4.3
6.8500000000000005      6.95\
CLVS1   9.5     20.6    7.0     33.8    12.5    16.6    18.7    23.6
        8.1     6.9     35.4    5.6     60.3    7.2     201.6   38.8
        9.3     74.0    5.4     14.9    15.6    67.3    29.6    52.1
        5.5     9.5     15.9    24.5    36.4    22.7    20.4    46.0
        20.0    70.8    61.3    7.2     4.1     33.5    52.6    19.0
        39.5    36.3    22.0    17.0    46.4    7.2     63.2    46.5
        9.6     8.4     16.4    57.4    7.3     36.8    86.7    20.2
        8.1     12.9    30.1    7.2     23.6    37.4    10.0    6.3
        7.6     7.6     44.7    12.7    15.3    69.0    123.8   7.7
        11.1    26.8    57.8    7.0     28.4    16.0    12.0    12.5
        94.3    38.5    35.2    53.4    67.8    35.2    45.3
115.2   25.2    6.3     11.1    37.5    3.9     3.0     3.7     13.8
        39.5    18.5    8.5     28.6    8.7     10.7    7.3\
RRM1    4424.049999999999       1056.55 449.85  3273.8  1052.1
2478.6499999999996      2722.45 975.9499999999999
1776.3999999999999      1484.55 1855.8500000000001      1437.1
2436.25 1403.0500000000002      1231.65 1293.3  1242.3000000000002
        1482.2  1492.9  1725.1499999999999      514.35  1625.05
449.45  1147.45 2477.0  1777.35 3864.2000000000003      1218.75
853.65  1981.6000000000001      1527.0  2243.5499999999997
588.75  928.1   1803.15 955.6999999999999       2092.15
1432.9499999999998      1738.25 1592.75 1581.6499999999999
1939.05 1354.1499999999999      987.7   1445.6000000000001
1388.55 2270.0  2146.85 1034.95 1129.65 1539.25 2265.35 1728.3
```

1201.95 1693.25 1204.95 1081.0 1485.55 1439.5500000000002
973.55 1953.35 1073.5 946.8499999999999   1370.1
732.1500000000001   721.15 873.85 653.9   730.85 1417.4
414.20000000000005   832.0500000000001   666.5999999999999
        612.95 844.35 2057.35 645.5999999999999   1235.4
1141.0 2148.2999999999997   937.4   2013.95 1238.95 889.85
649.1   617.7   3501.4 1034.4 911.6   453.8   1611.2 798.75
1342.6000000000001   1139.3 959.75 994.9000000000001
1188.8500000000001   1187.7 881.1999999999999   1073.75
949.95 1012.3 814.45\
RRM2   15438.95   2713.1 1230.95 10483.65   5138.0
6773.4 10651.15   9898.15 6563.900000000001
4055.3500000000004   4721.1 7070.0 8440.45 8227.9 2476.05
1578.8000000000002   6141.200000000001   5038.9 8496.85
5501.549999999999   391.45 9725.45 1439.35 3520.3999999999996
        17945.05   4878.3 16352.2 1702.85 1577.2 4518.75
1820.1 4258.85 1237.1 665.55 3764.85 1295.75 11850.800000000001
        9414.95 5155.9 4197.25 12437.0 5835.4 2601.1 1809.65
6351.45 9395.949999999999   15517.3 13535.400000000001
2281.8500000000004   1144.8 5220.45 9717.6 5081.700000000001
        6843.35 3466.65 1657.9499999999998   1130.15
4002.3500000000004   5973.15 1767.2 1104.9 1780.7 1633.95
3705.1499999999996   627.85 1420.0 2082.55 888.75 1006.2
3431.25 360.7   2311.6000000000004   361.9   4257.0 3339.2
4669.049999999999   697.15 3078.15 1553.8999999999999
11390.95   2004.15 14749.2 4896.45 2852.85 6356.950000000001
        7501.5 24200.95   2046.3 17153.649999999998
8787.3 9298.05 2462.35 524.2   286.6   65.65   470.8   209.9
199.5   311.1   228.45000000000002   195.0   3622.1499999999996
        228.64999999999998\
LOC101929910 /// LOC613037 /// NPIPA5 /// NPIPB3 /// NPIPB4 ///
NPIPB5 163.1   1011.4 3324.5 328.0   2233.9 57.3   278.1
132.9   193.0   960.8   275.1   2121.5 682.1   764.3   759.2
970.1   496.1   2214.4 419.6   1019.4 4019.0 250.8   1956.5
115.9   97.3   886.7   158.5   1428.0 615.5   681.9   319.0
112.7   4722.9 212.9   1302.6 306.3   10.5   3768.2 856.3
134.7   173.9   161.9   1353.2 829.8   233.7   551.8   144.4   53.1
        1920.7 1095.1 396.1   199.1   84.7   283.9   1457.3
1980.8 165.2   2514.3 585.3   1680.9 491.1   1164.1 1363.6
718.3   1552.6 538.6   229.2   533.0   664.4   475.6   1666.8
1730.6 1336.2 457.4   1572.5 14.1   153.1   659.5   299.0   96.1
        162.6   155.0   285.3   96.4   419.0   131.1   55.0
122.1   468.6   2410.4 56.0   242.6   94.0   441.8   719.4
118.0   94.3   81.8   1116.1 229.2   139.6   142.4   170.0\
LOC90246   24.35   130.65 149.25 154.85 166.4   65.7   8.55
        153.5   142.1   149.85 112.6   35.55   24.6   24.2
21.65   35.65   39.150000000000006   81.75   38.5   110.8
158.7   41.6   187.6   64.60000000000001   119.30000000000001
        30.549999999999997   12.399999999999999   93.45
145.8   67.75   126.75 44.449999999999996   116.0
204.54999999999998   78.2   101.65 52.55   150.55 172.75 59.1
        28.0   86.44999999999999   126.25 294.0   186.35
70.05   35.05   82.85   137.85 192.45000000000002   131.7
129.35 31.65   175.95 134.05 80.45   93.9   74.35   32.65

195.95	90.1	187.79999999999998	242.95	87.15	356.0
267.85	209.15	170.45	172.4	96.2	179.9	95.6	263.9
198.6	111.5	27.8	19.8	95.6	111.65	75.35	64.15
54.25	13.8	68.75	99.25	90.9	90.39999999999999
145.0	121.25	116.5	66.6	92.3	106.35	127.55	107.6
27.45	138.6	136.9	30.7	105.44999999999999	112.45
187.10000000000002	138.65\
CCK	24.4	18.3	7.6	3.8	5.1	10.4	9.1	12.8
	5.5	6.9	8.3	74.8	9.2	80.1	8.0	17.0
	5.9	3.9	6.9	10.2	27.6	17.3	131.5	9.3
	7.5	5.6	28.7	10.1	11.2	83.3	8.3	9.9
	27.5	49.3	24.8	16.8	2.7	29.4	5.3	15.9
	13.4	33.5	4.8	13.4	3.6	15.5	65.9	36.7
	70.0	18.5	57.7	8.1	13.8	26.2	9.0	10.8
	10.5	7.4	5.7	34.2	37.9	12.6	55.6	8.9
	49.0	108.6	17.6	52.7	14.9	22.5	32.3	5.1
	20.6	3.4	6.1	14.1	12.7	76.5	9.7	94.5
	11.6	82.5	53.0	11.5	33.3	28.7	118.2	15.1
	15.6	15.4	25.8	5.7	12.6	13.7	2.5	7.2
	30.2	13.1	32.2	11.5	5.4	17.9	5.3\
HS3ST5	75.8	144.8	21.2	58.3	78.4	15.9	36.7	21.7
	18.9	23.2	18.9	89.0	80.5	36.0	105.5	61.2
	137.9	121.0	104.4	42.8	259.0	76.4	39.0	12.1
	22.2	114.8	80.5	112.9	30.2	44.6	59.5	28.8
	35.5	9.6	278.4	104.2	102.8	115.1	12.7	19.3
	70.4	41.0	106.6	92.6	79.3	43.2	211.8	12.9
	51.7	470.9	22.6	55.1	14.5	25.0	134.6
121.2	59.6	188.8	75.3	76.8	682.2	78.2	76.5	60.6
	373.0	162.4	97.4	31.7	18.1	98.2	162.1
123.0	220.3	98.1	86.1	73.7	24.5	12.6	86.5	40.0
	33.8	87.0	61.2	136.1	30.8	250.7	23.6	33.3
	194.9	127.5	105.7	161.2	98.0	16.9	111.9	78.5
	71.1	39.5	138.7	188.3	53.9	159.6	73.1\
ZIC4	43.4	126.25	43.3	12.75	90.9	14.25	24.85
22.049999999999997	40.3	97.35	60.6	51.650000000000006
	59.35	87.85	157.9	41.7	36.4	22.75	47.25
90.05	110.9	66.2	52.55	39.75	53.900000000000006	32.5
	41.150000000000006	77.15	53.05	22.3	44.7
77.95	53.5	9.0	85.5	49.099999999999994	41.45
41.85	22.75	59.1	25.75	58.650000000000006
41.949999999999996	33.75	56.55	25.35	61.55
50.900000000000006	54.8	50.449999999999996
56.900000000000006	24.75	51.35	14.45	19.85	96.1
55.15	29.75	52.3	19.5	150.9	52.35	58.349999999999994
	28.25	28.25	44.5	38.65	34.849999999999994	27.3
	54.650000000000006	72.45	89.85	48.1	51.2
5.699999999999999	26.75	39.7	46.25	22.45	38.75	98.4
	54.2	11.0	46.2	122.85000000000001	120.9
51.55	101.9	41.349999999999994	58.75	44.5
57.349999999999994	26.45	3.25	39.2	43.5	65.3
20.35	38.05	89.25	14.600000000000001	51.25	50.05\
HS3ST6	27.7	4.9	10.2	51.3	5.6	13.6	23.7	29.7
	17.9	115.4	4.5	18.6	53.2	47.5	18.4
128.2	31.2	19.3	3.9	99.6	21.4	5.8	8.3	3.4

21.7 6.7 10.7 58.4 9.5 20.2 2.2 30.9
64.2 60.1 27.3 11.1 6.5 170.2 8.7 32.1
6.6 3.7 30.4 12.9 5.7 23.4 9.7 81.1
15.2 5.3 8.0 5.3 3.5 10.9 10.5
329.9 49.6 50.0 7.2 91.1 290.4 59.9 45.9 74.3
58.6 19.2 6.9 2.5 81.2 68.9 129.3 32.6
62.2 39.1 21.4 4.7 63.3 6.7 5.2 20.0
19.8 13.7 14.7 9.3 21.0 1020.2 20.9 21.9
105.7 14.6 7.9 10.6 4.6 4.6 4.6 5.7
6.1 32.4 15.3 67.9 8.8 7.5 14.9\
ZIC5 27.0 11.9 29.5 8.5 20.7 116.9 62.5 93.8
74.1 5.3 81.1 6.6 90.2 81.4 118.8 64.2
11.4 31.7 9.3 83.4 40.6 61.5 71.1 69.5
35.9 10.4 4.7 28.4 87.3 11.1 16.0 14.5
10.3 24.6 119.7 39.7 82.4 22.5 2.6 42.1
12.2 3.0 13.2 10.0 89.6 75.6 74.1 62.7
78.1 18.0 56.1 18.9 10.2 39.8 11.0 9.1
23.8 5.8 3.4 29.0 283.9 10.9 49.8 32.7
73.0 67.3 17.2 3.0 22.3 88.6 93.4 97.4
25.9 31.0 5.5 3.4 18.4 51.6 35.9 44.9
72.1 104.0 84.1 57.8 18.4 34.2 5.1
103.0 34.7 19.5 6.4 138.0 33.5 41.1 66.2 37.1
75.5 51.4 38.2 34.2 43.6 9.9 26.0\
AC079767.4 47.7 51.0 48.5 80.7 59.5 22.1 31.2
96.3 57.2 83.7 68.0 39.2 46.6 90.0 93.6
132.7 56.9 75.7 84.3 46.9 131.4 67.6 23.2
106.9 59.6 18.4 90.8 122.9 118.7 57.9 35.3
103.5 63.5 111.1 37.5 88.4 92.7 143.4 42.6
85.8 27.2 45.4 41.6 39.3 93.1 94.8 65.4
44.0 108.7 33.2 122.3 77.7 28.0 14.3 73.3
99.3 116.7 40.0 57.2 20.7 655.1 38.4 94.8
65.5 94.0 63.8 54.1 18.7 42.9 42.2
155.3 81.0 116.3 38.8 40.9 61.6 71.3 51.4 55.7
8.2 94.5 74.0 70.0 119.3 48.3 133.7
122.0 202.2 129.7 72.4 34.1 124.0 37.9 35.1 32.2
75.5 70.8 59.6 90.9 16.4 58.4 108.9
86.0\
SUOX 584.25 1851.8999999999999 803.5 721.35 1030.65
996.0 946.6999999999999 1044.8 977.5500000000001
1096.95 819.85 927.4 562.75 1488.35 753.8000000000001
533.0 767.25 986.35 1226.25 792.1 1139.45 638.95 684.9
1004.0500000000001 862.1 720.1 793.25 487.25 1592.75
863.0999999999999 436.35 1013.4499999999999 931.25
1135.4 1904.3999999999999 1231.55 675.0 966.85 825.6
638.3 772.8499999999999 1011.8 760.25 655.9000000000001
1188.9 803.3 887.15 414.45 2793.6 845.8499999999999
987.15 584.75 1065.5 735.55 1234.8000000000002
1005.4499999999999 1116.5 784.9000000000001 690.2
837.0 1313.75 1275.0500000000002 951.05 612.15 889.7
1523.6 1157.0 749.5500000000001 1535.5 1002.9 1368.75
2500.3 707.3000000000001 1364.65 1096.95 1086.85 825.35
1349.4 841.5 360.75 750.35 368.5 516.5 978.1 1158.0
1373.25 1030.8 1133.85 1099.95 1094.75 652.55 852.5999999999999
1399.45 917.95 1042.9 1038.1 1414.75 1058.05 1948.75

```
903.0   1047.15 796.65  885.4\
HS3ST4  73.5    26.9    29.9    18.9    50.5    24.2    12.4
187.8   85.9    23.1    16.0    22.6    69.6    340.0   176.9   80.6
        70.9    144.9   58.7    167.0   429.7   91.4    28.6    19.3
        67.6    33.4    105.3   211.1   78.6    110.2   54.1
183.6   147.6   143.1   77.9    106.7   68.2    17.6    47.8    63.6
        132.5   46.7    66.8    96.5    110.1   135.8   12.4    95.0
        286.6   152.1   19.2    120.8   102.5   176.7   66.0
146.0   88.7    80.4    38.2    40.2    196.3   92.0    33.4    67.4
        874.8   119.3   15.5    19.5    120.0   126.8   72.1
227.5   48.3    48.3    101.9   79.7    182.4   30.7    137.0   34.5
        51.6    103.7   24.0    106.6   205.5   296.4   54.2    70.3
        71.7    74.1    21.2    200.3   105.6   71.9    42.1    20.0
        84.5    53.5    45.6    43.3    26.2    93.5    146.6\
TBX19   314.6   763.7   958.4   155.7   647.8   361.4   299.1
1056.0  193.0   815.8   346.1   269.8   491.7   933.3   384.4
467.8   436.2   473.0   443.6   517.4   893.3   351.6   572.7
305.2   381.5   495.3   113.3   782.7   502.4   236.5   337.6
912.8   826.9   237.0   705.2   547.2   298.1   423.3   300.2
322.6   399.7   238.0   309.8   280.5   203.7   70.1    470.7
199.7   783.6   818.0   203.1   294.5   369.0   1424.7  552.1
1500.9  508.0   752.5   632.3   362.4   196.4   775.4   462.7
354.0   416.9   1122.9  458.9   264.0   408.2   344.6   912.8
449.7   184.7   371.2   835.1   40.7    439.2   397.0   439.2
436.1   571.4   587.2   245.0   339.5   925.6   784.5   614.1
491.1   595.8   230.6   448.6   374.7   350.2   312.0   576.4
431.4   430.4   301.6   593.4   543.1   371.6   476.3   529.6\
CFC1    7.6     69.55   63.75   55.050000000000004
80.35000000000001       15.35   25.950000000000003      21.9    65.3
        66.10000000000001       34.8    29.0    48.550000000000004
        21.0    56.45   37.25   31.25   75.55   14.049999999999999
        45.25   40.75   26.4    145.7   33.25   46.05   81.0
14.55   97.2    167.8   77.14999999999999       8.55
24.049999999999997      70.2    25.8    88.2    62.05   40.4
27.55   18.05   16.45   30.1    11.65   84.15   16.5
10.299999999999999      57.95   47.300000000000004      9.0
51.65   72.3    90.89999999999999       53.65   40.75   27.3
42.550000000000004      126.95  37.05   87.2    21.15   57.65
85.64999999999999       53.1    50.25   14.2    47.8    20.4
16.65   29.05   50.85   40.4    59.25   43.45   44.85   26.3    16.4
        12.8    26.25   25.25   36.6    75.1    21.549999999999997
        21.5    19.900000000000002      7.449999999999999
114.3   20.450000000000003      52.05   22.1    91.5    18.2
41.050000000000004      111.3   117.6   16.55   61.699999999999996
        76.75   71.2    13.9    51.75   33.05   110.7   19.15
22.5\
HS3ST1  293.3333333333333       114.83333333333333
163.46666666666667      95.76666666666667       280.03333333333336
        291.43333333333334      135.5   462.8666666666666
592.3000000000001       356.0666666666666       1795.3000000000002
        186.9333333333333       1124.3999999999999
194.76666666666668      76.96666666666667       201.20000000000002
        164.16666666666666      108.86666666666667
138.23333333333335      439.8333333333333       313.7666666666667
```

191.19999999999996	291.0	242.7666666666665
347.5	206.80000000000004	280.1666666666667
177.96666666666667	254.13333333333333	591.7333333333333
274.46666666666664	87.83333333333333
165.46666666666667	1908.3	160.26666666666668	73.2
683.7333333333332	160.23333333333332	1009.8666666666667
107.5	137.93333333333334	689.0666666666666
386.06666666666666	259.1666666666667	173.0666666666667
189.10000000000002	178.20000000000002
162.96666666666667	210.5666666666667	401.0999999999999
636.0	144.1	270.3666666666667	146.63333333333333
673.3000000000001	1270.6666666666667	1210.2
67.83333333333333	84.93333333333332	1260.1333333333332
708.3666666666667	281.8666666666667
331.93333333333334	396.6666666666667	287.7
225.9333333333333	300.1666666666667	334.0666666666666
258.90000000000003	409.3999999999999	332.0
114.60000000000001	143.5	643.1	241.9333333333333
352.1666666666667	498.2	1546.2	257.06666666666666
75.83333333333333	331.4666666666667	363.0
25.333333333333332	66.60000000000001	1337.9666666666667
190.43333333333337	240.4	176.16666666666666
410.8333333333333	124.7	191.86666666666667
303.3666666666667	287.59999999999997	402.0666666666666
230.39999999999998	193.26666666666668
225.73333333333332	226.4	231.4666666666667
319.43333333333334	255.66666666666666	113.0
271.3666666666667\
TBX18	14.350000000000001	76.32500000000002	131.8
3.4750000000000005	331.675	10.924999999999999	9.45
27.474999999999998	25.8	128.0	56.4	17.475	178.375
17.799999999999997	25.375	21.35	23.099999999999998
88.775	38.125	49.099999999999994	67.85	158.35	141.925
11.974999999999998	55.074999999999996	26.775	27.45
117.05	32.075	24.849999999999998	54.875	68.2	17.825
78.95	48.75	34.175000000000004	124.55	40.05
11.249999999999998	7.525	169.3	20.15	25.55	25.425
33.45	18.35	131.525	20.125	107.925	19.924999999999997
55.05	25.549999999999997	66.85	484.325	68.80000000000001
249.45	185.0	141.8	37.475	67.85000000000001
215.42499999999998	86.725	67.3	35.574999999999996
37.275	71.825	28.025	18.175	53.65	39.724999999999994
55.65	29.425	39.474999999999994	53.5	12.575
28.150000000000002	63.85	23.925	21.675	47.775	24.0
13.225000000000001	11.424999999999999	17.775	105.6
175.9	38.775	154.825	22.95	138.85	109.52499999999999
34.875	23.1	9.275	26.300000000000004	26.875	35.425
20.425	36.5	23.4	15.85	39.825	12.875\
HS3ST2	73.1	277.4	80.9	85.1	116.9	135.2	101.5
215.5	121.0	165.8	169.2	113.4	85.5	95.2	96.0	67.4
92.3	178.9	60.4	68.9	172.5	94.6	204.0
156.1	163.0	57.6	161.0	271.7	150.0	75.0	91.8
115.6	176.1	139.5	249.9	114.3	114.6	213.8	93.1
159.1	37.1	77.4	144.1	91.4	93.5	148.7	132.0

122.9	107.9	157.5	210.5	171.8	87.7	182.0	130.7
173.5	21.6	127.7	70.1	91.2	618.9	12.8	138.4	94.0
	128.4	121.0	123.6	122.2	109.9	158.8	250.8
260.0	173.0	208.2	134.8	109.5	229.7	74.1	142.5
183.1	253.9	146.8	84.9	124.8	301.3	272.6	106.3
289.8	174.2	110.0	86.6	192.1	166.5	133.0	123.2
205.7	192.3	172.0	148.2	184.3	148.4	149.2	125.9\
CD2	149.5	129.1	189.4	1859.7	268.4	63.5	441.4
1622.8	680.1	287.7	165.3	608.3	11.6	1637.4	31.0
588.8	536.0	281.0	474.0	328.5	1045.1	318.8	502.8
1666.0	401.2	293.8	173.4	835.6	411.0	1056.8	359.3
803.9	205.4	444.3	427.9	606.5	445.1	146.1	70.5
1085.8	27.9	185.8	787.3	18.4	213.1	573.3	670.0	73.5
	416.0	270.4	809.4	203.2	182.0	342.3	241.9
249.5	438.2	364.9	439.6	185.9	149.3	983.7	237.3
211.4	471.0	78.1	821.5	504.5	699.5	262.5	584.9
564.0	1829.4	704.8	1583.0	455.5	385.3	900.4	1028.6	21.6
	417.3	494.1	58.5	934.1	441.0	799.3	950.0
525.6	1195.0	64.7	288.3	480.2	250.8	399.6	364.9
121.6	153.5	428.8	114.9	234.7	322.3	195.1	330.0\
TBX15	373.8	103.1	95.5	373.0	115.8	172.5	313.9	79.8
	223.2	187.2	157.3	134.6	106.5	141.6	69.3
220.9	121.8	113.4	133.6	190.2	473.7	83.3	184.0
170.1	126.5	126.9	154.7	207.7	228.3	124.5	231.8
270.3	16.1	111.6	168.5	100.4	118.7	217.2	131.8	38.4
	266.0	4.2	21.6	201.7	110.4	94.7	127.1	59.2
	232.9	151.1	219.5	139.8	254.2	306.2	170.1
173.6	320.9	230.7	162.5	131.9	391.9	136.7	216.1
137.9	422.0	441.0	178.8	136.1	224.9	100.6	275.2
266.3	511.1	157.7	144.5	95.4	298.2	176.7	215.6
458.0	328.4	185.7	16.8	79.3	330.6	1054.1	128.9
339.1	245.1	147.6	82.0	70.2	54.9	93.5	17.8	93.3
	134.9	221.0	41.7	13.2	135.1	239.2	135.6\
CD4	29.35	77.10000000000001	92.4	77.2
79.85000000000001	76.89999999999999	49.050000000000004
	70.45	33.75	20.200000000000003	23.25	4.2
31.700000000000003	46.550000000000004	40.85	31.9
19.05	54.800000000000004	37.75	28.75	148.5	33.1
18.049999999999997	88.85000000000001	10.75	10.9
24.05	87.15	67.69999999999999	137.75	33.550000000000004
	22.25	94.94999999999999	29.650000000000002
48.050000000000004	22.55	38.75	106.14999999999999
24.75	49.5	48.0	19.200000000000003	27.8	32.7
100.15	106.60000000000001	8.05	55.05	71.55	6.25	40.0
	15.200000000000001	51.65	80.2	4.85	43.5
80.85	31.75	20.799999999999997	9.95	392.15	65.9
35.199999999999996	15.899999999999999	56.8	14.75	94.4
	62.25	83.60000000000001	8.5	84.9	108.95	26.8
	21.900000000000002	149.6	37.55	20.6	88.15	8.6
	46.199999999999996	10.2	44.2	11.3	45.3	51.8
	63.85	55.65	130.3	13.05	100.7	66.64999999999999
	48.9	39.05	10.45	23.35	15.75	31.5	5.5
56.65	21.3	62.050000000000004	14.6	55.15\
CCS	950.4	1715.4	774.6	711.1	800.6	644.6	1213.1

503.3	654.4	641.0	413.7	1453.5	665.8	1582.3	667.9
1641.4	682.4	369.4	775.7	982.7	1660.7	159.0	595.9
810.1	571.8	1230.0	533.1	450.7	587.3	646.5	648.7
745.6	877.7	405.1	44.0	581.4	707.3	1141.4	1284.2
957.7	831.9	931.0	680.9	892.8	384.1	785.5	435.6
304.8	1170.4	1241.3	613.2	144.4	688.6	750.3	601.8
708.4	1027.0	401.7	1182.4	937.6	368.7	875.2	1075.5
512.2	898.1	1590.0	946.1	1123.2	924.2	244.9	1130.8
899.8	924.8	521.4	1426.5	299.1	718.5	611.7	538.7
444.6	285.9	467.0	924.6	645.5	662.8	500.9	431.8
796.3	765.6	1141.7	247.2	1152.1	1018.6	815.2	1045.2
1076.7	1329.5	1197.3	1447.8	1592.3	1147.1	1258.5	1157.0\
CD6	33.67142857142857	106.07142857142858
32.957142857142856	93.62857142857142	85.47142857142856
	55.65714285714286	42.01428571428572
207.84285714285716	79.89999999999999	55.228571428571435
	56.08571428571429	113.04285714285713
33.214285714285715	96.54285714285713	75.34285714285714
	91.24285714285715	84.11428571428571	68.2	82.2
	40.328571428571436	282.32857142857137
51.48571428571429	90.6857142857143	137.24285714285716
	50.74285714285715	63.05714285714286
81.54285714285716	182.54285714285717	89.61428571428571
	56.15714285714286	49.44285714285714
115.61428571428571	75.54285714285713	47.0
100.72857142857141	80.78571428571429	53.699999999999996
	65.87142857142858	53.57142857142857
73.82857142857144	50.97142857142857	22.114285714285717
	132.05714285714285	15.757142857142858
65.98571428571428	102.5	170.22857142857143
21.685714285714287	125.97142857142856	62.32857142857143
	72.45714285714287	47.10000000000001
60.328571428571436	95.72857142857143	47.957142857142856
	80.78571428571429	44.442857142857136
38.81428571428571	101.08571428571427	57.07142857142858
	282.42857142857144	132.1285714285714
64.92857142857143	80.75714285714285	62.185714285714276
	68.01428571428572	67.6	37.92857142857143
125.79999999999998	64.92857142857143	128.34285714285713
	90.07142857142857	175.24285714285716
53.771428571428565	87.05714285714285	56.871428571428574
	52.800000000000004	70.95714285714287
67.78571428571429	62.32857142857143	110.17142857142858
	113.94285714285715	52.82857142857142
93.57142857142857	120.91428571428573	237.14285714285714
	118.55714285714285	70.67142857142858	107.9
110.89999999999999	45.67142857142858	66.47142857142858
	46.42857142857143	63.114285714285714
70.68571428571428	66.99999999999999	63.699999999999996
	65.42857142857143	64.21428571428571
49.15714285714286	85.42857142857143	91.47142857142858
	82.77142857142857\
CD5	82.45	132.05	114.19999999999999	91.25	167.75
48.15	39.65	81.8	43.3	135.6	74.25	74.69999999999999

47.5 81.5 62.25 119.75 130.3 133.6 30.65 98.1
276.35 15.5 209.95000000000002 111.80000000000001
24.349999999999998 41.65 60.0 92.05 119.15
76.69999999999999 86.4 96.7 106.44999999999999 94.1
209.7 95.65 81.45 77.85 66.85 110.15 40.25
15.45 233.9 271.7 57.599999999999994 86.6 65.75
10.65 177.05 213.0 80.8 33.95 82.3 155.75 84.95
134.6 105.39999999999999 31.799999999999997 117.75
93.25 638.15 81.65 155.9 90.05 199.5 109.95 166.5
427.65 130.3 108.3 56.25 44.050000000000004 346.4
113.8 116.7 116.45 46.449999999999996 26.35
25.299999999999997 98.5 86.80000000000001 72.1
34.15 121.0 148.0 173.55 87.19999999999999 120.5
40.55 40.3 82.9 58.800000000000004 55.300000000000004
40.9 36.0 52.199999999999996 49.25
28.349999999999998 76.75 99.6 38.1 160.7 80.9\
TBX10 56.650000000000006 18.950000000000003 81.65
48.25 116.05 43.35 47.65 38.3 53.1 73.5
112.19999999999999 44.599999999999994 38.2
81.89999999999999 68.9 89.05 129.0 31.950000000000003
29.15 66.8 273.4 58.8 105.39999999999999
23.25 55.5 65.95 78.35 124.9 76.35 100.35 25.4 46.7
79.05 73.25 177.45 79.5 50.25 98.65
42.800000000000004 77.65 33.05 39.05 45.0 34.1 55.7
113.4 62.9 35.3 69.0 58.65 83.95 83.5
45.05 87.89999999999999 68.8 61.550000000000004
118.5 71.85000000000001 124.94999999999999 12.95
295.2 53.2 78.19999999999999 63.1 54.15
73.05000000000001 41.75 15.2 80.19999999999999
151.2 102.65 77.7 68.7 50.65 99.4 44.6 91.0
42.85 82.65 22.15 100.55000000000001 41.400000000000006
86.80000000000001 59.55 129.89999999999998
165.89999999999998 43.25 176.10000000000002
119.10000000000001 19.9 10.7 52.1 100.0 50.75 55.6
78.4 84.6 25.9 92.8 127.9 50.949999999999996
131.3 109.05000000000001\
CD7 31.55 144.95 73.05 98.55 40.800000000000004 8.7
42.45 199.8 77.55 77.6 24.95 48.0 42.6
69.45 53.8 45.0 61.849999999999994 114.9 8.85
76.65 70.05000000000001 46.2 176.9 80.44999999999999
61.150000000000006 29.950000000000003 19.25
149.20000000000002 72.75 17.45 60.5 164.75 67.05 7.15
17.65 133.9 42.25 68.2 22.95 52.15
30.099999999999998 29.650000000000002 64.25
14.299999999999999 40.65 77.4 58.95 36.85 29.15 17.0
52.35 47.85 12.75 29.8 48.8 31.2 76.1
27.200000000000003 18.15 109.1 193.35 58.8 65.4
46.75 59.8 42.1 12.2 50.3 71.3 45.949999999999996
152.0 143.85 124.85 53.1 144.95 40.95 106.45 46.6
55.6 16.9 84.80000000000001 79.2 18.85
108.45 22.799999999999997 69.65 38.05 79.95 57.25 48.7
43.800000000000004 13.95 56.05 61.8 31.4
39.75 40.099999999999994 60.650000000000006 66.4 55.9
79.19999999999999 12.600000000000001 64.5\

ZNF259P1 17.0 9.0 9.0 41.2 82.2 21.4 4.5
42.6 34.6 8.4 4.6 15.7 53.9 8.8 6.1
7.0 26.1 6.2 5.7 4.3 22.4 14.3 22.1
17.1 7.2 5.0 4.7 99.2 77.5 10.9 2.0
11.5 36.0 5.9 57.4 14.3 3.8 5.5 28.3
24.6 20.9 24.2 22.3 2.7 5.9 8.8 14.1
7.5 25.9 19.4 37.0 32.7 44.6 17.4 6.8
11.5 7.9 13.2 10.4 5.8 32.3 46.9 13.8
6.0 12.5 53.3 6.7 8.9 30.1 8.1 96.3
68.0 8.9 19.6 28.6 6.0 14.7 7.1 3.3
11.4 96.1 47.6 17.1 6.4 28.5 30.2 15.1
13.0 15.7 23.2 31.6 13.6 22.6 21.0 50.4
11.4 9.4 15.2 93.5 17.5 24.6 16.2
53.3\
CD9 4321.733333333333 1037.4333333333334
1318.2666666666667 1499.3666666666666 660.1999999999999
2445.266666666667 4529.3 1917.2 3756.266666666667
5664.233333333333 6487.7666666666655 3454.4
5259.533333333334 6466.566666666667 2092.0
3941.233333333333 5156.333333333333 1456.7666666666667
5540.533333333333 4680.233333333334
445.7666666666665 3713.4 1659.6000000000004
3647.5000000000005 4576.833333333333 1315.6666666666667
2793.9 544.9333333333334 1543.7666666666667
4584.6 1274.9666666666665 940.8666666666667
495.29999999999995 2777.933333333334 2371.233333333333
2657.366666666667 5876.066666666666
1194.6666666666667 6093.0 3846.0333333333333
3257.5333333333333 5845.166666666665 1565.6333333333334
4946.2 6725.433333333333 1640.4666666666665
1748.3000000000002 3197.3999999999996 162.53333333333333
1284.4333333333334 2762.7666666666664
3067.7999999999997 3836.3333333333335 249.36666666666665
3207.6666666666665 2308.9333333333334
1292.466666666667 928.2333333333332 3008.56666666666
496.0333333333333 350.2 2681.4 2690.766666666667
2436.4666666666667 1214.1000000000001
1796.3000000000002 5556.566666666667 5530.299999999999
1542.1999999999998 2808.466666666667
509.90000000000003 424.20000000000005 2797.766666666667
2193.5333333333333 2507.5666666666666 5022.8
1853.5333333333335 2772.5333333333333 1842.7666666666667
707.1 392.86666666666673 1037.8333333333333
10666.733333333332 2458.3333333333335 189.53333333333333
220.4333333333333 795.2333333333332 1285.3
5025.633333333333 833.9666666666667 5553.033333333333
270.8 1211.4333333333332 2415.0666666666666
1079.3666666666666 1512.5 835.6999999999999
1628.0666666666666 560.8000000000001 765.3666666666667
993.0666666666666 1551.5 2137.266666666667\
RP11-307P5.2 44.1 246.1 118.0 43.9 192.9 67.4 64.7
84.7 108.5 177.1 106.0 84.8 51.1 113.2 46.0
136.1 135.5 4.0 21.8 12.8 31.2 107.4 67.4
86.1 66.5 137.9 76.6 20.5 89.3 106.9 66.2

119.4 92.3 78.7 69.8 89.6 98.8 63.4 44.5
70.6 69.8 73.7 18.1 66.0 163.3 28.4 60.4
156.8 149.0 73.0 127.6 150.7 26.3 32.3 22.5
133.7 188.3 30.4 68.2 61.4 66.5 70.7 14.4
114.4 122.9 121.6 33.6 12.2 171.9 24.1 55.0
33.3 77.1 86.8 85.1 85.6 135.8 88.0 82.5
146.6 325.3 239.8 86.2 106.1 79.5 365.4
132.7 181.7 98.5 79.8 82.5 34.3 123.0 37.7 14.3
134.8 152.5 71.9 117.2 178.3 100.3 110.4
21.3\
MNX1-AS1 3.2 5.8 4.6 3.2 5.4 2.0 5.8
4.8 3.7 4.3 4.7 2.3 5.2 3.1 5.0
5.1 5.5 11.9 4.2 3.8 15.3 13.8 5.1
1.9 5.6 7.8 6.7 13.1 9.5 5.6 6.1
9.1 38.8 7.4 12.2 29.1 3.1 6.0 4.2
3.0 4.2 7.2 3.3 4.1 10.0 13.4 7.0
8.5 12.9 6.8 3.9 9.3 14.4 6.6 4.9
9.2 9.4 4.2 4.9 6.2 15.7 3.6 3.8
3.6 4.0 5.5 1.9 4.8 4.6 16.0 6.3
6.3 7.5 17.8 7.4 7.4 2.9 3.3 4.6
5.3 7.0 5.7 2.5 3.9 11.8 20.0 8.2
7.0 3.8 5.0 15.4 16.5 5.8 11.8 6.5
8.7 14.9 6.8 6.6 5.6 8.3 7.6
12.0\
VPS29 3045.25 1179.25 1541.35 2134.9 826.25 1603.4
1421.6000000000001 1401.95 1321.45 907.25 1562.1499999999999
1385.6000000000001 1672.6499999999999
1895.3500000000001 1300.1 1116.1 1779.9499999999998
1035.95 2014.45 1464.6000000000001 1190.8 1166.2 1169.1
1288.5 1340.8500000000001 1229.8999999999999 2352.0
1198.85 1295.7 1433.6499999999999 1101.55 1022.35 1133.7
1089.0 1471.5 1654.45 1895.5 872.2 1683.55 1166.1499999999999
2068.65 1637.25 1572.75 1598.6 1602.65 1564.25 1466.1
2594.2999999999997 1507.6000000000001 1479.3 1186.5
1623.2 2134.75 1087.05 1048.8 1126.0 1232.2 1002.85 1532.45
1216.65 588.8 1513.4 1436.75 1253.6499999999999 1347.45
1281.95 1766.0 1473.95 816.5 1591.2 973.1500000000001
1898.3999999999999 1533.25 1381.95 1700.4 1953.9 1074.3
1217.5 1165.5 1696.2 636.9499999999999 1066.25
2124.2999999999997 1521.05 845.85 897.3 1661.4499999999998
874.1 1434.3000000000002 1257.05 2740.2999999999997
1164.3 1371.3500000000001 1219.95 1118.95 1351.4
1334.25 1242.5 1585.5 1653.75 1298.1 1631.75 1341.9\
CDA 18.4 12.7 17.5 61.8 35.6 12.7 6.2 13.6
63.7 12.8 17.0 8.8 9.9 86.8 44.1 66.9
58.1 9.0 28.9 21.6 38.3 15.0 99.1 52.1
12.9 41.4 468.6 14.4 12.9 17.5 61.3 98.9
114.1 44.9 197.6 82.5 91.2 97.9 54.9 14.0
7.1 5.3 8.5 32.1 164.0 128.6 157.4
102.2 100.2 37.8 39.2 141.4 55.5 60.1 43.2 45.3
50.1 28.9 18.9 5.8 84.4 26.0 6.3 22.6
39.3 118.1 68.9 43.7 129.5 107.0 100.3
104.4 26.5 8.7 29.0 77.6 108.2 5.3 23.3 14.5
35.0 39.5 107.8 9.4 185.3 464.7 18.1

150.6 328.3 432.0 37.1 11.2 91.4 104.0 17.6 26.0
24.7 49.6 9.0 8.2 16.1 32.3 4.5\
FRMPD3 125.8 139.1 61.1 86.4 135.0 18.0 109.3
100.8 66.8 18.7 39.5 70.9 43.8 52.7 137.4 34.3
70.8 149.6 54.9 221.2 386.5 126.1 100.8 13.1
102.3 49.8 82.4 35.6 54.0 21.0 41.7
291.7 50.0 33.5 94.3 184.7 26.7 61.3 38.7 55.9
205.0 74.5 65.1 80.2 49.8 91.5 69.2 83.4
35.4 76.3 63.2 60.8 99.8 179.3 66.9
103.6 63.4 121.9 33.1 147.6 253.8 153.7 96.1 84.4
82.6 162.1 59.9 63.2 34.0 140.7 167.3
177.4 128.7 58.5 215.0 127.9 45.3 106.3 107.8
157.5 59.3 75.1 73.9 11.8 114.6 196.3 73.9
118.8 131.2 22.4 29.0 47.9 110.0 50.5 48.1 12.6
77.3 37.4 132.2 71.2 28.6 95.8 85.5\
FRMPD4 31.8 44.150000000000006 21.0 41.199999999999996
83.4 28.3 39.35 47.150000000000006 22.3
24.25 35.1 17.200000000000003 32.0 53.2 70.3 36.1
24.1 12.9 47.85 14.8 59.6 27.85 39.3
50.05 24.25 23.8 33.050000000000004 19.45 29.2 14.0
49.7 24.85 13.95 17.65 25.75 50.050000000000004
24.95 55.7 26.299999999999997 256.6 15.35 23.7
47.75 34.75 33.0 38.15 13.899999999999999 7.9
22.35 72.6 17.3 39.449999999999996
71.39999999999999 22.7 28.95 54.550000000000004 20.7
23.900000000000002 19.15 65.45 65.95 25.8
67.75 37.75 85.05000000000001 71.05 45.5 13.6 39.4
44.2 90.05000000000001 126.30000000000001
50.75 10.350000000000001 11.8 36.300000000000004
21.45 44.0 30.4 96.85 29.25 17.55 30.25 66.85 86.2
108.60000000000001 24.85 10.649999999999999 22.0
53.5 39.2 125.65 21.95 23.0 9.65 14.25
89.69999999999999 19.35 41.2 39.800000000000004
28.85 18.55 34.55\
CLEC10A 36.7 163.1 146.5 388.9 43.6 40.1 38.2 51.5
91.2 149.4 74.3 75.9 57.4 106.4 69.1
165.6 59.7 256.0 87.9 82.6 365.9 212.1 112.6
150.0 91.3 102.2 88.0 214.6 225.0 175.5 101.9
245.2 363.3 418.6 81.1 110.5 148.6 164.4 68.6 51.8
8.3 103.1 212.4 51.2 220.0 24.5 117.9 72.6
112.1 8.4 61.7 25.6 9.6 92.8 245.6
212.6 115.5 152.0 9.2 344.9 121.6 269.4 77.0
188.4 59.6 76.9 131.4 84.3 248.2 197.7 169.2 83.4
176.3 59.3 76.6 53.0 26.9 185.0 206.6 19.3
22.3 83.0 9.4 155.9 158.0 74.8 85.5
136.8 41.1 95.6 82.4 74.0 33.2 78.9 47.4 44.6
113.3 85.0 78.6 51.0 110.4 109.4 128.7\
FRMPD1 18.9 92.8 42.4 12.8 33.2 27.8 35.9 21.8
91.2 15.3 8.8 90.1 76.1 66.5 181.4 62.6
19.8 118.7 29.6 130.7 808.7 21.7 92.5 43.8
47.3 14.3 14.3 128.6 120.9 194.4 49.9
170.6 72.8 39.0 129.3 10.1 23.1 20.4 198.0 70.4
31.9 68.8 18.1 271.9 43.8 93.3 4.5 70.1
16.1 59.1 98.4 111.4 43.3 25.1 33.1 15.2

149.9 111.6 12.5 120.3 416.3 82.1 107.5 28.1
432.7 125.9 183.0 256.9 85.2 64.4 50.8 88.7
437.9 23.5 120.7 57.5 35.5 90.0 96.9 96.4
71.7 129.4 50.3 59.4 122.6 162.7 103.4
172.1 37.8 127.3 9.1 122.1 84.6 12.8 49.7 54.0
77.7 38.5 35.4 71.2 29.4 60.2 52.8\
RRP1 225.04999999999998 387.04999999999995 331.55
339.15 527.25 266.65 284.0 244.35000000000002 235.05
412.45 225.85 456.2 335.5 319.2 338.7 275.04999999999995
274.2 464.85 223.0 418.2 798.85 278.7 377.75
121.0 209.0 339.35 263.2 416.9 256.1 231.8
244.10000000000002 468.90000000000003 595.85 263.75
351.6 260.65 158.3 468.29999999999995 250.55 154.75
95.80000000000001 225.45 360.55 264.15 166.15 321.15
196.5 186.3 521.7 309.8 289.7 202.8 186.8 335.25
281.75 312.55 277.7 260.2 269.6 315.2 328.6 314.3
255.45 247.8 301.5 253.95 212.20000000000002 214.95
464.65000000000003 214.05 390.20000000000005 328.65
346.35 203.75 236.5 152.35 244.55 241.25 118.0 272.45
174.05 170.45 216.25 165.8 362.65000000000003
513.0999999999999 210.5 293.2 298.55 475.04999999999995
238.65 259.0 214.85 179.55 172.60000000000002
192.05 194.55 230.75 329.85 291.25 157.95 317.35 177.2\
GRPR 41.4 66.0 56.1 120.5 258.5 45.8 50.9 73.7
93.2 111.4 97.0 70.5 81.4 70.1 180.1 38.7
153.1 105.9 117.3 59.4 77.0 94.8 120.2
129.9 200.9 59.5 43.2 194.5 57.7 145.2 35.3
119.9 169.8 152.6 288.2 118.8 129.1 39.8 136.3
137.7 66.3 65.2 65.8 33.2 148.9 219.7 139.4 97.8
80.4 124.8 101.6 153.1 62.2 147.0 106.2
178.7 162.3 125.6 202.7 90.7 371.5 229.9 20.7 48.8
138.2 107.9 100.3 110.8 126.7 169.3 117.8
141.2 19.6 68.8 120.7 107.1 125.1 94.5 96.5 67.4
70.0 112.3 59.2 110.6 336.2 148.5 158.6
179.9 191.9 236.8 83.5 26.5 207.9 124.2 27.9 35.1
80.9 81.4 117.9 208.9 28.0 55.2 173.2\
ELK1 103.36666666666667 304.3333333333333 69.3
155.76666666666665 115.53333333333335 185.9333333333333
172.29999999999998 55.0 177.1666666666666
174.70000000000002 180.5 155.1 157.36666666666667
138.0 279.59999999999997 117.13333333333334
281.3666666666666 95.66666666666667 195.03333333333333
151.36666666666667 69.33333333333333
106.76666666666665 207.53333333333333 144.66666666666666
177.83333333333334 202.1 137.6 270.2666666666665
126.46666666666665 209.4333333333333
251.03333333333333 159.13333333333335 189.96666666666667
248.16666666666663 218.63333333333335
111.16666666666667 83.46666666666667 169.79999999999998
161.36666666666667 288.06666666666666
191.79999999999998 165.36666666666667 116.16666666666667
162.6 62.6 194.13333333333333 115.16666666666667
230.23333333333335 199.46666666666667
207.33333333333334 123.73333333333333 221.2666666666665

236.4333333333333	192.86666666666665
123.63333333333333	248.03333333333333	211.56666666666663
223.70000000000002	197.63333333333335
122.16666666666667	159.20000000000002	168.23333333333332
153.33333333333334	135.86666666666667
253.53333333333333	245.53333333333333	85.60000000000001
166.06666666666666	266.8666666666667
192.36666666666667	259.1666666666667	89.76666666666667
156.86666666666667	181.1	162.53333333333333
279.3666666666666	194.26666666666665	196.20000000000002
205.96666666666667	188.26666666666665
137.53333333333333	132.5	300.76666666666665
290.8333333333333	116.96666666666665	221.4333333333333
164.73333333333332	188.83333333333334	177.5
142.33333333333334	79.66666666666667	162.66666666666666
416.09999999999997	202.89999999999998
158.96666666666667	263.7	320.9666666666667
214.39999999999998	105.86666666666667	220.9	264.5
282.26666666666665	313.8333333333333\
RRP8	123.46666666666668	231.13333333333333
164.70000000000002	133.26666666666665	89.63333333333334
259.43333333333334	124.76666666666667	122.5
104.80000000000001	126.33333333333336	200.39999999999998
168.93333333333334	174.63333333333333
161.46666666666667	141.70000000000002	118.56666666666666
153.76666666666668	101.16666666666667
221.83333333333334	170.0	246.73333333333335
197.5666666666667	167.6	174.33333333333334
126.26666666666667	171.9	172.86666666666665
101.96666666666665	202.29999999999998	126.06666666666668
188.70000000000002	251.53333333333333
174.5333333333333	110.83333333333333	87.5
185.4333333333333	151.79999999999998	164.96666666666667
184.53333333333333	143.5	122.19999999999999
230.60000000000002	106.83333333333333	153.83333333333334
175.4	126.76666666666667	180.0666666666667
132.76666666666668	303.09999999999997	158.89999999999998
199.36666666666667	148.33333333333334
143.06666666666666	327.99999999999994	187.46666666666667
167.29999999999998	166.83333333333334
123.26666666666667	169.03333333333333	84.0
310.53333333333336	109.83333333333333	73.73333333333333
168.26666666666668	94.5	145.9333333333333
153.53333333333333	105.0	166.66666666666666	164.9
178.33333333333334	109.56666666666665	126.53333333333335
96.13333333333333	116.66666666666667
156.56666666666666	154.4	168.36666666666665
159.86666666666667	171.63333333333333	166.20000000000002
141.06666666666666	181.20000000000002
142.16666666666666	267.0	142.86666666666665
147.13333333333335	110.2	199.4	101.26666666666667
72.10000000000001	293.7	199.26666666666665
187.89999999999998	112.89999999999999	221.0666666666667
228.8666666666667	230.5	204.10000000000002

189.7666666666665	189.63333333333333	310.3
183.96666666666667\
RRP9	269.8	291.2	166.4	331.0	171.2	267.0	374.1
228.9	366.8	318.6	299.3	190.1	238.7	106.0	164.0
268.1	233.4	269.8	322.9	240.0	48.9	243.9	120.2
148.1	174.5	112.9	428.9	190.9	195.1	319.6	255.0	42.5
	226.4	185.3	287.2	182.7	214.7	573.1	394.3
402.4	183.7	446.5	176.1	215.3	276.7	197.3	241.9
197.5	323.4	114.9	191.6	642.7	183.5	275.8	249.8
157.6	112.9	183.4	207.2	149.0	89.3	205.6	162.4
140.5	88.3	242.9	252.4	269.1	155.4	172.0	172.2
314.5	174.1	280.4	172.5	240.5	121.6	355.8	183.0
191.2	237.5	53.4	275.3	295.7	329.1	390.8	180.2
273.7	408.8	394.9	311.3	46.4	269.3	215.1	191.9
222.0	221.6	223.3	268.8	163.1	169.9	228.2	329.7\
ELK3	514.3000000000001	167.3	1299.9	1052.75	506.25
240.0	1081.6499999999999	899.55	1306.5	853.0	1024.0
420.0	1234.0500000000002	572.3000000000001
383.15000000000003	1205.55	1048.6499999999999	539.6
805.0500000000001	1511.1	304.05	927.4	611.9000000000001
	1859.05	1122.6	513.35	1048.95	963.05	526.4
1490.1999999999998	1742.3	380.05	279.5	1056.55	474.65
351.95	690.95	366.2	697.3	722.85	1860.3500000000001
606.4	1485.85	1008.5500000000001	475.5	277.35	1001.2
1364.75	194.8	398.40000000000003	1531.8999999999999
1033.6	585.45	537.4	739.75	157.25	876.4499999999999
501.84999999999997	525.9	574.5	892.05	777.4	816.8
1768.25	702.15	525.8	794.95	623.2	760.5	1737.65	584.5
440.85	814.4000000000001	539.45	910.5	945.2	2004.05
589.4499999999999	951.4499999999999	826.7	999.65
1599.1000000000001	406.84999999999997	1253.05	1053.3
319.29999999999995	361.65	1312.3	1514.35	235.2	1434.1
1393.1000000000001	643.0	1802.7	1175.8	580.7	690.1
1255.6	308.5	919.0500000000001	555.7	799.0	663.65\
ELK4	502.9666666666664	842.4333333333334
569.0166666666668	614.4	678.65	635.95	759.65
629.9500000000002	598.6166666666667	531.5833333333334
	389.09999999999997	714.5999999999999
726.1666666666666	574.3666666666667	520.3166666666667
	645.65	652.0333333333333	606.6333333333333
480.98333333333335	446.93333333333334	382.4666666666664
	486.15000000000003	547.25	432.45	308.74999999999994
	589.1833333333333	641.9833333333333
773.1166666666668	541.7333333333333	996.8166666666666
	1177.3000000000002	663.9833333333333
421.1666666666667	524.4833333333333	587.5
577.2833333333333	421.8666666666666	388.45
629.4499999999999	938.0	556.9	708.6833333333333
674.1	708.9166666666669	311.25	458.7666666666666
577.7	533.6333333333333	699.0833333333334	828.5
425.84999999999997	355.8666666666666	828.0333333333332
	547.6166666666667	442.3833333333334
1035.4833333333333	675.3166666666667	800.1
413.3333333333333	674.6999999999999	843.8499999999999

	772.9666666666667	798.8833333333336
630.6333333333333	1027.0666666666666	1176.75
864.9333333333334	720.2833333333334	503.90000000000003
	384.46666666666664	342.6000000000001
648.7666666666668	808.0333333333333	618.6666666666666
	556.9333333333333	301.71666666666664
532.0333333333334	417.3833333333334	812.5666666666666
	598.4166666666666	493.8333333333333
373.98333333333335	374.45000000000005	547.3166666666666
	440.46666666666664	789.8333333333335
353.9166666666667	459.5666666666666	938.3166666666667
	3088.4833333333336	467.73333333333335
492.9166666666667	517.5666666666667	584.4666666666666
	473.1833333333332	431.38333333333327
437.1000000000001	542.7666666666668	447.9833333333333
	397.25	536.2499999999999	807.2166666666666
525.4833333333333\
SHANK2-AS2 /// SHANK2-AS2	17.4	20.2	24.4	14.1
138.8	17.6	7.1	30.5	17.7	40.4	10.1	36.4	14.4
	22.3	18.4	9.3	101.5	26.1	24.3	144.6	85.3
	25.5	33.1	6.5	16.9	13.3	10.6	32.3	30.7
	92.5	9.5	62.0	44.4	20.0	52.2	23.9	10.6
	108.5	58.5	31.0	10.4	25.2	89.3	30.4	9.9
	42.7	23.2	7.2	107.0	16.7	10.1	13.9	17.2
	54.2	22.4	12.3	18.3	120.7	27.6	28.9
135.7	124.2	54.3	24.0	29.3	23.1	13.5	97.3	23.7
	23.1	24.9	74.5	11.1	8.9	30.7	11.6	25.0
	16.2	22.9	24.7	28.3	22.0	6.9	27.2	24.2
	92.9	34.7	83.9	48.0	112.8	3.4	20.8	16.0
	15.1	79.2	13.2	21.4	7.7	9.7	13.1	17.8
	28.3	11.7\
ALCAM	7504.333333333335	3692.6	2317.0	3259.5333333333333
	2887.566666666667	223.5666666666667	1272.2
486.79999999999995	1802.5333333333335	2371.4333333333334
	4925.366666666667	5919.233333333334
3987.0000000000005	1004.3666666666667	1157.8333333333335
	3978.8333333333335	4041.1	4700.233333333334
2811.433333333334	327.23333333333335	7309.966666666667
	1235.3999999999999	5400.0	476.26666666666665
431.40000000000003	4051.2000000000003	1450.0
2565.933333333333	2907.3333333333335	396.6666666666667
	3069.7666666666664	2859.733333333333
893.4666666666667	6110.766666666666	332.1666666666667
	6981.166666666667	177.9333333333333
332.73333333333335	1650.2666666666667	3431.366666666667
	823.4333333333334	2425.9666666666667
1812.5666666666666	1055.7666666666667	1481.3999999999999
	1391.9333333333334	4663.333333333333
3944.933333333333	1599.8666666666666	4790.5	2724.0
3477.7000000000003	2809.566666666666	1374.3666666666668
	800.6999999999999	1881.2	5335.233333333333
3023.2000000000003	3709.2666666666664	4299.200000000001
	2711.1666666666665	2819.066666666667
3847.2999999999997	5026.2	7183.133333333334

1526.1000000000001	3355.2999999999997	3702.533333333333
	839.6999999999999	2482.766666666667
2777.633333333333	377.0	5238.033333333334	2355.0
5477.866666666666	617.8666666666667	2674.0333333333333
	1929.8999999999999	6823.433333333333
480.4666666666667	2463.1	5540.433333333333
2143.6666666666665	6896.833333333332	4913.6
183.9666666666667	2165.9999999999995	3032.933333333333
	133.3	3033.2333333333336	2808.5	2422.0333333333333
	4262.133333333334	2448.066666666667
3598.8333333333335	5943.5	5380.766666666667
3180.933333333334	2850.566666666667	6337.566666666667
	4669.233333333334	5386.766666666666
5920.200000000001\
SCN11A	25.133333333333336	47.0	58.1	35.06666666666667
	88.3	40.233333333333334	42.93333333333333
55.833333333333336	52.96666666666666	54.199999999999996
	40.833333333333336	32.8	51.9	56.9
110.76666666666667	69.66666666666667	32.833333333333336
	57.5	50.43333333333334	51.133333333333326
92.36666666666667	69.3	130.4	30.866666666666664
25.96666666666667	22.266666666666666	37.800000000000004
	92.89999999999999	72.63333333333334
58.43333333333334	37.96666666666667	105.76666666666667
	68.10000000000001	62.4	62.699999999999996
23.566666666666666	18.933333333333334	68.26666666666667
	37.63333333333333	32.53333333333333
31.866666666666664	51.06666666666666	122.16666666666667
	49.73333333333333	29.433333333333334
59.63333333333333	75.76666666666667	18.366666666666664
	40.199999999999996	66.76666666666667
24.166666666666668	43.63333333333333	22.066666666666666
	195.33333333333334	62.5	34.199999999999996
49.199999999999996	40.699999999999996	39.86666666666667
	98.89999999999999	190.1	43.833333333333336
66.23333333333333	37.166666666666664	63.43333333333334
	50.166666666666664	30.033333333333335
38.266666666666666	82.33333333333333	104.5
354.3666666666666	74.60000000000001	62.03333333333333
	18.633333333333333	68.6	31.733333333333333
84.23333333333333	29.2	48.666666666666664
17.233333333333333	430.2333333333333	63.166666666666664
	20.666666666666668	35.1	93.7666666666665
182.5	65.53333333333335	146.2	65.23333333333333
52.4666666666666	54.93333333333334	105.6666666666667
	61.53333333333334	36.23333333333333
28.099999999999998	76.46666666666667	54.96666666666667
	55.6	106.76666666666665	119.66666666666667	55.6
	60.36666666666667	44.6\
VPS35	3079.4799999999996	2238.8599999999997	2885.1
2981.96 2600.14 4805.06 4100.24 2771.2999999999997	3407.88
3619.34 5003.36 3579.88 4246.24 5224.36 3922.7	4041.28
2735.4799999999996	3640.12 4283.8	3553.9800000000005
1489.3	4056.9800000000005	2615.56 3371.62 4162.82

5648.0199999999995	6725.860000000001	4263.4800000000005
	5544.039999999999	3713.0199999999995	3785.56
1947.8600000000001	2360.66 6406.32 10273.86
3666.1400000000003	4203.08 2487.62 3442.3399999999992
3601.8	4484.4400000000005	3025.1400000000003	4600.5
3536.7	4990.4	4964.9800000000005	4424.160000000001
4662.9800000000005	2956.98 3459.5800000000004	4530.76
6830.76 4234.42 1881.92 4233.24 3351.1	4396.08 3239.4799999999996
	4631.06 3437.54 3824.9199999999996	3641.2599999999998
	4138.58 3942.7	1593.48 2821.5	3174.0600000000004
3614.7	3691.16 5852.78 1829.94 3014.16 2396.5	4753.6	3973.2
6760.639999999999	3689.4800000000005	4588.36 3663.44
3611.9	5750.599999999999	5094.54 4502.08 2645.5
1152.0000000000002	1881.8799999999999	4428.46 2616.28
2914.0799999999995	5079.0599999999995	2859.9200000000005
	1507.4199999999998	5365.2	3746.6	3785.5800000000004
	5589.379999999999	5282.06 4873.159999999999
5211.18 4049.3199999999997	3640.4	2438.8599999999997
4173.820000000001\
VPS36	2184.85 310.5	1731.4	1913.2	470.0	993.5	1063.05
596.65	1043.25 525.75	1309.55 1054.8500000000001	1009.9
1309.1000000000001	1354.8000000000002	1033.5	811.6
892.6500000000001	1395.25 1186.35 680.75	811.9499999999999
	572.0500000000001	1046.05 1152.8999999999999
2023.6	1947.4499999999998	896.4	1664.25 1227.1
1263.3999999999999	1151.8	881.25	1332.8000000000002
467.5	1397.7	1337.4	1068.35 1261.1499999999999
630.1999999999999	967.25	1134.8999999999999	1027.0
916.55	770.35	988.4000000000001	1092.6000000000001
609.8000000000001	873.7	1600.3	1187.95 1012.1999999999999
	1463.75 909.0	985.1	729.6999999999999	1213.95
1532.9499999999998	1358.5	968.05	2192.65 869.3	862.75
1459.6499999999999	1788.8	1059.25 1233.15 1173.3	597.5
1513.25 455.29999999999995	582.4	1651.25 1398.95 2275.5
1059.4499999999998	1057.6499999999999	972.6	1350.3
638.45	1780.3	1408.9	471.75	1811.75 1338.8500000000001
501.34999999999997	1734.1000000000001	1171.45 1076.8
548.2	786.7	1898.1000000000001	2618.5499999999997
2005.45 2019.95 2219.9500000000003	2209.0	2004.3999999999999
	2123.25 2950.3	2201.15 2468.85 1717.55\
VPS39	576.4	693.4	762.7	484.9	673.2	762.0	938.0
718.9	655.6	643.8	765.5	900.4	466.1	758.8	636.7
446.8	503.4	672.3	472.3	624.4	1456.1	473.7	455.7
607.1	173.0	668.5	353.4	432.9	619.9	824.6	848.6
441.2	1222.3	740.7	294.6	617.7	280.1	943.2	723.6
687.6	585.0	835.5	535.2	998.9	625.3	299.7	613.4
338.3	884.2	737.8	297.3	578.8	462.8	1118.8	729.6
669.3	558.9	572.5	711.3	827.2	550.4	685.8	653.7
578.9	598.8	664.4	756.4	561.2	644.0	356.8	675.5
799.0	838.2	977.0	916.9	608.2	780.8	922.2	417.8
471.2	434.0	328.3	550.0	258.1	483.6	519.1	847.6
574.9	565.7	269.8	638.4	924.8	305.2	484.6	501.8
375.4	244.3	223.8	384.2	397.1	403.5	455.3	482.0\
VPS18	337.4	232.10000000000002	143.6	265.84999999999997

217.05	381.9	456.45	217.15	239.25	216.70000000000002
325.25	328.75	328.8	250.8	144.25	244.45000000000002
236.8	396.05	380.90000000000003	358.95
218.64999999999998	193.35	310.75	312.0	217.1
190.10000000000002	267.45	318.29999999999995	234.35
262.95	198.45	163.85	315.0	232.7	358.3	229.3	323.05
375.09999999999997	431.8	164.7	202.85	408.25	93.8
467.79999999999995	479.6	161.5	228.85000000000002
218.60000000000002	272.8	186.04999999999998	242.0
315.40000000000003	124.60000000000001	215.2	327.8
276.3	188.1	125.65	283.5	204.5	66.5	328.65000000000003
220.85	387.1	154.14999999999998	395.79999999999995
304.0	349.45000000000005	250.05	282.7	195.0
126.4	154.64999999999998	405.54999999999995	289.05
253.29999999999998	191.4	308.25	206.39999999999998
187.1	66.55	186.54999999999998	177.79999999999998
295.6	263.55	220.55	223.0	139.75	475.79999999999995
180.7	209.95000000000002	202.8	165.0	183.35	173.2
105.95	98.7	130.1	77.75	108.30000000000001	172.75
216.0	174.95\
SSTR5-AS1	12.5	11.4	23.1	12.8	17.5	9.3	10.9
15.3	19.1	50.2	11.7	62.6	16.6	62.7	14.4
40.0	7.0	80.4	145.8	16.2	25.5	71.9	27.7
7.8	44.6	1010.2	22.9	38.4	19.5	15.7	5.6
12.7	15.2	33.8	69.1	8.1	9.2	68.5	8.3
16.9	69.9	9.9	21.5	4.7	13.7	8.3	18.3
3.7	22.9	7.4	65.1	27.3	21.4	72.4	16.5
24.6	8.7	11.5	220.8	11.8	67.2	60.0	12.9
13.5	36.5	893.2	4.7	6.6	21.9	10.4	23.3
13.7	4.7	9.4	10.0	9.9	20.5	10.0	9.8
20.4	91.3	138.2	63.3	17.6	31.0	109.5	15.1
24.2	24.1	34.0	12.6	128.1	10.1	19.1	47.0
8.4	9.6	59.4	27.7	13.9	11.7	19.6
11.5\
AC008753.4	15.9	89.4	108.8	20.3	55.2	45.7	59.8
89.4	14.8	99.7	25.1	126.7	15.4	26.9
150.6	58.4	107.6	25.1	122.8	37.1	51.5	59.3
137.1	16.3	64.7	16.0	76.6	201.2	83.6	70.2	39.1
139.8	131.7	8.5	146.6	95.3	36.3	32.0	99.6
23.7	76.8	11.3	158.2	39.6	40.2	149.8
142.7	85.0	131.5	146.7	72.8	45.9	60.5	187.2	38.4
54.7	82.6	135.4	50.9	31.9	20.9	76.4	23.6
10.2	109.4	141.5	12.7	85.9	123.3	72.5
269.2	55.5	70.6	35.0	51.3	66.1	66.5	56.4	71.5
56.8	86.6	30.9	16.3	59.9	41.0	314.6
128.0	49.5	158.5	32.4	13.1	71.7	16.9	63.6	99.9
20.3	55.6	7.8	224.9	102.6	63.2	129.7
75.9\
CLEC11A	169.66666666666666	277.2	135.70000000000002
412.40000000000003	321.43333333333334	412.9333333333334
220.26666666666665	274.8	548.3333333333334
502.9666666666667	170.70000000000002	372.0333333333333
164.9	324.0	135.03333333333333	1962.8999999999999
326.8333333333333	336.06666666666666

342.6333333333334	368.8333333333333	633.4666666666666
	266.3333333333333	350.73333333333335
263.3333333333333	354.5333333333333	569.8000000000001
	356.6333333333334	468.5666666666666
409.0333333333333	607.6666666666666	663.5333333333334
	385.5333333333333	511.0	572.4333333333333
317.9666666666667	237.26666666666665	164.03333333333333
	215.86666666666667	335.2	400.1666666666667
388.26666666666665	225.66666666666666	558.3666666666667
	359.7666666666667	293.5	490.1333333333334
364.8666666666666	138.56666666666666	309.5
282.73333333333335	452.8333333333333	170.33333333333334
	216.83333333333334	241.23333333333335
1209.1333333333334	290.8666666666666	460.5333333333333
	209.36666666666667	212.26666666666665
992.3333333333334	712.9333333333334	196.23333333333335
	230.80000000000004	139.76666666666665
332.1333333333333	103.26666666666667	1001.9666666666666
	464.8333333333333	557.7333333333332
593.4333333333333	523.9666666666667	325.96666666666664
	315.93333333333334	495.56666666666666	520.2
358.56666666666666	575.9	361.3666666666666
395.3333333333333	64.63333333333334	491.2	542.1
383.8999999999999	431.7666666666667	506.6333333333334
	465.9333333333334	512.2666666666668
1013.2333333333332	283.8666666666667	370.90000000000003
	163.06666666666666	1226.4333333333334
689.1333333333332	642.8333333333334	608.0333333333333
	494.2333333333333	692.8333333333334	598.7
760.8000000000001	612.5333333333333	618.7666666666667
	842.3333333333334	420.5\
HUS1	425.3	523.9666666666667	379.3666666666666
340.8666666666666	355.73333333333335	378.26666666666665
	268.06666666666666	909.6	319.5333333333333
325.5	346.8333333333333	443.06666666666666	160.6
555.3000000000001	466.3	267.8333333333333
426.1666666666667	323.7	246.6	252.4666666666667
399.1666666666667	333.0	339.0333333333333
339.1333333333333	294.6666666666667	375.5333333333333
	345.76666666666665	326.8666666666666
638.3333333333334	464.9666666666667	352.73333333333335
	325.09999999999997	310.7	375.6666666666667
744.9333333333334	437.23333333333335	418.3999999999999
	260.93333333333334	268.6666666666667
525.3000000000001	413.3	365.7	260.26666666666665
195.5	92.83333333333333	409.0	496.4666666666667
524.0	444.23333333333335	490.0333333333333	296.0
302.09999999999997	405.93333333333334	568.2333333333335
	263.26666666666665	665.5	388.8999999999999
474.7333333333333	435.8333333333333	474.3333333333333
	873.6666666666666	379.3	349.8	296.1333333333334
	412.40000000000003	437.1333333333334
349.1333333333334	294.6666666666667	248.16666666666666
	266.7	243.0666666666667	324.23333333333335

334.3333333333333 239.33333333333334 225.20000000000002
378.3666666666666 495.4333333333334
284.93333333333334 418.9333333333334 366.8999999999999
662.0 609.3333333333334 370.06666666666666
633.2333333333333 479.5 569.1666666666666 565.4
517.8666666666667 233.86666666666665 364.20000000000005
426.1666666666667 549.6 437.59999999999997
275.5666666666666 263.6666666666667 400.8333333333333
352.73333333333335 298.2333333333333
314.73333333333335 473.8 438.73333333333335
440.2666666666665 159.70000000000002\
FLJ34503 3.4 8.7 10.3 3.4 7.3 5.2 1.2
7.9 2.9 5.4 3.8 5.5 4.0 9.0 28.1
4.5 2.3 11.0 2.4 3.4 7.8 7.3 6.1
8.3 4.2 3.5 4.4 9.3 4.4 5.1 6.7
5.4 127.2 3.0 3.1 5.2 15.5 3.2 3.1
4.3 3.5 10.0 15.5 7.8 8.7 4.1 1.9
4.0 8.6 15.8 4.6 4.7 5.3 3.9 6.9
5.2 6.4 7.1 3.5 8.8 15.9 2.5 8.7
2.9 69.7 3.6 59.2 4.0 17.6 9.8
186.3 93.8 9.3 3.3 3.3 3.8 4.0 5.6 1.6
9.7 5.9 3.4 5.1 2.1 6.3 7.7 2.9
3.3 3.8 93.5 2.3 5.0 20.2 6.5 29.4
165.7 13.2 8.0 12.1 11.2 337.5 6.3
74.4\
CEL 68.85 111.5 172.2 133.7 936.1 63.5 6.35
618.5 137.3 2118.1499999999996 2130.15 41.2 41.25 79.3
204.65 114.6 107.15 459.25 49.05 76.6 1741.0
279.15 414.25 79.9 13.85 153.5 21.4 556.7 411.95
164.95 43.15 504.45000000000005 628.2 274.35 21.95
341.4 312.04999999999995 659.7 78.5 11.55 90.85
356.20000000000005 586.0 118.85000000000001 1121.6
768.9000000000001 223.05 297.95000000000005
793.5999999999999 3924.0 2546.65 17.15 32.4 790.8
612.8 3519.75 452.75 133.15 151.75 1007.65 271.25 3160.65
390.1 202.3 955.4000000000001 252.39999999999998
255.0 225.35 1114.95 158.60000000000002 691.05 632.9
179.2 93.7 1502.4 272.1 648.8 11.100000000000001
2672.65 211.35 327.95000000000005 233.8 139.8 144.25
474.9 633.95 448.59999999999997 216.85000000000002
74.35000000000001 488.25 27.549999999999997 292.35
112.9 311.75 340.15 134.89999999999998 334.65 740.7
399.70000000000005 329.4 191.05 216.9 200.6\
CTD-2199O4.3 /// CTD-2199O4.7 55.8 123.9 147.2 42.9 92.0
79.4 26.0 94.4 12.0 141.7 91.3 79.2 43.9
113.4 77.8 109.1 155.1 100.6 8.3 37.3
144.6 66.1 201.7 132.5 81.3 87.1 24.7 151.5
118.1 170.2 4.4 52.5 117.8 66.1 151.6 137.2 62.5
125.1 90.5 93.9 68.5 93.2 144.0 29.5 58.8
120.4 131.0 69.2 206.5 44.9 61.4 100.0 85.4
214.2 49.9 215.1 14.9 13.7 56.8 13.7
449.5 11.1 101.3 53.1 93.6 105.8 45.9 42.3
122.7 79.3 170.0 52.3 186.2 71.7 103.3 11.1 32.4
69.3 91.4 75.9 169.7 77.5 90.1 105.6

195.9	389.1	162.2	104.9	113.1	152.5	38.1	219.2	82.7
	63.3	26.3	71.0	55.4	117.2	89.4	54.5	52.8
	92.5	95.2\
NUP93	686.0500000000001	625.9	301.84999999999997
548.3000000000001	298.75	728.8000000000001	756.7
370.84999999999997	791.8	587.35	574.5999999999999
388.4	576.0999999999999	426.45	430.5	572.15
549.6999999999999	478.7	485.7	533.05	470.04999999999995
	952.0	287.85	584.15	895.4	451.09999999999997
547.75	349.20000000000005	253.6	538.6999999999999
295.09999999999997	341.15000000000003	306.2	728.75
611.6	378.05	704.0	556.25	579.0	513.8000000000001
805.0	687.5	395.4	454.55	1020.3499999999999	554.35
483.6	619.4000000000001	391.55	273.95	482.15000000000003
	635.7	408.35	519.55	511.7	515.1	549.2	340.7
323.75	390.05	537.25	380.35	348.35	399.25	147.6
485.65000000000003	608.4	435.35	398.85	422.35
157.79999999999998	370.75	317.5	534.35	438.25	822.25
285.45	616.15	397.3	670.3	207.8	386.54999999999995
710.4	291.15000000000003	261.05	246.14999999999998
269.04999999999995	322.15000000000003	536.9499999999999
	389.79999999999995	991.0	392.84999999999997
369.45	299.8	188.5	288.0	277.75	232.89999999999998
272.15	235.4	220.4	283.9	340.20000000000005\
BCAN	49.85	91.325	112.0	83.35	160.275	44.22500000000001
	61.0	144.85	139.97500000000002	176.375	93.4
58.025000000000006	61.675	86.89999999999999	95.925
98.025	100.15	86.9	125.7	107.225	236.125	108.125
194.64999999999998	84.15	113.1	180.825	101.82499999999999
	165.35	179.825	119.95	68.975	151.75	150.575	126.1
112.175	100.375	97.275	109.95	39.975	106.85	95.025	74.85
89.19999999999999	75.5	72.55	155.775	90.9	132.575
133.975	109.02499999999999	117.05	157.525	34.175	151.6
115.94999999999999	70.4	128.375	182.05	51.824999999999996
	110.35	484.35	100.15	115.0	146.875	129.45	127.15
62.225	81.65	249.55	119.0	141.35	141.35	81.575
112.77499999999999	114.5	84.625	104.425	98.05000000000001
	77.82499999999999	122.82499999999999
233.22500000000002	161.6	51.6	127.075	299.825	229.45
115.5	159.54999999999998	74.275	109.125	62.10000000000001
	133.97500000000002	101.675	64.85	79.55	75.275
106.1	94.825	119.725	100.32499999999999	81.3
147.20000000000002	100.47500000000001\
RP11-532F12.5	1156.4	362.9	1750.6	790.5	1124.1	1549.8
1022.0	803.1	1118.0	1338.8	1555.9	1894.7	1276.6	1493.6
854.6	1524.6	1047.8	985.9	1188.6	2098.5	1339.7	1157.1
1683.2	876.6	1259.8	1972.8	2071.0	1605.2	1522.3	1167.2
1086.9	2500.5	1151.9	2685.6	860.4	3083.6	1992.9	1339.7
2296.4	704.5	932.4	1680.1	1187.6	1518.2	1926.5	997.6
2976.0	1023.7	2031.6	2481.2	1698.2	656.9	2270.9	1441.4
1765.8	1286.2	1650.1	1185.4	1970.2	1189.2	1136.9	1595.4
1405.4	2555.1	1870.2	2893.9	1194.3	1697.2	1158.7	2555.0
1789.9	1160.2	2236.2	1281.3	1629.2	2234.6	1553.7	1158.1
1820.9	189.9	722.2	922.6	395.6	2438.2	1177.0	194.1

```
3633.1  947.1   695.8   539.1   881.0   1645.2  2056.8  2116.8
1578.7  2406.6  1995.3  2809.7  3439.7  2777.6  2440.9  2037.4
3120.4\
BCAM    218.64999999999998      209.05  300.55  120.45
235.89999999999998      123.25  310.65  325.0   186.75  381.2
129.1   186.1   121.4   455.3   1654.3500000000001      195.15
277.35  148.2   125.35  437.15000000000003      893.5
165.95000000000002      269.84999999999997      162.95  275.6
300.65  153.1   500.7   419.6   156.60000000000002      80.9
340.45000000000005      266.7   214.85  548.0   342.70000000000005
        62.300000000000004      596.1999999999999       149.1
215.14999999999998      270.95  149.0   264.3   142.5   204.85
224.2   252.05  107.8   375.84999999999997      295.05  321.85
215.7   142.8   460.15  346.45000000000005      490.45  193.25
322.8   202.10000000000002      231.8   1207.55 403.2   245.25
178.29999999999998      333.95  436.1   137.45  136.45  268.55
245.79999999999998      273.1   214.55  185.60000000000002
146.3   288.05  139.64999999999998      500.59999999999997
163.10000000000002      275.2   145.0   565.8   643.8   513.85
672.15  976.6   1604.1  293.45  811.2   516.6999999999999
817.0999999999999       196.45000000000002      466.5   578.0
278.35  510.85  334.25  442.0   333.75  327.34999999999997
408.05  395.85  337.79999999999995      441.6\
SCN10A  328.7   308.0   142.1   83.7    224.5   115.2   118.0
289.9   236.3   271.0   224.7   132.5   169.5   527.6   300.0
154.7   223.4   180.7   123.5   215.3   549.6   150.4   61.8    35.3
        232.1   255.5   213.9   298.8   376.0   144.6   44.2    71.0
        220.7   346.9   361.1   430.3   120.5   292.6   182.8
214.1   105.0   22.1    237.3   132.9   156.6   234.3   302.2
143.3   411.1   222.7   197.4   344.8   221.3   709.0   268.6
289.4   327.2   309.8   152.1   214.3   1599.0  33.4    148.3   80.9
        197.3   340.8   115.4   189.4   195.3   436.0   356.1
230.6   245.0   228.8   382.2   212.8   347.7   170.1   201.4
282.4   362.0   225.1   181.0   351.1   319.8   1067.9  339.3
253.1   244.4   324.8   101.6   593.8   317.7   188.5   141.8
187.5   388.0   164.2   213.0   479.2   240.6   265.8   188.8\
KRTAP4-12       31.8    92.0    3.6     10.3    13.8    12.8    19.1
        63.5    10.1    5.1     9.0     74.1    7.2     2.5     27.5
        72.5    18.3    3.7     5.7     31.2    9.9     20.8    22.5
        6.6     16.1    6.7     6.0     47.9    59.7    7.2     9.2
        16.2    16.6    13.4    21.4    19.1    8.4     5.4     3.0
        17.8    17.8    34.4    115.7   30.7    10.5    33.4    30.5
        64.4    13.0    11.1    5.9     30.1    12.6    48.3    66.0
        6.1     9.9     73.7    33.6    70.0    73.0    32.0    23.4
        50.3    3.5     4.8     28.6    18.4    50.1    11.8    13.2
        3.6     35.3    8.0     3.2     23.5    36.9    12.4    24.8
        44.3    14.5    25.4    8.5     20.0    24.4    134.9   77.5
        15.5    19.9    73.9    4.9     13.8    33.7    6.2     44.7
        16.8    12.4    7.3     35.0    51.3    9.7     32.7
38.1\
KRTAP4-11       5.7     46.2    9.5     32.5    148.7   63.1    7.4
        8.6     11.3    8.5     2.8     2.8     106.0   18.5    9.1
        13.2    46.2    8.8     25.3    10.7    14.3    4.8     12.9
        25.7    19.6    12.3    1.7     13.8    9.2     13.9    4.4
```

28.0 9.6 25.7 7.0 7.0 6.4 2.6 12.2
21.3 38.6 25.6 3.6 11.3 4.4 3.6 28.0
7.2 10.1 31.4 54.6 8.6 3.6 22.9 14.0
42.7 45.8 5.5 10.2 26.7 22.5 10.5 12.8
4.9 12.3 58.8 4.8 4.8 10.0 5.1 11.8
6.4 5.1 22.9 67.5 3.9 3.7 1.6 1.5
8.8 10.7 6.9 37.4 74.6 12.0 7.3 2.7
2.9 11.9 5.6 22.5 16.0 10.2 2.3 3.8
11.3 19.4 4.3 3.2 8.4 5.7 7.0 6.5\
HECW2 42.699999999999996 79.56666666666668
107.93333333333334 78.86666666666666 168.93333333333334
27.399999999999995 154.36666666666667
152.76666666666668 176.36666666666667 265.0
45.666666666666664 225.53333333333333 35.93333333333333
108.16666666666667 47.63333333333335
79.10000000000001 133.66666666666669 65.8
54.06666666666667 149.93333333333334 409.76666666666665
79.16666666666667 90.69999999999999
100.80000000000001 93.86666666666667 104.46666666666668
89.13333333333333 220.73333333333335
82.33333333333333 104.86666666666666 92.56666666666668
237.23333333333332 121.56666666666666
94.49999999999999 153.83333333333334 116.16666666666664
48.46666666666666 528.9 80.96666666666665
96.60000000000001 164.23333333333332 118.36666666666667
112.33333333333333 240.43333333333337 60.0
112.03333333333332 122.8 22.866666666666664 77.6
143.43333333333334 223.83333333333334 123.3
189.29999999999998 175.33333333333334 55.833333333333336
69.3 98.8 90.30000000000001 133.9
48.833333333333336 395.4666666666667 60.46666666666667
142.83333333333334 95.90000000000002
198.9333333333333 148.33333333333334 98.16666666666667
79.73333333333333 57.199999999999996
37.53333333333333 44.96666666666666 120.16666666666667
122.03333333333332 46.26666666666666
78.43333333333334 35.43333333333333 103.03333333333335
106.43333333333334 74.3 282.03333333333336
97.30000000000001 240.96666666666667 46.866666666666674
249.89999999999998 782.5999999999999
820.9666666666667 203.03333333333333 220.46666666666667
99.03333333333332 197.5 88.53333333333335
178.60000000000002 81.8 24.3 54.800000000000004
58.800000000000004 51.26666666666667 44.43333333333333
69.0 69.1 45.9 88.3 20.533333333333335\
HECW1 61.666666666666664 119.46666666666665
82.06666666666666 38.93333333333334 108.06666666666668
81.66666666666667 556.9 107.7 115.86666666666667
68.93333333333334 80.2 66.23333333333333 41.9
84.43333333333334 59.366666666666674
59.93333333333334 74.46666666666665 71.93333333333334
83.36666666666667 111.53333333333332
269.93333333333334 48.23333333333333 149.33333333333334
53.6 67.96666666666665 35.199999999999996

60.900000000000006	170.96666666666667	93.26666666666667
	112.53333333333332	46.733333333333333
117.33333333333333	71.36666666666666	64.26666666666667
	143.0	78.3	57.56666666666666	66.13333333333334
	54.4	83.06666666666666	54.26666666666667
83.03333333333335	94.5	95.83333333333333
94.76666666666665	102.43333333333334	58.46666666666666
	37.666666666666664	133.36666666666667
122.86666666666667	68.86666666666666	65.23333333333333
	77.93333333333332	135.53333333333333	53.0
123.63333333333333	113.43333333333334	113.66666666666667
	90.06666666666666	145.56666666666666
130.56666666666666	135.56666666666666	65.5
107.56666666666666	105.13333333333333	161.99999999999997
	74.56666666666666	65.66666666666667
178.26666666666665	70.8	63.46666666666666
89.33333333333333	95.73333333333335	67.96666666666665
	91.86666666666667	81.86666666666666	100.2
101.63333333333333	62.20000000000001	62.599999999999994
	69.56666666666666	74.30000000000001
68.16666666666667	99.53333333333335	147.16666666666666
	89.53333333333335	72.06666666666666
168.83333333333334	72.63333333333333	130.5	51.6
135.36666666666667	69.83333333333333	56.300000000000004
	90.0	40.9	71.66666666666666	60.166666666666664
	76.93333333333334	75.06666666666666
84.86666666666666	107.46666666666665	92.56666666666666\
SRFBP1	288.1	161.2	309.0	388.6	301.2	276.7	347.7
286.6	264.1	312.0	340.9	164.0	341.5	198.3	136.7
216.5	166.9	217.4	257.8	274.4	80.0	196.7	181.0
284.1	272.5	194.5	238.9	277.3	125.4	149.9	234.3
174.6	81.2	229.3	181.0	182.3	224.6	107.9	244.8
270.8	259.0	126.8	197.6	202.9	128.6	153.4	307.2
187.4	150.3	176.0	248.8	349.9	197.8	246.2	159.5
143.0	152.3	291.7	191.0	181.4	484.9	246.0	272.6
292.0	188.1	103.2	192.7	161.6	124.2	228.6	132.9	95.0
	221.9	280.4	266.5	79.9	369.0	278.7	292.0
317.2	545.8	483.8	162.9	191.2	220.2	124.1	122.2
395.6	125.7	73.9	342.2	251.4	394.1	277.8	199.1
361.9	216.8	239.6	172.7	180.9	183.2	216.4	254.5\
DLX6-AS1		6498.8	17.1	163.3	2304.1	6815.6	297.5
138.4	16.1	4148.1	2732.4	3498.1	1097.8	6265.7	11.0
176.4	16.7	4807.6	444.5	564.7	2700.1	1176.8	729.7
815.3	578.5	646.5	1518.1	1175.4	2237.0	2583.5	2058.0
2227.2	776.8	1216.4	2623.3	14.5	4042.1	376.9	569.1
7489.9	1002.1	1360.9	2289.2	1536.8	3450.3	607.3	310.2
1481.6	454.9	1148.1	6116.6	2877.3	322.7	586.7	703.4
776.3	347.0	1195.9	5309.8	464.5	1355.0	1578.7	1105.6
7202.2	1770.2	3269.0	2267.9	2242.0	1393.9	540.5	236.2
1370.9	838.3	4739.9	1318.0	1901.1	696.5	710.2	988.5
3477.3	4.4	913.3	221.5	120.8	677.2	657.9	3093.1
1321.8	543.6	676.3	497.0	190.1	2641.7	4840.3	6088.2
4317.6	4652.2	2959.2	3581.0	3621.9	5983.4	5748.2	2281.4
4545.2\

NUP98 678.1 485.4333333333332 370.8333333333333
455.90000000000003 356.3333333333333 721.4333333333334
676.3333333333334 261.2666666666667
486.0333333333333 298.3666666666667 388.5666666666666
465.1000000000001 543.6666666666666
732.5333333333333 430.5 434.5666666666666 317.8
484.23333333333335 457.0666666666666 401.0666666666666
435.3999999999999 419.7 238.29999999999998
422.59999999999997 576.8000000000001 594.6999999999999
573.0 436.0 362.5333333333333 612.5333333333333
525.9666666666667 426.23333333333335 412.5
420.3 662.2666666666667 626.7333333333333
532.6666666666666 403.2 571.9333333333333
548.8333333333334 272.3333333333333 533.5
394.0666666666666 410.09999999999997 431.09999999999997
405.8666666666666 354.5 438.3333333333333
430.09999999999997 426.6666666666667 465.43333333333334
550.1999999999999 404.29999999999995
367.59999999999997 401.1333333333334 488.3666666666666
402.9666666666667 417.0333333333333
343.73333333333335 331.8333333333333 471.3333333333333
457.6333333333334 409.3666666666666
393.5333333333333 352.3 368.6333333333334
568.4333333333333 479.8999999999999 340.23333333333335
390.0666666666666 290.5333333333333 337.5
434.23333333333335 343.0333333333333 404.0666666666666
425.2 269.09999999999997 454.1000000000001
397.0 547.8000000000001 323.1666666666667
408.0666666666666 488.3999999999999 309.8333333333333
348.59999999999997 429.3333333333333
376.40000000000003 430.73333333333335 382.0333333333333
539.9 565.0 358.43333333333334 510.26666666666665
497.5666666666666 381.1333333333334
439.8333333333333 408.4666666666667 583.6666666666666
432.43333333333334 310.09999999999997
480.5333333333333 417.59999999999997 436.1333333333334\
RP11-480I12.10 34.4 165.1 79.6 69.0 169.4 21.4 16.7
161.2 62.0 37.0 121.4 31.2 17.7 96.3
112.8 126.8 199.6 130.2 63.1 101.2 70.4 68.2 54.1
66.9 78.3 75.0 114.0 167.4 99.5 173.0 46.8
164.6 28.3 117.8 131.0 74.9 129.1 47.2
122.2 141.0 138.6 75.3 76.6 68.1 88.7 82.7
171.6 98.3 159.2 92.1 155.3 163.0 109.1 147.4
132.8 142.1 112.6 130.6 74.1 50.6 213.7 34.0 75.3
79.0 102.0 251.6 75.1 78.5 173.2 176.3
149.6 6.5 66.7 131.8 49.8 90.5 148.1 107.7 99.5
57.1 123.3 41.8 48.0 211.0 140.4 264.6
227.4 122.5 180.4 138.7 49.3 124.2 119.8 95.7 88.8
141.1 132.4 187.5 98.4 246.6 157.7 143.1
139.1\
VPS25 1722.8 668.4 520.9 930.5 801.5 2332.0 1874.1
424.7 1434.5 759.6 1652.7 626.9 1306.6 1226.8 285.0
503.0 717.4 712.4 1949.2 801.6 396.5 1235.8 616.6
1310.3 907.9 785.4 1832.4 360.6 627.1 785.3 1099.8

501.7	404.3	1022.8	844.4	963.5	1874.2	701.5	1406.1
772.4	1353.3	1128.6	656.3	941.4	1607.4	729.2	704.5
1343.4	398.7	554.7	1171.1	1117.0	685.1	499.6	537.9
371.3	501.3	381.6	1009.6	532.7	467.8	724.4	841.2
1470.9	432.8	531.3	1092.1	1037.3	815.5	1100.1	343.8
366.3	566.5	1004.8	871.0	1842.9	453.0	895.7	880.0
503.2	413.1	454.3	2065.6	460.4	532.8	325.9	562.8
622.8	1281.9	477.2	916.8	489.7	846.8	880.8	732.7
840.8	580.2	942.4	482.0	471.3	505.2	680.9	976.6\
CFB	131.7	53.3	3047.8	722.0999999999999	800.35
15.95	719.5	768.1	1814.6000000000001	3493.4	583.5
88.55	1656.5	2438.8	134.3	4169.0	1043.55	331.8	4016.55
2352.5499999999997	2104.95	494.59999999999997	720.1
2636.8	1797.8500000000001	125.0	233.0	904.5999999999999
210.85000000000002	2693.75	1668.4	85.3	2813.8
1207.8500000000001	1161.2	619.85	126.85	1009.8
2926.5499999999997	1531.3	527.9000000000001
875.4499999999999	508.6	1392.1000000000001	1289.75
270.6	231.60000000000002	131.7	1674.0500000000002
569.5	152.25	2726.7000000000003	111.7	82.6	778.95
654.05	1621.15	362.5	139.65	619.8	950.0	2261.75	2686.55
2573.2999999999997	947.9000000000001	936.35	3727.65
2557.25	1471.85	1499.05	642.85	590.8000000000001	1796.2
1868.15	1745.3	737.1999999999999	553.1	934.4	2123.3	14.8
159.04999999999998	163.7	3075.0	8378.199999999999
1121.1499999999999	608.95	26.35	179.9	9588.65
555.35	970.4	72.75	319.5	608.4	472.6	453.75	428.2
388.15000000000003	207.85	591.35	729.6	117.25	1352.2\
VPS28	1063.9	2898.3	878.0	1946.2	1660.4	2821.4	1372.5
1010.8	1725.8	1258.6	2287.8	1770.7	2831.0	1782.4	1489.7
2191.8	1517.9	1481.1	2304.9	1350.1	1500.2	1278.5	1249.2
1628.1	829.6	3214.5	3476.8	2052.8	1081.6	2146.7	2021.5
617.6	1788.5	2114.3	488.1	2557.8	2000.9	1488.5	2110.3
1433.1	1797.2	2065.1	1579.1	2142.1	1774.2	2029.7	952.1
1912.1	1484.3	1921.9	1957.5	2165.6	1501.3	795.5	2282.2
1950.9	2390.1	1145.5	3189.8	1595.9	772.1	2021.1	1663.9
1706.7	1509.7	1790.9	2222.5	3000.8	2229.9	2590.3	733.9
1486.7	1731.1	1947.0	1427.4	2235.8	1224.3	1797.4	1692.7
1476.0	604.0	577.4	3992.8	2304.9	634.9	1376.5	969.9
1234.9	1342.1	847.9	1240.8	1751.5	2334.9	2858.9	2693.7
2789.7	2445.3	2973.3	2718.3	2544.5	2194.5	1572.0	2502.2\
AK055981	56.9	141.3	261.2	71.2	180.2	112.2	55.6
145.7	156.0	117.3	227.5	134.5	174.0	286.2
310.4	218.6	94.4	136.9	135.4	112.2	174.0	152.9	36.5
69.7	129.6	220.9	92.9	148.6	196.0	89.7
136.0	75.8	118.1	221.3	80.7	204.6	164.5	85.4
336.9	232.9	163.5	59.5	169.4	83.1	119.8	154.3
327.8	60.1	201.4	168.6	160.7	213.8	135.7	215.2
148.3	158.5	67.2	252.7	123.3	202.7	40.3	383.3
188.1	327.0	180.8	294.5	11.7	187.3	111.6	115.6
107.7	126.7	154.7	170.8	372.2	142.5	179.5	141.1
215.6	102.0	212.5	173.9	322.3	82.6	251.8	169.7
401.6	213.1	58.7	110.3	62.2	113.0	110.3	160.5
175.6	193.0	125.3	158.2	77.6	91.6	153.3	145.3

115.4\
DKFZP761C1711   38.699999999999996   182.6   13.15   28.8
135.35000000000002   3.3000000000000003   40.25   25.75
64.15   74.0   11.35   41.75   54.75   6.8500000000000005   70.1
        11.25   46.95   92.95   9.100000000000001
36.400000000000006   38.65   25.65   65.15   25.65   33.15
38.45   10.9   90.15   77.7   69.95   15.9   174.15 75.15   16.8
        60.2   65.55000000000001   4.65   66.65   19.2   29.6
        40.45   51.4   36.349999999999994   26.599999999999998
        7.2   111.15 53.7   10.3   42.85   120.35 74.4
18.65   74.8   86.15   11.3   64.9   41.95   21.1   29.15
92.85   172.85 41.95   21.6   7.300000000000001   119.5   67.6
        29.95   20.55   52.349999999999994   26.650000000000002
        108.05000000000001   65.45   70.65   21.65   9.3   51.5
        29.45   25.05   97.6   23.05   41.900000000000006
75.75   39.2   24.1   61.9   101.19999999999999   38.4   47.6
        18.599999999999998   43.699999999999996
7.949999999999999   32.15   25.25   54.400000000000006
57.05   21.55   59.300000000000004   45.25   33.3   55.4   26.5
        80.2   78.0\
CFD   417.0   23.8   75.2   694.1   140.2   1036.7 5.1
373.7   184.2   1170.0 87.8   768.8   222.3   1572.3 11.3
182.9   63.5   285.1   290.3   134.8   273.0   36.5   406.4
110.3   266.2   194.6   159.8   22.7   13.6   136.8   19.0
279.6   23.9   7.5   15.1   794.6   381.5   859.6   71.4   25.3
        4.3   520.6   125.2   8.0   319.3   276.5   407.6   67.1
        15.3   111.3   268.9   11.5   13.7   32.5   67.6
147.8   9.0   261.1   213.9   109.3   32.7   436.2   316.2   52.5
        138.8   28.7   54.6   184.9   559.9   9.5   251.2
321.8   452.0   213.1   11.4   309.2   1157.5 59.0   188.1
125.3   69.8   350.4   122.9   11.1   648.2   1659.2 179.9
493.8   648.9   101.8   100.7   308.1   434.1   193.3   522.2
229.9   439.6   338.8   684.5   101.1   61.4   461.6   359.4\
PLEKHA8P1   481.6   599.2   500.6   486.8   1108.8 128.0
272.2   544.6   268.5   593.0   238.6   845.7   958.9   418.1
1194.1 162.3   254.5   487.2   326.9   465.2   1723.3 605.6
344.8   299.5   584.2   512.3   465.4   683.3   603.8   406.4
281.1   738.8   833.0   135.2   395.2   306.9   247.5   891.7
862.5   213.0   520.4   482.8   454.6   309.9   425.2   640.6
428.0   414.9   3142.4 838.8   359.6   243.4   637.6   997.4
614.1   382.8   570.9   489.5   617.3   759.8   421.1   531.2
703.1   225.6   846.7   880.7   313.8   246.1   586.7   373.0
1425.6 560.9   286.8   137.0   270.2   175.3   437.8   369.5
285.8   358.2   286.3   349.3   342.2   811.0   187.8   385.6
477.5   356.7   169.8   431.3   474.5   446.1   421.2   246.1
530.2   175.8   241.2   291.7   391.6   403.0   170.8   342.8
265.4\
CFH   105.0   190.9   889.5   337.9   182.0   679.5   1069.6
2547.0 520.8   341.4   636.3   129.1   71.8   527.1   21.4
2322.4 707.3   118.2   210.9   237.5   319.3   151.7   154.8
442.5   180.3   88.1   84.3   488.2   91.6   437.7   3056.4 77.9
        126.0   5220.7 321.5   388.9   529.6   91.1   1583.7
1739.3 41.9   1043.0 2081.7 1011.9 274.1   38.3   461.5   85.9
        59.2   1557.5 240.8   153.6   261.9   275.7   992.6

1130.6 2850.3 306.1 141.9 793.0 52.2 185.5 827.5
2390.6 602.3 1178.4 3242.3 1205.6 104.4 234.9 933.3 81.5
622.0 938.2 2955.9 57.3 3567.0 581.2 418.2 11.5
186.3 508.2 40.3 2045.9 187.1 38.7 159.0
417.2 164.6 13.5 1834.2 249.4 2276.5 1142.6 747.8
1542.6 2118.3 1330.7 2321.5 426.3 769.1 200.4 2535.4\
DISC1 /// TSNAX-DISC1 224.9 338.9 140.6 142.25 358.6
330.8 297.45 223.25 156.0 326.25 112.75 202.8 434.2
118.6 136.05 178.95000000000002 241.15 134.35
84.55000000000001 173.1 289.20000000000005 68.6
377.4 122.0 58.9 377.15 63.45 169.8 98.15 206.75
384.5 121.15 154.05 203.7 288.95 307.20000000000005
82.80000000000001 114.55000000000001 163.45
214.79999999999998 178.2 111.85 216.4 187.6 105.4
64.55 111.75 43.8 161.25 337.6 247.35 125.15
399.04999999999995 253.8 107.05 179.45 284.2 187.1
208.1 199.15 518.05 201.70000000000002 386.95000000000005
149.5 558.75 292.3 373.6 240.55 262.55 82.05
123.7 226.6 398.6 191.55 241.7 74.05 161.65 246.3
300.5 80.0 148.35 110.25 114.9 106.45 37.55
310.45000000000005 215.75 111.3 71.44999999999999
101.65 180.55 182.75 143.3 196.95 113.4 130.6 168.0
133.5 166.85 144.55 143.35 114.55 172.5\
CFI 87.3 174.45 6302.5 2370.85 4448.65 172.45 6229.45
758.1 7798.5 6199.95 5482.849999999999 397.55 3691.15
3257.2000000000003 2230.5499999999997 3977.65 1291.1
2674.2000000000003 3917.7999999999997 4668.55
3123.8999999999996 6874.65 2336.15 11242.75 7877.0
161.05 1464.25 3848.3500000000004 3611.95 5755.3
6024.099999999999 356.09999999999997 3968.9 14976.5
368.5 758.3 584.5 1701.6000000000001 8899.35 962.55
1893.6 4376.599999999999 3788.0499999999997 8584.1
6204.5 1138.3 1482.1 1675.25 2629.7 3985.75 897.1 11412.75
222.25 294.35 9420.7 2247.55 7350.85 3322.5499999999997
191.0 5379.3 3253.2000000000003 4926.9 7317.7
5124.75 2249.8 6968.55 8168.95 7298.4 8302.7 9521.85 2209.35
547.5500000000001 2329.65 3846.2 10177.6 8546.599999999999
4066.5 7593.75 4822.4 284.55 3620.0 791.5500000000001
2455.7000000000003 6415.05 2549.8 2051.65 141.85
1532.95 8856.8 4793.75 4016.4500000000003 3976.5499999999997
4249.55 6535.049999999999 3956.15 7073.900000000001
5399.6 6424.2 5443.25 7163.65 4548.65 1135.55 6472.05\
LOC100507487 /// LOC101927184 361.4 1127.9 705.6 249.0
520.1 157.9 83.9 332.0 99.6 349.1 201.8 341.1
162.6 466.5 328.0 281.3 170.0 588.9 290.9 278.0
650.8 306.7 481.5 118.7 118.9 224.6 105.9 382.2
618.6 128.3 101.3 445.7 785.1 128.5 261.5 387.7
108.1 351.5 258.0 116.6 174.2 282.3 224.9 200.7
111.3 298.5 308.5 108.2 1549.3 594.6 388.4 265.7
489.5 438.2 375.0 608.2 230.4 441.5 636.3 367.5
771.7 657.8 272.5 232.7 1219.9 1159.8 187.8 175.5
462.7 403.5 680.3 794.3 393.0 177.6 377.8 114.7
426.3 95.5 212.8 203.7 348.1 12.8 554.6 288.1
1666.0 276.3 212.9 190.7 106.5 403.7 37.2 632.6

429.8 345.1 458.7 597.5 417.1 232.4 621.0 811.5
782.5 663.6 478.9\
CFP 12.6 38.5 21.8 11.3 10.9 2.9 11.2 9.1
3.6 13.2 6.4 8.4 17.5 9.5 11.6 9.4
12.2 20.3 11.8 16.7 32.8 54.5 44.2 15.2
18.7 6.6 14.2 59.3 20.5 7.8 4.9 24.9
44.4 35.0 13.9 7.1 6.0 11.2 47.2 3.4
12.1 6.6 44.9 6.4 123.7 67.3 14.5 8.0
24.1 36.3 8.6 6.9 14.4 17.3 108.8 21.0
11.9 6.3 8.5 94.7 65.3 18.0 2.7 16.2
11.0 24.5 6.9 13.3 38.6 51.4 13.1 7.4
6.0 5.9 6.3 18.8 40.5 11.7 8.6 4.7
14.9 24.4 20.3 14.8 17.3 24.8 15.4 26.6
7.3 9.1 4.7 25.9 21.5 14.5 9.7 17.7
18.8 4.9 3.8 32.5 15.2 66.6 15.2\
RRP7A 249.03333333333333 95.63333333333333
192.36666666666667 572.1666666666666 330.0
483.666666666667 520.2333333333333 278.4
455.0333333333333 353.2 310.3666666666667
415.43333333333334 553.5333333333333 236.3666666666667
146.1666666666666 289.93333333333334
215.4666666666667 479.1666666666667 289.3333333333333
283.43333333333334 223.6 269.1666666666667
194.26666666666665 358.90000000000003 299.3
319.40000000000003 631.9333333333334 381.3666666666666
330.2 450.5 246.5666666666667 105.39999999999999
410.59999999999997 298.1333333333334
420.90000000000003 404.0 304.1333333333334
380.23333333333335 278.7333333333333 211.53333333333333
622.8333333333334 244.76666666666665 342.2
304.5666666666666 424.4666666666667 408.3333333333333
265.23333333333335 579.8666666666667
381.9666666666667 401.59999999999997 375.6666666666667
448.5666666666666 468.2 196.16666666666666
344.7 400.1333333333334 389.7 355.40000000000003
527.4 331.06666666666666 194.93333333333337 486.8
225.13333333333335 501.3333333333333 369.4666666666667
286.8333333333333 326.00000000000006 374.8
565.9 298.3666666666667 146.26666666666668
366.4666666666667 266.3666666666666 176.76666666666665
167.5666666666667 368.8999999999999
167.70000000000002 201.23333333333335 207.96666666666667
217.1666666666666 126.33333333333333
171.3666666666667 300.90000000000003 405.9666666666667
302.5666666666666 351.3 148.8666666666667
367.3333333333333 377.93333333333334 386.56666666666666
345.5333333333333 235.1 436.76666666666665
362.9666666666667 227.53333333333333 186.79999999999998
285.8666666666666 205.9 238.5 238.70000000000002
289.4 334.8666666666666 301.6333333333334\
NUP85 1808.1 2117.1 856.0 1708.5 2139.8 1976.2 1285.9
673.9 1256.3 802.5 1520.5 1199.9 1607.8 1269.3 2271.1
1325.9 1663.8 1175.8 1692.2 1153.9 1011.3 2211.1 1156.9
908.3 1036.2 2556.5 2190.0 897.4 1314.2 928.9 1059.6

1052.2 2011.7 956.8 1070.3 1224.3 1308.3 1263.2 1538.2
1151.8 1167.3 2167.0 1134.1 942.6 1550.9 1593.3 899.7
1203.1 1287.9 1763.3 1251.9 2051.7 1370.6 1554.1 2113.3
1223.4 1004.5 1314.5 1198.0 1118.0 1402.5 1722.6 1072.7
962.5 479.7 953.2 891.5 930.5 931.7 1592.9 966.8
1618.6 1240.8 803.7 878.2 1495.2 1232.7 1249.9 997.1
2590.4 1215.0 1141.3 3160.7 2469.8 923.0 2483.0 1223.0
1230.6 1495.9 896.0 3694.7 1042.4 1087.4 2286.4 2172.4
1466.6 1354.7 2017.2 1673.7 1232.8 1807.8 1474.4 1739.3\
VPS11 526.1 747.8 581.3 805.1 889.8 723.8 777.6
539.7 652.9 539.1 674.1 697.4 574.8 370.1 1708.9
589.9 572.5 550.8 682.9 436.7 914.0 545.5 856.1
568.4 799.4 883.8 976.1 805.0 1139.6 707.6 534.0
1092.4 838.0 988.7 891.5 1220.0 563.0 970.3 571.4
639.9 734.2 595.4 818.1 394.5 499.7 1045.1 612.8
571.1 1562.8 1075.1 941.5 1322.7 871.4 1197.5 390.4
905.8 969.6 893.6 833.5 737.4 915.4 818.9 630.5
577.3 761.4 843.7 564.5 784.6 793.9 447.1 734.3
1257.1 601.4 992.8 793.1 735.8 745.7 644.9 565.5
562.1 514.5 747.3 606.5 1051.6 861.3 798.8 856.3
1330.1 835.1 1560.4 450.8 822.8 785.7 714.6 1423.7
780.9 1173.9 1299.6 1332.2 1134.8 1111.4 1169.3 826.9\
ST3GAL4 61.3 88.0 23.55 53.45 145.5 137.7 75.0
59.25 24.0 94.6 63.300000000000004 59.400000000000006
44.4 38.949999999999996 108.05 131.3 79.65 46.8
178.79999999999998 35.5 106.64999999999999 76.3
52.55 39.75 55.050000000000004 45.449999999999996
39.15 134.9 107.85 39.0 49.2 60.35
58.550000000000004 94.39999999999999 63.0 94.25 65.4
80.65 74.6 76.15 58.300000000000004 67.05
22.65 52.449999999999996 104.7 29.049999999999997
60.900000000000006 58.800000000000004 80.7
94.30000000000001 66.8 88.55 125.9 103.9 27.25 88.3
90.25 73.05 97.25 84.25 169.3 17.95 80.5
59.25 68.95 52.4 62.400000000000006 37.099999999999994
138.15 91.75 25.45 75.25 43.75 103.05 65.3 58.8
76.05 68.6 53.85 63.25 86.80000000000001 96.1
40.4 25.599999999999998 96.64999999999999
139.9 115.25 144.70000000000002 129.20000000000002
139.2 38.6 110.5 69.5 58.0 71.3 33.85
47.949999999999996 35.9 91.75 59.199999999999996
143.39999999999998 166.85 78.35\
RRS1 898.9 700.6 205.8 447.1 616.1 1190.7 1164.5
280.4 1543.3 885.6 779.7 1097.5 1504.4 1192.5 848.3
974.3 707.6 1503.1 678.8 1142.7 453.1 952.2 54.1
1090.7 1207.9 657.5 620.9 534.4 266.1 976.9 1555.5
341.4 439.5 687.8 1001.4 214.7 876.3 613.0 1304.5
1029.6 1002.8 864.5 232.7 954.9 1091.5 858.4 240.3
1129.9 120.5 725.7 796.3 1332.7 686.0 244.4 989.8
297.9 537.6 382.3 662.2 184.7 492.7 444.1 447.7
851.5 203.9 584.2 957.0 1395.2 118.0 206.3 82.2
112.5 409.9 1063.5 1080.7 504.8 50.4 1312.1 601.0
481.6 51.3 141.5 986.6 928.2 85.3 118.6 74.1
132.3 807.9 360.4 792.1 113.1 522.9 479.1 263.7

348.9	296.7	677.0	90.3	222.3	520.0	86.1	579.0\
ST3GAL5 1351.3	697.0	327.3	810.9	334.7	1130.0	1444.5
1722.6	1003.6	348.4	243.3	366.3	178.4	482.1	621.4
1120.4	596.0	361.3	361.4	347.9	1223.5	169.8	410.9
903.0	313.3	880.8	326.5	2199.1	749.6	502.2	1708.8
249.5	940.8	1071.6	388.9	601.1	737.8	109.8	494.1
500.3	571.9	420.0	1417.3	934.4	1260.0	774.7	669.5
925.2	776.3	3541.5	974.3	228.2	1137.6	2502.5	321.6
381.3	917.3	552.5	418.1	677.1	2246.9	592.3	261.2
372.4	2032.9	250.8	1690.1	1280.7	1523.8	452.8	2245.8
1075.4	1654.2	722.2	1174.7	284.0	876.2	1395.0	1059.4
1669.4	1076.2	1029.2	217.8	585.3	868.0	1036.6	4383.0
1597.9	375.3	236.0	459.3	3998.2	7010.4	2259.0	3722.7
4398.1	3968.7	3668.9	8078.3	5155.5	5501.8	1493.9	4631.1\
ST3GAL6 31.325000000000003	115.15	1112.325	167.35
235.52499999999998	142.825 60.1	183.77499999999998
187.65	261.4	122.475 47.95	384.125 1585.0749999999998
152.42499999999998	255.125 268.425 803.0	94.575
131.17499999999998	221.60000000000002	300.95000000000005
326.475 338.47499999999997	666.325 74.625	228.9
560.35	214.89999999999998	143.70000000000002
69.07499999999999	1090.5	1109.9	135.15	409.19999999999993
224.3	173.325 1361.875	40.625	38.925
218.14999999999998	59.400000000000006	264.6	72.025
177.9	187.04999999999998	73.27499999999999	193.65
239.09999999999997	75.625	294.04999999999995	1486.875
358.6	1114.7	187.875 127.05	163.15	933.8	873.45
194.9	219.475 340.725 514.6999999999999	821.425 189.5
887.2	100.30000000000001	38.275000000000006	515.5
717.3249999999999	571.325 118.625 294.35	129.65	166.7
175.14999999999998	405.825 95.975	331.925 93.475	564.675
492.225 60.875	270.45	756.2750000000001	859.75	283.225
237.45	422.15	218.0	267.0	205.225 152.75	195.5
216.15000000000003	343.725 296.225 299.725 296.3	131.225
116.325 521.0250000000001	390.69999999999993\
LOC101929864 /// SLX1A /// SLX1B	955.8	1142.7	959.8
708.0	548.6	855.0	887.7	784.3	1073.4	1059.0	785.7
637.1	1167.8	998.8	809.7	1018.3	534.4	600.7	1213.1
604.4	1255.7	582.5	693.0	776.4	981.6	833.8	1155.9
741.0	894.2	1090.0	814.8	1298.4	1619.9	618.3	2034.9
1070.3	506.5	820.5	896.4	933.7	674.4	1415.2	801.2
1022.6	971.1	584.7	658.9	539.9	984.3	807.2	851.3
1569.5	594.3	1460.5	624.6	670.1	642.7	1187.9	1039.1
556.2	1000.8	915.5	872.6	779.4	854.9	1255.4	696.4
826.9	773.6	822.0	197.6	932.1	494.6	863.8	728.5
935.8	842.4	766.0	639.8	382.1	746.4	829.7	1051.9
1073.9	1578.8	1237.2	710.6	1272.7	2352.4	1795.9	1765.9
687.2	651.1	660.7	518.4	226.2	431.1	570.6	617.6
526.4	577.4	814.1	349.9\
NUP88	1364.3500000000001	839.25	661.25	1434.6	1072.05
613.4	1196.0500000000002	310.05	1033.05 1074.6
1361.5500000000002	1042.1	1017.65 887.1	1606.5500000000002
873.3000000000001	900.4	850.5	865.6	877.95
907.3	895.35	638.1999999999999	1247.7	1152.6	1371.3

1621.8	804.5	842.4499999999999	920.55	1385.45	727.05
669.8000000000001	745.95	618.25	825.7	890.4000000000001
	801.25	920.4	736.6500000000001	883.4499999999999
	655.65	1050.95	1040.0	1067.8999999999999
1391.3500000000001	372.34999999999997	852.8	1003.25
725.55	1236.55	1684.05	848.9	556.25	898.6999999999999
815.3499999999999	1018.9499999999999	869.85	864.8
857.8499999999999	778.0	929.0	1342.45	1001.3	939.3
727.4	758.75	1211.65	858.0	1074.8	477.09999999999997
1029.65	720.75	1225.6	591.0	964.9000000000001	387.3
519.85	833.7	666.5	440.1	479.95	1186.05	726.4
208.70000000000002	312.04999999999995	510.7	614.1
1051.8	391.05	840.55	362.5	1025.6	1005.35	904.15	1259.1
894.25	1132.1999999999998	1295.5	1272.3	912.85
772.3499999999999	1191.65\
RP5-1065J22.8	98.1	166.9	155.3	119.4	305.7	95.4
227.7	822.9	111.9	334.8	258.4	486.4	150.3	606.8
164.1	113.4	134.4	277.2	249.8	153.7	629.2	217.8
302.4	183.9	150.0	181.7	131.3	100.8	426.6	103.3
115.0	224.3	289.2	141.8	343.9	156.6	123.6	196.8
133.2	142.0	119.5	120.7	121.8	180.9	166.6	154.0
397.8	134.1	840.9	179.0	228.5	127.0	198.0	336.1
156.2	235.5	153.2	118.1	173.5	199.5	157.1	294.6
140.0	41.3	300.8	335.9	115.1	186.0	214.3	261.6
278.3	530.3	187.3	74.6	218.7	117.7	246.8	124.8
215.8	420.1	406.8	264.8	214.0	204.8	738.3	197.9
300.0	305.8	174.4	104.0	111.8	349.6	236.7	190.2
174.3	279.7	257.6	120.4	355.2	284.2	136.7	231.3
195.9\
HOXA11-AS	16.5	101.05	55.0	52.65	94.45
27.599999999999998	1088.9	91.05	116.9	44.25	82.25
264.9	16.55	52.5	14.350000000000001	8.35	142.0
49.849999999999994	15.549999999999999	83.0
180.04999999999998	132.65	87.64999999999999	116.5
37.95	164.2	169.79999999999998	142.25	88.94999999999999
	87.35	111.5	276.6	104.3	82.60000000000001	33.4
	49.95	147.85	94.25	141.25	80.35000000000001
559.2	21.450000000000003	117.5	93.60000000000001
37.25	123.75	96.2	31.349999999999998	78.60000000000001
	10.15	136.45000000000002	43.7	56.95	14.05
121.75	84.9	31.05	69.3	30.9	91.5	52.5	75.25
54.25	84.0	30.35	206.6	72.6	58.65	177.3	74.95
147.75	97.55	97.05	53.1	136.4	67.0	66.85	76.3	54.5
	43.0	39.15	82.3	22.8	47.199999999999996
114.5	81.8	314.8	108.55	60.8	108.3	81.75	119.95
112.0	156.70000000000002	144.6	74.8	114.15	84.6
65.95	126.45	75.9	180.85000000000002	54.65\
CGA	14.0	43.0	30.3	23.8	35.25	3.9
20.299999999999997	47.5	25.549999999999997
24.400000000000002	21.5	18.5	21.150000000000002
32.25	35.15	17.75	23.95	41.95	32.4	51.75	80.25
18.85	50.6	7.5	24.799999999999997	18.5	16.1
18.549999999999997	51.75	14.6	6.25	96.75	40.85	12.8
	20.3	22.05	12.2	42.85	9.1	5.550000000000001

	36.8	8.8	7.05	20.65	42.099999999999994
45.35	29.15	22.9	54.15	15.200000000000001	39.85
44.25	8.55	19.35	11.8	8.25	37.7	11.3	48.9
38.15	160.15	91.95	99.9	5.8500000000000005	34.7
18.200000000000003	22.599999999999998	26.849999999999998
	17.2	3.95	27.1	67.65	40.050000000000004	9.35
	36.6	21.4	25.700000000000003	42.25	6.5
21.65	104.6	41.5	24.05	16.75	69.45	136.45	24.95	11.5
	14.95	43.5	55.650000000000006	13.899999999999999
	19.75	16.4	23.6	9.75	23.6	18.8	56.05	41.5
	13.75	34.35	22.65\
ST3GAL1 55.43333333333333	31.666666666666668
192.33333333333334	325.93333333333334	498.6666666666667
	202.5333333333333	429.40000000000003
191.96666666666667	324.53333333333336	302.0
72.23333333333333	280.50000000000006	433.3333333333333
	183.83333333333334	126.5	253.10000000000002
243.5666666666667	392.5333333333333	1320.3
403.23333333333335	378.59999999999997	101.60000000000001
	311.1666666666667	305.6	203.4666666666667
282.0	176.0333333333333	248.13333333333335
158.8666666666667	238.0666666666667	221.53333333333333
	137.26666666666668	1471.6666666666667
228.4333333333333	260.43333333333334	490.5
135.03333333333333	327.73333333333335	165.3	292.5
529.3000000000001	163.20000000000002	188.23333333333335
	318.8666666666666	662.1	164.83333333333334
342.6666666666667	231.4	454.3666666666666
262.8333333333333	193.46666666666667	1244.3333333333333
	312.5333333333333	186.46666666666667
158.8666666666667	342.8666666666666	260.7
371.2333333333333	363.1666666666667	228.9
254.33333333333334	512.4	719.2666666666668	534.4
278.0	1603.3	267.0	621.1999999999999	473.2333333333333
	190.26666666666665	211.26666666666668	98.7
414.5333333333333	210.03333333333333	136.83333333333334
	167.3666666666667	449.2	273.90000000000003
523.3333333333334	195.53333333333333	193.76666666666665
	338.6	166.2	447.29999999999995	468.8333333333333
	545.3666666666667	601.1666666666666
608.2666666666667	333.2333333333333	340.09999999999997
	167.9333333333333	193.73333333333335	108.0
235.63333333333333	204.73333333333335	96.06666666666666
	53.166666666666664	142.76666666666668
142.70000000000002	122.8666666666667	143.3666666666667
	111.46666666666665	183.6\
ST3GAL2 76.3	292.78000000000003	232.6	199.14
449.74000000000007	129.07999999999998	225.28000000000003
	218.28000000000003	238.66	360.6	316.86
140.66000000000003	176.98000000000002	281.54
888.4799999999999	584.6600000000001	168.3	168.62
198.6	316.17999999999995	1186.3200000000002
139.07999999999998	249.44000000000005	178.83999999999997
	112.22	299.54	254.75999999999993	325.28000000000003

203.64	261.8	187.52	333.82000000000005	243.0
337.0	420.82	184.16	128.14000000000001	519.3399999999999
151.23999999999998	197.38000000000002
221.11999999999998	99.78	187.4	297.03999999999996
92.36	186.4	131.66000000000003	191.12000000000003
367.43999999999994	314.43999999999994	226.94	80.28
243.53999999999996	461.82	247.11999999999998	513.2
197.17999999999998	213.3	131.1	277.66	248.85999999999999
665.0400000000001	222.73999999999995	150.94
253.43999999999997	356.18	130.16	362.23999999999995
212.28000000000003	177.84	288.14000000000004	211.66
216.47999999999996	173.82	175.56	163.1	236.6	164.22
273.0	406.1	179.02000000000004	246.51999999999998
220.58	229.32	483.48	338.36	355.48	365.52	209.6	256.88
118.4	321.64	283.99999999999994	295.76	450.84	277.4
206.45999999999998	290.84	279.22	209.76	324.2
243.13999999999996	230.8\
ST3GAL3 93.58571428571429	122.55714285714285
131.68571428571428	51.34285714285715	112.31428571428572
92.1857142857143	69.47142857142858
94.15714285714286	79.9	63.885714285714286
85.84285714285714	111.10000000000001	59.52857142857142
92.0	139.4857142857143	119.1	97.81428571428572
135.81428571428572	37.75714285714285
123.77142857142857	145.15714285714284	91.92857142857144
202.02857142857144	57.61428571428571
62.15714285714286	89.25714285714287	109.25714285714285
197.2428571428571	122.17142857142859
83.25714285714284	46.642857142857146	93.67142857142856
129.9	115.05714285714284	151.41428571428574
90.08571428571429	75.54285714285716	112.92857142857143
64.84285714285714	94.98571428571428	77.8
72.61428571428571	150.70000000000002	52.371428571428574
79.94285714285715	161.62857142857143
102.95714285714284	99.11428571428573	162.22857142857143
153.87142857142857	136.4142857142857	80.7
77.02857142857142	130.77142857142854	115.68571428571428
140.05714285714285	121.85714285714285
115.75714285714285	66.75714285714285	101.61428571428571
524.2	119.68571428571428	119.6857142857143
134.70000000000002	186.02857142857144	122.85714285714286
115.1857142857143	88.94285714285715
106.08571428571427	117.24285714285715	234.87142857142857
146.24285714285716	85.10000000000001
85.72857142857143	179.74285714285716	128.17142857142858
90.22857142857144	75.74285714285713
92.14285714285714	101.5	69.67142857142858
83.34285714285713	104.02857142857144	117.32857142857142
142.72857142857143	197.91428571428574
99.21428571428574	71.9	87.17142857142858
141.14285714285717	68.7	118.50000000000001
128.77142857142857	87.4	90.74285714285713
95.81428571428572	117.77142857142857	145.8
95.98571428571428	170.15714285714287	115.48571428571428

```
	148.02857142857144	104.85714285714286\
VPS16	572.5	2099.35	886.2	625.75	841.9499999999999
719.2	812.0500000000001	1035.8999999999999	560.0
720.7	678.15	1035.65	374.9	651.0	2113.5	881.7	648.15
464.85	867.9000000000001	587.9499999999999	1261.0
498.8	627.9	605.5999999999999	457.85	1250.6499999999999
	968.1	732.45	573.0	587.0	944.75	972.4000000000001
	1364.8999999999999	669.0	1213.4	871.25
713.9499999999999	809.1	693.1	456.55	429.45	1083.95
591.75	860.7	508.25	551.8000000000001	748.85	1181.55
1918.7	1176.6499999999999	586.1	671.9	1214.15
908.6999999999999	981.3000000000001	1971.4	974.2
719.0500000000001	895.9	899.55	261.6	1074.6999999999998
	660.5	525.4499999999999	1021.5	874.65
845.8000000000001	926.15	1009.3499999999999	715.05
928.4499999999999	813.5500000000001	1138.25
592.9000000000001	533.4	631.4	855.4499999999999
658.9000000000001	911.3	494.55	534.4	562.25
953.4499999999999	1027.0	1048.2	1109.9	742.15
682.9499999999999	530.45	829.05	719.4	1401.55	1224.45
1331.6499999999999	1653.8000000000002	1083.55	1089.85
1346.95	1340.15	1287.85	1130.75	1152.1	1058.75\
PLK4	736.7666666666668	135.76666666666665
273.43333333333334	415.43333333333334	261.73333333333335
	394.6333333333334	217.83333333333334
114.69999999999999	282.5	416.4666666666667	311.7
218.4	317.76666666666665	396.1000000000001
552.8000000000001	160.0	197.96666666666667
878.6999999999999	227.79999999999998	157.06666666666666
	161.70000000000002	751.5	91.23333333333333
215.1	938.9	244.5	388.23333333333335	191.33333333333334
	485.8	219.29999999999998	131.5666666666667
456.5333333333333	168.96666666666667	226.73333333333335
	258.2	122.53333333333335	218.1	425.6666666666667
	163.20000000000002	377.23333333333335
240.63333333333333	352.9666666666667	238.0666666666667
	95.13333333333333	464.7666666666667
1341.4666666666667	205.23333333333335	386.0
357.20000000000005	66.89999999999999	344.4666666666667
	1268.4333333333334	148.16666666666666
444.59999999999997	410.03333333333336	207.0
203.96666666666667	669.8000000000001	516.1333333333333
	262.3	316.5666666666666	230.6	263.23333333333335
	474.8333333333333	47.43333333333334
124.1666666666667	145.63333333333335	51.166666666666664
	166.23333333333335	365.5333333333333
143.76666666666665	282.43333333333334	52.03333333333334
	227.4666666666667	333.6	410.5666666666666
213.1	434.73333333333335	55.5	291.1666666666667
374.43333333333334	345.2	265.1666666666667	219.4
252.80000000000004	507.3333333333333	355.56666666666666
	189.86666666666667	321.8333333333333
119.23333333333333	369.3333333333333	169.79999999999998
	78.1	56.53333333333333	48.199999999999996
```

128.8666666666665	99.9666666666665	65.89999999999999
	100.53333333333335	135.0	110.03333333333335
160.79999999999998	109.1666666666667\
LRRC37A3	61.8666666666667	361.5333333333333
419.2333333333333	142.23333333333335	894.3333333333334
	112.23333333333333	106.9666666666665
142.5666666666667	154.13333333333335	312.90000000000003
	184.63333333333333	202.5666666666667
156.03333333333333	93.8666666666667	448.0333333333333
	216.4666666666667	235.0	225.8666666666665
170.53333333333333	294.6666666666663	737.0
203.7666666666665	258.2666666666665	108.5666666666666
	164.20000000000002	292.40000000000003
126.53333333333332	329.90000000000003	285.8333333333333
	143.9666666666667	132.9	357.2666666666665
425.866666666666	162.1666666666666	135.53333333333333
	196.9	71.7	484.8666666666673	198.29999999999998
	109.33333333333333	149.2666666666668
117.83333333333333	177.8666666666667	159.33333333333334
	134.4333333333333	276.43333333333334
115.80000000000001	80.0333333333333	530.1
229.8666666666667	151.83333333333334	186.4333333333333
	190.03333333333333	331.2	246.6666666666666
379.0333333333333	292.8333333333333	429.3
286.3333333333333	271.03333333333336	497.5333333333333
	402.2666666666667	194.5666666666667
193.60000000000002	362.90000000000003	275.5666666666666
	73.10000000000001	128.70000000000002
283.0666666666666	219.9666666666667	527.5000000000001
	291.23333333333335	338.6333333333334	150.9
393.09999999999997	103.93333333333332	132.93333333333334
	162.2	224.4666666666667	128.3666666666667
290.0333333333333	168.9666666666667	149.53333333333333
	156.1666666666666	304.5666666666666
401.3666666666673	111.0666666666668	215.9666666666667
	123.0666666666668	218.3666666666667
80.2666666666667	233.13333333333335	133.7
148.03333333333333	169.9666666666667	182.73333333333335
	120.60000000000001	208.2666666666665	316.2
211.83333333333334	145.13333333333333	265.6333333333333
	122.93333333333334\
C3orf18	307.8	34.0	263.6	216.4	562.4	325.4	218.1	43.9
	252.2	77.8	127.8	304.1	267.4	168.8	298.0
500.2	325.6	39.6	532.4	148.9	295.9	202.0	361.0
269.1	159.7	267.6	245.4	325.8	385.7	31.7	356.3	77.3
	252.6	549.3	575.5	91.3	165.2	374.6	98.2
392.8	151.1	372.7	431.9	274.9	243.0	205.1	137.0	41.2
	447.5	440.1	301.2	244.0	427.6	68.5	174.5
678.7	51.4	169.5	137.3	227.4	210.1	105.7	146.2
185.6	301.7	408.6	319.4	279.8	376.6	138.3	466.7
492.9	327.8	326.3	279.6	253.6	332.4	319.1	361.7
255.1	420.9	68.2	923.1	166.5	32.3	98.5	165.7
257.3	107.8	559.3	161.0	369.0	440.0	420.0	647.8
547.7	646.8	386.3	353.3	572.4	453.9	356.0	652.7\

NFE2 5.8 7.7 17.9 6.5 76.1 3.3 13.4 69.4
68.2 56.1 48.8 2.6 56.8 151.4 27.7 13.4
34.4 46.5 73.3 44.2 62.4 5.6 11.1 14.3
9.4 53.3 10.7 14.0 5.8 132.7 43.2 7.6
206.6 88.9 21.9 134.1 28.7 163.4 65.8 7.5
64.6 7.9 106.2 179.3 53.2 36.2 8.1 6.5
24.1 6.9 11.7 102.1 5.9 11.2 22.9 14.6
47.7 46.1 6.6 6.6 16.0 7.4 84.0 3.2
13.6 147.3 133.4 119.5 110.0 26.1 62.5 15.0
4.1 35.0 5.8 7.2 120.1 89.4 158.9 57.4
14.5 46.6 30.7 8.7 18.9 9.8 28.5 13.2
195.2 102.5 29.2 13.7 26.5 28.5 41.1 4.7
8.7 14.6 37.1 9.1 9.5 139.9 9.2\
LOC100422737 8.75 59.05 35.25 22.6 83.0 29.4 28.7
11.850000000000001 47.7 31.7 8.65
23.599999999999998 44.05 12.3 24.6 6.8999999999999995
24.35 10.85 33.75 65.05 19.55 21.5
46.349999999999994 28.95 22.8 44.9 37.35 19.7
30.25 52.400000000000006 22.650000000000002 29.75
39.400000000000006 30.799999999999997 33.7
70.39999999999999 29.549999999999997 53.85 7.65
19.200000000000003 34.4 24.2 62.400000000000006
12.100000000000001 14.05 22.700000000000003 46.65
38.45 38.599999999999994 23.450000000000003 68.45
44.15 72.3 49.6 47.65 11.9 36.3 38.7 51.95
12.850000000000001 57.5 55.0 28.75 23.0
42.050000000000004 44.75 30.9 21.55 72.95
52.849999999999994 129.8 40.300000000000004 24.0
20.85 47.45 23.0 14.25 25.75 8.9 43.2 55.25
36.05 19.0 21.25 366.45 547.9 11.0 42.5 265.1
892.05 12.3 40.05 7.199999999999999 24.799999999999997
4.75 20.700000000000003 24.25 16.900000000000002
45.949999999999996 33.3 54.5 29.150000000000002
15.350000000000001\
PLK3 100.03333333333335 105.73333333333335
145.76666666666668 135.33333333333334 196.73333333333332
67.06666666666666 106.23333333333333
264.56666666666666 113.89999999999999 90.06666666666666
108.36666666666666 180.5666666666667 75.0
147.43333333333334 143.2 268.43333333333334
105.8666666666667 190.8666666666667 151.6
148.70000000000002 160.4 143.79999999999998
255.16666666666666 121.39999999999999 80.0 180.4
105.73333333333333 111.3 130.4 196.73333333333335
80.56666666666666 158.23333333333335 337.6
113.03333333333335 219.19999999999996 125.56666666666668
66.03333333333333 302.50000000000006
91.43333333333334 96.7 134.03333333333333
136.79999999999998 137.66666666666666 171.79999999999998
76.3 82.0 62.96666666666667 79.8
149.66666666666666 86.5 91.16666666666667 116.5
83.63333333333334 239.29999999999998 197.20000000000002
139.76666666666668 87.3 161.83333333333334
216.4666666666667 79.73333333333333 191.4

186.73333333333335	162.4	273.8666666666673
106.63333333333333	170.9	116.76666666666665
81.63333333333334	166.26666666666668	132.1
200.4333333333333	163.49999999999997	168.06666666666666
	103.13333333333333	163.1	135.76666666666668
183.83333333333334	110.16666666666667	162.86666666666665
	79.60000000000001	179.33333333333334	179.1
142.33333333333334	164.79999999999998	304.1666666666667
	283.0	160.66666666666666	205.96666666666667
257.96666666666664	243.6	93.46666666666665
316.26666666666665	219.26666666666665	162.56666666666666
	247.9333333333333	139.43333333333334	114.3
121.23333333333333	150.36666666666667	125.46666666666665
	215.76666666666668	173.83333333333334
180.66666666666666\
LRRC37A2	813.5500000000001	2702.4	1907.5	909.9
3119.5	636.8000000000001	659.25	641.3000000000001
538.9	987.05	416.4	1492.8	1161.6000000000001	1169.85
433.25	1020.7	764.65	1072.45	732.05	698.5	1175.7	1593.3
2139.2999999999997	1004.5	1117.1000000000001	557.25
833.55	889.4499999999999	911.3	908.8499999999999
587.5	1269.65	1705.0500000000002	639.2	657.45
826.4000000000001	425.85	1644.8500000000001	1002.8
483.5	897.55	1430.85	922.25	1165.3500000000001
1060.8000000000002	1448.1	629.3	269.0	1275.3500000000001
	1037.3500000000001	1225.85	608.1	619.75
1433.3999999999999	601.1	792.5	1446.3500000000001
577.4	697.9	1452.6	538.5	580.3499999999999	1114.1
682.15	1419.25	1817.4	913.25	1174.0	1687.8	639.2	3259.9
1247.85	1437.85	664.7	1071.5	360.85	600.65	1270.1	624.8
799.9	915.1999999999999	952.7	961.1999999999999
596.35	893.7	513.45	969.4499999999999	544.15	1865.45
768.5	437.34999999999997	470.3	667.9000000000001
515.1	1166.35	625.45	445.34999999999997	502.95	408.25
595.4499999999999	1080.3	627.6999999999999
807.6999999999999\
ARL14EP	1166.3	822.5	1096.2	1056.5	894.5	1640.7	1304.5
924.8	1107.5	851.7	1384.6	1404.8	956.9	990.2	1601.8
1154.8	1079.5	983.9	1075.7	860.5	594.7	698.5	658.5
1295.1	862.4	1037.0	1393.1	1411.0	761.0	1281.4	1492.4
1279.3	1069.7	1114.0	686.1	927.6	862.5	511.6	909.4
980.5	790.7	953.1	1426.7	984.6	980.8	1133.4	1404.8
739.3	605.5	1486.5	896.2	902.3	756.8	1328.5	1131.4
1125.4	1029.2	786.6	1053.5	1040.6	3027.6	1184.4	1312.9
997.1	1280.5	926.2	1060.6	851.9	594.6	1223.6	664.1
1673.9	1003.8	923.9	935.4	1137.6	1165.8	818.8	1203.0
721.0	1176.7	1086.0	1358.5	1098.5	794.5	991.2	1099.9
1027.4	706.3	684.2	720.3	1514.3	1574.7	1489.9	1006.0
2377.6	1961.5	2146.8	1572.1	1981.9	1678.1	1350.8	1808.6\
C3orf17	3100.1	4579.650000000001	3842.7000000000003
3213.5499999999997	1805.2	2568.1	2822.65	2780.9	3531.0
2411.85	2007.55	1979.25	2771.9	2780.9	2907.5499999999997
1694.85	2657.9500000000003	3491.1499999999996	2498.4
2386.2999999999997	3939.95	3219.1	2579.1499999999996

3165.35 3952.3 2248.05 2308.1 3094.3 2805.35 2512.65
2156.7999999999997 3625.8 2762.85 2751.5 3237.55 2110.5
3661.9 3715.3999999999996 1983.9499999999998 2921.1
3896.5 2752.35 2141.55 2539.05 1754.75 2637.1499999999996
1438.45 2340.5499999999997 2425.0 2052.0 3210.3
2226.6499999999996 1889.9 2626.45 2733.45 2247.0499999999997
2784.3 2394.0 3637.4500000000003 2350.75 2757.5
2403.4500000000003 3042.35 3442.65 1786.0 2856.35 2640.0
2193.25 2620.75 1813.3999999999999 2486.15 2344.35 2396.85
3015.2 2609.0 1433.3 2007.25 1818.1999999999998 2400.9
2408.75 2606.35 1931.55 2515.75 3427.0 2980.9 1587.55
2386.3999999999996 2377.65 2659.55 4716.599999999999
3441.9500000000003 2499.2000000000003 2598.95 1328.05
2195.75 3385.35 2357.5 2487.5499999999997 3079.8
2649.6499999999996 2185.8999999999996 2743.65 1670.85\
NFE4 9.6 51.5 16.2 2.2 21.8 11.0 6.8 8.9
22.3 54.4 27.7 53.9 48.8 6.1 15.1 66.8
122.4 5.3 34.6 16.3 60.0 58.1 92.2 58.0
64.6 5.7 7.6 102.5 111.7 15.6 30.1 98.8
102.5 10.0 148.8 9.1 24.9 14.8 6.2 47.3
9.5 49.1 44.2 37.2 2.3 30.4 28.5 22.2
26.5 4.1 52.1 13.5 32.7 5.8 45.8 28.4
68.5 62.1 10.5 15.6 17.7 11.4 50.5 4.8
18.1 41.3 12.4 45.2 137.6 19.3 25.5 23.9
17.8 3.1 3.7 13.4 90.0 16.4 57.0 79.8
4.8 51.1 49.1 55.7 20.4 14.2 22.2 48.9
62.5 5.8 33.7 63.7 12.8 56.5 17.1 13.0
9.0 9.3 20.7 16.9 38.1 40.5 60.8\
PLK5 13.2 20.7 25.3 11.8 42.4 20.6 5.2 44.9
18.7 28.2 22.8 9.6 13.9 25.2 102.1 32.3
20.5 31.5 18.3 73.9 37.5 17.8 129.7 64.6
10.8 18.4 13.7 16.5 30.9 50.6 18.0 17.3
119.3 21.6 28.2 24.7 6.7 29.1 13.2 21.6
43.5 67.6 23.9 16.6 82.7 102.0 20.4 36.4
51.2 18.5 41.3 18.0 21.1 24.2 20.3
150.3 16.8 31.0 21.2 21.0 46.0 32.3 14.7 28.0
44.9 34.7 30.6 12.6 40.4 38.3 145.7 23.2
12.9 16.6 22.2 24.4 33.3 22.6 6.9 38.6
33.8 21.2 11.2 13.9 168.2 167.5 19.3
174.7 16.7 37.8 9.1 22.5 39.1 5.0 12.1 51.1
15.9 26.1 32.1 19.4 25.1 30.8 25.6\
APOOL 573.3499999999999 544.25 812.95 939.5 565.875
405.825 633.5500000000001 659.425 976.0500000000001
1030.3 689.6500000000001 466.725 822.0250000000001
661.55 708.55 615.15 702.9749999999999 948.85 799.3
804.45 329.675 893.35 850.125 985.525 1434.6750000000002
636.75 572.375 582.9 522.675 758.05 870.125 790.6750000000001
376.27500000000003 792.0250000000001
1089.3500000000001 662.075 882.975 287.275 723.3 1686.75
620.1750000000001 530.15 673.0999999999999 695.525
904.75 732.125 264.59999999999997 791.575 300.3 479.65
1008.3 959.05 725.75 637.175 511.0 494.05000000000007
851.35 644.8000000000001 598.2249999999999 461.475
901.525 579.8499999999999 951.65 722.675 604.45 405.525

735.925 582.7750000000001   804.3999999999999   633.675
371.90000000000003   471.75 459.475 972.9499999999999
850.125 637.175 670.65 1032.1750000000002   713.45 1471.4
930.05 771.05 347.79999999999995   1081.275   590.325
688.5250000000001   1010.925   1176.25 1222.0500000000002
        502.35 473.925 695.2249999999999   652.375 604.95
516.85 682.4749999999999   675.65 685.55 485.54999999999995
        565.475 682.125 638.1   641.0\
GTF2IRD2   452.85 519.15 470.1   586.35 659.2   730.85
394.65000000000003   936.85 646.95 509.3   667.3   896.2
528.55 494.20000000000005   1040.15 370.5   348.04999999999995
        578.25 514.75 617.5   517.9000000000001   701.65
784.25 498.4   459.75 675.75 1143.8999999999999   713.9
890.8   614.5500000000001   437.40000000000003   468.0
718.25 616.55 1401.4 877.4   842.75 872.85 570.8
315.04999999999995   469.9   1361.95 808.5   469.9   489.75
537.5999999999999   833.55 874.0   558.9   812.55 669.65
368.0   636.9   1054.35 713.0   1625.8 819.3   417.75
918.0999999999999   852.8499999999999   858.8499999999999
        714.15 622.05 878.4000000000001   438.54999999999995
        671.6500000000001   604.75 445.9   820.85 642.05
1876.7 1102.0 503.6   494.3   513.15 884.65 1119.15 610.45
493.9   768.35 874.3   1370.85 376.4   1526.75 588.0   389.0
881.45 561.95 622.5999999999999   373.25 964.1   1215.1
823.2   771.95 724.05 435.7   668.1   676.8499999999999
1174.7 710.9   669.25 688.2   594.85\
MCAM   299.0   453.7   518.9   686.7   826.3   326.9   1116.8
800.3   783.2   1246.0 355.1   789.3   390.6   616.9   796.3
322.0   613.4   349.5   238.1   1149.5 1605.2 263.6   385.8
480.7   212.3   502.8   469.8   707.4   606.4   812.7   779.0
663.7   756.7   561.7   1150.4 192.5   296.1   1035.6 425.5
388.3   1013.9 498.3   1108.1 1690.6 203.5   348.8   843.3
894.3   367.2   438.0   977.1   463.0   929.8   1160.2 823.0
523.5   418.4   487.6   430.8   497.9   789.7   378.3   589.4
423.9   954.7   541.4   364.9   816.9   836.2   158.5   760.9
497.5   919.8   344.4   581.8   338.8   807.9   414.0   459.8
933.8   333.1   451.1   375.9   305.2   823.5   370.4   878.0
1131.1 953.0   406.8   639.6   1064.0 513.2   544.6   630.3
227.9   351.6   320.8   248.6   260.0   284.9   923.2   295.0\
PLK2   1804.6 199.0   1795.8 8465.0 624.8   772.0   1034.0
309.8   4592.9 2694.5 2191.3 2660.4 10304.0 719.7   426.0
6583.7 1167.1 1371.6 400.8   3487.5 5522.8 2068.1 1215.9
1173.1 5811.9 820.1   561.1   1115.2 1273.0 3163.3 5070.6
950.5   2877.2 2369.5 398.0   3214.9 362.0   2258.8 4772.8
905.5   636.3   2362.1 2037.5 4075.3 324.4   277.9   487.3
483.6   3131.4 5371.6 2317.2 1609.9 327.3   1073.3 1376.4
819.2   3613.2 1306.2 2870.4 2117.8 1045.0 1296.1 3532.7
6337.7 2905.4 837.9   1832.3 2451.7 2149.5 1451.0 2020.3
304.1   2874.1 977.7   1406.1 886.1   6841.2 945.2   10345.8
511.8   602.9   2398.7 452.6   1312.1 14746.4 266.7   827.4
1022.1 1828.6 514.4   993.7   967.5   1305.1 3735.0 1923.0
2409.9 3286.9 1390.8 518.4   1921.7 2418.5 2277.7 1863.1\
PLK1   150.4   147.1   62.0   145.4   265.2   281.4   135.0   88.1
        97.6   90.1   57.7   149.0   273.0   138.5   126.4   97.8

116.5	115.1	130.9	53.6	133.8	148.0	125.7
137.0	95.9	117.6	239.3	161.9	103.3	172.7	56.1
157.3	183.1	45.2	178.5	59.3	203.3	141.1	164.8
201.2	156.4	133.5	129.6	91.4	95.4	171.6	78.5
164.5	166.8	7.3	177.8	181.3	189.8	191.7	204.8	35.0
116.8	102.2	87.9	100.2	225.1	142.6	132.2
105.4	118.2	129.7	101.8	112.3	79.7	156.5	34.6
271.7	67.2	134.2	105.8	163.6	167.1	85.7	62.7
248.5	138.5	186.8	217.1	139.6	72.0	262.3	186.0	93.1
214.2	189.4	206.0	83.2	98.7	75.3	48.4
109.4	72.4	83.9	94.8	54.9	31.6	98.6	119.3\
FAM170B-AS1	12.366666666666665	67.76666666666667
27.400000000000002	9.8	16.666666666666668
37.699999999999996	28.200000000000003	26.2
14.033333333333331	53.53333333333333	7.633333333333333
25.0	27.7	19.766666666666666	29.900000000000002
22.066666666666663	29.2	28.96666666666667
48.93333333333334	48.03333333333334	55.833333333333336
9.733333333333334	61.53333333333334
10.433333333333332	23.433333333333334	28.3
21.333333333333332	56.06666666666666	18.099999999999998
29.7	10.699999999999998	39.199999999999996
58.300000000000004	26.333333333333332	48.53333333333333
43.36666666666667	20.366666666666667	36.9
23.266666666666666	44.76666666666667	44.4
34.76666666666667	19.8	23.633333333333336
41.833333333333336	20.900000000000002	26.833333333333332
22.333333333333332	47.366666666666674
37.800000000000004	14.866666666666667	29.700000000000003
34.96666666666667	47.73333333333333	16.0
17.433333333333334	45.4	40.733333333333334	12.6
18.333333333333332	274.6	12.9	44.833333333333336
32.233333333333334	56.26666666666667	24.599999999999998
10.666666666666666	21.066666666666666
15.466666666666669	26.86666666666667	84.26666666666667
26.3	78.93333333333334	40.86666666666667
47.23333333333333	30.5	15.566666666666668
38.166666666666664	36.4	15.966666666666667
15.866666666666665	46.800000000000004	16.7
32.86666666666667	25.100000000000005	71.46666666666665
50.23333333333333	13.766666666666666
19.233333333333334	20.900000000000002	30.733333333333334
58.56666666666666	33.36666666666667	26.0
20.26666666666667	15.166666666666666	30.633333333333336
14.633333333333335	52.4	69.86666666666666	15.1
37.43333333333334	19.733333333333334\
RP11-288L9.1	100.6	154.4	128.5	52.1	291.5	80.5	61.9
118.1	34.7	154.8	94.9	50.3	43.0	50.1	35.8
41.3	37.3	101.1	65.7	100.4	334.7	61.6	78.3
93.2	124.5	30.0	40.7	148.9	103.9	246.1	11.4
255.7	139.4	79.5	92.5	29.1	84.6	31.0	10.1
93.6	124.2	12.5	46.1	33.4	55.1	25.8	81.0
95.8	14.2	92.2	114.6	17.9	14.9	125.3	59.3
110.7	117.2	86.4	109.0	119.8	393.7	160.3

123.3	83.9	29.2	69.8	63.1	84.0	107.9	49.7	65.8
	92.5	79.0	82.7	108.7	57.5	160.2	82.6	60.7
	70.5	208.6	113.1	85.1	100.3	222.0	241.2
106.3	64.5	144.5	198.8	82.8	127.8	92.3	52.0	14.3
	106.2	62.2	80.7	20.3	107.7	52.4	91.5
31.0\
LRRC75A-AS1 /// SNORD49A /// SNORD49B /// SNORD65	255.0
173.1	138.33333333333334	329.43333333333334
154.46666666666667	1222.3666666666666	437.20000000000005
	131.36666666666667	837.5999999999999
311.0333333333333	963.0	112.8	351.3333333333333
200.4666666666667	544.8333333333334	274.73333333333335
	183.30000000000004	205.96666666666667
210.8666666666667	308.8333333333333	147.53333333333333
	324.0	276.8333333333333	371.09999999999997
604.5666666666667	245.0	418.96666666666664
226.4333333333333	237.4666666666667	237.9666666666666
	464.0333333333333	253.79999999999998	145.5
588.5333333333333	245.86666666666665	190.5333333333333
	1030.8333333333333	202.5	543.9666666666667
449.2333333333333	347.5	528.6333333333333
309.6666666666667	190.96666666666667	904.6
549.8000000000001	213.33333333333334	489.8
123.83333333333333	249.23333333333332	664.6
471.8666666666666	1175.6666666666667	126.06666666666668
	392.3666666666666	146.73333333333335
427.40000000000003	163.70000000000002	217.1	173.6
1411.0333333333335	184.70000000000002	242.39999999999998
	268.5	105.26666666666667	229.26666666666665
703.3333333333334	191.36666666666667	320.93333333333334
	531.5	159.5666666666667	149.63333333333333
169.73333333333335	467.0666666666666	273.2
1154.6333333333334	198.1	426.8333333333333
269.6333333333334	168.6	138.70000000000002
153.2666666666668	887.4	226.36666666666665
146.5666666666667	228.4	177.36666666666667
138.0666666666667	372.1666666666667	109.2
896.5333333333333	170.43333333333334	846.6666666666666
	506.7333333333333	303.63333333333327
660.1666666666666	639.1333333333333	945.4666666666668
	353.8333333333333	668.4	298.8	369.40000000000003
	691.8333333333334\
KLF3-AS1	22.7	223.7	224.39999999999998
84.14999999999999	453.29999999999995	125.5	172.8
189.3	176.5	147.8	282.05	191.45	55.35	85.4	647.25
1174.2	118.60000000000001	196.85	150.85	398.85	842.35
105.95	256.25	257.75	105.25	208.9	70.4	520.3	384.8
268.4	803.3499999999999	416.45	484.0	684.65	74.75
192.3	369.45000000000005	175.60000000000002	379.3
163.65	175.65	128.95	471.15	460.25	79.15	342.6	78.75
56.05	397.79999999999995	2333.8	364.35	68.2	127.0
125.55	233.55	970.4000000000001	3263.0	208.35000000000002
	104.5	2028.85	665.95	406.95	462.6	326.65	512.5
337.15	605.7	560.05	756.15	419.29999999999995

1201.8999999999999	256.1	322.85	185.6	861.4	164.45
153.95000000000002	453.5	501.3	34.75	268.2	194.45
140.0	224.15	128.35	118.75	323.8	179.25	137.5	161.15
10.75	684.35	649.0	1932.3500000000001	3569.95	3001.1
3136.6000000000004	1726.0	2289.95	2337.8500000000004
3123.4	346.6	1736.75\
CGN	260.35	582.3	893.15	144.9	1032.45	314.1	306.25
901.4000000000001	233.25	1137.3	414.85	998.9000000000001
	771.2	540.3	899.4000000000001	274.05	532.85
676.25	1487.45	320.2	572.55	965.25	1287.45	277.6	533.0
847.65	484.15	495.5	563.75	423.6	64.85	1214.8	2058.65
340.05	720.95	1167.65	545.3	1735.5	492.4	370.25	441.65
621.55	347.1	276.04999999999995	629.55	569.75	223.45
275.45	1608.3000000000002	826.0	638.45	1134.3	896.6
365.15	885.35	1250.8	878.45	713.5999999999999	370.9
504.70000000000005	309.35	1049.45	998.3000000000001
444.1	514.15	488.2	662.7	413.95000000000005	793.2
754.5	1299.45	548.55	298.15	217.95	285.7	288.3	961.0
416.5	471.9	280.3	253.85000000000002	473.75
722.5999999999999	709.0999999999999	361.0	1557.4
687.6	1114.7	1449.55	788.9000000000001	281.5	314.85
420.6	408.9	341.95000000000005	344.75	334.85	502.65
1078.4	584.25	490.84999999999997	673.45	333.3\
C3orf14	15.05	34.3	29.549999999999997	148.3	100.15
42.55	49.75	304.9	81.0	167.8	39.0	89.65	47.0	75.7
	58.15	136.1	371.65000000000003	53.6	53.5
30.700000000000003	312.9	71.6	243.75	195.70000000000002
	34.0	29.95	69.80000000000001	97.75	252.15
112.35	187.04999999999998	103.1	60.099999999999994	62.1
	57.75	62.65	39.45	40.0	627.25	725.1999999999999
	339.35	27.55	334.35	214.5	76.85	57.400000000000006
	89.35	1009.45	25.0	117.25	66.5	221.95000000000002
	32.3	64.85	65.80000000000001	56.9	38.3
248.15	278.2	106.2	53.95	245.95	241.4	194.7
172.14999999999998	111.65	48.650000000000006	297.5
132.6	245.3	83.2	100.3	66.15	45.75	84.45	56.15
138.3	124.60000000000001	84.7	730.2	284.45
206.89999999999998	315.8	153.54999999999998	21.45	64.5
	50.1	102.6	337.65000000000003	7.4	404.55
211.14999999999998	504.45	223.65	131.1	448.8
236.10000000000002	255.15	109.75	287.5	132.20000000000002
	237.15	271.45\
ELL2	559.8	110.625	1016.9250000000001	208.92499999999998
	134.15	215.02499999999998	271.42499999999995
315.5250000000001	278.72499999999997	248.89999999999998
	195.35	436.55000000000007	261.15	329.12499999999994
	38.574999999999996	215.07500000000002
178.60000000000002	471.02500000000003	301.625
313.52500000000003	281.55	390.45	737.575	382.34999999999997
	235.62499999999997	304.84999999999997	413.2
180.675	280.4	154.0	178.875	646.575	309.25	172.55
401.20000000000005	452.25	147.1	171.1	299.09999999999997
	438.825	468.02500000000003	165.35	327.775
442.22499999999997	267.97499999999997	185.675	249.2

```
57.575  128.10000000000002      255.65  239.275 306.075 526.25
167.025 241.20000000000002      147.6   77.325  447.375 442.05
179.425 207.5   302.525 207.35  810.375 573.2749999999999
544.7750000000001       440.44999999999993      477.2   53.85
278.975 229.775 269.9   539.575 138.975 312.65000000000003
73.44999999999999       331.075 172.25  202.175 29.025000000000002
        147.2   289.27500000000003      51.6    328.6   318.5
184.15  169.475 141.825 448.675 205.25  174.67500000000004
159.0   174.47500000000002      244.87499999999997      220.125
226.97500000000002      175.65  193.25  264.325 174.27499999999998
        174.89999999999998      162.1   226.2\
MCAT    422.4   177.1   433.7   515.3   342.3   755.7   638.3
202.3   726.5   518.3   754.7   373.0   639.5   290.8   284.2
340.9   356.8   710.2   689.8   251.1   288.7   906.6   369.6
494.6   471.4   376.1   620.2   323.4   375.6   470.3   436.8
228.3   471.7   415.8   249.8   309.3   600.2   227.8   549.4
410.5   369.2   269.4   420.8   515.1   1146.2  462.9   449.7
359.6   258.1   340.0   472.6   1053.2  470.9   249.1   447.3
340.8   361.7   257.5   394.1   408.3   272.4   340.0   391.9
876.9   246.8   350.4   612.3   494.6   489.9   601.0   298.5
342.0   242.1   486.0   288.2   698.6   271.7   640.5   456.8
176.3   241.7   229.7   746.0   298.6   226.8   246.4   308.1
343.6   589.7   243.4   551.3   218.4   318.8   262.8   286.4
331.7   272.3   434.2   348.0   283.0   220.4   233.7   359.8\
ELL3    1790.15 2147.45 1192.4  398.3   1011.75 964.6500000000001
        722.85  528.5   340.55  612.3   579.15  631.15  270.75
1154.3500000000001      188.9   306.75  596.2   1249.35 707.5
818.0   1152.15 1070.7  518.75  365.75  330.45  1879.15
1690.8000000000002      671.2   682.0   623.45  563.35  647.9
904.05  390.35  695.7   602.85  326.65  1171.8  654.4
351.20000000000005      381.05  235.25  1010.3  152.15  656.4
916.05  1562.75 825.55  1031.6  360.75  725.2   1261.9  603.75
267.1   949.65  805.0999999999999       358.05  792.35  2445.3
638.05  1180.6  743.05  1049.05 792.9499999999999       498.3
721.05  263.65  215.35000000000002      375.7   1097.05 617.6
1171.9  483.65  790.85  372.0   790.4   1001.1  188.75  745.3
315.8   471.5   291.8   250.1   910.2   922.55  370.35  538.85
385.1   251.75  335.65  120.3   544.8   290.45  342.5   522.35
527.55  357.54999999999995      607.55  471.0   826.3000000000001
        543.25  529.35  352.0\
KIR2DL1 /// KIR2DL2 /// KIR2DL3 /// KIR2DS1 /// KIR2DS2 /// KIR2DS4
        87.4    33.3    88.0    62.8    169.5   66.5    22.5    62.5
        12.0    77.4    7.2     35.1    48.6    96.9    142.8   11.6
        32.0    129.8   7.8     37.5    122.8   95.9    100.0   5.1
        51.7    31.2    90.8    112.7   127.0   3.7     15.4    39.7
        25.1    63.9    65.0    72.1    64.9    21.1    58.8    64.1
        13.6    72.0    112.7   88.3    118.7   116.0   22.7    76.6
        36.4    30.5    38.6    60.1    15.2    42.0    49.7
106.3   27.0    48.9    27.8    26.8    210.6   20.0    20.0    12.1
        126.0   167.4   28.9    5.9     13.0    87.2    117.6   23.3
        41.0    41.0    82.8    41.2    15.0    78.0    8.8     18.5
        28.3    14.3    17.9    12.8    126.4   226.7   126.1   14.9
        25.2    35.6    42.9    52.5    42.3    14.1    7.7     79.3
        38.0    98.8    31.0    168.4   23.7    147.7   63.3\
```

FLJ34521	36.9	12.4	38.5	17.2	170.7	42.4	10.3
9.6	13.0	84.1	52.1	41.4	4.7	14.5	38.5
34.4	52.5	87.0	40.8	9.4	58.2	64.0	99.1
8.4	86.2	34.8	10.9	28.6	60.0	77.4	7.5
24.2	98.9	36.3	80.6	66.4	11.1	8.7	38.2
13.3	37.6	14.3	6.1	29.3	55.0	13.4	71.9
56.4	11.7	74.6	10.3	70.3	63.7	160.5	14.3
16.4	11.6	37.0	9.1	56.1	206.7	51.2	9.9
61.8	5.4	26.0	15.1	20.1	101.5	7.7	25.7
60.7	4.7	46.1	28.3	10.0	10.1	29.2	74.9
38.4	55.4	18.8	9.6	55.0	103.5	70.6	74.8
11.4	66.4	34.2	34.1	57.1	14.9	36.1	30.3
6.8	52.3	66.8	15.3	3.8	8.8	18.6
30.0\
AMIGO3 /// GMPPB	673.0666666666667	226.03333333333333
352.93333333333334	292.8	323.40000000000003
1063.6000000000001	448.09999999999997	553.4
685.8333333333334	380.6666666666667	449.5333333333333
352.5	424.83333333333326	1765.466666666667
114.60000000000002	238.23333333333335	244.23333333333335
476.63333333333327	384.3999999999999	372.5
363.0	696.9	535.0	731.8666666666668	469.40000000000003
559.8333333333334	395.3666666666666
350.43333333333334	398.93333333333334	320.8333333333333
167.33333333333334	371.90000000000003
238.56666666666663	589.4333333333333	572.4666666666667
616.5666666666667	754.6666666666666
320.3666666666667	370.2	561.0	241.83333333333337
292.49999999999994	505.2	226.83333333333334
1447.3000000000002	378.1666666666667	734.8666666666667
279.03333333333336	335.26666666666665
381.2666666666667	495.8333333333333	394.7
346.4666666666667	408.99999999999994	484.29999999999995
122.93333333333334	433.46666666666664	292.2
720.1333333333333	414.4666666666667	664.9
359.3666666666666	186.63333333333333	788.9666666666668
293.3333333333333	381.5	581.2666666666667
247.29999999999998	510.9666666666667	805.9
406.73333333333335	524.7	325.0	265.7	329.0666666666666
534.7666666666667	530.8333333333334
774.7666666666668	344.40000000000003	310.7
200.23333333333332	165.0	345.3666666666666
264.7666666666665	423.09999999999997	248.13333333333333
165.33333333333334	205.83333333333334
616.9999999999999	236.0333333333333	390.09999999999997
298.3666666666667	437.96666666666664
231.29999999999998	207.03333333333333	162.26666666666665
239.36666666666665	223.33333333333334
358.96666666666664	332.5	330.73333333333335
705.4000000000001	180.1\
GTF2IRD1	1163.5	397.3	464.8	856.0	690.1	795.3
654.3	1004.4	832.0	670.7	788.2	489.3	841.7	409.0
3725.3	795.7	681.4	424.5	789.9	784.4	1292.9	637.5
521.6	386.9	864.5	829.4	803.0	403.4	625.6	501.7

824.3 531.4 542.1 836.8 1074.2 739.4 812.8 537.3
983.1 743.6 1388.2 832.8 328.7 1204.5 672.9 923.3
778.6 1018.5 693.2 540.8 728.7 1625.4 817.0 1009.0
797.8 722.2 862.4 431.0 498.1 368.8 820.3 858.7
667.9 677.8 585.3 572.0 528.1 1741.4 667.9 658.3
462.9 604.8 694.4 813.7 367.6 875.5 1092.8 468.4
796.4 2063.8 1235.5 1286.1 1566.9 1917.9 1774.9 676.3
1426.8 1417.3 936.9 665.3 1009.5 408.5 554.1 470.0
591.9 865.9 562.5 953.9 505.6 517.1 645.5 271.7
637.1\
BPIFC 31.3 40.8 6.3 51.9 39.4 9.9 7.9 10.3
70.8 24.0 10.4 18.5 50.0 6.5 8.4 14.3
5.8 5.2 2.7 90.0 76.2 10.3 22.2 7.9
12.2 56.6 43.6 67.4 15.6 7.1 10.0 72.0
17.6 34.0 16.7 10.5 11.8 3.8 11.2 3.1
20.5 25.7 4.9 131.0 14.0 20.1 4.6 6.0
58.4 100.1 8.1 14.4 64.6 61.5 19.0 55.3
15.4 16.1 16.0 13.0 45.5 7.7 5.0 8.6
56.1 11.3 3.0 50.8 17.9 9.6 49.0 16.4
31.0 28.6 8.1 23.6 67.1 5.8 10.5 8.2
86.0 33.5 7.8 5.8 9.4 30.5 5.9 20.4
18.3 87.2 96.9 10.1 14.6 11.6 7.5 13.9
11.7 6.5 13.9 48.9 58.2 9.4 6.0\
MOCS2 314.5 216.35000000000002 250.5 443.29999999999995
315.4 352.9 210.85000000000002 173.95000000000002
440.40000000000003 182.65 259.15 446.79999999999995
344.7 313.35 72.65 272.25 231.0 326.8 279.3
326.8 244.60000000000002 305.1 204.9 401.25 422.5
353.70000000000005 268.7 139.5 156.95 412.15 324.5
352.6 201.35 334.25 213.15 293.85 418.2 176.64999999999998
229.0 295.85 368.95000000000005 292.25 241.25
310.6 326.15 278.04999999999995 341.15 376.75 149.75
267.9 252.75 198.14999999999998 373.15 223.89999999999998
128.25 244.45 356.20000000000005 245.60000000000002
259.9 294.75 327.35 253.3 240.95000000000002
236.29999999999998 271.55 225.7 299.75 277.40000000000003
225.35 180.85 84.2 213.35 314.2 331.65000000000003
392.05 254.25 187.3 281.65 331.6 328.7 301.5
307.7 129.9 205.25 178.2 87.35 212.64999999999998 85.0
220.45 304.85 219.75 222.2 425.6 220.4 208.2
327.4 217.05 312.2 208.75 192.0 329.45000000000005
342.35 329.95\
MOCS3 65.4 24.7 52.5 45.3 82.0 88.9 74.2 74.6
93.9 17.5 67.5 71.8 50.9 54.6 66.2 78.7
55.2 48.3 89.4 19.3 111.5 62.1 67.3 72.6
127.3 69.2 48.3 134.4 19.8 68.1 92.7
102.4 16.5 128.0 166.5 126.8 93.3 20.7 95.9 88.5
96.7 30.1 64.5 48.6 174.4 41.5 41.4 96.6
84.0 104.4 77.0 74.4 89.3 33.2 48.5 30.1
108.2 39.6 36.1 47.7 277.4 11.2 22.7 85.5
49.9 56.9 100.0 67.2 91.3 153.9 62.1 66.1
34.9 88.1 15.8 90.1 98.1 80.3 38.5 81.0
144.4 82.1 276.3 65.6 91.7 217.4 51.8 54.2
175.1 95.5 144.4 66.4 64.5 34.4 65.0 53.1

29.8 98.8 94.6 30.4 56.2 85.7 126.2\
STON1 72.3 41.6 119.75 160.55 223.85000000000002
61.25 226.0 588.75 214.54999999999998 123.35 154.8
173.2 115.0 104.05 850.0 712.6999999999999 629.75 88.7
44.85 230.0 183.35 80.85 150.65 276.6 12.35
183.8 123.75 178.7 168.8 137.9 610.1 194.64999999999998
179.05 180.20000000000002 109.1 233.95000000000002
74.5 79.7 79.05 256.95 529.3 113.2 237.2
283.0 139.05 118.3 150.04999999999998 156.2 121.45
131.5 146.45 108.80000000000001 316.0 230.95000000000002
113.9 116.25 122.6 389.3 118.2 93.14999999999999
280.05 136.75 257.3 58.65 218.95 190.95 94.25
194.1 150.45 88.0 160.4 163.7 176.95000000000002
180.65 50.25 132.55 232.5 127.25 197.64999999999998
1752.25 992.85 123.39999999999999 348.05 189.75
193.35000000000002 146.75 131.95 331.65000000000003
58.75 276.1 133.7 732.35 276.6 665.95 842.5999999999999
288.6 344.05 360.3 193.2 586.45 373.4 486.0
412.95\
CHM 1756.7 461.93333333333334 1177.9333333333334
908.9666666666667 270.46666666666664 532.0
937.3000000000001 1344.8666666666666 831.1999999999999
754.4 920.8666666666667 337.0333333333333
1027.4333333333334 1065.2666666666667 578.0333333333333
766.8666666666667 1197.2666666666667
685.2333333333332 501.93333333333334 641.2333333333332
296.3666666666667 1327.1333333333334
779.9666666666667 1204.2 1264.5 398.29999999999995
588.5666666666667 458.8333333333333 381.93333333333334
734.4333333333333 1175.0333333333333 596.0
245.4333333333333 501.1000000000001 743.9
437.43333333333334 1617.0333333333335 463.2666666666667
631.9666666666666 2014.966666666667
773.3333333333334 803.1999999999999 747.6999999999999
711.9333333333333 963.2333333333332
460.5333333333333 1416.3333333333333 1201.5
237.9333333333333 362.93333333333334 626.5333333333334
832.0333333333333 462.9666666666667
816.3666666666667 418.5666666666666 317.1333333333333
509.20000000000005 476.4666666666667 566.9
328.53333333333336 253.83333333333334 353.3333333333333
555.0666666666667 895.0333333333333
564.3000000000001 466.90000000000003 678.9
395.4666666666667 302.8666666666667 553.0333333333333
448.79999999999995 308.4 777.7333333333335
1028.2666666666667 1181.2333333333333 364.3333333333333
840.4333333333333 761.7333333333335 644.6
2043.6666666666667 1598.5 551.6 523.7333333333332
1146.1333333333334 703.4333333333334 621.4
1803.8666666666666 1236.8666666666668 1127.2
520.5666666666666 970.9333333333334 1499.9333333333332
660.2333333333332 531.4333333333333
376.93333333333334 761.1666666666666 804.9666666666666
535.1999999999999 407.1000000000001

722.6999999999999	696.1	653.2666666666667
742.2999999999998\
STON2	499.025	623.85	287.775	833.125	404.275	728.3000000000001
	917.075	183.82500000000002	547.075	862.3249999999999
	1394.2	555.975	934.2750000000001	144.825	926.575
429.05	259.15	552.925	674.2249999999999	392.925	291.4
850.875	405.175	230.95	771.675	384.675	304.775	368.85	426.475
414.925	260.4	842.175	332.75	575.625	62.6	249.7	434.55
696.9	381.5	564.925	1229.8	421.675	188.375	446.975	415.175
632.825	294.175	313.0	373.75	551.4	683.025	960.9000000000001
	244.97500000000002	666.45	568.475	727.025	342.75
601.675	480.2	361.475	555.9000000000001	606.575	685.875
529.85	321.95	379.325	513.325	265.0	290.57500000000005
425.275	349.025	142.95	266.325	347.475	387.25	290.925	335.75
266.3	385.825	179.7	971.525	895.05	1500.725	513.075
329.75	624.325	236.25	1048.025	289.775	910.1999999999999
	512.05	197.47500000000002	219.125	286.3	171.3
213.325	181.125	291.15000000000003	349.275	343.67499999999995
	221.6	278.07500000000005	234.64999999999998\
LINC00623 /// LINC00869 /// LINC01138 /// LOC103091866	726.4
134.9	424.3	1118.6	679.5	301.3	1093.6	644.4	1019.9
950.8	400.9	1159.0	675.9	643.2	817.3	533.8	895.3
628.6	886.8	544.2	687.9	773.8	537.5	942.8	1228.3
885.9	790.9	1168.4	491.9	631.2	1820.1	1064.1	1183.1
696.3	591.5	832.6	315.2	1379.5	1233.3	858.1	1315.0
888.5	636.9	986.5	1093.2	564.3	1232.8	335.7	1301.1
1272.8	501.5	545.0	1441.8	513.4	505.7	222.3	2015.7
436.7	639.5	848.2	1390.0	1295.2	1221.7	788.0	626.1
1326.5	1870.7	892.6	1521.7	672.4	723.9	512.2	545.8
517.6	838.1	652.9	1491.4	1357.2	995.5	646.0	796.4
1189.5	411.1	731.9	718.5	893.0	805.8	1135.4	731.8
453.9	893.0	461.6	713.5	570.1	622.1	896.4	911.0
919.5	1735.3	913.4	710.6	271.4	915.9\
MCEE	597.9	555.2	413.5	433.9	307.1	389.7	332.5
354.7	419.8	375.6	408.2	486.1	396.5	641.7	258.2
364.0	537.6	204.9	559.3	216.0	197.6	325.1	322.9
519.3	360.4	393.7	537.1	473.3	443.5	485.0	1015.6
386.2	291.7	427.9	116.8	624.7	382.0	457.1	684.4
523.2	385.1	250.5	400.8	196.7	428.0	570.9	683.4
343.4	467.7	503.9	640.3	362.8	761.4	226.8	452.9
141.8	357.7	261.7	476.3	463.8	324.3	240.8	728.8
178.1	304.0	345.9	601.3	548.4	357.4	474.3	248.5
408.5	308.5	632.6	354.4	433.1	463.4	452.7	465.6
443.0	216.8	268.1	242.2	281.0	330.5	354.6	548.4
341.5	319.8	307.8	331.0	497.6	718.2	610.6	617.2
761.9	876.0	738.9	652.8	515.9	653.2	606.5	1116.2\
FGD5-AS1	4676.8	1100.7	2253.2	2692.45	1061.0	2517.5
3387.65	1902.45	3336.0	1646.1000000000001	3787.1000000000004
	2265.2	1648.15	3092.3	2130.5499999999997	3297.05
2880.7000000000003	1425.3	2707.85	1970.5	986.1999999999999
	3614.8	1664.1	3073.65	4897.05	1842.45	2013.25
1544.8500000000001	1748.45	3285.4	2790.35	1330.3	1111.0
2385.15	2011.85	1588.15	4986.75	1053.05	2458.7	3371.0499999999997
	3000.6	3750.6	1781.95	2672.6	2991.25	2145.6

3798.6000000000004	2005.6999999999998	2011.5
1277.1000000000001	3117.3	2385.9	1449.1	2807.1499999999996
	2469.3999999999996	1392.1999999999998	1779.25
2051.95	1859.6499999999999	1449.45	1010.65	1361.1	1825.25
2547.55	1965.3	1528.9	2746.2000000000003	1655.25	709.7
2295.25	1799.7	1148.5	2089.7	3263.75	2685.35	4257.950000000001
	2496.25	2585.55	2385.1	2454.25	3534.15	5427.9	3019.7
2694.85	2170.35	1323.8999999999999	4816.05	2909.45	3408.45
1340.15	2805.95	3423.2999999999997	2486.45	1800.3	1951.05
2379.35	1868.25	2427.1	1731.45	2058.2	2528.6499999999996
3301.5	2335.9\
RP11-319G9.3	58.7666666666667	340.1666666666667
377.6333333333334	69.3	287.2	27.166666666666668
48.8666666666667	203.0	98.9666666666665
137.0333333333333	53.7333333333333	181.0666666666667
	72.8333333333333	56.2333333333333	126.7
231.5666666666663	121.7333333333333	172.0
70.5666666666666	104.5333333333335	392.2333333333333
	93.0	374.8333333333333	67.6666666666667
80.3666666666667	60.6	18.4666666666667
246.80000000000004	251.5666666666667	27.4666666666667
	20.3	209.80000000000004	235.5	42.5333333333334
	104.1333333333333	96.2666666666667
30.933333333333334	368.5666666666666	50.8666666666667
	27.066666666666666	106.60000000000001	55.5
117.1666666666667	92.0	42.4666666666667
107.39999999999999	42.333333333333336	34.199999999999996
	145.20000000000002	118.5666666666666	70.5
48.7666666666667	86.6666666666667	186.1333333333333
	156.4333333333334	144.7333333333335
45.699999999999996	327.0666666666666	123.6666666666667
	430.5999999999997	87.8999999999999
138.5666666666666	120.2333333333335	54.4666666666667
	99.5333333333335	226.5333333333333
46.2333333333333	44.1	318.9333333333334
67.3999999999999	355.2	331.4666666666667
150.7333333333332	97.7666666666667	426.7333333333333
	25.0333333333333	119.5999999999998	103.3
70.7666666666667	177.2666666666665	283.0333333333333
	144.20000000000002	46.2333333333333
36.4666666666667	422.4666666666667	223.2999999999998
	197.4	113.0	96.3666666666667	191.2999999999998
	19.366666666666667	215.2333333333335
33.1333333333333	53.5	100.5333333333335
70.10000000000001	34.5333333333333	54.2666666666667
	83.6666666666667	71.6333333333333
118.1999999999999	108.1666666666667	90.4666666666665\
RRP12	109.18000000000002	411.58000000000004
308.17999999999995	119.46000000000001	374.38
124.88000000000002	192.56	168.58	123.04	166.28
111.8399999999999	221.22000000000003	168.56	143.06
273.18	168.3	128.60000000000002	199.04000000000002
116.2	184.72	435.78000000000003	168.48	251.50000000000006
	83.08	69.6199999999999	280.08	149.52	278.92

167.14000000000001	150.3	195.66	172.71999999999997
233.53999999999996	123.13999999999999	218.07999999999998
	86.08000000000001	47.28000000000001	277.74
98.52	133.88000000000002	245.2	137.6	199.88	169.82
79.06	267.34	128.71999999999997	96.35999999999999
235.98000000000002	204.88000000000002	128.44	207.1
204.4	281.04	246.98000000000002	161.14	121.22	269.36
212.8	215.2	102.82000000000001	188.12	163.8
201.17999999999998	183.28	178.92000000000002
119.28000000000002	114.7	133.46	96.92	174.01999999999998
	178.42000000000002	205.66000000000003	134.94
143.11999999999998	120.78000000000002	164.12	158.42
185.17999999999998	176.61999999999998	204.02000000000004
	156.86	97.46	175.28	233.74	187.35999999999999
155.94	288.21999999999997	101.88000000000001	228.44
128.94	172.64000000000001	119.55999999999999	113.22
108.92	109.3	112.7	124.74000000000001	201.07999999999998
	144.3	189.66	150.57999999999998	140.2\
CIC	171.8	365.4	191.6	255.5	716.8	257.2	259.8
365.8	363.5	307.0	338.6	216.7	247.8	146.0	721.5
483.7	184.2	357.5	652.6	298.9	761.2	348.2	367.9
285.2	235.1	490.0	346.5	570.6	301.2	313.2	153.5
491.4	699.7	392.0	134.3	416.7	170.9	455.0	314.3
221.0	390.2	411.7	265.6	364.3	173.2	235.3	301.4	29.1
	624.5	310.1	393.1	342.6	217.4	601.6	500.1
684.5	350.8	467.3	351.4	502.1	576.7	655.3	490.4
404.5	299.7	431.5	189.8	339.8	307.5	187.9	656.4
536.8	250.3	277.0	235.4	255.8	305.9	271.9	282.4
140.7	404.3	773.0	989.9	718.7	89.8	788.0	288.2
382.2	362.9	453.8	292.3	507.9	290.1	466.6	591.3
274.5	308.5	431.0	507.3	275.4	221.7	208.5	306.5\
RRP15	1649.0	633.75	574.55	1140.55	1041.15	1217.85
1418.9499999999998	1256.3	956.45	657.45	963.95	629.0
1057.15	644.55	693.75	704.8	876.0	463.8	744.3	739.05
225.5	916.5	396.15	931.9499999999999	711.3000000000001
	443.75	1023.2	594.5	424.75	1528.15	2176.95	720.35
360.95	555.0	544.9	418.5	934.4499999999999	503.0
1044.35	1221.3	1143.7	653.5	535.0500000000001	513.5
632.95	662.8499999999999	930.75	770.35	547.4	878.15
722.35	967.8000000000001	1056.85	575.45	551.55	886.6
881.5500000000001	462.75	512.65	626.3	366.1	483.4
1048.0	778.85	700.5500000000001	769.1999999999999
1309.2	568.0	432.85	666.15	229.15	399.6	530.75	1376.45
573.55	602.35	605.5	514.75	929.3000000000001
1235.8000000000002	1083.7	1074.3	1021.8499999999999
825.85	611.7	393.95	679.1500000000001	775.25	431.8
346.7	1477.55	625.3	1216.0	781.1	544.4	810.65
827.0999999999999	965.55	516.3	661.45	720.85	652.45
794.3499999999999\
ZIK1	53.349999999999994	102.35000000000001	55.5
49.35	51.300000000000004	129.6	109.85	164.9	89.7
49.050000000000004	59.400000000000006	36.85	44.5
150.9	247.3	106.35	40.800000000000004	32.7	16.55
29.299999999999997	70.55	27.7	126.65	22.599999999999998

```
	33.75	51.7	49.75	122.80000000000001	69.25
66.95	37.15	124.0	83.19999999999999	97.10000000000001
	177.0	34.4	42.75	46.8	38.849999999999994
78.85	21.1	5.8999999999999995	82.14999999999999	53.5
	34.95	44.650000000000006	55.35	86.75	142.15
43.599999999999994	41.15	53.800000000000004
51.800000000000004	109.25	54.0	12.75	19.05	48.1
77.55	83.85	366.59999999999997	96.4	70.85
31.650000000000002	100.65	81.4	58.2	47.55	12.0
113.15	130.95	34.15	65.75	66.15	37.800000000000004
99.30000000000001	57.75	62.4	24.799999999999997
127.25	144.65	48.05	172.4	68.64999999999999	12.6
23.85	65.85	15.05	36.25	80.95	49.85	86.5	154.2	75.4
	169.35	118.1	129.05	64.7	56.75	144.75	85.15	50.6
	101.1\
EDARADD	56.6	14.3	25.2	43.7	37.2	29.3	10.8	68.2
	59.2	8.9	7.1	19.3	25.2	17.6	44.4	30.4
	86.3	21.0	62.5	10.4	21.5	3.6	233.6	88.3
	72.3	7.1	14.6	171.5	95.0	29.2	13.2
187.1	22.5	56.1	92.8	14.0	47.6	67.3	13.4	7.3
	13.1	2.6	6.0	45.7	43.3	28.8	60.1	77.3
	82.7	11.5	49.5	104.6	35.3	135.1	44.5	29.4
	69.5	100.6	14.7	11.9	478.6	23.2	8.6	88.1
	51.9	49.4	4.3	6.8	13.8	76.6	26.8	10.6
	31.1	20.9	35.0	9.4	95.7	31.4	74.5	8.1
	217.3	92.0	4.2	51.1	59.5	130.6	23.9	85.5
	15.8	71.1	3.9	65.7	11.5	6.3	37.8	51.1
	13.4	3.2	22.4	94.3	67.5	104.5	10.6\
RPS6KL1	140.3	112.7	161.8	126.3	76.1	170.9	77.1
146.1	142.1	49.0	105.7	205.0	165.3	19.3	35.1	73.1
	30.7	97.5	185.9	99.8	161.9	178.4	95.0
152.8	51.8	676.2	313.1	43.0	194.1	63.4	18.4
277.6	36.7	22.9	461.1	149.7	136.4	365.2	10.0
167.3	84.8	102.9	33.8	90.7	225.3	335.9	129.4
396.7	924.6	132.9	152.0	125.5	262.2	304.5	168.4
220.2	31.3	228.4	187.4	127.7	592.3	116.8	29.8	61.8
	30.4	217.3	88.7	19.0	190.8	171.1	58.4
433.7	85.3	201.0	23.9	169.5	14.8	355.1	37.6	46.3
	171.5	48.7	125.2	39.6	793.3	110.4	134.7
174.8	51.2	66.3	32.2	172.0	320.1	25.7	101.9	40.4
	192.1	107.3	1248.4	64.4	131.9	165.3	97.9\
EXOC8	1958.8	1082.1	1296.7	1769.3	2122.9	1248.5	1895.5
925.5	999.3	1258.3	1319.9	1035.1	2294.0	1463.4	941.1
1406.2	1678.0	1050.7	1438.6	950.9	463.1	1048.3	1331.8
1428.1	1166.2	840.7	1336.4	1143.1	919.4	1174.0	1768.1
927.1	702.7	1291.9	618.1	1472.8	1255.1	829.1	1699.1
1502.8	1257.4	798.6	1096.2	1028.7	1129.5	1074.3	895.0
1319.6	540.1	1377.4	976.5	1291.6	1335.3	879.8	692.7
1267.7	1460.8	1438.2	1118.3	558.9	352.6	1842.8	1939.4
1228.2	1333.8	1206.7	2199.4	1169.8	846.1	1368.5	520.5
714.3	1000.0	1641.8	1055.2	1223.8	510.3	1305.6	2018.6
812.7	505.5	578.1	1697.0	1849.8	487.5	1024.0	427.3
455.9	1210.8	1344.5	2071.4	422.0	1063.9	1271.9	965.5
1310.0	1318.2	1424.4	955.5	1018.8	1403.0	1018.4	1327.5\
```

EXOC7	339.23999999999995	1751.28 770.24	625.1200000000001
	2282.54 612.42	527.7	2265.1400000000003	515.2
653.4799999999999	485.26000000000005	826.78	482.14
517.36	958.6800000000001	888.0799999999999	528.7
936.0200000000001	700.64	400.96	1914.58 579.42	1031.4
611.54	507.41999999999996	1120.6200000000001	463.38
979.1	1013.1	430.53999999999996	489.9	1055.24 1519.52
532.2	997.3399999999999	897.6200000000001	386.3
926.82	713.32	473.7799999999999	445.8	684.62	566.24
614.54	567.46	925.0799999999999	837.0600000000001
409.2	2214.2400000000002	1024.9599999999998	498.82
496.96000000000004	659.24	1342.8	777.9200000000001
1221.94 878.22	726.72	943.1200000000001	1429.66 647.0
905.24	715.4	758.1	917.4799999999999	1146.3799999999999
	674.58	1152.94 1469.06 777.28	1550.54 1246.56 825.18
461.9799999999999	535.8	571.6	1362.3200000000002
723.2	618.4	731.6600000000001	986.5599999999998
1140.24 1050.6599999999999	1159.6799999999998
810.5600000000001	1601.1	1361.1799999999998	669.12
609.62	575.5	299.41999999999996	1164.3799999999999
933.5800000000002	996.18	1417.74 980.9	1273.94 1031.38
1496.54 1234.66 1062.06 906.18	851.1600000000001\
PLLP	123.9	1707.1	1594.3	432.9	633.7	2947.6	414.0
1494.9	1057.5	2391.4	1449.2	2157.8	606.6	1765.9	465.2
359.2	1739.5	2129.7	1223.2	1624.4	476.7	2538.7	2654.1
1735.1	2866.1	584.1	1101.0	884.3	2122.6	2035.2	460.1
2181.1	884.5	1161.7	3627.5	7799.7	2070.3	1408.5	419.5
804.2	442.6	2146.9	668.0	505.7	1537.9	1142.4	1592.4
242.7	544.5	891.1	2317.7	1865.7	1945.3	834.3	2021.9
3235.6	2764.4	2485.0	3254.5	448.5	1472.8	4475.2	1723.5
1094.4	565.2	1056.1	884.2	401.9	1803.9	3291.8	777.7
595.4	1429.5	1203.0	985.8	1716.4	1949.5	1352.6	1773.3
126.6	1397.6	654.7	815.3	2425.9	725.1	830.2	1490.8
1976.1	3328.1	1264.3	1340.3	775.4	1950.9	1477.1	626.3
2135.1	898.8	1721.1	2083.9	1853.3	1493.9	5203.6	1658.3\
AC079741.2	77.5	233.1	111.3	5.6	2.3	59.9	75.9
	38.1	67.2	126.8	66.1	100.4	21.6	21.6
118.3	38.4	114.0	7.3	44.7	20.5	119.2	58.3	72.0
	30.7	9.4	86.1	25.2	166.2	121.8	67.4	8.7
	98.8	95.5	72.0	66.7	118.7	23.8	145.2	32.0
	43.2	70.5	53.4	136.4	29.0	58.9	79.2	95.7
	9.8	116.5	102.6	12.8	58.4	47.3	39.3	83.0
	93.9	82.4	82.3	70.9	64.1	298.3	30.4	69.1
	99.0	144.5	81.9	55.3	45.2	107.3	53.6
180.0	141.1	76.5	23.3	124.7	64.2	75.8	29.0	18.2
	53.7	48.1	72.4	53.8	68.5	64.2	67.3
106.2	140.7	82.9	11.2	73.3	123.2	95.2	7.0
100.4	34.6	103.3	91.5	56.2	34.0	111.8	99.4
65.1\
EXOC4	484.0333333333333	612.4	483.5666666666666
424.5666666666666	536.3333333333334	913.8000000000001
	735.3333333333334	325.8666666666666
576.2666666666667	731.2666666666668	805.2666666666668
	565.6	699.5666666666666	703.3333333333334

736.6999999999999	878.6666666666666	545.4
569.8333333333334	463.09999999999997	546.2666666666667
	181.9333333333333	761.4666666666666
444.76666666666665	561.1333333333333	469.6666666666667
	470.7	616.9666666666667	406.8666666666666
461.2	629.4666666666667	1192.3999999999999
336.23333333333335	486.3666666666666	974.5	753.9
785.9666666666666	738.2999999999998	507.0	758.0
878.1666666666666	665.4666666666667	534.8333333333334
	644.2333333333333	747.6333333333333	855.6
659.9	456.5333333333333	698.5	297.1	1346.2
765.7666666666668	687.6666666666666	851.4333333333334
	194.70000000000002	542.0666666666667	584.0
695.6999999999999	382.3	652.8666666666667
588.8666666666667	427.1333333333334	551.9666666666667
	759.4666666666667	790.8666666666668
555.9666666666666	590.0666666666667	758.3666666666667
	662.1666666666666	619.5333333333334	530.2
514.6999999999999	566.6333333333333	504.1666666666667
	828.7000000000002	884.0333333333333
739.6333333333333	496.76666666666665	815.7666666666668
	733.3000000000001	457.96666666666664
324.23333333333335	423.36666666666673	1502.4666666666665
	974.3333333333334	247.4333333333333
170.83333333333334	462.1333333333334	263.53333333333336
	372.73333333333335	254.70000000000002	1071.2
336.9666666666667	701.9333333333334	569.2
411.09999999999997	846.0333333333334	724.4
740.1999999999999	631.5	807.8000000000001	759.0
695.6	938.6666666666666\
PLIN4	17.4	48.0	10.3	66.3	54.4	7.4	6.1	21.5
	17.9	97.1	7.4	7.2	31.8	10.2	17.1	13.1
	9.1	45.0	47.0	21.5	22.5	71.6	124.4	31.3
	9.7	7.2	6.4	17.1	18.8	18.5	66.6	16.1
	29.9	14.0	29.3	12.5	8.9	28.3	41.1	31.1
	7.1	21.1	7.1	10.3	62.3	61.8	16.1	16.3
	53.2	60.6	13.2	31.4	6.6	27.4	9.2	13.9
	10.4	83.6	14.6	202.5	200.2	67.4	22.9	6.8
	11.0	49.6	41.1	18.3	47.6	11.9	24.4	21.9
	11.5	9.0	14.5	9.0	126.7	17.2	8.9	29.8
	32.4	43.8	6.5	11.8	63.0	133.8	20.4	26.9
	213.4	38.5	19.7	19.4	18.3	30.2	41.8	14.7
	10.2	17.4	11.6	16.0	12.6	45.7	94.8\
EXOC3	735.3	1225.65	777.6500000000001	407.45	698.55
666.85	702.05	672.15	478.40000000000003	488.75	775.55
1044.3	443.75	726.15	9823.85	975.6999999999999	832.2
638.65	588.0	586.4	1137.15	633.1500000000001	471.65
554.5	310.8	722.7	670.15	624.45	651.6	712.5	916.4
673.5	1121.15	607.2	878.1999999999999	777.3000000000001
	502.75	824.1500000000001	764.55	534.25	653.3
1096.6	370.0	627.5	630.0	517.85	589.0	821.7	1012.0
816.8	491.8	658.1	615.55	607.55	1062.75	1777.8	708.45
609.85	919.95	697.6500000000001	213.20000000000002
563.75	584.45	817.4000000000001	624.3	682.7	978.2

714.2 646.25 577.55 964.25 1008.1500000000001 605.0
618.8 797.75 721.25 820.45 663.9 782.95 357.0
669.3499999999999 610.45 737.4 901.4000000000001
859.95 1346.75 528.8 903.4000000000001 474.5 397.4
632.65 953.55 1136.65 1047.95 985.6 1040.5 1226.6999999999998
929.8 1387.35 1331.9 1012.6500000000001 913.05
1075.9\
KB-1568E2.1 5.3 13.2 6.3 26.9 8.2 1.9 16.9
1.6 25.5 40.3 11.9 29.1 4.7 3.8 83.9
32.8 8.7 24.6 23.8 8.6 10.3 7.3 91.8
24.1 37.7 50.4 36.9 87.8 5.4 9.7 2.4
70.3 3.6 30.3 10.1 68.6 2.1 95.3 32.8
26.9 25.9 13.0 43.0 6.9 2.2 60.8 28.8
18.4 30.4 56.5 13.8 5.4 1.5 82.1 2.6
42.9 4.5 59.7 11.7 3.8 163.0 10.0 52.0
19.6 11.4 4.8 2.3 16.9 35.0 8.2 22.8
28.7 29.2 8.6 26.9 13.7 51.0 39.9 4.5
16.7 48.1 40.6 1.7 5.2 32.6 143.1 42.2
5.0 13.0 13.8 1.9 15.0 21.9 29.8 21.9
2.8 18.4 9.6 66.5 3.7 46.1 17.7 1.8\
PLIN3 709.4 423.9 443.5 1068.3 866.7 1367.4 1218.8
2357.0 1460.3 4238.8 573.1 513.6 1032.8 1413.6 240.8
464.0 1094.6 431.1 1286.0 913.0 473.4 1736.8 1207.4
2015.3 1103.4 634.3 1184.3 492.4 849.4 502.3 576.1
676.0 637.7 1052.1 1057.7 654.4 1152.6 419.8 1113.1
895.1 285.4 801.5 826.5 670.7 1265.8 1123.6 2810.8
472.9 365.4 661.0 826.9 1179.0 604.9 1299.9 1078.8
696.1 838.1 663.7 1263.3 697.9 143.3 939.5 1630.3
620.7 549.0 1339.8 1033.4 420.4 934.5 636.2 766.7
782.1 278.1 836.6 1021.0 1302.8 1444.2 1309.7 1414.9
1590.6 639.6 2059.5 488.2 345.5 1891.5 516.5 1684.3
1266.3 2494.7 1105.9 558.2 866.1 680.9 417.5 232.6
614.1 408.8 868.7 549.1 318.9 549.3 370.2 497.0\
EXOC6 451.63333333333327 370.1666666666667
366.9666666666667 501.7666666666667 229.53333333333333
728.4333333333334 447.8999999999999
216.89999999999998 372.90000000000003 204.5
631.7333333333332 420.03333333333336 693.5333333333333
282.9666666666664 315.5 290.0333333333333
469.43333333333334 386.93333333333334 454.7
259.8666666666667 254.4333333333333 543.1666666666666
268.8666666666667 513.7 511.1333333333334
331.40000000000003 530.8333333333334 397.29999999999995
202.53333333333333 572.2 415.0 190.43333333333337
223.63333333333333 446.29999999999995
190.79999999999998 519.3333333333334 1183.3666666666668
187.4666666666667 407.43333333333334
390.5333333333333 651.7333333333333 377.43333333333334
306.73333333333335 418.0333333333333
408.5333333333333 374.3333333333333 351.23333333333335
1913.2666666666667 129.53333333333333
280.59999999999997 281.93333333333334 427.79999999999995
569.7666666666668 43.26666666666667
387.0333333333333 213.93333333333337 128.83333333333334

273.2333333333333 339.6666666666667
322.2333333333333 134.46666666666667 267.43333333333334
460.5 551.3000000000001 311.5 197.16666666666666
378.63333333333327 452.73333333333335
263.40000000000003 346.79999999999995 240.93333333333337
165.29999999999998 547.4 456.43333333333334
293.3333333333333 362.0 310.53333333333336
490.6666666666667 596.2666666666667 315.06666666666666
76.16666666666667 350.5333333333333
288.96666666666664 553.0 174.5 50.5 263.5333333333333
195.23333333333335 188.79999999999998
201.63333333333333 259.5 168.03333333333333
322.1333333333333 338.7333333333333 266.76666666666665
300.5666666666666 216.79999999999998
333.93333333333334 171.79999999999998 228.06666666666663
290.43333333333334 249.2666666666668
344.6666666666667\
PLIN2 1572.4 423.15000000000003 244.95 591.35 463.65
3440.6 1247.3 1499.2 1400.6499999999999 916.3 292.6
224.4 381.7 2527.1 2333.95 386.9 812.35 218.45000000000002
728.75 1700.95 837.75 883.95 527.1 955.75
330.34999999999997 215.1 1910.15 487.65000000000003
397.15 2224.7 1767.35 708.7 529.75 1363.1 1314.8000000000002
6589.75 1114.45 3333.2000000000003 1626.4 1515.15
2693.0 670.75 659.5999999999999 3950.85 798.1 326.35
2910.65 2889.3 379.95 1361.8 742.85 1499.8 4126.6 1481.95
249.6 202.3 223.35 1421.95 703.9000000000001 406.75
894.8499999999999 3350.5 248.2 330.75 2731.1499999999996
987.75 1606.1000000000001 4312.9 367.0 180.4
222.5 318.84999999999997 3314.0 1110.3 226.35000000000002
359.85 453.35 520.25 4386.85 5359.200000000001
513.75 576.85 1099.3999999999999 1058.65 278.95
5512.849999999999 4241.95 643.3 1191.6000000000001
474.4 2252.75 801.3000000000001 663.2 696.95 1217.55
729.3000000000001 1346.2 686.8 494.05 852.3 854.5
2530.3999999999996 899.1\
EXOC5 1166.58 357.65999999999997 429.08000000000004
817.74 320.48 749.52 772.4399999999999 440.7200000000001
557.5 450.06000000000006 674.76 579.1199999999999
838.8399999999999 633.64 378.32 459.03999999999996
789.78 459.2 535.1 526.1400000000001 136.1
517.9 243.84 623.58 857.6 697.44 684.1000000000001
453.02 483.84000000000003 588.3199999999999 641.3
609.0600000000001 337.56 646.22 454.38 481.9599999999999
786.14 377.28 529.7 1313.6200000000001 343.8
817.6200000000001 654.72 671.96 584.18 357.28000000000003
551.34 812.94 251.32 333.46 469.14 612.94 369.66
291.6 329.4 401.34000000000003 466.91999999999996
460.8 474.91999999999996 445.3 457.4 405.5 505.68
575.6 404.21999999999997 300.28000000000003 638.58
529.6800000000001 350.53999999999996 523.88 332.72
408.86 477.64000000000004 591.3199999999999 481.3
664.44 476.06000000000006 556.3599999999999 567.38
785.72 445.28000000000003 766.8399999999999

462.9599999999999	298.4	267.28000000000003
302.08000000000004	550.08	444.14	317.47999999999996
405.32	679.02	330.82	497.23999999999995	531.12	341.44
380.5	358.5	470.28000000000003	419.41999999999996
436.84	367.2	394.78000000000003	414.14\
PLIN1	28.4	68.8	26.8	9.4	38.0	13.4	10.1
411.5	17.6	15.7	14.1	7.5	13.4	21.7	27.7	20.0
	20.0	15.2	11.3	17.2	139.2	27.2	67.0	13.1
	27.5	42.2	6.2	60.0	42.9	29.0	9.2
756.9	26.1	15.3	27.0	21.8	24.6	27.3	18.6	14.6
	13.0	16.3	20.4	23.7	18.1	18.7	34.2	7.8
	414.9	17.9	27.0	35.9	28.7	133.3	29.4
143.6	14.5	16.7	8.7	42.9	88.0	17.4	23.1	29.9
	35.4	26.8	14.1	14.3	22.2	46.8	70.1	39.7
	22.5	12.7	10.1	12.4	25.8	12.6	54.0	30.2
	103.5	17.6	21.9	9.7	94.5	99.5	54.7	46.1
	28.0	41.6	7.5	292.2	17.9	27.4	101.6	26.4
	51.3	24.4	50.2	24.3	57.4	43.8	23.1\
CTC-471J1.2	166.6	398.8	340.4	142.3	55.7	77.7	68.4
	192.1	74.9	56.8	102.8	148.8	128.4	372.4
234.2	165.4	219.3	95.5	100.1	169.2	458.8	105.0
184.5	223.3	127.7	451.4	79.0	292.8	272.6	102.3
158.9	401.4	106.7	152.8	60.4	202.8	78.2	237.1
147.3	105.5	55.0	112.4	245.8	102.4	65.2	140.5
248.0	31.3	326.2	285.8	198.7	73.7	142.1	154.0
127.7	161.3	245.3	298.8	185.3	228.3	1041.6	146.0
212.3	180.2	384.7	352.3	77.7	135.0	204.3	164.3
293.5	426.0	266.8	230.3	177.3	121.6	159.5	159.0
163.0	59.4	226.4	141.8	137.3	125.5	303.7	130.9
119.2	199.7	138.2	62.7	61.5	343.8	239.1	328.8
502.3	532.4	490.8	452.9	418.0	770.8	533.8	295.8
266.8\
RCCD1	1872.9	1327.05	805.5	661.75	837.5500000000001
1451.5	296.0	854.95	871.85	898.95	1147.7	850.5	1022.6
798.6999999999999	931.4	841.7	280.05	522.4	738.5
808.3	707.9	1189.3	771.6	776.8499999999999	1447.0
2069.35	1220.8	698.1500000000001	809.25	661.8
707.0500000000001	1329.6499999999999	1353.2	824.15
707.0999999999999	873.5	805.0	1671.7	902.8	394.55
1164.55	1182.75	640.3	600.6	1022.35	1322.15	980.2	712.45
1441.85	1152.8500000000001	856.2	1097.2	1261.1000000000001
	1415.9499999999998	1169.0	2400.2	888.5	1579.0
1058.6499999999999	1048.7	1253.2	1268.3500000000001
411.34999999999997	560.6	521.4499999999999
1051.5500000000002	702.5	732.6	1036.8999999999999
1240.2	659.25	921.55	465.7	643.0500000000001	697.2
802.45	934.8	789.9	786.15	1482.65	688.1	809.1	1842.65
2089.6	1322.3	1515.65	1921.55	889.7	1552.2	1257.1499999999999
	186.04999999999998	1538.05	1257.75	761.45
962.5999999999999	946.6999999999999	1098.8000000000002
	545.15	936.75	1195.55	1215.7	1295.85	892.9\
PRO2012	29.7	11.6	48.4	39.7	9.3	16.7	4.1	3.0
	47.4	12.9	2.2	4.0	46.7	5.6	22.4	69.3
	34.5	3.4	33.6	45.1	14.9	58.3	72.5	4.6

```
	8.6	53.8	2.9	10.7	68.1	70.0	32.3	52.5
	36.1	5.5	14.4	96.8	26.6	86.9	44.6	52.3
	24.1	1.8	33.8	39.2	4.1	19.0	100.4	80.7
	12.1	12.4	14.2	76.9	25.6	11.3	7.3	6.5
	53.5	27.6	4.3	21.9	36.0	41.4	39.6	5.5
	38.5	16.3	34.1	5.2	5.8	39.0	3.9	64.0
	2.2	40.6	22.7	30.8	47.5	60.7	29.8	5.4
	54.4	3.9	60.2	7.6	2.6	22.0	52.3	6.2
	31.5	17.4	21.8	16.4	1.4	2.1	20.2	65.1
	27.6	33.9	4.2	5.6	7.6	10.6	10.0\
MOCS1	86.10000000000001	55.03333333333333
153.96666666666667	128.33333333333334	200.29999999999998
	107.06666666666666	164.2	166.8	306.73333333333335
	163.46666666666667	135.53333333333333
346.23333333333335	241.66666666666666	122.73333333333333
	156.33333333333334	140.03333333333333	151.5
200.46666666666667	137.76666666666668	185.29999999999998
	277.2333333333333	203.33333333333337
96.10000000000001	172.36666666666667	217.66666666666666
	181.79999999999998	163.86666666666667
125.89999999999999	277.99999999999994	239.0666666666667
	350.56666666666666	124.80000000000001
267.73333333333335	282.8	305.1666666666667
280.8333333333333	242.96666666666667	344.73333333333335
	173.76666666666665	206.63333333333335
207.63333333333333	241.1666666666667	172.36666666666667
	174.36666666666667	239.70000000000002
139.13333333333335	105.2	202.26666666666665
311.43333333333334	480.7	256.43333333333334
235.5666666666667	119.63333333333333	58.86666666666667
	149.26666666666668	243.33333333333334
198.9333333333333	146.43333333333334	150.29999999999998
	223.4	223.03333333333333	80.63333333333334
162.03333333333333	96.93333333333334	166.4
257.3666666666667	386.33333333333326	180.46666666666667
	392.7666666666667	82.0	179.23333333333332
311.46666666666664	193.83333333333334	356.0
122.16666666666664	210.20000000000002	161.13333333333333
	212.63333333333333	203.5	99.46666666666665
160.39999999999998	103.86666666666667	176.0
127.06666666666668	77.1666666666667	474.8
423.8333333333333	390.1333333333334	375.23333333333335
	321.6666666666667	113.80000000000001
150.73333333333332	126.23333333333335	151.16666666666666
	173.66666666666666	156.83333333333334
238.73333333333332	126.5	180.5666666666667
149.43333333333334	219.1	145.76666666666668
148.36666666666665\
EXOC2	999.8	602.05	326.85	328.20000000000005	257.45
1008.1500000000001	867.3000000000001	420.1	566.2
269.85	323.05	583.65	372.65	413.15	483.45000000000005
533.3	706.9000000000001	407.45	451.09999999999997
514.25	212.55	441.65	461.65	497.90000000000003	535.3
395.4	588.3	488.65	697.05	634.05	595.45	399.15	256.9
```

539.65	931.65	439.15000000000003	403.6	387.75	574.4
1915.4	831.6500000000001	744.8	525.85	559.65	522.6
347.15	310.0	615.55	452.75	531.95	368.65	393.8	527.45
479.65000000000003	327.20000000000005	253.95000000000002
	355.3	420.84999999999997	567.4	216.5	170.85
356.65	402.8	483.8	467.45	266.25	810.45	430.65	273.45
372.55	371.9	582.35	450.6	805.3	497.5	579.1	334.4
854.3	344.2	1291.1000000000001	274.0	325.95	306.15
408.7	285.65	366.20000000000005	368.05	235.1
389.09999999999997	368.65	463.1	340.95	348.25	408.25
265.95000000000005	257.3	318.5	379.5	419.45	266.35
404.25	289.5	316.75\
PLIN5	57.36666666666667	155.55	152.08333333333331
90.11666666666667	499.8666666666673	136.11666666666667
	38.45	322.0	87.93333333333334	296.9
131.16666666666666	96.30000000000001	91.65000000000002
	125.23333333333333	189.43333333333333	173.5
95.83333333333333	139.86666666666667	92.26666666666667
	107.91666666666667	364.53333333333333	125.25
340.70000000000005	101.89999999999999	100.98333333333333
	116.26666666666667	85.73333333333333
255.33333333333334	169.95	138.31666666666663
61.95000000000001	187.36666666666667	238.56666666666667
	111.11666666666667	297.6	241.46666666666667
90.41666666666667	494.03333333333336	104.10000000000001
	94.46666666666665	82.43333333333334
101.78333333333332	134.71666666666667	71.45
84.81666666666668	101.78333333333335	282.166666666667
	69.1	246.01666666666668	201.53333333333333
176.53333333333333	159.51666666666668	99.51666666666667
	293.55	157.1	331.59999999999997	117.3
278.98333333333335	211.66666666666663	195.36666666666667
	377.65000000000003	293.0	120.86666666666667
143.0	225.76666666666665	345.4166666666667
93.06666666666666	106.2	205.03333333333333	152.95
387.76666666666665	123.76666666666667	124.7
105.06666666666666	110.33333333333333	133.98333333333332
	194.29999999999998	233.9	124.05000000000001
178.20000000000002	199.76666666666668	154.15
112.21666666666665	102.18333333333332	401.0
447.2333333333333	207.30000000000004	133.29999999999998
	232.38333333333335	279.53333333333336
66.18333333333332	232.03333333333333	212.73333333333332
	260.7833333333333	329.8666666666667
246.76666666666665	204.8166666666667	459.7833333333333
	357.0666666666666	229.31666666666663
236.63333333333335	186.1833333333333	174.58333333333334\
EXOC1	3704.3	2018.0	3735.5	3226.7	2455.3	2409.1	2644.6
2486.7	2318.5	2153.9	2958.8	2499.8	2412.5	3333.4	4446.9
3231.8	1538.5	2884.2	3826.4	3182.9	3102.9	3073.1	3852.9
2428.0	2011.7	4835.9	4001.0	3172.4	4858.8	3183.9	3999.4
2768.0	5630.0	2634.5	3487.8	4269.3	1979.7	4067.4	3485.2
2486.9	1470.7	1550.4	3934.9	2502.0	2098.3	2768.9	1964.5
1753.0	5658.2	5323.0	3410.6	4723.1	3573.2	2181.3	3653.4

```
3007.6  3084.3  5424.9  4457.4  3604.2  2119.9  4056.6  3662.9
3296.7  4136.0  2774.2  2569.8  3657.9  1393.0  3652.1  4661.7
3608.9  4297.6  3547.2  2659.3  3020.7  3294.8  3291.1  3519.0
2746.1  3654.9  1661.6  5620.9  3964.3  6338.0  5895.7  2336.3
1597.3  2045.5  4631.0  2683.1  3802.5  4056.3  4701.9  4872.3
5074.1  5252.9  4315.3  6092.5  6521.1  5904.3  5755.8  4601.9\
NFIX    110.55000000000001      89.30000000000001
48.650000000000006      87.25   112.39999999999999      210.9
30.799999999999997      164.20000000000002      189.175 309.85
132.375 385.79999999999995      25.800000000000004
105.69999999999999      130.42499999999998      295.775 213.45
101.25  208.27499999999998      40.4    118.70000000000002
204.95  192.1   165.075 89.25   358.025 61.474999999999994
349.25  76.35   99.25   258.05  97.19999999999999       138.05
254.59999999999997      56.3    327.25  242.5   12.35   319.35
104.525 49.825  101.275 100.625 135.625 163.45  253.7   466.85
55.825  270.75  589.0   303.7   47.75   503.425 235.22500000000002
        302.825 491.875 598.75  73.1    38.875  516.875 264.9
175.47500000000002      147.7   111.425 194.5   164.525
621.8000000000001       156.075 277.525 109.0   101.575
83.44999999999999       78.85   100.4   84.95000000000002
72.35   106.575 112.15  136.775 158.125 283.4   149.65
185.02499999999998      153.075 77.375  34.75   33.85   228.3
67.175  16.299999999999997      427.65  113.47500000000001
59.775  214.125 243.5   67.9    91.175  151.0   100.075 87.25
102.475 113.675 207.125\
C1orf198        1723.7  711.0   1228.2  1407.1  1207.2  3022.8
3422.4  1411.4  1621.6  1879.7  1091.3  895.9   1521.9  1338.6
1632.5  1845.1  1359.4  1044.4  1446.6  1992.4  1018.5  961.4
1114.0  2027.8  1576.6  857.7   1457.9  1632.4  1418.6  1356.4
2439.3  1138.8  598.8   2102.6  1424.1  664.9   1665.8  500.2
2169.8  2712.3  1837.9  2424.2  1711.0  1522.3  1619.9  1373.5
2242.3  1588.5  915.1   2226.9  1126.4  1973.2  1837.8  2178.2
1256.1  1758.9  935.7   997.1   767.5   1284.8  511.2   840.0
1280.0  1477.0  1551.6  1780.9  3121.3  1316.9  1495.3  1408.4
1125.7  758.0   943.4   2210.0  2607.4  2411.8  933.7   2174.6
2437.6  1273.0  1901.9  1369.7  1744.1  1009.1  981.9   795.5
1474.0  1304.0  995.8   1002.7  3703.7  985.2   615.7   1428.8
816.7   729.1   765.0   957.5   846.9   757.7   745.6   699.3
809.8\
DCAF12L2        22.4    62.9    72.1    31.1    71.8    49.0    11.6
        16.3    66.4    24.7    32.3    79.2    42.2    8.9
206.6   138.1   44.5    65.4    99.9    85.6    80.1    26.6    7.7
        71.7    6.1     67.3    26.1    102.6   8.6     13.2
122.7   221.9   50.1    60.3    24.8    83.2    60.2    37.6
107.5   95.9    26.0    34.0    76.9    65.0    52.0    62.1    61.9
        47.3    42.7    75.0    30.7    27.9    45.0    103.7   47.5
        9.4     134.4   30.0    30.4    22.0    232.4   7.1
122.3   12.7    23.2    27.7    18.7    6.8     5.6     3.5     65.7
        74.6    5.8     2.0     84.2    3.3     3.8     15.2    97.0
        1450.0  309.2   57.1    47.6    18.2    23.4    58.7    61.3
        62.0    49.5    10.6    9.8     136.7   45.9    69.2    28.0
        53.1    13.0    55.5    37.7    49.1    32.0    50.2
104.4\
```

PRUNE2	158.23333333333335	69.2	1148.0	72.36666666666667
	197.5666666666667	100.03333333333335
46.03333333333333	51.833333333333336	88.46666666666665
	155.7	82.9	159.5	210.6	108.06666666666666
618.0333333333334	92.96666666666665	85.53333333333332
	225.4333333333333	1322.8999999999999
60.366666666666674	138.16666666666666	489.03333333333336
	255.4333333333333	207.53333333333333
147.36666666666667	555.2333333333333	424.20000000000005
	120.16666666666667	200.0	103.50000000000001
246.86666666666667	90.0	122.96666666666665
62.56666666666666	30.86666666666667	136.7
77.10000000000001	133.29999999999998	609.5333333333334
	75.3	69.83333333333333	91.3	226.4333333333333
	52.73333333333333	68.23333333333333
86.53333333333335	97.19999999999999	55.666666666666664
	391.8333333333333	106.10000000000001
133.66666666666666	778.0333333333333	938.0333333333334
	140.4	103.5	78.66666666666667	55.099999999999994
	1042.1000000000001	243.46666666666667
67.16666666666667	256.73333333333335	730.6333333333333
	145.73333333333332	241.96666666666667
107.46666666666665	136.76666666666668	105.10000000000001
	135.3	86.83333333333333	306.36666666666673
160.46666666666667	121.76666666666665	88.63333333333334
	90.10000000000001	81.0	307.2333333333333
200.39999999999998	94.83333333333333	272.1666666666667
	347.4666666666667	435.23333333333335
162.23333333333335	1159.3	111.53333333333335
130.03333333333333	415.06666666666666	582.3000000000001
	398.5666666666666	34.166666666666664
360.3333333333333	95.73333333333333	116.83333333333333
	335.7	202.79999999999998	324.53333333333336
399.23333333333335	599.0	597.9	193.03333333333333
330.866666666667	352.1333333333334	288.46666666666664
	500.7333333333333\
DCAF12L1		36.3	104.5	52.3	82.8	15.3	6.6
169.9	71.7	95.9	35.2	114.6	65.2	55.3	36.1
424.2	32.1	56.9	112.5	72.5	9.3	20.2	839.4
115.1	13.5	56.2	109.1	108.1	47.3	72.0	12.4
652.5	13.7	37.2	678.8	86.5	138.2	97.1	15.5	36.4
	166.6	66.6	67.8	8.7	88.9	117.4	139.0	47.6
	79.1	143.9	20.6	93.0	2450.9	266.1	447.5	56.1
	152.5	81.8	32.7	32.6	87.7	345.5	240.0	74.8
	1281.8	3.9	386.1	258.6	11.9	45.3	5.7
164.5	91.4	59.6	69.4	3.1	71.5	2.6	70.6
158.6	13518.1	878.0	824.1	197.9	347.7	22.9	215.0	10.7
	413.9	538.6	80.6	906.8	224.0	440.4	585.3
128.4	946.9	515.2	582.3	399.5	512.6	329.5	219.9
982.5\
C1orf194		43.2	148.9	1605.6	101.5	18.9	153.9	5.4
	7.1	578.8	2324.7	728.0	125.0	4.8	128.8	21.5
	5.5	108.6	2092.3	5.3	179.2	28.0	2888.3
2701.4	295.8	551.5	38.5	14.4	1691.8	719.1	85.9	74.6

1796.1 948.8 1203.7 195.8 717.1 168.7 37.2 63.2
532.6 13.8 27.1 1964.5 65.4 1514.0 2595.6 24.3
21.0 96.4 814.8 1357.3 500.7 11.3 42.1
1070.5 1816.6 1422.8 476.7 1246.1 2009.8 2986.3 1434.0
258.1 1399.7 123.5 278.6 845.5 17.0 2812.0 1171.3
2456.9 1632.4 131.3 313.7 1254.9 801.6 1061.0 194.2
534.2 52.1 323.3 49.3 736.9 25.2 27.8 29.4 26.4
84.4 52.7 20.8 171.6 222.7 228.2 192.4
151.7 393.3 406.0 466.3 213.0 320.3 149.0 108.1
89.3\
C1orf192 8.5 25.5 2104.7 488.1 167.0 244.9 26.1
68.6 1064.3 4395.2 2935.9 17.1 66.3 393.4
391.3 22.8 72.0 2948.1 308.8 126.8 38.1 4731.3
1749.7 798.5 1618.8 29.6 41.6 2223.6 1426.0 144.0
169.8 426.5 933.4 2298.7 57.9 803.1 323.1 50.3 10.6
1043.9 177.2 49.8 1821.6 112.5 4174.8 2989.7
334.5 64.5 161.7 879.1 1713.8 4190.6 4.2 23.0
1613.1 2703.3 1807.1 596.7 2097.5 963.6 1770.3 2679.5
884.5 3808.5 151.0 759.1 1762.4 34.5 1920.3 4452.6
903.2 1135.2 104.9 499.8 1648.3 4292.1 2324.6 839.7
1634.9 6.2 254.6 283.8 2621.9 283.4 80.8 32.7
119.8 194.0 26.0 45.9 706.6 28.2 261.3 358.1 20.8
780.3 30.3 1118.1 149.5 547.1 107.6 19.3
23.4\
METTL21A 736.8000000000001 260.8333333333333
263.5666666666666 649.5000000000001 430.0
667.8333333333334 638.6 721.7999999999998
499.3333333333333 381.8 528.9666666666667
512.2333333333333 529.9666666666666 437.0666666666668
415.23333333333335 331.73333333333335
349.0666666666666 350.8 400.23333333333335
332.8333333333333 209.69999999999996 494.5
181.4333333333333 596.6333333333333 830.1333333333333
288.2666666666665 683.5333333333333
245.9666666666667 384.2666666666665 292.7666666666665
702.4 194.5666666666667 209.79999999999998
446.8 335.79999999999995 249.63333333333333
591.3333333333334 310.03333333333336 292.7333333333333
492.6333333333334 425.8 507.5 409.6666666666667
337.93333333333334 596.8666666666667
506.90000000000003 447.1666666666667 815.1333333333332
306.03333333333336 303.1666666666667
396.43333333333334 458.2 607.9 430.8999999999999
407.4666666666667 215.13333333333333 367.7
228.29999999999998 334.2 319.43333333333334
312.63333333333327 381.43333333333334 342.23333333333335
422.79999999999995 225.13333333333333
390.43333333333334 373.90000000000003 502.0333333333333
251.0333333333333 341.3333333333333 264.8
373.6333333333334 216.19999999999996 440.9666666666667
336.2333333333333 388.1333333333334 374.5
601.6333333333333 382.5333333333333 1308.6
617.0333333333333 680.2666666666667 271.1333333333333
555.5333333333334 317.3666666666667

242.63333333333333	703.9	328.0666666666666
202.4333333333333	137.79999999999998	780.4333333333334
	440.6666666666667	389.23333333333335
455.23333333333335	432.6333333333334	478.2666666666665
	401.5333333333333	560.9333333333333	193.6
305.40000000000003	400.5	299.73333333333335
386.5999999999997\
METTL21B	250.2	350.0	306.0	157.8	295.6	586.9
253.2	534.2	448.7	314.6	359.1	148.1	276.9	427.4
221.4	421.5	279.1	69.0	131.8	283.1	149.6	292.4
224.3	440.8	309.5	342.1	281.1	244.8	330.1	311.4
854.1	437.5	218.0	397.3	296.2	331.7	476.6	207.1
278.5	396.5	311.2	356.2	387.2	300.5	356.2	378.8
762.8	279.1	218.7	489.8	407.2	367.8	305.4	324.5
302.2	262.6	247.0	223.5	248.8	246.8	568.1	125.8
289.1	255.7	293.1	281.4	441.6	299.9	230.2	329.1
180.8	189.0	277.8	274.8	334.9	510.6	440.8	608.1
363.4	302.7	392.0	347.2	480.1	304.2	157.2	246.3
379.1	178.6	472.9	210.4	190.4	641.1	458.2	580.1
467.3	590.9	449.5	483.3	314.5	433.4	520.4	356.7
631.3\
SLC6A10P /// SLC6A8	81.8	7.4	12.3	8.0	48.3	47.3
	9.2	21.8	8.7	27.9	125.0	8.0	7.4	30.2
	7.1	20.8	5.1	19.2	38.9	32.0	15.8	25.4
	36.6	15.9	20.0	26.9	13.3	88.8	29.1	3.7
	22.8	57.8	24.8	15.6	18.5	36.8	32.5	6.1
	32.6	77.1	7.1	24.5	69.6	6.8	11.2
102.6	72.1	7.7	129.0	15.7	23.5	15.3	7.3	48.8
	7.8	53.6	21.1	72.1	22.4	92.5	306.9	15.9
	12.1	12.9	13.7	22.4	22.8	4.5	199.4	55.1
	52.8	25.2	4.3	91.4	79.1	10.8	27.9	31.4
	12.8	14.8	6.4	19.7	37.5	38.5	53.0
111.7	31.9	22.0	10.8	62.7	5.2	26.4	28.3	5.1
	3.4	18.8	4.8	17.1	5.3	10.0	25.6	94.2
	4.2\
EPPIN /// EPPIN-WFDC6	57.3	46.7	81.69999999999999
18.95	73.15	34.7	17.95	77.4	99.35000000000001
81.25	65.2	36.1	29.349999999999998	26.25	124.85
58.55	53.75	63.75	79.5	63.1	575.15	131.54999999999998
	251.55	95.0	33.8	26.6	22.9	309.15000000000003
	44.65	126.45	43.95	304.3	49.45	184.5	33.25
93.75	60.2	81.9	124.1	84.14999999999999
31.299999999999997	43.6	189.25	133.3	61.449999999999996
	100.45	149.2	25.7	643.3	257.3	159.35	44.5	17.8
	71.80000000000001	100.7	160.05	193.55	127.4
139.95	526.35	191.9	132.2	117.5	250.8	386.8	110.7
192.7	290.3	178.45	176.20000000000002	245.20000000000002
	129.4	447.0	43.0	160.1	118.44999999999999	78.9
	135.9	134.15	18.25	76.65	24.85	236.64999999999998
	57.4	122.25	117.55	52.0	64.55	23.950000000000003
	16.0	4.5	502.85	94.3	74.8	451.15	202.15
350.2	495.0	77.0	524.65	168.95000000000002
730.4499999999999	235.9\
APOL5	5.8	9.8	9.1	4.4	10.5	7.7	6.0	6.4

7.4	9.7	8.2	8.4	4.8	7.3	10.3	5.3
6.4	5.7	8.5	7.6	12.8	5.6	12.3	5.5
6.8	7.3	8.1	16.4	13.0	5.6	11.5	27.2
6.8	6.0	9.0	12.7	5.3	11.8	4.6	11.0
3.6	11.5	11.3	5.2	7.2	7.3	10.2	5.4
12.4	14.7	5.6	7.4	6.3	12.5	6.6	13.1
5.8	7.7	8.3	5.9	32.4	9.6	5.0	4.9
7.3	17.1	5.8	4.3	6.3	8.6	12.3	8.4
7.0	7.7	7.2	7.1	7.8	5.9	5.6	4.6
11.7	6.0	3.6	12.3	9.1	15.0	35.9	10.0
9.6	22.3	2.8	9.6	6.8	6.9	6.1	6.3
22.9	4.4	6.8	9.6	8.6	8.5	6.9\
APOL4	45.9	47.70000000000001	187.46666666666667
103.7	232.96666666666667	153.6	324.76666666666665	72.2
265.16666666666667	327.96666666666664
224.63333333333333	49.6	240.26666666666665
212.53333333333333	74.43333333333334	106.83333333333333
217.16666666666666	145.43333333333334
43.53333333333333	159.6	178.29999999999998
126.06666666666668	602.3333333333334	202.66666666666666
289.7	90.66666666666667	84.26666666666667
116.26666666666667	201.36666666666665	188.66666666666666
70.26666666666667	132.66666666666666
73.73333333333333	67.36666666666666	110.53333333333335
514.5666666666667	246.0	160.36666666666667
347.23333333333335	114.10000000000001	142.53333333333333
131.26666666666668	191.06666666666667
120.26666666666667	257.23333333333335	115.23333333333335
135.36666666666667	95.26666666666665	125.5
175.20000000000002	117.60000000000001	157.0
92.39999999999999	102.83333333333333	169.4	74.1
290.7666666666667	376.7666666666667	144.29999999999998
106.56666666666666	278.53333333333336	361.2
277.8333333333333	152.23333333333335	199.76666666666665
101.26666666666667	122.03333333333335	101.8
251.16666666666666	135.7	254.20000000000002
136.13333333333335	207.86666666666667	181.66666666666666
266.3666666666667	156.46666666666667
95.76666666666667	245.16666666666666	192.46666666666667
27.3	111.76666666666667	46.63333333333333
54.23333333333333	125.56666666666666	122.36666666666667
177.36666666666667	116.23333333333333
119.96666666666665	98.3	259.6666666666667
219.93333333333333	141.33333333333334	137.86666666666667
126.3	105.93333333333334	107.2	79.0
104.53333333333335	99.06666666666666	179.46666666666667
109.53333333333335	360.26666666666665	105.3\
METTL21C	3.5	52.9	65.3	30.0	61.2	62.8	44.0
57.8	49.7	88.3	18.7	46.5	13.6	26.4	8.7
6.3	47.6	60.1	30.0	46.6	38.3	17.7	27.3
78.3	10.6	48.3	68.9	113.8	42.1	64.3	18.0
44.0	44.0	17.0	45.0	27.1	22.1	10.1	56.9
30.5	3.6	62.8	18.9	24.5	5.7	80.7	14.5
61.3	22.2	19.1	25.0	73.4	30.9	106.4	79.0

```
        34.6    33.9    6.0     71.3    90.5    254.2   33.1    17.6
        44.4    77.9    4.8     44.6    23.1    90.8    55.4    90.8
        86.8    39.1    36.0    28.3    52.4    2.9     46.2    47.8
        57.2    75.9    32.8    23.4    2.3     41.7    143.8   91.5
        90.4    7.8     5.8     62.5    40.1    34.8    66.6    63.4
        9.8     32.1    71.0    7.6     7.3     57.0    73.1
88.5\
APOL6   212.625 23.95   293.775 342.875 279.67499999999995
33.575  443.775 527.2750000000001       326.025 398.475 239.3
205.225 235.65  706.625 61.125  499.2   545.55  235.275 628.35
200.075 437.07500000000005      180.35000000000002      332.65
542.45  466.225 115.2   226.975 566.725 206.325 324.375 145.925
243.60000000000002      261.1   190.025 430.175 387.275 97.775
270.2   215.3   590.15  273.0   126.64999999999999
391.70000000000005      113.85  237.57500000000002      354.725
675.2249999999999       166.475 423.125 200.75  165.95  364.975
93.95   230.05  199.725 230.175 359.525 327.375 79.625  156.875
651.55  692.45  325.7   364.625 249.85  196.9   337.55
240.27499999999998      286.125 244.775 208.425 593.45  364.975
359.9   719.925 315.82500000000005      418.85  382.15  324.125
56.75   318.725 197.0   83.125  802.725 534.55  541.5
179.89999999999998      351.925 817.4749999999999       398.625
250.675 217.04999999999998      170.775 238.77500000000003
223.75  208.2   145.125 167.65  135.1   229.15000000000003
201.05  107.22500000000001      296.65000000000003\
RASSF10 102.6   8.0     409.9   138.7   107.2   475.5   169.8
949.3   218.7   91.8    85.5    160.9   22.4    3.6     3099.1
261.0   514.4   419.1   425.4   345.8   219.9   282.4   485.1
362.6   146.9   753.1   170.1   193.1   139.8   161.9   96.2
1054.5  154.0   351.7   265.2   341.4   1993.4  151.8   532.1   9.5
        1112.2  53.4    683.0   172.0   15.0    382.1   16.8
159.8   73.3    130.1   393.8   39.3    130.8   765.3   69.1
157.0   439.1   217.4   760.6   205.7   813.8   210.8   1094.9
999.2   187.1   355.9   331.7   561.5   480.1   599.8   331.1
188.1   131.5   56.3    400.8   338.1   784.4   125.6   135.1   4.0
        424.6   726.5   2151.9  215.7   134.8   551.4   504.7
1614.1  271.9   184.0   1492.1  131.8   309.8   313.0   378.4
427.1   772.0   224.8   204.0   441.1   266.1   196.9   914.7\
MINOS1  4792.9  840.0500000000001       1628.9  4357.0
1359.3000000000002      6131.95 4645.2  1997.25 4850.4  4019.8
5213.25 3226.2  4154.65 3556.8  2668.65 3055.9  2907.5  4612.05
4069.6000000000004      2940.4500000000003      551.8   5075.1
1495.65 3841.8  4814.5  2412.1  6081.3  1961.1  1944.5  3351.1
2808.95 1642.05 1536.1000000000001      3650.85 4558.35 2828.85
5041.75 1650.8000000000002      4271.8  3691.7000000000003
4181.8  3451.75 4121.0  3630.35 7765.95 3231.3  4585.299999999999
        5258.450000000001       727.6999999999999       2565.2
3682.8  4891.85 4095.15 1303.7  3904.2  2686.5  2739.6  1859.65
2992.5499999999997      1612.85 3247.25 3440.45 2974.45
3366.2999999999997      1534.1499999999999      2471.1000000000004
        4161.5  2982.75 1720.1  3656.0  935.0   2165.7000000000003
        1770.6499999999999      2882.8500000000004
2756.4500000000003      7554.75 2547.0499999999997      5792.7
2471.0  3882.6499999999996      1711.25 3388.45 2574.1  3765.0
```

1569.6000000000001	637.85	3486.15	3742.15	2815.5499999999997
	1964.35	5210.45	1389.1	3244.0	2496.0	1523.3999999999999
	2394.15	2489.15	2834.25	2915.25	1268.1	1732.3999999999999
	1661.25	2519.1499999999996\
RRP36	725.5	411.45	424.6	906.25	550.2	969.85	819.15
649.85	736.8499999999999	510.59999999999997	780.1
504.0	599.6	930.0999999999999	520.6	512.0	899.75
463.2	744.3000000000001	567.55	454.35	589.75	487.2
677.6	462.15	724.8	1303.25	544.35	586.0	737.9	658.35
739.2	446.3	741.5	936.9	639.5	760.3	459.7
524.4000000000001	799.05	607.4	493.0	486.25	609.55
706.8	622.1	767.9	1345.8	708.8499999999999	416.55
770.8	832.8	720.55	466.8	556.2	487.3	641.4	427.25
658.8499999999999	565.3	972.3	389.1	548.55	563.5
502.35	545.8	475.35	602.65	453.15	533.85	456.6	437.5
533.05	646.6	461.15	924.2	535.8000000000001	514.95
631.0500000000001	638.0500000000001	476.54999999999995
	483.84999999999997	1005.45	1493.5	603.45
823.6999999999999	600.35	419.75	587.05	454.55	688.2
388.25	626.3	676.4000000000001	513.25	390.70000000000005
	436.7	595.15	636.2	469.90000000000003	381.25
475.85	430.95000000000005\
CKB	825.7	588.6	326.6	45.9	408.6	637.1	1145.7
377.5	566.5	2100.6	972.5	160.1	116.1	609.0	769.8
191.0	546.5	287.6	730.6	880.8	324.5	773.3	621.1
450.4	442.6	534.3	145.2	553.5	896.1	667.9	165.3
502.0	313.5	586.3	802.4	621.1	224.2	428.2	702.1
165.5	382.0	1107.5	313.8	664.2	2168.2	331.2	283.7
1746.6	242.5	215.6	575.7	474.2	296.4	889.8	1023.8
1878.8	274.2	529.7	924.1	383.4	628.6	3916.0	890.3
673.3	295.7	338.1	426.6	975.6	219.1	1633.7	326.9
584.9	293.2	295.4	305.6	925.9	611.5	282.8	392.3
321.9	603.2	366.5	2158.1	684.6	170.1	626.2	133.8
1710.8	329.1	132.5	329.6	343.9	919.2	355.8	515.2
228.2	222.7	396.8	976.9	397.2	242.1	1177.1	282.4\
PAIP2	2754.5	1325.8333333333333	1127.3333333333335
2180.433333333333	1316.6666666666665	3494.566666666667
	3927.4666666666667	1924.3	3104.033333333333
2466.366666666667	3181.2333333333336	2460.9999999999995
	2468.366666666667	2135.9	2010.3666666666668
3454.066666666667	2848.1333333333337	1938.1000000000001
	2038.2666666666667	2446.9333333333334	666.2
3891.2000000000003	1248.3333333333333	2713.9666666666667
	2748.2000000000003	1605.3999999999999
2075.3999999999996	1334.1666666666667	1166.5333333333333
	2458.1	4034.933333333334	1359.8333333333333
901.5333333333334	3080.0	1382.3666666666668
1540.9333333333334	4117.666666666667	839.1999999999999
	2817.1	2662.4666666666667	3893.1000000000004
2966.266666666667	1934.7	2816.1000000000004	4182.9
2493.8	3256.7999999999997	3296.233333333333	444.2
1523.5333333333335	2980.566666666666	1653.6333333333332
	2254.1	1408.0	2673.0666666666666	1205.8333333333333
	1839.96666666667	1162.8666666666666

1839.5333333333335	1305.4666666666665	1733.2333333333336
	1823.8333333333333	2016.8333333333333
2565.233333333333	1369.6333333333332	1285.4333333333334
	2777.5	2292.766666666667	1282.5666666666668
2390.4	729.9666666666667	1254.2666666666667
1800.8666666666668	3281.7999999999997	1938.733333333333
	3529.433333333333	1599.3999999999999
3114.733333333333	3120.433333333334	3983.8666666666663
	2782.2999999999997	2142.0333333333333
4607.833333333333	2061.266666666667	712.7666666666668
	1052.1666666666667	4095.7000000000003
1596.8999999999999	1618.7666666666664	1376.2666666666667
	3615.1666666666665	1925.6999999999998
3025.0333333333333	2398.233333333333	2254.8333333333335
	3273.6333333333328	2839.8333333333335
2888.2000000000003	1372.6999999999998	2082.2666666666664
	2428.2	2003.4333333333334	3916.933333333333\
SLC6A10P /// SLC6A10PB /// SLC6A8	350.4	1595.5	225.1
173.3	80.1	1368.2	929.0	718.6	676.7	560.7	572.4
252.4	480.8	85.9	375.3	282.7	354.4	952.9	1521.2
841.9	522.2	461.2	552.0	325.7	503.3	1034.0	188.0	68.1
	771.9	474.3	280.0	384.5	657.5	306.9	1179.5
289.0	1049.4	838.4	379.3	901.3	735.3	1807.6	190.3
882.6	757.1	359.8	296.6	1520.3	154.9	690.1	1072.2
583.0	143.5	2224.4	437.4	593.7	503.4	295.5	619.8
423.5	358.8	325.7	390.9	432.9	240.3	456.5	596.2
620.0	583.4	340.6	473.1	660.8	117.2	426.9	507.9
420.9	344.1	571.8	278.7	2348.2	784.4	194.5	699.2
1648.0	76.8	748.1	677.5	1205.1	555.5	568.1	1382.2
500.2	37.2	217.8	235.8	193.1	175.4	224.7	24.1
107.4	63.4	58.0	324.0\
APOL1	100.9	148.7	2088.3	249.0	425.5	91.7	429.5
502.3	600.6	188.1	478.7	288.3	2190.7	2600.4	43.9
661.6	741.1	1518.7	3641.7	364.5	519.4	470.4	789.6
1528.3	1710.9	116.1	310.9	701.6	355.2	665.4	413.9
664.8	1425.0	586.9	488.9	5686.8	149.8	1809.2	655.3
387.2	411.0	974.5	547.8	437.0	1215.9	472.9	289.8
172.0	71.6	348.4	192.7	6809.9	56.5	42.1	500.1
207.4	724.2	2389.7	180.5	272.3	399.5	1152.7	796.3
1386.1	441.4	304.3	1339.4	458.3	1209.2	1238.1	354.8
1256.6	1263.9	1466.9	3113.0	1319.8	1288.8	853.8	427.1	98.6
	432.0	299.4	265.5	459.2	638.0	1205.3	195.7
503.1	6842.3	2361.2	1365.7	87.1	259.6	531.0	226.1
193.3	149.9	300.4	269.7	173.5	160.0	367.9	632.1\
APOL3	119.6	20.3	375.4	824.0	272.4	47.3	285.0
332.3	317.4	358.4	120.8	382.5	414.8	818.0	14.7
292.5	415.1	314.9	491.7	355.8	1140.6	340.4	360.9
1031.0	660.5	105.5	74.0	571.7	190.0	257.5	336.1
355.4	83.2	198.5	372.4	352.4	184.2	227.4	175.8
575.1	101.5	116.3	199.6	170.8	198.7	214.7	385.6	21.8
	712.8	201.1	360.1	132.0	14.6	29.0	298.2
206.3	252.8	236.1	185.9	258.3	166.5	477.8	200.0
366.3	344.1	69.2	381.0	156.8	231.1	204.7	311.5
153.8	860.3	365.0	484.1	114.4	164.9	455.1	246.7	23.2

```
	309.0	151.9	22.6	357.5	284.2	218.4	337.3
371.8	900.7	401.5	305.0	205.3	222.0	295.6	356.2
179.6	243.7	95.0	17.0	60.6	395.8	178.1	295.3\
PAIP1	4697.22	2531.66	2084.5800000000004	3917.38
2030.1200000000001	3683.88	3803.2599999999998
2494.7200000000003	4016.6400000000003	2878.1600000000003
	3654.3200000000006	2723.78	2971.7799999999997
4105.82	3950.1799999999994	2133.48	5871.34	1939.48	2747.12
2952.86	946.4	2768.8200000000006	1412.64	3559.6800000000003
	4978.78	3260.1400000000003	3637.84	2711.12	2479.32
2888.46	2885.6600000000003	2612.7400000000002	1101.78
3693.56	2652.18	2304.7200000000003	3143.48	1962.4800000000002
	3095.42	4023.440000000001	3183.0	2440.3799999999997
	3145.1	2743.0	3106.7200000000003	3201.1200000000003
	3280.7599999999998	8836.619999999999
1247.1799999999998	2468.26	3353.3	2397.02	2377.44
2322.2799999999997	3162.1599999999994	1318.58
3481.1399999999994	1955.2599999999998	1949.8000000000004
	2146.44	3933.34	2510.44	2574.4	3318.38	1913.58
1758.2599999999998	3477.0	2304.58	1832.8200000000002
4253.019999999999	1171.8	2666.9399999999996	2168.16
3997.4	3560.5799999999995	4708.74	3422.1600000000008
3620.9800000000005	3411.4800000000005	2278.6
5532.4400000000005	3579.44	3324.7	3845.8799999999997
1440.7400000000002	1638.8200000000002	3725.7799999999997
	4182.84	1970.6799999999998	1482.56	4241.9	2656.7
5487.959999999999	3469.34	2288.5599999999995	5125.04
4128.46	4282.460000000001	3699.0	5706.9	4888.08	3602.7
3879.9399999999996\
APOL2	110.15	244.2	610.25	492.2	810.05	367.55	344.7
348.75	427.25	403.95000000000005	245.05	427.65	804.75
835.8	281.25	326.4	504.35	1218.4	852.95	422.04999999999995
	1115.2	467.15	1551.8	490.65	538.8	181.95	285.35
658.75	498.79999999999995	290.15	280.29999999999995
1077.35	1351.6	359.6	514.2	2466.1	340.85	2239.2	269.15
268.59999999999997	375.2	305.4	419.5	468.2
467.79999999999995	441.45	253.60000000000002
87.44999999999999	1061.1999999999998	345.5	300.8
933.2	271.75	251.14999999999998	614.7	346.7	586.2
1614.4499999999998	491.9	501.05	727.4	2595.2000000000003
	385.55	502.95	415.05	450.15	364.6	350.85	918.7
412.20000000000005	1103.25	1569.65	546.4000000000001
430.75	641.8	304.3	408.2	502.0	325.0	113.05
396.04999999999995	172.05	181.35	482.4	758.35	1125.7
164.5	466.9	1793.8999999999999	2032.7	243.0	303.9
265.75	345.35	317.6	361.6	260.05	330.65	751.85	387.2
307.45000000000005	741.7	356.5\
ZIM3	18.4	2.7	23.5	22.7	5.1	15.9	3.5	8.5
	5.1	4.0	11.0	31.7	2.1	26.3	2.4	1.8
	19.4	4.3	17.8	39.1	4.8	30.2	2.4	0.6
	20.7	2.6	4.0	2.2	5.8	7.4	14.8	13.0
	3.7	25.0	2.8	4.5	3.4	75.4	1.5	23.8
	7.5	6.1	3.9	4.1	7.4	5.9	20.9	25.8
	5.1	2.0	3.3	2.4	2.5	3.1	1.3	27.8
```

	35.8	33.6	2.3	90.2	15.2	2.4	35.3	1.8
	6.0	2.5	8.3	2.4	1.8	9.5	2.4	82.7
	25.9	2.2	5.9	15.1	46.3	3.6	1.5	21.8
	5.2	4.4	2.5	3.7	6.7	55.8	8.4	2.9
	3.4	44.2	7.8	37.3	2.2	4.3	1.5	55.9
	6.3	2.6	1.3	2.9	42.1	5.8	4.0\	
HIPK4	137.0	337.7	115.9	147.5	276.7	169.2	219.6	
148.9	192.6	38.5	111.1	147.9	127.9	124.9	148.6	
121.7	151.8	33.1	154.6	70.7	365.8	140.9	272.2	
136.8	81.9	115.3	107.0	307.2	121.7	73.6	124.9	
369.3	295.5	130.8	152.5	157.1	45.1	228.9	52.6	87.9
	92.9	91.0	109.8	106.1	153.7	141.4	191.8	
223.6	135.9	59.1	246.6	87.4	24.1	219.4	31.4	36.7
	194.1	207.8	148.4	165.4	86.7	34.2	339.1	
149.1	184.9	314.3	83.0	77.2	127.1	65.9	389.0	
168.5	106.9	123.6	258.5	144.0	72.2	225.5	44.2	96.3
	88.6	137.9	120.9	157.6	293.9	95.4	109.5	
170.9	78.8	297.3	135.3	28.8	173.9	42.0	97.5	
129.7	63.5	133.4	133.7	151.1	105.0	188.7	142.4\	
DIO2-AS1		87.3	116.9	36.6	70.5	28.9	29.5	37.7
	34.4	40.1	71.3	28.5	39.1	51.0	97.8	78.2
	57.5	78.8	68.0	70.8	49.4	89.1	25.4	95.8
	19.5	2.6	43.6	73.0	77.7	79.3	100.2	34.3
	163.7	60.8	68.2	16.6	29.9	10.7	104.6	42.6
	47.6	6.9	26.8	35.9	45.5	35.2	75.1	43.4
	60.8	130.0	50.5	49.5	65.3	26.8	23.4	77.0
	94.2	4.1	64.0	71.6	115.9	31.5	25.0	
112.7	37.5	46.3	88.8	23.8	42.2	44.8	61.5	
166.1	118.7	88.4	88.7	50.6	32.3	46.1	40.8	56.9
	38.1	62.4	95.4	57.7	62.9	134.8	187.6	50.8
	17.3	81.6	81.4	32.5	104.5	26.1	27.7	43.7
	30.4	51.9	54.0	66.3	127.6	39.4	26.6	
81.2\								
BCHE	38.9	9.0	13.8	124.2	10.3	31.7	22.5	3.5
	79.0	2.4	10.3	41.3	2.4	30.9	55.2	3.1
	59.2	73.9	45.3	168.3	91.6	40.1	14.9	
797.4	15.9	41.0	7.1	43.1	5.7	32.4	951.8	24.0
	8.5	51.8	8.7	41.5	23.7	4.1	82.0	
102.2	2.4	16.3	248.5	59.0	30.0	14.6	120.6	26.6
	13.8	35.5	51.4	30.4	33.7	36.4	56.6	12.4
	7.8	10.0	38.3	164.4	191.7	44.1	2.5	71.5
	22.4	5.6	39.2	12.1	15.7	31.1	267.6	6.5
	200.6	1.5	5.9	2.9	3.3	20.6	101.9	
4657.3	115.7	114.9	3.5	6.3	120.1	3.4	43.8	
814.8	49.1	19.7	72.4	1343.9	833.0	167.2	576.6	
191.1	534.5	217.9	87.2	104.4	278.4	562.0	149.1\	
AC145343.2		98.1	135.9	169.2	126.7	40.5	103.5	38.3
	277.1	52.4	182.5	90.5	126.2	63.7	338.6	39.6
	78.8	125.3	118.9	235.0	204.5	111.8	89.0	
288.1	59.1	82.9	58.9	146.6	53.3	117.1	11.3	68.0
	113.1	34.8	134.0	342.4	126.9	161.7	44.2	67.8
	14.4	98.0	136.5	236.7	60.7	80.1	285.0	
116.3	169.8	163.9	158.7	194.5	50.6	85.8	138.0	98.1
	167.0	210.3	128.9	151.1	156.1	332.1	59.3	83.8

	43.8	76.0	132.3	99.2	79.0	190.8	195.6
244.9	726.0	133.4	66.5	96.3	88.6	221.1	40.0	56.3
	125.7	214.5	85.6	131.5	69.8	150.2	446.5	47.2
	133.2	148.7	89.6	57.0	104.2	84.3	117.5	76.8
	69.5	124.8	81.8	199.4	121.7	16.5	70.1
115.3\
CKM	4.2	15.1	6.1	3.7	16.0	3.8	9.2	6.2
	8.9	6.9	10.6	4.7	6.1	5.3	10.9	5.9
	6.2	7.0	13.3	5.8	15.7	5.8	8.9	4.5
	11.1	12.5	9.1	12.6	9.7	8.1	3.6	12.6
	61.0	14.7	12.5	9.9	7.5	10.3	3.8	6.4
	4.6	7.9	5.3	5.1	55.6	21.3	6.3	11.0
	30.5	12.4	12.5	7.4	3.7	7.0	9.4	8.5
	8.7	7.0	7.2	11.1	207.3	10.9	5.9	10.0
	12.3	9.2	3.5	17.2	9.0	6.7	28.8	8.5
	6.1	5.7	8.3	6.0	10.5	4.1	4.1	13.4
	12.1	6.3	5.3	6.7	14.9	24.2	76.0	18.5
	11.8	9.1	2.9	11.6	8.2	12.5	5.5	11.4
	6.0	3.8	7.1	11.1	6.1	10.2	10.3\
MCF2	44.075	89.25	45.875	30.099999999999998
75.57499999999999	39.275	100.35	51.475	54.25	53.85
32.875	65.025	34.575	49.075	55.349999999999994
44.150000000000006	94.85	21.5	43.3	49.475	110.75
14.375	38.675000000000004	23.75	27.55	32.975	39.05
65.075	41.125	45.175	27.65	87.925	49.099999999999994
44.15	49.425	55.0	33.324999999999996	48.0	23.925
45.150000000000006	163.95	25.625	43.1	66.15	39.075
25.375	28.200000000000003	33.875	41.775000000000006
69.325	53.025	32.3	46.9	137.8	43.4	76.025
35.300000000000004	64.55000000000001	39.475
49.050000000000004	144.325	35.975	16.75	27.625000000000004
	7.175000000000001	22.1	27.625	27.225	45.025
50.325	83.7	36.025	31.575	27.675	31.025000000000002
24.349999999999998	24.025	38.650000000000006
45.324999999999996	62.900000000000006	46.875	51.45
19.825	70.9	73.2	143.64999999999998	967.0249999999999
	41.5	37.35	61.825	14.95	53.075	37.45	12.8
52.25	45.675	42.5	23.425	49.275	39.849999999999994
22.925	24.35	56.3\
MOCOS	702.95	430.05	427.29999999999995	224.35000000000002
	236.3	932.2	102.05000000000001	903.8
207.64999999999998	198.75	448.1	198.4	244.75	206.3	84.4
	101.3	602.8	212.55	369.84999999999997	314.7
335.05	359.04999999999995	459.8	644.95	667.95
397.70000000000005	405.79999999999995	446.3	325.1
256.05	359.4	274.9	206.95	385.25	265.04999999999995
953.6	640.15	329.9	611.9	501.59999999999997	153.0
842.85	485.95	175.8	425.55	328.9	425.55	495.15	375.9
185.6	262.1	492.05	175.05	761.3	606.2	564.4	578.25
232.4	347.25	340.1	363.2	283.25	432.6	355.9	135.5
630.2	283.85	189.65	395.85	802.9000000000001	230.5
445.79999999999995	217.85000000000002	365.8	241.95
662.5999999999999	457.25	714.9499999999999	315.25
297.15	380.1	326.05	180.20000000000002	386.15

463.34999999999997	251.8	272.05	89.0	925.65	676.75
183.25	354.85	223.65	301.15	147.25	236.15	135.5	262.25
406.29999999999995	163.35	224.25	333.20000000000005
195.05\
LOC642533	217.8	596.2	183.8	238.7	399.8	168.5
209.2	140.4	172.7	242.9	278.0	294.2	214.2	338.6	33.1
	257.7	125.7	257.3	160.8	234.1	137.5	122.7
332.8	244.9	102.1	385.2	122.1	178.0	382.2	136.3
251.0	70.5	282.5	196.1	290.7	359.9	131.5	341.0
218.3	329.1	177.8	237.9	205.4	189.6	118.7	157.5
193.3	41.1	319.2	276.5	125.6	108.7	229.9	834.3	88.0
	262.9	69.6	141.3	250.1	128.7	159.2	205.1
183.2	143.8	200.1	233.4	260.5	300.1	165.6	231.9
362.1	270.5	199.7	423.5	380.3	112.5	268.7	96.3
239.5	96.7	251.2	80.2	80.6	355.8	269.8	458.5
202.6	390.6	304.9	187.0	50.9	51.2	111.6	194.1
162.1	123.6	87.0	271.9	65.4	162.9	121.7	190.4
213.5\
ELP2	391.22	617.28	747.76	423.73999999999995	390.6
811.36	572.9399999999999	329.7	670.58	465.38	602.5
684.04	498.64	459.73999999999995	826.1	783.72	445.32
816.26	383.54	538.0	561.42	607.46	859.04	694.6	518.64
649.2	381.24000000000007	1132.48	878.3799999999999
482.5	946.6200000000001	890.3799999999999	732.64
1024.8600000000001	481.08000000000004	748.26	577.02
556.6800000000001	793.1	898.1	722.4	932.86
890.8399999999999	604.12	1085.52	554.8599999999999
334.26000000000005	411.82	839.36	818.94	533.5	518.68
574.12	708.7	779.14	771.42	653.32	622.9200000000001
784.16	991.8800000000001	466.03999999999996	533.26
541.74	575.0799999999999	734.36	804.72	755.6	517.22
804.8	701.8799999999999	958.9400000000002
783.5600000000001	857.8	841.32	595.5600000000001
532.1400000000001	512.4	939.1200000000001	688.14
287.44	796.88	300.30000000000007	757.86	558.0
385.46000000000004	473.16	343.28	506.76000000000005
451.66	533.1	214.58	732.3799999999999	845.1	964.4
1288.48	1058.54	1131.84	1031.56	1243.28	1128.48	1199.5400000000002
	892.4	1158.2\
G0S2	572.6	103.0	946.4	518.2	330.2	269.0	726.3
503.4	288.0	1912.3	379.4	401.2	4945.2	1352.9	187.9	58.2
	347.3	1253.4	281.0	854.0	702.4	389.0	932.9
324.3	146.7	1245.6	1689.1	1101.9	416.7	242.4	332.3
515.7	1685.3	652.2	1516.7	1046.7	641.3	1616.4	607.1
180.0	1144.5	277.6	530.0	223.7	383.5	283.2	833.3
131.5	919.0	1055.7	345.8	927.5	2644.7	490.2	776.5
614.8	781.4	446.7	337.9	330.2	928.6	1789.0	252.3
1767.8	463.4	1554.4	669.4	262.5	821.9	939.5	407.6
296.6	596.7	148.6	486.0	485.2	1525.5	402.9	671.7
167.6	405.6	334.3	120.1	292.4	453.1	464.8	225.1
398.1	586.5	592.8	137.3	266.0	258.0	452.4	518.3
646.7	1088.2	559.2	22.8	337.2	770.5	141.1	813.6\
ELP3	1270.6999999999998	4292.8	3548.05	2537.95	4914.75
4287.95	1516.15	1383.45	3904.7	1693.5	2451.1000000000004

2128.15 5849.9 1267.6 1089.1 1152.6000000000001   1226.25
9712.099999999999   1948.8 3915.9500000000003
2372.8999999999996   4438.85 7917.3 6866.2 4141.95
1725.8000000000002   2504.9 4227.05 3794.85 1168.65 4016.8
3518.0 2009.45 7561.8 1437.6 9541.9 5980.45 2193.25 7282.85
2598.95 3565.3 5229.75 3372.0 1442.05 9668.25 7997.65 3509.45
2416.85 2865.1 4048.6000000000004   5830.1 4345.55 2099.25
2589.25 7034.25 5285.6 4220.3 7060.2 2568.9 4556.8 5592.2
14466.35   2001.45 2488.25 4426.75 1276.25 3414.4 1074.8
3668.8500000000004   6584.450000000001   2900.05 4799.75
4013.45 6508.5 3215.65 6983.55 2612.2 10002.3 3488.8 1290.7
2066.2 1551.0 1215.7 880.4   1435.95 703.05 1541.1 2095.75
1249.55 683.65 1978.7 2064.5 3829.7000000000003   2540.1
1959.5 7054.0 5899.7 4521.55 5357.4 5102.799999999999
3804.0 14249.6 3786.2\
RRP1B   3096.25 1511.2 1886.2 3118.05 1683.1000000000001
1976.0 3427.7999999999997   1939.8500000000001   3328.8
2909.4 3257.4 2077.3 2732.6 2508.0 3024.4 2919.7000000000003
        2201.7 2405.2 1784.5 2484.65 2791.65 2845.35 1283.55
2259.0 4629.650000000001   1839.1499999999999   1984.4
1784.3 1484.4 2407.85 2074.65 1083.75 1500.8 1970.5
2324.8500000000004   1712.8000000000002   2294.55 1916.9
1665.8999999999999   2090.4 1786.0 2082.45 1607.3999999999999
        2037.8 2116.0 2368.8999999999996   2567.4 1542.3
2569.45 1633.8999999999999   2420.85 2825.7 1729.45 1847.95
1997.5500000000002   2566.0499999999997   2195.55 2069.1
1341.5 1692.45 1356.85 1415.95 1541.5 2027.8 2131.5 1364.55
1818.2 1518.35 1408.9 1397.25 1389.5 1557.7 1644.3999999999999
        2042.3500000000001   2301.9 2076.7000000000003
1122.75 1943.25 2184.25 5783.5 2599.6 3455.75 1123.3999999999999
        2820.6499999999996   2030.8500000000001   2126.75
2254.95 2216.95 3154.9500000000003   2382.05 3086.05 1815.7
1677.1 1504.1000000000001   1958.1 1882.85 1827.95 1516.35
1027.5 1738.15 1716.05 1818.4499999999998   1333.1000000000001\
ELP4   924.4   180.6   369.1   815.7   213.5   1727.5 890.7
742.6   810.0   782.2   1299.3 611.0   744.4   557.5   1195.9
806.9   955.0   638.2   1059.0 764.7   96.8   823.1   163.0
708.0   648.9   686.3   865.3   456.9   473.4   548.5   1008.7
386.4   344.3   1294.3 543.7   533.9   984.9   276.4   925.0
1019.0 440.9   524.2   773.1   681.8   871.1   739.0   528.0
824.8   256.7   494.7   830.3   1392.2 582.6   137.1   731.5
611.5   587.5   280.4   636.6   518.4   744.1   677.6   830.3
873.8   373.6   316.9   914.6   802.9   454.2   981.1   102.6
310.7   531.9   559.1   816.9   1300.8 532.7   807.6   709.0
430.0   268.1   448.1   803.6   472.6   284.5   82.4   498.5
362.4   782.1   644.0   433.5   235.4   729.1   878.4   642.1
1014.3 593.7   733.9   219.1   360.5   691.3   479.7   805.8\
ELP5   859.4   284.55 154.2   619.2   209.70000000000002
710.9   634.25 257.45000000000005   397.8   427.35 398.9
257.59999999999997   468.25 421.0   362.55 328.35
218.64999999999998   222.1   307.25 307.6   347.85 378.15
249.5   407.54999999999995   276.0   310.4   633.95 378.8
328.8   327.7   660.25 236.65 233.25 382.90000000000003
212.14999999999998   225.9   341.90000000000003   123.65

542.25	212.65	264.1	424.75	191.70000000000002	412.0
476.8	156.75	548.7	228.14999999999998	350.0	237.05
577.4	261.5	267.65000000000003	153.4	179.04999999999998
	355.95	340.0	171.45	383.75	219.6	482.15	206.45
287.20000000000005	230.85	265.6	349.65	553.1	386.8
384.0	276.05	156.25	145.85	241.95000000000002	627.95
358.9	414.15	360.35	401.9	268.35	417.59999999999997
161.15	267.05	769.9499999999999	132.0	133.25	268.5
372.05	344.9	256.75	182.3	239.95000000000002	327.25
330.1	405.25	362.3	332.4	382.3	324.3	287.1	287.75
314.15	261.75	345.05\
HIPK1	899.0	271.74999999999994	1235.45	510.97499999999997
	351.45	594.2249999999999	542.925	770.4	871.45
557.85	809.15	515.975	551.6750000000001	750.15	458.175
767.55	916.3	753.2	727.825	973.15	335.95	886.7	589.275
793.5500000000001	1056.425	822.1249999999999
475.625	777.2	1144.575	953.225	760.7250000000001
626.6	452.125	826.25	388.6	545.4	667.15	383.2	1055.075
	1155.875	506.9	1015.025	791.5000000000001
	674.55	924.925	600.9	601.6	770.0749999999999
241.97499999999997	630.875	671.275	913.9999999999999
507.975	482.27500000000003	767.55	682.5	770.975	461.925
802.2500000000001	495.425	842.2249999999999
728.3000000000001	547.6999999999999	1146.8750000000002
	775.75	489.975	958.975	684.5	644.8750000000001
1352.775	552.625	646.275	643.225	990.35	1232.3249999999998
	1251.65	1167.525	1133.05	863.1	1155.7500000000002
	656.575	532.525	564.575	609.45	256.425	324.8	604.275
609.85	440.5	347.325	663.8	449.125	378.04999999999995
601.6750000000001	397.62500000000006	374.75
403.32500000000005	674.725	282.15	314.0	376.27500000000003
	404.2	491.05\
ELP6	400.6	332.35	242.10000000000002	530.3000000000001
	271.15	641.725	514.825	306.725	577.8249999999999
430.87500000000006	421.825	390.7	399.75	435.95	561.95
315.77500000000003	291.425	385.95	414.675	335.575
474.1499999999999	482.025	326.45	386.72499999999997
438.6	345.05	515.9250000000001	304.22499999999997
368.275	356.72499999999997	336.5	386.25	254.175
348.12499999999994	493.62500000000006	346.29999999999995
	478.9	358.825	345.4	342.85	253.175	477.425	293.425
390.575	494.12499999999994	371.775	421.625	367.77500000000003
	456.97499999999997	311.8	370.3	281.07500000000005
	302.3	298.65	310.95	316.29999999999995	270.05
268.175	327.0	243.525	195.05	320.2	275.575	269.025	281.6
275.65	369.65	295.02500000000003	314.85	329.70000000000005
	289.625	329.85	325.975	418.425	352.8	508.12499999999994
	330.40000000000003	369.025	369.09999999999997
306.125	322.1	258.0	554.175	391.65000000000003	411.65
369.525	333.725	341.35	386.35	278.45000000000005
353.99999999999994	289.125	465.025	301.95	219.5
383.19999999999993	326.25	336.99999999999994	315.475
312.575	312.325	373.84999999999997	357.75\
HIPK3	2164.2750000000005	1738.025	3543.6500000000005

3736.2000000000003	1946.5500000000002	2320.725
	1800.2749999999996	2442.55 1803.4750000000001
2684.65 2594.375	2532.425	3075.4750000000004
2360.15 4085.975	2859.1	3010.625	3495.75 3143.0
2533.125	993.2	2899.85 3475.6749999999997	2271.425
	1142.125	3832.0249999999996	3113.675
4720.875	3794.425	2126.825	2413.7000000000003
	4682.599999999999	2896.0750000000003	3117.025
	2364.0	4892.825000000001	2820.6000000000004
1884.3	2056.3250000000003	2976.6749999999997	1217.2
2697.7999999999997	4131.900000000001	1626.5000000000002
	2028.5749999999998	3344.825	4572.000000000001
	1567.125	1676.7499999999998	2076.2250000000004
	3250.3250000000003	2890.1500000000005
3071.2999999999997	1732.625	3396.55 3780.75
2826.8250000000003	3131.5499999999997	3297.5	2458.2
4401.300000000001	3136.7	2389.85 3846.625
2677.3749999999995	2890.775	2143.8500000000004
2296.225	3804.6749999999997	5226.525000000001
2590.9	3107.95 2606.6	2113.2000000000003	2766.7000000000003
	3582.2749999999996	2891.1	2423.175
3586.2749999999996	2422.3	3811.175	3740.975
4983.575	4889.225	2052.4500000000003	3380.0
2871.375	3825.9	2035.0	2274.2500000000005	1836.425
	2875.3999999999996	3721.9999999999995
3971.2000000000003	3581.3	3669.45 4705.475
3475.8250000000003	4363.0	6325.95 3572.2250000000004
5693.85 3721.15\
HIPK2	564.75	515.8	1047.27 1291.29 1530.44 834.8
3907.3000000000006	1681.27 1661.25 2596.9299999999994
913.1599999999999	827.6099999999999	1967.2499999999995
	558.04	1385.2900000000002	1431.98 3134.7200000000003
	1348.9999999999998	1279.32 1420.33 620.24
1563.7999999999997	1153.97 1637.5700000000002	1918.14
748.83	653.3799999999999	1649.1799999999998
993.9200000000001	1494.6	1160.59 652.74	687.4300000000001
	3754.34 1103.7999999999997	550.2299999999999
1336.0499999999997	1440.37 2496.74 1414.2900000000002
2817.74 949.6400000000001	1763.2	2862.7699999999995
2607.27 2034.8199999999997	1381.8799999999997	1307.74
492.88	2102.04 1052.7199999999998	1321.87 527.62	1378.37
1152.54 989.9	1522.21 719.4599999999999	645.67	1241.66
2222.04 903.47	2117.71 1245.1200000000001	1011.74 910.04
1178.8700000000001	1810.3500000000004	1464.75 737.05
1294.4400000000003	1033.34 1456.9699999999998	1752.3
2299.33 1488.63 1035.8200000000002	3360.13 754.5699999999999
	267.51	1112.7400000000002	1948.9599999999998
1200.8500000000001	1002.47 2529.65 795.01	3002.4900000000002
	924.49	2067.55 526.4800000000001	1892.58 780.2
482.57	719.03	658.94	472.26000000000005	352.14
672.8399999999999	820.28	552.3200000000002	592.48
238.89000000000001	592.5899999999999\
LOC100132207	18.0	50.5	5.9	3.5	3.6	4.8	18.6
	48.9	28.9	9.9	19.9	69.0	3.8	10.2	25.8

51.0	36.7	9.9	36.3	3.8	13.2	84.3	7.8
5.8	32.8	47.2	26.9	143.6	12.2	49.1	1.9
155.0	62.9	4.1	20.7	89.7	8.1	35.3	2.1
33.4	16.5	4.6	65.4	42.6	2.4	16.1	2.3
44.6	7.0	3.9	67.2	34.1	40.8	33.3	14.6
29.5	8.2	6.2	25.8	3.8	411.9	4.8	6.8
32.7	63.1	4.1	3.6	4.6	13.7	4.7	7.1
3.4	10.3	34.9	14.9	60.7	71.2	3.8	52.8
27.5	53.8	25.6	2.6	31.7	98.0	229.7	42.5
59.5	57.8	42.5	23.9	54.9	17.7	6.2	66.5
41.8	42.8	39.3	86.7	104.0	66.8	20.4	7.6\
NFIA	1017.8285714285714	1232.4857142857143
2032.6142857142859	737.7142857142857	1658.142857142857
	2834.0142857142855	2512.2	1857.4714285714285
1950.7857142857142	2305.6428571428573	740.7428571428571
	2361.7428571428572	2177.7285714285713
1002.2714285714285	1591.7571428571428	1542.0142857142857
	2049.457142857143	2142.7714285714287
1244.0142857142857	2569.5	749.2285714285714
2393.828571428571	1786.7428571428572	3210.5714285714284
	2317.9285714285716	1760.2142857142858
1262.685714285714	1939.0571428571427	2063.628571428571
	1977.8428571428572	1435.8285714285714
5317.757142857142	843.5285714285714	3971.2999999999997
	1465.8428571428572	1316.742857142857
3720.8571428571427	429.04285714285714	2749.3142857142857
	997.0571428571429	1742.2571428571428	1121.8
1435.8428571428572	969.9142857142857	1276.5714285714287
	1760.9428571428573	1967.4142857142856
487.9142857142857	1719.4142857142856	2911.9428571428575
	3167.1857142857143	713.1857142857143
1733.1714285714286	4299.614285714286	1410.685714285714
	3049.128571428572	3507.7285714285717	1475.5
1771.2142857142858	2866.2999999999997	6705.642857142857
	1941.4428571428573	2995.1	2101.585714285714
1929.6571428571428	3087.5857142857144	1991.4428571428573
	1121.2857142857142	1951.2285714285717
2168.5857142857144	2374.8142857142857	2820.2
1812.4571428571428	3857.0142857142855	3052.2285714285713
	1212.957142857143	1400.3999999999999
3588.3285714285707	2984.4857142857145	2617.871428571429
	3712.3142857142857	1245.5428571428572
944.6285714285714	1645.4	748.6285714285715
165.05714285714285	3734.8142857142857	873.0285714285716
	348.67142857142863	110.87142857142858
1297.6000000000001	3143.4571428571426	2072.6	1030.6
1837.0571428571432	2173.9714285714285	2206.2285714285713
	1783.0857142857144	1956.3857142857141
2625.142857142858	1977.0714285714287	3977.028571428571
	1599.214285714286\
NFIB	1170.1125000000002	5721.2875	863.7625
151.0125	2633.5875	1803.975	242.2875
949.525	848.4749999999999	1870.6375	1395.5250000000003
	1949.3625	2218.2625000000003	1453.3625000000002

	2221.625	1092.0999999999997	1300.55
596.6125000000001	376.97499999999997	1850.425
842.15	1024.2749999999999	1012.2125000000002
628.0875000000001	1196.925	1537.8374999999999
631.3	814.1125	1032.9625	1371.6125	624.7625
	1621.0625	332.07499999999993	475.4625
1895.55	2997.3875	1186.65	265.875	928.1250000000001
489.23749999999995	711.7875000000001	790.775	787.6
365.38750000000005	83.175	859.1624999999998
734.8249999999999	722.9000000000001	855.725	712.9
2350.6375000000003	804.3124999999999	1298.3250000000003
	2149.8250000000003	956.95	849.6875	492.475
1521.5125	2966.9625000000005	955.0625
645.6999999999999	2653.8624999999997	958.95
1680.1250000000002	1169.0624999999998	1512.4874999999997
	1130.8999999999999	435.47499999999997	197.525
1049.6	1143.0125	175.39999999999998	2005.5874999999999
	799.5625000000001	1104.4874999999997
282.49999999999994	536.9875000000001	1071.5874999999999
	2048.025	1776.825	1579.1625000000001
1873.3625	1446.5250000000003	1466.2	580.5125
73.7875	1839.675	910.9625	79.2	137.1375
1557.3000000000002	827.1624999999998	968.7124999999999
	1566.4124999999997	1269.625	1110.9875
1081.1125000000002	1737.9624999999999	852.9499999999999
	1197.5500000000002	773.1250000000002	2515.525
	1024.1750000000002\
NFIC	418.65	665.2	1853.325	933.8	2156.325
810.1	571.85	2788.4750000000004	1601.4250000000002
3286.25	771.825	1886.175	643.725	1791.525	859.875
1641.0	1548.525	1516.225	656.5250000000001
1454.875	3691.65	1118.8	2648.1000000000004	1046.5
1708.625	1673.775	980.9	2275.9	2317.6499999999996
	1513.7	780.525	3907.875	2634.9750000000004
1640.475	1827.1750000000002	2094.575	764.825
2965.775	623.025	883.825	258.1	1007.1	1919.9	926.45
1155.45	2812.8999999999996	3722.0499999999997	571.975
3099.975	2452.9	2244.825	975.65	1200.825
5062.025	1731.7749999999999	3124.675
2221.7749999999996	2038.0749999999998	1787.175
2560.15	6686.25	2493.425	1994.55	1452.5	2430.4	2046.925
	1020.175	1094.9499999999998	6894.725
970.45	3494.275	2945.675	1317.0749999999998
1255.1	1828.3250000000003	1037.125	4521.625
1469.35	1660.275	935.175	2298.25	1902.175
337.42499999999995	735.95	3227.5	1614.3	3770.7	2599.775
	1711.125	1550.175	534.4000000000001
3023.05	923.95	1239.35	2360.675	900.5500000000001
908.1750000000001	845.425	1166.925	661.2750000000001
	1136.6	1695.1499999999999	762.4749999999999\
PLP1	26.2	46.5	14.4	14.8	14.0	75.1	35.7	50.5
	11.6	6.9	20.2	7.0	43.8	43.9	112.1	6.7
	35.4	5.5	64.1	18.6	16.9	7.4	79.6	47.7
	8.8	81.9	9.4	9.7	8.0	62.2	3.3	19.1

	6.1	15.5	14.6	20.4	47.4	8.3	24.2	
214.6	3.7	7.3	3.7	19.8	7.9	12.8	19.2	11.3
	14.0	36.4	17.7	6.8	6.6	21.4	13.6	13.3
	7.0	40.7	79.4	14.5	49.7	7.1	12.4	12.3
	56.2	6.0	6.0	8.0	8.2	52.2	45.5	11.9
	68.1	33.6	5.0	21.2	10.5	24.1	6.8	
139.7	71.9	11.6	113.0	35.2	18.4	225.9	27.5	35.7
	9.9	15.2	3.1	17.7	28.6	3.1	3.5	5.2
	8.2	50.3	4.6	98.5	8.7	40.4	53.4\	
FOSB	178.1	95.6	22.2	117.2	873.9	14.9	162.5	
8763.8	243.7	219.1	580.3	609.0	1625.5	321.0	68.0	
100.6	41.9	151.1	2351.3	813.4	967.4	142.0	171.3	
648.9	393.9	653.3	60.9	254.7	70.2	104.3	91.2	
332.8	26.5	65.4	375.6	601.3	1150.2	3154.4	161.3	12.6
	447.5	62.0	44.5	106.7	30.3	27.5	245.3	12.4
	39.5	675.1	19.0	279.3	704.5	417.4	53.1	31.5
	19.4	202.4	213.9	155.0	61.2	3231.6	356.2	
11127.3	445.2	72.8	1072.2	1977.2	123.0	253.9	636.5	
588.1	376.4	219.1	80.1	58.4	421.4	250.2	682.7	
152.8	467.0	552.8	2719.9	72.9	935.4	299.8	57.1	
999.3	1632.4	704.2	199.7	4608.1	18146.0	744.0	8981.5	
13057.7	14827.0	1698.8	6481.0	7587.3	16902.7	2882.2	13422.8\	
CLC	26.9	21.2	94.3	8.1	81.1	5.9	4.3	13.6
	13.4	3.9	29.7	3.6	5.1	8.1	6430.0	8.2
	10.2	4.8	43.7	34.5	71.7	5.2	18.5	45.3
	7.2	58.7	7.3	18.5	54.9	61.6	5.6	11.5
	51.4	6.8	20.3	15.5	3.7	13.6	19.7	37.4
	39.2	22.4	17.2	27.1	4.3	58.1	3.1	21.3
	9.5	20.9	7.2	21.7	43.7	22.8	8.4	24.5
	6.1	19.7	56.5	17.8	296.8	9.3	62.8	32.4
	16.3	8.8	31.4	44.4	73.2	8.1	24.3	35.1
	5.2	3.4	22.8	55.9	4.4	4.6	64.1	9.8
	35.4	9.9	69.7	35.3	27.6	12.2	104.8	13.9
	30.6	12.8	4.0	108.0	8.0	36.1	6.9	44.2
	16.0	22.0	5.6	68.4	28.3	5.3	5.9\	
PLP2	2437.6	402.2	470.5	1619.9	644.2	3252.8	1109.0	
251.9	2274.1	2557.7	1470.2	2727.0	2536.9	2206.1	1850.9	
1137.1	1969.0	1656.0	3633.3	962.9	505.9	1894.3	559.3	
1812.8	2630.2	1686.9	4011.2	624.5	786.2	1331.9	1196.4	
582.1	570.7	505.0	871.9	607.5	832.9	1094.8	1443.5	
3999.4	2592.6	4757.0	1341.5	734.4	2925.9	1901.8	1160.7	
2959.0	438.7	371.5	2009.3	1433.8	1168.6	529.4	2839.1	
731.9	460.3	816.8	184.7	561.4	159.1	812.9	1392.9	
956.8	605.5	451.2	1014.4	1111.5	1561.0	1409.7	47.7	
1568.2	862.6	696.7	810.9	1854.7	1152.9	533.0	681.6	
610.9	378.7	709.3	6014.5	1396.4	887.4	189.4	410.0	
597.1	5519.1	49.1	3626.4	247.4	464.2	842.0	455.0	
447.5	197.9	397.4	512.4	232.3	261.0	676.3	556.2\	
CCDC169	/// CCDC169-SOHLH2			3.2	57.6	11.2	33.8	87.8
	18.6	2.9	25.2	22.9	83.9	2.2	13.8	14.3
	4.8	38.4	31.4	11.0	36.3	20.6	5.2	5.0
	16.4	34.2	1.6	33.1	29.8	4.5	8.8	3.6
	5.6	41.9	105.0	2.3	28.2	5.0	6.0	0.8
	64.0	19.0	0.9	2.1	27.6	6.0	40.7	28.6

22.9	4.0	41.8	11.1	51.6	75.5	63.1	32.2
48.7	6.3	18.7	5.2	1.4	20.6	71.7	34.9
6.9	25.7	30.9	18.0	47.9	31.8	33.1	61.1
4.2	12.6	93.4	1.2	12.5	29.9	2.1	3.3
11.8	9.4	30.8	36.0	51.3	5.1	5.4	3.4
108.7	36.3	4.0	27.9	6.6	47.2	8.1	1.7
17.9	2.0	22.4	1.5	19.9	68.9	2.2	2.3
19.3	59.4\
TMEM229B	12.166666666666666	122.53333333333335
133.1	13.133333333333333	54.5666666666666	17.8	45.5
110.06666666666666	54.099999999999994
24.633333333333336	112.23333333333333	16.8
101.86666666666666	103.33333333333333	35.63333333333333
69.36666666666666	43.53333333333333
91.60000000000001	38.03333333333334	74.10000000000001
57.06666666666666	45.800000000000004
124.76666666666667	84.7	15.699999999999998
30.23333333333333	57.96666666666667	24.266666666666666
34.63333333333333	52.4	39.06666666666666
23.099999999999998	41.93333333333334	51.5
112.96666666666665	94.36666666666667	68.43333333333332
72.60000000000001	148.56666666666666
152.26666666666668	28.966666666666665	109.66666666666667
132.9	35.333333333333336	129.56666666666666
44.333333333333336	100.93333333333332	16.433333333333334
107.49999999999999	47.56666666666666
56.03333333333334	15.833333333333334	28.400000000000002
28.7	60.199999999999996	77.96666666666668
39.46666666666667	42.1	51.800000000000004	99.5	52.1
84.2	131.13333333333335	45.93333333333333
32.86666666666667	36.733333333333334	74.16666666666667
62.33333333333334	159.43333333333334
44.866666666666674	110.10000000000001	71.86666666666666
74.0	80.43333333333332	104.30000000000001
126.26666666666667	157.13333333333333	56.73333333333334
56.46666666666667	16.366666666666667
41.06666666666666	12.4	13.700000000000001
24.66666666666666	35.46666666666667	54.866666666666674
20.8	88.66666666666667	38.53333333333333
102.03333333333335	21.766666666666666	14.466666666666667
17.033333333333335	26.63333333333333
16.733333333333334	25.3	47.26666666666667
41.53333333333333	51.43333333333334	36.733333333333334
42.199999999999996	42.63333333333333
81.03333333333333\
FAM49A	47.9	93.06666666666666	107.56666666666666
171.66666666666666	46.5	59.9	93.36666666666667
366.1666666666667	45.93333333333333	309.6333333333334
62.6	97.76666666666667	65.76666666666667
174.0666666666667	150.5	120.09999999999998
121.83333333333333	34.5	92.96666666666665
88.16666666666667	151.63333333333333	79.03333333333333
128.06666666666666	174.73333333333335
65.73333333333333	57.53333333333333	38.4

192.1666666666666	82.03333333333333	124.96666666666665
	88.53333333333335	202.29999999999998	114.0
59.46666666666667	138.79999999999998	254.80000000000004
	221.03333333333333	93.66666666666667
43.26666666666667	180.46666666666667	55.20000000000001
	60.56666666666666	149.1666666666666
48.06666666666666	57.033333333333324	77.03333333333333
	92.36666666666667	35.400000000000006
92.73333333333333	110.36666666666667	131.53333333333333
	93.06666666666666	92.33333333333333
112.46666666666665	47.96666666666667	138.76666666666668
	70.96666666666667	48.9	78.39999999999999
88.36666666666666	131.63333333333333	122.03333333333335
	72.93333333333332	125.7	118.60000000000001
94.89999999999999	93.13333333333333	71.06666666666666
	137.79999999999998	60.79999999999999	123.2	96.2
	130.66666666666666	57.1	102.7	120.23333333333333
	100.3	108.56666666666666	86.0	334.8333333333333
	201.73333333333335	112.06666666666668
77.73333333333333	224.86666666666667	47.79999999999999
	110.96666666666665	96.86666666666667
264.8333333333333	95.83333333333333	139.4
124.63333333333333	173.73333333333335	147.70000000000002
	123.46666666666665	90.66666666666667
171.33333333333334	110.63333333333334	168.86666666666667
	199.4333333333333	180.29999999999998	169.6
157.73333333333332	96.76666666666667\
FAM49B	1117.6333333333334	564.7333333333332
772.4666666666666	1150.5333333333333	1498.6666666666667
	752.6	1118.5333333333335	1222.1333333333334
712.9666666666667	699.6333333333333	774.2333333333332
	531.3666666666667	1144.0666666666666
844.8000000000001	839.4666666666667	1027.1333333333332
	772.6333333333333	999.0333333333333
1051.3999999999999	851.4666666666667	818.7333333333332
	815.1666666666666	1129.3666666666668
881.1333333333332	895.0333333333334	1008.0333333333333
	836.0999999999999	882.2666666666668
905.8000000000001	566.1	820.1333333333333
879.8333333333334	844.6666666666666	647.4666666666667
	1004.2333333333332	676.0666666666667	1140.6
768.4333333333334	666.3333333333334	1438.9666666666665
	1336.1666666666667	1119.7666666666667
811.6999999999999	672.9333333333334	1050.4333333333332
	1169.0666666666666	1168.8666666666666
1623.0666666666666	711.0333333333333	530.0333333333333
	625.2333333333333	1112.7333333333333
760.3000000000001	1219.7666666666667	826.1333333333333
	740.1666666666666	703.4333333333334
873.8666666666667	520.5333333333333	676.6999999999999
	708.9666666666667	582.7666666666668
838.7000000000002	788.3333333333334	657.9
873.0333333333333	761.1	615.6333333333333
656.6333333333333	1033.0	685.8333333333334	854.9

704.9666666666667	821.6999999999999	873.9333333333334
	1114.9666666666667	869.1666666666666
822.4333333333333	810.3666666666667	2291.3666666666663
	1247.0	1440.5333333333335	1285.5666666666666
1332.8	515.7333333333333	1817.3999999999999
1379.3000000000002	1193.2	1123.7333333333333
1156.8999999999999	1009.2666666666668	729.3333333333334
	812.4666666666667	770.0999999999999
516.9666666666667	952.8333333333334	631.0	749.9
570.4333333333333	696.9666666666667	756.4666666666667
	910.1	815.0333333333333\
C1orf177	38.8	126.5	9.2	17.2	9.7	64.6	3.2
	21.0	97.7	128.2	20.8	6.3	38.7	15.0	7.8
	58.5	19.4	47.9	85.2	24.0	105.4	11.1	99.2
	36.7	72.8	14.4	20.0	49.9	12.6	157.7	62.4
	111.0	14.5	16.5	117.9	40.3	7.2	86.0	14.6
	19.0	42.8	36.6	40.4	9.7	18.8	17.8	6.6
	15.3	72.8	99.5	25.0	87.2	81.2	140.6	68.8
	50.4	94.1	121.1	60.7	145.8	392.3	73.0	7.9
	29.0	48.6	58.2	42.8	12.9	43.1	27.9
103.1	28.6	51.7	30.3	57.2	92.2	38.3	20.2	13.8
	18.7	15.8	20.1	20.8	92.9	88.4	106.9	75.2
	98.3	61.0	13.3	20.1	145.6	73.1	52.2	36.5
	43.5	39.0	32.5	111.2	49.9	19.9	23.1
34.7\
C1orf174	1286.15	138.0	846.55	1261.7	645.85	931.95
755.0	1738.5	1009.0999999999999	664.95	958.7	724.0
912.45	1011.95	813.1	939.9000000000001	531.2	512.4
938.75	908.1	663.25	852.7	672.3000000000001	1122.85
1452.0	749.0	1309.6000000000001	755.9000000000001
1348.2	550.55	847.3499999999999	2296.1	588.3000000000001
	778.1	808.45	775.25	1292.6999999999998	453.55
762.3	768.1	868.75	579.0	550.35	662.2	711.5	927.5
1710.65	1596.4	1167.1	1426.1999999999998	1109.45	769.5
1359.6	1396.05	719.4000000000001	622.3	701.65	568.9
718.9	767.6	856.55	894.7	882.65	836.75	866.1	667.75
919.35	605.75	655.3	1497.6	969.8000000000001	679.55
401.3	1104.5	715.0999999999999	1173.0	953.1
925.5500000000001	1128.6999999999998	1616.25	1938.65
1470.3999999999999	1231.4	1250.15	1261.1	1638.45	2353.4
1313.6	769.0	552.2	1203.5500000000002	1963.8
1691.1499999999999	1275.8999999999999	1133.4	1405.1
1604.3500000000001	1489.75	2214.8	1998.3	1802.3
797.8000000000001	1325.05\
LOC401324	7.9	17.3	31.2	5.1	81.2	3.8	6.3
	84.5	40.8	5.1	8.5	20.8	3.5	8.6	15.1
	4.7	6.0	1.8	20.4	5.5	13.3	7.4	67.2
	4.1	32.2	4.4	11.0	12.9	42.4	14.2	10.5
	9.6	12.1	10.8	14.3	13.6	56.5	8.4	34.5
	46.0	8.6	5.4	8.0	3.0	7.6	10.9	64.4
	14.1	31.9	24.0	37.7	8.5	7.3	5.5	4.5
	8.7	47.4	11.2	11.3	21.9	168.3	80.9	11.7
	63.1	94.8	12.4	40.9	44.8	11.2	54.7
112.7	10.3	114.2	13.6	16.3	6.3	14.1	7.5	4.1

13.2 19.1 5.8 3.7 6.8 32.8 26.5 9.6
16.5 43.5 19.0 40.9 14.0 19.6 11.7 19.7
19.6 7.3 25.4 11.3 4.9 52.1 6.9 4.2\
RP11-305L7.3 3.0 33.1 7.5 11.2 9.8 7.5 6.0
8.2 6.2 12.1 4.6 5.5 4.7 5.4 10.8
12.9 7.2 8.8 3.0 5.2 9.2 9.7 34.0
6.7 11.0 14.0 3.6 11.4 14.2 12.4 5.8
24.3 39.7 6.4 17.6 22.4 9.2 10.0 11.9
7.5 5.7 12.9 6.8 8.3 12.2 23.7 10.1
10.6 22.7 23.4 13.9 6.6 15.7 12.4 10.7
19.7 5.5 8.3 6.3 19.3 53.0 13.5 17.3
9.9 11.8 8.4 7.5 14.7 19.3 6.2 9.0
7.8 9.5 7.9 7.7 5.5 10.5 7.3 11.6
7.7 9.1 6.3 5.7 7.3 49.9 9.8 9.4
7.7 7.7 9.2 5.9 11.6 3.3 3.1 3.5
4.6 9.4 15.7 14.4 9.6 5.3 9.5 5.3\
DNM3OS 583.5 2657.3 262.05 456.1 301.85 54.900000000000006
53.25 162.6 622.85 2509.85 66.05 221.64999999999998
173.55 150.05 61.1 707.85 502.0 289.15 198.25
335.85 593.35 129.95 167.35 411.9 108.6 26.15 342.4
287.3 436.55 332.05 354.4 203.25 700.2 173.5 69.15
88.05 41.15 172.6 48.550000000000004 248.65 157.85
34.050000000000004 709.15 396.8 62.15 83.35
127.55000000000001 103.69999999999999 101.85000000000001
64.45 292.35 141.15 93.1 501.54999999999995
481.15 119.75 78.25 570.35 126.14999999999999 431.05
412.29999999999995 219.6 156.55 210.25 237.95 89.85
109.65 256.25 519.35 97.15 553.95 403.3 380.35 328.05
242.2 219.75 272.4 288.6 255.65 127.75 454.0 97.5 6.5
134.9 311.15 67.0 103.05 696.9 177.89999999999998
314.55 402.65 1416.3 470.35 1324.6 1473.35 426.5
751.75 653.75 463.8 568.6 474.55 1453.1999999999998
395.3\
CLU 96.64999999999999 227.0 476.425 1154.3500000000001
6273.125 561.05 1399.25 315.52500000000003
2282.9249999999997 14208.525 254.89999999999998
669.175 1688.8999999999999 493.77500000000003 510.25
1431.0249999999999 1555.8500000000001 281.05 3165.575
7642.5999999999985 497.675 1553.325
282.50000000000006 3443.8 326.79999999999995
624.6250000000001 1512.25 404.04999999999995 151.85
7326.3 1675.35 364.45000000000005 431.22499999999997
5933.075000000001 331.3 1846.6999999999998
160.02499999999998 436.47499999999997 17518.05 90.5
1669.375 349.35 1364.15 2239.725 4466.375
732.175 664.825 640.5500000000001 729.55
6586.299999999999 1863.3 515.125 225.47500000000002
147.275 1749.675 7217.775 435.975 465.20000000000005
1837.3749999999998 270.25 578.775 5278.55 4576.675
6312.8 1758.6250000000002 16188.4 6522.75
1200.1499999999999 696.0500000000001 1518.425
500.75 235.475 2411.825 964.5250000000001 227.35
885.8 514.8000000000001 4867.45 4883.725
120.42499999999998 322.07500000000005 141.82500000000002

```
        703.5   2010.15 1027.55 146.45000000000002
217.57500000000002      380.8   1562.825        789.8000000000001
        4198.625000000001       291.05  5265.05 6560.85 3896.3
3455.375        3846.5750000000007      3779.4999999999995
1860.6  580.3   4383.575000000001       343.325 10185.050000000001\
LCLAT1  5590.4  737.7   999.4   1587.8  1134.7  3187.9  2697.7
3780.9  3050.2  966.2   3513.6  1324.2  2117.1  1525.5  2459.1
2078.5  3017.6  1869.2  3329.8  2052.1  353.8   2021.6  771.2
2271.5  2143.2  1459.1  3990.5  1091.1  1276.7  2549.2  3269.3
665.1   564.4   2882.6  2328.2  1442.8  3679.7  550.8   2616.2
4024.3  2619.9  2726.1  1721.6  1953.4  2713.4  2236.9  2085.4
2717.6  633.9   2076.8  1756.5  2343.1  2549.8  706.9   2048.5
2031.6  1467.3  1036.9  1927.7  1348.4  814.3   1050.2  2067.1
2462.8  1342.0  836.3   2580.5  2632.5  913.5   2765.4  464.4
1064.2  1052.6  2935.2  1766.8  4445.7  1137.1  2409.6  1585.2
3829.0  756.2   1602.5  4062.0  2645.2  585.7   1099.3  2547.0
1235.5  964.3   960.3   6653.7  1136.2  1853.9  1915.1  1534.2
1915.6  1823.6  2026.1  1555.7  1693.6  1698.3  1094.2  1439.6\
RP11-111J6.2    39.2    107.9   54.8    69.4    112.9   21.8    29.5
        74.5    43.2    4.9     18.1    23.3    10.4    8.5     60.4
        77.9    68.2    37.8    66.4    61.6    115.7   28.0    4.4
        32.9    23.3    6.3     24.5    80.3    166.8   50.2    71.2
        4.1     80.8    50.4    63.6    0.9     45.6    106.9   54.8
        37.6    43.9    49.8    95.7    36.6    13.5    118.8   72.3
        46.2    138.2   42.1    29.6    20.6    27.8    120.1   34.1
        37.3    44.7    25.7    51.2    65.1    62.4    16.6    60.8
        39.3    20.4    3.1     53.3    34.0    3.9     26.7
113.6   96.8    80.6    38.5    61.6    42.0    104.4   20.3    50.1
        40.7    143.3   30.7    49.4    19.0    68.5    53.4    97.0
        13.8    8.8     3.3     104.5   180.6   66.1    119.6
125.3   143.8   96.7    95.8    115.9   240.1   95.3    86.8
103.4\
THG1L   885.4   74.1    326.2   503.0   293.6   873.9   1002.1
240.9   849.7   371.9   896.3   404.7   406.1   216.0   447.3
644.0   435.4   670.6   405.4   693.9   351.2   537.4   383.1
516.6   557.1   394.1   513.1   305.4   237.7   575.8   1179.7
392.2   133.2   545.1   476.0   310.4   387.2   490.4   409.6
819.5   889.1   445.4   414.2   775.3   1001.4  410.2   497.3
190.5   305.0   583.0   347.6   576.4   441.4   336.1   573.0
423.4   515.4   317.5   279.9   474.9   457.3   256.1   347.9
490.8   301.2   304.0   836.4   391.7   417.8   387.4   452.5
312.7   265.5   984.4   575.4   451.5   212.9   598.9   343.6
220.1   280.2   264.2   361.7   268.4   322.7   259.0   165.5
246.9   530.2   396.6   561.7   177.3   309.2   441.9   236.0
451.2   408.6   476.2   290.1   338.9   325.0   428.9   784.1\
LOC439938       49.6    117.0   102.9   146.9   314.4   46.1    57.0
        266.0   126.0   60.6    60.0    51.3    129.3   137.6
218.5   58.6    135.0   147.5   180.4   204.2   105.2   95.9
317.7   179.4   117.8   56.3    120.1   136.5   106.3   137.9
174.3   208.6   68.1    191.5   359.4   86.1    179.5   115.7   79.3
        168.6   218.3   58.6    228.2   127.5   289.0   111.8
111.4   102.4   168.4   131.5   200.5   144.0   99.5    341.1   56.0
        153.4   99.4    95.0    204.7   74.1    462.5   150.9
150.6   106.2   138.1   150.8   173.3   104.9   220.1   167.6
```

287.7	27.4	205.2	223.5	150.7	151.8	104.1	181.6
144.4	412.6	239.7	231.4	1395.6	113.7	218.0	250.0
266.7	346.6	106.9	144.8	81.8	387.3	317.7	180.5
220.5	332.7	454.0	173.7	164.1	445.1	278.2	245.3
207.1\
C20orf57 /// DUSP15	29.5	209.1	68.6	59.6	124.2	90.7
	38.5	159.6	88.2	109.7	75.0	33.4	12.9
133.2	161.6	53.0	158.2	164.7	31.4	95.5	87.0	86.7
	170.5	36.6	12.9	81.7	96.9	181.2	150.9	11.2
	28.5	32.2	29.1	75.7	209.6	58.6	89.3
239.2	14.9	85.8	75.2	78.3	77.2	103.6	17.9	53.3
	37.8	78.9	148.7	161.7	109.3	81.9	74.3
138.9	42.1	75.5	121.5	154.4	122.6	110.0	1095.7	82.9
	87.5	25.9	117.3	108.9	79.4	69.7	223.8	72.6
	114.4	69.7	9.6	110.1	146.9	88.0	33.9	80.8
	98.2	20.2	138.7	15.6	350.6	138.4	91.0
117.0	118.1	127.7	220.2	164.2	49.0	234.2	21.9	9.5
	196.4	196.6	13.6	46.3	15.2	177.8	49.5
101.1	13.7\
PODNL1	268.4	918.8	731.6	265.1	784.5	159.4	227.6
567.4	277.0	545.4	107.6	483.9	298.9	473.6	135.4
760.3	369.7	554.6	234.1	554.6	1699.4	232.9	783.3
246.9	244.0	400.8	237.6	755.5	431.3	452.9	266.1
212.3	1041.9	176.5	609.1	410.9	110.1	1035.7	297.4
130.9	332.7	266.5	537.5	340.2	95.5	538.5	274.6
172.7	896.5	710.5	302.9	207.9	314.3	932.7	520.1
694.8	335.2	737.6	392.0	1157.8	1011.7	399.7	471.9
285.7	528.6	513.3	204.2	428.9	498.9	246.5	783.8
949.1	504.5	267.5	390.0	144.4	440.9	395.4	349.6
481.6	585.3	469.9	340.6	326.6	865.9	852.4	451.2
493.0	290.1	872.7	160.9	680.7	244.2	348.1	379.5
353.0	281.8	289.1	555.8	443.1	357.8	314.3	265.8\
LOC100422781	24.7	67.5	15.7	37.1	12.4	77.0	68.8
	17.8	10.6	8.9	94.9	4.7	8.8	10.2	12.7
	46.8	7.0	39.1	19.4	11.1	116.0	6.5	53.3
	40.8	29.0	45.6	24.5	31.3	148.2	29.2	17.6
	39.3	14.0	7.9	41.6	56.3	10.8	9.2	51.3
	18.7	110.7	16.2	30.2	16.5	13.2	18.2
160.3	14.6	53.6	83.3	14.3	9.9	65.5	18.3	20.5
	60.2	12.7	17.7	16.0	128.9	41.2	75.0	56.4
	14.6	188.9	13.0	9.0	7.9	50.4	9.6	66.5
	20.8	15.9	18.5	11.4	9.5	32.8	49.7	11.8
	30.4	42.1	31.4	44.6	31.0	43.9	55.5	7.6
	31.9	109.2	13.0	18.3	19.9	13.2	64.9	7.6
	107.7	8.5	20.5	31.6	99.8	46.3	62.8
36.4\
CFL2	578.0333333333334	297.53333333333336
454.36666666666673	1050.6000000000001	322.06666666666666
	1224.6	369.3	581.1	473.6666666666667
225.10000000000002	749.1333333333332	468.6333333333334
	700.1333333333333	544.9333333333333
507.8666666666666	437.13333333333327	541.6333333333333
	528.7333333333333	354.3	288.09999999999997
114.6666666666667	492.9666666666664	375.6333333333334

799.8000000000002	560.0666666666667
366.93333333333334	682.4666666666667	351.40000000000003
527.3000000000001	426.26666666666665
966.8333333333331	419.06666666666666	168.76666666666668
509.9666666666667	577.8333333333334
337.59999999999997	813.1999999999999	282.2
157.83333333333334	1130.4333333333334	252.49999999999997
541.8333333333334	551.1333333333333
308.96666666666664	484.3666666666666	394.53333333333336
722.7000000000002	647.2333333333332
157.63333333333333	235.0	398.90000000000003
788.3666666666667	602.8000000000001	479.06666666666666
393.6333333333334	418.59999999999997
507.1666666666667	421.3	343.93333333333334
364.1666666666667	329.43333333333334	224.66666666666666
339.0333333333333	588.6333333333333
318.43333333333334	184.9333333333333	397.93333333333334
249.83333333333334	296.43333333333334
473.1333333333334	203.96666666666667	407.26666666666665
384.8	637.6666666666666	429.4666666666667
542.5666666666666	462.8666666666666	377.2
297.1666666666667	2354.0333333333333	768.5
696.7333333333332	670.1333333333333	711.9
265.93333333333334	390.0666666666666	497.9333333333332
537.6333333333333	360.6666666666667
417.09999999999997	584.1666666666666	704.6999999999999
748.4333333333334	840.5666666666667
933.0999999999999	768.1333333333333	935.0	826.0
471.63333333333327	737.3666666666667	667.5666666666666
768.5666666666667	701.6999999999999\
LOC285902 /// ZNF273 /// ZNF479 37.65	114.45	39.349999999999994
39.650000000000006	70.64999999999999	33.95	39.8
66.5	37.35	10.3	41.400000000000006	18.5
57.55	41.25	133.55	37.4	53.55	63.65	71.4	7.35
145.9	41.2	69.8	35.849999999999994	34.7
45.099999999999994	69.15	140.5	106.35	52.449999999999996
27.1	130.3	123.3	47.65	86.0	56.25	39.9
64.25	43.3	64.3	48.5	36.35	38.55	54.95
5.550000000000001	79.85	39.05	50.75	79.6
113.55000000000001	63.5	91.35	34.349999999999994
186.0	44.55	93.45	55.699999999999996	96.15	38.65
62.15	546.7	44.55	29.25	47.85	54.0	74.6	28.6	42.8
62.099999999999994	51.65	66.1	60.5	104.95	55.2
43.199999999999996	52.5	49.0	15.5	7.95
63.650000000000006	43.3	53.0	444.25	68.2	43.7
234.0	81.15	87.75	63.35	104.9	54.75	90.5	44.25	23.1
27.099999999999998	95.55	76.3	72.05	57.75	64.5
40.5	51.15	76.7\
CFL1	7028.475	20057.274999999998	12979.524999999998
10452.300000000001	16470.799999999996	8777.55
8133.099999999999	16873.5	10379.225	18858.125000000004
8426.175000000001	13894.0	14386.5	7759.35
12623.925000000001	11472.175	11913.425000000001
18586.425	12394.725	9016.975	14567.425

13124.3 24893.225000000002 7978.724999999999
13147.224999999999 12539.325 11969.075
11269.025000000001 15865.475 10003.65
5736.725000000001 18172.899999999998 14880.425
9493.675 18548.300000000003 16253.65 10881.825
16311.15 12439.775000000001 9291.175000000001
11129.325 12749.800000000001 15717.275
11880.05 14354.35 17226.349999999995
15650.949999999999 13719.55 11390.25 11563.525
12237.275 9868.925 9102.275
19776.850000000002 14247.65 11775.175000000001
11191.275000000001 13020.375 14860.300000000001
15079.025 29106.925 17176.7 13939.099999999999
10235.875 12519.675000000001 14469.2 8889.449999999999
11163.75 13583.900000000001 12019.225
12628.199999999999 17790.775 11768.925000000001
8099.200000000001 9809.150000000001 9547.4 15127.275
8819.225 10281.249999999998 11900.55
12225.725 12392.574999999999 12483.325 14493.4
10843.525 13868.9 21224.350000000002 17207.225
18077.050000000003 16966.975 8094.575 10909.2
6358.525000000001 4879.475 4919.45 1878.1499999999999
2888.6749999999997 3734.2 7173.075 3335.3
4843.7 15072.150000000001 2589.1749999999997\
PLS3 12551.1 1514.0 1852.7 11476.1 2732.9 3817.5 9824.5
5652.9 4576.3 2201.4 5275.7 4654.5 13940.4 830.1 520.9
1651.9 5506.0 3279.3 7877.8 7754.7 1237.3 7100.8 1952.1
7310.8 8838.0 11430.4 11851.8 2607.7 2124.4 6212.2 9122.1
3307.6 1161.2 4338.5 948.0 2390.0 14257.6 1771.2 6288.7
2616.1 2480.2 2036.4 2921.0 5214.0 5197.2 2974.2 8252.0
5252.3 1996.1 1943.6 5729.8 8077.5 7083.2 2964.5 4285.4
956.9 2396.7 3052.4 2882.0 1777.0 3587.2 1788.0 3699.7
6650.8 2388.6 2363.6 4673.6 3543.4 2406.2 5022.6 1536.6
1177.4 4772.5 3681.0 3829.7 5851.4 2753.2 6768.5 5260.0
11746.6 8344.9 2227.2 719.5 1481.5 6462.9 5224.1 3751.3
3872.1 5601.9 8633.6 4736.9 5541.8 4521.0 5476.8 4490.6
5117.2 3954.2 6249.6 1717.2 3845.8 3559.3 3303.6 4197.0\
OIP5 1250.2 199.5 161.9 809.5 198.4 812.4 708.1
180.1 507.6 319.5 507.8 206.3 480.5 406.1 348.1 67.8
352.8 118.9 858.2 144.9 6.5 694.5 76.2
292.1 1037.6 128.1 944.7 122.5 58.7 394.2 97.0 68.8
19.0 103.9 264.6 133.2 1291.9 171.4 232.9
274.7 305.3 458.9 156.0 106.8 753.4 488.7 642.6
1331.9 122.1 95.1 397.4 526.3 333.8 386.0 328.3 89.2
232.3 154.5 107.7 157.5 180.3 139.2 209.7 99.5
62.8 85.4 63.1 9.8 100.2 215.8 105.9
138.2 3.9 269.2 192.8 321.0 140.4 194.3 82.9
836.0 279.5 738.6 357.0 171.0 160.3 168.4 749.1 36.4
568.7 162.8 1113.1 77.3 108.4 43.9 7.6
111.6 37.9 89.2 9.7 117.3 77.4 198.9 71.7\
IL13RA2 6.2 8.9 6.4 4.2 16.7 16.3 58.7
111.0 234.4 359.0 54.4 97.4 54.4 63.8 26.0 41.0
44.2 8.5 6.4 221.0 20.9 46.8 75.7 66.8
18.0 27.9 81.2 80.8 284.4 89.6 74.8 15.9

	57.6	812.4	128.5	302.6	23.0	36.8	34.8
960.5	6.4	65.8	24.8	446.8	63.1	128.7	13.6
167.5	6.6	50.2	124.7	47.2	3.6	73.4	164.5	17.0
	2.6	145.5	52.8	314.8	24.3	44.0	9.8
398.3	92.2	16.4	70.5	110.4	34.6	336.5	24.1
127.4	95.9	81.0	1546.4	61.9	132.9	72.8	21.7
847.6	1201.9	134.6	167.1	27.0	72.4	23.6	85.0	14.0
	11.2	46.3	37.9	98.0	11.6	203.4	108.4	65.4
	19.2	44.0	12.1	15.0	71.3	132.5	8.9\
IL13RA1 1102.5000000000002	917.475 1024.625	803.025
755.6	1696.025	1387.65 1255.8249999999998
1542.3249999999998	1384.1750000000002	1680.625
2242.025	1953.1	1095.3500000000001	738.5000000000001
	1700.1499999999999	2090.975	671.2750000000001
	1752.25 1019.6000000000001	598.9250000000001
1086.5749999999998	676.275 1641.1999999999998
1035.9250000000002	1143.0000000000002	1290.9750000000001
	1611.675	1060.25 1958.4	1458.6749999999997
592.025 1046.45 1787.175	1287.45 947.625 1205.6
1136.6750000000002	2086.65 1012.1999999999999	1258.45
1279.875	1243.275	1458.625	1272.525
509.375 1353.7	1670.9250000000002	800.775 1129.2	924.2
2012.1000000000001	1207.0	557.75	1635.35 1234.5249999999999
	1164.125	811.0000000000001	789.6500000000001
	766.0	776.425 1306.875	1699.375
1770.2499999999998	1723.725	1310.8999999999999
1087.3	1413.6000000000001	938.275 1640.975
886.7249999999999	980.575 1115.65 1641.5749999999998
938.05	1612.0499999999997	1474.5500000000002	2097.4
1347.1250000000002	1559.8999999999999	812.975
1329.3999999999999	664.0749999999999	2251.775
1020.875	798.8000000000002	1349.6249999999998
1175.1	1065.4	950.0999999999999	911.1500000000001
800.55	882.65	1735.3	1062.4750000000001	912.975
833.9499999999999	1307.3999999999999	738.3499999999999
	911.2499999999999	861.1500000000001
706.5999999999999	896.225\
PLS1	10761.8 544.5	2221.5	1485.7	1081.5	2814.0	3090.4
3632.4	1670.6	1066.2	8066.1	2397.6	4454.4	9058.3	1392.2
283.0	1877.3	1758.1	5888.5	1210.7	35.9	5393.6	817.1
3671.1	9020.3	3049.4	3977.8	476.0	1424.2	2065.5	2157.5
908.5	572.3	2180.0	2175.0	1113.9	11824.3 523.7	2521.0
3783.1	1728.1	5291.3	1498.9	648.7	2910.4	1508.7	6054.0
5582.5	337.5	811.1	1849.0	9366.1	3381.9	930.0	1446.9
472.9	675.6	880.3	1089.1	433.8	1006.2	550.1	1282.9
4580.8	975.7	592.3	2401.7	817.0	391.5	5013.1	307.2
416.6	623.9	3328.6	975.3	6961.6	2044.4	4028.9	2187.2
5859.7	2109.1	4571.4	161.3	2284.4	1055.6	1496.0	3721.1
1071.4	3214.0	1241.3	3429.3	391.5	1092.6	1194.1	271.3
1410.6	815.1	1986.8	915.2	1060.4	728.8	379.5	1343.3\
KCMF1	3258.2250000000004	1402.075	1638.7749999999999
	2735.75 2060.3999999999996	3063.775	2565.925
	3624.25 2977.3	3312.3750000000005	4208.95 2466.9
4843.625000000001	3037.6	3301.2	2173.0	3465.725

2995.55 2672.8749999999995   3165.2 1301.3000000000002
3503.125   2172.675   2429.8500000000004   3959.7
2757.4999999999995   4043.275   2004.9000000000003
2074.9750000000004   2516.7000000000003   2641.6250000000005
        2405.025   1711.4 2353.675   3299.725
2574.8249999999994   4209.1 2290.675   3477.5750000000007
        3328.8749999999995   2707.05 2629.25 2123.4249999999997
        2681.3 3143.225   3246.675   3036.375
4384.025   2310.125   1782.3 3101.8 3599.625
4534.875   2801.625   2750.35 1604.725
2037.0000000000002   2085.275   2643.225   1274.325
        1011.9749999999999   2186.0 2955.275   3434.425
        2066.075   2321.475   2309.5 3445.0000000000005
        1442.0 2697.0499999999997   1427.15 1749.925
1895.55 2620.5249999999996   1670.1999999999998   2813.825
        2350.1749999999997   2901.425   2325.3
6628.175000000001   1859.575   3894.275
2482.0249999999996   3689.075   2694.225   2635.85
3363.375   2447.7250000000004   3455.65 4244.525
5090.925   1513.3500000000001   1965.8 1924.4750000000001
        1302.1750000000002   1668.125   1600.675
1624.6000000000001   1831.35 1391.7749999999999   1583.425
        2188.0 1754.0249999999999\
COL27A1 552.74 253.2   3271.54 695.98 3446.2200000000003
942.7   527.36 635.84 457.58000000000004   357.46 463.12
1404.2400000000002   383.82000000000005   275.28
672.8999999999999   1357.26 165.21999999999997
1698.2399999999998   626.9799999999999   1058.78 5304.4
755.68 832.0799999999999   819.0599999999998   581.9
1715.3800000000003   1079.3600000000001   6298.64 3202.04
398.49999999999994   876.46 3776.659999999999   4558.42
543.74 243.74 319.70000000000005   515.04 3957.2400000000002
        869.3000000000002   220.9   700.3999999999999
1533.06 2057.96 987.8999999999999   619.1800000000001
1607.7199999999998   714.5199999999999   556.5199999999999
        8874.52 3615.0799999999995   388.56000000000006
1097.56 1748.8799999999999   4485.28 2238.0 3520.5200000000004
        1459.94 862.46 660.5   4494.88 1688.8400000000001
306.35999999999996   1943.3 1154.8799999999997   863.14
4215.2 1717.56 735.08 4064.54 995.8399999999998   2698.36
1069.34 791.9399999999999   486.7   1236.8400000000001
248.8   1910.1799999999998   793.76 1172.2199999999998
632.74 2369.84 2218.3399999999997   188.26 546.3   10627.32
        17123.0 1400.3600000000001   1772.44 1055.68 8663.22
430.69999999999993   8176.1 5162.780000000001   3654.84
8506.5 6216.400000000001   6368.9400000000005   5010.14
4791.4400000000005   5097.06 7110.639999999999
1206.8199999999997   6349.58\
FKBPL   178.2   329.2   116.4   111.1   286.4   449.7   194.4   69.9
        161.1   216.2   206.1   85.5   154.0   162.2   90.0
111.0   228.1   100.1   302.4   64.7   108.8   109.6   220.8
194.2   267.7   35.3   258.2   37.7   33.3   106.4   197.7
144.5   104.4   121.1   60.6   289.2   168.5   104.7   199.1
294.2   299.8   89.7   25.5   64.6   255.6   166.7   159.3

```
287.0   114.1   237.5   140.9   171.5   233.5   167.3   116.0
115.9   138.4   76.6    93.3    249.2   70.3    192.9   179.9   55.3
        31.2    121.8   207.3   169.3   234.2   48.8    134.6   30.3
        42.1    133.7   43.9    173.9   148.8   107.0   129.2
169.8   32.0    45.2    452.6   445.5   44.5    175.9   197.7
405.7   149.2   13.8    346.8   28.6    58.1    102.1   110.3
199.6   60.6    190.1   40.9    265.7   132.4   167.6   117.8\
DIO1    98.5    52.2    100.5   10.2    63.0    54.2    15.7    31.0
        5.8     29.8    41.6    5.9     58.2    26.5    66.8    61.2
        51.0    108.8   44.9    56.7    102.3   34.4    101.0   2.8
        39.2    10.3    31.1    43.2    48.7    2.6     33.9    95.4
        6.6     38.4    167.8   122.2   10.2    72.6    18.9    56.4
        30.6    40.7    92.5    29.6    48.8    141.8   63.4    47.5
        78.2    30.7    15.4    55.5    30.4    13.3    31.6    77.0
        82.7    16.0    211.5   116.9   446.2   54.8    89.1    60.9
        11.5    67.0    30.4    48.2    109.9   123.4   45.0
182.8   15.3    9.2     44.7    8.8     7.1     31.3    48.7    4.2
        72.5    46.9    39.9    58.8    118.4   25.5    8.4
108.7   110.9   128.5   26.4    23.0    3.0     28.6    28.9    2.1
        8.1     53.2    60.8    30.7    31.4    46.3    28.2\
DIO2    73.05999999999999       152.83999999999997
165.28000000000003      196.46  197.26  84.9    91.66
554.4599999999999       3028.9800000000005      201.64000000000001
        191.84  299.6   249.2   279.82  91.74   403.62
284.82000000000005      543.8   128.28  232.17999999999998
476.94000000000005      523.4399999999999       457.06000000000006
        1079.84 284.0   84.46000000000001       1895.6799999999998
        1009.74 530.9   483.3   1814.3  378.46  799.72
872.3799999999999       147.3   986.5799999999999       540.28
217.82  236.06  741.6000000000001       300.5   146.8   2287.74
3436.94 602.22  121.06000000000002      536.76  385.82
219.45999999999998      1438.48 1046.02 187.82  57.9
688.6600000000001       1129.1799999999998      88.94000000000001
        256.76  796.62  218.73999999999995      628.78  780.28
440.26000000000005      251.18  377.36  1485.3400000000001
115.1   1096.2800000000002      270.34000000000003
2708.5800000000004      128.32  574.76  612.28  1024.9  1371.7
276.34000000000003      947.7400000000001       4111.360000000001
        1342.66 1114.5800000000002      62.54   830.3799999999999
        551.08  38.1    466.93999999999994      791.9199999999998
        257.06  160.6   1618.4  378.91999999999996      417.98
499.02  1365.72 180.85999999999999      646.1   653.22  134.68
235.37999999999997      332.56  145.32  154.59999999999997
261.92  392.64  477.2\
DIO3    81.2    30.2    32.7    52.1    21.8    9.7     41.2    90.8
        90.4    28.0    62.5    89.1    27.0    27.6    23.3    53.8
        34.4    49.4    85.6    100.3   44.9    109.4   38.7    21.2
        58.7    47.7    108.8   161.2   101.4   36.5    35.1    24.1
        10.8    88.6    22.7    28.1    24.3    97.2    14.2    42.2
        13.1    20.7    84.9    31.6    11.9    58.3    91.0    99.1
        81.4    90.8    97.9    94.0    57.4    145.0   137.0
199.9   96.0    21.6    100.0   210.8   26.4    81.8    46.0
134.6   65.8    25.4    25.5    40.5    153.8   5.2     107.2   85.7
        105.3   130.0   62.0    44.7    46.4    63.8    30.0
```

136.8	117.2	64.1	28.2	8.6	158.2	147.3	100.5
120.2	91.9	64.1	18.1	137.1	56.3	17.7	29.5	37.5
	45.8	27.9	23.2	48.3	18.5	124.8	114.4\
LOC439933		6.6	8.6	29.599999999999998		51.55	6.7
	22.45	25.049999999999997		18.0	19.1	9.65	7.65
	14.9	9.75	5.2	30.15	24.2	16.6	6.8
41.900000000000006		23.5	5.4	14.95	27.9
17.549999999999997		49.3	25.700000000000003		3.0
17.95	38.6	22.65	19.05	8.95	22.0	6.4	15.0	44.4
	2.65	63.7	22.2	13.850000000000001		28.5
5.6000000000000005		17.0	5.15	44.25	26.75	21.5	27.8
	31.95	22.200000000000003		9.7	6.75	11.0
53.449999999999996		34.5	21.6	9.8	73.45	3.05	31.2
	247.05	2.5999999999999996		35.05	3.25	11.9
10.700000000000001		20.1	4.2	5.15	5.7	11.2	69.4
	37.4	59.7	25.9	11.95	49.9	4.25	4.45
27.35	19.85	26.25	9.45	4.4	41.85	17.75	31.7
7.449999999999999		23.75	7.65	9.649999999999999
22.95	27.05	3.25	10.2	24.05	32.699999999999996		7.65
	15.75	10.0	16.95	57.8	33.6\
C1orf180		51.9	39.6	137.7	55.8	52.4	59.2	12.1
	91.5	8.8	141.4	53.0	20.3	23.9	65.8	84.7
	42.0	64.7	102.9	71.8	36.7	157.6	68.8	74.0
	42.2	85.7	93.5	24.5	99.1	117.0	111.6	49.8
	86.2	54.3	64.0	151.8	15.5	14.2	103.7	54.6
	50.5	8.2	5.8	72.1	61.5	47.3	52.2	7.3
	5.7	106.7	70.0	12.9	79.1	7.2	83.8	89.3
	164.3	18.1	6.4	21.7	17.0	446.2	105.8	46.8
	26.9	84.1	9.9	18.7	15.1	105.5	118.7	9.0
	44.7	79.8	54.0	87.9	46.7	15.6	44.1	46.5
	40.5	78.1	46.6	22.5	68.1	89.7	96.9	19.2
	25.8	86.9	113.6	2.1	48.7	14.5	7.5	71.3
	71.4	52.1	11.2	91.4	82.1	14.5	35.5
64.3\
APBB3 /// MIR6831		258.3	500.6	728.0	313.2	571.2
394.8	204.8	695.5	249.5	495.4	296.5	275.7	359.2
646.9	637.3	429.6	194.6	458.0	303.7	741.8	1865.8
518.8	903.2	354.7	217.1	610.3	384.7	835.6	948.6
460.1	277.2	984.3	1089.0	243.7	642.9	1169.3	475.9
711.0	293.3	314.5	308.8	534.0	295.6	309.7	304.5
577.0	898.8	515.2	1023.5	722.1	453.1	220.3	402.1
1215.5	367.4	648.6	649.4	671.6	800.9	495.1	1401.8
390.2	285.6	409.3	690.4	675.1	427.5	438.6	938.4
607.9	1213.3	738.4	385.9	322.6	606.4	321.3	1164.3
382.8	428.8	363.2	1015.1	299.6	347.6	558.4	1421.0
1602.0	643.7	798.9	721.5	631.9	305.5	1582.2	1055.1
722.2	477.5	764.2	668.1	806.7	895.7	957.5	897.6
915.6	877.7\
FAM91A1	2889.95	2142.1	2308.7	1012.5	995.4499999999999
1411.25	2091.7	1052.6	1609.0	874.5999999999999		2292.65
1026.95	1174.35	2266.3	1283.5	1502.15	2503.0	1508.05	1251.7
1627.6499999999999		562.8	1377.1499999999999		1051.5
2247.15	2429.35	1498.3500000000001		1195.3	1411.8	1427.7
1911.25	1350.2	933.25	967.75	697.8499999999999		826.85

```
1151.75 1900.45 1009.25 1449.5  4076.0  1910.85 2958.0
911.9499999999999       1472.0  1197.55 1626.7  793.95  2139.5
449.15  971.4   1284.65 1996.7  1495.3500000000001
826.9000000000001       1533.1999999999998      889.25
1605.1000000000001      1465.25 1507.6499999999999
785.5999999999999       582.05  966.75  894.0   2026.05
1164.8500000000001      1302.3  1515.5500000000002      1156.7
1075.6  1540.35 888.95  985.75  1317.45 2020.8999999999999
1932.2  1775.1  748.65  2033.8  1253.3  1189.45 820.15  951.15
2039.85 4263.15 948.9   695.0500000000001       590.7   933.55
1843.65 887.3499999999999       1994.8  1137.35 2054.95 1328.75
1118.0  2130.95 1418.1  2285.55 1378.55 2038.05 1836.8  2094.75
1259.25\
MIRLET7D        444.5   896.4   958.0   744.5   381.3   377.9
229.3   462.5   295.6   385.2   381.6   316.4   443.5   500.8
861.1   531.8   264.0   359.2   172.8   544.1   532.4   284.1
420.6   331.0   264.7   305.9   197.0   525.2   844.7   470.0
403.5   652.2   753.5   206.5   119.9   724.5   111.0   927.3
419.7   290.0   436.6   597.6   365.8   385.9   183.5   298.4
250.1   41.9    298.7   534.5   311.7   274.4   435.5   627.8
285.6   589.8   248.7   1110.0  504.2   217.7   636.5   654.4
803.3   738.3   560.2   1185.0  324.5   548.8   53.3    339.6
653.5   449.2   1108.9  382.5   730.4   182.4   450.6   333.2
276.9   153.4   572.1   459.5   484.4   1059.6  666.2   378.2
455.9   539.7   408.4   771.8   453.9   1125.4  1854.3  1089.2
1748.4  1139.0  1876.9  1064.8  728.2   1021.2  1503.5  889.6
1463.0\
LEMD3   6792.1  4080.6  3304.0  3714.3  2459.4  2913.5  4863.8
2630.4  3468.2  1828.0  3414.8  3709.9  4725.7  3544.0  3539.1
3585.8  4618.4  2696.1  3758.9  4831.6  2017.2  3393.8  2214.3
3624.8  4136.6  3627.1  3055.0  3674.1  3966.3  4878.1  4044.5
2937.7  2505.7  4127.1  2658.0  2041.1  2561.2  3294.3  5212.8
4366.4  5227.9  5672.1  3527.4  4842.3  3014.0  3988.3  3629.6
6075.1  3864.8  3319.0  3132.4  2984.8  3583.1  2681.0  3479.2
3144.0  3608.5  3615.0  4224.1  3896.7  1298.1  3060.9  3528.2
4280.9  4249.4  3348.4  4834.2  3546.1  1702.2  4314.1  2622.4
3043.7  4729.1  4115.7  4069.8  3752.7  3171.5  3139.1  4217.2
2558.2  3458.2  3955.2  2818.8  4489.9  3546.9  2356.6  4204.1
2890.5  2459.9  2156.6  5599.1  4313.1  4255.3  3522.1  3582.6
3858.6  4040.8  3513.9  2786.7  5397.4  4251.3  5236.6  3478.5\
LEMD2   134.1   358.4   380.2   338.0   505.6   288.6   315.6
174.9   297.7   320.0   212.9   442.6   350.4   304.2   548.0
303.7   292.6   373.5   430.9   399.3   406.7   163.1   300.1
149.4   204.6   259.8   195.1   232.8   238.4   281.5   233.6
105.1   544.5   75.8    278.0   211.0   233.4   414.6   276.2
580.6   501.4   407.4   168.8   362.0   236.1   114.4   163.5
509.6   350.2   123.9   206.7   159.3   174.6   116.0   396.9
123.6   337.5   301.8   380.9   166.8   132.3   230.0   189.5
256.5   186.0   425.3   388.0   290.4   221.6   98.1    289.0
333.4   87.3    376.5   333.2   230.8   120.7   278.0   193.9
170.0   131.2   80.6    697.4   289.6   219.5   382.7   43.3
191.3   382.0   435.0   272.2   226.5   90.1    112.4   102.4   98.3
        129.0   158.6   199.3   22.1    154.9   151.0   144.6\
LEMD1   41.0    80.4    244.6   69.8    322.0   25.5    739.4
```

```
105.3   641.1   193.1   150.6   64.0    91.8    70.0    168.0
183.3   319.6   210.5   582.8   201.8   212.1   86.2    60.8    12.9
        119.0   7.9     397.3   15.1    107.5   930.9   116.2   88.9
        259.4   188.5   92.7    55.9    108.3   151.1   658.6   54.1
        655.3   134.4   141.3   536.4   93.7    115.4   10.1    61.8
        213.1   174.0   276.5   440.7   73.0    7.1     212.7
113.8   147.7   107.6   50.8    150.0   357.5   167.4   1476.3
165.0   56.3    510.0   100.4   520.4   132.5   114.5   6.2
121.1   48.5    72.7    194.6   71.8    68.4    32.8    276.4   11.4
        130.6   238.8   194.3   245.6   230.8   27.8    556.5
134.8   153.7   5.7     350.1   87.5    74.0    316.9   14.9
114.3   9.2     114.8   12.3    199.4   101.6   116.3   129.1\
TOM1L1  1089.4499999999998      603.1   1228.55 714.95  2184.3
940.9   763.9499999999999       601.3   500.5   632.7
1401.3500000000001      863.95  985.4   839.0500000000001
554.9   574.85  587.3   808.75  1757.0  787.7   247.25  658.55
535.45  960.35  776.8   1550.25 1424.6  845.3   1753.25 720.15
1620.0  840.45  554.75  911.0   477.8   793.2   1393.2  633.05
1246.1  768.75  1110.25 655.85  1115.25 339.55  1124.0  701.15
1693.5  480.0   1235.1999999999998      882.7   959.3000000000001
        1190.75 1381.5  904.3   862.4   563.35  729.6999999999999
        1100.3999999999999      1273.35 811.9   1063.0  594.4
1175.25 1056.65 823.4   974.25  844.3   425.25  681.65
1647.3000000000002      1066.3  1247.55 368.2   1172.55 713.3
1432.8000000000002      993.65  898.15  664.9   956.5   1526.15
1138.0500000000002      709.55  312.3   586.8499999999999
409.1   1189.85 660.5   347.54999999999995      576.95  616.8
609.65  1528.5  1333.5500000000002      837.4   1937.7  1506.15
1633.65 1468.35 1588.0  1161.8500000000001      1011.15 1550.6\
ABI2 /// RP11-363J17.1  3.6     75.2    24.9    19.5    85.6    30.4
        40.9    40.5    14.0    7.6     29.8    9.9     32.9    35.3
        8.5     7.2     13.8    12.3    37.8    9.6     37.4    50.6
        110.3   61.2    61.8    14.8    63.3    109.7   20.0    12.2
        4.2     144.2   13.6    57.7    69.5    56.3    4.3     18.8
        30.5    12.9    7.8     44.9    18.4    36.0    12.4    3.8
        8.6     30.0    134.6   9.2     12.5    60.4    56.8
140.1   87.7    54.9    70.6    9.2     81.6    17.4    48.4    20.6
        4.7     47.6    15.0    10.4    10.9    12.0    22.0    32.7
        13.6    11.3    31.6    8.6     8.4     15.8    76.5    39.4
        6.2     50.5    93.5    83.5    59.2    28.0    71.3
147.2   57.4    126.4   3.7     102.1   29.4    112.4   43.6    6.9
        5.7     38.5    37.7    75.7    13.2    9.6     21.8    65.3
        4.0\
CTD-2587M23.1   9.5     10.2    6.8     4.4     17.3    14.4    3.5
        12.7    30.4    18.5    20.5    29.0    21.0    44.4    27.8
        28.6    37.1    2.3     44.8    52.6    17.5    19.3    43.6
        16.3    35.3    16.3    7.5     116.5   28.3    24.5    32.4
        34.7    6.0     34.8    30.1    8.4     13.4    7.1     5.2
        37.2    14.1    26.4    15.8    6.4     42.6    68.6    17.7
        19.7    39.8    11.6    21.1    20.4    11.1    101.0   7.2
        28.2    5.9     7.4     31.0    9.2     177.5   8.5     7.2
        16.3    21.2    7.3     21.5    25.2    10.3    82.2    12.9
        69.3    66.3    16.9    31.0    18.2    12.3    14.8    10.1
        6.3     17.2    35.6    6.6     5.9     68.8    51.6    18.9
```

	15.8	82.6	94.5	8.8	38.7	15.5	7.6	3.6
	20.1	23.2	15.1	5.9	29.7	17.9	25.1
28.5\
TOM1L2	174.5	378.59999999999997	162.86666666666667
137.66666666666666	235.93333333333333	73.03333333333333
	147.9	172.4	160.39999999999998	228.6
110.43333333333334	181.69999999999996	93.86666666666666
	131.33333333333334	160.33333333333334
150.43333333333333	131.06666666666667	122.0	117.2
237.96666666666666	439.13333333333334	122.93333333333335
	388.46666666666667	81.5	206.0	145.56666666666666
	131.36666666666667	232.56666666666667
177.16666666666666	193.39999999999998	123.16666666666664
	137.96666666666667	125.73333333333333	115.8
248.46666666666667	202.66666666666666	125.73333333333333
	262.06666666666666	72.6	115.66666666666667
85.63333333333333	82.66666666666667	113.2	162.4
169.33333333333334	110.46666666666665	190.5
128.63333333333333	357.09999999999997	224.73333333333335
	203.43333333333333	125.63333333333333	76.5
396.96666666666667	214.20000000000002	316.86666666666667
	102.06666666666668	217.5	175.5	253.33333333333334
	307.93333333333334	274.73333333333335
143.76666666666668	173.43333333333333	213.83333333333334
	155.7	126.06666666666666	152.33333333333334
196.69999999999996	171.63333333333335	193.29999999999998
	298.73333333333335	145.63333333333333
76.39999999999999	155.33333333333334	129.1
194.16666666666666	107.30000000000001	81.23333333333333
	61.400000000000006	257.96666666666664
154.56666666666667	148.43333333333334	92.66666666666667
	200.19999999999996	289.13333333333333
182.83333333333334	214.29999999999998	302.96666666666667
	191.20000000000002	102.16666666666667
241.06666666666667	163.50000000000003	97.63333333333334
	231.86666666666667	163.83333333333334	137.1
136.10000000000002	171.1	71.56666666666666
175.76666666666665	122.06666666666666	205.93333333333333\
MTNR1A	8.1	20.8	12.5	9.8	76.8	16.9	35.0	22.3
	3.0	13.8	16.4	35.9	49.8	20.0	16.8	81.9
	32.1	6.8	5.5	24.0	202.8	17.0	28.8	5.4
	6.9	4.7	50.2	9.1	17.6	11.6	1.8	11.0
	24.4	56.4	23.0	16.2	12.0	148.5	7.3	12.4
	3.3	9.4	20.6	68.6	52.6	2.5	20.2	24.7
	20.4	103.3	16.8	16.3	8.3	44.4	33.5	58.9
	83.1	23.5	20.4	30.7	106.8	6.5	10.1	3.7
	26.7	95.0	11.1	9.9	10.8	32.0	90.7	35.2
	71.7	3.9	23.9	31.4	6.0	64.4	40.1	15.2
	9.3	7.3	39.4	47.2	36.7	29.5	76.3	9.2
	84.2	77.8	30.8	113.7	4.8	5.2	31.1	83.2
	58.0	4.0	32.1	7.7	16.8	31.3	89.5\
OBSCN	64.76666666666667	309.43333333333334
75.26666666666667	81.3	854.7333333333335
58.06666666666666	159.4	125.8	54.0	165.43333333333337

```
	120.5666666666666	90.63333333333333
152.03333333333333	108.2	778.5333333333333
129.73333333333332	54.666666666666664	148.0666666666667
	176.4	96.66666666666667	498.5	26.03333333333333
	108.96666666666665	52.6	71.33333333333333
99.83333333333333	46.5	95.0666666666668
93.96666666666665	104.73333333333333	140.4
85.76666666666667	480.26666666666665	49.9	143.9
195.6	43.03333333333333	711.6999999999999
50.833333333333336	56.666666666666664	136.26666666666665
	63.4	82.86666666666666	82.39999999999999
62.633333333333326	166.5666666666666	73.3666666666666
	55.699999999999996	262.5	144.23333333333335
55.699999999999996	112.30000000000001	299.93333333333334
	176.6	147.03333333333333	397.1333333333334
28.066666666666666	95.33333333333333	51.93333333333334
	92.83333333333333	343.73333333333335
248.29999999999998	322.93333333333334	78.1666666666667
	142.63333333333333	578.9333333333334
59.2666666666666	151.4666666666667	158.83333333333334
	52.06666666666667	163.06666666666666
368.0333333333333	52.300000000000004	94.33333333333333
	97.39999999999999	39.86666666666667
113.69999999999999	41.199999999999996	101.5666666666666
	203.46666666666667	488.3666666666666
188.0666666666667	209.46666666666667	180.10000000000002
	414.76666666666665	1618.4666666666665
205.70000000000002	339.03333333333336	89.8666666666666
	553.9666666666667	66.3	327.6666666666667
243.36666666666665	211.46666666666667	226.26666666666665
	211.1	130.20000000000002	147.8	471.8333333333333
	280.3666666666667	176.0	95.3	246.93333333333337\
LOC101927382 /// PAX8-AS1	7.7	117.6	120.2	85.2	57.7
	10.9	33.9	38.2	59.1	31.1	13.0	107.7	40.5
	16.2	30.2	80.2	24.4	13.9	24.2	47.7	26.0
	19.7	26.3	60.4	20.3	34.5	97.8	47.5	57.8
	9.8	13.8	55.3	45.7	20.8	34.8	24.9	17.8
	188.5	9.7	20.9	13.5	16.5	22.1	85.4	8.3
	31.8	228.7	8.8	42.5	21.7	110.7	43.5	17.2
	417.9	19.5	54.8	105.6	26.9	16.4	116.4
161.8	9.6	16.4	18.7	26.1	35.3	15.9	27.7	31.0
	130.1	305.0	}
```
