# Supplementary material for: Identification and validation of hub genes in uterine corpus endometrioid carcinoma: An observational study from TCGA and GEO
Source: Medicine (Baltimore). 2025 May 2;104(18):e42338. doi: 10.1097/MD.0000000000042338 (PMC12055170; doi:10.1097/MD.0000000000042338)
Supplement: Supplementary file 3 [file medi-104-e42338-s003.pdf]

Tag	logFC	AveExpr	t	P.Value	adj.P.Val	B
ASPA	-4.87469201977401	1.25031492537313	-23.5049906993573	1.80536518481442e-59	3.70641472442399e-55	124.721439339277
TCF23	-5.42166814971752	1.21059104477612	-21.6089062277274	3.23252160062939e-54	3.31818342304606e-50	112.764540181259
ZNF695	4.58238813559322	6.23823731343284	20.1837374936551	3.8276747286698e-50	2.61940540598637e-46	103.481906628607
SKA3	4.23544442090396	7.73515721393035	19.7860050673053	5.47047917125949e-49	2.80772343464893e-45	100.84817112555
CLEC4M	-4.30389357344633	1.00426666666667	-19.7524410774057	6.85258685544011e-49	2.81367216284371e-45	100.625082436123
FGF10	-3.03905084745763	0.627954228855721	-19.6061485947327	1.83183969577841e-48	6.2679448257218e-45	99.6512325666186
MYBL2	5.83617069209039	10.1299686567164	19.4494851543924	5.26412940347604e-48	1.5438939521909e-44	98.6056749962299
TROAP	5.05481822033898	8.79639950248756	19.3538069449816	1.00433147690747e-47	2.5773656526138e-44	97.9657832794451
CCNB2	4.52514110169492	8.99311741293532	19.1508002338083	3.96784467537166e-47	9.05109457615335e-44	96.6047498099157
CDC25C	4.86741405367232	6.99164278606965	19.1180298767299	4.95509491877071e-47	1.01728098682363e-43	96.3846242883385
CDC20	5.01980310734463	10.1526736318408	19.0040263291402	1.07432494957985e-46	2.00508101953403e-43	95.6179365147939
SPAG5	3.62152676553672	9.69516815920398	18.7461865155038	6.21463905993949e-46	1.06322116583798e-42	93.8788337565372
CEP55	4.42087634180791	8.72336467661692	18.3997038033396	6.64317633579029e-45	1.04911084749057e-41	91.530983345345
KLF17	-3.71634879943503	1.00383184079602	-18.3732212102513	7.96589477143391e-45	1.16814156898241e-41	91.3510306231784
KIF4A	4.51339258474576	8.7442592039801	18.3212670629148	1.13770002479048e-44	1.55713210059658e-41	90.9977924252027
ZWINT	3.81223757062147	9.71962835820896	18.2911609634551	1.3988737790374e-44	1.79492991772736e-41	90.7929774824268
HJURP	4.5997311440678	8.47913034825871	18.2149301703635	2.3615847860895e-44	2.85196092108338e-41	90.2739723992395
NCAPG	4.31168425141243	8.70481044776119	18.2018166675987	2.58434465090487e-44	2.94758864905983e-41	

90.1846338547025
TPX2 4.68140430790961 10.3440348258706
18.1706088357742 3.20288833105974e-44 3.46080512824508e-41
89.9719564514697
CDCA2 4.49881843220339 7.32091691542289
18.1531207442256 3.61228084953822e-44 3.70800629205099e-41
89.852735970213
MYOZ2 -3.27957775423729
0.981076119402985 -18.1181261255431 4.59572250710486e-44
4.36305500205432e-41 89.6140804630284
SPC25 4.35770134180791 6.97597462686567
18.1156267613729 4.67546079129055e-44 4.36305500205432e-41
89.5970308331507
FOXM1 4.54097111581921 9.75521144278607
18.078769685962 6.0258763804419e-44 5.37874965610749e-41
89.3455373235916
ASF1B 4.15237394067797 9.34468059701492
17.8914253670552 2.19277994537762e-43 1.87574051160844e-40
88.0651986884711
RAD54L 4.33711991525424 7.95582985074627
17.8675444053945 2.58585361112571e-43 2.12350298545643e-40
87.9017555877918
PPP1R12B -4.33043460451978
9.37549054726368 -17.7846518121548 4.58498694759096e-43
3.62037623207855e-40 87.3340226101347
TUBA1C 2.60895600282485 12.6619597014925
17.7538767926297 5.6722130881832e-43 4.31298276668152e-40
87.1230830973062
MELK 4.87847542372881 8.24485422885572
17.7178370465476 7.27818650344567e-43 5.33647031841927e-40
86.8759480530427
UBE2C 5.05946751412429 9.99760199004975
17.6923744755768 8.68056728036416e-43 6.14524297468538e-40
86.701272729363
CENPA 4.62529731638418 7.38916467661692
17.6504384403001 1.16047684891289e-42 7.94152990272723e-40
86.4134605852655
EXO1 4.28119639830508 7.42451293532338
17.6362060405643 1.28069018439695e-42 8.48147402763529e-40
86.315745985947
CDC6 3.77039152542373 8.69732885572139
17.5704547498563 2.01979752846102e-42 1.29582635185327e-39
85.864086471772
SKA1 3.95475254237288 7.32480995024876
17.5501192456242 2.32560536404151e-42 1.41263271681805e-39
85.7243199831823
POC1A 3.59613305084746 8.55119402985075
17.5492614072727 2.3394794141166e-42 1.41263271681805e-39
85.718423234335
AURKB 4.84702973163842 8.58788407960199
17.5394823964456 2.50362166714342e-42 1.45578251817174e-39
85.6511980989322
PKMYT1 4.83895564971751 8.49201990049751
17.5366795758379 2.55276038257101e-42 1.45578251817174e-39

85.6319287506608
LOC134466 -4.8044718220339
3.02594228855721 -17.3956538534512 6.79388989261488e-42
3.67986886388118e-39 84.6614950587496
CDC45 5.04658255649718 7.7633263681592 17.3952864409029
6.81125264624865e-42 3.67986886388118e-39
84.6589645559139
KIF18B 4.64800692090396 7.95892935323383
17.3492612963972 9.37829571238709e-42 4.9368310506489e-39
84.3418816230082
BUB1 4.50397923728814 8.86179900497512
17.2968503700884 1.35016054255452e-41 6.92969898466109e-39
83.9805860280769
PLK1 4.07087846045198 9.14048258706468
17.2618964265932 1.72182107409595e-41 8.62170406126584e-39
83.7395020401183
CAMK2A -4.94119646892655
2.38033880597015 -17.244158821982 1.94801982543313e-41
9.5221064324148e-39 83.6171233915119
FAM54A 4.13223269774011 6.62841094527363
17.217956386199 2.33776538689758e-41 1.11614705565133e-38
83.4362947624116
MKI67 4.31326405367231 10.4992432835821
17.203147015718 2.59166577480721e-41 1.20924768992709e-38
83.3340670010083
SLITRK3 -5.6038572740113
1.33026417910448 -17.1934909397543 2.77191272544016e-41
1.26460818340636e-38 83.2674022350287
IQGAP3 4.5199490819209 8.71186616915423 17.1525760260766
3.68591155790515e-41 1.64503835399549e-38
82.9848441016469
MCM10 4.76545466101695 7.35517014925373
17.1256157908625 4.4476434080952e-41 1.94276849294031e-38
82.7985822277522
C16orf59 3.70884717514124 7.93374975124378
17.1023157494134 5.23189525231087e-41 2.23772519854046e-38
82.6375602971064
C9orf140 4.17524548022599 9.502992039801
17.0553960583894 7.25673369282164e-41 3.04042332068629e-38
82.3131750849199
RRM2 4.46299915254237 9.62438706467662
17.0042163022459 1.03707231554323e-40 4.2582189276205e-38
81.9591371164689
TK1 3.80493100282486 10.3428233830846
16.9925122911654 1.12533817851464e-40 4.53003780488343e-38
81.8781450476487
NEK2 4.63798269774012 8.42775621890547
16.9478124949905 1.53747155361795e-40 6.07005596072627e-38
81.5687223556472
BIRC5 5.09293742937853 9.0250671641791 16.9370011260923
1.65804288472712e-40 6.42256989121656e-38
81.4938600502083
ESPL1 4.34352923728814 8.36139054726368
16.9088928252969 2.01771849768493e-40 7.67106680693919e-38

81.2991843577845
UHRF1 4.07411101694915 8.21483333333333
16.8955204463356 2.21530744207281e-40 8.26913850650087e-38
81.2065469872778
CSRP1 -2.73279950564972
12.4494507462687 -16.8434051819752 3.18870153076805e-40
1.16900075761907e-37 80.8453868871736
SPARCL1 -4.54330098870057
11.1468577114428 -16.8389728846146 3.28905260824426e-40
1.17637320791614e-37 80.8146614060222
KIF2C 4.21471984463277 9.31925721393035
16.8374863758496 3.32341188792674e-40 1.17637320791614e-37
80.8043563267703
SGOL1 4.36499639830509 6.3351736318408 16.7962670873066
4.43366468279033e-40 1.54276501589297e-37
80.5185410904954
NCAPH 4.42847521186441 8.15122288557214
16.7737554717561 5.18980766715919e-40 1.77577919011297e-37
80.3623912890475
PGM5P2 -4.13254978813559
1.81760149253731 -16.7449842284188 6.34715895183575e-40
2.13618316854406e-37 80.1627671634452
TTK 4.51663905367232 8.10409303482587
16.7263436210831 7.23164607560915e-40 2.39460796664929e-37
80.0333999120607
CDCA5 3.8797770480226 9.37208258706468 16.6331305235757
1.38905513186444e-39 4.52655585034553e-37
79.3861124644813
ZBTB16 -5.36248453389831
2.06184129353234 -16.610615881439 1.62641679866015e-39
5.217240136952e-37 79.2296728439612
POLQ 3.97704632768362 7.18890845771144
16.5306074637492 2.84985570660369e-39 9.00115963947288e-37
78.6734552762847
CNRIP1 -3.60923516949153
6.48627661691542 -16.5015951312099 3.4930130564276e-39
1.08653875830998e-36 78.4716510631852
CBX7 -3.48949230225989
8.41863432835821 -16.4500148988616 5.01631857487559e-39
1.53708985585367e-36 78.1127255064802
PBK 4.61716483050847 8.03728656716418
16.4131458553986 6.49810647223485e-39 1.96185479227914e-36
77.8560579034719
GTSE1 4.07668601694916 8.06569253731343
16.386228685356 7.85006450493833e-39 2.33567861284614e-36
77.668613458092
PTTG1 4.05318799435028 9.47491691542289
16.3791150609496 8.25221131649521e-39 2.42025569039495e-36
77.6190678630747
KLHL33 -2.58757302259887
0.608182587064677 -16.34997375383 1.01267157494809e-38
2.92818977939216e-36 77.4160668510311
AURKA 3.55992951977401 8.66437164179104
16.3437178395826 1.05817135147162e-38 3.01725803412673e-36

77.3724802605775
LMOD1 -5.69245190677966
8.03046467661692 -16.3287432201911 1.17557253808347e-38
3.30609646669229e-36 77.2681377204751
TACC3 3.32639583333334 10.6552517412935
16.3079512544159 1.36051794017199e-38 3.77451801509878e-36
77.1232358126335
ORC6L 3.33492168079096 7.94871791044776
16.2906350450738 1.53659939623257e-38 4.20618474728729e-36
77.0025354354298
CDCA8 3.79514088983051 8.77265223880597
16.2859544840776 1.5879950080673e-38 4.28967598889759e-36
76.9699068624786
KANK2 -3.03811080508475
10.8497945273632 -16.2318833399124 2.32250781019412e-38
6.19234874588121e-36 76.5928705259782
DLGAP5 5.12103086158192 8.32550199004975
16.2189996521835 2.54278536067815e-38 6.69274146855417e-36
76.5030055243593
EME1 3.28560254237288 6.76434179104478
16.1986341604161 2.93443242243514e-38 7.62580982691057e-36
76.3609328549387
E2F2 3.9768645480226 7.33339004975124 16.1948738263883
3.01309448635303e-38 7.73235372560347e-36
76.334697364172
CCNB1 3.23505437853107 10.4163542288557
16.1675699612392 3.65126467256308e-38 9.25437823799013e-36
76.1441744549597
NDN -4.28010600282486
7.57561393034826 -16.1465477193859 4.23343625193421e-38
1.0599078811245e-35 75.9974524276542
UBE2T 3.3097468220339 8.64146567164179 16.1420333586717
4.37010289228911e-38 1.08094231781561e-35
75.96594147852
OMD -6.16536920903955
2.41504427860697 -16.1186254877619 5.1528671641901e-38
1.25938527239075e-35 75.8025308418257
KLHL4 -5.2248854519774
2.33317313432836 -16.0627874617269 7.63487272688144e-38
1.84404631862207e-35 75.4125908276854
DIXDC1 -3.00433679378532
7.89230248756219 -16.0045138675036 1.1510320033543e-37
2.74775430568183e-35 75.0054442556718
FBXL22 -3.7422384180791
5.04572935323383 -15.9919712125873 1.25739654135203e-37
2.96716678091462e-35 74.9177850883384
SHCBP1 3.28568206214689 7.49057014925373
15.9882842656669 1.29049503352763e-37 3.01066625435479e-35
74.8920157068653
SYNPO2 -4.94946913841808
8.54238308457711 -15.9794271571291 1.37361591497005e-37
3.16777900557635e-35 74.8301070189223
MYLK -4.72999279661017
10.1139741293532 -15.9778775381716 1.38870000244457e-37

3.16777900557635e-35	74.819275154794
NPAS4	-3.99153806497175
1.39740099502488	-15.9347668438856	1.88185700229001e-37
4.22831096206616e-35	74.5178755045186
KCNB1	-4.78686129943503
1.87552089552239	-15.9337938658479	1.89481056264046e-37
4.22831096206616e-35	74.5110719060132
KIF11	3.36562224576271	9.32115273631841
15.9233677025645	2.03934770460332e-37	4.50191487908669e-35
74.4381630718229
CKS2	2.81504371468927	9.83924825870647
15.8482180605561	3.46481839458117e-37	7.5673108128459e-35
73.9124717888219
FHL1	-5.08064081920904
8.31056119402985	-15.813449394352	4.42817240981443e-37
9.56951363931477e-35	73.6691511655286
MYOCD	-6.64929978813559
2.93280895522388	-15.7961321932406	5.00381486309224e-37
1.07008665770087e-34	73.5479363993844
TCEAL1	-2.28842803672317
8.29717960199005	-15.7814890361406	5.54865117793864e-37
1.17436916168124e-34	73.4454266085295
CACNB2	-4.08066016949153
4.03386616915423	-15.7747343899408	5.81961590085847e-37
1.21915014739413e-34	73.3981367116348
TNS1	-3.31514230225988
10.4976164179104	-15.7447377559974	7.19223351832418e-37
1.49148034475955e-34	73.1880985819567
MND1	4.03435197740113	6.72147164179104
15.7192666123895	8.60941974471541e-37	1.76751387359007e-34
73.0097114565393
FANCA	3.2496865819209 8.39773930348259	15.7115862363981
9.08927878769122e-37	1.84755340110199e-34
72.9559153982336
OGN	-8.66548382768362
4.00520497512438	-15.7056998273601	9.47509966418171e-37
1.90709604025148e-34	72.9146828736693
TOP2A	4.29480021186441	10.754984079602 15.6907158662072
1.05328260571369e-36	2.09940698012642e-34
72.8097167719511
NUF2	4.39159307909604	7.98324875621891
15.6657042388579	1.25682641442873e-36	2.48102368155979e-34
72.6344792820184
KIAA1462	-3.57843862994351
8.25904179104478	-15.657966650122	1.32744126572085e-36
2.59546373192849e-34	72.5802615186424
SEL1L2	-3.19977577683616
0.832899502487562	-15.6562937293659	1.34322337185899e-36
2.6015448890816e-34	72.5685388669916
HN1	2.83891398305084	11.3062641791045
15.6178495076924	1.76246615635701e-36	3.38162898972051e-34
72.2991106870837
C1orf135	3.35855868644068	6.73661592039801
15.6037206737829	1.9475351224554e-36	3.70212000592678e-34

72.2000735593969
GPRASP1 -4.14478672316384 6.5040631840796 -15.58434869419
2.23330956876226e-36 4.2064078391458e-34 72.0642685131772
CDK1 3.11521701977401 9.85553830845771
15.5729835039899 2.42013038196513e-36 4.51684334015855e-34
71.9845857675789
EZH2 2.93246186440678 8.79710049751244
15.5617766992298 2.61966021407485e-36 4.84519136891501e-34
71.9060074590908
KIFC1 3.75413622881355 9.63086865671642
15.5467317016368 2.9136600574983e-36 5.34084294468215e-34
71.8005077067147
CENPF 3.75416638418079 10.5225910447761
15.5403076325218 3.04905059906116e-36 5.53955830077219e-34
71.7554570864054
CACNA1H -4.66388778248588
7.10873184079602 -15.5344983029511 3.17689951008546e-36
5.72120587211005e-34 71.7147158407384
RAD51 3.30020120056497 7.58916815920398
15.5129797516534 3.69902171393061e-36 6.60355789452134e-34
71.5637910277492
IRS4 -3.65557577683616
1.06252338308458 -15.4982402091406 4.10541693024104e-36
7.26588013602143e-34 71.460399873509
LOC283856 -2.05204781073446
0.597768656716418 -15.4669979055549 5.12067939162685e-36
8.98526050513669e-34 71.241216693792
DPP3 2.02019872881355 10.4592393034826
15.4358552659708 6.38278916977648e-36 1.11049713267382e-33
71.0226891533715
BUB1B 4.25044625706215 8.60874278606965
15.4292823018951 6.68663613667505e-36 1.15358520912554e-33
70.9765612578572
CDCA3 3.50318855932204 8.34048905472637
15.3506807148003 1.16625731381156e-35 1.99527188771261e-33
70.424804670988
PDE2A -4.66706991525424
5.66863383084577 -15.323311574381 1.4156028626665e-35
2.4018451876482e-33 70.2326213783135
CCNA2 3.14777422316384 9.00301641791045
15.3166080470964 1.48440909867179e-35 2.49794416358458e-33
70.1855451973584
TBC1D7 2.12225395480226 8.96280049751244
15.2959663746801 1.71801951892602e-35 2.86755615638628e-33
70.0405755424463
MRGPRF -4.76451666666667
7.48366417910448 -15.2752011805582 1.99016862566259e-35
3.29501305523007e-33 69.8947210835282
RASL12 -3.79064406779661
7.26017014925373 -15.2667298894134 2.11322049591267e-35
3.47075334248697e-33 69.8352139183504
TGFB1I1 -3.7450895480226
8.60110199004975 -15.2565948415419 2.27048444612078e-35
3.69944806975077e-33 69.7640158650792

ORC1L 3.5383175141243 7.11066517412935 15.2539432415893
2.31352766520225e-35 3.73989944618915e-33
69.7453878829745
NKAPL -2.89977563559322
1.71165373134328 -15.2241617694843 2.85685929096521e-35
4.58213447214968e-33 69.5361487680656
KCTD8 -3.88571122881356
1.61144179104478 -15.2024779981212 3.33120976015812e-35
5.30152995163149e-33 69.3837812278917
ERCC6L 3.33521094632768 6.80660597014925
15.187156056361 3.71318573534387e-35 5.86397716512383e-33
69.2761063143575
KIAA0101 4.28819555084746 8.7293263681592
15.1843791650455 3.78696600014336e-35 5.93484060938498e-33
69.2565907789942
BAIAP2L1 2.30619209039548 10.0207258706468
15.1757419540233 4.02596165321736e-35 6.23535803133656e-33
69.1958880542355
ADH1B -7.59654138418079
3.55323333333333 -15.1752693069443 4.03946720977965e-35
6.23535803133656e-33 69.1925661888316
HMGB3 2.43608185028249 11.035252238806 15.1424322843047
5.0977724915332e-35 7.81024397396841e-33
68.9617605390844
DEPDC1 4.20456666666666 7.56346218905473
15.1327483514 5.45991768999888e-35 8.30311927227238e-33
68.8936864396431
FAM64A 3.99176588983051 7.52037462686567
15.1217204739446 5.903777141251e-35 8.9120988757267e-33
68.8161608890736
CDT1 3.52744378531074 8.72560646766169
15.114581520418 6.21018703725775e-35 9.30621458940887e-33
68.7659720296431
NUSAP1 3.49571596045197 9.475592039801 15.1056190780396
6.61749257152304e-35 9.84471902125856e-33
68.7029609957261
KIF14 4.13123043785311 7.11449502487562
15.0989749469685 6.93659994849786e-35 1.02452084131411e-32
68.6562471915051
FRMD7 -3.57906645480226
0.999480597014925 -15.0855775698944 7.62765302211598e-35
1.11854083245744e-32 68.5620477676207
ABCC9 -4.55625395480226
5.53579303482587 -15.078829445664 8.00140530262426e-35
1.16502731108423e-32 68.514598129394
MAMDC2 -6.50314230225989
4.58087313432836 -15.0555232242692 9.43895471495984e-35
1.36466014294455e-32 68.3507078502068
HPSE2 -4.55601235875706
1.47784328358209 -15.0496657910163 9.83919626112175e-35
1.41257831636944e-32 68.3095152773514
PRLHR -4.16854830508475
1.07247213930348 -15.0152160928232 1.25615776471376e-34
1.79089714649817e-32 68.067223867117

FANCI 2.24621200564972 9.69648258706468
15.0065942782074 1.33535537731714e-34 1.89067902733248e-32
68.006578940097
MLF1IP 2.8308436440678 8.68790746268657 14.9844102411426
1.56286670787977e-34 2.19764750087477e-32
67.8505278999975
FAM72B 3.06527436440678 7.15967562189055
14.9571957008458 1.89561308786275e-34 2.64741065944369e-32
67.6590693429344
ECE2 2.95708834745763 8.42439900497512
14.9501157131652 1.99323954456802e-34 2.7649464763501e-32
67.6092567902725
PRAME 4.82086144067797 11.5102945273632
14.9454772565513 2.05991143799354e-34 2.83825381355755e-32
67.5766212530923
ZCCHC24 -3.37992966101695
8.36269402985075 -14.9300621262857 2.29793551346218e-34
3.14510773942523e-32 67.4681578884162
TENC1 -2.69791709039548
9.67934726368159 -14.9207610788949 2.45466934243747e-34
3.33737494041333e-32 67.4027107835695
ADRB3 -3.58697683615819 1.1023855721393 -14.9183224469435
2.49750432606968e-34 3.37327393514543e-32
67.385550852865
KIF20A 4.36442711864407 8.84766815920398
14.9126277104061 2.60046863818499e-34 3.48938700274104e-32
67.3454779995026
TSHZ3 -4.18618629943503
6.69319850746269 -14.9064681962298 2.7166233301506e-34
3.62157642649298e-32 67.3021335227022
DIAPH3 4.03521016949153 7.28276069651741
14.9048866837122 2.74727561524171e-34 3.63881086328466e-32
67.2910042475812
CDC25A 3.22689173728814 7.80308706467662
14.8983333268091 2.87802222904491e-34 3.77930487518e-32
67.2448867937777
BLM 3.05758806497175 7.57531542288557
14.8977398935689 2.8901649556905e-34 3.77930487518e-32
67.2407106088206
RERG -5.40362422316384
6.26417213930348 -14.8907031529938 3.03811755366306e-34
3.94762996055079e-32 67.1911899804287
FOXI2 -2.50426793785311
0.653307462686567 -14.8578388627806 3.83597416303933e-34
4.95299053881744e-32 66.959891028635
TRAF4 2.37728509887006 11.1193009950249
14.8371909450592 4.44128615078189e-34 5.69872529222201e-32
66.8145556570052
LOC728264 -4.58390572033899
6.43817213930348 -14.8330735015206 4.57297219163178e-34
5.83124963318015e-32 66.7855726528823
MEF2C -2.96273898305085
7.70858756218905 -14.8078301670368 5.47018298278807e-34
6.9322751010271e-32 66.6078730226205

FLAD1	1.47885741525423	10.3277149253731
14.7785243686357	6.73496367024635e-34	8.48274872086857e-32
	66.4015550604709
PLCL1	-3.97940098870057
4.67549303482587	-14.7768263917686	6.81662594653597e-34
	8.53325187087704e-32	66.3896003373403
JPH4	-5.52836553672316
4.90730447761194	-14.7091287300816	1.10225025129577e-33
	1.37146652479408e-31	65.912913063315
DEPDC1B	4.16947902542373	7.33584328358209
14.7018775362025	1.16048176127712e-33	1.43522232283248e-31
	65.8618480011029
NUDT5	1.74867048022599	9.99939900497512
14.696538009308	1.20532007708106e-33	1.48174977140563e-31
65.8242447020602
SMTN	-2.54717740112995
10.2289631840796	-14.6806336322161	1.34941063496111e-33
	1.64901192474712e-31	65.7122352886131
TCEAL4	-1.67667210451978
11.2089900497512	-14.6506861060848	1.66914874224736e-33
	2.02211547392674e-31	65.5013094668109
ARHGAP20	-4.7246084039548
3.6728447761194	-14.6502415764952	1.67442586735288e-33
2.02211547392674e-31	65.4981784202989
TACC1	-3.36163538135593
9.42664975124378	-14.6251990455878	2.00031099712541e-33
	2.40154296906343e-31	65.3217845814613
HSPB7	-5.56679201977401
5.48498706467662	-14.623234351477	2.02841546443618e-33
	2.42112613284156e-31	65.3079451829041
ASPM	4.36897450564972	8.37235174129353
14.6106531410589	2.21799176304436e-33	2.62350094986221e-31
	65.2193207011476
LGI4	-4.07335374293786
4.89902935323383	-14.6103024587202	2.22352248064308e-33
	2.62350094986221e-31	65.216850381668
CD300LG	-3.84386052259887
1.35985373134328	-14.6005394153455	2.38316286709507e-33
	2.79579049494068e-31	65.148075383715
MYCT1	-3.18770536723164	6.2511328358209	-14.5937196185758
	2.50143107213233e-33	2.91786249493618e-31
65.1000327539114
NUP210	2.76119865819208	10.9556074626866
14.5680942162223	3.00075414333174e-33	3.48053573800003e-31
	64.9195045386007
ARHGAP6	-4.51995790960452	4.9353552238806	-14.5306521824104
	3.91498770255635e-33	4.51543244570123e-31
64.6557078136758
CENPN	2.28792528248588	8.62403184079602
14.5208699424105	4.19670205530489e-33	4.81331246901728e-31
	64.5867832910723
KLF2	-3.59754392655367
7.42942189054726	-14.5129298071613	4.44021089389099e-33
	5.06430720286567e-31	64.5308368340865

TBC1D2B -1.95077598870057
9.26990248756219 -14.4966522673165 4.9844994490792e-33
5.65368915412132e-31 64.4161414875767
DKFZp566F0947 -2.96390939265537
1.16651940298507 -14.4760721971812 5.76920519275509e-33
6.50779025314626e-31 64.2711234223589
PTGFR -5.96908834745763
3.32740945273632 -14.4716567148957 5.9530453035845e-33
6.67847104276447e-31 64.2400087370632
GSTM5 -5.79865607344633
4.55101243781094 -14.4702192556567 6.01414987370164e-33
6.71035309277689e-31 64.2298792940813
CCNF 2.68767810734463 8.69480298507463
14.4676821659604 6.12353324774843e-33 6.79546689601488e-31
64.2120009327741
ZAK -2.68939576271186
10.2024427860697 -14.4073689897088 9.39944521677205e-33
1.03747639946414e-30 63.7869586243231
MYH11 -7.08424110169492
9.73368009950249 -14.3786940542577 1.15236032836066e-32
1.26513141931788e-30 63.584861859792
LOC572558 -5.40597612994351
1.86728855721393 -14.363465092984 1.28405670158725e-32
1.40221723848863e-30 63.477526246036
FAM136A 1.53747048022599 10.6490169154229
14.3337691462613 1.58572571530906e-32 1.72248407065053e-30
63.2682178622701
SLC25A10 2.74207514124294 10.5476298507463
14.3089254289122 1.89191192305831e-32 2.04426062002038e-30
63.0931026055209
CFL2 -2.45578820621469
8.86575273631841 -14.3078756122663 1.90607913836526e-32
2.04878558694443e-30 63.0857026538993
BNC2 -4.96197005649718 6.7426039800995 -14.242563819699
3.03193291735439e-32 3.2419574371503e-30
62.6253131152384
DTL 2.83808820621469 8.32833283582089
14.2416030463728 3.05270646703162e-32 3.24725719006006e-30
62.6185402631816
CIT 2.54815225988701 8.71807860696517
14.218330047272 3.60179416790969e-32 3.81158939521577e-30
62.4544781212039
LDB2 -3.46959943502825
7.73084776119403 -14.21726666954 3.62911831948382e-32
3.82081021020528e-30 62.4469817900902
FGF7 -4.74802556497175
5.50532786069652 -14.204291186381 3.9797127413135e-32
4.16854604995745e-30 62.3555099248464
C15orf51 -2.93738820621469
1.25679054726368 -14.188192865445 4.46212858381823e-32
4.65012689471006e-30 62.2420218953515
RECQL4 3.26975056497175 9.37404925373134
14.1781878959638 4.7909824037367e-32 4.96761963377345e-30
62.1714893117796

DPT	-6.76453248587571
4.49085621890547	-14.1546476854374	5.66353391295848e-32
	5.84283172025314e-30	62.0055345911541
STAT5B	-2.01549964689265
10.1488945273632	-14.1494173607444	5.87803850272656e-32
	6.03380652304881e-30	61.9686612908024
LOC401093	-3.4351438559322
6.02745174129353	-14.134576177248	6.53197295949911e-32
	6.67171168450332e-30	61.8640317228363
CAV1	-3.41375091807909
9.50560696517413	-14.1278433883864	6.85215816750417e-32
	6.96409936529013e-30	61.8165656383368
RCC2	1.62052203389831	12.6773935323383
14.1005860538128	8.31700661552671e-32	8.41123871018539e-30
	61.624400256485
CASP12	-3.28736016949153
1.45311044776119	-14.0953469820821	8.63255743176652e-32
	8.68756882716503e-30	61.587464342263
CCL16	-2.42535077683616
0.621430845771144	-14.0929914416685	8.77830727478851e-32
	8.79115357811747e-30	61.5708575554283
PDZRN4	-4.48967563559322
2.06209253731343	-14.0579717429094	1.12593575045947e-31
	1.12210975519092e-29	61.3239641207333
RNFT2	2.81333439265537	7.0501263681592 14.0561567542669
	1.14055532405088e-31	1.131188444578e-29
61.3111681697712
KLHL38	-3.4911020480226
1.20880845771144	-14.0184005749913	1.49165212314583e-31
	1.47105364576401e-29	61.0449809312875
CNN1	-6.49760028248588
8.43221592039801	-14.0178439425407	1.49756557216112e-31
	1.47105364576401e-29	61.0410565819747
TCEAL7	-4.51367902542373	5.1866552238806 -14.0170936450523
	1.50557355785322e-31	1.47187738774888e-29
61.0357668629007
RERGL	-5.47410918079096
2.51697412935323	-14.0138780362816	1.54038189337994e-31
	1.49876968109432e-29	61.0130963028126
TNXB	-5.93790925141243
7.76263233830846	-14.0092769935436	1.59159145788343e-31
	1.54129116180881e-29	60.9806582221927
BRI3BP	2.52522740112994	6.95319104477612
13.9931457961232	1.78495723198857e-31	1.72043060904814e-29
	60.8669308858914
C20orf200	-4.18529837570622
1.82791791044776	-13.9808457729642	1.94803765842057e-31
	1.86884173492403e-29	60.7802141372586
RHOJ	-3.27508305084746
6.27853830845771	-13.9170277937049	3.06618600210995e-31
	2.92785109875894e-29	60.3302952475555
ZFPM2	-5.03212761299435	4.3136776119403 -13.9093222436317
	3.23880368710482e-31	3.07836294890101e-29
60.275971837513

ESCO2	4.01023128531074	6.74936019900498
13.9084150105256	3.25975635946011e-31	3.083999910586e-29
	60.2695759459891
KPNA2	2.68475183615818	11.5340094527363
13.8721750478592	4.21747246370726e-31	3.97177567339037e-29
	60.014091642742
HMMR	3.81122358757062	7.54931044776119
13.8705259725287	4.26719641815949e-31	4.00025308058513e-29
	60.0024661712001
SORBS1	-3.67269555084746
8.24204776119403	-13.8537800067718	4.80655442641718e-31
	4.48538919883385e-29	59.8844132367115
GSG2	3.18887429378531	5.90019651741293
13.8173338192775	6.22777695211403e-31	5.78535116863806e-29
	59.6274875905059
MRVI1	-3.9030634180791
8.06459900497512	-13.8013622845774	6.97640209790716e-31
	6.45160067883036e-29	59.5149002609024
TNFAIP8L3	-5.26860374293786
5.45744577114428	-13.7788974644873	8.18410149806055e-31
	7.53451137915619e-29	59.3565438921312
PABPC5	-3.87988672316384
3.04253333333333	-13.7558069482521	9.64361517653354e-31
	8.83854551670686e-29	59.1937819187391
STIL	2.7480061440678 8.09779850746269	13.7490194851435
	1.0120197259468e-30	9.23411776608344e-29
59.1459390232244
CENPM	3.4266543079096 8.25508905472637	13.722193312515
1.22457245907237e-30	1.11241029136087e-28	58.9568538671119
SOCS2	-3.31858651129943
7.55707512437811	-13.7028761938172	1.4047604551878e-30
	1.2704727817183e-28	58.8207016707547
FAM72D	3.03738333333333	5.89096069651741
13.6981876098013	1.45235313553728e-30	1.30775481897282e-28
	58.7876559691166
ACTA2	-4.5491656779661
11.7894766169154	-13.6779564032014	1.67690596632794e-30
	1.50335718291322e-28	58.6450672242513
E2F1	3.3322761299435 8.94651741293532	13.6768320791254
	1.69035716078654e-30	1.50882750047599e-28
58.6371431901052
PON3	-3.28478460451977	1.0319368159204 -13.6397729557049
	2.19959374647862e-30	1.95487703961931e-28
58.3759669680697
MASP1	-6.38396723163842
5.34229751243781	-13.6168239800031	2.58915904527673e-30
	2.29118255170393e-28	58.2142429050956
OR5K2	-1.19815776836158
0.379730845771144	-13.5757769369882	3.46588270363372e-30
	3.053844287794e-28	57.9250007268692
GPR124	-3.43005974576271
9.03544129353234	-13.5633488633282	3.78581839776301e-30
	3.32148938914849e-28	57.8374306973365
SDC1	3.36128086158192	11.1292213930348

13.5560696779276	3.98674686660974e-30	3.4828899221914e-28
	57.7861417693159
FANCD2	2.48577669491526	8.67519253731343
13.551730301034	4.11156351882949e-30	3.57671182379531e-28
57.7555671041764
ARHGAP11A	2.92382005649717	8.55285721393035
13.5498457253803	4.16698051241574e-30	3.60962489113482e-28
	57.7422887455324
VWC2	-4.62115685028249
1.70261890547264	-13.5321001223944	4.72683588496183e-30
	4.07739246715405e-28	57.6172598811579
NEIL3	3.7337125	5.43715024875622	13.5261546321311
	4.93075154858933e-30	4.23549494947861e-28
57.575371524983
KCNS2	-2.46733100282486
0.876029850746269	-13.5246424513552	4.9840035902701e-30
	4.26339973784355e-28	57.5647177158778
SDPR	-4.64082612994351
6.90932736318408	-13.4977614288061	6.0326191152242e-30
	5.13899047450427e-28	57.3753396633793
JAM3	-3.29091829096045
8.10606467661692	-13.4963950344941	6.09145520751e-30
	5.16766840537935e-28	57.3657137420584
PLK5P	-4.01474251412429
1.36972139303483	-13.4947194926712	6.16438653251718e-30
	5.20801874537357e-28	57.3539100029621
ST14	3.40538538135593	12.4493263681592
13.4835133531051	6.6751126836849e-30	5.61639604082176e-28
	57.2749672817649
TPM1	-2.5448375	11.6024945273632	-13.4802457107171
	6.83185177682899e-30	5.72481293788976e-28
57.2519485599599
FAM83D	3.95052175141243	8.08260099502488
13.4780432816956	6.93956597617931e-30	5.79143453215289e-28
	57.236433801449
MSRB3	-4.07950331920904
7.75098905472637	-13.4727197419884	7.20698731375465e-30
	5.9902611154406e-28	57.1989331656133
SYTL4	-3.13915275423729
7.64110895522388	-13.4705925575822	7.31670229454989e-30
	6.05693137528666e-28	57.1839488011294
OIP5	3.54625141242938	6.73945174129353
13.4680672801426	7.44911946449184e-30	6.14178404040231e-28
	57.1661603072477
AG2	-3.99350875706215
8.04067810945274	-13.4615897173469	7.79983808414479e-30
	6.4052270346997e-28	57.120531863585
ECM2	-4.62755663841808
5.72941243781095	-13.4601522141186	7.87988083564404e-30
	6.44517743250088e-28	57.1104061067604
CALD1	-2.90690861581922
11.4223766169154	-13.4411540391262	9.01817946957132e-30
	7.3469533535833e-28	56.9765875073939
VIP	-2.75786963276836

1.23388656716418	-13.4183829626068	1.06011033008723e-29
	8.60239726351416e-28	56.8162043934685
FLT4	-2.78984096045198
7.38155771144279	-13.4036372866445	1.17713990126345e-29
	9.51444180036953e-28	56.7123529940458
KIAA1644	-5.26845854519774
5.4958736318408 -13.4027114716552	1.18490438844972e-29
9.53964199798934e-28	56.7058328052895
EZH1	-1.66093785310734
9.24401641791045	-13.3988190371231	1.21811289014239e-29
	9.76869438852473e-28	56.6784199901087
AOC3	-4.2489509180791
7.28626567164179	-13.3949690441411	1.2518743222311e-29
	1.00003812589122e-27	56.6513064401985
CXorf36 -3.10979484463277
7.72987810945274	-13.3650441486124	1.54825502531719e-29
	1.23200293293651e-27	56.4405732665652
TMEM196 -3.57211913841808
0.92605671641791	-13.3635437236679	1.56483745098382e-29
	1.24039045825088e-27	56.4300077750278
C16orf45	-3.4618875
7.23179303482587	-13.3496131171231	1.72752647454667e-29
	1.36408148163243e-27	56.3319159167221
ADRA1D	-4.45683368644068
2.17482139303483	-13.3394704363963	1.85651590727443e-29
	1.46031691863387e-27	56.2604998491435
PRDM8	-4.1379009180791
4.11963930348259	-13.3370831107483	1.88825044239351e-29
	1.47440976877331e-27	56.2436907509322
EDNRA	-3.61278509887006
7.69042139303483	-13.3370424391115	1.88879575834087e-29
	1.47440976877331e-27	56.2434043843293
AKT3	-3.63617803672317
6.92810497512438	-13.3318834340338	1.95925837427976e-29
	1.52362024333195e-27	56.2070804986502
EMCN	-3.81205367231638
6.68535024875622	-13.3302593319127	1.98197982487693e-29
	1.53547342659333e-27	56.1956455569534
FHL5	-4.18491186440678
2.82511243781095	-13.3278918953425	2.01557323260869e-29
	1.55562851373896e-27	56.1789770926611
KIF15	3.67430790960452	7.76745124378109
13.3206585639291	2.12177935276306e-29	1.63146554727437e-27
	56.1280501722528
OLFML2A -3.21308947740113
8.31990049751244	-13.3158081608349	2.19611224862036e-29
	1.68232031582746e-27	56.0939012891197
SLC24A3 -4.36610381355932
6.84303034825871	-13.3095792759672	2.29540044372166e-29
	1.75184279217865e-27	56.050048282818
NPY5R	-2.61558686440678
0.79266368159204	-13.294271861162	2.55888035131836e-29
	1.94569680046541e-27	55.9422847048105
C7	-7.61982641242938

4.37991194029851	-13.2903152600709	2.63176684076413e-29
1.99373332992205e-27	55.9144314928825
RYR3	-4.56008043785311	3.9476776119403 -13.2784332384447
2.86336191799062e-29	2.16120662413042e-27
55.8307885984887
SYT15	-2.75397577683616
4.59736965174129	-13.2584906317138	3.29875197828993e-29
2.48070982103635e-27	55.6904130706706
CMA1	-2.4746886299435
0.511117412935323	-13.2568395873329	3.33763479933428e-29
2.50010277254184e-27	55.6787919484278
PTRF	-2.96699053672316
11.4324019900498	-13.2563650202193	3.34889557939117e-29
2.50010277254184e-27	55.6754516524022
HIC1	-3.49456320621468
6.90661890547264	-13.2387607929961	3.79456793955797e-29
2.82255361591033e-27	55.5515471532429
TEF	-2.08022217514124
8.58898756218905	-13.231113363759	4.00619818051484e-29
2.96172799233502e-27	55.4977249764668
C3orf70 -4.19964823446328
5.03273980099503	-13.2309613259337	4.01052304855887e-29
2.96172799233502e-27	55.4966549615278
PLN	-6.25022605932204
4.79918208955224	-13.2237261098723	4.22182201040281e-29
3.10659519260107e-27	55.4457356585169
CLIC2	-2.71856511299435
6.96606467661692	-13.1792154869556	5.78983369769993e-29
4.24518877906355e-27	55.132520575891
CCNE1	4.05753629943503	8.75972935323383
13.1735310200915	6.02811702073306e-29	4.40417232867081e-27
55.0925245301795
ANTXR2	-3.53967598870057
8.80302189054726	-13.1709834125167	6.13806505955112e-29
4.46859842810583e-27	55.0745998597459
C15orf42	2.79577012711864	7.57283432835821
13.146857311019 7.28392452553352e-29	5.28406256216265e-27
54.9048625468214
FEN1	2.17959830508475	10.0846810945274
13.1422930238959	7.52362833577999e-29	5.4387355539987e-27
54.8727531537236
FOXP2	-4.71041257062147
1.85018308457711	-13.1384993281549	7.72885073108326e-29
5.56748440382945e-27	54.8460653720942
FGF2	-4.19311038135593
6.20096368159204	-13.1225514266137	8.65455536152894e-29
6.21251823678983e-27	54.7338811794105
RSPO1	-6.43317055084746
4.16762338308458	-13.111531516003	9.35817118064567e-29
6.69419004664305e-27	54.6563679269947
FAM83H	3.11762711864406	10.8916064676617
13.1055573825792	9.76322288142421e-29	6.95968631095969e-27
54.6143481734267
PEG3	-6.15201370056497

5.32508009950249	-13.0911316232911	1.08150079782188e-28
	7.68277210355819e-27	54.5128884206452
GINS1	2.70762789548023	8.52482487562189
13.0873046700924	1.11125551610977e-28	7.8669226709426e-27
	54.4859738737138
NBLA00301	-5.64777309322034
4.88699203980099	-13.0855028280684	1.12554679814995e-28
	7.94071332165581e-27	54.473301906338
ECT2	2.86839540960452	9.88651293532338
13.0828336774334	1.14705536032758e-28	8.06474196833055e-27
	54.4545305709677
RTKN2	2.89816200564972	7.63673781094527
13.080198159729	1.16869585429921e-28	8.18884842619889e-27
54.435996032461
JAZF1	-2.65166285310734
8.15151194029851	-13.061237410097	1.33688923035112e-28
	9.30384267766387e-27	54.302660568748
TMEM63B	1.43260529661017	10.2256771144279
13.0312427951815	1.65370772264944e-28	1.14698039006733e-26
	54.0917617619244
CLEC3B	-4.40189166666666
6.45126567164179	-13.0203347150809	1.7866712325698e-28
	1.2350289698538e-26	54.0150735794119
FBXO40	-2.02063990112994
0.618226368159204	-13.0196650630246	1.79517351762383e-28
	1.23674202405427e-26	54.0103658145902
DBNDD1	2.74152725988701	9.5789223880597	13.0009028986272
	2.05054856599743e-28	1.40795190835877e-26
53.8784722043379
HIST1H2AM	3.17203086158192	3.84005174129353
12.9926730396781	2.17373030679643e-28	1.48755610661769e-26
	53.8206228510579
LRRN4CL	-4.36431186440678
5.96806666666667	-12.9890447620603	2.23035980954152e-28
	1.52123876710589e-26	53.7951198545306
HDGF	1.48631108757062	13.3276452736318
12.9602104446565	2.73608816242263e-28	1.85999635677273e-26
	53.5924647419049
FAM180A	-3.73884540960452
2.66468805970149	-12.9506696208474	2.92748381054082e-28
	1.98353936073938e-26	53.5254172707421
CRHBP	-3.91691687853107
2.17956218905473	-12.9363831191248	3.23937999959034e-28
	2.18764708524966e-26	53.4250273884264
SOX15	-3.41081490112994
4.36951741293532	-12.926555455184	3.47300028064974e-28
	2.33772772989309e-26	53.3559746203058
KCNMB1	-4.83047224576271
6.08962139303483	-12.9250856240975	3.50935912022314e-28
	2.35448178882945e-26	53.3456474221702
TLCD1	3.06552048022599	7.9565631840796	12.9162698610913
	3.73555392534132e-28	2.49807563802141e-26
53.28370893649
F10	-4.8122302259887

4.94404776119403	-12.9014126474756	4.15020145407603e-28
	2.7663518133825e-26	53.1793319845434
DGKB	-4.64664724576271
2.24557213930348	-12.8866419985602	4.60800356888882e-28
	3.0615635362229e-26	53.0755733054206
C17orf53	2.54498877118644	7.28256368159204
12.884268021918 4.68615047320207e-28	3.10344094241414e-26
53.0588978956806
DACT3	-4.12234865819209
6.55935124378109	-12.8815326861722	4.77783579998612e-28
	3.15398614063392e-26	53.0396845375278
CXCL12	-4.69483799435028
8.48253134328358	-12.8661036021987	5.3295989128563e-28
	3.49933322646543e-26	52.931315261913
HK2	2.68331511299435	10.2588776119403
12.8659585593606	5.33507695997896e-28	3.49933322646543e-26
	52.9302965781943
RELT	2.17324463276836	7.67621940298507
12.8397554336991	6.42297784563398e-28	4.199482011811e-26
	52.7462800547984
RACGAP1 2.25521454802259	9.43282686567164
12.825340520612 7.11324833319293e-28	4.63603137398256e-26
52.6450628623355
SYNE1	-3.65890225988701
8.36324825870647	-12.7943446625338	8.85886082907345e-28
	5.75545610192651e-26	52.4274544686768
SYNM	-3.15249399717514
7.00516069651741	-12.7919801878342	9.00839932236814e-28
	5.83414631193116e-26	52.410856526401
NEFM	-4.61922175141243
2.52297711442786	-12.7873736853006	9.30701290200672e-28
	6.0085841156666e-26	52.3785210089477
PCDHGA2 -4.15311355932204
2.96468059701493	-12.7671370653131	1.07404476888865e-27
	6.91226931200124e-26	52.2364823149247
LOC145820	-3.57998509887006
2.17007164179104	-12.7654641702053	1.0868381025074e-27
	6.97274570139906e-26	52.2247413979841
SLC1A7	-3.80890197740113
2.54380895522388	-12.7551896545741	1.16881916672551e-27
	7.47534501335659e-26	52.1526347716817
LEPR	-3.39682627118645	7.5956815920398 -12.7392155169173
	1.30871487674785e-27	8.34407342224635e-26
52.0405392986692
ZEB1	-3.59806730225989
7.94696517412935	-12.7306389485257	1.3906038365854e-27
	8.83872964863725e-26	51.9803605141358
GRID1	-3.53979286723164
4.13887313432836	-12.7080127296247	1.63204696853366e-27
	1.03413346493815e-25	51.8216194561049
KCNJ8	-2.68535621468926
6.48093333333333	-12.7037768887263	1.68169750725975e-27
	1.06231537920131e-25	51.7919047664832
FAM110A 2.15244950564971	8.87117810945274

12.6996255490614	1.73182125200509e-27	1.0906224019529e-25
	51.7627838242827
SAP30L	-1.22560974576271
9.30412338308458	-12.6908504953323	1.84273766760664e-27
	1.15692367938729e-25	51.7012314760824
E2F8	3.75886115819209	6.64861691542289
12.6875952215621	1.88566504917977e-27	1.1802653493799e-25
	51.678398553793
RUNX1T1	-4.40359343220339
2.97397960199005	-12.6854475959992	1.91453131397767e-27
	1.19469081689853e-25	51.6633351493313
CALHM2	-2.69665247175142
8.05619004975124	-12.670063480607	2.13462846129945e-27
	1.32799764577205e-25	51.5554388881044
RECK	-2.85177302259887
6.78976218905473	-12.6624763460786	2.25230822454223e-27
	1.39410046715322e-25	51.5022315990326
LTBP4	-3.9517311440678
10.4776298507463	-12.6623411132952	2.25446349291217e-27
	1.39410046715322e-25	51.5012832643725
NXPH3	-4.15119738700565
5.69443880597015	-12.6231686749015	2.97404588361712e-27
	1.82887155169434e-25	51.2266269828271
HAND2	-5.0714393361582
6.23650149253731	-12.6231058035309	2.97536823315104e-27
	1.82887155169434e-25	51.2261862346713
GPR182	-2.52754653954802
1.14825422885572	-12.617057324007	3.10536864243586e-27
	1.90308114117039e-25	51.1837855824095
C7orf58	-4.59986172316385	5.8980671641791	-12.6150172185477
	3.1504841582058e-27	1.92498332642753e-25
51.1694846611978
GLRA4	-2.12080416666667
0.430230348258706	-12.605968369064	3.35861725141179e-27
	2.04606564307074e-25	51.1060561962489
C1orf70	-3.41081377118644
3.78508507462687	-12.6052862886518	3.37485146409331e-27
	2.0498727975691e-25	51.1012753110533
CENPE	3.11751596045198	7.94395024875622
12.6036566728919	3.41395602951278e-27	2.06750788453975e-25
	51.0898530090031
MMRN2	-2.50897867231638	8.4369592039801	-12.5966054508586
	3.58844155311771e-27	2.16678544369137e-25
51.0404314344566
CTNNA3	-2.39149173728814
0.939330348258706	-12.5870991943007	3.8378592844908e-27
	2.31059387421103e-25	50.9738074508603
TIMP3	-3.76370141242938
10.5766124378109	-12.5684138249956	4.37970025511758e-27
	2.62910076718023e-25	50.8428683788586
CKAP2L	3.75794343220339	6.40307960199005
12.5509323394499	4.95559978980334e-27	2.96613596748287e-25
	50.7203852017327
GMIP	1.79639597457627	8.94664676616915

12.5442150370488	5.19648830462323e-27	3.10127630505567e-25
	50.6733258434803
KIF18A	3.35088156779661	7.20043880597015
12.542023468319 5.27758305591672e-27	3.14054435182523e-25
50.6579730018594
CDKN3	3.26470169491525	7.44035323383085
12.5407413665468	5.32560950895779e-27	3.1599642548816e-25
	50.6489914917606
HSPA12B -2.91725473163842
6.66756169154229	-12.5305653973695	5.72260330481132e-27
	3.38573619157857e-25	50.5777094561735
FYCO1	-1.93524519774011
9.51409552238806	-12.5297866936972	5.75417322220664e-27
	3.39463150149144e-25	50.5722549567095
ROBO4	-2.42711935028248
8.02221094527363	-12.5281292336724	5.82194970717086e-27
	3.4247744265965e-25	50.5606452588496
STAB2	-3.2748636299435
1.11219402985075	-12.500560956259	7.07366186989748e-27
	4.14920794825701e-25	50.3675688816022
LAMA4	-2.82348947740113
9.47438109452736	-12.4946380515625	7.37585393893569e-27
	4.31413907026638e-25	50.3260938439935
TRPC4	-3.89975776836158
4.11500995024876	-12.4925536103466	7.48524342501735e-27
	4.36568316805699e-25	50.3114981275179
OSR2	-4.65178778248588
8.04522039800995	-12.4759640068354	8.41563865465791e-27
	4.8944210079356e-25	50.1953442865485
CDH13	-3.64812620056497
6.71737512437811	-12.4450332172154	1.04696690151374e-26
	6.07181652205569e-25	49.9788282088965
PTGIS	-5.89513898305085
6.46005820895522	-12.4329154592775	1.14046870214758e-26
	6.59544294509573e-25	49.8940213622554
ADCY2	-4.56872570621469
3.70071044776119	-12.4261079352038	1.19660806455755e-26
	6.88322360083946e-25	49.8463829203236
ZCCHC12 -6.02565741525424
5.71708656716418	-12.4260690435389	1.19693659303443e-26
	6.88322360083946e-25	49.8461107691205
SGCA	-4.86231038135593
3.19953482587065	-12.418222203925	1.26509771911568e-26
	7.2548760261019e-25	49.7912032808723
GYPC	-3.23118538135593	7.6238736318408 -12.4150942988484
	1.29333654398429e-26	7.39615577938648e-25
49.7693172629024
AIM1L	4.18988855932203	5.54292835820895
12.4100421766912	1.34028274646316e-26	7.64333466246908e-25
	49.7339688955708
EPT1	1.42733651129944	9.77444577114428
12.4061828536239	1.37728826361938e-26	7.83261164878281e-25
	49.7069674295055
CPEB1	-4.28071744350282

1.98303383084577	-12.403963931406	1.39902480182496e-26
	7.93424839267027e-25	49.6914433776567
ILK	-1.3567011299435
11.3491597014925	-12.3817382824806	1.63657063342107e-26
	9.25586641987177e-25	49.5359671517325
JPH2	-4.89025261299435
5.78233731343284	-12.3695527454632	1.78348922383721e-26
	1.00590752102687e-24	49.4507399400577
ABCG2	-4.50343665254237	5.988707960199	-12.3665359289402
	1.82185221485842e-26	1.02472947865872e-24
49.4296415820471
C9orf100	2.25040218926554	7.70990995024876
12.362850793701	1.86983362555998e-26	1.04884383422804e-24
49.4038701708
EHD2	-2.86526617231639
9.65166766169154	-12.3599620551324	1.90832636762558e-26
	1.06751881000962e-24	49.383668919327
COL4A6	-5.22515586158192
4.97313084577114	-12.3583380686769	1.93031258792406e-26
	1.07688362581742e-24	49.3723124769234
RAC3	3.20299992937853	8.23736467661691
12.3567150956001	1.95253780137979e-26	1.08633065209558e-24
	49.3609633118648
VGLL3	-4.25070035310734
5.31247512437811	-12.3542512426732	1.98676752974811e-26
	1.10238749691159e-24	49.3437343871735
TPSG1	-2.1477656779661
0.782942288557214	-12.3469564059904	2.09166674767019e-26
	1.157464105921e-24	49.2927265477668
CRTAP	-1.39837062146893
11.6823323383085	-12.3355574579144	2.26676418576969e-26
	1.25098571865193e-24	49.2130292303107
NACC1	1.65691009887006	11.534815920398	12.3285092891831
	2.38227665381229e-26	1.31121017969883e-24
49.1637557657062
ADH1A	-2.89935896892655
0.796603980099503	-12.3252848880708	2.43706322128403e-26
	1.33777828697757e-24	49.1412153305741
KCNA4	-2.56951426553672
0.905118407960199	-12.3204799950611	2.52104730362107e-26
	1.38018936382242e-24	49.1076277699056
HSPA4	1.13494117231638	11.5728049751244
12.317335635766	2.57756540187631e-26	1.407378130333e-24
49.0856487383128
DSTN	-1.50549618644068
12.4024895522388	-12.3151350348357	2.61787087055995e-26
	1.42559387195214e-24	49.0702670038564
C6orf115	2.23699258474576	10.1267014925373
12.3129735538275	2.6580724293686e-26	1.44365679827876e-24
	49.0551590611848
SMOC2	-3.7169841101695	9.4077223880597	-12.3112746109426
	2.69010342922826e-26	1.45719850665056e-24
49.0432843318177
TMEM8A	1.67424484463277	11.1207308457711

12.3096453495131	2.7211826676202e-26	1.47015474121691e-24
	49.031896842541
MTUS2	-3.73778467514124
3.08220547263682	-12.2976255502313	2.96182340653752e-26
	1.59596416105552e-24	48.9478923371991
AQP1	-2.90606998587571	10.053344278607 -12.2969972477084
	2.97497097750643e-26	1.59885220335621e-24
48.9435015280251
NR2F2	-2.57179173728814	10.215592039801 -12.286750612332
	3.19780107444918e-26	1.71412156810552e-24
48.8718984891921
C14orf28	-1.68412252824859
6.38555422885572	-12.2757352519691	3.45596861854761e-26
	1.84768322236413e-24	48.7949325447784
HSPB3	-3.68810494350282
1.84290746268657	-12.268380340468	3.63983114662495e-26
	1.94092814130416e-24	48.7435478691024
CASQ2	-4.54152062146893
3.58680049751244	-12.26690125128 3.67796955452542e-26
1.9561843252437e-24	48.7332147982077
PPAPDC3 -3.25807860169492
4.51742487562189	-12.2493426903422	4.16238628543696e-26
	2.2081082801039e-24	48.6105617979994
ARHGEF15	-2.55945974576271
7.24108905472637	-12.2452415632239	4.28441540001818e-26
	2.26698577738075e-24	48.5819173724378
TPM3	1.1483604519774 12.8755094527363	12.2372401529626
	4.53287560531086e-26	2.39228627704452e-24
48.5260351116271
B3GNT4	3.39377761299435	4.50581293532338
12.2333495774658	4.6588335028201e-26	2.44976284737604e-24
	48.4988649496491
C10orf72	-3.55743177966102
6.89556119402985	-12.233142136135	4.66564672831969e-26
	2.44976284737604e-24	48.4974162991086
UCK2	1.72789357344632	9.04428208955224
12.2288109616048	4.81019473244602e-26	2.51921678206931e-24
	48.4671706513487
LRRC70	-2.4100220338983
4.70910845771144	-12.2227514184395	5.01995936808715e-26
	2.62238589890151e-24	48.4248578741788
CLIP3	-3.36454124293785
8.38889502487562	-12.2209983168021	5.08233301900506e-26
	2.6482308852836e-24	48.4126168015734
HLF	-4.65038354519774
5.51244029850746	-12.2202167398561	5.1103895868058e-26
	2.65610881562337e-24	48.4071595012327
SCARA5	-4.46620120056497
1.77675572139303	-12.2191224106845	5.14993317974076e-26
	2.66990222676964e-24	48.399518514382
FLNC	-4.88265953389831
7.87281542288557	-12.2102311621255	5.4827533032132e-26
	2.83528779130899e-24	48.3374402632334
KANK3	-2.59317231638418

6.39666019900497	-12.2064751400665	5.62972356383386e-26
	2.90397549662083e-24	48.3112178046012
DIO3OS	-3.14056906779661
1.42224925373134	-12.2022103295989	5.80137674422191e-26
	2.98501916187658e-24	48.2814446404414
PIF1	3.19201440677966	7.08452835820896
12.2001133666328	5.88768335090337e-26	3.02185347985116e-24
	48.266806021557
FAM138F	-2.40507266949153
0.883329353233831	-12.188358850535	6.39575543839467e-26
	3.27443539028036e-24	48.1847558839887
ASAM	-4.70569653954802
4.76349850746269	-12.1856205899191	6.5202653015491e-26
	3.32508625067343e-24	48.1656435903218
ZBTB4	-1.55585247175141
10.6301417910448	-12.1854722046835	6.52708114477055e-26
	3.32508625067343e-24	48.1646079202622
NEXN	-3.93655734463277	6.5697960199005	-12.1786662408289
	6.84746873902962e-26	3.47966666367025e-24
48.1171069207839
PDLIM3	-5.15088566384181
6.75084129353234	-12.1759011638308	6.98207592000292e-26
	3.53930910216445e-24	48.0978096435728
GAS1	-4.86097690677966
6.99945074626866	-12.1747753318063	7.03763663673367e-26
	3.55868670325474e-24	48.0899527204944
CCDC141	-3.11721038135593
1.95303134328358	-12.1739668652926	7.07780721804957e-26
	3.57020595052967e-24	48.0843106824351
DCN	-4.57798615819209
10.7045223880597	-12.1728627284531	7.13303895781533e-26
	3.58924729911639e-24	48.0766053390189
KLF9	-2.60383990112994
8.75219552238806	-12.1700920524744	7.27353736853401e-26
	3.65099565222502e-24	48.0572703045832
TYMS	2.79404653954802	9.65009154228856
12.1633497174277	7.62708564765151e-26	3.81912361820209e-24
	48.01022186683
NCRNA00028	-1.14714661016949
0.300976119402985	-12.1567738933245	7.98842204119072e-26
	3.99032371059965e-24	47.96433896471
CCBE1	-5.37631956214689
2.90155820895522	-12.1371636433734	9.17066624830214e-26
	4.56975189508842e-24	47.8275295049485
PLSCR4	-3.72516214689266	6.4773	-12.1052705082084
1.14777121188139e-25	5.70550677480021e-24	47.6050984584186
BCHE	-5.83907782485876
3.90410049751244	-12.0925608137963	1.25510973756058e-25
	6.22401036524606e-24	47.5164818857138
TCEAL6	-2.52098580508475
5.13751492537313	-12.0849922455352	1.32373141998607e-25
	6.54848338609976e-24	47.4637177082233
PGM5	-5.3704031779661
7.32913482587065	-12.0814890418192	1.35674961295151e-25

6.69568979660928e-24 47.4392968465991
E2F7 3.76948086158192 6.61214776119403
12.0767020040137 1.40320146169239e-25 6.90832757998676e-24
47.4059280901603
CLDN7 3.4982990819209 11.2537542288557 12.0762968217654
1.40720534744569e-25 6.91146549833973e-24
47.4031037987575
ITGA8 -4.53492111581921
2.67286616915423 -12.0684642194012 1.48688537947786e-25
7.28538349419583e-24 47.3485100660365
CCL14 -6.20217959039548 5.3985 -12.059237448241
1.58654460757987e-25 7.75518114133686e-24 47.2842057777829
CMTM5 -2.91461313559322
1.05755870646766 -12.0569392949447 1.61238750681593e-25
7.86278278264394e-24 47.2681903839155
GPRASP2 -2.79076391242938 7.2198407960199 -12.05654757392
1.61683416858107e-25 7.86578328932924e-24 47.2654606008975
CYBRD1 -3.10745324858757
9.79634427860696 -12.0551205326455 1.63313715965506e-25
7.91738335204103e-24 47.255516104753
TMEM132C -6.1567300141243
3.45554776119403 -12.0549450092181 1.6351536976451e-25
7.91738335204103e-24 47.2542929625939
IKBKE 2.15521235875706 8.48435273631841
12.0543820219914 1.64163848309665e-25 7.93007954305278e-24
47.2503697814986
PDE8B -3.8801947740113
5.35198706467662 -12.0511746700526 1.67907526517882e-25
8.09188150096741e-24 47.228019857217
PHYHIP -4.05869936440678
5.24637562189055 -12.0489748416927 1.7052435993171e-25
8.19874732880096e-24 47.2126912273483
LCN6 -3.8793781779661
1.11403532338308 -12.0341497284678 1.89253573871077e-25
9.07798100834864e-24 47.1093995196527
RDM1 3.38942838983051 4.11432885572139
12.0291550218749 1.96015284624864e-25 9.38040511270037e-24
47.0746040794449
GPR172A 1.68164357344633 10.7553378109453
12.0162695618409 2.14593821379232e-25 1.02456073323619e-23
46.9848483344895
ACTG2 -5.91166800847458 8.4689736318408 -11.994564546808
2.49947141903288e-25 1.19058348567854e-23
46.8336925206712
PALM2-AKAP2 -2.38575854519774
8.70563731343284 -11.9898758759668 2.58316941426497e-25
1.22760342765879e-23 46.8010458070286
TAGLN -4.15907443502825 12.076939800995 -11.9880364052093
2.61676498878446e-25 1.24069711823891e-23
46.788238319337
LMNB1 2.64998870056498 10.4734054726368
11.985302175946 2.66750982411801e-25 1.26184278085582e-23
46.7692015659314
LYVE1 -3.15991016949152 4.7536592039801 -11.9797246144129

2.77409028319916e-25	1.30924306928917e-23
46.7303705754088
SPINT2	2.51308898305085	13.6592686567164
11.97618704699	2.84388122467881e-25	1.3391027876756e-23
46.7057435137508
ANLN	3.497089759887	8.81097263681592	11.9709500381804
2.9504301780403e-25	1.38609454359651e-23
46.6692877789109
PSD	-3.67103488700565
7.72523930348259	-11.9694879007969	2.98088315681015e-25
1.39720390888841e-23	46.6591100386581
CSDC2	-4.79327464689266
5.76506915422886	-11.9521142597724	3.36766818642459e-25
1.57490268490426e-23	46.5381897148995
C10orf35	1.75521906779661	8.07686567164179
11.9462237925645	3.50986778094858e-25	1.63767239870169e-23
46.4971985486511
TIMELESS	2.00571228813559	10.0731492537313
11.9373734539563	3.73486594948354e-25	1.73870290119948e-23
46.4356160784368
PGD	1.78717111581921	11.8617567164179
11.9272866210316	4.00890273301741e-25	1.86205369024542e-23
46.3654388635103
PCDHGA3	-3.17170579096046
2.36439701492537	-11.9179277216493	4.28107899165422e-25
1.98398536565827e-23	46.3003347634715
NME1	1.839589759887	11.4830497512438	11.9138814750321
4.40439129010191e-25	2.03653498166198e-23
46.2721901088565
ABI3BP	-4.65081744350283
6.53112487562189	-11.8954034703917	5.01414742397105e-25
2.31326846323878e-23	46.1436818505156
CD34	-2.12637040960452
9.73112039800995	-11.8883593698477	5.26817469385007e-25
2.42501404629466e-23	46.0947012099073
PTPRN2	-3.33654322033898
5.99463333333333	-11.8838573577273	5.43721551915608e-25
2.49722672501732e-23	46.0633993233176
RTKN	1.6071581920904	10.2082587064677	11.8794493638337
5.60796998066251e-25	2.56990231479914e-23
46.032753048039
GGCT	1.54211892655367	10.7880353233831
11.8643897400079	6.23274609586253e-25	2.84985027501242e-23
45.9280664068948
FBXL7	-3.54099943502825
7.44527313432836	-11.8592792067338	6.46018160324212e-25
2.94727840699024e-23	45.8925457207559
PMP22	-3.05646956214689
8.99619452736318	-11.8581864870416	6.50987595311647e-25
2.96336481856943e-23	45.8849511235509
GCOM1	-3.40593446327684
5.35171094527363	-11.8573929436532	6.54620343074453e-25
2.97330877064569e-23	45.8794359291908
PRKG1	-4.1331177259887

3.00395970149254	-11.8509509734144	6.8486968262482e-25
	3.1038354490701e-23	45.8346659950267
GPIHBP1 -4.38318933615819
3.35648905472637	-11.8472221825662	7.03012187000042e-25
	3.17903969143411e-23	45.8087537997229
PRELP	-4.69338220338983
7.83457164179104	-11.8310171527345	7.87596301509058e-25
	3.55370375164417e-23	45.6961575673302
TAL1	-3.01270960451978
4.54088358208955	-11.8236324860386	8.29439534495162e-25
	3.73429685157581e-23	45.6448559594832
ZNF25	-1.89107507062147
7.45491243781095	-11.8190155244063	8.56720053194759e-25
	3.84867892605873e-23	45.6127845149568
RFTN2	-2.85217740112995
4.57098109452736	-11.8175328265259	8.65669523827293e-25
	3.8803919921778e-23	45.6024855009015
ITPR1	-3.15464632768362
7.79856119402985	-11.8075898865592	9.28143473867213e-25
	4.15136939400738e-23	45.5334262928955
PLK4	2.28315593220339	7.89094029850746
11.8031929124731	9.57186788676332e-25	4.27196625467937e-23
	45.502890085086
SLC22A3 -5.12247415254238
4.62062288557214	-11.7987930661341	9.87156915586926e-25
	4.39616734858993e-23	45.4723359041301
TRIP13	2.56650564971751	8.70685721393035
11.7957374983607	1.00851956791013e-24	4.48158154311581e-23
	45.4511180614838
CDC42BPG	2.79021857344633	8.3351039800995
11.79208176129	1.03468526509893e-24	4.57891247663839e-23
45.4257339038211
TRAIP	2.21490790960452	7.83187462686567
11.7920544478439	1.03488328746236e-24	4.57891247663839e-23
	45.4255442539777
TMEM200B	-4.55388375706215
6.21438855721393	-11.7824426395235	1.10697066966142e-24
	4.88733502110729e-23	45.3588098021412
PID1	-4.25690367231638	4.9683	-11.7719627568588
1.19129001851737e-24	5.24832276398319e-23	45.2860592040235
SRF	-1.16203072033898
10.5000567164179	-11.770193518341	1.20614445618377e-24
	5.3023866564139e-23	45.2737783989868
GRINL1A -1.50166391242938
9.48068507462687	-11.7660336314501	1.2418029621794e-24
	5.44563160918308e-23	45.2449046817099
HAPLN1	4.74104759887006	6.46179402985075
11.7657773700652	1.2440337188051e-24	5.44563160918308e-23
	45.2431260338484
CYP1B1	-4.11133700564972
7.69011890547264	-11.7600578716291	1.29487600885744e-24
	5.65612860890281e-23	45.2034301690926
SMPDL3B 3.30634689265537	9.62802587064677
11.7581489770913	1.31230211565275e-24	5.72007694996837e-23

45.1901823512464
PTGER3 -5.88788107344633
5.03386616915423 -11.7445644004695 1.44326098754235e-24
6.27757374454333e-23 45.0959157753887
ENO1 1.78882867231639 15.0612288557214
11.7421289396509 1.4680819994601e-24 6.37203455579615e-23
45.0790175895732
SLC2A4 -4.1193165960452
4.40414626865672 -11.7384286318181 1.50661137201851e-24
6.52547077374261e-23 45.0533445946051
CD302 -2.49496899717514
8.03121691542288 -11.7181802170905 1.73602741259331e-24
7.50329321695594e-23 44.9128853679039
C16orf75 2.25578241525424 8.5106144278607
11.7160164489588 1.76251722952872e-24 7.60178124416484e-23
44.8978783200358
SAMD10 2.00527033898305 8.09318507462687
11.712976538658 1.8004159510311e-24 7.74896005758248e-23
44.8767955392577
KCNAB1 -2.77568968926554 4.8418776119403 -11.6819153971545
2.23746381406924e-24 9.60986027256099e-23
44.6614332783847
PDIA4 1.55872514124294 13.253392039801 11.680356506578
2.26199558551431e-24 9.69494141348826e-23 44.6506274725898
OLFML1 -3.36811073446328
6.56098507462687 -11.6773114256843 2.3106919100422e-24
9.88302185690967e-23 44.6295205634094
C1QTNF7 -4.31915084745763
5.14360199004975 -11.6719077802644 2.39969527540575e-24
1.02423584208067e-22 44.5920678098882
C1orf150 -2.05582026836158
0.719638308457711 -11.6486257981971 2.82398153286575e-24
1.20282864874967e-22 44.4307366768565
IFI30 2.97098269774011 11.7447009950249
11.6477857260619 2.84061663017474e-24 1.20740909767054e-22
44.4249165641929
CRISPLD2 -2.71397846045198
8.63099950248756 -11.6410675142964 2.97721671406413e-24
1.26285659379621e-22 44.3783748666979
FLNA -2.42655819209039
13.8420467661692 -11.6380672070509 3.04032001486322e-24
1.28696432794107e-22 44.3575912845251
AGAP11 -3.33983199152542
3.33101641791045 -11.6233722698743 3.36919892498417e-24
1.42324390802315e-22 44.255811742688
KRT8 3.25711970338982 14.1273890547264
11.620399898011 3.43991914593015e-24 1.45013429293523e-22
44.2352275492716
CHRDL2 -6.47356271186441
3.67583333333333 -11.6132591350223 3.61592814012795e-24
1.52120911304973e-22 44.185780582084
ST5 -1.98676228813559
10.7087880597015 -11.6056049016971 3.81458248447679e-24
1.6015005809061e-22 44.1327844238651

FAM65C -3.41689371468927
5.80508109452736 -11.6024589697667 3.89935450841692e-24
1.63374996036325e-22 44.1110046344248
AOX1 -4.59873961864407
4.24970945273632 -11.6011530423302 3.93509446358242e-24
1.64536638161603e-22 44.1019638197722
SHE -3.0853311440678
6.00787910447761 -11.5949600753326 4.10908138076164e-24
1.71462277941131e-22 44.0590931211144
DUSP1 -3.25181468926553
10.7018950248756 -11.5895100585461 4.26853342585434e-24
1.77754546111135e-22 44.0213690940067
C5orf53 -1.99721878531074
7.35668009950249 -11.5882441133047 4.30644628705897e-24
1.78970328488503e-22 44.0126069358889
GRIA3 -3.67084851694915
2.14878258706468 -11.5805850381326 4.54309510545924e-24
1.88423722252683e-22 43.9595990521458
CBLN4 -3.93419858757062
2.25917313432836 -11.5780140707228 4.62540630725621e-24
1.91450789290262e-22 43.9418070871262
GPR133 -5.42490303672316
4.45738407960199 -11.575525832855 4.70648541053175e-24
1.94414779634239e-22 43.9245883615094
STXBP2 2.26504936440678 10.7837582089552
11.5746140736127 4.73654872344891e-24 1.95263745567081e-22
43.9182791219505
HSPE1 1.52312747175141 11.5826990049751
11.5700149154049 4.89114204167979e-24 2.01232757746866e-22
43.8864550841253
DARS2 1.62686320621469 9.08002338308458
11.567683370677 4.97142581867326e-24 2.04126744114724e-22
43.8703228093961
MEIS3 -4.02797146892655
6.55666368159204 -11.5587331898388 5.29201438459613e-24
2.16856397835845e-22 43.808401128104
C9orf3 -2.04202026836158
8.73459054726368 -11.5447256655026 5.83564519730511e-24
2.38656963945566e-22 43.7115089628793
MTP18 2.40121257062147 9.37274228855721
11.5436212767345 5.88080455955918e-24 2.40025681128728e-22
43.7038707112722
RFTN1 -2.47861532485876
8.48361791044776 -11.5383097240671 6.1029170137582e-24
2.48596996612016e-22 43.6671365682974
GRRP1 -2.71424406779661 5.0506447761194 -11.535756539811
6.21264227979585e-24 2.52565437632097e-22
43.6494801888484
ZEB2 -2.77019717514125
7.65463930348259 -11.5300318866491 6.46587070130592e-24
2.62340564224922e-22 43.6098945041833
NAALAD2 -3.63970508474576
4.26001990049751 -11.5178662712219 7.03872546264249e-24
2.85019790430079e-22 43.5257827123576

SYDE1	-2.51104555084746
7.85027661691542	-11.5143181311044	7.21514440402435e-24
	2.91588414595708e-22	43.5012545383339
EPCAM	3.52618552259887	13.0674776119403
11.512675134692 7.29832431762486e-24	2.94370526995753e-22
43.4898970646205
FERMT2	-2.09234653954802
9.64225671641791	-11.5056782969683	7.66339908307045e-24
	3.08015438833896e-22	43.4415339028345
C4orf49 -3.25842161016949
3.25836865671642	-11.5056178934877	7.66662879903169e-24
	3.08015438833896e-22	43.441116410531
ZNF835	-3.75156080508475
2.33498258706468	-11.4930187871474	8.37085251596301e-24
	3.35028327613399e-22	43.3540443583501
RHPN1	2.81772316384181	9.83777860696517
11.493005496817 8.37162844937524e-24	3.35028327613399e-22
43.3539525192922
NPR2	-2.75066603107345
7.45409253731343	-11.4924510764687	8.40406135266678e-24
	3.356719446892e-22	43.3501213736273
PLP1	-4.42492838983051
1.79397960199005	-11.4865690141438	8.75597042893943e-24
	3.49048685254615e-22	43.3094775220476
SLC45A1 -2.80856829096045	5.1834039800995 -11.4816669872188
	9.06045032660222e-24	3.60486521715394e-22
43.2756086925586
C13orf33	-3.67047090395481
4.96654427860696	-11.4804664881704	9.13661319626629e-24
	3.62813672958118e-22	43.2673147081497
PPP1R3C -3.46823439265537
5.39917064676617	-11.470764393757	9.77604724104425e-24
	3.87456080808182e-22	43.2002914540317
FAM48B1 -1.00813439265537
0.186294029850746	-11.4610784371858	1.04589411070814e-23
	4.13722660748327e-22	43.1333910585042
LRP1	-1.98694399717515	11.665623880597 -11.4605699511707
	1.04960777047475e-23	4.14393221689358e-22
43.1298792872375
PCDHB18 -2.93405134180791	2.7964039800995 -11.4579544016869
	1.06891895595885e-23	4.21207412012191e-22
43.11181594192
EID1	-1.43782902542373
11.4860228855721	-11.4510610509854	1.12152904530761e-23
	4.41091787359486e-22	43.0642135102421
KCNMA1	-4.04520261299435
8.23470248756219	-11.441856386007	1.19582790349994e-23
	4.69413897874834e-22	43.0006592308416
ATP8B4	-3.0158543079096
4.90846019900497	-11.4409798139459	1.20315456790149e-23
	4.71388612194991e-22	42.9946074174338
ALDH1A2 -5.25043029661017
7.21038258706468	-11.4391182251858	1.21886318016179e-23
	4.76633544547076e-22	42.981755407156

GIMAP8	-2.7798770480226
6.85155273631841	-11.4236168119711	1.35787045058204e-23
	5.29982516168237e-22	42.8747535635036
DNA2	2.45121723163842	7.22871791044776
11.4212672243422	1.38027783261044e-23	5.37705956423003e-22
	42.8585376230183
RHOV	3.76190451977401	7.8791552238806 11.4184267609777
	1.40785959750329e-23	5.47412074559516e-22
42.8389347638822
ESRP2	1.65906560734463	9.9334223880597 11.4176181492194
	1.41581152198095e-23	5.4946333735858e-22
42.8333544835777
PCDHGA5 -3.65880868644068
2.94333482587065	-11.410165139919	1.49124711087264e-23
	5.75585075592468e-22	42.7819246320029
CENPL	1.61805628531074	7.38039303482587
11.4101378335269	1.4915307365572e-23	5.75585075592468e-22
	42.7817362156492
ASB5	-2.23490600282486
0.661610945273632	-11.4068792196843	1.52576652692897e-23
	5.8769205999722e-22	42.75925218023
RIC3	-4.6264136299435
3.34460646766169	-11.4027129357636	1.57068267148293e-23
	6.03859836058885e-22	42.7305072658654
C12orf48	2.08283170903954	7.49608955223881
11.4008562343617	1.59112208923522e-23	6.1057451386914e-22
	42.7176978155457
ITM2A	-4.03735240112994
6.80492786069652	-11.3959195535536	1.64676708590639e-23
	6.3074866182198e-22	42.6836415781508
LAYN	-2.63531942090396
6.58941641791045	-11.389967660406	1.7164421869092e-23
	6.56211510190798e-22	42.6425858577247
SVIL	-2.6067845338983
10.2446358208955	-11.3849007243405	1.7780698852141e-23
	6.78508824227611e-22	42.6076380224192
SLMAP	-1.70614088983051
9.74994825870647	-11.3836870189771	1.79315655041647e-23
	6.82996363266235e-22	42.5992672955659
STRBP	1.4562375	9.93562089552239	11.3816948753157
	1.81819668511893e-23	6.91251443435029e-22
42.585528210973
MYL9	-3.47792196327684	11.693939800995 -11.3713900832786
	1.95339908791175e-23	7.41280652030099e-22
42.5144678616671
CHAF1A	1.68016843220338	9.21417213930348
11.3656272969636	2.03333395694831e-23	7.70190888120826e-22
	42.4747344042403
CYYR1	-2.75957231638418
7.54196218905473	-11.3574037540508	2.15308358843376e-23
	8.14047993932692e-22	42.4180417696148
FOXN3	-1.76359943502825
9.65154626865672	-11.3536518826675	2.21002980749932e-23
	8.340424990434e-22	42.3921794550444

LHFP	-2.75455882768362
8.33382288557214	-11.3515255751624	2.24296748995895e-23
	8.44919680162519e-22	42.3775232412397
TGFBR3	-3.56885437853107
8.39596915422886	-11.3441560606066	2.36096099643862e-23
	8.87738631078477e-22	42.3267311391456
HMGA1	2.80962281073446	12.144307960199 11.3417156464615
	2.40138335955769e-23	9.01287026905292e-22
42.3099128785597
PAFAH1B3	2.14681002824858	10.5837995024876
11.3385950527005	2.4540792843388e-23	9.19384082253203e-22
	42.2884082378031
SRL	-3.66662810734463
3.89778656716418	-11.3378260381143	2.46724141917672e-23
	9.22631445094683e-22	42.283108996226
RAB8B	-1.89264286723164
8.13043184079602	-11.3373160505771	2.47600896153902e-23
	9.24226617825381e-22	42.2795947393768
PRC1	2.49128177966101	9.6755736318408 11.3292001219878
	2.61979210598938e-23	9.76122176696226e-22
42.2236734723691
PNP	2.10497146892655	10.5756482587065
11.3283484897695	2.63535438779013e-23	9.80141767777742e-22
	42.217805956566
ABCA8	-5.28957076271186
4.88141144278607	-11.3162146956597	2.86736245843059e-23
	1.06324334437593e-21	42.1342176307449
SPC24	3.56921398305085	5.18209751243781
11.3161249847878	2.86915154790191e-23	1.06324334437593e-21
	42.1335996941494
PKM2	1.60166313559323	14.8031781094527
11.3096583131327	3.00109168230457e-23	1.11013355383266e-21
	42.0890594432191
H2AFY	1.03548848870056	12.279152238806 11.2855039111885
	3.54970200506258e-23	1.31070831230099e-21
41.922740597116
RPS6KA1 1.81776864406779	10.524871641791 11.2799757097256
	3.68871267638065e-23	1.35717083602647e-21
41.8846861409628
LRRC8E	2.51544879943503	8.25312587064677
11.2799740726882	3.68875463469445e-23	1.35717083602647e-21
	41.8846748726957
JAM2	-3.64754661016949
6.08369800995025	-11.2685229918108	3.99421355561937e-23
	1.46692673160761e-21	41.8058620273388
TSPAN18 -3.25271405367232
7.39212388059701	-11.2681092999133	4.00570887853915e-23
	1.46852148707873e-21	41.803015091345
NFKBIL2 2.25126221751413	9.11837562189055
11.261288130576 4.20007805648251e-23	1.53703391264859e-21
41.7560766093568
RUSC2	-1.92318926553673
8.82247860696517	-11.2585755574114	4.27996166067325e-23
	1.56348065647014e-21	41.73741232074

MOGS	1.04294632768361	11.0318154228856
11.2562835355845	4.34864018432583e-23	1.58574747751704e-21
	41.7216424703679
MREG	2.06738050847457	8.58157910447761
11.2559891253273	4.35754122917404e-23	1.58617591196708e-21
	41.7196168837608
RBM9	-1.62531129943503	10.472155721393 -11.2513386055441
	4.50057734910877e-23	1.63534253056997e-21
41.6876221552074
FXYD1	-5.66596384180791
4.88223582089552	-11.2459061687862	4.67360283097482e-23
	1.69521318233062e-21	41.6502516618505
PPP1R12C	-1.26567916666667
10.6368925373134	-11.2370861728909	4.96876315857695e-23
	1.799095372938e-21	41.5895861479086
GPM6A	-5.56449378531074
3.32622338308458	-11.2353224966546	5.02997808444756e-23
	1.81805369848078e-21	41.5774565276336
SPINT1	3.07080628531074	12.0031970149254
11.2303331631414	5.20725520652643e-23	1.87882160615092e-21
	41.5431448467277
SCRG1	-3.78889039548023
1.63704776119403	-11.228002460129	5.29218984366975e-23
	1.90611679807965e-21	41.5271177431773
GEN1	1.75887231638418	8.44275771144279
11.2255158561052	5.38432955158082e-23	1.93590692984158e-21
	41.5100193983307
NECAB1	-4.14480402542373
4.72034378109453	-11.2238821954848	5.44573296844033e-23
	1.95456115108531e-21	41.4987865062424
MAF	-2.90682358757063
8.45806815920398	-11.2230423542975	5.47757085180685e-23
	1.96255723538559e-21	41.4930119809813
TFAP2A	4.78189802259886	8.36434179104478
11.2208529902085	5.56144376590294e-23	1.98913659432034e-21
	41.4779589457349
LIMS2	-3.00168354519774
8.89208059701492	-11.2197663964741	5.60354470095834e-23
	2.00070909062043e-21	41.4704882820395
GIMAP6	-2.76940755649718
7.33833880597015	-11.2186547040649	5.64694705383711e-23
	2.01270526068187e-21	41.4628452239016
DES	-7.30325459039548
8.49630497512438	-11.216658798152	5.72571357976814e-23
	2.03724263072166e-21	41.4491234835942
KCNA1	-2.4159093220339	0.8330407960199 -11.2161291287564
	5.74679981530966e-23	2.04120761606068e-21
41.4454821277544
LOC283174	-4.73269265536723
3.82407164179104	-11.2038714577832	6.25700939660788e-23
	2.21859072387495e-21	41.361224168573
NMU	5.38880310734463	5.97321393034826
11.2033829858608	6.27825177538083e-23	2.22228463704428e-21
	41.357866890009

C1QTNF4 -3.18266108757062
3.17233731343284 -11.1994490258126 6.45197235250391e-23
2.27984496380216e-21 41.3308298892168
CCL23 -3.55289442090395
1.91051094527363 -11.197972651516 6.51839785037678e-23
2.29857430643055e-21 41.320683733366
C13orf34 1.56226235875706 7.66428606965174
11.1977744132514 6.52736882926942e-23 2.29857430643055e-21
41.319321394158
NRIP2 -2.36737168079096
6.02162039800995 -11.183761243063 7.19372587406134e-23
2.52889027730273e-21 41.2230333883288
MAD2L1 2.60589201977401 8.89298656716418
11.1765641132348 7.56190740439631e-23 2.65377707713259e-21
41.1735906716042
SLC25A39 1.54930084745763 12.1401930348259
11.1755740930944 7.61400260223381e-23 2.66749954648225e-21
41.1667900114765
CCDC80 -3.34697026836158
9.83740547263682 -11.1698332881798 7.9232179970227e-23
2.77110162655666e-21 41.1273578836557
BCL6B -2.19137245762712
6.98747562189055 -11.1682157574804 8.01258212431524e-23
2.7975903233366e-21 41.1162483063536
PARVA -1.45071412429379
10.4597388059701 -11.1633306987087 8.28861559040046e-23
2.88905395706148e-21 41.0826988033119
PEAR1 -3.14579992937853
5.88640547263682 -11.1561971714746 8.70882176790192e-23
3.02676627487102e-21 41.0337133135
HSD17B6 -3.67699788135593
4.59067164179104 -11.1561247545631 8.71319468314063e-23
3.02676627487102e-21 41.033216067621
MARS 1.05747761299435 11.0763741293532
11.1513167390173 9.00848036215922e-23 3.12405577424204e-21
41.0002037977845
TSTA3 1.74571257062147 11.175244278607 11.1450893325134
9.40581830946654e-23 3.25634822754381e-21
40.9574506935523
PLEKHA8 1.5717375 6.6317631840796 11.1274296751918
1.06301955244338e-22 3.67403895819234e-21 40.8362413806997
CDO1 -4.78278771186441
3.98234975124378 -11.1185070383996 1.13080008441194e-22
3.90173541730709e-21 40.7750165542618
GLI2 -3.59226235875706
5.10320199004975 -11.1134894804591 1.17079142140516e-22
4.03294427541074e-21 40.7405923555467
GNG11 -2.6130625 9.02020746268657 -11.1073295714965
1.22182093550815e-22 4.20167232931028e-21
40.698335697734
SH2D3C -2.07347895480226
7.74790895522388 -11.105813725483 1.23471475490202e-22
4.23891202644458e-21 40.6879379057295
PSRC1 2.21720649717514 8.45671542288557

11.1034887147098	1.25475547236568e-22	4.30052251213145e-21
	40.6719903707416
GSN	-2.21293128531074
12.9020034825871	-11.0990516360722	1.29390589557212e-22
	4.4273146726826e-21	40.641558057421
CRY2	-1.47041490112995
9.36512786069652	-11.0983571188916	1.30014323524728e-22
	4.43932538412352e-21	40.6367948722009
MGC16121	-4.13604096045198
3.88623134328358	-11.0981797661789	1.30174080917796e-22
	4.43932538412352e-21	40.6355785508267
C6orf129	2.02426235875706	9.1549552238806
11.0923381448756	1.35546986490591e-22	4.61489159643755e-21
	40.5955180432588
RAG1AP1 1.52564201977401	10.444571641791 11.0812165813784
	1.46394892056951e-22	4.97597207604173e-21
40.5192625184146
GNAO1	-3.76270939265537
4.99759850746269	-11.0779041046229	1.49790116854219e-22
	5.08296049424316e-21	40.4965538280014
FRMD6	-3.06415162429379
8.24952139303483	-11.0740466179414	1.53843036224056e-22
	5.2118771182836e-21	40.4701108294749
GHR	-3.67115995762712
4.41176666666667	-11.0729825498771	1.54980128717978e-22
	5.24174965828679e-21	40.4628170436779
ENPEP	-3.28731546610169
6.57643731343284	-11.0707244835473	1.57420996691925e-22
	5.31554779948228e-21	40.4473393954512
REEP4	1.63006927966102	9.57240497512438
11.0688899429889	1.59432243819436e-22	5.3746206331905e-21
	40.4347653013001
KATNAL1 -2.10127535310735
7.23639402985075	-11.0564901250279	1.73714866337141e-22
	5.8465019768877e-21	40.349788799806
GPR1	-3.01770572033898	1.8482631840796 -11.0533545261606
	1.77524300294925e-22	5.964932708764e-21
40.3283039672767
ZYX	-1.48915367231639
12.1664890547264	-11.0486671804871	1.8337481423765e-22
	6.15144597434471e-21	40.2961894012672
AHNAK	-2.29126320621469
12.9866746268657	-11.0448296233056	1.88307489808923e-22
	6.30661136342117e-21	40.2698994246443
ABCA9	-4.33514187853107
4.46586616915423	-11.0410596709921	1.93282054538642e-22
	6.46267195387347e-21	40.2440747016302
MAP3K3	-1.61965918079096
8.95264129353234	-11.0359828646084	2.00188685601273e-22
	6.68272148844575e-21	40.2093011519791
PTTG3P	2.30949858757062	2.52025422885572
11.0264071159323	2.13892509403503e-22	7.12859288645117e-21
	40.143722509975
TSPAN2	-4.30667408192091

6.08186815920398	-10.9994785425916	2.57650884993409e-22
	8.57305132725233e-21	39.9593777957979
KCNE4	-3.69690713276836
5.95494378109453	-10.9934944008891	2.68527687676001e-22
	8.92050716502962e-21	39.9184269544491
ABCA6	-3.64957471751413
3.48528706467662	-10.990325056616	2.74472502937503e-22
	9.10326411196597e-21	39.8967405950365
CDC42EP3	-2.30729823446328
9.04605422885572	-10.9894986391272	2.7604408397729e-22
	9.1406210387964e-21	39.8910860506598
GINS2	2.49036631355932	8.13997164179104
10.9881877223743	2.78555449458553e-22	9.20892653362979e-21
	39.8821166588068
MBNL1	-1.36956553672316
10.8042646766169	-10.9845360481568	2.85671821141823e-22
	9.42900721550101e-21	39.8571329949124
ZNF676	-3.45104223163842
1.51133084577114	-10.9821979768357	2.9032312907816e-22
	9.56714902082606e-21	39.8411376678739
WDR62	2.41570021186441	7.97675422885572
10.9755224616174	3.04022920332181e-22	1.00025489654161e-20
	39.7954733643717
GEFT	-2.89473961864407
8.05171343283582	-10.9726841873906	3.10041266497811e-22
	1.01842355219201e-20	39.7760600094672
TXNDC15	-1.07459053672316
9.85314378109453	-10.9718242858195	3.11887957953153e-22
	1.02285299948534e-20	39.7701786577815
PKD1	-1.50538149717514
10.4618572139303	-10.9708791586159	3.13930328589815e-22
	1.02790903444161e-20	39.7637145295873
SNED1	-3.07413855932204	7.9552144278607	-10.9678291934856
	3.2061252025468e-22	1.04811704471793e-20
39.7428554433855
FKBP7	-2.21986002824859
7.78628955223881	-10.9647689414882	3.27459758006813e-22
	1.0687994963243e-20	39.721927427438
TSSC1	1.12415466101695	9.42351890547264
10.9569550418683	3.45611751157429e-22	1.12625543670826e-20
	39.6684973232823
LOH3CR2A	-2.9594927259887
3.72339203980099	-10.9492808974423	3.64414953680126e-22
	1.18564801886735e-20	39.6160319303445
ADM2	3.33199420903955	7.26360248756219
10.9465623471398	3.71317749982271e-22	1.20619515935697e-20
	39.5974483359916
CALR	1.44252153954803	15.0631676616915
10.9420919576782	3.82953252919374e-22	1.24202690085857e-20
	39.566891905926
TRPM2	2.85517026836158	8.79941691542288
10.9392746418633	3.90472131563002e-22	1.26304893320742e-20
	39.547636301253
LYPLA2P1	1.46454463276836	6.83674029850746

10.9392029382348	3.90665403110915e-22	1.26304893320742e-20
	39.5471462419672
KIAA0408	-3.44475939265537
0.985534328358209	-10.937311048545	3.9579944090805e-22
	1.27763561664187e-20	39.5342163860833
SLC19A1 1.72790042372881	8.98844228855721
10.9342429262056	4.04268796370466e-22	1.30292596381251e-20
	39.5132489060208
PPP1CA	1.3691084039548 12.1829119402985	10.9337807433887
	4.0556018536203e-22	1.30503927985619e-20
39.5100904848227
COX7A1	-3.13336553672317
6.14203432835821	-10.9304877053005	4.14881094932263e-22
	1.33294348653511e-20	39.4875877880556
C14orf139	-2.8599418079096
4.72867711442786	-10.9271416774151	4.24570733847304e-22
	1.36194330716955e-20	39.4647247078158
SCN2B	-4.5329677259887
2.66946965174129	-10.9132234754719	4.67356502463008e-22
	1.49685319743612e-20	39.3696416339728
MCM2	2.07513290960452	10.6609641791045
10.911871003379 4.71736581163902e-22	1.50852835066899e-20
39.3604037414469
TMEM206 1.59897401129944	8.1786184079602 10.9084197732037
	4.83099968593669e-22	1.5424638188535e-20
39.3368318350856
KDR	-2.6844156779661
8.37435323383085	-10.9072127685511	4.87138170753876e-22
	1.55294202571073e-20	39.3285884304826
RAB3IP	1.59979258474576	9.01308507462686
10.8912664229549	5.43756899941953e-22	1.73074870632687e-20
	39.2197020678345
MAOB	-4.39504265536724	8.1963552238806 -10.8905582064334
	5.46418261049999e-22	1.73652738380131e-20
39.2148670811878
CHEK1	1.78055826271186	8.05600845771144
10.8900574321261	5.48307921037169e-22	1.73983950832969e-20
	39.2114483479282
FEM1B	-1.23083100282486
9.90118805970149	-10.8771359139781	5.99384815904126e-22
	1.89897689359749e-20	39.1232481721502
NOSTRIN -3.01009498587571
6.50206517412935	-10.8733514719832	6.1522299994613e-22
	1.94315818290678e-20	39.0974211805115
CHRDL1	-6.29768742937853
5.47295024875622	-10.8706851197215	6.26631725220346e-22
	1.97615196908966e-20	39.0792259739321
MPRIP	-1.28296490112995
10.8962457711443	-10.8681340524388	6.37744510536247e-22
	2.00811269958729e-20	39.0618185279753
LYPLA2	1.50772803672316	11.2758049751244
10.8670281492907	6.42622802580534e-22	2.02037459984355e-20
	39.0542726153937
TTLL4	1.20900303672316	9.0314736318408 10.8652672935472

6.50467054595552e-22	2.04190957658206e-20
39.0422581634266
ZBTB38	-2.09719901129944
9.68337114427861	-10.8643636523859	6.54529574485961e-22
2.05152552125142e-20	39.0360927426878
ARHGAP31	-2.30106115819209
7.51821741293532	-10.8600570424339	6.74241090225717e-22
2.1100868265753e-20	39.0067111129861
EMILIN1 -3.34457973163842
9.77725223880597	-10.8500296659906	7.22459725077889e-22
2.2565196654877e-20	38.9383112949039
SH3BGRL -1.74898361581921
10.8339835820896	-10.849875076467	7.23229391081788e-22
2.2565196654877e-20	38.9372569179012
PDGFRB	-2.41061737288136
10.3220925373134	-10.8459648840123	7.42971472893546e-22
2.31459853391571e-20	38.9105887430634
PGCP	-2.08625875706215
8.52538009950249	-10.8442585884897	7.51753825503397e-22
2.33841000569466e-20	38.8989522858313
C9orf44 -2.2345811440678
1.00363034825871	-10.8377068854463	7.86447774547333e-22
2.44262826194505e-20	38.8542758460833
LOC144571	-2.23402627118644
5.03940646766169	-10.8335081494403	8.0951624484237e-22
2.51047862637672e-20	38.8256480497523
PKD2	-2.3170656779661
9.17759054726368	-10.8281446876658	8.39967443660771e-22
2.60098516113961e-20	38.789083056099
VDAC1	1.13090169491525	11.9773353233831
10.8269916349122	8.46661587070147e-22	2.61776541905875e-20
38.7812228122398
ARHGEF16	2.78465903954802	8.95211243781094
10.8267519819346	8.48059572629653e-22	2.6181448159529e-20
38.779589149093
SNTG2	-3.19306800847458
1.22203134328358	-10.825176338018	8.57308357808946e-22
2.64272381168433e-20	38.7688485520578
GAPDH	1.70466800847458	16.3360213930348
10.8204508536115	8.85653438498318e-22	2.72600676047533e-20
38.7366390329001
GINS4	2.42023714689266	8.01121194029851
10.8191809680465	8.9342861150391e-22	2.7458217655951e-20
38.7279839427715
FZD4	-2.06179894067797
8.65749850746269	-10.8183061184758	8.98824626330603e-22
2.75827646914309e-20	38.722021429937
PLAC9	-4.77820953389831
3.54647711442786	-10.8137442865405	9.27492445178204e-22
2.8420029700759e-20	38.6909324014239
C1QTNF2 -2.91700077683616
3.79760199004975	-10.8087560503159	9.59883907204244e-22
2.93687281891254e-20	38.6569412799672
LIX1L	-2.06461998587571

8.62277263681592	-10.7974264165089	1.03770343771701e-21
	3.17024577028722e-20	38.5797532931628
RAB23	-2.06359583333333
8.76369950248756	-10.7963993203801	1.04506169529913e-21
	3.18798166485752e-20	38.572756795752
C3orf72 -4.22418820621469
5.16680497512438	-10.7874002832161	1.1117968223691e-21
	3.38573127046668e-20	38.5114634487868
CCDC58	1.30451278248588	8.41862089552239
10.7853941605749	1.12724342528153e-21	3.42341827234168e-20
	38.4978013547453
TCEAL3	-1.76991094632768
9.18939253731343	-10.7798429702302	1.17110991132964e-21
	3.55138648147673e-20	38.4600000899159
LOC644538	-2.63914392655367
6.18645273631841	-10.7791789349876	1.17646988026616e-21
	3.56237856074693e-20	38.4554786292892
MGC72080	1.98392266949152	7.41372388059701
10.7787610312293	1.17985564967406e-21	3.56736914400714e-20
	38.4526331316968
IFFO1	-2.46512485875707
6.73595174129353	-10.7782985731096	1.18361370970757e-21
	3.57346903827889e-20	38.4494842979352
H2AFX	1.88252951977401	10.9208915422886
10.7777631973249	1.18797922052345e-21	3.58138229035924e-20
	38.4458390186379
TEK	-3.08533368644068
6.69096019900497	-10.7752756539129	1.20847434752125e-21
	3.63782673821278e-20	38.4289023935398
OPCML	-3.90556906779661
2.10659253731343	-10.7750244949693	1.21056315735466e-21
	3.63877915380544e-20	38.4271924157877
C8orf51 2.44809639830509	5.48062388059702
10.77036132249	1.25000551114961e-21	3.75184402688617e-20
38.3954457999153
PCDHGA6 -3.64549011299435
3.40360895522388	-10.7678172895222	1.27206091285585e-21
	3.81246869210667e-20	38.3781276802989
DDX39	1.60999837570621	10.8209472636816
10.7649336903107	1.29752945029529e-21	3.88313113914904e-20
	38.3584993086805
GARNL3	-2.83234343220339
6.07383432835821	-10.7623756269803	1.32054781895292e-21
	3.94626589855945e-20	38.3410879792415
HSD17B13	-2.97658672316384
2.34737661691542	-10.7609781152306	1.33329456275595e-21
	3.97856647868889e-20	38.3315763436749
RCC1	2.03626793785311	10.2089731343284
10.7551422145247	1.38786264926667e-21	4.13538754563785e-20
	38.2918599922665
TIMP2	-2.35600296610169
12.1220024875622	-10.7523467133586	1.41478459640133e-21
	4.20949677740859e-20	38.2728371511725
FAM65B	-3.34799166666667

6.30526019900497	-10.7478393661701	1.45929273123458e-21
	4.33564106689522e-20	38.2421682697914
C2orf58 -2.75863368644068
2.48151791044776	-10.7466520218546	1.47124767971289e-21
	4.36484318851236e-20	38.2340899075102
EFNA4	1.78672415254237	9.17347562189055
10.7447993913905	1.49009640892961e-21	4.41438373381312e-20
	38.2214855918712
STARD13 -2.15874576271187	7.9613815920398 -10.7421368760566
	1.51760715208994e-21	4.48940559544761e-20
38.2033722519525
LIFR	-3.33729872881356
8.54269303482587	-10.7344560914241	1.59983866859788e-21
	4.72585436925388e-20	38.1511257979213
CACNA2D1	-3.69518778248588
1.96846616915423	-10.7239777061479	1.71922774461466e-21
	5.07122781565214e-20	38.0798653709042
HAPLN2	-3.08450882768362
1.58411990049751	-10.7213826349262	1.75014230437985e-21
	5.15501025952918e-20	38.0622199032457
CTSK	-2.85913538135593
9.35564975124378	-10.7177639438349	1.79417799780253e-21
	5.27714531445356e-20	38.0376161153115
SNRPA1	1.33211525423729	9.52369004975124
10.7075169274827	1.92495498235036e-21	5.6536946763452e-20
	37.967957773972
MEF2D	-1.22659223163842
10.0284248756219	-10.703823067565	1.97439154326418e-21
	5.79060834045908e-20	37.9428515866705
TBL1X	-1.9813216101695
9.83151243781094	-10.6997043261864	2.0310076784578e-21
	5.94815800837926e-20	37.914860321504
ABCB1	-3.38876151129944
6.17578756218906	-10.6984511290149	2.04855299007296e-21
	5.99099613763502e-20	37.9063440742241
ACBD7	3.03937867231638	7.59500945273632
10.6977006053436	2.05913288177411e-21	6.01337099044416e-20
	37.9012439310269
CHEK2	1.49041313559322	8.67109502487562
10.6972354223623	2.06571773238677e-21	6.02403196674722e-20
	37.8980828531956
CCDC69	-2.52438439265537
8.43783880597015	-10.6949232300551	2.09876041159768e-21
	6.11170939717736e-20	37.8823712599329
TMEM204 -2.35616984463277
7.35599303482587	-10.6915994261971	2.14718449907257e-21
	6.24386653908781e-20	37.8597872526923
SOX7	-2.82407514124294
5.97336119402985	-10.6862372880895	2.22766250073278e-21
	6.46872859123678e-20	37.8233574944274
HERC1	-1.40655847457627
9.38703880597015	-10.6849724323226	2.24708017050761e-21
	6.51589772606233e-20	37.8147649231556
TPI1	1.61522443502825	13.6849368159204

10.6845693060368	2.25330421246157e-21	6.52472996922934e-20
	37.8120264143241
PPM1F	-1.55582690677966
9.19447263681592	-10.6837737909551	2.26563695974613e-21
	6.55120095543493e-20	37.8066224198269
CCDC150 3.01604943502825	6.14147412935323
10.6796528585642	2.33060800431185e-21	6.72958963832944e-20
	37.7786303449432
RAB11FIP4	2.79848107344633	10.5211174129353
10.6745893574845	2.41298708235738e-21	6.95767202258384e-20
	37.7442397074412
PPIF	1.64972951977402	10.5729218905473
10.6717745351211	2.46003021628229e-21	7.08336891167958e-20
	37.725123703646
LOC729082	1.11558693502825	8.73809502487562
10.6676705962256	2.53026019035748e-21	7.27538399272255e-20
	37.6972554986867
NRK	-4.03619209039548
2.58790796019901	-10.6619123230622	2.6321811676819e-21
	7.55785725489642e-20	37.6581582583524
ANKRD29 -3.6866550141243
4.94408009950249	-10.6606950137368	2.65424579256351e-21
	7.61056789404035e-20	37.6498937629004
STON1	-3.22584992937853
7.48683034825871	-10.6546297765963	2.76695997127933e-21
	7.92269012696858e-20	37.6087197711509
TMEM132A	2.26134936440678	11.6023666666667
10.6383627679802	3.09336668501136e-21	8.8449607302623e-20
	37.4983222859629
ASB2	-3.07313382768362
6.36849502487562	-10.6322235790332	3.22628983069612e-21
	9.21220170016569e-20	37.4566700927636
GALE	1.9524031779661 10.0744179104478	10.6307777630865
	3.2584130670795e-21	9.29100281488085e-20
37.4468617064509
MYADM	-2.18068142655367	11.010844278607 -10.62885574276
3.30161059335396e-21	9.40111865208832e-20	37.433823324107
ZBTB47	-1.38096518361582
9.02376368159204	-10.6242073822221	3.40845618213407e-21
	9.69191210792415e-20	37.4022929740367
PI16	-4.44848446327684
3.02112786069652	-10.6216448033228	3.46882475747385e-21
	9.84992700842851e-20	37.3849123306205
LSR	2.07331836158192	11.903907960199 10.6147236715102
	3.63724824876302e-21	1.03139097440753e-19
37.3379756351685
FBXL3	-1.63690120056497
9.16486218905473	-10.6074383877964	3.82334140652348e-21
	1.08266481484037e-19	37.2885784775121
VAMP2	-1.65356574858757
9.98867910447761	-10.6033302953649	3.93242916306374e-21
	1.11202163523001e-19	37.2607280762324
PODN	-4.40816906779661
7.35270447761194	-10.6007154104709	4.00347532261877e-21

1.13055499825809e-19	37.2430022761483
WWC1	2.37273990112994	10.320631840796 10.5927829981348
4.22691755919005e-21	1.19201397651335e-19
37.1892373753861
SETD7	-2.65381235875706
9.06934825870647	-10.5923728517243	4.23880267730678e-21
1.19260668717045e-19	37.1864577569311
SH3BP5	-2.69209060734464
9.35905721393035	-10.5923096350185	4.24063751404983e-21
1.19260668717045e-19	37.1860293312994
PYCR1	2.40048241525423	10.7263368159204
10.5904054346585	4.29627855217578e-21	1.20660189707481e-19
37.1731247161361
MYOM1	-3.53320049435028
4.91366169154229	-10.5858550716483	4.43220450285391e-21
1.24307593502173e-19	37.1422898703587
PIWIL2	-2.30552803672316
1.53935472636816	-10.5769687830859	4.71012660271465e-21
1.31922099800453e-19	37.0820838899389
LONRF2	-3.85096631355932
8.00782139303483	-10.5767033539316	4.71868978818172e-21
1.31981881950096e-19	37.0802857825291
AP1M2	3.1677363700565 10.9439616915423	10.5759436736045
4.7432841592614e-21	1.32489284067533e-19
37.0751395188925
ABCD2	-3.42892111581921
2.74737313432836	-10.5743588691667	4.79500351515515e-21
1.33751932290945e-19	37.0644039903548
XRCC2	2.40207422316384	5.9541552238806 10.572285963611
4.86350080602498e-21	1.35478523131198e-19	37.05036272163
C15orf23	1.73727994350283	8.77109353233831
10.5690828934785	4.97126525829148e-21	1.3813519348848e-19
37.0286675502807
RGS2	-3.25341179378531
8.26084278606965	-10.5690516311625	4.97232868913721e-21
1.3813519348848e-19	37.0284558120597
SERPINF1	-3.22517337570622
10.1392427860697	-10.5671985482686	5.0357704210767e-21
1.3970860370906e-19	37.015905279452
GGH	2.68676412429378	9.42840646766169
10.5667741471151	5.05041325202255e-21	1.39925754472366e-19
37.0130309879802
TMEM43	-1.31604901129943
10.8406328358209	-10.5660098380868	5.07689076915808e-21
1.40469767507838e-19	37.007854723937
FBXO32	-2.6465875	7.93154378109453	-10.5637326203985
5.15660179063616e-21	1.42483223097928e-19
36.992432943145
CYR61	-2.77649392655368
11.0965527363184	-10.5555814196211	5.4522805960606e-21
1.50450699781081e-19	36.937238973595
SLC9A9	-2.43430374293786
5.57758855721393	-10.5471732192579	5.77498713749682e-21
1.59001814032547e-19	36.880317298924

ESAM	-1.74118298022599
8.68790696517413	-10.5471002431419	5.77786966594251e-21
	1.59001814032547e-19	36.8798233223219
SRPX	-4.10185211864407
5.79164626865672	-10.5469096563198	5.78540453396553e-21
	1.59001814032547e-19	36.8785332413272
STARD8	-2.29887902542373
6.36600149253731	-10.5458253126765	5.82846083417371e-21
	1.59970990542228e-19	36.8711934506706
DTWD1	-1.37503361581921
8.30693283582089	-10.5455995862843	5.83746387366505e-21
	1.60004183346253e-19	36.8696655627497
CCDC36	-2.58951214689265
2.08813333333333	-10.5399290105185	6.06823570106726e-21
	1.66107838590548e-19	36.831285809758
RAMP1	-4.00065988700565
8.72346417910448	-10.5348794519515	6.2813728707426e-21
	1.71713162498463e-19	36.7971141346142
FASTKD1 1.10649449152542	8.51915074626866
10.5267260794638	6.64137250453698e-21	1.81313002018809e-19
	36.7419478947058
PVRL4	3.8102104519774 8.97187661691542	10.5262530241513
	6.6628793327284e-21	1.81658582604136e-19
36.7387475424682
FAM69A	-2.36792944915254
6.58625422885572	-10.5247583328314	6.73129048945902e-21
	1.83280363061795e-19	36.7286358025148
DIO3	-4.03799336158192
3.36024676616915	-10.5236705981173	6.78151497684932e-21
	1.84403314536049e-19	36.7212774210064
SLC25A13	1.2092220338983 9.76647064676617
10.5192258016599	6.99065589892377e-21	1.89838843392731e-19
	36.6912111977086
SLC5A6	1.42627549435029	9.87737810945274
10.510873532283 7.4011904820497e-21	2.00721850193501e-19
36.6347231435344
RAB3IL1 -2.13075275423729
8.53293631840796	-10.4776275346247	9.28750709029108e-21
	2.51546860901947e-19	36.4100006297485
CLIC4	-1.85707570621469
11.3161313432836	-10.474364038044	9.49670829159637e-21
	2.56764071157567e-19	36.3879523810556
PLIN4	-3.86982210451978
6.72199203980099	-10.4742338836113	9.50514827470781e-21
	2.56764071157567e-19	36.3870730959856
HMBS	1.59824929378531	9.39348855721393
10.4683514762698	9.89450081555533e-21	2.66930488493234e-19
	36.3473365608749
SYT9	-3.37221624293785	1.6246447761194 -10.4676867347132
	9.93948679391272e-21	2.67792209814998e-19
36.3428465367642
MXRA7	-1.88378580508475
11.0153313432836	-10.4599923418787	1.04752662468611e-20
	2.81846820182466e-19	36.2908804231629

SCN7A	-4.48970261299435
2.28501044776119	-10.4598058848133	1.04886006146812e-20
	2.81846820182466e-19	36.2896212728705
PCDH18	-2.96915303672316
7.88106019900497	-10.4582307911473	1.06019193345427e-20
	2.84519482272107e-19	36.2789848754256
DENND2A	-2.88341405367232
7.67812835820896	-10.4562541508277	1.07458540246252e-20
	2.88005722096026e-19	36.2656375413134
REM1	-3.29022860169492
4.69343034825871	-10.4533770996712	1.09588401514959e-20
	2.93331145124134e-19	36.2462114535295
HCFC2	-1.67749194915255
7.19141393034826	-10.4526874882108	1.1010513247716e-20
	2.94330516895324e-19	36.2415553693956
SBK1	3.15540868644068	9.67537263681592
10.4494556526871	1.12559296189678e-20	3.00499655497281e-19
	36.2197360080671
FILIP1L	-3.41479964689266
8.48754179104478	-10.4477648072902	1.13864909825595e-20
	3.03590467366164e-19	36.2083212403248
S100A2	4.46349604519774	8.04587462686567
10.4427763740189	1.17805369709161e-20	3.13689265905199e-19
	36.1746478281701
TBC1D1	-1.48217711864407
10.6400492537313	-10.4413882143698	1.18925863128616e-20
	3.16262690418457e-19	36.1652781659447
MMP28	-3.36104908192091
4.68815771144279	-10.4351494294736	1.24094192861858e-20
	3.29580049088478e-19	36.1231727001065
CTSG	-3.73980670903955
1.76075820895522	-10.4271895312513	1.31014374308675e-20
	3.47329488314628e-19	36.069462092721
BMPER	-4.37125155367232
2.89584726368159	-10.427076216871	1.31115612978001e-20
	3.47329488314628e-19	36.068697573107
SLC25A25	-1.50563262711864
9.15605273631841	-10.4234446104363	1.34401802459558e-20
	3.55575902641074e-19	36.0441968082646
TIE1	-2.2830656779661
8.50966417910448	-10.4226342292085	1.35146228632507e-20
	3.57085208986533e-19	36.0387298815878
FLVCR1	1.66783255649717	7.12629253731343
10.4200288647879	1.37567488500751e-20	3.63015493434501e-19
	36.021154628702
HSPB2	-3.31399604519774
5.62197164179104	-10.417215924329	1.40230185128728e-20
	3.69566842194197e-19	36.0021805513649
MDFIC	-2.66833870056498
7.84695223880597	-10.416998083832	1.40438519408448e-20
	3.69641385058389e-19	36.0007112181861
TMPRSS4	4.84543947740113	10.2111587064677
10.416626092139	1.40794990937309e-20	3.7010514263034e-19
35.9982021566433

SOX10	-2.64114618644068
0.884489552238806	-10.4146412578608	1.427123034772e-20
	3.74665420765589e-19	35.9848150119606
FOXJ2	-1.54726553672317
8.82728258706468	-10.4137034423039	1.43627240704302e-20
	3.76585855895187e-19	35.9784899714043
PYGM	-3.65383100282486
4.83243482587065	-10.4079569623201	1.49362510711585e-20
	3.91124023585311e-19	35.9397368249305
OTX1	4.29041115819209	4.45961492537313
10.4068287405638	1.5051503784491e-20	3.93639965217326e-19
	35.9321290523003
CASP6	1.31774046610169	9.22370248756219
10.4024804493998	1.55040467505474e-20	4.04959389044197e-19
	35.9028101200202
DSP	2.50923757062147	12.1425104477612
10.3937303747426	1.64561832681678e-20	4.29282646118786e-19
	35.8438224872718
P4HB	1.36026765536723	14.6458726368159
10.3919307124873	1.66591189688964e-20	4.34025015775942e-19
	35.8316920797032
EHBP1	-1.66101207627119	9.3264552238806 -10.3910269303949
	1.67619706111e-20	4.36151149107582e-19
35.8256004782718
VAMP8	2.10397146892655	11.0520009950249
10.3896411251667	1.69209068578101e-20	4.39729389608661e-19
	35.8162602853622
AP1S3	2.22359872881356	7.33458606965174
10.3888984883043	1.7006695741784e-20	4.41400080377781e-19
	35.8112551359925
FAM13C	-4.35990346045198
4.85086119402985	-10.3844437458175	1.75304742903297e-20
	4.54419996439987e-19	35.7812337248261
EPM2A	-1.5258447740113
6.69783731343284	-10.3816996511478	1.78610838022147e-20
	4.62406116594537e-19	35.7627426072126
OSBPL5	-1.8034281779661
8.73709402985075	-10.380596508097	1.79957343220614e-20
	4.65305321954561e-19	35.7553094702529
MAPK13	2.33309816384181	9.21371094527363
10.378540095139 1.82494465141253e-20	4.7127187035848e-19
35.7414536864739
ABHD11	2.07315084745762	10.6175104477612
10.3749470501517	1.87013221274114e-20	4.8233435084894e-19
	35.7172462690759
PROS1	-2.64843594632768
7.80702437810945	-10.3747132012927	1.87311151830027e-20
	4.82496605655013e-19	35.7156708454382
KLHDC1	-2.88542323446328
4.60865074626866	-10.3744643623189	1.8762869941939e-20
	4.82708922190486e-19	35.7139944459768
FOXL2	-4.11426588983051
5.76995223880597	-10.3690294897204	1.94699546958532e-20
	5.00273053699457e-19	35.6773833077071

PRND	-4.08817930790961
2.43943233830846	-10.3663134588227	1.98331971586891e-20
	5.08969422084858e-19	35.6590893375923
RASA4	-2.35994103107345
7.26490547263682	-10.3661283873514	1.98581928638129e-20
	5.08974656047539e-19	35.6578428306382
SCD	2.61352401129943	11.8852781094527
10.3627880300364	2.03147728072406e-20	5.20027787696571e-19
	35.6353457469365
SMG6	-1.08801299435028
10.0572990049751	-10.3585078872647	2.09151209197612e-20
	5.34729056640967e-19	35.6065224202601
TLN1	-1.37045268361582
12.4281084577114	-10.3578462328958	2.10094908082687e-20
	5.36473689420096e-19	35.6020670251131
GIPC1	1.50589456214689	11.9876820895522
10.3566269642903	2.11845037940582e-20	5.40270637132939e-19
	35.5938570337509
DCHS1	-2.61463771186441
9.07533283582089	-10.3549433360115	2.1428559879571e-20
	5.45816791969718e-19	35.5825207318801
KIF23	2.7030488700565 8.56594676616916	10.3525337645273
	2.17827270346933e-20	5.54150416384453e-19
35.5662974228903
ZFYVE21 -1.04748128531074
10.0721452736318	-10.3463407859514	2.27199679856576e-20
	5.77278394487068e-19	35.5246061264585
C1orf97 1.98145981638418	7.13061343283582
10.3428952949991	2.3258683356933e-20	5.90235808798313e-19
	35.501414214227
LRRC4B	-3.78256927966102
5.70534328358209	-10.3394783265646	2.38055012534667e-20
	6.03366593498359e-19	35.4784165700384
PSMD14	1.17438813559323	10.6197293532338
10.3373260960031	2.41564756581179e-20	6.11507330778251e-19
	35.4639323141109
HAAO	-2.97909583333333
5.72931293532338	-10.3347026961312	2.45912621833085e-20
	6.21747059880941e-19	35.4462783656411
THSD1	-2.25074230225989
5.94962388059701	-10.3330767186462	2.48646450888313e-20
	6.27885810176762e-19	35.4353371609752
MIMT1	-1.90648566384181
0.570883582089552	-10.3309212852577	2.52317188623236e-20
	6.3637246712961e-19	35.4208340423603
GRK5	-2.14779032485876
7.00278308457711	-10.3181010929415	2.75292563326689e-20
	6.93467033754224e-19	35.3345904596902
C1orf31 1.25028107344633	9.05824378109453
10.3141622814961	2.82761245559256e-20	7.1140788864357e-19
	35.3080998855384
GPT2	1.75621822033898	10.6282955223881
10.304767340874 3.01400451723669e-20	7.57374696926183e-19
35.2449262975716

LPAR2	2.08232238700565	9.18805771144279
10.2988924119867	3.13672771267409e-20	7.87249632532996e-19
	35.2054308625846
WT1	-5.38164314971752
6.71391194029851	-10.296598353214	3.18598886030335e-20
	7.9791132204968e-19	35.1900104249262
MFAP4	-4.71943594632768
8.45054129353234	-10.2965524946038	3.18698141296024e-20
	7.9791132204968e-19	35.1897021784634
NAP1L3	-3.69459950564972	5.3529592039801 -10.2963391058082
	3.19160400009858e-20	7.98095372984455e-19
35.1882678547848
SFN	4.48509618644068	10.5137373134328
10.2957463703211	3.20447934079321e-20	8.00340156526576e-19
	35.1842837446673
ATP5J2	1.58523898305084	11.3738567164179
10.2944938233073	3.23185743286923e-20	8.06197242974549e-19
	35.175864897138
EPHA5	-3.1522261299435
1.30688407960199	-10.2845536287544	3.45755266011312e-20
	8.61450923690805e-19	35.1090640706531
MITF	-2.6922343220339
8.54950149253731	-10.2808556698742	3.54546409854967e-20
	8.82283369008786e-19	35.084217781019
CMAH	-3.13783566384181
6.05987711442786	-10.2799608894314	3.56706799783794e-20
	8.86584818348824e-19	35.0782062314517
C19orf46	2.97725444915254	8.25255721393035
10.2767719201224	3.64513526789887e-20	9.04892709189404e-19
	35.0567825541948
ADAMTSL5	-4.13734661016949
4.40114825870647	-10.2712089787175	3.78541260692716e-20
	9.38581169326263e-19	35.0194152480833
TM7SF2	2.59047683615819	10.2897661691542
10.2703365854709	3.80789363081056e-20	9.43016359958272e-19
	35.0135557813207
DIRAS1	-4.4234831920904
4.80051890547264	-10.2687354702854	3.84949984066936e-20
	9.52171466613758e-19	35.0028022177298
HTR2A	-2.22182549435028
1.00357960199005	-10.2673274674547	3.88646178846366e-20
	9.60157166271468e-19	34.9933460771013
OSBPL1A -1.99777881355933
9.00767860696517	-10.2659761608682	3.9222676813736e-20
	9.67838407435097e-19	34.984271080843
ELTD1	-2.1633415960452
7.81819701492537	-10.2649450027797	3.94981133120358e-20
	9.73464905517521e-19	34.9773463577963
TIPARP	-1.86530402542373
8.50929800995025	-10.2645482620883	3.9604600560716e-20
	9.74919004210432e-19	34.9746821096929
OTC	-2.3672281779661
0.754646766169154	-10.264326603713	3.96642192429725e-20
	9.75217270728415e-19	34.9731936123487

FAM48B2 -1.49458679378531
0.435133830845771	-10.263540118221	3.98764795416421e-20
	9.792633074042e-19	34.9679122239074
CLEC14A -2.03824194915254
8.37582189054726	-10.2617874666842	4.03535708499287e-20
	9.89795471384751e-19	34.9561433059247
MAGEE2	-1.99557245762712
0.823196517412935	-10.2586398719716	4.12246957446751e-20
	1.00995585159687e-18	34.9350089984565
DNMT3B	2.61446193502825	8.04305820895522
10.2577640690391	4.14703948514745e-20	1.01476425065646e-18
	34.9291288332792
AVPR1A	-4.08958608757062
3.45156019900497	-10.2527295972919	4.2911322361881e-20
	1.0487731524874e-18	34.895330235832
CD99L2	-1.49886857344633
9.76540497512438	-10.248916844816	4.40356582215842e-20
	1.07497272685984e-18	34.8697369594221
C5orf4	-3.10488961864407
7.73327263681592	-10.2476426534416	4.44179041038142e-20
	1.08301611787566e-18	34.8611845430846
PDE5A	-2.71365529661017
7.67475074626866	-10.245336309334	4.51182083050911e-20
	1.09878625919753e-18	34.8457051133509
HELLS	2.14116080508475	7.4829039800995 10.2376668481424
	4.75270100416703e-20	1.15607762577665e-18
34.7942379020443
AFAP1L1 -2.0947406779661
6.82754427860697	-10.2330240373491	4.90469960147566e-20
	1.19163884992065e-18	34.7630873099735
SPTBN2	2.55369653954802	9.98465671641791
10.2313910362794	4.95930432703866e-20	1.20348129827546e-18
	34.7521318478361
HVCN1	-2.24620240112994
6.33152587064677	-10.2186175305775	5.40784613940663e-20
	1.31078017995299e-18	34.6664557273168
F11R	1.77134025423729	12.2217786069652
10.2116535929773	5.66916910282879e-20	1.37250049152211e-18
	34.6197602666294
GNG7	-3.36834223163842
6.05515621890547	-10.2112642442034	5.68414508810582e-20
	1.37450528455609e-18	34.6171498484388
C20orf24	1.35654576271186	10.8076034825871
10.204520443951 5.9498797997542e-20	1.43707096810534e-18
34.5719404399262
TPM2	-2.72552740112994
11.9594587064677	-10.1997992798972	6.14323999176099e-20
	1.48202957733082e-18	34.5402960067828
PAPPA	-3.95627620056497
4.87746915422886	-10.1991655752724	6.16966587346348e-20
	1.48491489310909e-18	34.5360488372118
CGN	2.61744286723164	9.91262487562189
10.1984565189203	6.19936816164338e-20	1.49031649131778e-18
	34.5312967482048

FAM175A -1.71132302259887
7.40508905472637 -10.1950106565316 6.34575464778033e-20
1.52372330899333e-18 34.5082040777483
DNAJB11 1.25830444915254 11.2086383084577
10.1925991253715 6.45024303968e-20 1.54700338323166e-18
34.4920444934874
MS4A2 -3.68766497175141
2.31068656716418 -10.189686158531 6.57874476677337e-20
1.57598168100183e-18 34.4725263985879
DCAF12L2 -3.47430748587571
1.85889353233831 -10.1743818575387 7.29693630333759e-20
1.74599186838602e-18 34.3700098735898
GLT8D2 -3.00792895480226 6.5603184079602 -10.1713925618106
7.44607284445159e-20 1.77960274152027e-18
34.3499915868528
RANGAP1 1.36331871468926 11.6762552238806
10.1585363610054 8.12284019134235e-20 1.93909196660766e-18
34.2639191149104
C2orf84 -1.73882436440678
1.22530895522388 -10.1492548482735 8.64917381870954e-20
2.06234074910693e-18 34.201800571018
PRUNE2 -4.28960169491526
7.00878955223881 -10.1468282496069 8.79229218713172e-20
2.09403432252685e-18 34.1855629896564
PCDHB7 -2.82179187853108
5.25863432835821 -10.1418682095067 9.09220681775382e-20
2.16231317485849e-18 34.1523767111101
YPEL4 -2.77776899717514
4.25758407960199 -10.1417408273445 9.10004180749019e-20
2.16231317485849e-18 34.1515244993962
SCARF1 -1.7958218220339 7.1078671641791 -10.135053901915
9.52091748825324e-20 2.25970446281895e-18
34.1067924179332
TUB -3.8260229519774 6.6643447761194 -10.133266385811
9.636669820361e-20 2.28453615949205e-18
34.0948364428727
TXNIP -2.4752091101695 12.598584079602 -10.1297958779812
9.86542154131389e-20 2.33606809969059e-18
34.0716255268388
NEK6 1.52048213276836 9.77762786069652
10.128032014074 9.98374701742935e-20 2.36136320585051e-18
34.0598296923783
RABIF 1.09476822033898 8.54927462686567
10.1275266038607 1.00179111837395e-19 2.36671710704456e-18
34.0564498835194
CXorf57 -3.90236391242938
2.88100248756219 -10.1264950909002 1.00879998681958e-19
2.38053606085126e-18 34.0495520559504
KIF22 1.59366235875707 10.8739034825871
10.1175249196115 1.07184514859093e-19 2.52640423657541e-18
33.9895770799867
TOM1L2 -1.43509646892656
9.41863333333333 -10.1154697566933 1.0868330604456e-19
2.55879389116378e-18 33.975838552229

TUSC5	-1.8421261299435
0.744320895522388	-10.1063102718789	1.15620925305045e-19
	2.71590114017457e-18	33.9146192897391
DLC1	-2.23180685028249
8.45175671641791	-10.1058307889509	1.15996002215403e-19
	2.72159762912254e-18	33.9114150573614
ANO6	-1.77569710451977
9.79241393034826	-10.1029996467089	1.18235492903171e-19
	2.77097564988824e-18	33.8924964233167
ADAMTS5 -3.20217139830509	7.2672815920398 -10.1003738998222
	1.20350963048856e-19	2.81733782370925e-18
33.8749518266997
LLGL2	2.23903128531073	11.1044467661692
10.0943330331919	1.25362024328185e-19	2.93130109277635e-18
	33.8345937892065
TMOD2	-2.72423079096045	6.2492671641791 -10.0891163468345
	1.29856197815418e-19	3.03293258378899e-18
33.7997482020463
GALNTL2 -3.47351694915254
4.18226467661692	-10.08377702668 1.34622127012867e-19
3.13710813572549e-18	33.7640894628489
C1orf210	3.04001242937853	8.01762587064677
10.0818077005297	1.36423584276421e-19	3.17548320316884e-18
	33.7509388189361
ART4	-2.05996144067797
0.625112437810945	-10.0772245737662	1.40709487177143e-19
	3.27153541534172e-18	33.720337104172
NLGN3	-2.69996814971752
5.40007860696517	-10.0723369272663	1.45427932495409e-19
	3.37741567209361e-18	33.6877070454903
MAP7	2.06262281073446	9.54726865671642
10.0715298077452	1.46222123265761e-19	3.39202281428935e-18
	33.6823191857018
CEP68	-1.28215162429379
9.28631194029851	-10.0689646980959	1.48774920438114e-19
	3.44734663272515e-18	33.6651969340701
NEDD4	-2.60268870056497
6.36281343283582	-10.0655298942185	1.52262845000127e-19
	3.5241896368124e-18	33.6422716328709
TSPYL2	-1.67628185028249
9.43235870646766	-10.0528824491519	1.65822199556107e-19
	3.83370468117892e-18	33.557879198935
N4BP2L1 -2.28939604519774
6.80683134328358	-10.0523561143943	1.66411821717377e-19
	3.84300866125731e-18	33.5543678782435
MFSD3	2.15741334745762	8.94108258706468
10.0512952524278	1.67606593350047e-19	3.86625096795108e-18
	33.5472907642804
ZNF208	-3.94085077683616
3.05878606965174	-10.0479758058909	1.71400480412713e-19
	3.94932869009316e-18	33.5251479795949
DAB2	-2.14724844632768
8.72004776119403	-10.0453059660717	1.74513844561519e-19
	4.01655743144394e-18	33.5073402037157

ADARB1 -1.80772379943503
8.10116965174129   -10.042245608137   1.78151901477186e-19
        4.0956982500858e-18   33.4869295746325
PI15   -4.26812782485876
5.36409104477612   -10.0375749894552   1.83850224154769e-19
        4.22197438690986e-18   33.4557834422373
CPSF3   1.10193898305084   10.2299308457711
10.0335264384876   1.88936165458276e-19   4.33392120319375e-18
        33.4287894064195
ABRA   -1.8780384180791
0.852671641791045   -10.0181603434769   2.09546880694785e-19
        4.80133645163387e-18   33.3263671437895
SERINC1 -1.67183149717514
11.2259985074627   -10.0166556459777   2.11681676838861e-19
        4.84484372965643e-18   33.3163403895245
TXNDC17 1.41793347457627   10.1017696517413
10.0159061140465   2.12753141954291e-19   4.86394432552517e-18
        33.3113459669419
DTNB   1.60107881355933   9.08178855721393
10.0109819093979   2.19927835257614e-19   5.02237870727344e-18
        33.2785371274329
KIAA1543   2.7615336158192 9.38174825870647
10.0107290178367   2.20302737877795e-19   5.02535023181236e-18
        33.2768523123284
MRGPRE -2.24940755649718
0.884482587064677   -10.0082641118372   2.2399035216325e-19
        5.1037979244301e-18   33.2604313388109
FAM111B 3.02357789548023   6.94689950248756
10.0010224048246   2.35183478718754e-19   5.35290112870955e-18
        33.2121954572841
ERG   -2.46067944915255
7.44759552238806   -9.99954561385107   2.37533525707614e-19
        5.40040230650866e-18   33.202360193933
AXL   -2.32540275423729   8.7921447761194 -9.99920308196159
        2.38081934259469e-19   5.40688286542798e-18
33.2000790380799
PKP3   3.05609449152543   10.3056960199005
9.99809019730083   2.39872424840178e-19   5.44152583642967e-18
        33.1926677501148
PFKFB4 2.11102323446328   8.46864825870647
9.99720847419399   2.41300516208016e-19   5.46788035071807e-18
        33.1867960823436
PPP1R14B   1.78194131355932   11.2337263681592
9.98341868715109   2.64768684721539e-19   5.99305523410495e-18
        33.0949878604564
FBXO16 2.44992153954802   6.51418109452736
9.97921673885166   2.72361469567958e-19   6.15812882183939e-18
        33.0670208940589
TACR2   -3.01745487288136
4.77708407960199   -9.97530561037466   2.79623332817582e-19
        6.3153652615456e-18   33.0409930468601
PAQR4   2.40221016949152   9.49574577114428
9.97418889037672   2.81731908989849e-19   6.35599570501275e-18
        33.0335620997465

PLXDC1 -2.20755120056497
7.43876616915423   -9.97058952659112   2.88636479863386e-19
        6.50461792710791e-18   33.0096128702885
CALCOCO1   -1.610127259887
10.0050184079602   -9.96329556095434   3.03148984014955e-19
        6.8167016887481e-18   32.9610895297431
GRHL1   2.71166588983051   7.4172223880597 9.96221229797956
        3.05365374419671e-19   6.85902750200858e-18
32.9538840953201
RAD51AP1   2.81181115819209   7.58446417910448
9.95964466067556   3.10683470575305e-19   6.97085426329072e-18
        32.9368062351019
CYP2U1 -2.00054117231639
7.17351691542289   -9.95916504965368   3.11687010798966e-19
        6.9857361699812e-18   32.9336164104575
LSM4   1.59758298022599   11.6331726368159
9.94545744369232   3.41773553016668e-19   7.65170233743969e-18
        32.8424707377891
ZNF438 -1.02163057909605
7.79430099502488   -9.94417461486308   3.44732960509022e-19
        7.70955084885645e-18   32.8339429991112
C1orf172   2.73425896892656   8.65407611940299
9.94326704988181   3.468420474447e-19   7.74827773018464e-18
        32.8279100882991
SNRK   -1.35603968926553
9.40213383084577   -9.94039190487692   3.53608737348187e-19
        7.89085584538942e-18   32.808799181965
SLC7A5 2.83349237288135   10.5586149253731
9.93955989294101   3.55591285223772e-19   7.92648109190449e-18
        32.8032691966168
SSPN   -3.17878594632769
7.61358308457711   -9.93829908156683   3.58616694346598e-19
        7.98525025481091e-18   32.7948894819718
TRPC1   -3.40491511299435
5.70891691542289   -9.93514120292785   3.66307158103923e-19
        8.14765542348163e-18   32.7739028765614
FLI1   -2.2330656779661
7.39812736318408   -9.93207508685635   3.73931146519484e-19
        8.30823207580628e-18   32.7535282459655
PCDHB15 -3.0581572740113   4.6669 -9.93139649243828
3.75639719119834e-19   8.33717127949209e-18   32.7490192071899
LOC339524   -2.78049244350283
4.53793582089552   -9.9261854123621   3.89021765448049e-19
        8.62485620372402e-18   32.7143967257864
CACNA1C -3.41682266949153
6.81026865671642   -9.92535046771136   3.91209564412242e-19
        8.66400470052139e-18   32.7088499105288
CLEC1A -2.42784166666667
5.16719154228856   -9.92442291718785   3.93654377414158e-19
        8.70295752151596e-18   32.7026880675487
C11orf82   2.19582485875706   7.31407014925373
9.9243617073832 3.93816246346241e-19   8.70295752151596e-18
32.7022814493875
WSCD2   -4.35046765536723

3.49646616915423	-9.91807561163138	4.10797408903982e-19
	9.0684632309664e-18	32.6605272618961
CDCP1	2.52544449152542	10.1556905472637
9.91642481997054	4.15376369240374e-19	9.15969587594509e-18
	32.6495636711712
HIST1H3D	2.96149173728814	5.09550995024876
9.91571480628293	4.17361390568269e-19	9.19359372142335e-18
	32.6448483654956
LOC90586	-1.82884307909605
0.854851243781095	-9.91422232936348	4.21564815007014e-19
	9.27031784908748e-18	32.6349369770612
OVOL1	3.88023806497175	7.2315552238806	9.9141577891354
4.21747533903931e-19	9.27031784908748e-18	32.6345083832727
AKAP13	-1.41450515536723
10.7202368159204	-9.91069600891507	4.31664488741923e-19
	9.47815182232266e-18	32.6115210381173
USP31	-1.3691197740113
8.50155721393035	-9.90397248336065	4.51593164404073e-19
	9.90513639446114e-18	32.5668823891718
NGF	-3.11375557909605
3.40864029850746	-9.89294795502964	4.86270330598076e-19
	1.06543542018981e-17	32.4937108193637
CENPO	2.15531581920904	6.99219800995025
9.88020472718402	5.29666856741957e-19	1.15928151054503e-17
	32.4091664145025
CRB3	2.77563686440678	8.57312587064677
9.86901289334547	5.70946348290119e-19	1.24829909801876e-17
	32.3349452547322
INPP5A	-1.37123771186441
9.12500049751244	-9.86603805788215	5.8244711243385e-19
	1.27208927853904e-17	32.3152217916463
TBX3	-3.2845363700565
7.95795124378109	-9.86267151996092	5.95740469172339e-19
	1.29973983338025e-17	32.2929037508773
RNASEH2A	1.81277387005649	9.87101492537314
9.8618572832364	5.99000701432557e-19	1.30546543528773e-17
32.2875062615865
ESRP1	3.25589237288136	10.5004791044776
9.86137018155228	6.00959554474e-19	1.30834566843597e-17
	32.284277388774
ENPP2	-3.09236377118644
9.26669452736318	-9.85999960919455	6.06505503539214e-19
	1.31902097326907e-17	32.2751925068417
TDP1	1.08182224576271	9.12167860696517
9.85233744833525	6.38461402003166e-19	1.38704895059524e-17
	32.2244115954356
ATP1B2	-3.70449484463277
5.75948706467662	-9.85015537527674	6.47864674592698e-19
	1.40598961621439e-17	32.209952397637
S1PR1	-2.37503877118644
7.64027711442786	-9.84766921318173	6.58746416960233e-19
	1.42809545302994e-17	32.1934795356618
LRRK2	-3.62502577683616
5.31694228855721	-9.84702100838585	6.6161332582945e-19

        1.432797634945e-17   32.1891848819954
TCF4   -2.15343997175141
9.65752885572139   -9.84475710025647   6.71723985976351e-19
        1.45316053025232e-17   32.1741862150628
FAM55C -1.92083001412429
7.44980199004975   -9.83498596067545   7.17155443274046e-19
        1.54981065793854e-17   32.1094648251896
EPHA1   2.67531560734463   8.62594577114428
9.8341220741927 7.21316288455287e-19   1.55605272047482e-17
32.103743738396
RAMP2   -2.10463629943503
8.28548855721393   -9.83407167734867   7.215597612723e-19
        1.55605272047482e-17   32.1034099907226
IDH2   1.84820240112994   11.7788631840796
9.82922972476714   7.45337177951612e-19   1.60564242007834e-17
        32.0713474293303
CHMP4C 2.20227535310734   8.54507960199005
9.8279563558253 7.51718751190918e-19   1.61769244884167e-17
32.0629163074018
S1PR3   -2.38567351694915
7.95366218905473   -9.82661676104316   7.58490905936841e-19
        1.63055688993543e-17   32.0540471032807
SNAI2   -2.9142886299435   6.9053736318408 -9.8211470575166
        7.86778032696437e-19   1.68959759531986e-17
32.0178375602427
EPB41L3 -2.3354811440678   7.6838447761194 -9.82031516569421
        7.9117124627206e-19   1.69725660250422e-17
32.0123310304426
PSAT1   2.92005155367231   10.5001303482587
9.81372888178669   8.26825987027901e-19   1.77189326865165e-17
        31.9687402288802
UCHL3   1.28099223163842   8.65211293532338
9.80300574941822   8.88326812336488e-19   1.90170484434495e-17
        31.8977916977926
SHMT2   1.38792867231638   11.3248641791045
9.80214830838668   8.93436694509125e-19   1.9106515977367e-17
        31.8921196870245
THRA   -2.04081631355932
9.44128407960199   -9.80134444912749   8.98253815084566e-19
        1.9189543000714e-17   31.8868022785555
POU6F1 -1.91968213276836
7.16487114427861   -9.80087554451419   9.0107564634806e-19
        1.9229816028613e-17   31.8837006147915
SCN4B   -3.72672966101695
4.35090298507463   -9.79929175240397   9.10672148571873e-19
        1.94144332400629e-17   31.8732246848483
DPP6   -5.41510925141243
5.29897512437811   -9.79754193186455   9.21392920267948e-19
        1.96226106359969e-17   31.8616512506109
GNA14   -2.86169540960452
5.57982039800995   -9.79483704987506   9.38212733032353e-19
        1.9960111304823e-17   31.8437623879446
CKAP2   1.70661765536723   9.24605074626866
9.79361705610316   9.4589866792834e-19   2.01027946713963e-17

31.8356944647856
CCND2 -3.17028213276836
8.90539651741294 -9.78599674009557 9.95344385445363e-19
2.11317685968907e-17 31.7853085842796
ATP6V0B 1.30260529661017 11.5669328358209
9.77881805813037 1.04427387352719e-18 2.21476679994971e-17
31.7378553217268
POPDC2 -1.95452464689266
5.04248059701493 -9.77641018064786 1.06121598732302e-18
2.24837608046868e-17 31.7219412460806
BAK1 1.43624894067797 9.32012736318408
9.77477075050183 1.07290741439522e-18 2.27080301211689e-17
31.71110675476
C21orf34 -3.58836927966102
4.0710552238806 -9.76582562066792 1.13899253614949e-18
2.40818916242523e-17 31.6520023357496
C9orf5 -1.3037677259887
10.1548268656716 -9.76543005354802 1.14200662780302e-18
2.41207778485556e-17 31.6493890869649
ACTN1 -1.40831278248588
11.9271064676617 -9.76409466916965 1.15224055040108e-18
2.43119203491615e-17 31.6405673649547
GAS6 -2.98976504237288
10.2889676616915 -9.76114797847338 1.17514689732574e-18
2.47697800842889e-17 31.6211026420615
S100A11 1.97035515536723 13.4241930348259
9.75638996027162 1.21309384260156e-18 2.55434016293435e-17
31.5896773295659
CRTC3 -1.09167584745762
9.72989850746269 -9.75218963786419 1.24760462705766e-18
2.62431588048091e-17 31.5619399087573
PRSS8 3.76767542372881 11.97222039801 9.74225201192222
1.33319279966688e-18 2.80147883082509e-17
31.4963321380861
PROCR -2.31185035310735 7.4733039800995 -9.7371496299509
1.37938156045598e-18 2.89557294848275e-17
31.4626555997216
FIGF -3.35659329096045
2.73283980099502 -9.73589351516745 1.39099447923536e-18
2.91696799373871e-17 31.4543659963493
ARPC5L 1.23771991525423 10.1242074626866
9.7342094302923 1.40671677853024e-18 2.94692810849242e-17
31.4432526401572
ITGB1BP2 -2.79934096045198
3.69932537313433 -9.73351501017388 1.41325111579492e-18
2.95759892021098e-17 31.4386703265912
STOM -1.59738983050848
10.9086099502488 -9.73296555370407 1.41844276821412e-18
2.96544094006474e-17 31.4350446756249
MRPL15 1.18893827683616 10.3135671641791
9.72707252742666 1.47533133673048e-18 3.08123625056732e-17
31.3961634240756
C1orf21 -2.29350070621469
8.69627512437811 -9.71788524512314 1.56858103037704e-18

3.26933690899905e-17	31.3355638342403
BSPRY	2.95228008474576	8.60616069651741
9.71755510347379	1.57203889246051e-18	3.27322093937264e-17
31.3333865880093
WIT1	-4.66827627118644
4.42989651741293	-9.71695996277686	1.57829148775388e-18
3.28291025770893e-17	31.3294617698368
MUSTN1	-3.02986765536723
5.19171592039801	-9.71380215185891	1.61188391156173e-18
3.34939035469255e-17	31.3086381495462
CLDN4	3.26586638418079	12.4971955223881
9.70928003541967	1.66123181043912e-18	3.44844176626039e-17
31.278822048145
FBN1	-2.87703079096045
8.90651194029851	-9.70733907372069	1.68287094702484e-18
3.48983237802223e-17	31.2660260346674
FANCB	1.93110021186441	4.85621691542289
9.7060699812629 1.69717100445383e-18	3.51593549156782e-17
31.2576598887016
FAM8A1	-1.35495981638418
9.52604726368159	-9.70224136564136	1.74104811223313e-18
3.60319735324053e-17	31.2324231439585
GPR22	-3.52705501412429
1.78615671641791	-9.70165899461514	1.74782041716398e-18
3.61357030859783e-17	31.2285846914865
IGFBP4	-2.7730790960452
12.4274014925373	-9.70142188121675	1.75058527382762e-18
3.61564543980695e-17	31.2270218822594
UBL3	-1.6129070621469
9.63109552238806	-9.70047634719566	1.76165399009609e-18
3.63484989112289e-17	31.2207900235653
DLX4	3.25219639830509	4.41431194029851
9.69842449260848	1.78591367193546e-18	3.68120559084689e-17
31.2072673334063
ASPN	-4.31604357344633
6.53298407960199	-9.69519135149498	1.82481614018275e-18
3.75443087804102e-17	31.1859614781912
EBF1	-3.12387097457627
6.61112437810945	-9.69516834478806	1.82509596506816e-18
3.75443087804102e-17	31.1858098769522
PURG	-3.16488347457627
2.61695472636816	-9.69454456744036	1.83269910312796e-18
3.76629755627798e-17	31.1816995845435
SH3PXD2A	-1.82895564971752
10.2428104477612	-9.68837103848246	1.90966727268703e-18
3.92054691082648e-17	31.1410250845985
FBXO31	-1.17505868644068
9.34503432835821	-9.68790613812408	1.91559191668805e-18
3.92878142353703e-17	31.1379624481235
GPR146	-1.81570720338983
6.92306865671642	-9.68763182599452	1.91909629635763e-18
3.93204061519183e-17	31.1361553794529
C14orf80	2.00276440677966	9.05092835820895
9.68580152135102	1.9426425051106e-18	3.97631611464812e-17

31.1240984657314
SNCA -3.38055451977401
5.67927711442786 -9.68242343764494 1.98685749971422e-18
4.06276737740369e-17 31.101847882752
WISP2 -4.47456207627119
4.34726815920398 -9.67860435527082 2.03805121694368e-18
4.16330263520932e-17 31.0766959008411
DSN1 1.33947528248587 8.95174626865672
9.67461193148698 2.09297161772864e-18 4.27124327156749e-17
31.0504061178129
MAP3K9 2.01696779661017 5.95993582089552
9.66986923817241 2.16012880199902e-18 4.40391701142402e-17
31.0191809300039
ILF2 1.17711101694915 12.4663104477612
9.66817177744408 2.18468235467157e-18 4.44955642275866e-17
31.0080064371946
EDNRB -3.17708460451977
5.98774378109453 -9.66718881478149 2.19902749971955e-18
4.47433444690211e-17 31.0015358532873
MACF1 -1.29580656779661
11.5843457711443 -9.66277567644914 2.26459518295586e-18
4.60318208971127e-17 30.9724882432697
CDK5 1.36941144067797 8.84377462686567
9.66183481896813 2.27882338149271e-18 4.62752166390162e-17
30.9662960653514
NDC80 2.62288425141243 8.51389154228856
9.6547191012006 2.38935003152245e-18 4.84238461472418e-17
30.9194715731538
FOXO4 -1.59564357344633
8.54659353233831 -9.64112390505226 2.61555784547284e-18
5.29560183112007e-17 30.8300439382547
SNRPG 1.1040177259887 10.257223880597 9.64097218155681
2.6181989256544e-18 5.29572649691476e-17 30.8290461755854
KCTD7 -1.6122625 7.48510895522388 -9.63571889233135
2.7112982527001e-18 5.47863711889104e-17
30.7945030487043
XPO5 1.08317288135593 10.4456388059701
9.63455148247057 2.73243026182387e-18 5.51590887662183e-17
30.7868276438433
DDX11 1.62254187853107 9.5004263681592 9.6328392156775
2.76372174712158e-18 5.57359601850748e-17 30.7755705614181
CENPK 2.69267662429379 6.6949671641791 9.63023855022914
2.81193167518488e-18 5.66525586766886e-17
30.7584742017795
TMEM200A -3.30598142655367
7.12547512437811 -9.62689169811338 2.87520679919216e-18
5.78332378997693e-17 30.7364749950712
PJA2 -1.54308185028249
10.5293208955224 -9.62684140607495 2.87616833393397e-18
5.78332378997693e-17 30.7361444414476
FLRT2 -3.83898827683616
6.54285671641791 -9.62462643338666 2.91883518257314e-18
5.86337439317285e-17 30.7215867503276
GDF7 -2.42393968926554 1.0242776119403 -9.62419496696912

2.92721945157248e-18	5.86974516484254e-17
30.7187511211591
TRIM61	-1.82613248587571
0.892363184079602	-9.6241689994476	2.92772481675537e-18
5.86974516484254e-17	30.7185804621631
RASSF8	-3.26868594632769
7.25288407960199	-9.62302223407266	2.95012925932945e-18
5.90889304332035e-17	30.7110440673504
MAGI2	-2.20212394067796
6.34482039800995	-9.62248646582631	2.96065496739474e-18
5.92419556341268e-17	30.7075231790919
PTGDR	-2.3535709039548
2.70416915422886	-9.62211048748612	2.96806373563635e-18
5.93323743842399e-17	30.7050524187674
SFMBT2	-2.95241129943503
3.33428208955224	-9.62098559021288	2.99034047438627e-18
5.97195427423639e-17	30.697660310114
HSPB6	-4.09220275423729
8.76937263681592	-9.6155132153454	3.10110674895465e-18
6.18714495199601e-17	30.6617038323523
OLFML3	-3.23733848870057
9.20668855721393	-9.6118412173381	3.17770979069895e-18
6.32768011668763e-17	30.637580985699
PPM1K	-2.24357641242938
7.14120497512438	-9.60931867927828	3.2314210519885e-18
6.42839866253139e-17	30.6210113543337
PHF1	-1.07313757062147
10.2260482587065	-9.60501967874142	3.32504627348898e-18
6.60824782136773e-17	30.592776445681
LTC4S	-2.88776991525424
3.74511343283582	-9.604851703173 3.32875876910464e-18
6.60922800093987e-17	30.5916733119766
TCF21	-4.17033403954802
3.35717462686567	-9.60433255242495	3.34025880788719e-18
6.62565346144194e-17	30.5882639756452
KCTD12	-2.82130798022599
10.1027437810945	-9.59989024769173	3.44029269110024e-18
6.81749121122471e-17	30.559093485329
DBI	1.40511193502825	10.9784582089552
9.59572185186842	3.53686600421039e-18	7.00210791383215e-17
30.5317261108535
SIX4	1.96399632768362	8.59420597014925
9.58516436548674	3.79369299889676e-18	7.50332536294321e-17
30.4624309867671
CACNA1G -3.88173524011299
4.27955970149254	-9.58376034045448	3.82921672558006e-18
7.56629637884106e-17	30.4532176344669
FAM131B -3.03268531073446
4.83199303482587	-9.5805155322586	3.91258550516953e-18
7.72359427126254e-17	30.4319267751942
PTCHD1	-3.9248009180791
2.58849452736318	-9.57835609615009	3.96906571847107e-18
7.82756188282527e-17	30.417759069378
PDE7B	-2.73482281073446

6.25882736318408	-9.57752440283996	3.99103435182351e-18
	7.86333351659661e-17	30.4123027791677
RPN2	1.22738241525423	13.2256417910448
9.57612180460818	4.02835732306944e-18	7.92925942882221e-17
	30.4031014849453
GPI	1.42234703389831	13.0463895522388
9.57526375615262	4.05136080051683e-18	7.96690011825771e-17
	30.3974727785464
FOXO1	-1.89130925141243
9.46482288557214	-9.57314645662288	4.10868403068178e-18
	8.07189312439205e-17	30.3835843131133
GEM	-3.122810240113 7.68567512437811	-9.57157260868508
	4.15181594212498e-18	8.14883186346328e-17
30.3732613607811
SLC29A2 2.39794505649717	8.89961492537313
9.56450978631967	4.35098670816238e-18	8.53159093778163e-17
	30.3269436315261
FXYD6	-3.27935056497176
9.30763034825871	-9.55566712220073	4.61379304531373e-18
	9.03827969659264e-17	30.2689715479242
CAB39L	-1.85458679378532
7.57275223880597	-9.55212548164938	4.72342581613114e-18
	9.24422612060747e-17	30.2457582783066
SELP	-3.71838757062147
5.27636567164179	-9.55163594827499	4.73878189697774e-18
	9.25663642907899e-17	30.2425499392838
PER1	-1.90769759887005
9.07292885572139	-9.55163586064552	4.7387846502494e-18
	9.25663642907899e-17	30.242549364977
OLFM1	-4.3529084039548
5.76776268656716	-9.55121057979326	4.75216550567241e-18
	9.27395036420672e-17	30.2397621781401
SHANK3	-1.8075113700565
8.53942338308458	-9.54939717080291	4.80964478641027e-18
	9.37720868613513e-17	30.2278780539036
RASGRP2 -2.90505889830509
5.59243880597015	-9.54824900802813	4.84639478122931e-18
	9.43989419911174e-17	30.2203540336577
C16orf52	-1.27833997175142
7.68450597014925	-9.54154025835147	5.06677689942633e-18
	9.85980376731968e-17	30.1763976575202
GPR116	-2.01081264124294
7.98902189054726	-9.5348232325544	5.29741242593916e-18
	1.02988519985351e-16	30.1323985371187
DET1	-1.46299851694915
6.84879203980099	-9.52307115897266	5.72631642050332e-18
	1.11221642490949e-16	30.0554456102202
WHAMML1 -2.52642620056497
4.45807412935323	-9.52270615570068	5.74017648974837e-18
	1.1138546628973e-16	30.0530561236221
ADAM29	-1.38984837570622
0.469899502487562	-9.51961810009836	5.85878043932571e-18
	1.13579567912518e-16	30.0328415955313
NTRK3	-3.97397415254237

3.87262189054726	-9.51778607866916	5.93029182032138e-18
	1.1485744440679e-16	30.0208502696535
RASD2	-2.44499505649717
5.82940298507463	-9.51569296317946	6.013057600021e-18
	1.16350680988154e-16	30.007151030665
CSTF2	1.01732902542373	8.70418606965174
9.50650423601108	6.39019868059857e-18	1.23531806885771e-16
	29.9470250121388
PPAP2A	-1.97065607344633
9.27853781094527	-9.50590017861157	6.41579974191898e-18
	1.23910036407899e-16	29.9430731506909
BCAP29	-1.15448015536723
9.83265572139304	-9.5038672732939	6.50270946900007e-18
	1.25470512592642e-16	29.9297741773792
PRX	-2.10550169491525
6.89020149253731	-9.50039792811031	6.65374100702768e-18
	1.28264134154252e-16	29.9070806809915
AGTR1	-3.41934223163842
2.06291243781095	-9.49506387667151	6.89277981621701e-18
	1.32747438674423e-16	29.8721959235308
PDK4	-2.95906765536723
8.42819552238806	-9.49355455924374	6.96195458525685e-18
	1.33954009030293e-16	29.8623263038582
CLSPN	3.48437316384181	5.64107860696517
9.49326387157983	6.97535638897631e-18	1.34086204743149e-16
	29.8604255276569
HTR2B	-3.30592570621469
5.70279502487562	-9.49079828398784	7.09006573143003e-18
	1.36163750670027e-16	29.8443041869799
ENTPD6	1.29475649717514	11.0476587064677
9.48815778354451	7.21499327760334e-18	1.38433469148782e-16
	29.8270409155833
DNASE1L3	-3.1290904661017
2.40571144278607	-9.48585141727711	7.32590150136195e-18
	1.40430212719851e-16	29.8119636496229
KRT18	2.6859252118644 13.6427218905473	9.48294388688677
	7.46813685753289e-18	1.43023180676446e-16
29.7929583942641
KIAA1522	1.47148608757062	11.8095820895522
9.47544810245554	7.84761869974124e-18	1.50150616873893e-16
	29.7439718423638
ACACB	-2.23388396892655
7.79421094527363	-9.47483266130792	7.87961471144203e-18
	1.50622430191718e-16	29.7399504512727
PCDHB4	-2.9449063559322
5.35034925373134	-9.47198564208679	8.0293236787159e-18
	1.53341409417709e-16	29.7213488529091
TSPAN17	1.22156935028249	10.2531019900498
9.46541318630464	8.38582024822589e-18	1.60000826855091e-16
	29.6784143883487
SNCAIP	-3.40199604519774
6.97997910447761	-9.46091709958337	8.63871378477839e-18
	1.64568835468596e-16	29.6490502575308
FRY	-2.3483654661017

7.32779054726368	-9.46087238336729	8.64126666513132e-18
	1.64568835468596e-16	29.6487582405404
ATOH8	-3.50288615819209
6.70641890547264	-9.46024132541347	8.67737429632298e-18
	1.65103331143198e-16	29.6446372041171
FEZ2	-1.02176193502825
9.21296865671642	-9.45991447270969	8.69613495579339e-18
	1.65307083928184e-16	29.6425027787525
GLP2R	-1.19204145480226
0.294262686567164	-9.45763674230367	8.82799635305633e-18
	1.67658432126037e-16	29.6276294359028
C1QTNF1	-2.46185882768362
7.59574029850746	-9.45061075309434	9.24739506945361e-18
	1.75461202195825e-16	29.5817590014601
INTS7	1.14470127118644	8.86996218905473
9.44947766194636	9.31686270166204e-18	1.76616058416548e-16
	29.574362621873
C18orf56	2.48363290960452	4.65215472636816
9.44781019582471	9.42003643494296e-18	1.78407147610128e-16
	29.563478661404
GLIPR1	-2.40967048022599
7.96499303482587	-9.44655118409323	9.49868912867381e-18
	1.79730956508455e-16	29.5552612657835
F8	-2.16067083333333
7.00859054726368	-9.44460881056703	9.62131513353477e-18
	1.81883609292329e-16	29.5425844774298
SLC16A3	2.26084413841808	10.6611467661692
9.44256660175256	9.75194148758968e-18	1.84183402704891e-16
	29.5292571873125
ADAMTSL4	-2.77354131355933
7.32224029850746	-9.44216751506346	9.77767357030322e-18
	1.84499667645519e-16	29.5266529076118
GTF3C6	1.30960642655367	10.6332980099502
9.4408955130441	9.86014028981623e-18	1.85884922084414e-16
29.5183526115734
SAMD4A	-2.47270028248587
7.61509751243781	-9.43604357490207	1.01811010779257e-17
	1.91759637733775e-16	29.4866957769447
LRCH1	-1.85958884180791
8.27004328358209	-9.42502460815309	1.09491166928361e-17
	2.06036082221746e-16	29.4148247165559
CEP120	-1.1170384180791
8.53543930348259	-9.41791777435288	1.14747361910768e-17
	2.15729243592315e-16	29.3684874826689
VIPR2	-3.68486666666667
4.48705074626866	-9.41356661438254	1.18088492429186e-17
	2.21807570866531e-16	29.3401240964838
ZIM2	-1.55811475988701
0.452274626865672	-9.41041601993474	1.20567941680988e-17
	2.26257755275199e-16	29.3195898227239
HN1L	1.17161504237288	11.9901582089552
9.40409140616183	1.25702567419193e-17	2.35677964302834e-16
	29.2783765476052
EZR	1.68715070621469	12.9693248756219

9.40345889590574	1.26227875629057e-17	2.36446923965742e-16
	29.274255485662
CKMT1B	3.88885572033898	8.03971890547264
9.39461107253969	1.3380901990881e-17	2.50419250567718e-16
	29.2166194640467
PDGFRA	-3.60706617231639	8.5284407960199 -9.39441433383183
	1.33982638676952e-17	2.50515808018016e-16
29.2153381159863
C16orf88	1.02775402542373	8.97197462686567
9.39389597285025	1.34441157011334e-17	2.51144399767305e-16
	29.2119621096505
CSTB	1.58899844632768	12.2079089552239
9.39269035690933	1.35513631404044e-17	2.52917713884092e-16
	29.2041103930655
CCDC64	2.48057104519774	7.99897512437811
9.3907327320077 1.37273212367014e-17	2.55969032687993e-16
29.1913619543857
HAUS8	1.5980220338983 8.25164676616915	9.38924078750182
	1.38629457474409e-17	2.58263408525374e-16
29.1816468043491
HGF	-3.15104420903955
3.48569154228856	-9.38812413825711	1.39653250117577e-17
	2.59934834534348e-16	29.1743758668836
ZNF483	-2.29737987288136
3.40503830845771	-9.38565645882866	1.41942460636444e-17
	2.63717530938117e-16	29.1583090270559
KL	-2.78182980225989
4.07093731343284	-9.38422853333576	1.43284112215798e-17
	2.65969513905093e-16	29.1490126748352
PRICKLE2	-2.52807281073447
8.61440746268657	-9.37959275345231	1.47727302564409e-17
	2.73969423816378e-16	29.1188356956115
AP1S1	1.11200572033898	10.1155497512438
9.37928415819878	1.48027893045424e-17	2.7427911951467e-16
	29.1168270742333
C1orf112	1.67800183615819	7.71685174129353
9.37509614762857	1.52167892229035e-17	2.81695836561055e-16
	29.089570175292
PDS5B	-1.32328156779661
9.22865970149254	-9.37378690023943	1.53485593406555e-17
	2.8387921014744e-16	29.0810501427057
OCIAD2	1.8336395480226 10.8597641791045	9.37307636364487
	1.54205459712245e-17	2.84953923302645e-16
29.0764264610642
BOLA3	1.27195960451978	8.42480049751244
9.37280540681359	1.54480857414068e-17	2.85206115351692e-16
	29.0746632967109
SSC5D	-3.24356108757062
7.30927562189055	-9.36610089393831	1.61453163722897e-17
	2.97810732365776e-16	29.0310421116702
GDAP1L1 -2.61183086158192
1.49139203980099	-9.36412538470355	1.6356661313854e-17
	3.01167943294549e-16	29.0181912725304
WFIKKN2 -3.86032330508475

2.65116915422886	-9.35491389865779	1.73790106818774e-17
	3.19705277149591e-16	28.9582837112248
INMT	-3.02125600282486
5.67877014925373	-9.35242824461116	1.76656034093103e-17
	3.24686515660825e-16	28.9421219981006
NAV3	-3.0905979519774	4.0352328358209	-9.34273661051206
	1.88286096681357e-17	3.45443571480631e-16
28.8791229618818
UBE2S	2.13072740112994	9.64196218905473
9.34109641628372	1.90328298567813e-17	3.48878568714036e-16
	28.8684636340485
TRIM23	-1.36997761299435
7.49603930348259	-9.34062102588245	1.9092431071311e-17
	3.4965888482963e-16	28.8653742932915
C6orf132	2.4766020480226	7.3546592039801	9.33360480305881
	1.99939595225338e-17	3.65843127448859e-16
28.8197862500622
CCDC136	-2.77895430790961
4.21124029850746	-9.33213107546769	2.01886325991777e-17
	3.69076248674193e-16	28.8102123730246
INPP5F	-1.31270451977401
8.31243631840796	-9.33108750120586	2.0327622515301e-17
	3.7128655715225e-16	28.8034332869127
RBM24	-3.99061991525424
5.60546368159204	-9.33027279612462	2.04367908875603e-17
	3.72948726152544e-16	28.7981411466084
ZNF781	-2.71096511299435
4.18105721393035	-9.32717622653704	2.08570659914847e-17
	3.80280252935329e-16	28.7780281746341
FGD5	-2.31835543785311
8.10297562189055	-9.32477546720507	2.11888058743585e-17
	3.85985966815066e-16	28.7624364531067
APBB1	-2.58696843220339
8.45656915422886	-9.32063234290211	2.17736882523475e-17
	3.96288847358771e-16	28.7355326379327
GAB1	-1.74191334745763
8.42087213930348	-9.31225998617772	2.30050311626497e-17
	4.18328866048891e-16	28.6811801318485
DTYMK	1.54513100282485	9.75701691542289
9.31156180213664	2.31107881902859e-17	4.19880072165105e-16
	28.6766484545535
CCDC134	1.71001596045198	6.51930995024876
9.30652006098886	2.38889618063941e-17	4.33634293444094e-16
	28.6439281656264
NR1D1	-1.55218509887005
8.56204626865672	-9.30201962113206	2.46055551714969e-17
	4.46247391935364e-16	28.614726729337
PRR7	2.19866257062147	7.47279402985075
9.29581911292729	2.56279628726374e-17	4.6437959203464e-16
	28.5745033622299
DDR2	-3.56102005649718
5.06929303482587	-9.29537377967614	2.57029987957254e-17
	4.65328540807974e-16	28.5716148431104
LOC285830	-2.7305247881356

3.87109552238806	-9.29438915867955	2.58696773524552e-17
	4.67933459071282e-16	28.5652285907381
KIAA1755	-3.13819223163842
5.67460895522388	-9.29367839768466	2.59906635386166e-17
	4.69708030323767e-16	28.5606187598947
FAM72A	2.73515296610169	4.31369253731343
9.28986662490108	2.66491650362045e-17	4.81185011603587e-16
	28.5358988537615
SLC8A2	-3.57724512711865
3.68007611940298	-9.28934030346439	2.6741382862756e-17
	4.82206004743737e-16	28.5324858956857
LPAR4	-2.10634512711865
1.41157960199005	-9.28921148043815	2.67640024716894e-17
	4.82206004743737e-16	28.5316505478334
CCDC18	1.94002824858757	6.41478308457711
9.28914220789999	2.67761736681861e-17	4.82206004743737e-16
	28.5312013546679
PAGE4	-5.27190353107345
2.71550248756219	-9.28153417960627	2.81469353094395e-17
	5.0644748633023e-16	28.4818756369972
ZNF454	-2.66704096045198
2.92109502487562	-9.27857332437814	2.86990575915338e-17
	5.15929643042196e-16	28.4626836052262
ST6GALNAC6	-1.52574809322034
9.96090995024876	-9.26988380959192	3.03823436303069e-17
	5.45712611312512e-16	28.4063728196653
TPD52	1.70147959039548	10.768863681592	9.268002140869
3.0759579114162e-17	5.52005383928099e-16	28.3941817636045
GATA6	-3.48308276836158
6.23755771144279	-9.26423837334594	3.15281592528582e-17
	5.65304025730287e-16	28.3697998050569
AKNA	-1.69542387005649
9.57260646766169	-9.26088481295067	3.2229034383721e-17
	5.77366558375036e-16	28.3480785034559
MMRN1	-3.47392415254238
6.73478109452736	-9.25055441428153	3.44868144270856e-17
	6.17274891184018e-16	28.2811872255128
BCL2L12	1.46131461864407	9.05757512437811
9.25025372081503	3.45548352592813e-17	6.17953630551433e-16
	28.279240622734
SLC25A33	1.41345444915254	8.37934626865672
9.24237940024192	3.63843551581525e-17	6.49173089651099e-16
	28.2282735011677
ZDHHC12	1.5099615819209	9.97753432835821	9.24233293208478
	3.63954323520904e-17	6.49173089651099e-16
28.2279727838533
ALDH18A1	1.1469615819209	11.5396776119403
9.24002192485799	3.69505845163843e-17	6.585030382998e-16
	28.2130179280456
PALLD	-2.61658778248587
11.4329781094527	-9.23568147271531	3.80160934279673e-17
	6.76904074654093e-16	28.1849342739613
IGSF9B	-3.11246927966102
2.05709800995025	-9.2284965664094	3.98473734759273e-17

7.08896514264113e-16	28.138457961586
PNRC1	-1.12979484463277	10.779360199005 -9.2232747157723
4.12330667017798e-17	7.3291329817103e-16
28.1046889444494
FAM122A -1.15322662429379
8.20071791044776	-9.21908953246416	4.23781478126415e-17
7.52615375945961e-16	28.0776294359815
NOP2	1.17304456214689	10.3546587064677
9.21654442298277	4.30898922173729e-17	7.6459419811812e-16
28.0611763091868
SFRP4	-5.8541218220339
8.94410696517413	-9.21205236826692	4.43752058389068e-17
7.86721049976473e-16	28.0321413918071
NR3C1	-2.57045381355932
8.04499104477612	-9.21161221637252	4.45031777165926e-17
7.8830909277105e-16	28.0292967241129
SLC2A6	2.01991786723164	7.68310447761194
9.20790577928923	4.55954534347532e-17	8.06960912944382e-16
28.005344470013
ZNF165	1.52205494350282	7.31225870646766
9.20197788741048	4.73980148999825e-17	8.38140608007442e-16
27.9670444648595
MESTIT1 -2.16124180790961
1.37095024875622	-9.20181952656247	4.74471282592328e-17
8.38287042308132e-16	27.9660214340822
EDEM2	1.03457055084746	9.9292855721393 9.19963023677755
4.81313097018222e-17	8.49643841941883e-16
27.9518790748956
ZNF280D -1.40412161016949	8.8164263681592 -9.19688501212989
4.90030962994299e-17	8.64290006037196e-16
27.9341474036871
NOTCH4	-1.41167521186441
8.23029552238806	-9.1960569142237	4.92691381547194e-17
8.68236400271579e-16	27.9287990572468
FBLN5	-2.68235459039548	9.7973223880597 -9.19567638515213
4.93918709800779e-17	8.69652754048885e-16
27.9263414402461
PPP1R12A	-1.4896177259887
9.79955124378109	-9.18924542140535	5.15126156192156e-17
9.06215937157238e-16	27.8848137619425
FAM70A	-2.84031278248587
3.30895820895522	-9.18806506536533	5.19115759605348e-17
9.12452615128236e-16	27.8771929337178
CFC1B	-2.7338281779661
0.856019900497512	-9.18614562781917	5.2566911504376e-17
9.23181089123045e-16	27.8648011564964
C20orf151	3.13556603107345	7.49838258706468
9.18357798960703	5.34564256830392e-17	9.38000358352815e-16
27.8482262662272
ELF3	3.23909371468927	12.2447820895522
9.18185870217869	5.40603869199269e-17	9.4778799612818e-16
27.8371287849997
ST8SIA1 -2.89421518361582
5.04761691542289	-9.18115305125995	5.43102300317257e-17

9.51355821289529e-16   27.8325742641523
FAM198B -2.96587584745763
9.09648358208955   -9.18010071098405   5.46849548679405e-17
        9.57103259538635e-16   27.8257823500129
SMC4   1.62745310734463   10.6066970149254
9.17744023172687   5.56438134782647e-17   9.73055784249381e-16
        27.8086127412807
C19orf21   3.86534194915254   9.44475024875622
9.16931499886114   5.8676846089957e-17   1.02522182998027e-15
        27.7561883794996
CREB3L2 -1.49055607344632
10.2154124378109   -9.1606785358796   6.20809374731589e-17
        1.08377691013941e-15   27.7004861545921
ADAMTSL1   -3.38166362994351
6.68207960199005   -9.15998719797452   6.23617644725517e-17
        1.08739114967962e-15   27.6960281820946
COL15A1 -2.54564350282486
9.38391393034826   -9.15990828339358   6.23939003566778e-17
        1.08739114967962e-15   27.6955193237944
COPZ2   -2.53512033898305
6.07598059701492   -9.15956151399396   6.25353081674692e-17
        1.08893119311123e-15   27.6932833007214
PLEKHG6 2.79668050847458   7.24856517412935
9.15774950140571   6.32794173237918e-17   1.10095460818428e-15
        27.6815997201864
COLEC12 -3.55686673728814
6.56791293532338   -9.14488285062624   6.88222653269018e-17
        1.19637689006037e-15   27.5986645356737
ARHGEF6 -2.1407115819209
7.24337910447761   -9.1435726357345   6.94130682395468e-17
        1.20562630368688e-15   27.5902218821222
SEL1L3 2.03827168079096   10.7973084577114
9.14335725747976   6.95106677968768e-17   1.20630093818249e-15
        27.5888340928083
MEMO1   1.04260169491525   8.99341194029851
9.1410627290025 7.05589440132395e-17   1.22345871671605e-15
27.5740501300865
SLC23A2 -1.86810805084746
8.68190796019901   -9.13897301723854   7.1527305981226e-17
        1.23920303105027e-15   27.5605871450694
OSR1   -3.79931108757062
4.54460049751244   -9.137893511363 7.20327038930599e-17
1.24690675457379e-15   27.5536329111642
ZNF296 2.10280790960452   6.17059303482587
9.13676733881221   7.2563731309981e-17   1.2550407782594e-15
        27.5463784050521
RFC2   1.15689300847458   9.72296069651741
9.13638225286593   7.27462028577351e-17   1.25589909982302e-15
        27.5438978667576
BAI3   -3.73640480225989
2.39684975124378   -9.13632945366437   7.27712570737846e-17
        1.25589909982302e-15   27.5435577630415
GIMAP7 -2.85410783898305
6.58720199004975   -9.13627547644672   7.27968791422016e-17

1.25589909982302e-15	27.5432100720156
AGMAT	2.36240451977401	6.2043263681592	9.13300219449971
7.43675178392882e-17	1.28191867442535e-15
27.5221269881269
ALG3	1.35226518361582	10.2956631840796
9.13009574669944	7.57903281312976e-17	1.30534852058351e-15
27.5034092537629
SDS	2.93254117231639	6.18316616915423
9.12890409943233	7.63814692918134e-17	1.3144271287183e-15
27.495735662336
SLC16A10	3.15215903954802	5.11469850746269
9.1278839169445	7.68911896217418e-17	1.32209055522141e-15
27.4891665404362
MFSD2B	2.65137026836158	6.09865124378109
9.12704991851398	7.73103949719286e-17	1.32818611612861e-15
27.4837965110101
CAV2	-2.25805473163842
8.16091791044776	-9.12424657066942	7.8736197174234e-17
1.35042115955474e-15	27.4657475151797
MEOX2	-4.21636906779661
2.41533631840796	-9.1231321727176	7.93102168576462e-17
1.35831096797767e-15	27.4585732366057
C1orf182	2.42716122881356	2.90798009950249
9.12309673952248	7.93285363178388e-17	1.35831096797767e-15
27.4583451304252
ANGPTL1	-5.08756744350283	4.9974407960199	-9.12289969183255
7.94304894625324e-17	1.35892329055482e-15
27.4570766148006
LAPTM4A	-1.05087344632769
12.7260706467662	-9.11455100889441	8.38718367758705e-17
1.43371258035689e-15	27.4033413794941
STON1-GTF2A1L	-2.45708107344633
0.717218407960199	-9.10963094631381	8.66038572435482e-17
1.4779527757357e-15	27.3716834652595
ARHGEF17	-1.342352259887
10.1699452736318	-9.10831443194933	8.73497670768723e-17
1.48944411801345e-15	27.3632136039362
TNNI3K	-2.78962217514124	2.586492039801	-9.10302322198073
9.04125768863706e-17	1.54039021035451e-15
27.3291774083924
RNF144A	-1.66984265536724
9.15309154228856	-9.10207827450691	9.09706787731708e-17
1.54861362787164e-15	27.3230998018143
ABLIM3	-2.83340127118644	5.5835736318408	-9.10095259110958
9.16399946373471e-17	1.55871507034361e-15
27.3158600967756
CNPY2	1.107264759887	11.4213915422886	9.10034285869336
9.2004573276131e-17	1.5636207693369e-15
27.3119388256188
C2orf15	1.93189442090396	6.66027462686567
9.09639239485653	9.44018421910145e-17	1.60303541785073e-15
27.2865354795258
KLHL10	-1.52184251412429
1.05426169154229	-9.09467599717682	9.54626478857995e-17

1.61970922404584e-15	27.2754996471218
ERVFRDE1	-2.14704435028249
1.04540248756219	-9.09131772086166	9.75725719310336e-17
1.65414112447904e-15	27.2539095938314
BTG1	-1.2319625	11.1484333333333	-9.09036816687173
9.81775100754868e-17	1.66302333485952e-15
27.2478055931862
CASC5	3.57155197740113	5.98219800995025
9.08967406843346	9.86220592274456e-17	1.66917631981819e-15
27.2433438990339
RAPGEF4	-2.56633495762712
5.75978059701493	-9.08908989493313	9.89977539808424e-17
1.67415476872051e-15	27.2395889158549
ANXA13	-3.77246094632768
2.27926965174129	-9.08790807683222	9.97621598859595e-17
1.68569312136523e-15	27.2319926634149
PCDHGA12	-2.96526504237288
3.04427562189055	-9.08537343565025	1.01421406625654e-16
1.71232029442818e-15	27.2157023807556
CCDC50	-1.11589901129944
10.3685343283582	-9.07659427901735	1.07383246862322e-16
1.81148566810474e-15	27.1592927439837
ZCCHC5	-1.88524145480226
0.716632835820896	-9.07381626334527	1.09341313896976e-16
1.84300260616168e-15	27.1414475618418
MAL2	2.91475120056497	11.266592039801	9.07163849742478
1.10901062542799e-16	1.86775948646733e-15
27.1274597931319
NFASC	-3.5940884180791
6.20997313432836	-9.06799305079417	1.13561599656816e-16
1.91099970570035e-15	27.104048235907
MARVELD2	1.90948573446328	8.18284577114428
9.06479224492423	1.15949840326295e-16	1.94959068132583e-15
27.0834954356696
RPRM	-4.57813771186441
4.66244726368159	-9.06367869457263	1.16792345052786e-16
1.96214962678698e-15	27.0763458857251
PPP1R16B	-2.55236235875706
6.32476815920398	-9.06119833887968	1.18690885215768e-16
1.99241526858521e-15	27.0604220711124
PCBP3	-3.81871560734463
4.23097960199005	-9.06053267151853	1.1920560405975e-16
1.99942079358388e-15	27.0561488127836
DMRTC1B	-3.38278290960452
2.38793980099502	-9.05949673481854	1.20011042401875e-16
2.01128710245754e-15	27.0494988657489
PLCXD3	-4.19686666666667	3.2534184079602	-9.05318773220031
1.25034306205187e-16	2.09376370831362e-15
27.0090065524503
FBXL6	1.56771490112995	9.44412089552239
9.05286371818532	1.25297852534807e-16	2.09522499661344e-15
27.0069272885414
CDH1	2.76743912429379	12.2217069651741
9.05282954043658	1.25325684161778e-16	2.09522499661344e-15

27.0067079647715
CD93 -1.89515070621469
9.41724427860697 -9.05024561614691 1.27447703519421e-16
2.12896774064582e-15 26.9901275293137
TIMM17A 1.00296814971751 10.4454149253731
9.04733023928089 1.29884798647428e-16 2.16791456604203e-15
26.9714226034682
APOLD1 -2.24991440677966
7.53618208955224 -9.04567855809403 1.31285996055966e-16
2.18952193259868e-15 26.9608266043645
PPP4C 1.18026433615819 11.4156497512438
9.04500871769774 1.31858522476611e-16 2.19728528120521e-15
26.9565296193916
CMYA5 -2.9387195621469
6.20073482587065 -9.03826124184289 1.37765847137098e-16
2.29386280756254e-15 26.9132523679487
DHRS13 1.79621603107345 9.07070497512438
9.03713100236637 1.38780743135241e-16 2.30888870062115e-15
26.9060045053112
CDKN2A 4.28900303672316 8.63664577114428
9.01917986990202 1.55932249894568e-16 2.5921369152514e-15
26.7909404130802
PTPN6 1.48968234463277 10.0331348258706
9.01828267246 1.56842666492914e-16 2.60516176626176e-15
26.7851920211652
CORO2B -3.49431497175141
5.51906666666667 -9.01487334369177 1.60350631390173e-16
2.66127604077627e-15 26.7633504539149
STC2 2.80190967514124 9.56280746268657
9.01356954415651 1.61712653588616e-16 2.68171306799215e-15
26.754998690598
TWIST2 -3.78352923728814
4.84771044776119 -9.01191196611367 1.63460853896098e-16
2.70851600523558e-15 26.7443814553904
THBD -2.35376186440678
7.51770099502488 -9.00852644516589 1.67089996242443e-16
2.76641743778818e-15 26.7226988233472
DPYD -3.05923361581921
6.60943134328358 -9.00747499006699 1.68233315998788e-16
2.78310231865844e-15 26.7159654551875
PRRG2 2.28829279661017 7.73503880597015
9.0071887467768 1.68545911340169e-16 2.78602863108991e-15
26.7141324511922
CLEC2B -2.18966461864407 7.011792039801 -9.00318603375244
1.72978035021063e-16 2.85469377731705e-15
26.688503010101
FAM162B -2.66007288135593
4.05858109452736 -8.99967526988646 1.76960599861452e-16
2.91573123206709e-15 26.6660274678313
KIAA0427 -1.23947252824859
9.05629502487562 -8.99898976174409 1.77748792058052e-16
2.92636944743528e-15 26.6616393465357
CNNM1 -3.57159449152543
2.99428955223881 -8.99826803510621 1.78582395907926e-16

2.93773765063279e-15	26.6570195328289
PTPRB	-2.31660741525424
7.98841492537313	-8.99749105606509	1.79484157180778e-16
2.95020796390822e-15	26.6520462194866
KLC3	3.5574136299435 6.45840199004975	8.99374470921635
1.83896035851608e-16	3.01789417748482e-15
26.6280690136618
JSRP1	5.00657238700565	6.44606019900498
8.99338706617977	1.84322803524866e-16	3.02248175428554e-15
26.6257802620368
ROBLD3	1.51027584745762	10.5155527363184
8.99231930581404	1.85602799416924e-16	3.04104187711848e-15
26.6189473127362
COX5A	1.25837104519774	10.9970094527363
8.989938387846	1.88488840563405e-16	3.08586594638494e-15
26.6037122669923
HEYL	-2.1365747881356
7.95480945273632	-8.98833621172547	1.90455967522479e-16
3.11310590225835e-15	26.5934612003981
DMD	-3.30285847457627
7.34982935323383	-8.98770300223783	1.91239021411624e-16
3.12341854381912e-15	26.5894100025235
F12	3.10773241525424	6.21705572139303
8.98695384635545	1.9216958954469e-16	3.13612215687797e-15
26.5846171489751
STARD5	-1.69885925141243
5.76054378109453	-8.98185669174145	1.98621543198901e-16
3.23627006497892e-15	26.552011699413
C10orf47	1.72704851694915	8.72374626865672
8.97881130855499	2.02578560408841e-16	3.29812676066099e-15
26.5325347351528
WNT9B	-2.22466207627119
1.03289004975124	-8.97804514902318	2.03586333253996e-16
3.31190762417158e-15	26.5276351466104
MBNL2	-1.63880303672316
9.26164577114428	-8.9681614921223	2.17042029620467e-16
3.52800702146332e-15	26.4644450300186
SMARCA4 1.23218891242937	12.2919577114428
8.96802669450537	2.17231519486218e-16	3.52829358785765e-15
26.4635834192227
ESD	-1.12941235875706
10.4327303482587	-8.96596515360682	2.20150054846693e-16
3.57004788783776e-15	26.4504069711209
GNAL	-2.57576836158192
4.68471940298507	-8.9603983503009	2.28226990954676e-16
3.69810585974704e-15	26.4148328642634
E2F3	1.43720423728814	9.16344776119403
8.95819181923727	2.3150930213523e-16	3.74681996525492e-15
26.4007348376227
KIAA1210	-4.13949032485876
3.09886467661692	-8.95813237730378	2.31598369990672e-16
3.74681996525492e-15	26.4003550700204
ANO9	2.66727563559322	8.50240746268657
8.95722242288085	2.32966101892956e-16	3.76597958414362e-15

26.3945416104582
C6orf153 1.02945120056497 9.73333582089552
8.95605940362904 2.34725892800303e-16 3.7914418404329e-15
26.3871117510567
MID2 -2.09482994350283
7.19266567164179 -8.9557159466141 2.3524810564766e-16
3.79688962967489e-15 26.3849176805377
TMC6 1.71093884180791 10.0752393034826
8.95072214417951 2.42972618840076e-16 3.91848221899981e-15
26.3530203313648
CALCOCO2 -1.12182210451977
10.1970726368159 -8.95018577295631 2.4381712204206e-16
3.92901531830729e-15 26.34959477021
PENK -4.1848540960452
1.60550497512438 -8.94913850472212 2.45474430240418e-16
3.95261964928296e-15 26.3429065916721
NCRNA00087 -2.40101624293785
5.09078059701493 -8.94742957458418 2.4820287555236e-16
3.99342087389495e-15 26.3319935501536
EPR1 4.34182542372881 8.03526517412935
8.93921284761752 2.61747890212829e-16 4.20805339551243e-15
26.2795348099643
TNFSF12 -1.86011447740113
8.59678457711443 -8.93657882742761 2.66243442157577e-16
4.27697798708533e-15 26.2627225580111
IGFBP6 -3.27592217514125
7.72882437810945 -8.92996101500395 2.77879382165061e-16
4.46040947290752e-15 26.2204921391557
SETBP1 -2.50629809322034
7.47772388059701 -8.92857214464745 2.80384774223375e-16
4.4971089178171e-15 26.2116309993104
NEURL1B -1.89537923728813
9.16195970149254 -8.92723669218551 2.82814953879298e-16
4.53254566990008e-15 26.2031112262387
SLC25A22 1.19231793785311 9.71347661691542
8.92510611485502 2.86735427066264e-16 4.5917927594933e-15
26.1895199297097
CTSL2 3.83559540960452 9.01445970149254
8.92485604611966 2.87199102388011e-16 4.59563333751042e-15
26.1879247917824
ARRDC1 1.35593594632768 10.4425039800995
8.92240422716899 2.91784863304191e-16 4.66537635797122e-15
26.172286145913
HIGD1B -2.48816285310735
3.43665621890547 -8.92051521420216 2.95367501000718e-16
4.71898427668851e-15 26.1602385463074
MYO10 1.65200748587571 10.8630278606965
8.91835805273239 2.99512144003556e-16 4.78148080590435e-15
26.1464821044936
MEX3A 2.29082323446327 10.5248129353234
8.91311323851307 3.0983167333921e-16 4.94238092747008e-15
26.1130413221067
C1orf106 3.41841532485876 8.8346671641791
8.91293425701359 3.10189992437627e-16 4.94425508132336e-15

26.111900289936
FLJ34503 -1.44348008474576
0.608217412935323 -8.91195968093228 3.12148315668451e-16
4.97160971347812e-15 26.1056874029309
RAB33B -1.18291814971752
7.72900497512438 -8.91024648935288 3.156206226629e-16
5.02301657617778e-15 26.0947665747309
INSR -1.60397379943503
9.78710796019901 -8.90677352408776 3.22777757298355e-16
5.13294140769576e-15 26.0726307402097
APOD -4.83286836158192
7.00232388059701 -8.90478352099944 3.2695116413285e-16
5.1912663570359e-15 26.0599486134847
KNDC1 -4.51085685028248
4.24394875621891 -8.89724832738675 3.43245121304807e-16
5.44156165280903e-15 26.0119384683777
PODXL2 2.17223283898305 10.4082825870647
8.89547388667234 3.47197944272967e-16 5.49997978080556e-15
26.0006352402209
MAN1C1 -2.57815042372882
6.91778009950249 -8.89268308505112 3.53506488970949e-16
5.5955961592703e-15 25.9828597313335
NARF 1.20337584745762 10.5830308457711
8.88852870697102 3.63108752700197e-16 5.74039219643218e-15
25.9564036134128
CD248 -2.24542492937853
9.33666467661692 -8.88846407669991 3.63260164081583e-16
5.74039219643218e-15 25.9559920737369
FRAT2 1.51925861581921 9.17987960199005
8.88836477216437 3.63492930119914e-16 5.74039219643218e-15
25.9553597447468
CD24 2.44762620056497 14.0753014925373
8.88478020744102 3.71994894655814e-16 5.86563378439621e-15
25.9325368007993
C8orf46 -2.48022923728814
2.61642587064677 -8.88390321202396 3.74104858562602e-16
5.89437662800477e-15 25.9269535700127
EFHA2 -3.51846200564972
4.69066119402985 -8.88229204888087 3.78012169928877e-16
5.95137258331277e-15 25.9166970139802
HSPB8 -3.14351645480226
8.05080796019901 -8.88138087183527 3.8023981880658e-16
5.98185707287287e-15 25.9108968773888
YARS 1.04738778248588 11.006055721393 8.87728200119486
3.90423028154384e-16 6.13735433997665e-15
25.8848085151971
NCAM1 -4.56685261299435
7.10888905472637 -8.87371171645954 3.99513365114135e-16
6.27544711996418e-15 25.8620887163247
TGM2 -2.57594173728814
11.5906875621891 -8.86464782817566 4.23545870229008e-16
6.63264432936807e-15 25.8044276674722
RPS6KA2 -1.65879145480226
9.65166069651741 -8.86250474890553 4.29434771602567e-16

6.71973769893346e-15	25.7907979299147
IGSF9	3.04691292372881	9.4937815920398	8.86224417096217
4.30156325773309e-16	6.7259020320838e-15
25.7891407814734
PLAT	-2.69748185028249
9.25905970149254	-8.85664718261386	4.45948564782276e-16
6.96752209663632e-15	25.7535517658787
KIF26A	-3.48991793785311
5.51909701492537	-8.8552799026905	4.49893098232008e-16
7.0238063168845e-15	25.7448592596392
GJC1	-2.77823319209039
7.27567114427861	-8.85373081354075	4.54404008247549e-16
7.0888406453816e-15	25.7350116013439
PKIG	-1.38755607344633
10.0586805970149	-8.8530932664039	4.56273546584239e-16
7.11260129944907e-15	25.7309588907263
PLS1	2.82931101694915	8.5594328358209	8.85238855975284
4.58348908919696e-16	7.13953194242895e-15
25.7264794130154
ABL1	-1.30734293785311
10.9684910447761	-8.85163039247745	4.6059217843815e-16
7.16903519585688e-15	25.7216602848951
PAK3	-2.62838029661017
1.68756865671642	-8.84910583335895	4.68140721292727e-16
7.27549508564699e-15	25.7056147565023
ASIP	-2.30813093220339
1.20607512437811	-8.84771186375883	4.72361247873864e-16
7.33553435616522e-15	25.6967558518163
KCND3	-3.12461038135593
2.28054825870647	-8.84651447559288	4.76016728611442e-16
7.38671461707703e-15	25.6891467383215
ZNF366	-2.81303192090395
2.66100248756219	-8.84399196214008	4.83809808611714e-16
7.50197535558799e-15	25.6731182371832
PRKCZ	1.67261694915254	9.46493482587065
8.84263398474269	4.88057468361587e-16	7.56212817016105e-15
25.6644902290135
LOC255167	-3.16380388418079
2.03201791044776	-8.84025005502253	4.95603966915153e-16
7.67326503828665e-15	25.6493451518173
SPP1	3.52211927966102	11.6703885572139
8.83681730126049	5.06674514435045e-16	7.8387549218926e-15
25.6275400295709
EGR1	-2.82257429378531
11.3883661691542	-8.83164591080861	5.23817236755452e-16
8.0978673724318e-15	25.5946979134671
MUM1L1	-4.16646574858757
5.09766019900498	-8.82791338951921	5.36546801524222e-16
8.28841673084445e-15	25.5709988770199
TMEM55A	-2.11561610169492
6.61187810945274	-8.82739812980744	5.38328020929993e-16
8.30967990202463e-15	25.5677276625478
FAT4	-2.97510275423729
6.31565422885572	-8.8221583216183	5.56778409056222e-16

8.58157713057375e-15	25.534466576148
TULP2	-1.49914950564972
0.706982587064677	-8.82120781794288	5.60191954054636e-16
8.62771254069143e-15	25.5284339234128
NRXN2	-3.21678262711864
7.07226915422886	-8.81968079571451	5.65719498773397e-16
8.7063128259504e-15	25.5187428196909
C13orf39	-1.28396214689266
0.365613432835821	-8.81839319818734	5.70422418380386e-16
8.77211404445642e-15	25.5105717721684
HTR7P1	-1.74914597457627
5.44571144278607	-8.81563580096741	5.80624690556689e-16
8.92232402479702e-15	25.4930751830645
BPI	-2.61127337570621
1.26694129353234	-8.81350157238835	5.88645434180523e-16
9.03881134160519e-15	25.4795344462084
KLF4	-2.40840925141243
8.25669950248756	-8.81294377217226	5.90759795911371e-16
9.06449821379705e-15	25.4759956868591
C11orf95	-1.40488248587571
9.33729800995025	-8.81118166024598	5.97488793256068e-16
9.15416325900164e-15	25.4648172564965
RIPK4	2.70104809322034	9.3339223880597 8.81117996261347
5.97495312570005e-16	9.15416325900164e-15
25.4648064875804
SLC12A4 -1.42910692090396
9.35532437810945	-8.80380550136531	6.26491849428021e-16
9.5912585151061e-15	25.418035238154
RGMA	-2.89895628531073
8.26261691542289	-8.80292177426383	6.30059089592003e-16
9.63868338995815e-15	25.4124315029687
PCDHB5	-3.22955903954803
5.10120995024876	-8.80093166742999	6.38166320812087e-16
9.75543899201201e-15	25.3998130901692
LRRC32	-2.06233644067797	8.6520960199005 -8.80016058067414
6.41335307188937e-16	9.79658769091435e-15
25.3949242965934
CHAF1B	1.50190903954802	8.13224328358209
8.79986973903921	6.42534651038614e-16	9.80761069577899e-15
25.3930803703862
NR2F6	1.72382139830509	11.3740711442786
8.79023750332353	6.83538041245151e-16	1.04257325310275e-14
25.3320274402071
EFEMP1	-3.81534519774011	8.2362184079602 -8.78923294709107
6.87961094391189e-16	1.04854055440617e-14
25.3256618564958
SLC12A8 2.00706002824859	8.04678606965174
8.78372761423182	7.12710291718954e-16	1.08545565942063e-14
25.2907818305169
CENPI	2.64209265536723	5.2274855721393 8.77903676708817
7.34493863175995e-16	1.11780274358808e-14
25.2610697181914
LRRC2	-3.40020346045198	4.5042368159204 -8.77663593909955
7.45897310134072e-16	1.13431642792981e-14

25.245865433093
DUS1L	1.34706716101694	11.3190024875622
8.75963975472703	8.31809191526392e-16	1.2621613231365e-14
	25.1382822319928
GUCY1A2	-2.63719173728814
4.09520099502488	-8.75763551070322	8.42566528365198e-16
	1.27753994293482e-14	25.125601756927
C10orf116	-3.67885685028249
7.69023880597015	-8.75382899146853	8.63378731464474e-16
	1.30813028464691e-14	25.1015221628329
MAPKBP1	-1.18147464689265
8.47647611940299	-8.74968579854092	8.8661191128892e-16
	1.3423408951889e-14	25.0753180826739
ZNF34	-1.14854314971751	6.8523671641791	-8.74524706415178
	9.12191104888291e-16	1.38005035986416e-14
25.0472509223089
GP5	-1.5722761299435
0.910133333333333	-8.74384909703235	9.20397855793108e-16
	1.39144094104805e-14	25.0384125565105
FTO	-1.13470211864407	9.2391263681592	-8.74322367848187
	9.24093052725613e-16	1.39599929157151e-14
25.0344586766691
SLC39A11	1.49402485875706	9.3330671641791
8.74271992920043	9.27080086537195e-16	1.39948192475063e-14
	25.0312740780969
FEZ1	-2.40172824858757
6.46594527363184	-8.7417039529186	9.33133606073037e-16
	1.40758507955029e-14	25.0248515344678
TDRKH	1.73950338983051	7.94643482587065
8.74131118611346	9.35484339263447e-16	1.40936852331764e-14
	25.0223687287999
LMCD1	-1.86715233050848
8.67058706467662	-8.74127705506871	9.35688892977081e-16
	1.40936852331764e-14	25.0221529777966
NNAT	-2.60503234463277
3.35957462686567	-8.74065363142913	9.39433007101535e-16
	1.41397064778552e-14	25.0182122244626
DOCK4	-1.90757111581921
7.10911940298507	-8.73643555265552	9.65159244363315e-16
	1.45056510152115e-14	24.9915524042033
RASSF2	-2.18520374293785
8.40851194029851	-8.73584498514127	9.68816501226362e-16
	1.45499654500199e-14	24.9878202553178
BOLA2	1.54074738700565	10.5949900497512
8.73481280431825	9.75241629107846e-16	1.46357533958948e-14
	24.9812975575704
CREBL2	-1.04946327683616
9.43344577114428	-8.73361474281027	9.82752377031536e-16
	1.47356537421362e-14	24.9737270338929
ITPKB	-1.62382146892655
10.0407343283582	-8.73343804238415	9.83864973770076e-16
	1.47356537421362e-14	24.9726105069743
C1orf133	-2.71721158192091
5.1612815920398	-8.73340840725813	9.84051694129017e-16

1.47356537421362e-14	24.9724232508213
THBS1	-2.76987288135593
10.6646248756219	-8.73275200794857	9.88196459972325e-16
	1.47869339090611e-14	24.968275717876
RUNDC3B	-2.86570056497175
4.97230597014925	-8.71520548522255	1.10564785803119e-15
	1.65323747453608e-14	24.8574575340744
PSMA5	1.00142429378531	11.1449179104478
8.71076791272462	1.13748377267698e-15	1.6996027549533e-14
	24.8294470168616
SNTB2	-1.38478926553672
9.18559203980099	-8.70550699808306	1.17640678783048e-15
	1.75648228030253e-14	24.7962477235461
TMPRSS13	3.91466031073446	7.84672985074627
8.70525407444856	1.17831098971669e-15	1.75804684730259e-14
	24.7946518609972
CCDC137	1.25805550847457	9.79741492537313
8.70329523601113	1.19316256513393e-15	1.7787168968196e-14
	24.7822929560528
NEU1	1.24155798022599	11.1620164179104
8.70319891144098	1.1938976540757e-15	1.7787168968196e-14
	24.7816852472852
KIAA1383	-3.18462521186441
3.63515771144279	-8.70121081223854	1.20917034422203e-15
	1.80016440659016e-14	24.7691430627856
GEMIN8P4	1.69626814971751	4.94099950248756
8.701012514391	1.21070427167926e-15	1.8011419346069e-14
24.7678921451943
MRPL13	1.18111306497175	9.60550547263682
8.69917102239579	1.22504162884972e-15	1.82115167561801e-14
	24.756276115062
LDB3	-2.86812634180791
4.62553233830846	-8.69882327191471	1.22776797123119e-15
	1.82388396884055e-14	24.7540826478281
CDC7	1.99500960451978	7.7413328358209 8.69865270322253
	1.22910741931095e-15	1.82455352989543e-14
24.7530067849234
VAX2	3.12197485875706	6.19608507462687
8.6954744729673 1.25433245993315e-15	1.86065356953956e-14
24.7329618109042
LATS2	-1.64692754237288
8.20109552238806	-8.69533588838687	1.25544399297446e-15
	1.86095777442351e-14	24.7320878380961
PAK6	1.79641963276836	8.12414676616915
8.69409455153693	1.26544396197464e-15	1.87442745594079e-14
	24.7242597230047
B4GALT3	1.0659654661017 10.1891985074627	8.69349176057635
	1.2703283991906e-15	1.88030584249338e-14
24.7204585850949
PCNA	1.38763855932204	11.201092039801 8.69268155983359
	1.27692296902342e-15	1.8887052272371e-14
24.7153497286534
ARHGAP8	2.23636384180791	8.93020199004975
8.69140769974654	1.28736021886864e-15	1.90277215935013e-14

24.7073176226234
ATL1 -1.70995261299435 6.69 -8.69070058779128
1.29319036493791e-15 1.91001425842988e-14 24.7028592776331
COL6A2 -2.24195141242938 12.542144278607 -8.69019479847588
1.29737666906704e-15 1.91452287262111e-14
24.6996703729413
ZC3H12B -2.8773740819209
4.45090547263682 -8.69010659852885 1.2981080558639e-15
1.91452287262111e-14 24.699114297711
PKNOX2 -3.95520889830509
4.61709651741294 -8.68971368961656 1.30137117696953e-15
1.91795766426306e-14 24.6966371511127
AKNAD1 -2.05289201977401
1.03831691542289 -8.68876409349036 1.30929124970381e-15
1.92824600835145e-14 24.6906505034213
MIF 1.63101320621469 13.3837945273632
8.66573069091982 1.51675356075285e-15 2.2305838540298e-14
24.5455285519098
PCDHGA7 -2.99873947740113
3.02497313432836 -8.66040496277917 1.56918648730503e-15
2.30604141620417e-14 24.5119985194513
CNTN4 -3.47263234463277
5.40533532338308 -8.65151768524236 1.66071748760014e-15
2.4388075837218e-14 24.456066215817
TTC33 -1.26549124293785
8.05639552238806 -8.64889824466009 1.68869239095059e-15
2.47811685391105e-14 24.4395856575758
SERPIND1 -3.34547570621469
2.05658407960199 -8.64811307068879 1.69716855049308e-15
2.48877645297306e-14 24.4346460713619
MAGEL2 -3.50091151129944
3.55454527363184 -8.64672986613497 1.71220341547131e-15
2.50903184294261e-14 24.4259447255007
COL6A6 -3.46518509887006
2.36049751243781 -8.64596143202941 1.72061300116939e-15
2.519556698574e-14 24.4211109978016
ESPN 3.53904442090395 9.20287611940299
8.64499348008714 1.73126441448364e-15 2.53334700137912e-14
24.4150225079062
OAF -1.49777238700565
9.75617860696517 -8.6377402923653 1.81318840699963e-15
2.65133603957994e-14 24.36940925027
NOP16 1.18032210451977 8.79927562189055
8.63539106729544 1.84053849592774e-15 2.68941319013498e-14
24.3546393582862
UST -3.05750854519775
6.15817960199005 -8.63359309990869 1.86174690255608e-15
2.71846827236674e-14 24.3433365291517
FAM119B -1.04803156779661
7.61526069651741 -8.63251923989057 1.87452937400185e-15
2.7351874945457e-14 24.3365862734286
NR2F1 -3.3258488700565
7.20308407960199 -8.62742935555492 1.93631014660285e-15
2.82332722370429e-14 24.3045965850052

ZNF521 -3.35110289548023 6.325907960199 -8.62681616404972
1.9438880700035e-15 2.83236494515059e-14
24.3007432835465
LOC158696 -2.29514357344633
1.48084925373134 -8.62547829866058 1.96052391603914e-15
2.85457843945274e-14 24.2923365566185
HIPK4 -1.77494074858757
2.08040497512438 -8.62433467862748 1.97485631713542e-15
2.87340894335862e-14 24.2851508768352
GIMAP1 -2.1828540960452
6.18879353233831 -8.62333705112045 1.98744393092846e-15
2.88967591373663e-14 24.2788828623562
CALCRL -2.47432860169491
7.78334278606965 -8.62114477960778 2.0153853613358e-15
2.92822798784317e-14 24.2651101538133
SYPL2 -2.54583368644068
3.90244228855721 -8.62036054947159 2.02547506179433e-15
2.94080643696163e-14 24.2601836999999
MXRA8 -2.61955564971752
9.74387711442786 -8.61842556228885 2.050584979088e-15
2.97515969050719e-14 24.2480291807242
TTC39A 2.63233990112994 8.09953482587065
8.6182532687648 2.05283571246467e-15 2.97632183452682e-14
24.2469469883579
DARC -4.05951158192091
7.14282288557214 -8.61779942103593 2.05877622941935e-15
2.9828282279449e-14 24.2440963738474
MMP12 4.26180967514124 6.10195771144279
8.61347205399843 2.11628164771779e-15 3.06398182141369e-14
24.216919647042
RAB9B -2.30634576271186
2.37330547263682 -8.60472218124968 2.23746976270847e-15
3.23260057905735e-14 24.1619877301601
GLT1D1 -3.37251963276836
3.70244427860697 -8.60329531611383 2.25787483978902e-15
3.25978695224111e-14 24.1530322532889
SOX18 -2.23626624293785
7.32390895522388 -8.59341553045805 2.40431691403492e-15
3.46633611272029e-14 24.0910420869722
ZBTB46 -2.17157040960452
7.04915074626866 -8.59266192547604 2.41586634603123e-15
3.4805428830892e-14 24.0863149723471
BIK 2.89447379943503 7.03533582089552
8.58880553704365 2.47583472202303e-15 3.56443806754087e-14
24.0621280962314
ADAM33 -4.16968757062147
6.59835223880597 -8.58656204664053 2.51139889292777e-15
3.6131057653684e-14 24.0480594418142
GYLTL1B 2.79952923728814 9.77631542288557
8.58135965060455 2.59583012370714e-15 3.73196025488148e-14
24.0154423378101
TTYH3 1.5266052259887 11.3458990049751 8.57955151046029
2.62582950977533e-15 3.76980977871941e-14
24.0041080904458

PBX1 -2.08195423728814
11.0751706467662 -8.57906574946314 2.63394735474276e-15
3.77882174653171e-14 24.001063305756
MAGEH1 -2.64204166666667
7.73696766169154 -8.57874264034682 2.63936080812445e-15
3.78394395187116e-14 23.9990380785323
ORMDL2 1.15430141242938 9.64212437810945
8.57632781516564 2.68017041773455e-15 3.83976962149967e-14
23.9839032184519
SFRP1 -3.65705925141243
9.06105870646766 -8.57148028752006 2.76399056736461e-15
3.95709388758686e-14 23.9535273634925
USP27X -1.26641179378531
6.96085572139303 -8.56819316838538 2.82230348852814e-15
4.0377624125075e-14 23.9329339231906
P2RX7 -2.59287860169492
3.44053134328358 -8.56504828879894 2.87923485362227e-15
4.11634342234438e-14 23.9132350007193
FLJ10038 -1.19891151129944
8.00227960199005 -8.56404025747272 2.89772309460641e-15
4.13701356969885e-14 23.9069215905194
NUDT10 -3.40044668079096
4.67597263681592 -8.56179945752423 2.93924437822123e-15
4.19337644787226e-14 23.8928884420212
CKS1B 1.77905663841808 9.99200696517413
8.55758998682244 3.01884652413547e-15 4.30395271809036e-14
23.8665309470076
WASF2 -1.06137775423729
11.1943497512438 -8.55674458303942 3.03508931675525e-15
4.32410712512042e-14 23.8612381931365
FDPS 1.0222145480226 11.6018507462687 8.55614559944015
3.04665006300266e-15 4.33756766944831e-14
23.8574883289649
C20orf72 1.11752796610169 8.96890845771144
8.55159095222978 3.13599815254784e-15 4.46167997725621e-14
23.8289784750937
DDX12 1.49782676553672 7.19004427860697
8.55146114524641 3.13858229660696e-15 4.46226416546682e-14
23.8281660498497
KIF24 1.68291913841808 7.69111940298507
8.54876994058995 3.19263606601529e-15 4.53597359413798e-14
23.8113238430962
HEG1 -1.79698672316384
9.34512885572139 -8.54835724510316 3.20100645604517e-15
4.54472078441268e-14 23.8087413119128
CTGF -2.37882838983051
11.1286358208955 -8.54779658660746 3.21241275058494e-15
4.55776321834892e-14 23.8052329625121
PITX1 4.17462973163842 6.9307039800995 8.54533776845144
3.26291405245527e-15 4.62621723044935e-14
23.7898480371724
TBXA2R -1.73598072033898
5.05818805970149 -8.54498548174396 3.27021382115228e-15
4.63017170677629e-14 23.7876439324617

PNPO	1.07507464689265	10.099528358209 8.54364536536849
	3.29813082307601e-15	4.6632662395145e-14
23.7792597910458
RUVBL1	1.0823081920904 10.8283606965174	8.54341856993495
	3.30287870615889e-15	4.66676530195747e-14
23.7778409557875
SS18L2	1.03604470338983	8.94552537313433
8.54256130661135	3.32088653717528e-15	4.68898216012437e-14
	23.7724780623594
RAET1K	2.16143382768362	2.88823134328358
8.54125463705401	3.3485221891219e-15	4.7247533019019e-14
	23.7643042389192
TUFT1	1.36677810734463	9.22908905472637
8.53900083437685	3.39672648093331e-15	4.78947765477753e-14
	23.7502070186372
CNTNAP1 -2.04851673728814
7.56507810945274	-8.53878693871635	3.40133688225118e-15
	4.79268676682339e-14	23.7488692206798
SYNPO	-1.87911299435028
9.66709203980099	-8.53815166875331	3.41506639470611e-15
	4.80873203589276e-14	23.7448960531304
CTSO	-1.69265007062147
9.03595323383085	-8.53757285202105	3.42762368588513e-15
	4.82310584449772e-14	23.7412760800508
KIAA1614	-2.28257323446328
5.28770199004975	-8.53586868827829	3.46486164494127e-15
	4.87216503908523e-14	23.7306187430953
NMT2	-1.29993298022599
8.04132039800995	-8.53564852680226	3.46970160443979e-15
	4.87563134422648e-14	23.7292419900865
LOC728392	-2.57415692090396
7.66138009950249	-8.53503432718785	3.48323944952913e-15
	4.89130683302552e-14	23.7254012544629
FAM70B	-1.63567168079096
5.20741990049751	-8.53437366375911	3.49785991363336e-15
	4.90512732424132e-14	23.7212701125322
TGFB3	-1.85520042372882
8.11809104477612	-8.53362743045408	3.51444725102944e-15
	4.9216645336722e-14	23.716604078604
SBDS	-1.12269399717514
10.0522298507463	-8.53130738426083	3.5665173924149e-15
	4.99117941828751e-14	23.7020985440988
PDE1A	-2.67301101694915
5.27227114427861	-8.52877859599073	3.62414427412545e-15
	5.06492048657559e-14	23.6862899824316
SLC37A4 1.00589124293785	9.81923482587065
8.52707653549075	3.66345042888958e-15	5.1163698847009e-14
	23.6756508816067
CDK16	1.23893742937853	11.74597960199	8.52600992066503
	3.68829725972369e-15	5.14756918709227e-14
23.66898427487
ADCY3	-1.5748447740113
9.27622139303483	-8.52354708083379	3.74630956390612e-15
	5.22498202085548e-14	23.6535923948988

HYOU1	1.1402490819209 12.3460791044776	8.51761338665387
	3.88982136752338e-15	5.42145503565886e-14
23.616517382498
NBL1	-2.33089505649717
10.9701457711443	-8.51598480817964	3.93015480962085e-15
	5.47395374772836e-14	23.6063437684902
FNDC5	-2.92344138418079
4.72681791044776	-8.51382300599595	3.98433548058599e-15
	5.54565474009698e-14	23.5928405413568
LINGO2	-3.61137189265537
2.53176169154229	-8.51048080643803	4.06956153201823e-15
	5.66044026099826e-14	23.5719673580754
NAT2	-2.18235240112994
1.37596865671642	-8.50985051862755	4.08583532128992e-15
	5.67922810738538e-14	23.568031421904
MLL	-1.00709286723164
10.1524278606965	-8.50617999349306	4.18189734280994e-15
	5.80881951609527e-14	23.5451129196656
C12orf68	-2.47485812146893
5.29996815920398	-8.5046938951562	4.22142432350683e-15
	5.85975938888407e-14	23.5358351355951
CBLC	3.34418396892655	8.49298258706468
8.49820901502597	4.39828931143626e-15	6.10114051106665e-14
	23.4953585594625
TBRG4	1.03217549435028	10.684468159204 8.49545585442752
	4.47558174346197e-15	6.19997929779179e-14
23.4781785558
PCDHGB7 -3.7245729519774
4.69766467661692	-8.49466286000791	4.49809314839433e-15
	6.22696239625999e-14	23.4732306716076
LOC339674	2.09635120056497	2.96317810945274
8.49145941593035	4.59018079018567e-15	6.35016250825551e-14
	23.453244996415
PCDHGB6 -3.5709345338983
3.69072487562189	-8.48249221860515	4.8580165469913e-15
	6.71164735597116e-14	23.3973191079072
PRNP	-2.42431038135594
10.0276970149254	-8.48220078441158	4.86697645374384e-15
	6.71950414225696e-14	23.3955019786417
TM4SF18 -2.38418559322034
5.05214328358209	-8.47827509652781	4.9892777597788e-15
	6.883727984426e-14	23.3710276645995
PARM1	-2.90398707627119	8.2245368159204 -8.47410701739808
	5.12246735480694e-15	7.06274377395476e-14
23.3450479965382
SNRPD1	1.15613905367231	10.4548467661692
8.47173984835039	5.19967309962191e-15	7.15957670927148e-14
	23.3302960811872
TEX15	-4.35076991525424	2.6572	-8.47087187189358
5.22827054365606e-15	7.19412830169296e-14	23.3248874397464
LGI3	-2.8507697740113
1.84807064676617	-8.47066002622076	5.23527392366523e-15
	7.19893996335212e-14	23.3235674004578
CCL21	-5.8994834039548

5.95339502487562	-8.46810385143143	5.32051510712816e-15
	7.31125670343648e-14	23.307640747883
TLE4	-2.58620240112994
7.26375621890547	-8.46711599147958	5.35382491251547e-15
	7.35210872601623e-14	23.3014863342084
DHCR7	1.33174844632768	10.234892039801 8.46648524436881
	5.37520126276126e-15	7.37652954040699e-14
23.2975569265636
BDH1	1.55681574858757	9.17953980099503
8.46127417621069	5.55507606739694e-15	7.61828401226848e-14
	23.2650984463569
CC2D2B	-1.87959526836158
2.21769651741294	-8.45790924488729	5.6743847360903e-15
	7.77671018904766e-14	23.2441440962019
CDK15	-1.20712973163842
0.422400995024876	-8.45539218488968	5.76528889303522e-15
	7.89602274676538e-14	23.2284722365294
RHPN2	2.07949618644068	10.0548064676617
8.45472994721577	5.789444743665e-15	7.92382003916283e-14
	23.2243493400203
HIST1H3H	2.42903926553672	4.16848805970149
8.44570627444027	6.12876893018345e-15	8.38265330690648e-14
	23.1681857701109
NR1D2	-1.31661553672316
9.58230895522388	-8.44503100366348	6.15494068321275e-15
	8.41284502172821e-14	23.1639840042821
TMEM88	-2.01482662429379
5.78996218905473	-8.44485236567611	6.16188274723316e-15
	8.4167300599266e-14	23.1628724840986
LAMA2	-3.18639512711865
6.81977412935323	-8.4438250224359	6.20195738885021e-15
	8.46583678145577e-14	23.1564803705799
LEFTY2	-5.30476899717514
5.78408208955224	-8.44002111418311	6.3526032415344e-15
	8.6657106012426e-14	23.1328157074267
TRIM63	-2.70914816384181
1.83022388059702	-8.43940508305025	6.37733892915353e-15
	8.69367650833479e-14	23.1289837621807
C4orf32 -2.09774752824859
6.46637562189055	-8.43779477399039	6.44245019009363e-15
	8.7766093166969e-14	23.1189676581838
TRIM11	1.05601108757062	9.96152437810945
8.43526285014499	6.54616146838258e-15	8.90607653716993e-14
	23.1032209462132
TUBB3	2.91052641242938	10.4751592039801
8.43390344039469	6.60252613868332e-15	8.9768120282893e-14
	23.094767334368
ZWILCH	1.14085847457627	8.68411990049751
8.43306000070766	6.63773897800538e-15	9.01871483907681e-14
	23.0895226524652
RNASE4	-2.88897740112994
8.03924179104478	-8.42702889273698	6.89501704963954e-15
	9.35589557363514e-14	23.0520272075237
RAB31	-1.78940225988701

9.52929104477612	-8.42428179862405	7.01545812991863e-15
	9.50675613249039e-14	23.0349527096418
DNAJC27	-1.16333234463277
6.71289800995025	-8.42283493684727	7.07972930095638e-15
	9.58752259555637e-14	23.0259608344898
FAM26E	-2.20728940677966
4.51809253731343	-8.41726009846857	7.33287457793737e-15
	9.923791370142e-14	22.9913214843441
A2M	-1.77309018361582
12.7488845771144	-8.41396941271857	7.48649081899512e-15
	1.01117526424815e-13	22.9708798576642
LAD1	3.13376461864407	10.8002129353234
8.41388113691656	7.4906553689381e-15	1.01117526424815e-13
	22.9703315436383
MPL	-1.59183594632769
3.79689651741294	-8.41386400020824	7.49146408631969e-15
	1.01117526424815e-13	22.9702251014493
PASK	1.38691843220339	8.61265373134328
8.41368672064504	7.49983536425605e-15	1.01150915484353e-13
	22.9691239610791
DNAH14	1.80100501412429	6.95380547263682
8.41344050986811	7.51147701476143e-15	1.01188072908827e-13
	22.9675946850364
FASN	1.63556271186441	11.9250383084577
8.41254843819878	7.55380733996656e-15	1.0169158340296e-13
	22.9620539850718
EXTL1	-2.65943453389831	3.7292223880597	-8.41229218972884
	7.56601043621132e-15	1.01789118122817e-13
22.960462465068
POLR2H	1.22340480225988	10.7040432835821
8.41143564791238	7.60694281323734e-15	1.02272780586616e-13
	22.9551427820139
LIG1	1.21211701977401	9.9763407960199	8.40899815232733
	7.72463103166213e-15	1.03787091020958e-13
22.940005752597
LRCH2	-3.56582281073447
4.95325124378109	-8.40581783224177	7.88090274467077e-15
	1.05817484204114e-13	22.9202588605322
LOC92973	-2.2127145480226
4.63186517412935	-8.40563552937118	7.88995496186592e-15
	1.05869787821639e-13	22.9191270332386
AFAP1L2	-2.51542853107345
8.64777114427861	-8.40467467032577	7.93783687958942e-15
	1.06442711389922e-13	22.9131617330453
PCDHGB3	-2.31925247175141
1.96279402985075	-8.40293487651636	8.02526922948898e-15
	1.0754489378682e-13	22.902361397805
HAR1A	-2.21407111581921
1.72448308457711	-8.40105373099417	8.12087966136653e-15
	1.08755159457179e-13	22.8906847721021
NHSL1	1.91093968926554	8.61657611940299
8.39697787759578	8.33192907192683e-15	1.11508803029112e-13
	22.865389446663
CCDC85A	-3.32072274011299

4.26197313432836	-8.39603084136882	8.38174035249528e-15
	1.12102364453894e-13	22.8595128416745
BEST1	-1.77462111581921	4.5312447761194 -8.39545783104155
	8.41202222905288e-15	1.12434125235974e-13
22.8559573174607
NAALADL1	-2.45130331920904
5.60531044776119	-8.39431067316391	8.47297239406883e-15
	1.13175096454283e-13	22.8488395597963
DCT	-1.48619053672316
0.638823383084577	-8.39090180294916	8.65668620061972e-15
	1.15553815148714e-13	22.8276913148878
FLJ35390	-1.87546984463277
4.72656268656716	-8.39046825544469	8.68033260284803e-15
	1.15794170459045e-13	22.8250019286308
APEH	1.0623540960452 11.2658975124378	8.38869665711354
	8.77762582531724e-15	1.17016011814132e-13
22.8140130203132
MAN2B1	1.18205423728814	11.4308467661692
8.38708033182317	8.86733447621975e-15	1.18135221801941e-13
	22.8039882081276
TTC13	1.00520275423728	8.46339104477612
8.38682013192957	8.88186068456333e-15	1.18252010281508e-13
	22.8023744760805
ATAD3A	1.23303177966102	10.2113791044776
8.38580423268763	8.93880162055065e-15	1.18932985916983e-13
	22.7960742057261
TERT	3.11291553672317	3.54317412935323
8.38528086753666	8.96827722234024e-15	1.19247883014666e-13
	22.792828610633
RBM47	2.08016942090396	10.3295701492537
8.38477686714164	8.9967532843725e-15	1.19549090568393e-13
	22.7897031954243
DFNA5	-2.09475748587571
7.29037114427861	-8.38276820445781	9.11113615823854e-15
	1.20990702023698e-13	22.7772479352622
PCDHGC3 -1.57743679378532
10.6496930348259	-8.37982369940525	9.28142088974152e-15
	1.23172314716479e-13	22.7589923043091
RASGRF2 -2.3287738700565
4.64149701492537	-8.37592110159054	9.51198442254005e-15
	1.26150542761465e-13	22.7348013169166
PRKCDBP -2.18521348870056
7.87956169154229	-8.3744401008328	9.60095972228174e-15
	1.27239861884756e-13	22.7256224644855
ZDHHC17 -1.28753213276836
8.77959054726368	-8.37434805803181	9.60651660600937e-15
	1.27239861884756e-13	22.7250520330696
ZNF540	-1.7780509180791	5.3806328358209 -8.3719742183583
	9.75093872427698e-15	1.29069485499295e-13
22.7103412965514
TJP3	2.84490218926554	9.61352487562189
8.37123507758792	9.79634524317769e-15	1.29586963816004e-13
	22.7057612322251
UCP2	2.04506906779661	12.1941900497512

8.36870207301653	9.9535473415603e-15	1.31581665758038e-13
	22.6900670105809
SH3D19	-1.65664653954802
9.10549950248756	-8.36668271974284	1.00806608517854e-14
	1.33176298125582e-13	22.6775569452158
HAS1	-2.44937153954802
1.54995323383085	-8.36239901034703	1.03556623896547e-14
	1.36661083130009e-13	22.6510237878199
DHFRL1	-1.3460145480226
7.37391194029851	-8.36236689640753	1.03577518436577e-14
	1.36661083130009e-13	22.6508248997025
PDE1B	-2.46056440677967
5.52283432835821	-8.3621239084364	1.03735751078118e-14
	1.36781950522399e-13	22.649320037953
CCT5	1.01921581920904	12.5249184079602
8.3617931952009 1.03951495088528e-14	1.36978446352213e-13
22.6472719138647
ZNF575	-1.19160960451978
5.00940547263682	-8.36088936341324	1.0454339018509e-14
	1.37670032103907e-13	22.641674635125
LOC389033	-2.75365289548023
1.11053731343284	-8.35979054077993	1.05267481018166e-14
	1.38534704186086e-13	22.6348702014063
CDON	-2.38460572033898
7.63590248756219	-8.35444601050992	1.08860883327541e-14
	1.43171936881128e-13	22.6017804383871
RCBTB2	-1.50497358757062
8.21881641791045	-8.35422057727163	1.09015097429558e-14
	1.43282967364201e-13	22.6003849294589
AHCY	1.00785374293785	12.5781338308458
8.35282122800441	1.09977218649573e-14	1.44455041514762e-13
	22.5917228827221
ADAMTS10	-2.55531447740113
6.21452686567164	-8.35201202975782	1.10537418263006e-14
	1.45098030494854e-13	22.586714220221
PGP	1.60353926553672	9.22418855721393
8.34371085768438	1.16450125479632e-14	1.52761730102034e-13
	22.5353462464109
ADCY4	-2.1839438559322
6.96258805970149	-8.34192163833406	1.17765039168813e-14
	1.54388011119778e-13	22.5242777050046
MYO19	1.27687436440678	9.34158258706468
8.34002478067101	1.19175131807539e-14	1.56037337755662e-13
	22.5125445343
SMAD5	-1.20940515536723
9.92304726368159	-8.33553872832334	1.22576934728089e-14
	1.60389067556895e-13	22.4848007959008
PDZD4	-3.34693898305084
5.44116218905473	-8.33530880853419	1.22753854100246e-14
	1.60518256348921e-13	22.4833790635536
XYLT1	-2.92719576271187
6.00997014925373	-8.33076380145467	1.26303579333439e-14
	1.65054900300159e-13	22.4552784301176
NPY1R	-3.64771906779661

4.35933781094527	-8.32724454242011	1.2912190771752e-14
	1.68630583043301e-13	22.4335248146225
NXPH4	4.08419865819209	5.89637064676617
8.32625459917741	1.29925856899526e-14	1.69504340708338e-13
	22.427406471981
LOC338758	-1.81715127118644
4.74149701492537	-8.32621750244964	1.29956080017011e-14
	1.69504340708338e-13	22.4271772025364
TMEM229A	-2.09735084745763
0.810445771144279	-8.32433996857196	1.31494863932889e-14
	1.71402511526489e-13	22.4155740965725
C13orf36	-3.99709597457627
5.33582338308458	-8.31916992633477	1.35826074922469e-14
	1.76935870441516e-13	22.3836299128305
DPYSL2	-2.05396320621469
9.53740696517413	-8.31513038030162	1.39308359065697e-14
	1.81357045758958e-13	22.3586774093844
CENPH	1.38677824858757	8.03584278606965
8.30885397585402	1.44895255338167e-14	1.88391361120492e-13
	22.319919335775
GLI3	-2.98249639830509
6.99275174129353	-8.30806473981453	1.45613324216865e-14
	1.89205161150142e-13	22.3150466453796
PEX12	-1.08356596045198
7.09049004975124	-8.30289233263703	1.50407414925374e-14
	1.95310830386966e-13	22.2831181001331
ZNF187	-1.11520247175141
8.17506019900497	-8.29961065432075	1.53529841313992e-14
	1.99239421123657e-13	22.2628657566059
OTOP2	-1.20668467514124
0.359717910447761	-8.29811627783776	1.54972906156093e-14
	2.00985076650953e-13	22.2536447420434
C10orf54	-1.90264435028249
8.86735373134328	-8.29529101021744	1.57737995204678e-14
	2.04441984946467e-13	22.2362136968357
NID1	-2.29568615819209
9.69610895522388	-8.29446471044379	1.58555897094604e-14
	2.05372401725692e-13	22.2311162221053
FAM49B	1.28114668079096	9.82811144278607
8.29218756453213	1.60831716663345e-14	2.08188848871279e-13
	22.2170696983581
CBX8	1.27123905367232	8.00200348258706
8.29145036156249	1.61575404622548e-14	2.0901972633276e-13
	22.2125226796476
TMEM47	-2.70554872881355
8.58099353233831	-8.29023640751718	1.62807471567481e-14
	2.10480944035288e-13	22.2050355200823
DPYSL3	-2.89204519774011
10.1344069651741	-8.28972177645064	1.63332587375059e-14
	2.11026936363119e-13	22.2018616522002
RHOB	-1.86588446327683
11.9157243781095	-8.28412197726467	1.69156116536059e-14
	2.18276245913595e-13	22.1673323794456
TFDP2	1.23960310734463	8.82197412935323

8.28132860393169	1.7213755478002e-14	2.21983919574988e-13
	22.1501122277756
RAB26	3.10270141242938	5.39074626865672
8.27892467072793	1.74744988515464e-14	2.2520493497944e-13
	22.1352950987945
MBOAT7	1.00197471751413	11.1518109452736
8.27854467087677	1.75160723998587e-14	2.25599100607967e-13
	22.1329530844837
HDAC5	-1.22802323446328	10.283852238806 -8.26917663797752
	1.85725467991452e-14	2.39056041245423e-13
22.0752326083191
GIMAP5	-2.27549399717514
7.62911094527363	-8.26210775576223	1.94113183045479e-14
	2.49259934292343e-13	22.0316992938829
B3GNT1	-1.22742076271187
9.07994527363184	-8.26126941703082	1.95132502509696e-14
	2.50379392282754e-13	22.0265376377629
HIST1H2BO	2.23627330508475	2.43381393034826
8.25892102200467	1.9801619726334e-14	2.53920832593153e-13
	22.0120799194198
PCK2	1.57375049435029	9.50150248756219
8.2550673619719 2.0284005432271e-14	2.59944214434783e-13
21.9883595038611
ENOX1	-2.73703516949153	4.3293	-8.25360402330741
2.04702153325227e-14	2.62166887571236e-13	21.9793536406061
LPAR6	-1.8527854519774
8.15613432835821	-8.25319425259273	2.05226615920537e-14
	2.62674714766123e-13	21.9768319180128
RBPMS	-2.16290600282486
9.92381393034826	-8.25296807000553	2.05516675515336e-14
	2.62882077777561e-13	21.9754400201345
CDK17	-1.32150953389831
8.73987014925373	-8.24951941274975	2.09989932848559e-14
	2.68436694980132e-13	21.9542197497941
DNMT3A	1.28031694915254	10.0702099502488
8.2450721391011 2.15901199467958e-14	2.75821507472133e-13
21.9268612073987
LRRC8B	1.25517196327684	8.78652985074627
8.24322473216205	2.18404958702452e-14	2.78708028180556e-13
	21.9154985358474
C2orf34 1.20733093220339	7.75433034825871
8.24320477959997	2.18432156523387e-14	2.78708028180556e-13
	21.915375822319
CHTF18	1.65968933615819	9.58133830845771
8.24234770586192	2.19603628466284e-14	2.80028726236821e-13
	21.9101047298617
TAGAP	-2.67493389830508
5.63189452736318	-8.24176528259921	2.20403251292548e-14
	2.8087391365835e-13	21.9065229196903
IL33	-3.34786377118644
7.65063532338308	-8.24086742979368	2.21641590562208e-14
	2.82276789965393e-13	21.9010015112908
MTHFD2	1.48732288135593	10.2783487562189
8.24072894434779	2.21833205620579e-14	2.82345673365809e-13

21.9001499116499
FAM65A	-1.10782026836158	10.038344278607	-8.23527131220157
2.29516784876595e-14	2.91763442323003e-13
21.866594448593
GRHL2	2.86936468926554	9.8253039800995	8.23120846128315
2.35407398498018e-14	2.98881502236507e-13
21.8416216834532
TAB3	-1.16479689265536
8.80236666666667	-8.2262888860356	2.42740984174092e-14
3.08002002787027e-13	21.8113910730769
IGFBP5	-3.1247540960452
12.5214925373134	-8.22480878562015	2.44991338283268e-14
3.10665359787244e-13	21.8022976491224
ARHGEF5	1.3515781779661	8.89125820895522	8.21570516570129
2.59294258808443e-14	3.28599452675144e-13
21.7463846462252
MXD3	1.63065247175141	8.68399353233831
8.21550961793132	2.59610396268781e-14	3.28797127415057e-13
21.7451839571477
HOOK1	2.22724781073446	9.21817860696517
8.21461392097143	2.61063336392545e-14	3.30229839564938e-13
21.7396844397773
MYO5B	2.34380105932203	9.84102338308458
8.21121980401669	2.66642531007406e-14	3.37079505023525e-13
21.7188474788432
PDF	1.38042725988701	7.92187512437811
8.20944889751067	2.69600255208013e-14	3.40608814733569e-13
21.7079773208813
PAMR1	-3.12221617231638
8.26491492537313	-8.19987117437632	2.86169104815926e-14
3.60653881023386e-13	21.6492075098164
VARS	1.04007316384181	11.3760353233831
8.19619322368473	2.92796443013791e-14	3.68553707852429e-13
21.6266482665393
RSPO3	-4.40854145480226
6.18175572139303	-8.19320175678273	2.9829875946903e-14
3.75246980281103e-13	21.6083033625129
SLC7A3	-3.57050833333333
3.59766268656716	-8.19242097039588	2.99751669331382e-14
3.7661577548184e-13	21.6035158049139
AGRN	1.47269209039548	13.0451482587065
8.18416107538024	3.15557940145585e-14	3.96232691815832e-13
21.5528823429991
TMEM189	1.00136461864406	10.3464278606965
8.18101786312779	3.21787380737996e-14	4.03561082868116e-13
21.5336209848067
NGFR	-3.38363043785311
4.76073930348259	-8.17981873221908	3.24195876728761e-14
4.06333415704606e-13	21.5262737716045
GLI1	-3.37717337570621
4.80142537313433	-8.17886792883864	3.26118286000219e-14
4.0849349674097e-13	21.5204484695491
MRPL47	1.19697895480226	9.9408144278607	8.17852797605826
3.26808366684076e-14	4.09108278538053e-13

21.5183657570308
TMEM125 2.63458651129943 9.1471631840796 8.17653361564715 3.30886017532274e-14 4.13960386345983e-13 21.506148221356
RTN4IP1 1.02726624293785 7.64508258706468 8.17228105515063 3.39749850692611e-14 4.2427399237952e-13 21.4801017975997
PRRT2 -2.44093305084746 5.48333830845771 -8.16857743367068 3.47660890580071e-14 4.33889245204185e-13 21.4574230441773
ENG -1.35255148305085 11.2588263681592 -8.16732337950212 3.50380728621995e-14 4.37018004775793e-13 21.4497451277556
CSGALNACT1 -2.16317372881356 7.11256567164179 -8.16636763030048 3.52467737279753e-14 4.39354137604938e-13 21.4438939699025
MATN2 -2.82035981638418 9.71635373134328 -8.16597432686018 3.5333014138777e-14 4.40161881231245e-13 21.4414862399418
MEF2A -1.57866320621469 9.49675472636816 -8.16305744391036 3.59791623638715e-14 4.47939480491377e-13 21.4236314295336
LOC285629 3.74839816384181 4.52451990049751 8.16153911990012 3.63201203141245e-14 4.51910345484228e-13 21.4143387278155
NMUR1 -2.45705303672316 3.47638059701493 -8.16120397868922 3.63958104922195e-14 4.5257782520004e-13 21.4122876561856
EVPL 2.29958375706214 9.9093815920398 8.14996523774157 3.90265515679273e-14 4.84703631996096e-13 21.3435306130677
KIAA1328 -1.08001433615819 6.31601343283582 -8.14940230552248 3.91631648795842e-14 4.86106272658926e-13 21.3400879149535
MED13L -1.59434025423729 9.79145373134328 -8.14651970690873 3.98701774654602e-14 4.94582926505075e-13 21.3224608016709
SNX21 -1.26014399717514 8.78334179104478 -8.14535762734308 4.01587627330746e-14 4.97861955863539e-13 21.3153555563074
ENTPD7 1.48242768361582 8.44005472636816 8.14370215564604 4.05734515244905e-14 5.02699432587683e-13 21.3052344619923
THOC3 1.21243516949153 10.4413751243781 8.14086469761453 4.12941120931287e-14 5.11319735387172e-13 21.2878894214339
AIFM1 1.40111490112994 11.0704134328358 8.14018465206586 4.14687058777835e-14 5.13172110711812e-13 21.283732833218
RGAG4 -2.01066942090395 7.59916467661692 -8.13444160931467 4.29726303096142e-14 5.31462710997819e-13 21.2486370144693
CA3 -2.96965120056497 4.0621631840796 -8.13395555940773 4.31023641803383e-14 5.32746259254873e-13

21.2456673249427
MRPL14 1.24694879943503 10.4884552238806
8.12751852793385 4.48575403125046e-14 5.54106680274199e-13
21.2063464393031
MRPL12 1.46561560734463 11.0035208955224
8.12682713559159 4.50502211456813e-14 5.5615215882191e-13
21.2021239663692
C16orf61 1.00135685028249 8.7625960199005
8.1259781750323 4.52879355986111e-14 5.58660750994114e-13
21.1969394386124
LOC100130932 1.06105070621469 6.53681641791045
8.12590725606683 4.53078495082903e-14 5.58660750994114e-13
21.1965063549855
PCDHGA11 -2.16850854519774
2.84763034825871 -8.1256401685318 4.53829250525606e-14
5.59250571025852e-13 21.1948753380614
KCNAB3 -2.6527281779661
4.44063631840796 -8.1245397234505 4.5693550744494e-14
5.62740609948687e-13 21.1881555603522
NBEA -3.02953269774011
6.17782338308458 -8.12350803344129 4.59866810991552e-14
5.66011128876293e-13 21.1818560441546
SCN3B -2.95410713276836
3.71148457711443 -8.11864851802315 4.73926419306744e-14
5.82966410327589e-13 21.1521891579768
COL6A3 -2.61379675141243
11.4366233830846 -8.11464533534013 4.85827668492709e-14
5.9689060647249e-13 21.1277567943578
MICAL3 -1.00648940677966
10.1336467661692 -8.11352305234927 4.89216962023272e-14
6.00695229087187e-13 21.1209083235983
TUBA1B 1.06280459039547 14.5644567164179
8.10758063184893 5.07557200810851e-14 6.2284215975175e-13
21.0846540170029
KNTC1 1.48154463276836 8.58034378109453
8.10703119761694 5.09286939806531e-14 6.24591450073362e-13
21.0813026301192
HNMT -2.23188559322034
8.68072487562189 -8.10537169157793 5.14546956958973e-14
6.30665613514491e-13 21.0711808258376
ITGA11 -3.14503707627119
7.13181890547264 -8.10401047771748 5.18901641169075e-14
6.3562354971367e-13 21.0628791693599
TDRD10 -2.76673079096045
3.01760845771144 -8.09902540139655 5.35163399364198e-14
6.5515233088533e-13 21.0324825961126
H2AFZ 1.23794837570621 12.4598711442786
8.09786308112368 5.39026972698282e-14 6.59488900446707e-13
21.0253966856339
HRC -3.10433326271186
4.06948756218905 -8.09554856786175 5.46802921978399e-14
6.68604168446488e-13 21.0112881298735
ARMCX1 -2.97333516949153
7.56681492537313 -8.09515516339651 5.48135625205715e-14

6.69834784849603e-13 21.0088902601832
SIX1 4.48835748587571 6.18659253731343
8.09313759071656 5.55021108932864e-14 6.77845530421874e-13
20.996593721316
CGNL1 -2.27371348870057
8.91222885572139 -8.08947204961287 5.6775061781854e-14
6.92979796897421e-13 20.9742572321217
PTPN21 -1.83691490112995
8.25338905472637 -8.08484239251959 5.84242224976338e-14
7.12685257205242e-13 20.9460530630277
TSPYL5 -3.59461631355933
6.83677910447761 -8.07843408115002 6.07855186759226e-14
7.41049108323451e-13 20.9070266722771
AQP8 -2.28027288135593
1.42889850746269 -8.07814064983513 6.08958746999483e-14
7.4195389174477e-13 20.9052400602952
COL14A1 -3.1428550141243
8.88718208955224 -8.07091705575083 6.36760349805297e-14
7.75367140065406e-13 20.8612681932233
SUV39H2 1.13572846045197 8.00244825870647
8.07025047809893 6.39388305033648e-14 7.77977110421888e-13
20.8572115674921
RNF11 -1.02918516949153 10.520344278607 -8.07018130095614
6.3966164753636e-14 7.77977110421888e-13
20.8567905823279
ST6GALNAC3 -2.50078693502825
5.24770199004975 -8.06928920385378 6.43197014146247e-14
7.81813777408079e-13 20.8513617902958
SVEP1 -2.91302676553673
6.93240149253731 -8.06776477812728 6.49283131158981e-14
7.88278100691536e-13 20.8420857117233
CBX2 2.3917665960452 9.10706169154229 8.06519218156628
6.59683565568182e-14 8.0043165491222e-13
20.8264335624628
PDE7A 1.35071306497175 9.48035472636816
8.06441069055227 6.6287550861343e-14 8.03829544703704e-13
20.821679328655
EFNA3 1.9772093220339 7.84457462686567 8.06155950688042
6.74651196338838e-14 8.17626272776644e-13
20.8043360115901
STX3 1.02733072033898 9.83752686567164
8.05982784481081 6.81904016653094e-14 8.25928581822302e-13
20.7938040920967
DSCC1 1.51215289548023 7.79133482587065
8.05934176082387 6.83953753651257e-14 8.27922792597895e-13
20.7908479493596
TMEM102 1.53963481638418 8.03946865671642
8.05854442264152 6.87329220074555e-14 8.31518496648828e-13
20.7859990953651
KIFC2 1.96924230225989 9.43668805970149
8.05637679502957 6.96589311255464e-14 8.41487672095931e-13
20.772818328336
RNF38 -1.36137005649718 9.3909815920398 -8.05632796283695
6.96799338803255e-14 8.41487672095931e-13

20.7725214134841
PER3 -2.29568573446328
8.09523432835821 -8.05367975527526 7.08283706411323e-14
8.53986236695983e-13 20.7564208596379
DOLPP1 1.01229435028248 8.80959004975124
8.05365389647213 7.08396766241236e-14 8.53986236695983e-13
20.7562636567156
CNKSR1 1.9011699858757 9.21314378109453 8.0519478312559
7.1589564386817e-14 8.62519810364644e-13 20.7458925737349
HRNBP3 -4.12274837570622
3.36528258706468 -8.04795391234921 7.33759421337925e-14
8.83523807628599e-13 20.7216181107784
MMP15 1.60436701977401 10.669871641791 8.04744698202621
7.36058131095101e-14 8.85772182378805e-13
20.7185374991316
H6PD -1.08874032485876
10.8788626865672 -8.04595339804507 7.42872428788348e-14
8.9344879689659e-13 20.7094615743587
ATP13A2 1.10662669491525 10.4250338308458
8.04541866754032 7.45327228676889e-14 8.9587634688153e-13
20.706212434775
NAP1L2 -3.1084375 4.71875671641791 -8.03975115369933
7.71844112233917e-14 9.26664305506568e-13
20.6717821328566
KIAA0240 -1.26537669491525
8.60235472636816 -8.03827162940946 7.78918856872634e-14
9.34611579871138e-13 20.6627960148353
JUN -1.87890536723164
12.0789582089552 -8.03758571684879 7.82220482049283e-14
9.37477320284401e-13 20.658630305233
MGP -2.47226871468927
10.7404567164179 -8.03692930740949 7.85393070197365e-14
9.40730439390426e-13 20.6546439445966
PSD4 1.66052754237288 9.94641393034826
8.03543793304725 7.92648689942239e-14 9.48867498805491e-13
20.6455874760352
MCM4 1.91436016949152 10.1813298507463
8.02985468340082 8.20405728394132e-14 9.81522704191815e-13
20.6116904322931
NHEDC2 -2.00473220338983
6.38087213930348 -8.02885332103566 8.25484803325938e-14
9.87024054297118e-13 20.6056122282193
SIRT7 1.06072443502824 8.95931243781095
8.02874719158327 8.2602493088112e-14 9.87094984341642e-13
20.6049680520425
GPRIN1 1.96559131355932 7.08459800995025
8.02249094957746 8.58491084464238e-14 1.02410354236205e-12
20.5670020860589
ARHGAP10 -1.73301320621469
7.81758457711443 -8.02165883761424 8.62903376197591e-14
1.02876924003116e-12 20.561953558169
UNC5D -2.59138615819209
1.49648308457711 -8.0204951069787 8.69111767876268e-14
1.03556962243179e-12 20.5548935066887

MTHFD1L 1.35180861581921 9.47724029850746
8.01979635428122 8.7286077705578e-14 1.03943339634311e-12
20.5506546069839
RFC4 1.35928015536723 9.11369054726368
8.01643499776769 8.91120411128468e-14 1.06056243712855e-12
20.5302659770511
ISLR -2.74690204802261
10.7028820895522 -8.01616270537508 8.92616017844719e-14
1.06172693200186e-12 20.5286145527409
VIT -2.72079738700565
1.50041144278607 -8.0153878784086 8.96885483519838e-14
1.06618754931455e-12 20.5239154667653
CYB561D2 1.2390761299435 8.8645184079602 8.01513965877515
8.982574995942e-14 1.06720060571001e-12
20.5224101405541
TGFBR2 -1.91328608757062
10.0243034825871 -8.01439264128276 9.02399135671896e-14
1.07150111366941e-12 20.5178800020738
ZC3H6 -1.22892584745763
7.61965323383085 -8.01220199699954 9.14653908730405e-14
1.0854245518055e-12 20.5045965222689
ARTN 1.96893785310734 6.16227412935323
8.01055047029397 9.24001593565044e-14 1.09588403904624e-12
20.4945833352223
SPG20 -1.78568778248588
9.57869950248756 -8.00793820459976 9.38980578615982e-14
1.11300642488372e-12 20.4787473560394
VCL -1.39310021186441
11.5252776119403 -8.00761982253853 9.40822571387304e-14
1.11454630066828e-12 20.4768174530109
SUGT1P1 -1.80655324858757
1.30664626865672 -8.00697847409757 9.44543948836282e-14
1.11830953111931e-12 20.4729299779568
KCND2 -2.83101002824859
4.29986268656716 -8.00584058506169 9.51182369651823e-14
1.12552011809521e-12 20.4660331598096
RAB12 -1.12089406779661
9.22542736318408 -8.00339004340507 9.65636052532288e-14
1.14130732058076e-12 20.4511819752709
EMP3 -2.02573587570622
8.78305970149254 -8.00241530180771 9.71445497049931e-14
1.1475130065843e-12 20.4452753299647
NEGR1 -3.6112936440678
4.29494726368159 -7.99608094586534 1.010050419745e-13
1.1917433975497e-12 20.4068999967979
GIMAP4 -1.97042620056497
8.17178606965174 -7.995629868963 1.01285674883346e-13
1.19436812484497e-12 20.4041678389075
GRIN2A -3.4793895480226
2.62947661691542 -7.99549815930072 1.01367762229203e-13
1.19464991880915e-12 20.4033700927534
ACVRL1 -1.52904625706214
8.59044726368159 -7.99530289131058 1.0148958319698e-13
1.1953993935938e-12 20.402187395516

ZNF727	-2.36503467514124
1.32736169154229	-7.99365805388013	1.02521510313471e-13
	1.20686158643094e-12	20.3922255487331
CRIM1	-1.74825572033898
9.66135373134328	-7.98944665116192	1.05211223028825e-13
	1.23710561785898e-12	20.3667242691797
LMNB2	1.20244830508474	11.1902223880597
7.98697731894671	1.06820691156302e-13	1.25531127042867e-12
	20.3517749483509
BACH2	-2.59623276836158
5.83759850746269	-7.98548637031167	1.07804243056202e-13
	1.26614479973904e-12	20.3427499050751
RGL1	-1.62474950564972
8.76597960199005	-7.98458304295274	1.08404508061725e-13
	1.27246686707102e-12	20.3372822852645
CIRBP	-1.20798121468927	12.354063681592 -7.98419338675814
	1.08664456772475e-13	1.27478931287938e-12
20.3349238896899
BEND3	1.10593234463276	7.74188905472637
7.98290691350084	1.0952708312251e-13	1.2841753378099e-12
	20.3271379257525
CDCA4	1.12999639830509	9.30990895522388
7.98278511067849	1.09609106766392e-13	1.28440351707422e-12
	20.3264007869415
SEPP1	-2.13170670903955
11.1375462686567	-7.98139989161792	1.10546221248885e-13
	1.29464570578414e-12	20.3180179831929
PDE10A	-2.58234385593221
4.84149154228856	-7.97959738994995	1.11777516910373e-13
	1.30831951092928e-12	20.3071110689577
LRRC8C	-1.52701581920904	7.5901144278607 -7.97683735186145
	1.13689270403647e-13	1.32918036525449e-12
20.2904125713099
PRR19	1.65040783898305	6.14749004975124
7.97484870966231	1.15086742806654e-13	1.34426311989424e-12
	20.278382937011
CSPG5	2.83508834745763	6.54236616915423
7.97481538512196	1.15110305152171e-13	1.34426311989424e-12
	20.2781813643676
C2orf54 3.75156490112994	6.3624960199005 7.97276951535632
	1.16566035329642e-13	1.36048931513221e-12
20.2658071896744
XRCC3	1.25670670903955	8.70699800995025
7.97100698539712	1.17834760554914e-13	1.37373516989914e-12
	20.2551480703275
C4orf12 -1.73533629943503	3.2422263681592 -7.96878755623362
	1.19451838503034e-13	1.39179696053762e-12
20.2417275240334
CYP2A7	-1.06847302259887
0.273164179104478	-7.96849314909374	1.19667987626964e-13
	1.39352455245693e-12	20.2399474341522
PTH1R	-3.42379237288136
5.27736865671642	-7.96385078145042	1.2312798233259e-13
	1.43219120526237e-12	20.2118825194149

KIF4B	1.80601779661017	2.98169054726368
7.96244811806964	1.24192749012019e-13	1.4437582883447e-12
	20.2034045348463
C5orf41 -1.87772648305085
6.92090049751244	-7.96170550055067	1.24760157912779e-13
	1.44871382463198e-12	20.1989163146507
ZNF559	-1.26650423728814
7.93977014925373	-7.96128041540602	1.2508610525599e-13
	1.45167763759496e-12	20.1963472889315
TIMP4	-2.7264854519774
2.04935671641791	-7.96005187293923	1.26032875207045e-13
	1.46183894237324e-12	20.1889229223212
AP2S1	1.31696038135593	10.9691905472637
7.95896798762201	1.26874054096859e-13	1.47076472648702e-12
	20.1823732436613
STMN1	1.43037365819209	12.8081119402985
7.95793712669061	1.27679237809775e-13	1.47926340419564e-12
	20.1761444070419
DHCR24	1.97115367231638	12.0149253731343
7.95517735518595	1.29859821608697e-13	1.5028309682224e-12
	20.159470915958
EDIL3	-3.61262754237288
4.35683930348259	-7.95477013346507	1.30184686623133e-13
	1.50574175570305e-12	20.1570108900586
SPEG	-3.14923439265537
7.76004776119403	-7.94794768484296	1.35748560016512e-13
	1.56921055019088e-12	20.1158061722595
ECSCR	-2.05653870056497
5.95270149253731	-7.94315217479172	1.39799660560631e-13
	1.61513057473819e-12	20.0868542611664
EVC	-1.71809004237288
8.36989253731343	-7.94251220365887	1.40349261791675e-13
	1.62056824779701e-12	20.0829912507485
YDJC	1.19237443502825	9.49639303482587
7.9418614537654 1.4091031288989e-13	1.62613194133189e-12
20.079063342632
ARHGEF10	-1.79960896892656
8.40225721393035	-7.94150949023197	1.4121468662172e-13
	1.62872894176624e-12	20.0769389700888
MGAT4B	1.00746751412429	11.2802228855721
7.93416599325334	1.47715797661992e-13	1.70179872390611e-12
	20.0326264106516
SLC16A7 -2.22239505649718
2.33915721393035	-7.93391729827277	1.4794107490796e-13
	1.70343817602939e-12	20.0311260930397
SYTL1	2.20486278248587	9.50801641791045
7.9335423671188 1.48281344605972e-13	1.70639910580751e-12
20.028864268767
ABHD12	1.13670988700565	10.9182 7.93314596073939
1.48641946754158e-13	1.70892508440176e-12	20.0264729525303
FOXH1	2.45156200564972	3.3829631840796 7.93311809756109
	1.48667325900708e-13	1.70892508440176e-12
20.0263048706082
DENND2D 1.53649908192091	9.02423233830846

7.93296947831068	1.48802768061998e-13	1.70952480599486e-12
	20.0254083445613
UNC93B1 1.33152026836158	10.0912786069652
7.92778211815651	1.53607509241942e-13	1.76373722860015e-12
	19.9941217410963
FKBPL	1.17352464689266	8.10247313432836
7.92660608082298	1.54717978179889e-13	1.77549474121471e-12
	19.9870301672277
RORB	-3.09692224576271
2.81693034825871	-7.92631293853045	1.54996011670438e-13
	1.77769168692407e-12	19.9852625871609
ABCC8	-3.37371461864407
1.91240447761194	-7.92561442235573	1.55660520616297e-13
	1.78431629718178e-12	19.9810508334443
ENY2	1.01480331920904	9.79034527363184
7.92261117861795	1.58549825419306e-13	1.81453151437692e-12
	19.9629447560832
MARVELD3	2.25156405367231	8.19851641791045
7.92259905646023	1.58561594583156e-13	1.81453151437692e-12
	19.962871680756
P2RY14	-2.82540536723164
4.37647313432836	-7.92240312521415	1.58751940353189e-13
	1.81569767991697e-12	19.9616905674172
TNNT3	-3.61883721751413
2.28391691542289	-7.91853328798142	1.62558150466769e-13
	1.85819533913295e-12	19.9383655156691
ATP6V1G2	-2.09667161016949
3.8868855721393 -7.91652634801526	1.64567521079486e-13
1.88011753353469e-12	19.9262712230356
REEP1	-2.9378375	6.19528656716418	-7.91373076348012
	1.67407525016043e-13	1.91043718097797e-12
19.9094270320064
BCL2	-2.36007210451978
8.56629004975124	-7.91286050397655	1.6830147357788e-13
	1.91957180697437e-12	19.9041841034731
SGEF	-2.02972980225989
6.42743432835821	-7.9123953511197	1.68781224213744e-13
	1.92384805873273e-12	19.9013818864177
GAB2	-1.78236793785311
8.88544378109453	-7.91217168727864	1.69012388908228e-13
	1.92447273670877e-12	19.9000345004714
C1orf183	-1.88635141242938
4.63751791044776	-7.91097544317391	1.70254081012095e-13
	1.93753674233831e-12	19.8928284757688
C21orf63	-2.52825395480226
7.45007711442786	-7.91016788129503	1.71097424860633e-13
	1.94497792491074e-12	19.8879641450551
WDR12	1.04040790960452	9.24461343283582
7.90999051520827	1.71283202866599e-13	1.94601226057072e-12
	19.8868958190947
WNT2B	-2.63842620056497
4.00634726368159	-7.90303804295138	1.78724859757458e-13
	2.0271941275252e-12	19.8450289568277
NAA40	1.06279696327684	9.40713333333333

7.90287545914854	1.7890265874679e-13	2.02809032803512e-12
	19.8440501285509
C21orf45	1.13491398305085	8.83662686567164
7.90066951735668	1.81332413078015e-13	2.05450024309694e-12
	19.8307703962749
C9orf169	2.40799201977401	4.46438308457711
7.89800496396413	1.84310895892873e-13	2.08709470087186e-12
	19.8147324102019
GPR20	-2.12405670903955	1.7228328358209 -7.89745309663768
	1.84933808915296e-13	2.09299398954301e-12
19.8114110657168
SERP2	-2.24192782485876
5.14892338308458	-7.89064225029987	1.92795160210803e-13
	2.18076288657178e-12	19.7704308213403
C20orf203	-2.13583983050848
1.60340248756219	-7.88943860558707	1.94218445076698e-13
	2.19565235541003e-12	19.7631905219543
MSTN	-2.36111800847458
2.42839402985075	-7.88756289806124	1.96457186311505e-13
	2.21973914968366e-12	19.7519087060486
LGI2	-3.05467055084746
6.07252039800995	-7.88737848256812	1.96678668644562e-13
	2.22101928892896e-12	19.750799578153
NIPSNAP1	1.2369252118644 11.0761368159204
7.88570093158956	1.98704783479121e-13	2.24143362902547e-12
	19.7407109264077
TMEM97	1.28440254237288	9.82595771144279
7.8823131676798 2.0285965208886e-13	2.28704484205617e-12
19.7203406209801
C1GALT1 1.37017747175141	8.25309502487562
7.88086172307976	2.04665955716197e-13	2.30614273921708e-12
	19.7116146235961
ATP8B2	-2.00493255649718
9.14382487562189	-7.87358899748508	2.13959256654945e-13
	2.40689508993207e-12	19.6679041159005
ENPP1	-2.80783072033898
7.27140348258706	-7.86473582733276	2.25837235017725e-13
	2.53773313350515e-12	19.6147233969137
CWF19L2 -1.00631518361582
8.08096268656716	-7.86354771384423	2.27480161095683e-13
	2.55479633878248e-12	19.6075888267362
SNX18	-1.13837316384181
9.00843532338308	-7.86336464551717	2.27734357864856e-13
	2.5562527976848e-12	19.6064895596838
TOMM40	1.26744710451977	10.7728059701493
7.8587425762957 2.34246232566478e-13	2.62647468847067e-12
19.5787399611511
ZNF132	-1.92368651129944
5.48768656716418	-7.85450821948151	2.40373427245157e-13
	2.69370440029643e-12	19.5533256085003
TFCP2L1 3.22197874293785	8.43986069651741
7.85226676779885	2.43680775675865e-13	2.7277897080837e-12
	19.5398754686415
PYCRL	1.37977556497175	9.32155124378109

7.84919640665433	2.4828441375105e-13	2.77478443892709e-12
	19.5214546233929
NUDT11	-3.46083382768362
4.53342089552239	-7.84805141892846	2.50023128439217e-13
	2.79269577086895e-12	19.5145861610991
CA11	-2.28143594632769
8.21387014925373	-7.84759151228166	2.50724904050014e-13
	2.79901157158608e-12	19.5118274578648
NAP1L5	-2.06760741525424
6.38321691542289	-7.84697156404223	2.51673974099116e-13
	2.80807972187763e-12	19.508108896201
C11orf48	1.35614653954803	10.1108810945274
7.84052104494709	2.61762373751628e-13	2.91747097346412e-12
	19.4694267038606
FAM78B	-1.92959470338983
3.64970049751244	-7.8403776797199	2.6199107910524e-13
	2.91843562345664e-12	19.4685671680486
P2RY1	-2.47899689265537
3.80128606965174	-7.84003961064726	2.62531171633064e-13
	2.92286602691258e-12	19.4665403322901
SGOL2	1.87991094632769	7.80640646766169
7.83968042485303	2.63106206958825e-13	2.92768044924915e-12
	19.4643869453221
C14orf143	1.26709689265537	6.6065184079602
7.83743512954161	2.66729144536068e-13	2.96638642325324e-12
	19.4509271537473
SOCS5	-1.01987379943503
8.87926467661692	-7.83726434170836	2.67006734026559e-13
	2.96786586332715e-12	19.4499034212875
CYP46A1	-2.32928396892656
3.44506865671642	-7.83688161864616	2.67629830297692e-13
	2.97318204329633e-12	19.4476093543596
AASS	-2.63194717514124
7.22849850746269	-7.8323002471977	2.75201213398228e-13
	3.05546565851285e-12	19.4201529077012
HIST1H2BC	2.6406384180791	6.09640497512438
7.83222088776836	2.75334216670666e-13	3.05546565851285e-12
	19.4196773763684
SNRPF	1.09795494350282	10.6682975124378
7.83007650354906	2.78952298562441e-13	3.0939441866488e-12
	19.406828929542
HIST2H2AA3	2.3110604519774	9.74387960199005
7.82940789809891	2.80089984893641e-13	3.10488519971191e-12
	19.4028232451822
RASA3	-1.96952514124294
7.97172537313433	-7.82839272894645	2.81826160755682e-13
	3.12244526730392e-12	19.3967416074818
EPB41L4B	1.95799088983051	8.55144676616916
7.8278491962576	2.82760100991962e-13	3.13110295219254e-12
19.3934856033456
PRDX1	1.10100268361582	13.2130980099502
7.82680840648583	2.84557021795242e-13	3.14930224121635e-12
	19.3872511402101
LRRC8D	1.22374117231639	9.74613532338308

7.82247202176472	2.92166181700752e-13	3.23177355081705e-12
	19.3612803628167
STXBP5L	-2.93899449152542
2.42542189054726	-7.81595625483955	3.03979631125218e-13
	3.36063641734019e-12	19.3222715031554
CCDC64B	2.83604039548023	8.71675870646766
7.81325213182129	3.0902001770378e-13	3.41452150885824e-12
	19.3060874044351
ZNF181	-1.17881207627119
7.28861243781095	-7.81161358469374	3.1211435207563e-13
	3.44557213505644e-12	19.2962821909481
C20orf160	-2.09297153954803
6.29636467661692	-7.81158646982941	3.12165814476618e-13
	3.44557213505644e-12	19.2961199423284
SLC16A2	-2.34180098870057
7.51609203980099	-7.81141423651541	3.12492899160443e-13
	3.4473289735432e-12	19.2950893478881
FOXP1	-1.38728072033898
9.26745223880597	-7.81035946662601	3.14503400457193e-13
	3.4676449040742e-12	19.2887781721068
POLD1	1.19066956214689	9.94898756218906
7.80994957543232	3.15288144210535e-13	3.47443134763408e-12
	19.2863257255605
ABCA10	-2.85045762711864
3.74729552238806	-7.80632661412137	3.22309143051364e-13
	3.54609148276768e-12	19.2646519195907
BTBD19	-2.38892902542373
3.29262039800995	-7.7999443790996	3.35055646690547e-13
	3.68435587924849e-12	19.2264841692285
BCL2L2	-1.21395049435029
9.27373631840796	-7.7965471389811	3.42042663618758e-13
	3.75575035723086e-12	19.2061743792412
LRRC45	1.44221694915254	9.55894527363184
7.79652094819324	3.42097085631841e-13	3.75575035723086e-12
	19.2060178205838
PRKAR2B	-2.68962881355932
6.89625422885572	-7.79628779193511	3.42581940327202e-13
	3.75906319343531e-12	19.2046241126792
PPFIA2	-2.78022754237288
1.90681492537313	-7.79297791805855	3.49538691338729e-13
	3.83334900276929e-12	19.1848414993374
APOBEC3B	2.78406426553673	7.52666368159204
7.7908906288274	3.53997463516269e-13	3.88017508061346e-12
19.1723683857948
CLEC9A	-2.08311843220339	1.573307960199	-7.78983120352723
	3.56282041287763e-13	3.90313250140756e-12
19.1660382077444
CBWD6	1.32161144067797	7.11981542288557
7.78937338553036	3.57273802317674e-13	3.91190995284365e-12
	19.1633028386138
ACOX2	-2.83398333333333
5.93211940298507	-7.78916881767525	3.57717835550741e-13
	3.91443195779206e-12	19.1620806150693
ATXN1	-1.42299943502825

9.26111094527363	-7.78743980315066	3.61492694022967e-13
	3.95178115457482e-12	19.1517510222228
LOC100130776	-1.86232542372881
6.83414179104478	-7.78579893098705	3.65111553003999e-13
	3.98921776645668e-12	19.1419491443685
PANK1	1.44973594632768	7.61827064676617
7.785454302241	3.6587615608337e-13	3.99332136331292e-12
19.1398906174324
CCDC48	-2.20075790960452
5.39130248756219	-7.78457569189691	3.67832640594721e-13
	4.01041110536889e-12	19.1346427458835
CDKL1	-2.7148436440678
4.12487512437811	-7.78419864667938	3.68675413320716e-13
	4.01746615471035e-12	19.1323907807287
C16orf81	-1.28760289548023
0.639886567164179	-7.78390205694664	3.69339693793061e-13
	4.02256971542257e-12	19.1306193901396
ANGPTL2 -1.77407019774012
9.25475074626866	-7.78257284041071	3.72331352475831e-13
	4.05300247419344e-12	19.122681048352
NPTN	-1.11159371468927
10.7600880597015	-7.78045820174044	3.77140235308617e-13
	4.10100054602008e-12	19.1100535003703
FAM38B	-2.49110105932203
3.03739800995025	-7.77853059436902	3.81577298071537e-13
	4.14705237131215e-12	19.0985444018947
BZW2	1.11315882768362	10.6524651741294
7.77667855181242	3.85889054741298e-13	4.18947768050706e-12
	19.087487907951
PTGER2	-2.37709745762712	5.7539184079602 -7.77476617144661
	3.90391865474957e-13	4.23612314915479e-12
19.0760726774758
METTL7A -1.86787987288136
9.74308805970149	-7.77142211107084	3.98390903655852e-13
	4.31835546570995e-12	19.0561151755379
ADAMDEC1	3.57480346045198	5.13941542288557
7.76815924842624	4.06351937750094e-13	4.40232468707622e-12
	19.036646683566
RNASEH1 1.09649618644068	8.27131293532338
7.7677805678465 4.07286009554465e-13	4.41011697054492e-12
19.0343874964787
PARP1	1.03142838983051	12.1167810945274
7.76680754073547	4.09695877276067e-13	4.43387262017799e-12
	19.0285827411471
CLPB	1.04334611581921	8.23873830845771
7.76582859214533	4.12134644452275e-13	4.45791583277408e-12
	19.0227430523107
RGS5	-1.90634971751413
10.1344741293532	-7.76415618193232	4.16334237535437e-13
	4.50096992975383e-12	19.0127675903988
DNAJB4	-1.96391002824859
7.54246169154229	-7.75978593019757	4.27509282480348e-13
	4.61935029964292e-12	18.9867056766595
CLEC4G	-1.6475770480226

1.02601940298507	-7.75752452936351	4.33407819818341e-13
	4.68062206253053e-12	18.9732229369611
OAZ3	1.86344322033898	4.70619253731343
7.75506400012165	4.39917229793244e-13	4.74592786529443e-12
	18.9585553591035
PCDHB12 -2.10686228813559
4.91669502487562	-7.75397220303436	4.4283650257265e-13
	4.7749124988532e-12	18.9520477934184
ITGA7	-2.23432923728814
7.65869800995025	-7.7507706872928	4.51507728325575e-13
	4.86330202650789e-12	18.9329682542936
WNT4	-3.18748375706215
6.06340298507463	-7.74837040797606	4.58118791915378e-13
	4.93192385842827e-12	18.9186664790711
ANTXR1	-1.92227831920905
10.7622432835821	-7.74804116827526	4.5903304943482e-13
	4.93917636524993e-12	18.9167049291127
ANXA6	-1.60081292372882
11.0342497512438	-7.74574140267636	4.65469739493665e-13
	5.00581128957828e-12	18.9030045921471
TUBB2C	1.29526814971752	13.5106467661692
7.73723436159216	4.90065083849869e-13	5.26480176422701e-12
	18.8523447699764
HIP1R	1.2938572740113 10.4490626865672	7.73358199862434
	5.01015113713504e-13	5.37681143990499e-12
18.830603951254
GEMIN7	1.12199759887005	8.71731940298507
7.73216761881436	5.05320220754695e-13	5.41734941623701e-12
	18.8221862872781
EXOC3L2 -1.43288601694915	6.0601631840796 -7.72735538143829
	5.20243623066705e-13	5.5744267127137e-12
18.7935525150525
MANF	1.32086461864407	11.1446452736318
7.72673752334327	5.22190991314557e-13	5.59237404887212e-12
	18.789876830623
CBLN1	-3.17519653954802
3.36831890547264	-7.72349651312358	5.32524734462502e-13
	5.70006923801625e-12	18.7705984068022
FOSB	-3.83281610169491
8.04258358208955	-7.72193285254299	5.37582438257154e-13
	5.74521991536667e-12	18.7612988786644
ABCC1	1.03023940677966	10.9745507462687
7.72108748980034	5.40336543853047e-13	5.77164893095892e-12
	18.7562716914642
POLE	1.06897422316384	10.1014646766169
7.72091198410467	5.40910070496773e-13	5.77477053941693e-12
	18.7552280346569
HIST2H3C	1.93650847457627	2.68751691542289
7.71909998322049	5.46866733067521e-13	5.83229819733829e-12
	18.7444535924743
TUBG2	-1.06074336158192
8.02325422885572	-7.71793008495837	5.5074700494963e-13
	5.86758485294027e-12	18.7374979171307
C6orf208	-1.5383990819209

0.941427860696517	-7.71643703338279	5.55738673651689e-13
	5.91622080478385e-12	18.7286217463458
USP18	1.84900197740113	8.59660895522388
7.71639243842908	5.55888452626792e-13	5.91622080478385e-12
	18.7283566442009
CSPG4	-1.77634470338983
8.28173532338309	-7.71337777918633	5.66106767150544e-13
	6.02185074072573e-12	18.7104374141417
DLL4	-1.37686553672317
7.75700298507463	-7.71047851643001	5.76109108288422e-13
	6.12507508708508e-12	18.6932076661869
ATP2B4	-1.50396807909605
12.0291935323383	-7.70931634911081	5.80167436264362e-13
	6.16502974456902e-12	18.6863021155656
FBXL19	1.01136927966101	9.83497960199005
7.70604771881493	5.91733816463797e-13	6.28468455871792e-12
	18.6668830600006
SLC35F1 -2.82857916666667
3.68637412935323	-7.70185910580298	6.06889554897948e-13
	6.44231776734999e-12	18.6420048525408
NLGN1	-3.12774858757062
3.16698059701493	-7.70157947320409	6.0791491613899e-13
	6.44986730146432e-12	18.6403442388594
MAPK10	-3.07022471751412
6.38661094527363	-7.69124009168954	6.47054506771303e-13
	6.85804286216564e-12	18.5789661363589
SLC4A11 2.75401814971751	7.56303432835821
7.68734577499387	6.62433803152471e-13	7.01742310563479e-12
	18.5558597092677
FOS	-2.93648735875707
11.3608004975124	-7.68623416023586	6.66889692324125e-13
	7.05788117270502e-12	18.5492652487762
LIPG	3.07834371468926	7.40530945273632
7.68622151536554	6.66940549198624e-13	7.05788117270502e-12
	18.5491902382619
ESM1	2.67196151129944	7.48990049751244
7.68553318455857	6.69714767159992e-13	7.08358792879682e-12
	18.5451070985304
LRFN5	-3.92576610169492
3.61858208955224	-7.68446665360131	6.74035821801681e-13
	7.1256207114256e-12	18.5387808880301
PDLIM7	-1.49625720338983
10.7748253731343	-7.68151232091388	6.86149802965478e-13
	7.24622194181135e-12	18.5212595206778
TRANK1	-1.7908841101695
8.55394029850746	-7.67885533187275	6.9722829683903e-13
	7.35565104527507e-12	18.5055047398549
PLA2G4F 3.58304251412429	4.70652487562189
7.67686182947589	7.05656311477758e-13	7.44074169216147e-12
	18.4936860852143
VTN	-2.65569576271187	2.3713855721393 -7.67634122845139
	7.07873818414366e-13	7.45646459314876e-12
18.4905999303138
TMEM79	1.33148072033898	8.06107711442786

7.6743739732185 7.16315763240023e-13	7.54151929195778e-12
18.4789389457741
FHL3	-1.23732507062147
9.06988606965174	-7.67126833337088	7.29845848423813e-13
	7.68002832810911e-12	18.4605334431476
SMAD3	-1.43544187853108	9.543	-7.66985155292056
7.36101864403353e-13	7.73792692073775e-12	18.4521382703997
NCAPD2	1.17166610169491	10.9781990049751
7.66974141777888	7.36590398766016e-13	7.73909973729084e-12
	18.451485696775
MXI1	-1.22412344632768
9.54545721393035	-7.66840464786777	7.42545629119849e-13
	7.79767865259872e-12	18.4435654628601
MARVELD1	-1.89695889830509
9.04820199004975	-7.66711011991014	7.48358044069136e-13
	7.85469869362953e-12	18.4358962234294
DNASE2	1.03717916666666	10.9921577114428
7.66648889221902	7.51163302134834e-13	7.88011374186415e-12
	18.4322161017073
PACSIN1	2.64365303672317	7.90750547263682
7.66563821457225	7.55021555604796e-13	7.9125025709885e-12
	18.4271769928708
TTYH2	-2.36698700564972
5.78150497512438	-7.66536803424497	7.56251055514913e-13
	7.92134396414345e-12	18.425576605254
CAP2	-1.80060720338983
8.04349900497512	-7.6620784233994	7.71380714361237e-13
	8.07569916666813e-12	18.4060933666956
TUBG1	1.04733983050847	10.1794676616915
7.66136338491693	7.74708727556854e-13	8.10640681791142e-12
	18.4018590403918
KRTCAP3	2.52059187853107	8.92289651741294
7.66125509072084	7.75213999137327e-13	8.10756159057021e-12
	18.4012177608358
SLC24A2	-1.40200621468927
0.487930845771144	-7.6596864798323	7.82569364125604e-13
	8.1803202879321e-12	18.3919295603923
ANK2	-3.01667570621469
7.49199104477612	-7.65822620884455	7.89478717513049e-13
	8.24834507406763e-12	18.3832838021486
PLEKHH2	-3.04684032485876
6.72236567164179	-7.6568171193709	7.96203078064835e-13
	8.31436886707582e-12	18.3749419225129
TBX2	-2.11648439265537
7.97916766169154	-7.65643358290044	7.98043149351815e-13
	8.32934715617324e-12	18.3726715122482
PPIL5	1.0305238700565 7.68058457711443	7.65529304455932
	8.03539926667044e-13	8.38245665369635e-12
18.3659202647609
MCAM	-1.57120247175142	10.413607960199 -7.64969416057961
	8.31071226665958e-13	8.65207519444834e-12
18.3327864481585
CA2	2.82264117231638	6.8836552238806 7.64244813152848
	8.68090657063646e-13	9.02831873835697e-12

18.2899245603392
DENND4A -1.27127196327684
7.61329253731343 -7.63791654165113 8.92066054641733e-13
9.2682773794508e-12 18.2631304559235
SPIN2A -1.57117309322034
0.988467164179105 -7.63748510473435 8.94382517716764e-13
9.28522003752563e-12 18.2605799348299
MTX1 1.07321843220339 10.3194437810945
7.63744440912199 8.9460132655751e-13 9.28522003752563e-12
18.2603393590666
GLIPR1L2 -2.11281899717514
3.12368706467662 -7.63704041125795 8.96776382056511e-13
9.30309202810519e-12 18.257951127428
HPGDS -2.50656956214689
3.49756815920398 -7.63613634501836 9.01662680555527e-13
9.34905799586109e-12 18.2526069937433
MAMLD1 -2.31858707627119
6.69000497512438 -7.63518343477783 9.06841467966464e-13
9.39800875181803e-12 18.2469745066256
KRT15 3.48052994350282 5.50631094527363
7.6350322437468 9.07665845683153e-13 9.40180616139008e-12
18.2460808779725
C8orf30A 1.10115473163842 9.83601940298507
7.63285285215633 9.19631751141609e-13 9.52094798332689e-12
18.2332004556682
SCARA3 -2.1272459039548
10.2885452736318 -7.62941407166243 9.38830245800365e-13
9.71481096082736e-12 18.2128810074824
PPIH 1.00495579096045 9.35801940298508
7.62906803434631 9.40783956316002e-13 9.73012323585265e-12
18.2108365810365
NUAK2 2.00006377118644 8.97336268656716
7.62517939071846 9.63017377794251e-13 9.94504364492755e-12
18.1878655361206
SIGLECP3 -2.08894759887006
2.05257064676617 -7.62462247508543 9.66243796432084e-13
9.9733459732281e-12 18.1845762417102
TSPAN33 1.70155642655367 8.78356467661692
7.62286243797975 9.76510764356182e-13 1.00641395543335e-11
18.1741818509645
SGTB -1.18873305084746
7.14922189054726 -7.62261868248734 9.77941160152158e-13
1.00738243943421e-11 18.1727423883963
PDSS1 1.13804597457627 6.94798905472637
7.62251746240508 9.78535744131943e-13 1.00748940958018e-11
18.1721446553448
PNKD 1.1263304378531 11.1833223880597 7.62125634595619
9.8597379148781e-13 1.01463869369648e-11
18.164697772494
NR3C2 -2.40943213276836
6.38328208955224 -7.61847867539078 1.00255416755959e-12
1.03066785478210e-11 18.1482980297644
MCM7 1.13451433615819 11.8759124378109
7.61833008047704 1.00344888159158e-12 1.03107134830206e-11

18.1474207975894
RORA	-2.04458827683616
7.04476666666667	-7.61698886107757	1.01156038284236e-12
1.03888617607572e-11	18.1395033141537
psiTPTE22	-2.95020628531074
3.54296965174129	-7.61611983954012	1.01685067359463e-12
1.04379721644488e-11	18.1343737160024
A2BP1	-1.95230374293785
0.839302985074627	-7.61543601903492	1.02103274926551e-12
1.04756633395407e-11	18.1303375348027
HIST1H1C	2.54937337570621	9.90368308457711
7.61375748851528	1.03137045889065e-12	1.05722041032672e-11
18.1204310210633
PHKG2	1.05244329096045	9.71611592039801
7.61374104728504	1.0314722269286e-12	1.05722041032672e-11
18.1203339925426
NUMBL	-1.20847189265537
9.11524825870647	-7.61325438652204	1.03448906316102e-12
1.05978345642194e-11	18.1174619983127
PAQR6	2.13985762711865	6.69155223880597
7.61174968561176	1.04387207543958e-12	1.06886252911594e-11
18.1085827487641
IGFBP7	-1.71270586158192
12.3391004975124	-7.61087637850652	1.04935637839967e-12
1.07394249494244e-11	18.1034297995759
PMM2	1.01042733050848	10.0169751243781
7.60951844906504	1.05794070652257e-12	1.08170898084164e-11
18.0954179803223
C21orf7 -1.65336016949152
5.75187263681592	-7.60950928913438	1.05799884731126e-12
1.08170898084164e-11	18.0953639391552
CCDC125 1.23742379943503	8.39552587064677
7.6045281491652 1.0900881111268e-12	1.1134084040514e-11
18.0659818213533
RCAN2	-2.20126087570622
7.12218457711443	-7.60417508575816	1.09239877345037e-12
1.11521366578498e-11	18.0638996165809
CAPN13	4.55118891242938	7.57152587064677
7.60397987469067	1.09367843180507e-12	1.11596511953072e-11
18.0627483747362
NES	-1.89843587570622
10.2836263681592	-7.60255212554217	1.10308285522377e-12
1.12500203764253e-11	18.0543288305584
TXLNB	-2.90236271186441
4.10702139303483	-7.60144688396537	1.1104178144636e-12
1.13192044344278e-11	18.0478117342848
MMACHC	1.00348333333333	7.6077144278607 7.60111092518606
1.11265695279494e-12	1.13364006158214e-11
18.0458308445756
GFPT2	-2.64614449152543
6.55405472636816	-7.59813965026537	1.13265557126418e-12
1.15287153584798e-11	18.0283136237404
JAK2	-1.52000798022599
7.46983134328358	-7.59462371195209	1.15677954547007e-12

1.17684261984642e-11   18.0075901919796
CHPT1   -1.62050882768362
8.08418059701492   -7.5945082927944   1.15758002458475e-12
1.17707369513248e-11   18.0069099847752
WDHD1   1.47879597457627   7.83890248756219
7.58984773351632   1.19036446501391e-12   1.20981101320473e-11
17.9794483615519
CRHR1   -3.00846278248588
2.18430248756219   -7.58973715761606   1.19115334934628e-12
1.21001376853434e-11   17.9787969228915
HEPH   -2.44184668079096
8.09117960199005   -7.5853043664678   1.22320756811294e-12
1.24196099769331e-11   17.9526862108118
VDR   2.06865254237288   8.01691592039801
7.58383882218027   1.23399156421964e-12   1.25167227339077e-11
17.9440554837759
MFSD9   1.21349922316384   7.33667462686567
7.58275771346155   1.24200693101409e-12   1.25918036018367e-11
17.9376893227658
ZNF385D -2.34452980225989
2.76395572139303   -7.58193397068786   1.24814870654015e-12
1.26478247508733e-11   17.9328390094008
ARHGAP39   1.31901094632768   9.11181293532338
7.57880007956102   1.27179040965899e-12   1.28746829932441e-11
17.9143888729295
IQSEC3 -3.30745127118644
4.01544328358209   -7.57681971291177   1.2869577387789e-12
1.30218050158358e-11   17.9027320428992
C20orf194   -1.49389830508475
8.31334427860697   -7.57598278004256   1.29342130361565e-12
1.30807583070095e-11   17.8978061956775
DBF4   1.12491016949152   8.21592587064677
7.57506230149135   1.30056714197427e-12   1.31465501845061e-11
17.8923889792298
SLC8A1 -2.59726094632769
7.64544726368159   -7.57349176954311   1.31284966330553e-12
1.32641749939284e-11   17.8831468959874
MTL5   1.96739350282486   7.46583084577114
7.57078327692061   1.33430167741371e-12   1.34676565571797e-11
17.8672107603936
RBP7   -2.84417372881356
5.69844577114428   -7.56674719287124   1.36691298475954e-12
1.37832630535921e-11   17.8434692306008
RAB11FIP5   -1.35262450564972
8.99335373134328   -7.5658263857922   1.37446272014981e-12
1.38525869635128e-11   17.838053730755
PCDHGA4 -2.56405946327684
3.19622189054726   -7.56507205533889   1.38067820752239e-12
1.39084021591926e-11   17.8336175936223
FLJ36777   -1.86011094632769
2.50236268656716   -7.56491514794943   1.38197456999284e-12
1.3914633605666e-11   17.8326948686829
ISG15   2.79559957627119   11.503471641791 7.56417047164587
1.38814347852991e-12   1.39698949089309e-11

17.8283157966664
APBA1	-2.27354293785311
6.11479154228856	-7.56116011636528	1.41335993128972e-12
	1.42166973980294e-11	17.8106158201914
HES6	2.54301419491525	9.65392487562189
7.56042332155998	1.41960039449085e-12	1.42724760523493e-11
	17.8062842837789
LOC388152	1.26456588983051	9.49814378109453
7.55990181398598	1.42403388278453e-12	1.43100419058084e-11
	17.8032185387059
MMP1	3.85229025423729	5.88764029850746
7.55545127623096	1.46243021786475e-12	1.46886948986122e-11
	17.777060278515
DMPK	-1.32155677966102
9.61325771144279	-7.55536512728027	1.46318346137582e-12
	1.468907406457e-11	17.7765540178484
RPA3	1.12409519774011	8.61262487562189
7.55027365338843	1.50838848324866e-12	1.51280974895432e-11
	17.7466392749858
MARCKSL1	1.23624491525423	13.2519835820896
7.54834111586811	1.52590570757328e-12	1.52963106330466e-11
	17.7352876571483
SIGLEC6 -1.72736991525424
0.872624875621891	-7.54614861132329	1.54602291297963e-12
	1.54904101529877e-11	17.7224109582898
SNAP25	-3.4197947740113
4.43651393034826	-7.54541000302325	1.55285882488005e-12
	1.55437307044307e-11	17.7180735385756
LDHA	1.37295628531073	13.6280253731343
7.5432705202629 1.57282905107641e-12	1.57359553696874e-11
17.7055109218818
ESRRA	1.00669576271186	9.83677661691542
7.53831349160259	1.62007922021459e-12	1.61929047667992e-11
	17.6764118346391
LOC286002	-2.44905812146893
2.10338756218905	-7.53447375259215	1.6576412160833e-12
	1.65602793996059e-11	17.6538788385895
BEGAIN	-2.47173658192091
3.89850447761194	-7.53158182900427	1.68649830232099e-12
	1.68240088176141e-11	17.6369121827068
MPDZ	-2.1210018361582
8.18857711442786	-7.52695787266867	1.73367332595422e-12
	1.72811873388195e-11	17.6097913715984
CDH5	-1.67247662429379
8.89661791044776	-7.52692522469116	1.73401100428486e-12
	1.72811873388195e-11	17.6095999149189
SLC11A1 1.80476377118644	6.86984875621891
7.52597890605669	1.74382708416939e-12	1.73705822600667e-11
	17.6040506441352
PRSS35	-3.11068912429379
3.00587810945274	-7.5249692955617	1.75436023679982e-12
	1.74670299037344e-11	17.5981306540654
CYP2D6	2.4321197740113 5.71978407960199	7.52158022029579
	1.79017996206878e-12	1.78063927428643e-11

17.5782615778289
C3orf39 -1.19796800847458
9.38574378109453 -7.52131142003853 1.79305169973753e-12
1.78186588352358e-11 17.576685900256
WHSC1 1.08594350282486 10.8744119402985
7.521302319161 1.79314900894287e-12 1.78186588352358e-11
17.5766325524606
FCER1A -2.94143269774011
3.08933731343284 -7.52028476065391 1.80406201552488e-12
1.79184292108011e-11 17.5706680243853
NBEAL2 1.24401016949152 10.4541383084577
7.51922016247472 1.81554976655177e-12 1.80238088526634e-11
17.5644282493823
ATAD2 1.58821574858757 9.37879054726368
7.51906789672816 1.81719871757358e-12 1.8031459483705e-11
17.5635358364564
GMDS 1.45933079096045 9.07033233830846
7.51793639601947 1.82949867493798e-12 1.81447380659308e-11
17.5569045496701
EEA1 -1.25285692090396
8.45131293532338 -7.51484661069918 1.86350677610166e-12
1.84641863481501e-11 17.5387993492753
PDZRN3 -2.58099943502825
8.95737960199005 -7.51412167491755 1.87157591816232e-12
1.85351922816558e-11 17.5345520473939
ZNF624 -1.18244004237288
6.27103432835821 -7.51381593969619 1.87498934991516e-12
1.85600440471351e-11 17.5327608540729
PRDM5 -2.82511843220339
3.99310497512438 -7.50382969155781 1.98992906750105e-12
1.96409825749022e-11 17.4742773549046
TKT 1.26485621468927 12.9349119402985
7.50334212603145 1.99571571189987e-12 1.9688632179387e-11
17.4714230861616
CBFA2T3 -2.53237881355932 5.2671447761194 -7.50305728343459
1.99910404211965e-12 1.97074376936472e-11
17.4697556303946
ERBB3 2.17441129943503 10.8285800995025
7.50302051957712 1.9995417786589e-12 1.97074376936472e-11
17.4695404190006
TMEM119 -2.6840081920904
6.97565373134328 -7.50137035637818 2.0192876344935e-12
1.98925024645641e-11 17.4598811600299
BRCA1 1.46259491525424 8.14089004975124
7.50047571756454 2.03007330753322e-12 1.99891630712983e-11
17.4546448724197
ST3GAL3 -1.27663771186441
7.66497164179104 -7.49647555746049 2.07899931570217e-12
2.04512965746841e-11 17.4312363543617
C12orf53 -3.18613333333334
5.4777671641791 -7.49586462522138 2.08657349847123e-12
2.05159741013479e-11 17.4276618574456
GJC3 -1.52760939265537
0.962487562189055 -7.49058567141074 2.15316664965163e-12

2.11504838838986e-11   17.3967820711073
P2RY12 -2.09475508474576   1.6369815920398 -7.48762457353802
2.19143530986457e-12   2.15161008663413e-11
17.3794661532969
IL11RA -1.73739427966102
7.35300995024876   -7.48140569658911   2.27400824621585e-12
2.23054893907364e-11   17.3431118697917
KLF11   -1.25527831920904
8.83270149253731   -7.47991466605187   2.29425834747325e-12
2.24933733875959e-11   17.3343981261491
HIST1H2BG   2.55497959039548   4.17491990049751
7.4785358338507 2.31314317078938e-12   2.26676989481175e-11
17.3263409483934
TMEM8B -1.10989597457627
8.77851641791045   -7.47664367887532   2.33930882035326e-12
2.29131727489754e-11   17.3152855355613
PRICKLE1   -2.21115628531073
7.58132487562189   -7.47560658243446   2.35377415070643e-12
2.30328805119175e-11   17.3092266928031
XPNPEP2 -2.53139978813559
1.96998606965174   -7.47203024238144   2.40433779085146e-12
2.35052642124669e-11   17.2883368998184
MBLAC2 -1.39661814971751
6.91542139303483   -7.47105572419343   2.41830097532607e-12
2.36305183357659e-11   17.2826456054753
NRG2   -2.90755889830509
3.47933731343284   -7.47026888421413   2.42963348729838e-12
2.3729959797448e-11   17.2780506768356
PRKCA   -2.1971018361582
8.38696368159204   -7.46975160122832   2.43711225759278e-12
2.37916855199143e-11   17.2750300348206
C16orf5 -1.64940896892656
8.74758955223881   -7.46626280827649   2.48814992412524e-12
2.42783830524198e-11   17.2546605128312
GDF10   -3.21202570621469
2.82828308457711   -7.4653702613671   2.5013757680196e-12
2.43958406258633e-11   17.2494501843667
NRP1   -1.77355713276836
10.2311900497512   -7.45917433910975   2.5951218751579e-12
2.52861186981451e-11   17.2132905464095
C1orf53 1.79766610169492   5.91408606965174
7.4579627288721 2.61385556360691e-12   2.5456572448221e-11
17.206221517082
MRPS15 1.15506377118644   10.8186248756219
7.45457075540018   2.66701516572063e-12   2.59619826231601e-11
17.1864347948932
POLA2   1.17570402542373   9.18384228855721
7.45220351842656   2.70474634731771e-12   2.63167973983093e-11
17.1726287684812
C12orf56   3.21450776836158   4.39281094527363
7.45086777699965   2.72626884952704e-12   2.6513642577352e-11
17.1648396449331
TSEN54 1.05759901129943   9.67932686567164
7.44983599017576   2.7430095456436e-12   2.66638191155602e-11

17.1588235136557
FRK	2.00572443502825	6.00670995024876
7.44403150240285	2.83909724120053e-12	2.75717437851689e-11
17.1249875225699
PRR16	-2.82610826271187	4.6256	-7.44387766797388
2.84168854249775e-12	2.75838608876969e-11	17.1240909807126
CKMT1A	3.32026207627119	6.376392039801	7.43997420200218
2.90822895081961e-12	2.82164179396629e-11
17.1013452104761
RBPMS2	-2.07697754237288
7.33491144278607	-7.43593414625915	2.97871868640023e-12
2.88730380697812e-11	17.0778106136936
DLGAP2	-2.13584399717514
1.55320298507463	-7.43523830508044	2.99102876273578e-12
2.89786788574637e-11	17.0737578484078
MDGA1	-2.41485564971751
6.00680199004975	-7.4312058539169	3.06336242175996e-12
2.9651499537356e-11	17.0502759921208
QPCTL	1.30604505649718	8.95572437810945
7.43041664088815	3.07772011341053e-12	2.97764344619784e-11
17.0456810721579
SH2D3A	1.84466553672317	8.2370736318408 7.42971019798037
3.09062838843874e-12	2.98872354284726e-11
17.041568286577
TARBP2	1.02556320621469	9.69072039800995
7.42875960959531	3.10808217651169e-12	3.0041867741895e-11
17.036034478103
SORT1	1.109077259887	11.4627751243781	7.42619683693994
3.15562392905926e-12	3.04870396534525e-11
17.0211174046261
STXBP1	-1.90377831920904
8.64056766169154	-7.42284859388189	3.218820676443e-12
3.1082967303563e-11	17.0016327481573
RAB25	2.91853446327684	10.6259860696517
7.42211066937068	3.23291595148579e-12	3.12044026723099e-11
16.9973391630187
MOBKL2B -1.71808622881356
8.41388756218906	-7.41653897218206	3.34132882343314e-12
3.22185410531254e-11	16.9649282610221
EBF4	-2.54709392655368
7.84259502487562	-7.41646999361207	3.34269325100619e-12
3.22185410531254e-11	16.9645270948673
EIF2C2	1.24025437853108	7.79817263681592
7.40626216859114	3.55079582080268e-12	3.41922318016318e-11
16.9051836582905
IGDCC4	-2.42848488700565
5.00410845771144	-7.40255426769336	3.62951349150702e-12
3.49174845270099e-11	16.8836391686859
SESN1	-1.23124844632768
8.66337462686567	-7.40220581047323	3.63699914739009e-12
3.49731112393061e-11	16.8816147980995
CILP	-3.48339731638418
5.46565572139304	-7.39693151084178	3.75218308541403e-12
3.60132392442964e-11	16.8509802382362

C3orf18 -1.5138354519774
7.04202935323383 -7.39446667489717 3.80724013050328e-12
3.65245980744076e-11 16.8366680584876
TMOD1 -1.93165614406779
9.49222686567164 -7.39418602475066 3.81355939141798e-12
3.65681337252737e-11 16.8350386228612
TPTE2P3 -1.29014484463277
0.518406467661692 -7.39353682200544 3.82821689583644e-12
3.66915466253604e-11 16.8312695307125
CA5B -1.29226087570621
7.44364726368159 -7.3930717285053 3.83875183414356e-12
3.67581973670556e-11 16.8285694417781
LOC283267 -1.23540402542373
7.06661940298507 -7.39235031780617 3.85514935149863e-12
3.68980028840405e-11 16.8243815007316
C5orf34 1.47005790960452 6.18087860696517
7.39146017043736 3.87547759011691e-12 3.70752818849489e-11
16.8192143281232
SERINC2 2.30169173728814 11.8097253731343
7.38673975400649 3.98505975498859e-12 3.8105857834148e-11
16.7918189322633
RAP2C -1.20206610169492
9.14946417910448 -7.38620417655985 3.99768461280077e-12
3.82056822556257e-11 16.7887112856523
KCNIP4 -2.22961963276837
4.82464527363184 -7.38613910496705 3.9992211966556e-12
3.82056822556257e-11 16.7883337215196
GALNT6 2.17153573446328 9.07225621890547
7.38332365190763 4.06626616212786e-12 3.88281136318534e-11
16.7719994654106
NOXO1 2.37275529661017 4.08455373134328
7.38067730250326 4.13029577327253e-12 3.94211865296536e-11
16.7566495225031
DZIP1 -2.38143990112995 8.0385736318408 -7.37809014077283
4.1938556386898e-12 4.00092268876866e-11
16.7416459243844
TIMM8B 1.19273460451977 9.90609353233831
7.37533270224911 4.26266237164724e-12 4.06467526660092e-11
16.7256581498694
EBP 1.53056927966101 10.1612855721393
7.3722131863062 4.34184884196729e-12 4.13634137937766e-11
16.7075751406862
SLC4A5 1.07161899717514 7.72173532338308
7.36811701803455 4.44803675493103e-12 4.23357415756764e-11
16.6838373660713
PITPNM1 1.36876447740113 10.2800507462687
7.36751092273336 4.46396530244999e-12 4.24676587855877e-11
16.6803256125125
CLDN20 -1.4130031779661
1.04837611940299 -7.36657375076828 4.48870570161479e-12
4.26832459722796e-11 16.674895905085
C3orf54 -1.64618072033899
4.91803233830846 -7.36521871498365 4.524716902962e-12
4.29858574816335e-11 16.6670459112593

ADCYAP1 -2.9523688559322
1.71594029850746 -7.36191923144348 4.61360095681989e-12
4.38050773984898e-11 16.6479347923474
SLC27A4 1.05514442090396 9.88699452736318
7.36185986524857 4.61521589931483e-12 4.38050773984898e-11
16.6475909789812
HSPA1L -1.37600988700565
5.62180646766169 -7.36173234576892 4.61868670778963e-12
4.38177625281521e-11 16.6468524680564
DLEU2 1.44315395480226 5.30798706467662
7.35827615606931 4.71374480017968e-12 4.46989287518194e-11
16.6268392222884
CBX6 -1.9156761299435
7.89526965174129 -7.35497393737537 4.80637195964615e-12
4.55142141750625e-11 16.6077225746277
CHAC2 1.54993121468927 5.82327313432836
7.35454843955347 4.81843713638774e-12 4.56074294191057e-11
16.6052597114152
ZIK1 -2.89719971751413
3.74575124378109 -7.35351637691494 4.84782611646003e-12
4.58433303412826e-11 16.5992862731356
PDIA6 1.08771878531074 13.3523835820896
7.34936092572048 4.96795842505941e-12 4.69577285757227e-11
16.5752399423511
APH1B -1.50348495762712
7.56753980099502 -7.34658480152051 5.04984813690887e-12
4.76878483214072e-11 16.5591796941899
IL1RN 2.64096991525424 6.98872189054726
7.34648725025755 5.05274980317798e-12 4.76933119352846e-11
16.5586154103619
TPRN 1.26563870056497 9.19696766169154
7.34525319153964 5.08959939380645e-12 4.79970030109538e-11
16.551477386775
KLHDC5 -1.01842521186441
9.17704029850746 -7.34336783264395 5.1464101421849e-12
4.85104684201359e-11 16.5405734496413
KIF1A 4.78602556497175 9.15356815920398
7.34319982721998 5.15150286283506e-12 4.85361880559908e-11
16.5396018713397
LOC342346 -1.62825183615819
1.25914129353234 -7.3411780288086 5.21318097446915e-12
4.90947731219503e-11 16.5279107752951
ADAMTSL3 -3.43217231638418
5.14397263681592 -7.33525246078782 5.39818107951879e-12
5.07671358509028e-11 16.4936566788393
CCDC21 1.24441327683616 8.05597661691542
7.33516021843506 5.40111159464262e-12 5.07714382042184e-11
16.493123576358
ZBTB7B 1.05789766949153 10.4114606965174
7.32965364875548 5.57892950063696e-12 5.23710208724631e-11
16.4613060590144
C21orf71 -1.4426988700565
1.46383034825871 -7.32911211843338 5.59672567065327e-12
5.25140667360657e-11 16.4581777831755

HIST1H2BD	1.93701723163842	8.50364825870647
7.32836900481389	5.62123768632355e-12	5.27199678849806e-11
	16.4538852306893
RBM20	-2.93963757062147
4.19204726368159	-7.32197452750262	5.83659113409451e-12
	5.47147104945024e-11	16.4169582452283
ZDBF2	-3.28904209039548
5.32142935323383	-7.32151137299403	5.852501917948e-12
	5.48388244525205e-11	16.414284329075
OTUD7A	-2.08268100282486
2.50313482587065	-7.32058595097844	5.88442139533833e-12
	5.50876293872758e-11	16.4089419091088
CHD9	-1.42603333333333
8.84523930348259	-7.3158192207279	6.05157736360069e-12
	5.6600857983928e-11	16.3814299447154
GRIN2D	3.49897881355932	6.61284029850746
7.31419808952239	6.10948746445815e-12	5.70904768526745e-11
	16.3720756713526
COL16A1	-2.10738050847458
9.01023034825871	-7.31102073777574	6.22458057932063e-12
	5.81395083227719e-11	16.3537451366746
C4orf38	-1.74375049435028	3.4120960199005	-7.31016121809284
	6.25608108854078e-12	5.84071599580456e-11
16.3487872500352
TCEAL2	-4.11898290960452	5.2938776119403	-7.30882902107796
	6.30521601873476e-12	5.88391294839203e-11
16.3411035269878
OVOL2	2.30234258474576	7.69743184079602
7.30655975395445	6.38979130892059e-12	5.9578821372824e-11
	16.32801691614
CYBA	1.64401129943503	11.7833114427861
7.30654657714342	6.39028566307639e-12	5.9578821372824e-11
	16.3279409337797
LOC399959	-3.07956998587571
7.96116069651741	-7.30562973133405	6.42477582995279e-12
	5.98731946386431e-11	16.3226542561588
IRAK1	1.15032464689265	11.2972099502488
7.30554695135711	6.42789890175465e-12	5.98751199877599e-11
	16.3221769525789
CELF2	-2.39028305084746
8.00255721393035	-7.30533287448308	6.43598241351511e-12
	5.99232285485103e-11	16.3209426147006
CRYBG3	-2.50660628531074
6.66650248756219	-7.30292724629098	6.52751153140009e-12
	6.0720349678135e-11	16.3070735271637
EDA2R	-2.60477881355932
4.59415621890547	-7.30275234179383	6.53421617463232e-12
	6.07551893411239e-11	16.3060652588906
SORCS1	-3.17771899717514
3.14073930348259	-7.30175040075408	6.57275498399968e-12
	6.1085857773433e-11	16.300289658992
FREM1	-3.30072372881356
6.41279253731343	-7.29944255580891	6.66238010200635e-12
	6.18907979611721e-11	16.2869880322693

NT5DC3	-1.38056532485876
8.23367412935323	-7.29883367590803	6.68622641136356e-12
	6.20842280530502e-11	16.2834790620029
POLE2	1.90116765536723	6.20215422885572
7.29605596609036	6.79608671117435e-12	6.30757957415955e-11
	16.2674732855915
NR4A1	-2.17306186440678
10.3489248756219	-7.29544837173604	6.8203539865984e-12
	6.32438425225227e-11	16.2639726619506
TALDO1	1.36277535310734	12.2954850746269
7.29363233053381	6.89339766840293e-12	6.38634720813683e-11
	16.2535106378608
TP53I3	1.27368848870056	9.84221044776119
7.29269520176936	6.93139182024482e-12	6.41865016101155e-11
	16.2481125253695
VAV3	2.73609230225988	9.65179054726368
7.29250404083094	6.93916743607155e-12	6.42295344736469e-11
	16.2470114364223
SOBP	-2.37768834745763
6.48021890547264	-7.29232154746891	6.94659852593223e-12
	6.42693410263131e-11	16.2459602883654
MRPS17	1.15533050847458	8.71088059701493
7.29055594169325	7.01890052249889e-12	6.49090214986046e-11
	16.2357913168328
NCRNA00182	-1.09688305084746
4.58178805970149	-7.28971146225416	7.05374431705312e-12
	6.52018779059435e-11	16.2309280574285
CCDC81	-2.28085197740113
4.16868855721393	-7.28292423016473	7.34006213695005e-12
	6.78179458467978e-11	16.1918530066401
PLA2G4C	-2.06076701977401
6.02942388059702	-7.27999991860034	7.46693215365174e-12
	6.88971312874024e-11	16.1750238262581
RADIL	-3.02279463276837
5.06835273631841	-7.27969437525139	7.48031228120892e-12
	6.89895827193257e-11	16.1732656743856
SMPD2	1.26977330508474	8.68085024875622
7.27841904964878	7.53641643265262e-12	6.9444627182387e-11
	16.1659276808843
MTMR11	-1.6023261299435
7.39005472636816	-7.27821961149736	7.54522759931231e-12
	6.94946265652228e-11	16.1647802170565
CLDN5	-2.44698290960452
8.12966915422886	-7.27793597345215	7.55777623094964e-12
	6.95789892472628e-11	16.1631483420172
C9orf125	-3.33362740112994
5.55273432835821	-7.27779264425694	7.56412519290938e-12
	6.96062260019855e-11	16.162323729924
FMO2	-3.90779745762712	3.7759552238806	-7.27648993759505
	7.62207228516987e-12	7.01080394330365e-11
16.1548293332316
LOC284837	2.10884512711864	5.5538552238806
7.27558664198336	7.66250980522692e-12	7.04484219889425e-11
	16.1496331817327

PRRG3   -1.97164166666667
1.11801990049751   -7.27471506112456   7.70172829237881e-12
        7.07456294597481e-11   16.1446198217056
GRHL3   3.43110381355932   4.7007447761194 7.27054498229565
        7.89212826871327e-12   7.23650707265223e-11
16.1206382172351
SDF2L1 1.45760677966102   9.72383084577115
7.26807957905611   8.00687291049461e-12   7.33844200234171e-11
        16.1064637407761
CPE   -2.21857570621469
9.01549552238806   -7.26640173948248   8.08590295746399e-12
        7.40756750186237e-11   16.0968188416144
TFRC   1.75549350282485   11.6680656716418
7.26599252787588   8.10529425445418e-12   7.42202011792793e-11
        16.0944667241093
C1orf38 1.89835557909604   8.86251542288557
7.2638982536011 8.20525647507182e-12   7.50685897652515e-11
16.0824301968359
AMPH   -3.01965042372881
4.25279651741294   -7.26249087254582   8.27311513888586e-12
        7.56557032522613e-11   16.074342616941
GDF15   2.8264740819209 9.59147213930348   7.26237493249047
        8.27872996994084e-12   7.56733420671796e-11
16.0736764026516
PELI3   -1.14900162429378
7.98051343283582   -7.2601070174254   8.38932177663831e-12
        7.66160035918081e-11   16.060645760135
HOOK2   1.28255254237288   9.83871194029851
7.25692892095308   8.54675475683803e-12   7.79497446281141e-11
        16.0423895133224
PHLDB2 -2.51360120056497
7.46137611940298   -7.24716563602164   9.04886407906356e-12
        8.24559163529405e-11   15.9863344036798
NRXN1   -3.09616716101695
1.99371194029851   -7.24634797785513   9.09221273231874e-12
        8.28141647712971e-11   15.9816418781472
MCC   -2.41849286723164
8.02255323383085   -7.24419520691012   9.20732587118948e-12
        8.38254546055521e-11   15.969288638494
DENND5A -1.02381793785311
10.1042039800995   -7.24146271057201   9.35551176658945e-12
        8.50237523541751e-11   15.953611840744
AKAP11 -1.19213757062147
9.43149054726368   -7.24106656177759   9.37718976113109e-12
        8.51830556619563e-11   15.9513393534551
CRMP1   -2.26566525423729
7.68101194029851   -7.24035258564225   9.41638512454001e-12
        8.55012766947396e-11   15.9472438487975
PRR15L 2.91293997175141   8.42708457711443
7.23744667594417   9.57758695648163e-12   8.69265518198797e-11
        15.9305774191491
C4orf48 2.64630120056497   7.20181243781095
7.23402037511185   9.77115859801733e-12   8.86050733291943e-11
        15.9109313713339

HES2	3.08425812146893	6.66293432835821
7.23361966290998	9.79404784354288e-12	8.877342261719e-11
	15.9086340841519
SLC35F4	-1.53932634180791
0.78593631840796	-7.23220023823591	9.87555392474663e-12
	8.93937928020495e-11	15.900497105062
GPER	-2.05282916666667
5.99028059701493	-7.2321038860489	9.88111085199885e-12
	8.9404674213987e-11	15.8999447912831
AVL9	1.12293742937853	7.84409701492537
7.23058801628105	9.96894301820246e-12	9.01199472319227e-11
	15.8912560283646
ZSCAN16	1.18851313559322	7.58524278606965
7.2291173233758	1.00548938450471e-11	9.08569413022962e-11
15.8828272290594
ONECUT2	3.03873029661017	5.11260099502488
7.22873512998979	1.00773496135683e-11	9.10197921542269e-11
	15.8806369754741
SEC23A	-1.21278029661017
9.63180845771144	-7.2271527139395	1.01708523434062e-11
	9.17835598286281e-11	15.8715692688763
NDRG2	-2.17930127118644
10.0323054726368	-7.22326890934521	1.04039859567601e-11
	9.37637540352433e-11	15.8493188476413
C14orf132	-2.88714265536723
8.34680746268657	-7.22080073137473	1.05548761587271e-11
	9.50401787450292e-11	15.8351822208723
TLL1	-2.85866066384181
4.08650945273632	-7.22058319645862	1.05682780936008e-11
	9.51191360200023e-11	15.8339364129136
FLJ42709	-2.23250692090395
6.31738606965174	-7.21823496739868	1.07140216817564e-11
	9.63886350247412e-11	15.8204896583688
LOC148709	2.29198418079096	3.6632144278607
7.21646968222097	1.08248890416406e-11	9.73433955430928e-11
	15.8103827216993
PDLIM5	-1.36816031073447
10.6243104477612	-7.21515156015074	1.09084102677333e-11
	9.80515161105801e-11	15.8028369086534
DST	-2.3668434322034
10.4926189054726	-7.21329759505856	1.10269618390151e-11
	9.90737534157463e-11	15.7922249361987
MKL2	-1.06900741525424
9.37954776119403	-7.21302619055513	1.10444231236597e-11
	9.91872295401281e-11	15.7906715688274
FAM117A	-1.18730713276836
8.57892338308458	-7.21216247127299	1.11001738136563e-11
	9.96443237404304e-11	15.7857283510243
THOC4	1.20482789548023	10.701163681592	7.20958883494782
	1.12679468302042e-11	1.01062013291434e-10
15.771001030771
ZNF280A	2.5928072740113	3.13506417910448	7.20820283391179
	1.13593331569397e-11	1.01837165813089e-10
15.7630710836527

KRT80	2.88742937853107	7.63779502487562
7.20643145707695	1.1477194741066e-11	1.02848890455733e-10
	15.752937524777
DSCR6	3.31743587570621	5.47140746268657
7.20547380909268	1.1541415213381e-11	1.03379255816192e-10
	15.7474596920289
ZNF229	-3.61719449152542
3.83273432835821	-7.2053814486863	1.15476276773988e-11
	1.03389793378542e-10	15.7469314047326
SELE	-2.88534809322034
4.11745024875622	-7.20437148512521	1.16157768522227e-11
	1.03954620216274e-10	15.741154827649
TFPI	-2.62770868644068	7.6083184079602 -7.20339963242549
	1.16817290746532e-11	1.04499301918357e-10
15.7355966770184
IRS1	-2.44261292372882	9.0865631840796 -7.20052255496062
	1.18791482678804e-11	1.06126594403649e-10
15.7191448772858
PDK2	-1.13164392655367
9.43345273631841	-7.19878727712491	1.19998074021984e-11
	1.07157914731245e-10	15.7092240169708
SMPDL3A -1.82005812146893
6.43164975124378	-7.19857589885377	1.20145875571547e-11
	1.07231821455044e-10	15.7080156294697
TRH	-5.19503665254237
3.08785373134328	-7.19705003979574	1.21218140557442e-11
	1.08103987825146e-10	15.6992933586842
BRIP1	2.29958319209039	5.5127144278607 7.19527180187312
	1.22479678941713e-11	1.09136623640337e-10
15.6891297828672
C9orf106	-1.07781221751412
0.599410945273632	-7.19248198883773	1.24485013782516e-11
	1.10840856418555e-10	15.6731874871306
FAM195A 1.75480762711864	9.31256169154229
7.19246096136999	1.24500250804281e-11	1.10840856418555e-10
	15.6730673401301
DQX1	2.5944686440678 2.96829800995025	7.18859556801943
	1.27332701574195e-11	1.13264313835279e-10
15.6509847125424
OR2A9P	-1.33873481638419
6.89137661691542	-7.18345308036557	1.31199748925986e-11
	1.16653566281961e-10	15.6216169769915
FAM199X 1.14691002824859	8.91252388059701
7.18328360447249	1.31329144527854e-11	1.16718066543586e-10
	15.6206493437457
CHRNA1	2.8678938559322 3.21077960199005	7.18139795650527
	1.32777367427254e-11	1.17903086214599e-10
15.6098840238507
LOC284276	-2.69932379943503
4.86942935323383	-7.1756099506093	1.37321829557253e-11
	1.21780438911897e-10	15.5768502043118
PPM1M	-1.09358919491525
8.83844278606965	-7.17411866510091	1.38517366389065e-11
	1.22787630913969e-10	15.5683415472405

C14orf49 -1.96324738700565
3.37569104477612 -7.17088870942281 1.41142085356467e-11
1.25060294016757e-10 15.5499163288993
PCDHGA9 -3.02859985875706
3.60766567164179 -7.17081153879302 1.41205392074069e-11
1.25062411530658e-10 15.5494761703367
FAM172A -1.0686845338983
8.58888208955224 -7.17004738026109 1.41833781668985e-11
1.25564792482288e-10 15.5451177855581
C12orf45 1.14158792372881 8.54988308457711
7.166079615607 1.45141212147975e-11 1.28437460577497e-10
15.5224920054815
PRDM16 -2.83134512711865
3.35504676616915 -7.1580200107427 1.52095524106489e-11
1.34417611274482e-10 15.4765556538567
TXNL4A 1.16483940677965 10.3238791044776
7.1567329030303 1.53236152351965e-11 1.35367392761869e-10
15.4692225066751
PPP1R16A 1.16416673728814 10.4234492537313
7.15642542209634 1.53509886816283e-11 1.35550880702722e-10
15.4674707848425
EPN3 2.58110077683616 8.41257213930348
7.15335824617795 1.56266980925984e-11 1.37926101393398e-10
15.4499994867525
C1orf229 -1.77320826271187
2.92348059701493 -7.15286332635237 1.56716420180526e-11
1.3826334792893e-10 15.4471807307068
CDC42EP5 -2.11509745762712
6.46053482587065 -7.15246002750086 1.57083600758891e-11
1.3852776304038e-10 15.4448838760261
PBX3 -2.21028672316384 6.7579447761194 -7.1513189149879
1.58127122920253e-11 1.39388142273628e-10
15.4383854631178
KY -3.09770748587571 3.4507263681592 -7.15054338772809
1.58840225869887e-11 1.39956645369475e-10
15.433969336458
MYOC -3.02792125706215
4.11035074626866 -7.1495342976676 1.59772843088553e-11
1.40717995221278e-10 15.4282236442347
RASA4P -1.41486292372881
6.61201343283582 -7.14784386143103 1.61347290958919e-11
1.41982849695096e-10 15.4185994857115
ANKRD22 2.88122450564972 6.51042686567164
7.14654937785026 1.62563293572752e-11 1.4299162026772e-10
15.4112305134813
RASSF7 1.65983947740113 10.4164955223881
7.14243262349736 1.66490871777736e-11 1.46258348206971e-10
15.3878007399062
CYP27B1 1.66921214689266 4.72391990049751
7.14182467000918 1.67078766264783e-11 1.46712021874081e-10
15.3843413577584
EGR2 -2.26228086158192
6.40215472636816 -7.13939950407595 1.69444362959487e-11
1.48662084254627e-10 15.3705433917465

SEPHS2	1.05169336158192	11.0379263681592
7.13771001376666	1.71111850675456e-11	1.49996852876478e-10
	15.3609326867144
KRT7	3.77312210451978	11.8911542288557
7.13606989184522	1.72746094863249e-11	1.51364802712015e-10
	15.3516041031542
ACY1	1.23017252824859	9.55488208955224
7.1342993516076	1.74527574592968e-11	1.52860542081639e-10
15.3415351594466
ATG4D	1.17356694915254	9.13022288557214
7.13282752729201	1.76022277983064e-11	1.54103938890929e-10
	15.3331661197281
FAM108C1	1.15293474576271	9.05482089552239
7.13267006936542	1.76182929355061e-11	1.54178838007647e-10
	15.3322708481453
DUSP26	-2.66565635593221
3.25723930348259	-7.13250755781976	1.76348888516715e-11
	1.54258316201455e-10	15.3313468550613
PHLDA2	2.64973241525424	7.29147562189055
7.13162744086693	1.77250359509716e-11	1.54980829673529e-10
	15.3263429843846
TMEM134	1.02752379943503	9.64712189054726
7.1314497775571	1.7743288408883e-11	1.55074376770697e-10
15.3253329309395
OXER1	-2.24139053672316	4.4055631840796	-7.12993963865548
	1.78991848485927e-11	1.56370325507068e-10
15.316748074411
PVRL1	1.46669286723164	9.66859452736318
7.1296731170541	1.79268386550663e-11	1.56545298846666e-10
15.315233060953
ALDH1B1	-1.17343806497175	9.7256447761194	-7.12932241212873
	1.79632913986324e-11	1.56796927046736e-10
15.3132395675699
SEMA4A	1.66381652542373	9.13622686567164
7.12846490967211	1.80527296094709e-11	1.57510641259004e-10
	15.308365556739
C1orf159	1.11636970338983	8.53116766169154
7.12158904477782	1.87859509657693e-11	1.63629857160477e-10
	15.2692959799017
SYT1	-3.00264223163842
5.01809054726368	-7.11981599029053	1.89797399956105e-11
	1.65247693854912e-10	15.2592248775741
FYN	-1.58536603107344
9.33251890547264	-7.11778414481233	1.92042394839822e-11
	1.67060608731422e-10	15.2476856507874
PDLIM2	-1.36524166666667
8.30388059701493	-7.11760375921102	1.9224296501944e-11
	1.67164255478572e-10	15.2466613020703
STYXL1	1.06247973163842	9.7686039800995	7.11414459695056
	1.96129416495945e-11	1.70471503838347e-10
15.227020874778
PRKACB	-1.42849710451978
8.96285721393035	-7.113054163326	1.97370534674657e-11
1.71477658775739e-10	15.2208307880145

ARHGAP11B	2.49220247175141	3.9291552238806
7.10844284698908	2.02705290414761e-11	1.7596361996681e-10
	15.1946598709785
PLA2R1	-2.37533622881356
6.36313184079602	-7.10090729369209	2.1173073991682e-11
	1.83720713883868e-10	15.151914564089
ANGPTL7	-2.28695148305085	1.2099631840796	-7.10071561354176
	2.11965413319754e-11	1.83846638591236e-10
15.1508276134135
SLC26A6	1.43778933615819	8.5041671641791	7.0976701478815
2.15728569878211e-11	1.86952618809611e-10	15.1335601937527
TPSB2	-3.63850911016949	6.1609855721393	-7.09607831971016
	2.1772167095867e-11	1.88600249146899e-10
15.1245364776853
RANBP1	1.01067754237288	10.625907960199	7.09487842100162
	2.19236038173182e-11	1.89751933545339e-10
15.1177353187947
ZDHHC23	1.36519279661017	7.62499303482587
7.09470037827601	2.19461625533363e-11	1.89867137471553e-10
	15.1167262112841
IGF2BP3	3.7578375	7.00833631840796	7.09357883166098
	2.20887943234161e-11	1.90875964990533e-10
15.1103698755784
RDH10	2.37705557909605	10.586823880597	7.09356419236672
	2.20906620953486e-11	1.90875964990533e-10
15.1102869117264
MT1H	3.68449223163842	4.01069452736318
7.09323110495434	2.21332015699621e-11	1.91163074560927e-10
	15.1083992653294
PTCH1	-1.72278163841808	8.6234736318408	-7.09127080605062
	2.23851984033198e-11	1.93258251984927e-10
15.097291086497
PCDHB19P	-1.72302740112994
2.47522388059702	-7.08605427956475	2.30696505163931e-11
	1.99083617108681e-10	15.0677401735433
VRK1	1.02558566384181	8.86229850746269
7.08523345653169	2.31792125205864e-11	1.99945055902369e-10
	15.0630915041599
FAM89A	-1.76225487288135
6.46914278606965	-7.08195022924314	2.36226067823619e-11
	2.03598705811037e-10	15.0445004083671
HEATR7A	1.04679894067797	9.88399552238806
7.07950886507396	2.39577213951261e-11	2.06313766880008e-10
	15.030679652274
RGS13	-2.23860310734463
1.29180945273632	-7.07809162553145	2.41544055095731e-11
	2.07920312415738e-10	15.022657852713
SFXN5	1.04757874293785	8.62139054726368
7.07757042611346	2.42271372016987e-11	2.08458980197349e-10
	15.0197080230474
TAF9B	-1.00675826271187
8.98749751243781	-7.07598362911063	2.44499019329859e-11
	2.10287593918811e-10	15.0107280353939
EPDR1	-2.63807718926554

8.21867960199005	-7.07538537917656	2.45344114084802e-11
	2.10926074629857e-10	15.0073427370023
CCRL1	-2.27497775423729
3.42109054726368	-7.07160763969792	2.50747514667051e-11
	2.15481225454775e-10	14.9859697115809
YIF1B	1.13474427966102	9.9513815920398 7.06842995689016
	2.55383384685291e-11	2.19189836437668e-10
14.9679968671732
NCRNA00092	-2.60866327683616
3.5354855721393 -7.06690605028172	2.57636473229023e-11
2.21031207496525e-10	14.9593794282948
CCDC152 -1.93226108757062
5.37763582089552	-7.06418667329525	2.61705862109589e-11
	2.24334920630892e-10	14.9440045660091
GRIA1	-2.27992662429379
1.36914925373134	-7.06338399683227	2.62919090394291e-11
	2.25280839974741e-10	14.9394670566531
BOC	-1.97351638418079
9.21776069651741	-7.06249990967802	2.64261788397769e-11
	2.26336859232632e-10	14.9344696920818
LOC642587	3.65070713276836	4.38093930348259
7.06079726031496	2.66866748122704e-11	2.28472658004967e-10
	14.924846402868
GMNN	1.23443057909604	8.63695671641791
7.05891041286854	2.69783106006179e-11	2.30873162413791e-10
	14.9141836590384
SATB1	-2.52249661016949
7.25969701492537	-7.05628772377063	2.73889064626815e-11
	2.34289270699521e-10	14.8993654448885
SLC25A15	1.06066511299435	8.56212686567164
7.05490160736479	2.76083930369184e-11	2.35871955492274e-10
	14.891535209541
GPR44	-1.68491638418079
2.40853432835821	-7.05422247595123	2.7716563231488e-11
	2.36599186337817e-10	14.8876991015953
DSG2	1.64841108757063	10.9345567164179
7.0519335015268 2.80842319221668e-11	2.39538546473653e-10
14.8747713456473
MEIS2	-3.13972111581921
7.40564179104478	-7.05122704532253	2.81986752869544e-11
	2.40414785565271e-10	14.8707819039067
ZFP36	-2.2847415960452
10.9018925373134	-7.05091164163158	2.824991791895e-11
	2.40751687370711e-10	14.8690008593808
VPS13B	-1.15749300847457	8.8905776119403 -7.04996299387165
	2.84045957437542e-11	2.41969440090985e-10
14.8636442542323
HOXD8	-2.33262358757062
6.77304029850746	-7.04893451166172	2.85732341950562e-11
	2.43103397440739e-10	14.857837346952
PI3	4.82363064971751	6.70271641791045
7.04773225051007	2.87716187373671e-11	2.44492396848899e-10
	14.8510499119987
BCAN	-3.04653255649718

4.06139502487562	-7.04772863262068	2.87722177684822e-11
	2.44492396848899e-10	14.8510294880433
ENTPD1	-1.12049420903955
9.87403781094527	-7.04512066448673	2.92072466292195e-11
	2.47881262214913e-10	14.8363084465006
MTR	-1.10615303672316
9.69467910447761	-7.04405286271573	2.93872296610063e-11
	2.49305712785314e-10	14.830282033759
TMEM220	-1.53911829096045
6.26296119402985	-7.04311190203225	2.95467392011719e-11
	2.50555372077679e-10	14.8249719359109
APOBEC3C	-1.97019406779661
7.05690298507463	-7.04277729536795	2.96036664858709e-11
	2.50934464473546e-10	14.8230837619164
TUBB6	-1.47038283898305	10.310847761194	-7.04071914056978
	2.99562104328018e-11	2.53817994298564e-10
14.8114708498253
STEAP4	-2.54872238700565
6.03736517412935	-7.0378418315154	3.0456016737813e-11
	2.57733727793611e-10	14.7952393689559
TACR1	-3.05149350282486
4.96898059701493	-7.03684828889201	3.06305038378687e-11
	2.59103520309619e-10	14.7896355208868
C1orf114	-3.21992309322034
3.56666616915423	-7.03653944510477	3.06849438855647e-11
	2.59457124370117e-10	14.7878936556307
GABRB3	-3.31075162429379
4.15779950248756	-7.03458096797264	3.10323930493858e-11
	2.62286961426057e-10	14.7768490015993
ITPRIP	-1.23297528248587
9.22443731343284	-7.03162231111268	3.15646508988138e-11
	2.66566138606601e-10	14.7601674319171
PABPC1L	1.91342669491525	8.96659950248756
7.03106836149123	3.16653012660802e-11	2.67306182151573e-10
	14.7570446092058
B3GALTL	-1.09284639830509	8.0715552238806	-7.02972041111324
	3.19115420484231e-11	2.69274129985255e-10
14.749446323569
RBMS1	-1.34241292372881
10.1042293532338	-7.02892085161361	3.2058494989415e-11
	2.70403000054515e-10	14.7449396866098
PRSSL1	-1.42774435028249
0.78341592039801	-7.02703398889052	3.24079362984641e-11
	2.73238165177605e-10	14.734305797459
C2CD4C	-2.70711433615819
3.95579402985075	-7.0253745988927	3.27183521412813e-11
	2.75742105689863e-10	14.7249553087513
TCEAL5	-2.51636101694915
4.92653333333333	-7.02523938596344	3.27437746964073e-11
	2.75843124545442e-10	14.7241934566661
CYB561	1.20986709039548	11.425063681592	7.02378458243163
	3.30185400024702e-11	2.78043735131547e-10
14.71599698047
HOXD3	-2.81142026836158

4.86442587064677	-7.0226391534186	3.32364728513931e-11
	2.79764160573637e-10	14.7095442639159
EPS8L2	1.49563580508475	10.9837482587065
7.02167379340116	3.34212460252725e-11	2.81088972101124e-10
	14.704106449369
RAD54B	1.55989251412429	7.44089502487562
7.02092429371753	3.3565400848681e-11	2.82185781909673e-10
	14.6998848736694
COL6A1	-1.83971292372881
12.3271084577114	-7.02030411598885	3.36851459125729e-11
	2.83076563890758e-10	14.6963919124559
KIAA1524	2.02902662429378	7.42694228855721
7.01818996469247	3.40965238871819e-11	2.86299237383985e-10
	14.6844860002166
CPA3	-3.58353785310734
4.10194726368159	-7.01617006337495	3.44941875886679e-11
	2.89401582016899e-10	14.6731128795729
DSCAML1	-3.19536009887006
3.44757960199005	-7.01579580157603	3.45683700494472e-11
	2.89905489017627e-10	14.671005803033
LOC339290	-2.54320049435028
7.22495273631841	-7.01530947970394	3.46649989612405e-11
	2.90597153399048e-10	14.6682679347691
SMARCA2	-1.73739491525424
10.1781885572139	-7.01397116232966	3.4932290554882e-11
	2.92718336772134e-10	14.6607341398873
UTRN	-1.40204117231638
10.1975542288557	-7.00959243110993	3.58210986840604e-11
	2.99799085195173e-10	14.6360909938313
ZNF333	-1.15180670903955
7.47440995024876	-7.00917892280035	3.59061770751022e-11
	3.00388677812489e-10	14.6337642824592
MORC3	-1.22590826271187
8.67593134328358	-7.00821732893351	3.61047938399957e-11
	3.01927257651777e-10	14.6283539464923
CABYR	1.75686355932203	7.25403880597015
7.00602711069037	3.65612343461532e-11	3.05619764302331e-10
	14.6160325206831
TSTD1	1.80555776836158	9.56237711442786
6.99658793083943	3.85943174171592e-11	3.22220958346596e-10
	14.5629575244136
ST3GAL5	-1.85746645480226
7.65244975124378	-6.99396423817438	3.91789562492756e-11
	3.26969094226678e-10	14.5482126067827
LAMP3	2.50570600282486	7.10958805970149
6.99360807787444	3.92589896597435e-11	3.27503883671083e-10
	14.5462112757736
GRM7	-2.35625889830508
1.49317711442786	-6.99028823022591	4.00127934949036e-11
	3.33385815929534e-10	14.5275594014727
MYH14	2.72081723163842	11.5986268656716
6.98879887080344	4.03555861694314e-11	3.36105551342161e-10
	14.51919348727
TJP1	-1.16060240112994

10.8148353233831	-6.98095344468876	4.22096496024898e-11
	3.51119978257341e-10	14.4751425981706
PDLIM4	-1.94294279661017
8.65072537313433	-6.98037330896231	4.23500365711366e-11
	3.52071152074488e-10	14.4718864140467
EXOSC4	1.39421581920904	9.77180895522388
6.98028722274206	4.23709077413912e-11	3.52071152074488e-10
	14.4714032435399
CDH11	-2.45554230225989	8.6435776119403 -6.98026852755346
	4.23754416354632e-11	3.52071152074488e-10
14.4712983147532
PLA2G3	3.27291483050847	4.34499402985075
6.97964749650684	4.25263240852018e-11	3.53043277431607e-10
	14.4678128070715
ANGPT1	-2.34048629943503
4.57574975124378	-6.97964537675579	4.25268399945623e-11
	3.53043277431607e-10	14.46780091039
PNPLA3	2.50080402542373	4.61245472636816
6.97859726057003	4.27826897913436e-11	3.55023694994456e-10
	14.4619188349909
HOXB13	4.76122761299435	4.85726865671642
6.97802964446305	4.29218800951801e-11	3.56034827617797e-10
	14.4587335713932
LSAMP	-3.06473389830509
3.53340447761194	-6.97690880011893	4.31980447558435e-11
	3.58066754567337e-10	14.4524442454914
APOO	1.16827620056498	8.6221263681592 6.975541200252
4.35373805887719e-11	3.60703157178163e-10	14.4447711455117
SCN1B	-1.60326320621469	5.980192039801	-6.97539870752518
	4.35728873882346e-11	3.60851705558877e-10
14.4439717237198
CCDC46	-1.77941016949153
6.75545671641791	-6.97026797381277	4.4870573478339e-11
	3.712990219711e-10	14.4151935584422
QKI	-1.21992584745763
10.0929786069652	-6.96585981121352	4.60158737228554e-11
	3.80469547938067e-10	14.3904785490134
ITM2B	-1.12484209039548
13.5355507462687	-6.96525297595786	4.61757772731499e-11
	3.815727070967e-10	14.3870769821531
MAST1	2.73238848870056	5.82373731343284
6.96521248047458	4.61864674688407e-11	3.815727070967e-10
	14.3868499943458
NUDT14	1.31537775423728	9.5885552238806 6.96496326463943
	4.62523104029903e-11	3.81962965636923e-10
14.3854530918669
DNAJB5	-1.55105155367232	7.9842736318408 -6.9639084294271
	4.65320243379775e-11	3.84118399541085e-10
14.3795408749214
SLC37A1 1.40714837570622	9.19753482587065
6.96169440388351	4.71245578337634e-11	3.88853365083265e-10
	14.3671333143091
PHLPP2	-1.02847627118644
7.11119353233831	-6.95955594109627	4.77039266385368e-11

3.93160021633545e-10	14.3551514906019
KIAA1377	-2.4253020480226
6.29638208955224	-6.95787387290534	4.81645732628541e-11
3.96797226760191e-10	14.3457284226627
HIST1H2AE	2.48240692090396	5.96557512437811
6.95757318329355	4.82473799566191e-11	3.97231964283404e-10
14.344044083659
C8orf55 1.23339668079096	9.97417960199005
6.95754176299228	4.82560408632639e-11	3.97231964283404e-10
14.3438680826654
NEURL3	2.51615911016949	4.41619303482587
6.95718037895391	4.83557654610365e-11	3.97893332631294e-10
14.3418438227176
IRF7	1.67882937853108	9.71013880597015
6.95343568024233	4.94011537554392e-11	4.06169678253571e-10
14.3208719992887
NEK1	-1.23921991525424
7.89217164179104	-6.95213108247421	4.97705579528496e-11
4.09043056353884e-10	14.3135673399814
DHH	-1.8391529661017
2.51338905472637	-6.94712523319512	5.12134273520601e-11
4.20396506812393e-10	14.2855465070155
ATP1A2	-4.38364703389831
4.73333184079602	-6.9450456962616	5.18248752484405e-11
4.25075784598675e-10	14.2739096690475
CLVS2	-1.37318411016949
0.352165671641791	-6.94269546039727	5.25245800004442e-11
4.30470909145357e-10	14.2607605890012
INA	3.83328495762712	5.36090845771144
6.93968309693297	5.34350390831776e-11	4.37583307689524e-10
14.24391101175
PDE1C	-2.44595261299435
1.82214726368159	-6.93930554018562	5.35502433421347e-11
4.3818518751207e-10	14.2417994724023
C15orf48	2.64106483050847	6.67487213930348
6.93884456410686	5.3691233330976e-11	4.39155784974079e-10
14.2392214937102
TBC1D24 1.07627937853107	7.7865184079602 6.93751646726981
5.40994814514791e-11	4.42318739226947e-10
14.231794782888
WDR67	1.23022507062147	7.33162835820896
6.93561908484442	5.46880361772073e-11	4.46811496144812e-10
14.2211861350791
PDK3	1.3688479519774 7.21644925373134	6.93560473004125
5.4692512898778e-11	4.46811496144812e-10
14.2211058812303
GJD3	-1.94220783898305
5.67985174129353	-6.93547355786721	5.47334372823697e-11
4.46967966351253e-10	14.2203725375342
ZFP36L1 -1.24190656779661
12.9192099502488	-6.93513125505965	5.4840374454226e-11
4.4766317596233e-10	14.2184588666105
NOVA2	-1.57454053672317
5.01360597014925	-6.93411588020264	5.51587954960373e-11

4.50083494250257e-10	14.2127826723624
C2orf48 2.19736285310734	2.82706616915423
6.93298421763826	5.55158314945916e-11	4.5281685362891e-10
14.2064569993035
MRC1	-2.2084656779661
5.62735671641791	-6.93089418942266	5.61812259901381e-11
4.58062180134049e-10	14.194775991435
P2RX1	-2.41985176553672	3.4528815920398 -6.92936406102837
5.66733442591075e-11	4.61891130464262e-10
14.1862255849398
F2RL1	2.29395289548023	8.75871890547264
6.92733073383136	5.73338791099347e-11	4.66903823136437e-10
14.1748650723921
CA9	4.02755995762712	6.46297810945274
6.92691187950678	5.74708850606754e-11	4.67788325306648e-10
14.1725251217995
THBS2	-2.49342528248588
8.89270547263682	-6.92680986343693	5.75043030279168e-11
4.67788325306648e-10	14.1719552170291
SLC35A2 1.08177867231639	9.82835273631841
6.92678988672538	5.75108491511924e-11	4.67788325306648e-10
14.171843619298
PLEK2	2.00610451977401	6.86330298507463
6.92513094542574	5.80570341497328e-11	4.71857051106102e-10
14.162576810321
ULBP1	2.44372895480226	4.06544875621891
6.92474275134656	5.81855781946052e-11	4.72714649915016e-10
14.1604085627991
STX2	-1.68781581920904
7.55878109452736	-6.92243042381859	5.89570984220698e-11
4.78793208308976e-10	14.1474946599218
RCVRN	-1.67855677966102
1.48655124378109	-6.91936445916076	5.99956366081117e-11
4.8703456685035e-10	14.1303759072325
ALDOA	1.07445183615819	14.6171507462687
6.91738054308456	6.06772356165242e-11	4.92178446150629e-10
14.11930122414
C6orf176	-2.70621412429379
1.98284925373134	-6.91713889152137	6.0760777556299e-11
4.92661438874731e-10	14.117952401446
TMSB10	1.50639074858756	15.0666273631841
6.91682828648722	6.08683240472981e-11	4.93210199394281e-10
14.1162187446197
TMED3	1.13549244350283	11.5310487562189
6.91656575860578	6.09593701531569e-11	4.93512072276116e-10
14.1147534698769
CHAC1	1.78152507062147	7.10426815920398
6.91655863665634	6.09618419528607e-11	4.93512072276116e-10
14.1147137198586
PTPRF	1.09147838983051	12.9011109452736
6.91610517549563	6.11194272417912e-11	4.94592763608188e-10
14.1121828507156
FAM13B	-1.10191588983051
9.16120199004975	-6.91401351367388	6.18515258621959e-11

5.00319868380962e-10   14.1005101288477
ZNF658 -1.07559230225989
5.27060746268657   -6.91162774907118   6.26971152691867e-11
        5.06361831816052e-10   14.0871987776299
AGFG2   -1.10776165254237
8.68345422885572   -6.91136660955999   6.27903600541979e-11
        5.06915490331374e-10   14.0857419230454
CLCN6   -1.03249124293785   8.432407960199 -6.91065166298829
        6.30463449253232e-11   5.08782020957895e-10
14.0817535257068
KIAA0087   -2.13385169491525
1.47338656716418   -6.90555959541736   6.48995297661045e-11
        5.23531373712426e-10   14.0533542575923
LRP8   1.66695388418079   7.41091044776119
6.9048890104291 6.51475447855753e-11   5.25325645894682e-10
14.049615255106
KRT222 -1.44303008474577
4.89399701492537   -6.90349239942046   6.56670812560062e-11
        5.29307097835025e-10   14.041828840577
ZNF154 -1.38997330508475
3.15586865671642   -6.9028013946544   6.59256410516902e-11
        5.31182657296389e-10   14.0379766937661
PPP1R15A   -1.07422584745763
10.7579482587065   -6.90135953619605   6.64683893728392e-11
        5.35345639005252e-10   14.0299395218469
AFF3   -2.53165621468927
6.51438109452736   -6.89935715300904   6.72294410461792e-11
        5.41262911638455e-10   14.0187795955385
MRPS12 1.23167478813559   9.86883830845771
6.89731151491203   6.80157976979761e-11   5.47379195115425e-10
        14.0073806481342
C4orf31 -3.25083248587571
4.69260995024876   -6.89704322956537   6.81195982111355e-11
        5.47999745797262e-10   14.0058858305946
HIST1H4D   1.41601716101695   1.79253631840796
6.89390044497924   6.93472366749648e-11   5.57657175455161e-10
        13.9883776951296
ADCY9   -2.23406906779661
7.78894378109453   -6.89350331559905   6.95039068470531e-11
        5.58479533295499e-10   13.9861656762267
WBSCR17 -3.86474597457627
5.48155373134328   -6.89229255167119   6.99837167100678e-11
        5.62114907690802e-10   13.9794221791023
C17orf58   -1.13726355932203
8.03261243781095   -6.88936204875505   7.1158586097644e-11
        5.71104680447471e-10   13.9631033989331
SAMD3   -2.15648015536723
4.16429751243781   -6.88663278107243   7.22702434307237e-11
        5.7979995999717e-10   13.9479090523981
MOSC1   1.54529731638418   8.53149253731343
6.88237302858616   7.40395622586136e-11   5.93762583269273e-10
        13.9242016080156
CITED2 -1.85899540960452
9.61759502487562   -6.88165119154187   7.43435818692528e-11

5.9596787808503e-10	13.9201851533378
SAC3D1	1.41992245762712	8.77433781094527
6.8804090192002	7.48696353801351e-11	5.99950669146828e-10
13.9132740493936
EIF4EBP1	1.62418891242938	10.1468850746269
6.87707648594762	7.62991300140754e-11	6.11167046113527e-10
13.8947365482591
HTRA3	-2.17542690677966
8.81575472636816	-6.87536544628009	7.70434954376351e-11
6.16648328005711e-10	13.8852208922267
SGPP2	2.33337252824859	6.85335671641791
6.87406228050424	7.76152156120984e-11	6.20740310290759e-10
13.8779745357665
ELMOD1	-2.00858566384181
1.76727462686567	-6.8734653251307	7.78785035389516e-11
6.22603457030637e-10	13.8746554007293
EFHD1	-2.22207281073447
8.41007014925373	-6.87125225895095	7.88622787297438e-11
6.29977658490911e-10	13.8623520682729
HCG4	-2.78529668079096	2.6054552238806 -6.86838539500249
8.01549068917264e-11	6.39308561960817e-10
13.8464176393213
GREM2	-4.33295134180791	5.2425368159204 -6.86544307264144
8.15032589031217e-11	6.4981044865285e-10
13.8300680652323
LGALS7	3.43656687853107	4.3336263681592 6.86304142505801
8.26203820724503e-11	6.58461352464055e-10
13.816726056354
KDELC2	-1.36157245762712
9.53629502487562	-6.86279681574695	8.27350046035181e-11
6.59118992825079e-10	13.815367325991
IL17RE	1.77239456214689	7.36886119402985
6.86127382686579	8.34522006226698e-11	6.645747396367e-10
13.8069082580004
SLC26A4 -1.98996588983051
3.11900447761194	-6.86115411939013	8.35088313882241e-11
6.64767859015216e-10	13.8062434213164
OR2B6	2.1018375	2.04233432835821	6.85391299023732
8.70057668638878e-11	6.92068343167616e-10
13.7660406374112
CCNE2	2.31031751412429	6.54092537313433
6.85013713879376	8.88860434941835e-11	7.06477147865113e-10
13.7450874954996
LOC26102	-1.51632281073446
1.31130149253731	-6.84986601754486	8.90225846737056e-11
7.07288569408349e-10	13.7435832506475
ASXL3	-2.89824209039548
2.70883333333333	-6.84959905712901	8.91572322501185e-11
7.08084324214674e-10	13.7421021270446
ZDHHC15 -2.99222422316384
4.49487711442786	-6.84882226910337	8.95501651071257e-11
7.10929965061598e-10	13.7377926308864
GSPT2	-2.75116165254237
5.79104776119403	-6.84820910679774	8.98615332116208e-11

7.12850570647054e-10	13.7343911175554
PER2	-1.04096850282486
8.61621791044776	-6.84690795843164	9.0525802129158e-11
7.17842687412751e-10	13.7271736304941
CORO1C	-1.01741687853107
10.8442199004975	-6.84591180062306	9.10376324523735e-11
7.21622623261478e-10	13.7216485033219
FLT3LG	-1.51495176553673	6.6030671641791	-6.84524199928866
9.13833808883919e-11	7.24083677977107e-10
13.717933771564
SULF2	-2.24809936440678
10.8490179104478	-6.84351130719717	9.22827562165739e-11
7.30645964182901e-10	13.7083363593463
HIST1H2BJ	2.30328679378531	5.47643830845771
6.84266070809445	9.27279702489645e-11	7.33887906403717e-10
13.7036199815755
SLC2A13	-1.68828502824859
6.51775124378109	-6.84257161141547	9.27747267738034e-11
7.33975006037065e-10	13.7031259818279
ECHS1	1.07507923728813	12.0809532338308
6.84216008294198	9.29909919049094e-11	7.35402566952153e-10
13.7008442990316
FAM100B	1.1780593220339	10.3473955223881	6.84053665592592
9.38489837128847e-11	7.41616487923604e-10
13.6918441816412
PDCD4	-1.07389597457627
11.0001179104478	-6.84022201713998	9.4016172124212e-11
7.42651794424806e-10	13.6901000085457
MYLIP	-1.29027782485876
9.70812686567164	-6.83898955464355	9.46738907822131e-11
7.47272194447841e-10	13.6832684368378
PSMG3	1.23438573446328	10.1204213930348
6.83854505135152	9.49122156823405e-11	7.48865406594331e-10
13.6808047305358
MSI2	1.1080834039548	7.69227910447761	6.83452523314382
9.70944915478216e-11	7.65495357709976e-10
13.6585289690179
CHKA	1.05196391242938	9.75254378109453
6.83354573222772	9.76336861059642e-11	7.69155631525497e-10
13.6531023090748
IDH1	1.42770176553672	11.4185507462687
6.82879598802067	1.00290429633767e-10	7.87968817597106e-10
13.6267944727399
EIF2AK2	1.39106737288136	8.58101144278607
6.82802144149667	1.00730368303829e-10	7.91122594214846e-10
13.6225054975988
APOL3	-1.98756377118644	8.1334447761194	-6.82732545260447
1.01127307566747e-10	7.93632883924048e-10
13.6186517849745
GLB1	1.06733629943503	11.022068159204	6.82702026215595
1.01301850535772e-10	7.94698888612686e-10
13.6169620126007
GTF2IRD2P1	-1.43002966101695
7.71037014925373	-6.82513649488083	1.02385791560154e-10

8.02895454824279e-10	13.6065330448273
RASL11A -1.9125113700565
6.51759402985075	-6.82313987013606	1.03547142327194e-10
8.11692566619816e-10	13.5954812215991
TSPAN4	-1.32785035310734	9.3316447761194 -6.82108943938867
1.04753291387484e-10	8.20833997017191e-10
13.5841336572632
RAP1GAP 2.29148368644067	9.51419452736318
6.82050373920604	1.05100358162365e-10	8.23239356380521e-10
13.580892643931
LOC100271722	-1.64351059322034
5.09010447761194	-6.81964878440324	1.05609010351342e-10
8.26908078761653e-10	13.5761620013367
L2HGDH	1.12557450564972	7.69855671641791
6.81858743555553	1.06243829851445e-10	8.31370558277469e-10
13.5702898499077
ANGPT4	-2.0186938559322
1.15949004975124	-6.81598444385966	1.07816698615281e-10
8.43159272980183e-10	13.5558906156701
RRAS	-1.43511511299435
9.56665024875622	-6.815882907054 1.07878515138412e-10
8.43159272980183e-10	13.5553290033916
TMEM48	1.42542542372881	8.09732537313433
6.81524937288394	1.08265005525235e-10	8.45768859753833e-10
13.5518249671294
ANKDD1A -1.27334435028249
6.73701343283582	-6.81435172668991	1.08814954472708e-10
8.49741732721454e-10	13.5468604909694
ERO1L	1.56451165254237	9.83700845771144
6.81391108944408	1.09085919599909e-10	8.51533813454807e-10
13.544423673829
TOR1AIP1	-1.04568107344633
10.0208263681592	-6.80886136400389	1.12239035140562e-10
8.75149028270318e-10	13.516504618078
WDR4	1.01673834745763	7.99854925373134
6.80827132789544	1.12613267612351e-10	8.77733631010465e-10
13.5132432505704
NIPAL2	-1.96806814971751
5.00478756218905	-6.80744820077956	1.13137392432356e-10
8.81364063550893e-10	13.5086937887398
TMEM35	-2.82059420903955
5.43213482587065	-6.807251298967 1.13263124951261e-10
8.81460180155189e-10	13.5076055539812
DLL1	-2.0184802259887
6.82383233830846	-6.80515833987328	1.14608127226191e-10
8.90739523119782e-10	13.4960394211667
RASAL1	3.07235437853108	7.40414776119403
6.80512601677506	1.1462902192316e-10	8.90739523119782e-10
13.4958608142688
TNFRSF12A	1.64114731638418	10.0916810945274
6.8025500041492 1.16306358415155e-10	9.03431531692445e-10
13.4816283063924
GTF2IRD2B	-1.37189307909605
6.82085671641791	-6.80204531487936	1.16637800311761e-10

9.05320998261044e-10 13.4788402836306
SELM -1.89509378531074
10.0008527363184 -6.80104146519561 1.17299820446138e-10
9.10115386908242e-10 13.4732951636174
CYP11A1 -2.97515875706215
3.70471741293532 -6.79766713761964 1.19552364343474e-10
9.26542106444513e-10 13.4546596033532
RBP5 -1.79257231638418
6.17729950248756 -6.79716409419288 1.19891800902297e-10
9.28822140575154e-10 13.4518819147401
GNG12 -1.37362436440678
10.3975696517413 -6.79560397092981 1.20950565832386e-10
9.36671111482039e-10 13.4432680920186
PHF21B -3.51686744350283
3.59516119402985 -6.79466001514408 1.21595638686701e-10
9.41311637344631e-10 13.4380568791284
TNNI3 3.68706581920904 6.20336915422886
6.79354573920105 1.22361464312666e-10 9.46526323413352e-10
13.4319059754614
C18orf1 -1.81851158192091
7.98870746268657 -6.79277131710615 1.22896508796902e-10
9.50307090621619e-10 13.427631466022
PSME2 1.33671024011299 11.796423880597 6.79211345850458
1.23352829317067e-10 9.53476500707601e-10
13.4240005804487
ECM1 -2.25347175141243
8.93599950248756 -6.78717101930362 1.26834896435074e-10
9.8002274136698e-10 13.3967290393205
AHDC1 -1.00176087570622
9.77782736318408 -6.78670680491668 1.27166876106611e-10
9.8221819656461e-10 13.3941682192106
ANGPTL5 -1.56546765536723
0.660062189054726 -6.78639703110854 1.2738888449197e-10
9.83562917871437e-10 13.3924594251723
MPDU1 1.03502740112994 10.4522587064677
6.78500000613607 1.28394853670965e-10 9.90957272881549e-10
13.3847536715804
FBXO22OS 1.11355494350282 6.23066815920398
6.78458231505334 1.2869714054388e-10 9.92917059513661e-10
13.3824499504202
LRFN4 1.35489837570621 9.29014029850746
6.78442462774038 1.28811442419297e-10 9.93052539567465e-10
13.3815802694486
CDK2AP2 1.27333050847457 11.1860577114428
6.78270138558922 1.30067092207109e-10 1.00198026379436e-09
13.3720770239865
SLC2A1 1.6810052259887 11.7048631840796 6.7809169935842
1.31379999583731e-10 1.01133535487589e-09 13.3622381407884
NOVA1 -3.22650338983051
4.71251741293532 -6.78073549620525 1.31514269318327e-10
1.01198948617138e-09 13.3612374806727
GOLT1A 2.91102810734463 6.26360099502488
6.77804988417266 1.33516917922327e-10 1.0270147339623e-09
13.3464326937739

PRPH2	-2.20846080508475
3.72409850746269	-6.77776666813932	1.33729852950997e-10
	1.02805830286397e-09	13.3448716419486
PRKCD	1.20709823446328	10.2545462686567
6.77773625509173	1.33752738770076e-10	1.02805830286397e-09
	13.3447040114062
LOC154822	-2.07540875706215
2.77881791044776	-6.77640175136545	1.34760758834173e-10
	1.03503119299124e-09	13.3373489605769
PHF2	-1.18446956214689
9.80553880597015	-6.7719841560869	1.38151254583186e-10
	1.05988238288222e-09	13.3130080617253
CLDN3	2.66450353107344	11.8353621890547
6.76947696113343	1.40112736757616e-10	1.07452913172725e-09
	13.2991978611137
CYP2A6	-2.27114668079096
1.08039751243781	-6.76881382911176	1.40636101072204e-10
	1.07814008775667e-09	13.2955457137948
CENPW	1.61807492937853	8.91327611940299
6.76472104768073	1.43909112780567e-10	1.10281974071857e-09
	13.2730099970023
WISP1	-2.34361631355932
5.03963930348259	-6.75824812669458	1.49239234528139e-10
	1.14323936002339e-09	13.2373861502419
PPAP2B	-1.98860557909605
10.1996606965174	-6.75796258228098	1.49478789066299e-10
	1.14464734782959e-09	13.2358151429025
FLJ43663	-1.79532429378531
5.49384278606965	-6.7555190830541	1.5154427815443e-10
	1.16003133128652e-09	13.2223732074628
CADM3	-3.36639894067797
4.27553830845771	-6.75445130857185	1.52455665475002e-10
	1.16629170826432e-09	13.216500228937
RCAN3	1.6540895480226 6.56143631840796	6.75309387946314
	1.53622077800775e-10	1.17462244217874e-09
13.2090349307074
AVPR2	-2.83739442090396	3.8627447761194 -6.75276825840826
	1.53903181492598e-10	1.17633369919696e-09
13.2072442895692
MAP3K12 -1.47874519774011
7.83268805970149	-6.75220028859158	1.54394713618952e-10
	1.17965145909829e-09	13.204121064227
C6orf123	-2.23976949152542
1.65723233830846	-6.75127866509503	1.55195594906105e-10
	1.18532945067795e-09	13.1990534731316
AEBP1	-2.07264766949152
11.4834034825871	-6.75108693101311	1.55362723034973e-10
	1.18616463514615e-09	13.1979992685731
ZFP2	-1.42404060734463	5.4068815920398 -6.74595555566236
	1.59901984991581e-10	1.2199136944917e-09
13.169792590787
CHRD	-2.68957853107345
7.24409850746269	-6.74552058269	1.60292714892001e-10
1.2224403553985e-09	13.1674022048306

SLC25A42	-1.06567252824859
7.94887611940299	-6.74340898878062	1.62202925468848e-10
	1.23654885253452e-09	13.1557993542361
AKAP12	-2.36990621468926
9.31090099502488	-6.74256076483849	1.62976547200692e-10
	1.24152449500193e-09	13.1511391494909
TCTEX1D1	-1.87722196327684
2.76251144278607	-6.74227314992514	1.63239689776407e-10
	1.24306781569348e-09	13.1495590555571
FAM18A	-1.47322803672316
4.21264228855721	-6.74145423755031	1.63991216085126e-10
	1.24832764784117e-09	13.1450603604482
SYNGR3	2.41859025423729	5.33919502487562
6.74017675913095	1.65170380087856e-10	1.25637195376202e-09
	13.1380432180003
KLHL13	-2.66339837570622
7.57196517412935	-6.73990792508574	1.65419585677473e-10
	1.25780151628093e-09	13.1365666287551
RANBP3L	-1.75448792372881
1.23439900497512	-6.73827064279184	1.66945335601107e-10
	1.26846326420827e-09	13.1275745433867
DKFZp434J0226	-1.58572224576271
1.11862189054726	-6.73775747409313	1.6742639216539e-10
	1.27117745235039e-09	13.1247564623317
PLEKHN1	2.32214597457627	5.44567064676617
6.7339328518394	1.71054922456714e-10	1.29776702070818e-09
13.1037576850649
HOXD9	-1.7551320621469	8.0527776119403	-6.73352152021277
	1.71449744919386e-10	1.30028195906723e-09
13.1014997485952
FAM83F	3.03711843220339	6.14601492537313
6.73289544355625	1.72052415811139e-10	1.30437078899656e-09
	13.098063171482
BST1	-2.05073947740113
5.36014577114428	-6.73181854636435	1.73093935364915e-10
	1.31178238945799e-09	13.0921524799823
CA4	-2.8659895480226
2.08990149253731	-6.72737371201844	1.77458816451887e-10
	1.34287854838085e-09	13.0677627164422
FAAH2	1.97249562146893	7.61151542288557
6.72456714334643	1.80270515062139e-10	1.36365279079798e-09
	13.0523676905297
SEPT10	-1.39658036723164
9.42790049751244	-6.72228911272932	1.8258491086249e-10
	1.38014293814688e-09	13.0398748568857
PRKD1	-2.07393933615819
6.85219104477612	-6.72151815964946	1.83374775334739e-10
	1.38560328951866e-09	13.0356475153421
GFRA1	-3.54204618644068
5.48289303482587	-6.71897377102784	1.86005518660179e-10
	1.40496442166795e-09	13.0216981203406
ARVCF	1.05593163841808	9.74478358208955
6.71748768273891	1.87559191172547e-10	1.41617881381846e-09
	13.0135523064245

WDR17	-2.74510628531074
2.90658009950249	-6.71724329219341	1.87815916200535e-10
	1.41759586749889e-09	13.0122128178018
FGF13	-2.83695769774011
5.77386268656716	-6.7152550582224	1.89917389833303e-10
	1.43240411949953e-09	13.0013165785382
SPTBN1	-1.13055211864407
12.0227641791045	-6.71479500666044	1.90406930901046e-10
	1.43556896489111e-09	12.998795619696
ZMIZ1	-1.33252104519774	10.977344278607 -6.71407665524341
	1.9117381371438e-10	1.44082173111462e-09
12.994859464841
ACOT4	1.45372507062147	6.33504179104478
6.71373382157578	1.91540879849718e-10	1.4430584452531e-09
	12.9929810260348
LPCAT1	1.11384194915254	10.9077208955224
6.71190842339042	1.93507012810843e-10	1.45733638041328e-09
	12.982980404736
TEAD1	-1.17345254237288
10.2178945273632	-6.70920730831519	1.96452873838343e-10
	1.47843749996378e-09	12.9681852301226
IGF2AS	-3.05916362994351
2.40590547263682	-6.70700277760121	1.98889830165154e-10
	1.49622873334211e-09	12.9561128472437
KCNK3	-3.04176687853107
5.07313333333333	-6.70659693779185	1.99341689586135e-10
	1.4990787132613e-09	12.953890672708
CNN3	-1.21666906779661
11.6449631840796	-6.70522669594088	2.00874785084243e-10
	1.51005468245313e-09	12.9463885449864
MMP9	2.99558580508475	8.75084975124378
6.70202532517077	2.04502010757254e-10	1.53675925360411e-09
	12.928864686723
IGF1	-2.85652740112995
8.07500049751244	-6.70133201252422	2.05296000445294e-10
	1.5417632468341e-09	12.9250702858965
VAMP5	-1.70500925141243
8.31897810945274	-6.69590500248368	2.11616788835581e-10
	1.58616015874205e-09	12.895377605675
SLC6A1	-1.94031970338983
3.21278109452736	-6.69335721998782	2.14649852248833e-10
	1.60713401410231e-09	12.8814432240161
LMAN2	1.03828531073447	12.397744278607 6.69305205573708
	2.15015998450386e-10	1.60928853379016e-09
12.8797744385959
RAB41	-1.02653573446328
1.27832736318408	-6.69162553383685	2.16735755180345e-10
	1.62097816169489e-09	12.8719741664435
PLA2G5	-2.18023622881356
1.76970298507463	-6.69118788626829	2.17266073559423e-10
	1.62435269125089e-09	12.8695813048654
KLHL21	-1.07174491525424
9.67744129353234	-6.68978706905797	2.18972109931413e-10
	1.63591609057202e-09	12.8619229270917

EPAS1	-1.54789858757062
10.8009084577114	-6.688581597807 2.20450785746932e-10
1.64636399832103e-09	12.8553333331092
TPSAB1	-3.25998248587571
5.57493582089552	-6.68603529777551	2.23606545688301e-10
	1.66871769646704e-09	12.8414166961651
C1orf130	2.72688841807909	7.53963980099503
6.68586471865916	2.23819534754373e-10	1.66970023564945e-09
	12.8404845269771
EHBP1L1 -1.03346560734464
9.93911243781094	-6.68544439209249	2.24345216758144e-10
	1.67301391211213e-09	12.8381876191078
CSRNP1	-1.3486738700565
9.70000348258706	-6.68468141901507	2.25302536242167e-10
	1.67954287184157e-09	12.8340185257636
MESP1	3.19312323446328	5.95534228855721
6.68266299742602	2.27854521763739e-10	1.69795039267135e-09
	12.8229907714414
KLHL35	2.08304901129943	5.7810184079602 6.68221892815043
	2.28419785447032e-10	1.70154506358039e-09
12.8205648582182
CD36	-2.44045247175141
5.81143233830846	-6.67880591916815	2.32810570362308e-10
	1.73299528989782e-09	12.8019232846984
KRT37	-1.54675268361582
0.479025870646766	-6.67839420556247	2.33345815378584e-10
	1.73634997815235e-09	12.7996749467711
LOC541471	1.5728261299435 7.77960099502488
6.67659453541812	2.35699712201646e-10	1.75323010561587e-09
	12.7898481106553
TSPAN5	-2.41725656779661
4.87498358208955	-6.67550227394981	2.37139724224591e-10
	1.76330262163378e-09	12.7838847949038
MPHOSPH8	-1.02001906779661
9.04104925373134	-6.67302620723319	2.40436245893938e-10
	1.78652049518731e-09	12.7703687400382
FAM125B -1.0795822740113
8.15631890547264	-6.66905099490932	2.45823203579424e-10
	1.8245662941018e-09	12.74867598092
DMBX1	3.58469759887006	3.67143233830846
6.66696273448882	2.48700483727016e-10	1.84458848660247e-09
	12.7372836162814
NEURL	-3.16643516949153
4.33910497512438	-6.66563744810198	2.50543672288481e-10
	1.85758815170911e-09	12.7300547798412
HIST1H4E	1.73756264124294	2.91073830845771
6.66348033497854	2.53572535892592e-10	1.87936612342055e-09
	12.718290656671
LGI1	-1.79771038135593
0.785245771144279	-6.66311122324274	2.54094413732985e-10
	1.88255442581674e-09	12.7162778955725
KLF10	-1.28789307909604
9.76919353233831	-6.66227222599129	2.55284585277882e-10
	1.89000812684995e-09	12.7117031187524

ARHGAP23	-1.78751970338983
8.98166965174129	-6.66133435320659	2.56621511612505e-10
	1.89853680483053e-09	12.7065896391685
PCDHA3	-3.02385628531074
3.86209701492537	-6.66093399232241	2.57194317063773e-10
	1.9020890955761e-09	12.7044069267217
SLC39A4 1.92396596045198	9.80766368159204
6.65889816731954	2.60126537316236e-10	1.92238942084317e-09
	12.6933091783476
LOC100128239	-2.03680480225989
1.93237910447761	-6.65564509990997	2.64880372021135e-10
	1.95560822893231e-09	12.6755804374069
LYPLAL1 -2.5162145480226	6.1138039800995 -6.65564488818769
	2.64880684183904e-10	1.95560822893231e-09
12.6755792837307
SSTR3	-1.69991645480226
1.07267114427861	-6.65562684978958	2.64907281279141e-10
	1.95560822893231e-09	12.675480992456
CABP1	-1.79328403954802
3.42828109452736	-6.6549101244009	2.65966201114724e-10
	1.9619574493333e-09	12.67157569113
DLG2	-3.00705169491526
4.16784925373134	-6.65485079397296	2.66054044761028e-10
	1.9619574493333e-09	12.6712524228124
SGCE	-2.37832810734463
7.89585970149254	-6.65405590004027	2.6723370830138e-10
	1.96994902385182e-09	12.6669215331171
FOXF1	-1.61416334745763	5.5442184079602 -6.65222576100839
	2.6996932784246e-10	1.9886868678169e-09
12.6569514781855
TTC28	-1.22662252824859
9.46676069651741	-6.65053043191003	2.72528011384698e-10
	2.0068149475351e-09	12.6477173841171
PFDN6	1.0987720338983 10.0622587064677	6.64779797003001
	2.76702295689033e-10	2.03536299910277e-09
12.6328374018676
ADRB2	-2.31580466101695
3.98783432835821	-6.64618573995465	2.7919468217905e-10
	2.05222585933974e-09	12.624059615348
RGN	-3.03439894067797
4.41482985074627	-6.64218218650794	2.85479795472189e-10
	2.09692314885297e-09	12.602268131185
FA2H	2.31322570621469	6.22363631840796
6.64103582643487	2.87304941418396e-10	2.10957455197413e-09
	12.5960299924363
FAM71E1 1.84124456214689	6.8148736318408 6.6406771537125
2.87878346592191e-10	2.1130291224661e-09	12.5940783467197
WFS1	-1.37755381355932
10.9151885572139	-6.63862354890093	2.91183158898676e-10
	2.13575928981415e-09	12.5829053608277
ARL15	-1.58270903954802
7.31687114427861	-6.63710775884896	2.93646394077751e-10
	2.15305731086294e-09	12.5746598600567
ZHX1	-1.09601723163842

8.75503383084577	-6.63683716892623	2.94088267567935e-10
	2.15552735921803e-09	12.5731880479971
DNAJC18 -1.42860734463277
6.98917263681592	-6.6366754306592	2.94352698524537e-10
	2.15669553915373e-09	12.5723083279104
FLJ42289	-1.62998093220339
3.38073532338308	-6.63343537818815	2.9969951073889e-10
	2.19430490565956e-09	12.554688047994
VEGFC	-1.61340939265537
7.39639900497512	-6.62934233888694	3.06590697360688e-10
	2.24316002024766e-09	12.532436840234
SAMD5	-2.33310713276837
5.84726865671642	-6.62777862696675	3.09264367299236e-10
	2.26111020678536e-09	12.523938263011
TSPAN7	-2.59761031073447
8.28491044776119	-6.62411358548109	3.15621143905422e-10
	2.30676471497982e-09	12.5040242390523
FNBP1	-1.38228827683616
10.2885860696517	-6.62199478159887	3.19354520146468e-10
	2.3323900030619e-09	12.4925149143085
DCLK2	-1.87027930790961
6.33700845771144	-6.62026712378044	3.22430811567183e-10
	2.35402011432229e-09	12.4831320322784
SHC2	-2.44255670903955	7.8256960199005 -6.61675776575878
	3.2876963831736e-10	2.39859299028266e-09
12.4640775810482
C11orf80	1.15544653954802	7.89101194029851
6.61607066092119	3.30025003685454e-10	2.40689638567047e-09
	12.4603476245081
AMZ2P1	-1.0026800141243
7.40167213930348	-6.6134971151963	3.34769006966845e-10
	2.43716585568416e-09	12.4463793026552
DACT1	-2.4092593220339
6.31718009950249	-6.61255327431713	3.36525615549123e-10
	2.44908574520507e-09	12.4412573296253
ULBP3	1.97418651129944	5.65925024875622
6.61202531242257	3.37512171073972e-10	2.45452528237642e-09
	12.4383924249695
PIK3IP1 -1.21625444915255	9.1821407960199 -6.61153743890416
	3.38426345700727e-10	2.46030201035266e-09
12.4357451838359
DGAT2	2.02733446327684	7.26459800995025
6.60850725603215	3.44159072161377e-10	2.5010923014064e-09
	12.4193059651853
SEPT3	2.75061843220339	6.03179054726368
6.60618461179174	3.48617790104857e-10	2.53259845394646e-09
	12.4067085198573
GRASP	-1.66618255649718
7.64447711442786	-6.60552158943526	3.49900967553305e-10
	2.54102117575853e-09	12.4031129713734
MASTL	1.14171433615819	8.36191940298507
6.60429301272688	3.52290979287335e-10	2.55747305684901e-09
	12.3964510509026
RRAGB	-1.07928559322034

7.99923432835821	-6.60366543289555	3.53518030375285e-10
	2.56547372343747e-09	12.3930483241946
NIPSNAP3B	-1.61964512711865
4.97919950248756	-6.60231935129792	3.56164099193973e-10
	2.58376288213861e-09	12.3857505920636
TRIM9	-2.54581271186441
4.75311492537313	-6.59831089970733	3.64159652560387e-10
	2.63990030616693e-09	12.3640245629639
ZNF532	-1.27535812146893
10.1588776119403	-6.59598428355829	3.68881190639957e-10
	2.67129835761493e-09	12.3514180530108
PDGFD	-1.97011701977401
7.53003333333333	-6.59514713608568	3.70594750059287e-10
	2.68276100801028e-09	12.3468827600499
DLG4	-1.39059194915255
8.26945721393035	-6.59078934188451	3.79641965667693e-10
	2.74535031882977e-09	12.3232801299577
WDR34	1.31208008474576	11.3073388059701
6.59066030759221	3.7991313863696e-10	2.74634392120309e-09
	12.3225814085773
DNM1P35	-1.65363446327683	4.7646144278607	-6.58984263875286
	3.81635950330645e-10	2.75782684276246e-09
12.3181539314638
PTCH2	-2.34097323446327
5.47134179104478	-6.58898069288297	3.83460371722376e-10
	2.77003569016903e-09	12.3134870866317
GLIS1	-2.36570939265537	3.5836592039801	-6.58636806095739
	3.8904291245064e-10	2.80739929441534e-09
12.2993438726825
NPHP3	-1.37668057909605
8.20932835820896	-6.5811804606344	4.00365465978926e-10
	2.88808960525205e-09	12.2712720068642
FMOD	-2.4330988700565
10.0654203980099	-6.57928197933115	4.04589612691918e-10
	2.91548780223415e-09	12.261002231303
RNF24	1.02391652542373	7.8489328358209	6.57895960821326
	4.05311232887873e-10	2.91966302146948e-09
12.2592585638393
KERA	-1.6753540960452
1.22032985074627	-6.5779254554028	4.0763471331347e-10
	2.93537027861296e-09	12.2536653234671
OTUD1	-1.33937754237288
8.13337313432836	-6.57617959270076	4.11586983479966e-10
	2.96279129412472e-09	12.2442240653023
FAM198A	-2.36608029661017
6.11931592039801	-6.57374781442507	4.17154890327079e-10
	3.00181910214334e-09	12.2310762049432
ARRB1	-1.4683156779661
7.22954726368159	-6.57110204782475	4.2329690188238e-10
	3.04494933274186e-09	12.2167749289972
SPTBN4	-2.39300967514124
4.80363532338308	-6.5698655121817	4.26197865265482e-10
	3.06474331835389e-09	12.2100922995369
IRAK3	-1.92896320621469

6.01271492537313	-6.56899186412495	4.28259256508711e-10
	3.07848828295653e-09	12.2053713170846
MYO15B	-1.80726059322034
9.13975671641791	-6.56833943092246	4.2980507840875e-10
	3.08851881684692e-09	12.2018459896056
PRRX1	-2.69529322033899
9.16129950248756	-6.56223390179994	4.44539526336756e-10
	3.19104771877399e-09	12.1688665797052
HIST1H3B	1.74940798022599	1.82241094527363
6.56012549399041	4.49742228024177e-10	3.22726597040768e-09
	12.1574824667107
NCRNA00219	-1.22583728813559
9.01733134328358	-6.55917072043707	4.52117876085873e-10
	3.24317959330642e-09	12.1523280485681
RAX2	-2.05353396892655
0.885209950248756	-6.55893235663206	4.52712890214337e-10
	3.24631352989883e-09	12.1510412987171
CCDC89	-2.62580169491526
4.36576019900498	-6.55651485697221	4.58791232078346e-10
	3.28875139475155e-09	12.1379927091159
SPAG4	1.97229145480226	7.53753980099502
6.5560830697225	4.59885293407457e-10	3.2954433066859e-09
12.135662439619
OTUB2	1.40572175141243	6.98382437810945
6.55545924304862	4.6147047552582e-10	3.30564859125788e-09
	12.1322959464367
ATP6V0E2	1.64025176553672	10.3760049751244
6.55038809341457	4.74557505629792e-10	3.39702426449778e-09
	12.1049370505928
FRMD3	-1.9575979519774
2.86828258706468	-6.54784107323867	4.81267543685758e-10
	3.44265598322948e-09	12.0912010075111
ATAD3B	1.26221405367232	9.4457144278607 6.54660829623488
	4.84548668752658e-10	3.46371315093735e-09
12.084553896982
STX1B	-1.94625296610169
3.62431044776119	-6.54512327174356	4.88530383701092e-10
	3.48974557320231e-09	12.0765477454569
MAML3	-1.49130183615819
8.19768905472637	-6.54454713029634	4.90083806587053e-10
	3.49962453886337e-09	12.0734419334446
MCHR1	-2.28004025423729
2.42346169154229	-6.54403614222346	4.91465622656274e-10
	3.5082716387807e-09	12.0706874921467
C17orf108	-1.15798559322034
7.66519800995025	-6.54305911484902	4.94118376818412e-10
	3.52475687146699e-09	12.0654212890051
ZNF441	-1.31448898305085
6.85991492537313	-6.54051747576777	5.01085375239897e-10
	3.57197317835941e-09	12.0517241666244
UBE2QL1 -1.98460007062147
4.68541293532338	-6.53817635890059	5.0758811490124e-10
	3.61456260802028e-09	12.0391107183166
SLC6A3	-2.05702125706215

1.47238905472637	-6.53610631307269	5.13406986727836e-10
	3.65473142771237e-09	12.0279601749886
MPPED1	-2.16538107344633	1.2804552238806	-6.53557242686855
	5.14918341230251e-10	3.6642195998118e-09
12.02508470488
LOC100144603	1.72424053672316	3.55298606965174
6.53471499246521	5.17354772175697e-10	3.68028186859565e-09
	12.0204669467649
C18orf18	-2.22476101694915
4.13871542288557	-6.53414198733117	5.18989301390055e-10
	3.68935261687598e-09	12.0173812157995
DLX3	3.02607139830508	5.23927860696517
6.53359469495907	5.20555223468575e-10	3.69794299287276e-09
	12.0144341158221
ABO	-2.90659385593221
2.65947462686567	-6.53359373433446	5.20557976103374e-10
	3.69794299287276e-09	12.0144289431208
MNX1	3.63032733050847	4.38195124378109
6.52977123289529	5.31625306329473e-10	3.77311313538168e-09
	11.993849711516
EIF4E3	-1.36862528248588
8.84610099502487	-6.52968596972176	5.31874788786876e-10
	3.77311313538168e-09	11.9933907683091
TIMM8A	1.00352217514124	7.64024029850746
6.52954820082315	5.32278147745723e-10	3.77467024981682e-09
	11.992649212501
C6orf125	1.09850889830508	10.6867686567164
6.5263132398718	5.41836357747356e-10	3.8411258372076e-09
11.9752395997591
ANO4	-2.80799971751412
3.81203383084577	-6.52575110580945	5.43514394198019e-10
	3.85169158194178e-09	11.9722149282478
SH2B3	-1.21137153954803
8.39026567164179	-6.52505528005636	5.45598593339796e-10
	3.86505439093772e-09	11.9684711362119
GSDMC	2.30552733050847	3.01830298507463
6.52499598725656	5.45776555252238e-10	3.86505439093772e-09
	11.968152131639
C1orf116	2.98123255649717	7.26550199004975
6.52428194345123	5.47924179123529e-10	3.87892530945036e-09
	11.964310611232
HPCA	-1.73309724576271
2.88191890547264	-6.52398700858866	5.48813674874949e-10
	3.88120728390723e-09	11.9627239556695
FBXO30	-1.14825310734463
8.19885174129353	-6.52273751073016	5.52597815072172e-10
	3.90527819051005e-09	11.956002570589
C1S	-2.13280268361582
11.1864353233831	-6.5210724323766	5.57680443350958e-10
	3.93984153544225e-09	11.9470469620688
TMEM184A	2.38430798022599	8.89786865671642
6.51828806051703	5.6628256590813e-10	3.99648713581777e-09
	11.9320745530308
PCDHB10	-1.76114950564972	5.9613328358209	-6.51805314514246

5.6701425872187e-10	4.00027585276975e-09
11.9308115315751
HBEGF	-1.32139745762712
7.16186467661692	-6.51626099554983	5.72626955530709e-10
4.03571280365447e-09	11.9211770183036
C18orf34	-1.98445889830509
2.67331641791045	-6.51534274896569	5.75523866374973e-10
4.05473746625882e-09	11.9162412324866
CAPN11	-1.41512436440678
1.44269154228856	-6.51121415558738	5.88727877670128e-10
4.14450643362953e-09	11.8940546694908
GNG2	-1.65912697740113
7.18011940298508	-6.51116985022524	5.88871177150382e-10
4.14450643362953e-09	11.8938166272782
C19orf77	3.26940141242938	5.82544179104478
6.51025277468951	5.91845041075367e-10	4.16258262873494e-09
11.8888896336745
RHBDF2	1.33935783898305	9.10019701492537
6.50699212957032	6.02538602343504e-10	4.23634161168224e-09
11.8713754454418
GPR135	-1.63314901129944
3.75446218905473	-6.50673894256487	6.03376852111427e-10
4.24078287362123e-09	11.8700157181596
ELF5	4.05736687853108	3.97116766169154
6.50545528448606	6.0764444874027e-10	4.26931571958855e-09
11.8631224294498
CCDC99	1.16959661016949	8.57957661691542
6.50404451597807	6.12368857064029e-10	4.29956656481686e-09
11.8555475716282
ADHFE1	-2.61978792372882	4.9550184079602 -6.50233239461871
6.18150959142435e-10	4.33868006536553e-09
11.8463560887787
ZNF662	-2.66907379943503
5.87135074626866	-6.502168221849 6.18708207747024e-10
4.34110714458182e-09	11.8454748137265
HIST1H2BH	2.86890367231638	4.28489800995025
6.50204525842416	6.19125903618868e-10	4.34255374147433e-09
11.8448147588038
PRRT1	-1.41532203389831
6.39317164179104	-6.50096358489299	6.22812216424708e-10
4.36691762404346e-09	11.8390087966963
HSD17B11	-1.56840734463277
9.21993184079602	-6.50036313842157	6.24867824012072e-10
4.37983490166194e-09	11.8357861269729
CLDN9	3.32336461864407	5.7211407960199 6.49883190755522
6.30140175027149e-10	4.41377611508269e-09
11.8275686987831
TNFRSF18	2.65896440677966	6.69030945273632
6.49668919912875	6.37591445187289e-10	4.46444487370227e-09
11.8160718568874
FBXW9	1.32598940677966	8.91003731343284
6.49563723207471	6.41281290060353e-10	4.48875038695501e-09
11.8104283607652
EFS	-2.45928672316384

7.50427611940299	-6.49470998806739	6.44551058272502e-10
	4.51009994080929e-09	11.8054544599381
TSPYL4	-1.14394307909605
9.33250547263682	-6.49437957126262	6.45720172413557e-10
	4.51674110379909e-09	11.8036821573158
B3GNT3	3.78856264124293	7.26620945273632
6.49359603183064	6.48500908962186e-10	4.53464702349921e-09
	11.7994796120941
MAP6	-2.42441892655367
6.76498059701493	-6.49282797816221	6.51238109633273e-10
	4.55068699481657e-09	11.7953604453629
EFCAB4A	2.01045240112994	9.46672338308458
6.4908908166683	6.58192331890015e-10	4.59771642521334e-09
11.7849726162781
IFITM2	-1.5244268361582
11.3537776119403	-6.48912700673006	6.64587690663264e-10
	4.63923335236886e-09	11.7755161206813
GIPC3	-1.44024322033899
5.90154278606965	-6.48881979985324	6.6570781329062e-10
	4.64386395177089e-09	11.7738692318213
MYCL1	1.85720346045198	7.74016865671642
6.48875902199674	6.65929638286094e-10	4.64386395177089e-09
	11.7735434170824
GATA5	-3.80103389830509
3.49335422885572	-6.4864598198475	6.7437475723168e-10
	4.70115917350302e-09	11.7612194335048
OR52N4	-1.11775084745763
0.739378606965174	-6.48393607082263	6.83765855353333e-10
	4.76339090953645e-09	11.7476951259609
RPE65	-1.25642464689266
0.611025373134328	-6.48295794950137	6.87439990853697e-10
	4.78736194444586e-09	11.7424544747236
RAB20	1.14018411016949	9.0091552238806	6.48031560489228
	6.97462764950076e-10	4.8538679879407e-09
11.728299698171
BHMT2	-3.09906483050847
5.07204776119403	-6.47963153639519	7.00080846596111e-10
	4.87043706561104e-09	11.7246358239016
SPON2	-1.74377415254238
9.66476268656716	-6.47639374199368	7.12604230675038e-10
	4.95588240371224e-09	11.7072976001341
TIMM50	1.09850077683616	10.4950990049751
6.47332980371249	7.24657785416618e-10	5.0362980144222e-09
	11.6908955682597
SKI	-1.00590607344633
10.6414104477612	-6.47313074127813	7.25447793208381e-10
	5.04008229934621e-09	11.6898301120517
ARHGAP24	-1.62488396892655
6.54949452736318	-6.46945823511279	7.40175439179248e-10
	5.14066365573409e-09	11.6701773261562
SNAP91	-2.65338700564972
2.53250199004975	-6.46891314134999	7.42386293959276e-10
	5.15427481061344e-09	11.667260970524
RGS22	-3.646025	3.63864626865672	-6.46862126291119

7.43572795029891e-10	5.16076723528183e-09
11.665699431089
FBXO17	-2.8338886299435
7.02484029850746	-6.4635763109782	7.64377727991303e-10
5.30337098873317e-09	11.6387163805974
CCIN	-1.82196370056497
2.21779253731343	-6.4619161171891	7.71348510865349e-10
5.34992734056271e-09	11.6298397983701
HIST1H1B	1.88005805084746	2.03393184079602
6.45670905326537	7.9361951092363e-10	5.4988216534803e-09
11.6020087755411
TRPM3	-1.72195162429379
1.39892537313433	-6.4560166037349	7.96628368459027e-10
5.51780715400264e-09	11.5983088346636
C6orf223	2.74285564971752	5.51393383084577
6.45544281273158	7.99130116957824e-10	5.53248315364239e-09
11.5952431141428
PDGFRL	-2.18262846045198
7.17762139303483	-6.45540707994683	7.99286168227148e-10
5.53248315364239e-09	11.5950522025548
GTF2IRD2	-1.09155543785311
8.47905870646766	-6.45438036686612	8.03782815500144e-10
5.56173279481562e-09	11.5895670190384
TTYH1	-2.79593947740113
4.17212487562189	-6.4525704963794	8.11770029368642e-10
5.61390959659175e-09	11.5798992296591
SSBP2	-1.54470127118644
7.91773233830846	-6.45254788319003	8.11870316233848e-10
5.61390959659175e-09	11.5797784479452
EVC2	-2.24356299435028
6.36950845771144	-6.44970005986098	8.24597873193518e-10
5.69424632918363e-09	11.5645698479554
KANK1	-1.5020177259887
9.71733184079602	-6.44939665360914	8.25965365111001e-10
5.70088451402116e-09	11.5629497870551
MESP2	2.47423375706215	3.58636119402985
6.44936358678163	8.26114536250022e-10	5.70088451402116e-09
11.5627732272116
NLGN2	-1.43234074858757
9.59703084577114	-6.44901737462253	8.27677959639115e-10
5.70975420409645e-09	11.5609246679912
C17orf51	-1.54719540960452
7.08056517412935	-6.44645257391068	8.39350972521797e-10
5.7844496360767e-09	11.5472322374208
NDP	-3.1242947740113
6.26166368159204	-6.44587828699625	8.41986769735443e-10
5.80066724250626e-09	11.5441668408846
HIST1H1E	1.73690960451977	3.40538407960199
6.44501760917251	8.4595224628764e-10	5.82603140432246e-09
11.5395730976287
CYSLTR2	-2.13619491525424
2.42980646766169	-6.44381637792904	8.51517478755196e-10
5.86199049004407e-09	11.5331623710279
CAMK1	-1.00433192090396

8.65860497512438	-6.44370610164946	8.52030181309864e-10
	5.86199049004407e-09	11.5325738881733
IL17D	-1.8858697740113
4.66065870646766	-6.44176448736702	8.61107085099588e-10
	5.92245509450403e-09	11.522213658226
WHAMML2 -1.95606059322034
4.06339054726368	-6.44123470422489	8.63600237365046e-10
	5.93562533414945e-09	11.5193871514628
ROR1	-2.43050014124294
4.46500049751244	-6.44102576906928	8.64585432266578e-10
	5.93895422498457e-09	11.5182724794222
PANK3	1.05635861581921	8.33475124378109
6.4410093000453 8.64663135824593e-10	5.93895422498457e-09
11.5181846179507
FAM124B -1.99612598870056
3.81658407960199	-6.43962658606203	8.71211537795915e-10
	5.98193072607028e-09	11.5108084283432
AK3L1	1.64118185028248	8.21403233830846
6.4363740417948 8.86808005185931e-10	6.08537916659975e-09
11.4934615815967
ARID4A	-1.00417252824859
8.20916616915423	-6.43636109105709	8.86870651069968e-10
	6.08537916659975e-09	11.4933925226954
OSBPL8	-1.06146638418079
9.41592587064677	-6.43607287378099	8.88265954158005e-10
	6.0929168188653e-09	11.4918556478319
RHBDD3	1.02053735875706	9.58083631840796
6.43421911032043	8.97291989739086e-10	6.15071938208462e-09
	11.4819718136147
ZNF843	-1.47875353107345
2.85115572139303	-6.43356622195278	9.00492333162766e-10
	6.16965157487612e-09	11.4784912095546
DMRTA2	3.09893531073446	3.6948631840796 6.43163261790894
	9.10036452314321e-10	6.23183734690227e-09
11.4681843583944
GNGT1	2.10824555084746	2.3329855721393 6.43128961563623
	9.11739832514443e-10	6.24142006052735e-09
11.4663562368564
MEG3	-2.9572322740113
5.42318258706468	-6.43016764143388	9.17333568162179e-10
	6.27761938478984e-09	11.4603768245273
RRP1	1.02021165254237	10.2326328358209
6.42707313692885	9.32936917105146e-10	6.37589710658078e-09
	11.4438886205164
C7orf51 -2.40404329096045
4.34133582089552	-6.4265351883167	9.35675879999443e-10
	6.3924877924754e-09	11.4410228426592
RUNX1	1.46870783898305	9.53581890547264
6.4261522547783 9.37630396118437e-10	6.40370992425533e-09
11.4389829621888
COMTD1	1.66686490112994	8.08198208955224
6.424627760796	9.45451354654614e-10	6.45497715698677e-09
11.4308627994629
ARHGEF19	1.37315141242938	9.63589303482587

6.42425474431546	9.47374741957345e-10	6.46595859454265e-09
	11.4288761330445
TRABD	1.0212270480226	10.2335686567164	6.42416001676774
	9.47863797968311e-10	6.46714648464255e-09
11.4283716310953
AKAP2	-3.56187937853107
6.39733631840796	-6.42292427634235	9.54266401858052e-10
	6.50866751832087e-09	11.4217907463036
CAPG	1.44709957627119	11.1890532338308
6.41951083394313	9.72173683585331e-10	6.62640296281768e-09
	11.403616918438
LHFPL2	-1.01944943502825	9.0896592039801	-6.41886330940096
	9.75607725479374e-10	6.64654240360767e-09
11.4001700883915
RBMS3	-1.93536475988701
3.50661940298507	-6.41883165974858	9.75775879419071e-10
	6.64654240360767e-09	11.4000016203642
UQCRQ	1.14513149717514	11.4149910447761
6.41669256397262	9.8720708689557e-10	6.71994744494896e-09
	11.3886166859441
ABCA7	1.89674675141243	8.50232786069652
6.41413466236511	1.00104917449452e-09	6.80514554714319e-09
	11.3750060097609
ADAMTS1	-2.03525331920904
10.6724995024876	-6.4134889950165	1.00457323374853e-09
	6.8268416050504e-09	11.3715709569154
PPFIA4	1.97845331920904	6.69334925373134
6.41166040678969	1.01461995308489e-09	6.89283508829678e-09
	11.3618438106145
PDIA2	3.44785084745763	4.61080049751244
6.40966467718989	1.02569765051457e-09	6.96578655807614e-09
	11.3512296398122
CIDEB	-1.06432316384181
7.64072686567164	-6.40705447515367	1.04036557256679e-09
	7.06306389047495e-09	11.3373507133543
FLJ23867	1.83700925141243	10.1493273631841
6.4061208237295	1.04566206116823e-09	7.09667507959795e-09
11.3323872192403
GJA4	-1.90273898305085
7.75458706467662	-6.40351942426401	1.06055958775657e-09
	7.1954026228164e-09	11.3185601243833
SEC23B	1.11854498587571	10.4291582089552
6.40339982984063	1.06124946311171e-09	7.19770448552474e-09
	11.3179245386979
TET3	1.18062584745762	9.42961393034826
6.40122816665559	1.07385356100177e-09	7.28078388618439e-09
	11.3063845735523
ATRNL1	-3.37343778248588
5.01584825870647	-6.40050072922295	1.07810831563452e-09
	7.30721813138881e-09	11.302519631982
PNPLA7	-2.11552521186441
6.48387064676617	-6.40027205972651	1.07944921265718e-09
	7.31313975356862e-09	11.3013047497922
FOXK1	-1.10103163841808

9.40790149253731	-6.39990953451639	1.08157838529902e-09
	7.32348425138156e-09	11.2993787735072
IL4I1	2.29816574858757	9.14102039800995
6.39716680010772	1.09782115408893e-09	7.42856568669929e-09
	11.2848098627593
SGK269	-1.13476927966102
9.16212139303483	-6.39501229135891	1.11074819961511e-09
	7.51356195653977e-09	11.2733683857989
FAP	-2.34416800847458
7.27480348258706	-6.39353658869001	1.11968856598609e-09
	7.56905046417333e-09	11.2655331608128
ILDR1	1.95194470338983	7.70033084577114
6.39201053035399	1.12900834264861e-09	7.62953959005133e-09
	11.2574318265397
DMRT1	3.02814265536723	2.87705671641791
6.39126962154822	1.13356057454336e-09	7.65778170298626e-09
	11.2534990487378
BMPR2	-1.00847401129944
10.5955074626866	-6.39108264709302	1.13471221376708e-09
	7.66304004889413e-09	11.2525066272767
LOC286367	-1.47538799435028
5.77611940298507	-6.39015196770393	1.14046170279252e-09
	7.69933533651118e-09	11.247567059718
SDSL	1.48563418079096	8.64526169154229
6.388854442772	1.1485252466085e-09	7.7486767377169e-09
11.2406812563324
PGK1	1.10857584745763	12.9769502487562
6.38673809205427	1.16179774285418e-09	7.83307312341421e-09
	11.2294520252369
DKK3	-1.7044906779661
9.16951393034826	-6.38607574546566	1.16598245410855e-09
	7.85870642903758e-09	11.2259381578301
PARK2	-1.88191970338983
4.95514129353234	-6.38468313303705	1.174829335668e-09
	7.91573556326357e-09	11.2185508834304
USP51	-2.20543672316384
4.24599900497512	-6.38380358694176	1.18045078257925e-09
	7.95100215431499e-09	11.2138857767446
HIST1H2BL	1.52114286723164	1.94879303482587
6.38326213163219	1.18392451701838e-09	7.97178430120935e-09
	11.2110141122754
DUSP9	2.94660550847458	3.99049701492537
6.38313561766756	1.1847376197487e-09	7.97464371588221e-09
	11.2103431554928
CD200	-2.11659406779661
8.18848358208955	-6.38053262575132	1.20158925239753e-09
	8.08542358299619e-09	11.1965403424723
C14orf64	-2.0592581920904
2.38312338308458	-6.38028545824532	1.20320158677118e-09
	8.09362011022685e-09	11.1952298874067
LUM	-2.26457422316384	11.250860199005 -6.37889669164579
	1.21230044046348e-09	8.14948527921258e-09
11.1878674221678
SULT2B1 3.16963354519774	6.29336567164179

6.37750222405366 1.22150463152308e-09 8.20867105897506e-09
11.1804757983944
SLC9A7 2.02409230225989 4.81280547263682
6.37717766642056 1.22365670213033e-09 8.21775338395018e-09
11.1787555759874
LOC55908 -1.3263688559322
1.02932437810945 -6.37670429673668 1.22680219061713e-09
8.23618344452902e-09 11.1762467226892
FLJ45445 2.33140572033898 6.73604975124378
6.37388200270516 1.24572150034913e-09 8.35773281116592e-09
11.1612911538727
CCDC88C 1.14021186440678 10.0808004975124
6.37256999959398 1.25461382264766e-09 8.41464285493517e-09
11.1543402308013
PRTG -2.68024463276837
5.40871791044776 -6.37085657932802 1.26632073732515e-09
8.48761499748136e-09 11.145264044995
CXADR 1.8293238700565 7.81567213930348 6.36978673826133
1.27368474254461e-09 8.5341866071935e-09
11.1395977910617
NEK9 -1.23789096045198
10.3450169154229 -6.36795204166071 1.28641147903623e-09
8.61664850395232e-09 11.1298820584422
PALMD -1.96789187853108
8.56577611940298 -6.36395933705427 1.31454082622372e-09
8.80060371270063e-09 11.1087448788306
KIF13A -1.34297669491526
8.62718706467662 -6.36393246494855 1.31473217666112e-09
8.80060371270063e-09 11.1086026489531
C14orf156 1.01701666666667 10.076831840796
6.36308464179274 1.32078340702791e-09 8.8382279485929e-09
11.104115456877
HS6ST1 1.22438820621469 11.3578467661692
6.36162834182206 1.33124146511745e-09 8.90240627975937e-09
11.0964087615635
MICAL2 -1.51577944915254
9.41773830845771 -6.36139329954573 1.33293696720802e-09
8.9079413856708e-09 11.0951650343849
STRA13 1.11489971751412 9.65812736318408
6.36029132121394 1.34091452316954e-09 8.95251224737258e-09
11.0893343171051
COL5A1 -2.01414018361583
10.6683333333333 -6.35806006208294 1.35721112943506e-09
9.05542557273376e-09 11.0775304694657
C7orf68 1.50880487288136 8.96225323383084
6.35560473058424 1.37536911671702e-09 9.17359583047446e-09
11.0645443999358
MYOZ3 -2.30265755649718 3.6430407960199 -6.35446183482087
1.38390236694802e-09 9.2275139959217e-09
11.058500840692
IMPDH1 1.0203438559322 10.3629492537313 6.35420065933866
1.3858596779914e-09 9.23756467180634e-09
11.0571198626273
MEST 1.61200190677966 10.9851199004975

6.3520111027145 1.40237601298227e-09 9.33553162987222e-09
11.0455439548633
CHST6 2.50926108757062 7.86306865671642
6.3516523792915 1.40510031192992e-09 9.35063513903444e-09
11.0436476817528
TACSTD2 2.49383926553672 11.8790507462687
6.35030158918403 1.4154054955534e-09 9.4161616408656e-09
11.036507812079
ETS1 -1.3712290960452
9.77208308457711 -6.34983467823344 1.41898479233861e-09
9.43385938688849e-09 11.0340400955316
GLTSCR2 -1.08654788135594
12.8720308457711 -6.3493606129385 1.42262802653112e-09
9.45501890083651e-09 11.03153468993
C6orf174 -2.16865854519774
3.96234825870647 -6.34662238736782 1.4438519792776e-09
9.59297124096089e-09 11.0170657653173
GATA1 -1.04972895480226
0.743703482587065 -6.34509110344437 1.45585617091742e-09
9.66647063031519e-09 11.0089761870218
ZXDA -1.4173050141243
4.70667960199005 -6.34392077114707 1.46509677330142e-09
9.72468049009962e-09 11.0027943433229
HCG11 -2.06119788135594
7.46774029850746 -6.34291421822479 1.47309026796579e-09
9.77141945115915e-09 10.997478208232
KCNQ4 -2.33267733050848
3.32833880597015 -6.33959426937236 1.49976023677191e-09
9.93869517783324e-09 10.9799477816034
APOB -2.22185233050847
1.50140597014925 -6.33929030499992 1.50222561647237e-09
9.95182055701122e-09 10.97834305373
DSC2 2.37359858757062 9.25710049751244
6.33725067753445 1.5188717694376e-09 1.00588507827594e-08
10.9675765111407
CHSY3 -1.69552980225989
4.50229651741294 -6.33661751743644 1.52407596241213e-09
1.00900611120029e-08 10.9642347289476
OCLN 1.901052259887 7.65811094527363 6.33557097692134
1.53271630218206e-09 1.01341918466337e-08
10.958711635122
EPO -1.89856278248588
1.90711044776119 -6.33343629349743 1.55048982211935e-09
1.02484082576015e-08 10.9474477704052
FER1L4 2.02751257062147 7.08122985074627
6.33327398582138 1.55184945475251e-09 1.02540937579881e-08
10.946591441323
LAGE3 1.19445861581921 9.26182835820896
6.33006482506658 1.57897387884796e-09 1.041990798224e-08
10.9296630268489
KCNS1 2.74341617231639 4.08841492537313
6.32951651966848 1.58365458712082e-09 1.04440824521652e-08
10.9267712706339
FMO1 -2.61290536723164

3.08023333333333	-6.32626836321797	1.61166374790547e-09
	1.06253875223184e-08	10.9096439472976
MAB21L1 -1.6545540960452
1.11761293532338	-6.32468931430244	1.62545515741317e-09
	1.07128713905915e-08	10.9013198342629
ANAPC11 1.08478933615819	11.0057497512438
6.32450662317301	1.62705824008372e-09	1.07199954008083e-08
	10.9003568490508
RBL2	-1.21171581920904
9.93493333333333	-6.32361065242206	1.63494272663455e-09
	1.07684870637816e-08	10.8956343557192
TREM1	2.43038947740113	4.55212288557214
6.32324527909055	1.63816874534612e-09	1.07862746446299e-08
	10.8937086693639
SPARC	-1.34410155367232
13.4041965174129	-6.3223216104377	1.64635205798723e-09
	1.08366809074953e-08	10.8888408387094
AIMP2	1.02890981638419	9.26365074626866
6.31728153089831	1.69171698376867e-09	1.11245834967235e-08
	10.862287428231
SYK	1.43286836158192	9.99237960199005
6.31659266886619	1.69801187609306e-09	1.11624027589467e-08
	10.858659287675
C4orf21 1.28867175141243	6.75253383084577
6.31623995341687	1.70124392173271e-09	1.11800696905162e-08
	10.8568016864202
PM20D1	-1.91342747175141
1.30143731343284	-6.31517870306379	1.71100496772059e-09
	1.12394853018714e-08	10.8512129517887
TIMM10	1.01315197740113	9.27941194029851
6.31513804891503	1.71137998312956e-09	1.12394853018714e-08
	10.8509988721904
KLF1	1.39236200564972	1.97220149253731
6.31501400034714	1.71252477165956e-09	1.12434069594406e-08
	10.8503456538164
EID2B	-1.14393283898305
5.80020199004975	-6.31486573644888	1.71389401883893e-09
	1.12487992988374e-08	10.8495649328982
GPR56	1.47982436440678	11.5903830845771
6.31448806309068	1.71738676647601e-09	1.12681209062807e-08
	10.8475762538165
HECTD2	-1.75995988700565
7.30988457711443	-6.31416959056129	1.72033745069184e-09
	1.12838747165187e-08	10.845899364539
TRDN	-2.06208742937853
0.958873631840796	-6.31247940393163	1.73608071213752e-09
	1.1379864948973e-08	10.8370007774636
PCDH12	-1.10683855932203
7.25707960199005	-6.31238438389922	1.73696996398967e-09
	1.13820598023325e-08	10.8365005579165
WBSCR28 2.40853926553672	3.06433582089552
6.3116016996174 1.74431180226981e-09	1.14265224315887e-08
10.8323804178579
SCARF2	-1.48959646892656

7.79250746268657	-6.30941993668512	1.76493865650792e-09
	1.15542699675088e-08	10.8208971641406
ACTC1	-2.7090290960452
3.10645373134328	-6.30835294776455	1.77511319441783e-09
	1.16134716001906e-08	10.815282254177
TRIM4	-1.67816497175141
8.61261293532338	-6.30780063395291	1.78040251364732e-09
	1.16443655957883e-08	10.8123760131054
TFPT	1.14002846045198	9.39900149253731
6.30743249520114	1.78393664140276e-09	1.16637640917193e-08
	10.8104389840098
MUC1	2.42968411016949	12.7351850746269
6.30655397322478	1.79239829149044e-09	1.17153571869783e-08
	10.8058167851714
CX3CR1	-2.00079413841808
4.99723582089552	-6.30277976790472	1.82920068166455e-09
	1.19520973884702e-08	10.7859643094785
EPHA3	-2.99264562146893
4.19006019900498	-6.30146864073202	1.84215831357619e-09
	1.2032933559567e-08	10.7790695817983
ALDH1A3	-2.2172404661017
7.04700049751244	-6.30035124863452	1.85327237727261e-09
	1.20910010782505e-08	10.7731943898499
C2orf74	-2.31872874293785	6.2200552238806	-6.30033807311774
	1.85340381847318e-09	1.20910010782505e-08
10.7731251177866
FAM180B	-1.47861871468927
0.927111940298508	-6.2980411245805	1.87645897545513e-09
	1.22338983581824e-08	10.7610500775202
ASB16	-1.1936459039548
3.80033731343284	-6.29803705762677	1.87650004529549e-09
	1.22338983581824e-08	10.761028700183
FAM129A	-2.00792782485876
8.18628308457711	-6.29507643635558	1.90663297759397e-09
	1.24169679505018e-08	10.7454690803463
RHCG	3.46729378531073	3.63998855721393
6.29504070722744	1.90699951037176e-09	1.24169679505018e-08
	10.7452813348432
NUAK1	-1.40903199152542
8.21237313432836	-6.29299293544608	1.92812287264095e-09
	1.25386007523975e-08	10.7345221175843
FAM164A	-1.37394766949153
7.75660995024876	-6.29226339774449	1.93570361228272e-09
	1.25799288256297e-08	10.7306896114537
GGTLC1	1.70376518361582	2.48439303482587
6.29194636340816	1.93900707713449e-09	1.25974099030288e-08
	10.7290242163161
GCHFR	1.80958439265537	8.58437711442786
6.28965313777744	1.9630675836557e-09	1.2745660181041e-08
	10.7169794719531
BVES	-2.45111807909605
5.60751940298507	-6.28930151549076	1.96678262028202e-09
	1.27657436593076e-08	10.7151329002121
ZNF334	-3.12670240112995

4.50182686567164	-6.28888107682369	1.97123379008779e-09
	1.27905909325228e-08	10.7129250244719
CDS1	1.45423615819209	9.3363960199005	6.28846741066553
	1.97562290895568e-09	1.28150200065909e-08
10.7107528099037
TRPC3	-1.86741144067797
1.79062139303483	-6.2882982891902	1.97742009839198e-09
	1.28200101041309e-08	10.7098647586376
LOC113230	1.82680536723164	7.39566268656716
6.28827750163165	1.97764111055931e-09	1.28200101041309e-08
	10.7097556049577
GPR84	2.03043036723164	4.55901592039801
6.28675843010378	1.99385758623826e-09	1.29169757795744e-08
	10.7017797439875
ZYG11A	3.21124639830508	3.91363482587065
6.28558166669802	2.00650948085936e-09	1.2994839003799e-08
	10.695602051877
DYNC2H1	-2.01026144067797
7.48027810945274	-6.28157187516219	2.0502150153978e-09
	1.32611576137734e-08	10.6745575296983
FGF18	2.6463968220339	7.44450497512438	6.28005461658732
	2.06699519652704e-09	1.33654839006929e-08
10.666596869456
KLF6	-1.42188531073446
10.3928587064677	-6.27867392098013	2.08238214796443e-09
	1.34607385068355e-08	10.659353837746
PRSS22	2.60668735875706	7.48291194029851
6.27686014854283	2.10276640028925e-09	1.35839503454809e-08
	10.6498405353158
CELSR2	1.33539216101694	10.5410965174129
6.27673195576777	2.10421449365391e-09	1.35883977017575e-08
	10.6491682291549
SLCO2A1	-2.13501080508475
8.57406169154229	-6.27668204142007	2.10477860163609e-09
	1.35883977017575e-08	10.6489064561747
MT1G	3.14258079096045	7.23091393034826
6.27550660309727	2.11810582545585e-09	1.36701391375695e-08
	10.6427423390302
NSUN5P2	1.20538241525423	9.15180199004975
6.27417865328372	2.13326188643616e-09	1.3754983206198e-08
	10.6357793661221
ELK3	-1.95167146892656
7.35464726368159	-6.2739313361163	2.13609628227947e-09
	1.37689345918987e-08	10.63448269212
PCDHGB2	-2.69612436440678
3.79914676616915	-6.27059213791302	2.17472915099001e-09
	1.40047645764821e-08	10.616978761173
FUT3	3.16691398305085	6.12483333333333
6.2703248739752	2.17785073310814e-09	1.40204689716871e-08
10.6155780451017
DMRTC1	-1.01748968926554
0.249209950248756	-6.26691579472343	2.21805585458228e-09
	1.42658792902802e-08	10.5977147518631
RNF183	2.82353234463277	7.25856915422886

6.26311127562597	2.26378492623725e-09	1.45463237983257e-08
	10.5777870754073
C20orf132	-1.48933983050848
4.76024179104478	-6.26293493230755	2.26592680202879e-09
	1.45555310530823e-08	10.5768636041573
TRPC6	-2.26780734463277
5.50490895522388	-6.26151465370206	2.28325024890743e-09
	1.46622232124084e-08	10.5694265526697
DOK6	-2.40028093220339
3.97041144278607	-6.25870243997143	2.31793573919671e-09
	1.48803066684517e-08	10.5547042037926
ITGB6	2.98644329096045	8.16295721393035
6.25847865923637	2.32071792718107e-09	1.48935101735003e-08
	10.5535328696136
NACAD	-2.11297259887006
6.73590597014925	-6.25764559699717	2.3311039356806e-09
	1.49554886873508e-08	10.5491726239043
MT1F	2.1181688559322 8.41503233830846	6.25589687068056
	2.35305435792613e-09	1.50868850619061e-08
10.5400210617554
C14orf2 1.03884307909604	10.6900104477612
6.25211390618444	2.40123528518656e-09	1.53909960677115e-08
	10.5202296511575
KCTD1	1.36167076271186	10.3631915422886
6.25084473405623	2.41761567809877e-09	1.54863182125953e-08
	10.5135914990739
HTRA1	-1.32171355932203
11.1771029850746	-6.25049408467313	2.42216058371294e-09
	1.55105916355667e-08	10.5117576568884
NCAPG2	1.25553064971751	8.84185820895522
6.24945518506289	2.43567544609217e-09	1.55922721884229e-08
	10.5063247778509
TM6SF1	-1.24575233050847
5.72905671641791	-6.24668298271388	2.47210173400591e-09
	1.58129708707068e-08	10.4918306393908
MYOT	-1.82184505649718
1.28806368159204	-6.2466557963033	2.47246159254598e-09
	1.58129708707068e-08	10.4916885197933
STAC3	1.5635386299435 6.10830995024876	6.24213764552833
	2.53298665040664e-09	1.61899800538133e-08
10.4680752249695
RICTOR	-1.07409011299435
8.79844278606965	-6.24085330107846	2.55045546819097e-09
	1.62864232541091e-08	10.4613649287956
HM13	1.00611087570621	12.3130865671642
6.24049453778514	2.55535621817413e-09	1.63126440171377e-08
	10.4594906692522
SYT17	2.08108580508475	6.95507711442786
6.24015889130694	2.55994955475453e-09	1.63368866518839e-08
	10.4577372427273
MAOA	-1.76601031073447
8.43124825870647	-6.23995689460624	2.56271779206161e-09
	1.63475233475891e-08	10.4566820369273
ADAM11	-2.13168290960452

4.60445472636816	-6.23992108486895	2.56320884831414e-09
	1.63475233475891e-08	10.456494973683
LOC100128191	1.20799032485876	6.65116517412935
6.23911667171738	2.57426406795691e-09	1.6412932085452e-08
	10.4522930629562
SLC30A4	-1.66446652542373
4.47665074626866	-6.23880665873742	2.57853711930771e-09
	1.64350720457583e-08	10.4506737848425
HIST1H3J	1.31933975988701	1.32690248756219
6.23635254403467	2.61260980555347e-09	1.66367491650164e-08
	10.4378572184425
GPR112	-1.28810042372881
0.301177611940299	-6.23532038700972	2.62707178182355e-09
	1.67236538545233e-08	10.4324678121039
PECAM1	-1.28033820621469
9.40423383084577	-6.23189668894492	2.67560723602969e-09
	1.70167957111802e-08	10.4145952772543
RGS7BP	-2.78452055084746
3.28676368159204	-6.23176206580942	2.6775335663622e-09
	1.70237733407916e-08	10.4138926468548
ABCG1	1.40249618644067	9.46520895522388
6.22912726992214	2.71551010668166e-09	1.72438671482135e-08
	10.400143073625
FAM158A	1.32972048022599	8.32415621890547
6.22639659227784	2.75542622383963e-09	1.74865225271801e-08
	10.3858972786585
GADD45G	-2.0030320621469
7.86721094527363	-6.22600244413675	2.76123508398837e-09
	1.75162118236391e-08	10.3838413770777
GAS7	-1.90576052259887
8.90827213930348	-6.22596341800541	2.76181089494008e-09
	1.75162118236391e-08	10.383637819085
LOC654433	-1.86792641242938
4.40135124378109	-6.22413498539943	2.78892095526692e-09
	1.76826890709172e-08	10.3741017866813
SNHG3	1.29938121468927	7.06528955223881
6.22365124008896	2.79613698191996e-09	1.77229676563189e-08
	10.3715791705747
ARSI	-2.19113721751412
4.75551194029851	-6.22098836460127	2.83618822517341e-09
	1.79601925548458e-08	10.3576952781824
ZNF677	-2.30145720338983
4.38113532338308	-6.2209109524958	2.83736092852786e-09
	1.79620782801964e-08	10.357291721283
LOC100130872	-2.20047111581921
4.38802587064677	-6.21976919364546	2.8547125950581e-09
	1.80663531370354e-08	10.351340013703
BEND6	-2.30895466101695
4.49314975124378	-6.21918036008524	2.8637019266356e-09
	1.81176581059565e-08	10.3482708573857
VWF	-1.25996511299435
11.3966034825871	-6.21839925154385	2.87566945604818e-09
	1.81858206292703e-08	10.344199815417
CCDC147	-2.65295995762712

3.72955721393035	-6.21836160903077	2.87624742246666e-09
	1.81858206292703e-08	10.3440036359642
PNMAL2	-2.09071723163842
5.24877064676617	-6.21785397302955	2.88405284940271e-09
	1.82295581891126e-08	10.3413580950707
CPZ	-2.34800564971752
7.83675323383085	-6.21742051364475	2.89073417843963e-09
	1.82661657997432e-08	10.3390992403252
PRSS36	1.37138312146892	5.19692338308458
6.21595919121146	2.91337103249142e-09	1.83978798206856e-08
	10.3314847419406
PLXND1	-1.16572846045198
10.7524671641791	-6.21423477991365	2.940307050617e-09
	1.85508616315817e-08	10.3225009225068
TRERF1	-1.59388403954802
7.49080547263682	-6.21360347744409	2.95022922293258e-09
	1.86077437624595e-08	10.3192123903415
ASPRV1	-1.35332803672316
5.81909253731343	-6.21062709731295	2.99745317750688e-09
	1.88997892304104e-08	10.3037110985968
PMP2	-1.95618170903955
0.848621393034826	-6.20802210412724	3.03939254392422e-09
	1.91407143947129e-08	10.2901481434225
KSR1	-1.36257535310735
8.75802437810945	-6.2061940967785	3.06916588354544e-09
	1.93222862892327e-08	10.280632877118
RFXANK	1.28987923728813	11.0704721393035
6.20452449900365	3.09660901840273e-09	1.94771394447941e-08
	10.2719438292071
LGALS7B	3.4524770480226	4.05650348258706	6.20380219913663
	3.10855589939332e-09	1.95462948283445e-08
10.2681852707699
SLIT3	-2.08784752824859
8.28302686567164	-6.20328356725163	3.1171619749644e-09
	1.95884099620505e-08	10.2654867011167
PRKCH	-1.3104375	8.53260348258707	-6.19998324915561
	3.17247694176899e-09	1.99238151160959e-08
10.2483179047991
GJC2	-2.03679074858757
6.05393731343284	-6.19934281888926	3.18332187120549e-09
	1.99796997908434e-08	10.2449869971063
TPST1	-1.16689088983051
8.24576019900497	-6.19923816873361	3.18509745099292e-09
	1.99847343120063e-08	10.2444427288802
PFKM	-1.3381593220339
9.64845422885572	-6.19786340209001	3.20851325977452e-09
	2.01255048039019e-08	10.2372933708981
GBP2	-1.58170896892655
10.0987726368159	-6.19608537372749	3.23904819843096e-09
	2.03108306395198e-08	10.2280484748486
CTSF	-1.69590197740113
10.1484771144279	-6.18808419476307	3.38002076424782e-09
	2.1181876156901e-08	10.1864683908042
MEGF10	-2.34000713276836

2.92439253731343	-6.1860230276454	3.41730049483895e-09
	2.14089652606175e-08	10.1757629242951
LAPTM4B 1.41715967514124	13.1250174129353
6.18349853890486	3.46350994013737e-09	2.1678615570433e-08
	10.1626543098187
FIBCD1	2.48408149717514	3.24168308457711
6.18287727951607	3.47497540278412e-09	2.17437503868205e-08
	10.1594289254386
C9orf110	-2.03530360169492
4.73842189054726	-6.18221900598262	3.48716458567784e-09
	2.18133726215619e-08	10.156011614822
NDUFA7	1.02564717514124	9.89463184079602
6.18042997227474	3.52050417755042e-09	2.20152149756656e-08
	10.1467254085886
ELFN1	-1.73484738700565
5.41615273631841	-6.17314433881049	3.65953442463757e-09
	2.28637375951946e-08	10.1089272282325
LCA5	-1.93562139830508
6.68568507462687	-6.17239023311886	3.67422872364703e-09
	2.29415801996574e-08	10.1050166225902
CD97	-1.31404759887005
10.6268119402985	-6.1700898046904	3.71941264710174e-09
	2.3216643856795e-08	10.093089172643
RARRES2 -2.1308020480226
9.81471791044776	-6.16937963355188	3.73347124258883e-09
	2.32855491981778e-08	10.0894076286977
KLRG2	2.7475884180791 2.97403482587065	6.16936032210652
	3.73385426012671e-09	2.32855491981778e-08
10.0893075217265
TYRO3	-1.21153679378531
8.32647014925373	-6.16909057502611	3.7392083745469e-09
	2.33118578589274e-08	10.0879092248474
IGFN1	-3.34669548022599
4.01027661691542	-6.16741680017136	3.77259923433653e-09
	2.35128907956675e-08	10.0792337505191
MAGEE1	-1.92292485875706
6.19651144278607	-6.1670028350224	3.78090260261777e-09
	2.35574902676002e-08	10.0770883416816
APOC1	2.22232747175141	9.20437910447761
6.16334020154172	3.8551532366116e-09	2.40128325083847e-08
	10.0581106996718
CYP21A2 -2.31571264124294
5.28279452736318	-6.16025934439984	3.91871554369763e-09
	2.43865505038231e-08	10.0421534164205
CIDEC	-1.40157288135593
1.24337910447761	-6.15530979328655	4.02298941008643e-09
	2.50267447246078e-08	10.0165285979772
KIAA1109	-1.22882012711865
9.64013383084577	-6.15526115410642	4.02402748835511e-09
	2.50267447246078e-08	10.0162768526776
C15orf59	-2.3336790960452
4.83273582089552	-6.15445608106695	4.04124790438728e-09
	2.51262324279439e-08	10.0121101748181
ANO5	-2.53379717514124

4.62094577114428	-6.15380105605496	4.05531210596959e-09
	2.52060422450971e-08	10.0087203481777
GPR85	-1.41560451977401
4.01608955223881	-6.15344160201431	4.06305041445034e-09
	2.52464966733249e-08	10.0068602381825
FAM153A	-2.0649	1.54978855721393	-6.15046666487005
4.12765295365348e-09	2.56401558664163e-08	9.99146831120884
PROM2	2.39907351694915	9.57463781094527
6.1454966523108	4.23783349687492e-09	2.62627857352482e-08
9.96576543181583
EBF3	-2.47219576271186
3.65890845771144	-6.1454838826236	4.23812026989174e-09
	2.62627857352482e-08	9.96569941041715
MAST4	-1.85219378531074
6.95713034825871	-6.14466871039503	4.25646628693109e-09
	2.63685132379889e-08	9.96148502824674
KLF5	1.67999752824859	10.5457383084577
6.14284814175646	4.2977210467701e-09	2.66160522142354e-08
	9.95207419317562
HIST2H2AC	1.6062843220339	5.04186417910448
6.14105422626119	4.33875521692366e-09	2.68377959034175e-08
	9.94280299088764
RHBDL1	1.90640734463277	5.25704328358209
6.13932591344147	4.37865199151067e-09	2.707642330895e-08
	9.93387257724325
FAM43A	-1.5653288841808
7.59288855721393	-6.13843721390423	4.39930674112454e-09
	2.71959552530222e-08	9.92928121978771
ENAM	-2.31780459039548
1.53596069651741	-6.13715813749711	4.42920222881024e-09
	2.73642858132634e-08	9.92267382318864
C1orf113	1.26280042372881	7.01240995024876
6.13505536983756	4.47878303586845e-09	2.76539596169562e-08
	9.91181347854892
ATP6V1B1	2.67608312146893	7.47547164179104
6.13492370021647	4.48190569028253e-09	2.7664919970385e-08
	9.91113351756991
RAB3D	1.04938764124293	10.6237751243781
6.13447573571479	4.49254553285501e-09	2.77222602312935e-08
	9.90882023856632
WIPF1	-1.34799371468927
9.13581492537313	-6.13199170191862	4.55199604894261e-09
	2.80806727418244e-08	9.89599483219778
KRT17	3.3299988700565	9.15455373134328	6.1315901635628
4.56167822874559e-09	2.81319477429099e-08	9.89392196735996
KCNN2	-1.82253693502825
4.10348358208955	-6.13038653766924	4.59082204167368e-09
	2.82946792301293e-08	9.88770903405281
DMGDH	-2.11417168079096
4.01095422885572	-6.12982283164188	4.60453394054689e-09
	2.83536538090665e-08	9.88479955562139
FLJ42875	-2.31568686440678
1.55885472636816	-6.12445148449068	4.73721996094077e-09
	2.91532151673004e-08	9.85708536640008

SV2B	-2.32582528248588
2.58786019900498	-6.12432665057375	4.74034782646507e-09
	2.91637221688127e-08	9.85644146618925
ALDH1A1	-2.68411511299435
10.1887179104478	-6.12259400817837	4.78397091728967e-09
	2.93880679030391e-08	9.84750532680901
RBM43	-1.56355423728814
7.08867213930348	-6.11744441335068	4.91595933964813e-09
	3.01717922998434e-08	9.82095637881678
GNB5	-1.20230148305085
9.29548606965174	-6.11721380919295	4.92195268392192e-09
	3.01995482967474e-08	9.81976784664914
S100A9	3.35435564971751	8.68851890547264
6.11692794927121	4.92939205500931e-09	3.02271263110338e-08
	9.81829456917401
GREB1	-2.13126214689266
9.27465024875622	-6.11449750573394	4.99309006934085e-09
	3.05994445144978e-08	9.8057703429448
JAK1	-1.00768566384181
11.0917641791045	-6.11403707894872	5.0052476924332e-09
	3.06647971129972e-08	9.80339811849821
ARRDC4	-1.5205322740113
8.56633631840796	-6.11327788056341	5.02535781461124e-09
	3.07788174027353e-08	9.79948682093012
NR4A3	-2.10021165254237
5.55745572139304	-6.11212527470044	5.05604024625453e-09
	3.09575026112752e-08	9.79354937080469
LRRK1	-1.52869936440678
7.74228606965174	-6.11040277010788	5.10223596698625e-09
	3.12217300751796e-08	9.78467761281443
HOXD4	-2.62521581920904
4.34327611940298	-6.1092885047612	5.13233948070732e-09
	3.13872295319992e-08	9.77893950143561
FSTL4	2.75957196327684	4.56713333333333
6.10814449062558	5.1634278205163e-09	3.15679491230493e-08
	9.77304893808049
TMEM149	1.32067923728814	7.59606119402985
6.1067973761751	5.20027206766325e-09	3.17742814134305e-08
9.76611357273742
FAM82A1	-1.45136468926554
5.58348258706468	-6.10578140523952	5.22822971988252e-09
	3.19356013535222e-08	9.76088372992386
GNPDA2	-1.19540656779661
7.86985771144279	-6.10563758241577	5.23219934488178e-09
	3.19503428169016e-08	9.76014343130882
RABGAP1L	-1.10114406779661
7.27583582089552	-6.10334149166394	5.29597479861683e-09
	3.23107869437933e-08	9.74832638940334
KLHL20	-1.1602665960452
7.88046019900498	-6.10328615656596	5.29752113262582e-09
	3.23107869437933e-08	9.7480416397866
SCEL	3.44978940677966	4.39801592039801
6.10321034966167	5.29964026773768e-09	3.23141118790183e-08
	9.7476515468986

KANK4	3.33738361581921	6.84835671641791
6.10218353057877	5.32842634018362e-09	3.24799859750504e-08
	9.74236799056253
PRLR	-2.78949322033899
7.33151393034826	-6.09982779181463	5.39504799522144e-09
	3.28763239364488e-08	9.73024870231906
IVNS1ABP	1.03822507062147	11.97512039801
6.09669900978539	5.4847953525255e-09	3.34033961991541e-08
	9.7141573862651
NFATC4	-1.41336242937853
9.86638059701493	-6.09644904309912	5.49202830157461e-09
	3.34375269962416e-08	9.71287205238212
BMP8A	-1.34449851694915
6.85834278606965	-6.09591368367054	5.5075507418011e-09
	3.35220921224953e-08	9.71011934458964
CCL18	3.10081850282486	5.66919651741294
6.09509417227396	5.53139533060952e-09	3.36572454467734e-08
	9.70590590684172
ANKS1B	-2.22160593220339
3.81331044776119	-6.09372501878996	5.57145827873281e-09
	3.3890974359225e-08	9.69886740415011
ANKRD9	1.22888601694915	6.64476169154229
6.09329836246943	5.58400073402351e-09	3.39572082551844e-08
	9.69667428377323
CHD7	1.25334632768362	9.4928263681592 6.09306513129247
	5.59086874663557e-09	3.39821256614332e-08
9.69547546158938
MN1	-2.19000190677967
5.89986169154229	-6.09304680710677	5.59140869382959e-09
	3.39821256614332e-08	9.69538127551287
MYB	2.11294145480226	7.41414577114428
6.09255002595777	5.6060665200547e-09	3.40611262671568e-08
	9.69282790084793
SORBS3	-1.47899851694916
10.5915691542289	-6.08834601929133	5.73162547531345e-09
	3.47828173243231e-08	9.67122570293968
MST1R	1.91435692090396	7.64183532338308
6.08513573002748	5.82935747078988e-09	3.53237039183342e-08
	9.65473658784098
TSLP	-1.97903050847458
3.07943830845771	-6.08303597577348	5.89416253786772e-09
	3.56953265198892e-08	9.64395477860377
LOC151174	-1.9895334039548
2.71106865671642	-6.08120994002902	5.95109337019425e-09
	3.60082366313256e-08	9.63458053436112
PCDHGC5 -1.78095529661017
1.95555024875622	-6.08069830585395	5.96714114824542e-09
	3.60788950148069e-08	9.63195432565428
ATP5E	1.0067490819209 12.5482860696517	6.07957583179836
	6.00249708866431e-09	3.6276498448713e-08
9.62619321872709
DACH2	-2.11156002824859
1.54392487562189	-6.07855305120961	6.03489156382388e-09
	3.64615432034445e-08	9.62094442521776

AGTRAP 1.0032009180791 10.4033199004975   6.07719356073113
        6.07821596851297e-09   3.67124959792796e-08
9.61396861374617
PCDHGB4 -2.66455480225989
3.32363432835821   -6.07612500022977   6.1124827800129e-09
        3.69086092569603e-08   9.60848637314409
RAB11FIP2   -1.45248968926554
8.75065024875622   -6.07466808861148   6.15950849437475e-09
        3.71706964695807e-08   9.601012767496
JAKMIP3 -2.40947132768362   2.8058184079602 -6.0739148143248
        6.18396128787888e-09   3.73072951043648e-08
9.59714913479032
CNKSR2 -2.04005197740113
1.77720298507463   -6.07291731637867   6.21648851021331e-09
        3.74925114907988e-08   9.59203335678719
SIRT1   -1.06277062146893
8.87569402985075   -6.0708112923426   6.28571508283409e-09
        3.78655312941854e-08   9.58123427744102
ARHGEF3 -1.0617363700565
9.97614228855721   -6.07008585158072   6.30973533567358e-09
        3.79990807982924e-08   9.57751502419831
TTBK2   -1.45609901129944
4.99162189054726   -6.06782878123606   6.38504648153495e-09
        3.84188171939954e-08   9.56594523277278
FZD5   1.62217033898305   8.99412835820896
6.06737469230519   6.40030404334337e-09   3.84993384148372e-08
        9.56361792060599
ELANE   -1.21339293785311
0.601673134328358   -6.06601474422152   6.44621285095054e-09
        3.87641329320488e-08   9.55664858463099
HIST1H2AL   1.39760247175141   1.47705373134328
6.06517297791838   6.47479050958784e-09   3.89017995791157e-08
        9.55233531707758
FAM115C -1.29081031073446
6.40204129353234   -6.06505003707624   6.47897468232842e-09
        3.89155500960218e-08   9.55170539433618
HDAC4   -1.04141814971752
8.82714278606965   -6.06416200093061   6.50927692268272e-09
        3.90632725000515e-08   9.54715554678591
SLC7A11 2.2178793079096 7.74125373134328   6.06322753986958
        6.54131325580446e-09   3.92440564411647e-08
9.54236833642705
GRN   1.03795868644068   13.3750691542289
6.06252929508139   6.56535213496617e-09   3.93698675214519e-08
        9.5387915848968
ZFP36L2 -1.08288538135593   12.135260199005 -6.06250705691416
        6.56611916188268e-09   3.93698675214519e-08
9.53867767477836
HFM1   -2.31919844632768
2.73277910447761   -6.06154161694596   6.59950338391854e-09
        3.95584830574738e-08   9.53373269819388
TRPV4   1.67807521186441   6.88719552238806
6.05963575189168   6.66589577797036e-09   3.99447870174348e-08
        9.52397246229276

NAT8L	2.25291009887006	6.85105174129353
6.05571837686935	6.80442237938211e-09	4.07154740450932e-08
	9.50391760200999
ASPSCR1 1.11262422316384	9.87721940298507
6.05493557269617	6.83244019315323e-09	4.08712112952901e-08
	9.49991113495864
ACHE	-2.03017429378531
4.35965124378109	-6.05292140307208	6.90505116470679e-09
	4.1281508564773e-08	9.48960406115659
UNC80	-2.31306214689266
2.25472437810945	-6.05259325617739	6.91695228989062e-09
	4.13406202362313e-08	9.48792506479062
C14orf180	-1.73712789548023
1.11316119402985	-6.05175084437794	6.94759670455829e-09
	4.15116881095989e-08	9.48361506784974
SCNN1A	2.18711151129943	11.4885597014925
6.05158910398462	6.95349553742589e-09	4.15348453253866e-08
	9.48278760941189
NHSL2	-1.81056031073446
3.74206766169154	-6.05078578916926	6.98286601319705e-09
	4.1698149869382e-08	9.47867810352532
DIP2C	-1.38808305084746
8.81202487562189	-6.05039229923928	6.99729695062428e-09
	4.17721740029998e-08	9.47666527001503
SPRED1	-1.00857634180791
9.38062985074627	-6.05022741267382	7.00335272094767e-09
	4.17961719072836e-08	9.47582184652532
MAP2K6	2.10413206214689	8.79638407960199
6.04718449365253	7.1160358799913e-09	4.24316632634974e-08
	9.46025963027902
TRIM15	3.14143241525424	3.28343184079602
6.04642467775195	7.14444882571798e-09	4.25763525085602e-08
	9.45637459203678
B3GALT2 -1.97942351694915
1.89872139303483	-6.04628842317947	7.1495557387731e-09
	4.2594422320665e-08	9.45567794015123
COL3A1	-2.02804519774012	13.249976119403 -6.04299426100387
	7.27411737169649e-09	4.33113775060699e-08
9.43883861314414
TMEM139 1.9976759180791 9.05660995024876	6.04258113508017
	7.28988809763938e-09	4.33926943011124e-08
9.43672721406787
GRB7	2.40122824858757	9.46288109452736
6.04190091614306	7.31592788187705e-09	4.35224570892309e-08
	9.43325097615793
CES4	-2.53011483050848
1.78861343283582	-6.03939081906275	7.41280903164723e-09
	4.40732607644708e-08	9.42042553919817
MTSS1L	-1.10879081920904
10.2041104477612	-6.03893272777033	7.4306249032181e-09
	4.41663952701412e-08	9.41808530108906
CCNO	2.50417824858757	7.81628855721393
6.03680864707691	7.5137833471018e-09	4.46348298946759e-08
	9.40723567107033

DNMT1	1.13187973163842	10.8994507462687
6.03603648257948	7.5442392327187e-09	4.48027860710775e-08
	9.40329217118851
MYL3	-2.42210868644068
2.04450248756219	-6.0348013287026	7.59320806612885e-09
	4.50805556962479e-08	9.39698487527725
MRC2	-1.61221666666667
10.6836701492537	-6.03473238909748	7.59595039701028e-09
	4.50837992629722e-08	9.3966328624156
LOC100190938	-2.17970925141243
4.25262537313433	-6.03118557373283	7.73835491434669e-09
	4.59024635629984e-08	9.3785261981649
EBF2	-1.25108516949153
0.573373134328358	-6.02943240343105	7.80970721837682e-09
	4.62855915684977e-08	9.36957889482497
EPS8L1	1.95763834745763	10.3672029850746
6.02858949111725	7.84424194824827e-09	4.64768505620597e-08
	9.36527773231512
CCR10	-2.0181718220339
4.13701641791045	-6.02682508958753	7.91701576432387e-09
	4.68539445493137e-08	9.35627579529219
ADAMTS12	-2.02361094632768
3.79058507462687	-6.02610770028063	7.94679367313917e-09
	4.70166207808493e-08	9.35261621197854
GIMAP2	-1.7861581920904
6.42804776119403	-6.02504332461311	7.99117678786776e-09
	4.72383701281097e-08	9.34718711885861
SUSD3	-1.94160275423729
6.73235124378109	-6.02476656638356	8.00275697436527e-09
	4.72932068749911e-08	9.34577555841377
CILP2	2.56074053672316	6.41030547263682
6.02401090871847	8.03445916816001e-09	4.74668911431151e-08
	9.34192167742861
AURKAIP1	1.11800487288136	11.3072492537313
6.02372323701241	8.0465601945411e-09	4.75247067876665e-08
	9.34045462933747
WASF3	-2.16597012711864
7.12413233830846	-6.02345562169922	8.05783356899362e-09
	4.75776022926198e-08	9.33908990693758
CHST8	-2.58318057909605
2.41912139303483	-6.02085662791335	8.16812478884257e-09
	4.82010928183208e-08	9.32583834346887
RAB3C	-1.62115014124294
0.991128358208955	-6.02007732731034	8.20148249259468e-09
	4.83840332106232e-08	9.32186567228272
EPS8	-1.52841002824859
9.21722835820895	-6.01995773037659	8.2066135962624e-09
	4.84003956136935e-08	9.32125602980004
KLHDC7B	2.01297549435028	5.57945771144279
6.01596668647549	8.37965754703387e-09	4.93642380030432e-08
	9.30091658637338
LOC158572	-1.34959915254237
4.24699701492537	-6.01248959315677	8.533327037946e-09
	5.02406665010128e-08	9.28320398593206

DHFR	1.24028100282486	8.21705024875622
6.00888063698498	8.69574288447008e-09	5.11795588967505e-08
	9.2648271658428
MEIS3P1 -1.41736511299435
7.92649751243781	-6.0088357300846	8.69778280520032e-09
	5.11795588967505e-08	9.26459854786192
C1R	-1.48099724576271
11.5906174129353	-6.00314296354804	8.96021155655364e-09
	5.26633676656302e-08	9.23562664932008
RIMS3	-2.02390988700565
5.84620099502488	-6.00160184960933	9.03257855513235e-09
	5.304314580574e-08	9.22778681321215
HAPLN3	1.91011080508475	9.99535870646766
6.00013527322978	9.10197684945641e-09	5.3430612371379e-08
	9.22032745504699
MMP19	-1.12943672316384
8.72542139303483	-6.0000976433075	9.10376434851845e-09
	5.3430612371379e-08	9.22013607690024
UTS2D	-1.27749378531074
1.63967611940298	-5.99920559072086	9.14623950853631e-09
	5.36476442724483e-08	9.2155995222923
RAB17	1.80192966101695	8.97085721393035
5.99915649799933	9.14858268864304e-09	5.36476442724483e-08
	9.21534987367386
C11orf83	1.24274470338982	9.64691144278607
5.99792883884915	9.20737002027799e-09	5.3961549105426e-08
	9.20910738440475
USHBP1	-1.92040042372881
6.01784278606965	-5.99568508849535	9.31577106153379e-09
	5.45657003975146e-08	9.19770049221714
HTR1E	-2.39168262711864
1.50351641791045	-5.99404239333199	9.39592515551823e-09
	5.50194932808868e-08	9.18935115294571
SAA2	4.03185402542373	5.84214278606965
5.9936385818505 9.41573202512893e-09	5.51197543415731e-08
9.18729894039254
ANKRD34A	-1.36626002824859
4.34533383084577	-5.99262819529185	9.46547060174312e-09
	5.53793421070921e-08	9.18216446979683
SOD3	-2.05082831920904	9.3855592039801 -5.99152023614652
	9.52030820271396e-09	5.56843098010591e-08
9.17653485642368
METTL7B 2.66070360169492	6.42840348258706
5.99050962902433	9.57059867661835e-09	5.59625151896824e-08
	9.17140052536945
GALNT13 -2.86909138418079
3.12792835820896	-5.9898796404792	9.60208010302859e-09
	5.61146326544768e-08	9.16820020929724
FILIP1	-2.13088898305085
6.69036218905473	-5.98822983975103	9.68500429665158e-09
	5.65670378976549e-08	9.15982039661118
SHF	-1.41000847457627
7.35491741293532	-5.98801813727955	9.69569576920622e-09
	5.66133771734368e-08	9.15874521490245

VTCN1	3.22789555084746	9.97836268656716
5.98790281855793	9.70152449748026e-09	5.66313045019249e-08
	9.15815955226179
TPI1P2	1.23965282485876	3.90878457711443
5.98769066203471	9.71225680970065e-09	5.66778374937903e-08
	9.1570821054447
ARID5B	-1.15868940677966
10.3773532338308	-5.9870563265415	9.74441518693338e-09
	5.68493446398813e-08	9.15386076118196
DIRC3	-2.01316426553672
3.04202189054726	-5.98662535701678	9.76632315128948e-09
	5.69609699704469e-08	9.15167230504001
HAGHL	2.15514399717514	6.92152587064677
5.98493721706204	9.85260373648433e-09	5.74315601107392e-08
	9.1431010122701
REM2	1.40485275423729	5.34107164179104
5.98315677961892	9.9444103583143e-09	5.79338094938117e-08
	9.13406291209988
LYL1	-1.44892196327684
6.08410447761194	-5.98242536736105	9.98236723475622e-09
	5.81384395261121e-08	9.1303505587148
DAAM2	-1.91948403954802
8.07908308457711	-5.98215474208323	9.99644734757235e-09
	5.82039319471527e-08	9.12897705399789
IDO1	2.74701730225988	9.19131940298507
5.98204953327097	1.00019264079142e-08	5.82193221305581e-08
	9.12844309927725
CEP72	1.08080713276836	6.76109303482587
5.97989593207541	1.01147299442714e-08	5.88592419943001e-08
	9.11751459844385
STXBP6	2.157102259887	9.29457114427861	5.97801886395307
	1.02140643222013e-08	5.9420442203115e-08
9.10799160307157
BCL2L14	2.03349491525424	4.34432288557214
5.97714992124119	1.02603715846175e-08	5.96729259581296e-08
	9.1035838696696
CCL26	-1.74610741525424
1.25336218905473	-5.97687013395836	1.02753255738107e-08
	5.97260571999814e-08	9.10216473660605
DCTPP1	1.01034872881356	9.86305174129353
5.97470654410426	1.03916873237682e-08	6.03292754464001e-08
	9.09119216743936
PCDHGA8	-1.83466786723164
2.37887611940299	-5.97374681805282	1.04437152958705e-08
	6.06018866659754e-08	9.08632583578964
GULP1	-2.17799526836158
7.71345970149254	-5.97304180475575	1.04820973797772e-08
	6.08074199510672e-08	9.08275138254358
LOXL4	-2.19687097457627
6.74517114427861	-5.9722367069974	1.0526097161277e-08
	6.10454165878581e-08	9.07866985506232
LOC284440	-1.13449336158192
6.43466019900498	-5.97035165348245	1.06298267337586e-08
	6.16121803625253e-08	9.06911487665243

KIAA2026 -1.01833672316384
8.74675572139303 -5.96692509926589 1.08209503014273e-08
6.26492131100684e-08 9.05175170214014
CNTLN -1.22016370056497
7.55266915422886 -5.96599141304349 1.08736093642736e-08
6.29183820942588e-08 9.04702169290446
COL6A4P2 -2.11561673728814
2.54172338308458 -5.96526231181057 1.09149043999423e-08
6.31219682622016e-08 9.04332845981657
EPHB6 -2.44377281073446
7.33894179104478 -5.96452388201111 1.09568842852782e-08
6.33468978813746e-08 9.03958829373328
ZNF516 -1.76087394067797
8.99580049751244 -5.96441010894232 1.09633663462308e-08
6.33665290225559e-08 9.03901205886646
AMMECR1 1.0342168079096 8.70765323383084 5.96064485257902
1.11800175734685e-08 6.45823750093722e-08
9.01994620780525
BAG2 -1.60616631355932 8.6634855721393 -5.96006218014879
1.12139166770422e-08 6.47599745090511e-08
9.01699652207679
C2orf72 2.44187387005649 4.66139850746269
5.95877340129428 1.12892539513748e-08 6.51767108047595e-08
9.01047299979204
RNF112 -1.77229300847458
3.64895323383085 -5.95714066346975 1.13854096411187e-08
6.56949016110644e-08 9.00220984326817
C16orf86 -1.22288742937853
5.12733731343284 -5.95079538991349 1.17667688968037e-08
6.78572374863428e-08 8.97011191403818
HIST1H2AG 1.68458629943503 2.5504 5.94847577042862
1.19092793361268e-08 6.86597879165075e-08
8.95838394400418
RGL3 1.64822005649718 9.64653432835821
5.94824495036221 1.19235523145022e-08 6.87227762539947e-08
8.95721709493401
MCCC2 1.03840381355932 10.8994736318408
5.94757237138248 1.19652374302712e-08 6.8924333457763e-08
8.95381723214149
C19orf48 1.1260020480226 10.9190582089552
5.94648001892404 1.20332434729711e-08 6.92577764227912e-08
8.94829600130645
NUDT1 1.25281165254237 8.65765970149254
5.94147916842441 1.23494409925905e-08 7.09980463673715e-08
8.92302854173303
AMOTL2 -1.2721197740113
10.3290751243781 -5.94126094060984 1.23634229031565e-08
7.10585308515688e-08 8.92192625493356
RYR2 -2.09576087570622
4.20632089552239 -5.93987060501235 1.2452866600954e-08
7.1532554929375e-08 8.91490421869303
CTF1 -1.51572422316384
7.46894527363184 -5.9397504085649 1.24606288128606e-08
7.15571215463018e-08 8.91429720781623

MYEOV 3.13107189265537 4.88185422885572
5.93932021401135 1.24884493398381e-08 7.16968302424151e-08
8.91212472831024
AQP7 -2.35430268361582
2.83091641791045 -5.93924651327927 1.24932216363031e-08
7.17041767384128e-08 8.91175255115801
C1orf122 1.06197401129943 9.79473781094527
5.9390666431177 1.25048761500379e-08 7.17434333300153e-08
8.91084424856792
FOXA1 3.89482153954802 6.32208905472637
5.93123627954575 1.30226845430847e-08 7.45762102285992e-08
8.87132137203306
GSTP1 1.08981779661017 13.7709736318408
5.92517931301808 1.34375619931654e-08 7.68877223299013e-08
8.84077453872634
GPR21 -1.34081617231638
1.33054378109453 -5.92507059018892 1.34451260121783e-08
7.69095672973031e-08 8.84022642122803
LOC154761 -1.31314816384181
3.86547064676617 -5.92343040607328 1.35597431300091e-08
7.75220068112191e-08 8.83195841780669
PADI3 3.33082634180791 3.21198656716418
5.92248751483151 1.36260652941756e-08 7.78794878868109e-08
8.82720612327723
HYAL1 -1.83208128531074
5.85906218905473 -5.9219794722201 1.36619320895815e-08
7.80612280642428e-08 8.82464574204604
AFAP1 -1.11069908192091
9.47289253731343 -5.92192946081292 1.36654677867944e-08
7.80612280642428e-08 8.82439370798343
NT5E -2.41458679378531
9.06298457711443 -5.92104914866134 1.37278507552273e-08
7.8395765230825e-08 8.81995759120413
LGMN 1.0183115819209 11.4217721393035 5.92062238482747
1.37581934301524e-08 7.85253575538029e-08
8.81780718596649
RFX2 -2.0971384180791
7.46966616915423 -5.91882450467893 1.38867440601028e-08
7.92150196037542e-08 8.80874910325751
CAMK2N2 1.84956016949153 4.3715671641791 5.91789943203428
1.39533451725723e-08 7.95728267758079e-08
8.80408915170843
ZNF615 -1.00264992937853 7.3638736318408 -5.91665865270645
1.40431668178336e-08 8.00405926624439e-08
8.79783966387589
SFMBT1 1.27782189265537 7.79150447761194
5.91609205432287 1.40843715886394e-08 8.02531636732631e-08
8.79498615828688
ZNF502 -2.60068474576271 5.4270447761194 -5.91270954332995
1.43328311177945e-08 8.16235847013373e-08
8.77795512476143
NSUN5P1 1.31705056497175 7.88187412935323
5.9100879019568 1.45283474330381e-08 8.26682297118271e-08
8.76475978794031

ARL10 -1.63308594632768 3.7949552238806 -5.90848843476277
1.46489116521535e-08 8.33080765148787e-08
8.75671130971501
PLEKHJ1 1.1157761299435 10.3090407960199 5.90777584619228
1.47029397245835e-08 8.3592177387344e-08
8.75312607421934
NCS1 -1.23041483050847 9.630392039801 -5.90760763426701
1.4715721881384e-08 8.363770854323e-08
8.75227979650243
MYCBP2 -1.07903411016949
9.42177661691542 -5.90422901668931 1.49747725875709e-08
8.49964283170668e-08 8.73528548113781
MOV10L1 -2.25304865819209
3.11411791044776 -5.90120327614995 1.52105510797508e-08
8.63108384928923e-08 8.72007191551027
GGT1 2.01162740112994 8.56021641791045
5.9006849133979 1.52513069553364e-08 8.65181906032208e-08
8.7174661125569
LCN10 -2.96106553672317
4.42642786069652 -5.89950741595687 1.53442842737202e-08
8.70215900937781e-08 8.71154744516747
QRFPR -1.0358831920904
0.443829353233831 -5.89927474985751 1.53627215236744e-08
8.71020913783584e-08 8.71037805180479
HIST1H4H 1.97567189265537 6.24651243781094
5.89881422912864 1.53992786871656e-08 8.72852544029568e-08
8.70806354330673
PSMB3 1.1080472457627 11.6552805970149 5.89364400076839
1.58155906807917e-08 8.95213886618842e-08
8.68208745883627
NAP1L6 -1.91862789548023 1.1613328358209 -5.89203003595357
1.59477897212253e-08 9.02199291751876e-08
8.67398191012675
TWSG1 -1.17805042372881
10.2432039800995 -5.8907729551435 1.60515050425613e-08
9.0756650653755e-08 8.66766976198647
PCMTD1 -1.03740670903955
10.0791835820896 -5.88956657242847 1.61516579760346e-08
9.12475339152421e-08 8.66161307326965
SYT8 3.13010466101695 4.41830248756219
5.88614305713234 1.64392168463392e-08 9.28209906092807e-08
8.64442993935978
PTPRD -2.98456094632768
5.63435870646766 -5.88545684569864 1.64974550223278e-08
9.30986123167649e-08 8.64098658726507
ARHGAP28 -2.06697471751413
5.91611990049751 -5.88310820160738 1.66983143061773e-08
9.41803276664343e-08 8.62920342351213
KRT16 3.8605968220339 5.37012587064677 5.88207039500181
1.67878293397142e-08 9.46591970184928e-08
8.62399779478529
TNNT1 4.29732733050847 7.09880945273632
5.87794314170629 1.7148491103603e-08 9.66397261479468e-08
8.60330192623297

FOXP3	1.74333185028248	4.68273532338308
5.87718526278324	1.72155375489126e-08	9.69643308310493e-08
	8.59950269845945
C10orf10	-1.58938622881356
9.70685373134328	-5.87591517316717	1.73284726368926e-08
	9.75736542060901e-08	8.59313654500613
C7orf41	-1.32602217514124	9.5760671641791	-5.87310292076187
	1.75811207034346e-08	9.8941997818397e-08
8.57904395655654
KLF15	-1.88841998587571	6.4274671641791	-5.86947526455755
	1.79123512850182e-08	1.00750841611349e-07
8.56087228688526
KAAG1	2.41612026836158	4.63507412935323
5.86229702432769	1.85858652348164e-08	1.04424688908259e-07
	8.52493834686237
IGSF8	1.14611447740113	11.2178024875622
5.86206543312272	1.86080022797587e-08	1.04520461505731e-07
	8.52377952839995
WNT7B	2.62402718926554	6.13673134328358
5.86163650593648	1.86490701675298e-08	1.04693850297891e-07
	8.52163338046044
ABCA12	2.83991603107345	3.55548407960199
5.85740043788426	1.90594462532257e-08	1.06880751592112e-07
	8.50044405913719
BNIP3	1.25764420903955	10.4134860696517
5.8543368258368	1.93617299033484e-08	1.085166024886e-07
8.48512624175148
MAP1A	-1.54977535310735
8.37037661691542	-5.85382372211339	1.9412813763761e-08
	1.08743537945433e-07	8.48256131701336
LHX6	-1.4761415960452
5.80544328358209	-5.85242820672944	1.95524170451289e-08
	1.09465809090946e-07	8.47558615840615
KIF20B	1.18376673728814	8.16400895522388
5.8521861064452	1.95767358030285e-08	1.09572079072021e-07
8.47437619608337
ITIH3	-2.03489675141243
3.48073432835821	-5.84825672395616	1.99756006242076e-08
	1.11621959938754e-07	8.45474297796753
TMEM100	-2.62813220338983
4.77097462686567	-5.84783862663752	2.00185059485662e-08
	1.1183127268682e-07	8.45265449694445
LOC283314	-1.36339442090396
5.72961144278607	-5.84732554039808	2.00712822719224e-08
	1.12095599848359e-07	8.45009167117188
LBX2	2.04860953389831	4.07181194029851
5.84688113517692	2.01171039953796e-08	1.12320953229574e-07
	8.44787203017056
TMEM64	-1.50476313559322
8.09405174129353	-5.84628501628277	2.01787293145973e-08
	1.126037816876e-07	8.44489482228789
FLJ33360	-1.6487927259887
1.54555721393035	-5.84409023614777	2.04072157657716e-08
	1.13816935526023e-07	8.43393523971102

KAT2B	-1.31808608757062
8.04093482587065	-5.84249084914379	2.05753110566429e-08
	1.14629887650713e-07	8.42595057126672
PHF15	-1.30196652542373	8.8810671641791 -5.84172348268021
	2.06564411365495e-08	1.15050661023701e-07
8.42212017293057
GUCY1A3 -1.70650586158193
8.33612686567164	-5.84139125516354	2.06916629797733e-08
	1.15215579325941e-07	8.42046193127919
DDR1	1.12528665254237	12.1872865671642
5.83986283871694	2.08544596345475e-08	1.1605910986643e-07
	8.41283403114225
ACTL8	3.28055176553672	3.1742815920398 5.8397773018566
2.08636073682116e-08	1.1607855264753e-07	8.41240718225277
KCNH3	2.66140007062147	7.79057512437811
5.83896602260707	2.09505649291824e-08	1.16499213975112e-07
	8.40835892942601
JMJD1C	-1.22081998587571
9.63222039800995	-5.8380163249211	2.10528093766623e-08
	1.17036061874595e-07	8.40362047935736
YPEL2	-1.14731461864407
8.47516567164179	-5.83634808685826	2.12335946748752e-08
	1.17977185027114e-07	8.39529824283316
TMC4	1.71537641242938	10.398863681592 5.83293239840595
	2.16084989264294e-08	1.20027728073484e-07
8.37826386007212
RGS9	-2.35815423728814
4.13138308457711	-5.83115168005059	2.18065090178632e-08
	1.21029367433558e-07	8.3693860377574
ANKRD5	1.22455162429379	7.23743233830846
5.83046484606221	2.18833559912313e-08	1.21423053648643e-07
	8.36596232020675
PSENEN	1.12025218926554	11.0324532338308
5.82841540188008	2.21142372427748e-08	1.22571622730607e-07
	8.35574798591174
C21orf49	-1.28177718926554
2.27363333333333	-5.82735569556725	2.22345516384463e-08
	1.23205221359596e-07	8.35046745671346
GNAZ	-1.65152316384181
7.14824029850746	-5.82656861166204	2.23243277438357e-08
	1.23635945125694e-07	8.34654584847423
ADAM8	1.54775706214689	8.03958358208955
5.82385821835473	2.26362001516419e-08	1.25329339027294e-07
	8.33304431417122
DRP2	-1.73468516949152
3.50598308457711	-5.82044297633996	2.30352479747213e-08
	1.27435634848027e-07	8.31603799183792
ZC3H12C -1.47693050847458
7.19481940298507	-5.81851380771448	2.32636900232725e-08
	1.28664751125481e-07	8.30643475239088
CCDC121 -1.01389908192091
6.51210099502488	-5.81827081123924	2.3292621020556e-08
	1.28790064517106e-07	8.30522529643246
C17orf96	1.66018735875706	8.24246019900498

5.81612407154255	2.35497434490867e-08	1.30071625776096e-07
	8.2945419771847
IGF2	-3.25887478813559
10.7333820895522	-5.81551436309646	2.36232750143733e-08
	1.30442667037408e-07	8.29150825342145
ADC	-1.2293415960452
6.44307462686567	-5.81281314627124	2.39517545894126e-08
	1.32185355301248e-07	8.27807053476841
SREBF1	1.01989103107345	11.3789338308458
5.81150741201719	2.41121345645616e-08	1.33034701050914e-07
	8.27157649898023
FAM75C1 -1.48118481638418
1.12765223880597	-5.81124908836956	2.41439879814454e-08
	1.33174656974496e-07	8.27029185564782
DDN	1.75925847457627	3.0504144278607 5.81051043862292
	2.42352967334729e-08	1.33642396437873e-07
8.26661877459945
PGR	-3.77882966101695
9.64445721393035	-5.81015573397761	2.42792637331879e-08
	1.33848894855625e-07	8.26485505382962
ISG20	1.5365386299435 8.7747671641791 5.80986864540759
2.43149065602813e-08	1.34009404478544e-07	8.26342760079849
CYP4F12 -2.4811395480226
2.47451592039801	-5.80947647331529	2.43636784838842e-08
	1.34242168350548e-07	8.26147773560742
SLC16A11	-2.28140360169492
3.5634736318408 -5.80915352820004	2.44039128989348e-08
1.3442777886105e-07	8.25987213455626
MAP3K5	-1.37917083333333
8.25011044776119	-5.80905956411776	2.44156316970871e-08
	1.34456255027145e-07	8.25940498091767
DCLK1	-2.6595843220339
5.75696965174129	-5.80841333500246	2.44963758048153e-08
	1.34864734586446e-07	8.25619232115426
SYT12	2.50503961864407	5.50705124378109
5.80787037045128	2.4564419152072e-08	1.35166852101859e-07
	8.25349322645689
DSEL	-2.28857443502825
6.55589651741294	-5.8069236103229	2.46835077555648e-08
	1.35749374289243e-07	8.24878727923774
TUBBP5	2.16242175141243	5.90510547263682
5.80549725398491	2.48639891358638e-08	1.36705328591131e-07
	8.24169848938021
HIST1H2AD	1.62586370056497	1.72498905472637
5.80418066483066	2.50317260858729e-08	1.37553890937626e-07
	8.23515632481715
NKD2	2.37081087570622	6.21523731343284
5.80406688598105	2.50462736416182e-08	1.37597002371534e-07
	8.23459100434063
ZDHHC14 -1.40976892655367
7.87616169154229	-5.80274920362491	2.52153534743645e-08
	1.38488819376325e-07	8.22804455431035
IL16	-1.44448848870057
7.24955024875622	-5.80125006788557	2.54090739560957e-08

1.39478151956857e-07 8.22059790014907
PKD1L2 -2.17443396892656
3.57757910447761 -5.8007354825489 2.54759042789374e-08
1.39807622252495e-07 8.21804211660001
SCUBE1 -2.13333481638418
3.15605422885572 -5.79812406685304 2.58177124365359e-08
1.41607704066813e-07 8.20507452005266
ARID3A 1.48322358757062 6.80464726368159
5.79788624274965 2.58490630628156e-08 1.41741790779809e-07
8.20389375497282
COX5B 1.01694710451977 11.5339164179104
5.79735298893317 2.59194933939793e-08 1.42090039887422e-07
8.20124634578013
LEPREL2 -1.84108693502825
9.04393184079602 -5.79584788244085 2.61192963056703e-08
1.4303258286354e-07 8.1937749793007
TMEM22 -1.75710331920904
5.29628756218905 -5.79443465454772 2.63082726006958e-08
1.44029023064609e-07 8.18676095514599
ZFX -1.08686306497175
8.70710746268657 -5.79222163177566 2.66068857019311e-08
1.45586184291217e-07 8.17577989483555
ZNF501 -2.15566956214689
5.01953631840796 -5.79152347803538 2.67017764242827e-08
1.46066472153084e-07 8.17231626216817
MUM1 -1.23888622881356
9.54130298507463 -5.78886214718171 2.70665393788316e-08
1.47943571205381e-07 8.15911577407022
FAM66C -1.57055218926554
4.08518059701493 -5.78699804080399 2.73249307913374e-08
1.49276431385353e-07 8.14987217710007
LYSMD2 -1.3028581920904
6.87204029850746 -5.78651156219541 2.73927593849651e-08
1.49567380365248e-07 8.14746021058174
KCTD15 -1.7812154661017
8.68207114427861 -5.78554916640208 2.75274292536769e-08
1.5022278643753e-07 8.1426890664942
C15orf52 -1.52642358757062
7.5293776119403 -5.7853477497173 2.75556956638628e-08
1.50337079983817e-07 8.14169060077141
GPR39 1.88002507062147 7.1303736318408 5.7852490313353
2.75695599612748e-08 1.5037275929994e-07 8.1412012416089
GPR17 -1.86954844632768
2.39885621890547 -5.78505157922168 2.75973111749068e-08
1.50484143006863e-07 8.14022246503354
SCAPER -1.1416863700565
7.36558855721393 -5.78490306352604 2.76182025360994e-08
1.50558071711663e-07 8.13948628355508
GPR161 -1.56761892655367
7.19730945273632 -5.7845870885799 2.76627014535184e-08
1.5076062140715e-07 8.13792006355282
HS6ST3 -2.73293015536723
4.07012736318408 -5.78410339851605 2.7730955575056e-08
1.51092494149655e-07 8.13552263370977

KCNA5 -1.85226751412429
3.30304875621891 -5.78297465655617 2.78908747720442e-08
1.51923496702061e-07 8.12992853275415
KCNMB2 -2.53516723163842
3.96300597014925 -5.7828703601901 2.79056968093925e-08
1.51963913924888e-07 8.12941167405133
RACGAP1P 1.13788502824859 1.78823482587065
5.78123337900629 2.81393471093899e-08 1.53195650001531e-07
8.12130020029491
ACP6 1.05163594632768 8.87920497512438
5.77849368556025 2.85346756187609e-08 1.55265542129118e-07
8.10772829158382
TPSD1 -2.27084216101695
1.64669900497512 -5.77794121559702 2.86150504054966e-08
1.55661628199482e-07 8.1049920182022
SNPH -1.65607542372881
6.80931492537313 -5.77751629337643 2.86770197310457e-08
1.55957407967779e-07 8.10288759016547
STON2 1.65379201977401 8.76360348258707
5.77615952519809 2.88757659713268e-08 1.56955116598183e-07
8.0961689318344
PLEKHM3 -1.30114025423729 7.3809447761194 -5.77471777314566
2.90884365881359e-08 1.58027415494689e-07
8.08903066971102
SOX8 -2.03123559322034
3.49383333333333 -5.77433589762933 2.91450224399986e-08
1.58292939336818e-07 8.08714017771338
LPCAT2 -1.50460685028249
8.73806019900498 -5.77085501307545 2.96658007691482e-08
1.60993626695906e-07 8.06991201150739
FLJ10357 -1.19596518361582
9.5165407960199 -5.7707886864045 2.96758117475359e-08
1.61005395131319e-07 8.0695838085748
PYY2 1.68183665254237 3.28886616915423
5.77063668968358 2.96987657419698e-08 1.61051997307827e-07
8.06883169640407
NFIC -1.62438686440678
8.20291144278607 -5.76940028227396 2.98861293654719e-08
1.61932498251026e-07 8.06271421402212
LOC349196 -1.68691172316384
2.0715855721393 -5.76696548457634 3.02584794130349e-08
1.63906749960318e-07 8.05067008424591
SHISA9 3.14119668079096 4.15258258706468
5.76136343175933 3.11324949662538e-08 1.68463395270741e-07
8.02297238242426
CLK1 -1.01173177966102
10.2311970149254 -5.76115190700059 3.11659753250554e-08
1.68600124749246e-07 8.02192693703194
VAT1L -2.35471737288136
3.67855124378109 -5.75992753193886 3.13604631547266e-08
1.69607562846822e-07 8.01587609286408
FOXD4L1 1.30596596045198 3.72972139303483
5.75852912825497 3.15840461004085e-08 1.70726821074615e-07
8.00896632539199

DNAJC12 -2.17181899717514
5.58829850746269 -5.75571902158887 3.20380590965331e-08
1.72998777814788e-07 7.99508470054443
C19orf28 1.06842627118644 10.508236318408
5.75531722705204 3.21034937463303e-08 1.73286195535397e-07
7.99310027432667
GABBR1 -2.21574865819209
7.89919751243781 -5.75383296229499 3.23463495028996e-08
1.74518099550079e-07 7.98577048573949
AGPAT9 -2.33031553672317 5.3296144278607 -5.7533491826849
3.24258929134169e-08 1.74817116993816e-07
7.98338171454601
CPNE5 -1.64910374293786
5.70906666666667 -5.75258992630418 3.25511160063896e-08
1.75446156894507e-07 7.97963300399013
GGT6 2.80870141242938 7.82725074626866
5.75066967085073 3.28699339397003e-08 1.77025116417116e-07
7.97015361910254
RIMKLA 2.15798686440678 6.43340845771144
5.74817370140792 3.32889013778629e-08 1.79187505319226e-07
7.95783559136499
AGPAT2 1.16498290960452 11.1436054726368
5.74654895086228 3.35644275626856e-08 1.80595280147618e-07
7.94981923011878
SLAMF9 1.71240127118644 1.92701044776119
5.74597429527861 3.36624104941917e-08 1.81055616307508e-07
7.94698432340253
VASH1 -1.05176942090396
8.76236915422886 -5.74520166773435 3.37945897372046e-08
1.8171894376763e-07 7.94317309563768
MRAS -1.27361765536723
8.83648109452736 -5.74488686618918 3.38485905240936e-08
1.81950289449768e-07 7.94162034338593
LOC100302650 -1.73705854519774
5.36968059701493 -5.7448475610842 3.38553388065325e-08
1.81950289449768e-07 7.94142647602577
RPL23AP53 -1.21553735875707
6.65531393034826 -5.7440803652414 3.39873220308105e-08
1.82611808765386e-07 7.93764257154906
LOC441177 -1.12180586158192
0.561345273631841 -5.74289148133762 3.4192843515656e-08
1.83667995127268e-07 7.93177956396646
GABRG1 -1.66825685028249
1.01740049751244 -5.74079469181918 3.45582775858584e-08
1.85533849068429e-07 7.92144131637969
NFKBIE 1.11375798022599 9.07814278606965
5.74046628211852 3.46158583171012e-08 1.85794397712441e-07
7.9198223329158
EFEMP2 -1.64860098870056
10.0934552238806 -5.74017238563301 3.46674672585749e-08
1.86022766026802e-07 7.91837354779439
LOC390595 -1.31956800847458
3.6038631840796 -5.73616853634591 3.53780869488895e-08
1.89736709785972e-07 7.89864155813623

CRISP2 -2.29239088983051   1.1729815920398 -5.7342803348524
        3.57181325140264e-08   1.91460381334977e-07
7.88933944169485
MAP7D3 -1.72660098870057
7.03826865671642   -5.73231357373168   3.60757212277364e-08
        1.93276241337534e-07   7.87965263863594
CYGB   -1.47690762711865
7.75228955223881   -5.73147831490307   3.62286407218911e-08
        1.94044871907233e-07   7.8755394949572
ROMO1   1.28182104519773   10.9414094527363
5.72773401810455   3.69219670582271e-08   1.9765527606399e-07
        7.85710638119966
ROR2   -1.99580981638418
8.64936666666667   -5.72596113768543   3.7254754946849e-08
        1.99332843121921e-07   7.84838153164128
LIN7A   -2.22819498587571
3.15567064676617   -5.72387827226161   3.76494753427484e-08
        2.01339861627149e-07   7.83813363216544
LAMB3   2.62200769774011   9.80950298507463
5.72278704886461   3.78578993881611e-08   2.02401738135143e-07
        7.83276577498469
ZFP28   -1.36761398305085
6.99135820895522   -5.72202624072453   3.8003880526313e-08
        2.03129306744391e-07   7.82902370398467
CYP7B1 -1.77889201977401
3.14003383084577   -5.72005536603942   3.838460728907e-08
        2.05110876534255e-07   7.81933152325664
PITPNM2 -1.22091610169491
7.74514825870647   -5.71399031717309   3.95797597383726e-08
        2.11167481140538e-07   7.78952043657712
IL17B   -1.79794329096045
1.98049900497512   -5.71377465607157   3.96229195512487e-08
        2.11342826289201e-07   7.78846083155381
SYNGAP1 -1.27012316384181
8.51903333333333   -5.71339602295803   3.96988058666743e-08
        2.1163762255072e-07   7.78660056763557
LARGE   -1.27917429378531
8.65314626865672   -5.71327573858765   3.97229431757435e-08
        2.11711324869682e-07   7.78600961652722
ADCK5   1.15031031073446   8.41213482587065
5.71280309234368   3.98179274143415e-08   2.12162483731231e-07
        7.78368761565656
MPP6   1.51194124293785   6.25134427860697
5.70839741240157   4.07140448870295e-08   2.16712300111671e-07
        7.76205017502643
CYS1   -3.08227718926554   6.4147184079602 -5.70693817896435
        4.10151742072047e-08   2.18196366125433e-07
7.75488614425579
RCOR2   1.81645141242938   8.25146169154229
5.70672343671065   4.10596719479766e-08   2.18325580184398e-07
        7.75383198942314
DGKE   -1.23701532485876   7.6754671641791 -5.70469430724888
        4.14824721461507e-08   2.20467525812698e-07
7.74387254119785

HIST1H2BN	1.35926052259887	3.3758815920398
5.70468712017782	4.14839772145374e-08	2.20467525812698e-07
	7.74383726987507
TMEM133	-1.46289766949153
5.90139751243781	-5.70443105845413	4.15376347687151e-08
	2.20695559472495e-07	7.74258064032142
C5	-1.69411066384181	5.657792039801	-5.70332387098686
	4.17704265007076e-08	2.21874995099489e-07
7.73714755587307
C10orf140	-2.26857930790961
3.08283333333333	-5.70276262277363	4.18889180179184e-08
	2.22446840897016e-07	7.73439374193638
FAM114A1	-1.3814206920904
7.43595422885572	-5.70270628687274	4.19008298499223e-08
	2.22452556715517e-07	7.73411733562391
TEKT5	1.46753213276836	2.09045024875622
5.70262798532376	4.19173916601806e-08	2.22482950047442e-07
	7.73373316037586
IL6ST	-2.76665437853108
6.59518407960199	-5.70253799629848	4.19364334233378e-08
	2.22496275205044e-07	7.73329164689693
PRKCB	-1.99512874293786
5.72078308457711	-5.7021637157307	4.20157222423829e-08
	2.22832027289104e-07	7.73145536655217
SLC22A17	-1.80293411016949
8.48247910447761	-5.69998527446461	4.24801251530992e-08
	2.25236820607729e-07	7.72076930566876
F7	-1.93828213276836
2.31889203980099	-5.69868856832053	4.27589352038675e-08
	2.26613338304702e-07	7.71440987645471
LOC91450	-1.29350240112994
2.06887064676617	-5.69761913236982	4.29902232441247e-08
	2.27706213416378e-07	7.70916583052086
SYCE2	1.64708792372881	4.35569154228856
5.69521297903357	4.3515081309541e-08	2.30307970942221e-07
	7.69736969669347
EGR3	-2.06969639830508
6.82296069651741	-5.69070554064487	4.45151925816703e-08
	2.35479748441559e-07	7.6752816942874
ZNF582	-1.47126532485876
4.86377064676617	-5.69047862314475	4.45661302593071e-08
	2.35688473524878e-07	7.67417005318954
ABP1	4.14909399717514	7.58980895522388
5.68837314864689	4.50414828935977e-08	2.38018441133992e-07
	7.6638571132767
ZNF471	-1.90451207627118	6.2070631840796	-5.68690092863685
	4.53768039680395e-08	2.39728714735937e-07
7.65664758496467
MEOX1	-2.67284117231639
5.20882487562189	-5.68615025942348	4.55487178830655e-08
	2.40556530850827e-07	7.65297204120786
PPP1R13L	1.10831596045198	9.26788805970149
5.68589239098138	4.56079200824038e-08	2.40763846565119e-07
	7.65170950616436

C7orf31 -1.58897959039548
5.98267014925373 -5.68362384134774 4.6131989468623e-08
2.4334268853824e-07 7.64060436709311
FMN2 -3.23887302259887
3.64404975124378 -5.68318861840554 4.62332028268026e-08
2.43813936304715e-07 7.6384742029885
AR -2.83360183615819
5.07356368159204 -5.68214704117026 4.64763084177674e-08
2.45033028201532e-07 7.63337676335873
SAA1 3.88108813559322 6.71037462686567
5.68116637615891 4.67063378189264e-08 2.46119382808665e-07
7.62857804139849
GCNT4 -2.09288905367232
3.28523731343284 -5.67855897146201 4.73233646984814e-08
2.49115045451237e-07 7.61582204258209
MFAP5 -3.33474533898305
6.88645820895522 -5.67638199726444 4.78446245830043e-08
2.51426794326095e-07 7.60517503941896
SENP7 -1.26432824858758
8.23826965174129 -5.67582105977063 4.7979842742603e-08
2.52036370705635e-07 7.60243212008827
TCF7L2 -1.35482959039548
8.83997860696517 -5.67578393411663 4.79888053136059e-08
2.52036370705635e-07 7.60225058684201
NPR1 -2.55951546610169
8.16023582089552 -5.67545477749793 4.80683391464284e-08
2.5238951475094e-07 7.60064114775406
CDH23 -2.33906723163842
5.40392437810945 -5.6709920507353 4.91594516834504e-08
2.57986590762075e-07 7.57882691491702
ATM -1.02165579096045
9.23127462686567 -5.66932110374847 4.95741814017491e-08
2.6003013392384e-07 7.57066235376201
NUDT7 -1.31837203389831
5.76339552238806 -5.66322537812267 5.11163101891185e-08
2.6777694518566e-07 7.54089222222311
CPT1B 1.51920459039548 8.72092786069652
5.65483728351094 5.33151789448146e-08 2.78940016242875e-07
7.49996451777957
NDUFS8 1.06913566384181 11.2347651741294
5.65463857640744 5.33683724553434e-08 2.79147181275975e-07
7.49899550569847
HOMER2 2.04482062146893 9.57311293532338
5.65368562889249 5.36241956689011e-08 2.80271063412052e-07
7.49434871835667
APBA2 1.73738665254237 8.73813333333333
5.65320070289554 5.37548355861982e-08 2.80739449143894e-07
7.49198432710122
TUSC1 -1.63136299435028
7.70854328358209 -5.65283773651441 5.38528226601755e-08
2.81108174221562e-07 7.49021467977895
HRCT1 -2.05572379943503
3.87034825870647 -5.65219670534845 5.40263027573205e-08
2.81870392784699e-07 7.4870895247179

SLC9A3R1 1.33308587570622 10.7223781094527
5.65098754716147 5.43550196648601e-08 2.83513352062901e-07
7.48119533475868
PCDHB16 -1.86681758474577
6.42946517412935 -5.64972603176832 5.47000514570715e-08
2.85234077025947e-07 7.47504689562214
CARD14 1.63478594632768 5.25864179104478
5.6469005275035 5.5480619729526e-08 2.89164032253661e-07
7.46127941045686
UBL4B -1.28581320621469
1.09042089552239 -5.64664837552768 5.55508049441502e-08
2.8945635165061e-07 7.46005102296594
MPP1 -1.11825148305085
8.03535024875622 -5.64510460679289 5.59823988617287e-08
2.91631222692538e-07 7.45253124086942
TUBB8 1.35931038135593 4.36853383084577
5.64463851079557 5.61133482598663e-08 2.92239228760795e-07
7.45026115427519
RNASEL -1.19586475988701
7.47825422885572 -5.64335704493121 5.64749185722554e-08
2.93898625675134e-07 7.44402056763001
LOC646999 -1.49663799435028
3.04589004975124 -5.64030748730238 5.73445337557876e-08
2.98348524583456e-07 7.42917371041568
BTG2 -1.45051560734464
11.3215094527363 -5.64009697764175 5.740504290926e-08
2.98515864540475e-07 7.4281490526682
LMTK3 1.86584661016949 7.12627562189055
5.64009441977321 5.74057785292643e-08 2.98515864540475e-07
7.42813660238946
FCGR1A 1.90462676553672 5.76217114427861
5.63990690389005 5.74597314857397e-08 2.98720761560455e-07
7.42722389069795
C17orf93 2.94544992937853 2.70195024875622
5.63943760954231 5.75949761420261e-08 2.99348065872353e-07
7.42493975154237
LOC100128164 -1.17695437853107
1.17343582089552 -5.6390871728975 5.76961701429362e-08
2.99798120231455e-07 7.42323420378927
ZDHHC1 -1.25358961864407 7.8067552238806 -5.6365222644783
5.84421298661837e-08 3.03443835648142e-07
7.41075333294895
PHKG1 -1.08915423728814
2.37652736318408 -5.63607816876396 5.85722402004719e-08
3.04042500964776e-07 7.40859277649392
ZNF593 1.10427669491525 9.56144776119403
5.63327145925287 5.9401109957663e-08 3.08189231091944e-07
7.39494079416715
HIST1H2BF 1.4645084039548 1.85138756218905
5.63192676851206 5.98022665259473e-08 3.10147063879946e-07
7.38840189452649
PCDH20 -2.8994572740113
3.73768407960199 -5.63190533776438 5.98086812421192e-08
3.10147063879946e-07 7.38829769124531

MRPL52 1.00576574858757 10.1395701492537
5.62863317726081 6.07960355793738e-08 3.15187527890036e-07
7.3723907539573
AMZ1 -1.54023806497175
2.77370845771144 -5.62669085161437 6.13896268962911e-08
3.17943753829681e-07 7.36295170762855
SASS6 1.02012768361582 7.66428805970149
5.62544150879433 6.1774422447015e-08 3.19855962884544e-07
7.35688157274232
PPP1R14C 2.50832464689265 7.59309402985075
5.62258963093051 6.26616381975337e-08 3.2428621935855e-07
7.34302892622207
CES1 -3.67018926553672
6.63660895522388 -5.6220838427095 6.28202815482666e-08
3.25025297425885e-07 7.34057265433563
LARP6 -1.05794724576271
8.63977064676617 -5.62105999247417 6.31426189083273e-08
3.26528454959184e-07 7.33560099602265
MMP16 -1.88897768361582
3.68728855721393 -5.62100433175747 6.31601886855905e-08
3.2653706212923e-07 7.33533073503431
KIT -2.24410868644067
6.20788208955224 -5.61764849824685 6.42283488450337e-08
3.31808757370041e-07 7.31904005292926
ZSCAN18 -1.03577930790961 10.5733 -5.6147840085669
6.51540462976361e-08 3.36348069842765e-07 7.30514015828409
DHDH 1.92884519774011 3.58556865671642
5.6147774672194 6.5156175049424e-08 3.36348069842765e-07
7.30510842238391
ADAP1 1.44679110169492 8.7449815920398 5.61309152874396
6.57070997004472e-08 3.3885123256724e-07
7.29692984509778
MIOX 2.00478015536723 2.07074925373134
5.61092345458272 6.64222612064345e-08 3.42367316738162e-07
7.28641502539577
C1QL4 2.76799265536723 4.59568606965174
5.60973133078396 6.68187265946149e-08 3.44205173726953e-07
7.28063466994859
SLC10A6 -1.2522959039548 1.1548736318408 -5.60970017362981
6.6829119458141e-08 3.44205173726953e-07
7.28048360748876
ARHGAP25 -1.17538799435029
7.33913184079602 -5.60690300778525 6.77686061405337e-08
3.48781520196831e-07 7.26692430400698
PBX4 1.44111814971752 5.19843184079602
5.60631125344967 6.7969004746026e-08 3.49725229933813e-07
7.26405639615436
DTNA -2.50440529661017
5.79749900497512 -5.60590387827932 6.81072990220572e-08
3.50348997475027e-07 7.26208220065342
C21orf70 1.06138411016949 8.40353084577114
5.60424319703572 6.8673908140757e-08 3.53175183900235e-07
7.25403539301482
FLJ13197 -1.88777761299435

4.91209950248756	-5.60362106501653	6.88873561505889e-08
	3.54095498690934e-07	7.25102130756959
OSBP2	1.09530699152542	7.71198656716418
5.60335129211667	6.89801139681257e-08	3.54394829771177e-07
	7.24971439605244
ARNTL2	2.06204625706215	5.49341094527363
5.60323733003168	6.90193349234687e-08	3.54507617207609e-07
	7.24916232187591
SBF2	-1.10771207627119
8.84518955223881	-5.60122778106705	6.97145231480252e-08
	3.5780979005724e-07	7.23942867103995
DENND1C 1.15976122881356	7.6288447761194 5.60057573549488
	6.99415581812484e-08	3.58885326033749e-07
7.23627090428015
KISS1R	2.4868790960452 3.19747164179104	5.60049129488262
	6.99710121835651e-08	3.58946746658818e-07
7.23586198960778
TMEM52	1.84631899717514	5.2368671641791 5.60040006420168
	7.00028482551997e-08	3.59020353404759e-07
7.23542019818482
LAMB2L	-1.22397620056497
4.46799104477612	-5.59733185152188	7.10818094484563e-08
	3.64165504648466e-07	7.2205651982338
CLEC11A -1.51527104519774
8.10696517412935	-5.59729531212178	7.10947560950341e-08
	3.64165504648466e-07	7.22038832547567
TCERG1L -1.77311214689266
1.24905273631841	-5.59577436592101	7.16357041474432e-08
	3.66753368116461e-07	7.21302677327261
CAPN9	2.50546920903955	5.48502537313433
5.59504108418647	7.18979401039416e-08	3.68004166126632e-07
	7.20947812689656
OR51E2	-1.74230268361582
2.70790845771144	-5.59423157453944	7.21885245698574e-08
	3.69399404142366e-07	7.2055609761486
EMILIN3 -2.25696631355933	6.0489263681592 -5.59345170764429
	7.2469552050512e-08	3.70652691479076e-07
7.2017876546069
ADAMTS2 -1.94891158192091
6.12721094527363	-5.59338453290183	7.24938085565042e-08
	3.70684405894155e-07	7.20146265308664
MYO5A	-1.08901038135593
7.84682487562189	-5.59082018367923	7.3425731040594e-08
	3.74982651309302e-07	7.18905807118422
MYOM2	-1.88962337570621
4.50617412935323	-5.58923715050026	7.40068535069834e-08
	3.77574727261026e-07	7.18140249932023
MEX3B	-1.726922740113 6.62766865671642	-5.58904608864296
	7.40772940106182e-08	3.77746360168403e-07
7.18047862818329
C3orf64 -1.02838919491525
8.32569104477612	-5.58819597742802	7.43915061224982e-08
	3.79254437719118e-07	7.17636823210843
RAI2	-2.0812854519774

9.23631791044776	-5.58683599536553	7.48968793336795e-08
	3.81736080615799e-07	7.16979349306131
SORD	1.20762401129943	9.58494676616915
5.58652792567079	7.50118234285228e-08	3.82227037723398e-07
	7.16830431349329
CYTL1	-2.7118529661017
4.13892537313433	-5.58629735343036	7.50979649980709e-08
	3.8257102268248e-07	7.16718978840255
PKD2L1	1.50924752824859	1.6801263681592	5.58551419153166
	7.53912748493542e-08	3.83874720401101e-07
7.1634044417487
COL21A1	-2.99221002824859
6.21291044776119	-5.58367357675975	7.60850295124887e-08
	3.87023205126708e-07	7.15450950946569
MSH5	1.11664562146893	7.97360646766169
5.58174889147961	7.68171330933804e-08	3.90456980046323e-07
	7.14521058538586
NFE2L3	1.69273262711864	8.94984029850746
5.57626730656524	7.89400610996723e-08	4.00751596037654e-07
	7.11873967156689
GIPC2	-2.34000444915254
5.12541990049751	-5.57348578029625	8.00390845982663e-08
	4.05929448320753e-07	7.10531476730775
POM121L9P	-1.49440077683616
2.51507263681592	-5.57314307258112	8.01755222453655e-08
	4.06520985847704e-07	7.10366104312202
ZNF583	-1.02334209039548
5.77159800995025	-5.5714558150961	8.08505629107492e-08
	4.09640191647996e-07	7.09552032317176
PCDH17	-1.38801313559322
6.85751144278607	-5.56989514335948	8.14798933314451e-08
	4.124216494316e-07	7.08799195948911
LPP	-1.13371716101695
7.51462288557214	-5.56938602896551	8.16862221559751e-08
	4.13262232839371e-07	7.0855364281606
HSH2D	1.86009244350282	7.7726960199005	5.5675923493471
8.24172160765339e-08	4.1685771028609e-07	7.07688656178391
PCDHGB5	-3.17728827683616	4.0643144278607	-5.56634499961933
	8.29293173280368e-08	4.19172863493437e-07
7.07087252433118
CHST7	-1.34781306497175
6.51293233830846	-5.56631148964424	8.2943117625077e-08
	4.19172863493437e-07	7.07071097115676
AJAP1	-2.78189406779661
2.81175572139303	-5.56402520866117	8.38899782433932e-08
	4.23784757218716e-07	7.05969038060591
KCNQ5	-1.91997266949152
2.13794029850746	-5.56158117300344	8.49138333498754e-08
	4.28745941631318e-07	7.04791302216202
TEX19	1.93949293785311	2.01962835820896
5.56140308614592	8.49889120993974e-08	4.29019514482574e-07
	7.04705500217417
CDH4	-1.85314399717514
1.33364776119403	-5.56027936828194	8.54641503289199e-08

4.31312440081791e-07	7.04164140843915
ALDH3B2	2.79998100282486	8.39535074626866
5.55858607322819	8.61851717040544e-08	4.34684849924292e-07
7.03348534522488
TNRC6C	-1.12843474576271
8.39695373134328	-5.55670008954544	8.69952273214949e-08
4.38500372430712e-07	7.02440330063722
PCDHB6	-2.14827394067797
4.46349054726368	-5.55660137957748	8.70378284986352e-08
4.38607417544669e-07	7.02392802013992
PXK	-1.53550854519774	6.8608815920398 -5.5552336251124
8.76302216193128e-08	4.41375969049189e-07
7.01734302884719
PDZD2	-1.65801822033898
7.46274577114428	-5.55372640760876	8.82875731277081e-08
4.4446882695239e-07	7.01008797255547
TSC22D1	-1.09204675141243
12.5249218905473	-5.55291995871091	8.86412670895254e-08
4.45921395086488e-07	7.00620668759797
PCDHA10	-2.84871786723164
4.03235671641791	-5.55228257562923	8.89217893271586e-08
4.47223011976131e-07	7.00313937600297
DZIP1L	-2.00534166666667
5.76504378109453	-5.55116787757649	8.94144681524378e-08
4.49590749735378e-07	6.99777567619631
GALR2	1.90790628531073	2.51631741293532
5.55052830115931	8.96983518094377e-08	4.50907728366248e-07
6.99469852069137
ZNF665	-1.29171701977401
4.47373034825871	-5.5477730118053	9.09314039489965e-08
4.56994301853831e-07	6.98144513023615
C21orf58	1.59213757062147	7.88641492537313
5.54174865241686	9.36853059660827e-08	4.70489073259217e-07
6.95248376796409
PALM	-2.10525670903955
9.11088805970149	-5.53588382356883	9.64443444363184e-08
4.84108164126557e-07	6.92431147765878
ACSM5	-1.35348728813559
1.69460646766169	-5.53445691434127	9.71275098856077e-08
4.8716770344901e-07	6.91746048421887
IL6R	-1.79263354519774
6.59364975124378	-5.5307989726249	9.89004610530438e-08
4.95346783464013e-07	6.89990359083907
NECAB3	1.11564639830509	9.3118223880597 5.52946834172145
9.95531947917912e-08	4.98372857614112e-07
6.89351912079347
HIST1H3F	1.56711970338983	1.66978009950249
5.52939536369367	9.95891150271214e-08	4.98431138836373e-07
6.89316899916877
CAMKV	2.30961468926554	2.14820646766169
5.52914435680279	9.97127586868019e-08	4.98928329476004e-07
6.8919647866933
FIBIN	-2.53730430790961
5.23728656716418	-5.52698269873258	1.00783785456196e-07

5.03809048206831e-07	6.88159583273764
ZNF175	-1.36039597457627
6.50729353233831	-5.52697755007627	1.007863497801e-07
5.03809048206831e-07	6.88157113942319
GBAP1	1.11638368644068	9.49063830845771
5.52394795492958	1.0230634643219e-07	5.11033891059091e-07
6.86704391792063
LOC648691	1.38148947740113	1.89873134328358
5.52306444297402	1.02753812846752e-07	5.1301940120229e-07
6.86280848811872
KRT6A	3.96378418079096	5.16802985074627
5.5207599064582 1.03929976108198e-07	5.18765477632218e-07
6.85176321105659
DRD2	-2.4360311440678
3.23401741293532	-5.52069626162184	1.0396264401284e-07
5.18802401940595e-07	6.85145821951031
ICA1L	-1.51111348870057
7.22793880597015	-5.51962579488632	1.0451360072215e-07
5.21171781108994e-07	6.84632883753916
C3orf52 1.51952683615819	6.02817412935323
5.51782624176328	1.05446232198674e-07	5.25567163641365e-07
6.83770752079029
MYO6	1.2046199858757 10.9216472636816	5.51651331311683
1.06131781926756e-07	5.28727367861274e-07
6.83141883241612
LOC728554	1.10447768361582	8.82804975124378
5.51580085118506	1.06505613099614e-07	5.30203743194731e-07
6.8280067301454
ATP10D	-1.41800600282486	7.6701039800995 -5.51564078418353
1.06589777252259e-07	5.30365517932351e-07
6.82724018639688
LOC100240726	-1.62069929378531
1.01085870646766	-5.51353842544684	1.07701247106756e-07
5.35506564083727e-07	6.81717373271719
LYST	-1.18153502824859
8.15917711442786	-5.5132538481785	1.07852562367645e-07
5.36129081212529e-07	6.81581134475411
EML1	-1.60640508474576
7.95050447761194	-5.50813025985046	1.10612602355991e-07
5.49375701016471e-07	6.79129147327759
SNX22	1.04177281073446	8.28636119402985
5.50703224539981	1.11212993079832e-07	5.52165114372177e-07
6.78603890512468
NR5A2	-1.65107747175141
6.44441194029851	-5.50437156574221	1.12681051783431e-07
5.5891326240972e-07	6.77331421536897
C19orf23	1.18328771186441	4.58417462686567
5.50423113887393	1.12759056477258e-07	5.59165079584083e-07
6.77264275012434
ATAD5	1.45880451977401	6.37715223880597
5.50351426002356	1.1315809136045e-07	5.61008359244155e-07
6.76921511795957
HSPA12A -1.64574152542373	7.8132815920398 -5.50146347874426
1.14307231242643e-07	5.66158614574536e-07

6.75941147641102
ROCK2	-1.553925	8.48190995024876	-5.50079973333014
	1.14681587050386e-07	5.67875779581387e-07
6.75623905578817
C11orf93	-2.05491942090396
5.06231492537313	-5.50031576906884	1.149552985581e-07
	5.69093870122451e-07	6.75392609017852
KIAA1908	-1.30828269774011
6.17006766169154	-5.49902250796815	1.15689845633287e-07
	5.72454213268589e-07	6.74774606210236
INCENP	1.27531468926554	8.80077164179104
5.49794315969336	1.16306395678841e-07	5.7508918672606e-07
	6.74258906530934
TYMP	1.73792711864406	9.89278009950249
5.49743309889305	1.16598868222501e-07	5.76257767117944e-07
	6.74015231522943
CELSR1	1.39113234463277	10.9980353233831
5.4962780553836 1.17263831154837e-07	5.79265267952068e-07
6.73463485828564
NETO2	1.72926956214689	7.55487711442786
5.49560374502028	1.17653741756881e-07	5.80930370726275e-07
	6.73141418092515
LBXCOR1 -1.23556850282486
1.98015373134328	-5.49559725558543	1.17657500315628e-07
	5.80930370726275e-07	6.73138318715183
HIST1H2AB	1.06496836158192	1.07637462686567
5.49542804357333	1.17755546061617e-07	5.81274671951189e-07
	6.73057503403513
HOXA13	-2.43508036723164	2.3862671641791 -5.49489763829804
	1.18063392766834e-07	5.82654195553629e-07
6.72804194802584
KIAA1804	1.48202902542373	8.0947328358209
5.4926770136904 1.19360781045908e-07	5.8877386710055e-07
6.71743874287557
CDC42EP2	-1.05178290960452
8.52461144278607	-5.48770943211126	1.22313585034038e-07
	6.02470705553937e-07	6.69373059587374
ZNF367	1.3500759180791 7.00180199004975	5.48730379745544
	1.22557820543915e-07	6.03384186035148e-07
6.69179537299804
LOC202181	-1.49392648305085
4.54940696517413	-5.48430391845249	1.24378885750197e-07
	6.11762943088537e-07	6.67748666876858
FGD3	1.11297768361582	7.42103482587065
5.47730215535348	1.28732503077961e-07	6.32418829430613e-07
	6.64411239287801
SLC26A9 2.24191603107345	4.08252039800995
5.4733939147023 1.31226824687279e-07	6.44364197758872e-07
6.62549723970514
LIN28B	3.30982146892655	3.12105223880597
5.47296056117829	1.31506283985724e-07	6.45582020618582e-07
	6.62343375852219
KIAA1598	1.20763269774011	9.78658358208955
5.47252757317678	1.31786086821701e-07	6.46800947274567e-07

6.62137213831735
RASSF4 1.07335875706215 9.11582985074627
5.47194057438334 1.32166340208377e-07 6.48357219707998e-07
6.61857740660533
ZP2 -1.15120656779661
0.507946766169154 -5.47050178133985 1.3310290773275e-07
6.52639764927957e-07 6.61172817465364
LIPE -1.29497125706215
6.57576467661692 -5.47024046480642 1.33273701700794e-07
6.53321178585793e-07 6.61048434589025
C20orf141 -1.35419978813559
0.861924875621891 -5.46707975735054 1.3535650051113e-07
6.63214547850479e-07 6.59544331334633
RASIP1 -1.58423163841808
8.18439701492537 -5.46678384169477 1.35553114975952e-07
6.6389812112575e-07 6.59403545202691
CECR2 -2.68105875706215
3.48780099502488 -5.4640064349288 1.37412117292118e-07
6.72323824596564e-07 6.58082428437952
GJA9 1.16837288135593 3.00736567164179
5.46286430824221 1.38183765473141e-07 6.75938219004904e-07
6.57539302079867
HIST1H2AJ 1.44972951977401 1.62653582089552
5.46274765715381 1.38262815005318e-07 6.76163790390465e-07
6.57483834602877
HK3 1.86302521186441 5.56207860696517
5.46265073554451 1.38328528216798e-07 6.76324049604871e-07
6.57437749139615
HMGCR 1.22935769774011 10.7431562189055
5.46104570929039 1.39421177530672e-07 6.81341769746417e-07
6.56674659665894
PYGO1 -1.95858177966102
3.04578706467662 -5.46014704577648 1.40036626347671e-07
6.84186563283601e-07 6.56247473844645
WDR76 1.09191991525424 7.14561641791045
5.45677667561212 1.42368492324739e-07 6.95083269304851e-07
6.54645808588119
ZNF404 -1.28122528248588
5.10013184079602 -5.45663457340822 1.4246763569359e-07
6.95236643876731e-07 6.54578294935998
HHEX -1.44669201977401
6.19450149253731 -5.45609078805265 1.42847651308618e-07
6.96759867276297e-07 6.54319951099878
C14orf19 -1.01493721751413
2.25580348258706 -5.4557342904304 1.43097319752752e-07
6.97811870433255e-07 6.54150595045114
PAPPA2 -2.1819811440678
1.40081641791045 -5.45468172533768 1.43836953575348e-07
7.01085626045087e-07 6.5365061627895
PHGDH 1.46524314971751 11.2045179104478
5.45343909965205 1.44714937971477e-07 7.05152371436342e-07
6.53060448739146
C14orf72 1.52291377118644 3.03163582089552
5.4534037442295 1.44739994799452e-07 7.05152371436342e-07

6.53043658635686
YAP1 -1.06714710451977
11.2394776119403 -5.4521760856807 1.45612678448061e-07
7.09067430867812e-07 6.5246070003629
TIAM1 -1.53661447740113
8.02749104477612 -5.45078620862598 1.46606863940975e-07
7.1306299850941e-07 6.51800828569962
MAGIX 1.65692358757062 7.57514029850746
5.44961220197277 1.47451786576555e-07 7.1700264765909e-07
6.5124354284526
RBKS 1.07461638418079 7.1296039800995 5.44799640955113
1.4862242152853e-07 7.22352820544678e-07
6.50476692480693
SH2D2A 1.62600918079096 6.7350671641791 5.44695835411777
1.49379260691954e-07 7.25516021293073e-07
6.49984123239268
PDK1 1.09458220338983 8.63896915422885
5.44684440642375 1.49462567553653e-07 7.25748938475992e-07
6.49930057981631
NFE2 2.2928793079096 5.64829850746269 5.44614178181773
1.49977255595116e-07 7.27903796068019e-07
6.495966991371
CCNI -1.33333213276836
11.3145925373134 -5.44574838388332 1.50266181955652e-07
7.29133707291312e-07 6.49410066179274
PRKX 1.03002634180791 10.1932422885572
5.44384859149503 1.51669112094582e-07 7.35593402150194e-07
6.48508921185263
LOC648740 -1.70917683615819
2.7470039800995 -5.44208139958004 1.52985572942478e-07
7.41802978863738e-07 6.47670882836749
TMEM63A 1.07502076271187 10.5131184079602
5.43926470144407 1.55106903962811e-07 7.51733885353282e-07
6.46335564078928
TLR4 -1.6868300141243
7.25949502487562 -5.43915859659841 1.55187371930029e-07
7.51946364343522e-07 6.46285272716807
NLN 1.16191334745763 8.2757815920398 5.43865686533962
1.55568426900517e-07 7.53437085224728e-07
6.46047472988993
C12orf69 -1.33804703389831
2.3615447761194 -5.43692208034393 1.56892999556743e-07
7.59672943608474e-07 6.45225382439894
PAX8 2.43326016949153 12.125271641791 5.431351812216
1.61220811135814e-07 7.79525024168222e-07 6.42587024431523
IFI6 2.1802761299435 12.2690696517413 5.43109487774313
1.61423216938368e-07 7.80136215570787e-07
6.42465375791856
ARHGEF37 -1.33002507062147
7.92852139303483 -5.42781994817637 1.64024902067247e-07
7.92150373898042e-07 6.4091519604647
C19orf73 1.14073425141243 4.86829800995025
5.42555416367518 1.65848756619029e-07 8.00393740805988e-07
6.39843098729999

RNF125 -1.54376461864407
6.60280298507463 -5.42445445680808 1.66741077281322e-07
8.0432197288194e-07 6.39322872114535
PTPRR -2.37702959039548
3.74399900497512 -5.4230334341435 1.67901047380492e-07
8.09346913059754e-07 6.38650760006447
GPR137C 1.34095487288135 6.71139502487562
5.42008060634527 1.70336610693296e-07 8.20509295526365e-07
6.37254556333455
IGSF10 -2.44998721751413
4.78658606965174 -5.41925200615336 1.71026220868941e-07
8.23637887506299e-07 6.368628657061
ZNF442 -1.12573792372881
4.80872437810945 -5.41918259269213 1.71084114155871e-07
8.23723467077867e-07 6.36830055034271
ZCWPW2 -1.22855120056497
3.42607064676617 -5.41779006088667 1.72249575888381e-07
8.28946036799922e-07 6.36171892699159
RELN -3.3389790960452
4.37467164179104 -5.41726660492433 1.72689672481468e-07
8.30869223352364e-07 6.35924520435497
ANKAR -1.06922641242938
4.27295074626866 -5.41651213023098 1.73325926018963e-07
8.33344557650892e-07 6.35568005713106
HIPK3 -2.0780675141243
7.82734328358209 -5.41639214071913 1.73427324188438e-07
8.33636845138991e-07 6.35511310025061
PRR11 2.4755395480226 4.83888606965174 5.41494796875766
1.74652275904065e-07 8.38739467674959e-07
6.34829004244603
GABARAPL1 -1.26149555084746
9.44330995024876 -5.41233664928547 1.76888635975884e-07
8.4848684499647e-07 6.33595617515264
ATF3 -1.68274011299435
9.50078407960199 -5.41207938090659 1.77110466478846e-07
8.49154104813337e-07 6.33474127626813
SLPI 2.87249498587571 12.7762980099502
5.41015514837049 1.78778256158311e-07 8.56550198116714e-07
6.32565583139962
SSTR1 -2.78037026836158
3.91675174129353 -5.4087692907996 1.79988885274374e-07
8.61747158274929e-07 6.31911386277736
SGIP1 -1.88640621468927
5.29210298507463 -5.40696783793243 1.81574502058976e-07
8.68528547826371e-07 6.31061193143535
COL5A3 -1.39676200564972
7.38732388059702 -5.40606822584191 1.82371414567878e-07
8.72137232955636e-07 6.30636701358827
PTPN13 -2.06880218926554
8.20717313432836 -5.40297603304401 1.85136664291273e-07
8.84807056527915e-07 6.29178017198367
NDRG4 -1.62239449152543
7.70808258706468 -5.40291330891156 1.85193176906987e-07
8.84807056527915e-07 6.29148434687588

CELSR3 1.41604844632769   6.84619303482587
5.40281987895084   1.85277385729383e-07   8.85003426948404e-07
        6.2910437089605
PRR5-ARHGAP8   1.6325363700565 5.44350248756219
5.40259056463036   1.85484225817798e-07   8.85785335203394e-07
        6.28996223212604
SGPP1   -1.22415875706215
7.75280945273632   -5.4017519109011   1.86242603120514e-07
        8.89200149317247e-07   6.28600732053265
SMAD9   -2.36221031073446
4.49903631840796   -5.40150943506156   1.8646243120258e-07
        8.89835823474887e-07   6.28486394150004
SRCRB4D 1.89093495762712   6.03753084577114
5.40131478248149   1.86639085646519e-07   8.90471863426222e-07
        6.2839460975764
KLF14   -1.66082669491525
1.65247263681592   -5.40097089002065   1.86951579239889e-07
        8.91755558037854e-07   6.28232460407628
HMGCLL1 -1.86979244350283
1.56972885572139   -5.40086785063455   1.87045309851167e-07
        8.91995403308817e-07   6.28183877618389
CMTM7   1.32217697740113   10.3987134328358
5.40003352001247   1.87805952867906e-07   8.95414819409688e-07
        6.27790518419171
LOC344595   -1.41908806497175
6.02804278606965   -5.39711672323868   1.90488886841709e-07
        9.07784783393755e-07   6.26415701117846
PHACTR3 -2.22433008474576
2.15015323383085   -5.39679711073183   1.90785132581723e-07
        9.0898555857572e-07   6.26265087014608
C9orf47 -1.4031645480226
2.37966915422886   -5.39666090442623   1.90911517184018e-07
        9.09376670020391e-07   6.26200903213911
LPAR1   -1.72788693502825
7.62628208955224   -5.39614709602257   1.91389009647214e-07
        9.11228285727573e-07   6.25958794766801
NXPH2   -1.87055762711864
1.24099253731343   -5.39479643958819   1.92649755303224e-07
        9.17018195310733e-07   6.25322442273467
GYPE   -1.69938093220339   1.8578552238806 -5.39400480707285
        1.93392445504124e-07   9.20126745353341e-07
6.24949525209063
HOXD10 -1.72086242937853
7.04953034825871   -5.39360917222021   1.93764664574873e-07
        9.21684097247947e-07   6.24763167409962
CROCCL2 -1.09389209039548
5.71298855721393   -5.39271456024172   1.94608902687848e-07
        9.25485469581079e-07   6.24341811548044
PLD5   -2.64798693502825   2.1699447761194 -5.38937478349441
        1.97792384446393e-07   9.39536708163918e-07
6.2276926065299
GIYD2   1.11479004237288   9.39980995024876
5.38906480888213   1.98090411447459e-07   9.4073470900216e-07
        6.2262334434661

PTGDS	-2.44798644067797
9.76121044776119	-5.38744223519167	1.99657593972571e-07
	9.47958002834616e-07	6.21859642067663
OPLAH	1.36954018361582	8.5828671641791 5.38671899840601
	2.00360024773242e-07	9.51025762889595e-07
6.2151928898705
LNP1	-1.3851456920904
5.73639900497512	-5.38638844881052	2.00681865814558e-07
	9.52160551230155e-07	6.21363744672998
ZSCAN12 -1.35993326271187
6.40386766169154	-5.38592456915237	2.01134373281098e-07
	9.53866639746116e-07	6.21145472204136
CLIP4	-1.62235755649717
8.01001094527363	-5.38453479059778	2.02496036000176e-07
	9.60102452444252e-07	6.20491614097887
LOC100128640	-1.26537019774011
5.12624378109453	-5.3815183196653	2.05482436762434e-07
	9.73138276062921e-07	6.19072868506811
SEMA3G	-1.40328403954802
7.46124726368159	-5.38068305703576	2.06316925827945e-07
	9.76414589038198e-07	6.18680121830807
ARID5A	-1.02978968926554
9.38101194029851	-5.38010792948606	2.06893437958579e-07
	9.78917326870159e-07	6.18409719035372
C22orf45	-1.15078531073446
1.25972189054726	-5.3763633583778	2.10685558418934e-07
	9.96400487063053e-07	6.16649693469425
PEBP4	-2.07905798022599
1.84858358208955	-5.37487763113704	2.12208829014417e-07
	1.00291143178314e-06	6.15951624486871
HIST1H1D	1.36141617231639	1.96459651741294
5.37473044030601	2.12360321707134e-07	1.0033964107359e-06
	6.15882474711876
PTPRJ	1.09327161016949	9.51365671641791
5.37219013658687	2.14991519341785e-07	1.01559500508211e-06
	6.1468927102704
ITIH5	-2.14205550847458
8.33287064676617	-5.37158518484072	2.15622780491721e-07
	1.0183426923154e-06	6.144051818036
MCM8	1.10199053672316	7.85391791044776
5.36921720031212	2.18111150154696e-07	1.02867491676451e-06
	6.13293390880888
CCDC138 1.56763502824859	6.52005472636816
5.36910507727856	2.18229663971517e-07	1.02899747389418e-06
	6.13240757145274
ZNF486	-2.33222973163842	3.741407960199	-5.36859556576482
	2.18769006784957e-07	1.0313037219966e-06
6.13001588316272
TBX15	2.18047577683616	4.74287462686567
5.3672859414724 2.201612692257e-07	1.03739060298454e-06
6.12386917876582
FSTL1	-1.24667556497175
12.0571228855721	-5.36475906014279	2.22872017130085e-07
	1.04944094304602e-06	6.11201246121062

NCRNA00152 1.33661052259887 7.62571940298507
5.36470913061893 2.22925905735276e-07 1.04945398870562e-06
6.11177822225551
DOCK1 -1.21290473163842 10.030623880597 -5.36285258310853
2.24938694359971e-07 1.0584440511598e-06
6.10306958732191
SLC9A2 2.52606906779661 5.82885721393035
5.36178246531165 2.26106895611455e-07 1.06345350902707e-06
6.09805093592098
CCDC3 -1.69243015536723
8.50718855721393 -5.36157186824314 2.2633748979289e-07
1.0640505302148e-06 6.09706336341006
PDPN -2.00630861581921
7.62127562189055 -5.36038681999084 2.27639338204088e-07
1.06992573565245e-06 6.09150674665242
NUDT8 1.26486433615819 7.3421815920398 5.35876814606095
2.29429334724817e-07 1.07784536427929e-06
6.08391837037206
LOC100128788 1.30873460451977 4.24470945273632
5.35807992022211 2.30194547627032e-07 1.08119287640883e-06
6.08069247291204
BCL8 -1.21463072033898
0.971870646766169 -5.35614763449481 2.3235628800797e-07
1.09084715133858e-06 6.0716369924938
SUSD5 -1.93695416666667
5.38211144278607 -5.35591395862991 2.32619047887478e-07
1.09183105924324e-06 6.07054205751117
HIST1H3G 2.30793206214689 2.57124975124378
5.35353550199537 2.35310042082396e-07 1.10395684733811e-06
6.05939935663653
RPS6KA6 -2.48297514124294
2.20226616915423 -5.35347809793906 2.35375362270697e-07
1.10401100923405e-06 6.05913047329171
TIGD3 1.36841892655367 4.55840895522388
5.35255729762776 2.36425558045414e-07 1.10843039659108e-06
6.05481769546475
NDUFS6 1.05539901129944 10.6712562189055
5.35071226736012 2.38543603864547e-07 1.1178498487421e-06
6.04617774706993
GFRA2 -2.40247782485876
4.41049353233831 -5.34877835039664 2.40783511718958e-07
1.12731710275718e-06 6.03712395144716
ACSBG1 -1.9038438559322
3.31027661691542 -5.34848352868011 2.41126774444834e-07
1.12866682155779e-06 6.03574393391054
MASP2 -1.41019781073446 2.9194631840796 -5.34760802225915
2.42148941591978e-07 1.1331930182091e-06
6.03164615148688
HIST1H2AC 1.38355861581921 9.87664925373134
5.34363457966073 2.46841244829326e-07 1.15436235907655e-06
6.0130548824065
C17orf37 1.17403531073446 10.3375686567164
5.34232313687831 2.48409283971837e-07 1.16116634789203e-06
6.00692106833637

ANKRD34B 2.64252867231638 3.18369452736318
5.34147247804242 2.49431561233152e-07 1.16567947919796e-06
6.0029430128103
FTH1 1.04813008474577 15.4634885572139
5.34022311591468 2.50940405429576e-07 1.17193051034331e-06
5.9971013055087
TIGD7 -1.12854230225989
7.36739651741293 -5.33422001283083 2.58315061548877e-07
1.20527459399965e-06 5.96904657001778
KRBA2 -1.19698064971751
5.05278407960199 -5.33237423687216 2.60624641036639e-07
1.21577456952561e-06 5.96042532749095
NID2 -1.58571913841808
7.52634129353234 -5.33157317971079 2.61633228028884e-07
1.21992509003702e-06 5.95668444941747
ADRA2C -2.02792146892655
8.46505174129353 -5.32846830971566 2.65578489436864e-07
1.2377585444129e-06 5.94218891870296
ZFP37 -1.4647761299435
5.85643084577114 -5.32697074159893 2.67502038494571e-07
1.24587496603756e-06 5.93519957213388
NCCRP1 2.79812514124294 5.0793039800995 5.32542342095387
2.69503730122954e-07 1.25462847605992e-06
5.92797957268351
HYAL3 1.00988008474576 6.81968905472637
5.32515650741446 2.69850493626921e-07 1.25595797645901e-06
5.9267342785834
GCH1 1.03041172316384 7.92550248756219
5.32162400237177 2.7448088019247e-07 1.27614255004421e-06
5.91025767117858
CCDC102B -1.08721483050848
4.67719353233831 -5.32161099210337 2.74498076034839e-07
1.27614255004421e-06 5.91019700276198
TGM5 2.30167733050847 2.62276268656716
5.32098571332839 2.75325752102072e-07 1.27970063179885e-06
5.90728138536459
ANKRD34C -1.28466186440678
0.799747263681592 -5.31943788262438 2.77385061138143e-07
1.28839712786563e-06 5.90006509993517
CORO2A 1.22820423728814 8.65570298507463
5.31695544116953 2.80719151820544e-07 1.30358837070251e-06
5.88849477155932
POSTN -2.57965508474577
8.33628009950249 -5.3166763146076 2.81096467731158e-07
1.30504533752163e-06 5.88719405364392
FLJ46111 1.03828898305085 1.65306417910448
5.31591269894241 2.8213122639313e-07 1.30955326200564e-06
5.88363589926162
FAAH 1.23508799435028 9.00330646766169
5.31539919917852 2.82829141546884e-07 1.31249599366129e-06
5.88124340441573
HTATIP2 1.06340480225988 8.86424776119403
5.31144531383072 2.88259553656546e-07 1.33739404216246e-06
5.86282730454092

FOXG1 3.53903333333333 3.97556218905473
5.31078523990836 2.89175949996748e-07 1.34134257872419e-06
5.85975386688144
GRP 2.41278587570621 3.72422537313433
5.31005792676195 2.90188978262058e-07 1.34573745735714e-06
5.85636768232571
PIPOX -1.82483933615819
4.62248109452736 -5.30981977836429 2.90521429726051e-07
1.34697492147151e-06 5.85525899988582
DCXR 1.16234322033898 10.2092726368159
5.30750462492762 2.93772718353787e-07 1.36081992504586e-06
5.84448292096607
ADAMTS3 -1.8589718220339
4.68867412935323 -5.30424893656775 2.98404805036249e-07
1.3810954633143e-06 5.82933502228931
GGTLC2 1.9698438559322 6.57100945273632 5.30310432722992
3.00050137370705e-07 1.3883320532388e-06
5.82401110434279
C6orf52 1.8201886299435 4.93443184079602 5.30240858608432
3.01054547675088e-07 1.39235185036485e-06
5.820775428099
UPF0639 1.96236490112994 2.41199850746269
5.30214158637269 3.01440870991599e-07 1.39360376975309e-06
5.81953378008282
FAM184A -2.2539906779661
4.27119253731343 -5.30161487224859 3.02204391028089e-07
1.39641146698327e-06 5.81708450135732
WWC3 -1.00698877118644
9.16176119402985 -5.30073273194517 3.03487349442737e-07
1.40202414132749e-06 5.81298286187331
HRASLS5 -2.08598298022599
3.62594378109453 -5.29893601569825 3.06116845890136e-07
1.41297055478541e-06 5.80463035736451
FUT2 1.92214929378531 7.72581393034826
5.29868212554767 3.06490196938162e-07 1.41398735800909e-06
5.80345025459178
QSOX1 1.14196433615819 12.3513562189055
5.29677220752532 3.09313007499735e-07 1.42668974252293e-06
5.79457415956495
EID3 -1.42453241525424
4.86929850746269 -5.2963234590353 3.09979906407518e-07
1.42944462680736e-06 5.79248900899271
LILRB4 1.94345946327684 6.93517960199005
5.29482645112213 3.12214786725043e-07 1.43910407980807e-06
5.78553398800676
KALRN -1.24216475988701
7.89368258706468 -5.29397217071748 3.13497156132444e-07
1.44469059829384e-06 5.78156570918728
MARCH8 -1.4710093220339
6.33085024875622 -5.29370466111521 3.13899769933898e-07
1.4462213367915e-06 5.78032318040713
CD99 -1.07940988700565
11.0144582089552 -5.29247173253669 3.1576189209239e-07
1.45447423034704e-06 5.77459708419613

ZNF568 -1.29975388418079
6.09537810945274 -5.29168982214421 3.16948393595588e-07
1.45961205036281e-06 5.77096617457917
STAT1 1.23949978813559 12.0197706467662
5.29157585686212 3.17121690674701e-07 1.46008259913694e-06
5.77043699469155
CPA1 -1.22840353107345
0.744296517412935 -5.28865219679202 3.21599089016577e-07
1.47903882112686e-06 5.75686437417213
DEF6 1.09197987288136 8.53545671641791
5.28774281207298 3.2300424730812e-07 1.48450351404426e-06
5.75264385474483
SNORD116-4 -2.68111419491525
3.28506467661692 -5.28631460635999 3.25223136222761e-07
1.4943668278096e-06 5.74601655419609
ETV4 2.1708665960452 8.83187114427861 5.28572717744181
3.26140074109923e-07 1.49790955737734e-06
5.74329110044132
FEV -1.21726744350283
0.669682089552239 -5.28301691901052 3.30403273207354e-07
1.51647198724502e-06 5.73071945696057
C15orf2 -1.34272351694915
0.835830845771144 -5.28241466370169 3.31357946451175e-07
1.52051377752405e-06 5.72792653151829
CNGB3 -1.88021108757062
1.35072089552239 -5.28193060615324 3.32127201059236e-07
1.52358214511734e-06 5.7256819156536
TFR2 1.98840706214689 4.48953532338308
5.28190053589193 3.32175045374828e-07 1.52358214511734e-06
5.72554248242159
SACS -1.14432768361582
8.24760845771144 -5.2810668089595 3.33504247623718e-07
1.52933710156688e-06 5.72167680028508
ATF5 1.09356200564972 10.4163124378109
5.28044579098586 3.3449769028102e-07 1.53355015218163e-06
5.71879766967739
DCX -2.62891680790961
2.50912537313433 -5.2793590733291 3.36243041718906e-07
1.54120777997078e-06 5.7137601013587
TSPYL3 -1.79943121468927
4.20818457711443 -5.27844849997405 3.3771230893796e-07
1.54759680859293e-06 5.70953966656895
SYN3 -1.27118121468927
1.37060995024876 -5.27684581197899 3.40313526617109e-07
1.55916909204402e-06 5.70211266979669
PDE4D -1.68294802259887
7.17950099502488 -5.27566505409503 3.42242392252623e-07
1.56765647321427e-06 5.69664202293104
DOK3 1.07854915254237 6.94940049751244
5.27549528926657 3.42520589864162e-07 1.56858079632194e-06
5.69585555032283
PLXNC1 -2.25368516949153
5.10955323383085 -5.27422206000057 3.4461408180189e-07
1.57676110973764e-06 5.68995764675855

GPR87	2.18729745762712	2.6655776119403 5.27138375916936
	3.4932580900868e-07	1.59725141624681e-06
5.676813823902
PLD1	-1.10541525423729
7.41710796019901	-5.27126348237707	3.49526852169726e-07
	1.59781480183578e-06	5.67625695480513
SNCG	-2.47797923728814
6.60352388059701	-5.2710464148995	3.49889966166376e-07
	1.59911865658853e-06	5.6752519791351
GLDC	3.34433566384181	6.77376616915423
5.27003662455068	3.51583987953193e-07	1.60614581056499e-06
	5.67057727810367
LOC401052	1.0169790960452 6.27614875621891
5.26867410250497	3.53882397701148e-07	1.61592651797255e-06
	5.6642707207392
SLC5A4	-1.4197572740113	1.4773368159204 -5.26861130857849
	3.53988674900287e-07	1.61605236728995e-06
5.66398010301782
BHLHA15 1.57882450564972	2.27827064676617
5.26784278501206	3.55291899431288e-07	1.62164132844027e-06
	5.66042349695088
IRF6	1.50849964689265	8.76560945273632
5.26733438424219	3.5615658325082e-07	1.62486547869763e-06
	5.65807091359282
GUCY1B3 -1.33779583333333	7.7853368159204 -5.26665590527132
	3.57313722930378e-07	1.62978243318388e-06
5.65493157431064
FAM179B -1.30849371468927
7.84233184079602	-5.26612973741219	3.58213613412616e-07
	1.63352409670391e-06	5.65249719268845
P2RY2	1.53434830508474	6.32939253731343
5.26424502325093	3.61455108736336e-07	1.64793990281079e-06
	5.64377883401743
ZNF354C -2.04351278248588
3.79261791044776	-5.2623448963299	3.6475197824465e-07
	1.66149503291828e-06	5.63499156572487
PTN	-2.64894491525424
8.05289701492537	-5.26176712987087	3.65760232515908e-07
	1.66571818401765e-06	5.63232012010531
TNFAIP2 1.70714731638418	11.9097004975124
5.26149644116062	3.66233540309057e-07	1.66750378854401e-06
	5.63106860040029
MTMR9L	-1.21642330508475
6.84434626865672	-5.26040609755974	3.68146078543474e-07
	1.67584013137418e-06	5.62602792802387
RNF208	1.16161744350283	7.9660960199005 5.25585569187102
	3.76233170476609e-07	1.71113579749331e-06
5.6049998708465
ZNF214	-2.14698827683616
4.74036019900498	-5.25482771430824	3.78083893585339e-07
	1.71917216728837e-06	5.60025134918458
BAI1	-2.39491497175141
4.19302835820895	-5.2544470810644	3.78771410991448e-07
	1.72191697689425e-06	5.59849327385038

C8orf73 1.70037867231638 7.82249004975124
5.2541532180924 3.79303031213075e-07 1.72395201036184e-06
5.59713604042162
MYO9A -1.30369519774011
6.79971990049751 -5.25200270137503 3.83215618141037e-07
1.7413494113403e-06 5.58720542695244
GGT5 -1.42579858757062 8.1487960199005 -5.24803138470483
3.90544303110908e-07 1.77347368787148e-06
5.56887484528862
RAB19 1.5483761299435 2.54862039800995 5.24745022790144
3.91628136959512e-07 1.77760903200946e-06
5.56619325440175
KCNK5 1.33050162429379 8.7829815920398 5.24637919685881
3.93633231634901e-07 1.78631526203902e-06
5.56125186022919
ADCY5 -2.29002259887006
6.24004626865672 -5.24522438864631 3.95806348260729e-07
1.79538319261882e-06 5.55592480057751
CST6 2.53864180790961 4.90612039800995
5.2450320163088 3.96169485717421e-07 1.79661121600772e-06
5.5550374850215
GPR176 -1.69201998587571
4.20234975124378 -5.24438489204547 3.97393428225504e-07
1.8013881831463e-06 5.55205281144408
USP53 -1.39035155367232 8.8800855721393 -5.24289910250756
4.00217510687505e-07 1.81298885578431e-06
5.54520109191828
ICA1 1.09684209039548 9.45992587064677
5.24158012032142 4.0274087757515e-07 1.82361495734844e-06
5.53911983659738
LOC80054 -2.03096327683616
4.88990646766169 -5.24145008611932 4.029904840739e-07
1.82434280882848e-06 5.53852036799501
TMPRSS3 2.45009858757062 8.36289950248756
5.24132801689715 4.03224938278898e-07 1.82500175989104e-06
5.53795762887031
SERPING1 -1.51583855932203
12.4365487562189 -5.23736083027924 4.10917174301622e-07
1.85817832343883e-06 5.51967430313892
SAG -1.38844491525424
0.782022885572139 -5.23575248698051 4.14076167201729e-07
1.87205102678958e-06 5.51226502011374
MAPT -1.80732980225989
4.66317910447761 -5.23403555753002 4.17474486437934e-07
1.88658402081681e-06 5.5043574080283
PLCXD1 1.10796292372881 8.66799800995025
5.23376196236759 4.18018511651059e-07 1.88862677028967e-06
5.50309750033428
ARC -1.93773347457627
4.30882935323383 -5.23361780984959 4.18305426165682e-07
1.88950723854377e-06 5.5024336970327
ENC1 1.45735543785311 10.372163681592 5.2322226731499
4.21092151199787e-07 1.90167660891589e-06 5.49600999307649
OR7E91P 2.13861391242938 3.47126119402985

5.22984996263405	4.25873031677771e-07	1.9224215787917e-06
	5.48508817495951
EMID2	3.16890190677966	7.55082139303483
5.22873949553352	4.28128645431483e-07	1.93164198828815e-06
	5.47997787710905
C19orf45	1.81807535310735	4.10528756218905
5.22809030447642	4.29452675497317e-07	1.93645144475289e-06
	5.47699072305527
SLC7A2	-2.86458199152542
7.89820348258707	-5.22775773665893	4.30132490817903e-07
	1.93909091710398e-06	5.47546057172935
UPK2	2.37236447740113	3.57125124378109
5.22733572950241	4.30996638542558e-07	1.94256004155405e-06
	5.47351901446969
BOD1L	-1.08168439265537
9.38044278606965	-5.22707567457395	4.31529995324968e-07
	1.9445370509266e-06	5.47232262113986
BDKRB2	-1.40910324858757
7.36172288557214	-5.22655892490267	4.325917218579e-07
	1.94889107293792e-06	5.46994542715778
TRIB3	1.5097968220339 8.26508507462687	5.22482418986913
	4.36174551270603e-07	1.9641727434932e-06
5.46196646161729
ARL4C	1.54041087570621	10.1785228855721
5.22333932426704	4.39264208047811e-07	1.97591897266029e-06
	5.45513837445126
RARB	-1.48904943502825
6.68999751243781	-5.22297951660502	4.40016078897946e-07
	1.97843409981928e-06	5.45348403691394
ZNF578	-1.87835946327684
4.87144726368159	-5.22146241012129	4.43200073561584e-07
	1.99231388443602e-06	5.44650957747695
SHISA6	-2.0347709039548
1.66597014925373	-5.22020656551129	4.45852679903096e-07
	2.00379936917919e-06	5.44073735765955
PIM1	-1.0740843220339
9.64948955223881	-5.21851610807577	4.49447653875726e-07
	2.01907228316601e-06	5.43296919787422
CAMK2D	-1.05874173728814
9.01562935323383	-5.21721165587902	4.52240978878398e-07
	2.03073212956551e-06	5.42697615400058
IL17RD	-1.59476779661017
9.22485223880597	-5.21127632792372	4.65165226634192e-07
	2.08648505632509e-06	5.3997218506463
ART5	-1.94309293785311
3.88126815920398	-5.2102468668668	4.67443119570265e-07
	2.09624448335027e-06	5.39499708957075
FAM19A5 -2.39009618644068
6.30811343283582	-5.2097907493906	4.68455834472917e-07
	2.10032720719131e-06	5.39290394323845
C8orf48 -1.8793552259887
3.18118706467662	-5.20937236769089	4.69386639379333e-07
	2.10404098394273e-06	5.39098409077054
RNF43	1.70890748587571	8.82983383084577

5.20879248369844	4.7067972270968e-07	2.1089163481514e-06
	5.38832333710152
ANKRD56 1.81995536723164	5.50368059701493
5.20863764089159	4.71025591829844e-07	2.11000554227944e-06
	5.3876128907241
MAN1A1	-2.11604653954803
7.69591990049751	-5.20812072879975	4.72181996781377e-07
	2.11472434422375e-06	5.3852413222267
CYP27C1 -2.11774117231638	3.1938776119403 -5.20693402545649
	4.74847265253199e-07	2.12573361440213e-06
5.37979745968589
PRDM11	-1.28984823446328
4.02763432835821	-5.20520183152011	4.78763934376105e-07
	2.14245749190958e-06	5.37185291610756
KBTBD11 -2.05573594632768
5.99708756218905	-5.20261651347852	4.84668054275189e-07
	2.16780722315242e-06	5.35999933848087
VSIG2	-3.34233283898305
4.21159751243781	-5.2021402373317	4.85763421559918e-07
	2.17223329222939e-06	5.35781612035451
DPCD	1.03273827683616	9.43848457711443
5.20118546572427	4.87966509096309e-07	2.18160985011917e-06
	5.35343996879184
ETS2	-1.0375779661017
11.0717119402985	-5.19871929215307	4.93702100505673e-07
	2.20677207127835e-06	5.34213920395149
ABHD3	1.27729004237288	8.24300696517413
5.198390356337	4.94472041654004e-07	2.20973248044334e-06
5.34063222723186
ROBO3	-1.95041758474576
5.94004726368159	-5.19599618262347	5.00111346959183e-07
	2.23201868545044e-06	5.3296658175617
B3GNT9	-1.08696786723164
8.25089850746269	-5.19584027995209	5.00480723628814e-07
	2.2328784087346e-06	5.32895184542056
XDH	3.00291998587571	5.63649154228856
5.19582307547366	5.00521502045623e-07	2.2328784087346e-06
	5.3288730567606
CD1C	-2.00853947740113	3.2393736318408 -5.19326648189837
	5.06617211037877e-07	2.25909021342476e-06
5.31716723235914
JUP	1.02549053672317	12.974452238806 5.19290478253095
	5.07485416942244e-07	2.26247027357747e-06
5.31551148078513
ACOT7	1.0619531779661 9.4035736318408 5.19200278525425
5.09656830111912e-07	2.27165756018184e-06	5.31138278942618
FAM110B -2.01368269774012
6.94702786069652	-5.19142280793467	5.11057797137451e-07
	2.2774075483464e-06	5.30872836106965
TNMD	-1.43009209039548
1.10179850746269	-5.18955740499535	5.15589195329136e-07
	2.29660364072622e-06	5.30019235421126
ARSH	1.54836652542373	1.66917860696517
5.18856003430715	5.18027977034487e-07	2.30646592247192e-06

5.29562938571714
EPPK1 1.77943679378531 8.00082089552239
5.18771527058871 5.20102365144556e-07 2.31519981708971e-06
5.29176511630231
ZNF699 -1.49884830508475
3.00180199004975 -5.18599608957152 5.24348910055527e-07
2.33359703521352e-06 5.2839024111908
SLC5A7 -1.35646793785311
0.773990049751244 -5.18527561098674 5.26138561039047e-07
2.34054705484976e-06 5.28060787995262
TPO -1.18688898305085
0.568712437810945 -5.18470191726222 5.27567844331649e-07
2.34639684664834e-06 5.27798480104516
GCAT 1.19557040960452 8.99678656716418
5.18423459604776 5.2873490132278e-07 2.35056897448174e-06
5.27584824874746
TMEM106A -1.06858495762712
5.82367810945274 -5.18105227539659 5.3674917149748e-07
2.38568098957421e-06 5.26130285634877
ATP10B 2.73986313559322 3.98104029850746
5.18026562768856 5.38748361090036e-07 2.39353037290163e-06
5.25770838521164
WNT9A -1.87475353107345
3.63157263681592 -5.17990342143646 5.39671303677521e-07
2.3965935246592e-06 5.25605347686173
LTBP2 -1.34755360169492
9.96166915422886 -5.17737792801151 5.4614937067852e-07
2.42431277406054e-06 5.24451703364073
FAM5C -1.77530240112994
1.27563233830846 -5.17670777773454 5.47880994547174e-07
2.43094809121536e-06 5.24145651032394
RPP25 1.20580861581921 8.65960646766169
5.17181645376948 5.60682365390983e-07 2.48452600075046e-06
5.2191273738539
GJA5 -1.51815995762712
7.03829203980099 -5.17100742044815 5.62827546256915e-07
2.49349363932984e-06 5.21543564889201
GPR153 -1.4101218220339
7.82441343283582 -5.16949116288888 5.6686944816631e-07
2.50977566764165e-06 5.20851795430091
PARP11 -1.29514371468927
6.94763781094527 -5.16891066883809 5.68424327475818e-07
2.51557478833338e-06 5.20586994833826
FXYD3 2.52959943502825 9.26459104477612
5.16777523091919 5.71477632815411e-07 2.52854219864232e-06
5.2006911434257
MAL 3.32330706214689 7.74104427860697
5.16653247070409 5.74837797367355e-07 2.54121877259944e-06
5.19502383027967
C5orf25 -1.13528305084746
7.97560646766169 -5.16562961002143 5.77290970370552e-07
2.55151423502851e-06 5.1909072010028
EPHX2 -1.46057210451977
8.07245373134328 -5.16477602904745 5.79619593545574e-07

2.56125489786712e-06	5.18701576973452
FAT2	2.75334526836158	6.29737512437811
5.16217195337269	5.86780184842799e-07	2.5917808078362e-06
5.17514695893838
ADAMTS14	1.73635967514124	6.24167064676617
5.15999649647571	5.92827975089725e-07	2.61793037827319e-06
5.1652352048537
GLIPR2	-1.37380699152543
9.26080049751244	-5.15910771997617	5.953161611976e-07
2.62835285793263e-06	5.16118670539892
FAM101B	-1.16969117231638	7.9376776119403	-5.15876367288249
5.96282063141255e-07	2.63205133439905e-06
5.15961966653212
SRRM3	-1.78160303672316
5.73672985074627	-5.15835943796099	5.97418885294709e-07
2.6360426786352e-06	5.15777859087672
ENPP5	1.46735677966102	8.23533333333333
5.15835084023188	5.97443087369196e-07	2.6360426786352e-06
5.15773943397836
ITGA9	-2.01356313559322	6.2149039800995	-5.15757545000845
5.9962968223721e-07	2.64455367912566e-06
5.15420825585236
CG030	-1.03234809322034
4.65839651741294	-5.1572293900826	6.00608075151356e-07
2.64829978154152e-06	5.15263240709962
BHLHB9	-1.00326518361582
6.90833184079602	-5.15710462514919	6.00961195078661e-07
2.64871905001393e-06	5.15206428640365
FOXD4	1.24542838983051	4.46216218905473
5.15695618960769	6.01381572122693e-07	2.65000293532494e-06
5.15138839459904
FGR	-1.53846935028249
6.63530348258706	-5.15631822185983	6.03191582852041e-07
2.65740841114859e-06	5.1484836182905
MFSD6L	2.13714604519774	6.42648109452736
5.1548539411842	6.07365996585012e-07	2.67465120332267e-06
5.14181753665287
ITGA10	-1.70565190677966
4.70782885572139	-5.15235348031006	6.14559325079007e-07
2.70342895733277e-06	5.13043763067138
WFDC1	-1.67357895480226
6.42555970149254	-5.15070334629604	6.19351671895697e-07
2.72334329064439e-06	5.12292997964886
LEPREL1	-1.728372740113	7.99500845771144	-5.14976043204324
6.2210637086066e-07	2.7348701913853e-06
5.11864080767448
TTF2	1.16139583333333	8.32892587064677
5.1480949466029	6.2700113537794e-07	2.75461872658016e-06
5.11106623792883
NMNAT3	-1.75939872881356
6.44272039800995	-5.14799096672718	6.2730796325171e-07
2.75537708291776e-06	5.11059340323674
LYNX1	-2.00155148305085
7.68531890547264	-5.14785802476121	6.27700465961022e-07

2.7559218490547e-06	5.10998887792302
AKAP6	-1.97982874293786
6.35865621890547	-5.14495422056429	6.36333496161652e-07
2.79084099042912e-06	5.09678742586687
C9orf130	-1.00175028248588
7.28052835820896	-5.14115121821192	6.4781437459018e-07
2.83876821992239e-06	5.07950660639696
C2CD4D	1.27621002824859	3.61328507462687
5.13938354109489	6.53219037659227e-07	2.86123039111242e-06
5.071477622575
C5orf23	-1.81837768361582	6.671792039801	-5.13587030091097
6.64091023299289e-07	2.90761115554157e-06
5.05552637671595
KCNK6	-1.51875444915255
7.61402686567164	-5.13431966537921	6.68945273526874e-07
2.92761595939175e-06	5.04848864792544
CD22	-2.42850190677966
5.26330597014925	-5.13362394544104	6.71134396984929e-07
2.93657058186287e-06	5.04533157607856
ARSG	-1.42556468926554
6.24183532338308	-5.13281038424841	6.73703137266853e-07
2.94592660449169e-06	5.04164017421996
PMEPA1	-1.43217266949153
10.3703288557214	-5.132271364792	6.7541029289034e-07
2.95213398191158e-06	5.03919470782225
TGM7	2.51216920903955	2.90239203980099
5.13169313861866	6.77246290968571e-07	2.95952881089501e-06
5.03657158406148
CCDC111	-1.12076596045198
7.02629850746269	-5.13130097096524	6.78494267873196e-07
2.96435141933107e-06	5.03479264390574
GPR89C	1.05091179378531	6.56130895522388
5.12806965786672	6.88862458798779e-07	3.00709042720368e-06
5.02013881661407
GNE	-1.09795282485876	8.5857960199005	-5.1273788683984
6.91098840095872e-07	3.01621156189801e-06
5.0170070424021
SRMS	2.08079788135593	2.38411940298507
5.1251910169397	6.98228460745582e-07	3.04668019109602e-06
5.00709029867516
NFIX	-1.50947641242938
10.6661845771144	-5.1244584230845	7.00631705024663e-07
3.05651697920874e-06	5.00377043955137
ZNF536	-2.07967379943503
1.59777462686567	-5.12408140013127	7.01871641010298e-07
3.06100664541551e-06	5.00206204524284
APOBEC3A	1.67873220338983	4.42971990049751
5.12339924234761	7.04120506366467e-07	3.06912823687974e-06
4.99897124641759
HCN1	-1.71640967514124
1.47098059701493	-5.12236374154633	7.07547608465874e-07
3.08341167518667e-06	4.9942800836692
SPATA17	1.89181433615819	6.00988855721393
5.12012383385445	7.15016308689378e-07	3.11397641438119e-06

4.98413504605713
LRRC16A 1.04601751412429 8.93401990049751
5.11932457508522 7.17699820003466e-07 3.12500048879558e-06
4.9805158503404
C6orf122 -1.21914209039548
2.72141044776119 -5.11853253617155 7.20368733310106e-07
3.13595633902809e-06 4.97692977519316
SCXB 1.67311610169492 3.97562437810945
5.11496924468129 7.32495500205119e-07 3.1853701798795e-06
4.96080170539926
SYNGR1 -1.48234237288136
8.43872039800995 -5.11463016251356 7.33659751962681e-07
3.18975745611898e-06 4.95926741046304
SERTAD4 -2.37418015536723
4.67902487562189 -5.11429428500154 7.34814771028496e-07
3.19410274173513e-06 4.95774769308697
LOC728819 -2.01843728813559
4.03450348258706 -5.11205861725263 7.42547902632051e-07
3.22635099281185e-06 4.94763410350345
MTMR7 -1.11583495762712
4.99145323383085 -5.11183620732769 7.43321519996987e-07
3.22902894742661e-06 4.94662816353853
MAPRE2 -1.25117422316384
8.15615721393035 -5.11153072727642 7.44385358718834e-07
3.23296623958064e-06 4.94524655969241
EDN2 2.22768185028249 5.91466915422886
5.11089060949351 7.46619372921708e-07 3.24129746798111e-06
4.94235168532212
CNTFR -2.97690310734463
4.28663134328358 -5.10513618955424 7.66996684523533e-07
3.32694737656204e-06 4.91634035083654
CD109 -1.77572570621469
7.97018457711443 -5.10399397102068 7.71105176266772e-07
3.34335570617884e-06 4.91117993203519
LBH -1.09155296610169
10.5371567164179 -5.10363723103505 7.7239271982314e-07
3.34823111021306e-06 4.90956840154206
PLA2G2A -2.49927860169492
2.30692189054726 -5.10346358592665 7.73020193275961e-07
3.35024373399947e-06 4.90878401172534
C6orf204 -1.62132266949153
4.00018059701493 -5.10314162847593 7.74184908387643e-07
3.35458340422083e-06 4.90732971955722
C9orf102 -1.33955084745763
5.18042885572139 -5.10255911292569 7.76296549563458e-07
3.36231395834131e-06 4.90469865729025
PLCL2 -1.48944625706215
6.85701890547264 -5.10186114079139 7.78834086933998e-07
3.37188186519506e-06 4.90154641348601
FAM46A -1.26786052259887
8.33023830845771 -5.09851806407495 7.91100159930365e-07
3.42209993328496e-06 4.88645271013533
SEC14L2 1.07767838983051 8.43755870646766
5.09564377801365 8.01795897297072e-07 3.46398774652965e-06

4.8734816391344
GPLD1 -1.34626483050847
3.19239154228856 -5.09033965813372 8.21903049654275e-07
3.54861611133591e-06 4.84955999445365
SLC27A2 1.92370896892656 6.37095820895522
5.08975055014156 8.24166230869605e-07 3.55763934393461e-06
4.84690429324216
ZNF704 -1.6222470338983
7.30627910447761 -5.08895838759024 8.2721902278137e-07
3.56931621221134e-06 4.84333359420927
S100P 3.05184872881356 5.51142388059701
5.08862190998138 8.2851903881649e-07 3.57417437842037e-06
4.84181703965446
LRRC17 -1.99305762711864
6.84311940298507 -5.08689420471046 8.35225489927967e-07
3.60234859416411e-06 4.83403123125235
CDK14 -1.6192095338983
7.64388308457711 -5.08604871129819 8.38526615779341e-07
3.61506749725953e-06 4.83022180370959
LOC389333 -1.46338997175141
4.58333482587065 -5.08591247285363 8.39059725049318e-07
3.6166063731393e-06 4.82960801782858
RHBDL2 1.71694759887006 5.50354726368159
5.08258990205928 8.52163422967144e-07 3.6723163462459e-06
4.81464298348857
HSD17B14 -1.59757867231638
7.41893532338308 -5.08243654235802 8.52773018801788e-07
3.67403069366894e-06 4.81395242515321
CPEB2 -1.15359011299435
7.71526865671642 -5.08239979338334 8.52919156650082e-07
3.67403069366894e-06 4.8137869517923
IRS2 -1.91462415254238 8.0503960199005 -5.08176908671633
8.55431047124592e-07 3.68407791010444e-06
4.81094714812463
FBLN7 -1.6767947740113
5.94946119402985 -5.08074758373138 8.5951457946488e-07
3.7008880697177e-06 4.8063483309286
PPP1R3F -1.0256718220339
6.47404228855721 -5.08016493648431 8.61852217459027e-07
3.71017530392825e-06 4.80372556587929
LOC100133161 1.37062485875706 5.85403482587065
5.07759389118996 8.72241372792899e-07 3.75411223971451e-06
4.79215486840213
MMP2 -1.72496709039548
11.9450407960199 -5.07701665609048 8.74590527702762e-07
3.76343398317705e-06 4.78955770784497
KCNH2 -2.30742697740113
6.88782835820895 -5.07174522586788 8.96329391561136e-07
3.85052153353214e-06 4.7658504318562
NYNRIN -1.46052097457627
9.43558208955224 -5.06650217387956 9.18470762487498e-07
3.94398760800425e-06 4.74228962225964
CTAGE4 1.35128820621469 6.08552786069652
5.06625764514411 9.19516250697469e-07 3.94765132304873e-06

4.74119123734003
KCNH1 -1.76259745762712
3.01894527363184 -5.06262205040412 9.3519715642023e-07
4.00492232401071e-06 4.72486553894347
FAT3 -2.32781038135593
3.35850796019901 -5.06237254950286 9.36282753375255e-07
4.00873512550448e-06 4.72374548268901
CFD -1.9634686440678
6.73989203980099 -5.06183976066561 9.38605050691063e-07
4.0167817960252e-06 4.721353836501
P2RX6 -1.44798792372881
2.31380199004975 -5.06180675416052 9.38749101672133e-07
4.0167817960252e-06 4.72120567934913
ULBP2 2.02893425141243 3.56501094527363
5.06171524272083 9.39148599116507e-07 4.01765383201957e-06
4.72079491348624
MYLK2 1.26682761299435 3.79381044776119
5.0613379437526 9.40797457515194e-07 4.0230309941235e-06
4.71910139848471
LOC222699 1.24467365819209 4.96455174129353
5.06026464447104 9.45503320960236e-07 4.04147057657998e-06
4.71428440318389
C6orf227 1.32560995762712 2.12409900497512
5.05487257885158 9.69492331309967e-07 4.14142271364828e-06
4.6900966086615
KLF7 -1.47697422316384
5.08798507462687 -5.05226017523037 9.81326031383281e-07
4.18848719839891e-06 4.67838501974025
MFNG -1.24006412429378
7.43891990049751 -5.0520960326252 9.82074225783275e-07
4.19080936506561e-06 4.67764931310255
CDH19 -1.59847344632768
0.701555721393035 -5.05125916557225 9.85897439833697e-07
4.20362916714139e-06 4.67389866279161
NEK7 -1.15783382768362
9.42923582089552 -5.05075846473602 9.88191781218614e-07
4.21253680822636e-06 4.67165486359596
FAM49A -1.30460056497175
5.28373930348259 -5.0495180894664 9.93897827949003e-07
4.23510220170051e-06 4.66609708914377
GUCY1B2 2.34374491525424 3.3280447761194 5.04889776893527
9.96763435456175e-07 4.24643148576785e-06
4.66331800220921
CYP3A5 -2.66550692090396
3.93365422885572 -5.04624005741661 1.00913185559831e-06
4.29823174179113e-06 4.65141422416079
BRCA2 1.64823792372882 6.07852089552239
5.04553780681758 1.01242475824293e-06 4.31136284727802e-06
4.64826968265992
CRABP1 3.81773785310735 5.40528855721393
5.04537633890676 1.01318336473668e-06 4.31369856450517e-06
4.64754670862994
KIAA0748 -1.59895974576271
3.55310248756219 -5.04160014710453 1.03108262967041e-06

4.38717645329191e-06 4.63064387832443
GVIN1 -1.62119279661017 4.5681407960199 -5.04091983559734
1.03433976060892e-06 4.3983005976183e-06
4.62759973732344
PLS3 -1.18311483050847
11.0778109452736 -5.03830396853041 1.0469569494545e-06
4.45010893836458e-06 4.61589766557158
CDKN1B -1.20009110169492
9.97456169154229 -5.03799934516473 1.04843591366962e-06
4.45547284364257e-06 4.61453523958537
AMIGO1 -1.0775031779661
5.67154975124378 -5.03793612957068 1.04874308278641e-06
4.45585585463681e-06 4.61425251624635
RNASE2 1.45958488700565 3.1861144278607 5.03747327081781
1.0509948067897e-06 4.46449894131857e-06
4.6121825256558
TDO2 1.67088629943503 4.35393582089552
5.03559661744749 1.06017251518621e-06 4.50255311062741e-06
4.60379129033544
LRRC49 -1.10306885593221
7.57189502487562 -5.03366230350347 1.06971352190402e-06
4.53931761155218e-06 4.59514476540993
PCDHB14 -1.31513552259887
6.89394975124378 -5.03218492385336 1.07705672300471e-06
4.56812806971229e-06 4.58854250254772
EGFL7 -1.55668997175141
9.34364179104478 -5.0321621331991 1.07717038409533e-06
4.56812806971229e-06 4.58844066513105
TMEM132D -1.52246786723164
0.639698507462687 -5.03202044537554 1.0778772676838e-06
4.57018180618513e-06 4.58780755740297
PPFIA3 1.12223481638418 8.13260746268657
5.02546373265586 1.11108541377133e-06 4.70515337143676e-06
4.55852511508559
CADPS2 -1.60566814971751
8.30651592039801 -5.02505197490741 1.11320364225479e-06
4.71315132511668e-06 4.5566871817993
HIST2H2BE 1.42050105932203 9.02073482587065
5.02164498738959 1.13088125711029e-06 4.78602189414022e-06
4.54148413597888
MAMSTR -1.60559555084746 5.7420631840796 -5.02053625324673
1.13669254213935e-06 4.80863339998371e-06
4.5365383404938
ZNF302 -1.29464555084746
8.57700149253731 -5.02042491355332 1.13727771155248e-06
4.80925092882777e-06 4.53604172776363
CHST3 -1.12885826271186
9.18240995024876 -5.02041933723356 1.1373070267637e-06
4.80925092882777e-06 4.53601685571399
CLDN11 -2.06406878531074
5.22949552238806 -5.01947918222929 1.14225999276961e-06
4.82920050485175e-06 4.53182379013325
BARX1 2.98326461864407 3.58284975124378
5.01740929761611 1.15323845717493e-06 4.87059977901695e-06

4.52259430741443
ZNF311 -2.26575105932204
3.68822388059702 -5.0158501716245 1.16157537807966e-06
4.90296223830513e-06 4.51564421391546
HHIPL1 -1.41260741525424
5.48864129353234 -5.015163980635 1.16526304887255e-06
4.91531752483122e-06 4.51258592112557
RPS6KA3 -1.05865607344632
9.20700895522388 -5.01379025313673 1.17267975300504e-06
4.94558655077928e-06 4.50646431395858
ANKRD24 -1.54626829096045
4.31984825870647 -5.01294648773962 1.17725787371428e-06
4.96387433710291e-06 4.50270496988779
SYNGR4 1.48558651129943 1.61835721393035
5.01279986700649 1.17805517650991e-06 4.96519662774551e-06
4.50205176042104
UBE2Q2P1 -1.46232507062147
3.08878507462687 -5.01236247568063 1.18043675953444e-06
4.97421319237318e-06 4.50010322806639
C11orf53 -1.86033799435028
1.48485024875622 -5.01017692155391 1.19240713162234e-06
5.02156275122187e-06 4.49036879093853
PCSK9 2.58406490112994 6.06709900497512
5.00837077628016 1.20238818279281e-06 5.06151925215017e-06
4.48232672366992
TMPRSS2 2.2604177259887 8.20864626865672 5.00539868107111
1.21898897769163e-06 5.12719600737743e-06
4.46909803118569
FGL2 -1.72530190677966
7.69747512437811 -5.00493596124407 1.22159342807014e-06
5.13709813156081e-06 4.46703902978718
C8ORFK29 1.4465988700565 3.04017064676617
5.00430846090118 1.22513397458966e-06 5.1509319062719e-06
4.46424702748418
IL23A 1.63655275423729 4.79333930348259
5.00203704949717 1.23803331166987e-06 5.20196968657029e-06
4.45414287444992
SYN2 -2.58804300847458
4.54398955223881 -5.0015205150453 1.24098502698417e-06
5.21117255143894e-06 4.4518456183229
F13A1 -1.7415040960452
7.30669502487562 -4.99927401693038 1.25390207989297e-06
5.26218513904389e-06 4.4418565958062
ZNF853 -1.33354653954803
8.41432039800995 -4.99743040648449 1.26459984113914e-06
5.30491106223673e-06 4.43366161246883
REV3L -1.30010035310735
9.68793134328358 -4.996235787136 1.27157892658305e-06
5.33200885677085e-06 4.42835269614213
SIGLEC15 2.18298997175141 3.53440945273632
4.99398158639978 1.28484999893922e-06 5.38435812986776e-06
4.41833766226744
ARHGEF38 1.80367549435028 2.85324875621891
4.99349410899768 1.28773750760373e-06 5.39535735328663e-06

4.41617234435005
TMCC2 -1.37328516949152
6.12756417910448 -4.99296214640096 1.29089569396828e-06
5.40748594106686e-06 4.41380961586688
C17orf107 -1.30148608757062
5.53385273631841 -4.99074477134297 1.30414097356895e-06
5.46074121708555e-06 4.40396318336289
FCGR1B 1.57575120056497 5.87510597014925
4.99053978073699 1.30537209440428e-06 5.4645110218214e-06
4.40305307780132
C14orf149 -1.05076151129944
6.10175671641791 -4.98830939029481 1.31884024410206e-06
5.51666467225252e-06 4.39315259693681
SOAT2 -1.25746560734463
0.993092039800995 -4.98785563486719 1.32159666937986e-06
5.52369291986328e-06 4.39113884319132
TNFRSF25 1.13048043785311 7.73882686567164
4.98574740978214 1.33447688245815e-06 5.57412215602559e-06
4.38178446902432
TMEM98 -1.43478488700565
10.2988243781095 -4.9857003338938 1.33476587669313e-06
5.57419516853334e-06 4.38157562441592
AXIN2 -1.74784950564971 8.6516447761194 -4.98510055302492
1.33845318468967e-06 5.58845716527941e-06
4.37891492733449
SLC38A5 -2.27340056497175
6.13395024875622 -4.98472812940655 1.340747720201e-06
5.59689928745964e-06 4.37726293856155
LOC339535 -2.69076617231639
4.81683184079602 -4.97970615701009 1.37206324102231e-06
5.72529641020083e-06 4.35499596043292
RAB3B 1.91657577683616 2.6913184079602 4.97847482876119
1.37984895132251e-06 5.75597267856534e-06
4.34953902769912
IL20RB 1.17034653954802 4.59686616915423
4.97845486652977 1.37997552478805e-06 5.75597267856534e-06
4.34945056883553
PLCE1 -1.60090663841808
7.95018805970149 -4.97779421594031 1.3841708295051e-06
5.76995474715527e-06 4.34652317615686
MAP1B -1.67066991525424
9.11838805970149 -4.97666520196359 1.39136898445776e-06
5.79642963695573e-06 4.34152113012309
DNM3 -1.34602549435029
6.21053980099502 -4.97605375352901 1.39528246795512e-06
5.81155387849841e-06 4.33881250395502
FAM40B 1.61173665254237 4.79976965174129
4.9752365461772 1.4005295350807e-06 5.82986037210196e-06
4.33519280088826
INE2 -1.53252450564972
4.54780099502488 -4.97419550235795 1.40724148955595e-06
5.85661215904796e-06 4.33058231683782
SCUBE2 -2.0206290960452
7.52432338308458 -4.97199759669256 1.42151462119941e-06

5.91122041183389e-06	4.3208508923231
PPP4R4	-2.00796687853107
2.94182786069652	-4.9713109257952	1.42600252242272e-06
5.92748163299018e-06	4.31781128224611
LOC220930	-1.03437768361582
6.62404676616915	-4.9706478392225	1.43034930836236e-06
5.94314335168574e-06	4.31487638073047
HOXB9	2.97524371468927	8.47795323383085
4.97025708135808	1.43291688366078e-06	5.9526069651064e-06
4.31314698192325
DRD1	-1.53061631355932
1.30309800995025	-4.96812031163211	1.44703623742244e-06
6.0063999098833e-06	4.30369203508762
ARHGEF33	-1.40040628531074
3.68801890547264	-4.96694725092363	1.45484480034706e-06
6.03759121712659e-06	4.29850273104381
PLA2G10	2.24140077683616	4.04206666666667
4.96582202590124	1.46237328852103e-06	6.06638181720283e-06
4.29352593674678
FRMPD4	-1.06247810734463
0.603709950248756	-4.96532559462948	1.46570672204252e-06
6.07898161687535e-06	4.29133053420305
PDE4A	-1.29225360169491	7.7510328358209	-4.96388319551453
1.47543395085412e-06	6.11561861720878e-06
4.28495268317553
FCGR1C	1.54741144067797	4.39745422885572
4.96077258841638	1.49662451953334e-06	6.19719672973365e-06
4.27120344101645
SQLE	1.22897217514124	10.1956208955224
4.96037716784717	1.4993392940019e-06	6.20604550093028e-06
4.26945611803871
S100A8	2.84540077683616	6.95324726368159
4.96037326934683	1.4993660830304e-06	6.20604550093028e-06
4.26943889150601
LGALS1	-1.20775649717515
12.6254532338308	-4.95049550172162	1.5687523860192e-06
6.4862218528943e-06	4.22582534568331
CLEC18B	1.91592683615819	3.87927611940299
4.94997428075174	1.5724988015714e-06	6.49763616473224e-06
4.22352587104771
MTUS1	-1.10137394067796
10.0850875621891	-4.94995158112997	1.57266215794225e-06
6.49763616473224e-06	4.22342573124439
KRT75	2.12059336158192	1.9347631840796	4.94524852434848
1.60686423477705e-06	6.63093924421567e-06
4.20268584375188
SLC30A8	-1.56009816384181
1.00659054726368	-4.94264605591733	1.626098635496e-06
6.70761603108957e-06	4.19121590510054
GATS	-1.01252125706214
7.85463930348259	-4.94127413467995	1.63632782950765e-06
6.74709988748586e-06	4.18517129376898
LOC100302640	-1.38777330508475
1.86917263681592	-4.94096199785167	1.63866384232482e-06

6.75537523753587e-06 4.18379621877665
C19orf51 1.9941418079096 7.76183532338308
4.93999574852946 1.64591568267558e-06 6.78254696212962e-06
4.17953997335472
RAB30 -1.21698721751413
4.59919552238806 -4.93948410365202 1.64976822612058e-06
6.79705833478937e-06 4.17728648500874
CNFN 1.79552161016949 6.29343930348259
4.93943227019768 1.6501590043695e-06 6.79730424552686e-06
4.17705819995565
PCDH10 -2.75530889830509
4.18442487562189 -4.93644934077233 1.67279927971588e-06
6.88779968162194e-06 4.16392392833962
TPPP -1.98115353107345
6.33507711442786 -4.93613623124329 1.67519313380829e-06
6.89627331804374e-06 4.16254562133552
GRM6 -1.47393827683616
2.08852935323383 -4.93489728371924 1.68469798178625e-06
6.92984363175148e-06 4.15709244929087
TMEM191A 1.31084505649717 4.48528457711443
4.9313925032414 1.71186930771738e-06 7.03878968304382e-06
4.14167211302586
LRRTM2 -1.30341892655367
1.93462985074627 -4.92951218831414 1.72662111926213e-06
7.09518246165962e-06 4.1334026340949
SLC10A4 1.83271016949152 3.76557910447761
4.92771866152676 1.74080668968754e-06 7.1506125128622e-06
4.1255171420861
IQCD 1.32323580508475 6.91036666666667
4.9267298818988 1.74867544502201e-06 7.18149767679572e-06
4.12117079407566
SLC16A9 -2.04606765536723
7.62091393034826 -4.92024671559135 1.80112848387444e-06
7.38656967118302e-06 4.09268985682406
HOXA6 -2.19161744350283
3.19625572139303 -4.9185381312915 1.81520369032339e-06
7.44280642347496e-06 4.08518883360038
HGD 2.68348524011299 8.20831492537313
4.9183752449229 1.81655108360668e-06 7.44684379921028e-06
4.08447383674657
VIM -1.4325906779661
14.6997815920398 -4.91807725839325 1.81901852936692e-06
7.45547023515727e-06 4.08316585981825
PCP4 -3.0135020480226
4.98941492537313 -4.91791139409309 1.82039335633227e-06
7.45961588932165e-06 4.08243784481595
BBS10 -1.03830480225989
7.80662189054726 -4.91677555720125 1.82983522322044e-06
7.49531467133192e-06 4.07745292299304
FAM126A -1.17813792372882
7.79804776119403 -4.91654432679529 1.83176317082402e-06
7.50171512009118e-06 4.07643821746483
NLRP1 -1.60558573446328
7.26290099502488 -4.91519454483451 1.84305665516533e-06

7.54646053660633e-06	4.07051573215088
PITX2	2.88253997175141	3.3066144278607 4.91483528534409
1.84607388090019e-06	7.55730743267816e-06
4.06893961147417
GOLGA6L5	-1.5644831920904
4.37357910447761	-4.91358993607005	1.85656995385086e-06
7.59876019787842e-06	4.06347679220607
HHATL	-1.84892026836158
1.72033383084577	-4.91291842884996	1.86225351037464e-06
7.62050320270905e-06	4.06053162495016
PPP1R14A	-1.82232951977401
7.27695373134328	-4.91277310232719	1.86348575167277e-06
7.62384784248857e-06	4.05989427806895
GPR110	2.77140035310735	5.28089104477612
4.9126906874198 1.86418490839224e-06	7.62384784248857e-06
4.0595328441821
KLHL17	1.06221080508475	7.4629671641791 4.91215752154077
1.86871409413023e-06	7.64084850677029e-06
4.05719473861968
ZSWIM5	-1.56427125706215
6.25411990049751	-4.91083981316848	1.87995362390769e-06
7.67918649882818e-06	4.05141701267655
GTF2A1L -1.81755211864407
1.81396069651741	-4.90750417418074	1.90869855880267e-06
7.79037403821447e-06	4.03679674414081
KITLG	-1.66338622881356
7.94875621890547	-4.90655751257473	1.91693363366846e-06
7.82243043116943e-06	4.03264889914869
PPP4R1L -1.52983672316384
4.60226368159204	-4.90185813717769	1.95832556131633e-06
7.98340424420658e-06	4.01206764341791
AURKAPS1	1.07319569209039	3.95811542288557
4.90003370220865	1.97462711278875e-06	8.04506739939533e-06
4.00408156764186
FAM63A	-1.0063561440678
8.39697661691542	-4.89864186296814	1.98715160306578e-06
8.09127774909569e-06	3.99799065642195
CAND2	-1.58267401129944
7.14633532338308	-4.89501816132838	2.02012126138974e-06
8.2173745782309e-06	3.98213912978147
OASL	1.97971694915254	7.0950328358209 4.89496161910285
2.02063987272691e-06	8.21785590076929e-06
3.98189186434119
C21orf88	-2.04778693502825
2.82654577114428	-4.89062677285398	2.06078546052623e-06
8.36786501277758e-06	3.9629417772571
ABCA4	2.06884759887006	5.17326268656716
4.89025603606737	2.06425450830517e-06	8.38029366333898e-06
3.961321687268
TMEM150C	-1.98575007062147
5.48405024875622	-4.88750252567907	2.09019700891309e-06
8.48225827099936e-06	3.94929208745614
RPL22L1 1.17332810734463	10.1439875621891
4.88443602239418	2.11945973421221e-06	8.59421456515437e-06

3.93590134315257
KLHDC7A 2.41380911016949 6.18128059701493
4.88150535465105 2.14779643876772e-06 8.70052503707603e-06
3.9231099361475
ALS2CR8 -1.02836440677966 6.393292039801 -4.87735981951847
2.18850652750716e-06 8.85663415898605e-06
3.90502633868219
LHX2 2.27912196327683 2.54583383084577
4.87731793157577 2.18892166208939e-06 8.85663415898605e-06
3.90484367732366
SBSN 2.2102490819209 3.07681791044776 4.87683248779583
2.19373826166886e-06 8.87437369695796e-06
3.90272688561057
WNT2 -2.01116179378531 7.2058144278607 -4.87492549691446
2.21275898251941e-06 8.94782568087023e-06
3.8944130008189
ANO2 -1.60994830508475
3.72283432835821 -4.87492027113237 2.21281132411604e-06
8.94782568087023e-06 3.89439022155514
GPX1 1.07077118644068 12.4677845771144
4.87448898019163 2.21713527904515e-06 8.96195851128115e-06
3.89251028399391
LOC100270804 -1.29726963276836
4.43811990049751 -4.87306574965786 2.23146210617116e-06
9.01277140265473e-06 3.88630754739372
LEMD1 2.69452224576271 4.74495074626866
4.87053429140642 2.25716643962093e-06 9.1112125453043e-06
3.87527844472823
VCAM1 -1.66257528248588 7.1302328358209 -4.86997222118803
2.26291241820035e-06 9.13261095845354e-06
3.87283021903539
CPA4 2.46014781073446 3.54216965174129
4.86904621701227 2.27240972482011e-06 9.16553470541392e-06
3.86879728057876
PPP2R2C -2.66443149717514
7.70045124378109 -4.86864082454669 2.27657964602459e-06
9.18055001628066e-06 3.86703190308916
CLDND2 -1.1745927259887
1.92213631840796 -4.86806972599936 2.28246660369726e-06
9.20248220225939e-06 3.86454511555415
LOC100131193 1.21162379943503 5.12177313432836
4.86790552685576 2.28416191360919e-06 9.20750914714247e-06
3.86383017045428
TBC1D4 -1.12930459039548
8.51748358208955 -4.8676924089636 2.28636411243734e-06
9.21457699810335e-06 3.86290225487234
ZNF660 -1.57045677966102
3.44681044776119 -4.86666206866434 2.29703986905326e-06
9.25578577265229e-06 3.85841660297148
ISM2 -1.79707824858757 3.7371815920398 -4.86634614074409
2.30032296975108e-06 9.26719595152858e-06
3.85704134048521
ANG -1.62791737288136
6.99286915422886 -4.86547301379714 2.30942006884238e-06

9.30019498103844e-06   3.85324090643354
PDGFC   -1.0725261299435
9.33026169154229   -4.86517650248185   2.312517325206e-06
9.31084147606965e-06   3.85195041247362
FAM161B -1.54386596045198
5.63108457711443   -4.86131391401479   2.35323354783588e-06
9.46364049697759e-06   3.83514508742919
FES   -1.04076115819209   8.5628671641791 -4.86099077414645
2.35667108029844e-06   9.47560855435312e-06
3.83373964915543
NALCN   -2.14919442090395
2.60767164179104   -4.85600525304726   2.41032616693931e-06
9.68565202725857e-06   3.81206535861881
ZCCHC18 -1.43295685028249
4.47027164179104   -4.85547229938227   2.41613132716691e-06
9.70328171884519e-06   3.80974940766223
ACSS3   -2.41338064971751
6.55769253731343   -4.85352262545224   2.4374834547864e-06
9.78520440492076e-06   3.80127880398384
CKMT2   -2.01965798022599
5.32079104477612   -4.85183764175872   2.45608371144199e-06
9.85024391402697e-06   3.79396034101345
C1orf141   -1.10182189265537
0.225047263681592   -4.85124300484314   2.46268052241097e-06
9.87284341439116e-06   3.79137810696688
BCO2   -1.71150466101695
4.20694577114428   -4.84970244239153   2.47985109277892e-06
9.93973895641376e-06   3.78468931526123
ERO1LB -1.65895882768362
6.68923830845771   -4.84558969333014   2.52625941318945e-06
1.01119332721348e-05   3.76684084947857
GPR98   2.12486405367232   5.60411144278607
4.84448827399424   2.53882973845411e-06   1.01602679396614e-05
3.76206294941163
GAB3   -1.19105148305085
5.34604278606965   -4.84434309394123   2.54049116066224e-06
1.01649353982451e-05   3.76143322978718
TRY6   2.28777337570622   2.1740855721393 4.84368308265645
2.54805751079209e-06   1.01932230507719e-05
3.75857061337963
RAET1L 1.62988163841808   1.55165223880597
4.8433876947954 2.55145088332033e-06   1.02048093969543e-05
3.75728954993249
ALPPL2 3.56263516949153   6.04578756218906
4.84262853557486   2.56019205382716e-06   1.02298059293639e-05
3.75399744587933
MSC   -1.75090783898305
6.03884328358209   -4.84160996207301   2.57196569760476e-06
1.02748503155917e-05   3.74958102818976
SECISBP2L   -1.1174050141243
9.45197064676617   -4.84118384921298   2.57690663438035e-06
1.02925862264258e-05   3.74773366933058
PROC   1.99236920903955   4.17359104477612
4.83845225589182   2.60879917943764e-06   1.04138921162463e-05

3.73589423793477
RNF180 -1.65683742937853 7.1334855721393 -4.83633532358885
2.63377746444112e-06 1.05115574154308e-05
3.72672253511393
PROX1 -1.92123947740113
2.46211641791045 -4.83613729216541 2.63612588607945e-06
1.05188852169507e-05 3.72586471733442
FOXF2 1.99702683615819 4.1089592039801 4.8347749142392
2.65233706544322e-06 1.05794598705167e-05 3.71996402196096
SRD5A3 1.12445805084746 9.44398606965174
4.83311731838436 2.67219111185595e-06 1.06545122405132e-05
3.71278645732724
KRT6B 2.88894696327684 3.1662855721393 4.83150162089849
2.69168156708923e-06 1.07301403053091e-05
3.70579218833336
GRIN1 1.97204922316384 3.06246815920398
4.8314052926065 2.69284792436084e-06 1.07327058604403e-05
3.70537524661211
ABHD6 -1.05705896892655
7.08139452736318 -4.82852966648538 2.72789233459819e-06
1.0859730391565e-05 3.69293158046966
ENDOG 1.14994576271186 8.60980547263682
4.82756717394292 2.73972013314535e-06 1.09047022748108e-05
3.68876790463464
NRP2 -1.49689936440678
9.58728905472637 -4.82652460089151 2.75258795770904e-06
1.09537954587646e-05 3.68425854632977
C10orf125 1.31566765536723 7.31533134328358
4.82552283687822 2.76500713452419e-06 1.09959625270915e-05
3.67992642141628
KIAA1045 -1.89109675141243
3.21906417910448 -4.82549712760381 2.76532657220524e-06
1.09959625270915e-05 3.67981525110688
PRSS1 3.09158742937853 3.01740099502488
4.82442586705778 2.77866874472995e-06 1.10468763224837e-05
3.67518339481887
SAT1 1.23191052259887 13.2997542288557
4.82374494297844 2.78718174214618e-06 1.10764307329193e-05
3.67223967613259
VSIG1 1.80104992937853 3.12539502487562
4.82314943618082 2.79464750562479e-06 1.11018021072904e-05
3.66966549619136
PLAGL1 -1.66236504237288
7.63127213930348 -4.82011310050677 2.83301509308406e-06
1.12498645765988e-05 3.6565443239949
KRT78 1.89371709039548 1.92670995024876
4.81888006046602 2.84874091866963e-06 1.1310123972208e-05
3.65121775053479
MME -2.19842471751413
6.44084875621891 -4.81878085161522 2.85000986022026e-06
1.13129741744629e-05 3.65078922799203
MYO3A -1.97718248587571
1.23796567164179 -4.81067311327518 2.95558047993434e-06
1.17229650798014e-05 3.6157922774105

LONRF1 -1.02816942090396
8.51711791044776   -4.81000397727325   2.9644601754973e-06
        1.17536437626419e-05   3.61290604418841
CPXM1   -1.81300028248587
9.66646069651741   -4.80949342969217   2.97125267830324e-06
        1.1776026541615e-05   3.61070407579753
TSC22D3 -1.05809484463277
10.2813656716418   -4.80880170394441   2.98047964059545e-06
        1.18103159663047e-05   3.60772098950262
ZSWIM4 1.05734957627118   9.22942388059701
4.80492724230551   3.03267547910208e-06   1.20078741727996e-05
        3.59101854958398
ADM   1.42915007062147   8.94334278606965
4.80423550695716   3.04208685444572e-06   1.2042815873847e-05
        3.58803766543726
EIF5A2 -1.47145494350283
6.25139950248756   -4.80151620004641   3.07935860366621e-06
        1.21786230270213e-05   3.57632269516168
LRP12   -1.15662012711865
8.00001293532338   -4.79958793192663   3.10605512966521e-06
        1.22771104759389e-05   3.56801876210223
RUFY4   2.12092535310734   3.95965970149254
4.79818652253442   3.12559759725526e-06   1.23495994364224e-05
        3.56198536306315
PRDM1   -1.38157048022599
8.01400149253731   -4.79691576737812   3.14342079096096e-06
        1.24104670843132e-05   3.55651566886709
LRRC56 1.25481871468927   7.30197711442786
4.79594605408253   3.15708770098056e-06   1.24572382281628e-05
        3.55234251774821
PRIMA1 -2.15604060734463
6.60501791044776   -4.79305527452716   3.19817125832216e-06
        1.26120737482432e-05   3.53990604777174
RTBDN   1.98318043785311   1.89487860696517
4.79296264271616   3.19949624071998e-06   1.26148757100021e-05
        3.53950763327078
AQP5   3.02196327683616   9.14069850746269
4.78704050984298   3.28531486884947e-06   1.29333680263623e-05
        3.51404889372146
CHGB   -2.41495642655367
3.79488407960199   -4.78569509759352   3.30511925991413e-06
        1.3001360108457e-05   3.50826856382298
C1orf190   -1.67846031073446
4.52909452736318   -4.78485377883406   3.31756198879436e-06
        1.30453069584271e-05   3.5046546393134
DPY19L2 -2.28833771186441
5.69343830845771   -4.78417281620959   3.32766625213942e-06
        1.3080028366154e-05   3.50172990145908
KREMEN2 2.109975   5.11876268656716   4.78322437760799
        3.34178893838391e-06   1.31305123263199e-05
3.49765690356364
ZIC2   3.58928177966102   3.99810248756219
4.7826078958815 3.35099966920081e-06   1.31641835454827e-05
3.49500981314753

RNF169	-1.08956991525424
7.96806965174129	-4.78169373681605	3.36470310486769e-06
	1.32129599737823e-05	3.49108503454144
CNR1	-2.34593107344633
3.30511343283582	-4.77859111978583	3.41161685287516e-06
	1.33869445698637e-05	3.47776894494035
TGFA	1.83886186440678	8.03984626865672
4.77822798080758	3.41714890388578e-06	1.34060896229266e-05
	3.47621084125598
BAHCC1	-1.72575550847458
7.77479651741293	-4.7763949504028	3.44520578880165e-06
	1.35109980599996e-05	3.46834737772983
C6orf201	-1.36608199152543
2.51831144278607	-4.77518505630104	3.46384655802022e-06
	1.35763210836493e-05	3.46315840151672
PDPR	-1.09294590395481
8.49838009950249	-4.77501103756763	3.46653565655349e-06
	1.35842674229897e-05	3.46241215841361
ENTPD8	2.03441518361582	5.59054577114428
4.77445540990207	3.47513523608167e-06	1.36153676329688e-05
	3.46002960949754
RASSF6	2.15162203389831	5.94194527363184
4.77304061364619	3.49712552035219e-06	1.36962966296892e-05
	3.45396391326427
ITPR3	1.2824959039548 10.7713870646766	4.77186731965076
	3.51546402432769e-06	1.37602433592846e-05
3.44893470012259
FZD1	-1.04222874293786
9.08877064676617	-4.77148699727218	3.52142834781619e-06
	1.37757095999745e-05	3.44730469528984
ZIC1	3.89444646892656	4.44874626865672
4.767721711121	3.58100650908048e-06	1.39973549026507e-05
3.43117281422592
SIK1	-1.1768134180791
9.46300995024876	-4.76636875804646	3.60265115562642e-06
	1.4074677112276e-05	3.42537873455437
CRYAB	-1.82010854519774
8.94116368159204	-4.76313646865574	3.65487437296907e-06
	1.42651275431664e-05	3.41154161051858
CD79B	-1.66433764124294	5.6856184079602 -4.76305131472805
	3.65626001916126e-06	1.42667709881394e-05
3.41117717556949
USP43	1.63440812146893	8.10038407960199
4.76040004207672	3.69965692733865e-06	1.44316847270117e-05
	3.39983306440305
LEPROT	-1.84268057909605	7.5490368159204 -4.76025603759673
	3.7020282298152e-06	1.44381914054153e-05
3.39921705029194
SEMA5A	-1.73359463276836
8.43285174129353	-4.75958646826147	3.71307323263387e-06
	1.44785172774878e-05	3.39635299970763
TMC5	2.31595261299435	8.23897512437811
4.75945242733046	3.71528814940141e-06	1.44844029068004e-05
	3.39577968459054

PMCH 1.67966814971751 2.15864825870647
4.75824020433315 3.7353771822142e-06 1.45599570060485e-05
3.39059539087168
GNB3 -1.43741525423729
4.04818308457711 -4.75708538623177 3.75461244664982e-06
1.46273891419649e-05 3.38565757716525
GCK -2.10085861581921
3.28126517412935 -4.75638852911453 3.76626591204954e-06
1.46692163108285e-05 3.38267839135296
ID3 -1.27581885593221
10.2353552238806 -4.75577468666475 3.77656006349308e-06
1.47037318610872e-05 3.38005439550799
IL3RA -1.09405960451978
6.83165771144279 -4.75495872713676 3.79028579762182e-06
1.47543738007539e-05 3.3765668259571
GPR160 1.63470911016949 8.80117412935323
4.74899317462752 3.89210979683881e-06 1.51449988872443e-05
3.35108337192957
CDH8 -1.05564060734463
0.968893532338308 -4.7485029936413 3.90059298184307e-06
1.51751324459424e-05 3.3489905654128
C2orf40 -2.94655960451978
5.15358507462687 -4.74806951221282 3.90810978532324e-06
1.52014956219565e-05 3.34713997857359
PRAP1 2.82565098870057 4.03040149253731
4.74780029626787 3.91278517533207e-06 1.52135537994876e-05
3.34599072952476
BDNFOS -1.18860925141243
5.19008507462687 -4.74776302331522 3.91343290867482e-06
1.52135537994876e-05 3.3458316200632
KIF7 -1.24191998587571
7.62107313432836 -4.74739551328008 3.91982508338894e-06
1.52355185463792e-05 3.34426285950135
DNM1 -1.44718298022599
7.57176915422886 -4.7472402290764 3.92252900120878e-06
1.52431422288125e-05 3.34360003936955
FAM123A -1.35822888418079
0.830293532338308 -4.74672413893154 3.93152847224458e-06
1.52752232277027e-05 3.34139726754764
GRAP -1.22647556497175
7.49441094527363 -4.74545462197475 3.95375096637296e-06
1.53499446557558e-05 3.33597953737282
TNFSF11 1.83100642655367 2.96500796019901
4.74471906763161 3.96668205809292e-06 1.53972362738982e-05
3.33284104895884
TGFB1 -1.00257394067797
9.52239054726368 -4.74421062793495 3.97564432011517e-06
1.54291073519781e-05 3.33067184767841
PAPLN -1.66650459039548
8.06222686567164 -4.74352244081861 3.98780613641631e-06
1.54733812097197e-05 3.32773606912486
TP73 2.07922231638418 5.6759736318408 4.74294445752449
3.99804815097125e-06 1.55072602568373e-05
3.32527067787096

ENPP6	-1.36302994350283
1.85491990049751	-4.74230868178813	4.00934359820221e-06
	1.55432902639652e-05	3.32255904913555
PCOLCE2 -2.17924406779661
4.48463084577114	-4.74109151967712	4.03105421266577e-06
	1.56234742280589e-05	3.31736857445136
PELI2	-1.56111638418079
7.45455223880597	-4.73634140871459	4.11687330965761e-06
	1.59380367805526e-05	3.29712232959579
CLEC5A	1.55213114406779	4.96725621890547
4.73533917068864	4.13520453262322e-06	1.60029687190866e-05
	3.2928525902728
ZFYVE28 -1.13986963276836
6.89179253731343	-4.73524362058118	4.13695628054199e-06
	1.60067305766165e-05	3.29244556486631
GJB3	2.17433538135593	6.27868407960199
4.72763610456005	4.27875018747939e-06	1.65242177104876e-05
	3.26006001345859
KGFLP2	-1.00526257062147
5.78487711442786	-4.72707173526768	4.28945418676469e-06
	1.65593257717712e-05	3.25765912267627
TBX1	-2.47379830508475
4.13358059701493	-4.72655015810623	4.29936955807853e-06
	1.65913641028858e-05	3.25544047787733
PERP	1.11739597457627	12.1777880597015
4.72482357596135	4.33235060747829e-06	1.67092162260998e-05
	3.24809746983377
GNA11	-1.39513665254238
7.71870099502488	-4.72458362077342	4.33695349924544e-06
	1.67238270735366e-05	3.24707713019897
SAA4	2.45729639830509	3.208692039801	4.72441419119315
	4.34020639080524e-06	1.67300858436409e-05
3.24635670511452
MPV17L	-2.13639766949152
4.06411542288557	-4.72370129997036	4.35391906497606e-06
	1.67797932051734e-05	3.24332567340674
PABPC4L -1.86427238700565
5.88510646766169	-4.72281624506263	4.37100155563195e-06
	1.68361467049013e-05	3.23956315377579
DNAJC22 2.00941151129943	6.4087776119403 4.72151280970183
	4.39627707780917e-06	1.69301876673175e-05
3.23402305245681
C9orf170	-1.19760748587571
1.56714875621891	-4.72147225309116	4.39706578870612e-06
	1.69301876673175e-05	3.23385069086171
CCNB3	1.41772316384181	3.59477611940299
4.72078353410979	4.41048028843452e-06	1.69786537261505e-05
	3.230923883653
FBXO6	1.00691899717514	8.65016069651741
4.71746219028888	4.47572817937674e-06	1.71943674256371e-05
	3.21681415873798
C22orf31	-1.51918538135593
1.57168905472637	-4.7163678772555	4.49742931462828e-06
	1.72680426088122e-05	3.21216703827135

ASCL2 1.71754802259887 6.98726766169154
4.71625715695302 4.49963063896619e-06 1.72700349631662e-05
3.21169690033482
OLFML2B -1.16492973163842
8.60442885572139 -4.71584290327266 4.50787599595668e-06
1.72984475134562e-05 3.20993798435154
ZNF585B -1.08276024011299
5.44722039800995 -4.71481996799886 4.52829914134842e-06
1.73703253684385e-05 3.2055951427134
FAM115A -1.37714759887006
7.07977960199005 -4.71463023412949 4.53209702848526e-06
1.73816461787413e-05 3.20478971591844
TBC1D8B -1.72521588983051
6.03199253731343 -4.71444288293932 4.53585023824067e-06
1.73903762497043e-05 3.20399442909494
TSPAN13 1.03237394067797 10.5392179104478
4.71443204466527 4.5360674533447e-06 1.73903762497043e-05
3.20394842249234
MFRP -1.04981242937853
8.73230497512438 -4.71293236562153 4.56622024762186e-06
1.74929094390141e-05 3.19758335884914
SYTL5 -1.88658220338983
3.47089800995025 -4.71181848304889 4.58874125918545e-06
1.75726278774626e-05 3.19285677253528
ZNF469 -1.14862838983051
6.29274626865672 -4.71170552293495 4.59103111854566e-06
1.75781180275536e-05 3.1923774938509
C6orf127 2.31293403954802 2.19650447761194
4.70824169177606 4.66178565009056e-06 1.78357173679387e-05
3.17768525772989
AMOTL1 -1.07719336158192 9.6658184079602 -4.70725383999477
4.68215626691553e-06 1.79069799105395e-05
3.17349675470803
PLEKHH1 1.26257987288136 8.89735323383085
4.70651331534461 4.69748302513502e-06 1.79589062394827e-05
3.17035738252279
WFDC2 2.72913170903955 13.9575527363184
4.7059494989395 4.70918487756405e-06 1.80002914794992e-05
3.16796741022759
MYH3 -1.38888735875706
5.35677910447761 -4.70553718762782 4.71776008634045e-06
1.8026356704368e-05 3.16621980077824
C1QTNF9 -1.48679138418079
2.33221094527363 -4.70422695700999 4.74511027168857e-06
1.8124114209817e-05 3.16066711248822
PTK6 2.09614279661017 5.66321542288557
4.70177254540195 4.79675679593492e-06 1.83111597286248e-05
3.15026877465563
PTPLA -2.06982040960452
4.96040149253731 -4.69900848802732 4.85556840611212e-06
1.85253334654306e-05 3.13856379299799
SPESP1 -2.21062125706215
2.85812935323383 -4.69741717009467 4.8897422667219e-06
1.86522498580083e-05 3.13182752159793

C1orf170 1.47089992937853 5.24934925373134
4.69608190054827 4.91859639693831e-06 1.87588303973887e-05
3.12617654804742
AIFM3 1.51237718926554 4.72616567164179
4.69589722236323 4.92260005733385e-06 1.87706127743432e-05
3.12539507560328
MDK 1.16343601694915 13.7594099502488
4.69494164978351 4.94336630801726e-06 1.8846297178012e-05
3.12135192857044
MAP6D1 1.01064103107345 5.99007164179104
4.69389206061786 4.966273107712e-06 1.89265986451323e-05
3.11691174483614
MAPK15 2.12037069209039 7.89131293532338
4.69347467847129 4.97541071218001e-06 1.89579031033882e-05
3.11514627162144
TDRD6 -1.42718700564972
3.69259004975124 -4.69306553270698 4.98438374336025e-06
1.89885689833338e-05 3.11341575927058
PRSS23 -1.15770529661017
10.5484323383085 -4.69014119666039 5.04897388855403e-06
1.92239306253736e-05 3.10105058104355
CREB3L4 1.0750720338983 9.90882189054726 4.6900664399739
5.05063558753823e-06 1.92266917508177e-05 3.10073456294857
LOC100270710 -1.217660240113
8.08962786069652 -4.68914585764441 5.07114168581003e-06
1.93011751593771e-05 3.09684332646562
CXCR2P1 2.15898481638418 3.42248308457711
4.68827991964439 5.09050387074215e-06 1.93676880033981e-05
3.09318362622693
KIR2DL1 -1.18822429378531
0.762419900497512 -4.68175701345774 5.23866412269804e-06
1.99018827607311e-05 3.06563337697784
RIMBP2 -2.69407245762712 4.6002328358209 -4.67977719574068
5.28445035691222e-06 2.00683991541635e-05
3.05727747020822
HIST1H2AH 1.1916520480226 1.38331940298507
4.67970574406291 5.28610997952853e-06 2.00709890659739e-05
3.05697595827419
HIST3H2BB 1.35008644067797 2.46831542288557
4.67889566882311 5.30496095871211e-06 2.01388403258801e-05
3.05355785930241
FAM63B -1.02644477401131
6.12570746268657 -4.67869053374696 5.3097448654993e-06
2.0153274558828e-05 3.0526923705591
TRIM34 -1.14305247175141
6.50395223880597 -4.677857168107 5.32922241632671e-06
2.02234632545633e-05 3.0491766169206
GPR137B -1.14802902542373
7.68768656716418 -4.67708749645087 5.34727250687178e-06
2.02882100473254e-05 3.04593001783082
TNFRSF9 1.49921384180791 2.66798656716418
4.67465805711991 5.40463459115808e-06 2.0502059895875e-05
3.03568506203919
LOC389705 -1.25892316384181

2.3212960199005 -4.67085731640562 5.49556716379606e-06
2.08277633141468e-05 3.01966588922493
AQP6 2.13030713276836 4.28684626865672
4.6705913142843 5.50198620657616e-06 2.08482423073105e-05
3.01854514819531
ZMAT1 -1.62229350282486 7.5667631840796 -4.67031051336684
5.50877021301481e-06 2.08662458437627e-05
3.01736211129134
SRRM4 -1.96911984463277
1.91757810945274 -4.66763606486475 5.57378915122022e-06
2.10930675160463e-05 3.0060973053401
COL9A2 1.81545670903955 9.80014825870647
4.66571127391994 5.62104038930826e-06 2.12640426004235e-05
2.99799327054879
CLDN14 1.59485035310734 2.62740796019901
4.66444594183234 5.65231292516381e-06 2.13705311885107e-05
2.99266724735135
TSKS -1.55919759887006
1.93715024875622 -4.66405070794575 5.66211542721037e-06
2.14036512098378e-05 2.99100387056715
ZFP3 -1.66836723163842
6.59510746268657 -4.66278146663682 5.6937058492306e-06
2.15072274304883e-05 2.98566292160348
C1orf175 -1.82245677966102 5.243 -4.66233443217006
5.70487260799956e-06 2.1541481449739e-05
2.98378208506363
IBSP 1.83603312146893 2.16204278606965
4.66128651763647 5.73113197543267e-06 2.16366567590351e-05
2.97937369529949
ACOXL 1.63157733050848 3.15984129353234
4.65991481955415 5.76568112806749e-06 2.17630876189053e-05
2.97360440870449
GNA15 1.24035402542373 7.14368955223881
4.65785164916655 5.81802498608735e-06 2.19566273831569e-05
2.96492939659939
MCOLN3 -2.14178418079096
5.33881791044776 -4.65709981802072 5.83721306483221e-06
2.2024992505239e-05 2.96176893967603
LOC400696 1.93659731638418 2.60367014925373
4.65701806448369 5.83930323638417e-06 2.20288304746356e-05
2.96142529874746
PRSS3 2.10654088983051 3.04317611940299
4.64980990382994 6.02645684589775e-06 2.27014970727121e-05
2.93114574414057
ZNF425 -1.02310261299435
6.29278805970149 -4.64840274882155 6.06366105365517e-06
2.2837453940844e-05 2.92523906177056
NRGN -1.26878036723164
7.16390746268657 -4.64820343574752 6.06894862472421e-06
2.28531759474666e-05 2.92440254024758
RRAD -2.09592175141243
6.95822089552239 -4.64795662583168 6.07550239904077e-06
2.28736593163959e-05 2.92336671337165
SLC29A4 1.53224837570621 8.55979751243781

4.64696522415302	6.10189683320009e-06	2.29688195793175e-05
	2.91920638418948
OAT	-1.07839187853108
10.1638671641791	-4.64611421057994	6.12464181330063e-06
	2.30417622186296e-05	2.91563575059217
C10orf25	-1.04111384180791
5.7039815920398 -4.64589842870752	6.13042197764951e-06
2.30586214492128e-05	2.9147304690731
GRB14	2.51058305084746	6.01081194029851
4.64575096664947	6.13437507895787e-06	2.30656997016493e-05
	2.9141118328704
CLDN6	4.13231892655367	5.29522437810945
4.64461219535781	6.16498566188029e-06	2.31723097104362e-05
	2.90933496546946
WSCD1	-1.8197436440678
5.49583084577114	-4.64225107378663	6.228923341995e-06
	2.3404062264121e-05	2.89943363915109
ADCY8	-1.69074279661017
0.808945273631841	-4.64199419873188	6.23591778894506e-06
	2.34260552986353e-05	2.89835668197159
GALNT9	-1.90630077683616
2.90015472636816	-4.63840009386898	6.33457925331424e-06
	2.37679382791446e-05	2.88329330843194
SH2B2	1.13367379943503	6.84601393034826
4.63838381137891	6.33502962803115e-06	2.37679382791446e-05
	2.88322508770979
SIX3	2.3244688559322 2.45826616915423	4.63646221669174
	6.38839839099323e-06	2.39637893234225e-05
2.87517530347113
AMH	2.30718347457627	4.25935721393035
4.63602611535076	6.4005705046001e-06	2.40050625610961e-05
	2.8733487979752
ABCB5	-1.52862196327684
1.47025422885572	-4.63436287238128	6.44719916409644e-06
	2.41711100874543e-05	2.86638397505948
C6	-2.83981588983051
2.87151243781095	-4.63284851890557	6.48993838415937e-06
	2.43233171875953e-05	2.86004437665162
COX4I2	-1.69667641242938
4.08987014925373	-4.6301272398077	6.5674276128307e-06
	2.45859388934016e-05	2.84865637119549
ASTN1	-2.57657655367232
4.76602437810945	-4.62747060332401	6.64393591007934e-06
	2.48632891421671e-05	2.83754408209228
TUBB4	1.62366935028248	7.60199004975124
4.6251255624623 6.71218378853713e-06	2.50866799888344e-05
2.82773941854579
CXCL17	3.32327881355932	7.29678606965174
4.62333842736049	6.76464804546052e-06	2.52781624268847e-05
	2.82027005988939
CCL20	2.66792556497175	5.62234228855721
4.62065047927092	6.84430203236008e-06	2.55432686283135e-05
	2.80904012178026
ADRA2A	-1.90319435028249

6.97031940298507 -4.6199071362662 6.86648913441335e-06
2.56214143819531e-05 2.80593544961764
PCDHA13 -1.76570141242938
1.73527860696517 -4.61617350862026 6.97898189694364e-06
2.60080773904979e-05 2.79034754405662
SLC26A10 -1.62979011299435
3.56422835820896 -4.61289808568658 7.07912971136932e-06
2.63573690559326e-05 2.77668101588599
SLC16A14 -1.10539851694915
7.06199353233831 -4.60973549443087 7.17714076788863e-06
2.6693242747238e-05 2.76349269986725
TNFRSF13C 1.29942161016949 2.95990845771144
4.60541064082557 7.31328992263366e-06 2.71602464022556e-05
2.74546945504195
OSBPL3 1.20095946327683 9.06460298507463
4.60468817950536 7.33627468363101e-06 2.7240679915888e-05
2.74246002625148
UNC5C -1.88507718926554
3.02293582089552 -4.60428458931887 7.34914499468852e-06
2.72835346728672e-05 2.74077902828062
TNNC1 2.35869625706215 6.81542338308458
4.60421257964749 7.35144363670197e-06 2.72871339471147e-05
2.74047911250678
DPF1 1.79305861581921 3.75292039800995
4.60306108265505 7.38829514539644e-06 2.74140067476937e-05
2.73568371422218
CACNA1D -1.91345233050848
5.37368208955224 -4.60268967892801 7.40021913931833e-06
2.74532885670772e-05 2.7341372135433
PPP1R3E -1.02655882768362
5.70339452736318 -4.60251028269924 7.40598533079549e-06
2.74697161411439e-05 2.73339025554992
C11orf35 1.1417918079096 5.71347512437811
4.60216504119418 7.4170943389843e-06 2.750200160323e-05
2.73195282828287
C10orf105 -1.48519194915254
2.75065273631841 -4.60167393926402 7.43292443457464e-06
2.75476816494242e-05 2.72990825517212
NFATC2 -1.78190353107345
3.71988606965174 -4.6012444050103 7.44679664750969e-06
2.75911812260195e-05 2.72812014729774
TSPAN32 -1.37943799435028
3.51746268656716 -4.59983448476022 7.49250686775353e-06
2.77555333805449e-05 2.72225173886594
RIPPLY2 -1.08378368644068
0.639035820895522 -4.59668796768599 7.59549506641548e-06
2.81218239339062e-05 2.70916045992936
NPNT -1.79599548022599 8.2059184079602 -4.59633748393965
7.60705075016569e-06 2.81544532000904e-05
2.70770269868503
TMEM171 1.75729865819209 2.61984278606965
4.5929731506994 7.71884001558585e-06 2.85341322444514e-05
2.69371405345576
C10orf128 -1.63430303672316

2.86257512437811	-4.59188193267142	7.75543739791356e-06
	2.8655343333507e-05	2.68917862910747
GRAPL	-1.19240621468927
1.65513731343284	-4.59085576057626	7.7900057137304e-06
	2.87745263229372e-05	2.68491434980079
FCGR3A	1.42216151129943	8.99489701492537
4.590589221457	7.79900877995531e-06	2.88025994337979e-05
2.68380686706588
DENND5B -1.04118848870057
7.83149502487562	-4.58920819585764	7.84581730875931e-06
	2.89650475362037e-05	2.67806947256258
KIR2DS4 -1.43868919491525
1.19354875621891	-4.58806983401762	7.8846043239157e-06
	2.90873183773566e-05	2.67334126087231
GCNT2	1.4485165960452 8.89348855721393	4.58286615309811
	8.06427164009018e-06	2.97234285046771e-05
2.65173973676937
GTSF1L	1.02031207627119	1.07764626865672
4.58234544781025	8.08246549461966e-06	2.97851402987869e-05
	2.64957927621021
PPP1R3B -1.06982055084746
9.50079004975124	-4.58193458393765	8.09684929913894e-06
	2.98327918362029e-05	2.64787469935292
GOLGA2B 1.15454661016949	8.11620696517413
4.58149344437361	8.11232045923908e-06	2.98790705109756e-05
	2.6460446535191
CHD5	-1.58968241525424
2.23011791044776	-4.58099931622831	8.12968378083129e-06
	2.99376516628639e-05	2.64399495647236
EGLN3	1.50144936440678	9.91760099502488
4.5806492407302 8.14200684650472e-06	2.99776543326295e-05
2.64254291368515
CLEC10A -2.05525720338983
4.55933830845771	-4.57952779955166	8.18160411557743e-06
	3.01118148503173e-05	2.63789200581402
ANO8	1.05828312146893	8.18844179104478
4.57949867680639	8.18263488497214e-06	3.01118148503173e-05
	2.63777123850337
ANKRD58 1.11897351694915	4.07195223880597
4.57949273086718	8.18284535070241e-06	3.01118148503173e-05
	2.63774658173235
PRICKLE4	-1.5242093220339
6.04886915422886	-4.5791099437443	8.19640567038171e-06
	3.01563097514223e-05	2.63615928494615
GATA2	-1.80322323446328
9.35676616915423	-4.57884600423808	8.2057683889961e-06
	3.01853476126304e-05	2.63506487408842
SCN3A	-2.02569456214689
2.92218358208955	-4.5780554055934	8.23387494269358e-06
	3.0277888693086e-05	2.63178700522285
CKM	1.62378072033898	2.74984228855721
4.57718882839176	8.26478910680236e-06	3.03806840398661e-05
	2.62819465113587
TEPP	-1.35286913841808

1.39923830845771	-4.57607503345399	8.30468666446755e-06
	3.05164161842704e-05	2.623578276415
STMN3	-1.51188248587571	9.0257407960199 -4.57600665612805
	8.30714206076807e-06	3.05199761108748e-05
2.62329490077695
MGLL	-1.23472189265536
9.85934925373134	-4.57295358438575	8.41749167213578e-06
	3.09088005775259e-05	2.61064557307471
C2CD4A	2.88647394067797	6.5730328358209 4.56935909621506
	8.54921968677069e-06	3.13700590115107e-05
2.59576184163216
BMP6	-1.26884018361582
6.31614228855721	-4.56867058735524	8.57467713064658e-06
	3.1446609769949e-05	2.59291200844347
NOXA1	1.29991885593221	8.8155671641791 4.5684683211301
8.58216973432703e-06	3.14684666272073e-05	2.59207486674697
AP1S2	-1.10674943502825
9.05888308457711	-4.56599010208774	8.67448439359227e-06
	3.17842521150186e-05	2.58182042454635
PADI2	1.81436200564971	7.73754676616915
4.56381108139257	8.75644304315122e-06	3.20788322048348e-05
	2.5728077391468
NBPF14	-1.51986716101695
6.92239701492537	-4.56357341482717	8.76542726265311e-06
	3.21060163607972e-05	2.57182493324596
PTPRM	-1.57829152542373
9.24749452736318	-4.55813425740151	8.97347943065351e-06
	3.28270728281034e-05	2.54934410898062
PPP1R14D	1.67621631355932	2.45965024875622
4.55787285106774	8.9835972344637e-06	3.2858231110554e-05
	2.54826422634241
KLK4	-2.61009661016949
2.83079004975124	-4.55719395124122	9.00992556248777e-06
	3.29486590306152e-05	2.54545989183643
RHBG	1.51814774011299	2.0149263681592 4.55324162759989
	9.16468131359837e-06	3.34847672838894e-05
2.52914074018207
NKX2-8	1.75435331920904	1.87847412935323
4.55311570213511	9.16965382532874e-06	3.3496973849466e-05
	2.52862098253983
SLIT2	-2.21458629943503
6.48405024875622	-4.55184010212791	9.22017100087673e-06
	3.36695323102098e-05	2.52335659813988
BMX	-2.01125487288136
3.76767412935323	-4.54909613349753	9.32974902060637e-06
	3.40475391020037e-05	2.51203633312764
PKDREJ	-1.0575488700565
2.61130895522388	-4.54889920753287	9.33766111260224e-06
	3.40682748608004e-05	2.51122412653207
STAP2	1.2432429378531 9.6037960199005 4.54878315835861
9.34232676063558e-06	3.40792410085019e-05	2.51074550364979
GNG4	-2.29676673728814
6.07485323383085	-4.54616939714251	9.44800692860229e-06
	3.4446382923851e-05	2.49996816549046

IL1R1	-1.29439668079096
10.3994706467662	-4.54580020606943	9.46302656646154e-06
	3.44888932024597e-05	2.49844628295858
CP	2.69117930790961	11.0761402985075
4.54406882943149	9.53377052501251e-06	3.47282308159168e-05
	2.49131052661973
ARHGEF35	1.12218121468926	8.45564776119403
4.54399726954409	9.5367053847996e-06	3.4732758834475e-05
	2.49101564469562
WBP2NL	-1.29514152542373	2.2615855721393 -4.54277372918039
	9.58702074112341e-06	3.48974354282382e-05
2.48597429830065
SYT11	-1.3322406779661	8.9107328358209 -4.54173529798148
	9.62992423919317e-06	3.50411812532144e-05
2.48169652119244
RASGRP4 -1.14287231638418
3.40439004975124	-4.54089262584486	9.6648755723843e-06
	3.51558992737508e-05	2.47822574981799
GALNT3	1.64468665254237	9.25546865671642
4.53651009000659	9.84862653724693e-06	3.57821549743265e-05
	2.46018353662287
KCTD16	-1.22405748587571
1.80644029850746	-4.53649552116527	9.84924294982559e-06
	3.57821549743265e-05	2.46012358259395
LOC646851	1.11929668079096	6.8976368159204
4.53559172967557	9.88755534797377e-06	3.59086345823282e-05
	2.45640458513489
C12orf70	1.12162471751412	1.44699104477612
4.5348922761364 9.91730410739541e-06	3.60103030287987e-05
2.45352682809246
ATP4A	1.52590028248587	1.52525323383085
4.53426858860687	9.94390305473572e-06	3.61005003914632e-05
	2.45096109887376
KIRREL	-1.95777563559322
6.16234278606965	-4.53256935817348	1.00167203810766e-05
	3.63505192464731e-05	2.44397225068175
CGB	1.66576758474576	1.7304407960199 4.53253768107674
	1.00180827031926e-05	3.63505192464731e-05
2.44384198461583
CXorf50B	-1.27250918079096
2.20812388059701	-4.53161885592893	1.00576758639903e-05
	3.64877337847181e-05	2.44006381215865
KIF17	-1.35838192090395
5.39684626865672	-4.52679988699413	1.0267808435165e-05
	3.71732403709905e-05	2.4202586036059
SEC1	-1.01524936440678
2.61274527363184	-4.52622278019726	1.02932547663415e-05
	3.72567913175232e-05	2.41788793403954
C6orf105	1.69496009887006	3.09807263681592
4.52239484318987	1.04635821503339e-05	3.78399403816021e-05
	2.40216955831485
C19orf59	1.53247323446328	2.31339552238806
4.51577477962873	1.07645669440734e-05	3.88599541694791e-05
	2.37501162420831

RBM11	-1.74385324858757
4.18739303482587	-4.51566669570695	1.07695494714203e-05
	3.88711059508191e-05	2.37456849293573
SERPINB5	2.56686984463277	4.65624875621891
4.51499756395867	1.08004449858858e-05	3.89689166186707e-05
	2.37182532427863
PRRT4	-1.7202802259887
2.48379303482587	-4.51376555855028	1.08575529556357e-05
	3.9154323235412e-05	2.36677546782303
VILL	-1.29160939265537
7.13854179104478	-4.51366532752993	1.08622117863295e-05
	3.91642444631798e-05	2.36636468111408
C6orf145	-1.11766574858757
8.60469104477612	-4.51240895109195	1.09207726905767e-05
	3.93615630859446e-05	2.36121617940894
SNX32	-1.86036560734463
2.77834676616915	-4.51185770357013	1.09465626098288e-05
	3.94406687223209e-05	2.3589575920865
LOC283050	-1.07030367231639
4.06974378109453	-4.51037019485791	1.10164478402971e-05
	3.96855016952622e-05	2.35286404981109
ACR	-1.2273636299435
2.15784975124378	-4.5102611648343	1.10215870685014e-05
	3.96970495642689e-05	2.35241747542374
AKR1B15	2.23723573446328	2.41371144278607
4.50953715653946	1.10557723966176e-05	3.97992297567176e-05
	2.34945224413731
ZNF519	-1.07125197740113
5.57762537313433	-4.5044512406918	1.12988035875244e-05
	4.06384789158858e-05	2.32863341986805
ZNF750	2.39278947740113	5.23237910447761
4.50385504644634	1.13276270125959e-05	4.07136173964625e-05
	2.32619419609834
HSPA6	1.33899830508475	7.38255124378109
4.50365274305321	1.13374235701017e-05	4.07416954129509e-05
	2.32536656718919
FOXJ1	2.29269774011299	10.3670094527363
4.50313194340718	1.13626808370015e-05	4.08253128427792e-05
	2.32323610050959
KRT86	1.76728834745763	5.29773134328358
4.50272170919818	1.13826140949723e-05	4.08897755677656e-05
	2.32155807197055
LOC653786	1.24752789548023	1.84499850746269
4.50253118240584	1.13918832140163e-05	4.09159136430642e-05
	2.32077878052491
ME3	-1.1584697740113
7.76167164179104	-4.50147439610781	1.14434277593789e-05
	4.10938555011454e-05	2.31645680816198
LCAT	-1.16738848870057
6.58929701492537	-4.50058137219022	1.14871596098759e-05
	4.12364725984877e-05	2.31280522477902
SHANK1	-2.1731293079096
3.24346965174129	-4.49890105826273	1.15698815751572e-05
	4.14971468794509e-05	2.30593600645332

CCL7	1.57560338983051	2.08330497512438
4.49884472520856	1.15726647560741e-05	4.14998790292056e-05
	2.3057057499808
MAPK11	-1.20216257062147
7.68508756218905	-4.49875267776348	1.15772138214477e-05
	4.15089416266716e-05	2.30532951909285
MYRIP	-1.94270564971752
6.21839253731343	-4.49870666923722	1.15794882476531e-05
	4.15098469921982e-05	2.30514146811446
ZNF185	-1.07148206214689
9.38868109452736	-4.49786815480877	1.16210154693539e-05
	4.16441695908247e-05	2.30171447685966
P2RY6	1.31782634180791	6.13501144278607
4.49729642278718	1.16494124345806e-05	4.17386452499022e-05
	2.29937811853934
ATP2A1	1.08892662429379	3.9773263681592 4.49490475480578
	1.17689276277032e-05	4.21447905453944e-05
2.28960729641173
C10orf99	2.68206447740113	3.57793880597015
4.49367777112737	1.1830698549878e-05	4.23512190460325e-05
	2.28459627322991
KRT23	2.42712987288136	8.70026915422886
4.49339297967565	1.18450805254054e-05	4.23953108763203e-05
	2.28343333946402
ASAP3	-1.0526863700565
8.89752835820896	-4.49289346971833	1.18703464030843e-05
	4.24783356554506e-05	2.28139375705578
FAM50B	-1.5334625	7.1141	-4.49277830562819
1.18761789027157e-05	4.24918007794969e-05	2.28092354912458
SOX3	-2.26755981638418	1.5664144278607 -4.49246171985254
	1.18922266319718e-05	4.25343924659199e-05
2.27963099945491
PTPN3	1.11568057909605	9.32153582089552
4.49150291554021	1.19409557118437e-05	4.26938036858502e-05
	2.27571686699433
ZNF135	-1.90697076271187	6.4569855721393 -4.49049312179041
	1.19924837755594e-05	4.28705714630391e-05
2.27159531716126
WNT10A	2.44919293785311	5.66176865671642
4.48935248904059	1.20509454826244e-05	4.30679559630902e-05
	2.26694064766119
FAM167A 1.94762796610169	5.783892039801	4.4888277129659
1.20779341702246e-05	4.31534960867927e-05	2.26479947693528
PLEKHB1 1.48875416666666	9.46437164179105
4.48633475631433	1.22069401096012e-05	4.35992485125456e-05
	2.25463060391812
ERBB2	1.15284251412429	12.3556980099502
4.48343799976488	1.23585062309456e-05	4.41098979348597e-05
	2.24282040770254
KIAA1683	-1.47754173728814
7.78680199004975	-4.48298047733868	1.23826098820935e-05
	4.41882462853086e-05	2.24095563945164
LTBP1	-1.26140402542373
10.7855905472637	-4.48234823599353	1.24159926416476e-05

4.42996748232576e-05	2.23837900823104
KISS1	1.84897196327684	2.89482835820896
4.48226968754954	1.24201460887123e-05	4.43067939533038e-05
	2.23805891326999
C5orf46	1.44708439265537	1.43396069651741
4.48149229318763	1.2461324871102e-05	4.44459693543649e-05
	2.23489117880346
IYD	1.87875685028249	2.42056517412935
4.48061569884684	1.25079157482161e-05	4.4588906114061e-05
	2.23131976158029
NTN5	-1.36355423728814
3.63842487562189	-4.4799945191343	1.25410327036815e-05
	4.46992016330869e-05	2.22878929911308
CLSTN2	-1.86909392655368
5.86303980099502	-4.47990353416627	1.25458904574121e-05
	4.47087538779155e-05	2.22841868317873
SH3RF3	-1.13853947740113	7.3210263681592 -4.4777964384712
	1.26588971858539e-05	4.50567196993031e-05
2.2198374114898
L3MBTL3 -1.05587676553673
7.86851890547264	-4.47765206722084	1.26666757496868e-05
	4.50765909414231e-05	2.2192495717797
ZNF878	1.10094646892655	2.81819850746269
4.47758684950627	1.26701911147027e-05	4.50812865831623e-05
	2.21898402840277
KIAA1107	-1.0566593220339
6.06707562189055	-4.47653451189133	1.27270440375336e-05
	4.52678818590722e-05	2.21469972018581
C16orf93	1.15341956214689	6.76683532338308
4.47571182240413	1.277166085243e-05	4.54029778875131e-05
	2.21135093536977
RLTPR	1.61360098870057	5.44385721393035
4.475583723504	1.27786215381274e-05	4.54198580640158e-05
2.21082954980684
IFI16	-1.35817161016949	10.518739800995 -4.46954374230201
	1.31109959237805e-05	4.65689872517672e-05
2.18625958682193
SPRY1	-1.10709985875706	9.7050328358209 -4.4694777727849
	1.31146716348059e-05	4.65739852382919e-05
2.18599138001399
C1orf115	-1.15908714689266
9.16140298507463	-4.46917304757722	1.31316632713985e-05
	4.66181993708819e-05	2.18475252595552
MST1P2	1.29441843220339	6.44386517412935
4.46832901801603	1.3178837342748e-05	4.67614121407908e-05
	2.18132150174065
FAM83A	2.70135953389831	4.23477164179104
4.46715572649412	1.32446850243593e-05	4.69788154025737e-05
	2.17655289081954
MOCOS	1.35911461864407	6.58981144278607
4.46660923970738	1.32754628897839e-05	4.70798502551846e-05
	2.17433215315499
SLC24A5 1.46251560734463	4.24005771144279
4.46618424458056	1.32994459092622e-05	4.7147029442113e-05

2.1726052701457
HIST2H2BF 1.24284646892655 5.62468855721393
4.46318817801635 1.34697031883703e-05 4.77027784124964e-05
2.16043516442368
SLC25A27 -1.50237874293785
6.87464726368159 -4.46304608360115 1.34778297887772e-05
4.77233262441524e-05 2.15985813889947
DPY19L2P4 -1.43733156779661
2.27994427860697 -4.46253056008338 1.35073529570856e-05
4.7819616521636e-05 2.15776479625908
ARL13B -1.00432966101695
7.45260696517413 -4.46203226919692 1.35359483046471e-05
4.79125894300697e-05 2.15574161673877
ELN -1.71403262711865
8.95073631840796 -4.46072026154053 1.36115188308191e-05
4.81385842543869e-05 2.15041543774072
CYP2J2 1.6707615819209 6.57273432835821 4.45947242907704
1.36837690009017e-05 4.83774371600675e-05
2.14535097128219
CD44 -1.30274244350282
11.5579547263682 -4.45722235615459 1.38149818873931e-05
4.87993080089779e-05 2.13622173162341
SLC6A2 -2.57560995762712
2.79918805970149 -4.45332580614833 1.40450739000545e-05
4.95779517139132e-05 2.12042114846819
CYP27A1 -1.10783234463277
8.92597114427861 -4.45287844656468 1.40717250129378e-05
4.96634888285392e-05 2.11860782108297
SLC22A20 1.27884618644068 3.37593980099502
4.44799281791545 1.43659638862157e-05 5.06236248856862e-05
2.09881412571452
TUBA3D -2.48509668079096
2.98094378109453 -4.44331179218272 1.46534195803824e-05
5.16011499117069e-05 2.07986606136139
ELOVL6 1.18166652542373 8.30826368159204
4.44111376274069 1.47902966293617e-05 5.20385243874542e-05
2.07097442160224
CD80 1.31449230225989 3.00443383084577
4.43703962597571 1.50472587712168e-05 5.28701390677872e-05
2.05450294556033
RTN4RL1 -2.20235875706215
4.53587611940299 -4.43568791435474 1.51334567860265e-05
5.31457180665626e-05 2.04904080010064
ZFP82 -1.22973665254237
6.32928507462687 -4.43317226775811 1.52951427982884e-05
5.36768002818565e-05 2.038878931988
IGSF3 1.2972843220339 9.5344263681592 4.43159196780515
1.53975592454313e-05 5.4008524057527e-05 2.03249778303797
ZNF732 -1.11423700564972
1.93805621890547 -4.43114787242402 1.54264586389616e-05
5.40997432277132e-05 2.03070489060147
CDKN2B 1.35909724576271 7.61907114427861
4.43111138007455 1.54288356842796e-05 5.40997432277132e-05
2.03055757101825

B4GALT1 1.08582598870056 12.5613417910448
4.43006708534322 1.54970084221225e-05 5.43202292822564e-05
2.02634217595001
C16orf79 1.16826115819209 5.90994228855721
4.4245814919837 1.5859892109105e-05 5.55353206549421e-05
2.00421244864939
ADAT3 1.01200508474576 6.75973532338308
4.42409125460817 1.58927166747893e-05 5.56407696680463e-05
2.00223585131006
BNIPL 2.09946320621469 4.86396019900497
4.42368326570732 1.59200839055528e-05 5.57270797239556e-05
2.00059101024302
KLK2 -1.90606998587571
1.78334129353234 -4.42272022423687 1.59848627734533e-05
5.59442947049089e-05 1.99670892271824
IL15RA -1.25802881355932
7.58498358208955 -4.42153686436547 1.60648079949074e-05
5.62145062443238e-05 1.99193966667731
RPL36A 1.13544096045198 6.66771592039801
4.4164231162704 1.64147180405197e-05 5.73607083186161e-05
1.97134194995071
STK31 1.83998764124294 3.45764179104478
4.41619079367383 1.64307873486212e-05 5.74070905832526e-05
1.97040664045392
OPRK1 2.54768156779661 4.19867661691542
4.41375877065467 1.65999149790905e-05 5.79684052595217e-05
1.96061796528219
SYT5 2.4944063559322 4.22904179104478 4.41261385926106
1.66801118414579e-05 5.82088553637822e-05
1.95601133330284
TNFRSF1B -1.05973220338983
9.32686965174129 -4.41240041029228 1.66951042473102e-05
5.82512729771039e-05 1.95515261490142
KIR3DL1 -1.14441024011299
0.972948258706468 -4.40938793468115 1.6908081827511e-05
5.89442893392427e-05 1.94303688066169
KRT5 3.20496299435028 7.46439751243781
4.40506681894859 1.72181380963106e-05 5.99946325725147e-05
1.92566986633215
SEMA6B -1.08093820621469
7.77745820895522 -4.40146097639512 1.74810418475718e-05
6.08487265396148e-05 1.91118832847837
KCNG1 -2.09708919491526
6.84373034825871 -4.40047520644828 1.75535828994266e-05
6.10701672471153e-05 1.90723104479777
FAM101A -2.27323100282486
4.65032039800995 -4.40014326826107 1.75780746070351e-05
6.11346555450499e-05 1.90589867330041
LYPD1 2.37585254237288 8.78686865671642
4.3966485805803 1.78379250630916e-05 6.19439447809998e-05
1.891876314803
FOLR1 2.59565444915254 8.93365472636816
4.39643068766041 1.78542482033617e-05 6.19901430094732e-05
1.89100232784949

CCDC157 1.10954668079096 6.78240099502488
4.39612074884493 1.78774915356956e-05 6.20603485336204e-05
1.8897591984349
RNF150 -1.71088827683616
6.17022189054726 -4.39308849486698 1.81064275910744e-05
6.28232142039474e-05 1.87760097732025
CR1L 1.71526588983051 2.66816218905473
4.39279910315721 1.81284232474615e-05 6.28782783021432e-05
1.87644098420951
FAM189A2 -2.0475081920904
6.79768706467662 -4.3913568855011 1.82384241214944e-05
6.32384474268332e-05 1.87066096007402
DUSP19 -1.1805988700565
6.37960149253731 -4.39042782774947 1.83096242015971e-05
6.34746006178297e-05 1.86693837176387
EPM2AIP1 -1.62534653954802
8.86846218905473 -4.38830099478564 1.8473623774267e-05
6.40049904326313e-05 1.85841892869269
CMKLR1 -1.31044350282486
7.01279402985075 -4.38828207719022 1.84750888116792e-05
6.40049904326313e-05 1.85834316583977
NLRP7 2.17733728813559 3.44325671641791
4.38403041729653 1.88071945344616e-05 6.50786623617894e-05
1.84132256904806
RSPH9 -2.20731581920904
3.48891094527363 -4.38370341507547 1.88329732323043e-05
6.5156882450153e-05 1.84001404962151
CTXN2 1.21580939265537 1.72590895522388
4.38332651766877 1.88627274013515e-05 6.52397064146085e-05
1.83850597132012
SFRP5 -2.59908848870057
3.40957960199005 -4.3811702397983 1.90338240033034e-05
6.58074110454395e-05 1.82988011845247
GPD1 1.55773411016949 4.14919850746269
4.37980561125756 1.91428731283322e-05 6.61621524115588e-05
1.82442294248973
SH3GL2 -1.94262076271187
3.67264228855721 -4.37914283474538 1.91960524530228e-05
6.63347848612283e-05 1.82177299193878
MGAT4C -1.01568813559322
0.632854726368159 -4.37747463281794 1.93305324585416e-05
6.67545553194043e-05 1.81510455585492
C5orf36 -1.12811242937853
4.89838756218905 -4.37424096544968 1.95937893889098e-05
6.75840887356045e-05 1.80218432653303
C9orf172 -1.2042552259887
2.96807810945274 -4.37072762172203 1.98837133787937e-05
6.85228891721489e-05 1.78815556602039
RPS6KA5 -1.43520861581921
6.64501791044776 -4.37070022883452 1.98859899506899e-05
6.85228891721489e-05 1.78804622291427
ZNF423 -1.87709675141243 7.1358592039801 -4.37029247559819
1.99199071432389e-05 6.86282419282925e-05
1.78641867727924

ODF3L1 -1.41980564971752
2.52385671641791   -4.36937038693929   1.99968118881614e-05
        6.88816355812003e-05   1.78273862552588
C3orf45 -1.63534759887006
2.29740945273632   -4.36807062229946   2.01056997497746e-05
        6.92334813590862e-05   1.77755235865024
KCNA6   -1.83346758474576
2.93778308457711   -4.36698925753711   2.0196724412529e-05
        6.95352594649036e-05   1.7732385121431
ZNF703 -1.4343636299435
7.37346616915423   -4.36634283506607   2.02513261515739e-05
        6.96998702249477e-05   1.77066018496197
FHOD3   -1.61130896892655   6.6529736318408 -4.3662520830562
        2.02590030805069e-05   6.97146049686231e-05
1.77029823579518
ZIC4   2.57443898305085   2.57337661691542
4.36524215630387   2.03446238144894e-05   6.99857786379803e-05
        1.76627073135718
VGLL1   2.53795720338983   4.6424592039801 4.36447308028868
        2.04100581398907e-05   7.01873523638116e-05
1.76320423549838
RHBDL3 -1.96334766949152
4.24878606965174   -4.36308986211568   2.0528253158097e-05
        7.05583521405878e-05   1.75769012567572
RASSF3 -1.65755275423729
5.80173482587065   -4.36028995179003   2.07695163348296e-05
        7.13278973492892e-05   1.74653288071405
CH25H   -1.41205190677966
4.77907213930348   -4.35992247720864   2.08013821366147e-05
        7.14253847239838e-05   1.74506898568643
FGFBP1 2.33324618644068   5.27544129353234
4.35947088172407   2.0840606767978e-05   7.15481031683258e-05
        1.74327012082592
APOC2   1.94667641242938   5.55844577114428
4.35366586878196   2.13511781107055e-05   7.32152474716526e-05
        1.72016040119107
CNTD2   1.6791165960452 3.72955422885572   4.35329825990624
        2.13839113205687e-05   7.33152470626711e-05
1.71869780902022
APOBEC3G   -1.35305670903955
7.78817313432836   -4.35154999675769   2.15402427629202e-05
        7.38142520318398e-05   1.71174345412675
GLIS2   -1.15927062146893
9.12041641791045   -4.35056849849718   2.1628489120712e-05
        7.40548501748194e-05   1.7078401981949
DCHS2   -2.04097033898305
4.71851094527363   -4.34741922424534   2.19139869604197e-05
        7.49948578592127e-05   1.69532096987443
CDH15   1.65803403954802   3.01436815920398
4.34669305162612   2.19803291096098e-05   7.52093594367148e-05
        1.69243529784406
COL12A1 -1.6054415960452
11.1148154228856   -4.34574527830802   2.20672061721245e-05
        7.54814632978532e-05   1.68866962621511

ZBTB20	-1.00587853107345
2.80555970149254	-4.34473026274038	2.21606120762448e-05
	7.5775710513875e-05	1.68463754207025
GDA	2.48324816384181	8.25891592039801
4.34459831055066	2.21727826697182e-05	7.58047007842321e-05
	1.68411342767079
MTBP	1.09299124293785	6.75880149253731
4.3438904110307 2.22381851981301e-05	7.60156413782235e-05
1.68130187386938
MST4	-1.60688862994351
8.30206616915423	-4.34320623515285	2.23015716746641e-05
	7.62196215217004e-05	1.67858490290413
PLCH1	1.57180642655367	7.94785721393035
4.34244198002226	2.23725820552699e-05	7.63987208241337e-05
	1.67555034298206
LCN2	2.76647782485876	10.1914437810945
4.34194969437415	2.24184372772928e-05	7.65425772996543e-05
	1.67359590196842
ID4	-1.41593411016949
10.0668368159204	-4.33804569727352	2.27852899150494e-05
	7.77563168144886e-05	1.65810299223852
CCDC116 -1.14167803672316
2.35258756218905	-4.33600741803243	2.2979103966503e-05
	7.83916590947667e-05	1.65001871599948
C10orf108	-1.77996709039548
4.00147462686567	-4.33151171575648	2.34121904771826e-05
	7.97763104558604e-05	1.63219886022677
SGSM1	-1.99717803672316
5.50377014925373	-4.33110688772593	2.34515707120134e-05
	7.98839798768267e-05	1.63059497263055
ADAMTS19	-2.80671737288136
5.21875472636816	-4.32929603586969	2.362850259974e-05
	8.04599698743843e-05	1.62342208041282
TNFRSF8 -1.2241665960452
4.53129004975124	-4.32874237699917	2.36828535839949e-05
	8.06183027821945e-05	1.62122950033706
FLJ22536	-1.29829774011299
3.79275621890547	-4.32581540015013	2.39721831893762e-05
	8.15761513140881e-05	1.60964204991479
POU4F1	1.76638870056497	1.85900995024876
4.32535729374566	2.40177722214911e-05	8.16906666181596e-05
	1.60782906468651
HSPA2	-1.16740790960453
9.09288407960199	-4.32497137256208	2.40562421341261e-05
	8.17944105686665e-05	1.60630188047363
TSSK3	-1.02483509887006
4.09567910447761	-4.32386092092807	2.41672654243199e-05
	8.21582975925299e-05	1.60190818224941
HTR3A	2.60729731638418	3.64758656716418
4.32240326649554	2.43137478260179e-05	8.2601562612634e-05
	1.59614213338022
CXCL10	2.00752577683616	7.91225870646766
4.32204538476521	2.43498419308183e-05	8.27104988153044e-05
	1.59472670559621

C1orf54 -1.01453947740113
7.00875373134328 -4.32189566635491 2.43649569890733e-05
8.27264505667592e-05 1.5941345957717
FAHD2B -1.10722019774011
7.25612686567164 -4.32149498207284 2.44054529551128e-05
8.28445683148919e-05 1.59255004392151
C22orf41 1.78071511299435 2.82948407960199
4.32020420891065 2.45363466603808e-05 8.32475949326751e-05
1.58744636138186
LOC374491 -1.3291761299435
2.06143980099502 -4.32005644853246 2.45513734776169e-05
8.32848145233767e-05 1.58686220138503
GGT3P 1.7239043079096 4.94226915422886 4.31731815771386
2.48314515353869e-05 8.41931791943011e-05
1.57603956277822
LMO1 2.09542196327684 2.81616815920398
4.31285412305307 2.52946185078008e-05 8.57069678107195e-05
1.55840840399919
CDNF -1.06435353107345
3.95810845771144 -4.31186158847588 2.53987184006876e-05
8.60312966121295e-05 1.55449034271476
IL8 1.96236553672316 7.05701990049751
4.30911121647505 2.56893348528036e-05 8.69582925850054e-05
1.5436370706115
TRIM10 1.72010649717514 1.96783731343284
4.30696076544693 2.59187779399158e-05 8.76626871674582e-05
1.53515515424125
CELF6 -1.36311059322034
5.75035721393035 -4.30680688153201 2.59352717024269e-05
8.76989746899936e-05 1.53454833227292
NCAM2 -1.75145960451978
3.09831990049751 -4.30678096617959 2.59380503807911e-05
8.76989746899936e-05 1.53444614009214
GPR19 1.37221673728814 3.4963407960199 4.30641657918254
2.59771506529339e-05 8.7816713799561e-05
1.53300930444777
C9orf163 1.04061610169491 3.84741343283582
4.30499579883494 2.61301465151014e-05 8.82903074317037e-05
1.52740790583995
KCNIP1 -1.88953686440678
3.16360447761194 -4.304471220843 2.61868533145279e-05
8.84673520729401e-05 1.52534015504972
S100A14 2.22026419491526 8.85239552238806
4.30430828333596 2.62044908208887e-05 8.85123719237982e-05
1.52469794008774
GPR34 -1.2451168079096
4.98062885572139 -4.30349768529042 2.62924049745876e-05
8.87801108763626e-05 1.52150328372582
PCOLCE -1.38815939265536
10.2951034825871 -4.30094570030269 2.65710319709742e-05
8.96619471341388e-05 1.51144888983275
DENND1B 1.1488738700565 4.86061990049751 4.30072466859963
2.6595296993541e-05 8.97290792567619e-05
1.51057829482908

PTPRO	-1.19481723163842
5.42499751243781	-4.30050742606142	2.66191667253147e-05
	8.97801039708742e-05	1.50972266069536
TLX2	1.58448848870056	1.63143333333333
4.29902088730618	2.67830532076531e-05	9.02735318261563e-05
	1.50386872356923
PAR-SN	-1.52819420903955
6.04148507462687	-4.29786173748315	2.69115163544315e-05
	9.06767488522041e-05	1.49930519832265
SNAI1	-1.09067838983051
6.06669353233831	-4.2975629508372	2.69447250461702e-05
	9.07737455198349e-05	1.49812905318739
UPB1	-1.00942881355932
1.53603333333333	-4.29538377718555	2.71881197423849e-05
	9.15336337014041e-05	1.48955299728969
CEACAM5 3.10743757062147	4.18565024875622
4.29394925778918	2.73494911423837e-05	9.19899436608214e-05
	1.48390947172854
ATP8B3	-1.71217012711865
4.97901393034826	-4.29393994755151	2.73505414566807e-05
	9.19899436608214e-05	1.48387284954732
FCER2	-1.08321144067797
1.10835223880597	-4.29309338623611	2.74462060975252e-05
	9.22814626895173e-05	1.48054314338295
KLRA1	-1.38338262711865
4.55648905472637	-4.29298856814835	2.74580732156743e-05
	9.23062458028155e-05	1.48013090943061
CABP7	1.47690148305085	3.7746592039801 4.29092593230383
	2.7692599720471e-05	9.30641794501996e-05
1.47202057036344
KLK1	-2.27864194915254
3.12035970149254	-4.2879660227545	2.80325033125853e-05
	9.40706951111248e-05	1.46038778469922
NAT8B	-1.21462563559322
1.75120845771144	-4.284537743532 2.84311875081658e-05
9.52500456172723e-05	1.44692258964836
SLC9A4	2.01567676553672	3.5463447761194 4.2843116691475
2.84576683225135e-05	9.53232061773866e-05	1.44603495566474
C21orf82	-1.06250028248588
2.46958208955224	-4.28001456940196	2.89655158031953e-05
	9.6945229774959e-05	1.42917069244982
RIBC2	1.64331052259887	6.01923184079602
4.2792229001428 2.90600202186329e-05	9.72395771574585e-05
1.42606526700513
SARDH	-1.36297478813559
5.91710597014925	-4.27705411363021	2.9320430978067e-05
	9.80372065113543e-05	1.41756036743659
BLNK	1.26468100282486	7.3503368159204 4.27629824073816
	2.94117142420742e-05	9.83104027010392e-05
1.41459705343906
CDH22	-2.00244950564972
2.45987810945274	-4.2755775022383	2.94990079072778e-05
	9.85540492003927e-05	1.41177188558708
REC8	1.73313121468927	9.98673184079602

4.27344474375578	2.97587768530826e-05	9.9373404162945e-05
	1.403414168903
VEPH1	1.54975981638418	4.9652263681592 4.2721786645205
2.99140192924783e-05	9.98430850389496e-05	1.39845438019447
GALNT14 2.56975459039548	7.01445572139303
4.27002240199181	3.01801996095781e-05	0.000100649691030643
	1.39001016733543
NPTXR	-1.6142395480226
8.65570547263682	-4.26920655834316	3.02815017548386e-05
	0.0001009711273391	1.38681613945861
NPTX1	-1.91081899717514
3.76643432835821	-4.26863697788998	3.03524186519298e-05
	0.000101174728839766	1.38458653310267
CTHRC1	1.543475	8.35967114427861	4.26825471634341
	3.04001021285463e-05	0.000101267904705347
1.38309032008788
FAM3D	2.0721072740113 3.73077064676617	4.26517174199639
	3.07873042577915e-05	0.00010247460382822
1.37102730278793
TMTC1	-1.60435988700565
8.25253880597015	-4.26397260626756	3.09391798532896e-05
	0.000102913376926124	1.36633730213992
BMP4	-1.70445995762712
6.57310348258706	-4.26340833303946	3.10108953630309e-05
	0.000103135214936502	1.36413072439854
KCP	2.0306084039548 5.82756019900498	4.26271057775308
	3.1099795667585e-05	0.000103397377336926
1.36140250434608
RUNDC2C -1.17514300847458	3.3832184079602 -4.26121878825737
	3.12906822211896e-05	0.000104015172603793
1.35557086154237
GPC2	1.49511603107345	6.04825721393035
4.2610797842178 3.13085259284807e-05	0.000104040795939092
1.35502755918946
CNTN1	-2.24029406779661
5.12695373134328	-4.2592582004481	3.15432615780633e-05
	0.000104803877682091	1.34790919694829
CLIP2	-1.06052747175141
9.03066517412935	-4.25723725924435	3.18056583733955e-05
	0.00010559025976808	1.34001475519269
CLCA3P	1.13762245762712	1.27617860696517
4.25519025869383	3.20735680151591e-05	0.000106445255633885
	1.3320216996157
ANKRD44 -1.22648375706215
3.86388706467662	-4.25323783255488	3.23311115729771e-05
	0.000107265307141761	1.32440091977778
EGFLAM	-1.06956115819209
6.56280597014925	-4.25230309501558	3.24551118854079e-05
	0.000107647105576665	1.32075345047184
C20orf177	-1.21180748587571
6.37974427860697	-4.25229131702169	3.24566772293988e-05
	0.000107647105576665	1.32070749544729
ANKRD55 -1.02184879943503	1.0768671641791 -4.25105024560566
	3.26220263386163e-05	0.000108150655061673

1.315865714706
SPATA12 1.01374018361582 3.47848407960199
4.24999946311517 3.27626530753566e-05 0.000108539175026153
1.31176722748514
SLC5A9 -1.57410988700565
3.04292686567164 -4.24807109084173 3.30222388546466e-05
0.000109346219949338 1.30424797395717
PARD3B -1.4845238700565
4.58405771144279 -4.24741713637516 3.31107163361721e-05
0.000109603838500744 1.30169867182198
MYOF -1.00457563559322
10.8500278606965 -4.24510870593103 3.34248553992094e-05
0.000110590214560156 1.29270236445422
MCF2L2 -1.01824943502825
2.59814925373134 -4.24030719434122 3.40874221976706e-05
0.000112637176519906 1.27400320530592
ZNF626 -2.25096497175141 6.2231736318408 -4.2398400052698
3.41525569642371e-05 0.000112797939909232
1.27218471187059
CRIP1 1.54918156779661 11.6161671641791
4.23881281000134 3.4296185957489e-05 0.000113254093245496
1.26818703021394
CEACAM6 2.60971786723164 5.26641393034826
4.23814862463952 3.43893640821477e-05 0.000113543525990108
1.26560255605127
NHLH1 1.05103693502825 2.90816915422886
4.23775593497271 3.44445681480693e-05 0.00011370750668594
1.26407468332058
HOXD11 -2.03026906779661
4.99907611940298 -4.23620009109422 3.46641223420339e-05
0.000114340364987461 1.25802238415552
HMGA2 2.661325 7.27059651741294 4.23548624669642
3.47653046180307e-05 0.0001146372797636
1.25524611974118
SYTL3 1.13752803672316 7.82634129353234
4.23401918218353 3.4974138263778e-05 0.000115251855305837
1.24954167805045
C9orf116 1.29038983050847 7.89461343283582
4.23370809364205 3.50185750604601e-05 0.000115361255775232
1.24833227286973
GSDMB 1.38519032485876 7.63334029850746
4.23304835350097 3.51129930943718e-05 0.000115653737883435
1.24576767576566
TRHDE -2.62060995762712
3.51952786069652 -4.23175955631096 3.52981410803115e-05
0.000116244920817901 1.24075871809223
ANPEP 2.09929943502825 8.57890348258706
4.23163335993366 3.5316320514279e-05 0.00011628613635255
1.24026831968749
ZSCAN23 -2.00226553672316 3.0473776119403 -4.22767229754438
3.58915128286672e-05 0.00011806645703774
1.22488186179002
BOP1 1.274860240113 8.54207562189055 4.2242869241256
3.6390201171932e-05 0.000119534532809562 1.21174116525487

KCNJ11 1.14381935028249 6.70521890547264
4.22378932771161 3.64640568619917e-05 0.000119757972704638
1.20981043180429
SPAG6 -2.11611151129943
2.08982935323383 -4.22277528851886 3.66150095845727e-05
0.000120234508440703 1.2058764270289
TSPAN1 1.93557641242937 10.9266378109453
4.22268279975506 3.66288074290144e-05 0.000120260581563676
1.20551765252479
C6orf126 1.62181278248588 1.75883432835821
4.22110392538356 3.68651187775631e-05 0.000120959068004374
1.19939403097596
CCDC88A -1.17146235875706
8.20271791044776 -4.22087515632016 3.68994795395375e-05
0.000121052463238527 1.19850691509425
FAM23A -1.04753792372881
1.92122039800995 -4.22002240227237 3.70278320035239e-05
0.000121434727001972 1.19520047738513
FOXQ1 1.90706765536723 7.51631641791045
4.21898535621276 3.71844987108115e-05 0.000121890109936606
1.19118022495811
PKDCC -1.90181129943503
9.06227064676617 -4.21719869980996 3.74558973344554e-05
0.000122649185111883 1.18425594170472
KRTAP5-10 1.01767281073446 1.51990547263682
4.21718565674267 3.74578855651002e-05 0.000122649185111883
1.18420540160903
VIL1 2.71723149717514 2.91624427860697
4.21589721354173 3.76547887294844e-05 0.000123234945419467
1.1792135051285
RAPGEFL1 1.10153354519774 8.18200298507463
4.21567280326119 3.76891847472238e-05 0.00012330820125267
1.17834418868331
PCSK5 -1.72400925141243
5.90041343283582 -4.21379144831615 3.79787298281559e-05
0.000124215918969578 1.17105775226478
NTN4 -1.40463086158192
8.50441542288557 -4.21091609094509 3.84253681818981e-05
0.000125616689295282 1.15992682714069
SLC25A21 -1.40550402542373
2.55332985074627 -4.20993467601553 3.85789610227687e-05
0.000126078648487335 1.15612908250584
CC2D2A -1.11752161016949
8.33080547263682 -4.209263780543 3.86842947855626e-05
0.000126402764912876 1.15353336953813
CNIH2 1.60652351694915 4.96787064676617
4.20877833352627 3.87606837003933e-05 0.000126632214571781
1.15165537753921
NUDT9P1 -1.06641631355932
2.01407114427861 -4.20739196594008 3.89796345822405e-05
0.0001272867660209 1.14629309746055
KLHL23 -1.68211588983051
6.24807960199005 -4.20651673112141 3.91184700559009e-05
0.000127699505525146 1.14290857014126

MS4A15	1.89438206214689	2.24752885572139
4.20385000511937	3.9544404718238e-05	0.000129028389838752
	1.13259999101969
FRMD5	1.85045360169492	3.30971641791045
4.20302552201304	3.96769877480599e-05	0.000129419853585585
	1.12941395073614
THPO	-1.77616271186441
3.03204975124378	-4.2026250699994	3.97415366652158e-05
	0.000129609809013007	1.12786667750222
CUBN	-1.21774604519774
5.78120646766169	-4.20137417944698	3.99438151948588e-05
	0.000130186779798452	1.12303426035924
CTAGE9	1.09807507062147	7.38087412935323
4.20105235764089	3.99960152212036e-05	0.000130336221030367
	1.12179119910694
C8orf79 -2.05878806497175
5.79109154228856	-4.20043577336562	4.00962084485484e-05
	0.000130641986898698	1.11940981803289
FCHO1	1.79195911016949	7.12163134328358
4.2002615877102 4.01245565758417e-05	0.000130713606236438
1.11873712858384
TUBA3E	-2.25626970338983
2.67500646766169	-4.19707016492758	4.0647354194126e-05
	0.000132290770704725	1.10641627336984
GUCA1B	-1.15158672316384
4.54886616915423	-4.19689858921408	4.06756442596438e-05
	0.000132361860302819	1.10575410771099
BZRAP1	-1.18043615819209	8.7655447761194 -4.19612626588574
	4.08032211437844e-05	0.000132734927916637
1.1027737452056
PTPN20B -1.82732203389831
1.97589900497512	-4.19085537345466	4.16841670765393e-05
	0.000135450451104994	1.08244586646314
PADI1	2.34830155367232	3.57053233830846
4.18960211732	4.18962885817102e-05	0.00013611818398204
1.07761567215299
KIAA0754	-1.53072980225989
5.23761890547264	-4.18755486015026	4.22450208639397e-05
	0.000137207764331068	1.06972791008674
MACC1	1.61821539548023	7.47263731343284
4.18695877236127	4.23470795962413e-05	0.000137517485623352
	1.06743188432468
CCDC155 1.52432111581921	1.71324179104478
4.18276988297222	4.30709528424254e-05	0.000139823950324952
	1.05130474449301
PIWIL4	-1.31025868644068	4.8231776119403 -4.18234917912343
	4.31443038015333e-05	0.000140017792767227
1.0496857910458
KIAA0513	-1.02675331920904
7.38321492537313	-4.18202640292584	4.32006617064257e-05
	0.000140178534033969	1.04844377556341
TWIST1	-1.7861122881356
6.63542985074627	-4.18099013248756	4.33820742456202e-05
	0.000140722702522134	1.0444568379385

NQO1 1.83894201977401 10.2194293532338
4.17961464875901 4.3623995255721e-05 0.000141462742473535
1.03916609399334
LIPH 2.10212888418079 3.47151592039801
4.1795680893956 4.3632206665035e-05 0.000141467025084202
1.03898703078412
WBSCR26 1.77101850282486 4.22071194029851
4.17879097802107 4.37694796339801e-05 0.000141889691597286
1.03599857590894
IPW -1.43331080508475
8.41560995024876 -4.17861893959018 4.37999251412309e-05
0.000141965971447659 1.0353370489572
PSCA 2.10600798022599 4.25743184079602
4.17734443736425 4.40261039593747e-05 0.000142654026875941
1.03043700912547
KSR2 1.28904752824859 4.9662855721393 4.17707620508811
4.40738476111591e-05 0.000142786190856414
1.02940590439115
PIK3R1 -1.37084449152542
10.8326134328358 -4.17645855873592 4.41839729668859e-05
0.000143120379458846 1.02703183727361
TUBB4Q 1.07698552259887 2.34705273631841
4.17637053907048 4.41996881222878e-05 0.000143148698083384
1.02669353723531
COCH 1.74221285310735 6.13024577114428
4.17569516043373 4.43204490312711e-05 0.000143471904543046
1.02409794568507
NBPF10 -1.64733870056497
7.24604179104478 -4.17552507708184 4.43509104034315e-05
0.000143547878067547 1.02344434281053
IP6K3 -1.43736588983051 2.8615223880597 -4.17480062185651
4.44808821985015e-05 0.000143900490314408
1.02066062848354
FOXC2 -1.31661716101695
1.48309502487562 -4.17466696255631 4.45049012477477e-05
0.000143955510101821 1.0201470878048
KIR2DL3 -1.24579131355932
1.20105572139303 -4.17331188522726 4.47491142293322e-05
0.000144722639434182 1.01494144019136
TMEM158 -1.43567902542373
7.59286616915423 -4.17219271017381 4.49517777426161e-05
0.000145355173579447 1.01064310106465
EDN1 -1.16872973163842
7.14684925373134 -4.17068804278258 4.52256298646079e-05
0.000146171628010139 1.00486575270644
HERC2P4 -1.33003064971751
1.29627064676617 -4.16989111190381 4.53713176830077e-05
0.000146619416343798 1.00180655089763
ALOX12P2 2.28500473163842 3.76089751243781
4.16934176605752 4.54720051775856e-05 0.00014689854701744
0.999698046692599
TMEM45B 1.74370494350282 6.83379303482587
4.16920807018159 4.54965420641796e-05 0.000146954689832852
0.99918492925187

EXOC6B	-1.49207895480226
5.24658805970149	-4.16794161111967	4.57296006255569e-05
	0.000147682860122198	0.994325013967485
PTPN5	-1.75242281073446
2.06982587064677	-4.16532074939316	4.62155292997164e-05
	0.000149159694469923	0.984271645426883
E2F5	1.11879731638418	7.88021791044776
4.16467595416617	4.63358334268409e-05	0.00014952446718847
	0.981799088060212
LOC647946	1.69324456214689	3.54761194029851
4.16413426944886	4.64371306662238e-05	0.000149780721536147
	0.979722170799832
RNF175	1.64030120056497	3.46899850746269
4.16379024392764	4.6501574646347e-05	0.000149965021597472
	0.978403232477749
LY6D	2.43803474576271	3.33490945273632
4.16355011579035	4.65466068516919e-05	0.000150086671692356
	0.977482674188474
FLRT1	-1.50885211864407
5.07083432835821	-4.15944400837311	4.73231190466027e-05
	0.000152518623866052	0.961748349444614
MPZ	-1.2940593220339
3.69898855721393	-4.15912912993374	4.73831743048388e-05
	0.000152688207263905	0.960542295062121
CCDC68	-1.57941892655368	5.2464184079602 -4.1587572958485
	4.74541861873679e-05	0.0001528690479251
0.959118186679824
RCSD1	-1.07654484463277
6.65863532338308	-4.15659978322394	4.78682326507257e-05
	0.000154154480991278	0.950857118415337
PNPLA5	1.61453728813559	1.96939004975124
4.15414951178838	4.83426469377554e-05	0.000155560272983091
	0.941479452122865
LRRC16B 1.41109696327684	4.09994527363184
4.15344449637371	4.84799797777363e-05	0.000155953303797701
	0.938782081746918
SORCS2	-1.90353827683616
5.39772487562189	-4.15334405368632	4.84995757197526e-05
	0.000155991898719493	0.93839782199259
ST6GAL2 -1.77428036723164
5.81536567164179	-4.15128890710545	4.8902191010601e-05
	0.00015718834843395	0.930537241275843
PCDHB9	-1.18043382768362
5.83603432835821	-4.14913042018302	4.93284873639155e-05
	0.000158459371863744	0.922284923375344
COX6B2	1.4949040960452 2.80461641791045	4.14633630790598
	4.98855909137944e-05	0.000160048629701547
0.91160784525672
KLHL29	-1.32181087570622
7.06444228855721	-4.14310427621591	5.05375039517922e-05
	0.000161988283548836	0.899264901974869
SLC7A8	-1.56603079096045
9.32750796019901	-4.14305210136566	5.05480942435734e-05
	0.000161996936437802	0.89906571562873

FOLR2	-1.21689307909604
6.30788258706468	-4.14226501641796	5.07081116836674e-05
	0.000162484397200826	0.896061141302305
PPAP2C	1.41967351694915	10.7784144278607
4.14064731005287	5.10385177550608e-05	0.000163466578706926
	0.889887307031223
C12orf59	1.2213206920904 2.77306218905473
4.13784113190442	5.16165428779634e-05	0.000165240546590455
	0.879182588049117
HERC3	-1.13347323446328	7.6824960199005 -4.1371567250584
	5.17584642720197e-05	0.000165643222370158
0.876572709894045
KCNH8	-1.8113229519774
2.37288358208955	-4.1369490270006	5.18016070247851e-05
	0.000165755453899445	0.875780757798307
KRT14	2.51000805084746	3.33544726368159
4.13577111986638	5.20469305167564e-05	0.000166514490183732
	0.871290034405639
TDRD5	2.20874519774011	4.30564776119403
4.13243408451497	5.27479741338397e-05	0.000168543739616052
	0.858573567447648
ITGBL1	-1.85733990112994
4.35386119402985	-4.13081340478941	5.30916896390075e-05
	0.000169566332963414	0.852400748612078
TSHZ2	-1.56506426553672
5.49118407960199	-4.12888926808841	5.35025389469375e-05
	0.000170732365910815	0.845074775472785
MMP26	-2.92757881355932
2.31946616915423	-4.12886996842744	5.35066752201744e-05
	0.000170732365910815	0.845001308354714
KLHL14	1.46423008474576	8.80717164179105
4.12869204485845	5.35448219036399e-05	0.000170827535925676
	0.84432402863947
TMEM45A -1.13459131355932	7.6328447761194 -4.1268999250841
	5.39305015301602e-05	0.000171951109863983
0.837503558186061
EFCAB1	-2.76951172316384
5.68328457711443	-4.12438932389036	5.44752665618921e-05
	0.000173607144134686	0.82795287818875
ADRA1A	-1.05966588983051
0.596757213930348	-4.12408730779809	5.4541152618005e-05
	0.000173790138638453	0.826804296477553
MFGE8	-1.03875741525424
11.7356358208955	-4.12329627820006	5.47140796788954e-05
	0.00017423298523464	0.823796308825745
C17orf28	1.09431285310735	10.9733402985075
4.12283490597008	5.4815181807171e-05	0.000174527866392869
	0.822042108497699
SALL2	-1.30796843220339
8.50308606965174	-4.12230127926336	5.49323397034684e-05
	0.000174792457239958	0.820013393077009
C2orf82 1.46962768361582	2.55988308457711
4.12219436072325	5.49558424227525e-05	0.000174840143334745
	0.819606942111854

CAPS2 -1.39158057909605
4.36670746268657 -4.12125854483173 5.51619625052422e-05
0.000175408462585264 0.816049816024742
GOLGA6L10 -1.06846377118644
6.65520149253731 -4.11840835957201 5.57942916139458e-05
0.000177315295175589 0.80522018213585
GPR158 2.21908686440678 3.72504626865672
4.117575882547 5.59802826629582e-05 0.000177823797473392
0.802058273767329
SLCO2B1 -1.19109357344633
7.95308905472637 -4.11678918286037 5.61565898744032e-05
0.0001782922193739 0.799070731832483
LYG2 1.38344124293785 1.55415422885572
4.11582958500639 5.63723627407457e-05 0.000178903170052173
0.795427249761095
PIP5K1B -1.59712853107345
6.22509004975124 -4.11564769860129 5.64133503782664e-05
0.000179005577011717 0.794736728856449
DOCK10 -1.33791059322034
6.56954825870647 -4.11525757374552 5.65013599607005e-05
0.000179229437576202 0.793255729862183
DLK1 -2.49924004237288
1.88703084577114 -4.11511507044605 5.65335404210166e-05
0.000179303813508956 0.792714785792963
XKR5 -1.34229300847458
3.06947661691542 -4.11416959414944 5.67474937195764e-05
0.000179926802480757 0.789126147611322
KCNC4 -1.00568185028249
7.35172039800995 -4.1125654826044 5.71122593056729e-05
0.000181027433000689 0.783039192467399
MECOM 1.25627033898305 11.3196845771144
4.11186797948907 5.7271564419001e-05 0.000181476341645638
0.780393074531816
EPHA6 -1.63484201977401
2.58260049751244 -4.11173738416064 5.73014386203723e-05
0.000181542983777198 0.779897677042173
FAM171B -1.33296271186441
7.29569701492537 -4.10435415685433 5.90147768977136e-05
0.00018648197163461 0.751911893968025
ZNF641 -1.06948707627119 6.162992039801 -4.10373348087249
5.91610158770401e-05 0.000186915305625675
0.749561184365993
CLEC18A 1.41594964689265 4.67072686567164
4.10296392302457 5.93428133836531e-05 0.000187403162400615
0.746647025523721
SEPT5 -1.31248326271186
8.48943532338308 -4.10269506293371 5.94064534368032e-05
0.000187546438421893 0.745629015857837
ESPNP 1.18492987288136 1.85879154228856
4.09929296943464 6.02173856144284e-05 0.000189902139272537
0.732752221871936
GLUD2 -1.0358843220339
7.08722835820896 -4.09913207164827 6.02559979886041e-05
0.000189994722578105 0.732143453256644

KRT19	1.51509138418079	13.1501432835821
4.09845434655407	6.04188983994076e-05	0.000190479113043587
	0.72957945197116
GJB5	1.93513495762712	3.34391990049751
4.09837049209058	6.04390831377132e-05	0.000190513492525296
	0.729262234728774
WDR69	-1.04051687853107
0.56761144278607	-4.09828717116825	6.04591458178827e-05
	0.000190547476764067	0.728947041274369
GDAP1	-1.15475579096045
6.93423383084577	-4.09647796125643	6.08963517815528e-05
	0.000191807625356747	0.722104343011132
ELMO3	1.41424950564972	8.56148805970149
4.09507214584878	6.1238155633348e-05	0.000192825051403778
	0.716789103950433
SGK1	-1.09431906779661
9.39892089552239	-4.09466514380138	6.1337453446132e-05
	0.000193078491145215	0.715250559990222
ATP11B	-1.21594103107345
8.97124179104478	-4.09036457856599	6.2396106815559e-05
	0.000196200348127344	0.699001512701933
FGFR4	1.53839915254237	8.02589303482587
4.08949121855868	6.26132169389643e-05	0.000196822744412331
	0.695703414050236
LOC650623	-1.41228601694915
4.6678368159204 -4.0875061671765	6.31093674373312e-05
0.000198324740766224	0.68820941178716
PNMA2	-2.00822881355932
4.45945970149254	-4.08750224190664	6.31103522370065e-05
	0.000198324740766224	0.688194596082999
DNAJC5B 1.44357944915254	2.49944925373134
4.0873394531507 6.31512067501207e-05	0.000198422753991426
0.687580169926164
AMY2B	-1.46719103107345
6.89314179104478	-4.0870042077697	6.32354215587622e-05
	0.000198656955562569	0.686314892584241
PDCD1LG2	-1.27079060734463
4.58771641791045	-4.08506989945026	6.37234222085088e-05
	0.000200128783530777	0.67901617277391
PF4V1	1.48887422316384	1.47529402985075
4.08113061289426	6.47283801298242e-05	0.000203218608378936
	0.664161106987562
TMEM160 1.03282824858757	7.26637213930348
4.07964504369117	6.51112761122534e-05	0.000204268719221357
	0.65856216638592
CHI3L1	1.9477293079096 9.14667711442786	4.07740734636285
	6.56921076095633e-05	0.000205902132706005
0.650131795203734
LOC151009	1.10011242937853	6.69668905472637
4.07611508834684	6.60297814867105e-05	0.000206862941043844
	0.645265083950429
CD68	1.00650416666666	9.98378706467662
4.07608007551774	6.60389535119998e-05	0.000206862941043844
	0.645133241963979

ZNF167 -1.16299385593221
5.94618358208955 -4.07283525690672 6.68942652694048e-05
0.000209446281223255 0.632918927522146
ERN1 -1.44845451977401
5.26351194029851 -4.07156344349247 6.72323789981448e-05
0.000210472818059151 0.628133746917707
MAT1A 1.97915783898305 5.40556965174129
4.06950248666155 6.77837495834891e-05 0.000212101871505721
0.620382110585727
LRP2 2.69397471751413 4.24474776119403
4.06803610637653 6.81786740755923e-05 0.000213240124736732
0.614868808365149
FLG -1.92824675141243
3.26763333333333 -4.0675950973573 6.82978746992101e-05
0.0002135153597647 0.613211029971131
TMEM38B -1.21934519774011
6.22111492537313 -4.06739136903029 6.83530076272896e-05
0.000213646330544252 0.61244525479378
ATP2C2 1.93827627118644 7.6694736318408 4.06509671104519
6.89769248430684e-05 0.000215343106299908
0.603822324271394
ADARB2 -1.44655974576271
1.77067213930348 -4.0647753006691 6.90647485238725e-05
0.000215551731102935 0.602614848268215
LRIG3 -1.1683479519774
8.03717711442786 -4.06319799783451 6.94972853876065e-05
0.000216736938934766 0.596690401542794
RPL39L 1.36435586158192 8.13489353233831
4.06218999096979 6.97750577079834e-05 0.000217570160198192
0.592905285487122
ALOX15B 1.68884435028249 3.71060796019901
4.06156987494741 6.99464659427229e-05 0.000218071517965695
0.59057711407278
PROM1 2.1933438559322 9.73538955223881 4.05670884123025
7.13040924702718e-05 0.00022193344730362
0.572337219324918
LAMA1 2.27922394067797 8.25124029850746
4.0538036407788 7.21274297058945e-05 0.000224326031186489
0.561444969028352
FGF1 -1.26226836158192
5.62888358208955 -4.05286539133117 7.23952605626732e-05
0.000225056738242229 0.557928673510358
PCSK2 -1.8179438559322
1.52135323383085 -4.04941293979297 7.33889734513537e-05
0.000228042322530088 0.544995795040158
HDGFRP3 -1.07436186440678 9.0097368159204 -4.04870263316729
7.35950255253715e-05 0.000228613386902084
0.542336146454499
OAS3 1.21495628531074 10.0776243781095
4.04727347100626 7.40112811923589e-05 0.000229802117797811
0.536986037819605
SLC6A16 -1.25729378531073
4.73673084577114 -4.04608421982499 7.43593698167166e-05
0.000230778210481813 0.532535263693131

GJB4 1.72526391242938 2.71811641791045
4.04567647429128 7.44790738247844e-05 0.00023109110575149
0.531009530854943
GRIK5 -2.13435875706215
6.05572338308458 -4.04116935987382 7.58145412031544e-05
0.000234903792771017 0.514153161104147
RENBP 1.27890063559322 7.13651691542289
4.03825629537052 7.66897922692597e-05 0.000237472313014767
0.503266935263738
FAM132A 1.69407316384181 3.62061243781095
4.0375554805847 7.69017889533879e-05 0.000238056955249254
0.500648959524449
LOC643763 -1.76009371468927
2.11178756218905 -4.03734380688483 7.69659300049939e-05
0.000238219590381807 0.499858303443125
RIMS1 -1.24682937853107
1.21044577114428 -4.03719377101996 7.70114245102903e-05
0.000238324471690723 0.499297901941698
LOC151534 1.17875734463277 6.90486019900498
4.03573298413174 7.7455711361041e-05 0.000239591043278917
0.49384261592118
PRCD -1.09062570621469 3.3533815920398 -4.03549393943569
7.7528646986002e-05 0.000239780524649385
0.492950066747886
KLKP1 -1.17967210451977
0.772780099502488 -4.03542658452656 7.75492096496681e-05
0.000239807994292467 0.492698583940231
XCL2 -1.36725889830509
3.48192089552239 -4.03317694166149 7.82389912805361e-05
0.000241868166087849 0.484301146562033
RAPSN 1.67797747175141 2.25700945273632
4.03204671834054 7.85877401249287e-05 0.00024287314538082
0.480083764123087
OR2A7 1.03189851694915 6.92401592039801
4.03128020312133 7.88251029694874e-05 0.00024353338810588
0.477224114821439
ANKRD13B -1.00852344632769
7.46954726368159 -4.02943186577731 7.94002798109063e-05
0.000245162843212198 0.470330395090751
BDNF 1.5803729519774 4.21279253731343 4.02887581841791
7.95740946900578e-05 0.000245625644863462
0.468257037825915
ATP6V0A4 2.02173891242938 3.30514975124378
4.02822869739922 7.97768345713727e-05 0.000246140428877409
0.465844396335829
GPR35 1.46029484463277 6.89874378109453
4.02791730542492 7.9874567395891e-05 0.000246404938938789
0.464683560468951
PGAP1 -1.11836800847458
7.78543930348259 -4.02768618830235 7.99471790385659e-05
0.000246579815439247 0.463822029914085
FHL2 -1.13249781073447
10.0210049751244 -4.02727178352529 8.0077532858838e-05
0.000246919758124353 0.462277366871449

C6orf27 -1.59962111581921
5.52114925373134	-4.02530214836091	8.06998671371387e-05
	0.000248764004853672	0.454937543790013
GAL3ST3 -2.53116666666667
3.66266218905473	-4.02432234604762	8.10111625204465e-05
	0.000249648629022203	0.451287457687985
LOC150381	1.13145077683616	6.5791980099 5025
4.02406436382783	8.10933166685159e-05	0.000249864294042418
	0.450326514561938
THRB	-1.89176765536723
6.69935671641791	-4.0221449749587	8.1707039640097e-05
	0.000251528793493956	0.44317873680255
IL1R2	1.71885903954802	6.20433880597015
4.02201062357371	8.175016360608e-05	0.000251623816916465
	0.442678522603133
C12orf75	1.31236398305084	9.89693880597015
4.02169854176608	8.18504189559288e-05	0.000251894633662902
	0.441516641096738
C13orf18	-1.27718312146893
6.10139552238806	-4.0199940350942	8.24000558684795e-05
	0.000253472152679036	0.435172109653052
AKR1B10 2.87307789548023	4.30326965174129
4.01886288938951	8.27667443142763e-05	0.000254409531482571
	0.430963009820352
KRTAP5-9	1.10957040960452	2.8389552238806
4.01844314794378	8.29032085748842e-05	0.000254752712474536
	0.429401368435471
LYPD6B	1.88686193502825	7.05820248756219
4.01813694081171	8.3002896244816e-05	0.000255020870982651
	0.42826221712565 7
LILRB3	1.28441193502825	5.21960348258706
4.01545148203546	8.38820675772435e-05	0.000257644950233514
	0.418274938969034
PSD3	-1.27139244350283	8.2053447761194 -4.01447849242421
	8.42027896542447e-05	0.000258591364487905
0.414657770963783
ZNF418	-1.77824117231638
5.13110696517413	-4.01374555119316	8.44451563146284e-05
	0.000259180603848007	0.411933494529813
SMC1B	1.96560600282486	2.94761094527363
4.01363677151091	8.44811837843854e-05	0.00025925242198706
	0.411529206310247
CHN2	-1.18268502824859
6.71988109452736	-4.01275643227945	8.47732881223318e-05
	0.000260109939493569	0.408257699341378
SYCE1	-1.8975988700565
1.58318009950249	-4.01107817586226	8.53328142774075e-05
	0.000261709393055748	0.402022672034541
HDX	-1.73052118644068
4.08905820895522	-4.01101543421853	8.53538001962042e-05
	0.000261734655418682	0.401789618450638
IGSF5	-1.27392492937853
2.23866467661692	-4.01087426948843	8.54010351510982e-05
	0.000261840390031668	0.401265273962735

COL27A1 -1.82962937853108
7.90553383084577 -4.00915431966675 8.59785477623564e-05
0.000263571686659874 0.39487792607022
TMEM72 1.49360070621469 1.82376865671642
4.00856475549911 8.61773616357581e-05 0.000264102289055398
0.39268900813522
ACTN2 -1.53249074858757 2.3001223880597 -4.00832936145118
8.62568638192463e-05 0.000264267036891378
0.391815119960942
NPL 1.00061009887006 6.44244676616915
4.00565988935599 8.7163352749116e-05 0.000266924766100739
0.381907898559969
PLIN1 -1.13297125706215
2.84288805970149 -4.00554455988117 8.72027195135365e-05
0.000266991240361687 0.381480002413085
MGC16703 -1.3219175141243
2.99952338308458 -4.00552010385697 8.72110695501935e-05
0.000266991240361687 0.381389266864562
NTRK2 -2.03911398305085 6.0340039800995 -4.00473997270594
8.74778299604659e-05 0.000267767981077735
0.378495109665767
GPRC5A 1.56391581920904 9.19204626865672
4.0028408319122 8.81304784575188e-05 0.000269685306712306
0.371451617684686
STAC -1.75569230225989
3.68964676616915 -4.00174370238508 8.85096213932009e-05
0.000270684124415673 0.367383903401196
MAPRE3 -1.12388665254237
8.16761293532338 -4.0016940685114 8.85268103799573e-05
0.000270696368349795 0.367199903432565
TLR1 -1.11403742937853 6.0568736318408 -4.00032518047756
8.90021318157946e-05 0.000272028251626956
0.362126002536833
FABP3 -1.54323700564972
7.17014676616915 -3.99614281869433 9.04694742409598e-05
0.000276184134746008 0.346632875778787
ELOVL4 -1.62490353107345
4.88247313432836 -3.99583230222511 9.05793292254501e-05
0.000276478386708072 0.345483149923818
FAM150B -2.2468134180791
5.57817114427861 -3.99057617328644 9.24582218698318e-05
0.000281878125183791 0.326033224206169
B4GALT6 -1.26731384180791
6.15069054726368 -3.98969382462125 9.27772454521686e-05
0.000282808737807427 0.322770294455424
CLCN1 -1.02520473163842
1.18767860696517 -3.98668320629931 9.38736721967557e-05
0.000285820753500019 0.311641640544207
NR6A1 1.21299491525424 2.49279303482587
3.98667484708732 9.38767336020763e-05 0.000285820753500019
0.311610750951432
ITGB3 -1.87847231638418
6.29455621890547 -3.98660072335886 9.39038841896627e-05
0.000285861023489587 0.311336845754933

SIGLEC9 1.18171680790961 4.44117462686567
3.98352586821748 9.50367709442075e-05 0.000289052578886604
0.299978333112315
BAALC -1.54955875706215 5.1363631840796 -3.98121191487947
9.58978759732379e-05 0.000291585218265784
0.291435517622704
C12orf36 1.6304959039548 1.95570149253731
3.98105877607706 9.59551255022251e-05 0.000291693452924846
0.290870298331068
MICB 1.09690543785311 6.79960248756219
3.98041230374771 9.61971617453615e-05 0.000292365319116547
0.288484440922776
IL10 1.3134656779661 3.19317562189055 3.97487445223497
9.82944371033297e-05 0.000298341926926576
0.268060099311703
UGT3A2 1.84300360169491 2.65127263681592
3.97378252869411 9.87130676184863e-05 0.000299523984364103
0.264035800924633
GPR27 -1.27792853107345
3.49024328358209 -3.97324262846605 9.89206849570594e-05
0.000300109599847559 0.262046340101169
AQP2 1.57845550847458 2.63482487562189
3.97315060685479 9.89561130483657e-05 0.000300172724716748
0.261707275655328
IL2RA 1.35509484463277 4.24853830845771
3.96920145366902 0.000100487950344331 0.000304532173614364
0.247162470455924
SIGLEC1 1.33299540960452 7.12351094527363
3.9682849310405 0.000100846670788377 0.000305456204084595
0.243788665113096
FGF14 -1.93329943502825
3.69427562189055 -3.96732835822177 0.000101222365225508
0.000306458510260977 0.240268140459922
SLC38A1 1.03654929378531 11.245423880597 3.96518936946703
0.000102067275560071 0.000308788854590076
0.232398530152116
FAM7A3 -1.07894357344633
2.21406467661692 -3.96381508745904 0.000102613657269689
0.000310304666923953 0.227344284387058
TRIM36 1.45446264124294 5.84637462686567
3.96323627559262 0.000102844610434535 0.000310957268368336
0.225216015085318
FXYD5 -1.07035261299435
9.53622985074627 -3.96248738992405 0.000103144158701432
0.000311725243359398 0.222462784634804
KRT83 1.71446264124294 2.1459631840796 3.96175145300651
0.000103439335657556 0.000312571321908981
0.219757592241717
LYG1 1.09348411016949 4.69173134328358
3.96071519608623 0.000103856330011132 0.000313692872609761
0.215949196539814
DLX6 2.13956574858757 8.34612437810945
3.95791872845602 0.000104989632420663 0.000316882851160867
0.20567601538545

TAS1R1 1.61115543785311 3.7187328358209 3.95748992221882
0.000105164447638176 0.00031736382625485
0.204101288896426
PAR5 -1.29696920903955
2.67150945273632 -3.95713544463756 0.000105309169548248
0.000317660483518298 0.202799633265684
SLC18A3 1.83465402542373 1.80366169154229
3.9567355230314 0.000105472672119472 0.00031810694264915
0.201331224956269
S100A5 1.45710543785311 2.02319353233831
3.95548341987616 0.000105986139773457 0.000319514750300891
0.196734647094274
MOGAT1 1.33855918079096 2.01265621890547
3.95489692350625 0.000106227469094779 0.000320129672058799
0.194581996150037
MYADML2 1.01160854519774 1.65860696517413
3.95484730652029 0.000106247909255023 0.000320129672058799
0.194399896619426
TRIM22 -1.17217443502825
9.32089502487562 -3.95465084803532 0.000106328878800167
0.000320312821976146 0.19367889258593
KLF8 -1.40473488700565
6.47643631840796 -3.9496683455709 0.000108402114628833
0.000326141216742057 0.175403304724682
ZC4H2 -1.52608425141243
6.95324726368159 -3.94965764549239 0.000108406608039347
0.000326141216742057 0.175364078521851
VSTM2L -1.83470600282486
4.06502587064677 -3.94887406553346 0.000108736147692676
0.00032703678759605 0.172491742147065
KIAA1244 1.41204124293785 6.82027263681592
3.94745991913139 0.000109333287488017 0.000328715460844494
0.167309197597693
LOC284578 -1.33421038135593
2.16386915422886 -3.94743857871187 0.000109342322557577
0.000328715460844494 0.167231001671547
TRO -1.92582351694915
7.36672139303483 -3.94621804545588 0.000109860252452108
0.000330175813620521 0.162759304498437
TICAM2 -1.08754081920904
6.37570646766169 -3.94616799049913 0.000109881542830949
0.000330189391221942 0.162575942180737
NR2C2 -1.08624293785311
8.91865771144279 -3.94503263206653 0.000110365510866901
0.000331548717895445 0.158417408582934
SLC18A2 -1.7786709039548
2.87465771144279 -3.94424626810688 0.000110701900226354
0.000332413340887385 0.15553775407976
SDC2 -1.12817598870056
10.5919815920398 -3.94337555167625 0.000111075510557182
0.000333388922768851 0.152349774622829
ZNF876P -1.19239752824859 3.4890407960199 -3.94218571794934
0.000111587987021489 0.000334829198122064
0.147994374933714

COL4A5 -1.62166334745763
9.33128059701493   -3.94213070934544   0.000111611734187228
        0.000334851512913018   0.147793042507619
SEMA6D -1.60943912429379
5.58322437810945   -3.94193247005127   0.000111697353764408
        0.000335059420336544   0.14706750324999
CYP26B1 -1.69031221751413
5.07921194029851   -3.9416144752156   0.000111834826162516
        0.000335422787599188   0.145903733888948
CACNB4 -1.17808029661017
3.97723184079602   -3.94095382232198   0.000112120947348664
        0.000336182714921583   0.14348619159382
RICH2   -1.2326384180791
7.20157562189055   -3.94085685420857   0.000112163001628394
        0.000336259699683256   0.143131383120921
IL11   1.51416800847458   4.17193432835821
3.93442429859828   0.000114986474460857   0.000344221685721989
        0.119611201468597
C6orf138   -1.60435444915254
3.03218109452736   -3.93407396436287   0.000115142171625481
        0.000344637524926537   0.118331173311804
GAD1   2.43712026836158   6.43273333333333
3.93334154950092   0.000115468322238995   0.000345563360869761
        0.115655439263991
SLC38A8 1.50635586158192   1.45640497512438
3.93096701005228   0.000116531771923737   0.000348491955949645
        0.106983457284367
CPLX1   -1.03047443502825
6.58097562189055   -3.9282905224028   0.000117741605293405
        0.000351904958024983   0.0972141138754434
RANBP17 1.02798411016949   6.95601890547264
3.92700683404051   0.000118326086853635   0.000353600373086628
        0.0925305943445016
CBR3   -1.28181638418079
6.73120298507463   -3.92654859322688   0.000118535397216484
        0.000354174313033681   0.0908590264197917
SNHG4   1.08776193502825   3.48498407960199
3.92553787172973   0.000118998308027963   0.000355505713593435
        0.0871727135018245
CYP2B6 2.03290466101695   2.0383447761194 3.92297760410855
        0.000120178608723875   0.000358875176305622
0.0778385184155477
TREM2   1.35837881355932   6.68870099502488
3.9205899161272 0.000121289363638023   0.000362029660044756
0.0691382130359397
CAMP   1.8146311440678 1.90357960199005   3.92044441308823
        0.000121357366074535   0.000362131791498577
0.068608172830011
SLC6A10P   1.98567648305085   3.92204328358209
3.92040000051533   0.00012137812998919   0.000362141114471453
        0.0684463895367617
SH3TC2 -1.42282189265537
3.32787562189055   -3.91868904008282   0.000122180617918811
        0.00036432361450591   0.0622150072589056

FZD7	-1.50267196327684
8.16808905472637	-3.91798856605129	0.000122510611414961
	0.00036520151769263	0.0596645268906144
TMEM132B	-1.625397740113
3.67537960199005	-3.91592964648008	0.000123485476879514
	0.000367787152232181	0.0521701027959818
NKPD1	1.1926479519774 3.94558805970149	3.91326842390997
	0.000124756442094189	0.000371303240967484
0.0424883107166458
PARVG	1.29245098870056	8.5129368159204 3.91315153580421
	0.000124812550166167	0.000371389274777879
0.0420631895167842
ESRRG	1.67482881355932	6.03971592039801
3.91310585665064	0.000124834483367783	0.000371389274777879
	0.0418970577219397
ACTR3C	1.0042122881356 5.48379800995025	3.91214084173735
	0.000125298699177078	0.000372647007693092
0.0383877564559052
ALPP	2.80320296610169	6.06060049751244
3.91179751378619	0.000125464250462619	0.00037303129065859
	0.0371394145843054
ADAM22	-1.44308446327684	3.8486407960199 -3.91037550673507
	0.000126152150719413	0.000374913673171619
0.0319699911583839
AMHR2	-1.07125699152542
1.04267512437811	-3.90963654760292	0.000126511037171876
	0.000375871431713257	0.0292842887026366
FOLH1	1.35751031073446	8.08449452736318
3.90909949610544	0.000126772472319012	0.000376593670483189
	0.0273326804742418
C9orf4	-1.41929837570621
1.57971592039801	-3.90809418849622	0.000127263232782863
	0.00037788749913685	0.0236800796794148
CNGB1	1.54407118644068	2.84323781094527
3.90774188031679	0.000127435645353896	0.000378235333109076
	0.0224002231698064
GAS2L3	1.55512323446328	3.90719104477612
3.90737274017024	0.00012761653308488	0.000378717465196961
	0.0210593260559691
IGFBP1	-2.17042521186441
2.32162587064677	-3.90675805163828	0.000127918286671957
	0.00037944840707633	0.0188267184685884
LAMC2	1.58309456214689	11.0902975124378
3.90654958042454	0.000128020779963217	0.000379642728967912
	0.018069599473181
TRIM6	-1.2980386299435
5.57858855721393	-3.90585173851351	0.000128364436324379
	0.000380551895702454	0.0155354519827631
WIPF3	-1.32878785310735
4.15395671641791	-3.90456190045864	0.00012900193162554
	0.000382055634199703	0.0108525481505444
TNIK	-1.53389194915254
5.24173432835821	-3.90417494978195	0.000129193765271661
	0.000382568585176287	0.00944793839686309

FSTL3	-1.10752747175141
8.25254378109453	-3.90324119199808	0.000129657798582315
	0.000383735922398089	0.0060589405785878
EPC1	-1.2951072740113
7.81501990049751	-3.90070420236001	0.000130926565643903
	0.000387252901983768	-0.00314534486954265
C6orf15	2.36411221751413	2.56810099502488
3.89756344901886	0.000132513611063574
0.000391664905720583	-0.0145329918528407
WTIP	-1.1241095338983	8.1847	-3.8946868409229
0.000133983190331489	0.000395780560792154	-0.0249560015186923
TM4SF1	-1.17597351694915	10.67352039801	-3.89327521569212
	0.000134709996399875
0.000397755821384931	-0.0300684228168766
RUNX3	1.25275550847458	7.81368109452736
3.89305224496085	0.000134825139432305
0.000397924110486661	-0.03087580064118
KRT6C	2.16124350282486	2.33685124378109
3.89293544997571	0.000134885490135111
0.000398045006823894	-0.0312986998604643
IGF2BP1	2.52586829096045	3.34425124378109
3.88985838080448	0.000136484751599233
0.000402532962265803	-0.042436436571859
LRRC34	-1.25388298022599
5.66958855721393	-3.88874706865396	0.000137066750717455
	0.000404133332217342	-0.0464570747752857
SOX9	1.46353891242938	9.89820049751244
3.88726533878702	0.000137846403875811
0.000406373732276048	-0.0518163196944883
CXCL5	2.48802535310734	5.20539353233831
3.88542683140759	0.000138819637006194
0.000409022160130372	-0.0584635485271106
DLX6AS	2.18123262711865	5.76797860696517
3.88166904719493	0.000140829181572699
0.000414452852306122	-0.0720416282220917
BATF	1.36388460451978	5.88162437810945
3.87902788291665	0.000142258078645955
0.000418298246147445	-0.0815782454004825
FUT7	1.221214759887	3.5253855721393	3.87477697372694
0.000144586790026575	0.00042478059519828	-0.0969155519719527
FAM153B	-1.0217845338983
0.824382089552239	-3.87261869643554	0.000145782924593595
	0.000427967440147843	-0.104697095525261
CHST13	1.27768015536723	3.3177671641791	3.87259411125847
	0.00014579660381851
0.000427967440147843	-0.104785714478806
C11orf52	1.27639632768362	7.7541407960199
3.86875287797256	0.000147948893837748
0.000433789030346896	-0.118625758967739
KCNK4	-1.29183891242938
1.63426467661692	-3.86761827594387	0.000148590369134643
	0.000435483266000603	-0.122711493798428
IL34	-1.10640261299435
5.71550099502488	-3.86737479487504	0.000148728370574197

0.000435825499270377	-0.123588141835139
DLL3	1.92682118644068	3.37130298507463
3.86579726802669	0.000149625439125701
0.000438204032132759	-0.129266840854918
TMEM121 -1.24475621468926
5.60278805970149	-3.86518492334181	0.000149975032772788
0.000439165229328961	-0.131470590307903
SEMA3B	-1.18334745762711
9.58178109452736	-3.86463635397762	0.000150288874241646
0.000440021475781659	-0.133444565529605
IGF1R	-1.16285098870057
10.2529552238806	-3.86328696453827	0.000151063524905779
0.000441974371713788	-0.138299190472177
ZNF192	-1.71229696327684
6.85375223880597	-3.86292427121159	0.000151272382905939
0.000442504222777957	-0.139603783742165
MYC	-1.10938961864407
10.0780706467662	-3.85949675129575	0.000153259699571649
0.000447761723666708	-0.151927229140643
MSI1	1.41251942090395	8.66145621890547
3.85871985167318	0.000153713584665378
0.000449023889183298	-0.154719216761186
SULT1C4 -1.69989011299435
5.51383184079602	-3.85722448525416	0.000154590807391682
0.00045132953295666	-0.160091836625472
LHFPL5	1.28758509887006	1.23568109452736
3.85639977896826	0.000155076631535602
0.000452491960985672	-0.163054111455347
TNF	1.7219990819209 5.40101641791045	3.85639893748198
0.000155077127983204
0.000452491960985672	-0.163057133723961
C19orf57	1.26079110169492	6.04052388059702
3.85515322556567	0.000155813709451046
0.000454511999862172	-0.167530589995845
P2RY13	-1.29646984463277
4.44776169154229	-3.85387840707222	0.000156570935419612
0.00045646141780242	-0.172107281901579
EREG	2.01591257062147	2.94831492537313
3.85213311715714	0.000157613275671697
0.000459109045053908	-0.178370886670384
GRM8	1.41936094632768	3.11119751243781
3.85151844016241	0.000157981943623652
0.000459987136944203	-0.180576295732497
RNASE10 1.24036327683616	1.44269353233831
3.85143211914413	0.000158033782246974
0.000460072823245942	-0.180885983969256
SCN5A	-1.61749293785311
4.86062388059702	-3.8503599307641	0.000158679012524453
0.000461689360420495	-0.1847321053231
SV2A	-1.30115628531073
7.51423482587065	-3.84886833986403	0.000159580789698473
0.000463984366592502	-0.190081160199173
CDK5R2	1.45324533898305	1.76553184079602
3.84705464265189	0.000160683850359025

0.0004670592450617	-0.196582927920814
EDARADD 1.05989512711864	6.42190298507463
3.8467937551451 0.000160843111235514
0.000467455984380676	-0.197517943961567
C11orf87	-1.88239915254237
2.25931492537313	-3.84637715225798	0.00016109774013924
0.000468129738861798	-0.199010927779206
C15orf33	-1.09371984463277
4.23528606965174	-3.84518587400644	0.000161827963300461
0.000470052077894521	-0.203279354335775
RAB37	-1.09802097457627
4.81196517412935	-3.844072510219 0.000162513262643844
0.000471909092231699	-0.207267578241604
SLC30A2 2.06894124293785	5.05357014925373
3.84267392317631	0.000163378021897394
0.000474151935192749	-0.212276100897576
KIF5A	-1.75589194915254
3.01524179104478	-3.84260548446083	0.000163420449932927
0.000474208033515618	-0.212521148548986
BEX4	-1.24893086158192
9.63485771144279	-3.84162642485137	0.000164028554724717
0.000475838099265004	-0.216026300112216
TMEM26	-1.62943877118644
5.67398358208955	-3.84077382892555	0.000164559858168125
0.000477177102851921	-0.219078069222586
CCL19	-2.0786311440678
4.13097611940298	-3.8366343958694	0.000167162653773799
0.000484314039228914	-0.233886392893765
CTTNBP2 -1.70568601694916
6.97093631840796	-3.8361929962108	0.000167442488016224
0.000484950266131256	-0.235464634632415
HTR7	-1.06312853107345
2.26052885572139	-3.83617669560886	0.000167452830608408
0.000484950266131256	-0.235522915073738
KCNK9	1.72535586158192	2.7028223880597 3.83617624850385
0.000167453114301241
0.000484950266131256	-0.235524513629962
CACHD1	-1.038525	8.92136965174129	-3.83456038420694
0.000168481378239971
0.000487790536633282	-0.24130074185769
GJA3	-1.38025084745763
2.55798855721393	-3.83385718136413	0.000168930731383273
0.000488953604299817	-0.243813824778375
LOC399815	1.58629872881356	2.32551492537313
3.8326482633785 0.000169705897036278
0.000490782091302266	-0.248133286412633
SH2D1B	-1.11305098870056
2.63144228855721	-3.83155517973201	0.000170409689760742
0.00049274801842085	-0.252037860544814
KRT1	-1.38742019774011
1.24388358208955	-3.82960866915642	0.000171669823070224
0.00049618210159534	-0.258988558558467
HIST1H4J	1.09843495762712	5.61562437810945
3.82727349774982	0.000173193219093307

0.000500303473756239	-0.267323085058854
C3orf66 1.95352867231638	3.1106631840796 3.82665687173145
	0.000173597620165122
0.000501401117331169	-0.269523170304345
WNT7A	2.78789046610169	7.14112885572139
3.82424759740059	0.000175186284968577
0.000505705066142418	-0.278116385820923
RHOBTB3 -1.52665014124294
8.14201144278607	-3.82404554826995	0.000175320139451358
	0.000506020309705664	-0.278836826910601
TMEM150B	1.21130642655367	2.30796865671642
3.82214209475791	0.000176585913758881
0.000509458798407788	-0.285622306403543
FAM71F1 1.18753220338983	1.6131039800995 3.81894436013743
	0.000178731880777917
0.000515360324771157	-0.297015105877476
GPR109A 1.35095769774011	3.88107014925373
3.81875375014424	0.000178860574273951
0.000515658979054094	-0.29769394580106
DOC2B	-1.58553269774011
3.26777213930348	-3.81812622568866	0.000179284878298991
	0.000516737126418402	-0.299928609581958
SCML1	-1.1273052259887
7.39262338308458	-3.81655869335738	0.000180348943324451
	0.000519512250098356	-0.305509329442074
C21orf62	-1.18842803672316
1.35710298507463	-3.81642476366418	0.000180440133720032
	0.000519702012524165	-0.305986053412232
MST1	1.10852443502825	7.94683830845771
3.81628935730603	0.000180532373951652
0.000519894745017172	-0.306468018895652
TGFB2	-1.40456744350283
7.99057960199005	-3.81218242954463	0.000183351371971325
	0.000527273240870053	-0.321079201691675
PCDHA6	-1.96905416666667
3.89644427860697	-3.81042887205579	0.000184567672246104
	0.000530325305977958	-0.327313677805454
CTLA4	1.58215882768361	4.16376517412935
3.80991849490214	0.000184923112635805
0.000531272250547591	-0.329127771493421
EGFR	-1.25164046610169
7.58303830845771	-3.8087769197374	0.000185720484413228
	0.000533264551748752	-0.33318464654879
SCN2A	-1.70100536723164
3.34731641791045	-3.80869706014061	0.000185776386737747
	0.000533350471224437	-0.333468408432461
LRRN4	1.81446320621469	3.87552338308458
3.80864396498024	0.000185813562572596
0.000533382611803048	-0.333657066478161
CARD6	-1.08933531073446
6.93021791044776	-3.80774189399314	0.000186446246631713
	0.000535123926093817	-0.336861963221025
C9orf109	-1.1222436440678
3.26794726368159	-3.80688366226026	0.000187050076610254

0.000536781950350646	-0.339910497856002
XKR9	1.53530685028249	3.47510746268657
3.80629700552831	0.000187463898463022
0.000537894316624156	-0.341994026084963
ZNF487	-1.10574152542373
4.79103731343284	-3.8060182832304	0.000187660810077694
0.00053838407362983	-0.342983819070172
SHPRH	-1.44491031073447
6.69007462686567	-3.80534223110505	0.000188139241138154
0.000539681238027987	-0.345384341409184
ATP6V1C2	1.32828220338983	8.34193134328358
3.80476190269526	0.000188550851167737
0.000540786389281034	-0.347444674447391
KLRB1	-1.56511836158192
4.57377810945274	-3.80418082756309	0.000188963845117728
0.00054181951679706	-0.349507386390448
CDKN2BAS	1.37721264124294	2.98179850746269
3.80407932540488	0.000189036074695342
0.000541950930525815	-0.349867672806323
FOSL1	1.3814302259887 6.6315	3.79983190993806
0.000192082106988957	0.000550145878415638	-0.364936611112706
UPP1	1.032639759887	8.44256865671642	3.79928995964428
0.000192474092903393
0.000551114801576939	-0.366858289719668
GAL	2.01520374293785	3.66831990049751
3.79858746299298	0.000192983327243923
0.000552495845533082	-0.369348890064846
MUC21	1.60284519774011	1.63092736318408
3.79797758949354	0.000193426453942163
0.000553610079385558	-0.371510785392767
TPTE2P1 -1.22982097457627
3.74443582089552	-3.79665783289126	0.000194388673216765
0.000556208984131037	-0.376188064791698
PCDHA7	-1.35988721751412
3.30399154228856	-3.79549061324921	0.000195243454226451
0.00055849911039	-0.380323574248086
MAPK4	-1.89449201977401
3.95337014925373	-3.79434513141349	0.000196085774862269
0.00056075232733283	-0.384380996192406
LOC729799	-1.12680692090396
3.17212885572139	-3.79302484740426	0.000197060902365688
0.00056330553126811	-0.38905627204748
MYH7	1.25556320621469	1.30499104477612
3.79268324912714	0.000197313944480519
0.00056387183744224	-0.390265681154883
PRR22	1.02416687853107	6.45610746268657
3.79218767456955	0.000197681592819644
0.000564836090718085	-0.392020066900364
C1orf65 1.12179173728814	1.31604228855721
3.79042710074119	0.00019899294785114
0.000568353536363927	-0.398251078008893
PRR15	1.33168354519774	9.47988905472637
3.78808074848165	0.000200753420637794
0.000572823867365381	-0.406551379519627

OAS1 1.31102514124293 9.04483482587065
3.78610484168055 0.000202247371582299
0.000576846143176521 -0.413537766752436
CPS1 -1.89832274011299
4.52013432835821 -3.78535990354322 0.000202813333677719
0.000578139091974948 -0.416170890840596
NRADDP 1.07916292372881 2.6476368159204 3.78425854531236
0.000203652830656473
0.000580048919724943 -0.420063026958939
CPA5 1.09743877118644 1.06763880597015
3.78299723636139 0.000204618287731033
0.000582717914706354 -0.424519215004343
NPAS1 1.29458834745763 3.55717313432836
3.78249305996365 0.000205005415238619
0.000583658462744259 -0.426300103776137
NCRNA00164 -1.00662189265537
1.43024676616915 -3.77922310077128 0.000207533101340233
0.000590363665029097 -0.437845502713604
ANKLE1 1.55684929378531 5.17389850746269
3.77836740285821 0.000208199410140922
0.000592095011801237 -0.440865327894728
MMP23B -1.15256327683616
6.06198955223881 -3.77615636804816 0.000209930468375301
0.000596439595314825 -0.448665500590029
PRL -1.48048933615819
1.70409800995025 -3.7741808890522 0.000211488616991505
0.000600666889491924 -0.455631324372008
BST2 1.60788877118644 12.2683895522388
3.77346206710148 0.00021205829428505
0.000602068425068742 -0.458165210484366
LGSN 2.27595911016949 3.09100696517413
3.77112386108542 0.000213921416683367
0.000606938458334651 -0.466404619591245
GRIA4 -1.54259653954802
2.57661492537313 -3.77076888662911 0.000214205616759388
0.000607576859915755 -0.467655097473355
NFATC1 -1.06841836158192
7.76247064676617 -3.77008404842751 0.000214754922354306
0.000608811508304671 -0.470067306780348
KIAA1875 1.30311970338983 4.85480248756219
3.77004139987401 0.000214789174605491
0.000608811508304671 -0.470217515449934
GPR62 -1.13111539548023
2.42816815920398 -3.76980298121485 0.00021498075055018
0.000609270404306349 -0.47105720143086
LOC389458 1.50412168079096 3.33582089552239
3.76919240809688 0.000215472100156041
0.000610578635776883 -0.473207367231514
LOC84740 2.27484752824859 3.95361144278607
3.76817681632442 0.000216291736418906
0.000612563022303786 -0.476783159093678
IL18 1.26974992937853 7.33700049751244
3.76811540251045 0.000216341395064453
0.000612619150437686 -0.476999363892014

MT3	2.08006956214689	4.41695721393035
3.7670051498316 0.000217240997227808
0.000614742615173934	-0.480907435828134
SPDYC	1.47753877118644	1.47885373134328
3.76637935566889	0.000217749616366417
0.000615714739311681	-0.483109781420231
BCL2A1	1.24928813559322	5.53138606965174
3.76636501443968	0.000217761285505831
0.000615714739311681	-0.483160248519363
SLAMF8	1.17194053672316	7.28769900497512
3.76636109170046	0.00021776447745456
0.000615714739311681	-0.483174052695395
IL19	2.00945141242938	3.38915024875622
3.76584574195264	0.000218184204679672
0.000616816541183375	-0.484987467518137
FCRL6	-1.35046299435028
3.43030298507463	-3.76520260871968	0.000218709078747175
	0.000618215253570082	-0.487250225598272
KCNJ15	1.4668209039548 4.7632447761194 3.75770972661104
0.000224912843713835	0.000634440874064996	-0.513587992340788
GPNMB	-1.13054533898306
10.9024368159204	-3.75305612584345	0.000228849196624627
	0.00064501290591757	-0.529922662206899
PIK3AP1 1.12183919491525	7.73561741293532
3.75241662537799	0.000229395196331848
0.000646285629297768	-0.532166008968502
CYSLTR1 -1.13772733050847
3.05625820895522	-3.75142915859054	0.000230240706571289
	0.000648400782703507	-0.535629358483656
BEND5	-1.12939209039548
6.67136069651741	-3.74806419644574	0.000233144110073499
	0.000656217244284198	-0.547425372412499
SCUBE3	1.77242083333333	4.67800099502488
3.74729545647001	0.000233812245143791
0.000658007593255933	-0.550118932570238
SLC34A2 1.8754988700565 11.285192039801 3.74714919522319
0.000233939569997311	0.000658185469651199	-0.550631357735442
SP8	2.11514992937853	2.15589054726368
3.74577757003078	0.000235136805368365
0.000661100878418588	-0.555435991034966
FAM83C	1.33053686440678	1.80494179104478
3.7412719906011 0.000239110434202747
0.000671511619721283	-0.571207726803958
DKFZp686O24166	-1.21835699152542
3.37997263681592	-3.74124545812128	0.000239134021031773
	0.000671511619721283	-0.571300554651198
CCR8	1.03952238700565	1.54493781094527
3.74078716999044	0.00023954177699393
0.000672472676286801	-0.572903853942727
TFAP2B	2.1954479519774 2.58758407960199	3.73679368503246
	0.00024312284911191
0.000681593894888368	-0.586867649406457
GPRC5D	1.2293927259887 2.44425373134328	3.73402647826018
	0.000245633896512021

0.000688257662807669	-0.596535984373434
NCRNA00120	-1.02990423728814
4.29168606965174	-3.733337245268 0.00024626312925569
0.00068992658892185	-0.598943125020059
KCNJ10	1.2860311440678 3.99806268656716	3.73109175847856
0.000248323720413046
0.00069541481108714	-0.606782790339818
ATHL1	1.30126151129944	10.6726328358209
3.72946947659992	0.00024982254814922
0.000699326004022836	-0.612444109731163
CYP2D7P1	1.04455367231638	4.77463084577114
3.72862721455307	0.000250604082066549
0.000701322492479041	-0.615382529365346
IFI27L2 -1.08992083333333
8.24923980099502	-3.72595245360222	0.000253101335034137
0.000708021584446224	-0.624710204979207
ZNF285	-1.64719802259887
5.27903233830846	-3.72591139091202	0.00025313985526072
0.000708032864918609	-0.62485335725047
TMEM90B -2.07998813559322
5.15176865671642	-3.72433277706969	0.000254624933349749
0.000711701821874791	-0.630355662063455
FBXO2	1.55723213276836	7.53777313432836
3.72181844019471	0.00025700729920733
0.000718067481318247	-0.639115269033236
HRK	1.14608813559322	1.59047512437811
3.72157204255449	0.00025724189362657
0.000718625129426245	-0.639973407963951
CRB1	-1.03676073446328
1.41681492537313	-3.72133218262496	0.000257470457818024
0.000719165782177419	-0.640808730271462
STEAP3	1.00313672316384	9.48549253731343
3.72095095398072	0.000257834128069752
0.000720083614375189	-0.64213627869818
ECEL1	1.97164519774011	7.31322487562189
3.7201718666593 0.000258578842389467
0.000721967038522475	-0.644848918134063
LMO3	-2.64199548022599	5.7213	-3.71951320971304
0.000259210024323349	0.000723532535602767	-0.647141855518476
TLX3	1.82346144067797	1.7971263681592 3.71901766397617
0.000259685858136227
0.000724663676435604	-0.648866732133356
MOBP	-1.04876631355932
1.61296368159204	-3.71569412166416	0.000262898584489777
0.000733130662805639	-0.660430022178995
ZFP57	-1.21317853107345
1.64247860696517	-3.7144526401449	0.000264108274345467
0.000736132798492375	-0.66474708450489
DSCR8	2.40987838983051	2.23512338308458
3.71444209939475	0.000264118567642223
0.000736132798492375	-0.664783732977259
COL1A2	-1.13093566384181
13.6489139303483	-3.71203856716968	0.000266475594386935
0.000742399776464077	-0.673138059347738

SLC38A4 -1.63534717514124
3.31020149253731 -3.71133835689269 0.000267165984418066
0.000744222206255481 -0.675571002313605
HIST3H2A 1.1197988700565 6.31819154228856
3.71039021993021 0.000268103516740835
0.000746529933363536 -0.678864751736086
FXYD7 -1.37135042372881
1.88378457711443 -3.70916902207493 0.000269315634460921
0.000749803359843057 -0.683106012423287
CALCA 2.66929646892655 2.7847328358209 3.70527379669682
0.000273216569110145
0.000760045550654645 -0.696626135142671
EMX2OS -1.59624406779661 10.175552238806 -3.7049843653281
0.00027350854417797
0.000760754696108078 -0.697630242391527
PLXDC2 -1.21005240112995
7.40937313432836 -3.7017344118442 0.000276807352231789
0.000769304851945124 -0.708900423804732
ZNF506 -1.37444314971751
7.69480696517413 -3.70154561490846 0.000277000137158628
0.000769642209167289 -0.709554868578467
ANKRD35 -1.45861370056498
6.94620746268657 -3.70154211155621 0.000277003715710526
0.000769642209167289 -0.709567012306723
PDE4C -1.41672111581921
5.41164378109453 -3.70104507906173 0.000277511859656349
0.000770949726487801 -0.711289782827659
CYP4F11 2.00466758474576 5.98280845771144
3.70014611590392 0.000278433155789429
0.000773195277743402 -0.714405178234474
NOS2 -1.32396475988701
2.40931641791045 -3.69557680000871 0.000283160797648093
0.000785261539337478 -0.730230142468964
DDO -1.166735240113 4.96231393034826 -3.69456426650163
0.000284218622436036
0.000787875819418284 -0.733734554526976
C4orf39 -1.61542457627119
3.56347860696517 -3.68793445387178 0.000291237711603017
0.000805810002588939 -0.756659866156669
CALB1 2.25667288135593 2.88275273631841
3.68664872882989 0.000292617733833889
0.000809353384180699 -0.761101633609075
FAM107A -1.72705338983051
8.96397860696517 -3.68659925154771 0.000292670962975663
0.000809353384180699 -0.761272534749755
EGFL6 1.63762358757062 7.13261144278607
3.68659451367357 0.000292676060601925
0.000809353384180699 -0.761288899894832
CYP2C18 1.83344809322034 2.42881641791045
3.68481075157526 0.00029460123157048
0.000814128857738855 -0.767448909216841
ALPL 1.56443778248588 10.1817517412935
3.68467564248584 0.00029474753733639
0.000814313947182895 -0.767915386365911

NHS	-1.07445494350283	7.9772631840796	-3.68452186523662
	0.000294914141749981
0.000814555002035128	-0.768446298979044
KIF26B	1.40794901129943	7.35249402985075
3.68211435774587	0.000297534083367108
0.000821680754846211	-0.776755645859065
ACPP	1.26627584745763	5.66112039800995
3.6804711819023	0.000299334843766569
0.000826542615000358	-0.782424233503123
SLURP1	1.5901697740113	1.52044875621891	3.67931752946656
	0.000300605273481328
0.000829715819383123	-0.786402756033346
KLHL3	-1.05635635593221
6.95588407960199	-3.67807011450091	0.000301984680426262
	0.000833075179945062	-0.79070340617104
ODZ3	-2.06229350282486	6.4445671641791	-3.6746311465542
	0.00030581852619484
0.000843085046700694	-0.802553171755334
TNS4	1.73186193502825	5.35977661691542
3.67418486799022	0.00030631940127952
0.000844352484998462	-0.804090219211071
OAS2	1.30296793785311	9.59716467661691
3.67379390822218	0.000306758825869565
0.000845450220848728	-0.80543660685809
C1orf161	1.35926998587571	2.35431492537313
3.67279571789764	0.000307883454517056
0.000848322013318367	-0.808873608305655
ADAMTS8	-1.99447775423729
5.95869800995025	-3.67119464341278	0.000309695459410524
	0.000852971261295688	-0.814384777029626
SIM2	1.51819717514124	6.88748656716418
3.66890292704055	0.000312306605170242
0.000859701609566246	-0.822269600184607
C19orf26	1.32870515536723	4.14934129353234
3.6684294654835	0.000312848641808274
0.000861078243239559	-0.823898044446937
CITED4	1.1760261299435	10.5374467661692	3.66766397825951
	0.000313726877916801
0.000863356103008191	-0.826530506179119
RDH12	1.22507598870056	3.26711492537313
3.66763484763936	0.000313760345082519
0.000863356103008191	-0.826630675045714
ECT2L	-1.18088086158192
3.53786567164179	-3.66421469443732	0.000317713132786672
	0.000873647283165065	-0.838386419913836
ASRGL1	1.45137824858757	11.5783960199005
3.6635929184775	0.000318436766834371
0.000875519863815406	-0.840522557875733
CDKN1C	-1.14090635593221
9.38247960199005	-3.66287572984188	0.000319273374263289
	0.000877350070087715	-0.84298609679877
PRDM13	1.21882620056497	1.12669054726368
3.66098050646478	0.000321494157854143
0.000882861832630492	-0.849494149998985

MLXIPL 1.69848403954802   6.73970497512438
3.65899096494809   0.000323841129152504
0.000888939046535169   -0.856322917542192
SGCD   -1.75797690677966
7.33667661691542   -3.65653223826189   0.000326763898842799
        0.000896373976916443   -0.864757601887789
LPL   -1.02510670903955   5.9051815920398 -3.65597579739367
        0.000327428801094002
0.000897957959719459   -0.866665789606356
C16orf89   -2.5224875
4.97219253731343   -3.65495086652536   0.000328656851447311
        0.0009012054441316   -0.870179892989301
GPAT2   -1.39516857344633
3.99485621890547   -3.65479091841322   0.000328848889153838
        0.000901611604477602   -0.87072821734645
ETV7   1.31310261299435   6.20745174129353
3.65447629485279   0.000329226942838168
0.000902407094321441   -0.87180672941339
CXCL11 1.80722577683616   5.89170845771144
3.6541190142594 0.00032965675034798
0.000903223419811028   -0.873031368981678
TNNI1   1.22958898305085   5.83578905472637
3.65268526781537   0.000331386869973163
0.000907721473055241   -0.877944724006358
RAMP3   -1.1917145480226
8.30152139303483   -3.65179449406534   0.000332466085352531
        0.000910313247837753   -0.880996498023354
ZBED2   1.69874343220339   5.09712089552239
3.64863184959787   0.000336324607647052
0.00091952912438327   -0.891826399588669
USH1C   2.25928933615819   4.63304427860696
3.64791822889251   0.000337201064553087
0.000921679916825306   -0.894268929203193
HAPLN4 -1.55748629943503
4.26532388059702   -3.64644111483631   0.000339022076086935
        0.00092604087574039   -0.899323361062486
LY96   -1.04846694915255
5.40640646766169   -3.64454595904847   0.000341372034360006
        0.000932087759730141   -0.905805637371036
SPA17   1.03191878531073   8.09954626865672
3.64304756934165   0.000343240869037187
0.000936941236714992   -0.910928709852203
USP44   -1.6866375   3.75018955223881   -3.64221379525073
        0.000344284947358949
0.000939666308066901   -0.913778628612914
RGS4   -1.23599230225989
6.39849303482587   -3.64169850198268   0.000344931709699354
        0.000941181286566684   -0.915539664351884
WNT5B   -1.60658156779661
6.79828656716418   -3.6398666333912   0.000347240234149296
        0.00094685110998606   -0.921798384311159
AMIGO2 -1.51224378531074
8.42190597014925   -3.63939533317443   0.000347836518220646
        0.000948225165193182   -0.92340817197697

WNT5A	-1.19137888418079
9.37100447761194	-3.63699916111105	0.000350883083380638
	0.000955515280780541	-0.931589789540325
NR4A2	-1.17175572033899
7.40661791044776	-3.63681522269737	0.000351117984409584
	0.000956028145879145	-0.93221764370815
DLX5	2.12183142655367	9.91908258706468
3.63415329810585	0.000354534063771852
0.000964561930723048	-0.941300727050596
SLCO5A1 1.43364081920904	4.09373482587065
3.63369666556644	0.00035512320331574
0.00096603675156647	-0.942858274467943
H2BFXP	-1.14053615819209
3.00468059701493	-3.63159606711019	0.00035784526108723
	0.000972925865464287	-0.950021087669009
PDCL2	1.76271751412429	1.55224378109453
3.63034202212062	0.00035947967199555
0.000976981422566671	-0.954295516024436
ANO1	-1.4668811440678
9.87693134328358	-3.63021811060517	0.000359641548495486
	0.000977162650954516	-0.954717799813821
?|729884	1.49066631355932	1.43127213930348
3.62910240713933	0.00036110218613708
0.000980871643476351	-0.958519488684278
FPR1	1.28220211864407	5.41602835820896
3.62013627333257	0.00037304488658107
0.00101157198804773	-0.989033843399389
CALML5	2.15551560734463	2.22031144278607
3.61946413569923	0.000373954988259566
0.00101377207301847	-0.991318657344924
DOCK11	-1.1242843220339
6.90301691542289	-3.61827276230327	0.000375573296556485
	0.00101762172077401	-0.995367608341762
C6orf141	-1.64230183615819
4.36231890547264	-3.61642333092544	0.000378098553279431
	0.00102392340045202	-1.00165069217169
KRT81	1.5602074858757 4.79288905472637	3.61635298989193
	0.000378194913485209
0.00102396672638818	-1.00188960657253
ANKRD53 -1.3180947740113
4.20156467661692	-3.61525873656549	0.000379696912853075
	0.00102770964019428	-1.00560573009039
APLNR	-1.05153672316384
7.34505323383085	-3.61147801838205	0.000384929765746157
	0.00104104967603328	-1.01843759434812
CHRNA5	1.28000190677966	5.90702835820896
3.60773225718719	0.000390181142939764
0.00105469636136318	-1.0311391998469
EPHB2	1.18419011299435	8.56274427860697
3.60753823363685	0.000390454983596158
0.00105529763207334	-1.03179680470073
ITGAD	-1.31427634180791
1.87583134328358	-3.6070928580476	0.000391084261614856
	0.00105685927220653	-1.03330620073965

IGFL1	1.2768956920904 1.21295970149254	3.60416382797128
	0.000395246580478560
0.00106768582858222	-1.04322872930203
MPP3	-1.1430938559322
5.05417412935323	-3.6034955917046	0.000396202006971325
	0.00106970373528686	-1.04549148890894
RNLS	-1.46397231638418
5.65309701492537	-3.60260011759093	0.000397485741117688
	0.00107260545020322	-1.04852313648855
TM4SF19 1.04193170903955	2.20790945273632
3.6003129709673 0.000400782343057471
0.00108107495768885	-1.05626332077958
CPLX2	2.26019618644068	4.04139701492537
3.60010546790095	0.00040108269957833
0.00108174301397046	-1.0569653412333
PLD4	-1.46363418079096
4.48627810945274	-3.59896467393832	0.000402737763660342
	0.00108563632982495	-1.0608242193963
C21orf130	-1.28257853107345
2.83399850746269	-3.58998500062717	0.000415991715876559
	0.00111974694204087	-1.09116158913866
C20orf197	-1.01791935028249
1.6901815920398 -3.58947107918442	0.000416762543735541
0.00112138073694504	-1.09289583272555
ABCC3	1.55359999999999	8.78099552238806
3.58751091519402	0.000419714938386385
0.00112888087057153	-1.09950846333284
LY6H	-1.55721617231638
2.51961592039801	-3.58527601356424	0.000423105147397909
	0.00113725434355578	-1.10704405165631
TNIP3	1.31120847457627	2.60864626865672
3.58490612692898	0.000423668721486459
0.00113847105394202	-1.10829082865843
BNC1	-1.41058983050848
1.46987213930348	-3.58481693030071	0.000423804730685322
	0.00113868749129298	-1.10859146684608
MORN3	1.2889502118644 4.98435820895522	3.58424607164031
	0.000424676163082138
0.00114073029282694	-1.11051539638926
MPP2	-1.25792888418079
6.22461741293532	-3.58314864301474	0.000426356157082224
	0.00114468936878307	-1.11421323562442
SLC34A3 1.29951786723164	3.21695572139303
3.58099011255543	0.000429678792249741
0.00115240294704844	-1.12148360514324
GSTM2	-1.48364908192091
9.20229054726368	-3.58097942604577	0.000429695302467403
	0.00115240294704844	-1.12151958990103
TSHZ1	-1.0459136299435
9.52578407960199	-3.5805333506119	0.000430385003232695
	0.00115410189607722	-1.12302157858237
IL15	-1.06846235875706
5.88855273631841	-3.57874891365329	0.000433154443527611
	0.00116106490254129	-1.12902834349678

PRDM6	-1.44785911016949
4.29251542288557	-3.57866731584191	0.000433281482960794
	0.00116111067030219	-1.12930295479969
HIF3A	-2.16756857344633
5.84468308457711	-3.57770725155287	0.000434778838977808
	0.00116451527256548	-1.13253356559794
SLC12A3 1.72760790960452	2.86219303482587
3.57712979629585	0.000435681804753631
0.00116662937936508	-1.13447633215058
ERN2	2.05899576271186	3.51036218905473
3.57632353784965	0.000436945500556002
0.00116970806186135	-1.13718841341012
LXN	-1.06295649717514
7.63589701492537	-3.57616002879459	0.000437202197907022
	0.00117013512619488	-1.13773835739464
FBXO43	1.02130642655367	4.09668855721393
3.57614931449615	0.000437219023528539
0.00117013512619488	-1.13777439293877
GPA33	1.04495918079096	2.05341293532338
3.57608243812875	0.000437324059236917
0.00117026367780682	-1.13799931697959
SNORD116-20	-1.03293156779661
2.48129104477612	-3.57564139523408	0.000438017353696626
	0.00117196615031822	-1.13948257570074
AMN	1.79656370056497	5.01030696517413
3.57539209193479	0.000438409701519418
0.00117271025044868	-1.14032092931423
ATP6V0D2	1.07847492937853	1.94773930348259
3.57505893119272	0.000438934538267617
0.00117396118689867	-1.141441197304
HRASLS	1.68552740112994	4.59215373134328
3.57192032125598	0.000443907959213746
0.00118664458367945	-1.15199040781073
SPINK5	2.07442577683616	4.9570039800995 3.57014272455588
	0.000446748183677442
0.00119361533197526	-1.15796148928495
EHF	1.68697372881356	9.36006069651741
3.56860388055667	0.000449220722322825
0.00119975301538801	-1.16312847081126
SCHIP1	-1.04994533898305
7.47937960199005	-3.56585375135948	0.000453671569471736
	0.00121116740198371	-1.17235770719489
NRN1L	-1.07025395480226
2.42858756218905	-3.56271732972037	0.000458798193758226
	0.00122389902778799	-1.18287567059527
LILRA3	1.55804611581921	2.63854825870647
3.56078694281207	0.000461980522150792
0.00123174806750075	-1.18934515678033
NTSR1	1.52384661016949	2.34721144278607
3.56065298126617	0.000462202131878077
0.00123217890760381	-1.18979400010906
C6orf165	1.45444922316384	5.98867114427861
3.55918285418969	0.000464640706473563
0.00123787616193904	-1.19471874084542

ESR1	-1.75691709039548	11.494255721393	-3.55889017536428
	0.000465127629010933
0.00123901261497268	-1.1956989643734
BBOX1	2.10105211864407	4.1885552238806	3.55517437032573
	0.000471351368417507
0.00125379626618401	-1.20813755953682
CLLU1OS	1.04161306497175	1.13912885572139
3.555129425248	0.000471427125470657
0.00125379626618401	-1.20828794239371
ZNF829	-1.02768799435028	2.9725960199005	-3.55457537949276
	0.000472361936285225
0.00125600188472163	-1.21014160037444
HBQ1	1.32564964689265	1.56667562189055
3.55232647136308	0.000476174311839974
0.00126531952136612	-1.21766310730956
PCOTH	1.09382584745762	4.51461144278607
3.55194004928168	0.00047683228320551
0.00126674000701464	-1.21895507963198
IRF8	-1.18000141242938
7.00371094527363	-3.55125499917091	0.000478000837071893
	0.00126968006017415	-1.22124518736603
PLLP	1.06510677966101	9.5485631840796	3.55116727113194
	0.000478150677203618
0.00126991376494053	-1.22153843220075
HAP1	-1.39286744350283
4.16468258706468	-3.54813102259093	0.000483363894847446
	0.00128290331287288	-1.23168363993309
C3orf59	-1.036689759887	6.90441492537313	-3.54810056197047
	0.000483416465094232
0.00128290331287288	-1.23178538117848
FNIP2	-1.12525296610169
7.19604726368159	-3.5446157136966	0.000489466358430983
	0.00129811966652733	-1.24342001055174
BEST3	1.4025322740113	1.37345323383085	3.54439166672371
	0.000489857740308466
0.00129898984868675	-1.24416767645582
SH2D6	1.1304511299435	1.2486552238806	3.54422020986682
0.00049015745245092	0.00129961675046072	-1.24473981604289
NEB	1.19815812146893	4.9162736318408	3.54326214131958
	0.000491835360795607
0.00130342195949097	-1.24793637504844
HDC	-1.66049816384181
3.25527860696517	-3.54277348466307	0.000492693243707842
	0.00130532872542547	-1.2495664659292
PIK3CG	-1.17885501412429
4.02974129353234	-3.53960468592433	0.000498290576267031
	0.00131896667493065	-1.2601323296618
IGSF1	-2.01233728813559
3.93057512437811	-3.5394277271819	0.000498604908720947
	0.00131962856465657	-1.26072212517355
MYCNOS	1.26469950564972	1.85997810945274
3.5366087362644	0.000503637478306743
0.00133174619134949	-1.27011418874441
SYT13	2.2633324858757	5.93900049751244	3.53636956390119

0.000504066646405897
0.00133270936905513	-1.2709107383029
KCNT2	-1.52995381355932
2.71781890547264	-3.53490890828682	0.00050669509353342
0.00133896901406115	-1.27577433532981
ERMN	2.12833100282486	4.90964776119403
3.53412191616999	0.000508116615508111
0.0013422071688602	-1.27839407641796
TMEM40	1.61293269774011	2.48198955223881
3.53286004021109	0.000510403727193185
0.00134790179049088	-1.28259353805844
LTK	1.56818333333334	4.10698756218905
3.53122837452994	0.000513375406483064
0.00135540086099502	-1.28802168066817
ISL2	1.45766129943503	1.42156218905473
3.52732991948367	0.000520541405469631
0.00137308429324059	-1.30098190560203
CCR3	1.20669710451977	1.7541263681592 3.52537515559843
0.000524169836206088
0.00138230015893526	-1.3074756682915
LOC100133669	-1.18208884180791
2.3852144278607 -3.52480038487515	0.000525241234564576
0.00138459200637015	-1.30938446315605
FSD1	1.90078997175141	4.41613830845771
3.52231512553739	0.000529897579300924
0.00139614955121252	-1.31763476664905
MICALCL 1.13573933615819	2.6563328358209 3.52126043328835
0.00053188532441991
0.00140102716324618	-1.32113447094018
ERBB4	1.98697733050847	7.40753880597015
3.52115939353423	0.000532076117831155
0.00140134992932311	-1.32146969483259
IL21R	1.33960289548023	4.95590447761194
3.52052173181936	0.000533281697771249
0.00140434495321238	-1.3235850963347
C3orf30 1.21063771186441	1.15844228855721
3.51723858838017	0.000539529591180682
0.0014191598343292	-1.33447135789032
CXCL1	2.21516271186441	6.56179900497512
3.51714601620358	0.000539706750080877
0.00141944396351017	-1.33477817911855
MLC1	-1.33983757062147
2.72939303482587	-3.51244952494235	0.000548766599179499
0.0014412406653646	-1.35033487042761
LGR5	2.33018721751413	9.27768507462687
3.51123021845541	0.00055114195542794
0.00144673882431091	-1.35437070768853
CPNE8	-1.33296836158192
6.60092537313433	-3.510576942323 0.000552418582739194
0.00144971922582585	-1.35653250639012
GPT	1.27675007062147	5.19677313432836
3.50631398615921	0.000560817602401727
0.00147025735886955	-1.37063060605286
SORCS3	-1.09411271186441

1.29471094527363	-3.50319637235137	0.000567035728496265
	0.00148561045253041	-1.38093133916232
NTF3	-1.41251539548023
3.47936815920398	-3.5020559352719	0.000569326462101852
	0.00149123147064953	-1.38469737017731
PPARGC1A	-1.80547415254237
4.66949651741294	-3.5013505809853	0.000570747611250777
	0.00149476316608987	-1.38702609785282
HMX2	1.50812040960452	1.3760671641791 3.50032378513029
	0.000572822351239096
0.00149981416540475	-1.39041532386306
GBX2	1.01687457627119	0.99465671641791
3.50019817078164	0.000573076652313819
0.00150028866003605	-1.39082988871112
LY6K	1.71118361581921	6.3279631840796 3.49637205563816
	0.000580873398686193
0.00151973121894068	-1.40345090805901
FUT5	1.19701518361582	1.96713383084577
3.49400231286411	0.000585752172955876
0.00153171470013809	-1.41126174106337
FAM181A 1.83406419491525	5.66514228855721
3.49054269037506	0.000592943821178217
0.00154913930373998	-1.4226564800265
ZNF826	-1.52540367231638
4.34865074626866	-3.48873216202914	0.000596740361263913
	0.00155806684684575	-1.4286157215163
CCDC144B	-1.13382485875706
2.67799054726368	-3.48847439898609	0.000597282720807298
	0.0015584918985986	-1.42946391008558
DLX2	1.40937302259887	1.55653134328358
3.48806375737734	0.000598147707221457
0.00156055056922817	-1.43081504246643
C6orf154	1.25038072033898	5.93209402985075
3.48749817502258	0.000599340988217922
0.00156314251047745	-1.43267574531885
ALK	1.49655706214689	2.69966368159204
3.48608316274177	0.000602336205561238
0.00156988222675793	-1.4373298081795
NRG4	1.28188185028249	2.80360049751244
3.4850655754155 0.000604498846378795
0.00157531877585131	-1.44067568255705
GSC	1.62127627118644	4.22189104477612
3.48340682177291	0.000608039747957514
0.00158414416568119	-1.44612788826196
ZNF512	-1.072497740113 9.45536169154229	-3.47819302591505
	0.000619296551407547
0.00161122268412076	-1.46325030315547
NANOS3	1.05903799435028	3.79252736318408
3.47591084863345	0.000624285085193536
0.00162358408905793	-1.47073796290806
ADCYAP1R1	-1.88700854519774
2.8709184079602 -3.47494840653771	0.000626400125531345
0.00162867205891065	-1.47389436151384
NKAIN1	1.4796259180791 4.30163333333333	3.47491168814316

0.00062648094987598
0.00162867594035126 -1.47401476681897
LRMP -1.06722944915254
4.88041343283582 -3.47381102498237 0.000628908267100184
0.00163457231593452 -1.47762348869862
SYDE2 -1.02152026836158
4.94783980099503 -3.46576743474589 0.000646916395780659
0.00167882614149626 -1.5039650961572
KLRK1 -1.34399653954803
5.03373482587065 -3.46549600984625 0.000647532412486636
0.00168021238983198 -1.50485303009787
BEND7 -1.47295557909605
7.56599054726368 -3.46022416166939 0.000659606778760464
0.0017089509298274 -1.5220870217183
PKP1 2.08326038135593 4.21755870646766
3.45966645320445 0.000660896389280175
0.00171164411150776 -1.52390884405045
MPZL3 1.2042656779661 3.81528109452736 3.45927220507232
0.000661809448881944
0.00171379263188021 -1.52519654629983
CDX2 1.91650889830508 2.15106368159204
3.4548833735219 0.000672053885222577
0.00173922428635062 -1.53952266032046
LILRA6 1.1576938559322 4.39611592039801 3.4497067182343
0.000684328149923709 0.00176875952636708 -1.55639966867046
FBLN2 -1.34972598870056
8.99403532338308 -3.44848049281531 0.000687266225349988
0.00177568280977036 -1.56039413787733
SLC2A12 -1.00315494350283
8.08124975124378 -3.44770735530095 0.000689124755069382
0.00177981270871486 -1.56291201077386
CGB2 1.20125240112994 1.18101094527363
3.44537746880154 0.000694754023757876
0.00179209701064822 -1.57049671009698
MYBPC2 1.7113709039548 3.50881144278607 3.44472232524398
0.000696344663257059
0.00179597436390294 -1.5726286410519
TNFSF13B 1.08665946327684 4.38988407960199
3.44321963370929 0.000700005975058017
0.0018031521540704 -1.57751725521088
ASCL1 -1.54693778248588 1.952592039801 -3.44204392661158
0.000702883147792324
0.00181033634728094 -1.58134079074675
KLRF1 -1.11428495762712
2.95593482587065 -3.44009225748107 0.000707683699742413
0.00182201484270275 -1.58768528527756
SLC27A6 -1.75659096045198
5.59245124378109 -3.43780108555577 0.000713358495833969
0.00183570442710847 -1.59512936003379
LOC100133991 1.09503947740113 3.87373432835821
3.43575152212446 0.000718470942466759
0.00184793390739696 -1.60178471079861
C9orf173 1.12072620056497 2.1346039800995
3.43526910815151 0.000719679255571808

0.00185080986056485	-1.60335069496196
MUC2	1.49555564971751	2.17369850746269
3.43499041039926	0.000720378182659496
0.00185185271604823	-1.60425529838206
NXNL2	-1.52713481638418
4.96323880597015	-3.43492760461151	0.00072053577670693
	0.00185185271604823	-1.60445914581792
C6orf186	-1.48274689265537
3.52476417910448	-3.43326030911405	0.000724731219260519
	0.00186193616961813	-1.60986944158997
GAS2	-1.2959343220339
3.35474577114428	-3.43277731863718	0.000725950840311838
	0.00186483618013038	-1.61143628721038
CLEC18C	1.19265084745763	2.06141144278607
3.43204883412913	0.000727794003546452
0.00186910318899283	-1.61379915752241
AACSL	1.75081405367232	2.01707412935323
3.43039722456758	0.000731989030040924
0.00187964162435774	-1.61915457297986
PPTC7	-1.2425520480226
7.19305820895522	-3.42787331858697	0.000738443390927888
	0.00189479350278085	-1.62733402306145
KCNIP3	-1.19847648305085
7.12686268656716	-3.42733583113513	0.00073982476319941
	0.00189810077336714	-1.62907521629747
HEY2	-1.41347245762712	7.493192039801	-3.42650980971917
	0.000741952397876954
0.00190308379915216	-1.63175064375647
LPHN3	-1.89159802259887	5.6183144278607	-3.42511470086437
	0.000745558872405996
0.00191161779074498	-1.63626800552709
KRTAP5-1	1.03874894067797	2.97172338308458
3.42279316766159	0.000751596611691682
0.00192565561438041	-1.64378150463692
C11orf88	-2.39938050847458
3.4538960199005	-3.42243680780208	0.000752527456346974
0.00192755941095488	-1.64493444045932
ZNF711	-1.27661334745763
7.26861393034826	-3.42138212878737	0.000755288698357489
	0.00193342605701736	-1.64834603232396
NRCAM	1.58058820621469	8.72418208955224
3.42131059370381	0.000755476326308285
0.00193344155953461	-1.64857739447692
ZIC5	2.25427846045198	2.18655671641791
3.41919189650605	0.00076105322640892
0.00194575625631073	-1.65542783523474
CHRM2	-1.02273728813559
0.882880597014925	-3.41881472570643	0.000762050056261292
	0.00194781967816787	-1.65664695613322
CCNI2	1.12641511299435	2.78917611940299
3.41553729142763	0.00077076359459369
0.00196837624045384	-1.66723550035258
SPOCK3	-1.84263481638418
2.71314378109453	-3.41447123306552	0.000773617893838591

0.00197541982095849 -1.67067771311803
BTBD16 1.10086165254237 2.08510298507463
3.41333408452503 0.000776673427498319
0.00198248234073611 -1.67434841662621
SOX11 2.02518495762712 2.90393432835821
3.41104161961078 0.000782867656277997
0.0019970518120511 -1.68174516277909
IL7 -1.16146899717514
4.23459303482587 -3.41038885977946 0.000784639842251021
0.00200107527470975 -1.68385051453803
HEPHL1 1.32193594632768 2.00979701492537
3.41005702162515 0.000785542191567528
0.002002878936026 -1.68492065748283
ACSM1 -1.43482196327684
3.19452089552239 -3.40908829868579 0.000788181941384271
0.00200911041179775 -1.68804415648374
CRB2 -1.65380487288136
3.87989353233831 -3.4073844840032 0.000792844936409106
0.0020204948540813 -1.69353593066497
CDH18 2.11540536723164 2.36876965174129
3.40445805107137 0.000800914241648588
0.0020397927528899 -1.70296277388243
SERPINI1 -1.03254498587571
6.43450646766169 -3.40440600853647 0.000801058435471285
0.00203990693131053 -1.70313035192578
VAX1 1.70863495762712 1.52803134328358
3.40281152778685 0.00080548803162627
0.00205042396643364 -1.70826350796979
DPEP2 -1.08047316384181
4.11992189054726 -3.40256924763175 0.000806163106206011
0.002051811168918 -1.70904329985393
LGALS9C 1.05373502824859 5.20884925373134
3.40196325889654 0.000807853912810005
0.00205517234572359 -1.7109934912192
FAM133A -1.46128531073446
1.77202985074627 -3.40063312227654 0.000811576840836625
0.00206413188086917 -1.71527304915308
HRNR -1.20037323446328
2.44758407960199 -3.39565864691962 0.00082564255577818
0.00209782693937203 -1.73126463058
VGF 1.83506115819209 4.10899104477612
3.39491769035235 0.000827757061301838
0.00210241896183678 -1.7336448200215
C10orf91 1.33140564971751 2.06276517412935
3.39463629345569 0.000828561423233512
0.00210368117969132 -1.73454863563644
ALPK2 1.39151172316384 3.31478606965174
3.38955970691294 0.000843198704619891
0.00213950741365238 -1.7508426064949
LOC441204 -1.29712620056497
4.19989203980099 -3.38952578449874 0.000843297320568684
0.00213950741365238 -1.75095141187008
CCDC110 -1.17351920903955
3.81276915422886 -3.38893328941408 0.000845021503755009

0.00214335204745371	-1.75285166997651
MYCN	1.74888778248588	6.28899452736318
3.38304969327743	0.000862322671451635
0.00218426705057397	-1.77170555206585
GDF6	-1.34383644067797
2.22443830845771	-3.38102449842725	0.000868354078311961
0.00219873078783233	-1.77818849869384
ACCN3	-1.17340105932203
3.86836069651741	-3.38013983376233	0.00087100112187559
0.00220488939976644	-1.78101935439002
SPAG16	-1.43993043785311
7.68118905472637	-3.37815215566857	0.00087697605730158
0.00221919369609287	-1.78737735519605
HOXC13	1.74367895480226	1.6063368159204 3.37803358912588
0.00087733367402731
0.00221982503423474	-1.78775650949944
COL1A1	-1.0276613700565
13.9788736318408	-3.37636800527366	0.000882371774518598
0.00223037337242881	-1.79308149045305
BEND4	-1.29735494350283
1.87911144278607	-3.37410752147853	0.000889252574753742
0.00224665953232763	-1.80030465949097
ZNF300	-1.69096391242938
6.46168805970149	-3.37137515496347	0.000897636653130438
0.00226700461173181	-1.80902992680635
C10orf114	-1.2675261299435
4.04961194029851	-3.37082722479544	0.000899326799952119
0.00227044370433703	-1.81077887348863
KLRC1	-1.25694675141243	2.7982447761194 -3.37082284308616
0.000899340327753114
0.00227044370433703	-1.81079285851143
TFF1	1.91794053672316	2.26565771144279
3.37079031705287	0.000899440752429279
0.00227044370433703	-1.81089667076066
ACSL5	-1.31884420903955
9.91155472636816	-3.36533814618541	0.000916423202151749
0.00230933697559536	-1.82828555011796
CSMD1	-1.56733177966102	2.7695039800995 -3.3605059443043
0.000931724337915607
0.002345304151227	-1.84367613231718
ANXA9	-1.22885077683616
5.76843184079602	-3.35708501801229	0.000942700490785101
0.00237199646388496	-1.85455986062686
GPR97	-1.29743799435028
4.50193830845771	-3.35705708600796	0.000942790606200743
0.00237199646388496	-1.85464868598179
ZNF689	-1.0758979519774
6.28150497512438	-3.35676897983617	0.000943720575801173
0.00237404526665826	-1.85556484140965
HKDC1	1.79770911016949	7.48871144278607
3.35669422514802	0.000943962014154727
0.00237436169451073	-1.85580254408624
BICC1	-1.59248425141243
4.31320895522388	-3.35367128788444	0.000953773760342093

0.00239698559185006	-1.86541082704251
PAEP	-2.46149710451977
3.34650049751244	-3.35358320647564	0.000954061073839944
0.0023973770954724	-1.86569067429742
TIMD4	1.11067104519774	1.84914527363184
3.35191134555917	0.00095952987027628
0.00240938701526077	-1.87100117109163
TPRXL	1.25399399717514	3.78272587064677
3.35187546871471	0.000959647546385629
0.00240938781060254	-1.87111510432985
CD300LF 1.11334230225988	4.53418507462687
3.35177586816358	0.000959974306950236
0.00240991349005727	-1.87143139771206
SCML2	-1.12261885593221
5.26072935323383	-3.35172873843247	0.000960128962133593
0.00241000704152129	-1.87158106084588
OTOA	1.1010511299435 3.33609900497512	3.35066740679437
0.00096361786162654
0.00241846878963238	-1.87495088183775
SLC22A18AS	1.05844540960452	4.08649303482587
3.34929651599897	0.000968141879570549
0.00242863548235379	-1.87930216926354
SPATA13 -1.3145238700565	6.5743592039801 -3.34870618790787
0.00097009609361487
0.00243294317150175	-1.88117541509587
JAKMIP2 -1.27732358757062
3.27476766169154	-3.34797627724471	0.000972517468522782
0.00243842008167717	-1.88349118042122
TTC9	1.08746179378531	8.61857810945274
3.34627064308288	0.000978197668337497
0.00245236269763937	-1.88890083548822
FOXS1	-1.10734435028248
5.94772587064677	-3.33900520455657	0.0010027419154285
0.00251021723250178	-1.91191658096605
IL1F9	1.08779117231638	1.02866815920398
3.33760721784453	0.00100753002901132
0.00252097397874497	-1.9163400487214
LRRC31	1.81297302259887	3.32764029850746
3.33685053649206	0.00101013055327384
0.00252717283191712	-1.91873362640166
HGFAC	1.26702867231638	3.77723333333333
3.33465957709524	0.00101769567769197
0.00254300051886758	-1.92566145748551
AHNAK2	-1.39177379943503	9.7062039800995 -3.33434943447042
0.00101877082245648
0.00254537726482068	-1.92664180217741
NBPF16	-1.58777344632768
4.49501144278607	-3.33267299684286	0.00102460074369306
0.00255807530925678	-1.93193952255315
ZNF717	-1.29750939265537
3.95351293532338	-3.330777777751 0.00103122894762112
0.00257212128473595	-1.93792574584771
PLEKHG4B	1.52999406779661	8.54509154228856
3.33003130761317	0.00103385055618896

0.00257678182816065	-1.9402827041291
BDKRB1	-1.05319639830508
3.23622089552239	-3.32951603572637	0.00103566381840243
	0.00258067461971135	-1.94190938480514
AQP10	-1.00394526836158
0.783007960199005	-3.32757651623835	0.00104251568306838
	0.00259648756197911	-1.94803030572507
LRTM2	1.59538615819209	2.29474228855721
3.32671171151212	0.00104558443654918
0.00260300435441962	-1.95075851050534
HS6ST2	-1.46284710451977
6.20602537313433	-3.32638070224153	0.00104676124746293
	0.00260548113608317	-1.95180257958355
BIRC7	1.42937514124294	3.00970348258706
3.32575575674348	0.00104898643335206
0.00261007047348417	-1.95377352856088
HOXA5	-1.77043347457627
5.94911990049751	-3.32507598766591	0.0010514118307814
	0.00261547132993361	-1.95591700353285
MUC20	1.3919043079096 9.4643776119403 3.3247625817251
0.00105253181432658	0.00261762303429736	-1.95690511559292
ZNF667	-1.60730007062147
5.89226218905473	-3.32283040636024	0.00105946121107618
	0.00263421807719437	-1.96299507297197
HRH3	1.07944124293785	1.00571940298507
3.3215383572296 0.00106411861016042
0.00264483717513237	-1.96706567065677
LOC283392	-1.71809371468926
2.48760597014925	-3.3214881472317	0.00106429998479455
	0.00264496776272027	-1.96722382853332
HRH1	-1.03037372881356
8.02480597014925	-3.31834464392671	0.00107571287050131
	0.00266977577748934	-1.97712137629538
DPYSL4	-1.50736701977401
5.58311293532338	-3.31724857067994	0.00107971904749258
	0.00267907082971025	-1.98057046964118
PDX1	1.38730572033898	1.29057810945274
3.31713663674509	0.00108012895050918
0.00267976403068925	-1.98092264288039
STX19	1.3932188559322 5.57672686567164	3.31549313320663
	0.0010861641946751
0.00269278479853638	-1.98609230697162
MAGEA11 2.12208573446328	2.02519402985075
3.3150840188801 0.00108767141388131
0.00269601168709885	-1.98737882539693
ARHGDIG 1.57109258474576	2.14994527363184
3.31183149460727	0.00109972356716274
0.00272376943344808	-1.99760179434324
SPRR2A	1.86245056497175	1.98288507462687
3.30968157913814	0.00110775820837095
0.00274168487255643	-2.00435423328591
SPRR1B	2.07535466101695	1.94034278606965
3.30886333493231	0.00111083048382865
0.00274829474969899	-2.00692313687157

INSL3 1.07715310734463 2.48933781094527
3.30746467889093 0.00111610042871481
0.00276033511643358 -2.0113129441878
C3orf14 -1.40942012711864
5.15813134328358 -3.30636563296046 0.00112025781410601
0.00276961619985506 -2.01476123170186
FAM155B 1.15006278248588 6.6719960199005 3.30189907048582
0.00113730245794308
0.00281040195733888 -2.02876462990693
C14orf174 1.13325995762712 5.25896616915423
3.30155895010924 0.00113861022226288
0.00281261795969882 -2.0298302674038
MYOD1 1.46110925141243 1.34841094527363
3.30049806204036 0.00114269834388249
0.00282169797930087 -2.03315352355576
FADS6 1.3267904661017 1.28362189054726 3.30044647569329
0.0011428974794364
0.00282185030100173 -2.03331509456305
MEIS1 -1.2637645480226
10.5460910447761 -3.29973263313794 0.00114565640113161
0.00282798195445857 -2.0355506526086
GABRA2 -1.21958008474576
1.36938308457711 -3.29495104960342 0.00116429719440368
0.00287123380193485 -2.0505140764236
MATN4 1.25960402542373 2.49719104477612
3.2941606861338 0.00116740545416738
0.00287820751459784 -2.05298555569069
C3orf15 -1.57355882768362
6.78127462686567 -3.2914936087088 0.00117795137488069
0.00290281379501867 -2.06132162536049
SEMA6C -1.04090529661017
7.98388606965174 -3.29070514813873 0.00118108597949637
0.00290923268145636 -2.06378483445702
DDC 1.51541016949152 1.90683233830846
3.28952609712421 0.00118578790652183
0.00291932194758283 -2.06746728945106
IFI27 1.46183622881356 12.3665427860697
3.28797043853116 0.00119201836750904
0.00293219950694471 -2.07232416770647
PCDHA12 -1.689875 2.99974278606965 -3.28509706904449
0.00120360649222046
0.0029585777400965 -2.08128961527873
UPK1B 2.48236214689265 6.28116069651741
3.28136610076789 0.00121880973946325
0.00299343987931338 -2.0929204354824
MUC12 -1.3108020480226
3.13250746268657 -3.28132558554112 0.00121897581066495
0.00299348964030519 -2.09304667146362
LOC440905 1.60204244350282 6.37917462686567
3.27971953028359 0.00122557598984634
0.00300825861687533 -2.09804963762203
FAM83B 1.63837768361582 3.9180671641791 3.27893411585923
0.0012288157901876
0.003015302998630 8 -2.10049545402758

CASKIN1 1.18721603107345 5.14318805970149
3.27815267783508 0.00123204709507074
0.003022335626933 -2.10292836593263
ATG9B 1.13771271186441 6.54671641791045
3.27407181340372 0.00124905060612889
0.00306075542418551 -2.11562518133082
TGM3 1.88734449152542 4.8117815920398 3.27287231783812
0.00125408983835232
0.00307163736356157 -2.11935447799681
GCGR 1.78726878531073 2.2860552238806 3.27186230326998
0.00125834769726075
0.00308133089512919 -2.12249371635701
MAP4K1 1.12812923728814 6.75071890547264
3.27149653548301 0.00125989295683422
0.00308437901309403 -2.12363034898893
C10orf95 1.28822464689266 5.27351094527363
3.26981797685967 0.00126700702869696
0.00309957749036566 -2.1288450487359
THBS4 -1.52605346045198
7.37885771144279 -3.26977491079934 0.00126719004204435
0.00309965585168242 -2.12897880843136
CLU -1.49213580508475
13.8250671641791 -3.26863200815855 0.00127205591040849
0.00311044638407412 -2.13252799327686
CPXM2 -1.49392725988701
7.93634378109453 -3.26838655106105 0.00127310319984501
0.00311263650027606 -2.13329009359113
AGTR2 -2.29940247175141
3.33672388059701 -3.26686192705325 0.00127962627976757
0.00312783992423242 -2.13802262664956
MCCD1 1.42553771186441 1.48631592039801
3.26629458721795 0.00128206155983788
0.00313230082392855 -2.13978318037532
NRTN 1.40441483050847 5.83739850746269
3.26130899884059 0.00130364808208997
0.00318087653022429 -2.15524250951948
SEMA3D -1.76331871468926
5.83757263681592 -3.26105408737887 0.00130476082377417
0.00318321327535159 -2.156032369634
KCNQ3 1.19331730225989 2.74546915422886
3.25998631487441 0.00130943147738452
0.00319346973517512 -2.15934033078083
CCNJL 1.11356129943503 7.02898208955224
3.25961788041386 0.00131104668850436
0.00319664946733902 -2.16048151548493
C1orf187 1.01182104519774 2.39854825870647
3.25643981847608 0.00132505636633444
0.0032292743590748 -2.17032039548819
TMEM101 -1.57236694915254
9.46832835820896 -3.25619646453496 0.00132613484644653
0.00323151909763171 -2.17107343309784
ELAVL2 -1.1810938559322
2.16681144278607 -3.25437166572891 0.00133424790866016
0.003250420299714 -2.17671850232191

AIM2	1.34506927966102	3.69036815920398
3.25395602530621	0.00133610228739952
0.00325387662637155	-2.1780039005059
CSTA	1.06978665254237	4.86578009950249
3.25349061621327	0.00133818155190096
0.0032585538204871	-2.17944303673937
GREM1	-1.54705028248588
6.06863880597015	-3.25196413293788	0.00134502240692914
	0.0032736585671909	-2.1841619234689
CYP4F3	1.5796229519774 2.61518208955224	3.25011464563131
	0.00135335433331188
0.00329159631120637	-2.18987665920265
CRTAC1	-1.98369223163842
4.13019154228856	-3.2498082966583	0.00135473905516711
	0.00329457389274825	-2.19082296547116
HSD11B1 -1.26218149717514	3.6477184079602 -3.24869091282326
	0.00135980088734271
0.00330610045205421	-2.19427386339642
HAS2	-1.24523573446328
3.95310746268657	-3.24840927229054	0.00136107951047739
	0.00330881732979288	-2.19514350596702
OTOF	1.28923305084746	2.77707810945274
3.24567368969599	0.00137355707580713
0.00333826788413706	-2.20358684097763
HOXA11	-1.7328040960452
7.92402288557214	-3.24174647358001	0.00139165584409005
	0.0033791477799135	-2.21569694732167
FN1	-1.11318107344633
12.4920194029851	-3.23411468287354	0.0014274625895533
	0.00346199727862129	-2.23919287902412
ANO3	-1.07992379943503
1.83263482587065	-3.23232945246266	0.0014359611007669
	0.0034813747518593	-2.24468185887448
STEAP2	-1.24504830508475
7.51309751243781	-3.23201555270511	0.00143746025551475
	0.00348459783276867	-2.24564671241766
GABRE	-1.48183467514124
5.98973830845771	-3.23146866422181	0.00144007561446586
	0.00349052566292611	-2.24732751660937
ATP12A	1.23737274011299	1.39888507462687
3.22607849964693	0.00146609020135509
0.00354771709498113	-2.26387991694792
DNASE1L2	1.05021956214689	4.17182437810945
3.22376255935933	0.00147740109204633
0.0035734029712195	-2.27098418389775
DKK2	-1.38810826271186
4.05406517412935	-3.22370708602371	0.00147767301078195
	0.00357363964087095	-2.27115429487317
TLE6	1.26636673728813	5.54501293532338
3.2220931753401 0.00148560443339128
0.00359042285393645	-2.27610225552048
LPO	1.44442005649717	2.67813532338308
3.22075809379468	0.00149219542333229
0.0036045148889295	-2.28019368892022

ALX3 -1.07368651129944
4.53016069651741 -3.21930179134334 0.00149941579859248
0.00362110414599502 -2.28465486848469
EMX2 -1.35714194915255
10.4677631840796 -3.21849162544675 0.00150344664175072
0.00362998465896062 -2.28713591173189
GLOD5 1.10958912429378 2.95589253731343
3.2184411396938 0.00150369815765942
0.00363016500196942 -2.28729050012826
LOC220594 -1.26375628531073
5.38416915422886 -3.2175725811137 0.00150803136586384
0.0036401979942604 -2.28994970187241
EFNB3 -1.35107259887006
7.67266616915423 -3.21566824061467 0.00151757264604143
0.00366107713551476 -2.29577781685652
SOHLH1 1.59979392655367 1.68104875621891
3.21468350842078 0.00152252837696193
0.0036708758166798 -2.29879030830839
GPR162 -1.14574053672316
6.70827263681592 -3.2122044056825 0.00153507120246884
0.00369894504538561 -2.30637069363146
TNNI2 1.11759201977401 2.91582736318408
3.21210933708912 0.00153555409811184
0.00369967440843048 -2.30666128117077
TREML2 1.39117846045198 2.70044676616915
3.21155421019403 0.00153837665010134
0.00370517041606999 -2.30835793233799
H19 -1.78613064971751 11.816863681592 -3.21112628632028
0.00154055572118986
0.00370998345525254 -2.30966562860646
SLC13A5 -1.75149894067797
3.35724776119403 -3.20935440559808 0.00154960899599159
0.0037304728761383 -2.31507866213705
FAM131C 1.15816991525424 3.79723930348259
3.20844988448842 0.00155424957772449
0.00374076715482811 -2.31784090381865
ABCA3 -1.30638396892655
7.91509800995025 -3.20817748137055 0.00155564964679833
0.00374369795437459 -2.318672635454
GBP5 1.37766857344633 7.52923333333333
3.20807327187355 0.00155618556123911
0.00374454870748229 -2.31899080269343
IGFL2 1.15026673728814 1.52597562189055
3.20532606170814 0.00157037546667134
0.00377647983258318 -2.32737508621083
TNNT2 1.5300156779661 4.20801890547264 3.20246012628444
0.00158530646220459
0.00380970872867379 -2.336114805877
CSMD3 -1.03231737288136
1.24631940298507 -3.20153015062411 0.00159017968999011
0.00382097249947295 -2.3389492672855
SPATA4 -1.16364929378531
1.88401890547264 -3.19981392870239 0.0015992094779066
0.00383952410027161 -2.34417816905842

ISL1 -1.16418502824859
1.76897213930348 -3.19875667810368 0.00160479577850796
0.00385152010667495 -2.34739808976896
EPHA4 -1.29646334745763
7.49331741293532 -3.19461279629818 0.00162686614672414
0.00389908498625339 -2.36000927540979
MMP13 1.03505063559322 1.19253333333333
3.19432544164883 0.00162840698845448
0.00390186688526734 -2.36088324243932
LRRC55 -1.41154293785311
3.16926616915423 -3.19381394834461 0.00163115304602731
0.00390707875801432 -2.36243873436085
MCTP2 -1.25885346045198
6.27448656716418 -3.19328326953752 0.00163400664268392
0.0039130008601774 -2.36405233329076
C1orf125 1.10094505649717 1.97818059701493
3.1931080011734 0.00163495012215534
0.00391480359317112 -2.36458520671065
PPAPDC1A 1.39226186440678 4.39957164179105
3.19046955715781 0.00164921418316543
0.00394665662436021 -2.37260375321518
PDE3A -1.40034512711864 5.4179144278607 -3.18978839132896
0.00165291542029986
0.00395413115576278 -2.3746729256979
GPRIN2 1.92392189265537 4.42452487562189
3.18833262012868 0.00166085144358374
0.00397080239161224 -2.37909377406722
KATNAL2 1.0082229519774 6.43107562189055 3.18687114185507
0.00166885409426154
0.00398854185741437 -2.38353012065965
FABP5 1.08820155367232 7.240492039801 3.18675084104764
0.00166951441778862
0.00398965556945644 -2.38389521435897
BMP2 -1.41878665254237
6.32325820895522 -3.18396509003023 0.00168487312819091
0.00402354836824002 -2.39234604183446
CES8 -1.40781850282486
4.82152089552239 -3.18374295947561 0.00168610342295241
0.00402601805922458 -2.39301960761843
ARSE -1.84999625706215
5.44166965174129 -3.18169140485917 0.00169750560522207
0.0040527724241434 -2.39923852396124
SNURF -1.53202457627119
5.18248706467662 -3.18063282160381 0.0017034169341116
0.00406641275085014 -2.40244601120533
RD3 1.10705105932203 2.07862835820896
3.17729930722915 0.00172215675369214
0.0041082823789565 -2.41254019749159
PDCD1 1.31412937853107 4.90968507462687
3.17533605290993 0.00173328265519603
0.00413290277713989 -2.41848063611566
CEACAM21 -1.66440967514125
4.70220497512438 -3.1751041895516 0.00173460102891544
0.004135085824853 -2.41918199200385

ADH1C	-1.81312125706215
4.94206666666667	-3.1732277521473	0.00174530467288175
	0.00415776673044376	-2.42485626319556
FAM155A	-1.6079979519774
5.39610597014925	-3.17322324996568	0.00174533042781122
	0.00415776673044376	-2.42486987396608
RASA2	-1.19055762711865
5.21293184079602	-3.17177219344539	0.00175364962822149
	0.00417564681830053	-2.42925572621072
FRZB	-1.28113220338983
7.80307213930348	-3.17165048178125	0.00175434909287693
	0.00417682788783061	-2.42962352014176
TCEA3	-1.17674646892655
8.25231890547264	-3.17108561853866	0.00175759868902958
	0.00418407943944543	-2.43133028300217
SLC8A3	-1.15305882768362
1.62709900497512	-3.17104312814129	0.00175784335695254
	0.00418417670936065	-2.43145865877499
KCNN3	-1.04445557909605
5.04369303482587	-3.17069173682387	0.0017598679456845
	0.0041875392819776	-2.43252025392036
TEKT3	-1.31681687853107
3.44870248756219	-3.17012245908036	0.00176315250111518
	0.0041943824852717	-2.43423988429272
CENPV	-1.14690254237288
7.13099104477612	-3.16934708702922	0.00176763529052323
	0.00420455943858672	-2.43658161949234
CGB5	1.05685826271187	1.13115373134328
3.16865774100864	0.0017716295852788
0.00421308414059698	-2.43866310885269
LRGUK	1.27114124293785	4.29849651741294
3.16803099831949	0.00177526838961924
0.00422075970340279	-2.44055521137234
DSG3	1.80014336158192	1.7887592039801 3.16634477724838
	0.00178509280976542
0.00424166150283381	-2.44564414228163
KLRD1	-1.19716680790961
3.82377014925373	-3.16622880802377	0.00178577032766188
	0.004242780329464	-2.44599404157466
GABRQ	-1.15233163841808
1.51009353233831	-3.16340886311582	0.00180231854893664
	0.00427962061180536	-2.45449873824089
MMP10	2.3312438559322 5.96019054726368	3.16157075436802
	0.00181318140423184
0.00430441885162809	-2.4600386172497
HORMAD1 1.60641433615819	2.05379950248756
3.15961710646084	0.00182479350567405
0.00433048325875485	-2.46592352728433
CCL14-CCL15	-1.0280322740113
0.726397014925373	-3.15842487100778	0.00183191370935576
	0.00434587340571687	-2.46951324263157
LOC400931	-1.01790430790961
5.13683233830846	-3.15720149688587	0.00183924660710674
	0.00436125350472411	-2.47319543973303

RASGRF1 -1.61792634180791
4.73205472636816 -3.1561960171378 0.00184529378073711
0.00437357207556371 -2.47622083683527
OLR1 1.20590572033898 5.43927114427861
3.15612394197524 0.00184572796256785
0.0043740961643216 -2.47643767095507
FAM43B -1.19658262711864
4.43321791044776 -3.15543768708955 0.00184986671127954
0.00438238675081572 -2.47850200622247
NKX3-1 -1.04355225988701
5.77493432835821 -3.15447434829488 0.00185569102560652
0.00439517036867811 -2.48139915713489
DLX1 1.39387372881356 1.84115771144279
3.15326761976321 0.00186301081381388
0.00441047186434489 -2.48502715044224
TRIM29 1.57225812146892 7.56803830845771
3.15276354426442 0.00186607634650959
0.00441721980789137 -2.48654226643058
PXDN -1.09079745762711
10.0720970149254 -3.1503095889583 0.00188106686030775
0.00444962583732205 -2.49391506782309
ICAM5 1.32632048022599 3.40239552238806
3.14757941533603 0.00189787563114999
0.00448628517069768 -2.50211164860393
VSIG8 1.68480988700565 3.17888855721393
3.14616500530114 0.00190663823327314
0.00450544232609319 -2.50635548844287
CES3 1.54361320621469 7.08689701492537
3.14584757720096 0.0019086099111648
0.00450958240030076 -2.50730767232184
HERC5 -1.11301800847458
7.93023383084577 -3.13690511614448 0.00196493647993655
0.00463413508708757 -2.53409647608694
KIAA2022 -1.4805643361582
4.8503736318408 -3.13548930135322 0.00197399408679413
0.00465282417932072 -2.53833147870306
IGFBP2 1.22261052259887 12.7254313432836
3.13387220512522 0.00198438661175894
0.00467517295609492 -2.54316643788672
C19orf33 1.75406108757062 9.09844179104478
3.13350146922579 0.00198677632344243
0.00467972899498313 -2.54427458344972
ELOVL3 1.4099668079096 3.37982089552239 3.13122315333772
0.00200152055305119
0.00471175518336669 -2.55108195957547
LOC440356 1.22963806497175 2.14411144278607
3.12954540136764 0.00201244281822809
0.00473475258517335 -2.55609204315944
TMEM61 1.29527860169492 4.39990597014925
3.12796914670237 0.00202275451174776
0.00475737772095103 -2.56079681946475
YBX2 1.69210353107344 5.96946965174129
3.12654538037935 0.00203211060853019
0.00477761559880904 -2.56504460468081

RGPD5 -1.33666087570621
4.48934875621891 -3.12651811610794 0.00203229016192885
0.00477761559880904 -2.56512593011537
CFB 1.57091285310734 11.3436328358209
3.12586866840036 0.00203657154595846
0.0047865842974845 -2.56706294999752
COL9A1 2.37719173728814 4.71357711442786
3.12457870497389 0.00204510016908815
0.00480464861646409 -2.57090926768124
GLYATL3 1.64175423728814 1.73419054726368
3.12456460356817 0.00204519358301411
0.00480464861646409 -2.5709513062589
C14orf23 1.20115310734463 1.08319850746269
3.11614915524642 0.00210165009497429
0.0049276926050505 -2.5960084355422
PP14571 1.23504830508475 3.26032985074627
3.11307534504164 0.00212262786756483
0.00497517412045964 -2.60514547097615
IQCA1 -1.78503375706215
4.96621293532338 -3.11258680026251 0.00212597979163373
0.00498189306269153 -2.60659693831555
ARL14 1.19706730225989 2.13155472636816
3.10845388432814 0.00215453216782957 0.005043044738974
-2.6188675678925
DSCR4 1.25055692090395 1.12246169154229
3.10750343507261 0.00216114827565868
0.00505680124222391 -2.62168735981772
MAP3K15 -1.34919724576271
2.16488009950249 -3.10685201899095 0.00216569363661898
0.00506512763269397 -2.62361952842461
C6orf97 -1.54956807909605
7.22700497512438 -3.10652058143733 0.00216800968331097
0.00506996682974988 -2.62460246595479
KBTBD7 -1.07863580508475
5.96472736318408 -3.10593851744476 0.00217208261533458
0.0050789129946263 -2.62632845081511
CXorf49B 1.3250406779661 1.94822686567164
3.09978853095159 0.00221555039565704
0.00517407002876111 -2.64454696608228
NEK5 1.15838467514124 3.27688258706468
3.09792604026099 0.00222887198268881
0.00520340479924962 -2.65005787864692
TRIM17 1.47909625706215 6.20398358208955
3.09508997430666 0.00224929922737644
0.00524632051102457 -2.6584437199346
ACY3 1.34803580508474 5.80907213930348
3.09387234822186 0.00225812229783921
0.00526450724218023 -2.66204192570417
CNTNAP3 -1.46592966101695
4.32519004975124 -3.09054051384222 0.00228242875298897
0.00531694795176031 -2.67188125201241
FABP4 -1.8600436440678
4.68434278606965 -3.08810833914884 0.00230032421647248
0.00535498992676949 -2.67905768820393

C11orf41 1.43296320621469 4.52990895522388
3.08494682530147 0.00232377956606601
0.00540713980407289 -2.68837845687965
HCN4 1.26339653954802 3.21418905472637
3.08375457063663 0.0023326820884O473
0.00542539518238915 -2.69189120667067
FAM3B 1.9444113700565 5.64679701492537 3.08178157817912
0.00234748350213254
0.00545734755959473 -2.69770153961965
GPC6 -1.30831207627119
6.82377512437811 -3.08106448495718 0.00235288457821612
0.00546928446453543 -2.6998124944384
B3GNT7 1.02976052259887 8.51832487562189
3.08031157245807 0.00235856777688188
0.00548063344192247 -2.702028412254
CLUL1 1.39024039548022 6.05092487562189
3.07879289725563 0.00237006972184864
0.00550362305050929 -2.70649656958983
ADAMTS6 -1.34037648305085
5.66113930348259 -3.07742160882074 0.00238049983647791
0.00552659297103827 -2.71052937307286
AREG 1.59978213276836 4.89019253731343
3.07730582173388 0.00238138245654698
0.0055280169417582 -2.71086981495295
FOXD2 -1.01898276836158
3.60227014925373 -3.07644012103575 0.00238799107776625
0.00554210454742722 -2.71341481411527
MMEL1 1.24309456214689 4.55596119402985
3.07502082205538 0.00239886238881545
0.00556356132426357 -2.71758588052404
ST8SIA5 1.20343714689266 3.39680149253731
3.07294808730916 0.00241482079933714
0.00559614753475466 -2.72367413336811
USH1G -1.45282535310734
3.47693383084577 -3.07224862479022 0.00242022815953105
0.0056078329538415 -2.72572782513588
STK33 -1.1327238700565
7.39435174129353 -3.07121899990773 0.00242820823516815
0.00562399763853815 -2.72875013288922
MS4A8B -2.4416529661017 4.7074960199005 -3.07050043917702
0.00243379178460228
0.0056358074757176 -2.73085881304622
CCL28 1.10397415254237 6.19556467661692
3.07049215017262 0.00243385626301569
0.0056358074757176 -2.73088313524581
IL20RA -1.4675834039548
7.61511293532338 -3.07026071191225 0.00243565720840025
0.00563934165878618 -2.73156221410809
SIGLEC10 1.05361779661017 6.04236268656716
3.06835725776952 0.00245051568312113
0.00566799087139215 -2.73714550303459
NKX2-5 1.53155953389831 1.61828258706468
3.0667315828457 0.00246327191471737
0.0056953944397012 -2.74191150463348

UBD	1.69177408192091	8.294792039801	3.06457973761617
	0.0024802510106896
0.00573160212173093	-2.74821654884436
FAIM2	-1.57402055084746
6.02969303482587	-3.05965661765208	0.00251950333042716
	0.00581385314248622	-2.76262645723592
NR0B2	1.36127514124294	1.70336815920398
3.05877375328999	0.0025266027068363
0.0058288744321 1025	-2.7652083599791
KLHDC8A -1.40405261299435
5.03197462686567	-3.05765626782914	0.00253561517573995
	0.0058476948503641	-2.76847543046009
CLRN3	1.03576038135593	1.6995	3.05694762090346
0.00254134572020102	0.0058589362869991	-2.77054666115992
ZNF462	-1.0364668079096
7.32834875621891	-3.05038007462785	0.0025950254860773
	0.00597531103961047	-2.78972146288694
CHRNA9	1.17093524011299	1.58568457711443
3.04898713103426	0.0026065441198904 1
0.0059985852807509	-2.79378351455955
BTBD11	1.35552252824859	6.47984825870647
3.0489810851196 0.00260659421770963
0.0059985852807509	-2.79380114175372
CAMK2N1 -1.08401447740113
9.20708258706468	-3.04700793891646	0.00262299161410662
	0.00603158801944544	-2.79955225643223
MAGEA9B 2.10033714689266	2.22630895522388
3.04576586111449	0.00263336229806463
0.00605272368778178	-2.80317079203066
AZGP1	-1.90846596045198
4.07881890547264	-3.04482223737716	0.00264126627807684
	0.0060668154720203	-2.80591894529245
S100A7	1.89814851694915	2.20598308457711
3.04394538030239	0.0026486305850608
0.00608100938395193	-2.80847195589944
PRODH	1.81554110169492	6.35908507462687
3.04211976837631	0.00266402373651151
0.00611429930805828	-2.81378516129649
UPK1A	1.28152372881356	1.98547263681592
3.04010484204287	0.00268110874909995
0.00614938695330375	-2.81964596951605
GPC3	-1.5391375	6.14241094527363	-3.03883931131628
	0.00269189098761003
0.00617201334507899	-2.82332520437708
IL1F5	1.05353672316384	1.00137462686567
3.03483034856716	0.00272631117056735
0.00624608507217361	-2.83497110911927
VNN3	1.69318601694915	3.55452288557214
3.03467931365617	0.00272761581141756
0.0062477040746289	-2.8354095866254
PKIA	-1.4206311440678
5.02840895522388	-3.0345762618188	0.00272850630385699
	0.0062490221350049	-2.8357087504936
COL10A1 1.64027097457627	3.77408855721393

3.03375835213304	0.00273558352841695
0.00626383335248717	-2.83808284864011
C2orf70 1.44407104519774	4.03710298507463
3.03323868540877	0.00274008888448249
0.00627275031204567	-2.83959095143697
TRPM8	1.46012733050847	2.80497960199005
3.03297196655217	0.00274240390892507
0.00627665019512058	-2.84036489346775
DGCR9	1.15187605932203	2.67737611940299
3.03123455431319	0.00275752813714719
0.00630704686448661	-2.84540484950504
UPK3BL	1.43148566384181	7.62676616915423
3.03025180876719	0.00276611692091062
0.00632387309424221	-2.84825447173755
LOC338651	1.22851970338983	3.79730995024876
3.02765769511356	0.00278890678298263
0.00637313627055136	-2.85577245643721
CCNA1	1.84788622881356	7.65548059701493
3.02554590579315	0.00280758675537869
0.00641439529133369	-2.86188828390469
F3	-1.1452759180791	9.1060776119403 -3.02490923975081
0.00281324097025822
0.00642588307959515	-2.86373133095658
CCDC8	-1.10875374293785
9.34391393034826	-3.02240031221767	0.00283562483013347
0.00647197084631908	-2.87099083305351
C8orf42 -1.02204300847458
8.01730447761194	-3.02086561162827	0.00284939758599193
0.00650051477279858	-2.87542873152105
C21orf15	-1.18082224576271
1.72827562189055	-3.0197953090429	0.00285903911926409
0.00652106133968357	-2.87852251157639
PACRG	-1.56397528248588
5.23905970149254	-3.01907585118246	0.00286553701757705
0.00653515607319004	-2.88060158963943
TRIM43	1.2362613700565 1.2610855721393 3.01822010558599
0.00287328350581844	0.00655136721173396	-2.88307392125915
MMP3	1.78334265536723	3.82828955223881
3.01726946746375	0.00288191157154404
0.00657031033468063	-2.88581965664318
DGKG	-1.05653107344633
3.86372189054726	-3.01060898259276	0.00294303412458238
0.00668960258831668	-2.90503504286311
GAMT	-1.39168411016949
7.21817462686567	-3.01038264102679	0.00294513202421183
0.00669363007384798	-2.90568735341148
ANKRD2	1.29343149717514	2.22921691542289
3.00971097238726	0.00295136563806083
0.00670705518589649	-2.90762282149886
HNF4G	1.49032987288136	5.11362587064677
3.00703666784975	0.0029763057724112
0.00675625359438323	-2.91532513780178
IL12RB2 1.13283968926554	3.23051691542289
3.00682626385085	0.00297827616375334

0.00675997895432349	-2.91593086113518
FRAS1	1.07605218926554	9.03359751243781
3.00666362702997	0.00297980004659379
0.00676269013448711	-2.91639904300245
CXorf22 1.28321214689265	2.79597611940299
3.00637814163227	0.0029824767357123
0.00676726872062041	-2.9172208125766
APOE	1.03435091807909	11.8089004975124
3.00611427348864	0.00298495270836558
0.00677064181888691	-2.9179802935268
LRRC48	-1.22155515536723
6.39023134328358	-3.00186272434205	0.00302510792028029
	0.00685532480291203	-2.93020896104986
MYH15	1.01798199152542	2.57078606965174
3.00180823001004	0.00302562581778792
0.00685532480291203	-2.93036559967605
MUC16	1.69820416666666	10.0180626865672
3.00176014937364	0.00302608282914792
0.00685560367274407	-2.93050380060476
XIST	-1.53562083333333
11.4755243781095	-3.00124666867351	0.0030309674735512
	0.00686591219596229	-2.93197960150032
KBTBD12 -1.70798961864407
3.42634527363184	-2.99939992510394	0.00304859514650982
	0.00690431973059533	-2.93728544242673
TRPM5	1.31377302259887	2.67554626865672
2.99895408513144	0.00305286490388409
0.00691170230224309	-2.93856592898232
TAGLN3	1.12837040960452	1.73114378109453
2.99728494092982	0.00306889894276	0.0069434092235908
	-2.9433582946584
TSPAN10 1.04219258474576	5.73506417910448
2.99276645699292	0.00311269316332604
0.00703474137418358	-2.95631931272508
LRRC3B	-1.55630798022599
3.45226567164179	-2.9872665766673	0.00316677370014993
	0.00714909435496791	-2.97207129409405
UGT3A1	1.41441094632768	1.35578855721393
2.98628881709372	0.00317647790427738
0.00716863706439647	-2.97486888201505
SPOCK1	-1.25854717514125
8.33247711442786	-2.9861760883533	0.00317759848404271
	0.00717037776185941	-2.97519137021558
NPR3	-1.22665084745763	4.6076815920398 -2.98513064406026
	0.0031880080271976
0.00718833660608092	-2.97818158965416
CEACAM7 1.9133865819209 2.87833084577114	2.98484497258821
	0.00319085791491085
0.00719397243500108	-2.97899851149339
IL22RA1 1.13229138418079	4.51698407960199
2.98411397205009	0.00319816110547927
0.00720885457789739	-2.98108859528266
ANK1	1.2433334039548 4.95129651741294	2.98324689610157
	0.0032068436790336

0.00722208213367263	-2.98356714024378
EPHA8	1.50268389830509	1.87007562189055
2.98311522418917	0.00320816408339628
0.00722349852412191	-2.98394346806867
SLC22A4 -1.01788672316384
5.18106865671642	-2.98311400566597	0.00320817630506301
	0.00722349852412191	-2.98394695062495
GDF5	1.52901144067797	6.05061791044776
2.98180440494979	0.00322133620875889
0.00725074359892774	-2.98768905568101
TMEM213 1.42939957627119	2.7009	2.98156077747148
0.00322378983080067	0.00725547086454044	-2.98838504072401
GLT25D2 -1.22106638418079
5.47361592039801	-2.98006485768452	0.0032388931923537
	0.00728467103845547	-2.9926573832159
KLK12	1.60174470338983	2.19091243781095
2.97994919770054	0.00324006363947699
0.00728570717617334	-2.99298762611656
SLC1A1	-1.02665014124294
6.84380995024876	-2.97972905822523	0.0032422924630854
	0.00728992051989302	-2.99361615596679
SPRR1A	1.66174646892655	1.5897039800995 2.9789931400715
0.00324975355888691	0.00730349650398996	-2.99571699978398
ZNF215	-1.10784689265537
4.19846666666667	-2.97725899881409	0.00326739749215305
	0.00733993549774616	-3.00066561870934
GLP1R	1.16935550847458	1.51250597014925
2.97556440719287	0.00328472392850625
0.00737724343641104	-3.00549883051427
KCNJ1	1.00301956214689	1.96339353233831
2.97527863205644	0.00328765414621336
0.00738250425458673	-3.00631365301334
UPK3B	1.55014399717514	4.95226467661692
2.97526582659778	0.00328778550412501
0.00738250425458673	-3.00635016317647
ENDOU	-1.3021884180791
2.75124676616915	-2.97321821031346	0.00330885194562496
	0.00742574665978142	-3.01218635553448
SLCO4C1 1.30699879943503	3.00248507462687
2.9715285953249 0.00332632836939806
0.00746069726789549	-3.01699939069229
TCHH	1.23358361581921	3.76182338308458
2.96954013162452	0.00334700438954062
0.00750316664307369	-3.02266051872091
FOXE1	1.31876617231638	1.5002223880597 2.96930619311902
	0.00334944461583054
0.00750781722491549	-3.02332631032308
DMKN	1.05866942090396	10.0058069651741
2.96876794513972	0.00335506531158802
0.0075195950706225	-3.02485798779391
C10orf107	-1.56521052259887
4.92564278606965	-2.96257406233074	0.003420371067707
	0.00765009456585955	-3.04246545708164
C1QB	1.04551179378531	10.2149537313433

2.96206381346492	0.00342580256584487
0.00765890522452305	-3.04391445031233
MAG	-1.55865981638418
2.42136965174129	-2.96162536285161	0.00343047610365607
	0.00766851855488449	-3.04515936992819
SOX17	1.19471673728813	11.6413348258706
2.96108366688518	0.00343625823001613
0.00768060767144597	-3.04669720735411
MPP7	-1.12270459039548
7.51582885572139	-2.96001009487636	0.00344774408173595
	0.00770460280810266	-3.04974424297736
TAC3	1.23868312146893	1.66813482587065
2.95986219892823	0.003449291360223
0.00770730596022398	-3.05016392520534
CHRM3	-1.40249011299435
3.00068805970149	-2.95945332801366	0.00345371463129224
	0.0077162652498019	-3.05132407245347
SGK2	1.35475918079096	5.5272552238806 2.9593060356994
0.00345529572146032	0.00771895768896412	-3.05174196979903
SLC16A4 -1.13617019774011
5.97935024875622	-2.9592507994822	0.0034558888185478
	0.00771944265529173	-3.05189868093041
WDR86	-1.16645303672316
6.88284577114428	-2.95652497412949	0.00348527351605665
	0.00777776300994977	-3.05962881461815
FMO5	-1.17336504237288
5.20041741293532	-2.95492357328679	0.00350264344174448
	0.00781197934372777	-3.06416716438308
ZBP1	1.24490416666667	4.38165472636816
2.95452591859838	0.00350696896876321
0.00781907829373466	-3.06529376366058
CAPS	1.47574865819209	12.0990273631841
2.95156560618168	0.00353932429662464
0.00788693452835167	-3.07367628693755
TARP	-1.02041786723164
2.57955223880597	-2.95089410376012	0.00354670158756391
	0.00790027220288464	-3.07557666523042
NBPF15	-1.08971299435028
6.83370199004975	-2.94968498138892	0.00356002085227537
	0.00792617157544879	-3.07899753018053
CYP39A1 -1.06853199152542
6.28771641791045	-2.94933790023501	0.00356385263547804
	0.00793384239930211	-3.07997925945204
EMR1	1.06830268361582	3.03030248756219
2.9473863883502 0.0035854678343522
0.00797936635655834	-3.08549719732954
ESPNL	-1.36334392655367	4.6797184079602 -2.94498330097563
	0.00361224978254245
0.00803374369359728	-3.09228737505981
ADORA1	1.07760939265536	6.50171044776119
2.94283301072682	0.00363636962362117
0.00808213363353283	-3.09835894422033
CHRFAM7A	-1.00481405367232
3.76465124378109	-2.9420164806997	0.00364556724462316

0.00809906888130219	-3.10066343711365
PDE11A	-1.28515148305085
4.44891194029851	-2.94128638026296	0.00365380934274125
0.00811650138568252	-3.10272350329963
CLEC2L	-1.34493156779661
2.33321144278607	-2.93864219072417	0.00368380265470213
0.00817781882580394	-3.11018047890729
CBS	1.4419281779661 8.93620348258706	2.93773631956265
0.00369412979953914
0.00819985779917164	-3.11273374506844
CLIC3	1.23278015536723	6.25012835820896
2.93581275356707	0.0037161469006531
0.00824605446070126	-3.11815306701343
CCL25	1.13227125706215	2.20829552238806
2.93313379027394	0.00374701053856455
0.00831094709990604	-3.12569516912721
CCR2	-1.16329039548023
4.28914328358209	-2.93150265631092	0.00376591719822925
0.00834927430665729	-3.13028421755295
DEFB4A	1.37504258474576	1.58538756218905
2.93096448499617	0.00377217434288506
0.00836224373819568	-3.13179780032093
SLC3A1	1.77501539548022	5.3674815920398 2.92820375428364
0.00380442239666553
0.00843009085845045	-3.13955822383556
PDZK1IP1	1.83606518361582	8.3534960199005
2.92804195929082	0.00380632012819628
0.00843302801803918	-3.14001282199073
CA12	-1.30016151129944
9.41400248756219	-2.92751037535928	0.00381256130410716
0.00844539097683644	-3.14150626019293
CREB3L1 -1.32270063559322
8.72374626865672	-2.92529504810141	0.00383867183515464
0.00850047813350498	-3.14772734791886
CLCA4	1.2835718220339 1.89443034825871	2.9241695118716
0.00385200036729321	0.00852815351456159	-3.1508864260913
HPGD	-2.07647733050847
7.12814776119403	-2.92187661140947	0.00387928388964657
0.00858578032066021	-3.15731853086488
KHDC1	-1.03718785310735
4.65677213930348	-2.91713436476763	0.0039362744765069
0.00870346957487201	-3.17060691133403
GABRB2	-1.09292598870057
2.13973930348259	-2.91645460850992	0.00394450602195703
0.00871791459045946	-3.1725100514122
CUZD1	-1.15462281073446
3.09358009950249	-2.91407562544441	0.00397343861318809
0.00877430297179213	-3.17916737647336
CLCA2	1.23972966101695	1.66925223880597
2.91266171516702	0.00399072608259112
0.00880517658309715	-3.1831216869661
GRIK1	-1.12645918079096
1.77248507462687	-2.91265153043364	0.00399085085756059
0.00880517658309715	-3.18315016441607

PIK3C2G -1.37494978813559
1.89790248756219 -2.90361599624612 0.00410296333526261
0.00903699573789737 -3.20837831235977
ZNF671 -1.00531073446328
5.84799303482587 -2.90187365354002 0.00412491062951315
0.00908338680938591 -3.21323483176372
KRT4 1.74880586158192 2.8040223880597 2.9011533491853
0.00413401520756313 0.00910002815208641 -3.2152417897131
LOC286467 1.23680882768361 2.72733432835821
2.9011245730635 0.00413437931632109
0.00910002815208641 -3.21532195806908
POU3F3 1.7349343220339 1.80843631840796 2.90025497419829
0.0041453963253737
0.00911969423059602 -3.21774425780551
OSTalpha 1.14167330508474 5.27241243781094
2.89936625386672 0.00415668330807357
0.00914241780753613 -3.22021913156773
SLC13A2 1.11574894067797 1.19852935323383
2.89590930528225 0.00420085513183696
0.00923477415747006 -3.2298392775144
VAV1 1.03727337570621 7.01996567164179
2.89534768867976 0.00420807167070681
0.00924865768114879 -3.23140117232273
TH -1.14945473163842
2.18628109452736 -2.89438299371474 0.00422049404790471
0.00927298189249611 -3.23408340608146
CLDN19 1.52137697740113 1.70949502487562
2.89284586596937 0.00424035689704987
0.00930666314907354 -3.23835553172175
NKX2-2 1.33241179378531 1.22226368159204
2.89228900390521 0.00424757375163188
0.00932050973931193 -3.23990269872313
WNT3A 1.19821624293785 2.87663731343284
2.8914475178269 0.00425850058940888
0.0093424895384232 -3.24224013602237
ANKRD1 1.34337641242938 3.23534825870647
2.89058747457911 0.00426969492885003
0.00936604732228775 -3.24462847427092
PGBD5 -1.20104286723164
6.32978606965174 -2.88686889219392 0.0043184061602613
0.00946177998614349 -3.25494744992277
CALCB 1.76428968926554 2.81821990049751
2.88585353933037 0.00433179462860815
0.0094870646175939 -3.25776290620762
PAX2 -2.56094406779661 5.2740736318408 -2.88534224027357
0.00433855097458188
0.00950084816087104 -3.25918033461815
EFHC2 -1.25245042372881
5.67845422885572 -2.88500181308843 0.00434305474248181
0.00950969644444876 -3.26012394219487
KCTD14 1.00565148305085 8.48969452736318
2.88362348834305 0.00436133331900309
0.00954462989437517 -3.26394338257585
?|652919 -1.26037266949153

1.25634875621891	-2.88167359776761	0.00438731170033949
	0.00959432352023537	-3.26934380666011
LCN15	1.58408474576271	1.44074029850746
2.88068971254148	0.0044004735685130 8
0.00962208141032842	-3.27206750311291
CD1A	1.26894187853107	3.03608059701493
2.87792494606721	0.00443765232372223
0.00969857903716882	-3.27971664462606
LOC441046	-1.01691631355932
3.31076069651741	-2.87762881373883	0.00444165147334271
	0.0097048855627635	-3.28053553846472
DPPA2	1.45276871468927	1.383907960199	2.87705565886092
	0.00444940105136249
0.00971974926414897	-3.28212026165049
IVL	1.73319265536723	1.80484427860697
2.87579640946142	0.00446647062660621
0.00975496191108782	-3.28560095605329
PCDHA11	-1.57666384180791
3.20562786069652	-2.86913361860098	0.00455778619533695
	0.00993537381506345	-3.30399424971163
AQP12B	1.51344484463277	2.14073084577114
2.86896132506507	0.00456016996846479
0.00993923770409855	-3.30446936214112
AGR2	1.85028855932203	9.51525024875622
2.86516811200893	0.00461294019445983
0.0100428061709714	-3.31492277758844
LRRC15	-1.50621440677966
3.57764875621891	-2.86149057446971	0.00466463250404813
	0.0101456621790558	-3.32504525259514
GFRA3	1.69325515536723	4.92697960199005
2.86065636494162	0.00467643162670827
0.0101691707760111	-3.32733976017526
SPRR2D	1.80122966101695	1.99210746268657
2.86022956268205	0.00468247887147816
0.0101801642731597	-3.32851344880498
SLC39A5	1.34182994350282	2.4656447761194	2.86011354743561
	0.00468412389233243
0.0101826623792445	-3.32883245806975
ATP1A3	1.13420586158192	2.91687412935323
2.85989691397489	0.00468719702533045
0.0101871855737915	-3.32942810718822
FGF8	1.36143213276836	1.6596855721393	2.84965069971149
	0.00483466597741468
0.0104799590873533	-3.35755332090998
CD38	1.24397422316384	4.92740248756219
2.84922467368178	0.00484088817397857
0.0104920503704743	-3.35872072120246
TREH	1.22335670903955	1.84004676616915
2.84581163834654	0.00489100034785359
0.0105897740077446	-3.36806734335221
SGCG	-1.16098679378531
1.92810099502488	-2.84478684939632	0.00490613892376985
	0.0106147678619753	-3.37087171939651
GALNT5	1.19189103107345	2.9766631840796	2.84382832004215

0.0049203373244868
0.0106409486223232 -3.37349393056411
ZNF883 -1.34365776836158 4.9320039800995 -2.84266392507053
0.00493763551614839
0.0106738609188719 -3.37667822420853
OGDHL 1.18516666666667 8.32555422885572
2.84073234817313 0.00496645317060115
0.0107327666939412 -3.38195789342173
KHDRBS2 -1.17158771186441
1.71832835820896 -2.83947131303113 0.00498534944414979
0.0107684549915684 -3.38540295408297
KIAA1324L -1.27680769774011
6.18874278606965 -2.83798080501504 0.00500776875896606
0.0108129462159837 -3.38947309964926
RXRG -1.46965444915254
4.20803084577114 -2.83788342510433 0.00500923667976311
0.0108149783400501 -3.38973894738164
BHLHE41 -1.30351292372882
8.36345124378109 -2.83705353004739 0.00502176256815254
0.010840881758588 -3.39200422441525
STAR -1.09326235875706
3.21421393034826 -2.83638252788953 0.00503191108526541
0.0108579352812324 -3.3938353414663
DCDC2 1.32815218926554 8.59332935323383
2.83281974590246 0.00508610969855137
0.0109671076684444 -3.40355122670667
LOC284233 -1.00573050847458
2.40912338308458 -2.83107601406452 0.00511282942485738
0.0110189363943231 -3.40830236460795
ACCN1 -1.72908820621469
4.58091592039801 -2.82797575501181 0.00516065148451335
0.0111103371410507 -3.41674295977913
LRRC26 1.77148043785311 3.68198955223881
2.82584174784317 0.00519380518608468
0.0111793688897377 -3.42254793149968
AGT -1.32412252824859
5.56583582089552 -2.81678201233863 0.00533672592820417
0.0114689608820299 -3.44714737506799
PAPL 1.22772464689266 1.82860149253731
2.81587268069125 0.00535126681085568
0.0114953968428238 -3.44961240987428
MYH6 1.13185868644068 1.57225721393035
2.81576134960829 0.00535304955142235
0.0114980233616552 -3.4499141579481
HCN2 1.00703093220339 3.02221343283582
2.81364005285738 0.00538712119576706
0.0115615302267508 -3.45566154529715
LASS1 1.17279894067797 5.80081741293532
2.81292054395635 0.0053987224651659
0.0115815853928794 -3.45761005583069
GRAMD2 1.07687959039548 6.21797611940299
2.81151519280694 0.00542144773601384
0.0116279066047183 -3.46141457633772
DLK2 -1.18504314971751

3.49782537313433	-2.81009620586022	0.00544448175269151
	0.0116736512149093	-3.46525422943249
GDF1	1.24712860169492	3.99008855721393
2.80966558247366	0.00545148952498697
0.0116874561349188	-3.46641910406144
RFPL1S	-1.17595896892656
2.65635820895522	-2.80877139898551	0.00546606727662876
	0.0117138164080572	-3.46883742330597
CCDC160 1.32020275423729	4.80944577114428
2.80809839083961	0.00547706260984	0.0117349295950757
	-3.4706571048057
LOC441666	-1.60674223163842
3.92784925373134	-2.80386407757117	0.00554670369661944
	0.0118766506314527	-3.48209661226036
TSPAN11 -1.1086063559322
6.66565621890547	-2.80280698890846	0.00556421458580605
	0.011910470800396	-3.48494997645017
DMRT3	1.58351518361582	2.79941791044776
2.80129193274452	0.00558939944074678
0.0119606389950523	-3.48903778321276
UGT1A6	1.87425105932203	3.18578905472637
2.7986204080571 0.00563406091507838
0.0120524404070605	-3.4962409064597
FOXI1	1.2786668079096 1.53439850746269	2.79540274382832
	0.00568828299138287
0.0121600717973558	-3.50490812952735
RGR	1.22012083333333	1.36424726368159
2.79481757297086	0.0056981947371556
0.0121782154855095	-3.50648337744823
MGC4473 1.39216101694915	2.3460328358209 2.79274730944143
	0.00573338739922634
0.0122470547608071	-3.51205396592412
POLR2J2 -1.05467203389831
7.69790895522388	-2.79162743339448	0.00575250650555758
	0.012281505673783	-3.51506569615759
AQP9	1.18394858757062	5.52334825870647
2.7890175292825 0.00579728914126223
0.012369397845574	-3.52208028697744
DAPL1	1.61330755649718	4.53180298507463
2.78834147024666	0.00580894098020697	0.01239297083276
	-3.52389632882782
CD1E	-1.12632139830509
2.85195223880597	-2.7878500474302	0.00581742397116084
	0.0124075294086138	-3.52521614200276
COMP	2.04106073446328	5.67983532338308
2.78603774755812	0.00584880543018142
0.0124676539800254	-3.53008157184944
BEX1	-1.68843601694916
6.25117661691542	-2.78055554487996	0.00594467249068219
	0.0126562404058597	-3.54478166638903
FGF3	1.65195663841808	1.65128308457711
2.77698968689004	0.00600779196872275
0.0127704957922825	-3.55432885298599
UMODL1	1.40365713276836	3.33857711442786

2.77684881141918	0.00601029806025172
0.0127734388381954	-3.55470579852188
LOC100132354	1.17855473163842	1.5930776119403
2.77664397201491	0.00601394372545206
0.0127772614360051	-3.5552538630668
GZMK	-1.21306052259887
5.22856169154229	-2.77117288930352	0.00611206208284095
	0.012973597452515	-3.56987834314534
RASL11B -1.05143396892655
7.07402288557214	-2.76972949015548	0.0061381890297876
	0.0130169425453506	-3.57373217027468
RNF212	-1.69273396892656
4.05962935323383	-2.76858216342393	0.00615902901540922
	0.0130557424559991	-3.57679416792226
GRIA2	-1.95136652542373
5.89867412935323	-2.76748932852502	0.00617893888477584
	0.0130925395091803	-3.57970964488642
C6orf222	1.02087330508475	1.70223631840796
2.76646679794478	0.00619762076749546
0.0131267052880101	-3.58243659802856
SST	2.37187733050847	4.75211393034826
2.76551678238621	0.00621502368518816
0.0131567783312964	-3.58496932698083
HOXB8	1.66179604519774	8.74753930348259
2.76517046399505	0.00622137875640782
0.0131675160689745	-3.58589240688716
MSLNL	1.04902959039548	1.65026815920398
2.76276428907098	0.00626569595400295
0.0132517500706377	-3.59230288939648
RXFP4	1.04465218926554	1.66271343283582
2.7623586122039 0.00627319589795245
0.0132648791621139	-3.59338317631435
KRTAP3-1	1.14878778248588	1.31751492537313
2.76105589202621	0.00629733499754775	0.0133076981471596
	-3.596851228412
ME1	-1.11714731638418	6.5199184079602 -2.76044335565229
	0.00630871424166585
0.013327629489751	-3.59848137568506
STYK1	1.14020021186441	5.25388606965174
2.75233666923802	0.00646108057621983
0.0136286450122992	-3.6200241905872
SPINK1	1.42231391242938	2.03575621890547
2.75129316066211	0.00648093389521707
0.0136661434746104	-3.6227929588275
MUC4	1.60653382768361	6.22598805970149
2.74982764436997	0.00650890949321404
0.0137152737242825	-3.62667980806523
MYO3B	1.30168036723164	5.06376417910448
2.74873512116587	0.00652983608609571
0.0137551338854448	-3.62957615440968
GJB2	1.26955388418079	7.60522786069652
2.7485166135937 0.00653402876979658
0.0137625536723016	-3.63015530342652
PROK2	1.22437535310734	2.18561044776119

2.74607560457231	0.00658103252478574
0.0138530296046192	-3.63662223707125
C9orf70 1.05763382768362	2.07702835820896
2.74485358939146	0.00660467837614412
0.0138999535686559	-3.6398577052345
MYT1	1.32661779661017	2.36866567164179
2.73538385295244	0.00679054178399519
0.0142604155917984	-3.66488497707066
HTR6	1.02110303672316	1.43444278606965
2.73479718336325	0.00680221070747645
0.0142819989593467	-3.66643282896489
FBN2	1.67225953389831	8.32221592039801
2.73278296879541	0.0068424119535444
0.0143605313234785	-3.67174472979913
S100A1	1.63051490112994	8.3878223880597 2.73174185341783
	0.0068632755438262
0.0144013743780409	-3.6744889431873
MMP17	-1.17998206214689
5.44226965174129	-2.73105108075909	0.00687715014922997
	0.0144290130366573	-3.67630917388678
CRISP3	-2.06632535310735
3.84945422885572	-2.73090481151156	0.00688009131466598
	0.0144337088381456	-3.67669454803341
SFTPB	1.44109646892655	1.79958358208955
2.72815410121122	0.00693561473715319
0.0145397907233488	-3.68393825063452
ZMYND10 1.48441398305085	7.72566218905473
2.72534893784104	0.00699265463522431
0.0146503928626549	-3.69131837273427
PCDH8	-1.00764724576271
1.44169800995025	-2.72498837991719	0.00700001688604503
	0.014664321088827	-3.69226645580855
FAM110C 1.13109512711865	5.09110149253731
2.72439929616374	0.00701206050899891
0.0146865539940571	-3.6938151945295
MUC6	-1.91351059322034
5.02157661691542	-2.72420402785721	0.00701605684049574
	0.0146934251693744	-3.69432849876527
HBB	-1.20509668079096	7.767207960199	-2.7190963068672
	0.00712132533984084
0.0149000876325863	-3.70774309779146
EPHB1	-1.23686214689266
4.99722288557214	-2.71906402748824	0.00712199512608717
	0.0149000876325863	-3.70782780001549
CAPN6	-1.83355550847458
7.80566815920398	-2.71796891445638	0.0071447521370795
	0.0149426334655378	-3.71070086195481
MCTP1	-1.07629060734463	4.5912592039801 -2.71435144640253
	0.00722039316747042
0.0150874983947244	-3.72018375970008
ODZ1	-1.60015960451977
3.80052089552239	-2.71228987939881	0.00726382360671997
	0.0151720723009422	-3.72558274289075
NXF2	2.05581278248588	3.04634676616915

2.71168323998598	0.00727664840348668
0.0151973135018903	-3.72717072926717
COL4A4	-1.3037947740113
5.12875721393035	-2.71074341338674	0.00729655744528499
	0.0152373435410132	-3.72963024075464
CDH17	-1.13217598870057	2.2548592039801	-2.71036830814514
	0.007304517316455
0.0152493126405147	-3.73061166418428
FABP6	1.70014378531073	5.03882487562189
2.70617799507861	0.00739397243401508
0.0154219500223844	-3.74156660057789
COL4A3	-1.47692916666667
3.04208308457711	-2.70406950953793	0.00743935793666513
	0.0155024379252675	-3.74707295983758
ASPG	-1.39392365819209
3.13256915422886	-2.70286945642659	0.00746530154651299
	0.0155485538219563	-3.75020514575455
LTB	1.22215755649717	7.43807412935323
2.69993376986286	0.0075291122603306
0.0156640327021268	-3.75786195685059
TCAM1P	1.33641334745763	1.88214577114428
2.69800887165629	0.00757121929457117
0.0157420632081777	-3.76287824797224
SPRR3	1.66198163841808	1.74481940298507
2.69475493688008	0.00764288285184765
0.0158782012698272	-3.77135045034549
HOXA7	-1.3899781779661
6.37599353233831	-2.69440537191496	0.00765061787741216
	0.0158878398769241	-3.77226004000412
FIGN	1.08801299435029	5.02680099502488
2.69433238984672	0.00765223368549356
0.0158895881018694	-3.77244993001666
CSAG3	1.37708700564972	2.63852537313433
2.69418021582163	0.00765560377855319
0.015894978314492	-3.77284585199797
TP53AIP1	1.17504131355932	3.74055870646766
2.69393977934725	0.00766093129104144
0.0159044311260068	-3.77347137029502
FOLR3	1.34773474576271	1.91632786069652
2.69274770533375	0.00768739435527014
0.0159513044384168	-3.77657189734041
PCDHGA10	-1.29007132768362
4.54992537313433	-2.691761682314	0.0077093455988218
0.0159936201640877	-3.7791355315161
LUZP2	-1.00270021186441
1.76252935323383	-2.68984319364381	0.00775221802349603
	0.0160760642446842	-3.78412105021202
L3MBTL4	-1.09341271186441
6.33469402985075	-2.6894945548936	0.00776003211776228
	0.0160873936562314	-3.7850266923107
FMO6P	1.36100028248588	1.79780945273632
2.68859338438724	0.00778026314640667
0.0161260779803866	-3.78736711377537
CPNE7	1.15178912429379	5.77185373134328

2.68490151815886	0.0078636425231932
0.0162808169626015	-3.79694760914433
LOC731789	1.21943834745763	1.50737960199005
2.68426575487806	0.00787808211434205
0.0163041356660728	-3.79859619663746
ZNF257	-1.36356433615819	4.0668368159204 -2.68368001760974
0.00789140667773677
0.016326054719932	-3.80011474105927
SCGB1D1 1.43837323446328	1.81683134328358
2.68366035970779	0.00789185421532419
0.016326054719932	-3.80016569951329
TPPP3	-1.42495324858757
9.19811542288557	-2.68167776644582	0.00793710833344967
0.0164080992936987	-3.80530331935188
C20orf103	-1.51050734463277
6.64048507462687	-2.68163285853147	0.00793813609538974
0.0164085716913362	-3.80541965117178
CHRNA3	-1.1799490819209
3.51596119402985	-2.68076969778899	0.00795791373013073
0.0164477971287208	-3.80765527588676
DGCR5	1.26459343220339	3.2476263681592 2.68045050358088
0.00796523869204854
0.016461017845111	-3.80848183349162
PLA1A	-1.19533622881356
6.43453631840796	-2.68042109405943	0.00796591389630674
0.016461017845111	-3.8085579852275
CCBP2	1.08614081920904	6.62302736318408
2.67881515265395	0.00800286271073273
0.0165307145036063	-3.81271515980178
SALL4	1.29643241525424	4.03469701492537
2.67618163995911	0.0080637882072273
0.0166448393217752	-3.81952730957095
CPLX3	1.28406066384181	2.13920248756219
2.67543577386147	0.00808111944680206
0.0166772599761607	-3.82145552040554
EVPLL	1.21479548022599	2.20284527363184
2.67531876653273	0.00808384132192541
0.0166799299499838	-3.82175796201808
CDSN	1.03285692090395	4.8666223880597 2.67151478297881
0.00817278288573348
0.0168444164887168	-3.83158382732339
JAKMIP1 1.11383912429379	3.8256184079602 2.6713485291932
0.00817669014148585	0.0168507778161719	-3.83201297172765
CGA	1.09002316384181	1.23163781094527
2.66754393809522	0.00826656625530235
0.0170249085843396	-3.84182681951513
PNOC	1.89245925141243	4.31540646766169
2.66751506258156	0.00826725177542245
0.0170249085843396	-3.8419012533919
RGS6	-1.17821426553672
4.32199303482587	-2.66577645100823	0.00830862189847882
0.017105496146788	-3.84638157823198
ACADL	-1.51390812146893
3.71149452736318	-2.6623906388593	0.00838972295522677

0.0172568893167824	-3.85509885939514
CELF4	1.15746836158192	3.8187631840796 2.66028441335072
0.00844053304334867
0.0173527081293759	-3.86051644730132
APLP1	1.15506278248588	7.42056567164179
2.66021507629852	0.00844221041813392
0.0173544187327816	-3.86069472668058
RHCE	1.00208305084746	3.58379800995025
2.65668833595163	0.0085279251130037
0.0175165885512722	-3.86975696995411
TF	-1.12001666666667	3.6904	-2.65380175748811
0.0085986637261769	0.0176442345125849	-3.87716593087688
SMOC1	-1.6213104519774
5.40436218905473	-2.64929181219714	0.00871024289621537
0.0178535629651859	-3.88872655321326
CT45A5	1.33245197740113	1.21945323383085
2.64502009404797	0.00881712856816482
0.018052822330151	-3.89965961381697
CHIT1	1.44791581920904	4.25129701492537
2.64423748531882	0.00883683824871687
0.0180895691740111	-3.90166084480076
RALGPS2 -1.05068742937853
7.61064278606965	-2.64346563954301	0.00885631570126127
0.0181222128323427	-3.90363401280868
SLC6A14 1.67012528248587	4.35373233830846
2.64151726323788	0.00890565477039534
0.0182119177515495	-3.90861250736524
CT45A1	1.64086320621469	1.8687184079602 2.63458265571092
0.00908327422855212
0.0185293739976327	-3.92630405736539
NTN1	-1.04229971751412	8.9969039800995 -2.63384657425109
0.00910231367980738
0.0185626800284539	-3.92817940002603
LIX1	1.73946320621469	3.97887860696517
2.63220075789569	0.00914501420893869
0.0186405025525726	-3.93237074057519
HAVCR1	1.17368644067797	1.4671447761194 2.63072101334206
0.00918355973187164
0.0187153545061867	-3.9361370657538
OLFM2	-1.0599488700565
8.58362885572139	-2.63066214105641	0.00918509629984948
0.0187166279936387	-3.9362868697867
PDE6G	1.10361207627119	3.97163333333333
2.63029683968438	0.00919463584279489
0.0187342074089499	-3.93721633094999
CALHM3	1.07940190677966	1.98454726368159
2.6267115556976 0.0092887364461476
0.0188996788146095	-3.94633222228569
KCNG3	1.03143001412429	3.85972835820896
2.62491065854691	0.00933632931502123
0.0189889876002958	-3.95090678002342
C11orf92	-1.30946716101695
6.051507960199	-2.62259571602513	0.00939782894965636
0.0190989337098045	-3.95678279202718

KLK3	-1.14798100282486
1.37816218905473	-2.61990746464732	0.00946970275646843
	0.0192259689072683	-3.96360030092619
LRG1	1.23971631355932	8.74253631840796
2.61871966842949	0.00950161703554004
0.0192846315877684	-3.96661051904066
HSD17B2 1.26153057909604	4.13268756218906
2.61786303390145	0.00952469344827244
0.0193185098293848	-3.9687806870834
EYA4	-1.6085302259887
2.97831641791045	-2.61711270970781	0.00954494737913826
	0.0193557654774505	-3.97068098770814
CAMK2B	1.28551765536723	5.37389800995025
2.61617023283532	0.00957044301915856
0.019405550141563	-3.97306722122294
ADAMTS16	-1.46309187853108
4.04176815920398	-2.61544116827738	0.00959020743400972
	0.0194398655825651	-3.97491257102977
IGDCC3	-1.31948820621469
3.71357512437811	-2.61352179896157	0.00964241559236745
	0.0195360497494625	-3.97976842870074
TMEM130 -1.01091299435028	4.8680263681592 -2.60623395943832
	0.00984298245301202
0.0199011650345023	-3.99817578780708
RSPO4	1.64059491525424	4.16836965174129
2.60451830716608	0.0098907397625578
0.0199878814179852	-4.00250213842242
FBLN1	-1.01769985875707
13.3987248756219	-2.60374839543951	0.0099122387421462
	0.0200293564346714	-4.00444275535255
EPS8L3	1.66290564971751	3.02724975124378
2.60141609485805	0.00997762201537097
0.0201436306397449	-4.01031821257239
C1orf173	-1.79321744350283
5.0794855721393 -2.59754431796432	0.010087017615582
0.0203444809556831	-4.02006100702622
CXCL6	1.35179420903955	3.19794427860697
2.59518578883242	0.0101541832109739
0.0204658728962589	-4.02598927508922
OTX2	1.31327019774011	1.37870348258706
2.59326013412477	0.0102093187094655
0.0205648855087643	-4.03082576174294
NRAP	1.32794484463277	2.87264676616915
2.58774053065345	0.0103688473263244	0.020859621323806
	-4.04467023692986
CAPN8	1.30246998587571	3.09709701492537
2.58199422910854	0.0105372990321771
0.0211777532188542	-4.05905402001952
CDH12	-1.00296850282486
3.04161393034826	-2.58145527446645	0.0105532235036767
	0.02120560619854	-4.06040156374229
MX1	1.00368912429378	10.9709373134328
2.57903735176522	0.0106249314993572
0.0213392529526318	-4.06644383730178

MATK   -1.02244865819209
5.20461393034826   -2.57303990667373   0.0108046848250365
        0.0216790636686864   -4.08140830278227
HOXA3   -1.29778163841808
6.73756069651741   -2.56760977559393   0.0109697790806514
        0.0219888268429772   -4.09492911946264
SAGE1   1.22568086158192   1.18005174129353
2.56552156203456   0.0110338666504418
0.0221043405868042   -4.10012157333815
BMP5   1.10092351694915   1.1305407960199 2.56447851672804
        0.011066003197598
0.0221622325282107   -4.10271367994643
TMEM145 1.01603057909604   2.68283432835821
2.56430538759449   0.0110713454588616
0.0221686064830225   -4.10314383343876
CYP26A1 1.20340593220339   3.14056567164179
2.56236004418101   0.011131531978714   0.0222717426686482
        -4.10797532929616
LDOC1   -1.11256221751413
9.28996019900497   -2.55802723086766   0.0112666374003911
        0.0225069636888225   -4.11872405738204
FAM83E 1.29011998587571   6.54153830845771
2.55345175305994   0.0114109003746039   0.02277078000492
        -4.1300562882706
PCDHA2 -1.0621718220339
3.43340049751244   -2.55162997930548   0.0114687984880422
        0.0228685346697268   -4.13456304400012
OLIG3   1.01908361581921   1.40093532338308
2.548566887125 0.011566739531792
0.0230326684697174   -4.14213381639973
ALDH1L1 -1.32338283898305
5.77499552238806   -2.54796797414535   0.0115859766794577
        0.0230618674839313   -4.14361310060031
TLX1   1.07394435028249   1.18032885572139
2.54704597375141   0.0116156473396449   0.0231074844847781
        -4.1458897573573
ST8SIA2 1.36630995762712   4.28977263681592
2.54649873519388   0.0116332899887319
0.0231336152139351   -4.14724066540069
SFTA2   1.70561949152542   4.22408009950249
2.54290211503859   0.0117498401357473
0.0233495516394243   -4.15611248029563
OTOS   1.09555946327684   1.25288805970149
2.53866994444059   0.0118883211311692   0.0236047472886681
        -4.1665369631941
RPSAP52 1.06827429378531   3.01090248756219
2.52814056376553   0.0122391997779334
0.0242328837343016   -4.19240180300266
CD164L2 1.28018700564972   4.95402885572139
2.52737137554916   0.0122651907551907
0.0242786543131324   -4.19428732023792
MORN5   -1.56378905367232
4.58296119402985   -2.5273514495421   0.0122658647119245
        0.0242786543131324   -4.19433615786821

ADAMTS17 -1.00822337570622
5.49799900497512 -2.52721389441023 0.0122705181368015
0.0242855237008131 -4.19467328865203
SHISA3 -1.1100154661017
2.27529452736318 -2.52592866390192 0.0123140730779227
0.0243670284616629 -4.19782239963847
CTCFL 1.26903778248588 5.02702338308458
2.52345502783899 0.0123982902032285
0.0245203075307274 -4.20387914920912
SLITRK4 -1.4358884180791 3.3340776119403 -2.52310369921567
0.0124102930873048
0.0245408704567874 -4.20473893323067
SUSD4 1.2208677259887 7.01863830845771 2.52219400591541
0.0124414202864086
0.0245929480531455 -4.20696464549135
TNNC2 -1.00145028248588 3.8802592039801 -2.52006926395071
0.012514394625032
0.0247229139387901 -4.21216023822663
C14orf73 1.11626617231639 3.85026815920398
2.51709769981586 0.012617093531082 0.0249162110612845
-4.21941965898034
PSORS1C2 1.1493490819209 2.49172587064677
2.51502297213796 0.0126892422348507
0.0250466438882304 -4.22448337686142
LIMS3 -1.44028495762712
6.23886019900498 -2.51430671483188 0.0127142353998636
0.0250911518561185 -4.22623061201336
PCYT1B -1.23060155367232 5.4802776119403 -2.51329230136571
0.0127497075702763
0.0251514842334749 -4.22870436891717
IHH -2.1337186440678
5.79432039800995 -2.51294866732257 0.0127617438139404
0.0251679731508353 -4.22954214523781
PALM3 1.16755550847457 7.37568756218905
2.51137070060137 0.0128171443936683
0.0252699485644877 -4.23338783167685
DPY19L2P2 -1.07474548022599
4.56109900497512 -2.5081270247771 0.0129316999260603
0.0254688986456272 -4.24128592164997
C1orf95 -1.19978615819209 4.4507552238806 -2.5077123087819
0.0129464118808563
0.0254926130082563 -4.24229503086835
C14orf105 -1.47953269774011
2.93371542288557 -2.50510483560459 0.0130392536193088
0.0256585715330595 -4.2486360854504
HMGCS2 1.31155077683616 2.55889353233831
2.49970109658862 0.0132335502277124 0.026001032268632
-4.26175759421911
XAGE2 1.37167507062147 1.33210547263682
2.49905975310808 0.0132567807075717
0.0260367079237011 -4.26331315569052
GLB1L3 -1.01982153954802
1.54829402985075 -2.4984041230748 0.0132805662065808
0.0260717502335619 -4.26490298129621

GLYATL2 1.98585296610169 4.90706119402985
2.49597485841996 0.0133690287839578 0.0262245519715893
-4.2707902464271
DPP4 -1.44552655367232 7.3551736318408 -2.49215635860936
0.0135091417426716
0.0264741007996418 -4.2800334148197
LHFPL3 -1.06256165254237
2.36002388059701 -2.48359239473644 0.0138281444876633
0.0270527736165168 -4.30071523064013
SLC6A20 1.23310437853108 6.5392263681592 2.47969801334869
0.0139754117603893
0.0273200536508086 -4.31009795686695
IGLON5 1.24933347457627 3.35663432835821
2.47858317766136 0.0140178256402653 0.0273899267530833
-4.3127813800733
NKX2-1 1.51918926553672 1.52772686567164
2.47788126832585 0.0140445884402609
0.0274343863633259 -4.31447030246144
ENPP3 -1.76247309322034
5.07317562189055 -2.4759845272446 0.0141171358815179
0.0275734753731865 -4.31903195896923
AP3B2 -1.32539964689265
4.22116119402985 -2.46993486009293 0.0143507574521115
0.0279978191097453 -4.33355944214985
LOC100190940 1.34955374293785 2.69000049751244
2.46658443192623 0.0144816146803927
0.0282343351745927 -4.34159067372174
PLAG1 -1.17373100282486 5.7405368159204 -2.466225184161
0.014495708509778 0.0282564466108757 -4.34245120922721
DIRAS2 1.02553877118644 4.88689054726368
2.46586617200995 0.0145098052680929
0.0282758711109584 -4.34331106254594
CYP4Z1 1.05847330508475 2.27891343283582
2.46432235643867 0.0145705626965791
0.0283834948439861 -4.34700724140092
JPH3 -1.28291984463277
3.52191044776119 -2.46045352732504 0.0147238157537121
0.0286412675216704 -4.35626036270929
SLC14A2 1.14907330508475 1.87462885572139
2.45740515360625 0.0148455752158933
0.0288507818233898 -4.36354155501173
HOXC6 -1.44519138418079
4.42997711442786 -2.4548352384513 0.0149489174720039
0.0290213972293372 -4.36967332503223
STK32B -1.0761613700565
5.57499800995025 -2.44919717236149 0.0151778771015544
0.0294213782357579 -4.38310448622061
CHST4 1.14464901129944 3.76176616915423
2.44903715069231 0.0151844206785812
0.0294312389655598 -4.38348527005447
BMP3 1.41996603107345 4.74353034825871
2.44884773611241 0.0151921694197239
0.0294379649067419 -4.38393596629834
PRPH 1.39189837570621 4.63438905472637

2.44860808789157	0.015201978190485	0.0294514119326845
	-4.3845061422807
C1orf87 -1.52081553672316
2.48945771144279	-2.44510703278404	0.0153459178672733
	0.0297050437313898	-4.39282993360878
ADD2	1.22449555084746	5.54480796019901
2.44076148395312	0.0155262578169208
0.0300258169726247	-4.40314591522448
CLGN	1.19873206214689	7.01205820895522
2.43323089560731	0.0158432295580905
0.0305926921395409	-4.42098198719117
CHGA	1.99918665254237	6.86884029850746
2.42155789084004	0.0163458976272983
0.0314656613491265	-4.44852663986104
MOXD1	-1.19472351694916	8.8901592039801 -2.41938105710864
	0.0164411838335079
0.0316283268461317	-4.45364947417656
NKX6-1	1.03278206214689	1.84777313432836
2.41912798350217	0.0164522934219144	0.031646733247625
	-4.45424476105617
C13orf30	-1.85355875706215
3.90632835820896	-2.41290292931225	0.0167276645499513
	0.0321372780470241	-4.46886902061709
CCDC60	-1.35602563559322
2.07785870646766	-2.41031471411542	0.0168433514501678
	0.0323197281647101	-4.47493894609502
FXYD4	1.30540211864407	2.40679154228856
2.41028528780963	0.0168446707921285
0.0323197281647101	-4.47500792182846
ANKRD36BP1	-1.03253418079096
5.05387014925373	-2.40741418690056	0.0169738382178085
	0.032543229231566	-4.48173400832553
SDR42E1 -1.31850762711864
5.29904278606965	-2.40671045606391	0.0170056315564116
	0.0325950532959696	-4.48338147422877
TMEM215 -1.16593403954802
4.10654029850746	-2.40191464525559	0.0172237033844772
	0.0329828424247914	-4.49459658319279
TMEM108 -1.24676398305085
5.06239502487562	-2.39677582479223	0.0174601116370518
	0.0333944561122298	-4.50659039597935
PHYHD1	-1.05458086158192
8.91358507462687	-2.3896966835672	0.017790475724341
	0.0339661923761482	-4.52307312988509
TMEM146 -1.24104816384181
1.93679502487562	-2.38961279025468	0.0177944236587344
	0.0339666635629129	-4.52326818705627
DOK5	-1.09584505649718
4.92711044776119	-2.38866417869205	0.0178391182046989
	0.0340432326401253	-4.52547331801505
FSTL5	1.11850466101695	1.71626019900498
2.37811666666748	0.0183427883541707	0.0349200152922038
	-4.5499362184084
HOXA10	-1.13129378531074

9.31361940298507	-2.36973745506007	0.0187518141105594
	0.0356128347539116	-4.569297259287
CCDC78	1.25227337570621	6.98882736318408
2.36509964793421	0.0189816441123545
0.0359960422710731	-4.57998562604736
FCRL2	1.00223785310735	2.49710099502488
2.36348760117374	0.0190621095154912	0.0361386192956907
	-4.5836961396115
MAGED4B	-1.00738672316385
8.80454825870647	-2.36337288840827	0.0190678468435862
	0.0361428211336741	-4.58396008748771
OCA2	1.10240338983051	4.24727313432836
2.3625834894543	0.0191073696838698
0.0362084826350676	-4.58577612335924
ZDHHC8P1	-1.00954724576271
5.58542487562189	-2.36257532355997	0.0191077789024999
	0.0362084826350676	-4.58579490624554
PTX3	-1.10728587570622
4.60892736318408	-2.36015398770402	0.0192294606626386
	0.036415536150168	-4.59136166519132
UNC93A	1.09538721751413	3.12959054726368
2.35985302163357	0.0192446329827717
0.0364409071330293	-4.59205322244315
LRRC4C	-1.12097139830509
4.41484676616915	-2.35961916630974	0.0192564293882839
	0.0364585125733924	-4.59259051562188
DHRS2	1.32698877118644	4.01619651741294
2.35547346662044	0.0194666111133278
0.0368103091237561	-4.60210708896496
MS4A1	-1.10195423728814
3.05825572139304	-2.35110658784131	0.0196901875637988
	0.0371919726455781	-4.61211426726502
HOXD13	1.19868290960452	1.83324825870647
2.34940326985854	0.0197780050508005
0.0373372362016492	-4.61601284354415
HOXA4	-1.28255303672316
5.08824179104478	-2.34193119274268	0.0201673245928405
	0.038019758851333	-4.63308344199656
FAM21A	-1.11107605932203
7.39765223880597	-2.33419490322314	0.0205774938115267
	0.0387361037915499	-4.65070342499074
MAGEA4	1.59441468926554	1.7700368159204	2.33343164543564
	0.0206183553618666
0.0388094650755589	-4.6524388108596
ANKS4B	1.07142761299435	1.51100845771144
2.33238624042967	0.0206744375324834
0.0389114597123106	-4.65481483048995
DLGAP3	1.08019456214689	4.23794527363184
2.33031831840124	0.0207857688207379	0.0390815856662468
	-4.659511879887
ADCY1	-1.10247881355932
6.67081044776119	-2.33015113340162	0.0207947925675859
	0.0390949717410749	-4.65989144920559
CCDC39	-1.10703968926554

4.13353582089552	-2.32927315387588	0.0208422376761026
	0.0391734083576294	-4.66188435119394
CYP2B7P1	-1.18743735875706
3.20672537313433	-2.32736366073718	0.0209457530292217
	0.0393571581264802	-4.66621620246258
CXorf30 1.26854724576271	4.01854975124378
2.3239701522345 0.02113083250367
0.0396613632565685	-4.67390636821056
C3orf55 1.41805670903955	2.83656567164179
2.32241668648511	0.0212160352816457
0.0398012155719309	-4.67742319277697
CCDC19	1.23073036723164	7.32403582089552
2.32239957781749	0.0212169753151102
0.0398012155719309	-4.67746191195504
MEGF11	1.12823608757062	4.4102671641791 2.32130886577622
	0.0212769798393815
0.0399064860316556	-4.67992977987432
SERPINA1	1.21050748587571	11.006944278607
2.32093924766798	0.0212973477970672	0.039941038665734
	-4.68076583640575
CASQ1	-1.05135014124294
2.94252587064677	-2.31796691122018	0.0214617625483535
	0.0402273336179766	-4.68748451804979
P2RX2	-1.04078524011299
2.32267512437811	-2.31464817467814	0.0216466531366224
	0.0405183979663438	-4.69497656434847
C2orf65 1.17819350282486	5.64163034825871
2.31307933643802	0.0217345401005
0.0406532533038689	-4.69851467512348
HOXA2	-1.12541320621469
4.17816865671642	-2.30771989805171	0.0220371425830708
	0.0411667458808411	-4.71058435785532
TCTE1	-1.11921009887006
2.56584776119403	-2.30160915078037	0.0223866646373072
	0.0417626737849993	-4.72431363569299
NLRP2	1.89712379943503	7.3213223880597 2.29842385607292
	0.022570774921493
0.0420688560615785	-4.73145648822326
LOC90246	-1.01236560734463
6.87209303482587	-2.29841508932993	0.0225712834641153
	0.0420688560615785	-4.73147613422951
SLC47A1 -1.3006511299435
8.15410049751244	-2.29767412566447	0.0226143016797819
	0.0421413827254173	-4.73313635459085
ARMC4	-1.17828050847458
4.64215970149254	-2.29625909862086	0.022696653563842
	0.0422871674077209	-4.73630548645052
CLDN22	1.56595649717514	1.64127661691542
2.29469988458096	0.0227877007917756
0.0424413950154362	-4.73979539898937
IDO2	1.04831631355932	2.70789004975124
2.28896110301651	0.023125565153597	0.0430160236117918
	-4.75262086369822
PRSS50	1.37002330508475	4.34551343283582

2.28736341660241	0.023220403670454	0.0431767853776307
	-4.75618607257586
ECHDC3	-1.06515176553673
6.69525970149254	-2.2862585084271	0.0232861897589738
	0.0432873484020041	-4.75865027399051
STAC2	-1.21540974576271
4.27890547263682	-2.28471602479711	0.0233783016551856
	0.0434379957676525	-4.76208848062823
ALX1	-1.02939322033898
2.18898606965174	-2.28450679264742	0.0233908208016772
	0.0434543073982837	-4.7625546909306
OVGP1	-1.57853248587571
7.16636567164179	-2.28429863875446	0.0234032812492394
	0.0434656562372793	-4.76301845846056
SNTN	-1.71044251412429	4.2530144278607 -2.28390988367101
	0.0234265683375173
0.0435049704178408	-4.76388449862658
GCNT3	1.15152951977401	6.14497512437811
2.27917874665405	0.0237115993986133
0.0439882979222712	-4.77441296899994
PCDHB3	-1.03401892655367
5.44816268656716	-2.27384390142016	0.0240366332998306
	0.0445130225701168	-4.78626004612348
CLDN10	-1.65556490112994
6.79953532338308	-2.2691781903621	0.0243240831345206
	0.0449764412097368	-4.79659957113718
AMBP	1.50525607344633	4.04856567164179
2.26782468628635	0.0244080309374789	0.045115411465422
	-4.79959525213374
SLC46A2 -1.11505416666667
3.32220746268657	-2.25852390672299	0.0249917606010129
	0.0460989079190292	-4.82013454776939
SLC15A1 1.1167843220339 6.20624527363184	2.25637442348517
	0.0251283849354209
0.0463395759291364	-4.8248699326153
FBN3	1.54002012711864	5.61387462686567
2.25306009623403	0.0253403250768499
0.0466915162293778	-4.83216311226354
CXCL3	1.12532747175142	4.85739751243781
2.25029897607282	0.0255180778574719
0.0469853038936231	-4.83823118001469
MAGEA2	1.09336228813559	1.43428656716418
2.24149014095178	0.0260924490163458
0.0479225244503113	-4.85754293087759
LOC723809	1.09251560734463	3.0929368159204
2.23589281860091	0.0264632389248615
0.0485427354474094	-4.86977660964544
DBC1	-1.13486052259887
2.74325771144279	-2.23098782100804	0.0267919284584856
	0.0490522684900287	-4.88047320573322
FUT9	-1.14066588983051
2.91146218905473	-2.22533204871028	0.0271753319884643
	0.0496891312542903	-4.8927793164904
UGT8	1.01764046610169	6.57093532338308

2.22336881558638	0.0273095300575314
0.0498989544394019	−4.89704406781519
