# Supplementary material for: Identification and validation of hub genes in uterine corpus endometrioid carcinoma: An observational study from TCGA and GEO
Source: Medicine (Baltimore). 2025 May 2;104(18):e42338. doi: 10.1097/MD.0000000000042338 (PMC12055170; doi:10.1097/MD.0000000000042338)
Supplement: Supplementary file 4 [file medi-104-e42338-s004.pdf]

Tag TCGA-A5-A2K7-01 TCGA-EO-A3AS-01 TCGA-EY-A549-01 TCGA-EO-
A3B0-01 TCGA-KJ-A3U4-01 TCGA-EY-A2OQ-01 TCGA-PG-A7D5-01 TCGA-B5-
A0JN-01 TCGA-EO-A3KW-01 TCGA-PG-A915-01 TCGA-D1-A3JQ-01 TCGA-AX-
A2HH-01 TCGA-AJ-A5DV-01 TCGA-5S-A9Q8-01 TCGA-DI-A2QY-01 TCGA-DI-
A1BU-01 TCGA-B5-A5OE-01 TCGA-AX-A3G3-01 TCGA-PG-A916-01 TCGA-AJ-
A3NC-01 TCGA-DF-A2KY-01 TCGA-AX-A3FV-01 TCGA-QS-A5YR-01 TCGA-DF-
A2KZ-01 TCGA-A5-A2K2-01 TCGA-AX-A3FZ-01 TCGA-B5-A0JR-01 TCGA-AJ-
A3TW-01 TCGA-PG-A914-01 TCGA-AX-A3GI-01 TCGA-D1-A2G0-01 TCGA-EY-
A2OO-01 TCGA-A5-A7WJ-01 TCGA-AX-A3G9-01 TCGA-BK-A4ZD-01 TCGA-B5-
A3FC-01 TCGA-AP-A3K1-01 TCGA-EY-A3QX-01 TCGA-AX-A05W-01 TCGA-AJ-
A3BI-01 TCGA-EY-A2ON-01 TCGA-AX-A3FS-01 TCGA-SL-A6JA-01 TCGA-B5-
A0K9-01 TCGA-AX-A2H4-01 TCGA-AJ-A3EJ-01 TCGA-EY-A1GP-01 TCGA-EO-
A3KX-01 TCGA-AJ-A3BH-01 TCGA-D1-A3JP-01 TCGA-AJ-A3NF-01 TCGA-FI-
A3PV-01 TCGA-EY-A54A-01 TCGA-EO-A22U-01 TCGA-D1-A3DH-01 TCGA-SJ-
A6ZI-01 TCGA-DF-A2KR-01 TCGA-AJ-A3NH-01 TCGA-EY-A1GL-01 TCGA-AX-
A3G4-01 TCGA-SL-A6J9-01 TCGA-BG-A3EW-01 TCGA-A5-A2K3-01 TCGA-B5-
A5OD-01 TCGA-B5-A5OC-01 TCGA-EY-A1GX-01 TCGA-E6-A8L9-01 TCGA-BG-
A0MK-01 TCGA-AX-A3GB-01 TCGA-E6-A2P8-01 TCGA-QF-A5YT-01 TCGA-5B-
A90C-01 TCGA-AJ-A3OK-01 TCGA-AX-A3G1-01 TCGA-SJ-A6ZJ-01 TCGA-K6-
A3WQ-01 TCGA-EY-A3L3-01 TCGA-AJ-A3BK-01 TCGA-KP-A3W0-01 TCGA-AX-
A2IN-01 TCGA-AJ-A3QS-01 TCGA-EY-A210-01 TCGA-DI-A1C3-01 TCGA-AJ-
A3EL-01 TCGA-A5-A3LP-01 TCGA-EO-A22Y-01 TCGA-AX-A3FT-01 TCGA-A5-
A2K5-01 TCGA-DF-A2KN-01 TCGA-AJ-A2QM-01 TCGA-AX-A3FX-01 TCGA-AJ-
A3I9-01 TCGA-AJ-A3EM-01 TCGA-KP-A3W4-01 TCGA-PG-A5BC-01 TCGA-B5-
A3FD-01 TCGA-E6-A2P9-01 TCGA-B5-A3FB-01 TCGA-QS-A8F1-01 TCGA-FI-
A2EY-01 TCGA-BK-A139-02 TCGA-EO-A3KU-01 TCGA-B5-A11R-01 TCGA-B5-
A1MS-01 TCGA-A5-A3LO-01 TCGA-KP-A3W1-01 TCGA-A5-A1OH-01 TCGA-AJ-
A3OJ-01 TCGA-A5-A2K4-01 TCGA-AJ-A2QO-01 TCGA-EY-A4KR-01 TCGA-BG-
A3PP-01 TCGA-AX-A1C7-01 TCGA-A5-A7WK-01 TCGA-AX-A3FW-01 TCGA-AJ-
A8CV-01 TCGA-AP-A5FX-01 TCGA-JU-AAVI-01 TCGA-EO-A3AZ-01 TCGA-DF-
A2KU-01 TCGA-BS-A0V7-01 TCGA-BK-A6W4-01 TCGA-EO-A1Y7-01 TCGA-BK-
A13B-01 TCGA-QS-A5YQ-01 TCGA-EO-A3AU-01 TCGA-AJ-A3NE-01 TCGA-B5-
A3FH-01 TCGA-B5-A3FA-01 TCGA-AX-A3G8-01 TCGA-A5-AB3J-01 TCGA-DF-
A2L0-01 TCGA-4E-A92E-01 TCGA-AJ-A3BF-01 TCGA-EY-A2OP-01 TCGA-AJ-
A23N-01 TCGA-QS-A744-01 TCGA-2E-A9G8-01 TCGA-PG-A6IB-01 TCGA-AJ-
A3BG-01 TCGA-EO-A3AV-01 TCGA-B5-A3F9-01 TCGA-QF-A5YS-01 TCGA-EY-
A72D-01 TCGA-AJ-A5DW-01 TCGA-AX-A3G7-01 TCGA-AJ-A6NU-01 TCGA-EO-
A3L0-01 TCGA-AX-A3G6-01 TCGA-B5-A1MW-01 TCGA-FI-A3PX-01 TCGA-D1-
A3DG-01 TCGA-EY-A1GO-01 TCGA-BS-A0V4-01 TCGA-AJ-A3OL-01 TCGA-AJ-
A3NG-01 TCGA-AJ-A8CT-01 TCGA-EY-A5W2-01 TCGA-DI-A2QT-01 TCGA-KP-
A3VZ-01 TCGA-EY-A548-01 TCGA-B5-A3S1-01 TCGA-PG-A917-01 TCGA-EO-
A3B1-01 TCGA-AJ-A3BD-01 TCGA-DF-A2KV-01 TCGA-EO-A22X-01 TCGA-AJ-
A3EK-01 TCGA-D1-A3DA-01 TCGA-KP-A3W3-01 TCGA-AJ-A3IA-01 TCGA-AJ-
A8CW-01 TCGA-H5-A2HR-01 TCGA-EY-A547-01 TCGA-BK-A6W3-01 TCGA-BK-
A56F-01 TCGA-EO-A3AY-01 TCGA-FL-A1YI-11 TCGA-FL-A1YU-11 TCGA-FL-
A1YF-11 TCGA-DI-A2QY-11 TCGA-FL-A1YG-11 TCGA-AJ-A3NE-11 TCGA-AX-
A0IZ-11 TCGA-AJ-A3NC-11 TCGA-AX-A05Y-11 TCGA-FL-A1YQ-11 TCGA-BG-
A2AD-11 TCGA-AX-A2HC-11 TCGA-FL-A1YT-11 TCGA-FL-A1YH-11 TCGA-AJ-
A3NH-11 TCGA-AX-A0J0-11 TCGA-BK-A4ZD-11 TCGA-FL-A1YL-11 TCGA-BG-
A3PP-11 TCGA-AX-A2HD-11 TCGA-FL-A1YN-11 TCGA-FL-A3WE-11 TCGA-BG-
A3EW-11 TCGA-FL-A1YV-11
ASPA 0 0 0 0.9133 0.4059 0 0.4748 0
0.4291 0.4849 1.0422 0.7759 0 0 0.6221
1.9468 0 0 0 0.5262 0.6062 1.0809 1.021 0

| | | | | | | | | |
|---|---|---|---|---|---|---|---|---|
| | 0 | 2.2152 | 1.537 | 0 | 0 | 0.5853 | 0.6819 | |
| 1.5255 | 0.3648 | 0 | 0.7666 | 0.6649 | 0 | 0 | 0 | 0 |
| | 0 | 1.2792 | 0.4764 | 0.453 | 0 | 1.0956 | 0.4645 | |
| 0.5608 | 0 | 1.9711 | 2.1468 | 2.7727 | 3.2053 | 0 | 0 | 0 |
| | 1.0195 | 0 | 0 | 1.6464 | 0 | 0.9537 | 0 | |
| 1.569 | 3.6687 | 0 | 0.9546 | 1.2414 | 1.5048 | 1.8728 | 0 | 0 |
| | 3.2356 | 0 | 0.911 | 0.6199 | 0.5376 | 0 | 1.6807 | |
| 0.9113 | 1.1363 | 0 | 1.7895 | 1.2026 | 0 | 0.8845 | 1.2591 | 0 |
| | 0 | 0 | 1.583 | 0.8449 | 1.5391 | 1.0326 | 3.6341 | |
| 0.5877 | 0 | 0 | 0 | 0 | 2.1456 | 0 | 0 | |
| 0.7641 | 0 | 0 | 1.0794 | 0.5886 | 0 | 0 | 0.9214 | |
| 1.3754 | 0.7843 | 0 | 0.6684 | 0.9511 | 1.2511 | 0.8107 | 0 | 0 |
| | 0 | 4.9362 | 0 | 0 | 1.0973 | 0 | 0 | 0 |
| | 0 | 0 | 4.6276 | 0 | 0 | 2.4626 | 0.9437 | |
| 1.2732 | 0.3855 | 0 | 0 | 0 | 0.6138 | 1.3167 | 0.4033 | 0 |
| | 0.5023 | 0 | 2.7594 | 1.4679 | 0.5466 | 0 | 0.529 | |
| 2.214 | 0.6229 | 0 | 1.1272 | 0.7148 | 1.6765 | 0 | 1.1551 | 0 |
| | 0.8792 | 0 | 0.6248 | 0 | 0 | 0 | 0 | 0 |
| | 0 | 0 | 1.0222 | 0.8682 | 0 | 0 | 0.3736 | |
| 0.8824 | 0 | 3.2435 | 5.3088 | 2.904 | 2.5783 | 6.1345 | 5.5967 | |
| 6.4771 | 7.5431 | 6.3449 | 5.3558 | 7.4601 | 5.2055 | 5.0907 | 4.4239 | |
| 5.3925 | 6.0393 | 6.5633 | 5.5851 | 6.0876 | 6.6863 | 4.8449 | 5.4684 | |
| 7.1616 | 5.535 | | | | | | | |
| TCF23 | 0 | 0 | 0.4935 | 3.2977 | 0.7223 | 0.5431 | 1.1171 | 0 |
| | 0.4291 | 0 | 1.2705 | 0 | 0 | 0 | 0 | 0 |
| | 0 | 0 | 0 | 0 | 1.0317 | 0 | 0 | |
| 0.8532 | 0 | 0 | 2.4004 | 0 | 0 | 0 | 0 | |
| 0.9556 | 0 | 0 | 0 | 0 | 0 | 0 | 0 | 0 |
| | 0 | 1.0498 | 0 | 0 | 0 | 1.942 | 0.4645 | |
| 0.5608 | 1.682 | 0 | 4.1538 | 0.5715 | 0 | 0 | 0.5968 | |
| 1.1659 | 0 | 0.4832 | 0 | 3.2513 | 0 | 0 | 0 | 0 |
| | 0.5367 | 0 | 1.2673 | 0 | 0 | 2.8855 | 0.5659 | 0 |
| | 2.6279 | 0 | 2.029 | 0 | 0.9284 | 0 | 1.6807 | 0 |
| | 2.1998 | 0 | 0 | 0 | 0 | 2.7914 | 0.5504 | 0 |
| | 1.7978 | 0 | 0 | 0 | 3.2882 | 0.7631 | 0 | 0 |
| | 0 | 0 | 0 | 0 | 0 | 0.6116 | 0 | |
| 1.4572 | 1.9462 | 0 | 0.8008 | 0 | 0 | 0 | 0 | |
| 1.3754 | 2.205 | 0.9567 | 0 | 0.5526 | 0 | 0 | 2.0117 | |
| 2.5939 | 0 | 6.4753 | 0 | 0 | 0 | 0 | 0 | 0 |
| | 0 | 0 | 5.9963 | 0 | 0.5414 | 0 | 0 | |
| 0.4374 | 0.3855 | 0 | 0 | 0 | 0 | 0 | 0 | 0 |
| | 0 | 0 | 0 | 2.1803 | 0 | 0 | 0 | 0 |
| | 0 | 0 | 0 | 2.0726 | 2.2762 | 0 | 0 | |
| 3.9132 | 0 | 0.7366 | 1.0594 | 0 | 0.7058 | 0 | 0 | 0 |
| | 0 | 0 | 1.9066 | 0 | 0 | 0 | 0.3736 | |
| 3.1721 | 0 | 4.269 | 3.6379 | 4.3187 | 4.6616 | 6.8536 | 4.3419 | |
| 6.6948 | 7.7978 | 7.1399 | 3.8994 | 7.7323 | 7.5088 | 6.8272 | 2.1046 | |
| 5.1008 | 6.1691 | 7.2981 | 6.3201 | 7.3062 | 6.9449 | 6.3544 | 5.6426 | |
| 8.2028 | 6.511 | | | | | | | |
| ZNF695 | 7.0712 | 5.7304 | 6.5923 | 6.9975 | 7.4954 | 6.0334 | 8.0875 | |
| 7.3145 | 6.2585 | 6.1673 | 6.3192 | 7.4918 | 6.425 | 5.2184 | 6.6849 | |
| 6.658 | 7.1434 | 6.9338 | 5.6039 | 6.7725 | 6.8765 | 8.6682 | 6.6187 | |
| 5.6023 | 6.6947 | 6.5255 | 7.7034 | 6.9449 | 7.0329 | 7.9088 | 7.0728 | |
| 6.2336 | 7.9929 | 7.2832 | 6.8278 | 6.119 | 6.7179 | 7.458 | 6.7615 | |

| | | | | | | | | |
|---|---|---|---|---|---|---|---|---|
| 7.4671 | 8.0177 | 7.0282 | 5.9551 | 7.5844 | 5.9947 | 7.3089 | 6.7834 | |
| 6.9773 | 5.7409 | 5.3595 | 7.4011 | 7.4835 | 7.0106 | 7.6031 | 6.9542 | |
| 6.2644 | 4.4632 | 6.9579 | 3.5039 | 8.2507 | 7.4564 | 7.4698 | 7.183 | |
| 6.6124 | 6.6532 | 6.5876 | 7.3779 | 3.9162 | 5.2387 | 7.2147 | 6.9088 | |
| 7.9505 | 3.2396 | 7.273 | 6.7197 | 7.5198 | 7.3835 | 6.5191 | 6.353 | |
| 5.7629 | 7.9607 | 6.7864 | 5.2188 | 7.1974 | 5.4411 | 6.601 | 6.3543 | |
| 7.4868 | 7.0394 | 7.8258 | 7.2419 | 7.1457 | 8.8819 | 7.1486 | 7.7768 | |
| 6.9833 | 7.497 | 6.9034 | 7.2404 | 6.3399 | 8.2385 | 8.1372 | 7.7382 | |
| 5.7505 | 6.7147 | 5.8773 | 6.8768 | 5.9184 | 7.0813 | 6.014 | 6.5143 | |
| 7.9177 | 7.6461 | 6.8247 | 6.538 | 6.1985 | 7.2816 | 6.7167 | 6.9784 | |
| 7.8589 | 3.371 | 6.5604 | 7.9277 | 6.3629 | 6.0575 | 5.4267 | 6.2247 | |
| 7.0036 | 6.1494 | 8.0197 | 5.2406 | 6.9175 | 6.8923 | 7.6262 | 7.3744 | |
| 7.227 | 8.0411 | 6.9461 | 7.3146 | 5.291 | 5.7837 | 6.2016 | 5.6636 | |
| 5.4363 | 6.6231 | 4.6023 | 7.4888 | 7.7163 | 8.269 | 7.2601 | 8.4087 | |
| 6.3093 | 7.7736 | 5.6358 | 7.5196 | 7.7112 | 3.0258 | 7.47 | 7.4077 | |
| 7.3003 | 5.246 | 7.282 | 7.7676 | 6.7978 | 7.5917 | 6.9449 | 7.4339 | |
| 7.241 | 6.5711 | 7.3946 | 8.5401 | 5.5265 | 7.776 | 7.1767 | 5.5356 | |
| 5.7254 | 7.4981 | 1.8777 | 5.449 | 3.1701 | 2.1093 | 1.522 | 1.9318 | |
| 1.026 | 2.3895 | 0.5422 | 4.013 | 0 | 0.6266 | 2.3603 | 1.8642 | |
| 2.6149 | 3.1077 | 3.0295 | 0.8792 | 1.9011 | 2.225 | 2.4071 | 4.8125 | |
| 0.6293 | 2.384 | | | | | | | |
| SKA3 | 9.1124 | 7.6897 | 7.5511 | 9.1454 | 7.9693 | 9.0196 | 8.7377 | |
| 9.1977 | 9.5928 | 8.7667 | 8.2997 | 8.5608 | 8.2899 | 7.2051 | 7.7591 | |
| 8.6783 | 7.7797 | 7.6616 | 7.0119 | 7.5381 | 8.4205 | 8.7257 | 8.3875 | |
| 6.3513 | 8.5311 | 9.2635 | 8.9782 | 9.0804 | 7.1261 | 8.5654 | 8.9635 | |
| 6.4282 | 9.1155 | 8.7477 | 8.4623 | 8.7136 | 8.4059 | 8.8964 | 8.8179 | |
| 7.8931 | 8.1524 | 9.4036 | 7.6351 | 7.7152 | 7.9414 | 7.7466 | 7.2307 | |
| 8.9789 | 7.5547 | 6.4984 | 8.3211 | 7.8238 | 8.7489 | 8.9496 | 8.9442 | |
| 7.8945 | 6.7595 | 8.1566 | 4.9095 | 8.6981 | 7.8085 | 8.1568 | 8.0975 | |
| 8.1721 | 8.0476 | 6.7466 | 7.955 | 5.567 | 6.3771 | 8.8402 | 8.6882 | |
| 9.4248 | 6.1623 | 8.34 | 7.9959 | 8.1766 | 8.3429 | 8.3443 | 7.6584 | |
| 9.2773 | 8.73 | 8.3977 | 7.3393 | 9.5957 | 7.2444 | 8.6009 | 8.9008 | |
| 8.8041 | 9.0557 | 8.0554 | 9.4256 | 8.386 | 9.762 | 8.4216 | 8.067 | |
| 9.066 | 8.6705 | 8.5178 | 8.4898 | 9.5626 | 8.5655 | 8.8886 | 8.2828 | |
| 8.6949 | 8.1324 | 8.5106 | 8.614 | 8.7228 | 7.8298 | 8.3044 | 8.1608 | |
| 8.3204 | 7.3866 | 7.4284 | 9.4315 | 8.3356 | 8.8964 | 8.6939 | 8.309 | |
| 9.1191 | 6.8919 | 7.8683 | 8.2905 | 6.8029 | 7.5855 | 8.4259 | 8.4273 | |
| 7.182 | 8.1576 | 8.658 | 6.592 | 7.5023 | 7.359 | 8.519 | 8.5224 | |
| 9.2382 | 8.5196 | 8.14 | 9.2585 | 8.1494 | 8.5659 | 7.4895 | 7.6403 | |
| 8.0779 | 8.2382 | 7.7252 | 7.0202 | 8.7384 | 9.4077 | 8.7475 | 7.7734 | |
| 7.8164 | 9.2575 | 6.9805 | 8.6927 | 9.1581 | 6.4202 | 8.9952 | 8.5158 | |
| 8.4448 | 7.0991 | 8.9294 | 7.5243 | 9.1144 | 8.9432 | 8.5072 | 8.782 | |
| 9.1451 | 7.067 | 8.004 | 9.2472 | 8.3295 | 9.4125 | 8.527 | 8.1111 | |
| 7.9041 | 8.7906 | 2.4485 | 8.4508 | 4.3205 | 4.1354 | 3.5567 | 2.3266 | |
| 2.0387 | 2.6047 | 2.5892 | 7.1943 | 2.8267 | 2.5596 | 3.4734 | 6.0085 | |
| 4.7071 | 4.1815 | 5.5814 | 3.0974 | 2.5884 | 5.5959 | 3.3344 | 7.012 | |
| 1.6723 | 3.8265 | | | | | | | |
| CLEC4M | 0 | 3.0624 | 0 | 0.00E+00 | | 7.22E-01 | | 0 |
| | 1.3555 | 0.4327 | 0.4291 | 0 | 1.2705 | 0 | 0 | |
| 1.4442 | 0 | 0 | 2.368 | 0 | 0.8427 | 0 | 0.6062 | |
| 0.4561 | 3.4974 | 0 | 0 | 0.7334 | 0 | 0.5159 | 0 | 0 |
| | 0 | 0.9556 | 0 | 0 | 0 | 1.4628 | 0 | |
| 0.4326 | 0 | 0 | 0 | 0 | 0 | 0 | 0 | 0 |
| | 0.4645 | 0 | 0 | 0.8754 | 0.4252 | 1.5581 | 0 | 0 |

| | | | | | | | | |
|---|---|---|---|---|---|---|---|---|
| | 0.5968 | 0 | 0 | 0.4832 | 0 | 0 | 0 | 0 |
| | 0.9449 | 2.1829 | 1.889 | 0 | 1.5242 | 2.3485 | 0.6894 | 0 |
| | 0 | 0 | 0 | 0 | 0.5263 | 0 | 0 | 0 |
| | 0.5273 | 0.5265 | 1.7639 | 1.6676 | 4.3249 | 0.5198 | 0 | |
| 0.8845 | 0 | 0 | 1.4068 | 0 | 0 | 0 | 0.4659 | |
| 0.4315 | 2.2182 | 0 | 0 | 0 | 2.0513 | 0 | 0 | 0 |
| | 0 | 0.4321 | 0.3974 | 0.6089 | 4.0837 | 0 | 0 | 0.47 |
| | 2.4516 | 0 | 0 | 0 | 0 | 0 | 0 | |
| 0.8107 | 0 | 2.1237 | 0 | 4.8516 | 0 | 0 | 0 | 0 |
| | 0 | 0 | 0 | 0 | 3.3441 | 0 | 0 | |
| 0.5862 | 0 | 1.2732 | 0 | 0.6 | 0.5416 | 0 | 0.6138 | |
| 2.1631 | 0.4033 | 0 | 0.5023 | 0 | 2.8261 | 0 | 0 | 0 |
| | 0 | 0 | 0 | 0 | 0 | 0.9723 | 1.9052 | |
| 0.5545 | 0.6902 | 0.5257 | 0 | 0 | 0 | 0 | 0 | 0 |
| | 0 | 1.18 | 0 | 0 | 0 | 0 | 0 | 0 |
| | 0 | 0 | 0 | 6.1314 | 3.3648 | 5.389 | 3.2986 | |
| 5.0365 | 6.5878 | 3.3683 | 4.3262 | 3.7837 | 4.7238 | 1.4126 | 6.5631 | |
| 6.2631 | 2.1046 | 5.7766 | 4.6873 | 5.8021 | 6.0271 | 7.1436 | 4.2061 | |
| 7.2446 | 3.863 | 2.6934 | 5.265 | | | | | |
| FGF10 | 0 | 1.5051 | 0 | 0 | 0 | 0 | 0 | |
| 0.4327 | 0 | 0 | 1.0422 | 0 | 0 | 0 | 0 | 0 |
| | 0 | 0 | 0 | 0 | 0 | 0 | 0 | 0 |
| | 0 | 0 | 0 | 0 | 0 | 0 | 0 | 0 |
| | 0 | 0 | 0 | 0 | 0 | 0 | 0 | 0 |
| | 0.6741 | 0.7769 | 0 | 0 | 0 | 0 | 0.4645 | |
| 0.5608 | 0 | 0.503 | 1.2457 | 0.5715 | 1.4031 | 0 | 0 | |
| 0.3906 | 0 | 1.3736 | 1.9829 | 1.0463 | 0 | 0.3921 | 0 | |
| 0.4786 | 2.0547 | 0 | 0 | 0 | 0 | 0 | 0 | 0 |
| | 0.7204 | 0 | 0 | 0 | 0 | 0 | 0.5273 | 0 |
| | 0 | 0.9036 | 0 | 0 | 0 | 0 | 0 | 0 |
| | 0 | 0 | 0 | 0 | 0 | 1.0326 | 1.2448 | 0 |
| | 0 | 0 | 0 | 0 | 0.8924 | 1.0399 | 0 | |
| 0.4321 | 0 | 0 | 1.3128 | 0.5886 | 0.6224 | 0 | 0 | |
| 0.6149 | 0 | 0 | 0 | 0.5526 | 0 | 0 | 0 | 0 |
| | 0 | 3.2795 | 0 | 0 | 0 | 0.9357 | 1.3147 | 0 |
| | 0 | 0 | 1.4139 | 0 | 1.2425 | 0.5862 | 0 | 0 |
| | 1.6526 | 0 | 0.5416 | 0 | 0 | 0.9957 | 0 | 0 |
| | 0 | 0 | 0.9722 | 0 | 0 | 0 | 0 | 0 |
| | 0 | 0 | 2.8712 | 0.4012 | 1.9052 | 0 | 0 | 0 |
| | 0 | 0 | 0.6248 | 0 | 0 | 0 | 0 | 0 |
| | 0 | 0 | 0.4262 | 0 | 0 | 0 | 0.3736 | 0 |
| | 0 | 1.8777 | 4.0342 | 2.904 | 0 | 0.953 | 3.6608 | |
| 2.0387 | 3.0357 | 3.2033 | 4.621 | 4.7272 | 2.4199 | 4.343 | 2.6511 | |
| 1.018 | 2.9388 | 3.5282 | 5.8806 | 5.5697 | 3.0618 | 4.3982 | 3.4082 | |
| 4.2513 | 4.7748 | | | | | | | |
| MYBL2 | 12.0786 | 9.2326 | 8.4899 | 12.5397 | 10.8028 | 10.1618 | 10.9263 | |
| 11.7914 | 11.5019 | 10.9427 | 10.1846 | 10.3757 | 10.4386 | 9.2695 | 10.9923 | |
| 10.7552 | 11.662 | 12.1689 | 9.4978 | 8.8429 | 11.0413 | 13.6086 | 10.9587 | |
| 9.2466 | 12.0936 | 11.2746 | 11.2205 | 11.8619 | 11.0193 | 11.7147 | 12.3365 | |
| 9.0648 | 11.3036 | 10.1972 | 10.6321 | 10.4605 | 11.6629 | 10.6457 | 11.0171 | |
| 13.3628 | 10.9772 | 11.5371 | 9.9452 | 10.9361 | 11.1935 | 11.6237 | 10.6643 | |
| 10.9103 | 9.5353 | 8.5094 | 10.3871 | 11.6816 | 10.7306 | 10.9269 | 12.1965 | |
| 10.2161 | 8.4028 | 11.4913 | 6.7521 | 12.0521 | 12.7899 | 10.5381 | 12.1495 | |
| 10.0536 | 8.8176 | 9.7697 | 10.6277 | 8.4541 | 6.79 | 11.5041 | 11.1215 | |

| | | | | | | | |
|---|---|---|---|---|---|---|---|
| 12.2496 | 6.718 | 12.0158 | 10.5237 | 9.8141 | 10.3781 | 11.6596 | 9.4149 |
| 11.1408 | 11.2476 | 10.5764 | 9.2936 | 11.6562 | 10.4117 | 10.1759 | 9.6686 |
| 11.1073 | 11.1083 | 10.9209 | 12.0405 | 11.7576 | 14.3924 | 10.0248 | 11.6219 |
| 11.7469 | 10.4692 | 10.2375 | 12.1426 | 11.1687 | 10.9742 | 10.8608 | 9.9584 |
| 10.6578 | 11.6275 | 11.2012 | 11.4336 | 9.7304 | 11.2054 | 10.9241 | 11.6114 |
| 11.4287 | 11.0736 | 11.1496 | 10.7272 | 10.9477 | 11.3324 | 11.516 | 11.1833 |
| 12.2905 | 8.1554 | 9.4998 | 11.9607 | 9.886 | 9.8643 | 10.7735 | 10.4144 |
| 9.2587 | 10.3168 | 11.7893 | 8.5907 | 12.7151 | 9.1771 | 11.394 | 11.1027 |
| 11.5429 | 10.7931 | 11.7628 | 12.5133 | 9.9906 | 12.3845 | 10.1786 | 9.1886 |
| 11.2982 | 10.4755 | 10.9341 | 10.7096 | 11.1168 | 12.6314 | 11.2255 | 11.8886 |
| 12.1289 | 11.5005 | 9.9299 | 10.3783 | 10.6703 | 7.9258 | 11.1252 | 11.3476 |
| 11.7237 | 8.5061 | 10.2299 | 9.2608 | 11.5972 | 12.654 | 11.0004 | 11.1153 |
| 11.5581 | 8.7667 | 11.807 | 12.1417 | 10.7088 | 12.281 | 14.2906 | 10.2664 |
| 9.4596 | 12.2614 | 3.8 | 10.1404 | 6.604 | 4.4949 | 4.8384 | 4.5785 |
| 1.026 | 3.9449 | 4.0048 | 8.6126 | 4.0932 | 5.0484 | 4.2762 | 7.3982 |
| 4.9889 | 4.6604 | 6.8 | 3.3552 | 2.2162 | 6.7493 | 4.4622 | 8.0944 |
| 2.2714 | 3.3172 | | | | | | |
| TROAP | 10.7512 | 8.326 | 8.3069 | 9.9225 | 10.4827 | 9.1676 | 9.9762 |
| 9.8595 | 10.0756 | 9.6229 | 9.8379 | 8.8896 | 8.789 | 9.2935 | 10.1197 |
| 9.5761 | 10.1359 | 10.0925 | 8.237 | 7.7681 | 9.5644 | 10.7282 | 9.3119 |
| 7.1011 | 9.5814 | 10.8958 | 9.9761 | 10.6752 | 9.0737 | 10.0704 | 9.9559 |
| 8.1049 | 10.1985 | 9.7088 | 9.1418 | 9.4788 | 9.5039 | 10.6778 | 9.727 |
| 10.5052 | 9.6238 | 9.707 | 8.9113 | 8.8951 | 9.7562 | 8.5641 | 9.4881 |
| 10.3821 | 8.1539 | 7.6649 | 8.9414 | 10.0791 | 9.354 | 9.9097 | 9.6527 |
| 8.7589 | 9.2842 | 9.4287 | 5.387 | 11.1048 | 9.6252 | 8.8497 | 9.636 |
| 8.8919 | 8.8824 | 8.1138 | 9.4957 | 6.801 | 6.7162 | 9.335 | 9.1566 |
| 10.559 | 6.2747 | 10.2602 | 9.2056 | 9.9666 | 10.2535 | 9.4701 | 8.7371 |
| 10.5474 | 9.3557 | 9.9502 | 9.6094 | 10.1055 | 8.5075 | 8.7396 | 9.8377 |
| 9.8484 | 9.5905 | 9.7017 | 12.1224 | 10.2681 | 10.2903 | 9.2149 | 9.7069 |
| 10.5184 | 8.913 | 9.1208 | 9.9763 | 11.6667 | 9.9844 | 9.5267 | 8.525 |
| 9.6895 | 9.4927 | 9.1985 | 10.8481 | 8.9883 | 9.0354 | 8.404 | 9.1995 |
| 9.8619 | 10.4458 | 10.007 | 10.4913 | 8.8665 | 10.6029 | 9.287 | 9.0586 |
| 9.6742 | 6.1326 | 8.1279 | 10.8004 | 7.2641 | 8.3578 | 9.0938 | 8.6467 |
| 7.7572 | 8.7069 | 10.148 | 7.6312 | 10.4767 | 7.7514 | 9.41 | 9.5281 |
| 9.9636 | 9.2797 | 9.6676 | 10.315 | 8.6115 | 9.9208 | 8.9367 | 8.7241 |
| 9.0975 | 9.5368 | 9.4156 | 8.6844 | 9.7179 | 11.1255 | 8.881 | 10.9358 |
| 10.1694 | 10.2599 | 8.2302 | 9.4162 | 9.3794 | 7.2739 | 10.4954 | 10.1934 |
| 10.251 | 8.612 | 9.4889 | 8.1576 | 9.1294 | 10.8828 | 9.8234 | 10.4734 |
| 9.7713 | 7.2813 | 8.888 | 9.5702 | 9.2906 | 10.7399 | 10.817 | 8.5276 |
| 8.6408 | 10.1164 | 3.3733 | 9.2626 | 5.126 | 4.3063 | 4.7904 | 3.5661 |
| 1.026 | 3.7315 | 1.9018 | 7.9576 | 2.7094 | 2.0921 | 3.7932 | 6.7977 |
| 4.2433 | 3.5794 | 6.311 | 3.4689 | 3.3387 | 5.9458 | 3.7478 | 7.9463 |
| 1.066 | 4.2022 | | | | | | |
| CCNB2 | 10.993 | 7.9962 | 8.6726 | 10.1002 | 10.5976 | 9.5909 | 10.1104 |
| 10.1134 | 10.3478 | 9.924 | 9.6395 | 9.4983 | 9.6659 | 8.3727 | 8.375 |
| 9.6252 | 10.5588 | 10.2387 | 8.624 | 8.0835 | 9.7377 | 10.0657 | 9.8312 |
| 7.7134 | 9.8244 | 10.038 | 9.9099 | 10.8392 | 8.8159 | 9.7177 | 10.0843 |
| 8.0213 | 9.8018 | 10.1215 | 8.6364 | 10.1715 | 9.2814 | 11.1174 | 9.1133 |
| 10.3426 | 9.2632 | 10.6342 | 8.1689 | 9.357 | 9.1298 | 9.2348 | 8.3727 |
| 9.8615 | 8.4072 | 8.4331 | 9.3496 | 9.3071 | 10.4215 | 10.1609 | 9.7563 |
| 8.8444 | 8.0526 | 9.9522 | 6.2993 | 10.2889 | 10.4863 | 9.4671 | 9.4746 |
| 9.5852 | 8.8989 | 8.7656 | 8.97 | 7.1262 | 6.4391 | 9.0214 | 10.1698 |
| 10.7794 | 6.4017 | 9.9088 | 9.4738 | 8.832 | 10.3791 | 10.2063 | 8.9112 |
| 10.9094 | 9.704 | 9.2803 | 7.8954 | 10.9571 | 9.0151 | 9.5007 | 9.3336 |

| | | | | | | | |
|---|---|---|---|---|---|---|---|
| 10.6778 | 9.7941 | 9.3461 | 10.0556 | 11.0523 | 10.6065 | 9.5748 | 9.7006 |
| 10.6029 | 9.3244 | 9.9223 | 9.6631 | 9.4346 | 10.6698 | 9.886 | 9.5791 |
| 9.5574 | 10.2588 | 10.095 | 10.631 | 9.8713 | 9.0175 | 9.5931 | 10.3056 |
| 9.9537 | 10.592 | 9.5571 | 9.945 | 9.8165 | 9.3934 | 9.6295 | 9.1596 |
| 10.9883 | 5.331 | 8.729 | 10.654 | 8.5969 | 8.8211 | 9.942 | 9.7895 |
| 8.8199 | 9.1654 | 10.2755 | 7.4576 | 11.121 | 8.4085 | 9.0345 | 9.7852 |
| 9.9321 | 9.8186 | 10.5077 | 10.555 | 9.288 | 9.838 | 9.0493 | 8.9851 |
| 8.7236 | 10.2158 | 8.8621 | 8.5806 | 10.2787 | 10.6741 | 10.0992 | 10.9925 |
| 9.5364 | 9.4484 | 7.6241 | 9.605 | 10.3442 | 7.2058 | 10.1409 | 10.115 |
| 10.577 | 8.5487 | 9.2852 | 8.8286 | 9.5584 | 10.6763 | 9.143 | 10.8492 |
| 10.3967 | 8.7063 | 9.1758 | 9.3493 | 9.6682 | 11.4244 | 10.5243 | 9.3545 |
| 8.8713 | 10.3747 | 3.8896 | 9.4373 | 6.019 | 5.5671 | 4.5807 | 4.4519 |
| 2.6279 | 4.1989 | 3.9632 | 7.8855 | 3.7213 | 4.1588 | 4.8591 | 5.4125 |
| 5.3568 | 5.217 | 7.0127 | 3.4689 | 3.1292 | 6.9977 | 4.5823 | 6.8245 |
| 2.2714 | 4.5657 | | | | | | |
| CDC25C | 8.6723 | 6.3771 | 6.5419 | 8.0696 | 8.6485 | 7.7628 | 8.7615 |
| 8.1743 | 8.9503 | 7.7492 | 8.4301 | 6.919 | 6.3943 | 7.0423 | 7.941 |
| 8.1979 | 7.2846 | 7.8532 | 6.178 | 6.0378 | 7.7937 | 9.5842 | 7.7068 |
| 4.9839 | 7.7868 | 7.7853 | 7.4198 | 8.0022 | 7.6756 | 8.8805 | 7.4514 |
| 6.071 | 7.8218 | 8.1819 | 6.5318 | 8.0941 | 8.2812 | 7.4465 | 7.8596 |
| 9.0863 | 7.8548 | 8.1922 | 7.1122 | 7.0758 | 6.7259 | 7.3689 | 5.7896 |
| 7.7626 | 6.7712 | 5.3449 | 8.3936 | 8.1642 | 8.6683 | 7.9352 | 8.4967 |
| 7.2927 | 6.5141 | 6.9532 | 3.564 | 9.0409 | 8.2861 | 7.1766 | 7.5464 |
| 6.6114 | 6.5027 | 6.2122 | 6.5812 | 4.5127 | 5.1166 | 7.7234 | 7.6464 |
| 8.2572 | 3.9306 | 8.1524 | 7.2054 | 7.4194 | 7.9982 | 7.9337 | 7.3243 |
| 8.5937 | 7.8115 | 8.0177 | 6.9667 | 8.4717 | 6.9062 | 6.4197 | 7.4256 |
| 8.4487 | 7.9293 | 8.3454 | 8.9619 | 8.7947 | 9.1516 | 7.8886 | 10.0652 |
| 8.249 | 7.2834 | 7.6534 | 7.8391 | 7.3923 | 8.1099 | 7.8793 | 7.0216 |
| 8.0033 | 7.5816 | 7.546 | 8.4241 | 7.5959 | 7.6046 | 6.8835 | 7.8588 |
| 7.4842 | 8.8997 | 7.6426 | 8.1339 | 7.4168 | 8.2641 | 6.8905 | 6.0815 |
| 9.0886 | 4.8598 | 6.6914 | 8.8009 | 6.5319 | 7.1178 | 7.24 | 7.3631 |
| 6.3767 | 7.0446 | 8.5485 | 4.7442 | 8.0716 | 6.293 | 8.5907 | 7.3493 |
| 8.2253 | 7.4047 | 8.4582 | 8.8144 | 7.0286 | 6.8457 | 6.9913 | 7.0855 |
| 7.0846 | 8.0181 | 7.0759 | 7.4173 | 8.4303 | 8.8706 | 7.8593 | 8.7487 |
| 9.8981 | 7.6611 | 7.012 | 7.8404 | 8.1714 | 5.2814 | 8.7818 | 7.5695 |
| 8.9033 | 6.62 | 7.8085 | 6.6229 | 8.315 | 8.786 | 7.8446 | 7.8964 |
| 8.1199 | 7.1054 | 7.1952 | 8.0135 | 7.7384 | 8.9123 | 10.4088 | 6.7392 |
| 7.0463 | 7.503 | 2.0429 | 7.5221 | 3.5205 | 2.2832 | 3.0035 | 0.4871 |
| 1.026 | 1.8243 | 1.9018 | 5.929 | 1.6854 | 1.0621 | 2.3603 | 4.2843 |
| 3.1191 | 2.5267 | 4.8277 | 1.1757 | 0.9349 | 3.8402 | 1.2837 | 5.578 |
| 1.6723 | 1.0391 | | | | | | |
| CDC20 | 11.737 | 10.1828 | 9.2869 | 11.5598 | 11.0441 | 10.5874 | 10.8989 |
| 12.1493 | 11.6987 | 11.381 | 11.8739 | 10.0285 | 10.3166 | 9.6276 | 10.9548 |
| 10.821 | 11.7509 | 11.5977 | 9.3425 | 8.6635 | 10.3935 | 11.6647 | 10.8591 |
| 8.8825 | 10.7715 | 11.0319 | 10.7727 | 11.8434 | 10.0972 | 11.4425 | 12.7236 |
| 8.7158 | 10.9389 | 11.0474 | 10.3707 | 10.6015 | 11.3402 | 10.2097 | 11.2958 |
| 12.495 | 10.7476 | 11.5066 | 9.914 | 10.2877 | 10.6401 | 10.2485 | 8.6731 |
| 10.4355 | 9.7559 | 8.8282 | 10.8057 | 10.7343 | 10.5911 | 11.0245 | 11.4259 |
| 10.2164 | 9.2445 | 11.2247 | 7.2252 | 11.595 | 11.6652 | 10.1803 | 11.4513 |
| 10.6106 | 9.0947 | 9.1252 | 10.4523 | 8.0386 | 7.5675 | 10.9017 | 10.7759 |
| 10.8533 | 8.2606 | 11.5128 | 10.4379 | 11.305 | 10.581 | 11.5698 | 9.943 |
| 11.4851 | 10.948 | 11.3283 | 8.8698 | 11.4666 | 11.2568 | 10.4729 | 10.8785 |
| 11.6516 | 10.7552 | 11.4962 | 11.4274 | 11.4655 | 12.5833 | 10.2145 | 10.8522 |
| 11.5668 | 10.2562 | 10.4096 | 12.3921 | 12.2866 | 11.4242 | 11.0332 | 10.0303 |

| | | | | | | | |
|---|---|---|---|---|---|---|---|
| 10.505 | 11.2082 | 11.8729 | 10.9806 | 10.7538 | 10.6299 | 10.6882 | 11.5647 |
| 11.2279 | 12.101 | 11.0432 | 10.8195 | 11.2191 | 11.7887 | 12.3389 | 12.2858 |
| 11.5379 | 8.3078 | 9.2919 | 11.8304 | 9.3125 | 10.4256 | 10.5776 | 10.7679 |
| 8.9848 | 9.9694 | 11.9784 | 8.0307 | 12.7105 | 9.1204 | 11.1263 | 11.4629 |
| 12.1043 | 10.1757 | 11.8281 | 12.1924 | 10.478 | 11.0144 | 9.7628 | 9.5623 |
| 9.7546 | 10.6332 | 10.4045 | 10.3558 | 10.7273 | 12.1906 | 11.1179 | 11.0287 |
| 11.6427 | 9.9278 | 10.2416 | 10.2956 | 11.0976 | 8.2744 | 11.9934 | 11.8479 |
| 12.119 | 8.8885 | 11.2123 | 9.0245 | 11.3851 | 13.4334 | 10.9626 | 11.5631 |
| 11.0977 | 9.402 | 11.1886 | 10.7722 | 10.1518 | 13.1606 | 12.7375 | 9.9822 |
| 9.4954 | 12.4203 | 4.3966 | 10.0371 | 6.6405 | 6.3418 | 5.6068 | 4.5292 |
| 2.3632 | 4.652 | 5.2109 | 8.7928 | 4.2693 | 4.7894 | 5.7626 | 7.7207 |
| 6.8684 | 5.2535 | 7.1172 | 4.1528 | 3.9194 | 7.7176 | 5.3967 | 8.1752 |
| 3.1136 | 4.7467 | | | | | | |
| SPAG5 | 10.8232 | 8.507 | 9.4053 | 10.5081 | 11.5907 | 11.174 | 9.6374 |
| 9.9491 | 12.51 | 11.1481 | 10.4959 | 9.9657 | 9.9732 | 9.5829 | 10.0258 |
| 10.3617 | 10.5925 | 9.7183 | 9.0471 | 9.0631 | 9.9815 | 11.3444 | 9.8426 |
| 8.5359 | 11.099 | 10.6739 | 10.4726 | 10.5292 | 9.0293 | 11.2791 | 10.4873 |
| 8.978 | 10.9744 | 10.4076 | 9.1274 | 10.1918 | 10.3854 | 11.4544 | 9.6864 |
| 9.866 | 10.5072 | 10.9374 | 9.641 | 10.1767 | 10.4856 | 10.6954 | 9.8694 |
| 10.2877 | 9.2251 | 8.4978 | 10.6218 | 10.3077 | 10.6144 | 11.1703 | 9.8247 |
| 10.1716 | 8.3124 | 11.6906 | 7.0107 | 11.1498 | 10.8423 | 10.2577 | 9.7735 |
| 9.5576 | 9.7773 | 9.3644 | 9.4606 | 8.4965 | 8.5041 | 10.2501 | 10.1277 |
| 12.1837 | 8.4054 | 10.2227 | 9.5545 | 10.278 | 10.8538 | 10.2364 | 8.8899 |
| 11.2146 | 10.3072 | 10.6077 | 9.2002 | 11.2728 | 9.9959 | 9.6094 | 10.0149 |
| 10.6461 | 10.0995 | 10.6529 | 10.4933 | 10.0546 | 11.0292 | 10.2652 | 10.3908 |
| 10.4783 | 9.9606 | 9.8643 | 9.7072 | 10.4419 | 10.6995 | 10.3744 | 9.4599 |
| 10.2155 | 10.3464 | 10.2273 | 10.8376 | 9.9718 | 10.6265 | 9.9573 | 10.848 |
| 10.845 | 10.1232 | 9.7886 | 10.4578 | 9.943 | 11.3301 | 9.3163 | 9.8771 |
| 10.7272 | 6.9584 | 9.2347 | 10.0693 | 9.161 | 9.6229 | 10.1757 | 10.0741 |
| 9.0134 | 9.5562 | 10.1197 | 8.3977 | 9.4983 | 9.1929 | 9.5027 | 10.1536 |
| 10.872 | 10.2337 | 10.9051 | 11.064 | 10.1972 | 10.5458 | 9.2032 | 9.1481 |
| 9.5984 | 10.6593 | 11.0307 | 9.8731 | 10.5242 | 11.0171 | 10.6522 | 11.7622 |
| 10.9049 | 10.6793 | 7.8585 | 10.334 | 11.1359 | 9.193 | 10.5962 | 10.7938 |
| 10.8248 | 8.8418 | 10.9951 | 9.4336 | 10.3867 | 11.16 | 10.1673 | 10.9332 |
| 10.7512 | 9.5249 | 10.6038 | 9.5182 | 10.3821 | 10.8529 | 10.0711 | 9.9137 |
| 9.2882 | 10.4843 | 5.6831 | 9.6628 | 6.2634 | 6.9043 | 5.9503 | 5.8387 |
| 5.004 | 6.2393 | 5.7029 | 8.5038 | 5.6686 | 4.64 | 6.3791 | 7.3581 |
| 6.8234 | 6.2526 | 7.6401 | 6.143 | 6.2876 | 7.1824 | 6.0763 | 8.1947 |
| 5.0558 | 6.6912 | | | | | | |
| CEP55 | 9.5898 | 8.1316 | 8.2109 | 9.9179 | 10.1162 | 9.709 | 9.4483 |
| 9.4063 | 11.0823 | 10.475 | 9.445 | 9.7392 | 9.5541 | 7.4919 | 8.6181 |
| 9.0418 | 9.7894 | 9.8138 | 8.5357 | 8.503 | 8.775 | 10.7081 | 8.8698 |
| 7.1346 | 9.7769 | 10.1902 | 9.3487 | 10.851 | 7.86 | 9.7211 | 9.0458 |
| 7.7898 | 9.852 | 9.9302 | 9.7199 | 9.3501 | 9.8623 | 10.3227 | 10.3241 |
| 9.1115 | 8.8405 | 10.3102 | 8.1492 | 9.6371 | 9.7727 | 9.1622 | 8.9249 |
| 10.0665 | 8.9583 | 7.9148 | 9.9222 | 9.6729 | 10.5834 | 9.8969 | 8.7058 |
| 9.4829 | 7.5061 | 9.4253 | 6.2348 | 9.6552 | 9.8184 | 9.4 | 8.7978 |
| 8.7633 | 8.8934 | 9.0797 | 8.8709 | 6.747 | 6.9051 | 10.6851 | 9.1961 |
| 10.4224 | 6.9166 | 10.0381 | 9.0549 | 7.8802 | 8.2839 | 9.4734 | 9.3452 |
| 10.2474 | 9.6915 | 8.958 | 8.3099 | 10.4442 | 8.7273 | 9.1645 | 9.9699 |
| 8.5095 | 9.8266 | 8.8155 | 10.5824 | 10.4955 | 10.1223 | 9.5183 | 10.5131 |
| 9.2561 | 9.5194 | 9.2438 | 8.0421 | 8.7682 | 9.7979 | 9.4666 | 9.6362 |
| 7.8611 | 10.7777 | 9.4894 | 9.0542 | 9.6303 | 9.7331 | 9.382 | 9.8727 |
| 9.3894 | 8.3581 | 8.7044 | 10.4871 | 9.4114 | 10.4973 | 8.9643 | 7.8594 |

| | | | | | | | | |
|---|---|---|---|---|---|---|---|---|
| 8.8079 | 6.2435 | 8.6762 | 11.0116 | 8.0355 | 7.9426 | 9.461 | 9.732 | |
| 8.2832 | 8.5648 | 9.7314 | 6.9803 | 8.9403 | 8.3654 | 8.8047 | 10.0393 | |
| 10.6007 | 9.8892 | 9.3677 | 9.6957 | 8.8276 | 9.793 | 9.0302 | 7.9069 | |
| 9.3233 | 10.0318 | 9.7852 | 9.3834 | 11.1421 | 10.1279 | 9.4995 | 10.7008 | |
| 8.7963 | 9.9358 | 6.4013 | 9.2917 | 9.9595 | 7.279 | 9.4185 | 9.209 | |
| 10.8376 | 8.3645 | 9.7012 | 8.4976 | 9.6512 | 9.9051 | 9.4635 | 9.9123 | |
| 10.1908 | 8.1701 | 9.1069 | 8.9153 | 9.1426 | 9.5748 | 10.0241 | 9.5094 | |
| 8.5463 | 9.2841 | 4.1289 | 9.2482 | 5.6971 | 4.5298 | 4.0393 | 4.5292 | |
| 3.0451 | 4.1653 | 3.7837 | 7.7582 | 3.3023 | 4.0682 | 4.4681 | 6.7364 | |
| 4.6491 | 5.7384 | 7.1389 | 3.4131 | 1.2434 | 6.4566 | 3.6413 | 7.7955 | |
| 3.1136 | 3.2388 | | | | | | | |
| KLF17 | 0 | 0.6896 | 0 | 2.0327 | 0 | 0.9367 | 0.8314 | 0 |
| | 0.4291 | 1.1366 | 0.4363 | 0 | 0 | 0 | 0 | 0 |
| | 0 | 0 | 0 | 0 | 0 | 1.9907 | 0 | |
| 1.3857 | 0 | 0 | 1.756 | 0 | 0 | 0 | 0 | |
| 0.5555 | 0 | 0.541 | 0 | 0 | 0 | 0.4326 | 0 | 0 |
| | 0 | 1.4771 | 0 | 0.453 | 0 | 1.0956 | 0.8153 | 0 |
| | 2.5776 | 0.503 | 3.738 | 0 | 0 | 0.5573 | 0.5968 | 0 |
| | 0 | 0.8444 | 0.5773 | 3.4751 | 0 | 0 | 0 | 0 |
| | 1.889 | 0 | 0 | 0 | 0 | 2.241 | 0.5659 | |
| 0.3965 | 2.0835 | 1.1284 | 0.911 | 0.6199 | 0.5376 | 0 | 1.8667 | |
| 1.2149 | 0.847 | 0 | 0.6909 | 0 | 0 | 2.1324 | 1.2591 | 0 |
| | 1.6155 | 0 | 1.2208 | 0 | 2.8278 | 1.4556 | 0 | 0 |
| | 0 | 0 | 1.3631 | 0 | 1.4396 | 0 | 0 | |
| 0.4321 | 1.9462 | 0.6089 | 1.0794 | 0.5886 | 0 | 0.8239 | 1.2272 | |
| 0.6149 | 0 | 0 | 0 | 0.5526 | 1.9109 | 0 | 2.2603 | 0 |
| | 0 | 4.1334 | 0 | 0 | 0 | 1.2444 | 0 | 0 |
| | 0 | 0 | 2.2465 | 0 | 0.5414 | 0 | 0 | |
| 2.1834 | 1.3399 | 0.6 | 0 | 0 | 0 | 0.5821 | 0 | |
| 0.4418 | 0.5023 | 0 | 0.4012 | 1.8676 | 0 | 0 | 0.9151 | 0 |
| | 0.6229 | 0 | 0 | 1.5479 | 1.4046 | 0 | 0.6902 | |
| 0.5257 | 0 | 0 | 1.0594 | 0 | 0.7058 | 0 | 0 | |
| 0.5078 | 0 | 0 | 2.2571 | 0 | 0 | 0 | 0 | |
| 2.5996 | 0 | 2.7646 | 2.7493 | 3.0845 | 3.2153 | 6.1818 | 4.0586 | |
| 4.857 | 6.1037 | 6.6567 | 3.4175 | 5.2733 | 5.1403 | 4.6897 | 2.1046 | |
| 2.938 | 4.7397 | 6.005 | 5.129 | 0.5418 | 4.0548 | 4.8921 | 4.6712 | |
| 6.1272 | 3.2388 | | | | | | | |
| KIF4A | 10.063 | 9.0089 | 8.1059 | 10.2011 | 9.8185 | 8.8279 | 10.121 | |
| 9.9039 | 10.8688 | 10.2469 | 9.0497 | 9.2365 | 9.0802 | 8.0082 | 8.659 | |
| 9.5653 | 9.5362 | 9.0081 | 8.6975 | 7.9331 | 9.6239 | 11.6454 | 8.8148 | |
| 6.872 | 9.5052 | 10.004 | 9.5534 | 9.7704 | 8.2806 | 10.5708 | 9.5302 | |
| 7.3901 | 10.2671 | 9.4557 | 9.297 | 9.6197 | 8.7243 | 10.1638 | 8.5332 | |
| 9.695 | 9.2249 | 10.3118 | 8.4432 | 9.2021 | 9.0447 | 9.5035 | 8.593 | |
| 9.255 | 8.8961 | 8.3265 | 9.8692 | 9.7499 | 10.1254 | 10.367 | 10.4724 | |
| 9.0623 | 7.7145 | 10.3302 | 5.7324 | 10.4254 | 9.8089 | 9.4414 | 8.6952 | |
| 7.8866 | 8.4373 | 8.338 | 9.1487 | 9.6512 | 6.4891 | 9.8851 | 9.3042 | |
| 10.7521 | 6.7703 | 10.371 | 8.9159 | 8.6873 | 10.1756 | 9.3684 | 8.8669 | |
| 9.9744 | 10.1086 | 9.0709 | 7.2891 | 10.2558 | 9.1186 | 10.1477 | 8.9599 | |
| 9.8743 | 9.5241 | 10.0788 | 9.4504 | 9.9615 | 10.7578 | 9.2947 | 9.9494 | |
| 9.8426 | 9.5485 | 9.0726 | 8.4643 | 8.4676 | 10.0008 | 9.459 | 9.1474 | |
| 9.7748 | 10.6323 | 9.8362 | 9.8509 | 8.8071 | 9.6791 | 10.0912 | 10.0016 | |
| 9.7979 | 9.613 | 9.721 | 9.353 | 9.4035 | 9.9197 | 9.2588 | 9.0064 | |
| 10.2467 | 4.4303 | 8.3405 | 9.956 | 8.3553 | 7.8243 | 9.7156 | 9.4773 | |
| 8.638 | 8.8519 | 9.1018 | 6.9171 | 9.1254 | 8.4296 | 9.8232 | 9.8188 | |

| | | | | | | | |
|---|---|---|---|---|---|---|---|
| 10.6103 | 9.3306 | 9.4551 | 10.1839 | 9.5088 | 8.892 | 8.407 | 7.7209 |
| 9.6828 | 10.1353 | 8.9066 | 9.3498 | 9.173 | 10.47 | 10.158 | 10.3739 |
| 10.6317 | 9.7814 | 6.8473 | 9.5657 | 10.3133 | 8.1376 | 9.2905 | 9.3821 |
| 10.2793 | 7.8697 | 9.945 | 7.7847 | 9.2957 | 9.3657 | 8.5468 | 9.9846 |
| 10.1887 | 7.8962 | 9.3711 | 9.3092 | 9.2705 | 10.0215 | 9.6963 | 9.2179 |
| 8.8184 | 9.4364 | 2.9425 | 9.0753 | 4.7247 | 7.861 | 4.0798 | 4.1266 |
| 2.3632 | 3.5346 | 3.5787 | 7.578 | 3.7806 | 3.5027 | 4.0144 | 4.8108 |
| 6.9181 | 4.2189 | 7.0995 | 3.1658 | 1.9011 | 6.5933 | 4.9822 | 7.0974 |
| 2.8104 | 3.7151 | | | | | | |
| ZWINT | 10.9006 | 10.4329 | 8.9594 | 11.2131 | 9.3704 | 9.7448 | 11.1778 |
| 10.7457 | 11.2501 | 10.2191 | 10.4868 | 10.7666 | 10.2024 | 9.2816 | 9.9086 |
| 10.78 | 9.5293 | 9.3945 | 9.1555 | 9.9615 | 10.5066 | 11.7945 | 9.8832 |
| 9.0189 | 10.538 | 11.6369 | 10.9791 | 10.5929 | 9.5616 | 10.01 | 11.2923 |
| 9.5274 | 11.4325 | 10.5325 | 10.7945 | 11.0684 | 10.4697 | 11.1859 | 9.925 |
| 9.4857 | 9.7589 | 10.755 | 10.3308 | 10.088 | 9.0679 | 10.2805 | 10.1674 |
| 11.1974 | 9.7467 | 8.8661 | 10.5813 | 11.1536 | 11.0272 | 11.1821 | 8.7817 |
| 9.3386 | 8.8668 | 10.6269 | 7.6461 | 9.7204 | 10.7905 | 10.539 | 9.6526 |
| 9.4481 | 10.35 | 9.702 | 9.6418 | 7.6517 | 8.1184 | 10.7981 | 10.2781 |
| 10.3668 | 7.5756 | 10.2156 | 9.9308 | 9.2786 | 10.5522 | 10.3737 | 9.413 |
| 11.3378 | 11.0655 | 10.4352 | 8.7617 | 11.0096 | 9.66 | 9.9692 | 10.5029 |
| 10.2099 | 10.3623 | 11.3492 | 11.2429 | 10.6061 | 11.8777 | 10.1598 | 10.5305 |
| 10.0124 | 10.8283 | 10.3531 | 8.3228 | 10.0861 | 10.8859 | 10.2143 | 10.6325 |
| 9.8444 | 11.1554 | 10.3149 | 10.4873 | 10.5752 | 10.2552 | 10.1882 | 10.4159 |
| 10.3114 | 10.0445 | 9.3314 | 10.6808 | 10.0466 | 10.8023 | 9.8003 | 9.1805 |
| 10.4075 | 7.3706 | 9.4028 | 11.3531 | 10.0535 | 9.1307 | 10.572 | 10.6771 |
| 8.7931 | 9.496 | 10.5975 | 8.499 | 10.4668 | 9.3602 | 10.2759 | 10.7638 |
| 10.5539 | 10.8287 | 10.3519 | 11.2272 | 10.4231 | 10.6172 | 9.7012 | 9.6793 |
| 10.1581 | 10.6954 | 9.7805 | 9.9635 | 11.7573 | 11.2165 | 10.5443 | 11.0179 |
| 11.683 | 10.4733 | 7.7319 | 10.7921 | 10.6352 | 7.3242 | 11.6667 | 10.6337 |
| 10.6582 | 8.8418 | 10.3273 | 9.3023 | 10.862 | 10.5922 | 10.1759 | 10.5603 |
| 11.2431 | 9.563 | 9.9742 | 9.2664 | 10.5068 | 10.5662 | 10.5161 | 10.3482 |
| 9.4518 | 10.0855 | 4.7944 | 10.0854 | 6.662 | 6.7275 | 5.5211 | 5.8982 |
| 4.9566 | 5.7305 | 4.9378 | 8.8469 | 5.1209 | 5.7774 | 5.5527 | 7.4536 |
| 7.0355 | 6.0077 | 7.703 | 5.6225 | 5.137 | 7.6466 | 5.9255 | 8.9975 |
| 4.6785 | 5.8832 | | | | | | |
| HJURP | 9.6149 | 8.6348 | 7.4185 | 9.513 | 10.3554 | 8.7881 | 8.9887 |
| 10.3221 | 9.8836 | 9.4174 | 9.8974 | 9.1351 | 8.4142 | 8.0178 | 9.6878 |
| 9.4105 | 9.1729 | 9.5901 | 7.6218 | 7.5884 | 8.5116 | 11.4792 | 8.5827 |
| 7.0754 | 8.8211 | 10.3728 | 9.8804 | 9.9181 | 8.712 | 10.1746 | 9.5868 |
| 7.7651 | 9.4946 | 9.131 | 9.2849 | 9.3885 | 9.0642 | 9.8513 | 10.0058 |
| 8.1904 | 9.1588 | 9.8847 | 7.751 | 9.3398 | 8.6556 | 9.1961 | 9.188 |
| 9.6491 | 8.7795 | 8.4844 | 9.5244 | 9.4087 | 8.5966 | 9.4132 | 9.2519 |
| 8.8178 | 8.6009 | 9.3367 | 5.1477 | 10.223 | 8.9677 | 8.9848 | 9.2908 |
| 8.8507 | 8.4871 | 7.4126 | 9.6843 | 6.2048 | 6.3766 | 9.0991 | 9.0869 |
| 9.9497 | 6.4559 | 9.6859 | 8.0552 | 9.4411 | 9.5955 | 9.5085 | 8.5579 |
| 10.374 | 9.1172 | 8.9529 | 8.5248 | 10.2378 | 9.232 | 8.0405 | 9.9545 |
| 9.0376 | 9.293 | 9.8473 | 9.7996 | 8.5683 | 9.9744 | 9.0501 | 10.4935 |
| 9.677 | 8.8191 | 8.2513 | 8.3577 | 9.8704 | 9.365 | 9.7197 | 8.4395 |
| 9.5674 | 9.7341 | 9.6442 | 10.1227 | 8.3751 | 9.2551 | 8.6114 | 9.91 |
| 9.6483 | 9.5753 | 9.8807 | 9.7084 | 8.438 | 9.9552 | 10.0911 | 8.7813 |
| 9.0125 | 4.2995 | 7.9334 | 9.7049 | 7.3999 | 7.8243 | 9.1735 | 9.0655 |
| 7.9673 | 8.4891 | 9.1968 | 7.2166 | 8.0664 | 7.6955 | 9.4468 | 8.7229 |
| 10.8564 | 9.0843 | 9.6116 | 9.7301 | 8.5705 | 9.2383 | 8.4711 | 7.7729 |
| 8.9028 | 9.1766 | 8.9812 | 8.7144 | 9.2895 | 10.619 | 9.606 | 10.4531 |

| | | | | | | | |
|---|---|---|---|---|---|---|---|
| 9.9428 | 9.8353 | 7.0275 | 10.0274 | 10.2417 | 6.6653 | 9.2937 | 9.1447 |
| 10.4823 | 7.938 | 9.5009 | 7.5166 | 9.7215 | 9.6717 | 9.0659 | 9.4476 |
| 9.8453 | 7.5687 | 9.598 | 8.3688 | 9.1624 | 9.8678 | 9.434 | 8.6388 |
| 8.1476 | 8.4098 | 3.7046 | 8.9804 | 5.126 | 3.2153 | 4.5807 | 3.4113 |
| 1.026 | 3.4251 | 3.3396 | 7.8367 | 2.7094 | 3.1952 | 4.3753 | 6.5148 |
| 4.6491 | 5.0194 | 6.4427 | 3.0966 | 2.2162 | 5.9241 | 3.9842 | 8.004 |
| 2.2714 | 3.2388 | | | | | | |
| NCAPG | 9.8626 | 9.6028 | 7.8777 | 10.3168 | 9.8674 | 9.476 | 9.4451 |
| 9.9453 | 10.1707 | 9.3089 | 9.2273 | 9.4617 | 8.9293 | 7.9624 | 8.9981 |
| 10.1269 | 8.547 | 9.6535 | 7.9215 | 8.1866 | 9.978 | 9.8152 | 8.9535 |
| 7.498 | 9.5581 | 10.9546 | 9.8755 | 9.5244 | 8.7766 | 9.7462 | 10.4321 |
| 7.429 | 10.067 | 9.819 | 9.6464 | 9.1503 | 8.792 | 9.6755 | 9.1311 |
| 10.0001 | 9.0708 | 9.9741 | 8.2487 | 9.0546 | 8.7716 | 8.9202 | 7.8134 |
| 9.8184 | 8.8281 | 7.624 | 9.1463 | 9.5648 | 10.1054 | 10.2317 | 10.0873 |
| 9.1554 | 7.1822 | 8.9302 | 5.9989 | 9.6885 | 9.4708 | 9.2341 | 9.1562 |
| 8.3926 | 8.6119 | 8.2418 | 8.7855 | 6.4624 | 6.5489 | 9.5002 | 9.5849 |
| 10.3199 | 7.1038 | 9.7558 | 8.3556 | 7.7715 | 9.1202 | 9.7496 | 8.2924 |
| 10.5141 | 9.9239 | 9.0715 | 8.4281 | 11.0032 | 7.662 | 8.0896 | 10.1342 |
| 10.39 | 9.8796 | 9.9371 | 10.3334 | 9.9747 | 11.1179 | 9.2951 | 9.204 |
| 9.7099 | 9.2479 | 9.3817 | 9.0968 | 10.5267 | 10.2386 | 9.346 | 9.0382 |
| 9.192 | 9.8474 | 8.8515 | 10.0306 | 9.4157 | 9.004 | 8.7969 | 8.8741 |
| 9.591 | 8.7611 | 9.4328 | 10.0887 | 9.0638 | 9.9834 | 9.151 | 9.7698 |
| 10.636 | 5.0468 | 8.5819 | 9.5099 | 8.1346 | 8.2362 | 9.3966 | 9.0764 |
| 8.5881 | 7.9862 | 10.2179 | 7.0266 | 9.1564 | 8.2183 | 9.548 | 9.882 |
| 10.0426 | 9.6623 | 9.5916 | 10.8376 | 9.3718 | 9.9153 | 8.5374 | 8.2912 |
| 9.3158 | 9.5931 | 8.8287 | 9.2768 | 9.8013 | 10.5413 | 10.3093 | 9.9479 |
| 10.1565 | 9.3364 | 7.983 | 9.594 | 10.1798 | 7.308 | 9.8659 | 9.1698 |
| 9.6555 | 7.8926 | 9.1205 | 8.5095 | 9.6001 | 9.2115 | 9.2886 | 10.0144 |
| 10.5125 | 8.1368 | 9.3118 | 9.3234 | 9.2715 | 10.1404 | 10.4345 | 8.6986 |
| 8.4595 | 9.8141 | 3.1495 | 9.0091 | 5.7053 | 4.6162 | 4.4241 | 3.8611 |
| 2.1303 | 4.3656 | 4.4559 | 8.0398 | 4.2589 | 4.4409 | 4.5581 | 6.9076 |
| 5.2447 | 5.1919 | 7.2074 | 2.9993 | 2.6347 | 6.4631 | 3.6621 | 7.7157 |
| 3.0626 | 3.687 | | | | | | |
| TPX2 | 11.6929 | 10.2343 | 9.1527 | 10.7239 | 11.5557 | 11.2496 | 12.3757 |
| 11.7995 | 11.863 | 11.6948 | 11.5935 | 10.5174 | 10.2167 | 9.3478 | 11.2794 |
| 10.9572 | 11.4194 | 11.4944 | 9.6826 | 9.2363 | 10.7461 | 12.8133 | 10.2166 |
| 8.6847 | 11.3855 | 12.6613 | 10.9641 | 12.2242 | 10.9581 | 12.1797 | 11.576 |
| 9.2206 | 11.2056 | 10.7955 | 10.5087 | 10.9686 | 11.5282 | 11.7773 | 10.4663 |
| 11.1584 | 11.4325 | 12.4359 | 9.6586 | 10.9534 | 11.3738 | 11.1166 | 10.112 |
| 10.7093 | 10.3298 | 9.0027 | 11.4445 | 11.5357 | 11.4733 | 11.106 | 11.7125 |
| 10.474 | 9.2579 | 11.7064 | 7.7454 | 12.6444 | 11.2603 | 10.7479 | 11.4302 |
| 11.1087 | 9.9197 | 10.2239 | 10.9604 | 7.8121 | 7.5249 | 10.9057 | 10.6581 |
| 12.0153 | 7.6473 | 11.6799 | 9.9321 | 10.4233 | 11.4086 | 10.8022 | 10.4954 |
| 11.8407 | 11.6213 | 11.6949 | 9.3517 | 11.5852 | 11.0044 | 10.7041 | 11.118 |
| 10.8046 | 11.0152 | 11.4202 | 11.2831 | 10.9097 | 12.9497 | 10.6 | 11.8977 |
| 11.3368 | 10.7427 | 10.6388 | 11.2819 | 11.0861 | 12.542 | 11.9964 | 10.4128 |
| 11.7462 | 11.9164 | 11.7577 | 12.2469 | 10.3403 | 11.2383 | 11.0222 | 11.5906 |
| 11.2775 | 12.1128 | 11.5261 | 11.7333 | 10.2956 | 10.6358 | 11.4999 | 11.1978 |
| 11.0937 | 6.5899 | 9.626 | 11.5878 | 9.5785 | 9.2967 | 11.0246 | 10.7959 |
| 10.4829 | 10.161 | 10.6344 | 8.2258 | 10.6841 | 9.6581 | 11.4332 | 11.1819 |
| 12.2021 | 10.7634 | 10.8783 | 11.4397 | 10.469 | 11.2765 | 10.0627 | 9.4397 |
| 11.2823 | 11.0311 | 11.5287 | 10.5093 | 10.9667 | 12.808 | 11.5577 | 12.5728 |
| 11.5294 | 10.9599 | 9.0269 | 10.8467 | 11.5598 | 8.0295 | 10.7392 | 11.6749 |
| 12.423 | 9.2964 | 12.3989 | 9.488 | 11.7608 | 11.6701 | 10.4911 | 11.4924 |

| | | | | | | | | |
|---|---|---|---|---|---|---|---|---|
| 11.3306 | 9.4532 | 11.6993 | 11.6274 | 10.8121 | 11.5385 | 12.5785 | 10.4034 | |
| 10.0511 | 10.8272 | 4.3657 | 10.6709 | 6.704 | 6.24 | 5.9933 | 5.4185 | |
| 3.8551 | 5.1761 | 5.7887 | 9.475 | 5.4299 | 5.4937 | 5.8571 | 7.7738 | |
| 6.5353 | 6.4144 | 8.1617 | 4.9643 | 3.1292 | 8.2402 | 5.2043 | 8.5276 | |
| 4.8248 | 5.0749 | | | | | | | |
| CDCA2 | 8.4989 | 7.7266 | 7.0875 | 9.7732 | 9.297 | 6.679 | 7.6672 | |
| 8.4942 | 9.4712 | 7.942 | 7.645 | 7.8365 | 7.8054 | 6.229 | 6.9964 | |
| 8.2731 | 7.5863 | 6.9401 | 6.2247 | 6.6316 | 8.3617 | 9.7836 | 7.7489 | |
| 5.9421 | 7.9282 | 9.4754 | 8.7286 | 7.8784 | 6.6197 | 8.8881 | 9.6326 | |
| 6.7624 | 8.8965 | 8.3938 | 8.9988 | 8.3665 | 7.3106 | 8.1597 | 8.0036 | |
| 8.8019 | 7.4988 | 9.2142 | 7.1363 | 8.0842 | 7.0625 | 7.8848 | 7.1528 | |
| 8.273 | 7.0787 | 6.9696 | 8.0939 | 6.5819 | 8.9687 | 8.5932 | 6.8867 | |
| 7.9981 | 5.3954 | 9.0292 | 4.5928 | 7.9046 | 7.9297 | 8.0879 | 7.5956 | |
| 6.2654 | 7.6387 | 7.0928 | 7.8151 | 5.1887 | 4.8669 | 8.2975 | 8.0613 | |
| 7.8257 | 5.2447 | 9.2474 | 6.7127 | 6.5526 | 7.6866 | 9.0683 | 6.7158 | |
| 8.5772 | 7.2475 | 7.5326 | 6.4223 | 9.2682 | 8.5785 | 8.1542 | 8.8953 | |
| 8.7433 | 8.0711 | 8.902 | 8.2902 | 8.9755 | 9.9038 | 7.2571 | 8.0066 | |
| 8.409 | 7.8784 | 7.6182 | 6.9316 | 6.3219 | 8.433 | 7.7003 | 8.17 | |
| 7.6109 | 9.812 | 7.4189 | 8.3107 | 7.7858 | 8.0805 | 8.0778 | 7.7875 | |
| 8.3778 | 6.7436 | 7.5394 | 8.5124 | 7.9562 | 9.0842 | 7.606 | 8.8108 | |
| 8.788 | 5.8813 | 7.4349 | 8.3851 | 6.336 | 7.0262 | 8.1168 | 8.5711 | |
| 7.3418 | 7.2036 | 8.3765 | 5.3411 | 8.8789 | 7.2711 | 8.086 | 8.3529 | |
| 7.6367 | 8.9945 | 7.5363 | 8.8419 | 7.7385 | 8.3349 | 7.0412 | 6.7122 | |
| 6.2458 | 8.3737 | 8.1868 | 8.3278 | 9.2659 | 9.6835 | 9.9834 | 9.3409 | |
| 8.7785 | 8.1077 | 6.0231 | 8.8994 | 9.8827 | 5.6243 | 8.6931 | 7.3484 | |
| 7.6946 | 6.1514 | 8.1706 | 6.4459 | 6.8895 | 8.9551 | 8.0624 | 7.8334 | |
| 8.8574 | 6.8237 | 8.3351 | 8.6188 | 9.0126 | 7.9511 | 8.6178 | 7.936 | |
| 7.3558 | 8.597 | 2.3255 | 7.4619 | 3.5819 | 3.5909 | 2.8244 | 3.2379 | 0 |
| | 2.5005 | 2.4755 | 6.2719 | 1.6854 | 2.0921 | 3.4124 | 4.7052 | |
| 3.1191 | 3.9785 | 5.2985 | 1.9774 | 2.4747 | 4.789 | 2.1203 | 5.454 | |
| 2.2714 | 2.9741 | | | | | | | |
| MYOZ2 | 1.737 | 0 | 0 | 0.5278 | 0.7223 | 0 | 0.8314 | 0 |
| | 0.4291 | 0 | 0.4363 | 0.4395 | 0 | 0 | 1.3881 | |
| 1.2795 | 0.3755 | 0 | 0.8427 | 0 | 0 | 0 | 1.3471 | |
| 0.8532 | 0 | 0 | 0.5612 | 0 | 0 | 0 | 0 | 0 |
| | 0.6557 | 0.541 | 0 | 1.1184 | 0 | 0 | 0.9285 | 0 |
| | 0 | 0.7769 | 0 | 0 | 0 | 0.6494 | 0.8153 | |
| 0.5608 | 0.6343 | 1.171 | 3.2061 | 0.9799 | 1.1594 | 0.5573 | 0 | |
| 0.6977 | 0 | 0 | 0.5773 | 2.0689 | 0.4334 | 0 | 0.9449 | |
| 1.1245 | 0.927 | 0 | 2.3844 | 0.5408 | 1.5048 | 0.6159 | 0 | |
| 0.3965 | 1.1987 | 1.4748 | 0.5263 | 0 | 0 | 0 | 1.6807 | |
| 0.9113 | 0.847 | 0 | 2.0255 | 0.9013 | 1.4589 | 0.8845 | 0.5504 | 0 |
| | 1.7978 | 0 | 0.7358 | 0 | 1.1001 | 1.0326 | 1.7151 | |
| 1.0041 | 0.5136 | 0 | 0 | 0 | 1.1918 | 0.6116 | 0 | 0 |
| | 0.7085 | 1.0358 | 5.1501 | 0 | 1.0557 | 0.47 | 1.2272 | |
| 1.8708 | 0 | 1.811 | 0 | 0 | 0 | 0.8107 | 0 | 0 |
| | 0 | 2.0174 | 1.3507 | 0 | 0 | 0.9357 | 0 | |
| 1.4775 | 0 | 1.224 | 2.7711 | 2.7757 | 0.5414 | 0 | 0 | |
| 0.7727 | 0.3855 | 1.3489 | 0.5416 | 0 | 0 | 0 | 0 | |
| 0.4418 | 0.5023 | 0 | 1.1906 | 0 | 0 | 0 | 0 | 0 |
| | 0.6229 | 0 | 0 | 0.7148 | 1.9052 | 2.0978 | 0 | 0 |
| | 0.8792 | 0 | 0.6248 | 0 | 0 | 0 | 0 | |
| 0.5078 | 0.9557 | 0 | 0.4262 | 0 | 0 | 0.6064 | 0 | |
| 1.9827 | 0.6959 | 3.9323 | 2.7493 | 4.8296 | 1.4101 | 3.6666 | 4.093 | |

| | | | | | | | |
|---|---|---|---|---|---|---|---|
| 5.4425 | 5.2741 | 5.8585 | 1.7745 | 4.4263 | 5.5946 | 2.4839 | 5.0013 |
| 2.3506 | 4.2908 | 4.6694 | 3.7193 | 3.1292 | 3.9299 | 2.6463 | 3.0757 |
| 5.5356 | 2.9741 | | | | | | |
| SPC25 | 8.6739 | 7.6495 | 5.92 | 8.1139 | 7.6286 | 7.6024 | 8.0742 |
| 7.8614 | 7.8819 | 7.4885 | 7.9917 | 7.5868 | 6.8263 | 6.7042 | 7.6664 |
| 7.9949 | 7.4628 | 7.4655 | 6.2625 | 6.418 | 8.3776 | 10.2871 | 7.4078 |
| 5.9421 | 7.7227 | 8.6937 | 7.8196 | 8.5476 | 6.3084 | 8.9681 | 7.0588 |
| 5.8421 | 7.9262 | 7.672 | 7.046 | 7.3451 | 8.2509 | 8.0479 | 8.0633 |
| 7.7885 | 7.3005 | 8.6082 | 6.9203 | 7.3284 | 6.6663 | 7.7805 | 7.5595 |
| 8.0166 | 7.4778 | 6.6601 | 7.1765 | 6.971 | 8.3055 | 7.7736 | 7.9717 |
| 6.9043 | 5.7104 | 8.3574 | 4.7593 | 8.4463 | 7.6616 | 7.3714 | 8.2367 |
| 7.424 | 6.8847 | 6.2923 | 7.1516 | 3.959 | 4.7069 | 7.7769 | 8.5515 |
| 8.7277 | 4.7498 | 8.0398 | 7.1705 | 7.6217 | 8.1133 | 7.9462 | 7.2341 |
| 9.4913 | 7.3286 | 6.9616 | 6.5881 | 9.0014 | 7.1432 | 6.4825 | 7.8771 |
| 7.8565 | 7.6258 | 7.7406 | 7.894 | 8.1839 | 9.1505 | 7.792 | 8.6735 |
| 7.2655 | 7.3976 | 6.9888 | 7.8192 | 7.8887 | 7.8537 | 7.9579 | 6.9768 |
| 7.5095 | 8.0879 | 7.0757 | 8.6191 | 7.5959 | 7.8128 | 7.2836 | 8.0893 |
| 8.4299 | 8.0554 | 7.9873 | 6.9002 | 7.1866 | 6.5782 | 8.106 | 7.8067 |
| 7.5964 | 4.1556 | 6.3729 | 7.6436 | 7.2174 | 5.8716 | 7.8434 | 8.0297 |
| 5.8458 | 6.9837 | 7.1828 | 5.7171 | 7.7897 | 6.6673 | 7.8462 | 7.5473 |
| 8.7891 | 7.7773 | 7.7622 | 8.9321 | 7.267 | 8.2241 | 6.7328 | 6.3849 |
| 7.9966 | 7.5092 | 7.3769 | 7.2235 | 7.9962 | 8.5149 | 7.8751 | 8.291 |
| 7.7623 | 8.7418 | 4.983 | 8.0157 | 7.8887 | 4.3377 | 7.5866 | 7.0675 |
| 9.0789 | 6.4224 | 7.7018 | 6.2646 | 8.1101 | 7.6913 | 7.4085 | 8.2651 |
| 8.527 | 6.5548 | 8.0186 | 8.9608 | 7.6493 | 9.2883 | 8.4726 | 6.9913 |
| 6.2332 | 8.0936 | 2.0429 | 7.6093 | 4.2822 | 2.8232 | 1.522 | 1.9303 |
| 1.026 | 3.0357 | 0.5422 | 6.3381 | 2.4416 | 1.8952 | 2.225 | 4.3324 |
| 3.0314 | 3.4596 | 5.4028 | 1.8148 | 1.4974 | 5.0711 | 1.9521 | 5.425 |
| 3.7631 | 1.8629 | | | | | | |
| FOXM1 | 11.6751 | 10.0018 | 8.9046 | 11.8064 | 11.0094 | 10.6007 | 10.3556 |
| 11.2516 | 11.0842 | 11.1518 | 10.5699 | 10.1783 | 9.8558 | 8.7656 | 9.2936 |
| 10.5312 | 10.5623 | 12.4484 | 9.465 | 8.8625 | 9.9724 | 12.8488 | 10.3237 |
| 8.3241 | 9.9774 | 11.6103 | 10.6381 | 11.4263 | 10.2988 | 11.8186 | 10.8113 |
| 8.9411 | 11.2317 | 10.1563 | 9.8151 | 10.6745 | 9.6502 | 12.0489 | 9.2648 |
| 9.5573 | 9.6957 | 11.4084 | 9.6249 | 10.3329 | 9.4348 | 10.1553 | 10.3813 |
| 10.5348 | 9.61 | 9.5643 | 10.472 | 10.0389 | 10.9349 | 11.1301 | 11.1351 |
| 9.9269 | 9.7811 | 11.5457 | 7.5629 | 11.1849 | 10.7018 | 10.6178 | 9.9299 |
| 10.7623 | 10.1462 | 9.3031 | 9.8716 | 7.7411 | 7.1185 | 10.8484 | 10.5957 |
| 11.9644 | 6.9746 | 11.5092 | 9.3445 | 9.6262 | 10.8552 | 10.309 | 9.8579 |
| 11.5487 | 10.1687 | 10.6438 | 8.9374 | 11.2319 | 10.7599 | 10.0571 | 9.8515 |
| 9.8517 | 10.7725 | 10.6733 | 10.9137 | 11.4541 | 12.1802 | 9.2223 | 10.8245 |
| 10.8372 | 10.3029 | 10.1361 | 11.1979 | 9.946 | 11.7583 | 10.4199 | 10.675 |
| 10.2473 | 11.6419 | 9.8571 | 11.0867 | 10.0829 | 9.9315 | 10.5808 | 10.7808 |
| 10.9697 | 9.8296 | 10.7541 | 9.9917 | 10.1205 | 10.8367 | 10.2225 | 8.8833 |
| 10.371 | 7.312 | 9.4354 | 11.0546 | 9.0864 | 9.0931 | 11.0545 | 10.8417 |
| 9.203 | 9.8607 | 8.8929 | 7.9155 | 9.2183 | 9.3759 | 9.6023 | 11.1668 |
| 11.3387 | 10.6646 | 10.3793 | 11.3389 | 10.144 | 11.0492 | 9.4277 | 9.015 |
| 11.3568 | 11.0649 | 10.2188 | 10.151 | 10.9736 | 11.9685 | 11.1125 | 11.3869 |
| 11.2429 | 11.1963 | 7.1689 | 10.4976 | 11.0896 | 7.9656 | 10.3919 | 10.2705 |
| 11.3956 | 8.8085 | 10.4322 | 8.8752 | 12.6219 | 11.1252 | 10.1747 | 11.5857 |
| 11.1071 | 8.9442 | 9.6396 | 11.9539 | 10.1858 | 10.2573 | 10.1505 | 10.3444 |
| 9.6478 | 10.0179 | 4.8656 | 10.32 | 5.9504 | 6.0808 | 5.2745 | 5.5045 |
| 2.2357 | 5.0582 | 4.7557 | 8.7255 | 4.1132 | 4.3587 | 5.6662 | 6.4715 |
| 6.6241 | 5.5977 | 7.6686 | 4.6817 | 3.6581 | 7.9274 | 5.2356 | 7.9505 |

4.2849 5.1456
ASF1B 11.2024 8.7986 8.6523 10.6795 10.0497 9.9157 11.5708
10.3122 11.2158 11.1808 11.2335 9.7695 9.867 8.7697 9.5781
10.4615 10.1144 10.2969 9.2279 8.496 9.9057 11.7583 9.4752
8.7537 9.7885 9.8582 10.4865 11.1643 9.5157 10.1107 11.098
8.7972 10.5622 9.9555 8.6262 10.1404 8.8961 10.1133 10.0096
10.8815 10.2479 8.9733 9.2195 9.5239 9.2547 10.2518 8.9136
10.4756 9.1302 8.2534 10.9314 10.1744 9.7707 10.7138 10.2706
9.4817 8.5265 9.3143 7.2252 10.6596 10.071 9.3798 9.6789
8.2456 9.513 8.9754 9.6477 7.655 7.3786 10.5233 10.1577
9.8875 7.4097 11.05 9.8022 8.8303 10.8127 10.4707 8.3552
11.3667 10.6254 9.567 8.4709 10.8469 9.7703 10.1139 10.2844
10.2982 10.4708 10.3998 10.631 10.3989 11.1182 9.4552 10.4885
11.2846 9.6066 9.6634 9.949 9.388 10.4753 10.4207 9.2824
10.6386 10.3492 9.9991 10.0694 9.8713 10.0206 9.941 10.6702
9.9057 10.7474 9.8279 10.0909 9.8015 10.3029 8.4205 9.2287
10.2001 5.1904 8.9214 10.6203 8.5105 9.1424 10.3073 10.2619
8.3558 9.6905 10.0423 7.725 9.7574 8.4728 9.7704 10.3327
10.1133 9.5303 11.213 11.1271 9.7864 10.307 9.3053 9.5394
9.8195 10.0249 10.7434 8.9783 10.6293 10.6802 9.9798 11.0865
10.7798 9.9812 7.8323 9.8991 9.6962 7.9029 10.9483 9.5414
11.705 8.7092 9.2303 9.437 10.6409 10.2057 9.8489 10.4615
10.4999 8.4668 9.6326 9.3993 9.889 11.5801 11.2383 9.7433
9.1408 10.4849 5.0488 9.7535 6.4805 5.8278 5.8249 4.7605
2.8515 3.9841 4.0048 8.4746 4.5337 4.7607 5.2559 7.2745
5.9894 5.7255 7.0407 4.9049 4.8483 7.3256 4.9822 8.1376
3.364 5.3607
RAD54L 9.7833 8.0202 7.1302 9.902 8.7802 7.2022 9.1675
9.2335 8.8225 8.587 8.8566 8.3335 8.2441 6.7964 8.024
8.4096 8.7562 8.984 7.416 6.4401 9.3352 9.2296 8.6266
5.8239 9.2309 9.2396 8.4766 8.7178 8.8147 9.2249 8.9696
7.4687 8.8241 8.0981 7.8866 8.2767 7.9536 8.9777 7.2972
10.2118 8.2877 8.8987 7.3828 7.8249 7.5204 7.7805 7.4031
8.7402 8.1651 7.3403 8.1525 7.8933 9.1529 8.7356 9.4393
8.2144 6.865 8.6986 5.2815 9.9676 9.4763 8.1125 8.9859
8.5542 7.3388 7.6931 8.5928 5.466 5.0648 8.993 8.7269
8.6366 4.9138 8.7396 8.3694 9.5266 8.8306 9.0825 8.0797
9.2329 8.635 9.6678 7.2372 9.1073 8.3267 8.215 8.5926
9.2427 8.3056 9.2632 9.7592 9.3651 9.7046 7.7553 9.0788
9.3039 7.8811 8.6401 9.1026 7.6582 9.2999 8.7979 7.9532
9.2996 8.7385 9.5351 8.9639 8.069 8.4399 8.3108 9.0236
9.0348 10.5065 9.6814 8.8731 8.2115 9.4684 8.7536 8.3431
10.1036 6.0124 7.7558 9.1509 6.9238 7.9359 8.595 8.3356
7.1913 7.819 8.3619 6.1223 9.8101 7.1281 9.5689 9.1248
9.1265 8.4531 9.1387 9.9007 8.8109 9.1427 7.6778 7.618
7.9824 9.1735 8.0745 8.4452 8.8884 10.3427 9.1417 8.8779
10.7461 8.102 7.1876 8.2338 8.7686 6.343 9.0954 8.371
9.7354 6.557 8.6465 7.2551 8.5238 9.8637 8.8737 9.3861
8.9985 6.6791 8.0266 8.9548 8.4855 10.2612 10.1946 8.2282
7.9077 9.8057 4.0917 8.2749 4.2447 3.6556 3.7692 3.792
1.6194 3.4809 2.9713 6.8879 3.3023 2.8039 3.2124 5.8524
4.6193 3.0257 5.7121 2.9476 2.9704 5.6217 3.113 7.0218
2.6934 3.5942
PPP1R12B 8.1369 8.8065 8.6884 9.9505 10.0134 8.5415

| | | | | | | | |
|---|---|---|---|---|---|---|---|
| 9.5136 | 7.9293 | 9.2229 | 8.147 | 8.6791 | 8.5628 | 8.3716 | 8.7468 |
| 8.0768 | 8.5175 | 6.8734 | 7.6341 | 9.519 | 9.3052 | 7.0164 | 9.2061 |
| 9.0132 | 9.7748 | 7.5699 | 11.688 | 10.1832 | 7.1367 | 8.5944 | 8.2863 |
| 7.9337 | 9.6463 | 8.9241 | 7.8418 | 8.8167 | 9.068 | 8.4733 | 8.5499 |
| 7.6549 | 7.7418 | 8.2849 | 9.7791 | 8.0483 | 8.8405 | 8.01 | 9.2443 |
| 9.4185 | 8.1039 | 10.2935 | 9.2412 | 12.3943 | 8.4108 | 9.8698 | 8.734 |
| 7.4529 | 9.4583 | 8.4837 | 8.8343 | 7.791 | 12.0665 | 8.0239 | 9.0208 |
| 7.4807 | 7.7975 | 11.2011 | 9.4378 | 8.7551 | 9.0433 | 9.1582 | 10.5517 |
| 8.0031 | 8.6661 | 11.7942 | 9.4963 | 10.4162 | 8.5243 | 8.7781 | 7.6812 |
| 10.2054 | 10.5702 | 9.8372 | 7.6654 | 8.8961 | 9.1276 | 8.225 | 10.6957 |
| 8.3677 | 7.0259 | 9.4061 | 8.5143 | 9.4143 | 8.4906 | 10.4463 | 10.3878 |
| 9.5284 | 8.8235 | 9.3539 | 7.7395 | 8.5696 | 8.7616 | 9.3126 | 8.4729 |
| 9.1563 | 8.0033 | 9.8151 | 8.5643 | 9.5069 | 8.4704 | 9.2829 | 7.8583 |
| 8.4257 | 10.3216 | 6.943 | 8.4279 | 7.8133 | 9.2681 | 9.7727 | 8.7934 |
| 9.2066 | 7.7241 | 8.8422 | 12.7061 | 9.2362 | 8.7622 | 7.6754 | 9.1849 |
| 7.9646 | 8.7717 | 7.6511 | 7.9972 | 12.7027 | 7.6842 | 9.3124 | 8.7934 |
| 7.5003 | 8.615 | 8.991 | 8.1504 | 8.8144 | 6.9812 | 8.2369 | 8.2463 |
| 7.6919 | 7.8294 | 9.4389 | 9.6493 | 9.7438 | 9.7823 | 8.5294 | 8.024 |
| 8.6455 | 8.1862 | 8.5556 | 8.7567 | 8.9195 | 10.3925 | 10.6335 | 8.5362 |
| 7.9733 | 7.9076 | 8.0172 | 8.719 | 9.9436 | 9.0918 | 7.8536 | 7.5305 |
| 7.7706 | 9.0974 | 8.8557 | 8.7414 | 10.3447 | 9.0377 | 7.3981 | 8.1212 |
| 8.9243 | 11.0397 | 7.3024 | 12.8774 | 11.3701 | 13.4685 | 10.357 | 14.7107 |
| 13.8893 | 14.6381 | 14.6208 | 15.0394 | 11.4633 | 13.8652 | 14.7299 | 13.6443 |
| 11.1329 | 11.345 | 14.1876 | 14.5393 | 13.3634 | 11.5428 | 12.5154 | 13.363 |
| 12.7614 | 14.4201 | 12.6877 | | | | | |
| TUBA1C | 13.7267 | 12.3685 | 11.7232 | 12.7216 | 13.2948 | 12.5124 | 13.0914 |
| 13.5251 | 13.6598 | 12.8129 | 12.8355 | 11.8592 | 12.563 | 11.9367 | 12.9067 |
| 12.7682 | 14.0109 | 13.3188 | 11.7218 | 11.677 | 13.2978 | 13.3172 | 13.2845 |
| 13.492 | 13.5168 | 12.5085 | 12.4723 | 14.0987 | 12.4531 | 12.7715 | 13.2048 |
| 12.3837 | 12.4965 | 13.9209 | 13.0725 | 13.2066 | 13.7537 | 12.9798 | 13.5077 |
| 14.7641 | 13.1456 | 12.1719 | 12.8832 | 12.5422 | 13.7418 | 12.8764 | 12.5577 |
| 12.6734 | 12.287 | 13.0521 | 12.4508 | 12.5403 | 12.8871 | 13.7662 | 13.1895 |
| 12.9773 | 12.5682 | 12.3373 | 13.2003 | 14.17 | 13.9122 | 12.9479 | 13.365 |
| 12.6436 | 12.287 | 11.902 | 12.8949 | 12.069 | 11.7888 | 12.5546 | 13.3126 |
| 13.4138 | 11.2886 | 13.2788 | 12.6182 | 12.1317 | 11.9674 | 13.9465 | 12.3225 |
| 12.6268 | 12.8965 | 12.4611 | 11.9368 | 13.5456 | 12.5917 | 12.214 | 13.036 |
| 14.1245 | 12.7261 | 13.2419 | 13.4774 | 13.4347 | 13.8836 | 12.985 | 12.3337 |
| 13.5934 | 12.9673 | 12.707 | 12.944 | 13.3999 | 13.2276 | 12.9274 | 12.7868 |
| 12.6196 | 13.6356 | 13.6202 | 13.5327 | 12.9317 | 13.1315 | 13.3463 | 12.9448 |
| 13.375 | 13.9331 | 13.365 | 13.0592 | 13.0053 | 13.4863 | 14.0483 | 13.8285 |
| 13.4197 | 11.1999 | 11.6645 | 13.5713 | 12.3214 | 12.7666 | 12.8194 | 13.0613 |
| 12.5445 | 12.7987 | 13.5062 | 11.2062 | 14.4528 | 11.9515 | 13.4647 | 13.4639 |
| 13.1799 | 12.5475 | 13.1332 | 13.5984 | 13.2429 | 13.5515 | 12.4518 | 12.4881 |
| 13.0476 | 12.7205 | 13.4514 | 12.6144 | 13.4433 | 14.0511 | 13.4063 | 13.3492 |
| 12.6659 | 13.0144 | 12.972 | 12.2293 | 12.807 | 11.0645 | 13.836 | 13.8584 |
| 13.2085 | 12.0348 | 13.0731 | 12.347 | 12.3851 | 13.8496 | 12.9303 | 13.7657 |
| 13.5183 | 12.7226 | 12.8356 | 12.7682 | 12.5824 | 14.226 | 14.0656 | 12.5567 |
| 11.9264 | 14.4401 | 10.4613 | 11.7395 | 10.6053 | 10.7237 | 9.7925 | 11.6256 |
| 9.9717 | 9.1064 | 9.9644 | 10.7735 | 10.3141 | 10.7106 | 9.2465 | 11.1124 |
| 10.3928 | 9.9157 | 9.9675 | 9.5877 | 10.7784 | 10.7072 | 9.9596 | 11.3072 |
| 10.3686 | 9.6163 | | | | | | |
| MELK | 9.6286 | 8.3093 | 8.1313 | 9.993 | 9.4082 | 8.6957 | 9.0871 |
| 9.9567 | 9.2445 | 9.2448 | 9.1793 | 8.8434 | 9.1947 | 7.0977 | 8.7896 |
| 9.8086 | 9.084 | 9.6237 | 8.3795 | 7.6965 | 9.4113 | 10.7395 | 8.4949 |

| | | | | | | | |
|---|---|---|---|---|---|---|---|
| 6.7475 | 9.5172 | 10.3185 | 9.4918 | 10.408 | 8.5393 | 10.0405 | 9.7557 |
| 7.2767 | 9.6265 | 9.6005 | 8.7264 | 8.9687 | 8.8394 | 10.5925 | 8.8593 |
| 7.9688 | 9.7399 | 10.155 | 8.1689 | 8.9118 | 9.2015 | 8.1958 | 8.5262 |
| 8.7766 | 8.6781 | 7.8511 | 9.0264 | 9.8034 | 9.1096 | 9.6702 | 9.2807 |
| 8.8522 | 6.3144 | 8.3094 | 5.8723 | 8.6184 | 10.0164 | 9.118 | 8.6561 |
| 8.3203 | 8.7361 | 8.1297 | 8.0655 | 6.0149 | 6.243 | 9.8762 | 9.0754 |
| 11.6924 | 5.8578 | 10.1635 | 8.1962 | 8.1162 | 9.3541 | 9.0409 | 9.0676 |
| 9.9347 | 9.6782 | 8.037 | 6.9738 | 10.1677 | 8.9566 | 8.8951 | 9.3887 |
| 8.4231 | 9.4806 | 9.4591 | 8.909 | 9.1942 | 9.8535 | 9.5367 | 8.8778 |
| 8.4469 | 8.7634 | 8.7048 | 6.2569 | 8.0928 | 9.3575 | 10.0145 | 8.8322 |
| 8.6417 | 9.7518 | 9.4037 | 9.3663 | 8.9013 | 9.4073 | 9.0895 | 10.165 |
| 9.8636 | 7.7514 | 9.5898 | 9.6941 | 8.9988 | 8.6876 | 9.0794 | 8.0365 |
| 8.7603 | 3.0835 | 8.0565 | 9.1257 | 7.7896 | 8.0977 | 9.3843 | 9.34 |
| 8.0666 | 8.2205 | 8.2681 | 6.83 | 6.1533 | 8.053 | 8.1837 | 9.6308 |
| 9.2508 | 8.9496 | 10.0083 | 10.1315 | 8.6192 | 8.908 | 8.1562 | 8.0187 |
| 8.5838 | 9.7089 | 8.6744 | 9.1485 | 9.5351 | 9.6721 | 9.1583 | 11.3508 |
| 9.4778 | 8.7599 | 3.2253 | 9.3592 | 9.7875 | 6.5684 | 9.5251 | 9.0677 |
| 10.0183 | 7.2994 | 9.1513 | 7.9519 | 9.626 | 9.0679 | 8.3795 | 10.0065 |
| 10.1028 | 7.4223 | 9.0594 | 9.3606 | 8.4052 | 8.9303 | 9.0829 | 9.3867 |
| 8.5528 | 9.166 | 2.6668 | 8.5543 | 4.9036 | 2.9316 | 3.865 | 3.4113 |
| 1.026 | 2.9592 | 2.4755 | 7.1866 | 2.4416 | 2.6869 | 2.9802 | 5.9786 |
| 4.7349 | 4.1037 | 5.9372 | 2.3776 | 2.6938 | 5.4804 | 3.8942 | 7.0458 |
| 1.6723 | 2.7661 | | | | | | |
| UBE2C | 11.3692 | 9.1741 | 8.97 | 10.0227 | 10.9368 | 10.4747 | 11.0742 |
| 11.2597 | 12.0413 | 10.8183 | 10.2349 | 9.5965 | 9.9028 | 9.6617 | 11.2981 |
| 10.4767 | 11.6384 | 11.7583 | 8.9015 | 8.7052 | 11.2124 | 12.4746 | 10.245 |
| 8.9382 | 12.0209 | 11.975 | 10.661 | 12.3465 | 10.7248 | 10.7186 | 10.9525 |
| 8.5137 | 10.6318 | 10.841 | 9.7054 | 10.7264 | 12.6193 | 10.4875 | 11.6236 |
| 12.022 | 10.5826 | 11.4443 | 9.6389 | 9.8659 | 11.535 | 10.4872 | 9.6551 |
| 11.2802 | 9.1316 | 8.3853 | 10.7206 | 11.5286 | 11.1751 | 10.5447 | 10.7456 |
| 9.9921 | 8.8492 | 11.4635 | 7.2719 | 11.7691 | 11.2863 | 9.6181 | 12.0456 |
| 9.5171 | 9.4039 | 9.7485 | 11.6255 | 7.5538 | 8.1837 | 10.4658 | 10.7398 |
| 11.3059 | 8.1266 | 11.5357 | 10.0512 | 11.078 | 11.4052 | 10.736 | 9.6674 |
| 12.122 | 10.7125 | 10.7575 | 10.253 | 11.491 | 9.66 | 10.3036 | 11.2879 |
| 10.9904 | 10.6367 | 10.7097 | 13.2314 | 11.4691 | 12.3097 | 10.3517 | 11.3927 |
| 11.8614 | 9.9179 | 10.0391 | 12.1773 | 10.9571 | 11.6342 | 10.9919 | 9.8124 |
| 11.6481 | 11.158 | 12.2219 | 11.7259 | 10.4488 | 10.7737 | 8.9914 | 11.1502 |
| 10.9173 | 12.795 | 10.8698 | 11.5069 | 10.0978 | 11.2925 | 11.9045 | 10.2838 |
| 11.5398 | 6.1326 | 8.6839 | 12.0346 | 9.0568 | 9.5236 | 10.0284 | 10.364 |
| 9.4202 | 9.4841 | 11.1933 | 8.3152 | 11.6686 | 8.3554 | 10.7436 | 10.6066 |
| 11.2025 | 10.411 | 11.6103 | 11.9701 | 9.8252 | 11.7321 | 10.1347 | 9.5387 |
| 10.9054 | 9.9221 | 11.2116 | 9.1272 | 11.1739 | 12.0901 | 10.5733 | 12.3325 |
| 11.083 | 10.7864 | 8.6357 | 10.4017 | 10.5588 | 7.8962 | 11.0158 | 11.2886 |
| 11.3729 | 9.2897 | 10.2925 | 9.1344 | 10.2843 | 11.978 | 10.7511 | 11.2369 |
| 10.254 | 8.9312 | 11.9274 | 11.7282 | 10.2223 | 13.0727 | 12.7253 | 9.7177 |
| 9.2204 | 11.0157 | 4.6736 | 10.2213 | 6.7246 | 5.6003 | 5.6876 | 4.5039 |
| 2.0387 | 4.8129 | 4.4281 | 9.097 | 3.8925 | 4.3247 | 5.4504 | 8.3911 |
| 5.4944 | 5.1984 | 6.8055 | 4.0469 | 2.884 | 7.2536 | 5.1664 | 9.0895 |
| 3.202 | 4.0266 | | | | | | |
| CENPA | 8.7895 | 6.9704 | 6.3169 | 7.7962 | 8.6843 | 8.7005 | 8.8198 |
| 8.1045 | 9.337 | 9.2333 | 8.3988 | 8.4216 | 7.5753 | 6.5537 | 9.3468 |
| 8.1732 | 9.4567 | 7.6263 | 6.6521 | 6.766 | 8.2045 | 9.7994 | 7.6302 |
| 5.6023 | 8.1549 | 8.9108 | 8.0674 | 9.9777 | 7.7568 | 8.6555 | 8.191 |
| 5.5411 | 8.3273 | 8.2653 | 8.3381 | 8.8196 | 8.631 | 8.5633 | 7.8023 |

| | | | | | | | |
|---|---|---|---|---|---|---|---|
| 9.4492 | 7.2449 | 8.8846 | 7.0495 | 8.2242 | 8.2109 | 7.9559 | 6.8871 |
| 8.3506 | 7.3902 | 7.0215 | 8.623 | 9.0536 | 8.6148 | 8.0241 | 8.6898 |
| 7.8543 | 6.7933 | 8.8443 | 3.8367 | 8.4551 | 8.9121 | 7.2073 | 8.4767 |
| 7.7509 | 6.8171 | 6.0789 | 8.7722 | 4.8676 | 4.8051 | 7.8685 | 7.9895 |
| 9.0599 | 4.4836 | 8.9236 | 7.4646 | 7.9191 | 7.802 | 8.8551 | 7.5584 |
| 8.9644 | 8.3701 | 8.3516 | 6.6155 | 8.9517 | 7.6685 | 6.6859 | 8.118 |
| 8.1046 | 7.7828 | 9.098 | 8.9562 | 8.6406 | 9.1915 | 7.639 | 9.0824 |
| 7.924 | 7.2874 | 7.3964 | 8.932 | 8.3399 | 9.2016 | 8.0528 | 7.5764 |
| 7.9835 | 8.5029 | 8.9623 | 9.1666 | 7.3508 | 8.1313 | 7.4454 | 9.1774 |
| 8.7726 | 9.0606 | 8.5195 | 8.7929 | 7.7116 | 8.4291 | 9.28 | 7.8405 |
| 8.5609 | 4.661 | 6.5102 | 9.2595 | 6.2551 | 6.8733 | 7.686 | 7.6055 |
| 6.5787 | 6.7641 | 8.5485 | 5.5981 | 7.463 | 6.1798 | 7.7043 | 7.61 |
| 9.3418 | 7.8765 | 9.2866 | 8.9628 | 7.655 | 7.1121 | 7.6848 | 6.6254 |
| 8.6302 | 8.1551 | 6.7547 | 7.4199 | 8.1227 | 9.9686 | 7.9301 | 9.7207 |
| 9.3718 | 7.7793 | 5.7889 | 8.0266 | 7.7981 | 4.8583 | 8.3014 | 7.7895 |
| 9.2871 | 7.0344 | 7.7694 | 6.7948 | 8.8757 | 8.9104 | 7.8943 | 8.2165 |
| 8.5884 | 5.9338 | 8.5581 | 9.0078 | 7.1301 | 7.8662 | 10.0154 | 7.5327 |
| 6.865 | 8.4462 | 2.6668 | 7.7927 | 4.2822 | 2.706 | 2.5058 | 3.1751 |
| 2.3632 | 2.6047 | 2.4755 | 6.6391 | 2.2864 | 1.396 | 3.3488 | 4.5101 |
| 3.0314 | 3.69 | 5.1523 | 2.1235 | 1.2434 | 4.4069 | 2.6463 | 5.8536 |
| 0.6293 | 2.0583 | | | | | | |
| EXO1 | 8.868 | 7.5865 | 7.0962 | 9.1963 | 7.8108 | 8.32 | 8.93 |
| 8.3204 | 8.3175 | 7.1822 | 7.7646 | 8.496 | 7.289 | 6.7964 | 7.2371 |
| 8.5034 | 7.4604 | 8.2321 | 6.7457 | 7.2138 | 8.3198 | 10.3806 | 6.9301 |
| 6.8317 | 7.9955 | 9.3083 | 9.5405 | 8.9106 | 7.5549 | 9.0963 | 9.0687 |
| 6.7498 | 9.7736 | 7.6394 | 6.9511 | 8.2325 | 7.6149 | 8.1419 | 6.6749 |
| 8.1998 | 7.7349 | 9.5072 | 7.5786 | 9.2003 | 6.3505 | 8.2879 | 8.3136 |
| 8.481 | 7.2767 | 6.769 | 8.3624 | 7.9903 | 8.5703 | 8.4853 | 8.8312 |
| 6.9117 | 5.6078 | 8.8293 | 4.2262 | 8.6627 | 8.7872 | 8.7359 | 7.721 |
| 7.0184 | 8.6465 | 7.3148 | 7.1372 | 3.9162 | 5.4953 | 8.6942 | 7.7816 |
| 9.2054 | 4.4836 | 8.7575 | 7.2054 | 7.9063 | 8.2328 | 6.9856 | 7.6016 |
| 9.8771 | 8.4164 | 7.6372 | 5.9061 | 8.9643 | 7.9027 | 8.0429 | 7.8997 |
| 7.7854 | 7.8971 | 8.3513 | 8.2466 | 9.1188 | 10.4018 | 7.5929 | 7.7152 |
| 8.305 | 8.4169 | 7.8379 | 7.9163 | 7.3487 | 8.5147 | 9.1949 | 8.6397 |
| 7.2792 | 8.1179 | 7.659 | 8.7342 | 7.4908 | 8.2814 | 7.8342 | 8.2714 |
| 8.0792 | 7.6821 | 8.1266 | 8.7142 | 7.393 | 7.2752 | 7.4653 | 7.9284 |
| 8.6819 | 4.1556 | 7.4672 | 7.893 | 8.0166 | 5.8716 | 8.3076 | 8.4335 |
| 7.3937 | 7.3202 | 8.8886 | 6.7763 | 7.6426 | 7.6373 | 8.1986 | 8.774 |
| 8.2287 | 8.5867 | 7.9958 | 8.4341 | 8.1474 | 7.7319 | 7.5687 | 7.1492 |
| 7.326 | 8.8558 | 6.0553 | 8.1983 | 8.2989 | 9.0901 | 8.8608 | 9.02 |
| 8.5188 | 8.8605 | 7.1598 | 8.726 | 8.8063 | 5.1975 | 8.7174 | 7.6554 |
| 9.4124 | 6.6261 | 8.258 | 7.6116 | 8.1434 | 7.9039 | 7.6677 | 8.2674 |
| 8.9178 | 7.1586 | 8.3199 | 9.1297 | 9.5115 | 8.7458 | 8.7602 | 7.5892 |
| 7.1181 | 7.6836 | 1.8777 | 7.7211 | 3.4564 | 2.1093 | 3.4976 | 2.2062 |
| 2.0387 | 2.5005 | 3.2731 | 6.558 | 2.9352 | 3.1952 | 3.2822 | 5.0307 |
| 3.3542 | 3.6357 | 5.9764 | 3.0241 | 2.2162 | 4.4681 | 2.4071 | 6.5359 |
| 2.6934 | 3.7151 | | | | | | |
| CDC6 | 9.7617 | 7.5445 | 7.5933 | 1.07E+01 | | 8.44E+00 | |
| 8.1951 | 8.8322 | 10.9355 | 9.0741 | 8.8191 | 8.1453 | 9.628 | 9.5671 |
| 7.721 | 9.3563 | 10.3091 | 9.2601 | 8.4571 | 8.7235 | 8.2068 | 9.2059 |
| 10.1588 | 9.2812 | 7.91 | 8.6807 | 9.9942 | 9.959 | 10.5828 | 8.4349 |
| 10.1852 | 10.885 | 7.8789 | 9.9535 | 9.2789 | 8.1227 | 9.1916 | 8.9204 |
| 10.0476 | 9.3752 | 8.8224 | 9.5129 | 10.4787 | 9.1073 | 9.3398 | 9.2664 |
| 8.6746 | 9.3327 | 9.9487 | 9.4806 | 9.8477 | 9.2279 | 9.354 | 9.8811 |

| | | | | | | | |
|---|---|---|---|---|---|---|---|
| 10.8379 | 8.7817 | 8.2851 | 7.2084 | 8.9432 | 6.866 | 8.7312 | 9.4184 |
| 9.4639 | 9.0826 | 8.0877 | 9.322 | 8.5865 | 9.1806 | 6.9831 | 6.1589 |
| 9.7045 | 9.659 | 9.8514 | 6.7001 | 9.1663 | 8.8459 | 9.0953 | 10.0312 |
| 9.6806 | 8.3878 | 9.4922 | 11.4776 | 8.2417 | 7.7873 | 10.4284 | 8.6942 |
| 8.1369 | 9.1477 | 8.9164 | 9.4905 | 8.8141 | 8.5194 | 9.3995 | 9.8416 |
| 8.3966 | 9.3246 | 9.506 | 9.067 | 9.132 | 7.8091 | 11.0768 | 7.9592 |
| 9.9975 | 9.1064 | 8.1488 | 9.9018 | 9.8939 | 10.0295 | 9.4425 | 8.722 |
| 9.2776 | 9.9711 | 9.178 | 9.2975 | 9.3474 | 8.386 | 8.9492 | 8.394 |
| 8.7958 | 9.0338 | 10.1087 | 6.5899 | 9.2969 | 8.4095 | 8.6168 | 8.8463 |
| 9.886 | 10.1773 | 8.3105 | 9.9163 | 9.5087 | 7.67 | 9.2461 | 9.2207 |
| 8.5963 | 9.9356 | 8.6561 | 9.3878 | 10.6002 | 10.1186 | 9.4475 | 9.7041 |
| 8.9828 | 8.6996 | 9.0554 | 9.7451 | 7.5096 | 9.2409 | 10.0454 | 9.6283 |
| 9.8132 | 10.3094 | 10.0276 | 9.9904 | 7.0729 | 9.4564 | 9.7189 | 6.889 |
| 10.7124 | 8.5516 | 10.1893 | 7.7313 | 9.8198 | 9.1063 | 9.7691 | 10.4041 |
| 9.025 | 9.944 | 10.4516 | 8.0601 | 8.6388 | 9.3162 | 9.4523 | 9.4386 |
| 8.689 | 9.2658 | 9.2174 | 9.7434 | 3.9323 | 8.7894 | 5.9036 | 4.4224 |
| 5.8833 | 4.8027 | 3.3683 | 4.471 | 4.1964 | 7.8519 | 4.7863 | 4.3633 |
| 4.3753 | 6.2277 | 5.9056 | 5.0611 | 7.2509 | 4.6634 | 5.4694 | 6.0987 |
| 4.4622 | 7.8916 | 4.5156 | 4.3588 | | | | |
| SKA1 | 8.7431 | 7.9143 | 6.5035 | 8.6416 | 7.2912 | 7.9795 | 8.2396 |
| 7.5422 | 8.757 | 8.4941 | 8.5532 | 7.6673 | 7.4468 | 7.1685 | 7.3979 |
| 8.0465 | 7.8928 | 7.5543 | 6.5761 | 6.7543 | 8.7437 | 9.6491 | 7.7454 |
| 6.0689 | 8.2442 | 8.115 | 8.5571 | 9.0316 | 6.6529 | 7.8713 | 9.0458 |
| 5.9113 | 8.2612 | 8.2198 | 6.2082 | 8.1397 | 8.7768 | 8.0131 | 8.4904 |
| 8.5543 | 6.5804 | 8.9723 | 7.2144 | 7.7254 | 8.3933 | 7.4225 | 6.8257 |
| 8.543 | 7.0963 | 5.8723 | 8.2629 | 8.023 | 8.1047 | 8.9855 | 7.7075 |
| 7.2193 | 5.8194 | 8.4341 | 4.9778 | 7.882 | 7.3662 | 7.6812 | 8.058 |
| 6.9603 | 7.619 | 6.7134 | 7.4552 | 4.8676 | 5.1166 | 8.5884 | 7.8849 |
| 8.7575 | 5.5137 | 7.7843 | 7.0318 | 7.1242 | 8.1389 | 7.9306 | 7.2214 |
| 8.9542 | 7.8115 | 8.1251 | 7.1711 | 9.2591 | 6.9218 | 7.1181 | 7.9815 |
| 8.0265 | 8.0887 | 7.6596 | 8.6446 | 9.0662 | 10.2085 | 8.2207 | 7.6674 |
| 8.4859 | 7.5436 | 7.4147 | 8.1578 | 9.2969 | 8.1903 | 8.2231 | 7.4546 |
| 7.9935 | 8.2165 | 7.4365 | 8.4504 | 7.7625 | 8.2814 | 8.2075 | 8.4201 |
| 7.9519 | 8.8866 | 7.9048 | 8.4937 | 7.4973 | 8.7257 | 8.4205 | 7.9595 |
| 8.9272 | 5.331 | 6.7968 | 9.2312 | 6.8704 | 6.5262 | 8.0313 | 8.3037 |
| 6.6316 | 7.5362 | 8.3233 | 5.8991 | 8.4266 | 6.553 | 6.772 | 8.0017 |
| 8.5318 | 7.7467 | 8.5284 | 9.2572 | 7.3432 | 8.262 | 6.7792 | 6.907 |
| 5.9688 | 7.7347 | 6.7351 | 8.0084 | 8.2847 | 9.4873 | 8.2007 | 8.9571 |
| 8.8081 | 7.9274 | 7.0275 | 8.0718 | 8.5784 | 5.762 | 8.7365 | 7.8674 |
| 8.7526 | 6.3938 | 7.9239 | 6.4637 | 8.7468 | 7.7601 | 8.0993 | 8.5861 |
| 8.9587 | 5.9989 | 8.5818 | 8.0439 | 7.6757 | 9.4963 | 8.0518 | 7.671 |
| 6.8702 | 8.6866 | 2.3255 | 7.8204 | 4.2063 | 3.1268 | 3.2362 | 3.5661 |
| 3.0451 | 2.7928 | 3.0528 | 6.6565 | 2.7094 | 3.2784 | 3.1391 | 5.6009 |
| 3.492 | 4.0215 | 5.359 | 2.2561 | 1.7133 | 4.9477 | 3.031 | 6.2347 |
| 3.364 | 3.2388 | | | | | | |
| POC1A | 10.1721 | 7.9143 | 8.0952 | 9.86E+00 | | 9.23E+00 | |
| 8.177 | 9.1812 | 8.6149 | 9.135 | 8.8229 | 10.027 | 8.0723 | 9.0814 |
| 7.3469 | 8.9772 | 9.2857 | 9.7777 | 9.8275 | 8.2445 | 7.7147 | 9.8931 |
| 9.0398 | 9.8658 | 7.9387 | 9.2716 | 8.5079 | 9.0375 | 9.7409 | 9.3202 |
| 8.0724 | 9.4761 | 8.4199 | 8.7528 | 8.9701 | 7.4506 | 8.6886 | 8.8342 |
| 8.4824 | 8.9726 | 11.5693 | 8.5278 | 8.8448 | 8.8972 | 8.1827 | 8.7471 |
| 9.5414 | 7.6899 | 8.4617 | 8.3595 | 7.7259 | 8.5203 | 9.1678 | 8.3553 |
| 8.5023 | 9.6342 | 8.9525 | 8.4607 | 8.9374 | 6.5623 | 9.3857 | 8.9947 |
| 8.8251 | 9.3856 | 9.5386 | 7.4802 | 8.7669 | 7.9983 | 7.4859 | 7.4101 |

| | | | | | | | |
|---|---|---|---|---|---|---|---|
| 8.8736 | 9.3633 | 9.0752 | 6.992 | 8.7575 | 9.0381 | 9.2534 | 8.6314 |
| 9.8192 | 8.6994 | 9.3259 | 9.5972 | 8.8244 | 6.9808 | 9.2662 | 8.3883 |
| 9.2391 | 8.2927 | 9.6535 | 9.3184 | 9.5808 | 8.9849 | 10.6989 | 10.1184 |
| 7.8389 | 9.5645 | 9.5415 | 8.2138 | 9.218 | 10.7292 | 8.4878 | 9.147 |
| 9.2422 | 9.4162 | 9.0374 | 9.2476 | 8.7464 | 9.5681 | 9.0649 | 9.2653 |
| 9.3147 | 8.534 | 10.6464 | 10.4555 | 9.1734 | 8.5717 | 9.4015 | 10.6747 |
| 8.3522 | 11.0612 | 10.3078 | 5.9263 | 7.9179 | 9.9364 | 7.9381 | 8.9646 |
| 8.6484 | 8.9001 | 8.0952 | 8.7358 | 11.0139 | 6.6018 | 11.1559 | 7.9236 |
| 8.4775 | 8.7006 | 9.4778 | 9.4742 | 9.371 | 10.3381 | 9.118 | 9.3147 |
| 8.244 | 8.4391 | 9.2039 | 8.7114 | 9.5687 | 7.4885 | 9.8314 | 10.4223 |
| 8.8171 | 9.5134 | 10.5526 | 8.5536 | 8.352 | 8.469 | 9.1889 | 7.7603 |
| 9.8876 | 9.3458 | 9.5651 | 7.8749 | 8.9169 | 7.8335 | 10.0074 | 10.1792 |
| 9.6848 | 9.6675 | 9.1562 | 8.4378 | 7.6545 | 9.0792 | 8.6619 | 11.2025 |
| 11.1057 | 8.179 | 8.1815 | 10.9983 | 4.9059 | 8.2981 | 5.5203 | 6.2816 |
| 5.1165 | 4.8027 | 3.6322 | 4.1989 | 5.3137 | 6.9315 | 5.4299 | 5.2267 |
| 4.6105 | 5.3438 | 6.235 | 4.7902 | 5.7237 | 4.8001 | 4.3962 | 6.5656 |
| 4.9822 | 6.1749 | 4.4449 | 5.5017 | | | | |
| AURKB | 10.5757 | 7.1616 | 7.4117 | 9.9873 | 8.8087 | 8.4081 | 8.963 |
| 9.3181 | 9.7772 | 8.8506 | 9.0206 | 7.9939 | 8.6499 | 8.474 | 10.0184 |
| 9.433 | 10.7011 | 9.6821 | 7.9619 | 7.3367 | 10.3206 | 10.942 | 9.6719 |
| 7.5304 | 9.6374 | 9.8196 | 9.6716 | 10.6649 | 8.0774 | 8.5727 | 10.4498 |
| 7.8468 | 9.4395 | 9.7597 | 7.9329 | 9.4646 | 9.9625 | 10.4601 | 9.4683 |
| 10.8731 | 9.5188 | 9.7156 | 8.1689 | 9.1001 | 9.6346 | 9.9656 | 9.1909 |
| 9.8681 | 8.0793 | 7.1835 | 8.885 | 9.0323 | 8.7532 | 9.5922 | 10.3148 |
| 8.4949 | 6.2477 | 9.0281 | 6.0649 | 9.8847 | 9.742 | 8.6193 | 10.0222 |
| 9.2158 | 7.8078 | 7.4701 | 9.3653 | 6.3883 | 6.5564 | 9.6501 | 10.3664 |
| 9.6256 | 6.0674 | 9.4904 | 9.3797 | 7.6177 | 9.1502 | 10.6586 | 8.0608 |
| 10.5043 | 9.2452 | 8.8613 | 8.6991 | 10.4415 | 8.2037 | 8.0794 | 9.5222 |
| 10.0666 | 9.3052 | 11.5303 | 9.669 | 8.8505 | 11.8191 | 9.1615 | 8.9999 |
| 10.0222 | 8.7158 | 9.5003 | 9.5527 | 10.1485 | 9.3519 | 8.5317 | 8.4505 |
| 9.3484 | 9.3928 | 10.4347 | 10.0011 | 8.6958 | 9.016 | 8.6241 | 8.8859 |
| 8.9318 | 10.2492 | 9.3824 | 8.5007 | 9.2339 | 9.1522 | 9.8856 | 10.4202 |
| 10.246 | 6.3793 | 7.9864 | 9.1961 | 7.7508 | 8.8355 | 9.3413 | 9.2792 |
| 7.1913 | 8.6295 | 10.1564 | 6.83 | 10.2736 | 7.5488 | 8.5333 | 10.0927 |
| 9.1155 | 9.5463 | 8.683 | 11.3524 | 9.1167 | 10.123 | 8.8051 | 8.8043 |
| 9.1301 | 9.1194 | 8.9998 | 7.4387 | 10.2694 | 10.8428 | 9.4634 | 8.9194 |
| 10.4375 | 10.4662 | 8.5579 | 8.9042 | 9.8293 | 6.862 | 10.1307 | 10.486 |
| 10.4662 | 7.9055 | 8.3956 | 8.0228 | 9.5555 | 10.4775 | 9.7701 | 9.8332 |
| 9.6613 | 7.5178 | 9.7775 | 10.4488 | 8.7068 | 11.1053 | 10.3077 | 8.7745 |
| 8.2788 | 11.3521 | 2.8563 | 9.1159 | 5.6405 | 4.4949 | 4.1575 | 3.792 |
| 1.6194 | 4.0593 | 3.3396 | 7.6185 | 2.5817 | 2.5596 | 3.8402 | 7.034 |
| 4.2038 | 4.2908 | 6.1144 | 2.6906 | 0.9349 | 6.8155 | 3.6413 | 7.5518 |
| 1.4008 | 3.3172 | | | | | | |
| PKMYT1 | 10.9639 | 8.9542 | 7.2177 | 10.9373 | 8.5182 | 8.9142 | 9.0199 |
| 9.5477 | 10.2549 | 8.6409 | 8.5005 | 8.9573 | 8.5587 | 8.2508 | 8.8522 |
| 9.6371 | 10.0037 | 9.8149 | 7.8373 | 7.3965 | 9.8846 | 10.6752 | 9.5085 |
| 7.8504 | 10.6167 | 10.0858 | 9.865 | 9.5533 | 9.5012 | 9.4796 | 10.4709 |
| 7.8276 | 9.6587 | 8.8908 | 7.8371 | 9.2925 | 9.7416 | 8.2822 | 9.1338 |
| 10.7907 | 9.1116 | 9.6261 | 9.4976 | 9.2971 | 8.661 | 8.8096 | 9.0915 |
| 9.9245 | 8.8786 | 6.9494 | 8.897 | 9.0196 | 9.6563 | 10.17 | 9.1081 |
| 8.2519 | 7.6397 | 8.3676 | 6.7577 | 10.4907 | 9.7367 | 8.3111 | 10.1693 |
| 9.9081 | 7.8229 | 8.9291 | 7.7054 | 6.9087 | 6.0078 | 9.8058 | 9.9404 |
| 9.531 | 5.7723 | 9.5159 | 9.4522 | 8.6786 | 8.6996 | 10.2769 | 8.9364 |
| 9.3396 | 9.0622 | 8.2611 | 7.7516 | 9.762 | 8.8115 | 8.5923 | 9.2222 |

9.644 9.7956 8.8585 10.2105 10.13 11.0158 8.1106 7.9124
10.0835 9.1327 9.2258 9.5864 9.5269 8.7935 9.6117 8.3407
8.9114 8.842 10.1332 8.8122 8.4819 8.9317 9.2347 9.5686
9.4067 10.4234 9.4873 9.5011 8.9244 9.5424 9.4947 9.501
10.8825 1.5206 7.9029 8.8725 8.5296 8.4245 9.0187 9.2954
7.356 9.7391 9.9999 7.0811 10.4551 7.2921 8.7818 10.0657
9.3967 8.8799 9.6342 10.9531 9.3766 10.163 8.6844 8.8887
9.3683 8.7599 8.6663 8.4932 10.6659 9.8333 9.5031 9.2409
9.0615 9.8583 8.0045 8.7598 9.4174 6.7908 10.4566 9.7041
8.082 7.359 7.8369 8.2261 9.4703 10.5819 9.7444 9.784
9.3584 7.1566 9.7146 9.0574 9.4612 9.5452 10.513 8.4852
7.5948 11.5909 3.8106 8.8689 5.8144 4.184 4.0216 3.8619
2.533 3.001 2.7612 7.4326 2.6095 3.2945 3.6788 6.2551
4.4814 3.9597 5.8037 2.5745 1.0537 6.6833 3.0411 7.2048
1.7635 2.8476
LOC134466 0 2.8336 2.3445 4.1524 1.9724 0.9367
1.8984 2.4718 2.4603 1.5838 3.7774 1.4754 1.489 0
3.0955 3.999 1.3142 4.3761 1.7571 0.911 1.627 1.0809
4.6839 2.8976 1.5953 0.4127 2.9368 4.9181 4.3004 1.5856
1.492 2.6245 2.8159 2.9681 2.0552 2.35 2.5514 1.6313
2.2058 3.8516 1.992 2.5837 1.359 1.5081 1.946 2.472
3.5404 0.9635 3.0318 2.3708 5.1687 3.2117 2.2329 1.9369
1.8326 3.279 1.8352 3.0286 2.1522 4.1734 0.4334 1.523
2.2328 2.6121 3.1877 1.1076 2.5085 3.3991 3.6599 2.241
2.7579 0.9627 4.1569 3.3909 4.2845 3.0901 1.7039 2.5297
3.5345 3.7836 2.9615 0 4.7106 3.5663 0.9065 2.6033
1.921 1.418 3.6507 0.5352 4.343 3.2866 3.2307 4.4537
3.1314 3.3293 1.6498 1.0545 4.9483 3.1699 3.9593 1.0399
1.217 2.5579 1.9462 3.7089 1.0794 2.0083 2.9016 1.8858
2.4516 3.0773 1.9593 1.5269 1.1234 2.6167 3.7466 2.6505
2.8207 2.5939 4.4303 7.4707 3.1296 0.5748 2.3186 3.4039
1.991 1.1308 0.9947 1.8766 6.2222 5.1339 1.7123 1.3242
0.5476 3.9564 1.1539 0 1.2429 0.6063 2.0641 3.4515
1.9645 2.3049 2.3687 2.9165 4.2687 3.1615 0.942 0.7903
0.9151 0.541 1.3895 0 1.5724 5.9146 4.9888 2.2477
3.6618 3.0059 2.1234 1.8738 2.7995 3.1597 2.4374 1.725
3.4201 2.1293 0.9557 1.5498 3.2618 1.9594 2.8198 0
2.6602 3.899 0.6959 6.4802 7.1529 6.2728 5.0702 6.8061
6.3598 7.8904 8.1686 6.8402 8.2231 6.4683 5.4405 7.6199
8.9176 7.353 7.5189 7.0268 8.3701 7.9493 7.0833 7.0911
8.4158 7.6442 7.9988
CDC45 9.8373 6.9984 7.1551 10.0967 7.8192 8.0182 9.3621
8.5423 9.2682 8.2203 7.9917 8.5519 8.2484 7.1857 7.9969
9.2199 9.2685 8.6498 7.2379 6.8674 9.261 9.7763 8.6266
7.2613 8.1421 8.9993 8.3179 9.5994 8.1176 9.9974 9.9777
7.0242 8.7217 8.7469 7.5699 8.7663 8.8529 8.3679 7.5123
9.2453 7.6982 9.2868 8.4625 8.4218 8.7732 7.3388 7.6952
9.1707 8.374 7.9297 8.6844 8.4812 9.0626 9.3406 9.1147
7.9057 5.9689 8.4107 5.4854 8.2227 8.5421 8.2885 9.3976
8.7182 8.0818 7.8979 7.6191 4.8676 5.2619 8.9345 9.2184
9.3025 4.1036 8.612 8.4442 8.7096 9.336 9.2249 8.6191
9.5711 8.6161 8.6803 6.8563 9.949 7.107 7.9222 8.3411
9.282 8.7957 8.6846 7.8654 8.3231 10.65 8.5654 8.6343
8.2655 8.0135 8.321 8.3298 9.3859 9.0443 8.6502 7.9035

| | | | | | | | |
|---|---|---|---|---|---|---|---|
| 7.5178 | 8.2812 | 9.9729 | 9.3293 | 8.1117 | 8.7673 | 8.2826 | 9.2701 |
| 9.174 | 8.4828 | 8.7206 | 7.6064 | 8.1932 | 8.1354 | 8.746 | 8.6594 |
| 9.8153 | 0 | 7.6409 | 7.3328 | 7.5086 | 7.4305 | 8.7635 | 9.1098 |
| 7.5066 | 7.9977 | 9.2309 | 6.1309 | 8.7991 | 7.2626 | 10.1338 | 8.9103 |
| 7.9516 | 8.7392 | 8.3755 | 10.1385 | 8.3294 | 9.1602 | 7.8524 | 8.5928 |
| 8.8399 | 8.893 | 6.3409 | 8.0544 | 9.0486 | 9.1369 | 9.286 | 8.2726 |
| 9.9494 | 9.9052 | 6.3773 | 8.8607 | 9.2532 | 5.6402 | 9.0257 | 8.4439 |
| 9.405 | 6.7324 | 8.4626 | 7.1211 | 8.645 | 10.4235 | 8.7497 | 9.4131 |
| 9.3299 | 7.1538 | 7.5765 | 8.2598 | 8.5599 | 9.4003 | 8.0347 | 7.989 |
| 7.7185 | 10.3141 | 2.4485 | 8.2749 | 3.3189 | 3.0325 | 3.0035 | 2.6372 |
| 1.026 | 2.8784 | 1.498 | 6.7887 | 2.7094 | 3.3571 | 0.9402 | 5.901 |
| 3.9403 | 3.0257 | 4.8905 | 1.1757 | 0.5418 | 5.1848 | 2.7525 | 6.879 |
| 1.4008 | 2.0583 | | | | | | |
| KIF18B | 9.6547 | 8.2438 | 6.961 | 9.6406 | 9.196 | 8.3323 | 9.8923 |
| 10.5401 | 8.5777 | 9.0595 | 8.3836 | 9.3753 | 7.96 | 7.5592 | 8.7175 |
| 8.9758 | 9.3873 | 8.536 | 7.2493 | 6.889 | 8.775 | 10.4965 | 7.9841 |
| 5.9978 | 8.6718 | 9.6437 | 9.0414 | 9.2713 | 8.0569 | 10.1683 | 9.8503 |
| 7.6551 | 9.3945 | 8.2131 | 7.8715 | 8.7468 | 8.4355 | 9.8103 | 8.13 |
| 7.7801 | 8.2458 | 10.0355 | 7.0664 | 8.3437 | 9.3317 | 8.3843 | 9.0522 |
| 9.2403 | 8.3051 | 7.8973 | 8.7153 | 8.9957 | 9.2938 | 9.5816 | 9.0038 |
| 8.2458 | 6.6531 | 8.7818 | 5.1275 | 9.6457 | 8.8877 | 8.6239 | 8.2717 |
| 7.9951 | 7.8675 | 7.5845 | 7.8389 | 6.0939 | 4.8363 | 9.5085 | 8.3832 |
| 9.1012 | 5.6315 | 9.0709 | 7.6874 | 8.2107 | 9.2732 | 9.016 | 8.752 |
| 9.8449 | 9.0814 | 7.9482 | 7.3116 | 9.7121 | 8.4014 | 7.9121 | 9.1298 |
| 8.0958 | 9.1175 | 9.2872 | 9.4408 | 8.1898 | 10.0884 | 8.8813 | 9.6283 |
| 9.6274 | 8.4925 | 8.6026 | 7.3202 | 8.4471 | 10.002 | 9.0729 | 7.8901 |
| 9.5654 | 8.8918 | 9.6068 | 9.8653 | 7.8248 | 9.1617 | 8.2428 | 8.7531 |
| 8.7172 | 7.6039 | 8.6184 | 8.053 | 7.9777 | 8.8147 | 8.3884 | 7.6684 |
| 9.2538 | 4.2995 | 7.7732 | 8.9842 | 6.5471 | 7.3217 | 8.9441 | 8.7636 |
| 7.3835 | 8.1314 | 7.9395 | 6.3306 | 7.0822 | 7.4395 | 9.1434 | 9.0663 |
| 9.2848 | 9.1857 | 7.8931 | 9.7721 | 7.9143 | 9.0403 | 7.4007 | 7.4763 |
| 8.7002 | 8.9989 | 7.5437 | 8.4676 | 9.1293 | 10.7389 | 9.6668 | 10.1111 |
| 9.7693 | 9.692 | 6.3282 | 8.8184 | 9.2787 | 6.3233 | 8.3415 | 8.2336 |
| 10.3265 | 7.2041 | 8.6272 | 6.7151 | 9.8261 | 9.6469 | 8.3569 | 10.0261 |
| 9.0292 | 6.9164 | 7.9071 | 9.2856 | 8.4017 | 10.0873 | 8.1076 | 8.5323 |
| 7.9178 | 8.5524 | 2.0429 | 8.8842 | 5.0411 | 3.4522 | 3.6666 | 3.3558 |
| 1.026 | 2.8784 | 2.6946 | 7.4288 | 2.9352 | 3.0128 | 3.3488 | 6.3259 |
| 4.3194 | 3.5794 | 5.5785 | 1.9774 | 1.2434 | 5.9022 | 3.113 | 6.6904 |
| 1.9008 | 2.384 | | | | | | |
| BUB1 | 9.7917 | 8.8816 | 8.5834 | 1.00E+01 | | 1.07E+01 | |
| 10.328 | 10.079 | 10.7253 | 10.3382 | 10.2723 | 10.5006 | 9.9277 | 9.5918 |
| 7.5056 | 9.0799 | 9.3721 | 10.5794 | 9.7319 | 8.5526 | 8.0599 | 8.9649 |
| 11.1621 | 8.9973 | 6.7036 | 10.6993 | 10.2213 | 9.4493 | 10.7727 | 8.4711 |
| 11.2015 | 10.0161 | 7.9408 | 10.1624 | 9.5946 | 9.618 | 9.3227 | 9.6004 |
| 10.6769 | 9.0896 | 8.7492 | 9.4868 | 10.2207 | 8.1904 | 10.128 | 9.2067 |
| 9.8645 | 8.8497 | 9.3568 | 9.4717 | 8.731 | 10.1111 | 9.4673 | 10.2054 |
| 9.6036 | 10.3464 | 9.8047 | 8.6441 | 10.2613 | 5.7719 | 10.1686 | 9.5954 |
| 9.7038 | 9.2052 | 9.1434 | 8.9312 | 8.4739 | 9.5272 | 6.1869 | 6.0827 |
| 9.3807 | 9.1925 | 10.7587 | 6.1086 | 10.0663 | 8.2659 | 10.0406 | 10.3961 |
| 9.6666 | 9.1783 | 10.2099 | 10.1937 | 8.7722 | 7.2431 | 10.1861 | 9.456 |
| 9.7199 | 9.2414 | 8.2358 | 9.6436 | 10.3229 | 9.6819 | 10.4799 | 10.4055 |
| 9.9373 | 10.2907 | 9.4614 | 9.5616 | 9.4243 | 8.1175 | 9.4838 | 11.0193 |
| 10.4704 | 9.6671 | 10.4448 | 10.4718 | 9.9916 | 10.5092 | 9.4151 | 10.0858 |
| 9.6633 | 10.3299 | 10.4483 | 8.4019 | 10.2845 | 9.5034 | 9.2659 | 9.4824 |

| | | | | | | | | |
|---|---|---|---|---|---|---|---|---|
| 9.9331 | 8.2741 | 9.1396 | 3.816 | 8.726 | 10.0623 | 8.1592 | 8.168 | |
| 9.7754 | 9.9132 | 8.4691 | 8.7934 | 8.9619 | 7.1631 | 8.276 | 8.9998 | |
| 9.9617 | 10.0437 | 10.9963 | 9.6407 | 9.1544 | 9.981 | 9.1207 | 9.4792 | |
| 8.5148 | 7.6829 | 9.2572 | 10.2384 | 8.0822 | 10.5429 | 9.361 | 10.5765 | |
| 10.2333 | 10.765 | 9.2369 | 9.8164 | 4.5406 | 9.8282 | 10.571 | 7.1732 | |
| 9.2105 | 9.545 | 10.63 | 7.8749 | 10.127 | 8.5127 | 10.5659 | 8.9805 | |
| 9.055 | 10.5313 | 10.3881 | 8.5897 | 9.4064 | 11.3442 | 9.3179 | 9.7876 | |
| 9.8472 | 9.3694 | 9.1504 | 8.7145 | 3.8896 | 8.6992 | 5.5045 | 3.6556 | |
| 4.5807 | 4.2535 | 3.0451 | 4.0222 | 4.4281 | 7.4639 | 3.7806 | 3.756 | |
| 4.1324 | 6.4618 | 5.5107 | 4.8149 | 7.0682 | 3.8084 | 3.5778 | 6.3469 | |
| 4.1094 | 6.9724 | 2.8104 | 4.8023 | | | | | |
| PLK1 | 10.8045 | 9.0661 | 8.2208 | 10.772 | 9.8086 | 10.5753 | 10.1486 | |
| 10.7664 | 11.3765 | 11.5533 | 9.8357 | 9.7337 | 9.0461 | 8.5945 | 8.7594 | |
| 9.6739 | 9.7981 | 10.5837 | 8.5663 | 7.8112 | 10.563 | 10.8924 | 9.2705 | |
| 7.9003 | 10.7037 | 9.8084 | 10.0414 | 10.4784 | 9.5321 | 10.8417 | 10.9834 | |
| 8.283 | 10.411 | 9.2338 | 8.8455 | 9.396 | 8.8944 | 10.205 | 8.5119 | |
| 10.2979 | 10.0643 | 10.9176 | 8.8429 | 10.0388 | 9.4918 | 9.8052 | 9.0818 | |
| 8.7937 | 9.4468 | 8.5094 | 9.8306 | 9.5843 | 10.0591 | 10.5948 | 10.4446 | |
| 9.8433 | 8.6104 | 10.2401 | 6.3524 | 10.339 | 11.1125 | 9.7316 | 9.9184 | |
| 9.1374 | 8.7648 | 8.6528 | 8.7597 | 7.5292 | 7.0588 | 10.2142 | 9.6988 | |
| 11.5057 | 6.2747 | 10.6845 | 8.9381 | 8.7623 | 9.0857 | 9.9316 | 9.1166 | |
| 10.9027 | 10.0451 | 9.3609 | 7.3004 | 10.2138 | 10.103 | 9.1824 | 8.941 | |
| 10.1304 | 10.1585 | 9.6844 | 9.2009 | 10.2856 | 11.7681 | 8.4494 | 9.1559 | |
| 10.4783 | 9.505 | 9.7784 | 8.5815 | 9.4491 | 10.7487 | 10.3483 | 9.1807 | |
| 10.5621 | 10.3337 | 10.1633 | 10.1275 | 9.13 | 9.2032 | 10.2916 | 10.6467 | |
| 10.0016 | 9.5835 | 10.2878 | 9.3425 | 9.8527 | 9.4962 | 9.7252 | 9.7225 | |
| 10.5543 | 6.2786 | 8.7364 | 10.3056 | 8.3335 | 8.3402 | 10.0017 | 10.0183 | |
| 8.8294 | 9.2735 | 9.8215 | 7.2366 | 10.1954 | 8.3554 | 10.1163 | 10.4203 | |
| 10.71 | 9.8757 | 10.2449 | 10.6415 | 10.2747 | 10.0685 | 8.5188 | 8.2969 | |
| 9.651 | 9.9952 | 8.5761 | 9.7465 | 9.7531 | 10.3309 | 10.3854 | 10.7944 | |
| 10.2503 | 10.1545 | 6.9484 | 9.5604 | 10.3545 | 7.2636 | 10.2173 | 10.7043 | |
| 10.2263 | 8.2597 | 9.4371 | 8.0961 | 9.7325 | 11.3955 | 9.6091 | 10.6658 | |
| 10.665 | 8.4591 | 8.4608 | 10.3197 | 9.9108 | 11.802 | 10.5063 | 9.3649 | |
| 8.7409 | 10.3358 | 4.3657 | 9.364 | 5.9155 | 5.6003 | 4.6361 | 4.5785 | |
| 2.0387 | 4.9177 | 4.8029 | 7.6837 | 5.0742 | 4.9503 | 5.6217 | 6.7364 | |
| 6.0687 | 5.0611 | 7.0951 | 4.9249 | 4.9999 | 7.2493 | 5.147 | 6.6658 | |
| 4.5156 | 5.3232 | | | | | | | |
| CAMK2A | 0 | 1.5051 | 1.6038 | 2.7531 | 0 | 0 | 2.0418 | |
| 1.4589 | 1.2544 | 0.8472 | 1.6409 | 0 | 0.5377 | 0 | 1.0553 | |
| 1.2795 | 3.585 | 1.8761 | 1.9172 | 1.6784 | 2.2189 | 0 | 1.8373 | |
| 1.3857 | 0 | 1.4094 | 2.7455 | 0 | 0.8184 | 0 | 1.1431 | |
| 2.3861 | 1.8437 | 1.2418 | 1.037 | 1.1184 | 1.39 | 1.0349 | 1.8914 | 0 |
| | 0 | 1.2792 | 0.8339 | 0.7972 | 2.5833 | 3.5609 | 2.1436 | |
| 1.5362 | 2.7053 | 1.171 | 4.3115 | 1.7785 | 0.8658 | 1.2717 | 2.3502 | |
| 2.3341 | 0 | 1.5796 | 2.98 | 3.8395 | 0.4334 | 0.9537 | 0 | |
| 2.0521 | 3.0375 | 0 | 2.9196 | 3.2691 | 1.7868 | 3.4039 | 5.2474 | |
| 0.9627 | 3.0222 | 2.6641 | 3.0846 | 3.0901 | 1.8912 | 0 | 3.2962 | |
| 6.0955 | 1.9243 | 2.6379 | 4.7441 | 1.8492 | 1.209 | 2.4992 | 2.4969 | |
| 1.6914 | 2.2374 | 2.457 | 4.1402 | 0.4835 | 3.4479 | 2.3737 | 2.3532 | |
| 1.3268 | 0.8916 | 0.6215 | 2.5153 | 2.3219 | 3.0492 | 2.3852 | 1.6815 | |
| 2.3757 | 2.525 | 1.6324 | 4.6927 | 0 | 1.3885 | 0.8239 | 2.1941 | |
| 2.6587 | 0.7843 | 1.5269 | 0.6684 | 1.5194 | 4.1345 | 3.8654 | 2.8207 | |
| 1.0837 | 5.1146 | 6.5775 | 1.6169 | 0.9848 | 1.9445 | 1.4986 | 0.8022 | |
| 1.9907 | 2.576 | 0 | 5.9191 | 0.9816 | 1.7123 | 1.3242 | 0.9437 | |

| | | | | | | | |
|---|---|---|---|---|---|---|---|
| 2.8119 | 2.1273 | 0.6 | 1.4969 | 0 | 1.0431 | 1.3167 | 0.4033 |
| 2.1964 | 0.8742 | 2.6075 | 1.3802 | 1.217 | 1.5072 | 1.829 | 1.4707 |
| 5.2423 | 0.6229 | 0 | 1.1272 | 3.9773 | 2.2762 | 1.5234 | 1.5061 |
| 1.6771 | 0.8792 | 0.7366 | 3.0083 | 2.45 | 2.0548 | 0 | 0.5707 |
| 1.6366 | 0 | 2.2796 | 2.7745 | 0.4985 | 0 | 0.6064 | 2.2759 |
| 2.5996 | 0 | 5.3142 | 5.4572 | 5.1465 | 4.1801 | 8.6478 | 5.523 |
| 6.3175 | 8.007 | 6.4615 | 6.2259 | 8.4537 | 8.7883 | 7.2922 | 5.6396 |
| 5.9894 | 6.7248 | 8.0024 | 8.584 | 4.4574 | 6.3222 | 6.7629 | 6.5154 |
| 8.5275 | 8.2165 | | | | | | |
| FAM54A | 8.0198 | 6.9845 | 5.7664 | 7.9328 | 7.6498 | 5.024 | 6.8478 |
| 7.6541 | 8.469 | 7.6407 | 7.1841 | 6.0559 | 7.0245 | 5.569 | 7.3124 |
| 7.5102 | 8.2336 | 6.4162 | 5.7904 | 5.9887 | 7.0734 | 8.17 | 6.9362 |
| 5.6724 | 7.9725 | 6.794 | 7.3331 | 8.2463 | 6.7066 | 8.0371 | 7.6938 |
| 5.1218 | 7.5546 | 8.0933 | 7.2592 | 7.6611 | 7.554 | 8.5593 | 7.2466 |
| 7.7461 | 7.2896 | 7.4782 | 7.7246 | 7.0235 | 8.4881 | 6.9385 | 6.8013 |
| 7.4927 | 6.7491 | 6.7412 | 8.1261 | 7.1496 | 8.6669 | 7.159 | 8.2348 |
| 6.9192 | 5.5925 | 7.7321 | 4.1077 | 7.7418 | 8.5743 | 6.3556 | 8.5779 |
| 6.6683 | 7.1319 | 5.3036 | 6.284 | 3.46 | 4.527 | 7.2701 | 6.8375 |
| 8.2954 | 4.3526 | 8.6337 | 6.6571 | 7.184 | 8.2393 | 7.2193 | 6.5959 |
| 7.7484 | 7.3986 | 6.1338 | 5.355 | 7.9809 | 6.3861 | 6.9799 | 7.3981 |
| 7.1946 | 7.3472 | 6.9909 | 8.4715 | 8.0055 | 8.9996 | 7.5771 | 7.7584 |
| 7.9535 | 6.4133 | 6.7855 | 6.6893 | 6.5999 | 8.9625 | 8.425 | 7.357 |
| 7.0777 | 7.5281 | 8.2774 | 7.2966 | 6.8564 | 8.1005 | 6.5035 | 8.1404 |
| 7.2871 | 6.8558 | 7.973 | 7.9875 | 6.9126 | 8.0886 | 7.9866 | 6.5351 |
| 7.1098 | 2.7241 | 6.4963 | 7.6473 | 6.2081 | 6.4436 | 7.2744 | 7.4607 |
| 5.6716 | 6.9141 | 7.7331 | 4.7442 | 7.2111 | 5.6878 | 6.9107 | 6.8859 |
| 8.4391 | 6.7077 | 7.7449 | 7.908 | 6.7347 | 7.0786 | 6.5459 | 6.2629 |
| 7.1072 | 7.1559 | 7.9008 | 7.3872 | 7.3255 | 8.0793 | 6.815 | 8.7337 |
| 6.5517 | 7.8852 | 2.9928 | 6.593 | 7.4679 | 4.9888 | 8.356 | 7.7694 |
| 7.9847 | 6.0905 | 7.7472 | 6.6229 | 7.1735 | 8.4818 | 7.2262 | 8.1091 |
| 7.2523 | 5.5701 | 6.7612 | 8.3138 | 7.6223 | 7.7626 | 8.0086 | 6.759 |
| 6.3423 | 7.9308 | 3.4924 | 6.2368 | 4.0843 | 2.706 | 3.0035 | 1.9303 |
| 1.026 | 1.4299 | 2.217 | 4.7564 | 1.4126 | 2.2653 | 2.3603 | 4.2346 |
| 3.492 | 3.6357 | 4.1923 | 2.4897 | 1.9011 | 3.7446 | 3.1906 | 5.2371 |
| 1.6723 | 1.0391 | | | | | | |
| MKI67 | 11.7649 | 11.0353 | 9.7184 | 11.9379 | 11.1949 | 12.05 | 11.1556 |
| 11.817 | 11.4905 | 12.0028 | 11.2655 | 11.5718 | 10.8366 | 10.0421 | 10.7884 |
| 11.1892 | 10.9204 | 11.108 | 9.9006 | 10.2351 | 11.158 | 12.7333 | 11.0963 |
| 9.3643 | 11.9202 | 12.6323 | 11.0364 | 11.0011 | 10.1052 | 12.3988 | 11.7858 |
| 9.475 | 12.5023 | 10.9295 | 11.2515 | 11.3345 | 9.9197 | 11.475 | 11.5927 |
| 11.7766 | 11.1303 | 11.9112 | 9.8548 | 11.0157 | 10.9706 | 10.9338 | 10.474 |
| 11.6422 | 10.8379 | 9.4891 | 11.2221 | 11.3364 | 11.7054 | 11.6799 | 10.7477 |
| 10.9929 | 9.098 | 11.8063 | 8.2718 | 10.8896 | 10.9705 | 12.1538 | 10.7182 |
| 10.4262 | 10.4868 | 10.5227 | 9.8664 | 8.2145 | 8.4696 | 11.6446 | 10.8378 |
| 10.4769 | 8.4727 | 11.2399 | 10.7533 | 9.0682 | 10.3752 | 10.8052 | 11.0295 |
| 12.2892 | 12.1979 | 11.0828 | 10.1176 | 11.2364 | 10.1692 | 10.8786 | 11.4348 |
| 11.6519 | 11.7518 | 12.1924 | 11.657 | 11.2894 | 13.3903 | 10.5738 | 11.6783 |
| 10.3091 | 11.2972 | 10.9808 | 10.5365 | 11.8273 | 11.7647 | 10.869 | 10.9812 |
| 10.9337 | 12.596 | 10.825 | 11.4237 | 10.2625 | 12.0762 | 11.8089 | 12.0145 |
| 10.7296 | 10.8848 | 10.5646 | 12.1255 | 10.9309 | 11.6891 | 10.7359 | 10.2447 |
| 12.0241 | 7.5682 | 11.0149 | 11.8566 | 9.0582 | 9.5 | 11.8208 | 11.4652 |
| 10.1293 | 10.5681 | 11.2271 | 8.9353 | 11.3304 | 10.658 | 10.7008 | 11.9871 |
| 12.6301 | 11.9887 | 9.7777 | 11.5895 | 10.9626 | 11.6312 | 10.4685 | 9.5224 |
| 11.2687 | 12.2368 | 10.9347 | 11.2917 | 12.2517 | 12.4518 | 12.4368 | 11.3146 |

| | | | | | | | | |
|---|---|---|---|---|---|---|---|---|
| 11.7009 | 11.4474 | 10.1667 | 11.0666 | 12.0462 | 8.7878 | 10.8079 | 11.2011 | |
| 12.4955 | 9.1686 | 11.0703 | 9.201 | 10.9496 | 11.7286 | 10.9361 | 11.8589 | |
| 11.5372 | 9.4895 | 9.808 | 11.3592 | 11.1462 | 12.0579 | 11.9083 | 11.3031 | |
| 10.6005 | 11.0213 | 5.4675 | 11.49 | 7.5928 | 6.6101 | 6.1911 | 6.4815 | |
| 3.7479 | 5.9736 | 5.9465 | 9.9292 | 6.0526 | 5.719 | 6.6863 | 8.9038 | |
| 6.9839 | 6.9692 | 9.4987 | 4.9049 | 3.1292 | 8.6246 | 5.3636 | 9.3201 | |
| 4.0753 | 5.1625 | | | | | | | |
| SLITRK3 | 0 | 0 | 0 | 0 | 0 | 0 | 1.3555 | |
| 0.4327 | 0 | 0 | 0 | 0 | 0 | 0 | 0 | 0 |
| | 0 | 0.6175 | 0 | 0 | 0 | 1.0809 | 0.599 | |
| 1.7737 | 0 | 0 | 1.946 | 0 | 0 | 0 | 0 | 0 |
| | 1.1048 | 0.541 | 0 | 0 | 0 | 0 | 0 | 0 |
| | 0 | 0 | 0 | 0 | 0 | 2.472 | 0 | 0 |
| | 1.4095 | 0 | 4.8972 | 0.9799 | 0.4969 | 0 | 0.5968 | |
| 0.3906 | 0 | 1.3736 | 0 | 0 | 0.4334 | 0 | 0 | |
| 0.8374 | 4.5287 | 0 | 2.0987 | 0 | 0 | 1.0461 | 0.5659 | 0 |
| | 1.1987 | 1.1284 | 0 | 0.6199 | 0 | 0 | 3.2962 | 0 |
| | 0.847 | 0 | 0 | 1.4518 | 0 | 2.2653 | 0 | 0 |
| | 1.1625 | 0 | 2.1133 | 0 | 2.1477 | 3.38 | 1.7151 | 0 |
| | 0 | 0 | 4.3124 | 0 | 2.4008 | 3.3929 | 0 | 0 |
| | 0.3974 | 0 | 8.8042 | 1.3285 | 0.6224 | 0 | 0 | |
| 3.8366 | 0 | 1.5269 | 0.6684 | 0 | 0 | 0 | 1.7113 | |
| 0.6413 | 0 | 5.6247 | 0 | 0 | 1.438 | 0 | 0 | 0 |
| | 0 | 0 | 5.3262 | 0 | 0 | 0 | 0 | |
| 1.2732 | 0.9402 | 0 | 0 | 0 | 0 | 0.5821 | 0 | |
| 0.4418 | 0 | 1.6706 | 0.4012 | 0.5276 | 1.7238 | 0.4486 | 0.9151 | 0 |
| | 0.6229 | 0 | 0 | 1.3803 | 0 | 0 | 0 | |
| 2.4318 | 0 | 0.7366 | 1.3929 | 0 | 0 | 0 | 0.5707 | |
| 0.5078 | 0 | 0 | 2.4509 | 0 | 0 | 0 | 0.3736 | |
| 1.1797 | 1.5159 | 4.771 | 2.1946 | 3.6973 | 6.4277 | 6.6735 | 5.1514 | |
| 8.6512 | 9.2337 | 8.0102 | 2.6888 | 11.317 | 4.845 | 5.738 | 0 | |
| 8.3372 | 5.4705 | 6.9001 | 6.9538 | 3.8298 | 10.0117 | 5.4291 | 5.4825 | |
| 10.8544 | 7.6916 | | | | | | | |
| IQGAP3 | 10.1697 | 9.6173 | 8.5477 | 9.334 | 10.0838 | 9.465 | 8.9056 | |
| 10.4831 | 10.5214 | 9.1394 | 9.4376 | 9.1514 | 8.7365 | 9.689 | 9.6859 | |
| 9.7855 | 9.4152 | 9.3644 | 7.8646 | 8.8346 | 8.0764 | 10.8915 | 8.0044 | |
| 7.604 | 10.4343 | 9.9734 | 10.3952 | 10.0556 | 8.4076 | 9.8026 | 10.2344 | |
| 8.7819 | 11.3166 | 9.37 | 9.21 | 8.727 | 10.4375 | 10.5046 | 9.688 | |
| 8.1998 | 9.6815 | 9.6778 | 9.052 | 10.4069 | 10.215 | 8.8359 | 9.7117 | |
| 9.9507 | 9.7336 | 8.4521 | 9.8323 | 9.5218 | 10.7219 | 9.4824 | 9.7408 | |
| 9.3442 | 8.4331 | 9.634 | 6.3261 | 10.6259 | 9.4897 | 10.1333 | 8.9647 | |
| 8.0603 | 9.2618 | 8.5484 | 8.5135 | 5.9945 | 7.0388 | 9.7746 | 7.9647 | |
| 9.7949 | 6.3906 | 9.7559 | 7.8722 | 8.9557 | 9.3451 | 8.048 | 9.5695 | |
| 10.7004 | 10.9418 | 8.6129 | 8.7308 | 9.2498 | 10.2548 | 9.2936 | 10.6966 | |
| 8.0449 | 9.6665 | 11.1192 | 10.3609 | 8.3863 | 11.0007 | 9.2875 | 10.1383 | |
| 9.577 | 9.7475 | 8.4354 | 8.6843 | 10.3332 | 10.3327 | 10.1956 | 8.3329 | |
| 11.582 | 9.5663 | 10.6513 | 9.4559 | 8.6799 | 10.8235 | 8.8052 | 9.4662 | |
| 8.539 | 7.7896 | 10.5054 | 9.8542 | 8.4951 | 10.6238 | 9.3943 | 6.9072 | |
| 7.6537 | 6.7488 | 8.1808 | 9.7192 | 8.5144 | 6.845 | 9.5638 | 9.6006 | |
| 8.2582 | 7.6758 | 8.7432 | 8.0826 | 7.113 | 8.6035 | 9.7069 | 10.0817 | |
| 11.01 | 10.3377 | 8.1426 | 8.8801 | 9.3035 | 9.476 | 8.2974 | 7.7362 | |
| 8.4497 | 9.4689 | 9.2871 | 11.4785 | 9.5004 | 11.1883 | 9.7208 | 10.0406 | |
| 10.0538 | 10.0679 | 8.1757 | 9.5702 | 10.0482 | 7.0411 | 8.9443 | 9.6795 | |
| 10.3926 | 7.8277 | 9.2868 | 8.2323 | 9.515 | 10.0296 | 8.9547 | 8.9211 | |

| | | | | | | | | |
|---|---|---|---|---|---|---|---|---|
| 9.9251 | 7.4613 | 9.062 | 10.2152 | 10.4699 | 8.8505 | 8.4053 | 8.8252 | |
| 8.2808 | 7.4167 | 4.0142 | 9.4747 | 5.6691 | 4.346 | 4.7904 | 4.3702 | |
| 3.2157 | 3.8633 | 2.7929 | 8.0059 | 3.1304 | 3.5703 | 4.4069 | 6.429 | |
| 5.1641 | 4.2189 | 6.8848 | 2.5936 | 1.9011 | 6.7055 | 3.9842 | 8.235 | |
| 2.9187 | 2.8738 | | | | | | | |
| MCM10 | 8.8196 | 6.1839 | 6.1856 | 9.70E+00 | | 8.30E+00 | | |
| 8.8959 | 9.3125 | 8.6316 | 8.4914 | 7.9112 | 7.9532 | 8.8106 | 7.5267 | |
| 5.8094 | 7.4917 | 7.9868 | 8.4721 | 7.9561 | 6.5641 | 7.0414 | 7.7277 | |
| 10.414 | 7.0129 | 6.0159 | 9.0433 | 9.2277 | 8.0442 | 9.7936 | 8.3698 | |
| 8.8558 | 9.9356 | 6.8295 | 8.787 | 8.0369 | 8.0075 | 8.0179 | 8.3545 | |
| 8.3877 | 7.3699 | 7.3419 | 8.9366 | 9.7501 | 7.1562 | 8.1381 | 7.9405 | |
| 8.0205 | 8.2502 | 8.53 | 7.9272 | 6.3784 | 8.0461 | 8.4128 | 9.089 | |
| 8.4566 | 9.038 | 7.2608 | 6.305 | 9.3116 | 4.5017 | 7.6299 | 8.5106 | |
| 8.0729 | 8.276 | 6.9694 | 7.6485 | 7.0682 | 6.4786 | 3.46 | 3.5128 | |
| 9.0302 | 7.8613 | 9.8797 | 3.6619 | 8.2954 | 7.3641 | 7.9255 | 8.524 | |
| 7.4469 | 7.7714 | 8.3072 | 9.3047 | 8.2867 | 5.0418 | 9.0352 | 8.9102 | |
| 7.3713 | 8.1108 | 7.4551 | 8.06 | 8.3192 | 7.544 | 8.5112 | 9.8642 | |
| 7.8168 | 8.3289 | 8.0607 | 8.2867 | 8.2152 | 7.3483 | 7.6147 | 9.0064 | |
| 9.8517 | 8.2391 | 8.825 | 8.6249 | 9.1298 | 8.8149 | 7.2155 | 8.9165 | |
| 7.691 | 8.582 | 8.6153 | 8.2077 | 8.2565 | 8.3655 | 7.1586 | 7.5548 | |
| 7.7031 | 7.095 | 8.4841 | 5.1146 | 7.2696 | 8.7075 | 6.8213 | 5.506 | |
| 8.4118 | 8.8672 | 6.4073 | 8.0172 | 7.5903 | 5.4821 | 6.7447 | 6.797 | |
| 8.9566 | 9.1729 | 8.767 | 8.7659 | 8.2901 | 9.2711 | 7.4505 | 8.0474 | |
| 7.0788 | 6.6672 | 8.2037 | 8.7854 | 6.9877 | 7.9347 | 8.5041 | 8.9263 | |
| 9.0344 | 9.618 | 7.9833 | 9.1247 | 5.0451 | 8.1135 | 9.2295 | 4.6214 | |
| 7.8288 | 8.484 | 9.2344 | 5.2768 | 8.1806 | 6.3911 | 8.8818 | 8.6723 | |
| 7.1106 | 9.3339 | 8.8495 | 6.4029 | 7.4863 | 9.3831 | 8.528 | 8.9658 | |
| 7.5866 | 8.0123 | 7.3997 | 7.6704 | 1.4768 | 7.5108 | 3.0845 | 2.706 | 2.62 |
| | 3.2379 | 1.6194 | 2.9592 | 2.352 | 6.2605 | 2.2864 | 2.5596 | |
| 2.225 | 4.5913 | 3.0314 | 3.1077 | 5.4171 | 1.4215 | 1.2434 | 3.8858 | |
| 2.5317 | 5.8966 | 1.4008 | 2.384 | | | | | |
| C16orf59 | | 9.8772 | 7.6886 | 6.6918 | 9.7453 | 7.5127 | 7.5042 | |
| 8.219 | 9.0313 | 8.9432 | 7.3039 | 7.5061 | 7.5598 | 7.9442 | 7.1857 | |
| 8.7193 | 8.76 | 8.62 | 8.6401 | 7.5288 | 7.166 | 9.5271 | 8.4128 | |
| 9.421 | 7.6453 | 9.0391 | 9.4868 | 8.781 | 8.5771 | 9.2295 | 8.6555 | 9.58 |
| | 8.4894 | 8.5716 | 7.9062 | 7.1706 | 8.3144 | 7.6848 | 8.3402 | |
| 8.3695 | 9.8441 | 8.6202 | 8.4727 | 8.8343 | 8.5047 | 7.6439 | 8.6726 | |
| 8.8282 | 8.9065 | 7.7124 | 6.3418 | 8.3351 | 8.2146 | 8.5178 | 8.8388 | |
| 8.6182 | 7.6006 | 6.6383 | 8.997 | 5.719 | 9.6244 | 9.405 | 8.1109 | |
| 8.9322 | 8.9754 | 7.2086 | 7.0723 | 8.2961 | 7.024 | 6.1712 | 8.9702 | |
| 9.159 | 8.8265 | 5.4722 | 8.6699 | 9.0525 | 9.0782 | 8.3393 | 9.7274 | |
| 7.9312 | 9.6444 | 8.4464 | 8.3496 | 7.6876 | 9.7518 | 8.074 | 7.884 | |
| 8.1035 | 9.148 | 8.2002 | 7.9489 | 9.3554 | 9.2434 | 10.1243 | 7.5098 | |
| 7.7121 | 9.8179 | 8.2628 | 8.299 | 9.2918 | 9.4179 | 7.6443 | 9.2597 | |
| 8.0621 | 8.369 | 7.9706 | 8.2277 | 8.9563 | 8.0663 | 7.0627 | 7.8511 | |
| 8.4125 | 8.2476 | 9.768 | 8.2873 | 8.5263 | 8.2046 | 8.9253 | 7.0051 | |
| 9.2806 | 9.881 | 7.5682 | 7.3255 | 8.2545 | 7.4207 | 8.7136 | 7.8689 | |
| 8.2676 | 6.3133 | 8.0767 | 9.7661 | 6.5387 | 10.1038 | 7.0852 | 8.7335 | |
| 8.835 | 8.1625 | 7.9209 | 9.1956 | 10.3335 | 8.1919 | 8.7067 | 8.1486 | |
| 8.5375 | 8.3598 | 7.9852 | 7.0204 | 7.6561 | 8.7709 | 8.6973 | 8.0639 | |
| 8.7911 | 8.9248 | 8.9994 | 7.7507 | 8.376 | 9.0093 | 6.0034 | 9.7569 | |
| 7.7734 | 9.2459 | 7.3223 | 7.4337 | 7.9955 | 8.1255 | 9.675 | 9.1605 | |
| 9.3458 | 8.5868 | 7.2682 | 9.4939 | 8.3658 | 8.1876 | 8.9716 | 10.3711 | |
| 7.551 | 7.4033 | 10.6098 | 4.2006 | 8.0165 | 5.3717 | 5.8418 | 4.0393 | |

| | | | | | | | |
|---|---|---|---|---|---|---|---|
| 3.3558 | 3.2157 | 2.9592 | 2.5892 | 6.6651 | 3.1304 | 3.3571 | 4.1697 |
| 5.6586 | 5.635 | 4.0632 | 4.4916 | 4.7782 | 3.8298 | 5.9022 | 4.5823 |
| 7.3307 | 3.5773 | 5.265 | | | | | |
| C9orf140 | | 11.5822 | 10.0672 | 9.8783 | 9.7615 | 7.8087 | 10.8681 |
| 9.3782 | 10.2473 | 9.1649 | 11.3627 | 9.4853 | 10.0107 | 9.8423 | 9.5031 |
| 9.3903 | 10.5103 | 9.8182 | 9.4703 | 9.6149 | 9.5322 | 9.5584 | 11.6056 |
| 10.962 | 9.9661 | 10.0147 | 9.8535 | 11.6726 | 10.0918 | 9.5761 | 11.1098 |
| 11.688 | 9.5547 | 11.2858 | 11.0628 | 10.2567 | 10.8867 | 10.0687 | 10.906 |
| 11.0469 | 10.1027 | 8.5848 | 9.0665 | 10.2107 | 10.4268 | 8.8533 | 9.4899 |
| 9.2433 | 11.2961 | 9.8616 | 7.9098 | 10.2254 | 9.4004 | 9.6774 | 10.5194 |
| 10.0645 | 9.8496 | 9.1859 | 9.5385 | 10.3385 | 10.8477 | 9.5463 | 10.2702 |
| 9.3472 | 9.6684 | 9.4905 | 9.7179 | 9.2117 | 9.261 | 7.722 | 10.6473 |
| 10.5504 | 9.8011 | 8.9761 | 9.5959 | 9.5945 | 8.7207 | 9.3208 | 11.2105 |
| 9.6783 | 10.8351 | 9.9764 | 9.8605 | 8.9274 | 9.746 | 9.1699 | 8.7859 |
| 10.3926 | 10.8533 | 11.1584 | 11.2695 | 10.6461 | 9.9911 | 11.2156 | 9.1132 |
| 8.9896 | 10.1147 | 10.9443 | 10.5473 | 7.1707 | 9.5507 | 10.4218 | 9.435 |
| 9.1884 | 9.4783 | 9.9479 | 9.4778 | 9.8584 | 10.286 | 8.0485 | 11.7997 |
| 9.371 | 11.6826 | 9.9799 | 11.1562 | 11.0792 | 10.6058 | 10.9429 | 9.7405 |
| 9.4052 | 10.0275 | 6.98 | 10.5537 | 10.7709 | 10.2946 | 8.5278 | 10.4221 |
| 10.6227 | 9.1435 | 9.9081 | 10.9742 | 8.403 | 9.1355 | 9.1703 | 10.0659 |
| 10.7438 | 9.8002 | 10.7042 | 9.1413 | 10.4123 | 11.5122 | 10.5931 | 8.7661 |
| 8.6634 | 11.1296 | 9.451 | 9.2565 | 8.9117 | 10.7771 | 11.1496 | 11.4356 |
| 9.9583 | 10.4652 | 10.1297 | 10.5061 | 10.5764 | 10.8592 | 8.2407 | 11.5355 |
| 9.7418 | 9.7227 | 10.7385 | 9.618 | 10.3481 | 9.6423 | 9.4278 | 10.7149 |
| 11.1514 | 10.8329 | 8.7526 | 9.7639 | 10.0374 | 10.7042 | 11.185 | 11.2261 |
| 9.4032 | 9.8066 | 11.084 | 5.3775 | 9.9563 | 6.5665 | 7.5653 | 5.6342 |
| 5.6895 | 1.6194 | 3.8633 | 4.4879 | 8.8526 | 5.0503 | 3.5027 | 5.4042 |
| 6.8062 | 8.1778 | 5.4392 | 7.3879 | 4.9049 | 1.2434 | 7.0636 | 6.3203 |
| 9.3387 | 4.0278 | 5.5513 | | | | | |
| RRM2 | 11.2677 | 9.4068 | 8.642 | 10.8862 | 9.8289 | 10.1543 | 9.5113 |
| 11.6756 | 11.1033 | 9.7441 | 9.1527 | 10.6772 | 10.2139 | 8.6136 | 8.6121 |
| 10.9572 | 10.1698 | 9.5079 | 9.2581 | 8.8567 | 11.5778 | 11.7921 | 9.9342 |
| 9.1557 | 11.1386 | 11.5091 | 11.0328 | 9.9485 | 8.6129 | 9.8774 | 13.3506 |
| 8.3088 | 11.4357 | 10.8704 | 10.0584 | 10.3842 | 11.3347 | 10.2881 | 10.6175 |
| 10.7185 | 11.0184 | 11.88 | 10.0691 | 11.018 | 11.433 | 10.498 | 9.765 |
| 11.1727 | 10.8778 | 10.089 | 8.7809 | 10.4158 | 10.5553 | 11.721 | 9.5714 |
| 9.9295 | 9.2819 | 10.7019 | 7.5734 | 9.813 | 10.8993 | 11.4977 | 10.8317 |
| 9.0231 | 10.3481 | 9.196 | 9.4596 | 7.3437 | 6.6385 | 11.3951 | 11.0808 |
| 10.9605 | 6.7617 | 10.7965 | 9.1077 | 8.9743 | 10.9215 | 10.7833 | 9.0533 |
| 11.5689 | 10.8728 | 9.0027 | 8.1475 | 11.4367 | 9.6593 | 9.2218 | 10.8624 |
| 11.0451 | 10.8156 | 10.3897 | 10.17 | 10.3003 | 10.8166 | 10.0876 | 8.9999 |
| 9.5996 | 9.9643 | 9.803 | 9.0522 | 11.0587 | 10.6965 | 10.3921 | 9.4105 |
| 9.4207 | 11.1749 | 11.6885 | 10.4407 | 10.4357 | 11.8669 | 10.9109 | 10.6345 |
| 10.711 | 9.2909 | 9.8721 | 10.2389 | 10.2394 | 9.2601 | 11.4518 | 9.7367 |
| 10.8024 | 6.3793 | 9.6116 | 9.4988 | 9.6204 | 8.6658 | 11.3807 | 10.9708 |
| 8.556 | 10.0123 | 10.3512 | 8.0238 | 10.2588 | 9.2284 | 10.9729 | 10.6697 |
| 10.1289 | 10.6599 | 9.8688 | 12.1149 | 10.7933 | 11.4929 | 9.3641 | 8.8813 |
| 11.2086 | 10.9432 | 9.3285 | 9.6398 | 11.032 | 11.2874 | 11.401 | 10.5146 |
| 10.8786 | 10.9924 | 8.4003 | 10.1741 | 10.5123 | 7.9581 | 10.9196 | 10.8136 |
| 11.1772 | 8.8245 | 9.9882 | 9.1204 | 10.2376 | 10.6092 | 9.9474 | 11.212 |
| 11.4487 | 9.4762 | 9.3489 | 8.9913 | 10.3376 | 10.7141 | 10.5662 | 9.8208 |
| 9.1025 | 10.4347 | 4.9482 | 10.3455 | 6.1766 | 5.2694 | 5.4611 | 5.523 |
| 3.0451 | 4.8557 | 5.0612 | 9.0549 | 4.6337 | 5.5282 | 5.1291 | 7.804 |
| 6.0125 | 5.5312 | 7.7714 | 3.8923 | 2.0673 | 7.2005 | 5.0252 | 8.0967 |

| | | | | | | | |
|---|---|---|---|---|---|---|---|
| 4.0278 | 4.2022 | | | | | | |
| TK1 | 12.5997 | 11.1279 | 9.0925 | 11.7231 | 10.8589 | 10.9078 | 11.436 |
| 10.8617 | 12.133 | 10.9514 | 10.9568 | 10.4558 | 10.3777 | 10.2501 | 10.6578 |
| 11.0597 | 11.531 | 12.3323 | 9.5392 | 9.2829 | 11.5216 | 12.3182 | 10.9255 |
| 10.0073 | 11.2933 | 8.9937 | 11.2136 | 10.848 | 9.5005 | 10.4433 | 11.8665 |
| 10.0798 | 11.109 | 11.1105 | 9.2362 | 10.6316 | 11.7578 | 10.9753 | 11.0586 |
| 11.919 | 11.4535 | 10.7659 | 10.9035 | 10.7769 | 10.9655 | 10.3285 | 10.3209 |
| 10.8743 | 10.2038 | 10.2244 | 10.4261 | 11.473 | 11.2043 | 10.8549 | 11.0631 |
| 10.0444 | 9.9834 | 10.8516 | 8.9444 | 12.7494 | 12.5233 | 10.122 | 11.7821 |
| 10.4104 | 9.7942 | 10.4227 | 8.5875 | 9.466 | 8.4393 | 11.1166 | 10.6895 |
| 10.7113 | 9.5919 | 11.4115 | 10.6504 | 10.7877 | 10.5759 | 11.8912 | 9.6477 |
| 10.5681 | 11.1256 | 10.4746 | 9.7175 | 11.4977 | 9.9443 | 9.3859 | 11.206 |
| 11.6064 | 11.3394 | 10.8095 | 11.6291 | 11.9621 | 11.3554 | 10.5337 | 11.37 |
| 10.8086 | 10.0236 | 10.4119 | 11.1633 | 11.7444 | 11.1204 | 10.639 | 9.4245 |
| 10.1957 | 10.1251 | 11.7043 | 11.743 | 10.724 | 10.8132 | 10.3188 | 11.8365 |
| 10.9374 | 11.7225 | 11.4647 | 11.6391 | 10.584 | 12.1722 | 10.8534 | 12.6902 |
| 12.1246 | 8.2821 | 9.0923 | 10.7751 | 10.4154 | 10.6657 | 11.2592 | 11.0976 |
| 9.6798 | 10.2729 | 11.889 | 8.3994 | 12.898 | 9.3632 | 11.589 | 11.1436 |
| 10.9253 | 10.1004 | 10.7861 | 12.2863 | 10.9044 | 11.915 | 9.7251 | 10.6793 |
| 10.4141 | 10.0506 | 10.9997 | 10.1546 | 11.3489 | 11.214 | 10.7299 | 11.674 |
| 10.7689 | 11.6082 | 9.9825 | 10.0915 | 10.205 | 9.9577 | 11.7984 | 11.5839 |
| 11.7308 | 8.6426 | 9.5466 | 9.1997 | 11.2768 | 12.7091 | 11.2655 | 10.8162 |
| 11.0183 | 9.5549 | 10.3236 | 11.2929 | 9.9207 | 13.7033 | 10.9276 | 10.4097 |
| 9.2882 | 13.2545 | 6.3123 | 10.4347 | 7.5241 | 8.3939 | 7.0998 | 6.4084 |
| 4.6343 | 6.1213 | 5.4097 | 9.411 | 6.2856 | 6.3097 | 6.6991 | 8.4711 |
| 7.7567 | 6.8787 | 7.2145 | 5.7404 | 4.516 | 8.5111 | 7.0024 | 8.8832 |
| 5.5692 | 6.2259 | | | | | | |
| NEK2 | 9.3154 | 9.2458 | 9.6476 | 9.6173 | 10.4791 | 9.1139 | 10.1466 |
| 9.0274 | 10.1505 | 9.3197 | 9.8368 | 9.0011 | 9.6562 | 7.4066 | 8.5198 |
| 9.1086 | 9.4737 | 8.7372 | 8.9027 | 8.6287 | 8.4244 | 11.1213 | 9.3602 |
| 7.0754 | 8.7123 | 9.6515 | 10.2382 | 10.01 | 8.8281 | 9.9846 | 9.2167 |
| 8.2786 | 10.3642 | 9.344 | 8.2192 | 9.1296 | 8.6794 | 9.7825 | 9.0848 |
| 7.9428 | 8.339 | 9.6759 | 8.5881 | 9.6284 | 8.235 | 8.6047 | 8.8604 |
| 9.397 | 7.8739 | 7.9949 | 9.7218 | 9.4474 | 10.443 | 8.7578 | 9.6304 |
| 9.405 | 7.739 | 9.9482 | 5.37 | 9.0262 | 9.2983 | 9.6476 | 8.8565 |
| 8.6841 | 9.0864 | 9.0673 | 9.5697 | 6.7652 | 8.2168 | 8.7753 | 9.1663 |
| 10.2974 | 7.1777 | 10.1372 | 8.6901 | 8.1817 | 9.238 | 9.1978 | 8.8407 |
| 9.9969 | 9.3326 | 8.1474 | 6.7696 | 9.7706 | 8.6446 | 10.4878 | 8.851 |
| 7.4132 | 9.4519 | 9.6837 | 9.2443 | 10.2676 | 12.7304 | 8.606 | 9.3133 |
| 10.0166 | 9.9413 | 9.24 | 6.7952 | 6.4919 | 10.4218 | 9.3628 | 10.7007 |
| 8.3475 | 9.5166 | 8.8858 | 9.8085 | 9.0417 | 8.8008 | 9.8288 | 9.558 |
| 9.2236 | 7.5438 | 9.2144 | 9.8085 | 9.6149 | 9.659 | 8.4827 | 7.8309 |
| 8.1479 | 0 | 8.183 | 9.507 | 8.4103 | 8.6122 | 9.0914 | 9.0893 |
| 9.6693 | 8.0473 | 9.7902 | 7.3254 | 7.3726 | 8.7156 | 8.6745 | 9.3394 |
| 9.3156 | 9.5505 | 10.0347 | 9.0459 | 8.2326 | 8.3537 | 8.3356 | 8.3521 |
| 8.7812 | 9.5352 | 9.0482 | 8.5526 | 9.1873 | 10.7047 | 9.2716 | 9.8503 |
| 9.9367 | 10.2529 | 5.217 | 9.9192 | 10.1376 | 7.7567 | 10.1204 | 8.1109 |
| 10.014 | 7.9649 | 9.4274 | 8.2504 | 9.2716 | 8.9859 | 8.5401 | 9.3099 |
| 8.6729 | 8.4725 | 8.822 | 9.7117 | 9.9408 | 8.4449 | 8.9228 | 8.8751 |
| 9.1579 | 8.1607 | 2.6668 | 8.5788 | 4.4886 | 6.4552 | 3.4365 | 3.1095 |
| 2.3632 | 3.6364 | 3.9204 | 6.8731 | 2.9352 | 3.1069 | 4.0144 | 4.7052 |
| 6.8234 | 3.7927 | 6.2323 | 2.3776 | 2.0673 | 5.6875 | 4.1094 | 6.2514 |
| 3.0195 | 3.5942 | | | | | | |
| BIRC5 | 10.8318 | 9.4794 | 7.7332 | 10.4869 | 9.6456 | 10.2516 | 10.027 |

| | | | | | | | |
|---|---|---|---|---|---|---|---|
| 9.9224 | 11.4792 | 10.9591 | 9.6573 | 9.6492 | 8.7092 | 8.7409 | 10.3271 |
| 9.7459 | 10.8594 | 9.3089 | 8.1698 | 7.5584 | 10.8633 | 11.1145 | 10.383 |
| 7.8578 | 8.9727 | 10.2479 | 9.6851 | 9.6062 | 7.8232 | 10.556 | 10.5712 |
| 8.083 | 9.7133 | 10.3576 | 9.0423 | 9.8114 | 10.1449 | 9.7899 | 9.6924 |
| 12.752 | 9.6684 | 10.1842 | 9.7497 | 9.4018 | 9.4748 | 8.723 | 9.0294 |
| 9.846 | 7.6438 | 8.4639 | 8.9403 | 10.0903 | 10.287 | 10.403 | 8.622 |
| 8.3277 | 8.1166 | 9.7574 | 6.4762 | 11.592 | 11.6511 | 8.7886 | 9.3791 |
| 9.9116 | 8.2152 | 7.8757 | 8.4197 | 6.5847 | 7.0104 | 9.6863 | 10.5546 |
| 9.5655 | 6.4361 | 10.6154 | 9.045 | 9.4793 | 9.8102 | 10.7439 | 8.2451 |
| 10.7661 | 9.4402 | 9.711 | 9.1181 | 11.0653 | 8.9424 | 7.8743 | 9.7564 |
| 11.5893 | 9.4532 | 10.0259 | 10.7313 | 10.5244 | 10.6408 | 9.127 | 10.1786 |
| 10.0859 | 8.2524 | 9.6438 | 12.2264 | 9.6889 | 10.0662 | 9.5318 | 8.7302 |
| 9.6281 | 9.3627 | 9.5128 | 10.2843 | 9.4491 | 9.5887 | 9.2953 | 10.9594 |
| 10.168 | 11.9687 | 9.521 | 10.3975 | 9.065 | 10.6835 | 9.3878 | 12.2238 |
| 11.7403 | 7.8858 | 7.7195 | 10.2828 | 8.1287 | 9.2352 | 8.6252 | 9.4528 |
| 8.3802 | 9.069 | 11.8593 | 6.9418 | 13.4797 | 7.51 | 10.382 | 9.996 |
| 10.2546 | 9.3601 | 9.7586 | 11.6371 | 9.66 | 9.9752 | 8.3477 | 8.8122 |
| 8.7487 | 9.5538 | 9.1022 | 8.8758 | 10.191 | 10.6372 | 9.6062 | 11.0155 |
| 9.9042 | 10.2937 | 9.6389 | 9.0437 | 9.4693 | 6.7608 | 10.8282 | 9.4823 |
| 10.7787 | 8.2695 | 8.9671 | 8.0705 | 8.9571 | 11.3843 | 10.4328 | 10.814 |
| 9.5797 | 7.7802 | 9.1628 | 9.792 | 8.9676 | 13.3907 | 12.3322 | 8.3769 |
| 8.282 | 11.9987 | 2.3648 | 9.2741 | 4.8833 | 4.9301 | 4.5989 | 3.096 |
| 1.7848 | 3.9497 | 3.4315 | 8.0483 | 3.6345 | 4.4695 | 4.639 | 7.3347 |
| 4.4418 | 4.6205 | 6.117 | 2.8653 | 1.327 | 6.7979 | 4.2703 | 7.8371 |
| 1.5073 | 2.7424 | | | | | | |
| ESPL1 | 9.5915 | 8.5781 | 7.5214 | 9.6985 | 9.7124 | 9.3198 | 9.7163 |
| 10.0937 | 9.7125 | 9.7727 | 9.5273 | 9.4572 | 8.2071 | 7.478 | 9.0272 |
| 8.2929 | 9.9026 | 9.6383 | 6.9723 | 7.5209 | 8.5841 | 9.9823 | 8.1157 |
| 6.0861 | 9.9041 | 10.022 | 9.9706 | 9.7753 | 9.3063 | 10.6913 | 9.7846 |
| 8.4969 | 10.5495 | 8.382 | 9.1337 | 7.6569 | 8.9663 | 9.2211 | 8.4312 |
| 8.2185 | 9.4999 | 10.104 | 8.3494 | 9.1793 | 9.6397 | 9.1536 | 9.0092 |
| 8.9292 | 8.0969 | 7.4407 | 8.6065 | 9.0283 | 9.4889 | 8.9154 | 9.0999 |
| 8.4558 | 8.9738 | 8.9351 | 5.5775 | 10.1786 | 9.4095 | 9.2348 | 8.7857 |
| 9.1781 | 9.2145 | 8.0482 | 9.1689 | 5.6602 | 5.4756 | 9.1378 | 8.5607 |
| 10.2192 | 4.8825 | 9.253 | 8.0384 | 9.5578 | 9.7396 | 8.6456 | 8.2315 |
| 9.9591 | 9.4987 | 9.8503 | 7.7383 | 8.36 | 8.9076 | 8.2455 | 8.8261 |
| 7.5822 | 8.9898 | 10.5207 | 10.0401 | 8.9197 | 10.8251 | 8.9439 | 8.6092 |
| 9.2608 | 8.8767 | 8.9725 | 7.8782 | 8.96 | 9.5228 | 9.5503 | 8.26 |
| 10.5597 | 9.2408 | 9.1738 | 9.7631 | 8.1984 | 9.7468 | 8.6354 | 9.3573 |
| 9.4188 | 9.0819 | 10.2975 | 10.6356 | 8.3081 | 8.7583 | 8.1488 | 7.5682 |
| 8.342 | 5.1146 | 8.4216 | 9.1535 | 7.9178 | 7.2587 | 9.1633 | 9.1744 |
| 7.8789 | 7.5892 | 8.1778 | 6.7763 | 6.6776 | 7.8372 | 10.9028 | 9.9491 |
| 9.9126 | 9.7581 | 9.1413 | 9.3925 | 8.4805 | 9.1615 | 7.7361 | 7.4893 |
| 9.7102 | 8.9681 | 9.1008 | 9.4163 | 8.9909 | 11.1648 | 9.6248 | 10.3329 |
| 9.7954 | 10.0147 | 6.4708 | 9.4499 | 9.8196 | 6.1667 | 9.3845 | 10.5086 |
| 10.4836 | 7.2801 | 9.4218 | 7.7045 | 8.8071 | 9.844 | 8.7361 | 8.8934 |
| 9.6822 | 7.5067 | 8.2577 | 10.1844 | 9.2763 | 9.5594 | 8.9807 | 8.3298 |
| 7.8323 | 8.5453 | 3.3733 | 8.7029 | 4.6972 | 4.8938 | 4.0393 | 5.5967 |
| 2.6279 | 3.5346 | 3.4032 | 6.7966 | 3.5947 | 2.9121 | 4.3753 | 5.9634 |
| 5.012 | 3.9785 | 6.0608 | 2.8669 | 3.2722 | 6.1083 | 3.2643 | 7.2082 |
| 3.202 | 3.3916 | | | | | | |
| UHRF1 | 9.8743 | 8.8646 | 7.699 | 10.3776 | 7.6167 | 9.8378 | 10.2994 |
| 8.6239 | 9.517 | 8.3672 | 7.5825 | 9.1323 | 9.4064 | 6.904 | 7.3511 |
| 8.7999 | 6.8022 | 8.6459 | 9.5711 | 7.8721 | 9.047 | 9.9261 | 9.9433 |

| | | | | | | | |
|---|---|---|---|---|---|---|---|
| 7.4856 | 9.1187 | 10.1629 | 9.8679 | 8.3993 | 7.3808 | 9.1015 | 9.6059 |
| 7.9436 | 9.9812 | 8.6424 | 8.1901 | 9.2798 | 7.6772 | 9.6538 | 9.1823 |
| 7.2478 | 7.5684 | 10.222 | 7.506 | 9.4259 | 8.0648 | 8.248 | 8.5658 |
| 9.1394 | 8.592 | 7.9735 | 8.1902 | 7.5596 | 9.2681 | 10.1638 | 8.8962 |
| 8.9641 | 7.8903 | 9.2347 | 6.8228 | 8.2733 | 7.3723 | 9.3866 | 8.0729 |
| 8.5436 | 8.3018 | 8.8058 | 8.214 | 6.537 | 6.547 | 9.7746 | 9.6374 |
| 8.7406 | 5.9514 | 8.8099 | 8.424 | 7.9878 | 8.6149 | 9.3273 | 7.4387 |
| 9.8366 | 8.239 | 8.8189 | 7.8577 | 10.9929 | 7.4186 | 8.6497 | 9.7909 |
| 7.911 | 9.2564 | 8.2271 | 8.733 | 8.6348 | 9.3482 | 8.345 | 7.7885 |
| 8.9739 | 9.4646 | 9.4644 | 6.4247 | 7.5699 | 9.9323 | 8.4984 | 9.4236 |
| 7.708 | 9.6179 | 8.2847 | 9.1459 | 8.8184 | 8.2201 | 10.6507 | 8.6494 |
| 10.2088 | 7.9995 | 9.0678 | 9.3659 | 9.0543 | 8.636 | 7.9909 | 8.6567 |
| 9.9512 | 5.9263 | 8.6777 | 8.2859 | 7.2687 | 8.2727 | 9.4897 | 9.4353 |
| 8.2749 | 8.9185 | 8.4527 | 6.9659 | 7.9404 | 8.5814 | 9.9331 | 9.2493 |
| 8.8992 | 9.412 | 7.2293 | 10.0093 | 8.7173 | 9.6384 | 8.9885 | 8.9212 |
| 8.7026 | 9.9916 | 8.2537 | 7.2173 | 9.9169 | 9.6216 | 9.2877 | 8.8379 |
| 9.3204 | 9.8455 | 6.0836 | 9.695 | 9.9209 | 7.0229 | 8.943 | 8.511 |
| 9.4199 | 7.6702 | 7.982 | 8.4514 | 8.7748 | 9.1567 | 9.7068 | 9.9983 |
| 9.8459 | 7.6005 | 8.4187 | 8.6573 | 10.0499 | 7.7218 | 10.1568 | 10.0577 |
| 9.4387 | 8.6974 | 4.4854 | 8.7201 | 6.0841 | 4.2654 | 3.9977 | 3.9124 |
| 2.0387 | 2.7928 | 3.5787 | 7.7501 | 3.7806 | 3.6967 | 4.0942 | 6.6251 |
| 4.2038 | 4.3255 | 6.1576 | 3.5226 | 2.2162 | 5.5668 | 4.6662 | 7.9283 |
| 3.1136 | 3.5298 | | | | | | |
| SPARCL1 | 9.9593 | 10.033 | 9.8689 | 11.3795 | 10.5415 | 10.3699 | 11.1315 |
| 10.6231 | 10.3861 | 10.1674 | 11.476 | 9.3201 | 9.8049 | 10.0701 | 9.8115 |
| 10.3404 | 9.7471 | 10.6557 | 12.2557 | 10.7546 | 9.8291 | 8.7168 | 11.1911 |
| 10.8585 | 9.5032 | 9.0243 | 10.9658 | 9.4045 | 10.3008 | 9.3092 | 9.7856 |
| 10.7027 | 9.8525 | 10.5196 | 9.7336 | 10.5629 | 9.9068 | 10.1475 | 7.582 |
| 7.8617 | 11.5963 | 11.2995 | 10.8695 | 9.521 | 11.5985 | 10.902 | 11.7588 |
| 11.5951 | 11.2945 | 11.4601 | 14.6635 | 10.8622 | 11.6379 | 10.1609 | 8.6754 |
| 11.2153 | 9.4775 | 11.1517 | 10.465 | 13.4203 | 9.4946 | 10.5041 | 10.0067 |
| 10.6037 | 12.8492 | 10.7529 | 12.4317 | 11.8816 | 11.5209 | 11.8123 | 10.3227 |
| 11.0058 | 13.9108 | 11.5262 | 11.8521 | 10.6402 | 10.8481 | 9.1419 | 12.0982 |
| 12.7655 | 11.779 | 8.8077 | 10.5809 | 12.3811 | 11.6775 | 11.9574 | 10.3174 |
| 8.9197 | 11.6624 | 9.1918 | 11.5267 | 9.4063 | 12.2988 | 12.7733 | 12.2174 |
| 11.599 | 10.7606 | 9.3548 | 10.5025 | 8.8887 | 11.5201 | 9.472 | 12.1867 |
| 10.6314 | 10.6026 | 11.0992 | 11.0898 | 10.5277 | 11.3188 | 10.0467 | 10.9671 |
| 11.4693 | 8.0318 | 10.4408 | 8.0878 | 11.2132 | 10.3707 | 10.5437 | 9.9596 |
| 9.9132 | 8.4457 | 14.4394 | 8.8223 | 10.7363 | 9.6801 | 11.4052 | 9.202 |
| 10.5724 | 10.074 | 9.5746 | 14.6819 | 10.006 | 9.6326 | 10.4786 | 9.5716 |
| 10.0515 | 9.8804 | 9.752 | 9.9487 | 10.0016 | 10.6147 | 10.4269 | 8.8595 |
| 10.3161 | 12.7867 | 11.6539 | 11.8925 | 11.1032 | 9.3018 | 9.5972 | 11.8723 |
| 9.5222 | 9.7209 | 7.76 | 11.7182 | 11.6717 | 13.1933 | 9.3946 | 10.367 |
| 9.1319 | 10.8074 | 10.1909 | 12.0223 | 11.6922 | 9.6423 | 8.6946 | 10.0994 |
| 10.8185 | 10.7473 | 12.1521 | 12.2103 | 9.4633 | 8.5262 | 9.232 | 10.8699 |
| 12.6513 | 9.1488 | 15.1628 | 13.5654 | 14.9623 | 13.6525 | 16.2942 | 15.3936 |
| 16.2195 | 16.4351 | 16.4177 | 14.198 | 15.7315 | 16.0725 | 15.3867 | 13.9894 |
| 14.4988 | 15.6799 | 15.6886 | 15.0635 | 13.069 | 15.2497 | 15.455 | 14.5287 |
| 16.2746 | 14.5552 | | | | | | |
| KIF2C | 10.5783 | 9.4326 | 8.011 | 11.0559 | 10.6905 | 9.5172 | 10.3851 |
| 10.8221 | 10.8813 | 10.6552 | 10.5679 | 9.5444 | 9.3121 | 8.4024 | 9.6981 |
| 10.235 | 10.9223 | 10.5491 | 8.2198 | 7.7652 | 10.2325 | 11.2561 | 9.4773 |
| 7.6854 | 10.4532 | 10.7488 | 9.8103 | 11.0994 | 9.6159 | 10.5196 | 10.9182 |
| 7.8644 | 10.1573 | 9.7337 | 9.9949 | 10.0971 | 10.329 | 11.0352 | 9.2502 |

| | | | | | | | |
|---|---|---|---|---|---|---|---|
| 11.1344 | 10.1264 | 11.0218 | 9.023 | 9.678 | 9.2257 | 9.2003 | 8.1933 |
| 9.8444 | 8.9061 | 8.2904 | 10.2924 | 10.1137 | 10.4336 | 10.6664 | 10.3258 |
| 9.3695 | 8.3217 | 10.4388 | 6.2442 | 11.2396 | 11.0892 | 9.2023 | 9.6822 |
| 10.17 | 8.4871 | 8.4801 | 9.1559 | 6.6718 | 6.3872 | 10.1124 | 10.0494 |
| 10.5657 | 6.5081 | 10.7301 | 9.0891 | 9.628 | 10.0975 | 10.2572 | 9.1794 |
| 10.586 | 9.7642 | 10.9421 | 8.4833 | 10.9498 | 9.5465 | 8.8133 | 9.6669 |
| 10.6022 | 10.0373 | 10.4193 | 10.8303 | 11.0639 | 11.5759 | 9.6028 | 10.4816 |
| 10.8042 | 9.1044 | 9.3619 | 10.2779 | 9.8642 | 10.689 | 10.4848 | 9.0889 |
| 10.0943 | 10.4543 | 10.6466 | 10.4457 | 9.2837 | 10.4193 | 9.8301 | 10.5316 |
| 10.1207 | 10.7997 | 10.2578 | 10.1705 | 9.3744 | 10.3061 | 11.1529 | 10.0039 |
| 10.9919 | 5.1904 | 8.6778 | 10.9902 | 8.2244 | 8.5674 | 9.6176 | 9.6402 |
| 8.6445 | 9.3837 | 10.2369 | 7.0857 | 10.6266 | 8.329 | 10.241 | 9.9505 |
| 10.1892 | 9.8298 | 10.438 | 11.1212 | 9.7299 | 10.2985 | 8.857 | 8.9478 |
| 9.1907 | 9.9922 | 9.9482 | 9.9751 | 10.3512 | 11.7538 | 10.1772 | 10.7323 |
| 11.2554 | 9.4945 | 8.1757 | 9.6819 | 10.4147 | 6.9415 | 10.398 | 10.3804 |
| 11.139 | 8.2318 | 10.2894 | 8.4933 | 10.4943 | 11.1675 | 9.9448 | 10.9759 |
| 10.3555 | 7.7931 | 9.9995 | 10.1002 | 9.8662 | 12.146 | 11.9311 | 9.2885 |
| 8.7136 | 10.8936 | 3.8 | 9.304 | 5.5966 | 4.5972 | 5.5211 | 5.1514 |
| 4.7499 | 4.6026 | 5.1192 | 8.0723 | 4.757 | 4.6082 | 5.3564 | 6.6632 |
| 5.3925 | 5.3908 | 7.0174 | 5.5206 | 4.5724 | 6.8618 | 4.796 | 7.407 |
| 4.7967 | 4.9326 | | | | | | |
| SGOL1 | 7.8551 | 7.2744 | 5.7664 | 7.9966 | 8.3295 | 6.98 | 7.6194 |
| 7.9042 | 8.5526 | 7.6465 | 7.9614 | 6.649 | 6.9993 | 5.1466 | 6.8758 |
| 7.0245 | 6.6736 | 7.3802 | 5.9378 | 6.2859 | 6.1703 | 9.1307 | 6.2891 |
| 4.655 | 7.4416 | 7.5378 | 7.5682 | 7.6318 | 7.3062 | 7.6733 | 7.129 |
| 5.5411 | 7.6954 | 7.4683 | 6.9388 | 6.2689 | 6.6804 | 6.9952 | 7.0442 |
| 5.5168 | 7.1634 | 7.6572 | 5.991 | 7.2606 | 6.9588 | 6.3628 | 5.6438 |
| 7.6096 | 6.5166 | 5.4857 | 7.8042 | 7.8755 | 7.8405 | 7.8013 | 7.4144 |
| 7.1324 | 5.1874 | 7.2532 | 3.2274 | 7.6944 | 6.274 | 7.4764 | 5.7723 |
| 5.8194 | 6.459 | 5.5954 | 6.5669 | 3.959 | 3.9155 | 7.4031 | 6.3522 |
| 8.7743 | 3.2356 | 8.0878 | 5.5065 | 6.7881 | 7.3092 | 6.7712 | 7.2962 |
| 8.1528 | 8.4596 | 6.2457 | 4.8706 | 7.9072 | 6.9879 | 7.2878 | 6.6628 |
| 6.3933 | 7.7361 | 7.9411 | 7.1091 | 8.111 | 8.4058 | 6.7963 | 7.5806 |
| 7.168 | 6.7762 | 7.007 | 3.3832 | 6.6724 | 9.0184 | 8.7859 | 7.6826 |
| 7.837 | 8.0625 | 6.6846 | 7.6511 | 7.0222 | 7.6441 | 6.7033 | 7.9344 |
| 7.2822 | 4.4556 | 7.4645 | 7.9875 | 6.3774 | 7.5118 | 5.9207 | 5.2536 |
| 7.3113 | 3.816 | 6.7274 | 7.8867 | 5.1199 | 5.3535 | 7.5021 | 7.3761 |
| 5.8157 | 6.9666 | 6.4568 | 4.474 | 5.6916 | 6.4739 | 6.4216 | 6.6674 |
| 8.3163 | 7.817 | 6.8961 | 7.1382 | 6.9872 | 7.009 | 6.0999 | 5.7171 |
| 6.9064 | 7.8972 | 6.6748 | 6.6216 | 7.2812 | 8.9667 | 7.5692 | 8.922 |
| 7.3842 | 6.4494 | 2.9928 | 7.5526 | 8.1091 | 3.9843 | 6.8732 | 6.8079 |
| 8.3461 | 5.0397 | 7.377 | 5.9346 | 8.1049 | 8.1545 | 6.5517 | 8.0012 |
| 7.5408 | 5.6261 | 6.3987 | 7.0624 | 7.6102 | 6.6143 | 6.7907 | 7.0179 |
| 6.4917 | 6.1634 | 2.3255 | 6.1522 | 2.6976 | 1.9117 | 1.522 | 2.6372 |
| 1.026 | 2.1337 | 1.714 | 5.0213 | 1.9147 | 1.8952 | 1.5047 | 4.0742 |
| 2.0269 | 3.1077 | 4.4916 | 0.8792 | 1.7133 | 3.0618 | 1.5425 | 4.1737 |
| 0.6293 | 1.6369 | | | | | | |
| NCAPH | 9.6999 | 7.9927 | 7.2702 | 9.8676 | 9.5468 | 8.9727 | 9.509 |
| 9.1283 | 9.8065 | 9.3927 | 9.2063 | 9.1756 | 8.7622 | 7.0795 | 8.4898 |
| 8.9552 | 8.7503 | 9.2576 | 7.8121 | 7.4244 | 8.6772 | 10.3505 | 7.9119 |
| 6.2479 | 9.4911 | 9.4449 | 9.272 | 10.038 | 8.858 | 10.1029 | 9.9356 |
| 7.1819 | 9.5248 | 8.9767 | 8.9738 | 8.4702 | 8.9075 | 9.8159 | 8.7363 |
| 7.0942 | 8.6202 | 10.0257 | 7.6223 | 9.1884 | 8.577 | 9.1636 | 8.4182 |
| 9.154 | 8.2823 | 8.1321 | 9.4457 | 9.4928 | 9.7488 | 9.2926 | 9.3537 |

| | | | | | | | | |
|---|---|---|---|---|---|---|---|---|
| 8.8772 | 6.9393 | 9.6927 | 4.9086 | 9.3247 | 9.0289 | 9.0016 | 8.7144 | |
| 8.3467 | 8.0528 | 7.8385 | 8.584 | 5.1706 | 5.1166 | 9.1486 | 8.5533 | |
| 10.5148 | 5.534 | 9.7489 | 8.0214 | 8.3359 | 9.4679 | 9.4207 | 8.3493 | |
| 9.3832 | 9.836 | 8.4572 | 6.7284 | 9.3861 | 8.6477 | 8.3962 | 8.6421 | |
| 8.5358 | 9.0528 | 9.4387 | 9.5727 | 9.6214 | 9.8553 | 8.8225 | 9.1617 | 9.5 |
| | 8.6559 | 8.7379 | 6.398 | 8.4094 | 10.1556 | 9.4996 | 8.2786 | |
| 8.9039 | 9.4977 | 9.3674 | 10.0699 | 8.6072 | 9.1481 | 8.5652 | 9.0086 | |
| 9.158 | 8.4622 | 9.3481 | 9.3067 | 8.7727 | 8.6209 | 8.9556 | 7.1879 | |
| 8.9272 | 4.1556 | 8.1368 | 9.2943 | 7.2174 | 7.0262 | 9.1633 | 9.3792 | |
| 7.7651 | 7.7902 | 7.8421 | 6.5192 | 7.1629 | 8.023 | 9.0939 | 9.2882 | 9.69 |
| | 9.077 | 8.2589 | 9.3244 | 8.3011 | 9.205 | 7.8647 | 7.2565 | |
| 9.5567 | 9.4604 | 8.407 | 8.8408 | 8.8111 | 9.9282 | 9.4575 | 9.8675 | |
| 9.1905 | 9.3622 | 3.4255 | 8.7981 | 10.0682 | 6.4374 | 8.5507 | 8.4816 | |
| 10.4622 | 7.3852 | 9.0223 | 7.8506 | 9.5958 | 9.3435 | 8.4597 | 10.0672 | |
| 9.5391 | 7.4223 | 9.1454 | 10.4114 | 9.1128 | 8.7925 | 8.1925 | 8.9903 | |
| 8.1195 | 8.569 | 3.1739 | 8.4869 | 4.9509 | 3.8887 | 3.6124 | 4.093 | |
| 1.6194 | 3.6848 | 4.2319 | 6.9738 | 2.7094 | 3.8677 | 3.5318 | 5.6586 | |
| 4.4264 | 4.2553 | 6.4357 | 2.6906 | 2.6938 | 5.4804 | 3.8471 | 6.5893 | |
| 1.6723 | 3.4624 | | | | | | | |
| PGM5P2 | 1.5194 | 3.9734 | 0.4935 | 1.87E+00 | | 4.06E-01 | | |
| 2.8873 | 3.4253 | 0.7652 | 0 | 1.9247 | 2.1799 | 1.0485 | 0 | 0 |
| | 0 | 1.6514 | 0.6732 | 1.0485 | 1.131 | 2.0289 | 0 | |
| 0.8021 | 2.0314 | 0 | 2.0116 | 1.8679 | 2.1139 | 0.8951 | 1.3374 | |
| 1.808 | 0.6819 | 2.8291 | 1.5916 | 1.4957 | 0.4337 | 1.4628 | 0.6231 | |
| 2.4713 | 1.8914 | 0 | 0.6741 | 2.1914 | 0.4764 | 0.7972 | 1.2789 | |
| 3.3357 | 2.3724 | 1.2781 | 4.0886 | 1.6258 | 3.6618 | 1.2977 | 0.8658 | |
| 1.2717 | 0.5968 | 1.6675 | 0 | 1.3736 | 0 | 3.4042 | 0 | |
| 2.1491 | 0 | 2.1829 | 3.5065 | 2.388 | 2.2486 | 0.9332 | 0.6894 | |
| 1.3771 | 0 | 0.9627 | 2.7716 | 0.6718 | 1.2145 | 1.6542 | 1.8912 | |
| 1.0433 | 2.0314 | 0.9113 | 1.5834 | 0.9036 | 0 | 0.9013 | 0.5233 | |
| 2.8769 | 2.8152 | 1.0805 | 1.6155 | 0.5352 | 1.2208 | 0 | 2.6639 | |
| 2.6388 | 1.7151 | 0 | 0.8916 | 2.7915 | 0 | 2.3219 | 0.8924 | |
| 2.2315 | 0 | 1.9235 | 0.7085 | 0.6089 | 2.8695 | 1.3285 | 0 | |
| 0.8239 | 0.9214 | 1.0447 | 0 | 2.4304 | 0 | 0 | 1.2511 | |
| 1.3267 | 0 | 0 | 0 | 5.6374 | 1.024 | 1.7855 | 1.0973 | |
| 2.4761 | 0.8022 | 2.9153 | 0.9947 | 1.224 | 6.1309 | 0 | 1.7123 | 0 |
| | 0.9437 | 1.7989 | 0.9402 | 0 | 1.2429 | 1.8525 | 0 | |
| 2.3149 | 0.4033 | 1.0531 | 2.2474 | 0 | 1.5478 | 2.655 | 0 | |
| 0.4486 | 1.2195 | 1.8991 | 0.6229 | 0 | 2.056 | 1.834 | 1.9052 | |
| 0.5545 | 0 | 2.6495 | 0.8792 | 1.8738 | 2.4158 | 0 | 0 | |
| 1.1063 | 0.9785 | 1.4263 | 2.1004 | 0.5674 | 2.7745 | 0.4985 | 0 | 0 |
| | 1.4718 | 2.5996 | 0 | 4.8843 | 2.587 | 4.8794 | 3.8887 | |
| 6.5493 | 5.7871 | 6.6802 | 6.8447 | 6.5832 | 3.4175 | 7.1582 | 7.3447 | |
| 5.6745 | 3.5314 | 3.5563 | 6.1787 | 6.4635 | 6.4225 | 4.0038 | 4.969 | |
| 6.3116 | 4.5686 | 6.5027 | 6.1742 | | | | | |
| TTK | 8.7431 | 8.9039 | 7.422 | 9.1821 | 8.9556 | 8.452 | 8.8819 | |
| 9.6687 | 10.0494 | 9.9364 | 9.5655 | 8.3851 | 8.6883 | 6.6802 | 9.1321 | |
| 8.148 | 8.9369 | 9.2863 | 7.4622 | 7.5278 | 7.7628 | 10.2077 | 7.8359 | |
| 5.2207 | 9.4099 | 9.6449 | 8.9007 | 9.1608 | 8.6989 | 10.2267 | 9.4326 | |
| 7.5469 | 9.0725 | 9.2608 | 9.7758 | 8.1576 | 8.9156 | 9.9798 | 8.6153 | |
| 7.5968 | 9.6347 | 9.8483 | 7.0324 | 8.2903 | 8.5304 | 8.7921 | 8.2175 | |
| 8.3838 | 8.0435 | 7.8998 | 10.0302 | 9.6124 | 10.2712 | 9.382 | 9.3806 | |
| 8.4533 | 7.6235 | 9.7317 | 4.9551 | 9.333 | 9.0681 | 8.5356 | 7.663 | |
| 8.5191 | 8.2059 | 6.9016 | 7.8883 | 5.9314 | 5.5717 | 8.6063 | 8.0691 | |

| | | | | | | | | |
|---|---|---|---|---|---|---|---|---|
| 9.8199 | 5.5933 | 10.0027 | 7.2225 | 9.1178 | 10.0571 | 8.2501 | 8.67 | |
| 9.2858 | 9.8867 | 8.1781 | 7.2016 | 9.1085 | 9.1873 | 8.6329 | 8.7472 | |
| 7.0259 | 8.5317 | 9.5186 | 9.5043 | 9.5702 | 10.3476 | 9.4145 | 10.2018 | |
| 9.0876 | 8.2788 | 8.6126 | 7.9806 | 9.9571 | 10.261 | 9.9083 | 9.3908 | |
| 9.5119 | 9.6721 | 8.9982 | 10.0478 | 8.5656 | 9.2928 | 8.3842 | 9.633 | |
| 9.2876 | 7.2055 | 9.7065 | 9.7875 | 8.1955 | 10.0606 | 8.6518 | 7.3576 | |
| 7.7985 | 3.9958 | 8.0423 | 9.577 | 6.2365 | 7.2905 | 8.7994 | 8.771 | |
| 7.9775 | 7.8316 | 8.0527 | 6.2619 | 7.4061 | 7.9715 | 8.939 | 8.5363 | |
| 9.8918 | 8.8828 | 9.6211 | 8.7894 | 7.8732 | 8.2744 | 7.9072 | 7.4552 | |
| 8.1001 | 9.1704 | 8.4646 | 9.168 | 9.1098 | 10.0219 | 9.1029 | 10.2519 | |
| 9.1285 | 9.2166 | 5.7522 | 8.868 | 9.7536 | 6.0874 | 8.4956 | 8.9165 | |
| 10.5095 | 7.6033 | 9.6826 | 7.977 | 9.5565 | 9.2146 | 8.571 | 9.6641 | |
| 9.4531 | 7.7433 | 9.2335 | 9.0345 | 9.4378 | 9.1412 | 8.7357 | 8.4949 | |
| 8.0338 | 7.2563 | 2.7646 | 8.3192 | 5.0628 | 3.7175 | 3.7692 | 3.1095 | |
| 2.0387 | 2.3883 | 3.7351 | 7.2682 | 2.4416 | 3.6967 | 3.4124 | 5.5811 | |
| 4.8159 | 5.1605 | 6.3338 | 3.5226 | 1.2434 | 5.4654 | 3.7478 | 6.852 | |
| 1.066 | 3.5298 | | | | | | | |
| CDCA5 | 11.0039 | 9.2252 | 8.0735 | 1.12E+01 | | 9.61E+00 | | |
| 10.3813 | 10.6407 | 10.4506 | 11.0012 | 10.511 | 10.1243 | 9.5757 | 9.2967 | |
| 7.9591 | 9.4954 | 10.0512 | 11.0874 | 10.6369 | 8.5388 | 8.1273 | 10.715 | |
| 9.987 | 9.9731 | 8.2528 | 9.8332 | 10.3136 | 9.7196 | 11.5277 | 9.713 | |
| 10.4076 | 11.0437 | 8.1955 | 10.0428 | 9.357 | 9.6141 | 10.3063 | 9.4176 | |
| 11.3458 | 8.9224 | 11.4267 | 9.3045 | 10.7397 | 9.2476 | 9.623 | 9.3835 | |
| 9.5035 | 8.8185 | 10.1204 | 9.1556 | 8.3321 | 10.1958 | 9.5898 | 10.1222 | |
| 10.3905 | 10.7767 | 8.9186 | 8.296 | 10.5216 | 6.3783 | 10.1839 | 11.9181 | |
| 9.3428 | 9.8492 | 9.6974 | 8.6817 | 8.382 | 9.5454 | 7.1769 | 7.0048 | |
| 10.3387 | 10.342 | 9.7985 | 6.3681 | 10.2426 | 9.3806 | 9.7436 | 10.1099 | |
| 10.2389 | 9.5811 | 10.6761 | 10.0806 | 9.8435 | 7.7832 | 10.8431 | 9.8981 | |
| 9.1047 | 9.1953 | 10.7636 | 9.7594 | 10.1654 | 10.3388 | 11.1135 | 11.1209 | |
| 9.3727 | 9.9836 | 10.4531 | 8.9219 | 9.8792 | 11.4776 | 9.0715 | 9.7854 | |
| 10.326 | 9.4134 | 9.8405 | 10.8056 | 9.1764 | 10.893 | 9.2613 | 9.2841 | |
| 9.5852 | 10.4079 | 10.7255 | 11.0692 | 9.9687 | 9.5597 | 9.3642 | 9.9682 | |
| 9.5721 | 11.3485 | 11.7602 | 6.5033 | 8.9097 | 10.3853 | 8.3137 | 9.2175 | |
| 9.7543 | 9.8022 | 10.533 | 9.7391 | 10.8909 | 7.1756 | 11.2321 | 8.5831 | |
| 10.7741 | 10.2508 | 11.2863 | 9.8888 | 10.6835 | 11.6665 | 10.0838 | 10.0613 | |
| 8.9757 | 9.3413 | 9.7486 | 10.0853 | 9.4415 | 9.6357 | 10.4727 | 10.9203 | |
| 10.6842 | 10.6183 | 10.0438 | 9.5433 | 8.752 | 9.5245 | 10.3071 | 7.4012 | |
| 10.4708 | 10.1583 | 11.3178 | 8.291 | 9.8923 | 8.4425 | 10.3133 | 10.7789 | |
| 9.7078 | 10.9766 | 10.2894 | 8.3921 | 10.0489 | 10.3868 | 9.4834 | 12.7315 | |
| 12.0586 | 9.3545 | 8.8371 | 11.3192 | 4.9892 | 9.2751 | 5.6405 | 5.9871 | |
| 5.6205 | 5.3491 | 4.0482 | 5.5854 | 5.3464 | 7.742 | 5.2733 | 5.3664 | |
| 5.9458 | 6.5958 | 6.1851 | 6.0183 | 6.895 | 5.094 | 5.0603 | 7.4138 | |
| 5.5953 | 7.0742 | 4.768 | 6.0647 | | | | | |
| ZBTB16 | 0.5526 | 0.6896 | 0 | 2.5505 | 1.2014 | 1.4999 | 2.505 | 0 |
| | 2.9226 | 0.4849 | 1.0422 | 0 | 0.9286 | 0 | 0 | |
| 0.7772 | 0 | 3.4789 | 2.5262 | 1.465 | 0.6062 | 2.4493 | 0.599 | 0 |
| | 0.5901 | 1.2172 | 1.756 | 0.5159 | 0.8184 | 2.3226 | 1.1431 | |
| 2.8291 | 1.5916 | 1.2418 | 0.4337 | 0 | 0 | 2.9342 | 0 | 0 |
| | 3.1281 | 3.0861 | 0 | 0.453 | 0 | 1.0956 | 1.7133 | |
| 0.5608 | 1.9111 | 0.503 | 3.8103 | 0 | 3.3286 | 0.9583 | 0 | |
| 2.1442 | 3.7908 | 0 | 1.791 | 4.1734 | 1.2639 | 1.357 | 0 | |
| 0.4786 | 3.8144 | 1.8854 | 2.7287 | 1.4952 | 3.062 | 2.5341 | 0 | |
| 0.3965 | 7.5658 | 1.1284 | 2.029 | 0.6199 | 0 | 0 | 1.6807 | |
| 1.4655 | 1.7639 | 0.9036 | 2.0255 | 1.2026 | 4.8572 | 3.6349 | 1.2591 | |

| | | | | | | | | |
|---|---|---|---|---|---|---|---|---|
| 1.0805 | 2.6706 | 1.231 | 2.6605 | 0.4835 | 3.0433 | 1.0326 | 3.4654 | |
| 0.5877 | 0 | 0 | 4.8729 | 1 | 2.1456 | 0 | 0 | |
| 2.2752 | 1.6868 | 0 | 1.0794 | 1.0055 | 1.0557 | 1.7271 | 1.6938 | 0 |
| | 0.7843 | 0.7024 | 0 | 1.5194 | 0.7571 | 3.622 | 4.4243 | |
| 1.0837 | 5.8348 | 6.1521 | 1.8416 | 0.5748 | 0.6506 | 1.4986 | 0.4562 | |
| 1.1308 | 2.576 | 0 | 6.9755 | 0 | 0.9341 | 1.8101 | 0 | 0 |
| | 0.6894 | 0 | 0 | 1.0317 | 4.9149 | 1.3167 | 0 | 0 |
| | 0 | 1.6706 | 2.3696 | 1.4679 | 0.942 | 0 | 0.529 | |
| 2.0651 | 0 | 2.9928 | 0 | 1.9582 | 5.7909 | 1.5234 | 0 | 0.91 |
| | 0.5055 | 0 | 0.6248 | 0.5811 | 1.5325 | 1.1063 | 1.2961 | |
| 1.4263 | 0 | 0.9737 | 2.9126 | 0.4985 | 1.5946 | 0.6064 | 2.3614 | |
| 0.5077 | 1.5159 | 6.2878 | 2.963 | 5.7246 | 3.9415 | 5.906 | 9.355 | |
| 6.2206 | 9.9537 | 8.0751 | 4.8502 | 7.2345 | 7.9283 | 6.7554 | 7.4536 | |
| 4.7894 | 8.154 | 7.1646 | 7.9986 | 8.3407 | 7.7762 | 7.375 | 4.4866 | |
| 7.5547 | 6.5276 | | | | | | | |
| POLQ | 7.9804 | 7.9996 | 6.3747 | 8.7477 | 8.7554 | 7.3348 | 8.1788 | |
| 8.1191 | 9.2527 | 8.0568 | 8.0658 | 7.5323 | 7.84 | 6.1261 | 7.3124 | |
| 7.3864 | 7.7225 | 8.7939 | 6.3492 | 6.4619 | 6.8236 | 8.8236 | 6.9956 | |
| 5.0202 | 8.619 | 8.2917 | 7.8586 | 8.1514 | 7.6997 | 9.2417 | 8.6861 | |
| 6.5107 | 8.4652 | 7.688 | 7.7686 | 7.046 | 8.3991 | 9.6619 | 7.1211 | |
| 6.4385 | 8.5324 | 8.7643 | 6.1897 | 8.0404 | 8.7872 | 7.7427 | 6.7256 | |
| 8.8979 | 7.2137 | 7.5183 | 8.1986 | 8.7277 | 8.7311 | 8.6637 | 8.3042 | |
| 7.596 | 6.6604 | 7.7103 | 3.9328 | 8.7348 | 8.1432 | 7.8253 | 7.4428 | |
| 7.0615 | 7.4017 | 6.3204 | 7.6764 | 5.436 | 4.9263 | 8.23 | 7.1999 | |
| 8.873 | 5.2447 | 7.6113 | 6.7307 | 8.0737 | 8.5416 | 7.3281 | 7.4461 | |
| 7.7125 | 9.2423 | 6.3183 | 6.175 | 7.9932 | 8.0069 | 7.0414 | 8.3778 | |
| 6.6146 | 8.0488 | 8.3334 | 8.9868 | 8.5175 | 9.4445 | 7.7966 | 8.4125 | |
| 7.7394 | 7.2992 | 7.3964 | 6.8552 | 8.6511 | 9.2881 | 9.0544 | 7.6256 | |
| 8.0629 | 8.0504 | 9.4873 | 8.1597 | 7.4581 | 8.7256 | 6.7349 | 8.4664 | |
| 8.0961 | 5.0345 | 8.033 | 9.1389 | 6.9237 | 8.6107 | 7.9737 | 6.2775 | |
| 7.3953 | 6.0124 | 7.7932 | 8.4291 | 6.3706 | 6.7102 | 7.8941 | 8.5325 | |
| 6.2583 | 6.4892 | 6.7171 | 5.5605 | 6.072 | 6.9051 | 8.235 | 8.2451 | |
| 8.4869 | 8.7361 | 7.7518 | 8.362 | 6.0953 | 7.8427 | 6.8862 | 6.4341 | |
| 8.0346 | 8.5572 | 8.2118 | 8.203 | 8.0716 | 8.5824 | 8.6582 | 9.6204 | |
| 7.5405 | 7.8315 | 5.1619 | 7.439 | 8.4199 | 5.8608 | 7.0314 | 8.3575 | |
| 8.3921 | 6.2261 | 7.8171 | 6.3628 | 8.3082 | 8.3568 | 7.4987 | 7.9226 | |
| 8.5548 | 6.1966 | 8.0789 | 8.7832 | 8.2805 | 7.2905 | 7.3298 | 7.7203 | |
| 7.6012 | 6.9221 | 2.9425 | 7.4323 | 4.1667 | 3.4522 | 2.7258 | 2.9686 | |
| 2.3632 | 2.7928 | 2.217 | 6.0915 | 1.9147 | 2.4199 | 3.6421 | 5.7849 | |
| 4.3194 | 3.5207 | 5.1693 | 3.0241 | 1.9011 | 3.8402 | 3.031 | 7.0695 | |
| 2.0979 | 3.5942 | | | | | | | |
| CNRIP1 | 5.2953 | 6.2435 | 5.8298 | 5.8899 | 5.134 | 5.488 | 6.0462 | |
| 5.3655 | 5.679 | 5.0427 | 6.9773 | 4.7162 | 5.6891 | 6.229 | 6.8824 | |
| 6.8746 | 5.1249 | 5.3409 | 5.9563 | 6.076 | 5.3465 | 4.9068 | 7.8744 | |
| 6.7367 | 4.1438 | 8.42 | 6.286 | 6.0141 | 5.3469 | 3.8588 | 4.6878 | |
| 6.1778 | 4.9233 | 5.1714 | 5.369 | 7.5301 | 6.7105 | 4.9574 | 6.1033 | |
| 6.2573 | 5.8916 | 5.5877 | 5.2691 | 5.428 | 6.363 | 6.7069 | 7.4941 | |
| 5.0781 | 5.9737 | 6.9552 | 8.168 | 6.0475 | 6.597 | 5.0675 | 5.2241 | |
| 6.4033 | 5.082 | 6.1089 | 6.9139 | 7.7877 | 5.5407 | 5.4212 | 6.7341 | |
| 6.5092 | 7.2562 | 5.1081 | 6.6298 | 6.941 | 7.4101 | 6.5492 | 6.5938 | |
| 5.6629 | 8.6806 | 5.9836 | 7.0221 | 5.7004 | 5.5564 | 6.0614 | 6.4652 | |
| 7.3336 | 6.0552 | 5.0348 | 8.4508 | 6.7628 | 5.44 | 6.3009 | 6.8312 | |
| 5.7082 | 6.709 | 4.8502 | 7.2918 | 5.5105 | 5.9718 | 7.337 | 7.292 | |
| 6.796 | 5.0286 | 4.4595 | 7.7989 | 5.9542 | 7.212 | 5.7915 | 5.1109 | |

| | | | | | | | |
|---|---|---|---|---|---|---|---|
| 5.4229 | 5.5635 | 6.721 | 8.4701 | 6.3685 | 6.4209 | 5.29 | 6.1352 |
| 6.2866 | 5.2415 | 5.3843 | 4.5839 | 6.3693 | 6.4923 | 6.1828 | 7.7366 |
| 5.9928 | 9.4818 | 8.911 | 4.3307 | 5.2739 | 6.1045 | 5.9789 | 5.3502 |
| 5.6716 | 4.771 | 4.6466 | 8.9989 | 6.2489 | 5.5544 | 5.6185 | 4.0211 |
| 7.6521 | 5.6528 | 4.3644 | 6.335 | 4.5837 | 5.9289 | 6.8555 | 4.8769 |
| 6.2043 | 5.6718 | 5.5274 | 6.7474 | 6.3619 | 5.2939 | 5.1993 | 5.2387 |
| 6.6916 | 5.4302 | 7.4853 | 5.5962 | 6.2905 | 7.662 | 6.4155 | 5.7649 |
| 4.0725 | 6.2261 | 5.2845 | 6.3145 | 6.7765 | 5.4354 | 5.2656 | 6.0017 |
| 6.0252 | 5.4356 | 6.2874 | 7.3627 | 5.536 | 4.7689 | 4.5525 | 5.6796 |
| 6.7224 | 4.8835 | 9.3807 | 9.3064 | 9.229 | 6.5347 | 9.8854 | 9.2684 |
| 10.5036 | 10.5219 | 10.2546 | 9.801 | 10.2181 | 9.8879 | 10.3647 | 9.7133 |
| 8.0525 | 10.1653 | 9.6319 | 9.8904 | 10.4892 | 9.5991 | 9.8277 | 9.3619 |
| 10.1991 | 9.8626 | | | | | | |
| PBK | 10.148 | 8.4089 | 8.275 | 1.03E+01 | | 1.01E+01 | |
| 6.7234 | 8.0468 | 8.2624 | 10.1153 | 8.7337 | 8.1774 | 8.3078 | 8.7456 |
| 7.061 | 7.7805 | 9.307 | 9.0997 | 7.8632 | 7.9176 | 7.7505 | 9.3164 |
| 10.3838 | 8.6171 | 6.769 | 9.0069 | 10.9312 | 9.307 | 8.87 | 7.1828 |
| 8.5027 | 10.6594 | 7.2233 | 9.6597 | 9.8421 | 9.7496 | 9.6154 | 8.836 |
| 8.3182 | 7.7244 | 9.0812 | 7.6347 | 9.7732 | 8.6777 | 9.1664 | 7.9695 |
| 8.5201 | 8.4639 | 9.6058 | 7.7535 | 6.9258 | 8.222 | 7.0472 | 9.7863 |
| 9.7081 | 7.5781 | 8.7328 | 6.1981 | 9.5576 | 5.5923 | 7.9519 | 9.6417 |
| 8.7888 | 8.4232 | 6.8317 | 7.9902 | 8.2495 | 8.0955 | 6.2136 | 5.3294 |
| 9.1567 | 9.5111 | 8.3227 | 4.5648 | 9.6157 | 7.883 | 7.3873 | 9.0712 |
| 9.3822 | 7.4785 | 10.041 | 7.9437 | 8.475 | 7.1526 | 9.7976 | 9.447 |
| 9.2769 | 9.6898 | 7.8143 | 9.4613 | 9.5586 | 9.5613 | 10.5475 | 10.6626 |
| 8.7566 | 8.4842 | 9.6502 | 8.7143 | 8.5517 | 7.073 | 5.8329 | 9.5261 |
| 8.9443 | 9.119 | 7.7945 | 10.2194 | 7.6813 | 8.012 | 9.2217 | 8.942 |
| 9.1543 | 8.1877 | 9.1593 | 8.8051 | 8.0889 | 9.5313 | 8.5554 | 9.5017 |
| 7.8936 | 7.7671 | 9.5167 | 5.331 | 8.0518 | 9.2399 | 8.1568 | 7.5228 |
| 9.1894 | 9.6354 | 8.0277 | 8.6457 | 9.4424 | 6.4034 | 8.4307 | 7.4395 |
| 8.5718 | 9.5163 | 7.4709 | 9.615 | 8.4916 | 10.0539 | 8.8008 | 8.8529 |
| 8.1712 | 7.4684 | 7.738 | 9.4232 | 9.2005 | 8.461 | 10.2397 | 10.6903 |
| 10.8731 | 9.6057 | 9.4109 | 9.3202 | 5.7144 | 9.6368 | 10.212 | 5.1312 |
| 9.3235 | 8.5516 | 8.3145 | 8.2054 | 8.3842 | 7.9893 | 7.5698 | 9.1898 |
| 8.5776 | 8.4725 | 9.94 | 7.469 | 9.4987 | 7.57 | 9.75 | 8.6402 |
| 9.3418 | 8.4819 | 8.0292 | 9.081 | 2.4485 | 8.7754 | 4.5203 | 3.9925 |
| 3.8179 | 3.2981 | 1.026 | 3.3671 | 3.3396 | 7.3614 | 2.8267 | 2.8039 |
| 3.3488 | 4.8108 | 4.5578 | 4.3255 | 6.6507 | 2.1235 | 0.9349 | 6.5012 |
| 3.113 | 6.8245 | 1.6723 | 2.8738 | | | | |
| GTSE1 | 9.7733 | 8.7481 | 7.3262 | 9.933 | 8.9443 | 9.0887 | 8.2745 |
| 9.5673 | 9.3417 | 9.0078 | 8.2661 | 8.7922 | 8.4123 | 7.5723 | 8.7843 |
| 9.0535 | 9.8129 | 9.8535 | 7.7967 | 7.1615 | 8.5623 | 10.4752 | 8.4531 |
| 6.6582 | 9.4088 | 10.1094 | 9.5661 | 8.7089 | 7.9089 | 10.2718 | 9.2312 |
| 7.1678 | 10.0424 | 8.6861 | 8.5075 | 8.997 | 9.6292 | 8.9817 | 8.5297 |
| 7.4981 | 8.8273 | 9.9983 | 7.7484 | 8.8416 | 8.8286 | 6.9716 | 7.7649 |
| 9.2494 | 7.6542 | 7.6349 | 8.9957 | 9.552 | 9.5215 | 9.3152 | 10.0443 |
| 8.858 | 7.3239 | 8.9397 | 5.6069 | 9.0276 | 8.3197 | 9.0993 | 8.9102 |
| 7.6779 | 7.7088 | 7.5552 | 7.9471 | 6.5228 | 5.5717 | 9.1144 | 8.7072 |
| 9.7774 | 5.9514 | 9.1558 | 8.1589 | 6.4142 | 7.4976 | 8.8402 | 8.3277 |
| 10.2163 | 8.7849 | 7.5989 | 7.6876 | 9.6635 | 7.7875 | 8.217 | 9.1453 |
| 8.7033 | 9.1514 | 9.928 | 9.1976 | 7.8298 | 10.7991 | 7.7599 | 8.7763 |
| 8.7189 | 8.8273 | 8.7141 | 7.4426 | 9.0251 | 8.3041 | 8.6215 | 8.3929 |
| 8.8082 | 9.2796 | 9.9894 | 9.4095 | 8.399 | 9.5819 | 8.6326 | 8.2818 |
| 8.1831 | 7.0092 | 8.5134 | 8.1003 | 8.7273 | 9.1592 | 8.2162 | 6.3328 |

| | | | | | | | |
|---|---|---|---|---|---|---|---|
| 8.9745 | 5.0346 | 8.0892 | 8.7549 | 7.2079 | 7.482 | 9.0597 | 8.8358 |
| 8.2694 | 8.3965 | 8.0122 | 6.5515 | 7.455 | 7.9742 | 8.3099 | 9.5236 |
| 9.0514 | 9.3141 | 7.8583 | 9.815 | 8.6811 | 9.2942 | 8.0601 | 7.5625 |
| 7.6221 | 9.3422 | 7.3705 | 8.5661 | 9.1992 | 10.2486 | 9.783 | 9.6155 |
| 9.3316 | 9.4858 | 5.1047 | 9.1525 | 9.1905 | 6.5088 | 9.0035 | 8.4515 |
| 9.3516 | 7.5845 | 8.9049 | 7.4421 | 9.8031 | 9.0881 | 8.9463 | 9.81 |
| 9.7267 | 7.0906 | 8.4003 | 9.3986 | 8.876 | 9.6709 | 8.292 | 8.8587 |
| 7.8873 | 8.3287 | 4.0142 | 8.8832 | 5.4561 | 3.5909 | 4.3353 | 3.2379 |
| 1.026 | 3.1083 | 3.5787 | 7.4835 | 3.8377 | 3.3571 | 4.4977 | 6.6632 |
| 3.8913 | 4.4248 | 6.3857 | 3.5743 | 3.052 | 5.891 | 3.7983 | 6.8355 |
| 2.5659 | 3.93 | | | | | | |
| PTTG1 | 10.6416 | 8.2525 | 8.718 | 1.05E+01 | | 9.97E+00 | |
| 10.1744 | 10.5736 | 10.2281 | 11.2747 | 9.7448 | 10.1916 | 9.418 | 9.4274 |
| 9.5369 | 10.1993 | 9.7464 | 9.9437 | 10.7451 | 8.4285 | 8.4622 | 10.9411 |
| 11.875 | 10.3523 | 8.6092 | 8.8577 | 10.1604 | 10.0058 | 10.7628 | 8.8256 |
| 10.0037 | 10.287 | 9.0406 | 9.9943 | 11.0245 | 8.0728 | 9.8937 | 10.3204 |
| 10.5497 | 10.6672 | 12.5284 | 8.8866 | 10.3271 | 9.2166 | 9.3456 | 9.8561 |
| 9.6227 | 8.5513 | 10.0601 | 9.1116 | 7.959 | 10.1285 | 10.0654 | 9.9802 |
| 10.1339 | 9.74 | 9.9161 | 8.9486 | 9.6195 | 8.4869 | 11.3328 | 10.9312 |
| 9.0852 | 10.5873 | 9.8467 | 8.4355 | 9.094 | 9.3363 | 7.3598 | 8.0501 |
| 9.5263 | 10.811 | 9.5491 | 7.9826 | 11.0248 | 9.7596 | 10.1985 | 9.5096 |
| 10.9246 | 9.2616 | 11.903 | 9.3504 | 11.1729 | 9.3812 | 10.9195 | 10.3746 |
| 8.9504 | 10.6331 | 11.5606 | 10.0951 | 11.0796 | 10.9841 | 11.033 | 10.7613 |
| 9.5428 | 11.0478 | 10.466 | 9.2207 | 9.111 | 10.5487 | 10.5392 | 10.263 |
| 9.3789 | 9.3562 | 9.7908 | 9.9681 | 9.3731 | 10.3144 | 9.4705 | 8.722 |
| 9.5351 | 9.744 | 10.8933 | 11.9716 | 10.1913 | 10.3732 | 9.6861 | 10.8435 |
| 10.9003 | 12.024 | 11.8493 | 5.4591 | 8.0255 | 11.3625 | 9.1393 | 9.6881 |
| 9.5655 | 9.5137 | 8.4348 | 9.6268 | 12.2812 | 7.6463 | 12.7681 | 7.9128 |
| 10.3892 | 10.1085 | 9.2449 | 9.5487 | 10.7512 | 11.6079 | 9.6374 | 10.9675 |
| 9.407 | 9.5026 | 9.3926 | 9.3851 | 11.115 | 9.3848 | 10.3306 | 11.3059 |
| 9.553 | 11.1593 | 11.1559 | 11.4643 | 9.4277 | 9.6116 | 9.7106 | 7.8071 |
| 11.2912 | 10.6309 | 10.3636 | 9.1739 | 9.9901 | 8.7991 | 9.834 | 11.2794 |
| 10.2786 | 10.5774 | 10.1215 | 9.0266 | 9.7389 | 9.8588 | 9.3401 | 12.1454 |
| 12.685 | 9.1331 | 8.5167 | 11.7533 | 5.3142 | 9.5267 | 6.8104 | 6.1076 |
| 5.7263 | 5.0325 | 3.2157 | 4.9963 | 4.5454 | 8.3192 | 5.4112 | 5.0951 |
| 5.3884 | 7.6794 | 6.0125 | 5.8498 | 6.2484 | 5.0025 | 4.0836 | 7.4446 |
| 4.7196 | 8.0805 | 5.3927 | 5.734 | | | | |
| AURKA | 9.3611 | 8.5142 | 7.7353 | 9.84E+00 | | 1.03E+01 | |
| 9.1269 | 8.9743 | 9.8843 | 10.0972 | 9.7542 | 8.8522 | 8.6688 | 8.6676 |
| 7.3315 | 9.0655 | 8.7695 | 10.0767 | 9.8393 | 7.6276 | 7.5618 | 9.3629 |
| 11.4217 | 8.5962 | 7.2988 | 10.5128 | 10.2436 | 9.4135 | 11.044 | 9.3046 |
| 9.8866 | 10.5421 | 7.5577 | 9.4062 | 9.369 | 8.8043 | 8.8656 | 9.9889 |
| 8.9837 | 8.7117 | 9.0571 | 10.3802 | 10.4679 | 8.2857 | 8.9051 | 10.0818 |
| 9.123 | 8.5788 | 9.0466 | 8.6084 | 8.4945 | 9.1877 | 9.8337 | 10.1216 |
| 9.4776 | 9.0645 | 8.9036 | 9.2036 | 9.7458 | 5.9082 | 9.5194 | 10.1891 |
| 8.9267 | 9.8126 | 7.6357 | 8.4336 | 8.1631 | 8.951 | 6.558 | 6.2661 |
| 8.9839 | 8.7269 | 10.1127 | 6.4017 | 9.8488 | 7.8695 | 8.4242 | 10.4747 |
| 8.9134 | 8.3237 | 10.2594 | 10.0153 | 8.7865 | 7.4898 | 9.6658 | 9.9315 |
| 8.3351 | 9.1999 | 8.9114 | 8.9542 | 9.4066 | 9.8274 | 9.4215 | 10.7286 |
| 9.0867 | 9.8515 | 9.6438 | 8.5197 | 8.3331 | 9.7812 | 9.2923 | 9.9691 |
| 9.864 | 8.1722 | 9.7239 | 10.3184 | 10.1725 | 10.4625 | 8.8376 | 10.1645 |
| 9.1484 | 9.9267 | 9.2937 | 9.2759 | 9.2303 | 9.2973 | 9.0517 | 9.1505 |
| 9.8729 | 8.9654 | 9.532 | 5.9263 | 8.0085 | 10.0228 | 7.6595 | 7.6993 |
| 9.1035 | 9.0238 | 6.9279 | 8.7508 | 8.9444 | 6.7374 | 9.1529 | 7.5697 |

| | | | | | | | | |
|---|---|---|---|---|---|---|---|---|
| 9.5756 | 9.1461 | 9.8896 | 8.8715 | 10.0446 | 10.2141 | 8.9331 | 10.0051 | |
| 8.1983 | 7.497 | 9.9029 | 9.1557 | 9.0039 | 8.6223 | 9.2132 | 9.7273 | |
| 9.6281 | 10.6725 | 10.3043 | 9.0634 | 6.8987 | 8.8297 | 9.1315 | 6.8483 | |
| 9.3214 | 10.564 | 9.8608 | 7.7227 | 9.3964 | 8.2683 | 9.4273 | 10.0843 | |
| 8.5622 | 9.8363 | 9.7036 | 8.0525 | 9.8181 | 10.1301 | 8.8709 | 9.8008 | |
| 8.7071 | 8.7608 | 8.093 | 8.7356 | 4.6986 | 8.3385 | 5.626 | 5.3498 | |
| 5.5788 | 4.6026 | 3.7479 | 4.3564 | 4.9801 | 7.1926 | 5.0503 | 5.268 | 5.11 |
| | 6.4618 | 5.6644 | 5.6036 | 6.3857 | 5.0398 | 5.9022 | 6.4184 | |
| 5.0874 | 6.7561 | 4.166 | 5.3232 | | | | | |
| LMOD1 | 8.1488 | 7.5865 | 5.14 | 9.48E+00 | | 6.25E+00 | | |
| 5.333 | 7.941 | 5.8496 | 5.609 | 6.0815 | 6.5848 | 6.9732 | 5.8671 | |
| 5.6204 | 7.3511 | 7.0864 | 6.5092 | 7.0662 | 6.6349 | 5.9787 | 4.4172 | |
| 5.3366 | 6.9179 | 9.5118 | 5.0593 | 4.4938 | 9.356 | 4.5189 | 5.5835 | |
| 4.8835 | 5.2425 | 8.586 | 5.7986 | 7.0597 | 6.5097 | 6.7332 | 7.2505 | |
| 7.255 | 5.7628 | 7.4461 | 5.9759 | 8.6485 | 6.5262 | 7.0797 | 7.4456 | |
| 9.5058 | 9.2905 | 5.87 | 8.7222 | 8.3057 | 11.6119 | 6.0369 | 8.348 | |
| 6.8989 | 6.8097 | 7.4001 | 6.9453 | 8.4958 | 7.6283 | 11.4108 | 6.4918 | |
| 7.2012 | 7.0783 | 5.5912 | 10.5942 | 5.3588 | 8.5769 | 7.7796 | 8.3446 | |
| 10.0369 | 6.9426 | 6.9695 | 10.5055 | 5.7962 | 10.175 | 7.1733 | 7.4425 | |
| 6.3973 | 9.1472 | 10.1316 | 8.3367 | 6.1158 | 9.436 | 8.6381 | 6.8081 | |
| 9.9801 | 7.3619 | 8.0929 | 9.1324 | 6.8797 | 8.5453 | 6.7613 | 9.6447 | |
| 9.6462 | 8.0912 | 7.1218 | 5.5601 | 4.8311 | 9.7234 | 8.2621 | 8.4839 | |
| 6.0999 | 5.5791 | 6.2949 | 8.5549 | 7.7776 | 8.5693 | 8.6781 | 7.535 | |
| 6.2786 | 6.5054 | 9.8503 | 5.9961 | 6.4313 | 4.929 | 8.1055 | 8.2897 | |
| 7.6391 | 10.1439 | 7.3564 | 9.6591 | 12.2366 | 5.8412 | 5.8402 | 6.3858 | |
| 8.0612 | 6.5655 | 4.9141 | 6.626 | 6.542 | 12.5944 | 8.9056 | 6.5944 | |
| 6.4467 | 5.1918 | 8.6712 | 7.7302 | 4.4001 | 7.1613 | 5.6314 | 8.267 | |
| 5.7596 | 5.5081 | 5.5723 | 7.608 | 8.72 | 8.1841 | 8.6252 | 3.8893 | |
| 5.1258 | 7.6202 | 5.3427 | 7.7722 | 8.9065 | 6.7388 | 9.1889 | 9.7424 | |
| 8.3846 | 6.6582 | 6.1113 | 5.911 | 6.5485 | 9.4967 | 7.7991 | 6.5985 | |
| 5.6767 | 7.8787 | 8.2595 | 7.3618 | 7.0026 | 9.9889 | 6.4876 | 4.7689 | |
| 6.5384 | 7.6541 | 10.0534 | 7.7639 | 12.7292 | 10.539 | 12.6361 | 10.3019 | |
| 14.1993 | 13.135 | 14.3111 | 14.3638 | 14.305 | 11.2189 | 14.0837 | 14.6417 | |
| 13.3275 | 12.4612 | 11.9188 | 13.4135 | 13.5135 | 13.4607 | 11.6451 | 13.172 | |
| 13.3558 | 12.2011 | 14.7965 | 13.3069 | | | | | |
| TACC3 | 12.0932 | 10.5251 | 9.4764 | 1.22E+01 | | 1.17E+01 | | |
| 10.9146 | 11.0184 | 12.233 | 13.1689 | 11.3506 | 11.0215 | 11.0947 | 10.4586 | |
| 10.0701 | 12.0882 | 11.064 | 11.7663 | 12.4196 | 9.8181 | 9.2436 | 11.4453 | |
| 10.9903 | 11.2141 | 9.9176 | 12.1037 | 11.8217 | 11.5583 | 11.8056 | 11.1348 | |
| 11.6515 | 11.9981 | 9.8176 | 11.3185 | 11.1149 | 10.5699 | 11.4573 | 10.8263 | |
| 11.544 | 11.1284 | 12.7112 | 10.1117 | 11.0888 | 10.8192 | 10.9986 | 10.0979 | |
| 10.0469 | 9.7719 | 11.2029 | 10.6284 | 9.598 | 11.0455 | 10.9932 | 11.3571 | |
| 11.0001 | 12.2459 | 10.6549 | 9.1795 | 10.6186 | 8.1817 | 12.2526 | 12.148 | |
| 10.5287 | 11.9135 | 11.1357 | 10.2425 | 9.9286 | 10.747 | 8.89 | 8.7165 | |
| 11.1832 | 11.5123 | 11.2703 | 8.532 | 11.2975 | 11.0807 | 10.113 | 10.1592 | |
| 11.9303 | 9.9196 | 12.106 | 11.169 | 11.0225 | 10.2472 | 12.1966 | 9.8032 | |
| 10.5089 | 11.2471 | 12.4559 | 11.065 | 11.4467 | 11.604 | 11.7287 | 12.5002 | |
| 11.7842 | 10.4509 | 12.0778 | 10.6715 | 10.9026 | 11.9808 | 10.6786 | 11.5709 | |
| 11.2514 | 10.2281 | 10.9974 | 10.8107 | 10.699 | 11.3524 | 10.6313 | 11.088 | |
| 10.6845 | 10.3577 | 10.7893 | 12.487 | 12.7881 | 11.824 | 10.8515 | 11.0231 | |
| 11.4943 | 12.3153 | 12.0405 | 9.7928 | 9.7472 | 10.8694 | 9.9155 | 10.6157 | |
| 10.7097 | 10.8145 | 10.0367 | 10.8284 | 12.0911 | 9.3233 | 11.9995 | 9.8504 | |
| 11.968 | 11.541 | 11.8897 | 10.8607 | 11.8102 | 12.3985 | 11.446 | 11.5752 | |
| 10.2847 | 10.5567 | 10.8443 | 10.8818 | 11.2396 | 10.6527 | 10.6381 | 12.6918 | |

| | | | | | | | | |
|---|---|---|---|---|---|---|---|---|
| 11.1165 | 11.7539 | 11.3662 | 10.9434 | 9.7735 | 10.4903 | 11.2826 | 9.5069 | |
| 12.2218 | 11.1064 | 11.298 | 9.8716 | 11.4311 | 10.2241 | 12.225 | 11.6851 | |
| 11.4496 | 11.9356 | 11.4572 | 9.4753 | 10.4547 | 10.9338 | 10.7715 | 12.8269 | |
| 13.3002 | 10.8751 | 9.9133 | 12.8207 | 7.2577 | 10.7167 | 8.4313 | 7.8087 | |
| 7.3438 | 7.267 | 6.2405 | 7.3242 | 7.1306 | 9.6186 | 7.5439 | 7.1921 | |
| 7.338 | 8.9925 | 7.3024 | 7.422 | 8.3012 | 7.0934 | 6.352 | 8.633 | |
| 7.2761 | 9.126 | 6.8422 | 6.871 | | | | | |
| ORC6L | 9.2746 | 7.8958 | 7.5963 | 9.7542 | 8.2471 | 7.4486 | 8.7006 | |
| 8.1008 | 8.5176 | 8.3567 | 7.8641 | 9.3225 | 8.3989 | 7.1336 | 8.0682 | |
| 8.8676 | 8.212 | 7.8161 | 7.9129 | 7.7826 | 7.7277 | 8.9374 | 8.472 | |
| 7.0047 | 8.0012 | 8.9038 | 8.4881 | 9.4326 | 7.8974 | 8.3001 | 9.1134 | |
| 7.685 | 8.8558 | 8.7636 | 7.8887 | 8.9937 | 9.4199 | 7.9655 | 8.4611 | |
| 7.8092 | 8.1673 | 9.345 | 6.1742 | 7.8366 | 8.3593 | 8.3293 | 9.4727 | |
| 9.3651 | 7.843 | 7.8195 | 8.6342 | 8.3795 | 9.3144 | 9.8439 | 7.8743 | |
| 8.5724 | 7.5021 | 8.5303 | 5.664 | 8.9521 | 7.8617 | 8.17 | 7.9595 | |
| 7.889 | 8.0281 | 7.6578 | 8.6745 | 6.5012 | 5.7132 | 8.6043 | 8.8596 | |
| 9.4868 | 5.7749 | 8.2623 | 8.832 | 8.8656 | 8.377 | 8.6456 | 8.487 | |
| 8.8271 | 8.9064 | 7.3289 | 8.3238 | 9.9883 | 8.2949 | 7.991 | 8.5196 | |
| 9.2427 | 8.6491 | 8.3473 | 8.9981 | 10.1375 | 10.1258 | 8.371 | 9.649 | |
| 8.6283 | 7.8223 | 8.7792 | 7.073 | 8.5236 | 9.7861 | 8.7205 | 8.2949 | |
| 7.9954 | 8.6004 | 8.9331 | 8.7178 | 8.1195 | 8.1313 | 8.4713 | 8.5236 | |
| 9.8073 | 7.0956 | 8.6469 | 9.3659 | 8.5228 | 9.3963 | 8.7761 | 7.0461 | |
| 9.025 | 6.3465 | 7.6659 | 10.3645 | 6.8583 | 8.2102 | 8.011 | 8.6224 | |
| 7.897 | 8.3424 | 7.6539 | 6.5643 | 7.9799 | 8.1762 | 9.4917 | 8.5978 | |
| 8.3713 | 8.9406 | 9.0558 | 9.1234 | 7.4462 | 8.3975 | 8.4811 | 7.8952 | |
| 9.3297 | 9.1704 | 7.4326 | 7.2811 | 9.1361 | 8.7538 | 8.9854 | 9.5394 | |
| 7.1911 | 8.9002 | 5.1619 | 8.5803 | 9.4525 | 6.2627 | 8.2038 | 8.5326 | |
| 7.7338 | 7.9206 | 8.0705 | 8.5297 | 7.6143 | 8.9179 | 8.9053 | 9.7689 | |
| 8.9464 | 7.1491 | 9.4458 | 8.2071 | 8.7125 | 7.6798 | 8.5946 | 8.3258 | |
| 8.192 | 7.7598 | 4.4268 | 8.3128 | 5.1465 | 4.5298 | 5.4144 | 4.6949 | |
| 2.3632 | 3.8207 | 3.8762 | 7.1985 | 3.8377 | 4.4009 | 4.3753 | 6.6056 | 5.41 |
| | 5.0404 | 5.9956 | 4.2514 | 4.4271 | 4.8361 | 4.3312 | 7.7813 | |
| 4.5829 | 4.6286 | | | | | | | |
| CDCA8 | 9.9794 | 7.8846 | 8.254 | 10.2656 | 10.1376 | 8.822 | 9.441 | |
| 10.2728 | 10.5643 | 10.1549 | 10.6293 | 9.4187 | 8.9505 | 7.939 | 8.7843 | |
| 9.6574 | 9.9696 | 9.7721 | 8.0766 | 7.9614 | 9.3258 | 11.316 | 8.7386 | |
| 7.1914 | 9.5399 | 10.4411 | 9.8532 | 10.2727 | 8.9444 | 10.0937 | 10.2279 | |
| 8.0056 | 10.0459 | 9.4168 | 9.3257 | 9.7441 | 9.2574 | 9.3033 | 8.721 | |
| 8.6856 | 9.0118 | 10.4917 | 8.2096 | 9.3899 | 8.7988 | 9.2225 | 8.2823 | |
| 9.5502 | 8.6246 | 8.4173 | 10.0712 | 9.6617 | 9.5151 | 9.9501 | 9.3412 | |
| 9.4544 | 8.1314 | 10.1137 | 5.9317 | 10.4437 | 10.1017 | 9.3303 | 8.8216 | |
| 9.2263 | 8.5526 | 7.8578 | 9.2276 | 6.8473 | 6.4186 | 9.499 | 9.092 | |
| 10.1693 | 6.8774 | 10.4167 | 8.2091 | 9.1819 | 10.0168 | 9.3057 | 8.8435 | |
| 10.6946 | 9.6383 | 10.1809 | 7.6261 | 10.035 | 9.5236 | 8.6694 | 9.3274 | |
| 9.016 | 9.738 | 10.1603 | 9.6795 | 9.8985 | 11.3648 | 9.1367 | 10.2091 | |
| 10.0896 | 8.8648 | 9.218 | 7.6601 | 8.4263 | 9.9023 | 10.0138 | 8.7113 | |
| 9.5063 | 10.4147 | 9.2824 | 9.7965 | 8.9104 | 9.8269 | 9.7497 | 9.7877 | |
| 10.0392 | 9.3351 | 9.5433 | 9.5876 | 8.941 | 9.6749 | 10.009 | 7.8309 | |
| 9.3298 | 3.6106 | 8.6373 | 10.2546 | 7.7409 | 7.9088 | 9.3178 | 9.4407 | |
| 8.1656 | 8.7086 | 8.7319 | 7.0989 | 8.148 | 8.3167 | 9.9854 | 9.3156 | |
| 10.0248 | 9.5025 | 9.6547 | 9.8245 | 9.2513 | 9.3897 | 8.7445 | 7.8189 | |
| 9.1134 | 9.7444 | 9.0282 | 8.3449 | 9.6928 | 10.8985 | 10.2135 | 10.0913 | |
| 10.0927 | 9.1835 | 6.3282 | 9.475 | 9.9204 | 6.585 | 9.0954 | 9.5928 | |
| 10.895 | 8.0032 | 10.2323 | 8.5381 | 9.9237 | 10.8521 | 9.0706 | 10.2684 | |

| | | | | | | | |
|---|---|---|---|---|---|---|---|
| 10.0933 | 8.2635 | 9.5663 | 9.9327 | 9.5445 | 9.678 | 9.6436 | 8.9426 |
| 8.4694 | 8.7145 | 4.5959 | 8.9639 | 5.6691 | 5.4625 | 5.1165 | 4.313 |
| 3.9549 | 4.7461 | 4.3333 | 7.7582 | 3.8377 | 4.9247 | 4.8361 | 6.1358 |
| 5.5586 | 5.8014 | 6.8163 | 4.9835 | 4.3324 | 6.8096 | 4.8687 | 7.0022 |
| 4.5829 | 4.9326 | | | | | | |
| KANK2 | 10.6311 | 10.5064 | 9.5002 | 11.3029 | 10.0673 | 12.7575 | 11.0942 |
| 10.6225 | 10.4367 | 10.473 | 11.7102 | 10.5386 | 9.4758 | 9.9771 | 10.756 |
| 11.1719 | 8.6985 | 9.9035 | 9.4256 | 9.6372 | 10.3584 | 10.5131 | 10.2426 |
| 10.4135 | 10.429 | 11.1381 | 11.1443 | 10.2143 | 9.9946 | 9.7798 | 10.1348 |
| 12.2203 | 10.4864 | 9.8421 | 9.9248 | 9.8348 | 9.8024 | 10.1287 | 9.2243 |
| 8.9266 | 9.6519 | 10.2531 | 9.4215 | 10.0019 | 10.0837 | 11.0404 | 11.2548 |
| 10.2197 | 11.4977 | 10.3712 | 12.6645 | 10.1671 | 10.275 | 10.1423 | 11.0782 |
| 11.225 | 10.9419 | 11.1762 | 10.9342 | 12.2521 | 8.5502 | 10.3531 | 9.7395 |
| 9.6802 | 11.1228 | 11.0707 | 11.2269 | 10.5758 | 10.4228 | 11.0876 | 10.4862 |
| 10.297 | 11.5088 | 10.0575 | 10.7024 | 11.1568 | 12.1396 | 9.6703 | 10.117 |
| 11.349 | 10.0619 | 11.0328 | 11.8132 | 10.411 | 10.0186 | 10.8604 | 11.082 |
| 10.0992 | 11.1982 | 10.755 | 10.7858 | 9.603 | 11.5096 | 12.0612 | 11.1815 |
| 11.4289 | 9.9612 | 9.1347 | 10.1095 | 9.7279 | 10.3139 | 10.6207 | 9.2628 |
| 13.1536 | 10.5018 | 10.256 | 12.6653 | 11.4712 | 10.898 | 11.9657 | 10.7633 |
| 10.2623 | 9.3303 | 10.5329 | 9.6063 | 10.0434 | 10.2751 | 10.2052 | 9.8736 |
| 10.2782 | 11.895 | 12.7249 | 8.993 | 9.4278 | 9.3443 | 10.8036 | 10.4969 |
| 9.145 | 10.8742 | 9.0877 | 13.0886 | 8.6704 | 10.6371 | 9.9904 | 9.3239 |
| 10.8867 | 11.1745 | 10.5838 | 10.2388 | 9.6029 | 10.9287 | 10.6101 | 9.8006 |
| 11.1477 | 10.2349 | 10.6273 | 10.569 | 10.9812 | 9.2547 | 9.2674 | 9.5047 |
| 11.5493 | 10.9049 | 9.8538 | 10.72 | 10.4255 | 10.9385 | 10.7855 | 9.0889 |
| 11.4222 | 9.04 | 11.0276 | 10.3737 | 10.5267 | 10.124 | 9.8003 | 10.5482 |
| 11.3658 | 10.5988 | 10.8338 | 11.1681 | 10.5491 | 8.9716 | 9.6445 | 10.7358 |
| 10.9395 | 9.8324 | 13.6607 | 12.9636 | 13.2379 | 10.885 | 14.7627 | 13.0515 |
| 13.6742 | 14.0552 | 13.8047 | 13.4722 | 13.829 | 14.3921 | 14.2648 | 13.0575 |
| 11.8582 | 13.8589 | 14.2441 | 13.794 | 13.5346 | 13.1939 | 13.734 | 13.6088 |
| 14.0252 | 13.6407 | | | | | | |
| DLGAP5 | 9.4533 | 7.9252 | 7.8545 | 9.89E+00 | | 1.06E+01 | |
| 8.758 | 9.9978 | 9.1426 | 10.1749 | 9.6561 | 9.8041 | 8.716 | 9.1322 |
| 6.9449 | 8.7558 | 9.1123 | 9.7386 | 9.0407 | 8.167 | 7.6081 | 9.0006 |
| 10.8477 | 8.6341 | 6.8418 | 9.5132 | 10.6826 | 9.3189 | 10.6422 | 8.6227 |
| 9.6275 | 9.6182 | 7.2188 | 9.3884 | 9.7406 | 9.8489 | 9.3985 | 9.187 |
| 10.0313 | 8.2418 | 8.8265 | 9.1145 | 10.6287 | 8.1729 | 9.2236 | 9.3092 |
| 8.3036 | 8.2717 | 9.0165 | 8.5178 | 7.5604 | 10.4728 | 9.4821 | 9.2623 |
| 8.7966 | 9.9814 | 9.2456 | 7.3739 | 9.4681 | 5.1275 | 9.7741 | 9.1611 |
| 8.8369 | 8.6856 | 8.1349 | 8.136 | 7.6412 | 8.3427 | 6.0842 | 5.534 |
| 9.1675 | 9.23 | 10.8975 | 5.7919 | 10.0234 | 8.1045 | 9.0478 | 9.9078 |
| 9.0654 | 8.4709 | 10.1013 | 9.8347 | 8.3027 | 6.9308 | 10.405 | 9.5321 |
| 9.9471 | 9.2645 | 8.7985 | 8.5807 | 9.9133 | 9.1065 | 10.0861 | 10.9856 |
| 10.1087 | 10.349 | 9.5424 | 8.8635 | 9.8751 | 7.6145 | 8.96 | 9.6711 |
| 9.3766 | 9.2207 | 9.6956 | 10.4173 | 9.451 | 10.6696 | 9.1339 | 9.5717 |
| 9.0669 | 9.1447 | 10.5148 | 8.1243 | 8.5306 | 8.8713 | 8.8866 | 9.1382 |
| 8.5797 | 8.3262 | 9.5472 | 0 | 8.427 | 9.5848 | 7.7075 | 7.7875 |
| 9.3136 | 9.4415 | 8.4372 | 7.6862 | 8.7567 | 6.9319 | 8.66 | 8.2341 |
| 9.5087 | 9.5317 | 9.7109 | 9.2364 | 10.034 | 10.0808 | 8.5999 | 8.8904 |
| 8.3576 | 7.7319 | 8.9306 | 10.2108 | 6.8305 | 8.3263 | 9.4951 | 10.1154 |
| 9.8266 | 10.501 | 10.1148 | 8.4791 | 2.7155 | 9.5019 | 10.2371 | 6.3815 |
| 8.9594 | 9.2179 | 10.7034 | 7.9889 | 9.47 | 7.6742 | 10.0157 | 9.3629 |
| 8.8719 | 9.8538 | 10.0348 | 7.5829 | 9.0827 | 7.8009 | 9.2024 | 8.5881 |
| 10.1118 | 9.3322 | 8.5397 | 9.0442 | 2.4485 | 8.8589 | 4.974 | 4.0893 |

| | | | | | | | |
|---|---|---|---|---|---|---|---|
| 3.2362 | 2.8926 | 0 | 3.0357 | 3.4032 | 7.5734 | 2.2864 | 2.6869 |
| 3.6943 | 5.367 | 4.2433 | 3.6357 | 6.9551 | 1.1757 | 1.9011 | 6.0495 |
| 3.4012 | 6.3781 | 1.066 | 2.2304 | | | | |
| EME1 | 8.1359 | 6.2903 | 6.0419 | 8.22E+00 | | 7.92E+00 | |
| 6.9277 | 7.201 | 7.675 | 7.7872 | 7.3733 | 7.705 | 7.3751 | 6.6798 |
| 6.4339 | 8.7793 | 7.9574 | 7.5773 | 7.2255 | 5.8984 | 5.9525 | 6.8723 |
| 8.0912 | 7.195 | 6.0581 | 7.1364 | 7.4862 | 7.5592 | 9.1351 | 7.0732 |
| 7.3555 | 9.0209 | 5.7267 | 7.2842 | 7.4291 | 6.2761 | 7.2237 | 7.6795 |
| 7.2501 | 7.2619 | 7.3081 | 6.8654 | 7.7735 | 6.5248 | 7.1506 | 7.2394 |
| 6.6926 | 7.4275 | 8.2453 | 6.6359 | 6.6821 | 6.7197 | 7.8027 | 8.3176 |
| 7.8843 | 7.3833 | 6.2503 | 5.2277 | 8.1158 | 4.1952 | 7.4912 | 8.6798 |
| 6.2816 | 7.3225 | 7.3093 | 6.7674 | 6.4173 | 6.6313 | 4.6767 | 5.4873 |
| 7.1514 | 6.7948 | 8.6337 | 5.0771 | 6.7061 | 6.8997 | 7.9776 | 8.042 |
| 6.9923 | 6.3379 | 7.6153 | 7.9423 | 6.1572 | 7.1607 | 8.1698 | 6.3878 |
| 5.7981 | 7.5263 | 7.9539 | 6.948 | 6.7731 | 8.5353 | 7.1669 | 8.2411 |
| 7.1458 | 7.588 | 7.7664 | 6.3542 | 6.932 | 8.4105 | 8.1261 | 9.3132 |
| 8.2103 | 6.5 | 7.5528 | 6.4232 | 7.6249 | 8.5292 | 6.8487 | 7.0293 |
| 6.0199 | 7.6607 | 7.4168 | 7.161 | 7.0595 | 7.9199 | 6.8113 | 8.5901 |
| 7.8744 | 6.3605 | 8.1039 | 7.74 | 6.4348 | 7.7582 | 5.5172 | 6.8226 |
| 6.9058 | 7.0623 | 5.7077 | 6.4506 | 7.1935 | 5.2952 | 7.3804 | 6.5641 |
| 8.0107 | 7.0291 | 6.8814 | 7.0556 | 8.3192 | 7.7083 | 7.0478 | 6.6713 |
| 6.2789 | 6.7895 | 6.7709 | 6.9871 | 6.2979 | 6.7162 | 7.0926 | 8.1379 |
| 7.6041 | 8.5207 | 6.4116 | 7.4652 | 8.349 | 7.0724 | 7.8724 | 5.9627 |
| 8.2574 | 6.7837 | 7.9618 | 6.1743 | 6.8257 | 6.3782 | 7.6702 | 8.2476 |
| 7.0676 | 8.3816 | 7.6514 | 5.5926 | 6.3627 | 6.7454 | 7.1079 | 7.8855 |
| 5.6318 | 7.1502 | 6.5822 | 6.2906 | 3.193 | 6.9057 | 4.2583 | 3.4287 |
| 3.1665 | 3.2827 | 1.0405 | 3.8616 | 2.7188 | 5.9525 | 2.9936 | 2.3166 |
| 3.0948 | 5.516 | 4.3008 | 3.5039 | 4.5543 | 4.0571 | 3.9661 | 4.5479 |
| 2.9618 | 6.3852 | 2.7578 | 4.141 | | | | |
| E2F2 | 9.3703 | 6.6479 | 6.3315 | 9.5509 | 7.6262 | 9.085 | 7.3184 |
| 8.0636 | 7.6846 | 7.5544 | 6.9934 | 8.5924 | 7.0145 | 7.2845 | 7.1576 |
| 8.2564 | 8.4329 | 8.3501 | 5.4929 | 6.657 | 9.4234 | 9.3626 | 7.8163 |
| 6.153 | 8.8656 | 9.2732 | 8.0977 | 8.5608 | 7.4199 | 8.7608 | 9.3075 |
| 7.6314 | 9.3728 | 8.2653 | 8.2725 | 7.4599 | 8.1639 | 6.8277 | 7.9414 |
| 9.093 | 7.2105 | 8.4886 | 7.4554 | 7.9721 | 7.885 | 7.0664 | 7.2131 |
| 8.7766 | 7.3021 | 6.4559 | 7.7751 | 7.4988 | 8.2166 | 8.2971 | 8.4677 |
| 7.1356 | 6.2671 | 7.3688 | 5.1275 | 8.138 | 8.2113 | 7.7911 | 7.9966 |
| 7.4074 | 7.2217 | 6.7344 | 6.4166 | 4.9548 | 4.9833 | 8.8472 | 8.4038 |
| 8.7722 | 5.3168 | 8.3903 | 6.8998 | 6.2611 | 7.5048 | 8.4004 | 8.1687 |
| 10.0149 | 7.3986 | 6.9103 | 6.66 | 8.3939 | 7.5252 | 6.9505 | 8.1156 |
| 8.1046 | 7.9797 | 9.1238 | 6.9934 | 9.0015 | 9.8476 | 6.349 | 7.7058 |
| 7.9968 | 7.5135 | 8.3331 | 8.9458 | 8.0056 | 7.3993 | 7.8164 | 7.0555 |
| 8.3644 | 8.6581 | 8.4627 | 7.693 | 6.9298 | 8.345 | 7.9488 | 7.0095 |
| 7.5834 | 8.2698 | 8.5232 | 8.1034 | 7.5628 | 8.1425 | 6.1059 | 7.6684 |
| 9.5612 | 6.4426 | 6.6668 | 9.2716 | 6.6135 | 6.6043 | 8.4631 | 8.4037 |
| 6.1657 | 7.3944 | 8.6164 | 6.0875 | 8.2805 | 6.8029 | 8.3983 | 8.2798 |
| 8.0455 | 8.777 | 8.9915 | 9.7067 | 7.8175 | 8.1262 | 7.3177 | 7.0261 |
| 7.9722 | 7.2615 | 8.4998 | 6.5143 | 8.1364 | 9.859 | 9.1087 | 7.9754 |
| 8.4972 | 7.9962 | 7.2549 | 8.5133 | 8.8032 | 5.6716 | 8.1714 | 8.3025 |
| 8.7408 | 5.7403 | 6.2533 | 7.424 | 8.2832 | 8.4919 | 8.1529 | 9.2921 |
| 8.3958 | 5.6666 | 6.6409 | 9.7708 | 8.3606 | 8.9063 | 9.0175 | 7.2569 |
| 6.8064 | 8.6154 | 2.8563 | 8.1016 | 5.0628 | 3.5232 | 2.62 | 2.8926 |
| 2.0387 | 2.1337 | 2.4755 | 6.7808 | 2.8267 | 4.0682 | 3.3488 | 5.6773 |
| 4.3916 | 3.1853 | 5.5134 | 2.4897 | 1.2434 | 5.1291 | 3.1906 | 6.4375 |

| | | | | | | | |
|---|---|---|---|---|---|---|---|
| 2.8104 | 3.1558 | | | | | | |
| CCNB1 | 11.6056 | 9.3924 | 9.6029 | 11.7373 | 10.7085 | 11.2722 | 11.1698 |
| 11.6284 | 12.4873 | 10.9622 | 11.5106 | 10.5731 | 10.9092 | 9.701 | 9.935 |
| 11.0702 | 10.9983 | 11.682 | 9.8525 | 9.6672 | 11.0623 | 11.9059 | 10.7777 |
| 9.5277 | 11.6086 | 11.2113 | 11.1696 | 11.752 | 10.5364 | 11.1433 | 11.6476 |
| 9.6472 | 11.4942 | 11.5479 | 10.1702 | 11.2846 | 11.042 | 10.8936 | 10.7013 |
| 11.049 | 10.9501 | 12.18 | 10.4283 | 10.8858 | 10.1994 | 10.6348 | 9.3622 |
| 11.178 | 10.1182 | 9.3411 | 10.9696 | 10.4967 | 11.4356 | 11.9331 | 11.2061 |
| 10.7804 | 8.5583 | 11.1265 | 8.6389 | 11.0007 | 11.0507 | 10.9586 | 11.1748 |
| 10.0445 | 10.4754 | 9.2608 | 10.2687 | 8.5253 | 8.1499 | 10.753 | 11.3656 |
| 11.5745 | 8.1166 | 11.7319 | 10.3117 | 10.0567 | 11.3268 | 11.5828 | 10.2447 |
| 11.5436 | 10.9023 | 10.8265 | 9.4776 | 11.9199 | 10.3656 | 10.2876 | 11.1514 |
| 11.4829 | 11.2116 | 11.231 | 11.3166 | 12.1089 | 11.6785 | 11.3374 | 10.5099 |
| 11.1525 | 10.4024 | 11.1168 | 10.0315 | 9.6421 | 11.8587 | 11.1027 | 10.5885 |
| 11.0172 | 11.3797 | 10.8167 | 11.026 | 11.0503 | 11.1114 | 11.2252 | 11.2645 |
| 11.0777 | 11.3103 | 11.5704 | 11.3154 | 11.0078 | 11.447 | 11.0899 | 10.4074 |
| 11.2344 | 6.4426 | 9.9102 | 11.6962 | 9.8489 | 9.9639 | 11.0703 | 11.1823 |
| 9.6954 | 10.6376 | 11.2722 | 8.6077 | 10.4963 | 9.7901 | 11.4018 | 11.5857 |
| 11.3646 | 10.9583 | 11.7401 | 11.9681 | 10.7929 | 10.6561 | 10.2147 | 10.1526 |
| 10.6988 | 11.3532 | 10.9475 | 10.4169 | 11.5891 | 11.9342 | 11.6054 | 11.6605 |
| 11.7619 | 10.9812 | 8.7659 | 10.8352 | 11.6512 | 8.4086 | 11.7619 | 11.3755 |
| 11.4961 | 9.8163 | 11.3151 | 9.9958 | 10.9899 | 11.8827 | 10.764 | 11.9738 |
| 11.7774 | 10.0838 | 9.7838 | 9.9811 | 10.8651 | 12.1115 | 11.9865 | 10.7958 |
| 10.0693 | 11.5491 | 6.6627 | 10.4481 | 7.363 | 7.9889 | 7.1097 | 6.2269 |
| 6.5103 | 6.7626 | 6.8799 | 9.1716 | 6.9861 | 6.5124 | 7.5078 | 7.5173 |
| 8.2836 | 7.5226 | 8.3505 | 7.4212 | 7.1483 | 8.5907 | 7.2536 | 8.8163 |
| 6.8491 | 7.7386 | | | | | | |
| NDN | 6.9457 | 7.5772 | 6.3676 | 7.58E+00 | | 6.04E+00 | |
| 7.4674 | 7.4625 | 7.181 | 6.1422 | 6.1642 | 8.6553 | 4.9335 | 6.1501 |
| 6.4999 | 8.3633 | 8.1624 | 5.792 | 6.478 | 6.594 | 6.418 | 6.9201 |
| 4.7954 | 9.1318 | 8.6988 | 5.2818 | 4.4064 | 7.2544 | 9.1737 | 5.7564 |
| 4.1301 | 5.4759 | 6.8175 | 5.2191 | 6.6918 | 6.6011 | 8.252 | 7.9878 |
| 5.8046 | 6.1221 | 7.4981 | 6.5624 | 6.177 | 6.2051 | 6.4723 | 6.891 |
| 7.7655 | 8.2412 | 6.4584 | 7.7085 | 8.4556 | 9.6892 | 7.7443 | 7.611 |
| 6.2651 | 6.4011 | 7.2869 | 3.9431 | 6.5497 | 7.7716 | 9.6008 | 7.9705 |
| 6.0971 | 7.6093 | 7.9749 | 8.3916 | 4.4955 | 9.0666 | 8.7338 | 9.0869 |
| 7.8913 | 7.9111 | 6.6071 | 9.9139 | 6.844 | 8.7023 | 7.0848 | 5.8554 |
| 7.2142 | 7.712 | 8.669 | 6.873 | 5.6987 | 9.6529 | 8.3485 | 5.4545 |
| 7.618 | 7.2431 | 6.9103 | 7.8385 | 6.4014 | 7.6241 | 6.4026 | 7.7319 |
| 8.7225 | 9.2036 | 8.0796 | 5.6488 | 5.8193 | 8.7008 | 6.1898 | 8.121 |
| 5.0304 | 6.0424 | 6.7197 | 6.6744 | 7.578 | 6.8162 | 7.8022 | 8.0631 |
| 6.4473 | 7.4903 | 7.8291 | 5.1269 | 6.2823 | 5.1606 | 7.3358 | 6.3285 |
| 8.1449 | 8.7307 | 6.7309 | 10.1959 | 9.6629 | 5.1548 | 6.0887 | 7.6096 |
| 7.365 | 6.7809 | 6.4174 | 6.1693 | 5.3604 | 10.1137 | 6.9291 | 7.2014 |
| 7.5156 | 4.3498 | 8.6447 | 5.9743 | 6.8961 | 7.7672 | 4.7042 | 7.1177 |
| 8.7004 | 6.2385 | 7.5604 | 7.0511 | 7.4326 | 8.0262 | 7.6289 | 5.4235 |
| 5.3744 | 5.9671 | 5.3747 | 6.7245 | 8.3458 | 5.0261 | 8.7057 | 9.0494 |
| 7.6917 | 6.735 | 4.2159 | 7.2565 | 7.1041 | 7.482 | 8.2317 | 6.808 |
| 6.4696 | 7.0014 | 6.9416 | 5.9989 | 6.9303 | 8.6087 | 6.9541 | 6.6437 |
| 4.9439 | 6.9085 | 7.7834 | 7.8283 | 11.3392 | 11.2083 | 11.3081 | 10.0834 |
| 12.5928 | 10.6859 | 11.2602 | 11.5301 | 10.9749 | 11.7017 | 11.4013 | 11.6935 |
| 11.698 | 10.8111 | 10.2908 | 11.4264 | 11.6225 | 11.2715 | 12.1416 | 11.0831 |
| 11.3214 | 11.5792 | 11.5369 | 11.71 | | | | |
| UBE2T | 9.7359 | 8.5167 | 8.5382 | 9.60E+00 | | 9.85E+00 | |

| | | | | | | | | |
|---|---|---|---|---|---|---|---|---|
| 8.295 | 9.9343 | 8.643 | 10.4461 | 8.6515 | 8.6853 | 9.2585 | 8.8411 | |
| 8.146 | 8.659 | 9.0379 | 9.2447 | 8.5484 | 8.4791 | 8.9674 | 9.912 | |
| 11.1368 | 8.9973 | 8.3805 | 9.1002 | 10.2317 | 9.9507 | 9.8511 | 8.7147 | |
| 8.8805 | 9.4489 | 8.7586 | 10.1843 | 9.8139 | 8.3145 | 9.0102 | 9.7733 | |
| 9.6002 | 9.3273 | 9.1607 | 8.3417 | 9.5255 | 9.3236 | 9.4405 | 8.061 | |
| 9.0993 | 10.2722 | 9.363 | 7.9723 | 7.5919 | 8.9967 | 8.6426 | 9.5955 | |
| 9.7608 | 8.6388 | 8.7998 | 7.7631 | 9.7938 | 7.0962 | 8.9223 | 9.7319 | |
| 9.2855 | 9.5098 | 8.2531 | 9.4239 | 9.2499 | 9.1594 | 6.8068 | 7.4049 | |
| 9.341 | 9.3118 | 9.9033 | 6.7957 | 9.5058 | 8.9865 | 8.9681 | 9.5683 | |
| 8.9526 | 8.6175 | 11.1873 | 9.075 | 9.4134 | 7.3771 | 10.029 | 8.6525 | |
| 9.2611 | 9.0775 | 9.615 | 9.3552 | 8.5768 | 9.4762 | 9.0446 | 11.0244 | |
| 8.767 | 9.0191 | 9.8777 | 9.5135 | 8.4554 | 8.4449 | 7.0112 | 10.0377 | |
| 9.0544 | 10.1365 | 7.577 | 9.435 | 8.8858 | 9.536 | 8.9299 | 8.4754 | |
| 8.6506 | 9.2438 | 9.7806 | 9.3367 | 9.5607 | 9.4252 | 8.7649 | 9.0011 | |
| 8.7662 | 8.9119 | 10.1058 | 4.2995 | 7.9411 | 10.539 | 9.197 | 8.0364 | |
| 8.8644 | 9.1463 | 8.7834 | 8.7982 | 10.927 | 8.1475 | 9.3006 | 8.0653 | |
| 8.8256 | 9.432 | 8.6395 | 9.8423 | 9.4175 | 10.4597 | 8.3846 | 8.618 | |
| 8.669 | 8.6414 | 8.9724 | 8.7565 | 8.5314 | 8.2705 | 9.5368 | 9.8747 | |
| 9.0553 | 9.6016 | 9.2183 | 10.6731 | 5.7144 | 9.7758 | 9.8871 | 7.5524 | |
| 10.1045 | 8.9545 | 9.2375 | 8.0835 | 8.8096 | 8.886 | 8.0384 | 9.7371 | |
| 9.0138 | 9.6212 | 8.9241 | 8.7791 | 9.2801 | 9.5089 | 8.7364 | 9.973 | |
| 9.8381 | 8.3364 | 7.8822 | 9.0794 | 5.2815 | 8.7372 | 5.7649 | 5.3301 | |
| 8.1901 | 5.0675 | 4.9566 | 4.4148 | 4.654 | 7.4639 | 4.7272 | 5.4762 | |
| 5.3564 | 6.2529 | 6.0352 | 5.2535 | 7.0221 | 5.0762 | 3.3387 | 6.0089 | |
| 5.5221 | 7.643 | 4.5497 | 5.3232 | | | | | |
| OMD | 0.5526 | 0.6896 | 0.4935 | 3.96E+00 | | 0.00E+00 | | |
| 4.7577 | 2.7752 | 0 | 0.4291 | 0 | 1.0422 | 0 | 1.7043 | |
| 1.4442 | 0 | 2.4013 | 0 | 1.0485 | 2.7123 | 0 | 2.2189 | |
| 0.8021 | 2.2025 | 0 | 0.5901 | 0.9954 | 3.5773 | 0.5159 | 0 | |
| 0.5853 | 0.6819 | 0.5555 | 0 | 1.4957 | 3.8047 | 3.0046 | 1.0569 | |
| 1.4585 | 1.8914 | 0 | 0 | 2.8917 | 0.4764 | 2.111 | 0 | 0 |
| | 3.2259 | 1.5362 | 1.4095 | 1.171 | 5.7403 | 0.5715 | 1.1594 | |
| 1.9369 | 0 | 2.971 | 0 | 0 | 0.9886 | 4.3345 | 0.4334 | |
| 0.3921 | 0 | 0.8374 | 5.0442 | 0.4698 | 4.3046 | 4.7261 | 4.527 | |
| 3.6684 | 0.9714 | 1.9437 | 9.3151 | 3.021 | 3.9151 | 4.267 | 0 | 0 |
| | 2.8432 | 0.9113 | 3.7845 | 0 | 5.4593 | 4.5582 | 0.5233 | |
| 4.4416 | 3.855 | 0 | 2.6706 | 0 | 3.3666 | 3.8615 | 3.1085 | |
| 3.4275 | 2.0692 | 2.4661 | 1.4385 | 1.0545 | 0 | 0 | 6.1882 | 0 |
| | 1.217 | 2.1672 | 0.9644 | 1.0358 | 1.6899 | 1.0055 | 1.0557 | 0 |
| | 0 | 2.6587 | 0 | 0.7024 | 0 | 1.737 | 2.362 | 0 |
| | 2.4722 | 1.4217 | 3.6106 | 6.3188 | 1.024 | 1.5646 | 0.6506 | |
| 1.9025 | 1.8493 | 0.6735 | 0 | 0 | 6.9659 | 0 | 1.7123 | |
| 1.3242 | 0 | 4.4528 | 0.6894 | 0.6 | 1.9005 | 0 | 1.6421 | |
| 2.7999 | 0 | 0 | 1.4147 | 0 | 1.3802 | 3.2973 | 0 | 0 |
| | 0.529 | 1.7114 | 0 | 0 | 0 | 7.4038 | 6.6574 | |
| 0.954 | 2.2268 | 0 | 3.0239 | 0 | 3.0083 | 0.5811 | 0 | 0 |
| | 3.8665 | 2.4959 | 0 | 0 | 3.1545 | 2.4698 | 0 | 0 |
| | 3.427 | 5.5537 | 0 | 6.9906 | 6.9757 | 7.2012 | 4.7531 | |
| 8.6991 | 7.5802 | 10.2011 | 9.5634 | 9.7933 | 8.6215 | 8.335 | 7.66 | |
| 6.6472 | 7.6979 | 6.9603 | 8.3266 | 7.34 | 8.0174 | 8.6318 | 7.4099 | |
| 6.8492 | 8.235 | 9.0961 | 6.6764 | | | | | |
| KLHL4 | 1.926 | 0 | 0.4935 | 3.1619 | 0.7223 | 0 | 2.0418 | |
| 0.4327 | 2.3666 | 0.4849 | 3.1323 | 0.7759 | 0 | 4.3171 | 1.0553 | |
| 1.6514 | 0.6732 | 1.0485 | 1.577 | 3.0845 | 6.5458 | 1.3145 | 1.6129 | |

| | | | | | | | | |
|---|---|---|---|---|---|---|---|---|
| 2.7323 | 0.5901 | 2.2152 | 2.5248 | 1.1952 | 1.3374 | 1.0004 | 1.1431 | |
| 1.2685 | 0.6557 | 0.541 | 1.037 | 1.4628 | 0 | 1.0349 | 0 | |
| 0.7135 | 0 | 4.9692 | 0 | 0.7972 | 2.191 | 4.8508 | 2.3724 | |
| 1.5362 | 1.9111 | 3.4055 | 6.1965 | 2.6662 | 1.6116 | 0.5573 | 0 | |
| 1.9254 | 0.598 | 3.6479 | 0.5773 | 3.6688 | 0 | 1.6718 | 0 | |
| 1.569 | 3.904 | 0.8237 | 2.2486 | 1.2414 | 3.973 | 3.2509 | 0.5659 | |
| 1.5351 | 4.1569 | 1.9875 | 2.1768 | 0 | 2.3403 | 0 | 3.0874 | |
| 0.9113 | 2.3201 | 0.5215 | 2.4059 | 0.9013 | 0 | 2.9577 | 0.5504 | 0 |
| | 1.6155 | 0.9246 | 0.7358 | 1.3743 | 3.3434 | 3.1726 | 4.7318 | |
| 1.8132 | 0.5136 | 0.6215 | 4.6197 | 0 | 2.4008 | 4.6604 | 0.5276 | |
| 1.9235 | 1.1815 | 1.0358 | 6.7679 | 1.3285 | 2.5495 | 0 | 1.2272 | |
| 2.7753 | 0 | 0.7024 | 0 | 1.926 | 2.9821 | 2.8142 | 0 | |
| 1.4217 | 0 | 6.1164 | 0 | 1.9771 | 0.6506 | 0 | 0.4562 | |
| 1.4775 | 0 | 1.8766 | 5.9582 | 0.9816 | 1.7123 | 2.325 | 0.5476 | |
| 4.6284 | 1.3399 | 0.6 | 1.7128 | 0 | 0 | 1.5791 | 0 | |
| 1.2829 | 1.8069 | 3.6605 | 5.061 | 2.5502 | 0.942 | 0.7903 | 1.4707 | |
| 0.541 | 1.0565 | 3.2253 | 1.7522 | 2.4565 | 2.6986 | 0.5545 | 2.2268 | |
| 1.2133 | 1.1756 | 1.8738 | 2.6826 | 2.3128 | 0 | 0 | 2.7703 | |
| 3.0307 | 3.5613 | 1.5498 | 3.455 | 1.4064 | 0 | 0 | 2.185 | |
| 3.1721 | 0 | 6.3123 | 4.6002 | 6.0301 | 4.5298 | 8.1645 | 7.3757 | |
| 8.8326 | 8.0583 | 8.8089 | 5.4784 | 8.5592 | 8.603 | 6.2802 | 3.9564 | |
| 6.2545 | 6.9247 | 7.7908 | 5.4805 | 8.9623 | 7.1218 | 6.8129 | 6.4375 | |
| 9.2837 | 5.7623 | | | | | | | |
| DIXDC1 | 7.591 | 8.4219 | 8.4349 | 7.126 | 7.929 | 9.1584 | 8.4695 | |
| 7.391 | 6.3828 | 7.8943 | 8.6477 | 7.0872 | 8.6724 | 7.061 | 6.9903 | |
| 6.287 | 7.4701 | 5.7801 | 8.6355 | 8.1363 | 7.1444 | 8.4954 | 7.4208 | |
| 7.0581 | 5.91 | 7.7788 | 7.7544 | 6.7727 | 7.2091 | 7.5007 | 7.2656 | |
| 8.1816 | 6.7893 | 7.3922 | 8.0896 | 5.9516 | 6.5113 | 7.3935 | 7.0541 | |
| 5.3905 | 6.6927 | 9.6076 | 5.6299 | 7.7724 | 7.0548 | 7.8883 | 8.1839 | |
| 7.6477 | 8.5458 | 8.4961 | 9.6034 | 6.914 | 7.6675 | 6.6303 | 7.5819 | |
| 7.6728 | 7.4897 | 7.8032 | 8.2968 | 8.3245 | 4.8595 | 7.6207 | 5.6971 | |
| 7.3459 | 9.0852 | 7.5163 | 8.0245 | 8.1397 | 7.6402 | 7.9515 | 6.7882 | |
| 7.4406 | 9.3894 | 6.9653 | 7.8 | 8.068 | 7.3457 | 6.8262 | 7.859 | |
| 8.1235 | 8.0432 | 7.1008 | 7.7832 | 6.8248 | 7.4075 | 8.0101 | 7.7365 | |
| 5.6771 | 7.9607 | 7.1635 | 7.7587 | 8.1234 | 7.585 | 8.4011 | 8.4694 | |
| 7.0902 | 7.661 | 8.1888 | 7.1227 | 6.6147 | 7.7798 | 7.8399 | 8.311 | |
| 7.5012 | 7.6309 | 7.0067 | 8.8651 | 8.2542 | 8.0218 | 7.7541 | 6.9894 | |
| 7.4969 | 7.0956 | 6.9052 | 7.4499 | 7.6592 | 7.5914 | 7.1803 | 7.3032 | |
| 6.2897 | 6.7235 | 10.158 | 6.4134 | 6.8644 | 7.0325 | 7.5486 | 7.1902 | |
| 8.0045 | 6.5818 | 7.0141 | 10.0312 | 6.2854 | 8.192 | 8.001 | 7.4966 | |
| 8.2236 | 7.9605 | 8.3183 | 8.4284 | 7.1446 | 6.3876 | 7.8115 | 6.9032 | |
| 7.9346 | 6.8728 | 7.0837 | 8.7727 | 7.3686 | 6.8193 | 8.1535 | 7.8673 | |
| 6.4352 | 7.2239 | 6.8987 | 7.2424 | 8.6538 | 9.4377 | 6.2387 | 5.4974 | |
| 8.4458 | 7.9181 | 8.407 | 7.3445 | 8.0492 | 6.3755 | 6.711 | 7.8364 | |
| 7.5827 | 7.7114 | 6.9971 | 8.4632 | 7.0515 | 7.0912 | 7.2178 | 7.3116 | |
| 9.2215 | 6.2244 | 10.7823 | 9.0147 | 10.2575 | 9.1423 | 10.4571 | 10.6376 | |
| 11.8192 | 11.4355 | 11.5763 | 9.0696 | 11.2981 | 11.1624 | 10.7116 | 9.3789 | |
| 10.1912 | 11.0324 | 10.8772 | 10.9086 | 9.9382 | 10.5848 | 10.75 | 9.5703 | |
| 11.4908 | 10.8233 | | | | | | | |
| SHCBP1 | 8.5247 | 7.9143 | 7.6412 | 8.6012 | 8.1824 | 6.8939 | 7.0223 | |
| 7.7274 | 8.9753 | 8.8876 | 7.797 | 7.9633 | 8.0635 | 6.6309 | 6.6925 | |
| 8.3382 | 7.6645 | 8.4875 | 6.9723 | 6.8176 | 8.3056 | 9.3618 | 7.7175 | |
| 6.3079 | 8.1369 | 8.7458 | 7.9108 | 8.6391 | 6.8942 | 7.5284 | 8.9218 | |
| 6.7116 | 8.8186 | 7.9333 | 8.6288 | 8.1247 | 7.2454 | 8.2048 | 7.2466 | |

| | | | | | | | |
|---|---|---|---|---|---|---|---|
| 7.6846 | 8.7396 | 9.8955 | 6.8968 | 8.9424 | 7.9818 | 7.9592 | 7.5301 |
| 8.2818 | 7.5261 | 8.3799 | 8.1119 | 7.5371 | 8.823 | 9.1442 | 6.9538 |
| 7.9946 | 7.2769 | 7.3963 | 5.2815 | 7.7128 | 7.7251 | 8.1076 | 7.9966 |
| 6.7284 | 7.9005 | 8.2514 | 5.5961 | 6.0047 | 8.9842 | 8.2902 | 7.6805 |
| 8.3869 | 6.9166 | 8.4354 | 7.1393 | 7.5737 | 7.3092 | 7.7144 | 7.595 |
| 8.8299 | 8.6566 | 5.9326 | 6.5602 | 8.1903 | 8.6095 | 7.2321 | 7.639 |
| 7.5483 | 8.6236 | 7.8846 | 9.3138 | 10.2727 | 9.0993 | 7.4449 | 8.2205 |
| 7.4047 | 8.9682 | 7.5452 | 5.9976 | 7.4998 | 9.2672 | 8.3287 | 7.8072 |
| 7.3703 | 7.987 | 8.3064 | 7.9995 | 8.2229 | 7.4574 | 8.0901 | 8.6185 |
| 8.7496 | 5.8044 | 8.3371 | 9.0726 | 7.7652 | 10.3797 | 8.1753 | 7.5449 |
| 7.9661 | 6.0536 | 7.2283 | 9.9249 | 7.1344 | 7.0007 | 8.406 | 8.5397 |
| 7.9638 | 7.604 | 7.7148 | 6.3081 | 7.381 | 8.0628 | 7.9017 | 7.7651 |
| 8.0986 | 7.5812 | 7.7622 | 9.1913 | 8.1394 | 8.9333 | 7.4007 | 6.4502 |
| 8.3723 | 8.7786 | 6.0866 | 7.6946 | 9.0748 | 7.5896 | 8.4255 | 9.6886 |
| 7.616 | 7.8247 | 5.5948 | 7.9107 | 8.1874 | 7.2739 | 8.9319 | 8.623 |
| 7.0915 | 6.2098 | 7.0325 | 7.2449 | 7.9486 | 8.9624 | 8.1441 | 8.0416 |
| 9.2249 | 7.469 | 8.6952 | 8.2727 | 7.5222 | 7.6218 | 7.9397 | 8.4333 |
| 6.8545 | 8.0537 | 3.8455 | 8.3638 | 5.1465 | 4.0417 | 4.4323 | 4.5541 |
| 3.0451 | 4.4148 | 4.0048 | 7.1128 | 3.8377 | 4.2441 | 3.8858 | 5.2468 |
| 4.2433 | 4.932 | 5.7351 | 3.2947 | 3.1292 | 5.6081 | 3.9842 | 6.0661 |
| 3.2853 | 3.8792 | | | | | | |
| SYNPO2 | 8.3867 | 8.9582 | 8.1016 | 9.7008 | 7.5127 | 6.6531 | 8.3745 |
| 7.3789 | 7.0248 | 6.148 | 7.7194 | 7.0909 | 7.9255 | 6.7042 | 8.3844 |
| 8.5062 | 5.5753 | 5.766 | 7.5412 | 8.1498 | 5.1902 | 5.0912 | 7.8457 |
| 8.9382 | 6.1445 | 4.5762 | 9.4542 | 5.4732 | 6.8709 | 5.4438 | 6.1998 |
| 8.1509 | 6.8337 | 7.3805 | 8.7629 | 8.9014 | 8.4908 | 7.5578 | 7.3218 |
| 7.5776 | 7.4456 | 9.2305 | 6.9203 | 7.3218 | 8.1065 | 8.8991 | 9.3446 |
| 7.4889 | 9.2423 | 8.2711 | 11.9502 | 6.7672 | 9.1139 | 7.2908 | 7.1383 |
| 9.1207 | 5.1874 | 7.724 | 6.445 | 11.8069 | 6.274 | 7.6253 | 7.7769 |
| 6.9234 | 11.2556 | 5.5839 | 8.9524 | 8.5812 | 8.7248 | 9.8531 | 8.1223 |
| 8.2148 | 11.3847 | 7.9248 | 9.197 | 6.5609 | 7.2203 | 6.4332 | 9.4635 |
| 10.3258 | 9.0836 | 5.7695 | 9.4474 | 9.1932 | 8.4935 | 10.2062 | 8.3286 |
| 7.4132 | 8.8275 | 7.2469 | 9.073 | 7.3169 | 9.9361 | 11.1549 | 8.7055 |
| 8.1477 | 7.3902 | 6.4721 | 7.9163 | 7.1898 | 7.5017 | 6.7504 | 7.3096 |
| 7.1397 | 8.7027 | 8.661 | 8.28 | 8.622 | 9.007 | 7.7203 | 8.0533 |
| 9.0709 | 4.8661 | 8.5269 | 6.2998 | 8.184 | 8.7924 | 7.0135 | 8.5901 |
| 7.1894 | 9.4931 | 11.6118 | 6.2662 | 7.6595 | 7.5494 | 9.3704 | 7.9009 |
| 7.1795 | 7.0337 | 6.8977 | 12.3995 | 7.6705 | 9.1996 | 7.2846 | 8.2363 |
| 6.6567 | 8.0382 | 9.9783 | 7.9485 | 5.5851 | 8.7589 | 7.5109 | 6.5968 |
| 9.1467 | 10.2118 | 9.1235 | 9.0481 | 8.7839 | 5.1885 | 6.7962 | 7.2731 |
| 6.2833 | 7.2548 | 9.1697 | 7.4225 | 9.1124 | 9.9617 | 8.7206 | 6.4813 |
| 6.6038 | 7.6002 | 8.2359 | 8.8827 | 9.1281 | 6.8161 | 6.9914 | 7.4447 |
| 9.2158 | 6.8766 | 8.8448 | 9.3644 | 8.4226 | 6.5221 | 5.5698 | 8.7929 |
| 10.4442 | 6.2244 | 12.3017 | 11.2646 | 12.9982 | 9.8895 | 14.5753 | 13.3828 |
| 14.0683 | 13.8609 | 14.6479 | 11.6417 | 13.8802 | 15.2234 | 12.7412 | 11.8517 |
| 11.3947 | 13.6579 | 14.2735 | 12.5965 | 11.5468 | 12.3815 | 12.8077 | 12.4325 |
| 14.3296 | 11.8728 | | | | | | |
| MYLK | 9.3835 | 10.0721 | 9.4243 | 11.0817 | 10.0686 | 8.3055 | 10.061 |
| 8.5584 | 8.7958 | 8.9303 | 9.8461 | 9.3695 | 9.5782 | 8.1328 | 9.3775 |
| 9.439 | 8.032 | 10.7224 | 9.7899 | 9.2634 | 7.8437 | 8.993 | 9.0584 |
| 10.9162 | 9.2089 | 9.0588 | 10.7135 | 6.6171 | 8.9264 | 8.2677 | 8.868 |
| 10.2239 | 9.416 | 9.105 | 9.9398 | 9.4122 | 10.3106 | 9.84 | 8.8508 |
| 7.5184 | 10.1078 | 11.4922 | 6.726 | 9.2888 | 10.2752 | 11.6905 | 10.3145 |
| 10.5759 | 11.2533 | 10.5302 | 13.6104 | 8.8268 | 10.47 | 9.0265 | 10.3119 |

| | | | | | | | | |
|---|---|---|---|---|---|---|---|---|
| 9.669 | 7.6122 | 9.9111 | 9.3971 | 12.8876 | 7.6262 | 9.2751 | 8.5918 | |
| 7.4372 | 12.3695 | 7.7841 | 11.7167 | 10.5632 | 9.6936 | 11.4133 | 8.8371 | |
| 10.3616 | 12.0938 | 9.0272 | 10.5206 | 8.9855 | 9.0307 | 8.0364 | 11.3007 | |
| 11.452 | 9.9423 | 6.9965 | 10.5213 | 10.0445 | 9.6028 | 11.1562 | 11.0918 | |
| 7.5738 | 10.4737 | 9.5706 | 10.6425 | 8.174 | 12.0389 | 11.5925 | 10.7041 | |
| 9.2737 | 9.2349 | 8.6459 | 9.1231 | 9.2574 | 10.8092 | 9.351 | 10.4345 | |
| 8.089 | 10.3272 | 10.3617 | 10.8086 | 9.7857 | 11.0588 | 9.3294 | 10.1267 | |
| 10.9545 | 7.4336 | 8.7881 | 9.0599 | 10.1008 | 10.706 | 9.7595 | 9.1861 | |
| 7.2805 | 10.0305 | 13.1369 | 8.312 | 7.5842 | 8.1366 | 10.2721 | 9.2102 | |
| 8.9278 | 8.4008 | 9.4424 | 13.5099 | 7.2851 | 10.231 | 8.6332 | 8.57 | |
| 10.6301 | 9.7876 | 8.8806 | 8.4623 | 6.6621 | 9.8091 | 9.2321 | 6.6119 | |
| 8.5715 | 10.2718 | 10.6133 | 9.9888 | 10.2874 | 6.6576 | 9.1476 | 9.4627 | |
| 8.4053 | 8.3604 | 8.8335 | 9.7377 | 11.0718 | 11.5159 | 8.8122 | 8.6541 | |
| 9.7842 | 8.3942 | 9.3978 | 10.6689 | 10.4272 | 8.2798 | 6.9108 | 9.2932 | |
| 10.6695 | 9.5957 | 10.1062 | 11.9769 | 9.011 | 5.9916 | 6.811 | 9.6465 | |
| 11.7945 | 8.4741 | 13.9933 | 12.5181 | 13.9416 | 11.9847 | 16.7929 | 13.9326 | |
| 14.6834 | 15.2323 | 15.3115 | 13.2235 | 15.3187 | 15.9627 | 14.1872 | 12.9418 | |
| 12.9907 | 14.9698 | 15.9164 | 13.9358 | 12.1874 | 14.5387 | 14.2006 | 14.3018 | |
| 15.9166 | 13.7185 | | | | | | | |
| NPAS4 | 0.5526 | 0 | 1.3959 | 0.5278 | 0.7223 | 0 | 1.7391 | |
| 1.2625 | 0 | 0 | 0 | 0 | 0 | 0 | 1.6582 | |
| 0.7772 | 0 | 0 | 0.482 | 0 | 1.3599 | 0 | 0 | 0 |
| | 0 | 0.7334 | 0.5612 | 0 | 0 | 0 | 0 | |
| 0.5555 | 0 | 0.541 | 0 | 1.1184 | 0.6231 | 0 | 1.4889 | 2.07 |
| | 0.6741 | 3.0861 | 0 | 0 | 1.2789 | 0.6494 | 1.8714 | 0 |
| | 1.0734 | 0.8754 | 4.4982 | 0.5715 | 1.4031 | 0.9583 | 0.5968 | |
| 1.3532 | 0 | 2.1953 | 0 | 2.3953 | 0.4334 | 0 | 0 | |
| 1.1245 | 1.4868 | 0 | 2.2486 | 0 | 1.1539 | 1.0461 | 0 | |
| 0.3965 | 0 | 0 | 2.749 | 0 | 0 | 0 | 1.4671 | |
| 6.5669 | 1.3771 | 0.5215 | 3.4367 | 1.2026 | 0.5233 | 0.509 | 1.515 | |
| 0.6391 | 0.8684 | 0.9246 | 2.1133 | 0.4835 | 1.8753 | 3.1726 | 1.903 | |
| 1.3268 | 0.8916 | 0 | 2.5153 | 2 | 1.9988 | 1.0399 | 0 | |
| 2.0505 | 1.1815 | 0.6089 | 2.3453 | 0 | 1.8863 | 0 | 0.5331 | 0 |
| | 0.7843 | 0.3935 | 0 | 0.5526 | 2.5437 | 1.3267 | 2.8207 | |
| 0.6413 | 4.5503 | 3.9825 | 0 | 0 | 1.7133 | 0.5424 | 0 | 0 |
| | 0.9947 | 0 | 4.0716 | 2.9676 | 0 | 0 | 0 | |
| 1.0445 | 0.9402 | 0 | 2.0666 | 0 | 1.3735 | 0 | 0.4033 | 0 |
| | 0 | 0 | 0.4012 | 1.217 | 0 | 1.298 | 0 | |
| 0.9335 | 0 | 4.0279 | 0 | 2.5385 | 0 | 1.7413 | 1.7883 | 0.91 |
| | 0 | 0.7366 | 1.8914 | 0.9943 | 2.0548 | 0.6567 | 0 | |
| 0.8827 | 0 | 1.2903 | 0 | 1.7976 | 0 | 1.8529 | 0.6701 | |
| 1.8199 | 0 | 4.771 | 3.0277 | 5.1052 | 3.1268 | 6.4369 | 4.739 | |
| 3.6322 | 5.9441 | 4.8719 | 4.5104 | 6.4409 | 6.5548 | 4.6638 | 7.0194 | |
| 2.8381 | 4.3255 | 6.6806 | 5.5337 | 1.2434 | 4.4378 | 5.1274 | 4.9817 | |
| 7.525 | 4.3588 | | | | | | | |
| KIF11 | 10.7359 | 8.6437 | 8.7849 | 10.7362 | 9.778 | 10.1914 | 10.5927 | |
| 9.842 | 10.8099 | 10.9104 | 10.2256 | 10.7871 | 9.853 | 8.7116 | 8.9818 | |
| 10.0666 | 9.9584 | 9.2513 | 8.7727 | 9.4682 | 9.5891 | 10.9763 | 9.2572 | |
| 7.9482 | 10.5947 | 11.1646 | 10.4369 | 10.3455 | 8.5569 | 10.2321 | 10.0394 | |
| 8.8496 | 10.7649 | 10.2494 | 10.2886 | 10.0219 | 9.8292 | 10.8747 | 9.825 | |
| 10.2388 | 9.5908 | 11.088 | 8.6484 | 9.9683 | 9.5643 | 9.7501 | 9.6913 | |
| 10.5423 | 9.1996 | 8.3943 | 9.7707 | 9.9922 | 10.6275 | 10.511 | 9.4446 | |
| 9.9824 | 7.7459 | 9.6626 | 6.9996 | 9.534 | 9.4798 | 10.0634 | 9.2277 | |
| 9.5447 | 9.17 | 9.3914 | 9.1499 | 7.2083 | 7.1363 | 10.3321 | 9.8581 | |

| | | | | | | | |
|---|---|---|---|---|---|---|---|
| 10.7879 | 7.3131 | 10.2076 | 9.3662 | 8.5556 | 10.4535 | 9.6769 | 9.6261 |
| 10.6769 | 10.4586 | 9.9317 | 8.4149 | 10.5898 | 8.8837 | 9.5778 | 10.1716 |
| 8.6402 | 9.9291 | 9.6582 | 10.366 | 10.409 | 11.0475 | 9.6278 | 10.6725 |
| 9.2078 | 10.673 | 9.6285 | 8.5333 | 9.6129 | 9.3873 | 10.7153 | 10.3887 |
| 9.9129 | 11.2105 | 10.0978 | 10.3361 | 9.8272 | 10.1523 | 9.9579 | 9.7899 |
| 9.8107 | 9.1403 | 9.4019 | 10.3929 | 9.3395 | 9.4371 | 8.574 | 8.6621 |
| 10.1109 | 5.331 | 9.2263 | 10.8339 | 8.8702 | 8.4547 | 10.2832 | 10.0904 |
| 9.2346 | 8.9361 | 9.8172 | 8.0284 | 9.2917 | 9.1298 | 9.5245 | 10.8231 |
| 10.6676 | 10.7748 | 9.9432 | 10.8406 | 9.1288 | 10.0382 | 9.4102 | 8.7865 |
| 9.8839 | 10.4801 | 9.7405 | 10.2088 | 11.0087 | 10.6717 | 10.5584 | 11.0771 |
| 9.4109 | 10.508 | 7.2549 | 10.2448 | 11.0608 | 7.3584 | 9.9456 | 10.0696 |
| 10.8022 | 8.7236 | 9.9371 | 9.0275 | 9.823 | 9.9342 | 9.6396 | 10.8332 |
| 10.7967 | 8.6768 | 9.4428 | 9.0117 | 9.5947 | 10.09 | 11.0096 | 9.7821 |
| 9.4193 | 9.6859 | 5.5927 | 9.9364 | 6.5589 | 6.0486 | 6.0039 | 5.1016 |
| 5.298 | 5.5981 | 5.6515 | 8.6908 | 4.976 | 5.3081 | 6.3387 | 6.7977 |
| 6.9181 | 6.7442 | 7.9881 | 5.6101 | 5.3932 | 7.2273 | 6.0453 | 7.8594 |
| 5.0076 | 5.8832 | | | | | | |
| CKS2 | 10.6123 | 9.5351 | 9.8293 | 10.498 | 10.0306 | 9.7525 | 10.7231 |
| 10.0144 | 10.89 | 10.76 | 10.1688 | 9.9795 | 10.1845 | 9.8618 | 9.7496 |
| 10.3115 | 10.2526 | 9.698 | 10.913 | 9.4031 | 11.3544 | 11.3557 | 10.3369 |
| 9.3957 | 10.1803 | 10.8248 | 10.3253 | 10.7628 | 10.2237 | 9.2018 | 11.539 |
| 8.3899 | 10.5224 | 10.9268 | 9.7205 | 11.5619 | 10.4051 | 11.2523 | 10.7849 |
| 10.6034 | 9.5672 | 10.3975 | 9.7473 | 9.9972 | 10.1738 | 9.3078 | 9.9939 |
| 10.7651 | 8.9758 | 9.1306 | 10.1125 | 9.7136 | 10.4887 | 11.1468 | 9.2385 |
| 10.1242 | 8.6879 | 9.7658 | 9.1878 | 10.119 | 10.3103 | 9.6089 | 12.0701 |
| 9.7933 | 9.8268 | 9.4298 | 11.2139 | 7.8524 | 8.3144 | 10.2006 | 10.3468 |
| 10.2814 | 7.9752 | 9.8414 | 10.8487 | 9.869 | 10.1164 | 10.3725 | 9.8196 |
| 11.0373 | 10.597 | 9.7727 | 9.8013 | 11.1116 | 9.2362 | 9.7307 | 10.4348 |
| 11.3345 | 10.3668 | 9.811 | 10.1947 | 10.6389 | 10.5042 | 9.4969 | 10.1016 |
| 10.4339 | 9.631 | 10.4584 | 10.1136 | 9.6724 | 11.0912 | 10.9486 | 10.6083 |
| 10.2969 | 10.0695 | 9.9646 | 10.5611 | 10.5574 | 9.622 | 10.3404 | 10.5643 |
| 11.2431 | 11.4013 | 10.8538 | 11.3221 | 10.6289 | 9.7975 | 10.9583 | 9.2876 |
| 11.7546 | 7.7294 | 9.6574 | 11.1473 | 10.4461 | 9.5589 | 9.7649 | 9.8082 |
| 9.5768 | 9.8062 | 10.922 | 8.6291 | 10.666 | 9.5092 | 9.4227 | 9.5725 |
| 10.3665 | 10.4071 | 10.8403 | 12.589 | 9.7075 | 9.5806 | 10.2545 | 9.5524 |
| 10.7278 | 10.1826 | 10.7915 | 9.4889 | 11.0971 | 10.8707 | 10.4323 | 10.4296 |
| 10.5429 | 10.5133 | 8.4471 | 9.7562 | 10.6162 | 8.4086 | 11.0775 | 10.8686 |
| 10.8237 | 9.7061 | 9.9094 | 10.2024 | 9.08 | 10.7458 | 10.8041 | 10.822 |
| 10.5511 | 9.4686 | 10.0846 | 9.0911 | 9.8675 | 12.3013 | 10.4319 | 9.7398 |
| 9.5378 | 10.1895 | 7.5149 | 9.8811 | 7.9595 | 7.0498 | 6.8995 | 7.6191 |
| 5.9332 | 5.5069 | 6.708 | 9.2682 | 5.9658 | 6.5038 | 7.5187 | 8.1575 |
| 7.701 | 7.5485 | 7.5197 | 6.8155 | 6.5262 | 8.2292 | 7.2262 | 9.1181 |
| 6.779 | 6.6985 | | | | | | |
| FHL1 | 7.8667 | 8.0371 | 5.9678 | 8.16E+00 | | 7.40E+00 | |
| 7.351 | 9.2418 | 6.8058 | 7.5391 | 6.8133 | 9.3443 | 7.5378 | 7.0929 |
| 6.7042 | 7.9754 | 7.7728 | 7.4701 | 8.601 | 8.1946 | 7.1165 | 8.2122 |
| 6.6315 | 7.5884 | 7.7731 | 7.7261 | 11.1182 | 8.252 | 5.1801 | 7.4102 |
| 6.3846 | 5.0455 | 7.484 | 5.5467 | 7.1317 | 6.8755 | 8.434 | 7.554 |
| 7.2012 | 6.5131 | 5.4759 | 8.1094 | 8.1601 | 4.996 | 6.9523 | 7.4278 |
| 9.0319 | 9.3927 | 6.4739 | 8.8206 | 7.6197 | 11.1329 | 6.6672 | 10.1259 |
| 6.5086 | 8.0519 | 7.4394 | 4.7922 | 9.0004 | 7.4119 | 10.992 | 6.579 |
| 6.4667 | 7.1076 | 6.7861 | 10.032 | 6.2994 | 8.7753 | 8.9862 | 7.9284 |
| 8.8866 | 6.8192 | 6.9176 | 10.7206 | 7.2046 | 9.1828 | 8.2392 | 8.3731 |
| 7.8566 | 8.704 | 10.6869 | 8.2275 | 9.4992 | 9.4986 | 8.5456 | 9.4959 |

| | | | | | | | | |
|---|---|---|---|---|---|---|---|---|
| 9.3776 | 8.1132 | 6.1545 | 8.1318 | 6.8742 | 8.6964 | 6.2823 | 9.021 | |
| 8.8598 | 8.9973 | 7.5511 | 10.8602 | 5.6594 | 8.4579 | 6.7415 | 8.5264 | |
| 10.2473 | 6.8882 | 6.6054 | 7.2592 | 7.4145 | 10.9493 | 8.3056 | 8.3402 | |
| 6.0207 | 7.501 | 8.6539 | 8.7957 | 7.2133 | 6.3424 | 7.7836 | 8.7766 | |
| 8.9246 | 7.2752 | 6.7832 | 8.911 | 11.4156 | 5.4895 | 6.3791 | 6.5873 | |
| 7.6892 | 6.898 | 6.2471 | 5.9319 | 3.9723 | 11.6337 | 6.2114 | 7.1789 | |
| 8.4957 | 4.6174 | 8.3464 | 7.2126 | 11.2121 | 6.8556 | 4.8936 | 7.2986 | |
| 7.7954 | 5.5484 | 6.5068 | 7.1432 | 10.7372 | 10.4374 | 7.9761 | 4.9078 | |
| 4.862 | 6.5478 | 6.7839 | 6.8648 | 6.8987 | 7.0793 | 9.266 | 10.6558 | |
| 7.3666 | 7.6154 | 9.7167 | 7.0843 | 8.0453 | 8.6973 | 8.1101 | 8.0101 | |
| 5.0566 | 6.9904 | 7.5505 | 5.9227 | 6.6956 | 8.9845 | 6.4472 | 7.6511 | |
| 5.6471 | 6.9191 | 10.0285 | 5.73 | 12.7692 | 10.6944 | 13.51 | 10.5707 | |
| 13.8797 | 12.9726 | 13.9 | 14.3915 | 14.3881 | 11.5304 | 13.9377 | 14.216 | |
| 12.391 | 11.9549 | 11.6638 | 13.0992 | 13.0578 | 12.2895 | 11.3449 | 12.9665 | |
| 12.4102 | 12.212 | 14.6776 | 12.0017 | | | | | |
| MYOCD | 1.926 | 0 | 1.3959 | 5.81E+00 | | 4.06E-01 | | 0 |
| | 3.2591 | 0.7652 | 0.4291 | 0.4849 | 1.9354 | 1.2777 | 2.0573 | |
| 2.6237 | 0.6221 | 1.2795 | 0 | 0 | 3.0938 | 1.465 | 0.6062 | |
| 2.5443 | 0.599 | 2.7323 | 0 | 1.2172 | 4.6029 | 0 | 0 | 0 |
| | 0 | 3.311 | 0.3648 | 0.541 | 5.2936 | 0.6649 | 1.0569 | |
| 1.0349 | 0 | 1.5457 | 0.6741 | 4.3153 | 0.4764 | 1.0749 | 2.191 | |
| 3.3357 | 4.1154 | 1.2781 | 4.7816 | 1.9711 | 7.6551 | 0 | 2.7563 | |
| 2.2545 | 0.5968 | 3.6799 | 0.598 | 1.9201 | 3.3716 | 8.0067 | 0 | |
| 1.523 | 0.9449 | 2.3027 | 7.0557 | 0 | 3.8681 | 1.2414 | 1.1539 | |
| 5.515 | 1.2875 | 2.5997 | 6.7091 | 1.4748 | 5.143 | 0.6199 | 2.0569 | |
| 0.614 | 5.3781 | 3.9158 | 4.3052 | 0.5215 | 3.7317 | 3.0661 | 0.5233 | |
| 5.5053 | 1.7322 | 0 | 3.9875 | 1.231 | 1.583 | 0.4835 | 6.1528 | |
| 4.8522 | 1.903 | 0.5877 | 4.9309 | 0 | 2.0513 | 2 | 1.4396 | |
| 1.3697 | 2.5502 | 2.7941 | 4.2772 | 1.0358 | 4.4449 | 1.5923 | 4.0567 | 0.47 |
| | 1.8805 | 4.9678 | 0 | 1.5269 | 1.4687 | 3.2327 | 3.5049 | |
| 0.8107 | 3.9453 | 1.9254 | 2.7241 | 8.8068 | 1.024 | 0 | 0.6506 | |
| 3.877 | 1.991 | 0.6735 | 0.9947 | 0 | 8.9907 | 2.5542 | 3.7814 | |
| 0.5862 | 1.9146 | 1.2732 | 2.4027 | 0 | 2.2156 | 1.8525 | 3.1626 | |
| 0.5821 | 0 | 1.0531 | 2.8575 | 4.9804 | 1.3802 | 4.7576 | 0 | |
| 1.0663 | 0.9151 | 0 | 2.4113 | 0 | 3.3889 | 5.019 | 5.6402 | |
| 1.7413 | 1.1551 | 0 | 0 | 1.2218 | 4.9453 | 2.161 | 0.7058 | |
| 0.6567 | 0 | 3.0307 | 0 | 0.5674 | 5.1583 | 2.3581 | 0 | 0 |
| | 4.9337 | 6.5707 | 0 | 7.1932 | 7.1493 | 6.8608 | 6.0606 | |
| 10.9485 | 8.5065 | 9.0084 | 9.7679 | 10.5981 | 8.1358 | 10.1309 | 10.0177 | |
| 9.873 | 8.248 | 6.8364 | 8.9509 | 10.9801 | 9.5225 | 6.7985 | 8.0331 | |
| 9.3912 | 8.4612 | 10.2165 | 9.2268 | | | | | |
| CACNB2 | 2.5026 | 4.2769 | 3.6813 | 3.30E+00 | | 4.14E+00 | | |
| 2.6971 | 4.3837 | 4.1243 | 3.1647 | 1.7643 | 3.8498 | 3.8962 | 1.489 | |
| 4.9193 | 3.42 | 2.1918 | 2.4488 | 3.8962 | 3.4486 | 4.2177 | 1.0317 | |
| 2.7174 | 2.9431 | 2.8976 | 2.9203 | 7.7679 | 4.2168 | 3.7046 | 4.4591 | |
| 3.0008 | 2.2096 | 4.1626 | 3.5768 | 3.3363 | 3.4815 | 2.7771 | 1.888 | |
| 3.4777 | 2.4638 | 0 | 3.9349 | 4.1219 | 2.6955 | 3.5929 | 3.4576 | |
| 4.5343 | 4.4771 | 3.5761 | 2.931 | 3.9247 | 5.5737 | 3.9163 | 5.3679 | |
| 3.828 | 3.9398 | 3.7145 | 4.0808 | 3.1628 | 2.3036 | 5.3743 | 2.1712 | |
| 2.9765 | 2.4919 | 4.6208 | 5.28 | 3.2432 | 4.9331 | 3.8266 | 2.7035 | |
| 3.4747 | 1.7663 | 4.5127 | 4.8825 | 3.307 | 4.6809 | 4.0504 | 3.04 | |
| 2.6565 | 3.7859 | 4.1839 | 4.1837 | 5.7923 | 5.5185 | 2.9894 | 2.8322 | |
| 4.0577 | 4.1091 | 1.6914 | 3.0033 | 3.9837 | 5.8301 | 1.5803 | 4.2124 | |
| 4.0903 | 3.7851 | 4.5928 | 4.3392 | 3.2654 | 4.3124 | 3.3219 | 4.9122 | |

| | | | | | | | |
|---|---|---|---|---|---|---|---|
| 4.3968 | 3.1615 | 4.0324 | 5.9214 | 4.4937 | 6.24 | 2.1785 | 4.0567 |
| 1.8858 | 2.3286 | 4.0367 | 1.6629 | 2.9233 | 2.1813 | 2.8211 | 5.0332 |
| 3.5308 | 2.2603 | 4.2831 | 5.1146 | 6.8358 | 3.2906 | 2.9732 | 2.3186 |
| 3.6335 | 1.6921 | 2.6677 | 4.4491 | 1.5869 | 6.9368 | 0 | 4.3632 |
| 4.3256 | 2.7093 | 4.1123 | 3.761 | 2.8458 | 2.474 | 2.2191 | 2.9807 |
| 3.1622 | 3.7633 | 3.2081 | 2.5845 | 4.3681 | 2.8899 | 3.9193 | 3.5353 |
| 2.6939 | 5.0199 | 2.0651 | 2.5508 | 0 | 3.1531 | 4.406 | 4.7739 |
| 3.3748 | 3.2614 | 3.2915 | 3.7191 | 3.2722 | 3.8082 | 4.237 | 3.6991 |
| 1.4486 | 3.8665 | 3.7263 | 3.2412 | 3.1228 | 4.7612 | 4.0956 | 1.5946 |
| 6.7633 | 3.5413 | 4.9318 | 2.8479 | 7.1666 | 5.7147 | 7.8701 | 5.5838 |
| 8.7949 | 7.9495 | 7.3549 | 8.1233 | 7.5013 | 6.5946 | 8.7042 | 8.5683 |
| 7.5259 | 8.715 | 6.4104 | 6.9964 | 8.5421 | 7.6065 | 8.9715 | 7.258 |
| 7.6674 | 7.1823 | 8.536 | 7.7171 | | | | |
| TNS1 | 10.1551 | 11.4574 | 9.8377 | 10.5516 | 11.0764 | 10.3078 | 10.0806 |
| 10.6488 | 10.653 | 10.0115 | 10.6021 | 9.2917 | 9.6586 | 9.4928 | 9.88 |
| 9.129 | 8.9395 | 10.7428 | 10.5424 | 10.2863 | 8.3708 | 11.3063 | 9.3893 |
| 10.4472 | 9.1421 | 9.4045 | 10.3788 | 9.1157 | 10.4214 | 11.1376 | 8.3938 |
| 10.7655 | 8.7992 | 8.0518 | 10.4916 | 9.4465 | 9.6233 | 11.1933 | 9.3354 |
| 9.0414 | 10.3041 | 10.502 | 8.6157 | 9.9915 | 9.755 | 11.6096 | 11.0651 |
| 8.5721 | 10.6544 | 11.382 | 12.4768 | 9.1907 | 10.37 | 9.1728 | 8.9086 |
| 10.174 | 11.7806 | 10.148 | 10.0909 | 12.2994 | 10.3662 | 10.3604 | 9.0094 |
| 10.4446 | 11.6364 | 9.4095 | 10.0646 | 10.3651 | 9.942 | 11.5178 | 9.493 |
| 10.3816 | 12.0093 | 9.6859 | 10.5485 | 11.1996 | 10.0741 | 9.9604 | 10.9033 |
| 11.2771 | 10.3876 | 10.1727 | 10.7018 | 9.8439 | 10.8754 | 11.1703 | 9.2067 |
| 9.3575 | 10.4099 | 10.2503 | 10.1524 | 8.9537 | 10.5271 | 11.2303 | 10.5653 |
| 10.2096 | 8.9744 | 9.5247 | 9.9212 | 10.2107 | 10.7807 | 10.3938 | 9.5741 |
| 11.139 | 10.1555 | 9.8795 | 10.5999 | 10.2423 | 10.4193 | 8.8648 | 10.4688 |
| 11.1477 | 9.3796 | 10.8356 | 9.4302 | 9.7939 | 10.2185 | 10.5239 | 9.9341 |
| 8.9155 | 10.6615 | 13.066 | 8.8741 | 9.5402 | 9.4397 | 10.2565 | 10.265 |
| 9.6766 | 10.7861 | 6.542 | 13.2333 | 8.7246 | 10.9802 | 10.5377 | 8.9513 |
| 10.7167 | 10.9162 | 9.4813 | 8.5491 | 8.0766 | 9.9153 | 9.7311 | 9.2887 |
| 10.1747 | 10.1035 | 11.6519 | 11.1454 | 10.1517 | 9.0278 | 9.238 | 9.7253 |
| 9.2238 | 9.7636 | 8.8379 | 9.7997 | 10.6349 | 11.0017 | 9.1782 | 9.5343 |
| 11.7209 | 10.1279 | 10.7658 | 10.4115 | 10.6494 | 9.5567 | 8.7361 | 9.5436 |
| 10.288 | 10.2076 | 10.1533 | 11.4007 | 8.7586 | 9.6922 | 8.7792 | 10.0925 |
| 11.6568 | 9.4169 | 12.9615 | 11.7067 | 13.2608 | 11.1774 | 14.1809 | 13.3083 |
| 14.4848 | 14.4795 | 14.869 | 11.8221 | 14.2069 | 15.1445 | 13.5301 | 11.9224 |
| 12.2806 | 13.8767 | 14.5905 | 13.0925 | 13.6251 | 13.6622 | 13.2841 | 12.8566 |
| 14.9909 | 12.692 | | | | | | |
| MND1 | 8.7587 | 4.4486 | 5.4129 | 7.8358 | 7.1029 | 8.0682 | 7.8093 |
| 6.2845 | 8.4676 | 7.0921 | 7.0521 | 7.2513 | 6.5761 | 6.1955 | 7.4699 |
| 7.9119 | 7.2595 | 8.0608 | 5.9095 | 6.1583 | 7.5622 | 8.397 | 6.9662 |
| 7.691 | 7.364 | 7.6681 | 8.4216 | 8.6266 | 6.7013 | 7.0288 | 7.955 |
| 5.4816 | 7.3176 | 8.6956 | 6.2554 | 7.9783 | 8.2328 | 6.8232 | 7.0442 |
| 8.0371 | 6.4291 | 8.1051 | 10.0528 | 7.0479 | 6.3878 | 5.6732 | 6.434 |
| 8.0006 | 6.3702 | 5.8306 | 7.9149 | 6.3437 | 7.8611 | 7.6092 | 6.3202 |
| 6.5919 | 4.8447 | 7.0645 | 4.4376 | 7.3049 | 8.3525 | 7.2375 | 8.007 |
| 5.5674 | 8.6103 | 6.3547 | 7.6564 | 4.3614 | 4.5648 | 7.745 | 8.1396 |
| 7.6996 | 4.2581 | 7.7687 | 7.5001 | 7.056 | 7.585 | 7.6587 | 7.2717 |
| 7.8538 | 7.3106 | 7.1325 | 6.5602 | 8.6599 | 5.3807 | 6.1109 | 7.2694 |
| 7.7183 | 7.2875 | 6.5049 | 8.0793 | 8.7764 | 8.8751 | 7.4592 | 7.3045 |
| 7.4634 | 7.2432 | 7.2356 | 5.3882 | 6.0875 | 7.7048 | 7.2778 | 6.8555 |
| 8.9268 | 6.3152 | 7.582 | 7.659 | 7.5345 | 6.0735 | 6.9922 | 7.7962 |
| 7.1907 | 8.1756 | 7.4721 | 7.9707 | 7.1058 | 6.7171 | 7.119 | 5.7967 |

| | | | | | | | | |
|---|---|---|---|---|---|---|---|---|
| 9.3473 | 1.5206 | 6.5577 | 8.604 | 6.8338 | 6.8234 | 8.1473 | 8.1629 | |
| 7.1436 | 6.4732 | 10.2273 | 5.4954 | 5.6916 | 5.675 | 7.2984 | 8.3427 | |
| 6.4675 | 6.9733 | 9.046 | 9.4042 | 6.8371 | 7.7814 | 7.1154 | 7.1687 | |
| 7.3547 | 7.8143 | 7.3449 | 5.735 | 7.7639 | 8.3537 | 7.0396 | 8.3451 | |
| 6.1324 | 7.7969 | 6.6812 | 7.1531 | 8.0614 | 4.7739 | 9.2753 | 8.1077 | |
| 8.241 | 6.9922 | 6.215 | 7.1977 | 7.2507 | 7.4576 | 7.2317 | 8.3997 | |
| 7.345 | 7.4341 | 6.9015 | 5.8333 | 7.5543 | 8.219 | 8.573 | 7.1766 | |
| 6.5707 | 8.1419 | 2.7646 | 7.0081 | 3.7518 | 2.706 | 2.9167 | 0.8506 | |
| 1.6194 | 1.9872 | 3.2731 | 5.377 | 1.9147 | 2.5596 | 3.3488 | 3.8937 | |
| 3.5563 | 3.6357 | 4.4365 | 1.9774 | 2.0673 | 4.7402 | 1.9521 | 5.2864 | |
| 2.5659 | 1.8629 | | | | | | | |
| FANCA | 9.557 | 7.4716 | 7.6069 | 10.2857 | 7.7934 | 6.9452 | 7.8823 | |
| 8.545 | 7.5539 | 7.8774 | 8.3497 | 9.5409 | 8.7811 | 8.6701 | 8.0687 | |
| 9.5966 | 8.1329 | 9.1206 | 8.0882 | 7.3423 | 9.6788 | 10.1445 | 9.3672 | |
| 6.9908 | 9.0029 | 9.9489 | 9.7285 | 9.2156 | 8.5073 | 8.8858 | 9.7604 | |
| 7.8052 | 9.709 | 8.2652 | 8.1867 | 8.7389 | 9.9644 | 8.843 | 8.9437 | |
| 9.5382 | 8.9813 | 9.4227 | 6.4409 | 9.0957 | 9.209 | 8.8406 | 9.1493 | |
| 9.3703 | 9.0719 | 7.0649 | 7.623 | 8.338 | 8.7998 | 9.4199 | 9.0101 | |
| 8.8276 | 8.1691 | 7.5134 | 6.8366 | 9.8539 | 8.572 | 8.0958 | 10.004 | |
| 8.7631 | 8.6159 | 9.1402 | 8.3571 | 7.0289 | 6.4865 | 9.2264 | 8.7073 | 8.64 |
| | 6.6297 | 8.8878 | 8.6582 | 9.2977 | 8.2398 | 9.5562 | 8.1974 | |
| 10.6767 | 8.9594 | 8.4768 | 8.5594 | 8.9322 | 8.1956 | 8.2181 | 9.9414 | |
| 9.5683 | 9.4601 | 8.1148 | 9.4824 | 9.6644 | 8.901 | 7.2743 | 8.3032 | |
| 9.3099 | 8.3622 | 9.1115 | 8.609 | 9.6467 | 9.4264 | 8.1642 | 9.0048 | |
| 8.3309 | 9.5833 | 9.9416 | 8.8738 | 8.7161 | 8.638 | 8.699 | 8.4067 | |
| 9.3554 | 8.7028 | 9.4096 | 7.6068 | 8.8872 | 10.1377 | 9.7616 | 8.3714 | |
| 10.5905 | 9.4612 | 8.6549 | 10.0702 | 7.2511 | 8.4755 | 9.1579 | 9.1916 | |
| 8.872 | 8.7347 | 9.0995 | 7.4824 | 9.4486 | 8.388 | 7.9331 | 9.1348 | |
| 8.1613 | 9.1179 | 9.3404 | 10.2749 | 8.8935 | 10.0326 | 7.8474 | 8.8783 | |
| 9.106 | 9.321 | 7.4247 | 7.5858 | 9.3515 | 9.0901 | 9.3619 | 9.9318 | |
| 9.9101 | 9.3671 | 7.6206 | 10.0304 | 9.5055 | 8.3902 | 9.4222 | 9.2292 | |
| 8.9555 | 6.9036 | 8.6874 | 8.0068 | 7.9704 | 9.1723 | 9.4878 | 9.9255 | |
| 9.6541 | 6.8449 | 7.8155 | 8.9588 | 9.1106 | 8.6809 | 9.1779 | 9.5721 | |
| 8.5303 | 10.1238 | 5.7961 | 8.6656 | 6.2678 | 6.1204 | 5.065 | 5.2672 | |
| 3.1515 | 3.7629 | 4.4189 | 7.3441 | 4.9031 | 4.3972 | 4.6214 | 7.6167 | |
| 5.9142 | 4.6921 | 7.1322 | 5.1292 | 4.3054 | 6.827 | 4.7545 | 8.0414 | |
| 3.9716 | 4.7003 | | | | | | | |
| OGN | 0 | 0 | 0.8605 | 5.98E+00 | | 2.55E+00 | | 0 |
| | 5.6128 | 0.4327 | 2.1584 | 1.1366 | 5.415 | 0.4395 | 0.9286 | |
| 3.5017 | 1.3881 | 6.8026 | 0.3755 | 0.6175 | 5.9095 | 1.465 | 3.8656 | |
| 2.4493 | 6.1179 | 2.0792 | 1.3312 | 1.2172 | 3.8394 | 0.8951 | 2.2682 | 0 |
| | 2.2096 | 1.7435 | 0.6557 | 1.7115 | 6.2219 | 6.082 | 1.6604 | |
| 2.4713 | 1.8914 | 0 | 0 | 6.0113 | 0.4764 | 1.5081 | 1.6507 | |
| 2.6127 | 1.8714 | 2.1129 | 4.1793 | 3.8845 | 8.9088 | 4.5158 | 2.3538 | |
| 2.8337 | 0.5968 | 4.0485 | 0 | 3.2255 | 1.791 | 7.1833 | 0.7662 | |
| 0.3921 | 1.9166 | 2.3027 | 9.1054 | 0.4698 | 5.1582 | 9.4445 | 5.8875 | |
| 4.215 | 0 | 3.391 | 11.2346 | 1.9875 | 7.7329 | 3.1783 | 3.39 | |
| 1.0433 | 6.4138 | 3.2276 | 6.0201 | 3.0706 | 7.5666 | 6.4268 | 0 | |
| 7.1442 | 3.2266 | 0 | 5.3591 | 0 | 4.1937 | 6.0332 | 6.4007 | |
| 4.9035 | 8.5716 | 6.4999 | 2.7134 | 0 | 2.8658 | 0 | 4.1798 | |
| 4.1616 | 2.1803 | 0.4321 | 2.937 | 0 | 6.4444 | 1.0055 | 1.3885 | |
| 1.1079 | 0.5331 | 3.5397 | 0 | 0.3935 | 0 | 4.9261 | 3.59 | 0 |
| | 4.5695 | 2.1237 | 10.4808 | 9.7124 | 1.6169 | 1.7855 | 4.3884 | |
| 2.4761 | 0.8022 | 0 | 0.5814 | 0 | 10.4266 | 2.5542 | 2.4734 | |

| | | | | | | | | |
|---|---|---|---|---|---|---|---|---|
| 3.1733 | 0.9437 | 2.3925 | 1.3399 | 1.0225 | 4.1947 | 0 | 2.2364 | |
| 7.4912 | 0.7182 | 0 | 1.4147 | 3.387 | 5.4476 | 5.0736 | 0.5466 | 0 |
| | 2.4406 | 2.214 | 0 | 5.27 | 1.1272 | 6.5593 | 9.8251 | |
| 1.5234 | 4.3668 | 1.2133 | 7.3707 | 0.7366 | 6.3817 | 0 | 0 | 0 |
| | 4.5732 | 1.8202 | 0 | 1.7696 | 4.0413 | 4.4075 | 3.1828 | |
| 0.6064 | 1.6179 | 7.0691 | 1.1634 | 11.393 | 12.1966 | 11.4205 | 8.8528 | |
| 12.8193 | 11.5201 | 13.4731 | 12.9182 | 13.0423 | 12.9168 | 12.0959 | 11.2864 | |
| 11.2705 | 10.4374 | 10.5156 | 11.7177 | 12.2924 | 11.3392 | 11.2987 | 10.5392 | |
| 10.7735 | 13.0148 | 11.8289 | 10.3012 | | | | | |
| TOP2A | 12.1862 | 10.2054 | 10.7998 | 1.22E+01 | | 1.19E+01 | | |
| 11.076 | 11.2688 | 13.5349 | 12.0378 | 12.8737 | 11.3007 | 12.118 | 11.6603 | |
| 10.0816 | 10.2026 | 11.6491 | 11.2105 | 10.8987 | 10.5852 | 10.3803 | 9.8716 | |
| 12.9352 | 10.3876 | 9.0565 | 11.5055 | 12.682 | 11.4936 | 11.606 | 10.3807 | |
| 13.0474 | 12.4084 | 10.1194 | 12.791 | 11.6858 | 11.222 | 12.0993 | 11.348 | |
| 12.7216 | 10.9289 | 9.906 | 11.6284 | 13.0982 | 9.6189 | 11.5532 | 10.721 | |
| 11.7669 | 11.5284 | 12.2529 | 10.9525 | 11.1378 | 12.3704 | 12.0582 | 12.7772 | |
| 11.8165 | 11.2526 | 11.8362 | 9.4775 | 11.6752 | 8.5356 | 11.1461 | 11.4324 | |
| 12.0792 | 10.6509 | 9.7959 | 10.9733 | 10.3982 | 12.4592 | 8.5019 | 8.447 | |
| 11.4636 | 11.1794 | 11.8582 | 8.743 | 11.8102 | 10.2686 | 10.8814 | 12.4974 | |
| 11.1742 | 10.4402 | 12.62 | 13.5521 | 9.9716 | 10.4572 | 12.5615 | 11.204 | |
| 11.2931 | 11.8028 | 10.1489 | 11.8893 | 12.2312 | 11.2349 | 11.0585 | 12.5091 | |
| 10.5489 | 12.3238 | 12.0883 | 11.8192 | 11.5335 | 9.2477 | 12.4732 | 11.6549 | |
| 11.7991 | 11.6421 | 12.0674 | 11.9798 | 10.9223 | 12.869 | 11.6766 | 11.1954 | |
| 11.3643 | 12.3861 | 11.4136 | 11.2796 | 11.3325 | 11.2946 | 11.1402 | 11.2745 | |
| 10.2235 | 9.8055 | 10.81 | 5.97 | 10.9686 | 11.1174 | 10.4308 | 9.661 | |
| 12.0141 | 12.3575 | 10.9536 | 9.8126 | 10.8035 | 9.3919 | 8.8179 | 11.1075 | |
| 10.795 | 12.2512 | 12.0165 | 12.1708 | 10.7696 | 11.381 | 10.8165 | 10.8748 | |
| 10.8739 | 9.8182 | 11.4706 | 12.6616 | 10.5801 | 11.5764 | 11.8193 | 12.7481 | |
| 12.7348 | 12.8952 | 12.2164 | 12.3813 | 8.2032 | 12.1816 | 12.8185 | 8.9758 | |
| 11.0769 | 10.8505 | 13.0162 | 10.5579 | 12.5883 | 10.5478 | 12.5048 | 11.6258 | |
| 10.6569 | 11.7153 | 12.122 | 10.5467 | 11.0823 | 11.8273 | 11.555 | 11.2807 | |
| 10.9217 | 10.8835 | 11.2225 | 10.0454 | 5.8925 | 11.4772 | 7.6584 | 6.302 | |
| 7.1482 | 6.6057 | 3.8551 | 5.9038 | 6.2871 | 10.4542 | 6.0526 | 6.0265 | |
| 6.8272 | 8.4247 | 7.3485 | 7.4732 | 9.8193 | 5.3381 | 3.1292 | 8.7805 | |
| 6.1751 | 9.6525 | 4.6785 | 6.0418 | | | | | |
| NUF2 | 9.1534 | 8.0065 | 7.7869 | 9.45E+00 | | 9.98E+00 | | |
| 8.758 | 8.6733 | 9.1909 | 9.6573 | 8.8011 | 8.6704 | 9.0552 | 8.1714 | |
| 7.611 | 8.8716 | 8.5861 | 8.9456 | 8.7154 | 7.1153 | 7.9991 | 7.8306 | |
| 11.1756 | 7.9333 | 6.6697 | 8.9696 | 10.0266 | 9.6792 | 10.0556 | 8.4819 | |
| 9.048 | 8.5578 | 7.7959 | 9.6149 | 8.8544 | 8.7963 | 8.5802 | 9.1776 | |
| 8.9656 | 8.8252 | 7.7504 | 8.6547 | 9.1657 | 7.5727 | 9.1571 | 8.4025 | |
| 8.5021 | 9.1069 | 9.1802 | 7.5936 | 6.7242 | 8.7467 | 9.2848 | 9.9184 | |
| 8.9406 | 9.3321 | 8.1079 | 7.147 | 9.2517 | 5.1275 | 9.4197 | 9.2191 | |
| 8.7243 | 8.5603 | 8.0793 | 8.6153 | 7.5902 | 8.4511 | 5.5242 | 6.0827 | |
| 7.9822 | 8.2021 | 9.5696 | 5.8738 | 9.7035 | 7.5176 | 8.2747 | 9.9863 | |
| 8.3044 | 8.6855 | 10.6211 | 9.0173 | 8.1496 | 7.7047 | 9.2794 | 7.8407 | |
| 9.0308 | 9.0837 | 7.7068 | 8.3773 | 9.7596 | 9.6923 | 9.3227 | 10.1419 | |
| 7.9345 | 9.4775 | 8.921 | 9.3652 | 7.8581 | 5.5422 | 9.1674 | 10.1132 | |
| 9.2219 | 9.1807 | 9.1103 | 8.4671 | 8.6907 | 9.9715 | 8.4246 | 8.8061 | |
| 7.8439 | 8.879 | 9.3535 | 6.7041 | 9.4493 | 9.2796 | 8.1816 | 9.9 | |
| 8.2771 | 6.6808 | 8.3789 | 0 | 7.4018 | 9.5484 | 7.9779 | 7.1471 | |
| 8.2697 | 8.2252 | 7.9431 | 8.0061 | 9.1985 | 6.9944 | 5.8216 | 7.8199 | |
| 8.7249 | 8.6942 | 9.0533 | 9.1189 | 9.1492 | 8.8953 | 7.9544 | 8.2495 | |
| 7.8083 | 7.497 | 8.2054 | 8.4042 | 8.3011 | 10.1803 | 8.3467 | 10.5386 | |

| | | | | | | | | |
|---|---|---|---|---|---|---|---|---|
| 8.5402 | 10.3618 | 9.1001 | 9.7726 | 4.918 | 9.2521 | 9.7314 | 6.0279 | |
| 9.1284 | 8.9437 | 9.5801 | 7.284 | 8.5853 | 7.9135 | 8.9543 | 8.9477 | |
| 8.2787 | 9.0521 | 8.6699 | 7.8052 | 9.4298 | 9.0929 | 9.5745 | 9.2264 | |
| 8.1427 | 7.9114 | 7.3182 | 7.6572 | 3.3733 | 8.0244 | 4.974 | 3.2986 | |
| 3.8179 | 2.6372 | 3.3683 | 3.6364 | 2.8848 | 7.2453 | 1.4126 | 2.4199 | |
| 3.5318 | 6.3259 | 4.2038 | 4.2553 | 5.7005 | 3.1658 | 2.884 | 5.4351 | |
| 3.1906 | 7.1293 | 2.0979 | 3.7719 | | | | | |
| KIAA1462 | | 6.8143 | 9.3483 | 7.2354 | 8.06E+00 | | 7.74E+00 | |
| | 7.0363 | 8.6843 | 6.6884 | 5.9854 | 6.2348 | 8.0847 | 9.209 | |
| 7.8765 | 7.6609 | 7.483 | 8.1444 | 6.5139 | 6.8955 | 7.3474 | 7.9916 | |
| 8.4353 | 7.1787 | 8.0217 | 8.7429 | 5.9577 | 8.3703 | 8.3645 | 6.9386 | |
| 7.5343 | 6.3496 | 6.1269 | 8.7142 | 7.6833 | 7.0937 | 8.5674 | 7.6694 | |
| 7.2607 | 7.2451 | 7.8983 | 6.3953 | 7.5455 | 7.0897 | 6.5505 | 7.7965 | 7.57 |
| | 9.0645 | 11.741 | 7.5884 | 8.3202 | 8.6494 | 9.7943 | 6.7412 | |
| 7.5988 | 7.6402 | 8.0181 | 7.5271 | 6.9632 | 7.7877 | 7.2013 | 9.5688 | |
| 6.1655 | 8.1527 | 6.5753 | 7.5075 | 9.4705 | 6.592 | 8.1417 | 8.6432 | |
| 8.2491 | 9.1446 | 8.1494 | 6.9251 | 10.3877 | 7.2891 | 8.6808 | 8.3784 | |
| 7.2802 | 6.7429 | 7.8341 | 9.1492 | 7.8063 | 8.4247 | 8.6798 | 6.9964 | |
| 9.986 | 8.3816 | 8.9437 | 7.5696 | 8.1276 | 6.1869 | 7.798 | 6.4493 | |
| 8.0939 | 8.2882 | 8.3083 | 7.4385 | 6.9001 | 6.5816 | 7.1864 | 7.2384 | |
| 9.0802 | 8.0413 | 6.8445 | 7.6186 | 8.4384 | 8.3901 | 9.0998 | 7.633 | |
| 7.7563 | 7.0053 | 8.0164 | 7.6986 | 6.6739 | 8.2873 | 5.9332 | 7.8139 | |
| 7.7979 | 9.6514 | 9.0749 | 6.8823 | 8.8185 | 11.6078 | 5.6466 | 7.2031 | |
| 7.5841 | 8.9481 | 7.7168 | 7.0879 | 6.5129 | 6.6028 | 10.9538 | 6.9291 | |
| 8.6744 | 7.5352 | 10.7144 | 8.8579 | 9.0219 | 7.6029 | 8.7654 | 6.7417 | |
| 8.1667 | 7.4751 | 6.3223 | 7.1981 | 7.608 | 8.1466 | 9.4338 | 7.5255 | |
| 6.4528 | 9.2605 | 7.2141 | 8.4416 | 7.5281 | 8.6255 | 8.4865 | 8.8915 | |
| 8.9081 | 7.454 | 6.6406 | 8.058 | 7.0482 | 7.2374 | 9.0003 | 8.2786 | |
| 6.707 | 5.3082 | 8.5399 | 8.428 | 7.9383 | 7.0506 | 9.3711 | 6.9966 | |
| 7.5458 | 5.8952 | 8.6096 | 8.5083 | 7.4832 | 11.355 | 10.0821 | 10.9035 | |
| 9.6946 | 12.1075 | 11.1476 | 12.3594 | 12.0707 | 12.6122 | 10.4966 | 11.6187 | |
| 12.0024 | 12.3361 | 9.8812 | 10.574 | 11.4961 | 11.7196 | 11.9098 | 11.0804 | |
| 11.9355 | 12.0138 | 10.6247 | 11.7889 | 12.0345 | | | | |
| C1orf135 | | 8.2584 | 5.0914 | 6.1529 | 8.8228 | 7.5102 | 6.5157 | |
| 7.8778 | 8.2508 | 7.9133 | 7.3751 | 7.198 | 6.6022 | 6.9207 | 5.4029 | |
| 7.4567 | 7.291 | 8.9342 | 6.9401 | 5.3875 | 5.6397 | 7.2221 | 7.6697 | |
| 6.5882 | 6.3079 | 7.7901 | 6.9721 | 7.1664 | 7.7518 | 6.7533 | 8.5323 | |
| 8.185 | 5.7569 | 7.6671 | 7.7953 | 6.9834 | 7.2484 | 8 | 6.8541 | |
| 7.8369 | 6.7269 | 7.6433 | 8.0692 | 6.7153 | 7.0914 | 7.0235 | 7.3029 | |
| 5.7289 | 7.8334 | 7.4138 | 6.2259 | 8.5108 | 7.3347 | 7.4708 | 7.4432 | |
| 7.6681 | 6.874 | 6.1467 | 6.7812 | 4.1077 | 7.7912 | 8.3556 | 7.2553 | |
| 7.5886 | 5.6713 | 6.7703 | 5.7886 | 7.4513 | 4.3929 | 4.0282 | 7.6256 | |
| 7.1373 | 8.3566 | 3.2356 | 7.8963 | 6.6944 | 6.9282 | 7.1486 | 7.2944 | |
| 7.2962 | 6.3268 | 7.8166 | 5.9831 | 5.2424 | 8.477 | 7.0367 | 5.9424 | |
| 7.2519 | 7.9441 | 7.8043 | 7.3656 | 8.0969 | 8.7271 | 8.9659 | 7.3739 | |
| 7.6544 | 7.5079 | 6.2446 | 7.5781 | 6.7111 | 7.2946 | 7.8617 | 8.8923 | |
| 6.4807 | 7.5205 | 7.8067 | 8.0927 | 7.9699 | 7.5656 | 7.4707 | 6.8598 | |
| 7.307 | 8.0361 | 7.0092 | 7.1918 | 7.9084 | 6.7779 | 7.2816 | 7.7187 | |
| 6.8796 | 7.8201 | 3.816 | 6.9622 | 8.1922 | 6.0572 | 5.6765 | 7.0788 | |
| 7.8919 | 5.5302 | 7.0873 | 7.4813 | 4.6276 | 7.3208 | 6.1978 | 8.7283 | |
| 7.9078 | 7.5385 | 6.484 | 8.9257 | 7.7882 | 6.4715 | 7.7569 | 6.6061 | |
| 5.8681 | 6.9021 | 7.6018 | 7.6625 | 6.5641 | 7.4905 | 8.2483 | 7.1622 | |
| 8.4291 | 6.3889 | 6.7097 | 5.4645 | 7.0666 | 7.8828 | 3.9334 | 8.0567 | |
| 7.9122 | 7.7922 | 5.8907 | 7.3885 | 7.4907 | 7.7267 | 7.8097 | 7.4894 | |

| | | | | | | | |
|---|---|---|---|---|---|---|---|
| 8.1976 | 7.8193 | 6.1497 | 7.3901 | 7.5303 | 7.2445 | 7.6798 | 7.9366 |
| 6.4677 | 6.2413 | 8.4098 | 3.0238 | 5.8728 | 4.0412 | 3.9415 | 3.0035 |
| 3.1095 | 3.3683 | 3.7315 | 3.4641 | 4.3049 | 3.3811 | 3.4317 | 3.4124 |
| 4.2346 | 3.7335 | 3.9343 | 4.4916 | 3.851 | 3.2722 | 4.4378 | 3.5262 |
| 4.5144 | 3.6419 | 2.9741 | | | | | |
| GPRASP1 | 5.2606 | 5.6446 | 6.4372 | 6.7592 | 5.4664 | 5.1958 | 6.1528 |
| 6.3663 | 5.0983 | 4.9148 | 7.0132 | 5.0466 | 5.2148 | 7.5592 | 6.7658 |
| 5.8427 | 3.3103 | 4.5506 | 6.4896 | 6.1223 | 3.3041 | 5.1069 | 6.4925 |
| 5.7822 | 5.3004 | 6.8069 | 6.3547 | 4.9586 | 6.3629 | 6.2297 | 4.9358 |
| 6.4027 | 5.1738 | 5.7119 | 6.8712 | 6.119 | 5.6329 | 6.7312 | 5.6111 |
| 2.7566 | 4.8277 | 6.1911 | 4.3343 | 5.1418 | 5.9947 | 6.6011 | 8.031 |
| 6.6684 | 6.2808 | 7.6679 | 8.4442 | 6.2174 | 6.0966 | 5.1843 | 4.8669 |
| 6.2289 | 5.2472 | 8.2672 | 5.9433 | 8.1516 | 6.0481 | 5.8772 | 4.5362 |
| 6.1422 | 7.8786 | 5.4505 | 7.2348 | 7.054 | 7.3189 | 6.6748 | 5.3529 |
| 5.7671 | 8.1597 | 6.0104 | 6.5254 | 7.4639 | 6.5056 | 4.9653 | 6.9755 |
| 6.4188 | 7.2399 | 6.7089 | 8.4097 | 6.2572 | 5.2376 | 7.1951 | 6.3379 |
| 4.1475 | 6.0849 | 5.9243 | 7.4428 | 5.7954 | 6.1215 | 7.6565 | 7.4359 |
| 5.8153 | 6.9206 | 5.0105 | 4.8729 | 5.3576 | 6.6173 | 7.5087 | 4.8266 |
| 6.7851 | 6.164 | 5.444 | 7.3336 | 5.8563 | 7.3278 | 4.036 | 6.275 |
| 6.5547 | 4.4556 | 5.4375 | 6.6267 | 6.1873 | 6.4244 | 5.828 | 4.7832 |
| 5.2242 | 8.3498 | 10.3512 | 6.1131 | 5.6423 | 6.3858 | 6.183 | 5.4145 |
| 5.6202 | 5.0339 | 1.224 | 10.0957 | 4.0526 | 6.6993 | 6.5116 | 4.911 |
| 7.0451 | 6.102 | 4.2518 | 5.6366 | 3.1431 | 4.7821 | 6.0789 | 5.844 |
| 5.9354 | 5.8281 | 6.601 | 7.7013 | 6.5229 | 4.6675 | 4.7686 | 7.0357 |
| 5.6992 | 6.2794 | 5.9919 | 6.1148 | 6.5094 | 8.3022 | 4.3374 | 6.0584 |
| 5.9255 | 6.5635 | 7.1723 | 6.5245 | 6.1586 | 5.4142 | 4.5176 | 6.4728 |
| 5.7591 | 5.0825 | 6.6269 | 7.0056 | 6.5776 | 2.8198 | 2.7543 | 5.4743 |
| 7.8296 | 2.8479 | 9.6453 | 9.371 | 9.1336 | 7.9604 | 9.8868 | 10.2771 |
| 11.0322 | 11.139 | 11.0655 | 9.2711 | 10.6419 | 9.767 | 10.6403 | 9.3427 |
| 9.2354 | 10.6793 | 10.7421 | 10.9221 | 11.4431 | 9.5767 | 10.5725 | 9.8321 |
| 10.6735 | 10.8441 | | | | | | |
| CDK1 | 10.6692 | 9.606 | 9.9732 | 10.5414 | 9.8914 | 9.4556 | 11.0206 |
| 10.3706 | 10.8475 | 10.6849 | 10.5726 | 10.3788 | 10.6247 | 8.931 | 9.8054 |
| 10.165 | 10.8167 | 9.3408 | 9.5831 | 9.9129 | 11.0417 | 12.7007 | 10.0689 |
| 8.0873 | 10.5463 | 11.4437 | 10.5786 | 10.5085 | 9.1513 | 10.1308 | 10.6696 |
| 8.6035 | 10.9229 | 10.967 | 10.812 | 10.8214 | 10.5146 | 11.2413 | 10.9415 |
| 10.4468 | 9.9342 | 11.0121 | 9.9256 | 10.0713 | 9.7868 | 9.8271 | 9.8428 |
| 10.9689 | 9.5577 | 8.5991 | 10.23 | 9.9867 | 10.9877 | 11.1516 | 9.6079 |
| 10.4769 | 8.8794 | 10.9745 | 7.2487 | 10.2409 | 10.2806 | 9.8437 | 10.0388 |
| 9.8405 | 10.2754 | 11.0195 | 9.0691 | 7.8237 | 7.9247 | 10.3195 | 10.5941 |
| 10.9059 | 8.0227 | 10.2953 | 10.4088 | 8.985 | 9.7927 | 10.4523 | 9.6509 |
| 10.9339 | 10.5005 | 9.9603 | 9.179 | 11.2333 | 9.2539 | 10.3655 | 10.571 |
| 9.6242 | 10.4402 | 11.1977 | 11.1743 | 11.5187 | 11.8009 | 10.4361 | 11.2634 |
| 10.2726 | 10.8327 | 10.2161 | 9.0607 | 9.9773 | 11.1207 | 10.3336 | 11.0256 |
| 10.3798 | 10.9446 | 10.5498 | 11.6465 | 9.9455 | 10.2603 | 10.3179 | 10.5749 |
| 11.2466 | 9.8894 | 9.8565 | 11.2499 | 10.4143 | 11.2956 | 10.4504 | 8.9478 |
| 10.2691 | 7.3039 | 9.4823 | 12.0362 | 9.5822 | 9.7265 | 10.3468 | 10.658 |
| 9.8456 | 9.5448 | 10.1997 | 8.4874 | 9.3956 | 9.249 | 9.5362 | 10.8892 |
| 10.6278 | 10.8261 | 10.8831 | 11.0038 | 9.5556 | 10.3771 | 10.186 | 9.3683 |
| 10.7916 | 10.7921 | 10.6386 | 9.8367 | 10.7603 | 11.3416 | 10.2542 | 10.807 |
| 11.0791 | 10.6659 | 7.9425 | 10.4759 | 11.2455 | 8.4635 | 10.827 | 10.5924 |
| 10.5025 | 9.5741 | 10.6323 | 9.3256 | 10.7963 | 10.5738 | 10.1571 | 10.951 |
| 10.7691 | 8.7959 | 10.289 | 10.7416 | 10.6604 | 10.6878 | 11.26 | 10.3247 |
| 9.9684 | 9.8596 | 7.4538 | 10.1912 | 8.2326 | 6.3806 | 6.4369 | 6.2269 |

| | | | | | | | | |
|---|---|---|---|---|---|---|---|---|
| 5.9813 | 6.6876 | 6.7143 | 9.3292 | 5.3923 | 6.2073 | 6.7307 | 7.9613 | |
| 6.8935 | 8.2269 | 8.5934 | 6.3649 | 5.5276 | 7.3664 | 6.9193 | 8.9813 | |
| 5.2956 | 6.6 | | | | | | | |
| EZH2 | 9.9634 | 7.568 | 8.9271 | 9.7348 | 9.0337 | 9.6804 | 8.7769 | |
| 9.0684 | 9.1908 | 9.2121 | 9.9601 | 9.5285 | 9.0534 | 8.4809 | 8.8036 | |
| 9.3264 | 9.3378 | 9.2181 | 8.1307 | 8.4183 | 8.8715 | 10.8512 | 9.2091 | |
| 8.0614 | 8.4205 | 11.2577 | 9.7106 | 9.9283 | 9.1658 | 9.3047 | 9.7777 | |
| 8.4434 | 9.1038 | 8.8614 | 9.2446 | 9.1099 | 9.5566 | 9.88 | 8.0188 | |
| 9.6031 | 8.0261 | 9.1853 | 7.8424 | 8.5551 | 9.8064 | 9.517 | 9.4032 | |
| 9.5756 | 8.4893 | 7.9469 | 8.8618 | 7.8207 | 9.7863 | 9.2351 | 8.5626 | |
| 9.1422 | 8.7252 | 9.5795 | 8.2626 | 9.2944 | 8.876 | 9.7151 | 9.1452 | |
| 9.0534 | 8.3018 | 9.6771 | 8.9248 | 6.8301 | 6.6296 | 9.19 | 9.6565 | |
| 9.2177 | 6.5783 | 10.337 | 8.9612 | 9.1247 | 9.2711 | 9.1807 | 9.8544 | |
| 10.4289 | 9.2612 | 10.3783 | 8.8295 | 10.3714 | 7.7528 | 9.8807 | 9.706 | |
| 9.7667 | 9.3715 | 9.5842 | 9.8975 | 9.5496 | 10.1898 | 9.3711 | 8.5854 | |
| 10.1094 | 9.2754 | 9.5194 | 8.918 | 9.6239 | 9.4991 | 8.3662 | 9.1167 | |
| 9.7023 | 10.3506 | 9.5442 | 8.8042 | 8.7559 | 9.4557 | 8.7952 | 9.3807 | |
| 9.6319 | 8.9149 | 9.6032 | 8.5851 | 9.171 | 9.0675 | 8.5854 | 7.4729 | |
| 10.8015 | 7.8037 | 8.4755 | 9.7058 | 8.1764 | 8.6413 | 8.9785 | 8.8775 | |
| 8.9065 | 9.27 | 8.4987 | 7.8681 | 8.8179 | 8.9608 | 9.7189 | 8.9963 | |
| 9.4252 | 10.0666 | 9.8399 | 9.7402 | 8.2574 | 8.7464 | 8.4931 | 9.1997 | |
| 9.522 | 9.4908 | 8.2911 | 8.7243 | 9.267 | 10.3273 | 10.0643 | 9.5055 | |
| 11.1183 | 10.0124 | 6.8987 | 10.3198 | 10.8252 | 7.5396 | 8.7986 | 8.9437 | |
| 10.6017 | 8.1638 | 9.7218 | 8.2427 | 8.4724 | 9.2161 | 9.4564 | 9.7983 | |
| 9.4303 | 8.609 | 8.6159 | 9.3644 | 9.0354 | 9.9047 | 9.8907 | 8.4842 | |
| 9.1136 | 9.9172 | 7.202 | 8.9946 | 7.5627 | 5.6167 | 5.3985 | 6.6923 | |
| 4.6343 | 5.5467 | 5.5981 | 8.0059 | 4.9243 | 4.0682 | 6.0792 | 7.8419 | |
| 6.3208 | 6.0077 | 6.9354 | 6.1515 | 5.7996 | 6.1649 | 5.3124 | 8.3477 | |
| 3.9786 | 5.9708 | | | | | | | |
| KIFC1 | 11.0889 | 10.0771 | 8.39 | 1.12E+01 | | 1.09E+01 | | |
| 10.4723 | 10.699 | 10.716 | 11.1782 | 11.0351 | 11.0448 | 9.9118 | 9.4921 | 8.62 |
| | 10.302 | 10.432 | 10.6654 | 11.7469 | 8.7529 | 8.4978 | 10.4185 | |
| 12.0197 | 9.6999 | 8.1825 | 10.748 | 10.9827 | 10.562 | 11.2458 | 9.6465 | |
| 12.0817 | 10.5 | 8.7957 | 10.7154 | 9.9927 | 9.9819 | 10.4563 | 10.5199 | |
| 11.3793 | 9.8703 | 10.6592 | 10.134 | 10.6484 | 9.2852 | 9.8682 | 9.3492 | |
| 9.8334 | 9.798 | 10.1807 | 9.2671 | 8.7395 | 10.7422 | 10.5997 | 10.7303 | |
| 9.7741 | 11.276 | 9.6569 | 8.6123 | 11.6588 | 6.866 | 10.7155 | 10.6389 | |
| 9.7065 | 10.765 | 10.4808 | 8.9259 | 8.8937 | 9.7015 | 7.3836 | 7.3997 | |
| 10.5481 | 10.3003 | 11.0093 | 6.8933 | 10.8756 | 9.5854 | 9.6503 | 10.8036 | |
| 10.7211 | 10.1594 | 11.0494 | 10.628 | 9.8304 | 8.7535 | 11.0078 | 9.7847 | |
| 10.0236 | 10.2196 | 10.7811 | 10.0582 | 10.719 | 10.5849 | 10.3679 | 12.3748 | |
| 9.6028 | 10.767 | 10.6819 | 9.9306 | 9.9021 | 9.1393 | 10.3663 | 9.5964 | |
| 10.7211 | 10.0788 | 10.6471 | 10.9325 | 11.0418 | 10.9123 | 9.4847 | 10.2879 | |
| 9.9612 | 10.557 | 10.2629 | 11.0164 | 9.7162 | 10.1542 | 9.7128 | 10.4897 | |
| 10.2125 | 9.8067 | 10.9212 | 3.816 | 8.874 | 10.3388 | 9.0395 | 8.7749 | |
| 10.0584 | 9.8325 | 8.9261 | 9.6131 | 9.6377 | 7.9161 | 9.6582 | 8.7798 | |
| 11.1507 | 10.6155 | 11.4617 | 10.2884 | 9.9754 | 10.9336 | 9.7787 | 10.1953 | |
| 9.0787 | 9.4517 | 9.5906 | 9.8895 | 10.1539 | 9.3363 | 10.6345 | 12.1763 | |
| 10.6539 | 11.4109 | 11.5194 | 10.8955 | 7.3192 | 10.3032 | 10.643 | 7.3871 | |
| 10.4784 | 10.3569 | 11.4935 | 8.9777 | 10.3228 | 8.6494 | 10.9074 | 11.1723 | |
| 10.1615 | 10.9286 | 10.6216 | 8.595 | 10.4861 | 10.0825 | 10.3233 | 11.0732 | |
| 10.7407 | 9.7343 | 8.9777 | 10.9552 | 5.0488 | 9.8789 | 6.5512 | 5.5157 | |
| 6.1152 | 6.8002 | 4.857 | 5.5069 | 5.1192 | 8.6501 | 5.7891 | 4.9755 | |
| 6.1179 | 6.9897 | 6.1333 | 5.8258 | 7.5971 | 5.3381 | 5.6505 | 7.6793 | |

| | | | | | | | | |
|---|---|---|---|---|---|---|---|---|
| 5.8196 | 8.1465 | 5.448 | 6.2461 | | | | | |
| CENPF | 11.5091 | 11.0042 | 10.7085 | 1.16E+01 | | 1.22E+01 | | |
| 11.8392 | 11.9955 | 11.288 | 11.9957 | 12.0363 | 11.1923 | 11.7567 | 10.8049 | |
| 10.6392 | 10.8792 | 10.7782 | 11.1278 | 10.9742 | 9.7983 | 10.6166 | 9.8423 | |
| 13.3073 | 10.2116 | 9.03 | 11.9285 | 12.6376 | 12.2958 | 11.5016 | 10.6535 | |
| 12.7921 | 10.8688 | 10.299 | 12.8655 | 10.6641 | 11.1304 | 11.482 | 11.3834 | |
| 11.8188 | 11.2865 | 9.3705 | 10.9523 | 11.4476 | 9.7427 | 11.7903 | 10.566 | |
| 10.98 | 10.9482 | 11.6099 | 10.5295 | 9.7829 | 12.0715 | 12.2098 | 12.0035 | |
| 10.899 | 11.6538 | 10.8379 | 9.3538 | 12.3744 | 8.0023 | 11.8438 | 10.9771 | |
| 12.0518 | 10.3255 | 10.0547 | 10.7761 | 9.9297 | 11.0649 | 8.2451 | 8.9998 | |
| 10.9073 | 10.6291 | 13.0529 | 8.5913 | 12.2511 | 9.8818 | 10.6479 | 11.1508 | |
| 10.7636 | 10.8872 | 12.6649 | 12.0129 | 10.0829 | 9.8636 | 10.5668 | 10.7251 | |
| 11.3983 | 11.3508 | 9.5446 | 11.4652 | 11.781 | 11.2727 | 11.3417 | 12.9592 | |
| 11.1499 | 11.5436 | 11.3039 | 11.3824 | 11.1445 | 9.2846 | 10.2021 | 12.5249 | |
| 11.3522 | 11.7702 | 10.6696 | 11.6314 | 11.2709 | 12.0508 | 10.8188 | 11.5851 | |
| 10.3052 | 11.9176 | 11.3821 | 10.1006 | 11.4626 | 11.6093 | 10.7192 | 11.6782 | |
| 10.491 | 8.0155 | 10.8033 | 6.3793 | 10.6546 | 11.7591 | 10.0021 | 9.2326 | |
| 11.1105 | 11.0067 | 11.0933 | 10.0465 | 10.4441 | 9.8315 | 8.2485 | 10.9103 | |
| 11.1703 | 11.8675 | 12.1169 | 12.0068 | 11.3185 | 10.9576 | 10.0866 | 9.924 | |
| 10.2784 | 9.4936 | 10.3149 | 11.6235 | 11.4171 | 10.7272 | 11.3093 | 13.0232 | |
| 12.0665 | 12.461 | 11.1628 | 11.8399 | 8.2956 | 11.8126 | 12.261 | 8.7533 | |
| 10.9898 | 10.363 | 12.1789 | 9.7545 | 11.4518 | 10.8037 | 11.5062 | 11.1331 | |
| 10.3402 | 11.2681 | 11.5086 | 10.2911 | 10.9658 | 12.4362 | 11.954 | 10.7467 | |
| 10.1482 | 10.2853 | 10.4403 | 10.1269 | 5.8816 | 11.0305 | 7.6333 | 7.3815 | |
| 6.7879 | 6.1877 | 5.1792 | 6.7052 | 6.3927 | 9.6752 | 6.0646 | 5.763 | |
| 7.2964 | 7.8868 | 7.7533 | 7.2203 | 9.1247 | 6.5181 | 6.3028 | 7.7942 | |
| 6.4522 | 9.3052 | 6.046 | 6.818 | | | | | |
| CACNA1H | 6.9781 | 6.8833 | 5.4671 | 7.9171 | 5.7275 | 6.4493 | 6.1685 | |
| 5.9346 | 5.0387 | 4.3907 | 6.9487 | 5.6065 | 5.2665 | 6.7278 | 6.542 | |
| 6.9763 | 5.1493 | 5.2426 | 6.0106 | 6.7425 | 9.9635 | 5.1528 | 7.5172 | |
| 7.8406 | 6.3461 | 4.7461 | 7.4632 | 4.8336 | 5.0304 | 3.0883 | 6.5367 | 9.81 |
| | 6.0321 | 5.0966 | 5.9708 | 6.547 | 6.3957 | 5.5593 | 5.8781 | |
| 5.9787 | 11.1655 | 6.5563 | 5.8016 | 7.4881 | 5.9947 | 7.9526 | 8.5556 | |
| 5.6142 | 7.6263 | 9.5206 | 8.6179 | 5.855 | 8.7751 | 5.502 | 4.4931 | |
| 7.3652 | 4.4292 | 5.7943 | 8.3413 | 9.7626 | 4.6133 | 7.0222 | 6.1745 | |
| 5.859 | 8.8391 | 3.8932 | 6.7666 | 7.7555 | 6.9558 | 8.2604 | 6.6842 | |
| 5.9773 | 8.7732 | 6.0104 | 8.0647 | 5.1219 | 4.3833 | 6.9427 | 6.8321 | |
| 9.3009 | 6.3518 | 4.8069 | 9.0856 | 6.5786 | 8.6208 | 7.7372 | 6.5287 | |
| 6.2632 | 7.5324 | 5.7666 | 8.0934 | 5.3224 | 7.4149 | 7.3859 | 6.9942 | |
| 6.921 | 5.9575 | 5.3137 | 6.8939 | 6.5999 | 6.3635 | 3.7164 | 4.8708 | |
| 5.4346 | 6.478 | 7.7141 | 9.3696 | 7.0053 | 6.1522 | 6.1199 | 6.1352 | |
| 7.3115 | 2.7607 | 5.8123 | 4.548 | 6.9402 | 6.8781 | 7.0713 | 6.8981 | |
| 6.4827 | 8.7944 | 10.4909 | 4.8789 | 6.1397 | 6.7102 | 7.2997 | 6.3391 | |
| 6.4274 | 5.9552 | 4.4839 | 10.7018 | 6.8459 | 7.2626 | 6.824 | 5.9636 | |
| 5.512 | 6.7161 | 5.6136 | 9.1281 | 6.128 | 7.198 | 6.5536 | 5.3686 | |
| 7.0922 | 6.1991 | 6.2052 | 6.8711 | 7.0651 | 3.6978 | 5.387 | 4.9595 | |
| 4.4245 | 6.016 | 7.3564 | 6.1392 | 7.3165 | 7.7676 | 6.7403 | 6.6227 | |
| 4.4073 | 5.6942 | 6.215 | 7.087 | 6.9134 | 5.5172 | 5.5224 | 5.7519 | |
| 7.4773 | 5.2522 | 4.9486 | 7.7254 | 6.8649 | 4.6578 | 9.6417 | 6.5336 | |
| 7.895 | 6.7499 | 11.012 | 9.8947 | 11.0328 | 9.8929 | 12.3454 | 11.2384 | |
| 11.2718 | 11.5563 | 11.1057 | 9.7713 | 12.2466 | 12.2297 | 11.5903 | 10.4947 | |
| 10.1364 | 10.7706 | 11.3443 | 11.7436 | 12.9005 | 11.2269 | 11.1456 | 10.475 | |
| 12.4561 | 11.2961 | | | | | | | |
| RAD51 | 9.2604 | 6.8682 | 6.2795 | 9.70E+00 | | 8.5668 | 8.6765 | |

| | | | | | | | | |
|---|---|---|---|---|---|---|---|---|
| 8.5148 | 7.788 | 9.3699 | 7.5328 | 8.0292 | 7.734 | 7.9012 | 6.7739 | |
| 7.5216 | 8.986 | 8.2435 | 8.7974 | 7.4816 | 6.418 | 8.7027 | 8.4939 | |
| 8.2776 | 7.1179 | 7.9608 | 7.7414 | 8.5859 | 9.2192 | 7.3808 | 8.0562 | |
| 9.3061 | 6.2685 | 8.7576 | 8.2524 | 6.7958 | 8.7349 | 6.9811 | 8.6336 | |
| 7.5614 | 8.8745 | 7.743 | 8.8448 | 7.9352 | 7.8202 | 7.316 | 7.4695 | |
| 7.0655 | 8.7672 | 7.7009 | 7.2718 | 8.2237 | 8.3017 | 8.9436 | 8.9639 | |
| 8.3683 | 6.6732 | 6.228 | 7.9096 | 5.1676 | 7.6757 | 8.5435 | 7.5647 | |
| 8.3596 | 7.9727 | 7.4766 | 6.7292 | 7.1032 | 5.1706 | 5.1911 | 8.7199 | |
| 8.4659 | 9.0582 | 5.061 | 7.4592 | 7.7683 | 6.7089 | 8.1799 | 8.5488 | |
| 8.3668 | 8.2911 | 7.9853 | 7.3367 | 5.7988 | 9.1454 | 7.3926 | 7.3639 | |
| 7.8483 | 8.6582 | 8.9409 | 7.5324 | 7.902 | 9.4981 | 8.991 | 6.8496 | |
| 8.2979 | 8.2889 | 7.6082 | 7.9267 | 8.3298 | 7.9307 | 8.8331 | 8.2358 | |
| 7.8658 | 7.9994 | 8.3487 | 8.8237 | 8.461 | 7.9997 | 8.2044 | 8.5856 | |
| 8.6983 | 8.4672 | 9.1964 | 7.5754 | 8.0909 | 7.6692 | 7.3924 | 8.591 | |
| 8.0448 | 9.5515 | 6.5899 | 7.3098 | 8.1768 | 7.6383 | 6.9748 | 8.4157 | |
| 8.5076 | 7.0816 | 8.0873 | 8.7477 | 5.5605 | 9.0689 | 6.6737 | 9.2811 | |
| 8.3812 | 8.9099 | 8.2961 | 9.1387 | 9.3437 | 7.9785 | 8.4333 | 7.7792 | |
| 7.8991 | 8.7248 | 8.4919 | 8.3788 | 7.0282 | 8.9922 | 8.7645 | 8.4603 | |
| 8.9952 | 7.4192 | 8.1631 | 6.0836 | 7.8868 | 9.0656 | 5.3606 | 9.0386 | |
| 8.1888 | 8.8004 | 6.5047 | 7.3537 | 7.3777 | 8.29 | 8.5682 | 8.382 | |
| 9.0744 | 8.886 | 6.562 | 7.6125 | 7.3011 | 8.6126 | 9.9526 | 7.8737 | |
| 7.9149 | 7.4033 | 9.7117 | 3.5484 | 7.9842 | 4.974 | 4.7531 | 3.6666 | |
| 4.3979 | 3.2157 | 4.3564 | 4.0452 | 6.6478 | 3.6594 | 4.2441 | 4.1324 | |
| 6.0946 | 4.3194 | 5.0611 | 5.5001 | 3.8923 | 4.487 | 6.0792 | 4.1094 | |
| 6.1124 | 3.2853 | 3.8265 | | | | | | |
| BUB1B | 10.0637 | 8.2514 | 8.252 | 10.178 | 10.4229 | 9.7667 | 9.8261 | |
| 10.4691 | 10.9037 | 9.6175 | 9.5291 | 9.2 | 9.5765 | 7.9352 | 8.6838 | |
| 9.5915 | 8.6222 | 9.1364 | 8.048 | 8.503 | 8.7683 | 9.8564 | 9.0212 | |
| 6.7797 | 9.6169 | 10.1612 | 8.968 | 10.3974 | 7.9474 | 10.1476 | 9.8648 | |
| 7.7047 | 10.2399 | 10.0648 | 9.6655 | 9.4071 | 8.1567 | 10.6337 | 9.0126 | |
| 6.8498 | 9.1452 | 10.7475 | 8.0855 | 9.3556 | 8.7194 | 8.7156 | 8.327 | |
| 9.8941 | 8.7556 | 8.3633 | 9.5088 | 9.1481 | 10.88 | 10.1538 | 9.5507 | |
| 8.6826 | 6.9453 | 9.6711 | 6.2884 | 9.3857 | 8.6796 | 9.6713 | 7.9913 | |
| 8.9768 | 8.7708 | 7.8713 | 8.972 | 6.4645 | 5.9603 | 9.648 | 9.3656 | |
| 11.2302 | 5.9103 | 9.6214 | 8.4557 | 8.8449 | 10.1094 | 8.7638 | 8.6896 | |
| 10.2501 | 9.7795 | 7.9825 | 7.8987 | 10.0947 | 8.7975 | 8.9205 | 9.4349 | |
| 8.4253 | 9.7974 | 9.4155 | 10.115 | 10.2776 | 10.4246 | 8.924 | 9.8495 | |
| 9.7033 | 9.2669 | 10.0162 | 6.398 | 8.3661 | 10.897 | 10.2049 | 8.8796 | |
| 9.5857 | 10.3606 | 9.0605 | 10.635 | 9.4946 | 10.1677 | 9.3696 | 10.0919 | |
| 10.4803 | 8.4884 | 9.1991 | 10.4197 | 9.2524 | 10.1968 | 9.1729 | 6.0322 | |
| 8.7807 | 4.5503 | 9.148 | 9.7575 | 8.0233 | 8.0538 | 9.5962 | 10.0774 | |
| 8.4969 | 8.5181 | 7.5008 | 7.1836 | 7.0718 | 8.8724 | 10.4795 | 9.8897 | |
| 11.0696 | 9.9342 | 9.5713 | 9.9024 | 8.872 | 9.1224 | 8.8087 | 7.9319 | |
| 9.7712 | 10.3137 | 9.3071 | 8.8634 | 10.0715 | 10.5493 | 10.5198 | 10.3348 | |
| 8.8494 | 9.3233 | 5.0451 | 9.393 | 10.8924 | 6.6062 | 9.2818 | 9.2333 | |
| 10.4569 | 8.2307 | 9.5804 | 8.6538 | 9.0148 | 9.3064 | 9.3773 | 10.1235 | |
| 10.1031 | 8.5034 | 8.9423 | 8.7744 | 9.7466 | 9.1664 | 7.5822 | 9.1061 | |
| 8.6968 | 8.3576 | 4.2773 | 9.2855 | 5.2635 | 4.6034 | 4.4323 | 4.3419 | |
| 1.026 | 3.0652 | 3.8609 | 7.7383 | 3.3811 | 3.4895 | 4.4874 | 5.7675 | |
| 5.8592 | 5.217 | 7.5673 | 4.4857 | 3.052 | 6.4566 | 3.5262 | 8.1501 | |
| 2.9456 | 4.5 | | | | | | | |
| CDCA3 | 9.8314 | 8.0981 | 7.2262 | 9.50E+00 | | 8.94E+00 | | |
| 8.2707 | 8.633 | 9.2255 | 9.4417 | 8.7447 | 8.437 | 8.3511 | 8.1573 | 8.07 |
| | 9.3436 | 8.9388 | 9.1166 | 9.905 | 7.5855 | 7.0775 | 9.4759 | |

| | | | | | | | |
|---|---|---|---|---|---|---|---|
| 11.1592 | 8.9993 | 7.0856 | 8.6945 | 10.058 | 9.4037 | 9.5683 | 8.7571 |
| 9.0296 | 9.8535 | 7.1109 | 9.3548 | 9.4363 | 7.696 | 8.8872 | 9.236 |
| 9.1925 | 9.2623 | 10.7402 | 8.3947 | 9.4959 | 8.036 | 8.7004 | 8.8915 |
| 7.6409 | 8.3288 | 9.4928 | 7.4238 | 7.1089 | 8.1666 | 9.1015 | 8.3182 |
| 9.1238 | 10.0246 | 8.3829 | 6.9365 | 9.5455 | 6.1587 | 9.9208 | 8.9456 |
| 8.186 | 9.6741 | 9.2965 | 8.0118 | 7.0673 | 8.1594 | 6.4881 | 6.4117 |
| 8.7809 | 9.1458 | 10.0822 | 6.1921 | 9.295 | 8.7881 | 7.8112 | 9.4141 |
| 9.264 | 7.6879 | 10.2806 | 8.3598 | 8.9567 | 8.8801 | 9.478 | 8.8624 |
| 7.5909 | 9.3988 | 9.7491 | 8.8714 | 8.5513 | 10.4008 | 9.461 | 10.1417 |
| 7.3911 | 9.1724 | 9.6807 | 8.5417 | 8.6792 | 9.4559 | 11.0676 | 9.6375 |
| 8.4198 | 8.2717 | 8.0018 | 9.0367 | 9.1224 | 9.1103 | 8.5306 | 8.0076 |
| 8.4522 | 9.0086 | 9.6972 | 9.5688 | 9.0061 | 9.0022 | 8.6191 | 9.2433 |
| 9.4545 | 8.8751 | 9.0412 | 7.083 | 7.6711 | 10.3816 | 7.6914 | 8.1703 |
| 8.4833 | 8.3997 | 6.6712 | 7.9376 | 9.6099 | 7.0815 | 9.9201 | 6.83 |
| 7.7297 | 9.0902 | 8.8849 | 8.3813 | 9.0618 | 10.1287 | 8.9256 | 9.9504 |
| 8.1006 | 7.7547 | 9.7735 | 8.337 | 8.9581 | 7.8372 | 9.0832 | 10.0929 |
| 8.4148 | 9.369 | 9.6598 | 9.1557 | 7.6663 | 8.4816 | 8.2513 | 6.4113 |
| 10.1013 | 9.1444 | 10.1325 | 7.9928 | 8.1816 | 7.2926 | 9.6122 | 10.4017 |
| 8.7889 | 9.1698 | 9.136 | 6.5417 | 8.3365 | 10.0408 | 8.3198 | 10.23 |
| 10.8199 | 8.0211 | 7.5515 | 10.1894 | 4.1341 | 8.7953 | 6.1222 | 4.6856 |
| 4.724 | 4.2925 | 3.3941 | 5.0849 | 4.3656 | 7.5947 | 4.3065 | 4.0206 |
| 5.0392 | 6.4805 | 4.6748 | 4.6093 | 6.7726 | 4.2505 | 5.5772 | 6.1759 |
| 4.5114 | 7.5051 | 4.3591 | 4.6585 | | | | |
| PDE2A | 3.7656 | 3.8562 | 3.854 | 4.6352 | 5.7711 | 3.3422 | 6.184 |
| 4.388 | 4.3505 | 4.6422 | 6.9026 | 5.3535 | 3.8176 | 4.3171 | 5.6762 |
| 5.8063 | 5.1613 | 6.361 | 5.2439 | 4.3789 | 7.4286 | 7.2684 | 6.5648 |
| 4.7445 | 3.7692 | 6.3544 | 4.3573 | 4.8336 | 4.5323 | 2.4602 | 4.3882 |
| 7.0138 | 4.8389 | 3.5192 | 4.3915 | 5.615 | 3.6259 | 3.6075 | 4.6139 |
| 2.8871 | 5.6563 | 3.6342 | 4.1238 | 2.9335 | 6.2728 | 5.705 | 7.1719 |
| 5.1181 | 4.4583 | 5.6861 | 8.6577 | 5.0481 | 4.8145 | 5.517 | 6.8163 |
| 4.489 | 2.7333 | 6.2209 | 7.9856 | 7.7346 | 4.9275 | 4.3915 | 3.982 |
| 6.2051 | 7.3063 | 4.1016 | 4.9331 | 6.5992 | 7.1612 | 6.2695 | 7.5199 |
| 4.7174 | 8.2997 | 5.2165 | 7.0415 | 4.0504 | 3.4508 | 5.1705 | 5.9718 |
| 5.1792 | 5.6006 | 4.1328 | 6.4929 | 4.5041 | 6.3479 | 6.0751 | 4.8744 |
| 4.4346 | 5.402 | 2.6756 | 6.6069 | 3.6025 | 5.9091 | 6.6785 | 5.7541 |
| 5.3117 | 3.876 | 1.8846 | 5.8481 | 5.2095 | 5.6131 | 4.1184 | 4.3525 |
| 4.9716 | 4.5937 | 6.609 | 6.1895 | 3.8683 | 6.0024 | 2.8403 | 4.866 |
| 4.2929 | 3.0394 | 4.4395 | 3.6084 | 4.2276 | 6.1008 | 4.9619 | 7.1422 |
| 4.7107 | 6.7736 | 10.1563 | 3.7445 | 4.5853 | 4.9098 | 3.8315 | 3.6893 |
| 4.1422 | 4.6335 | 3.547 | 10.2745 | 5.8462 | 5.5544 | 6.3961 | 6.4098 |
| 5.1295 | 4.5075 | 2.9457 | 4.5153 | 3.9656 | 3.5368 | 4.8245 | 3.18 |
| 5.0707 | 3.2219 | 6.7153 | 7.1341 | 4.2887 | 2.8049 | 6.2777 | 5.6985 |
| 6.1785 | 3.0975 | 4.7036 | 5.7523 | 6.3023 | 7.0709 | 4.4027 | 4.7751 |
| 5.9359 | 4.2827 | 3.2722 | 5.1574 | 5.2176 | 4.4888 | 2.876 | 5.4006 |
| 4.3208 | 5.122 | 4.7302 | 6.335 | 4.2907 | 5.3717 | 6.9936 | 5.0018 |
| 5.2362 | 4.4234 | 9.9419 | 8.3401 | 9.4824 | 6.0725 | 10.2088 | 9.9028 |
| 11.4803 | 11.4665 | 11.1171 | 8.4246 | 11.0887 | 11.3473 | 10.6444 | 7.9226 |
| 8.0691 | 10.4777 | 10.2934 | 9.9062 | 7.4296 | 11.1791 | 11.1303 | 8.4268 |
| 10.5305 | 9.7999 | | | | | | |
| CCNA2 | 10.0548 | 7.9883 | 8.6594 | 1.03E+01 | | 9.96E+00 | |
| 9.7688 | 9.7728 | 9.4412 | 10.8274 | 10.2895 | 9.3303 | 9.392 | 9.3457 |
| 8.2268 | 9.0845 | 9.7339 | 9.9593 | 10.3084 | 8.4709 | 8.2887 | 9.57 |
| 10.9005 | 9.2135 | 7.3427 | 9.9925 | 10.6387 | 10.0743 | 11.1004 | 8.3515 |
| 10.42 | 10.5077 | 7.5373 | 10.3084 | 10.3823 | 9.7254 | 9.9303 | 9.7339 |

| | | | | | | | |
|---|---|---|---|---|---|---|---|
| 9.4933 | 8.3543 | 9.1956 | 9.6281 | 11.0412 | 8.7295 | 9.6857 | 9.1209 |
| 8.3495 | 8.1209 | 10.3974 | 8.9386 | 8.212 | 10.2629 | 8.9194 | 10.1358 |
| 10.2375 | 8.9985 | 9.5882 | 8.2484 | 9.0898 | 6.5158 | 10.1255 | 9.8703 |
| 9.3935 | 9.6855 | 8.6122 | 9.2219 | 7.6907 | 8.85 | 6.8674 | 7.2422 |
| 10.0924 | 9.7681 | 10.4013 | 7.4191 | 10.0186 | 8.514 | 8.7305 | 9.1178 |
| 9.3093 | 8.0055 | 10.3838 | 10.0985 | 9.0321 | 8.1289 | 10.3109 | 9.0302 |
| 8.5831 | 9.9771 | 8.6214 | 9.658 | 9.5296 | 9.8783 | 10.63 | 11.5298 |
| 9.2324 | 9.5251 | 9.2639 | 9.4005 | 9.3841 | 9.1445 | 7.6633 | 10.8815 |
| 9.9432 | 9.1136 | 9.0617 | 9.7626 | 9.2451 | 10.066 | 9.6479 | 8.9957 |
| 10.1458 | 10.1501 | 9.2608 | 8.8213 | 9.8996 | 10.0616 | 9.4397 | 9.5671 |
| 9.0911 | 7.7577 | 9.3216 | 8.4577 | 9.1548 | 10.1056 | 8.5558 | 8.4537 |
| 10.1531 | 10.0708 | 8.4191 | 9.0641 | 9.5384 | 7.5636 | 8.1277 | 8.4128 |
| 9.616 | 9.8213 | 9.66 | 10.0222 | 10.1418 | 10.7412 | 9.2014 | 9.5343 |
| 9.0867 | 8.3144 | 8.6179 | 10.5196 | 9.131 | 9.4512 | 9.7679 | 10.4357 |
| 10.2626 | 10.1608 | 9.1715 | 9.8214 | 7.0935 | 9.7191 | 10.0662 | 7.7845 |
| 9.8518 | 9.4039 | 10.5555 | 8.663 | 9.5925 | 8.4626 | 10.3761 | 9.8927 |
| 8.894 | 10.4403 | 10.5195 | 8.0858 | 9.0968 | 9.9283 | 10.0432 | 10.2787 |
| 10.4425 | 9.265 | 8.9487 | 8.7191 | 5.7675 | 9.3566 | 6.6037 | 5.5934 |
| 5.3122 | 5.2421 | 5.2766 | 5.9862 | 5.4726 | 8.3917 | 4.8553 | 4.828 |
| 6.1653 | 7.7311 | 6.5893 | 6.1686 | 7.863 | 5.8896 | 4.8855 | 7.0897 |
| 5.9327 | 7.6217 | 4.7905 | 6.1334 | | | | |
| MRGPRF | 6.8843 | 6.9634 | 5.654 | 8.4599 | 5.2246 | 7.0463 | 7.5963 |
| 7.7034 | 5.7269 | 6.3872 | 8.2825 | 4.2868 | 5.3647 | 2.6237 | 8.7138 |
| 7.6449 | 6.1103 | 7.1391 | 5.727 | 5.1429 | 4.7327 | 4.4959 | 8.9058 |
| 8.4751 | 4.5693 | 5.7898 | 8.1027 | 4.7439 | 7.179 | 5.1506 | 5.1474 |
| 6.2773 | 4.0475 | 5.9429 | 6.3452 | 7.8078 | 7.5077 | 5.7681 | 5.8331 |
| 7.7332 | 5.8021 | 6.7695 | 5.4987 | 6.1212 | 6.5613 | 8.6235 | 6.6729 |
| 5.0159 | 8.1734 | 7.5249 | 10.4379 | 6.8053 | 7.3007 | 5.7994 | 5.829 |
| 7.6144 | 4.4292 | 7.6743 | 7.6354 | 10.3696 | 6.399 | 5.0243 | 6.118 |
| 7.3906 | 8.3079 | 3.356 | 6.996 | 8.6821 | 8.2892 | 8.4237 | 7.3978 |
| 6.6801 | 8.0859 | 5.8115 | 9.4977 | 7.8537 | 5.8097 | 6.75 | 8.1054 |
| 8.9631 | 6.5976 | 6.8408 | 9.8862 | 9.2723 | 5.483 | 7.9323 | 6.0651 |
| 6.7477 | 7.7828 | 7.0941 | 7.2482 | 5.3924 | 8.2429 | 9.393 | 8.1032 |
| 8.047 | 4.9708 | 6.1499 | 9.8952 | 6.585 | 7.0904 | 5.5032 | 5.5385 |
| 6.5201 | 7.128 | 7.0184 | 8.3473 | 6.8438 | 7.8804 | 6.6926 | 7.5431 |
| 9.0216 | 8.7957 | 6.7954 | 3.2094 | 7.4747 | 6.5251 | 7.5663 | 10.4282 |
| 7.1838 | 10.3526 | 10.1408 | 4.6307 | 3.8783 | 6.9617 | 6.5976 | 6.1174 |
| 5.9043 | 6.0338 | 5.5368 | 10.5342 | 8.5215 | 5.9861 | 7.6903 | 5.2629 |
| 7.2061 | 7.4099 | 5.3655 | 6.9383 | 3.7586 | 6.3506 | 7.8524 | 5.884 |
| 7.9304 | 7.2963 | 7.2307 | 7.4091 | 8.1544 | 5.5423 | 4.1519 | 6.9811 |
| 7.2951 | 6.502 | 8.5472 | 5.7836 | 7.796 | 8.1178 | 7.507 | 6.0716 |
| 6.6862 | 6.0454 | 7.4882 | 7.2345 | 8.3958 | 7.0539 | 7.0174 | 6.3322 |
| 7.0328 | 5.0213 | 6.9917 | 8.7374 | 6.0217 | 7.9273 | 5.945 | 7.1678 |
| 8.2511 | 8.6603 | 11.1128 | 11.4947 | 11.8196 | 9.4059 | 13.4135 | 11.241 |
| 11.6443 | 11.7768 | 11.8218 | 12.0612 | 12.5472 | 13.2365 | 11.9947 | 11.2197 |
| 10.4526 | 11.1529 | 12.2712 | 11.6033 | 10.1688 | 12.127 | 12.0366 | 11.7305 |
| 12.8661 | 11.1041 | | | | | | |
| RASL12 | 5.7478 | 5.7304 | 5.1566 | 7.2442 | 5.5488 | 5.601 | 7.2388 |
| 6.6285 | 5.5674 | 5.5893 | 7.65 | 5.2358 | 5.0277 | 6.6056 | 7.0444 |
| 7.4701 | 7.7606 | 5.6934 | 6.255 | 6.657 | 7.0337 | 4.8146 | 8.6077 |
| 7.4461 | 3.8723 | 5.6253 | 6.9338 | 5.5808 | 5.3469 | 5.1708 | 7.2936 |
| 7.382 | 6.0448 | 5.3906 | 6.3942 | 8.0437 | 6.9436 | 6.5166 | 6.1591 |
| 7.3701 | 6.0295 | 6.8341 | 5.6069 | 7.1413 | 7.9571 | 7.4649 | 8.0331 |
| 5.1377 | 8.4534 | 8.4928 | 9.2669 | 7.0096 | 8.2809 | 6.5455 | 5.905 |

| | | | | | | | | |
|---|---|---|---|---|---|---|---|---|
| 6.9985 | 3.4947 | 6.3531 | 6.4931 | 9.2446 | 6.1006 | 6.4462 | 7.8188 | |
| 6.8613 | 8.544 | 4.3935 | 7.0983 | 8.2473 | 6.8216 | 7.8553 | 7.3813 | |
| 6.006 | 8.6714 | 6.3621 | 8.4222 | 6.2509 | 5.9214 | 6.4768 | 7.1335 | |
| 8.5038 | 6.4539 | 6.2865 | 8.3457 | 7.011 | 6.14 | 7.7543 | 6.4417 | |
| 6.0732 | 7.6674 | 5.8019 | 7.798 | 6.2525 | 7.3148 | 7.9138 | 7.7615 | |
| 7.8315 | 5.586 | 4.5285 | 8.3577 | 7.5157 | 6.2943 | 7.2177 | 5.7663 | |
| 5.4691 | 6.366 | 6.8382 | 8.9925 | 5.6833 | 7.9323 | 5.8386 | 8.1788 | |
| 7.1642 | 9.8773 | 8.0399 | 4.9008 | 7.096 | 7.1335 | 8.3422 | 9.2287 | |
| 6.5702 | 9.8019 | 9.1685 | 5.2362 | 6.4209 | 6.6541 | 7.1547 | 6.1705 | |
| 5.5302 | 6.3817 | 4.9256 | 9.5501 | 7.533 | 5.7623 | 6.9047 | 5.5738 | |
| 7.4121 | 6.1772 | 7.3976 | 7.8579 | 5.1485 | 7.5962 | 7.1051 | 5.9607 | |
| 6.2927 | 6.8365 | 7.2167 | 7.1308 | 7.1968 | 6.3908 | 5.1107 | 6.4562 | |
| 5.7242 | 5.6164 | 8.1687 | 6.3068 | 7.2633 | 8.4016 | 7.0621 | 6.8314 | |
| 7.9872 | 6.6684 | 5.6525 | 7.1544 | 7.6106 | 7.1273 | 5.9566 | 5.7124 | |
| 6.9662 | 6.0094 | 6.8422 | 7.7115 | 6.4472 | 6.8599 | 5.7902 | 6.6739 | |
| 7.4562 | 7.6349 | 10.5583 | 9.9306 | 11.1246 | 7.7506 | 12.5945 | 10.056 | |
| 10.4166 | 11.0611 | 11.4215 | 10.593 | 11.542 | 10.9531 | 10.8761 | 10.147 | |
| 9.5435 | 10.401 | 11.3321 | 10.8816 | 8.164 | 11.5192 | 10.5515 | 10.569 | |
| 12.0615 | 10.3084 | | | | | | | |
| TGFB1I1 | 9.0339 | 9.4466 | 7.8797 | 9.26E+00 | | 6.53E+00 | | |
| 8.9674 | 8.2672 | 7.3698 | 6.8364 | 8.7868 | 8.942 | 6.8583 | 6.3866 | |
| 8.0833 | 8.4234 | 9.4255 | 6.8587 | 9.2949 | 7.0215 | 7.2518 | 8.0311 | |
| 6.4836 | 8.9166 | 10.0331 | 7.3099 | 9.472 | 8.8088 | 6.5055 | 6.6028 | |
| 5.067 | 5.8975 | 8.261 | 6.9557 | 8.7636 | 6.9914 | 8.647 | 9.0017 | |
| 7.2616 | 8.153 | 8.0266 | 6.3887 | 10.0666 | 7.4097 | 8.6215 | 7.4098 | |
| 9.5148 | 9.522 | 7.5884 | 8.4142 | 7.838 | 10.1903 | 7.0787 | 8.3297 | |
| 7.4156 | 8.2591 | 7.915 | 6.7799 | 7.8832 | 9.0202 | 10.7942 | 7.737 | |
| 7.4636 | 8.178 | 7.5168 | 9.1495 | 5.7375 | 8.4665 | 8.9362 | 8.866 | |
| 9.2755 | 8.7783 | 8.0472 | 9.2259 | 7.2046 | 10.1161 | 7.3687 | 6.548 | |
| 8.2247 | 8.7564 | 10.6118 | 7.0267 | 7.3444 | 10.3376 | 8.5305 | 7.1521 | |
| 9.0226 | 8.5874 | 8.3136 | 8.6785 | 7.1407 | 8.5708 | 6.2525 | 8.705 | |
| 8.2241 | 8.1802 | 8.7377 | 6.9951 | 5.9125 | 9.6936 | 8.8765 | 8.9834 | |
| 8.4448 | 7.0793 | 9.1952 | 8.5909 | 8.0899 | 9.5674 | 7.9911 | 8.123 | |
| 7.2729 | 7.6107 | 8.8477 | 8.7038 | 7.724 | 5.8619 | 8.0094 | 7.652 | |
| 8.7205 | 10.2338 | 7.5754 | 11.1873 | 10.869 | 6.2069 | 6.5695 | 8.0735 | |
| 8.4705 | 7.3401 | 6.6142 | 7.6223 | 7.0868 | 11.0762 | 7.9347 | 7.6207 | |
| 7.3789 | 8.8596 | 9.7636 | 8.9629 | 5.3835 | 9.6399 | 6.7834 | 9.5826 | |
| 8.2368 | 8.8198 | 7.6506 | 7.3672 | 7.8787 | 7.7536 | 9.0736 | 6.0432 | |
| 8.8743 | 5.9671 | 7.7036 | 8.4659 | 9.8363 | 8.7111 | 8.1984 | 8.8731 | |
| 8.6865 | 7.8406 | 7.3659 | 8.1012 | 7.3999 | 9.0305 | 9.1023 | 8.9459 | |
| 8.1084 | 9.2888 | 8.3274 | 7.246 | 8.0078 | 9.1929 | 7.3321 | 7.776 | 7.97 |
| | 7.967 | 8.9595 | 8.7932 | 11.8011 | 11.1541 | 12.0836 | 8.9703 | |
| 13.8223 | 11.4368 | 11.7353 | 12.6121 | 12.3942 | 11.693 | 12.4844 | 13.2723 | |
| 12.3928 | 12.2282 | 9.9449 | 11.5759 | 12.2816 | 12.0751 | 10.8096 | 12.1363 | |
| 12.0105 | 11.7832 | 12.7981 | 12.0807 | | | | | |
| ORC1L | 8.9506 | 7.4963 | 5.5573 | 9.491 | 7.738 | 7.9611 | 7.6837 | |
| 8.9054 | 7.7159 | 7.6349 | 7.205 | 7.8409 | 7.7908 | 6.229 | 6.491 | |
| 8.2698 | 7.6729 | 7.8229 | 5.9746 | 6.5661 | 8.1605 | 8.8495 | 7.7104 | |
| 6.4072 | 8.2199 | 8.0624 | 7.9912 | 7.9554 | 7.3474 | 8.2583 | 9.5697 | |
| 6.4027 | 8.8122 | 7.5823 | 7.7381 | 6.4245 | 7.0416 | 9.1241 | 6.8204 | |
| 7.4827 | 8.8006 | 8.8177 | 6.5262 | 7.8937 | 6.7452 | 7.8174 | 7.1098 | |
| 7.5191 | 7.3021 | 6.8338 | 7.7339 | 6.8053 | 8.1985 | 8.327 | 8.2807 | |
| 7.0228 | 5.7385 | 8.0088 | 4.4376 | 7.7596 | 8.3541 | 8.3213 | 7.8937 | |
| 7.1992 | 7.2261 | 5.5839 | 6.4862 | 4.7507 | 3.7934 | 8.3432 | 7.6635 | |

| | | | | | | | | |
|---|---|---|---|---|---|---|---|---|
| 8.7038 | 3.5862 | 7.7004 | 7.0123 | 6.694 | 7.828 | 8.001 | 7.475 | |
| 7.8176 | 8.5407 | 8.9605 | 5.5185 | 8.4682 | 7.1741 | 6.5557 | 7.1039 | |
| 8.3899 | 8.1716 | 7.8513 | 6.5259 | 8.3638 | 8.6663 | 7.13 | 7.5457 | |
| 8.183 | 7.1893 | 7.5943 | 6.5018 | 7.1997 | 7.9642 | 8.4857 | 7.0887 | |
| 7.1504 | 8.618 | 8.2602 | 7.8169 | 7.708 | 8.0128 | 7.8487 | 8.0337 | |
| 7.6306 | 8.263 | 7.9603 | 7.9399 | 7.397 | 7.3564 | 7.8798 | 7.0869 | |
| 8.5481 | 5.6856 | 7.5399 | 7.1663 | 6.8276 | 6.3049 | 8.3606 | 8.3894 | |
| 6.0668 | 7.4931 | 7.5394 | 4.6754 | 8.002 | 6.4959 | 8.1045 | 8.1982 | |
| 7.9516 | 7.9693 | 7.4538 | 8.9389 | 8.0905 | 7.9763 | 6.7529 | 6.9263 | |
| 7.4405 | 8.3592 | 4.9123 | 7.9347 | 7.2354 | 8.3249 | 8.6266 | 7.7799 | |
| 9.2766 | 7.7288 | 5.9919 | 7.8077 | 8.2141 | 4.8026 | 8.005 | 8.1395 | |
| 9.0239 | 5.7958 | 8.1572 | 6.5989 | 7.1179 | 8.8913 | 7.1106 | 8.9095 | |
| 9.1626 | 6.4346 | 6.6685 | 8.0307 | 7.6071 | 8.9002 | 8.4448 | 7.4473 | |
| 7.1823 | 8.6425 | 3.1008 | 7.6612 | 4.0412 | 3.2986 | 3.3061 | 3.7059 | |
| 1.6194 | 3.4809 | 3.6327 | 5.929 | 2.8267 | 3.756 | 3.6421 | 5.0596 | |
| 3.9403 | 3.2589 | 5.359 | 3.6243 | 3.4632 | 4.8591 | 3.1906 | 5.6551 | |
| 3.4387 | 4.0266 | | | | | | | |
| NKAPL | 0.5526 | 2.2256 | 1.9468 | 9.13E-01 | | 1.39E+00 | | |
| 1.4999 | 1.1171 | 0.7652 | 1.0281 | 1.3775 | 2.0628 | 0.4395 | 2.206 | |
| 1.4442 | 1.6582 | 0.7772 | 0.3755 | 1.6493 | 0.482 | 2.0289 | 2.6374 | |
| 1.3145 | 2.3554 | 1.3857 | 0 | 0.7334 | 0.9642 | 2.2837 | 0.8184 | 0 |
| | 0.6819 | 0.9556 | 0.8977 | 1.7115 | 1.928 | 2.1741 | 2.2575 | |
| 1.4585 | 0 | 0 | 0.6741 | 1.2792 | 0.8339 | 1.5081 | 2.7455 | |
| 1.0956 | 2.4746 | 0.5608 | 0.6343 | 2.3708 | 2.7715 | 0.5715 | 1.4031 | |
| 0.9583 | 1.6085 | 1.9254 | 0 | 1.1331 | 2.3036 | 3.0801 | 1.6333 | |
| 0.9537 | 2.4919 | 1.7486 | 3.1877 | 0.8237 | 2.2486 | 1.711 | 1.5048 | |
| 2.0686 | 0.9714 | 0.7073 | 3.2356 | 2.1886 | 1.8644 | 2.0776 | 0.5376 | |
| 1.0433 | 1.2163 | 1.8648 | 1.7639 | 1.6676 | 3.5917 | 2.4164 | 1.209 | |
| 0.8845 | 2.7167 | 1.418 | 1.1625 | 0.5352 | 2.8049 | 1.7606 | 1.8753 | |
| 1.6282 | 0.5426 | 2.3284 | 1.1907 | 0.6215 | 0 | 1.585 | 0.8924 | 0 |
| | 0.5276 | 1.7841 | 1.1815 | 1.6324 | 1.0794 | 0.5886 | 1.3885 | |
| 0.8239 | 2.0458 | 1.0447 | 0.7843 | 1.1727 | 0 | 0 | 0 | 0 |
| | 3.7148 | 1.9254 | 4.8598 | 2.5337 | 0.601 | 1.9771 | 1.0973 | 0 |
| | 0.8022 | 0.6735 | 0.5814 | 1.224 | 3.5631 | 1.9723 | 0.5414 | |
| 1.0019 | 0 | 1.9388 | 0.6894 | 1.0225 | 1.9005 | 1.0317 | 2.3902 | |
| 1.3167 | 0.7182 | 1.0531 | 1.1696 | 2.7703 | 0.9722 | 0.9131 | 0 | |
| 1.298 | 1.2195 | 1.7114 | 1.0565 | 0 | 1.3669 | 1.3803 | 3.0248 | |
| 1.5234 | 1.5061 | 0 | 1.4215 | 1.2218 | 2.0881 | 1.315 | 2.4374 | |
| 1.1063 | 0.5707 | 2.2621 | 0.5556 | 2.1288 | 1.768 | 0.8682 | 0 | 0 |
| | 1.4718 | 2.2618 | 0 | 3.9323 | 3.0897 | 4.423 | 2.4383 | |
| 4.4632 | 4.2535 | 5.2199 | 5.071 | 4.3972 | 4.1163 | 5.5022 | 4.7315 | |
| 4.6638 | 3.8282 | 1.609 | 4.2189 | 4.7394 | 3.9326 | 5.4988 | 4.2762 | |
| 4.3982 | 4.2082 | 5.0319 | 4.3212 | | | | | |
| KCTD8 | 0.9511 | 0.6896 | 0.4935 | 5.28E-01 | | 9.82E-01 | | |
| 1.716 | 1.56 | 0.4327 | 1.45 | 0.4849 | 1.9354 | 0.4395 | 0 | |
| 1.4442 | 1.8857 | 0.7772 | 0.3755 | 0.6175 | 1.131 | 0.5262 | 0 | |
| 0.4561 | 2.3554 | 0 | 0 | 0 | 0.9642 | 0 | 1.101 | 0 |
| | 0.6819 | 1.7435 | 1.2859 | 0.9336 | 0 | 0 | 0 | |
| 0.4326 | 0.9285 | 0 | 1.758 | 1.2792 | 1.359 | 0 | 0.7768 | |
| 0.6494 | 1.8714 | 0.9635 | 1.4095 | 2.117 | 3.406 | 1.2977 | 1.4031 | |
| 1.529 | 0 | 1.3532 | 1.0195 | 1.1331 | 1.3082 | 2.9862 | 0 | |
| 1.1694 | 0 | 1.569 | 2.8697 | 0.4698 | 1.5242 | 2.0646 | 2.7035 | |
| 2.8855 | 0.5659 | 0 | 3.8679 | 1.1284 | 1.6785 | 0 | 0 | 0 |
| | 3.4785 | 2.434 | 2.3201 | 2.2981 | 2.0255 | 0 | 0 | |

| | | | | | | | | |
|---|---|---|---|---|---|---|---|---|
| 1.986 | 0.9478 | 0 | 2.6706 | 0 | 2.1133 | 0.8449 | 1.8753 | |
| 2.6388 | 1.7151 | 0.5877 | 1.1907 | 0.6215 | 1.3631 | 0 | 0 | |
| 1.0399 | 0.9131 | 0.4321 | 1.6868 | 1.8581 | 6.0184 | 0.5886 | 2.0828 | 0.47 |
| | 2.0458 | 2.7753 | 0 | 0.9567 | 0 | 2.2429 | 1.2511 | |
| 0.8107 | 0 | 2.298 | 6.6448 | 4.0599 | 0.601 | 0.5748 | 0 | |
| 2.0686 | 0 | 0 | 0.5814 | 0.7381 | 3.6131 | 0 | 2.2151 | |
| 1.0019 | 0 | 1.2732 | 1.1539 | 0.6 | 0 | 0.6063 | 0 | |
| 0.9957 | 0.4033 | 0.7796 | 1.1696 | 2.2138 | 1.5478 | 1.8676 | 1.2521 | 0 |
| | 0.529 | 1.4956 | 2.5508 | 0 | 1.7522 | 2.5385 | 2.6986 | |
| 0.954 | 1.1551 | 0.91 | 1.4215 | 0.7366 | 1.3929 | 1.315 | 0 | 0 |
| | 0 | 0.5078 | 0.9557 | 0 | 3.1545 | 0.4985 | 0 | |
| 2.2195 | 0.3736 | 1.9827 | 0 | 5.7079 | 5.3725 | 5.126 | 1.0738 | |
| 5.3161 | 5.4976 | 3.7479 | 5.0341 | 4.5734 | 6.3381 | 5.2733 | 4.3633 | |
| 6.4338 | 5.885 | 1.018 | 5.2354 | 6.0789 | 7.6158 | 6.7258 | 2.4839 | |
| 5.7184 | 5.9688 | 3.364 | 6.8447 | | | | | |
| ERCC6L | 7.1727 | 6.9491 | 6.5608 | 8.1299 | 8.4894 | 6.3877 | 8.097 | |
| 7.8701 | 8.4348 | 8.0438 | 7.1123 | 7.1857 | 7.6871 | 4.9991 | 7.1685 | |
| 7.1888 | 7.7684 | 6.5538 | 6.558 | 5.9378 | 7.411 | 9.2976 | 7.2213 | |
| 5.1892 | 7.8 | 7.5301 | 7.5718 | 8.0497 | 6.4219 | 9.683 | 8.3518 | |
| 6.159 | 7.7073 | 7.6525 | 6.6673 | 7.584 | 7.0056 | 7.7363 | 7.0042 | |
| 6.8115 | 7.6773 | 2.4952 | 6.52 | 6.8452 | 7.3976 | 7.6801 | 6.9638 | |
| 7.7845 | 6.4903 | 6.7012 | 8.1919 | 7.4675 | 7.4808 | 8.1152 | 8.1823 | |
| 7.2132 | 5.9329 | 8.1366 | 4.3705 | 7.408 | 7.7905 | 7.1355 | 6.8193 | |
| 5.7152 | 6.552 | 6.1114 | 8.6579 | 4.4837 | 4.6017 | 7.9453 | 6.8851 | |
| 8.4971 | 4.8825 | 8.1219 | 6.3497 | 7.2868 | 7.9384 | 6.9427 | 6.9603 | |
| 7.8288 | 8.4079 | 6.1067 | 5.1203 | 8.4893 | 7.5286 | 8.0567 | 7.3372 | |
| 6.647 | 7.4598 | 7.8315 | 7.6999 | 8.4922 | 7.5354 | 7.4362 | 7.7675 | |
| 7.5473 | 7.2715 | 7.1175 | 5.7248 | 6.375 | 8.2845 | 8.0002 | 7.1794 | |
| 7.9391 | 8.8348 | 7.7776 | 7.8751 | 7.2398 | 7.6245 | 7.0053 | 7.9105 | |
| 7.753 | 7.1031 | 7.5024 | 7.571 | 7.1491 | 7.3625 | 7.4405 | 5.1649 | |
| 7.7241 | 5.8813 | 6.8577 | 7.8835 | 5.3451 | 6.356 | 7.4199 | 7.732 | |
| 6.6743 | 6.8348 | 6.9217 | 5.4686 | 6.4712 | 6.9589 | 7.9375 | 7.4592 | |
| 7.8687 | 7.4809 | 8.0944 | 7.8997 | 6.7696 | 6.7776 | 6.8115 | 6.2988 | |
| 7.4787 | 7.8302 | 7.3578 | 7.5976 | 7.9812 | 8.2152 | 7.7166 | 8.7634 | |
| 7.7379 | 7.6062 | 4.6244 | 7.2081 | 8.2691 | 5.1757 | 7.0314 | 6.735 | |
| 7.8511 | 6.0816 | 8.0343 | 6.7151 | 7.5622 | 7.8418 | 7.2966 | 7.9398 | |
| 8.2435 | 5.8773 | 7.9013 | 7.5032 | 7.4007 | 7.482 | 7.3576 | 7.3798 | |
| 6.9658 | 6.1881 | 3.3098 | 6.7545 | 4.5203 | 3.1268 | 3.2362 | 3.2379 | |
| 2.3632 | 2.9592 | 2.5892 | 5.7459 | 2.2864 | 2.5596 | 4.0144 | 4.5101 | |
| 3.8406 | 4.0632 | 5.0642 | 4.6634 | 3.052 | 4.0942 | 4.2246 | 5.4825 | |
| 2.4262 | 4.7467 | | | | | | | |
| KIAA0101 | | 10.6174 | 7.9325 | 8.2049 | 11.3781 | 8.601 | 8.5851 | |
| 8.9619 | 9.4646 | 9.8407 | 9.2552 | 8.1668 | 9.0426 | 9.8288 | 8.4599 | |
| 8.6377 | 10.398 | 9.2761 | 8.9026 | 8.7582 | 8.9585 | 11.2851 | 11.0319 | |
| 9.6828 | 8.3272 | 9.8459 | 9.8525 | 9.5661 | 9.6711 | 7.4038 | 8.4145 | |
| 11.1822 | 7.8789 | 10.0568 | 11.1199 | 8.0405 | 10.7053 | 10.0334 | 9.7606 | |
| 10.5625 | 8.0889 | 9.4443 | 9.7309 | 8.9323 | 8.6939 | 8.6859 | 8.3941 | |
| 8.4577 | 10.5511 | 8.5774 | 8.0413 | 8.4958 | 9.6124 | 9.7721 | 8.9922 | |
| 8.6754 | 8.1992 | 8.1047 | 8.9857 | 6.1573 | 11.3024 | 9.4925 | 9.3642 | |
| 9.4576 | 8.0303 | 9.7428 | 8.8714 | 7.8211 | 7.049 | 7.5581 | 10.0051 | |
| 9.5064 | 10.4533 | 7.5805 | 11.425 | 9.2163 | 8.3596 | 8.0532 | 10.3154 | |
| 8.1442 | 9.7382 | 9.5349 | 7.6529 | 8.4252 | 10.8496 | 8.8997 | 8.4231 | |
| 10.922 | 8.881 | 8.4726 | 7.7284 | 12.7364 | 11.8587 | 11.2624 | 9.2508 | |
| 8.9435 | 11.1911 | 10.3371 | 9.496 | 6.8939 | 7.6221 | 10.9807 | 8.8713 | |

| | | | | | | | | |
|---|---|---|---|---|---|---|---|---|
| 9.1156 | 8.8481 | 9.6905 | 8.9347 | 9.7692 | 10.3724 | 8.2201 | 9.7171 | |
| 9.6117 | 8.1266 | 9.2418 | 9.6758 | 11.9241 | 9.7764 | 9.7772 | 7.4405 | |
| 6.8981 | 9.3936 | 5.4591 | 8.8319 | 10.3769 | 8.9609 | 8.6247 | 10.0491 | |
| 9.9009 | 8.6097 | 8.3794 | 9.51 | 7.7335 | 9.6858 | 8.1602 | 8.5642 | |
| 10.221 | 7.9516 | 9.3245 | 9.5485 | 11.5314 | 8.6719 | 10.499 | 9.4133 | |
| 9.1717 | 8.3582 | 9.731 | 9.3301 | 7.4118 | 10.9975 | 10.0065 | 9.5992 | |
| 10.4245 | 10.9893 | 8.5348 | 7.2149 | 10.7153 | 9.278 | 7.5481 | 10.1688 | |
| 10.379 | 8.6139 | 8.0032 | 8.2003 | 8.904 | 8.5546 | 10.0968 | 9.6695 | |
| 10.1428 | 10.474 | 8.5541 | 9.7727 | 8.2501 | 9.8076 | 9.7097 | 8.7268 | |
| 9.2748 | 9.3209 | 10.2392 | 4.0917 | 9.2726 | 6.3276 | 5.0463 | 4.4007 | |
| 4.4781 | 1.026 | 3.9449 | 4.0048 | 8.4598 | 3.1304 | 3.5703 | 5.1665 | |
| 7.0121 | 5.6498 | 5.6316 | 6.3263 | 4.1183 | 0.5418 | 6.1369 | 4.9152 | |
| 8.1839 | 2.6934 | 4.7467 | | | | | | |
| ADH1B | 0.9511 | 0.6896 | 0.8605 | 4.15E+00 | | 4.06E-01 | | 0 |
| | 5.3068 | 1.0352 | 1.7761 | 0.8472 | 0 | 1.6493 | 2.5775 | |
| 1.4442 | 0.6221 | 7.5326 | 1.7564 | 0.6175 | 4.0067 | 2.0289 | 0 | |
| 2.3476 | 2.4936 | 2.7323 | 0 | 1.5789 | 4.9299 | 1.1952 | 1.3374 | 0 |
| | 0.6819 | 1.9329 | 1.2859 | 0.541 | 2.0552 | 3.1062 | 0 | |
| 3.4316 | 2.4638 | 0 | 1.1317 | 4.0921 | 1.359 | 0 | 0.7768 | |
| 1.4359 | 2.2625 | 1.755 | 3.0318 | 4.8557 | 7.8796 | 1.9697 | 3.3286 | |
| 2.2545 | 0.5968 | 3.4933 | 4.5606 | 2.8806 | 1.5697 | 5.4109 | 0 | |
| 2.9765 | 2.2328 | 3.2112 | 5.5673 | 1.1076 | 1.9314 | 6.7592 | 3.973 | |
| 4.0393 | 0.9714 | 2.354 | 10.2425 | 4.5201 | 7.1303 | 1.6542 | 1.2355 | |
| 1.3738 | 6.6914 | 0.5265 | 5.7037 | 1.2055 | 7.0692 | 5.4578 | 2.4256 | |
| 6.4197 | 1.2591 | 0 | 5.7171 | 0.9246 | 4.1937 | 4.4397 | 5.2759 | |
| 5.1781 | 1.7151 | 3.8149 | 2.1442 | 2.081 | 5.5422 | 0 | 3.3205 | 0 |
| | 0.5276 | 2.7941 | 3.4368 | 0 | 1.3128 | 0.5886 | 1.8863 | |
| 2.4909 | 1.4793 | 6.1742 | 0.7843 | 3.1441 | 2.1813 | 4.1912 | 4.6126 | 0 |
| | 5.5851 | 0.6413 | 8.5644 | 10.5823 | 2.0359 | 3.1427 | 5.9131 | 0 |
| | 0 | 2.5258 | 1.5778 | 0.7381 | 10.9343 | 1.9723 | 2.2151 | |
| 1.0019 | 1.7261 | 1.2732 | 1.1539 | 1.6149 | 4.6782 | 1.36 | 0 | |
| 0.9957 | 2.284 | 1.2829 | 1.1696 | 4.1489 | 5.3145 | 4.2558 | 0.5466 | |
| 0.4486 | 1.4707 | 2.349 | 0 | 3.2253 | 1.7522 | 5.4156 | 7.9644 | |
| 1.2666 | 4.6747 | 0.5257 | 2.256 | 1.8738 | 4.396 | 1.315 | 1.1775 | |
| 1.725 | 1.2961 | 1.4263 | 3.9158 | 1.7696 | 6.4416 | 2.6702 | 5.0585 | 0 |
| | 2.4421 | 7.4769 | 1.799 | 8.9421 | 4.923 | 9.159 | 7.4332 | |
| 9.0144 | 10.2757 | 13.6153 | 12.8814 | 13.1462 | 5.1743 | 14.2472 | 10.5757 | |
| 8.2181 | 9.2488 | 11.1773 | 12.7514 | 10.4053 | 9.0309 | 10.1796 | 12.9623 | |
| 9.4227 | 7.8756 | 13.8146 | 11.3513 | | | | | |
| HMGB3 | 11.7613 | 9.2296 | 10.3091 | 1.17E+01 | | 1.03E+01 | | |
| 10.0904 | 11.2326 | 11.8814 | 11.4418 | 11.722 | 11.3077 | 11.3555 | 11.4546 | |
| 10.7842 | 12.0575 | 11.573 | 11.404 | 11.0324 | 11.8043 | 10.5971 | 10.9725 | |
| 12.276 | 11.9563 | 10.8839 | 11.504 | 11.1581 | 11.2075 | 11.137 | 10.96 | |
| 10.5807 | 11.4367 | 10.6138 | 11.167 | 11.8191 | 11.3976 | 11.0617 | 11.3997 | |
| 11.2129 | 11.3848 | 12.8218 | 10.4555 | 11.9981 | 10.4482 | 10.6712 | 10.8791 | |
| 11.265 | 11.7119 | 11.6609 | 11.4297 | 9.765 | 11.4219 | 10.7624 | 11.0506 | |
| 10.7799 | 11.6279 | 10.7178 | 10.4608 | 12.3955 | 10.3325 | 11.6946 | 11.0869 | |
| 11.4765 | 11.4992 | 10.7473 | 10.3903 | 9.7767 | 11.8119 | 11.5219 | 9.8045 | |
| 11.1307 | 11.8223 | 12.1015 | 10.4098 | 12.8604 | 11.4635 | 11.4247 | 12.3629 | |
| 11.3778 | 11.0347 | 12.5544 | 12.4836 | 11.698 | 10.9911 | 11.6566 | 12.0342 | |
| 12.2039 | 10.4435 | 12.3435 | 11.0335 | 11.1897 | 12.2945 | 12.7049 | 11.542 | |
| 11.3198 | 11.6873 | 11.5159 | 10.9069 | 11.3519 | 12.3674 | 10.769 | 11.0001 | |
| 11.3612 | 11.0904 | 11.1806 | 12.3081 | 10.9559 | 11.4419 | 11.5861 | 10.6116 | |
| 10.9327 | 11.1766 | 12.1006 | 12.0082 | 10.5517 | 11.6524 | 11.3717 | 12.1866 | |

| | | | | | | | |
|---|---|---|---|---|---|---|---|
| 11.2432 | 11.8808 | 12.0042 | 9.3681 | 10.5851 | 13.1119 | 11.3592 | 11.1639 |
| 11.0797 | 11.6603 | 10.9449 | 10.8249 | 11.5707 | 10.0397 | 13.2192 | 10.7963 |
| 10.9861 | 11.4024 | 11.9004 | 11.6549 | 10.9894 | 11.4766 | 10.9313 | 11.4862 |
| 11.5326 | 10.7102 | 11.8524 | 11.1992 | 9.8662 | 10.108 | 12.081 | 12.269 |
| 11.6292 | 12.4702 | 12.0383 | 11.5005 | 10.6555 | 11.8088 | 12.0807 | 9.7135 |
| 11.4194 | 11.1203 | 11.7248 | 10.7178 | 11.3132 | 11.5387 | 10.9629 | 11.5302 |
| 11.5084 | 11.5444 | 11.1015 | 10.8106 | 11.5808 | 11.0708 | 11.0798 | 11.3028 |
| 13.06 | 10.9955 | 10.8849 | 12.0071 | 9.9354 | 10.8757 | 9.8008 | 9.8632 |
| 8.9476 | 9.43 | 7.8127 | 7.0976 | 7.3602 | 10.0748 | 7.414 | 7.7863 |
| 8.9708 | 9.269 | 9.7654 | 9.2552 | 8.8834 | 8.6593 | 7.642 | 9.0802 |
| 8.8017 | 10.0991 | 7.6363 | 8.9004 | | | | |
| DEPDC1 | 8.6557 | 8.1473 | 7.4759 | 8.57E+00 | | 9.46E+00 | |
| 8.5885 | 8.5362 | 9.131 | 9.6247 | 8.8544 | 8.4123 | 8.0818 | 8.2333 |
| 6.5799 | 7.6237 | 8.1767 | 9.4929 | 8.4636 | 7.0477 | 7.1302 | 8.0792 |
| 9.5374 | 7.505 | 5.8032 | 8.8033 | 9.589 | 8.4136 | 9.0771 | 7.8671 |
| 9.3759 | 8.399 | 5.8421 | 9.0617 | 8.7697 | 8.8792 | 9.0696 | 7.4598 |
| 9.4916 | 6.5131 | 7.1143 | 9.0349 | 9.9175 | 7.3896 | 8.837 | 8.165 |
| 7.6761 | 6.5261 | 8.5319 | 7.6971 | 6.8805 | 9.0063 | 8.3858 | 9.7287 |
| 8.7308 | 8.6916 | 8.1337 | 7.0384 | 8.8431 | 4.5928 | 8.6589 | 8.7072 |
| 8.4402 | 7.1076 | 7.6556 | 8.0985 | 6.421 | 6.9906 | 4.9335 | 5.0648 |
| 8.3526 | 7.8642 | 9.6087 | 5.3855 | 9.2318 | 6.9825 | 6.9782 | 8.5573 |
| 7.4901 | 7.6372 | 9.3485 | 8.8834 | 7.3521 | 6.2584 | 9.0902 | 8.745 |
| 7.7628 | 8.2734 | 7.2001 | 8.5445 | 9.1537 | 8.1519 | 9.1834 | 10.2744 |
| 8.3434 | 8.8031 | 8.173 | 8.2985 | 8.2791 | 7.1389 | 7.1189 | 10.3398 |
| 8.7151 | 8.3986 | 8.7368 | 9.375 | 8.4367 | 8.9883 | 7.4119 | 8.9887 |
| 7.5966 | 8.9088 | 9.6587 | 6.9848 | 8.6604 | 8.3578 | 8.0173 | 9.1399 |
| 8.1138 | 5.7568 | 8.2469 | 0 | 7.7146 | 8.8306 | 7.1091 | 6.6043 |
| 8.4908 | 8.7917 | 6.9279 | 7.4851 | 8.2172 | 5.929 | 7.2761 | 7.2279 |
| 7.9167 | 8.7291 | 9.3379 | 8.7309 | 8.7276 | 9.1199 | 7.6362 | 7.4895 |
| 7.2345 | 6.7122 | 7.9155 | 8.3989 | 7.4326 | 8.6164 | 8.7144 | 9.2774 |
| 8.9454 | 9.2887 | 7.8531 | 7.4186 | 5.3211 | 8.2109 | 9.6206 | 5.5752 |
| 8.6415 | 8.2828 | 9.6417 | 7.2486 | 8.7371 | 7.2397 | 8.2809 | 8.4944 |
| 7.9186 | 9.551 | 9.0186 | 6.865 | 8.0504 | 8.3912 | 8.1164 | 8.3409 |
| 8.5511 | 8.1597 | 8.0591 | 7.0558 | 1.8777 | 8.2137 | 4.5203 | 3.5232 |
| 3.4365 | 3.0408 | 2.8515 | 3.5346 | 3.8307 | 6.6303 | 2.4416 | 3.3571 |
| 3.6943 | 3.7595 | 4.2433 | 4.1037 | 6.4908 | 2.3776 | 1.4974 | 5.2559 |
| 3.2643 | 5.8536 | 2.0979 | 2.7661 | | | | |
| FAM64A | 9.7446 | 6.9202 | 6.2719 | 8.681 | 7.8192 | 8.2063 | 8.0742 |
| 8.3731 | 8.2253 | 8.4085 | 7.6876 | 7.4929 | 7.1213 | 6.8186 | 8.734 |
| 8.6021 | 8.9715 | 8.6536 | 6.1937 | 6.5322 | 9.1802 | 10.305 | 7.6961 |
| 6.6466 | 8.5172 | 9.5073 | 8.5221 | 8.8188 | 7.6567 | 8.9522 | 9.2123 |
| 6.2239 | 8.6178 | 8.3094 | 7.3519 | 8.5934 | 7.8594 | 8.6662 | 7.5052 |
| 9.0675 | 7.4797 | 9.9138 | 7.4229 | 7.8037 | 8.4621 | 8.6625 | 8.5716 |
| 8.4791 | 7.2767 | 6.126 | 8.6638 | 7.0942 | 7.4842 | 8.8447 | 10.0464 |
| 7.6837 | 7.0774 | 8.4894 | 4.9551 | 8.4151 | 7.4724 | 8.1542 | 8.1359 |
| 7.3872 | 6.3357 | 6.7551 | 7.8969 | 5.1706 | 4.8363 | 9.4559 | 8.6514 |
| 8.5924 | 5.1159 | 7.5257 | 8.268 | 7.1517 | 9.1964 | 7.9708 | 7.2214 |
| 10.4459 | 8.4113 | 9.3192 | 6.3804 | 9.3559 | 7.9079 | 7.087 | 8.2316 |
| 9.1151 | 8.2283 | 11.4765 | 7.8859 | 8.2608 | 10.8099 | 8.141 | 7.7246 |
| 7.9911 | 7.8889 | 8.1619 | 7.7576 | 7.4757 | 8.0896 | 7.9732 | 7.7958 |
| 7.2662 | 8.2664 | 8.1945 | 9.2621 | 8.1454 | 7.4261 | 7.715 | 8.4517 |
| 8.465 | 8.6189 | 8.6537 | 7.3699 | 7.401 | 7.2161 | 7.3566 | 8.6621 |
| 9.384 | 5.1146 | 7.0922 | 8.0699 | 7.1692 | 6.5701 | 8.1885 | 8.5223 |
| 6.1894 | 8.5798 | 8.2587 | 5.4133 | 7.7897 | 6.6412 | 8.0095 | 8.5857 |

| | | | | | | | |
|---|---|---|---|---|---|---|---|
| 8.4332 | 8.4031 | 7.796 | 9.4575 | 8.3778 | 7.5601 | 7.0999 | 6.815 |
| 7.3704 | 8.3573 | 5.4575 | 6.7517 | 8.2868 | 9.5175 | 9.4219 | 8.1853 |
| 9.5105 | 9.7911 | 6.1416 | 8.685 | 8.744 | 5.2609 | 8.6931 | 7.8787 |
| 9.4152 | 6.8938 | 8.1971 | 7.4331 | 9.3755 | 8.1672 | 7.8079 | 9.6923 |
| 9.2651 | 6.6922 | 8.4389 | 9.8503 | 7.6522 | 8.9889 | 8.6706 | 6.9401 |
| 6.5834 | 8.459 | 3.3733 | 8.5882 | 4.2063 | 3.5232 | 3.1627 | 3.4648 |
| 1.6194 | 2.6047 | 3.13 | 7.9002 | 2.9352 | 3.1069 | 3.1391 | 4.4239 |
| 4.1631 | 4.1431 | 5.4453 | 2.4897 | 3.5778 | 6.2717 | 3.5849 | 6.0567 |
| 2.5659 | 2.6496 | | | | | | |
| CDT1 | 10.9009 | 7.667 | 8.148 | 1.07E+01 | | 8.11E+00 | |
| 8.4837 | 8.5482 | 9.4048 | 8.1847 | 8.907 | 8.6741 | 9.2777 | 8.693 |
| 8.4809 | 8.2115 | 9.9329 | 9.3271 | 9.9266 | 8.0004 | 8.0503 | 10.6244 |
| 10.8555 | 9.5295 | 7.7624 | 9.2064 | 9.4901 | 9.7358 | 10.0338 | 8.4413 |
| 8.7491 | 11.1572 | 8.3046 | 9.7798 | 8.7831 | 7.8866 | 8.8746 | 9.305 |
| 9.7808 | 9.6167 | 10.8473 | 9.4369 | 9.9207 | 8.0398 | 8.9283 | 8.2039 |
| 9.3435 | 9.0666 | 9.1873 | 8.7165 | 7.7485 | 7.8542 | 8.6088 | 8.5703 |
| 10.2507 | 9.452 | 8.557 | 7.5503 | 8.499 | 6.4931 | 8.8087 | 9.5196 |
| 8.7006 | 10.3492 | 9.2479 | 8.4504 | 9.4136 | 7.1892 | 7.3275 | 6.8677 |
| 10.4311 | 9.9394 | 8.6469 | 6.7788 | 9.2669 | 9.9282 | 8.5327 | 8.235 |
| 10.1946 | 8.2251 | 10.3337 | 9.0426 | 10.1841 | 8.4175 | 10.3562 | 8.1268 |
| 9.039 | 9.6336 | 10.3765 | 9.533 | 8.2655 | 8.8767 | 10.5932 | 11.3682 |
| 7.5639 | 8.1847 | 10.1381 | 9.1333 | 9.3619 | 10.1054 | 10.0993 | 7.7195 |
| 8.5501 | 9.3745 | 7.9411 | 9.4818 | 9.8963 | 9.0247 | 8.6109 | 8.6827 |
| 9.8489 | 9.1229 | 9.4856 | 9.921 | 9.1425 | 8.6287 | 9.2494 | 9.3513 |
| 9.8659 | 10.7435 | 10.6489 | 8.117 | 8.7334 | 11.0062 | 8.1115 | 9.1453 |
| 9.5751 | 9.1956 | 7.773 | 8.9651 | 10.2273 | 7.2003 | 10.6617 | 7.7696 |
| 9.1202 | 9.7516 | 8.6357 | 9.1093 | 10.5315 | 11.2452 | 9.7084 | 10.1482 |
| 8.4931 | 10.0835 | 9.2256 | 8.554 | 7.7578 | 8.2077 | 9.5598 | 9.2848 |
| 9.4679 | 9.2557 | 10.5734 | 10.0606 | 8.4643 | 9.6203 | 9.6035 | 7.6062 |
| 10.614 | 9.5766 | 9.2594 | 8.1826 | 7.5965 | 8.7582 | 8.7764 | 9.8975 |
| 9.441 | 10.6554 | 10.1193 | 6.9982 | 8.132 | 9.1612 | 9.3728 | 10.2754 |
| 11.3149 | 9.1414 | 8.7337 | 11.5292 | 4.0535 | 9.0453 | 5.7649 | 6.5854 |
| 5.0771 | 4.0234 | 3.0451 | 4.3564 | 3.9632 | 7.7379 | 4.7863 | 4.64 |
| 5.9672 | 6.566 | 6.7428 | 5.1796 | 5.9956 | 5.1967 | 4.5998 | 7.689 |
| 6.2404 | 7.3996 | 3.82 | 6.3892 | | | | |
| NUSAP1 | 11.1515 | 8.5965 | 9.3259 | 10.8493 | 11.0512 | 10.2303 | 10.6816 |
| 9.9838 | 11.6076 | 10.0468 | 9.7448 | 10.3278 | 10.0813 | 9.0102 | 9.2945 |
| 10.4961 | 9.2147 | 10.126 | 9.1342 | 8.9938 | 9.839 | 10.9379 | 9.1645 |
| 7.9945 | 9.9977 | 11.4368 | 10.9279 | 10.6329 | 8.9841 | 10.3374 | 10.2974 |
| 8.4218 | 11.0102 | 10.3189 | 9.9889 | 10.1404 | 8.9443 | 11.2571 | 9.7254 |
| 9.0309 | 9.3381 | 10.7833 | 8.6013 | 10.0055 | 9.3958 | 9.4761 | 9.1138 |
| 11.2608 | 9.3365 | 8.9525 | 9.6565 | 10.0249 | 10.8681 | 11.1567 | 10.0548 |
| 9.3071 | 8.296 | 10.5141 | 7.2204 | 11.082 | 8.9453 | 10.3743 | 9.203 |
| 9.3269 | 9.5559 | 9.449 | 9.4197 | 7.1073 | 7.2097 | 10.3575 | 9.8195 |
| 11.3635 | 7.6886 | 10.5676 | 9.4746 | 8.5821 | 10.544 | 9.9527 | 9.4102 |
| 11.829 | 9.7368 | 9.5207 | 9.0105 | 11.003 | 9.14 | 9.6751 | 10.6812 |
| 10.1825 | 10.4957 | 10.2556 | 10.9237 | 10.6393 | 11.4315 | 9.1944 | 9.9461 |
| 11.0281 | 10.3896 | 10.3237 | 8.1578 | 9.5255 | 11.5453 | 9.7633 | 10.2061 |
| 10.6002 | 10.8477 | 10.457 | 10.8714 | 10.0932 | 9.5676 | 10.1958 | 9.8568 |
| 10.884 | 9.7882 | 9.6411 | 11.3204 | 9.9042 | 10.7792 | 9.9409 | 8.0198 |
| 10.3817 | 5.8813 | 9.2869 | 11.0289 | 9.4752 | 8.4618 | 10.6462 | 10.2866 |
| 9.553 | 9.4197 | 9.6182 | 8.3451 | 9.5666 | 9.2937 | 9.9968 | 10.3749 |
| 10.4605 | 10.5158 | 10.2283 | 10.4751 | 9.4356 | 9.7394 | 9.4196 | 8.9712 |
| 10.1921 | 11.0305 | 10.3225 | 8.9783 | 10.7702 | 11.3793 | 11.0059 | 10.8148 |

| | | | | | | | |
|---|---|---|---|---|---|---|---|
| 10.2088 | 10.0917 | 6.9963 | 11.1299 | 11.3773 | 7.7857 | 10.1397 | 10.1481 |
| 10.8867 | 9.3787 | 9.8781 | 9.5581 | 9.7846 | 10.0094 | 9.8721 | 10.7364 |
| 10.9259 | 8.7868 | 9.4832 | 9.6088 | 10.5311 | 10.6429 | 9.8667 | 9.8488 |
| 9.5005 | 9.0238 | 4.5691 | 10.2922 | 6.6691 | 5.9489 | 5.9612 | 5.7553 |
| 4.7499 | 6.0585 | 5.3625 | 9.2468 | 4.8431 | 4.2849 | 6.0395 | 7.6177 |
| 6.4017 | 6.5452 | 8.0853 | 5.4669 | 8.0143 | 7.5684 | 5.0874 | 8.683 |
| 4.5497 | 5.734 | | | | | | |
| KIF14 | 7.6791 | 8.5382 | 7.5718 | 8.61E+00 | | 9.89E+00 | |
| 8.3138 | 7.6136 | 7.6466 | 9.2304 | 8.4503 | 8.1236 | 8.1267 | 7.7937 |
| 6.2617 | 7.0025 | 7.3925 | 7.0424 | 7.2085 | 6.7188 | 7.5618 | 6.338 |
| 9.4549 | 6.9897 | 5.5782 | 8.1978 | 9.2085 | 8.3222 | 7.9148 | 7.7076 |
| 9.2909 | 7.2599 | 7.6784 | 9.7366 | 8.1682 | 7.5911 | 7.2373 | 7.2708 |
| 8.5445 | 7.338 | 5.9035 | 7.4797 | 9.7519 | 6.7314 | 9.0213 | 7.0392 |
| 7.6761 | 8.0804 | 8.4906 | 7.1534 | 6.9454 | 8.8175 | 7.6636 | 8.9781 |
| 7.6871 | 7.5973 | 7.3266 | 6.7249 | 8.9292 | 4.3002 | 8.6145 | 7.6465 |
| 8.9378 | 7.1363 | 6.3303 | 7.8869 | 6.6261 | 7.7443 | 4.7011 | 5.9316 |
| 8.0003 | 7.0511 | 9.3599 | 5.2199 | 8.7456 | 6.0476 | 7.4057 | 8.4644 |
| 6.6845 | 7.3123 | 8.8793 | 9.0503 | 6.762 | 6.304 | 8.02 | 7.5149 |
| 8.1369 | 8.3431 | 4.7356 | 7.6137 | 8.3982 | 8.6032 | 8.056 | 9.4326 |
| 8.2291 | 8.4279 | 7.5119 | 8.566 | 7.6261 | 5.8481 | 5.6439 | 9.7406 |
| 8.0756 | 8.7234 | 7.0128 | 8.8146 | 7.3181 | 8.9103 | 7.8119 | 8.2331 |
| 7.6108 | 8.3327 | 7.8723 | 5.373 | 8.7577 | 8.3237 | 7.2186 | 8.7328 |
| 6.284 | 6.8131 | 6.4736 | 1.5206 | 7.2737 | 8.2545 | 6.5621 | 5.8994 |
| 8.0212 | 8.3942 | 7.6672 | 6.8159 | 7.9629 | 5.8991 | 5.0933 | 7.5628 |
| 7.7249 | 8.5909 | 8.6318 | 8.2562 | 7.2093 | 7.9774 | 6.1388 | 7.4767 |
| 6.9161 | 6.169 | 7.166 | 8.595 | 7.5096 | 8.114 | 8.1159 | 8.9493 |
| 8.4163 | 8.9889 | 7.6326 | 8.8076 | 4.918 | 8.3553 | 9.4832 | 5.5585 |
| 7.8407 | 7.0475 | 8.4476 | 5.911 | 8.2454 | 7.4732 | 7.6324 | 7.2279 |
| 6.5248 | 7.3535 | 8.8795 | 7.3535 | 7.5582 | 8.5517 | 8.6968 | 7.5767 |
| 7.6291 | 8.1081 | 7.3521 | 5.6609 | 3.3098 | 7.7309 | 3.951 | 2.9316 |
| 2.3817 | 3.4648 | 1.026 | 3.0357 | 3.2033 | 6.4321 | 2.1125 | 1.6671 |
| 3.1391 | 5.5811 | 3.3542 | 3.8886 | 6.005 | 2.3776 | 2.0673 | 3.9299 |
| 3.031 | 6.0472 | 1.4008 | 1.3688 | | | | |
| ABCC9 | 3.8143 | 4.3653 | 5.2053 | 5.20E+00 | | 4.82E+00 | |
| 4.9619 | 4.6972 | 4.569 | 5.1695 | 3.2311 | 5.8891 | 3.6727 | 4.7168 |
| 5.3429 | 4.461 | 5.0182 | 4.5605 | 3.4081 | 4.6091 | 7.166 | 3.2258 |
| 4.8522 | 5.8091 | 4.608 | 2.472 | 5.576 | 5.6155 | 3.7046 | 4.0902 |
| 2.5857 | 4.1139 | 5.5555 | 4.6698 | 4.5135 | 3.9752 | 6.0052 | 4.4989 |
| 4.9079 | 4.9453 | 4.548 | 6.1311 | 4.8132 | 3.0077 | 5.2295 | 5.5657 |
| 5.7964 | 6.59 | 4.8356 | 5.2052 | 6.449 | 8.4986 | 5.17 | 5.9119 |
| 4.5039 | 3.4915 | 6.8892 | 4.3944 | 5.4967 | 4.8848 | 7.6869 | 3.7294 |
| 5.6627 | 5.4137 | 5.1223 | 7.2981 | 4.1016 | 5.0804 | 5.9314 | 6.2776 |
| 6.0319 | 2.9495 | 4.5905 | 8.1233 | 5.6333 | 5.5478 | 5.655 | 4.7888 |
| 2.7731 | 6.3134 | 6.4336 | 5.7147 | 3.7214 | 5.8611 | 5.1405 | 6.2601 |
| 5.9816 | 5.2362 | 1.418 | 5.4161 | 3.558 | 6.7829 | 4.811 | 6.0569 |
| 7.2035 | 6.1837 | 5.3117 | 4.4841 | 3.0001 | 4.6197 | 4.9069 | 6.3484 |
| 3.5311 | 5.4683 | 4.9227 | 4.8531 | 5.5927 | 5.7466 | 5.4537 | 5.6148 |
| 4.9012 | 4.5175 | 6.4445 | 2.4149 | 3.7258 | 3.8633 | 6.0313 | 6.6981 |
| 3.8654 | 0 | 3.5403 | 6.313 | 9.511 | 3.5671 | 5.8646 | 3.6966 |
| 6.1921 | 4.1179 | 5.8754 | 2.4506 | 4.3485 | 9.3857 | 4.2129 | 5.4227 |
| 4.396 | 4.4137 | 5.6405 | 5.4454 | 8.1426 | 5.1731 | 4.868 | 3.8863 |
| 4.8495 | 3.3264 | 5.7841 | 5.5743 | 7.5267 | 6.9923 | 5.1829 | 2.897 |
| 3.6212 | 4.6893 | 3.8272 | 4.1921 | 5.1047 | 4.2895 | 6.0672 | 7.2993 |
| 4.3374 | 3.4351 | 4.2159 | 4.687 | 2.8065 | 5.6839 | 6.2554 | 2.5964 |

| | | | | | | | | |
|---|---|---|---|---|---|---|---|---|
| 3.8337 | 5.4654 | 6.3427 | 5.2162 | 5.6062 | 6.994 | 4.8405 | 3.1828 | |
| 2.5115 | 4.3993 | 7.1049 | 1.5159 | 9.3004 | 7.2203 | 8.7279 | 6.3806 | |
| 10.0389 | 9.9656 | 11.3534 | 11.6221 | 10.9277 | 7.742 | 10.8747 | 10.0734 | |
| 9.5583 | 8.4108 | 8.2006 | 10.7827 | 10.4222 | 9.8859 | 9.907 | 9.2671 | |
| 9.8922 | 8.8752 | 10.0389 | 9.6845 | | | | | |
| MAMDC2 | 1.5194 | 5.0386 | 3.7702 | 3.012 | 7.3205 | 4.1628 | 6.304 | |
| 3.9393 | 4.4905 | 2.6757 | 5.0191 | 3.0838 | 3.8631 | 6.3857 | 4.6276 | |
| 2.1918 | 1.1305 | 7.3569 | 3.7755 | 3.2932 | 0.6062 | 4.356 | 3.8969 | |
| 3.1802 | 3.0135 | 0.7334 | 3.1057 | 1.1952 | 5.1215 | 2.1706 | 1.1431 | |
| 5.3876 | 3.9101 | 2.7898 | 3.9723 | 5.4701 | 5.1511 | 2.1688 | 1.4889 | 0 |
| | 4.4094 | 5.0335 | 2.6955 | 0.453 | 1.6507 | 3.7556 | 3.7985 | |
| 1.5895 | 5.9089 | 5.3413 | 8.6514 | 4.3876 | 4.9162 | 2.9261 | 2.1974 | |
| 4.4884 | 1.8352 | 5.4184 | 4.906 | 7.6906 | 1.9272 | 2.7272 | 1.511 | |
| 5.3341 | 6.227 | 2.1585 | 6.2467 | 4.6756 | 5.3512 | 4.8154 | 2.1251 | |
| 4.1461 | 8.5912 | 2.7931 | 6.8777 | 5.9957 | 4.0315 | 2.2368 | 6.575 | |
| 4.8003 | 5.5757 | 3.4611 | 6.7938 | 5.7874 | 5.6556 | 5.5964 | 2.4969 | |
| 0.6391 | 5.7058 | 3.2529 | 4.8782 | 2.6265 | 6.3477 | 6.1079 | 6.7406 | |
| 5.2933 | 1.4385 | 3.0001 | 2.5153 | 3.1699 | 3.2573 | 4.5671 | 1.6815 | |
| 4.4095 | 2.0603 | 0.6089 | 10.237 | 1.5923 | 4.1028 | 2.3885 | 6.5872 | |
| 4.4057 | 2.7607 | 3.3449 | 1.7472 | 3.0001 | 5.2318 | 4.3449 | 0 | |
| 0.6413 | 2.2444 | 9.5254 | 0 | 2.6341 | 2.6155 | 3.3403 | 1.1088 | 4.19 |
| | 2.7981 | 1.8766 | 9.6822 | 0 | 3.8284 | 3.0909 | 3.1454 | |
| 6.0648 | 2.2251 | 2.0337 | 4.5437 | 0.6063 | 2.0641 | 4.7729 | 2.8974 | |
| 4.5552 | 3.3986 | 6.9199 | 5.3478 | 3.6917 | 1.7238 | 2.2332 | 5.8279 | |
| 5.0176 | 1.8874 | 0 | 3.9902 | 5.7933 | 8.1373 | 2.0978 | 1.5061 | |
| 8.3016 | 4.0096 | 4.8167 | 5.9578 | 5.0549 | 2.4374 | 2.758 | 1.9676 | 1.18 |
| | 3.7744 | 3.1228 | 5.6059 | 2.7609 | 1.5946 | 2.638 | 4.6577 | |
| 6.7328 | 1.5159 | 10.2021 | 7.9817 | 9.6159 | 7.6824 | 11.2879 | 10.2933 | |
| 11.0507 | 11.9471 | 11.2789 | 9.3799 | 11.4186 | 11.2645 | 10.1269 | 8.824 | |
| 8.7849 | 11.0226 | 11.4623 | 10.9385 | 10.8313 | 10.1997 | 10.8238 | 9.3882 | |
| 11.589 | 9.9863 | | | | | | | |
| FANCI | 10.5988 | 10.3203 | 9.4567 | 11.2119 | 10.5989 | 9.5006 | 10.1258 | |
| 9.9902 | 10.8533 | 9.1136 | 9.8069 | 9.4729 | 10.2529 | 9.3897 | 10.0445 | |
| 10.5612 | 9.7365 | 9.395 | 9.2837 | 9.2434 | 9.1684 | 10.7746 | 10.1344 | |
| 8.6432 | 9.8006 | 10.6328 | 10.779 | 10.172 | 9.9582 | 9.9606 | 10.5839 | |
| 8.7121 | 10.9106 | 10.3751 | 9.1795 | 9.7548 | 9.3914 | 10.2082 | 9.9018 | |
| 10.3832 | 9.2611 | 10.5527 | 10.2981 | 9.5836 | 9.6101 | 9.9271 | 9.6908 | |
| 10.723 | 9.5971 | 9.3203 | 9.7017 | 10.1143 | 10.9308 | 10.5698 | 9.4212 | |
| 9.0969 | 8.1645 | 10.1856 | 7.9583 | 10.7963 | 9.6908 | 10.2566 | 9.9687 | |
| 9.8864 | 9.9829 | 9.4279 | 9.3745 | 8.1058 | 8.1541 | 10.1664 | 10.4329 | |
| 10.9986 | 8.4425 | 10.601 | 9.7851 | 9.3258 | 10.3171 | 10.3337 | 9.4782 | |
| 10.819 | 9.7739 | 9.9059 | 9.4275 | 10.8673 | 8.7525 | 9.5834 | 10.209 | |
| 10.5568 | 10.1083 | 9.5144 | 10.8391 | 10.6448 | 10.4205 | 10.0773 | 9.5824 | |
| 10.6922 | 10.1399 | 9.7331 | 10.4592 | 10.5987 | 11.1982 | 10.8003 | 10.1628 | |
| 9.7581 | 10.4756 | 10.0463 | 10.7628 | 9.8403 | 9.5897 | 10.0545 | 9.2816 | |
| 9.955 | 9.887 | 9.3642 | 10.1486 | 10.0341 | 10.0011 | 10.0838 | 9.1461 | |
| 10.5623 | 9.0242 | 9.6529 | 10.3771 | 9.6056 | 9.8802 | 10.4003 | 10.8395 | |
| 8.8815 | 9.4994 | 10.5854 | 8.5393 | 9.6196 | 9.5502 | 10.6717 | 10.454 | |
| 9.5841 | 10.3763 | 10.3547 | 10.9489 | 9.808 | 10.4596 | 9.6732 | 9.7817 | |
| 10.452 | 10.8304 | 9.7228 | 9.2536 | 10.428 | 10.4367 | 9.9444 | 10.2721 | |
| 11.4422 | 10.1442 | 8.838 | 10.5374 | 11.0489 | 8.5533 | 10.8375 | 9.891 | |
| 10.3598 | 8.9385 | 9.5446 | 9.6723 | 9.5161 | 9.2683 | 10.175 | 10.4848 | |
| 10.8109 | 8.9321 | 9.4527 | 11.0074 | 10.4464 | 10.8101 | 9.7624 | 10.2801 | |
| 9.9636 | 10.6535 | 7.3419 | 10.0585 | 7.8012 | 7.2631 | 7.4789 | 7.6113 | |

| | | | | | | | |
|---|---|---|---|---|---|---|---|
| 6.9518 | 7.3876 | 7.4908 | 8.8194 | 6.5425 | 7.1733 | 7.5247 | 8.4586 |
| 7.8401 | 7.3911 | 8.5748 | 7.1024 | 7.8986 | 7.8313 | 7.1589 | 9.3265 |
| 6.5945 | 7.6216 | | | | | | |
| MLF1IP | 10.1131 | 6.8754 | 8.6911 | 10.2928 | 9.2944 | 9.1273 | 8.6194 |
| 8.3675 | 9.191 | 8.6191 | 9.4742 | 9.1391 | 8.824 | 7.53 | 9.2528 |
| 9.7306 | 7.9044 | 8.0024 | 8.4444 | 8.5358 | 8.2406 | 9.866 | 9.5073 |
| 7.9198 | 9.8907 | 10.43 | 9.3297 | 9.5684 | 8.4737 | 9.4203 | 9.7459 |
| 6.8877 | 9.2762 | 9.6532 | 8.293 | 9.3207 | 10.2629 | 9.8385 | 9.4799 |
| 9.4016 | 8.9939 | 9.4307 | 8.5902 | 8.0695 | 8.2971 | 8.8484 | 8.1481 |
| 10.2699 | 8.6611 | 8.7614 | 8.8015 | 8.5425 | 10.2505 | 9.4024 | 9.2738 |
| 8.8802 | 7.1731 | 9.2455 | 7.0696 | 8.3088 | 8.3926 | 8.9573 | 9.5308 |
| 7.3354 | 8.9596 | 8.7804 | 9.4064 | 6.589 | 7.888 | 8.9057 | 9.5529 |
| 8.3467 | 8.0322 | 9.9431 | 8.7838 | 9.6693 | 8.3829 | 9.6912 | 9.5711 |
| 10.5291 | 8.5821 | 10.166 | 8.674 | 9.6902 | 8.9002 | 8.7665 | 9.8018 |
| 9.5502 | 9.6689 | 8.9683 | 10.1092 | 10.1203 | 10.6959 | 9.5506 | 8.9231 |
| 8.6447 | 9.1825 | 8.8641 | 7.7784 | 9.2746 | 10.4058 | 9.2212 | 8.7198 |
| 8.2668 | 9.559 | 10.4388 | 9.2335 | 9.8915 | 8.6441 | 9.4285 | 8.2672 |
| 9.4275 | 9.0647 | 9.3147 | 9.6033 | 9.4988 | 9.0777 | 8.8417 | 7.6877 |
| 10.0622 | 6.0257 | 7.8997 | 9.6885 | 8.6269 | 8.7206 | 9.8396 | 9.3542 |
| 9.2942 | 8.7009 | 9.6187 | 7.669 | 8.9288 | 8.5053 | 8.9408 | 9.0263 |
| 8.9118 | 8.5683 | 9.6997 | 10.5852 | 9.3358 | 8.9383 | 9.4145 | 9.3916 |
| 8.5744 | 9.98 | 8.7035 | 8.333 | 9.9019 | 10.1879 | 8.9116 | 9.4099 |
| 9.6278 | 9.825 | 6.8974 | 9.9134 | 10.0827 | 8.1806 | 9.6636 | 8.5472 |
| 10.029 | 8.2339 | 8.7019 | 8.058 | 9.5857 | 9.409 | 9.187 | 9.2757 |
| 10.1949 | 8.4791 | 8.7811 | 8.2647 | 8.4587 | 7.8477 | 10.2615 | 8.8428 |
| 8.9227 | 8.9632 | 5.3263 | 9.2269 | 6.4013 | 6.2896 | 6.526 | 4.9667 |
| 4.8856 | 6.0491 | 5.5565 | 7.7653 | 4.7344 | 4.9722 | 6.0254 | 7.1618 |
| 6.9158 | 6.641 | 6.0411 | 5.3038 | 5.7149 | 7.0269 | 6.1267 | 8.2576 |
| 4.9139 | 5.853 | | | | | | |
| FAM72B | 7.4364 | 6.9634 | 7.4356 | 8.2352 | 8.966 | 7.351 | 7.9905 |
| 8.3957 | 8.1446 | 9.1697 | 7.7646 | 7.5071 | 7.0043 | 7.1158 | 7.2576 |
| 7.4047 | 7.6834 | 7.8195 | 6.6406 | 7.2138 | 8.0454 | 9.9981 | 7.0967 |
| 5.3114 | 7.7967 | 8.5301 | 8.4708 | 8.464 | 7.9913 | 8.343 | 8.3677 |
| 5.8539 | 8.4522 | 8.5154 | 7.1494 | 7.8153 | 7.5077 | 8.046 | 7.4011 |
| 7.5581 | 6.9506 | 8.2149 | 6.4247 | 7.7869 | 7.809 | 6.9385 | 7.1719 |
| 7.7938 | 6.7785 | 6.5869 | 7.9271 | 8.0013 | 8.8269 | 7.478 | 7.4862 |
| 7.2402 | 6.188 | 7.9543 | 4.1077 | 7.9014 | 8.565 | 7.2073 | 8.1548 |
| 6.8267 | 7.5303 | 6.2709 | 7.9956 | 4.7011 | 6.1465 | 8.2951 | 7.4423 |
| 9.2307 | 5.451 | 8.3304 | 6.8891 | 7.6982 | 8.0895 | 7.0214 | 7.2042 |
| 9.4179 | 8.648 | 6.884 | 6.9089 | 8.445 | 7.1207 | 6.9054 | 8.0814 |
| 6.9368 | 6.9303 | 7.6435 | 8.8747 | 7.5057 | 9.3372 | 6.8053 | 8.8104 |
| 8.1374 | 6.9206 | 7.3918 | 6.5986 | 7.9366 | 8.3709 | 8.0355 | 7.4975 |
| 7.7531 | 8.152 | 7.7982 | 9.0339 | 7.3553 | 7.1142 | 6.9298 | 8.1877 |
| 8.2577 | 7.0173 | 7.7868 | 8.1786 | 7.4286 | 8.646 | 7.9475 | 6.9432 |
| 8.0231 | 5.1146 | 7.0406 | 8.727 | 6.6561 | 6.4717 | 7.2658 | 7.31 |
| 6.3559 | 6.1693 | 7.4038 | 6.4315 | 7.4308 | 6.398 | 7.8304 | 7.4131 |
| 8.1318 | 7.6154 | 8.7995 | 9.0335 | 6.2617 | 7.5232 | 7.606 | 6.5627 |
| 7.8112 | 7.3562 | 7.7626 | 7.0318 | 7.5996 | 8.4763 | 7.2408 | 9.0613 |
| 7.512 | 8.0113 | 4.918 | 7.2158 | 8.4333 | 5.6081 | 8.2038 | 7.443 |
| 9.5867 | 6.997 | 7.3942 | 6.8364 | 6.9544 | 7.6692 | 6.9914 | 7.7545 |
| 7.1701 | 6.0912 | 7.848 | 7.5726 | 7.5856 | 8.3409 | 8.1346 | 7.5958 |
| 6.6576 | 6.0996 | 4.0142 | 7.1852 | 4.7516 | 3.8887 | 4.0798 | 4.0234 |
| 2.8515 | 4.9771 | 4.1601 | 6.2375 | 3.9454 | 3.9714 | 4.4069 | 6.1358 |
| 3.9403 | 3.69 | 5.7465 | 3.2317 | 3.9194 | 4.9261 | 4.0688 | 6.2094 |

| | | | | | | | |
|---|---|---|---|---|---|---|---|
| 4.166 | 2.5229 | | | | | | |
| ZCCHC24 | 6.4408 | 7.9069 | 6.7366 | 8.5663 | 6.5026 | 8.5038 | 8.1846 |
| 7.7627 | 7.2614 | 7.0665 | 8.3308 | 7.0411 | 6.4174 | 8.0649 | 8.2245 |
| 7.9246 | 6.7711 | 7.746 | 7.4622 | 7.305 | 7.7348 | 6.1923 | 8.7712 |
| 8.5328 | 7.2051 | 10.5573 | 8.1324 | 5.6192 | 6.5971 | 6.6375 | 7.4364 |
| 8.4589 | 6.8264 | 7.3201 | 7.1423 | 8.1064 | 8.2534 | 7.8324 | 6.8432 |
| 7.9047 | 6.4877 | 9.9334 | 6.8388 | 7.4851 | 7.6894 | 8.9059 | 9.5566 |
| 6.2847 | 8.7502 | 8.6037 | 9.8671 | 7.2077 | 8.2265 | 7.1155 | 6.5895 |
| 8.0155 | 7.5738 | 7.5389 | 8.6425 | 9.5288 | 6.954 | 6.2291 | 7.7769 |
| 8.9156 | 9.3402 | 6.5452 | 8.8694 | 9.4963 | 8.5776 | 9.065 | 7.9024 |
| 6.9767 | 10.1859 | 7.2503 | 8.4351 | 9.0227 | 8.6199 | 7.0096 | 8.4547 |
| 9.2621 | 7.814 | 6.8075 | 9.8569 | 8.3561 | 7.8462 | 8.2026 | 7.4021 |
| 7.7221 | 8.0238 | 10.1806 | 7.6813 | 7.647 | 7.996 | 9.3787 | 9.2378 |
| 8.2151 | 6.8205 | 6.6057 | 10.5547 | 7.6582 | 8.0828 | 10.0996 | 7.2812 |
| 8.1328 | 7.761 | 8.101 | 11.1707 | 7.8814 | 8.5541 | 6.4663 | 8.0014 |
| 8.5266 | 7.353 | 6.6543 | 6.8495 | 7.6824 | 7.6371 | 8.2015 | 10.2617 |
| 7.9466 | 10.9611 | 10.3859 | 8.1243 | 7.3746 | 7.715 | 8.4594 | 7.4968 |
| 7.0816 | 7.3902 | 5.9336 | 10.6195 | 8.3725 | 8.2406 | 8.2254 | 6.3941 |
| 9.4393 | 7.7893 | 6.7308 | 8.2564 | 6.7557 | 7.639 | 8.7986 | 6.658 |
| 7.9026 | 7.8169 | 7.9183 | 8.6375 | 8.1963 | 6.5049 | 7.6021 | 6.2022 |
| 7.209 | 7.4186 | 8.8024 | 6.593 | 8.3564 | 9.3261 | 7.6144 | 6.993 |
| 7.9947 | 7.1709 | 7.6113 | 8.2219 | 9.2353 | 7.7933 | 7.0491 | 9.6787 |
| 8.0319 | 7.9019 | 8.5654 | 9.5688 | 7.0738 | 7.7892 | 7.97 | 7.7583 |
| 8.8726 | 7.8362 | 11.5242 | 10.505 | 11.5698 | 8.809 | 11.6142 | 11.2346 |
| 12.0793 | 11.8086 | 12.2861 | 11.2425 | 12.6157 | 11.9075 | 11.6652 | 11.3318 |
| 9.7319 | 11.1568 | 11.5903 | 11.4944 | 11.5867 | 11.3373 | 11.0834 | 10.6831 |
| 12.1318 | 11.148 | | | | | | |
| TENC1 | 9.2439 | 9.3098 | 9.9732 | 8.9168 | 10.6457 | 8.4984 | 8.6257 |
| 8.0215 | 9.556 | 8.7077 | 10.3514 | 8.0159 | 8.09 | 9.896 | 10.1726 |
| 9.4463 | 8.5301 | 10.9811 | 9.0341 | 9.0913 | 9.6992 | 8.9505 | 9.4825 |
| 9.6701 | 9.2114 | 9.6663 | 9.9325 | 9.3523 | 9.212 | 8.3138 | 7.4414 |
| 10.4745 | 9.3236 | 8.5028 | 7.893 | 9.782 | 8.8119 | 9.9168 | 9.3849 |
| 8.7768 | 9.4724 | 8.48 | 9.9458 | 9.7686 | 9.9149 | 8.8323 | 10.0611 |
| 8.932 | 9.1387 | 10.1443 | 10.2267 | 8.5294 | 8.8151 | 8.4929 | 8.4466 |
| 9.5046 | 12.4915 | 8.8481 | 9.5394 | 10.7476 | 8.6618 | 8.5123 | 8.8008 |
| 9.2692 | 9.8311 | 9.9954 | 9.823 | 10.3872 | 10.6182 | 9.47 | 8.9619 |
| 7.8712 | 11.3229 | 9.2016 | 9.7006 | 10.8636 | 8.2543 | 8.6302 | 9.8011 |
| 10.1434 | 8.4759 | 8.8189 | 11.3006 | 8.6063 | 9.2886 | 10.7005 | 9.2829 |
| 9.688 | 9.6591 | 9.4526 | 10.9147 | 8.2035 | 9.4276 | 10.1876 | 9.5435 |
| 10.1992 | 10.1292 | 8.3805 | 9.8663 | 9.9084 | 9.2531 | 9.3026 | 8.4865 |
| 10.9385 | 9.6172 | 9.4711 | 10.4667 | 8.7097 | 9.7746 | 9.7504 | 8.3085 |
| 9.3594 | 8.9084 | 8.6748 | 9.7652 | 9.434 | 9.5714 | 8.6023 | 9.8225 |
| 9.3948 | 12.0938 | 11.386 | 8.3352 | 10.3633 | 9.4644 | 9.7304 | 9.0635 |
| 8.5606 | 9.1654 | 8.2042 | 12.0016 | 8.2063 | 9.2117 | 9.659 | 8.3832 |
| 9.5901 | 8.7485 | 8.0016 | 8.4169 | 9.4181 | 9.1827 | 9.1398 | 9.1116 |
| 8.6749 | 8.6765 | 10.5559 | 9.6944 | 9.4143 | 8.7492 | 8.2892 | 8.8069 |
| 9.2553 | 8.6556 | 10.679 | 9.3407 | 9.1282 | 11.0589 | 8.7206 | 8.5634 |
| 8.4183 | 9.928 | 8.361 | 9.3665 | 9.2053 | 9.481 | 8.7765 | 9.1204 |
| 9.3991 | 9.0421 | 9.5962 | 9.6364 | 9.0646 | 8.9363 | 8.33 | 9.6023 |
| 9.5271 | 7.7306 | 12.0938 | 10.8004 | 11.908 | 10.9657 | 11.8318 | 12.1092 |
| 12.5641 | 12.9622 | 12.4697 | 11.0794 | 12.8758 | 12.0104 | 12.3274 | 12.6873 |
| 11.1721 | 12.3453 | 11.7193 | 12.234 | 12.8496 | 12.153 | 12.2273 | 11.2059 |
| 12.5449 | 12.1864 | | | | | | |
| KIF20A | 10.1402 | 9.4107 | 8.0952 | 10.2724 | 10.2244 | 10.47 | 9.6598 |

| | | | | | | | |
|---|---|---|---|---|---|---|---|
| 10.5127 | 11.1169 | 10.4734 | 10.509 | 9.1442 | 9.307 | 8.417 | 9.4371 |
| 10.257 | 8.3734 | 9.8734 | 8.7126 | 8.3204 | 8.5321 | 12.304 | 8.3903 |
| 7.7244 | 10.1834 | 10.658 | 9.7817 | 9.8383 | 9.4929 | 10.4776 | 9.9923 |
| 8.042 | 10.3096 | 10.2039 | 8.8256 | 9.8731 | 10.1423 | 9.7865 | 9.7872 |
| 7.7113 | 9.9586 | 11.3624 | 7.8668 | 9.6371 | 8.9228 | 8.4538 | 8.0099 |
| 9.8043 | 9.0468 | 7.6972 | 10.2609 | 10.5126 | 10.0652 | 10.7612 | 10.0303 |
| 9.7727 | 8.6534 | 10.0545 | 6.1672 | 10.9232 | 8.8356 | 9.9004 | 8.5318 |
| 7.8253 | 8.9615 | 7.7354 | 8.6067 | 7.0391 | 6.0564 | 9.341 | 8.531 |
| 11.0649 | 6.8774 | 10.7306 | 8.5463 | 8.8387 | 9.6089 | 9.7056 | 9.0652 |
| 10.8428 | 9.6513 | 9.021 | 8.7637 | 10.2548 | 9.2077 | 8.9942 | 10.374 |
| 9.0709 | 10.2122 | 10.546 | 10.5986 | 10.1718 | 11.0793 | 9.927 | 11.377 |
| 9.4469 | 9.4905 | 9.2743 | 5.5422 | 10.29 | 10.0389 | 10.063 | 9.0413 |
| 10.2158 | 10.0801 | 9.2726 | 10.5826 | 9.2095 | 10.2308 | 9.8781 | 9.539 |
| 9.1634 | 8.4142 | 10.2189 | 10.3302 | 9.4543 | 11.0145 | 8.786 | 6.5351 |
| 8.8241 | 4.7638 | 8.6514 | 10.9307 | 8.3574 | 7.9325 | 9.8515 | 9.5734 |
| 8.818 | 8.6757 | 7.9078 | 7.2681 | 5.6361 | 8.7265 | 9.3426 | 9.9606 |
| 10.5062 | 9.6065 | 9.3733 | 9.3508 | 9.3331 | 9.3759 | 8.857 | 7.6761 |
| 8.6675 | 10.3434 | 9.7685 | 9.4557 | 10.0382 | 10.8326 | 10.2751 | 10.7461 |
| 10.5181 | 9.6431 | 5.9274 | 10.1722 | 10.3299 | 7.8896 | 9.191 | 9.9699 |
| 11.1432 | 8.4893 | 9.7929 | 8.0846 | 10.7066 | 10.075 | 8.9259 | 10.0834 |
| 10.0572 | 8.6314 | 9.2381 | 10.4654 | 9.6229 | 9.7576 | 8.5571 | 9.8654 |
| 8.5397 | 8.4229 | 3.4924 | 10.0972 | 5.804 | 4.7531 | 5.2107 | 3.8333 |
| 2.3632 | 3.9047 | 3.6848 | 9.3401 | 3.8377 | 3.9714 | 5.1848 | 6.9363 |
| 4.9172 | 5.3066 | 7.1856 | 3.2317 | 2.0673 | 7.164 | 4.1094 | 8.3726 |
| 2.0979 | 3.2388 | | | | | | |
| TSHZ3 | 6.0001 | 7.0121 | 5.5065 | 6.2156 | 5.5488 | 5.7432 | 6.1917 |
| 6.2047 | 5.9373 | 5.3417 | 8.1236 | 4.0591 | 5.543 | 5.4604 | 7.2525 |
| 7.3553 | 3.6203 | 7.8023 | 6.3632 | 5.8525 | 7.1707 | 6.9763 | 7.1499 |
| 6.7367 | 4.3722 | 5.3603 | 5.8357 | 5.9352 | 5.4 | 4.1708 | 4.1634 |
| 5.7443 | 4.2355 | 5.1156 | 6.5041 | 7.3242 | 7.0881 | 4.7293 | 5.8558 |
| 4.6618 | 5.6218 | 5.6608 | 3.7983 | 5.6978 | 6.8823 | 7.4273 | 7.3095 |
| 6.9212 | 7.7864 | 7.4511 | 8.5549 | 7.1196 | 7.0596 | 5.6174 | 4.892 |
| 6.6466 | 4.6811 | 5.7523 | 7.8258 | 8.3364 | 4.7322 | 5.7065 | 6.4125 |
| 5.6145 | 8.2147 | 3.3007 | 7.2122 | 7.8092 | 7.8385 | 7.4212 | 6.041 |
| 5.7503 | 8.7668 | 6.5196 | 6.8233 | 6.2711 | 4.4444 | 5.4929 | 7.0447 |
| 7.851 | 6.6154 | 4.6901 | 8.792 | 6.704 | 6.2846 | 6.4619 | 6.1988 |
| 4.8234 | 7.0787 | 5.4184 | 6.8593 | 5.4461 | 5.8814 | 8.2882 | 8.9687 |
| 7.1374 | 5.4515 | 5.1483 | 5.8869 | 5.6724 | 7.9164 | 4.3226 | 4.7097 |
| 7.5423 | 6.0581 | 6.9649 | 8.8834 | 6.8054 | 7.7563 | 5.5493 | 7.274 |
| 6.6796 | 3.8798 | 5.2717 | 4.5112 | 5.8795 | 5.334 | 7.2676 | 5.7967 |
| 4.8848 | 10.0149 | 9.4449 | 4.1324 | 4.7253 | 5.6274 | 7.1361 | 6.4414 |
| 6.0668 | 4.8225 | 2.9411 | 9.3612 | 4.2129 | 7.3294 | 6.3875 | 4.6707 |
| 8.0319 | 6.6205 | 3.7963 | 5.9123 | 3.5167 | 6.0608 | 8.6351 | 5.0271 |
| 6.4781 | 6.3467 | 6.1322 | 7.7472 | 6.7049 | 4.5304 | 4.4694 | 4.6644 |
| 7.636 | 7.149 | 5.7144 | 6.8417 | 7.239 | 8.5099 | 5.3351 | 4.7089 |
| 3.9132 | 6.5951 | 6.1756 | 6.7077 | 6.8895 | 5.4354 | 5.1297 | 6.3147 |
| 6.9015 | 4.7712 | 6.4393 | 7.6212 | 6.1446 | 5.2995 | 5.4713 | 6.2828 |
| 7.2518 | 4.6892 | 10.7066 | 10.615 | 10.7334 | 7.655 | 10.957 | 10.3016 |
| 10.082 | 9.9908 | 10.7178 | 11.2707 | 10.1571 | 10.4223 | 11.3381 | 10.5299 |
| 8.5534 | 10.2518 | 11.077 | 10.9058 | 11.5496 | 9.0924 | 10.3458 | 11.2609 |
| 10.2557 | 10.3393 | | | | | | |
| DIAPH3 | 9.4696 | 6.8682 | 6.8118 | 8.9506 | 8.3936 | 7.9398 | 8.7019 |
| 9.3102 | 9.4977 | 8.6269 | 9.3676 | 7.3553 | 7.991 | 4.3171 | 7.8292 |
| 8.0925 | 7.29 | 8.078 | 7.6534 | 6.6506 | 8.2816 | 7.8618 | 7.8359 |

| | | | | | | | | |
|---|---|---|---|---|---|---|---|---|
| 6.3225 | 8.3685 | 8.2871 | 7.908 | 8.5021 | 7.4005 | 8.1907 | 7.7435 | 6.45 |
| | 8.4804 | 9.8563 | 8.1987 | 8.4413 | 7.5664 | 7.3322 | 6.2475 | |
| 7.5968 | 7.6731 | 9.8184 | 7.6534 | 8.3968 | 8.061 | 7.4925 | 6.2446 | |
| 8.7608 | 7.7609 | 6 | 8.6868 | 7.2726 | 9.0095 | 8.6753 | 7.942 | |
| 8.5688 | 5.1037 | 7.6488 | 5.2065 | 7.8457 | 7.3352 | 8.1158 | 7.4807 | |
| 7.5626 | 8.2789 | 6.9652 | 6.0589 | 4.9965 | 4.0282 | 8.3943 | 8.2552 | |
| 8.3142 | 4.9138 | 7.9561 | 7.0024 | 7.113 | 8.6988 | 7.5112 | 7.3123 | |
| 8.7376 | 10.3458 | 5.9428 | 5.355 | 7.759 | 6.9008 | 9.1245 | 7.6983 | |
| 7.1836 | 8.1297 | 7.6944 | 7.6906 | 9.0315 | 9.3667 | 8.3771 | 7.6478 | |
| 6.7762 | 7.0704 | 8.0255 | 7.2019 | 9.3083 | 9.5568 | 9.0856 | 7.5081 | |
| 8.0247 | 7.8087 | 7.7948 | 8.966 | 8.3374 | 9.589 | 8.5899 | 9.4161 | |
| 8.6634 | 6.6113 | 7.8411 | 7.3122 | 7.7465 | 7.6911 | 7.6768 | 7.2539 | |
| 7.3856 | 2.7241 | 7.6283 | 7.036 | 6.8151 | 6.284 | 9.0938 | 9.6858 | |
| 7.6373 | 7.4931 | 7.5549 | 5.3262 | 6.4869 | 7.7361 | 7.7008 | 9.3051 | |
| 9.062 | 9.0827 | 6.3929 | 8.2325 | 7.0511 | 8.4748 | 7.4751 | 6.3454 | |
| 7.2156 | 9.4655 | 6.9626 | 7.7233 | 8.2454 | 9.0405 | 10.0056 | 8.0154 | |
| 7.5753 | 8.0501 | 3.8992 | 8.329 | 9.3357 | 5.9659 | 7.9293 | 8.072 | |
| 8.3692 | 6.8577 | 8.2674 | 6.9356 | 9.0438 | 8.3373 | 7.9759 | 8.9915 | |
| 9.1162 | 6.3035 | 6.7991 | 7.5195 | 8.292 | 8.0751 | 7.5905 | 8.4554 | |
| 7.3816 | 9.1566 | 2.6668 | 7.802 | 3.951 | 2.8232 | 3.4365 | 3.9503 | |
| 3.0451 | 3.0357 | 3.4641 | 6.472 | 3.3023 | 5.0951 | 3.2822 | 3.6115 | |
| 3.6178 | 4.0215 | 6.0239 | 2.4897 | 0.5418 | 4.9261 | 2.7525 | 5.0594 | |
| 2.2714 | 1.8629 | | | | | | | |
| CDC25A | 9.0955 | 7.7919 | 6.9032 | 9.5069 | 7.9519 | 8.2589 | 8.7679 | |
| 8.5942 | 7.9461 | 8.4387 | 7.7386 | 8.5963 | 7.5788 | 6.8619 | 7.6316 | |
| 8.6994 | 9.2961 | 7.5868 | 6.8585 | 6.9722 | 8.4782 | 8.9879 | 8.0245 | |
| 6.2479 | 8.5192 | 9.4565 | 8.3456 | 8.3024 | 8.5184 | 8.4928 | 9.5697 | |
| 6.8413 | 8.749 | 8.2969 | 8.3767 | 8.3381 | 8.077 | 7.8456 | 7.6678 | |
| 8.537 | 7.7668 | 9.4377 | 7.6393 | 6.8591 | 8.6884 | 7.6478 | 7.5122 | |
| 8.2685 | 7.4138 | 7.1752 | 8.4793 | 8.2838 | 7.8483 | 8.9612 | 8.2759 | |
| 7.5125 | 6.7457 | 8.858 | 5.3527 | 8.6417 | 7.7275 | 8.0253 | 8.1072 | |
| 8.4671 | 7.8049 | 6.5862 | 9.1052 | 5.0569 | 5.4353 | 8.5724 | 9.359 | |
| 9.9184 | 4.9138 | 8.6509 | 7.618 | 8.2107 | 7.8508 | 7.6924 | 7.6308 | |
| 8.9192 | 8.6765 | 8.8189 | 7.0626 | 9.5361 | 7.1915 | 7.7486 | 7.8425 | |
| 9.3215 | 8.1612 | 8.3354 | 8.911 | 9.1595 | 10.2726 | 7.3525 | 8.7657 | |
| 8.7944 | 7.4828 | 7.9042 | 9.5314 | 8.6582 | 8.9749 | 8.7331 | 7.9351 | |
| 8.7262 | 9.1679 | 8.1919 | 9.3663 | 7.5959 | 9.1371 | 7.5169 | 7.454 | |
| 8.1778 | 9.2572 | 8.587 | 8.8548 | 7.8109 | 8.0403 | 8.2841 | 9.1842 | |
| 9.3007 | 5.9263 | 7.5766 | 8.7635 | 7.3916 | 7.2905 | 8.3303 | 8.9767 | |
| 6.9137 | 8.2466 | 8.9735 | 5.9963 | 9.6033 | 6.7911 | 8.206 | 9.0836 | |
| 9.0857 | 9.7214 | 7.5719 | 9.3638 | 7.3662 | 8.5441 | 8.2534 | 7.5073 | |
| 8.9359 | 8.562 | 7.9736 | 6.7517 | 8.6634 | 9.2372 | 9.612 | 9.2389 | |
| 9.4667 | 8.8232 | 7.5617 | 8.2895 | 8.6142 | 4.9888 | 8.6314 | 8.9687 | |
| 8.433 | 7.0573 | 8.5343 | 7.567 | 7.7732 | 8.7227 | 8.1989 | 9.4813 | |
| 8.4936 | 7.4144 | 8.3775 | 8.9913 | 8.3804 | 9.7304 | 9.0262 | 7.7504 | |
| 7.4176 | 8.6016 | 4.3657 | 7.5987 | 5.3005 | 3.8339 | 4.6086 | 5.1514 | |
| 3.0451 | 3.8633 | 4.7303 | 6.5764 | 4.1393 | 3.3571 | 4.5268 | 6.2149 | |
| 4.2819 | 4.5765 | 5.5267 | 4.0469 | 7.6714 | 4.8361 | 5.147 | 6.8189 | |
| 4.8794 | 3.9791 | | | | | | | |
| BLM | 8.8951 | 8.1566 | 6.5482 | 9.0074 | 8.7456 | 8.2284 | 8.6992 | |
| 8.219 | 8.8873 | 8.5273 | 7.6526 | 8.0159 | 8.2823 | 7.0233 | 8.5779 | |
| 8.2119 | 8.3721 | 6.9084 | 6.9212 | 7.4894 | 7.4759 | 9.6269 | 7.9272 | |
| 6.337 | 8.1498 | 8.1692 | 8.2086 | 8.4427 | 7.8811 | 9.5821 | 9.1507 | |
| 6.4807 | 8.5933 | 8.1886 | 7.2492 | 7.3911 | 7.755 | 8.7377 | 8.1112 | |

| | | | | | | | | |
|---|---|---|---|---|---|---|---|---|
| 6.9449 | 7.6647 | 8.4727 | 6.6037 | 7.591 | 8.4708 | 7.9592 | 7.271 | |
| 8.7859 | 7.3272 | 7.7457 | 8.4751 | 8.3237 | 9.1839 | 8.3973 | 8.0931 | |
| 6.9558 | 5.1462 | 8.7069 | 5.1676 | 8.6165 | 7.9766 | 8.2342 | 8.3433 | |
| 7.8403 | 7.8869 | 6.6091 | 7.6396 | 5.3094 | 5.3939 | 8.4437 | 8.3303 | |
| 9.4573 | 5.9514 | 8.7255 | 7.6905 | 7.1946 | 8.5187 | 7.9021 | 7.9102 | |
| 8.1947 | 8.3187 | 7.7869 | 7.5239 | 8.963 | 6.9829 | 7.525 | 8.2001 | |
| 8.1306 | 8.1147 | 7.5974 | 8.6277 | 9.1157 | 8.7259 | 8.5773 | 9.3154 | |
| 8.2584 | 7.8889 | 8.3546 | 7.2474 | 8.9658 | 9.1644 | 9.5764 | 8.1854 | |
| 8.0459 | 7.7964 | 8.9377 | 8.8006 | 7.9179 | 8.0037 | 7.7746 | 7.7339 | |
| 7.5633 | 7.2473 | 7.2345 | 9.2492 | 7.7558 | 9.3635 | 8.3456 | 6.3463 | |
| 8.5758 | 4.8598 | 7.6874 | 8.5029 | 6.635 | 7.5538 | 7.4541 | 8.8626 | |
| 6.8922 | 7.5966 | 7.7644 | 6.3156 | 6.0079 | 7.5453 | 9.1176 | 8.3406 | |
| 7.9842 | 8.4954 | 8.4326 | 8.92 | 6.7205 | 7.2492 | 7.375 | 7.515 | |
| 8.0658 | 8.5683 | 8.0469 | 7.626 | 8.5975 | 8.9385 | 8.5888 | 8.8055 | |
| 8.0979 | 8.3585 | 6.3773 | 8.1461 | 9.2287 | 6.4824 | 8.1017 | 7.9943 | |
| 8.348 | 6.6504 | 8.1673 | 7.7776 | 8.4663 | 7.6781 | 8.2158 | 9.1698 | |
| 8.727 | 7.0706 | 7.5208 | 8.6176 | 8.2159 | 8.7991 | 6.0848 | 7.8989 | |
| 7.8847 | 8.1855 | 4.6482 | 7.8653 | 5.3543 | 4.9204 | 4.5807 | 4.9784 | |
| 3.5062 | 4.5514 | 4.517 | 6.6391 | 3.4559 | 3.813 | 4.4378 | 5.948 | |
| 5.4273 | 5.0611 | 6.0699 | 4.2829 | 2.6938 | 5.0711 | 3.8942 | 7.4364 | |
| 3.8748 | 4.1603 | | | | | | | |
| RERG | 5.1512 | 4.2305 | 4.6239 | 7.63E+00 | | 9.22E+00 | | |
| 3.6875 | 6.6549 | 5.2771 | 4.9117 | 4.2156 | 7.145 | 3.9636 | 3.3269 | |
| 5.7644 | 5.5324 | 5.6915 | 3.6203 | 8.513 | 4.1746 | 5.5878 | 3.3041 | |
| 3.5585 | 5.4158 | 6.5382 | 4.441 | 9.1692 | 7.0574 | 6.0706 | 4.8513 | |
| 5.7151 | 4.0627 | 6.2773 | 3.0343 | 5.832 | 4.1277 | 5.3918 | 5.2986 | |
| 7.7197 | 3.0403 | 3.0068 | 6.4973 | 6.692 | 3.9521 | 5.0322 | 5.476 | |
| 5.9369 | 5.687 | 3.6867 | 6.3508 | 8.9849 | 9.5589 | 4.3537 | 6.9678 | |
| 4.8489 | 4.4259 | 6.0711 | 3.4274 | 6.158 | 6.0218 | 8.9112 | 7.3289 | |
| 3.9707 | 5.1386 | 6.1342 | 8.2642 | 5.5368 | 5.9093 | 5.4054 | 5.4756 | 7.14 |
| | 4.4992 | 4.7685 | 9.3427 | 7.6026 | 7.3091 | 5.5922 | 6.2212 | |
| 2.0646 | 7.3361 | 7.4689 | 6.4928 | 3.403 | 6.9808 | 6.6613 | 4.6212 | |
| 7.9121 | 5.8496 | 3.5349 | 7.0294 | 5.6808 | 6.9475 | 3.649 | 7.5384 | |
| 7.5015 | 7.1692 | 4.7357 | 6.5902 | 2.2538 | 6.1003 | 5.3219 | 8.6713 | |
| 7.4001 | 5.1829 | 5.3257 | 5.9513 | 4.9509 | 8.6354 | 6.8997 | 5.5499 | |
| 3.4611 | 6.1443 | 7.8756 | 1.2894 | 6.3941 | 3.5369 | 6.1403 | 7.1754 | |
| 7.055 | 7.2176 | 4.9118 | 5.0346 | 10.1178 | 2.9483 | 4.8033 | 3.8753 | |
| 6.8751 | 5.9626 | 4.7323 | 4.0343 | 3.0614 | 10.2823 | 4.4882 | 4.3632 | |
| 5.9811 | 5.2629 | 3.6656 | 5.4146 | 7.738 | 5.3924 | 3.3043 | 5.3682 | |
| 3.6358 | 3.4163 | 4.2415 | 4.7895 | 7.3642 | 6.3875 | 6.5367 | 3.7481 | |
| 2.2163 | 6.976 | 5.2248 | 5.8093 | 5.8598 | 4.8982 | 7.3281 | 7.6737 | |
| 5.5379 | 4.6396 | 7.6256 | 4.2827 | 6.9494 | 7.0163 | 5.8342 | 4.2224 | |
| 3.3538 | 3.7148 | 5.9484 | 4.3068 | 5.8182 | 8.2598 | 4.4626 | 3.1828 | |
| 2.2195 | 5.3954 | 8.3439 | 4.5045 | 10.7093 | 7.8829 | 10.7313 | 10.4636 | |
| 12.1188 | 11.5684 | 12.8994 | 12.5668 | 12.66 | 8.9458 | 11.6008 | 12.576 | |
| 10.3521 | 9.4944 | 10.4568 | 12.2127 | 11.7415 | 10.7984 | 9.8794 | 10.7975 | |
| 10.8875 | 10.0985 | 12.1316 | 10.9686 | | | | | |
| LOC728264 | | 5.17 | 6.0164 | 5.2368 | 6.96E+00 | | 3.8401 | |
| 3.0554 | 6.2146 | 6.0834 | 4.082 | 4.0515 | 7.0975 | 4.5724 | 5.1243 | |
| 7.3919 | 7.8663 | 6.4839 | 4.7636 | 4.9749 | 4.7646 | 6.057 | 3.7016 | |
| 3.9883 | 7.6599 | 6.4208 | 3.8217 | 4.9298 | 7.3331 | 3.8834 | 4.9614 | |
| 2.9077 | 4.964 | 6.7686 | 4.686 | 6.3002 | 5.7722 | 5.9516 | 5.8376 | |
| 6.2969 | 5.5288 | 5.5168 | 5.1516 | 8.5856 | 4.3892 | 5.0802 | 6.4597 | |
| 6.5669 | 8.0373 | 5.2679 | 6.7116 | 6.4763 | 8.8745 | 5.7931 | 5.756 | |

| | | | | | | | | |
|---|---|---|---|---|---|---|---|---|
| 5.0263 | 4.2032 | 6.149 | 4.4964 | 6.5125 | 5.387 | 8.3577 | 3.8394 | |
| 4.9678 | 5.6971 | 5.1059 | 8.7407 | 3.6494 | 5.6101 | 6.8185 | 6.3766 | |
| 7.7234 | 5.2835 | 5.187 | 6.2986 | 5.3692 | 8.377 | 6.2811 | 3.9495 | |
| 5.9169 | 7.3826 | 8.7917 | 6.712 | 3.9417 | 9.4449 | 7.011 | 4.4001 | |
| 6.8901 | 6.4027 | 6.4123 | 6.7428 | 4.9373 | 7.6719 | 4.1793 | 6.2427 | |
| 7.852 | 6.3545 | 6.1979 | 4.0721 | 4.7467 | 8.1418 | 7.2668 | 6.3408 | |
| 3.8279 | 4.9347 | 5.5995 | 7.1571 | 6.2985 | 7.5461 | 5.7922 | 5.5664 | |
| 4.1961 | 5.4857 | 7.547 | 2.7607 | 4.4185 | 4.4346 | 7.1155 | 6.7545 | |
| 7.1033 | 7.8735 | 5.2456 | 10.0175 | 10.9595 | 5.0235 | 4.9926 | 5.8856 | |
| 5.515 | 4.6105 | 4.826 | 5.199 | 4.4402 | 10.6822 | 6.7447 | 5.6359 | |
| 5.0698 | 3.9795 | 6.8814 | 6.6604 | 4.6868 | 6.8725 | 3.5167 | 6.4933 | |
| 5.515 | 5.5081 | 6.1325 | 6.7216 | 6.0395 | 6.6769 | 7.098 | 3.6978 | |
| 4.2928 | 4.9799 | 5.1529 | 5.1521 | 7.3686 | 6.4309 | 7.5615 | 7.8489 | |
| 6.1174 | 6.2566 | 4.0343 | 5.7515 | 5.6525 | 6.2022 | 6.4885 | 5.2325 | |
| 5.5042 | 5.4171 | 6.6214 | 4.0446 | 6.186 | 7.1134 | 5.8257 | 5.7956 | |
| 3.2264 | 5.6879 | 8.3791 | 4.9716 | 9.9883 | 9.898 | 10.3089 | 6.6182 | |
| 11.604 | 10.5331 | 9.1335 | 11.9186 | 10.9226 | 10.424 | 10.7774 | 10.1193 | |
| 10.9916 | 11.5063 | 9.7205 | 10.4485 | 12.9696 | 10.6786 | 10.0497 | 9.8673 | |
| 10.3814 | 10.7977 | 11.0303 | 10.7065 | | | | | |
| MEF2C | 7.1958 | 7.7718 | 6.8429 | 7.2315 | 7.7002 | 9.8153 | 6.8575 | |
| 6.5499 | 7.9175 | 6.1561 | 8.4093 | 6.4577 | 7.668 | 7.2359 | 7.5174 | |
| 7.6294 | 6.0007 | 7.8092 | 6.9259 | 8.0503 | 7.1444 | 6.1015 | 8.1317 | |
| 7.2988 | 5.7833 | 6.3129 | 7.4825 | 7.2056 | 6.7886 | 5.2677 | 5.9827 | 8.07 |
| | 7.3019 | 6.4504 | 6.6871 | 8.1094 | 7.0995 | 7.5389 | 7.9307 | |
| 6.2693 | 7.8842 | 6.9173 | 6.489 | 6.5875 | 7.9654 | 7.7312 | 8.4246 | |
| 6.3108 | 7.6343 | 8.7437 | 9.7611 | 6.9198 | 9.0084 | 6.4161 | 5.3919 | |
| 8.0932 | 6.8132 | 6.5435 | 6.614 | 9.1558 | 7.4495 | 7.4687 | 8.8765 | |
| 7.5383 | 9.0792 | 5.932 | 7.5376 | 7.8916 | 7.8577 | 7.566 | 7.1665 | |
| 7.2825 | 9.5464 | 8.3903 | 7.5619 | 6.8498 | 6.8026 | 6.6696 | 7.9102 | |
| 9.5253 | 8.0692 | 7.016 | 8.6381 | 7.2102 | 7.1915 | 7.85 | 8.1655 | |
| 6.1317 | 7.4011 | 6.2479 | 8.4079 | 6.042 | 6.7718 | 9.8877 | 8.696 | |
| 7.5277 | 6.7362 | 6.6909 | 7.0561 | 7.5392 | 8.0342 | 7.0439 | 6.5906 | |
| 7.0962 | 6.7988 | 8.0448 | 8.1333 | 7.1247 | 7.6518 | 6.621 | 7.253 | |
| 7.3913 | 5.6676 | 6.7499 | 6.2223 | 7.3063 | 8.3641 | 7.5606 | 7.4973 | |
| 6.9094 | 9.5908 | 9.7206 | 5.9295 | 7.8643 | 7.3522 | 7.8462 | 6.7513 | |
| 6.9489 | 7.2181 | 5.9017 | 10.424 | 6.3033 | 7.5937 | 7.0482 | 6.2873 | |
| 7.6773 | 6.4741 | 6.552 | 7.9297 | 6.4284 | 7.5601 | 7.4951 | 7.9049 | |
| 6.9321 | 7.2056 | 8.844 | 8.7989 | 7.3055 | 6.651 | 6.3697 | 7.3767 | |
| 5.6992 | 6.2074 | 8.1476 | 7.2272 | 7.8084 | 8.6308 | 7.021 | 7.0609 | |
| 6.0456 | 7.154 | 7.2182 | 7.4421 | 7.7797 | 7.0332 | 6.4219 | 7.6115 | |
| 7.9942 | 7.2856 | 7.7768 | 8.057 | 6.9684 | 6.7286 | 5.3286 | 8.1922 | |
| 8.2886 | 6.0464 | 10.2016 | 9.5028 | 10.1653 | 9.8365 | 11.3019 | 10.3807 | |
| 11.3362 | 10.6288 | 11.334 | 9.9837 | 10.4862 | 11.3973 | 10.2088 | 9.1733 | |
| 9.2525 | 10.9316 | 11.0762 | 9.6546 | 10.5784 | 9.6236 | 10.4687 | 10.1163 | |
| 10.4761 | 9.5065 | | | | | | | |
| PLCL1 | 2.7225 | 4.0286 | 4.1139 | 4.56E+00 | | 4.14E+00 | | |
| 3.5251 | 3.6208 | 4.3139 | 4.4222 | 3.4044 | 5.0655 | 2.6649 | 4.0713 | |
| 4.7451 | 5.1715 | 4.4863 | 2.5253 | 5.6785 | 3.3952 | 4.9914 | 4.4861 | |
| 7.7392 | 2.4936 | 4.0984 | 3.1836 | 6.4721 | 4.3234 | 3.6563 | 5.1438 | |
| 4.5555 | 3.2417 | 4.9127 | 4.2789 | 2.3491 | 3.2878 | 4.8338 | 4.3916 | |
| 4.0946 | 2.6826 | 0 | 6.1554 | 4.9191 | 4.8665 | 3.7593 | 4.7376 | |
| 4.9337 | 5.7494 | 3.739 | 4.1793 | 4.6067 | 6.5585 | 3.6631 | 5.7001 | |
| 3.5075 | 2.3502 | 4.5477 | 2.0292 | 2.7156 | 3.3716 | 6.508 | 4.8063 | |
| 4.9239 | 5.0608 | 3.6783 | 6.0421 | 1.8854 | 6.1185 | 3.7796 | 4.5648 | |

| | | | | | | | | |
|---|---|---|---|---|---|---|---|---|
| 4.895 | 3.4708 | 3.5159 | 6.6911 | 4.9657 | 4.0744 | 2.6709 | 4.1463 | |
| 3.9377 | 4.1146 | 5.8642 | 5.0246 | 3.6729 | 5.6301 | 4.0165 | 3.8194 | |
| 4.5235 | 5.9066 | 3.2267 | 4.164 | 3.1832 | 5.6506 | 3.1638 | 3.6366 | |
| 5.3233 | 6.3219 | 3.7624 | 4.1782 | 2.8996 | 4.7091 | 2.585 | 6.8058 | |
| 4.7481 | 4.8924 | 5.2484 | 3.6703 | 3.9238 | 7.6404 | 3.5951 | 2.7935 | |
| 2.8403 | 4.3056 | 5.9935 | 4.4074 | 2.2519 | 4.0798 | 2.7225 | 4.2436 | |
| 3.622 | 4.0808 | 4.3638 | 2.7241 | 5.9434 | 4.6896 | 4.4302 | 3.1663 | |
| 4.5741 | 3.809 | 3.4742 | 2.3133 | 2.1177 | 7.4542 | 0 | 4.9354 | |
| 4.048 | 2.8077 | 5.1 | 3.6943 | 4.3644 | 4.9574 | 2.6375 | 4.4384 | |
| 4.1617 | 2.0791 | 4.705 | 4.176 | 5.2246 | 5.1806 | 3.7871 | 3.2857 | |
| 3.7829 | 5.6356 | 4.9342 | 2.5508 | 2.3719 | 5.0261 | 5.0332 | 5.7175 | |
| 2.6218 | 3.9175 | 4.7977 | 3.9324 | 3.1691 | 4.3584 | 5.4482 | 3.0995 | |
| 2.332 | 4.2443 | 5.2367 | 4.8672 | 4.9259 | 5.4353 | 5.6471 | 3.1828 | |
| 4.6154 | 4.7714 | 4.7173 | 1.5159 | 8.9342 | 6.1594 | 8.7796 | 7.1653 | |
| 8.271 | 9.2361 | 9.706 | 7.9439 | 8.1017 | 6.7568 | 8.0988 | 10.2667 | |
| 7.5927 | 10.9936 | 7.4909 | 8.137 | 8.576 | 7.7315 | 8.3141 | 6.7977 | |
| 8.3934 | 7.102 | 8.2708 | 7.4946 | | | | | |
| JPH4 | 1.737 | 3.1646 | 2.2237 | 4.2561 | 2.7758 | 2.8873 | 5.6801 | |
| 4.0037 | 2.1584 | 4.1842 | 6.2784 | 2.7437 | 2.5775 | 4.5469 | 5.8481 | |
| 4.5754 | 2.7962 | 3.4081 | 4.0419 | 3.0076 | 1.8524 | 5.6244 | 3.9933 | |
| 5.3404 | 2.472 | 3.361 | 4.4227 | 2.7201 | 3.2323 | 2.5857 | 2.2096 | 5 |
| | 2.5584 | 4.1567 | 1.4612 | 3.5998 | 4.3153 | 3.4777 | 3.0403 | 2.07 |
| | 2.1933 | 7.1988 | 2.5098 | 1.3077 | 3.4576 | 4.5693 | 5.6438 | |
| 3.2557 | 5.515 | 6.5097 | 8.5081 | 6.1399 | 5.5688 | 3.828 | 6.8867 | |
| 3.7482 | 2.8406 | 4.8895 | 3.7337 | 7.93 | 3.0632 | 1.8067 | 3.2212 | |
| 4.7738 | 5.6983 | 1.3447 | 4.9108 | 5.4054 | 5.553 | 5.9473 | 4.6992 | |
| 3.959 | 7.0495 | 2.3651 | 7.0749 | 4.1842 | 4.2864 | 3.2461 | 6.2728 | |
| 7.5423 | 5.4637 | 4.3331 | 6.8332 | 4.4763 | 3.4668 | 6.3391 | 3.2964 | |
| 3.6027 | 5.9565 | 4.7332 | 4.9514 | 2.8815 | 6.7413 | 6.5609 | 7.5064 | |
| 4.9148 | 2.3994 | 2.081 | 6.0327 | 3.7004 | 2.1456 | 7.9144 | 2.1803 | |
| 4.6289 | 4.5937 | 4.154 | 9.9906 | 4.3324 | 5.2356 | 1.7271 | 3.7081 | |
| 5.2755 | 2.9068 | 3.9176 | 1.7472 | 4.2631 | 5.0025 | 5.2461 | 5.8162 | |
| 3.9061 | 8.6608 | 9.243 | 2.4986 | 2.5594 | 3.9832 | 5.022 | 2.12 | |
| 1.9907 | 3.5137 | 3.1725 | 9.4053 | 2.9676 | 3.1278 | 5.772 | 4.1392 | |
| 3.0771 | 2.4027 | 2.2048 | 3.4015 | 1.36 | 2.2364 | 6.2582 | 3.0171 | |
| 3.0297 | 1.4147 | 5.9908 | 6.4576 | 5.4968 | 2.3633 | 2.0984 | 3.0923 | |
| 7.7036 | 4.4632 | 3.7579 | 3.2158 | 7.2143 | 6.1886 | 3.6145 | 3.8577 | |
| 6.5165 | 3.5742 | 4.1955 | 6.0214 | 5.4317 | 3.0995 | 2.4884 | 2.7703 | |
| 4.054 | 2.6247 | 5.6484 | 6.6485 | 2.8461 | 3.7138 | 3.3048 | 2.7261 | |
| 6.2652 | 3.744 | 9.7174 | 9.1389 | 9.5919 | 6.6661 | 11.8839 | 9.7611 | |
| 9.5847 | 10.3713 | 10.1357 | 9.6018 | 10.3677 | 11.3406 | 10.2681 | 9.5938 | |
| 7.8104 | 10.0133 | 11.0096 | 10.4558 | 7.5746 | 9.2703 | 10.2368 | 10.1259 | |
| 10.1549 | 9.939 | | | | | | | |
| DEPDC1B | 8.3644 | 5.9175 | 7.0701 | 9.3 | 8.063 | 8.2459 | 8.8567 | |
| 8.3867 | 8.8081 | 8.4893 | 8.219 | 8.2734 | 7.6158 | 6.1612 | 7.6664 | |
| 8.1075 | 9.8291 | 7.8764 | 6.7404 | 7.256 | 7.6623 | 9.6002 | 7.7592 | |
| 5.3967 | 9.9498 | 8.139 | 8.379 | 9.3226 | 7.4454 | 8.779 | 9.4326 | 6.45 |
| | 8.5889 | 9.1228 | 8.0989 | 8.5239 | 8.3305 | 9.2446 | 6.994 | |
| 7.0461 | 8.2877 | 9.3057 | 6.5684 | 8.1643 | 9.4003 | 7.995 | 7.2131 | |
| 8.9884 | 7.7387 | 7.0982 | 8.5013 | 7.7011 | 8.8858 | 8.1128 | 7.6011 | |
| 7.8755 | 4.7377 | 7.9795 | 3.8367 | 7.5085 | 7.9109 | 8.3478 | 8.0173 | |
| 7.0868 | 6.8569 | 6.3682 | 2.0987 | 5.0371 | 4.4071 | 8.4125 | 8.1712 | |
| 9.2458 | 4.4836 | 8.6337 | 6.6944 | 7.4329 | 8.5276 | 7.67 | 8.5081 | |
| 9.1402 | 9.1036 | 7.5859 | 6.5602 | 8.7844 | 7.0746 | 7.0274 | 8.1701 | |

| | | | | | | | | |
|---|---|---|---|---|---|---|---|---|
| 6.6146 | 8.01 | 8.1379 | 7.5846 | 8.9432 | 9.0178 | 7.985 | 9.0153 | |
| 7.8754 | 7.7836 | 9.1026 | 7.8685 | 6.8455 | 8.6126 | 8.3311 | 7.3452 | |
| 8.2745 | 8.7093 | 10.4714 | 8.6515 | 7.4539 | 8.9165 | 8.0839 | 8.7813 | |
| 8.0678 | 7.8896 | 8.8488 | 8.4771 | 7.4325 | 8.5509 | 8.4827 | 6.6701 | |
| 7.506 | 4.2995 | 7.5962 | 8.8485 | 6.5621 | 6.9551 | 8.0783 | 8.2342 | |
| 6.9967 | 7.2839 | 7.4151 | 5.2335 | 5.9178 | 6.749 | 8.7146 | 8.7891 | |
| 9.0847 | 8.5076 | 7.3843 | 9.134 | 6.9388 | 7.958 | 7.821 | 7.0508 | |
| 7.9283 | 8.6824 | 6.9025 | 7.7145 | 8.5198 | 8.9344 | 8.94 | 9.5248 | |
| 8.9016 | 8.5242 | 4.4516 | 8.518 | 8.8359 | 5.3798 | 7.5971 | 8.6867 | |
| 9.6055 | 7.378 | 8.7573 | 7.0928 | 8.3979 | 7.8575 | 8.0499 | 8.8256 | |
| 8.8066 | 7.1868 | 6.7803 | 8.1953 | 8.0461 | 7.1333 | 8.1076 | 7.6198 | |
| 7.4318 | 6.987 | 4.6986 | 7.4203 | 5.1465 | 3.4522 | 4.3014 | 3.1751 | 0 |
| | 2.5005 | 2.4755 | 5.7295 | 2.2864 | 1.6671 | 2.9802 | 6.189 | |
| 3.6768 | 3.69 | 5.845 | 3.0966 | 0.5418 | 4.5268 | 3.3344 | 6.2179 | |
| 1.6723 | 3.3172 | | | | | | | |
| ARHGAP20 | | 2.0932 | 5.3299 | 2.7464 | 2.5505 | 2.5543 | 3.9659 | |
| 3.2591 | 2.4718 | 2.4603 | 2.2003 | 4.8171 | 0 | 2.6827 | 1.4442 | |
| 1.3881 | 3.8641 | 0.9198 | 3.6727 | 3.0938 | 3.5854 | 1.627 | 7.2857 | |
| 4.2489 | 4.5595 | 1.5953 | 6.525 | 2.7455 | 3.6062 | 2.0192 | 1.3224 | |
| 1.1431 | 3.0901 | 1.5916 | 1.7115 | 3.2351 | 4.3859 | 2.9036 | 2.6427 | |
| 1.8914 | 0 | 3.7557 | 4.1799 | 1.359 | 2.44 | 1.946 | 4.6368 | |
| 3.6317 | 1.755 | 4.1793 | 4.9754 | 4.7395 | 4.2095 | 4.6351 | 2.5147 | |
| 0.5968 | 3.9358 | 1.6109 | 3.2856 | 2.1522 | 4.7882 | 2.3798 | 3.7871 | |
| 2.9022 | 6.2946 | 3.8599 | 0.8237 | 4.0425 | 5.1337 | 4.4482 | 4.7019 | |
| 1.2875 | 2.354 | 6.0812 | 2.9116 | 2.3108 | 5.144 | 2.9586 | 2.0646 | |
| 4.3513 | 4.7061 | 3.8257 | 2.017 | 4.93 | 2.9084 | 4.0271 | 2.1324 | |
| 2.611 | 0 | 3.6507 | 3.3193 | 2.8049 | 2.5303 | 2.2668 | 4.1763 | |
| 6.3383 | 4.7083 | 1.6498 | 2.081 | 2.8658 | 1.585 | 4.0738 | 2.5241 | |
| 0.5276 | 3.7623 | 2.876 | 3.8204 | 6.1373 | 3.7651 | 3.9601 | 1.5487 | |
| 3.8931 | 4.4763 | 0 | 2.5892 | 1.9805 | 3.1593 | 2.362 | 3.8654 | |
| 3.1012 | 1.0837 | 6.6977 | 6.4682 | 0.601 | 2.7811 | 3.255 | 2.5899 | |
| 2.6338 | 3.4742 | 1.9919 | 0.7381 | 6.6445 | 0 | 3.6825 | 3.8109 | |
| 1.7261 | 5.3334 | 2.6338 | 3.5644 | 2.474 | 1.0317 | 2.2364 | 6.6638 | 1.84 |
| | 2.5889 | 3.8407 | 4.9123 | 5.9297 | 2.655 | 0.942 | 1.298 | |
| 2.7564 | 2.4724 | 1.6598 | 1.2582 | 1.1272 | 3.6853 | 6.6416 | 1.9305 | |
| 3.166 | 3.3548 | 3.9715 | 3.3684 | 3.498 | 3.7442 | 2.8698 | 1.1063 | |
| 3.2092 | 3.1725 | 5.3716 | 2.7619 | 5.4122 | 2.8461 | 2.8198 | 4.0595 | |
| 2.7261 | 4.0165 | 1.1634 | 7.2577 | 8.76 | 7.6077 | 5.936 | 7.7659 | |
| 6.5325 | 8.6325 | 7.4237 | 8.033 | 9.5492 | 8.5096 | 7.4517 | 8.9918 | |
| 9.3471 | 6.8234 | 7.0851 | 7.9468 | 8.4467 | 5.2622 | 7.309 | 7.9994 | |
| 9.0507 | 8.8703 | 7.4077 | | | | | | |
| TACC1 | 8.7384 | 10.8412 | 9.9502 | 9.2496 | 10.9219 | 8.4763 | 8.4063 | |
| 7.5556 | 8.9227 | 7.4981 | 9.0526 | 7.6521 | 9.6497 | 9.2452 | 8.325 | |
| 9.3486 | 8.0992 | 8.7834 | 8.9347 | 9.6866 | 8.5703 | 9.5828 | 8.6077 | |
| 8.3594 | 7.8702 | 10.4974 | 8.6019 | 8.9157 | 8.446 | 7.5896 | 8.2835 | |
| 8.7158 | 8.2907 | 7.3283 | 11.4474 | 9.02 | 8.0361 | 9.2487 | 8.4831 | |
| 7.2172 | 8.6547 | 9.5648 | 7.8788 | 9.6047 | 9.6307 | 9.2101 | 9.8938 | |
| 8.5935 | 9.2684 | 11.2289 | 11.1087 | 8.2972 | 9.3117 | 10.2668 | 6.9176 | |
| 9.786 | 11.1913 | 9.4295 | 10.3876 | 10.3983 | 8.5462 | 8.8565 | 9.3141 | |
| 8.6978 | 10.8342 | 8.1669 | 9.0047 | 10.0149 | 9.4713 | 9.9045 | 9.1234 | |
| 7.9927 | 11.2531 | 9.2972 | 9.2983 | 9.8244 | 8.3553 | 8.0247 | 9.6541 | |
| 10.4085 | 9.1285 | 8.3876 | 10.0837 | 9.3758 | 8.764 | 9.9692 | 9.0625 | |
| 7.7448 | 10.0554 | 8.7628 | 9.0802 | 9.5264 | 9.2773 | 9.5031 | 9.4683 | |
| 8.0081 | 7.8466 | 8.2047 | 9.8169 | 7.1799 | 11.4242 | 8.7755 | 9.3533 | |

| | | | | | | | | |
|---|---|---|---|---|---|---|---|---|
| 9.4833 | 10.8415 | 8.7847 | 8.4731 | 8.7842 | 9.2753 | 7.184 | 9.0347 | |
| 9.1753 | 5.4702 | 9.2189 | 8.7541 | 9.2158 | 9.2172 | 8.591 | 9.3033 | |
| 7.7203 | 9.692 | 11.9602 | 7.4093 | 9.2513 | 8.3553 | 9.6735 | 10.1824 | |
| 8.3531 | 8.2654 | 6.3427 | 11.9589 | 7.6705 | 9.9626 | 8.662 | 10.2013 | |
| 9.2108 | 9.175 | 7.4195 | 9.156 | 6.2909 | 10.2906 | 9.4373 | 8.5574 | |
| 8.5607 | 9.4415 | 9.4685 | 10.9584 | 9.6514 | 8.0744 | 9.0953 | 8.6165 | |
| 9.4769 | 9.5485 | 8.1476 | 9.5679 | 9.5259 | 10.5403 | 7.8199 | 7.5173 | |
| 6.9804 | 8.7005 | 8.3985 | 9.322 | 8.8833 | 8.7076 | 8.2017 | 8.9929 | |
| 9.8741 | 7.9076 | 8.5909 | 10.0997 | 8.876 | 6.7009 | 9.213 | 10.1526 | |
| 10.3881 | 7.5515 | 12.347 | 10.938 | 12.3161 | 11.1492 | 13.5322 | 12.9842 | |
| 13.1901 | 13.28 | 13.4024 | 11.1822 | 13.0608 | 13.2911 | 12.4321 | 10.7466 | |
| 11.6521 | 12.6993 | 13.4763 | 12.6206 | 11.566 | 11.99 | 12.5037 | 11.8192 | |
| 13.1147 | 11.9916 | | | | | | | |
| HSPB7 | 5.9465 | 4.8055 | 3.8127 | 6.78E+00 | | 4.1351 | 4.303 | |
| 5.7019 | 2.7217 | 4.5343 | 4.0163 | 4.1658 | 3.8612 | 3.8176 | 2.1513 | |
| 4.8326 | 3.8641 | 2.7962 | 5.3785 | 4.0762 | 5.3122 | 2.7536 | 0.8021 | |
| 4.6839 | 6.3795 | 2.472 | 1.2172 | 7.0366 | 3.0546 | 5.1282 | 3.5858 | |
| 1.492 | 7.2767 | 3.1787 | 3.7318 | 2.3807 | 5.0275 | 4.2348 | 4.5471 | |
| 3.5656 | 2.8871 | 3.8179 | 6.4719 | 2.6955 | 3.8726 | 2.5833 | 8.2587 | |
| 5.9039 | 2.8432 | 6.5846 | 4.7023 | 8.9313 | 3.5478 | 4.3452 | 4.5039 | |
| 4.3911 | 4.2026 | 4.6519 | 5.8747 | 9.3684 | 8.9675 | 3.6511 | 4.0271 | |
| 3.3577 | 3.5387 | 6.459 | 1.1076 | 7.2747 | 3.46 | 4.6727 | 8.2223 | |
| 3.3401 | 4.2261 | 7.495 | 2.7931 | 7.1121 | 7.3546 | 4.6136 | 5.1705 | |
| 6.6914 | 7.2937 | 5.6242 | 2.017 | 5.8913 | 6.5047 | 5.1155 | 7.2956 | |
| 3.9447 | 5.9997 | 5.7943 | 5.7182 | 6.1469 | 1.9209 | 7.4841 | 6.5925 | 5.53 |
| | 4.2556 | 2.9711 | 3.4186 | 7.5063 | 5.3923 | 5.1741 | 4.5671 | |
| 4.0404 | 7.1397 | 5.7017 | 3.062 | 8.4037 | 4.8933 | 4.4967 | 4.3402 | |
| 4.3694 | 7.4362 | 2.4149 | 7.0958 | 1.4687 | 5.3123 | 5.475 | 4.1368 | |
| 7.4912 | 8.0898 | 7.0221 | 9.1609 | 2.9483 | 4.141 | 4.7044 | 5.062 | |
| 3.6471 | 3.9333 | 5.3989 | 3.1725 | 9.6867 | 6.5025 | 5.1356 | 4.8111 | |
| 2.8077 | 5.8231 | 6.3604 | 3.3622 | 4.3639 | 2.2191 | 3.0745 | 3.946 | |
| 5.2994 | 4.0675 | 3.9614 | 7.4863 | 4.4276 | 5.7895 | 2.3633 | 2.6939 | |
| 4.1562 | 2.6914 | 4.4275 | 6.1698 | 2.5204 | 6.6542 | 6.6885 | 5.0179 | |
| 3.166 | 5.7481 | 3.6724 | 4.247 | 6.7446 | 5.0763 | 5.106 | 2.985 | |
| 5.6717 | 4.7177 | 4.1245 | 4.3071 | 8.0625 | 4.4075 | 2.8198 | 2.2195 | |
| 3.6809 | 7.8162 | 5.8588 | 10.5168 | 8.1406 | 10.2552 | 7.5986 | 12.5195 | |
| 9.8155 | 10.8286 | 11.5987 | 11.272 | 9.2001 | 11.2023 | 12.1317 | 10.4899 | |
| 8.3653 | 8.8734 | 10.6633 | 11.8918 | 10.376 | 9.9798 | 10.8487 | 9.9979 | |
| 10.29 | 11.8278 | 10.6066 | | | | | | |
| ASPM | 9.5245 | 9.395 | 8.4685 | 9 | 10.689 | 9.5422 | 9.3509 | |
| 9.4423 | 9.7036 | 9.6185 | 9.3078 | 9.5011 | 8.7801 | 8.1977 | 8.545 | |
| 9.2708 | 8.4217 | 9.1972 | 7.5319 | 8.5065 | 8.1525 | 11.4115 | 7.6961 | |
| 6.6114 | 9.2609 | 10.4626 | 10.5579 | 9.702 | 8.6613 | 10.599 | 9.0225 | |
| 8.9988 | 10.7206 | 9.0366 | 9.4415 | 8.2658 | 8.8647 | 10.1594 | 8.3336 | |
| 6.7005 | 10.0506 | 10.4318 | 7.6478 | 10.088 | 9.1316 | 9.1186 | 9.2542 | |
| 9.4577 | 8.3978 | 8.1213 | 9.9237 | 9.3324 | 10.2387 | 9.5031 | 9.3717 | |
| 8.8483 | 7.8001 | 9.958 | 5.9317 | 9.7502 | 9.4176 | 10.1599 | 7.7829 | |
| 8.5617 | 9.2597 | 7.4448 | 8.8651 | 5.2759 | 5.9316 | 9.1701 | 8.2933 | |
| 10.6505 | 6.4665 | 10.1739 | 7.4059 | 8.7534 | 9.1582 | 8.1075 | 8.8709 | |
| 11.0574 | 10.0286 | 7.8536 | 7.4799 | 8.9454 | 8.9452 | 8.6887 | 9.5899 | |
| 7.7448 | 8.9445 | 9.9706 | 9.3258 | 9.51 | 11.4011 | 9.1772 | 9.9895 | |
| 8.7051 | 10.1544 | 8.9028 | 6.9682 | 8.2574 | 10.8506 | 9.4709 | 9.4291 | 8.69 |
| | 9.8936 | 8.8616 | 10.1461 | 8.7842 | 9.4401 | 8.5971 | 9.6476 | |
| 9.9264 | 6.6325 | 10.2036 | 9.7326 | 8.3664 | 9.4314 | 8.4978 | 7.7315 | |

| | | | | | | | | |
|---|---|---|---|---|---|---|---|---|
| 8.5307 | 0 | 8.8346 | 9.3661 | 8.1789 | 7.1758 | 9.3813 | 9.4523 | |
| 8.4691 | 7.1592 | 8.8452 | 7.4712 | 6.8088 | 8.9274 | 8.6459 | 9.6722 | |
| 9.8613 | 10.1875 | 8.9072 | 9.1675 | 7.267 | 8.9734 | 8.1662 | 7.3846 | |
| 8.6612 | 9.345 | 8.3914 | 9.5739 | 9.2261 | 10.6449 | 9.67 | 10.2654 | |
| 9.4583 | 10.0788 | 4.983 | 9.7263 | 10.5114 | 6.2627 | 9.2687 | 8.2742 | |
| 10.0035 | 7.4245 | 9.6768 | 8.4603 | 9.6306 | 8.7076 | 8.5153 | 9.1515 | |
| 9.516 | 8.5102 | 8.6177 | 10.1261 | 9.7312 | 9.6709 | 8.7017 | 9.0829 | |
| 8.4026 | 6.4041 | 3.0238 | 8.9546 | 5.126 | 3.8339 | 3.0853 | 4.1593 | |
| 2.8515 | 4.0593 | 3.2033 | 7.8594 | 3.9963 | 3.5027 | 4.4378 | 6.0085 | |
| 5.0791 | 5.217 | 7.3512 | 2.8669 | 3.4632 | 5.7748 | 2.7525 | 6.7792 | |
| 2.5659 | 2.6496 | | | | | | | |
| CD300LG | 0 | 0.6896 | 0 | 5.28E-01 | | 1.20E+00 | | 0 |
| | 1.56 | 0.7652 | 0 | 0 | 1.2705 | 1.2777 | 0.5377 | |
| 1.4442 | 2.5489 | 0 | 0 | 0.6175 | 1.131 | 0 | 3.3041 | 0 |
| | 1.8373 | 0.8532 | 0 | 4.3836 | 0 | 0 | 0 | 0 |
| | 0.6819 | 2.8291 | 0.3648 | 0 | 0.4337 | 3.1062 | 0 | 0 |
| | 0 | 0 | 0 | 0 | 0 | 0 | 0 | |
| 1.0956 | 1.7133 | 0 | 0.6343 | 1.8088 | 2.6189 | 1.2977 | 1.1594 | 0 |
| | 0 | 0.6977 | 0 | 0 | 1.9829 | 3.9922 | 0 | |
| 1.1694 | 0 | 2.0521 | 2.2033 | 2.388 | 0.5548 | 2.7891 | 1.5048 | 0 |
| | 3.0365 | 0 | 5.5137 | 0.6718 | 2.9265 | 0 | 0.9284 | |
| 0.614 | 2.3134 | 1.679 | 0.847 | 1.2055 | 2.0255 | 0.5198 | 0 | |
| 1.8231 | 0 | 0 | 2.2374 | 0.5352 | 0 | 1.7606 | 1.717 | |
| 1.7824 | 0.5426 | 2.0061 | 1.1907 | 0 | 0 | 0 | 0 | |
| 1.3697 | 2.5502 | 1.261 | 1.1815 | 0.6089 | 3.6007 | 0 | 0 | 0 |
| | 0 | 0 | 0 | 0 | 0 | 0.5526 | 2.8502 | |
| 0.8107 | 0 | 0 | 0 | 4.8297 | 0.601 | 0.5748 | 0.6506 | |
| 0.5424 | 0 | 0.6735 | 0 | 0 | 3.708 | 0 | 0.5414 | |
| 2.0029 | 0 | 1.0445 | 0.3855 | 0 | 0 | 0.6063 | 0 | |
| 1.3167 | 0 | 1.0531 | 0 | 2.4241 | 3.4931 | 1.4679 | 0.5466 | |
| 0.4486 | 0 | 2.4724 | 0 | 0 | 0 | 1.834 | 2.6986 | |
| 1.2666 | 0 | 0.91 | 0.5055 | 0 | 0 | 2.161 | 0 | |
| 1.4486 | 0.9785 | 0 | 0.5556 | 0.9737 | 0.7547 | 0.8682 | 0 | |
| 3.0556 | 0.3736 | 0 | 0 | 2.1912 | 2.0772 | 4.3187 | 2.2832 | |
| 3.9977 | 5.6438 | 8.0227 | 6.5179 | 7.226 | 2.5466 | 7.0049 | 5.3081 | |
| 5.2559 | 2.9263 | 4.2433 | 3.9785 | 4.8061 | 5.7629 | 3.1292 | 7.663 | |
| 3.6955 | 4.5686 | 5.9599 | 4.7467 | | | | | |
| MYCT1 | 4.8581 | 5.7633 | 5.6423 | 4.6352 | 5.9934 | 4.6304 | 6.2884 | |
| 5.3777 | 6.1563 | 5.5156 | 6.8373 | 5.3024 | 5.8559 | 5.2804 | 5.807 | |
| 6.0277 | 4.8405 | 5.2221 | 6.5881 | 6.6819 | 4.8678 | 5.2962 | 6.6779 | |
| 5.5286 | 5.3188 | 5.3369 | 5.8595 | 4.9979 | 5.8453 | 4.6733 | 4.6536 | |
| 6.7372 | 5.7684 | 5.7244 | 4.9615 | 6.8254 | 6.0288 | 5.5593 | 5.067 | |
| 5.8565 | 7.1634 | 5.5332 | 4.6809 | 5.1113 | 6.5504 | 6.0402 | 7.4509 | |
| 5.157 | 5.841 | 7.0536 | 8.4986 | 5.9488 | 6.7203 | 5.0675 | 4.2813 | |
| 6.3216 | 5.2665 | 5.2325 | 5.7589 | 7.6981 | 4.1553 | 5.9735 | 5.176 | |
| 6.3856 | 7.3104 | 5.7059 | 6.716 | 7.4185 | 7.9027 | 5.6588 | 5.8135 | |
| 6.0131 | 8.4754 | 6.5834 | 6.6124 | 5.6703 | 6.2727 | 4.726 | 6.4063 | |
| 6.8951 | 6.5181 | 4.6656 | 7.1773 | 5.6816 | 6.4739 | 6.7326 | 6.5241 | |
| 4.7356 | 6.4339 | 5.2583 | 7.0232 | 5.0722 | 6.2571 | 7.3829 | 7.5244 | |
| 6.1165 | 5.1023 | 4.4595 | 5.9616 | 5.7814 | 6.1539 | 5.2861 | 6.6988 | |
| 5.7936 | 5.8198 | 6.8774 | 8.0306 | 5.3145 | 6.7878 | 5.3181 | 5.1646 | |
| 5.2556 | 4.8661 | 5.7295 | 4.8721 | 6.0918 | 7.0903 | 5.3565 | 5.7769 | |
| 4.7711 | 7.9955 | 9.0865 | 4.3307 | 6.0678 | 5.5593 | 6.07 | 5.0916 | |
| 5.7212 | 4.8476 | 4.3004 | 9.3054 | 4.8711 | 5.2947 | 6.3076 | 3.9795 | |

| | | | | | | | |
|---|---|---|---|---|---|---|---|
| 5.6709 | 5.4655 | 5.453 | 4.7036 | 5.3467 | 5.7698 | 5.8618 | 4.324 |
| 5.6464 | 5.386 | 5.9403 | 7.4988 | 6.2575 | 5.5705 | 5.496 | 6.8376 |
| 4.624 | 4.8072 | 7.2417 | 6.3563 | 6.2726 | 8.7386 | 5.0386 | 6.136 |
| 5.0289 | 5.9009 | 4.754 | 6.1259 | 6.1686 | 5.4972 | 4.1933 | 5.8278 |
| 6.2814 | 6.6591 | 5.9135 | 6.503 | 4.7549 | 4.872 | 5.149 | 5.9992 |
| 6.1755 | 3.9316 | 9.2558 | 8.3401 | 8.9095 | 6.6101 | 9.6842 | 9.5746 |
| 10.6002 | 9.9944 | 10.6074 | 8.8045 | 9.7234 | 10.2436 | 9.3768 | 8.2912 |
| 7.5902 | 9.842 | 9.97 | 8.5489 | 6.0583 | 8.9787 | 9.9081 | 8.7446 |
| 9.5447 | 8.1959 | | | | | | |
| ARHGAP6 | 3.7656 | 3.4337 | 3.5366 | 6.70E+00 | | 3.51E+00 | |
| 4.3999 | 5.4551 | 3.9061 | 3.51 | 3.0341 | 4.6647 | 2.2979 | 3.4513 |
| 3.5017 | 4.9877 | 4.7766 | 3.785 | 3.8962 | 3.8167 | 3.9968 | 3.7016 |
| 4.8335 | 4.2875 | 3.4165 | 5.7294 | 3.2143 | 4.5453 | 3.7968 | 3.844 |
| 2.4602 | 2.0076 | 4.867 | 3.084 | 3.6294 | 3.9752 | 4.8631 | 4.5988 |
| 7.1237 | 5.0276 | 1.5457 | 7.9378 | 6.5672 | 3.5316 | 3.3005 | 4.0611 |
| 4.8508 | 5.913 | 4.8114 | 6.5166 | 4.6067 | 7.8218 | 3.8203 | 5.8618 |
| 3.7288 | 2.0265 | 4.2268 | 2.9405 | 4.0799 | 2.98 | 6.7557 | 3.0014 |
| 4.3256 | 5.176 | 8.622 | 6.4064 | 1.3447 | 4.1607 | 4.6756 | 5.2619 |
| 4.7019 | 3.7537 | 3.6309 | 6.9011 | 4.4037 | 5.7263 | 4.3067 | 3.4508 |
| 4.0803 | 4.9335 | 6.0386 | 4.9142 | 4.7613 | 6.2232 | 4.0547 | 6.0666 |
| 5.5837 | 5.388 | 0.6391 | 4.2604 | 5.3877 | 5.912 | 3.6025 | 5.9805 |
| 7.0142 | 5.2474 | 4.8152 | 2.5118 | 3.8543 | 3.7633 | 3.3219 | 4.513 |
| 3.8279 | 3.3607 | 4.9227 | 4.1541 | 3.4563 | 7.2052 | 3.9645 | 4.6899 |
| 3.5107 | 4.5175 | 8.9365 | 1.2894 | 8.2069 | 3.2985 | 4.1539 | 4.6528 |
| 5.3565 | 4.2627 | 2.7218 | 6.0536 | 7.8757 | 3.2906 | 3.6721 | 3.6966 |
| 4.6807 | 2.9437 | 4.7323 | 3.9441 | 5.1593 | 8.3743 | 2.2924 | 5.1356 |
| 3.5271 | 4.933 | 3.9889 | 2.952 | 2.7383 | 4.0031 | 2.9615 | 4.2094 |
| 4.2012 | 4.2526 | 4.5146 | 4.3917 | 4.9467 | 5.9523 | 3.1615 | 3.1425 |
| 2.848 | 2.848 | 3.7316 | 2.4113 | 1.2582 | 4.6711 | 8.1455 | 6.1333 |
| 3.7208 | 3.4351 | 2.3092 | 3.7644 | 4.6429 | 4.4327 | 3.3116 | 2.4374 |
| 2.6295 | 4.385 | 4.3208 | 7.5544 | 7.0558 | 4.9005 | 4.1636 | 2.8198 |
| 4.0136 | 4.5343 | 5.6294 | 3.6757 | 8.5882 | 7.301 | 8.5684 | 6.8009 |
| 10.1916 | 9.0376 | 9.4586 | 10.0004 | 9.8628 | 8.2521 | 9.166 | 9.932 |
| 9.1715 | 7.6841 | 7.4702 | 9.0998 | 9.8565 | 8.8314 | 9.5115 | 9.0291 |
| 8.8125 | 8.6967 | 9.8831 | 8.7688 | | | | |
| CENPN | 9.8052 | 8.6016 | 7.8526 | 10.0939 | 8.2902 | 7.9536 | 8.1759 |
| 9.4465 | 8.8051 | 8.4138 | 8.574 | 9.1752 | 8.6469 | 8.5463 | 8.1125 |
| 8.9702 | 8.311 | 9.0713 | 7.6617 | 8.4212 | 9.7477 | 9.8868 | 8.9123 |
| 8.223 | 8.8046 | 8.9987 | 9.1209 | 10.1114 | 8.191 | 8.5057 | 9.7038 |
| 8.0154 | 9.3385 | 9.4581 | 7.8841 | 9.6151 | 9.1093 | 8.7369 | 9.468 |
| 10.5817 | 8.7857 | 9.7736 | 8.4456 | 8.7945 | 8.9409 | 9.0338 | 9.2581 |
| 9.3343 | 8.3414 | 8.3239 | 8.3593 | 8.4881 | 8.8205 | 9.996 | 8.8229 |
| 8.8738 | 7.8861 | 8.7522 | 7.5009 | 8.9764 | 8.799 | 8.7011 | 9.3839 |
| 8.5173 | 9.4548 | 8.1658 | 8.1769 | 7.5353 | 7.2038 | 9.2716 | 9.5379 |
| 9.1705 | 7.69 | 9.1476 | 8.8826 | 9.2301 | 8.2884 | 9.2506 | 7.9818 |
| 9.7638 | 9.2289 | 7.7037 | 8.606 | 10.2349 | 8.2231 | 8.1604 | 8.929 |
| 8.9034 | 9.48 | 8.8071 | 9.3976 | 10.2734 | 10.0064 | 8.525 | 10.7325 |
| 8.4681 | 8.5717 | 8.6793 | 8.9458 | 9.3601 | 9.2353 | 8.8778 | 8.4441 |
| 8.1427 | 8.7107 | 9.5304 | 9.304 | 9.0157 | 8.3663 | 8.8829 | 9.6675 |
| 9.6203 | 8.2226 | 8.7546 | 8.8444 | 8.5668 | 9.4749 | 9.2221 | 9.304 |
| 10.3433 | 7.4368 | 8.1357 | 10.4945 | 8.7152 | 8.5113 | 9.3824 | 9.3758 |
| 7.9485 | 9.357 | 10.2967 | 7.7738 | 9.6051 | 8.145 | 8.0961 | 8.9292 |
| 8.8085 | 9.3642 | 9.1334 | 9.8615 | 9.2457 | 9.8856 | 8.2102 | 7.8186 |
| 8.5976 | 9.0459 | 8.3883 | 8.4408 | 9.8177 | 8.8124 | 8.8787 | 9.3406 |

| | | | | | | | | |
|---|---|---|---|---|---|---|---|---|
| 8.5115 | 9.3693 | 8.4869 | 8.6265 | 9.1628 | 7.0329 | 9.7545 | 9.223 | |
| 8.7808 | 8.1164 | 8.3385 | 8.5676 | 8.1242 | 9.7938 | 9.2879 | 9.9445 | |
| 9.8646 | 8.2027 | 8.8524 | 9.8255 | 9.0229 | 9.4332 | 8.9103 | 8.8689 | |
| 8.313 | 9.7707 | 6.7384 | 9.055 | 7.1089 | 5.8845 | 6.3655 | 7.1742 | |
| 5.0943 | 6.4601 | 6.6251 | 8.2054 | 6.1339 | 6.4862 | 6.2327 | 7.6537 | |
| 5.8211 | 6.555 | 7.0464 | 5.8553 | 5.9069 | 6.9567 | 6.0898 | 7.8288 | |
| 5.7878 | 5.5573 | | | | | | | |
| TBC1D2B | 8.2932 | 9.794 | 8.8717 | 8.4689 | 9.3052 | 8.5183 | 8.8223 | |
| 8.3654 | 8.3253 | 8.3301 | 8.8774 | 8.4644 | 8.7772 | 8.9859 | 8.6705 | |
| 9.2607 | 9.3083 | 9.1546 | 8.0935 | 9.335 | 8.6772 | 10.889 | 8.9243 | |
| 9.1038 | 8.7141 | 8.9824 | 9.336 | 8.9785 | 8.8147 | 8.143 | 9.4072 | |
| 9.5601 | 9.2582 | 8.1382 | 8.7204 | 8.7875 | 8.0943 | 9.4604 | 8.4499 | |
| 8.4709 | 8.9225 | 10.1849 | 8.8793 | 9.2905 | 9.9548 | 9.0397 | 9.6666 | |
| 8.5485 | 10.1002 | 10.2999 | 9.7871 | 8.8082 | 9.2776 | 9.3332 | 9.2592 | |
| 8.9269 | 8.8857 | 8.7314 | 8.8855 | 10.0819 | 8.8356 | 8.6821 | 9.8081 | |
| 8.7396 | 10.2812 | 8.2997 | 9.5409 | 9.6771 | 9.2009 | 10.2142 | 8.195 | |
| 8.1663 | 10.4431 | 9.1914 | 8.4926 | 8.1763 | 8.6607 | 9.3045 | 9.1074 | |
| 10.2744 | 9.4033 | 9.2497 | 9.889 | 8.7772 | 9.0453 | 8.5829 | 9.5002 | |
| 8.7263 | 9.7112 | 8.9447 | 8.8013 | 8.7433 | 9.0647 | 9.5197 | 9.5895 | |
| 9.2784 | 8.8395 | 8.5476 | 9.0902 | 9.2503 | 9.1173 | 9.8964 | 8.8322 | |
| 9.1139 | 8.2856 | 9.4947 | 9.5765 | 8.9869 | 9.4788 | 8.3437 | 9.1707 | |
| 9.0216 | 7.3011 | 8.1185 | 7.3175 | 9.1474 | 9.2698 | 9.8016 | 9.5954 | |
| 8.8834 | 9.7589 | 10.5537 | 8.7235 | 9.2408 | 8.9041 | 9.2207 | 8.9102 | |
| 8.625 | 9.0171 | 7.8753 | 10.7069 | 8.3597 | 9.3721 | 8.9493 | 8.1333 | |
| 9.7387 | 9.1181 | 8.5183 | 9.7615 | 8.22 | 9.3255 | 8.9205 | 8.8229 | |
| 9.2765 | 8.5651 | 7.8876 | 9.5457 | 8.7083 | 8.5168 | 8.7281 | 8.6311 | |
| 9.8597 | 8.539 | 8.7427 | 9.1665 | 9.2675 | 10.3878 | 8.5398 | 9.4621 | |
| 8.3499 | 8.7604 | 8.56 | 9.306 | 9.4116 | 8.0891 | 8.2435 | 9.1662 | |
| 9.5634 | 7.9019 | 8.8895 | 10.687 | 9.3282 | 8.7114 | 8.7946 | 9.0599 | |
| 9.3228 | 8.4046 | 10.7972 | 10.661 | 11.2227 | 9.9512 | 10.5072 | 10.8929 | |
| 11.2975 | 11.3064 | 11.0977 | 11.0761 | 11.7198 | 10.4535 | 11.7515 | 10.1554 | |
| 10.3795 | 11.1224 | 11.0944 | 11.4783 | 12.1555 | 10.8393 | 11.1364 | 10.0338 | |
| 11.1421 | 11.4342 | | | | | | | |
| PTGFR | 3.433 | 1.5051 | 3.3181 | 5.8899 | 0 | 3.0554 | 2.505 | |
| 0.4327 | 0.7594 | 1.3775 | 2.483 | 0.4395 | 2.206 | 1.4442 | 2.4094 | |
| 4.1804 | 1.3142 | 1.0485 | 1.131 | 2.3107 | 0 | 0.4561 | 2.0314 | |
| 2.8976 | 1.5953 | 2.8666 | 3.9322 | 0 | 1.101 | 0.5853 | 1.1431 | |
| 2.7304 | 2.3299 | 0.541 | 3.3387 | 3.4533 | 0.6231 | 1.2622 | 0.9285 | 0 |
| | 0 | 5.0799 | 2.86 | 1.5081 | 2.7455 | 3.4148 | 4.4771 | |
| 0.9635 | 6.6093 | 2.5866 | 7.3102 | 4.7423 | 3.2053 | 2.2545 | 2.6144 | |
| 3.279 | 0 | 3.2255 | 6.4531 | 8.2432 | 0 | 3.7871 | 2.4919 | |
| 1.9083 | 3.5065 | 0 | 1.9314 | 1.711 | 4.9551 | 4.2955 | 2.7579 | |
| 2.0578 | 8.3607 | 1.1284 | 5.6525 | 1.3843 | 2.7804 | 0 | 4.4419 | |
| 5.3908 | 4.895 | 2.7354 | 4.4108 | 2.9084 | 0.5233 | 5.5187 | 7.4295 | 0 |
| | 3.5526 | 0.5352 | 4.2949 | 3.6941 | 5.0118 | 5.4204 | 2.2182 | |
| 2.8143 | 3.1897 | 1.3871 | 1.3631 | 3.585 | 4.9526 | 4.3226 | 6.6168 | |
| 0.7641 | 3.9621 | 3.7657 | 5.1196 | 0.5886 | 2.6767 | 0.8239 | 1.8805 | |
| 4.6694 | 0 | 0 | 0.6684 | 4.0357 | 4.5288 | 2.4659 | 4.144 | |
| 3.7942 | 4.2995 | 8.1721 | 0.601 | 0 | 0 | 3.204 | 3.1391 | |
| 1.7568 | 1.3155 | 0 | 7.6342 | 0 | 2.6925 | 2.4626 | 0 | |
| 3.1934 | 2.6338 | 0 | 3.6832 | 1.36 | 2.8804 | 1.8011 | 0 | |
| 0.4418 | 2.9381 | 2.7703 | 3.4514 | 4.1875 | 2.0787 | 0.7903 | 0.9151 | |
| 2.7896 | 2.084 | 2.7155 | 4.8982 | 4.0348 | 5.6871 | 3.6145 | 2.4045 | |
| 2.4318 | 2.4895 | 2.5017 | 4.3584 | 1.7992 | 0 | 2.1566 | 2.4241 | |

| | | | | | | | |
|---|---|---|---|---|---|---|---|
| 3.8154 | 0.5556 | 1.7696 | 3.8135 | 2.9266 | 0 | 0 | 0.3736 |
| 5.6171 | 0.6959 | 6.5977 | 6.9128 | 7.1666 | 5.2486 | 10.2134 | 7.3861 |
| 11.6725 | 8.9656 | 10.5707 | 7.7582 | 9.8953 | 10.4663 | 8.5734 | 6.1493 |
| 7.7186 | 10.4035 | 10.1632 | 9.6702 | 8.3648 | 7.6063 | 8.1768 | 8.099 |
| 9.3408 | 8.8908 | | | | | | |
| GSTM5 | 2.2429 | 2.2256 | 2.988 | 4.6352 | 3.4259 | 4.1992 | 5.2037 |
| 3.6885 | 2.987 | 2.886 | 6.792 | 1.2777 | 3.4513 | 4.0436 | 4.6587 |
| 5.3054 | 2.7331 | 2.2447 | 3.8167 | 2.8404 | 0 | 2.8719 | 6.3811 |
| 4.3473 | 1.5953 | 2.7995 | 3.5773 | 2.2837 | 1.3374 | 0.5853 | 1.7727 |
| 2.6245 | 1.5916 | 2.7898 | 3.8754 | 5.4509 | 4.808 | 1.7856 | 4.3198 |
| 3.9141 | 3.3964 | 3.7911 | 2.4071 | 2.44 | 3.9981 | 5.7361 | 5.2127 |
| 1.5362 | 3.4514 | 6.5229 | 6.9188 | 6.9932 | 4.6588 | 3.6766 | 1.6085 |
| 3.7812 | 3.034 | 4.1783 | 1.791 | 6.8386 | 1.6333 | 3.1387 | 5.5338 |
| 5.7472 | 5.3294 | 0.8237 | 4.6119 | 6.4116 | 7.9284 | 3.7276 | 4.2306 |
| 2.354 | 8.4462 | 3.3909 | 5.9988 | 5.3668 | 3.6714 | 3.8869 | 5.6807 |
| 4.7773 | 4.2758 | 3.403 | 8.798 | 4.7332 | 2.3031 | 5.051 | 5.3719 |
| 4.1922 | 4.4898 | 4.0633 | 4.9514 | 5.4722 | 4.6449 | 6.1928 | 6.3545 |
| 5.9738 | 1.9975 | 0 | 6.0327 | 4.7549 | 6.008 | 0.6116 | 2.9304 |
| 3.4758 | 3.2084 | 4.9011 | 3.806 | 4.2199 | 5.8216 | 3.2437 | 4.8437 |
| 5.4258 | 1.2894 | 2.6625 | 3.1145 | 3.663 | 4.0766 | 5.1267 | 5.491 |
| 1.0837 | 10.1867 | 7.4883 | 2.0359 | 3.4956 | 3.7587 | 3.6856 | 2.7971 |
| 1.9907 | 2.1615 | 0 | 8.3001 | 5.0515 | 4.571 | 5.5429 | 1.5093 |
| 2.3925 | 2.1273 | 0 | 4.2986 | 1.36 | 1.0431 | 6.5149 | 3.4163 |
| 3.7206 | 3.7543 | 0 | 6.6678 | 5.1109 | 1.5072 | 1.829 | 0.9151 |
| 2.214 | 3.4221 | 6.9963 | 1.3669 | 5.5794 | 7.3536 | 4.7429 | 4.1841 |
| 3.2252 | 5.1461 | 2.5017 | 4.1094 | 5.3107 | 4.4477 | 2.6295 | 1.7765 |
| 2.3837 | 0.9557 | 3.9503 | 5.7802 | 4.1964 | 5.3717 | 2.7543 | 3.259 |
| 5.2682 | 3.744 | 7.9013 | 8.6266 | 7.1311 | 5.9996 | 8.636 | 8.1341 |
| 10.0443 | 10.8958 | 10.348 | 9.5233 | 10.4671 | 9.5201 | 9.8681 | 9.6037 |
| 9.6179 | 9.9128 | 9.6212 | 10.1812 | 13.4606 | 10.1061 | 9.6655 | 10.3727 |
| 11.5855 | 10.5524 | | | | | | |
| CCNF | 9.6547 | 8.1933 | 8.0018 | 10.0655 | 8.4389 | 8.7319 | 8.8518 |
| 10.1823 | 9.3608 | 8.6254 | 8.1417 | 9.1396 | 8.3892 | 7.7443 | 8.5324 |
| 9.2624 | 8.8752 | 8.8945 | 7.6816 | 7.6016 | 9.5117 | 10.3676 | 9.2955 |
| 8.1169 | 10.4327 | 10.9165 | 9.6203 | 9.5801 | 9.9113 | 9.4776 | 10.7833 |
| 7.9709 | 9.9114 | 8.8009 | 8.603 | 9.0118 | 8.5688 | 9.0547 | 8.8137 |
| 10.033 | 9.2134 | 9.9729 | 8.6638 | 9.103 | 8.4967 | 8.6131 | 8.7075 |
| 9.2806 | 8.7557 | 6.3784 | 8.9494 | 8.6408 | 9.116 | 9.6926 | 9.1913 |
| 8.8886 | 7.6008 | 9.8207 | 6.8102 | 10.0114 | 9.368 | 8.8882 | 8.8651 |
| 9.3225 | 8.089 | 8.2422 | 8.4968 | 6.9303 | 6.4793 | 9.7444 | 9.449 |
| 9.3737 | 6.6729 | 9.8395 | 7.9641 | 8.1329 | 7.7903 | 9.3937 | 8.632 |
| 10.2718 | 9.2366 | 8.7285 | 7.968 | 9.4376 | 8.8997 | 7.8788 | 9.1942 |
| 9.8131 | 9.4279 | 8.8677 | 9.8316 | 9.745 | 11.3648 | 7.8301 | 8.5785 |
| 9.8074 | 8.8327 | 8.9879 | 9.4265 | 8.9571 | 8.7708 | 9.1858 | 8.0234 |
| 9.3561 | 9.6631 | 8.5782 | 9.7525 | 8.3642 | 8.4754 | 9.501 | 9.026 |
| 9.6329 | 10.0825 | 9.6377 | 8.6287 | 9.1307 | 10.0559 | 8.9643 | 9.7659 |
| 9.7493 | 7.9307 | 8.5377 | 8.904 | 8.0111 | 8.3553 | 9.3116 | 9.2093 |
| 8.0762 | 8.6058 | 9.5696 | 7.2485 | 10.2414 | 8.3949 | 8.9434 | 9.4407 |
| 9.8723 | 9.5213 | 9.4688 | 10.3119 | 9.326 | 9.1332 | 7.7661 | 8.0664 |
| 9.5581 | 8.7256 | 8.4254 | 8.7427 | 9.127 | 9.7218 | 10.092 | 9.5539 |
| 9.8639 | 9.76 | 9.0611 | 9.2257 | 10.3863 | 7.5095 | 9.8345 | 9.4746 |
| 9.3331 | 8.0835 | 8.7924 | 8.0107 | 9.1421 | 9.8685 | 9.1893 | 9.8424 |
| 9.5634 | 7.8412 | 9.6918 | 9.7046 | 9.2705 | 9.3873 | 10.8258 | 8.554 |
| 7.7862 | 9.426 | 5.7079 | 8.7202 | 6.6405 | 6.3418 | 5.5788 | 5.9646 |

| | | | | | | | |
|---|---|---|---|---|---|---|---|
| 5.0498 | 5.3659 | 6.2701 | 7.5362 | 5.9004 | 5.4937 | 7.136 | 7.5634 |
| 6.6721 | 5.686 | 6.4772 | 6.1515 | 6.0284 | 7.1687 | 5.9141 | 7.1427 |
| 4.9829 | 6.3801 | | | | | | |
| MYH11 | 10.9445 | 8.911 | 7.3588 | 12.1111 | 7.7541 | 4.9829 | 10.7283 |
| 6.9457 | 6.6586 | 6.3569 | 7.3661 | 9.235 | 7.4067 | 5.8632 | 7.6214 |
| 7.933 | 5.0815 | 6.9268 | 8.78 | 8.7547 | 4.6922 | 6.9483 | 7.6051 |
| 11.8838 | 5.6165 | 6.6381 | 12.2744 | 6.1167 | 6.504 | 5.2811 | 5.9711 |
| 10.3562 | 7.7257 | 9.2463 | 6.9079 | 8.3525 | 9.1919 | 8.7608 | 7.8592 |
| 8.9532 | 5.9728 | 11.1751 | 7.7036 | 7.5926 | 8.2415 | 11.4425 | 10.4927 |
| 7.0901 | 11.9018 | 9.1921 | 14.5688 | 7.7737 | 10.6676 | 9.5216 | 6.8933 |
| 10.326 | 5.6336 | 10.5245 | 7.6772 | 14.6062 | 4.9232 | 9.2585 | 7.2383 |
| 7.2823 | 13.8979 | 6.3921 | 10.4801 | 8.1478 | 9.1161 | 12.9445 | 8.6584 |
| 9.8558 | 13.159 | 8.0213 | 13.0993 | 7.6321 | 8.5595 | 7.7731 | 12.1324 |
| 12.8055 | 11.2093 | 10.4044 | 10.1887 | 10.7981 | 8.1388 | 12.8973 | 8.8634 |
| 10.4662 | 11.5281 | 7.6151 | 11.4687 | 5.6165 | 12.9137 | 11.1526 | 9.6217 |
| 7.6727 | 8.5797 | 6.131 | 11.1847 | 9.0881 | 8.9288 | 6.0379 | 7.0411 |
| 7.186 | 11.5781 | 9.4997 | 11.209 | 10.1654 | 9.1169 | 8.0042 | 7.1735 |
| 12.3687 | 5.2846 | 8.4286 | 5.9766 | 10.8939 | 10.793 | 8.8989 | 12.9167 |
| 9.7112 | 11.5929 | 15.4214 | 7.1204 | 7.8899 | 7.0366 | 10.8579 | 7.1184 |
| 6.792 | 7.9899 | 6.8626 | 15.4332 | 10.6105 | 8.523 | 7.6853 | 7.2132 |
| 7.4282 | 8.8869 | 4.7468 | 8.7612 | 7.2533 | 10.1751 | 5.9453 | 6.0205 |
| 7.5885 | 8.9415 | 12.5861 | 8.8933 | 11.3094 | 5.0977 | 7.4306 | 7.0922 |
| 7.0511 | 9.0252 | 11.2787 | 9.503 | 11.8818 | 12.2483 | 10.1559 | 8.267 |
| 6.6089 | 6.5553 | 7.9081 | 12.0477 | 10.087 | 7.4994 | 7.2574 | 9.5856 |
| 11.2356 | 7.334 | 6.8256 | 12.194 | 7.9253 | 5.4545 | 5.0214 | 9.6179 |
| 12.9982 | 9.5386 | 15.189 | 13.8695 | 15.1864 | 13.5352 | 18.1628 | 16.056 |
| 16.7204 | 17.067 | 16.9465 | 14.3001 | 17.4981 | 17.6842 | 16.5946 | 14.4379 |
| 14.8595 | 16.2748 | 17.1526 | 16.2791 | 12.6808 | 16.5397 | 16.3365 | 15.6917 |
| 18.2009 | 16.0657 | | | | | | |
| BNC2 | 7.2762 | 7.6318 | 4.2746 | 7.21E+00 | | 7.41E+00 | |
| 6.7725 | 7.329 | 8.5718 | 7.7511 | 4.9525 | 7.6475 | 4.5724 | 4.8583 |
| 6.6802 | 4.5958 | 7.2168 | 5.918 | 8.6688 | 4.537 | 5.0501 | 4.8153 |
| 3.0115 | 4.8732 | 6.9015 | 6.6735 | 2.7292 | 6.6964 | 4.1856 | 6.7786 |
| 6.7491 | 4.4691 | 6.45 | 5.7531 | 6.1786 | 5.7441 | 4.9476 | 6.5862 |
| 7.2909 | 4.987 | 3.2199 | 8.789 | 5.9708 | 3.482 | 5.951 | 5.8945 |
| 8.0854 | 7.0448 | 6.8635 | 7.79 | 6.1685 | 9.5971 | 5.2273 | 7.3669 |
| 5.2394 | 5.0114 | 5.7922 | 4.4632 | 5.9694 | 7.3126 | 8.8223 | 4.55 |
| 4.3915 | 5.0203 | 4.0305 | 8.4634 | 2.586 | 7.9525 | 6.3725 | 5.7465 |
| 7.2899 | 5.01 | 5.1995 | 8.5694 | 4.7913 | 7.252 | 5.7588 | 4.5594 |
| 3.8342 | 7.344 | 10.1729 | 6.4604 | 4.1328 | 7.9394 | 6.3522 | 7.8017 |
| 6.9895 | 8.2294 | 4.6739 | 7.3834 | 7.1904 | 6.0775 | 4.4397 | 7.7626 |
| 9.5918 | 6.9091 | 4.9621 | 4.2769 | 5.6898 | 6.575 | 4.2479 | 9.4247 |
| 7.0949 | 5.3167 | 5.0799 | 6.0021 | 6.9342 | 9.7772 | 7.1759 | 7.8571 |
| 4.4459 | 8.9731 | 6.9515 | 5.6879 | 7.5369 | 3.8037 | 5.7097 | 5.7949 |
| 7.2535 | 5.7568 | 4.9645 | 7.3542 | 11.0467 | 3.2124 | 6.1792 | 3.6966 |
| 6.9622 | 6.2 | 3.6209 | 4.5746 | 6.2696 | 10.9282 | 2.5542 | 5.8785 |
| 5.3436 | 6.7189 | 8.215 | 6.2944 | 6.9691 | 4.9784 | 3.3786 | 5.7271 |
| 5.2583 | 5.3459 | 4.6023 | 5.4561 | 6.5002 | 5.7945 | 6.5504 | 5.2769 |
| 3.3899 | 7.8087 | 6.9838 | 5.2978 | 5.4645 | 4.0697 | 7.2542 | 7.1732 |
| 5.3845 | 7.1755 | 9.0505 | 4.3436 | 6.8361 | 6.9356 | 6.4398 | 6.7244 |
| 3.1812 | 6.8759 | 6.5838 | 8.1841 | 6.4232 | 8.5088 | 4.5414 | 3.4726 |
| 2.8619 | 6.6863 | 7.5272 | 5.5884 | 10.7786 | 9.4079 | 10.8236 | 9.8929 |
| 12.4051 | 11.5671 | 11.9382 | 12.6869 | 11.6275 | 10.0437 | 11.5225 | 11.9942 |
| 11.2556 | 8.733 | 10.4662 | 11.3972 | 12.0483 | 11.0871 | 11.6667 | 10.13 |

| | | | | | | | |
|---|---|---|---|---|---|---|---|
| 11.334 | 10.5883 | 11.7459 | 11.5499 | | | | |
| DTL | 9.6564 | 8.7461 | 7.5872 | 9.95E+00 | | 9.26E+00 | |
| 8.502 | 9.1459 | 8.4298 | 9.2851 | 8.0129 | 8.705 | 9.3975 | 8.5733 |
| 7.316 | 8.1416 | 8.8498 | 7.7566 | 7.9184 | 7.4751 | 8.4978 | 8.5958 |
| 10.6556 | 8.204 | 7.674 | 8.9193 | 10.0889 | 9.9795 | 8.5442 | 8.6694 |
| 9.4124 | 9.6048 | 8.5431 | 10.6621 | 8.6956 | 7.7663 | 8.3355 | 8.6506 |
| 9.1286 | 8.5402 | 8.2792 | 8.5668 | 10.1703 | 8.3267 | 9.4596 | 7.2226 |
| 9.1449 | 9.485 | 9.2426 | 7.8568 | 7.8432 | 9.1612 | 8.1739 | 10.0428 |
| 9.3479 | 8.9747 | 8.1127 | 7.3193 | 9.1664 | 5.65 | 8.6532 | 9.0942 |
| 9.6498 | 8.9593 | 7.6724 | 9.2933 | 9.0285 | 8.8104 | 5.9097 | 7.1173 |
| 9.0679 | 8.4501 | 9.3881 | 6.0392 | 8.9907 | 7.8254 | 8.6395 | 8.8853 |
| 8.0335 | 8.6384 | 10.1162 | 9.2096 | 8.587 | 6.7858 | 9.4194 | 8.6046 |
| 8.9858 | 9.1069 | 8.4043 | 8.7549 | 8.785 | 8.1952 | 8.762 | 10.3104 |
| 8.3786 | 8.7986 | 8.5968 | 9.3801 | 8.294 | 7.7989 | 8.0768 | 9.2782 |
| 9.343 | 9.5256 | 7.5954 | 9.1053 | 8.5703 | 9.9172 | 8.5029 | 8.6884 |
| 8.5871 | 8.8416 | 8.6747 | 8.1465 | 8.5379 | 9.4153 | 7.9724 | 8.2802 |
| 7.8185 | 8.7021 | 9.267 | 6.2435 | 7.9564 | 8.4654 | 8.5786 | 7.2095 |
| 9.4251 | 9.2495 | 8.9401 | 8.1865 | 9.5746 | 7.891 | 7.9855 | 8.5165 |
| 9.165 | 9.4663 | 8.4567 | 9.4282 | 8.7081 | 9.4871 | 8.3411 | 8.6199 |
| 8.4049 | 8.1242 | 8.4601 | 9.3887 | 8.2944 | 8.7663 | 8.7839 | 9.6729 |
| 9.3551 | 9.1916 | 9.6913 | 10.1809 | 7.0878 | 9.7719 | 9.4709 | 6.0639 |
| 9.4642 | 7.9556 | 9.6614 | 7.1876 | 8.7144 | 8.0016 | 8.5622 | 8.7735 |
| 8.1441 | 8.8741 | 9.5537 | 8.3112 | 8.5525 | 9.7702 | 9.7535 | 8.4532 |
| 8.8914 | 8.5102 | 8.192 | 8.4842 | 5.956 | 8.7918 | 5.0842 | 4.1801 |
| 5.6744 | 4.7818 | 4.9566 | 6.287 | 6.7391 | 7.6051 | 5.0742 | 6.6438 |
| 6.9012 | 6.8646 | 5.0571 | 5.4705 | 7.3019 | 4.5646 | 3.052 | 5.8798 |
| 5.8558 | 7.3268 | 4.2513 | 5.5992 | | | | |
| CIT | 8.8993 | 7.6362 | 7.6876 | 9.299 | 10.4426 | 8.3527 | 9.6296 |
| 8.7035 | 9.7789 | 9.567 | 9.131 | 9.0465 | 8.7319 | 9.1782 | 8.3867 |
| 9.2438 | 9.4791 | 8.7587 | 8.0574 | 8.7413 | 8.5158 | 10.797 | 8.3859 |
| 6.9956 | 9.6754 | 11.8805 | 9.2303 | 9.8165 | 8.4788 | 9.5774 | 8.63 |
| 9.8893 | 9.8752 | 8.2738 | 8.5432 | 8.4437 | 9.2043 | 10.6463 | 9.1367 |
| 9.1656 | 8.9471 | 9.3771 | 7.8788 | 8.7357 | 9.6462 | 9.0241 | 9.507 |
| 8.9022 | 8.6043 | 9.9047 | 9.0461 | 9.2146 | 8.9615 | 9.5842 | 8.7732 |
| 9.0856 | 9.2735 | 9.0215 | 6.9716 | 9.9444 | 8.83 | 8.935 | 8.8963 |
| 7.8102 | 8.6285 | 8.4471 | 8.8239 | 7.024 | 7.5201 | 9.3303 | 8.4911 |
| 9.3495 | 7.7636 | 9.5058 | 7.9488 | 9.0405 | 10.2503 | 8.7183 | 7.8672 |
| 10.1751 | 9.7905 | 9.23 | 9.2226 | 9.1016 | 9.7315 | 7.9886 | 9.845 |
| 8.451 | 8.7754 | 9.447 | 10.593 | 9.0747 | 10.6002 | 9.4365 | 9.6383 |
| 9.1718 | 8.8286 | 8.5538 | 8.8848 | 9.42 | 9.5411 | 9.7677 | 8.9707 |
| 9.9388 | 9.6219 | 10.0493 | 9.1231 | 8.5208 | 10.7826 | 8.7572 | 8.991 |
| 9.4222 | 8.5088 | 9.7865 | 9.5198 | 8.3229 | 10.0267 | 8.3355 | 7.762 |
| 8.7843 | 7.4343 | 8.4913 | 9.8837 | 8.3423 | 8.2513 | 8.9048 | 9.0774 |
| 8.8313 | 7.7837 | 8.5277 | 7.7165 | 8.0075 | 9.0675 | 9.5727 | 9.39 |
| 9.6092 | 9.4114 | 8.5992 | 9.0657 | 8.1474 | 9.2204 | 8.2129 | 8.0061 |
| 9.0602 | 9.6681 | 8.9791 | 9.7171 | 8.6365 | 10.3836 | 9.8415 | 10.2196 |
| 9.909 | 9.9484 | 8.1045 | 8.8496 | 9.8555 | 8.146 | 8.8405 | 8.708 |
| 9.5157 | 7.3961 | 8.1335 | 8.2657 | 9.1559 | 9.4052 | 8.6906 | 9.6388 |
| 9.838 | 9.296 | 7.9499 | 9.1118 | 9.0091 | 9.7777 | 9.392 | 9.1883 |
| 8.4694 | 8.3091 | 6.9339 | 9.4755 | 7.0627 | 6.3418 | 4.9075 | 6.2875 |
| 5.4765 | 7.3985 | 5.4097 | 8.082 | 6.2013 | 5.6582 | 6.1832 | 7.3811 |
| 6.2737 | 6.7946 | 6.8109 | 5.9702 | 4.3962 | 7.3664 | 6.2034 | 7.3958 |
| 5.5525 | 5.8173 | | | | | | |
| LDB2 | 6.2855 | 7.0053 | 7.251 | 6.9822 | 6.9671 | 7.3101 | 7.5934 |

| | | | | | | | |
|---|---|---|---|---|---|---|---|
| 6.7831 | 6.9089 | 6.394 | 8.2511 | 6.3294 | 8.6369 | 8.2719 | 7.7483 |
| 7.4816 | 5.925 | 6.5782 | 8.5265 | 9.2778 | 5.8164 | 6.1923 | 8.0016 |
| 7.592 | 6.2631 | 6.3947 | 7.5826 | 5.7517 | 7.1129 | 5.3228 | 5.7104 |
| 7.832 | 6.4046 | 7.2571 | 7.3237 | 8.0972 | 7.8185 | 6.5439 | 7.0042 |
| 5.3458 | 6.8647 | 9.5924 | 6.0348 | 6.5819 | 7.5371 | 7.8462 | 9.3216 |
| 6.8337 | 8.0037 | 8.0595 | 10.0822 | 8.0364 | 8.6433 | 8.4929 | 5.3381 |
| 7.797 | 6.6677 | 7.5512 | 7.5086 | 9.1504 | 5.6353 | 7.0866 | 6.9438 |
| 7.7614 | 8.7871 | 7.4191 | 8.2096 | 8.4856 | 9.1427 | 7.5124 | 8.0509 |
| 6.844 | 9.9755 | 8.1493 | 8.2069 | 7.1242 | 6.5892 | 6.5923 | 7.7656 |
| 8.6784 | 7.8791 | 7.3674 | 9.1244 | 7.1409 | 7.5388 | 7.957 | 7.2909 |
| 5.4008 | 7.9096 | 6.5191 | 8.9658 | 6.5385 | 7.5414 | 9.0727 | 9.0678 |
| 7.909 | 6.2034 | 5.5966 | 5.7671 | 6.4094 | 8.076 | 7.382 | 8.0974 |
| 7.504 | 7.0228 | 7.8905 | 7.6323 | 7.4867 | 8.1033 | 6.9613 | 7.3432 |
| 7.6801 | 6.2361 | 7.361 | 6.1282 | 7.4518 | 7.3195 | 7.8936 | 5.282 |
| 5.1578 | 8.8998 | 10.5872 | 5.8282 | 7.3873 | 6.5873 | 8.0734 | 6.6798 |
| 7.0688 | 6.0874 | 5.0761 | 10.7318 | 5.1339 | 7.3294 | 7.3167 | 7.5544 |
| 8.5713 | 6.4791 | 6.3015 | 6.4966 | 6.1705 | 6.9359 | 8.6133 | 6.4608 |
| 7.5848 | 7.683 | 7.3254 | 8.7926 | 7.8382 | 5.9815 | 6.4197 | 7.021 |
| 5.9212 | 6.9927 | 7.4046 | 7.3889 | 7.9442 | 8.5336 | 6.2828 | 6.4411 |
| 6.5165 | 9.7326 | 6.7495 | 7.8232 | 8.3127 | 6.4913 | 5.7403 | 7.5493 |
| 7.5111 | 7.2053 | 7.7863 | 8.3231 | 6.9396 | 5.9916 | 4.7048 | 7.2927 |
| 9.5964 | 4.8529 | 10.772 | 10.4272 | 10.3146 | 7.5986 | 11.412 | 11.1694 |
| 12.0731 | 12.3867 | 12.1491 | 10.8704 | 10.6597 | 11.5642 | 11.1133 | 10.5636 |
| 9.1689 | 11.6855 | 11.4183 | 11.0951 | 8.7872 | 10.0384 | 11.4004 | 10.7246 |
| 10.5224 | 10.9533 | | | | | | |
| LOC401093 | | 5.9403 | 6.2531 | 5.4028 | 6.694 | 6.7542 | 4.5383 |
| 5.688 | 4.5972 | 6.611 | 4.5998 | 6.8166 | 4.7602 | 4.2203 | 5.3294 |
| 5.6486 | 5.7651 | 2.6399 | 7.1445 | 5.5822 | 5.9964 | 4.0434 | 3.0155 |
| 4.4307 | 5.4116 | 4.4053 | 4.3777 | 6.4157 | 4.9151 | 5.3569 | 5.2328 |
| 3.3905 | 5.2108 | 4.5646 | 7.8107 | 6.0759 | 5.6313 | 4.9173 | 6.5064 |
| 3.7474 | 4.8438 | 4.8644 | 6.4842 | 5.4283 | 5.2391 | 4.7809 | 6.154 |
| 6.6175 | 5.4584 | 5.195 | 6.2287 | 8.5148 | 6.22 | 6.2638 | 6.0925 |
| 4.9527 | 5.4513 | 5.4963 | 5.9931 | 5.8711 | 8.5639 | 6.1245 | 5.0401 |
| 4.2268 | 5.4335 | 7.5919 | 4.3609 | 6.0103 | 5.0456 | 6.1644 | 6.6697 |
| 5.673 | 5.7497 | 8.6001 | 6.523 | 6.7295 | 5.7185 | 5.6257 | 4.3487 |
| 6.4788 | 6.5676 | 6.0391 | 4.6552 | 5.4916 | 6.4597 | 4.9945 | 8.2333 |
| 6.0155 | 5.7059 | 5.6242 | 6.0831 | 6.1851 | 5.4339 | 7.0724 | 7.11 |
| 6.0426 | 5.7279 | 4.4143 | 5.0646 | 3.9258 | 4.9864 | 5.367 | 6.6249 |
| 6.785 | 5.9941 | 5.956 | 5.0125 | 7.1532 | 5.399 | 4.5474 | 4.7175 |
| 5.6834 | 6.4705 | 4.3574 | 4.2384 | 5.6113 | 5.3332 | 6.1774 | 4.8984 |
| 3.7148 | 4.3313 | 5.8854 | 7.4116 | 6.0917 | 4.9526 | 5.1044 | 6.9696 |
| 5.5067 | 4.7229 | 5.5144 | 4.6439 | 9.2405 | 4.5495 | 5.5763 | 4.7408 |
| 4.6709 | 5.5287 | 6.518 | 5.6353 | 4.9919 | 4.8582 | 5.7344 | 5.1368 |
| 5.2523 | 6.145 | 5.7684 | 6.9804 | 5.4921 | 5.7997 | 4.3989 | 6.8125 |
| 5.1874 | 5.7724 | 5.1884 | 4.6834 | 7.2074 | 5.7563 | 6.7614 | 4.731 |
| 3.1531 | 7.2474 | 5.6983 | 4.5651 | 6.7064 | 5.4991 | 5.5498 | 4.5922 |
| 5.3913 | 5.4679 | 4.7777 | 5.8021 | 5.6813 | 6.0166 | 5.7421 | 6.8748 |
| 5.829 | 6.3421 | 3.9263 | 8.9336 | 7.7118 | 9.3947 | 6.5282 | 11.5343 |
| 10.1125 | 10.9652 | 10.3165 | 10.2757 | 8.1628 | 8.8392 | 11.8607 | 8.7234 |
| 8.8111 | 6.6647 | 9.28 | 10.3103 | 8.8936 | 5.5405 | 7.3877 | 9.2524 |
| 8.7419 | 10.4036 | 8.6139 | | | | | |
| CAV1 | 8.1863 | 9.5399 | 8.6998 | 8.93E+00 | | 8.86E+00 | |
| 8.3425 | 9.5335 | 9.6208 | 8.2119 | 8.698 | 9.7136 | 8.3897 | 9.8173 |
| 9.1214 | 8.3538 | 9.0082 | 9.3096 | 10.3181 | 8.1368 | 9.1695 | 9.0036 |

| | | | | | | | | |
|---|---|---|---|---|---|---|---|---|
| 10.591 | 9.4221 | 9.7096 | 7.4788 | 7.2764 | 8.4976 | 11.4148 | 8.348 | |
| 7.6193 | 10.6924 | 8.9598 | 7.7996 | 9.9979 | 8.7501 | 10.3183 | 9.187 | |
| 8.7353 | 7.8255 | 8.1552 | 9.5581 | 9.2168 | 7.8151 | 8.0302 | 10.4391 | |
| 9.4138 | 10.2032 | 10.0355 | 9.9901 | 11.0864 | 11.3765 | 8.7523 | 9.9196 | |
| 7.5451 | 7.2971 | 9.6078 | 6.865 | 8.3039 | 10.6178 | 10.9677 | 7.853 | |
| 10.0676 | 10.5916 | 10.0179 | 10.4895 | 8.7541 | 9.5847 | 10.3849 | 10.2579 | |
| 10.1868 | 9.3249 | 9.0418 | 11.1857 | 9.3894 | 9.3272 | 9.849 | 9.2261 | |
| 8.135 | 9.2333 | 10.8834 | 9.2771 | 8.2847 | 9.5761 | 10.2016 | 11.7135 | |
| 9.2916 | 11.1369 | 7.4085 | 9.0449 | 7.9594 | 9.146 | 7.7862 | 9.4865 | |
| 9.9187 | 9.9434 | 9.5646 | 8.5264 | 7.9171 | 7.9534 | 9.0224 | 9.6703 | |
| 7.7788 | 9.1361 | 8.7321 | 9.1325 | 9.6732 | 11.0937 | 8.7235 | 9.6628 | |
| 8.3471 | 9.8287 | 8.7299 | 8.6962 | 8.8951 | 7.5302 | 9.2757 | 8.6485 | |
| 13.077 | 9.4745 | 7.5711 | 8.9497 | 11.1385 | 6.9839 | 8.5506 | 8.5543 | |
| 8.8869 | 9.3158 | 8.1476 | 8.0952 | 7.1828 | 12.0353 | 7.0293 | 8.7618 | |
| 8.5294 | 7.9729 | 9.7683 | 9.5463 | 8.1968 | 9.2014 | 7.5782 | 9.5215 | |
| 8.6078 | 8.21 | 8.6675 | 10.022 | 8.7324 | 9.4766 | 9.2493 | 7.3128 | |
| 7.4777 | 10.8948 | 6.6789 | 7.7434 | 8.3884 | 9.4061 | 9.1132 | 9.4794 | |
| 8.3035 | 8.7558 | 9.1364 | 8.56 | 8.5239 | 8.2504 | 11.0147 | 7.5624 | |
| 7.7588 | 8.6693 | 9.7685 | 9.2511 | 8.8464 | 9.0938 | 8.1998 | 7.8786 | |
| 10.2402 | 9.674 | 9.376 | 10.6708 | 11.8106 | 11.2172 | 12.2131 | 10.0311 | |
| 13.6895 | 12.2601 | 14.2037 | 13.9744 | 14.0666 | 11.2396 | 13.3662 | 14.2824 | |
| 12.016 | 11.2599 | 11.141 | 12.9041 | 13.0676 | 11.9564 | 12.1475 | 13.3684 | |
| 12.6583 | 11.6471 | 14.1746 | 11.5865 | | | | | |
| RCC2 | 12.7548 | 11.8978 | 12.2802 | 13.1761 | 13.2492 | 13.3769 | 12.9406 | |
| 12.8749 | 12.9309 | 13.4056 | 12.4784 | 13.0486 | 12.8168 | 12.7429 | 12.8874 | |
| 12.653 | 13.1793 | 12.4807 | 12.6801 | 13.0466 | 12.2102 | 14.6724 | 12.784 | |
| 12.6881 | 13.1744 | 13.1916 | 13.0873 | 12.5771 | 13.2741 | 13.6059 | 12.5728 | |
| 12.8844 | 12.8371 | 12.7055 | 12.7882 | 12.6015 | 13.2822 | 13.5867 | 13.1641 | |
| 12.8661 | 13.27 | 13.1036 | 11.8247 | 12.5268 | 12.5362 | 13.2698 | 12.7372 | |
| 12.844 | 12.6271 | 11.5758 | 13.2134 | 13.767 | 12.4457 | 12.8989 | 12.8435 | |
| 13.1866 | 11.7318 | 12.9721 | 12.6472 | 13.6835 | 13.3031 | 13.0532 | 12.1914 | |
| 12.3341 | 12.5487 | 12.5526 | 13.7372 | 12.3218 | 12.5532 | 13.0748 | 12.8873 | |
| 13.4745 | 12.0376 | 13.5124 | 12.9859 | 12.4085 | 12.7725 | 13.6832 | 12.8335 | |
| 13.0416 | 12.7687 | 12.4642 | 13.082 | 13.3746 | 12.9226 | 12.752 | 12.8101 | |
| 12.9136 | 12.9562 | 13.5931 | 12.431 | 13.1805 | 13.6858 | 12.9944 | 13.4172 | |
| 13.3574 | 12.3999 | 13.2173 | 11.5527 | 13.3994 | 12.9505 | 13.2142 | 13.0899 | |
| 12.6549 | 13.2846 | 12.8582 | 12.6181 | 12.9488 | 12.5363 | 12.6749 | 12.6188 | |
| 13.2347 | 12.2717 | 13.1047 | 13.3129 | 12.9763 | 13.8381 | 12.6535 | 12.0639 | |
| 12.5838 | 11.4718 | 12.6312 | 13.7975 | 12.542 | 12.7413 | 13.0046 | 12.9189 | |
| 13.0439 | 12.6635 | 12.0829 | 12.1151 | 11.9843 | 12.9955 | 13.6474 | 13.1715 | |
| 13.0721 | 12.6743 | 13.9828 | 12.4778 | 12.1062 | 12.6766 | 13.1784 | 12.5045 | |
| 12.7152 | 12.9851 | 13.4173 | 12.4127 | 13.1708 | 13.7837 | 13.348 | 12.9112 | |
| 12.197 | 12.6075 | 11.9452 | 13.1014 | 13.067 | 11.6341 | 12.5298 | 12.768 | |
| 12.8349 | 12.2888 | 12.6309 | 12.8446 | 13.3789 | 12.7875 | 13.4938 | 12.9058 | |
| 12.9691 | 12.7986 | 13.0409 | 13.6479 | 12.8733 | 12.8156 | 12.7827 | 12.7445 | |
| 12.6238 | 13.3796 | 12.1626 | 12.5004 | 12.2358 | 11.5414 | 10.9786 | 11.3979 | |
| 10.0157 | 10.1238 | 10.4699 | 12.3995 | 10.8605 | 10.4097 | 11.7117 | 11.821 | |
| 11.2204 | 11.2517 | 10.9534 | 11.37 | 10.5959 | 11.3161 | 11.1917 | 12.2948 | |
| 10.0253 | 11.161 | | | | | | | |
| CASP12 | 0 | 0.6896 | 1.1529 | 0 | 1.3921 | 0 | 1.3555 | |
| 1.2625 | 0.4291 | 0.4849 | 2.0628 | 0.4395 | 1.2358 | 0 | 2.4094 | |
| 1.2795 | 1.1305 | 2.2447 | 1.7571 | 1.465 | 0 | 0.4561 | 3.1243 | |
| 1.3857 | 1.0077 | 0.9954 | 0 | 0 | 0 | 0.5853 | 0.6819 | |
| 0.9556 | 1.5916 | 0 | 0.7666 | 1.7407 | 1.39 | 0 | 0 | 0 |

| | | | | | | | | |
|---|---|---|---|---|---|---|---|---|
| | 1.758 | 0.4402 | 1.5638 | 0 | 0 | 2.472 | 2.4746 | 0 |
| | 0.6343 | 1.6258 | 3.7004 | 2.1385 | 0.4969 | 0.9583 | 0.5968 | |
| 1.5189 | 0 | 1.7598 | 0 | 3.9922 | 0.7662 | 0 | 0.9449 | |
| 1.7486 | 1.889 | 0.8237 | 2.0987 | 2.3485 | 1.5048 | 2.0686 | 0.5659 | |
| 0.9627 | 3.5862 | 0.6718 | 3.0077 | 2.4045 | 3.1903 | 0 | 0.5273 | |
| 0.5265 | 1.9243 | 1.2055 | 2.5641 | 1.8492 | 0.5233 | 1.4288 | 0 | 0 |
| | 1.1625 | 1.4835 | 1.8724 | 0 | 1.5391 | 1.6282 | 1.2448 | |
| 2.1763 | 0.5136 | 1.0545 | 0 | 0 | 0 | 1.0399 | 0.9131 | |
| 0.7641 | 0 | 0.6089 | 1.847 | 0.5886 | 1.6588 | 0.47 | 1.6938 | 0 |
| | 0 | 0.7024 | 0.6684 | 0.5526 | 1.6184 | 1.7059 | 0 | 0 |
| | 5.1146 | 4.8074 | 0 | 1.7855 | 0 | 0.9357 | 0 | |
| 0.6735 | 0 | 0.7381 | 5.9869 | 0 | 0.9341 | 1.0019 | 0 | 0 |
| | 0.9402 | 1.3489 | 1.2429 | 0.6063 | 0.6138 | 1.3167 | 0.9764 | 0 |
| | 0.5023 | 0 | 2.2772 | 1.217 | 0 | 0 | 2.4406 | |
| 1.2418 | 0.6229 | 0 | 0.8396 | 0 | 2.1026 | 0.5545 | 1.1551 | 0 |
| | 2.1234 | 1.5843 | 3.8082 | 0 | 0 | 1.725 | 0 | |
| 0.8827 | 0 | 1.2903 | 1.2477 | 0.8682 | 0 | 1.032 | 1.126 | |
| 1.8199 | 0 | 4.1652 | 3.261 | 3.6973 | 1.0738 | 4.8618 | 4.2228 | |
| 5.2595 | 5.4934 | 5.5285 | 2.8183 | 3.6594 | 4.0206 | 5.9458 | 2.4909 | |
| 3.7881 | 5.4705 | 5.2027 | 4.8639 | 5.88 | 3.7446 | 5.4608 | 4.0267 | |
| 5.213 | 4.2022 | | | | | | | |
| PDZRN4 | 0.5526 | 1.7871 | 2.8315 | 1.68E+00 | | 3.08E+00 | | |
| 0.9367 | 2.1723 | 1.4589 | 1.2544 | 1.1366 | 1.9354 | 1.6493 | 0.5377 | |
| 1.4442 | 0 | 1.2795 | 0 | 0.6175 | 1.131 | 2.0289 | 2.6374 | |
| 1.6918 | 0.599 | 0 | 0 | 0.4127 | 0 | 1.8398 | 0 | 0 |
| | 0.6819 | 2.3861 | 1.7231 | 0 | 0.4337 | 1.9736 | 0.6231 | |
| 2.052 | 0 | 0 | 0 | 4.7186 | 2.2965 | 0.7972 | 0 | |
| 2.472 | 7.2496 | 0.5608 | 1.682 | 3.1582 | 3.6222 | 0.5715 | 3.781 | 0 |
| | 1.0177 | 1.3532 | 0 | 2.1953 | 3.2274 | 3.2513 | 0.4334 | |
| 0.7001 | 1.511 | 2.7021 | 4.4114 | 0.4698 | 4.3385 | 2.0646 | 2.561 | |
| 1.0461 | 1.2875 | 1.6845 | 6.4344 | 1.7538 | 3.0077 | 3.2615 | 1.2355 | 0 |
| | 1.6807 | 5.8642 | 1.3771 | 0 | 3.0654 | 0.9013 | 4.744 | |
| 1.8231 | 0.5504 | 0 | 1.6155 | 0.5352 | 1.2208 | 0.8449 | 2.5744 | |
| 2.1653 | 3.6341 | 2.1763 | 2.8045 | 2.081 | 1.3631 | 0 | 2.806 | |
| 2.7673 | 0.9131 | 2.4697 | 0.3974 | 0 | 6.1822 | 1.5923 | 1.8863 | |
| 0.8239 | 1.6938 | 0.6149 | 0 | 0.3935 | 1.1234 | 0.9511 | 2.154 | |
| 1.3267 | 0 | 0 | 0 | 3.8141 | 1.024 | 3.4316 | 1.0973 | |
| 1.7146 | 0.8022 | 1.1308 | 0 | 0.7381 | 5.4954 | 0 | 2.0661 | |
| 2.4626 | 0 | 0.7727 | 1.1539 | 4.4689 | 0 | 0 | 0 | 0 |
| | 0.9764 | 0.7796 | 0.5023 | 1.6706 | 3.0096 | 1.8676 | 0 | 0 |
| | 1.2195 | 1.7114 | 1.0565 | 0 | 1.3669 | 2.7595 | 3.9334 | |
| 0.5545 | 1.1551 | 2.5448 | 0 | 2.5017 | 1.0594 | 0 | 1.5325 | 0 |
| | 3.2092 | 0.8827 | 2.7306 | 0.9737 | 2.0332 | 0.8682 | 0 | |
| 0.6064 | 1.6179 | 2.2618 | 0 | 5.3775 | 4.0958 | 3.3189 | 5.3301 | |
| 7.2359 | 4.9599 | 6.1592 | 7.1065 | 5.4851 | 3.8994 | 9.4333 | 5.6736 | |
| 5.3725 | 3.2604 | 6.6129 | 6.9247 | 6.3263 | 5.706 | 8.3588 | 8.0926 | |
| 6.066 | 4.1383 | 10.4855 | 4.9573 | | | | | |
| CNN1 | 10.127 | 7.7056 | 6.0677 | 11.0955 | 5.8217 | 4.7331 | 9.9818 | |
| 5.2901 | 5.1695 | 5.3837 | 7.5799 | 7.0758 | 6.5274 | 5.569 | 7.9908 | 5.5 |
| | 7.8053 | 8.4788 | 7.6391 | 6.0947 | 7.9663 | 5.646 | 6.9483 | |
| 10.9665 | 4.0145 | 4.8156 | 10.6062 | 8.4797 | 5.7046 | 2.8081 | 3.6416 | |
| 8.353 | 3.3513 | 8.0788 | 5.4522 | 6.0569 | 7.6886 | 7.1201 | 6.1591 | |
| 7.801 | 7.747 | 9.7597 | 5.9089 | 6.9306 | 7.316 | 10.3329 | 9.5733 | |
| 4.8828 | 9.9203 | 7.2718 | 13.4896 | 6.6878 | 8.7926 | 7.0707 | 7.1435 | |

| | | | | | | | | |
|---|---|---|---|---|---|---|---|---|
| 7.3706 | 4.0808 | 8.0911 | 7.3215 | 12.8291 | 5.3921 | 7.145 | 7.028 | |
| 6.051 | 11.6123 | 3.9301 | 9.132 | 8.2602 | 8.2138 | 11.3145 | 7.8702 | |
| 7.4087 | 11.0933 | 6.2086 | 12.0555 | 7.8802 | 7.4046 | 6.4332 | 10.5414 | |
| 11.6573 | 9.2173 | 5.163 | 8.7012 | 10.0727 | 5.8321 | 11.2471 | 7.6323 | |
| 9.5975 | 9.537 | 6.2046 | 9.3451 | 4.2414 | 11.6269 | 9.2533 | 8.5991 | |
| 6.8907 | 6.3767 | 6.2655 | 10.2334 | 8.9278 | 8.6622 | 7.5781 | 6.9717 | |
| 5.3751 | 9.7723 | 7.374 | 10.1838 | 8.4477 | 8.1313 | 6.0035 | 6.0007 | |
| 10.7876 | 9.214 | 6.8196 | 3.3824 | 9.0568 | 9.7658 | 7.6661 | 12.1134 | |
| 9.1903 | 8.9606 | 12.5893 | 4.4717 | 6.362 | 7.0387 | 8.7267 | 6.0216 | |
| 3.876 | 6.4569 | 6.4347 | 13.0186 | 9.8819 | 6.3014 | 5.5429 | 5.3795 | |
| 7.9139 | 7.8877 | 6.0664 | 9.1537 | 5.8562 | 9.3409 | 5.1208 | 4.8769 | |
| 5.4711 | 6.3831 | 10.7421 | 6.9292 | 9.3542 | 3.5915 | 6.0184 | 6.2617 | |
| 5.7731 | 8.0705 | 9.9884 | 7.4192 | 9.9729 | 10.6573 | 9.9667 | 6.6582 | |
| 8.3883 | 5.0024 | 8.0047 | 10.5378 | 8.076 | 7.3048 | 5.1533 | 8.6864 | |
| 8.7198 | 6.2949 | 7.214 | 10.1625 | 5.1398 | 4.1011 | 5.3656 | 6.7392 | |
| 10.6472 | 9.1968 | 14.4033 | 11.9802 | 14.2544 | 11.5179 | 16.5486 | 14.517 | |
| 14.8019 | 14.9589 | 15.3933 | 12.5805 | 15.4783 | 16.608 | 14.0579 | 14.0893 | |
| 12.5108 | 14.3241 | 15.3008 | 14.2592 | 9.8093 | 14.6751 | 14.1243 | 14.0155 | |
| 16.1142 | 13.3728 | | | | | | | |
| TCEAL7 | 3.663 | 5.0114 | 3.7264 | 4.71E+00 | | 3.38E+00 | | |
| 4.9829 | 5.6012 | 4.7857 | 3.1647 | 4.4179 | 6.5741 | 2.9572 | 3.9072 | |
| 4.6494 | 6.2466 | 5.5226 | 2.368 | 3.3336 | 3.9707 | 5.0112 | 3.9655 | |
| 3.689 | 6.27 | 6.0338 | 3.1011 | 2.3146 | 4.7881 | 2.4058 | 3.7241 | |
| 2.4602 | 1.7727 | 4.2719 | 3.2239 | 5.0177 | 4.7141 | 6.6089 | 5.7389 | |
| 4.1524 | 4.183 | 4.0317 | 2.9168 | 5.3049 | 4.1871 | 3.1287 | 2.8913 | |
| 5.8256 | 6.1705 | 4.1397 | 5.7403 | 6.3343 | 8.6292 | 4.4208 | 6.0704 | |
| 4.4736 | 3.9398 | 5.0725 | 1.3453 | 5.5467 | 6.1067 | 7.4438 | 3.3377 | |
| 3.2376 | 4.5926 | 4.6437 | 6.3832 | 2.2778 | 6.2488 | 5.1149 | 5.7132 | |
| 5.2381 | 6.1028 | 4.5325 | 7.3479 | 4.1859 | 6.5046 | 5.8011 | 5.5702 | |
| 3.3991 | 6.2562 | 5.6148 | 5.1915 | 3.0706 | 7.0223 | 6.2653 | 4.6962 | |
| 5.3642 | 5.0865 | 2.7168 | 5.402 | 4.0633 | 5.4675 | 4.413 | 6.1984 | |
| 7.337 | 7.1274 | 5.9028 | 2.5118 | 3.0941 | 6.315 | 3.585 | 5.7798 | |
| 4.4325 | 3.7871 | 4.7649 | 4.2534 | 5.3167 | 8.5437 | 5.4865 | 5.953 | |
| 3.8558 | 5.1646 | 6.0525 | 0.7843 | 4.5206 | 3.4618 | 4.948 | 4.6126 | |
| 5.5076 | 4.8977 | 3.2319 | 9.0899 | 7.9765 | 2.6248 | 3.6721 | 4.5016 | |
| 5.7037 | 3.8836 | 3.876 | 4.1597 | 2.3242 | 7.821 | 2.9676 | 4.8469 | |
| 3.8109 | 2.8077 | 6.1453 | 2.833 | 1.6149 | 5.7508 | 1.6272 | 4.2899 | |
| 6.4426 | 3.6192 | 5.2307 | 5.636 | 0 | 5.6174 | 5.6442 | 2.9836 | |
| 3.0521 | 4.2599 | 2.4724 | 4.661 | 4.7787 | 3.9272 | 5.8276 | 6.4556 | |
| 4.9096 | 3.7953 | 6.81 | 4.3135 | 5.3799 | 5.6057 | 5.7302 | 3.8329 | |
| 3.5079 | 4.0884 | 3.858 | 3.3112 | 5.0982 | 6.5084 | 4.0603 | 3.1828 | |
| 3.0556 | 3.8967 | 6.0887 | 4.2463 | 9.7647 | 9.4565 | 9.4804 | 4.4591 | |
| 9.8389 | 9.4426 | 10.0806 | 9.9506 | 10.2876 | 9.8707 | 8.6985 | 9.0115 | |
| 9.9852 | 8.6238 | 6.8103 | 10.0358 | 9.2277 | 9.555 | 9.6374 | 8.1403 | 9.5 |
| | 9.2168 | 9.3001 | 9.4992 | | | | | |
| RERGL | 0 | 0 | 3.854 | 3.9611 | 0 | 1.2456 | 2.1723 | |
| 0.4327 | 0.7594 | 0.4849 | 2.8789 | 0.4395 | 0 | 2.1513 | 5.6914 | |
| 0.7772 | 0 | 0 | 0.8427 | 0.5262 | 0.6062 | 0.4561 | 2.3554 | |
| 2.3312 | 0 | 0.4127 | 0 | 0.5159 | 0 | 0.5853 | 0.6819 | |
| 2.8291 | 0 | 0 | 0.7666 | 2.35 | 0 | 1.2622 | 1.4889 | 0 |
| | 0 | 2.9595 | 2.6056 | 0.453 | 1.2789 | 2.472 | 1.0973 | |
| 1.5362 | 1.4095 | 2.5866 | 6.288 | 0.5715 | 0 | 6.1063 | 0 | |
| 2.3341 | 0 | 3.6479 | 0.9886 | 4.1734 | 0.4334 | 1.357 | 0 | |
| 7.8427 | 3.1877 | 0.8237 | 0 | 2.4718 | 3.4332 | 4.5787 | 1.2875 | |

| | | | | | | | | |
|---|---|---|---|---|---|---|---|---|
| 0.9627 | 6.5081 | 1.7538 | 5.2302 | 6.1779 | 3.04 | 0 | 3.0874 | |
| 2.3114 | 3.6067 | 1.2055 | 5.5939 | 0.5198 | 2.1693 | 3.1069 | 0 | |
| 1.6914 | 3.6025 | 0 | 3.2704 | 1.7606 | 2.5744 | 4.4989 | 2.8861 | |
| 5.4003 | 3.1204 | 1.0545 | 0 | 0 | 0.8924 | 1.6379 | 3.1615 | |
| 8.1165 | 1.6868 | 1.0358 | 2.3453 | 0.5886 | 0.6224 | 0.47 | 0.9214 | |
| 3.5397 | 0 | 1.3606 | 1.1234 | 3.3693 | 2.154 | 0.8107 | 0 | |
| 0.6413 | 5.97 | 6.7214 | 3.6878 | 5.2181 | 4.2222 | 1.9025 | 0.4562 | |
| 1.7568 | 1.5778 | 0 | 5.7948 | 0 | 1.9 | 2.1729 | 0 | 0 |
| | 0.3855 | 0.6 | 3.2017 | 0 | 0 | 3.7468 | 3.2305 | |
| 1.8107 | 1.1696 | 2.6075 | 6.0392 | 4.2558 | 0 | 0.4486 | 0.9151 | |
| 0.541 | 1.0565 | 0 | 1.7522 | 3.0661 | 5.0378 | 2.3835 | 0.6902 | |
| 0.5257 | 1.4215 | 0.7366 | 3.2738 | 2.161 | 1.5325 | 0.6567 | 0 | |
| 0.5078 | 0 | 0.5674 | 3.6254 | 2.2371 | 1.5946 | 0 | 2.5185 | |
| 4.226 | 1.5159 | 7.9147 | 5.9247 | 6.6477 | 3.6556 | 8.7517 | 6.6468 | |
| 10.5847 | 8.1792 | 8.0921 | 5.9433 | 8.3644 | 6.7202 | 6.9609 | 6.1222 | |
| 7.5073 | 8.0228 | 6.6567 | 8.3955 | 6.9761 | 7.8061 | 7.0078 | 6.1749 | |
| 10.0905 | 6.9531 | | | | | | | |
| TNXB | 4.3754 | 5.518 | 5.8624 | 6.8693 | 8.7255 | 6.7885 | 8.2684 | |
| 6.1114 | 7.6875 | 7.2292 | 8.3509 | 5.1431 | 4.1552 | 5.5298 | 8.9218 | |
| 8.2276 | 5.6602 | 9.031 | 8.6287 | 5.6517 | 4.8972 | 6.1926 | 9.8888 | |
| 6.4433 | 5.7164 | 2.992 | 7.5665 | 4.7836 | 5.7304 | 8.4609 | 8.6653 | 8.48 |
| | 4.537 | 5.0734 | 6.2824 | 8.0879 | 4.8097 | 3.7028 | 6.1967 | |
| 5.1985 | 5.3475 | 6.6668 | 4.6543 | 8.3917 | 6.6067 | 6.7102 | 8.069 | |
| 2.4541 | 8.5317 | 9.3899 | 11.2771 | 6.7992 | 7.0987 | 7.7179 | 5.2008 | |
| 8.0024 | 5.6374 | 7.7499 | 4.5639 | 10.3217 | 8.2979 | 4.4508 | 6.4336 | |
| 8.6534 | 8.911 | 4.7462 | 9.5484 | 10.5239 | 10.7715 | 9.6963 | 6.1871 | |
| 4.4166 | 11.6783 | 6.9732 | 9.932 | 5.1402 | 4.6797 | 7.0389 | 9.017 | |
| 7.2111 | 8.1076 | 5.5547 | 11.734 | 8.4846 | 4.853 | 9.2114 | 5.1931 | |
| 4.1297 | 8.0524 | 5.0767 | 8.0473 | 7.5003 | 8.3007 | 9.4922 | 9.2776 | |
| 9.1696 | 5.9139 | 4.8759 | 10.2972 | 6.6263 | 4.4361 | 6.5072 | 5.4861 | |
| 7.1335 | 7.1255 | 8.3131 | 9.2246 | 4.9909 | 9.8751 | 4.2993 | 6.9523 | |
| 7.5042 | 8.0256 | 5.4389 | 5.2807 | 7.7231 | 7.2165 | 6.7246 | 10.5335 | |
| 7.4772 | 12.917 | 11.7821 | 5.4849 | 5.459 | 8.1765 | 5.5004 | 4.5738 | |
| 4.2921 | 6.0105 | 3.8844 | 12.3817 | 7.7287 | 5.9422 | 8.0848 | 3.1515 | |
| 5.0261 | 4.2876 | 8.8565 | 8.2074 | 5.4706 | 6.3658 | 8.8408 | 6.5007 | |
| 4.4616 | 6.7476 | 9.2669 | 9.0202 | 8.0096 | 3.9058 | 3.7916 | 5.1531 | |
| 8.7784 | 6.8271 | 9.9181 | 3.9952 | 8.3319 | 11.1366 | 5.6111 | 7.9493 | |
| 8.093 | 7.7266 | 5.6153 | 9.4627 | 7.2819 | 7.8947 | 5.9258 | 6.6818 | |
| 5.9583 | 5.3835 | 6.0919 | 9.0772 | 5.7276 | 9.5068 | 6.4875 | 6.7278 | |
| 9.5784 | 7.337 | 13.3961 | 12.2907 | 13.9851 | 10.3238 | 12.6286 | 13.0211 | |
| 14.1213 | 13.743 | 13.6374 | 13.0736 | 14.3334 | 13.8468 | 12.9874 | 13.6087 | |
| 11.4537 | 13.0554 | 12.8119 | 12.0372 | 13.1542 | 12.8032 | 12.7858 | 12.4289 | |
| 14.1025 | 12.1671 | | | | | | | |
| RHOJ | 4.6592 | 6.3007 | 5.5448 | 5.0927 | 5.7187 | 4.897 | 6.252 | |
| 6.1417 | 7.3375 | 5.1602 | 7.2985 | 4.7923 | 5.6764 | 6.0901 | 6.7369 | |
| 5.8246 | 4.447 | 5.3969 | 5.4279 | 7.0841 | 4.5518 | 4.6534 | 6.3988 | |
| 7.3061 | 5.922 | 5.1611 | 6.0476 | 4.8336 | 5.572 | 3.955 | 4.3882 | |
| 6.1871 | 5.6659 | 4.7269 | 4.9452 | 6.4728 | 6.1435 | 5.3649 | 5.4712 | |
| 5.3458 | 6.6927 | 6.8296 | 4.4931 | 5.1568 | 7.2364 | 6.2379 | 7.7973 | |
| 4.356 | 6.6255 | 6.984 | 8.6699 | 6.0045 | 6.8338 | 5.1843 | 3.9872 | |
| 6.5825 | 4.8703 | 5.7091 | 5.387 | 7.5905 | 5.3679 | 5.6715 | 5.5903 | |
| 6.1818 | 7.3227 | 5.4378 | 6.6768 | 7.0088 | 7.3892 | 6.5976 | 5.498 | |
| 5.8565 | 8.0511 | 7.2726 | 6.3729 | 5.6082 | 5.6372 | 4.6054 | 6.436 | |
| 7.9999 | 6.6445 | 4.6152 | 7.5666 | 5.6694 | 6.2013 | 7.3111 | 5.8841 | |

| | | | | | | | | |
|---|---|---|---|---|---|---|---|---|
| 4.6095 | 6.6803 | 4.6837 | 7.0596 | 4.4659 | 6.3877 | 7.7692 | 7.5633 | |
| 6.1979 | 4.8041 | 5.6898 | 5.2756 | 6.1085 | 5.8131 | 6.9668 | 5.1652 | |
| 6.3077 | 5.3669 | 7.0813 | 9.2133 | 5.5186 | 6.9413 | 5.0925 | 5.6743 | |
| 5.9692 | 4.7928 | 5.9357 | 4.8128 | 5.5608 | 7.5495 | 5.8087 | 5.3098 | |
| 4.4402 | 8.0576 | 8.6467 | 4.215 | 5.981 | 4.7356 | 6.5562 | 4.1179 | |
| 5.9467 | 4.8965 | 3.7031 | 8.6531 | 7.6142 | 7.1789 | 5.824 | 4.3168 | 5.1 |
| | 5.0646 | 4.6868 | 5.1912 | 4.4174 | 5.6222 | 6.4832 | 4.7268 | |
| 6.2321 | 5.8281 | 6.1471 | 7.1407 | 6.2575 | 5.1885 | 5.5754 | 5.9874 | |
| 6.3492 | 5.9795 | 6.8473 | 5.9402 | 7.0874 | 6.7922 | 4.4344 | 5.2171 | |
| 4.2496 | 5.1965 | 5.4694 | 6.4901 | 6.9942 | 5.9143 | 4.5535 | 5.4968 | |
| 6.1843 | 6.2241 | 6.186 | 6.9425 | 5.0527 | 4.4061 | 1.8529 | 6.7312 | |
| 6.281 | 3.8093 | 9.583 | 8.725 | 9.2254 | 6.7793 | 9.6256 | 9.2712 | |
| 9.3822 | 9.9251 | 9.969 | 9.0777 | 9.9649 | 9.5787 | 10.2049 | 8.3064 | |
| 7.6974 | 9.7356 | 9.6623 | 9.5655 | 8.1268 | 9.3137 | 9.4751 | 8.5395 | |
| 9.5904 | 8.5769 | | | | | | | |
| ZFPM2 | 2.8211 | 5.9321 | 1.9468 | 4.7813 | 3.7384 | 4.0479 | 5.3625 | |
| 4.339 | 4.7353 | 2.8059 | 4.0476 | 6.0789 | 2.8727 | 4.9193 | 3.2668 | |
| 6.4954 | 3.1743 | 4.8199 | 2.8772 | 3.8723 | 1.3599 | 3.6033 | 3.1243 | |
| 5.3689 | 3.1011 | 8.7086 | 5.0995 | 2.2837 | 4.4338 | 1.808 | 0.6819 | |
| 3.3777 | 3.3513 | 2.5862 | 2.2802 | 3.9118 | 4.4989 | 3.4777 | 0.9285 | |
| 1.1888 | 3.8776 | 3.2574 | 1.359 | 3.4047 | 3.7143 | 5.9634 | 5.3263 | |
| 2.2632 | 4.5964 | 4.679 | 6.9671 | 3.7698 | 5.2943 | 2.8337 | 2.7303 | |
| 3.232 | 3.6795 | 3.2255 | 4.1483 | 5.5154 | 3.0014 | 2.9181 | 0.9449 | |
| 7.1116 | 5.5256 | 1.1076 | 5.4947 | 3.3356 | 4.7069 | 5.5482 | 2.5369 | |
| 4.099 | 6.2867 | 3.617 | 3.0846 | 3.553 | 3.8626 | 0 | 4.9125 | |
| 4.6053 | 4.1837 | 0.9036 | 5.2885 | 3.139 | 2.8322 | 4.9559 | 3.4878 | |
| 2.1192 | 3.6507 | 3.4436 | 4.0848 | 2.1962 | 4.3536 | 6.0932 | 5.2474 | |
| 3.1772 | 5.334 | 0.6215 | 0 | 2.585 | 6.9919 | 4.5346 | 1.8676 | |
| 5.9409 | 4.3006 | 4.7155 | 7.0523 | 4.1397 | 4.5307 | 2.2783 | 3.3768 | |
| 5.6254 | 3.6501 | 3.7258 | 1.9805 | 3.0001 | 4.3933 | 3.2165 | 4.3186 | |
| 0.6413 | 5.5192 | 7.1014 | 1.024 | 4.0586 | 1.438 | 3.6335 | 2.6338 | |
| 1.7568 | 2.7981 | 3.0614 | 7.0224 | 1.9723 | 4.1205 | 3.5885 | 1.7261 | |
| 7.5302 | 4.1325 | 6.5036 | 2.3506 | 3.0551 | 2.3902 | 3.0798 | 3.5414 | |
| 2.5003 | 2.6813 | 6.0395 | 5.54 | 4.3525 | 0.5466 | 1.97 | 2.848 | |
| 0.541 | 5.9419 | 0 | 2.5204 | 6.4365 | 4.298 | 3.3748 | 4.232 | |
| 4.9074 | 1.4215 | 4.0288 | 5.3614 | 4.3814 | 2.7395 | 1.9569 | 6.6575 | |
| 4.3805 | 3.4417 | 2.4161 | 7.6901 | 3.0753 | 4.9682 | 0 | 2.9076 | |
| 5.2839 | 1.5159 | 7.5497 | 6.7593 | 7.6187 | 9.0187 | 7.9574 | 8.2922 | |
| 10.8937 | 10.3038 | 10.2098 | 7.1254 | 9.9407 | 9.5083 | 8.1309 | 6.9595 | |
| 9.6075 | 8.6915 | 8.27 | 7.8905 | 10.7826 | 9.9487 | 7.44 | 7.7435 | |
| 10.325 | 8.9115 | | | | | | | |
| ESCO2 | 8.8607 | 7.7718 | 7.3408 | 9.4174 | 8.3895 | 5.9914 | 7.2971 | |
| 7.4586 | 8.579 | 6.7343 | 6.8283 | 7.9674 | 8.4704 | 5.718 | 4.9877 | |
| 7.7053 | 6.2179 | 5.3598 | 6.9447 | 6.7834 | 7.5502 | 8.989 | 6.978 | |
| 5.9795 | 7.9869 | 8.7132 | 8.1421 | 6.8819 | 6.4347 | 8.0777 | 8.5624 | |
| 6.1108 | 9.3379 | 7.9704 | 8.1245 | 7.6188 | 6.4591 | 7.2451 | 6.6361 | |
| 6.5111 | 6.8499 | 8.6727 | 7.1991 | 8.2524 | 6.7452 | 7.7542 | 7.2816 | |
| 8.0609 | 7.0247 | 6.568 | 6.8562 | 6.0045 | 9.1224 | 7.9352 | 6.164 | |
| 7.556 | 4.9682 | 8.3713 | 4.3002 | 6.4564 | 7.8086 | 8.6159 | 6.7466 | |
| 5.2321 | 8.1103 | 6.3883 | 6.8334 | 3.8266 | 4.23 | 8.0989 | 7.4816 | |
| 7.2227 | 3.9306 | 8.3357 | 6.5595 | 6.6714 | 8.063 | 7.7751 | 6.654 | |
| 7.5925 | 6.8482 | 6.3025 | 4.9006 | 7.8405 | 8.4254 | 8.0521 | 7.9302 | |
| 5.6454 | 7.9021 | 8.371 | 7.8777 | 8.4517 | 9.1792 | 7.611 | 6.9994 | |
| 7.5277 | 8.072 | 7.9139 | 3.7633 | 4.8074 | 7.6163 | 7.927 | 8.2049 | |

| | | | | | | | |
|---|---|---|---|---|---|---|---|
| 6.1392 | 9.0364 | 6.7281 | 6.8444 | 8.0306 | 7.5096 | 7.6137 | 7.1295 |
| 7.4582 | 6.8377 | 7.4999 | 8.0721 | 7.2892 | 7.5601 | 6.4173 | 6.0815 |
| 7.3415 | 0 | 7.6597 | 6.9602 | 7.104 | 5.9267 | 8.6468 | 8.9999 |
| 6.4174 | 6.6404 | 6.2058 | 5.5981 | 5.6361 | 7.2497 | 7.7317 | 9.5873 |
| 6.9656 | 8.8158 | 6.8645 | 7.9431 | 7.2864 | 8.0358 | 7.2392 | 6.5379 |
| 6.4665 | 8.7426 | 6.4288 | 8.2839 | 8.8844 | 8.5402 | 9.7038 | 8.3073 |
| 8.1817 | 7.7614 | 4.85 | 8.3726 | 9.0896 | 5.1537 | 7.6247 | 6.8392 |
| 7.1054 | 6.1081 | 7.8549 | 6.4726 | 6.7029 | 7.7001 | 7.328 | 7.055 |
| 8.754 | 6.4502 | 7.451 | 7.742 | 8.5865 | 6.3542 | 6.7281 | 7.7971 |
| 7.5469 | 7.1595 | 2.1912 | 7.7138 | 3.951 | 1.9117 | 3.2362 | 3.2981 |
| 2.6279 | 2.7928 | 2.352 | 5.8095 | 1.4126 | 2.0921 | 3.3488 | 3.4466 |
| 3.6768 | 2.6412 | 5.9667 | 2.1235 | 0.5418 | 4.8361 | 2.4071 | 5.3184 |
| 1.6723 | 1.8629 | | | | | | |
| KPNA2 | 12.5952 | 11.2718 | 10.8246 | 11.905 | 12.8642 | 12.2353 | 12.6916 |
| 13.4526 | 13.0989 | 13.2143 | 12.3819 | 12.3286 | 12.3346 | 10.4355 | 10.8976 |
| 11.5632 | 12.3551 | 12.3392 | 11.2671 | 11.0596 | 12.2694 | 11.7533 | 12.0893 |
| 11.0032 | 13.0376 | 12.012 | 11.4694 | 13.3095 | 12.3485 | 12.1465 | 13.2479 |
| 10.7795 | 12.2516 | 11.952 | 11.654 | 12.5466 | 11.4981 | 12.0387 | 11.3332 |
| 11.265 | 12.3734 | 12.3964 | 11.573 | 11.8885 | 13.2176 | 11.6652 | 11.337 |
| 11.8547 | 10.9059 | 12.6438 | 11.7037 | 11.6679 | 12.5627 | 12.4892 | 11.1837 |
| 11.9985 | 10.5682 | 13.1315 | 11.4047 | 13.3456 | 13.135 | 12.048 | 11.61 |
| 11.3159 | 11.2884 | 11.6335 | 12.0838 | 10.0319 | 9.8377 | 11.6573 | 12.4502 |
| 12.84 | 9.4733 | 12.818 | 11.1118 | 11.4895 | 12.7933 | 12.3759 | 10.8209 |
| 12.1404 | 12.5434 | 11.1718 | 9.5899 | 12.6689 | 11.4981 | 11.2353 | 11.4593 |
| 11.9762 | 12.6868 | 11.9559 | 11.433 | 11.7352 | 12.2432 | 12.823 | 12.0833 |
| 11.6041 | 11.3865 | 12.5986 | 10.608 | 10.7748 | 13.2071 | 12.3297 | 11.7066 |
| 11.8301 | 12.2682 | 11.7243 | 12.6098 | 12.1576 | 12.1314 | 13.1884 | 13.1757 |
| 12.8682 | 11.2875 | 11.3435 | 12.2436 | 12.3476 | 10.5659 | 12.7251 | 12.1555 |
| 11.9985 | 7.7793 | 10.8432 | 11.1711 | 11.2223 | 10.6189 | 11.6916 | 12.1553 |
| 12.8396 | 11.5984 | 12.5124 | 10.0277 | 11.6082 | 11.4357 | 11.7213 | 11.2351 |
| 12.6875 | 12.4774 | 12.4389 | 12.2615 | 11.4484 | 11.1124 | 10.987 | 10.9653 |
| 11.1624 | 12.2557 | 11.891 | 11.5012 | 12.589 | 12.9884 | 12.3578 | 13.0977 |
| 11.947 | 11.9012 | 9.0153 | 11.3584 | 12.3786 | 9.5561 | 12.2822 | 11.7624 |
| 13.1422 | 11.1699 | 11.5876 | 10.6876 | 12.29 | 13.1206 | 12.3356 | 12.4347 |
| 12.357 | 11.6044 | 11.1527 | 12.4973 | 11.3855 | 12.4087 | 11.3491 | 12.3638 |
| 10.7543 | 12.8094 | 9.5204 | 11.0134 | 9.555 | 8.3579 | 9.6342 | 10.1349 |
| 8.2361 | 8.7639 | 9.5717 | 10.4029 | 9.3014 | 9.9408 | 8.2855 | 10.1361 |
| 8.1905 | 9.1719 | 9.9467 | 8.3609 | 7.2118 | 8.9918 | 8.6958 | 9.9991 |
| 8.6366 | 8.0165 | | | | | | |
| HMMR | 8.7509 | 7.3577 | 7.5839 | 8.4064 | 9.7875 | 8.013 | 8.5893 |
| 9.0187 | 9.0888 | 8.8727 | 9.2224 | 9.3516 | 8.0012 | 6.9449 | 6.456 |
| 8.2494 | 7.084 | 8.4982 | 7.2714 | 7.4016 | 8.2523 | 9.9138 | 6.345 |
| 5.9978 | 8.3839 | 9.8561 | 8.7023 | 8.9524 | 7.8043 | 8.2296 | 8.74 |
| 7.0853 | 9.1021 | 9.0404 | 7.1094 | 7.4308 | 6.8656 | 8.2943 | 6.9206 |
| 6.7182 | 8.4828 | 8.6108 | 6.6267 | 7.6634 | 7.7859 | 8.011 | 7.224 |
| 8.2435 | 8.0099 | 7.3254 | 9.1842 | 8.6616 | 10.4866 | 8.5575 | 8.7816 |
| 8.7935 | 7.9089 | 9.4575 | 5.7589 | 8.634 | 8.3415 | 8.6158 | 7.4956 |
| 6.0338 | 8.1079 | 7.2901 | 7.696 | 5.1495 | 5.534 | 8.6023 | 8.0082 |
| 8.6204 | 5.7228 | 8.8859 | 7.3131 | 8.5138 | 8.7435 | 7.4248 | 8.3492 |
| 9.6228 | 9.1233 | 7.92 | 7.4392 | 8.7308 | 7.9995 | 6.9846 | 9.2269 |
| 7.3157 | 9.0761 | 9.1525 | 6.851 | 7.994 | 8.4009 | 8.4507 | 9.458 |
| 7.6375 | 8.3636 | 8.399 | 5.8481 | 8.0112 | 9.9542 | 8.46 | 7.7639 |
| 8.4066 | 9.3007 | 8.2725 | 8.8616 | 9.2264 | 9.0835 | 7.9284 | 8.5235 |
| 9.6756 | 5.4224 | 8.772 | 8.0497 | 7.7806 | 7.8635 | 8.5739 | 5.8918 |

| | | | | | | | |
|---|---|---|---|---|---|---|---|
| 8.5631 | 1.5206 | 7.5926 | 8.814 | 7.5506 | 6.1282 | 8.5622 | 9.0298 |
| 7.497 | 8.0846 | 7.2349 | 6.3891 | 7.1231 | 7.5382 | 8.1459 | 8.8866 |
| 9.5854 | 9.2651 | 9.3986 | 8.6585 | 8.6645 | 7.3179 | 7.7361 | 6.6942 |
| 8.4242 | 8.7853 | 7.4387 | 8.9028 | 8.9694 | 8.3718 | 9.367 | 8.9338 |
| 6.2664 | 8.6103 | 3.6013 | 8.3289 | 9.604 | 5.8743 | 8.0337 | 8.8199 |
| 9.2217 | 7.6492 | 7.7383 | 7.0042 | 8.5296 | 7.9265 | 7.793 | 9.1102 |
| 9.4703 | 7.6005 | 6.9189 | 7.7065 | 7.2207 | 6.8088 | 7.4639 | 8.6714 |
| 7.5005 | 5.9629 | 2.4485 | 8.3885 | 5.1465 | 3.8887 | 3.7692 | 3.7496 |
| 3.2157 | 3.1775 | 3.0528 | 7.1253 | 3.7213 | 2.6869 | 3.9728 | 5.6773 |
| 4.8675 | 4.3594 | 6.7725 | 2.1235 | 1.7133 | 5.9241 | 3.5262 | 6.3392 |
| 1.6723 | 3.3172 | | | | | | |
| SORBS1 | 7.7496 | 8.6958 | 7.7046 | 8.79E+00 | | 8.71E+00 | |
| 9.6347 | 7.8268 | 5.7216 | 8.1464 | 7.6203 | 7.025 | 8.7629 | 7.2381 |
| 9.7508 | 7.1134 | 7.1312 | 6.7069 | 7.9499 | 8.0423 | 10.166 | 6.6394 |
| 6.2357 | 6.231 | 8.5822 | 7.1133 | 7.082 | 8.3456 | 7.8625 | 8.1136 |
| 6.3672 | 5.31 | 7.6718 | 7.9682 | 6.9094 | 5.8619 | 7.2595 | 7.9723 |
| 6.9097 | 7.3299 | 6.3953 | 6.6591 | 8.5829 | 4.8273 | 8.2524 | 7.9858 |
| 9.2156 | 10.0358 | 6.9212 | 9.0453 | 8.3996 | 10.305 | 6.2079 | 8.5451 |
| 9.253 | 8.0958 | 7.9715 | 7.7528 | 8.414 | 10.4908 | 10.072 | 6.686 |
| 6.7355 | 5.9118 | 6.4059 | 10.5631 | 7.8956 | 7.8478 | 6.7832 | 8.4085 |
| 9.391 | 9.4371 | 8.6695 | 9.357 | 7.6713 | 8.5581 | 6.8363 | 6.737 |
| 6.792 | 9.4742 | 9.6696 | 8.2654 | 8.6193 | 7.7953 | 7.1931 | 7.4729 |
| 9.6265 | 7.3659 | 5.9355 | 8.0077 | 7.3967 | 7.9725 | 8.1898 | 9.3432 |
| 8.8147 | 8.1665 | 7.168 | 8.2405 | 9.4026 | 8.1093 | 6.3038 | 7.7937 |
| 7.667 | 7.7872 | 7.5369 | 8.5229 | 8.2022 | 8.3685 | 7.9797 | 8.0718 |
| 5.7791 | 8.2568 | 8.4365 | 5.6469 | 7.8431 | 7.7317 | 6.9834 | 8.4606 |
| 7.2246 | 8.333 | 7.1783 | 6.0936 | 11.0321 | 7.7021 | 7.5619 | 6.9551 |
| 8.765 | 8.8626 | 7.0168 | 6.6187 | 5.4291 | 11.3414 | 6.4228 | 9.1646 |
| 6.3257 | 6.8517 | 9.0562 | 8.3701 | 7.1992 | 6.1344 | 7.1339 | 7.9824 |
| 7.3965 | 7.7835 | 8.4438 | 9.2003 | 8.2047 | 9.1542 | 9.7538 | 6.4828 |
| 8.4913 | 7.8618 | 6.6595 | 7.0348 | 6.4933 | 7.8552 | 8.8052 | 8.7496 |
| 7.2952 | 6.0451 | 8.241 | 8.1785 | 8.0159 | 7.977 | 8.594 | 6.0849 |
| 6.2577 | 6.8878 | 8.3499 | 6.6922 | 6.884 | 8.8033 | 6.1697 | 6.4573 |
| 7.9023 | 8.1372 | 9.5337 | 5.6251 | 10.7517 | 9.605 | 11.5669 | 9.0656 |
| 13.1931 | 11.4327 | 12.3753 | 12.221 | 13.0947 | 9.9497 | 12.5618 | 13.5177 |
| 11.5332 | 10.5032 | 10.1252 | 12.1853 | 13.5505 | 11.3221 | 8.7813 | 11.278 |
| 11.7098 | 11.7331 | 13.599 | 9.7732 | | | | |
| GSG2 | 7.4247 | 6.4994 | 5.2523 | 7.98E+00 | | 6.58E+00 | |
| 6.4718 | 6.8331 | 7.0115 | 6.0011 | 6.4145 | 6.285 | 5.617 | 6.339 |
| 5.2804 | 5.3538 | 6.5292 | 6.7632 | 6.284 | 5.2133 | 5.4781 | 6.1278 |
| 7.3724 | 5.6702 | 4.4573 | 7.2101 | 6.264 | 7.2364 | 7.1145 | 5.694 |
| 6.9434 | 7.9655 | 5.0418 | 7.4493 | 6.6532 | 6.0106 | 6.5738 | 5.6485 |
| 7.3005 | 5.3486 | 4.7329 | 7.4307 | 7.6105 | 5.5953 | 6.8077 | 7.6994 |
| 6.216 | 6.4715 | 6.5044 | 6.4903 | 5.068 | 6.0172 | 5.4361 | 6.4823 |
| 7.3711 | 7.8262 | 6.8154 | 5.6962 | 6.5312 | 3.6228 | 6.4652 | 6.1795 |
| 6.7731 | 5.9338 | 5.6145 | 6.4664 | 5.14 | 5.8401 | 3.5184 | 2.9521 |
| 7.461 | 5.9318 | 6.7454 | 3.7339 | 6.5289 | 5.3749 | 5.0767 | 7.1714 |
| 6.3323 | 6.1967 | 6.9578 | 6.9909 | 6.5571 | 3.7969 | 7.5002 | 7.4 |
| 6.2696 | 6.2437 | 6.5645 | 7.0874 | 7.3967 | 6.1057 | 6.1017 | 8.6878 |
| 6.4387 | 5.6265 | 5.6945 | 6.8635 | 7.2458 | 5.8082 | 5.4263 | 6.3332 |
| 7.0439 | 6.4373 | 6.382 | 6.8273 | 7.3694 | 7.0401 | 6.0983 | 6.2164 |
| 6.428 | 6.6148 | 6.5465 | 5.066 | 7.2284 | 4.9838 | 6.574 | 4.6528 |
| 6.0246 | 6.0652 | 6.5095 | 0 | 6.6481 | 5.6165 | 5.2181 | 4.8253 |
| 7.0788 | 7.5664 | 5.2881 | 6.3902 | 5.3836 | 4.5008 | 6.3385 | 6.4813 |

| | | | | | | | |
|---|---|---|---|---|---|---|---|
| 6.0261 | 7.4131 | 7.1205 | 7.2738 | 5.9982 | 7.1099 | 6.2715 | 6.4848 |
| 5.4509 | 4.8926 | 6.8803 | 7.4382 | 5.5942 | 6.3426 | 6.7822 | 7.0118 |
| 7.6526 | 6.6721 | 7.2266 | 7.0643 | 3.4255 | 6.2024 | 7.4548 | 5.6081 |
| 6.485 | 7.6901 | 7.0346 | 5.5595 | 6.5689 | 6.1808 | 7.6959 | 6.7244 |
| 6.109 | 8.0601 | 7.6448 | 5.8773 | 5.959 | 7.0698 | 7.0738 | 5.5688 |
| 5.0839 | 6.9879 | 6.3119 | 6.5544 | 2.4485 | 6.1951 | 4.3891 | 3.2986 |
| 1.9289 | 3.2981 | 2.0387 | 2.3883 | 3.0528 | 4.3049 | 2.1125 | 2.5596 |
| 3.0618 | 3.5314 | 2.938 | 3.3957 | 4.1587 | 2.4897 | 2.4747 | 3.6421 |
| 2.944 | 3.7267 | 1.066 | 2.7661 | | | | |
| MRVI1 | 7.6289 | 9.2355 | 7.1133 | 8.7872 | 6.9407 | 8.3806 | 7.0136 |
| 7.7463 | 6.0089 | 6.777 | 7.6167 | 6.1238 | 7.0394 | 6.9649 | 7.7042 |
| 8.3882 | 6.0206 | 7.6724 | 7.2901 | 7.7207 | 6.3838 | 7.7367 | 8.5573 |
| 9.2562 | 5.7294 | 4.7986 | 8.0699 | 5.3869 | 6.9126 | 5.1506 | 6.1393 |
| 7.6618 | 6.4669 | 6.778 | 7.5884 | 8.7028 | 8.6172 | 6.7073 | 7.0541 |
| 7.4981 | 7.0769 | 6.9045 | 6.0938 | 7.1031 | 7.6179 | 8.6706 | 8.878 |
| 7.0142 | 9.081 | 8.1385 | 10.265 | 6.7864 | 8.3963 | 6.9383 | 5.9654 |
| 8.638 | 5.4129 | 7.3722 | 9.167 | 10.9745 | 6.2003 | 7.053 | 7.7769 |
| 6.372 | 9.403 | 4.0354 | 7.5948 | 8.5001 | 7.7754 | 8.6755 | 8.2665 |
| 7.6368 | 9.4455 | 6.9024 | 9.3093 | 8.2263 | 6.8654 | 7.94 | 8.2399 |
| 9.9288 | 8.0256 | 5.4769 | 10.4489 | 8.8414 | 6.6475 | 8.5077 | 7.9921 |
| 7.7522 | 8.5171 | 7.8258 | 8.7216 | 5.6543 | 8.2556 | 9.7184 | 8.1802 |
| 8.4634 | 6.6772 | 6.2755 | 8.0248 | 8.0056 | 8.4805 | 5.8975 | 6.2407 |
| 6.388 | 7.7819 | 8.783 | 7.9995 | 8.2778 | 8.709 | 7.6193 | 8.214 |
| 8.669 | 4.1383 | 7.2613 | 5.7869 | 8.0224 | 7.5965 | 8.4109 | 9.1615 |
| 9.2774 | 10.8295 | 10.7294 | 6.1022 | 6.1792 | 7.746 | 8.1658 | 7.6301 |
| 7.0234 | 7.1642 | 5.9493 | 10.9692 | 7.864 | 8.6631 | 7.0482 | 5.2629 |
| 9.4488 | 7.9552 | 5.3655 | 7.2503 | 4.9677 | 7.8658 | 7.6778 | 6.4395 |
| 8.3847 | 8.4042 | 8.0189 | 8.0648 | 8.3777 | 5.8144 | 6.6881 | 7.1023 |
| 5.8772 | 6.7245 | 8.8158 | 6.1148 | 8.5304 | 9.5966 | 8.1477 | 7.6199 |
| 5.2943 | 6.9827 | 8.2003 | 8.4447 | 9.015 | 7.1848 | 7.2482 | 7.4922 |
| 8.4084 | 6.4424 | 7.4863 | 9.3493 | 7.6873 | 5.8471 | 4.9439 | 7.8646 |
| 9.518 | 8.2189 | 11.2178 | 11.0956 | 11.0841 | 8.2415 | 14.1922 | 10.9938 |
| 11.2802 | 11.8224 | 11.8567 | 12.123 | 12.0519 | 12.4807 | 12.0475 | 10.3489 |
| 9.7108 | 12.0786 | 13.7736 | 11.377 | 9.6933 | 11.1341 | 11.7727 | 12.1385 |
| 12.5333 | 10.9908 | | | | | | |
| PABPC5 | 1.2631 | 2.7039 | 3.6346 | 2.31E+00 | | 1.56E+00 | |
| 1.4999 | 4.8801 | 2.4718 | 1.7761 | 2.2003 | 4.0165 | 1.0485 | 2.0573 |
| 2.1513 | 3.0015 | 2.8922 | 2.0944 | 2.8895 | 2.3124 | 2.6512 | 0 |
| 1.3145 | 3.739 | 2.7323 | 1.5953 | 1.4094 | 2.9368 | 1.8398 | 2.1491 |
| 1.3224 | 2.2096 | 3.1675 | 1.2859 | 2.5862 | 2.7248 | 3.1062 | 3.3483 |
| 1.0349 | 1.4889 | 0 | 2.5272 | 1.4771 | 0 | 1.8408 | 3.6337 |
| 2.472 | 4.5491 | 3.2557 | 3.1261 | 3.7115 | 5.141 | 2.6662 | 3.4421 |
| 0.5573 | 1.3432 | 2.9126 | 0.598 | 2.6255 | 2.3036 | 4.7023 | 0.7662 |
| 2.2472 | 3.2212 | 3.7219 | 3.9468 | 0 | 3.3091 | 3.5184 | 3.349 |
| 3.1679 | 2.5369 | 2.1634 | 5.1426 | 1.9875 | 3.0077 | 4.7711 | 5.6886 |
| 1.3738 | 2.9293 | 2.434 | 2.8054 | 0.5215 | 4.3685 | 2.5291 | 0.9065 |
| 3.5331 | 2.373 | 0 | 3.2105 | 2.457 | 2.9361 | 2.1962 | 2.5744 |
| 4.309 | 4.3348 | 2.8143 | 1.9975 | 1.3871 | 3.9213 | 1 | 3.381 |
| 1.0399 | 1.8676 | 2.7195 | 2.1659 | 3.5882 | 5.5663 | 2.8168 | 4.1028 |
| 1.5487 | 2.9459 | 2.7753 | 0 | 1.811 | 0 | 2.0932 | 4.2952 |
| 3.2165 | 0.8141 | 1.6954 | 6.0936 | 5.2152 | 0.601 | 1.3036 | 3.1663 |
| 3.1308 | 1.8493 | 3.4742 | 1.5778 | 0 | 5.3991 | 0 | 3.4009 |
| 2.325 | 1.9146 | 3.4487 | 1.5047 | 1.6149 | 2.474 | 1.0317 | 2.8804 |
| 4.1617 | 1.9645 | 3.0916 | 2.7721 | 3.9594 | 3.7553 | 2.4371 | 0.5466 |

| | | | | | | | | |
|---|---|---|---|---|---|---|---|---|
| 1.4975 | 2.0357 | 0.541 | 2.2569 | 2.7155 | 6.5389 | 3.0661 | 4.5214 | |
| 1.7413 | 2.7052 | 4.9695 | 2.8668 | 4.0288 | 5.263 | 3.8473 | 2.4374 | |
| 1.9569 | 3.484 | 3.8994 | 3.724 | 1.9604 | 4.1003 | 2.4698 | 1.5946 | 0 |
| | 2.9633 | 4.0536 | 0 | 6.1854 | 6.1449 | 6.1052 | 3.9415 | 7.07 |
| | 5.9175 | 7.3549 | 6.9815 | 6.9719 | 7.0216 | 6.3654 | 6.7642 | 6.89 |
| | 6.0522 | 5.8165 | 7.125 | 7.0268 | 6.2343 | 7.7681 | 5.5242 | |
| 6.5064 | 6.6904 | 6.3563 | 6.2054 | | | | | |
| STIL | 8.6656 | 9.0072 | 8.4866 | 8.9377 | 10.173 | 6.315 | 8.7432 | |
| 9.3228 | 9.2633 | 8.7146 | 8.8434 | 8.3821 | 9.1461 | 7.5592 | 7.6158 | |
| 8.5861 | 8.1799 | 8.6612 | 7.8373 | 8.3124 | 7.7628 | 9.2813 | 8.137 | |
| 7.3135 | 9.9739 | 8.8164 | 9.641 | 8.4657 | 8.4586 | 9.4282 | 8.7299 | |
| 7.307 | 9.6312 | 8.261 | 8.586 | 8.3328 | 8.2987 | 9.1527 | 7.6549 | |
| 7.4619 | 8.8739 | 8.7678 | 7.645 | 8.6469 | 9.2962 | 8.2534 | 7.2205 | |
| 8.7577 | 8.3692 | 7.8871 | 8.7943 | 8.4634 | 8.8059 | 9.5374 | 8.679 | |
| 8.5414 | 7.4648 | 8.8934 | 5.5474 | 9.4073 | 8.4938 | 8.7306 | 7.4352 | |
| 7.7323 | 9.2953 | 7.9164 | 8.0579 | 7.1994 | 6.4693 | 8.3073 | 7.5161 | |
| 9.0041 | 6.8854 | 9.4259 | 7.6212 | 8.7836 | 8.6149 | 8.1753 | 7.9102 | |
| 8.3623 | 8.7954 | 8.3726 | 7.0426 | 9.0075 | 9.1948 | 8.8322 | 8.6116 | |
| 7.1098 | 9.4485 | 9.4199 | 9.3123 | 9.2613 | 9.4231 | 8.8214 | 8.9961 | |
| 8.7993 | 9.0214 | 8.3283 | 6.3709 | 7.9542 | 9.412 | 9.4173 | 8.828 | |
| 9.1239 | 9.3245 | 9.5432 | 8.4926 | 8.266 | 10.075 | 9.0418 | 9.5364 | |
| 8.5224 | 7.3402 | 8.8537 | 8.7242 | 8.532 | 9.767 | 8.7333 | 4.6588 | |
| 7.9168 | 5.5768 | 8.2485 | 9.3616 | 7.7671 | 7.1984 | 8.9307 | 8.9301 | |
| 8.4248 | 8.2976 | 7.1695 | 7.3254 | 6.5483 | 8.9843 | 8.541 | 8.8292 | |
| 8.9204 | 8.6095 | 9.4 | 8.0903 | 7.9574 | 8.1479 | 7.3793 | 7.4392 | |
| 7.8135 | 9.1097 | 7.8148 | 10.4193 | 8.325 | 9.8095 | 8.9347 | 9.1673 | |
| 9.1644 | 7.4412 | 5.5948 | 8.6199 | 9.0854 | 7.4012 | 8.8405 | 8.7332 | |
| 9.2871 | 6.7772 | 9.0892 | 7.3636 | 8.9998 | 8.727 | 8.196 | 8.6999 | |
| 9.5794 | 7.4144 | 8.3437 | 9.1429 | 8.2515 | 8.3938 | 8.344 | 8.5102 | |
| 8.7136 | 7.7348 | 5.5793 | 7.7478 | 6.019 | 5.4072 | 5.1359 | 5.6322 | |
| 4.6343 | 5.071 | 4.7791 | 7.1254 | 4.1393 | 4.2021 | 5.4042 | 7.7207 | |
| 6.1333 | 5.7255 | 6.6567 | 5.0762 | 5.2276 | 5.6217 | 5.7698 | 7.0788 | |
| 4.768 | 5.6148 | | | | | | | |
| SOCS2 | 6.3364 | 5.9465 | 6.1446 | 6.31E+00 | | 6.13E+00 | | |
| 7.4674 | 7.2276 | 6.8763 | 6.0166 | 7.7862 | 7.9695 | 7.9426 | 6.8936 | |
| 7.2194 | 7.4344 | 7.3426 | 6.6048 | 6.795 | 7.2568 | 7.5174 | 8.6228 | |
| 5.4015 | 8.1579 | 6.461 | 6.3461 | 8.5595 | 5.1963 | 5.3417 | 6.4411 | |
| 5.5707 | 6.6277 | 7.8261 | 6.1711 | 6.9787 | 5.7059 | 7.265 | 8.2962 | |
| 7.792 | 5.8781 | 5.0157 | 6.8939 | 9.4265 | 5.5478 | 7.1563 | 6.7924 | |
| 6.5755 | 8.0784 | 7.2974 | 7.67 | 7.5379 | 9.6577 | 7.1981 | 7.8788 | |
| 7.5703 | 5.2241 | 7.4952 | 5.4808 | 8.1057 | 7.8901 | 8.5646 | 7.0449 | |
| 7.6922 | 7.7083 | 6.4967 | 8.6594 | 3.8164 | 7.9498 | 7.2649 | 7.7262 | |
| 8.2529 | 7.6973 | 6.9475 | 8.7099 | 6.4232 | 8.9014 | 7.49 | 6.6682 | |
| 6.8126 | 7.3826 | 8.7257 | 6.9261 | 8.2355 | 8.8431 | 8.9567 | 5.6804 | |
| 7.4113 | 8.2647 | 5.9997 | 7.8795 | 8.4712 | 7.0158 | 5.5355 | 7.2331 | |
| 8.6679 | 9.4268 | 6.866 | 4.951 | 8.1673 | 7.849 | 6.4429 | 6.4718 | |
| 5.9843 | 5.9629 | 6.5311 | 6.0374 | 6.8249 | 7.5404 | 7.3102 | 8.6598 | |
| 6.0035 | 7.3707 | 6.7946 | 7.4515 | 6.7749 | 4.4734 | 5.9789 | 6.6192 | |
| 7.3764 | 7.0626 | 5.9801 | 8.521 | 9.523 | 5.2163 | 6.099 | 7.8206 | |
| 7.6273 | 7.9099 | 7.0365 | 7.0821 | 6.9375 | 10.0929 | 5.6361 | 7.3171 | |
| 7.455 | 7.23 | 7.3695 | 7.8248 | 4.9012 | 8.3256 | 4.8936 | 7.2986 | |
| 8.5246 | 6.3339 | 8.0047 | 7.3153 | 7.5771 | 8.0048 | 7.3991 | 6.3987 | |
| 6.2845 | 8.2135 | 8.1929 | 8.9964 | 7.5075 | 6.6509 | 8.5796 | 8.3564 | |
| 7.2426 | 6.5863 | 5.6378 | 6.3124 | 8.223 | 7.4688 | 8.0165 | 7.5382 | |

| | | | | | | | |
|---|---|---|---|---|---|---|---|
| 6.8616 | 7.8148 | 7.4704 | 8.0828 | 6.4553 | 7.4812 | 6.2501 | 6.6437 |
| 5.8567 | 6.9085 | 7.414 | 7.4271 | 11.3978 | 10.5071 | 10.7237 | 7.1983 |
| 12.0201 | 12.1086 | 11.3665 | 12.3532 | 10.4208 | 10.7034 | 9.807 | 11.2205 |
| 10.9027 | 10.9326 | 8.0187 | 10.7323 | 11.5326 | 11.1285 | 7.3791 | 9.1909 |
| 10.8487 | 10.6815 | 9.8294 | 10.5019 | | | | |
| FAM72D | 6.3935 | 6.0701 | 6.1194 | 7.0415 | 8.1248 | 6.3955 | 6.4255 |
| 7.069 | 7.4843 | 8.2126 | 6.0591 | 6.4102 | 5.9643 | 5.569 | 5.3726 |
| 5.7878 | 6.2577 | 6.5122 | 5.5306 | 6.3955 | 6.3657 | 8.7142 | 5.6107 |
| 4.0984 | 6.491 | 7.4088 | 7.7512 | 7.2223 | 6.8376 | 7.1557 | 7.1165 |
| 5.4816 | 7.6486 | 7.218 | 6.2554 | 6.4728 | 6.1324 | 6.7073 | 5.7866 |
| 6.0639 | 6.0163 | 6.9593 | 4.362 | 7.1261 | 5.8053 | 5.8684 | 6.7411 |
| 6.6274 | 5.4251 | 5.4722 | 6.8172 | 7.0259 | 7.8687 | 6.5956 | 6.2047 |
| 5.6829 | 4.9444 | 6.0838 | 1.9829 | 6.4825 | 7.4144 | 6.3989 | 6.3484 |
| 5.0726 | 6.6717 | 5.6517 | 6.3435 | 2.9674 | 5.3512 | 7.2948 | 5.6437 |
| 8.1711 | 4.1036 | 7.3375 | 5.2636 | 6.1564 | 5.8777 | 5.7563 | 5.9514 |
| 8.5655 | 7.5291 | 5.2808 | 4.93 | 7.0958 | 6.5766 | 6.0931 | 7.2994 |
| 5.4568 | 6.3267 | 6.8797 | 7.0379 | 6.3542 | 7.7952 | 5.0166 | 7.8948 |
| 6.7829 | 6.3767 | 5.9756 | 4.7934 | 6.8202 | 7.439 | 7.0206 | 7.098 |
| 6.9243 | 7.0713 | 6.521 | 7.8682 | 6.1089 | 6.3841 | 5.63 | 7.1882 |
| 6.8946 | 5.539 | 6.6898 | 6.6691 | 6.178 | 6.928 | 6.6078 | 5.7568 |
| 6.0549 | 3.0835 | 5.4489 | 7.0015 | 5.0787 | 4.854 | 6.2624 | 6.2145 |
| 5.3099 | 5.0981 | 5.8016 | 5.6227 | 5.4557 | 5.2085 | 6.3701 | 5.9312 |
| 6.6156 | 6.5842 | 7.5323 | 7.2242 | 4.9677 | 6.2636 | 5.6208 | 4.8121 |
| 6.0014 | 6.3017 | 6.4288 | 6.2362 | 6.1717 | 6.97 | 6.2295 | 7.8917 |
| 6.1785 | 7.3581 | 3.8992 | 6.1632 | 7.5953 | 4.4865 | 6.9463 | 6.9507 |
| 8.2049 | 5.5072 | 5.6525 | 5.5231 | 6.5278 | 6.408 | 5.1765 | 6.504 |
| 5.968 | 4.8672 | 6.2144 | 5.8333 | 6.9151 | 6.8599 | 5.5377 | 6.4773 |
| 5.7474 | 3.744 | 2.9425 | 5.6129 | 3.5819 | 2.1093 | 2.7258 | 2.8926 |
| 2.6279 | 4.3564 | 3.13 | 4.9096 | 1.4126 | 3.1952 | 2.8016 | 3.7595 |
| 2.938 | 2.4022 | 4.7164 | 2.2561 | 2.792 | 3.3488 | 3.031 | 4.6712 |
| 3.1136 | 1.8629 | | | | | | |
| ACTA2 | 12.9444 | 11.3655 | 9.4179 | 1.38E+01 | | 9.93E+00 | |
| 8.7206 | 11.5298 | 8.923 | 10.089 | 8.9525 | 9.9694 | 11.2453 | 9.7507 |
| 11.3639 | 9.7541 | 11.651 | 9.5057 | 11.3226 | 10.5181 | 10.6576 | 10.9471 |
| 10.146 | 11.736 | 13.862 | 8.321 | 11.4219 | 13.4216 | 8.6733 | 9.3348 |
| 7.5929 | 9.4227 | 11.7469 | 9.089 | 11.5031 | 10.3828 | 11.8887 | 11.2521 |
| 11.3046 | 10.7727 | 12.3838 | 9.6806 | 12.6978 | 10.518 | 11.1765 | 10.9291 |
| 13.4991 | 13.3518 | 9.9097 | 12.0331 | 10.8746 | 15.0395 | 9.7893 | 12.1276 |
| 10.7596 | 9.938 | 10.9401 | 8.9614 | 11.5393 | 10.4752 | 15.175 | 9.7536 |
| 11.3783 | 11.1907 | 10.3589 | 13.6492 | 9.4734 | 11.5576 | 11.1693 | 11.3049 |
| 13.4585 | 12.1813 | 11.4666 | 13.4108 | 10.6584 | 13.6604 | 10.057 | 10.8113 |
| 10.9007 | 12.4452 | 15.4525 | 10.8728 | 10.0815 | 12.5633 | 12.4479 | 10.4263 |
| 13.1017 | 12.1107 | 13.1288 | 12.3541 | 10.0948 | 12.6587 | 9.3716 | 13.0808 |
| 12.5635 | 11.8885 | 11.4545 | 9.7498 | 9.4952 | 12.7898 | 12.5614 | 12.8654 |
| 9.0538 | 10.9552 | 9.1463 | 12.2033 | 11.9735 | 12.1841 | 12.381 | 11.2991 |
| 10.267 | 10.032 | 13.6785 | 9.5195 | 10.5803 | 8.6197 | 12.0408 | 12.2208 |
| 11.7179 | 14.5322 | 11.8739 | 13.0304 | 14.4606 | 9.5388 | 10.6201 | 10.8806 |
| 11.8452 | 10.2748 | 8.9203 | 10.5225 | 10.8065 | 14.9535 | 13.0841 | 10.5734 |
| 9.8273 | 10.3099 | 13.2271 | 11.5725 | 8.8316 | 12.6412 | 10.1525 | 12.6012 |
| 10.085 | 10.2247 | 10.7461 | 11.0625 | 13.0089 | 10.6971 | 12.7136 | 8.1628 |
| 10.0262 | 9.6751 | 9.6427 | 11.2534 | 13.3001 | 11.7453 | 12.6381 | 12.2055 |
| 13.2876 | 11.1546 | 8.5203 | 10.3849 | 10.0616 | 12.5024 | 12.2592 | 10.3615 |
| 9.4091 | 12.7924 | 12.5603 | 10.7463 | 10.5736 | 13.078 | 10.5467 | 9.5391 |
| 9.5535 | 11.3388 | 13.4014 | 12.3403 | 15.3721 | 14.3431 | 15.4832 | 13.0436 |

| | | | | | | | | |
|---|---|---|---|---|---|---|---|---|
| 18.3933 | 15.6109 | 16.0863 | 16.7209 | 16.7726 | 14.7228 | 16.7489 | 17.9465 | |
| 15.9157 | 15.4897 | 14.1902 | 16.3682 | 17.2692 | 15.5043 | 12.4315 | 16.2625 | |
| 15.8225 | 15.6779 | 17.4983 | 15.4168 | | | | | |
| E2F1 | 9.9721 | 8.5311 | 7.0657 | 10.8774 | 9.3725 | 9.9765 | 10.1441 | |
| 9.8013 | 10.2275 | 9.2024 | 9.0961 | 9.3369 | 7.9962 | 7.845 | 9.5862 | |
| 8.8654 | 9.8977 | 10.8224 | 6.9677 | 7.5381 | 10.5231 | 11.4703 | 9.7611 | |
| 7.6218 | 8.9941 | 10.4095 | 8.9035 | 9.8027 | 9.6124 | 10.5092 | 10.1034 | |
| 9.0431 | 9.0947 | 8.6652 | 8.0055 | 9.8096 | 9.9213 | 10.1568 | 8.1253 | |
| 11.1765 | 9.3167 | 9.4001 | 9.367 | 8.737 | 9.382 | 9.5816 | 9.3122 | |
| 9.0659 | 8.3498 | 7.12 | 9.0976 | 9.2553 | 9.6548 | 10.0199 | 10.1286 | |
| 8.2038 | 8.4607 | 9.9888 | 7.3523 | 9.9684 | 10.9226 | 8.8151 | 10.3003 | |
| 9.481 | 7.8841 | 9.1262 | 9.1653 | 6.9622 | 6.4492 | 9.6152 | 9.9708 | |
| 9.8434 | 6.8372 | 9.9859 | 8.7693 | 8.9257 | 9.5683 | 9.116 | 9.0841 | |
| 10.9737 | 9.1988 | 10.1139 | 7.5191 | 9.9933 | 9.6004 | 8.1818 | 8.6538 | |
| 9.6967 | 8.9733 | 10.0044 | 10.2127 | 9.7383 | 11.7724 | 9.0765 | 9.2247 | |
| 9.8715 | 7.94 | 10.5084 | 11.3822 | 9.1799 | 10.4119 | 9.1766 | 8.5975 | |
| 9.0507 | 9.6068 | 10.079 | 10.7668 | 8.218 | 8.7833 | 9.3986 | 9.1973 | |
| 10.9745 | 11.5501 | 9.0821 | 9.0212 | 8.6236 | 10.6934 | 9.7633 | 10.0303 | |
| 11.1374 | 8.1363 | 8.2912 | 9.953 | 7.9149 | 8.2185 | 8.138 | 8.8923 | |
| 8.8885 | 10.1821 | 10.0404 | 6.887 | 11.394 | 7.2711 | 9.3019 | 8.7417 | |
| 9.5788 | 9.9756 | 10.1334 | 10.7141 | 8.7855 | 9.647 | 8.3132 | 9.3225 | |
| 9.9334 | 8.8336 | 8.9304 | 8.5189 | 10.4396 | 10.9366 | 8.8517 | 9.9975 | |
| 11.1619 | 9.2592 | 8.5364 | 9.2512 | 9.8565 | 6.5 | 10.2218 | 9.7367 | |
| 10.8698 | 7.9281 | 8.8643 | 7.7488 | 8.9913 | 10.2218 | 10.1716 | 10.109 | |
| 9.0863 | 7.5435 | 9.6552 | 8.4073 | 8.6693 | 11.6018 | 12.6752 | 9.5634 | |
| 7.6565 | 11.1154 | 4.84 | 9.0695 | 5.6549 | 5.9618 | 6.0454 | 4.5292 | |
| 3.8551 | 5.351 | 4.5454 | 7.6964 | 5.6528 | 5.5451 | 5.5098 | 6.9671 | |
| 6.4617 | 5.3409 | 6.1996 | 5.6468 | 7.1249 | 7.4969 | 5.3467 | 7.7376 | |
| 5.8817 | 5.8307 | | | | | | | |
| MASP1 | 4.4286 | 5.1171 | 4.9244 | 5.38E+00 | | 3.14E+00 | | 0 |
| | 6.1685 | 2.4718 | 2.7843 | 4.4179 | 4.5606 | 2.5815 | 3.6718 | |
| 2.9791 | 4.8326 | 4.8499 | 2.6671 | 1.38 | 4.9426 | 3.0076 | 0.6062 | |
| 6.2569 | 5.6847 | 6.6697 | 3.3358 | 0.4127 | 5.2332 | 1.8398 | 2.3782 | |
| 0.5853 | 3.3311 | 6.4958 | 2.8736 | 3.6294 | 4.6947 | 3.374 | 3.0982 | |
| 2.3773 | 5.8558 | 2.2746 | 2.3699 | 5.6709 | 5.5717 | 3.6785 | 4.5305 | |
| 4.2624 | 5.7494 | 3.3925 | 5.0212 | 8.1192 | 8.7282 | 6.0045 | 7.2817 | |
| 3.6224 | 5.3197 | 7.3787 | 2.8406 | 5.5094 | 8.3802 | 8.9843 | 2.2792 | |
| 4.5148 | 6.1929 | 5.2471 | 8.8948 | 0.8237 | 7.5156 | 8.09 | 7.1917 | |
| 7.2701 | 3.27 | 2.2618 | 9.0781 | 3.3909 | 7.9254 | 2.2503 | 4.0315 | |
| 3.7795 | 6.3837 | 3.4763 | 6.9955 | 2.5333 | 7.8729 | 6.9363 | 4.1741 | |
| 8.0289 | 1.921 | 1.6914 | 6.5143 | 2.8654 | 5.8301 | 6.0679 | 7.8629 | |
| 5.2326 | 6.5778 | 5.7495 | 3.7441 | 3.2654 | 7.2172 | 1 | 6.3332 | |
| 5.0304 | 4.0011 | 4.1505 | 5.5243 | 3.3856 | 5.9157 | 6.4539 | 6.8363 | |
| 2.0289 | 4.4891 | 5.8007 | 6.9848 | 3.4225 | 2.1813 | 5.2069 | 5.1217 | |
| 4.2555 | 7.0869 | 3.0489 | 6.0124 | 11.0438 | 3.4355 | 2.6745 | 3.255 | |
| 5.8706 | 5.883 | 0.6735 | 1.9919 | 3.2756 | 11.3143 | 3.773 | 3.5202 | |
| 3.8616 | 4.4749 | 2.0663 | 8.5051 | 2.2048 | 4.9784 | 3.4493 | 3.9371 | |
| 3.9918 | 3.1277 | 1.4811 | 3.7982 | 6.971 | 8.8327 | 5.801 | 2.7065 | |
| 2.848 | 2.848 | 3.3362 | 4.598 | 5.1619 | 2.056 | 6.2846 | 8.7625 | |
| 4.7174 | 3.6618 | 3.5836 | 3.4688 | 4.7179 | 6.1915 | 1.9914 | 4.8774 | |
| 1.9569 | 0 | 4.2265 | 5.122 | 6.5399 | 7.9265 | 3.6503 | 0 | |
| 1.3603 | 6.4236 | 7.3333 | 3.1794 | 11.9103 | 8.0922 | 11.7205 | 8.6112 | |
| 10.7381 | 12.2301 | 13.0473 | 12.4033 | 12.4843 | 10.1044 | 10.5982 | 13.0727 | |
| 12.0976 | 10.7521 | 8.4408 | 12.6916 | 10.3616 | 12.7213 | 7.0867 | 9.769 | |

| | | | | | | | |
|---|---|---|---|---|---|---|---|
| 12.2579 | 9.6759 | 9.6516 | 12.6173 | | | | |
| GPR124 | 8.4801 | 9.0003 | 9.0025 | 9.31E+00 | | 7.86E+00 | |
| 8.7427 | 8.5911 | 8.7347 | 7.7787 | 7.4691 | 10.1776 | 7.9045 | 7.1988 |
| 9.1825 | 9.3599 | 9.336 | 6.8439 | 7.5747 | 8.2502 | 8.6109 | 8.4655 |
| 8.5407 | 9.5204 | 10.1277 | 6.3728 | 12.7738 | 9.4034 | 6.8446 | 7.4798 |
| 5.9667 | 7.4955 | 9.7702 | 8.1676 | 7.7745 | 7.9676 | 9.2566 | 8.6032 |
| 7.792 | 7.9728 | 7.4724 | 8.4513 | 10.9023 | 7.3486 | 8.9628 | 8.948 |
| 9.7463 | 10.2969 | 7.8894 | 9.1259 | 10.0857 | 11.1984 | 9.1357 | 9.1705 |
| 7.9572 | 7.2971 | 9.074 | 6.4813 | 8.6566 | 9.578 | 11.0591 | 6.6814 |
| 9.0761 | 8.058 | 8.8641 | 10.258 | 6.5804 | 9.1841 | 9.5655 | 9.6871 |
| 9.7711 | 8.7831 | 8.6616 | 11.4649 | 8.4912 | 9.0561 | 7.9287 | 7.5884 |
| 7.8828 | 9.3627 | 10.8331 | 8.3631 | 7.7466 | 10.5664 | 8.7985 | 8.3188 |
| 9.1256 | 9.0824 | 7.6913 | 9.2199 | 7.8315 | 9.6295 | 7.6355 | 9.184 |
| 10.8295 | 10.8532 | 9.2102 | 7.5236 | 6.4367 | 8.4319 | 8.8948 | 9.6123 |
| 8.6634 | 7.9937 | 8.0909 | 8.3543 | 9.0887 | 11.5748 | 9.1759 | 10.0693 |
| 8.0083 | 8.1151 | 8.9842 | 5.8592 | 7.9123 | 6.5288 | 8.8031 | 8.9577 |
| 8.2771 | 9.095 | 7.4517 | 11.083 | 11.3678 | 6.5707 | 8.2078 | 7.9756 |
| 9.3595 | 8.0622 | 7.3835 | 7.6617 | 8.1745 | 11.3764 | 7.3726 | 9.0138 |
| 9.1215 | 7.1572 | 9.411 | 8.6072 | 6.4192 | 8.415 | 7.4023 | 8.8529 |
| 9.0787 | 6.4555 | 8.8766 | 8.7703 | 8.7833 | 10.3523 | 9.3067 | 6.4221 |
| 7.504 | 8.2178 | 8.6022 | 7.2548 | 9.0649 | 8.7827 | 9.2683 | 9.8146 |
| 8.1596 | 9.5295 | 6.9293 | 8.6667 | 7.4225 | 9.0791 | 9.5995 | 7.5479 |
| 7.0365 | 8.8726 | 9.2925 | 7.7746 | 8.3921 | 10.0251 | 8.3588 | 7.6069 |
| 9.5293 | 8.5642 | 9.2921 | 7.2446 | 11.6408 | 12.3849 | 12.3405 | 9.2299 |
| 12.3743 | 11.1814 | 11.5867 | 12.3981 | 11.7945 | 13.0026 | 12.726 | 12.122 |
| 13.1717 | 12.0816 | 10.8027 | 12.5324 | 12.3375 | 12.3116 | 11.5593 | 12.1609 |
| 12.2234 | 12.6845 | 12.5458 | 12.1495 | | | | |
| FANCD2 | 9.9382 | 9.0628 | 8.0133 | 10.1749 | 9.9342 | 8.5038 | 9.5627 |
| 9.101 | 9.8863 | 9.6725 | 10.0172 | 9.0397 | 9.0424 | 8.2799 | 9.2051 |
| 9.6174 | 9.4076 | 9.3301 | 8.0787 | 8.2984 | 8.1446 | 10.3652 | 8.749 |
| 7.3061 | 9.6069 | 9.4394 | 9.0531 | 9.0552 | 9.7537 | 9.5057 | 9.2181 |
| 8.3634 | 9.8115 | 9.5017 | 8.6962 | 8.8326 | 8.0567 | 8.6637 | 8.1667 |
| 8.8823 | 8.7416 | 9.6922 | 9.1634 | 8.7884 | 8.5877 | 8.1416 | 7.7649 |
| 9.529 | 8.947 | 7.9854 | 8.7409 | 9.2495 | 9.2201 | 9.7355 | 10.1127 |
| 9.0369 | 7.6606 | 9.5151 | 5.719 | 10.3337 | 8.7024 | 9.4466 | 8.5709 |
| 8.4461 | 8.8046 | 8.3936 | 9.0424 | 6.8185 | 6.528 | 9.1701 | 8.9632 |
| 10.2898 | 6.2747 | 9.1811 | 8.6993 | 9.2823 | 9.515 | 8.6088 | 9.4371 |
| 8.9413 | 9.8794 | 8.3839 | 7.9358 | 10.0254 | 8.352 | 8.6237 | 8.7689 |
| 8.8377 | 8.3808 | 9.4498 | 9.3568 | 9.9592 | 10.1728 | 8.345 | 9.097 |
| 9.0429 | 8.8109 | 9.24 | 8.9732 | 9.8734 | 10.813 | 10.3755 | 9.1949 |
| 9.8072 | 9.6068 | 9.0492 | 9.6437 | 9.2719 | 9.9374 | 8.9334 | 9.6958 |
| 8.5916 | 7.8036 | 9.5126 | 8.9881 | 8.497 | 9.0913 | 8.2665 | 7.317 |
| 9.6811 | 7.1977 | 9.0911 | 9.4559 | 8.1518 | 8.2101 | 8.7236 | 9.5115 |
| 7.9431 | 9.1147 | 9.318 | 7.5725 | 8.2158 | 8.9568 | 8.5372 | 9.4416 |
| 9.5801 | 10.1188 | 8.5876 | 9.3397 | 8.4268 | 8.3884 | 8.5129 | 8.6553 |
| 9.7164 | 9.8826 | 7.777 | 9.1494 | 9.4374 | 10.2869 | 9.8767 | 9.2651 |
| 8.8837 | 8.8755 | 6.9155 | 8.9018 | 10.4796 | 7.2214 | 9.2522 | 9.24 |
| 9.8655 | 7.9454 | 9.057 | 8.6783 | 10.0123 | 9.7572 | 8.8341 | 9.1939 |
| 9.7108 | 8.2983 | 7.857 | 8.8747 | 10.0499 | 9.5083 | 9.0189 | 9.2824 |
| 9.0005 | 8.1824 | 5.9456 | 8.907 | 6.5966 | 6.4732 | 5.5928 | 6.5757 |
| 4.9076 | 6.1731 | 5.6383 | 7.4884 | 5.3732 | 4.6082 | 6.1832 | 6.8315 |
| 7.2499 | 6.1787 | 7.3916 | 6.8262 | 6.7258 | 7.0234 | 6.5512 | 7.7669 |
| 5.4298 | 7.2315 | | | | | | |
| ARHGAP11A | | 9.5135 | 7.8544 | 8.0735 | 9.998 | 10.0954 | 9.0217 |

| | | | | | | | | |
|---|---|---|---|---|---|---|---|---|
| 9.8057 | 9.5653 | 9.7772 | 9.7917 | 8.9808 | 8.9542 | 9.7363 | 8.0556 | |
| 8.5698 | 8.7929 | 8.7201 | 8.8367 | 8.0893 | 8.4477 | 8.8875 | 9.7836 | |
| 8.4339 | 6.9493 | 9.4056 | 9.636 | 9.4424 | 10.098 | 7.2817 | 9.9471 | |
| 9.8081 | 7.5433 | 9.8695 | 9.3061 | 8.9597 | 9.0151 | 7.9441 | 9.8986 | |
| 8.6416 | 7.8814 | 8.8739 | 10.3845 | 7.058 | 9.4512 | 8.8599 | 8.3216 | |
| 8.0142 | 9.5047 | 8.8606 | 7.6884 | 9.1818 | 9.2904 | 9.6904 | 9.9693 | |
| 9.606 | 8.8867 | 7.3423 | 10.3637 | 6.0218 | 8.2457 | 8.337 | 9.1985 | |
| 8.2144 | 8.2697 | 8.7483 | 7.7967 | 8.4079 | 6.4039 | 6.3977 | 9.7903 | |
| 8.6358 | 10.4151 | 7.0282 | 10.0567 | 8.1589 | 7.8835 | 9.5406 | 8.4004 | |
| 8.3859 | 10.0222 | 9.8751 | 7.3938 | 7.666 | 9.9066 | 8.4199 | 8.741 | |
| 9.3695 | 7.4459 | 9.4123 | 10.2892 | 10.0982 | 10.2441 | 10.0909 | 7.981 | |
| 9.5113 | 9.1893 | 8.9805 | 9.3631 | 7.5188 | 8.0334 | 10.0908 | 9.6216 | |
| 9.1316 | 9.5878 | 9.3632 | 8.8203 | 9.4189 | 9.2095 | 8.7511 | 9.1937 | |
| 9.8891 | 10.0494 | 7.7706 | 9.33 | 10.1286 | 9.0415 | 9.3205 | 8.8605 | |
| 6.8324 | 8.646 | 6.0536 | 9.0538 | 9.773 | 7.9381 | 7.8671 | 9.4601 | |
| 9.6192 | 9.267 | 8.3578 | 8.4664 | 7.3366 | 8.2439 | 8.6035 | 9.2834 | |
| 9.4915 | 10.7076 | 9.4786 | 9.412 | 9.6099 | 8.1152 | 9.5513 | 8.7545 | |
| 7.7009 | 9.3512 | 10.0278 | 9.01 | 8.3994 | 9.361 | 9.9612 | 9.9614 | |
| 10.3878 | 8.8627 | 8.1953 | 5.6756 | 9.3382 | 10.1069 | 7.029 | 8.7617 | |
| 8.8394 | 10.1243 | 8.2013 | 8.7967 | 8.5734 | 8.6539 | 8.9733 | 8.6601 | |
| 9.9239 | 10.4551 | 7.8934 | 8.9996 | 9.6618 | 9.3189 | 9.0229 | 8.7017 | |
| 9.2186 | 8.7323 | 8.6177 | 5.5104 | 9.1426 | 6.2728 | 5.936 | 5.3824 | |
| 5.4454 | 4.2185 | 5.3806 | 5.5142 | 8.0161 | 5.3141 | 4.9755 | 5.9882 | |
| 6.8147 | 6.3115 | 6.323 | 7.4203 | 5.094 | 4.487 | 6.5444 | 5.5371 | |
| 7.6079 | 4.4082 | 5.8307 | | | | | | |
| NEIL3 | 7.0254 | 5.4958 | 5.173 | 7.73E+00 | | 7.31E+00 | | |
| 6.7905 | 6.0547 | 6.0609 | 7.5944 | 6.8185 | 6.7595 | 6.4222 | 5.6245 | |
| 4.5469 | 6.0137 | 6.2738 | 4.9555 | 5.9642 | 4.7646 | 5.0308 | 6.2417 | |
| 8.5805 | 4.7951 | 2.5456 | 6.5544 | 8.0426 | 6.9615 | 6.098 | 5.2197 | |
| 6.1608 | 6.5083 | 4.771 | 6.778 | 6.5236 | 6.5097 | 6.3121 | 6.6651 | |
| 6.0375 | 5.7866 | 4.8007 | 6.0685 | 7.1812 | 4.3343 | 5.8346 | 5.7298 | |
| 5.7666 | 5.38 | 6.9268 | 4.629 | 8.009 | 6.722 | 5.6607 | 6.5785 | |
| 7.1057 | 5.4939 | 5.7759 | 4.7097 | 6.6388 | 2.98 | 6.8913 | 6.4635 | |
| 6.6618 | 5.8437 | 4.5254 | 6.165 | 3.6494 | 6.0487 | 2.5855 | 2.7035 | |
| 6.6521 | 5.9765 | 6.2293 | 2.7716 | 7.0836 | 5.3902 | 6.6329 | 6.8654 | |
| 5.8118 | 6.1347 | 6.7194 | 6.4473 | 5.9631 | 4.5685 | 6.1555 | 6.7624 | |
| 5.2882 | 6.7338 | 5.5627 | 6.6331 | 6.2816 | 7.2918 | 6.888 | 8.9626 | |
| 6.3552 | 6.3545 | 5.1164 | 6.22 | 5.644 | 4.4223 | 5.4594 | 7.0629 | |
| 6.5935 | 5.4683 | 6.3013 | 6.615 | 6.1249 | 7.146 | 5.8935 | 5.6148 | 5.63 |
| | 6.8227 | 6.634 | 5.6047 | 7.059 | 7.1836 | 5.6304 | 6.2781 | |
| 5.6225 | 3.629 | 5.9013 | 0 | 5.232 | 7.5393 | 4.5252 | 4.7044 | |
| 6.6312 | 6.148 | 4.0415 | 5.3818 | 4.4402 | 4.1413 | 4.6083 | 4.7767 | |
| 6.7317 | 6.4176 | 6.8857 | 6.3923 | 6.9751 | 7.0762 | 6.4108 | 5.8647 | |
| 5.5306 | 4.8121 | 5.3496 | 5.4145 | 5.9057 | 5.54 | 6.7171 | 7.3294 | |
| 6.7033 | 7.3728 | 5.567 | 6.6096 | 1.2582 | 6.4242 | 7.0105 | 3.5829 | |
| 5.9081 | 6.3022 | 7.2869 | 5.2923 | 6.0651 | 4.4327 | 6.9015 | 6.0849 | |
| 5.2217 | 6.601 | 6.74 | 4.3406 | 5.7028 | 6.2134 | 6.3038 | 5.2995 | |
| 6.1605 | 6.2098 | 5.0831 | 3.5284 | 1.6912 | 6.1738 | 2.4567 | 0.6346 | |
| 2.246 | 1.1407 | 0 | 2.1337 | 1.9018 | 5.048 | 1.4126 | 1.396 | |
| 1.5047 | 3.3565 | 1.609 | 2.1156 | 3.7703 | 0.8792 | 0 | 3.9299 | |
| 1.2837 | 4.4582 | 1.4008 | 1.0391 | | | | | |
| SDPR | 4.8581 | 4.6381 | 5.7984 | 5.61E+00 | | 5.31E+00 | | |
| 4.4907 | 8.0721 | 10.4056 | 6.1064 | 7.1214 | 8.6464 | 5.427 | 5.8102 | |
| 7.6972 | 6.7 | 5.6714 | 7.8873 | 6.2337 | 6.2699 | 6.926 | 5.3831 | |

| | | | | | | | | |
|---|---|---|---|---|---|---|---|---|
| 6.8237 | 6.5491 | 5.5536 | 5.3549 | 6.8154 | 5.405 | 8.7455 | 5.5249 | |
| 5.5244 | 4.8171 | 7.1441 | 3.8884 | 4.4845 | 5.2544 | 6.7092 | 4.9408 | |
| 7.1977 | 6.8989 | 3.1173 | 6.5804 | 6.0033 | 5.131 | 4.9993 | 7.1302 | |
| 10.1305 | 7.007 | 4.9286 | 7.1421 | 7.377 | 9.7971 | 7.7212 | 7.8327 | |
| 10.3976 | 2.9375 | 5.3831 | 4.9201 | 5.2923 | 6.592 | 8.3364 | 4.0974 | |
| 4.8787 | 7.1363 | 8.073 | 8.3404 | 7.2937 | 9.2699 | 6.5228 | 8.7922 | |
| 7.3382 | 5.8011 | 4.4721 | 10.0837 | 7.3322 | 7.7856 | 6.4412 | 4.9237 | |
| 5.4585 | 6.8152 | 6.932 | 7.2513 | 7.4737 | 7.6484 | 8.8414 | 5.6927 | |
| 7.3639 | 6.0948 | 3.9007 | 7.0294 | 4.024 | 7.2795 | 6.1266 | 7.3724 | |
| 9.5675 | 8.1317 | 5.9028 | 5.0659 | 5.2741 | 6.6672 | 6 | 5.9592 | |
| 6.3997 | 3.9608 | 6.2359 | 4.5158 | 6.5293 | 9.6583 | 4.988 | 7.4619 | |
| 4.9913 | 7.0732 | 5.1078 | 6.9682 | 5.1873 | 4.8128 | 5.7352 | 6.6493 | 7.21 |
| | 5.0375 | 8.2282 | 6.8457 | 10.8764 | 3.9024 | 6.4774 | 5.9667 | |
| 6.2188 | 4.8149 | 5.6202 | 4.6901 | 3.2756 | 11.4235 | 3.1371 | 5.6621 | |
| 6.6111 | 6.3941 | 8.119 | 4.8668 | 3.9486 | 6.4142 | 4.8155 | 6.128 | |
| 7.3793 | 5.2017 | 5.6256 | 6.9956 | 7.0041 | 8.7373 | 5.4826 | 4.3467 | |
| 3.1141 | 7.6523 | 5.2072 | 5.3369 | 6.4013 | 5.8738 | 6.3427 | 8.5315 | |
| 4.1213 | 6.0451 | 9.2281 | 7.1283 | 7.7782 | 6.4548 | 6.9544 | 5.8208 | |
| 4.1933 | 8.4526 | 5.5014 | 9.2445 | 5.8547 | 6.5139 | 5.728 | 4.2617 | |
| 5.6769 | 5.4055 | 6.167 | 4.2463 | 10.1178 | 9.3007 | 10.1162 | 8.1236 | |
| 11.1613 | 10.6026 | 13.3316 | 13.1207 | 11.8008 | 9.8194 | 12.0122 | 12.1206 | |
| 11.1458 | 9.1913 | 9.9761 | 11.6091 | 12.0273 | 10.8082 | 11.6217 | 11.1808 | |
| 12.1436 | 9.8182 | 12.2674 | 10.4876 | | | | | |
| JAM3 | 7.501 | 7.7225 | 6.7199 | 8.15E+00 | | 6.3616 | 7.659 | |
| 7.9133 | 6.931 | 6.453 | 7.0449 | 7.65 | 6.6075 | 6.7252 | 7.362 | |
| 8.1138 | 8.062 | 5.7843 | 7.0489 | 7.0259 | 6.926 | 7.5299 | 10.531 | |
| 8.0611 | 8.3452 | 5.7965 | 11.1251 | 8.0416 | 7.0077 | 6.2225 | 5.6447 | |
| 6.1393 | 7.6751 | 6.5622 | 7.5193 | 6.9874 | 8.3536 | 7.9504 | 6.778 | |
| 6.8658 | 7.1538 | 6.8939 | 11.8176 | 6.911 | 7.1748 | 7.9023 | 8.6173 | |
| 9.3902 | 7.1555 | 8.0998 | 7.6349 | 9.6577 | 6.7214 | 7.626 | 7.3336 | |
| 8.6645 | 7.2898 | 6.1467 | 6.5919 | 8.7387 | 9.7933 | 6.025 | 7.0866 | |
| 6.997 | 6.9466 | 9.1689 | 7.3218 | 8.4119 | 7.9325 | 7.8842 | 8.5042 | |
| 7.9424 | 7.1345 | 9.75 | 6.888 | 8.4479 | 7.6334 | 7.3215 | 7.2797 | |
| 7.957 | 12.1158 | 6.9818 | 9.3348 | 9.0293 | 8.5672 | 6.7031 | 8.0429 | |
| 7.4717 | 7.9571 | 7.9914 | 6.5948 | 8.7509 | 7.0441 | 8.3051 | 8.8291 | |
| 8.0936 | 7.6006 | 6.5774 | 6.1278 | 8.5756 | 8.224 | 8.5951 | 7.1656 | |
| 6.4592 | 7.5557 | 7.6263 | 7.509 | 11.6173 | 7.6034 | 7.9544 | 7.1255 | |
| 7.4795 | 8.387 | 5.8044 | 6.9127 | 6.1282 | 7.1866 | 7.4275 | 7.7844 | |
| 10.0092 | 7.8658 | 8.7575 | 10.1218 | 6.3958 | 7.0833 | 7.4258 | 7.9323 | |
| 7.1605 | 7.1795 | 6.9493 | 6.4677 | 10.3828 | 7.455 | 7.4732 | 7.3525 | |
| 6.0863 | 8.7775 | 7.3489 | 6.4536 | 7.8089 | 6.1494 | 7.5722 | 7.6602 | |
| 6.1231 | 7.313 | 7.3924 | 8.1317 | 7.29 | 7.9736 | 6.083 | 6.6467 | |
| 10.1666 | 9.4041 | 8.7087 | 8.7751 | 7.3335 | 8.0049 | 8.4432 | 7.5689 | |
| 7.3484 | 7.2162 | 7.1709 | 7.0251 | 9.7208 | 7.9914 | 8.1416 | 6.695 | |
| 7.7349 | 7.8696 | 6.8995 | 9.1818 | 8.7234 | 7.2247 | 7.2528 | 10.4696 | |
| 7.0727 | 8.4608 | 7.7137 | 11.1941 | 10.1929 | 11.0414 | 8.5657 | 11.8666 | |
| 10.9637 | 11.5337 | 11.8028 | 11.6862 | 10.6216 | 11.5706 | 11.693 | 11.4131 | |
| 10.1933 | 9.1612 | 11.457 | 11.254 | 11.2558 | 11.306 | 10.4935 | 11.2393 | |
| 10.5014 | 12.0026 | 11.0874 | | | | | | |
| FAM83D | 9.6063 | 8.6569 | 7.1878 | 9.4479 | 10.0581 | 9.1813 | 9.003 | |
| 9.5792 | 9.6423 | 9.8934 | 8.8903 | 8.4483 | 8.1043 | 6.4151 | 7.5837 | |
| 8.7337 | 9.1877 | 10.1675 | 7.2303 | 7.2929 | 8.4462 | 11.3754 | 7.8064 | |
| 6.2782 | 9.9667 | 9.5281 | 9.1481 | 9.523 | 8.4492 | 9.9931 | 9.7302 | |
| 7.645 | 9.4508 | 9.2988 | 8.4865 | 9.1145 | 8.3183 | 9.2628 | 6.2646 | |

| | | | | | | | |
|---|---|---|---|---|---|---|---|
| 7.611 | 9.5796 | 10.4613 | 6.9841 | 8.8578 | 8.6116 | 8.8214 | 9.1918 |
| 8.3981 | 8.2115 | 7.5216 | 9.1903 | 9.0243 | 9.1767 | 10.2401 | 9.2748 |
| 9.0049 | 8.405 | 10.0251 | 5.1676 | 9.4563 | 9.5016 | 8.8892 | 8.2846 |
| 7.4631 | 7.954 | 7.6877 | 8.7983 | 5.736 | 4.0282 | 9.0317 | 8.2866 |
| 10.1847 | 4.9745 | 9.6828 | 7.0367 | 8.4151 | 10.0675 | 8.3706 | 8.1687 |
| 9.3794 | 9.1681 | 8.0298 | 6.1872 | 9.2354 | 8.8513 | 8.6175 | 8.3978 |
| 6.8278 | 8.9839 | 10.0369 | 8.6131 | 9.6474 | 10.7704 | 8.4041 | 9.2367 |
| 8.9029 | 8.4683 | 8.6303 | 6.6447 | 7.1799 | 9.7427 | 8.9914 | 8.3661 |
| 10.3752 | 10.6785 | 9.3431 | 9.9527 | 8.2446 | 8.9902 | 8.9357 | 9.4123 |
| 9.5748 | 8.523 | 9.5535 | 8.1816 | 8.4745 | 8.69 | 8.949 | 6.9784 |
| 8.5109 | 1.5206 | 7.865 | 9.2024 | 7.3489 | 6.5873 | 8.8445 | 9.1947 |
| 6.5787 | 7.9977 | 7.6003 | 6.3156 | 6.7315 | 7.6891 | 8.6441 | 9.0995 |
| 9.9316 | 9.1666 | 9.5345 | 8.8815 | 8.2939 | 9.1277 | 7.4092 | 6.6064 |
| 9.8132 | 9.2584 | 7.8241 | 8.6989 | 9.2814 | 9.6425 | 9.9055 | 10.4315 |
| 9.8196 | 8.9515 | 4.7036 | 8.9772 | 10.7495 | 5.0135 | 8.5579 | 9.3458 |
| 9.9704 | 7.2245 | 9.5834 | 7.2755 | 9.6288 | 8.7735 | 7.9117 | 9.7753 |
| 9.6478 | 7.3368 | 9.2138 | 9.7793 | 8.6472 | 8.4532 | 7.2178 | 8.7539 |
| 7.8847 | 7.0355 | 2.3255 | 8.2032 | 4.423 | 3.5909 | 3.7692 | 4.5292 |
| 3.6322 | 3.6364 | 4.0048 | 6.422 | 5.5197 | 4.4734 | 4.4681 | 3.8937 |
| 5.0791 | 4.6873 | 6.1996 | 3.0241 | 3.9623 | 6.5373 | 3.6413 | 5.7043 |
| 5.6964 | 3.0678 | | | | | | |
| MSRB3 | 7.4518 | 8.3774 | 6.5793 | 8.8589 | 7.0823 | 7.124 | 8.1981 |
| 6.0457 | 6.5361 | 6.8185 | 7.0937 | 6.8315 | 5.9538 | 7.6609 | 6.4736 |
| 7.3925 | 5.1493 | 7.8263 | 6.6291 | 7.6655 | 10.1705 | 5.2962 | 7.2164 |
| 8.6299 | 5.1849 | 8.9354 | 8.463 | 5.0919 | 6.6529 | 5.2103 | 4.9917 |
| 7.5827 | 6.013 | 7.1176 | 6.9263 | 7.5392 | 7.822 | 6.4605 | 4.987 |
| 4.9275 | 5.7866 | 8.3938 | 5.5111 | 7.3084 | 6.9421 | 8.8269 | 9.2433 |
| 5.7349 | 8.2142 | 7.3514 | 10.9349 | 6.0581 | 8.5323 | 6.7234 | 6.4357 |
| 7.4081 | 5.4973 | 6.8122 | 8.1471 | 10.1951 | 5.3556 | 7.2845 | 6.1929 |
| 6.7444 | 9.7556 | 5.0756 | 8.0271 | 7.4223 | 8.3365 | 9.2148 | 8.5987 |
| 7.5023 | 9.7369 | 6.6274 | 8.1983 | 7.2718 | 7.0924 | 5.8913 | 8.3415 |
| 10.0446 | 7.462 | 5.2147 | 8.7182 | 8.1201 | 6.2601 | 8.4993 | 7.6187 |
| 6.8136 | 7.8043 | 7.001 | 7.9648 | 5.8057 | 8.5478 | 8.9003 | 8.0569 |
| 7.3961 | 5.6488 | 5.5309 | 6.8552 | 6.2288 | 9.114 | 8.1999 | 6.8828 |
| 6.6462 | 8.4158 | 7.4923 | 9.9948 | 8.3531 | 7.6086 | 6.0794 | 8.0164 |
| 8.7935 | 5.0345 | 7.4874 | 4.8128 | 7.1155 | 7.4953 | 7.4152 | 8.4376 |
| 5.928 | 7.2517 | 10.456 | 5.0462 | 6.8644 | 6.2082 | 8.6648 | 7.2583 |
| 5.5302 | 6.3731 | 6.9991 | 10.8591 | 6.4552 | 7.929 | 6.1123 | 9.7124 |
| 9.5402 | 8.3767 | 4.9975 | 10.0321 | 5.1485 | 7.2492 | 7.0841 | 4.8121 |
| 6.771 | 7.4486 | 7.6933 | 7.3105 | 8.0974 | 4.5868 | 9.5446 | 6.7334 |
| 5.8203 | 6.9109 | 5.7889 | 7.8404 | 8.3134 | 8.3804 | 7.0672 | 6.5395 |
| 7.295 | 6.3866 | 7.6596 | 7.7561 | 8.4211 | 5.2325 | 4.8415 | 8.7928 |
| 8.3198 | 7.0956 | 7.2093 | 9.1118 | 6.8902 | 5.6289 | 4.6154 | 7.3329 |
| 9.3464 | 6.456 | 11.0994 | 9.6385 | 11.4174 | 8.1322 | 13.0128 | 11.6651 |
| 12.8937 | 12.8656 | 13.0525 | 9.9283 | 12.253 | 13.4878 | 11.2124 | 9.9751 |
| 9.6931 | 11.8878 | 12.7771 | 10.9426 | 9.1395 | 11.2928 | 11.3198 | 11.1836 |
| 12.7872 | 10.584 | | | | | | |
| OIP5 | 8.6747 | 5.6377 | 6.3296 | 8.50E+00 | | 7.77E+00 | |
| 7.2385 | 8.442 | 7.4569 | 8.4944 | 7.5583 | 7.386 | 6.4383 | 6.896 |
| 5.8352 | 6.588 | 8.5087 | 7.2108 | 7.1738 | 6.3101 | 6.1238 | 8.0123 |
| 7.7018 | 7.443 | 5.5065 | 6.6424 | 7.0981 | 8.0192 | 8.445 | 6.6585 |
| 7.6946 | 7.8543 | 5.0524 | 8.1481 | 8.5103 | 6.7969 | 8.7451 | 5.6364 |
| 8.2964 | 6.9398 | 6.3613 | 6.6636 | 7.8442 | 7.1292 | 7.0831 | 6.0314 |
| 6.7052 | 6.2678 | 7.91 | 6.4509 | 6.5482 | 6.5404 | 7.4121 | 8.0455 |

| | | | | | | | |
|---|---|---|---|---|---|---|---|
| 8.0325 | 6.8136 | 5.8511 | 4.9805 | 7.5645 | 3.6671 | 8.7072 | 7.692 |
| 7.0681 | 7.0899 | 7.5723 | 6.7374 | 6.3361 | 7.2883 | 4.0402 | 4.0113 |
| 8.066 | 7.7162 | 8.8591 | 3.1466 | 7.6875 | 7.5136 | 7.1614 | 7.2487 |
| 7.9235 | 6.6827 | 8.1759 | 6.8999 | 6.1986 | 5.2045 | 8.9275 | 6.1044 |
| 6.1695 | 7.3243 | 7.7311 | 7.5763 | 6.9403 | 8.547 | 9.2067 | 7.9559 |
| 6.3691 | 7.3233 | 8.1835 | 7.3758 | 7.4066 | 6.1023 | 6.2221 | 8.5218 |
| 7.7427 | 7.287 | 7.654 | 7.7195 | 6.2767 | 7.7797 | 7.6666 | 5.9407 |
| 7.656 | 7.4707 | 8.5911 | 7.7606 | 7.3026 | 8.6779 | 7.3956 | 7.9061 |
| 8.033 | 5.5388 | 8.3018 | 0 | 6.5796 | 8.7402 | 6.7895 | 7.0332 |
| 7.6568 | 7.7949 | 5.3207 | 7.1196 | 8.0011 | 4.7442 | 7.9423 | 6.1343 |
| 8.1295 | 7.5369 | 7.2373 | 7.3531 | 7.9466 | 8.9168 | 7.1186 | 7.0452 |
| 6.6417 | 6.853 | 8.2753 | 7.901 | 7.3702 | 6.019 | 7.7171 | 8.0109 |
| 7.9658 | 8.6177 | 7.1804 | 7.282 | 4.1289 | 7.515 | 7.9503 | 4.6648 |
| 8.1967 | 7.4299 | 8.308 | 6.7078 | 6.94 | 7.0628 | 6.9501 | 8.2216 |
| 7.375 | 8.3594 | 7.8849 | 6.1529 | 7.2728 | 6.5418 | 7.6406 | 8.71 |
| 8.0251 | 7.1234 | 6.7221 | 7.5865 | 1.8387 | 6.713 | 3.405 | 1.5308 |
| 3.3438 | 2.8973 | 2.0387 | 3.1714 | 2.3049 | 5.9654 | 3.1102 | 1.6296 |
| 3.815 | 4.2989 | 4.0745 | 3.6637 | 4.484 | 3.6942 | 5.2786 | 4.5126 |
| 3.5208 | 4.9657 | 2.7342 | 3.8082 | | | | |
| ECM2 | 5.6304 | 5.3517 | 4.9437 | 6.5909 | 6.0652 | 6.0221 | 6.0462 |
| 4.153 | 5.1272 | 3.2912 | 6.8283 | 3.8612 | 3.3904 | 5.67 | 5.193 |
| 6.4252 | 2.7962 | 3.4081 | 6.2625 | 5.9274 | 4.8678 | 4.5195 | 6.4506 |
| 5.9421 | 2.472 | 4.0212 | 6.3378 | 3.2628 | 4.5083 | 2.5857 | 3.7749 |
| 5.2341 | 4.1678 | 4.3299 | 6.4239 | 7.4012 | 4.0114 | 4.8213 | 4.183 |
| 1.5457 | 4.8576 | 5.2786 | 2.6955 | 4.9826 | 5.3302 | 5.5208 | 7.9602 |
| 4.484 | 5.7987 | 5.9028 | 8.6987 | 4.8435 | 6.4213 | 5.2749 | 3.0309 |
| 6.6466 | 2.0292 | 4.8502 | 4.0229 | 8.0681 | 3.2868 | 6.5981 | 5.3166 |
| 4.753 | 8.3181 | 3.4092 | 6.6229 | 7.7555 | 6.782 | 6.6597 | 4.6169 |
| 5.2241 | 9.476 | 6.4818 | 6.2614 | 3.553 | 3.9495 | 3.4699 | 5.643 |
| 7.2896 | 5.7895 | 2.9127 | 8.52 | 6.4048 | 4.1741 | 6.2931 | 6.143 |
| 3.7293 | 6.0313 | 3.8552 | 7.31 | 6.4093 | 5.627 | 7.5902 | 7.4397 |
| 6.8025 | 5.2715 | 4.2712 | 2.8658 | 4.0875 | 7.5683 | 3.0691 | 6.2904 |
| 4.3114 | 4.7378 | 5.3908 | 4.3949 | 5.4031 | 6.5175 | 4.1342 | 4.866 |
| 6.8083 | 1.2894 | 2.9233 | 2.7851 | 6.1403 | 5.6034 | 4.7356 | 4.8977 |
| 4.153 | 8.2201 | 8.0984 | 3.8517 | 5.4935 | 5.4878 | 6.3294 | 5.5 |
| 4.7642 | 3.7449 | 3.1725 | 9.1723 | 4.0526 | 6.6412 | 5.3613 | 3.8469 |
| 7.0451 | 3.5127 | 2.4961 | 5.0396 | 3.7018 | 6.0377 | 7.507 | 3.1277 |
| 3.9719 | 5.8386 | 4.5131 | 6.9555 | 5.3793 | 2.6009 | 3.7441 | 4.2928 |
| 2.968 | 4.3908 | 3.4255 | 5.0606 | 7.5857 | 8.488 | 4.3704 | 4.5289 |
| 2.8387 | 6.6564 | 3.2722 | 5.9473 | 5.8713 | 4.1728 | 2.1566 | 7.2049 |
| 6.5965 | 4.82 | 6.2605 | 6.6826 | 5.927 | 2.3335 | 3.7591 | 6.5655 |
| 8.0907 | 3.2749 | 10.4799 | 8.3716 | 10.6513 | 6.6817 | 9.4427 | 9.8391 |
| 11.6349 | 10.4595 | 10.931 | 9.439 | 10.248 | 9.4623 | 9.9373 | 9.2705 |
| 8.5435 | 10.8822 | 9.6142 | 9.9194 | 11.3819 | 9.2703 | 9.4847 | 9.1847 |
| 10.7922 | 9.3843 | | | | | | |
| CALD1 | 11.3909 | 12.5127 | 11.3111 | 11.6585 | 11.1179 | 10.8769 | 10.4412 |
| 10.4781 | 9.9744 | 9.897 | 10.8369 | 11.8321 | 10.9038 | 11.0833 | 10.6977 |
| 10.6692 | 10.1179 | 12.023 | 10.1062 | 11.6743 | 10.6755 | 9.2179 | 10.7675 |
| 11.8203 | 9.7176 | 11.1375 | 11.5515 | 9.9851 | 11.0354 | 9.4675 | 11.1154 |
| 10.9078 | 10.983 | 11.3765 | 11.442 | 11.3164 | 10.995 | 11.9661 | 10.007 |
| 9.5828 | 9.9953 | 12.7048 | 11.1724 | 11.0971 | 10.6174 | 11.8819 | 12.3954 |
| 11.586 | 12.005 | 12.3284 | 13.0344 | 9.2633 | 12.2529 | 10.042 | 10.1391 |
| 11.5257 | 10.0987 | 10.8426 | 11.7997 | 13.0396 | 8.9225 | 11.7484 | 10.468 |
| 10.4903 | 13.2291 | 9.3401 | 12.1731 | 10.8695 | 10.9777 | 12.4593 | 10.3254 |

| | | | | | | | | |
|---|---|---|---|---|---|---|---|---|
| 12.2384 | 12.9445 | 10.3237 | 11.5003 | 12.0821 | 10.2945 | 10.3754 | 12.4525 | |
| 13.2544 | 11.509 | 10.069 | 11.7994 | 11.5869 | 11.6823 | 11.2079 | 11.2468 | |
| 10.5372 | 11.4094 | 11.1944 | 11.2277 | 10.0301 | 11.4723 | 12.399 | 11.4983 | |
| 11.1006 | 10.8485 | 9.8151 | 11.1161 | 10.6987 | 12.3396 | 10.6071 | 10.5584 | |
| 11.0589 | 11.5005 | 11.3 | 12.0965 | 12.1271 | 11.7167 | 9.8349 | 11.6196 | |
| 11.8706 | 9.8011 | 11.4871 | 11.1211 | 10.7453 | 10.8017 | 10.8452 | 12.1748 | |
| 9.3134 | 11.5213 | 13.0357 | 9.1574 | 11.1936 | 10.754 | 11.5726 | 11.9335 | |
| 9.142 | 10.8086 | 9.4644 | 13.558 | 9.9728 | 11.872 | 10.4826 | 11.0705 | |
| 12.721 | 11.4495 | 9.9568 | 11.2121 | 9.4827 | 11.554 | 11.4568 | 10.2905 | |
| 10.1545 | 12.2071 | 10.4837 | 11.8316 | 11.1064 | 8.9263 | 13.0286 | 10.7665 | |
| 10.3156 | 10.7056 | 10.8033 | 11.1403 | 12.0818 | 11.6051 | 11.0759 | 10.2836 | |
| 10.4946 | 9.6202 | 11.9611 | 11.398 | 11.9014 | 10.224 | 10.1959 | 11.2013 | |
| 11.9077 | 10.6583 | 11.249 | 12.7425 | 10.9202 | 9.3114 | 8.2231 | 11.0575 | |
| 12.7255 | 10.025 | 13.9255 | 12.4949 | 14.2664 | 11.2374 | 14.859 | 14.0924 | |
| 15.1271 | 14.9786 | 15.447 | 12.757 | 14.5439 | 15.2315 | 13.6767 | 13.116 | |
| 12.5508 | 14.7501 | 14.8811 | 13.8921 | 14.1102 | 13.7968 | 13.582 | 13.492 | |
| 15.2147 | 13.5494 | | | | | | | |
| FLT4 | 6.3529 | 7.2339 | 5.2523 | 5.97E+00 | | 6.8542 | 5.7183 | |
| 7.7027 | 6.4894 | 7.0849 | 6.4675 | 8.1826 | 6.2574 | 5.9111 | 7.2685 | |
| 7.5958 | 8.4425 | 6.4564 | 7.8058 | 6.6175 | 7.5449 | 8.2792 | 7.9072 | |
| 7.9811 | 7.0316 | 5.5069 | 6.4117 | 7.6074 | 5.445 | 6.9883 | 6.3846 | |
| 6.1879 | 8.4969 | 7.3433 | 6.4201 | 5.4865 | 7.3451 | 6.8723 | 7.4436 | |
| 6.4987 | 6.0912 | 8.339 | 5.946 | 7.5904 | 7.0875 | 8.3561 | 7.4225 | |
| 8.573 | 5.8816 | 7.4044 | 7.8007 | 9.192 | 7.4273 | 7.7049 | 5.9166 | |
| 6.2827 | 7.0869 | 5.6818 | 7.5014 | 6.8962 | 8.6853 | 7.1973 | 7.456 | |
| 6.5753 | 8.2642 | 8.4448 | 6.4339 | 7.5807 | 7.6484 | 8.9435 | 8.17 | |
| 7.1075 | 6.9138 | 8.9536 | 7.6459 | 7.959 | 6.8159 | 6.904 | 6.4508 | |
| 6.9293 | 8.4135 | 7.1535 | 7.3406 | 8.8411 | 6.4698 | 7.2753 | 7.1699 | |
| 7.3037 | 6.2099 | 7.5673 | 6.3782 | 7.9219 | 5.0551 | 7.5205 | 7.4477 | |
| 8.2489 | 7.2842 | 6.0902 | 5.0344 | 6.8355 | 8.2992 | 7.0064 | 5.9722 | |
| 5.6819 | 6.3329 | 6.0718 | 7.5132 | 8.4941 | 6.5808 | 7.4306 | 6.2127 | |
| 5.7361 | 6.3537 | 6.0284 | 7.4999 | 5.36 | 6.89 | 7.1823 | 7.0051 | |
| 7.9058 | 6.6924 | 9.3803 | 9.6574 | 5.9172 | 7.0464 | 5.8432 | 7.6372 | |
| 5.3502 | 6.6316 | 6.5592 | 5.5368 | 10.4197 | 6.0079 | 6.6866 | 8.1228 | |
| 6.362 | 7.0795 | 6.6604 | 7.1477 | 7.4018 | 7.1446 | 6.7279 | 6.7124 | |
| 4.9236 | 6.6781 | 6.0162 | 7.8334 | 9.0741 | 6.198 | 6.2582 | 6.5823 | 6.94 |
| | 7.0839 | 6.8173 | 8.3334 | 7.5855 | 7.3566 | 7.7196 | 6.9353 | |
| 7.4732 | 6.5165 | 6.3425 | 5.2085 | 6.7077 | 7.5393 | 6.998 | 5.8161 | |
| 7.7382 | 7.2642 | 7.3157 | 6.1571 | 7.5933 | 6.6799 | 7.7892 | 8.5531 | |
| 7.0013 | 6.4781 | 6.1126 | 10.5089 | 9.5204 | 10.2646 | 8.7911 | 10.3407 | |
| 10.4671 | 9.7535 | 10.6168 | 10.4107 | 9.8782 | 10.3659 | 10.7329 | 10.4387 | |
| 9.8933 | 9.1727 | 9.5148 | 10.4481 | 9.4594 | 6.8677 | 10.4657 | 10.6376 | |
| 9.5137 | 9.6893 | 8.367 | | | | | | |
| KIAA1644 | | 3.8143 | 7.9788 | 4.3341 | 5.1113 | 3.0829 | 6.1296 | |
| 5.4551 | 4.9084 | 3.0487 | 4.1522 | 5.735 | 1.6493 | 3.95 | 0 | |
| 6.5586 | 4.5754 | 3.2664 | 4.0896 | 3.7755 | 3.8723 | 4.4861 | 4.2643 | |
| 5.8739 | 5.124 | 2.7135 | 8.5468 | 6.396 | 1.8398 | 6.5031 | 4.2103 | |
| 4.6185 | 4.5283 | 9.4755 | 3.3363 | 3.9752 | 4.1205 | 6.2801 | 2.6427 | |
| 4.183 | 0.7135 | 1.992 | 5.8776 | 5.4607 | 4.3611 | 5.3555 | 4.8508 | |
| 5.4568 | 3.1045 | 6.8354 | 4.9168 | 8.986 | 8.224 | 6.8234 | 5.3929 | |
| 4.3911 | 6.2585 | 3.3568 | 5.5094 | 5.7977 | 7.9796 | 2.2792 | 3.189 | |
| 4.6992 | 4.8148 | 7.9644 | 0 | 5.7168 | 5.7111 | 3.7934 | 6.5897 | |
| 4.6992 | 4.9857 | 7.0066 | 3.9844 | 7.4097 | 3.0901 | 7.9803 | 4.2904 | |
| 7.0105 | 6.8014 | 6.0112 | 5.0155 | 6.6423 | 5.7645 | 6.0853 | 7.4467 | |

| | | | | | | | |
|---|---|---|---|---|---|---|---|
| 3.9876 | 2.589 | 6.7372 | 5.3722 | 4.6791 | 2.3164 | 6.7413 | 6.2468 |
| 7.0198 | 4.8905 | 3.9969 | 4.3495 | 5.02 | 3.7004 | 4.0366 | 3.9805 |
| 2.655 | 5.3507 | 5.1392 | 5.3167 | 6.6923 | 5.5958 | 5.7071 | 4.7022 |
| 5.1646 | 5.461 | 2.5982 | 5.0021 | 2.5146 | 5.17 | 4.1901 | 5.4835 |
| 4.8224 | 4.3638 | 7.3542 | 10.1981 | 3.0418 | 2.1461 | 5.0152 | 5.6653 |
| 4.5659 | 4.597 | 5.0556 | 1.224 | 10.3292 | 1.9723 | 6.7118 | 4.4955 |
| 3.0681 | 4.8747 | 5.5434 | 4.5021 | 3.338 | 4.1467 | 3.0745 | 5.3838 |
| 3.2792 | 6.3445 | 4.2724 | 6.4885 | 5.8597 | 5.3484 | 1.9122 | 4.4211 |
| 2.7564 | 4.2629 | 7.5197 | 2.9928 | 3.2158 | 6.0323 | 7.2376 | 5.4006 |
| 1.7883 | 1.8629 | 3.2946 | 5.8979 | 6.4726 | 6.6033 | 4.3168 | 5.3699 |
| 4.2443 | 6.5711 | 3.5613 | 4.1593 | 6.6084 | 1.9594 | 1.5946 | 5.9327 |
| 5.5672 | 7.0508 | 4.0975 | 11.2435 | 8.9956 | 11.1728 | 6.1753 | 12.3017 |
| 10.9237 | 11.4933 | 11.3648 | 11.7129 | 9.7204 | 8.1699 | 11.8219 | 11.326 |
| 9.0848 | 7.2401 | 10.7913 | 12.0091 | 11.1006 | 6.1164 | 8.1888 | 11.1438 |
| 10.4401 | 9.8662 | 10.8433 | | | | | |
| AOC3 | 7.8018 | 4.7409 | 5.9297 | 1.35E+01 | | 6.36E+00 | |
| 4.0872 | 6.4895 | 6.5117 | 6.0845 | 6.331 | 7.5229 | 6.4041 | 6.425 |
| 4.1868 | 6.7222 | 6.5404 | 6.7313 | 5.2426 | 7.0391 | 6.5992 | 5.7894 |
| 6.7509 | 7.2509 | 8.0036 | 4.9922 | 6.0926 | 8.4136 | 6.3392 | 6.5799 |
| 9.8526 | 8.6669 | 7.484 | 6.5311 | 5.4962 | 5.7906 | 8.2903 | 5.5015 |
| 7.1805 | 5.5568 | 5.5761 | 6.7336 | 6.8021 | 5.9089 | 6.803 | 7.8718 |
| 8.7044 | 8.1025 | 5.25 | 7.292 | 7.0169 | 12.4569 | 6.1597 | 7.3488 |
| 6.0458 | 5.6344 | 7.2869 | 6.0716 | 6.6502 | 5.7589 | 9.6185 | 4.9275 |
| 5.8145 | 5.1386 | 7.4007 | 9.5481 | 7.0133 | 7.5119 | 7.9432 | 7.4049 |
| 9.0679 | 6.3858 | 6.3793 | 9.4667 | 6.2315 | 8.1252 | 6.1886 | 6.3704 |
| 5.6837 | 7.4053 | 9.0169 | 6.8076 | 4.6152 | 8.4957 | 8.1468 | 6.4668 |
| 8.1818 | 6.1712 | 5.8682 | 7.4632 | 5.3405 | 7.398 | 5.2182 | 7.3591 |
| 7.3494 | 8.0643 | 6.6591 | 5.8114 | 5.0344 | 6.7326 | 6.8704 | 8.6484 |
| 5.9722 | 8.1499 | 6.1815 | 7.729 | 6.3458 | 7.0523 | 6.1606 | 7.2482 |
| 6.0293 | 4.626 | 8.9016 | 4.5476 | 5.3625 | 4.9838 | 7.2543 | 7.3319 |
| 6.0246 | 7.2823 | 5.9147 | 9.364 | 9.5609 | 5.6013 | 6.0251 | 6.1858 |
| 6.9831 | 6.6319 | 6.2012 | 6.0006 | 4.3485 | 10.3697 | 5.0933 | 4.7526 |
| 7.1536 | 5.4573 | 7.2868 | 6.1893 | 4.7154 | 5.7988 | 7.1762 | 7.1664 |
| 6.7395 | 5.2514 | 6.4606 | 6.354 | 8.334 | 8.275 | 7.9937 | 4.6934 |
| 5.7711 | 5.8938 | 5.8319 | 6.2283 | 7.0428 | 6.2178 | 7.6992 | 7.7893 |
| 7.0417 | 6.4714 | 5.4032 | 6.1599 | 5.2845 | 7.1762 | 7.7233 | 5.5564 |
| 4.7822 | 9.5275 | 8.3312 | 5.7319 | 7.3693 | 8.4131 | 8.7531 | 4.5374 |
| 5.1059 | 7.6499 | 8.8504 | 5.6251 | 10.3069 | 10.0687 | 9.9073 | 8.7097 |
| 13.0105 | 11.2969 | 12.4735 | 12.4425 | 12.0955 | 9.8068 | 13.1329 | 13.0732 |
| 10.5018 | 10.4368 | 10.5652 | 10.7866 | 12.579 | 9.5101 | 7.2895 | 12.6949 |
| 10.7903 | 10.6263 | 13.6448 | 8.9194 | | | | |
| CXorf36 | 6.8556 | 6.8757 | 6.5229 | 6.6991 | 7.479 | 5.8269 | 7.8945 |
| 6.7693 | 7.181 | 6.5559 | 8.5867 | 6.7903 | 6.9839 | 6.8619 | 8.1138 |
| 7.8729 | 6.8022 | 6.9834 | 7.5164 | 7.7681 | 6.9013 | 6.9199 | 8.6284 |
| 7.1179 | 6.1547 | 6.7811 | 7.2318 | 6.3545 | 7.2957 | 5.9316 | 6.4194 |
| 8.0751 | 6.8942 | 6.0458 | 6.3074 | 8.2876 | 6.6497 | 6.6781 | 7.2805 |
| 7.698 | 8.705 | 6.7975 | 6.3847 | 6.3795 | 8.5957 | 7.7466 | 8.8197 |
| 6.3194 | 7.049 | 8.1639 | 10.208 | 7.8874 | 7.777 | 5.9718 | 5.6195 |
| 7.6304 | 6.554 | 7.3548 | 6.829 | 9.5015 | 6.3126 | 7.4611 | 6.9438 |
| 8.3292 | 8.9165 | 7.5224 | 7.6797 | 8.3291 | 9.2277 | 7.5124 | 7.6601 |
| 7.1442 | 10.4043 | 8.3224 | 8.4479 | 7.0146 | 7.078 | 6.8463 | 7.5851 |
| 8.2098 | 7.7566 | 6.4534 | 8.7308 | 6.9158 | 7.3812 | 7.8179 | 7.6557 |
| 6.8207 | 7.7882 | 5.9028 | 8.4983 | 6.3401 | 7.7448 | 8.7693 | 8.8424 |
| 8.0661 | 6.3542 | 5.86 | 8.816 | 8.1033 | 7.8484 | 6.7991 | 6.6233 |

| | | | | | | | |
|---|---|---|---|---|---|---|---|
| 7.7624 | 7.1442 | 8.4125 | 7.0114 | 6.1504 | 8.2663 | 6.6323 | 6.8565 |
| 6.5133 | 7.0725 | 7.2345 | 5.9608 | 7.5268 | 7.7844 | 6.8344 | 8.746 |
| 6.746 | 10.4406 | 11.0565 | 5.8923 | 7.7541 | 7.2853 | 7.1822 | 6.0862 |
| 7.1436 | 6.3644 | 6.2188 | 11.0826 | 7.9518 | 6.6543 | 8.1637 | 5.8868 |
| 7.1278 | 6.6907 | 6.7585 | 6.8269 | 6.8567 | 7.0842 | 7.3533 | 6.4395 |
| 7.5131 | 6.9183 | 8.1832 | 9.1477 | 7.4178 | 6.6443 | 6.0663 | 7.8229 |
| 7.1455 | 5.9419 | 9.4248 | 7.3649 | 7.4548 | 8.4455 | 6.2025 | 7.4531 |
| 5.7946 | 7.6402 | 5.6902 | 7.2704 | 8.7715 | 7.6329 | 6.7731 | 7.6983 |
| 7.2878 | 7.8762 | 7.3223 | 7.812 | 6.5776 | 7.1124 | 6.272 | 7.3089 |
| 7.5404 | 6.2599 | 10.6331 | 9.6937 | 10.4297 | 6.0365 | 11.045 | 11.074 |
| 11.7189 | 12.0791 | 11.905 | 10.152 | 11.4151 | 11.8967 | 10.9527 | 10.1018 |
| 8.4703 | 11.2322 | 11.3575 | 10.2563 | 7.2206 | 10.7463 | 11.8745 | 10.0718 |
| 10.982 | 9.8957 | | | | | | |
| C16orf45 | | 5.7971 | 6.9491 | 5.8504 | 6.5975 | 6.7921 | 7.3101 |
| 8.1494 | 6.727 | 7.5147 | 9.2561 | 7.2724 | 4.6765 | 6.0454 | 5.7644 |
| 6.8824 | 6.7558 | 4.8405 | 6.9401 | 7.3333 | 6.7125 | 6.1703 | 5.0912 |
| 7.3407 | 7.6218 | 8.344 | 9.0389 | 6.1574 | 4.6727 | 5.947 | 4.3228 |
| 4.6878 | 8.139 | 6.0321 | 5.4816 | 6.6822 | 7.6058 | 7.8012 | 5.1685 |
| 5.8558 | 5.5168 | 5.0253 | 5.7206 | 5.1628 | 5.7282 | 6.517 | 8.0268 |
| 8.7387 | 5.3874 | 7.1021 | 7.2041 | 9.0749 | 6.7672 | 7.9903 | 6.4 |
| 6.1743 | 7.164 | 7.6235 | 7.8412 | 8.0161 | 9.0582 | 7.7058 | 5.8225 |
| 7.0979 | 6.3373 | 8.013 | 3.3007 | 8.0088 | 7.8581 | 6.5375 | 7.0901 |
| 7.3391 | 6.2414 | 8.4164 | 5.765 | 7.4718 | 6.9966 | 9.3249 | 5.273 |
| 6.8432 | 9.3553 | 6.3797 | 11.1318 | 8.3182 | 7.6003 | 7.1297 | 6.7833 |
| 8.2755 | 5.7977 | 7.1049 | 7.0798 | 6.8258 | 5.0377 | 6.8066 | 8.2207 |
| 7.6478 | 7.047 | 4.5645 | 5.5309 | 7.7681 | 5.7549 | 6.2785 | 8.8467 |
| 6.4151 | 9.7498 | 6.5135 | 6.9649 | 8.6791 | 7.2541 | 6.8295 | 5.8091 |
| 7.2274 | 6.7877 | 6.1653 | 6.8236 | 5.0365 | 6.5454 | 5.258 | 7.1953 |
| 7.0042 | 5.6671 | 7.9084 | 9.0969 | 4.9287 | 5.3099 | 5.9401 | 6.5976 |
| 6.5427 | 6.0922 | 7.875 | 7.4486 | 9.5332 | 5.3214 | 7.0108 | 6.9463 |
| 4.5336 | 7.8243 | 6.7161 | 5.0433 | 6.0073 | 4.7885 | 9.3885 | 7.1562 |
| 4.7954 | 7.4105 | 6.7992 | 6.7836 | 8.4007 | 7.0068 | 6.0024 | 5.7121 |
| 6.6658 | 7.512 | 8.1217 | 7.1456 | 5.0943 | 7.9831 | 8.8612 | 6.184 |
| 6.8158 | 5.8832 | 5.4251 | 6.5068 | 6.7299 | 7.581 | 6.707 | 5.6102 |
| 6.8518 | 6.437 | 5.5119 | 7.061 | 7.7853 | 5.9561 | 8.5341 | 7.0797 |
| 7.0472 | 7.4423 | 6.1881 | 10.7528 | 9.6692 | 10.4002 | 8.751 | 11.4653 |
| 10.0005 | 10.5311 | 11.0184 | 10.4088 | 10.1907 | 10.9982 | 10.7499 | 10.5477 |
| 9.1297 | 9.0187 | 10.2608 | 10.7505 | 10.1607 | 10.5856 | 10.0591 | 10.1014 |
| 9.6193 | 11.2042 | 10.3539 | | | | | |
| PRDM8 | 2.0932 | 4.5274 | 2.0919 | 3.54E+00 | | 3.47E+00 | |
| 3.2059 | 3.2591 | 2.0525 | 2.4603 | 1.9247 | 3.8847 | 1.2777 | 3.6198 |
| 2.1513 | 3.5585 | 4.4863 | 2.5253 | 3.4789 | 2.6223 | 3.1574 | 4.4172 |
| 1.5155 | 3.6823 | 4.5093 | 5.5697 | 1.5789 | 4.3904 | 3.3866 | 2.3782 |
| 2.0007 | 2.8161 | 3.0901 | 2.4102 | 2.4725 | 1.6341 | 4.2145 | 3.9114 |
| 1.9249 | 1.8914 | 0 | 2.5272 | 6.9088 | 2.4071 | 4.0437 | 4.8127 |
| 3.9801 | 4.3213 | 3.1045 | 7.7719 | 5.0858 | 6.0019 | 3.0518 | 3.5972 |
| 2.735 | 3.4242 | 4.5668 | 2.2002 | 4.5652 | 4.9086 | 4.9951 | 3.4344 |
| 3.0326 | 6.0592 | 6.8166 | 5.5114 | 0.8237 | 4.3385 | 3.1261 | 5.3512 |
| 4.2955 | 3.6471 | 2.5997 | 4.6787 | 3.8124 | 5.1066 | 4.3453 | 1.4887 |
| 4.2904 | 3.7859 | 6.8184 | 4.3341 | 3.5171 | 5.8611 | 4.2317 | 2.1693 |
| 3.5849 | 5.5823 | 2.589 | 3.9875 | 3.7628 | 4.1402 | 2.0651 | 4.5066 |
| 4.4765 | 3.9218 | 3.3997 | 3.3794 | 2.6747 | 4.4223 | 1 | 4.4295 |
| 2.9753 | 2.3144 | 4.2062 | 4.1282 | 6.5697 | 5.4239 | 3.2578 | 4.1906 |
| 2.159 | 3.5519 | 3.4014 | 0.7843 | 3.7926 | 2.6562 | 3.3693 | 4.0164 |

| | | | | | | | |
|---|---|---|---|---|---|---|---|
| 4.5622 | 5.7157 | 4.153 | 5.3965 | 6.9264 | 2.9483 | 2.4344 | 3.6318 |
| 4.4887 | 2.7971 | 3.9333 | 3.2383 | 3.547 | 7.9383 | 2.9676 | 3.9609 |
| 2.4626 | 2.2306 | 5.6709 | 4.7236 | 2.3578 | 7.8059 | 1.36 | 5.6979 |
| 3.3141 | 2.284 | 3.0297 | 2.5845 | 3.283 | 4.5707 | 5.1652 | 2.228 |
| 1.0663 | 1.8707 | 4.4547 | 2.678 | 0 | 5.191 | 5.3827 | 5.656 |
| 3.5582 | 2.2268 | 0.91 | 2.1234 | 2.6622 | 3.9157 | 4.0336 | 2.2587 |
| 3.5793 | 3.6605 | 4.2265 | 3.96 | 2.7619 | 5.1302 | 2.6702 | 1.5946 |
| 6.9936 | 4.0332 | 4.7628 | 2.5752 | 7.6265 | 6.5327 | 7.8421 | 4.4949 |
| 8.9707 | 7.9307 | 8.0227 | 8.3851 | 7.9635 | 6.9099 | 7.4826 | 8.2085 |
| 8.2489 | 7.9304 | 5.9541 | 8.185 | 8.7499 | 8.3973 | 8.4349 | 6.8956 |
| 8.1981 | 7.6014 | 8.3979 | 8.9597 | | | | |
| EDNRA | 7.3316 | 7.9397 | 6.6803 | 7.78E+00 | | 7.22E+00 | |
| 7.4373 | 7.0223 | 6.8805 | 6.6147 | 6.3803 | 8.4168 | 5.3282 | 5.6377 |
| 6.6558 | 7.4071 | 8.3851 | 6.3341 | 6.788 | 7.4327 | 7.8112 | 6.4017 |
| 5.6673 | 7.6926 | 7.598 | 4.8478 | 10.8597 | 7.5573 | 5.2792 | 6.3084 |
| 5.4103 | 5.9688 | 7.1101 | 5.8499 | 7.0937 | 7.7663 | 8.0405 | 8.1611 |
| 7.9695 | 6.0053 | 6.1047 | 6.5441 | 7.6366 | 5.5478 | 7.4822 | 6.8381 |
| 8.3794 | 8.5037 | 6.8926 | 8.3978 | 8.2711 | 9.7047 | 6.3778 | 8.2501 |
| 6.644 | 6.0347 | 7.6967 | 4.7652 | 6.9013 | 7.2856 | 9.7439 | 5.5729 |
| 7.3769 | 6.5182 | 6.6458 | 9.6316 | 3.1833 | 8.1249 | 8.7658 | 7.5297 |
| 7.566 | 7.7721 | 7.438 | 10.1899 | 7.1271 | 7.6997 | 8.2133 | 6.785 |
| 6.9917 | 7.3711 | 9.2318 | 6.7009 | 7.0257 | 9.1276 | 8.1622 | 8.0167 |
| 7.7084 | 6.9498 | 5.475 | 7.4496 | 7.0111 | 8.4607 | 7.9875 | 7.349 |
| 9.1205 | 8.5594 | 7.8877 | 6.1522 | 6.1823 | 7.849 | 6.7279 | 8.9224 |
| 8.0528 | 6.674 | 7.5531 | 7.2441 | 8.215 | 9.8293 | 9.1143 | 8.4151 |
| 6.3279 | 8.2031 | 8.9223 | 4.3574 | 6.8356 | 6.1159 | 7.2935 | 7.1335 |
| 7.7392 | 7.448 | 5.6349 | 9.762 | 8.836 | 5.5223 | 6.7575 | 6.6127 |
| 8.6809 | 8.8591 | 6.1657 | 6.4812 | 4.4839 | 10.2461 | 5.9859 | 7.5276 |
| 7.7181 | 5.6421 | 8.8177 | 8.0894 | 6.1209 | 7.8147 | 5.3651 | 7.003 |
| 8.2651 | 5.7259 | 7.6761 | 7.5578 | 8.176 | 8.124 | 7.9141 | 5.3439 |
| 6.1649 | 6.4634 | 5.2934 | 6.6254 | 6.8647 | 6.2331 | 8.2826 | 8.6407 |
| 7.4579 | 5.6982 | 4.9695 | 6.7606 | 7.9278 | 7.058 | 8.2436 | 5.7379 |
| 5.3291 | 7.9625 | 8.4102 | 6.8823 | 8.1711 | 8.8297 | 7.4728 | 5.506 |
| 6.7839 | 7.3976 | 8.6177 | 7.0491 | 10.474 | 12.2653 | 10.5778 | 6.9109 |
| 13.0212 | 10.8902 | 10.8129 | 11.4779 | 10.2595 | 12.3729 | 11.0579 | 11.3815 |
| 11.1742 | 10.5867 | 8.7275 | 11.3146 | 12.3712 | 10.2509 | 11.1083 | 10.1175 |
| 10.8062 | 11.9774 | 10.8768 | 10.1106 | | | | |
| AKT3 | 5.532 | 9.7587 | 5.6656 | 6.9309 | 6.0998 | 6.9104 | 6.0631 |
| 6.278 | 6.3341 | 5.5406 | 6.7406 | 5.5307 | 5.9111 | 6.4722 | 5.5981 |
| 5.5882 | 4.4859 | 9.5372 | 6.0017 | 6.889 | 4.9675 | 6.1552 | 6.3173 |
| 6.8317 | 7.1342 | 9.3196 | 6.9834 | 6.8976 | 6.3014 | 4.6733 | 4.6185 |
| 6.8175 | 5.4644 | 5.5392 | 6.4588 | 6.78 | 6.8386 | 5.8491 | 5.067 |
| 3.7862 | 6.6246 | 7.1598 | 4.4931 | 6.4111 | 7.1807 | 7.4273 | 8.4262 |
| 5.5127 | 9.2397 | 7.3254 | 8.7775 | 5.5302 | 7.5458 | 5.961 | 8.904 |
| 7.0331 | 4.7652 | 6.1976 | 7.6138 | 8.466 | 5.2265 | 7.6115 | 8.0828 |
| 5.3757 | 8.6674 | 5.0422 | 7.9498 | 7.2391 | 6.7739 | 8.1239 | 5.4029 |
| 6.8598 | 9.0307 | 6.5289 | 6.3497 | 6.0897 | 6.4471 | 5.2124 | 6.9806 |
| 10.3854 | 7.2013 | 5.4484 | 8.1286 | 6.7219 | 6.5227 | 6.6918 | 7.1136 |
| 5.3623 | 7.4359 | 5.6553 | 7.0009 | 4.9288 | 6.9995 | 8.2207 | 7.4012 |
| 6.256 | 6.8529 | 4.6572 | 6.6893 | 5.5236 | 9.2078 | 6.5856 | 5.6055 |
| 6.3391 | 6.2203 | 6.9029 | 8.3878 | 7.0278 | 8.6789 | 5.8288 | 6.5125 |
| 7.0181 | 3.3768 | 6.0697 | 4.3538 | 5.8795 | 6.669 | 8.0079 | 6.2052 |
| 5.5684 | 7.1792 | 9.8698 | 4.6307 | 6.0466 | 5.4507 | 7.3409 | 6.5598 |
| 5.9878 | 4.9896 | 4.4402 | 9.8295 | 5.2493 | 7.6039 | 5.6037 | 4.6443 |

| | | | | | | | | |
|---|---|---|---|---|---|---|---|---|
| 7.8153 | 6.616 | 4.6868 | 5.9553 | 4.3817 | 7.1611 | 6.5536 | 5.5484 | |
| 6.5238 | 6.5269 | 7.0991 | 7.7558 | 6.4807 | 4.2097 | 5.1849 | 6.8432 | |
| 8.0467 | 5.7086 | 6.1129 | 6.508 | 6.9852 | 7.9095 | 6.3671 | 6.784 | |
| 4.8426 | 5.5466 | 6.1076 | 6.4096 | 7.3593 | 5.0522 | 4.6226 | 6.9285 | |
| 7.2035 | 5.9227 | 6.3226 | 8.1243 | 5.8568 | 3.4726 | 5.8697 | 6.5789 | |
| 7.7779 | 4.3376 | 10.3777 | 8.7516 | 10.217 | 8.6153 | 10.3943 | 10.7811 | |
| 11.1235 | 11.2435 | 11.14 | 8.9997 | 10.5695 | 10.7329 | 10.7195 | 8.6116 | |
| 8.9366 | 10.5199 | 10.2847 | 10.3117 | 10.8154 | 9.9121 | 10.2448 | 9.0328 | |
| 10.7752 | 10.0123 | | | | | | | |
| EMCN | 4.9697 | 4.8055 | 6.0411 | 5.5525 | 6.5782 | 2.6971 | 7.2049 | |
| 5.9991 | 5.6983 | 5.3559 | 6.9321 | 5.6785 | 6.2304 | 5.4604 | 6.6925 | |
| 6.5292 | 4.6656 | 5.2628 | 7.2827 | 6.6695 | 6.2317 | 6.0376 | 8.1939 | |
| 6.2325 | 6.0039 | 5.4829 | 5.5141 | 5.1971 | 5.7867 | 4.8082 | 4.964 | |
| 7.6245 | 5.4644 | 6.3086 | 5.1585 | 8.1277 | 5.8512 | 4.9896 | 5.6635 | |
| 6.6737 | 7.1989 | 6.376 | 5.7501 | 4.1967 | 6.9168 | 6.6011 | 8.0014 | |
| 2.7443 | 6.1297 | 7.4057 | 9.2805 | 6.3862 | 7.1994 | 5.502 | 3.4242 | 6.44 |
| | 5.957 | 6.166 | 3.8855 | 8.7366 | 5.6353 | 5.5128 | 4.6468 | |
| 7.0487 | 8.3059 | 6.0287 | 7.1372 | 7.4749 | 8.452 | 6.2595 | 7.1324 | |
| 5.7838 | 10.2441 | 6.8661 | 7.9893 | 4.6218 | 6.0251 | 5.8388 | 7.3902 | |
| 7.1579 | 6.9955 | 4.9959 | 7.9285 | 5.6694 | 5.44 | 7.1137 | 6.1712 | |
| 4.0538 | 6.9971 | 3.612 | 7.5285 | 6.1266 | 7.0502 | 8.4479 | 8.0295 | |
| 7.2795 | 5.1724 | 5.3331 | 8.5272 | 5.8826 | 5.366 | 5.6316 | 6.1805 | |
| 6.6106 | 6.4151 | 7.6401 | 8.9258 | 5.5807 | 7.6323 | 4.8443 | 4.7268 | |
| 4.2532 | 5.0345 | 5.2241 | 5.9471 | 7.1107 | 6.928 | 5.9386 | 7.5154 | |
| 5.928 | 9.8812 | 9.984 | 5.4728 | 7.0302 | 6.4809 | 6.0402 | 4.9072 | |
| 5.7049 | 4.4491 | 4.3004 | 9.7819 | 6.4552 | 5.2947 | 6.5963 | 3.7998 | |
| 5.7202 | 5.0244 | 5.2915 | 6.3103 | 4.8155 | 5.1699 | 6.3489 | 5.7862 | |
| 6.568 | 6.3243 | 7.2653 | 8.0351 | 6.8555 | 4.4717 | 3.9624 | 6.3583 | |
| 5.3905 | 5.7526 | 8.9839 | 5.6651 | 7.0069 | 7.8246 | 6.0269 | 5.6811 | |
| 4.4659 | 6.4224 | 4.6429 | 6.7591 | 7.3015 | 6.4502 | 5.7247 | 5.4968 | |
| 6.3276 | 7.125 | 7.2419 | 7.533 | 4.9409 | 6.2431 | 5.5214 | 6.657 | |
| 7.1697 | 5.6431 | 10.0009 | 8.8733 | 10.0431 | 6.7865 | 10.8378 | 11.0844 | |
| 11.4084 | 11.6812 | 11.7935 | 9.0761 | 11.1634 | 11.2693 | 10.0001 | 9.104 | |
| 8.2378 | 10.8949 | 11.0046 | 9.5534 | 7.5918 | 10.0856 | 10.7156 | 9.5162 | |
| 10.7308 | 9.5609 | | | | | | | |
| KIF15 | 9.3591 | 7.5726 | 7.1797 | 9.2232 | 9.0355 | 9.0911 | 9.0183 | |
| 7.9681 | 9.7262 | 9.4433 | 9.2353 | 8.3774 | 8.4142 | 6.3857 | 8.0624 | |
| 7.8237 | 8.342 | 8.1035 | 7.3684 | 7.3563 | 8.0454 | 9.7278 | 7.8995 | |
| 5.2818 | 8.8593 | 9.4088 | 8.9666 | 8.8607 | 8.348 | 9.1146 | 8.6669 | |
| 6.8175 | 8.9728 | 8.73 | 8.7442 | 8.4486 | 8.1693 | 8.5034 | 7.2466 | |
| 6.5111 | 7.5864 | 10.1787 | 6.2941 | 8.5997 | 8.136 | 7.8137 | 7.4819 | |
| 9.4301 | 7.5174 | 6.8285 | 9.2677 | 8.9468 | 8.6448 | 9.1633 | 9.4812 | |
| 8.336 | 5.957 | 9.1604 | 5.0863 | 8.4061 | 7.9645 | 8.7579 | 6.4125 | |
| 7.6527 | 7.4582 | 7.0515 | 7.9471 | 5.1706 | 4.527 | 8.9455 | 8.6685 | |
| 10.3424 | 5.0039 | 9.2488 | 7.618 | 7.9632 | 9.2445 | 8.5851 | 9.1483 | |
| 9.8723 | 9.7143 | 8.4995 | 6.6245 | 8.9655 | 8.2417 | 8.4461 | 8.1817 | |
| 7.9144 | 8.2063 | 9.5749 | 8.9406 | 9.1423 | 10.3203 | 7.6187 | 9.3781 | |
| 7.8567 | 8.639 | 8.5393 | 6.5266 | 7.3837 | 9.6801 | 9.1352 | 9.2721 | |
| 9.3985 | 9.2521 | 8.2749 | 9.4016 | 8.3817 | 9.0175 | 7.1954 | 8.4462 | |
| 8.8038 | 6.684 | 8.8231 | 9.1966 | 8.1955 | 8.5928 | 6.786 | 5.2246 | |
| 8.0898 | 4.661 | 8.1301 | 9.2087 | 7.0728 | 6.8234 | 8.9267 | 8.4748 | |
| 7.6068 | 7.6475 | 7.529 | 6.0426 | 6.5781 | 7.9689 | 7.752 | 9.1648 | |
| 9.2782 | 10.006 | 7.6105 | 9.1293 | 7.5584 | 8.3443 | 7.8051 | 7.1059 | |
| 8.715 | 9.386 | 6.9112 | 7.6142 | 8.7723 | 10.5985 | 9.6975 | 9.4167 | |

| | | | | | | | | |
|---|---|---|---|---|---|---|---|---|
| 9.4291 | 7.7066 | 3.4255 | 9.3884 | 10.119 | 5.9403 | 8.0155 | 7.8054 | |
| 10.2348 | 7.1239 | 8.682 | 7.9424 | 9.7139 | 7.8097 | 7.7473 | 9.9581 | |
| 9.2045 | 7.0245 | 8.5507 | 8.5557 | 8.0992 | 8.5341 | 7.1236 | 8.4346 | |
| 8.1731 | 7.3415 | 3.0238 | 8.3979 | 3.9968 | 4.6616 | 4.3353 | 2.8124 | |
| 2.8515 | 4.9963 | 5.1749 | 7.7338 | 3.5947 | 3.4317 | 3.5318 | 4.5913 | |
| 4.9172 | 4.1431 | 6.8899 | 3.2947 | 2.3512 | 5.5668 | 3.9399 | 6.8465 | |
| 3.4387 | 4.243 | | | | | | | |
| OLFML2A | 8.291 | 8.4219 | 6.5793 | 7.7045 | 7.3355 | 7.2245 | 8.8211 | |
| 8.3639 | 6.8495 | 7.5967 | 8.2809 | 7.8873 | 7.0493 | 9.0482 | 8.0653 | |
| 8.8654 | 7.4457 | 9.7481 | 8.0336 | 8.1386 | 8.5116 | 7.8386 | 8.219 | |
| 8.5233 | 5.1235 | 8.8807 | 7.3915 | 9.6105 | 8.9411 | 4.9078 | 6.4194 | |
| 9.1304 | 7.8218 | 8.2065 | 9.9006 | 8.4985 | 7.8357 | 7.3723 | 7.0442 | |
| 6.5899 | 7.3328 | 7.02 | 6.911 | 7.7404 | 8.8775 | 8.3165 | 10.1674 | |
| 7.0714 | 8.4072 | 8.9489 | 10.1351 | 6.3263 | 8.4966 | 8.3647 | 7.1487 | |
| 8.5438 | 5.2076 | 6.9898 | 8.9912 | 10.6467 | 6.1795 | 9.169 | 6.8885 | |
| 7.795 | 9.6316 | 6.0202 | 7.7123 | 9.017 | 7.486 | 9.4948 | 8.4402 | |
| 8.7909 | 9.6027 | 7.4592 | 8.3998 | 7.2718 | 7.3537 | 7.1934 | 8.208 | |
| 9.5322 | 7.3033 | 7.0824 | 9.4373 | 8.5389 | 8.6256 | 8.6902 | 8.13 | |
| 7.001 | 8.0375 | 8.028 | 8.7774 | 7.2841 | 8.6125 | 7.9909 | 8.7149 | |
| 8.5258 | 7.4157 | 6.5735 | 7.5063 | 7.858 | 8.7652 | 7.0724 | 7.3647 | |
| 8.5838 | 7.9743 | 7.5581 | 9.3574 | 7.6294 | 8.1697 | 8.4364 | 9.0639 | |
| 7.2372 | 10.8947 | 6.9534 | 6.2998 | 7.7621 | 7.9097 | 9.4928 | 7.1954 | |
| 6.5095 | 8.247 | 11.2531 | 6.0469 | 8.2173 | 7.0819 | 8.8602 | 7.6675 | |
| 7.2823 | 7.7442 | 6.865 | 11.2178 | 6.3897 | 8.0677 | 8.1279 | 8.1568 | |
| 7.2671 | 9.1619 | 6.384 | 8.2841 | 6.6621 | 7.3744 | 8.4771 | 5.844 | |
| 8.8534 | 7.3489 | 8.3209 | 8.2705 | 8.0787 | 5.9279 | 7.8156 | 6.8432 | |
| 6.931 | 6.4847 | 8.4529 | 8.5743 | 8.5916 | 9.1957 | 7.3292 | 6.6137 | |
| 9.7598 | 7.6402 | 7.4169 | 7.6742 | 8.2623 | 6.6356 | 6.4411 | 8.4426 | |
| 8.5693 | 7.4536 | 6.9189 | 8.3522 | 7.9506 | 8.7526 | 8.2129 | 7.9272 | |
| 7.764 | 6.0599 | 11.8231 | 10.4624 | 11.3256 | 8.7565 | 12.8606 | 11.4037 | |
| 12.0803 | 11.7811 | 11.7963 | 11.048 | 10.6488 | 11.8674 | 12.4829 | 8.6019 | |
| 8.5094 | 12.2604 | 11.6575 | 12.1362 | 10.2193 | 9.772 | 12.0243 | 10.8736 | |
| 11.1358 | 12.057 | | | | | | | |
| C7 | 0 | 1.7871 | 1.1529 | 6.2241 | 3.4689 | 0 | 6.7203 | |
| 2.8671 | 4.8084 | 0.8472 | 3.7397 | 3.2002 | 1.7043 | 9.5535 | 2.4094 | |
| 1.9468 | 0.6732 | 2.0721 | 2.7123 | 2.5463 | 1.0317 | 2.7174 | 3.4974 | |
| 3.0458 | 1.0077 | 5.9218 | 5.0382 | 1.4434 | 1.7182 | 0 | 0.6819 | |
| 3.1675 | 0.3648 | 2.0653 | 2.172 | 3.8546 | 0.6231 | 1.7856 | 0 | 0 |
| | 1.4786 | 5.8688 | 0.8339 | 2.2292 | 6.4242 | 1.4359 | 6.7462 | |
| 3.1821 | 4.866 | 5.7546 | 9.9673 | 2.6662 | 5.4104 | 3.7792 | 0.5968 | |
| 3.7482 | 2.2002 | 6.7655 | 2.4407 | 5.2983 | 1.6333 | 1.1694 | 2.4919 | |
| 3.2712 | 7.4802 | 0.4698 | 3.3759 | 5.3258 | 6.0957 | 6.8449 | 2.1251 | |
| 3.103 | 8.8542 | 5.1933 | 7.3251 | 1.0522 | 0.9284 | 3.1631 | 9.0605 | |
| 1.2149 | 7.1454 | 2.5333 | 7.4498 | 5.5137 | 1.4589 | 8.797 | 5.7903 | 0 |
| | 5.0351 | 0.5352 | 4.4784 | 0.8449 | 7.87 | 7.961 | 6.8244 | |
| 3.708 | 1.6498 | 3.0941 | 7.6374 | 1 | 4.4295 | 2.8751 | 3.4797 | |
| 2.997 | 4.4753 | 2.0533 | 9.6009 | 0 | 5.9019 | 2.159 | 0.5331 | |
| 5.6561 | 1.6629 | 0.7024 | 1.1234 | 2.3786 | 4.939 | 1.3267 | 8.1564 | |
| 2.8392 | 7.3542 | 10.5113 | 0 | 3.7265 | 2.1437 | 2.5899 | 1.3147 | |
| 3.4742 | 2.3133 | 5.2633 | 10.5643 | 5.3901 | 3.2708 | 3.9105 | 0 | |
| 2.8829 | 1.1539 | 2.2048 | 1.7128 | 2.5111 | 0.6138 | 4.1617 | 0.7182 | |
| 1.4811 | 0 | 4.6862 | 7.29 | 6.3925 | 0.5466 | 0.4486 | 2.1836 | |
| 5.7118 | 1.0565 | 4.6244 | 3.3889 | 7.8146 | 6.4004 | 1.7413 | 8.8962 | 0.91 |
| | 1.4215 | 0.7366 | 5.9346 | 4.0336 | 0.7058 | 0.6567 | 4.7396 | |

| | | | | | | | | |
|---|---|---|---|---|---|---|---|---|
| 1.4263 | 5.1978 | 2.4161 | 8.7865 | 1.7976 | 2.8198 | 0 | 2.7261 | |
| 8.8622 | 3.5284 | 9.5309 | 6.3423 | 9.7431 | 6.982 | 12.0583 | 11.4009 | |
| 13.1656 | 14.6826 | 14.8279 | 8.2635 | 15.2814 | 12.3381 | 8.2727 | 9.72 | |
| 11.2864 | 12.2387 | 12.2741 | 8.3628 | 13.6349 | 14.6485 | 9.1417 | 8.4121 | |
| 13.932 | 9.6173 | | | | | | | |
| SYT15 | 4.1912 | 2.7039 | 5.4935 | 3.7874 | 4.3799 | 3.5813 | 5.4161 | |
| 3.1771 | 3.5952 | 4.8761 | 5.154 | 5.0621 | 5.0864 | 4.7451 | 4.4253 | |
| 5.3312 | 4.4272 | 4.7356 | 4.3532 | 4.4677 | 3.3041 | 4.1781 | 5.4501 | |
| 3.9557 | 3.5376 | 4.7813 | 3.6337 | 2.5183 | 3.2323 | 4.2103 | 3.8373 | |
| 5.451 | 4.0723 | 2.5862 | 4.212 | 4.2593 | 3.1865 | 4.2081 | 4.9453 | |
| 4.8007 | 3.2232 | 3.0861 | 3.3218 | 4.5546 | 3.2569 | 4.0312 | 4.8252 | |
| 3.739 | 5.1162 | 4.3056 | 6.4439 | 2.2896 | 4.705 | 3.8751 | 3.4915 | |
| 5.4045 | 4.3944 | 4.5652 | 3.7861 | 5.4985 | 4.4378 | 3.8818 | 3.7033 | |
| 5.0894 | 5.2115 | 4.7846 | 4.9108 | 4.5412 | 4.8669 | 5.1757 | 3.4708 | |
| 4.0165 | 5.705 | 4.3205 | 3.6876 | 4.3829 | 4.1091 | 2.2368 | 4.1509 | |
| 4.3174 | 5.0246 | 2.7354 | 5.355 | 3.8956 | 2.8322 | 4.7216 | 4.5983 | |
| 3.2267 | 4.516 | 3.7628 | 5.7251 | 4.1473 | 4.1824 | 4.7249 | 5.3479 | |
| 3.3293 | 5.6365 | 3.907 | 6.0669 | 4.6439 | 3.4391 | 5.2263 | 4.0011 | |
| 4.5876 | 3.9911 | 4.4937 | 6.0909 | 4.0104 | 4.6283 | 5.187 | 3.849 | |
| 3.1654 | 3.2729 | 3.29 | 4.7823 | 4.1155 | 4.2436 | 2.8142 | 2.2603 | |
| 2.7218 | 7.1792 | 7.5733 | 4.2931 | 3.7789 | 4.1774 | 4.1236 | 3.6893 | |
| 3.8163 | 4.416 | 3.547 | 7.9721 | 2.2924 | 4.9354 | 3.6474 | 4.9759 | |
| 3.6656 | 5.1165 | 3.5001 | 4.1584 | 3.9166 | 3.6629 | 3.6358 | 4.6548 | |
| 4.7247 | 4.8942 | 5.6786 | 5.1933 | 3.231 | 4.136 | 4.0914 | 4.1562 | |
| 3.0495 | 3.9615 | 4.5406 | 3.8102 | 5.0046 | 5.1312 | 3.5582 | 4.8689 | |
| 4.4659 | 3.8083 | 4.0288 | 4.8401 | 4.5126 | 3.6991 | 3.5079 | 3.604 | 3.42 |
| | 4.614 | 4.4088 | 5.3769 | 3.9871 | 3.7138 | 3.9662 | 4.8767 | 5.33 |
| | 1.799 | 6.6878 | 5.5034 | 6.6549 | 4.7233 | 7.2224 | 7.1206 | |
| 8.5709 | 8.8218 | 7.643 | 6.1905 | 8.71 | 8.0128 | 6.6669 | 5.8688 | |
| 6.4017 | 7.1348 | 7.6282 | 6.7884 | 6.3276 | 6.9609 | 7.6156 | 6.5827 | |
| 8.941 | 5.7623 | | | | | | | |
| HIC1 | 6.0313 | 5.888 | 5.5321 | 6.8448 | 5.7008 | 6.8199 | 5.9126 | |
| 6.7079 | 5.7079 | 5.3975 | 6.7642 | 3.9636 | 5.5569 | 7.7443 | 7.7301 | |
| 7.9868 | 5.4533 | 5.8751 | 6.3209 | 6.6251 | 7.5176 | 5.1678 | 8.2363 | |
| 7.9854 | 4.7962 | 5.9607 | 7.176 | 4.8552 | 5.3737 | 3.5858 | 6.1143 | |
| 5.988 | 5.2079 | 5.4962 | 5.8532 | 7.479 | 7.7111 | 5.5048 | 7.5683 | |
| 5.4123 | 5.8624 | 7.392 | 5.4218 | 5.8064 | 7.1807 | 8.0362 | 7.7649 | |
| 5.9274 | 7.2818 | 7.2041 | 7.8775 | 6.5598 | 6.9098 | 5.8709 | 4.5573 | |
| 7.6706 | 3.1217 | 5.5094 | 6.5623 | 8.3947 | 5.1574 | 6.1361 | 8.0276 | |
| 6.781 | 7.173 | 4.7228 | 7.9823 | 8.0535 | 7.3626 | 7.8046 | 7.2599 | |
| 5.3857 | 8.1984 | 6.5834 | 7.8749 | 6.5277 | 4.4142 | 6.9176 | 7.0543 | |
| 8.9478 | 6.0809 | 5.0155 | 9.7984 | 7.1974 | 5.9076 | 6.6377 | 7.3901 | |
| 6.5301 | 7.4325 | 6.0951 | 7.6672 | 5.5478 | 5.872 | 7.3245 | 8.0066 | |
| 7.6043 | 5.2228 | 4.9363 | 7.0561 | 7.4094 | 7.0064 | 6.6325 | 5.0353 | |
| 5.8471 | 5.966 | 7.4923 | 7.9549 | 6.3328 | 7.0093 | 6.1977 | 6.794 | |
| 7.0589 | 4.1963 | 6.3615 | 4.4346 | 6.8143 | 6.4127 | 8.1099 | 7.792 | |
| 6.1603 | 11.7522 | 9.0935 | 5.456 | 6.0678 | 6.9683 | 6.3457 | 6.2073 | |
| 5.8458 | 5.8842 | 4.4839 | 8.9243 | 5.771 | 7.0209 | 6.9522 | 5.1549 | |
| 7.8489 | 6.5085 | 4.6576 | 7.9774 | 4.5195 | 6.7914 | 7.8524 | 5.9607 | |
| 7.496 | 6.467 | 4.6862 | 6.9063 | 6.9717 | 5.2066 | 4.9153 | 5.4138 | |
| 6.2319 | 6.3091 | 8.1334 | 5.2521 | 6.5593 | 8.3637 | 6.8789 | 6.3022 | |
| 3.5836 | 6.0905 | 5.6138 | 6.8635 | 6.8895 | 6.3422 | 6.4507 | 6.4248 | |
| 6.1245 | 5.2343 | 5.2702 | 8.5688 | 6.454 | 6.0804 | 5.0615 | 6.326 | |
| 6.8805 | 7.0491 | 10.4576 | 11.0634 | 11.0887 | 7.0255 | 9.8782 | 9.73 | |

| | | | | | | | | |
|---|---|---|---|---|---|---|---|---|
| 8.563 | 9.0278 | 9.6499 | 11.1129 | 10.4483 | 9.0957 | 11.4262 | 11.3538 | |
| 7.9928 | 9.5214 | 9.1844 | 10.7605 | 10.4308 | 10.1587 | 10.1027 | 10.756 | |
| 10.5595 | 10.2263 | | | | | | | |
| C3orf70 | 4.1155 | 5.3732 | 2.988 | 6.1363 | 4.4242 | 2.7953 | 7.329 | |
| 4.1243 | 3.2721 | 3.5094 | 4.4249 | 5.1659 | 3.8631 | 4.4366 | 5.5981 | |
| 4.3913 | 2.0944 | 3.172 | 2.5262 | 4.7995 | 2.7536 | 8.6014 | 4.3616 | |
| 6.2325 | 2.5978 | 6.3309 | 5.405 | 3.0546 | 4.8702 | 5.8713 | 3.0446 | |
| 6.2946 | 4.3001 | 4.0014 | 3.3387 | 4.1682 | 5.015 | 4.2877 | 1.8914 | |
| 3.1173 | 4.2379 | 4.8314 | 3.1417 | 3.0665 | 4.2351 | 6.0526 | 5.913 | |
| 2.5237 | 5.515 | 3.8006 | 8.1936 | 3.8203 | 6.4823 | 3.828 | 2.8376 | |
| 4.7446 | 2.7333 | 4.7471 | 3.564 | 8.1407 | 4.0676 | 4.3479 | 5.7476 | |
| 3.7643 | 6.7032 | 3.356 | 6.8915 | 2.3485 | 3.8558 | 6.1229 | 5.5132 | |
| 5.0283 | 6.5977 | 3.7501 | 6.1315 | 4.2262 | 7.1161 | 2.2368 | 5.199 | |
| 8.2033 | 5.127 | 1.2055 | 4.9006 | 5.292 | 3.6787 | 5.6581 | 4.5983 | |
| 3.3101 | 3.6025 | 7.0702 | 5.1804 | 1.5803 | 6.6836 | 6.5715 | 4.2679 | |
| 4.4001 | 3.1897 | 1.8846 | 5.5901 | 4.6439 | 5.5486 | 5.5848 | 4.0011 | |
| 5.4346 | 5.5827 | 3.9729 | 7.7238 | 5.7113 | 3.8564 | 3.605 | 4.1326 | |
| 5.1298 | 1.6629 | 4.3755 | 2.3575 | 4.6856 | 5.3585 | 4.9964 | 5.4161 | |
| 4.153 | 4.2995 | 7.6059 | 2.3602 | 4.0156 | 2.6155 | 5.3792 | 4.1485 | |
| 3.0248 | 3.5751 | 2.6654 | 8.3743 | 5.4873 | 4.7767 | 3.5271 | 2.0813 | |
| 5.3078 | 4.9115 | 3.4328 | 3.5208 | 2.6375 | 4.4732 | 4.121 | 2.8974 | |
| 4.908 | 5.2971 | 5.6157 | 5.2059 | 5.268 | 2.6009 | 3.0521 | 3.4836 | |
| 5.7366 | 4.661 | 3.6013 | 4.8397 | 5.8914 | 5.5585 | 4.9756 | 4.5289 | |
| 4.7276 | 3.355 | 5.2343 | 5.2832 | 5.8835 | 3.8329 | 1.9569 | 3.8177 | |
| 5.1014 | 4.6415 | 4.1593 | 5.4804 | 3.3334 | 2.3335 | 8.1157 | 4.4587 | |
| 5.9381 | 4.5045 | 8.0736 | 7.8587 | 8.4039 | 5.5157 | 11.4073 | 8.9205 | |
| 9.1066 | 9.1447 | 9.0452 | 8.0949 | 10.0397 | 10.8784 | 8.4687 | 7.7607 | |
| 6.5432 | 8.9633 | 10.7346 | 8.0858 | 7.5712 | 9.0802 | 9.0034 | 9.0134 | |
| 10.1897 | 7.6391 | | | | | | | |
| PLN | 6.0618 | 4.3653 | 3.1949 | 6.3776 | 3.3816 | 2.0702 | 5.2339 | |
| 2.0525 | 1.45 | 2.6308 | 2.6546 | 4.1488 | 2.3407 | 3.7058 | 0.6221 | |
| 3.0246 | 1.4771 | 2.3988 | 4.0419 | 4.2177 | 2.5109 | 3.6468 | 2.4936 | |
| 8.1865 | 1.0077 | 1.9932 | 7.5826 | 1.4434 | 1.8766 | 0 | 0.6819 | |
| 5.216 | 1.7231 | 5.225 | 2.8702 | 3.6678 | 2.6786 | 5.4597 | 0.9285 | |
| 3.4901 | 1.1317 | 6.7882 | 3.5316 | 4.8412 | 3.6337 | 7.2173 | 7.3403 | |
| 2.8432 | 6.1635 | 3.5666 | 9.5244 | 1.5581 | 6.1053 | 4.2415 | 3.4242 | |
| 4.3855 | 0.598 | 5.5711 | 3.7337 | 7.7454 | 2.3798 | 4.0813 | 3.3577 | |
| 1.7486 | 8.3323 | 2.8398 | 4.7937 | 3.3991 | 3.7282 | 8.0182 | 5.3698 | |
| 3.8681 | 8.7451 | 3.617 | 7.618 | 2.6709 | 4.2864 | 1.0433 | 6.9906 | |
| 8.4079 | 6.4928 | 0.5215 | 4.7106 | 5.4144 | 4.1741 | 7.1268 | 5.2183 | |
| 5.5796 | 6.808 | 2.9516 | 6.7476 | 1.9209 | 7.5821 | 6.9828 | 3.9646 | |
| 3.7624 | 3.2557 | 1.3871 | 5.0883 | 3.3219 | 7.0722 | 1.0399 | 4.6602 | |
| 1.7841 | 6.5962 | 5.6839 | 5.5549 | 5.2006 | 3.6237 | 2.0289 | 1.6938 | |
| 7.8624 | 2.5982 | 3.4641 | 1.1234 | 4.948 | 5.4063 | 4.7759 | 6.7331 | |
| 4.4769 | 4.9498 | 9.3246 | 1.8416 | 4.4942 | 2.1437 | 6.4772 | 2.4496 | |
| 1.1308 | 2.9906 | 1.8766 | 9.6393 | 4.6083 | 3.7814 | 1.3242 | 2.2306 | |
| 3.4011 | 1.3399 | 1.0225 | 4.4566 | 2.8614 | 5.7975 | 1.8011 | 0.9764 | |
| 2.965 | 2.6813 | 6.4649 | 3.3186 | 6.145 | 0.942 | 1.829 | 3.1654 | |
| 3.0495 | 4.7791 | 5.4182 | 5.5724 | 6.7083 | 7.4012 | 6.3589 | 3.3509 | |
| 4.1097 | 2.6905 | 2.5017 | 6.3817 | 5.415 | 1.5325 | 0.6567 | 6.2052 | 7.77 |
| | 3.5027 | 2.1288 | 7.0549 | 3.2101 | 0 | 2.0478 | 2.9076 | |
| 8.0175 | 3.4488 | 10.2211 | 7.2272 | 10.1527 | 6.8362 | 12.0064 | 10.5599 | |
| 12.2428 | 12.356 | 12.4216 | 7.922 | 11.8004 | 12.7291 | 10.3455 | 8.7486 | |
| 8.987 | 11.5096 | 10.8827 | 10.718 | 6.3358 | 10.4161 | 10.9813 | 9.0268 | |

| | | | | | | | |
|---|---|---|---|---|---|---|---|
| 12.524 | 10.3239 | | | | | | |
| CCNE1 | 9.6853 | 8.3177 | 7.7243 | 8.7193 | 10.0039 | 10.1681 | 9.6074 |
| 9.9743 | 10.8581 | 10.9584 | 9.8742 | 7.8632 | 8.851 | 5.4029 | 9.6981 |
| 7.954 | 11.276 | 10.4606 | 7.3579 | 6.2777 | 9.1151 | 9.2296 | 8.9885 |
| 8.5106 | 11.3252 | 9.1432 | 7.4437 | 11.8025 | 8.5944 | 10.3529 | 9.0703 |
| 9.2719 | 7.5479 | 9.2554 | 6.7867 | 9.0899 | 9.9804 | 8.4481 | 8.6416 |
| 13.2802 | 10.0747 | 9.3544 | 9.0262 | 9.1393 | 10.6593 | 10.1844 | 8.4086 |
| 7.9599 | 8.0192 | 9.6386 | 8.2352 | 11.2707 | 10.4434 | 9.0664 | 10.4719 |
| 8.2204 | 7.0992 | 10.3537 | 8.0699 | 10.1259 | 10.8623 | 8.255 | 11.1111 |
| 9.9223 | 8.0722 | 7.7406 | 8.3406 | 6.2657 | 5.8875 | 8.9915 | 8.2044 |
| 9.872 | 5.3855 | 10.7295 | 8.5343 | 9.625 | 10.6332 | 9.5489 | 9.9176 |
| 9.0448 | 9.9798 | 8.958 | 7.0493 | 10.6799 | 10.305 | 8.9117 | 8.5769 |
| 9.9065 | 10.0682 | 10.327 | 9.1679 | 7.925 | 11.5512 | 9.6138 | 10.269 |
| 9.4552 | 6.8582 | 10.943 | 10.2633 | 8.5774 | 10.8236 | 10.146 | 6.6168 |
| 9.2317 | 8.2664 | 11.0091 | 9.73 | 9.6174 | 9.6734 | 9.7065 | 11.6662 |
| 10.232 | 9.9123 | 10.4235 | 6.7184 | 8.3786 | 10.6741 | 11.0794 | 9.9803 |
| 9.435 | 6.3465 | 8.9623 | 7.9663 | 7.0356 | 8.3876 | 7.6504 | 9.2583 |
| 7.8079 | 8.667 | 10.9723 | 5.4549 | 10.5002 | 7.7726 | 10.2852 | 9.1763 |
| 10.3141 | 6.9592 | 10.537 | 9.9467 | 8.4507 | 8.3584 | 8.1102 | 8.7052 |
| 9.7353 | 8.9426 | 9.2444 | 8.9534 | 10.6517 | 13.7417 | 9.7836 | 10.6126 |
| 10.1631 | 9.5671 | 8.0296 | 9.1109 | 11.7334 | 6.372 | 9.936 | 9.6967 |
| 10.7886 | 7.2285 | 8.983 | 8.5816 | 9.2269 | 11.6298 | 9.8543 | 9.0988 |
| 7.6387 | 9.2545 | 10.1697 | 9.1895 | 8.1525 | 11.5089 | 10.0327 | 9.6051 |
| 7.1948 | 11.8057 | 5.1059 | 7.8453 | 5.6114 | 5.6646 | 4.4632 | 5.5104 |
| 2.8515 | 2.9592 | 3.5225 | 6.7648 | 4.757 | 4.64 | 5.2902 | 6.9671 |
| 5.3748 | 4.932 | 5.359 | 5.094 | 5.0203 | 5.5668 | 4.5823 | 7.1472 |
| 4.166 | 5.2847 | | | | | | |
| ANTXR2 | 8.3166 | 9.547 | 8.6464 | 8.8479 | 7.1663 | 8.0484 | 7.5218 |
| 8.322 | 7.1032 | 7.0361 | 8.326 | 8.426 | 8.592 | 8.9157 | 8.0969 |
| 8.7624 | 6.6652 | 7.2895 | 6.6464 | 9.7396 | 6.85 | 4.9936 | 8.2065 |
| 10.4663 | 7.9372 | 9.0845 | 9.3445 | 7.881 | 7.0807 | 6.7558 | 5.8828 |
| 8.283 | 9.8143 | 7.0399 | 7.3426 | 8.7309 | 8.7408 | 7.5684 | 7.1581 |
| 7.5532 | 7.295 | 9.6454 | 8.502 | 9.143 | 8.2787 | 9.3536 | 9.7098 |
| 7.574 | 10.523 | 9.7726 | 10.5156 | 7.6107 | 8.6103 | 7.7767 | 7.4986 |
| 9.8866 | 5.871 | 8.787 | 9.1744 | 10.1128 | 7.3662 | 10.1353 | 8.6327 |
| 9.7498 | 10.2 | 4.4454 | 9.9008 | 9.2032 | 9.0204 | 9.721 | 9.6504 |
| 8.9218 | 10.1584 | 8.0688 | 9.4136 | 7.9319 | 8.6166 | 7.3841 | 8.5098 |
| 10.0191 | 8.7002 | 8.3086 | 10.217 | 9.2914 | 6.8415 | 9.0132 | 8.2427 |
| 7.9144 | 8.6712 | 9.1004 | 8.4248 | 6.9635 | 8.8503 | 9.9707 | 8.9206 |
| 8.5531 | 8.6467 | 7.3871 | 7.6601 | 6.7682 | 9.3443 | 7.4619 | 7.4066 |
| 8.2383 | 8.5365 | 9.2909 | 8.0469 | 9.3773 | 8.9671 | 7.7229 | 8.5042 |
| 9.672 | 3.4738 | 8.3088 | 8.3316 | 8.8151 | 7.7616 | 7.9866 | 8.8751 |
| 7.7692 | 8.6741 | 11.0045 | 5.456 | 9.7201 | 7.9156 | 9.9184 | 8.9367 |
| 7.8642 | 8.2419 | 8.3383 | 11.8169 | 6.8702 | 9.6589 | 7.6549 | 6.9193 |
| 9.875 | 9.6122 | 5.329 | 7.6802 | 8.7666 | 9.401 | 9.2141 | 8.0973 |
| 8.1754 | 8.1987 | 7.8562 | 9.9486 | 9.0474 | 8.0495 | 7.2581 | 6.634 |
| 7.9087 | 9.367 | 7.9011 | 9.4524 | 9.4299 | 9.4251 | 9.0153 | 6.4714 |
| 4.9491 | 8.0312 | 8.8118 | 8.4648 | 8.661 | 6.4502 | 8.3467 | 9.0188 |
| 9.7006 | 8.8231 | 8.4288 | 9.6094 | 9.302 | 6.4573 | 7.6888 | 9.298 |
| 9.4095 | 7.0149 | 12.1858 | 10.2542 | 11.9622 | 8.7821 | 13.1256 | 12.2927 |
| 13.0556 | 12.6837 | 12.8056 | 10.9321 | 11.9914 | 13.4347 | 12.2435 | 10.949 |
| 10.0706 | 12.4882 | 12.7105 | 12.2394 | 12.4969 | 11.0314 | 12.2916 | 11.1708 |
| 12.6528 | 12.2308 | | | | | | |
| C15orf42 | | 8.893 | 7.9441 | 6.7083 | 9.5398 | 8.8727 | 8.3352 |

| | | | | | | | |
|---|---|---|---|---|---|---|---|
| 8.4037 | 8.6512 | 8.8676 | 7.2631 | 7.4645 | 8.1498 | 7.5796 | 6.5944 |
| 8.4947 | 8.0186 | 8.681 | 7.3986 | 6.947 | 6.4921 | 7.8599 | 8.6735 |
| 7.6447 | 6.3715 | 8.4911 | 8.3299 | 8.6876 | 8.2472 | 8.1288 | 8.8355 |
| 9.474 | 6.3644 | 8.9051 | 7.3301 | 7.1304 | 7.5744 | 7.5694 | 8.4766 |
| 7.5223 | 8.0016 | 7.7734 | 9.2722 | 6.3104 | 7.6372 | 8.4953 | 7.8503 |
| 7.0165 | 8.9185 | 7.1493 | 6.2607 | 8.3068 | 8.768 | 8.3793 | 8.9015 |
| 8.3228 | 6.4452 | 6.1909 | 8.5169 | 4.1894 | 9.5558 | 8.422 | 7.9003 |
| 7.5832 | 7.5428 | 7.1024 | 6.693 | 7.9462 | 5.3792 | 4.6751 | 8.8813 |
| 7.7729 | 8.6976 | 5.1484 | 8.7984 | 7.4857 | 7.1314 | 7.3927 | 8.1706 |
| 7.3659 | 8.4133 | 8.4436 | 7.9553 | 6.7327 | 8.3485 | 7.1847 | 7.0008 |
| 8.5372 | 8.2586 | 8.2744 | 8.0905 | 9.5632 | 8.8639 | 9.5219 | 7.3777 |
| 8.2623 | 9.0173 | 7.7712 | 7.9628 | 9.3276 | 8.7357 | 9.6425 | 8.9955 |
| 7.3161 | 8.4357 | 8.3682 | 8.0982 | 9.0539 | 7.2577 | 7.4574 | 7.7581 |
| 7.7019 | 7.4513 | 8.114 | 7.508 | 8.46 | 7.4124 | 8.3612 | 8.1908 |
| 7.1954 | 9.4326 | 6.9584 | 8.0139 | 9.2006 | 6.1509 | 6.883 | 8.2203 |
| 8.128 | 6.9501 | 6.997 | 7.9601 | 6.0586 | 8.3425 | 7.1296 | 8.8825 |
| 8.4855 | 8.0975 | 8.6096 | 8.1486 | 9.1614 | 6.9692 | 8.2504 | 7.432 |
| 6.4814 | 8.4158 | 8.8137 | 7.4567 | 7.1589 | 8.0088 | 9.2947 | 9.173 |
| 9.1275 | 8.9145 | 7.6091 | 6.9155 | 8.4946 | 8.8935 | 5.4965 | 8.4343 |
| 8.6229 | 8.0723 | 5.8067 | 8.5316 | 6.9251 | 7.783 | 8.4496 | 8.2211 |
| 8.3066 | 8.4483 | 5.7528 | 7.4127 | 9.5699 | 7.8997 | 9.0291 | 7.7678 |
| 7.9486 | 7.7374 | 8.3544 | 4.6181 | 7.6939 | 5.0907 | 3.4522 | 4.9393 |
| 4.5636 | 4.2297 | 4.7164 | 4.5221 | 6.6342 | 4.6191 | 3.9288 | 5.177 |
| 5.631 | 4.8266 | 4.8212 | 5.9234 | 5.3633 | 4.2768 | 5.141 | 5.1371 |
| 6.9716 | 5.2253 | 5.1589 | | | | | |
| FEN1 | 11.5329 | 10.0263 | 8.6017 | 11.6055 | 9.6705 | 10.4242 | 10.4517 |
| 11.2622 | 10.931 | 10.6567 | 10.104 | 10.4529 | 10.2604 | 9.1952 | 9.3775 |
| 10.9742 | 10.2207 | 10.7124 | 9.4642 | 9.5236 | 10.7978 | 11.4694 | 10.9952 |
| 9.7507 | 9.9417 | 11.4467 | 10.764 | 11.1703 | 10.2775 | 9.907 | 11.3482 |
| 9.4068 | 11.1291 | 10.1361 | 9.3106 | 10.7457 | 9.4833 | 10.2384 | 9.6034 |
| 10.8761 | 10.4635 | 10.7108 | 10.5168 | 10.8016 | 9.6767 | 9.8601 | 9.5295 |
| 10.6232 | 9.564 | 8.9329 | 10.1399 | 10.2786 | 10.341 | 10.8933 | 9.8458 |
| 9.7794 | 9.5415 | 10.7002 | 8.9108 | 10.3938 | 11.4497 | 10.3586 | 10.1892 |
| 10.1361 | 9.6768 | 9.5028 | 9.7852 | 8.9456 | 8.5683 | 10.7073 | 11.065 |
| 10.178 | 8.0227 | 10.8987 | 10.4264 | 9.8312 | 10.5158 | 11.1835 | 10.1793 |
| 11.783 | 10.4013 | 11.0539 | 8.6536 | 11.4148 | 9.7854 | 9.3895 | 9.8916 |
| 11.5364 | 10.3224 | 10.2493 | 10.8165 | 10.9091 | 11.3558 | 9.8642 | 9.7799 |
| 11.0607 | 10.1769 | 10.4255 | 10.6594 | 9.9944 | 10.1332 | 10.6173 | 10.0026 |
| 9.9946 | 11.6124 | 9.663 | 11.1189 | 10.1499 | 9.3464 | 10.3417 | 10.7755 |
| 11.3598 | 11.663 | 9.8696 | 10.0074 | 10.3559 | 9.8328 | 9.5821 | 11.3589 |
| 11.9133 | 7.4803 | 9.8597 | 10.0134 | 9.943 | 10.0907 | 10.4497 | 10.68 |
| 9.718 | 10.8062 | 10.8046 | 8.8364 | 12.0371 | 9.691 | 10.7168 | 10.8566 |
| 10.9863 | 10.572 | 9.9189 | 11.671 | 10.7602 | 11.0632 | 10.0978 | 10.7843 |
| 9.5311 | 10.3341 | 9.754 | 9.9874 | 11.216 | 10.4978 | 10.7243 | 11.1551 |
| 11.2321 | 10.4072 | 9.5559 | 10.7361 | 10.4117 | 8.6328 | 11.3796 | 9.6457 |
| 11.5685 | 9.5717 | 10.2933 | 9.867 | 10.8438 | 10.8477 | 10.6605 | 10.4871 |
| 10.8756 | 9.6734 | 11.035 | 9.6458 | 10.5883 | 11.5434 | 11.561 | 10.5928 |
| 9.8593 | 11.9093 | 8.1457 | 10.1559 | 8.4081 | 8.2907 | 7.9628 | 7.5589 |
| 7.7446 | 7.1884 | 7.6198 | 8.9208 | 8.0544 | 8.1709 | 8.0076 | 8.0892 |
| 8.188 | 7.9441 | 8.4957 | 7.9406 | 7.5039 | 8.5523 | 7.9584 | 8.9975 |
| 7.7615 | 8.3082 | | | | | | |
| FGF2 | 4.4292 | 5.731 | 6.0019 | 4.9111 | 4.2248 | 5.4244 | 7.0736 |
| 4.6365 | 5.0221 | 4.3807 | 6.0428 | 3.959 | 4.4461 | 4.3911 | 5.7242 |
| 7.0691 | 2.8049 | 4.7973 | 5.3013 | 5.0913 | 7.2685 | 6.9243 | 6.5494 |

| | | | | | | | | |
|---|---|---|---|---|---|---|---|---|
| 5.6863 | 4.1418 | 7.1889 | 5.2452 | 3.8246 | 4.3104 | 4.0887 | 4.158 | |
| 5.7796 | 3.5572 | 9.7366 | 5.1431 | 6.2091 | 6.4635 | 5.4219 | 4.3824 | |
| 6.0069 | 4.3249 | 6.295 | 3.8288 | 3.4365 | 6.3349 | 5.4886 | 7.0506 | |
| 5.5661 | 6.4022 | 9.1266 | 8.7056 | 5.9037 | 6.9455 | 5.1321 | 4.2432 | |
| 5.5097 | 6.0311 | 5.2731 | 7.5167 | 7.4883 | 3.3033 | 4.615 | 5.5881 | |
| 4.9982 | 7.6608 | 2.4635 | 7.1574 | 6.0539 | 6.8588 | 7.6469 | 6.0956 | |
| 5.2527 | 9.1181 | 5.3596 | 6.5166 | 6.0557 | 4.9844 | 4.67 | 6.5286 | |
| 7.6606 | 6.0019 | 4.6913 | 7.1019 | 6.334 | 6.039 | 5.8373 | 5.9353 | |
| 2.7637 | 5.9807 | 4.8602 | 6.363 | 3.8559 | 6.2001 | 7.5389 | 8.6 | |
| 5.1017 | 3.9162 | 5.1231 | 6.4242 | 3.3964 | 6.0615 | 6.9914 | 5.0498 | |
| 6.612 | 8.484 | 6.0896 | 7.7035 | 5.6199 | 7.0978 | 4.344 | 7.2521 | |
| 5.7785 | 3.3237 | 4.1413 | 4.1562 | 4.5947 | 6.0204 | 7.3139 | 5.0953 | |
| 2.8946 | 6.0892 | 9.5382 | 3.0977 | 4.2257 | 4.834 | 6.8178 | 4.7214 | |
| 4.3866 | 7.0776 | 3.1214 | 9.1562 | 4.581 | 5.8943 | 5.5913 | 7.1325 | |
| 5.1819 | 5.9527 | 4.251 | 7.9402 | 1.6822 | 9.8824 | 7.0441 | 4.1925 | |
| 5.1386 | 6.2385 | 6.1432 | 7.2903 | 6.0785 | 2.4153 | 10.2854 | 6.0094 | |
| 4.7598 | 5.3509 | 3.0351 | 7.545 | 6.3275 | 7.7285 | 5.172 | 3.8218 | |
| 7.0841 | 5.5784 | 6.4145 | 6.0659 | 6.1799 | 4.9925 | 3.8308 | 4.8466 | |
| 5.7166 | 6.0431 | 5.6375 | 6.6432 | 5.1889 | 4.1756 | 7.6789 | 5.4115 | |
| 7.3376 | 4.9659 | 10.3113 | 7.998 | 9.8945 | 8.9732 | 10.3353 | 10.2992 | |
| 10.4304 | 11.0004 | 10.7454 | 8.82 | 9.9454 | 10.2623 | 10.0159 | 8.9226 | |
| 9.5811 | 10.6316 | 10.4612 | 10.7495 | 9.7979 | 8.9965 | 9.7828 | 9.1444 | |
| 9.9251 | 10.4177 | | | | | | | |
| RSPO1 | 3.907 | 1.1543 | 1.6038 | 5.3643 | 2.2925 | 0 | 3.6208 | |
| 1.0352 | 1.2544 | 1.7643 | 6.5085 | 0 | 0 | 0 | 5.6141 | |
| 4.6995 | 0.9198 | 9.8198 | 4.7646 | 0 | 0.6062 | 0.4561 | 5.2105 | |
| 5.0202 | 1.0077 | 1.4094 | 5.6569 | 0 | 3.4495 | 3.0008 | 2.0076 | |
| 3.3777 | 0 | 3.5192 | 3.7304 | 3.795 | 0 | 2.1688 | 0.9285 | 0 |
| | 1.1317 | 4.6588 | 1.1202 | 1.0749 | 4.1794 | 3.0688 | 2.5699 | |
| 1.2781 | 5.0212 | 5.2211 | 7.9005 | 3.7698 | 4.9357 | 3.2459 | 5.0565 | |
| 4.6935 | 0 | 4.3569 | 4.9086 | 8.7931 | 4.006 | 0 | 0.9449 | |
| 3.4376 | 6.3357 | 0.4698 | 3.821 | 6.5992 | 4.2764 | 6.4821 | 1.9568 | |
| 3.7027 | 6.4978 | 2.3651 | 6.5595 | 4.4902 | 4.765 | 1.3738 | 6.9188 | |
| 4.7061 | 5.7037 | 5.3897 | 7.3877 | 5.9881 | 2.8322 | 5.5319 | 1.2591 | |
| 2.2933 | 5.6594 | 2.5704 | 4.4784 | 1.7606 | 5.963 | 5.8792 | 5.2647 | |
| 4.5314 | 1.8341 | 1.0545 | 7.5311 | 0 | 2.6176 | 2.6508 | 0.9131 | |
| 1.0339 | 4.5553 | 1.6324 | 7.4575 | 1.0055 | 4.4967 | 0.8239 | 3.4959 | |
| 5.8413 | 9.5393 | 1.1727 | 0 | 5.0932 | 4.1901 | 2.9611 | 6.1447 | |
| 4.324 | 9.1338 | 8.6561 | 2.2071 | 0.9848 | 3.0717 | 4.3664 | 0.8022 | |
| 1.1308 | 2.4506 | 0 | 9.3167 | 4.7192 | 2.4734 | 2.325 | 1.254 | |
| 1.4705 | 3.728 | 3.9486 | 3.6311 | 0 | 2.3902 | 5.2394 | 0 | |
| 1.0531 | 3.2849 | 5.4089 | 4.3616 | 5.7779 | 0.5466 | 1.0663 | 2.1836 | 0 |
| | 3.0035 | 4.7787 | 0.4801 | 5.6639 | 7.4382 | 3.1647 | 2.0241 | |
| 7.5328 | 3.8083 | 0 | 6.5663 | 3.6333 | 3.6273 | 2.4884 | 0.9785 | |
| 3.2385 | 0.5556 | 4.0375 | 6.0878 | 2.6702 | 5.3717 | 4.4868 | 4.6407 | |
| 6.3793 | 2.8479 | 10.8577 | 7.396 | 9.7598 | 10.3515 | 11.07 | 9.8251 | |
| 10.0957 | 10.0503 | 10.2357 | 8.9687 | 9.2472 | 11.0283 | 9.9031 | 7.999 | |
| 10.7547 | 10.2741 | 10.8455 | 9.688 | 7.2597 | 11.0757 | 9.6983 | 9.6344 | |
| 10.5416 | 9.4236 | | | | | | | |
| PEG3 | 1.5194 | 7.2451 | 5.7555 | 6.0258 | 7.1105 | 3.8708 | 6.1289 | |
| 3.7271 | 3.9906 | 7.1245 | 7.2084 | 1.9445 | 3.6574 | 6.1261 | 5.9522 | |
| 5.2251 | 2.2486 | 1.0485 | 4.6553 | 4.3166 | 2.3723 | 6.7742 | 6.517 | |
| 4.608 | 1.8184 | 2.3563 | 3.9322 | 6.0599 | 2.9048 | 1.0004 | 2.0076 | |
| 3.3777 | 2.5328 | 2.8817 | 7.3979 | 5.5077 | 4.1438 | 2.7212 | 2.4638 | |

| | | | | | | | |
|---|---|---|---|---|---|---|---|
| 2.8871 | 4.9439 | 3.9985 | 2.6056 | 2.3384 | 5.0108 | 4.2624 | 4.8057 |
| 2.8432 | 5.3293 | 6.4898 | 8.3503 | 7.7987 | 4.8964 | 3.7792 | 6.9538 |
| 5.8238 | 7.686 | 5.1541 | 4.4376 | 6.7269 | 1.2016 | 3.2846 | 2.7116 |
| 4.4233 | 7.1195 | 1.3447 | 5.2851 | 7.0784 | 6.7979 | 4.6416 | 4.3026 |
| 3.8042 | 7.538 | 3.347 | 6.8511 | 9.1413 | 6.8694 | 4.6365 | 5.2667 |
| 4.3174 | 6.9627 | 1.4549 | 8.7034 | 5.4999 | 11.8025 | 5.379 | 2.9074 |
| 2.4487 | 5.0427 | 2.8138 | 4.8552 | 3.5047 | 7.8242 | 6.0483 | 9.1262 |
| 5.4674 | 2.7134 | 4.007 | 5.7671 | 2.585 | 4.7882 | 3.6966 | 2.655 |
| 4.8719 | 3.8396 | 3.1961 | 11.587 | 4.6545 | 5.9404 | 3.9306 | 4.888 |
| 5.0395 | 1.2894 | 4.3086 | 1.9805 | 4.4286 | 4.2794 | 4.6075 | 3.5378 |
| 1.9254 | 9.3434 | 11.2 | 1.8416 | 4.4302 | 4.6399 | 4.398 | 3.4154 |
| 4.2797 | 1.7997 | 0.7381 | 10.1472 | 0 | 4.9275 | 8.8775 | 1.7261 |
| 4.6891 | 3.97 | 0.6 | 4.2302 | 0.6063 | 3.2456 | 7.4218 | 2.284 |
| 5.1161 | 5.905 | 4.7265 | 8.2208 | 5.1473 | 3.6978 | 2.3253 | 2.7564 |
| 1.4956 | 3.4721 | 0 | 2.3068 | 6.1214 | 7.1787 | 2.9186 | 7.276 |
| 2.1878 | 4.4583 | 3.6996 | 6.5497 | 4.6766 | 6.3645 | 3.2701 | 3.4201 |
| 3.7263 | 2.2504 | 2.9536 | 7.1126 | 3.9871 | 4.1011 | 8.885 | 4.3981 |
| 6.1238 | 3.1794 | 10.5865 | 9.6338 | 9.3618 | 7.9246 | 11.4005 | 11.4443 |
| 10.7825 | 10.405 | 11.2457 | 10.7688 | 10.5077 | 10.4286 | 12.0598 | 9.8267 |
| 9.617 | 10.5119 | 11.6116 | 12.0547 | 14.9232 | 8.7014 | 11.4151 | 10.4064 |
| 10.4397 | 11.7633 | | | | | | |
| GINS1 | 9.6455 | 7.8696 | 7.6646 | 10.4243 | 8.6497 | 8.4652 | 9.7727 |
| 9.3268 | 9.3199 | 9.0067 | 8.5064 | 9.0868 | 8.738 | 7.721 | 9.2908 |
| 9.5281 | 8.9175 | 8.8299 | 7.9247 | 7.9665 | 8.9558 | 10.3335 | 9.0103 |
| 7.3208 | 9.3519 | 10.1499 | 9.2003 | 9.597 | 9.0655 | 9.3964 | 9.9611 |
| 8.7093 | 9.4673 | 9.1086 | 7.6727 | 8.721 | 9.3427 | 9.365 | 8.4462 |
| 8.9622 | 8.3364 | 10.2766 | 8.3751 | 8.6038 | 8.6959 | 9.5103 | 9.2905 |
| 9.5675 | 8.2007 | 7.1752 | 9.654 | 8.4634 | 9.4764 | 8.8696 | 9.2176 |
| 8.6615 | 8.5083 | 9.788 | 6.1272 | 8.1543 | 9.6512 | 8.9258 | 9.4802 |
| 9.2101 | 8.6432 | 7.4126 | 9.4872 | 6.3405 | 6.0296 | 9.3679 | 9.4589 |
| 10.1291 | 5.996 | 8.217 | 8.4129 | 9.7024 | 10.1705 | 8.8351 | 8.0868 |
| 9.3368 | 9.3574 | 8.668 | 7.4294 | 10.1425 | 8.523 | 8.699 | 9.4154 |
| 7.7145 | 8.953 | 9.2642 | 8.6374 | 9.7708 | 10.6489 | 8.8192 | 9.5023 |
| 9.787 | 8.891 | 8.7233 | 7.4029 | 8.4676 | 9.8288 | 9.9397 | 8.2114 |
| 8.5759 | 10.5435 | 9.4059 | 10.6154 | 9.0744 | 9.186 | 8.3368 | 8.8189 |
| 9.1925 | 9.4363 | 8.9474 | 8.9627 | 8.582 | 8.5295 | 8.4918 | 7.9239 |
| 8.8919 | 4.9498 | 8.1678 | 9.458 | 7.796 | 8.4071 | 9.1154 | 9.5536 |
| 8.3479 | 8.7273 | 8.75 | 6.9466 | 8.3554 | 8.123 | 9.3669 | 9.2945 |
| 9.6416 | 9.0211 | 8.3276 | 9.8924 | 7.6437 | 9.3747 | 8.925 | 8.6622 |
| 8.5148 | 9.4773 | 8.8553 | 8.6164 | 9.21 | 9.5157 | 9.3202 | 10.156 |
| 9.226 | 9.0221 | 4.4516 | 9.2651 | 9.7167 | 6.391 | 8.791 | 8.9365 |
| 9.5363 | 7.4455 | 9.2914 | 8.4357 | 9.5013 | 9.5996 | 8.3131 | 9.7584 |
| 10.0486 | 8.1174 | 9.3012 | 9.6296 | 9.0922 | 8.766 | 9.7707 | 8.7687 |
| 8.6131 | 8.866 | 5.606 | 8.6409 | 6.1362 | 6.1191 | 6.4677 | 5.246 |
| 4.6343 | 5.466 | 5.193 | 7.6274 | 5.3341 | 5.1403 | 6.2972 | 6.2898 |
| 6.6053 | 6.0289 | 6.7444 | 5.9702 | 5.8461 | 6.833 | 5.9812 | 7.4401 |
| 4.9829 | 6.7418 | | | | | | |
| NBLA00301 | | 3.4941 | 5.1423 | 4.0795 | 3.9611 | 1.7112 | 4.1628 |
| 5.3896 | 4.7857 | 2.5484 | 3.6988 | 7.4016 | 1.0485 | 2.4639 | 3.2639 |
| 7.8189 | 5.43 | 1.8781 | 2.2447 | 5.1177 | 3.3566 | 1.0317 | 1.3145 |
| 7.3589 | 5.1569 | 2.1819 | 10.9209 | 3.3937 | 0.8951 | 3.0448 | 1.0004 |
| 2.3867 | 4.6138 | 1.2859 | 3.0497 | 4.5725 | 5.6987 | 5.3182 | 2.5595 |
| 2.4638 | 3.9741 | 2.1933 | 4.531 | 2.86 | 2.2292 | 3.0237 | 4.5343 |
| 5.0235 | 3.0225 | 4.9196 | 6.7127 | 7.8649 | 6.1399 | 5.2006 | 3.6766 |

| | | | | | | | |
|---|---|---|---|---|---|---|---|
| 3.0309 | 5.7841 | 0.598 | 4.8895 | 4.1483 | 7.7382 | 1.7877 | 2.3391 |
| 3.982 | 5.7043 | 6.552 | 0 | 7.5303 | 6.6973 | 6.984 | 4.9948 |
| 4.1932 | 4.4091 | 7.5105 | 3.307 | 7.3871 | 4.3453 | 3.04 | 2.7731 |
| 6.281 | 5.4502 | 6.0894 | 2.017 | 8.1753 | 5.753 | 1.6718 | 5.6581 |
| 3.1532 | 2.9428 | 5.3879 | 3.6641 | 5.307 | 4.0114 | 6.3745 | 7.989 |
| 7.6112 | 6.4999 | 2.7134 | 2.4082 | 6.551 | 1.585 | 3.6506 | 2.8751 |
| 2.7528 | 6.132 | 3.1581 | 5.0226 | 6.6609 | 3.4695 | 6.6 | 3.7359 |
| 5.2518 | 4.8398 | 0 | 3.29 | 2.9034 | 5.1321 | 4.6126 | 4.467 |
| 2.9678 | 3.7348 | 8.877 | 10.3444 | 3.2906 | 1.3036 | 4.5374 | 5.062 |
| 1.6921 | 3.0248 | 3.7973 | 1.5869 | 9.4489 | 3.1371 | 5.2261 | 4.4955 |
| 2.0813 | 4.4054 | 3.8556 | 2.7383 | 3.9615 | 0 | 3.3241 | 6.7328 |
| 4.1774 | 5.5394 | 5.6239 | 3.9594 | 7.1275 | 4.781 | 2.3633 | 2.0984 |
| 1.8707 | 0.541 | 4.0582 | 1.92 | 1.1272 | 5.3491 | 8.4658 | 4.1213 |
| 1.1551 | 3.4155 | 5.1631 | 4.8235 | 6.1481 | 5.2926 | 4.4888 | 4.0994 |
| 2.2874 | 3.5812 | 0.9557 | 3.0407 | 6.1449 | 3.9491 | 4.6578 | 3.3048 |
| 5.2573 | 6.0154 | 4.3811 | 9.9588 | 9.6787 | 9.1349 | 6.963 | 11.3075 |
| 9.7551 | 8.9062 | 10.274 | 9.4214 | 10.6913 | 8.4491 | 10.408 | 11.3206 |
| 11.4689 | 8.1258 | 10.7643 | 10.8382 | 11.5282 | 8.4042 | 8.5889 | 11.121 |
| 10.2615 | 7.9262 | 11.3539 | | | | | |
| ECT2 | 10.319 | 10.7017 | 9.9502 | 10.6895 | 10.7989 | 10.2516 | 11.7144 |
| 11.1396 | 12.6033 | 10.8657 | 10.8781 | 9.6228 | 10.7635 | 8.741 | 11.2487 |
| 9.8404 | 11.5306 | 11.073 | 9.6937 | 9.769 | 8.9421 | 11.3516 | 9.8189 |
| 9.0166 | 10.8581 | 10.1951 | 10.6783 | 11.6654 | 9.5013 | 11.5364 | 9.8821 |
| 9.0572 | 11.7256 | 10.161 | 9.7543 | 10.5455 | 10.0556 | 12.2913 | 8.416 |
| 9.6019 | 10.3489 | 10.4131 | 10.279 | 10.204 | 11.7454 | 9.7997 | 9.2622 |
| 10.2644 | 10.6598 | 10.1857 | 10.957 | 10.4518 | 11.7937 | 10.6057 | 11.1846 |
| 10.6347 | 9.4513 | 10.186 | 8.5221 | 10.6409 | 10.2716 | 10.2455 | 9.7333 |
| 10.4137 | 10.2947 | 9.3781 | 10.3289 | 9.0667 | 7.7094 | 10.6084 | 9.5888 |
| 12.4122 | 7.8308 | 11.0268 | 8.989 | 10.0975 | 10.9481 | 10.0005 | 10.7215 |
| 9.7337 | 11.0671 | 8.515 | 7.5525 | 10.1915 | 10.0594 | 10.2934 | 10.2699 |
| 7.8214 | 9.612 | 11.7586 | 10.9827 | 10.7588 | 11.2584 | 9.8642 | 12.1345 |
| 10.4798 | 10.5672 | 9.8004 | 8.6224 | 9.7814 | 11.5868 | 11.2561 | 10.5856 |
| 10.6528 | 10.979 | 11.1272 | 10.6752 | 10.352 | 11.1135 | 10.6033 | 12.0812 |
| 11.845 | 9.4303 | 10.5985 | 10.9593 | 10.0183 | 9.9602 | 9.6621 | 9.1519 |
| 8.5416 | 6.9584 | 9.9444 | 9.6079 | 9.4602 | 9.1011 | 10.6584 | 11.0093 |
| 9.7442 | 9.1654 | 8.8784 | 9.1279 | 10.0845 | 10.2299 | 9.5661 | 11.6575 |
| 11.4923 | 10.5953 | 10.5148 | 10.3642 | 9.7873 | 10.096 | 9.6078 | 8.5916 |
| 10.645 | 11.2829 | 8.8417 | 10.4746 | 10.0917 | 11.18 | 10.4773 | 13.5761 |
| 10.3037 | 10.2523 | 6.7574 | 9.6798 | 10.6911 | 8.1982 | 10.4718 | 9.4857 |
| 11.7792 | 9.4445 | 11.1021 | 9.6388 | 11.182 | 10.3947 | 9.495 | 10.9112 |
| 11.6352 | 9.3899 | 10.2213 | 10.8814 | 10.8801 | 9.426 | 10.0492 | 9.9519 |
| 9.6451 | 9.0459 | 6.8748 | 9.4499 | 7.1615 | 7.4469 | 6.8061 | 8.2065 |
| 6.8323 | 7.2215 | 7.5962 | 8.1726 | 6.9347 | 7.2653 | 6.5444 | 6.7094 |
| 7.9395 | 7.3268 | 8.6625 | 6.7439 | 5.9348 | 7.6199 | 7.5286 | 7.9101 |
| 6.9418 | 6.8247 | | | | | | |
| RTKN2 | 8.7956 | 8.5118 | 7.1047 | 9.08E+00 | | 7.51E+00 | |
| 8.0998 | 9.1782 | 8.5025 | 9.0545 | 8.6472 | 8.1033 | 9.0329 | 7.8285 |
| 6.8619 | 9.1041 | 8.2962 | 7.8964 | 6.9401 | 7.8597 | 7.8721 | 7.7628 |
| 9.5301 | 7.5413 | 5.8644 | 8.0679 | 8.6586 | 8.3435 | 7.6002 | 7.1599 |
| 8.4766 | 7.9049 | 7.2635 | 8.7861 | 9.3926 | 8.7156 | 8.066 | 8.2277 |
| 8.9278 | 8.1253 | 7.3756 | 6.8794 | 7.8811 | 6.7472 | 7.0836 | 7.7905 |
| 8.6686 | 7.8576 | 8.5356 | 7.4867 | 6.7909 | 9.197 | 7.5446 | 8.8541 |
| 7.3955 | 8.1899 | 7.6167 | 5.7659 | 9.0661 | 5.107 | 7.2147 | 7.1695 |
| 7.9938 | 6.7834 | 7.6499 | 7.1998 | 7.1642 | 7.4121 | 6.537 | 6.0564 |

| | | | | | | | | |
|---|---|---|---|---|---|---|---|---|
| 9.5573 | 7.8004 | 8.9377 | 6.7703 | 8.325 | 8.1567 | 7.9909 | 8.026 | 7.85 |
| | 7.8229 | 8.6101 | 8.6087 | 8.1518 | 7.1773 | 8.747 | 6.5633 | |
| 8.3521 | 7.8913 | 7.1272 | 7.1005 | 8.0816 | 8.5453 | 8.4713 | 9.2083 | |
| 8.5865 | 8.9055 | 7.0955 | 8.8608 | 8.0105 | 7.7258 | 7.6724 | 8.121 | |
| 8.5481 | 8.5181 | 8.8492 | 8.8879 | 8.2478 | 8.2609 | 7.5694 | 8.3593 | |
| 7.6501 | 8.2334 | 11.3898 | 8.2596 | 7.4438 | 9.6106 | 7.9535 | 8.9109 | |
| 7.7494 | 5.7364 | 8.2092 | 6.1326 | 7.5229 | 9.257 | 5.9006 | 7.045 | |
| 8.1519 | 8.7747 | 7.4876 | 7.1134 | 8.296 | 6.6975 | 7.5925 | 7.6072 | |
| 8.0038 | 9.4906 | 8.9109 | 8.9979 | 7.249 | 8.9915 | 6.3475 | 7.1066 | |
| 7.6917 | 6.4765 | 7.2901 | 9.0083 | 7.5437 | 8.2387 | 8.9541 | 9.3666 | |
| 8.8788 | 9.0213 | 8.6634 | 8.32 | 5.7522 | 8.4528 | 9.2448 | 6.2627 | |
| 7.4149 | 8.1109 | 8.1876 | 7.2994 | 8.9229 | 7.5504 | 9.0879 | 8.5778 | |
| 7.8978 | 9.1879 | 8.2172 | 7.4341 | 8.3586 | 8.5451 | 7.7796 | 8.0751 | |
| 8.3509 | 7.3356 | 8.0338 | 7.7925 | 6.5228 | 7.9842 | 6.5816 | 4.6928 | |
| 4.8384 | 5.2612 | 3.6322 | 3.2435 | 3.5787 | 7.4033 | 2.9352 | 2.9121 | |
| 6.0987 | 4.7765 | 5.8165 | 6.2068 | 6.4145 | 5.5206 | 2.4747 | 4.9261 | |
| 5.3297 | 7.3846 | 2.2714 | 5.2249 | | | | | |
| LRRN4CL | 5.1321 | 7.0121 | 5.7984 | 4.6093 | 3.8725 | 6.9214 | 5.6128 | |
| 6.4262 | 5.5987 | 5.7691 | 7.4402 | 3.0219 | 5.5569 | 4.0436 | 8.7522 | |
| 5.6714 | 3.6548 | 4.8469 | 4.5121 | 4.8664 | 4.1033 | 1.0809 | 6.6035 | |
| 6.2782 | 4.6295 | 3.361 | 4.9959 | 4.4064 | 4.6684 | 2.5857 | 3.7749 | |
| 5.1218 | 4.2355 | 5.7852 | 4.2391 | 7.0332 | 7.856 | 3.2841 | 4.987 | |
| 4.1918 | 3.8179 | 4.5086 | 4.9419 | 3.7195 | 3.9981 | 6.0151 | 5.1369 | |
| 5.8344 | 6.5339 | 6.9696 | 8.7375 | 6.4598 | 6.1808 | 4.8009 | 3.9872 | |
| 5.9673 | 2.6173 | 6.5374 | 7.4284 | 8.5687 | 4.0974 | 2.8572 | 4.5926 | |
| 6.3788 | 6.459 | 1.1076 | 7.0734 | 6.0745 | 8.0798 | 5.5967 | 8.2324 | |
| 5.6539 | 8.7143 | 4.1859 | 8.116 | 6.3107 | 4.8122 | 7.6849 | 6.9956 | |
| 6.7551 | 5.3269 | 3.7214 | 8.786 | 7.2311 | 3.348 | 6.9456 | 4.828 | |
| 5.8263 | 6.6089 | 6.3937 | 6.5259 | 4.3857 | 6.4329 | 7.8323 | 7.5244 | |
| 6.7428 | 3.599 | 4.5285 | 7.004 | 4.6439 | 6.0365 | 4.4325 | 4.2558 | |
| 5.4229 | 5.0032 | 5.4613 | 5.5549 | 4.9176 | 5.8078 | 5.5844 | 6.3948 | |
| 6.4089 | 1.2894 | 6.0832 | 5.0622 | 6.0819 | 4.6126 | 6.9268 | 6.2915 | |
| 4.9384 | 8.1363 | 8.493 | 3.6287 | 2.4344 | 7.237 | 5.5005 | 4.7958 | |
| 5.6716 | 5.2563 | 3.3718 | 8.4891 | 5.8462 | 6.6543 | 4.8617 | 2.4897 | |
| 6.6818 | 6.1711 | 3.4328 | 4.5153 | 1.6272 | 4.6048 | 7.1103 | 6.2261 | |
| 7.219 | 6.3168 | 6.3666 | 6.3369 | 6.584 | 3.7481 | 3.8206 | 3.1654 | |
| 4.081 | 4.4275 | 5.6756 | 1.3669 | 6.0038 | 7.5608 | 6.0269 | 5.5921 | |
| 2.4318 | 5.6825 | 6.8529 | 6.6774 | 7.1485 | 6.0983 | 6.4121 | 4.3164 | |
| 4.3208 | 3.6176 | 5.3576 | 6.8975 | 5.7831 | 4.4061 | 2.8619 | 4.9475 | |
| 6.5448 | 6.5449 | 10.5656 | 8.7348 | 10.5372 | 7.619 | 9.6694 | 9.8064 | |
| 10.5392 | 10.0979 | 10.0569 | 9.1701 | 10.276 | 9.7751 | 10.6286 | 9.0403 | |
| 8.3165 | 10.2896 | 9.4613 | 10.859 | 10.7417 | 9.8808 | 9.9809 | 8.548 | |
| 10.6209 | 10.2552 | | | | | | | |
| C17orf53 | | 8.6389 | 7.079 | 6.1024 | 8.62E+00 | | 7.73E+00 | |
| | 6.5372 | 9.544 | 9.4437 | 8.1983 | 7.5697 | 7.6551 | 7.1963 | |
| 7.2467 | 6.2938 | 8.5155 | 7.7775 | 8.7423 | 7.8829 | 6.2247 | 6.0085 | |
| 8.0166 | 7.9749 | 8.1343 | 5.4241 | 8.1186 | 6.9019 | 7.494 | 8.2787 | |
| 7.6594 | 8.6393 | 8.7154 | 7.0346 | 7.6382 | 7.472 | 5.8087 | 7.3399 | |
| 8.5334 | 8.0929 | 7.6614 | 8.5834 | 8.4488 | 7.5348 | 6.9886 | 6.9565 | |
| 7.4748 | 8.011 | 7.5152 | 7.7046 | 7.2083 | 6.8071 | 7.9533 | 8.2972 | |
| 8.0895 | 8.088 | 7.5703 | 7.1919 | 5.7385 | 7.5202 | 4.2637 | 8.0537 | |
| 8.5202 | 7.192 | 7.8881 | 7.3494 | 6.5099 | 6.8351 | 7.3881 | 5.4511 | |
| 5.3939 | 7.6178 | 7.8342 | 7.8397 | 5.4931 | 7.1753 | 7.6147 | 8.179 | |
| 8.6607 | 8.1726 | 6.6728 | 7.2733 | 8.522 | 6.6031 | 6.4429 | 8.7729 | |

| | | | | | | | |
|---|---|---|---|---|---|---|---|
| 7.5624 | 7.1699 | 7.1136 | 8.3111 | 7.4496 | 7.6691 | 7.785 | 7.7278 |
| 9.005 | 7.13 | 8.2789 | 8.5473 | 6.7592 | 7.807 | 8.831 | 8.2288 |
| 8.9675 | 8.6728 | 7.5289 | 8.0723 | 7.1507 | 8.1038 | 8.3798 | 7.2964 |
| 7.8771 | 7.2621 | 7.501 | 7.7846 | 8.3581 | 7.1446 | 6.9764 | 7.4973 |
| 7.7103 | 7.5256 | 8.4533 | 8.5695 | 7.1415 | 6.8951 | 7.6585 | 5.5847 |
| 7.618 | 7.0038 | 7.4577 | 6.2357 | 7.7442 | 8.2775 | 5.2174 | 7.7379 |
| 6.6012 | 10.1293 | 7.8191 | 7.5051 | 7.338 | 7.508 | 8.8462 | 6.8761 |
| 7.4018 | 6.9513 | 7.2102 | 6.7662 | 7.5925 | 6.5002 | 7.7211 | 8.2908 |
| 8.7963 | 7.6913 | 8.5919 | 7.8417 | 8.9002 | 6.5587 | 7.0234 | 8.3493 |
| 5.5245 | 8.4595 | 7.4582 | 8.5185 | 6.0362 | 7.711 | 7.1042 | 9.2923 |
| 9.7253 | 8.1352 | 8.2674 | 7.8112 | 6.5183 | 7.061 | 7.8598 | 7.6522 |
| 8.0966 | 7.6555 | 7.3089 | 6.8702 | 8.8013 | 4.6986 | 7.2912 | 4.423 |
| 4.4591 | 4.5231 | 4.6495 | 4.857 | 4.6275 | 4.5454 | 5.999 | 4.2693 |
| 4.2021 | 5.5527 | 7.5583 | 4.6491 | 4.7138 | 4.6931 | 4.8639 | 4.5998 |
| 5.4804 | 4.6662 | 6.0941 | 4.5497 | 5.029 | | | |
| DACT3 | 6.581 | 6.2894 | 3.485 | 7.6829 | 3.6664 | 6.4265 | 6.0547 |
| 5.4602 | 5.5674 | 4.6422 | 6.5525 | 4.6563 | 5.486 | 6.2617 | 7.5131 |
| 6.4954 | 3.2664 | 5.766 | 5.0673 | 5.213 | 7.2619 | 7.4634 | 6.4675 |
| 8.0036 | 2.472 | 9.7222 | 7.303 | 3.7514 | 4.5791 | 4.0883 | 4.3024 |
| 5.6664 | 3.5768 | 6.1696 | 4.7522 | 5.9101 | 6.4049 | 4.5032 | 5.8781 |
| 4.6248 | 3.7557 | 9.0127 | 4.9234 | 4.8225 | 4.6583 | 8.1958 | 8.3239 |
| 4.7363 | 6.7192 | 6.8599 | 9.1629 | 6.4194 | 7.7359 | 5.6312 | 5.6784 |
| 5.6113 | 3.8434 | 6.1418 | 7.2299 | 8.7687 | 4.55 | 5.0243 | 4.4163 |
| 5.7578 | 7.7273 | 4.2837 | 6.8095 | 5.9097 | 6.782 | 7.3803 | 6.987 |
| 5.2956 | 7.9863 | 4.9929 | 8.3751 | 7.3593 | 5.8097 | 4.3666 | 7.3557 |
| 8.9896 | 5.5887 | 4.8292 | 8.0438 | 6.9363 | 6.502 | 7.4778 | 5.6372 |
| 6.3837 | 6.8292 | 6.8576 | 7.3221 | 4.7694 | 7.556 | 7.7875 | 7.7215 |
| 6.1476 | 4.2769 | 3.5571 | 7.2622 | 4.9542 | 6.7028 | 7.3728 | 3.4797 |
| 6.8477 | 6.5724 | 5.444 | 10.4922 | 6.971 | 6.2977 | 4.496 | 9.75 |
| 7.6726 | 5.626 | 5.4165 | 2.3575 | 6.2681 | 6.0715 | 6.7561 | 8.1602 |
| 5.6186 | 9.7464 | 9.1587 | 4.1743 | 5.6841 | 5.2708 | 6.7944 | 5.4881 |
| 4.4476 | 5.8842 | 3.7031 | 9.581 | 6.6638 | 5.5118 | 5.5583 | 4.0615 |
| 7.0451 | 5.8107 | 3.685 | 7.1335 | 3.7586 | 5.9661 | 6.2952 | 4.7954 |
| 5.875 | 5.2817 | 6.1909 | 6.5738 | 6.7049 | 4.6411 | 4.3455 | 4.5047 |
| 6.1602 | 5.8504 | 6.2512 | 6.2704 | 7.0942 | 6.7113 | 6.7148 | 4.9283 |
| 4.1097 | 4.5123 | 5.9139 | 7.0283 | 6.4398 | 5.9589 | 5.4858 | 6.3409 |
| 5.7478 | 4.1628 | 8.0712 | 8.7187 | 5.2534 | 5.0585 | 6.5546 | 5.7206 |
| 7.7069 | 6.5449 | 10.4267 | 9.6546 | 10.2302 | 7.0795 | 11.8479 | 9.5562 |
| 9.8441 | 10.651 | 10.7264 | 10.0683 | 11.6062 | 11.0687 | 11.2488 | 9.2107 |
| 8.8906 | 10.4529 | 10.923 | 10.3595 | 8.0716 | 10.7598 | 10.3642 | 9.7828 |
| 11.6194 | 10.1044 | | | | | | |
| CXCL12 | 7.2321 | 9.0174 | 6.3676 | 9.5346 | 6.0998 | 7.6393 | 7.3872 |
| 6.2047 | 6.3888 | 6.2947 | 8.9979 | 6.0481 | 6.339 | 7.1857 | 8.1025 |
| 8.6541 | 7.9529 | 8.0866 | 7.7282 | 6.5661 | 6.6166 | 12.079 | 8.5454 |
| 8.5973 | 5.5229 | 7.0022 | 7.9363 | 7.746 | 7.4454 | 4.7288 | 6.0631 |
| 8.9407 | 5.5377 | 5.6994 | 8.4652 | 9.292 | 7.9346 | 7.1237 | 6.5273 |
| 6.836 | 5.6733 | 7.4283 | 6.1742 | 7.591 | 9.4542 | 10.1407 | 9.6144 |
| 5.3709 | 7.7085 | 9.6003 | 10.5765 | 8.127 | 8.1197 | 7.6705 | 6.1743 |
| 8.1337 | 7.1882 | 6.9013 | 4.6784 | 11.2871 | 9.097 | 7.3741 | 10.314 |
| 10.0795 | 9.9337 | 3.1208 | 9.1594 | 9.3986 | 10.0766 | 9.2264 | 7.8849 |
| 7.2227 | 10.8003 | 8.3277 | 9.4042 | 6.2611 | 9.7608 | 8.1911 | 9.4973 |
| 8.7465 | 8.1566 | 8.7737 | 11.3716 | 8.051 | 9.7811 | 8.5442 | 9.1429 |
| 7.5989 | 8.1982 | 5.1347 | 8.8644 | 8.1357 | 8.604 | 9.6041 | 9.5714 |
| 8.8785 | 7.741 | 7.2356 | 9.0394 | 5.4263 | 9.5203 | 7.9207 | 7.1925 |

| | | | | | | | | |
|---|---|---|---|---|---|---|---|---|
| 6.916 | 8.2885 | 9.3101 | 10.7763 | 7.2446 | 10.0139 | 6.0035 | 8.4755 | |
| 9.6017 | 8.7236 | 4.6523 | 5.6749 | 7.8346 | 7.7432 | 7.9118 | 7.7315 | |
| 8.7843 | 11.444 | 11.4202 | 6.6321 | 6.9353 | 7.8846 | 8.4481 | 6.285 | |
| 6.0668 | 6.5592 | 10.4864 | 12.6354 | 6.6638 | 7.9949 | 8.3658 | 4.6966 | |
| 11.5867 | 6.076 | 4.6576 | 7.8408 | 5.1904 | 7.6769 | 9.0466 | 4.7786 | |
| 5.9269 | 8.1551 | 8.2083 | 8.9127 | 8.2369 | 5.9169 | 6.4011 | 7.0883 | |
| 8.614 | 6.8378 | 7.9011 | 7.732 | 8.7244 | 10.2126 | 7.5653 | 5.8285 | |
| 10.2686 | 8.5839 | 6.4197 | 8.7104 | 7.5584 | 6.1115 | 4.8983 | 10.8833 | |
| 8.5994 | 6.2862 | 8.2667 | 11.6803 | 8.0729 | 6.2041 | 7.6778 | 7.8627 | |
| 9.6744 | 6.6643 | 12.2967 | 11.9826 | 12.4279 | 8.4474 | 15.4146 | 12.4388 | |
| 13.8856 | 13.678 | 14.0118 | 12.5043 | 13.7522 | 13.6901 | 13.6407 | 11.1772 | |
| 10.2574 | 12.9055 | 14.3132 | 12.7209 | 9.0653 | 12.3169 | 13.1712 | 12.94 | |
| 12.8529 | 12.9118 | | | | | | | |
| RACGAP1 | 10.2811 | 9.786 | 8.8205 | 1.04E+01 | | 1.08E+01 | | |
| 10.0437 | 10.5847 | 9.8649 | 10.4017 | 10.1195 | 10.1411 | 10.051 | 9.7109 | |
| 8.5355 | 9.2038 | 9.6802 | 9.9017 | 9.7721 | 8.8786 | 8.9418 | 9.9807 | |
| 11.5001 | 9.229 | 7.8253 | 9.7509 | 11.0699 | 10.1826 | 10.6925 | 8.9556 | |
| 10.47 | 10.4684 | 8.8422 | 10.5439 | 9.6907 | 9.9799 | 10.3671 | 8.9818 | |
| 10.9097 | 8.1712 | 9.8759 | 9.31 | 10.5567 | 8.9878 | 10.2809 | 9.6666 | |
| 9.7017 | 9.6557 | 10.0171 | 9.2023 | 9.255 | 9.3159 | 9.5843 | 10.3258 | |
| 10.5017 | 9.5869 | 9.5346 | 9.816 | 9.8991 | 8.0726 | 9.9935 | 10.063 | |
| 10.0063 | 9.6191 | 9.0871 | 9.5858 | 9.2204 | 9.4548 | 7.3193 | 7.0185 | |
| 9.792 | 9.8438 | 11.0326 | 7.2408 | 10.4575 | 9.0235 | 9.4687 | 10.73 | |
| 9.638 | 9.6148 | 10.6331 | 10.0997 | 9.2341 | 8.1286 | 10.2337 | 10.1365 | |
| 9.6388 | 9.3614 | 9.1955 | 9.8331 | 10.6448 | 9.738 | 10.4779 | 10.9948 | |
| 9.4189 | 9.3997 | 9.9825 | 9.7323 | 9.766 | 8.6621 | 9.8392 | 10.2835 | |
| 10.3128 | 10.4263 | 10.0989 | 10.9304 | 9.3065 | 10.5615 | 9.7734 | 9.9827 | |
| 10.0143 | 9.7589 | 10.693 | 9.4632 | 10.5959 | 9.7855 | 9.3925 | 9.6203 | |
| 9.152 | 8.9957 | 9.8216 | 7.5395 | 9.2849 | 9.755 | 9.153 | 8.5455 | |
| 9.7747 | 10.1829 | 9.1631 | 9.1729 | 9.883 | 8.0238 | 9.1529 | 9.1974 | |
| 9.7025 | 10.1348 | 10.8451 | 10.2516 | 9.9357 | 10.0176 | 9.1275 | 9.7938 | |
| 9.3267 | 8.7674 | 9.7795 | 10.4918 | 8.6087 | 9.883 | 10.2045 | 10.9247 | |
| 10.7654 | 10.807 | 10.651 | 10.3997 | 6.6812 | 10.1825 | 10.7785 | 7.3776 | 9.58 |
| | 9.4304 | 10.9763 | 8.6903 | 10.4451 | 8.5318 | 9.7592 | 9.9127 | |
| 9.3121 | 10.0214 | 10.4341 | 8.7014 | 9.1157 | 9.2387 | 10.0566 | 10.4851 | |
| 10.7951 | 9.6917 | 9.2627 | 9.5707 | 6.7549 | 9.7184 | 6.8915 | 6.5174 | |
| 7.1765 | 6.9854 | 7.2008 | 7.1673 | 7.3919 | 8.4941 | 7.2815 | 6.8139 | |
| 7.5473 | 7.1791 | 7.5355 | 7.5776 | 8.4355 | 7.1794 | 6.1903 | 8.1659 | |
| 7.432 | 7.9941 | 7.5879 | 7.5072 | | | | | |
| SYNE1 | 6.3693 | 8.9582 | 7.8922 | 7.9354 | 9.1339 | 8.8133 | 8.5557 | |
| 6.7175 | 7.4361 | 7.9088 | 8.5022 | 7.1751 | 7.9833 | 9.0898 | 7.5003 | |
| 8.2831 | 5.5753 | 7.5337 | 10.2672 | 8.7442 | 6.039 | 6.7258 | 7.4927 | |
| 7.7299 | 7.9312 | 8.5921 | 8.159 | 6.8766 | 6.6364 | 6.1302 | 7.2712 | |
| 8.4454 | 8.1734 | 5.8996 | 6.5695 | 7.7501 | 8.3329 | 9.4909 | 7.6678 | |
| 5.252 | 7.6982 | 7.2471 | 5.194 | 7.0317 | 8.9809 | 8.551 | 8.7953 | |
| 6.938 | 9.6676 | 10.2918 | 10.5749 | 7.0366 | 9.577 | 7.3628 | 6.574 | |
| 8.539 | 7.1206 | 7.542 | 7.8506 | 9.676 | 7.6516 | 9.219 | 8.5136 | |
| 7.1198 | 9.639 | 4.7437 | 8.9023 | 10.5563 | 8.6651 | 8.5582 | 6.505 | |
| 7.0646 | 11.152 | 9.1407 | 8.3655 | 8.171 | 8.4773 | 6.9176 | 8.4727 | |
| 9.7284 | 8.6807 | 10.5507 | 9.7854 | 8.0928 | 6.8305 | 8.6528 | 10.1044 | |
| 6.8278 | 8.4742 | 7.5566 | 9.1187 | 7.3872 | 7.5443 | 10.1751 | 9.4277 | |
| 7.5968 | 8.5676 | 7.5618 | 6.7326 | 6.0661 | 8.2725 | 9.0019 | 9.3484 | |
| 10.1307 | 7.1762 | 8.5257 | 9.1086 | 7.8814 | 8.9116 | 6.621 | 9.3154 | |
| 8.0419 | 5.9631 | 7.0991 | 6.5288 | 8.6219 | 10.418 | 9.7633 | 7.9149 | |

| | | | | | | | | |
|---|---|---|---|---|---|---|---|---|
| 7.0921 | 8.5063 | 11.1732 | 6.2169 | 6.9525 | 7.6305 | 8.293 | 7.853 | |
| 8.8407 | 7.0661 | 4.5676 | 11.051 | 5.2858 | 9.302 | 8.3437 | 5.4421 | |
| 9.0193 | 7.1517 | 6.8058 | 8.4246 | 5.9926 | 7.8789 | 8.118 | 8.5697 | |
| 7.1768 | 7.5674 | 8.7249 | 9.6668 | 8.0356 | 5.6119 | 5.6919 | 6.9243 | |
| 7.0397 | 5.7953 | 6.3529 | 6.6164 | 8.2781 | 11.8426 | 6.601 | 7.3912 | |
| 7.5565 | 6.8314 | 8.5651 | 8.1981 | 8.6179 | 7.3162 | 6.7424 | 8.0202 | |
| 8.6995 | 7.6486 | 7.9778 | 9.6043 | 7.2007 | 5.6866 | 3.9171 | 8.032 | |
| 9.9476 | 5.7796 | 11.435 | 10.7925 | 11.6344 | 11.3114 | 11.5256 | 11.4221 | |
| 12.1073 | 12.3477 | 12.0072 | 10.8885 | 11.6674 | 11.3679 | 11.6737 | 10.5156 | |
| 11.5374 | 11.7197 | 12.22 | 11.7338 | 12.8623 | 11.4351 | 11.6074 | 10.8648 | |
| 11.7831 | 11.5865 | | | | | | | |
| LEPR | 6.3196 | 6.3912 | 6.5641 | 6.65E+00 | | 7.4572 | 7.0062 | 7.27 |
| | 8.5544 | 7.4858 | 7.8871 | 8.3533 | 7.098 | 6.6758 | 7.0423 | |
| 7.8529 | 7.6888 | 7.8895 | 6.5215 | 6.4532 | 9.0192 | 5.8164 | 5.7804 | |
| 7.8214 | 6.7576 | 7.0245 | 9.8923 | 7.1186 | 5.463 | 6.5637 | 6.5182 | |
| 5.3377 | 7.5972 | 5.0837 | 7.2207 | 5.7388 | 7.5871 | 9.4703 | 6.2868 | |
| 5.2832 | 4.2894 | 7.7663 | 7.091 | 6.655 | 8.02 | 6.9309 | 6.4778 | |
| 7.0078 | 6.5446 | 8.7078 | 8.385 | 10.2985 | 8.3095 | 10.0108 | 5.7346 | |
| 7.3451 | 8.2991 | 7.8017 | 8.5636 | 7.6628 | 7.7837 | 7.0427 | 6.5591 | |
| 6.4738 | 7.4229 | 7.6684 | 6.1116 | 7.1482 | 8.3753 | 8.8565 | 7.2353 | |
| 5.8193 | 7.751 | 10.6796 | 7.0365 | 7.0221 | 8.097 | 7.6058 | 4.5736 | |
| 8.4223 | 6.1099 | 7.8522 | 5.3412 | 7.0344 | 7.1771 | 6.4872 | 6.2965 | |
| 7.4151 | 5.6292 | 7.457 | 7.3419 | 7.4206 | 5.0938 | 6.926 | 10.1961 | |
| 9.8022 | 6.3831 | 6.4349 | 8.2334 | 7.7887 | 6.2957 | 8.5335 | 8.1819 | |
| 6.6908 | 8.1828 | 5.5793 | 7.5445 | 9.2827 | 7.8312 | 9.0923 | 6.6862 | |
| 7.9406 | 6.8792 | 4.5997 | 8.347 | 4.8428 | 6.8013 | 7.3873 | 7.2854 | |
| 5.0704 | 5.4775 | 6.9584 | 10.921 | 5.0152 | 6.7078 | 6.252 | 7.769 | |
| 7.7883 | 7.3479 | 6.7442 | 7.1421 | 10.7444 | 5.6361 | 6.9766 | 7.0036 | |
| 7.4638 | 7.0105 | 7.6912 | 6.9365 | 5.2615 | 6.5779 | 6.2883 | 7.6468 | |
| 7.225 | 7.7946 | 7.6215 | 8.1317 | 8.6891 | 7.2702 | 6.2051 | 6.0423 | |
| 6.5824 | 6.3557 | 5.2458 | 7.4853 | 6.8892 | 7.9076 | 8.5664 | 6.8322 | |
| 7.6772 | 7.3689 | 6.6408 | 6.9742 | 7.2383 | 9.0938 | 6.9765 | 5.3291 | |
| 6.243 | 6.3749 | 7.6889 | 8.944 | 8.0897 | 6.8467 | 5.6866 | 5.8691 | |
| 7.9413 | 7.6601 | 6.1993 | 10.6755 | 8.9006 | 10.1599 | 7.2877 | 11.0701 | |
| 10.9014 | 11.5037 | 11.8877 | 12.1594 | 9.083 | 10.6829 | 11.03 | 11.8263 | |
| 8.9353 | 9.3747 | 12.3857 | 11.4165 | 11.4111 | 10.7551 | 9.3001 | 12.1785 | |
| 9.2692 | 10.3913 | 11.5003 | | | | | | |
| ZEB1 | 5.9789 | 8.0407 | 7.299 | 8.07E+00 | | 7.71E+00 | | |
| 7.9373 | 7.4701 | 6.8596 | 6.8026 | 6.8401 | 7.8042 | 6.4294 | 7.2172 | |
| 7.7111 | 7.2058 | 8.1592 | 5.3728 | 6.3889 | 7.4639 | 8.5507 | 7.7524 | |
| 6.1794 | 7.3858 | 7.8558 | 6.2741 | 11.657 | 7.7741 | 5.6071 | 6.8217 | |
| 5.458 | 6.0761 | 7.5547 | 6.7229 | 7.0695 | 7.9785 | 8.0213 | 9.0819 | |
| 6.6543 | 6.9837 | 5.8079 | 7.2696 | 10.1788 | 5.9104 | 7.2686 | 7.4456 | |
| 8.5663 | 9.0492 | 7.8632 | 9.065 | 8.2639 | 10.3604 | 7.0695 | 9.009 | 7.04 |
| | 5.5281 | 8.3806 | 5.6527 | 7.3143 | 8.0818 | 9.3578 | 5.9857 | |
| 7.1474 | 7.8423 | 6.0765 | 10.0949 | 5.3057 | 8.6821 | 7.8779 | 7.6043 | |
| 8.5583 | 7.1373 | 7.4889 | 10.5248 | 7.7569 | 7.4321 | 7.6739 | 6.8286 | |
| 6.4939 | 8.1329 | 8.9387 | 8.219 | 5.266 | 9.4193 | 8.5773 | 8.2547 | |
| 8.0839 | 9.1133 | 4.7654 | 8.1083 | 7.0409 | 9.9919 | 6.912 | 7.7243 | |
| 10.1518 | 8.6314 | 7.3119 | 6.9358 | 5.4969 | 8.1093 | 6.6153 | 8.8955 | |
| 9.1629 | 6.5202 | 8.336 | 7.2711 | 8.7165 | 10.3056 | 8.3602 | 8.5746 | |
| 6.7245 | 8.0586 | 8.0106 | 4.0148 | 6.9511 | 6.0912 | 7.2151 | 7.9303 | |
| 8.1413 | 7.2102 | 5.2667 | 9.3722 | 10.878 | 5.6466 | 6.5849 | 7.3722 | |
| 8.3546 | 8.0056 | 7.0942 | 6.4323 | 5.3127 | 10.7853 | 5.4233 | 9.1026 | 7.2 |

| | | | | | | | |
|---|---|---|---|---|---|---|---|
| | 6.6746 | 8.9106 | 8.1818 | 5.4566 | 8.9429 | 5.016 | 8.7417 |
| 7.8685 | 6.1887 | 7.5726 | 8.9068 | 7.4447 | 8.4011 | 8.1138 | 5.9177 |
| 6.1836 | 6.9251 | 6.9523 | 6.4225 | 6.8818 | 6.6005 | 8.3061 | 9.1825 |
| 6.8904 | 7.1001 | 6.028 | 7.0848 | 7.7036 | 8.0756 | 8.4025 | 6.6807 |
| 5.9159 | 7.7284 | 8.2948 | 6.6456 | 7.6266 | 8.8278 | 7.7565 | 4.872 |
| 5.5539 | 8.1721 | 8.9753 | 6.1126 | 11.2024 | 10.5132 | 11.4218 | 7.4338 |
| 11.5979 | 11.6934 | 12.1893 | 12.4058 | 12.1904 | 10.5844 | 11.3212 | 12.0025 |
| 11.3433 | 12.0033 | 9.2018 | 11.8198 | 12.0223 | 11.4613 | 9.4229 | 9.907 |
| 11.4763 | 10.8738 | 11.5356 | 11.1464 | | | | |
| GRID1 | 3.7152 | 3.4337 | 0.8605 | 2.66E+00 | | 3.3816 | 2.8873 |
| 4.9526 | 4.4119 | 2.7843 | 2.6308 | 4.5606 | 3.1368 | 2.0573 | 1.4442 |
| 4.9122 | 4.5136 | 3.0242 | 4.9749 | 3.4433 | 2.1767 | 6.1598 | 1.3145 |
| 3.4974 | 4.3473 | 2.5978 | 7.9095 | 2.8443 | 1.6552 | 3.7652 | 1.5856 |
| 3.7749 | 4.1626 | 2.8159 | 1.7115 | 4.4144 | 4.4645 | 4.8891 | 2.8667 |
| 4.3198 | 0 | 2.9168 | 3.0242 | 2.5098 | 3.1287 | 3.5483 | 4.0805 |
| 5.2274 | 3.3257 | 4.4941 | 4.0018 | 6.8788 | 3.962 | 5.4785 | 3.5075 |
| 2.9375 | 3.7482 | 1.6109 | 3.0972 | 4.6784 | 6.0785 | 2.8693 | 4.8296 |
| 3.2212 | 5.1059 | 5.6191 | 2.0284 | 4.9767 | 4.3291 | 4.4071 | 4.8154 |
| 4.0331 | 4.1251 | 6.0105 | 2.7931 | 4.4093 | 4.0028 | 5.5564 | 3.4699 |
| 3.3596 | 8.0097 | 3.1679 | 2.6379 | 6.5973 | 5.292 | 5.0216 | 4.4132 |
| 3.5463 | 2.2933 | 3.7426 | 2.0509 | 5.2577 | 3.0982 | 3.7224 | 4.9035 |
| 5.5722 | 4.6225 | 1.9975 | 3.0941 | 4.7934 | 5.319 | 4.9122 | 3.1571 |
| 2.8443 | 3.6861 | 3.3497 | 3.7455 | 5.1651 | 3.654 | 5.2723 | 3.1213 |
| 3.9774 | 2.6587 | 3.0394 | 3.9176 | 2.6562 | 3.3693 | 3.5049 | 5.4086 |
| 2.0117 | 0.6413 | 6.2786 | 5.162 | 2.2071 | 2.7811 | 4.2656 | 4.6807 |
| 3.6037 | 3.4742 | 1.7997 | 1.8766 | 6.7029 | 2.5542 | 4.0822 | 4.2516 |
| 1.7261 | 4.3012 | 3.3048 | 2.0337 | 4.8011 | 1.6272 | 4.0337 | 3.6924 |
| 2.6967 | 3.5098 | 3.2849 | 3.283 | 3.6487 | 4.1521 | 2.3633 | 2.4089 |
| 4.4177 | 3.2697 | 2.7948 | 4.4516 | 3.9272 | 3.8224 | 5.4719 | 3.3748 |
| 4.1792 | 6.0165 | 4.4021 | 3.4586 | 4.0158 | 5.0112 | 2.9892 | 1.725 |
| 2.7703 | 3.6795 | 2.7867 | 3.7058 | 4.5232 | 3.5522 | 2.3335 | 4.1891 |
| 3.3454 | 4.4374 | 2.0355 | 7.1025 | 6.3101 | 7.41 | 5.4805 | 7.5488 |
| 7.0028 | 7.9092 | 7.1841 | 6.1731 | 7.1746 | 8.33 | 8.4917 | 7.4856 |
| 7.1049 | 7.0854 | 7.5776 | 7.7147 | 8.3536 | 3.9623 | 8.5522 | 7.8727 |
| 6.7792 | 8.6872 | 6.8513 | | | | | |
| E2F8 | 9.0568 | 7.5302 | 6.2017 | 8.23E+00 | | 9.00E+00 | |
| 7.8512 | 8.4663 | 7.674 | 8.7466 | 9.0476 | 8.2121 | 6.6944 | 8.2137 |
| 5.718 | 6.1623 | 7.6653 | 6.1165 | 6.8955 | 6.9814 | 6.5458 | 6.2615 |
| 8.6675 | 6.5648 | 3.7973 | 7.9898 | 7.5759 | 8.0416 | 8.3956 | 6.7223 |
| 9.4614 | 7.8303 | 5.8302 | 8.7672 | 7.0695 | 7.5646 | 7.4647 | 6.2701 |
| 9.1756 | 5.6375 | 5.3683 | 7.354 | 8.1438 | 5.1148 | 7.7893 | 6.3121 |
| 7.1496 | 6.7153 | 7.6235 | 6.6653 | 6.2734 | 7.1523 | 8.4614 | 8.3278 |
| 7.3628 | 6.8423 | 8.3193 | 6.5852 | 6.7549 | 5.387 | 6.6138 | 6.9294 |
| 8.471 | 5.6971 | 6.0163 | 7.006 | 7.6715 | 6.3518 | 5.8427 | 3.8558 |
| 7.6977 | 7.0141 | 8.7712 | 5.451 | 7.6797 | 6.1849 | 6.8632 | 8.4699 |
| 6.5191 | 6.9755 | 6.8736 | 8.7967 | 6.2865 | 5.0147 | 8.0103 | 6.8902 |
| 7.228 | 6.9169 | 6.2099 | 7.9559 | 9.1985 | 7.914 | 8.0538 | 9.3355 |
| 7.5666 | 7.0892 | 7.3345 | 7.7439 | 7.8945 | 5.5901 | 5.2095 | 8.169 |
| 7.8096 | 7.6701 | 8.1292 | 8.8318 | 7.1035 | 7.0999 | 7.5227 | 7.6787 |
| 7.5439 | 8.1404 | 7.6801 | 6.8008 | 7.4823 | 8.2585 | 7.4049 | 8.2046 |
| 6.1216 | 2.0117 | 6.7758 | 3.0835 | 7.4129 | 7.3738 | 5.3099 | 5.3932 |
| 7.8232 | 8.3749 | 5.9186 | 7.3944 | 6.0689 | 4.7216 | 5.1339 | 6.4959 |
| 7.2181 | 7.8911 | 8.3018 | 7.6729 | 7.3252 | 7.1382 | 6.9141 | 7.101 |
| 6.2202 | 5.4978 | 7.3673 | 7.58 | 4.4181 | 7.4036 | 7.6795 | 8.6468 |

| | | | | | | | | |
|---|---|---|---|---|---|---|---|---|
| 7.2854 | 7.6299 | 6.9576 | 7.3534 | 1.2582 | 8.1032 | 8.5354 | 5.8608 | |
| 6.8083 | 6.4714 | 8.9819 | 5.4109 | 7.8918 | 5.4355 | 6.5811 | 7.2932 | |
| 7.3638 | 6.9343 | 8.6921 | 7.7402 | 7.3135 | 7.9163 | 7.3541 | 6.9092 | |
| 6.7702 | 7.8237 | 7.414 | 5.8433 | 2.1912 | 7.5277 | 3.8042 | 5.6957 | |
| 2.9167 | 3.2379 | 1.6194 | 2.9592 | 1.9018 | 5.4784 | 2.5817 | 1.6671 | |
| 2.7033 | 4.3789 | 5.679 | 2.6412 | 6.5374 | 1.1757 | 0.5418 | 3.3488 | |
| 1.7618 | 6.4805 | 1.066 | 2.2304 | | | | | |
| RUNX1T1 | 0.5526 | 2.9525 | 2.3445 | 3.4218 | 2.4722 | 2.5916 | 1.7391 | |
| 1.786 | 1.0281 | 1.3775 | 3.6614 | 1.0485 | 2.5775 | 0 | 3.42 | |
| 2.5841 | 0.3755 | 1.6493 | 3.8167 | 2.3107 | 2.0473 | 0.4561 | 2.2025 | 0 |
| | 1.5953 | 5.0356 | 2.8443 | 0.8951 | 1.101 | 1.0004 | 1.1431 | |
| 3.0082 | 1.2859 | 0.541 | 5.5089 | 3.374 | 3.1865 | 1.4585 | 2.4638 | |
| 1.8316 | 1.4786 | 5.0017 | 0 | 2.8621 | 0.7768 | 3.8151 | 3.3917 | |
| 2.3993 | 3.888 | 3.8431 | 5.3126 | 2.2896 | 2.5691 | 1.7473 | 1.3432 | |
| 4.7108 | 0 | 0.8444 | 1.9829 | 5.9098 | 1.0365 | 2.3391 | 1.511 | |
| 1.7486 | 4.6372 | 0 | 4.4967 | 5.2242 | 1.5048 | 4.2955 | 0.9714 | |
| 3.0489 | 5.5137 | 1.9875 | 1.6785 | 1.6542 | 1.4887 | 1.3738 | 2.4361 | |
| 5.8751 | 3.5089 | 3.1434 | 3.0654 | 3.3984 | 1.8574 | 3.0342 | 2.9074 | 0 |
| | 3.5526 | 2.5704 | 4.027 | 2.1962 | 3.0433 | 5.8446 | 3.8776 | |
| 1.5904 | 2.8045 | 0.6215 | 1.3631 | 1 | 3.2573 | 4.934 | 1.217 | |
| 2.997 | 1.3703 | 3.3113 | 6.9215 | 2.5942 | 4.7776 | 2.9876 | 2.67 | |
| 1.0447 | 0 | 0.3935 | 0.6684 | 2.7225 | 2.362 | 2.2542 | 3.4404 | |
| 0.6413 | 6.0536 | 7.5161 | 2.6248 | 1.5646 | 0 | 3.0536 | 2.8723 | |
| 2.192 | 0.9947 | 0 | 7.3254 | 0 | 4.4257 | 2.8106 | 0.5476 | |
| 4.8747 | 3.3489 | 1.0225 | 0.5416 | 0 | 2.9807 | 4.3136 | 0.4033 | |
| 3.4637 | 4.8739 | 2.9165 | 5.1549 | 2.3144 | 0 | 1.6727 | 1.2195 | |
| 2.7896 | 1.6598 | 2.7155 | 2.056 | 4.804 | 5.0853 | 1.2666 | 1.7883 | |
| 0.5257 | 0.8792 | 2.1147 | 2.5553 | 3.8962 | 1.1775 | 0.6567 | 1.5562 | |
| 3.3015 | 0.5556 | 0.5674 | 4.815 | 3.5005 | 1.5946 | 0.6064 | 3.3454 | |
| 4.7851 | 0 | 6.4657 | 7.6329 | 6.7448 | 3.8887 | 7.6664 | 6.1797 | |
| 7.0622 | 7.3166 | 7.2521 | 7.6051 | 7.3951 | 7.4427 | 7.7972 | 5.9169 | |
| 4.8675 | 7.4616 | 9.5812 | 7.4387 | 5.7878 | 4.9041 | 7.217 | 7.3656 | |
| 6.5371 | 6.9158 | | | | | | | |
| RECK | 5.8091 | 6.208 | 5.9865 | 5.7666 | 7.2671 | 6.7109 | 7.3184 | |
| 6.3785 | 6.032 | 6.777 | 7.0598 | 5.7374 | 4.8127 | 5.4029 | 7.3173 | |
| 6.6366 | 4.5422 | 5.9011 | 5.7162 | 6.1583 | 7.9842 | 7.5258 | 5.8739 | |
| 6.1854 | 5.5542 | 9.1245 | 6.1085 | 4.572 | 5.7359 | 6.5323 | 4.4292 | |
| 6.7933 | 5.8499 | 4.7756 | 5.2676 | 5.9241 | 6.3099 | 6.2379 | 6.2814 | |
| 5.5566 | 6.3365 | 9.5997 | 4.1871 | 6.1589 | 7.0702 | 7.282 | 8.1611 | |
| 6.1656 | 6.3312 | 7.3697 | 8.207 | 6.4357 | 6.982 | 4.619 | 5.8926 | |
| 6.3493 | 5.9806 | 6.8475 | 6.5319 | 8.2533 | 6.4577 | 6.4149 | 6.428 | |
| 6.309 | 7.5685 | 4.4197 | 7.2258 | 6.0149 | 6.4693 | 7.371 | 5.4353 | |
| 7.2011 | 8.6599 | 6.6531 | 6.5862 | 6.883 | 7.6053 | 6.0499 | 7.2551 | |
| 7.6219 | 6.9355 | 7.0496 | 7.5944 | 7.0681 | 7.1252 | 7.28 | 7.2563 | |
| 3.6027 | 5.9078 | 6.4467 | 6.851 | 6.5385 | 7.8606 | 7.8011 | 7.7183 | |
| 6.0304 | 4.826 | 6.0122 | 6.7952 | 5.2095 | 7.7136 | 6.5372 | 6.0616 | |
| 6.878 | 5.9062 | 5.9398 | 9.4537 | 6.7725 | 6.9476 | 4.5205 | 7.111 | |
| 7.8525 | 5.5613 | 5.6774 | 6.0275 | 6.0415 | 6.2652 | 6.1216 | 6.9956 | |
| 4.2831 | 7.2517 | 8.6341 | 6.3044 | 6.3791 | 6.4901 | 6.1086 | 5.7793 | |
| 6.3767 | 5.7968 | 4.8936 | 8.9645 | 6.4869 | 6.7305 | 5.824 | 4.8196 | |
| 7.629 | 7.6489 | 5.3103 | 6.5882 | 5.3467 | 5.0595 | 6.9338 | 5.4978 | |
| 5.5614 | 6.5269 | 6.9626 | 7.2389 | 6.8389 | 6.1121 | 6.4501 | 8.6911 | |
| 6.9093 | 6.1092 | 4.7787 | 6.2778 | 7.2872 | 7.0649 | 4.7679 | 5.8745 | |
| 6.0741 | 5.5723 | 7.0543 | 7.4057 | 7.2554 | 6.285 | 5.5582 | 6.1372 | |

| | | | | | | | |
|---|---|---|---|---|---|---|---|
| 6.053 | 6.1592 | 7.4589 | 7.3445 | 4.7549 | 5.6866 | 8.2456 | 5.5491 |
| 7.1441 | 5.3461 | 9.2953 | 8.1147 | 9.3792 | 6.2081 | 9.8092 | 9.6659 |
| 10.769 | 10.5604 | 10.6276 | 8.5604 | 9.9785 | 9.7388 | 9.6764 | 7.9613 |
| 7.9723 | 10.0726 | 10.0386 | 9.4525 | 9.2666 | 8.8921 | 9.383 | 8.7104 |
| 9.696 | 9.3957 | | | | | | |
| HAND2 | 5.575 | 6.3118 | 6.0141 | 5.4248 | 3.1372 | 6.0322 | 6.3448 |
| 6.6638 | 4.7723 | 4.2763 | 8.8467 | 2.3988 | 5.0671 | 3.2639 | 8.7772 |
| 7.0166 | 3.0242 | 4.6765 | 5.603 | 4.2844 | 3.7016 | 3.73 | 8.4166 |
| 7.1673 | 3.1011 | 11.4182 | 5.4527 | 2.8112 | 4.4839 | 2.0007 | 3.9544 |
| 5.1411 | 3.741 | 4.7514 | 5.8792 | 6.9739 | 7.0117 | 2.9342 | 5.1053 |
| 4.3808 | 2.7982 | 5.1684 | 4.6133 | 4.4119 | 4.1214 | 5.7361 | 6.2944 |
| 4.3891 | 6.2498 | 8.3321 | 8.7639 | 7.2218 | 5.9119 | 4.6465 | 4.892 |
| 6.8431 | 3.1217 | 5.5589 | 6.7455 | 8.689 | 3.9417 | 4.1077 | 5.4447 |
| 6.7654 | 7.7696 | 0 | 10.0026 | 7.6287 | 8.2864 | 5.9841 | 6.3858 |
| 5.187 | 8.5421 | 4.7913 | 8.6045 | 4.8267 | 4.3517 | 4.168 | 7.5718 |
| 7.4399 | 6.3797 | 3.403 | 9.1987 | 7.1931 | 2.7408 | 6.2775 | 5.4506 |
| 4.2776 | 6.6686 | 5.3722 | 5.9436 | 4.811 | 8.0979 | 8.6176 | 8.5169 |
| 7.4427 | 3.6974 | 4.5943 | 7.3895 | 4.1699 | 4.6174 | 5.2664 | 5.268 |
| 8.2416 | 4.7548 | 5.8375 | 8.8078 | 5.1192 | 7.8197 | 6.0546 | 6.5611 |
| 6.064 | 0 | 4.9445 | 3.2094 | 6.1211 | 5.334 | 6.0078 | 5.9811 |
| 4.9118 | 9.5191 | 9.9322 | 6.0011 | 3.6157 | 5.7394 | 6.4013 | 3.9195 |
| 4.597 | 4.8225 | 1.8766 | 10.309 | 5.3901 | 6.1615 | 5.9226 | 2.0813 |
| 6.4384 | 5.3832 | 4.3278 | 5.3447 | 1.0317 | 4.9149 | 8.32 | 4.7443 |
| 7.0143 | 6.5399 | 6.0395 | 7.5366 | 6.5639 | 3.3522 | 3.6634 | 3.9648 |
| 1.4956 | 5.4481 | 6.0231 | 2.7911 | 6.9179 | 8.4792 | 6.006 | 4.0833 |
| 6.6738 | 6.1933 | 6.3741 | 6.1481 | 7.0493 | 6.0715 | 6.0474 | 5.8278 |
| 4.9323 | 2.6247 | 5.1962 | 7.2758 | 5.0706 | 5.6289 | 7.2329 | 7.1884 |
| 6.1328 | 5.1585 | 11.0976 | 10.6642 | 11.0396 | 7.732 | 12.0657 | 9.4649 |
| 10.4597 | 10.7394 | 9.811 | 11.4374 | 9.8843 | 11.1144 | 11.6469 | 12.8028 |
| 8.9229 | 10.7607 | 11.0058 | 11.5734 | 10.0391 | 10.2464 | 11.4476 | 11.4636 |
| 10.1897 | 11.2484 | | | | | | |
| C7orf58 | 3.907 | 6.8453 | 5.6185 | 5.50E+00 | | 5.05E+00 | |
| 5.9278 | 5.4161 | 3.8014 | 4.6582 | 4.0163 | 6.8638 | 2.3988 | 5.6891 |
| 6.7278 | 5.779 | 6.9007 | 2.368 | 5.3218 | 6.2015 | 5.7141 | 4.9914 |
| 3.3107 | 6.1601 | 4.1647 | 3.0135 | 8.8175 | 5.0995 | 4.9181 | 3.8818 |
| 1.3224 | 3.4947 | 5 | 3.3101 | 3.4608 | 6.4239 | 6.8694 | 6.4413 |
| 4.6907 | 5.3486 | 4.0317 | 3.6225 | 6.4187 | 3.7983 | 4.8412 | 5.6294 |
| 6.1366 | 7.6658 | 6.2491 | 7.4507 | 8.0572 | 8.6951 | 5.4198 | 6.8799 |
| 5.7747 | 2.6144 | 7.2072 | 4.124 | 5.6645 | 6.9257 | 7.8949 | 4.5714 |
| 4.8787 | 6.1559 | 5.1546 | 8.0305 | 1.1076 | 6.716 | 7.407 | 6.782 |
| 6.2893 | 5.8973 | 4.7174 | 9.2734 | 5.0195 | 6.1041 | 5.3093 | 3.5653 |
| 5.1916 | 6.9552 | 6.0483 | 6.6445 | 4.0594 | 8.1159 | 6.2324 | 4.4586 |
| 6.2617 | 5.9614 | 2.4487 | 6.621 | 3.2529 | 6.5466 | 5.0892 | 5.6045 |
| 8.8445 | 7.8612 | 6.3118 | 4.3392 | 3.0941 | 2.0513 | 3.3219 | 6.7947 |
| 4.2443 | 4.1875 | 5.3507 | 3.5966 | 5.8767 | 7.5433 | 6.9652 | 6.7808 |
| 5.6633 | 6.5474 | 5.0626 | 0 | 4.1133 | 4.5112 | 5.9465 | 6.1296 |
| 6.284 | 4.5695 | 4.324 | 5.7867 | 9.6076 | 4.1743 | 4.9476 | 5.7698 |
| 6.8179 | 6.8146 | 5.7212 | 4.3473 | 0.7381 | 9.2959 | 2.7757 | 7.7696 |
| 5.3613 | 4.7221 | 5.6405 | 6.0891 | 3.5644 | 6.9701 | 2.9615 | 5.7837 |
| 6.9397 | 4.0982 | 6.568 | 7.2615 | 5.1962 | 7.865 | 5.0736 | 3.5353 |
| 3.5778 | 3.7911 | 3.5752 | 4.3146 | 5.4182 | 3.8892 | 6.1607 | 8.5379 |
| 4.1213 | 3.166 | 3.7342 | 6.6802 | 6.3028 | 7.0403 | 4.4153 | 4.0681 |
| 3.3538 | 5.4334 | 6.2009 | 2.7306 | 5.5179 | 6.7018 | 6.2887 | 2.8198 |
| 1.8529 | 6.2883 | 7.6504 | 3.6039 | 9.6292 | 8.5012 | 9.3197 | 7.0558 |

| | | | | | | | |
|---|---|---|---|---|---|---|---|
| 9.9129 | 10.0268 | 11.152 | 11.1971 | 11.4136 | 9.2893 | 10.6735 | 10.6332 |
| 10.8484 | 7.5431 | 8.1675 | 10.7396 | 10.6371 | 10.7871 | 11.642 | 8.6822 |
| 10.2846 | 9.524 | 11.0701 | 10.0386 | | | | |
| CENPE | 8.4801 | 9.3497 | 7.9457 | 9.1578 | 9.7136 | 8.4689 | 8.5853 |
| 8.5315 | 9.8082 | 9.3927 | 8.6853 | 8.9666 | 8.4142 | 7.316 | 6.5167 |
| 8.5426 | 8.6428 | 8.3215 | 7.1277 | 8.0139 | 7.8005 | 10.0023 | 7.4886 |
| 6.2013 | 8.7836 | 10.1305 | 9.0505 | 9.4353 | 7.3541 | 9.2512 | 8.8424 |
| 7.1003 | 9.2976 | 9.4101 | 9.7901 | 9.1371 | 9.0244 | 9.066 | 7.9985 |
| 7.2838 | 8.5301 | 10.0237 | 6.102 | 8.9965 | 8.6299 | 7.5329 | 7.271 |
| 9.0633 | 8.0675 | 7.1286 | 9.1525 | 8.6088 | 9.306 | 8.9639 | 8.2711 |
| 8.6593 | 6.387 | 8.9007 | 5.2256 | 8.9934 | 8.1802 | 9.0778 | 6.976 |
| 6.7861 | 8.1337 | 7.5284 | 8.3999 | 5.6859 | 4.9551 | 8.9809 | 8.0192 |
| 9.9071 | 5.8897 | 9.0788 | 7.2267 | 8.3596 | 8.9274 | 8.0964 | 7.375 |
| 9.8283 | 9.5611 | 7.8179 | 7.7174 | 9.1879 | 7.9921 | 7.4077 | 9.5057 |
| 8.2907 | 8.5057 | 9.0413 | 9.5266 | 9.0576 | 9.9523 | 8.7762 | 8.9502 |
| 7.6375 | 8.5595 | 8.5309 | 6.95 | 6.8948 | 10.0668 | 9.2143 | 8.4632 |
| 8.5452 | 9.2759 | 9.1271 | 9.0071 | 7.8659 | 8.9795 | 8.0675 | 9.1043 |
| 8.7549 | 6.136 | 8.5416 | 9.3307 | 7.8725 | 9.9682 | 7.6768 | 6.1447 |
| 8.5631 | 4.5503 | 8.2214 | 9.9945 | 7.5582 | 6.7102 | 8.9226 | 9.2255 |
| 7.6714 | 7.7172 | 7.8463 | 6.5257 | 5.4873 | 7.8487 | 9.0179 | 8.9432 |
| 9.2692 | 8.8458 | 8.4174 | 9.1456 | 7.068 | 8.3325 | 8.1983 | 6.9863 |
| 7.8609 | 8.9402 | 8.1015 | 9.3604 | 8.3948 | 9.772 | 9.3226 | 10.1317 |
| 8.0392 | 8.6805 | 4.7036 | 8.9405 | 9.8674 | 6.2102 | 7.8843 | 8.1888 |
| 9.3113 | 7.2285 | 9.0874 | 8.1075 | 9.1204 | 7.9451 | 8.2517 | 9.3252 |
| 9.5043 | 6.9497 | 7.9722 | 8.9174 | 8.4603 | 8.3228 | 8.3806 | 8.6716 |
| 8.2085 | 7.0758 | 4.2352 | 8.841 | 5.4561 | 3.2986 | 4.6086 | 4.4781 |
| 4.5728 | 5.3659 | 4.9801 | 7.7214 | 5.5371 | 4.4376 | 4.0548 | 5.4565 |
| 4.4264 | 5.1216 | 6.8483 | 4.4857 | 4.7294 | 5.5386 | 4.261 | 7.2707 |
| 4.6473 | 4.3954 | | | | | | |
| MMRN2 | 7.5628 | 7.6539 | 7.6204 | 9.2391 | 8.9883 | 6.8828 | 8.3262 |
| 7.8679 | 8.2567 | 7.5817 | 8.7892 | 8.0818 | 7.7245 | 8.1635 | 7.1467 |
| 7.4874 | 7.4628 | 9.3229 | 7.6334 | 8.6744 | 7.6398 | 7.566 | 9.0833 |
| 8.4946 | 7.6078 | 7.1099 | 8.2247 | 7.6749 | 7.9607 | 7.5362 | 7.2542 |
| 9.4355 | 7.8723 | 7.3119 | 7.0536 | 9.4965 | 7.822 | 7.2616 | 6.9837 |
| 7.9798 | 9.3566 | 8.0557 | 7.3312 | 7.8226 | 9.0329 | 8.747 | 9.9121 |
| 7.3403 | 8.2823 | 9.11 | 10.2944 | 7.2312 | 8.4634 | 7.3462 | 6.5506 |
| 8.5653 | 7.9028 | 8.0155 | 7.6354 | 9.8207 | 7.6863 | 8.1383 | 8.2009 |
| 8.6506 | 9.3015 | 8.672 | 9.0754 | 8.7152 | 9.3438 | 8.6239 | 8.3811 |
| 7.6391 | 10.7712 | 9.1286 | 8.624 | 7.6373 | 7.8925 | 7.6396 | 8.2997 |
| 9.2073 | 8.4096 | 7.5859 | 8.2265 | 7.2681 | 8.9193 | 8.4942 | 7.9868 |
| 7.3653 | 8.7997 | 7.1453 | 8.7642 | 7.1651 | 8.1472 | 8.6875 | 8.6041 |
| 8.0498 | 7.4337 | 6.8203 | 8.4579 | 7.585 | 8.0964 | 8.2282 | 7.2647 |
| 8.0591 | 8.0676 | 9.2714 | 8.4549 | 7.828 | 8.4754 | 10.4065 | 8.3973 |
| 7.5592 | 9.6263 | 7.7003 | 6.5471 | 8.3123 | 8.0739 | 7.6874 | 7.7969 |
| 7.8864 | 9.4629 | 11.1482 | 6.547 | 8.5373 | 7.5969 | 8.5709 | 7.4759 |
| 7.8494 | 7.4197 | 6.7353 | 11.1155 | 7.6705 | 7.7049 | 8.7164 | 7.4855 |
| 7.2966 | 7.4099 | 7.9604 | 7.546 | 8.0988 | 7.8951 | 7.1411 | 7.0889 |
| 7.8363 | 7.9637 | 9.3318 | 9.3952 | 8.408 | 7.1016 | 7.8548 | 8.7382 |
| 7.6059 | 7.9684 | 9.8297 | 8.2822 | 8.1488 | 8.8882 | 7.1926 | 8.1077 |
| 7.0728 | 8.2637 | 6.7315 | 7.9232 | 9.3646 | 8.1735 | 6.8327 | 7.9139 |
| 8.4525 | 8.536 | 9.3858 | 8.7888 | 7.6132 | 8.7793 | 6.2325 | 7.9636 |
| 8.3736 | 6.8541 | 10.707 | 9.7331 | 10.4724 | 8.0947 | 11.0716 | 11.1132 |
| 11.9233 | 12.0029 | 12.5091 | 9.3839 | 11.8145 | 11.9055 | 10.9305 | 9.0421 |
| 9.1232 | 11.5065 | 11.2269 | 10.5625 | 8.2529 | 11.107 | 11.7642 | 9.5528 |

| | | | | | | | | |
|---|---|---|---|---|---|---|---|---|
| 11.3729 | 10.3399 | | | | | | | |
| TIMP3 | 9.2494 | 10.7133 | 8.367 | 1.01E+01 | | 8.94E+00 | | |
| 9.6247 | 11.6809 | 9.5713 | 9.0676 | 9.2961 | 10.993 | 8.591 | 8.5085 | |
| 10.0492 | 10.5285 | 8.9153 | 9.3705 | 11.012 | 10.0365 | 9.8863 | 11.9445 | |
| 10.3235 | 9.5295 | 11.286 | 8.3031 | 11.64 | 9.7723 | 8.8406 | 10.1403 | |
| 9.3363 | 9.9275 | 11.9371 | 9.1994 | 8.8699 | 8.4523 | 8.95 | 9.0259 | |
| 9.327 | 7.92 | 8.1808 | 10.2359 | 14.4282 | 8.6694 | 10.567 | 9.9138 | |
| 12.008 | 10.9686 | 8.5521 | 12.5234 | 10.2612 | 12.8511 | 10.0939 | 10.6609 | |
| 8.1444 | 8.9791 | 9.4627 | 7.8935 | 9.485 | 9.887 | 11.8063 | 8.8099 | |
| 8.9812 | 9.4231 | 11.4926 | 11.6173 | 10.1957 | 13.6525 | 9.7429 | 11.4871 | |
| 10.8668 | 9.3934 | 8.8554 | 13.6884 | 9.4999 | 10.8991 | 12.1327 | 10.1558 | |
| 9.0234 | 10.9815 | 12.592 | 10.4724 | 8.8894 | 10.8373 | 10.1628 | 11.823 | |
| 11.1058 | 10.2015 | 10.4461 | 10.4007 | 10.2979 | 10.3594 | 9.3555 | 12.1243 | |
| 10.962 | 12.3574 | 9.3199 | 8.6312 | 7.574 | 11.8144 | 9.5098 | 12.9695 | |
| 11.0437 | 9.5682 | 11.6739 | 10.094 | 9.7261 | 12.6453 | 10.0541 | 11.1994 | |
| 9.1775 | 12.609 | 10.716 | 11.7302 | 10.6116 | 7.5621 | 9.164 | 9.1132 | |
| 9.1327 | 11.0217 | 9.9479 | 11.278 | 13.5412 | 7.7199 | 9.6853 | 8.3851 | |
| 10.6966 | 8.4164 | 8.6315 | 10.4322 | 7.9706 | 13.4443 | 8.9517 | 10.2714 | |
| 10.6042 | 9.4483 | 13.8196 | 9.6172 | 8.8455 | 8.9077 | 9.0851 | 8.9901 | 9.34 |
| | 7.6554 | 8.2332 | 9.6952 | 11.0481 | 11.2333 | 9.6234 | 8.2241 | |
| 8.872 | 10.55 | 9.7466 | 9.374 | 9.856 | 10.7177 | 10.6079 | 11.8413 | |
| 9.333 | 10.7301 | 11.1493 | 9.2848 | 11.4501 | 10.2462 | 10.3559 | 10.0581 | |
| 7.9693 | 12.7724 | 11.3165 | 9.1546 | 10.7587 | 11.8889 | 8.5787 | 8.9002 | |
| 9.3089 | 9.5757 | 10.6776 | 8.9334 | 14.2437 | 12.0906 | 14.1341 | 10.6913 | |
| 14.4375 | 14.4356 | 15.1855 | 15.274 | 15.1776 | 12.7489 | 14.6094 | 14.7353 | |
| 14.5779 | 13.1858 | 12.0625 | 14.9723 | 14.4188 | 14.6221 | 11.8928 | 14.0416 | |
| 14.1793 | 12.4937 | 15.0868 | 14.0849 | | | | | |
| CKAP2L | 7.5805 | 6.8063 | 6.005 | 7.53E+00 | | 8.74E+00 | | |
| 8.1283 | 8.0319 | 7.5258 | 8.5539 | 7.5817 | 7.65 | 7.9088 | 6.6941 | |
| 5.2804 | 5.793 | 6.9598 | 6.6986 | 7.3475 | 5.6607 | 5.4638 | 6.4628 | |
| 9.3504 | 5.2694 | 4.8288 | 7.4991 | 8.642 | 7.3996 | 8.2846 | 6.0252 | |
| 9.1532 | 7.184 | 4.771 | 8.3012 | 6.5796 | 7.5911 | 6.4146 | 7.5289 | |
| 8.4568 | 6.6097 | 4.548 | 6.7813 | 9.8213 | 5.4862 | 7.7153 | 7.2634 | |
| 7.6478 | 6.8257 | 7.3275 | 7.1866 | 7.2041 | 7.3288 | 8.6284 | 7.9739 | |
| 7.6092 | 8.78 | 7.0502 | 6.4729 | 7.6488 | 3.8367 | 7.5253 | 6.0558 | |
| 7.4965 | 6.428 | 5.9718 | 6.9908 | 5.6517 | 6.5454 | 3.8266 | 3.6599 | |
| 7.3429 | 6.1909 | 8.1647 | 4.5248 | 7.871 | 5.0693 | 6.2097 | 7.4976 | |
| 6.6003 | 7.2717 | 8.0983 | 7.1856 | 6.3025 | 5.2885 | 7.3239 | 7.3075 | |
| 6.7268 | 7.1184 | 5.5627 | 7.5547 | 8.1987 | 7.2545 | 7.5214 | 8.5682 | |
| 7.2896 | 7.5493 | 6.8723 | 7.2594 | 6.5488 | 5.02 | 6.1293 | 9.0016 | |
| 7.7472 | 7.3215 | 7.5095 | 8.4555 | 7.6627 | 8.4985 | 7.1299 | 7.7988 | |
| 7.7281 | 8.0411 | 7.8156 | 5.2687 | 7.4516 | 6.7264 | 6.2238 | 7.2558 | |
| 6.6838 | 5.2246 | 6.2897 | 0 | 6.4177 | 7.515 | 5.16 | 4.8253 | |
| 7.8203 | 7.6806 | 5.7534 | 6.5818 | 6.0832 | 4.474 | 2.2924 | 6.7428 | |
| 7.0427 | 7.3815 | 8.7914 | 7.6729 | 6.4192 | 6.6153 | 6.6547 | 6.6614 | |
| 5.6639 | 4.8121 | 6.9982 | 7.9541 | 5.0769 | 7.8944 | 7.0507 | 8.2657 | |
| 8.1882 | 8.111 | 6.6595 | 7.4277 | 2.7155 | 7.4714 | 8.2234 | 4.6214 | |
| 6.2741 | 6.0451 | 8.4923 | 5.6942 | 7.0687 | 5.8422 | 7.862 | 5.7882 | |
| 5.4095 | 7.9139 | 8.4577 | 6.6252 | 6.8053 | 8.3793 | 7.306 | 5.8471 | |
| 6.7281 | 7.5958 | 6.2965 | 5.7796 | 0.9197 | 7.2713 | 2.6976 | 2.8232 | |
| 2.9167 | 2.6372 | 2.3632 | 1.6405 | 2.217 | 6.0787 | 2.2864 | 2.8039 | |
| 2.3603 | 4.4239 | 3.28 | 3.69 | 5.3291 | 1.1757 | 1.9011 | 4.8818 | |
| 2.2708 | 4.5686 | 1.066 | 2.6496 | | | | | |
| KIF18A | 8.2606 | 6.6031 | 6.4774 | 8.88E+00 | | 9.02E+00 | | |

| | | | | | | | | |
|---|---|---|---|---|---|---|---|---|
| 8.3303 | 8.2636 | 8.0078 | 9.3759 | 8.8656 | 8.7542 | 7.7243 | 8.0063 | |
| 6.4999 | 7.5999 | 6.8658 | 6.5781 | 7.4168 | 6.8732 | 7.5381 | 7.4417 | |
| 9.8485 | 6.9662 | 5.9231 | 7.67 | 8.9334 | 8.0852 | 9.0017 | 7.3406 | |
| 8.7322 | 7.9194 | 6.7435 | 8.358 | 8.2131 | 7.5457 | 7.7103 | 7.6999 | |
| 8.6773 | 7.7427 | 6.3953 | 7.9095 | 9.1376 | 6.3847 | 7.5405 | 6.82 | |
| 7.4129 | 6.9327 | 8.273 | 7.4461 | 6.8957 | 7.7316 | 8.1195 | 8.5935 | |
| 8.5247 | 7.0293 | 7.9608 | 6.7595 | 7.866 | 5.664 | 7.5745 | 7.2679 | |
| 8.1431 | 6.1559 | 6.3651 | 7.4055 | 5.8858 | 7.142 | 4.7261 | 5.2619 | |
| 7.9853 | 7.4303 | 8.7277 | 5.1688 | 8.0333 | 6.5862 | 7.0325 | 8.5205 | |
| 7.8265 | 7.6875 | 8.3623 | 8.0926 | 7.1635 | 6.7031 | 8.8139 | 7.2834 | |
| 6.8483 | 8.4195 | 6.8558 | 7.5736 | 9.481 | 8.3503 | 8.3846 | 8.2823 | |
| 8.7201 | 8.2315 | 7.256 | 7.3717 | 7.9203 | 5.6813 | 6.4757 | 8.9044 | |
| 10.3378 | 8.0739 | 8.0286 | 9.5028 | 7.5741 | 8.8913 | 7.8596 | 7.6245 | |
| 7.8269 | 8.8722 | 8.7969 | 6.5459 | 6.9571 | 8.2048 | 7.2141 | 8.9294 | |
| 7.4342 | 7.0127 | 7.6617 | 0 | 7.2325 | 8.4759 | 6.099 | 6.4717 | |
| 8.0488 | 7.8366 | 7.0879 | 7.5817 | 7.0436 | 6.1223 | 6.2854 | 7.6891 | |
| 7.5274 | 7.8627 | 8.6523 | 7.5904 | 7.816 | 8.1142 | 7.0736 | 8.2318 | |
| 7.052 | 7.116 | 7.3228 | 8.361 | 8.031 | 8.1888 | 8.3369 | 8.5077 | |
| 8.3241 | 9.31 | 7.4719 | 7.481 | 0 | 8.4066 | 9.1257 | 5.1757 | |
| 7.3542 | 7.0741 | 9.1992 | 6.32 | 8.6537 | 7.508 | 7.8834 | 7.6098 | |
| 7.1637 | 8.7544 | 8.7198 | 7.1774 | 8.4349 | 7.7535 | 7.9042 | 8.9063 | |
| 7.967 | 8.3071 | 7.5501 | 6.6555 | 2.9425 | 7.3525 | 4.4562 | 4.4224 | |
| 3.9106 | 3.7496 | 3.5062 | 3.6364 | 4.0845 | 6.3489 | 3.2189 | 3.5703 | |
| 4.3753 | 4.0165 | 4.5261 | 4.3255 | 5.6529 | 4.083 | 3.052 | 4.0548 | |
| 4.3312 | 5.6042 | 2.2714 | 4.5 | | | | | |
| CDKN3 | 9.0428 | 6.3448 | 6.7476 | 8.8465 | 8.5356 | 7.7259 | 8.3829 | |
| 7.8219 | 8.9613 | 8.4569 | 7.875 | 7.6289 | 7.2932 | 6.8831 | 8.9925 | |
| 8.0348 | 8.2881 | 8.4372 | 6.2846 | 6.2859 | 8.4929 | 10.9599 | 7.6227 | |
| 6.0338 | 8.057 | 9.3854 | 8.8891 | 9.5938 | 7.2638 | 7.3628 | 8.5647 | |
| 5.1218 | 8.4199 | 8.8558 | 8.7688 | 8.5713 | 8.3687 | 9.0613 | 8.13 | |
| 8.7189 | 7.4505 | 7.9638 | 7.6976 | 7.9529 | 8.2553 | 6.9452 | 7.7084 | |
| 8.4362 | 6.3508 | 7.3403 | 8.5848 | 8.217 | 7.9477 | 8.7843 | 7.6825 | |
| 7.4107 | 6.8132 | 7.6715 | 6.0218 | 8.6532 | 8.8043 | 6.9475 | 8.1501 | 7.14 |
| | 7.4471 | 7.0219 | 6.6229 | 4.4541 | 5.2151 | 8.2402 | 8.2415 | |
| 8.4259 | 5.1426 | 8.7052 | 7.5314 | 7.6058 | 7.5714 | 7.782 | 7.3902 | |
| 9.3669 | 7.6516 | 8.1607 | 6.6155 | 8.7586 | 8.5518 | 8.1348 | 8.8276 | |
| 8.6301 | 8.613 | 8.5665 | 8.7642 | 8.9374 | 10.6314 | 7.2669 | 8.9634 | |
| 8.4407 | 7.573 | 7.2252 | 6.8355 | 7.4838 | 8.4348 | 7.4402 | 7.6029 | |
| 7.5557 | 8.6051 | 7.7811 | 9.7256 | 7.8502 | 8.4242 | 8.0654 | 7.6007 | |
| 9.7479 | 7.481 | 7.5635 | 8.6265 | 7.7928 | 8.1565 | 8.4887 | 8.1716 | |
| 9.1778 | 0 | 7.0019 | 9.2037 | 7.9002 | 6.8592 | 8.1427 | 8.1999 | |
| 6.7717 | 7.1939 | 8.6383 | 6.7926 | 8.1579 | 5.8673 | 7.2084 | 8.1661 | |
| 8.2773 | 7.5812 | 8.7691 | 9.096 | 7.9604 | 7.856 | 7.2056 | 6.8191 | |
| 7.6736 | 7.8485 | 6.4649 | 6.3196 | 8.4865 | 8.6166 | 8.02 | 8.0687 | |
| 9.1541 | 6.9492 | 5.0451 | 7.8103 | 7.7191 | 5.3016 | 8.8581 | 8.511 | |
| 8.9152 | 6.904 | 7.3942 | 6.9098 | 8.5603 | 8.8603 | 7.5078 | 8.6502 | |
| 8.6548 | 5.8423 | 8.0105 | 7.2499 | 7.4286 | 8.6547 | 10.1816 | 7.3874 | |
| 6.6933 | 7.8899 | 3.7046 | 8.146 | 4.8041 | 2.9316 | 3.9548 | 4.1266 | |
| 3.6322 | 4.8344 | 3.13 | 7.1378 | 5.3732 | 4.7315 | 4.31 | 5.1957 | |
| 2.4888 | 4.5474 | 5.2985 | 3.4689 | 4.231 | 5.8456 | 2.8514 | 5.9587 | |
| 4.2093 | 4.6591 | | | | | | | |
| ROBO4 | 7.6824 | 8.4167 | 6.6396 | 6.86E+00 | | 7.37E+00 | | |
| 6.0523 | 7.8243 | 7.47 | 7.5364 | 7.3576 | 8.2578 | 7.6495 | 7.2765 | |
| 8.0178 | 8.2426 | 8.126 | 6.986 | 7.394 | 7.4879 | 7.8502 | 7.4066 | |

| | | | | | | | |
|---|---|---|---|---|---|---|---|
| 6.8611 | 9.086 | 8.0787 | 6.319 | 7.4721 | 7.8378 | 6.4569 | 7.6837 |
| 6.3318 | 6.7049 | 9.2011 | 7.9296 | 7.1223 | 6.8927 | 8.2876 | 7.7036 |
| 7.0342 | 7.6419 | 7.8657 | 8.705 | 7.2806 | 7.3963 | 7.2076 | 8.4881 |
| 8.0487 | 9.3547 | 6.4109 | 7.5433 | 7.8248 | 9.3617 | 7.6876 | 8.1761 |
| 6.671 | 6.8293 | 8.1353 | 6.3958 | 7.6344 | 8.0406 | 9.7295 | 6.6958 |
| 8.1431 | 7.3881 | 8.775 | 9.0077 | 7.9595 | 8.7008 | 8.3832 | 9.0473 |
| 8.1156 | 8.7432 | 8.2194 | 9.7672 | 8.317 | 8.4479 | 7.687 | 7.1759 |
| 7.4292 | 7.4642 | 9.1922 | 7.6856 | 6.9767 | 8.6292 | 6.375 | 7.9747 |
| 7.9121 | 8.437 | 7.5906 | 7.7692 | 7.2469 | 9.0533 | 6.5751 | 7.8487 |
| 8.2274 | 8.4952 | 8.6247 | 7.1637 | 6.3818 | 8.4384 | 8.8329 | 8.4648 |
| 6.2069 | 7.1026 | 7.2662 | 7.5816 | 8.2378 | 8.0037 | 7.5997 | 8.1061 |
| 7.2657 | 6.8454 | 7.024 | 7.3208 | 8.1395 | 6.3735 | 7.8198 | 8.1212 |
| 7.4405 | 8.5701 | 6.8122 | 9.271 | 10.2537 | 6.7683 | 7.7832 | 7.5712 |
| 7.6892 | 6.9639 | 6.885 | 7.2747 | 6.5727 | 10.4831 | 8.1128 | 7.5133 |
| 8.3918 | 6.8459 | 7.8818 | 7.5224 | 6.9572 | 7.7763 | 7.6056 | 7.8293 |
| 7.3222 | 6.3793 | 8.2672 | 7.3039 | 8.1614 | 8.9561 | 7.6826 | 7.3459 |
| 7.1841 | 7.7296 | 7.173 | 6.8581 | 9.6981 | 8.4722 | 8.1504 | 8.2692 |
| 7.5653 | 7.3859 | 6.0647 | 7.7455 | 6.6855 | 7.0697 | 8.7797 | 7.8378 |
| 7.2038 | 8.1808 | 7.7811 | 8.5231 | 7.8051 | 7.9942 | 7.5825 | 7.1943 |
| 7.0742 | 8.1357 | 7.0042 | 7.5799 | 10.3295 | 9.8052 | 10.4401 | 6.0606 |
| 10.246 | 10.849 | 10.8253 | 11.3768 | 11.3627 | 10.0445 | 10.9682 | 11.5324 |
| 10.7559 | 10.0515 | 8.0964 | 10.892 | 10.4671 | 9.9716 | 7.2162 | 11.5617 |
| 11.1604 | 9.6858 | 10.2902 | 9.8395 | | | | |
| TRPC4 | 2.7225 | 2.2256 | 3.1292 | 6.41E+00 | | 4.38E+00 | |
| 3.4667 | 4.2718 | 3.872 | 3.7518 | 2.6308 | 4.1079 | 1.0485 | 2.0573 |
| 2.9791 | 2.676 | 3.6346 | 3.3529 | 2.6649 | 3.7331 | 4.5782 | 2.5109 |
| 2.4493 | 4.6839 | 3.7111 | 2.7135 | 5.5251 | 3.0237 | 3.326 | 2.7497 |
| 1.5856 | 2.5445 | 4.0027 | 2.9292 | 4.0418 | 3.9752 | 4.3859 | 4.6617 |
| 1.4585 | 4.0318 | 0.7135 | 3.6225 | 3.5488 | 3.2043 | 1.3077 | 3.9322 |
| 3.0688 | 5.6438 | 3.6325 | 5.0456 | 6.0372 | 6.2299 | 2.7727 | 6.1309 |
| 3.8751 | 3.9398 | 4.9326 | 2.0292 | 4.0455 | 4.7059 | 7.1129 | 2.6452 |
| 3.5766 | 4.5362 | 3.8455 | 6.2528 | 1.1076 | 5.7424 | 4.2277 | 3.9155 |
| 4.6105 | 3.4069 | 3.4754 | 6.3794 | 4.5201 | 5.2963 | 3.4854 | 2.7804 |
| 2.5297 | 4.3821 | 6.0578 | 4.0509 | 2.017 | 6.3151 | 4.4189 | 2.6431 |
| 4.8328 | 3.0758 | 2.4487 | 3.9875 | 2.3339 | 4.4347 | 1.1338 | 4.4321 |
| 6.0559 | 5.7783 | 3.5924 | 2.5118 | 2.4082 | 4.7091 | 3.585 | 3.3205 |
| 4.284 | 2.7528 | 4.0013 | 3.9324 | 4.1108 | 4.4201 | 4.8435 | 5.7219 |
| 3.4097 | 4.2726 | 4.2929 | 0 | 4.0869 | 2.5146 | 3.6089 | 2.7051 |
| 4.0736 | 2.9678 | 2.1237 | 3.371 | 7.7205 | 2.6248 | 2.1461 | 2.9704 |
| 5.264 | 4.2924 | 3.3108 | 2.576 | 1.8766 | 7.1206 | 0 | 4.703 |
| 5.7982 | 2.4897 | 2.3925 | 3.3489 | 2.0337 | 3.338 | 2.0474 | 4.8632 |
| 3.9918 | 2.1852 | 3.9044 | 3.9994 | 0.7889 | 4.8843 | 4.7339 | 4.2798 |
| 1.298 | 2.9341 | 1.7114 | 3.8031 | 4.5406 | 4.23 | 5.4688 | 5.9403 |
| 3.0872 | 2.8349 | 2.8387 | 3.5224 | 2.3211 | 3.3525 | 4.4809 | 3.202 |
| 1.9569 | 2.4241 | 3.2385 | 2.1004 | 3.1228 | 5.1443 | 3.2101 | 2.8198 |
| 4.8941 | 4.3583 | 3.8576 | 3.6757 | 8.613 | 5.6023 | 9.1628 | 3.2986 |
| 8.2819 | 9.9615 | 9.6419 | 8.5685 | 7.8077 | 6.6036 | 6.0765 | 9.6903 |
| 8.2467 | 8.0962 | 3.8913 | 8.7325 | 8.5776 | 9.413 | 6.6161 | 4.6105 |
| 8.5995 | 7.0169 | 5.5012 | 8.5689 | | | | |
| ABCG2 | 2.8211 | 3.8562 | 5.8298 | 4.9557 | 5.1071 | 3.7378 | 7.2573 |
| 6.6285 | 6.0697 | 5.5156 | 8.0331 | 4.3637 | 5.7141 | 5.3429 | 6.9021 |
| 7.5547 | 4.4272 | 5.3785 | 5.7591 | 6.7246 | 4.1884 | 4.0863 | 7.9782 |
| 4.7004 | 5.7965 | 4.5961 | 5.3216 | 2.978 | 5.4762 | 5.4603 | 5.9407 |
| 6.3032 | 5.9936 | 4.3937 | 4.3424 | 6.8766 | 5.4489 | 2.9342 | 4.5034 |

| | | | | | | | | |
|---|---|---|---|---|---|---|---|---|
| 3.7862 | 7.5177 | 5.5108 | 4.4931 | 2.9335 | 4.0611 | 5.2244 | 6.7765 | |
| 2.1129 | 6.0357 | 7.1415 | 9.7011 | 7.7541 | 6.5661 | 3.6766 | 3.4915 | |
| 6.1616 | 5.2076 | 6.3946 | 3.6793 | 7.2655 | 4.3904 | 3.417 | 3.982 | |
| 7.1521 | 6.8513 | 5.9679 | 5.9427 | 7.7525 | 7.5297 | 5.088 | 4.8022 | |
| 5.0283 | 9.281 | 6.2429 | 6.8837 | 4.5242 | 8.5521 | 1.3738 | 6.3053 | |
| 5.4789 | 7.1041 | 2.9938 | 6.6774 | 5.2098 | 6.2183 | 5.9325 | 4.5704 | |
| 3.1381 | 6.4339 | 2.8654 | 5.378 | 5.6881 | 7.0377 | 8.4708 | 7.9003 | |
| 6.9506 | 9.2155 | 4.6572 | 6.3709 | 4.5236 | 6.1712 | 4.8573 | 5.3945 | |
| 6.4527 | 3.3939 | 5.2386 | 6.432 | 3.3319 | 7.4574 | 4.2843 | 4.4003 | |
| 3.327 | 5.8592 | 5.2241 | 4.0798 | 7.3687 | 6.2781 | 4.8903 | 4.5695 | |
| 3.8512 | 6.8457 | 10.8535 | 4.4029 | 5.9922 | 6.0435 | 4.5741 | 3.3111 | |
| 5.5302 | 3.1603 | 1.224 | 9.919 | 2.2924 | 4.8695 | 6.5963 | 3.2188 | |
| 4.4988 | 3.5509 | 5.4699 | 7.0615 | 2.2191 | 2.5292 | 7.0788 | 4.7786 | |
| 6.4606 | 6.2709 | 6.441 | 8.465 | 5.2513 | 4.9727 | 3.0521 | 7.6364 | |
| 5.5105 | 2.9029 | 6.1698 | 4.5303 | 6.1864 | 8.2485 | 3.3748 | 6.6582 | |
| 5.586 | 7.3816 | 5.0723 | 6.4459 | 6.1076 | 6.1884 | 3.7742 | 2.9621 | |
| 4.6221 | 5.7061 | 6.8053 | 6.0878 | 4.8405 | 5.1434 | 4.3456 | 6.3884 | |
| 6.5185 | 1.1634 | 9.692 | 9.2457 | 9.5319 | 5.6488 | 11.3971 | 10.8702 | |
| 10.8906 | 11.6511 | 11.3725 | 9.9184 | 10.0336 | 10.9957 | 10.649 | 7.9575 | |
| 7.6017 | 11.36 | 11.5703 | 10.1145 | 8.7155 | 8.5075 | 11.2172 | 10.2346 | |
| 10.2506 | 9.4801 | | | | | | | |
| C9orf100 | | 8.4949 | 6.6162 | 7.7238 | 8.5058 | 9.5047 | 7.3942 | |
| 7.8905 | 8.5767 | 8.8576 | 8.0958 | 7.9 | 7.8315 | 7.852 | 8.2169 | |
| 8.2688 | 8.3582 | 7.9183 | 9.4645 | 7.6119 | 7.3956 | 8.4215 | 10.4562 | |
| 7.776 | 6.2942 | 7.443 | 8.7402 | 8.5848 | 8.897 | 8.1916 | 8.4766 | |
| 9.4768 | 7.1069 | 8.6864 | 8.8903 | 7.0299 | 8.0707 | 8.1232 | 10.0011 | |
| 8.1664 | 7.9382 | 9.3521 | 7.6565 | 7.887 | 8.1543 | 8.316 | 6.8151 | |
| 7.7213 | 9.3305 | 7.2073 | 6.8168 | 7.4043 | 8.6663 | 8.3208 | 7.3603 | |
| 8.6023 | 7.4669 | 7.6277 | 7.0942 | 6.0595 | 8.5683 | 9.309 | 7.3223 | |
| 8.4084 | 7.3268 | 7.2201 | 6.1699 | 7.6747 | 7.0199 | 7.127 | 8.5378 | |
| 8.1562 | 9.7282 | 6.6185 | 8.4549 | 7.8747 | 7.2881 | 8.3063 | 8.8977 | |
| 7.2027 | 9.2218 | 8.4158 | 7.3628 | 8.2738 | 8.9236 | 6.8947 | 6.6326 | |
| 8.9859 | 8.3581 | 7.8233 | 7.5631 | 8.1867 | 6.6099 | 9.6692 | 7.5131 | |
| 7.2879 | 8.0122 | 7.5719 | 7.7753 | 7.202 | 9.3556 | 8.0565 | 7.6597 | |
| 7.1306 | 8.0033 | 8.0798 | 8.679 | 8.2911 | 7.9479 | 7.7171 | 7.6057 | |
| 8.1266 | 7.6924 | 7.8842 | 9.0435 | 8.2971 | 7.7635 | 8.5133 | 7.0335 | |
| 7.4592 | 7.7141 | 6.7937 | 6.9632 | 8.3173 | 6.7716 | 7.9259 | 8.0286 | |
| 8.1476 | 5.5205 | 7.2574 | 8.1726 | 6.9181 | 6.8483 | 6.6968 | 6.933 | |
| 8.5942 | 7.5755 | 8.0561 | 9.3368 | 9.4288 | 8.1835 | 8.1638 | 7.5673 | |
| 8.0155 | 7.9789 | 7.744 | 8.5582 | 8.1668 | 8.613 | 8.6737 | 7.8193 | |
| 9.8117 | 8.679 | 7.2762 | 7.0584 | 8.2855 | 7.8618 | 7.2793 | 9.0646 | |
| 8.6589 | 9.0093 | 7.7267 | 7.8283 | 7.4021 | 7.9869 | 8.707 | 7.8963 | |
| 9.1624 | 7.7631 | 7.0698 | 7.1275 | 7.5112 | 7.4689 | 8.7771 | 9.4298 | |
| 7.7687 | 7.5588 | 7.6185 | 5.2166 | 7.9006 | 5.7861 | 5.1684 | 6.3458 | |
| 4.8619 | 4.6961 | 5.7042 | 5.3821 | 6.8654 | 5.5103 | 4.9411 | 5.232 | |
| 6.8659 | 5.3383 | 5.0278 | 7.0595 | 5.008 | 4.2768 | 6.05 | 5.0364 | |
| 7.736 | 6.3149 | 5.1529 | | | | | | |
| VGLL3 | 4.6049 | 7.1555 | 4.7604 | 6.4595 | 4.8551 | 5.6931 | 5.4029 | |
| 2.9347 | 3.2721 | 4.4966 | 4.2215 | 3.3079 | 4.6664 | 5.6204 | 3.4909 | |
| 5.7691 | 2.368 | 5.137 | 5.2133 | 5.4781 | 4.2292 | 3.689 | 4.5946 | |
| 5.9795 | 3.658 | 11.1107 | 6.5721 | 2.897 | 4.4338 | 2.1706 | 2.6866 | |
| 4.3404 | 3.3915 | 4.8458 | 6.1666 | 6.1668 | 5.8512 | 4.5253 | 3.1906 | 3.57 |
| | 3.8776 | 5.3307 | 3.1417 | 5.5914 | 5.6501 | 6.6911 | 5.9926 | |
| 5.0159 | 5.6949 | 5.5642 | 7.5795 | 2.2896 | 6.4689 | 4.0495 | 4.3188 | |

| | | | | | | | |
|---|---|---|---|---|---|---|---|
| 5.5552 | 2.2002 | 3.9373 | 4.1077 | 8.2881 | 3.0632 | 5.2542 | 4.8913 |
| 3.4376 | 7.2304 | 1.8854 | 6.5882 | 6.606 | 4.1822 | 9.1621 | 4.8268 |
| 5.056 | 7.9678 | 4.7281 | 4.8881 | 4.8803 | 3.5653 | 3.7227 | 5.0724 |
| 7.1535 | 4.5707 | 1.2055 | 5.9782 | 5.7058 | 4.1025 | 5.4782 | 5.4506 |
| 2.2933 | 5.6114 | 5.1347 | 5.896 | 4.2414 | 5.2759 | 6.349 | 6.7468 |
| 4.8905 | 3.599 | 3.4186 | 3.5857 | 4.7004 | 8.4648 | 6.0775 | 3.6416 |
| 4.5665 | 4.9889 | 6.6549 | 5.1349 | 5.8053 | 5.9779 | 4.1961 | 5.0329 |
| 7.1208 | 1.9593 | 4.9445 | 3.3824 | 4.1912 | 5.5619 | 4.0736 | 3.5378 |
| 3.1433 | 4.4303 | 7.1596 | 1.8416 | 2.6745 | 4.4271 | 6.2963 | 5.427 |
| 5.2435 | 3.5137 | 2.8098 | 8.4974 | 2.5542 | 7.2014 | 4.4631 | 2.9864 |
| 8.7302 | 6.5326 | 2.7383 | 4.4566 | 1.6272 | 6.0377 | 5.0359 | 2.0791 |
| 4.3968 | 6.9331 | 5.0769 | 4.8843 | 5.3793 | 3.0652 | 4.0283 | 5.6356 |
| 4.0012 | 4.1921 | 3.7579 | 4.9544 | 5.9449 | 5.8332 | 5.2128 | 4.1841 |
| 3.5298 | 3.7644 | 5.5536 | 3.863 | 5.5123 | 2.5964 | 1.1063 | 6.3834 |
| 7.3893 | 4.0851 | 3.9046 | 6.095 | 4.9214 | 1.5946 | 3.2264 | 5.8146 |
| 7.1398 | 2.5752 | 8.6583 | 8.9452 | 9.4824 | 6.3516 | 10.0937 | 9.6551 |
| 11.2963 | 9.7367 | 10.5564 | 8.7609 | 8.916 | 11.0658 | 7.8267 | 8.6595 |
| 7.4365 | 9.6989 | 9.9105 | 7.9257 | 9.9455 | 7.7239 | 8.2306 | 8.8644 |
| 9.8137 | 7.7808 | | | | | | |
| AQP1 | 8.8447 | 10.6343 | 9.0726 | 8.69E+00 | | 9.11E+00 | |
| 9.9169 | 10.3392 | 9.9637 | 9.1036 | 9.5371 | 9.3602 | 9.7908 | 9.0861 |
| 8.9512 | 9.3551 | 9.6015 | 8.5248 | 9.1287 | 10.6756 | 9.2457 | 8.9467 |
| 8.6872 | 10.5774 | 10.433 | 9.1611 | 9.2722 | 9.5156 | 8.0021 | 9.6229 |
| 8.123 | 8.8817 | 11.6219 | 8.7452 | 9.7981 | 8.6288 | 10.6496 | 9.5493 |
| 7.5189 | 9.2023 | 9.5437 | 9.5089 | 9.2476 | 9.2969 | 9.069 | 10.0187 |
| 9.9916 | 11.5785 | 7.3942 | 10.1852 | 10.2309 | 12.3252 | 7.642 | 9.879 |
| 9.1015 | 8.0706 | 9.7432 | 8.9142 | 9.6194 | 9.0593 | 12.0973 | 7.9109 |
| 8.742 | 9.2823 | 9.7339 | 11.3324 | 8.6134 | 10.9306 | 11.0918 | 11.2508 |
| 10.486 | 10.2539 | 9.8628 | 12.6244 | 9.4358 | 11.1622 | 9.5182 | 8.6215 |
| 10.0648 | 11.3726 | 11.0361 | 9.589 | 7.9558 | 11.2389 | 9.489 | 9.1742 |
| 10.6244 | 9.9203 | 9.5575 | 10.6377 | 10.2322 | 10.7332 | 9.1854 | 10.1089 |
| 11.8195 | 10.3442 | 10.0459 | 9.2236 | 8.9956 | 11.4584 | 9.6348 | 9.851 |
| 8.5823 | 9.7022 | 9.419 | 9.7772 | 10.8325 | 9.6615 | 10.3166 | 10.3074 |
| 9.1201 | 9.5876 | 8.7299 | 8.9363 | 8.3399 | 8.5263 | 10.4657 | 10.3095 |
| 9.7405 | 10.8302 | 9.2604 | 10.7643 | 12.3349 | 8.7549 | 9.5739 | 10.1663 |
| 9.8742 | 8.8647 | 9.3479 | 11.1589 | 8.6788 | 12.415 | 9.8349 | 9.7922 |
| 9.9113 | 7.61 | 9.7152 | 9.9101 | 8.9679 | 9.1969 | 8.6207 | 9.4279 |
| 9.1063 | 8.4892 | 9.8395 | 8.9043 | 10.5213 | 10.0454 | 10.3556 | 8.5909 |
| 9.2588 | 9.1662 | 10.1123 | 9.8765 | 12.0264 | 8.5164 | 10.4614 | 11.6168 |
| 9.4954 | 9.1576 | 8.1092 | 9.6959 | 8.4976 | 10.2793 | 10.5092 | 9.5125 |
| 8.8305 | 9.9181 | 9.631 | 9.3057 | 10.7535 | 10.9887 | 9.0172 | 8.9482 |
| 9.1131 | 10.2013 | 10.4295 | 9.8451 | 12.5118 | 11.2079 | 12.9567 | 9.7114 |
| 12.9693 | 13.4478 | 14.8205 | 14.4624 | 14.1295 | 11.0725 | 15.1089 | 14.3401 |
| 12.1948 | 10.7084 | 11.2497 | 13.222 | 12.4697 | 12.053 | 8.8571 | 13.7872 |
| 12.9545 | 11.5082 | 14.2547 | 12.7 | | | | |
| C10orf72 | | 5.7097 | 6.4087 | 5.6185 | 6.62E+00 | | 5.85E+00 |
| | 6.5653 | 6.0881 | 6.9997 | 5.7642 | 4.7317 | 7.2323 | 5.6378 |
| 4.7168 | 7.1511 | 6.4649 | 7.9204 | 4.447 | 5.137 | 6.5458 | 6.8344 |
| 8.4908 | 4.6954 | 8.075 | 6.461 | 3.5991 | 8.8499 | 5.8945 | 3.4447 |
| 5.1282 | 3.7013 | 5.3745 | 9.7052 | 5.016 | 5.0773 | 6.2554 | 7.521 |
| 5.9802 | 8.6884 | 5.067 | 7.1921 | 5.8916 | 6.0033 | 5.9089 | 5.985 |
| 5.8053 | 10.4513 | 8.0352 | 5.3542 | 6.8493 | 7.3734 | 8.4944 | 6.2268 |
| 6.1644 | 4.9403 | 5.0786 | 6.9701 | 3.8941 | 5.4581 | 7.5845 | 8.088 |
| 6.6612 | 6.7142 | 6.3484 | 6.7758 | 8.0405 | 6.6867 | 8.9578 | 9.0582 |

| | | | | | | | | |
|---|---|---|---|---|---|---|---|---|
| 7.6223 | 7.0674 | 6.2375 | 5.2603 | 8.7517 | 6.8365 | 6.9469 | 5.5761 | |
| 5.2143 | 6.5603 | 6.3134 | 8.4173 | 6.0552 | 4.0212 | 9.8946 | 7.4723 | |
| 8.3029 | 6.4126 | 5.7285 | 5.6129 | 6.3118 | 5.7305 | 6.892 | 6.1671 | |
| 6.5314 | 8.954 | 8.1688 | 7.677 | 5.0659 | 4.3109 | 6.8355 | 5.7549 | |
| 7.4461 | 4.7481 | 6.9768 | 6.1605 | 5.6929 | 5.9398 | 7.5084 | 5.5958 | |
| 6.935 | 5.5253 | 6.5679 | 6.4089 | 3.1609 | 4.4602 | 4.7191 | 6.1687 | |
| 6.3652 | 6.5157 | 7.5967 | 5.1351 | 10.7889 | 8.6762 | 5.9048 | 4.8772 | |
| 6.6127 | 6.8179 | 6.9639 | 6.2806 | 6.978 | 7.5133 | 8.8481 | 4.8222 | |
| 6.5738 | 7.8399 | 4.7221 | 7.5521 | 6.5703 | 5.0206 | 7.0317 | 3.9656 | |
| 6.7134 | 8.0053 | 4.7615 | 7.3065 | 6.6636 | 5.9908 | 7.9516 | 6.4446 | |
| 3.8893 | 4.7095 | 5.9152 | 7.5475 | 7.5324 | 7.5403 | 6.9238 | 6.7083 | |
| 8.1792 | 5.7412 | 6.6046 | 4.9896 | 8.5011 | 5.6333 | 6.1259 | 6.7029 | |
| 4.8141 | 5.2439 | 6.9457 | 6.4717 | 8.205 | 5.3576 | 6.9705 | 6.1362 | |
| 6.3542 | 9.1977 | 6.4036 | 7.1004 | 5.8893 | 9.3932 | 11.0702 | 10.0647 | |
| 6.6182 | 10.4383 | 9.3354 | 10.3879 | 10.478 | 10.2945 | 11.1591 | 10.5716 | |
| 10.1015 | 10.7556 | 8.8114 | 8.3326 | 10.0739 | 10.5747 | 10.104 | 11.7937 | |
| 9.7575 | 9.5734 | 10.6375 | 10.8566 | 9.4939 | | | | |
| CLIP3 | 6.9347 | 7.735 | 7.2197 | 7.83E+00 | | 6.70E+00 | | 8.32 |
| | 7.7557 | 7.3698 | 6.0243 | 9.7734 | 8.4724 | 7.7579 | 7.0444 | |
| 8.4948 | 9.5072 | 8.2564 | 6.9518 | 10.5074 | 7.5006 | 7.1256 | 6.7555 | |
| 5.827 | 8.2437 | 8.957 | 7.1752 | 10.1423 | 8.8906 | 9.0954 | 8.4429 | |
| 6.277 | 6.848 | 7.2413 | 7.4349 | 6.9525 | 7.5511 | 8.1064 | 10.1984 | |
| 6.4775 | 7.7847 | 6.836 | 8.3575 | 6.5994 | 6.1974 | 6.7297 | 7.4632 | |
| 8.7278 | 10.0305 | 7.4064 | 8.1056 | 8.3961 | 9.4717 | 7.5484 | 8.0203 | |
| 7.0707 | 6.6047 | 7.803 | 6.8198 | 8.897 | 8.7136 | 10.2617 | 9.0859 | |
| 6.8532 | 7.3229 | 8.1227 | 8.8879 | 5.7684 | 10.4171 | 7.8439 | 8.5234 | |
| 8.8304 | 7.5791 | 7.2499 | 9.7662 | 7.0582 | 8.7488 | 9.2786 | 5.6886 | |
| 6.5108 | 10.3288 | 10.3802 | 7.35 | 7.375 | 10.6421 | 8.3408 | 6.9928 | |
| 8.2293 | 7.8628 | 7.9044 | 8.315 | 8.7065 | 8.909 | 6.3682 | 7.5025 | |
| 9.5655 | 9.7465 | 8.4778 | 7.3976 | 5.9507 | 10.6817 | 8.5469 | 8.4114 | |
| 9.5194 | 5.3945 | 7.4123 | 7.4147 | 9.1632 | 11.1098 | 7.9209 | 8.7034 | |
| 6.6709 | 8.2963 | 8.0125 | 10.0465 | 7.7474 | 6.8495 | 7.6323 | 8.8732 | |
| 8.7486 | 10.1593 | 7.2908 | 12.0726 | 10.1375 | 6.9898 | 6.3447 | 7.3268 | |
| 8.0488 | 6.8098 | 6.1416 | 6.8348 | 6.139 | 10.3542 | 7.1231 | 8.104 | |
| 10.4201 | 6.0863 | 9.2432 | 7.5556 | 8.4218 | 7.1886 | 5.3092 | 6.9729 | |
| 8.4911 | 6.6944 | 7.6168 | 7.7205 | 8.4998 | 8.1888 | 7.8465 | 5.4841 | |
| 6.1576 | 9.2367 | 5.6865 | 8.0353 | 9.0649 | 7.137 | 8.5613 | 9.5155 | |
| 7.6949 | 7.8094 | 11.6985 | 7.378 | 8.4406 | 7.9676 | 8.0492 | 7.4628 | |
| 6.8032 | 7.3705 | 7.6808 | 5.9558 | 10.3563 | 9.5224 | 7.6699 | 8.8879 | |
| 5.6471 | 7.5825 | 8.6754 | 7.2853 | 11.9607 | 10.7077 | 12.1449 | 9.3187 | |
| 11.7723 | 11.1764 | 11.9843 | 11.8063 | 12.0068 | 11.3914 | 11.3486 | 12.2119 | |
| 12.3609 | 11.291 | 9.5435 | 11.5974 | 11.0666 | 11.5156 | 11.6033 | 11.1335 | |
| 11.1219 | 10.8929 | 11.3504 | 11.1338 | | | | | |
| PIF1 | 8.5608 | 5.5906 | 6.4304 | 8.18E+00 | | 8.79E+00 | | |
| 6.8258 | 7.8707 | 7.8219 | 9.0489 | 8.3139 | 7.5284 | 7.2813 | 6.9421 | |
| 7.0977 | 8.4746 | 7.2374 | 8.841 | 8.1984 | 6.328 | 6.6819 | 7.5741 | |
| 9.4396 | 8.5072 | 4.9084 | 6.4331 | 7.4613 | 8.5644 | 8.2175 | 7.7695 | |
| 8.1683 | 8.0923 | 6.1205 | 7.5612 | 8.3642 | 7.1494 | 8.4242 | 7.1052 | |
| 8.9867 | 7.8539 | 8.4206 | 6.5162 | 7.8072 | 6.2941 | 7.8202 | 7.9488 | |
| 5.157 | 6.6729 | 8.392 | 6.551 | 4.8348 | 6.8604 | 8.0524 | 7.8585 | |
| 7.7294 | 7.836 | 6.6195 | 6.3958 | 6.9298 | 3.4386 | 9.6638 | 8.4726 | |
| 6.8648 | 7.9105 | 7.7296 | 6.2865 | 6.151 | 7.5629 | 4.8899 | 5.5717 | |
| 7.394 | 7.9396 | 8.4857 | 4.0482 | 8.408 | 7.4896 | 7.2156 | 6.541 | |
| 8.5385 | 6.576 | 8.9152 | 6.9166 | 7.6654 | 8.3807 | 8.4396 | 5.1865 | |

| | | | | | | | | |
|---|---|---|---|---|---|---|---|---|
| 6.1458 | 8.7488 | 7.5611 | 7.5705 | 7.3656 | 10.2744 | 8.6492 | 7.6412 | |
| 7.0335 | 7.2793 | 9.1193 | 7.5633 | 7.574 | 8.3013 | 8.3083 | 9.9728 | |
| 6.1861 | 6.5298 | 7.7177 | 7.5599 | 7.6515 | 8.4021 | 6.6256 | 6.7594 | |
| 5.5493 | 7.3352 | 7.227 | 8.6451 | 8.1634 | 8.607 | 7.7775 | 9.3033 | |
| 7.8088 | 7.0544 | 8.5652 | 7.4343 | 6.6481 | 9.931 | 5.3451 | 7.4065 | |
| 7.0592 | 6.7312 | 6.0795 | 5.9667 | 7.6053 | 5.1344 | 9.0689 | 5.9007 | |
| 6.7385 | 7.9243 | 7.797 | 7.4809 | 8.6974 | 9.4368 | 6.2419 | 7.4375 | |
| 6.8679 | 6.6854 | 6.2321 | 7.3303 | 6.774 | 5.608 | 7.6385 | 10.1397 | |
| 8.0098 | 9.0826 | 8.6056 | 7.7324 | 5.4182 | 7.9225 | 8.1504 | 6.4824 | |
| 8.5633 | 8.3763 | 8.2638 | 6.5441 | 7.318 | 6.7373 | 7.3766 | 8.4638 | |
| 8.5927 | 8.0229 | 7.5538 | 4.6948 | 7.2556 | 6.7113 | 7.2947 | 9.6013 | |
| 9.4231 | 6.9331 | 6.8908 | 7.3634 | 2.8563 | 7.5907 | 5.0628 | 3.3774 | |
| 4.4323 | 3.4648 | 2.3632 | 4.1653 | 3.2731 | 6.3701 | 4.3886 | 4.7315 | |
| 3.8402 | 6.5355 | 3.8913 | 3.3957 | 4.762 | 3.4689 | 2.6938 | 5.0711 | |
| 3.113 | 6.7026 | 4.2513 | 2.7661 | | | | | |
| ASAM | 3.7656 | 5.8578 | 3.9331 | 4.8043 | 3.7029 | 4.6035 | 4.1817 | |
| 4.569 | 2.7843 | 3.9802 | 5.1104 | 1.2777 | 3.8176 | 2.1513 | 4.3124 | |
| 3.999 | 5.7043 | 3.6109 | 3.8167 | 2.9264 | 2.3723 | 1.0809 | 4.4661 | |
| 5.5032 | 3.1011 | 8.3775 | 4.4543 | 1.4434 | 2.8293 | 2.8081 | 2.3867 | |
| 3.6174 | 1.5916 | 3.7803 | 4.6144 | 4.6775 | 5.0624 | 3.2841 | 2.6826 | 0 |
| | 1.992 | 6.2396 | 1.9026 | 4.4119 | 4.1214 | 6.2912 | 5.6105 | |
| 4.1397 | 6.4176 | 5.2371 | 7.9946 | 3.3557 | 5.5308 | 3.5661 | 4.3188 | |
| 5.5456 | 1.3453 | 3.8994 | 6.6568 | 6.6976 | 3.4804 | 3.499 | 4.7984 | |
| 2.7868 | 7.2261 | 1.3447 | 7.2393 | 6.045 | 4.3648 | 6.4026 | 3.532 | |
| 4.4721 | 5.9208 | 3.021 | 4.4093 | 4.267 | 3.5092 | 3.6018 | 5.4083 | |
| 6.626 | 5.5767 | 3.1434 | 6.1627 | 5.6445 | 6.8902 | 5.0325 | 4.1474 | |
| 3.6674 | 5.1736 | 4.5518 | 2.8049 | 2.8014 | 5.5817 | 6.9342 | 6.1746 | |
| 3.2552 | 2.1442 | 2.5476 | 3.3832 | 1.585 | 7.0112 | 6.2773 | 4.1521 | |
| 6.0355 | 4.4122 | 4.4937 | 7.1384 | 5.8182 | 6.509 | 3.8169 | 5.6869 | |
| 5.1941 | 1.2894 | 4.3536 | 1.9805 | 3.5526 | 1.9109 | 4.6075 | 2.657 | |
| 3.2319 | 6.6176 | 8.6639 | 1.3507 | 2.2974 | 3.0717 | 6.1086 | 5.8462 | |
| 3.5494 | 3.078 | 1.224 | 8.1954 | 1.5604 | 5.8099 | 3.7584 | 6.244 | |
| 7.4938 | 6.489 | 1.3489 | 3.0513 | 0 | 3.7221 | 5.4833 | 1.7039 | |
| 5.4357 | 5.9301 | 2.4241 | 6.996 | 4.0786 | 0.5466 | 3.2858 | 3.4836 | |
| 5.8203 | 4.6914 | 0 | 2.7911 | 5.5986 | 6.0874 | 3.5582 | 2.4045 | |
| 1.4637 | 1.9773 | 5.1283 | 5.3422 | 4.9889 | 3.3874 | 2.6295 | 5.3671 | |
| 5.9385 | 0.9557 | 4.1198 | 7.3747 | 4.6872 | 0 | 4.6154 | 5.3954 | |
| 5.1702 | 3.744 | 9.3456 | 8.3917 | 9.7389 | 5.4982 | 9.4173 | 9.5235 | |
| 9.9747 | 10.1959 | 10.1969 | 8.8488 | 9.0844 | 10.1733 | 9.5073 | 6.7453 | |
| 6.2052 | 9.7169 | 10.2591 | 9.4122 | 7.5291 | 7.288 | 9.0678 | 8.9813 | |
| 9.4074 | 9.2669 | | | | | | | |
| NEXN | 6.3935 | 7.1922 | 4.2125 | 7.7584 | 5.4234 | 4.4907 | 6.1763 | |
| 6.1274 | 5.7642 | 5.144 | 6.7548 | 3.9961 | 4.2186 | 5.4604 | 6.226 | |
| 5.5448 | 7.1029 | 6.9084 | 3.9337 | 6.403 | 6.8435 | 3.73 | 5.3445 | |
| 7.2151 | 4.0589 | 6.6728 | 7.5204 | 5.0919 | 6.1233 | 5.6447 | 4.1139 | |
| 5.5841 | 2.8736 | 6.6335 | 9.432 | 6.3643 | 6.703 | 6.4888 | 5.3163 | |
| 4.087 | 7.1146 | 8.7595 | 3.3218 | 5.512 | 7.4748 | 7.5851 | 6.8591 | |
| 6.1656 | 7.2083 | 6.984 | 9.568 | 6.1597 | 6.3431 | 4.2415 | 5.4267 | |
| 5.5835 | 3.2826 | 6.9578 | 6.2628 | 9.1891 | 7.3067 | 6.0971 | 5.176 | |
| 5.2767 | 9.0303 | 3.1833 | 5.9967 | 5.0169 | 5.2151 | 8.0003 | 5.3865 | |
| 6.7831 | 8.8338 | 6.2429 | 6.795 | 6.291 | 5.7136 | 4.9896 | 7.2085 | |
| 8.9452 | 6.2033 | 5.8148 | 7.056 | 7.0207 | 7.234 | 7.6579 | 6.553 | |
| 6.8901 | 6.2891 | 5.9136 | 6.8342 | 3.7378 | 7.1504 | 8.2671 | 6.5569 | |
| 4.8407 | 4.0721 | 3.8543 | 5.4414 | 7.1699 | 8.6345 | 7.2973 | 5.0157 | |

| | | | | | | | |
|---|---|---|---|---|---|---|---|
| 5.8027 | 7.428 | 8.4345 | 8.6818 | 7.0166 | 6.5428 | 6.0629 | 6.895 |
| 7.911 | 3.0394 | 4.6702 | 4.5839 | 6.1687 | 6.6091 | 7.2956 | 6.6265 |
| 5.2456 | 6.2435 | 7.3944 | 3.4355 | 4.3284 | 5.0152 | 6.5771 | 5.7397 |
| 4.2811 | 5.4324 | 3.2756 | 9.1074 | 4.5495 | 5.9964 | 5.0481 | 2.7093 |
| 8.4769 | 6.0629 | 5.436 | 8.7132 | 4.1886 | 8.4705 | 4.7195 | 3.4163 |
| 4.7633 | 6.2709 | 6.453 | 6.4148 | 6.8989 | 3.8438 | 4.8438 | 8.1063 |
| 4.0012 | 4.8884 | 5.1047 | 6.179 | 7.3734 | 7.4699 | 6.0678 | 5.4576 |
| 5.4032 | 4.7558 | 7.8756 | 6.3145 | 6.2366 | 6.4398 | 4.5176 | 7.3492 |
| 7.3783 | 5.5413 | 8.4207 | 8.3628 | 5.2377 | 4.872 | 4.2299 | 6.8357 |
| 7.8162 | 6.2363 | 9.3564 | 7.9699 | 10.4799 | 7.0677 | 11.8379 | 10.5789 |
| 11.7699 | 11.6374 | 11.8528 | 8.3938 | 10.9686 | 12.1139 | 9.4918 | 8.7965 |
| 8.3822 | 10.8479 | 11.521 | 9.1196 | 8.218 | 10.3526 | 9.6866 | 9.9705 |
| 12.152 | 8.3058 | | | | | | |
| DCN | 9.8053 | 11.5129 | 10.272 | 1.06E+01 | | 8.85E+00 | |
| 12.1119 | 10.8631 | 9.733 | 9.8154 | 9.778 | 11.1986 | 6.889 | 9.3773 |
| 10.78 | 10.5792 | 11.4809 | 8.1287 | 10.1815 | 10.2214 | 9.8254 | 10.1776 |
| 9.6591 | 11.1914 | 11.4228 | 7.7568 | 12.1452 | 10.5335 | 8.2886 | 9.3133 |
| 7.2103 | 8.2946 | 9.0787 | 7.6361 | 9.4696 | 10.4477 | 11.8522 | 10.851 |
| 8.6537 | 8.9726 | 9.1363 | 9.2321 | 13.2184 | 8.4942 | 10.2834 | 9.8793 |
| 11.3173 | 11.5711 | 10.1018 | 10.6794 | 11.5979 | 13.4979 | 9.6504 | 11.4127 |
| 9.4487 | 10.4714 | 11.2241 | 7.8715 | 9.4345 | 12.2439 | 12.1631 | 8.7169 |
| 8.7178 | 10.4595 | 10.4768 | 12.5075 | 2.1585 | 13.656 | 12.3217 | 11.7796 |
| 11.2582 | 10.6735 | 9.8077 | 14.9426 | 9.7997 | 11.5225 | 10.2214 | 9.5016 |
| 10.2421 | 11.2569 | 10.7653 | 10.4153 | 8.0201 | 13.1469 | 12.2576 | 8.1132 |
| 11.3308 | 10.5249 | 9.492 | 10.8968 | 9.9147 | 10.6804 | 10.4715 | 10.9516 |
| 13.0527 | 11.9622 | 11.5324 | 8.8314 | 8.8466 | 10.9016 | 7.6582 | 13.8064 |
| 9.3324 | 9.0689 | 11.8744 | 10.1414 | 10.7149 | 10.1555 | 11.1652 | 11.6827 |
| 10.2005 | 11.1736 | 11.9609 | 5.3979 | 9.5595 | 8.431 | 10.7311 | 9.0432 |
| 10.2736 | 9.6698 | 9.6229 | 12.386 | 13.5361 | 7.8867 | 7.9918 | 10.9091 |
| 11.0058 | 10.4879 | 10.1392 | 9.1275 | 5.9493 | 13.4131 | 8.143 | 10.9938 |
| 10.6886 | 6.9357 | 13.6143 | 11.1834 | 7.7927 | 9.1092 | 4.9677 | 9.5585 |
| 11.7117 | 9.1701 | 10.8691 | 11.7531 | 9.6994 | 10.4398 | 11.1105 | 7.5225 |
| 8.243 | 8.7737 | 8.9739 | 9.8421 | 9.4479 | 6.8367 | 10.8198 | 12.8784 |
| 10.4478 | 10.6387 | 6.7583 | 11.1965 | 10.4326 | 10.916 | 11.8236 | 9.2222 |
| 9.2884 | 11.6311 | 11.2451 | 8.3449 | 10.1466 | 12.6877 | 10.1012 | 9.59 |
| 6.1069 | 10.471 | 11.9545 | 9.4011 | 14.7013 | 13.255 | 15.0975 | 11.4709 |
| 13.7022 | 14.8851 | 16.9807 | 16.4128 | 16.5686 | 13.744 | 16.3065 | 14.6298 |
| 14.51 | 13.6928 | 13.9141 | 15.3109 | 14.0569 | 14.9022 | 15.9147 | 15.17 |
| 13.7313 | 13.237 | 16.6218 | 14.8451 | | | | |
| TYMS | 11.9488 | 7.8826 | 8.931 | 11.6882 | 10.8188 | 10.1367 | 10.2573 |
| 9.6831 | 10.06 | 10.1884 | 10.3972 | 10.03 | 10.1715 | 8.0291 | 9.7711 |
| 10.6629 | 12.0223 | 10.6715 | 9.3246 | 8.5634 | 10.9726 | 10.9811 | 9.9798 |
| 8.8249 | 10.2151 | 11.5775 | 9.7869 | 11.3726 | 9.0904 | 11.3003 | 12.3667 |
| 8.0109 | 10.6583 | 10.1509 | 9.1947 | 10.8769 | 9.7939 | 10.5214 | 8.3617 |
| 9.3122 | 10.6172 | 12.1214 | 9.5132 | 9.6072 | 9.8335 | 9.398 | 8.9866 |
| 10.721 | 9.8591 | 8.7268 | 10.5631 | 9.4817 | 10.0752 | 10.0373 | 11.2367 |
| 10.0371 | 8.9421 | 10.3662 | 7.6589 | 9.7734 | 10.2453 | 9.8989 | 10.0992 |
| 10.237 | 9.4247 | 8.9597 | 9.1993 | 7.8991 | 8.1512 | 11.2618 | 10.8343 |
| 10.8688 | 7.2722 | 9.7174 | 10.3072 | 11.7916 | 9.8642 | 10.7882 | 8.7915 |
| 11.7528 | 11.2007 | 10.5169 | 8.3113 | 11.2092 | 9.8137 | 9.3375 | 10.3429 |
| 9.9824 | 11.1105 | 9.5203 | 9.2202 | 10.2419 | 11.876 | 9.1413 | 11.0714 |
| 11.3513 | 10.011 | 10.2238 | 9.2972 | 8.3221 | 9.4198 | 9.9915 | 9.9974 |
| 9.0434 | 10.5693 | 9.4314 | 11.024 | 10.0005 | 9.8918 | 10.669 | 10.3794 |
| 10.1198 | 10.1644 | 10.0783 | 9.5437 | 9.968 | 8.9552 | 9.6766 | 9.6075 |

| | | | | | | | |
|---|---|---|---|---|---|---|---|
| 10.9813 | 6.5438 | 9.3843 | 9.6315 | 9.4161 | 9.3789 | 10.4422 | 10.3389 |
| 9.5933 | 10.1312 | 9.5457 | 7.6599 | 8.234 | 8.7928 | 9.3576 | 10.5024 |
| 9.3703 | 10.574 | 11.0685 | 12.1967 | 10.2146 | 10.5072 | 9.6007 | 10.8018 |
| 10.6746 | 10.2034 | 9.0219 | 9.5875 | 10.1375 | 10.3971 | 10.6625 | 10.0572 |
| 11.6061 | 10.5128 | 7.1365 | 10.8291 | 10.1069 | 8.1397 | 10.4651 | 9.4085 |
| 10.969 | 8.9266 | 9.834 | 9.5839 | 9.847 | 10.8174 | 10.0998 | 11.5629 |
| 10.7163 | 8.9009 | 9.3733 | 11.0296 | 10.3584 | 10.6277 | 10.6493 | 9.9031 |
| 9.5622 | 11.001 | 5.7455 | 10.7625 | 6.9299 | 7.2172 | 7.216 | 6.259 |
| 5.928 | 6.4259 | 6.4982 | 9.6058 | 6.2419 | 6.3631 | 6.6444 | 7.768 |
| 7.7063 | 6.7521 | 8.6747 | 7.2328 | 5.3521 | 8.7224 | 6.8395 | 8.8318 |
| 5.8387 | 6.9961 | | | | | | |
| PALM2-AKAP2 | | 7.6791 | 8.9603 | 8.4203 | 8.01E+00 | | 9.26E+00 |
| | 8.7941 | 8.2423 | 8.1471 | 8.0041 | 9.0169 | 7.8944 | 7.6844 |
| 7.8287 | 8.387 | 8.015 | 8.31 | 7.8849 | 9.1059 | 7.6183 | 8.0767 |
| 8.3269 | 7.9003 | 8.3635 | 9.2389 | 8.6346 | 7.1652 | 8.676 | 7.209 |
| 8.767 | 7.6616 | 7.3923 | 8.4787 | 8.1888 | 8.9073 | 7.3983 | 8.764 |
| 8.8461 | 7.4421 | 8.1067 | 6.6662 | 8.3026 | 10.4462 | 6.8764 | 7.477 |
| 9.9221 | 8.9081 | 9.6841 | 7.8413 | 8.2278 | 8.7947 | 10.6864 | 7.1238 |
| 9.7983 | 7.0398 | 7.8182 | 7.963 | 8.9955 | 8.0404 | 8.5178 | 9.6727 |
| 8.1999 | 8.817 | 9.8548 | 7.7458 | 9.7558 | 6.7651 | 8.8073 | 7.656 |
| 8.557 | 9.5782 | 7.825 | 8.2641 | 10.0893 | 9.0401 | 8.2721 | 8.6752 |
| 11.225 | 7.5123 | 9.4013 | 10.4394 | 8.5579 | 8.8168 | 8.6777 | 8.9088 |
| 8.6758 | 8.3547 | 9.7461 | 7.8285 | 8.2532 | 7.231 | 8.9507 | 6.203 |
| 8.4213 | 9.4209 | 9.2333 | 7.991 | 7.3992 | 8.4582 | 8.22 | 8.3456 |
| 8.9651 | 9.4929 | 7.8852 | 8.8244 | 8.6655 | 10.1154 | 10.639 | 7.8205 |
| 9.0928 | 7.2129 | 9.6239 | 9.0002 | 6.8825 | 9.031 | 7.0029 | 7.9359 |
| 9.0724 | 9.6977 | 9.302 | 7.873 | 8.8597 | 10.3188 | 7.1559 | 7.8925 |
| 7.7912 | 8.6717 | 8.1377 | 8.5334 | 7.7288 | 7.6539 | 10.4018 | 6.6358 |
| 8.5253 | 8.5763 | 7.5024 | 9.0122 | 7.9252 | 8.8155 | 8.4361 | 7.5383 |
| 8.6099 | 7.7192 | 7.5291 | 7.9623 | 8.2235 | 9.5325 | 8.7718 | 8.5554 |
| 7.4388 | 8.5083 | 7.954 | 7.7287 | 8.5005 | 8.1895 | 8.0396 | 9.3243 |
| 9.2108 | 7.0646 | 7.3757 | 9.2581 | 7.5603 | 9.1791 | 8.4726 | 8.7856 |
| 7.7806 | 8.7244 | 8.6114 | 9.0577 | 6.7223 | 9.4065 | 9.2665 | 7.2874 |
| 8.5652 | 7.2966 | 8.3827 | 8.9652 | 6.8922 | 11.2846 | 9.6158 | 11.1577 |
| 9.5407 | 11.5166 | 11.6292 | 12.2755 | 12.1382 | 12.0429 | 9.6584 | 10.8602 |
| 12.3086 | 10.6594 | 9.5888 | 9.6888 | 11.7103 | 11.575 | 9.9719 | 8.8256 |
| 10.6224 | 11.3606 | 9.9964 | 11.6297 | 9.6994 | | | |
| LMNB1 | 11.6913 | 9.8894 | 9.7002 | 11.6418 | 11.6458 | 11.6627 | 11.5522 |
| 11.2502 | 12.4005 | 11.2626 | 11.0675 | 11.3409 | 10.915 | 9.701 | 10.3439 |
| 11.311 | 10.651 | 10.7383 | 10.6737 | 10.317 | 10.5363 | 11.8958 | 10.7146 |
| 9.3447 | 11.7418 | 11.8924 | 11.5542 | 10.7266 | 10.9942 | 12.3363 | 11.5314 |
| 10.233 | 12.0445 | 11.3194 | 10.0749 | 11.3246 | 11.2544 | 11.2235 | 10.5907 |
| 8.806 | 10.2884 | 12.0023 | 9.3738 | 11.0351 | 9.9621 | 10.4466 | 10.0237 |
| 11.7257 | 10.7725 | 9.2018 | 11.1571 | 11.675 | 11.5979 | 11.3826 | 11.226 |
| 11.1899 | 10.1609 | 11.2443 | 8.6443 | 11.5732 | 10.8224 | 11.2037 | 10.2198 |
| 10.1356 | 10.9196 | 9.7874 | 10.2116 | 8.63 | 8.6564 | 11.3596 | 11.3145 |
| 11.5661 | 8.5816 | 11.6349 | 10.419 | 10.5034 | 11.8231 | 10.9368 | 10.7476 |
| 12.4133 | 11.1685 | 10.6388 | 9.6639 | 12.1039 | 10.6304 | 10.5165 | 11.7546 |
| 9.9856 | 11.2121 | 11.1066 | 11.5339 | 11.3319 | 11.8176 | 10.9539 | 12.0097 |
| 11.8058 | 11.0155 | 11.3286 | 7.2474 | 10.0014 | 11.7567 | 11.2823 | 10.8379 |
| 10.9485 | 11.8782 | 10.7972 | 11.1281 | 11.3005 | 10.9919 | 11.0225 | 10.913 |
| 10.5376 | 9.6679 | 12.0765 | 11.5605 | 11.1036 | 10.7179 | 9.6835 | 7.0042 |
| 10.4572 | 5.8348 | 10.5143 | 11.184 | 10.257 | 9.8412 | 11.6093 | 11.3624 |
| 10.4385 | 9.9118 | 9.7539 | 9.3964 | 8.2531 | 10.1905 | 11.2387 | 11.3918 |

| | | | | | | | | |
|---|---|---|---|---|---|---|---|---|
| 11.6735 | 11.3058 | 11.4362 | 11.5676 | 10.4395 | 11.3419 | 10.7747 | 10.6716 | |
| 10.4163 | 11.7428 | 10.7304 | 10.0042 | 11.5928 | 11.6536 | 11.3507 | 11.4147 | |
| 11.9607 | 11.4155 | 7.8498 | 12.054 | 11.8118 | 8.8781 | 10.8703 | 10.8869 | |
| 12.1809 | 10.2567 | 11.1091 | 10.5566 | 11.187 | 10.8477 | 10.6864 | 11.5956 | |
| 11.8162 | 9.7642 | 10.1613 | 10.0059 | 11.2261 | 10.4502 | 10.6093 | 11.2801 | |
| 10.7539 | 10.333 | 8.3586 | 10.7518 | 9.1182 | 8.0593 | 6.8767 | 8.3178 | |
| 6.2795 | 8.175 | 7.3682 | 9.6859 | 7.4555 | 6.6438 | 7.867 | 8.0538 | |
| 8.3372 | 9.3801 | 8.6819 | 7.4141 | 7.4065 | 9.1188 | 7.6809 | 9.5059 | |
| 6.7426 | 8.0769 | | | | | | | |
| ANLN | 9.7625 | 9.7226 | 9.6874 | 9.92E+00 | | 1.11E+01 | | |
| 9.1688 | 8.831 | 10.3736 | 10.621 | 10.3 | 10.3941 | 8.9271 | 9.9823 | |
| 7.4919 | 8.9585 | 8.9067 | 9.756 | 9.7748 | 8.9075 | 8.3959 | 8.9205 | |
| 12.4488 | 8.4741 | 7.0926 | 10.4432 | 11.469 | 9.943 | 9.9116 | 9.9357 | |
| 10.247 | 9.7536 | 7.7713 | 9.9747 | 10.112 | 10.6602 | 9.216 | 9.3439 | |
| 10.4522 | 9.248 | 7.8854 | 9.7084 | 11.0764 | 8.5763 | 9.8453 | 10.2202 | |
| 8.9906 | 9.215 | 9.9064 | 9.5137 | 9.5041 | 10.0159 | 8.419 | 10.248 | |
| 10.6283 | 8.453 | 9.6519 | 7.7935 | 10.2552 | 7.3082 | 10.0204 | 8.5716 | |
| 9.7027 | 8.8067 | 8.096 | 9.2776 | 9.0528 | 7.9983 | 7.1073 | 6.3002 | |
| 10.1808 | 8.8341 | 11.2874 | 7.1647 | 10.1687 | 7.6935 | 9.1765 | 9.0988 | |
| 8.9118 | 9.2459 | 9.8669 | 9.2499 | 7.3328 | 7.0426 | 9.9254 | 9.5832 | |
| 9.6743 | 10.4712 | 6.4859 | 10.369 | 10.1085 | 9.5914 | 9.9371 | 10.8129 | |
| 10.0297 | 9.7171 | 9.5395 | 9.5875 | 8.9286 | 6.315 | 8.1699 | 11.5729 | |
| 9.6475 | 9.0182 | 9.6956 | 10.8982 | 9.6788 | 10.246 | 9.6666 | 10.6196 | |
| 10.1496 | 10.2522 | 9.8302 | 6.9174 | 9.5219 | 9.8487 | 9.3825 | 10.2235 | |
| 9.1634 | 8.0654 | 7.6006 | 0 | 8.6561 | 9.4697 | 7.7008 | 7.3217 | |
| 9.938 | 10.5095 | 8.9815 | 7.9022 | 8.8494 | 7.5598 | 6.8337 | 8.5985 | |
| 10.2753 | 9.7547 | 10.0015 | 10.116 | 10.1087 | 9.6555 | 8.9935 | 10.072 | |
| 8.3576 | 7.5124 | 8.9221 | 10.2108 | 9.3924 | 9.3255 | 10.0835 | 10.4072 | |
| 9.8983 | 10.5957 | 9.2249 | 9.7088 | 4.7036 | 9.6721 | 11.3387 | 7.4743 | |
| 8.8167 | 9.0857 | 9.9672 | 7.9576 | 9.2109 | 8.0348 | 9.6242 | 9.1375 | |
| 8.5732 | 9.0362 | 10.1693 | 8.1295 | 9.2079 | 10.9079 | 9.362 | 8.4615 | |
| 7.7249 | 9.705 | 8.8971 | 8.3119 | 4.5139 | 9.4402 | 5.7516 | 5.0702 | |
| 5.739 | 4.9029 | 4.8044 | 4.8344 | 5.138 | 8.4196 | 5.4299 | 5.0719 | 5.11 |
| | 6.0085 | 5.6644 | 5.6316 | 7.7348 | 3.9717 | 5.6893 | 7.0685 | |
| 4.7196 | 7.6207 | 5.1025 | 4.1171 | | | | | |
| CSDC2 | 5.1512 | 4.4886 | 3.0603 | 6.2326 | 3.6664 | 5.4732 | 6.2297 | |
| 5.2241 | 2.7843 | 4.1842 | 7.6876 | 6.7287 | 2.4639 | 3.7058 | 7.9785 | |
| 5.4537 | 4.2582 | 5.5026 | 4.5855 | 5.3748 | 5.7619 | 1.5155 | 7.6227 | |
| 6.6582 | 4.9218 | 1.8679 | 6.823 | 4.5189 | 5.9648 | 3.3227 | 1.492 | |
| 7.1772 | 1.8437 | 4.0811 | 5.6566 | 4.9748 | 5.6012 | 3.4316 | 3.863 | |
| 4.8968 | 4.4881 | 5.0799 | 3.3218 | 4.0111 | 6.1331 | 6.6011 | 7.5566 | |
| 4.8594 | 6.3112 | 6.3565 | 8.0423 | 3.5478 | 5.4785 | 4.2771 | 5.8028 | |
| 4.7777 | 1.3453 | 6.7864 | 5.9433 | 9.0247 | 2.0543 | 2.4255 | 3.8023 | |
| 4.8545 | 6.2698 | 0 | 7.4161 | 6.0939 | 5.5717 | 7.3476 | 5.467 | |
| 5.0142 | 6.6637 | 2.7931 | 7.0843 | 7.392 | 4.6659 | 3.7227 | 7.8808 | |
| 6.0386 | 5.2072 | 7.4488 | 8.6668 | 6.249 | 2.6431 | 6.3541 | 3.9876 | |
| 5.6771 | 6.8654 | 7.6107 | 6.1604 | 3.5544 | 6.1136 | 6.8925 | 6.4097 | |
| 6.6875 | 2.3994 | 3.7426 | 8.4187 | 5.7004 | 4.1453 | 8.5823 | 3.3607 | |
| 6.1745 | 5.3669 | 4.7403 | 8.6607 | 5.0107 | 6.592 | 3.5586 | 6.6082 | |
| 7.3066 | 8.4828 | 5.0301 | 1.7472 | 6.2417 | 4.939 | 6.4299 | 6.9697 | |
| 5.0644 | 9.3267 | 8.5595 | 1.8416 | 2.7811 | 4.4271 | 5.691 | 5.3633 | |
| 1.4775 | 6.6758 | 2.3242 | 9.7557 | 5.3562 | 3.9181 | 5.0261 | 1.5093 | |
| 6.6768 | 6.4692 | 3.626 | 6.0276 | 0 | 3.7789 | 5.2953 | 4.3471 | |
| 4.1571 | 4.7895 | 6.7836 | 4.8999 | 5.7187 | 2.0787 | 3.2308 | 5.3049 | |

| | | | | | | | |
|---|---|---|---|---|---|---|---|
| 2.214 | 4.8618 | 4.7787 | 2.1868 | 6.3427 | 7.8106 | 6.1463 | 4.5289 |
| 7.5599 | 3.6241 | 6.6477 | 5.9848 | 7.0054 | 5.7208 | 4.05 | 5.4968 |
| 4.7855 | 1.7437 | 5.7292 | 6.9979 | 5.3441 | 5.3717 | 2.3729 | 4.8767 |
| 7.0554 | 6.3283 | 11.7777 | 7.9822 | 11.2869 | 6.5433 | 11.0687 | 11.0569 |
| 10.02 | 10.6419 | 10.1031 | 8.9652 | 9.6438 | 11.0496 | 10.9291 | 9.5889 |
| 7.3887 | 9.92 | 9.9541 | 10.9823 | 12.4707 | 8.5506 | 10.3033 | 9.1181 |
| 9.8654 | 10.4538 | | | | | | |
| TIMELESS | | 11.0182 | 9.6529 | 9.2784 | 11.6274 | 11.2803 | 9.8839 |
| 11.0553 | 10.3015 | 10.2831 | 10.5461 | 10.8271 | 10.6143 | 10.2062 | 9.5634 |
| 10.6641 | 10.8436 | 11.0905 | 10.5852 | 10.0646 | 9.6436 | 9.9104 | 12.4352 |
| 10.0508 | 8.9894 | 10.3215 | 11.1118 | 10.5284 | 11.3492 | 9.6299 | 11.3626 |
| 10.587 | 9.9099 | 11.1195 | 10.0687 | 10.5898 | 10.301 | 9.8938 | 11.1129 |
| 9.092 | 9.5413 | 10.3905 | 10.6393 | 9.9521 | 10.1304 | 10.9624 | 11.0137 |
| 10.6353 | 10.6969 | 9.8761 | 10.2006 | 10.7648 | 9.7343 | 10.1066 | 10.8713 |
| 12.1639 | 10.0282 | 10.3825 | 11.3773 | 8.9644 | 10.8242 | 10.7497 | 10.7676 |
| 9.8764 | 9.7419 | 10.0308 | 9.803 | 10.6464 | 8.7568 | 8.5186 | 10.7422 |
| 10.3286 | 10.3658 | 7.9068 | 10.7133 | 9.7464 | 10.5711 | 10.2347 | 10.3118 |
| 9.5157 | 11.1838 | 10.7524 | 11.1149 | 9.1292 | 10.7011 | 9.7061 | 9.8205 |
| 10.1087 | 9.6841 | 10.4966 | 10.7464 | 10.9713 | 10.9085 | 11.7404 | 10.0397 |
| 10.3735 | 11.0846 | 10.2842 | 10.2961 | 8.7595 | 9.9556 | 10.3317 | 10.0652 |
| 11.1854 | 10.6237 | 10.9576 | 10.3507 | 10.7891 | 9.9962 | 10.5615 | 9.9257 |
| 10.4098 | 10.4428 | 11.1365 | 10.5791 | 10.0206 | 9.9498 | 9.8545 | 9.2991 |
| 8.5873 | 10.4663 | 7.6102 | 9.8624 | 10.1278 | 9.8125 | 9.0492 | 10.8995 |
| 10.8463 | 9.7769 | 9.4517 | 9.5683 | 9.2083 | 8.1919 | 10.0156 | 10.7164 |
| 10.8811 | 10.6259 | 10.8497 | 10.8021 | 10.9269 | 10.1316 | 10.928 | 10.1662 |
| 10.3491 | 10.4976 | 10.7052 | 9.3154 | 11.1234 | 10.757 | 11.473 | 11.0986 |
| 10.6686 | 11.6171 | 11.396 | 8.3944 | 10.9308 | 11.7416 | 8.3946 | 9.8918 |
| 10.0125 | 11.7166 | 9.3887 | 10.5831 | 9.7236 | 10.611 | 10.4921 | 9.8907 |
| 11.7222 | 11.4004 | 9.7728 | 9.6273 | 10.5669 | 10.4475 | 10.3034 | 10.3528 |
| 10.3859 | 9.9672 | 10.2024 | 8.1251 | 10.3707 | 8.555 | 7.8542 | 8.7391 |
| 8.5049 | 7.364 | 7.8196 | 7.8905 | 9.4059 | 7.7621 | 8.3451 | 8.2314 |
| 8.3739 | 8.0243 | 8.3935 | 9.0102 | 7.965 | 6.9121 | 8.0998 | 8.3975 |
| 9.5137 | 7.5336 | 8.175 | | | | | |
| TAL1 | 2.8211 | 3.7286 | 4.1139 | 3.8769 | 3.8725 | 2.2192 | 4.6307 |
| 3.5228 | 3.372 | 2.5346 | 4.9383 | 3.0838 | 3.2604 | 4.4366 | 4.2727 |
| 4.1224 | 3.3942 | 3.172 | 4.6091 | 4.3481 | 2.9613 | 4.0218 | 5.1903 |
| 4.4573 | 3.658 | 3.7641 | 3.9322 | 2.8112 | 4.4338 | 2.1706 | 3.4947 |
| 5.7818 | 3.8601 | 3.5753 | 3.4354 | 4.6099 | 3.9622 | 6.5918 | 2.6826 |
| 3.7177 | 4.4094 | 3.9985 | 3.5796 | 3.5019 | 5.9117 | 3.4897 | 5.7697 |
| 2.3993 | 4.629 | 5.2996 | 6.505 | 4.2095 | 4.4302 | 3.6766 | 2.3502 |
| 4.2969 | 4.3222 | 5.4581 | 3.1496 | 6.101 | 2.7984 | 3.912 | 4.7984 |
| 4.9857 | 5.6473 | 3.9301 | 4.9331 | 5.1522 | 6.1085 | 4.7311 | 4.5296 |
| 3.5936 | 7.3875 | 4.4824 | 4.8881 | 3.9537 | 3.26 | 4.0343 | 4.7798 |
| 4.8229 | 4.9705 | 3.8576 | 6.2814 | 3.2745 | 4.4001 | 4.2302 | 4.5704 |
| 1.6914 | 4.7554 | 1.8854 | 5.3547 | 3.0296 | 4.4064 | 4.9852 | 5.3799 |
| 4.052 | 2.9711 | 2.8996 | 5.0883 | 5.1699 | 4.4006 | 5.3805 | 4.1521 |
| 4.2332 | 3.8396 | 5.156 | 8.4549 | 4.0979 | 4.8057 | 2.6761 | 4.2388 |
| 3.327 | 2.9068 | 3.5438 | 2.6562 | 4.1912 | 5.1778 | 5.2172 | 5.2536 |
| 4.2831 | 6.9144 | 7.3944 | 1.8416 | 4.141 | 3.8753 | 3.6856 | 2.5446 |
| 3.689 | 3.3826 | 2.6654 | 8.2615 | 5.1339 | 3.6825 | 4.4631 | 4.9546 |
| 3.889 | 3.6598 | 3.4328 | 4.0828 | 2.9615 | 3.4693 | 3.9918 | 2.9585 |
| 3.3158 | 3.5125 | 4.6023 | 5.8437 | 4.0404 | 2.8049 | 3.0521 | 3.9233 |
| 3.6292 | 3.0035 | 4.6244 | 4.23 | 4.316 | 5.7765 | 3.4385 | 4.6747 |
| 3.3548 | 4.2827 | 3.3684 | 3.863 | 4.7963 | 4.2224 | 3.1812 | 4.4821 |

| | | | | | | | |
|---|---|---|---|---|---|---|---|
| 4.1936 | 4.3737 | 4.6768 | 5.0123 | 3.0753 | 3.4726 | 8.0632 | 4.2726 |
| 4.5714 | 2.0355 | 7.5219 | 6.4227 | 7.0788 | 3.8339 | 7.2973 | 8.2318 |
| 8.3366 | 9.5472 | 8.3437 | 6.472 | 8.1133 | 8.2478 | 7.1687 | 6.2653 |
| 5.4444 | 7.7147 | 8.2076 | 6.9924 | 4.9152 | 7.8613 | 8.0602 | 6.6153 |
| 7.8185 | 6.1422 | | | | | | |
| RFTN2 | 2.9134 | 5.7304 | 3.9331 | 4.0015 | 5.8217 | 2.9738 | 3.9467 |
| 4.153 | 4.6778 | 3.5094 | 5.485 | 3.3589 | 4.3522 | 4.7451 | 5.3726 |
| 4.7766 | 3.3529 | 4.3385 | 4.2059 | 3.9968 | 4.3815 | 5.6673 | 4.1686 |
| 4.0288 | 4.1845 | 3.7641 | 3.6879 | 2.897 | 4.214 | 2.8081 | 4.6878 |
| 4.4684 | 3.8312 | 3.7803 | 4.1845 | 5.1032 | 5.6944 | 3.4777 | 5.067 |
| 1.8316 | 4.1438 | 4.7948 | 3.2642 | 3.4047 | 4.3404 | 3.4148 | 6.035 |
| 3.9752 | 4.8384 | 6.2966 | 6.5373 | 3.5478 | 7.3119 | 3.6766 | 3.733 |
| 4.6406 | 2.9405 | 3.7791 | 4.4376 | 6.7977 | 3.3377 | 3.1387 | 3.0705 |
| 4.4753 | 5.468 | 2.7601 | 4.9767 | 4.5691 | 4.0814 | 4.6105 | 3.7537 |
| 4.3874 | 6.7443 | 4.277 | 4.4963 | 4.2262 | 4.1091 | 4.3666 | 4.3513 |
| 5.0699 | 4.6645 | 1.8528 | 6.1251 | 4.4479 | 4.0653 | 4.0939 | 4.9844 |
| 2.8342 | 4.2911 | 5.4184 | 5.5921 | 3.5544 | 3.6802 | 5.7261 | 5.5722 |
| 5.0303 | 3.4375 | 2.8996 | 5.8869 | 5.4919 | 7.7283 | 4.1616 | 3.0885 |
| 4.2858 | 2.6758 | 4.5269 | 6.5904 | 4.4368 | 4.5307 | 3.1838 | 4.626 |
| 4.1272 | 2.205 | 3.8874 | 3.2094 | 3.994 | 4.7298 | 4.8903 | 3.336 |
| 4.5818 | 5.3965 | 6.2544 | 4.0447 | 5.4294 | 3.8753 | 4.1972 | 3.6893 |
| 3.4742 | 3.4496 | 1.224 | 6.9803 | 3.1371 | 5.1906 | 4.3256 | 2.4897 |
| 5.3954 | 4.2299 | 2.7383 | 3.6311 | 2.6375 | 4.5405 | 4.7195 | 2.8335 |
| 5.3994 | 4.3333 | 5.8881 | 4.6091 | 5.1109 | 2.6009 | 3.1737 | 3.6957 |
| 2.0651 | 2.678 | 4.2552 | 3.5915 | 5.55 | 4.8307 | 2.6218 | 3.9749 |
| 3.2915 | 6.0082 | 4.2968 | 3.4271 | 5.1786 | 3.8329 | 1.9569 | 4.0467 |
| 4.1936 | 3.3112 | 3.9946 | 4.9495 | 4.6872 | 1.5946 | 3.7023 | 3.6472 |
| 4.9318 | 3.2749 | 7.633 | 6.6245 | 7.406 | 3.5232 | 6.9441 | 7.1248 |
| 8.206 | 8.6331 | 7.3481 | 7.1806 | 7.4871 | 7.3639 | 7.3942 | 6.6056 |
| 5.012 | 8.0307 | 7.0817 | 6.8838 | 8.3708 | 6.8618 | 7.6606 | 6.7964 |
| 7.2104 | 6.6 | | | | | | |
| PLK4 | 8.9397 | 6.8063 | 8.554 | 8.8115 | 9.2993 | 8.545 | 8.9642 |
| 7.9681 | 9.5533 | 8.7645 | 9.1237 | 7.9654 | 9.2674 | 6.904 | 7.8154 |
| 7.7491 | 8.074 | 8.4593 | 8.3199 | 7.6592 | 7.8405 | 8.9264 | 8.4804 |
| 5.6951 | 8.7089 | 8.7929 | 8.5662 | 8.7089 | 7.4102 | 8.2817 | 8.5203 |
| 6.8354 | 9.1038 | 8.5578 | 8.3381 | 8.0879 | 8.4355 | 8.1859 | 8.5261 |
| 7.8051 | 8.0096 | 8.918 | 7.263 | 7.7228 | 7.7043 | 7.0355 | 7.4064 |
| 9.0138 | 7.7609 | 7.3842 | 8.531 | 7.6601 | 9.61 | 8.792 | 7.3158 |
| 8.8098 | 6.6531 | 7.9497 | 5.2256 | 8.2608 | 8.0736 | 7.9434 | 7.5103 |
| 6.8807 | 7.6256 | 8.3294 | 8.0114 | 6.4345 | 6.7658 | 8.6661 | 8.3065 |
| 9.0349 | 6.7957 | 8.6783 | 8.0092 | 8.2846 | 8.5293 | 8.5219 | 8.2399 |
| 8.6907 | 8.2803 | 7.1852 | 7.393 | 8.7411 | 7.7082 | 9.5722 | 8.5233 |
| 7.2953 | 9.0717 | 8.305 | 9.6038 | 10.053 | 9.3132 | 8.3558 | 7.9911 |
| 7.971 | 8.7663 | 8.4861 | 7.3621 | 7.1799 | 9.2521 | 9.0063 | 9.4638 |
| 9.7227 | 8.4528 | 7.621 | 8.3424 | 8.2398 | 7.8023 | 8.956 | 8.8458 |
| 8.5203 | 7.4693 | 8.5999 | 9.2783 | 9.1873 | 9.2985 | 7.0794 | 6.2346 |
| 7.2596 | 5.5192 | 7.9839 | 9.696 | 6.5695 | 7.9789 | 8.5881 | 8.7299 |
| 8.2722 | 7.1237 | 7.2914 | 6.7542 | 5.5184 | 8.2384 | 8.0886 | 8.3116 |
| 8.511 | 8.9567 | 9.9545 | 8.8144 | 7.6773 | 7.7355 | 8.022 | 7.0717 |
| 6.9655 | 8.87 | 7.8334 | 8.4492 | 8.5926 | 9.2774 | 8.8113 | 9.1164 |
| 7.6718 | 8.3867 | 5.3211 | 8.6697 | 9.7785 | 7.2111 | 8.3332 | 8.0687 |
| 8.6025 | 6.997 | 8.2829 | 7.7488 | 8.7959 | 8.9532 | 8.5401 | 8.8302 |
| 9.1129 | 7.0192 | 8.4588 | 7.7139 | 8.5312 | 8.1894 | 8.1157 | 8.2619 |
| 8.9632 | 7.5705 | 4.9059 | 8.3369 | 5.6971 | 5.7559 | 6.0248 | 5.1836 |

| | | | | | | | |
|---|---|---|---|---|---|---|---|
| 4.5085 | 5.6718 | 5.3625 | 6.9528 | 5.1882 | 5.0245 | 5.8571 | 6.202 |
| 6.4017 | 5.9421 | 6.9204 | 5.5468 | 5.4842 | 6.1832 | 5.9368 | 7.307 |
| 4.9321 | 5.8037 | | | | | | |
| TRIP13 | 9.3885 | 8.719 | 7.9995 | 9.9936 | 9.3015 | 9.2737 | 9.5097 |
| 9.9753 | 10.3846 | 9.5978 | 9.5574 | 8.66 | 8.7592 | 7.3004 | 9.1238 |
| 9.4029 | 9.733 | 9.8519 | 8.5693 | 8.1204 | 9.1447 | 9.9425 | 8.6322 |
| 7.3918 | 8.8857 | 10.7466 | 8.5806 | 10.3673 | 9.3072 | 10.5994 | 10.3384 |
| 7.3942 | 8.9043 | 9.5585 | 7.8343 | 8.9067 | 8.8697 | 9.8321 | 8.8788 |
| 7.8458 | 9.2206 | 10.1622 | 7.2813 | 9.4709 | 8.5277 | 8.7239 | 7.4192 |
| 9.0988 | 8.6839 | 7.3027 | 9.3112 | 8.5871 | 9.5886 | 10.2712 | 11.0267 |
| 7.915 | 7.0271 | 9.9598 | 5.9433 | 10.2255 | 9.9032 | 8.4761 | 8.6192 |
| 8.2734 | 8.1882 | 8.3397 | 9.002 | 8.5622 | 8.4291 | 9.5096 | 8.6922 |
| 10.6933 | 8.7232 | 9.6817 | 8.7355 | 9.4085 | 8.7722 | 8.9448 | 8.5663 |
| 8.9218 | 9.7602 | 7.4808 | 7.6217 | 9.5294 | 9.0236 | 7.9644 | 9.1571 |
| 8.4348 | 9.2229 | 9.2387 | 9.483 | 10.0063 | 10.7767 | 8.808 | 9.625 |
| 9.062 | 8.5247 | 9.158 | 7.2915 | 9.7381 | 10.9807 | 11.1511 | 8.581 |
| 8.9956 | 8.8034 | 8.4946 | 10.4896 | 8.3193 | 9.6648 | 8.6956 | 10.3498 |
| 9.3652 | 9.0508 | 8.6848 | 9.3399 | 8.7618 | 9.075 | 9.7942 | 8.4533 |
| 9.0496 | 4.4303 | 8.5428 | 9.8437 | 7.7109 | 8.1651 | 9.0781 | 9.2047 |
| 8.2356 | 8.388 | 8.0886 | 7.3329 | 7.5779 | 7.7846 | 10.2898 | 9.8823 |
| 9.1777 | 8.5257 | 9.7372 | 9.5785 | 8.2794 | 8.8611 | 8.7445 | 8.1024 |
| 8.7753 | 9.3151 | 8.9645 | 9.6921 | 9.3464 | 9.5382 | 9.7752 | 9.9653 |
| 8.8342 | 9.0517 | 6.0231 | 8.3466 | 9.7417 | 10.2173 | 8.4672 | 10.0925 |
| 11.2299 | 7.8541 | 9.3097 | 9.1619 | 11.1178 | 9.4475 | 8.7066 | 9.8711 |
| 9.9959 | 7.1491 | 9.062 | 10.0308 | 9.1489 | 9.6163 | 10.3095 | 8.538 |
| 8.4881 | 9.2315 | 6.0069 | 8.5125 | 6.0628 | 8.382 | 6.9926 | 5.6554 |
| 4.2185 | 5.9638 | 5.824 | 7.5409 | 5.4112 | 5.2267 | 6.4028 | 6.2149 |
| 8.2476 | 6.3652 | 6.8536 | 6.1852 | 6.1721 | 6.982 | 6.5512 | 7.2543 |
| 5.3353 | 6.3617 | | | | | | |
| OLFML1 | 5.0328 | 7.2162 | 5.4129 | 6.6744 | 5.9179 | 7.0513 | 8.0059 |
| 6.3102 | 6.6648 | 5.8907 | 7.9511 | 4.1195 | 4.9455 | 6.8619 | 7.119 |
| 7.6704 | 4.0667 | 7.8023 | 5.727 | 6.1404 | 6.3473 | 4.3975 | 6.924 |
| 7.2384 | 4.102 | 4.882 | 6.1184 | 4.0796 | 5.6287 | 3.7013 | 4.2576 |
| 5.4816 | 4.6368 | 4.8684 | 6.1524 | 7.3294 | 7.6542 | 5.1963 | 5.4712 |
| 5.1775 | 6.1793 | 7.0475 | 4.4679 | 4.9826 | 6.2046 | 6.884 | 7.8995 |
| 5.9386 | 7.1975 | 7.3103 | 8.6392 | 5.8181 | 9.5955 | 5.5317 | 4.9167 |
| 6.5919 | 3.7362 | 6.0495 | 4.1483 | 8.4551 | 4.1553 | 6.0229 | 6.2813 |
| 5.6713 | 7.5091 | 3.6494 | 8.0955 | 6.565 | 6.8828 | 6.2794 | 6.5427 |
| 6.1671 | 8.7689 | 5.8712 | 6.5323 | 6.5933 | 4.8578 | 5.4929 | 6.7635 |
| 7.5217 | 5.8901 | 4.6406 | 9.0545 | 7.0588 | 4.5691 | 6.2696 | 7.4178 |
| 5.1294 | 6.5718 | 6.1869 | 7.4094 | 6.2674 | 6.0235 | 8.8456 | 6.9091 |
| 6.9564 | 5.0096 | 4.1452 | 6.398 | 5.3576 | 8.3312 | 6.1328 | 4.781 |
| 6.6562 | 5.5538 | 7.0126 | 11.1252 | 7.6834 | 7.6634 | 6.1515 | 6.8731 |
| 6.142 | 8.2965 | 5.5672 | 4.9838 | 6.021 | 5.964 | 6.6078 | 4.7429 |
| 4.8848 | 9.2535 | 7.8044 | 4.2546 | 5.0363 | 5.9798 | 7.4801 | 6.8429 |
| 4.6996 | 5.4652 | 3.0614 | 8.4518 | 4.6649 | 7.4952 | 6.3875 | 4.4137 |
| 8.4436 | 4.4277 | 3.7963 | 5.8567 | 3.8658 | 5.9036 | 7.1959 | 4.4781 |
| 6.3572 | 6.7386 | 5.5045 | 6.5738 | 6.5298 | 3.5915 | 5.4725 | 6.9861 |
| 5.3747 | 5.7381 | 6.6615 | 4.879 | 6.8108 | 7.9193 | 5.9849 | 6.6317 |
| 7.8064 | 6.2179 | 6.3028 | 5.7866 | 7.246 | 6.2009 | 4.9797 | 6.7836 |
| 6.4786 | 4.7712 | 6.003 | 7.3074 | 6.5397 | 7.1743 | 2.962 | 6.2718 |
| 6.6933 | 4.2463 | 8.8793 | 9.4268 | 10.1502 | 6.4184 | 9.6032 | 8.3412 |
| 10.678 | 10.4784 | 10.4049 | 10.1312 | 10.716 | 9.1629 | 9.9264 | 9.2566 |
| 8.4992 | 10.6872 | 9.2206 | 8.236 | 10.7904 | 10.0059 | 8.5562 | 9.4875 |

| | | | | | | | | |
|---|---|---|---|---|---|---|---|---|
| 10.658 | 8.9319 | | | | | | | |
| C1QTNF7 | 3.8143 | 7.1178 | 4.6938 | 6.315 | 4.665 | 4.6827 | 5.7339 | |
| 5.8584 | 4.3987 | 4.9148 | 7.6802 | 3.255 | 5.3164 | 3.7058 | 7.2474 | |
| 6.647 | 2.7962 | 3.255 | 4.7 | 4.4387 | 0.6062 | 1.3145 | 7.4761 | |
| 4.8692 | 2.472 | 1.5789 | 4.0611 | 1.8398 | 2.9765 | 0.5853 | 0.6819 | |
| 4.2364 | 3.8884 | 2.9681 | 6.6969 | 6.3745 | 5.5687 | 3.7642 | 4.9024 | |
| 5.1255 | 3.3124 | 6.089 | 4.8857 | 3.3536 | 3.145 | 4.0805 | 5.6217 | |
| 4.9509 | 4.7523 | 6.9982 | 8.5135 | 4.9831 | 5.7114 | 3.4464 | 1.6085 | |
| 6.3927 | 1.3453 | 4.1783 | 4.7853 | 8.5667 | 3.0632 | 4.3698 | 3.8949 | |
| 5.859 | 5.8526 | 0 | 4.8651 | 6.9831 | 7.6665 | 2.5341 | 6.4664 | |
| 5.7671 | 8.6413 | 4.3627 | 6.8177 | 4.1842 | 3.8626 | 4.8638 | 5.2667 | |
| 6.0386 | 5.7791 | 4.1328 | 8.3321 | 5.9782 | 4.3075 | 5.8919 | 4.0292 | |
| 4.1475 | 4.841 | 4.8045 | 6.6458 | 3.9384 | 5.5702 | 7.0752 | 6.6186 | |
| 6.4259 | 3.7441 | 2.081 | 2.5153 | 2.3219 | 3.6006 | 3.3185 | 3.4797 | |
| 6.2359 | 3.6703 | 5.0457 | 8.3782 | 5.2962 | 6.4026 | 4.6367 | 5.3966 | |
| 4.639 | 1.9593 | 4.5206 | 3.1145 | 4.737 | 4.8382 | 2.6505 | 2.9678 | |
| 3.7348 | 6.4426 | 7.75 | 3.3649 | 3.2943 | 5.7547 | 5.7895 | 3.3642 | 4.19 |
| | 3.9899 | 1.224 | 8.7273 | 2.2924 | 6.1244 | 5.9925 | 2.366 | |
| 6.0012 | 3.4734 | 3.996 | 2.9697 | 0.6063 | 3.5368 | 7.4507 | 3.9555 | |
| 6.535 | 6.5655 | 6.1171 | 6.0671 | 5.2175 | 1.7238 | 4.2928 | 2.848 | |
| 2.8815 | 3.6869 | 5.3211 | 3.2158 | 5.8678 | 7.916 | 3.9997 | 3.5147 | |
| 5.8939 | 5.8387 | 4.0866 | 5.3422 | 4.8715 | 4.1214 | 4.2814 | 3.7671 | |
| 3.3015 | 3.0084 | 4.0375 | 5.5771 | 5.7722 | 5.1434 | 2.0478 | 5.7367 | |
| 6.2005 | 2.5752 | 8.5406 | 7.872 | 8.1865 | 6.8774 | 10.1188 | 8.6705 | |
| 10.0358 | 9.5755 | 9.6581 | 8.5765 | 8.6927 | 9.5445 | 9.52 | 7.3049 | |
| 7.4827 | 10.7464 | 9.638 | 8.7195 | 9.5913 | 8.502 | 9.1466 | 9.1944 | |
| 9.6178 | 8.9163 | | | | | | | |
| CRISPLD2 | | 9.0881 | 8.6979 | 7.4288 | 9.0087 | 7.9174 | 7.4636 | |
| 9.6289 | 7.833 | 7.284 | 7.0361 | 8.1471 | 8.6174 | 7.1072 | 8.2476 | |
| 8.5134 | 7.8192 | 7.7326 | 8.1312 | 7.8597 | 8.2302 | 6.9139 | 8.9031 | |
| 8.7729 | 9.0054 | 6.8608 | 7.9966 | 9.3518 | 7.2952 | 7.8718 | 7.1506 | |
| 7.6248 | 9.7483 | 7.9549 | 8.0294 | 7.1776 | 7.7063 | 8.6291 | 7.7783 | |
| 7.4541 | 7.7968 | 7.639 | 9.2661 | 6.3711 | 8.2801 | 9.3492 | 9.2737 | |
| 9.4949 | 8.308 | 8.8793 | 9.1035 | 9.1412 | 8.3795 | 9.19 | 8.6637 | |
| 7.823 | 8.1289 | 6.1043 | 7.8636 | 8.5771 | 8.8206 | 6.6052 | 8.3143 | |
| 9.0452 | 9.1742 | 10.3879 | 6.9519 | 11.5636 | 8.3494 | 9.748 | 9.9307 | |
| 7.2187 | 7.904 | 9.8085 | 8.1851 | 7.7828 | 9.3336 | 8.8462 | 7.1292 | |
| 9.5407 | 9.8939 | 7.9623 | 8.5656 | 9.9204 | 9.189 | 8.1887 | 8.943 | |
| 8.4695 | 8.3111 | 8.7508 | 8.5476 | 8.7926 | 7.2084 | 7.9873 | 8.2978 | |
| 11.0905 | 7.127 | 8.3063 | 6.7571 | 9.9986 | 8.0168 | 10.41 | 7.3956 | |
| 7.429 | 9.3445 | 8.5229 | 8.3765 | 8.928 | 8.1759 | 10.0078 | 7.5908 | |
| 8.659 | 9.3745 | 6.7141 | 7.7537 | 5.6915 | 8.1606 | 7.1192 | 9.4088 | |
| 9.1345 | 8.0355 | 10.5669 | 10.3717 | 7.5982 | 8.4692 | 7.0325 | 9.822 | |
| 8.3587 | 8.374 | 6.8159 | 7.6682 | 10.3797 | 7.267 | 8.4182 | 8.6405 | |
| 8.3324 | 9.8308 | 8.1071 | 6.7096 | 8.0109 | 7.7491 | 7.846 | 8.3858 | |
| 6.9186 | 9.0814 | 7.1347 | 8.334 | 9.2794 | 8.3369 | 7.8905 | 7.3926 | |
| 7.848 | 8.5559 | 8.1953 | 8.4356 | 8.5951 | 8.9394 | 8.8561 | 8.3987 | |
| 6.7433 | 7.1373 | 7.559 | 7.711 | 7.4331 | 8.6838 | 6.9546 | 7.4941 | |
| 9.5778 | 9.2321 | 8.1795 | 7.866 | 10.2752 | 6.5713 | 6.1228 | 7.8673 | |
| 8.2367 | 8.6961 | 8.1947 | 11.1911 | 11.0673 | 11.8579 | 7.4378 | 11.3231 | |
| 14.4868 | 10.3823 | 12.439 | 11.8211 | 11.2732 | 11.7726 | 12.1771 | 11.0696 | |
| 11.9818 | 8.3326 | 11.5712 | 11.9276 | 10.8373 | 8.4981 | 10.8343 | 10.2763 | |
| 11.1906 | 11.1864 | 9.567 | | | | | | |
| SHE | 3.663 | 7.4616 | 6.3315 | 4.91E+00 | | 5.99E+00 | | |

| | | | | | | | |
|---|---|---|---|---|---|---|---|
| 3.6875 | 5.8457 | 5.4136 | 5.2105 | 4.6422 | 5.8276 | 5.3908 | 5.4417 |
| 4.9193 | 4.7769 | 5.2251 | 5.9461 | 5.137 | 5.4543 | 6.1404 | 4.7327 |
| 7.445 | 6.3266 | 5.5782 | 5.9694 | 8.4734 | 5.5872 | 4.7669 | 5.7151 |
| 8.1506 | 4.6185 | 6.7748 | 4.7336 | 4.2285 | 4.8606 | 6.5649 | 5.3376 |
| 8.6169 | 4.6139 | 4.1403 | 6.4685 | 5.4995 | 4.3892 | 5.6667 | 7.0702 |
| 5.4094 | 8.0601 | 4.2519 | 5.9221 | 6.7012 | 8.0665 | 5.1104 | 6.2526 |
| 5.3096 | 3.4915 | 6.1741 | 5.8194 | 5.5344 | 5.2631 | 7.3 | 5.0239 |
| 5.4728 | 5.5623 | 5.2767 | 7.5957 | 5.2315 | 6.4937 | 6.472 | 6.8601 |
| 6.4116 | 5.2835 | 5.5408 | 8.43 | 7.9969 | 5.8636 | 4.0964 | 5.0272 |
| 5.441 | 6.1616 | 6.6578 | 6.2342 | 7.0401 | 6.5221 | 4.2968 | 8.024 |
| 5.6581 | 5.1254 | 3.3101 | 6.5208 | 5.1711 | 5.8798 | 4.3857 | 5.9453 |
| 7.176 | 7.0844 | 5.7084 | 5.1724 | 4.5943 | 4.9483 | 4.7549 | 5.4811 |
| 6.165 | 5.0924 | 7.0814 | 5.3446 | 7.0301 | 8.0709 | 5.14 | 7.1907 |
| 5.1087 | 5.2518 | 4.5103 | 3.7308 | 4.7396 | 4.1293 | 6.0001 | 5.964 |
| 5.5547 | 4.4743 | 5.1578 | 5.7867 | 9.4341 | 4.3672 | 5.0995 | 4.8253 |
| 5.9997 | 4.2078 | 5.1007 | 4.9896 | 3.1725 | 9.4744 | 2.7757 | 6.2505 |
| 5.9345 | 4.0211 | 5.6709 | 4.6196 | 4.2122 | 4.7774 | 4.842 | 5.6831 |
| 4.7729 | 4.6173 | 5.5939 | 5.1525 | 5.6989 | 8.7089 | 5.6314 | 4.379 |
| 4.9834 | 5.5966 | 9.3648 | 5.13 | 4.85 | 5.8143 | 6.1672 | 6.6808 |
| 4.197 | 4.8689 | 5.2282 | 5.7515 | 4.2968 | 5.758 | 6.7634 | 4.8141 |
| 4.1471 | 5.2246 | 6.4229 | 6.251 | 6.3738 | 6.6033 | 5.2843 | 2.8198 |
| 5.0387 | 5.9791 | 6.2965 | 3.6039 | 8.8036 | 7.459 | 8.9125 | 5.8137 |
| 8.7827 | 9.021 | 10.0085 | 10.0398 | 10.6686 | 7.5961 | 9.7936 | 9.6855 |
| 9.0291 | 7.0047 | 7.0299 | 9.4921 | 9.6111 | 8.7295 | 8.1594 | 8.7283 |
| 9.8299 | 7.8916 | 9.3396 | 7.9657 | | | | |
| ZEB2 | 6.4866 | 7.6362 | 7.2432 | 8.0954 | 8.056 | 6.7604 | 6.1208 |
| 6.4204 | 7.7016 | 6.3664 | 8.0959 | 6.0559 | 7.8765 | 7.2845 | 6.7514 |
| 7.4407 | 5.7368 | 8.0637 | 6.9539 | 8.2218 | 7.4021 | 6.8332 | 7.8713 |
| 7.3135 | 5.8606 | 10.8801 | 7.8378 | 8.7222 | 7.552 | 5.3584 | 6.2117 |
| 7.536 | 6.255 | 5.7612 | 6.8625 | 8.1693 | 7.2658 | 7.2384 | 7.4466 |
| 6.8197 | 7.5363 | 7.3579 | 5.194 | 7.3514 | 8.8331 | 8.2426 | 9.2817 |
| 6.4427 | 7.9403 | 8.9658 | 9.0101 | 6.824 | 8.8946 | 6.5086 | 6.0791 |
| 8.3277 | 6.8066 | 6.5374 | 7.0541 | 8.4551 | 7.4781 | 7.5285 | 9.221 |
| 7.3424 | 9.3472 | 4.0689 | 7.5413 | 8.6399 | 8.0299 | 8.8031 | 5.9089 |
| 7.2288 | 9.8875 | 7.5848 | 6.5323 | 6.7163 | 6.0645 | 7.2038 | 7.7301 |
| 9.4948 | 8.2914 | 6.7849 | 8.9862 | 7.7 | 7.7499 | 6.4197 | 8.6099 |
| 5.0344 | 8.3683 | 6.4614 | 7.8489 | 6.5811 | 6.1528 | 8.368 | 8.393 |
| 7.5039 | 7.0612 | 6.6682 | 6.8355 | 6.585 | 9.004 | 7.4919 | 6.3146 |
| 7.0475 | 6.8787 | 8.6173 | 8.2731 | 7.8783 | 8.3258 | 6.9747 | 8.261 |
| 7.2473 | 4.1963 | 6.5806 | 5.8765 | 7.5341 | 8.1354 | 7.1803 | 8.1017 |
| 6.5702 | 8.8185 | 8.9227 | 5.9776 | 6.8338 | 6.8942 | 7.8968 | 7.2758 |
| 7.3258 | 6.2561 | 4.8272 | 9.9289 | 6.2672 | 8.8384 | 6.9345 | 5.9847 |
| 9.5026 | 7.3216 | 6.1733 | 7.9956 | 6.0843 | 7.9425 | 8.3466 | 5.5583 |
| 7.2121 | 7.6202 | 7.498 | 9.3456 | 6.6615 | 7.0674 | 5.9939 | 7.2895 |
| 5.9104 | 5.3175 | 6.1129 | 6.7912 | 7.3706 | 8.7478 | 6.4075 | 6.4513 |
| 5.7831 | 7.473 | 7.2814 | 7.6076 | 8.5565 | 6.4187 | 6.1909 | 8.1192 |
| 8.9612 | 6.9442 | 8.0994 | 8.4979 | 7.2484 | 5.6289 | 5.7627 | 8.1342 |
| 8.6162 | 5.8433 | 9.8941 | 10.4617 | 9.8585 | 7.2775 | 10.0078 | 9.6969 |
| 10.9171 | 10.7416 | 10.4275 | 10.8268 | 10.9521 | 10.2926 | 10.2155 | 9.7178 |
| 8.5826 | 10.4116 | 10.5858 | 9.8371 | 11.46 | 9.8452 | 9.7344 | 10.4666 |
| 10.7088 | 9.338 | | | | | | |
| SYDE1 | 8.3582 | 7.8078 | 6.7255 | 7.78E+00 | | 6.00E+00 | |
| 7.636 | 8.0721 | 6.8193 | 5.8447 | 7.8299 | 8.7062 | 6.2165 | 5.8102 |
| 7.5853 | 8.3225 | 7.5818 | 7.3905 | 9.3169 | 6.4639 | 7.0365 | 8.9589 |

| | | | | | | | | |
|---|---|---|---|---|---|---|---|---|
| 6.8425 | 8.327 | 8.5201 | 6.5927 | 9.2709 | 7.5426 | 9.1434 | 8.4711 | |
| 6.6375 | 6.2693 | 7.7898 | 6.142 | 5.9746 | 6.4873 | 7.5618 | 8.1666 | |
| 5.9174 | 6.2128 | 6.637 | 7.6129 | 9.785 | 6.6772 | 7.3317 | 7.1736 | |
| 8.1761 | 9.0989 | 6.9155 | 8.1342 | 7.9854 | 8.8639 | 6.78 | 7.3488 | 6.56 |
| | 9.2664 | 7.6506 | 6.7663 | 7.4199 | 8.547 | 9.2784 | 7.4524 | |
| 8.0645 | 7.5029 | 8.2077 | 8.1452 | 5.1556 | 7.8211 | 8.4046 | 8.1248 | |
| 8.1566 | 7.822 | 6.9176 | 8.557 | 6.61 | 7.7914 | 8.018 | 6.5269 | |
| 7.2895 | 7.4387 | 9.8325 | 6.5917 | 7.9122 | 9.1805 | 7.5545 | 9.0188 | |
| 7.1399 | 8.3012 | 6.8765 | 7.6408 | 8.7806 | 7.7408 | 6.1101 | 7.1387 | |
| 8.3357 | 7.9859 | 7.9939 | 5.8967 | 5.5476 | 9.1593 | 6.7142 | 8.2924 | |
| 8.868 | 6.584 | 7.3794 | 6.5579 | 7.4625 | 9.9916 | 7.386 | 8.1033 | |
| 6.8788 | 8.3915 | 7.4451 | 6.568 | 7.7283 | 4.7823 | 6.7594 | 6.8781 | |
| 7.8424 | 10.1849 | 6.0055 | 9.9039 | 8.89 | 5.1961 | 6.0678 | 7.1871 | |
| 7.3245 | 6.7513 | 6.7874 | 7.2514 | 7.5903 | 9.6618 | 6.5927 | 7.7964 | |
| 7.9638 | 7.627 | 8.7972 | 8.0264 | 7.921 | 10.1588 | 5.7896 | 8.679 | |
| 7.565 | 7.116 | 7.6009 | 6.7554 | 7.7134 | 7.7057 | 7.44 | 5.0349 | |
| 7.0555 | 7.0836 | 7.0196 | 6.6096 | 8.302 | 8.8826 | 8.0049 | 8.1149 | |
| 8.0337 | 7.7734 | 8.6379 | 6.6624 | 7.626 | 7.6196 | 8.3172 | 7.6098 | |
| 6.0348 | 7.3957 | 7.3562 | 7.1868 | 6.9641 | 8.8176 | 7.047 | 7.1943 | |
| 6.5221 | 7.2087 | 7.6136 | 7.147 | 10.1015 | 9.9615 | 10.439 | 7.8051 | |
| 10.6969 | 9.7039 | 10.0286 | 10.1728 | 10.2427 | 9.9992 | 10.4495 | 10.6837 | |
| 10.71 | 9.9957 | 8.3888 | 10.2325 | 9.935 | 10.3632 | 10.5123 | 10.176 | |
| 10.1767 | 9.891 | 10.4368 | 10.3735 | | | | | |
| LRP1 | 11.0487 | 13.3178 | 10.9246 | 11.5697 | 11.6134 | 12.1559 | 11.5904 | |
| 11.5814 | 10.9929 | 11.8543 | 11.6867 | 11.1059 | 10.8529 | 11.8302 | 12.4263 | |
| 11.0902 | 10.241 | 12.4989 | 10.6663 | 10.5424 | 11.2696 | 10.4249 | 11.1978 | |
| 12.5915 | 9.9498 | 11.6974 | 11.501 | 11.1165 | 11.0161 | 11.1845 | 9.9409 | |
| 11.9826 | 10.7068 | 9.6081 | 11.6444 | 10.8326 | 11.0208 | 10.7274 | 11.0321 | |
| 11.1224 | 11.2505 | 12.4298 | 10.4372 | 11.0126 | 11.6047 | 12.8797 | 12.3919 | |
| 11.0299 | 11.6181 | 13.1155 | 12.7258 | 10.3699 | 11.1416 | 11.313 | 12.4326 | |
| 11.0355 | 10.9893 | 11.5267 | 12.6385 | 12.6658 | 11.1331 | 12.0956 | 11.1852 | |
| 12.339 | 12.1843 | 11.4828 | 12.1338 | 11.7815 | 11.2709 | 12.5818 | 9.8187 | |
| 10.987 | 12.5687 | 10.9924 | 11.1542 | 12.9616 | 10.7727 | 11.3633 | 11.7727 | |
| 12.1436 | 10.7083 | 12.1034 | 12.2718 | 11.5765 | 12.3772 | 11.7079 | 10.6402 | |
| 11.399 | 11.5731 | 13.112 | 10.9761 | 10.2578 | 10.6255 | 12.1243 | 12.2514 | |
| 11.4823 | 10.7191 | 10.4435 | 12.704 | 9.4737 | 12.0994 | 11.3804 | 11.2248 | |
| 12.0636 | 10.6963 | 11.4396 | 12.2192 | 11.2587 | 11.8682 | 10.5115 | 12.0173 | |
| 12.5592 | 12.0457 | 12.4338 | 9.2932 | 11.1185 | 10.9282 | 10.8357 | 11.6781 | |
| 11.1153 | 12.5415 | 12.661 | 10.4932 | 11.3659 | 10.8305 | 12.1286 | 11.6665 | |
| 11.1649 | 11.5081 | 9.486 | 13.164 | 11.1367 | 12.547 | 11.7466 | 11.3287 | |
| 13.3357 | 12.6376 | 10.5599 | 11.3373 | 10.6346 | 10.3292 | 11.3908 | 11.3356 | |
| 10.5616 | 11.1482 | 10.651 | 12.4052 | 10.8302 | 11.9172 | 11.121 | 11.302 | |
| 10.7549 | 11.1245 | 9.7053 | 11.1312 | 11.4477 | 12.0584 | 10.2659 | 10.3529 | |
| 10.2494 | 11.3081 | 11.8465 | 11.4551 | 12.192 | 11.0254 | 10.7593 | 12.3148 | |
| 11.5139 | 11.8595 | 11.8337 | 12.937 | 11.5471 | 10.6356 | 10.8015 | 11.2948 | |
| 11.702 | 10.6375 | 13.0054 | 12.986 | 13.5867 | 11.657 | 13.2297 | 13.4462 | |
| 13.8185 | 14.3306 | 13.5089 | 13.0961 | 14.4363 | 13.3876 | 13.8288 | 12.9672 | |
| 12.2782 | 13.6454 | 13.547 | 13.6993 | 14.6747 | 13.4492 | 13.1533 | 12.6153 | |
| 13.8975 | 13.7228 | | | | | | | |
| DNA2 | 7.5121 | 6.7744 | 6.7747 | 8.52E+00 | | 7.33E+00 | | 6.98 |
| | 8.6257 | 8.2037 | 8.1357 | 8.1183 | 8.1126 | 8.6764 | 7.8903 | |
| 6.6558 | 7.3653 | 7.571 | 7.8525 | 6.0246 | 6.5519 | 8.1938 | 6.7694 | |
| 9.8886 | 6.6924 | 5.3967 | 8.2704 | 8.4734 | 8.252 | 7.7431 | 7.0766 | |
| 8.4624 | 8.4818 | 7.6003 | 8.6125 | 7.8979 | 8.2361 | 6.6599 | 7.6189 | |

| | | | | | | | | |
|---|---|---|---|---|---|---|---|---|
| 8.6625 | 6.7971 | 5.9341 | 7.7747 | 8.7416 | 6.7099 | 7.7601 | 8.0763 | |
| 7.8354 | 7.7874 | 8.6355 | 7.7237 | 6.3042 | 8.0479 | 7.589 | 8.8971 | |
| 7.5916 | 7.0903 | 7.0869 | 5.3777 | 8.4207 | 4.6504 | 6.3284 | 7.2971 | |
| 8.3712 | 7.1363 | 6.4777 | 7.9566 | 8.0936 | 6.4786 | 4.6497 | 5.779 | |
| 8.3193 | 7.0406 | 8.5196 | 5.4722 | 7.8782 | 7.0982 | 7.056 | 7.7271 | |
| 6.6159 | 7.8781 | 8.1551 | 8.3701 | 8.1607 | 6.6334 | 8.6722 | 6.7389 | |
| 7.5578 | 8.0839 | 5.5796 | 7.7078 | 7.1453 | 8.6057 | 8.758 | 9.0872 | |
| 8.1814 | 8.0269 | 7.1061 | 8.1126 | 7.52 | 6.3709 | 7.5392 | 8.2402 | |
| 8.5152 | 8.1364 | 7.3207 | 8.5092 | 8.3395 | 7.7136 | 7.3947 | 8.0337 | |
| 6.9922 | 8.063 | 7.3163 | 7.0333 | 7.1446 | 8.5515 | 7.2892 | 7.9384 | |
| 7.1112 | 4.9339 | 7.0743 | 4.8598 | 7.6535 | 8.6604 | 6.1792 | 6.1515 | |
| 7.9643 | 8.1629 | 7.5576 | 6.8902 | 7.0509 | 7.0362 | 6.1334 | 7.3537 | |
| 7.9522 | 8.6926 | 7.8016 | 9.0786 | 7.8454 | 8.1609 | 5.5372 | 6.917 | |
| 7.1562 | 7.0225 | 8.0087 | 8.4953 | 6.2333 | 7.8124 | 8.3872 | 8.7305 | |
| 8.2203 | 9.3191 | 8.3779 | 8.4121 | 4.4516 | 7.9913 | 9.0156 | 5.9403 | |
| 6.9948 | 7.9698 | 9.2677 | 6.2016 | 7.8715 | 6.9033 | 8.1127 | 7.3612 | |
| 6.9651 | 8.0336 | 8.8296 | 7.1201 | 7.451 | 8.0116 | 8.125 | 7.0256 | |
| 6.9394 | 7.4906 | 7.7944 | 6.1758 | 4.8843 | 7.3076 | 5.4724 | 3.8887 | |
| 3.9977 | 4.9029 | 4.5728 | 5.3054 | 4.5734 | 6.3911 | 4.3886 | 2.8039 | 4.31 |
| | 6.7977 | 5.4273 | 4.6604 | 6.2801 | 4.9835 | 4.8019 | 4.6897 | |
| 4.3651 | 6.7792 | 4.7967 | 5.304 | | | | | |
| CENPL | 7.737 | 7.9143 | 7.2197 | 7.9328 | 8.2624 | 8.0258 | 7.8292 | |
| 8.4956 | 8.3803 | 7.4659 | 7.888 | 7.9898 | 7.0043 | 6.6558 | 7.5384 | |
| 7.5978 | 7.8034 | 7.4699 | 6.4311 | 7.6624 | 7.2521 | 8.6911 | 6.6924 | |
| 6.8917 | 7.733 | 8.0819 | 8.5425 | 8.1138 | 6.9398 | 8.8227 | 7.3482 | |
| 7.9214 | 8.9736 | 7.3805 | 7.3673 | 7.0396 | 7.7332 | 8.1076 | 7.6614 | |
| 6.9449 | 7.2393 | 8.3629 | 7.112 | 8.1075 | 6.6042 | 7.839 | 7.995 | |
| 7.8092 | 7.0963 | 6.8071 | 8.119 | 7.9483 | 7.9621 | 7.5775 | 7.5742 | |
| 7.5296 | 6.6531 | 8.5412 | 5.5923 | 8.0824 | 7.7882 | 8.5173 | 7.4807 | |
| 7.4696 | 7.8786 | 6.9155 | 7.7411 | 6.0351 | 6.528 | 7.371 | 6.7756 | |
| 8.7082 | 5.9208 | 8.1463 | 6.3729 | 7.4239 | 8.7855 | 6.7989 | 7.6049 | |
| 8.1098 | 7.9761 | 7.1503 | 6.5602 | 8.051 | 7.2794 | 7.6518 | 7.649 | |
| 6.7477 | 7.3834 | 8.8677 | 7.8489 | 7.8988 | 9.1697 | 7.6314 | 7.6511 | |
| 7.6981 | 8.4805 | 7.0131 | 7.4684 | 8.1396 | 8.5214 | 7.5336 | 8.2049 | |
| 7.9534 | 8.0522 | 7.6055 | 8.069 | 6.8875 | 7.7087 | 7.4131 | 8.0014 | |
| 7.717 | 7.6039 | 8.6161 | 7.5844 | 6.838 | 7.8332 | 7.0386 | 6.7019 | |
| 7.6171 | 6.2074 | 7.0117 | 8.2447 | 6.8644 | 6.3459 | 7.2443 | 7.2618 | |
| 8.0602 | 6.9949 | 8.1543 | 6.9563 | 6.4063 | 7.6667 | 7.7112 | 7.8882 | |
| 8.3066 | 7.8229 | 7.8805 | 7.3901 | 7.1709 | 7.0149 | 6.6848 | 6.5479 | |
| 6.9363 | 7.5223 | 6.9199 | 7.3954 | 7.5596 | 8.9956 | 7.7849 | 8.6149 | |
| 7.616 | 8.0053 | 6.0836 | 8.2357 | 8.1125 | 5.9143 | 7.5107 | 7.7775 | |
| 8.6332 | 6.6322 | 7.6879 | 7.1816 | 7.1932 | 7.5479 | 6.8544 | 8.1735 | |
| 7.9407 | 7.85 | 8.0608 | 8.4262 | 8.2574 | 7.7355 | 8.1021 | 7.6263 | |
| 6.6333 | 6.8847 | 5.8596 | 7.0937 | 5.9274 | 5.1397 | 6.0351 | 5.5967 | |
| 6.073 | 5.9038 | 5.7526 | 6.6036 | 5.3923 | 6.0503 | 6.2887 | 6.0665 | |
| 5.3017 | 6.4144 | 6.6263 | 6.0995 | 5.4242 | 5.5668 | 5.8317 | 6.6596 | |
| 5.2549 | 5.9708 | | | | | | | |
| C12orf48 | | 8.5093 | 7.609 | 6.6118 | 8.71E+00 | | 8.89E+00 | |
| | 7.1505 | 8.8496 | 7.2431 | 8.7954 | 8.023 | 7.9668 | 7.442 | |
| 8.1877 | 6.8944 | 7.072 | 7.7172 | 7.5629 | 7.3853 | 7.2825 | 7.7748 | |
| 6.7965 | 9.0361 | 7.4883 | 5.7654 | 8.0652 | 9.565 | 8.2253 | 8.366 | |
| 7.3781 | 8.5865 | 7.3694 | 6.3189 | 8.5652 | 8.5884 | 8.3564 | 7.7622 | |
| 7.2366 | 9.1233 | 7.2858 | 7.1411 | 6.9083 | 9.3456 | 6.5128 | 7.7197 | |
| 8.122 | 7.8731 | 7.4593 | 8.2879 | 7.0931 | 6.9884 | 7.9051 | 7.5957 | |

| | | | | | | | | |
|---|---|---|---|---|---|---|---|---|
| 8.5536 | 8.5927 | 7.3693 | 8.0249 | 6.0935 | 7.3916 | 5.3252 | 8.1685 | |
| 8.6058 | 8.0278 | 7.6739 | 7.402 | 8.203 | 6.2418 | 7.8134 | 5.4798 | |
| 6.3596 | 8.3098 | 7.654 | 8.4409 | 5.4608 | 8.4515 | 7.3943 | 7.3729 | |
| 8.6217 | 7.8876 | 6.9005 | 7.7341 | 8.3897 | 7.1482 | 7.3679 | 8.2553 | |
| 7.7905 | 7.5396 | 8.1946 | 7.275 | 7.2823 | 8.4729 | 8.1614 | 8.7288 | |
| 8.438 | 7.6577 | 8.2743 | 8.0043 | 7.4996 | 7.8918 | 6.398 | 7.3597 | |
| 8.9209 | 8.9113 | 8.3118 | 8.4581 | 8.1016 | 8.1491 | 8.3506 | 8.1599 | 7.86 |
| | 7.3917 | 8.2265 | 9.0894 | 7.8937 | 8.2852 | 8.378 | 7.4543 | |
| 7.8749 | 6.9242 | 5.2831 | 7.7328 | 6.1611 | 7.7643 | 7.9898 | 7.5845 | |
| 7.3036 | 8.5262 | 8.3164 | 7.0222 | 7.5231 | 7.3414 | 6.2221 | 7.1788 | |
| 7.5294 | 8.1604 | 7.784 | 8.2693 | 7.7011 | 8.0769 | 8.0667 | 7.7006 | |
| 8.1471 | 8.1826 | 6.9519 | 8.4709 | 8.8543 | 8.2012 | 8.054 | 8.2322 | |
| 8.684 | 8.1799 | 8.8456 | 8.263 | 8.2845 | 4.7823 | 7.7933 | 8.7777 | |
| 5.4438 | 7.7507 | 6.549 | 8.4126 | 7.6052 | 8.5206 | 6.927 | 7.9764 | |
| 7.9774 | 7.6156 | 8.0528 | 9.1039 | 7.5013 | 7.3749 | 6.5436 | 8.0512 | |
| 8.0201 | 8.1044 | 7.5633 | 7.5194 | 7.3403 | 5.4717 | 7.8211 | 5.6663 | |
| 4.5972 | 5.333 | 5.4509 | 5.3721 | 5.3018 | 4.9378 | 7.0318 | 4.7935 | |
| 5.6279 | 5.5731 | 7.9343 | 5.7906 | 5.9861 | 6.5308 | 5.3078 | 4.0933 | |
| 5.469 | 5.0039 | 6.9317 | 4.7967 | 5.0645 | | | | |
| CHAF1A | 11.0049 | 9.2443 | 8.7594 | 10.7022 | 9.0638 | 9.7865 | 9.2893 | |
| 9.2047 | 10.5153 | 9.4499 | 9.097 | 9.3899 | 9.3142 | 8.5488 | 8.6648 | |
| 9.9507 | 9.2967 | 9.8113 | 8.8871 | 8.5664 | 10.2512 | 10.1958 | 9.845 | |
| 8.5484 | 9.4769 | 9.9236 | 9.6018 | 9.9104 | 8.1797 | 9.848 | 10.6803 | |
| 8.8169 | 10.2805 | 9.3082 | 8.9423 | 8.7798 | 8.1557 | 9.3802 | 9.1823 | |
| 9.9828 | 8.9575 | 10.3545 | 9.7328 | 8.9934 | 8.601 | 9.2948 | 8.7578 | |
| 9.9431 | 9.5703 | 8.5405 | 8.8723 | 8.5687 | 8.7764 | 9.9231 | 10.5719 | |
| 9.5213 | 8.57 | 9.8478 | 8.1218 | 9.2809 | 9.7741 | 9.7065 | 9.4784 | |
| 9.6947 | 9.2319 | 9.4029 | 9.1759 | 8.1139 | 7.5105 | 10.1775 | 10.1234 | |
| 9.5976 | 7.7288 | 9.1855 | 9.5813 | 8.8251 | 9.3127 | 10.2224 | 8.6715 | |
| 10.3854 | 9.4851 | 10.2711 | 8.2702 | 10.4902 | 8.8279 | 9.3442 | 9.4271 | |
| 9.8631 | 9.9174 | 8.6783 | 8.829 | 10.2625 | 10.2977 | 9.4602 | 8.7212 | |
| 9.2935 | 9.4103 | 9.5088 | 8.6843 | 9.0168 | 9.7391 | 9.1594 | 9.3128 | |
| 9.1338 | 9.5908 | 8.5298 | 9.7692 | 9.2082 | 8.5949 | 9.5193 | 9.131 | |
| 10.5632 | 9.1767 | 8.9782 | 9.5347 | 9.4095 | 8.5214 | 9.0854 | 9.4776 | |
| 10.7007 | 6.9584 | 8.9798 | 9.7679 | 9.1837 | 9.1726 | 10.0265 | 9.9768 | |
| 8.995 | 9.7475 | 9.3286 | 8.3779 | 9.6738 | 8.9315 | 9.6176 | 10.4052 | |
| 9.1599 | 9.9584 | 8.9988 | 10.8903 | 9.3631 | 10.341 | 9.1995 | 9.7273 | |
| 9.1329 | 9.7618 | 8.3566 | 8.5722 | 10.0568 | 10.1057 | 10.0731 | 9.3576 | |
| 10.1029 | 9.9492 | 7.9507 | 9.7706 | 10.1849 | 8.0743 | 10.478 | 9.5138 | |
| 9.8985 | 8.9608 | 8.8684 | 9.2493 | 10.0653 | 10.3667 | 9.8362 | 10.6082 | |
| 10.184 | 8.6568 | 8.9413 | 9.2935 | 9.8783 | 9.5862 | 10.3954 | 9.6429 | |
| 9.1504 | 10.8818 | 7.2322 | 9.5091 | 7.5891 | 7.7395 | 7.5048 | 7.7104 | |
| 7.3085 | 7.4058 | 7.6561 | 8.4423 | 7.2077 | 7.5002 | 7.7732 | 7.7563 | |
| 8.1416 | 7.3053 | 7.5197 | 7.7371 | 7.1202 | 8.1403 | 7.3625 | 8.3341 | |
| 7.6481 | 7.9868 | | | | | | | |
| LHFP | 7.7116 | 8.4089 | 7.366 | 8.7162 | 7.0614 | 8.8191 | 8.354 | |
| 8.2722 | 7.6552 | 6.9641 | 9.0157 | 7.7908 | 6.8715 | 8.4599 | 8.5881 | |
| 8.354 | 7.8225 | 6.8694 | 7.2303 | 8.5273 | 6.6841 | 8.0955 | 8.3107 | |
| 8.3162 | 6.4075 | 10.5886 | 8.9021 | 8.5608 | 7.1483 | 6.2006 | 6.0761 | |
| 8.3899 | 7.8741 | 8.6158 | 8.5594 | 8.3893 | 8.0241 | 8.2151 | 7.2118 | |
| 5.8405 | 7.9928 | 9.2585 | 6.7525 | 7.335 | 8.4266 | 7.9918 | 9.8338 | |
| 8.035 | 7.9108 | 8.2808 | 10.361 | 7.3818 | 8.9592 | 6.5956 | 9.3171 | |
| 8.3985 | 5.1669 | 9.0303 | 8.526 | 9.659 | 6.1298 | 8.7349 | 7.4807 | |
| 8.288 | 9.365 | 5.9679 | 9.4167 | 8.1234 | 8.729 | 9.0636 | 7.5971 | |

| | | | | | | | |
|---|---|---|---|---|---|---|---|
| 7.8377 | 10.531 | 8.5078 | 8.1655 | 7.9413 | 7.2376 | 6.2642 | 8.5028 |
| 10.2651 | 8.5789 | 8.0724 | 9.2062 | 8.6428 | 8.1865 | 8.0475 | 8.1701 |
| 6.3545 | 8.0307 | 7.8625 | 9.1255 | 6.7129 | 7.7977 | 10.3087 | 8.3124 |
| 7.6627 | 7.3186 | 7.7683 | 7.5911 | 8.2336 | 9.4776 | 8.7313 | 6.9768 |
| 8.6007 | 7.8087 | 8.5339 | 9.4824 | 7.4704 | 8.709 | 7.0687 | 8.5752 |
| 9.6329 | 3.8798 | 8.7055 | 6.4139 | 8.1321 | 8.4774 | 7.4714 | 7.2682 |
| 6.8616 | 8.7003 | 9.9815 | 7.3872 | 8.109 | 7.4305 | 8.2611 | 7.5378 |
| 8.0762 | 8.9434 | 5.9953 | 10.4696 | 6.3557 | 7.8741 | 9.1536 | 8.2517 |
| 9.4289 | 7.2738 | 6.3661 | 7.8804 | 6.31 | 7.7319 | 8.251 | 7.7209 |
| 7.8042 | 7.8897 | 7.4081 | 8.3549 | 9.2793 | 10.0366 | 7.6102 | 7.3996 |
| 7.7162 | 7.459 | 7.1598 | 8.0354 | 10.241 | 8.7806 | 7.4921 | 8.3412 |
| 5.6121 | 7.4559 | 7.6113 | 7.8741 | 8.581 | 7.442 | 5.7862 | 8.7083 |
| 8.2929 | 7.7559 | 9.7559 | 9.5623 | 7.038 | 5.945 | 4.9921 | 8.9339 |
| 9.5798 | 6.5544 | 10.9678 | 9.7061 | 11.4076 | 7.97 | 10.6581 | 10.4626 |
| 12.4541 | 12.3261 | 11.9095 | 10.2319 | 12.0821 | 11.7095 | 10.7923 | 10.5548 |
| 9.4388 | 11.5806 | 10.697 | 10.2243 | 9.9152 | 10.9587 | 10.5423 | 10.3297 |
| 11.6999 | 9.6085 | | | | | | |
| PRC1 | 11.0639 | 10.3861 | 9.2803 | 10.571 | 11.2055 | 10.145 | 10.4708 |
| 10.1966 | 11.8258 | 10.6958 | 9.8013 | 10.2765 | 10.0758 | 9.0529 | 9.875 |
| 10.4527 | 10.1857 | 9.9179 | 9.1902 | 9.154 | 9.9604 | 11.7393 | 9.1748 |
| 8.2102 | 10.1337 | 11.0225 | 10.4634 | 10.5089 | 9.6159 | 11.0415 | 10.3173 |
| 8.6683 | 10.9875 | 10.5606 | 9.7792 | 10.3268 | 9.4713 | 11.0366 | 9.595 |
| 8.6901 | 9.7961 | 11.1008 | 10.2813 | 10.4194 | 10.3346 | 9.7319 | 9.1538 |
| 10.9199 | 9.8513 | 9.2501 | 10.1495 | 10.5093 | 11.0742 | 11.0657 | 10.0125 |
| 9.2625 | 8.3918 | 11.1527 | 8.0857 | 11.1935 | 9.7816 | 10.1437 | 9.0016 |
| 9.406 | 10.124 | 9.8639 | 9.823 | 7.4262 | 7.7262 | 10.601 | 10.025 |
| 11.1713 | 7.5054 | 11.3318 | 9.4764 | 8.3992 | 10.5466 | 9.9619 | 9.2173 |
| 12.1063 | 9.8067 | 9.9349 | 8.8924 | 10.7474 | 9.2404 | 9.7134 | 10.4531 |
| 9.5273 | 9.7449 | 10.0214 | 10.8764 | 11.4988 | 11.228 | 9.9465 | 11.3489 |
| 10.5917 | 10.4304 | 10.0611 | 7.5793 | 10.3848 | 11.542 | 10.8087 | 10.2104 |
| 10.0278 | 10.3842 | 10.146 | 11.0577 | 10.0717 | 9.6686 | 10.5214 | 9.9471 |
| 10.3495 | 9.2942 | 9.9167 | 10.5048 | 9.8858 | 11.0121 | 10.386 | 7.6302 |
| 10.1441 | 4.5503 | 9.4158 | 10.9832 | 9.2924 | 8.4144 | 10.5449 | 10.9199 |
| 9.6068 | 9.9984 | 9.4548 | 8.5875 | 8.1078 | 9.0785 | 10.0753 | 10.7407 |
| 10.3332 | 10.7578 | 10.1148 | 10.5043 | 10.2327 | 10.207 | 9.2451 | 8.8474 |
| 10.4023 | 10.6827 | 10.0014 | 9.4577 | 10.1595 | 11.3427 | 10.9835 | 10.7792 |
| 11.0788 | 10.5429 | 7.0428 | 10.2411 | 11.4791 | 7.6384 | 10.2173 | 10.3207 |
| 10.4986 | 9.0673 | 9.3777 | 9.6339 | 9.4993 | 8.8913 | 9.6583 | 11.2633 |
| 11.0151 | 8.7368 | 9.2173 | 11.3223 | 10.3861 | 10.9176 | 8.337 | 10.15 |
| 9.3027 | 9.5325 | 6.7187 | 10.4332 | 7.1716 | 6.9175 | 6.8594 | 7.2481 |
| 7.1385 | 7.2337 | 7.3761 | 9.2597 | 6.4131 | 6.0621 | 7.2229 | 7.5684 |
| 7.2204 | 7.434 | 8.3542 | 7.4141 | 6.8103 | 8.3438 | 7.3242 | 8.479 |
| 7.0107 | 7.5485 | | | | | | |
| SPC24 | 7.584 | 4.7735 | 2.6559 | 7.1305 | 5.9712 | 8.5272 | 7.6222 |
| 6.4144 | 7.3063 | 7.382 | 7.198 | 6.2506 | 3.5658 | 4.3171 | 4.0085 |
| 6.9432 | 5.5842 | 6.4252 | 3.8957 | 3.4755 | 5.537 | 7.9095 | 5.8354 |
| 3.1802 | 5.9459 | 5.719 | 7.2589 | 6.85 | 6.0913 | 7.0884 | 7.2485 |
| 3.9156 | 7.1784 | 5.225 | 5.1585 | 5.4117 | 3.9622 | 6.88 | 4.5034 |
| 5.7576 | 3.6225 | 6.4307 | 5.771 | 7.1895 | 5.0172 | 6.0151 | 4.3751 |
| 5.3709 | 4.5964 | 3.5149 | 6.9867 | 5.3698 | 5.3093 | 6.6776 | 7.1277 |
| 6.1616 | 3.3568 | 6.6785 | 3.1496 | 5.8709 | 6.452 | 6.1361 | 5.4137 |
| 4.9309 | 3.5065 | 6.2564 | 5.6909 | 2.7891 | 0 | 5.8574 | 6.891 |
| 6.5977 | 0.7204 | 6.7909 | 5.7966 | 4.7425 | 6.785 | 5.4929 | 3.9181 |
| 6.7492 | 5.6588 | 5.9428 | 2.2282 | 6.7395 | 5.1691 | 5.7742 | 5.0865 |

| | | | | | | | | |
|---|---|---|---|---|---|---|---|---|
| 5.7387 | 5.9275 | 6.2479 | 5.4009 | 6.0679 | 8.2061 | 6.0407 | 5.3317 | |
| 6.7495 | 5.586 | 5.3898 | 6.8355 | 4.1699 | 7.0112 | 6.0547 | 5.4826 | |
| 8.7427 | 5.9214 | 5.1979 | 6.6395 | 5.9177 | 5.5827 | 7.2291 | 7.5292 | |
| 5.1298 | 5.6676 | 7.1155 | 5.1122 | 5.603 | 4.8382 | 3.7078 | 3.629 | |
| 6.4553 | 0 | 4.8297 | 5.0462 | 4.5556 | 4.7044 | 5.2293 | 5.9365 | |
| 3.3948 | 5.5894 | 5.1043 | 4.0354 | 6.3897 | 4.9354 | 4.8617 | 6.6674 | |
| 6.8374 | 6.8214 | 7.4367 | 7.4326 | 6.1705 | 5.6222 | 4.2012 | 5.0831 | |
| 6.4606 | 6.0709 | 4.2069 | 5.8028 | 6.7049 | 6.8826 | 6.1127 | 5.529 | |
| 7.3076 | 6.6411 | 3.6013 | 5.5844 | 5.8357 | 2.9241 | 7.5936 | 5.0401 | |
| 8.2659 | 4.0096 | 5.7804 | 4.1973 | 6.1883 | 6.5985 | 4.6226 | 5.644 | |
| 7.1359 | 4.5285 | 4.5038 | 5.4467 | 7.056 | 4.872 | 6.4973 | 6.2607 | |
| 3.4754 | 7.7432 | 1.6912 | 6.0854 | 1.8049 | 1.9117 | 1.7397 | 2.0748 | 0 |
| | 1.1831 | 0 | 4.9383 | 2.2864 | 1.8952 | 1.9094 | 2.3106 | |
| 2.3506 | 1.2802 | 2.8723 | 0.5056 | 0.9349 | 4.6638 | 1.7618 | 2.3027 | |
| 1.066 | 1.3688 | | | | | | | |
| NFKBIL2 | 10.341 | 9.2774 | 8.3125 | 11.1512 | 9.6842 | 10.0819 | 8.0359 | |
| 9.6156 | 9.1472 | 9.1954 | 9.0068 | 9.3748 | 8.5936 | 8.8078 | 10.2416 | |
| 8.854 | 10.6192 | 9.8978 | 8.0702 | 8.1581 | 9.2696 | 10.725 | 9.1077 | |
| 8.146 | 10.2633 | 10.2201 | 10.14 | 9.8909 | 10.9338 | 9.6582 | 11.3914 | |
| 8.7086 | 10.2139 | 8.4735 | 9.4254 | 9.3527 | 9.5038 | 7.7575 | 7.9031 | |
| 9.7951 | 10.1755 | 9.6715 | 9.6331 | 9.1956 | 8.4994 | 9.434 | 8.9832 | |
| 9.4761 | 10.0034 | 8.1926 | 9.2046 | 9.8566 | 8.7532 | 10.0821 | 10.8998 | |
| 8.8772 | 7.7827 | 9.8079 | 7.0316 | 10.3764 | 10.1039 | 9.5924 | 9.0274 | |
| 9.847 | 8.3504 | 9.237 | 9.7627 | 6.7409 | 6.4186 | 10.2096 | 9.2333 | |
| 9.4002 | 6.3568 | 9.5187 | 8.9061 | 9.1845 | 8.6862 | 9.4273 | 10.2176 | |
| 9.007 | 10.2815 | 9.8026 | 8.0803 | 9.5428 | 8.8553 | 9.4803 | 10.171 | |
| 9.735 | 9.5313 | 9.5629 | 10.8967 | 10.3805 | 10.3412 | 8.4217 | 9.2787 | |
| 10.1159 | 9.1882 | 9.3583 | 10.1738 | 9.2312 | 9.0813 | 8.7151 | 9.0807 | |
| 10.1335 | 9.5221 | 10.6958 | 9.2637 | 8.653 | 9.7948 | 9.0119 | 9.4422 | |
| 8.0361 | 10.3894 | 10.1368 | 9.0274 | 8.5906 | 8.7834 | 8.9729 | 10.5047 | |
| 10.1842 | 8.8655 | 8.8753 | 10.7853 | 8.1982 | 8.5499 | 9.3443 | 9.4885 | |
| 7.9431 | 8.8657 | 9.9883 | 7.891 | 9.9245 | 8.345 | 10.542 | 9.8002 | |
| 9.4648 | 9.9551 | 10.2563 | 10.5567 | 8.8956 | 9.0605 | 8.6405 | 9.3814 | |
| 10.0115 | 9.1577 | 6.633 | 9.5979 | 10.4794 | 10.3746 | 10.4006 | 9.7477 | |
| 10.129 | 9.9617 | 8.2698 | 10.1029 | 10.8439 | 7.2271 | 10.3814 | 8.9016 | |
| 9.5767 | 7.8357 | 9.0749 | 8.3729 | 10.0397 | 10.3374 | 9.1591 | 10.5691 | |
| 9.7473 | 7.9466 | 8.5376 | 10.662 | 10.2698 | 11.3825 | 11.2572 | 9.0019 | |
| 8.5801 | 10.3847 | 6.9123 | 8.6904 | 7.0735 | 7.3013 | 6.8241 | 6.3669 | |
| 6.0506 | 6.5894 | 6.3444 | 7.7657 | 7.0234 | 6.6594 | 6.992 | 7.7207 | |
| 7.4182 | 6.39 | 7.2429 | 7.1537 | 6.9814 | 8.3041 | 7.2716 | 8.0451 | |
| 6.7199 | 7.4211 | | | | | | | |
| MAD2L1 | 10.0453 | 8.1379 | 8.9886 | 1.02E+01 | | 9.5864 | 9.4696 | |
| 10.1426 | 9.38 | 11.2228 | 10.0473 | 9.6867 | 9.2046 | 9.7062 | 7.316 | |
| 8.2089 | 9.6278 | 9.9472 | 9.5771 | 8.7248 | 8.6698 | 9.4322 | 10.7107 | |
| 9.3726 | 8.0261 | 9.4258 | 10.7449 | 9.1625 | 10.4316 | 8.5048 | 9.9609 | |
| 10.6685 | 7.5176 | 9.8748 | 9.8634 | 9.2833 | 10.3497 | 9.4909 | 9.1105 | |
| 7.4316 | 8.7977 | 8.6048 | 10.5055 | 8.7646 | 9.193 | 7.7433 | 8.5066 | |
| 8.6239 | 9.987 | 8.8451 | 8.711 | 9.6675 | 8.491 | 10.6365 | 10.3629 | |
| 8.7304 | 9.3233 | 8.0554 | 9.227 | 6.445 | 9.0654 | 10.2539 | 9.3435 | |
| 9.4709 | 8.7329 | 8.7782 | 8.0012 | 9.662 | 7.6967 | 6.9051 | 9.5347 | |
| 9.5523 | 10.0781 | 7.2834 | 9.7035 | 8.8222 | 9.1275 | 9.8955 | 9.4755 | |
| 8.2251 | 9.8757 | 10.4375 | 8.6304 | 6.1502 | 10.0006 | 8.9781 | 9.23 | |
| 9.1606 | 8.1249 | 9.3387 | 9.1907 | 9.589 | 11.0045 | 10.9224 | 9.3366 | |
| 9.3602 | 9.0823 | 9.0202 | 9.5987 | 8.2575 | 5.7004 | 10.9776 | 10.4531 | |

| | | | | | | | |
|---|---|---|---|---|---|---|---|
| 9.5623 | 9.4861 | 9.1398 | 8.6554 | 9.7563 | 9.6257 | 8.7637 | 9.4926 |
| 10.5545 | 9.9452 | 8.7721 | 9.7706 | 10.0016 | 9.5766 | 9.18 | 8.7049 |
| 7.9595 | 9.6573 | 2.7241 | 9.0562 | 9.4029 | 8.6078 | 8.953 | 9.8286 |
| 9.836 | 8.1626 | 8.9376 | 9.6712 | 7.0132 | 8.2485 | 8.4505 | 10.08 |
| 9.7912 | 9.2057 | 9.3113 | 10.5061 | 10.1611 | 9.2587 | 9.2509 | 9.3211 |
| 8.4696 | 8.502 | 10.0174 | 8.8978 | 9.6334 | 9.8159 | 9.8468 | 10.1566 |
| 9.9066 | 8.6356 | 9.7973 | 4.85 | 9.4524 | 10.0465 | 7.2636 | 9.9018 |
| 9.3363 | 10.6384 | 8.725 | 10.0302 | 9.4028 | 10.6023 | 9.2494 | 8.7784 |
| 10.8469 | 10.1204 | 8.6568 | 9.2587 | 9.4147 | 9.8482 | 9.761 | 9.6351 |
| 8.8642 | 9.2357 | 8.6713 | 5.4675 | 8.8744 | 5.4396 | 7.7872 | 5.7263 |
| 5.7337 | 6.095 | 6.3026 | 6.5554 | 7.7131 | 5.8035 | 5.7485 | 6.3139 |
| 5.3201 | 8.344 | 6.6447 | 7.2903 | 6.4711 | 6.6427 | 7.205 | 6.3878 |
| 6.9724 | 6.7352 | 6.7839 | | | | | |
| PEAR1 | 4.6856 | 6.0569 | 5.173 | 5.016 | 5.9637 | 4.3683 | 5.5542 |
| 5.643 | 4.6383 | 5.0251 | 6.8058 | 5.137 | 4.6141 | 6.2938 | 5.2554 |
| 5.2791 | 3.9044 | 4.8995 | 5.5792 | 5.9685 | 4.5835 | 7.7861 | 5.9479 |
| 5.9421 | 5.4074 | 5.0501 | 5.5584 | 3.8834 | 5.2048 | 3.6447 | 4.6536 |
| 7.0754 | 5.9673 | 6.0157 | 5.4977 | 5.8673 | 7.1443 | 5.1258 | 3.863 |
| 1.1888 | 6.6071 | 5.2786 | 7.015 | 7.6452 | 7.9236 | 5.4846 | 7.2532 |
| 4.8594 | 6.7343 | 7.1918 | 7.8413 | 5.17 | 6.9196 | 5.4717 | 4.7343 |
| 6.2585 | 4.6811 | 5.6185 | 4.6784 | 7.0212 | 5.3679 | 6.0435 | 5.0203 |
| 5.8194 | 7.0943 | 5.14 | 6.157 | 7.0341 | 6.5658 | 6.0082 | 4.7771 |
| 5.2363 | 8.1629 | 5.5073 | 5.464 | 6.1345 | 6.3782 | 3.8869 | 5.4526 |
| 6.3812 | 5.8407 | 3.8136 | 7.3447 | 5.0863 | 6.8795 | 5.694 | 8.5269 |
| 2.9428 | 6.0494 | 4.8045 | 6.0775 | 5.7102 | 5.1576 | 6.0559 | 6.2458 |
| 5.7222 | 4.3694 | 3.7995 | 2.8658 | 7.4179 | 6.5197 | 5.9228 | 4.5558 |
| 6.5365 | 4.8531 | 7.4838 | 8.3291 | 4.6253 | 6.5511 | 4.8635 | 6.0306 |
| 5.4783 | 3.5647 | 5.5088 | 3.5369 | 5.2606 | 5.7769 | 5.6002 | 5.63 |
| 3.3943 | 7.5824 | 9.0227 | 3.9514 | 6.2081 | 5.0404 | 5.7776 | 5.337 |
| 4.7642 | 4.7445 | 3.3718 | 9.0865 | 0 | 5.7623 | 5.8618 | 4.2129 |
| 5.8849 | 5.1542 | 4.1715 | 5.1731 | 5.4877 | 4.989 | 5.7057 | 4.861 |
| 5.3496 | 5.7742 | 2.9165 | 7.6213 | 4.9554 | 3.5353 | 4.6479 | 6.1402 |
| 3.7802 | 4.5322 | 5.4182 | 6.2331 | 5.9146 | 7.2942 | 6.1653 | 5.5737 |
| 4.9074 | 5.5466 | 4.9533 | 6.1146 | 6.2175 | 5.4142 | 3.9989 | 4.4821 |
| 5.9084 | 5.7061 | 4.5038 | 6.5982 | 5.0527 | 2.8198 | 5.9693 | 5.8593 |
| 6.5642 | 3.0771 | 8.1615 | 8.4176 | 8.662 | 3.4522 | 8.7857 | 8.5223 |
| 9.5788 | 9.9709 | 9.7327 | 8.3192 | 9.6929 | 9.0899 | 9.0789 | 8.4924 |
| 7.0634 | 9.1503 | 9.5556 | 8.25 | 8.8851 | 9.2585 | 9.7335 | 8.1817 |
| 9.5116 | 8.2114 | | | | | | |
| PSRC1 | 9.3353 | 8.0168 | 7.7708 | 9.3419 | 10.1782 | 8.2416 | 9.3492 |
| 9.5519 | 9.0713 | 8.8618 | 8.8006 | 8.5422 | 8.1394 | 8.5816 | 9.2858 |
| 8.949 | 9.8234 | 9.6451 | 7.7282 | 7.4823 | 8.7716 | 10.9081 | 8.8745 |
| 7.2151 | 7.8259 | 10.0955 | 9.5387 | 9.8206 | 9.5089 | 9.203 | 8.8743 |
| 8.9988 | 9.5083 | 8.8727 | 8.6017 | 8.9119 | 8.5123 | 8.1437 | 8.1112 |
| 10.4329 | 7.8362 | 9.8869 | 8.4071 | 8.5409 | 8.2553 | 8.5792 | 7.5939 |
| 8.5612 | 7.5687 | 7.2081 | 8.207 | 9.1617 | 9.5985 | 8.2797 | 9.0408 |
| 8.3958 | 8.9753 | 9.4009 | 6.5009 | 9.352 | 9.8077 | 8.1076 | 7.9913 |
| 9.0651 | 7.7395 | 6.7957 | 8.5264 | 5.9206 | 6.6474 | 8.8371 | 9.1925 |
| 9.1045 | 5.7577 | 8.5744 | 8.6336 | 8.9865 | 9.1398 | 9.1554 | 8.3859 |
| 10.1718 | 9.9311 | 8.9743 | 7.7258 | 10.0675 | 7.7903 | 8.1239 | 8.3898 |
| 9.4116 | 7.9867 | 9.357 | 9.2315 | 9.3918 | 10.4172 | 7.5425 | 9.24 |
| 9.2273 | 8.4187 | 8.756 | 9.5004 | 8.9009 | 8.3158 | 8.4448 | 8.6571 |
| 9.0268 | 9.3701 | 8.1418 | 9.2303 | 7.5997 | 8.1341 | 8.323 | 8.651 |
| 8.836 | 10.3137 | 11.2478 | 9.0195 | 8.3438 | 9.38 | 8.8487 | 7.8874 |

| | | | | | | | |
|---|---|---|---|---|---|---|---|
| 8.9105 | 7.3376 | 7.6566 | 10.1331 | 8.975 | 8.1185 | 8.2993 | 8.0521 |
| 6.7796 | 7.5128 | 8.7655 | 7.2326 | 9.1279 | 7.7299 | 9.3167 | 8.9647 |
| 9.2542 | 8.6552 | 9.2638 | 9.8128 | 9.1342 | 8.8856 | 8.0466 | 8.6179 |
| 8.3801 | 8.7662 | 8.6898 | 7.5808 | 8.828 | 10.2288 | 8.5274 | 9.2483 |
| 10.4643 | 8.7087 | 7.6139 | 9.1375 | 8.6595 | 6.3233 | 9.4861 | 8.6003 |
| 10.0401 | 8.0358 | 8.9248 | 8.214 | 7.7765 | 9.3726 | 8.9846 | 9.9411 |
| 9.4374 | 7.2053 | 9.3285 | 8.7409 | 8.588 | 10.308 | 10.5869 | 7.6751 |
| 7.8847 | 9.3554 | 5.1609 | 8.3209 | 5.6549 | 6.1972 | 7.6122 | 5.135 |
| 5.5421 | 5.8519 | 5.9357 | 7.746 | 6.2546 | 5.2882 | 7.0183 | 6.4067 |
| 7.1751 | 6.0601 | 6.96 | 6.5572 | 6.4073 | 8.2336 | 6.0865 | 6.8575 |
| 6.5027 | 7.1374 | | | | | | |
| MCM2 | 11.991 | 10.4258 | 9.335 | 1.25E+01 | | 1.13E+01 | |
| 11.7574 | 11.8194 | 11.2135 | 11.5936 | 11.6782 | 11.3654 | 10.9856 | 10.2757 |
| 9.4754 | 10.5184 | 11.0334 | 11.1413 | 11.9573 | 9.6495 | 9.6966 | 11.0985 |
| 11.9968 | 10.6087 | 9.7655 | 10.9988 | 12.0418 | 10.7011 | 10.8947 | 12.0719 |
| 11.7443 | 12.075 | 10.4427 | 11.3495 | 10.0632 | 9.9284 | 10.5895 | 10.2182 |
| 12.3274 | 9.699 | 10.9531 | 11.2604 | 11.5056 | 9.9577 | 10.4022 | 10.5289 |
| 11.7395 | 9.8102 | 10.8466 | 10.8323 | 10.6033 | 11.6098 | 11.2701 | 11.227 |
| 10.6781 | 12.397 | 10.4089 | 10.3049 | 11.1557 | 8.7957 | 10.6624 | 11.8821 |
| 11.0778 | 10.9142 | 10.8893 | 10.0808 | 10.323 | 11.4708 | 9.3672 | 8.9272 |
| 11.1766 | 11.0015 | 11.7641 | 8.6319 | 10.8066 | 10.3719 | 11.1071 | 12.1678 |
| 10.9282 | 11.0677 | 11.8927 | 11.8308 | 12.3574 | 8.8736 | 11.6705 | 10.8898 |
| 10.4284 | 10.1232 | 10.7677 | 10.8697 | 11.2888 | 10.606 | 11.3006 | 12.6184 |
| 10.5843 | 11.0068 | 11.6375 | 10.4308 | 10.8756 | 8.749 | 11.679 | 10.9804 |
| 11.3878 | 11.4607 | 11.3407 | 11.4996 | 11.2162 | 12.3868 | 10.5034 | 10.7138 |
| 10.5483 | 11.4798 | 11.3941 | 11.7129 | 11.2851 | 10.8279 | 10.5783 | 9.8197 |
| 10.5825 | 10.1244 | 12.0813 | 6.7736 | 10.148 | 10.4248 | 9.6114 | 10.0587 |
| 11.2025 | 11.1008 | 9.8906 | 10.8954 | 10.0622 | 8.9451 | 9.7704 | 10.4845 |
| 11.9401 | 11.3779 | 11.0664 | 11.8457 | 11.5863 | 11.5702 | 10.9768 | 10.7665 |
| 10.357 | 11.2458 | 11.3355 | 10.8368 | 10.2766 | 11.0371 | 10.958 | 11.5206 |
| 11.6593 | 11.7082 | 12.6938 | 11.135 | 8.4586 | 11.7361 | 11.4855 | 9.6128 |
| 11.1918 | 10.6732 | 12.4698 | 9.7118 | 10.9588 | 10.706 | 11.8515 | 11.8067 |
| 10.3807 | 11.9311 | 11.381 | 9.9066 | 10.815 | 11.1614 | 11.4418 | 11.953 |
| 11.4996 | 11.1095 | 10.0493 | 11.7008 | 7.8986 | 10.4425 | 7.8453 | 9.5415 |
| 8.6827 | 8.0404 | 8.3911 | 8.5426 | 8.466 | 9.1286 | 8.3956 | 8.4743 |
| 9.1472 | 7.8501 | 9.6179 | 8.9467 | 8.9227 | 8.9791 | 8.6966 | 9.9244 |
| 8.9666 | 8.8819 | 8.4371 | 9.7871 | | | | |
| ARHGAP31 | | 6.581 | 8.48 | 7.2393 | 7.0072 | 7.4417 | 6.2899 |
| 7.494 | 6.7365 | 6.9377 | 6.196 | 7.1414 | 8.0059 | 7.4051 | 6.8619 |
| 6.8558 | 7.4168 | 6.7474 | 7.1059 | 6.7981 | 8.464 | 7.4242 | 7.7189 |
| 6.5959 | 7.8708 | 5.5069 | 8.7502 | 8.3666 | 7.4163 | 7.031 | 5.6874 |
| 5.7913 | 8.1746 | 7.8899 | 6.1786 | 6.262 | 6.9604 | 6.9436 | 7.4808 |
| 7.8426 | 6.1703 | 8.7655 | 6.4187 | 5.4479 | 7.9572 | 8.6934 | 7.9691 |
| 8.3324 | 5.4824 | 7.3663 | 8.7577 | 9.0178 | 5.6882 | 7.6906 | 6.2018 |
| 7.397 | 7.8269 | 6.8586 | 6.8624 | 7.3781 | 8.3149 | 6.7247 | 7.5064 |
| 7.9649 | 6.914 | 8.7069 | 5.7986 | 8.1584 | 7.5985 | 7.8308 | 8.5643 |
| 6.3522 | 7.5783 | 9.5389 | 7.8855 | 7.0318 | 6.4051 | 6.1769 | 7.0506 |
| 7.6563 | 8.5838 | 7.6459 | 8.056 | 8.0201 | 6.249 | 9.7609 | 7.228 |
| 7.6119 | 6.7327 | 8.0865 | 7.1499 | 7.5896 | 5.6769 | 7.2799 | 8.3891 |
| 7.5943 | 6.5158 | 7.3902 | 6.0479 | 7.2172 | 7.0553 | 7.8828 | 7.1867 |
| 6.7764 | 6.9119 | 6.9056 | 8.7464 | 8.5636 | 6.9535 | 7.9854 | 6.2127 |
| 7.5907 | 6.0292 | 5.1269 | 6.1611 | 5.3392 | 6.8321 | 7.3442 | 7.6115 |
| 8.0897 | 7.0622 | 9.5043 | 9.7428 | 5.9295 | 7.3958 | 6.1744 | 7.8996 |
| 7.6596 | 6.7238 | 6.9608 | 6.0252 | 9.9122 | 6.6912 | 7.9317 | 7.1018 |

| | | | | | | | |
|---|---|---|---|---|---|---|---|
| 5.9636 | 8.0685 | 8.1167 | 6.0098 | 6.5882 | 5.7482 | 7.2592 | 6.8242 |
| 5.9074 | 7.1443 | 6.8574 | 8.2468 | 8.8599 | 6.6803 | 7.4215 | 8.0401 |
| 6.8208 | 5.7731 | 7.6649 | 8.0972 | 7.3718 | 7.084 | 7.8694 | 6.1653 |
| 7.1065 | 5.5593 | 6.6444 | 6.589 | 7.508 | 8.1204 | 6.8241 | 6.168 |
| 7.4122 | 8.2455 | 7.8083 | 7.8926 | 8.5142 | 6.6023 | 6.0366 | 4.8685 |
| 7.6068 | 7.8081 | 6.2124 | 9.4966 | 8.8589 | 9.4754 | 6.5687 | 9.9024 |
| 10.4057 | 10.6127 | 10.9035 | 11.2578 | 9.2222 | 10.3671 | 10.3658 | 9.7953 |
| 8.5392 | 7.4909 | 10.2077 | 10.2441 | 9.4967 | 8.9793 | 9.265 | 10.1058 |
| 8.7837 | 9.7782 | 8.9459 | | | | | |
| PDGFRB | 9.7962 | 10.7368 | 9.2085 | 1.04E+01 | | 9.42E+00 | |
| 10.4616 | 10.2655 | 10.3687 | 8.9248 | 9.7367 | 11.5656 | 8.7735 | 8.8858 |
| 10.7715 | 10.4863 | 11.1376 | 8.8088 | 9.5881 | 9.6552 | 10.4373 | 10.4086 |
| 8.5479 | 10.9864 | 10.6884 | 8.1472 | 9.0435 | 10.0104 | 8.1601 | 9.3055 |
| 7.5245 | 8.9397 | 10.6301 | 9.6871 | 9.6123 | 10.1188 | 10.6209 | 10.8442 |
| 9.186 | 9.3068 | 8.5543 | 10.0723 | 9.11 | 9.0541 | 10.1318 | 10.5762 |
| 11.1295 | 12.3774 | 9.2584 | 11.645 | 11.036 | 11.5723 | 9.4474 | 11.1334 |
| 9.801 | 9.3872 | 11.0109 | 7.5463 | 9.8028 | 10.4252 | 12.1768 | 8.3479 |
| 10.4549 | 10.017 | 10.3621 | 11.5761 | 7.616 | 11.7055 | 11.4288 | 10.3756 |
| 10.8028 | 10.7636 | 9.837 | 12.114 | 10.2156 | 10.0542 | 10.2849 | 9.357 |
| 9.2172 | 9.9629 | 12.5657 | 9.9747 | 8.8947 | 11.6974 | 11.1313 | 9.3889 |
| 10.514 | 10.807 | 9.329 | 10.54 | 10.1811 | 10.9804 | 9.8351 | 9.8876 |
| 11.9981 | 11.5815 | 10.9259 | 9.3586 | 8.6709 | 9.0479 | 9.4939 | 11.4516 |
| 9.6682 | 8.8503 | 10.6358 | 9.5621 | 10.3698 | 10.6663 | 10.3386 | 11.4553 |
| 10.183 | 10.6067 | 10.378 | 10.3651 | 10.0353 | 8.2333 | 9.9126 | 10.0419 |
| 10.9026 | 9.8701 | 8.6561 | 11.835 | 11.2263 | 8.5233 | 9.6672 | 9.8204 |
| 9.9553 | 10.8089 | 9.7953 | 9.5891 | 7.8957 | 12.2838 | 8.4388 | 10.8592 |
| 10.9725 | 9.4311 | 11.5607 | 10.4911 | 7.8868 | 10.6345 | 8.7475 | 11.157 |
| 10.8067 | 8.54 | 10.8029 | 10.4868 | 9.364 | 11.1215 | 10.227 | 8.3778 |
| 9.2293 | 9.8003 | 8.6585 | 9.6235 | 10.344 | 9.2162 | 10.9793 | 10.8926 |
| 9.7052 | 9.1649 | 7.8997 | 9.6596 | 10.1509 | 9.6476 | 11.5624 | 9.2672 |
| 8.9714 | 10.6855 | 10.7905 | 9.1546 | 10.0103 | 11.3951 | 10.3932 | 8.9303 |
| 7.7033 | 10.2084 | 10.6828 | 10.1124 | 13.1003 | 12.7707 | 13.4756 | 9.6907 |
| 12.0547 | 12.3763 | 12.7013 | 12.8249 | 12.4226 | 12.9319 | 12.7493 | 12.4638 |
| 13.3096 | 12.6171 | 10.7367 | 12.7973 | 12.5037 | 12.4886 | 12.8133 | 12.4186 |
| 12.2771 | 12.1688 | 12.6935 | 12.2906 | | | | |
| GINS4 | 9.6738 | 8.0304 | 6.9372 | 9.70E+00 | | 7.99E+00 | |
| 8.133 | 9.3213 | 8.1813 | 9.0751 | 8.8717 | 8.6247 | 8.4817 | 8.4959 |
| 6.6056 | 8.6989 | 8.7695 | 9.0689 | 9.1801 | 7.2827 | 7.2223 | 7.9633 |
| 9.3861 | 7.7104 | 7.6797 | 8.6718 | 9.6861 | 8.5571 | 8.0093 | 11.0596 |
| 8.2677 | 10.0118 | 7.7526 | 8.893 | 8.6125 | 8.284 | 7.7343 | 9.2587 |
| 7.3844 | 8.153 | 7.9725 | 9.0051 | 9.0607 | 6.4763 | 7.5938 | 8.1864 |
| 9.2087 | 8.243 | 9.0309 | 8.1623 | 7.5857 | 9.5391 | 8.7669 | 9.3756 |
| 9.4728 | 9.9346 | 8.5747 | 7.6197 | 9.581 | 5.37 | 8.4638 | 7.7558 |
| 8.3833 | 6.7088 | 7.4437 | 7.8251 | 7.2099 | 8.8578 | 5.736 | 5.2387 |
| 9.008 | 7.5754 | 8.2542 | 5.8086 | 8.4155 | 7.7507 | 9.7042 | 8.2242 |
| 9.0797 | 9.8224 | 7.3098 | 9.6303 | 7.4416 | 6.7615 | 8.5254 | 8.5213 |
| 8.2694 | 8.8068 | 7.901 | 8.4557 | 8.9951 | 7.9533 | 9.0435 | 8.654 |
| 8.6137 | 9.4794 | 7.9882 | 7.3939 | 8.4969 | 8.8703 | 8.4717 | 8.6142 |
| 9.158 | 8.2433 | 9.2451 | 8.8802 | 9.0928 | 8.6515 | 8.2446 | 10.6006 |
| 8.1519 | 8.0893 | 9.0977 | 8.3454 | 8.1005 | 8.155 | 7.9205 | 8.5078 |
| 8.6078 | 7.5624 | 7.7087 | 6.2435 | 7.8677 | 9.8191 | 6.8276 | 7.0635 |
| 8.274 | 8.9444 | 5.4539 | 8.4155 | 7.6151 | 6.0517 | 7.876 | 7.8199 |
| 8.6459 | 8.8978 | 10.3451 | 8.7649 | 7.0778 | 8.3976 | 7.2423 | 8.37 |
| 7.5266 | 7.5699 | 10.3217 | 8.6378 | 8.2944 | 8.4624 | 9.9687 | 8.8082 |

| | | | | | | | | |
|---|---|---|---|---|---|---|---|---|
| 9.638 | 8.6833 | 8.3917 | 8.6204 | 4.6244 | 8.4049 | 9.7363 | 5.5752 | |
| 8.3226 | 9.1833 | 8.8575 | 6.3275 | 9.0962 | 7.9519 | 9.1383 | 9.1358 | |
| 8.2894 | 8.8069 | 8.8756 | 7.4262 | 8.4207 | 8.8286 | 8.3642 | 7.3631 | |
| 7.353 | 8.1716 | 7.8323 | 8.1066 | 5.5658 | 8.683 | 6.1362 | 5.4982 | |
| 6.5989 | 5.9078 | 4.5085 | 5.1591 | 4.7791 | 7.5916 | 5.026 | 5.0245 | |
| 6.3056 | 5.5611 | 5.7494 | 6.1107 | 6.4908 | 5.6826 | 5.4394 | 6.3949 | |
| 5.4608 | 7.3958 | 4.166 | 5.8832 | | | | | |
| TEK | 4.8811 | 6.8221 | 7.0657 | 5.55E+00 | | 7.48E+00 | | |
| 4.1255 | 6.8816 | 6.3229 | 5.7079 | 6.196 | 6.0591 | 6.5969 | 7.9255 | 5.67 |
| | 5.1715 | 6.0118 | 4.2805 | 5.415 | 7.8172 | 6.9722 | 4.2292 | |
| 6.8285 | 7.0187 | 6.5129 | 6.1751 | 4.2393 | 6.138 | 5.2632 | 6.4025 | |
| 4.9551 | 3.897 | 7.5649 | 6.142 | 5.832 | 6.1945 | 6.9873 | 6.11 | |
| 4.891 | 5.2493 | 4.5869 | 7.1453 | 6.8296 | 4.8857 | 6.3274 | 6.7063 | |
| 6.9781 | 8.5098 | 6.3947 | 7.556 | 7.5857 | 8.9242 | 5.3003 | 7.5741 | |
| 6.3425 | 4.8153 | 7.4186 | 6.0494 | 6.9807 | 6.0964 | 8.6027 | 4.4143 | |
| 6.5508 | 5.9555 | 5.7259 | 8.2642 | 6.6867 | 8.2788 | 8.1833 | 8.2578 | |
| 6.997 | 6.0723 | 6.836 | 9.7347 | 7.2836 | 6.8007 | 5.7004 | 5.2829 | |
| 5.6228 | 7.0253 | 7.6788 | 6.9542 | 3.7214 | 7.5383 | 5.292 | 7.2875 | |
| 7.2157 | 5.8612 | 3.6674 | 7.3509 | 5.8476 | 7.3576 | 5.308 | 7.478 | |
| 8.0828 | 7.6213 | 6.6805 | 6.6281 | 5.5309 | 5.4927 | 4.6439 | 5.9887 | |
| 5.7915 | 7.7403 | 7.2397 | 6.3935 | 7.8517 | 8.2539 | 6.6108 | 7.5804 | |
| 6.4409 | 5.0525 | 5.7157 | 4.5024 | 5.6506 | 4.8428 | 7.1058 | 5.6238 | |
| 5.6002 | 4.6588 | 5.0401 | 5.737 | 9.8891 | 4.7183 | 6.1889 | 5.8143 | |
| 7.8319 | 6.1328 | 6.4472 | 6.0768 | 4.3004 | 9.8978 | 4.5495 | 7.1281 | |
| 6.4301 | 4.933 | 5.8849 | 5.2732 | 6.0213 | 5.4537 | 4.4174 | 6.2026 | |
| 7.6917 | 5.0554 | 6.7277 | 7.5642 | 6.0072 | 7.8847 | 6.8052 | 4.6411 | |
| 5.3227 | 7.0976 | 4.081 | 6.3949 | 7.0579 | 6.5204 | 6.9557 | 8.1955 | |
| 5.4632 | 4.6037 | 5.6755 | 7.3261 | 5.6525 | 6.6621 | 7.6716 | 4.8774 | |
| 5.0566 | 5.6579 | 7.2602 | 6.5834 | 7.2464 | 7.5436 | 7.365 | 5.8969 | |
| 2.962 | 6.947 | 9.2696 | 4.2463 | 10.2141 | 9.1911 | 9.691 | 4.3847 | |
| 10.1824 | 10.0829 | 10.9287 | 10.8143 | 11.0664 | 9.6373 | 9.8043 | 10.6775 | |
| 9.9359 | 8.1169 | 6.8234 | 10.4983 | 11.0167 | 10.3002 | 6.1538 | 8.159 | |
| 10.6021 | 9.0531 | 9.2698 | 9.1856 | | | | | |

| Gene | Module | MM_R | MM_pvalue | GS_R | GS_pvalue |
|---|---|---|---|---|---|
| ASPA | black | 0.88758767 | 6.49E-69 | -0.8569432 | 3.44E-59 |
| TCF23 | black | 0.8487794 | 5.61E-57 | -0.836774 | 5.95E-54 |
| ZNF695 | blue | 0.91747406 | 1.32E-81 | 0.8190416 | 6.62E-50 |
| SKA3 | blue | 0.92180605 | 7.71E-84 | 0.81363971 | 9.26E-49 |
| CLEC4M | black | 0.80219323 | 1.88E-46 | -0.813167 | 1.16E-48 |
| FGF10 | black | 0.80629474 | 2.92E-47 | -0.8112642 | 2.87E-48 |
| MYBL2 | blue | 0.92471097 | 2.06E-85 | 0.80880226 | 9.14E-48 |
| TROAP | blue | 0.92068973 | 2.98E-83 | 0.80744629 | 1.72E-47 |
| CCNB2 | blue | 0.94682489 | 5.91E-100 | 0.80449285 | 6.66E-47 |
| CDC25C | blue | 0.92516487 | 1.16E-85 | 0.8039875 | 8.38E-47 |
| CDC20 | blue | 0.91004804 | 4.79E-78 | 0.802274 | 1.82E-46 |
| SPAG5 | blue | 0.93865806 | 5.85E-94 | 0.79844027 | 9.97E-46 |
| CEP55 | blue | 0.93383252 | 8.59E-91 | 0.79292035 | 1.09E-44 |
| KLF17 | black | 0.84820369 | 7.94E-57 | -0.7925522 | 1.27E-44 |
| KIF4A | blue | 0.94720098 | 2.97E-100 | 0.79165364 | 1.86E-44 |
| ZWINT | blue | 0.93735864 | 4.41E-93 | 0.7912191 | 2.24E-44 |
| HJURP | blue | 0.95003366 | 1.42E-102 | 0.78992276 | 3.86E-44 |
| NCAPG | blue | 0.93676415 | 1.10E-92 | 0.78972579 | 4.19E-44 |
| TPX2 | blue | 0.96055356 | 1.46E-112 | 0.78919346 | 5.24E-44 |
| CDCA2 | blue | 0.91844083 | 4.30E-82 | 0.78891642 | 5.88E-44 |
| MYOZ2 | black | 0.8339504 | 2.82E-53 | -0.7884548 | 7.13E-44 |
| SPC25 | blue | 0.9393754 | 1.88E-94 | 0.78830791 | 7.58E-44 |
| FOXM1 | blue | 0.95385416 | 6.27E-106 | 0.78768814 | 9.80E-44 |
| ASF1B | blue | 0.93312578 | 2.39E-90 | 0.78458187 | 3.51E-43 |
| RAD54L | blue | 0.92747881 | 5.72E-87 | 0.78416591 | 4.16E-43 |
| PPP1R12B | black | 0.90040668 | 7.35E-74 | -0.7827581 | 7.35E-43 |
| TUBA1C | blue | 0.8209122 | 2.60E-50 | 0.78247193 | 8.25E-43 |
| MELK | blue | 0.95769828 | 1.32E-109 | 0.78158536 | 1.18E-42 |
| UBE2C | blue | 0.91614321 | 6.07E-81 | 0.78113987 | 1.41E-42 |
| CENPA | blue | 0.94108173 | 1.20E-95 | 0.78043559 | 1.87E-42 |
| EXO1 | blue | 0.93603771 | 3.29E-92 | 0.78020906 | 2.04E-42 |
| CDC6 | blue | 0.90665699 | 1.60E-76 | 0.77904904 | 3.24E-42 |
| SKA1 | blue | 0.91802121 | 7.01E-82 | 0.77873461 | 3.67E-42 |
| POC1A | blue | 0.83965097 | 1.18E-54 | 0.77875204 | 3.64E-42 |
| AURKB | blue | 0.89201412 | 1.50E-70 | 0.77849461 | 4.03E-42 |
| PKMYT1 | blue | 0.89775703 | 8.74E-73 | 0.77844592 | 4.11E-42 |
| LOC134466 | black | 0.8223326 | 1.27E-50 | -0.775961 | 1.09E-41 |
| CDC45 | blue | 0.92952571 | 3.67E-88 | 0.77594432 | 1.09E-41 |
| KIF18B | blue | 0.95687784 | 8.59E-109 | 0.77514242 | 1.50E-41 |
| BUB1 | blue | 0.96690869 | 5.12E-120 | 0.77423171 | 2.13E-41 |
| PLK1 | blue | 0.94888389 | 1.29E-101 | 0.77361092 | 2.70E-41 |

| | | | | | |
|---|---|---|---|---|---|
| CAMK2A | black | 0.88309433 | 2.54E-67 | -0.7732432 | 3.11E-41 |
| FAM54A | blue | 0.93606484 | 3.16E-92 | 0.77281327 | 3.67E-41 |
| MKI67 | blue | 0.92163287 | 9.52E-84 | 0.77253323 | 4.08E-41 |
| SLITRK3 | black | 0.80391366 | 8.66E-47 | -0.7723042 | 4.46E-41 |
| IQGAP3 | blue | 0.8954488 | 7.16E-72 | 0.77160259 | 5.83E-41 |
| MCM10 | blue | 0.96667658 | 1.02E-119 | 0.77110181 | 7.05E-41 |
| C16orf59 | blue | 0.85216001 | 7.06E-58 | 0.7707428 | 8.08E-41 |
| C9orf140 | blue | 0.82147705 | 1.96E-50 | 0.7698439 | 1.13E-40 |
| RRM2 | blue | 0.91282131 | 2.45E-79 | 0.76888213 | 1.63E-40 |
| TK1 | blue | 0.84521103 | 4.73E-56 | 0.76871131 | 1.74E-40 |
| NEK2 | blue | 0.90946915 | 8.81E-78 | 0.76782658 | 2.42E-40 |
| BIRC5 | blue | 0.8564476 | 4.73E-59 | 0.76760609 | 2.63E-40 |
| ESPL1 | blue | 0.9500922 | 1.27E-102 | 0.7671166 | 3.15E-40 |
| UHRF1 | blue | 0.87466916 | 1.67E-64 | 0.76688403 | 3.44E-40 |
| SPARCL1 | black | 0.88042248 | 2.10E-66 | -0.7657944 | 5.15E-40 |
| KIF2C | blue | 0.94616532 | 1.95E-99 | 0.76578526 | 5.17E-40 |
| SGOL1 | blue | 0.94980136 | 2.22E-102 | 0.76499848 | 6.91E-40 |
| NCAPH | blue | 0.97068397 | 3.60E-125 | 0.7645688 | 8.09E-40 |
| PGM5P2 | black | 0.82440138 | 4.43E-51 | -0.7640385 | 9.83E-40 |
| TTK | blue | 0.95353295 | 1.23E-105 | 0.76366339 | 1.13E-39 |
| CDCA5 | blue | 0.91690013 | 2.56E-81 | 0.76187166 | 2.17E-39 |
| ZBTB16 | black | 0.81359912 | 9.44E-49 | -0.7614124 | 2.56E-39 |
| POLQ | blue | 0.92892508 | 8.29E-88 | 0.75993064 | 4.36E-39 |
| CNRIP1 | black | 0.91129176 | 1.28E-78 | -0.7593975 | 5.28E-39 |
| PBK | blue | 0.90592899 | 3.34E-76 | 0.75757172 | 1.01E-38 |
| GTSE1 | blue | 0.93146785 | 2.51E-89 | 0.75709535 | 1.20E-38 |
| PTTG1 | blue | 0.85249376 | 5.74E-58 | 0.75695017 | 1.26E-38 |
| AURKA | blue | 0.94423389 | 5.90E-98 | 0.75625216 | 1.62E-38 |
| LMOD1 | black | 0.87438727 | 2.06E-64 | -0.7558965 | 1.83E-38 |
| TACC3 | blue | 0.87219135 | 1.03E-63 | 0.75559768 | 2.04E-38 |
| ORC6L | blue | 0.90688109 | 1.27E-76 | 0.75526767 | 2.29E-38 |
| CDCA8 | blue | 0.96866133 | 2.49E-122 | 0.75512712 | 2.40E-38 |
| KANK2 | black | 0.87125892 | 2.02E-63 | -0.7541347 | 3.40E-38 |
| DLGAP5 | blue | 0.95509607 | 4.42E-107 | 0.75369627 | 3.96E-38 |
| EME1 | blue | 0.85781154 | 1.97E-59 | 0.75342571 | 4.35E-38 |
| E2F2 | blue | 0.89260005 | 8.97E-71 | 0.75328742 | 4.57E-38 |
| CCNB1 | blue | 0.94808486 | 5.79E-101 | 0.75281054 | 5.39E-38 |
| NDN | black | 0.85682252 | 3.72E-59 | -0.7522976 | 6.44E-38 |
| UBE2T | blue | 0.89998952 | 1.09E-73 | 0.75228335 | 6.47E-38 |
| OMD | black | 0.84441426 | 7.56E-56 | -0.7516636 | 8.02E-38 |
| KLHL4 | black | 0.86550794 | 1.16E-61 | -0.7505488 | 1.18E-37 |
| DIXDC1 | black | 0.84767692 | 1.09E-56 | -0.7495225 | 1.67E-37 |

| | | | | | |
|---|---|---|---|---|---|
| SHCBP1 | blue | 0.85501757 | 1.18E-58 | 0.74914777 | 1.90E-37 |
| SYNPO2 | black | 0.86826026 | 1.71E-62 | -0.748849 | 2.11E-37 |
| MYLK | black | 0.89355053 | 3.89E-71 | -0.7488258 | 2.12E-37 |
| NPAS4 | black | 0.82049377 | 3.21E-50 | -0.7479761 | 2.83E-37 |
| KIF11 | blue | 0.94740038 | 2.06E-100 | 0.74779713 | 3.01E-37 |
| CKS2 | blue | 0.86251229 | 8.86E-61 | 0.7463177 | 4.96E-37 |
| FHL1 | black | 0.90559229 | 4.69E-76 | -0.7453963 | 6.76E-37 |
| MYOCD | black | 0.85375581 | 2.61E-58 | -0.7450023 | 7.71E-37 |
| CACNB2 | black | 0.849704 | 3.20E-57 | -0.744624 | 8.75E-37 |
| TNS1 | black | 0.88981642 | 9.92E-70 | -0.7440658 | 1.05E-36 |
| MND1 | blue | 0.86192031 | 1.32E-60 | 0.7434622 | 1.29E-36 |
| FANCA | blue | 0.82391243 | 5.69E-51 | 0.74337258 | 1.33E-36 |
| OGN | black | 0.85605092 | 6.10E-59 | -0.7430731 | 1.47E-36 |
| TOP2A | blue | 0.93198785 | 1.21E-89 | 0.74285371 | 1.58E-36 |
| NUF2 | blue | 0.93722112 | 5.45E-93 | 0.74230138 | 1.89E-36 |
| KIAA1462 | black | 0.86730126 | 3.34E-62 | -0.742193 | 1.96E-36 |
| C1orf135 | blue | 0.92038583 | 4.30E-83 | 0.74106023 | 2.85E-36 |
| GPRASP1 | black | 0.90275861 | 7.69E-75 | -0.7405784 | 3.34E-36 |
| CDK1 | blue | 0.921025 | 1.99E-83 | 0.74043153 | 3.50E-36 |
| EZH2 | blue | 0.88676891 | 1.28E-68 | 0.74021908 | 3.75E-36 |
| KIFC1 | blue | 0.95472315 | 9.88E-107 | 0.73972693 | 4.41E-36 |
| CENPF | blue | 0.93153785 | 2.28E-89 | 0.73968732 | 4.46E-36 |
| CACNA1H | black | 0.82632806 | 1.64E-51 | -0.7394786 | 4.78E-36 |
| RAD51 | blue | 0.89683144 | 2.04E-72 | 0.73911091 | 5.39E-36 |
| BUB1B | blue | 0.94250004 | 1.14E-96 | 0.737215 | 9.96E-36 |
| CDCA3 | blue | 0.87543412 | 9.44E-65 | 0.73554909 | 1.70E-35 |
| PDE2A | black | 0.8636329 | 4.16E-61 | -0.7348689 | 2.12E-35 |
| CCNA2 | blue | 0.92341985 | 1.05E-84 | 0.734847 | 2.13E-35 |
| MRGPRF | black | 0.8518642 | 8.48E-58 | -0.7338004 | 2.97E-35 |
| RASL12 | black | 0.85033214 | 2.18E-57 | -0.7336663 | 3.10E-35 |
| TGFB1I1 | black | 0.85027042 | 2.26E-57 | -0.7334394 | 3.33E-35 |
| ORC1L | blue | 0.92592432 | 4.36E-86 | 0.73340382 | 3.37E-35 |
| NKAPL | black | 0.80288386 | 1.38E-46 | -0.732824 | 4.05E-35 |
| KCTD8 | black | 0.83961858 | 1.20E-54 | -0.7322274 | 4.89E-35 |
| ERCC6L | blue | 0.90383392 | 2.69E-75 | 0.7319342 | 5.36E-35 |
| KIAA0101 | blue | 0.84955655 | 3.50E-57 | 0.73179753 | 5.60E-35 |
| ADH1B | black | 0.81161625 | 2.43E-48 | -0.7315176 | 6.12E-35 |
| HMGB3 | blue | 0.81686237 | 1.94E-49 | 0.73097092 | 7.26E-35 |
| DEPDC1 | blue | 0.94704734 | 3.94E-100 | 0.73064044 | 8.05E-35 |
| FAM64A | blue | 0.91257487 | 3.20E-79 | 0.73040678 | 8.67E-35 |
| CDT1 | blue | 0.8145838 | 5.87E-49 | 0.73028518 | 9.00E-35 |
| NUSAP1 | blue | 0.92543356 | 8.20E-86 | 0.73008308 | 9.59E-35 |

| | | | | | |
|---|---|---|---|---|---|
| KIF14 | blue | 0.9085993 | 2.18E-77 | 0.729877 | 1.02E-34 |
| ABCC9 | black | 0.85853484 | 1.23E-59 | -0.7294081 | 1.18E-34 |
| MAMDC2 | black | 0.88930488 | 1.53E-69 | -0.7288278 | 1.42E-34 |
| FANCI | blue | 0.87323152 | 4.82E-64 | 0.72807419 | 1.79E-34 |
| MLF1IP | blue | 0.86195421 | 1.29E-60 | 0.72741545 | 2.20E-34 |
| FAM72B | blue | 0.91967065 | 1.01E-82 | 0.72675385 | 2.69E-34 |
| ZCCHC24 | black | 0.89677721 | 2.15E-72 | -0.7260914 | 3.30E-34 |
| TENC1 | blue | -0.8221864 | 1.37E-50 | -0.7259792 | 3.42E-34 |
| KIF20A | blue | 0.92072562 | 2.86E-83 | 0.72561815 | 3.82E-34 |
| TSHZ3 | black | 0.90402616 | 2.22E-75 | -0.7254854 | 3.98E-34 |
| DIAPH3 | blue | 0.89818117 | 5.91E-73 | 0.72545783 | 4.01E-34 |
| CDC25A | blue | 0.8908912 | 3.95E-70 | 0.72537724 | 4.11E-34 |
| BLM | blue | 0.91943153 | 1.34E-82 | 0.72538584 | 4.10E-34 |
| RERG | black | 0.85522139 | 1.03E-58 | -0.725077 | 4.50E-34 |
| LOC728264 | black | 0.86523081 | 1.40E-61 | -0.7237622 | 6.72E-34 |
| MEF2C | black | 0.87523648 | 1.09E-64 | -0.7233131 | 7.71E-34 |
| PLCL1 | black | 0.8342133 | 2.44E-53 | -0.7224809 | 9.91E-34 |
| JPH4 | black | 0.9031011 | 5.51E-75 | -0.7208364 | 1.63E-33 |
| DEPDC1B | blue | 0.91956013 | 1.15E-82 | 0.7207152 | 1.69E-33 |
| ARHGAP20 | black | 0.87212881 | 1.08E-63 | -0.7194692 | 2.45E-33 |
| TACC1 | black | 0.8377803 | 3.39E-54 | -0.7189618 | 2.85E-33 |
| HSPB7 | black | 0.84367827 | 1.16E-55 | -0.718807 | 2.98E-33 |
| ASPM | blue | 0.92443377 | 2.93E-85 | 0.71854405 | 3.22E-33 |
| CD300LG | black | 0.80738499 | 1.77E-47 | -0.7183347 | 3.43E-33 |
| MYCT1 | black | 0.88850959 | 3.00E-69 | -0.7182318 | 3.54E-33 |
| ARHGAP6 | black | 0.84689471 | 1.74E-56 | -0.7166226 | 5.69E-33 |
| CENPN | blue | 0.83150372 | 1.06E-52 | 0.71666964 | 5.61E-33 |
| TBC1D2B | black | 0.83314456 | 4.38E-53 | -0.7162272 | 6.39E-33 |
| PTGFR | black | 0.85178464 | 8.91E-58 | -0.7151636 | 8.73E-33 |
| GSTM5 | black | 0.83482255 | 1.75E-53 | -0.7151321 | 8.81E-33 |
| CCNF | blue | 0.87630747 | 4.90E-65 | 0.7152835 | 8.43E-33 |
| MYH11 | black | 0.82553838 | 2.46E-51 | -0.7128916 | 1.69E-32 |
| BNC2 | black | 0.84092392 | 5.72E-55 | -0.7095777 | 4.39E-32 |
| DTL | blue | 0.88360625 | 1.69E-67 | 0.70971384 | 4.23E-32 |
| CIT | blue | 0.85540724 | 9.19E-59 | 0.70920291 | 4.89E-32 |
| LDB2 | black | 0.88668739 | 1.37E-68 | -0.7090412 | 5.12E-32 |
| LOC401093 | black | 0.81507117 | 4.64E-49 | -0.7069828 | 9.19E-32 |
| CAV1 | black | 0.81885975 | 7.25E-50 | -0.7068158 | 9.63E-32 |
| RCC2 | blue | 0.82025818 | 3.61E-50 | 0.70668836 | 9.98E-32 |
| CASP12 | black | 0.83071758 | 1.62E-52 | -0.7060131 | 1.21E-31 |
| PDZRN4 | black | 0.826127 | 1.82E-51 | -0.704997 | 1.61E-31 |
| CNN1 | black | 0.81493935 | 4.95E-49 | -0.7039277 | 2.17E-31 |

| | | | | | |
|---|---|---|---|---|---|
| TCEAL7 | black | 0.89315769 | 5.50E-71 | -0.7039552 | 2.15E-31 |
| RERGL | black | 0.80725456 | 1.88E-47 | -0.7038453 | 2.22E-31 |
| TNXB | black | 0.82312162 | 8.52E-51 | -0.7037193 | 2.30E-31 |
| RHOJ | black | 0.88319335 | 2.35E-67 | -0.7014877 | 4.27E-31 |
| ZFPM2 | black | 0.81940774 | 5.52E-50 | -0.7011911 | 4.63E-31 |
| ESCO2 | blue | 0.87902688 | 6.18E-66 | 0.70120964 | 4.61E-31 |
| KPNA2 | blue | 0.87670252 | 3.64E-65 | 0.7004192 | 5.73E-31 |
| HMMR | blue | 0.8990678 | 2.59E-73 | 0.70024833 | 6.00E-31 |
| SORBS1 | black | 0.80911191 | 7.91E-48 | -0.6998226 | 6.75E-31 |
| GSG2 | blue | 0.89813368 | 6.18E-73 | 0.69892695 | 8.62E-31 |
| MRVI1 | black | 0.84895569 | 5.04E-57 | -0.6984551 | 9.81E-31 |
| PABPC5 | black | 0.86541901 | 1.23E-61 | -0.697273 | 1.35E-30 |
| STIL | blue | 0.87041657 | 3.70E-63 | 0.69721479 | 1.37E-30 |
| SOCS2 | black | 0.83821777 | 2.65E-54 | -0.6959316 | 1.94E-30 |
| FAM72D | blue | 0.89031983 | 6.45E-70 | 0.69584148 | 1.99E-30 |
| ACTA2 | black | 0.80323228 | 1.18E-46 | -0.6952265 | 2.35E-30 |
| E2F1 | blue | 0.84301878 | 1.71E-55 | 0.69524772 | 2.34E-30 |
| MASP1 | black | 0.81755456 | 1.38E-49 | -0.6935488 | 3.69E-30 |
| GPR124 | black | 0.89363727 | 3.60E-71 | -0.6922402 | 5.23E-30 |
| FANCD2 | blue | 0.89891286 | 2.99E-73 | 0.69206463 | 5.48E-30 |
| ARHGAP11A | blue | 0.92348761 | 9.65E-85 | 0.69193489 | 5.67E-30 |
| NEIL3 | blue | 0.92024016 | 5.12E-83 | 0.69121843 | 6.86E-30 |
| SDPR | black | 0.83854493 | 2.21E-54 | -0.6904194 | 8.47E-30 |
| JAM3 | black | 0.84475401 | 6.19E-56 | -0.6904579 | 8.39E-30 |
| FAM83D | blue | 0.92902158 | 7.27E-88 | 0.68992131 | 9.66E-30 |
| MSRB3 | black | 0.87157074 | 1.61E-63 | -0.6897714 | 1.00E-29 |
| OIP5 | blue | 0.89726347 | 1.38E-72 | 0.68967552 | 1.03E-29 |
| ECM2 | black | 0.85518278 | 1.06E-58 | -0.6894102 | 1.11E-29 |
| CALD1 | black | 0.8511771 | 1.30E-57 | -0.6890242 | 1.22E-29 |
| FLT4 | black | 0.82445351 | 4.31E-51 | -0.6880238 | 1.59E-29 |
| KIAA1644 | black | 0.83436706 | 2.25E-53 | -0.6878425 | 1.67E-29 |
| AOC3 | black | 0.80035469 | 4.28E-46 | -0.6877129 | 1.72E-29 |
| CXorf36 | black | 0.85332305 | 3.42E-58 | -0.6869384 | 2.11E-29 |
| C16orf45 | black | 0.83973983 | 1.12E-54 | -0.6864842 | 2.37E-29 |
| PRDM8 | black | 0.81911895 | 6.37E-50 | -0.6860977 | 2.62E-29 |
| EDNRA | black | 0.87934423 | 4.84E-66 | -0.6861275 | 2.60E-29 |
| AKT3 | black | 0.8735835 | 3.72E-64 | -0.6859878 | 2.70E-29 |
| EMCN | black | 0.8429767 | 1.75E-55 | -0.6859318 | 2.74E-29 |
| KIF15 | blue | 0.92333409 | 1.17E-84 | 0.68567897 | 2.93E-29 |
| OLFML2A | black | 0.82081186 | 2.74E-50 | -0.6855858 | 3.00E-29 |
| C7 | black | 0.83210799 | 7.67E-53 | -0.6847518 | 3.72E-29 |
| SYT15 | black | 0.80160027 | 2.46E-46 | -0.6840755 | 4.43E-29 |

| | | | | | |
|---|---|---|---|---|---|
| HIC1 | black | 0.81400503 | 7.77E-49 | -0.6834474 | 5.20E-29 |
| C3orf70 | black | 0.83552818 | 1.19E-53 | -0.6831896 | 5.56E-29 |
| PLN | black | 0.80440091 | 6.94E-47 | -0.6829372 | 5.93E-29 |
| CCNE1 | blue | 0.81409089 | 7.45E-49 | 0.68160945 | 8.32E-29 |
| ANTXR2 | black | 0.80621728 | 3.03E-47 | -0.6815711 | 8.40E-29 |
| C15orf42 | blue | 0.88556347 | 3.45E-68 | 0.68098254 | 9.76E-29 |
| FEN1 | blue | 0.83963786 | 1.19E-54 | 0.68099499 | 9.73E-29 |
| FGF2 | black | 0.83327375 | 4.08E-53 | -0.6801861 | 1.19E-28 |
| RSPO1 | black | 0.8046 | 6.34E-47 | -0.6798238 | 1.31E-28 |
| PEG3 | black | 0.82291996 | 9.44E-51 | -0.679258 | 1.51E-28 |
| GINS1 | blue | 0.92779972 | 3.74E-87 | 0.67933533 | 1.48E-28 |
| NBLA00301 | black | 0.8601043 | 4.40E-60 | -0.6791089 | 1.57E-28 |
| ECT2 | blue | 0.86096421 | 2.49E-60 | 0.67918527 | 1.54E-28 |
| RTKN2 | blue | 0.85909102 | 8.55E-60 | 0.67910448 | 1.57E-28 |
| LRRN4CL | black | 0.80392419 | 8.62E-47 | -0.676432 | 3.07E-28 |
| C17orf53 | blue | 0.83593192 | 9.50E-54 | 0.6736141 | 6.18E-28 |
| DACT3 | black | 0.86770437 | 2.52E-62 | -0.6733866 | 6.54E-28 |
| CXCL12 | black | 0.85107397 | 1.38E-57 | -0.6729232 | 7.33E-28 |
| RACGAP1 | blue | 0.92204179 | 5.77E-84 | 0.67194432 | 9.32E-28 |
| SYNE1 | black | 0.84874489 | 5.72E-57 | -0.6709083 | 1.20E-27 |
| LEPR | black | 0.82298272 | 9.14E-51 | -0.6693324 | 1.76E-27 |
| ZEB1 | black | 0.88981163 | 9.96E-70 | -0.6690618 | 1.88E-27 |
| GRID1 | black | 0.84273904 | 2.01E-55 | -0.6684169 | 2.20E-27 |
| E2F8 | blue | 0.8742123 | 2.34E-64 | 0.66780419 | 2.55E-27 |
| RUNX1T1 | black | 0.85973607 | 5.60E-60 | -0.6677113 | 2.61E-27 |
| RECK | black | 0.87393988 | 2.86E-64 | -0.6671535 | 2.98E-27 |
| HAND2 | black | 0.84384746 | 1.05E-55 | -0.665866 | 4.07E-27 |
| C7orf58 | black | 0.85294701 | 4.33E-58 | -0.6656405 | 4.29E-27 |
| CENPE | blue | 0.89928856 | 2.11E-73 | 0.66539504 | 4.55E-27 |
| MMRN2 | black | 0.80349694 | 1.05E-46 | -0.6652755 | 4.68E-27 |
| TIMP3 | black | 0.86364021 | 4.14E-61 | -0.6643163 | 5.89E-27 |
| CKAP2L | blue | 0.90208843 | 1.47E-74 | 0.66378293 | 6.68E-27 |
| KIF18A | blue | 0.90133658 | 3.03E-74 | 0.66355832 | 7.05E-27 |
| CDKN3 | blue | 0.87384741 | 3.06E-64 | 0.66352218 | 7.11E-27 |
| ROBO4 | black | 0.80936036 | 7.04E-48 | -0.6632649 | 7.56E-27 |
| TRPC4 | black | 0.83111743 | 1.31E-52 | -0.6620406 | 1.01E-26 |
| ABCG2 | black | 0.81335418 | 1.06E-48 | -0.6582434 | 2.46E-26 |
| C9orf100 | blue | 0.80096651 | 3.26E-46 | 0.65835822 | 2.39E-26 |
| VGLL3 | black | 0.82231692 | 1.28E-50 | -0.6578817 | 2.67E-26 |
| AQP1 | black | 0.81807362 | 1.07E-49 | -0.6562443 | 3.91E-26 |
| C10orf72 | black | 0.80304482 | 1.28E-46 | -0.6542366 | 6.20E-26 |
| CLIP3 | black | 0.8221947 | 1.36E-50 | -0.6538747 | 6.74E-26 |

| | | | | | |
|---|---|---|---|---|---|
| PIF1 | blue | 0.80299266 | 1.31E-46 | 0.6532472 | 7.77E-26 |
| ASAM | black | 0.85000021 | 2.67E-57 | -0.6527256 | 8.76E-26 |
| NEXN | black | 0.81969459 | 4.79E-50 | -0.6525404 | 9.13E-26 |
| DCN | black | 0.84348017 | 1.31E-55 | -0.6523001 | 9.65E-26 |
| TYMS | blue | 0.87532304 | 1.03E-64 | 0.65216285 | 9.95E-26 |
| PALM2-AKAP2 | black | 0.82490279 | 3.42E-51 | -0.6468185 | 3.31E-25 |
| LMNB1 | blue | 0.88543952 | 3.82E-68 | 0.64664033 | 3.45E-25 |
| ANLN | blue | 0.87456445 | 1.80E-64 | 0.64604853 | 3.93E-25 |
| CSDC2 | black | 0.81397507 | 7.88E-49 | -0.6454533 | 4.49E-25 |
| TIMELESS | blue | 0.88640838 | 1.72E-68 | 0.64528283 | 4.66E-25 |
| TAL1 | black | 0.84096045 | 5.60E-55 | -0.6414709 | 1.08E-24 |
| RFTN2 | black | 0.83438537 | 2.22E-53 | -0.6412939 | 1.12E-24 |
| PLK4 | blue | 0.83791187 | 3.15E-54 | 0.64093201 | 1.21E-24 |
| TRIP13 | blue | 0.85927407 | 7.59E-60 | 0.64063675 | 1.29E-24 |
| OLFML1 | black | 0.84461268 | 6.73E-56 | -0.6367289 | 3.01E-24 |
| C1QTNF7 | black | 0.82028861 | 3.56E-50 | -0.6365059 | 3.16E-24 |
| CRISPLD2 | black | 0.81144921 | 2.63E-48 | -0.6356163 | 3.82E-24 |
| SHE | black | 0.82347605 | 7.11E-51 | -0.6340634 | 5.32E-24 |
| ZEB2 | black | 0.8238561 | 5.85E-51 | -0.6319711 | 8.28E-24 |
| SYDE1 | black | 0.81036399 | 4.40E-48 | -0.6314956 | 9.16E-24 |
| LRP1 | black | 0.80260936 | 1.56E-46 | -0.6298383 | 1.30E-23 |
| DNA2 | blue | 0.85703673 | 3.24E-59 | 0.62840644 | 1.75E-23 |
| CENPL | blue | 0.84268883 | 2.07E-55 | 0.6283295 | 1.78E-23 |
| C12orf48 | blue | 0.8622724 | 1.04E-60 | 0.62781601 | 1.98E-23 |
| CHAF1A | blue | 0.80864699 | 9.83E-48 | 0.6268022 | 2.44E-23 |
| LHFP | black | 0.83826847 | 2.58E-54 | -0.6260364 | 2.86E-23 |
| PRC1 | blue | 0.88631575 | 1.86E-68 | 0.62532453 | 3.31E-23 |
| SPC24 | blue | 0.85588773 | 6.77E-59 | 0.62477646 | 3.70E-23 |
| NFKBIL2 | blue | 0.80094336 | 3.29E-46 | 0.62307809 | 5.25E-23 |
| MAD2L1 | blue | 0.86220589 | 1.09E-60 | 0.62011363 | 9.58E-23 |
| PEAR1 | black | 0.80793559 | 1.37E-47 | -0.6193744 | 1.11E-22 |
| PSRC1 | blue | 0.81261892 | 1.51E-48 | 0.61769355 | 1.56E-22 |
| MCM2 | blue | 0.86333228 | 5.10E-61 | 0.61093092 | 5.95E-22 |
| ARHGAP31 | black | 0.80112504 | 3.04E-46 | -0.6091837 | 8.36E-22 |
| PDGFRB | black | 0.82284531 | 9.80E-51 | -0.6086474 | 9.28E-22 |
| GINS4 | blue | 0.84237692 | 2.48E-55 | 0.60771888 | 1.11E-21 |
| TEK | black | 0.80073107 | 3.62E-46 | -0.6060845 | 1.52E-21 |

Table S5. hub_Gene list.
